# A Transcontinental Challenge — A Test of DNA Barcode Performance for 1,541 Species of Canadian Noctuoidea (Lepidoptera)

**DOI:** 10.1371/journal.pone.0092797

**Published:** 2014-03-25

**Authors:** Reza Zahiri, J. Donald Lafontaine, B. Christian Schmidt, Jeremy R. deWaard, Evgeny V. Zakharov, Paul D. N. Hebert

**Affiliations:** 1 Biodiversity Institute of Ontario, University of Guelph, Guelph, Ontario, Canada; 2 Canadian National Collection of Insects, Arachnids, and Nematodes, Agriculture and Agri-Food Canada, Ottawa, Ontario, Canada; 3 Canadian National Collection of Insects, Arachnids, and Nematodes, Canadian Food Inspection Agency, Ottawa, Ontario, Canada; National Center for Biotechnology Information, United States of America

## Abstract

This study provides a first, comprehensive, diagnostic use of DNA barcodes for the Canadian fauna of noctuoids or “owlet” moths (Lepidoptera: Noctuoidea) based on vouchered records for 1,541 species (99.1% species coverage), and more than 30,000 sequences. When viewed from a Canada-wide perspective, DNA barcodes unambiguously discriminate 90% of the noctuoid species recognized through prior taxonomic study, and resolution reaches 95.6% when considered at a provincial scale. Barcode sharing is concentrated in certain lineages with 54% of the cases involving 1.8% of the genera. Deep intraspecific divergence exists in 7.7% of the species, but further studies are required to clarify whether these cases reflect an overlooked species complex or phylogeographic variation in a single species. Non-native species possess higher Nearest-Neighbour (NN) distances than native taxa, whereas generalist feeders have lower NN distances than those with more specialized feeding habits. We found high concordance between taxonomic names and sequence clusters delineated by the Barcode Index Number (BIN) system with 1,082 species (70%) assigned to a unique BIN. The cases of discordance involve both BIN mergers and BIN splits with 38 species falling into both categories, most likely reflecting bidirectional introgression. One fifth of the species are involved in a BIN merger reflecting the presence of 158 species sharing their barcode sequence with at least one other taxon, and 189 species with low, but diagnostic COI divergence. A very few cases (13) involved species whose members fell into both categories. Most of the remaining 140 species show a split into two or three BINs per species, while *Virbia ferruginosa* was divided into 16. The overall results confirm that DNA barcodes are effective for the identification of Canadian noctuoids. This study also affirms that BINs are a strong proxy for species, providing a pathway for a rapid, accurate estimation of animal diversity.

## Introduction

DNA barcoding has established itself as a powerful tool for species identification and discovery [1] with varied applications, especially in species-rich groups. Prior work on DNA barcoding of butterflies and moths (Lepidoptera) has investigated taxa with high morphological variability [2], [3], has linked immature stages with adults [4], has examined species of biosecurity concern [5]–[7] and sexual dimorphisms [8]. DNA barcoding has also aided the discovery of new species [9], [10] and is accelerating their description [11]–[14]. Although there are situations in which DNA barcoding does not deliver species-level resolution [15]–[18], they seem infrequent, and most cases involve a small group of closely allied species.

**Figure 1:**
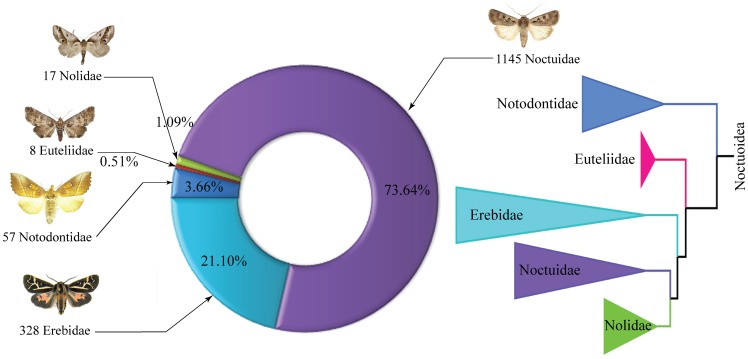
Phylogenetic hypothesis and species richness of Canadian Noctuoidea. Number of species known from Canada for five noctuoid families, as well as the family-level phylogeny [64].

Because of the effectiveness of DNA barcoding and its diverse applications, efforts are underway to assemble comprehensive DNA barcode reference libraries at both national and continental scales. Although these libraries are complete for some groups of vertebrates in certain geographic realms (e.g., the birds of North America), no major invertebrate group has seen similar analysis. The present study begins to address this gap by providing barcode coverage for Canadian Noctuoidea (hereafter noctuoids), the most diverse superfamily of Lepidoptera. With nearly 50,000 described species [19], noctuoids are an important component of terrestrial ecosystems. They are also one of the most destructive groups of agricultural pests [20]. Although knowledge of global noctuoid diversity is relatively poor, the fauna of North America [21]–[25], especially Canada [26]–[30], is well known. Among the 3700 noctuoid species from North America [25], 1555 occur in Canada including representatives from five of the six noctuoid families (Fig. 1, Table 1). The taxonomic maturity and high diversity of Canadian noctuoids provide an excellent system for assessing the performance of DNA barcodes in species discrimination.

**Table 1:** Summary of barcode coverage for Canadian noctuoid species including the source of specimens, Nearest-Neighbour distances, and the percentage of species in each family identifiable with barcodes.

Family	CAN species/barcode coverage	Origin of specimens (Canada/USA/other)	# DNA sequences	Mean Nearest-Neighbour Distance	% ID success	Species sharing barcodes
Notodontidae	57 / 57	53/4/0	1650	4.73	100	0
Euteliidae	8 / 8	5/3/0	90	5.80	100	0
Nolidae	17 / 17	16/1/0	220	4.08	100	0
Noctuidae	1145 / 1133	1001/132/0	21726	3.01	91.10	101
Erebidae	328 / 326	258/64/4	6839	3.49	82.5	57
Total	1555 / 1541	1333/204/4	30525	3.19*	90.0*	158

Asterisks indicate weighted means.

Prior barcode studies on Lepidoptera have demonstrated that DNA barcode libraries deliver high species resolution, but most investigations have examined small geographic areas or only a fraction of the species in a target assembly. For example, prior work on North American Lepidoptera examined just 20% of the species known from the eastern third of the continent [31]. Although this study reported 99% success in species identification, cases of incomplete resolution might well rise with increasing taxon coverage. Other taxonomically comprehensive studies have revealed 90–99% success [32]–[35], but they targeted relatively small areas so they do not rule out the possibility that resolution may drop with increasing geographic scope. The present study examines the impacts of increasing taxon coverage and geographic scale by examining barcode resolution for nearly all Canadian species of noctuoids.

Aside from enabling a test of barcode performance in a diverse species assemblage at a large geographic scale, the present results provide a good opportunity to examine the performance of the Barcode Index Number (BIN) System, an interim taxonomy that assigns specimens to sequence clusters termed BINs [36]. The BIN system aggregates individuals sharing similar COI sequences using single linkage clustering and a graph analytical approach, and the members of a BIN often correspond to recognized species in groups with strong taxonomy. It has been proposed that the BIN system can accelerate taxonomic progress in groups that have seen little investigation by providing a tool for aggregating specimens that are likely to be conspecific [36]. Although the BIN system has been recently implemented, its performance needs further evaluation. By testing the concordance between BIN membership and morphospecies boundaries in well-studied lineages, such as Canadian noctuoids, the utility and constraints of the BIN system for species delineation in lesser-known groups can be evaluated. Furthermore, the rich biological data available for this economically important taxon allow for the investigation of the link between feeding habits (i.e., specialized versus generalist) and barcode divergences (i.e., Nearest-Neighbour distances).

## Materials and Methods

### Sampling strategy and geographic coverage

With a surface area of 9.984 million km^2^ and a maximum breadth of 9306 km, Canada is the world's second largest country. It includes four biomes: tundra (arctic and alpine), forests (temperate and boreal), deserts (cold and semiarid), and grasslands (mixed and fescue Prairie; tallgrass Prairie; and bunchgrass/sagebrush). About 50,000 insect species occur in Canada, and Lepidoptera comprise nearly 10% of this total [29] with one third (1555 out of 4700) of these species being noctuoids (Fig. 1). The present study involved the analysis of 30,525 specimens with 86.8% derived from Canada (1333 species; about 28,000 sequences) (Data set S1). The Canadian National Collection of Insects, Arachnids, and Nematodes made the largest contribution of museum specimens (5976), while the Biodiversity Institute of Ontario provided 19,993 freshly collected individuals. Specimens were analyzed from the full geographic and habitat range of each species within Canada whenever possible (Data sets S1 and S2). However, coverage for some taxa could only be gained by analyzing specimens from other nations (Table 1). Most of these ‘extra-territorials’ derived from the USA (204 species, 2419 specimens), but 69 Eurasian specimens were analyzed for three introduced species that are very rare (*Parascotia fuliginaria*) or extirpated (*Euproctis chrysorrhoea*, *Euproctis similis*). Finally, barcodes were obtained from 23 Neotropical specimens for two species (*Eudocima apta*, *Hypocala andremona*) that are extremely rare migrants to Canada and the USA (Data set S1). The inclusion of extra-territorial specimens was justified by examining sequence variation in other species with barcode records from both Canada and United States; this analysis did not reveal significant sequence divergence linked to their nation of origin. All specimens were identified and validated by co-authors JDL and BCS; genitalia dissections were made when necessary. Taxonomy (see Data set S2) follows the most recent checklist of the Noctuoidea of North America north of Mexico [23]–[25]).

### Data acquisition and analysis

DNA extraction, PCR amplification, and sequencing of the COI barcode region were performed at the Canadian Centre for DNA Barcoding (CCDB) and followed standard protocols [37]–[41]. PCR and sequencing generally used a single pair of primers: LepF1 (ATTCAACCAATCATAAAGATATTGG) and LepR1 (TAAACTTCTGGATGTCCAAAAAATCA) [2] which recovers a 658 bp region near the 5′ end of COI including the 648 bp barcode region for the animal kingdom [1]. For museum specimens older than ten years, primer pairs designed to amplify smaller overlapping fragments (307 bp, 407 bp) were employed [41].

Data set S1 provides details (e.g., voucher codes, higher taxonomy, repository institutions, COI sequence length, collection dates and collection data) on all barcoded specimens; residual DNA extracts are stored in the DNA Archive at the CCDB. All new sequences are deposited in GenBank with accession numbers available in Data set S3. Specimen data including images, details on the voucher repositories, GPS coordinates for collection sites, sequence records, trace files, and GenBank accession numbers are available in the Barcode of Life Data Systems (BOLD, www.boldsystems.org) in two public datasets: DS-CANNOC1 (dx.doi.org/10.5883/DS-CANNOC1) and DS-CANNOC2 (dx.doi.org/10.5883/DS-CANNOC2). The number of barcode sequences per species varies from 1 to 508 (mean = 19.8) (Data set S2). Only sequence records greater than 500 bp (range 500 bp–658 bp), those that meet length and quality requirements of the BARCODE data standard [42], are included. Of the 1555 species known from Canada (Data set S1), only 14 extremely rare species now lack barcode coverage. They include two Erebidae (*Grammia philipiana*, *Hypena modestoides*) and 12 Noctuidae (*Acronicta falcula, Agrotis kingi, Annaphila danistica, Eupsilia fringata, Lasionycta illima, Lasionycta macleani, Melaporphyria immortua, Papaipema aerata, Papaipema pertincta, Pyreferra ceromatica*, *Xestia fergusoni*, and *Xestia staudingeri*).

Tests of barcode performance were made at a national level using the species list for Canada and for three regions (British Columbia, Ontario, New Brunswick/Nova Scotia) based on current barcode coverage for each area. Coverage was available for 668 of the 800 species known from British Columbia, for 617 of the 867 species from Ontario and for 387 of the 585 species known from New Brunswick and Nova Scotia. Patterns of intra- and interspecific sequence variation were explored at various taxonomic levels using the Kimura-2-Parameter (K2P) distance model and the neighbor-joining (NJ) algorithm calculated using analytical tools on BOLD. For a few taxa with either low or deep sequence divergence, model-based phylogenetic analysis (i.e., maximum likelihood, ML) was employed to examine patterns of intraspecific variation and relationships with sister species in more detail. For the study of association between host plant use and barcode divergences, we divided host plant types into four major categories 1) monocots (primarily grasses) & herbaceous dicots, 2) trees & shrubs, 3) detritus, fungi & lichens, and 4) generalists. Generalist feeders are those species that consume a broad range of monocots and dicots, often both herbaceous and woody plants. The significance of differences in interspecific (i.e., NN distances) and intraspecific variation among the four categories was assessed using nonparametric tests (e.g., Mood's Median Test). To dealing with the problem of unequal variances and sample sizes in NN distances and intraspecific data, unequal variance t-test and random sample of cases was also employed. And finally, to assess the correlation between genus size and barcode-sharing incidence, we performed a nonparametric correlation test (Spearman) in SPSS v18 (IBM).

## Results

### Barcode Performance

**Figure 2:**
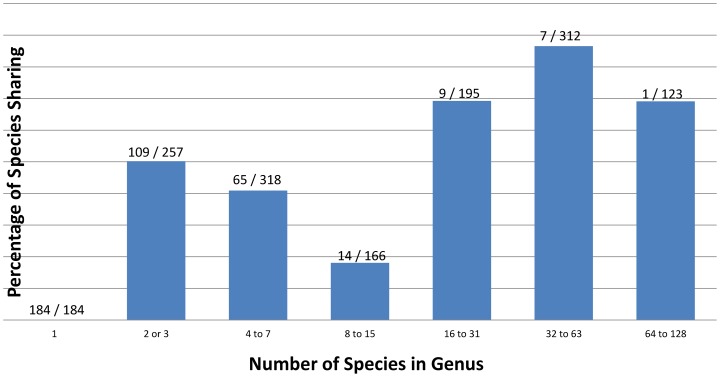
Impact of genus size on DNA barcode performance. The relationship between the number of species in a genus (plotted on a log2 scale) and the incidence of barcode sharing. Values above the bars indicate the number of genera and the number of species in each log2 category.

DNA barcodes were obtained for 1541 of the 1555 noctuoid species known from Canada. No indels causing frameshifts or stop codons were detected among the 30,525 sequences recovered from these taxa suggesting that they derive from COI rather than a pseudogene. Most species (90%) have diagnostic barcode sequences when considered from a Canada-wide perspective (Trees S1, S2, S3, S4, and S5). Identification success was even higher when analysis was restricted to a particular region with 95.3% success for New Brunswick and Nova Scotia (369/387), 96% for Ontario (592/617), and 95.4% for British Columbia (637/668) (Table S1). Mean Nearest-Neighbour (NN) distances showed modest variation among the families with more than 50 species, ranging from a low of 3.01% in Noctuidae to a high of 4.73% in the Notodontidae (Table 1); the Euteliidae had a slightly higher NN distance (5.8%), but the family was represented by only a few species. There was significant variation in barcode performance among families (X^2^ = 38.3, p<0.0001). Species in three families (Euteliidae –8 species, Nolidae –17 species, Notodontidae –57 species) were perfectly discriminated by barcode sequences, but 8.9% (101/1133 species) of the Noctuidae, and 17.5% (57 out of 326) of the Erebidae could not be discriminated because of barcode sharing by two or more species (Table S2; Tree S4 and S5). The incidence of barcode sharing seemed to be associated with the number of species in a genus (Fig. 2), however, statistical tests reject this hypothesis (Spearman Correlation Coefficient  =  0.22; p = 0.24): 15.6% of those in the 17 most diverse genera (16–123 species) and 8.1% of the species in genera with two to fifteen species shared their barcode with at least one other taxon.

### Cases of Barcode Sharing

The 57 cases of barcode sharing among the 326 species of Erebidae involved taxa in 11 of its 109 genera (Table S2). Twenty-two of these cases involved assemblages of two to four species in nine genera (*Arctia* –3, *Dasychira* –2, *Dodia* –2, *Haploa* –4, *Idia* –2, *Pararctia* –2, *Spilosoma* –3, *Virbia* –2, *Zanclognatha* –2), while the other 35 cases involved members of just two genera – *Grammia* and *Catocala*. The 11 cases in *Grammia* involved three haplotype clusters shared by two to seven species, while the 24 cases in *Catocala* included five sequence clusters with two to eight species. The most dramatic cases of sequence sharing in *Catocala* involved assemblages of species which feed on the same food plant. For example, eight hickory-feeding species (*Carya*, Juglandaceae) (*C*. *flebilis, C*. *habilis*, *C*. *judith*, *C*. *obscura*, *C*. *residua*, *C*. *retecta*, *C*. *robinsonii*, *C*. *vidua*) possess closely similar or identical barcodes, while another barcode-sharing assemblage of six species (*C. californica*, *C*. *briseis*, *C*. *faustina*, *C*. *grotiana*, *C*. *hermia*, *C*. *semirelicta*) feeds on willows and poplars (Salicaceae) [43]–[45].

The 101 cases of barcode sharing among the 1133 species of Noctuidae (Table S2) involved taxa in 29 of its 248 genera (*Abagrotis* –8, *Acronicta* –2, *Agrotis* –*2, Agriopodes* –2, *Alypia* –2, *Amphipoea* –2, *Apamea* –2, *Bellura* –2, *Copablepharon* –2, *Dargida* –2, *Epidemas* –2, *Eremobina* –2, *Eupsilia* –2, *Euxoa* –17, *Hyppa* –2, *Ipimorpha* –3, *Lasionycta* –7, *Lithophane* –5, *Mythimna* –2, *Panthea* –3, *Papaipema* –2, *Polia* –2, *Resapamea* –2, *Rhyacia* –2, *Sunira* –2, *Sympistis* –2, *Syngrapha* –4, *Trichordestra* –2, *Xestia* –12). Some large genera, such as *Acronicta* and *Sympistis*, which include 48 and 52 Canadian species respectively, showed a very low incidence of barcode sharing (just two species each). By contrast, nearly half of the cases of barcode sharing in this family involved just four genera (8/26 species of *Abagrotis*, 17/123 species of *Euxoa*, 7/34 species of *Lasionycta*, 12/45 species of *Xestia*). Most of these cases of barcode sharing involved very morphologically similar species, but there were exceptions. For example, *Lasionycta taigata* and *L. skraelingia* are morphologically distinct sister species, but they share barcodes.

### Cases of Low Barcode Divergence

**Figure 3:**
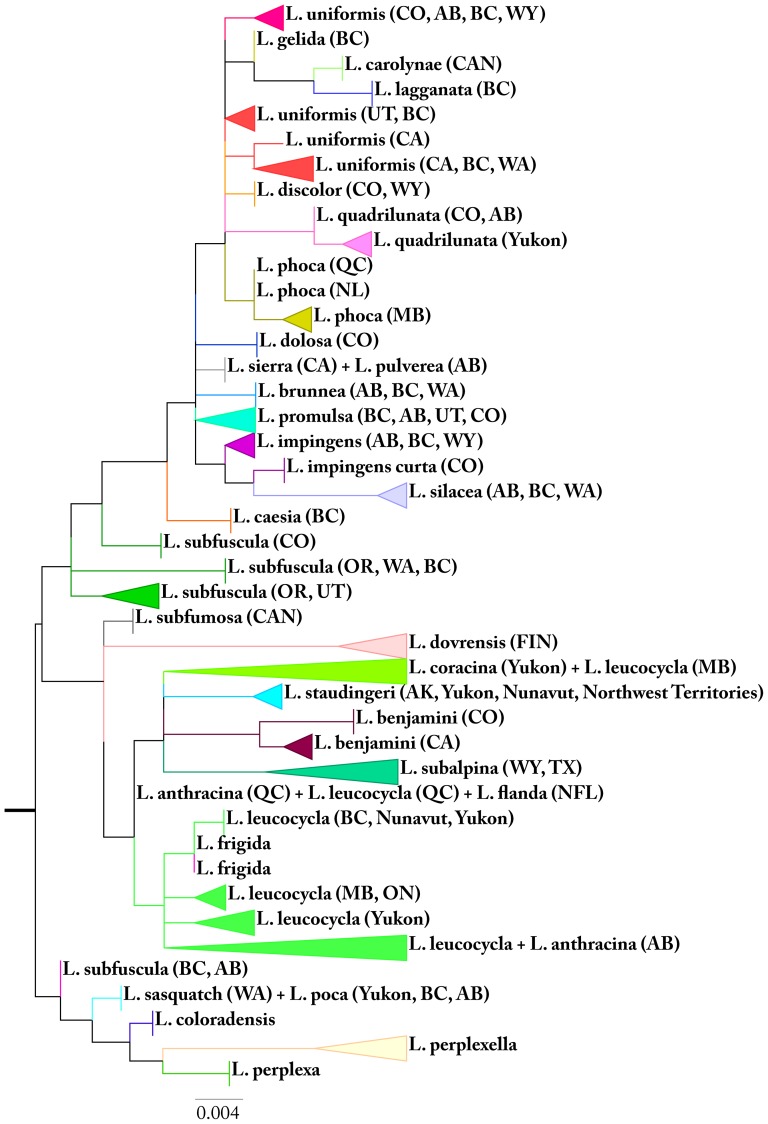
Low sequence divergence in *Lasionycta*. Maximum likelihood tree (COI barcode) for *Lasionycta* demonstrating very low sequence divergences and cases of overlapping or shared haplotypes. Terminals with vertical bars indicate one or few samples shared identical haplotype, those with trianglesrepresenting collapsed haplotypes with less than 2% sequence divergence. Geographic origin is given in brackets as standard abbreviations for provinces (Canada) or states (USA); FIN  =  Finland.

Twenty-seven genera (*Anarta*, *Caradrina*, *Cissusa*, *Cosmia*, *Cucullia*, *Dasychira*, *Datana*, *Diarsia*, *Egira*, *Enargia*, *Euclidia*, *Eupsilia*, *Feltia*, *Feralia*, *Hadena*, *Hypoprepia*, *Leucania*, *Neoarctia*, *Papestra*, *Phragmatobia*, *Schinia*, *Setagrotis*, *Spaelotis*, *Symmerista*, *Sympistis*, *Xylena*, *Zale*) included two or more species with low divergence, but with no evidence of shared sequences (Table S3). Species of *Lasionycta* provide a key example of low divergence coupled with a few cases of sequence sharing (Fig. 3).

### Cases of Deep Intraspecific Sequence Divergence

Deep (>2%) barcode divergence was detected in 119 (7.7%) species and another 21 species showed sufficient divergence (1.2%–1.9%) for their members to be assigned to two BINs. These 140 taxa included representatives from 83 of the 387 genera of noctuoids and most were partitioned into two (100) or three (30) BINs, but 10 were placed in four or more (Table S4). *Virbia ferruginosa* showed exceptional diversity with its members assigned to 16 BINs. The cause of this remarkable molecular variation is currently not clear, but taxonomic study (BCS) suggests that this variation is not linked to cryptic species. Although many of the 140 cases require more investigation, Table 2 lists 12 species where biological covariates are associated with barcode clusters, indicating that unrecognized species are known or probable. For example, specimens in the 11 barcode lineages of *Idia lubricalis* show differences in external and genitalic morphology, and include a number of unrecognized species (BCS, in prep.).

**Table 2:** Twelve Canadian noctuoids with deep (>2%) intraspecific barcode variation that also show morphological divergence between their barcode clusters.

Family	Subfamily	Species Auth	# of clusters	%Sequence divergence	Condition
Notodontidae	Notodontinae	*Furcula cinerea* (Walker, 1865)	5	2.7	taxonomic status under revision
Notodontidae	Notodontinae	*Furcula occidentalis* (Lintner, 1878)	5	2.7	taxonomic status under revision
Notodontidae	Notodontinae	*Pheosia rimosa* Packard, 1864	8	5.1	one haplotype seems to be a good species (*P. portlandia*) - under revision
Nolidae	Chloephorinae	*Nycteola* n. sp.	2	3	a possible new species from BC and CO 3% diverged from sister species *N. fletcheri*
Erebidae	Herminiinae	*Idia lubricalis* (Geyer, 1832)	11	3.8	species complex includes various form (size, colour, maculationsand etc.) - needs to be studied
Erebidae	Herminiinae	*Idia americalis* (Guenée, 1854)	3	1.8	biological evidence for cryptic species (i.e., pheromones), despite low intraspecific barcode divergence
Erebidae	Hypenodinae	*Hypenodes* n. sp.	5	2.1	five undescribed species
Erebidae	Erebinae	*Melipotis perpendicularis* (Guenée, 1852)	4	2.3	species complex with various haplotypes of 1.45% intraspecific variation
Erebidae	Erebinae	*Caenurgina crassiuscula* (Haworth, 1809)	5	2.95	species complex with various diverged haplotypes of 2.95% intraspecific variation
Noctuidae	Noctuinae	*Anarta crotchii* (Grote, 1880)	2	4.3	two distinct barcode clusters of 3.6% sequence divergence - barcode clusters do not match the morphotypes
Noctuidae	Noctuinae	*Papaipema pterisii* Bird, 1907	6	1.95	one diverged haplotype seems related to *P. pterisii* but with different feeding habits (ostrich fern)
Noctuidae	Noctuinae	*Lacinipolia strigicollis* (Wallengren, 1860)	4	5.4	one diverged haplotype of 5.4% different - no obvious difference in external or internal morphology, distribution

#### Factors Influencing Nearest-Neighbour Distances

**Figure 4:**
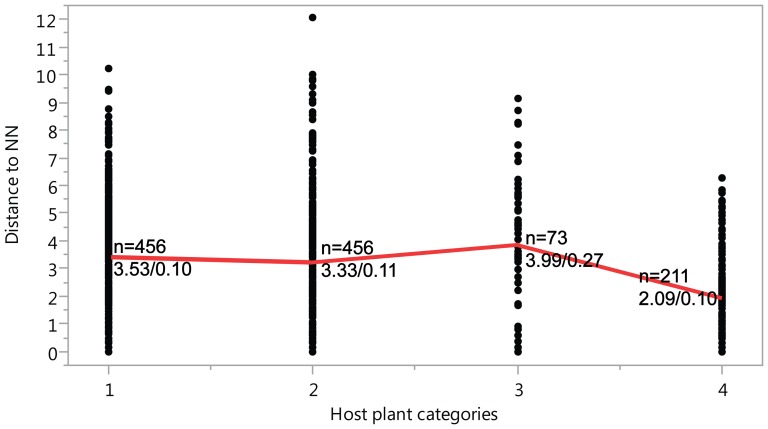
Impact of host plant type on NN distances. Nearest-Neighbour (NN) distances for species of Canadian noctuoids using four food plant categories: 1) monocots or herbaceous dicots, 2) trees or shrubs, 3) detritus, fungi and lichens, and 4) generalist. Values above the bars indicate the number of species in each food plant category (n), average of NN/standard errors (SE).

Two factors were found to impact Nearest-Neighbour (NN) distance. Firstly, the 26 species of non-native Canadian noctuoids [23]–[25] possess a significantly (X^2^ = 17.53; Median = 2.95; p<0.0001) higher NN distance (x = 5.9%) (Table 3) than native species (x = 3.02%). Secondly, there is evidence of an association between food plant usage and interspecific (i.e., NN distance) divergences. Records on host plants are available for about 80% of Canadian noctuoids [43]–[45], permitting their assignment to one of four host plant categories 1) monocots & herbaceous dicots, 2) trees & shrubs, 3) detritus, fungi & lichens, and 4) generalists. Generalist feeders possessed a lower NN distance (2.09%) than species in the other feeding categories (Fig. 4), and both nonparametric test and analysis of variance of random samples with equal size indicated that this difference was significant (X^2^ = 94.89; Median = 3.09; p<0.0001) (Tables 4–7). The levels of intraspecific variation was significantly (X^2^ = 10.03; Median = 0.16; p<0.018) lower among grass/herbaceous feeders (first category = 0.27%) than in other categories (category 2 = 0.33%; 3 = 0.51%; 4 = 0.48%). As discussed under ‘Barcode Performance’, genus size can also affects NN distance, with large genera presumably having higher rates of barcode-sharing (Fig. 2) which would intuitively mark a decrease in NN values. Nevertheless, statistical tests revealed that this association is not significantly supported.

**Table 3:** A list of introduced noctuoid species into Canada.

Introduced species to Canada	Approximate dates of introduction	Barcode coverage	NN Distance
*Agrochola lota*	1976	x	6.40
*Amphipyra tragopoginis*		x	6.28
*Apamea unanimis*	1991	x	4.07
*Calophasia lunula*	1965 — bio-control agent	x	6.51
*Caradrina morpheus*	1944–1955	x	4.14
*Cerapteryx graminis*	1966	x	4.22
*Chrysodeixis chalcites*	2008	x	5.39
*Cucullia umbratica*	1998	x	4.68
*Euproctis chrysorrhoea*	1897		
*Euproctis similis*	1933		
*Garella nilotica*		x	7.85
*Hydraecia micacea*	1902	x	1.72
*Lateroligia ophiogramma*	1989	x	4.67
*Leucoma salicis*	1920	x	12.07
*Lymantria dispar*	1868	x	10.08
*Noctua comes*	1982	x	5.23
*Noctua pronuba*	1979	x	4.54
*Nola cucullatella*	2008		
*Oligia strigilis*	1990	x	4.82
*Parascotia fuliginaria*	<1980		
*Rhizedra lutosa*	1991	x	6.21
*Spodoptera exigua*		x	5.72
*Tathorhynchus exsiccata*		x	6.86
*Trichoplusia ni*		x	6.22
*Tyria jacobaeae*	1965 — bio-control agent	x	7.73
*Xestia xanthographa*	1907–1950	x	4.13
**Total**		**22**	**5.89**

Their NN distance and approximate date of introduction are shown.

**Table 4:** Summary of analysis of variance (ANOVA) of the relationship between NN distances at COI and larval food plant categories for 1196 species of Canadian noctuoids.

*Groups*	*Count (1/2)*	*Sum (1/2)*	*Average (1/2)*	*Variance (1/2)*
Grass/herbaceous	456/73	1608.29/243.43	3.53/3.33	4.75/4.20
Tree/shrubs	456/73	1518.13/259.92	3.33/3.56	5.73/6.43
Detritivore/fungivore/lichenivore	73/73	291.51/291.51	3.99/3.99	5.29/5.29
Generalist	211/73	440.53/175.18	2.09/2.40	2.22/2.28

Host plant data set was analyzed in two different ways: 1) actual data set with unequal sample size (non-normal distributed data) and 2) re-sampled data set with equal sample size (73 samples).

**Table 5:** Statistical results of analysis of variance (ANOVA) of the relationship between NN distances at COI and larval food plant categories for 1196 species of Canadian noctuoids.

*Source of Variation*	*SS (1/2)*	*df (1/2)*	*MS (1/2)*	*F (1/2)*	*P-value (1/2)*
Between Groups	362.48/99.15	3/3	120.83/33.05	25.66/7.26	0.00/0.00
Within Groups	5613.26/1310.91	1192/288	4.71/4.55		
					
Total	5975.74/1410.06	1195/291			

Host plant data set was analyzed in two different ways: 1) actual data set with unequal sample size (non-normal distributed data) and 2) re-sampled data set with equal sample size (73 samples).

**Table 6:** Summary of nonparametric test (Mood's Median) of the relationship between NN distances at COI and larval food plant categories for 1196 species of Canadian noctuoids.

*Groups*	*> Median*	*< = Median*
Grass/herbaceous	271	185
Tree/shrubs	230	226
Detritivore/fungivore/lichenivore	51	22
Generalist	46	165

**Table 7:** Statistical results of nonparametric test (Mood's Median) of the relationship between NN distances at COI and larval food plant categories for 1196 species of Canadian noctuoids.

N	1196
Median	3.09
Chi-Square	94.89
df	3
*P-value*	0.00

#### Congruence Between Species Boundaries of Recognized Species and BINs

We found close correspondence between the number of species (1541) analyzed and the number of BIN (1515) assignments (Table 8). However, the strength of this congruence was partially a consequence of the counterbalancing effects of BIN splits and mergers. In actuality, perfect correspondence between the assignment of specimens to a particular species and their placement in a unique BIN was only evident for 1082 of the 1541 species (70%). Another 140 species (including all 119 species with >2% intraspecific sequence divergence) were involved in splits with their members assigned to two (100 species), three (30 species), or more BINs (10 species). Finally, 348 species were involved in a merger where they were placed in a BIN that included at least one other species. Some mergers involved species (158) that shared barcodes with at least one other taxon (Table S2), but most (189) involved species with diagnostic but low barcode divergence (Table S3). A very few cases (13) involved species whose members fell into both categories.

**Table 8:** The correspondence between the number of BINs and current species counts for five families of Canadian noctuoids.

Superfamily	Family	CDN Species richness	Species coverage	BINs	Species count on BOLD	Notes
**Noctuoidea**	Notodontidae	57	57	63	62	3 subsp. +2 sp. under study
	Euteliidae	8	8	8	8	-
	Nolidae	17	17	20	19	2 new sp.
	Noctuidae	1145	1133	1090	1159	species complex + new sp. + subsp.
	Erebidae	328	326	337	357	species complex +16 new sp. + subsp.
**Total**		1555	1541	1518	1605	

## Discussion

As revealed by this study and other investigations, the results of large-scale DNA barcode analyses never perfectly replicate existing taxonomic systems; they reveal both instances of deep intraspecific sequence divergence and other cases where members of different species share the same barcode sequence. In the present study, DNA barcodes differentiated more than 95% of currently recognized noctuoid species when considered at a provincial level (Table S1), and 90% when examined for the whole of Canada. The modest decline in identification success with increased geographic scale reinforces an earlier conclusion, based on a much smaller dataset, that increased geographic sampling does not seriously diminish the performance of DNA barcodes [46]. Moreover, the resolution obtained for Canadian noctuoids is similar to that observed for other groups of Lepidoptera in other geographic regions. For example, deWaard et al. [35] found 93% resolution in a study on 400 species of Geometridae from British Columbia, while Hebert et al. [31] observed 99% resolution for 1200 species in diverse families of Lepidoptera from eastern North America. Results from Europe show similar performance with 90% for 185 species of Romanian butterflies [32], 98.5% for 400 species of Bavarian geometrids [33] and 99% for 957 species from a broad range of macro-Lepidoptera in the same region [34].

This study revealed that 7.7% of Canadian noctuoids possess more than 2% intraspecific divergence with this variation falling into two or more discrete sequence clusters. So long as these clusters are ‘private’ to a particular species, their presence does not complicate the assignment of specimens to a known taxon although they may signal overlooked species. The incidence of such cases of deep divergence in Canadian noctuoids is similar to the 5–8% reported in earlier work on other Lepidoptera faunas with well-studied taxonomy [3], [31], [33], [34]. Such cases of deep divergence can arise in three ways and it is important to determine the causal factor for each case to understand its significance. Deep divergences can arise through the presence of cryptic species, the recovery of a pseudogene, or high intraspecific variation. The simplest initial step to discriminate among these alternatives lies in examining barcode groups for diagnostic differences in external or genitalic morphology. Any covariation between barcode clusters and other traits provides strong evidence that the current taxonomic system has overlooked species in the group under investigation [2], [47], [48]. For example, such covariation was noted in 12 of the 140 species with deep ‘intraspecific’ divergence in this study (Table 2). In cases where such variation is not apparent, it is important to rule out the possibility that the clusters reflect the recovery of the authentic COI gene from some individuals, and a pseudogene from others. If the analysis of a second mitochondrial gene (e.g., cytochrome b) also reveals deep intraspecific divergence and its sequence clusters correspond with those at COI, the deep barcode divergence is likely to be real rather than an artifact of variable pseudogene recovery. Subsequent analysis can then focus on determining if the sequence divergence at COI reflects the presence of sibling species or an unusually high level of intraspecific diversity. Such cases are best resolved through multi-loci analysis (e.g., a nuclear loci) [49], [50] of specimens from geographic settings where the component lineages are sympatric. If an exhaustive examination of nuclear markers shows no differentiation between lineages, the variation at COI likely reflects deep intraspecific divergence, such as that reported in European populations of the geometrid *Epirrita autumnata* [51]. The factor(s) responsible for divergence can then be analyzed; it may reflect selective sweeps driven by *Wolbachia* [52] or secondary contact between lineages formerly isolated in different glacial refugia. Our study indicates the need for detailed analyses of this sort to better understand the cause and taxonomic implications of the deep sequence divergences in 140 species of Canadian noctuoids (including the 12 taxa where barcode divergence was linked to morphological differentiation). *Virbia feruginosa* should be a priority target, given its assignment to 16 BINs and the long-standing taxonomic uncertainty surrounding this genus [53].

Because the standard criterion for the evaluation of barcode success involves its capacity to discriminate known species, cases of barcode sharing attract particular attention. This study revealed that 10% of Canadian noctuoids (158/1541) share their barcode sequence with at least one other species and that the incidence of such cases varies significantly among the five noctuoid families. These cases of barcode sharing can have three causes; the species involved may be young; they may be older, but have experienced recent introgression; or they may actually represent a single species (i.e., wrong taxonomy). Lineages undergoing active speciation should include more species that are so young that they lack diagnostic COI sequences. Viewed from this perspective, the Notodontidae, which lacked any case of barcode sharing, has seen less recent speciation than the Erebidae where 17.5% of species share barcodes. Aside from this divergence between families, there was also a link to generic diversity. As might be expected, no case of barcode sharing involved species in monotypic genera, while its incidence reached 15.6% in the 17 most diverse genera (>16 species). Genera with an intermediate species count (2–15) also showed an intermediate level of barcode sharing (8.1%), although there was evidence of an unexpected trend toward lower barcode sharing in these genera as the species count rose. Viewed from an overall perspective, the ‘taxonomic localization’ of compromised resolution was striking; seven of the 387 genera of noctuoids accounted for 54% of all cases of barcode sharing. Although each of these genera included a substantial number of species (range 20–123), they only account for 21.7% of all Canadian noctuoids, meaning that they include a high proportion of taxa that share barcodes, suggestive of active or recent speciation. Cases of sequence sharing can also be due to oversplitting of species, especially in species-rich genera. A recent study that utilized both DNA barcoding and morphological approaches resolved several taxonomic issues in North American Erebidae and Noctuidae through the synonymization previous oversplit species [54]. Most current taxonomy is based on traditional morphological studies, so there is no correct taxonomy to act as reference system. Indeed, correct designation of species boundaries in high diversity genera usually requires comprehensive examination of reproductive compatibility, host plant associations, morphological characters and sequence divergences. Consequently, some cases of discordance between traditional taxonomy and results of DNA barcoding could reflect incorrect taxonomy arising as a result of intraspecific polymorphism or overly exhaustive morphological studies of charismatic taxa.

Other cases of barcode sharing may arise as a consequence of limited or biased sampling. In cases where only one or a few specimens were barcoded, it is likely that some cases of barcode sharing be associated with this artifact. A single individual would not reflect the intraspecific diversity (either morphological or genetic variation) of species as a whole. The collection sites (e.g., hybrid zones) and extreme specimens with intermediate characteristics (e.g., hybrids) can dramatically impact results. In addition, a single species can be assigned to a unique BIN over part of its geographic range, but share a BIN with a second species in another region [33]. Further studies (e.g., increasing taxon sampling and genetic markers) are needed to identify the possible reasons and causes for barcode sharing. As expected, NN distances were significantly (p<0.0001) higher for introduced than native species, undoubtedly reflecting the fact that many of them have left their sister taxon behind in Eurasia. By contrast, introduced species had lower intraspecific divergence (x = 0.11%) than native species (x = 0.39%), reflecting the expected loss of diversity as a consequence of founder effects. However, nonparametric tests indicated that this difference was not significant (X^2^ = 4.30; Median  = 0.15; p = 0.065).

Nearest-Neighbour distances werefound to be significantly lower among generalist feeders than among species with specialized feeding habits. This result is counterintuitive as host-plant specialization should foster diversification, creating assemblages of closely related species. Comparison of intraspecific divergences revealed that species feeding on grass/herbaceous possess significantly lower intraspecific barcode divergence than species with other feeding behavior. This result conflicts with the usual expectation that species with wide niches (e.g., generalists) should be more variable than species with narrow niches (e.g., host-plant specialists) [55]–[56]. Taking into account that mtDNA markers such as the barcode region are poor candidates for assessing this association because of the selective sweeps on mitochondria regularly deletes variation [57]. However, an equally likely scenario is that polyphagy (generalist feeding) is actually more difficult from an evolutionary perspective – those species that are able to switch to a broad diet could equally undergo a species radiation (polyphagy is basically a specialized feeding strategy). Other results suggest the needfor a deeper investigation into the linkage between host plant use and barcode divergences.

The potential causes of barcode-sharing in the genus *Catocala* appear to be particularly complex, and may include larval hostplant-mediated mechanisms, such as those documented in sawflies [58]. Dramatic cases of barcode sharing were detected among two groups of taxa, those feeding on hickories (*Carya* spp.) and those on poplars / willows (Salicaceae). The latter group includes parapatric species pairs that are morphologically very similar (the *C*. *briseis* / *californica* / *grotiana* complex) and might therefore exhibit incomplete lineage sorting due to recent or incomplete speciation. However, barcode sharing or overlap is equally prevalent among sympatric species of a second Salicaceae-group, and a *Carya*-group. Species in both groups show strident phenotypic differences in both adults and larvae, and their status as bona species has not been questioned [59], [60]; for example, *C*. *parta* / *luciana* / *junctura* / *meskei* and *C*. *briseis* / *semirelicta* have closely similar barcodes, but shared host plants, habitats and similar genitalic morphologies may facilitate hybridization. Further study of the 16 North American species in the Salicaceae-group [59], [60] is needed to resolve the evolutionary history of this complex, particularly through nuclear gene markers and biogeographical analysis. The same is true of the 23 species of the *Carya-*group, where at least *Catocala insolabilis*, *C*. *dejecta*, *C*. *lacrymosa*, *C*. *palaeogama*, *C*. *retecta*, *C*. *judith*, *C*. *robinsonii*, *C*. *obscura*, *C*. *habilis*, *C*. *residua*, *C*. *vidua*, *C*. *flebilis*, and *C*. *robinsonii* form a series with overlapping and identical barcodes.

Species of *Grammia*, a grass- and herb-feeding genus, possessed a particularly unusual pattern of barcode variation where species not only share barcodes, but often very divergent ones, suggesting that past hybridization events, sometimes between distantly related species, have led to the bidirectional introgression of mitochondrial genomes [18]. Broad zones of sympatry, weak divergence in genitalia, and overlap in pheromone usage have apparently facilitated such hybridization [18]. All of these cases of barcode sharing require more detailed study to evaluate causal factors [33], [34].

Aside from probing the efficacy of DNA barcodes as a tool for species identification, the present study has examined the correspondence between sequence clusters recognized by the BIN system and known species. The results of this analysis indicate the strong capacity of the BIN system to estimate species diversity (1515 BINs versus 1541 species), supporting the conclusion of an earlier investigation [36]. These results suggest that DNA barcoding is poised to resolve a long-standing question – how many animal species are there on the planet [11], [61]? Moreover, the BIN system has the capacity to do more than just to deliver a species count when it is coupled with a well-parameterized barcode reference library. In this situation, in most cases, each BIN can be automatically assigned to a higher-level taxon. Automated phylum-level assignments are now secure and class and ordinal placements are correct in more than 90% of cases for terrestrial animals (pers. obs.). Further parameterization of the barcode library will undoubtedly lead to robust familial assignments [62]. Although Ekrem et al. [63] correctly pointed out that DNA barcodes can only deliver a species-level assignment when a fully parameterized reference library is in place, the BIN system will provide a species count for each major compartment of biodiversity long before all species gain description. However, this capacity will require more large-scale reference libraries such as the one assembled in this study.

## Supporting Information

Table S1 **List of 158 species that cannot be discriminated from one or more of their congeners with DNA barcodes when considered on a Canada-wide basis.** Because the species assemblage varies regionally, the incidence of barcode sharing decreases when considered regionally. This table presents data for three regions: New Brunswick/Nova Scotia, Ontario, and British Columbia.
(XLS)

Table S2 **Fifty-seven assemblages of Canadian noctuoids where two or more species share their barcode sequence(s).**
(XLS)

Table S3 **Seventy-six assemblages of Canadian noctuoids where two or more species possess low sequence divergence (<2%), but with no evidence of sequence sharing.** Asterisks indicate cases where a species shows slight sequence divergence from two or more species which share barcodes.
(XLS)

Table S4 **Canadian noctuoids with a maximum intraspecific barcode divergence >2% (121 species) or that were partitioned into two or more BINs (140 species).** Asterisks indicate species with less than 2% maximum divergence.
(XLS)

Tree S1 **NJ tree for Canadian species in the family Notodontidae.**
(PDF)

Tree S2 **NJ tree for Canadian species in the family Euteliidae.**
(PDF)

Tree S3 **NJ tree for Canadian species in the family Nolidae.**
(PDF)

Tree S4 **NJ tree for Canadian species in the family Erebidae.**
(PDF)

Tree S5 **NJ tree for Canadian species in the family Noctuidae.**
(PDF)

Data set S1 **Data set for Canadian Noctuoidea: families Notodontidae, Euteliidae, Nolidae, Erebidae and Noctuidae.**
(XLS)

Data set S2 **List of Canadian species of Noctuoidea and the number of barcode records for each taxon.** Species with specimen barcoded from out-of-Canada are marked in green. Missing species (14 taxa) are in red.
(XLS)

Data set S3 **GenBank accession numbers.**
(XLS)

## References

[1] Hebert PDN, Cywinska A, Ball SL, deWaard JR (2003) Biological identifications through DNA barcodes. Proceedings of the Royal Society B: Biological Sciences 270: 313–321. 12614582 10.1098/rspb.2002.2218 PMC1691236

[2] Hebert PDN, Penton EH, Burns JM, Janzen DH, Hallwachs W (2004) Ten species in one: DNA barcoding reveals cryptic species in the neotropical skipper butterfly *Astraptes fulgerator* . Proceedings of the National Academy of Sciences of the United States of America 101: 14812–14817. 15465915 10.1073/pnas.0406166101 PMC522015

[3] Hajibabaei M, Janzen DH, Burns JM, Hallwachs W, Hebert PDN (2006) DNA barcodes distinguish species of tropical Lepidoptera. Proceedings of the National Academy of Science USA 103: 968–971. 10.1073/pnas.0510466103 PMC1327734 16418261

[4] Miller SE, Hrcek J, Novotny V, Weiblen GD, Hebert PDN (2013) DNA barcodes of caterpillars (Lepidoptera) from Papua New Guinea. Proceedings of the Entomological Society of Washington 115: 107–109.

[5] Gwiazdowski RA, Elkinton JS, deWaard JR, Sremac M (2013) Phylogeographic diversity of the winter moths *Operophtera brumata* and *O. bruceata* (Lepidoptera: Geometridae) in Europe and North America. Annals of the Entomological Society of America 106: 143–151.

[6] deWaard JR, Mitchell A, Keena MA, Gopurenko D, Boykin LM, et al (2010) Towards a global barcode library for *Lymantria* (Lepidoptera: Lymantriinae) tussock moths of biosecurity concern. PLoS ONE 5: e14280. 21151562 10.1371/journal.pone.0014280 PMC3000334

[7] Valade R, Kenis M, Hernandez-Lopez A, Augustin S, Mari Mena S, et al (2009) Mitochondrial and microsatellite DNA markers reveal a Balkan origin for the highly invasive horse-chestnut leaf miner *Cameraria ohridella* (Lepidoptera, Gracillariidae). Molecular Ecology 18: 3458–3470. 19627490 10.1111/j.1365-294X.2009.04290.x

[8] Rougerie R, Laguerre M (2010) Les codes barres ADN révèlent un cas remarquable de dimorphisme sexuel chez une arctiide de Guyane Française: *Senecauxia coraliae* de Toulgoët, 1990 (Lepidoptera: Arctiidae). Annales de la Société Entomologique de France 46: 477–480.

[9] Huemer P (2011) Pseudo-endemism and cryptic diversity in Lepidoptera – case studies from the Alps and the Abruzzi. Journal on Protected Mountain Areas Research and Management 3: 11–18.

[10] Dinca V, Lukhtanov VA, Talavera G, Vila R (2011) Unexpected layers of cryptic diversity in wood white *Leptidea* butterflies. Nature Communications 2: 324. 10.1038/ncomms1329 21610727

[11] Mora C, Tittensor DP, Adl S, Simpson AGB, Worm B (2011) How many species are there on Earth and in the ocean? PLoS Biology 9: e1001127. 21886479 10.1371/journal.pbio.1001127 PMC3160336

[12] Butcher BA, Smith MA, Sharkey MJ, Quicke DLJ (2012) A turbo-taxonomic study of Thai *Aleiodes* (*Aleiodes*) and *Aleiodes* (Arcaleiodes) (Hymenoptera: Braconidae: Rogadiniae) based largely on COI barcoded specimens, with rapid descriptions of 179 new species. Zootaxa 3457: 1–232.

[13] Riedel A, Sagata K, Suhardjono YR, Tänzler R, Balke M (2013a) Integrative taxonomy on the fast track —towards more sustainability in biodiversity research. Frontiers in Zoology 10: 15. 23537182 10.1186/1742-9994-10-15 PMC3626550

[14] Riedel A, Sagata K, Surbakti S, Tänzler R, Balke M (2013b) One hundred and one new species of *Trigonopterus* weevils from New Guinea. ZooKeys 280: 1–150. 10.3897/zookeys.280.3906 PMC3677382 23794832

[15] Collins RA, Cruickshank RH (2012) The seven deadly sins of DNA barcoding. Molecular Ecology Resources. DOI: 10.1111/1755-0998.12046. 10.1111/1755-0998.12046 23280099

[16] Dupuis JR, Roe A, Sperling FAH (2012) Multi-locus species delimitation in closely related animals and fungi: one marker is not enough. Molecular Ecology 21: 4422–4436. 22891635 10.1111/j.1365-294X.2012.05642.x

[17] Fujita MK, Leache AD, Burbrink FT, McGuire JA, Moritz C (2012) Coalescent-based species delimitation in an integrative taxonomy. Trends in Ecology and Evolution 27: 480–488. 22633974 10.1016/j.tree.2012.04.012

[18] Schmidt BC, Sperling FAH (2008) Widespread decoupling of mtDNA variation and species integrity in *Grammia* tiger moths (Lepidoptera: Noctuidae). Systematic Entomology 33: 613–634.

[19] Nieukerken EJ van, Kaila L, Kitching IJ, Kristensen NP, Lees DC, et al (2011) Animal biodiversity: An outline of higher-level classification and survey of taxonomic richness. Zootaxa 3148: 212–221. 10.11646/zootaxa.3703.1.1 26146682

[20] Mitchell A, Mitter C, Regier JC (2006) Systematics and evolution of the cutworm moths (Lepidoptera: Noctuidae): evidence from two protein-coding nuclear genes. Systematic Entomology 31: 21–46.

[21] Franclemont JG, Todd EL (1983) pp. 120–159. *In* Hodges RW, et al. Check list of the Lepidoptera of America North of Mexico. University Press. Cambridge. xxiv,. 284 pp.

[22] Poole RW (1995) Noctuoidea: Noctuidae (part), Cuculliinae, Stiriinae, Psaphidinae (part). In: Dominick RB, et al. (Eds) The Moths of America North of Mexico. Fascicle 26. The Wedge Entomological Research Foundation, Washington, 249 pp.

[23] Lafontaine JD, Schmidt BC (2010) Annotated check list of the Noctuoidea (Insecta, Lepidoptera) of North America north of Mexico. ZooKeys 40: 1–239. 10.3897/zookeys.149.1805 PMC3234417 22207802

[24] Lafontaine JD, Schmidt BC (2011) Additions and correction to the check list of the Noctuoidea (Insecta, Lepidoptera) of North America north of Mexico. ZooKeys 149: 145–161. 10.3897/zookeys.149.1805 PMC3234417 22207802

[25] Lafontaine JD, Schmidt BC (2013) Comments on differences in classification of the superfamily Noctuoidea (Insecta, Lepidoptera) between Eurasia and North America. ZooKeys 264: 209–217. 10.3897/zookeys.264.4441 PMC3668380 23730182

[26] Pohl GR, Anweiler GG, Schmidt BC, Kondla NG (2010) An annotated list of the Lepidoptera of Alberta, Canada. ZooKeys 38: 1–549.

[27] Handfield L (2011) Les Papillons du Québec. Sant-Constant, Québec: Broquet. 672 pp., 166 plates.

[28] Riotte JCE (1992) *Annotated list of Ontario Lepidoptera*. Royal Ontario Museum, Toronto, 208 pp.

[29] Lafontaine JD, Troubridge JT, Thomas AW (2010b) Moths and butterflies (Lepidoptera) of the Atlantic Maritime Ecozone. In *Assessment of Species Diversity in the Atlantic Maritime Ecozone*. Edited by McAlpine DF and Smith IM. NRC Research Press, Ottawa, Canada. pp. 489–537.

[30] Lafontaine JD, Wood DM (1997) Butterflies and moths (Lepidoptera) of the Yukon. In *Insects of the Yukon*. Danks HV and Downes JA (Eds), Biological Survey of Canada (Terrestrial Arthropods), Ottawa, Canada, pp. 723–785.

[31] Hebert PDN, deWaard JR, Landry JF (2010) DNA barcodes for 1/1000 of the animal kingdom. Biology Letters 6: 359–362. 20015856 10.1098/rsbl.2009.0848 PMC2880045

[32] Dinca V, Zakharov EV, Hebert PDN, Vila R (2010) Complete DNA barcode reference library for a country's butterfly fauna reveals high performance for temperate Europe. Proceedings of the Royal Society of London B 278: 347–355. 10.1098/rspb.2010.1089 PMC3013404 20702462

[33] Hausmann A, Haszprunar G, Hebert PDN (2011a) DNA barcoding the geometrid fauna of Bavaria (Lepidoptera): successes, surprises, and questions. PLoS ONE 6: e17134. 21423340 10.1371/journal.pone.0017134 PMC3040642

[34] Hausmann A, Haszprunar G, Segerer AH, Speidel W, Behounek G, et al (2011b) Now DNA-barcoded: The butterflies and larger moths of Germany (Lepidoptera: Rhopalocera, Macroheterocera). Spixiana 34: 47–58.

[35] deWaard JR, Hebert PDN, Humble LM (2011) A comprehensive DNA barcode library for the looper moths (Lepidoptera: Geometridae) of British Columbia, Canada. PLoS ONE 6: e18290. 21464900 10.1371/journal.pone.0018290 PMC3065486

[36] Ratnasingham S, Hebert PDN (2013) A DNA-based registry for all animal species: The Barcode Index Number (BIN) System. PLoS ONE 8: e66213. 23861743 10.1371/journal.pone.0066213 PMC3704603

[37] Hajibabaei M, deWaard JR, Ivanova NV, Ratnasingham S, Dooh R, et al (2005) Critical factors for the high volume assembly of DNA barcodes. Philosophical Transactions of the Royal Society B 360: 1959–1967. 10.1098/rstb.2005.1727 PMC1609220 16214753

[38] Ivanova NV, deWaard JR, Hebert PDN (2006) An inexpensive, automation-friendly protocol for recovering high-quality DNA. Molecular Ecology Notes 6: 998–1002.

[39] deWaard JR, Ivanova NV, Hajibabaei M, Hebert PDN (2008) Assembling DNA barcodes: Analytical protocols. In: Martin Cristofre, editor. In Methods in Molecular Biology: Environmental Genetics. Totowa, USA: Humana Press Inc. pp. 275–293. 10.1007/978-1-59745-548-0_15 18642605

[40] Hebert PDN, deWaard JR, Zakharov EV, Prosser SWJ, Sones JE, et al (2013) A DNA ‘Barcode Blitz’: Rapid digitization and sequencing of a natural history collection. PLoS ONE 8: e68535. 23874660 10.1371/journal.pone.0068535 PMC3707885

[41] The Canadian Centre for DNA Barcoding (CCDB) (2013) Available: http://www.ccdb.ca/resources.php.

[42] Hanner R (2005) Consortium for the Barcode of Life: Data Standards for BARCODE Records in INSDC (BRIs). Available: http://barcoding.si.edu/pdf/dwg_data_standards-final.pdf.

[43] Prentice RM (1962) Forest Lepidoptera of Canada reported by the forest insect survey, Volume 2: Nycteolidae, Notodontidae, Noctuidae, Liparidae. pp. 77–281. Publication 1013. Canada Department of Forestry, Forest Entomology and Pathology Branch. Ottawa, Ontario.

[44] Wagner DL, Schweitzer DF, Sullivan JB, Reardon RC (2011) Owlet caterpillars of eastern North America. Princeton: Princeton University Press. 576 pp.

[45] Powell JA, Opler PA (2009) Moths of Western North America. Berkeley: University of California Press. 369 pp.

[46] Lukhtanov VA, Sourakov A, Zakharov EV, Hebert PDN (2009) DNA barcoding Central Asian butterflies: increasing geographical dimension does not significantly reduce success of species identification. Molecular Ecology Resources 9: 1302–1310. 21564901 10.1111/j.1755-0998.2009.02577.x

[47] Smith MA, Rodriguez JJ, Whitfield JB, Deans AR, Janzen DH, et al (2008) Extreme diversity of tropical parasitoid wasps exposed by iterative integration of natural history, DNA barcoding, morphology, and collections. PNAS 105(35): 12359–12364. 18716001 10.1073/pnas.0805319105 PMC2518452

[48] Rougerie R, Kitching IJ, Haxaire J, Miller SE, Hausmann A, et al.. (2013) Australian Sphingidae – DNA barcodes challenge current species boundaries and distributions. Journal of Biogeography: in review. 10.1371/journal.pone.0101108 PMC4079597 24987846

[49] Dupuis JR, Roe AD, Sperling FAH (2012) Multi-locus species delimitation in closely related animals and fungi: one marker is not enough. Molecular Ecology: no-no. doi: 10.1111/j.1365-294X.2012.05642.x. 10.1111/j.1365-294X.2012.05642.x 22891635

[50] Nieukerken EJ van, Doorenweerd C, Stokvis FR, Groenenberg DSJ (2012) DNA barcoding of the leaf-mining moth subgenus *Ectoedemia* s. str. (Lepidoptera: Nepticulidae) with COI and EF1- α: two are better than one in recognising cryptic species. Contributions to Zoology 81: 1–24.

[51] Kvie KS, Hogner S, Aarvik L, Lifjeld JT, Johnsen A (2012) Deep sympatric mtDNA divergence in the autumnal moth (*Epirrita autumnata*). Ecology and Evolution 3: 126–144. 23404314 10.1002/ece3.434 PMC3568849

[52] Smith MA, Bertrand C, Crosby K, Eveleigh ES, Fernandez-Triana J, et al (2012) *Wolbachia* and DNA barcoding insects: Patterns, potential, and problems. PLoS ONE 7: e36514. 22567162 10.1371/journal.pone.0036514 PMC3342236

[53] Zaspel JM, Weller SJ, Carde R (2008) A review of *Virbia* (formerly *Holomelina*) of America north of Mexico (Arctiidae: Arctiinae: Arctiini). Bulletin of the Florida Museum of Natural History 48: 59–118.

[54] Crabo LG, Davis M, Hammond P, Mustelin T, Shepard J (2013) Five new species and three new subspecies of Erebidae and Noctuidae (Insecta, Lepidoptera) from Northwestern North America, with notes on *Chytolita* Grote (Erebidae) and *Hydraecia* Guenée (Noctuidae). ZooKeys 264: 85–123. 10.3897/zookeys.264.4304 PMC3668377 23730179

[55] Van Valen L (1965) Morphological variation and width of ecological niche. The American Naturalist 99: 377–390.

[56] Steiner WWM (1977) Niche width and genetic variation in Hawaiian *Drosophila* . The American Naturalist 111: 1037–1045.

[57] Hurst GDD, Jiggins FM (2005) Problems with mitochondrial DNA as a marker in population, phylogeographic and phylogenetic studies: the effects of inherited symbionts. Proceedings of the Royal Society of London B 272: 1525–1534. 10.1098/rspb.2005.3056 PMC1559843 16048766

[58] Linnen CR, Farrell BD (2007) Mito-nuclear discordance is caused by rampant mitochondrial introgression in *Neodiprion* (Hymenoptera: Diprionidae) sawflies. Evolution 61: 1417–1438. 17542850 10.1111/j.1558-5646.2007.00114.x

[59] Barnes W, McDunnough JH (1918) Illustrations of the North American species of the genus *Catocala* . Memoirs of the American Museum of Natural History 3: 1–47.

[60] Gall L, Hawks D (2010) Systematics of moths in the genus *Catocala* (Lepidoptera, Erebidae) IV. Nomenclatorial stabilization of the Nearctic fauna, with a revised synonymic check list. ZooKeys 39: 37–83.

[61] Scheffers BR, Joppa LN, Pimm SL, Laurance WF (2012) What we know and don't know about Earth's missing biodiversity. Trends in Ecology and Evolution 27: 501–510. 22784409 10.1016/j.tree.2012.05.008

[62] Wilson JJ, Rougerie R, Schonfeld J, Janzen DH, Hallwachs W, et al. (2011) When species matches are unavailable are DNA barcodes correctly assigned to higher taxa? An assessment using sphingid moths. BMC Ecology 11: , 18. 10.1186/1472-6785-11-18 PMC3161832 21806794

[63] Ekrem T, Willassen E, Stur E (2007) A comprehensive DNA sequence library is essential for identification with DNA barcodes. Molecular Phylogenetics and Evolution 43: 530–542. 17208018 10.1016/j.ympev.2006.11.021

[64] Zahiri R, Kitching IJ, Lafontaine JD, Mutanen M, Kaila L, et al. (2011) A new molecular phylogeny offers hope for a stable family-level classification of the Noctuoidea (Lepidoptera). Zoologica Scripta 40: , 158–173.

