# Supplementary material for: A Transcontinental Challenge — A Test of DNA Barcode Performance for 1,541 Species of Canadian Noctuoidea (Lepidoptera)
Source: PLoS One. 2014 Mar 25;9(3):e92797. doi: 10.1371/journal.pone.0092797 (PMC3965468; doi:10.1371/journal.pone.0092797)
Supplement: Tree S1 — NJ tree for Canadian species in the family Notodontidae. (PDF) [file pone.0092797.s005.pdf]

BOLD TaxonID Tree

```
Title           : SEARCH: Sample ids(1650 ids) [SEARCH2]
Date            : 17-October-2013
Data Type       : Nucleotide
Distance Model  : Kimura 2 Parameter
Marker          : COI-5P
Codon Positions :
Labels          : Country & Province, SampleID, ProcessID, Sequence Length, BIN URI
Filters         : Length > 200
Colorization    : [blue]=Stop Codons [red]=Contamination or misidentification
Attachment      : Photographs & Spreadsheet

Sequence Count  : 1650
Species count   : 62
Genus count     : 22
Family count    : 1
Unidentified    : 0

BIN Count       : 65
```

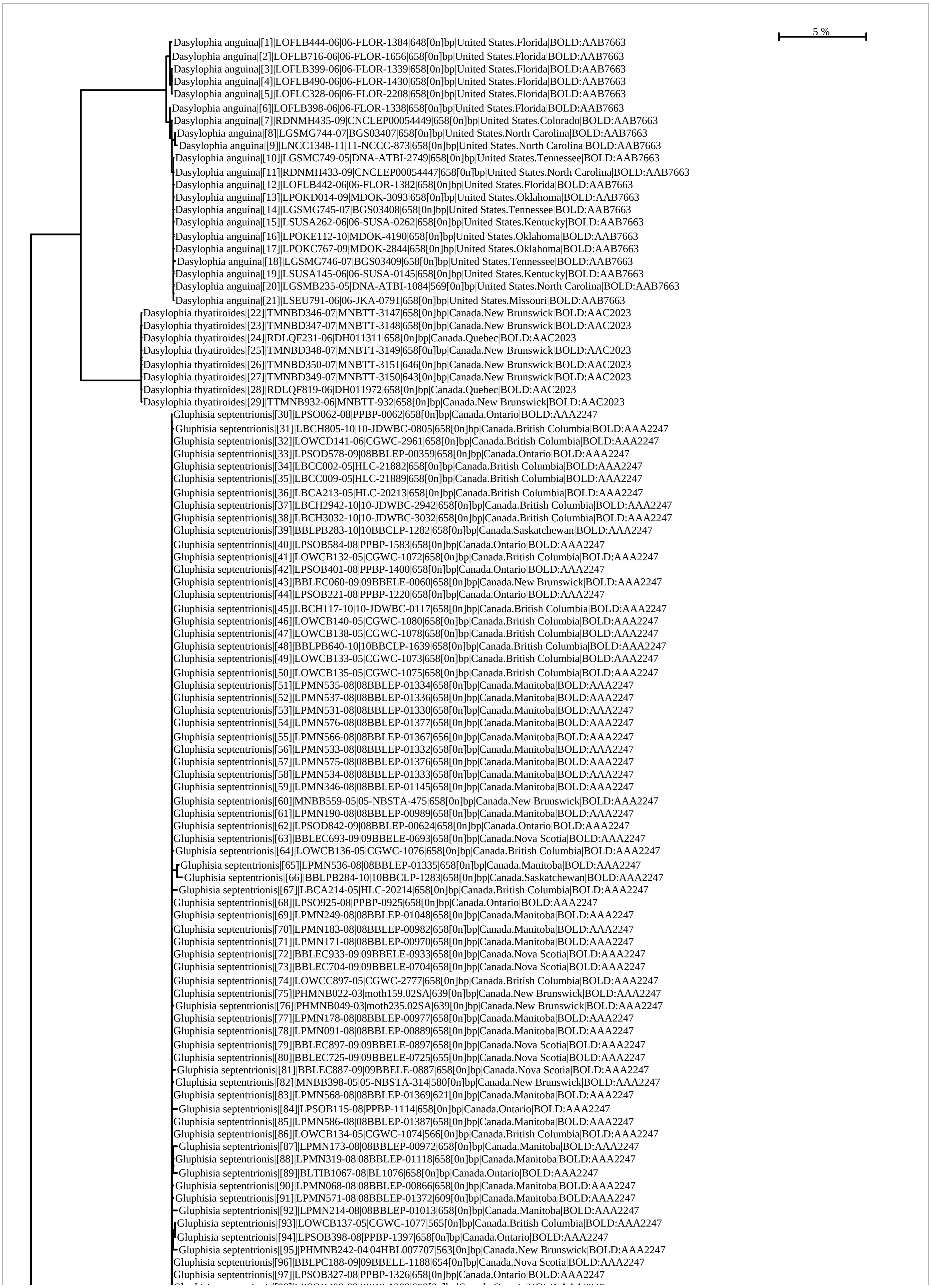
5 %
Dasylophia anguina|[1]|LOFLB444-06|06-FLOR-1384|648[0n]bp|United States.Florida|BOLD:AAB7663
Dasylophia anguina|[2]|LOFLB716-06|06-FLOR-1656|658[0n]bp|United States.Florida|BOLD:AAB7663
Dasylophia anguina|[3]|LOFLB399-06|06-FLOR-1339|658[0n]bp|United States.Florida|BOLD:AAB7663
Dasylophia anguina|[4]|LOFLB490-06|06-FLOR-1430|658[0n]bp|United States.Florida|BOLD:AAB7663
Dasylophia anguina|[5]|LOFLC328-06|06-FLOR-2208|658[0n]bp|United States.Florida|BOLD:AAB7663
Dasylophia anguina|[6]|LOFLB398-06|06-FLOR-1338|658[0n]bp|United States.Florida|BOLD:AAB7663
Dasylophia anguina|[7]|RDNMH435-09|CNCLEP00054449|658[0n]bp|United States.Colorado|BOLD:AAB7663
Dasylophia anguina|[8]|LGSMG744-07|BGS03407|658[0n]bp|United States.North Carolina|BOLD:AAB7663
Dasylophia anguina|[9]|LNCC1348-11|11-NCCC-873|658[0n]bp|United States.North Carolina|BOLD:AAB7663
Dasylophia anguina|[10]|LGSMC749-05|DNA-ATBI-2749|658[0n]bp|United States.Tennessee|BOLD:AAB7663
Dasylophia anguina|[11]|RDNMH433-09|CNCLEP00054447|658[0n]bp|United States.North Carolina|BOLD:AAB7663
Dasylophia anguina|[12]|LOFLB442-06|06-FLOR-1382|658[0n]bp|United States.Florida|BOLD:AAB7663
Dasylophia anguina|[13]|LPOKD014-09|MDOK-3093|658[0n]bp|United States.Oklahoma|BOLD:AAB7663
Dasylophia anguina|[14]|LGSMG745-07|BGS03408|658[0n]bp|United States.Tennessee|BOLD:AAB7663
Dasylophia anguina|[15]|LSUSA262-06|06-SUSA-0262|658[0n]bp|United States.Kentucky|BOLD:AAB7663
Dasylophia anguina|[16]|LPOKE112-10|MDOK-4190|658[0n]bp|United States.Oklahoma|BOLD:AAB7663
Dasylophia anguina|[17]|LPOKC767-09|MDOK-2844|658[0n]bp|United States.Oklahoma|BOLD:AAB7663
Dasylophia anguina|[18]|LGSMG746-07|BGS03409|658[0n]bp|United States.Tennessee|BOLD:AAB7663
Dasylophia anguina|[19]|LSUSA145-06|06-SUSA-0145|658[0n]bp|United States.Kentucky|BOLD:AAB7663
Dasylophia anguina|[20]|LGSMB235-05|DNA-ATBI-1084|569[0n]bp|United States.North Carolina|BOLD:AAB7663
Dasylophia anguina|[21]|LSEU791-06|06-JKA-0791|658[0n]bp|United States.Missouri|BOLD:AAB7663
Dasylophia thyatiroides|[22]|TMNBD346-07|MNBTT-3147|658[0n]bp|Canada.New Brunswick|BOLD:AAC2023
Dasylophia thyatiroides|[23]|TMNBD347-07|MNBTT-3148|658[0n]bp|Canada.New Brunswick|BOLD:AAC2023
Dasylophia thyatiroides|[24]|RDLQF231-06|DH011311|658[0n]bp|Canada.Quebec|BOLD:AAC2023
Dasylophia thyatiroides|[25]|TMNBD348-07|MNBTT-3149|658[0n]bp|Canada.New Brunswick|BOLD:AAC2023
Dasylophia thyatiroides|[26]|TMNBD350-07|MNBTT-3151|646[0n]bp|Canada.New Brunswick|BOLD:AAC2023
Dasylophia thyatiroides|[27]|TMNBD349-07|MNBTT-3150|643[0n]bp|Canada.New Brunswick|BOLD:AAC2023
Dasylophia thyatiroides|[28]|RDLQF819-06|DH011972|658[0n]bp|Canada.Quebec|BOLD:AAC2023
Dasylophia thyatiroides|[29]|TTMNB932-06|MNBTT-932|658[0n]bp|Canada.New Brunswick|BOLD:AAC2023
Gluphisia septentrionis|[30]|LPSO062-08|PPBP-0062|658[0n]bp|Canada.Ontario|BOLD:AAA2247
Gluphisia septentrionis|[31]|LBCH805-10|10-JDWBC-0805|658[0n]bp|Canada.British Columbia|BOLD:AAA2247
Gluphisia septentrionis|[32]|LOWCD141-06|CGWC-2961|658[0n]bp|Canada.British Columbia|BOLD:AAA2247
Gluphisia septentrionis|[33]|LPSOD578-09|08BBLEP-00359|658[0n]bp|Canada.Ontario|BOLD:AAA2247
Gluphisia septentrionis|[34]|LBCC002-05|HLC-21882|658[0n]bp|Canada.British Columbia|BOLD:AAA2247
Gluphisia septentrionis|[35]|LBCC009-05|HLC-21889|658[0n]bp|Canada.British Columbia|BOLD:AAA2247
Gluphisia septentrionis|[36]|LBCA213-05|HLC-20213|658[0n]bp|Canada.British Columbia|BOLD:AAA2247
Gluphisia septentrionis|[37]|LBCH2942-10|10-JDWBC-2942|658[0n]bp|Canada.British Columbia|BOLD:AAA2247
Gluphisia septentrionis|[38]|LBCH3032-10|10-JDWBC-3032|658[0n]bp|Canada.British Columbia|BOLD:AAA2247
Gluphisia septentrionis|[39]|BBLPB283-10|10BBCLP-1282|658[0n]bp|Canada.Saskatchewan|BOLD:AAA2247
Gluphisia septentrionis|[40]|LPSOB584-08|PPBP-1583|658[0n]bp|Canada.Ontario|BOLD:AAA2247
Gluphisia septentrionis|[41]|LOWCB132-05|CGWC-1072|658[0n]bp|Canada.British Columbia|BOLD:AAA2247
Gluphisia septentrionis|[42]|LPSOB401-08|PPBP-1400|658[0n]bp|Canada.Ontario|BOLD:AAA2247
Gluphisia septentrionis|[43]|BBLEC060-09|09BBELE-0060|658[0n]bp|Canada.New Brunswick|BOLD:AAA2247
Gluphisia septentrionis|[44]|LPSOB221-08|PPBP-1220|658[0n]bp|Canada.Ontario|BOLD:AAA2247
Gluphisia septentrionis|[45]|LBCH117-10|10-JDWBC-0117|658[0n]bp|Canada.British Columbia|BOLD:AAA2247
Gluphisia septentrionis|[46]|LOWCB140-05|CGWC-1080|658[0n]bp|Canada.British Columbia|BOLD:AAA2247
Gluphisia septentrionis|[47]|LOWCB138-05|CGWC-1078|658[0n]bp|Canada.British Columbia|BOLD:AAA2247
Gluphisia septentrionis|[48]|BBLPB640-10|10BBCLP-1639|658[0n]bp|Canada.British Columbia|BOLD:AAA2247
Gluphisia septentrionis|[49]|LOWCB133-05|CGWC-1073|658[0n]bp|Canada.British Columbia|BOLD:AAA2247
Gluphisia septentrionis|[50]|LOWCB135-05|CGWC-1075|658[0n]bp|Canada.British Columbia|BOLD:AAA2247
Gluphisia septentrionis|[51]|LPMN535-08|08BBLEP-01334|658[0n]bp|Canada.Manitoba|BOLD:AAA2247
Gluphisia septentrionis|[52]|LPMN537-08|08BBLEP-01336|658[0n]bp|Canada.Manitoba|BOLD:AAA2247
Gluphisia septentrionis|[53]|LPMN531-08|08BBLEP-01330|658[0n]bp|Canada.Manitoba|BOLD:AAA2247
Gluphisia septentrionis|[54]|LPMN576-08|08BBLEP-01377|658[0n]bp|Canada.Manitoba|BOLD:AAA2247
Gluphisia septentrionis|[55]|LPMN566-08|08BBLEP-01367|656[0n]bp|Canada.Manitoba|BOLD:AAA2247
Gluphisia septentrionis|[56]|LPMN533-08|08BBLEP-01332|658[0n]bp|Canada.Manitoba|BOLD:AAA2247
Gluphisia septentrionis|[57]|LPMN575-08|08BBLEP-01376|658[0n]bp|Canada.Manitoba|BOLD:AAA2247
Gluphisia septentrionis|[58]|LPMN534-08|08BBLEP-01333|658[0n]bp|Canada.Manitoba|BOLD:AAA2247
Gluphisia septentrionis|[59]|LPMN346-08|08BBLEP-01145|658[0n]bp|Canada.Manitoba|BOLD:AAA2247
Gluphisia septentrionis|[60]|MNBB559-05|05-NBSTA-475|658[0n]bp|Canada.New Brunswick|BOLD:AAA2247
Gluphisia septentrionis|[61]|LPMN190-08|08BBLEP-00989|658[0n]bp|Canada.Manitoba|BOLD:AAA2247
Gluphisia septentrionis|[62]|LPSOD842-09|08BBLEP-00624|658[0n]bp|Canada.Ontario|BOLD:AAA2247
Gluphisia septentrionis|[63]|BBLEC693-09|09BBELE-0693|658[0n]bp|Canada.Nova Scotia|BOLD:AAA2247
Gluphisia septentrionis|[64]|LOWCB136-05|CGWC-1076|658[0n]bp|Canada.British Columbia|BOLD:AAA2247
Gluphisia septentrionis|[65]|LPMN536-08|08BBLEP-01335|658[0n]bp|Canada.Manitoba|BOLD:AAA2247
Gluphisia septentrionis|[66]|BBLPB284-10|10BBCLP-1283|658[0n]bp|Canada.Saskatchewan|BOLD:AAA2247
Gluphisia septentrionis|[67]|LBCA214-05|HLC-20214|658[0n]bp|Canada.British Columbia|BOLD:AAA2247
Gluphisia septentrionis|[68]|LPSO925-08|PPBP-0925|658[0n]bp|Canada.Ontario|BOLD:AAA2247
Gluphisia septentrionis|[69]|LPMN249-08|08BBLEP-01048|658[0n]bp|Canada.Manitoba|BOLD:AAA2247
Gluphisia septentrionis|[70]|LPMN183-08|08BBLEP-00982|658[0n]bp|Canada.Manitoba|BOLD:AAA2247
Gluphisia septentrionis|[71]|LPMN171-08|08BBLEP-00970|658[0n]bp|Canada.Manitoba|BOLD:AAA2247
Gluphisia septentrionis|[72]|BBLEC933-09|09BBELE-0933|658[0n]bp|Canada.Nova Scotia|BOLD:AAA2247
Gluphisia septentrionis|[73]|BBLEC704-09|09BBELE-0704|658[0n]bp|Canada.Nova Scotia|BOLD:AAA2247
Gluphisia septentrionis|[74]|LOWCC897-05|CGWC-2777|658[0n]bp|Canada.British Columbia|BOLD:AAA2247
Gluphisia septentrionis|[75]|PHMNB022-03|moth159.02SA|639[0n]bp|Canada.New Brunswick|BOLD:AAA2247
Gluphisia septentrionis|[76]|PHMNB049-03|moth235.02SA|639[0n]bp|Canada.New Brunswick|BOLD:AAA2247
Gluphisia septentrionis|[77]|LPMN178-08|08BBLEP-00977|658[0n]bp|Canada.Manitoba|BOLD:AAA2247
Gluphisia septentrionis|[78]|LPMN091-08|08BBLEP-00889|658[0n]bp|Canada.Manitoba|BOLD:AAA2247
Gluphisia septentrionis|[79]|BBLEC897-09|09BBELE-0897|658[0n]bp|Canada.Nova Scotia|BOLD:AAA2247
Gluphisia septentrionis|[80]|BBLEC725-09|09BBELE-0725|655[0n]bp|Canada.Nova Scotia|BOLD:AAA2247
Gluphisia septentrionis|[81]|BBLEC887-09|09BBELE-0887|658[0n]bp|Canada.Nova Scotia|BOLD:AAA2247
Gluphisia septentrionis|[82]|MNBB398-05|05-NBSTA-314|580[0n]bp|Canada.New Brunswick|BOLD:AAA2247
Gluphisia septentrionis|[83]|LPMN568-08|08BBLEP-01369|621[0n]bp|Canada.Manitoba|BOLD:AAA2247
Gluphisia septentrionis|[84]|LPSOB115-08|PPBP-1114|658[0n]bp|Canada.Ontario|BOLD:AAA2247
Gluphisia septentrionis|[85]|LPMN586-08|08BBLEP-01387|658[0n]bp|Canada.Manitoba|BOLD:AAA2247
Gluphisia septentrionis|[86]|LOWCB134-05|CGWC-1074|566[0n]bp|Canada.British Columbia|BOLD:AAA2247
Gluphisia septentrionis|[87]|LPMN173-08|08BBLEP-00972|658[0n]bp|Canada.Manitoba|BOLD:AAA2247
Gluphisia septentrionis|[88]|LPMN319-08|08BBLEP-01118|658[0n]bp|Canada.Manitoba|BOLD:AAA2247
Gluphisia septentrionis|[89]|BLTIB1067-08|BL1076|658[0n]bp|Canada.Ontario|BOLD:AAA2247
Gluphisia septentrionis|[90]|LPMN068-08|08BBLEP-00866|658[0n]bp|Canada.Manitoba|BOLD:AAA2247
Gluphisia septentrionis|[91]|LPMN571-08|08BBLEP-01372|609[0n]bp|Canada.Manitoba|BOLD:AAA2247
Gluphisia septentrionis|[92]|LPMN214-08|08BBLEP-01013|658[0n]bp|Canada.Manitoba|BOLD:AAA2247
Gluphisia septentrionis|[93]|LOWCB137-05|CGWC-1077|565[0n]bp|Canada.British Columbia|BOLD:AAA2247
Gluphisia septentrionis|[94]|LPSOB398-08|PPBP-1397|658[0n]bp|Canada.Ontario|BOLD:AAA2247
Gluphisia septentrionis|[95]|PHMNB242-04|04HBL007707|563[0n]bp|Canada.New Brunswick|BOLD:AAA2247
Gluphisia septentrionis|[96]|BBLPC188-09|09BBELE-1188|654[0n]bp|Canada.Nova Scotia|BOLD:AAA2247
Gluphisia septentrionis|[97]|LPSOB327-08|PPBP-1326|658[0n]bp|Canada.Ontario|BOLD:AAA2247

Gluphisia septentrionis|[95]|PHMNB242-04|04HBL00[illegible]|563[0n]bp|Canada.New Brunswick|BOLD:AAA2247
Gluphisia septentrionis|[96]|BBLPC188-09|09BBELE-1188|654[0n]bp|Canada.Nova Scotia|BOLD:AAA2247
Gluphisia septentrionis|[97]|LPSOB327-08|PPBP-1326|658[0n]bp|Canada.Ontario|BOLD:AAA2247
Gluphisia septentrionis|[98]|LPSOB400-08|PPBP-1399|658[0n]bp|Canada.Ontario|BOLD:AAA2247
Gluphisia septentrionis|[99]|BBLEC223-09|09BBELE-0223|643[0n]bp|Canada.Nova Scotia|BOLD:AAA2247
Gluphisia septentrionis|[100]|LPMN180-08|08BBLEP-00979|658[0n]bp|Canada.Manitoba|BOLD:AAA2247
Gluphisia septentrionis|[101]|LPMN197-08|08BBLEP-00996|658[0n]bp|Canada.Manitoba|BOLD:AAA2247
Gluphisia septentrionis|[102]|PHMNB573-04|04HBL00799|658[0n]bp|Canada.New Brunswick|BOLD:AAA2247
Gluphisia septentrionis|[103]|BBLEC701-09|09BBELE-0701|655[0n]bp|Canada.Nova Scotia|BOLD:AAA2247
Gluphisia septentrionis|[104]|BBLEC735-09|09BBELE-0735|658[0n]bp|Canada.Nova Scotia|BOLD:AAA2247
Gluphisia septentrionis|[105]|BBLEC929-09|09BBELE-0929|658[0n]bp|Canada.Nova Scotia|BOLD:AAA2247
Gluphisia septentrionis|[106]|PHMO055-03|moth360.01|617[0n]bp|Canada.Ontario|BOLD:AAA2247
Gluphisia septentrionis|[107]|MNAF481-08|CNCLEP00040466|658[0n]bp|Canada.Manitoba|BOLD:AAA2247
Gluphisia septentrionis|[108]|LPSOD717-09|08BBLEP-00499|658[0n]bp|Canada.Ontario|BOLD:AAA2247
Gluphisia septentrionis|[109]|PHMNB571-04|04HBL00797|658[0n]bp|Canada.New Brunswick|BOLD:AAA2247
Gluphisia septentrionis|[110]|LPMN349-08|08BBLEP-01148|658[0n]bp|Canada.Manitoba|BOLD:AAA2247
Gluphisia septentrionis|[111]|LPSOB402-08|PPBP-1401|658[0n]bp|Canada.Ontario|BOLD:AAA2247
Gluphisia septentrionis|[112]|TMNBD292-07|MNBTT-3093|626[0n]bp|Canada.New Brunswick|BOLD:AAA2247
Gluphisia septentrionis|[113]|BBLPB282-10|10BBCLP-1281|658[0n]bp|Canada.Saskatchewan|BOLD:AAA2247
Gluphisia septentrionis|[114]|MNBB326-05|05-NBSTA-242|562[1n]bp|Canada.New Brunswick|BOLD:AAA2247
Gluphisia septentrionis|[115]|TTMNB247-06|MNBTT-247|657[0n]bp|Canada.New Brunswick|BOLD:AAA2247
Gluphisia septentrionis|[116]|BBLPB280-10|10BBCLP-1279|658[0n]bp|Canada.Saskatchewan|BOLD:AAA2247
Gluphisia septentrionis|[117]|LPMN179-08|08BBLEP-00978|658[0n]bp|Canada.Manitoba|BOLD:AAA2247
Gluphisia septentrionis|[118]|LPMN186-08|08BBLEP-00985|658[0n]bp|Canada.Manitoba|BOLD:AAA2247
Gluphisia septentrionis|[119]|LPMN198-08|08BBLEP-00997|658[0n]bp|Canada.Manitoba|BOLD:AAA2247
Gluphisia septentrionis|[120]|LPMN184-08|08BBLEP-00983|658[0n]bp|Canada.Manitoba|BOLD:AAA2247
Gluphisia septentrionis|[121]|LPMN316-08|08BBLEP-01115|658[0n]bp|Canada.Manitoba|BOLD:AAA2247
Gluphisia septentrionis|[122]|LPMN532-08|08BBLEP-01331|658[0n]bp|Canada.Manitoba|BOLD:AAA2247
Gluphisia septentrionis|[123]|LPMN538-08|08BBLEP-01337|658[0n]bp|Canada.Manitoba|BOLD:AAA2247
Gluphisia septentrionis|[124]|BBLPB279-10|10BBCLP-1278|658[0n]bp|Canada.Saskatchewan|BOLD:AAA2247
Gluphisia septentrionis|[125]|BBLEC222-09|09BBELE-0222|658[0n]bp|Canada.Nova Scotia|BOLD:AAA2247
Gluphisia septentrionis|[126]|LPMN574-08|08BBLEP-01375|658[0n]bp|Canada.Manitoba|BOLD:AAA2247
Gluphisia septentrionis|[127]|BBLPB281-10|10BBCLP-1280|658[0n]bp|Canada.Saskatchewan|BOLD:AAA2247
Gluphisia septentrionis|[128]|LPMN185-08|08BBLEP-00984|658[0n]bp|Canada.Manitoba|BOLD:AAA2247
Gluphisia septentrionis|[129]|LPMN252-08|08BBLEP-01051|658[0n]bp|Canada.Manitoba|BOLD:AAA2247
Gluphisia septentrionis|[130]|LMIS023-05|05-ONMIS-0023|658[0n]bp|Canada.Ontario|BOLD:AAA2247
Gluphisia septentrionis|[131]|LPMN200-08|08BBLEP-00999|658[0n]bp|Canada.Manitoba|BOLD:AAA2247
Gluphisia septentrionis|[132]|LPMN251-08|08BBLEP-01050|658[0n]bp|Canada.Manitoba|BOLD:AAA2247
Gluphisia septentrionis|[133]|LPMN567-08|08BBLEP-01368|657[0n]bp|Canada.Manitoba|BOLD:AAA2247
Gluphisia septentrionis|[134]|RDMAB365-05|UASM77830|657[0n]bp|Canada.Alberta|BOLD:AAA2247
Gluphisia septentrionis|[135]|RDMAB353-05|UASM7383|658[0n]bp|Canada.Alberta|BOLD:AAA2247
Gluphisia septentrionis|[136]|LPMN530-08|08BBLEP-01329|658[0n]bp|Canada.Manitoba|BOLD:AAA2247
Gluphisia septentrionis|[137]|TMNBD294-07|MNBTT-3095|631[0n]bp|Canada.New Brunswick|BOLD:AAA2247
Gluphisia septentrionis|[138]|BBLPC239-09|09BBELE-1239|658[0n]bp|Canada.Nova Scotia|BOLD:AAA2247
Gluphisia septentrionis|[139]|BBLPC204-09|09BBELE-1204|658[0n]bp|Canada.Nova Scotia|BOLD:AAA2247
Gluphisia septentrionis|[140]|BBLPC178-09|09BBELE-1178|658[0n]bp|Canada.Nova Scotia|BOLD:AAA2247
Gluphisia septentrionis|[141]|RDMAB029-05|UASM57574|634[0n]bp|Canada.Alberta|BOLD:AAA2247
Gluphisia septentrionis|[142]|XAD667-05|2005-ONT-82|658[0n]bp|Canada.Ontario|BOLD:AAA2247
Gluphisia septentrionis|[143]|XAG086-05|2005-ONT-670|658[0n]bp|Canada.Ontario|BOLD:AAA2247
Gluphisia septentrionis|[144]|XAG087-05|2005-ONT-671|658[0n]bp|Canada.Ontario|BOLD:AAA2247
Gluphisia septentrionis|[145]|XAB605-04|04HBL005605|658[0n]bp|Canada.Ontario|BOLD:AAA2247
Gluphisia septentrionis|[146]|BLTIB916-08|BL1336|658[0n]bp|Canada.Ontario|BOLD:AAA2247
Gluphisia septentrionis|[147]|BLTIB718-08|BL1004|658[0n]bp|Canada.Ontario|BOLD:AAA2247
Gluphisia septentrionis|[148]|BLTIB1055-08|BL1063|658[0n]bp|Canada.Ontario|BOLD:AAA2247
Gluphisia septentrionis|[149]|LPSOB381-08|PPBP-1380|658[0n]bp|Canada.Ontario|BOLD:AAA2247
Gluphisia septentrionis|[150]|LPSOB116-08|PPBP-1115|658[0n]bp|Canada.Ontario|BOLD:AAA2247
Gluphisia septentrionis|[151]|TTMNB246-06|MNBTT-246|658[0n]bp|Canada.New Brunswick|BOLD:AAA2247
Gluphisia septentrionis|[152]|XAB330-04|04HBL005330|658[0n]bp|Canada.Ontario|BOLD:AAA2247
Gluphisia septentrionis|[153]|LPMN539-08|08BBLEP-01338|658[0n]bp|Canada.Manitoba|BOLD:AAA2247
Gluphisia septentrionis|[154]|LPMN608-08|08BBLEP-01409|658[0n]bp|Canada.Manitoba|BOLD:AAA2247
Gluphisia septentrionis|[155]|LPMN570-08|08BBLEP-01371|658[0n]bp|Canada.Manitoba|BOLD:AAA2247
Gluphisia septentrionis|[156]|XAF532-05|2005-ONT-181|658[0n]bp|Canada.Ontario|BOLD:AAA2247
Gluphisia septentrionis|[157]|LPMN342-08|08BBLEP-01141|658[0n]bp|Canada.Manitoba|BOLD:AAA2247
Gluphisia septentrionis|[158]|LPSOB399-08|PPBP-1398|658[0n]bp|Canada.Ontario|BOLD:AAA2247
Gluphisia septentrionis|[159]|XAE440-04|Moth4440.03|658[0n]bp|Canada.Ontario|BOLD:AAA2247
Gluphisia septentrionis|[160]|BBLEC631-09|09BBELE-0631|658[0n]bp|Canada.Nova Scotia|BOLD:AAA2247
Gluphisia septentrionis|[161]|BBLPC240-09|09BBELE-1240|658[0n]bp|Canada.Nova Scotia|BOLD:AAA2247
Gluphisia septentrionis|[162]|BBLPC218-09|09BBELE-1218|658[0n]bp|Canada.Nova Scotia|BOLD:AAA2247
Gluphisia septentrionis|[163]|BBLEC629-09|09BBELE-0629|658[0n]bp|Canada.Nova Scotia|BOLD:AAA2247
Gluphisia septentrionis|[164]|BBLPC181-09|09BBELE-1181|614[0n]bp|Canada.Nova Scotia|BOLD:AAA2247
Gluphisia septentrionis|[165]|BBLPC235-09|09BBELE-1235|631[0n]bp|Canada.Nova Scotia|BOLD:AAA2247
Gluphisia septentrionis|[166]|BBLPC216-09|09BBELE-1216|655[0n]bp|Canada.Nova Scotia|BOLD:AAA2247
Gluphisia septentrionis|[167]|BBLPC209-09|09BBELE-1209|658[0n]bp|Canada.Nova Scotia|BOLD:AAA2247
Gluphisia septentrionis|[168]|LPMN328-08|08BBLEP-01127|655[0n]bp|Canada.Manitoba|BOLD:AAA2247
Gluphisia septentrionis|[169]|LPMN315-08|08BBLEP-01114|658[0n]bp|Canada.Manitoba|BOLD:AAA2247
Gluphisia septentrionis|[170]|LPSOB403-08|PPBP-1402|657[0n]bp|Canada.Ontario|BOLD:AAA2247
Gluphisia septentrionis|[171]|LPMN170-08|08BBLEP-00969|658[0n]bp|Canada.Manitoba|BOLD:AAA2247
Gluphisia septentrionis|[172]|BBLPE512-09|09BBELE-2512|626[0n]bp|Canada.Newfoundland and Labrador|BOLD...
Gluphisia septentrionis|[173]|XAJ254-06|2006-ONT-0254|658[0n]bp|Canada.Ontario|BOLD:AAA2247
Gluphisia septentrionis|[174]|XAF671-05|2005-ONT-320|658[0n]bp|Canada.Ontario|BOLD:AAA2247
Gluphisia septentrionis|[175]|XAE434-04|Moth4434.03|613[1n]bp|Canada.Ontario|BOLD:AAA2247
Gluphisia septentrionis|[176]|PHMO074-03|moth374.01|639[0n]bp|Canada.Ontario|BOLD:AAA2247
Gluphisia septentrionis|[177]|XAG011-05|2005-ONT-595|612[0n]bp|Canada.Ontario|BOLD:AAA2247
Gluphisia septentrionis|[178]|RDLQB563-05|DH010649|658[0n]bp|Canada.Quebec|BOLD:AAA2247
Gluphisia septentrionis|[179]|BLTIB846-08|BL1265|658[1n]bp|Canada.Ontario|BOLD:AAA2247
Gluphisia septentrionis|[180]|XAB255-04|04HBL005255|658[0n]bp|Canada.Ontario|BOLD:AAA2247
Gluphisia septentrionis|[181]|KPOEC078-08|08OEC-237|652[0n]bp|Canada.Ontario|BOLD:AAA2247
Gluphisia septentrionis|[182]|XAK329-06|2006-ONT-1324|658[0n]bp|Canada.Ontario|BOLD:AAA2247
Gluphisia septentrionis|[183]|LPSOD418-09|08BBLEP-00197|658[0n]bp|Canada.Ontario|BOLD:AAA2247
Gluphisia septentrionis|[184]|BLGSM019-09|BL328|658[0n]bp|Canada.Ontario|BOLD:AAA2247
Gluphisia septentrionis|[185]|TMNBD296-07|MNBTT-3097|658[0n]bp|Canada.New Brunswick|BOLD:AAA2247
Gluphisia septentrionis|[186]|LPSOB382-08|PPBP-1381|658[0n]bp|Canada.Ontario|BOLD:AAA2247
Gluphisia septentrionis|[187]|LPMN318-08|08BBLEP-01117|658[0n]bp|Canada.Manitoba|BOLD:AAA2247
Gluphisia septentrionis|[188]|LPMN196-08|08BBLEP-00995|658[0n]bp|Canada.Manitoba|BOLD:AAA2247
Gluphisia septentrionis|[189]|LPMN199-08|08BBLEP-00998|658[0n]bp|Canada.Manitoba|BOLD:AAA2247
Gluphisia septentrionis|[190]|LPMN189-08|08BBLEP-00988|658[0n]bp|Canada.Manitoba|BOLD:AAA2247
Gluphisia septentrionis|[191]|BBLPE616-09|09BBELE-2616|655[0n]bp|Canada.Nova Scotia|BOLD:AAA2247
Gluphisia septentrionis|[192]|LPMN799-08|08BBLEP-01602|658[0n]bp|Canada.Manitoba|BOLD:AAA2247
Gluphisia septentrionis|[193]|LBCB072-05|HLC-21012|658[0n]bp|Canada.British Columbia|BOLD:AAA2247
Gluphisia septentrionis|[194]|LBCA212-05|HLC-20212|658[0n]bp|Canada.British Columbia|BOLD:AAA2247
Gluphisia septentrionis|[195]|LPABB451-08|08BBLEP-03716|658[0n]bp|Canada.Alberta|BOLD:AAA2247

Gluphisia septentrionis|[192]|[illegible]|Canada.Manitoba|BOLD:AAA2247
Gluphisia septentrionis|[193]|LBCB072-05|HLC-21012|658[0n]bp|Canada.British Columbia|BOLD:AAA2247
Gluphisia septentrionis|[194]|LBCA212-05|HLC-20212|658[0n]bp|Canada.British Columbia|BOLD:AAA2247
Gluphisia septentrionis|[195]|LPABB451-08|08BBLEP-03716|658[0n]bp|Canada.Alberta|BOLD:AAA2247
Gluphisia septentrionis|[196]|LBCB201-05|HLC-21141|658[0n]bp|Canada.British Columbia|BOLD:AAA2247
Gluphisia septentrionis|[197]|LPMN529-08|08BBLEP-01328|658[0n]bp|Canada.Manitoba|BOLD:AAA2247
Gluphisia septentrionis|[198]|LPMN314-08|08BBLEP-01113|658[0n]bp|Canada.Manitoba|BOLD:AAA2247
Gluphisia septentrionis|[199]|LPABC917-09|08BBLEP-05328|658[0n]bp|Canada.Alberta|BOLD:AAA2247
Gluphisia septentrionis|[200]|LPMN191-08|08BBLEP-00990|658[0n]bp|Canada.Manitoba|BOLD:AAA2247
Gluphisia septentrionis|[201]|LBCA609-05|HLC-20609|649[0n]bp|Canada.British Columbia|BOLD:AAA2247
Gluphisia septentrionis|[202]|LPMNB564-09|08BBLEP-05642|634[0n]bp|Canada.Manitoba|BOLD:AAA2247
Gluphisia septentrionis|[203]|LPVIB846-08|PFC-2006-2353|658[0n]bp|Canada.British Columbia|BOLD:AAA2247
Gluphisia septentrionis|[204]|LBCH5288-10|10-JDWBC-5288|658[0n]bp|Canada.British Columbia|BOLD:AAA2247
Gluphisia septentrionis|[205]|RDMAB028-05|UASM57573|658[0n]bp|Canada.Alberta|BOLD:AAA2247
Gluphisia septentrionis|[206]|LBCA372-05|HLC-20372|658[0n]bp|Canada.British Columbia|BOLD:AAA2247
Gluphisia septentrionis|[207]|BBLEC224-09|09BBELE-0224|658[0n]bp|Canada.Nova Scotia|BOLD:AAA2247
Gluphisia septentrionis|[208]|LOWCB142-05|CGWC-1082|603[4n]bp|Canada.British Columbia|BOLD:AAA2247
Gluphisia septentrionis|[209]|LOWCE855-06|CGWC-4615|658[0n]bp|Canada.British Columbia|BOLD:AAA2247
Gluphisia septentrionis|[210]|LPMN182-08|08BBLEP-00981|658[0n]bp|Canada.Manitoba|BOLD:AAA2247
Gluphisia septentrionis|[211]|LOWCB143-05|CGWC-1083|658[0n]bp|Canada.British Columbia|BOLD:AAA2247
Gluphisia septentrionis|[212]|LBCH4428-10|10-JDWBC-4428|658[0n]bp|Canada.British Columbia|BOLD:AAA2247
Clostera albosigma|[213]|XAB594-04|04HBL005594|658[0n]bp|Canada.Ontario|BOLD:AAA5417
Clostera albosigma|[214]|BBLPB266-10|10BBCLP-1265|572[0n]bp|Canada.Ontario|BOLD:AAA5417
Clostera albosigma|[215]|XAB221-04|04HBL005221|658[0n]bp|Canada.Ontario|BOLD:AAA5417
Clostera albosigma|[216]|BBLPB261-10|10BBCLP-1260|658[0n]bp|Canada.Saskatchewan|BOLD:AAA5417
Clostera albosigma|[217]|LPSOB695-08|PPBP-1694|656[0n]bp|Canada.Ontario|BOLD:AAA5417
Clostera albosigma|[218]|BBLPB262-10|10BBCLP-1261|658[0n]bp|Canada.Saskatchewan|BOLD:AAA5417
Clostera albosigma|[219]|BBLPB257-10|10BBCLP-1256|658[0n]bp|Canada.Saskatchewan|BOLD:AAA5417
Clostera albosigma|[220]|LPSOB593-08|PPBP-1592|658[0n]bp|Canada.Ontario|BOLD:AAA5417
Clostera albosigma|[221]|LPMNB234-09|08BBLEP-05078|658[0n]bp|Canada.Manitoba|BOLD:AAA5417
Clostera albosigma|[222]|XAF320-05|HLC-10361|658[0n]bp|Canada.Ontario|BOLD:AAA5417
Clostera albosigma|[223]|BBLPB267-10|10BBCLP-1266|658[0n]bp|Canada.Ontario|BOLD:AAA5417
Clostera albosigma|[224]|LPMN326-08|08BBLEP-01125|658[0n]bp|Canada.Manitoba|BOLD:AAA5417
Clostera albosigma|[225]|LPSOD798-09|08BBLEP-00580|658[0n]bp|Canada.Ontario|BOLD:AAA5417
Clostera albosigma|[226]|BBLPB263-10|10BBCLP-1262|658[0n]bp|Canada.Saskatchewan|BOLD:AAA5417
Clostera albosigma|[227]|TMG54-03|CLOS2.00|639[0n]bp|Canada.Ontario|BOLD:AAA5417
Clostera albosigma|[228]|TMG55-03|moth246.01|639[0n]bp|Canada.Ontario|BOLD:AAA5417
Clostera albosigma|[229]|BBLPB260-10|10BBCLP-1259|658[0n]bp|Canada.Saskatchewan|BOLD:AAA5417
Clostera albosigma|[230]|LMIS021-05|05-ONMIS-0021|658[0n]bp|Canada.Ontario|BOLD:AAA5417
Clostera albosigma|[231]|XAG022-05|2005-ONT-606|658[0n]bp|Canada.Ontario|BOLD:AAA5417
Clostera albosigma|[232]|XAG089-05|2005-ONT-673|658[0n]bp|Canada.Ontario|BOLD:AAA5417
Clostera albosigma|[233]|XAF408-05|HLC-10449|658[0n]bp|Canada.Ontario|BOLD:AAA5417
Clostera albosigma|[234]|LPABC665-09|08BBLEP-04884|632[0n]bp|Canada.Alberta|BOLD:AAA5417
Clostera albosigma|[235]|LPABC701-09|08BBLEP-04920|658[0n]bp|Canada.Alberta|BOLD:AAA5417
Clostera albosigma|[236]|LPMN906-08|08BBLEP-02264|658[0n]bp|Canada.Alberta|BOLD:AAA5417
Clostera albosigma|[237]|LPMN896-08|08BBLEP-02254|658[0n]bp|Canada.Alberta|BOLD:AAA5417
Clostera albosigma|[238]|LPSOB514-08|PPBP-1513|658[0n]bp|Canada.Ontario|BOLD:AAA5417
Clostera albosigma|[239]|LBCG3219-09|08-JDWBC-3219|632[0n]bp|Canada.British Columbia|BOLD:AAA5417
Clostera albosigma|[240]|LOWCB111-05|CGWC-1051|615[0n]bp|Canada.British Columbia|BOLD:AAA5417
Clostera albosigma|[241]|BBLPE528-09|09BBELE-2528|658[0n]bp|Canada.Newfoundland and Labrador|BOLD:AA...
Clostera albosigma|[242]|BBLPC747-09|09BBELE-1747|658[0n]bp|Canada.Newfoundland and Labrador|BOLD:AA...
Clostera albosigma|[243]|BBLPC041-09|09BBELE-1041|658[0n]bp|Canada.New Brunswick|BOLD:AAA5417
Clostera albosigma|[244]|TMNBB042-06|MNBTT-982|658[0n]bp|Canada.New Brunswick|BOLD:AAA5417
Clostera albosigma|[245]|MNBB172-05|05-NBSTA-088|658[0n]bp|Canada.New Brunswick|BOLD:AAA5417
Clostera albosigma|[246]|XAK330-06|2006-ONT-1325|658[0n]bp|Canada.Ontario|BOLD:AAA5417
Clostera albosigma|[247]|RDLQB512-05|DH010598|658[0n]bp|Canada.Quebec|BOLD:AAA5417
Clostera albosigma|[248]|TMNBD254-07|MNBTT-3055|658[0n]bp|Canada.New Brunswick|BOLD:AAA5417
Clostera albosigma|[249]|TMNBD252-07|MNBTT-3053|658[0n]bp|Canada.New Brunswick|BOLD:AAA5417
Clostera albosigma|[250]|LPABB207-08|08BBLEP-03472|658[0n]bp|Canada.Alberta|BOLD:AAA5417
Clostera albosigma|[251]|LPMN921-08|08BBLEP-02279|658[0n]bp|Canada.Alberta|BOLD:AAA5417
Clostera albosigma|[252]|XAB634-04|04HBL005634|658[0n]bp|Canada.Ontario|BOLD:AAA5417
Clostera albosigma|[253]|XAG090-05|2005-ONT-674|658[0n]bp|Canada.Ontario|BOLD:AAA5417
Clostera albosigma|[254]|LBCH3028-10|10-JDWBC-3028|658[0n]bp|Canada.British Columbia|BOLD:AAA5417
Clostera albosigma|[255]|LBCH2956-10|10-JDWBC-2956|658[0n]bp|Canada.British Columbia|BOLD:AAA5417
Clostera albosigma|[256]|LBCH2919-10|10-JDWBC-2919|658[0n]bp|Canada.British Columbia|BOLD:AAA5417
Clostera albosigma|[257]|XAJ139-06|2006-ONT-0139|658[0n]bp|Canada.Ontario|BOLD:AAA5417
Clostera albosigma|[258]|LBCG181-08|08-JDWBC-0181|658[0n]bp|Canada.British Columbia|BOLD:AAA5417
Clostera albosigma|[259]|LBCA027-05|HLC-20027|658[0n]bp|Canada.British Columbia|BOLD:AAA5417
Clostera albosigma|[260]|LPSOB513-08|PPBP-1512|658[0n]bp|Canada.Ontario|BOLD:AAA5417
Clostera albosigma|[261]|BBLPB269-10|10BBCLP-1268|658[0n]bp|Canada.British Columbia|BOLD:AAA5417
Clostera albosigma|[262]|BLTIB1018-08|BL1458|658[0n]bp|Canada.Ontario|BOLD:AAA5417
Clostera albosigma|[263]|XAE065-04|Moth4065.03|658[0n]bp|Canada.Ontario|BOLD:AAA5417
Clostera albosigma|[264]|XAF409-05|HLC-10450|658[0n]bp|Canada.Ontario|BOLD:AAA5417
Clostera albosigma|[265]|BBLEC050-09|09BBELE-0050|658[0n]bp|Canada.New Brunswick|BOLD:AAA5417
Clostera albosigma|[266]|XAF432-05|HLC-10473|658[0n]bp|Canada.Ontario|BOLD:AAA5417
Clostera albosigma|[267]|LBCH119-10|10-JDWBC-0119|658[0n]bp|Canada.British Columbia|BOLD:AAA5417
Clostera albosigma|[268]|LBCH806-10|10-JDWBC-0806|658[0n]bp|Canada.British Columbia|BOLD:AAA5417
Clostera albosigma|[269]|LBCH011-10|10-JDWBC-0011|658[0n]bp|Canada.British Columbia|BOLD:AAA5417
Clostera albosigma|[270]|LBCH653-10|10-JDWBC-0653|658[0n]bp|Canada.British Columbia|BOLD:AAA5417
Clostera albosigma|[271]|XAF346-05|HLC-10387|658[0n]bp|Canada.Ontario|BOLD:AAA5417
Clostera albosigma|[272]|BBLPB265-10|10BBCLP-1264|658[0n]bp|Canada.Alberta|BOLD:AAA5417
Clostera albosigma|[273]|TTMNB915-06|MNBTT-915|658[0n]bp|Canada.New Brunswick|BOLD:AAA5417
Clostera albosigma|[274]|BBLPC111-09|09BBELE-1111|658[0n]bp|Canada.New Brunswick|BOLD:AAA5417
Clostera albosigma|[275]|XAB283-04|04HBL005283|658[0n]bp|Canada.Ontario|BOLD:AAA5417
Clostera albosigma|[276]|MNBB665-05|05-NBSTA-581|658[0n]bp|Canada.New Brunswick|BOLD:AAA5417
Clostera albosigma|[277]|LPABC916-09|08BBLEP-05327|658[0n]bp|Canada.Alberta|BOLD:AAA5417
Clostera albosigma|[278]|TMNBD253-07|MNBTT-3054|658[0n]bp|Canada.New Brunswick|BOLD:AAA5417
Clostera albosigma|[279]|TMNBD255-07|MNBTT-3056|658[0n]bp|Canada.New Brunswick|BOLD:AAA5417
Clostera albosigma|[280]|TMNBD256-07|MNBTT-3057|658[0n]bp|Canada.New Brunswick|BOLD:AAA5417
Clostera albosigma|[281]|MNBB439-05|05-NBSTA-355|658[0n]bp|Canada.New Brunswick|BOLD:AAA5417
Clostera albosigma|[282]|BBLEC698-09|09BBELE-0698|658[0n]bp|Canada.Nova Scotia|BOLD:AAA5417
Clostera albosigma|[283]|BBLPB264-10|10BBCLP-1263|658[0n]bp|Canada.Alberta|BOLD:AAA5417
Clostera albosigma|[284]|XAE600-04|Moth4600.03|562[0n]bp|Canada.Ontario|BOLD:AAA5417
Clostera albosigma|[285]|PHMNB576-04|04HBL00802|658[0n]bp|Canada.New Brunswick|BOLD:AAA5417
Clostera albosigma|[286]|XAH082-05|2005-ONT-1665|658[0n]bp|Canada.Ontario|BOLD:AAA5417
Clostera albosigma|[287]|LPSOB580-08|PPBP-1579|658[0n]bp|Canada.Ontario|BOLD:AAA5417
Clostera albosigma|[288]|PMG171-03|moth232.01|617[0n]bp|Canada.Ontario|BOLD:AAA5417
Clostera albosigma|[289]|MNBB607-05|05-NBSTA-523|556[1n]bp|Canada.New Brunswick|BOLD:AAA5417
Clostera albosigma|[290]|LOWCB112-05|CGWC-1052|556[0n]bp|Canada.British Columbia|BOLD:AAA5417
Clostera albosigma|[291]|LBCA365-05|HLC-20365|600[2n]bp|Canada.British Columbia|BOLD:AAA5417
Clostera albosigma|[292]|XAJ500-06|2006-ONT-0500|658[0n]bp|Canada.Ontario|BOLD:AAA5417

[illegible]
Clostera albosigma|[290]|LOWCB112-05|CGWC-1052|556[0n]bp|Canada.British Columbia|BOLD:AAA5417
Clostera albosigma|[291]|LBCA365-05|HLC-20365|600[2n]bp|Canada.British Columbia|BOLD:AAA5417
Clostera albosigma|[292]|XAJ500-06|2006-ONT-0500|658[0n]bp|Canada.Ontario|BOLD:AAA5417
Clostera brucei|[293]|LBCA026-05|HLC-20026|658[0n]bp|Canada.British Columbia|BOLD:AAB0492
Clostera brucei|[294]|LOWCE258-06|CGWC-4018|658[0n]bp|Canada.British Columbia|BOLD:AAB0492
Clostera brucei|[295]|LOWCE263-06|CGWC-4023|658[0n]bp|Canada.British Columbia|BOLD:AAB0492
Clostera brucei|[296]|LBCH2959-10|10-JDWBC-2959|641[0n]bp|Canada.British Columbia|BOLD:AAB0492
Clostera brucei|[297]|LCH216-04|04HBL003216|658[0n]bp|Canada.Manitoba|BOLD:AAB0492
Clostera brucei|[298]|RDLQ782-07|DH008582|655[0n]bp|Canada.Newfoundland and Labrador|BOLD:AAB0492
Clostera brucei|[299]|CHLEP240-09|09PROBE-09535|658[0n]bp|Canada.Manitoba|BOLD:AAB0492
Clostera brucei|[300]|CHLEP243-09|09PROBE-09538|658[0n]bp|Canada.Manitoba|BOLD:AAB0492
Clostera brucei|[301]|CHLEP239-09|09PROBE-09534|658[0n]bp|Canada.Manitoba|BOLD:AAB0492
Clostera brucei|[302]|CHLEP244-09|09PROBE-09539|658[0n]bp|Canada.Manitoba|BOLD:AAB0492
Clostera brucei|[303]|CHLEP238-09|09PROBE-09533|658[0n]bp|Canada.Manitoba|BOLD:AAB0492
Clostera brucei|[304]|CHLEP241-09|09PROBE-09536|658[0n]bp|Canada.Manitoba|BOLD:AAB0492
Clostera brucei|[305]|LCH581-04|04HBL003581|658[0n]bp|Canada.Manitoba|BOLD:AAB0492
Clostera brucei|[306]|LCH215-04|04HBL003215|658[0n]bp|Canada.Manitoba|BOLD:AAB0492
Clostera brucei|[307]|LCHP980-07|07PROBE-10742|658[0n]bp|Canada.Manitoba|BOLD:AAB0492
Clostera brucei|[308]|CHLEP250-09|09PROBE-09545|643[0n]bp|Canada.Manitoba|BOLD:AAB0492
Clostera brucei|[309]|CHLEP252-09|09PROBE-09547|658[0n]bp|Canada.Manitoba|BOLD:AAB0492
Clostera brucei|[310]|CHLEP251-09|09PROBE-09546|658[0n]bp|Canada.Manitoba|BOLD:AAB0492
Clostera brucei|[311]|CHLEP288-09|09PROBE-09583|658[0n]bp|Canada.Manitoba|BOLD:AAB0492
Clostera brucei|[312]|CHLEP289-09|09PROBE-09584|658[0n]bp|Canada.Manitoba|BOLD:AAB0492
Clostera brucei|[313]|CHLEP080-09|09PROBE-09375|658[0n]bp|Canada.Manitoba|BOLD:AAB0492
Clostera brucei|[314]|CHLEP073-09|09PROBE-09368|658[0n]bp|Canada.Manitoba|BOLD:AAB0492
Clostera brucei|[315]|CHLEP079-09|09PROBE-09374|658[0n]bp|Canada.Manitoba|BOLD:AAB0492
Clostera brucei|[316]|CHLEP072-09|09PROBE-09367|658[0n]bp|Canada.Manitoba|BOLD:AAB0492
Clostera brucei|[317]|CHLEP070-09|09PROBE-09365|658[0n]bp|Canada.Manitoba|BOLD:AAB0492
Clostera brucei|[318]|CHLEP071-09|09PROBE-09366|658[0n]bp|Canada.Manitoba|BOLD:AAB0492
Clostera brucei|[319]|CHLEP088-09|09PROBE-09383|658[0n]bp|Canada.Manitoba|BOLD:AAB0492
Clostera brucei|[320]|CHLEP089-09|09PROBE-09384|658[0n]bp|Canada.Manitoba|BOLD:AAB0492
Clostera brucei|[321]|CHLEP104-09|09PROBE-09399|658[0n]bp|Canada.Manitoba|BOLD:AAB0492
Clostera apicalis|[322]|LOWCE374-06|CGWC-4134|658[0n]bp|Canada.British Columbia|BOLD:AAA4924
Clostera apicalis|[323]|LPVIB845-08|PFC-2006-2352|658[0n]bp|Canada.British Columbia|BOLD:AAA4924
Clostera apicalis|[324]|LBCH5289-10|10-JDWBC-5289|658[0n]bp|Canada.British Columbia|BOLD:AAA4924
Clostera apicalis|[325]|LALPA847-11|AVBC 1020-11|658[0n]bp|Canada.British Columbia|BOLD:AAA4924
Clostera apicalis|[326]|LALPA132-10|AVBC 132-10|658[0n]bp|Canada.British Columbia|BOLD:AAA4924
Clostera apicalis|[327]|LOWCB114-05|CGWC-1054|658[0n]bp|Canada.British Columbia|BOLD:AAA4924
Clostera apicalis|[328]|LALPA306-10|AVBC 308-10|658[0n]bp|Canada.British Columbia|BOLD:AAA4924
Clostera apicalis|[329]|XAG675-05|2005-ONT-1259|658[0n]bp|Canada.Ontario|BOLD:AAA4924
Clostera apicalis|[330]|LALPA248-10|AVBC 249-10|658[0n]bp|Canada.British Columbia|BOLD:AAA4924
Clostera apicalis|[331]|LOWCE343-06|CGWC-4103|658[0n]bp|Canada.British Columbia|BOLD:AAA4924
Clostera apicalis|[332]|LALPA231-10|AVBC 232-10|658[0n]bp|Canada.British Columbia|BOLD:AAA4924
Clostera apicalis|[333]|LOWCB113-05|CGWC-1053|578[0n]bp|Canada.British Columbia|BOLD:AAA4924
Clostera apicalis|[334]|LOWCE386-06|CGWC-4146|609[0n]bp|Canada.British Columbia|BOLD:AAA4924
Clostera apicalis|[335]|LOWCE257-06|CGWC-4017|658[0n]bp|Canada.British Columbia|BOLD:AAA4924
Clostera apicalis|[336]|BBLPB253-10|10BBCLP-1252|658[0n]bp|Canada.Alberta|BOLD:AAA4924
Clostera apicalis|[337]|BBLPB254-10|10BBCLP-1253|658[0n]bp|Canada.Alberta|BOLD:AAA4924
Clostera apicalis|[338]|BBLPB255-10|10BBCLP-1254|658[0n]bp|Canada.Alberta|BOLD:AAA4924
Clostera apicalis|[339]|BBLPB252-10|10BBCLP-1251|658[0n]bp|Canada.Alberta|BOLD:AAA4924
Clostera apicalis|[340]|BBLPB256-10|10BBCLP-1255|658[0n]bp|Canada.Alberta|BOLD:AAA4924
Clostera apicalis|[341]|LBCA548-05|HLC-20548|658[0n]bp|Canada.British Columbia|BOLD:AAA4924
Clostera apicalis|[342]|LBCA308-05|HLC-20308|658[0n]bp|Canada.British Columbia|BOLD:AAA4924
Clostera apicalis|[343]|LPSOB239-08|PPBP-1238|658[0n]bp|Canada.Ontario|BOLD:AAA4924
Clostera apicalis|[344]|LPSOB451-08|PPBP-1450|658[0n]bp|Canada.Ontario|BOLD:AAA4924
Clostera apicalis|[345]|XAE173-04|Moth4173.03|658[0n]bp|Canada.Ontario|BOLD:AAA4924
Clostera apicalis|[346]|PHMNB496-04|04HBL00722|658[0n]bp|Canada.New Brunswick|BOLD:AAA4924
Clostera apicalis|[347]|TMNBD260-07|MNBTT-3061|658[0n]bp|Canada.New Brunswick|BOLD:AAA4924
Clostera apicalis|[348]|TMNBD259-07|MNBTT-3060|658[0n]bp|Canada.New Brunswick|BOLD:AAA4924
Clostera apicalis|[349]|TTMNB914-06|MNBTT-914|658[0n]bp|Canada.New Brunswick|BOLD:AAA4924
Clostera apicalis|[350]|LBCG3220-09|08-JDWBC-3220|641[0n]bp|Canada.British Columbia|BOLD:AAA4924
Clostera apicalis|[351]|LPSOB114-08|PPBP-1113|655[0n]bp|Canada.Ontario|BOLD:AAA4924
Clostera apicalis|[352]|BBLPB258-10|10BBCLP-1257|658[0n]bp|Canada.Saskatchewan|BOLD:AAA4924
Clostera apicalis|[353]|BLTIB841-08|BL1260|658[0n]bp|Canada.Ontario|BOLD:AAA4924
Clostera apicalis|[354]|BLTIB917-08|BL1337|658[0n]bp|Canada.Ontario|BOLD:AAA4924
Clostera apicalis|[355]|BLTIB825-08|BL1243|658[0n]bp|Canada.Ontario|BOLD:AAA4924
Clostera apicalis|[356]|XAE116-04|Moth4116.03|658[0n]bp|Canada.Ontario|BOLD:AAA4924
Clostera apicalis|[357]|XAE296-04|Moth4296.03|658[0n]bp|Canada.Ontario|BOLD:AAA4924
Clostera apicalis|[358]|LPSOB453-08|PPBP-1452|658[0n]bp|Canada.Ontario|BOLD:AAA4924
Clostera apicalis|[359]|LPSOB340-08|PPBP-1339|658[0n]bp|Canada.Ontario|BOLD:AAA4924
Clostera apicalis|[360]|LPSOB349-08|PPBP-1348|658[0n]bp|Canada.Ontario|BOLD:AAA4924
Clostera apicalis|[361]|LPSOB178-08|PPBP-1177|658[0n]bp|Canada.Ontario|BOLD:AAA4924
Clostera apicalis|[362]|BBLPB268-10|10BBCLP-1267|658[0n]bp|Canada.Ontario|BOLD:AAA4924
Clostera apicalis|[363]|XAJ877-06|2006-ONT-0877|658[0n]bp|Canada.Ontario|BOLD:AAA4924
Clostera apicalis|[364]|LPMN732-08|08BBLEP-01535|658[0n]bp|Canada.Manitoba|BOLD:AAA4924
Clostera apicalis|[365]|XAG606-05|2005-ONT-1190|658[0n]bp|Canada.Ontario|BOLD:AAA4924
Clostera apicalis|[366]|TMNBD262-07|MNBTT-3063|658[0n]bp|Canada.New Brunswick|BOLD:AAA4924
Clostera apicalis|[367]|BLTIB045-08|BL0075|658[0n]bp|Canada.Ontario|BOLD:AAA4924
Clostera apicalis|[368]|TMG57-03|CLOS1.00|639[0n]bp|Canada.Ontario|BOLD:AAA4924
Clostera apicalis|[369]|PMG172-03|moth284.01|617[0n]bp|Canada.Ontario|BOLD:AAA4924
Clostera apicalis|[370]|XAF805-05|2005-ONT-454|588[0n]bp|Canada.Ontario|BOLD:AAA4924
Clostera apicalis|[371]|TMG56-03|moth297.01|639[0n]bp|Canada.Ontario|BOLD:AAA4924
Clostera apicalis|[372]|LBCG3204-09|08-JDWBC-3204|639[0n]bp|Canada.British Columbia|BOLD:AAA4924
Clostera apicalis|[373]|BBLPB259-10|10BBCLP-1258|658[0n]bp|Canada.Saskatchewan|BOLD:AAA4924
Clostera apicalis|[374]|XAG088-05|2005-ONT-672|658[0n]bp|Canada.Ontario|BOLD:AAA4924
Clostera apicalis|[375]|TMNBD261-07|MNBTT-3062|658[0n]bp|Canada.New Brunswick|BOLD:AAA4924
Clostera apicalis|[376]|LPSOD380-09|08BBLEP-00159|658[0n]bp|Canada.Ontario|BOLD:AAA4924
Clostera apicalis|[377]|RDLQ780-07|DH009490|658[0n]bp|Canada.Quebec|BOLD:AAA4924
Clostera apicalis|[378]|XAG257-05|2005-ONT-841|658[0n]bp|Canada.Ontario|BOLD:AAA4924
Clostera apicalis|[379]|XAG349-05|2005-ONT-933|658[0n]bp|Canada.Ontario|BOLD:AAA4924
Clostera apicalis|[380]|XAB007-04|04HBL005007|606[0n]bp|Canada.Ontario|BOLD:AAA4924
Clostera inclusa|[381]|LPSO270-08|PPBP-0270|658[0n]bp|Canada.Ontario|BOLD:AAC4151
Clostera inclusa|[382]|LPSO366-08|PPBP-0366|658[0n]bp|Canada.Ontario|BOLD:AAC4151
Clostera inclusa|[383]|LPSO350-08|PPBP-0350|658[0n]bp|Canada.Ontario|BOLD:AAC4151
Clostera inclusa|[384]|LPSO365-08|PPBP-0365|658[0n]bp|Canada.Ontario|BOLD:AAC4151
Clostera inclusa|[385]|LPSO737-08|PPBP-0737|648[0n]bp|Canada.Ontario|BOLD:AAC4151
Clostera inclusa|[386]|LPSO837-08|PPBP-0837|658[0n]bp|Canada.Ontario|BOLD:AAC4151
Clostera inclusa|[387]|LPSO867-08|PPBP-0867|658[0n]bp|Canada.Ontario|BOLD:AAC4151
Clostera inclusa|[388]|LPSOB092-08|PPBP-1091|658[0n]bp|Canada.Ontario|BOLD:AAC4151
Clostera inclusa|[389]|LPSOB095-08|PPBP-1094|658[0n]bp|Canada.Ontario|BOLD:AAC4151

Clostera inclusa|[387]|LPSO867-08|PPBP-0867|658[0n]bp|Canada.Ontario|BOLD:AAC4151
Clostera inclusa|[388]|LPSOB092-08|PPBP-1091|658[0n]bp|Canada.Ontario|BOLD:AAC4151
Clostera inclusa|[389]|LPSOB095-08|PPBP-1094|658[0n]bp|Canada.Ontario|BOLD:AAC4151
Clostera inclusa|[390]|LPSO156-08|PPBP-0156|658[0n]bp|Canada.Ontario|BOLD:AAC4151
Clostera strigosa|[391]|TTMNB916-06|MNBTT-916|658[0n]bp|Canada.New Brunswick|BOLD:ABY6578
Clostera strigosa|[392]|TTMNB917-06|MNBTT-917|658[0n]bp|Canada.New Brunswick|BOLD:ABY6578
Clostera strigosa|[393]|TMNBD258-07|MNBTT-3059|658[0n]bp|Canada.New Brunswick|BOLD:ABY6578
Clostera strigosa|[394]|TMNBD257-07|MNBTT-3058|658[0n]bp|Canada.New Brunswick|BOLD:ABY6578
Clostera strigosa|[395]|RDLQ781-07|DH005160|643[0n]bp|Canada.Quebec|BOLD:ABY6578
Clostera strigosa|[396]|TTMNB918-06|MNBTT-918|658[0n]bp|Canada.New Brunswick|BOLD:ABY6578
Clostera strigosa|[397]|TTMNB919-06|MNBTT-919|658[0n]bp|Canada.New Brunswick|BOLD:ABY6578
Clostera strigosa|[398]|LPSOB137-08|PPBP-1136|655[0n]bp|Canada.Ontario|BOLD:ABY6578
Clostera strigosa|[399]|LPSOB348-08|PPBP-1347|657[0n]bp|Canada.Ontario|BOLD:ABY6578
Clostera strigosa|[400]|XAB380-04|04HBL005380|658[0n]bp|Canada.Ontario|BOLD:ABY6578
Clostera strigosa|[401]|XAK616-07|HLC-16169|593[0n]bp|Canada.Ontario|BOLD:ABY6578
Clostera strigosa|[402]|XAJ507-06|2006-ONT-0507|626[0n]bp|Canada.Ontario|BOLD:ABY6578
Gluphisia avimacula|[403]|LPSOB343-08|PPBP-1342|658[0n]bp|Canada.Ontario|BOLD:ABZ0941
Gluphisia avimacula|[404]|LPSOB456-08|PPBP-1455|658[0n]bp|Canada.Ontario|BOLD:ABZ0941
Gluphisia avimacula|[405]|TTMNB927-06|MNBTT-927|658[0n]bp|Canada.New Brunswick|BOLD:ABZ0941
Gluphisia avimacula|[406]|TTMNB922-06|MNBTT-922|658[0n]bp|Canada.New Brunswick|BOLD:ABZ0941
Gluphisia avimacula|[407]|TTMNB921-06|MNBTT-921|658[0n]bp|Canada.New Brunswick|BOLD:ABZ0941
Gluphisia avimacula|[408]|TTMNB924-06|MNBTT-924|658[0n]bp|Canada.New Brunswick|BOLD:ABZ0941
Gluphisia avimacula|[409]|TTMNB923-06|MNBTT-923|658[0n]bp|Canada.New Brunswick|BOLD:ABZ0941
Gluphisia avimacula|[410]|TTMNB925-06|MNBTT-925|658[0n]bp|Canada.New Brunswick|BOLD:ABZ0941
Gluphisia avimacula|[411]|LPSOB388-08|PPBP-1387|658[0n]bp|Canada.Ontario|BOLD:ABZ0941
Gluphisia avimacula|[412]|BBLPB639-10|10BBCLP-1638|658[0n]bp|Canada.Saskatchewan|BOLD:ABZ0941
Gluphisia avimacula|[413]|RDLQ773-07|DH009248|632[0n]bp|Canada.Quebec|BOLD:ABZ0941
Gluphisia lintneri|[414]|XAD683-05|2005-ONT-98|658[0n]bp|Canada.Ontario|BOLD:AAB5830
Gluphisia lintneri|[415]|TMNBD299-07|MNBTT-3100|658[0n]bp|Canada.New Brunswick|BOLD:AAB5830
Gluphisia lintneri|[416]|TTMNB928-06|MNBTT-928|658[0n]bp|Canada.New Brunswick|BOLD:AAB5830
Gluphisia lintneri|[417]|TTMNB929-06|MNBTT-929|658[0n]bp|Canada.New Brunswick|BOLD:AAB5830
Gluphisia lintneri|[418]|PMG178-03|moth156.01|617[0n]bp|Canada.Ontario|BOLD:AAB5830
Gluphisia lintneri|[419]|PHMO023-03|moth72.02|639[0n]bp|Canada.Ontario|BOLD:AAB5830
Gluphisia lintneri|[420]|TMG65-03|moth111.02|639[0n]bp|Canada.Ontario|BOLD:AAB5830
Gluphisia lintneri|[421]|XAJ127-06|2006-ONT-0127|658[0n]bp|Canada.Ontario|BOLD:AAB5830
Gluphisia lintneri|[422]|XAJ165-06|2006-ONT-0165|658[0n]bp|Canada.Ontario|BOLD:AAB5830
Gluphisia lintneri|[423]|XAJ137-06|2006-ONT-0137|633[0n]bp|Canada.Ontario|BOLD:AAB5830
Gluphisia lintneri|[424]|XAE130-04|Moth4130.03|658[0n]bp|Canada.Ontario|BOLD:AAB5830
Gluphisia lintneri|[425]|XAJ067-06|2006-ONT-0067|658[0n]bp|Canada.Ontario|BOLD:AAB5830
Gluphisia lintneri|[426]|XAF133-05|HBL008949|658[0n]bp|Canada.Ontario|BOLD:AAB5830
Gluphisia lintneri|[427]|XAJ055-06|2006-ONT-0055|658[0n]bp|Canada.Ontario|BOLD:AAB5830
Gluphisia lintneri|[428]|TTMNB926-06|MNBTT-926|658[0n]bp|Canada.New Brunswick|BOLD:AAB5830
Gluphisia lintneri|[429]|XAE131-04|Moth4131.03|658[0n]bp|Canada.Ontario|BOLD:AAB5830
Gluphisia lintneri|[430]|XAJ092-06|2006-ONT-0092|658[0n]bp|Canada.Ontario|BOLD:AAB5830
Gluphisia lintneri|[431]|XAF193-05|HLC-10234|658[0n]bp|Canada.Ontario|BOLD:AAB5830
Gluphisia lintneri|[432]|XAJ113-06|2006-ONT-0113|658[0n]bp|Canada.Ontario|BOLD:AAB5830
Gluphisia lintneri|[433]|XAJ170-06|2006-ONT-0170|658[0n]bp|Canada.Ontario|BOLD:AAB5830
Gluphisia avimacula|[434]|TTMNB920-06|MNBTT-920|658[0n]bp|Canada.New Brunswick|BOLD:ABZ7450
Gluphisia avimacula|[435]|TMNBD298-07|MNBTT-3099|658[0n]bp|Canada.New Brunswick|BOLD:ABZ7450
Gluphisia avimacula|[436]|TMNBD297-07|MNBTT-3098|658[0n]bp|Canada.New Brunswick|BOLD:ABZ7450
Gluphisia severa|[437]|RDMAB694-06|UASM2598|658[0n]bp|Canada.Alberta|BOLD:ABZ7451
Gluphisia severa|[438]|RDMAB695-06|UASM2638|621[0n]bp|Canada.Alberta|BOLD:AAC5383
Gluphisia severa|[439]|LBCH5281-10|10-JDWBC-5281|658[0n]bp|Canada.British Columbia|BOLD:AAC5383
Gluphisia severa|[440]|LBCA020-05|HLC-20020|658[0n]bp|Canada.British Columbia|BOLD:AAC5383
Gluphisia severa|[441]|LBCA019-05|HLC-20019|658[0n]bp|Canada.British Columbia|BOLD:AAC5383
Gluphisia severa|[442]|LBCA144-05|HLC-20144|658[0n]bp|Canada.British Columbia|BOLD:AAC5383
Gluphisia severa|[443]|LBCA279-05|HLC-20279|658[0n]bp|Canada.British Columbia|BOLD:AAC5383
Gluphisia severa|[444]|DUNLP191-08|Dun-08-191|658[0n]bp|Canada.British Columbia|BOLD:AAC5383
Macrurocampa marthesia|[445]|XAE426-04|Moth4426.03|591[0n]bp|Canada.Ontario|BOLD:AAA9644
Macrurocampa marthesia|[446]|XAF570-05|2005-ONT-219|658[0n]bp|Canada.Ontario|BOLD:AAA9644
Macrurocampa marthesia|[447]|XAE425-04|Moth4425.03|658[0n]bp|Canada.Ontario|BOLD:AAA9644
Macrurocampa marthesia|[448]|XAE441-04|Moth4441.03|658[0n]bp|Canada.Ontario|BOLD:AAA9644
Macrurocampa marthesia|[449]|TMNBD352-07|MNBTT-3153|630[0n]bp|Canada.New Brunswick|BOLD:AAA9644
Macrurocampa marthesia|[450]|RDLQ771-07|DH007822|653[0n]bp|Canada.Quebec|BOLD:AAA9644
Misogada unicolor|[451]|LGSMB255-05|DNA-ATBI-1104|571[0n]bp|United States.Tennessee|BOLD:AAF7371
Misogada unicolor|[452]|LGSMC762-05|DNA-ATBI-2762|658[0n]bp|United States.Tennessee|BOLD:AAF7371
Misogada unicolor|[453]|LGSMB254-05|DNA-ATBI-1103|535[1n]bp|United States.Tennessee|BOLD:AAF7371
Cerura scitiscripta|[454]|RDLQ775-07|DH001233|656[0n]bp|Canada.Quebec|BOLD:AAE9718
Cerura scitiscripta|[455]|LPSOB723-08|PPBP-1722|609[0n]bp|Canada.Ontario|BOLD:AAE9718
Cerura scitiscripta|[456]|RDNMF622-08|NOC14708|640[0n]bp|Canada.British Columbia|BOLD:AAE9718
Cerura scitiscripta|[457]|RDNMF551-08|NOC14637|640[0n]bp|Canada.Ontario|BOLD:AAE9718
Cerura scitiscripta|[458]|XAJ421-06|2006-ONT-0421|658[0n]bp|Canada.Ontario|BOLD:AAE9718
Furcula occidentalis|[459]|LBCW050-08|08-JDWWI-0050|658[0n]bp|Canada.British Columbia|BOLD:AAA8926
Furcula occidentalis|[460]|PHMNB731-05|Moth 424.03SA|658[0n]bp|Canada.New Brunswick|BOLD:AAA8926
Furcula occidentalis|[461]|BBLPB295-10|10BBCLP-1294|658[0n]bp|Canada.Ontario|BOLD:AAA8926
Furcula occidentalis|[462]|RDLQH087-06|DH013325|658[0n]bp|Canada.Quebec|BOLD:AAA8926
Furcula occidentalis|[463]|TMNBD225-07|MNBTT-3026|658[0n]bp|Canada.New Brunswick|BOLD:AAA8926
Furcula occidentalis|[464]|LPSO653-08|PPBP-0653|657[0n]bp|Canada.Ontario|BOLD:AAA8926
Furcula occidentalis|[465]|LPSO425-08|PPBP-0425|658[0n]bp|Canada.Ontario|BOLD:AAA8926
Furcula occidentalis|[466]|LPSO422-08|PPBP-0422|657[0n]bp|Canada.Ontario|BOLD:AAA8926
Furcula occidentalis|[467]|LPSO424-08|PPBP-0424|658[0n]bp|Canada.Ontario|BOLD:AAA8926
Furcula occidentalis|[468]|LPSO423-08|PPBP-0423|657[0n]bp|Canada.Ontario|BOLD:AAA8926
Furcula occidentalis|[469]|LPSO421-08|PPBP-0421|658[0n]bp|Canada.Ontario|BOLD:AAA8926
Furcula occidentalis|[470]|PHMNB050-03|moth236.02SA|639[0n]bp|Canada.New Brunswick|BOLD:AAA8926
Furcula occidentalis|[471]|XAK535-07|HLC-16088|658[0n]bp|Canada.Ontario|BOLD:AAA8926
Furcula occidentalis|[472]|LPSOD458-09|08BBLEP-00237|658[0n]bp|Canada.Ontario|BOLD:AAA8926
Furcula occidentalis|[473]|LPMN585-08|08BBLEP-01386|658[0n]bp|Canada.Manitoba|BOLD:AAA8926
Furcula occidentalis|[474]|LPMN715-08|08BBLEP-01518|658[0n]bp|Canada.Manitoba|BOLD:AAA8926
Furcula occidentalis|[475]|LCHP167-07|07PROBE-00569|656[0n]bp|Canada.Manitoba|BOLD:AAA8926
Furcula occidentalis|[476]|BBLPB299-10|10BBCLP-1298|658[0n]bp|Canada.Saskatchewan|BOLD:AAA8926
Furcula occidentalis|[477]|BBLPB296-10|10BBCLP-1295|658[0n]bp|Canada.Saskatchewan|BOLD:AAA8926
Furcula occidentalis|[478]|CHLEP242-09|09PROBE-09537|658[0n]bp|Canada.Manitoba|BOLD:AAA8926
Furcula occidentalis|[479]|JGLL035-10|10PROBE-18811|658[0n]bp|Canada.Manitoba|BOLD:AAA8926
Furcula occidentalis|[480]|JGLL031-10|10PROBE-18807|658[0n]bp|Canada.Manitoba|BOLD:AAA8926
Furcula occidentalis|[481]|PHLCH737-10|10PROBE-26547|658[0n]bp|Canada.Manitoba|BOLD:AAA8926
Furcula occidentalis|[482]|JGLL028-10|10PROBE-18772|658[0n]bp|Canada.Manitoba|BOLD:AAA8926
Furcula occidentalis|[483]|JGLL011-10|10PROBE-18733|658[0n]bp|Canada.Manitoba|BOLD:AAA8926
Furcula occidentalis|[484]|LCH217-04|04HBL003217|658[0n]bp|Canada.Manitoba|BOLD:AAA8926
Furcula occidentalis|[485]|CHLEP249-09|09PROBE-09544|644[0n]bp|Canada.Manitoba|BOLD:AAA8926
Furcula occidentalis|[486]|CHLEP290-09|09PROBE-09585|658[0n]bp|Canada.Manitoba|BOLD:AAA8926

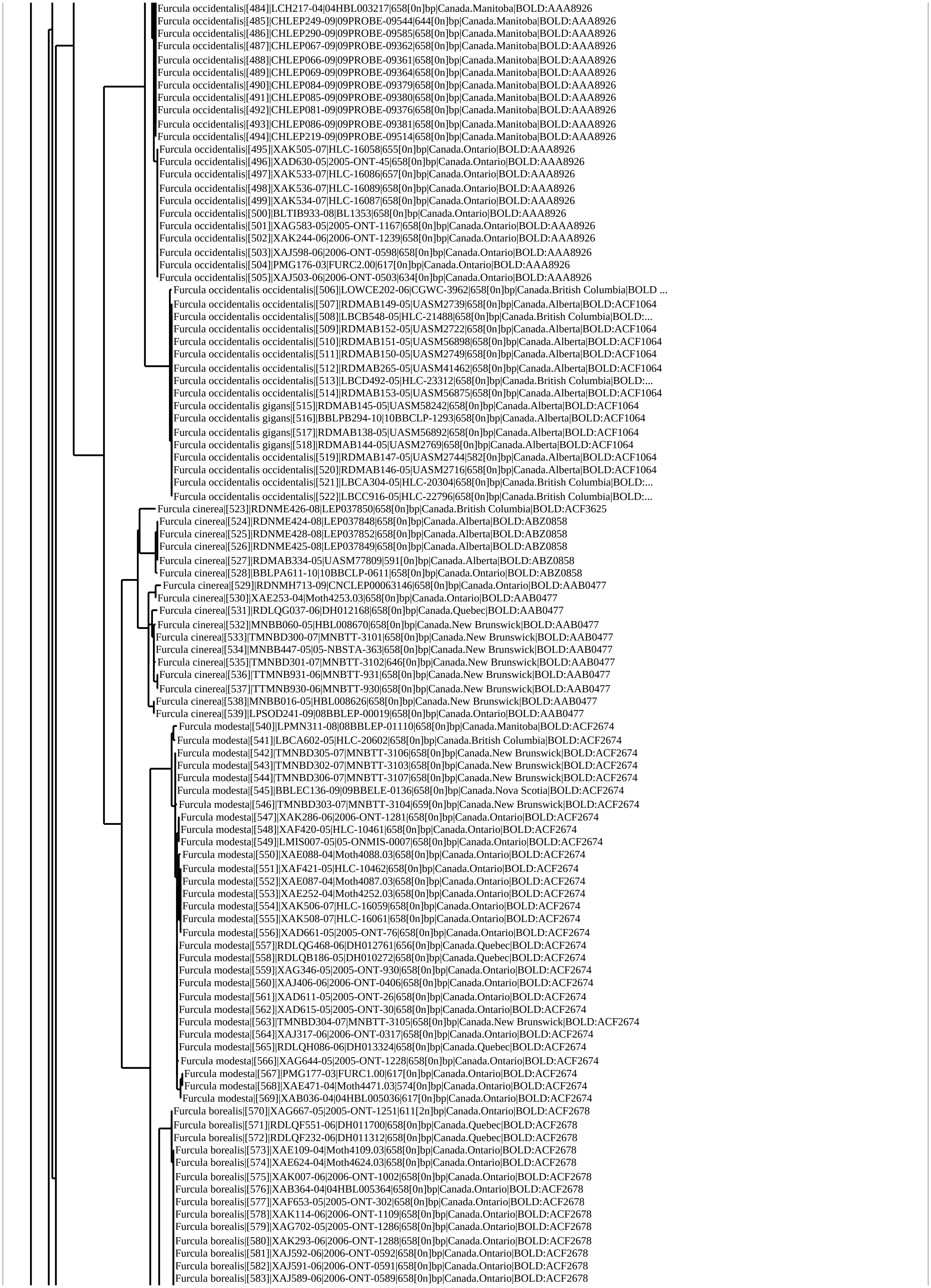
Furcula occidentalis|[484]|LCH217-04|04HBL003217|658[0n]bp|Canada.Manitoba|BOLD:AAA8926
Furcula occidentalis|[485]|CHLEP249-09|09PROBE-09544|644[0n]bp|Canada.Manitoba|BOLD:AAA8926
Furcula occidentalis|[486]|CHLEP290-09|09PROBE-09585|658[0n]bp|Canada.Manitoba|BOLD:AAA8926
Furcula occidentalis|[487]|CHLEP067-09|09PROBE-09362|658[0n]bp|Canada.Manitoba|BOLD:AAA8926
Furcula occidentalis|[488]|CHLEP066-09|09PROBE-09361|658[0n]bp|Canada.Manitoba|BOLD:AAA8926
Furcula occidentalis|[489]|CHLEP069-09|09PROBE-09364|658[0n]bp|Canada.Manitoba|BOLD:AAA8926
Furcula occidentalis|[490]|CHLEP084-09|09PROBE-09379|658[0n]bp|Canada.Manitoba|BOLD:AAA8926
Furcula occidentalis|[491]|CHLEP085-09|09PROBE-09380|658[0n]bp|Canada.Manitoba|BOLD:AAA8926
Furcula occidentalis|[492]|CHLEP081-09|09PROBE-09376|658[0n]bp|Canada.Manitoba|BOLD:AAA8926
Furcula occidentalis|[493]|CHLEP086-09|09PROBE-09381|658[0n]bp|Canada.Manitoba|BOLD:AAA8926
Furcula occidentalis|[494]|CHLEP219-09|09PROBE-09514|658[0n]bp|Canada.Manitoba|BOLD:AAA8926
Furcula occidentalis|[495]|XAK505-07|HLC-16058|655[0n]bp|Canada.Ontario|BOLD:AAA8926
Furcula occidentalis|[496]|XAD630-05|2005-ONT-45|658[0n]bp|Canada.Ontario|BOLD:AAA8926
Furcula occidentalis|[497]|XAK533-07|HLC-16086|657[0n]bp|Canada.Ontario|BOLD:AAA8926
Furcula occidentalis|[498]|XAK536-07|HLC-16089|658[0n]bp|Canada.Ontario|BOLD:AAA8926
Furcula occidentalis|[499]|XAK534-07|HLC-16087|658[0n]bp|Canada.Ontario|BOLD:AAA8926
Furcula occidentalis|[500]|BLTIB933-08|BL1353|658[0n]bp|Canada.Ontario|BOLD:AAA8926
Furcula occidentalis|[501]|XAG583-05|2005-ONT-1167|658[0n]bp|Canada.Ontario|BOLD:AAA8926
Furcula occidentalis|[502]|XAK244-06|2006-ONT-1239|658[0n]bp|Canada.Ontario|BOLD:AAA8926
Furcula occidentalis|[503]|XAJ598-06|2006-ONT-0598|658[0n]bp|Canada.Ontario|BOLD:AAA8926
Furcula occidentalis|[504]|PMG176-03|FURC2.00|617[0n]bp|Canada.Ontario|BOLD:AAA8926
Furcula occidentalis|[505]|XAJ503-06|2006-ONT-0503|634[0n]bp|Canada.Ontario|BOLD:AAA8926
Furcula occidentalis occidentalis|[506]|LOWCE202-06|CGWC-3962|658[0n]bp|Canada.British Columbia|BOLD ...
Furcula occidentalis occidentalis|[507]|RDMAB149-05|UASM2739|658[0n]bp|Canada.Alberta|BOLD:ACF1064
Furcula occidentalis occidentalis|[508]|LBCB548-05|HLC-21488|658[0n]bp|Canada.British Columbia|BOLD:...
Furcula occidentalis occidentalis|[509]|RDMAB152-05|UASM2722|658[0n]bp|Canada.Alberta|BOLD:ACF1064
Furcula occidentalis occidentalis|[510]|RDMAB151-05|UASM56898|658[0n]bp|Canada.Alberta|BOLD:ACF1064
Furcula occidentalis occidentalis|[511]|RDMAB150-05|UASM2749|658[0n]bp|Canada.Alberta|BOLD:ACF1064
Furcula occidentalis occidentalis|[512]|RDMAB265-05|UASM41462|658[0n]bp|Canada.Alberta|BOLD:ACF1064
Furcula occidentalis occidentalis|[513]|LBCD492-05|HLC-23312|658[0n]bp|Canada.British Columbia|BOLD:...
Furcula occidentalis occidentalis|[514]|RDMAB153-05|UASM56875|658[0n]bp|Canada.Alberta|BOLD:ACF1064
Furcula occidentalis gigans|[515]|RDMAB145-05|UASM58242|658[0n]bp|Canada.Alberta|BOLD:ACF1064
Furcula occidentalis gigans|[516]|BBLPB294-10|10BBCLP-1293|658[0n]bp|Canada.Alberta|BOLD:ACF1064
Furcula occidentalis gigans|[517]|RDMAB138-05|UASM56892|658[0n]bp|Canada.Alberta|BOLD:ACF1064
Furcula occidentalis gigans|[518]|RDMAB144-05|UASM2769|658[0n]bp|Canada.Alberta|BOLD:ACF1064
Furcula occidentalis occidentalis|[519]|RDMAB147-05|UASM2744|582[0n]bp|Canada.Alberta|BOLD:ACF1064
Furcula occidentalis occidentalis|[520]|RDMAB146-05|UASM2716|658[0n]bp|Canada.Alberta|BOLD:ACF1064
Furcula occidentalis occidentalis|[521]|LBCA304-05|HLC-20304|658[0n]bp|Canada.British Columbia|BOLD:...
Furcula occidentalis occidentalis|[522]|LBCC916-05|HLC-22796|658[0n]bp|Canada.British Columbia|BOLD:...
Furcula cinerea|[523]|RDNME426-08|LEP037850|658[0n]bp|Canada.British Columbia|BOLD:ACF3625
Furcula cinerea|[524]|RDNME424-08|LEP037848|658[0n]bp|Canada.Alberta|BOLD:ABZ0858
Furcula cinerea|[525]|RDNME428-08|LEP037852|658[0n]bp|Canada.Alberta|BOLD:ABZ0858
Furcula cinerea|[526]|RDNME425-08|LEP037849|658[0n]bp|Canada.Alberta|BOLD:ABZ0858
Furcula cinerea|[527]|RDMAB334-05|UASM77809|591[0n]bp|Canada.Alberta|BOLD:ABZ0858
Furcula cinerea|[528]|BBLPA611-10|10BBCLP-0611|658[0n]bp|Canada.Ontario|BOLD:ABZ0858
Furcula cinerea|[529]|RDNMH713-09|CNCLEP00063146|658[0n]bp|Canada.Ontario|BOLD:AAB0477
Furcula cinerea|[530]|XAE253-04|Moth4253.03|658[0n]bp|Canada.Ontario|BOLD:AAB0477
Furcula cinerea|[531]|RDLQG037-06|DH012168|658[0n]bp|Canada.Quebec|BOLD:AAB0477
Furcula cinerea|[532]|MNBB060-05|HBL008670|658[0n]bp|Canada.New Brunswick|BOLD:AAB0477
Furcula cinerea|[533]|TMNBD300-07|MNBTT-3101|658[0n]bp|Canada.New Brunswick|BOLD:AAB0477
Furcula cinerea|[534]|MNBB447-05|05-NBSTA-363|658[0n]bp|Canada.New Brunswick|BOLD:AAB0477
Furcula cinerea|[535]|TMNBD301-07|MNBTT-3102|646[0n]bp|Canada.New Brunswick|BOLD:AAB0477
Furcula cinerea|[536]|TTMNB931-06|MNBTT-931|658[0n]bp|Canada.New Brunswick|BOLD:AAB0477
Furcula cinerea|[537]|TTMNB930-06|MNBTT-930|658[0n]bp|Canada.New Brunswick|BOLD:AAB0477
Furcula cinerea|[538]|MNBB016-05|HBL008626|658[0n]bp|Canada.New Brunswick|BOLD:AAB0477
Furcula cinerea|[539]|LPSOD241-09|08BBLEP-00019|658[0n]bp|Canada.Ontario|BOLD:AAB0477
Furcula modesta|[540]|LPMN311-08|08BBLEP-01110|658[0n]bp|Canada.Manitoba|BOLD:ACF2674
Furcula modesta|[541]|LBCA602-05|HLC-20602|658[0n]bp|Canada.British Columbia|BOLD:ACF2674
Furcula modesta|[542]|TMNBD305-07|MNBTT-3106|658[0n]bp|Canada.New Brunswick|BOLD:ACF2674
Furcula modesta|[543]|TMNBD302-07|MNBTT-3103|658[0n]bp|Canada.New Brunswick|BOLD:ACF2674
Furcula modesta|[544]|TMNBD306-07|MNBTT-3107|658[0n]bp|Canada.New Brunswick|BOLD:ACF2674
Furcula modesta|[545]|BBLEC136-09|09BBELE-0136|658[0n]bp|Canada.Nova Scotia|BOLD:ACF2674
Furcula modesta|[546]|TMNBD303-07|MNBTT-3104|659[0n]bp|Canada.New Brunswick|BOLD:ACF2674
Furcula modesta|[547]|XAK286-06|2006-ONT-1281|658[0n]bp|Canada.Ontario|BOLD:ACF2674
Furcula modesta|[548]|XAF420-05|HLC-10461|658[0n]bp|Canada.Ontario|BOLD:ACF2674
Furcula modesta|[549]|LMIS007-05|05-ONMIS-0007|658[0n]bp|Canada.Ontario|BOLD:ACF2674
Furcula modesta|[550]|XAE088-04|Moth4088.03|658[0n]bp|Canada.Ontario|BOLD:ACF2674
Furcula modesta|[551]|XAF421-05|HLC-10462|658[0n]bp|Canada.Ontario|BOLD:ACF2674
Furcula modesta|[552]|XAE087-04|Moth4087.03|658[0n]bp|Canada.Ontario|BOLD:ACF2674
Furcula modesta|[553]|XAE252-04|Moth4252.03|658[0n]bp|Canada.Ontario|BOLD:ACF2674
Furcula modesta|[554]|XAK506-07|HLC-16059|658[0n]bp|Canada.Ontario|BOLD:ACF2674
Furcula modesta|[555]|XAK508-07|HLC-16061|658[0n]bp|Canada.Ontario|BOLD:ACF2674
Furcula modesta|[556]|XAD661-05|2005-ONT-76|658[0n]bp|Canada.Ontario|BOLD:ACF2674
Furcula modesta|[557]|RDLQG468-06|DH012761|656[0n]bp|Canada.Quebec|BOLD:ACF2674
Furcula modesta|[558]|RDLQB186-05|DH010272|658[0n]bp|Canada.Quebec|BOLD:ACF2674
Furcula modesta|[559]|XAG346-05|2005-ONT-930|658[0n]bp|Canada.Ontario|BOLD:ACF2674
Furcula modesta|[560]|XAJ406-06|2006-ONT-0406|658[0n]bp|Canada.Ontario|BOLD:ACF2674
Furcula modesta|[561]|XAD611-05|2005-ONT-26|658[0n]bp|Canada.Ontario|BOLD:ACF2674
Furcula modesta|[562]|XAD615-05|2005-ONT-30|658[0n]bp|Canada.Ontario|BOLD:ACF2674
Furcula modesta|[563]|TMNBD304-07|MNBTT-3105|658[0n]bp|Canada.New Brunswick|BOLD:ACF2674
Furcula modesta|[564]|XAJ317-06|2006-ONT-0317|658[0n]bp|Canada.Ontario|BOLD:ACF2674
Furcula modesta|[565]|RDLQH086-06|DH013324|658[0n]bp|Canada.Quebec|BOLD:ACF2674
Furcula modesta|[566]|XAG644-05|2005-ONT-1228|658[0n]bp|Canada.Ontario|BOLD:ACF2674
Furcula modesta|[567]|PMG177-03|FURC1.00|617[0n]bp|Canada.Ontario|BOLD:ACF2674
Furcula modesta|[568]|XAE471-04|Moth4471.03|574[0n]bp|Canada.Ontario|BOLD:ACF2674
Furcula modesta|[569]|XAB036-04|04HBL005036|617[0n]bp|Canada.Ontario|BOLD:ACF2674
Furcula borealis|[570]|XAG667-05|2005-ONT-1251|611[2n]bp|Canada.Ontario|BOLD:ACF2678
Furcula borealis|[571]|RDLQF551-06|DH011700|658[0n]bp|Canada.Quebec|BOLD:ACF2678
Furcula borealis|[572]|RDLQF232-06|DH011312|658[0n]bp|Canada.Quebec|BOLD:ACF2678
Furcula borealis|[573]|XAE109-04|Moth4109.03|658[0n]bp|Canada.Ontario|BOLD:ACF2678
Furcula borealis|[574]|XAE624-04|Moth4624.03|658[0n]bp|Canada.Ontario|BOLD:ACF2678
Furcula borealis|[575]|XAK007-06|2006-ONT-1002|658[0n]bp|Canada.Ontario|BOLD:ACF2678
Furcula borealis|[576]|XAB364-04|04HBL005364|658[0n]bp|Canada.Ontario|BOLD:ACF2678
Furcula borealis|[577]|XAF653-05|2005-ONT-302|658[0n]bp|Canada.Ontario|BOLD:ACF2678
Furcula borealis|[578]|XAK114-06|2006-ONT-1109|658[0n]bp|Canada.Ontario|BOLD:ACF2678
Furcula borealis|[579]|XAG702-05|2005-ONT-1286|658[0n]bp|Canada.Ontario|BOLD:ACF2678
Furcula borealis|[580]|XAK293-06|2006-ONT-1288|658[0n]bp|Canada.Ontario|BOLD:ACF2678
Furcula borealis|[581]|XAJ592-06|2006-ONT-0592|658[0n]bp|Canada.Ontario|BOLD:ACF2678
Furcula borealis|[582]|XAJ591-06|2006-ONT-0591|658[0n]bp|Canada.Ontario|BOLD:ACF2678
Furcula borealis|[583]|XAJ589-06|2006-ONT-0589|658[0n]bp|Canada.Ontario|BOLD:ACF2678

Furcula borealis|[581]|XAJ592-06|2006-ONT-0592|658[0n]bp|Canada.Ontario|BOLD:ACF2678
Furcula borealis|[582]|XAJ591-06|2006-ONT-0591|658[0n]bp|Canada.Ontario|BOLD:ACF2678
Furcula borealis|[583]|XAJ589-06|2006-ONT-0589|658[0n]bp|Canada.Ontario|BOLD:ACF2678
Furcula borealis|[584]|XAJ588-06|2006-ONT-0588|658[0n]bp|Canada.Ontario|BOLD:ACF2678
Furcula borealis|[585]|XAJ590-06|2006-ONT-0590|658[0n]bp|Canada.Ontario|BOLD:ACF2678
Furcula borealis|[586]|PMG175-03|moth576.01|617[0n]bp|Canada.Ontario|BOLD:ACF2678
Furcula borealis|[587]|XAJ593-06|2006-ONT-0593|658[0n]bp|Canada.Ontario|BOLD:ACF2678
Furcula borealis|[588]|XAE586-04|Moth4586.03|576[0n]bp|Canada.Ontario|BOLD:ACF2678
Furcula borealis|[589]|TMG66-03|FURC3.00|639[0n]bp|Canada.Ontario|BOLD:ACF2678
Furcula borealis|[590]|PHMO225-03|moth1149.01|639[0n]bp|Canada.Ontario|BOLD:ACF2678
Furcula scolopendrina|[591]|RDMAB328-05|UASM77820|658[0n]bp|Canada.Alberta|BOLD:AAA6237
Furcula scolopendrina|[592]|LPSOB127-08|PPBP-1126|658[0n]bp|Canada.Ontario|BOLD:AAA6237
Furcula scolopendrina|[593]|BBLPB297-10|10BBCLP-1296|658[0n]bp|Canada.Saskatchewan|BOLD:AAA6237
Furcula scolopendrina|[594]|BBLPB298-10|10BBCLP-1297|658[0n]bp|Canada.Saskatchewan|BOLD:AAA6237
Furcula scolopendrina|[595]|LPSK243-08|08BBLEP-01811|658[0n]bp|Canada.Saskatchewan|BOLD:AAA6237
Furcula scolopendrina|[596]|LPSK244-08|08BBLEP-01812|658[0n]bp|Canada.Saskatchewan|BOLD:AAA6237
Furcula scolopendrina|[597]|LPSK242-08|08BBLEP-01810|658[0n]bp|Canada.Saskatchewan|BOLD:AAA6237
Furcula scolopendrina|[598]|RDMAB066-05|UASM57593|635[0n]bp|Canada.Alberta|BOLD:AAA6237
Furcula scolopendrina|[599]|RDMAB496-06|UASM58442|658[0n]bp|Canada.Alberta|BOLD:AAA6237
Furcula scolopendrina|[600]|RDMAB142-05|UASM2814|658[0n]bp|Canada.Alberta|BOLD:AAA6237
Furcula scolopendrina|[601]|LPSK241-08|08BBLEP-01809|658[0n]bp|Canada.Saskatchewan|BOLD:AAA6237
Furcula scolopendrina|[602]|LOWCB149-05|CGWC-1089|658[0n]bp|Canada.British Columbia|BOLD:AAA6237
Furcula scolopendrina|[603]|RDMAB120-05|UASM41264|658[0n]bp|Canada.Alberta|BOLD:AAA6237
Furcula scolopendrina|[604]|LOWCB146-05|CGWC-1086|658[0n]bp|Canada.British Columbia|BOLD:AAA6237
Furcula scolopendrina|[605]|LOWC381-05|CGWC-0381|658[0n]bp|Canada.British Columbia|BOLD:AAA6237
Furcula scolopendrina|[606]|LOWCB150-05|CGWC-1090|658[0n]bp|Canada.British Columbia|BOLD:AAA6237
Furcula scolopendrina|[607]|LOWCB148-05|CGWC-1088|658[0n]bp|Canada.British Columbia|BOLD:AAA6237
Furcula scolopendrina|[608]|LOWCB151-05|CGWC-1091|658[0n]bp|Canada.British Columbia|BOLD:AAA6237
Furcula scolopendrina|[609]|LOWCB147-05|CGWC-1087|658[0n]bp|Canada.British Columbia|BOLD:AAA6237
Furcula scolopendrina|[610]|RDMAB259-05|UASM41461|658[0n]bp|Canada.Alberta|BOLD:AAA6237
Furcula scolopendrina|[611]|RDMAB065-05|UASM57592|593[0n]bp|Canada.Alberta|BOLD:AAA6237
Furcula scolopendrina|[612]|RDMAB140-05|UASM56888|578[1n]bp|Canada.Alberta|BOLD:AAA6237
Furcula scolopendrina|[613]|RDMAB119-05|UASM41263|630[0n]bp|Canada.Alberta|BOLD:AAA6237
Furcula scolopendrina|[614]|RDMAB143-05|UASM58305|636[0n]bp|Canada.Alberta|BOLD:AAA6237
Furcula scolopendrina|[615]|RDMAB141-05|UASM56901|658[0n]bp|Canada.Alberta|BOLD:AAA6237
Furcula scolopendrina|[616]|LOWCB145-05|CGWC-1085|658[0n]bp|Canada.British Columbia|BOLD:AAA6237
Furcula scolopendrina|[617]|RDMAB139-05|UASM58110|658[0n]bp|Canada.Alberta|BOLD:AAA6237
Furcula scolopendrina|[618]|LBCG3227-09|08-JDWBC-3227|570[11n]bp|Canada.British Columbia|
Furcula scolopendrina|[619]|LBCG165-08|08-JDWBC-0165|658[0n]bp|Canada.British Columbia|BOLD:AAA6237
Furcula scolopendrina|[620]|LBCA546-05|HLC-20546|658[0n]bp|Canada.British Columbia|BOLD:AAA6237
Furcula scolopendrina|[621]|LALPA331-10|AVBC 333-10|658[0n]bp|Canada.British Columbia|BOLD:AAA6237
Furcula scolopendrina|[622]|LBCB077-05|HLC-21017|658[0n]bp|Canada.British Columbia|BOLD:AAA6237
Furcula scolopendrina|[623]|LBCB356-05|HLC-21296|658[0n]bp|Canada.British Columbia|BOLD:AAA6237
Furcula scolopendrina|[624]|LBCG166-08|08-JDWBC-0166|658[0n]bp|Canada.British Columbia|BOLD:AAA6237
Furcula scolopendrina|[625]|LBCG138-08|08-JDWBC-0138|658[0n]bp|Canada.British Columbia|BOLD:AAA6237
Furcula scolopendrina|[626]|LBCG3226-09|08-JDWBC-3226|658[0n]bp|Canada.British Columbia|BOLD:AAA6237
Furcula scolopendrina|[627]|LBCG3224-09|08-JDWBC-3224|658[0n]bp|Canada.British Columbia|BOLD:AAA6237
Furcula scolopendrina|[628]|LBCG3222-09|08-JDWBC-3222|658[0n]bp|Canada.British Columbia|BOLD:AAA6237
Furcula scolopendrina|[629]|LBCG3225-09|08-JDWBC-3225|658[0n]bp|Canada.British Columbia|BOLD:AAA6237
Furcula scolopendrina|[630]|LBCG3223-09|08-JDWBC-3223|658[0n]bp|Canada.British Columbia|BOLD:AAA6237
Furcula scolopendrina|[631]|LBCG2540-09|08-JDWBC-2540|658[0n]bp|Canada.British Columbia|BOLD:AAA6237
Furcula scolopendrina|[632]|LALPA399-10|AVBC 401-10|658[0n]bp|Canada.British Columbia|BOLD:AAA6237
Furcula scolopendrina|[633]|LALPA400-10|AVBC 402-10|658[0n]bp|Canada.British Columbia|BOLD:AAA6237
Furcula scolopendrina|[634]|LBCA210-05|HLC-20210|658[0n]bp|Canada.British Columbia|BOLD:AAA6237
Furcula scolopendrina|[635]|LBCC025-05|HLC-21905|658[0n]bp|Canada.British Columbia|BOLD:AAA6237
Furcula scolopendrina|[636]|LBCC026-05|HLC-21906|658[0n]bp|Canada.British Columbia|BOLD:AAA6237
Furcula scolopendrina|[637]|LBCC411-05|HLC-22291|658[0n]bp|Canada.British Columbia|BOLD:AAA6237
Datana drexelii|[638]|RDLQG125-06|DH012289|658[0n]bp|Canada.Quebec|BOLD:AAA7653
Datana drexelii|[639]|RDLQG124-06|DH012288|632[0n]bp|Canada.Quebec|BOLD:AAA7653
Datana contracta|[640]|RDNMI040-10|CNCLEP 69830|658[0n]bp|Canada.Ontario|BOLD:AAA7653
Datana integerrima|[641]|LPSOD1049-09|08MZPP-115|658[0n]bp|Canada.Ontario|BOLD:AAA7653
Datana integerrima|[642]|XAK253-06|2006-ONT-1248|658[0n]bp|Canada.Ontario|BOLD:AAA7653
Datana integerrima|[643]|RDNMI035-10|CNCLEP 69825|658[0n]bp|Canada.Ontario|BOLD:AAA7653
Datana integerrima|[644]|LPSOB059-08|PPBP-1058|658[0n]bp|Canada.Ontario|BOLD:AAA7653
Datana integerrima|[645]|XAD750-05|2005-ONT-549|658[0n]bp|Canada.Ontario|BOLD:AAA7653
Datana integerrima|[646]|BBLPA578-10|10BBCLP-0578|658[0n]bp|Canada.Ontario|BOLD:AAA7653
Datana integerrima|[647]|BBLPA580-10|10BBCLP-0580|658[0n]bp|Canada.Ontario|BOLD:AAA7653
Datana integerrima|[648]|BBLPA581-10|10BBCLP-0581|658[0n]bp|Canada.Ontario|BOLD:AAA7653
Datana integerrima|[649]|BBLPA577-10|10BBCLP-0577|658[0n]bp|Canada.Ontario|BOLD:AAA7653
Datana integerrima|[650]|BBLPA579-10|10BBCLP-0579|658[0n]bp|Canada.Ontario|BOLD:AAA7653
Datana angusii|[651]|RDNMI030-10|CNCLEP 69820|658[0n]bp|Canada.Ontario|BOLD:ACH9674
Datana angusii|[652]|RDNMI034-10|CNCLEP 69824|658[0n]bp|Canada.Ontario|BOLD:ACH9674
Datana angusii|[653]|RDNMI029-10|CNCLEP 69819|658[0n]bp|Canada.Ontario|BOLD:ACH9674
Datana ministra|[654]|TMNBD224-07|MNBTT-3025|658[0n]bp|Canada.New Brunswick|BOLD:ACH9674
Datana ministra|[655]|PHMNB063-03|moth44.02SA|639[0n]bp|Canada.New Brunswick|BOLD:ACH9674
Datana ministra|[656]|PHMNB067-03|moth55.02SA|639[0n]bp|Canada.New Brunswick|BOLD:ACH9674
Datana ministra|[657]|PHMNB012-03|moth135.02SA|639[0n]bp|Canada.New Brunswick|BOLD:ACH9674
Datana ministra|[658]|PHMNB010-03|moth130.02SA|639[0n]bp|Canada.New Brunswick|BOLD:ACH9674
Datana ministra|[659]|TMG58-03|moth972.01|639[0n]bp|Canada.Ontario|BOLD:ACH9674
Datana ministra|[660]|RDNMI032-10|CNCLEP 69822|658[0n]bp|Canada.Ontario|BOLD:ACH9674
Datana ministra|[661]|XAE411-04|Moth4411.03|658[0n]bp|Canada.Ontario|BOLD:ACH9674
Datana ministra|[662]|XAJ825-06|2006-ONT-0825|658[0n]bp|Canada.Ontario|BOLD:ACH9674
Datana ministra|[663]|LPSO866-08|PPBP-0866|658[0n]bp|Canada.Ontario|BOLD:ACH9674
Datana ministra|[664]|RDNMI039-10|CNCLEP 69829|658[0n]bp|Canada.Ontario|BOLD:ACH9674
Datana ministra|[665]|LPMN063-08|08BBLEP-00861|658[0n]bp|Canada.Manitoba|BOLD:ACH9674
Datana ministra|[666]|RDNMI033-10|CNCLEP 69823|658[0n]bp|Canada.Ontario|BOLD:ACH9674
Datana ministra|[667]|RDNMI031-10|CNCLEP 69821|658[0n]bp|Canada.Ontario|BOLD:ACH9674
Datana ministra|[668]|PMG173-03|DATA1.00|617[0n]bp|Canada.Ontario|BOLD:ACH9674
Datana ministra|[669]|PHMO172-03|moth907.01|639[0n]bp|Canada.Ontario|BOLD:ACH9674
Datana ministra|[670]|PHMO129-03|moth758.02|639[0n]bp|Canada.Ontario|BOLD:ACH9674
Datana ministra|[671]|LPSOD319-09|08BBLEP-00097|658[0n]bp|Canada.Ontario|BOLD:ACH9674
Datana ministra|[672]|RDLQB198-05|DH010284|658[0n]bp|Canada.Quebec|BOLD:ACH9674
Datana ministra|[673]|XAJ824-06|2006-ONT-0824|658[0n]bp|Canada.Ontario|BOLD:ACH9674
Datana ministra|[674]|LPSOB426-08|PPBP-1425|658[0n]bp|Canada.Ontario|BOLD:ACH9674
Datana ministra|[675]|LPSOD442-09|08BBLEP-00221|658[0n]bp|Canada.Ontario|BOLD:ACH9674
Datana perspicua|[676]|XAJ922-06|2006-ONT-0922|630[0n]bp|Canada.Ontario|BOLD:ACH9676
Datana perspicua|[677]|RDNMF511-08|NOC14597|658[0n]bp|Canada.Ontario|BOLD:ACH9676
Datana perspicua|[678]|RDNMF510-08|NOC14596|658[0n]bp|Canada.Ontario|BOLD:ACH9676
Datana perspicua|[679]|RDNMF509-08|NOC14595|640[0n]bp|Canada.Ontario|BOLD:ACH9676
Heterocampa biundata|[680]|PHMO372-03|moth1058.02|639[1n]bp|Canada.Ontario|BOLD:AAB0601
[illegible]

Datana perspicua|[678]|RDNMF510-08|NOC14596|658[0n]bp|Canada.Ontario|BOLD:ACH9676
Datana perspicua|[679]|RDNMF509-08|NOC14595|640[0n]bp|Canada.Ontario|BOLD:ACH9676
Heterocampa biundata|[680]|PHMO372-03|moth1058.02|639[1n]bp|Canada.Ontario|BOLD:AAB0601
Heterocampa biundata|[681]|MNBB067-05|HBL008677|658[0n]bp|Canada.New Brunswick|BOLD:AAB0601
Heterocampa biundata|[682]|XAE599-04|Moth4599.03|658[0n]bp|Canada.Ontario|BOLD:AAB0601
Heterocampa biundata|[683]|XAE413-04|Moth4413.03|658[0n]bp|Canada.Ontario|BOLD:AAB0601
Heterocampa biundata|[684]|XAB143-04|04HBL005143|658[0n]bp|Canada.Ontario|BOLD:AAB0601
Heterocampa biundata|[685]|XAB508-04|04HBL005508|658[0n]bp|Canada.Ontario|BOLD:AAB0601
Heterocampa biundata|[686]|PHMO376-03|moth1322.02|639[0n]bp|Canada.Ontario|BOLD:AAB0601
Heterocampa biundata|[687]|RDLQ772-07|DH011572|633[0n]bp|Canada.Quebec|BOLD:AAB0601
Heterocampa biundata|[688]|MNBB022-05|HBL008632|596[0n]bp|Canada.New Brunswick|BOLD:AAB0601
Heterocampa biundata|[689]|BBLEC841-09|09BBELE-0841|633[0n]bp|Canada.Newfoundland and Labrador|BOLD:...
Heterocampa biundata|[690]|BBLEC847-09|09BBELE-0847|633[0n]bp|Canada.Newfoundland and Labrador|BOLD:...
Heterocampa biundata|[691]|BBLEC404-09|09BBELE-0404|658[0n]bp|Canada.Newfoundland and Labrador|BOLD:...
Heterocampa biundata|[692]|BBLPE421-09|09BBELE-2421|658[0n]bp|Canada.Newfoundland and Labrador|BOLD:...
Heterocampa biundata|[693]|BBLEC862-09|09BBELE-0862|658[0n]bp|Canada.Newfoundland and Labrador|BOLD:...
Heterocampa biundata|[694]|BBLEC876-09|09BBELE-0876|658[0n]bp|Canada.Newfoundland and Labrador|BOLD:...
Heterocampa biundata|[695]|BBLEC802-09|09BBELE-0802|658[0n]bp|Canada.Newfoundland and Labrador|BOLD:...
Heterocampa biundata|[696]|BBLEC803-09|09BBELE-0803|658[0n]bp|Canada.Newfoundland and Labrador|BOLD:...
Heterocampa biundata|[697]|BBLPC198-09|09BBELE-1198|658[0n]bp|Canada.Nova Scotia|BOLD:AAB0601
Heterocampa biundata|[698]|PHMNB037-03|moth207.02SA|639[0n]bp|Canada.New Brunswick|BOLD:AAB0601
Heterocampa biundata|[699]|BBLPC242-09|09BBELE-1242|658[0n]bp|Canada.Nova Scotia|BOLD:AAB0601
Heterocampa biundata|[700]|TTMNB937-06|MNBTT-937|658[0n]bp|Canada.New Brunswick|BOLD:AAB0601
Heterocampa obliqua|[701]|BBLEC138-09|09BBELE-0138|658[0n]bp|Canada.Nova Scotia|BOLD:AAB0486
Heterocampa subrotata|[702]|LPSOB087-08|PPBP-1086|653[0n]bp|Canada.Ontario|BOLD:AAA6576
Heterocampa subrotata|[703]|LPSO420-08|PPBP-0420|631[1n]bp|Canada.Ontario|BOLD:AAA6576
Heterocampa subrotata|[704]|LPSO413-08|PPBP-0413|656[0n]bp|Canada.Ontario|BOLD:AAA6576
Heterocampa subrotata|[705]|LPSO537-08|PPBP-0537|658[0n]bp|Canada.Ontario|BOLD:AAA6576
Heterocampa subrotata|[706]|LPSO534-08|PPBP-0534|658[0n]bp|Canada.Ontario|BOLD:AAA6576
Heterocampa subrotata|[707]|LPSO533-08|PPBP-0533|658[0n]bp|Canada.Ontario|BOLD:AAA6576
Heterocampa subrotata|[708]|LPSO528-08|PPBP-0528|658[0n]bp|Canada.Ontario|BOLD:AAA6576
Heterocampa subrotata|[709]|LPSO541-08|PPBP-0541|658[0n]bp|Canada.Ontario|BOLD:AAA6576
Heterocampa subrotata|[710]|LPSO426-08|PPBP-0426|658[0n]bp|Canada.Ontario|BOLD:AAA6576
Heterocampa subrotata|[711]|LPSO414-08|PPBP-0414|658[0n]bp|Canada.Ontario|BOLD:AAA6576
Heterocampa subrotata|[712]|LPSO416-08|PPBP-0416|658[0n]bp|Canada.Ontario|BOLD:AAA6576
Heterocampa subrotata|[713]|LPSO411-08|PPBP-0411|658[0n]bp|Canada.Ontario|BOLD:AAA6576
Heterocampa subrotata|[714]|LPSO419-08|PPBP-0419|658[0n]bp|Canada.Ontario|BOLD:AAA6576
Heterocampa subrotata|[715]|LPSO375-08|PPBP-0375|658[0n]bp|Canada.Ontario|BOLD:AAA6576
Heterocampa subrotata|[716]|LPSO358-08|PPBP-0358|658[0n]bp|Canada.Ontario|BOLD:AAA6576
Heterocampa subrotata|[717]|LPSO559-08|PPBP-0559|658[0n]bp|Canada.Ontario|BOLD:AAA6576
Heterocampa subrotata|[718]|LPSO529-08|PPBP-0529|658[0n]bp|Canada.Ontario|BOLD:AAA6576
Heterocampa subrotata|[719]|LPSOB098-08|PPBP-1097|658[0n]bp|Canada.Ontario|BOLD:AAA6576
Heterocampa subrotata|[720]|LPSOB090-08|PPBP-1089|658[0n]bp|Canada.Ontario|BOLD:AAA6576
Heterocampa subrotata|[721]|LPSOB083-08|PPBP-1082|658[0n]bp|Canada.Ontario|BOLD:AAA6576
Heterocampa subrotata|[722]|LPSO488-08|PPBP-0488|658[0n]bp|Canada.Ontario|BOLD:AAA6576
Heterocampa subrotata|[723]|LPSO530-08|PPBP-0530|658[0n]bp|Canada.Ontario|BOLD:AAA6576
Heterocampa subrotata|[724]|LPSO548-08|PPBP-0548|658[0n]bp|Canada.Ontario|BOLD:AAA6576
Heterocampa subrotata|[725]|LPSO539-08|PPBP-0539|658[0n]bp|Canada.Ontario|BOLD:AAA6576
Heterocampa subrotata|[726]|LPSO540-08|PPBP-0540|658[0n]bp|Canada.Ontario|BOLD:AAA6576
Heterocampa subrotata|[727]|LPSO490-08|PPBP-0490|658[0n]bp|Canada.Ontario|BOLD:AAA6576
Heterocampa subrotata|[728]|LPSO535-08|PPBP-0535|658[0n]bp|Canada.Ontario|BOLD:AAA6576
Heterocampa subrotata|[729]|LPSO531-08|PPBP-0531|658[0n]bp|Canada.Ontario|BOLD:AAA6576
Heterocampa subrotata|[730]|LPSO532-08|PPBP-0532|658[0n]bp|Canada.Ontario|BOLD:AAA6576
Heterocampa subrotata|[731]|LPSO410-08|PPBP-0410|658[0n]bp|Canada.Ontario|BOLD:AAA6576
Heterocampa subrotata|[732]|LPSO412-08|PPBP-0412|658[0n]bp|Canada.Ontario|BOLD:AAA6576
Heterocampa subrotata|[733]|LPSO469-08|PPBP-0469|658[0n]bp|Canada.Ontario|BOLD:AAA6576
Heterocampa subrotata|[734]|LPSO415-08|PPBP-0415|658[0n]bp|Canada.Ontario|BOLD:AAA6576
Heterocampa subrotata|[735]|LPSO418-08|PPBP-0418|658[0n]bp|Canada.Ontario|BOLD:AAA6576
Heterocampa subrotata|[736]|LPSO356-08|PPBP-0356|658[0n]bp|Canada.Ontario|BOLD:AAA6576
Heterocampa subrotata|[737]|LPSO373-08|PPBP-0373|658[0n]bp|Canada.Ontario|BOLD:AAA6576
Heterocampa subrotata|[738]|LPSO536-08|PPBP-0536|658[0n]bp|Canada.Ontario|BOLD:AAA6576
Heterocampa subrotata|[739]|LPSO538-08|PPBP-0538|658[0n]bp|Canada.Ontario|BOLD:AAA6576
Heterocampa subrotata|[740]|LPSO501-08|PPBP-0501|658[0n]bp|Canada.Ontario|BOLD:AAA6576
Heterocampa subrotata|[741]|LPSO884-08|PPBP-0884|658[0n]bp|Canada.Ontario|BOLD:AAA6576
Heterocampa subrotata|[742]|LPSO956-08|PPBP-0956|658[0n]bp|Canada.Ontario|BOLD:AAA6576
Heterocampa subrotata|[743]|LPSO957-08|PPBP-0957|658[0n]bp|Canada.Ontario|BOLD:AAA6576
Heterocampa subrotata|[744]|LPSOB086-08|PPBP-1085|658[0n]bp|Canada.Ontario|BOLD:AAA6576
Heterocampa subrotata|[745]|LPSOB091-08|PPBP-1090|658[0n]bp|Canada.Ontario|BOLD:AAA6576
Heterocampa subrotata|[746]|LPSOB089-08|PPBP-1088|658[0n]bp|Canada.Ontario|BOLD:AAA6576
Heterocampa subrotata|[747]|LPSOB085-08|PPBP-1084|658[0n]bp|Canada.Ontario|BOLD:AAA6576
Heterocampa subrotata|[748]|LPSOB084-08|PPBP-1083|658[0n]bp|Canada.Ontario|BOLD:AAA6576
Heterocampa subrotata|[749]|LPSOB073-08|PPBP-1072|658[0n]bp|Canada.Ontario|BOLD:AAA6576
Heterocampa subrotata|[750]|LPSOB088-08|PPBP-1087|658[0n]bp|Canada.Ontario|BOLD:AAA6576
Heterocampa subrotata|[751]|LPSO164-08|PPBP-0164|658[0n]bp|Canada.Ontario|BOLD:AAA6576
Heterocampa subrotata|[752]|LPSO176-08|PPBP-0176|658[0n]bp|Canada.Ontario|BOLD:AAA6576
Heterocampa subrotata|[753]|LPSO163-08|PPBP-0163|658[0n]bp|Canada.Ontario|BOLD:AAA6576
Heterocampa guttivitta|[754]|BLTIB162-08|BL237|658[0n]bp|Canada.Ontario|BOLD:AAA3773
Heterocampa guttivitta|[755]|XAB396-04|04HBL005396|658[0n]bp|Canada.Ontario|BOLD:AAA3773
Heterocampa guttivitta|[756]|XAE163-04|Moth4163.03|658[0n]bp|Canada.Ontario|BOLD:AAA3773
Heterocampa guttivitta|[757]|XAB556-04|04HBL005556|658[0n]bp|Canada.Ontario|BOLD:AAA3773
Heterocampa guttivitta|[758]|XAB555-04|04HBL005555|658[0n]bp|Canada.Ontario|BOLD:AAA3773
Heterocampa guttivitta|[759]|PMG180-03|moth782.01|617[1n]bp|Canada.Ontario|BOLD:AAA3773
Heterocampa guttivitta|[760]|PHMNB359-04|04HBL00585|658[0n]bp|Canada.New Brunswick|BOLD:AAA3773
Heterocampa guttivitta|[761]|XAB393-04|04HBL005393|658[0n]bp|Canada.Ontario|BOLD:AAA3773
Heterocampa guttivitta|[762]|PHMNB323-04|04HBL00549|658[0n]bp|Canada.New Brunswick|BOLD:AAA3773
Heterocampa guttivitta|[763]|KPOEC083-08|08OEC-242|658[0n]bp|Canada.Ontario|BOLD:AAA3773
Heterocampa guttivitta|[764]|KPOEC128-08|08OEC-107|658[0n]bp|Canada.Ontario|BOLD:AAA3773
Heterocampa guttivitta|[765]|XAD607-05|2005-ONT-22|651[0n]bp|Canada.Ontario|BOLD:AAA3773
Heterocampa guttivitta|[766]|KPOEC038-08|08OEC-141|658[0n]bp|Canada.Ontario|BOLD:AAA3773
Heterocampa guttivitta|[767]|PHMNB426-04|04HBL00652|658[0n]bp|Canada.New Brunswick|BOLD:AAA3773
Heterocampa guttivitta|[768]|PHMNB570-04|04HBL00796|658[0n]bp|Canada.New Brunswick|BOLD:AAA3773
Heterocampa guttivitta|[769]|PHMNB439-04|04HBL00665|658[0n]bp|Canada.New Brunswick|BOLD:AAA3773
Heterocampa guttivitta|[770]|PHMNB627-04|04HBL00853|658[0n]bp|Canada.New Brunswick|BOLD:AAA3773
Heterocampa guttivitta|[771]|TTMNB542-06|MNBTT-542|658[0n]bp|Canada.New Brunswick|BOLD:AAA3773
Heterocampa guttivitta|[772]|PMG179-03|moth286.01|617[0n]bp|Canada.Ontario|BOLD:AAA3773
Heterocampa guttivitta|[773]|XAE522-04|Moth4522.03|658[4n]bp|Canada.Ontario|BOLD:AAA3773
Heterocampa guttivitta|[774]|KPOEC122-08|08OEC-068|658[0n]bp|Canada.Ontario|BOLD:AAA3773
Heterocampa guttivitta|[775]|TTMNB059-06|MNBTT-059|658[0n]bp|Canada.New Brunswick|BOLD:AAA3773
Heterocampa guttivitta|[776]|XAG897-05|2005-ONT-1481|658[0n]bp|Canada.Ontario|BOLD:AAA3773
Heterocampa guttivitta|[777]|XAE239-04|Moth4239.03|658[0n]bp|Canada.Ontario|BOLD:AAA3773
[illegible]

Heterocampa guttivitta|[775]|TTMNB039-06|MNBTT-039|658[0n]bp|Canada.New Brunswick|BOLD:AAA3773
Heterocampa guttivitta|[776]|XAG897-05|2005-ONT-1481|658[0n]bp|Canada.Ontario|BOLD:AAA3773
Heterocampa guttivitta|[777]|XAE239-04|Moth4239.03|658[0n]bp|Canada.Ontario|BOLD:AAA3773
Heterocampa guttivitta|[778]|XAE222-04|Moth4222.03|658[0n]bp|Canada.Ontario|BOLD:AAA3773
Heterocampa guttivitta|[779]|XAF405-05|HLC-10446|658[0n]bp|Canada.Ontario|BOLD:AAA3773
Heterocampa guttivitta|[780]|XAB367-04|04HBL005367|658[0n]bp|Canada.Ontario|BOLD:AAA3773
Heterocampa guttivitta|[781]|XAK285-06|2006-ONT-1280|658[0n]bp|Canada.Ontario|BOLD:AAA3773
Heterocampa guttivitta|[782]|PHMNB526-04|04HBL00752|658[0n]bp|Canada.New Brunswick|BOLD:AAA3773
Heterocampa guttivitta|[783]|PHMNB456-04|04HBL00682|658[0n]bp|Canada.New Brunswick|BOLD:AAA3773
Heterocampa guttivitta|[784]|PHMNB440-04|04HBL00666|658[0n]bp|Canada.New Brunswick|BOLD:AAA3773
Heterocampa guttivitta|[785]|PHMNB577-04|04HBL00803|658[0n]bp|Canada.New Brunswick|BOLD:AAA3773
Heterocampa guttivitta|[786]|PHMNB572-04|04HBL00798|658[0n]bp|Canada.New Brunswick|BOLD:AAA3773
Heterocampa guttivitta|[787]|PHMNB442-04|04HBL00668|658[0n]bp|Canada.New Brunswick|BOLD:AAA3773
Heterocampa guttivitta|[788]|TMNBD354-07|MNBTT-3155|630[0n]bp|Canada.New Brunswick|BOLD:AAA3773
Heterocampa guttivitta|[789]|KPOEC152-08|08OEC-193|658[0n]bp|Canada.Ontario|BOLD:AAA3773
Heterocampa guttivitta|[790]|TMNBD355-07|MNBTT-3156|631[0n]bp|Canada.New Brunswick|BOLD:AAA3773
Heterocampa guttivitta|[791]|TMNBD356-07|MNBTT-3157|590[0n]bp|Canada.New Brunswick|BOLD:AAA3773
Heterocampa guttivitta|[792]|TMG69-03|moth468.01|639[0n]bp|Canada.Ontario|BOLD:AAA3773
Heterocampa guttivitta|[793]|XAJ423-06|2006-ONT-0423|658[0n]bp|Canada.Ontario|BOLD:AAA3773
Heterocampa guttivitta|[794]|XAJ459-06|2006-ONT-0459|658[0n]bp|Canada.Ontario|BOLD:AAA3773
Heterocampa guttivitta|[795]|TTMNB248-06|MNBTT-248|658[0n]bp|Canada.New Brunswick|BOLD:AAA3773
Heterocampa guttivitta|[796]|XAF765-05|2005-ONT-414|535[0n]bp|Canada.Ontario|BOLD:AAA3773
Heterocampa guttivitta|[797]|LPSOD251-09|08BBLEP-00029|658[0n]bp|Canada.Ontario|BOLD:AAA3773
Heterocampa guttivitta|[798]|LPSOD318-09|08BBLEP-00096|658[0n]bp|Canada.Ontario|BOLD:AAA3773
Heterocampa guttivitta|[799]|LPSOD352-09|08BBLEP-00130|658[0n]bp|Canada.Ontario|BOLD:AAA3773
Heterocampa guttivitta|[800]|LPSOD272-09|08BBLEP-00050|608[0n]bp|Canada.Ontario|BOLD:AAA3773
Heterocampa guttivitta|[801]|LPSO232-08|PPBP-0232|658[0n]bp|Canada.Ontario|BOLD:AAA3773
Heterocampa guttivitta|[802]|LPSO239-08|PPBP-0239|658[0n]bp|Canada.Ontario|BOLD:AAA3773
Heterocampa guttivitta|[803]|XAB547-04|04HBL005547|658[0n]bp|Canada.Ontario|BOLD:AAA3773
Heterocampa guttivitta|[804]|XAE240-04|Moth4240.03|658[0n]bp|Canada.Ontario|BOLD:AAA3773
Heterocampa guttivitta|[805]|LPSO030-08|PPBP-0030|658[0n]bp|Canada.Ontario|BOLD:AAA3773
Heterocampa guttivitta|[806]|LPSOB788-08|PPBP-1787|658[0n]bp|Canada.Ontario|BOLD:AAA3773
Heterocampa guttivitta|[807]|XAF472-05|2005-ONT-121|658[0n]bp|Canada.Ontario|BOLD:AAA3773
Heterocampa guttivitta|[808]|XAE241-04|Moth4241.03|658[0n]bp|Canada.Ontario|BOLD:AAA3773
Heterocampa guttivitta|[809]|XAE221-04|Moth4221.03|658[0n]bp|Canada.Ontario|BOLD:AAA3773
Heterocampa guttivitta|[810]|XAB394-04|04HBL005394|658[0n]bp|Canada.Ontario|BOLD:AAA3773
Heterocampa guttivitta|[811]|XAB395-04|04HBL005395|658[0n]bp|Canada.Ontario|BOLD:AAA3773
Heterocampa guttivitta|[812]|XAB361-04|04HBL005361|658[0n]bp|Canada.Ontario|BOLD:AAA3773
Heterocampa guttivitta|[813]|XAF328-05|HLC-10369|658[0n]bp|Canada.Ontario|BOLD:AAA3773
Heterocampa guttivitta|[814]|XAB509-04|04HBL005509|658[0n]bp|Canada.Ontario|BOLD:AAA3773
Heterocampa guttivitta|[815]|LPSO526-08|PPBP-0526|622[0n]bp|Canada.Ontario|BOLD:AAA3773
Heterocampa guttivitta|[816]|TMG68-03|moth140.01|639[0n]bp|Canada.Ontario|BOLD:AAA3773
Heterocampa guttivitta|[817]|XAE397-04|Moth4397.03|567[0n]bp|Canada.Ontario|BOLD:AAA3773
Heterocampa guttivitta|[818]|LPSO361-08|PPBP-0361|658[0n]bp|Canada.Ontario|BOLD:AAA3773
Heterocampa guttivitta|[819]|LPSO527-08|PPBP-0527|658[0n]bp|Canada.Ontario|BOLD:AAA3773
Heterocampa guttivitta|[820]|XAF736-05|2005-ONT-385|596[1n]bp|Canada.Ontario|BOLD:AAA3773
Heterocampa guttivitta|[821]|LPSOB967-08|PPBP-1966|658[0n]bp|Canada.Ontario|BOLD:AAA3773
Heterocampa guttivitta|[822]|LPSOC079-08|PPBP-2078|658[0n]bp|Canada.Ontario|BOLD:AAA3773
Heterocampa guttivitta|[823]|LPSO009-08|PPBP-0009|658[0n]bp|Canada.Ontario|BOLD:AAA3773
Heterocampa umbrata|[824]|XAK153-06|2006-ONT-1148|658[0n]bp|Canada.Ontario|BOLD:AAB3178
Heterocampa umbrata|[825]|LPSOD505-09|08BBLEP-00284|658[0n]bp|Canada.Ontario|BOLD:AAB3178
Heterocampa umbrata|[826]|LPSOD363-09|08BBLEP-00141|658[0n]bp|Canada.Ontario|BOLD:AAB3178
Heterocampa umbrata|[827]|TTMNB936-06|MNBTT-936|658[0n]bp|Canada.New Brunswick|BOLD:AAB3178
Heterocampa umbrata|[828]|TTMNB935-06|MNBTT-935|658[0n]bp|Canada.New Brunswick|BOLD:AAB3178
Heterocampa umbrata|[829]|TMNBD209-07|MNBTT-3010|658[0n]bp|Canada.New Brunswick|BOLD:AAB3178
Heterocampa umbrata|[830]|TMNBD208-07|MNBTT-3009|658[0n]bp|Canada.New Brunswick|BOLD:AAB3178
Heterocampa umbrata|[831]|TMNBD211-07|MNBTT-3012|658[0n]bp|Canada.New Brunswick|BOLD:AAB3178
Heterocampa umbrata|[832]|TMNBD210-07|MNBTT-3011|658[0n]bp|Canada.New Brunswick|BOLD:AAB3178
Heterocampa umbrata|[833]|TMNBD212-07|MNBTT-3013|658[0n]bp|Canada.New Brunswick|BOLD:AAB3178
Heterocampa umbrata|[834]|PHMNB668-04|04HBL00894|658[0n]bp|Canada.New Brunswick|BOLD:AAB3178
Heterocampa umbrata|[835]|PHMNB018-03|moth148.02SA|639[0n]bp|Canada.New Brunswick|BOLD:AAB3178
Heterocampa umbrata|[836]|MNBB243-05|05-NBSTA-159|589[3n]bp|Canada.New Brunswick|BOLD:AAB3178
Heterocampa umbrata|[837]|PMG181-03|HETEROC1.00|617[0n]bp|Canada.Ontario|BOLD:AAB3178
Heterocampa umbrata pulverea|[838]|RDLQB213-05|DH010299|597[0n]bp|Canada.Quebec|BOLD:AAB3178
Heterocampa umbrata pulverea|[839]|RDLQB211-05|DH010297|558[0n]bp|Canada.Quebec|BOLD:AAB3178
Lochmaeus bilineata|[840]|XAG879-05|2005-ONT-1463|658[0n]bp|Canada.Ontario|BOLD:AAB3762
Lochmaeus bilineata|[841]|XAE308-04|Moth4308.03|658[0n]bp|Canada.Ontario|BOLD:AAB3762
Lochmaeus bilineata|[842]|XAG104-05|2005-ONT-688|658[0n]bp|Canada.Ontario|BOLD:AAB3762
Lochmaeus bilineata|[843]|XAE394-04|Moth4394.03|658[0n]bp|Canada.Ontario|BOLD:AAB3762
Lochmaeus bilineata|[844]|XAK263-06|2006-ONT-1258|658[0n]bp|Canada.Ontario|BOLD:AAB3762
Lochmaeus bilineata|[845]|XAK115-06|2006-ONT-1110|658[0n]bp|Canada.Ontario|BOLD:AAB3762
Lochmaeus bilineata|[846]|XAF820-05|2005-ONT-469|658[0n]bp|Canada.Ontario|BOLD:AAB3762
Lochmaeus bilineata|[847]|XAG110-05|2005-ONT-694|658[0n]bp|Canada.Ontario|BOLD:AAB3762
Lochmaeus bilineata|[848]|XAB282-04|04HBL005282|658[0n]bp|Canada.Ontario|BOLD:AAB3762
Lochmaeus bilineata|[849]|XAE627-04|Moth4627.03|658[0n]bp|Canada.Ontario|BOLD:AAB3762
Lochmaeus bilineata|[850]|XAE354-04|Moth4354.03|658[0n]bp|Canada.Ontario|BOLD:AAB3762
Lochmaeus bilineata|[851]|XAG760-05|2005-ONT-1344|658[0n]bp|Canada.Ontario|BOLD:AAB3762
Lochmaeus bilineata|[852]|XAG766-05|2005-ONT-1350|658[0n]bp|Canada.Ontario|BOLD:AAB3762
Lochmaeus bilineata|[853]|PHMO157-03|moth861.02|639[0n]bp|Canada.Ontario|BOLD:AAB3762
Lochmaeus bilineata|[854]|XAE584-04|Moth4584.03|569[0n]bp|Canada.Ontario|BOLD:AAB3762
Lochmaeus bilineata|[855]|XAK602-07|HLC-16155|594[0n]bp|Canada.Ontario|BOLD:AAB3762
Lochmaeus bilineata|[856]|XAK603-07|HLC-16156|658[0n]bp|Canada.Ontario|BOLD:AAB3762
Lochmaeus manteo|[857]|BBLPE589-09|09BBELE-2589|658[0n]bp|Canada.Nova Scotia|BOLD:AAB1511
Lochmaeus manteo|[858]|XAE623-04|Moth4623.03|658[0n]bp|Canada.Ontario|BOLD:AAB1511
Lochmaeus manteo|[859]|XAK507-07|HLC-16060|658[0n]bp|Canada.Ontario|BOLD:AAB1511
Lochmaeus manteo|[860]|XAG847-05|2005-ONT-1431|658[0n]bp|Canada.Ontario|BOLD:AAB1511
Lochmaeus manteo|[861]|TMNBD358-07|MNBTT-3159|656[0n]bp|Canada.New Brunswick|BOLD:AAB1511
Lochmaeus manteo|[862]|TMNBD353-07|MNBTT-3154|657[0n]bp|Canada.New Brunswick|BOLD:AAB1511
Lochmaeus manteo|[863]|BBLEC904-09|09BBELE-0904|658[0n]bp|Canada.Nova Scotia|BOLD:AAB1511
Lochmaeus manteo|[864]|TMNBD359-07|MNBTT-3160|658[0n]bp|Canada.New Brunswick|BOLD:AAB1511
Lochmaeus manteo|[865]|PHMNB040-03|moth212.02SA|639[0n]bp|Canada.New Brunswick|BOLD:AAB1511
Lochmaeus manteo|[866]|RDLQB677-05|DH010780|658[0n]bp|Canada.Quebec|BOLD:AAB1511
Lochmaeus manteo|[867]|PMG182-03|HETEROC2.00|617[0n]bp|Canada.Ontario|BOLD:AAB1511
Lochmaeus manteo|[868]|XAG249-05|2005-ONT-833|505[2n]bp|Canada.Ontario|
Lochmaeus manteo|[869]|MNBB234-05|05-NBSTA-150|658[0n]bp|Canada.New Brunswick|BOLD:AAB1511
Schizura ipomoeae|[870]|XAK529-07|HLC-16082|658[0n]bp|Canada.Ontario|BOLD:AAA7094
Schizura ipomoeae|[871]|PHMO125-03|moth738.02|639[0n]bp|Canada.Ontario|BOLD:AAA7094
Schizura ipomoeae|[872]|RDLQB192-05|DH010278|571[0n]bp|Canada.Quebec|BOLD:AAA7094
Schizura ipomoeae|[873]|RDLQB189-05|DH010275|658[3n]bp|Canada.Quebec|BOLD:AAA7094
Schizura ipomoeae|[874]|LHLEP067-06|UBC-2006-0271|658[0n]bp|Canada.British Columbia|BOLD:AAA7094
Schizura ipomoeae|[875]|LMH030-06|RFC-2006-0135|658[0n]bp|Canada.British Columbia|BOLD:AAA7094

Schizura ipomoeae|[872]|RDLQB192-05|DH010278|571[0n]bp|Canada.Quebec|BOLD:AAA7094
Schizura ipomoeae|[873]|RDLQB189-05|DH010275|658[3n]bp|Canada.Quebec|BOLD:AAA7094
Schizura ipomoeae|[874]|LHLEP067-06|UBC-2006-0271|658[0n]bp|Canada.British Columbia|BOLD:AAA7094
Schizura ipomoeae|[875]|LMH030-06|PFC-2006-0135|658[0n]bp|Canada.British Columbia|BOLD:AAA7094
Schizura ipomoeae|[876]|TTMNB938-06|MNBTT-938|658[0n]bp|Canada.New Brunswick|BOLD:AAA7094
Schizura ipomoeae|[877]|TMNBD213-07|MNBTT-3014|658[0n]bp|Canada.New Brunswick|BOLD:AAA7094
Schizura ipomoeae|[878]|TMNBD216-07|MNBTT-3017|658[0n]bp|Canada.New Brunswick|BOLD:AAA7094
Schizura ipomoeae|[879]|TMNBD217-07|MNBTT-3018|658[0n]bp|Canada.New Brunswick|BOLD:AAA7094
Schizura ipomoeae|[880]|TMNBD214-07|MNBTT-3015|658[0n]bp|Canada.New Brunswick|BOLD:AAA7094
Schizura ipomoeae|[881]|TMNBD215-07|MNBTT-3016|630[0n]bp|Canada.New Brunswick|BOLD:AAA7094
Schizura ipomoeae|[882]|LALPA475-10|AVBC 477-10|658[0n]bp|Canada.British Columbia|BOLD:AAA7094
Schizura ipomoeae|[883]|LBCC344-05|HLC-22224|658[0n]bp|Canada.British Columbia|BOLD:AAA7094
Schizura ipomoeae|[884]|LBCC422-05|HLC-22302|658[0n]bp|Canada.British Columbia|BOLD:AAA7094
Schizura ipomoeae|[885]|LBCC419-05|HLC-22299|658[0n]bp|Canada.British Columbia|BOLD:AAA7094
Schizura ipomoeae|[886]|LBCC420-05|HLC-22300|658[0n]bp|Canada.British Columbia|BOLD:AAA7094
Schizura ipomoeae|[887]|LALPA366-10|AVBC 368-10|658[0n]bp|Canada.British Columbia|BOLD:AAA7094
Schizura ipomoeae|[888]|LBCB179-05|HLC-21119|658[0n]bp|Canada.British Columbia|BOLD:AAA7094
Schizura ipomoeae|[889]|LALPA336-10|AVBC 338-10|658[0n]bp|Canada.British Columbia|BOLD:AAA7094
Schizura ipomoeae|[890]|LBCA791-05|HLC-20791|658[0n]bp|Canada.British Columbia|BOLD:AAA7094
Schizura ipomoeae|[891]|LHLEP153-06|UBC-2006-0741|658[0n]bp|Canada.British Columbia|BOLD:AAA7094
Schizura ipomoeae|[892]|LHLEP154-06|UBC-2006-1513|658[0n]bp|Canada.British Columbia|BOLD:AAA7094
Schizura ipomoeae|[893]|LBCA795-05|HLC-20795|658[0n]bp|Canada.British Columbia|BOLD:AAA7094
Schizura ipomoeae|[894]|LBCA793-05|HLC-20793|658[0n]bp|Canada.British Columbia|BOLD:AAA7094
Schizura ipomoeae|[895]|RDLQB190-05|DH010276|584[2n]bp|Canada.Quebec|BOLD:AAA7094
Schizura ipomoeae|[896]|LBCA792-05|HLC-20792|600[0n]bp|Canada.British Columbia|BOLD:AAA7094
Schizura ipomoeae|[897]|RDLQB191-05|DH010277|593[0n]bp|Canada.Quebec|BOLD:AAA7094
Schizura ipomoeae|[898]|LBCS260-07|UBC-2007-0768|658[0n]bp|Canada.British Columbia|BOLD:AAA7094
Oligocentria lignicolor|[899]|XAB468-04|04HBL005468|558[1n]bp|Canada.Ontario|BOLD:AAA8725
Oligocentria lignicolor|[900]|MNBB251-05|05-NBSTA-167|658[0n]bp|Canada.New Brunswick|BOLD:AAA8725
Oligocentria lignicolor|[901]|TMNBD201-07|MNBTT-3002|659[0n]bp|Canada.New Brunswick|BOLD:AAA8725
Oligocentria lignicolor|[902]|MNBB361-05|05-NBSTA-277|655[0n]bp|Canada.New Brunswick|BOLD:AAA8725
Oligocentria lignicolor|[903]|TMNBD199-07|MNBTT-3000|658[0n]bp|Canada.New Brunswick|BOLD:AAA8725
Oligocentria lignicolor|[904]|TMNBD200-07|MNBTT-3001|658[0n]bp|Canada.New Brunswick|BOLD:AAA8725
Oligocentria lignicolor|[905]|RDLQF347-06|DH011414|658[0n]bp|Canada.Quebec|BOLD:AAA8725
Oligocentria lignicolor|[906]|TMNBB003-06|MNBTT-943|657[0n]bp|Canada.New Brunswick|BOLD:AAA8725
Oligocentria lignicolor|[907]|TMNBD202-07|MNBTT-3003|658[0n]bp|Canada.New Brunswick|BOLD:AAA8725
Oligocentria lignicolor|[908]|XAE357-04|Moth4357.03|658[0n]bp|Canada.Ontario|BOLD:AAA8725
Oligocentria lignicolor|[909]|TTMNB251-06|MNBTT-251|658[0n]bp|Canada.New Brunswick|BOLD:AAA8725
Oligocentria lignicolor|[910]|TTMNB041-06|MNBTT-041|658[0n]bp|Canada.New Brunswick|BOLD:AAA8725
Oligocentria lignicolor|[911]|MNBB614-05|05-NBSTA-530|658[0n]bp|Canada.New Brunswick|BOLD:AAA8725
Oligocentria lignicolor|[912]|TTMNB252-06|MNBTT-252|658[0n]bp|Canada.New Brunswick|BOLD:AAA8725
Oligocentria lignicolor|[913]|MNBB431-05|05-NBSTA-347|658[0n]bp|Canada.New Brunswick|BOLD:AAA8725
Oligocentria lignicolor|[914]|PHMNB773-05|Moth 466.03SA|658[0n]bp|Canada.New Brunswick|BOLD:AAA8725
Oligocentria lignicolor|[915]|TMNBD203-07|MNBTT-3004|658[0n]bp|Canada.New Brunswick|BOLD:AAA8725
Oligocentria lignicolor|[916]|PHMO219-03|moth1120.01|639[0n]bp|Canada.Ontario|BOLD:AAA8725
Oligocentria lignicolor|[917]|TMNBB004-06|MNBTT-944|656[0n]bp|Canada.New Brunswick|BOLD:AAA8725
Oligocentria lignicolor|[918]|MNBB177-05|05-NBSTA-093|658[0n]bp|Canada.New Brunswick|BOLD:AAA8725
Oligocentria lignicolor|[919]|RDLQB203-05|DH010289|658[0n]bp|Canada.Quebec|BOLD:AAA8725
Oligocentria lignicolor|[920]|RDLQB201-05|DH010287|658[0n]bp|Canada.Quebec|BOLD:AAA8725
Oligocentria lignicolor|[921]|RDLQB204-05|DH010290|658[0n]bp|Canada.Quebec|BOLD:AAA8725
Oligocentria lignicolor|[922]|RDLQB202-05|DH010288|658[0n]bp|Canada.Quebec|BOLD:AAA8725
Oligocentria lignicolor|[923]|RDLQB200-05|DH010286|658[0n]bp|Canada.Quebec|BOLD:AAA8725
Oligocentria lignicolor|[924]|BBLEC970-09|09BBELE-0970|658[0n]bp|Canada.Nova Scotia|BOLD:AAA8725
Oligocentria pallida|[925]|LALPA337-10|AVBC 339-10|658[0n]bp|Canada.British Columbia|BOLD:AAB7833
Oligocentria pallida|[926]|LBCH645-10|10-JDWBC-0645|658[0n]bp|Canada.British Columbia|BOLD:AAB7833
Oligocentria pallida|[927]|LBCH004-10|10-JDWBC-0004|658[0n]bp|Canada.British Columbia|BOLD:AAB7833
Oligocentria pallida|[928]|LPABC961-09|08BBLEP-05372|658[0n]bp|Canada.Alberta|BOLD:AAB7833
Oligocentria pallida|[929]|LBCD315-05|HLC-23135|658[0n]bp|Canada.British Columbia|BOLD:AAB7833
Oligocentria pallida|[930]|LBCD314-05|HLC-23134|648[0n]bp|Canada.British Columbia|BOLD:AAB7833
Oligocentria pallida|[931]|LMH034-06|PFC-2006-0439|632[0n]bp|Canada.British Columbia|BOLD:AAB7833
Oligocentria pallida|[932]|LBCA794-05|HLC-20794|658[0n]bp|Canada.British Columbia|BOLD:AAB7833
Oligocentria pallida|[933]|LBCC023-05|HLC-21903|658[0n]bp|Canada.British Columbia|BOLD:AAB7833
Oligocentria semirufescens|[934]|LBCC427-05|HLC-22307|658[0n]bp|Canada.British Columbia|BOLD:AAA8308
Oligocentria semirufescens|[935]|LBCA797-05|HLC-20797|658[0n]bp|Canada.British Columbia|BOLD:AAA8308
Oligocentria semirufescens|[936]|LBCA586-05|HLC-20586|649[0n]bp|Canada.British Columbia|BOLD:AAA8308
Oligocentria semirufescens|[937]|LHLEP152-06|UBC-2006-0740|658[0n]bp|Canada.British Columbia|BOLD:AA...
Oligocentria semirufescens|[938]|LBCA589-05|HLC-20589|658[0n]bp|Canada.British Columbia|BOLD:AAA8308
Oligocentria semirufescens|[939]|LBCA588-05|HLC-20588|658[0n]bp|Canada.British Columbia|BOLD:AAA8308
Oligocentria semirufescens|[940]|LBCD233-05|HLC-23053|616[0n]bp|Canada.British Columbia|BOLD:AAA8308
Oligocentria semirufescens|[941]|LBCA579-05|HLC-20579|645[0n]bp|Canada.British Columbia|BOLD:AAA8308
Oligocentria semirufescens|[942]|LHLEP150-06|UBC-2006-0738|628[0n]bp|Canada.British Columbia|BOLD:AA...
Oligocentria semirufescens|[943]|LHLEP149-06|UBC-2006-0737|658[0n]bp|Canada.British Columbia|BOLD:AA...
Oligocentria semirufescens|[944]|LBCB180-05|HLC-21120|658[0n]bp|Canada.British Columbia|BOLD:AAA8308
Oligocentria semirufescens|[945]|LBCC022-05|HLC-21902|658[0n]bp|Canada.British Columbia|BOLD:AAA8308
Oligocentria semirufescens|[946]|LBCC020-05|HLC-21900|658[0n]bp|Canada.British Columbia|BOLD:AAA8308
Oligocentria semirufescens|[947]|LBCC424-05|HLC-22304|658[0n]bp|Canada.British Columbia|BOLD:AAA8308
Oligocentria semirufescens|[948]|LBCC423-05|HLC-22303|658[0n]bp|Canada.British Columbia|BOLD:AAA8308
Oligocentria semirufescens|[949]|LBCC421-05|HLC-22301|658[0n]bp|Canada.British Columbia|BOLD:AAA8308
Oligocentria semirufescens|[950]|LBCD024-05|HLC-22844|658[0n]bp|Canada.British Columbia|BOLD:AAA8308
Oligocentria semirufescens|[951]|TMNBD191-07|MNBTT-2992|643[0n]bp|Canada.New Brunswick|BOLD:AAA8308
Oligocentria semirufescens|[952]|TMNBD193-07|MNBTT-2994|649[0n]bp|Canada.New Brunswick|BOLD:AAA8308
Oligocentria semirufescens|[953]|PHSEP272-11|BIOUG01146-H01|658[0n]bp|Canada.Ontario|BOLD:AAA8308
Oligocentria semirufescens|[954]|TMNBD190-07|MNBTT-2991|658[0n]bp|Canada.New Brunswick|BOLD:AAA8308
Oligocentria semirufescens|[955]|RDLQB530-05|DH010616|658[0n]bp|Canada.Quebec|BOLD:AAA8308
Oligocentria semirufescens|[956]|PHMNB774-05|Moth 467.03SA|658[0n]bp|Canada.New Brunswick|BOLD:AAA8308
Oligocentria semirufescens|[957]|LPMN339-08|08BBLEP-01138|658[0n]bp|Canada.Manitoba|BOLD:AAA8308
Oligocentria semirufescens|[958]|LPMN031-08|08BBLEP-00829|658[0n]bp|Canada.Manitoba|BOLD:AAA8308
Oligocentria semirufescens|[959]|PHMNB060-03|moth38.02SA|639[0n]bp|Canada.New Brunswick|BOLD:AAA8308
Oligocentria semirufescens|[960]|TMNBD189-07|MNBTT-2990|651[0n]bp|Canada.New Brunswick|BOLD:AAA8308
Oligocentria semirufescens|[961]|TMNBD192-07|MNBTT-2993|646[0n]bp|Canada.New Brunswick|BOLD:AAA8308
Oligocentria semirufescens|[962]|XAB169-04|04HBL005169|555[1n]bp|Canada.Ontario|BOLD:AAA8308
Oligocentria semirufescens|[963]|XAB467-04|04HBL005467|578[0n]bp|Canada.Ontario|BOLD:AAA8308
Oligocentria semirufescens|[964]|LHLEP151-06|UBC-2006-0739|624[0n]bp|Canada.British Columbia|BOLD:AA...
Oligocentria semirufescens|[965]|LBCA587-05|HLC-20587|597[0n]bp|Canada.British Columbia|BOLD:AAA8308
Oligocentria semirufescens|[966]|RDNMB447-05|CNCNoctuoidea10213|581[1n]bp|Canada.British Columbia|BOLD...
Oligocentria semirufescens|[967]|LBCC016-05|HLC-21896|587[0n]bp|Canada.British Columbia|BOLD:AAA8308
Oligocentria semirufescens|[968]|XAG176-05|2005-ONT-760|658[0n]bp|Canada.Ontario|BOLD:AAA8308
Oligocentria semirufescens|[969]|XAG238-05|2005-ONT-822|658[0n]bp|Canada.Ontario|BOLD:AAA8308
Oligocentria semirufescens|[970]|XAK419-06|2006-ONT-1414|658[0n]bp|Canada.Ontario|BOLD:AAA8308
Oligocentria semirufescens|[971]|MNBB297-05|05-NBSTA-213|658[0n]bp|Canada.New Brunswick|BOLD:AAA8308
Schizura apicalis|[972]|RDNMF401-08|NOC14487|658[0n]bp|Canada.Ontario|BOLD:AAF2054

[illegible]
Oligocentria semirufescens|[970]|XAK419-06|2006-ONT-1414|658[0n]bp|Canada.Ontario|BOLD:AAA8308
Oligocentria semirufescens|[971]|MNBB297-05|05-NBSTA-213|658[0n]bp|Canada.New Brunswick|BOLD:AAA8308
Schizura apicalis|[972]|RDNMF401-08|NOC14487|658[0n]bp|Canada.Ontario|BOLD:AAF2054
Schizura apicalis|[973]|RDNMF402-08|NOC14488|658[0n]bp|Canada.Ontario|BOLD:AAF2054
Schizura concinna|[974]|LSEU605-06|06-JKA-0605|658[0n]bp|United States.Georgia|BOLD:AAE3774
Schizura concinna|[975]|LPOKC770-09|MDOK-2847|658[0n]bp|United States.Oklahoma|BOLD:AAE3774
Schizura concinna|[976]|LGSMB241-05|DNA-ATBI-1090|584[0n]bp|United States.Tennessee|BOLD:AAE3774
Schizura concinna|[977]|LGSMB240-05|DNA-ATBI-1089|578[0n]bp|United States.Tennessee|BOLD:AAE3774
Schizura concinna|[978]|LPOKA065-08|MDOK-0065|658[0n]bp|United States.Oklahoma|BOLD:AAE3774
Hyparpax aurora|[979]|MMNA069-08|HLC-17631|658[0n]bp|United States.Georgia|BOLD:AAD3007
Hyparpax aurora|[980]|LNCB054-06|06-NCC-1010|658[0n]bp|United States.North Carolina|BOLD:AAD3007
Hyparpax aurora|[981]|LNCB052-06|06-NCC-1008|658[0n]bp|United States.North Carolina|BOLD:AAD3007
Hyparpax aurora|[982]|MMNA068-08|HLC-17630|658[0n]bp|United States.Georgia|BOLD:AAD3007
Hyparpax aurora|[983]|LNCB053-06|06-NCC-1009|658[0n]bp|United States.North Carolina|BOLD:AAD3007
Hyparpax aurora|[984]|LSEU239-06|06-JKA-0239|658[0n]bp|United States.Georgia|BOLD:AAD3007
Hyparpax aurora|[985]|HKONS530-08|3059-COI-08|658[0n]bp|United States.Florida|BOLD:AAD3007
Schizura badia|[986]|RDLQF508-06|DH011657|658[0n]bp|Canada.Quebec|BOLD:AAC4502
Schizura badia|[987]|RDLQF365-06|DH011432|658[0n]bp|Canada.Quebec|BOLD:AAC4502
Schizura badia|[988]|RDLQF698-06|DH011848|639[0n]bp|Canada.Quebec|BOLD:AAC4502
Schizura badia|[989]|PMG188-03|moth603.01|617[0n]bp|Canada.Ontario|BOLD:AAC4502
Schizura badia|[990]|RDLQF697-06|DH011847|637[1n]bp|Canada.Quebec|BOLD:AAC4502
Schizura badia|[991]|XAB597-04|04HBL005597|658[0n]bp|Canada.Ontario|BOLD:AAC4502
Schizura badia|[992]|TTMNB939-06|MNBTT-939|658[0n]bp|Canada.New Brunswick|BOLD:AAC4502
Schizura badia|[993]|BBLPE021-09|09BBELE-2021|658[0n]bp|Canada.Nova Scotia|BOLD:AAC4502
Schizura leptinoides|[994]|RDMAB068-05|UASM57595|636[0n]bp|Canada.Alberta|BOLD:AAB0904
Schizura leptinoides|[995]|LPMN176-08|08BBLEP-00975|658[0n]bp|Canada.Manitoba|BOLD:AAB0904
Schizura leptinoides|[996]|LPMN344-08|08BBLEP-01143|658[0n]bp|Canada.Manitoba|BOLD:AAB0904
Schizura leptinoides|[997]|LPMN725-08|08BBLEP-01528|658[0n]bp|Canada.Manitoba|BOLD:AAB0904
Schizura leptinoides|[998]|LPSO344-08|PPBP-0344|658[0n]bp|Canada.Ontario|BOLD:AAB0904
Schizura leptinoides|[999]|TMNBD198-07|MNBTT-2999|658[0n]bp|Canada.New Brunswick|BOLD:AAB0904
Schizura leptinoides|[1000]|TMNBD195-07|MNBTT-2996|648[0n]bp|Canada.New Brunswick|BOLD:AAB0904
Schizura leptinoides|[1001]|TMNBD194-07|MNBTT-2995|646[0n]bp|Canada.New Brunswick|BOLD:AAB0904
Schizura leptinoides|[1002]|TMNBD197-07|MNBTT-2998|658[0n]bp|Canada.New Brunswick|BOLD:AAB0904
Schizura leptinoides|[1003]|TMNBD196-07|MNBTT-2997|658[0n]bp|Canada.New Brunswick|BOLD:AAB0904
Schizura leptinoides|[1004]|XAG542-05|2005-ONT-1126|564[0n]bp|Canada.Ontario|BOLD:AAB0904
Schizura leptinoides|[1005]|XAG703-05|2005-ONT-1287|654[0n]bp|Canada.Ontario|BOLD:AAB0904
Schizura leptinoides|[1006]|PHMO198-03|moth1015.02|639[0n]bp|Canada.Ontario|BOLD:AAB0904
Schizura leptinoides|[1007]|PHMO197-03|moth1011.02|639[0n]bp|Canada.Ontario|BOLD:AAB0904
Schizura leptinoides|[1008]|XAB170-04|04HBL005170|538[0n]bp|Canada.Ontario|BOLD:AAB0904
Schizura leptinoides|[1009]|XAK481-07|HLC-16034|581[1n]bp|Canada.Ontario|BOLD:AAB0904
Schizura leptinoides|[1010]|MNBB669-05|05-NBSTA-585|658[0n]bp|Canada.New Brunswick|BOLD:AAB0904
Schizura leptinoides|[1011]|RDLQF939-06|DH012119|658[0n]bp|Canada.Quebec|BOLD:AAB0904
Schizura leptinoides|[1012]|RDLQB194-05|DH010280|658[0n]bp|Canada.Quebec|BOLD:AAB0904
Schizura leptinoides|[1013]|TMNBB002-06|MNBTT-942|656[0n]bp|Canada.New Brunswick|BOLD:AAB0904
Schizura leptinoides|[1014]|RDLQB195-05|DH010281|658[0n]bp|Canada.Quebec|BOLD:AAB0904
Schizura leptinoides|[1015]|RDLQB193-05|DH010279|658[1n]bp|Canada.Quebec|BOLD:AAB0904
Schizura sp.|[1016]|LBCG1105-09|08-JDWBC-1105|658[0n]bp|Canada.British Columbia|BOLD:ABY7736
Schizura sp.|[1017]|RDNMB448-05|CNCNoctuoidea10214|579[0n]bp|Canada.British Columbia|BOLD:ABY7736
Schizura unicornis|[1018]|XAE337-04|Moth4337.03|658[0n]bp|Canada.Ontario|BOLD:AAA3873
Schizura unicornis|[1019]|BBLPB681-10|10BBCLP-1680|658[0n]bp|Canada.Ontario|BOLD:AAA3873
Schizura unicornis|[1020]|TMNBD185-07|MNBTT-2986|658[0n]bp|Canada.New Brunswick|BOLD:AAA3873
Schizura unicornis|[1021]|PHMNB575-04|04HBL00801|658[0n]bp|Canada.New Brunswick|BOLD:AAA3873
Schizura unicornis|[1022]|RDLQF520-06|DH011669|658[0n]bp|Canada.Quebec|BOLD:AAA3873
Schizura unicornis|[1023]|TMNBD188-07|MNBTT-2989|658[0n]bp|Canada.New Brunswick|BOLD:AAA3873
Schizura unicornis|[1024]|XAJ357-06|2006-ONT-0357|614[0n]bp|Canada.Ontario|BOLD:AAA3873
Schizura unicornis|[1025]|XAE291-04|Moth4291.03|658[0n]bp|Canada.Ontario|BOLD:AAA3873
Schizura unicornis|[1026]|LALPA500-10|AVBC 502-10|658[0n]bp|Canada.British Columbia|BOLD:AAA3873
Schizura unicornis|[1027]|PHMNB574-04|04HBL00800|658[0n]bp|Canada.New Brunswick|BOLD:AAA3873
Schizura unicornis|[1028]|MNBB487-05|05-NBSTA-403|658[0n]bp|Canada.New Brunswick|BOLD:AAA3873
Schizura unicornis|[1029]|LALPA274-10|AVBC 275-10|658[0n]bp|Canada.British Columbia|BOLD:AAA3873
Schizura unicornis|[1030]|LPVIB979-08|PFC-2006-2529|658[0n]bp|Canada.British Columbia|BOLD:AAA3873
Schizura unicornis|[1031]|TMNBB001-06|MNBTT-941|658[0n]bp|Canada.New Brunswick|BOLD:AAA3873
Schizura unicornis|[1032]|LPVIA797-08|PFC-2006-1087|658[0n]bp|Canada.British Columbia|BOLD:AAA3873
Schizura unicornis|[1033]|LPVIA796-08|PFC-2006-1086|658[0n]bp|Canada.British Columbia|BOLD:AAA3873
Schizura unicornis|[1034]|TMNBD186-07|MNBTT-2987|655[0n]bp|Canada.New Brunswick|BOLD:AAA3873
Schizura unicornis|[1035]|TMNBD184-07|MNBTT-2985|657[0n]bp|Canada.New Brunswick|BOLD:AAA3873
Schizura unicornis|[1036]|TTMNB940-06|MNBTT-940|658[0n]bp|Canada.New Brunswick|BOLD:AAA3873
Schizura unicornis|[1037]|LPVIA093-08|PFC-2006-0133|658[0n]bp|Canada.British Columbia|BOLD:AAA3873
Schizura unicornis|[1038]|MNBB302-05|05-NBSTA-218|658[0n]bp|Canada.New Brunswick|BOLD:AAA3873
Schizura unicornis|[1039]|XAJ501-06|2006-ONT-0501|658[0n]bp|Canada.Ontario|BOLD:AAA3873
Schizura unicornis|[1040]|XAG183-05|2005-ONT-767|611[0n]bp|Canada.Ontario|BOLD:AAA3873
Schizura unicornis|[1041]|LPMN265-08|08BBLEP-01064|658[0n]bp|Canada.Manitoba|BOLD:AAA3873
Schizura unicornis|[1042]|TMNBD187-07|MNBTT-2988|658[0n]bp|Canada.New Brunswick|BOLD:AAA3873
Schizura unicornis|[1043]|XAJ413-06|2006-ONT-0413|658[0n]bp|Canada.Ontario|BOLD:AAA3873
Schizura unicornis|[1044]|LPSK238-08|08BBLEP-01806|658[0n]bp|Canada.Saskatchewan|BOLD:AAA3873
Schizura unicornis|[1045]|XAE568-04|Moth4568.03|597[0n]bp|Canada.Ontario|BOLD:AAA3873
Schizura unicornis|[1046]|BBLPC003-09|09BBELE-1003|658[0n]bp|Canada.New Brunswick|BOLD:AAA3873
Schizura unicornis|[1047]|LPSO958-08|PPBP-0958|609[0n]bp|Canada.Ontario|BOLD:AAA3873
Schizura unicornis|[1048]|LPSOC368-08|PPBP-2367|655[0n]bp|Canada.Ontario|BOLD:AAA3873
Schizura unicornis|[1049]|XAE474-04|Moth4474.03|658[0n]bp|Canada.Ontario|BOLD:AAA3873
Schizura unicornis|[1050]|LPSOB097-08|PPBP-1096|658[0n]bp|Canada.Ontario|BOLD:AAA3873
Schizura unicornis|[1051]|PMG189-03|moth609.01|617[0n]bp|Canada.Ontario|BOLD:AAA3873
Schizura unicornis|[1052]|TMG70-03|moth323.01|617[0n]bp|Canada.Ontario|BOLD:AAA3873
Schizura unicornis|[1053]|XAG018-05|2005-ONT-602|629[0n]bp|Canada.Ontario|BOLD:AAA3873
Schizura unicornis|[1054]|MNBB008-05|HBL008618|658[0n]bp|Canada.New Brunswick|BOLD:AAA3873
Schizura unicornis|[1055]|XAB118-04|04HBL005118|658[0n]bp|Canada.Ontario|BOLD:AAA3873
Schizura unicornis|[1056]|XAK009-06|2006-ONT-1004|658[0n]bp|Canada.Ontario|BOLD:AAA3873
Schizura unicornis|[1057]|BLTIB791-08|BL1208|658[0n]bp|Canada.Ontario|BOLD:AAA3873
Schizura unicornis|[1058]|TTMNB543-06|MNBTT-543|658[0n]bp|Canada.New Brunswick|BOLD:AAA3873
Schizura unicornis|[1059]|LPSO265-08|PPBP-0265|658[0n]bp|Canada.Ontario|BOLD:AAA3873
Schizura unicornis|[1060]|LPSO928-08|PPBP-0928|658[0n]bp|Canada.Ontario|BOLD:AAA3873
Schizura unicornis|[1061]|XAB284-04|04HBL005284|658[0n]bp|Canada.Ontario|BOLD:AAA3873
Schizura unicornis|[1062]|TTMNB249-06|MNBTT-249|658[0n]bp|Canada.New Brunswick|BOLD:AAA3873
Schizura unicornis|[1063]|XAG263-05|2005-ONT-847|658[0n]bp|Canada.Ontario|BOLD:AAA3873
Schizura unicornis|[1064]|MNBB248-05|05-NBSTA-164|658[0n]bp|Canada.New Brunswick|BOLD:AAA3873
Schizura unicornis|[1065]|RDMAB069-05|UASM57596|591[1n]bp|Canada.Alberta|BOLD:AAA3873
Schizura unicornis|[1066]|LOWCB160-05|CGWC-1100|658[0n]bp|Canada.British Columbia|BOLD:AAA3873
Schizura unicornis|[1067]|BLGSM093-09|BL1646|630[0n]bp|Canada.Ontario|BOLD:AAA3873
Schizura unicornis|[1068]|XAG668-05|2005-ONT-1252|603[2n]bp|Canada.Ontario|BOLD:AAA3873
Schizura unicornis|[1069]|XAJ670-06|2006-ONT-0670|658[0n]bp|Canada.Ontario|BOLD:AAA3873

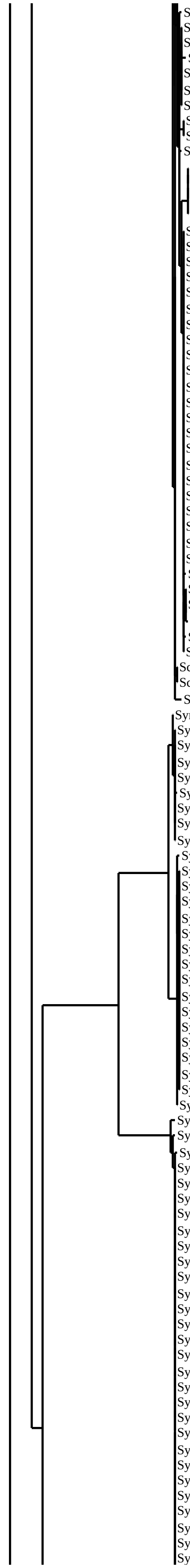

Schizura unicornis|[1067]|BLGSM093-09|BL1646|630[0n]bp|Canada.Ontario|BOLD:AAA3873
Schizura unicornis|[1068]|XAG668-05|2005-ONT-1252|603[2n]bp|Canada.Ontario|BOLD:AAA3873
Schizura unicornis|[1069]|XAJ670-06|2006-ONT-0670|658[0n]bp|Canada.Ontario|BOLD:AAA3873
Schizura unicornis|[1070]|XAF559-05|2005-ONT-208|658[0n]bp|Canada.Ontario|BOLD:AAA3873
Schizura unicornis|[1071]|BLGSM015-09|BL324|658[0n]bp|Canada.Ontario|BOLD:AAA3873
Schizura unicornis|[1072]|XAB559-04|04HBL005559|658[0n]bp|Canada.Ontario|BOLD:AAA3873
Schizura unicornis|[1073]|LPSOC358-08|PPBP-2357|658[0n]bp|Canada.Ontario|BOLD:AAA3873
Schizura unicornis|[1074]|MNBB352-05|05-NBSTA-268|658[0n]bp|Canada.New Brunswick|BOLD:AAA3873
Schizura unicornis|[1075]|TTMNB250-06|MNBTT-250|658[0n]bp|Canada.New Brunswick|BOLD:AAA3873
Schizura unicornis|[1076]|LOWCB154-05|CGWC-1094|658[0n]bp|Canada.British Columbia|BOLD:AAA3873
Schizura unicornis|[1077]|LOWCB157-05|CGWC-1097|658[0n]bp|Canada.British Columbia|BOLD:AAA3873
Schizura unicornis|[1078]|LOWCB567-05|CGWC-1507|658[0n]bp|Canada.British Columbia|BOLD:AAA3873
Schizura unicornis|[1079]|LPSK143-08|08BBLEP-01711|658[0n]bp|Canada.Saskatchewan|BOLD:AAA3873
Schizura unicornis|[1080]|LOWCB159-05|CGWC-1099|658[0n]bp|Canada.British Columbia|BOLD:AAA3873
Schizura unicornis|[1081]|LOWCB152-05|CGWC-1092|658[0n]bp|Canada.British Columbia|BOLD:AAA3873
Schizura unicornis|[1082]|LOWCB161-05|CGWC-1101|658[0n]bp|Canada.British Columbia|BOLD:AAA3873
Schizura unicornis|[1083]|LBCC004-05|HLC-21884|658[0n]bp|Canada.British Columbia|BOLD:AAA3873
Schizura unicornis|[1084]|LBCC767-05|HLC-22647|658[0n]bp|Canada.British Columbia|BOLD:AAA3873
Schizura unicornis|[1085]|LALPA478-10|AVBC 480-10|658[0n]bp|Canada.British Columbia|BOLD:AAA3873
Schizura unicornis|[1086]|LBCD023-05|HLC-22843|658[0n]bp|Canada.British Columbia|BOLD:AAA3873
Schizura unicornis|[1087]|LBCB207-05|HLC-21147|658[0n]bp|Canada.British Columbia|BOLD:AAA3873
Schizura unicornis|[1088]|LBCB141-05|HLC-21081|658[0n]bp|Canada.British Columbia|BOLD:AAA3873
Schizura unicornis|[1089]|BBLPC183-09|09BBELE-1183|658[0n]bp|Canada.Nova Scotia|BOLD:AAA3873
Schizura unicornis|[1090]|BBLPC248-09|09BBELE-1248|658[0n]bp|Canada.Nova Scotia|BOLD:AAA3873
Schizura unicornis|[1091]|LPSOB458-08|PPBP-1457|658[0n]bp|Canada.Ontario|BOLD:AAA3873
Schizura unicornis|[1092]|LOWCB156-05|CGWC-1096|658[0n]bp|Canada.British Columbia|BOLD:AAA3873
Schizura unicornis|[1093]|LOWCB153-05|CGWC-1093|658[0n]bp|Canada.British Columbia|BOLD:AAA3873
Schizura unicornis|[1094]|LOWCB155-05|CGWC-1095|658[0n]bp|Canada.British Columbia|BOLD:AAA3873
Schizura unicornis|[1095]|BBLPB682-10|10BBCLP-1681|658[0n]bp|Canada.British Columbia|BOLD:AAA3873
Schizura unicornis|[1096]|LBCA810-05|HLC-20810|658[0n]bp|Canada.British Columbia|BOLD:AAA3873
Schizura unicornis|[1097]|LBCA809-05|HLC-20809|658[0n]bp|Canada.British Columbia|BOLD:AAA3873
Schizura unicornis|[1098]|LBCA804-05|HLC-20804|658[0n]bp|Canada.British Columbia|BOLD:AAA3873
Schizura unicornis|[1099]|DUNLP192-08|Dun-08-192|658[0n]bp|Canada.British Columbia|BOLD:AAA3873
Schizura unicornis|[1100]|BBLPB683-10|10BBCLP-1682|658[0n]bp|Canada.British Columbia|BOLD:AAA3873
Schizura unicornis|[1101]|BBLPB680-10|10BBCLP-1679|658[0n]bp|Canada.British Columbia|BOLD:AAA3873
Schizura unicornis|[1102]|LPABC702-09|08BBLEP-04921|658[0n]bp|Canada.Alberta|BOLD:AAA3873
Schizura unicornis|[1103]|LBCC765-05|HLC-22645|608[0n]bp|Canada.British Columbia|BOLD:AAA3873
Schizura unicornis|[1104]|LBCA606-05|HLC-20606|633[0n]bp|Canada.British Columbia|BOLD:AAA3873
Schizura unicornis|[1105]|LOWCB158-05|CGWC-1098|648[0n]bp|Canada.British Columbia|BOLD:AAA3873
Schizura unicornis|[1106]|LPVIA630-08|PFC-2006-0863|624[0n]bp|Canada.British Columbia|BOLD:AAA3873
Schizura unicornis|[1107]|MNBB247-05|05-NBSTA-163|570[0n]bp|Canada.New Brunswick|BOLD:AAA3873
Schizura unicornis|[1108]|LBCD022-05|HLC-22842|658[0n]bp|Canada.British Columbia|BOLD:AAA3873
Schizura unicornis|[1109]|MNBB126-05|05-NBSTA-042|658[0n]bp|Canada.New Brunswick|BOLD:AAA3873
Schizura unicornis|[1110]|BBLEC205-09|09BBELE-0205|658[0n]bp|Canada.Nova Scotia|BOLD:AAA3873
Schizura unicornis|[1111]|LPSO089-08|PPBP-0089|648[0n]bp|Canada.Ontario|BOLD:AAA3873
Symmerista albifrons|[1112]|LPSO744-08|PPBP-0744|658[0n]bp|Canada.Ontario|BOLD:AAB3596
Symmerista albifrons|[1113]|TMNBD325-07|MNBTT-3126|655[0n]bp|Canada.New Brunswick|BOLD:AAB3596
Symmerista albifrons|[1114]|TMNBD318-07|MNBTT-3119|658[0n]bp|Canada.New Brunswick|BOLD:AAB3596
Symmerista albifrons|[1115]|TMNBD321-07|MNBTT-3122|658[0n]bp|Canada.New Brunswick|BOLD:AAB3596
Symmerista albifrons|[1116]|RDLQF233-06|DH011313|658[0n]bp|Canada.Quebec|BOLD:AAB3596
Symmerista albifrons|[1117]|TMNBD327-07|MNBTT-3128|635[0n]bp|Canada.New Brunswick|BOLD:AAB3596
Symmerista albifrons|[1118]|RDLQ104-05|DH003038|658[0n]bp|Canada.Quebec|BOLD:AAB3596
Symmerista albifrons|[1119]|LPSOC216-08|PPBP-2215|658[0n]bp|Canada.Ontario|BOLD:AAB3596
Symmerista albifrons|[1120]|RDLQF458-06|DH011565|658[0n]bp|Canada.Quebec|BOLD:AAB3596
Symmerista canicosta|[1121]|TMNBD316-07|MNBTT-3117|658[0n]bp|Canada.New Brunswick|BOLD:AAB3596
Symmerista canicosta|[1122]|TMNBD330-07|MNBTT-3131|610[0n]bp|Canada.New Brunswick|BOLD:AAB3596
Symmerista canicosta|[1123]|TMNBD317-07|MNBTT-3118|658[0n]bp|Canada.New Brunswick|BOLD:AAB3596
Symmerista canicosta|[1124]|TMNBD220-07|MNBTT-3021|658[0n]bp|Canada.New Brunswick|BOLD:AAB3596
Symmerista canicosta|[1125]|TMNBD219-07|MNBTT-3020|658[0n]bp|Canada.New Brunswick|BOLD:AAB3596
Symmerista canicosta|[1126]|MNBB488-05|05-NBSTA-404|658[0n]bp|Canada.New Brunswick|BOLD:AAB3596
Symmerista canicosta|[1127]|RDNMG365-08|NOC15212|658[0n]bp|Canada.New Brunswick|BOLD:AAB3596
Symmerista canicosta|[1128]|RDNMG367-08|NOC15214|658[0n]bp|Canada.Ontario|BOLD:AAB3596
Symmerista canicosta|[1129]|RDNMG366-08|NOC15213|658[0n]bp|Canada.Ontario|BOLD:AAB3596
Symmerista canicosta|[1130]|RDNMG363-08|NOC15210|658[0n]bp|Canada.Ontario|BOLD:AAB3596
Symmerista canicosta|[1131]|TMNBD335-07|MNBTT-3136|638[0n]bp|Canada.New Brunswick|BOLD:AAB3596
Symmerista canicosta|[1132]|TMNBD223-07|MNBTT-3024|647[0n]bp|Canada.New Brunswick|BOLD:AAB3596
Symmerista canicosta|[1133]|MNBB436-05|05-NBSTA-352|615[0n]bp|Canada.New Brunswick|BOLD:AAB3596
Symmerista canicosta|[1134]|TMNBD218-07|MNBTT-3019|594[0n]bp|Canada.New Brunswick|BOLD:AAB3596
Symmerista canicosta|[1135]|TMNBD313-07|MNBTT-3114|631[0n]bp|Canada.New Brunswick|BOLD:AAB3596
Symmerista canicosta|[1136]|TMNBD319-07|MNBTT-3120|658[0n]bp|Canada.New Brunswick|BOLD:AAB3596
Symmerista canicosta|[1137]|TMNBD309-07|MNBTT-3110|656[0n]bp|Canada.New Brunswick|BOLD:AAB3596
Symmerista leucitys|[1138]|RDLQ099-05|DH001503|579[0n]bp|Canada.Quebec|BOLD:AAA7013
Symmerista leucitys|[1139]|TMNBD341-07|MNBTT-3142|658[0n]bp|Canada.New Brunswick|BOLD:AAA7013
Symmerista leucitys|[1140]|LPSOC287-08|PPBP-2286|658[0n]bp|Canada.Ontario|BOLD:AAA7013
Symmerista leucitys|[1141]|TMNBD333-07|MNBTT-3134|655[0n]bp|Canada.New Brunswick|BOLD:AAA7013
Symmerista leucitys|[1142]|TMNBD222-07|MNBTT-3023|655[0n]bp|Canada.New Brunswick|BOLD:AAA7013
Symmerista leucitys|[1143]|TMNBD345-07|MNBTT-3146|658[0n]bp|Canada.New Brunswick|BOLD:AAA7013
Symmerista leucitys|[1144]|PMG190-03|moth1151.01|617[0n]bp|Canada.Ontario|BOLD:AAA7013
Symmerista leucitys|[1145]|TMNBD339-07|MNBTT-3140|659[0n]bp|Canada.New Brunswick|BOLD:AAA7013
Symmerista leucitys|[1146]|LPSOC309-08|PPBP-2308|658[0n]bp|Canada.Ontario|BOLD:AAA7013
Symmerista leucitys|[1147]|LPSOC308-08|PPBP-2307|658[0n]bp|Canada.Ontario|BOLD:AAA7013
Symmerista leucitys|[1148]|TMNBD322-07|MNBTT-3123|657[0n]bp|Canada.New Brunswick|BOLD:AAA7013
Symmerista leucitys|[1149]|LPSOC310-08|PPBP-2309|658[0n]bp|Canada.Ontario|BOLD:AAA7013
Symmerista leucitys|[1150]|TMNBD337-07|MNBTT-3138|658[0n]bp|Canada.New Brunswick|BOLD:AAA7013
Symmerista leucitys|[1151]|LPSOB969-08|PPBP-1968|658[0n]bp|Canada.Ontario|BOLD:AAA7013
Symmerista leucitys|[1152]|TMNBD323-07|MNBTT-3124|658[0n]bp|Canada.New Brunswick|BOLD:AAA7013
Symmerista leucitys|[1153]|TMNBD332-07|MNBTT-3133|658[0n]bp|Canada.New Brunswick|BOLD:AAA7013
Symmerista leucitys|[1154]|TMNBD334-07|MNBTT-3135|658[0n]bp|Canada.New Brunswick|BOLD:AAA7013
Symmerista leucitys|[1155]|TMNBD338-07|MNBTT-3139|658[0n]bp|Canada.New Brunswick|BOLD:AAA7013
Symmerista leucitys|[1156]|TMNBD343-07|MNBTT-3144|658[0n]bp|Canada.New Brunswick|BOLD:AAA7013
Symmerista leucitys|[1157]|TMNBD328-07|MNBTT-3129|658[0n]bp|Canada.New Brunswick|BOLD:AAA7013
Symmerista leucitys|[1158]|RDLQF853-06|DH012014|658[0n]bp|Canada.Quebec|BOLD:AAA7013
Symmerista leucitys|[1159]|TMNBD331-07|MNBTT-3132|658[0n]bp|Canada.New Brunswick|BOLD:AAA7013
Symmerista leucitys|[1160]|TMNBD326-07|MNBTT-3127|657[0n]bp|Canada.New Brunswick|BOLD:AAA7013
Symmerista leucitys|[1161]|XAI067-05|0102-ONT-0067|658[0n]bp|Canada.Ontario|BOLD:AAA7013
Symmerista leucitys|[1162]|XAF801-05|2005-ONT-450|658[0n]bp|Canada.Ontario|BOLD:AAA7013
Symmerista leucitys|[1163]|XAE466-04|Moth4466.03|658[0n]bp|Canada.Ontario|BOLD:AAA7013
Symmerista leucitys|[1164]|XAE414-04|Moth4414.03|658[0n]bp|Canada.Ontario|BOLD:AAA7013
Symmerista leucitys|[1165]|TMNBD221-07|MNBTT-3022|658[0n]bp|Canada.New Brunswick|BOLD:AAA7013
Symmerista leucitys|[1166]|TMNBD307-07|MNBTT-3108|658[0n]bp|Canada.New Brunswick|BOLD:AAA7013

Symmerista leucitys|[1164]|XAE414-04|Moth4414.03|658[0n]bp|Canada.Ontario|BOLD:AAA7013
Symmerista leucitys|[1165]|TMNBD221-07|MNBTT-3022|658[0n]bp|Canada.New Brunswick|BOLD:AAA7013
Symmerista leucitys|[1166]|TMNBD307-07|MNBTT-3108|658[0n]bp|Canada.New Brunswick|BOLD:AAA7013
Symmerista leucitys|[1167]|RDLQ103-05|DH005439|658[0n]bp|Canada.Quebec|BOLD:AAA7013
Symmerista leucitys|[1168]|RDLQ194-05|05HBLT0194|658[0n]bp|Canada.Quebec|BOLD:AAA7013
Symmerista leucitys|[1169]|RDNMG364-08|NOC15211|658[0n]bp|Canada.New Brunswick|BOLD:AAA7013
Symmerista leucitys|[1170]|TMNBD344-07|MNBTT-3145|645[0n]bp|Canada.New Brunswick|BOLD:AAA7013
Symmerista leucitys|[1171]|TMNBD324-07|MNBTT-3125|621[0n]bp|Canada.New Brunswick|BOLD:AAA7013
Symmerista leucitys|[1172]|TMNBD315-07|MNBTT-3116|632[0n]bp|Canada.New Brunswick|BOLD:AAA7013
Symmerista leucitys|[1173]|TMNBD336-07|MNBTT-3137|588[0n]bp|Canada.New Brunswick|BOLD:AAA7013
Symmerista leucitys|[1174]|TMNBD311-07|MNBTT-3112|578[0n]bp|Canada.New Brunswick|BOLD:AAA7013
Symmerista leucitys|[1175]|TMNBD340-07|MNBTT-3141|621[0n]bp|Canada.New Brunswick|BOLD:AAA7013
Symmerista leucitys|[1176]|TMNBD310-07|MNBTT-3111|618[0n]bp|Canada.New Brunswick|BOLD:AAA7013
Symmerista leucitys|[1177]|TMNBD312-07|MNBTT-3113|618[0n]bp|Canada.New Brunswick|BOLD:AAA7013
Symmerista leucitys|[1178]|RDLQF940-06|DH012120|620[0n]bp|Canada.Quebec|BOLD:AAA7013
Symmerista leucitys|[1179]|PHMO217-03|moth1100.02|639[0n]bp|Canada.Ontario|BOLD:AAA7013
Symmerista leucitys|[1180]|TMG67-03|moth1183.01|639[0n]bp|Canada.Ontario|BOLD:AAA7013
Symmerista leucitys|[1181]|PHMO371-03|moth874.02|639[0n]bp|Canada.Ontario|BOLD:AAA7013
Ellida caniplaga|[1182]|LPSO868-08|PPBP-0868|658[0n]bp|Canada.Ontario|BOLD:AAA9083
Ellida caniplaga|[1183]|LPSO153-08|PPBP-0153|658[0n]bp|Canada.Ontario|BOLD:AAA9083
Ellida caniplaga|[1184]|LPSO545-08|PPBP-0545|658[0n]bp|Canada.Ontario|BOLD:AAA9083
Ellida caniplaga|[1185]|LMIS012-05|05-ONMIS-0012|612[1n]bp|Canada.Ontario|BOLD:AAA9083
Ellida caniplaga|[1186]|LPSOB980-08|PPBP-1979|658[0n]bp|Canada.Ontario|BOLD:AAA9083
Ellida caniplaga|[1187]|XAB112-04|04HBL005112|658[1n]bp|Canada.Ontario|BOLD:AAA9083
Ellida caniplaga|[1188]|XAE349-04|Moth4349.03|658[0n]bp|Canada.Ontario|BOLD:AAA9083
Ellida caniplaga|[1189]|XAE307-04|Moth4307.03|658[0n]bp|Canada.Ontario|BOLD:AAA9083
Ellida caniplaga|[1190]|XAB133-04|04HBL005133|658[0n]bp|Canada.Ontario|BOLD:AAA9083
Ellida caniplaga|[1191]|XAF426-05|HLC-10467|658[0n]bp|Canada.Ontario|BOLD:AAA9083
Ellida caniplaga|[1192]|XAJ837-06|2006-ONT-0837|658[0n]bp|Canada.Ontario|BOLD:AAA9083
Ellida caniplaga|[1193]|XAB323-04|04HBL005323|658[0n]bp|Canada.Ontario|BOLD:AAA9083
Ellida caniplaga|[1194]|XAB252-04|04HBL005252|658[0n]bp|Canada.Ontario|BOLD:AAA9083
Ellida caniplaga|[1195]|XAF385-05|HLC-10426|658[0n]bp|Canada.Ontario|BOLD:AAA9083
Ellida caniplaga|[1196]|XAF427-05|HLC-10468|658[0n]bp|Canada.Ontario|BOLD:AAA9083
Ellida caniplaga|[1197]|PMG174-03|moth326.01|617[0n]bp|Canada.Ontario|BOLD:AAA9083
Ellida caniplaga|[1198]|TMG64-03|moth358.01|639[0n]bp|Canada.Ontario|BOLD:AAA9083
Ellida caniplaga|[1199]|MNBB073-05|HBL008683|658[0n]bp|Canada.New Brunswick|BOLD:AAA9083
Ellida caniplaga|[1200]|XAB043-04|04HBL005043|658[0n]bp|Canada.Ontario|BOLD:AAA9083
Ellida caniplaga|[1201]|LPSO256-08|PPBP-0256|658[0n]bp|Canada.Ontario|BOLD:AAA9083
Ellida caniplaga|[1202]|LPSO133-08|PPBP-0133|658[0n]bp|Canada.Ontario|BOLD:AAA9083
Ellida caniplaga|[1203]|LPSOC212-08|PPBP-2211|658[0n]bp|Canada.Ontario|BOLD:AAA9083
Ellida caniplaga|[1204]|LPSOC214-08|PPBP-2213|658[0n]bp|Canada.Ontario|BOLD:AAA9083
Ellida caniplaga|[1205]|KPOEC172-08|08OEC-213|658[0n]bp|Canada.Ontario|BOLD:AAA9083
Ellida caniplaga|[1206]|LPSOC083-08|PPBP-2082|658[0n]bp|Canada.Ontario|BOLD:AAA9083
Ellida caniplaga|[1207]|LPSOC089-08|PPBP-2088|658[0n]bp|Canada.Ontario|BOLD:AAA9083
Ellida caniplaga|[1208]|XAJ491-06|2006-ONT-0491|658[0n]bp|Canada.Ontario|BOLD:AAA9083
Notodonta pacifica|[1209]|RDNMF110-08|NOC14196|609[0n]bp|Canada.British Columbia|BOLD:AAF2709
Notodonta pacifica|[1210]|RDNMF111-08|NOC14197|658[0n]bp|Canada.British Columbia|BOLD:AAF2709
Notodonta scitipennis|[1211]|TMNBD285-07|MNBTT-3086|613[0n]bp|Canada.New Brunswick|BOLD:ABZ2129
Notodonta scitipennis|[1212]|BBLPB309-10|10BBCLP-1308|658[0n]bp|Canada.Ontario|BOLD:ABZ2129
Notodonta scitipennis|[1213]|RDLQB188-05|DH010274|605[0n]bp|Canada.Quebec|BOLD:ABZ2129
Notodonta scitipennis|[1214]|RDLQB187-05|DH010273|580[0n]bp|Canada.Quebec|BOLD:ABZ2129
Notodonta scitipennis|[1215]|TMNBD284-07|MNBTT-3085|647[0n]bp|Canada.New Brunswick|BOLD:AAB8177
Notodonta scitipennis|[1216]|TTMNB934-06|MNBTT-934|658[0n]bp|Canada.New Brunswick|BOLD:AAB8177
Notodonta scitipennis|[1217]|TTMNB933-06|MNBTT-933|658[0n]bp|Canada.New Brunswick|BOLD:AAB8177
Notodonta scitipennis|[1218]|XAG669-05|2005-ONT-1253|658[0n]bp|Canada.Ontario|BOLD:AAB8177
Notodonta scitipennis|[1219]|MECD404-06|jflandry2976|658[0n]bp|Canada.Quebec|BOLD:AAB8177
Notodonta scitipennis|[1220]|TTMNB080-06|MNBTT-080|657[0n]bp|Canada.New Brunswick|BOLD:AAB8177
Notodonta scitipennis|[1221]|XAG645-05|2005-ONT-1229|658[0n]bp|Canada.Ontario|BOLD:AAB8177
Notodonta scitipennis|[1222]|TMNBD286-07|MNBTT-3087|646[0n]bp|Canada.New Brunswick|BOLD:AAB8177
Notodonta scitipennis|[1223]|TMNBD283-07|MNBTT-3084|647[0n]bp|Canada.New Brunswick|BOLD:AAB8177
Notodonta scitipennis|[1224]|TMNBD282-07|MNBTT-3083|646[0n]bp|Canada.New Brunswick|BOLD:AAB8177
Notodonta scitipennis|[1225]|PHMNB039-03|moth211.02SA|639[0n]bp|Canada.New Brunswick|BOLD:AAB8177
Notodonta scitipennis|[1226]|XAJ351-06|2006-ONT-0351|614[0n]bp|Canada.Ontario|BOLD:AAB8177
Notodonta scitipennis|[1227]|XAB046-04|04HBL005046|587[0n]bp|Canada.Ontario|BOLD:AAB8177
Notodonta scitipennis|[1228]|XAJ492-06|2006-ONT-0492|656[0n]bp|Canada.Ontario|BOLD:AAB8177
Notodonta torva|[1229]|RDLQ778-07|DH004493|658[0n]bp|Canada.Quebec|BOLD:AAB4660
Notodonta torva|[1230]|TMNBD288-07|MNBTT-3089|617[0n]bp|Canada.New Brunswick|BOLD:AAB4660
Notodonta torva|[1231]|XAE313-04|Moth4313.03|658[0n]bp|Canada.Ontario|BOLD:AAB4660
Notodonta torva|[1232]|TMNBD290-07|MNBTT-3091|658[0n]bp|Canada.New Brunswick|BOLD:AAB4660
Notodonta torva|[1233]|LBCH104-10|10-JDWBC-0104|658[0n]bp|Canada.British Columbia|BOLD:AAB4660
Notodonta torva|[1234]|XAG799-05|2005-ONT-1383|658[0n]bp|Canada.Ontario|BOLD:AAB4660
Notodonta torva|[1235]|MNBB606-05|05-NBSTA-522|658[0n]bp|Canada.New Brunswick|BOLD:AAB4660
Notodonta torva|[1236]|XAF569-05|2005-ONT-218|658[0n]bp|Canada.Ontario|BOLD:AAB4660
Notodonta torva|[1237]|PMG184-03|moth206.01|617[0n]bp|Canada.Ontario|BOLD:AAB4660
Notodonta torva|[1238]|TMG63-03|moth236.01|639[0n]bp|Canada.Ontario|BOLD:AAB4660
Notodonta torva|[1239]|XAE172-04|Moth4172.03|602[1n]bp|Canada.Ontario|BOLD:AAB4660
Notodonta torva|[1240]|XAG541-05|2005-ONT-1125|552[0n]bp|Canada.Ontario|BOLD:AAB4660
Notodonta torva|[1241]|PHMNB754-05|Moth 447.03SA|614[0n]bp|Canada.New Brunswick|BOLD:AAB4660
Notodonta torva|[1242]|BBLPB557-10|10BBCLP-1556|658[0n]bp|Canada.Saskatchewan|BOLD:AAB4660
Notodonta torva|[1243]|LPMN645-08|08BBLEP-01446|658[0n]bp|Canada.Manitoba|BOLD:AAB4660
Notodonta torva|[1244]|RDMAB067-05|UASM57594|658[0n]bp|Canada.Alberta|BOLD:AAB4660
Notodonta torva|[1245]|MNBB613-05|05-NBSTA-529|658[0n]bp|Canada.New Brunswick|BOLD:AAB4660
Notodonta torva|[1246]|LBCH696-10|10-JDWBC-0696|658[0n]bp|Canada.British Columbia|BOLD:AAB4660
Notodonta torva|[1247]|LBCH650-10|10-JDWBC-0650|658[0n]bp|Canada.British Columbia|BOLD:AAB4660
Notodonta torva|[1248]|LBCH798-10|10-JDWBC-0798|658[0n]bp|Canada.British Columbia|BOLD:AAB4660
Notodonta torva|[1249]|LBCH646-10|10-JDWBC-0646|658[0n]bp|Canada.British Columbia|BOLD:AAB4660
Notodonta torva|[1250]|LBCH226-10|10-JDWBC-0226|658[0n]bp|Canada.British Columbia|BOLD:AAB4660
Notodonta torva|[1251]|LBCG167-08|08-JDWBC-0167|658[0n]bp|Canada.British Columbia|BOLD:AAB4660
Notodonta torva|[1252]|LBCH2954-10|10-JDWBC-2954|658[0n]bp|Canada.British Columbia|BOLD:AAB4660
Hyperaeschra georgica|[1253]|RDLQ779-07|DH001276|658[0n]bp|Canada.Quebec|BOLD:AAB4104
Hyperaeschra georgica|[1254]|MECC506-06|jflandry2526|658[0n]bp|Canada.Ontario|BOLD:AAB4103
Nerice bidentata|[1255]|XAJ688-06|2006-ONT-0688|658[0n]bp|Canada.Ontario|BOLD:AAB3168
Nerice bidentata|[1256]|XAE082-04|Moth4082.03|658[0n]bp|Canada.Ontario|BOLD:AAB3168
Nerice bidentata|[1257]|XAG582-05|2005-ONT-1166|658[0n]bp|Canada.Ontario|BOLD:AAB3168
Nerice bidentata|[1258]|XAJ981-06|2006-ONT-0981|658[0n]bp|Canada.Ontario|BOLD:AAB3168
Nerice bidentata|[1259]|XAK113-06|2006-ONT-1108|658[0n]bp|Canada.Ontario|BOLD:AAB3168
Nerice bidentata|[1260]|XAK120-06|2006-ONT-1115|658[0n]bp|Canada.Ontario|BOLD:AAB3168
Nerice bidentata|[1261]|XAJ587-06|2006-ONT-0587|658[0n]bp|Canada.Ontario|BOLD:AAB3168
Nerice bidentata|[1262]|XAK443-06|2006-ONT-1438|658[0n]bp|Canada.Ontario|BOLD:AAB3168
Nerice bidentata|[1263]|XAK438-06|2006-ONT-1433|658[0n]bp|Canada.Ontario|BOLD:AAB3168

Nerice bidentata|[1261]|XAJ587-06|2006-ONT-0587|658[0n]bp|Canada.Ontario|BOLD:AAB3168
Nerice bidentata|[1262]|XAK443-06|2006-ONT-1438|658[0n]bp|Canada.Ontario|BOLD:AAB3168
Nerice bidentata|[1263]|XAK438-06|2006-ONT-1433|658[0n]bp|Canada.Ontario|BOLD:AAB3168
Nerice bidentata|[1264]|XAK607-07|HLC-16160|573[0n]bp|Canada.Ontario|BOLD:AAB3168
Nerice bidentata|[1265]|LPSOB666-08|PPBP-1665|644[0n]bp|Canada.Ontario|BOLD:AAB3168
Nerice bidentata|[1266]|XAB589-04|04HBL005589|658[0n]bp|Canada.Ontario|BOLD:AAB3168
Nerice bidentata|[1267]|LPSOC351-08|PPBP-2350|656[0n]bp|Canada.Ontario|BOLD:AAB3168
Nerice bidentata|[1268]|RDLQF808-06|DH011958|658[1n]bp|Canada.Quebec|BOLD:AAB3168
Nerice bidentata|[1269]|XAK349-06|2006-ONT-1344|615[0n]bp|Canada.Ontario|BOLD:AAB3168
Nerice bidentata|[1270]|LPSOD320-09|08BBLEP-00098|658[0n]bp|Canada.Ontario|BOLD:AAB3168
Nerice bidentata|[1271]|XAB025-04|04HBL005025|658[0n]bp|Canada.Ontario|BOLD:AAB3168
Nerice bidentata|[1272]|LPSO026-08|PPBP-0026|658[0n]bp|Canada.Ontario|BOLD:AAB3168
Nadata gibbosa|[1273]|LPSOB062-08|PPBP-1061|658[0n]bp|Canada.Ontario|BOLD:AAA2279
Nadata gibbosa|[1274]|LPMN033-08|08BBLEP-00831|658[0n]bp|Canada.Manitoba|BOLD:AAA2279
Nadata gibbosa|[1275]|LPSOB288-08|PPBP-1287|658[0n]bp|Canada.Ontario|BOLD:AAA2279
Nadata gibbosa|[1276]|LPSOD800-09|08BBLEP-00582|658[0n]bp|Canada.Ontario|BOLD:AAA2279
Nadata gibbosa|[1277]|LPMN562-08|08BBLEP-01363|658[0n]bp|Canada.Manitoba|BOLD:AAA2279
Nadata gibbosa|[1278]|XAB618-04|04HBL005618|658[0n]bp|Canada.Ontario|BOLD:AAA2279
Nadata gibbosa|[1279]|PHMNB437-04|04HBL00663|658[0n]bp|Canada.New Brunswick|BOLD:AAA2279
Nadata gibbosa|[1280]|MNBB453-05|05-NBSTA-369|658[0n]bp|Canada.New Brunswick|BOLD:AAA2279
Nadata gibbosa|[1281]|LPMN037-08|08BBLEP-00835|658[0n]bp|Canada.Manitoba|BOLD:AAA2279
Nadata gibbosa|[1282]|LPMN306-08|08BBLEP-01105|658[0n]bp|Canada.Manitoba|BOLD:AAA2279
Nadata gibbosa|[1283]|XAE280-04|Moth4280.03|658[0n]bp|Canada.Ontario|BOLD:AAA2279
Nadata gibbosa|[1284]|PHMNB728-05|Moth 421.03SA|658[0n]bp|Canada.New Brunswick|BOLD:AAA2279
Nadata gibbosa|[1285]|LBCS116-07|UBC-2007-0106|658[0n]bp|Canada.British Columbia|BOLD:AAA2279
Nadata gibbosa|[1286]|XAB619-04|04HBL005619|658[0n]bp|Canada.Ontario|BOLD:AAA2279
Nadata gibbosa|[1287]|LBCH106-10|10-JDWBC-0106|658[0n]bp|Canada.British Columbia|BOLD:AAA2279
Nadata gibbosa|[1288]|RDLQB196-05|DH010282|658[0n]bp|Canada.Quebec|BOLD:AAA2279
Nadata gibbosa|[1289]|XAB106-04|04HBL005106|658[1n]bp|Canada.Ontario|BOLD:AAA2279
Nadata gibbosa|[1290]|LBCA771-05|HLC-20771|658[0n]bp|Canada.British Columbia|BOLD:AAA2279
Nadata gibbosa|[1291]|BBLEC411-09|09BBELE-0411|658[0n]bp|Canada.Newfoundland and Labrador|BOLD:AAA2279
Nadata gibbosa|[1292]|TTMNB076-06|MNBTT-076|658[0n]bp|Canada.New Brunswick|BOLD:AAA2279
Nadata gibbosa|[1293]|LBCB031-05|HLC-20971|658[0n]bp|Canada.British Columbia|BOLD:AAA2279
Nadata gibbosa|[1294]|LBCA237-05|HLC-20237|658[0n]bp|Canada.British Columbia|BOLD:AAA2279
Nadata gibbosa|[1295]|PHMNB569-04|04HBL00795|658[0n]bp|Canada.New Brunswick|BOLD:AAA2279
Nadata gibbosa|[1296]|LPSOB060-08|PPBP-1059|658[0n]bp|Canada.Ontario|BOLD:AAA2279
Nadata gibbosa|[1297]|LBCA768-05|HLC-20768|658[0n]bp|Canada.British Columbia|BOLD:AAA2279
Nadata gibbosa|[1298]|LALPA256-10|AVBC 257-10|658[0n]bp|Canada.British Columbia|BOLD:AAA2279
Nadata gibbosa|[1299]|XAB204-04|04HBL005204|658[0n]bp|Canada.Ontario|BOLD:AAA2279
Nadata gibbosa|[1300]|LBCA777-05|HLC-20777|658[0n]bp|Canada.British Columbia|BOLD:AAA2279
Nadata gibbosa|[1301]|LBCA240-05|HLC-20240|632[0n]bp|Canada.British Columbia|BOLD:AAA2279
Nadata gibbosa|[1302]|XAE321-04|Moth4321.03|658[0n]bp|Canada.Ontario|BOLD:AAA2279
Nadata gibbosa|[1303]|LBCS657-07|UBC-2007-0360|658[0n]bp|Canada.British Columbia|BOLD:AAA2279
Nadata gibbosa|[1304]|LBCS655-07|UBC-2007-0358|658[0n]bp|Canada.British Columbia|BOLD:AAA2279
Nadata gibbosa|[1305]|LALPA367-10|AVBC 369-10|658[0n]bp|Canada.British Columbia|BOLD:AAA2279
Nadata gibbosa|[1306]|LPSOD339-09|08BBLEP-00117|658[0n]bp|Canada.Ontario|BOLD:AAA2279
Nadata gibbosa|[1307]|XAJ415-06|2006-ONT-0415|658[0n]bp|Canada.Ontario|BOLD:AAA2279
Nadata gibbosa|[1308]|LPSOC220-08|PPBP-2219|658[0n]bp|Canada.Ontario|BOLD:AAA2279
Nadata gibbosa|[1309]|RDLQ097-05|DH001306|658[0n]bp|Canada.Quebec|BOLD:AAA2279
Nadata gibbosa|[1310]|LBCS114-07|UBC-2007-0104|658[0n]bp|Canada.British Columbia|BOLD:AAA2279
Nadata gibbosa|[1311]|LBCS115-07|UBC-2007-0105|658[0n]bp|Canada.British Columbia|BOLD:AAA2279
Nadata gibbosa|[1312]|LBCB019-05|HLC-20959|658[0n]bp|Canada.British Columbia|BOLD:AAA2279
Nadata gibbosa|[1313]|TMNBD265-07|MNBTT-3066|658[0n]bp|Canada.New Brunswick|BOLD:AAA2279
Nadata gibbosa|[1314]|PHMNB729-05|Moth 422.03SA|658[0n]bp|Canada.New Brunswick|BOLD:AAA2279
Nadata gibbosa|[1315]|XAE257-04|Moth4257.03|658[0n]bp|Canada.Ontario|BOLD:AAA2279
Nadata gibbosa|[1316]|LPSOB428-08|PPBP-1427|658[0n]bp|Canada.Ontario|BOLD:AAA2279
Nadata gibbosa|[1317]|XAB084-04|04HBL005084|658[0n]bp|Canada.Ontario|BOLD:AAA2279
Nadata gibbosa|[1318]|LBCS656-07|UBC-2007-0359|658[0n]bp|Canada.British Columbia|BOLD:AAA2279
Nadata gibbosa|[1319]|LPMN561-08|08BBLEP-01362|658[0n]bp|Canada.Manitoba|BOLD:AAA2279
Nadata gibbosa|[1320]|LPMN560-08|08BBLEP-01361|658[0n]bp|Canada.Manitoba|BOLD:AAA2279
Nadata gibbosa|[1321]|LPMN307-08|08BBLEP-01106|658[0n]bp|Canada.Manitoba|BOLD:AAA2279
Nadata gibbosa|[1322]|TMNBD263-07|MNBTT-3064|658[0n]bp|Canada.New Brunswick|BOLD:AAA2279
Nadata gibbosa|[1323]|LPSOD826-09|08BBLEP-00608|658[0n]bp|Canada.Ontario|BOLD:AAA2279
Nadata gibbosa|[1324]|LPSOD825-09|08BBLEP-00607|658[0n]bp|Canada.Ontario|BOLD:AAA2279
Nadata gibbosa|[1325]|LBCA232-05|HLC-20232|633[0n]bp|Canada.British Columbia|BOLD:AAA2279
Nadata gibbosa|[1326]|LBCA778-05|HLC-20778|658[0n]bp|Canada.British Columbia|BOLD:AAA2279
Nadata gibbosa|[1327]|RDMAB064-05|UASM57591|649[0n]bp|Canada.Alberta|BOLD:AAA2279
Nadata gibbosa|[1328]|XAE518-04|Moth4518.03|589[0n]bp|Canada.Ontario|BOLD:AAA2279
Nadata gibbosa|[1329]|TMNBD264-07|MNBTT-3065|646[0n]bp|Canada.New Brunswick|BOLD:AAA2279
Nadata gibbosa|[1330]|TMNBD266-07|MNBTT-3067|648[0n]bp|Canada.New Brunswick|BOLD:AAA2279
Nadata gibbosa|[1331]|LBCA233-05|HLC-20233|621[0n]bp|Canada.British Columbia|BOLD:AAA2279
Nadata gibbosa|[1332]|PMG183-03|moth665.01|617[0n]bp|Canada.Ontario|BOLD:AAA2279
Nadata gibbosa|[1333]|LBCB018-05|HLC-20958|631[0n]bp|Canada.British Columbia|BOLD:AAA2279
Nadata gibbosa|[1334]|BBLPC621-09|09BBELE-1621|614[0n]bp|Canada.Nova Scotia|BOLD:AAA2279
Nadata gibbosa|[1335]|TMG59-03|moth908.01|639[0n]bp|Canada.Ontario|BOLD:AAA2279
Nadata gibbosa|[1336]|RDLQ098-05|DH001310|588[0n]bp|Canada.Quebec|BOLD:AAA2279
Nadata gibbosa|[1337]|RDLQB197-05|DH010283|658[0n]bp|Canada.Quebec|BOLD:AAA2279
Nadata gibbosa|[1338]|LPMN305-08|08BBLEP-01104|658[0n]bp|Canada.Manitoba|BOLD:AAA2279
Nadata gibbosa|[1339]|LBCB033-05|HLC-20973|658[0n]bp|Canada.British Columbia|BOLD:AAA2279
Nadata gibbosa|[1340]|LPSOD431-09|08BBLEP-00210|658[0n]bp|Canada.Ontario|BOLD:AAA2279
Nadata gibbosa|[1341]|LBCB135-05|HLC-21075|658[0n]bp|Canada.British Columbia|BOLD:AAA2279
Nadata gibbosa|[1342]|LBCB025-05|HLC-20965|658[0n]bp|Canada.British Columbia|BOLD:AAA2279
Nadata gibbosa|[1343]|LBCB034-05|HLC-20974|658[0n]bp|Canada.British Columbia|BOLD:AAA2279
Nadata gibbosa|[1344]|LBCC377-05|HLC-22257|658[0n]bp|Canada.British Columbia|BOLD:AAA2279
Nadata gibbosa|[1345]|LPSO326-08|PPBP-0326|658[0n]bp|Canada.Ontario|BOLD:AAA2279
Nadata gibbosa|[1346]|LPSOB061-08|PPBP-1060|658[0n]bp|Canada.Ontario|BOLD:AAA2279
Nadata gibbosa|[1347]|LPSOD526-09|08BBLEP-00305|658[0n]bp|Canada.Ontario|BOLD:AAA2279
Nadata gibbosa|[1348]|XAF804-05|2005-ONT-453|658[0n]bp|Canada.Ontario|BOLD:AAA2279
Nadata gibbosa|[1349]|LBCH5082-10|10-JDWBC-5082|658[0n]bp|Canada.British Columbia|BOLD:AAA2279
Nadata gibbosa|[1350]|LBCH5144-10|10-JDWBC-5144|658[0n]bp|Canada.British Columbia|BOLD:AAA2279
Nadata gibbosa|[1351]|LBCB021-05|HLC-20961|658[0n]bp|Canada.British Columbia|BOLD:AAA2279
Nadata gibbosa|[1352]|LBCB131-05|HLC-21071|658[0n]bp|Canada.British Columbia|BOLD:AAA2279
Nadata gibbosa|[1353]|LBCB032-05|HLC-20972|658[0n]bp|Canada.British Columbia|BOLD:AAA2279
Nadata gibbosa|[1354]|LBCH217-10|10-JDWBC-0217|658[0n]bp|Canada.British Columbia|BOLD:AAA2279
Nadata gibbosa|[1355]|LBCH799-10|10-JDWBC-0799|658[0n]bp|Canada.British Columbia|BOLD:AAA2279
Nadata gibbosa|[1356]|LBCH008-10|10-JDWBC-0008|658[0n]bp|Canada.British Columbia|BOLD:AAA2279
Nadata gibbosa|[1357]|LBCH216-10|10-JDWBC-0216|658[0n]bp|Canada.British Columbia|BOLD:AAA2279
Nadata gibbosa|[1358]|LBCA765-05|HLC-20765|658[0n]bp|Canada.British Columbia|BOLD:AAA2279
Nadata gibbosa|[1359]|LBCA767-05|HLC-20767|658[0n]bp|Canada.British Columbia|BOLD:AAA2279
Nadata gibbosa|[1360]|LBCA774-05|HLC-20774|658[0n]bp|Canada.British Columbia|BOLD:AAA2279
[illegible]

Nadata gibbosa|[1358]|LBCA765-05|HLC-20765|658[0n]bp|Canada.British Columbia|BOLD:AAA2279
Nadata gibbosa|[1359]|LBCA767-05|HLC-20767|658[0n]bp|Canada.British Columbia|BOLD:AAA2279
Nadata gibbosa|[1360]|LBCA774-05|HLC-20774|658[0n]bp|Canada.British Columbia|BOLD:AAA2279
Nadata gibbosa|[1361]|LBCA776-05|HLC-20776|658[0n]bp|Canada.British Columbia|BOLD:AAA2279
Nadata gibbosa|[1362]|LBCA766-05|HLC-20766|658[0n]bp|Canada.British Columbia|BOLD:AAA2279
Nadata gibbosa|[1363]|LBCA773-05|HLC-20773|658[0n]bp|Canada.British Columbia|BOLD:AAA2279
Nadata gibbosa|[1364]|LBCB030-05|HLC-20970|658[0n]bp|Canada.British Columbia|BOLD:AAA2279
Nadata gibbosa|[1365]|LBCB029-05|HLC-20969|658[0n]bp|Canada.British Columbia|BOLD:AAA2279
Nadata gibbosa|[1366]|LBCB125-05|HLC-21065|658[0n]bp|Canada.British Columbia|BOLD:AAA2279
Nadata gibbosa|[1367]|LBCB022-05|HLC-20962|658[0n]bp|Canada.British Columbia|BOLD:AAA2279
Nadata gibbosa|[1368]|LBCB133-05|HLC-21073|658[0n]bp|Canada.British Columbia|BOLD:AAA2279
Nadata gibbosa|[1369]|LBCB129-05|HLC-21069|658[0n]bp|Canada.British Columbia|BOLD:AAA2279
Nadata gibbosa|[1370]|LBCB124-05|HLC-21064|658[0n]bp|Canada.British Columbia|BOLD:AAA2279
Nadata gibbosa|[1371]|LBCB035-05|HLC-20975|658[0n]bp|Canada.British Columbia|BOLD:AAA2279
Nadata gibbosa|[1372]|LBCB134-05|HLC-21074|658[0n]bp|Canada.British Columbia|BOLD:AAA2279
Nadata gibbosa|[1373]|LBCB126-05|HLC-21066|658[0n]bp|Canada.British Columbia|BOLD:AAA2279
Nadata gibbosa|[1374]|LBCB127-05|HLC-21067|658[0n]bp|Canada.British Columbia|BOLD:AAA2279
Nadata gibbosa|[1375]|LBCB027-05|HLC-20967|658[0n]bp|Canada.British Columbia|BOLD:AAA2279
Nadata gibbosa|[1376]|LBCB024-05|HLC-20964|658[0n]bp|Canada.British Columbia|BOLD:AAA2279
Nadata gibbosa|[1377]|LBCB023-05|HLC-20963|658[0n]bp|Canada.British Columbia|BOLD:AAA2279
Nadata gibbosa|[1378]|LBCB128-05|HLC-21068|658[0n]bp|Canada.British Columbia|BOLD:AAA2279
Nadata gibbosa|[1379]|LBCB026-05|HLC-20966|658[0n]bp|Canada.British Columbia|BOLD:AAA2279
Nadata gibbosa|[1380]|LBCA043-05|HLC-20043|658[0n]bp|Canada.British Columbia|BOLD:AAA2279
Nadata gibbosa|[1381]|LALPA149-10|AVBC 149-10|658[0n]bp|Canada.British Columbia|BOLD:AAA2279
Nadata gibbosa|[1382]|LBCA235-05|HLC-20235|658[0n]bp|Canada.British Columbia|BOLD:AAA2279
Nadata gibbosa|[1383]|LPAB265-08|08BBLEP-02587|658[0n]bp|Canada.Alberta|BOLD:AAA2279
Nadata gibbosa|[1384]|LBCC375-05|HLC-22255|658[0n]bp|Canada.British Columbia|BOLD:AAA2279
Nadata gibbosa|[1385]|LBCC373-05|HLC-22253|658[0n]bp|Canada.British Columbia|BOLD:AAA2279
Nadata gibbosa|[1386]|LBCC376-05|HLC-22256|658[0n]bp|Canada.British Columbia|BOLD:AAA2279
Nadata gibbosa|[1387]|LBCC374-05|HLC-22254|658[0n]bp|Canada.British Columbia|BOLD:AAA2279
Nadata gibbosa|[1388]|LBCC339-05|HLC-22219|658[0n]bp|Canada.British Columbia|BOLD:AAA2279
Nadata gibbosa|[1389]|LMH027-06|PFC-2006-0159|658[0n]bp|Canada.British Columbia|BOLD:AAA2279
Nadata gibbosa|[1390]|LBCB020-05|HLC-20960|658[0n]bp|Canada.British Columbia|BOLD:AAA2279
Nadata gibbosa|[1391]|LBCB130-05|HLC-21070|658[0n]bp|Canada.British Columbia|BOLD:AAA2279
Nadata gibbosa|[1392]|LBCG097-08|08-JDWBC-0097|658[0n]bp|Canada.British Columbia|BOLD:AAA2279
Nadata gibbosa|[1393]|LBCB017-05|HLC-20957|658[0n]bp|Canada.British Columbia|BOLD:AAA2279
Nadata gibbosa|[1394]|LBCB136-05|HLC-21076|658[0n]bp|Canada.British Columbia|BOLD:AAA2279
Nadata gibbosa|[1395]|LBCB132-05|HLC-21072|658[0n]bp|Canada.British Columbia|BOLD:AAA2279
Nadata gibbosa|[1396]|LBCA769-05|HLC-20769|658[0n]bp|Canada.British Columbia|BOLD:AAA2279
Nadata gibbosa|[1397]|LBCA775-05|HLC-20775|658[0n]bp|Canada.British Columbia|BOLD:AAA2279
Nadata gibbosa|[1398]|LBCA764-05|HLC-20764|658[0n]bp|Canada.British Columbia|BOLD:AAA2279
Nadata gibbosa|[1399]|LBCA772-05|HLC-20772|658[0n]bp|Canada.British Columbia|BOLD:AAA2279
Nadata gibbosa|[1400]|LBCA770-05|HLC-20770|658[0n]bp|Canada.British Columbia|BOLD:AAA2279
Nadata gibbosa|[1401]|LBCH5929-10|10-JDWBC-5929|658[0n]bp|Canada.British Columbia|BOLD:AAA2279
Nadata gibbosa|[1402]|LBCH5852-10|10-JDWBC-5852|658[0n]bp|Canada.British Columbia|BOLD:AAA2279
Nadata gibbosa|[1403]|LBCH2237-10|10-JDWBC-2237|658[0n]bp|Canada.British Columbia|BOLD:AAA2279
Nadata gibbosa|[1404]|LALPA300-10|AVBC 302-10|658[0n]bp|Canada.British Columbia|BOLD:AAA2279
Nadata gibbosa|[1405]|LOWCE821-06|CGWC-4581|658[0n]bp|Canada.British Columbia|BOLD:AAA2279
Nadata gibbosa|[1406]|LBCA238-05|HLC-20238|658[1n]bp|Canada.British Columbia|BOLD:AAA2279
Nadata gibbosa|[1407]|LBCA491-05|HLC-20491|658[1n]bp|Canada.British Columbia|BOLD:AAA2279
Nadata gibbosa|[1408]|LBCG044-08|08-JDWBC-0044|658[0n]bp|Canada.British Columbia|BOLD:AAA2279
Nadata gibbosa|[1409]|LBCA080-05|HLC-20080|651[0n]bp|Canada.British Columbia|BOLD:AAA2279
Nadata gibbosa|[1410]|LBCB028-05|HLC-20968|650[0n]bp|Canada.British Columbia|BOLD:AAA2279
Nadata gibbosa|[1411]|LBCA575-05|HLC-20575|622[0n]bp|Canada.British Columbia|BOLD:AAA2279
Nadata gibbosa|[1412]|LBCH5986-10|10-JDWBC-5986|623[0n]bp|Canada.British Columbia|BOLD:AAA2279
Nadata gibbosa|[1413]|LBCA239-05|HLC-20239|622[0n]bp|Canada.British Columbia|BOLD:AAA2279
Nadata gibbosa|[1414]|LBCA234-05|HLC-20234|636[0n]bp|Canada.British Columbia|BOLD:AAA2279
Nadata gibbosa|[1415]|LBCA236-05|HLC-20236|636[0n]bp|Canada.British Columbia|BOLD:AAA2279
Nadata gibbosa|[1416]|LBCC746-05|HLC-22626|658[0n]bp|Canada.British Columbia|BOLD:AAA2279
Nadata gibbosa|[1417]|LOWCE805-06|CGWC-4565|658[0n]bp|Canada.British Columbia|BOLD:AAA2279
Nadata gibbosa|[1418]|LOWCB117-05|CGWC-1057|658[0n]bp|Canada.British Columbia|BOLD:AAA2279
Nadata gibbosa|[1419]|LOWCB118-05|CGWC-1058|658[0n]bp|Canada.British Columbia|BOLD:AAA2279
Nadata gibbosa|[1420]|LPSO013-08|PPBP-0013|658[0n]bp|Canada.Ontario|BOLD:AAA2279
Odontosia elegans|[1421]|LOWCB124-05|CGWC-1064|658[0n]bp|Canada.British Columbia|BOLD:AAB3986
Odontosia elegans|[1422]|XAE513-04|Moth4513.03|658[0n]bp|Canada.Ontario|BOLD:AAB3986
Odontosia elegans|[1423]|LOWCB128-05|CGWC-1068|658[0n]bp|Canada.British Columbia|BOLD:AAB3986
Odontosia elegans|[1424]|LOWCB126-05|CGWC-1066|658[0n]bp|Canada.British Columbia|BOLD:AAB3986
Odontosia elegans|[1425]|LOWCB125-05|CGWC-1065|658[0n]bp|Canada.British Columbia|BOLD:AAB3986
Odontosia elegans|[1426]|LOWCB127-05|CGWC-1067|658[0n]bp|Canada.British Columbia|BOLD:AAB3986
Odontosia elegans|[1427]|LPMN558-08|08BBLEP-01359|658[0n]bp|Canada.Manitoba|BOLD:AAB3986
Odontosia elegans|[1428]|LBCD264-05|HLC-23084|658[0n]bp|Canada.British Columbia|BOLD:AAB3986
Odontosia elegans|[1429]|LBCD263-05|HLC-23083|658[0n]bp|Canada.British Columbia|BOLD:AAB3986
Odontosia elegans|[1430]|LPMN289-08|08BBLEP-01088|643[0n]bp|Canada.Manitoba|BOLD:AAB3986
Odontosia elegans|[1431]|LBCB877-05|HLC-21817|658[0n]bp|Canada.British Columbia|BOLD:AAB3986
Odontosia elegans|[1432]|PMG185-03|moth749.01|617[0n]bp|Canada.Ontario|BOLD:AAB3986
Odontosia elegans|[1433]|TMG62-03|moth670.01|639[0n]bp|Canada.Ontario|BOLD:AAB3986
Odontosia elegans|[1434]|TMNBD280-07|MNBTT-3081|644[0n]bp|Canada.New Brunswick|BOLD:AAB3986
Odontosia elegans|[1435]|RDLQF807-06|DH011957|658[0n]bp|Canada.Quebec|BOLD:AAB3986
Odontosia elegans|[1436]|TMNBD277-07|MNBTT-3078|656[0n]bp|Canada.New Brunswick|BOLD:AAB3986
Odontosia elegans|[1437]|LPSOB431-08|PPBP-1430|658[0n]bp|Canada.Ontario|BOLD:AAB3986
Odontosia elegans|[1438]|LPSOB292-08|PPBP-1291|658[0n]bp|Canada.Ontario|BOLD:AAB3986
Odontosia elegans|[1439]|LPSOB301-08|PPBP-1300|658[0n]bp|Canada.Ontario|BOLD:AAB3986
Odontosia elegans|[1440]|TMNBD279-07|MNBTT-3080|639[0n]bp|Canada.New Brunswick|BOLD:AAB3986
Odontosia elegans|[1441]|PHMNB014-03|moth137.02SA|639[0n]bp|Canada.New Brunswick|BOLD:AAB3986
Odontosia elegans|[1442]|TMNBD281-07|MNBTT-3082|617[0n]bp|Canada.New Brunswick|BOLD:AAB3986
Odontosia elegans|[1443]|MNBB180-05|05-NBSTA-096|550[0n]bp|Canada.New Brunswick|BOLD:AAB3986
Pheosia rimosa|[1444]|LPSOD639-09|08BBLEP-00420|658[0n]bp|Canada.Ontario|BOLD:AAA3484
Pheosia portlandia|[1445]|LBCH007-10|10-JDWBC-0007|658[0n]bp|Canada.British Columbia|BOLD:ABZ4471
Pheosia portlandia|[1446]|RDNMG925-08|CNC LEP00053049|658[0n]bp|Canada.British Columbia|BOLD:ABZ4471
Pheosia portlandia|[1447]|RDNMF113-08|NOC14199|658[0n]bp|Canada.British Columbia|BOLD:ABZ4471
Pheosia portlandia|[1448]|LOWCE103-06|CGWC-3863|658[0n]bp|Canada.British Columbia|BOLD:ABZ4471
Pheosia portlandia|[1449]|LOWCE104-06|CGWC-3864|658[0n]bp|Canada.British Columbia|BOLD:ABZ4471
Pheosia portlandia|[1450]|LBCH221-10|10-JDWBC-0221|658[0n]bp|Canada.British Columbia|BOLD:ABZ4471
Pheosia portlandia|[1451]|LBCH096-10|10-JDWBC-0096|658[0n]bp|Canada.British Columbia|BOLD:ABZ4471
Pheosia portlandia|[1452]|LBCH100-10|10-JDWBC-0100|658[0n]bp|Canada.British Columbia|BOLD:ABZ4471
Pheosia portlandia|[1453]|LBCH098-10|10-JDWBC-0098|658[0n]bp|Canada.British Columbia|BOLD:ABZ4471
Pheosia portlandia|[1454]|LBCH797-10|10-JDWBC-0797|658[0n]bp|Canada.British Columbia|BOLD:ABZ4471
Pheosia portlandia|[1455]|LBCH102-10|10-JDWBC-0102|658[0n]bp|Canada.British Columbia|BOLD:ABZ4471
Pheosia portlandia|[1456]|LBCH642-10|10-JDWBC-0642|658[0n]bp|Canada.British Columbia|BOLD:ABZ4471
Pheosia portlandia|[1457]|LOWCB120-05|CGWC-1060|658[0n]bp|Canada.British Columbia|BOLD:ABZ4471
Pheosia portlandia|[1458]|LOWCB119-05|CGWC-1059|658[0n]bp|Canada.British Columbia|BOLD:ABZ4471

Pheosia portlandia|[1455]|LBCH102-10|10-JDWBC-0102|658[0n]bp|Canada.British Columbia|BOLD:ABZ4471
Pheosia portlandia|[1456]|LBCH642-10|10-JDWBC-0642|658[0n]bp|Canada.British Columbia|BOLD:ABZ4471
Pheosia portlandia|[1457]|LOWCB120-05|CGWC-1060|658[0n]bp|Canada.British Columbia|BOLD:ABZ4471
Pheosia portlandia|[1458]|LOWCB119-05|CGWC-1059|658[0n]bp|Canada.British Columbia|BOLD:ABZ4471
Pheosia portlandia|[1459]|LOWCB121-05|CGWC-1061|658[0n]bp|Canada.British Columbia|BOLD:ABZ4471
Pheosia portlandia|[1460]|LBCH1301-10|10-JDWBC-1301|658[0n]bp|Canada.British Columbia|BOLD:ABZ4471
Pheosia portlandia|[1461]|LOWCD184-06|CGWC-3004|658[0n]bp|Canada.British Columbia|BOLD:ABZ4471
Pheosia portlandia|[1462]|LALPA973-11|AVBC 1146-11|658[0n]bp|Canada.British Columbia|BOLD:ABZ4471
Pheosia portlandia|[1463]|LOWCB122-05|CGWC-1062|658[0n]bp|Canada.British Columbia|BOLD:ABZ4471
Pheosia portlandia|[1464]|LOWCC871-05|CGWC-2751|658[0n]bp|Canada.British Columbia|BOLD:ABZ4471
Pheosia portlandia|[1465]|LOWCC844-05|CGWC-2724|573[2n]bp|Canada.British Columbia|BOLD:ABZ4471
Pheosia portlandia|[1466]|LBCG3202-09|08-JDWBC-3202|641[0n]bp|Canada.British Columbia|BOLD:ABZ4471
Pheosia portlandia|[1467]|RDNMF112-08|NOC14198|550[0n]bp|Canada.British Columbia|BOLD:ABZ4471
Pheosia portlandia|[1468]|LBCH099-10|10-JDWBC-0099|658[0n]bp|Canada.British Columbia|BOLD:ABZ4471
Pheosia portlandia|[1469]|LBCH101-10|10-JDWBC-0101|658[0n]bp|Canada.British Columbia|BOLD:ABZ4471
Pheosia portlandia|[1470]|LBCH103-10|10-JDWBC-0103|658[0n]bp|Canada.British Columbia|BOLD:ABZ4471
Pheosia portlandia|[1471]|LBCH097-10|10-JDWBC-0097|658[0n]bp|Canada.British Columbia|BOLD:ABZ4471
Pheosia portlandia|[1472]|LBCH487-10|10-JDWBC-0487|658[0n]bp|Canada.British Columbia|BOLD:ABZ4471
Pheosia portlandia|[1473]|LBCG2495-09|08-JDWBC-2495|658[0n]bp|Canada.British Columbia|BOLD:ABZ4471
Pheosia portlandia|[1474]|LBCG2496-09|08-JDWBC-2496|658[0n]bp|Canada.British Columbia|BOLD:ABZ4471
Pheosia portlandia|[1475]|LBCH2441-10|10-JDWBC-2441|658[0n]bp|Canada.British Columbia|BOLD:ABZ4471
Pheosia portlandia|[1476]|LBCH4668-10|10-JDWBC-4668|658[0n]bp|Canada.British Columbia|BOLD:ABZ4471
Pheosia portlandia|[1477]|LBCH4406-10|10-JDWBC-4406|658[0n]bp|Canada.British Columbia|BOLD:ABZ4471
Pheosia portlandia|[1478]|LBCH3063-10|10-JDWBC-3063|658[0n]bp|Canada.British Columbia|BOLD:ABZ4471
Pheosia portlandia|[1479]|LBCH4112-10|10-JDWBC-4112|658[0n]bp|Canada.British Columbia|BOLD:ABZ4471
Pheosia portlandia|[1480]|LPMN878-08|08BBLEP-02236|658[0n]bp|Canada.Alberta|BOLD:ABZ4471
Pheosia portlandia|[1481]|LPMN876-08|08BBLEP-02234|658[0n]bp|Canada.Alberta|BOLD:ABZ4471
Pheosia portlandia|[1482]|LPMN905-08|08BBLEP-02263|658[0n]bp|Canada.Alberta|BOLD:ABZ4471
Pheosia portlandia|[1483]|LBCD219-05|HLC-23039|658[0n]bp|Canada.British Columbia|BOLD:ABZ4471
Pheosia portlandia|[1484]|LPABC486-09|08BBLEP-04705|658[0n]bp|Canada.Alberta|BOLD:ABZ4471
Pheosia portlandia|[1485]|LBCB878-05|HLC-21818|641[0n]bp|Canada.British Columbia|BOLD:ABZ4471
Pheosia portlandia|[1486]|LBCA929-05|HLC-20929|621[0n]bp|Canada.British Columbia|BOLD:ABZ4471
Pheosia portlandia|[1487]|LBCA243-05|HLC-20243|634[0n]bp|Canada.British Columbia|BOLD:ABZ4471
Pheosia portlandia|[1488]|LBCC378-05|HLC-22258|622[0n]bp|Canada.British Columbia|BOLD:ABZ4471
Pheosia portlandia|[1489]|LPAB309-08|08BBLEP-02631|658[0n]bp|Canada.Alberta|BOLD:ABZ4471
Pheosia portlandia|[1490]|LBCD051-05|HLC-22871|658[0n]bp|Canada.British Columbia|BOLD:ABZ4471
Pheosia rimosa|[1491]|CHLEP237-09|09PROBE-09532|658[0n]bp|Canada.Manitoba|BOLD:AAA3482
Pheosia rimosa|[1492]|MHCOL477-09|CHU-APH-003.1|658[0n]bp|Canada.Manitoba|BOLD:AAA3482
Pheosia rimosa|[1493]|JGLL033-10|10PROBE-18809|658[0n]bp|Canada.Manitoba|BOLD:AAA3482
Pheosia rimosa|[1494]|LCH206-04|04HBL003206|658[0n]bp|Canada.Manitoba|BOLD:AAA3482
Pheosia rimosa|[1495]|LCH210-04|04HBL003210|658[0n]bp|Canada.Manitoba|BOLD:AAA3482
Pheosia rimosa|[1496]|LCH213-04|04HBL003213|658[0n]bp|Canada.Manitoba|BOLD:AAA3482
Pheosia rimosa|[1497]|MHCOL475-09|CHU-APH-001.1|632[0n]bp|Canada.Manitoba|BOLD:AAA3482
Pheosia rimosa|[1498]|DSSAW062-06|CHU-APH-001|650[0n]bp|Canada.Manitoba|
Pheosia rimosa|[1499]|DSSAW064-06|CHU-APH-003|650[0n]bp|Canada.Manitoba|
Pheosia rimosa|[1500]|MHCOL481-09|CHU-APH-007.1|658[0n]bp|Canada.Manitoba|BOLD:AAA3482
Pheosia rimosa|[1501]|MHCOL480-09|CHU-APH-006.1|658[0n]bp|Canada.Manitoba|BOLD:AAA3482
Pheosia rimosa|[1502]|MHCOL479-09|CHU-APH-005.1|658[0n]bp|Canada.Manitoba|BOLD:AAA3482
Pheosia rimosa|[1503]|LCH207-04|04HBL003207|658[0n]bp|Canada.Manitoba|BOLD:AAA3482
Pheosia rimosa|[1504]|LCH214-04|04HBL003214|658[0n]bp|Canada.Manitoba|BOLD:AAA3482
Pheosia rimosa|[1505]|LCH212-04|04HBL003212|658[0n]bp|Canada.Manitoba|BOLD:AAA3482
Pheosia rimosa|[1506]|MHCOL478-09|CHU-APH-004.1|638[0n]bp|Canada.Manitoba|BOLD:AAA3482
Pheosia rimosa|[1507]|DSSAW065-06|CHU-APH-004|650[0n]bp|Canada.Manitoba|
Pheosia rimosa|[1508]|DSSAW068-06|CHU-APH-007|650[0n]bp|Canada.Manitoba|
Pheosia rimosa|[1509]|DSSAW066-06|CHU-APH-005|650[0n]bp|Canada.Manitoba|
Pheosia rimosa|[1510]|DSSAW067-06|CHU-APH-006|650[0n]bp|Canada.Manitoba|
Pheosia rimosa|[1511]|DSSAW063-06|CHU-APH-002|650[0n]bp|Canada.Manitoba|
Pheosia rimosa|[1512]|PHLCH736-10|10PROBE-26546|658[0n]bp|Canada.Manitoba|BOLD:AAA3482
Pheosia rimosa|[1513]|LCH208-04|04HBL003208|658[0n]bp|Canada.Manitoba|BOLD:AAA3482
Pheosia rimosa|[1514]|MHCOL476-09|CHU-APH-002.1|638[0n]bp|Canada.Manitoba|BOLD:AAA3482
Pheosia rimosa|[1515]|LCHQ114-07|07PROBE-10883|658[0n]bp|Canada.Manitoba|BOLD:AAA3482
Pheosia rimosa|[1516]|CHLEP293-09|09PROBE-09588|658[0n]bp|Canada.Manitoba|BOLD:AAA3482
Pheosia rimosa|[1517]|LCH211-04|04HBL003211|658[0n]bp|Canada.Manitoba|BOLD:AAA3482
Pheosia rimosa|[1518]|LCH209-04|04HBL003209|658[0n]bp|Canada.Manitoba|BOLD:AAA3482
Pheosia rimosa|[1519]|LCH580-04|04HBL003580|658[0n]bp|Canada.Manitoba|BOLD:AAA3482
Pheosia rimosa|[1520]|MNBB583-05|05-NBSTA-499|658[0n]bp|Canada.New Brunswick|BOLD:AAA3482
Pheosia rimosa|[1521]|MNBB659-05|05-NBSTA-575|658[0n]bp|Canada.New Brunswick|BOLD:AAA3482
Pheosia rimosa|[1522]|MNBB229-05|05-NBSTA-145|658[0n]bp|Canada.New Brunswick|BOLD:AAA3482
Pheosia rimosa|[1523]|MNBB581-05|05-NBSTA-497|658[0n]bp|Canada.New Brunswick|BOLD:AAA3482
Pheosia rimosa|[1524]|LPMN243-08|08BBLEP-01042|658[0n]bp|Canada.Manitoba|BOLD:AAA3482
Pheosia rimosa|[1525]|LPSO345-08|PPBP-0345|658[0n]bp|Canada.Ontario|BOLD:AAA3482
Pheosia rimosa|[1526]|MNBB584-05|05-NBSTA-500|658[0n]bp|Canada.New Brunswick|BOLD:AAA3482
Pheosia rimosa|[1527]|XAB273-04|04HBL005273|658[0n]bp|Canada.Ontario|BOLD:AAA3482
Pheosia rimosa|[1528]|BBLEC041-09|09BBELE-0041|658[0n]bp|Canada.New Brunswick|BOLD:AAA3482
Pheosia rimosa|[1529]|XAB017-04|04HBL005017|658[0n]bp|Canada.Ontario|BOLD:AAA3482
Pheosia rimosa|[1530]|TMNBD276-07|MNBTT-3077|655[0n]bp|Canada.New Brunswick|BOLD:AAA3482
Pheosia rimosa|[1531]|TMNBD274-07|MNBTT-3075|658[0n]bp|Canada.New Brunswick|BOLD:AAA3482
Pheosia rimosa|[1532]|PHAUG1786-11|BIOUG01497-D08|658[0n]bp|Canada.Ontario|BOLD:AAA3482
Pheosia rimosa|[1533]|BBLPC028-09|09BBELE-1028|658[0n]bp|Canada.New Brunswick|BOLD:AAA3482
Pheosia rimosa|[1534]|MNBB656-05|05-NBSTA-572|658[0n]bp|Canada.New Brunswick|BOLD:AAA3482
Pheosia rimosa|[1535]|MNBB653-05|05-NBSTA-569|658[0n]bp|Canada.New Brunswick|BOLD:AAA3482
Pheosia rimosa|[1536]|MNBB580-05|05-NBSTA-496|658[0n]bp|Canada.New Brunswick|BOLD:AAA3482
Pheosia rimosa|[1537]|MNBB586-05|05-NBSTA-502|658[0n]bp|Canada.New Brunswick|BOLD:AAA3482
Pheosia rimosa|[1538]|TTMNB245-06|MNBTT-245|658[0n]bp|Canada.New Brunswick|BOLD:AAA3482
Pheosia rimosa|[1539]|MNBB549-05|05-NBSTA-465|658[0n]bp|Canada.New Brunswick|BOLD:AAA3482
Pheosia rimosa|[1540]|MNBB519-05|05-NBSTA-435|658[0n]bp|Canada.New Brunswick|BOLD:AAA3482
Pheosia rimosa|[1541]|MNBB479-05|05-NBSTA-395|658[0n]bp|Canada.New Brunswick|BOLD:AAA3482
Pheosia rimosa|[1542]|TMNBD273-07|MNBTT-3074|657[0n]bp|Canada.New Brunswick|BOLD:AAA3482
Pheosia rimosa|[1543]|MNBB318-05|05-NBSTA-234|658[0n]bp|Canada.New Brunswick|BOLD:AAA3482
Pheosia rimosa|[1544]|MNBB548-05|05-NBSTA-464|632[1n]bp|Canada.New Brunswick|BOLD:AAA3482
Pheosia rimosa|[1545]|XAE629-04|Moth4629.03|612[0n]bp|Canada.Ontario|BOLD:AAA3482
Pheosia rimosa|[1546]|PMG187-03|moth979.01|617[0n]bp|Canada.Ontario|BOLD:AAA3482
Pheosia rimosa|[1547]|MNBB432-05|05-NBSTA-348|658[0n]bp|Canada.New Brunswick|BOLD:AAA3482
Pheosia rimosa|[1548]|XAD686-05|2005-ONT-101|622[0n]bp|Canada.Ontario|BOLD:AAA3482
Pheosia rimosa|[1549]|XAD687-05|2005-ONT-102|634[0n]bp|Canada.Ontario|BOLD:AAA3482
Pheosia rimosa|[1550]|PHMNB183-04|04HBL007648|541[0n]bp|Canada.New Brunswick|BOLD:AAA3482
Pheosia rimosa|[1551]|XAB015-04|04HBL005015|658[0n]bp|Canada.Ontario|BOLD:AAA3482
Pheosia rimosa|[1552]|MNBB289-05|05-NBSTA-205|658[0n]bp|Canada.New Brunswick|BOLD:AAA3482
Pheosia rimosa|[1553]|MNBB547-05|05-NBSTA-463|658[0n]bp|Canada.New Brunswick|BOLD:ABZ4474
Pheosia rimosa|[1554]|MNBB161-05|05-NBSTA-077|658[0n]bp|Canada.New Brunswick|BOLD:ABZ4474
Pheosia rimosa|[1555]|TMNBD275-07|MNBTT-3076|658[0n]bp|Canada.New Brunswick|BOLD:ABZ4474

[illegible]
Pheosia rimosa|[1553]|MNBB547-05|05-NBSTA-463|658[0n]bp|Canada.New Brunswick|BOLD:ABZ4474
Pheosia rimosa|[1554]|MNBB161-05|05-NBSTA-077|658[0n]bp|Canada.New Brunswick|BOLD:ABZ4474
Pheosia rimosa|[1555]|TMNBD275-07|MNBTT-3076|658[0n]bp|Canada.New Brunswick|BOLD:ABZ4474
Pheosia rimosa|[1556]|MNBB657-05|05-NBSTA-573|658[0n]bp|Canada.New Brunswick|BOLD:ABZ4474
Pheosia rimosa|[1557]|MNBB658-05|05-NBSTA-574|658[0n]bp|Canada.New Brunswick|BOLD:ABZ4474
Pheosia rimosa|[1558]|MNBB654-05|05-NBSTA-570|658[0n]bp|Canada.New Brunswick|BOLD:ABZ4474
Pheosia rimosa|[1559]|MNBB582-05|05-NBSTA-498|658[0n]bp|Canada.New Brunswick|BOLD:ABZ4474
Pheosia rimosa|[1560]|MNBB545-05|05-NBSTA-461|658[0n]bp|Canada.New Brunswick|BOLD:ABZ4474
Pheosia rimosa|[1561]|MNBB520-05|05-NBSTA-436|658[0n]bp|Canada.New Brunswick|BOLD:ABZ4474
Pheosia rimosa|[1562]|MNBB370-05|05-NBSTA-286|658[0n]bp|Canada.New Brunswick|BOLD:ABZ4474
Pheosia rimosa|[1563]|MNBB372-05|05-NBSTA-288|658[0n]bp|Canada.New Brunswick|BOLD:ABZ4474
Pheosia rimosa|[1564]|MNBB319-05|05-NBSTA-235|658[0n]bp|Canada.New Brunswick|BOLD:ABZ4474
Pheosia rimosa|[1565]|MNBB232-05|05-NBSTA-148|607[0n]bp|Canada.New Brunswick|BOLD:ABZ4474
Pheosia rimosa|[1566]|MNBB230-05|05-NBSTA-146|658[0n]bp|Canada.New Brunswick|BOLD:ABZ5199
Pheosia rimosa|[1567]|MNBB585-05|05-NBSTA-501|658[0n]bp|Canada.New Brunswick|BOLD:ABZ5199
Pheosia rimosa|[1568]|MNBB546-05|05-NBSTA-462|658[0n]bp|Canada.New Brunswick|BOLD:ABZ5199
Pheosia rimosa|[1569]|MNBB373-05|05-NBSTA-289|658[0n]bp|Canada.New Brunswick|BOLD:ABZ5199
Pheosia rimosa|[1570]|MNBB655-05|05-NBSTA-571|658[0n]bp|Canada.New Brunswick|BOLD:ABZ5199
Pheosia rimosa|[1571]|MNBB317-05|05-NBSTA-233|658[0n]bp|Canada.New Brunswick|BOLD:ABZ5199
Pheosia rimosa|[1572]|TMNBD272-07|MNBTT-3073|631[0n]bp|Canada.New Brunswick|BOLD:ABZ5199
Pheosia rimosa|[1573]|XAB039-04|04HBL005039|574[0n]bp|Canada.Ontario|BOLD:ABZ5199
Pheosia rimosa|[1574]|XAD642-05|2005-ONT-57|658[0n]bp|Canada.Ontario|BOLD:ABZ5199
Pheosia rimosa|[1575]|LPSO486-08|PPBP-0486|658[0n]bp|Canada.Ontario|BOLD:ABZ5199
Pheosia rimosa|[1576]|TMG61-03|moth222.01|639[0n]bp|Canada.Ontario|BOLD:ABZ5199
Pheosia rimosa|[1577]|RDLQ777-07|DH007410|646[0n]bp|Canada.Quebec|BOLD:ABZ5199
Pheosia rimosa|[1578]|XAB549-04|04HBL005549|658[0n]bp|Canada.Ontario|BOLD:ABZ5199
Pheosia rimosa|[1579]|LPSO008-08|PPBP-0008|658[0n]bp|Canada.Ontario|BOLD:ABZ5199
Pheosia rimosa|[1580]|LPSO229-08|PPBP-0229|658[0n]bp|Canada.Ontario|BOLD:ABZ5199
Pheosia rimosa|[1581]|LPSO873-08|PPBP-0873|658[0n]bp|Canada.Ontario|BOLD:ABZ5199
Pheosia rimosa|[1582]|LPSO007-08|PPBP-0007|658[0n]bp|Canada.Ontario|BOLD:ABZ5199
Peridea angulosa|[1583]|LPSO934-08|PPBP-0934|658[0n]bp|Canada.Ontario|BOLD:AAB2020
Peridea angulosa|[1584]|LPSOB096-08|PPBP-1095|658[0n]bp|Canada.Ontario|BOLD:AAB2020
Peridea angulosa|[1585]|XAJ975-06|2006-ONT-0975|658[0n]bp|Canada.Ontario|BOLD:AAB2020
Peridea angulosa|[1586]|MNBB379-05|05-NBSTA-295|658[0n]bp|Canada.New Brunswick|BOLD:AAB2020
Peridea angulosa|[1587]|MNBB552-05|05-NBSTA-468|658[0n]bp|Canada.New Brunswick|BOLD:AAB2020
Peridea angulosa|[1588]|RDLQB678-05|DH010781|544[1n]bp|Canada.Quebec|BOLD:AAB2020
Peridea angulosa|[1589]|XAK524-07|HLC-16077|658[0n]bp|Canada.Ontario|BOLD:AAB2020
Peridea basitriens|[1590]|XAE617-04|Moth4617.03|616[0n]bp|Canada.Ontario|BOLD:ABZ5648
Peridea basitriens|[1591]|XAE415-04|Moth4415.03|658[0n]bp|Canada.Ontario|BOLD:ABZ5648
Peridea basitriens|[1592]|BBLEC612-09|09BBELE-0612|658[0n]bp|Canada.Nova Scotia|BOLD:ABZ5648
Peridea basitriens|[1593]|XAG102-05|2005-ONT-686|658[0n]bp|Canada.Ontario|BOLD:ABZ5648
Peridea basitriens|[1594]|XAK525-07|HLC-16078|658[0n]bp|Canada.Ontario|BOLD:ABZ5648
Peridea basitriens|[1595]|LPSOC319-08|PPBP-2318|658[0n]bp|Canada.Ontario|BOLD:ABZ5648
Peridea basitriens|[1596]|TMNBD204-07|MNBTT-3005|658[0n]bp|Canada.New Brunswick|BOLD:ABZ5648
Peridea basitriens|[1597]|RDLQG442-06|DH012721|657[0n]bp|Canada.Quebec|BOLD:ABZ5648
Peridea basitriens|[1598]|TMNBD205-07|MNBTT-3006|641[0n]bp|Canada.New Brunswick|BOLD:ABZ5648
Peridea basitriens|[1599]|TMG60-03|PERI1.00|639[0n]bp|Canada.Ontario|BOLD:ABZ5648
Peridea basitriens|[1600]|TMNBD206-07|MNBTT-3007|595[0n]bp|Canada.New Brunswick|BOLD:ABZ5648
Peridea basitriens|[1601]|PMG186-03|moth1110.01|617[0n]bp|Canada.Ontario|BOLD:ABZ5648
Peridea basitriens|[1602]|XAJ493-06|2006-ONT-0493|647[0n]bp|Canada.Ontario|BOLD:ABZ5648
Peridea ferruginea|[1603]|BBLPE152-09|09BBELE-2152|636[0n]bp|Canada.Nova Scotia|BOLD:AAA7026
Peridea ferruginea|[1604]|BBLPE049-09|09BBELE-2049|658[0n]bp|Canada.Nova Scotia|BOLD:AAA7026
Peridea ferruginea|[1605]|BBLPE180-09|09BBELE-2180|658[0n]bp|Canada.Nova Scotia|BOLD:AAA7026
Peridea ferruginea|[1606]|BBLPE012-09|09BBELE-2012|658[0n]bp|Canada.Nova Scotia|BOLD:AAA7026
Peridea ferruginea|[1607]|BBLEC562-09|09BBELE-0562|639[0n]bp|Canada.Nova Scotia|BOLD:AAA7026
Peridea ferruginea|[1608]|BBLPC993-09|09BBELE-1993|636[0n]bp|Canada.Nova Scotia|
Peridea ferruginea|[1609]|BBLEC900-09|09BBELE-0900|658[0n]bp|Canada.Nova Scotia|BOLD:AAA7026
Peridea ferruginea|[1610]|MNBB237-05|05-NBSTA-153|658[0n]bp|Canada.New Brunswick|BOLD:AAA7026
Peridea ferruginea|[1611]|MNBB286-05|05-NBSTA-202|658[0n]bp|Canada.New Brunswick|BOLD:AAA7026
Peridea ferruginea|[1612]|BBLEC098-09|09BBELE-0098|658[0n]bp|Canada.Nova Scotia|BOLD:AAA7026
Peridea ferruginea|[1613]|BBLPE063-09|09BBELE-2063|658[0n]bp|Canada.Nova Scotia|BOLD:AAA7026
Peridea ferruginea|[1614]|BBLPE011-09|09BBELE-2011|658[0n]bp|Canada.Nova Scotia|BOLD:AAA7026
Peridea ferruginea|[1615]|BBLPE019-09|09BBELE-2019|658[0n]bp|Canada.Nova Scotia|BOLD:AAA7026
Peridea ferruginea|[1616]|BBLPE297-09|09BBELE-2297|658[0n]bp|Canada.Nova Scotia|BOLD:AAA7026
Peridea ferruginea|[1617]|TTMNB017-06|MNBTT-017|658[0n]bp|Canada.New Brunswick|BOLD:AAA7026
Peridea ferruginea|[1618]|TTMNB014-06|MNBTT-014|658[0n]bp|Canada.New Brunswick|BOLD:AAA7026
Peridea ferruginea|[1619]|TTMNB018-06|MNBTT-018|658[0n]bp|Canada.New Brunswick|BOLD:AAA7026
Peridea ferruginea|[1620]|TTMNB244-06|MNBTT-244|658[0n]bp|Canada.New Brunswick|BOLD:AAA7026
Peridea ferruginea|[1621]|TMNBD269-07|MNBTT-3070|656[0n]bp|Canada.New Brunswick|BOLD:AAA7026
Peridea ferruginea|[1622]|TMNBD268-07|MNBTT-3069|648[0n]bp|Canada.New Brunswick|BOLD:AAA7026
Peridea ferruginea|[1623]|TMNBD267-07|MNBTT-3068|658[0n]bp|Canada.New Brunswick|BOLD:AAA7026
Peridea ferruginea|[1624]|MNBB440-05|05-NBSTA-356|658[0n]bp|Canada.New Brunswick|BOLD:AAA7026
Peridea ferruginea|[1625]|MNBB441-05|05-NBSTA-357|658[0n]bp|Canada.New Brunswick|BOLD:AAA7026
Peridea ferruginea|[1626]|MNBB527-05|05-NBSTA-443|658[0n]bp|Canada.New Brunswick|BOLD:AAA7026
Peridea ferruginea|[1627]|MNBB551-05|05-NBSTA-467|658[0n]bp|Canada.New Brunswick|BOLD:AAA7026
Peridea ferruginea|[1628]|TMNBD270-07|MNBTT-3071|658[0n]bp|Canada.New Brunswick|BOLD:AAA7026
Peridea ferruginea|[1629]|TMNBD271-07|MNBTT-3072|658[0n]bp|Canada.New Brunswick|BOLD:AAA7026
Peridea ferruginea|[1630]|PHMNB734-05|Moth 427.03SA|658[0n]bp|Canada.New Brunswick|BOLD:AAA7026
Peridea ferruginea|[1631]|PHMNB733-05|Moth 426.03SA|658[0n]bp|Canada.New Brunswick|BOLD:AAA7026
Peridea ferruginea|[1632]|PHMNB095-04|04HBL007560|658[0n]bp|Canada.New Brunswick|BOLD:AAA7026
Peridea ferruginea|[1633]|PHMNB743-05|Moth 436.03SA|658[0n]bp|Canada.New Brunswick|BOLD:AAA7026
Peridea ferruginea|[1634]|BBLEC279-09|09BBELE-0279|640[0n]bp|Canada.Nova Scotia|BOLD:AAA7026
Peridea ferruginea|[1635]|XAE463-04|Moth4463.03|658[0n]bp|Canada.Ontario|BOLD:AAA7026
Peridea ferruginea|[1636]|BBLEC937-09|09BBELE-0937|588[0n]bp|Canada.Nova Scotia|BOLD:AAA7026
Peridea ferruginea|[1637]|RDLQF829-06|DH011982|612[0n]bp|Canada.Quebec|BOLD:AAA7026
Peridea ferruginea|[1638]|XAE442-04|Moth4442.03|591[0n]bp|Canada.Ontario|BOLD:AAA7026
Peridea ferruginea|[1639]|BBLPE295-09|09BBELE-2295|650[0n]bp|Canada.Nova Scotia|BOLD:AAA7026
Peridea ferruginea|[1640]|LPSOB220-08|PPBP-1219|658[0n]bp|Canada.Ontario|BOLD:AAA7026
Peridea ferruginea|[1641]|XAG872-05|2005-ONT-1456|658[0n]bp|Canada.Ontario|BOLD:AAA7026
Peridea ferruginea|[1642]|XAE368-04|Moth4368.03|658[0n]bp|Canada.Ontario|BOLD:AAA7026
Peridea ferruginea|[1643]|XAE457-04|Moth4457.03|658[0n]bp|Canada.Ontario|BOLD:AAA7026
Peridea ferruginea|[1644]|XAE447-04|Moth4447.03|658[0n]bp|Canada.Ontario|BOLD:AAA7026
Peridea ferruginea|[1645]|XAJ976-06|2006-ONT-0976|658[0n]bp|Canada.Ontario|BOLD:AAA7026
Peridea ferruginea|[1646]|XAJ577-06|2006-ONT-0577|658[0n]bp|Canada.Ontario|BOLD:AAA7026
Peridea ferruginea|[1647]|XAJ576-06|2006-ONT-0576|658[0n]bp|Canada.Ontario|BOLD:AAA7026
Peridea ferruginea|[1648]|LPSOD365-09|08BBLEP-00143|658[0n]bp|Canada.Ontario|BOLD:AAA7026
Peridea ferruginea|[1649]|MNBB238-05|05-NBSTA-154|658[0n]bp|Canada.New Brunswick|BOLD:AAA7026
Peridea ferruginea|[1650]|MNBB284-05|05-NBSTA-200|658[0n]bp|Canada.New Brunswick|BOLD:AAA7026
