# Supplementary material for: A Transcontinental Challenge — A Test of DNA Barcode Performance for 1,541 Species of Canadian Noctuoidea (Lepidoptera)
Source: PLoS One. 2014 Mar 25;9(3):e92797. doi: 10.1371/journal.pone.0092797 (PMC3965468; doi:10.1371/journal.pone.0092797)
Supplement: Tree S2 — NJ tree for Canadian species in the family Euteliidae. (PDF) [file pone.0092797.s006.pdf]

BOLD TaxonID Tree

Title	:	SEARCH: Sample ids(90 ids) [SEARCH4]
Date	:	17-October-2013
Data Type	:	Nucleotide
Distance Model	:	Kimura 2 Parameter
Marker	:	COI-5P
Codon Positions	:	
Labels	:	Country & Province, SampleID, ProcessID, Sequence Length, BIN URI
Filters	:	Length > 200
Colorization	:	[blue]=Stop Codons [red]=Contamination or misidentification
Attachment	:	Photographs & Spreadsheet

Sequence Count	:	90
Species count	:	8
Genus count	:	3
Family count	:	1
Unidentified	:	0

BIN Count	:	8

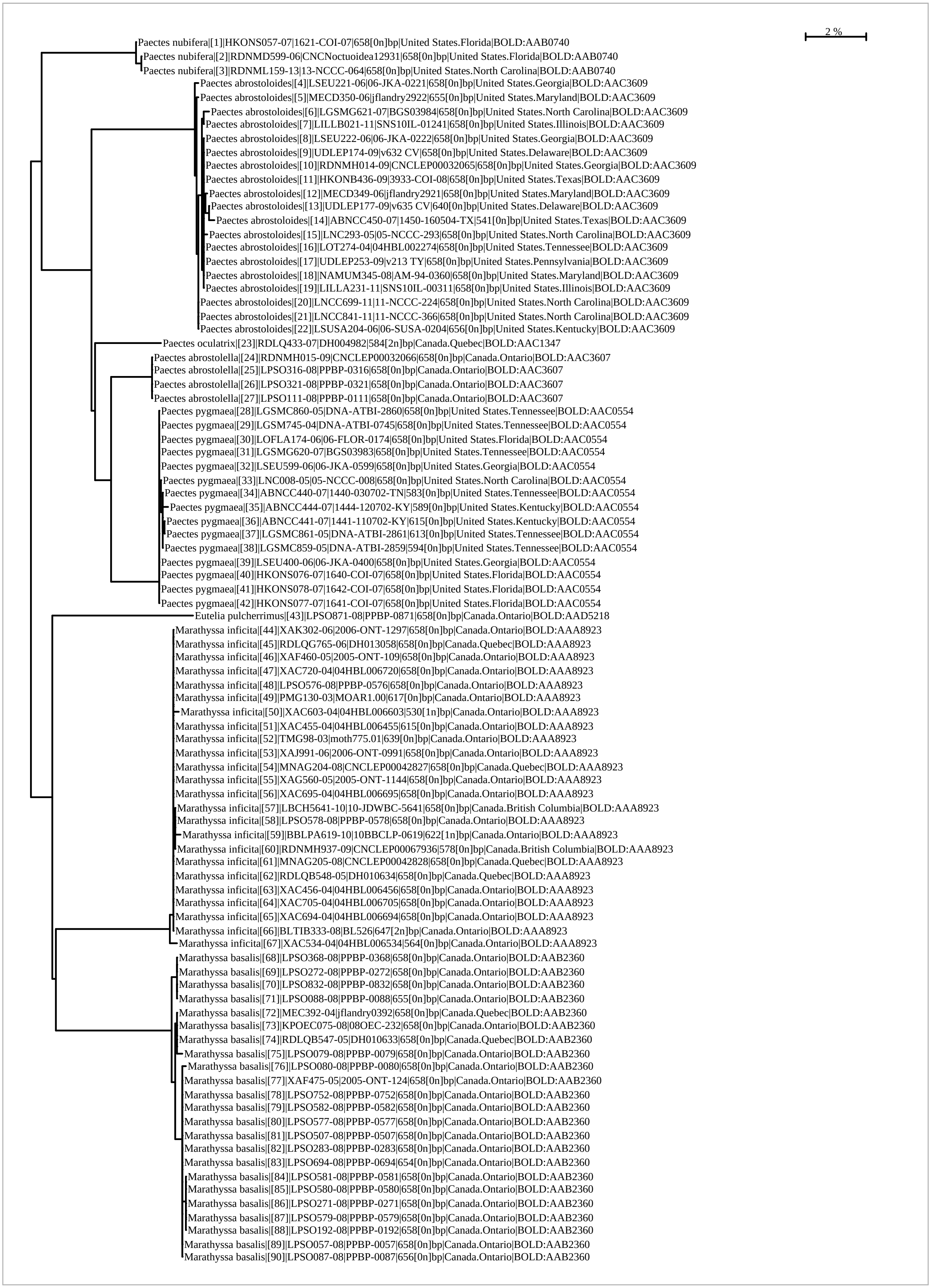
2 %
Paectes nubifera|[1]|HKONS057-07|1621-COI-07|658[0n]bp|United States.Florida|BOLD:AAB0740
Paectes nubifera|[2]|RDNMD599-06|CNCNoctuoidea12931|658[0n]bp|United States.Florida|BOLD:AAB0740
Paectes nubifera|[3]|RDNML159-13|13-NCCC-064|658[0n]bp|United States.North Carolina|BOLD:AAB0740
Paectes abrostoloides|[4]|LSEU221-06|06-JKA-0221|658[0n]bp|United States.Georgia|BOLD:AAC3609
Paectes abrostoloides|[5]|MECD350-06|jflandry2922|655[0n]bp|United States.Maryland|BOLD:AAC3609
Paectes abrostoloides|[6]|LGSMG621-07|BGS03984|658[0n]bp|United States.North Carolina|BOLD:AAC3609
Paectes abrostoloides|[7]|LILLB021-11|SNS10IL-01241|658[0n]bp|United States.Illinois|BOLD:AAC3609
Paectes abrostoloides|[8]|LSEU222-06|06-JKA-0222|658[0n]bp|United States.Georgia|BOLD:AAC3609
Paectes abrostoloides|[9]|UDLEP174-09|v632 CV|658[0n]bp|United States.Delaware|BOLD:AAC3609
Paectes abrostoloides|[10]|RDNMH014-09|CNCLEP00032065|658[0n]bp|United States.Georgia|BOLD:AAC3609
Paectes abrostoloides|[11]|HKONB436-09|3933-COI-08|658[0n]bp|United States.Texas|BOLD:AAC3609
Paectes abrostoloides|[12]|MECD349-06|jflandry2921|658[0n]bp|United States.Maryland|BOLD:AAC3609
Paectes abrostoloides|[13]|UDLEP177-09|v635 CV|640[0n]bp|United States.Delaware|BOLD:AAC3609
Paectes abrostoloides|[14]|ABNCC450-07|1450-160504-TX|541[0n]bp|United States.Texas|BOLD:AAC3609
Paectes abrostoloides|[15]|LNC293-05|05-NCCC-293|658[0n]bp|United States.North Carolina|BOLD:AAC3609
Paectes abrostoloides|[16]|LOT274-04|04HBL002274|658[0n]bp|United States.Tennessee|BOLD:AAC3609
Paectes abrostoloides|[17]|UDLEP253-09|v213 TY|658[0n]bp|United States.Pennsylvania|BOLD:AAC3609
Paectes abrostoloides|[18]|NAMUM345-08|AM-94-0360|658[0n]bp|United States.Maryland|BOLD:AAC3609
Paectes abrostoloides|[19]|LILLA231-11|SNS10IL-00311|658[0n]bp|United States.Illinois|BOLD:AAC3609
Paectes abrostoloides|[20]|LNCC699-11|11-NCCC-224|658[0n]bp|United States.North Carolina|BOLD:AAC3609
Paectes abrostoloides|[21]|LNCC841-11|11-NCCC-366|658[0n]bp|United States.North Carolina|BOLD:AAC3609
Paectes abrostoloides|[22]|LSUSA204-06|06-SUSA-0204|656[0n]bp|United States.Kentucky|BOLD:AAC3609
Paectes oculatrix|[23]|RDLQ433-07|DH004982|584[2n]bp|Canada.Quebec|BOLD:AAC1347
Paectes abrostolella|[24]|RDNMH015-09|CNCLEP00032066|658[0n]bp|Canada.Ontario|BOLD:AAC3607
Paectes abrostolella|[25]|LPSO316-08|PPBP-0316|658[0n]bp|Canada.Ontario|BOLD:AAC3607
Paectes abrostolella|[26]|LPSO321-08|PPBP-0321|658[0n]bp|Canada.Ontario|BOLD:AAC3607
Paectes abrostolella|[27]|LPSO111-08|PPBP-0111|658[0n]bp|Canada.Ontario|BOLD:AAC3607
Paectes pygmaea|[28]|LGSMC860-05|DNA-ATBI-2860|658[0n]bp|United States.Tennessee|BOLD:AAC0554
Paectes pygmaea|[29]|LGSM745-04|DNA-ATBI-0745|658[0n]bp|United States.Tennessee|BOLD:AAC0554
Paectes pygmaea|[30]|LOFLA174-06|06-FLOR-0174|658[0n]bp|United States.Florida|BOLD:AAC0554
Paectes pygmaea|[31]|LGSMG620-07|BGS03983|658[0n]bp|United States.Tennessee|BOLD:AAC0554
Paectes pygmaea|[32]|LSEU599-06|06-JKA-0599|658[0n]bp|United States.Georgia|BOLD:AAC0554
Paectes pygmaea|[33]|LNC008-05|05-NCCC-008|658[0n]bp|United States.North Carolina|BOLD:AAC0554
Paectes pygmaea|[34]|ABNCC440-07|1440-030702-TN|583[0n]bp|United States.Tennessee|BOLD:AAC0554
Paectes pygmaea|[35]|ABNCC444-07|1444-120702-KY|589[0n]bp|United States.Kentucky|BOLD:AAC0554
Paectes pygmaea|[36]|ABNCC441-07|1441-110702-KY|615[0n]bp|United States.Kentucky|BOLD:AAC0554
Paectes pygmaea|[37]|LGSMC861-05|DNA-ATBI-2861|613[0n]bp|United States.Tennessee|BOLD:AAC0554
Paectes pygmaea|[38]|LGSMC859-05|DNA-ATBI-2859|594[0n]bp|United States.Tennessee|BOLD:AAC0554
Paectes pygmaea|[39]|LSEU400-06|06-JKA-0400|658[0n]bp|United States.Georgia|BOLD:AAC0554
Paectes pygmaea|[40]|HKONS076-07|1640-COI-07|658[0n]bp|United States.Florida|BOLD:AAC0554
Paectes pygmaea|[41]|HKONS078-07|1642-COI-07|658[0n]bp|United States.Florida|BOLD:AAC0554
Paectes pygmaea|[42]|HKONS077-07|1641-COI-07|658[0n]bp|United States.Florida|BOLD:AAC0554
Eutelia pulcherrimus|[43]|LPSO871-08|PPBP-0871|658[0n]bp|Canada.Ontario|BOLD:AAD5218
Marathyssa inficita|[44]|XAK302-06|2006-ONT-1297|658[0n]bp|Canada.Ontario|BOLD:AAA8923
Marathyssa inficita|[45]|RDLQG765-06|DH013058|658[0n]bp|Canada.Quebec|BOLD:AAA8923
Marathyssa inficita|[46]|XAF460-05|2005-ONT-109|658[0n]bp|Canada.Ontario|BOLD:AAA8923
Marathyssa inficita|[47]|XAC720-04|04HBL006720|658[0n]bp|Canada.Ontario|BOLD:AAA8923
Marathyssa inficita|[48]|LPSO576-08|PPBP-0576|658[0n]bp|Canada.Ontario|BOLD:AAA8923
Marathyssa inficita|[49]|PMG130-03|MOAR1.00|617[0n]bp|Canada.Ontario|BOLD:AAA8923
Marathyssa inficita|[50]|XAC603-04|04HBL006603|530[1n]bp|Canada.Ontario|BOLD:AAA8923
Marathyssa inficita|[51]|XAC455-04|04HBL006455|615[0n]bp|Canada.Ontario|BOLD:AAA8923
Marathyssa inficita|[52]|TMG98-03|moth775.01|639[0n]bp|Canada.Ontario|BOLD:AAA8923
Marathyssa inficita|[53]|XAJ991-06|2006-ONT-0991|658[0n]bp|Canada.Ontario|BOLD:AAA8923
Marathyssa inficita|[54]|MNAG204-08|CNCLEP00042827|658[0n]bp|Canada.Quebec|BOLD:AAA8923
Marathyssa inficita|[55]|XAG560-05|2005-ONT-1144|658[0n]bp|Canada.Ontario|BOLD:AAA8923
Marathyssa inficita|[56]|XAC695-04|04HBL006695|658[0n]bp|Canada.Ontario|BOLD:AAA8923
Marathyssa inficita|[57]|LBCH5641-10|10-JDWBC-5641|658[0n]bp|Canada.British Columbia|BOLD:AAA8923
Marathyssa inficita|[58]|LPSO578-08|PPBP-0578|658[0n]bp|Canada.Ontario|BOLD:AAA8923
Marathyssa inficita|[59]|BBLPA619-10|10BBCLP-0619|622[1n]bp|Canada.Ontario|BOLD:AAA8923
Marathyssa inficita|[60]|RDNMH937-09|CNCLEP00067936|578[0n]bp|Canada.British Columbia|BOLD:AAA8923
Marathyssa inficita|[61]|MNAG205-08|CNCLEP00042828|658[0n]bp|Canada.Quebec|BOLD:AAA8923
Marathyssa inficita|[62]|RDLQB548-05|DH010634|658[0n]bp|Canada.Quebec|BOLD:AAA8923
Marathyssa inficita|[63]|XAC456-04|04HBL006456|658[0n]bp|Canada.Ontario|BOLD:AAA8923
Marathyssa inficita|[64]|XAC705-04|04HBL006705|658[0n]bp|Canada.Ontario|BOLD:AAA8923
Marathyssa inficita|[65]|XAC694-04|04HBL006694|658[0n]bp|Canada.Ontario|BOLD:AAA8923
Marathyssa inficita|[66]|BLTIB333-08|BL526|647[2n]bp|Canada.Ontario|BOLD:AAA8923
Marathyssa inficita|[67]|XAC534-04|04HBL006534|564[0n]bp|Canada.Ontario|BOLD:AAA8923
Marathyssa basalis|[68]|LPSO368-08|PPBP-0368|658[0n]bp|Canada.Ontario|BOLD:AAB2360
Marathyssa basalis|[69]|LPSO272-08|PPBP-0272|658[0n]bp|Canada.Ontario|BOLD:AAB2360
Marathyssa basalis|[70]|LPSO832-08|PPBP-0832|658[0n]bp|Canada.Ontario|BOLD:AAB2360
Marathyssa basalis|[71]|LPSO088-08|PPBP-0088|655[0n]bp|Canada.Ontario|BOLD:AAB2360
Marathyssa basalis|[72]|MEC392-04|jflandry0392|658[0n]bp|Canada.Quebec|BOLD:AAB2360
Marathyssa basalis|[73]|KPOEC075-08|08OEC-232|658[0n]bp|Canada.Ontario|BOLD:AAB2360
Marathyssa basalis|[74]|RDLQB547-05|DH010633|658[0n]bp|Canada.Quebec|BOLD:AAB2360
Marathyssa basalis|[75]|LPSO079-08|PPBP-0079|658[0n]bp|Canada.Ontario|BOLD:AAB2360
Marathyssa basalis|[76]|LPSO080-08|PPBP-0080|658[0n]bp|Canada.Ontario|BOLD:AAB2360
Marathyssa basalis|[77]|XAF475-05|2005-ONT-124|658[0n]bp|Canada.Ontario|BOLD:AAB2360
Marathyssa basalis|[78]|LPSO752-08|PPBP-0752|658[0n]bp|Canada.Ontario|BOLD:AAB2360
Marathyssa basalis|[79]|LPSO582-08|PPBP-0582|658[0n]bp|Canada.Ontario|BOLD:AAB2360
Marathyssa basalis|[80]|LPSO577-08|PPBP-0577|658[0n]bp|Canada.Ontario|BOLD:AAB2360
Marathyssa basalis|[81]|LPSO507-08|PPBP-0507|658[0n]bp|Canada.Ontario|BOLD:AAB2360
Marathyssa basalis|[82]|LPSO283-08|PPBP-0283|658[0n]bp|Canada.Ontario|BOLD:AAB2360
Marathyssa basalis|[83]|LPSO694-08|PPBP-0694|654[0n]bp|Canada.Ontario|BOLD:AAB2360
Marathyssa basalis|[84]|LPSO581-08|PPBP-0581|658[0n]bp|Canada.Ontario|BOLD:AAB2360
Marathyssa basalis|[85]|LPSO580-08|PPBP-0580|658[0n]bp|Canada.Ontario|BOLD:AAB2360
Marathyssa basalis|[86]|LPSO271-08|PPBP-0271|658[0n]bp|Canada.Ontario|BOLD:AAB2360
Marathyssa basalis|[87]|LPSO579-08|PPBP-0579|658[0n]bp|Canada.Ontario|BOLD:AAB2360
Marathyssa basalis|[88]|LPSO192-08|PPBP-0192|658[0n]bp|Canada.Ontario|BOLD:AAB2360
Marathyssa basalis|[89]|LPSO057-08|PPBP-0057|658[0n]bp|Canada.Ontario|BOLD:AAB2360
Marathyssa basalis|[90]|LPSO087-08|PPBP-0087|656[0n]bp|Canada.Ontario|BOLD:AAB2360
