# Supplementary material for: A Transcontinental Challenge — A Test of DNA Barcode Performance for 1,541 Species of Canadian Noctuoidea (Lepidoptera)
Source: PLoS One. 2014 Mar 25;9(3):e92797. doi: 10.1371/journal.pone.0092797 (PMC3965468; doi:10.1371/journal.pone.0092797)
Supplement: Tree S3 — NJ tree for Canadian species in the family Nolidae. (PDF) [file pone.0092797.s007.pdf]

BOLD TaxonID Tree

Title	:	SEARCH: Sample ids(220 ids) [SEARCH3]
Date	:	17-October-2013
Data Type	:	Nucleotide
Distance Model	:	Kimura 2 Parameter
Marker	:	COI-5P
Codon Positions	:	
Labels	:	Country & Province, SampleID, ProcessID, Sequence Length, BIN URI
Filters	:	Length > 200
Colorization	:	[blue]=Stop Codons [red]=Contamination or misidentification
Attachment	:	Photographs & Spreadsheet

Sequence Count	:	220
Species count	:	19
Genus count	:	5
Family count	:	1
Unidentified	:	0

BIN Count	:	20

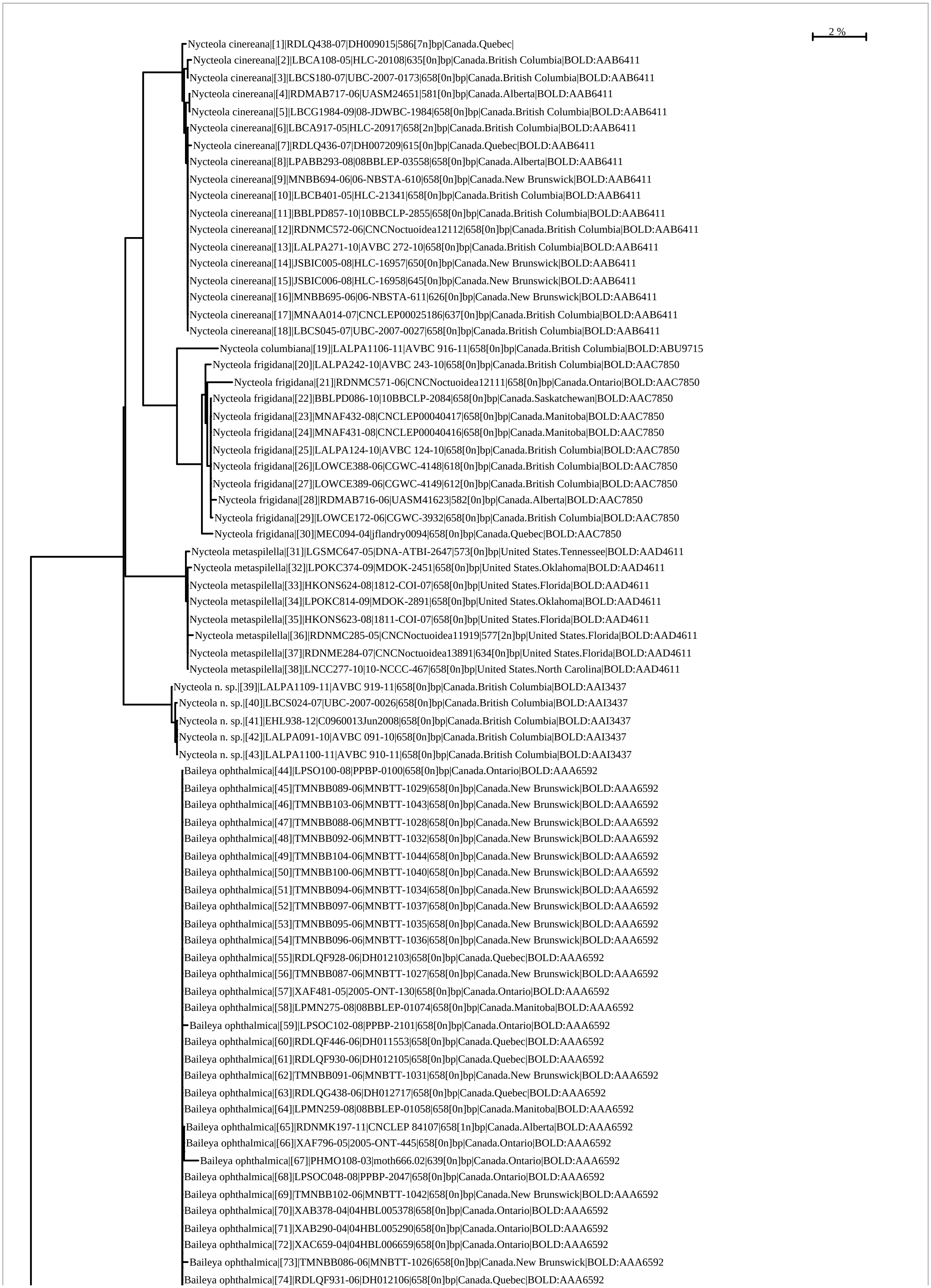
2 %
Nycteola cinereana|[1]|RDLQ438-07|DH009015|586[7n]bp|Canada.Quebec|
Nycteola cinereana|[2]|LBCA108-05|HLC-20108|635[0n]bp|Canada.British Columbia|BOLD:AAB6411
Nycteola cinereana|[3]|LBCS180-07|UBC-2007-0173|658[0n]bp|Canada.British Columbia|BOLD:AAB6411
Nycteola cinereana|[4]|RDMAB717-06|UASM24651|581[0n]bp|Canada.Alberta|BOLD:AAB6411
Nycteola cinereana|[5]|LBCG1984-09|08-JDWBC-1984|658[0n]bp|Canada.British Columbia|BOLD:AAB6411
Nycteola cinereana|[6]|LBCA917-05|HLC-20917|658[2n]bp|Canada.British Columbia|BOLD:AAB6411
Nycteola cinereana|[7]|RDLQ436-07|DH007209|615[0n]bp|Canada.Quebec|BOLD:AAB6411
Nycteola cinereana|[8]|LPABB293-08|08BBLEP-03558|658[0n]bp|Canada.Alberta|BOLD:AAB6411
Nycteola cinereana|[9]|MNBB694-06|06-NBSTA-610|658[0n]bp|Canada.New Brunswick|BOLD:AAB6411
Nycteola cinereana|[10]|LBCB401-05|HLC-21341|658[0n]bp|Canada.British Columbia|BOLD:AAB6411
Nycteola cinereana|[11]|BBLPD857-10|10BBCLP-2855|658[0n]bp|Canada.British Columbia|BOLD:AAB6411
Nycteola cinereana|[12]|RDNMC572-06|CNCNoctuoidea12112|658[0n]bp|Canada.British Columbia|BOLD:AAB6411
Nycteola cinereana|[13]|LALPA271-10|AVBC 272-10|658[0n]bp|Canada.British Columbia|BOLD:AAB6411
Nycteola cinereana|[14]|JSBIC005-08|HLC-16957|650[0n]bp|Canada.New Brunswick|BOLD:AAB6411
Nycteola cinereana|[15]|JSBIC006-08|HLC-16958|645[0n]bp|Canada.New Brunswick|BOLD:AAB6411
Nycteola cinereana|[16]|MNBB695-06|06-NBSTA-611|626[0n]bp|Canada.New Brunswick|BOLD:AAB6411
Nycteola cinereana|[17]|MNAA014-07|CNCLEP00025186|637[0n]bp|Canada.British Columbia|BOLD:AAB6411
Nycteola cinereana|[18]|LBCS045-07|UBC-2007-0027|658[0n]bp|Canada.British Columbia|BOLD:AAB6411
Nycteola columbiana|[19]|LALPA1106-11|AVBC 916-11|658[0n]bp|Canada.British Columbia|BOLD:ABU9715
Nycteola frigidana|[20]|LALPA242-10|AVBC 243-10|658[0n]bp|Canada.British Columbia|BOLD:AAC7850
Nycteola frigidana|[21]|RDNMC571-06|CNCNoctuoidea12111|658[0n]bp|Canada.Ontario|BOLD:AAC7850
Nycteola frigidana|[22]|BBLPD086-10|10BBCLP-2084|658[0n]bp|Canada.Saskatchewan|BOLD:AAC7850
Nycteola frigidana|[23]|MNAF432-08|CNCLEP00040417|658[0n]bp|Canada.Manitoba|BOLD:AAC7850
Nycteola frigidana|[24]|MNAF431-08|CNCLEP00040416|658[0n]bp|Canada.Manitoba|BOLD:AAC7850
Nycteola frigidana|[25]|LALPA124-10|AVBC 124-10|658[0n]bp|Canada.British Columbia|BOLD:AAC7850
Nycteola frigidana|[26]|LOWCE388-06|CGWC-4148|618[0n]bp|Canada.British Columbia|BOLD:AAC7850
Nycteola frigidana|[27]|LOWCE389-06|CGWC-4149|612[0n]bp|Canada.British Columbia|BOLD:AAC7850
Nycteola frigidana|[28]|RDMAB716-06|UASM41623|582[0n]bp|Canada.Alberta|BOLD:AAC7850
Nycteola frigidana|[29]|LOWCE172-06|CGWC-3932|658[0n]bp|Canada.British Columbia|BOLD:AAC7850
Nycteola frigidana|[30]|MEC094-04|jflandry0094|658[0n]bp|Canada.Quebec|BOLD:AAC7850
Nycteola metaspilella|[31]|LGSMC647-05|DNA-ATBI-2647|573[0n]bp|United States.Tennessee|BOLD:AAD4611
Nycteola metaspilella|[32]|LPOKC374-09|MDOK-2451|658[0n]bp|United States.Oklahoma|BOLD:AAD4611
Nycteola metaspilella|[33]|HKONS624-08|1812-COI-07|658[0n]bp|United States.Florida|BOLD:AAD4611
Nycteola metaspilella|[34]|LPOKC814-09|MDOK-2891|658[0n]bp|United States.Oklahoma|BOLD:AAD4611
Nycteola metaspilella|[35]|HKONS623-08|1811-COI-07|658[0n]bp|United States.Florida|BOLD:AAD4611
Nycteola metaspilella|[36]|RDNMC285-05|CNCNoctuoidea11919|577[2n]bp|United States.Florida|BOLD:AAD4611
Nycteola metaspilella|[37]|RDNME284-07|CNCNoctuoidea13891|634[0n]bp|United States.Florida|BOLD:AAD4611
Nycteola metaspilella|[38]|LNCC277-10|10-NCCC-467|658[0n]bp|United States.North Carolina|BOLD:AAD4611
Nycteola n. sp.|[39]|LALPA1109-11|AVBC 919-11|658[0n]bp|Canada.British Columbia|BOLD:AAI3437
Nycteola n. sp.|[40]|LBCS024-07|UBC-2007-0026|658[0n]bp|Canada.British Columbia|BOLD:AAI3437
Nycteola n. sp.|[41]|EHL938-12|C0960013Jun2008|658[0n]bp|Canada.British Columbia|BOLD:AAI3437
Nycteola n. sp.|[42]|LALPA091-10|AVBC 091-10|658[0n]bp|Canada.British Columbia|BOLD:AAI3437
Nycteola n. sp.|[43]|LALPA1100-11|AVBC 910-11|658[0n]bp|Canada.British Columbia|BOLD:AAI3437
Baileya ophthalmica|[44]|LPSO100-08|PPBP-0100|658[0n]bp|Canada.Ontario|BOLD:AAA6592
Baileya ophthalmica|[45]|TMNBB089-06|MNBTT-1029|658[0n]bp|Canada.New Brunswick|BOLD:AAA6592
Baileya ophthalmica|[46]|TMNBB103-06|MNBTT-1043|658[0n]bp|Canada.New Brunswick|BOLD:AAA6592
Baileya ophthalmica|[47]|TMNBB088-06|MNBTT-1028|658[0n]bp|Canada.New Brunswick|BOLD:AAA6592
Baileya ophthalmica|[48]|TMNBB092-06|MNBTT-1032|658[0n]bp|Canada.New Brunswick|BOLD:AAA6592
Baileya ophthalmica|[49]|TMNBB104-06|MNBTT-1044|658[0n]bp|Canada.New Brunswick|BOLD:AAA6592
Baileya ophthalmica|[50]|TMNBB100-06|MNBTT-1040|658[0n]bp|Canada.New Brunswick|BOLD:AAA6592
Baileya ophthalmica|[51]|TMNBB094-06|MNBTT-1034|658[0n]bp|Canada.New Brunswick|BOLD:AAA6592
Baileya ophthalmica|[52]|TMNBB097-06|MNBTT-1037|658[0n]bp|Canada.New Brunswick|BOLD:AAA6592
Baileya ophthalmica|[53]|TMNBB095-06|MNBTT-1035|658[0n]bp|Canada.New Brunswick|BOLD:AAA6592
Baileya ophthalmica|[54]|TMNBB096-06|MNBTT-1036|658[0n]bp|Canada.New Brunswick|BOLD:AAA6592
Baileya ophthalmica|[55]|RDLQF928-06|DH012103|658[0n]bp|Canada.Quebec|BOLD:AAA6592
Baileya ophthalmica|[56]|TMNBB087-06|MNBTT-1027|658[0n]bp|Canada.New Brunswick|BOLD:AAA6592
Baileya ophthalmica|[57]|XAF481-05|2005-ONT-130|658[0n]bp|Canada.Ontario|BOLD:AAA6592
Baileya ophthalmica|[58]|LPMN275-08|08BBLEP-01074|658[0n]bp|Canada.Manitoba|BOLD:AAA6592
Baileya ophthalmica|[59]|LPSOC102-08|PPBP-2101|658[0n]bp|Canada.Ontario|BOLD:AAA6592
Baileya ophthalmica|[60]|RDLQF446-06|DH011553|658[0n]bp|Canada.Quebec|BOLD:AAA6592
Baileya ophthalmica|[61]|RDLQF930-06|DH012105|658[0n]bp|Canada.Quebec|BOLD:AAA6592
Baileya ophthalmica|[62]|TMNBB091-06|MNBTT-1031|658[0n]bp|Canada.New Brunswick|BOLD:AAA6592
Baileya ophthalmica|[63]|RDLQG438-06|DH012717|658[0n]bp|Canada.Quebec|BOLD:AAA6592
Baileya ophthalmica|[64]|LPMN259-08|08BBLEP-01058|658[0n]bp|Canada.Manitoba|BOLD:AAA6592
Baileya ophthalmica|[65]|RDNMK197-11|CNCLEP 84107|658[1n]bp|Canada.Alberta|BOLD:AAA6592
Baileya ophthalmica|[66]|XAF796-05|2005-ONT-445|658[0n]bp|Canada.Ontario|BOLD:AAA6592
Baileya ophthalmica|[67]|PHMO108-03|moth666.02|639[0n]bp|Canada.Ontario|BOLD:AAA6592
Baileya ophthalmica|[68]|LPSOC048-08|PPBP-2047|658[0n]bp|Canada.Ontario|BOLD:AAA6592
Baileya ophthalmica|[69]|TMNBB102-06|MNBTT-1042|658[0n]bp|Canada.New Brunswick|BOLD:AAA6592
Baileya ophthalmica|[70]|XAB378-04|04HBL005378|658[0n]bp|Canada.Ontario|BOLD:AAA6592
Baileya ophthalmica|[71]|XAB290-04|04HBL005290|658[0n]bp|Canada.Ontario|BOLD:AAA6592
Baileya ophthalmica|[72]|XAC659-04|04HBL006659|658[0n]bp|Canada.Ontario|BOLD:AAA6592
Baileya ophthalmica|[73]|TMNBB086-06|MNBTT-1026|658[0n]bp|Canada.New Brunswick|BOLD:AAA6592
Baileya ophthalmica|[74]|RDLQF931-06|DH012106|658[0n]bp|Canada.Quebec|BOLD:AAA6592

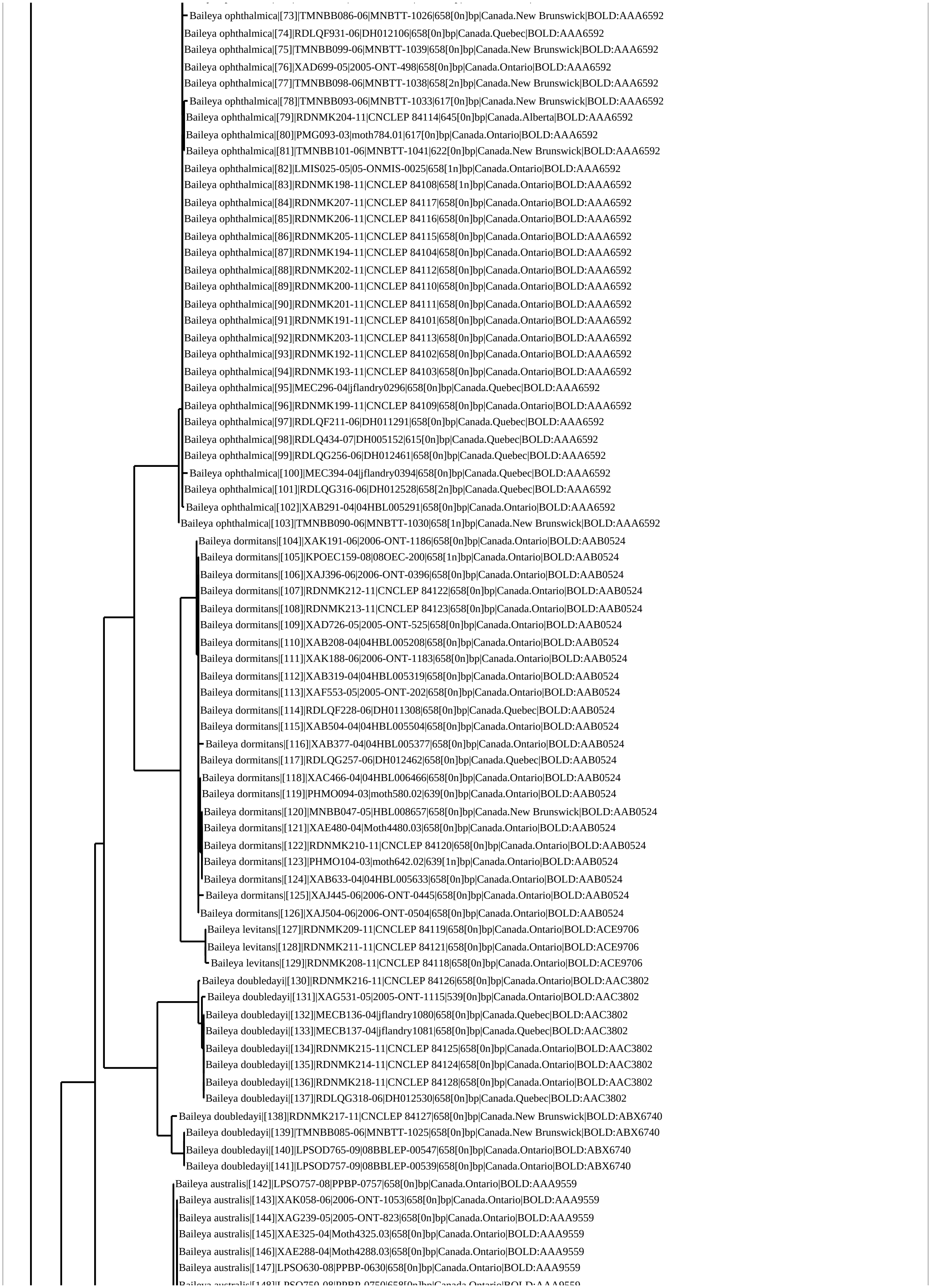
Baileya ophthalmica|[73]|TMNBB086-06|MNBTT-1026|658[0n]bp|Canada.New Brunswick|BOLD:AAA6592
Baileya ophthalmica|[74]|RDLQF931-06|DH012106|658[0n]bp|Canada.Quebec|BOLD:AAA6592
Baileya ophthalmica|[75]|TMNBB099-06|MNBTT-1039|658[0n]bp|Canada.New Brunswick|BOLD:AAA6592
Baileya ophthalmica|[76]|XAD699-05|2005-ONT-498|658[0n]bp|Canada.Ontario|BOLD:AAA6592
Baileya ophthalmica|[77]|TMNBB098-06|MNBTT-1038|658[2n]bp|Canada.New Brunswick|BOLD:AAA6592
Baileya ophthalmica|[78]|TMNBB093-06|MNBTT-1033|617[0n]bp|Canada.New Brunswick|BOLD:AAA6592
Baileya ophthalmica|[79]|RDNMK204-11|CNCLEP 84114|645[0n]bp|Canada.Alberta|BOLD:AAA6592
Baileya ophthalmica|[80]|PMG093-03|moth784.01|617[0n]bp|Canada.Ontario|BOLD:AAA6592
Baileya ophthalmica|[81]|TMNBB101-06|MNBTT-1041|622[0n]bp|Canada.New Brunswick|BOLD:AAA6592
Baileya ophthalmica|[82]|LMIS025-05|05-ONMIS-0025|658[1n]bp|Canada.Ontario|BOLD:AAA6592
Baileya ophthalmica|[83]|RDNMK198-11|CNCLEP 84108|658[1n]bp|Canada.Ontario|BOLD:AAA6592
Baileya ophthalmica|[84]|RDNMK207-11|CNCLEP 84117|658[0n]bp|Canada.Ontario|BOLD:AAA6592
Baileya ophthalmica|[85]|RDNMK206-11|CNCLEP 84116|658[0n]bp|Canada.Ontario|BOLD:AAA6592
Baileya ophthalmica|[86]|RDNMK205-11|CNCLEP 84115|658[0n]bp|Canada.Ontario|BOLD:AAA6592
Baileya ophthalmica|[87]|RDNMK194-11|CNCLEP 84104|658[0n]bp|Canada.Ontario|BOLD:AAA6592
Baileya ophthalmica|[88]|RDNMK202-11|CNCLEP 84112|658[0n]bp|Canada.Ontario|BOLD:AAA6592
Baileya ophthalmica|[89]|RDNMK200-11|CNCLEP 84110|658[0n]bp|Canada.Ontario|BOLD:AAA6592
Baileya ophthalmica|[90]|RDNMK201-11|CNCLEP 84111|658[0n]bp|Canada.Ontario|BOLD:AAA6592
Baileya ophthalmica|[91]|RDNMK191-11|CNCLEP 84101|658[0n]bp|Canada.Ontario|BOLD:AAA6592
Baileya ophthalmica|[92]|RDNMK203-11|CNCLEP 84113|658[0n]bp|Canada.Ontario|BOLD:AAA6592
Baileya ophthalmica|[93]|RDNMK192-11|CNCLEP 84102|658[0n]bp|Canada.Ontario|BOLD:AAA6592
Baileya ophthalmica|[94]|RDNMK193-11|CNCLEP 84103|658[0n]bp|Canada.Ontario|BOLD:AAA6592
Baileya ophthalmica|[95]|MEC296-04|jflandry0296|658[0n]bp|Canada.Quebec|BOLD:AAA6592
Baileya ophthalmica|[96]|RDNMK199-11|CNCLEP 84109|658[0n]bp|Canada.Ontario|BOLD:AAA6592
Baileya ophthalmica|[97]|RDLQF211-06|DH011291|658[0n]bp|Canada.Quebec|BOLD:AAA6592
Baileya ophthalmica|[98]|RDLQ434-07|DH005152|615[0n]bp|Canada.Quebec|BOLD:AAA6592
Baileya ophthalmica|[99]|RDLQG256-06|DH012461|658[0n]bp|Canada.Quebec|BOLD:AAA6592
Baileya ophthalmica|[100]|MEC394-04|jflandry0394|658[0n]bp|Canada.Quebec|BOLD:AAA6592
Baileya ophthalmica|[101]|RDLQG316-06|DH012528|658[2n]bp|Canada.Quebec|BOLD:AAA6592
Baileya ophthalmica|[102]|XAB291-04|04HBL005291|658[0n]bp|Canada.Ontario|BOLD:AAA6592
Baileya ophthalmica|[103]|TMNBB090-06|MNBTT-1030|658[1n]bp|Canada.New Brunswick|BOLD:AAA6592
Baileya dormitans|[104]|XAK191-06|2006-ONT-1186|658[0n]bp|Canada.Ontario|BOLD:AAB0524
Baileya dormitans|[105]|KPOEC159-08|08OEC-200|658[1n]bp|Canada.Ontario|BOLD:AAB0524
Baileya dormitans|[106]|XAJ396-06|2006-ONT-0396|658[0n]bp|Canada.Ontario|BOLD:AAB0524
Baileya dormitans|[107]|RDNMK212-11|CNCLEP 84122|658[0n]bp|Canada.Ontario|BOLD:AAB0524
Baileya dormitans|[108]|RDNMK213-11|CNCLEP 84123|658[0n]bp|Canada.Ontario|BOLD:AAB0524
Baileya dormitans|[109]|XAD726-05|2005-ONT-525|658[0n]bp|Canada.Ontario|BOLD:AAB0524
Baileya dormitans|[110]|XAB208-04|04HBL005208|658[0n]bp|Canada.Ontario|BOLD:AAB0524
Baileya dormitans|[111]|XAK188-06|2006-ONT-1183|658[0n]bp|Canada.Ontario|BOLD:AAB0524
Baileya dormitans|[112]|XAB319-04|04HBL005319|658[0n]bp|Canada.Ontario|BOLD:AAB0524
Baileya dormitans|[113]|XAF553-05|2005-ONT-202|658[0n]bp|Canada.Ontario|BOLD:AAB0524
Baileya dormitans|[114]|RDLQF228-06|DH011308|658[0n]bp|Canada.Quebec|BOLD:AAB0524
Baileya dormitans|[115]|XAB504-04|04HBL005504|658[0n]bp|Canada.Ontario|BOLD:AAB0524
Baileya dormitans|[116]|XAB377-04|04HBL005377|658[0n]bp|Canada.Ontario|BOLD:AAB0524
Baileya dormitans|[117]|RDLQG257-06|DH012462|658[0n]bp|Canada.Quebec|BOLD:AAB0524
Baileya dormitans|[118]|XAC466-04|04HBL006466|658[0n]bp|Canada.Ontario|BOLD:AAB0524
Baileya dormitans|[119]|PHMO094-03|moth580.02|639[0n]bp|Canada.Ontario|BOLD:AAB0524
Baileya dormitans|[120]|MNBB047-05|HBL008657|658[0n]bp|Canada.New Brunswick|BOLD:AAB0524
Baileya dormitans|[121]|XAE480-04|Moth4480.03|658[0n]bp|Canada.Ontario|BOLD:AAB0524
Baileya dormitans|[122]|RDNMK210-11|CNCLEP 84120|658[0n]bp|Canada.Ontario|BOLD:AAB0524
Baileya dormitans|[123]|PHMO104-03|moth642.02|639[1n]bp|Canada.Ontario|BOLD:AAB0524
Baileya dormitans|[124]|XAB633-04|04HBL005633|658[0n]bp|Canada.Ontario|BOLD:AAB0524
Baileya dormitans|[125]|XAJ445-06|2006-ONT-0445|658[0n]bp|Canada.Ontario|BOLD:AAB0524
Baileya dormitans|[126]|XAJ504-06|2006-ONT-0504|658[0n]bp|Canada.Ontario|BOLD:AAB0524
Baileya levitans|[127]|RDNMK209-11|CNCLEP 84119|658[0n]bp|Canada.Ontario|BOLD:ACE9706
Baileya levitans|[128]|RDNMK211-11|CNCLEP 84121|658[0n]bp|Canada.Ontario|BOLD:ACE9706
Baileya levitans|[129]|RDNMK208-11|CNCLEP 84118|658[0n]bp|Canada.Ontario|BOLD:ACE9706
Baileya doubledayi|[130]|RDNMK216-11|CNCLEP 84126|658[0n]bp|Canada.Ontario|BOLD:AAC3802
Baileya doubledayi|[131]|XAG531-05|2005-ONT-1115|539[0n]bp|Canada.Ontario|BOLD:AAC3802
Baileya doubledayi|[132]|MECB136-04|jflandry1080|658[0n]bp|Canada.Quebec|BOLD:AAC3802
Baileya doubledayi|[133]|MECB137-04|jflandry1081|658[0n]bp|Canada.Quebec|BOLD:AAC3802
Baileya doubledayi|[134]|RDNMK215-11|CNCLEP 84125|658[0n]bp|Canada.Ontario|BOLD:AAC3802
Baileya doubledayi|[135]|RDNMK214-11|CNCLEP 84124|658[0n]bp|Canada.Ontario|BOLD:AAC3802
Baileya doubledayi|[136]|RDNMK218-11|CNCLEP 84128|658[0n]bp|Canada.Ontario|BOLD:AAC3802
Baileya doubledayi|[137]|RDLQG318-06|DH012530|658[0n]bp|Canada.Quebec|BOLD:AAC3802
Baileya doubledayi|[138]|RDNMK217-11|CNCLEP 84127|658[0n]bp|Canada.New Brunswick|BOLD:ABX6740
Baileya doubledayi|[139]|TMNBB085-06|MNBTT-1025|658[0n]bp|Canada.New Brunswick|BOLD:ABX6740
Baileya doubledayi|[140]|LPSOD765-09|08BBLEP-00547|658[0n]bp|Canada.Ontario|BOLD:ABX6740
Baileya doubledayi|[141]|LPSOD757-09|08BBLEP-00539|658[0n]bp|Canada.Ontario|BOLD:ABX6740
Baileya australis|[142]|LPSO757-08|PPBP-0757|658[0n]bp|Canada.Ontario|BOLD:AAA9559
Baileya australis|[143]|XAK058-06|2006-ONT-1053|658[0n]bp|Canada.Ontario|BOLD:AAA9559
Baileya australis|[144]|XAG239-05|2005-ONT-823|658[0n]bp|Canada.Ontario|BOLD:AAA9559
Baileya australis|[145]|XAE325-04|Moth4325.03|658[0n]bp|Canada.Ontario|BOLD:AAA9559
Baileya australis|[146]|XAE288-04|Moth4288.03|658[0n]bp|Canada.Ontario|BOLD:AAA9559
Baileya australis|[147]|LPSO630-08|PPBP-0630|658[0n]bp|Canada.Ontario|BOLD:AAA9559
Baileya australis|[148]|LPSO750-08|PPBP-0750|658[0n]bp|Canada.Ontario|BOLD:AAA9559

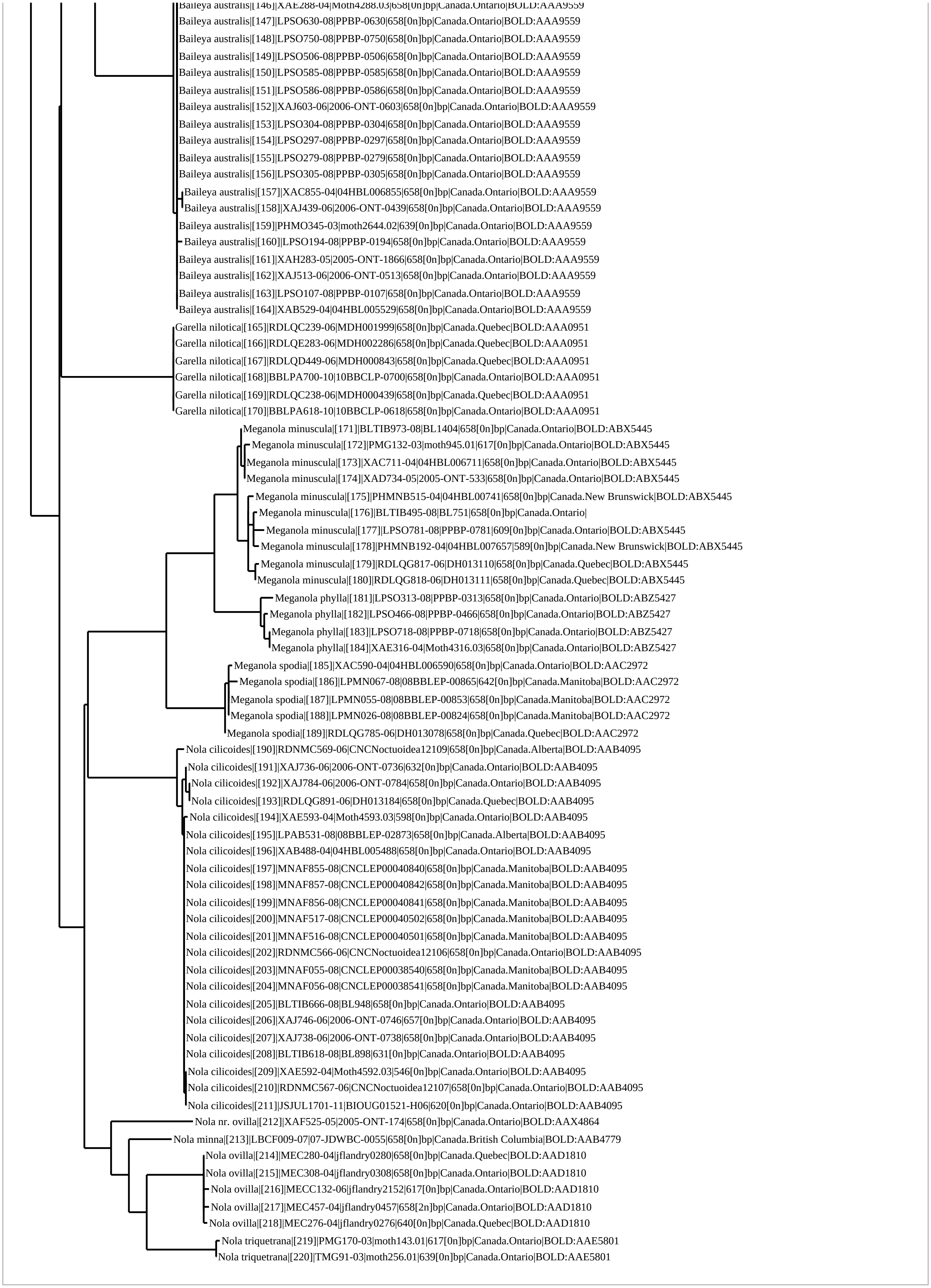
Baileya australis|[146]|XAE288-04|Moth4288.03|658[0n]bp|Canada.Ontario|BOLD:AAA9559
Baileya australis|[147]|LPSO630-08|PPBP-0630|658[0n]bp|Canada.Ontario|BOLD:AAA9559
Baileya australis|[148]|LPSO750-08|PPBP-0750|658[0n]bp|Canada.Ontario|BOLD:AAA9559
Baileya australis|[149]|LPSO506-08|PPBP-0506|658[0n]bp|Canada.Ontario|BOLD:AAA9559
Baileya australis|[150]|LPSO585-08|PPBP-0585|658[0n]bp|Canada.Ontario|BOLD:AAA9559
Baileya australis|[151]|LPSO586-08|PPBP-0586|658[0n]bp|Canada.Ontario|BOLD:AAA9559
Baileya australis|[152]|XAJ603-06|2006-ONT-0603|658[0n]bp|Canada.Ontario|BOLD:AAA9559
Baileya australis|[153]|LPSO304-08|PPBP-0304|658[0n]bp|Canada.Ontario|BOLD:AAA9559
Baileya australis|[154]|LPSO297-08|PPBP-0297|658[0n]bp|Canada.Ontario|BOLD:AAA9559
Baileya australis|[155]|LPSO279-08|PPBP-0279|658[0n]bp|Canada.Ontario|BOLD:AAA9559
Baileya australis|[156]|LPSO305-08|PPBP-0305|658[0n]bp|Canada.Ontario|BOLD:AAA9559
Baileya australis|[157]|XAC855-04|04HBL006855|658[0n]bp|Canada.Ontario|BOLD:AAA9559
Baileya australis|[158]|XAJ439-06|2006-ONT-0439|658[0n]bp|Canada.Ontario|BOLD:AAA9559
Baileya australis|[159]|PHMO345-03|moth2644.02|639[0n]bp|Canada.Ontario|BOLD:AAA9559
Baileya australis|[160]|LPSO194-08|PPBP-0194|658[0n]bp|Canada.Ontario|BOLD:AAA9559
Baileya australis|[161]|XAH283-05|2005-ONT-1866|658[0n]bp|Canada.Ontario|BOLD:AAA9559
Baileya australis|[162]|XAJ513-06|2006-ONT-0513|658[0n]bp|Canada.Ontario|BOLD:AAA9559
Baileya australis|[163]|LPSO107-08|PPBP-0107|658[0n]bp|Canada.Ontario|BOLD:AAA9559
Baileya australis|[164]|XAB529-04|04HBL005529|658[0n]bp|Canada.Ontario|BOLD:AAA9559
Garella nilotica|[165]|RDLQC239-06|MDH001999|658[0n]bp|Canada.Quebec|BOLD:AAA0951
Garella nilotica|[166]|RDLQE283-06|MDH002286|658[0n]bp|Canada.Quebec|BOLD:AAA0951
Garella nilotica|[167]|RDLQD449-06|MDH000843|658[0n]bp|Canada.Quebec|BOLD:AAA0951
Garella nilotica|[168]|BBLPA700-10|10BBCLP-0700|658[0n]bp|Canada.Ontario|BOLD:AAA0951
Garella nilotica|[169]|RDLQC238-06|MDH000439|658[0n]bp|Canada.Quebec|BOLD:AAA0951
Garella nilotica|[170]|BBLPA618-10|10BBCLP-0618|658[0n]bp|Canada.Ontario|BOLD:AAA0951
Meganola minuscula|[171]|BLTIB973-08|BL1404|658[0n]bp|Canada.Ontario|BOLD:ABX5445
Meganola minuscula|[172]|PMG132-03|moth945.01|617[0n]bp|Canada.Ontario|BOLD:ABX5445
Meganola minuscula|[173]|XAC711-04|04HBL006711|658[0n]bp|Canada.Ontario|BOLD:ABX5445
Meganola minuscula|[174]|XAD734-05|2005-ONT-533|658[0n]bp|Canada.Ontario|BOLD:ABX5445
Meganola minuscula|[175]|PHMNB515-04|04HBL00741|658[0n]bp|Canada.New Brunswick|BOLD:ABX5445
Meganola minuscula|[176]|BLTIB495-08|BL751|658[0n]bp|Canada.Ontario|
Meganola minuscula|[177]|LPSO781-08|PPBP-0781|609[0n]bp|Canada.Ontario|BOLD:ABX5445
Meganola minuscula|[178]|PHMNB192-04|04HBL007657|589[0n]bp|Canada.New Brunswick|BOLD:ABX5445
Meganola minuscula|[179]|RDLQG817-06|DH013110|658[0n]bp|Canada.Quebec|BOLD:ABX5445
Meganola minuscula|[180]|RDLQG818-06|DH013111|658[0n]bp|Canada.Quebec|BOLD:ABX5445
Meganola phylla|[181]|LPSO313-08|PPBP-0313|658[0n]bp|Canada.Ontario|BOLD:ABZ5427
Meganola phylla|[182]|LPSO466-08|PPBP-0466|658[0n]bp|Canada.Ontario|BOLD:ABZ5427
Meganola phylla|[183]|LPSO718-08|PPBP-0718|658[0n]bp|Canada.Ontario|BOLD:ABZ5427
Meganola phylla|[184]|XAE316-04|Moth4316.03|658[0n]bp|Canada.Ontario|BOLD:ABZ5427
Meganola spodia|[185]|XAC590-04|04HBL006590|658[0n]bp|Canada.Ontario|BOLD:AAC2972
Meganola spodia|[186]|LPMN067-08|08BBLEP-00865|642[0n]bp|Canada.Manitoba|BOLD:AAC2972
Meganola spodia|[187]|LPMN055-08|08BBLEP-00853|658[0n]bp|Canada.Manitoba|BOLD:AAC2972
Meganola spodia|[188]|LPMN026-08|08BBLEP-00824|658[0n]bp|Canada.Manitoba|BOLD:AAC2972
Meganola spodia|[189]|RDLQG785-06|DH013078|658[0n]bp|Canada.Quebec|BOLD:AAC2972
Nola cilicoides|[190]|RDNMC569-06|CNCNoctuoidea12109|658[0n]bp|Canada.Alberta|BOLD:AAB4095
Nola cilicoides|[191]|XAJ736-06|2006-ONT-0736|632[0n]bp|Canada.Ontario|BOLD:AAB4095
Nola cilicoides|[192]|XAJ784-06|2006-ONT-0784|658[0n]bp|Canada.Ontario|BOLD:AAB4095
Nola cilicoides|[193]|RDLQG891-06|DH013184|658[0n]bp|Canada.Quebec|BOLD:AAB4095
Nola cilicoides|[194]|XAE593-04|Moth4593.03|598[0n]bp|Canada.Ontario|BOLD:AAB4095
Nola cilicoides|[195]|LPAB531-08|08BBLEP-02873|658[0n]bp|Canada.Alberta|BOLD:AAB4095
Nola cilicoides|[196]|XAB488-04|04HBL005488|658[0n]bp|Canada.Ontario|BOLD:AAB4095
Nola cilicoides|[197]|MNAF855-08|CNCLEP00040840|658[0n]bp|Canada.Manitoba|BOLD:AAB4095
Nola cilicoides|[198]|MNAF857-08|CNCLEP00040842|658[0n]bp|Canada.Manitoba|BOLD:AAB4095
Nola cilicoides|[199]|MNAF856-08|CNCLEP00040841|658[0n]bp|Canada.Manitoba|BOLD:AAB4095
Nola cilicoides|[200]|MNAF517-08|CNCLEP00040502|658[0n]bp|Canada.Manitoba|BOLD:AAB4095
Nola cilicoides|[201]|MNAF516-08|CNCLEP00040501|658[0n]bp|Canada.Manitoba|BOLD:AAB4095
Nola cilicoides|[202]|RDNMC566-06|CNCNoctuoidea12106|658[0n]bp|Canada.Ontario|BOLD:AAB4095
Nola cilicoides|[203]|MNAF055-08|CNCLEP00038540|658[0n]bp|Canada.Manitoba|BOLD:AAB4095
Nola cilicoides|[204]|MNAF056-08|CNCLEP00038541|658[0n]bp|Canada.Manitoba|BOLD:AAB4095
Nola cilicoides|[205]|BLTIB666-08|BL948|658[0n]bp|Canada.Ontario|BOLD:AAB4095
Nola cilicoides|[206]|XAJ746-06|2006-ONT-0746|657[0n]bp|Canada.Ontario|BOLD:AAB4095
Nola cilicoides|[207]|XAJ738-06|2006-ONT-0738|658[0n]bp|Canada.Ontario|BOLD:AAB4095
Nola cilicoides|[208]|BLTIB618-08|BL898|631[0n]bp|Canada.Ontario|BOLD:AAB4095
Nola cilicoides|[209]|XAE592-04|Moth4592.03|546[0n]bp|Canada.Ontario|BOLD:AAB4095
Nola cilicoides|[210]|RDNMC567-06|CNCNoctuoidea12107|658[0n]bp|Canada.Ontario|BOLD:AAB4095
Nola cilicoides|[211]|JSJUL1701-11|BIOUG01521-H06|620[0n]bp|Canada.Ontario|BOLD:AAB4095
Nola nr. ovilla|[212]|XAF525-05|2005-ONT-174|658[0n]bp|Canada.Ontario|BOLD:AAX4864
Nola minna|[213]|LBCF009-07|07-JDWBC-0055|658[0n]bp|Canada.British Columbia|BOLD:AAB4779
Nola ovilla|[214]|MEC280-04|jflandry0280|658[0n]bp|Canada.Quebec|BOLD:AAD1810
Nola ovilla|[215]|MEC308-04|jflandry0308|658[0n]bp|Canada.Ontario|BOLD:AAD1810
Nola ovilla|[216]|MECC132-06|jflandry2152|617[0n]bp|Canada.Ontario|BOLD:AAD1810
Nola ovilla|[217]|MEC457-04|jflandry0457|658[2n]bp|Canada.Ontario|BOLD:AAD1810
Nola ovilla|[218]|MEC276-04|jflandry0276|640[0n]bp|Canada.Quebec|BOLD:AAD1810
Nola triquetrana|[219]|PMG170-03|moth143.01|617[0n]bp|Canada.Ontario|BOLD:AAE5801
Nola triquetrana|[220]|TMG91-03|moth256.01|639[0n]bp|Canada.Ontario|BOLD:AAE5801
