# Supplementary material for: A Transcontinental Challenge — A Test of DNA Barcode Performance for 1,541 Species of Canadian Noctuoidea (Lepidoptera)
Source: PLoS One. 2014 Mar 25;9(3):e92797. doi: 10.1371/journal.pone.0092797 (PMC3965468; doi:10.1371/journal.pone.0092797)
Supplement: Tree S4 — NJ tree for Canadian species in the family Erebidae. (PDF) [file pone.0092797.s008.pdf]

BOLD TaxonID Tree

Title	:	SEARCH: Marker(COI-5P), Sample ids(6839 ids) [SEARCH1]
Date	:	28-October-2013
Data Type	:	Nucleotide
Distance Model	:	Kimura 2 Parameter
Marker	:	COI-5P
Codon Positions	:	
Labels	:	Country & Province, SampleID, ProcessID, Sequence Length, BIN URI
Filters	:	Length > 200
Colorization	:	[blue]=Stop Codons [red]=Contamination or misidentification
Attachment	:	Photographs & Spreadsheet
Sequence Count	:	6839
Species count	:	357
Genus count	:	110
Family count	:	1
Unidentified	:	1
BIN Count	:	337

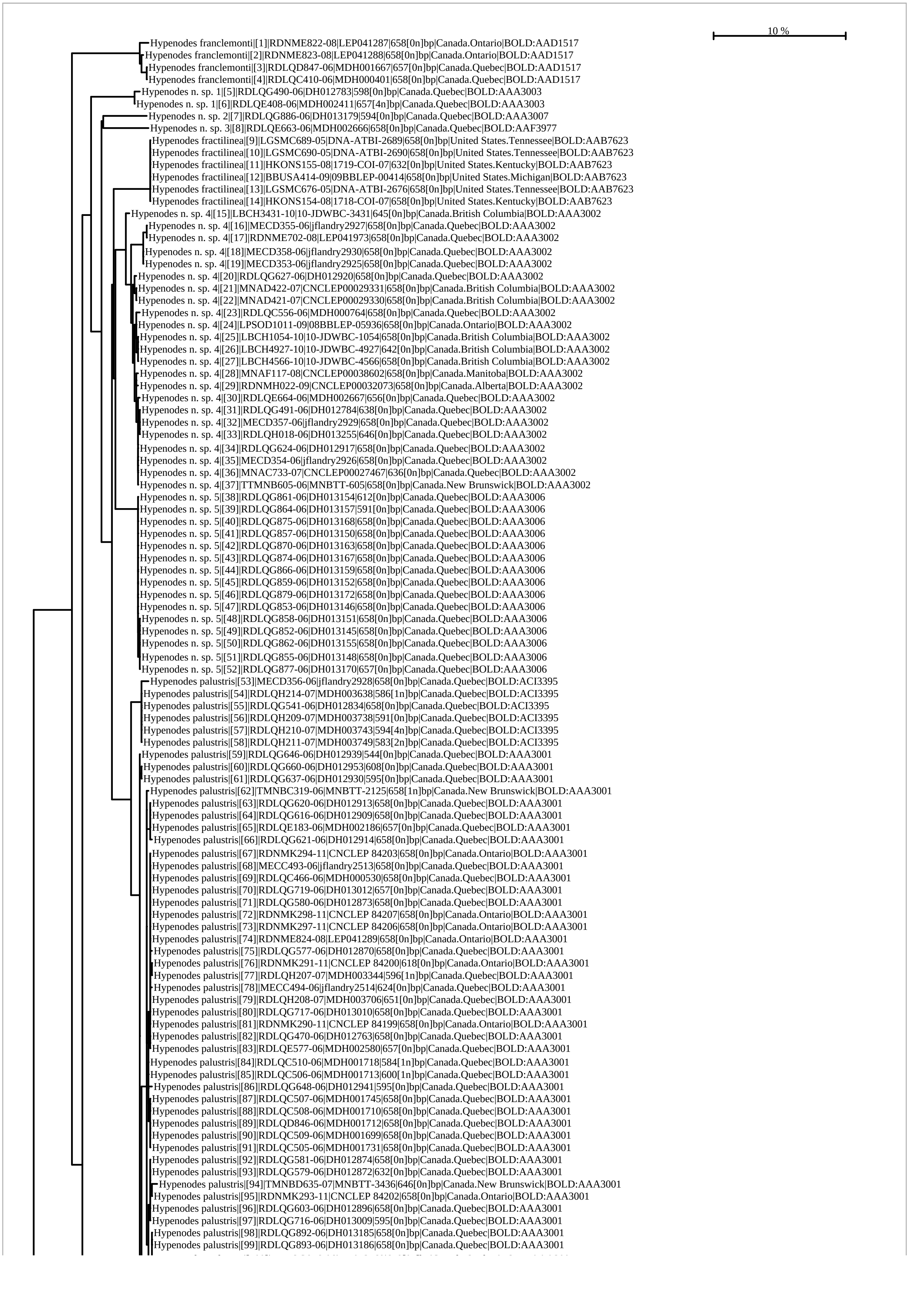
10 %
Hypenodes franclemonti|[1]|RDNME822-08|LEP041287|658[0n]bp|Canada.Ontario|BOLD:AAD1517
Hypenodes franclemonti|[2]|RDNME823-08|LEP041288|658[0n]bp|Canada.Ontario|BOLD:AAD1517
Hypenodes franclemonti|[3]|RDLQD847-06|MDH001667|657[0n]bp|Canada.Quebec|BOLD:AAD1517
Hypenodes franclemonti|[4]|RDLQC410-06|MDH000401|658[0n]bp|Canada.Quebec|BOLD:AAD1517
Hypenodes n. sp. 1|[5]|RDLQG490-06|DH012783|598[0n]bp|Canada.Quebec|BOLD:AAA3003
Hypenodes n. sp. 1|[6]|RDLQE408-06|MDH002411|657[4n]bp|Canada.Quebec|BOLD:AAA3003
Hypenodes n. sp. 2|[7]|RDLQG886-06|DH013179|594[0n]bp|Canada.Quebec|BOLD:AAA3007
Hypenodes n. sp. 3|[8]|RDLQE663-06|MDH002666|658[0n]bp|Canada.Quebec|BOLD:AAF3977
Hypenodes fractilinea|[9]|LGSMC689-05|DNA-ATBI-2689|658[0n]bp|United States.Tennessee|BOLD:AAB7623
Hypenodes fractilinea|[10]|LGSMC690-05|DNA-ATBI-2690|658[0n]bp|United States.Tennessee|BOLD:AAB7623
Hypenodes fractilinea|[11]|HKONS155-08|1719-COI-07|632[0n]bp|United States.Kentucky|BOLD:AAB7623
Hypenodes fractilinea|[12]|BBUSA414-09|09BBLEP-00414|658[0n]bp|United States.Michigan|BOLD:AAB7623
Hypenodes fractilinea|[13]|LGSMC676-05|DNA-ATBI-2676|658[0n]bp|United States.Tennessee|BOLD:AAB7623
Hypenodes fractilinea|[14]|HKONS154-08|1718-COI-07|658[0n]bp|United States.Kentucky|BOLD:AAB7623
Hypenodes n. sp. 4|[15]|LBCH3431-10|10-JDWBC-3431|645[0n]bp|Canada.British Columbia|BOLD:AAA3002
Hypenodes n. sp. 4|[16]|MECD355-06|jflandry2927|658[0n]bp|Canada.Quebec|BOLD:AAA3002
Hypenodes n. sp. 4|[17]|RDNME702-08|LEP041973|658[0n]bp|Canada.Quebec|BOLD:AAA3002
Hypenodes n. sp. 4|[18]|MECD358-06|jflandry2930|658[0n]bp|Canada.Quebec|BOLD:AAA3002
Hypenodes n. sp. 4|[19]|MECD353-06|jflandry2925|658[0n]bp|Canada.Quebec|BOLD:AAA3002
Hypenodes n. sp. 4|[20]|RDLQG627-06|DH012920|658[0n]bp|Canada.Quebec|BOLD:AAA3002
Hypenodes n. sp. 4|[21]|MNAD422-07|CNCLEP00029331|658[0n]bp|Canada.British Columbia|BOLD:AAA3002
Hypenodes n. sp. 4|[22]|MNAD421-07|CNCLEP00029330|658[0n]bp|Canada.British Columbia|BOLD:AAA3002
Hypenodes n. sp. 4|[23]|RDLQC556-06|MDH000764|658[0n]bp|Canada.Quebec|BOLD:AAA3002
Hypenodes n. sp. 4|[24]|LPSOD1011-09|08BBLEP-05936|658[0n]bp|Canada.Ontario|BOLD:AAA3002
Hypenodes n. sp. 4|[25]|LBCH1054-10|10-JDWBC-1054|658[0n]bp|Canada.British Columbia|BOLD:AAA3002
Hypenodes n. sp. 4|[26]|LBCH4927-10|10-JDWBC-4927|642[0n]bp|Canada.British Columbia|BOLD:AAA3002
Hypenodes n. sp. 4|[27]|LBCH4566-10|10-JDWBC-4566|658[0n]bp|Canada.British Columbia|BOLD:AAA3002
Hypenodes n. sp. 4|[28]|MNAF117-08|CNCLEP00038602|658[0n]bp|Canada.Manitoba|BOLD:AAA3002
Hypenodes n. sp. 4|[29]|RDNMH022-09|CNCLEP00032073|658[0n]bp|Canada.Alberta|BOLD:AAA3002
Hypenodes n. sp. 4|[30]|RDLQE664-06|MDH002667|656[0n]bp|Canada.Quebec|BOLD:AAA3002
Hypenodes n. sp. 4|[31]|RDLQG491-06|DH012784|638[0n]bp|Canada.Quebec|BOLD:AAA3002
Hypenodes n. sp. 4|[32]|MECD357-06|jflandry2929|658[0n]bp|Canada.Quebec|BOLD:AAA3002
Hypenodes n. sp. 4|[33]|RDLQH018-06|DH013255|646[0n]bp|Canada.Quebec|BOLD:AAA3002
Hypenodes n. sp. 4|[34]|RDLQG624-06|DH012917|658[0n]bp|Canada.Quebec|BOLD:AAA3002
Hypenodes n. sp. 4|[35]|MECD354-06|jflandry2926|658[0n]bp|Canada.Quebec|BOLD:AAA3002
Hypenodes n. sp. 4|[36]|MNAC733-07|CNCLEP00027467|636[0n]bp|Canada.Quebec|BOLD:AAA3002
Hypenodes n. sp. 4|[37]|TTMNB605-06|MNBTT-605|658[0n]bp|Canada.New Brunswick|BOLD:AAA3002
Hypenodes n. sp. 5|[38]|RDLQG861-06|DH013154|612[0n]bp|Canada.Quebec|BOLD:AAA3006
Hypenodes n. sp. 5|[39]|RDLQG864-06|DH013157|591[0n]bp|Canada.Quebec|BOLD:AAA3006
Hypenodes n. sp. 5|[40]|RDLQG875-06|DH013168|658[0n]bp|Canada.Quebec|BOLD:AAA3006
Hypenodes n. sp. 5|[41]|RDLQG857-06|DH013150|658[0n]bp|Canada.Quebec|BOLD:AAA3006
Hypenodes n. sp. 5|[42]|RDLQG870-06|DH013163|658[0n]bp|Canada.Quebec|BOLD:AAA3006
Hypenodes n. sp. 5|[43]|RDLQG874-06|DH013167|658[0n]bp|Canada.Quebec|BOLD:AAA3006
Hypenodes n. sp. 5|[44]|RDLQG866-06|DH013159|658[0n]bp|Canada.Quebec|BOLD:AAA3006
Hypenodes n. sp. 5|[45]|RDLQG859-06|DH013152|658[0n]bp|Canada.Quebec|BOLD:AAA3006
Hypenodes n. sp. 5|[46]|RDLQG879-06|DH013172|658[0n]bp|Canada.Quebec|BOLD:AAA3006
Hypenodes n. sp. 5|[47]|RDLQG853-06|DH013146|658[0n]bp|Canada.Quebec|BOLD:AAA3006
Hypenodes n. sp. 5|[48]|RDLQG858-06|DH013151|658[0n]bp|Canada.Quebec|BOLD:AAA3006
Hypenodes n. sp. 5|[49]|RDLQG852-06|DH013145|658[0n]bp|Canada.Quebec|BOLD:AAA3006
Hypenodes n. sp. 5|[50]|RDLQG862-06|DH013155|658[0n]bp|Canada.Quebec|BOLD:AAA3006
Hypenodes n. sp. 5|[51]|RDLQG855-06|DH013148|658[0n]bp|Canada.Quebec|BOLD:AAA3006
Hypenodes n. sp. 5|[52]|RDLQG877-06|DH013170|657[0n]bp|Canada.Quebec|BOLD:AAA3006
Hypenodes palustris|[53]|MECD356-06|jflandry2928|658[0n]bp|Canada.Quebec|BOLD:ACI3395
Hypenodes palustris|[54]|RDLQH214-07|MDH003638|586[1n]bp|Canada.Quebec|BOLD:ACI3395
Hypenodes palustris|[55]|RDLQG541-06|DH012834|658[0n]bp|Canada.Quebec|BOLD:ACI3395
Hypenodes palustris|[56]|RDLQH209-07|MDH003738|591[0n]bp|Canada.Quebec|BOLD:ACI3395
Hypenodes palustris|[57]|RDLQH210-07|MDH003743|594[4n]bp|Canada.Quebec|BOLD:ACI3395
Hypenodes palustris|[58]|RDLQH211-07|MDH003749|583[2n]bp|Canada.Quebec|BOLD:ACI3395
Hypenodes palustris|[59]|RDLQG646-06|DH012939|544[0n]bp|Canada.Quebec|BOLD:AAA3001
Hypenodes palustris|[60]|RDLQG660-06|DH012953|608[0n]bp|Canada.Quebec|BOLD:AAA3001
Hypenodes palustris|[61]|RDLQG637-06|DH012930|595[0n]bp|Canada.Quebec|BOLD:AAA3001
Hypenodes palustris|[62]|TMNBC319-06|MNBTT-2125|658[1n]bp|Canada.New Brunswick|BOLD:AAA3001
Hypenodes palustris|[63]|RDLQG620-06|DH012913|658[0n]bp|Canada.Quebec|BOLD:AAA3001
Hypenodes palustris|[64]|RDLQG616-06|DH012909|658[0n]bp|Canada.Quebec|BOLD:AAA3001
Hypenodes palustris|[65]|RDLQE183-06|MDH002186|657[0n]bp|Canada.Quebec|BOLD:AAA3001
Hypenodes palustris|[66]|RDLQG621-06|DH012914|658[0n]bp|Canada.Quebec|BOLD:AAA3001
Hypenodes palustris|[67]|RDNMK294-11|CNCLEP 84203|658[0n]bp|Canada.Ontario|BOLD:AAA3001
Hypenodes palustris|[68]|MECC493-06|jflandry2513|658[0n]bp|Canada.Quebec|BOLD:AAA3001
Hypenodes palustris|[69]|RDLQC466-06|MDH000530|658[0n]bp|Canada.Quebec|BOLD:AAA3001
Hypenodes palustris|[70]|RDLQG719-06|DH013012|657[0n]bp|Canada.Quebec|BOLD:AAA3001
Hypenodes palustris|[71]|RDLQG580-06|DH012873|658[0n]bp|Canada.Quebec|BOLD:AAA3001
Hypenodes palustris|[72]|RDNMK298-11|CNCLEP 84207|658[0n]bp|Canada.Ontario|BOLD:AAA3001
Hypenodes palustris|[73]|RDNMK297-11|CNCLEP 84206|658[0n]bp|Canada.Ontario|BOLD:AAA3001
Hypenodes palustris|[74]|RDNME824-08|LEP041289|658[0n]bp|Canada.Ontario|BOLD:AAA3001
Hypenodes palustris|[75]|RDLQG577-06|DH012870|658[0n]bp|Canada.Quebec|BOLD:AAA3001
Hypenodes palustris|[76]|RDNMK291-11|CNCLEP 84200|618[0n]bp|Canada.Ontario|BOLD:AAA3001
Hypenodes palustris|[77]|RDLQH207-07|MDH003344|596[1n]bp|Canada.Quebec|BOLD:AAA3001
Hypenodes palustris|[78]|MECC494-06|jflandry2514|624[0n]bp|Canada.Quebec|BOLD:AAA3001
Hypenodes palustris|[79]|RDLQH208-07|MDH003706|651[0n]bp|Canada.Quebec|BOLD:AAA3001
Hypenodes palustris|[80]|RDLQG717-06|DH013010|658[0n]bp|Canada.Quebec|BOLD:AAA3001
Hypenodes palustris|[81]|RDNMK290-11|CNCLEP 84199|658[0n]bp|Canada.Ontario|BOLD:AAA3001
Hypenodes palustris|[82]|RDLQG470-06|DH012763|658[0n]bp|Canada.Quebec|BOLD:AAA3001
Hypenodes palustris|[83]|RDLQE577-06|MDH002580|657[0n]bp|Canada.Quebec|BOLD:AAA3001
Hypenodes palustris|[84]|RDLQC510-06|MDH001718|584[1n]bp|Canada.Quebec|BOLD:AAA3001
Hypenodes palustris|[85]|RDLQC506-06|MDH001713|600[1n]bp|Canada.Quebec|BOLD:AAA3001
Hypenodes palustris|[86]|RDLQG648-06|DH012941|595[0n]bp|Canada.Quebec|BOLD:AAA3001
Hypenodes palustris|[87]|RDLQC507-06|MDH001745|658[0n]bp|Canada.Quebec|BOLD:AAA3001
Hypenodes palustris|[88]|RDLQC508-06|MDH001710|658[0n]bp|Canada.Quebec|BOLD:AAA3001
Hypenodes palustris|[89]|RDLQD846-06|MDH001712|658[0n]bp|Canada.Quebec|BOLD:AAA3001
Hypenodes palustris|[90]|RDLQC509-06|MDH001699|658[0n]bp|Canada.Quebec|BOLD:AAA3001
Hypenodes palustris|[91]|RDLQC505-06|MDH001731|658[0n]bp|Canada.Quebec|BOLD:AAA3001
Hypenodes palustris|[92]|RDLQG581-06|DH012874|658[0n]bp|Canada.Quebec|BOLD:AAA3001
Hypenodes palustris|[93]|RDLQG579-06|DH012872|632[0n]bp|Canada.Quebec|BOLD:AAA3001
Hypenodes palustris|[94]|TMNBD635-07|MNBTT-3436|646[0n]bp|Canada.New Brunswick|BOLD:AAA3001
Hypenodes palustris|[95]|RDNMK293-11|CNCLEP 84202|658[0n]bp|Canada.Ontario|BOLD:AAA3001
Hypenodes palustris|[96]|RDLQG603-06|DH012896|658[0n]bp|Canada.Quebec|BOLD:AAA3001
Hypenodes palustris|[97]|RDLQG716-06|DH013009|595[0n]bp|Canada.Quebec|BOLD:AAA3001
Hypenodes palustris|[98]|RDLQG892-06|DH013185|658[0n]bp|Canada.Quebec|BOLD:AAA3001
Hypenodes palustris|[99]|RDLQG893-06|DH013186|658[0n]bp|Canada.Quebec|BOLD:AAA3001

Hypenodes palustris|[97]|RDLQG716-06|DH013009|595[0n]bp|Canada.Quebec|BOLD:AAA3001
Hypenodes palustris|[98]|RDLQG892-06|DH013185|658[0n]bp|Canada.Quebec|BOLD:AAA3001
Hypenodes palustris|[99]|RDLQG893-06|DH013186|658[0n]bp|Canada.Quebec|BOLD:AAA3001
Hypenodes palustris|[100]|RDLQG876-06|DH013169|658[0n]bp|Canada.Quebec|BOLD:AAA3001
Hypenodes palustris|[101]|RDLQG881-06|DH013174|658[0n]bp|Canada.Quebec|BOLD:AAA3001
Hypenodes palustris|[102]|RDLQG860-06|DH013153|658[0n]bp|Canada.Quebec|BOLD:AAA3001
Hypenodes palustris|[103]|RDLQG863-06|DH013156|658[0n]bp|Canada.Quebec|BOLD:AAA3001
Hypenodes palustris|[104]|RDLQG856-06|DH013149|658[0n]bp|Canada.Quebec|BOLD:AAA3001
Hypenodes palustris|[105]|RDLQG867-06|DH013160|658[0n]bp|Canada.Quebec|BOLD:AAA3001
Hypenodes palustris|[106]|RDLQG873-06|DH013166|632[0n]bp|Canada.Quebec|BOLD:AAA3001
Hypenodes palustris|[107]|RDLQG869-06|DH013162|658[0n]bp|Canada.Quebec|BOLD:AAA3001
Hypenodes palustris|[108]|RDLQG871-06|DH013164|658[0n]bp|Canada.Quebec|BOLD:AAA3001
Hypenodes palustris|[109]|RDLQG854-06|DH013147|658[0n]bp|Canada.Quebec|BOLD:AAA3001
Hypenodes palustris|[110]|RDLQG878-06|DH013171|654[0n]bp|Canada.Quebec|BOLD:AAA3001
Hypenodes palustris|[111]|RDLQG851-06|DH013144|595[0n]bp|Canada.Quebec|BOLD:AAA3001
Hypenodes palustris|[112]|RDLQG872-06|DH013165|658[0n]bp|Canada.Quebec|BOLD:AAA3001
Hypenodes palustris|[113]|RDLQG868-06|DH013161|658[0n]bp|Canada.Quebec|BOLD:AAA3001
Hypenodes palustris|[114]|RDLQG880-06|DH013173|658[0n]bp|Canada.Quebec|BOLD:AAA3001
Hypenodes palustris|[115]|RDLQG865-06|DH013158|658[0n]bp|Canada.Quebec|BOLD:AAA3001
Hypenodes palustris|[116]|RDLQG652-06|DH012945|587[0n]bp|Canada.Quebec|BOLD:AAA3001
Hypenodes palustris|[117]|RDLQE778-06|MDH002781|575[0n]bp|Canada.Quebec|BOLD:AAA3001
Hypenodes palustris|[118]|RDLQE780-06|MDH002783|577[0n]bp|Canada.Quebec|BOLD:AAA3001
Hypenodes palustris|[119]|RDLQG484-06|DH012777|634[0n]bp|Canada.Quebec|BOLD:AAA3001
Hypenodes palustris|[120]|RDLQG617-06|DH012910|658[0n]bp|Canada.Quebec|BOLD:AAA3001
Hypenodes palustris|[121]|RDLQG618-06|DH012911|658[0n]bp|Canada.Quebec|BOLD:AAA3001
Hypenodes palustris|[122]|RDLQG619-06|DH012912|658[0n]bp|Canada.Quebec|BOLD:AAA3001
Hypenodes palustris|[123]|RDLQG623-06|DH012916|658[0n]bp|Canada.Quebec|BOLD:AAA3001
Hypenodes palustris|[124]|RDLQG658-06|DH012951|582[2n]bp|Canada.Quebec|BOLD:AAA3001
Hypenodes palustris|[125]|RDLQG643-06|DH012936|590[0n]bp|Canada.Quebec|BOLD:AAA3001
Hypenodes palustris|[126]|RDLQG659-06|DH012952|633[0n]bp|Canada.Quebec|BOLD:AAA3001
Hypenodes palustris|[127]|RDLQG638-06|DH012931|595[0n]bp|Canada.Quebec|BOLD:AAA3001
Hypenodes palustris|[128]|RDLQG645-06|DH012938|595[0n]bp|Canada.Quebec|BOLD:AAA3001
Hypenodes palustris|[129]|RDLQG641-06|DH012934|595[0n]bp|Canada.Quebec|BOLD:AAA3001
Hypenodes palustris|[130]|RDLQG651-06|DH012944|595[0n]bp|Canada.Quebec|BOLD:AAA3001
Hypenodes palustris|[131]|RDLQG885-06|DH013178|595[0n]bp|Canada.Quebec|BOLD:AAA3001
Hypenodes palustris|[132]|RDLQG639-06|DH012932|595[0n]bp|Canada.Quebec|BOLD:AAA3001
Hypenodes palustris|[133]|RDLQG634-06|DH012927|595[0n]bp|Canada.Quebec|BOLD:AAA3001
Hypenodes palustris|[134]|RDLQG630-06|DH012923|599[0n]bp|Canada.Quebec|BOLD:AAA3001
Hypenodes palustris|[135]|RDLQG631-06|DH012924|594[0n]bp|Canada.Quebec|BOLD:AAA3001
Hypenodes palustris|[136]|RDLQG632-06|DH012925|603[0n]bp|Canada.Quebec|BOLD:AAA3001
Hypenodes palustris|[137]|RDLQG655-06|DH012948|605[0n]bp|Canada.Quebec|BOLD:AAA3001
Hypenodes palustris|[138]|RDLQG636-06|DH012929|631[0n]bp|Canada.Quebec|BOLD:AAA3001
Hypenodes palustris|[139]|RDLQG657-06|DH012950|621[0n]bp|Canada.Quebec|BOLD:AAA3001
Hypenodes palustris|[140]|RDLQG650-06|DH012943|656[0n]bp|Canada.Quebec|BOLD:AAA3001
Hypenodes palustris|[141]|RDLQG629-06|DH012922|658[0n]bp|Canada.Quebec|BOLD:AAA3001
Hypenodes palustris|[142]|TMNBD634-07|MNBTT-3435|658[0n]bp|Canada.New Brunswick|BOLD:AAA3001
Hypenodes palustris|[143]|RDLQG640-06|DH012933|658[0n]bp|Canada.Quebec|BOLD:AAA3001
Hypenodes palustris|[144]|RDLQG642-06|DH012935|658[0n]bp|Canada.Quebec|BOLD:AAA3001
Hypenodes palustris|[145]|RDLQG653-06|DH012946|658[0n]bp|Canada.Quebec|BOLD:AAA3001
Hypenodes palustris|[146]|RDLQG626-06|DH012919|658[0n]bp|Canada.Quebec|BOLD:AAA3001
Hypenodes palustris|[147]|RDLQE229-06|MDH002232|657[0n]bp|Canada.Quebec|BOLD:AAA3001
Hypenodes palustris|[148]|RDLQG628-06|DH012921|658[0n]bp|Canada.Quebec|BOLD:AAA3001
Hypenodes palustris|[149]|RDLQG661-06|DH012954|649[0n]bp|Canada.Quebec|BOLD:AAA3001
Hypenodes palustris|[150]|RDLQG647-06|DH012940|646[0n]bp|Canada.Quebec|BOLD:AAA3001
Hypenodes palustris|[151]|RDLQG649-06|DH012942|648[0n]bp|Canada.Quebec|BOLD:AAA3001
Hypenodes palustris|[152]|RDLQG654-06|DH012947|646[1n]bp|Canada.Quebec|BOLD:AAA3001
Hypenodes palustris|[153]|RDLQG656-06|DH012949|608[0n]bp|Canada.Quebec|BOLD:AAA3001
Hypenodes caducus|[154]|RDNME654-08|LEP038078|658[0n]bp|Canada.Ontario|BOLD:AAA3004
Hypenodes caducus|[155]|RDLQC409-06|MDH000426|658[0n]bp|Canada.Quebec|BOLD:AAA3004
Hypenodes caducus|[156]|RDNME656-08|LEP038080|658[0n]bp|Canada.Ontario|BOLD:AAA3004
Hypenodes caducus|[157]|RDNME641-08|LEP038065|658[0n]bp|Canada.Quebec|BOLD:AAA3004
Hypenodes caducus|[158]|RDLQG578-06|DH012871|658[0n]bp|Canada.Quebec|BOLD:AAA3004
Hypenodes caducus|[159]|RDNMK288-11|CNCLEP 84197|658[0n]bp|Canada.Ontario|BOLD:AAA3004
Hypenodes caducus|[160]|RDLQC560-06|MDH001696|658[0n]bp|Canada.Quebec|BOLD:AAA3004
Hypenodes caducus|[161]|RDLQC562-06|MDH000334|658[0n]bp|Canada.Quebec|BOLD:AAA3004
Hypenodes caducus|[162]|RDNMK296-11|CNCLEP 84205|658[0n]bp|Canada.Ontario|BOLD:AAA3004
Hypenodes caducus|[163]|RDLQG715-06|DH013008|658[0n]bp|Canada.Quebec|BOLD:AAA3004
Hypenodes caducus|[164]|RDNMK289-11|CNCLEP 84198|658[0n]bp|Canada.Ontario|BOLD:AAA3004
Hypenodes caducus|[165]|RDLQC503-06|MDH000860|658[0n]bp|Canada.Quebec|BOLD:AAA3004
Hypenodes caducus|[166]|RDLQC501-06|MDH000826|658[0n]bp|Canada.Quebec|BOLD:AAA3004
Hypenodes caducus|[167]|RDLQC561-06|MDH001673|658[0n]bp|Canada.Quebec|BOLD:AAA3004
Hypenodes caducus|[168]|RDLQG718-06|DH013011|658[0n]bp|Canada.Quebec|BOLD:AAA3004
Hypenodes caducus|[169]|RDLQC502-06|MDH001689|658[0n]bp|Canada.Quebec|BOLD:AAA3004
Hypenodes caducus|[170]|RDLQC563-06|MDH000277|658[0n]bp|Canada.Quebec|BOLD:AAA3004
Hypenodes caducus|[171]|RDLQC408-06|MDH000410|658[0n]bp|Canada.Quebec|BOLD:AAA3004
Hypenodes caducus|[172]|RDLQC504-06|MDH000789|658[0n]bp|Canada.Quebec|BOLD:AAA3004
Hypenodes caducus|[173]|RDNMK295-11|CNCLEP 84204|658[0n]bp|Canada.Ontario|BOLD:AAA3004
Hypenodes caducus|[174]|RDLQG771-06|DH013064|658[0n]bp|Canada.Quebec|BOLD:AAA3004
Hypenodes caducus|[175]|RDNMK292-11|CNCLEP 84201|658[0n]bp|Canada.Ontario|BOLD:AAA3004
Hypenodes caducus|[176]|RDNMK286-11|CNCLEP 84195|658[0n]bp|Canada.Ontario|BOLD:AAA3004
Hypenodes caducus|[177]|RDNMK287-11|CNCLEP 84196|658[0n]bp|Canada.Ontario|BOLD:AAA3004
Hypenodes caducus|[178]|RDNME655-08|LEP038079|658[0n]bp|Canada.Ontario|BOLD:AAA3004
Hypenodes sombrus|[179]|LBCH3839-10|10-JDWBC-3839|658[0n]bp|Canada.British Columbia|BOLD:AAA3005
Hypenodes sombrus|[180]|TMNBD639-07|MNBTT-3440|658[0n]bp|Canada.New Brunswick|BOLD:AAA3005
Hypenodes sombrus|[181]|TMNBD640-07|MNBTT-3441|658[0n]bp|Canada.New Brunswick|BOLD:AAA3005
Hypenodes sombrus|[182]|RDLQE184-06|MDH002187|658[0n]bp|Canada.Quebec|BOLD:AAA3005
Hypenodes sombrus|[183]|RDLQC558-06|MDH002049|573[2n]bp|Canada.Quebec|BOLD:AAA3005
Hypenodes sombrus|[184]|RDLQG662-06|DH012955|658[0n]bp|Canada.Quebec|BOLD:AAA3005
Hypenodes sombrus|[185]|RDLQE185-06|MDH002188|657[0n]bp|Canada.Quebec|BOLD:AAA3005
Hypenodes sombrus|[186]|MECD359-06|jflandry2931|658[0n]bp|Canada.Quebec|BOLD:AAA3005
Hypenodes sombrus|[187]|RDLQC557-06|MDH002041|658[0n]bp|Canada.Quebec|BOLD:AAA3005
Hypenodes sombrus|[188]|RDLQE228-06|MDH002231|657[0n]bp|Canada.Quebec|BOLD:AAA3005
Hypenodes sombrus|[189]|RDLQG663-06|DH012956|658[0n]bp|Canada.Quebec|BOLD:AAA3005
Hypenodes sombrus|[190]|RDLQC559-06|MDH000676|658[0n]bp|Canada.Quebec|BOLD:AAA3005
Hypenodes sombrus|[191]|RDNME640-08|LEP038064|658[0n]bp|Canada.Quebec|BOLD:AAA3005
Hypenodes sombrus|[192]|RDLQG622-06|DH012915|658[0n]bp|Canada.Quebec|BOLD:AAA3005
Hypenodes sombrus|[193]|MNAF482-08|CNCLEP00040467|658[1n]bp|Canada.Manitoba|BOLD:AAA3005
Dasychira dorsipennata|[194]|JSSEP1056-11|BIOUG01630-E10|609[0n]bp|Canada.Ontario|BOLD:AAB8421
Dasychira dorsipennata|[195]|RDLQB692-05|DH010795|658[0n]bp|Canada.Quebec|BOLD:AAB8421
Dasychira obliquata|[196]|PHMO293-03|moth2037.02|639[2n]bp|Canada.Ontario|BOLD:AAB8420
Dasychira obliquata|[197]|RDNMF632-08|NOC14718|658[0n]bp|Canada.Ontario|BOLD:AAB8420
Dasychira obliquata|[198]|RDNMF631-08|NOC14717|658[0n]bp|Canada.Ontario|BOLD:AAB8420
Dasychira obliquata|[199]|[illegible]|[illegible]|[illegible]|Canada.Ontario|BOLD:AAB8420

Dasychira obliquata|[196]|PHMO293-03|moth2037.02|639[2n]bp|Canada.Ontario|BOLD:AAB8420
Dasychira obliquata|[197]|RDNMF632-08|NOC14718|658[0n]bp|Canada.Ontario|BOLD:AAB8420
Dasychira obliquata|[198]|RDNMF631-08|NOC14717|658[0n]bp|Canada.Ontario|BOLD:AAB8420
Dasychira obliquata|[199]|RDNML069-13|CNCLEP 94293|658[0n]bp|Canada.Ontario|BOLD:AAB8420
Dasychira obliquata|[200]|RDLQF809-06|DH011959|658[0n]bp|Canada.Quebec|BOLD:AAB8420
Dasychira obliquata|[201]|RDLQB828-05|DH010915|615[0n]bp|Canada.Quebec|BOLD:AAB8420
Dasychira obliquata|[202]|RDLQB829-05|DH010916|658[0n]bp|Canada.Quebec|BOLD:AAB8420
Dasychira grisefacta|[203]|LBCB617-05|HLC-21557|639[1n]bp|Canada.British Columbia|BOLD:ABZ5347
Dasychira grisefacta ella|[204]|LPVIA113-08|PFC-2006-0179|658[0n]bp|Canada.British Columbia|BOLD:ABZ...
Dasychira grisefacta|[205]|RDNMJ798-11|CNCLEP 80326|658[0n]bp|Canada.Quebec|BOLD:ABZ5347
Dasychira grisefacta|[206]|RDNMJ799-11|CNCLEP 80327|658[0n]bp|Canada.Quebec|BOLD:ABZ5347
Dasychira grisefacta|[207]|LBCD252-05|HLC-23072|658[0n]bp|Canada.British Columbia|BOLD:ABZ5347
Dasychira grisefacta|[208]|RDNMJ409-11|CNCLEP 79937|658[0n]bp|Canada.Quebec|BOLD:ABZ5347
Dasychira grisefacta|[209]|LPAB219-08|08BBLEP-02541|656[1n]bp|Canada.Alberta|BOLD:ABZ5347
Dasychira grisefacta|[210]|LBCH506-10|10-JDWBC-0506|658[0n]bp|Canada.British Columbia|BOLD:ABZ5347
Dasychira grisefacta|[211]|LBCH801-10|10-JDWBC-0801|658[0n]bp|Canada.British Columbia|BOLD:ABZ5347
Dasychira grisefacta|[212]|DUNLP142-08|Dun-08-142|658[0n]bp|Canada.British Columbia|BOLD:ABZ5347
Dasychira grisefacta|[213]|LBCD324-05|HLC-23144|642[0n]bp|Canada.British Columbia|BOLD:ABZ5347
Dasychira grisefacta|[214]|LBCB183-05|HLC-21123|613[0n]bp|Canada.British Columbia|BOLD:ABZ5347
Dasychira grisefacta|[215]|RDNMK656-11|CNCLEP 81833|618[0n]bp|Canada.British Columbia|BOLD:ABZ5347
Dasychira grisefacta|[216]|LBCD100-05|HLC-22920|658[0n]bp|Canada.British Columbia|BOLD:ABZ5347
Dasychira grisefacta|[217]|LPABC077-09|08BBLEP-04296|658[1n]bp|Canada.Alberta|BOLD:ABZ5347
Dasychira grisefacta ella|[218]|LMH018-06|PFC-2006-0160|656[0n]bp|Canada.British Columbia|BOLD:ABZ5347
Dasychira grisefacta ella|[219]|LMH019-06|PFC-2006-0161|658[0n]bp|Canada.British Columbia|BOLD:ABZ5347
Dasychira grisefacta|[220]|LPMN964-08|08BBLEP-02322|658[0n]bp|Canada.Alberta|BOLD:ABZ5347
Dasychira grisefacta|[221]|BBLPB641-10|10BBCLP-1640|658[0n]bp|Canada.British Columbia|BOLD:ABZ5347
Dasychira grisefacta|[222]|LBCD272-05|HLC-23092|658[0n]bp|Canada.British Columbia|BOLD:ABZ5347
Dasychira grisefacta|[223]|RDNMK657-11|CNCLEP 81834|658[0n]bp|Canada.British Columbia|BOLD:ABZ5347
Dasychira grisefacta|[224]|RDNMJ408-11|CNCLEP 79936|658[0n]bp|Canada.Quebec|BOLD:ABZ5347
Dasychira grisefacta|[225]|LPVIB264-08|PFC-2006-1654|658[0n]bp|Canada.British Columbia|BOLD:ABZ5347
Dasychira grisefacta|[226]|LBCC870-05|HLC-22750|658[0n]bp|Canada.British Columbia|BOLD:ABZ5347
Dasychira grisefacta|[227]|LPVIB848-08|PFC-2006-2355|658[0n]bp|Canada.British Columbia|BOLD:ABZ5347
Dasychira grisefacta|[228]|LBCH698-10|10-JDWBC-0698|658[0n]bp|Canada.British Columbia|BOLD:ABZ5347
Dasychira grisefacta|[229]|LPABC071-09|08BBLEP-04290|658[0n]bp|Canada.Alberta|BOLD:ABZ5347
Dasychira grisefacta|[230]|LBCH2129-10|10-JDWBC-2129|658[0n]bp|Canada.British Columbia|BOLD:ABZ5347
Dasychira grisefacta|[231]|LBCH222-10|10-JDWBC-0222|658[0n]bp|Canada.British Columbia|BOLD:ABZ5347
Dasychira grisefacta|[232]|LPVIB265-08|PFC-2006-1655|658[0n]bp|Canada.British Columbia|BOLD:ABZ5347
Dasychira grisefacta|[233]|LBCB148-05|HLC-21088|658[0n]bp|Canada.British Columbia|BOLD:ABZ5347
Dasychira grisefacta|[234]|RDLQB199-05|DH010285|658[0n]bp|Canada.Quebec|BOLD:ABZ5347
Dasychira grisefacta|[235]|LBCH116-10|10-JDWBC-0116|658[0n]bp|Canada.British Columbia|BOLD:ABZ5347
Dasychira grisefacta|[236]|LALPA840-11|AVBC 1013-11|658[0n]bp|Canada.British Columbia|BOLD:ABZ5347
Dasychira grisefacta|[237]|LBCD281-05|HLC-23101|658[0n]bp|Canada.British Columbia|BOLD:ABZ5347
Dasychira grisefacta|[238]|LBCB203-05|HLC-21143|658[0n]bp|Canada.British Columbia|BOLD:ABZ5347
Dasychira grisefacta|[239]|LPVIB448-08|PFC-2006-1855|658[0n]bp|Canada.British Columbia|BOLD:ABZ5347
Dasychira grisefacta|[240]|RDLQF859-06|DH012020|658[0n]bp|Canada.Quebec|BOLD:ABZ5347
Dasychira grisefacta|[241]|LOWCE828-06|CGWC-4588|658[0n]bp|Canada.British Columbia|BOLD:ABZ5347
Dasychira grisefacta|[242]|LPABC343-09|08BBLEP-04562|658[0n]bp|Canada.Alberta|BOLD:ABZ5347
Dasychira pinicola|[243]|RDNML068-13|CNCLEP 94292|658[0n]bp|Canada.Ontario|BOLD:ABZ5347
Dasychira grisefacta|[244]|LBCG2301-09|08-JDWBC-2301|658[0n]bp|Canada.British Columbia|BOLD:ABZ5347
Dasychira grisefacta|[245]|LBCD251-05|HLC-23071|645[0n]bp|Canada.British Columbia|BOLD:ABZ5347
Dasychira grisefacta|[246]|LPABC092-09|08BBLEP-04311|606[2n]bp|Canada.Alberta|BOLD:ABZ5347
Dasychira pinicola|[247]|RDNML065-13|CNCLEP 94289|658[0n]bp|Canada.Ontario|BOLD:ABZ5347
Dasychira plagiata|[248]|RDLQB698-05|DH010801|658[0n]bp|Canada.Quebec|BOLD:AAB2274
Dasychira plagiata|[249]|RDNMF634-08|NOC14720|658[0n]bp|Canada.Ontario|BOLD:AAB2274
Dasychira plagiata|[250]|XAG403-05|2005-ONT-987|658[0n]bp|Canada.Ontario|BOLD:AAB2274
Dasychira plagiata|[251]|PHMO239-03|moth1262.02|639[0n]bp|Canada.Ontario|BOLD:AAB2274
Dasychira plagiata|[252]|RDNML066-13|CNCLEP 94290|658[0n]bp|Canada.Ontario|BOLD:AAB2274
Dasychira plagiata|[253]|XAG161-05|2005-ONT-745|658[0n]bp|Canada.Ontario|BOLD:AAB2274
Dasychira plagiata|[254]|XAK261-06|2006-ONT-1256|658[0n]bp|Canada.Ontario|BOLD:AAB2274
Dasychira plagiata|[255]|RDNML067-13|CNCLEP 94291|658[0n]bp|Canada.Nova Scotia|BOLD:AAB2274
Dasychira plagiata|[256]|MNBB059-05|HBL008669|658[0n]bp|Canada.New Brunswick|BOLD:AAB2274
Dasychira plagiata|[257]|XAG163-05|2005-ONT-747|658[0n]bp|Canada.Ontario|BOLD:AAB2274
Dasychira plagiata|[258]|XAG162-05|2005-ONT-746|658[0n]bp|Canada.Ontario|BOLD:AAB2274
Dasychira plagiata|[259]|PHMO001-03|DASY1|639[0n]bp|Canada.Ontario|BOLD:AAB2274
Dasychira plagiata|[260]|XAG114-05|2005-ONT-698|658[0n]bp|Canada.Ontario|BOLD:AAB2274
Dasychira plagiata|[261]|PHMO237-03|moth1244.02|639[0n]bp|Canada.Ontario|BOLD:AAB2274
Dasychira plagiata|[262]|XAB485-04|04HBL005485|658[0n]bp|Canada.Ontario|BOLD:AAB2274
Dasychira plagiata|[263]|XAK015-06|2006-ONT-1010|658[0n]bp|Canada.Ontario|BOLD:AAB2274
Dasychira plagiata|[264]|MNBB002-05|HBL008612|658[0n]bp|Canada.New Brunswick|BOLD:AAB2274
Dasychira plagiata|[265]|RDLQF463-06|DH011570|658[0n]bp|Canada.Quebec|BOLD:AAB2274
Dasychira plagiata|[266]|PHMO391-03|moth1156.02|639[0n]bp|Canada.Ontario|BOLD:AAB2274
Dasychira plagiata|[267]|XAK010-06|2006-ONT-1005|658[0n]bp|Canada.Ontario|BOLD:AAB2274
Dasychira plagiata|[268]|XAB003-04|04HBL005003|658[0n]bp|Canada.Ontario|BOLD:AAB2274
Dasychira plagiata|[269]|XAG179-05|2005-ONT-763|658[0n]bp|Canada.Ontario|BOLD:AAB2274
Dasychira plagiata|[270]|XAG097-05|2005-ONT-681|658[0n]bp|Canada.Ontario|BOLD:AAB2274
Dasychira plagiata|[271]|RDLQG142-06|DH012313|658[2n]bp|Canada.Quebec|BOLD:AAB2274
Dasychira basiflava|[272]|LOFLA661-06|06-FLOR-0661|658[0n]bp|United States.Florida|BOLD:AAA7428
Dasychira basiflava|[273]|LNCB212-06|06-NCCC-1168|658[0n]bp|United States.North Carolina|BOLD:AAA7428
Dasychira basiflava|[274]|LSEU721-06|06-JKA-0721|658[0n]bp|United States.Georgia|BOLD:ACE7279
Dasychira basiflava|[275]|HKONS746-08|3462-COI-08|658[0n]bp|United States.Tennessee|BOLD:ACE7279
Dasychira basiflava|[276]|LSEU720-06|06-JKA-0720|658[0n]bp|United States.Georgia|BOLD:ACE7279
Dasychira basiflava|[277]|LGSM615-04|DNA-ATBI-0615|658[1n]bp|United States.Tennessee|BOLD:ACE7279
Dasychira basiflava|[278]|LOT554-04|04HBL002554|595[0n]bp|United States.Tennessee|BOLD:ACE7279
Dasychira basiflava|[279]|HKONS732-08|3448-COI-08|658[0n]bp|United States.Florida|BOLD:ACE7279
Dasychira basiflava|[280]|HKONS068-07|1632-COI-07|658[0n]bp|United States.Florida|BOLD:ACE7279
Dasychira basiflava|[281]|HKONS731-08|3447-COI-08|658[0n]bp|United States.Florida|BOLD:ACE7279
Dasychira basiflava|[282]|HKONS733-08|3449-COI-08|658[0n]bp|United States.Florida|BOLD:ACE7279
Dasychira basiflava|[283]|HKONS069-07|1633-COI-07|658[0n]bp|United States.Florida|BOLD:ACE7279
Dasychira basiflava|[284]|BBLSZ168-09|09BBLEP-04094|658[0n]bp|United States.Texas|BOLD:ACE7279
Dasychira basiflava|[285]|BBLSZ169-09|09BBLEP-04095|658[0n]bp|United States.Texas|BOLD:ACE7279
Dasychira basiflava|[286]|BBLSY926-09|09BBLEP-03853|658[0n]bp|United States.Texas|BOLD:ACE7279
Dasychira basiflava|[287]|HKONS738-08|3454-COI-08|658[0n]bp|United States.Texas|BOLD:ACE7279
Dasychira basiflava|[288]|HKONB394-09|3891-COI-08|658[0n]bp|United States.Kentucky|BOLD:ACE7279
Dasychira basiflava|[289]|HKONS741-08|3457-COI-08|658[0n]bp|United States.Texas|BOLD:ACE7279
Dasychira basiflava|[290]|HKONS740-08|3456-COI-08|627[0n]bp|United States.Texas|BOLD:ACE7279
Dasychira basiflava|[291]|LTOL618-07|CWM-94-0313|632[0n]bp|United States.Maryland|BOLD:ACE7279
Dasychira basiflava|[292]|HKONS739-08|3455-COI-08|658[0n]bp|United States.Texas|BOLD:ACE7279
Dasychira basiflava|[293]|HKONS745-08|3461-COI-08|658[0n]bp|United States.Arkansas|BOLD:ACE7279
Dasychira basiflava|[294]|HKONS748-08|3464-COI-08|605[0n]bp|United States.Virginia|BOLD:ACE7279
Dasychira basiflava|[295]|LNCB213-06|06-NCCC-1169|658[0n]bp|United States.North Carolina|BOLD:ACE7279
Dasychira basiflava|[296]|LNCB211-06|06-NCCC-1167|658[0n]bp|United States.North Carolina|BOLD:ACE7279
Dasychira vagans|[297]|LPABC841-09|08BBLEP-05060|658[0n]bp|Canada.Alberta|BOLD:ACE7279
Dasychira vagans|[298]|LPABB076-08|08BBLEP-03341|658[0n]bp|Canada.Alberta|BOLD:ACE7279

[illegible]
Dasychira basiflava|[296]|LNCB211-06|06-NCCC-1167|658[0n]bp|United States.North Carolina|BOLD:ACE7279
Dasychira vagans|[297]|LPABC841-09|08BBLEP-05060|658[0n]bp|Canada.Alberta|BOLD:ACE7279
Dasychira vagans|[298]|LPABB076-08|08BBLEP-03341|658[0n]bp|Canada.Alberta|BOLD:ACE7279
Dasychira vagans|[299]|LPABB503-08|08BBLEP-03768|658[0n]bp|Canada.Alberta|BOLD:ACE7279
Dasychira vagans|[300]|LPMN922-08|08BBLEP-02280|654[0n]bp|Canada.Alberta|BOLD:ACE7279
Dasychira vagans|[301]|RDLQ754-07|DH006772|658[0n]bp|Canada.Quebec|BOLD:ACE7279
Dasychira vagans|[302]|BBLPB636-10|10BBCLP-1635|658[0n]bp|Canada.British Columbia|BOLD:ACE7279
Dasychira vagans|[303]|LBCC019-05|HLC-21899|658[0n]bp|Canada.British Columbia|BOLD:ACE7279
Dasychira vagans|[304]|LBCD014-05|HLC-22834|658[0n]bp|Canada.British Columbia|BOLD:ACE7279
Dasychira vagans|[305]|BBLPB637-10|10BBCLP-1636|658[0n]bp|Canada.British Columbia|BOLD:ACE7279
Dasychira vagans|[306]|LBCB202-05|HLC-21142|658[0n]bp|Canada.British Columbia|BOLD:ACE7279
Dasychira vagans|[307]|LBCD015-05|HLC-22835|658[0n]bp|Canada.British Columbia|BOLD:ACE7279
Dasychira vagans|[308]|LBCA798-05|HLC-20798|658[0n]bp|Canada.British Columbia|BOLD:ACE7279
Dasychira vagans|[309]|LOWCB661-05|CGWC-1601|571[0n]bp|Canada.British Columbia|BOLD:ACE7279
Dasychira vagans|[310]|RDNMJ656-11|CNC LEP 70118|642[0n]bp|Canada.Alberta|BOLD:ACE7279
Dasychira vagans|[311]|LBCC015-05|HLC-21895|658[0n]bp|Canada.British Columbia|BOLD:ACE7279
Dasychira vagans|[312]|RDLQG370-06|DH012604|658[0n]bp|Canada.Quebec|BOLD:ACE7279
Dasychira vagans|[313]|RDLQG369-06|DH012603|658[0n]bp|Canada.Quebec|BOLD:ACE7279
Dasychira vagans|[314]|XAE448-04|Moth4448.03|573[0n]bp|Canada.Ontario|BOLD:ACE7279
Dasychira vagans|[315]|RDNMF633-08|NOC14719|652[0n]bp|Canada.Ontario|BOLD:ACE7279
Gynaephora groenlandica|[316]|RDNMJ572-11|acrorev gga2|643[0n]bp|Canada.Yukon Territory|BOLD:AAE6832
Gynaephora groenlandica|[317]|RDNMJ571-11|acrorev gga1|658[0n]bp|Canada.Yukon Territory|BOLD:AAE6832
Gynaephora groenlandica|[318]|MNAD827-07|CNCLEP00028425|658[0n]bp|Canada.Nunavut|BOLD:AAE6832
Gynaephora groenlandica|[319]|MNAD825-07|CNCLEP00028423|654[0n]bp|Canada.Nunavut|BOLD:AAE6832
Gynaephora groenlandica|[320]|MNAD826-07|CNCLEP00028424|658[0n]bp|Canada.Nunavut|BOLD:AAE6832
Gynaephora groenlandica|[321]|RDNME645-08|LEP038069|649[1n]bp|Canada|BOLD:AAE6832
Gynaephora rossii|[322]|LCHP021-07|07PROBE-00081|658[0n]bp|Canada.Manitoba|BOLD:AAD4553
Gynaephora rossii|[323]|RDMAB751-06|BCSC420|643[0n]bp|Canada.British Columbia|BOLD:AAD4553
Gynaephora rossii|[324]|MNAD824-07|CNCLEP00028422|658[0n]bp|Canada.Nunavut|BOLD:AAD4553
Gynaephora rossii|[325]|MNAD823-07|CNCLEP00028421|658[0n]bp|Canada.Nunavut|BOLD:AAD4553
Gynaephora rossii|[326]|RDNME644-08|LEP038068|658[0n]bp|Canada|BOLD:AAD4553
Gynaephora rossii|[327]|RDNME642-08|LEP038066|658[0n]bp|Canada.Yukon Territory|BOLD:AAD4553
Zanclognatha protumnusalis|[328]|PMG169-03|PHAEL1.00|617[0n]bp|Canada.Ontario|BOLD:AAA5206
Zanclognatha protumnusalis|[329]|RDLQF781-06|DH011931|597[1n]bp|Canada.Quebec|BOLD:AAA5206
Zanclognatha protumnusalis|[330]|XAH227-05|2005-ONT-1810|627[0n]bp|Canada.Ontario|BOLD:AAA5206
Zanclognatha protumnusalis|[331]|BBLPC464-09|09BBELE-1464|658[0n]bp|Canada.New Brunswick|BOLD:AAA5206
Zanclognatha protumnusalis|[332]|RDLQF410-06|DH011517|658[0n]bp|Canada.Quebec|BOLD:AAA5206
Zanclognatha protumnusalis|[333]|RDLQF578-06|DH011728|658[0n]bp|Canada.Quebec|BOLD:AAA5206
Zanclognatha protumnusalis|[334]|RDLQG048-06|DH012179|658[0n]bp|Canada.Quebec|BOLD:AAA5206
Zanclognatha protumnusalis|[335]|RDLQF765-06|DH011915|658[0n]bp|Canada.Quebec|BOLD:AAA5206
Zanclognatha protumnusalis|[336]|BBLEC743-09|09BBELE-0743|658[0n]bp|Canada.Nova Scotia|BOLD:AAA5206
Zanclognatha protumnusalis|[337]|RDLQG170-06|DH012341|658[0n]bp|Canada.Quebec|BOLD:AAA5206
Zanclognatha protumnusalis|[338]|XAE548-04|Moth4548.03|586[1n]bp|Canada.Ontario|BOLD:AAA5206
Zanclognatha protumnusalis|[339]|RDMAB964-09|UASM99717|622[0n]bp|Canada.Ontario|BOLD:AAA5206
Zanclognatha protumnusalis|[340]|RDLQG117-06|DH012274|606[2n]bp|Canada.Quebec|BOLD:AAA5206
Zanclognatha protumnusalis|[341]|RDLQF418-06|DH011525|621[0n]bp|Canada.Quebec|BOLD:AAA5206
Zanclognatha protumnusalis|[342]|XAC045-04|04HBL006045|658[0n]bp|Canada.Ontario|BOLD:AAA5206
Zanclognatha protumnusalis|[343]|RDLQF784-06|DH011934|658[0n]bp|Canada.Quebec|BOLD:AAA5206
Zanclognatha protumnusalis|[344]|RDLQF409-06|DH011516|658[0n]bp|Canada.Quebec|BOLD:AAA5206
Zanclognatha protumnusalis|[345]|XAG720-05|2005-ONT-1304|658[0n]bp|Canada.Ontario|BOLD:AAA5206
Zanclognatha protumnusalis|[346]|RDLQF344-06|DH011503|658[0n]bp|Canada.Quebec|BOLD:AAA5206
Zanclognatha protumnusalis|[347]|XAE604-04|Moth4604.03|658[0n]bp|Canada.Ontario|BOLD:AAA5206
Zanclognatha protumnusalis|[348]|RDLQG845-06|DH013138|658[0n]bp|Canada.Quebec|BOLD:AAA5206
Zanclognatha protumnusalis|[349]|XAG595-05|2005-ONT-1179|658[0n]bp|Canada.Ontario|BOLD:AAA5206
Zanclognatha protumnusalis|[350]|RDLQG790-06|DH013083|658[0n]bp|Canada.Quebec|BOLD:AAA5206
Zanclognatha protumnusalis|[351]|XAG299-05|2005-ONT-883|658[0n]bp|Canada.Ontario|BOLD:AAA5206
Zanclognatha protumnusalis|[352]|RDLQG679-06|DH012972|658[0n]bp|Canada.Quebec|BOLD:AAA5206
Zanclognatha protumnusalis|[353]|HEJUL264-12|BIOUG02385-F12|625[0n]bp|Canada.Ontario|BOLD:AAA5206
Zanclognatha protumnusalis|[354]|RDLQG789-06|DH013082|658[0n]bp|Canada.Quebec|BOLD:AAA5206
Zanclognatha protumnusalis|[355]|RDLQF407-06|DH011514|658[0n]bp|Canada.Quebec|BOLD:AAA5206
Zanclognatha protumnusalis|[356]|XAC666-04|04HBL006666|658[0n]bp|Canada.Ontario|BOLD:AAA5206
Zanclognatha protumnusalis|[357]|RDLQF414-06|DH011521|621[0n]bp|Canada.Quebec|BOLD:AAA5206
Zanclognatha protumnusalis|[358]|RDLQF785-06|DH011935|658[0n]bp|Canada.Quebec|BOLD:AAA5206
Zanclognatha protumnusalis|[359]|TMNBB050-06|MNBTT-990|658[0n]bp|Canada.New Brunswick|BOLD:AAA5206
Zanclognatha protumnusalis|[360]|RDLQG029-06|DH012160|658[0n]bp|Canada.Quebec|BOLD:AAA5206
Zanclognatha protumnusalis|[361]|RDLQF780-06|DH011930|658[0n]bp|Canada.Quebec|BOLD:AAA5206
Zanclognatha protumnusalis|[362]|RDLQF413-06|DH011520|658[0n]bp|Canada.Quebec|BOLD:AAA5206
Zanclognatha protumnusalis|[363]|XAD689-05|2005-ONT-488|658[0n]bp|Canada.Ontario|BOLD:AAA5206
Zanclognatha protumnusalis|[364]|RDLQG774-06|DH013067|658[0n]bp|Canada.Quebec|BOLD:AAA5206
Zanclognatha protumnusalis|[365]|XAG331-05|2005-ONT-915|658[0n]bp|Canada.Ontario|BOLD:AAA5206
Zanclognatha protumnusalis|[366]|XAK066-06|2006-ONT-1061|658[0n]bp|Canada.Ontario|BOLD:AAA5206
Zanclognatha protumnusalis|[367]|BLTIB1138-08|BL1152|658[0n]bp|Canada.Ontario|BOLD:AAA5206
Zanclognatha protumnusalis|[368]|RDLQF411-06|DH011518|658[0n]bp|Canada.Quebec|BOLD:AAA5206
Zanclognatha protumnusalis|[369]|XAH218-05|2005-ONT-1801|658[0n]bp|Canada.Ontario|BOLD:AAA5206
Zanclognatha marcidilinea|[370]|XAG122-05|2005-ONT-706|658[0n]bp|Canada.Ontario|BOLD:AAA5206
Zanclognatha marcidilinea|[371]|RDNMJ346-11|CNCLEP 80254|658[0n]bp|Canada.Quebec|BOLD:AAA5206
Zanclognatha marcidilinea|[372]|RDLQF257-06|DH011337|658[0n]bp|Canada.Quebec|BOLD:AAA5206
Zanclognatha marcidilinea|[373]|PHMO221-03|moth1127.01|639[0n]bp|Canada.Ontario|BOLD:AAA5206
Zanclognatha marcidilinea|[374]|XAG135-05|2005-ONT-719|636[0n]bp|Canada.Ontario|BOLD:AAA5206
Zanclognatha marcidilinea|[375]|RDLQH139-06|DH006884|636[0n]bp|Canada.Quebec|BOLD:AAA5206
Zanclognatha marcidilinea|[376]|BLGSM006-09|BL1615|644[0n]bp|Canada.Ontario|BOLD:AAA5206
Zanclognatha marcidilinea|[377]|RDNMJ347-11|CNCLEP 80255|658[0n]bp|Canada.Quebec|BOLD:AAA5206
Zanclognatha protumnusalis|[378]|RDLQF442-06|DH011549|658[0n]bp|Canada.Quebec|BOLD:AAA5206
Zanclognatha protumnusalis|[379]|MNBB029-05|HBL008639|658[0n]bp|Canada.New Brunswick|BOLD:AAA5206
Zanclognatha protumnusalis|[380]|RDLQG169-06|DH012340|658[0n]bp|Canada.Quebec|BOLD:AAA5206
Zanclognatha protumnusalis|[381]|XAG099-05|2005-ONT-683|658[0n]bp|Canada.Ontario|BOLD:AAA5206
Zanclognatha protumnusalis|[382]|XAG323-05|2005-ONT-907|658[0n]bp|Canada.Ontario|BOLD:AAA5206
Zanclognatha protumnusalis|[383]|BLTIB757-08|BL1047|658[0n]bp|Canada.Ontario|BOLD:AAA5206
Zanclognatha protumnusalis|[384]|BLTIB692-08|BL977|658[0n]bp|Canada.Ontario|
Zanclognatha protumnusalis|[385]|RDLQF345-06|DH011504|658[0n]bp|Canada.Quebec|BOLD:AAA5206
Zanclognatha protumnusalis|[386]|TMNBB053-06|MNBTT-993|658[0n]bp|Canada.New Brunswick|BOLD:AAA5206
Zanclognatha protumnusalis|[387]|RDLQG028-06|DH012159|658[0n]bp|Canada.Quebec|BOLD:AAA5206
Zanclognatha protumnusalis|[388]|RDLQF560-06|DH011709|658[0n]bp|Canada.Quebec|BOLD:AAA5206
Zanclognatha protumnusalis|[389]|RDLQB688-05|DH010791|658[0n]bp|Canada.Quebec|BOLD:AAA5206
Zanclognatha protumnusalis|[390]|BBLPE153-09|09BBELE-2153|658[0n]bp|Canada.Nova Scotia|BOLD:AAA5206
Zanclognatha protumnusalis|[391]|RDLQG597-06|DH012890|658[0n]bp|Canada.Quebec|BOLD:AAA5206
Zanclognatha protumnusalis|[392]|RDLQG909-06|DH013202|658[0n]bp|Canada.Quebec|BOLD:AAA5206
Zanclognatha protumnusalis|[393]|RDLQG609-06|DH012902|658[0n]bp|Canada.Quebec|BOLD:AAA5206
Zanclognatha protumnusalis|[394]|MNBB080-05|HBL008690|658[0n]bp|Canada.New Brunswick|BOLD:AAA5206
Zanclognatha protumnusalis|[395]|PHMO196-03|moth1009.01|639[0n]bp|Canada.Ontario|BOLD:AAA5206
Zanclognatha protumnusalis|[396]|RDLQG045-06|DH012176|658[0n]bp|Canada.Quebec|BOLD:AAA5206
Zanclognatha protumnusalis|[397]|RDLQG168-06|DH012339|594[2n]bp|Canada.Quebec|BOLD:AAA5206

Zanclognatha protumnusalis|[395]|PHMO196-03|moth1009.01|639[0n]bp|Canada.Ontario|BOLD:AAA5206
Zanclognatha protumnusalis|[396]|RDLQG045-06|DH012176|658[0n]bp|Canada.Quebec|BOLD:AAA5206
Zanclognatha protumnusalis|[397]|RDLQG168-06|DH012339|594[2n]bp|Canada.Quebec|BOLD:AAA5206
Zanclognatha protumnusalis|[398]|BLTIB575-08|BL853|644[0n]bp|Canada.Ontario|BOLD:AAA5206
Zanclognatha protumnusalis|[399]|RDMAB965-09|UASM99718|618[0n]bp|Canada.Ontario|BOLD:AAA5206
Zanclognatha protumnusalis|[400]|BBLPC513-09|09BBELE-1513|658[0n]bp|Canada.New Brunswick|BOLD:AAA5206
Zanclognatha protumnusalis|[401]|RDLQB709-05|DH010812|606[3n]bp|Canada.Quebec|BOLD:AAA5206
Zanclognatha cruralis|[402]|XAC295-04|04HBL006295|658[0n]bp|Canada.Ontario|BOLD:AAA5206
Zanclognatha cruralis|[403]|RDLQF436-06|DH011543|658[0n]bp|Canada.Quebec|BOLD:AAA5206
Zanclognatha cruralis|[404]|RDLQG471-06|DH012764|658[0n]bp|Canada.Quebec|BOLD:AAA5206
Zanclognatha cruralis|[405]|RDLQF437-06|DH011544|659[0n]bp|Canada.Quebec|BOLD:AAA5206
Zanclognatha cruralis|[406]|RDLQF536-06|DH011685|658[0n]bp|Canada.Quebec|BOLD:AAA5206
Zanclognatha jacchusalis|[407]|XAC836-04|04HBL006836|658[0n]bp|Canada.Ontario|BOLD:AAA5206
Zanclognatha jacchusalis|[408]|RDNMF096-08|NOC14182|628[0n]bp|Canada.New Brunswick|BOLD:AAA5206
Zanclognatha jacchusalis|[409]|XAD007-04|04HBL007007|554[0n]bp|Canada.Ontario|BOLD:AAA5206
Zanclognatha jacchusalis|[410]|BLTIB920-08|BL1340|658[0n]bp|Canada.Ontario|BOLD:AAA5206
Zanclognatha jacchusalis|[411]|RDLQF763-06|DH011913|621[0n]bp|Canada.Quebec|BOLD:AAA5206
Zanclognatha jacchusalis|[412]|RDLQF764-06|DH011914|658[0n]bp|Canada.Quebec|BOLD:AAA5206
Zanclognatha jacchusalis|[413]|RDLQF637-06|DH011787|602[3n]bp|Canada.Quebec|BOLD:AAA5206
Zanclognatha jacchusalis|[414]|RDLQF647-06|DH011797|637[0n]bp|Canada.Quebec|BOLD:AAA5206
Zanclognatha jacchusalis|[415]|RDLQF688-06|DH011838|637[0n]bp|Canada.Quebec|BOLD:AAA5206
Zanclognatha jacchusalis|[416]|RDLQF644-06|DH011794|643[0n]bp|Canada.Quebec|BOLD:AAA5206
Zanclognatha jacchusalis|[417]|XAE630-04|Moth4630.03|658[0n]bp|Canada.Ontario|BOLD:AAA5206
Zanclognatha jacchusalis|[418]|XAD011-04|04HBL007011|658[0n]bp|Canada.Ontario|BOLD:AAA5206
Zanclognatha jacchusalis|[419]|RDLQF586-06|DH011736|658[0n]bp|Canada.Quebec|BOLD:AAA5206
Zanclognatha jacchusalis|[420]|RDLQF640-06|DH011790|637[0n]bp|Canada.Quebec|BOLD:AAA5206
Zanclognatha jacchusalis|[421]|RDLQF769-06|DH011919|658[0n]bp|Canada.Quebec|BOLD:AAA5206
Zanclognatha jacchusalis|[422]|RDLQG175-06|DH012346|658[0n]bp|Canada.Quebec|BOLD:AAA5206
Zanclognatha jacchusalis|[423]|RDLQG174-06|DH012345|658[0n]bp|Canada.Quebec|BOLD:AAA5206
Zanclognatha jacchusalis|[424]|XAD001-04|04HBL007001|658[0n]bp|Canada.Ontario|BOLD:AAA5206
Zanclognatha jacchusalis|[425]|RDNMF779-08|UASM99493|658[0n]bp|Canada.British Columbia|BOLD:AAA5206
Zanclognatha jacchusalis|[426]|LBCS240-07|UBC-2007-0621|658[0n]bp|Canada.British Columbia|BOLD:AAA5206
Zanclognatha jacchusalis|[427]|RDNMF778-08|UASM99492|658[0n]bp|Canada.British Columbia|BOLD:AAA5206
Zanclognatha jacchusalis|[428]|LALPA549-10|AVBC 551-10|658[0n]bp|Canada.British Columbia|BOLD:AAA5206
Zanclognatha jacchusalis|[429]|LALPA557-10|AVBC 559-10|658[0n]bp|Canada.British Columbia|BOLD:AAA5206
Zanclognatha jacchusalis|[430]|LALPA491-10|AVBC 493-10|658[0n]bp|Canada.British Columbia|BOLD:AAA5206
Zanclognatha jacchusalis|[431]|RDNMF780-08|UASM99494|658[0n]bp|Canada.British Columbia|BOLD:AAA5206
Zanclognatha jacchusalis|[432]|RDNMF099-08|NOC14185|658[0n]bp|Canada.Ontario|BOLD:AAA5206
Zanclognatha jacchusalis|[433]|XAC856-04|04HBL006856|591[0n]bp|Canada.Ontario|BOLD:AAA5206
Zanclognatha jacchusalis|[434]|RDLQF645-06|DH011795|637[0n]bp|Canada.Quebec|BOLD:AAA5206
Zanclognatha jacchusalis|[435]|RDLQG090-06|DH012247|592[0n]bp|Canada.Quebec|BOLD:AAA5206
Zanclognatha jacchusalis|[436]|RDLQF539-06|DH011688|658[0n]bp|Canada.Quebec|BOLD:AAA5206
Zanclognatha jacchusalis|[437]|RDLQF766-06|DH011916|658[0n]bp|Canada.Quebec|BOLD:AAA5206
Zanclognatha jacchusalis|[438]|RDLQG089-06|DH012246|621[0n]bp|Canada.Quebec|BOLD:AAA5206
Zanclognatha jacchusalis|[439]|RDLQF638-06|DH011788|600[0n]bp|Canada.Quebec|BOLD:AAA5206
Zanclognatha jacchusalis|[440]|RDLQF636-06|DH011786|637[0n]bp|Canada.Quebec|BOLD:AAA5206
Zanclognatha jacchusalis|[441]|RDLQF635-06|DH011785|622[0n]bp|Canada.Quebec|BOLD:AAA5206
Zanclognatha jacchusalis|[442]|RDLQF422-06|DH011529|658[0n]bp|Canada.Quebec|BOLD:AAA5206
Zanclognatha jacchusalis|[443]|XAG164-05|2005-ONT-748|658[0n]bp|Canada.Ontario|BOLD:AAA5206
Zanclognatha jacchusalis|[444]|BLTIB572-08|BL850|609[2n]bp|Canada.Ontario|BOLD:AAA5206
Zanclognatha jacchusalis|[445]|BBLEC553-09|09BBELE-0553|658[0n]bp|Canada.Nova Scotia|BOLD:AAA5206
Zanclognatha jacchusalis|[446]|BBLPE131-09|09BBELE-2131|658[0n]bp|Canada.Nova Scotia|BOLD:AAA5206
Zanclognatha jacchusalis|[447]|BBLPE144-09|09BBELE-2144|658[0n]bp|Canada.Nova Scotia|BOLD:AAA5206
Zanclognatha jacchusalis|[448]|BBLPE098-09|09BBELE-2098|658[0n]bp|Canada.Nova Scotia|BOLD:AAA5206
Zanclognatha jacchusalis|[449]|BBLEC255-09|09BBELE-0255|658[0n]bp|Canada.Nova Scotia|BOLD:AAA5206
Zanclognatha jacchusalis|[450]|BBLEC552-09|09BBELE-0552|658[0n]bp|Canada.Nova Scotia|BOLD:AAA5206
Zanclognatha jacchusalis|[451]|RDLQF641-06|DH011791|637[0n]bp|Canada.Quebec|BOLD:AAA5206
Zanclognatha jacchusalis|[452]|BBLEC207-09|09BBELE-0207|658[0n]bp|Canada.Nova Scotia|BOLD:AAA5206
Zanclognatha jacchusalis|[453]|BBLEC914-09|09BBELE-0914|658[0n]bp|Canada.Nova Scotia|BOLD:AAA5206
Zanclognatha jacchusalis|[454]|RDLQG911-06|DH013204|658[0n]bp|Canada.Quebec|BOLD:AAA5206
Zanclognatha jacchusalis|[455]|BLTIB1137-08|BL1151|656[0n]bp|Canada.Ontario|BOLD:AAA5206
Zanclognatha jacchusalis|[456]|XAH594-05|2005-ONT-2177|658[0n]bp|Canada.Ontario|BOLD:AAA5206
Zanclognatha obscuripennis|[457]|LMEM189-09|RBMIS-0189|658[0n]bp|United States.Mississippi|BOLD:AAA5206
Zanclognatha obscuripennis|[458]|LILLA147-11|SNS10IL-00193|658[0n]bp|United States.Illinois|BOLD:AAA...
Zanclognatha obscuripennis|[459]|LNCC1213-11|11-NCCC-738|658[0n]bp|United States.North Carolina|BOLD ...
Zanclognatha obscuripennis|[460]|LMEM190-09|RBMIS-0190|658[0n]bp|United States.Mississippi|BOLD:AAA5206
Zanclognatha jacchusalis|[461]|RDLQF258-06|DH011338|658[0n]bp|Canada.Quebec|BOLD:AAA5206
Zanclognatha jacchusalis|[462]|BLTIB768-08|BL1162|658[0n]bp|Canada.Ontario|BOLD:AAA5206
Zanclognatha jacchusalis|[463]|BLTIB1099-08|BL1109|658[0n]bp|Canada.Ontario|BOLD:AAA5206
Zanclognatha jacchusalis|[464]|BLTIB1098-08|BL1108|658[0n]bp|Canada.Ontario|BOLD:AAA5206
Zanclognatha jacchusalis|[465]|RDLQH140-06|DH006275|615[0n]bp|Canada.Quebec|BOLD:AAA5206
Zanclognatha jacchusalis|[466]|LPSOD929-09|08BBLEP-05469|658[0n]bp|Canada.Ontario|BOLD:AAA5206
Zanclognatha jacchusalis|[467]|BBLPB977-10|10BBCLP-1976|658[0n]bp|Canada.Alberta|BOLD:AAA5206
Zanclognatha jacchusalis|[468]|XAC025-04|04HBL006025|582[0n]bp|Canada.Ontario|BOLD:AAA5206
Zanclognatha jacchusalis|[469]|XAE637-04|Moth4637.03|658[0n]bp|Canada.Ontario|BOLD:AAA5206
Zanclognatha jacchusalis|[470]|MNBB027-05|HBL008637|589[0n]bp|Canada.New Brunswick|BOLD:AAA5206
Zanclognatha jacchusalis|[471]|RDLQH141-06|DH006898|597[2n]bp|Canada.Quebec|BOLD:AAA5206
Zanclognatha jacchusalis|[472]|BBLPB993-10|10BBCLP-1992|658[0n]bp|Canada.Alberta|BOLD:AAA5206
Zanclognatha jacchusalis|[473]|BBLEC548-09|09BBELE-0548|658[0n]bp|Canada.Nova Scotia|BOLD:AAA5206
Zanclognatha jacchusalis|[474]|TMNBB052-06|MNBTT-992|658[0n]bp|Canada.New Brunswick|BOLD:AAA5206
Zanclognatha jacchusalis|[475]|RDLQF584-06|DH011734|622[1n]bp|Canada.Quebec|BOLD:AAA5206
Zanclognatha jacchusalis|[476]|BBLEC195-09|09BBELE-0195|621[0n]bp|Canada.Nova Scotia|BOLD:AAA5206
Zanclognatha jacchusalis|[477]|RDLQF771-06|DH011921|585[3n]bp|Canada.Quebec|BOLD:AAA5206
Zanclognatha jacchusalis|[478]|BBLPE108-09|09BBELE-2108|658[0n]bp|Canada.Nova Scotia|BOLD:AAA5206
Zanclognatha jacchusalis|[479]|RDLQF443-06|DH011550|658[0n]bp|Canada.Quebec|BOLD:AAA5206
Zanclognatha jacchusalis|[480]|BBLPE107-09|09BBELE-2107|658[0n]bp|Canada.Nova Scotia|BOLD:AAA5206
Zanclognatha jacchusalis|[481]|BBLPE106-09|09BBELE-2106|658[0n]bp|Canada.Nova Scotia|BOLD:AAA5206
Zanclognatha jacchusalis|[482]|BBLEC889-09|09BBELE-0889|658[0n]bp|Canada.Nova Scotia|BOLD:AAA5206
Zanclognatha jacchusalis|[483]|BBLPE105-09|09BBELE-2105|658[0n]bp|Canada.Nova Scotia|BOLD:AAA5206
Zanclognatha jacchusalis|[484]|RDLQF768-06|DH011918|658[0n]bp|Canada.Quebec|BOLD:AAA5206
Zanclognatha jacchusalis|[485]|BBLPE072-09|09BBELE-2072|658[0n]bp|Canada.Nova Scotia|BOLD:AAA5206
Zanclognatha jacchusalis|[486]|RDLQF574-06|DH011724|658[0n]bp|Canada.Quebec|BOLD:AAA5206
Zanclognatha jacchusalis|[487]|BBLPE084-09|09BBELE-2084|658[0n]bp|Canada.Nova Scotia|BOLD:AAA5206
Zanclognatha jacchusalis|[488]|BBLPE032-09|09BBELE-2032|658[0n]bp|Canada.Nova Scotia|BOLD:AAA5206
Zanclognatha jacchusalis|[489]|BBLEC141-09|09BBELE-0141|658[0n]bp|Canada.Nova Scotia|BOLD:AAA5206
Zanclognatha jacchusalis|[490]|RDLQF770-06|DH011920|658[0n]bp|Canada.Quebec|BOLD:AAA5206
Zanclognatha jacchusalis|[491]|BBLPE055-09|09BBELE-2055|658[0n]bp|Canada.Nova Scotia|BOLD:AAA5206
Zanclognatha jacchusalis|[492]|RDLQB418-05|DH010504|658[0n]bp|Canada.Quebec|BOLD:AAA5206
Zanclognatha jacchusalis|[493]|BBLPE090-09|09BBELE-2090|658[0n]bp|Canada.Nova Scotia|BOLD:AAA5206
Zanclognatha jacchusalis|[494]|BBLPE073-09|09BBELE-2073|658[0n]bp|Canada.Nova Scotia|BOLD:AAA5206
Zanclognatha jacchusalis|[495]|TMNBB047-06|MNBTT-987|658[0n]bp|Canada.New Brunswick|BOLD:AAA5206
Zanclognatha jacchusalis|[496]|BBLPE137-09|09BBELE-2137|658[0n]bp|Canada.Nova Scotia|BOLD:AAA5206

Zanclognatha jacchusalis|[494]|BBLPE073-09|09BBELE-2073|658[0n]bp|Canada.Nova Scotia|BOLD:AAA5206
Zanclognatha jacchusalis|[495]|TMNBB047-06|MNBTT-987|658[0n]bp|Canada.New Brunswick|BOLD:AAA5206
Zanclognatha jacchusalis|[496]|BBLPE137-09|09BBELE-2137|658[0n]bp|Canada.Nova Scotia|BOLD:AAA5206
Zanclognatha jacchusalis|[497]|BBLEC292-09|09BBELE-0292|658[0n]bp|Canada.Nova Scotia|BOLD:AAA5206
Zanclognatha jacchusalis|[498]|RDNMJ348-11|CNCLEP 80256|658[0n]bp|Canada.Quebec|BOLD:AAA5206
Zanclognatha jacchusalis|[499]|RDLQF642-06|DH011792|637[0n]bp|Canada.Quebec|BOLD:AAA5206
Zanclognatha jacchusalis|[500]|BBLEC254-09|09BBELE-0254|624[0n]bp|Canada.Nova Scotia|BOLD:AAA5206
Zanclognatha jacchusalis|[501]|RDLQF585-06|DH011735|637[0n]bp|Canada.Quebec|BOLD:AAA5206
Zanclognatha jacchusalis|[502]|BBLEC948-09|09BBELE-0948|644[0n]bp|Canada.Nova Scotia|BOLD:AAA5206
Zanclognatha jacchusalis|[503]|RDLQF703-06|DH011853|637[0n]bp|Canada.Quebec|BOLD:AAA5206
Zanclognatha jacchusalis|[504]|RDLQF701-06|DH011851|637[0n]bp|Canada.Quebec|BOLD:AAA5206
Zanclognatha jacchusalis|[505]|RDLQF702-06|DH011852|637[0n]bp|Canada.Quebec|BOLD:AAA5206
Zanclognatha jacchusalis|[506]|RDLQF704-06|DH011854|637[0n]bp|Canada.Quebec|BOLD:AAA5206
Zanclognatha jacchusalis|[507]|RDLQF687-06|DH011837|637[0n]bp|Canada.Quebec|BOLD:AAA5206
Zanclognatha jacchusalis|[508]|RDLQF643-06|DH011793|637[0n]bp|Canada.Quebec|BOLD:AAA5206
Zanclognatha jacchusalis|[509]|BBLEC227-09|09BBELE-0227|658[0n]bp|Canada.Nova Scotia|BOLD:AAA5206
Zanclognatha jacchusalis|[510]|RDLQF423-06|DH011530|658[0n]bp|Canada.Quebec|BOLD:AAA5206
Zanclognatha jacchusalis|[511]|LOWCB630-05|CGWC-1570|601[1n]bp|Canada.British Columbia|BOLD:AAA5206
Zanclognatha jacchusalis|[512]|BBLPB976-10|10BBCLP-1975|658[0n]bp|Canada.Alberta|BOLD:AAA5206
Zanclognatha jacchusalis|[513]|LOWCB636-05|CGWC-1576|585[0n]bp|Canada.British Columbia|BOLD:AAA5206
Zanclognatha jacchusalis|[514]|LOWCB633-05|CGWC-1573|584[0n]bp|Canada.British Columbia|BOLD:AAA5206
Zanclognatha jacchusalis|[515]|RDNMF098-08|NOC14184|658[0n]bp|Canada.Alberta|BOLD:AAA5206
Zanclognatha jacchusalis|[516]|LOWCB638-05|CGWC-1578|658[0n]bp|Canada.British Columbia|BOLD:AAA5206
Zanclognatha jacchusalis|[517]|BBLPB980-10|10BBCLP-1979|658[0n]bp|Canada.Alberta|BOLD:AAA5206
Zanclognatha jacchusalis|[518]|RDNME671-08|LEP031946|658[0n]bp|Canada.Ontario|BOLD:AAA5206
Zanclognatha jacchusalis|[519]|BBLPB145-10|10BBCLP-1144|658[0n]bp|Canada.British Columbia|BOLD:AAA5206
Zanclognatha jacchusalis|[520]|RDNMF097-08|NOC14183|658[0n]bp|Canada.British Columbia|BOLD:AAA5206
Zanclognatha jacchusalis|[521]|BBLPB146-10|10BBCLP-1145|658[0n]bp|Canada.British Columbia|BOLD:AAA5206
Zanclognatha jacchusalis|[522]|BBLPB143-10|10BBCLP-1142|658[0n]bp|Canada.British Columbia|BOLD:AAA5206
Zanclognatha jacchusalis|[523]|RDLQF767-06|DH011917|658[0n]bp|Canada.Quebec|BOLD:AAA5206
Zanclognatha jacchusalis|[524]|BBLPB446-10|10BBCLP-1445|658[0n]bp|Canada.Alberta|BOLD:AAA5206
Zanclognatha jacchusalis|[525]|BBLPD782-10|10BBCLP-2780|658[0n]bp|Canada.British Columbia|BOLD:AAA5206
Zanclognatha jacchusalis|[526]|BBLPB144-10|10BBCLP-1143|658[0n]bp|Canada.British Columbia|BOLD:AAA5206
Zanclognatha jacchusalis|[527]|BBLPD774-10|10BBCLP-2772|658[0n]bp|Canada.British Columbia|BOLD:AAA5206
Zanclognatha jacchusalis|[528]|BBLPB142-10|10BBCLP-1141|658[0n]bp|Canada.British Columbia|BOLD:AAA5206
Zanclognatha jacchusalis|[529]|LOWCB634-05|CGWC-1574|658[0n]bp|Canada.British Columbia|BOLD:AAA5206
Zanclognatha jacchusalis|[530]|RDLQF639-06|DH011789|637[0n]bp|Canada.Quebec|BOLD:AAA5206
Zanclognatha jacchusalis|[531]|RDLQF646-06|DH011796|637[0n]bp|Canada.Quebec|BOLD:AAA5206
Zanclognatha jacchusalis|[532]|BBLPB992-10|10BBCLP-1991|658[0n]bp|Canada.Alberta|BOLD:AAA5206
Zanclognatha jacchusalis|[533]|BBLPA864-10|10BBCLP-0864|658[0n]bp|Canada.British Columbia|BOLD:AAA5206
Zanclognatha jacchusalis|[534]|LOWCB635-05|CGWC-1575|585[0n]bp|Canada.British Columbia|BOLD:AAA5206
Zanclognatha obscuripennis|[535]|LGSMC415-05|DNA-ATBI-2415|658[0n]bp|United States.Tennessee|BOLD:AA...
Zanclognatha obscuripennis|[536]|RDNMH149-09|CNCLEP00054367|638[0n]bp|United States.Florida|BOLD:AAA...
Zanclognatha obscuripennis|[537]|RDNMD501-06|CNCNoctuoidea12833|658[0n]bp|United States.Florida|BOLD ...
Zanclognatha obscuripennis|[538]|ABNCC067-07|1067-160505-FL|598[0n]bp|United States.Florida|BOLD:AAA...
Zanclognatha obscuripennis|[539]|ABNCC068-07|1068-120505-FL|593[0n]bp|United States.Florida|BOLD:AAA...
Zanclognatha obscuripennis|[540]|LNCC1214-11|11-NCCC-739|658[0n]bp|United States.North Carolina|BOLD ...
Zanclognatha obscuripennis|[541]|LGSMC413-05|DNA-ATBI-2413|658[0n]bp|United States.Tennessee|BOLD:AA...
Zanclognatha obscuripennis|[542]|LNCB203-06|06-NCCC-1159|658[0n]bp|United States.North Carolina|BOLD ...
Zanclognatha obscuripennis|[543]|LNC833-06|06-NCCC-833|658[0n]bp|United States.North Carolina|BOLD:A...
Zanclognatha obscuripennis|[544]|LGSMC417-05|DNA-ATBI-2417|658[0n]bp|United States.Tennessee|BOLD:AA...
Zanclognatha obscuripennis|[545]|MILEP346-10|10-MISC-251|658[0n]bp|United States.Alabama|BOLD:AAA5206
Zanclognatha obscuripennis|[546]|LNCB537-09|09-NCCC-007|658[0n]bp|United States.North Carolina|BOLD:...
Zanclognatha obscuripennis|[547]|HKONS327-08|3232-COI-08|658[0n]bp|United States.Florida|BOLD:AAA5206
Zanclognatha obscuripennis|[548]|LGSMC418-05|DNA-ATBI-2418|658[0n]bp|United States.Tennessee|BOLD:AA...
Zanclognatha obscuripennis|[549]|LGSMC877-05|DNA-ATBI-2877|658[0n]bp|United States.Tennessee|BOLD:AA...
Zanclognatha obscuripennis|[550]|LNCB848-09|09-MISC-033|658[0n]bp|United States.Alabama|BOLD:AAA5206
Zanclognatha obscuripennis|[551]|LNCC959-11|11-NCCC-484|658[0n]bp|United States.North Carolina|BOLD:...
Zanclognatha obscuripennis|[552]|LNCB538-09|09-NCCC-008|658[0n]bp|United States.North Carolina|BOLD:...
Zanclognatha obscuripennis|[553]|LNC830-06|06-NCCC-830|658[0n]bp|United States.North Carolina|BOLD:A...
Zanclognatha obscuripennis|[554]|LGSMC416-05|DNA-ATBI-2416|658[0n]bp|United States.Tennessee|BOLD:AA...
Zanclognatha cruralis|[555]|BLTIB574-08|BL852|631[0n]bp|Canada.Ontario|
Zanclognatha cruralis|[556]|RDLQG035-06|DH012166|658[0n]bp|Canada.Quebec|BOLD:AAA5206
Zanclognatha cruralis|[557]|XAC293-04|04HBL006293|658[0n]bp|Canada.Ontario|BOLD:AAA5206
Zanclognatha cruralis|[558]|RDLQF919-06|DH012094|658[0n]bp|Canada.Quebec|BOLD:AAA5206
Zanclognatha cruralis|[559]|RDLQF916-06|DH012091|658[0n]bp|Canada.Quebec|BOLD:AAA5206
Zanclognatha cruralis|[560]|LPSOC136-08|PPBP-2135|658[0n]bp|Canada.Ontario|BOLD:AAA5206
Zanclognatha cruralis|[561]|RDLQF915-06|DH012090|658[0n]bp|Canada.Quebec|BOLD:AAA5206
Zanclognatha cruralis|[562]|XAB621-04|04HBL005621|658[0n]bp|Canada.Ontario|BOLD:AAA5206
Zanclognatha cruralis|[563]|PHJUN4021-11|BIOUG01497-C08|658[0n]bp|Canada.Ontario|BOLD:AAA5206
Zanclognatha cruralis|[564]|PHJUN4022-11|BIOUG01497-C09|658[0n]bp|Canada.Ontario|BOLD:AAA5206
Zanclognatha dentata|[565]|RDLQF533-06|DH011682|645[0n]bp|Canada.Quebec|BOLD:AAA5206
Zanclognatha dentata|[566]|TMNBB049-06|MNBTT-989|658[0n]bp|Canada.New Brunswick|BOLD:AAA5206
Zanclognatha dentata|[567]|TMNBB048-06|MNBTT-988|658[0n]bp|Canada.New Brunswick|BOLD:AAA5206
Zanclognatha dentata|[568]|RDLQG027-06|DH012158|658[0n]bp|Canada.Quebec|BOLD:AAA5206
Zanclognatha dentata|[569]|BLTIB802-08|BL1220|658[0n]bp|Canada.Ontario|BOLD:AAA5206
Zanclognatha dentata|[570]|PHMO226-03|moth1150.01|639[0n]bp|Canada.Ontario|BOLD:AAA5206
Zanclognatha dentata|[571]|RDLQF415-06|DH011522|635[0n]bp|Canada.Quebec|BOLD:AAA5206
Zanclognatha dentata|[572]|RDLQF342-06|DH011501|658[0n]bp|Canada.Quebec|BOLD:AAA5206
Zanclognatha dentata|[573]|BBLPC014-09|09BBELE-1014|658[0n]bp|Canada.New Brunswick|BOLD:AAA5206
Zanclognatha dentata|[574]|XAG192-05|2005-ONT-776|587[0n]bp|Canada.Ontario|BOLD:AAA5206
Zanclognatha dentata|[575]|BLGSM091-09|BL1644|630[0n]bp|Canada.Ontario|BOLD:AAA5206
Zanclognatha dentata|[576]|BBLEC067-09|09BBELE-0067|621[0n]bp|Canada.New Brunswick|BOLD:AAA5206
Zanclognatha dentata|[577]|RDLQF332-06|DH011491|632[0n]bp|Canada.Quebec|BOLD:AAA5206
Zanclognatha dentata|[578]|RDLQF333-06|DH011492|631[0n]bp|Canada.Quebec|BOLD:AAA5206
Zanclognatha dentata|[579]|RDLQF416-06|DH011523|598[0n]bp|Canada.Quebec|BOLD:AAA5206
Zanclognatha dentata|[580]|RDLQF337-06|DH011496|658[0n]bp|Canada.Quebec|BOLD:AAA5206
Zanclognatha dentata|[581]|RDLQF537-06|DH011686|658[0n]bp|Canada.Quebec|BOLD:AAA5206
Zanclognatha dentata|[582]|RDLQF338-06|DH011497|658[0n]bp|Canada.Quebec|BOLD:AAA5206
Zanclognatha dentata|[583]|BLTIB804-08|BL1222|658[0n]bp|Canada.Ontario|BOLD:AAA5206
Zanclognatha dentata|[584]|RDLQG345-06|DH012562|658[0n]bp|Canada.Quebec|BOLD:AAA5206
Zanclognatha dentata|[585]|MNBB565-05|05-NBSTA-481|658[0n]bp|Canada.New Brunswick|BOLD:AAA5206
Zanclognatha dentata|[586]|BBLPC616-09|09BBELE-1616|655[0n]bp|Canada.Nova Scotia|BOLD:AAA5206
Zanclognatha dentata|[587]|RDLQF335-06|DH011494|658[0n]bp|Canada.Quebec|BOLD:AAA5206
Zanclognatha dentata|[588]|RDLQF343-06|DH011502|658[0n]bp|Canada.Quebec|BOLD:AAA5206
Zanclognatha dentata|[589]|RDLQG167-06|DH012338|658[0n]bp|Canada.Quebec|BOLD:AAA5206
Zanclognatha dentata|[590]|BBLPC516-09|09BBELE-1516|658[0n]bp|Canada.New Brunswick|BOLD:AAA5206
Zanclognatha dentata|[591]|RDLQF837-06|DH011990|658[0n]bp|Canada.Quebec|BOLD:AAA5206
Zanclognatha dentata|[592]|RDLQF339-06|DH011498|658[0n]bp|Canada.Quebec|BOLD:AAA5206
Zanclognatha dentata|[593]|RDLQF782-06|DH011932|658[0n]bp|Canada.Quebec|BOLD:AAA5206
Zanclognatha dentata|[594]|RDLQF331-06|DH011490|658[0n]bp|Canada.Quebec|BOLD:AAA5206
Zanclognatha dentata|[595]|BBLPC546-09|09BBELE-1546|658[0n]bp|Canada.New Brunswick|BOLD:AAA5206

Zanclognatha dentata|[593]|RDLQF782-06|DH011932|658[0n]bp|Canada.Quebec|BOLD:AAA5206
Zanclognatha dentata|[594]|RDLQF331-06|DH011490|658[0n]bp|Canada.Quebec|BOLD:AAA5206
Zanclognatha dentata|[595]|BBLPC546-09|09BBELE-1546|658[0n]bp|Canada.New Brunswick|BOLD:AAA5206
Zanclognatha dentata|[596]|TMNBB051-06|MNBTT-991|658[0n]bp|Canada.New Brunswick|BOLD:AAA5206
Zanclognatha dentata|[597]|RDLQG026-06|DH012157|658[0n]bp|Canada.Quebec|BOLD:AAA5206
Zanclognatha dentata|[598]|RDLQF340-06|DH011499|658[0n]bp|Canada.Quebec|BOLD:AAA5206
Zanclognatha dentata|[599]|BBLEC058-09|09BBELE-0058|658[0n]bp|Canada.New Brunswick|BOLD:AAA5206
Zanclognatha dentata|[600]|RDLQF580-06|DH011730|658[0n]bp|Canada.Quebec|BOLD:AAA5206
Zanclognatha dentata|[601]|RDLQF534-06|DH011683|658[0n]bp|Canada.Quebec|BOLD:AAA5206
Zanclognatha dentata|[602]|XAJ983-06|2006-ONT-0983|658[0n]bp|Canada.Ontario|BOLD:AAA5206
Zanclognatha dentata|[603]|RDLQF408-06|DH011515|658[0n]bp|Canada.Quebec|BOLD:AAA5206
Zanclognatha dentata|[604]|RDLQF417-06|DH011524|658[0n]bp|Canada.Quebec|BOLD:AAA5206
Zanclognatha dentata|[605]|RDLQG171-06|DH012342|658[0n]bp|Canada.Quebec|BOLD:AAA5206
Zanclognatha dentata|[606]|XAJ876-06|2006-ONT-0876|658[0n]bp|Canada.Ontario|BOLD:AAA5206
Zanclognatha dentata|[607]|RDLQF330-06|DH011489|658[0n]bp|Canada.Quebec|BOLD:AAA5206
Zanclognatha dentata|[608]|RDLQF419-06|DH011526|658[0n]bp|Canada.Quebec|BOLD:AAA5206
Zanclognatha dentata|[609]|RDLQF406-06|DH011513|658[1n]bp|Canada.Quebec|BOLD:AAA5206
Zanclognatha dentata|[610]|RDLQF412-06|DH011519|654[0n]bp|Canada.Quebec|BOLD:AAA5206
Zanclognatha dentata|[611]|RDLQF336-06|DH011495|622[0n]bp|Canada.Quebec|BOLD:AAA5206
Zanclognatha dentata|[612]|RDLQF705-06|DH011855|637[0n]bp|Canada.Quebec|BOLD:AAA5206
Zanclognatha dentata|[613]|PHMO223-03|moth1133.01|639[0n]bp|Canada.Ontario|BOLD:AAA5206
Zanclognatha dentata|[614]|RDLQF579-06|DH011729|649[0n]bp|Canada.Quebec|BOLD:AAA5206
Zanclognatha dentata|[615]|RDLQF341-06|DH011500|658[0n]bp|Canada.Quebec|BOLD:AAA5206
Zanclognatha dentata|[616]|RDLQF783-06|DH011933|589[2n]bp|Canada.Quebec|BOLD:AAA5206
Zanclognatha theralis|[617]|BBLEC234-09|09BBELE-0234|658[0n]bp|Canada.Nova Scotia|BOLD:AAA5206
Zanclognatha theralis|[618]|RDLQG680-06|DH012973|658[0n]bp|Canada.Quebec|BOLD:AAA5206
Zanclognatha theralis|[619]|RDLQB256-05|DH010342|570[0n]bp|Canada.Quebec|BOLD:AAA5206
Zanclognatha theralis|[620]|RDLQH148-06|DH006635|631[0n]bp|Canada.Quebec|BOLD:AAA5206
Zanclognatha theralis|[621]|BBLPE174-09|09BBELE-2174|658[0n]bp|Canada.Nova Scotia|BOLD:AAA5206
Zanclognatha theralis|[622]|BBLEC750-09|09BBELE-0750|632[0n]bp|Canada.Nova Scotia|BOLD:AAA5206
Zanclognatha theralis|[623]|TTMNB025-06|MNBTT-025|656[0n]bp|Canada.New Brunswick|BOLD:AAA5206
Zanclognatha theralis|[624]|BBLEC556-09|09BBELE-0556|658[0n]bp|Canada.Nova Scotia|BOLD:AAA5206
Zanclognatha theralis|[625]|RDLQB257-05|DH010343|658[0n]bp|Canada.Quebec|BOLD:AAA5206
Zanclognatha theralis|[626]|RDLQH144-06|DH006637|606[5n]bp|Canada.Quebec|BOLD:AAA5206
Zanclognatha theralis|[627]|RDLQH146-06|DH006639|599[3n]bp|Canada.Quebec|BOLD:AAA5206
Zanclognatha theralis|[628]|RDLQH149-06|DH006638|595[0n]bp|Canada.Quebec|BOLD:AAA5206
Zanclognatha sp. 1|[629]|BBLPB981-10|10BBCLP-1980|658[0n]bp|Canada.Alberta|BOLD:AAA5206
Zanclognatha sp. 1|[630]|BBLPB978-10|10BBCLP-1977|658[0n]bp|Canada.Alberta|BOLD:AAA5206
Zanclognatha pedipilalis|[631]|RDLQF917-06|DH012092|658[0n]bp|Canada.Quebec|BOLD:ABZ2935
Zanclognatha pedipilalis|[632]|RDLQG436-06|DH012715|658[0n]bp|Canada.Quebec|BOLD:ABZ2935
Zanclognatha pedipilalis|[633]|RDLQG437-06|DH012716|658[0n]bp|Canada.Quebec|BOLD:ABZ2935
Zanclognatha pedipilalis|[634]|BLTIB442-08|BL689|658[0n]bp|Canada.Ontario|BOLD:ABZ2935
Zanclognatha pedipilalis|[635]|PHMNB420-04|04HBL00646|658[0n]bp|Canada.New Brunswick|BOLD:ABZ2935
Zanclognatha pedipilalis|[636]|RDLQF424-06|DH011531|658[0n]bp|Canada.Quebec|BOLD:ABZ2935
Zanclognatha pedipilalis|[637]|RDLQF438-06|DH011545|658[0n]bp|Canada.Quebec|BOLD:ABZ2935
Zanclognatha pedipilalis|[638]|RDLQF921-06|DH012096|658[0n]bp|Canada.Quebec|BOLD:ABZ2935
Zanclognatha pedipilalis|[639]|RDLQF922-06|DH012097|658[0n]bp|Canada.Quebec|BOLD:ABZ2935
Zanclognatha pedipilalis|[640]|RDLQF923-06|DH012098|658[0n]bp|Canada.Quebec|BOLD:ABZ2935
Zanclognatha pedipilalis|[641]|RDLQF918-06|DH012093|658[0n]bp|Canada.Quebec|BOLD:ABZ2935
Zanclognatha pedipilalis|[642]|RDLQF920-06|DH012095|658[0n]bp|Canada.Quebec|BOLD:ABZ2935
Zanclognatha pedipilalis|[643]|LPSOB799-08|PPBP-1798|658[0n]bp|Canada.Ontario|BOLD:ABZ2935
Zanclognatha pedipilalis|[644]|BLTIB267-08|BL451|658[0n]bp|Canada.Ontario|BOLD:ABZ2935
Zanclognatha pedipilalis|[645]|XAB520-04|04HBL005520|658[0n]bp|Canada.Ontario|BOLD:ABZ2935
Zanclognatha pedipilalis|[646]|BLTIB345-08|BL541|643[0n]bp|Canada.Ontario|BOLD:ABZ2935
Zanclognatha pedipilalis|[647]|XAC610-04|04HBL006610|582[0n]bp|Canada.Ontario|BOLD:ABZ2935
Zanclognatha pedipilalis|[648]|BLTIB440-08|BL687|658[0n]bp|Canada.Ontario|BOLD:ABZ2935
Zanclognatha pedipilalis|[649]|LPSO565-08|PPBP-0565|658[0n]bp|Canada.Ontario|BOLD:ABZ2935
Zanclognatha pedipilalis|[650]|LPSO571-08|PPBP-0571|658[0n]bp|Canada.Ontario|BOLD:ABZ2935
Zanclognatha pedipilalis|[651]|LPSO563-08|PPBP-0563|658[0n]bp|Canada.Ontario|BOLD:ABZ2935
Chytolita morbidalis|[652]|RDLQF216-06|DH011296|655[0n]bp|Canada.Quebec|BOLD:AAA2868
Chytolita morbidalis|[653]|RDLQF212-06|DH011292|658[0n]bp|Canada.Quebec|BOLD:AAA2868
Chytolita morbidalis|[654]|BLTIB253-08|BL435|658[0n]bp|Canada.Ontario|BOLD:AAA2868
Chytolita morbidalis|[655]|LPSOB669-08|PPBP-1668|647[0n]bp|Canada.Ontario|BOLD:AAA2868
Chytolita morbidalis|[656]|LPSOB647-08|PPBP-1646|647[0n]bp|Canada.Ontario|BOLD:AAA2868
Chytolita morbidalis|[657]|RDLQF219-06|DH011299|658[0n]bp|Canada.Quebec|BOLD:AAA2868
Chytolita morbidalis|[658]|RDLQF213-06|DH011293|658[0n]bp|Canada.Quebec|BOLD:AAA2868
Chytolita morbidalis|[659]|LPSOC038-08|PPBP-2037|658[0n]bp|Canada.Ontario|BOLD:AAA2868
Chytolita morbidalis|[660]|LPSOC039-08|PPBP-2038|658[0n]bp|Canada.Ontario|BOLD:AAA2868
Chytolita morbidalis|[661]|LPSOB965-08|PPBP-1964|658[0n]bp|Canada.Ontario|BOLD:AAA2868
Chytolita morbidalis|[662]|XAI058-05|0102-ONT-0058|658[0n]bp|Canada.Ontario|BOLD:AAA2868
Chytolita morbidalis|[663]|BLTIB246-08|BL428|658[0n]bp|Canada.Ontario|BOLD:AAA2868
Chytolita morbidalis|[664]|LPSOC234-08|PPBP-2233|658[0n]bp|Canada.Ontario|BOLD:AAA2868
Chytolita morbidalis|[665]|LPSOB956-08|PPBP-1955|658[0n]bp|Canada.Ontario|BOLD:AAA2868
Chytolita morbidalis|[666]|LPSOB627-08|PPBP-1626|658[0n]bp|Canada.Ontario|BOLD:AAA2868
Chytolita morbidalis|[667]|LPSOB655-08|PPBP-1654|658[0n]bp|Canada.Ontario|BOLD:AAA2868
Chytolita morbidalis|[668]|LPSOC240-08|PPBP-2239|658[0n]bp|Canada.Ontario|BOLD:AAA2868
Chytolita morbidalis|[669]|LPSOC148-08|PPBP-2147|658[0n]bp|Canada.Ontario|BOLD:AAA2868
Chytolita morbidalis|[670]|RDLQF214-06|DH011294|658[0n]bp|Canada.Quebec|BOLD:AAA2868
Chytolita morbidalis|[671]|RDLQF440-06|DH011547|639[0n]bp|Canada.Quebec|BOLD:AAA2868
Chytolita morbidalis|[672]|LPMN354-08|08BBLEP-01153|658[0n]bp|Canada.Manitoba|BOLD:AAA2868
Chytolita morbidalis|[673]|RDNML266-13|CNCLEP 83713|602[1n]bp|Canada.Ontario|BOLD:AAA2868
Chytolita morbidalis|[674]|RDNM909-05|CNCNoctuoidea7749|658[0n]bp|Canada.British Columbia|BOLD:AAA2868
Chytolita morbidalis|[675]|RDLQF220-06|DH011300|658[0n]bp|Canada.Quebec|BOLD:AAA2868
Chytolita morbidalis|[676]|RDLQF218-06|DH011298|658[0n]bp|Canada.Quebec|BOLD:AAA2868
Chytolita morbidalis|[677]|RDLQF215-06|DH011295|658[0n]bp|Canada.Quebec|BOLD:AAA2868
Chytolita morbidalis|[678]|RDNML264-13|CNCLEP 83711|658[0n]bp|Canada.Ontario|BOLD:AAA2868
Chytolita morbidalis|[679]|RDLQF925-06|DH012100|658[0n]bp|Canada.Quebec|BOLD:AAA2868
Chytolita morbidalis|[680]|LPMN655-08|08BBLEP-01456|658[0n]bp|Canada.Manitoba|BOLD:AAA2868
Chytolita morbidalis|[681]|RDLQF848-06|DH012001|658[0n]bp|Canada.Quebec|BOLD:AAA2868
Chytolita morbidalis|[682]|BBLPD119-10|10BBCLP-2117|658[0n]bp|Canada.Saskatchewan|BOLD:AAA2868
Chytolita morbidalis|[683]|RDNM910-05|CNCNoctuoidea7750|573[0n]bp|Canada.British Columbia|BOLD:AAA2868
Chytolita morbidalis|[684]|LOWCC895-05|CGWC-2775|523[1n]bp|Canada.British Columbia|BOLD:AAA2868
Chytolita morbidalis|[685]|RDLQF221-06|DH011301|658[0n]bp|Canada.Quebec|BOLD:AAA2868
Chytolita morbidalis|[686]|RDLQH137-06|DH006915|589[1n]bp|Canada.Quebec|BOLD:AAA2868
Chytolita morbidalis|[687]|RDLQB242-05|DH010328|658[0n]bp|Canada.Quebec|BOLD:AAA2868
Chytolita morbidalis|[688]|LPSOD882-09|08BBLEP-00664|658[0n]bp|Canada.Ontario|BOLD:AAA2868
Chytolita morbidalis|[689]|LPMN821-08|08BBLEP-01624|658[0n]bp|Canada.Manitoba|BOLD:AAA2868
Chytolita morbidalis|[690]|LPSOB660-08|PPBP-1659|658[0n]bp|Canada.Ontario|BOLD:AAA2868
Chytolita morbidalis|[691]|LPMN470-08|08BBLEP-01269|658[0n]bp|Canada.Manitoba|BOLD:AAA2868
Chytolita morbidalis|[692]|RDLQF439-06|DH011546|621[0n]bp|Canada.Quebec|BOLD:AAA2868
Chytolita morbidalis|[693]|LPMN824-08|08BBLEP-01627|658[0n]bp|Canada.Manitoba|BOLD:AAA2868
Chytolita morbidalis|[694]|LPMN163-08|08BBLEP-00962|658[0n]bp|Canada.Manitoba|BOLD:AAA2868

Chytolita morbidalis|[692]|RDLQF439-06|DH011546|621[0n]bp|Canada.Quebec|BOLD:AAA2868
Chytolita morbidalis|[693]|LPMN824-08|08BBLEP-01627|658[0n]bp|Canada.Manitoba|BOLD:AAA2868
Chytolita morbidalis|[694]|LPMN163-08|08BBLEP-00962|658[0n]bp|Canada.Manitoba|BOLD:AAA2868
Chytolita morbidalis|[695]|RDLQF441-06|DH011548|658[0n]bp|Canada.Quebec|BOLD:AAA2868
Chytolita morbidalis|[696]|RDLQF222-06|DH011302|658[0n]bp|Canada.Quebec|BOLD:AAA2868
Chytolita morbidalis|[697]|RDNML267-13|CNCLEP 83714|658[0n]bp|Canada.Ontario|BOLD:AAA2868
Chytolita morbidalis|[698]|RDLQG439-06|DH012718|658[0n]bp|Canada.Quebec|BOLD:AAA2868
Chytolita morbidalis|[699]|LPSOB668-08|PPBP-1667|645[0n]bp|Canada.Ontario|BOLD:AAA2868
Chytolita morbidalis|[700]|RDNML265-13|CNCLEP 83712|614[0n]bp|Canada.Ontario|BOLD:AAA2868
Chytolita morbidalis|[701]|LPSOB659-08|PPBP-1658|646[0n]bp|Canada.Ontario|BOLD:AAA2868
Chytolita morbidalis|[702]|RDLQG248-06|DH012453|607[0n]bp|Canada.Quebec|BOLD:AAA2868
Chytolita morbidalis|[703]|RDLQG441-06|DH012720|621[0n]bp|Canada.Quebec|BOLD:AAA2868
Chytolita morbidalis|[704]|BBLPD437-10|10BBCLP-2435|658[0n]bp|Canada.Ontario|BOLD:AAA2868
Chytolita morbidalis|[705]|LPMN415-08|08BBLEP-01214|655[0n]bp|Canada.Manitoba|BOLD:AAA2868
Chytolita morbidalis|[706]|LPSOC165-08|PPBP-2164|658[0n]bp|Canada.Ontario|BOLD:AAA2868
Chytolita morbidalis|[707]|LPMN474-08|08BBLEP-01273|658[0n]bp|Canada.Manitoba|BOLD:AAA2868
Chytolita morbidalis|[708]|RDLQF217-06|DH011297|658[0n]bp|Canada.Quebec|BOLD:AAA2868
Chytolita morbidalis|[709]|LPMN475-08|08BBLEP-01274|658[0n]bp|Canada.Manitoba|BOLD:AAA2868
Chytolita morbidalis|[710]|BBLPD150-10|10BBCLP-2148|658[0n]bp|Canada.Saskatchewan|BOLD:AAA2868
Chytolita morbidalis|[711]|BBLPC147-09|09BBELE-1147|658[0n]bp|Canada.Nova Scotia|BOLD:AAA2868
Chytolita morbidalis|[712]|LPMN471-08|08BBLEP-01270|658[0n]bp|Canada.Manitoba|BOLD:AAA2868
Chytolita morbidalis|[713]|BBLPD459-10|10BBCLP-2457|658[0n]bp|Canada.Ontario|BOLD:AAA2868
Chytolita morbidalis|[714]|RDLQG247-06|DH012452|658[0n]bp|Canada.Quebec|BOLD:AAA2868
Chytolita morbidalis|[715]|RDNM911-05|CNCNoctuoidea7751|658[0n]bp|Canada.Ontario|BOLD:AAA2868
Chytolita morbidalis|[716]|LPMN848-08|08BBLEP-01651|657[0n]bp|Canada.Manitoba|BOLD:AAA2868
Chytolita morbidalis|[717]|LPMN613-08|08BBLEP-01414|658[0n]bp|Canada.Manitoba|BOLD:AAA2868
Chytolita morbidalis|[718]|LPMN801-08|08BBLEP-01604|658[0n]bp|Canada.Manitoba|BOLD:AAA2868
Chytolita morbidalis|[719]|PHMNB224-04|04HBL007689|580[0n]bp|Canada.New Brunswick|BOLD:AAA2868
Chytolita morbidalis|[720]|LPMN122-08|08BBLEP-00920|658[0n]bp|Canada.Manitoba|BOLD:AAA2868
Chytolita morbidalis|[721]|LPMN385-08|08BBLEP-01184|658[0n]bp|Canada.Manitoba|BOLD:AAA2868
Chytolita morbidalis|[722]|RDLQG440-06|DH012719|658[0n]bp|Canada.Quebec|BOLD:AAA2868
Chytolita sp. 1|[723]|LPSOC151-08|PPBP-2150|658[0n]bp|Canada.Ontario|BOLD:AAA2868
Chytolita sp. 1|[724]|RDLQF924-06|DH012099|658[0n]bp|Canada.Quebec|BOLD:AAA2868
Chytolita sp. 1|[725]|RDLQF538-06|DH011687|658[0n]bp|Canada.Quebec|BOLD:AAA2868
Chytolita sp. 1|[726]|LPSOB810-08|PPBP-1809|658[0n]bp|Canada.Ontario|BOLD:AAA2868
Chytolita sp. 1|[727]|LPSOC156-08|PPBP-2155|658[0n]bp|Canada.Ontario|BOLD:AAA2868
Chytolita sp. 1|[728]|MNAE841-13|CNCLEP00098206|658[0n]bp|Canada.Ontario|BOLD:AAA2868
Chytolita sp. 1|[729]|BBLPA641-10|10BBCLP-0641|658[0n]bp|Canada.Ontario|BOLD:AAA2868
Chytolita sp. 1|[730]|RDNML263-13|CNCLEP 83710|658[0n]bp|Canada.Ontario|BOLD:AAA2868
Chytolita sp. 1|[731]|RDNML262-13|CNCLEP 83709|658[0n]bp|Canada.Ontario|BOLD:AAA2868
Zanclognatha laevigata|[732]|RDLQG198-06|DH012374|658[0n]bp|Canada.Quebec|BOLD:ACH2253
Zanclognatha laevigata|[733]|RDLQG077-06|DH012234|621[0n]bp|Canada.Quebec|BOLD:ABZ7767
Zanclognatha laevigata|[734]|RDLQF706-06|DH011856|617[0n]bp|Canada.Quebec|BOLD:ABZ7767
Zanclognatha laevigata|[735]|RDLQG033-06|DH012164|658[0n]bp|Canada.Quebec|BOLD:ABZ7767
Zanclognatha laevigata|[736]|RDLQF747-06|DH011897|658[0n]bp|Canada.Quebec|BOLD:ABZ7767
Zanclognatha laevigata|[737]|XAD088-04|04HBL007088|580[0n]bp|Canada.Ontario|BOLD:ABZ7767
Zanclognatha laevigata|[738]|RDLQF632-06|DH011782|637[0n]bp|Canada.Quebec|BOLD:ABZ7767
Zanclognatha laevigata|[739]|RDLQF630-06|DH011780|637[0n]bp|Canada.Quebec|BOLD:ABZ7767
Zanclognatha laevigata|[740]|RDLQF715-06|DH011865|637[0n]bp|Canada.Quebec|BOLD:ABZ7767
Zanclognatha laevigata|[741]|RDLQF716-06|DH011866|637[0n]bp|Canada.Quebec|BOLD:ABZ7767
Zanclognatha laevigata|[742]|RDLQF631-06|DH011781|637[0n]bp|Canada.Quebec|BOLD:ABZ7767
Zanclognatha laevigata|[743]|RDLQF717-06|DH011867|637[0n]bp|Canada.Quebec|BOLD:ABZ7767
Zanclognatha laevigata|[744]|RDLQG034-06|DH012165|658[0n]bp|Canada.Quebec|BOLD:ABZ7767
Zanclognatha laevigata|[745]|RDLQF745-06|DH011895|658[0n]bp|Canada.Quebec|BOLD:ABZ7767
Zanclognatha laevigata|[746]|RDLQF746-06|DH011896|658[0n]bp|Canada.Quebec|BOLD:ABZ7767
Zanclognatha laevigata|[747]|RDLQG197-06|DH012373|658[0n]bp|Canada.Quebec|BOLD:ABZ7767
Zanclognatha laevigata|[748]|RDLQF743-06|DH011893|658[0n]bp|Canada.Quebec|BOLD:ABZ7767
Zanclognatha laevigata|[749]|RDLQF530-06|DH011679|658[0n]bp|Canada.Quebec|BOLD:ABZ7767
Zanclognatha laevigata|[750]|BBLEC182-09|09BBELE-0182|658[0n]bp|Canada.Nova Scotia|BOLD:ABZ7767
Zanclognatha laevigata|[751]|XAE636-04|Moth4636.03|658[0n]bp|Canada.Ontario|BOLD:ABZ7767
Zanclognatha laevigata|[752]|RDLQG076-06|DH012233|658[0n]bp|Canada.Quebec|BOLD:ABZ7767
Zanclognatha laevigata|[753]|RDLQF744-06|DH011894|658[0n]bp|Canada.Quebec|BOLD:ABZ7767
Zanclognatha lituralis|[754]|RDLQH151-06|DH005643|566[3n]bp|Canada.Quebec|BOLD:AAB3774
Zanclognatha lituralis|[755]|RDLQF535-06|DH011684|658[0n]bp|Canada.Quebec|BOLD:AAB3774
Zanclognatha sp. 2|[756]|BLTIB415-08|BL662|658[0n]bp|Canada.Ontario|BOLD:ABX5357
Zanclognatha sp. 2|[757]|PHJUN3397-11|BIOUG01486-D05|658[0n]bp|Canada.Ontario|BOLD:ABX5357
Zanclognatha sp. 2|[758]|RDLQH150-06|DH005360|609[4n]bp|Canada.Quebec|BOLD:ABX5357
Idia rotundalis|[759]|LMIS070-06|05-ONMIS-0070|658[0n]bp|Canada.Ontario|BOLD:AAA3326
Idia rotundalis|[760]|XAC718-04|04HBL006718|658[0n]bp|Canada.Ontario|BOLD:AAA3326
Idia rotundalis|[761]|RDLQB543-05|DH010629|658[0n]bp|Canada.Quebec|BOLD:AAA3326
Idia rotundalis|[762]|RDLQG101-06|DH012258|614[0n]bp|Canada.Quebec|BOLD:ACE4734
Idia rotundalis|[763]|BBLEC959-09|09BBELE-0959|636[0n]bp|Canada.Nova Scotia|BOLD:ACE4734
Idia rotundalis|[764]|RDLQG102-06|DH012259|614[0n]bp|Canada.Quebec|BOLD:ACE4734
Idia rotundalis|[765]|RDLQG103-06|DH012260|614[0n]bp|Canada.Quebec|BOLD:ACE4734
Idia rotundalis|[766]|RDLQG100-06|DH012257|620[1n]bp|Canada.Quebec|BOLD:ACE4734
Idia rotundalis|[767]|RDLQB710-05|DH010813|587[1n]bp|Canada.Quebec|BOLD:ACE4734
Idia rotundalis|[768]|BBLPC386-09|09BBELE-1386|658[0n]bp|Canada.New Brunswick|BOLD:ACE4734
Idia rotundalis|[769]|BBLEC720-09|09BBELE-0720|658[0n]bp|Canada.Nova Scotia|BOLD:ACE4734
Idia rotundalis|[770]|BBLEC936-09|09BBELE-0936|649[0n]bp|Canada.Nova Scotia|BOLD:ACE4734
Idia rotundalis|[771]|BBLPE561-09|09BBELE-2561|658[0n]bp|Canada.Nova Scotia|BOLD:ACE4734
Idia rotundalis|[772]|BBLEC910-09|09BBELE-0910|658[0n]bp|Canada.Nova Scotia|BOLD:ACE4734
Idia rotundalis|[773]|BBLEC994-09|09BBELE-0994|658[0n]bp|Canada.Nova Scotia|BOLD:ACE4734
Idia rotundalis|[774]|BBLEC925-09|09BBELE-0925|658[0n]bp|Canada.Nova Scotia|BOLD:ACE4734
Idia rotundalis|[775]|BBLEC973-09|09BBELE-0973|632[0n]bp|Canada.Nova Scotia|BOLD:ACE4734
Idia rotundalis|[776]|RDLQG099-06|DH012256|632[0n]bp|Canada.Quebec|BOLD:ACE4734
Idia rotundalis|[777]|TMNBD514-07|MNBTT-3315|658[0n]bp|Canada.New Brunswick|BOLD:ACE4734
Idia rotundalis|[778]|TMNBD513-07|MNBTT-3314|656[0n]bp|Canada.New Brunswick|BOLD:ACE4734
Idia rotundalis|[779]|RDLQG907-06|DH013200|658[0n]bp|Canada.Quebec|BOLD:ACE4734
Idia rotundalis|[780]|BBLEC963-09|09BBELE-0963|658[0n]bp|Canada.Nova Scotia|BOLD:ACE4734
Idia rotundalis|[781]|BBLEC986-09|09BBELE-0986|658[0n]bp|Canada.Nova Scotia|BOLD:ACE4734
Idia rotundalis|[782]|BBLEC546-09|09BBELE-0546|658[0n]bp|Canada.Nova Scotia|BOLD:ACE4734
Idia rotundalis|[783]|RDLQF793-06|DH011943|658[0n]bp|Canada.Quebec|BOLD:ACE4734
Idia rotundalis|[784]|BBLEC990-09|09BBELE-0990|658[0n]bp|Canada.Nova Scotia|BOLD:ACE4734
Idia rotundalis|[785]|BBLEC057-09|09BBELE-0057|658[0n]bp|Canada.New Brunswick|BOLD:ACE4734
Idia rotundalis|[786]|RDLQF384-06|DH011451|658[0n]bp|Canada.Quebec|BOLD:ACE4734
Idia rotundalis|[787]|BBLEC557-09|09BBELE-0557|656[0n]bp|Canada.Nova Scotia|BOLD:ACE4734
Idia rotundalis|[788]|RDLQH014-06|DH013251|658[0n]bp|Canada.Quebec|BOLD:ACE4734
Idia rotundalis|[789]|RDLQG905-06|DH013198|658[0n]bp|Canada.Quebec|BOLD:ACE4734
Idia rotundalis|[790]|TMNBD515-07|MNBTT-3316|658[0n]bp|Canada.New Brunswick|BOLD:ACE4734
Idia rotundalis|[791]|RDLQH013-06|DH013250|658[0n]bp|Canada.Quebec|BOLD:ACE4734
Idia rotundalis|[792]|RDLQG681-06|DH012974|658[0n]bp|Canada.Quebec|BOLD:ACE4734
Idia rotundalis|[793]|MNBB692-05|05-NBSTA-608|658[0n]bp|Canada.New Brunswick|BOLD:ACE4734
[illegible]

Idia rotundalis|[791]|RDLQH013-06|DH013250|658[0n]bp|Canada.Quebec|BOLD:ACE4734
Idia rotundalis|[792]|RDLQG681-06|DH012974|658[0n]bp|Canada.Quebec|BOLD:ACE4734
Idia rotundalis|[793]|MNBB692-05|05-NBSTA-608|658[0n]bp|Canada.New Brunswick|BOLD:ACE4734
Idia rotundalis|[794]|RDLQG683-06|DH012976|658[0n]bp|Canada.Quebec|BOLD:ACE4734
Idia rotundalis|[795]|RDLQG564-06|DH012857|658[0n]bp|Canada.Quebec|BOLD:ACE4734
Idia rotundalis|[796]|RDLQG599-06|DH012892|658[0n]bp|Canada.Quebec|BOLD:ACE4734
Idia rotundalis|[797]|RDLQG165-06|DH012336|658[0n]bp|Canada.Quebec|BOLD:ACE4734
Idia rotundalis|[798]|RDLQG906-06|DH013199|658[0n]bp|Canada.Quebec|BOLD:ACE4734
Idia rotundalis|[799]|RDLQF655-06|DH011805|637[0n]bp|Canada.Quebec|BOLD:ACE4734
Idia rotundalis|[800]|BBLEC233-09|09BBELE-0233|658[0n]bp|Canada.Nova Scotia|BOLD:ACE4734
Idia rotundalis|[801]|BBLEC551-09|09BBELE-0551|658[0n]bp|Canada.Nova Scotia|BOLD:ACE4734
Idia rotundalis|[802]|BBLEC763-09|09BBELE-0763|658[0n]bp|Canada.Nova Scotia|BOLD:ACE4734
Idia rotundalis|[803]|BBLPE596-09|09BBELE-2596|658[0n]bp|Canada.Nova Scotia|BOLD:ACE4734
Idia rotundalis|[804]|BBLPC533-09|09BBELE-1533|658[0n]bp|Canada.New Brunswick|BOLD:ACE4734
Idia rotundalis|[805]|BBLEC992-09|09BBELE-0992|658[0n]bp|Canada.Nova Scotia|BOLD:ACE4734
Idia rotundalis|[806]|RDLQG097-06|DH012254|658[0n]bp|Canada.Quebec|BOLD:ACE4734
Idia rotundalis|[807]|RDLQF794-06|DH011944|658[0n]bp|Canada.Quebec|BOLD:ACE4734
Idia rotundalis|[808]|BBLPE597-09|09BBELE-2597|658[0n]bp|Canada.Nova Scotia|BOLD:ACE4734
Idia rotundalis|[809]|RDLQG608-06|DH012901|658[0n]bp|Canada.Quebec|BOLD:ACE4734
Idia rotundalis|[810]|BBLEC246-09|09BBELE-0246|658[0n]bp|Canada.Nova Scotia|BOLD:ACE4734
Idia rotundalis|[811]|RDLQG098-06|DH012255|650[0n]bp|Canada.Quebec|BOLD:ACE4734
Idia rotundalis|[812]|TMNBB044-06|MNBTT-984|658[0n]bp|Canada.New Brunswick|BOLD:ACE4734
Idia rotundalis|[813]|RDLQF714-06|DH011864|637[0n]bp|Canada.Quebec|BOLD:ACE4734
Idia rotundalis|[814]|RDLQF657-06|DH011807|637[0n]bp|Canada.Quebec|BOLD:ACE4734
Idia rotundalis|[815]|RDLQF654-06|DH011804|637[0n]bp|Canada.Quebec|BOLD:ACE4734
Idia rotundalis|[816]|RDLQF658-06|DH011808|637[0n]bp|Canada.Quebec|BOLD:ACE4734
Idia rotundalis|[817]|RDLQF656-06|DH011806|637[0n]bp|Canada.Quebec|BOLD:ACE4734
Idia rotundalis|[818]|RDLQG598-06|DH012891|643[0n]bp|Canada.Quebec|BOLD:ACE4734
Idia rotundalis|[819]|RDLQF713-06|DH011863|637[0n]bp|Canada.Quebec|BOLD:ACE4734
Idia rotundalis|[820]|RDLQG684-06|DH012977|658[0n]bp|Canada.Quebec|BOLD:ACE4734
Idia rotundalis|[821]|BBLEC902-09|09BBELE-0902|658[0n]bp|Canada.Nova Scotia|BOLD:ACE4734
Idia lubricalis|[822]|XAJ842-06|2006-ONT-0842|658[0n]bp|Canada.Ontario|BOLD:ACF3661
Idia lubricalis|[823]|LBCC768-05|HLC-22648|658[0n]bp|Canada.British Columbia|BOLD:ACF3661
Idia lubricalis complex|[824]|MNAE846-13|CNCLEP00098211|658[0n]bp|Canada.Ontario|BOLD:ACF3661
Idia lubricalis complex|[825]|MNAE844-13|CNCLEP00098209|658[0n]bp|Canada.Ontario|BOLD:ACF3661
Idia lubricalis complex|[826]|MNAE845-13|CNCLEP00098210|658[0n]bp|Canada.Ontario|BOLD:ACF3661
Idia lubricalis|[827]|RDLQF593-06|DH011743|658[0n]bp|Canada.Quebec|BOLD:ACF3661
Idia lubricalis|[828]|RDLQF710-06|DH011860|637[0n]bp|Canada.Quebec|BOLD:ACF3661
Idia lubricalis complex|[829]|MNAE835-13|CNCLEP00098200|656[0n]bp|Canada.Ontario|BOLD:ACF3661
Idia lubricalis complex|[830]|MNAE848-13|CNCLEP00098213|658[0n]bp|Canada.Ontario|BOLD:ACF3661
Idia denticulalis|[831]|RDLQG085-06|DH012242|624[0n]bp|Canada.Quebec|BOLD:ABY9096
Idia denticulalis|[832]|RDLQG083-06|DH012240|624[0n]bp|Canada.Quebec|BOLD:ABY9096
Idia denticulalis|[833]|RDLQG087-06|DH012244|658[1n]bp|Canada.Quebec|BOLD:ABY9096
Idia denticulalis|[834]|RDLQF729-06|DH011879|658[1n]bp|Canada.Quebec|BOLD:ABY9096
Idia denticulalis|[835]|RDLQG086-06|DH012243|658[1n]bp|Canada.Quebec|BOLD:ABY9096
Idia denticulalis|[836]|RDLQG088-06|DH012245|632[0n]bp|Canada.Quebec|BOLD:ABY9096
Idia denticulalis|[837]|RDLQG082-06|DH012239|630[0n]bp|Canada.Quebec|BOLD:ABY9096
Idia denticulalis|[838]|RDLQF601-06|DH011751|658[0n]bp|Canada.Quebec|BOLD:ABY9096
Idia denticulalis|[839]|RDLQF602-06|DH011752|658[0n]bp|Canada.Quebec|BOLD:ABY9096
Idia denticulalis|[840]|RDLQG084-06|DH012241|653[0n]bp|Canada.Quebec|BOLD:ABY9096
Idia lubricalis complex|[841]|MNAE849-13|CNCLEP00098214|658[0n]bp|Canada.Ontario|BOLD:ABY9096
Idia lubricalis|[842]|TMNBC559-06|MNBTT-2365|658[0n]bp|Canada.New Brunswick|BOLD:ABY9096
Idia lubricalis|[843]|RDLQG067-06|DH012224|658[0n]bp|Canada.Quebec|BOLD:ABY9096
Idia lubricalis|[844]|RDLQG075-06|DH012232|658[0n]bp|Canada.Quebec|BOLD:ABY9096
Idia denticulalis|[845]|RDLQF603-06|DH011753|658[0n]bp|Canada.Quebec|BOLD:ABY9096
Idia lubricalis|[846]|RDLQ109-05|DH007896|658[0n]bp|Canada.Quebec|BOLD:ABY9096
Idia lubricalis|[847]|MNBB627-05|05-NBSTA-543|658[0n]bp|Canada.New Brunswick|BOLD:ABY9096
Idia lubricalis complex|[848]|MNAE836-13|CNCLEP00098201|658[0n]bp|Canada.Ontario|BOLD:ABY9096
Idia lubricalis complex|[849]|MNAE842-13|CNCLEP00098207|658[0n]bp|Canada.Ontario|BOLD:ABY9096
Idia lubricalis complex|[850]|MNAE834-13|CNCLEP00098199|658[0n]bp|Canada.Ontario|BOLD:ABY9096
Idia lubricalis complex|[851]|MNAE843-13|CNCLEP00098208|658[0n]bp|Canada.Ontario|BOLD:ABY9096
Idia lubricalis|[852]|RDLQG069-06|DH012226|630[0n]bp|Canada.Quebec|BOLD:ABY9096
Idia lubricalis|[853]|XAJ821-06|2006-ONT-0821|658[0n]bp|Canada.Ontario|BOLD:ABY9096
Idia lubricalis complex|[854]|MNAE831-13|CNCLEP00098196|658[0n]bp|Canada.Ontario|BOLD:ABY9096
Idia lubricalis complex|[855]|MNAE840-13|CNCLEP00098205|658[0n]bp|Canada.Ontario|BOLD:ABY9096
Idia lubricalis complex|[856]|MNAE851-13|CNCLEP00098216|658[0n]bp|Canada.Quebec|BOLD:ABY9096
Idia lubricalis|[857]|BBLPE573-09|09BBELE-2573|658[0n]bp|Canada.Nova Scotia|BOLD:ABY9096
Idia lubricalis|[858]|RDLQF591-06|DH011741|658[0n]bp|Canada.Quebec|BOLD:ABY9096
Idia lubricalis|[859]|RDLQF748-06|DH011898|658[0n]bp|Canada.Quebec|BOLD:ABY9096
Idia lubricalis|[860]|RDLQF565-06|DH011714|658[0n]bp|Canada.Quebec|BOLD:ABY9096
Idia lubricalis|[861]|RDLQG068-06|DH012225|658[0n]bp|Canada.Quebec|BOLD:ABY9096
Idia lubricalis|[862]|RDLQF532-06|DH011681|658[0n]bp|Canada.Quebec|BOLD:ABY9096
Idia lubricalis|[863]|RDLQG072-06|DH012229|658[0n]bp|Canada.Quebec|BOLD:ABY9096
Idia lubricalis|[864]|RDLQF590-06|DH011740|658[0n]bp|Canada.Quebec|BOLD:ABY9096
Idia lubricalis|[865]|RDLQG071-06|DH012228|658[0n]bp|Canada.Quebec|BOLD:ABY9096
Idia lubricalis|[866]|TTMNB098-06|MNBTT-098|658[0n]bp|Canada.New Brunswick|BOLD:ABY9096
Idia lubricalis|[867]|RDLQF749-06|DH011899|658[0n]bp|Canada.Quebec|BOLD:ABY9096
Idia lubricalis|[868]|RDLQG073-06|DH012230|658[0n]bp|Canada.Quebec|BOLD:ABY9096
Idia lubricalis|[869]|RDLQG074-06|DH012231|658[0n]bp|Canada.Quebec|BOLD:ABY9096
Idia lubricalis|[870]|RDLQF564-06|DH011713|658[0n]bp|Canada.Quebec|BOLD:ABY9096
Idia lubricalis|[871]|RDLQ108-05|DH006882|658[0n]bp|Canada.Quebec|BOLD:ABY9096
Idia lubricalis|[872]|RDLQF576-06|DH011726|658[0n]bp|Canada.Quebec|BOLD:ABY9096
Idia lubricalis|[873]|RDLQF577-06|DH011727|658[0n]bp|Canada.Quebec|BOLD:ABY9096
Idia lubricalis|[874]|RDLQF589-06|DH011739|658[0n]bp|Canada.Quebec|BOLD:ABY9096
Idia lubricalis complex|[875]|MNAE850-13|CNCLEP00098215|658[0n]bp|Canada.Quebec|BOLD:ABY9096
Idia lubricalis complex|[876]|PHSEP348-11|BIOUG01292-B07|652[0n]bp|Canada.Ontario|BOLD:ABY9096
Idia lubricalis complex|[877]|PHSEP342-11|BIOUG01292-B01|652[0n]bp|Canada.Ontario|BOLD:ABY9096
Idia lubricalis|[878]|RDMAB513-06|UASM58185|658[0n]bp|Canada.Alberta|BOLD:AAA2230
Idia lubricalis|[879]|RDMAB512-06|UASM58184|658[0n]bp|Canada.Alberta|BOLD:AAA2230
Idia lubricalis|[880]|RDMAB514-06|UASM58183|658[0n]bp|Canada.Alberta|BOLD:AAA2230
Idia lubricalis|[881]|RDLQF588-06|DH011738|658[2n]bp|Canada.Quebec|BOLD:AAA2230
Idia lubricalis|[882]|PHSEP351-11|BIOUG01292-B10|652[0n]bp|Canada.Ontario|BOLD:AAA2230
Idia lubricalis complex|[883]|MNAE838-13|CNCLEP00098203|658[0n]bp|Canada.Ontario|BOLD:AAA2230
Idia lubricalis|[884]|RDLQF075-06|DH006883|658[0n]bp|Canada.Quebec|BOLD:AAA2230
Idia lubricalis|[885]|RDLQF076-06|DH006816|658[0n]bp|Canada.Quebec|BOLD:AAA2230
Idia lubricalis|[886]|PHSEP345-11|BIOUG01292-B04|652[0n]bp|Canada.Ontario|BOLD:AAA2230
Idia lubricalis|[887]|RDLQF592-06|DH011742|658[0n]bp|Canada.Quebec|BOLD:AAA2230
Idia lubricalis complex|[888]|MNAE832-13|CNCLEP00098197|639[0n]bp|Canada.Ontario|BOLD:AAA2230
Idia lubricalis complex|[889]|MNAE837-13|CNCLEP00098202|658[0n]bp|Canada.Ontario|BOLD:AAA2230
Idia lubricalis complex|[890]|MNAE847-13|CNCLEP00098212|658[0n]bp|Canada.Ontario|BOLD:AAA2230
Idia lubricalis complex|[891]|MNAE833-13|CNCLEP00098198|658[0n]bp|Canada.Ontario|BOLD:AAA2230
Idia lubricalis complex|[892]|MNAE839-13|CNCLEP00098204|658[0n]bp|Canada.Ontario|BOLD:AAA2230
[illegible]

Idia lubricalis complex|[890]|MNAE847-13|CNCLEP00098212|658[0n]bp|Canada.Ontario|BOLD:AAA2230
Idia lubricalis complex|[891]|MNAE833-13|CNCLEP00098198|658[0n]bp|Canada.Ontario|BOLD:AAA2230
Idia lubricalis complex|[892]|MNAE839-13|CNCLEP00098204|658[0n]bp|Canada.Ontario|BOLD:AAA2230
Idia lubricalis complex|[893]|LBCH6242-10|10-JDWBC-6242|658[0n]bp|Canada.British Columbia|BOLD:ACE7853
Idia lubricalis complex|[894]|LBCH6234-10|10-JDWBC-6234|658[0n]bp|Canada.British Columbia|BOLD:ACE7853
Idia lubricalis complex|[895]|LBCH7267-10|10-JDWBC-7267|634[0n]bp|Canada.British Columbia|BOLD:ACE7853
Idia lubricalis complex|[896]|LBCH7268-10|10-JDWBC-7268|635[0n]bp|Canada.British Columbia|BOLD:ACE7853
Idia lubricalis complex|[897]|LBCG1107-09|08-JDWBC-1107|658[0n]bp|Canada.British Columbia|BOLD:ACE7853
Idia lubricalis|[898]|RDLQF709-06|DH011859|637[0n]bp|Canada.Quebec|BOLD:ACF3662
Idia denticulalis|[899]|RDLQG081-06|DH012238|632[0n]bp|Canada.Quebec|BOLD:ACF4863
Idia denticulalis|[900]|RDLQF600-06|DH011750|658[0n]bp|Canada.Quebec|BOLD:ACF4863
Idia lubricalis|[901]|RDLQG070-06|DH012227|658[0n]bp|Canada.Quebec|BOLD:ACF4863
Idia lubricalis|[902]|RDLQF726-06|DH011876|658[0n]bp|Canada.Quebec|BOLD:ACF4863
Idia majoralis|[903]|LMEM152-09|RBMIS-0152|658[0n]bp|United States.Mississippi|BOLD:ABY9095
Idia immaculalis|[904]|RDMAB508-06|UASM2608|658[0n]bp|Canada.Alberta|BOLD:ACF2450
Idia immaculalis|[905]|LPSK497-08|08BBLEP-02065|658[0n]bp|Canada.Saskatchewan|BOLD:ACF2450
Idia immaculalis|[906]|RDMAB509-06|UASM19703|622[1n]bp|Canada.Alberta|BOLD:ACF2450
Idia occidentalis|[907]|RDMAB510-06|UASM2609|632[0n]bp|Canada.Alberta|BOLD:ABZ4706
Idia occidentalis|[908]|RDMAB511-06|UASM2654|656[0n]bp|Canada.Alberta|BOLD:ABZ4706
Idia occidentalis|[909]|LBCH7324-10|10-JDWBC-7324|658[0n]bp|Canada.British Columbia|BOLD:ABZ4706
Idia occidentalis|[910]|LBCH7491-10|10-JDWBC-7491|658[0n]bp|Canada.British Columbia|BOLD:ABZ4706
Idia occidentalis|[911]|LBCH7408-10|10-JDWBC-7408|658[0n]bp|Canada.British Columbia|BOLD:ABZ4706
Idia occidentalis|[912]|LBCH6135-10|10-JDWBC-6135|658[0n]bp|Canada.British Columbia|BOLD:ABZ4706
Idia occidentalis|[913]|LBCH7407-10|10-JDWBC-7407|658[0n]bp|Canada.British Columbia|BOLD:ABZ4706
Idia occidentalis|[914]|LBCH7448-10|10-JDWBC-7448|658[0n]bp|Canada.British Columbia|BOLD:ABZ4706
Idia occidentalis|[915]|LBCH7447-10|10-JDWBC-7447|638[0n]bp|Canada.British Columbia|BOLD:ABZ4706
Idia occidentalis|[916]|LBCH6243-10|10-JDWBC-6243|658[0n]bp|Canada.British Columbia|BOLD:ABZ4706
Idia diminuendis|[917]|MECB117-04|jflandry1061|658[0n]bp|Canada.Quebec|BOLD:AAB5887
Idia aemula|[918]|PHSEP350-11|BIOUG01292-B09|652[0n]bp|Canada.Ontario|BOLD:AAA2229
Idia aemula|[919]|MNAF485-08|CNCLEP00040470|658[1n]bp|Canada.Manitoba|BOLD:AAA2229
Idia aemula|[920]|RDLQF088-06|DH006858|658[0n]bp|Canada.Quebec|BOLD:AAA2229
Idia aemula|[921]|RDLQF077-06|DH007025|658[0n]bp|Canada.Quebec|BOLD:AAA2229
Idia aemula|[922]|LOWCC514-05|CGWC-2394|658[0n]bp|Canada.British Columbia|BOLD:AAA2229
Idia aemula|[923]|BBLPF076-10|10BBCLP-3073|658[0n]bp|Canada.Alberta|BOLD:AAA2229
Idia aemula|[924]|BBLPD107-10|10BBCLP-2105|658[0n]bp|Canada.British Columbia|BOLD:AAA2229
Idia aemula|[925]|LPMNB405-09|08BBLEP-05249|636[0n]bp|Canada.Manitoba|BOLD:AAA2229
Idia aemula|[926]|RDLQF787-06|DH011937|658[0n]bp|Canada.Quebec|BOLD:AAA2229
Idia aemula|[927]|RDLQG584-06|DH012877|658[0n]bp|Canada.Quebec|BOLD:AAA2229
Idia aemula|[928]|RDLQF607-06|DH011757|658[0n]bp|Canada.Quebec|BOLD:AAA2229
Idia aemula|[929]|XAJ860-06|2006-ONT-0860|658[0n]bp|Canada.Ontario|BOLD:AAA2229
Idia aemula|[930]|RDLQF090-06|DH006875|658[0n]bp|Canada.Quebec|BOLD:AAA2229
Idia aemula|[931]|RDLQF791-06|DH011941|658[0n]bp|Canada.Quebec|BOLD:AAA2229
Idia aemula|[932]|MNAF866-08|CNCLEP00040851|658[0n]bp|Canada.Manitoba|BOLD:AAA2229
Idia aemula|[933]|RDLQF788-06|DH011938|658[0n]bp|Canada.Quebec|BOLD:AAA2229
Idia aemula|[934]|RDLQF079-06|DH007027|658[0n]bp|Canada.Quebec|BOLD:AAA2229
Idia aemula|[935]|RDLQG096-06|DH012253|632[0n]bp|Canada.Quebec|BOLD:AAA2229
Idia aemula|[936]|BBLPB935-10|10BBCLP-1934|658[0n]bp|Canada.Alberta|BOLD:AAA2229
Idia aemula|[937]|RDLQH019-06|DH013256|658[0n]bp|Canada.Quebec|BOLD:AAA2229
Idia aemula|[938]|RDLQG573-06|DH012866|658[0n]bp|Canada.Quebec|BOLD:AAA2229
Idia aemula|[939]|RDLQF732-06|DH011882|658[0n]bp|Canada.Quebec|BOLD:AAA2229
Idia aemula|[940]|MECB135-04|jflandry1079|658[0n]bp|Canada.Quebec|BOLD:AAA2229
Idia aemula|[941]|RDLQG602-06|DH012895|632[0n]bp|Canada.Quebec|BOLD:AAA2229
Idia aemula|[942]|RDLQF730-06|DH011880|658[0n]bp|Canada.Quebec|BOLD:AAA2229
Idia aemula|[943]|RDLQG787-06|DH013080|658[0n]bp|Canada.Quebec|BOLD:AAA2229
Idia aemula|[944]|RDLQF606-06|DH011756|658[0n]bp|Canada.Quebec|BOLD:AAA2229
Idia aemula|[945]|RDLQF731-06|DH011881|658[0n]bp|Canada.Quebec|BOLD:AAA2229
Idia aemula|[946]|BBLPE614-09|09BBELE-2614|658[0n]bp|Canada.Nova Scotia|BOLD:AAA2229
Idia aemula|[947]|RDLQF608-06|DH011758|658[0n]bp|Canada.Quebec|BOLD:AAA2229
Idia aemula|[948]|XAJ875-06|2006-ONT-0875|658[0n]bp|Canada.Ontario|BOLD:AAA2229
Idia aemula|[949]|XAC833-04|04HBL006833|658[0n]bp|Canada.Ontario|BOLD:AAA2229
Idia aemula|[950]|BBLPB940-10|10BBCLP-1939|658[0n]bp|Canada.Alberta|BOLD:AAA2229
Idia aemula|[951]|BBLPB939-10|10BBCLP-1938|658[0n]bp|Canada.Alberta|BOLD:AAA2229
Idia aemula|[952]|BBLPD054-10|10BBCLP-2052|658[0n]bp|Canada.Alberta|BOLD:AAA2229
Idia aemula|[953]|RDLQH020-06|DH013257|658[0n]bp|Canada.Quebec|BOLD:AAA2229
Idia aemula|[954]|RDLQF789-06|DH011939|658[0n]bp|Canada.Quebec|BOLD:AAA2229
Idia aemula|[955]|BBLPB937-10|10BBCLP-1936|658[0n]bp|Canada.Alberta|BOLD:AAA2229
Idia aemula|[956]|BBLPD056-10|10BBCLP-2054|658[0n]bp|Canada.Alberta|BOLD:AAA2229
Idia aemula|[957]|BBLPB934-10|10BBCLP-1933|658[0n]bp|Canada.Alberta|BOLD:AAA2229
Idia aemula|[958]|RDLQG677-06|DH012970|658[0n]bp|Canada.Quebec|BOLD:AAA2229
Idia aemula|[959]|RDLQF792-06|DH011942|658[0n]bp|Canada.Quebec|BOLD:AAA2229
Idia aemula|[960]|BBLPD055-10|10BBCLP-2053|658[0n]bp|Canada.Alberta|BOLD:AAA2229
Idia aemula|[961]|BBLPB938-10|10BBCLP-1937|658[0n]bp|Canada.Alberta|BOLD:AAA2229
Idia aemula|[962]|RDLQF790-06|DH011940|616[0n]bp|Canada.Quebec|BOLD:AAA2229
Idia aemula|[963]|XAE606-04|Moth4606.03|658[0n]bp|Canada.Ontario|BOLD:AAA2229
Idia aemula|[964]|TTMNB266-06|MNBTT-266|658[0n]bp|Canada.New Brunswick|BOLD:AAA2229
Idia aemula|[965]|MEC612-04|jflandry0612|615[4n]bp|Canada.Quebec|BOLD:AAA2229
Idia aemula|[966]|RDLQF085-06|DH007033|595[1n]bp|Canada.Quebec|BOLD:AAA2229
Idia aemula|[967]|RDLQF084-06|DH007032|658[0n]bp|Canada.Quebec|BOLD:AAA2229
Idia aemula|[968]|RDLQF082-06|DH007030|658[0n]bp|Canada.Quebec|BOLD:AAA2229
Idia aemula|[969]|RDLQF086-06|DH007034|658[0n]bp|Canada.Quebec|BOLD:AAA2229
Idia aemula|[970]|RDLQF091-06|DH006191|658[0n]bp|Canada.Quebec|BOLD:AAA2229
Idia aemula|[971]|RDLQG755-06|DH013048|658[0n]bp|Canada.Quebec|BOLD:AAA2229
Idia aemula|[972]|RDLQG764-06|DH013057|658[0n]bp|Canada.Quebec|BOLD:AAA2229
Idia aemula|[973]|RDLQG884-06|DH013177|658[0n]bp|Canada.Quebec|BOLD:AAA2229
Idia aemula|[974]|RDLQF081-06|DH007029|658[0n]bp|Canada.Quebec|BOLD:AAA2229
Idia aemula|[975]|TTMNB267-06|MNBTT-267|658[0n]bp|Canada.New Brunswick|BOLD:AAA2229
Idia aemula|[976]|RDLQF379-06|DH011446|658[0n]bp|Canada.Quebec|BOLD:AAA2229
Idia aemula|[977]|RDLQF080-06|DH007028|658[0n]bp|Canada.Quebec|BOLD:AAA2229
Idia aemula|[978]|LPSK135-08|08BBLEP-01703|658[0n]bp|Canada.Saskatchewan|BOLD:AAA2229
Idia aemula|[979]|XAH478-05|2005-ONT-2061|658[0n]bp|Canada.Ontario|BOLD:AAA2229
Idia aemula|[980]|XAH714-05|2005-ONT-2297|658[0n]bp|Canada.Ontario|BOLD:AAA2229
Idia aemula|[981]|LPSK258-08|08BBLEP-01826|658[0n]bp|Canada.Saskatchewan|BOLD:AAA2229
Idia aemula|[982]|XAH490-05|2005-ONT-2073|658[0n]bp|Canada.Ontario|BOLD:AAA2229
Idia aemula|[983]|XAH583-05|2005-ONT-2166|658[0n]bp|Canada.Ontario|BOLD:AAA2229
Idia aemula|[984]|XAD499-04|04HBL007499|658[0n]bp|Canada.Ontario|BOLD:AAA2229
Idia aemula|[985]|XAH426-05|2005-ONT-2009|658[0n]bp|Canada.Ontario|BOLD:AAA2229
Idia aemula|[986]|XAC831-04|04HBL006831|658[0n]bp|Canada.Ontario|BOLD:AAA2229
Idia aemula|[987]|XAH575-05|2005-ONT-2158|658[0n]bp|Canada.Ontario|BOLD:AAA2229
Idia aemula|[988]|XAH568-05|2005-ONT-2151|658[0n]bp|Canada.Ontario|BOLD:AAA2229
Idia aemula|[989]|XAH713-05|2005-ONT-2296|658[0n]bp|Canada.Ontario|BOLD:AAA2229
Idia aemula|[990]|XAB659-04|04HBL005659|658[1n]bp|Canada.Ontario|BOLD:AAA2229
Idia aemula|[991]|XAH715-05|2005-ONT-2298|597[0n]bp|Canada.Ontario|BOLD:AAA2229
Idia aemula|[992]|XAH215-05|2005-ONT-1798|649[0n]bp|Canada.Ontario|BOLD:AAA2229

Idia aemula|[illegible]|Canada.Ontario|BOLD:AAA2229
Idia aemula|[990]|XAB659-04|04HBL005659|658[1n]bp|Canada.Ontario|BOLD:AAA2229
Idia aemula|[991]|XAH715-05|2005-ONT-2298|597[0n]bp|Canada.Ontario|BOLD:AAA2229
Idia aemula|[992]|XAH215-05|2005-ONT-1798|649[0n]bp|Canada.Ontario|BOLD:AAA2229
Idia aemula|[993]|RDMAB492-06|UASM58426|658[0n]bp|Canada.Alberta|BOLD:AAA2229
Idia aemula|[994]|RDMAB493-06|UASM58428|612[3n]bp|Canada.Alberta|BOLD:AAA2229
Idia aemula|[995]|RDLQF092-06|DH006192|658[0n]bp|Canada.Quebec|BOLD:AAA2229
Idia aemula|[996]|XAE332-04|Moth4332.03|658[0n]bp|Canada.Ontario|BOLD:AAA2229
Idia aemula|[997]|XAH420-05|2005-ONT-2003|658[0n]bp|Canada.Ontario|BOLD:AAA2229
Idia aemula|[998]|XAH327-05|2005-ONT-1910|658[0n]bp|Canada.Ontario|BOLD:AAA2229
Idia aemula|[999]|XAH576-05|2005-ONT-2159|658[0n]bp|Canada.Ontario|BOLD:AAA2229
Idia aemula|[1000]|XAH393-05|2005-ONT-1976|658[0n]bp|Canada.Ontario|BOLD:AAA2229
Idia aemula|[1001]|XAH593-05|2005-ONT-2176|658[0n]bp|Canada.Ontario|BOLD:AAA2229
Idia aemula|[1002]|LPSK157-08|08BBLEP-01725|658[0n]bp|Canada.Saskatchewan|BOLD:AAA2229
Idia aemula|[1003]|XAB486-04|04HBL005486|658[0n]bp|Canada.Ontario|BOLD:AAA2229
Idia aemula|[1004]|RDLQB417-05|DH010503|658[0n]bp|Canada.Quebec|BOLD:AAA2229
Idia aemula|[1005]|RDLQF609-06|DH011759|658[0n]bp|Canada.Quebec|BOLD:AAA2229
Idia aemula|[1006]|BBLPE281-09|09BBELE-2281|648[0n]bp|Canada.Nova Scotia|BOLD:AAA2229
Idia aemula|[1007]|BBLPC565-09|09BBELE-1565|658[0n]bp|Canada.Nova Scotia|BOLD:AAA2229
Idia aemula|[1008]|BBLPE118-09|09BBELE-2118|658[0n]bp|Canada.Nova Scotia|BOLD:AAA2229
Idia aemula|[1009]|BBLPC197-09|09BBELE-1197|658[0n]bp|Canada.Nova Scotia|BOLD:AAA2229
Idia aemula|[1010]|BBLPB936-10|10BBCLP-1935|658[0n]bp|Canada.Alberta|BOLD:AAA2229
Idia aemula|[1011]|RDLQG797-06|DH013090|658[0n]bp|Canada.Quebec|BOLD:AAA2229
Idia aemula|[1012]|RDLQG933-06|DH013230|658[0n]bp|Canada.Quebec|BOLD:AAA2229
Idia americalis|[1013]|LBCA885-05|HLC-20885|603[0n]bp|Canada.British Columbia|BOLD:AAA2087
Idia americalis|[1014]|LBCA884-05|HLC-20884|658[0n]bp|Canada.British Columbia|BOLD:AAA2087
Idia americalis|[1015]|BBLEC755-09|09BBELE-0755|614[0n]bp|Canada.Nova Scotia|BOLD:AAA2087
Idia americalis|[1016]|RDNMJ805-11|CNCLEP 80333|658[0n]bp|Canada.Quebec|BOLD:AAA2087
Idia americalis|[1017]|BBLEC713-09|09BBELE-0713|658[0n]bp|Canada.Nova Scotia|BOLD:AAA2087
Idia americalis|[1018]|RDLQG546-06|DH012839|658[0n]bp|Canada.Quebec|BOLD:AAA2087
Idia americalis|[1019]|RDLQG544-06|DH012837|658[0n]bp|Canada.Quebec|BOLD:AAA2087
Idia americalis|[1020]|BBLPC045-09|09BBELE-1045|658[0n]bp|Canada.New Brunswick|BOLD:AAA2087
Idia americalis|[1021]|RDLQG540-06|DH012833|658[0n]bp|Canada.Quebec|BOLD:AAA2087
Idia americalis|[1022]|RDLQG545-06|DH012838|658[0n]bp|Canada.Quebec|BOLD:AAA2087
Idia americalis|[1023]|LOWCB644-05|CGWC-1584|573[3n]bp|Canada.British Columbia|BOLD:AAA2087
Idia americalis|[1024]|LOWCB654-05|CGWC-1594|601[0n]bp|Canada.British Columbia|BOLD:AAA2087
Idia americalis|[1025]|LOWCB655-05|CGWC-1595|601[0n]bp|Canada.British Columbia|BOLD:AAA2087
Idia americalis|[1026]|LOWCB641-05|CGWC-1581|608[1n]bp|Canada.British Columbia|BOLD:AAA2087
Idia americalis|[1027]|LOWCB652-05|CGWC-1592|573[0n]bp|Canada.British Columbia|BOLD:AAA2087
Idia americalis|[1028]|RDNM912-05|CNCNoctuoidea7752|546[3n]bp|Canada.British Columbia|BOLD:AAA2087
Idia americalis|[1029]|RDNM913-05|CNCNoctuoidea7753|535[2n]bp|Canada.British Columbia|BOLD:AAA2087
Idia americalis|[1030]|RDLQG610-06|DH012903|658[0n]bp|Canada.Quebec|BOLD:AAA2087
Idia americalis|[1031]|MNAF867-08|CNCLEP00040852|658[0n]bp|Canada.Manitoba|BOLD:AAA2087
Idia americalis|[1032]|RDLQG899-06|DH013192|634[0n]bp|Canada.Quebec|BOLD:AAA2087
Idia americalis|[1033]|RDLQG894-06|DH013187|658[0n]bp|Canada.Quebec|BOLD:AAA2087
Idia americalis|[1034]|BBLPB418-10|10BBCLP-1417|658[0n]bp|Canada.Alberta|BOLD:AAA2087
Idia americalis|[1035]|LOWCB650-05|CGWC-1590|564[1n]bp|Canada.British Columbia|BOLD:AAA2087
Idia americalis|[1036]|LOWCE842-06|CGWC-4602|615[0n]bp|Canada.British Columbia|BOLD:AAA2087
Idia americalis|[1037]|LOWCB649-05|CGWC-1589|601[0n]bp|Canada.British Columbia|BOLD:AAA2087
Idia americalis|[1038]|LOWCB651-05|CGWC-1591|601[1n]bp|Canada.British Columbia|BOLD:AAA2087
Idia americalis|[1039]|BBLPC047-09|09BBELE-1047|658[0n]bp|Canada.New Brunswick|BOLD:AAA2087
Idia americalis|[1040]|LOWCB645-05|CGWC-1585|599[1n]bp|Canada.British Columbia|BOLD:AAA2087
Idia americalis|[1041]|LBCH4653-10|10-JDWBC-4653|658[0n]bp|Canada.British Columbia|BOLD:AAA2087
Idia americalis|[1042]|LOWCB643-05|CGWC-1583|592[2n]bp|Canada.British Columbia|BOLD:AAA2087
Idia americalis|[1043]|LOWCD875-06|CGWC-3695|590[0n]bp|Canada.British Columbia|BOLD:AAA2087
Idia americalis|[1044]|LOWCE861-06|CGWC-4621|616[0n]bp|Canada.British Columbia|BOLD:AAA2087
Idia americalis|[1045]|LOWCD872-06|CGWC-3692|617[0n]bp|Canada.British Columbia|BOLD:AAA2087
Idia americalis|[1046]|BBLEC334-09|09BBELE-0334|658[0n]bp|Canada.Nova Scotia|BOLD:AAA2087
Idia americalis|[1047]|LOWCD871-06|CGWC-3691|616[0n]bp|Canada.British Columbia|BOLD:AAA2087
Idia americalis|[1048]|BBLEC324-09|09BBELE-0324|632[0n]bp|Canada.Nova Scotia|BOLD:AAA2087
Idia americalis|[1049]|LOWCD869-06|CGWC-3689|608[0n]bp|Canada.British Columbia|BOLD:AAA2087
Idia americalis|[1050]|LOWCB642-05|CGWC-1582|658[0n]bp|Canada.British Columbia|BOLD:AAA2087
Idia americalis|[1051]|LOWCB648-05|CGWC-1588|601[2n]bp|Canada.British Columbia|BOLD:AAA2087
Idia americalis|[1052]|LOWCD865-06|CGWC-3685|605[0n]bp|Canada.British Columbia|BOLD:AAA2087
Idia americalis|[1053]|LPMN056-08|08BBLEP-00854|658[0n]bp|Canada.Manitoba|BOLD:AAA2087
Idia americalis|[1054]|LBCH3226-10|10-JDWBC-3226|658[0n]bp|Canada.British Columbia|BOLD:AAA2087
Idia americalis|[1055]|BBLPE139-09|09BBELE-2139|658[0n]bp|Canada.Nova Scotia|BOLD:AAA2087
Idia americalis|[1056]|LPAB817-08|08BBLEP-03159|658[0n]bp|Canada.Alberta|BOLD:AAA2087
Idia americalis|[1057]|BBLPC044-09|09BBELE-1044|658[0n]bp|Canada.New Brunswick|BOLD:AAA2087
Idia americalis|[1058]|BBLPE155-09|09BBELE-2155|658[0n]bp|Canada.Nova Scotia|BOLD:AAA2087
Idia americalis|[1059]|BBLPE184-09|09BBELE-2184|658[0n]bp|Canada.Nova Scotia|BOLD:AAA2087
Idia americalis|[1060]|LPSK057-08|08BBLEP-00760|658[0n]bp|Canada.Saskatchewan|BOLD:AAA2087
Idia americalis|[1061]|LPMN447-08|08BBLEP-01246|658[0n]bp|Canada.Manitoba|BOLD:AAA2087
Idia americalis|[1062]|RDLQG542-06|DH012835|658[0n]bp|Canada.Quebec|BOLD:AAA2087
Idia americalis|[1063]|RDLQB243-05|DH010329|658[0n]bp|Canada.Quebec|BOLD:AAA2087
Idia americalis|[1064]|LPMNB513-09|08BBLEP-05551|658[0n]bp|Canada.Manitoba|BOLD:AAA2087
Idia americalis|[1065]|RDNM918-05|CNCNoctuoidea7758|658[0n]bp|Canada.British Columbia|BOLD:AAA2087
Idia americalis|[1066]|LPVIA081-08|PFC-2006-0119|658[0n]bp|Canada.British Columbia|BOLD:AAA2087
Idia americalis|[1067]|LOWCB657-05|CGWC-1597|658[0n]bp|Canada.British Columbia|BOLD:AAA2087
Idia americalis|[1068]|TMNBB043-06|MNBTT-983|658[0n]bp|Canada.New Brunswick|BOLD:AAA2087
Idia americalis|[1069]|LPMN352-08|08BBLEP-01151|658[0n]bp|Canada.Manitoba|BOLD:AAA2087
Idia americalis|[1070]|LPABC666-09|08BBLEP-04885|658[0n]bp|Canada.Alberta|BOLD:AAA2087
Idia americalis|[1071]|RDNMJ804-11|CNCLEP 80332|658[0n]bp|Canada.Quebec|BOLD:AAA2087
Idia americalis|[1072]|LPMN665-08|08BBLEP-01466|658[0n]bp|Canada.Manitoba|BOLD:AAA2087
Idia americalis|[1073]|LOWCE834-06|CGWC-4594|656[0n]bp|Canada.British Columbia|BOLD:AAA2087
Idia americalis|[1074]|LPMN370-08|08BBLEP-01169|658[0n]bp|Canada.Manitoba|BOLD:AAA2087
Idia americalis|[1075]|BBLPB159-10|10BBCLP-1158|658[0n]bp|Canada.Alberta|BOLD:AAA2087
Idia americalis|[1076]|LPMN353-08|08BBLEP-01152|658[0n]bp|Canada.Manitoba|BOLD:AAA2087
Idia americalis|[1077]|RDLQG547-06|DH012840|658[0n]bp|Canada.Quebec|BOLD:AAA2087
Idia americalis|[1078]|LPABC870-09|08BBLEP-05281|658[0n]bp|Canada.Alberta|BOLD:AAA2087
Idia americalis|[1079]|LOWCD868-06|CGWC-3688|658[0n]bp|Canada.British Columbia|BOLD:AAA2087
Idia americalis|[1080]|LOWCB656-05|CGWC-1596|658[0n]bp|Canada.British Columbia|BOLD:AAA2087
Idia americalis|[1081]|RDLQG678-06|DH012971|658[0n]bp|Canada.Quebec|BOLD:AAA2087
Idia americalis|[1082]|LPMNB523-09|08BBLEP-05561|658[0n]bp|Canada.Manitoba|BOLD:AAA2087
Idia americalis|[1083]|LOWCB646-05|CGWC-1586|565[2n]bp|Canada.British Columbia|BOLD:AAA2087
Idia americalis|[1084]|LOWCD864-06|CGWC-3684|597[0n]bp|Canada.British Columbia|BOLD:AAA2087
Idia americalis|[1085]|LOWCB653-05|CGWC-1593|658[0n]bp|Canada.British Columbia|BOLD:AAA2087
Idia americalis|[1086]|LOWCB659-05|CGWC-1599|658[0n]bp|Canada.British Columbia|BOLD:AAA2087
Idia americalis|[1087]|LOWCD870-06|CGWC-3690|658[0n]bp|Canada.British Columbia|BOLD:AAA2087
Idia americalis|[1088]|LOWCD866-06|CGWC-3686|596[0n]bp|Canada.British Columbia|BOLD:AAA2087
Idia americalis|[1089]|HEJUL247-12|BIOUG02385-E07|618[0n]bp|Canada.Ontario|BOLD:AAA2087
Idia americalis|[1090]|XAH044-05|2005-ONT-1627|658[0n]bp|Canada.Ontario|BOLD:AAA2087
Idia americalis|[1091]|XAB192-04|04HBL005192|658[0n]bp|Canada.Ontario|BOLD:AAA2087

[illegible]
Idia americalis|[1089]|HEJUL247-12|BIOUG02385-E07|618[0n]bp|Canada.Ontario|BOLD:AAA2087
Idia americalis|[1090]|XAH044-05|2005-ONT-1627|658[0n]bp|Canada.Ontario|BOLD:AAA2087
Idia americalis|[1091]|XAB192-04|04HBL005192|658[0n]bp|Canada.Ontario|BOLD:AAA2087
Idia americalis|[1092]|JSAUG484-11|BIOUG01602-D04|658[0n]bp|Canada.Ontario|BOLD:AAA2087
Idia americalis|[1093]|XAH306-05|2005-ONT-1889|658[0n]bp|Canada.Ontario|BOLD:AAA2087
Idia americalis|[1094]|XAF740-05|2005-ONT-389|658[0n]bp|Canada.Ontario|BOLD:AAA2087
Idia americalis|[1095]|XAH301-05|2005-ONT-1884|658[0n]bp|Canada.Ontario|BOLD:AAA2087
Idia americalis|[1096]|LPMNB355-09|08BBLEP-05199|658[0n]bp|Canada.Manitoba|BOLD:AAA2087
Idia americalis|[1097]|TTMNB262-06|MNBTT-262|658[0n]bp|Canada.New Brunswick|BOLD:AAA2087
Idia americalis|[1098]|LPMNB406-09|08BBLEP-05250|658[0n]bp|Canada.Manitoba|BOLD:AAA2087
Idia americalis|[1099]|LPMNB337-09|08BBLEP-05181|658[0n]bp|Canada.Manitoba|BOLD:AAA2087
Idia americalis|[1100]|RDLQG549-06|DH012842|658[0n]bp|Canada.Quebec|BOLD:AAA2087
Idia americalis|[1101]|XAG297-05|2005-ONT-881|658[0n]bp|Canada.Ontario|BOLD:AAA2087
Idia americalis|[1102]|XAH286-05|2005-ONT-1869|658[0n]bp|Canada.Ontario|BOLD:AAA2087
Idia americalis|[1103]|TTMNB261-06|MNBTT-261|658[0n]bp|Canada.New Brunswick|BOLD:AAA2087
Idia americalis|[1104]|XAC463-04|04HBL006463|658[0n]bp|Canada.Ontario|BOLD:AAA2087
Idia americalis|[1105]|LPABB308-08|08BBLEP-03573|658[0n]bp|Canada.Alberta|BOLD:AAA2087
Idia americalis|[1106]|LPSOC227-08|PPBP-2226|658[0n]bp|Canada.Ontario|BOLD:AAA2087
Idia americalis|[1107]|RDLQG092-06|DH012249|658[0n]bp|Canada.Quebec|BOLD:AAA2087
Idia americalis|[1108]|RDLQG488-06|DH012781|658[0n]bp|Canada.Quebec|BOLD:AAA2087
Idia americalis|[1109]|LPMNB415-09|08BBLEP-05415|658[0n]bp|Canada.Manitoba|BOLD:AAA2087
Idia americalis|[1110]|LPMNB539-09|08BBLEP-05577|658[0n]bp|Canada.Manitoba|BOLD:AAA2087
Idia americalis|[1111]|XAC041-04|04HBL006041|658[0n]bp|Canada.Ontario|BOLD:AAA2087
Idia americalis|[1112]|PHMNB009-03|moth127.02SA|639[0n]bp|Canada.New Brunswick|BOLD:AAA2087
Idia americalis|[1113]|BBLEC026-09|09BBELE-0026|658[0n]bp|Canada.New Brunswick|BOLD:AAA2087
Idia americalis|[1114]|RDMAB006-05|UASM57551|658[0n]bp|Canada.Alberta|BOLD:AAA2087
Idia americalis|[1115]|RDNM916-05|CNCNoctuoidea7756|658[0n]bp|Canada.British Columbia|BOLD:AAA2087
Idia americalis|[1116]|RDMAB074-05|UASM57601|658[0n]bp|Canada.Alberta|BOLD:AAA2087
Idia americalis|[1117]|BBLPB340-10|10BBCLP-1339|658[0n]bp|Canada.Alberta|BOLD:AAA2087
Idia americalis|[1118]|PMG121-03|IDIA1.00|617[0n]bp|Canada.Ontario|BOLD:AAA2087
Idia americalis|[1119]|RDLQG705-06|DH012998|658[0n]bp|Canada.Quebec|BOLD:AAA2087
Idia americalis|[1120]|MNBB469-05|05-NBSTA-385|658[0n]bp|Canada.New Brunswick|BOLD:AAA2087
Idia americalis|[1121]|LPSOC224-08|PPBP-2223|658[0n]bp|Canada.Ontario|BOLD:AAA2087
Idia americalis|[1122]|RDLQG780-06|DH013073|655[0n]bp|Canada.Quebec|BOLD:AAA2087
Idia americalis|[1123]|BBLEC181-09|09BBELE-0181|658[0n]bp|Canada.Nova Scotia|BOLD:AAA2087
Idia americalis|[1124]|RDLQF604-06|DH011754|658[0n]bp|Canada.Quebec|BOLD:AAA2087
Idia americalis|[1125]|PHMNB617-04|04HBL00843|658[0n]bp|Canada.New Brunswick|BOLD:AAA2087
Idia americalis|[1126]|RDLQG935-06|DH013232|658[0n]bp|Canada.Quebec|BOLD:AAA2087
Idia americalis|[1127]|PHMNB109-04|04HBL007574|573[0n]bp|Canada.New Brunswick|BOLD:AAA2087
Idia americalis|[1128]|RDLQF772-06|DH011922|658[0n]bp|Canada.Quebec|BOLD:AAA2087
Idia americalis|[1129]|RDNM917-05|CNCNoctuoidea7757|578[0n]bp|Canada.Ontario|BOLD:AAA2087
Idia americalis|[1130]|BLTIB458-08|BL705|658[0n]bp|Canada.Ontario|BOLD:AAA2087
Idia americalis|[1131]|XAK316-06|2006-ONT-1311|658[0n]bp|Canada.Ontario|BOLD:AAA2087
Idia americalis|[1132]|LOWCB658-05|CGWC-1598|593[2n]bp|Canada.British Columbia|BOLD:AAA2087
Idia americalis|[1133]|LPABC663-09|08BBLEP-04882|658[0n]bp|Canada.Alberta|BOLD:AAA2087
Idia americalis|[1134]|LOWCD874-06|CGWC-3694|591[0n]bp|Canada.British Columbia|BOLD:AAA2087
Idia americalis|[1135]|RDLQG478-06|DH012771|640[0n]bp|Canada.Quebec|BOLD:AAA2087
Idia americalis|[1136]|RDNM914-05|CNCNoctuoidea7754|658[0n]bp|Canada.Ontario|BOLD:AAA2087
Idia americalis|[1137]|MNAD043-07|CNCLEP00027703|658[0n]bp|Canada.Quebec|BOLD:AAA2087
Idia americalis|[1138]|RDLQG612-06|DH012905|658[0n]bp|Canada.Quebec|BOLD:AAA2087
Idia americalis|[1139]|RDLQG474-06|DH012767|658[0n]bp|Canada.Quebec|BOLD:AAA2087
Idia americalis|[1140]|LBCH4439-10|10-JDWBC-4439|658[0n]bp|Canada.British Columbia|BOLD:AAA2087
Idia americalis|[1141]|LOWCB647-05|CGWC-1587|658[0n]bp|Canada.British Columbia|BOLD:AAA2087
Idia americalis|[1142]|LBCH093-10|10-JDWBC-0093|658[0n]bp|Canada.British Columbia|BOLD:AAA2087
Idia americalis|[1143]|RDLQF380-06|DH011447|658[0n]bp|Canada.Quebec|BOLD:AAA2087
Idia americalis|[1144]|LBCH351-10|10-JDWBC-0351|658[0n]bp|Canada.British Columbia|BOLD:AAA2087
Idia americalis|[1145]|RDLQF381-06|DH011448|658[0n]bp|Canada.Quebec|BOLD:AAA2087
Idia americalis|[1146]|LBCB741-05|HLC-21681|658[0n]bp|Canada.British Columbia|BOLD:AAA2087
Idia americalis|[1147]|MNAD087-07|CNCLEP00027753|658[0n]bp|Canada.Ontario|BOLD:AAA2087
Idia americalis|[1148]|BLTIB1037-08|BL1480|658[0n]bp|Canada.Ontario|BOLD:AAA2087
Idia americalis|[1149]|RDLQF447-06|DH011554|658[0n]bp|Canada.Quebec|BOLD:AAA2087
Idia americalis|[1150]|LPSOC233-08|PPBP-2232|658[0n]bp|Canada.Ontario|BOLD:AAA2087
Idia americalis|[1151]|RDLQG199-06|DH012375|658[0n]bp|Canada.Quebec|BOLD:AAA2087
Idia americalis|[1152]|RDLQF573-06|DH011723|658[1n]bp|Canada.Quebec|BOLD:AAA2087
Idia americalis|[1153]|LOWCD867-06|CGWC-3687|605[0n]bp|Canada.British Columbia|BOLD:AAA2087
Idia americalis|[1154]|RDLQF725-06|DH011875|654[0n]bp|Canada.Quebec|BOLD:AAA2087
Idia americalis|[1155]|RDLQG496-06|DH012789|644[1n]bp|Canada.Quebec|BOLD:AAA2087
Idia americalis|[1156]|RDLQG473-06|DH012766|658[0n]bp|Canada.Quebec|BOLD:AAA2087
Idia americalis|[1157]|RDLQG472-06|DH012765|658[0n]bp|Canada.Quebec|BOLD:AAA2087
Idia americalis|[1158]|RDLQF605-06|DH011755|658[0n]bp|Canada.Quebec|BOLD:AAA2087
Idia americalis|[1159]|RDLQG091-06|DH012248|658[0n]bp|Canada.Quebec|BOLD:AAA2087
Idia americalis|[1160]|RDLQG263-06|DH012468|658[0n]bp|Canada.Quebec|BOLD:AAA2087
Idia americalis|[1161]|RDLQF572-06|DH011722|658[0n]bp|Canada.Quebec|BOLD:AAA2087
Idia americalis|[1162]|RDLQG607-06|DH012900|658[0n]bp|Canada.Quebec|BOLD:AAA2087
Idia americalis|[1163]|XAH486-05|2005-ONT-2069|658[0n]bp|Canada.Ontario|BOLD:AAA2087
Idia americalis|[1164]|RDLQG475-06|DH012768|658[0n]bp|Canada.Quebec|BOLD:AAA2087
Idia americalis|[1165]|RDLQF540-06|DH011689|658[0n]bp|Canada.Quebec|BOLD:AAA2087
Idia americalis|[1166]|RDLQF723-06|DH011873|658[0n]bp|Canada.Quebec|BOLD:AAA2087
Idia americalis|[1167]|LPSOC397-08|PPBP-2396|658[0n]bp|Canada.Ontario|BOLD:AAA2087
Idia americalis|[1168]|LBCH4051-10|10-JDWBC-4051|658[0n]bp|Canada.British Columbia|BOLD:AAA2087
Idia americalis|[1169]|RDLQG936-06|DH013233|658[0n]bp|Canada.Quebec|BOLD:AAA2087
Idia americalis|[1170]|RDLQF724-06|DH011874|658[0n]bp|Canada.Quebec|BOLD:AAA2087
Idia americalis|[1171]|RDLQG934-06|DH013231|658[0n]bp|Canada.Quebec|BOLD:AAA2087
Idia americalis|[1172]|PHMNB333-04|04HBL00559|658[0n]bp|Canada.New Brunswick|BOLD:AAA2087
Idia americalis|[1173]|RDLQG497-06|DH012790|658[0n]bp|Canada.Quebec|BOLD:AAA2087
Idia majoralis|[1174]|ABCNA932-08|932-230707-WI|658[0n]bp|United States.Wisconsin|BOLD:AAD8378
Idia majoralis|[1175]|LMEM153-09|RBMIS-0153|658[0n]bp|United States.Mississippi|BOLD:AAD8378
Idia majoralis|[1176]|LPOKD420-09|MDOK-3499|658[0n]bp|United States.Oklahoma|BOLD:AAD8378
Idia majoralis|[1177]|LMEM155-09|RBMIS-0155|658[0n]bp|United States.Alabama|BOLD:AAD8378
Idia majoralis|[1178]|LMEM156-09|RBMIS-0156|658[0n]bp|United States.Mississippi|BOLD:AAD8378
Idia majoralis|[1179]|LMEM154-09|RBMIS-0154|624[0n]bp|United States.Mississippi|BOLD:AAD8378
Idia julia|[1180]|RDLQG106-06|DH012263|587[0n]bp|Canada.Quebec|BOLD:AAA3327
Idia julia|[1181]|LPMNB478-09|08BBLEP-05516|658[0n]bp|Canada.Manitoba|BOLD:AAA3327
Idia julia|[1182]|LPMNB364-09|08BBLEP-05208|658[0n]bp|Canada.Manitoba|BOLD:AAA3327
Idia julia|[1183]|RDLQG929-06|DH013226|658[0n]bp|Canada.Quebec|BOLD:AAA3327
Idia julia|[1184]|LPMNB350-09|08BBLEP-05194|658[0n]bp|Canada.Manitoba|BOLD:AAA3327
Idia julia|[1185]|RDLQG682-06|DH012975|658[0n]bp|Canada.Quebec|BOLD:AAA3327
Idia julia|[1186]|LPMNB392-09|08BBLEP-05236|658[0n]bp|Canada.Manitoba|BOLD:AAA3327
Idia julia|[1187]|LPMNB403-09|08BBLEP-05247|658[0n]bp|Canada.Manitoba|BOLD:AAA3327
Idia julia|[1188]|MNAF433-08|CNCLEP00040418|649[0n]bp|Canada.Manitoba|BOLD:AAA3327
Idia forbesii|[1189]|RDLQG122-06|DH012279|656[0n]bp|Canada.Quebec|BOLD:ABZ1974
Idia forbesii|[1190]|RDLQG123-06|DH012280|636[0n]bp|Canada.Quebec|BOLD:ABZ1974

Idia julia|[1188]|MNAF433-08|CNCLEP00040418|649[0n]bp|Canada.Manitoba|BOLD:AAA3327
Idia forbesii|[1189]|RDLQG122-06|DH012279|656[0n]bp|Canada.Quebec|BOLD:ABZ1974
Idia forbesii|[1190]|RDLQG123-06|DH012280|636[0n]bp|Canada.Quebec|BOLD:ABZ1974
Idia forbesii|[1191]|RDLQF661-06|DH011811|620[0n]bp|Canada.Quebec|BOLD:ABZ1974
Idia forbesii|[1192]|RDLQF610-06|DH011760|658[0n]bp|Canada.Quebec|BOLD:ABZ1974
Idia forbesii|[1193]|RDLQF659-06|DH011809|642[0n]bp|Canada.Quebec|BOLD:ABZ1974
Idia forbesii|[1194]|RDLQF660-06|DH011810|639[0n]bp|Canada.Quebec|BOLD:ABZ1974
Idia forbesii|[1195]|RDLQG105-06|DH012262|632[0n]bp|Canada.Quebec|BOLD:ABZ1974
Idia scobialis|[1196]|XAJ859-06|2006-ONT-0859|658[0n]bp|Canada.Ontario|BOLD:AAC0879
Idia scobialis|[1197]|XAJ862-06|2006-ONT-0862|658[0n]bp|Canada.Ontario|BOLD:AAC0879
Panopoda carneicosta|[1198]|XAJ811-06|2006-ONT-0811|658[0n]bp|Canada.Ontario|BOLD:AAA9857
Panopoda carneicosta|[1199]|RDLQ753-07|DH007831|658[0n]bp|Canada.Quebec|BOLD:AAA9857
Panopoda carneicosta|[1200]|PMG147-03|moth1128.01|617[0n]bp|Canada.Ontario|BOLD:AAA9857
Panopoda carneicosta|[1201]|XAJ809-06|2006-ONT-0809|632[0n]bp|Canada.Ontario|BOLD:AAA9857
Panopoda rufimargo|[1202]|PHMO163-03|moth882.02|639[0n]bp|Canada.Ontario|BOLD:AAA7227
Panopoda rufimargo|[1203]|RDLQF932-06|DH012107|658[0n]bp|Canada.Quebec|BOLD:AAA7227
Panopoda rufimargo|[1204]|PHMO107-03|moth665.02|639[0n]bp|Canada.Ontario|BOLD:AAA7227
Palthis angulalis|[1205]|XAD002-04|04HBL007002|510[0n]bp|Canada.Ontario|BOLD:AAA3933
Palthis angulalis|[1206]|XAK276-06|2006-ONT-1271|576[0n]bp|Canada.Ontario|BOLD:AAA3933
Palthis angulalis|[1207]|RDLQG740-06|DH013033|658[0n]bp|Canada.Quebec|BOLD:AAA3933
Palthis angulalis|[1208]|XAE102-04|Moth4102.03|658[0n]bp|Canada.Ontario|BOLD:AAA3933
Palthis angulalis|[1209]|XAB301-04|04HBL005301|613[0n]bp|Canada.Ontario|BOLD:AAA3933
Palthis angulalis|[1210]|XAD295-04|04HBL007295|559[0n]bp|Canada.Ontario|BOLD:AAA3933
Palthis angulalis|[1211]|RDLQG615-06|DH012908|655[0n]bp|Canada.Quebec|BOLD:AAA3933
Palthis angulalis|[1212]|PHMNB485-04|04HBL00711|658[0n]bp|Canada.New Brunswick|BOLD:AAA3933
Palthis angulalis|[1213]|RDLQG820-06|DH013113|658[0n]bp|Canada.Quebec|BOLD:AAA3933
Palthis angulalis|[1214]|LPSOD1065-09|08MZPP-166|658[0n]bp|Canada.Ontario|BOLD:AAA3933
Palthis angulalis|[1215]|BLTIB1016-08|BL1455|658[0n]bp|Canada.Ontario|BOLD:AAA3933
Palthis angulalis|[1216]|RDLQF445-06|DH011552|658[0n]bp|Canada.Quebec|BOLD:AAA3933
Palthis angulalis|[1217]|BBLEC636-09|09BBELE-0636|658[0n]bp|Canada.Nova Scotia|BOLD:AAA3933
Palthis angulalis|[1218]|LPSOC445-08|PPBP-2444|658[0n]bp|Canada.Ontario|BOLD:AAA3933
Palthis angulalis|[1219]|LPSO085-08|PPBP-0085|658[0n]bp|Canada.Ontario|BOLD:AAA3933
Palthis angulalis|[1220]|RDLQG041-06|DH012172|658[0n]bp|Canada.Quebec|BOLD:AAA3933
Palthis angulalis|[1221]|MEC715-04|jflandry0715|658[0n]bp|Canada.Quebec|BOLD:AAA3933
Palthis angulalis|[1222]|RDLQF492-06|DH011641|658[0n]bp|Canada.Quebec|BOLD:AAA3933
Palthis angulalis|[1223]|RDLQG040-06|DH012171|658[0n]bp|Canada.Quebec|BOLD:AAA3933
Palthis angulalis|[1224]|XAE223-04|Moth4223.03|658[0n]bp|Canada.Ontario|BOLD:AAA3933
Palthis angulalis|[1225]|PHAUG1516-11|BIOUG01521-A04|658[0n]bp|Canada.Ontario|BOLD:AAA3933
Palthis angulalis|[1226]|BBLPC687-09|09BBELE-1687|658[0n]bp|Canada.Newfoundland and Labrador|BOLD:AAA...
Palthis angulalis|[1227]|BLTIB745-08|BL1035|658[0n]bp|Canada.Ontario|BOLD:AAA3933
Palthis angulalis|[1228]|RDLQF444-06|DH011551|658[0n]bp|Canada.Quebec|BOLD:AAA3933
Palthis angulalis|[1229]|RDLQG756-06|DH013049|658[0n]bp|Canada.Quebec|BOLD:AAA3933
Palthis angulalis|[1230]|BBLEC867-09|09BBELE-0867|658[0n]bp|Canada.Newfoundland and Labrador|BOLD:AAA...
Palthis angulalis|[1231]|BLTIB425-08|BL672|658[0n]bp|Canada.Ontario|BOLD:AAA3933
Palthis angulalis|[1232]|RDMAB021-05|UASM57566|658[0n]bp|Canada.Alberta|BOLD:AAA3933
Palthis angulalis|[1233]|RDLQG594-06|DH012887|658[0n]bp|Canada.Quebec|BOLD:AAA3933
Palthis angulalis|[1234]|PHMNB186-04|04HBL007651|658[0n]bp|Canada.New Brunswick|BOLD:AAA3933
Palthis angulalis|[1235]|XAG980-05|2005-ONT-1564|658[0n]bp|Canada.Ontario|BOLD:AAA3933
Palthis angulalis|[1236]|LPSOC056-08|PPBP-2055|658[0n]bp|Canada.Ontario|BOLD:AAA3933
Palthis angulalis|[1237]|XAK554-07|HLC-16107|658[0n]bp|Canada.Ontario|BOLD:AAA3933
Palthis angulalis|[1238]|RDLQG809-06|DH013102|658[0n]bp|Canada.Quebec|BOLD:AAA3933
Palthis angulalis|[1239]|LPSO284-08|PPBP-0284|658[0n]bp|Canada.Ontario|BOLD:AAA3933
Palthis angulalis|[1240]|TMNBC180-06|MNBTT-1986|658[0n]bp|Canada.New Brunswick|BOLD:AAA3933
Palthis angulalis|[1241]|BBLPB892-10|10BBCLP-1891|658[0n]bp|Canada.Manitoba|BOLD:AAA3933
Palthis angulalis|[1242]|TTMNB270-06|MNBTT-270|658[0n]bp|Canada.New Brunswick|BOLD:AAA3933
Palthis angulalis|[1243]|BBLPE505-09|09BBELE-2505|658[0n]bp|Canada.Newfoundland and Labrador|BOLD:AAA...
Palthis angulalis|[1244]|BLTIB965-08|BL1394|658[0n]bp|Canada.Ontario|BOLD:AAA3933
Palthis angulalis|[1245]|BBLPC735-09|09BBELE-1735|658[0n]bp|Canada.Newfoundland and Labrador|BOLD:AAA...
Palthis angulalis|[1246]|RDLQG254-06|DH012459|658[0n]bp|Canada.Quebec|BOLD:AAA3933
Palthis angulalis|[1247]|BBLPC850-09|09BBELE-1850|658[0n]bp|Canada.Newfoundland and Labrador|BOLD:AAA...
Palthis angulalis|[1248]|LPSO273-08|PPBP-0273|658[0n]bp|Canada.Ontario|BOLD:AAA3933
Palthis angulalis|[1249]|RDLQF727-06|DH011877|658[0n]bp|Canada.Quebec|BOLD:AAA3933
Palthis angulalis|[1250]|BLTIB969-08|BL1398|658[0n]bp|Canada.Ontario|BOLD:AAA3933
Palthis angulalis|[1251]|RDLQG279-06|DH012491|658[0n]bp|Canada.Quebec|BOLD:AAA3933
Palthis angulalis|[1252]|BLTIB1073-08|BL1082|658[0n]bp|Canada.Ontario|BOLD:AAA3933
Palthis angulalis|[1253]|LPSOB591-08|PPBP-1590|621[0n]bp|Canada.Ontario|BOLD:AAA3933
Palthis angulalis|[1254]|RDLQB680-05|DH010783|552[1n]bp|Canada.Quebec|BOLD:AAA3933
Palthis angulalis|[1255]|RDMAB020-05|UASM57565|631[0n]bp|Canada.Alberta|BOLD:AAA3933
Palthis angulalis|[1256]|XAK611-07|HLC-16164|603[1n]bp|Canada.Ontario|BOLD:AAA3933
Palthis angulalis|[1257]|LOWCB640-05|CGWC-1580|577[1n]bp|Canada.British Columbia|BOLD:AAA3933
Palthis angulalis|[1258]|PHMNB062-03|moth42.02SA|639[0n]bp|Canada.New Brunswick|BOLD:AAA3933
Palthis angulalis|[1259]|LPSOC164-08|PPBP-2163|646[0n]bp|Canada.Ontario|BOLD:AAA3933
Palthis angulalis|[1260]|LALPA190-10|AVBC 190-10|658[0n]bp|Canada.British Columbia|BOLD:AAA3933
Palthis angulalis|[1261]|LALPA249-10|AVBC 250-10|658[0n]bp|Canada.British Columbia|BOLD:AAA3933
Palthis angulalis|[1262]|LHLEP330-06|UBC-2006-0800|657[0n]bp|Canada.British Columbia|BOLD:AAA3933
Palthis angulalis|[1263]|BBLPD755-10|10BBCLP-2753|658[0n]bp|Canada.British Columbia|BOLD:AAA3933
Palthis angulalis|[1264]|LHLEP331-06|UBC-2006-0801|657[0n]bp|Canada.British Columbia|BOLD:AAA3933
Palthis angulalis|[1265]|PMG146-03|moth379.01|617[0n]bp|Canada.Ontario|BOLD:AAA3933
Palthis angulalis|[1266]|LBCA530-05|HLC-20530|658[0n]bp|Canada.British Columbia|BOLD:AAA3933
Palthis angulalis|[1267]|LHLEP333-06|UBC-2006-1019|657[0n]bp|Canada.British Columbia|BOLD:AAA3933
Palthis angulalis|[1268]|LALPA243-10|AVBC 244-10|658[0n]bp|Canada.British Columbia|BOLD:AAA3933
Palthis angulalis|[1269]|LBCA531-05|HLC-20531|658[0n]bp|Canada.British Columbia|BOLD:AAA3933
Palthis angulalis|[1270]|LHLEP332-06|UBC-2006-1018|657[0n]bp|Canada.British Columbia|BOLD:AAA3933
Palthis angulalis|[1271]|BBLPD992-10|10BBCLP-2990|658[0n]bp|Canada.Alberta|BOLD:AAA3933
Palthis angulalis|[1272]|RDLQG566-06|DH012859|658[0n]bp|Canada.Quebec|BOLD:AAA3933
Palthis asopialis|[1273]|LPSOD1061-09|08MZPP-158|658[0n]bp|Canada.Ontario|BOLD:AAB6251
Palthis asopialis|[1274]|BBLPA623-10|10BBCLP-0623|658[0n]bp|Canada.Ontario|BOLD:AAB6251
Palthis asopialis|[1275]|BBLPA750-10|10BBCLP-0750|658[0n]bp|Canada.Ontario|BOLD:AAB6251
Redectis vitrea|[1276]|BBLPA624-10|10BBCLP-0624|658[0n]bp|Canada.Ontario|BOLD:AAC7139
Macrochilo bivittata|[1277]|RDNMF781-08|UASM29931|658[0n]bp|Canada.Alberta|BOLD:AAD6368
Macrochilo bivittata|[1278]|RDNME947-08|CNC LEP 00047422|658[0n]bp|Canada.Alberta|BOLD:AAD6368
Macrochilo bivittata|[1279]|RDNME948-08|CNC LEP 00047423|658[0n]bp|Canada.Ontario|BOLD:AAD6368
Macrochilo bivittata|[1280]|RDNMF783-08|UASM110767|658[0n]bp|Canada.Ontario|BOLD:AAD6368
Macrochilo bivittata|[1281]|RDNMF782-08|UASM56917|658[0n]bp|Canada.Alberta|BOLD:AAD6368
Macrochilo bivittata|[1282]|RDNMF784-08|UASM7333|658[0n]bp|Canada.Alberta|BOLD:AAD6368
Macrochilo absorptalis|[1283]|RDLQG910-06|DH013203|653[0n]bp|Canada.Quebec|BOLD:ACF4191
Macrochilo absorptalis|[1284]|XAJ776-06|2006-ONT-0776|636[0n]bp|Canada.Ontario|BOLD:ACF4191
Macrochilo absorptalis|[1285]|RDLQB399-05|DH010485|658[0n]bp|Canada.Quebec|BOLD:AAB3885
Macrochilo absorptalis|[1286]|BLTIB743-08|BL1033|657[0n]bp|Canada.Ontario|BOLD:AAB3885
Macrochilo absorptalis|[1287]|BLTIB1074-08|BL1083|658[0n]bp|Canada.Ontario|BOLD:AAB3885
Macrochilo absorptalis|[1288]|BLTIB357-08|BL575|632[0n]bp|Canada.Ontario|BOLD:AAB3885
Macrochilo absorptalis|[1289]|RDLQG779-06|DH013072|658[0n]bp|Canada.Quebec|BOLD:AAB3885

Macrochilo absorptalis|[1287]|BLTIB1074-08|BL1083|658[0n]bp|Canada.Ontario|BOLD:AAB3885
Macrochilo absorptalis|[1288]|BLTIB357-08|BL575|632[0n]bp|Canada.Ontario|BOLD:AAB3885
Macrochilo absorptalis|[1289]|RDLQG779-06|DH013072|658[0n]bp|Canada.Quebec|BOLD:AAB3885
Macrochilo absorptalis|[1290]|RDLQG166-06|DH012337|658[0n]bp|Canada.Quebec|BOLD:AAB3885
Macrochilo absorptalis|[1291]|RDLQG588-06|DH012881|658[0n]bp|Canada.Quebec|BOLD:AAB3885
Macrochilo absorptalis|[1292]|RDLQG902-06|DH013195|658[0n]bp|Canada.Quebec|BOLD:AAB3885
Macrochilo absorptalis|[1293]|RDLQF559-06|DH011708|658[0n]bp|Canada.Quebec|BOLD:AAB3885
Macrochilo absorptalis|[1294]|RDLQF427-06|DH011534|658[0n]bp|Canada.Quebec|BOLD:AAB3885
Macrochilo absorptalis|[1295]|RDLQG903-06|DH013196|658[0n]bp|Canada.Quebec|BOLD:AAB3885
Macrochilo absorptalis|[1296]|RDLQG924-06|DH013217|658[0n]bp|Canada.Quebec|BOLD:AAB3885
Macrochilo absorptalis|[1297]|RDLQG667-06|DH012960|658[0n]bp|Canada.Quebec|BOLD:AAB3885
Macrochilo absorptalis|[1298]|RDLQG938-06|DH013235|658[0n]bp|Canada.Quebec|BOLD:AAB3885
Macrochilo absorptalis|[1299]|PHMNB216-04|04HBL007681|658[0n]bp|Canada.New Brunswick|BOLD:AAB3885
Macrochilo absorptalis|[1300]|BLTIB630-08|BL910|635[0n]bp|Canada.Ontario|BOLD:AAB3885
Macrochilo absorptalis|[1301]|BLTIB446-08|BL693|658[0n]bp|Canada.Ontario|BOLD:AAB3885
Macrochilo absorptalis|[1302]|PHMO255-03|moth1473.02|639[0n]bp|Canada.Ontario|BOLD:AAB3885
Macrochilo absorptalis|[1303]|RDLQG668-06|DH012961|658[0n]bp|Canada.Quebec|BOLD:AAB3885
Macrochilo absorptalis|[1304]|XAJ898-06|2006-ONT-0898|658[0n]bp|Canada.Ontario|BOLD:AAB3885
Macrochilo absorptalis|[1305]|PHMNB672-04|04HBL00898|658[0n]bp|Canada.New Brunswick|BOLD:AAB3885
Macrochilo litophora|[1306]|XAB489-04|04HBL005489|658[0n]bp|Canada.Ontario|BOLD:AAC6075
Macrochilo louisiana|[1307]|RDLQG781-06|DH013074|658[0n]bp|Canada.Quebec|BOLD:AAC0807
Macrochilo louisiana|[1308]|RDLQG937-06|DH013234|658[0n]bp|Canada.Quebec|BOLD:AAC0807
Macrochilo louisiana|[1309]|RDLQG625-06|DH012918|658[0n]bp|Canada.Quebec|BOLD:AAC0807
Macrochilo louisiana|[1310]|RDLQH154-06|AC000654|606[0n]bp|Canada.Quebec|BOLD:AAC0807
Macrochilo louisiana|[1311]|RDLQB405-05|DH010491|581[0n]bp|Canada.Quebec|BOLD:AAC0807
Macrochilo orciferalis|[1312]|LPSO759-08|PPBP-0759|656[0n]bp|Canada.Ontario|BOLD:AAB1328
Macrochilo orciferalis|[1313]|MNAF487-08|CNCLEP00040472|658[0n]bp|Canada.Manitoba|BOLD:AAB1327
Macrochilo orciferalis|[1314]|RDLQG913-06|DH013206|658[0n]bp|Canada.Quebec|BOLD:AAB1327
Macrochilo orciferalis|[1315]|RDLQG671-06|DH012964|658[0n]bp|Canada.Quebec|BOLD:AAB1327
Macrochilo orciferalis|[1316]|RDLQB463-05|DH010549|658[0n]bp|Canada.Quebec|BOLD:AAB1327
Macrochilo orciferalis|[1317]|MNAF865-08|CNCLEP00040850|658[0n]bp|Canada.Manitoba|BOLD:AAB1327
Macrochilo orciferalis|[1318]|RDLQG172-06|DH012343|658[0n]bp|Canada.Quebec|BOLD:AAB1327
Macrochilo orciferalis|[1319]|RDLQG670-06|DH012963|658[0n]bp|Canada.Quebec|BOLD:AAB1327
Macrochilo orciferalis|[1320]|RDLQG672-06|DH012965|658[0n]bp|Canada.Quebec|BOLD:AAB1327
Macrochilo orciferalis|[1321]|RDLQG669-06|DH012962|658[0n]bp|Canada.Quebec|BOLD:AAB1327
Macrochilo orciferalis|[1322]|XAB004-04|04HBL005004|565[0n]bp|Canada.Ontario|BOLD:AAB1327
Macrochilo orciferalis|[1323]|RDLQH156-06|DH005421|594[4n]bp|Canada.Quebec|BOLD:AAB1327
Macrochilo orciferalis|[1324]|RDLQH155-06|DH003070|600[0n]bp|Canada.Quebec|BOLD:AAB1327
Phalaenostola eumelusalis|[1325]|BLTIB1068-08|BL1077|656[0n]bp|Canada.Ontario|BOLD:AAB8468
Phalaenostola eumelusalis|[1326]|XAG442-05|2005-ONT-1026|658[0n]bp|Canada.Ontario|BOLD:AAB8468
Phalaenostola eumelusalis|[1327]|BLTIB888-08|BL1307|658[0n]bp|Canada.Ontario|BOLD:AAB8468
Phalaenostola eumelusalis|[1328]|BLTIB1070-08|BL1079|658[0n]bp|Canada.Ontario|BOLD:AAB8468
Phalaenostola eumelusalis|[1329]|BLTIB677-08|BL960|658[0n]bp|Canada.Ontario|BOLD:AAB8468
Phalaenostola eumelusalis|[1330]|BLTIB823-08|BL1241|658[0n]bp|Canada.Ontario|BOLD:AAB8468
Phalaenostola eumelusalis|[1331]|BLTIB674-08|BL957|658[0n]bp|Canada.Ontario|BOLD:AAB8468
Phalaenostola eumelusalis|[1332]|BLTIB1069-08|BL1078|658[0n]bp|Canada.Ontario|BOLD:AAB8468
Phalaenostola eumelusalis|[1333]|BLTIB793-08|BL1210|658[0n]bp|Canada.Ontario|BOLD:AAB8468
Phalaenostola eumelusalis|[1334]|RDLQF634-06|DH011784|643[0n]bp|Canada.Quebec|BOLD:AAB8468
Phalaenostola eumelusalis|[1335]|RDLQF633-06|DH011783|606[3n]bp|Canada.Quebec|BOLD:AAB8468
Phalaenostola eumelusalis|[1336]|BLTIB659-08|BL940|644[0n]bp|Canada.Ontario|BOLD:AAB8468
Phalaenostola hanhami|[1337]|RDNMF103-08|NOC14189|658[0n]bp|Canada.British Columbia|BOLD:AAD3686
Phalaenostola hanhami|[1338]|RDNMF604-08|NOC14690|609[0n]bp|Canada.Alberta|BOLD:AAD3686
Phalaenostola hanhami|[1339]|RDNMF101-08|NOC14187|658[0n]bp|Canada.Ontario|BOLD:AAD3686
Phalaenostola hanhami|[1340]|RDNMF104-08|NOC14190|658[0n]bp|Canada.Ontario|BOLD:AAD3686
Phalaenostola hanhami|[1341]|RDNMF523-08|NOC14609|658[0n]bp|Canada.Manitoba|BOLD:AAD3686
Phalaenostola hanhami|[1342]|RDNMF605-08|NOC14691|658[0n]bp|Canada.Alberta|BOLD:AAD3686
Phalaenostola hanhami|[1343]|RDNMF606-08|NOC14692|658[0n]bp|Canada.Alberta|BOLD:AAD3686
Phalaenostola larentioides|[1344]|RDLQG485-06|DH012778|658[0n]bp|Canada.Quebec|BOLD:AAA7226
Phalaenostola larentioides|[1345]|BLTIB836-08|BL1254|658[0n]bp|Canada.Ontario|BOLD:AAA7226
Phalaenostola larentioides|[1346]|RDLQG761-06|DH013054|658[0n]bp|Canada.Quebec|BOLD:AAA7226
Phalaenostola larentioides|[1347]|RDLQG778-06|DH013071|658[0n]bp|Canada.Quebec|BOLD:AAA7226
Phalaenostola larentioides|[1348]|BLTIB576-08|BL854|643[1n]bp|Canada.Ontario|BOLD:AAA7226
Phalaenostola larentioides|[1349]|BLTIB795-08|BL1213|658[0n]bp|Canada.Ontario|BOLD:AAA7226
Phalaenostola larentioides|[1350]|PMG149-03|moth1142.01|617[0n]bp|Canada.Ontario|BOLD:AAA7226
Phalaenostola larentioides|[1351]|RDLQG798-06|DH013091|658[0n]bp|Canada.Quebec|BOLD:AAA7226
Phalaenostola larentioides|[1352]|RDLQG918-06|DH013211|658[0n]bp|Canada.Quebec|BOLD:AAA7226
Phalaenostola larentioides|[1353]|RDLQG916-06|DH013209|658[0n]bp|Canada.Quebec|BOLD:AAA7226
Phalaenostola larentioides|[1354]|RDLQG762-06|DH013055|658[0n]bp|Canada.Quebec|BOLD:AAA7226
Phalaenostola larentioides|[1355]|RDLQB719-05|DH010822|658[0n]bp|Canada.Quebec|BOLD:AAA7226
Phalaenostola larentioides|[1356]|RDLQG904-06|DH013197|658[0n]bp|Canada.Quebec|BOLD:AAA7226
Phalaenostola larentioides|[1357]|RDLQG486-06|DH012779|658[0n]bp|Canada.Quebec|BOLD:AAA7226
Phalaenostola larentioides|[1358]|RDLQB718-05|DH010821|658[0n]bp|Canada.Quebec|BOLD:AAA7226
Phalaenostola larentioides|[1359]|RDLQG763-06|DH013056|658[0n]bp|Canada.Quebec|BOLD:AAA7226
Phalaenostola larentioides|[1360]|BLTIB974-08|BL1405|658[0n]bp|Canada.Ontario|BOLD:AAA7226
Phalaenostola larentioides|[1361]|RDLQG890-06|DH013183|658[0n]bp|Canada.Quebec|BOLD:AAA7226
Phalaenostola larentioides|[1362]|RDLQG919-06|DH013212|658[0n]bp|Canada.Quebec|BOLD:AAA7226
Phalaenostola larentioides|[1363]|RDLQG095-06|DH012252|658[0n]bp|Canada.Quebec|BOLD:AAA7226
Phalaenostola larentioides|[1364]|BLTIB887-08|BL1306|658[0n]bp|Canada.Ontario|BOLD:AAA7226
Phalaenostola larentioides|[1365]|RDLQG758-06|DH013051|658[1n]bp|Canada.Quebec|BOLD:AAA7226
Phalaenostola larentioides|[1366]|RDLQG481-06|DH012774|643[0n]bp|Canada.Quebec|BOLD:AAA7226
Phalaenostola larentioides|[1367]|RDLQG482-06|DH012775|642[1n]bp|Canada.Quebec|BOLD:AAA7226
Phalaenostola larentioides|[1368]|RDLQG477-06|DH012770|618[0n]bp|Canada.Quebec|BOLD:AAA7226
Phalaenostola metonalis|[1369]|RDLQG109-06|DH012266|618[0n]bp|Canada.Quebec|BOLD:ACF3696
Phalaenostola metonalis|[1370]|BLTIB638-08|BL918|644[0n]bp|Canada.Ontario|BOLD:ACF3696
Phalaenostola metonalis|[1371]|LPMN384-08|08BBLEP-01183|632[0n]bp|Canada.Manitoba|BOLD:AAA7565
Phalaenostola metonalis|[1372]|BBLPB991-10|10BBCLP-1990|658[0n]bp|Canada.Alberta|BOLD:AAA7565
Phalaenostola metonalis|[1373]|LPMN591-08|08BBLEP-01392|658[0n]bp|Canada.Manitoba|BOLD:AAA7565
Phalaenostola metonalis|[1374]|BBLPB985-10|10BBCLP-1984|658[0n]bp|Canada.Alberta|BOLD:AAA7565
Phalaenostola metonalis|[1375]|BBLPB986-10|10BBCLP-1985|658[0n]bp|Canada.Alberta|BOLD:AAA7565
Phalaenostola metonalis|[1376]|BBLPB975-10|10BBCLP-1974|658[0n]bp|Canada.Alberta|BOLD:AAA7565
Phalaenostola metonalis|[1377]|BBLEC380-09|09BBELE-0380|658[0n]bp|Canada.Newfoundland and Labrador|BOLD...
Phalaenostola metonalis|[1378]|LPMN601-08|08BBLEP-01402|658[0n]bp|Canada.Manitoba|BOLD:AAA7565
Phalaenostola metonalis|[1379]|LPMNB255-09|08BBLEP-05099|658[0n]bp|Canada.Manitoba|BOLD:AAA7565
Phalaenostola metonalis|[1380]|BBLPB974-10|10BBCLP-1973|658[0n]bp|Canada.Alberta|BOLD:AAA7565
Phalaenostola metonalis|[1381]|LPMN593-08|08BBLEP-01394|658[0n]bp|Canada.Manitoba|BOLD:AAA7565
Phalaenostola metonalis|[1382]|BBLEC109-09|09BBELE-0109|658[0n]bp|Canada.Nova Scotia|BOLD:AAA7565
Phalaenostola metonalis|[1383]|BBLEC608-09|09BBELE-0608|658[0n]bp|Canada.Nova Scotia|BOLD:AAA7565
Phalaenostola metonalis|[1384]|LOWCB629-05|CGWC-1569|658[0n]bp|Canada.British Columbia|BOLD:AAA7565
Phalaenostola metonalis|[1385]|LBCB693-05|HLC-21633|658[0n]bp|Canada.British Columbia|BOLD:AAA7565
Phalaenostola metonalis|[1386]|BBLPD615-10|10BBCLP-2613|658[0n]bp|Canada.British Columbia|BOLD:AAA7565
Phalaenostola metonalis|[1387]|LOWCB166-05|CGWC-1106|658[0n]bp|Canada.British Columbia|BOLD:AAA7565
Phalaenostola metonalis|[1388]|LOWCB628-05|CGWC-1568|658[0n]bp|Canada.British Columbia|BOLD:AAA7565

Phalaenostola metonalis|[1386]|BBLPD615-10|10BBCLP-2613|658[0n]bp|Canada.British Columbia|BOLD:AAA7565
Phalaenostola metonalis|[1387]|LOWCB166-05|CGWC-1106|658[0n]bp|Canada.British Columbia|BOLD:AAA7565
Phalaenostola metonalis|[1388]|LOWCB628-05|CGWC-1568|658[0n]bp|Canada.British Columbia|BOLD:AAA7565
Phalaenostola metonalis|[1389]|BBLPC298-09|09BBELE-1298|658[0n]bp|Canada.Newfoundland and Labrador|BOLD...
Phalaenostola metonalis|[1390]|RDLQF581-06|DH011731|658[0n]bp|Canada.Quebec|BOLD:AAA7565
Phalaenostola metonalis|[1391]|LOWCB167-05|CGWC-1107|658[0n]bp|Canada.British Columbia|BOLD:AAA7565
Phalaenostola metonalis|[1392]|LBCA401-05|HLC-20401|658[0n]bp|Canada.British Columbia|BOLD:AAA7565
Phalaenostola metonalis|[1393]|LPMN647-08|08BBLEP-01448|658[0n]bp|Canada.Manitoba|BOLD:AAA7565
Phalaenostola metonalis|[1394]|LPMN653-08|08BBLEP-01454|658[0n]bp|Canada.Manitoba|BOLD:AAA7565
Phalaenostola metonalis|[1395]|LPABB508-08|08BBLEP-03773|658[0n]bp|Canada.Alberta|BOLD:AAA7565
Phalaenostola metonalis|[1396]|BBLPD614-10|10BBCLP-2612|658[0n]bp|Canada.British Columbia|BOLD:AAA7565
Phalaenostola metonalis|[1397]|BBLPC317-09|09BBELE-1317|658[0n]bp|Canada.Newfoundland and Labrador|BOLD...
Phalaenostola metonalis|[1398]|LPAB082-08|08BBLEP-02404|582[0n]bp|Canada.Alberta|BOLD:AAA7565
Phalaenostola metonalis|[1399]|LOWCB165-05|CGWC-1105|658[0n]bp|Canada.British Columbia|BOLD:AAA7565
Phalaenostola metonalis|[1400]|LBCA423-05|HLC-20423|658[0n]bp|Canada.British Columbia|BOLD:AAA7565
Phalaenostola metonalis|[1401]|BBLPD613-10|10BBCLP-2611|658[0n]bp|Canada.British Columbia|BOLD:AAA7565
Phalaenostola metonalis|[1402]|LBCA524-05|HLC-20524|658[0n]bp|Canada.British Columbia|BOLD:AAA7565
Phalaenostola metonalis|[1403]|BBLPC150-09|09BBELE-1150|658[0n]bp|Canada.Nova Scotia|BOLD:AAA7565
Phalaenostola metonalis|[1404]|LPMN525-08|08BBLEP-01324|658[0n]bp|Canada.Manitoba|BOLD:AAA7565
Phalaenostola metonalis|[1405]|LOWCB627-05|CGWC-1567|658[0n]bp|Canada.British Columbia|BOLD:AAA7565
Phalaenostola metonalis|[1406]|LPMN610-08|08BBLEP-01411|658[0n]bp|Canada.Manitoba|BOLD:AAA7565
Phalaenostola metonalis|[1407]|LOWCB177-05|CGWC-1117|658[0n]bp|Canada.British Columbia|BOLD:AAA7565
Spargaloma sexpunctata|[1408]|RDMAB048-05|UASM57531|658[0n]bp|Canada.Alberta|BOLD:AAB3581
Spargaloma sexpunctata|[1409]|LBCH5642-10|10-JDWBC-5642|658[0n]bp|Canada.British Columbia|BOLD:AAB3581
Spargaloma sexpunctata|[1410]|RDLQF847-06|DH012000|658[0n]bp|Canada.Quebec|BOLD:AAB3581
Spargaloma sexpunctata|[1411]|TMNBB064-06|MNBTT-1004|658[0n]bp|Canada.New Brunswick|BOLD:AAB3581
Spargaloma sexpunctata|[1412]|BBLPA981-10|10BBCLP-0981|658[0n]bp|Canada.British Columbia|BOLD:AAB3581
Spargaloma sexpunctata|[1413]|XAC607-04|04HBL006607|658[0n]bp|Canada.Ontario|BOLD:AAB3581
Spargaloma sexpunctata|[1414]|LBCH5776-10|10-JDWBC-5776|658[0n]bp|Canada.British Columbia|BOLD:AAB3581
Spargaloma sexpunctata|[1415]|RDLQG358-06|DH012592|658[0n]bp|Canada.Quebec|BOLD:AAB3581
Spargaloma sexpunctata|[1416]|LPSOB517-08|PPBP-1516|658[0n]bp|Canada.Ontario|BOLD:AAB3581
Spargaloma sexpunctata|[1417]|LBCB644-05|HLC-21584|658[0n]bp|Canada.British Columbia|BOLD:AAB3581
Spargaloma sexpunctata|[1418]|LPMN065-08|08BBLEP-00863|658[0n]bp|Canada.Manitoba|BOLD:AAB3581
Spargaloma sexpunctata|[1419]|LBCC489-05|HLC-22369|658[0n]bp|Canada.British Columbia|BOLD:AAB3581
Spargaloma sexpunctata|[1420]|LBCA675-05|HLC-20675|654[0n]bp|Canada.British Columbia|BOLD:AAB3581
Spargaloma sexpunctata|[1421]|LBCA130-05|HLC-20130|658[0n]bp|Canada.British Columbia|BOLD:AAB3581
Spargaloma sexpunctata|[1422]|LBCB267-05|HLC-21207|658[0n]bp|Canada.British Columbia|BOLD:AAB3581
Spargaloma sexpunctata|[1423]|LOWCB162-05|CGWC-1102|658[0n]bp|Canada.British Columbia|BOLD:AAB3581
Spargaloma sexpunctata|[1424]|LOWCB631-05|CGWC-1571|548[0n]bp|Canada.British Columbia|BOLD:AAB3581
Spargaloma sexpunctata|[1425]|LOWCB163-05|CGWC-1103|564[0n]bp|Canada.British Columbia|BOLD:AAB3581
Bleptina caradrinalis|[1426]|BBLPA660-10|10BBCLP-0660|658[0n]bp|Canada.Ontario|BOLD:AAA2867
Bleptina caradrinalis|[1427]|LPSO689-08|PPBP-0689|658[0n]bp|Canada.Ontario|BOLD:AAA2867
Bleptina caradrinalis|[1428]|RDLQF821-06|DH011974|658[0n]bp|Canada.Quebec|BOLD:AAA2867
Bleptina caradrinalis|[1429]|RDLQF434-06|DH011541|658[0n]bp|Canada.Quebec|BOLD:AAA2867
Bleptina caradrinalis|[1430]|XAC662-04|04HBL006662|581[0n]bp|Canada.Ontario|BOLD:AAA2867
Bleptina caradrinalis|[1431]|RDLQG173-06|DH012344|658[0n]bp|Canada.Quebec|BOLD:AAA2867
Bleptina caradrinalis|[1432]|XAC722-04|04HBL006722|658[0n]bp|Canada.Ontario|BOLD:AAA2867
Bleptina caradrinalis|[1433]|XAB186-04|04HBL005186|572[0n]bp|Canada.Ontario|BOLD:AAA2867
Bleptina caradrinalis|[1434]|XAE558-04|Moth4558.03|658[0n]bp|Canada.Ontario|BOLD:AAA2867
Bleptina caradrinalis|[1435]|XAC460-04|04HBL006460|658[0n]bp|Canada.Ontario|BOLD:AAA2867
Bleptina caradrinalis|[1436]|XAC047-04|04HBL006047|658[0n]bp|Canada.Ontario|BOLD:AAA2867
Bleptina caradrinalis|[1437]|XAC658-04|04HBL006658|658[0n]bp|Canada.Ontario|BOLD:AAA2867
Bleptina caradrinalis|[1438]|TMNBB058-06|MNBTT-998|658[0n]bp|Canada.New Brunswick|BOLD:AAA2867
Bleptina caradrinalis|[1439]|LALPA726-10|AVBC 728-10|630[0n]bp|Canada.British Columbia|BOLD:AAA2867
Bleptina caradrinalis|[1440]|PHMNB193-04|04HBL007658|658[0n]bp|Canada.New Brunswick|BOLD:AAA2867
Bleptina caradrinalis|[1441]|RDLQB241-05|DH010327|658[0n]bp|Canada.Quebec|BOLD:AAA2867
Bleptina caradrinalis|[1442]|BBLPD859-10|10BBCLP-2857|658[0n]bp|Canada.British Columbia|BOLD:AAA2867
Bleptina caradrinalis|[1443]|LALPA408-10|AVBC 410-10|658[0n]bp|Canada.British Columbia|BOLD:AAA2867
Bleptina caradrinalis|[1444]|RDLQB259-05|DH010345|658[0n]bp|Canada.Quebec|BOLD:AAA2867
Bleptina caradrinalis|[1445]|LPMN116-08|08BBLEP-00914|658[0n]bp|Canada.Manitoba|BOLD:AAA2867
Bleptina caradrinalis|[1446]|LPSK050-08|08BBLEP-00753|658[0n]bp|Canada.Saskatchewan|BOLD:AAA2867
Bleptina caradrinalis|[1447]|PHMNB215-04|04HBL007680|658[0n]bp|Canada.New Brunswick|BOLD:AAA2867
Bleptina caradrinalis|[1448]|TMNBB055-06|MNBTT-995|658[0n]bp|Canada.New Brunswick|BOLD:AAA2867
Bleptina caradrinalis|[1449]|LPSK132-08|08BBLEP-01700|658[0n]bp|Canada.Saskatchewan|BOLD:AAA2867
Bleptina caradrinalis|[1450]|TMNBB056-06|MNBTT-996|658[0n]bp|Canada.New Brunswick|BOLD:AAA2867
Bleptina caradrinalis|[1451]|LALPA434-10|AVBC 436-10|658[0n]bp|Canada.British Columbia|BOLD:AAA2867
Bleptina caradrinalis|[1452]|LPSK047-08|08BBLEP-00750|658[0n]bp|Canada.Saskatchewan|BOLD:AAA2867
Bleptina caradrinalis|[1453]|TMNBB057-06|MNBTT-997|658[0n]bp|Canada.New Brunswick|BOLD:AAA2867
Bleptina caradrinalis|[1454]|RDLQF435-06|DH011542|658[0n]bp|Canada.Quebec|BOLD:AAA2867
Bleptina caradrinalis|[1455]|TMNBB059-06|MNBTT-999|658[0n]bp|Canada.New Brunswick|BOLD:AAA2867
Bleptina caradrinalis|[1456]|TMNBB060-06|MNBTT-1000|658[0n]bp|Canada.New Brunswick|BOLD:AAA2867
Bleptina caradrinalis|[1457]|RDLQF432-06|DH011539|658[0n]bp|Canada.Quebec|BOLD:AAA2867
Bleptina caradrinalis|[1458]|LPMN097-08|08BBLEP-00895|658[0n]bp|Canada.Manitoba|BOLD:AAA2867
Bleptina caradrinalis|[1459]|TTMNB092-06|MNBTT-092|657[0n]bp|Canada.New Brunswick|BOLD:AAA2867
Bleptina caradrinalis|[1460]|TTMNB268-06|MNBTT-268|658[0n]bp|Canada.New Brunswick|BOLD:AAA2867
Bleptina caradrinalis|[1461]|TTMNB096-06|MNBTT-096|658[0n]bp|Canada.New Brunswick|BOLD:AAA2867
Bleptina caradrinalis|[1462]|MNBB005-05|HBL008615|658[0n]bp|Canada.New Brunswick|BOLD:AAA2867
Bleptina caradrinalis|[1463]|RDLQF433-06|DH011540|658[0n]bp|Canada.Quebec|BOLD:AAA2867
Bleptina caradrinalis|[1464]|RDLQG359-06|DH012593|658[0n]bp|Canada.Quebec|BOLD:AAA2867
Bleptina caradrinalis|[1465]|LALPA1214-11|AVBC 1216-11|658[0n]bp|Canada.British Columbia|BOLD:AAA2867
Bleptina caradrinalis|[1466]|PHMNB157-04|04HBL007622|658[0n]bp|Canada.New Brunswick|BOLD:AAA2867
Bleptina caradrinalis|[1467]|LALPA536-10|AVBC 538-10|658[0n]bp|Canada.British Columbia|BOLD:AAA2867
Bleptina caradrinalis|[1468]|LALPA497-10|AVBC 499-10|658[0n]bp|Canada.British Columbia|BOLD:AAA2867
Bleptina caradrinalis|[1469]|TMNBB054-06|MNBTT-994|658[0n]bp|Canada.New Brunswick|BOLD:AAA2867
Bleptina caradrinalis|[1470]|RDLQG920-06|DH013213|658[0n]bp|Canada.Quebec|BOLD:AAA2867
Bleptina caradrinalis|[1471]|RDNMF102-08|NOC14188|658[0n]bp|Canada.Saskatchewan|BOLD:AAA2867
Bleptina caradrinalis|[1472]|LPMN092-08|08BBLEP-00890|647[0n]bp|Canada.Manitoba|BOLD:AAA2867
Bleptina caradrinalis|[1473]|PHMO201-03|moth1030.02|639[0n]bp|Canada.Ontario|BOLD:AAA2867
Bleptina caradrinalis|[1474]|RDMAB049-05|UASM57532|638[0n]bp|Canada.Alberta|BOLD:AAA2867
Bleptina caradrinalis|[1475]|XAC840-04|04HBL006840|615[0n]bp|Canada.Ontario|BOLD:AAA2867
Bleptina caradrinalis|[1476]|TTMNB269-06|MNBTT-269|658[0n]bp|Canada.New Brunswick|BOLD:AAA2867
Bleptina caradrinalis|[1477]|RDMAB131-05|UASM41276|631[0n]bp|Canada.Alberta|BOLD:AAA2867
Lascoria ambigualis|[1478]|RDLQG575-06|DH012868|643[0n]bp|Canada.Quebec|BOLD:AAA4458
Lascoria ambigualis|[1479]|RDLQF391-06|DH011458|658[0n]bp|Canada.Quebec|BOLD:AAA4458
Lascoria ambigualis|[1480]|RDLQG226-06|DH012409|658[0n]bp|Canada.Quebec|BOLD:AAA4458
Lascoria ambigualis|[1481]|RDLQG586-06|DH012879|658[0n]bp|Canada.Quebec|BOLD:AAA4458
Lascoria ambigualis|[1482]|RDLQG261-06|DH012466|658[0n]bp|Canada.Quebec|BOLD:AAA4458
Lascoria ambigualis|[1483]|JSCOL076-11|BIOUG00874-H05|658[0n]bp|Canada.Ontario|BOLD:AAA4458
Lascoria ambigualis|[1484]|BLTIB082-08|BL0124|658[0n]bp|Canada.Ontario|BOLD:AAA4458
Lascoria ambigualis|[1485]|XAE238-04|Moth4238.03|570[3n]bp|Canada.Ontario|BOLD:AAA4458
Lascoria ambigualis|[1486]|BLTIB013-08|BL0013|658[0n]bp|Canada.Ontario|BOLD:AAA4458
Lascoria ambigualis|[1487]|LPSO287-08|PPBP-0287|658[0n]bp|Canada.Ontario|BOLD:AAA4458
[illegible]

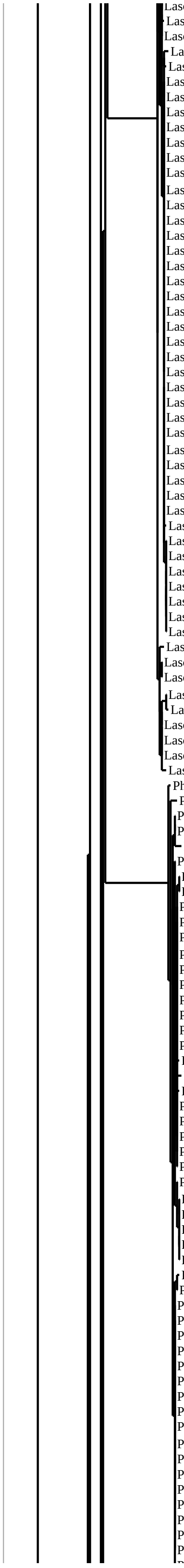

Lascoria ambigualis|[1485]|XAE238-04|Moth4238.03|570[3n]bp|Canada.Ontario|BOLD:AAA4458
Lascoria ambigualis|[1486]|BLTIB013-08|BL0013|658[0n]bp|Canada.Ontario|BOLD:AAA4458
Lascoria ambigualis|[1487]|LPSO287-08|PPBP-0287|658[0n]bp|Canada.Ontario|BOLD:AAA4458
Lascoria ambigualis|[1488]|LPSO947-08|PPBP-0947|657[0n]bp|Canada.Ontario|BOLD:AAA4458
Lascoria ambigualis|[1489]|BLTIB122-08|BL189|541[2n]bp|Canada.Ontario|
Lascoria ambigualis|[1490]|BLTIB182-08|BL262|658[0n]bp|Canada.Ontario|BOLD:AAA4458
Lascoria ambigualis|[1491]|LPSO098-08|PPBP-0098|658[0n]bp|Canada.Ontario|BOLD:AAA4458
Lascoria ambigualis|[1492]|XAJ333-06|2006-ONT-0333|658[0n]bp|Canada.Ontario|BOLD:AAA4458
Lascoria ambigualis|[1493]|XAE245-04|Moth4245.03|658[0n]bp|Canada.Ontario|BOLD:AAA4458
Lascoria ambigualis|[1494]|XAJ358-06|2006-ONT-0358|658[0n]bp|Canada.Ontario|BOLD:AAA4458
Lascoria ambigualis|[1495]|LPSO300-08|PPBP-0300|658[0n]bp|Canada.Ontario|BOLD:AAA4458
Lascoria ambigualis|[1496]|BLTIB056-08|BL0089|658[0n]bp|Canada.Ontario|BOLD:AAA4458
Lascoria ambigualis|[1497]|LPSO700-08|PPBP-0700|658[0n]bp|Canada.Ontario|BOLD:AAA4458
Lascoria ambigualis|[1498]|LPSO298-08|PPBP-0298|658[0n]bp|Canada.Ontario|BOLD:AAA4458
Lascoria ambigualis|[1499]|LPSO463-08|PPBP-0463|658[0n]bp|Canada.Ontario|BOLD:AAA4458
Lascoria ambigualis|[1500]|BLTIB181-08|BL261|658[0n]bp|Canada.Ontario|BOLD:AAA4458
Lascoria ambigualis|[1501]|BLTIB187-08|BL268|658[0n]bp|Canada.Ontario|BOLD:AAA4458
Lascoria ambigualis|[1502]|LPSO109-08|PPBP-0109|658[0n]bp|Canada.Ontario|BOLD:AAA4458
Lascoria ambigualis|[1503]|BLTIB185-08|BL265|658[0n]bp|Canada.Ontario|BOLD:AAA4458
Lascoria ambigualis|[1504]|BLTIB164-08|BL242|658[0n]bp|Canada.Ontario|BOLD:AAA4458
Lascoria ambigualis|[1505]|LPSO946-08|PPBP-0946|658[0n]bp|Canada.Ontario|BOLD:AAA4458
Lascoria ambigualis|[1506]|BLTIB062-08|BL0101|658[0n]bp|Canada.Ontario|BOLD:AAA4458
Lascoria ambigualis|[1507]|BLTIB183-08|BL263|658[0n]bp|Canada.Ontario|BOLD:AAA4458
Lascoria ambigualis|[1508]|BLTIB230-08|BL411|658[0n]bp|Canada.Ontario|BOLD:AAA4458
Lascoria ambigualis|[1509]|BLTIB159-08|BL234|658[0n]bp|Canada.Ontario|BOLD:AAA4458
Lascoria ambigualis|[1510]|BLTIB120-08|BL187|658[0n]bp|Canada.Ontario|BOLD:AAA4458
Lascoria ambigualis|[1511]|BLTIB154-08|BL228|658[0n]bp|Canada.Ontario|BOLD:AAA4458
Lascoria ambigualis|[1512]|LPSO110-08|PPBP-0110|658[0n]bp|Canada.Ontario|BOLD:AAA4458
Lascoria ambigualis|[1513]|LPSO082-08|PPBP-0082|656[0n]bp|Canada.Ontario|BOLD:AAA4458
Lascoria ambigualis|[1514]|LPSO299-08|PPBP-0299|658[0n]bp|Canada.Ontario|BOLD:AAA4458
Lascoria ambigualis|[1515]|BLTIB074-08|BL0114|658[0n]bp|Canada.Ontario|BOLD:AAA4458
Lascoria ambigualis|[1516]|BLTIB257-08|BL439|658[0n]bp|Canada.Ontario|BOLD:AAA4458
Lascoria ambigualis|[1517]|BLTIB949-08|BL1378|658[0n]bp|Canada.Ontario|BOLD:AAA4458
Lascoria ambigualis|[1518]|LPSO061-08|PPBP-0061|658[0n]bp|Canada.Ontario|BOLD:AAA4458
Lascoria ambigualis|[1519]|BLGSM062-09|BL381|658[0n]bp|Canada.Ontario|BOLD:AAA4458
Lascoria ambigualis|[1520]|BLTIB078-08|BL0118|577[1n]bp|Canada.Ontario|BOLD:AAA4458
Lascoria ambigualis|[1521]|XAJ631-06|2006-ONT-0631|658[0n]bp|Canada.Ontario|BOLD:AAA4458
Lascoria ambigualis|[1522]|TMG92-03|moth380.01|639[0n]bp|Canada.Ontario|BOLD:AAA4458
Lascoria ambigualis|[1523]|PMG125-03|moth333.01|617[0n]bp|Canada.Ontario|BOLD:AAA4458
Lascoria ambigualis|[1524]|BLTIB204-08|BL296|609[0n]bp|Canada.Ontario|BOLD:AAA4458
Lascoria ambigualis|[1525]|BLTIB118-08|BL185|609[0n]bp|Canada.Ontario|BOLD:AAA4458
Lascoria ambigualis|[1526]|BLTIB206-08|BL300|614[0n]bp|Canada.Ontario|BOLD:AAA4458
Lascoria ambigualis|[1527]|RDLQG576-06|DH012869|658[0n]bp|Canada.Quebec|BOLD:AAA4458
Lascoria ambigualis|[1528]|RDLQG601-06|DH012894|647[0n]bp|Canada.Quebec|BOLD:AAA4458
Lascoria ambigualis|[1529]|RDLQG587-06|DH012880|658[0n]bp|Canada.Quebec|BOLD:AAA4458
Lascoria ambigualis|[1530]|RDLQG280-06|DH012492|601[0n]bp|Canada.Quebec|BOLD:AAA4458
Lascoria ambigualis|[1531]|RDLQG258-06|DH012463|616[0n]bp|Canada.Quebec|BOLD:AAA4458
Lascoria ambigualis|[1532]|RDLQG259-06|DH012464|658[0n]bp|Canada.Quebec|BOLD:AAA4458
Lascoria ambigualis|[1533]|RDLQG260-06|DH012465|658[0n]bp|Canada.Quebec|BOLD:AAA4458
Lascoria ambigualis|[1534]|RDLQG227-06|DH012410|658[0n]bp|Canada.Quebec|BOLD:AAA4458
Lascoria ambigualis|[1535]|RDLQG585-06|DH012878|658[0n]bp|Canada.Quebec|BOLD:AAA4458
Phalaenophana pyramusalis|[1536]|PHJUN3375-11|BIOUG01486-B07|658[0n]bp|Canada.Ontario|BOLD:AAA5643
Phalaenophana pyramusalis|[1537]|LPMN473-08|08BBLEP-01272|658[0n]bp|Canada.Manitoba|BOLD:AAA5643
Phalaenophana pyramusalis|[1538]|PHJUN3372-11|BIOUG01486-B04|658[0n]bp|Canada.Ontario|BOLD:AAA5643
Phalaenophana pyramusalis|[1539]|LPSO907-08|PPBP-0907|658[0n]bp|Canada.Ontario|BOLD:AAA5643
Phalaenophana pyramusalis|[1540]|LPSO949-08|PPBP-0949|658[0n]bp|Canada.Ontario|BOLD:AAA5643
Phalaenophana pyramusalis|[1541]|TMNBB045-06|MNBTT-985|622[0n]bp|Canada.New Brunswick|BOLD:AAA5643
Phalaenophana pyramusalis|[1542]|LPSOB828-08|PPBP-1827|658[0n]bp|Canada.Ontario|BOLD:AAA5643
Phalaenophana pyramusalis|[1543]|BBLPD439-10|10BBCLP-2437|658[0n]bp|Canada.Ontario|BOLD:AAA5643
Phalaenophana pyramusalis|[1544]|RDLQG811-06|DH013104|658[0n]bp|Canada.Quebec|BOLD:AAA5643
Phalaenophana pyramusalis|[1545]|TTMNB546-06|MNBTT-546|658[0n]bp|Canada.New Brunswick|BOLD:AAA5643
Phalaenophana pyramusalis|[1546]|XAF810-05|2005-ONT-459|658[0n]bp|Canada.Ontario|BOLD:AAA5643
Phalaenophana pyramusalis|[1547]|RDLQG426-06|DH012705|658[0n]bp|Canada.Quebec|BOLD:AAA5643
Phalaenophana pyramusalis|[1548]|LPSOD820-09|08BBLEP-00602|658[0n]bp|Canada.Ontario|BOLD:AAA5643
Phalaenophana pyramusalis|[1549]|RDLQG425-06|DH012704|658[0n]bp|Canada.Quebec|BOLD:AAA5643
Phalaenophana pyramusalis|[1550]|LPSOD817-09|08BBLEP-00599|658[0n]bp|Canada.Ontario|BOLD:AAA5643
Phalaenophana pyramusalis|[1551]|PHMNB671-04|04HBL00897|658[0n]bp|Canada.New Brunswick|BOLD:AAA5643
Phalaenophana pyramusalis|[1552]|RDLQG427-06|DH012706|658[0n]bp|Canada.Quebec|BOLD:AAA5643
Phalaenophana pyramusalis|[1553]|LPSOD604-09|08BBLEP-00385|658[0n]bp|Canada.Ontario|BOLD:AAA5643
Phalaenophana pyramusalis|[1554]|RDLQG430-06|DH012709|658[0n]bp|Canada.Quebec|BOLD:AAA5643
Phalaenophana pyramusalis|[1555]|BBLPD149-10|10BBCLP-2147|658[0n]bp|Canada.Saskatchewan|BOLD:AAA5643
Phalaenophana pyramusalis|[1556]|BBLPE047-09|09BBELE-2047|658[0n]bp|Canada.Nova Scotia|BOLD:AAA5643
Phalaenophana pyramusalis|[1557]|RDLQG428-06|DH012707|632[0n]bp|Canada.Quebec|BOLD:AAA5643
Phalaenophana pyramusalis|[1558]|LPSOB657-08|PPBP-1656|658[1n]bp|Canada.Ontario|BOLD:AAA5643
Phalaenophana pyramusalis|[1559]|LPSOD533-09|08BBLEP-00312|658[0n]bp|Canada.Ontario|BOLD:AAA5643
Phalaenophana pyramusalis|[1560]|LPSOD791-09|08BBLEP-00573|658[0n]bp|Canada.Ontario|BOLD:AAA5643
Phalaenophana pyramusalis|[1561]|RDLQG423-06|DH012702|658[0n]bp|Canada.Quebec|BOLD:AAA5643
Phalaenophana pyramusalis|[1562]|BLTIB700-08|BL985|658[0n]bp|Canada.Ontario|BOLD:AAA5643
Phalaenophana pyramusalis|[1563]|LPSO320-08|PPBP-0320|658[0n]bp|Canada.Ontario|BOLD:AAA5643
Phalaenophana pyramusalis|[1564]|LPSO902-08|PPBP-0902|658[0n]bp|Canada.Ontario|BOLD:AAA5643
Phalaenophana pyramusalis|[1565]|LPSO699-08|PPBP-0699|658[0n]bp|Canada.Ontario|BOLD:AAA5643
Phalaenophana pyramusalis|[1566]|LPSO193-08|PPBP-0193|658[0n]bp|Canada.Ontario|BOLD:AAA5643
Phalaenophana pyramusalis|[1567]|LPSO385-08|PPBP-0385|658[0n]bp|Canada.Ontario|BOLD:AAA5643
Phalaenophana pyramusalis|[1568]|RDLQG107-06|DH012264|616[0n]bp|Canada.Quebec|BOLD:AAA5643
Phalaenophana pyramusalis|[1569]|LPSO391-08|PPBP-0391|637[0n]bp|Canada.Ontario|BOLD:AAA5643
Phalaenophana pyramusalis|[1570]|LPSO896-08|PPBP-0896|658[0n]bp|Canada.Ontario|BOLD:AAA5643
Phalaenophana pyramusalis|[1571]|BLTIB541-08|BL812|658[0n]bp|Canada.Ontario|BOLD:AAA5643
Phalaenophana pyramusalis|[1572]|LPSO672-08|PPBP-0672|658[0n]bp|Canada.Ontario|BOLD:AAA5643
Phalaenophana pyramusalis|[1573]|BLTIB643-08|BL923|658[0n]bp|Canada.Ontario|BOLD:AAA5643
Phalaenophana pyramusalis|[1574]|LPSO941-08|PPBP-0941|658[0n]bp|Canada.Ontario|BOLD:AAA5643
Phalaenophana pyramusalis|[1575]|RDLQG043-06|DH012174|658[0n]bp|Canada.Quebec|BOLD:AAA5643
Phalaenophana pyramusalis|[1576]|LPSO944-08|PPBP-0944|658[0n]bp|Canada.Ontario|BOLD:AAA5643
Phalaenophana pyramusalis|[1577]|LPSO893-08|PPBP-0893|658[0n]bp|Canada.Ontario|BOLD:AAA5643
Phalaenophana pyramusalis|[1578]|RDLQG422-06|DH012701|658[0n]bp|Canada.Quebec|BOLD:AAA5643
Phalaenophana pyramusalis|[1579]|RDLQG429-06|DH012708|658[0n]bp|Canada.Quebec|BOLD:AAA5643
Phalaenophana pyramusalis|[1580]|RDLQG421-06|DH012700|658[1n]bp|Canada.Quebec|BOLD:AAA5643
Phalaenophana pyramusalis|[1581]|RDLQH153-06|DH005646|616[3n]bp|Canada.Quebec|BOLD:AAA5643
Phalaenophana pyramusalis|[1582]|PHMNB490-04|04HBL00716|658[0n]bp|Canada.New Brunswick|BOLD:AAA5643
Phalaenophana pyramusalis|[1583]|LPSO702-08|PPBP-0702|658[0n]bp|Canada.Ontario|BOLD:AAA5643
Phalaenophana pyramusalis|[1584]|RDLQG424-06|DH012703|658[0n]bp|Canada.Quebec|BOLD:AAA5643
Phalaenophana pyramusalis|[1585]|LPSO908-08|PPBP-0908|658[0n]bp|Canada.Ontario|BOLD:AAA5643
Phalaenophana pyramusalis|[1586]|TMNBB046-06|MNBTT-986|658[0n]bp|Canada.New Brunswick|BOLD:AAA5643
[illegible]

Phalaenophana pyramusalis|[1584]|RDLQG424-06|DH012703|658[0n]bp|Canada.Quebec|BOLD:AAA5643
Phalaenophana pyramusalis|[1585]|LPSO908-08|PPBP-0908|658[0n]bp|Canada.Ontario|BOLD:AAA5643
Phalaenophana pyramusalis|[1586]|TMNBB046-06|MNBTT-986|658[0n]bp|Canada.New Brunswick|BOLD:AAA5643
Phalaenophana pyramusalis|[1587]|RDLQG420-06|DH012699|658[0n]bp|Canada.Quebec|BOLD:AAA5643
Renia flavipunctalis|[1588]|RDLQF820-06|DH011973|658[0n]bp|Canada.Quebec|BOLD:AAA7213
Renia flavipunctalis|[1589]|MNAF484-08|CNCLEP00040469|658[0n]bp|Canada.Manitoba|BOLD:AAA7213
Renia flavipunctalis|[1590]|PMG158-03|RENI1.00|617[0n]bp|Canada.Ontario|BOLD:AAA7213
Renia flavipunctalis|[1591]|RDLQF622-06|DH011772|658[0n]bp|Canada.Quebec|BOLD:AAA7213
Renia flavipunctalis|[1592]|XAK324-06|2006-ONT-1319|658[0n]bp|Canada.Ontario|BOLD:AAA7213
Renia flavipunctalis|[1593]|RDNMD184-06|CNCNoctuoidea12520|658[0n]bp|Canada.Quebec|BOLD:AAA7213
Renia flavipunctalis|[1594]|RDLQF628-06|DH011778|658[0n]bp|Canada.Quebec|BOLD:AAA7213
Renia flavipunctalis|[1595]|RDLQF718-06|DH011868|644[0n]bp|Canada.Quebec|BOLD:AAA7213
Renia flavipunctalis|[1596]|LPMNB351-09|08BBLEP-05195|658[0n]bp|Canada.Manitoba|BOLD:AAA7213
Renia flavipunctalis|[1597]|LPMNB413-09|08BBLEP-05257|658[0n]bp|Canada.Manitoba|BOLD:AAA7213
Renia flavipunctalis|[1598]|LPMNB377-09|08BBLEP-05221|658[0n]bp|Canada.Manitoba|BOLD:AAA7213
Renia flavipunctalis|[1599]|LPMNB384-09|08BBLEP-05228|658[0n]bp|Canada.Manitoba|BOLD:AAA7213
Renia flavipunctalis|[1600]|LPMNB336-09|08BBLEP-05180|658[0n]bp|Canada.Manitoba|BOLD:AAA7213
Renia flavipunctalis|[1601]|LPMN1005-09|08BBLEP-04046|658[0n]bp|Canada.Manitoba|BOLD:AAA7213
Renia flavipunctalis|[1602]|RDNMD185-06|CNCNoctuoidea12521|654[0n]bp|Canada.Quebec|BOLD:AAA7213
Renia flavipunctalis|[1603]|RDLQF626-06|DH011776|649[0n]bp|Canada.Quebec|BOLD:AAA7213
Renia flavipunctalis|[1604]|LPMNB562-09|08BBLEP-05640|658[0n]bp|Canada.Manitoba|BOLD:AAA7213
Renia flavipunctalis|[1605]|LPMNB360-09|08BBLEP-05204|658[0n]bp|Canada.Manitoba|BOLD:AAA7213
Renia flavipunctalis|[1606]|LPMNB378-09|08BBLEP-05222|658[0n]bp|Canada.Manitoba|BOLD:AAA7213
Renia flavipunctalis|[1607]|RDLQF623-06|DH011773|658[0n]bp|Canada.Quebec|BOLD:AAA7213
Renia flavipunctalis|[1608]|LPMNB342-09|08BBLEP-05186|658[0n]bp|Canada.Manitoba|BOLD:AAA7213
Renia flavipunctalis|[1609]|LPMNB340-09|08BBLEP-05184|658[0n]bp|Canada.Manitoba|BOLD:AAA7213
Renia flavipunctalis|[1610]|LPMNB343-09|08BBLEP-05187|658[0n]bp|Canada.Manitoba|BOLD:AAA7213
Renia flavipunctalis|[1611]|LPMNB484-09|08BBLEP-05522|658[0n]bp|Canada.Manitoba|BOLD:AAA7213
Renia flavipunctalis|[1612]|MNAF483-08|CNCLEP00040468|658[0n]bp|Canada.Manitoba|BOLD:AAA7213
Renia flavipunctalis|[1613]|RDLQF624-06|DH011774|658[0n]bp|Canada.Quebec|BOLD:AAA7213
Renia flavipunctalis|[1614]|LPMNB368-09|08BBLEP-05212|658[0n]bp|Canada.Manitoba|BOLD:AAA7213
Renia flavipunctalis|[1615]|RDLQF722-06|DH011872|642[0n]bp|Canada.Quebec|BOLD:AAA7213
Renia flavipunctalis|[1616]|RDLQF720-06|DH011870|637[0n]bp|Canada.Quebec|BOLD:AAA7213
Renia flavipunctalis|[1617]|RDLQF629-06|DH011779|643[0n]bp|Canada.Quebec|BOLD:AAA7213
Renia flavipunctalis|[1618]|RDLQB714-05|DH010817|593[0n]bp|Canada.Quebec|BOLD:AAA7213
Renia flavipunctalis|[1619]|RDLQG094-06|DH012251|658[0n]bp|Canada.Quebec|BOLD:AAA7213
Renia flavipunctalis|[1620]|LPMNB559-09|08BBLEP-05594|658[0n]bp|Canada.Manitoba|BOLD:AAA7213
Renia flavipunctalis|[1621]|RDLQF796-06|DH011946|658[0n]bp|Canada.Quebec|BOLD:AAA7213
Renia flavipunctalis|[1622]|LPMNB397-09|08BBLEP-05241|658[0n]bp|Canada.Manitoba|BOLD:AAA7213
Renia flavipunctalis|[1623]|RDLQF721-06|DH011871|639[0n]bp|Canada.Quebec|BOLD:AAA7213
Renia flavipunctalis|[1624]|RDLQF719-06|DH011869|642[0n]bp|Canada.Quebec|BOLD:AAA7213
Renia flavipunctalis|[1625]|RDLQF795-06|DH011945|658[0n]bp|Canada.Quebec|BOLD:AAA7213
Renia flavipunctalis|[1626]|RDLQF797-06|DH011947|658[0n]bp|Canada.Quebec|BOLD:AAA7213
Renia flavipunctalis|[1627]|LPMN1000-09|08BBLEP-04041|658[0n]bp|Canada.Manitoba|BOLD:AAA7213
Renia flavipunctalis|[1628]|RDLQF575-06|DH011725|658[0n]bp|Canada.Quebec|BOLD:AAA7213
Renia flavipunctalis|[1629]|RDLQF627-06|DH011777|658[0n]bp|Canada.Quebec|BOLD:AAA7213
Renia adspergillus|[1630]|RDLQG193-06|DH012369|601[0n]bp|Canada.Quebec|BOLD:AAA6692
Renia adspergillus|[1631]|RDLQG489-06|DH012782|636[1n]bp|Canada.Quebec|BOLD:AAA6692
Renia adspergillus|[1632]|RDLQF525-06|DH011674|658[0n]bp|Canada.Quebec|BOLD:AAA6692
Renia adspergillus|[1633]|RDLQG923-06|DH013216|658[0n]bp|Canada.Quebec|BOLD:AAA6692
Renia adspergillus|[1634]|RDLQG922-06|DH013215|658[0n]bp|Canada.Quebec|BOLD:AAA6692
Renia adspergillus|[1635]|RDLQF527-06|DH011676|658[0n]bp|Canada.Quebec|BOLD:AAA6692
Renia adspergillus|[1636]|RDLQG195-06|DH012371|658[0n]bp|Canada.Quebec|BOLD:AAA6692
Renia adspergillus|[1637]|RDLQG192-06|DH012368|658[0n]bp|Canada.Quebec|BOLD:AAA6692
Renia adspergillus|[1638]|RDLQF526-06|DH011675|658[0n]bp|Canada.Quebec|BOLD:AAA6692
Renia adspergillus|[1639]|RDLQG194-06|DH012370|658[0n]bp|Canada.Quebec|BOLD:AAA6692
Renia adspergillus|[1640]|RDLQF074-06|DH006954|658[0n]bp|Canada.Quebec|BOLD:AAA6692
Renia adspergillus|[1641]|RDLQG019-06|DH012139|658[0n]bp|Canada.Quebec|BOLD:AAA6692
Renia adspergillus|[1642]|BLTIB360-08|BL578|609[2n]bp|Canada.Ontario|BOLD:AAA6692
Renia discoloralis|[1643]|LGSM717-04|DNA-ATBI-0717|658[0n]bp|United States.Tennessee|BOLD:AAA6434
Renia discoloralis|[1644]|LNCC1372-11|11-NCCC-897|658[0n]bp|United States.North Carolina|BOLD:AAA6434
Renia discoloralis|[1645]|LGSMG539-07|BGS03902|658[0n]bp|United States.Tennessee|BOLD:AAA6434
Renia discoloralis|[1646]|LNC604-06|06-NCCC-604|658[0n]bp|United States.North Carolina|BOLD:AAA6434
Renia discoloralis|[1647]|LNCC1374-11|11-NCCC-899|658[0n]bp|United States.North Carolina|BOLD:AAA6434
Renia discoloralis|[1648]|LNCNW112-06|06-NCNW-0112|658[0n]bp|United States.North Carolina|BOLD:AAA6434
Renia discoloralis|[1649]|LGSM718-04|DNA-ATBI-0718|658[0n]bp|United States.Tennessee|BOLD:AAA6434
Renia discoloralis|[1650]|LNCC1371-11|11-NCCC-896|658[0n]bp|United States.North Carolina|BOLD:AAA6434
Renia discoloralis|[1651]|LNCB553-09|09-NCCC-023|658[0n]bp|United States.North Carolina|BOLD:AAA6434
Renia discoloralis|[1652]|LNC601-06|06-NCCC-601|658[0n]bp|United States.North Carolina|BOLD:AAA6434
Renia discoloralis|[1653]|LGSMG518-07|BGS03881|658[0n]bp|United States.Tennessee|BOLD:AAA6434
Renia discoloralis|[1654]|LGSMG517-07|BGS03880|658[0n]bp|United States.Tennessee|BOLD:AAA6434
Renia discoloralis|[1655]|LOT503-04|04HBL002503|658[0n]bp|United States.Tennessee|BOLD:AAA6434
Renia discoloralis|[1656]|LNCC1370-11|11-NCCC-895|658[1n]bp|United States.North Carolina|BOLD:AAA6434
Renia discoloralis|[1657]|LOT152-04|04HBL002152|609[0n]bp|United States.Tennessee|BOLD:AAA6434
Renia discoloralis|[1658]|LOT158-04|04HBL002158|609[0n]bp|United States.Tennessee|BOLD:AAA6434
Renia discoloralis|[1659]|LOT190-04|04HBL002190|609[0n]bp|United States.Tennessee|BOLD:AAA6434
Renia discoloralis|[1660]|LOT155-04|04HBL002155|609[0n]bp|United States.Tennessee|BOLD:AAA6434
Renia discoloralis|[1661]|LOT156-04|04HBL002156|609[0n]bp|United States.Tennessee|BOLD:AAA6434
Renia discoloralis|[1662]|LTOLB758-11|CWM-94-0303|658[0n]bp|United States.Maryland|BOLD:AAA6434
Renia discoloralis|[1663]|RDNME708-08|LEP041979|658[6n]bp|United States.Florida|BOLD:AAA6434
Renia discoloralis|[1664]|RDNMD550-06|CNCNoctuoidea12882|658[8n]bp|United States.Florida|
Renia discoloralis|[1665]|LNC605-06|06-NCCC-605|658[0n]bp|United States.North Carolina|BOLD:AAA6434
Renia discoloralis|[1666]|LNCB693-09|09-NCCC-163|632[0n]bp|United States.North Carolina|BOLD:AAA6434
Renia discoloralis|[1667]|LNCB832-09|09-MISC-017|649[0n]bp|United States.Alabama|BOLD:AAA6434
Renia discoloralis|[1668]|LNCC462-10|10-NCCC-557|658[0n]bp|United States.North Carolina|BOLD:AAA6434
Renia discoloralis|[1669]|LNCB196-06|06-NCCC-1152|658[0n]bp|United States.North Carolina|BOLD:AAA6434
Renia discoloralis|[1670]|LNCB694-09|09-NCCC-164|658[0n]bp|United States.North Carolina|BOLD:AAA6434
Renia discoloralis|[1671]|MILEQ288-11|11-MISC-763|658[0n]bp|United States.Alabama|BOLD:AAA6434
Renia discoloralis|[1672]|LMEM290-09|RBMIS-0290|658[0n]bp|United States.Alabama|BOLD:AAA6434
Renia discoloralis|[1673]|RDNMD177-06|CNCNoctuoidea12513|588[0n]bp|United States.Florida|BOLD:AAA6434
Renia discoloralis|[1674]|LNCB814-09|09-NCCC-284|614[0n]bp|United States.North Carolina|BOLD:AAA6434
Renia discoloralis|[1675]|LNCB800-09|09-NCCC-270|658[0n]bp|United States.North Carolina|BOLD:AAA6434
Renia discoloralis|[1676]|LNCB707-09|09-NCCC-177|658[0n]bp|United States.North Carolina|BOLD:AAA6434
Renia discoloralis|[1677]|LNCB798-09|09-NCCC-268|614[0n]bp|United States.North Carolina|BOLD:AAA6434
Renia discoloralis|[1678]|RDNME369-07|CNCNoctuoidea13976|623[0n]bp|United States.Florida|BOLD:AAA6434
Renia discoloralis|[1679]|RDNMD176-06|CNCNoctuoidea12512|608[0n]bp|United States.Florida|BOLD:AAA6434
Renia discoloralis|[1680]|LNCB750-09|09-NCCC-220|631[0n]bp|United States.North Carolina|BOLD:AAA6434
Renia discoloralis|[1681]|LNCB799-09|09-NCCC-269|631[0n]bp|United States.North Carolina|BOLD:AAA6434
Renia discoloralis|[1682]|LNCB797-09|09-NCCC-267|658[0n]bp|United States.North Carolina|BOLD:AAA6434
Renia discoloralis|[1683]|MILEP003-09|09-NCCC-288|658[0n]bp|United States.North Carolina|BOLD:AAA6434
Renia discoloralis|[1684]|LNCC131-10|10-NCCC-226|658[0n]bp|United States.North Carolina|BOLD:AAA6434
Renia discoloralis|[1685]|LOFLA316-06|06-FLOR-0316|658[0n]bp|United States.Florida|BOLD:AAA6434
Renia discoloralis|[1686]|RDNMD174-06|CNCNoctuoidea12510|658[0n]bp|United States.Florida|BOLD:AAA6434

Renia discoloralis|[1683]|MILEP[illegible]|09-NCCC-[illegible]|658[0n]bp|United States.North Carolina|BOLD:AAA6434
Renia discoloralis|[1684]|LNCC131-10|10-NCCC-226|658[0n]bp|United States.North Carolina|BOLD:AAA6434
Renia discoloralis|[1685]|LOFLA316-06|06-FLOR-0316|658[0n]bp|United States.Florida|BOLD:AAA6434
Renia discoloralis|[1686]|RDNMD174-06|CNCNoctuoidea12510|658[0n]bp|United States.Florida|BOLD:AAA6434
Renia discoloralis|[1687]|LNCB692-09|09-NCCC-162|658[0n]bp|United States.North Carolina|BOLD:AAA6434
Renia discoloralis|[1688]|LNCB195-06|06-NCCC-1151|658[0n]bp|United States.North Carolina|BOLD:AAA6434
Renia discoloralis|[1689]|LNCB193-06|06-NCCC-1149|658[0n]bp|United States.North Carolina|BOLD:AAA6434
Renia discoloralis|[1690]|LNCB229-06|06-NCCC-1185|658[0n]bp|United States.North Carolina|BOLD:AAA6434
Renia discoloralis|[1691]|LNCB230-06|06-NCCC-1186|658[0n]bp|United States.North Carolina|BOLD:AAA6434
Renia discoloralis|[1692]|LNCC130-10|10-NCCC-225|658[0n]bp|United States.North Carolina|BOLD:AAA6434
Renia discoloralis|[1693]|LNCB194-06|06-NCCC-1150|658[0n]bp|United States.North Carolina|BOLD:AAA6434
Renia discoloralis|[1694]|LNC603-06|06-NCCC-603|658[0n]bp|United States.North Carolina|BOLD:AAA6434
Renia discoloralis|[1695]|LNCB753-09|09-NCCC-223|658[0n]bp|United States.North Carolina|BOLD:AAA6434
Renia discoloralis|[1696]|LNCB198-06|06-NCCC-1154|658[0n]bp|United States.North Carolina|BOLD:AAA6434
Renia discoloralis|[1697]|LMEM289-09|RBMIS-0289|658[0n]bp|United States.Alabama|BOLD:AAA6434
Renia discoloralis|[1698]|RDNME709-08|LEP041980|658[0n]bp|United States.Florida|BOLD:AAA6434
Renia discoloralis|[1699]|LNCB695-09|09-NCCC-165|658[0n]bp|United States.North Carolina|BOLD:AAA6434
Renia discoloralis|[1700]|LNCB326-06|06-NCCC-1282|658[0n]bp|United States.North Carolina|BOLD:AAA6434
Renia discoloralis|[1701]|MILEP040-09|09-NCCC-325|658[0n]bp|United States.North Carolina|BOLD:AAA6434
Renia discoloralis|[1702]|MILEP039-09|09-NCCC-324|658[0n]bp|United States.North Carolina|BOLD:AAA6434
Renia discoloralis|[1703]|HKONS354-08|3259-COI-08|658[0n]bp|United States.Florida|BOLD:AAA6434
Renia discoloralis|[1704]|LNCB637-09|09-NCCC-107|658[0n]bp|United States.North Carolina|BOLD:AAA6434
Renia discoloralis|[1705]|HKONS355-08|3260-COI-08|658[0n]bp|United States.Florida|BOLD:AAA6434
Renia discoloralis|[1706]|LNC602-06|06-NCCC-602|657[0n]bp|United States.North Carolina|BOLD:AAA6434
Renia discoloralis|[1707]|RDNMD175-06|CNCNoctuoidea12511|601[0n]bp|United States.Florida|BOLD:AAA6434
Renia discoloralis|[1708]|LNCB691-09|09-NCCC-161|658[1n]bp|United States.North Carolina|BOLD:AAA6434
Renia discoloralis|[1709]|HKONS356-08|3261-COI-08|658[0n]bp|United States.Florida|BOLD:AAA6434
Renia discoloralis|[1710]|LNCB197-06|06-NCCC-1153|658[0n]bp|United States.North Carolina|BOLD:AAA6434
Renia discoloralis|[1711]|LNCB462-07|07-NCNW-0146|658[0n]bp|United States.North Carolina|BOLD:AAA6434
Renia factiosalis|[1712]|RDLQF612-06|DH011762|658[0n]bp|Canada.Quebec|BOLD:AAA8624
Renia factiosalis|[1713]|RDNMD186-06|CNCNoctuoidea12522|658[0n]bp|Canada.Quebec|BOLD:AAA8624
Renia factiosalis|[1714]|XAG121-05|2005-ONT-705|658[0n]bp|Canada.Ontario|BOLD:AAA8624
Renia factiosalis|[1715]|RDLQF728-06|DH011878|658[0n]bp|Canada.Quebec|BOLD:AAA8624
Renia factiosalis|[1716]|RDLQF611-06|DH011761|658[0n]bp|Canada.Quebec|BOLD:AAA8624
Renia factiosalis|[1717]|RDNMD187-06|CNCNoctuoidea12523|658[0n]bp|Canada.Quebec|BOLD:AAA8624
Renia factiosalis|[1718]|RDLQF773-06|DH011923|658[0n]bp|Canada.Quebec|BOLD:AAA8624
Renia factiosalis|[1719]|RDLQF616-06|DH011766|658[0n]bp|Canada.Quebec|BOLD:AAA8624
Renia factiosalis|[1720]|RDLQF776-06|DH011926|658[0n]bp|Canada.Quebec|BOLD:AAA8624
Renia factiosalis|[1721]|RDLQF621-06|DH011771|658[0n]bp|Canada.Quebec|BOLD:AAA8624
Renia factiosalis|[1722]|RDLQF615-06|DH011765|658[0n]bp|Canada.Quebec|BOLD:AAA8624
Renia factiosalis|[1723]|RDLQF778-06|DH011928|658[0n]bp|Canada.Quebec|BOLD:AAA8624
Renia factiosalis|[1724]|RDLQF777-06|DH011927|658[0n]bp|Canada.Quebec|BOLD:AAA8624
Renia factiosalis|[1725]|RDLQF775-06|DH011925|658[0n]bp|Canada.Quebec|BOLD:AAA8624
Renia factiosalis|[1726]|RDLQF617-06|DH011767|658[0n]bp|Canada.Quebec|BOLD:AAA8624
Renia factiosalis|[1727]|RDLQF620-06|DH011770|658[0n]bp|Canada.Quebec|BOLD:AAA8624
Renia factiosalis|[1728]|RDLQF774-06|DH011924|658[0n]bp|Canada.Quebec|BOLD:AAA8624
Renia factiosalis|[1729]|RDLQF614-06|DH011764|658[0n]bp|Canada.Quebec|BOLD:AAA8624
Renia factiosalis|[1730]|RDLQF613-06|DH011763|658[1n]bp|Canada.Quebec|BOLD:AAA8624
Renia factiosalis|[1731]|RDLQF618-06|DH011768|658[1n]bp|Canada.Quebec|BOLD:AAA8624
Renia sobrialis|[1732]|RDLQF094-06|DH006866|658[0n]bp|Canada.Quebec|BOLD:AAA8623
Renia sobrialis|[1733]|TMNBB061-06|MNBTT-1001|656[0n]bp|Canada.New Brunswick|BOLD:AAA8622
Renia sobrialis|[1734]|RDNMD188-06|CNCNoctuoidea12524|583[0n]bp|Canada.Quebec|BOLD:AAA8622
Renia sobrialis|[1735]|RDLQG164-06|DH012335|658[0n]bp|Canada.Quebec|BOLD:AAA8622
Renia sobrialis|[1736]|RDLQF388-06|DH011455|658[0n]bp|Canada.Quebec|BOLD:AAA8622
Renia sobrialis|[1737]|BBLPC539-09|09BBELE-1539|658[0n]bp|Canada.New Brunswick|BOLD:AAA8622
Renia sobrialis|[1738]|RDLQF389-06|DH011456|658[0n]bp|Canada.Quebec|BOLD:AAA8622
Renia sobrialis|[1739]|RDLQG163-06|DH012334|658[0n]bp|Canada.Quebec|BOLD:AAA8622
Renia sobrialis|[1740]|RDLQF619-06|DH011769|658[0n]bp|Canada.Quebec|BOLD:AAA8622
Renia sobrialis|[1741]|RDLQF583-06|DH011733|658[0n]bp|Canada.Quebec|BOLD:AAA8622
Renia sobrialis|[1742]|RDLQF582-06|DH011732|658[0n]bp|Canada.Quebec|BOLD:AAA8622
Renia sobrialis|[1743]|XAG258-05|2005-ONT-842|658[0n]bp|Canada.Ontario|BOLD:AAA8622
Renia sobrialis|[1744]|RDLQG160-06|DH012331|658[0n]bp|Canada.Quebec|BOLD:AAA8622
Renia sobrialis|[1745]|RDLQF387-06|DH011454|658[0n]bp|Canada.Quebec|BOLD:AAA8622
Renia sobrialis|[1746]|RDLQG159-06|DH012330|658[0n]bp|Canada.Quebec|BOLD:AAA8622
Renia sobrialis|[1747]|RDLQF390-06|DH011457|658[0n]bp|Canada.Quebec|BOLD:AAA8622
Renia sobrialis|[1748]|RDLQG161-06|DH012332|658[0n]bp|Canada.Quebec|BOLD:AAA8622
Renia sobrialis|[1749]|RDLQG162-06|DH012333|658[0n]bp|Canada.Quebec|BOLD:AAA8622
Renia sobrialis|[1750]|RDLQF392-06|DH011459|658[0n]bp|Canada.Quebec|BOLD:AAA8622
Renia sobrialis|[1751]|PHMO180-03|moth952.01|639[0n]bp|Canada.Ontario|BOLD:AAA8622
Renia sobrialis|[1752]|RDLQG155-06|DH012326|638[0n]bp|Canada.Quebec|BOLD:AAA8622
Renia sobrialis|[1753]|RDLQG157-06|DH012328|632[0n]bp|Canada.Quebec|BOLD:AAA8622
Renia sobrialis|[1754]|RDLQG158-06|DH012329|594[0n]bp|Canada.Quebec|BOLD:AAA8622
Renia sobrialis|[1755]|RDLQG153-06|DH012324|643[0n]bp|Canada.Quebec|BOLD:AAA8622
Renia sobrialis|[1756]|RDLQG154-06|DH012325|642[0n]bp|Canada.Quebec|BOLD:AAA8622
Renia sobrialis|[1757]|RDLQF708-06|DH011858|611[0n]bp|Canada.Quebec|BOLD:AAA8622
Renia sobrialis|[1758]|RDLQF093-06|DH006813|622[0n]bp|Canada.Quebec|BOLD:AAA8622
Renia sobrialis|[1759]|RDLQG156-06|DH012327|640[0n]bp|Canada.Quebec|BOLD:AAA8622
Renia sobrialis|[1760]|BBLEC975-09|09BBELE-0975|658[0n]bp|Canada.Nova Scotia|BOLD:AAA8622
Renia sp.|[1761]|RDLQF625-06|DH011775|658[0n]bp|Canada.Quebec|BOLD:AAA7214
Tetanolita mynesalis|[1762]|LOFLB301-06|06-FLOR-1241|626[4n]bp|United States.Florida|BOLD:AAA9813
Tetanolita mynesalis|[1763]|LOFLB459-06|06-FLOR-1399|658[0n]bp|United States.Florida|BOLD:AAA9813
Tetanolita mynesalis|[1764]|BBLSU039-09|09BBLEP-04408|658[0n]bp|United States.Arkansas|BOLD:AAA9813
Tetanolita mynesalis|[1765]|BBLOC721-11|BIOUG01465-D02|658[0n]bp|United States.Arkansas|BOLD:AAA9813
Tetanolita mynesalis|[1766]|LMEM243-09|RBMIS-0243|658[0n]bp|United States.Alabama|BOLD:AAA9813
Tetanolita mynesalis|[1767]|LOFLB861-06|06-FLOR-1801|658[0n]bp|United States.Florida|BOLD:AAA9813
Tetanolita mynesalis|[1768]|LOFLB546-06|06-FLOR-1486|658[0n]bp|United States.Florida|BOLD:AAA9813
Tetanolita mynesalis|[1769]|LPOKA420-09|MDOK-0420|658[0n]bp|United States.Oklahoma|BOLD:AAA9813
Tetanolita mynesalis|[1770]|LGSMG509-07|BGS03872|658[0n]bp|United States.Tennessee|BOLD:AAA9813
Tetanolita mynesalis|[1771]|LPOKA254-08|MDOK-0254|658[0n]bp|United States.Oklahoma|BOLD:AAA9813
Tetanolita mynesalis|[1772]|LPOKB614-09|MDOK-1656|562[0n]bp|United States.Oklahoma|BOLD:AAA9813
Tetanolita mynesalis|[1773]|LOFLA619-06|06-FLOR-0619|658[0n]bp|United States.Florida|BOLD:AAA9813
Tetanolita mynesalis|[1774]|LOFLC346-06|06-FLOR-2226|658[0n]bp|United States.Florida|BOLD:AAA9813
Tetanolita mynesalis|[1775]|LPOKD423-09|MDOK-3502|658[0n]bp|United States.Oklahoma|BOLD:AAA9813
Tetanolita mynesalis|[1776]|LNC611-06|06-NCCC-611|658[0n]bp|United States.North Carolina|BOLD:AAA9813
Tetanolita mynesalis|[1777]|LOFLB175-06|06-FLOR-1115|658[0n]bp|United States.Florida|BOLD:AAA9813
Tetanolita mynesalis|[1778]|LGSMC489-05|DNA-ATBI-2489|601[0n]bp|United States.Tennessee|BOLD:AAA9813
Tetanolita mynesalis|[1779]|LNC610-06|06-NCCC-610|658[1n]bp|United States.North Carolina|BOLD:AAA9813
Tetanolita mynesalis|[1780]|LGSMC487-05|DNA-ATBI-2487|616[0n]bp|United States.Tennessee|BOLD:AAA9813
Tetanolita mynesalis|[1781]|LILLB062-11|SNS10IL-01282|658[0n]bp|United States.Illinois|BOLD:AAA9813
Tetanolita mynesalis|[1782]|LGSM760-04|DNA-ATBI-0760|658[0n]bp|United States.North Carolina|BOLD:AAA9813
Tetanolita mynesalis|[1783]|MJMSL115-10|TDWG-0032|658[0n]bp|United States.Massachusetts|BOLD:AAA9813
Tetanolita mynesalis|[1784]|USLEP777-10|10BBLEP-00777|658[0n]bp|United States.Arkansas|BOLD:AAA9813
Tetanolita mynesalis|[1785]|LGSMC488-05|DNA-ATBI-2488|658[0n]bp|United States.Tennessee|BOLD:AAA9813

Tetanolita mynesalis|[1783]|MJMSL115-10|TDWG-0032|658[0n]bp|United States.Massachusetts|BOLD:AAA9813
Tetanolita mynesalis|[1784]|USLEP777-10|10BBLEP-00777|658[0n]bp|United States.Arkansas|BOLD:AAA9813
Tetanolita mynesalis|[1785]|LGSMC488-05|DNA-ATBI-2488|658[0n]bp|United States.Tennessee|BOLD:AAA9813
Tetanolita mynesalis|[1786]|LILLA745-11|SNS10IL-00940|658[0n]bp|United States.Illinois|BOLD:AAA9813
Tetanolita mynesalis|[1787]|BBLOC1105-11|BIOUG01539-D01|658[0n]bp|United States.Texas|BOLD:AAA9813
Tetanolita mynesalis|[1788]|LMEM244-09|RBMIS-0244|658[0n]bp|United States.Alabama|BOLD:AAA9813
Tetanolita mynesalis|[1789]|LNC584-06|06-NCCC-584|658[0n]bp|United States.North Carolina|BOLD:AAA9813
Tetanolita mynesalis|[1790]|LOFLB884-06|06-FLOR-1824|658[0n]bp|United States.Florida|BOLD:AAA9813
Tetanolita mynesalis|[1791]|LMEM241-09|RBMIS-0241|658[0n]bp|United States.Alabama|BOLD:AAA9813
Tetanolita mynesalis|[1792]|HKONS391-08|1861-COI-07|658[0n]bp|United States.Florida|BOLD:AAA9813
Tetanolita mynesalis|[1793]|LOFLB190-06|06-FLOR-1130|658[0n]bp|United States.Florida|BOLD:AAA9813
Tetanolita mynesalis|[1794]|LNCB279-06|06-NCCC-1235|658[0n]bp|United States.North Carolina|BOLD:AAA9813
Tetanolita mynesalis|[1795]|USLEP762-10|10BBLEP-00762|658[0n]bp|United States.Texas|BOLD:AAA9813
Tetanolita mynesalis|[1796]|LNCB383-06|06-NCCC-1339|658[0n]bp|United States.North Carolina|BOLD:AAA9813
Tetanolita mynesalis|[1797]|LPOKB337-09|MDOK-1356|658[0n]bp|United States.Oklahoma|BOLD:AAA9813
Tetanolita mynesalis|[1798]|LMEM246-09|RBMIS-0246|658[0n]bp|United States.Texas|BOLD:AAA9813
Tetanolita mynesalis|[1799]|LMEM242-09|RBMIS-0242|658[0n]bp|United States.Mississippi|BOLD:AAA9813
Tetanolita mynesalis|[1800]|BBLOC046-11|BIOUG01452-E03|658[0n]bp|United States.Florida|BOLD:AAA9813
Tetanolita mynesalis|[1801]|HKONS392-08|1862-COI-07|658[0n]bp|United States.Florida|BOLD:AAA9813
Tetanolita mynesalis|[1802]|LGSMG510-07|BGS03873|625[0n]bp|United States.Tennessee|BOLD:AAA9813
Tetanolita mynesalis|[1803]|LOFLB300-06|06-FLOR-1240|585[0n]bp|United States.Florida|BOLD:AAA9813
Tetanolita mynesalis|[1804]|LPOKA582-09|MDOK-0582|636[0n]bp|United States.Oklahoma|BOLD:AAA9813
Tetanolita mynesalis|[1805]|BBLOE1387-12|BIOUG01986-G12|658[1n]bp|United States.Arkansas|BOLD:AAA9813
Tetanolita mynesalis|[1806]|LOFLD028-07|HLC-16602|658[0n]bp|United States.Florida|BOLD:AAA9813
Tetanolita palligera|[1807]|RDNMF736-08|NOC14822|609[0n]bp|Canada.British Columbia|BOLD:ACF1365
Dodia kononenkoi|[1808]|TIPSY578-12|STG104|658[0n]bp|Canada.Yukon Territory|BOLD:AAF5396
Dodia kononenkoi|[1809]|RDNMF522-08|NOC14608|652[0n]bp|Canada.Yukon Territory|BOLD:AAF5396
Dodia kononenkoi|[1810]|RDNMF520-08|NOC14606|652[0n]bp|Canada.Yukon Territory|BOLD:AAF5396
Dodia albertae|[1811]|RDMAB421-05|BCSC94|601[0n]bp|Canada.Yukon Territory|BOLD:ABZ1341
Dodia albertae|[1812]|RDMAB418-05|BCSC91|658[0n]bp|Canada.Alberta|BOLD:ABZ1341
Dodia albertae|[1813]|RDNME215-07|CNCNoctuoidea13822|658[0n]bp|Canada.Alberta|BOLD:ABZ1341
Dodia albertae|[1814]|RDNME214-07|CNCNoctuoidea13821|658[0n]bp|Canada.Alberta|BOLD:ABZ1341
Dodia albertae|[1815]|RDNME217-07|CNCNoctuoidea13824|658[0n]bp|Canada.Alberta|BOLD:ABZ1341
Dodia albertae|[1816]|RDMAB419-05|BCSC92|658[0n]bp|Canada.Alberta|BOLD:ABZ1341
Dodia albertae|[1817]|RDNME216-07|CNCNoctuoidea13823|658[0n]bp|Canada.Alberta|BOLD:ABZ1341
Dodia albertae|[1818]|TIPSY588-12|STG114|658[0n]bp|Canada.Yukon Territory|BOLD:ABZ1341
Dodia albertae|[1819]|RDNME837-08|LEP041302|598[0n]bp|Canada.Newfoundland and Labrador|BOLD:ABZ1341
Dodia albertae|[1820]|RDNME838-08|LEP041303|609[0n]bp|Canada.Newfoundland and Labrador|BOLD:ABZ1341
Dodia tarandus|[1821]|RDNME213-07|CNCNoctuoidea13820|617[0n]bp|Canada.Alberta|BOLD:AAC2146
Dodia tarandus|[1822]|RDMAB420-05|BCSC93|658[0n]bp|Canada.Alberta|BOLD:AAC2146
Dodia albertae|[1823]|RDMAB417-05|BCSC90|546[0n]bp|Canada.Alberta|BOLD:AAC2146
Dodia albertae|[1824]|RDMAB416-05|BCSC89|583[0n]bp|Canada.Yukon Territory|BOLD:AAC2146
Dodia verticalis|[1825]|RDNMG902-08|CNC LEP00019115|658[0n]bp|Canada.Yukon Territory|BOLD:AAC2146
Dodia verticalis|[1826]|TIPSY589-12|STG115|658[0n]bp|Canada.Yukon Territory|BOLD:AAC2146
Dodia verticalis|[1827]|TIPSY590-12|STG116|658[0n]bp|Canada.Yukon Territory|BOLD:AAC2146
Hypoprepia fucosa tricolor|[1828]|RDLQB676-05|DH010779|516[0n]bp|Canada.Quebec|BOLD:AAA4714
Hypoprepia fucosa|[1829]|MECB142-04|jflandry1086|603[0n]bp|Canada.Quebec|BOLD:AAA4714
Hypoprepia fucosa|[1830]|RDMAB403-05|BCSC76|577[0n]bp|Canada.Saskatchewan|BOLD:AAA4714
Hypoprepia fucosa|[1831]|TMNBD361-07|MNBTT-3162|597[0n]bp|Canada.New Brunswick|BOLD:AAA4714
Hypoprepia fucosa|[1832]|XAE433-04|Moth4433.03|658[0n]bp|Canada.Ontario|BOLD:AAA4714
Hypoprepia fucosa|[1833]|TMG71-03|HYPO1.00|639[0n]bp|Canada.Ontario|BOLD:AAA4714
Hypoprepia fucosa|[1834]|XAK452-06|2006-ONT-1447|658[0n]bp|Canada.Ontario|BOLD:AAA4714
Hypoprepia fucosa|[1835]|XAG138-05|2005-ONT-722|658[1n]bp|Canada.Ontario|BOLD:AAA4714
Hypoprepia fucosa|[1836]|RDMAB384-05|BCSC58|555[0n]bp|Canada.Nova Scotia|BOLD:AAA4714
Hypoprepia fucosa|[1837]|MNAC816-07|CNCLEP00027557|658[0n]bp|Canada.Quebec|BOLD:AAA4714
Hypoprepia fucosa|[1838]|MNAC814-07|CNCLEP00027555|658[0n]bp|Canada.Quebec|BOLD:AAA4714
Hypoprepia fucosa|[1839]|TMNBD362-07|MNBTT-3163|636[0n]bp|Canada.New Brunswick|BOLD:AAA4714
Hypoprepia fucosa|[1840]|TMNBD363-07|MNBTT-3164|649[0n]bp|Canada.New Brunswick|BOLD:AAA4714
Hypoprepia fucosa|[1841]|XAJ926-06|2006-ONT-0926|658[0n]bp|Canada.Ontario|BOLD:AAA4714
Hypoprepia fucosa|[1842]|XAB188-04|04HBL005188|658[0n]bp|Canada.Ontario|BOLD:AAA4714
Hypoprepia fucosa|[1843]|TMG72-03|MOTH2.00|639[0n]bp|Canada.Ontario|BOLD:AAA4714
Hypoprepia fucosa|[1844]|MNAC815-07|CNCLEP00027556|658[0n]bp|Canada.Quebec|BOLD:AAA4714
Hypoprepia fucosa|[1845]|TMNBD365-07|MNBTT-3166|658[0n]bp|Canada.New Brunswick|BOLD:AAA4714
Hypoprepia fucosa tricolor|[1846]|RDLQB691-05|DH010794|658[0n]bp|Canada.Quebec|BOLD:AAA4714
Hypoprepia fucosa|[1847]|LPMNB358-09|08BBLEP-05202|658[0n]bp|Canada.Manitoba|BOLD:AAA4714
Hypoprepia fucosa|[1848]|LPMNB325-09|08BBLEP-05169|658[0n]bp|Canada.Manitoba|BOLD:AAA4714
Hypoprepia fucosa|[1849]|LPMNB324-09|08BBLEP-05168|658[0n]bp|Canada.Manitoba|BOLD:AAA4714
Hypoprepia fucosa|[1850]|LPMNB389-09|08BBLEP-05233|627[0n]bp|Canada.Manitoba|BOLD:AAA4714
Hypoprepia fucosa|[1851]|HEOCT1021-12|BIOUG02500-E05|614[0n]bp|Canada.Ontario|BOLD:AAA4714
Hypoprepia fucosa|[1852]|PMG012-03|moth958.01|617[0n]bp|Canada.Ontario|BOLD:AAA4714
Hypoprepia fucosa|[1853]|BLTIB649-08|BL929|636[0n]bp|Canada.Ontario|BOLD:AAA4714
Hypoprepia fucosa|[1854]|BLTIB1096-08|BL1106|658[1n]bp|Canada.Ontario|BOLD:AAA4714
Hypoprepia fucosa|[1855]|BLTIB877-08|BL1296|658[0n]bp|Canada.Ontario|BOLD:AAA4714
Hypoprepia fucosa|[1856]|BLTIB986-08|BL1423|658[0n]bp|Canada.Ontario|BOLD:AAA4714
Hypoprepia fucosa|[1857]|LPMN1001-09|08BBLEP-04042|658[0n]bp|Canada.Manitoba|BOLD:AAA4714
Hypoprepia fucosa|[1858]|TMNBB005-06|MNBTT-945|658[0n]bp|Canada.New Brunswick|BOLD:AAA4714
Hypoprepia fucosa|[1859]|BLTIB782-08|BL1199|658[0n]bp|Canada.Ontario|BOLD:AAA4714
Hypoprepia fucosa|[1860]|LPSOD1052-09|08MZPP-119|658[0n]bp|Canada.Ontario|BOLD:AAA4714
Hypoprepia fucosa|[1861]|LPMNB382-09|08BBLEP-05226|658[0n]bp|Canada.Manitoba|BOLD:AAA4714
Hypoprepia fucosa|[1862]|TMG73-03|moth1164.01|639[0n]bp|Canada.Ontario|BOLD:AAA4714
Hypoprepia fucosa|[1863]|TMNBD364-07|MNBTT-3165|658[0n]bp|Canada.New Brunswick|BOLD:AAA4714
Hypoprepia fucosa|[1864]|MNAF435-08|CNCLEP00040420|648[0n]bp|Canada.Manitoba|BOLD:AAA4714
Hypoprepia fucosa|[1865]|BLTIB931-08|BL1351|658[0n]bp|Canada.Ontario|BOLD:AAA4714
Hypoprepia fucosa|[1866]|LPMNB338-09|08BBLEP-05182|658[0n]bp|Canada.Manitoba|BOLD:AAA4714
Hypoprepia fucosa|[1867]|LPMN992-09|08BBLEP-04033|658[0n]bp|Canada.Manitoba|BOLD:AAA4714
Hypoprepia fucosa|[1868]|XAB151-04|04HBL005151|658[0n]bp|Canada.Ontario|BOLD:AAA4714
Hypoprepia fucosa|[1869]|BLTIB932-08|BL1352|658[0n]bp|Canada.Ontario|BOLD:AAA4714
Hypoprepia fucosa|[1870]|BLTIB987-08|BL1424|658[0n]bp|Canada.Ontario|BOLD:AAA4714
Hypoprepia fucosa|[1871]|MNAF862-08|CNCLEP00040847|658[0n]bp|Canada.Manitoba|BOLD:AAA4714
Hypoprepia fucosa|[1872]|MNAF864-08|CNCLEP00040849|658[0n]bp|Canada.Manitoba|BOLD:AAA4714
Hypoprepia fucosa|[1873]|LPMN1003-09|08BBLEP-04044|658[0n]bp|Canada.Manitoba|BOLD:AAA4714
Hypoprepia fucosa|[1874]|LPMN079-08|08BBLEP-00877|658[0n]bp|Canada.Manitoba|BOLD:AAA4714
Hypoprepia fucosa|[1875]|MNAF863-08|CNCLEP00040848|658[0n]bp|Canada.Manitoba|BOLD:AAA4714
Hypoprepia fucosa|[1876]|LPMNB404-09|08BBLEP-05248|658[0n]bp|Canada.Manitoba|BOLD:AAA4714
Hypoprepia fucosa|[1877]|LPMNB352-09|08BBLEP-05196|658[0n]bp|Canada.Manitoba|BOLD:AAA4714
Hypoprepia fucosa|[1878]|MNAF476-08|CNCLEP00040461|658[0n]bp|Canada.Manitoba|BOLD:AAA4714
Hypoprepia fucosa|[1879]|BLTIB781-08|BL1198|658[0n]bp|Canada.Ontario|BOLD:AAA4714
Hypoprepia fucosa|[1880]|LPMNB479-09|08BBLEP-05517|658[0n]bp|Canada.Manitoba|BOLD:AAA4714
Hypoprepia fucosa tricolor|[1881]|RDLQB749-05|DH010664|658[0n]bp|Canada.Quebec|BOLD:AAA4714
Hypoprepia fucosa tricolor|[1882]|XAJ881-06|2006-ONT-0881|658[0n]bp|Canada.Ontario|BOLD:AAA4714
Hypoprepia fucosa tricolor|[1883]|BLTIB780-08|BL1197|658[0n]bp|Canada.Ontario|BOLD:AAA4714
Hypoprepia miniata|[1884]|PHMO381-03|moth1885.02|639[0n]bp|Canada.Ontario|BOLD:AAA4714

Hypoprepia fucosa tricolor|[1882]|XAJ881-06|2006-ONT-0881|658[0n]bp|Canada.Ontario|BOLD:AAA4714
Hypoprepia fucosa tricolor|[1883]|BLTIB780-08|BL1197|658[0n]bp|Canada.Ontario|BOLD:AAA4714
Hypoprepia miniata|[1884]|PHMO381-03|moth1885.02|639[0n]bp|Canada.Ontario|BOLD:AAA4714
Hypoprepia miniata|[1885]|PMG013-03|HYPO2.00|617[0n]bp|Canada.Ontario|BOLD:AAA4714
Hypoprepia miniata|[1886]|LNEL012-06|CNCLEP00007384|658[0n]bp|Canada.Alberta|BOLD:AAA4714
Hypoprepia miniata|[1887]|RDLQ769-07|DH006568|658[0n]bp|Canada.Quebec|BOLD:AAA4714
Hypoprepia miniata|[1888]|BBLPA586-10|10BBCLP-0586|658[0n]bp|Canada.Ontario|BOLD:AAA4714
Hypoprepia miniata|[1889]|BBLPA585-10|10BBCLP-0585|658[0n]bp|Canada.Ontario|BOLD:AAA4714
Hypoprepia miniata|[1890]|MNAF436-08|CNCLEP00040421|658[0n]bp|Canada.Manitoba|BOLD:AAA4714
Hypoprepia miniata|[1891]|LPSOD1062-09|08MZPP-159|658[0n]bp|Canada.Ontario|BOLD:AAA4714
Hypoprepia miniata|[1892]|RDMAB378-05|BCSC52|658[0n]bp|Canada.Alberta|BOLD:AAA4714
Hypoprepia miniata|[1893]|BBLPA589-10|10BBCLP-0589|658[0n]bp|Canada.Ontario|BOLD:AAA4714
Hypoprepia miniata|[1894]|LPSK629-08|08BBLEP-02197|658[0n]bp|Canada.Saskatchewan|BOLD:AAA4714
Hypoprepia miniata|[1895]|RDMAB401-05|BCSC74|658[0n]bp|Canada.Alberta|BOLD:AAA4714
Hypoprepia miniata|[1896]|MNAF058-08|CNCLEP00038543|658[0n]bp|Canada.Manitoba|BOLD:AAA4714
Hypoprepia miniata|[1897]|BBLPA588-10|10BBCLP-0588|658[0n]bp|Canada.Ontario|BOLD:AAA4714
Hypoprepia miniata|[1898]|BBLPA587-10|10BBCLP-0587|658[0n]bp|Canada.Ontario|BOLD:AAA4714
Hypoprepia miniata|[1899]|RDMAB400-05|BCSC73|658[0n]bp|Canada.Alberta|BOLD:AAA4714
Hypoprepia miniata|[1900]|RDMAB380-05|BCSC54|580[1n]bp|Canada.Alberta|BOLD:AAA4714
Hypoprepia miniata|[1901]|LOWCB563-05|CGWC-1503|658[6n]bp|Canada.British Columbia|BOLD:AAA4714
Hypoprepia miniata|[1902]|LOWCB566-05|CGWC-1506|600[0n]bp|Canada.British Columbia|BOLD:AAA4714
Hypoprepia miniata|[1903]|LBCB631-05|HLC-21571|658[0n]bp|Canada.British Columbia|BOLD:AAA4714
Hypoprepia miniata|[1904]|LBCC354-05|HLC-22234|658[0n]bp|Canada.British Columbia|BOLD:AAA4714
Hypoprepia miniata|[1905]|LOWCB562-05|CGWC-1502|586[0n]bp|Canada.British Columbia|BOLD:AAA4714
Hypoprepia miniata|[1906]|LBCC462-05|HLC-22342|658[0n]bp|Canada.British Columbia|BOLD:AAA4714
Hypoprepia miniata|[1907]|LBCB632-05|HLC-21572|658[1n]bp|Canada.British Columbia|BOLD:AAA4714
Hypoprepia miniata|[1908]|LOWCB564-05|CGWC-1504|658[0n]bp|Canada.British Columbia|BOLD:AAA4714
Hypoprepia miniata|[1909]|LALPA865-11|AVBC 1038-11|658[0n]bp|Canada.British Columbia|BOLD:AAA4714
Hypoprepia miniata|[1910]|LBCC460-05|HLC-22340|658[0n]bp|Canada.British Columbia|BOLD:AAA4714
Hypoprepia miniata|[1911]|RDMAB381-05|BCSC55|658[0n]bp|Canada.British Columbia|BOLD:AAA4714
Hypoprepia miniata|[1912]|RDMAB402-05|BCSC75|658[0n]bp|Canada.British Columbia|BOLD:AAA4714
Hypoprepia miniata|[1913]|LBCC769-05|HLC-22649|658[0n]bp|Canada.British Columbia|BOLD:AAA4714
Hypoprepia miniata|[1914]|LOWCB565-05|CGWC-1505|658[0n]bp|Canada.British Columbia|BOLD:AAA4714
Hypoprepia miniata|[1915]|RDMAB382-05|BCSC56|658[0n]bp|Canada.British Columbia|BOLD:AAA4714
Hypoprepia miniata|[1916]|LBCC459-05|HLC-22339|658[0n]bp|Canada.British Columbia|BOLD:AAA4714
Hypoprepia miniata|[1917]|LBCC461-05|HLC-22341|658[0n]bp|Canada.British Columbia|BOLD:AAA4714
Gnophaela vermiculata|[1918]|LPAB437-08|08BBLEP-02759|658[0n]bp|Canada.Alberta|BOLD:AAD5697
Gnophaela vermiculata|[1919]|BBLPA343-10|10BBCLP-0343|658[0n]bp|Canada.Alberta|BOLD:AAD5697
Gnophaela vermiculata|[1920]|BBLPA344-10|10BBCLP-0344|658[0n]bp|Canada.Alberta|BOLD:AAD5697
Gnophaela vermiculata|[1921]|RDMAB929-06|UASM43016|658[0n]bp|Canada.Alberta|BOLD:AAD5697
Gnophaela vermiculata|[1922]|LPMNB269-09|08BBLEP-05113|658[0n]bp|Canada.Manitoba|BOLD:AAD5697
Gnophaela vermiculata|[1923]|BBLPA345-10|10BBCLP-0345|658[0n]bp|Canada.Alberta|BOLD:AAD5697
Gnophaela vermiculata|[1924]|RDMAB966-09|UASM99695|658[0n]bp|Canada.British Columbia|BOLD:AAD5697
Gnophaela vermiculata|[1925]|BBLPA342-10|10BBCLP-0342|658[0n]bp|Canada.British Columbia|BOLD:AAD5697
Gnophaela vermiculata|[1926]|LPMNB268-09|08BBLEP-05112|658[0n]bp|Canada.Manitoba|BOLD:AAD5697
Gnophaela vermiculata|[1927]|RDMAB967-09|UASM99696|658[0n]bp|Canada.British Columbia|BOLD:AAD5697
Gnophaela vermiculata|[1928]|BBLPA346-10|10BBCLP-0346|658[0n]bp|Canada.British Columbia|BOLD:AAD5697
Gnophaela vermiculata|[1929]|RDMAB968-09|UASM99697|606[0n]bp|Canada.British Columbia|BOLD:AAD5697
Catocala antinympha|[1930]|QUNOE279-12|10278-060811-MN|658[0n]bp|United States.Minnesota|BOLD:AAB9017
Catocala badia|[1931]|BBLEC533-09|09BBELE-0533|658[0n]bp|Canada.New Brunswick|BOLD:AAB9017
Catocala badia|[1932]|BBLEC421-09|09BBELE-0421|658[0n]bp|Canada.New Brunswick|BOLD:AAB9017
Catocala badia|[1933]|BBLEC417-09|09BBELE-0417|658[0n]bp|Canada.New Brunswick|BOLD:AAB9017
Catocala antinympha|[1934]|QUNOE407-12|10408-310811-MI|658[0n]bp|United States.Michigan|BOLD:AAB9017
Catocala badia|[1935]|BBLEC686-09|09BBELE-0686|642[0n]bp|Canada.Nova Scotia|BOLD:AAB9017
Catocala badia|[1936]|BBLEC531-09|09BBELE-0531|658[0n]bp|Canada.New Brunswick|BOLD:AAB9017
Catocala badia|[1937]|BBLEC532-09|09BBELE-0532|658[0n]bp|Canada.New Brunswick|BOLD:AAB9017
Catocala badia|[1938]|BBLPC038-09|09BBELE-1038|622[0n]bp|Canada.New Brunswick|BOLD:AAB9017
Catocala antinympha|[1939]|QUNOD341-10|9055-100809-MI|639[0n]bp|United States.Michigan|BOLD:AAB9017
Catocala antinympha|[1940]|QUNO024-07|2024-100807-WI|658[0n]bp|United States.Wisconsin|BOLD:AAB9017
Catocala badia coelebs|[1941]|RDLQB569-05|DH010672|581[1n]bp|Canada.Quebec|BOLD:AAB9017
Catocala piatrix|[1942]|LPSOD203-09|08MZPP-033|658[0n]bp|Canada.Ontario|BOLD:AAB7092
Catocala retecta|[1943]|RDLQ113-05|DH003387|544[15n]bp|Canada.Quebec|
Catocala lacrymosa|[1944]|MILEQ188-11|11-MISC-663|658[0n]bp|United States.North Carolina|BOLD:ACE7470
Catocala lacrymosa|[1945]|LPOKA378-08|MDOK-0378|658[0n]bp|United States.Oklahoma|BOLD:ACE7470
Catocala lacrymosa|[1946]|QUNO593-08|5126-280808-IN|658[0n]bp|United States.Indiana|BOLD:ACE7470
Catocala lacrymosa|[1947]|MILEQ186-11|11-MISC-661|658[0n]bp|United States.Florida|BOLD:ACE7470
Catocala lacrymosa|[1948]|MILEQ183-11|11-MISC-658|658[0n]bp|United States.Florida|BOLD:ACE7470
Catocala lacrymosa|[1949]|MILEQ182-11|11-MISC-657|658[0n]bp|United States.Florida|BOLD:ACE7470
Catocala lacrymosa|[1950]|LNCC446-10|10-NCCC-541|658[0n]bp|United States.North Carolina|BOLD:ACE7470
Catocala lacrymosa|[1951]|QUNO638-08|5171-300608-MS|658[0n]bp|United States.Mississippi|BOLD:ACE7470
Catocala lacrymosa|[1952]|QUNO565-08|5098-220808-IN|658[0n]bp|United States.Indiana|BOLD:ACE7470
Catocala lacrymosa|[1953]|MILEQ189-11|11-MISC-664|658[0n]bp|United States.North Carolina|BOLD:ACE7470
Catocala lacrymosa|[1954]|MILEQ187-11|11-MISC-662|658[0n]bp|United States.Florida|BOLD:ACE7470
Catocala lacrymosa|[1955]|MILEQ184-11|11-MISC-659|658[0n]bp|United States.Florida|BOLD:ACE7470
Catocala lacrymosa|[1956]|LNCC445-10|10-NCCC-540|658[0n]bp|United States.North Carolina|BOLD:ACE7470
Catocala lacrymosa|[1957]|MILEQ185-11|11-MISC-660|658[0n]bp|United States.Florida|BOLD:ACE7470
Catocala lacrymosa|[1958]|JRLAA032-09|JRLAA-032|630[0n]bp|United States.Alabama|BOLD:ACE7470
Catocala lacrymosa|[1959]|ABCNA146-06|146-94-160702-MS|617[0n]bp|United States.Mississippi|BOLD:ACE7470
Catocala lacrymosa|[1960]|ABCNA150-06|150-94-150801-IN|594[0n]bp|United States.Indiana|BOLD:ACE7470
Catocala lacrymosa|[1961]|ABCNA147-06|147-94-150802-IN|614[0n]bp|United States.Indiana|BOLD:ACE7470
Catocala lacrymosa|[1962]|ABCNA149-06|149-94-040602-FL|599[0n]bp|United States.Florida|BOLD:ACE7470
Catocala lacrymosa|[1963]|ABCNA408-07|502-8794-160702-MS|595[0n]bp|United States.Mississippi|BOLD:ACE...
Catocala lacrymosa|[1964]|QUNO564-08|5097-220808-IN|658[0n]bp|United States.Indiana|BOLD:ACE7470
Catocala lacrymosa|[1965]|LGSMG597-07|BGS03960|658[0n]bp|United States.Tennessee|BOLD:ACE7470
Catocala lacrymosa|[1966]|QUNO566-08|5099-220808-IN|658[0n]bp|United States.Indiana|BOLD:ACE7470
Catocala dejecta|[1967]|ABCNA291-06|291-90-070802-KY|609[0n]bp|United States.Kentucky|BOLD:ACE7470
Catocala dejecta|[1968]|LGSM641-04|DNA-ATBI-0641|606[1n]bp|United States.Tennessee|BOLD:ACE7470
Catocala dejecta|[1969]|ABCNA398-07|492-8790-020702-TN|573[0n]bp|United States.Tennessee|BOLD:ACE7470
Catocala dejecta|[1970]|QUNO660-08|5193-030708-KY|658[0n]bp|United States.Kentucky|BOLD:ACE7470
Catocala dejecta|[1971]|LNCC1293-11|11-NCCC-818|633[0n]bp|United States.North Carolina|BOLD:ACE7470
Catocala dejecta|[1972]|ABCNA779-07|779-100702-VA|583[0n]bp|United States.Virginia|BOLD:ACE7470
Catocala dejecta|[1973]|ABCNA397-07|491-8790-090802-KY|584[0n]bp|United States.Kentucky|BOLD:ACE7470
Catocala dejecta|[1974]|ABCNA399-07|493-8790-170702-TN|595[0n]bp|United States.Tennessee|BOLD:ACE7470
Catocala dejecta|[1975]|QUNOD275-10|7373-COI-09|658[0n]bp|United States.Georgia|BOLD:ACE7470
Catocala palaeogama|[1976]|RDLQB468-05|DH010554|658[0n]bp|Canada.Quebec|BOLD:ACE7470
Catocala serena|[1977]|QUNO754-08|5287-070808-WI|658[0n]bp|United States.Wisconsin|BOLD:ACE7468
Catocala serena|[1978]|QUNO074-07|2074-250807-WI|658[0n]bp|United States.Wisconsin|BOLD:ACE7468
Catocala serena|[1979]|QUNO756-08|5289-070808-WI|658[0n]bp|United States.Wisconsin|BOLD:ACE7468
Catocala serena|[1980]|QUNO751-08|5284-070808-WI|658[0n]bp|United States.Wisconsin|BOLD:ACE7468
Catocala serena|[1981]|QUNOC005-09|5835-270808-IN|658[0n]bp|United States.Indiana|BOLD:ACE7468
Catocala serena|[1982]|QUNO757-08|5290-070808-WI|658[0n]bp|United States.Wisconsin|BOLD:ACE7468
Catocala serena|[1983]|QUNO755-08|5288-070808-WI|658[0n]bp|United States.Wisconsin|BOLD:ACE7468

Catocala serena|[1981]|QUNOC005-09|5835-270808-IN|658[0n]bp|United States.Indiana|BOLD:ACE7468
Catocala serena|[1982]|QUNO757-08|5290-070808-WI|658[0n]bp|United States.Wisconsin|BOLD:ACE7468
Catocala serena|[1983]|QUNO755-08|5288-070808-WI|658[0n]bp|United States.Wisconsin|BOLD:ACE7468
Catocala serena|[1984]|QUNO073-07|2073-010807-WI|658[0n]bp|United States.Wisconsin|BOLD:ACE7468
Catocala serena|[1985]|QUNO758-08|5291-070808-WI|658[0n]bp|United States.Wisconsin|BOLD:ACE7468
Catocala serena|[1986]|QUNO752-08|5285-070808-WI|658[0n]bp|United States.Wisconsin|BOLD:ACE7468
Catocala serena|[1987]|QUNO760-08|5293-070808-WI|658[0n]bp|United States.Wisconsin|BOLD:ACE7468
Catocala serena|[1988]|ABCNA636-07|448-8779-090802-KY|577[0n]bp|United States.Kentucky|BOLD:ACE7468
Catocala serena|[1989]|QUNO761-08|5294-070808-WI|658[0n]bp|United States.Wisconsin|BOLD:ACE7468
Catocala serena|[1990]|QUNO072-07|2072-010807-WI|658[0n]bp|United States.Wisconsin|BOLD:ACE7468
Catocala serena|[1991]|QUNO759-08|5292-070808-WI|658[0n]bp|United States.Wisconsin|BOLD:ACE7468
Catocala serena|[1992]|QUNO753-08|5286-070808-WI|658[0n]bp|United States.Wisconsin|BOLD:ACE7468
Catocala retecta|[1993]|RDLQ176-05|LH 0002|577[0n]bp|Canada.Quebec|BOLD:ACE7470
Catocala judith|[1994]|ABCNA285-06|285-81-020702-TN|585[3n]bp|United States.Tennessee|BOLD:ACE7470
Catocala judith|[1995]|QUNO770-08|5303-070808-WI|658[0n]bp|United States.Wisconsin|BOLD:ACE7470
Catocala judith|[1996]|QUNO769-08|5302-070808-WI|658[0n]bp|United States.Wisconsin|BOLD:ACE7470
Catocala judith|[1997]|QUNO767-08|5300-070808-WI|658[0n]bp|United States.Wisconsin|BOLD:ACE7470
Catocala judith|[1998]|QUNO768-08|5301-070808-WI|658[0n]bp|United States.Wisconsin|BOLD:ACE7470
Catocala judith|[1999]|QUNO764-08|5297-070808-WI|658[0n]bp|United States.Wisconsin|BOLD:ACE7470
Catocala judith|[2000]|QUNO763-08|5296-070808-WI|658[0n]bp|United States.Wisconsin|BOLD:ACE7470
Catocala judith|[2001]|QUNO762-08|5295-070808-WI|658[0n]bp|United States.Wisconsin|BOLD:ACE7470
Catocala judith|[2002]|ABCNA284-06|284-81-020702-TN|598[1n]bp|United States.Tennessee|BOLD:ACE7470
Catocala judith|[2003]|ABCNA645-07|457-8781-020702-TN|564[0n]bp|United States.Tennessee|BOLD:ACE7470
Catocala judith|[2004]|QUNO766-08|5299-070808-WI|658[0n]bp|United States.Wisconsin|BOLD:ACE7470
Catocala judith|[2005]|QUNO765-08|5298-070808-WI|658[0n]bp|United States.Wisconsin|BOLD:ACE7470
Catocala judith|[2006]|QUNO771-08|5304-070808-WI|658[0n]bp|United States.Wisconsin|BOLD:ACE7470
Catocala robinsonii|[2007]|QUNO598-08|5131-220808-IN|658[0n]bp|United States.Indiana|BOLD:ACE7470
Catocala obscura|[2008]|RDLQB668-05|DH010771|608[0n]bp|Canada.Quebec|BOLD:ACE7470
Catocala retecta|[2009]|QUNOD397-10|9111-230707-QU|658[0n]bp|Canada.Quebec|BOLD:ACE7470
Catocala retecta|[2010]|RDLQB467-05|DH010553|658[0n]bp|Canada.Quebec|BOLD:ACE7470
Catocala habilis|[2011]|RDLQB457-05|DH010543|658[0n]bp|Canada.Quebec|BOLD:ACE7470
Catocala habilis|[2012]|RDLQB456-05|DH010542|658[0n]bp|Canada.Quebec|BOLD:ACE7470
Catocala habilis|[2013]|RDLQB671-05|DH010774|658[0n]bp|Canada.Quebec|BOLD:ACE7470
Catocala habilis|[2014]|RDLQB461-05|DH010547|658[0n]bp|Canada.Quebec|BOLD:ACE7470
Catocala habilis|[2015]|RDLQB460-05|DH010546|658[0n]bp|Canada.Quebec|BOLD:ACE7470
Catocala habilis|[2016]|RDLQB670-05|DH010773|586[0n]bp|Canada.Quebec|BOLD:ACE7470
Catocala habilis|[2017]|RDLQB669-05|DH010772|584[0n]bp|Canada.Quebec|BOLD:ACE7470
Catocala habilis|[2018]|RDLQB672-05|DH010775|595[0n]bp|Canada.Quebec|BOLD:ACE7470
Catocala habilis|[2019]|RDLQB462-05|DH010548|592[0n]bp|Canada.Quebec|BOLD:ACE7470
Catocala residua|[2020]|RDLQB455-05|DH010541|578[0n]bp|Canada.Quebec|BOLD:ACE7470
Catocala retecta|[2021]|RDLQB663-05|DH010766|585[0n]bp|Canada.Quebec|BOLD:ACE7470
Catocala flebilis|[2022]|QUNO587-08|5120-220808-IN|658[0n]bp|United States.Indiana|BOLD:ACE7470
Catocala retecta|[2023]|RDLQ111-05|DH003374|658[1n]bp|Canada.Quebec|BOLD:ACE7470
Catocala retecta|[2024]|QUNOD396-10|9110-230807-QU|658[0n]bp|Canada.Quebec|BOLD:ACE7470
Catocala retecta|[2025]|QUNOD362-10|9076-070706-QU|658[0n]bp|Canada.Quebec|BOLD:ACE7470
Catocala retecta|[2026]|RDLQB454-05|DH010540|658[0n]bp|Canada.Quebec|BOLD:ACE7470
Catocala retecta|[2027]|RDLQ114-05|DH003371|658[0n]bp|Canada.Quebec|BOLD:ACE7470
Catocala obscura|[2028]|RDLQB827-05|DH010914|658[0n]bp|Canada.Quebec|BOLD:ACE7470
Catocala obscura|[2029]|RDLQ115-05|DH003379|658[0n]bp|Canada.Quebec|BOLD:ACE7470
Catocala obscura|[2030]|RDLQB667-05|DH010770|658[0n]bp|Canada.Quebec|BOLD:ACE7470
Catocala obscura|[2031]|RDLQB753-05|DH010840|658[0n]bp|Canada.Quebec|BOLD:ACE7470
Catocala flebilis|[2032]|LGSM418-04|DNA-ATBI-0418|658[0n]bp|United States.Tennessee|BOLD:ACE7470
Catocala flebilis|[2033]|QUNOB211-08|4020-COI-08|658[0n]bp|United States.Indiana|BOLD:ACE7470
Catocala flebilis|[2034]|QUNOB210-08|4019-COI-08|658[0n]bp|United States.Indiana|BOLD:ACE7470
Catocala flebilis|[2035]|LGSM419-04|DNA-ATBI-0419|658[0n]bp|United States.Tennessee|BOLD:ACE7470
Catocala flebilis|[2036]|LNCC1278-11|11-NCCC-803|658[0n]bp|United States.North Carolina|BOLD:ACE7470
Catocala flebilis|[2037]|QUNOB212-08|4021-COI-08|658[0n]bp|United States.Indiana|BOLD:ACE7470
Catocala flebilis|[2038]|QUNOB207-08|4016-COI-08|658[0n]bp|United States.Indiana|BOLD:ACE7470
Catocala flebilis|[2039]|QUNOB035-08|5410-220808-IN|658[0n]bp|United States.Indiana|BOLD:ACE7470
Catocala flebilis|[2040]|QUNOB209-08|4018-COI-08|658[0n]bp|United States.Indiana|BOLD:ACE7470
Catocala flebilis|[2041]|LNCC1279-11|11-NCCC-804|658[0n]bp|United States.North Carolina|BOLD:ACE7470
Catocala flebilis|[2042]|QUNO585-08|5118-220808-IN|658[0n]bp|United States.Indiana|BOLD:ACE7470
Catocala flebilis|[2043]|QUNOB208-08|4017-COI-08|658[0n]bp|United States.Indiana|BOLD:ACE7470
Catocala flebilis|[2044]|QUNO586-08|5119-220808-IN|658[0n]bp|United States.Indiana|BOLD:ACE7470
Catocala flebilis|[2045]|LNCC389-10|10-NCCC-484|658[0n]bp|United States.North Carolina|BOLD:ACE7470
Catocala nr. obscura|[2046]|RDLQ116-05|DH003384|524[0n]bp|Canada.Quebec|BOLD:ACE7470
Catocala retecta|[2047]|RDLQB660-05|DH010763|593[0n]bp|Canada.Quebec|BOLD:ACE7470
Catocala retecta|[2048]|RDLQB661-05|DH010764|586[0n]bp|Canada.Quebec|BOLD:ACE7470
Catocala flebilis|[2049]|ABCNA652-07|464-8782-020702-TN|577[0n]bp|United States.Tennessee|BOLD:ACE7470
Catocala residua|[2050]|RDLQB665-05|DH010768|582[0n]bp|Canada.Quebec|BOLD:ACE7470
Catocala residua|[2051]|RDLQB666-05|DH010769|594[0n]bp|Canada.Quebec|BOLD:ACE7470
Catocala obscura|[2052]|RDLQ112-05|DH003375|590[0n]bp|Canada.Quebec|BOLD:ACE7470
Catocala retecta|[2053]|RDLQB659-05|DH010762|588[0n]bp|Canada.Quebec|BOLD:ACE7470
Catocala retecta|[2054]|RDLQB664-05|DH010767|591[0n]bp|Canada.Quebec|BOLD:ACE7470
Catocala retecta|[2055]|RDLQB662-05|DH010765|591[0n]bp|Canada.Quebec|BOLD:ACE7470
Catocala retecta|[2056]|RDLQ179-05|LH 0005|525[0n]bp|Canada.Quebec|BOLD:ACE7470
Catocala robinsonii|[2057]|ABCNA648-07|460-8780-150802-IN|574[0n]bp|United States.Indiana|BOLD:ACE7470
Catocala robinsonii|[2058]|ABCNA012-06|012-80-150802-IN|607[0n]bp|United States.Indiana|BOLD:ACE7470
Catocala robinsonii|[2059]|QUNOB241-08|4050-COI-08|658[0n]bp|United States.Indiana|BOLD:ACE7470
Catocala robinsonii|[2060]|QUNO596-08|5129-260808-IN|658[0n]bp|United States.Indiana|BOLD:ACE7470
Catocala robinsonii|[2061]|QUNO597-08|5130-200808-IN|658[0n]bp|United States.Indiana|BOLD:ACE7470
Catocala robinsonii|[2062]|HKONB476-09|2589-COI-08|658[0n]bp|United States.Louisiana|BOLD:ACE7470
Catocala robinsonii|[2063]|LNCC420-10|10-NCCC-515|658[0n]bp|United States.North Carolina|BOLD:ACE7470
Catocala robinsonii|[2064]|QUNO595-08|5128-260808-IN|658[0n]bp|United States.Indiana|BOLD:ACE7470
Catocala robinsonii|[2065]|QUNO574-08|5107-240808-IN|658[0n]bp|United States.Indiana|BOLD:ACE7470
Catocala robinsonii|[2066]|QUNOB244-08|4053-COI-08|658[0n]bp|United States.Indiana|BOLD:ACE7470
Catocala robinsonii|[2067]|ABCNA011-06|011-80-170999-SC|579[0n]bp|United States.South Carolina|BOLD:A...
Catocala robinsonii|[2068]|QUNOB240-08|4049-COI-08|658[0n]bp|United States.Indiana|BOLD:ACE7470
Catocala robinsonii|[2069]|QUNOB125-08|5500-250808-IN|658[0n]bp|United States.Indiana|BOLD:ACE7470
Catocala robinsonii|[2070]|QUNO599-08|5132-260808-IN|658[0n]bp|United States.Indiana|BOLD:ACE7470
Catocala robinsonii|[2071]|LNCC447-10|10-NCCC-542|658[0n]bp|United States.North Carolina|BOLD:ACE7470
Catocala robinsonii|[2072]|QUNOB239-08|4048-COI-08|658[0n]bp|United States.Indiana|BOLD:ACE7470
Catocala robinsonii|[2073]|LNCC419-10|10-NCCC-514|658[0n]bp|United States.North Carolina|BOLD:ACE7470
Catocala robinsonii|[2074]|QUNOB243-08|4052-COI-08|658[0n]bp|United States.Indiana|BOLD:ACE7470
Catocala robinsonii|[2075]|QUNOB242-08|4051-COI-08|658[0n]bp|United States.Indiana|BOLD:ACE7470
Catocala vidua|[2076]|LNCC1325-11|11-NCCC-850|658[0n]bp|United States.North Carolina|BOLD:ACE7470
Catocala nebulosa|[2077]|ABCNA296-06|296-96-080802-KY|658[0n]bp|United States.Kentucky|BOLD:ACE8372
Catocala nebulosa|[2078]|ABCNA765-07|765-120702-KY|647[0n]bp|United States.Kentucky|BOLD:ACE8372
Catocala nebulosa|[2079]|LNCC1110-11|11-NCCC-635|658[0n]bp|United States.North Carolina|BOLD:ACE8372
Catocala nebulosa|[2080]|ABCNA295-06|295-96-090802-KY|605[1n]bp|United States.Kentucky|BOLD:ACE8372
Catocala nebulosa|[2081]|QUNO050-07|2050-310807-WI|658[0n]bp|United States.Wisconsin|BOLD:ACE8372
Catocala nebulosa|[2082]|QUNOB201-08|4010-COI-08|658[0n]bp|United States.Indiana|BOLD:ACE8372

Catocala nebulosa|[2080]|ABCNA295-06|295-96-090802-KY|605[1n]bp|United States.Kentucky|BOLD:ACE8372
Catocala nebulosa|[2081]|QUNO050-07|2050-310807-WI|658[0n]bp|United States.Wisconsin|BOLD:ACE8372
Catocala nebulosa|[2082]|QUNOB201-08|4010-COI-08|658[0n]bp|United States.Indiana|BOLD:ACE8372
Catocala nebulosa|[2083]|QUNOB202-08|4011-COI-08|658[0n]bp|United States.Indiana|BOLD:ACE8372
Catocala nebulosa|[2084]|QUNO049-07|2049-310807-WI|658[0n]bp|United States.Wisconsin|BOLD:ACE8372
Catocala insolabilis|[2085]|QUNO738-08|5271-070808-WI|658[0n]bp|United States.Wisconsin|BOLD:ACF2562
Catocala insolabilis|[2086]|ABCNA405-07|499-8791-170702-TN|593[0n]bp|United States.Tennessee|BOLD:ACF...
Catocala insolabilis|[2087]|QUNO651-08|5184-230608-MS|658[0n]bp|United States.Mississippi|BOLD:ACF2562
Catocala insolabilis|[2088]|QUNOB025-08|5400-220808-IN|658[0n]bp|United States.Indiana|BOLD:ACF2562
Catocala insolabilis|[2089]|QUNO736-08|5269-070808-WI|658[0n]bp|United States.Wisconsin|BOLD:ACF2562
Catocala insolabilis|[2090]|QUNO740-08|5273-070808-WI|658[0n]bp|United States.Wisconsin|BOLD:ACF2562
Catocala insolabilis|[2091]|LNCC391-10|10-NCCC-486|658[0n]bp|United States.North Carolina|BOLD:ACF2562
Catocala insolabilis|[2092]|ABCNA292-06|292-91-120802-IN|658[0n]bp|United States.Indiana|BOLD:ACF2562
Catocala insolabilis|[2093]|QUNO739-08|5272-070808-WI|658[0n]bp|United States.Wisconsin|BOLD:ACF2562
Catocala insolabilis|[2094]|QUNOB213-08|4022-COI-08|658[0n]bp|United States.Indiana|BOLD:ACF2562
Catocala insolabilis|[2095]|QUNOB214-08|4023-COI-08|658[0n]bp|United States.Indiana|BOLD:ACF2562
Catocala insolabilis|[2096]|QUNO737-08|5270-070808-WI|658[0n]bp|United States.Wisconsin|BOLD:ACF2562
Catocala insolabilis|[2097]|QUNO659-08|5192-020708-KY|658[0n]bp|United States.Kentucky|BOLD:ACF2562
Catocala neogama|[2098]|XAH429-05|2005-ONT-2012|658[0n]bp|Canada.Ontario|BOLD:AAA9558
Catocala neogama|[2099]|PHMO354-03|moth2710.02|639[0n]bp|Canada.Ontario|BOLD:AAA9558
Catocala neogama|[2100]|XAH202-05|2005-ONT-1785|658[0n]bp|Canada.Ontario|BOLD:AAA9558
Catocala neogama|[2101]|XAB447-04|04HBL005447|658[0n]bp|Canada.Ontario|BOLD:AAA9558
Catocala neogama|[2102]|MECD409-06|jflandry2981|658[0n]bp|Canada.Quebec|BOLD:AAA9558
Catocala neogama|[2103]|RDLQ118-05|DH003263|658[0n]bp|Canada.Quebec|BOLD:AAA9558
Catocala neogama|[2104]|RDLQ117-05|DH003261|658[0n]bp|Canada.Quebec|BOLD:AAA9558
Catocala neogama|[2105]|MECD406-06|jflandry2978|658[0n]bp|Canada.Quebec|BOLD:AAA9558
Catocala neogama|[2106]|PHMO359-03|moth2750.02|639[0n]bp|Canada.Ontario|BOLD:AAA9558
Catocala neogama|[2107]|RDLQB469-05|DH010555|540[0n]bp|Canada.Quebec|BOLD:AAA9558
Catocala neogama|[2108]|RDLQB453-05|DH010539|591[0n]bp|Canada.Quebec|BOLD:AAA9558
Catocala neogama|[2109]|MECD407-06|jflandry2979|658[0n]bp|Canada.Quebec|BOLD:AAA9558
Catocala subnata|[2110]|QUNO784-08|5317-070808-WI|658[0n]bp|United States.Wisconsin|BOLD:AAA9558
Catocala subnata|[2111]|QUNOB192-08|4001-COI-08|658[0n]bp|United States.Indiana|BOLD:AAA9558
Catocala subnata|[2112]|ABCNA416-07|510-8797-070802-KY|575[0n]bp|United States.Kentucky|BOLD:AAA9558
Catocala subnata|[2113]|QUNO785-08|5318-070808-WI|658[0n]bp|United States.Wisconsin|BOLD:AAA9558
Catocala subnata|[2114]|LNCC224-10|10-NCCC-414|658[0n]bp|United States.North Carolina|BOLD:AAA9558
Catocala subnata|[2115]|LGSM738-04|DNA-ATBI-0738|658[0n]bp|United States.North Carolina|BOLD:AAA9558
Catocala subnata|[2116]|LGSMG596-07|BGS03959|658[0n]bp|United States.Tennessee|BOLD:AAA9558
Catocala subnata|[2117]|ABCNA298-06|298-97-120702-KY|658[0n]bp|United States.Kentucky|BOLD:AAA9558
Catocala subnata|[2118]|QUNO080-07|2080-230707-WI|658[0n]bp|United States.Wisconsin|BOLD:AAA9558
Catocala subnata|[2119]|QUNO789-08|5322-070808-WI|658[0n]bp|United States.Wisconsin|BOLD:AAA9558
Catocala subnata|[2120]|QUNO788-08|5321-070808-WI|658[0n]bp|United States.Wisconsin|BOLD:AAA9558
Catocala subnata|[2121]|QUNOB191-08|4000-COI-08|658[0n]bp|United States.Indiana|BOLD:AAA9558
Catocala subnata|[2122]|QUNO786-08|5319-070808-WI|658[0n]bp|United States.Wisconsin|BOLD:AAA9558
Catocala subnata|[2123]|QUNO081-07|2081-310807-WI|658[0n]bp|United States.Wisconsin|BOLD:AAA9558
Catocala subnata|[2124]|LGSMG595-07|BGS03958|658[0n]bp|United States.Tennessee|BOLD:AAA9558
Catocala subnata|[2125]|QUNO787-08|5320-070808-WI|658[0n]bp|United States.Wisconsin|BOLD:AAA9558
Catocala vidua|[2126]|QUNOB124-08|5499-250808-IN|658[0n]bp|United States.Indiana|BOLD:ACE7470
Catocala vidua|[2127]|ABCNA145-06|145-92-020906-WI|658[0n]bp|United States.Wisconsin|BOLD:ACE7470
Catocala vidua|[2128]|QUNOB033-08|5408-220808-IN|658[0n]bp|United States.Indiana|BOLD:ACE7470
Catocala vidua|[2129]|QUNO591-08|5124-240808-IN|658[0n]bp|United States.Indiana|BOLD:ACE7470
Catocala vidua|[2130]|LNCC1324-11|11-NCCC-849|658[0n]bp|United States.North Carolina|BOLD:ACE7470
Catocala vidua|[2131]|LGSMG593-07|BGS03956|658[0n]bp|United States.North Carolina|BOLD:ACE7470
Catocala vidua|[2132]|ABCNA407-07|501-8792-150802--IN|570[1n]bp|United States.Indiana|BOLD:ACE7470
Catocala vidua|[2133]|QUNO592-08|5125-290808-IN|658[0n]bp|United States.Indiana|BOLD:ACE7470
Catocala ilia|[2134]|RDNMB026-05|CNCNoctuoidea7866|658[0n]bp|Canada.Ontario|BOLD:AAB0903
Catocala ilia|[2135]|RDLQB485-05|DH010571|658[0n]bp|Canada.Quebec|BOLD:AAB0903
Catocala ilia|[2136]|RDLQB476-05|DH010562|658[0n]bp|Canada.Quebec|BOLD:AAB0903
Catocala ilia|[2137]|RDLQB471-05|DH010557|616[0n]bp|Canada.Quebec|BOLD:AAB0903
Catocala ilia|[2138]|BBLPC575-09|09BBELE-1575|638[0n]bp|Canada.Nova Scotia|BOLD:AAB0903
Catocala umbrosa|[2139]|LPMNB308-09|08BBLEP-05152|658[0n]bp|Canada.Manitoba|BOLD:AAB0903
Catocala illecta|[2140]|QUNO489-08|5022-240508-TX|658[0n]bp|United States.Texas|BOLD:AAE4366
Catocala illecta|[2141]|QUNOD207-10|7305-COI-09|623[0n]bp|United States.Texas|BOLD:AAE4366
Catocala illecta|[2142]|ABCNA465-07|559-8840-150602-MO|621[0n]bp|United States.Missouri|BOLD:AAE4366
Catocala illecta|[2143]|QUNO490-08|5023-250508-TX|658[0n]bp|United States.Texas|BOLD:AAE4366
Catocala illecta|[2144]|LPOKB982-09|MDOK-2024|658[0n]bp|United States.Oklahoma|BOLD:AAE4366
Catocala minuta|[2145]|XAK046-06|2006-ONT-1041|658[0n]bp|Canada.Ontario|BOLD:AAD7106
Catocala gracilis|[2146]|ABCNA221-06|221-47-160504-TX|572[3n]bp|United States.Texas|BOLD:AAA6134
Catocala gracilis|[2147]|QUNOB110-08|5485-290508-LA|658[0n]bp|United States.Louisiana|BOLD:AAA6134
Catocala gracilis|[2148]|QUNO706-08|5239-070708-KY|658[0n]bp|United States.Kentucky|BOLD:AAA6134
Catocala gracilis|[2149]|QUNO704-08|5237-050708-KY|658[0n]bp|United States.Kentucky|BOLD:AAA6134
Catocala gracilis|[2150]|BBLOB1059-11|BIOUG01415-B02|658[0n]bp|United States.Florida|BOLD:AAA6134
Catocala gracilis|[2151]|QUNO698-08|5231-050708-KY|658[1n]bp|United States.Kentucky|BOLD:AAA6134
Catocala gracilis|[2152]|QUNO711-08|5244-060708-KY|658[0n]bp|United States.Kentucky|BOLD:AAA6134
Catocala gracilis|[2153]|LNCC112-10|10-NCCC-207|658[0n]bp|United States.North Carolina|BOLD:AAA6134
Catocala gracilis|[2154]|ABCNA908-08|908-190507-FL|658[0n]bp|United States.Florida|BOLD:AAA6134
Catocala gracilis|[2155]|QUNO314-08|2317-090507-FL|658[0n]bp|United States.Florida|BOLD:AAA6134
Catocala gracilis|[2156]|QUNO313-08|2316-220507-FL|658[0n]bp|United States.Florida|BOLD:AAA6134
Catocala gracilis|[2157]|QUNOB095-08|5470-300508-LA|658[0n]bp|United States.Louisiana|BOLD:AAA6134
Catocala gracilis|[2158]|ABCNA066-06|066-47-160506-TX|600[0n]bp|United States.Texas|BOLD:AAA6134
Catocala gracilis|[2159]|LGSM780-04|DNA-ATBI-0780|658[0n]bp|United States.Tennessee|BOLD:AAA6134
Catocala gracilis|[2160]|QUNO077-07|2077-220507-FL|658[0n]bp|United States.Florida|BOLD:AAA6134
Catocala gracilis|[2161]|LGSM781-04|DNA-ATBI-0781|658[0n]bp|United States.North Carolina|BOLD:AAA6134
Catocala gracilis|[2162]|QUNO710-08|5243-020708-KY|658[0n]bp|United States.Kentucky|BOLD:AAA6134
Catocala gracilis|[2163]|QUNO712-08|5245-030708-KY|658[0n]bp|United States.Kentucky|BOLD:AAA6134
Catocala gracilis|[2164]|QUNOB088-08|5463-190508-TX|658[0n]bp|United States.Texas|BOLD:AAA6134
Catocala gracilis|[2165]|QUNOB046-08|5421-250508-TX|658[0n]bp|United States.Texas|BOLD:AAA6134
Catocala gracilis|[2166]|QUNO544-08|5077-300508-LA|658[0n]bp|United States.Louisiana|BOLD:AAA6134
Catocala gracilis|[2167]|QUNOB100-08|5475-280508-LA|658[0n]bp|United States.Louisiana|BOLD:AAA6134
Catocala gracilis|[2168]|QUNO541-08|5074-290508-LA|658[0n]bp|United States.Louisiana|BOLD:AAA6134
Catocala gracilis|[2169]|QUNO713-08|5246-020708-KY|658[0n]bp|United States.Kentucky|BOLD:AAA6134
Catocala gracilis|[2170]|QUNO705-08|5238-020708-KY|658[0n]bp|United States.Kentucky|BOLD:AAA6134
Catocala gracilis|[2171]|QUNO708-08|5241-020708-KY|658[0n]bp|United States.Kentucky|BOLD:AAA6134
Catocala gracilis|[2172]|QUNO707-08|5240-030708-KY|649[0n]bp|United States.Kentucky|BOLD:AAA6134
Catocala gracilis|[2173]|QUNO709-08|5242-050708-KY|658[0n]bp|United States.Kentucky|BOLD:AAA6134
Catocala gracilis|[2174]|QUNO701-08|5234-040708-KY|658[0n]bp|United States.Kentucky|BOLD:AAA6134
Catocala gracilis|[2175]|QUNO700-08|5233-060708-KY|658[0n]bp|United States.Kentucky|BOLD:AAA6134
Catocala gracilis|[2176]|QUNO702-08|5235-070708-KY|658[0n]bp|United States.Kentucky|BOLD:AAA6134
Catocala gracilis|[2177]|ABCNA065-06|065-47-010506-FL|658[0n]bp|United States.Florida|BOLD:AAA6134
Catocala gracilis|[2178]|LNCC113-10|10-NCCC-208|658[0n]bp|United States.North Carolina|BOLD:AAA6134
Catocala gracilis|[2179]|LNCC946-11|11-NCCC-471|658[0n]bp|United States.North Carolina|BOLD:AAA6134
Catocala gracilis|[2180]|LNCC1281-11|11-NCCC-806|658[0n]bp|United States.North Carolina|BOLD:AAA6134
Catocala sordida|[2181]|RDLQB587-05|DH010690|658[0n]bp|Canada.Quebec|BOLD:AAA6134
[illegible]

Catocala gracilis|[2179]|LNCC946-11|11-NCCC-471|658[0n]bp|United States.North Carolina|BOLD:AAA6134
Catocala gracilis|[2180]|LNCC1281-11|11-NCCC-806|658[0n]bp|United States.North Carolina|BOLD:AAA6134
Catocala sordida|[2181]|RDLQB587-05|DH010690|658[0n]bp|Canada.Quebec|BOLD:AAA6134
Catocala sordida|[2182]|RDLQB577-05|DH010680|539[0n]bp|Canada.Quebec|BOLD:AAA6134
Catocala sordida|[2183]|RDLQF353-06|DH011420|658[0n]bp|Canada.Quebec|BOLD:AAA6134
Catocala sordida|[2184]|RDLQF736-06|DH011886|658[0n]bp|Canada.Quebec|BOLD:AAA6134
Catocala sordida|[2185]|RDLQB584-05|DH010687|658[0n]bp|Canada.Quebec|BOLD:AAA6134
Catocala sordida|[2186]|TTMNB286-06|MNBTT-286|600[1n]bp|Canada.New Brunswick|BOLD:AAA6134
Catocala sordida|[2187]|TTMNB285-06|MNBTT-285|613[0n]bp|Canada.New Brunswick|BOLD:AAA6134
Catocala sordida|[2188]|BBLEC526-09|09BBELE-0526|644[0n]bp|Canada.New Brunswick|BOLD:AAA6134
Catocala sordida|[2189]|BBLEC529-09|09BBELE-0529|648[0n]bp|Canada.New Brunswick|BOLD:AAA6134
Catocala sordida|[2190]|BBLPC034-09|09BBELE-1034|647[0n]bp|Canada.New Brunswick|BOLD:AAA6134
Catocala sordida|[2191]|RDLQB592-05|DH010695|540[0n]bp|Canada.Quebec|BOLD:AAA6134
Catocala sordida|[2192]|TMNBB076-06|MNBTT-1016|610[0n]bp|Canada.New Brunswick|BOLD:AAA6134
Catocala sordida|[2193]|RDLQB594-05|DH010697|658[0n]bp|Canada.Quebec|BOLD:AAA6134
Catocala sordida|[2194]|BBLEC538-09|09BBELE-0538|658[0n]bp|Canada.New Brunswick|BOLD:AAA6134
Catocala sordida|[2195]|RDLQB585-05|DH010688|658[0n]bp|Canada.Quebec|BOLD:AAA6134
Catocala sordida|[2196]|TTMNB287-06|MNBTT-287|658[0n]bp|Canada.New Brunswick|BOLD:AAA6134
Catocala sordida|[2197]|RDLQF355-06|DH011422|658[0n]bp|Canada.Quebec|BOLD:AAA6134
Catocala sordida|[2198]|TTMNB284-06|MNBTT-284|658[0n]bp|Canada.New Brunswick|BOLD:AAA6134
Catocala sordida|[2199]|RDLQB578-05|DH010681|658[0n]bp|Canada.Quebec|BOLD:AAA6134
Catocala sordida|[2200]|RDLQB590-05|DH010693|658[0n]bp|Canada.Quebec|BOLD:AAA6134
Catocala sordida|[2201]|TTMNB282-06|MNBTT-282|658[0n]bp|Canada.New Brunswick|BOLD:AAA6134
Catocala sordida|[2202]|RDLQB586-05|DH010689|658[0n]bp|Canada.Quebec|BOLD:AAA6134
Catocala sordida|[2203]|RDLQB595-05|DH010698|658[0n]bp|Canada.Quebec|BOLD:AAA6134
Catocala sordida|[2204]|RDLQB583-05|DH010686|658[0n]bp|Canada.Quebec|BOLD:AAA6134
Catocala sordida|[2205]|RDLQB582-05|DH010685|658[0n]bp|Canada.Quebec|BOLD:AAA6134
Catocala sordida|[2206]|RDLQB579-05|DH010682|658[0n]bp|Canada.Quebec|BOLD:AAA6134
Catocala sordida|[2207]|RDLQF356-06|DH011423|658[0n]bp|Canada.Quebec|BOLD:AAA6134
Catocala sordida|[2208]|TTMNB283-06|MNBTT-283|658[0n]bp|Canada.New Brunswick|BOLD:AAA6134
Catocala sordida|[2209]|RDLQB580-05|DH010683|658[0n]bp|Canada.Quebec|BOLD:AAA6134
Catocala sordida|[2210]|RDLQB737-05|DH010652|658[0n]bp|Canada.Quebec|BOLD:AAA6134
Catocala sordida|[2211]|RDLQB589-05|DH010692|658[0n]bp|Canada.Quebec|BOLD:AAA6134
Catocala sordida|[2212]|RDLQF354-06|DH011421|658[0n]bp|Canada.Quebec|BOLD:AAA6134
Catocala sordida|[2213]|RDLQB593-05|DH010696|658[0n]bp|Canada.Quebec|BOLD:AAA6134
Catocala sordida|[2214]|RDLQB581-05|DH010684|658[0n]bp|Canada.Quebec|BOLD:AAA6134
Catocala clintonii|[2215]|ABCNA515-07|609-8872-190506-FL|568[2n]bp|United States.Florida|BOLD:AAC4282
Catocala clintonii|[2216]|ABCNA514-07|608-8872-200506-FL|534[0n]bp|United States.Florida|BOLD:AAC4282
Catocala clintonii|[2217]|QUNO727-08|5260-070708-KY|658[0n]bp|United States.Kentucky|BOLD:AAC4282
Catocala clintonii|[2218]|LNCB165-06|06-NCCC-1121|658[0n]bp|United States.North Carolina|BOLD:AAC4282
Catocala clintonii|[2219]|QUNOB561-09|5821-060508-TX|658[0n]bp|United States.Texas|BOLD:AAC4282
Catocala clintonii|[2220]|LNCC947-11|11-NCCC-472|658[0n]bp|United States.North Carolina|BOLD:AAC4282
Catocala clintonii|[2221]|QUNO450-08|3358-COI-08|658[0n]bp|United States.Florida|BOLD:AAC4282
Catocala clintonii|[2222]|QUNO451-08|3359-COI-08|658[0n]bp|United States.Florida|BOLD:AAC4282
Catocala clintonii|[2223]|QUNO447-08|3355-COI-08|658[0n]bp|United States.Florida|BOLD:AAC4282
Catocala clintonii|[2224]|QUNO448-08|3356-COI-08|658[0n]bp|United States.Florida|BOLD:AAC4282
Catocala clintonii|[2225]|ABCNA270-06|270-72-300406-FL|658[0n]bp|United States.Florida|BOLD:AAC4282
Catocala clintonii|[2226]|QUNO449-08|3357-COI-08|658[0n]bp|United States.Florida|BOLD:AAC4282
Catocala clintonii|[2227]|QUNOE398-12|10399-220711-WI|658[0n]bp|United States.Wisconsin|BOLD:ACE7289
Catocala clintonii|[2228]|BBLSX362-09|09BBLEP-02290|658[0n]bp|United States.Oklahoma|BOLD:ACE7289
Catocala clintonii|[2229]|ABCNA516-07|610-8872-180506-FL|568[1n]bp|United States.Florida|BOLD:ACE7289
Catocala innubens|[2230]|XAK294-06|2006-ONT-1289|658[0n]bp|Canada.Ontario|BOLD:AAC3067
Catocala innubens|[2231]|RDLQB473-05|DH010559|658[0n]bp|Canada.Quebec|BOLD:AAC3067
Catocala innubens|[2232]|PHMO320-03|moth2409.02|639[0n]bp|Canada.Ontario|BOLD:AAC3067
Catocala connubialis|[2233]|QUNOB065-08|5440-230508-TX|658[0n]bp|United States.Texas|BOLD:ACE7219
Catocala connubialis|[2234]|LNCC711-11|11-NCCC-236|658[0n]bp|United States.North Carolina|BOLD:ACE7219
Catocala connubialis|[2235]|QUNOB024-08|5399-050308-LA|658[0n]bp|United States.Louisiana|BOLD:ACE7219
Catocala connubialis|[2236]|QUNOB165-08|5540-030507-FL|658[0n]bp|United States.Florida|BOLD:ACE7219
Catocala connubialis|[2237]|QUNOB167-08|5542-080507-FL|658[0n]bp|United States.Florida|BOLD:ACE7219
Catocala connubialis|[2238]|LNCC111-10|10-NCCC-206|658[0n]bp|United States.North Carolina|BOLD:ACE7219
Catocala connubialis|[2239]|ABCNA092-06|092-77-020506-FL|656[0n]bp|United States.Florida|BOLD:ACE7219
Catocala connubialis|[2240]|QUNOB166-08|5541-080507-FL|658[0n]bp|United States.Florida|BOLD:ACE7219
Catocala connubialis|[2241]|QUNO315-08|2318-100507-FL|658[0n]bp|United States.Florida|BOLD:ACE7219
Catocala connubialis|[2242]|ABCNA346-06|346-77-110702-KY|658[0n]bp|United States.Kentucky|BOLD:ACE7219
Catocala connubialis|[2243]|QUNOB164-08|5539-050507-FL|658[0n]bp|United States.Florida|BOLD:ACE7219
Catocala connubialis|[2244]|LNCC052-10|10-NCCC-147|658[0n]bp|United States.North Carolina|BOLD:ACE7219
Catocala connubialis|[2245]|QUNOB163-08|5538-110507-FL|658[0n]bp|United States.Florida|BOLD:ACE7219
Catocala connubialis|[2246]|LNCC110-10|10-NCCC-205|658[0n]bp|United States.North Carolina|BOLD:ACE7219
Catocala micronympha|[2247]|QUNO530-08|5063-160508-TX|658[0n]bp|United States.Texas|BOLD:AAB3488
Catocala micronympha|[2248]|LPOKB993-09|MDOK-2035|658[0n]bp|United States.Oklahoma|BOLD:AAB3488
Catocala micronympha|[2249]|ABCNA091-06|091-76-180505-FL|656[0n]bp|United States.Florida|BOLD:AAB3488
Catocala micronympha|[2250]|BBLOC194-11|BIOUG01454-A09|658[0n]bp|United States.Florida|BOLD:AAB3488
Catocala micronympha|[2251]|ABCNA282-06|282-76-020506-FL|658[0n]bp|United States.Florida|BOLD:AAB3488
Catocala micronympha|[2252]|QUNO381-08|3289-COI-08|658[0n]bp|United States.Florida|BOLD:AAB3488
Catocala micronympha|[2253]|QUNO388-08|3296-COI-08|658[0n]bp|United States.Florida|BOLD:AAB3488
Catocala micronympha|[2254]|LNCC696-11|11-NCCC-221|658[0n]bp|United States.North Carolina|BOLD:AAB3488
Catocala micronympha|[2255]|LOT526-04|04HBL002526|658[0n]bp|United States.Tennessee|BOLD:AAB3488
Catocala micronympha|[2256]|QUNOB007-08|5382-150508-TX|658[0n]bp|United States.Texas|BOLD:AAB3488
Catocala micronympha|[2257]|QUNOB099-08|5474-280508-LA|658[0n]bp|United States.Louisiana|BOLD:AAB3488
Catocala micronympha|[2258]|ABCNA277-06|277-76-030506-FL|658[0n]bp|United States.Florida|BOLD:AAB3488
Catocala micronympha|[2259]|LOT249-04|04HBL002249|609[0n]bp|United States.Tennessee|BOLD:AAB3488
Catocala micronympha|[2260]|JRLAA025-09|JRLAA-025|632[0n]bp|United States.Alabama|BOLD:AAB3488
Catocala micronympha|[2261]|LSEU597-06|06-JKA-0597|658[0n]bp|United States.Georgia|BOLD:AAB3488
Catocala micronympha|[2262]|QUNOB009-08|5384-170508-TX|658[0n]bp|United States.Texas|BOLD:AAB3488
Catocala micronympha|[2263]|QUNO735-08|5268-050708-KY|658[0n]bp|United States.Kentucky|BOLD:AAB3488
Catocala micronympha|[2264]|ABCNA278-06|278-76-300406-FL|658[0n]bp|United States.Florida|BOLD:AAB3488
Catocala micronympha|[2265]|QUNO681-08|5214-070708-KY|658[0n]bp|United States.Kentucky|BOLD:AAB3488
Catocala micronympha|[2266]|LSEU596-06|06-JKA-0596|658[0n]bp|United States.Georgia|BOLD:AAB3488
Catocala micronympha|[2267]|QUNO682-08|5215-010708-KY|658[0n]bp|United States.Kentucky|BOLD:AAB3488
Catocala micronympha|[2268]|ABCNA529-07|623-8876-170506-FL|560[0n]bp|United States.Florida|BOLD:AAB3488
Catocala micronympha|[2269]|LPOKB969-09|MDOK-2011|658[0n]bp|United States.Oklahoma|BOLD:AAB3488
Catocala micronympha|[2270]|ABCNA281-06|281-76-040506-FL|658[0n]bp|United States.Florida|BOLD:AAB3488
Catocala micronympha|[2271]|BBLOB1541-11|BIOUG01420-B09|658[0n]bp|United States.Florida|BOLD:AAB3488
Catocala micronympha|[2272]|ABCNA578-07|390-COI-06|573[0n]bp|United States.Florida|BOLD:AAB3488
Catocala micronympha|[2273]|ABCNA587-07|399-COI-06|564[0n]bp|United States.Florida|BOLD:AAB3488
Catocala micronympha|[2274]|QUNO457-08|3365-COI-08|658[0n]bp|United States.Florida|BOLD:AAB3488
Catocala micronympha|[2275]|ABCNA276-06|276-76-030506-FL|658[0n]bp|United States.Florida|BOLD:AAB3488
Catocala similis|[2276]|QUNOB015-08|5390-140508-TX|658[0n]bp|United States.Texas|BOLD:ABY7551
Catocala similis|[2277]|LNCB441-07|07-NCNW-0125|658[0n]bp|United States.North Carolina|BOLD:ABY7551
Catocala similis|[2278]|LNCB440-07|07-NCNW-0124|658[0n]bp|United States.North Carolina|BOLD:ABY7551
Catocala similis|[2279]|LNCB439-07|07-NCNW-0123|658[0n]bp|United States.North Carolina|BOLD:ABY7551
Catocala similis|[2280]|QUNOB013-08|5388-140508-TX|658[0n]bp|United States.Texas|BOLD:ABY7551
Catocala similis|[2281]|[illegible]

Catocala similis|[2278]|LNCB440-07|07-NCNW-0124|658[0n]bp|United States.North Carolina|BOLD:ABY7551
Catocala similis|[2279]|LNCB439-07|07-NCNW-0123|658[0n]bp|United States.North Carolina|BOLD:ABY7551
Catocala similis|[2280]|QUNOB013-08|5388-140508-TX|658[0n]bp|United States.Texas|BOLD:ABY7551
Catocala similis|[2281]|QUNOB005-08|5380-150508-TX|658[0n]bp|United States.Texas|BOLD:ABY7551
Catocala similis|[2282]|QUNOB014-08|5389-140508-TX|658[0n]bp|United States.Texas|BOLD:ABY7551
Catocala similis|[2283]|ABCNA524-07|618-8873-200506-FL|568[0n]bp|United States.Florida|BOLD:ABY7551
Catocala similis|[2284]|ABCNA525-07|619-8873-210506-FL|568[0n]bp|United States.Florida|BOLD:ABY7551
Catocala similis|[2285]|QUNOB006-08|5381-150508-TX|658[0n]bp|United States.Texas|BOLD:ABY7551
Catocala similis|[2286]|LOFLA236-06|06-FLOR-0236|658[0n]bp|United States.Florida|BOLD:ABY7551
Catocala similis|[2287]|ABCNA265-06|265-73-010506-FL|658[0n]bp|United States.Florida|BOLD:ABY7551
Catocala similis|[2288]|QUNO453-08|3361-COI-08|658[0n]bp|United States.Florida|BOLD:ABY7551
Catocala similis|[2289]|QUNO454-08|3362-COI-08|658[0n]bp|United States.Florida|BOLD:ABY7551
Catocala similis|[2290]|QUNO456-08|3364-COI-08|658[0n]bp|United States.Florida|BOLD:ABY7551
Catocala similis|[2291]|BBLOB1672-11|BIOUG01421-E09|658[0n]bp|United States.Florida|BOLD:ABY7551
Catocala similis|[2292]|QUNO323-08|2326-150507-FL|658[0n]bp|United States.Florida|BOLD:ABY7551
Catocala similis|[2293]|QUNO455-08|3363-COI-08|658[0n]bp|United States.Florida|BOLD:ABY7551
Catocala similis|[2294]|QUNO452-08|3360-COI-08|658[0n]bp|United States.Florida|BOLD:ABY7551
Catocala similis|[2295]|LOFLA533-06|06-FLOR-0533|658[0n]bp|United States.Florida|BOLD:ABY7551
Catocala similis|[2296]|LSEU032-06|06-JKA-0032|537[1n]bp|United States.Florida|BOLD:ABY7551
Catocala similis|[2297]|LSEU031-06|06-JKA-0031|576[2n]bp|United States.Florida|BOLD:ABY7551
Catocala similis|[2298]|ABCNA266-06|266-73-050506-FL|658[0n]bp|United States.Florida|BOLD:ABY7551
Catocala aholibah|[2299]|ABCNA300-06|300-00-110703-UT|658[0n]bp|United States.Utah|BOLD:AAE9541
Catocala aholibah|[2300]|ABCNA302-06|302-00-110703-UT|596[0n]bp|United States.Utah|BOLD:AAE9541
Catocala aholibah|[2301]|ABCNA301-06|301-00-110703-UT|658[0n]bp|United States.Utah|BOLD:AAE9541
Catocala aholibah|[2302]|RDNMJ287-11|CNCLEP 80195|658[0n]bp|United States.Arizona|BOLD:AAE9541
Catocala aholibah|[2303]|LTOLB1333-11|RR-99-1308|658[0n]bp|United States.California|BOLD:AAE9541
Catocala aholibah|[2304]|GMLC1053-12|2011GM-0749|658[0n]bp|United States.California|BOLD:AAE9541
Catocala aholibah|[2305]|ABCNA303-06|303-00-250705-CA|658[0n]bp|United States.California|BOLD:AAE9541
Catocala aholibah|[2306]|QUNOE163-11|1160-250811-CA|658[0n]bp|United States.California|BOLD:AAE9541
Catocala coccinata|[2307]|LPMNB313-09|08BBLEP-05157|603[0n]bp|Canada.Manitoba|BOLD:ABZ3835
Catocala coccinata|[2308]|LPMNB309-09|08BBLEP-05153|658[0n]bp|Canada.Manitoba|BOLD:ABZ3835
Catocala coccinata|[2309]|LPMNB501-09|08BBLEP-05539|658[0n]bp|Canada.Manitoba|BOLD:ABZ3835
Catocala coccinata|[2310]|LPMNB311-09|08BBLEP-05155|658[0n]bp|Canada.Manitoba|BOLD:ABZ3835
Catocala coccinata|[2311]|LPMNB310-09|08BBLEP-05154|658[0n]bp|Canada.Manitoba|BOLD:ABZ3835
Catocala coccinata|[2312]|XAJ960-06|2006-ONT-0960|658[0n]bp|Canada.Ontario|BOLD:ABZ3835
Catocala coccinata|[2313]|LPMNB303-09|08BBLEP-05147|658[0n]bp|Canada.Manitoba|BOLD:ABZ3835
Catocala coccinata|[2314]|XAJ961-06|2006-ONT-0961|658[0n]bp|Canada.Ontario|BOLD:ABZ3835
Catocala coccinata|[2315]|LPMNB304-09|08BBLEP-05148|658[0n]bp|Canada.Manitoba|BOLD:ABZ3835
Catocala coccinata|[2316]|LPMNB302-09|08BBLEP-05146|658[0n]bp|Canada.Manitoba|BOLD:ABZ3835
Catocala coccinata|[2317]|LPMNB305-09|08BBLEP-05149|658[0n]bp|Canada.Manitoba|BOLD:ABZ3835
Catocala coccinata|[2318]|XAJ962-06|2006-ONT-0962|658[0n]bp|Canada.Ontario|BOLD:ABZ3835
Catocala amatrix|[2319]|RDLQB466-05|DH010552|658[0n]bp|Canada.Quebec|BOLD:AAB8332
Catocala amatrix|[2320]|XAB645-04|04HBL005645|658[0n]bp|Canada.Ontario|BOLD:AAB8332
Catocala cara|[2321]|JSAUG1674-11|BIOUG01497-F02|658[0n]bp|Canada.Ontario|BOLD:ABZ7619
Catocala cara|[2322]|XAH084-05|2005-ONT-1667|621[0n]bp|Canada.Ontario|BOLD:ABZ7619
Catocala cara|[2323]|PHMO353-03|moth2701.02|639[5n]bp|Canada.Ontario|BOLD:ABZ7619
Catocala cara|[2324]|XAB465-04|04HBL005465|658[0n]bp|Canada.Ontario|BOLD:ABZ7619
Catocala concumbens|[2325]|BBLEC457-09|09BBELE-0457|658[0n]bp|Canada.New Brunswick|BOLD:AAB6567
Catocala concumbens|[2326]|RDLQB491-05|DH010577|658[0n]bp|Canada.Quebec|BOLD:AAB6567
Catocala concumbens|[2327]|RDLQB472-05|DH010558|658[0n]bp|Canada.Quebec|BOLD:AAB6567
Catocala concumbens|[2328]|RDLQF804-06|DH011954|657[0n]bp|Canada.Quebec|BOLD:AAB6567
Catocala concumbens|[2329]|XAH053-05|2005-ONT-1636|658[0n]bp|Canada.Ontario|BOLD:AAB6567
Catocala concumbens|[2330]|PHMNB274-04|04HBL007739|658[0n]bp|Canada.New Brunswick|BOLD:AAB6567
Catocala concumbens|[2331]|RDLQB565-05|DH010668|535[0n]bp|Canada.Quebec|BOLD:AAB6567
Catocala concumbens|[2332]|PHMNB273-04|04HBL007738|658[0n]bp|Canada.New Brunswick|BOLD:AAB6567
Catocala concumbens|[2333]|PHMO313-03|moth2356.02|639[0n]bp|Canada.Ontario|BOLD:AAB6567
Catocala concumbens|[2334]|PHMNB275-04|04HBL007740|658[0n]bp|Canada.New Brunswick|BOLD:AAB6567
Catocala crataegi|[2335]|RDLQB732-05|DH010835|658[0n]bp|Canada.Quebec|BOLD:AAB2593
Catocala crataegi|[2336]|XAK425-06|2006-ONT-1420|658[0n]bp|Canada.Ontario|BOLD:AAB2593
Catocala crataegi|[2337]|MNBB574-05|05-NBSTA-490|658[0n]bp|Canada.New Brunswick|BOLD:AAB2593
Catocala crataegi|[2338]|RDLQG021-06|DH012152|658[0n]bp|Canada.Quebec|BOLD:AAB2593
Catocala crataegi|[2339]|RDLQF681-06|DH011831|637[0n]bp|Canada.Quebec|BOLD:AAB2593
Catocala crataegi|[2340]|RDLQF680-06|DH011830|637[0n]bp|Canada.Quebec|BOLD:AAB2593
Catocala crataegi|[2341]|RDLQF682-06|DH011832|637[0n]bp|Canada.Quebec|BOLD:AAB2593
Catocala crataegi|[2342]|RDLQB416-05|DH010502|658[0n]bp|Canada.Quebec|BOLD:AAB2593
Catocala crataegi|[2343]|XAK456-06|2006-ONT-1451|658[0n]bp|Canada.Ontario|BOLD:AAB2593
Catocala crataegi|[2344]|XAK516-07|HLC-16069|658[0n]bp|Canada.Ontario|BOLD:AAB2593
Catocala crataegi|[2345]|XAE622-04|Moth4622.03|658[0n]bp|Canada.Ontario|BOLD:AAB2593
Catocala crataegi|[2346]|XAK045-06|2006-ONT-1040|658[0n]bp|Canada.Ontario|BOLD:AAB2593
Catocala crataegi|[2347]|XAC796-04|04HBL006796|658[0n]bp|Canada.Ontario|BOLD:AAB2593
Catocala crataegi|[2348]|PHSEP338-11|BIOUG01292-A09|657[0n]bp|Canada.Ontario|BOLD:AAB2593
Catocala crataegi|[2349]|XAJ977-06|2006-ONT-0977|658[0n]bp|Canada.Ontario|BOLD:AAB2593
Catocala crataegi|[2350]|PMG098-03|moth1146.01|617[0n]bp|Canada.Ontario|BOLD:AAB2593
Catocala crataegi|[2351]|BLTIB1056-08|BL1064|658[0n]bp|Canada.Ontario|BOLD:AAB2593
Catocala crataegi|[2352]|XAK044-06|2006-ONT-1039|658[0n]bp|Canada.Ontario|BOLD:AAB2593
Catocala crataegi|[2353]|MNBB001-05|HBL008611|658[0n]bp|Canada.New Brunswick|BOLD:AAB2593
Catocala blandula|[2354]|XAK521-07|HLC-16074|658[0n]bp|Canada.Ontario|BOLD:ABZ4961
Catocala blandula|[2355]|RDLQF679-06|DH011829|637[0n]bp|Canada.Quebec|BOLD:ABZ4961
Catocala blandula|[2356]|XAK431-06|2006-ONT-1426|657[0n]bp|Canada.Ontario|BOLD:ABZ4961
Catocala blandula|[2357]|BBLPC522-09|09BBELE-1522|658[0n]bp|Canada.New Brunswick|BOLD:ABZ4961
Catocala blandula|[2358]|RDLQF737-06|DH011887|658[0n]bp|Canada.Quebec|BOLD:ABZ4961
Catocala blandula|[2359]|BBLPC078-09|09BBELE-1078|658[0n]bp|Canada.New Brunswick|BOLD:ABZ4961
Catocala blandula|[2360]|RDLQF806-06|DH011956|592[0n]bp|Canada.Quebec|BOLD:ABZ4961
Catocala blandula|[2361]|RDLQF805-06|DH011955|658[0n]bp|Canada.Quebec|BOLD:ABZ4961
Catocala blandula manitoba|[2362]|RDMAB168-05|UASM41351|658[0n]bp|Canada.Alberta|BOLD:ABZ4961
Catocala blandula manitoba|[2363]|RDMAB185-05|UASM24720|565[0n]bp|Canada.Alberta|BOLD:ABZ4961
Catocala blandula manitoba|[2364]|RDMAB186-05|UASM2301|658[0n]bp|Canada.Alberta|BOLD:ABZ4961
Catocala blandula manitoba|[2365]|RDMAB184-05|UASM24719|559[1n]bp|Canada.Alberta|BOLD:ABZ4961
Catocala grynea|[2366]|XAK522-07|HLC-16075|659[0n]bp|Canada.Ontario|BOLD:AAA9713
Catocala grynea|[2367]|XAG861-05|2005-ONT-1445|658[0n]bp|Canada.Ontario|BOLD:AAA9713
Catocala grynea|[2368]|MNBB068-05|HBL008678|658[0n]bp|Canada.New Brunswick|BOLD:AAA9713
Catocala grynea|[2369]|XAK264-06|2006-ONT-1259|658[0n]bp|Canada.Ontario|BOLD:AAA9713
Catocala grynea|[2370]|XAK520-07|HLC-16073|658[0n]bp|Canada.Ontario|BOLD:AAA9713
Catocala grynea|[2371]|PHMO286-03|moth1883.02|639[0n]bp|Canada.Ontario|BOLD:AAA9713
Catocala grynea|[2372]|XAK565-07|HLC-16118|581[1n]bp|Canada.Ontario|BOLD:AAA9713
Catocala grynea|[2373]|PHMO316-03|moth2384.02|639[0n]bp|Canada.Ontario|BOLD:AAA9713
Catocala grynea|[2374]|XAD348-04|04HBL007348|565[0n]bp|Canada.Ontario|BOLD:AAA9713
Catocala grynea|[2375]|XAC857-04|04HBL006857|591[0n]bp|Canada.Ontario|BOLD:AAA9713
Catocala grynea|[2376]|XAC859-04|04HBL006859|599[1n]bp|Canada.Ontario|BOLD:AAA9713
Catocala grynea|[2377]|XAK290-06|2006-ONT-1285|658[0n]bp|Canada.Ontario|BOLD:AAA9713
Catocala grynea|[2378]|XAG007-05|2005-ONT-591|658[0n]bp|Canada.Ontario|BOLD:AAA9713
Catocala grynea|[2379]|XAK430-06|2006-ONT-1425|658[0n]bp|Canada.Ontario|BOLD:AAA9713
Catocala mira|[2380]|XAD392-04|04HBL007392|658[0n]bp|Canada.Ontario|BOLD:AAA7679

Catocala grynea|[2377]|XAK250-06|2006-ONT-1285|658[0n]bp|Canada.Ontario|BOLD:AAA9713
Catocala grynea|[2378]|XAG007-05|2005-ONT-591|658[0n]bp|Canada.Ontario|BOLD:AAA9713
Catocala grynea|[2379]|XAK430-06|2006-ONT-1425|658[0n]bp|Canada.Ontario|BOLD:AAA9713
Catocala mira|[2380]|XAD392-04|04HBL007392|658[0n]bp|Canada.Ontario|BOLD:AAA7679
Catocala mira|[2381]|XAK288-06|2006-ONT-1283|658[0n]bp|Canada.Ontario|BOLD:AAA7679
Catocala mira|[2382]|XAK289-06|2006-ONT-1284|658[0n]bp|Canada.Ontario|BOLD:AAA7679
Catocala mira|[2383]|XAK211-06|2006-ONT-1206|658[0n]bp|Canada.Ontario|BOLD:AAA7679
Catocala mira|[2384]|XAK432-06|2006-ONT-1427|658[0n]bp|Canada.Ontario|BOLD:AAA7679
Catocala mira|[2385]|XAK515-07|HLC-16068|658[0n]bp|Canada.Ontario|BOLD:AAA7679
Catocala mira|[2386]|XAD090-04|04HBL007090|571[0n]bp|Canada.Ontario|BOLD:AAA7679
Catocala mira|[2387]|XAD345-04|04HBL007345|567[0n]bp|Canada.Ontario|BOLD:AAA7679
Catocala mira|[2388]|XAJ949-06|2006-ONT-0949|658[0n]bp|Canada.Ontario|BOLD:AAA7679
Catocala mira|[2389]|XAG800-05|2005-ONT-1384|658[0n]bp|Canada.Ontario|BOLD:AAA7679
Catocala mira|[2390]|XAB679-04|04HBL005679|658[0n]bp|Canada.Ontario|BOLD:AAA7679
Catocala mira|[2391]|XAG544-05|2005-ONT-1128|658[0n]bp|Canada.Ontario|BOLD:AAA7679
Catocala mira|[2392]|XAK519-07|HLC-16072|658[0n]bp|Canada.Ontario|BOLD:AAA7679
Catocala praeclara|[2393]|RDLQF351-06|DH011418|658[0n]bp|Canada.Quebec|BOLD:ABX6316
Catocala praeclara|[2394]|RDLQF350-06|DH011417|658[0n]bp|Canada.Quebec|BOLD:ABX6316
Catocala praeclara|[2395]|RDLQF349-06|DH011416|658[0n]bp|Canada.Quebec|BOLD:ABX6316
Catocala praeclara|[2396]|RDLQF683-06|DH011833|619[0n]bp|Canada.Quebec|BOLD:ABX6316
Catocala praeclara|[2397]|RDLQF352-06|DH011419|658[0n]bp|Canada.Quebec|BOLD:ABX6316
Catocala praeclara|[2398]|BBLEC535-09|09BBELE-0535|658[0n]bp|Canada.New Brunswick|BOLD:ABX6316
Catocala praeclara|[2399]|QUNOE282-12|10281-000000-QU|658[0n]bp|Canada.Quebec|BOLD:ABX6316
Catocala praeclara|[2400]|QUNOB321-09|5601-280808-SK|658[0n]bp|Canada.Saskatchewan|BOLD:ACE4888
Catocala praeclara|[2401]|QUNOB320-09|5600-160808-SK|658[0n]bp|Canada.Saskatchewan|BOLD:ACE4888
Catocala praeclara|[2402]|RDMAB187-05|UASM19705|658[0n]bp|Canada.Alberta|BOLD:ACE4888
Catocala praeclara|[2403]|QUNOB319-09|5599-160808-SK|658[0n]bp|Canada.Saskatchewan|BOLD:ACE4888
Catocala sp.|[2404]|PMG099-03|CATO1.00|617[0n]bp|Canada.Ontario|BOLD:ACE4888
Catocala ultronia|[2405]|PHMO282-03|moth1845.02|639[0n]bp|Canada.Ontario|BOLD:AAA8724
Catocala ultronia|[2406]|XAG093-05|2005-ONT-677|658[0n]bp|Canada.Ontario|BOLD:AAA8724
Catocala ultronia|[2407]|QUNOB338-09|5618-280805-SK|658[0n]bp|Canada.Saskatchewan|BOLD:AAA8724
Catocala ultronia|[2408]|RDLQF803-06|DH011953|658[0n]bp|Canada.Quebec|BOLD:AAA8724
Catocala ultronia|[2409]|XAG174-05|2005-ONT-758|617[1n]bp|Canada.Ontario|BOLD:AAA8724
Catocala ultronia|[2410]|PHMO300-03|moth2200.02|639[0n]bp|Canada.Ontario|BOLD:AAA8724
Catocala ultronia|[2411]|XAD574-04|04HBL006989|602[0n]bp|Canada.Ontario|BOLD:AAA8724
Catocala ultronia|[2412]|XAG009-05|2005-ONT-593|629[0n]bp|Canada.Ontario|BOLD:AAA8724
Catocala ultronia|[2413]|PHMO285-03|moth1876.02|639[0n]bp|Canada.Ontario|BOLD:AAA8724
Catocala ultronia|[2414]|XAK513-07|HLC-16066|658[0n]bp|Canada.Ontario|BOLD:AAA8724
Catocala ultronia|[2415]|RDLQB474-05|DH010560|658[0n]bp|Canada.Quebec|BOLD:AAA8724
Catocala ultronia|[2416]|RDLQB475-05|DH010561|658[0n]bp|Canada.Quebec|BOLD:AAA8724
Catocala ultronia|[2417]|RDLQF738-06|DH011888|658[0n]bp|Canada.Quebec|BOLD:AAA8724
Catocala ultronia|[2418]|RDLQB567-05|DH010670|593[0n]bp|Canada.Quebec|BOLD:AAA8724
Catocala ultronia|[2419]|XAD436-04|04HBL007436|580[0n]bp|Canada.Ontario|BOLD:AAA8724
Catocala ultronia|[2420]|XAK512-07|HLC-16065|658[0n]bp|Canada.Ontario|BOLD:AAA8724
Catocala ultronia|[2421]|RDLQF802-06|DH011952|658[0n]bp|Canada.Quebec|BOLD:AAA8724
Catocala relicta|[2422]|RDBBC377-05|CSG22840 C BC|594[12n]bp|Canada.British Columbia|
Catocala relicta|[2423]|XAD179-04|04HBL007179|564[0n]bp|Canada.Ontario|BOLD:AAB3451
Catocala relicta|[2424]|RDLQB451-05|DH010537|591[0n]bp|Canada.Quebec|BOLD:AAB3451
Catocala relicta|[2425]|XAH235-05|2005-ONT-1818|623[0n]bp|Canada.Ontario|BOLD:AAB3451
Catocala relicta|[2426]|PHMO333-03|moth2589.02|639[0n]bp|Canada.Ontario|BOLD:AAB3451
Catocala relicta|[2427]|XAG757-05|2005-ONT-1341|658[1n]bp|Canada.Ontario|BOLD:AAB3451
Catocala relicta|[2428]|XAH341-05|2005-ONT-1924|658[0n]bp|Canada.Ontario|BOLD:AAB3451
Catocala relicta|[2429]|RDLQB452-05|DH010538|658[0n]bp|Canada.Quebec|BOLD:AAB3451
Catocala relicta|[2430]|BBLPA326-10|10BBCLP-0326|658[0n]bp|Canada.Saskatchewan|BOLD:AAB3451
Catocala relicta|[2431]|LPMNB500-09|08BBLEP-05538|658[0n]bp|Canada.Manitoba|BOLD:AAB3451
Catocala relicta|[2432]|LPMNB306-09|08BBLEP-05150|658[0n]bp|Canada.Manitoba|BOLD:AAB3451
Catocala relicta|[2433]|LPMNB307-09|08BBLEP-05151|658[0n]bp|Canada.Manitoba|BOLD:AAB3451
Catocala relicta|[2434]|XAB652-04|04HBL005652|658[0n]bp|Canada.Ontario|BOLD:AAB3451
Catocala relicta|[2435]|XAH146-05|2005-ONT-1729|658[0n]bp|Canada.Ontario|BOLD:AAB3451
Catocala relicta|[2436]|BBLPA325-10|10BBCLP-0325|658[0n]bp|Canada.Saskatchewan|BOLD:AAB3451
Catocala relicta|[2437]|XAH331-05|2005-ONT-1914|658[0n]bp|Canada.Ontario|BOLD:AAB3451
Catocala relicta|[2438]|XAH236-05|2005-ONT-1819|658[0n]bp|Canada.Ontario|BOLD:AAB3451
Catocala relicta|[2439]|XAG659-05|2005-ONT-1243|650[0n]bp|Canada.Ontario|BOLD:AAB3451
Catocala cerogama|[2440]|RDLQB479-05|DH010565|658[0n]bp|Canada.Quebec|BOLD:AAB3383
Catocala cerogama|[2441]|RDLQB480-05|DH010566|658[0n]bp|Canada.Quebec|BOLD:AAB3383
Catocala cerogama|[2442]|RDLQB478-05|DH010564|658[0n]bp|Canada.Quebec|BOLD:AAB3383
Catocala cerogama|[2443]|XAG310-05|2005-ONT-894|658[0n]bp|Canada.Ontario|BOLD:AAB3383
Catocala cerogama|[2444]|XAH174-05|2005-ONT-1757|658[0n]bp|Canada.Ontario|BOLD:AAB3383
Catocala cerogama|[2445]|XAG871-05|2005-ONT-1455|658[0n]bp|Canada.Ontario|BOLD:AAB3383
Catocala cerogama|[2446]|XAG661-05|2005-ONT-1245|658[0n]bp|Canada.Ontario|BOLD:AAB3383
Catocala cerogama|[2447]|RDLQB486-05|DH010572|658[0n]bp|Canada.Quebec|BOLD:AAB3383
Catocala cerogama|[2448]|RDLQB570-05|DH010673|571[0n]bp|Canada.Quebec|BOLD:AAB3383
Catocala cerogama|[2449]|XAK249-06|2006-ONT-1244|643[0n]bp|Canada.Ontario|BOLD:AAB3383
Catocala cerogama|[2450]|XAG755-05|2005-ONT-1339|636[0n]bp|Canada.Ontario|BOLD:AAB3383
Catocala cerogama|[2451]|RDLQB477-05|DH010563|658[0n]bp|Canada.Quebec|BOLD:AAB3383
Catocala cerogama|[2452]|LPSOD1070-09|08MZPP-181|631[0n]bp|Canada.Ontario|BOLD:AAB3383
Catocala cerogama|[2453]|XAK270-06|2006-ONT-1265|658[0n]bp|Canada.Ontario|BOLD:AAB3383
Catocala cerogama|[2454]|XAG422-05|2005-ONT-1006|658[0n]bp|Canada.Ontario|BOLD:AAB3383
Catocala cerogama|[2455]|RDLQB481-05|DH010567|658[0n]bp|Canada.Quebec|BOLD:AAB3383
Catocala cerogama|[2456]|XAH293-05|2005-ONT-1876|658[0n]bp|Canada.Ontario|BOLD:AAB3383
Catocala faustina|[2457]|QUNO134-08|2135-190901-WA|658[0n]bp|United States.Oregon|BOLD:AAB0100
Catocala faustina|[2458]|QUNOE222-12|10220-130897-UT|658[0n]bp|United States.Utah|BOLD:AAB0100
Catocala faustina|[2459]|ABCNA019-06|019-11-180703-UT|589[0n]bp|United States.Utah|BOLD:AAB0100
Catocala faustina|[2460]|QUNOE170-11|1167-240808-CA|615[0n]bp|United States.California|BOLD:AAB0100
Catocala faustina|[2461]|QUNOE277-12|10276-230897-WA|658[0n]bp|United States.Washington|BOLD:AAB0100
Catocala faustina|[2462]|QUNOE508-12|10532-230706-CA|658[0n]bp|United States.California|BOLD:AAB0100
Catocala faustina|[2463]|QUNOE220-12|10218-040911-CA|658[0n]bp|United States.California|BOLD:AAB0100
Catocala faustina|[2464]|QUNOE223-12|10221-130897-UT|658[0n]bp|United States.Utah|BOLD:AAB0100
Catocala faustina|[2465]|QUNOE224-12|10222-130897-UT|658[0n]bp|United States.Utah|BOLD:AAB0100
Catocala junctura|[2466]|RDMAB178-05|UASM24314|658[0n]bp|Canada.Alberta|BOLD:AAB0100
Catocala junctura|[2467]|QUNOB334-09|5614-200808-SK|658[0n]bp|Canada.Saskatchewan|BOLD:AAB0100
Catocala meskei|[2468]|RDMAB171-05|UASM24343|658[0n]bp|Canada.Alberta|BOLD:AAB0100
Catocala meskei|[2469]|RDMAB159-05|UASM19695|531[0n]bp|Canada.Alberta|BOLD:AAB0100
Catocala meskei|[2470]|RDMAB158-05|UASM19825|658[0n]bp|Canada.Alberta|BOLD:AAB0100
Catocala meskei|[2471]|QUNOB329-09|5609-160808-SK|658[0n]bp|Canada.Saskatchewan|BOLD:AAB0100
Catocala meskei|[2472]|QUNOB327-09|5607-160808-SK|658[0n]bp|Canada.Saskatchewan|BOLD:AAB0100
Catocala meskei|[2473]|QUNOC077-09|5908-270703-CA|658[0n]bp|Canada.Alberta|BOLD:AAB0100
Catocala meskei|[2474]|QUNOC074-09|5905-270703-CA|641[0n]bp|Canada.Alberta|BOLD:AAB0100
Catocala meskei|[2475]|RDMAB155-05|UASM19693|605[2n]bp|Canada.Alberta|BOLD:AAB0100
Catocala parta|[2476]|RDMAB172-05|UASM24689|658[0n]bp|Canada.Alberta|BOLD:AAB0100
Catocala meskei|[2477]|RDMAB160-05|UASM24325|658[0n]bp|Canada.Alberta|BOLD:AAB0100
Catocala meskei|[2478]|RDMAB163-05|UASM24690|658[0n]bp|Canada.Alberta|BOLD:AAB0100
Catocala meskei|[2479]|RDMAB542-06|UASM58527|621[0n]bp|Canada.Alberta|BOLD:AAB0100

[illegible]
Catocala meskei|[2477]|RDMAB160-05|UASM24325|658[0n]bp|Canada.Alberta|BOLD:AAB0100
Catocala meskei|[2478]|RDMAB163-05|UASM24690|658[0n]bp|Canada.Alberta|BOLD:AAB0100
Catocala meskei|[2479]|RDMAB542-06|UASM58527|621[0n]bp|Canada.Alberta|BOLD:AAB0100
Catocala meskei|[2480]|RDMAB162-05|UASM24326|658[0n]bp|Canada.Alberta|BOLD:AAB0100
Catocala luciana|[2481]|QUNOB324-09|5604-250808-SK|658[0n]bp|Canada.Saskatchewan|BOLD:AAB0100
Catocala luciana|[2482]|QUNOB325-09|5605-250808-SK|658[0n]bp|Canada.Saskatchewan|BOLD:AAB0100
Catocala luciana|[2483]|RDMAB176-05|UASM24315|658[0n]bp|Canada.Alberta|BOLD:AAB0100
Catocala meskei|[2484]|RDMAB161-05|UASM24714|658[0n]bp|Canada.Alberta|BOLD:AAB0100
Catocala meskei|[2485]|RDMAB156-05|UASM24317|658[0n]bp|Canada.Alberta|BOLD:AAB0100
Catocala parta|[2486]|QUNOB333-09|5613-150808-SK|658[0n]bp|Canada.Saskatchewan|BOLD:AAB0100
Catocala luciana|[2487]|RDMAB177-05|UASM19740|542[0n]bp|Canada.Alberta|BOLD:AAB0100
Catocala meskei|[2488]|QUNOB337-09|5617-280805-SK|658[0n]bp|Canada.Saskatchewan|BOLD:AAB0100
Catocala meskei|[2489]|RDMAB157-05|UASM24713|658[0n]bp|Canada.Alberta|BOLD:AAB0100
Catocala luciana|[2490]|QUNOB322-09|5602-250808-SK|658[0n]bp|Canada.Saskatchewan|BOLD:AAB0100
Catocala meskei|[2491]|QUNOB336-09|5616-250808-SK|658[0n]bp|Canada.Saskatchewan|BOLD:AAB0100
Catocala meskei|[2492]|QUNOC075-09|5906-270703-CA|658[0n]bp|Canada.Alberta|BOLD:AAB0100
Catocala parta|[2493]|QUNOB331-09|5611-200808-SK|658[0n]bp|Canada.Saskatchewan|BOLD:AAB0100
Catocala parta|[2494]|QUNOB332-09|5612-200808-SK|658[0n]bp|Canada.Saskatchewan|BOLD:AAB0100
Catocala parta|[2495]|QUNOB330-09|5610-160808-SK|658[0n]bp|Canada.Saskatchewan|BOLD:AAB0100
Catocala parta|[2496]|QUNOB335-09|5615-250808-SK|658[0n]bp|Canada.Saskatchewan|BOLD:AAB0100
Catocala parta|[2497]|QUNOB326-09|5606-200808-SK|658[0n]bp|Canada.Saskatchewan|BOLD:AAB0100
Catocala briseis|[2498]|RDLQB568-05|DH010671|592[0n]bp|Canada.Quebec|BOLD:AAB0100
Catocala briseis|[2499]|XAG218-05|2005-ONT-802|658[0n]bp|Canada.Ontario|BOLD:AAB0100
Catocala briseis|[2500]|RDMAB183-05|UASM24723|658[0n]bp|Canada.Alberta|BOLD:AAB0100
Catocala briseis|[2501]|RDLQB482-05|DH010568|658[0n]bp|Canada.Quebec|BOLD:AAB0100
Catocala hermia|[2502]|RDMAB686-06|UASM58799|658[0n]bp|Canada.Alberta|BOLD:AAB0100
Catocala hermia|[2503]|RDMAB181-05|UASM24331|658[0n]bp|Canada.Alberta|BOLD:AAB0100
Catocala hermia|[2504]|QUNOB323-09|5603-200808-SK|658[0n]bp|Canada.Saskatchewan|BOLD:AAB0100
Catocala hermia|[2505]|QUNOD450-10|9164-060903-SK|658[0n]bp|Canada.Saskatchewan|BOLD:AAB0100
Catocala hermia|[2506]|RDMAB179-05|UASM57106|658[0n]bp|Canada.Alberta|BOLD:AAB0100
Catocala californica|[2507]|RDMAB167-05|UASM41350|658[0n]bp|Canada.Alberta|BOLD:AAB0100
Catocala californica|[2508]|LALPA707-10|AVBC 709-10|658[0n]bp|Canada.British Columbia|BOLD:AAB0100
Catocala briseis|[2509]|LBCH6620-10|10-JDWBC-6620|658[0n]bp|Canada.British Columbia|BOLD:AAB0100
Catocala faustina allusa|[2510]|RDMAB169-05|UASM41352|528[0n]bp|Canada.British Columbia|BOLD:AAB0100
Catocala grotiana|[2511]|QUNOB305-09|5585-090908-WY|658[0n]bp|United States.Wyoming|BOLD:AAB0100
Catocala grotiana|[2512]|QUNO819-08|5352-290808-WY|658[0n]bp|United States.Wyoming|BOLD:AAB0100
Catocala grotiana|[2513]|QUNO818-08|5351-010908-WY|658[0n]bp|United States.Wyoming|BOLD:AAB0100
Catocala grotiana|[2514]|RDNMJ286-11|CNCLEP 80194|658[0n]bp|United States.Arizona|BOLD:AAB0100
Catocala grotiana|[2515]|QUNO837-08|5370-150808-WY|658[0n]bp|United States.Wyoming|BOLD:AAB0100
Catocala grotiana|[2516]|QUNOB307-09|5587-090908-WY|658[0n]bp|United States.Wyoming|BOLD:AAB0100
Catocala grotiana|[2517]|ABCNA180-06|180-18-030803-AZ|658[0n]bp|United States.Arizona|BOLD:AAB0100
Catocala grotiana|[2518]|QUNOB300-09|5580-300808-WY|658[0n]bp|United States.Wyoming|BOLD:AAB0100
Catocala grotiana|[2519]|ABCNA179-06|179-18-030803-AZ|620[0n]bp|United States.Arizona|BOLD:AAB0100
Catocala grotiana|[2520]|ABCNA032-06|032-18-300703-AZ|604[0n]bp|United States.Arizona|BOLD:AAB0100
Catocala grotiana|[2521]|ABCNA178-06|178-18-300703-AZ|597[0n]bp|United States.Arizona|BOLD:AAB0100
Catocala grotiana|[2522]|ABCNA440-07|534-8818-030803-AZ|595[0n]bp|United States.Arizona|BOLD:AAB0100
Catocala n. sp. nr. amica|[2523]|QUNO831-08|5364-120808-WY|658[0n]bp|United States.Wyoming|BOLD:AAB0100
Catocala briseis|[2524]|QUNOC078-09|5909-270703-CA|641[0n]bp|Canada.Alberta|BOLD:AAB0100
Catocala meskei|[2525]|QUNOC292-10|5910-270703-CA.1|658[0n]bp|Canada.Alberta|BOLD:AAB0100
Catocala meskei|[2526]|QUNOC079-09|5910-270703-CA|658[0n]bp|Canada.Alberta|BOLD:AAB0100
Catocala semirelicta|[2527]|RDMAB228-05|UASM41396|658[0n]bp|Canada.Alberta|BOLD:AAB0100
Catocala semirelicta|[2528]|RDMAB166-05|UASM41349|658[0n]bp|Canada.Alberta|BOLD:AAB0100
Catocala semirelicta|[2529]|RDMAB173-05|UASM19788|658[0n]bp|Canada.Alberta|BOLD:AAB0100
Catocala hermia|[2530]|RDMAB180-05|UASM57104|658[0n]bp|Canada.Alberta|BOLD:AAB0100
Catocala semirelicta|[2531]|RDMAB165-05|UASM41348|658[0n]bp|Canada.Alberta|BOLD:AAB0100
Catocala semirelicta|[2532]|LBCH3449-10|10-JDWBC-3449|658[0n]bp|Canada.British Columbia|BOLD:AAB0100
Catocala semirelicta|[2533]|RDMAB175-05|UASM57105|658[0n]bp|Canada.Alberta|BOLD:AAB0100
Catocala briseis|[2534]|BBLPA334-10|10BBCLP-0334|658[0n]bp|Canada.Saskatchewan|BOLD:AAB0100
Catocala briseis|[2535]|XAK523-07|HLC-16076|658[0n]bp|Canada.Ontario|BOLD:AAB0100
Catocala briseis|[2536]|RDMAB182-05|UASM24722|658[0n]bp|Canada.Alberta|BOLD:AAB0100
Catocala briseis|[2537]|BBLPA323-10|10BBCLP-0323|658[0n]bp|Canada.Saskatchewan|BOLD:AAB0100
Catocala briseis|[2538]|BBLPA322-10|10BBCLP-0322|658[0n]bp|Canada.Alberta|BOLD:AAB0100
Catocala briseis|[2539]|RDMAB690-06|UASM58800|658[0n]bp|Canada.Alberta|BOLD:AAB0100
Catocala semirelicta|[2540]|RDNMK560-11|CNCLEP 81989|635[0n]bp|Canada.New Brunswick|BOLD:AAB0100
Catocala semirelicta|[2541]|RDNMK559-11|CNCLEP 81988|606[0n]bp|Canada.New Brunswick|BOLD:AAB0100
Catocala unijuga|[2542]|LBCD218-05|HLC-23038|658[0n]bp|Canada.British Columbia|BOLD:AAB0100
Catocala unijuga|[2543]|RDLQB566-05|DH010669|658[0n]bp|Canada.Quebec|BOLD:AAB0100
Catocala unijuga|[2544]|LPAB310-08|08BBLEP-02632|658[0n]bp|Canada.Alberta|BOLD:AAB0100
Catocala unijuga|[2545]|MNBB573-05|05-NBSTA-489|658[0n]bp|Canada.New Brunswick|BOLD:AAB0100
Catocala unijuga|[2546]|QUNOB328-09|5608-160808-SK|658[0n]bp|Canada.Saskatchewan|BOLD:AAB0100
Catocala unijuga|[2547]|BBLPA324-10|10BBCLP-0324|658[0n]bp|Canada.Alberta|BOLD:AAB0100
Catocala unijuga|[2548]|LPMNB312-09|08BBLEP-05156|658[0n]bp|Canada.Manitoba|BOLD:AAB0100
Catocala unijuga|[2549]|XAH340-05|2005-ONT-1923|658[0n]bp|Canada.Ontario|BOLD:AAB0100
Catocala unijuga|[2550]|BBLPA321-10|10BBCLP-0321|658[0n]bp|Canada.Alberta|BOLD:AAB0100
Catocala unijuga|[2551]|DSCNI031-07|06-PROBE-0256|658[0n]bp|Canada.Manitoba|BOLD:AAB0100
Catocala whitneyi|[2552]|QUNO088-07|2088-070707-WI|658[0n]bp|United States.Wisconsin|BOLD:AAE3940
Catocala whitneyi|[2553]|QUNOB507-09|5769-250708-NE|658[0n]bp|United States.Nebraska|BOLD:AAE3940
Catocala whitneyi|[2554]|QUNO086-07|2086-070707-WI|658[0n]bp|United States.Wisconsin|BOLD:AAE3940
Catocala whitneyi|[2555]|QUNO087-07|2087-070707-WI|658[0n]bp|United States.Wisconsin|BOLD:AAE3940
Catocala whitneyi|[2556]|QUNO089-07|2089-070707-WI|530[0n]bp|United States.Wisconsin|BOLD:AAE3940
Catocala amica|[2557]|LNCC716-11|11-NCCC-241|658[0n]bp|United States.North Carolina|BOLD:AAA5644
Catocala amica|[2558]|LNCC712-11|11-NCCC-237|658[0n]bp|United States.North Carolina|BOLD:AAA5644
Catocala amica|[2559]|LNCB438-07|07-NCNW-0122|658[0n]bp|United States.North Carolina|BOLD:AAA5644
Catocala amica|[2560]|LNCC713-11|11-NCCC-238|658[0n]bp|United States.North Carolina|BOLD:AAA5644
Catocala amica|[2561]|ABCNA535-07|629-8878-210506-FL|642[0n]bp|United States.Florida|BOLD:AAA5644
Catocala amica|[2562]|MILEQ284-11|11-MISC-759|658[0n]bp|United States.Alabama|BOLD:AAA5644
Catocala amica|[2563]|MILEP326-10|10-MISC-231|658[0n]bp|United States.Alabama|BOLD:AAA5644
Catocala amica|[2564]|LNCC553-11|11-NCCC-0078|658[0n]bp|United States.North Carolina|BOLD:AAA5644
Catocala amica|[2565]|LSEU577-06|06-JKA-0577|658[0n]bp|United States.Georgia|BOLD:AAA5644
Catocala amica|[2566]|ABCNA099-06|099-78-270504-TX|658[0n]bp|United States.Texas|BOLD:AAA5644
Catocala amica|[2567]|LNCC1282-11|11-NCCC-807|658[0n]bp|United States.North Carolina|BOLD:AAA5644
Catocala amica|[2568]|LGSMG605-07|BGS03968|658[0n]bp|United States.Tennessee|BOLD:AAA5644
Catocala amica|[2569]|ABCNA545-07|639-8878-270503-TX|649[0n]bp|United States.Texas|BOLD:AAA5644
Catocala amica|[2570]|LGSM465-04|DNA-ATBI-0465|589[0n]bp|United States.Tennessee|BOLD:AAA5644
Catocala amica|[2571]|ABCNA702-07|702-8878-110681-OK|615[0n]bp|United States.Oklahoma|BOLD:AAA5644
Catocala amica|[2572]|LOFLA234-06|06-FLOR-0234|658[0n]bp|United States.Florida|BOLD:AAA5644
Catocala amica|[2573]|BBLOB241-11|BIOUG01369-E03|658[0n]bp|United States.Florida|BOLD:AAA5644
Catocala amica|[2574]|LGSMG604-07|BGS03967|658[0n]bp|United States.Tennessee|BOLD:AAA5644
Catocala amica|[2575]|LNCC1163-11|11-NCCC-688|658[0n]bp|United States.North Carolina|BOLD:AAA5644
Catocala amica|[2576]|ABCNA533-07|627-8878-210506-FL|649[0n]bp|United States.Florida|BOLD:AAA5644
Catocala amica|[2577]|LGSM464-04|DNA-ATBI-0464|616[0n]bp|United States.Tennessee|BOLD:AAA5644
Catocala amica|[2578]|LOFLA222-06|06-FLOR-0222|658[0n]bp|United States.Florida|BOLD:AAA5644

Catocala amica|[2576]|ABCNA533-07|627-8878-210506-FL|649[0n]bp|United States.Florida|BOLD:AAA5644
Catocala amica|[2577]|LGSM464-04|DNA-ATBI-0464|616[0n]bp|United States.Tennessee|BOLD:AAA5644
Catocala amica|[2578]|LOFLA222-06|06-FLOR-0222|658[0n]bp|United States.Florida|BOLD:AAA5644
Catocala lineella|[2579]|XAK427-06|2006-ONT-1422|658[0n]bp|Canada.Ontario|BOLD:AAA5644
Catocala epione|[2580]|QUNO797-08|5330-070808-WI|658[0n]bp|United States.Wisconsin|BOLD:ABZ2889
Catocala epione|[2581]|QUNO796-08|5329-070808-WI|658[0n]bp|United States.Wisconsin|BOLD:ABZ2889
Catocala epione|[2582]|QUNO723-08|5256-030708-KY|658[0n]bp|United States.Kentucky|BOLD:ABZ2889
Catocala epione|[2583]|LOT229-04|04HBL002229|609[0n]bp|United States.Tennessee|BOLD:ABZ2889
Catocala epione|[2584]|LOT230-04|04HBL002230|609[0n]bp|United States.Tennessee|BOLD:ABZ2889
Catocala epione|[2585]|LSEU594-06|06-JKA-0594|658[0n]bp|United States.Georgia|BOLD:ABZ2889
Catocala epione|[2586]|ABCNA271-06|271-73-030506-FL|658[0n]bp|United States.Florida|BOLD:ABZ2889
Catocala epione|[2587]|QUNOB045-08|5420-250508-TX|658[0n]bp|United States.Texas|BOLD:ABZ2889
Catocala epione|[2588]|ABCNA272-06|272-73-020506-FL|582[0n]bp|United States.Florida|BOLD:ABZ2889
Catocala epione|[2589]|LNCC120-10|10-NCCC-215|658[0n]bp|United States.North Carolina|BOLD:ABZ2889
Catocala epione|[2590]|QUNO798-08|5331-070808-WI|658[0n]bp|United States.Wisconsin|BOLD:ABZ2889
Catocala epione|[2591]|QUNO724-08|5257-070708-KY|658[0n]bp|United States.Kentucky|BOLD:ABZ2889
Catocala epione|[2592]|LNCC1275-11|11-NCCC-800|658[0n]bp|United States.North Carolina|BOLD:ABZ2889
Catocala epione|[2593]|LNCC1276-11|11-NCCC-801|658[0n]bp|United States.North Carolina|BOLD:ABZ2889
Catocala epione|[2594]|QUNO795-08|5328-070808-WI|658[0n]bp|United States.Wisconsin|BOLD:ABZ2889
Catocala epione|[2595]|QUNO725-08|5258-030708-KY|658[0n]bp|United States.Kentucky|BOLD:ABZ2889
Catocala epione|[2596]|LOT232-04|04HBL002232|609[0n]bp|United States.Tennessee|BOLD:ABZ2889
Catocala epione|[2597]|LOT231-04|04HBL002231|609[0n]bp|United States.Tennessee|BOLD:ABZ2889
Catocala epione|[2598]|LOT233-04|04HBL002233|609[0n]bp|United States.Tennessee|BOLD:ABZ2889
Catocala epione|[2599]|JRLAA026-09|JRLAA-026|635[0n]bp|United States.Alabama|BOLD:ABZ2889
Euparthenos nubilis|[2600]|XAB190-04|04HBL005190|658[0n]bp|Canada.Ontario|BOLD:AAB1002
Zale horrida|[2601]|RDLQG308-06|DH012520|658[0n]bp|Canada.Quebec|BOLD:AAB2002
Zale horrida|[2602]|RDLQG306-06|DH012518|583[0n]bp|Canada.Quebec|BOLD:AAB2002
Zale horrida|[2603]|XAJ578-06|2006-ONT-0578|658[0n]bp|Canada.Ontario|BOLD:AAB2002
Zale horrida|[2604]|XAJ403-06|2006-ONT-0403|658[0n]bp|Canada.Ontario|BOLD:AAB2002
Zale horrida|[2605]|XAJ683-06|2006-ONT-0683|658[0n]bp|Canada.Ontario|BOLD:AAB2002
Zale horrida|[2606]|PHMO138-03|moth791.01|639[0n]bp|Canada.Ontario|BOLD:AAB2002
Zale horrida|[2607]|PHMO140-03|moth795.02|639[0n]bp|Canada.Ontario|BOLD:AAB2002
Zale horrida|[2608]|XAE348-04|Moth4348.03|658[0n]bp|Canada.Ontario|BOLD:AAB2002
Zale horrida|[2609]|RDLQF499-06|DH011648|658[0n]bp|Canada.Quebec|BOLD:AAB2002
Zale horrida|[2610]|RDLQB551-05|DH010637|658[0n]bp|Canada.Quebec|BOLD:AAB2002
Zale horrida|[2611]|RDLQG307-06|DH012519|658[0n]bp|Canada.Quebec|BOLD:AAB2002
Zale horrida|[2612]|RDLQG304-06|DH012516|658[0n]bp|Canada.Quebec|BOLD:AAB2002
Zale horrida|[2613]|RDLQG305-06|DH012517|658[0n]bp|Canada.Quebec|BOLD:AAB2002
Zale horrida|[2614]|RDLQF500-06|DH011649|658[0n]bp|Canada.Quebec|BOLD:AAB2002
Zale horrida|[2615]|LPSOC324-08|PPBP-2323|657[0n]bp|Canada.Ontario|BOLD:AAB2002
Zale intenta|[2616]|TMNBB067-06|MNBTT-1007|658[0n]bp|Canada.New Brunswick|BOLD:AAB1413
Zale intenta|[2617]|TTMNB277-06|MNBTT-277|658[0n]bp|Canada.New Brunswick|BOLD:AAB1413
Zale intenta|[2618]|RDLQH063-06|DH013300|659[0n]bp|Canada.Quebec|BOLD:AAB1413
Zale intenta|[2619]|RDLQH062-06|DH013299|658[0n]bp|Canada.Quebec|BOLD:AAB1413
Zale intenta|[2620]|RDLQG303-06|DH012515|658[0n]bp|Canada.Quebec|BOLD:AAB1413
Zale lunata|[2621]|LALPA090-10|AVBC 090-10|658[0n]bp|Canada.British Columbia|BOLD:AAB8832
Zale lunata|[2622]|LALPA095-10|AVBC 095-10|658[0n]bp|Canada.British Columbia|BOLD:AAB8832
Zale lunata|[2623]|LALPA615-10|AVBC 617-10|658[0n]bp|Canada.British Columbia|BOLD:AAB8832
Zale lunata|[2624]|DUNLP362-08|Dun-08-362|658[0n]bp|Canada.British Columbia|BOLD:AAB8832
Zale lunata|[2625]|RDLQH030-06|DH013267|658[0n]bp|Canada.Quebec|BOLD:AAB8831
Zale lunata|[2626]|XAH650-05|2005-ONT-2233|658[0n]bp|Canada.Ontario|BOLD:AAB8831
Zale lunata|[2627]|RDLQB465-05|DH010551|550[3n]bp|Canada.Quebec|BOLD:AAB8831
Zale lunata|[2628]|BLTIB243-08|BL425|658[0n]bp|Canada.Ontario|BOLD:AAB8831
Zale phaeocapna|[2629]|RDLQH088-06|DH013326|658[0n]bp|Canada.Quebec|BOLD:AAE4850
Zale phaeocapna|[2630]|RDNMG928-08|CNC LEP00053052|658[0n]bp|Canada.Ontario|BOLD:AAE4850
Zale galbanata|[2631]|XAK116-06|2006-ONT-1111|658[0n]bp|Canada.Ontario|BOLD:AAA9400
Zale galbanata|[2632]|XAJ268-06|2006-ONT-0268|658[0n]bp|Canada.Ontario|BOLD:AAA9400
Zale galbanata|[2633]|RDLQF834-06|DH011987|658[0n]bp|Canada.Quebec|BOLD:AAA9400
Zale galbanata|[2634]|RDLQF678-06|DH011828|637[0n]bp|Canada.Quebec|BOLD:AAA9400
Zale galbanata|[2635]|RDLQF735-06|DH011885|658[0n]bp|Canada.Quebec|BOLD:AAA9400
Zale galbanata|[2636]|XAJ720-06|2006-ONT-0720|658[0n]bp|Canada.Ontario|BOLD:AAA9400
Zale galbanata|[2637]|RDLQG066-06|DH012223|592[0n]bp|Canada.Quebec|BOLD:AAA9400
Zale galbanata|[2638]|RDLQF689-06|DH011839|637[0n]bp|Canada.Quebec|BOLD:AAA9400
Zale galbanata|[2639]|RDLQH075-06|DH013313|658[0n]bp|Canada.Quebec|BOLD:AAA9400
Zale galbanata|[2640]|RDLQF677-06|DH011827|609[0n]bp|Canada.Quebec|BOLD:AAA9400
Zale galbanata|[2641]|XAC717-04|04HBL006717|658[0n]bp|Canada.Ontario|BOLD:AAA9400
Zale galbanata|[2642]|XAE580-04|Moth4580.03|658[0n]bp|Canada.Ontario|BOLD:AAA9400
Zale galbanata|[2643]|XAJ816-06|2006-ONT-0816|658[0n]bp|Canada.Ontario|BOLD:AAA9400
Zale galbanata|[2644]|LPSO887-08|PPBP-0887|658[0n]bp|Canada.Ontario|BOLD:AAA9400
Zale galbanata|[2645]|RDLQF676-06|DH011826|609[0n]bp|Canada.Quebec|BOLD:AAA9400
Zale galbanata|[2646]|PHMO126-03|moth739.01|639[0n]bp|Canada.Ontario|BOLD:AAA9400
Zale galbanata|[2647]|PMG166-03|moth242.01|617[0n]bp|Canada.Ontario|BOLD:AAA9400
Zale galbanata|[2648]|PHMO093-03|moth580.01|639[0n]bp|Canada.Ontario|BOLD:AAA9400
Zale galbanata|[2649]|XAB137-04|04HBL005137|658[0n]bp|Canada.Ontario|BOLD:AAA9400
Zale galbanata|[2650]|MNBB025-05|HBL008635|658[0n]bp|Canada.New Brunswick|BOLD:AAA9400
Zale galbanata|[2651]|LPSO237-08|PPBP-0237|658[0n]bp|Canada.Ontario|BOLD:AAA9400
Zale galbanata|[2652]|XAE236-04|Moth4236.03|658[0n]bp|Canada.Ontario|BOLD:AAA9400
Zale galbanata|[2653]|XAJ319-06|2006-ONT-0319|658[0n]bp|Canada.Ontario|BOLD:AAA9400
Zale aeruginosa|[2654]|RDLQG298-06|DH012510|658[0n]bp|Canada.Quebec|BOLD:AAB4323
Zale aeruginosa|[2655]|TTMNB055-06|MNBTT-055|599[0n]bp|Canada.New Brunswick|BOLD:AAB4323
Zale aeruginosa|[2656]|TMNBB066-06|MNBTT-1006|656[0n]bp|Canada.New Brunswick|BOLD:AAB4323
Zale minerea|[2657]|LPMN325-08|08BBLEP-01124|658[0n]bp|Canada.Manitoba|BOLD:ABY9488
Zale minerea|[2658]|RDLQG208-06|DH012384|658[0n]bp|Canada.Quebec|BOLD:ABY9488
Zale minerea|[2659]|LOWCE247-06|CGWC-4007|658[0n]bp|Canada.British Columbia|BOLD:ABY9488
Zale minerea|[2660]|LOWCB608-05|CGWC-1548|572[0n]bp|Canada.British Columbia|BOLD:ABY9488
Zale minerea|[2661]|LOWCB611-05|CGWC-1551|585[0n]bp|Canada.British Columbia|BOLD:ABY9488
Zale minerea|[2662]|LOWCB610-05|CGWC-1550|584[0n]bp|Canada.British Columbia|BOLD:ABY9488
Zale minerea|[2663]|LOWCB609-05|CGWC-1549|584[0n]bp|Canada.British Columbia|BOLD:ABY9488
Zale minerea|[2664]|LBCS029-07|UBC-2007-0052|658[0n]bp|Canada.British Columbia|BOLD:ABY9488
Zale minerea|[2665]|LALPA073-10|AVBC 073-10|658[0n]bp|Canada.British Columbia|BOLD:ABY9488
Zale minerea|[2666]|LALPA172-10|AVBC 172-10|658[0n]bp|Canada.British Columbia|BOLD:ABY9488
Zale minerea|[2667]|LBCS023-07|UBC-2007-0025|658[0n]bp|Canada.British Columbia|BOLD:ABY9488
Zale minerea|[2668]|XAB218-04|04HBL005218|658[0n]bp|Canada.Ontario|BOLD:AAA4886
Zale minerea|[2669]|BLTIB066-08|BL0105|658[0n]bp|Canada.Ontario|BOLD:AAA4886
Zale minerea|[2670]|LPMN175-08|08BBLEP-00974|658[0n]bp|Canada.Manitoba|BOLD:AAA4886
Zale minerea|[2671]|XAJ543-06|2006-ONT-0543|658[0n]bp|Canada.Ontario|BOLD:AAA4886
Zale minerea|[2672]|XAJ348-06|2006-ONT-0348|656[0n]bp|Canada.Ontario|BOLD:AAA4886
Zale minerea|[2673]|XAB016-04|04HBL005016|605[0n]bp|Canada.Ontario|BOLD:AAA4886
Zale minerea|[2674]|XAE229-04|Moth4229.03|658[0n]bp|Canada.Ontario|BOLD:AAA4886
Zale minerea|[2675]|XAK122-06|2006-ONT-1117|658[0n]bp|Canada.Ontario|BOLD:AAA4886
Zale minerea|[2676]|RDLQG209-06|DH012385|658[0n]bp|Canada.Quebec|BOLD:AAA4886
Zale minerea|[2677]|XAJ461-06|2006-ONT-0461|658[0n]bp|Canada.Ontario|BOLD:AAA4886

Zale minerea|[2675]|XAK122-06|2006-ONT-1117|658[0n]bp|Canada.Ontario|BOLD:AAA4886
Zale minerea|[2676]|RDLQG209-06|DH012385|658[0n]bp|Canada.Quebec|BOLD:AAA4886
Zale minerea|[2677]|XAJ461-06|2006-ONT-0461|658[0n]bp|Canada.Ontario|BOLD:AAA4886
Zale minerea|[2678]|XAJ290-06|2006-ONT-0290|658[0n]bp|Canada.Ontario|BOLD:AAA4886
Zale minerea|[2679]|RDLQG211-06|DH012387|658[0n]bp|Canada.Quebec|BOLD:AAA4886
Zale minerea|[2680]|XAB050-04|04HBL005050|624[0n]bp|Canada.Ontario|BOLD:AAA4886
Zale minerea|[2681]|PHMO089-03|moth568.01|639[2n]bp|Canada.Ontario|BOLD:AAA4886
Zale minerea|[2682]|XAE494-04|Moth4494.03|575[0n]bp|Canada.Ontario|BOLD:AAA4886
Zale minerea|[2683]|XAB029-04|04HBL005029|573[0n]bp|Canada.Ontario|BOLD:AAA4886
Zale minerea|[2684]|RDLQG207-06|DH012383|583[3n]bp|Canada.Quebec|BOLD:AAA4886
Zale minerea|[2685]|RDLQH061-06|DH013298|658[0n]bp|Canada.Quebec|BOLD:AAA4886
Zale minerea|[2686]|TTMNB275-06|MNBTT-275|658[0n]bp|Canada.New Brunswick|BOLD:AAA4886
Zale minerea|[2687]|RDLQH059-06|DH013296|658[0n]bp|Canada.Quebec|BOLD:AAA4886
Zale minerea|[2688]|RDLQH057-06|DH013294|658[0n]bp|Canada.Quebec|BOLD:AAA4886
Zale minerea|[2689]|RDLQG210-06|DH012386|658[0n]bp|Canada.Quebec|BOLD:AAA4886
Zale minerea|[2690]|XAJ466-06|2006-ONT-0466|656[0n]bp|Canada.Ontario|BOLD:AAA4886
Zale minerea|[2691]|RDLQH056-06|DH013293|658[0n]bp|Canada.Quebec|BOLD:AAA4886
Zale minerea|[2692]|KPOEC149-08|08OEC-190|658[0n]bp|Canada.Ontario|BOLD:AAA4886
Zale minerea|[2693]|XAJ277-06|2006-ONT-0277|658[0n]bp|Canada.Ontario|BOLD:AAA4886
Zale minerea|[2694]|TTMNB276-06|MNBTT-276|658[0n]bp|Canada.New Brunswick|BOLD:AAA4886
Zale minerea|[2695]|XAE502-04|Moth4502.03|575[0n]bp|Canada.Ontario|BOLD:AAA4886
Zale minerea|[2696]|RDLQH058-06|DH013295|658[0n]bp|Canada.Quebec|BOLD:AAA4886
Zale minerea|[2697]|XAE207-04|Moth4207.03|658[0n]bp|Canada.Ontario|BOLD:AAA4886
Zale minerea|[2698]|XAE364-04|Moth4364.03|658[0n]bp|Canada.Ontario|BOLD:AAA4886
Zale minerea|[2699]|RDLQH080-06|DH013318|658[0n]bp|Canada.Quebec|BOLD:AAA4886
Zale minerea|[2700]|XAJ460-06|2006-ONT-0460|658[0n]bp|Canada.Ontario|BOLD:AAA4886
Zale minerea|[2701]|RDLQG206-06|DH012382|658[0n]bp|Canada.Quebec|BOLD:AAA4886
Zale minerea|[2702]|RDLQH060-06|DH013297|658[0n]bp|Canada.Quebec|BOLD:AAA4886
Zale helata|[2703]|XAJ579-06|2006-ONT-0579|658[0n]bp|Canada.Ontario|BOLD:ABY8158
Zale helata|[2704]|XAB034-04|04HBL005034|658[0n]bp|Canada.Ontario|BOLD:ABY8158
Zale helata|[2705]|RDLQF498-06|DH011647|658[0n]bp|Canada.Quebec|BOLD:ABY8158
Zale helata|[2706]|RDLQH118-06|DH009933|615[1n]bp|Canada.Quebec|BOLD:ABY8158
Zale helata|[2707]|XAJ541-06|2006-ONT-0541|658[0n]bp|Canada.Ontario|BOLD:ABY8158
Zale helata|[2708]|RDNMG064-08|NOC15005|658[0n]bp|Canada.Ontario|BOLD:ABY8158
Zale helata|[2709]|RDNMJ190-10|NOC 7590|658[0n]bp|Canada.Quebec|BOLD:ABY8158
Zale helata|[2710]|RDNM744-05|CNCNoctuoidea7590|608[0n]bp|Canada.Ontario|BOLD:ABY8158
Zale helata|[2711]|RDLQG299-06|DH012511|593[0n]bp|Canada.Quebec|BOLD:ABY8158
Zale helata|[2712]|RDLQG205-06|DH012381|595[0n]bp|Canada.Quebec|BOLD:ABY8158
Zale helata|[2713]|XAJ619-06|2006-ONT-0619|643[0n]bp|Canada.Ontario|BOLD:ABY8158
Zale helata|[2714]|LPSOB210-08|PPBP-1209|658[0n]bp|Canada.Ontario|BOLD:ABY8158
Zale helata|[2715]|RDLQB550-05|DH010636|658[0n]bp|Canada.Quebec|BOLD:ABY8158
Zale helata|[2716]|RDNMG065-08|NOC15006|658[0n]bp|Canada.Ontario|BOLD:ABY8158
Zale helata|[2717]|XAE310-04|Moth4310.03|658[0n]bp|Canada.Ontario|BOLD:ABY8158
Zale helata|[2718]|XAE311-04|Moth4311.03|619[0n]bp|Canada.Ontario|BOLD:ABY8158
Zale helata|[2719]|RDLQF497-06|DH011646|658[0n]bp|Canada.Quebec|BOLD:ABY8158
Zale helata|[2720]|RDLQG300-06|DH012512|658[0n]bp|Canada.Quebec|BOLD:ABY8158
Zale helata|[2721]|RDLQG301-06|DH012513|658[0n]bp|Canada.Quebec|BOLD:ABY8158
Zale duplicata|[2722]|RDNMG173-08|NOC14926|658[0n]bp|Canada.New Brunswick|BOLD:ABY8158
Zale duplicata|[2723]|RDNMG359-08|NOC15206|658[0n]bp|Canada.Alberta|BOLD:ABY8158
Zale duplicata|[2724]|LOWCB607-05|CGWC-1547|658[0n]bp|Canada.British Columbia|BOLD:ABY8158
Zale duplicata|[2725]|RDNMG358-08|NOC15205|658[0n]bp|Canada.British Columbia|BOLD:ABY8158
Zale duplicata|[2726]|LALPA1120-11|AVBC 930-11|658[0n]bp|Canada.British Columbia|BOLD:ABY8158
Zale duplicata|[2727]|RDNMJ797-11|CNCLEP 80325|658[0n]bp|Canada.Quebec|BOLD:ABY8158
Zale duplicata|[2728]|RDLQG295-06|DH012507|593[0n]bp|Canada.Quebec|BOLD:ABY8158
Zale duplicata|[2729]|RDNMG174-08|NOC14927|658[0n]bp|Canada.New Brunswick|BOLD:ABY8158
Zale duplicata|[2730]|RDLQG297-06|DH012509|658[0n]bp|Canada.Quebec|BOLD:ABY8158
Zale duplicata|[2731]|XAD646-05|2005-ONT-61|658[0n]bp|Canada.Ontario|BOLD:ABY8158
Zale duplicata|[2732]|XAD612-05|2005-ONT-27|658[0n]bp|Canada.Ontario|BOLD:ABY8158
Zale duplicata|[2733]|XAJ353-06|2006-ONT-0353|658[0n]bp|Canada.Ontario|BOLD:ABY8158
Zale duplicata|[2734]|RDLQH042-06|DH013279|657[0n]bp|Canada.Quebec|BOLD:ABY8158
Zale duplicata|[2735]|XAJ349-06|2006-ONT-0349|658[0n]bp|Canada.Ontario|BOLD:ABY8158
Zale duplicata|[2736]|RDLQH078-06|DH013316|658[0n]bp|Canada.Quebec|BOLD:ABY8158
Zale duplicata|[2737]|RDLQH077-06|DH013315|658[0n]bp|Canada.Quebec|BOLD:ABY8158
Zale duplicata|[2738]|XAF370-05|HLC-10411|658[0n]bp|Canada.Ontario|BOLD:ABY8158
Zale duplicata|[2739]|RDLQH055-06|DH013292|658[0n]bp|Canada.Quebec|BOLD:ABY8158
Zale duplicata|[2740]|XAB045-04|04HBL005045|658[1n]bp|Canada.Ontario|BOLD:ABY8158
Zale duplicata|[2741]|RDLQH052-06|DH013289|649[0n]bp|Canada.Quebec|BOLD:ABY8158
Zale duplicata|[2742]|RDLQH051-06|DH013288|649[0n]bp|Canada.Quebec|BOLD:ABY8158
Zale duplicata|[2743]|RDLQH041-06|DH013278|658[0n]bp|Canada.Quebec|BOLD:ABY8158
Zale duplicata|[2744]|RDLQH049-06|DH013286|658[0n]bp|Canada.Quebec|BOLD:ABY8158
Zale duplicata|[2745]|RDLQH043-06|DH013280|658[0n]bp|Canada.Quebec|BOLD:ABY8158
Zale duplicata|[2746]|XAF332-05|HLC-10373|658[0n]bp|Canada.Ontario|BOLD:ABY8158
Zale duplicata|[2747]|XAE171-04|Moth4171.03|658[0n]bp|Canada.Ontario|BOLD:ABY8158
Zale duplicata|[2748]|RDNMG383-08|CNC LEP00052207|658[0n]bp|Canada.Ontario|BOLD:ABY8158
Zale duplicata|[2749]|RDLQH048-06|DH013285|658[0n]bp|Canada.Quebec|BOLD:ABY8158
Zale duplicata|[2750]|RDLQG302-06|DH012514|658[0n]bp|Canada.Quebec|BOLD:ABY8158
Zale duplicata|[2751]|RDLQH053-06|DH013290|658[0n]bp|Canada.Quebec|BOLD:ABY8158
Zale duplicata|[2752]|RDLQH054-06|DH013291|658[0n]bp|Canada.Quebec|BOLD:ABY8158
Zale duplicata|[2753]|RDLQG296-06|DH012508|658[0n]bp|Canada.Quebec|BOLD:ABY8158
Zale duplicata|[2754]|RDLQH079-06|DH013317|658[0n]bp|Canada.Quebec|BOLD:ABY8158
Zale duplicata|[2755]|RDLQH103-06|DH013341|658[0n]bp|Canada.Quebec|BOLD:ABY8158
Zale duplicata|[2756]|RDLQH046-06|DH013283|658[0n]bp|Canada.Quebec|BOLD:ABY8158
Zale duplicata|[2757]|KPOEC170-08|08OEC-211|658[0n]bp|Canada.Ontario|BOLD:ABY8158
Zale duplicata|[2758]|XAB030-04|04HBL005030|649[0n]bp|Canada.Ontario|BOLD:ABY8158
Zale duplicata|[2759]|XAE161-04|Moth4161.03|658[0n]bp|Canada.Ontario|BOLD:ABY8158
Zale duplicata|[2760]|RDLQH044-06|DH013281|658[0n]bp|Canada.Quebec|BOLD:ABY8158
Zale duplicata|[2761]|RDLQH045-06|DH013282|654[0n]bp|Canada.Quebec|BOLD:ABY8158
Zale duplicata|[2762]|RDLQH050-06|DH013287|654[0n]bp|Canada.Quebec|BOLD:ABY8158
Zale duplicata|[2763]|PHMO366-03|moth187.01|639[0n]bp|Canada.Ontario|BOLD:ABY8158
Zale duplicata|[2764]|RDLQG204-06|DH012380|593[1n]bp|Canada.Quebec|BOLD:ABY8158
Zale duplicata|[2765]|XAB028-04|04HBL005028|573[0n]bp|Canada.Ontario|BOLD:ABY8158
Zale duplicata|[2766]|PMG167-03|moth186.01|617[0n]bp|Canada.Ontario|BOLD:ABY8158
Zale duplicata|[2767]|RDLQH047-06|DH013284|658[0n]bp|Canada.Quebec|BOLD:ABY8158
Zale duplicata|[2768]|RDLQG220-06|DH012403|658[0n]bp|Canada.Quebec|BOLD:ABY8158
Zale metatoides|[2769]|PSAT140-10|CNCLEP 70024|658[0n]bp|Canada.Ontario|BOLD:ABY8158
Zale metatoides|[2770]|RDNMG382-08|CNC LEP00052206|658[0n]bp|Canada.Ontario|BOLD:ABY8158
Zale metatoides|[2771]|RDNMG063-08|NOC15004|658[0n]bp|Canada.Ontario|BOLD:ABY8158
Zale submediana|[2772]|RDNMG381-08|CNC LEP00052205|658[0n]bp|Canada.Ontario|BOLD:ABY9489
Zale submediana|[2773]|RDLQG294-06|DH012506|658[0n]bp|Canada.Quebec|BOLD:ABY9489
Zale submediana|[2774]|LPSOB304-08|PPBP-1303|658[0n]bp|Canada.Ontario|BOLD:ABY9489
Zale submediana|[2775]|PSAT141-10|CNCLEP 70025|658[0n]bp|Canada.Ontario|BOLD:ABY9489
Zale calycanthata|[2776]|LSEU037-06|06-JKA-0037|658[0n]bp|United States.Georgia|BOLD:ACF5274

Zale submediana|[2774]|LPSOB304-08|PPBP-1303|658[0n]bp|Canada.Ontario|BOLD:ABY9489
Zale submediana|[2775]|PSAT141-10|CNCLEP 70025|658[0n]bp|Canada.Ontario|BOLD:ABY9489
Zale calycanthata|[2776]|LSEU037-06|06-JKA-0037|658[0n]bp|United States.Georgia|BOLD:ACF5274
Zale calycanthata|[2777]|HKONS001-07|1565-COI-07|658[0n]bp|United States.Florida|BOLD:ACF5274
Zale calycanthata|[2778]|LSEU497-06|06-JKA-0497|658[0n]bp|United States.Georgia|BOLD:ACF5274
Zale undularis|[2779]|RDLQH117-06|DH009161|658[0n]bp|Canada.Quebec|BOLD:AAC5348
Zale undularis|[2780]|XAD588-05|2005-ONT-3|658[0n]bp|Canada.Ontario|BOLD:AAC5348
Zale undularis|[2781]|PHMO137-03|moth788.01|639[0n]bp|Canada.Ontario|BOLD:AAC5348
Zale unilineata|[2782]|TTMNB279-06|MNBTT-279|658[0n]bp|Canada.New Brunswick|BOLD:AAC1852
Zale unilineata|[2783]|TTMNB278-06|MNBTT-278|658[0n]bp|Canada.New Brunswick|BOLD:AAC1852
Zale unilineata|[2784]|XAJ465-06|2006-ONT-0465|658[0n]bp|Canada.Ontario|BOLD:AAC1852
Zale unilineata|[2785]|XAJ422-06|2006-ONT-0422|658[0n]bp|Canada.Ontario|BOLD:AAC1852
Zale unilineata|[2786]|XAJ485-06|2006-ONT-0485|658[0n]bp|Canada.Ontario|BOLD:AAC1852
Zale unilineata|[2787]|XAD587-05|2005-ONT-2|658[0n]bp|Canada.Ontario|BOLD:AAC1852
Zale unilineata|[2788]|PMG168-03|moth321.01|617[0n]bp|Canada.Ontario|BOLD:AAC1852
Zale unilineata|[2789]|TMG93-03|moth301.01|639[0n]bp|Canada.Ontario|BOLD:AAC1852
Melipotis acontioides|[2790]|BBLOD504-11|BIOUG01565-A11|658[0n]bp|United States.Texas|BOLD:AAC4750
Melipotis acontioides|[2791]|BBLOD1685-11|BIOUG01830-E04|633[0n]bp|United States.Texas|BOLD:AAC4750
Melipotis acontioides|[2792]|HKONB102-08|3599-COI-08|658[0n]bp|United States.Texas|BOLD:AAC4750
Melipotis acontioides|[2793]|HKONB100-08|3597-COI-08|658[0n]bp|United States.Texas|BOLD:AAC4750
Melipotis acontioides|[2794]|BBLOD507-11|BIOUG01565-B02|658[0n]bp|United States.Texas|BOLD:AAC4750
Melipotis acontioides|[2795]|BBLOB615-11|BIOUG01398-D09|658[0n]bp|United States.Texas|BOLD:AAC4750
Melipotis acontioides|[2796]|BBLOC601-11|BIOUG01458-C12|622[0n]bp|United States.Texas|BOLD:AAC4750
Melipotis acontioides|[2797]|LMEMB131-09|RBMIS-1224|658[0n]bp|United States.Texas|BOLD:AAC4750
Melipotis acontioides|[2798]|IAWL141-09|IAWAZ-0140|658[0n]bp|United States.Arizona|BOLD:AAC4750
Melipotis acontioides|[2799]|BBLSW312-09|09BBLEP-01240|658[0n]bp|United States.Arizona|BOLD:AAC4750
Melipotis acontioides|[2800]|HKONB101-08|3598-COI-08|658[0n]bp|United States.Texas|BOLD:AAC4750
Melipotis acontioides|[2801]|BBLOD503-11|BIOUG01565-A10|658[0n]bp|United States.Texas|BOLD:AAC4750
Melipotis acontioides|[2802]|HKONB104-08|3601-COI-08|658[0n]bp|United States.Texas|BOLD:AAC4750
Melipotis acontioides|[2803]|BBLOC605-11|BIOUG01458-D04|658[0n]bp|United States.Texas|BOLD:AAC4750
Melipotis acontioides|[2804]|IAWL140-09|IAWAZ-0139|658[0n]bp|United States.Arizona|BOLD:AAC4750
Melipotis acontioides|[2805]|HKONB099-08|3596-COI-08|658[0n]bp|United States.Texas|BOLD:AAC4750
Melipotis acontioides|[2806]|HKONB103-08|3600-COI-08|658[0n]bp|United States.Texas|BOLD:AAC4750
Melipotis acontioides|[2807]|IAWL139-09|IAWAZ-0138|658[0n]bp|United States.Arizona|BOLD:AAC4750
Melipotis acontioides|[2808]|BBLOD192-11|BIOUG01552-G07|658[0n]bp|United States.Texas|BOLD:AAC4750
Bulia deducta|[2809]|AWCLB589-11|AWC-07661|561[3n]bp|United States.Arizona|BOLD:AAB5985
Bulia deducta|[2810]|LMEMB136-09|RBMIS-1229|658[0n]bp|United States.Texas|BOLD:AAB5985
Bulia deducta|[2811]|LPOKC729-09|MDOK-2806|658[0n]bp|United States.Oklahoma|BOLD:AAB5985
Bulia deducta|[2812]|LPOKB1022-09|MDOK-2064|658[0n]bp|United States.Oklahoma|BOLD:AAB5985
Bulia deducta|[2813]|BBLOD924-11|BIOUG01569-E03|658[0n]bp|United States.Texas|BOLD:AAB5985
Bulia deducta|[2814]|BBLOD1285-11|BIOUG01825-C08|658[0n]bp|United States.Texas|BOLD:AAB5985
Bulia deducta|[2815]|BBLSW828-09|09BBLEP-01756|658[0n]bp|United States.Texas|BOLD:AAB5985
Bulia deducta|[2816]|BBLOD1097-11|BIOUG01822-C10|658[0n]bp|United States.Texas|BOLD:AAB5985
Bulia deducta|[2817]|BBLOE1390-12|BIOUG01986-H03|658[0n]bp|United States.Texas|BOLD:AAB5985
Bulia deducta|[2818]|IAWLB538-11|IAWAZ-1446|658[0n]bp|United States.Arizona|BOLD:AAB5985
Bulia deducta|[2819]|AWCLB615-11|AWC-07961|617[0n]bp|United States.Arizona|BOLD:AAB5985
Bulia deducta|[2820]|BBLSW280-09|09BBLEP-01208|658[0n]bp|United States.Arizona|BOLD:AAB5985
Bulia deducta|[2821]|LMEMB139-09|RBMIS-1232|658[0n]bp|United States.Texas|BOLD:AAB5985
Bulia deducta|[2822]|AWCLB485-11|AWC-08305|658[0n]bp|United States.Arizona|BOLD:AAB5985
Bulia deducta|[2823]|CMAZA348-10|CMAZ-0348|658[0n]bp|United States.Arizona|BOLD:AAB5985
Bulia deducta|[2824]|AWCLB612-11|AWC-07954|658[0n]bp|United States.Arizona|BOLD:AAB5985
Bulia deducta|[2825]|BBLSX767-09|09BBLEP-02695|658[0n]bp|United States.Texas|BOLD:AAB5985
Bulia deducta|[2826]|BBLOD877-11|BIOUG01569-A04|658[0n]bp|United States.California|BOLD:AAB5985
Bulia deducta|[2827]|BBLOE1986-12|BIOUG01995-B06|658[0n]bp|United States.Texas|BOLD:AAB5985
Bulia deducta|[2828]|BBLOB693-11|BIOUG01399-C04|658[0n]bp|United States.Texas|BOLD:AAB5985
Bulia deducta|[2829]|ABNCC291-07|1291-120603-TX|653[0n]bp|United States.Texas|BOLD:AAB5985
Bulia deducta|[2830]|BBLOD1100-11|BIOUG01822-D01|658[0n]bp|United States.Texas|BOLD:AAB5985
Bulia deducta|[2831]|BBLOE1984-12|BIOUG01995-B04|658[0n]bp|United States.Texas|BOLD:AAB5985
Bulia deducta|[2832]|BBLOE1710-12|BIOUG01990-C03|658[0n]bp|United States.Texas|BOLD:AAB5985
Bulia deducta|[2833]|USLEP1171-10|10BBLEP-01171|658[0n]bp|United States.Arizona|BOLD:AAB5985
Bulia deducta|[2834]|BBLOE1459-12|BIOUG01987-F01|658[0n]bp|United States.Texas|BOLD:AAB5985
Bulia deducta|[2835]|BBLOC1986-11|BIOUG01550-F03|658[0n]bp|United States.Texas|BOLD:AAB5985
Bulia deducta|[2836]|IAWLB540-11|IAWAZ-1448|658[0n]bp|United States.Arizona|BOLD:AAB5985
Bulia deducta|[2837]|BBLOD919-11|BIOUG01569-D10|658[0n]bp|United States.Texas|BOLD:AAB5985
Bulia deducta|[2838]|BBLOE1469-12|BIOUG01987-F11|658[0n]bp|United States.Texas|BOLD:AAB5985
Bulia deducta|[2839]|BBLSW056-09|09BBLEP-00984|658[0n]bp|United States.Texas|BOLD:AAB5985
Bulia deducta|[2840]|USLEP1167-10|10BBLEP-01167|658[0n]bp|United States.Arizona|BOLD:AAB5985
Bulia deducta|[2841]|BBLOC234-11|BIOUG01454-E01|658[0n]bp|United States.Arizona|BOLD:AAB5985
Bulia deducta|[2842]|BBLOD140-11|BIOUG01552-C03|658[0n]bp|United States.Texas|BOLD:AAB5985
Bulia deducta|[2843]|BBLOE1991-12|BIOUG01995-B11|658[0n]bp|United States.Texas|BOLD:AAB5985
Bulia deducta|[2844]|BBLOB011-11|BIOUG01367-A11|658[0n]bp|United States.Arizona|BOLD:AAB5985
Bulia deducta|[2845]|BBLOD1272-11|BIOUG01825-B07|658[0n]bp|United States.Texas|BOLD:AAB5985
Bulia deducta|[2846]|BBLOC634-11|BIOUG01458-F09|658[0n]bp|United States.Arizona|BOLD:AAB5985
Bulia deducta|[2847]|BBLOD925-11|BIOUG01569-E04|658[0n]bp|United States.Texas|BOLD:AAB5985
Bulia deducta|[2848]|IAWLB537-11|IAWAZ-1445|658[0n]bp|United States.Arizona|BOLD:AAB5985
Bulia deducta|[2849]|BBLWU090-09|09BBLEP-04248|658[0n]bp|United States.Colorado|BOLD:AAB5985
Bulia deducta|[2850]|LTOLB1362-11|RFD-93-0055|658[0n]bp|United States.Texas|BOLD:AAB5985
Bulia deducta|[2851]|BBLOE1442-12|BIOUG01987-D08|658[0n]bp|United States.Texas|BOLD:AAB5985
Bulia deducta|[2852]|BBLOC152-11|BIOUG01453-F02|658[0n]bp|United States.Arizona|BOLD:AAB5985
Bulia deducta|[2853]|BBLSX183-09|09BBLEP-02111|658[0n]bp|United States.Texas|BOLD:AAB5985
Bulia deducta|[2854]|BBLOD1299-11|BIOUG01825-D10|658[0n]bp|United States.Texas|BOLD:AAB5985
Bulia deducta|[2855]|BBLSY676-09|09BBLEP-03603|658[0n]bp|United States.Arizona|BOLD:AAB5985
Bulia deducta|[2856]|BBLSY915-09|09BBLEP-03842|658[0n]bp|United States.Texas|BOLD:AAB5985
Bulia deducta|[2857]|BBLOE2022-12|BIOUG01995-E06|658[0n]bp|United States.Texas|BOLD:AAB5985
Bulia deducta|[2858]|BBLOB1528-11|BIOUG01420-A08|658[0n]bp|United States.Texas|BOLD:AAB5985
Bulia deducta|[2859]|BBLOD918-11|BIOUG01569-D09|658[0n]bp|United States.Texas|BOLD:AAB5985
Bulia deducta|[2860]|AWCLB412-10|AWC-08101|658[0n]bp|United States.Arizona|BOLD:AAB5985
Bulia deducta|[2861]|BBLOD1276-11|BIOUG01825-B11|658[0n]bp|United States.Texas|BOLD:AAB5985
Bulia deducta|[2862]|BBLOC627-11|BIOUG01458-F02|658[0n]bp|United States.Arizona|BOLD:AAB5985
Bulia deducta|[2863]|BBLOD1102-11|BIOUG01822-D03|658[0n]bp|United States.Texas|BOLD:AAB5985
Bulia deducta|[2864]|AWCLB476-11|AWC-08438|658[0n]bp|United States.Arizona|BOLD:AAB5985
Bulia deducta|[2865]|USLEP952-10|10BBLEP-00952|658[0n]bp|United States.Arizona|BOLD:AAB5985
Bulia deducta|[2866]|BBLSY909-09|09BBLEP-03836|658[0n]bp|United States.Texas|BOLD:AAB5985
Bulia deducta|[2867]|BBLOD880-11|BIOUG01569-A07|658[0n]bp|United States.California|BOLD:AAB5985
Bulia deducta|[2868]|BBLSY917-09|09BBLEP-03844|658[0n]bp|United States.Texas|BOLD:AAB5985
Bulia deducta|[2869]|BBLSX747-09|09BBLEP-02675|658[0n]bp|United States.Arizona|BOLD:AAB5985
Bulia deducta|[2870]|BBLSW080-09|09BBLEP-01008|658[0n]bp|United States.Texas|BOLD:AAB5985
Bulia deducta|[2871]|BBLOD1279-11|BIOUG01825-C02|658[0n]bp|United States.Texas|BOLD:AAB5985
Bulia deducta|[2872]|BBLOD1284-11|BIOUG01825-C07|658[0n]bp|United States.Texas|BOLD:AAB5985
Bulia deducta|[2873]|BBLOD1286-11|BIOUG01825-C09|658[0n]bp|United States.Texas|BOLD:AAB5985
Bulia deducta|[2874]|BBLOD899-11|BIOUG01569-C02|658[0n]bp|United States.California|BOLD:AAB5985
Bulia deducta|[2875]|BBLOC666-11|CCDB-11714-G06|658[0n]bp|United States.Texas|BOLD:AAB5985
[illegible]

Bulia deducta|[2873]|BBLOD1286-11|BIOUG01825-C09|658[0n]bp|United States.Texas|BOLD:AAB5985
Bulia deducta|[2874]|BBLOD899-11|BIOUG01569-C02|658[0n]bp|United States.California|BOLD:AAB5985
Bulia deducta|[2875]|BBLOC666-11|CCDB-11714-G06|658[0n]bp|United States.Texas|BOLD:AAB5985
Bulia deducta|[2876]|BBLSZ178-09|09BBLEP-04104|658[0n]bp|United States.Texas|BOLD:AAB5985
Bulia deducta|[2877]|USLEP1172-10|10BBLEP-01172|658[0n]bp|United States.Arizona|BOLD:AAB5985
Bulia deducta|[2878]|BBLOD875-11|BIOUG01569-A02|658[0n]bp|United States.California|BOLD:AAB5985
Bulia deducta|[2879]|IAWLB536-11|IAWAZ-1444|658[0n]bp|United States.Arizona|BOLD:AAB5985
Bulia deducta|[2880]|BBLSY665-09|09BBLEP-03592|658[0n]bp|United States.Arizona|BOLD:AAB5985
Bulia deducta|[2881]|IAWL110-09|IAWAZ-0109|658[0n]bp|United States.Arizona|BOLD:AAB5985
Bulia deducta|[2882]|IAWL111-09|IAWAZ-0110|658[0n]bp|United States.California|BOLD:AAB5985
Bulia deducta|[2883]|AWCLB568-11|AWC-00850|658[0n]bp|United States.Arizona|BOLD:AAB5985
Bulia deducta|[2884]|BBLOC633-11|BIOUG01458-F08|658[0n]bp|United States.Arizona|BOLD:AAB5985
Bulia deducta|[2885]|BBLOC665-11|CCDB-11714-G05|658[0n]bp|United States.Texas|BOLD:AAB5985
Bulia deducta|[2886]|AWCLB594-11|AWC-07666|658[0n]bp|United States.Arizona|BOLD:AAB5985
Bulia deducta|[2887]|BBLSY914-09|09BBLEP-03841|658[0n]bp|United States.Texas|BOLD:AAB5985
Bulia deducta|[2888]|BBLSZ179-09|09BBLEP-04105|658[0n]bp|United States.Texas|BOLD:AAB5985
Bulia deducta|[2889]|CMAZA281-09|CMAZ-0281|658[0n]bp|United States.Arizona|BOLD:AAB5985
Bulia deducta|[2890]|CMAZA1021-12|BIOUG02041-H03|658[0n]bp|United States.Arizona|BOLD:AAB5985
Bulia deducta|[2891]|BBLOD1098-11|BIOUG01822-C11|658[0n]bp|United States.Texas|BOLD:AAB5985
Bulia deducta|[2892]|BBLOE1988-12|BIOUG01995-B08|658[0n]bp|United States.Texas|BOLD:AAB5985
Bulia deducta|[2893]|BBLOD137-11|BIOUG01552-B12|658[0n]bp|United States.Texas|BOLD:AAB5985
Bulia deducta|[2894]|BBLOE1441-12|BIOUG01987-D07|658[0n]bp|United States.Texas|BOLD:AAB5985
Bulia deducta|[2895]|BBLOC272-11|BIOUG01454-H03|658[0n]bp|United States.Arizona|BOLD:AAB5985
Bulia deducta|[2896]|USLEP1166-10|10BBLEP-01166|658[0n]bp|United States.Texas|BOLD:AAB5985
Bulia deducta|[2897]|BBLSW031-09|09BBLEP-00959|658[0n]bp|United States.Texas|BOLD:AAB5985
Bulia deducta|[2898]|AWCLB414-10|AWC-08099|658[0n]bp|United States.Arizona|BOLD:AAB5985
Bulia deducta|[2899]|AWCLB600-11|AWC-07672|658[0n]bp|United States.Arizona|BOLD:AAB5985
Bulia deducta|[2900]|IAWLB541-11|IAWAZ-1449|658[0n]bp|United States.Arizona|BOLD:AAB5985
Bulia deducta|[2901]|BBLSW313-09|09BBLEP-01241|658[0n]bp|United States.Arizona|BOLD:AAB5985
Bulia deducta|[2902]|BBLSW057-09|09BBLEP-00985|658[0n]bp|United States.Texas|BOLD:AAB5985
Bulia deducta|[2903]|IAWL109-09|IAWAZ-0108|658[0n]bp|United States.Arizona|BOLD:AAB5985
Bulia deducta|[2904]|AWCLB618-11|AWC-07965|658[0n]bp|United States.Arizona|BOLD:AAB5985
Bulia deducta|[2905]|BBLOC1649-11|BIOUG01547-A11|658[0n]bp|United States.Arizona|BOLD:AAB5985
Bulia deducta|[2906]|IAWLB539-11|IAWAZ-1447|658[0n]bp|United States.Arizona|BOLD:AAB5985
Bulia deducta|[2907]|BBLSZ195-09|09BBLEP-04121|658[0n]bp|United States.Texas|BOLD:AAB5985
Bulia deducta|[2908]|AWCLB601-11|AWC-07673|658[0n]bp|United States.Arizona|BOLD:AAB5985
Bulia deducta|[2909]|BBLOD1697-11|BIOUG01830-F04|658[0n]bp|United States.Texas|BOLD:AAB5985
Bulia deducta|[2910]|BBLOB674-11|BIOUG01399-A09|658[0n]bp|United States.Texas|BOLD:AAB5985
Bulia deducta|[2911]|CMAZA796-10|CMAZ-0796|658[0n]bp|United States.Arizona|BOLD:AAB5985
Bulia deducta|[2912]|BBLOD142-11|BIOUG01552-C05|658[0n]bp|United States.Texas|BOLD:AAB5985
Bulia deducta|[2913]|BBLSY732-09|09BBLEP-03659|658[0n]bp|United States.Arizona|BOLD:AAB5985
Bulia deducta|[2914]|BBLOE1425-12|BIOUG01987-C03|658[0n]bp|United States.Arizona|BOLD:AAB5985
Bulia deducta|[2915]|BBLSY838-09|09BBLEP-03765|658[0n]bp|United States.Arizona|BOLD:AAB5985
Bulia deducta|[2916]|BBLOC227-11|BIOUG01454-D06|658[0n]bp|United States.Arizona|BOLD:AAB5985
Bulia deducta|[2917]|BBLOC154-11|BIOUG01453-F04|658[0n]bp|United States.Arizona|BOLD:AAB5985
Bulia deducta|[2918]|BBLSY230-09|09BBLEP-03157|658[0n]bp|United States.Texas|BOLD:AAB5985
Bulia deducta|[2919]|BBLOD1096-11|BIOUG01822-C09|658[0n]bp|United States.Texas|BOLD:AAB5985
Bulia deducta|[2920]|IAWL114-09|IAWAZ-0113|658[0n]bp|United States.Arizona|BOLD:AAB5985
Bulia deducta|[2921]|BBLOB019-11|BIOUG01367-B07|658[0n]bp|United States.Arizona|BOLD:AAB5985
Bulia deducta|[2922]|AWCLB507-11|AWC-70018|658[0n]bp|United States.Arizona|BOLD:AAB5985
Bulia deducta|[2923]|BBLOC226-11|BIOUG01454-D05|658[0n]bp|United States.Arizona|BOLD:AAB5985
Bulia deducta|[2924]|BBLOD1612-11|BIOUG01829-G02|658[0n]bp|United States.Texas|BOLD:AAB5985
Bulia deducta|[2925]|BBLOE1456-12|BIOUG01987-E10|658[0n]bp|United States.Texas|BOLD:AAB5985
Bulia deducta|[2926]|IAWL106-09|IAWAZ-0105|658[0n]bp|United States.Arizona|BOLD:AAB5985
Bulia deducta|[2927]|BBLOD1275-11|BIOUG01825-B10|658[0n]bp|United States.Texas|BOLD:AAB5985
Bulia deducta|[2928]|BBLOE1460-12|BIOUG01987-F02|658[0n]bp|United States.Texas|BOLD:AAB5985
Bulia deducta|[2929]|BBLOD1274-11|BIOUG01825-B09|658[0n]bp|United States.Texas|BOLD:AAB5985
Bulia deducta|[2930]|BBLSW324-09|09BBLEP-01252|658[0n]bp|United States.Arizona|BOLD:AAB5985
Bulia deducta|[2931]|IAWL108-09|IAWAZ-0107|658[0n]bp|United States.Arizona|BOLD:AAB5985
Bulia deducta|[2932]|BBLOE2020-12|BIOUG01995-E04|658[0n]bp|United States.Texas|BOLD:AAB5985
Bulia deducta|[2933]|BBLOE1707-12|BIOUG01990-B12|658[0n]bp|United States.Texas|BOLD:AAB5985
Bulia deducta|[2934]|BBLSY294-09|09BBLEP-03221|658[0n]bp|United States.Arizona|BOLD:AAB5985
Bulia deducta|[2935]|BBLOD1106-11|BIOUG01822-D07|658[0n]bp|United States.Texas|BOLD:AAB5985
Bulia deducta|[2936]|BBLOB711-11|BIOUG01399-D10|658[0n]bp|United States.Texas|BOLD:AAB5985
Bulia deducta|[2937]|BBLOD518-11|BIOUG01565-C01|658[0n]bp|United States.Texas|BOLD:AAB5985
Bulia deducta|[2938]|BBLOD1664-11|BIOUG01830-C07|658[0n]bp|United States.Texas|BOLD:AAB5985
Bulia deducta|[2939]|BBLOD917-11|BIOUG01569-D08|658[0n]bp|United States.Texas|BOLD:AAB5985
Bulia deducta|[2940]|BBLOB686-11|BIOUG01399-B09|658[0n]bp|United States.Texas|BOLD:AAB5985
Bulia deducta|[2941]|LPOKB131-09|MDOK-1253|658[0n]bp|United States.Oklahoma|BOLD:AAB5985
Bulia deducta|[2942]|BBLOC626-11|BIOUG01458-F01|658[0n]bp|United States.Arizona|BOLD:AAB5985
Bulia deducta|[2943]|BBLOB023-11|BIOUG01367-B11|658[0n]bp|United States.Arizona|BOLD:AAB5985
Bulia deducta|[2944]|BBLOD921-11|BIOUG01569-D12|658[0n]bp|United States.Texas|BOLD:AAB5985
Bulia deducta|[2945]|BBLSY906-09|09BBLEP-03833|658[0n]bp|United States.Texas|BOLD:AAB5985
Bulia deducta|[2946]|BBLOC1995-11|BIOUG01550-F12|658[0n]bp|United States.Texas|BOLD:AAB5985
Bulia deducta|[2947]|AWCLB492-11|AWC-07985|658[0n]bp|United States.Arizona|BOLD:AAB5985
Bulia deducta|[2948]|BBLSZ177-09|09BBLEP-04103|658[0n]bp|United States.Texas|BOLD:AAB5985
Bulia deducta|[2949]|BBLOD1280-11|BIOUG01825-C03|658[0n]bp|United States.Texas|BOLD:AAB5985
Bulia deducta|[2950]|BBLOD879-11|BIOUG01569-A06|658[0n]bp|United States.California|BOLD:AAB5985
Bulia deducta|[2951]|BBLOC886-11|BIOUG01467-B01|658[0n]bp|United States.Arizona|BOLD:AAB5985
Bulia deducta|[2952]|BBLOC1414-11|BIOUG01542-F01|648[0n]bp|United States.Texas|BOLD:AAB5985
Bulia deducta|[2953]|ABNCC289-07|1289-110603-TX|643[0n]bp|United States.Texas|BOLD:AAB5985
Bulia deducta|[2954]|ABNCC287-07|1287-130603-TX|641[0n]bp|United States.Texas|BOLD:AAB5985
Bulia deducta|[2955]|BBLSY295-09|09BBLEP-03222|642[0n]bp|United States.Arizona|BOLD:AAB5985
Bulia deducta|[2956]|BBLOD1081-11|BIOUG01822-B06|658[0n]bp|United States.Texas|BOLD:AAB5985
Bulia deducta|[2957]|BBLSY829-09|09BBLEP-03756|643[0n]bp|United States.Arizona|BOLD:AAB5985
Bulia deducta|[2958]|BBLOD1614-11|BIOUG01829-G04|556[1n]bp|United States.Texas|BOLD:AAB5985
Bulia deducta|[2959]|USLEP1170-10|10BBLEP-01170|634[0n]bp|United States.Arizona|BOLD:AAB5985
Bulia deducta|[2960]|LPOKB1023-09|MDOK-2065|658[0n]bp|United States.Oklahoma|BOLD:AAB5985
Bulia deducta|[2961]|BBLSZ181-09|09BBLEP-04107|648[0n]bp|United States.Texas|BOLD:AAB5985
Bulia deducta|[2962]|ABNCC288-07|1288-280503-TX|638[0n]bp|United States.Texas|BOLD:AAB5985
Bulia deducta|[2963]|BBLOD127-11|BIOUG01552-B02|636[0n]bp|United States.California|BOLD:AAB5985
Bulia deducta|[2964]|USLEP089-10|10BBLEP-00089|636[0n]bp|United States.Arizona|BOLD:AAB5985
Bulia deducta|[2965]|LPOKD666-10|MDOK-3745|658[0n]bp|United States.Oklahoma|BOLD:AAB5985
Bulia deducta|[2966]|BBLSW146-09|09BBLEP-01074|638[0n]bp|United States.Arizona|BOLD:AAB5985
Bulia deducta|[2967]|ABNCC290-07|1290-260503-TX|642[0n]bp|United States.Texas|BOLD:AAB5985
Bulia deducta|[2968]|LMEMB140-09|RBMIS-1233|658[0n]bp|United States.Texas|BOLD:AAB5985
Bulia deducta|[2969]|BBLOC150-11|BIOUG01453-E12|658[0n]bp|United States.Arizona|BOLD:AAB5985
Bulia deducta|[2970]|BBLOE2021-12|BIOUG01995-E05|658[0n]bp|United States.Texas|BOLD:AAB5985
Bulia deducta|[2971]|BBLSY125-09|09BBLEP-03052|658[0n]bp|United States.Texas|BOLD:AAB5985
Melipotis indomita|[2972]|BBLOB1053-11|BIOUG01415-A08|658[0n]bp|United States.Arizona|BOLD:AAD4517
Melipotis indomita|[2973]|LPOKD787-10|MDOK-3866|658[0n]bp|United States.Oklahoma|BOLD:AAD4517
Melipotis indomita|[2974]|RDNMG913-08|CNC LEP00053037|658[0n]bp|United States.Arizona|BOLD:AAD4517
[illegible]

Melipotis indomita|[2972]|BBLOB1033-11|BIOUG01415-A08|658[0n]bp|United States.Arizona|BOLD:AAD4517
Melipotis indomita|[2973]|LPOKD787-10|MDOK-3866|658[0n]bp|United States.Oklahoma|BOLD:AAD4517
Melipotis indomita|[2974]|RDNMG913-08|CNC LEP00053037|658[0n]bp|United States.Arizona|BOLD:AAD4517
Melipotis indomita|[2975]|QUNOB022-08|5397-060608-TX|658[0n]bp|United States.Texas|BOLD:AAD4517
Melipotis indomita|[2976]|AWCLB620-11|AWC-07967|631[0n]bp|United States.Arizona|BOLD:AAD4517
Melipotis indomita|[2977]|BBLOE1440-12|BIOUG01987-D06|658[0n]bp|United States.Texas|BOLD:AAD4517
Melipotis indomita|[2978]|BBLOB595-11|BIOUG01398-C01|658[0n]bp|United States.Arizona|BOLD:AAD4517
Melipotis indomita|[2979]|BBLOE1423-12|BIOUG01987-C01|658[0n]bp|United States.Arizona|BOLD:AAD4517
Melipotis indomita|[2980]|IAWLB533-11|IAWAZ-1441|658[0n]bp|United States.Arizona|BOLD:AAD4517
Melipotis indomita|[2981]|BBLSW343-09|09BBLEP-01271|658[0n]bp|United States.Arizona|BOLD:AAD4517
Melipotis indomita|[2982]|LPOKD784-10|MDOK-3863|658[0n]bp|United States.Oklahoma|BOLD:AAD4517
Melipotis indomita|[2983]|IAWL092-09|IAWAZ-0091|658[0n]bp|United States.Arizona|BOLD:AAD4517
Melipotis indomita|[2984]|BBLOC1029-11|BIOUG01468-F01|658[0n]bp|United States.Texas|BOLD:AAD4517
Melipotis indomita|[2985]|IAWL090-09|IAWAZ-0089|658[0n]bp|United States.Arizona|BOLD:AAD4517
Melipotis indomita|[2986]|BBLSW802-09|09BBLEP-01730|658[0n]bp|United States.Oklahoma|BOLD:AAD4517
Melipotis indomita|[2987]|LPOKB153-09|MDOK-1355|658[0n]bp|United States.Oklahoma|BOLD:AAD4517
Melipotis indomita|[2988]|BBLOD1662-11|BIOUG01830-C05|658[0n]bp|United States.Texas|BOLD:AAD4517
Melipotis indomita|[2989]|BBLOD145-11|BIOUG01552-C08|658[0n]bp|United States.Texas|BOLD:AAD4517
Melipotis indomita|[2990]|BBLOE1977-12|BIOUG01995-A09|658[0n]bp|United States.Texas|BOLD:AAD4517
Melipotis indomita|[2991]|BBLOE1450-12|BIOUG01987-E04|658[0n]bp|United States.Texas|BOLD:AAD4517
Melipotis indomita|[2992]|BBLSW704-09|09BBLEP-01632|658[0n]bp|United States.Texas|BOLD:AAD4517
Melipotis indomita|[2993]|BBLOE1463-12|BIOUG01987-F05|658[0n]bp|United States.Texas|BOLD:AAD4517
Melipotis indomita|[2994]|BBLOD1698-11|BIOUG01830-F05|658[0n]bp|United States.Texas|BOLD:AAD4517
Melipotis indomita|[2995]|BBLOE1978-12|BIOUG01995-A10|658[0n]bp|United States.Texas|BOLD:AAD4517
Melipotis indomita|[2996]|BBLOC609-11|BIOUG01458-D08|658[0n]bp|United States.Arizona|BOLD:AAD4517
Melipotis indomita|[2997]|CMAZA877-12|BIOUG02040-C04|658[0n]bp|United States.Arizona|BOLD:AAD4517
Melipotis indomita|[2998]|BBLOE1992-12|BIOUG01995-B12|658[0n]bp|United States.Arizona|BOLD:AAD4517
Melipotis indomita|[2999]|BBLOE1976-12|BIOUG01995-A08|658[0n]bp|United States.Texas|BOLD:AAD4517
Melipotis indomita|[3000]|BBLOD1283-11|BIOUG01825-C06|658[0n]bp|United States.Texas|BOLD:AAD4517
Melipotis indomita|[3001]|BBLOC1413-11|BIOUG01542-E12|658[0n]bp|United States.Texas|BOLD:AAD4517
Melipotis indomita|[3002]|BBLOD897-11|BIOUG01569-B12|658[0n]bp|United States.California|BOLD:AAD4517
Melipotis indomita|[3003]|BBLOE1457-12|BIOUG01987-E11|658[0n]bp|United States.Texas|BOLD:AAD4517
Melipotis indomita|[3004]|BBLSX678-09|09BBLEP-02606|658[0n]bp|United States.Arizona|BOLD:AAD4517
Melipotis indomita|[3005]|IAWLB534-11|IAWAZ-1442|658[0n]bp|United States.Arizona|BOLD:AAD4517
Melipotis indomita|[3006]|ABNCC265-07|1265-070603-TX|635[1n]bp|United States.Texas|BOLD:AAD4517
Melipotis indomita|[3007]|BBLOE1449-12|BIOUG01987-E03|658[0n]bp|United States.Texas|BOLD:AAD4517
Melipotis indomita|[3008]|USLEP297-10|10BBLEP-00297|658[0n]bp|United States.Texas|BOLD:AAD4517
Melipotis indomita|[3009]|BBLOD1658-11|BIOUG01830-C01|658[0n]bp|United States.Texas|BOLD:AAD4517
Melipotis indomita|[3010]|BBLOD883-11|BIOUG01569-A10|658[0n]bp|United States.California|BOLD:AAD4517
Melipotis indomita|[3011]|BBLSX679-09|09BBLEP-02607|658[0n]bp|United States.Arizona|BOLD:AAD4517
Melipotis indomita|[3012]|IAWL067-09|IAWAZ-0066|658[0n]bp|United States.Arizona|BOLD:AAD4517
Melipotis indomita|[3013]|RDNMG912-08|CNC LEP00053036|658[0n]bp|United States.Arizona|BOLD:AAD4517
Melipotis indomita|[3014]|LPOKC613-09|MDOK-2690|658[0n]bp|United States.Oklahoma|BOLD:AAD4517
Melipotis indomita|[3015]|BBLOE1464-12|BIOUG01987-F06|658[0n]bp|United States.Texas|BOLD:AAD4517
Melipotis indomita|[3016]|BBLOE1418-12|BIOUG01987-B08|658[0n]bp|United States.Texas|BOLD:AAD4517
Melipotis indomita|[3017]|BBLOB610-11|BIOUG01398-D04|658[0n]bp|United States.Texas|BOLD:AAD4517
Melipotis indomita|[3018]|BBLOD194-11|BIOUG01552-G09|658[0n]bp|United States.Texas|BOLD:AAD4517
Melipotis indomita|[3019]|BBLOD1271-11|BIOUG01825-B06|658[0n]bp|United States.Texas|BOLD:AAD4517
Melipotis indomita|[3020]|BBLOD885-11|BIOUG01569-A12|658[0n]bp|United States.California|BOLD:AAD4517
Melipotis indomita|[3021]|BBLOE1427-12|BIOUG01987-C05|658[0n]bp|United States.Arizona|BOLD:AAD4517
Melipotis indomita|[3022]|BBLOC224-11|BIOUG01454-D03|658[0n]bp|United States.Arizona|BOLD:AAD4517
Melipotis indomita|[3023]|BBLOD1287-11|BIOUG01825-C10|658[0n]bp|United States.Texas|BOLD:AAD4517
Melipotis indomita|[3024]|BBLOB681-11|BIOUG01399-B04|658[0n]bp|United States.Texas|BOLD:AAD4517
Melipotis indomita|[3025]|BBLOB594-11|BIOUG01398-B12|658[0n]bp|United States.Arizona|BOLD:AAD4517
Melipotis indomita|[3026]|BBLOD1273-11|BIOUG01825-B08|658[0n]bp|United States.Texas|BOLD:AAD4517
Melipotis indomita|[3027]|ABNCC266-07|1266-240504-TX|636[0n]bp|United States.Texas|BOLD:AAD4517
Melipotis indomita|[3028]|HKONB113-08|3610-COI-08|658[0n]bp|United States.Texas|BOLD:AAD4517
Melipotis indomita|[3029]|BBLSW525-09|09BBLEP-01453|658[0n]bp|United States.Oklahoma|BOLD:AAD4517
Melipotis indomita|[3030]|BBLOC223-11|BIOUG01454-D02|658[0n]bp|United States.Arizona|BOLD:AAD4517
Drasteria hudsonica|[3031]|RDNM752-05|CNCNoctuoidea7598|658[0n]bp|Canada.Saskatchewan|BOLD:AAB3338
Drasteria hudsonica|[3032]|LPMN899-08|08BBLEP-02257|658[0n]bp|Canada.Alberta|BOLD:AAB3338
Drasteria hudsonica|[3033]|LPABB280-08|08BBLEP-03545|658[0n]bp|Canada.Alberta|BOLD:AAB3338
Drasteria hudsonica|[3034]|BBLPB331-10|10BBCLP-1330|658[0n]bp|Canada.British Columbia|BOLD:AAB3338
Drasteria hudsonica|[3035]|LPAB043-08|08BBLEP-02365|658[0n]bp|Canada.Alberta|BOLD:AAB3338
Drasteria hudsonica|[3036]|LOWCE348-06|CGWC-4108|656[0n]bp|Canada.British Columbia|BOLD:AAB3338
Drasteria hudsonica|[3037]|BBLPB328-10|10BBCLP-1327|658[0n]bp|Canada.Alberta|BOLD:AAB3338
Drasteria hudsonica|[3038]|LPABB377-08|08BBLEP-03642|658[0n]bp|Canada.Alberta|BOLD:AAB3338
Drasteria hudsonica|[3039]|LPABB401-08|08BBLEP-03666|658[0n]bp|Canada.Alberta|BOLD:AAB3338
Drasteria hudsonica|[3040]|LPABB201-08|08BBLEP-03466|658[0n]bp|Canada.Alberta|BOLD:AAB3338
Drasteria hudsonica|[3041]|LPAB052-08|08BBLEP-02374|658[0n]bp|Canada.Alberta|BOLD:AAB3338
Drasteria hudsonica|[3042]|LOWCE087-06|CGWC-3847|658[0n]bp|Canada.British Columbia|BOLD:AAB3338
Drasteria hudsonica|[3043]|LPABB626-08|08BBLEP-03891|658[0n]bp|Canada.Alberta|BOLD:AAB3338
Drasteria hudsonica|[3044]|BBLPB326-10|10BBCLP-1325|658[0n]bp|Canada.British Columbia|BOLD:AAB3338
Drasteria hudsonica|[3045]|BBLPB324-10|10BBCLP-1323|658[0n]bp|Canada.British Columbia|BOLD:AAB3338
Drasteria hudsonica|[3046]|LPABB264-08|08BBLEP-03529|658[0n]bp|Canada.Alberta|BOLD:AAB3338
Drasteria hudsonica|[3047]|LPMN952-08|08BBLEP-02310|658[0n]bp|Canada.Alberta|BOLD:AAB3338
Drasteria hudsonica|[3048]|LOWCE350-06|CGWC-4110|592[0n]bp|Canada.British Columbia|BOLD:AAB3338
Drasteria hudsonica|[3049]|LALPA848-11|AVBC 1021-11|658[0n]bp|Canada.British Columbia|BOLD:AAB3338
Drasteria hudsonica|[3050]|RDNM751-05|CNCNoctuoidea7597|658[0n]bp|Canada.British Columbia|BOLD:AAB3338
Drasteria hudsonica|[3051]|LBCG3441-09|08-JDWBC-3441|658[0n]bp|Canada.British Columbia|BOLD:AAB3338
Drasteria hudsonica|[3052]|LPAB042-08|08BBLEP-02364|658[0n]bp|Canada.Alberta|BOLD:AAB3338
Drasteria hudsonica|[3053]|BBLPB325-10|10BBCLP-1324|658[0n]bp|Canada.British Columbia|BOLD:AAB3338
Drasteria hudsonica|[3054]|BBLPB327-10|10BBCLP-1326|658[0n]bp|Canada.Alberta|BOLD:AAB3338
Drasteria hudsonica|[3055]|BBLPB329-10|10BBCLP-1328|658[0n]bp|Canada.British Columbia|BOLD:AAB3338
Drasteria hudsonica|[3056]|LPABC642-09|08BBLEP-04861|658[0n]bp|Canada.Alberta|BOLD:AAB3338
Drasteria hudsonica|[3057]|LPABB576-08|08BBLEP-03841|658[0n]bp|Canada.Alberta|BOLD:AAB3338
Drasteria hudsonica|[3058]|LPAB293-08|08BBLEP-02615|658[0n]bp|Canada.Alberta|BOLD:AAB3338
Drasteria hudsonica|[3059]|LPABB295-08|08BBLEP-03560|658[0n]bp|Canada.Alberta|BOLD:AAB3338
Drasteria hudsonica|[3060]|LPABC988-09|08BBLEP-05399|658[0n]bp|Canada.Alberta|BOLD:AAB3338
Drasteria grandirena|[3061]|ABNCC308-07|1308-130602-AR|612[0n]bp|United States.Arkansas|BOLD:AAC3386
Drasteria grandirena|[3062]|HKONS432-08|1902-COI-07|658[0n]bp|United States.Florida|BOLD:AAC3386
Drasteria grandirena|[3063]|LNC948-06|06-NCCC-948|605[0n]bp|United States.North Carolina|BOLD:AAC3386
Drasteria grandirena|[3064]|LNC927-06|06-NCCC-927|658[0n]bp|United States.North Carolina|BOLD:AAC3386
Drasteria grandirena|[3065]|MMNA115-08|HLC-17677|642[0n]bp|United States.Georgia|BOLD:AAC3386
Drasteria grandirena|[3066]|LMEMB194-09|RBMIS-1287|612[0n]bp|United States.Alabama|BOLD:AAC3386
Drasteria grandirena|[3067]|BBLCU203-09|09BBLEP-04690|658[0n]bp|United States.Michigan|BOLD:AAC3386
Drasteria grandirena|[3068]|ABNCC309-07|1309-150504-TX|642[0n]bp|United States.Texas|BOLD:AAC3386
Drasteria grandirena|[3069]|BBLCU191-09|09BBLEP-04678|658[0n]bp|United States.Michigan|BOLD:AAC3386
Drasteria grandirena|[3070]|LNC947-06|06-NCCC-947|573[0n]bp|United States.North Carolina|BOLD:AAC3386
Drasteria grandirena|[3071]|LMEMB195-09|RBMIS-1288|658[0n]bp|United States.Georgia|BOLD:AAC3386
Drasteria grandirena|[3072]|LMEMB193-09|RBMIS-1286|621[0n]bp|United States.Alabama|BOLD:AAC3386
Drasteria grandirena|[3073]|HKONS433-08|1903-COI-07|658[0n]bp|United States.Florida|BOLD:AAC3386
Drasteria grandirena|[3074]|MMNA114-08|HLC-17676|658[0n]bp|United States.Georgia|BOLD:AAC3386

Drasteria grandirena|[3071]|LMEMB19[illegible]-09|RBMIS-128[illegible]|658[0n]bp|United States.Georgia|BOLD:AAC3386
Drasteria grandirena|[3072]|LMEMB193-09|RBMIS-1286|621[0n]bp|United States.Alabama|BOLD:AAC3386
Drasteria grandirena|[3073]|HKONS433-08|1903-COI-07|658[0n]bp|United States.Florida|BOLD:AAC3386
Drasteria grandirena|[3074]|MMNA114-08|HLC-17676|658[0n]bp|United States.Georgia|BOLD:AAC3386
Drasteria howlandii|[3075]|RDMAB219-05|UASM57366|658[0n]bp|Canada.Alberta|BOLD:ABZ1548
Drasteria petricola|[3076]|RDMAB224-05|UASM41314|658[0n]bp|Canada.Alberta|BOLD:AAE0309
Drasteria petricola|[3077]|LPABC649-09|08BBLEP-04868|658[0n]bp|Canada.Alberta|BOLD:AAE0309
Drasteria petricola|[3078]|RDMAB227-05|UASM58307|525[1n]bp|Canada.Alberta|BOLD:AAE0309
Drasteria petricola|[3079]|RDMAB226-05|UASM34361|629[0n]bp|Canada.Alberta|BOLD:AAE0309
Drasteria petricola|[3080]|RDMAB225-05|UASM34353|631[0n]bp|Canada.Alberta|BOLD:AAE0309
Drasteria petricola|[3081]|QUNOE512-12|10536-240605-AL|658[0n]bp|Canada.Yukon Territory|BOLD:AAE0309
Drasteria pallescens|[3082]|LOCBB124-06|06-BLLOC-1064|658[0n]bp|United States.California|BOLD:AAB4995
Drasteria pallescens|[3083]|IAWL076-09|IAWAZ-0075|658[0n]bp|United States.Arizona|BOLD:AAB4995
Drasteria pallescens|[3084]|LOCBB115-06|06-BLLOC-1055|658[0n]bp|United States.California|BOLD:AAB4995
Drasteria pallescens|[3085]|IAWL127-09|IAWAZ-0126|658[0n]bp|United States.California|BOLD:AAB4995
Drasteria pallescens|[3086]|LOCBB125-06|06-BLLOC-1065|658[0n]bp|United States.California|BOLD:AAB4995
Drasteria pallescens|[3087]|LOCBB123-06|06-BLLOC-1063|658[0n]bp|United States.California|BOLD:AAB4995
Drasteria pallescens|[3088]|LOCBD154-06|06-BLLOC-2974|658[0n]bp|United States.California|BOLD:AAB4995
Drasteria pallescens|[3089]|LOCBB122-06|06-BLLOC-1062|658[0n]bp|United States.California|BOLD:AAB4995
Drasteria pallescens|[3090]|LOCBB114-06|06-BLLOC-1054|658[0n]bp|United States.California|BOLD:AAB4995
Drasteria pallescens|[3091]|ABNCC303-07|1303-290603-UT|632[1n]bp|United States.Utah|BOLD:AAB4995
Drasteria pallescens|[3092]|IAWL128-09|IAWAZ-0127|658[0n]bp|United States.California|BOLD:AAB4995
Drasteria pallescens|[3093]|BBLSW727-09|09BBLEP-01655|658[0n]bp|United States.Texas|BOLD:AAB4995
Drasteria pallescens|[3094]|BBLOE1987-12|BIOUG01995-B07|658[0n]bp|United States.Texas|BOLD:AAB4995
Drasteria pallescens|[3095]|ABNCC302-07|1302-080603-TX|644[1n]bp|United States.Texas|BOLD:AAB4995
Drasteria pallescens|[3096]|LMEMB184-09|RBMIS-1277|657[0n]bp|United States.Arizona|BOLD:AAB4995
Drasteria pallescens|[3097]|ABNCC304-07|1304-270603-UT|630[1n]bp|United States.Utah|BOLD:AAB4995
Drasteria pallescens|[3098]|LMEMB185-09|RBMIS-1278|657[0n]bp|United States.Arizona|BOLD:AAB4995
Drasteria pallescens|[3099]|BBLOB375-11|BIOUG01370-H06|658[0n]bp|United States.Arizona|BOLD:AAB4995
Drasteria pallescens|[3100]|ABNCC012-07|1012-120703-UT|648[1n]bp|United States.Utah|BOLD:AAB4995
Drasteria pallescens|[3101]|LMEMB183-09|RBMIS-1276|657[0n]bp|United States.Arizona|BOLD:AAB4995
Drasteria pallescens|[3102]|BBLSX994-09|09BBLEP-02922|658[0n]bp|United States.Arizona|BOLD:AAB4995
Drasteria pallescens|[3103]|BBLSY424-09|09BBLEP-03351|658[0n]bp|United States.Arizona|BOLD:AAB4995
Drasteria pallescens|[3104]|IAWL129-09|IAWAZ-0128|658[0n]bp|United States.Arizona|BOLD:AAB4995
Drasteria pallescens|[3105]|BBLSX993-09|09BBLEP-02921|658[0n]bp|United States.Arizona|BOLD:AAB4995
Drasteria pallescens|[3106]|RDNMJ301-11|CNCLEP 80209|658[0n]bp|United States.Arizona|BOLD:AAB4995
Drasteria pallescens|[3107]|BBLOE1996-12|BIOUG01995-C04|658[0n]bp|United States.New Mexico|BOLD:ACF2882
Drasteria pallescens|[3108]|RDNME949-08|CNC LEP 00047424|658[0n]bp|United States.Texas|BOLD:ACF2882
Drasteria pallescens|[3109]|BBLOE1416-12|BIOUG01987-B06|658[0n]bp|United States.Texas|BOLD:ACF2882
Drasteria pallescens|[3110]|USLEP1063-10|10BBLEP-01063|658[0n]bp|United States.Colorado|BOLD:ACF2882
Drasteria pallescens|[3111]|USLEP1065-10|10BBLEP-01065|658[0n]bp|United States.Colorado|BOLD:ACF2882
Drasteria pallescens|[3112]|USLEP1064-10|10BBLEP-01064|658[0n]bp|United States.Colorado|BOLD:ACF2882
Drasteria pallescens|[3113]|BBLSW718-09|09BBLEP-01646|658[0n]bp|United States.Texas|BOLD:ACF2882
Drasteria pallescens|[3114]|LMEMB186-09|RBMIS-1279|657[0n]bp|United States.Wyoming|BOLD:ACF2882
Drasteria pallescens|[3115]|BBLOE1745-12|BIOUG01990-F02|658[0n]bp|United States.Texas|BOLD:ACF2882
Drasteria pallescens|[3116]|BBLOD922-11|BIOUG01569-E01|658[0n]bp|United States.Texas|BOLD:ACF2882
Drasteria pallescens|[3117]|BBLOE1709-12|BIOUG01990-C02|658[0n]bp|United States.Texas|BOLD:ACF2882
Drasteria pallescens|[3118]|BBLOD878-11|BIOUG01569-A05|658[0n]bp|United States.California|BOLD:ACF2882
Drasteria pallescens|[3119]|BBLOD1118-11|BIOUG01822-E07|658[0n]bp|United States.Texas|BOLD:ACF2882
Drasteria divergens|[3120]|RDNMC267-05|CNCNoctuoidea11901|597[1n]bp|Canada.British Columbia|BOLD:ABZ6755
Drasteria divergens|[3121]|LBCH5792-10|10-JDWBC-5792|658[0n]bp|Canada.British Columbia|BOLD:ABZ6755
Drasteria divergens|[3122]|LPAB004-08|08BBLEP-02326|658[0n]bp|Canada.Alberta|BOLD:ABZ6755
Drasteria divergens|[3123]|LALPA1288-11|AVBC 1290-11|658[0n]bp|Canada.British Columbia|BOLD:ABZ6755
Drasteria divergens|[3124]|LPABB326-08|08BBLEP-03591|658[0n]bp|Canada.Alberta|BOLD:ABZ6755
Drasteria divergens|[3125]|LPMN951-08|08BBLEP-02309|658[0n]bp|Canada.Alberta|BOLD:ABZ6755
Drasteria hastingsii|[3126]|RDNM749-05|CNCNoctuoidea7595|658[0n]bp|United States.Washington|BOLD:ACE6786
Drasteria hastingsii|[3127]|RDNMG546-08|CNC LEP00052370|658[0n]bp|United States.Washington|BOLD:ACE6786
Drasteria hastingsii|[3128]|RDMAB1011-09|UASM99702|635[0n]bp|United States.Washington|BOLD:ACE6786
Drasteria hastingsii|[3129]|RDNM748-05|CNCNoctuoidea7594|658[0n]bp|United States.Washington|BOLD:ACE6786
Drasteria hastingsii|[3130]|RDMAB1012-09|UASM99703|639[0n]bp|United States.Washington|BOLD:ACE6786
Drasteria hastingsii|[3131]|RDNMG545-08|CNC LEP00052369|658[0n]bp|United States.Washington|BOLD:ACE6786
Drasteria hastingsii|[3132]|RDMAB1013-09|UASM99704|658[0n]bp|United States.Washington|BOLD:ACE6786
Drasteria hastingsii|[3133]|RDNMG547-08|CNC LEP00052371|658[0n]bp|United States.Washington|BOLD:ACE6786
Drasteria adumbrata|[3134]|LOWCE349-06|CGWC-4109|658[0n]bp|Canada.British Columbia|BOLD:AAB6890
Drasteria adumbrata|[3135]|RDMAB222-05|UASM41395|658[0n]bp|Canada.Manitoba|BOLD:AAB6890
Drasteria adumbrata|[3136]|RDMAB220-05|UASM19821|658[0n]bp|Canada.Alberta|BOLD:AAB6890
Drasteria adumbrata|[3137]|LOWCE038-06|CGWC-3798|658[0n]bp|Canada.British Columbia|BOLD:AAB6890
Drasteria adumbrata|[3138]|LPAB053-08|08BBLEP-02375|658[0n]bp|Canada.Alberta|BOLD:AAB6890
Drasteria adumbrata|[3139]|LPABB368-08|08BBLEP-03633|658[0n]bp|Canada.Alberta|BOLD:AAB6890
Drasteria adumbrata|[3140]|LPAB003-08|08BBLEP-02325|658[0n]bp|Canada.Alberta|BOLD:AAB6890
Drasteria adumbrata|[3141]|LPABB518-08|08BBLEP-03783|658[0n]bp|Canada.Alberta|BOLD:AAB6890
Drasteria adumbrata|[3142]|LPAB002-08|08BBLEP-02324|634[0n]bp|Canada.Alberta|BOLD:AAB6890
Drasteria adumbrata|[3143]|LPABB363-08|08BBLEP-03628|658[0n]bp|Canada.Alberta|BOLD:AAB6890
Drasteria adumbrata|[3144]|LPABB254-08|08BBLEP-03519|658[0n]bp|Canada.Alberta|BOLD:AAB6890
Drasteria adumbrata|[3145]|LBCH5590-10|10-JDWBC-5590|658[0n]bp|Canada.British Columbia|BOLD:AAB6890
Drasteria adumbrata|[3146]|LPABC823-09|08BBLEP-05042|658[0n]bp|Canada.Alberta|BOLD:AAB6890
Drasteria adumbrata|[3147]|LOWCE351-06|CGWC-4111|658[0n]bp|Canada.British Columbia|BOLD:AAB6890
Drasteria adumbrata|[3148]|LPABB360-08|08BBLEP-03625|658[0n]bp|Canada.Alberta|BOLD:AAB6890
Drasteria adumbrata|[3149]|LPMN948-08|08BBLEP-02306|658[0n]bp|Canada.Alberta|BOLD:AAB6890
Drasteria adumbrata alleni|[3150]|RDLQB248-05|DH010334|658[0n]bp|Canada.Quebec|BOLD:AAB6890
Drasteria adumbrata alleni|[3151]|RDLQB252-05|DH010338|658[0n]bp|Canada.Quebec|BOLD:AAB6890
Drasteria adumbrata alleni|[3152]|RDLQB249-05|DH010335|658[0n]bp|Canada.Quebec|BOLD:AAB6890
Drasteria adumbrata alleni|[3153]|RDLQB251-05|DH010337|588[0n]bp|Canada.Quebec|BOLD:AAB6890
Drasteria adumbrata alleni|[3154]|RDLQB250-05|DH010336|561[1n]bp|Canada.Quebec|BOLD:AAB6890
Drasteria perplexa|[3155]|RDMAB094-05|UASM41973|615[1n]bp|Canada.Alberta|BOLD:AAE0308
Drasteria ochracea|[3156]|LBCH5695-10|10-JDWBC-5695|658[0n]bp|Canada.British Columbia|BOLD:AAD3446
Drasteria sabulosa|[3157]|LBCH5688-10|10-JDWBC-5688|658[0n]bp|Canada.British Columbia|BOLD:AAA9524
Drasteria sabulosa|[3158]|LBCH5687-10|10-JDWBC-5687|658[0n]bp|Canada.British Columbia|BOLD:AAA9524
Drasteria sabulosa|[3159]|LBCH5587-10|10-JDWBC-5587|658[0n]bp|Canada.British Columbia|BOLD:AAA9524
Drasteria sabulosa|[3160]|LBCH5585-10|10-JDWBC-5585|658[0n]bp|Canada.British Columbia|BOLD:AAA9524
Drasteria sabulosa|[3161]|LBCH5152-10|10-JDWBC-5152|658[0n]bp|Canada.British Columbia|BOLD:AAA9524
Drasteria sabulosa|[3162]|LBCH5931-10|10-JDWBC-5931|658[0n]bp|Canada.British Columbia|BOLD:AAA9524
Drasteria sabulosa|[3163]|LBCH5789-10|10-JDWBC-5789|658[0n]bp|Canada.British Columbia|BOLD:AAA9524
Drasteria sabulosa|[3164]|LBCH5694-10|10-JDWBC-5694|658[0n]bp|Canada.British Columbia|BOLD:AAA9524
Drasteria sabulosa|[3165]|LBCH5689-10|10-JDWBC-5689|658[0n]bp|Canada.British Columbia|BOLD:AAA9524
Drasteria sabulosa|[3166]|LBCH5100-10|10-JDWBC-5100|658[0n]bp|Canada.British Columbia|BOLD:AAA9524
Drasteria sabulosa|[3167]|LBCH5683-10|10-JDWBC-5683|658[0n]bp|Canada.British Columbia|BOLD:AAA9524
Drasteria sabulosa|[3168]|LBCH5098-10|10-JDWBC-5098|658[0n]bp|Canada.British Columbia|BOLD:AAA9524
Drasteria sabulosa|[3169]|LBCH5148-10|10-JDWBC-5148|658[0n]bp|Canada.British Columbia|BOLD:AAA9524
Drasteria sabulosa|[3170]|LBCH5583-10|10-JDWBC-5583|658[0n]bp|Canada.British Columbia|BOLD:AAA9524
Drasteria sabulosa|[3171]|LBCH5684-10|10-JDWBC-5684|658[0n]bp|Canada.British Columbia|BOLD:AAA9524
Drasteria sabulosa|[3172]|LBCH5102-10|10-JDWBC-5102|658[0n]bp|Canada.British Columbia|BOLD:AAA9524
Drasteria sabulosa|[3173]|LBCH5101-10|10-JDWBC-5101|658[0n]bp|Canada.British Columbia|BOLD:AAA9524

[illegible]
Drasteria sabulosa|[3171]|LBCH5684-10|10-JDWBC-5684|658[0n]bp|Canada.British Columbia|BOLD:AAA9524
Drasteria sabulosa|[3172]|LBCH5102-10|10-JDWBC-5102|658[0n]bp|Canada.British Columbia|BOLD:AAA9524
Drasteria sabulosa|[3173]|LBCH5101-10|10-JDWBC-5101|658[0n]bp|Canada.British Columbia|BOLD:AAA9524
Drasteria sabulosa|[3174]|LBCH5282-10|10-JDWBC-5282|658[0n]bp|Canada.British Columbia|BOLD:AAA9524
Drasteria sabulosa|[3175]|LBCH5099-10|10-JDWBC-5099|658[0n]bp|Canada.British Columbia|BOLD:AAA9524
Drasteria sabulosa|[3176]|LBCH5153-10|10-JDWBC-5153|658[0n]bp|Canada.British Columbia|BOLD:AAA9524
Drasteria sabulosa|[3177]|LBCH6219-10|10-JDWBC-6219|658[0n]bp|Canada.British Columbia|BOLD:AAA9524
Drasteria sabulosa|[3178]|LBCH6017-10|10-JDWBC-6017|658[0n]bp|Canada.British Columbia|BOLD:AAA9524
Drasteria sabulosa|[3179]|LBCH5105-10|10-JDWBC-5105|658[0n]bp|Canada.British Columbia|BOLD:AAA9524
Drasteria sabulosa|[3180]|LBCH5582-10|10-JDWBC-5582|658[0n]bp|Canada.British Columbia|BOLD:AAA9524
Drasteria sabulosa|[3181]|LBCH5151-10|10-JDWBC-5151|658[0n]bp|Canada.British Columbia|BOLD:AAA9524
Drasteria sabulosa|[3182]|LBCH5786-10|10-JDWBC-5786|658[0n]bp|Canada.British Columbia|BOLD:AAA9524
Drasteria sabulosa|[3183]|LBCH5784-10|10-JDWBC-5784|658[0n]bp|Canada.British Columbia|BOLD:AAA9524
Drasteria sabulosa|[3184]|LBCH5791-10|10-JDWBC-5791|658[0n]bp|Canada.British Columbia|BOLD:AAA9524
Drasteria sabulosa|[3185]|LBCH5150-10|10-JDWBC-5150|658[0n]bp|Canada.British Columbia|BOLD:AAA9524
Drasteria sabulosa|[3186]|LBCH5588-10|10-JDWBC-5588|658[0n]bp|Canada.British Columbia|BOLD:AAA9524
Drasteria sabulosa|[3187]|LBCH5790-10|10-JDWBC-5790|658[0n]bp|Canada.British Columbia|BOLD:AAA9524
Drasteria sabulosa|[3188]|LBCH5872-10|10-JDWBC-5872|658[0n]bp|Canada.British Columbia|BOLD:AAA9524
Drasteria sabulosa|[3189]|LBCH5418-10|10-JDWBC-5418|658[0n]bp|Canada.British Columbia|BOLD:AAA9524
Drasteria sabulosa|[3190]|LBCH5149-10|10-JDWBC-5149|658[0n]bp|Canada.British Columbia|BOLD:AAA9524
Drasteria sabulosa|[3191]|LBCH5685-10|10-JDWBC-5685|658[0n]bp|Canada.British Columbia|BOLD:AAA9524
Drasteria sabulosa|[3192]|LBCH5787-10|10-JDWBC-5787|658[0n]bp|Canada.British Columbia|BOLD:AAA9524
Drasteria sabulosa|[3193]|RDNM920-05|CNCNoctuoidea7760|658[0n]bp|Canada.British Columbia|BOLD:AAA9524
Drasteria sabulosa|[3194]|LBCH5146-10|10-JDWBC-5146|658[0n]bp|Canada.British Columbia|BOLD:AAA9524
Drasteria sabulosa|[3195]|LBCH5785-10|10-JDWBC-5785|632[0n]bp|Canada.British Columbia|BOLD:AAA9524
Drasteria sabulosa|[3196]|LBCH5103-10|10-JDWBC-5103|658[0n]bp|Canada.British Columbia|BOLD:AAA9524
Drasteria sabulosa|[3197]|LBCH5589-10|10-JDWBC-5589|658[0n]bp|Canada.British Columbia|BOLD:AAA9524
Drasteria sabulosa|[3198]|LBCH5104-10|10-JDWBC-5104|658[0n]bp|Canada.British Columbia|BOLD:AAA9524
Drasteria sabulosa|[3199]|LBCH5584-10|10-JDWBC-5584|658[0n]bp|Canada.British Columbia|BOLD:AAA9524
Drasteria sabulosa|[3200]|LBCH5441-10|10-JDWBC-5441|658[0n]bp|Canada.British Columbia|BOLD:AAA9524
Drasteria sabulosa|[3201]|LBCH5586-10|10-JDWBC-5586|658[0n]bp|Canada.British Columbia|BOLD:AAA9524
Drasteria sabulosa|[3202]|LBCH5147-10|10-JDWBC-5147|658[0n]bp|Canada.British Columbia|BOLD:AAA9524
Drasteria sabulosa|[3203]|LBCH5442-10|10-JDWBC-5442|658[0n]bp|Canada.British Columbia|BOLD:AAA9524
Drasteria sabulosa|[3204]|LBCH5788-10|10-JDWBC-5788|658[0n]bp|Canada.British Columbia|BOLD:AAA9524
Drasteria sabulosa|[3205]|LBCH5686-10|10-JDWBC-5686|658[0n]bp|Canada.British Columbia|BOLD:AAA9524
Melipotis jucunda|[3206]|LBCH5801-10|10-JDWBC-5801|658[0n]bp|Canada.British Columbia|BOLD:AAB3598
Melipotis jucunda|[3207]|LALPA173-10|AVBC 173-10|658[0n]bp|Canada.British Columbia|BOLD:AAB3598
Melipotis jucunda|[3208]|LALPA294-10|AVBC 296-10|658[0n]bp|Canada.British Columbia|BOLD:AAB3598
Melipotis jucunda|[3209]|LALPA142-10|AVBC 142-10|658[0n]bp|Canada.British Columbia|BOLD:AAB3598
Melipotis jucunda|[3210]|LALPA143-10|AVBC 143-10|658[0n]bp|Canada.British Columbia|BOLD:AAB3598
Melipotis jucunda|[3211]|LALPA642-10|AVBC 644-10|658[0n]bp|Canada.British Columbia|BOLD:AAB3598
Melipotis jucunda|[3212]|LPAB013-08|08BBLEP-02335|658[0n]bp|Canada.Alberta|BOLD:AAB3598
Melipotis jucunda|[3213]|LPABB041-08|08BBLEP-03306|658[0n]bp|Canada.Alberta|BOLD:AAB3598
Melipotis jucunda|[3214]|LPABC962-09|08BBLEP-05373|658[0n]bp|Canada.Alberta|BOLD:AAB3598
Cissusa indiscreta|[3215]|RDNMF504-08|NOC14590|658[0n]bp|Canada.British Columbia|BOLD:AAB4232
Cissusa indiscreta|[3216]|RDNMF507-08|NOC14593|609[0n]bp|Canada.British Columbia|BOLD:AAB4232
Cissusa indiscreta|[3217]|RDNMF506-08|NOC14592|658[0n]bp|Canada.British Columbia|BOLD:AAB4232
Cissusa spadix|[3218]|BBLSW789-09|09BBLEP-01717|658[0n]bp|United States.Oklahoma|BOLD:AAB4232
Cissusa spadix|[3219]|LOCT285-05|05-CTATBI-0285|658[0n]bp|United States.Connecticut|BOLD:AAB4232
Cissusa spadix|[3220]|LPOKB277-09|MDOK-1283|658[0n]bp|United States.Oklahoma|BOLD:AAB4232
Phoberia atomaris|[3221]|RDLQ367-05|DH010050|658[0n]bp|Canada.Quebec|BOLD:AAA6714
Phoberia atomaris|[3222]|MECB886-05|jflandry1918|658[0n]bp|Canada.Quebec|BOLD:AAA6714
Phoberia atomaris|[3223]|RDLQ364-05|DH010047|573[0n]bp|Canada.Quebec|BOLD:AAA6714
Phoberia atomaris|[3224]|RDLQ369-05|DH010052|658[0n]bp|Canada.Quebec|BOLD:AAA6714
Phoberia atomaris|[3225]|RDLQ385-05|DH010068|579[0n]bp|Canada.Quebec|BOLD:AAA6714
Phoberia atomaris|[3226]|RDLQ366-05|DH010049|658[0n]bp|Canada.Quebec|BOLD:AAA6714
Phoberia atomaris|[3227]|RDLQ375-05|DH010058|658[0n]bp|Canada.Quebec|BOLD:AAA6714
Phoberia atomaris|[3228]|RDLQ388-05|DH010071|658[0n]bp|Canada.Quebec|BOLD:AAA6714
Phoberia atomaris|[3229]|RDLQ362-05|DH010045|658[0n]bp|Canada.Quebec|BOLD:AAA6714
Phoberia atomaris|[3230]|MECB887-05|jflandry1919|658[0n]bp|Canada.Quebec|BOLD:AAA6714
Phoberia atomaris|[3231]|RDLQ383-05|DH010066|658[0n]bp|Canada.Quebec|BOLD:AAA6714
Phoberia atomaris|[3232]|RDLQ361-05|DH010044|658[0n]bp|Canada.Quebec|BOLD:AAA6714
Phoberia atomaris|[3233]|RDLQ363-05|DH010046|658[0n]bp|Canada.Quebec|BOLD:AAA6714
Phoberia atomaris|[3234]|RDLQ377-05|DH010060|658[0n]bp|Canada.Quebec|BOLD:AAA6714
Phoberia atomaris|[3235]|RDLQ365-05|DH010048|658[0n]bp|Canada.Quebec|BOLD:AAA6714
Phoberia atomaris|[3236]|RDLQ381-05|DH010064|658[0n]bp|Canada.Quebec|BOLD:AAA6714
Phoberia atomaris|[3237]|RDLQ382-05|DH010065|658[0n]bp|Canada.Quebec|BOLD:AAA6714
Phoberia atomaris|[3238]|RDLQ370-05|DH010053|638[0n]bp|Canada.Quebec|BOLD:AAA6714
Phoberia atomaris|[3239]|RDLQ384-05|DH010067|579[0n]bp|Canada.Quebec|BOLD:AAA6714
Phoberia atomaris|[3240]|MEC133-04|jflandry0133|600[0n]bp|Canada.Quebec|BOLD:AAA6714
Phoberia atomaris|[3241]|RDLQ371-05|DH010054|658[0n]bp|Canada.Quebec|BOLD:AAA6714
Phoberia atomaris|[3242]|RDLQ372-05|DH010055|658[0n]bp|Canada.Quebec|BOLD:AAA6714
Phoberia atomaris|[3243]|RDLQ387-05|DH010070|602[0n]bp|Canada.Quebec|BOLD:AAA6714
Phoberia atomaris|[3244]|RDLQ374-05|DH010057|658[0n]bp|Canada.Quebec|BOLD:AAA6714
Phoberia atomaris|[3245]|RDLQ386-05|DH010069|658[0n]bp|Canada.Quebec|BOLD:AAA6714
Phoberia atomaris|[3246]|RDLQ368-05|DH010051|658[0n]bp|Canada.Quebec|BOLD:AAA6714
Phoberia atomaris|[3247]|RDLQ373-05|DH010056|658[0n]bp|Canada.Quebec|BOLD:AAA6714
Euclidia ardita|[3248]|NAMUM264-08|RR-98-0900|658[0n]bp|United States.California|BOLD:AAB9610
Euclidia cuspidea|[3249]|KPOEC109-08|08OEC-031|658[0n]bp|Canada.Ontario|BOLD:AAB9610
Euclidia cuspidea|[3250]|TMNBB074-06|MNBTT-1014|658[0n]bp|Canada.New Brunswick|BOLD:AAB9610
Euclidia cuspidea|[3251]|KPOEC108-08|08OEC-030|658[0n]bp|Canada.Ontario|BOLD:AAB9610
Euclidia cuspidea|[3252]|BBLPB817-10|10BBCLP-1816|658[0n]bp|Canada.Saskatchewan|BOLD:AAB9610
Euclidia cuspidea|[3253]|BBLPB818-10|10BBCLP-1817|658[0n]bp|Canada.Saskatchewan|BOLD:AAB9610
Euclidia cuspidea|[3254]|LPMN466-08|08BBLEP-01265|658[0n]bp|Canada.Manitoba|BOLD:AAB9610
Euclidia cuspidea|[3255]|RDLQG276-06|DH012488|658[0n]bp|Canada.Quebec|BOLD:AAB9610
Euclidia cuspidea|[3256]|LPMN465-08|08BBLEP-01264|658[0n]bp|Canada.Manitoba|BOLD:AAB9610
Euclidia cuspidea|[3257]|LPMN467-08|08BBLEP-01266|658[0n]bp|Canada.Manitoba|BOLD:AAB9610
Euclidia cuspidea|[3258]|LPMN464-08|08BBLEP-01263|658[0n]bp|Canada.Manitoba|BOLD:AAB9610
Euclidia cuspidea|[3259]|LPMN201-08|08BBLEP-01000|658[0n]bp|Canada.Manitoba|BOLD:AAB9610
Euclidia cuspidea|[3260]|RDMAB133-05|UASM41285|658[0n]bp|Canada.Alberta|BOLD:AAB9610
Allotria elonympha|[3261]|LGSMC356-05|DNA-ATBI-2356|658[0n]bp|United States.Tennessee|BOLD:AAB0988
Allotria elonympha|[3262]|LOT241-04|04HBL002241|658[0n]bp|United States.Tennessee|BOLD:AAB0988
Allotria elonympha|[3263]|HKONB258-09|3755-COI-08|583[0n]bp|United States.Texas|BOLD:AAB0988
Allotria elonympha|[3264]|QUNO017-07|2017-105007-FL|658[0n]bp|United States.Florida|BOLD:AAB0988
Allotria elonympha|[3265]|ABCNA617-07|429-8721-160504-TX|577[0n]bp|United States.Texas|BOLD:AAB0988
Allotria elonympha|[3266]|ABCNA729-07|729-8721-050603-MD|600[0n]bp|United States.Maryland|BOLD:AAB0988
Allotria elonympha|[3267]|LOTB087-05|05-TN-00087|658[0n]bp|United States.Tennessee|BOLD:AAB0988
Allotria elonympha|[3268]|LSUSA249-06|06-SUSA-0249|658[0n]bp|United States.Kentucky|BOLD:AAB0988
Allotria elonympha|[3269]|LNC065-05|05-NCCC-065|658[0n]bp|United States.North Carolina|BOLD:AAB0988
Allotria elonympha|[3270]|LNCC666-11|11-NCCC-191|658[0n]bp|United States.North Carolina|BOLD:AAB0988
Allotria elonympha|[3271]|LNC066-05|05-NCCC-066|658[0n]bp|United States.North Carolina|BOLD:AAB0988
Allotria elonympha|[3272]|LOT541-04|04HBL002541|611[1n]bp|United States.Tennessee|BOLD:AAB0988

Allotria elonympha|[3270]|LNCC666-11|11-NCCC-191|658[0n]bp|United States.North Carolina|BOLD:AAB0988
Allotria elonympha|[3271]|LNC066-05|05-NCCC-066|658[0n]bp|United States.North Carolina|BOLD:AAB0988
Allotria elonympha|[3272]|LOT541-04|04HBL002541|611[1n]bp|United States.Tennessee|BOLD:AAB0988
Allotria elonympha|[3273]|LGSMC359-05|DNA-ATBI-2359|658[0n]bp|United States.Tennessee|BOLD:AAB0988
Allotria elonympha|[3274]|LOTB089-05|05-TN-00089|658[0n]bp|United States.Tennessee|BOLD:AAB0988
Allotria elonympha|[3275]|LGSM425-04|DNA-ATBI-0425|658[0n]bp|United States.Tennessee|BOLD:AAB0988
Allotria elonympha|[3276]|LGSMC357-05|DNA-ATBI-2357|658[0n]bp|United States.Tennessee|BOLD:AAB0988
Allotria elonympha|[3277]|LOCT063-05|05-CTATBI-0063|658[0n]bp|United States.Connecticut|BOLD:AAB0988
Allotria elonympha|[3278]|LOTB322-05|05-TN-00322|658[0n]bp|United States.Tennessee|BOLD:AAB0988
Allotria elonympha|[3279]|LGSM424-04|DNA-ATBI-0424|658[0n]bp|United States.Tennessee|BOLD:AAB0988
Allotria elonympha|[3280]|LOT243-04|04HBL002243|658[0n]bp|United States.Tennessee|BOLD:AAB0988
Allotria elonympha|[3281]|LGSMC358-05|DNA-ATBI-2358|658[0n]bp|United States.Tennessee|BOLD:AAB0988
Allotria elonympha|[3282]|LOTB090-05|05-TN-00090|658[0n]bp|United States.Tennessee|BOLD:AAB0988
Allotria elonympha|[3283]|BBLOC1033-11|BIOUG01468-F05|658[0n]bp|United States.Texas|BOLD:AAB0988
Allotria elonympha|[3284]|LOT246-04|04HBL002246|658[0n]bp|United States.Tennessee|BOLD:AAB0988
Allotria elonympha|[3285]|LGSMG579-07|BGS03942|658[0n]bp|United States.Tennessee|BOLD:AAB0988
Allotria elonympha|[3286]|LOCT064-05|05-CTATBI-0064|658[0n]bp|United States.Connecticut|BOLD:AAB0988
Allotria elonympha|[3287]|LSEU321-06|06-JKA-0321|658[0n]bp|United States.Georgia|BOLD:AAB0988
Allotria elonympha|[3288]|LOT242-04|04HBL002242|658[0n]bp|United States.Tennessee|BOLD:AAB0988
Allotria elonympha|[3289]|LOTB088-05|05-TN-00088|616[0n]bp|United States.Tennessee|BOLD:AAB0988
Allotria elonympha|[3290]|LOT245-04|04HBL002245|587[0n]bp|United States.Tennessee|BOLD:AAB0988
Allotria elonympha|[3291]|LOT244-04|04HBL002244|597[0n]bp|United States.Tennessee|BOLD:AAB0988
Allotria elonympha|[3292]|LOTB488-05|05-TN-00488|658[0n]bp|United States.Tennessee|BOLD:AAB0988
Allotria elonympha|[3293]|LOT499-04|04HBL002499|658[0n]bp|United States.Tennessee|BOLD:AAB0988
Argyrostrotis anilis|[3294]|RDNMG1024-08|CNC LEP00053148|658[0n]bp|Canada.Ontario|BOLD:AAC8698
Argyrostrotis anilis|[3295]|MNAG041-08|CNCLEP00041026|658[0n]bp|Canada.Manitoba|BOLD:AAC8698
Argyrostrotis anilis|[3296]|RDNMG1025-08|CNC LEP00053149|658[0n]bp|Canada.Ontario|BOLD:AAC8698
Parallelia bistriaris|[3297]|PHMTV421-10|10PHMAL-2521|658[0n]bp|Canada.Ontario|BOLD:AAA8563
Parallelia bistriaris|[3298]|XAB562-04|04HBL005562|658[0n]bp|Canada.Ontario|BOLD:AAA8563
Parallelia bistriaris|[3299]|BBLEC001-09|09BBELE-0001|658[0n]bp|Canada.New Brunswick|BOLD:AAA8563
Parallelia bistriaris|[3300]|TMNBB072-06|MNBTT-1012|658[0n]bp|Canada.New Brunswick|BOLD:AAA8563
Parallelia bistriaris|[3301]|TMNBB073-06|MNBTT-1013|658[0n]bp|Canada.New Brunswick|BOLD:AAA8563
Parallelia bistriaris|[3302]|RDLQB253-05|DH010339|658[0n]bp|Canada.Quebec|BOLD:AAA8563
Parallelia bistriaris|[3303]|LPSOC307-08|PPBP-2306|658[0n]bp|Canada.Ontario|BOLD:AAA8563
Parallelia bistriaris|[3304]|LPSO991-08|PPBP-0991|658[0n]bp|Canada.Ontario|BOLD:AAA8563
Parallelia bistriaris|[3305]|XAE628-04|Moth4628.03|658[0n]bp|Canada.Ontario|BOLD:AAA8563
Parallelia bistriaris|[3306]|TMNBB071-06|MNBTT-1011|658[0n]bp|Canada.New Brunswick|BOLD:AAA8563
Parallelia bistriaris|[3307]|BBLPE016-09|09BBELE-2016|658[0n]bp|Canada.Nova Scotia|BOLD:AAA8563
Parallelia bistriaris|[3308]|MNBB054-05|HBL008664|658[0n]bp|Canada.New Brunswick|BOLD:AAA8563
Parallelia bistriaris|[3309]|XAG545-05|2005-ONT-1129|658[0n]bp|Canada.Ontario|BOLD:AAA8563
Parallelia bistriaris|[3310]|TTMNB280-06|MNBTT-280|658[0n]bp|Canada.New Brunswick|BOLD:AAA8563
Parallelia bistriaris|[3311]|MNBB201-05|05-NBSTA-117|658[0n]bp|Canada.New Brunswick|BOLD:AAA8563
Parallelia bistriaris|[3312]|RDLQF734-06|DH011884|658[0n]bp|Canada.Quebec|BOLD:AAA8563
Parallelia bistriaris|[3313]|TMNBB069-06|MNBTT-1009|658[0n]bp|Canada.New Brunswick|BOLD:AAA8563
Parallelia bistriaris|[3314]|XAJ680-06|2006-ONT-0680|658[0n]bp|Canada.Ontario|BOLD:AAA8563
Parallelia bistriaris|[3315]|TMNBB070-06|MNBTT-1010|658[0n]bp|Canada.New Brunswick|BOLD:AAA8563
Parallelia bistriaris|[3316]|PHMO076-03|moth504.01|639[0n]bp|Canada.Ontario|BOLD:AAA8563
Spiloloma lunilinea|[3317]|ABNCC433-07|1433-150602-MO|606[0n]bp|United States.Missouri|BOLD:AAC7835
Spiloloma lunilinea|[3318]|QUNOB292-09|5572-240808-IN|658[0n]bp|United States.Indiana|BOLD:AAC7835
Spiloloma lunilinea|[3319]|QUNOB291-09|5571-270808-IN|658[0n]bp|United States.Indiana|BOLD:AAC7835
Spiloloma lunilinea|[3320]|LILLA805-11|SNS10IL-01011|658[0n]bp|United States.Illinois|BOLD:AAC7835
Spiloloma lunilinea|[3321]|HKONB255-09|3752-COI-08|637[0n]bp|United States.Louisiana|BOLD:AAC7835
Spiloloma lunilinea|[3322]|ABNCC432-07|1432-110602-OK|594[0n]bp|United States.Oklahoma|BOLD:AAC7835
Spiloloma lunilinea|[3323]|BBLSW441-09|09BBLEP-01369|658[0n]bp|United States.Oklahoma|BOLD:AAC7835
Spiloloma lunilinea|[3324]|LSUSA224-06|06-SUSA-0224|658[0n]bp|United States.Kentucky|BOLD:AAC7835
Spiloloma lunilinea|[3325]|QUNOB190-08|5565-210808-IN|658[0n]bp|United States.Indiana|BOLD:AAC7835
Spiloloma lunilinea|[3326]|ABNCC434-07|1434-110802-IN|592[0n]bp|United States.Indiana|BOLD:AAC7835
Spiloloma lunilinea|[3327]|QUNO577-08|5110-260808-IN|658[0n]bp|United States.Indiana|BOLD:AAC7835
Spiloloma lunilinea|[3328]|QUNOB290-09|5570-210808-IN|658[0n]bp|United States.Indiana|BOLD:AAC7835
Ascalapha odorata|[3329]|DSCNI024-07|06-PROBE-0249|658[0n]bp|Canada.Manitoba|BOLD:AAA5595
Thysania zenobia|[3330]|HKONB001-08|3469-COI-08|658[0n]bp|United States.Texas|BOLD:AAB8451
Thysania zenobia|[3331]|QUNOD463-10|9177-250710-WI|658[0n]bp|United States.Wisconsin|BOLD:AAB8451
Celiptera frustulum|[3332]|LPSO885-08|PPBP-0885|658[0n]bp|Canada.Ontario|BOLD:AAC2193
Celiptera frustulum|[3333]|LPSO644-08|PPBP-0644|658[0n]bp|Canada.Ontario|BOLD:AAC2193
Celiptera frustulum|[3334]|LPSO875-08|PPBP-0875|658[0n]bp|Canada.Ontario|BOLD:AAC2193
Caenurgina caerulea|[3335]|RDNMF106-08|NOC14192|658[0n]bp|United States.California|BOLD:ABX5276
Caenurgina caerulea|[3336]|RDMAB1010-09|UASM99701|658[0n]bp|United States.California|BOLD:ABX5276
Caenurgina caerulea|[3337]|RDNMF105-08|NOC14191|618[0n]bp|United States.California|BOLD:ABX5276
Caenurgina caerulea|[3338]|JMMMB312-11|BIOUG00850-C03|658[0n]bp|United States.California|BOLD:ABX5276
Caenurgina annexa|[3339]|LALPA1093-11|AVBC 903-11|658[0n]bp|Canada.British Columbia|BOLD:AAE0528
Caenurgina annexa|[3340]|LALPA1094-11|AVBC 904-11|658[0n]bp|Canada.British Columbia|BOLD:AAE0528
Caenurgina annexa|[3341]|RDNMF365-08|NOC14451|658[0n]bp|Canada.Alberta|BOLD:AAE0528
Caenurgina annexa|[3342]|LOWCE091-06|CGWC-3851|658[0n]bp|Canada.British Columbia|BOLD:AAE0528
Caenurgina annexa|[3343]|RDNMF364-08|NOC14450|658[0n]bp|Canada.Alberta|BOLD:AAE0528
Caenurgina annexa|[3344]|RDNMF107-08|NOC14193|658[0n]bp|Canada.Alberta|BOLD:AAE0528
Caenurgina annexa|[3345]|RDNMF363-08|NOC14449|658[0n]bp|Canada.Alberta|BOLD:AAE0528
Caenurgina crassiuscula|[3346]|LOWCB179-05|CGWC-1119|654[0n]bp|Canada.British Columbia|BOLD:ABZ0302
Caenurgina crassiuscula|[3347]|BBLPB162-10|10BBCLP-1161|658[0n]bp|Canada.Saskatchewan|BOLD:ABZ0302
Caenurgina crassiuscula|[3348]|LOWCE090-06|CGWC-3850|658[0n]bp|Canada.British Columbia|BOLD:ABZ0302
Caenurgina crassiuscula|[3349]|LALPA845-11|AVBC 1018-11|658[0n]bp|Canada.British Columbia|BOLD:ABZ0302
Caenurgina crassiuscula|[3350]|RDNME520-08|LEP037944|658[0n]bp|Canada.Yukon Territory|BOLD:ABZ0302
Caenurgina crassiuscula|[3351]|RDNME526-08|LEP037950|658[0n]bp|Canada.Yukon Territory|BOLD:ABZ0302
Caenurgina crassiuscula|[3352]|XAD291-04|04HBL007291|658[0n]bp|Canada.Ontario|BOLD:AAA4171
Caenurgina crassiuscula|[3353]|BBLPC509-09|09BBELE-1509|658[0n]bp|Canada.New Brunswick|BOLD:AAA4171
Caenurgina crassiuscula|[3354]|XAG314-05|2005-ONT-898|658[0n]bp|Canada.Ontario|BOLD:AAA4171
Caenurgina crassiuscula|[3355]|MEC139-04|jflandry0139|649[0n]bp|Canada.Quebec|BOLD:AAA4171
Caenurgina crassiuscula|[3356]|XAC089-04|04HBL006089|591[0n]bp|Canada.Ontario|BOLD:AAA4171
Caenurgina crassiuscula|[3357]|KPOEC182-08|08OEC-225|658[0n]bp|Canada.Ontario|BOLD:AAA4171
Caenurgina crassiuscula|[3358]|XAG677-05|2005-ONT-1261|658[0n]bp|Canada.Ontario|BOLD:AAA4171
Caenurgina crassiuscula|[3359]|NCCH104-11|BIOUG01862-E06|655[0n]bp|Canada.Ontario|BOLD:AAA4171
Caenurgina crassiuscula|[3360]|RDLQ423-07|DH001746|611[0n]bp|Canada.Quebec|BOLD:AAA4171
Caenurgina crassiuscula|[3361]|BBLEC736-09|09BBELE-0736|655[1n]bp|Canada.Nova Scotia|BOLD:AAA4171
Caenurgina crassiuscula|[3362]|LPSO651-08|PPBP-0651|658[0n]bp|Canada.Ontario|BOLD:AAA4171
Caenurgina crassiuscula|[3363]|XAH415-05|2005-ONT-1998|657[0n]bp|Canada.Ontario|BOLD:AAA4171
Caenurgina crassiuscula|[3364]|XAD575-04|04HBL006990|658[0n]bp|Canada.Ontario|BOLD:AAA4171
Caenurgina crassiuscula|[3365]|MEC140-04|jflandry0140|658[0n]bp|Canada.Quebec|BOLD:AAA4171
Caenurgina crassiuscula|[3366]|BBLEC691-09|09BBELE-0691|658[0n]bp|Canada.Nova Scotia|BOLD:AAA4171
Caenurgina crassiuscula|[3367]|XAH318-05|2005-ONT-1901|657[0n]bp|Canada.Ontario|BOLD:AAA4171
Caenurgina crassiuscula|[3368]|XAF343-05|HLC-10384|658[0n]bp|Canada.Ontario|BOLD:AAA4171
Caenurgina crassiuscula|[3369]|XAC034-04|04HBL006034|658[0n]bp|Canada.Ontario|BOLD:AAA4171
Caenurgina crassiuscula|[3370]|XAG092-05|2005-ONT-676|658[0n]bp|Canada.Ontario|BOLD:AAA4171
Caenurgina crassiuscula|[3371]|BBLPC357-09|09BBELE-1357|658[0n]bp|Canada.New Brunswick|BOLD:AAA4171

Caenurgina crassiuscula|[3369]|XAC034-04|04HBL006034|658[0n]bp|Canada.Ontario|BOLD:AAA4171
Caenurgina crassiuscula|[3370]|XAG092-05|2005-ONT-676|658[0n]bp|Canada.Ontario|BOLD:AAA4171
Caenurgina crassiuscula|[3371]|BBLPC357-09|09BBELE-1357|658[0n]bp|Canada.New Brunswick|BOLD:AAA4171
Caenurgina crassiuscula|[3372]|XAF325-05|HLC-10366|658[0n]bp|Canada.Ontario|BOLD:AAA4171
Caenurgina crassiuscula|[3373]|BLTIB717-08|BL1003|658[0n]bp|Canada.Ontario|BOLD:AAA4171
Caenurgina crassiuscula|[3374]|XAC070-04|04HBL006070|658[0n]bp|Canada.Ontario|BOLD:AAA4171
Caenurgina crassiuscula|[3375]|BLTIB731-08|BL1021|658[0n]bp|Canada.Ontario|BOLD:AAA4171
Caenurgina crassiuscula|[3376]|TMNBB075-06|MNBTT-1015|658[0n]bp|Canada.New Brunswick|BOLD:AAA4171
Caenurgina crassiuscula|[3377]|XAE153-04|Moth4153.03|658[0n]bp|Canada.Ontario|BOLD:AAA4171
Caenurgina crassiuscula|[3378]|LPSOB229-08|PPBP-1228|658[0n]bp|Canada.Ontario|BOLD:AAA4171
Caenurgina crassiuscula|[3379]|KPOEC107-08|08OEC-029|655[0n]bp|Canada.Ontario|BOLD:AAA4171
Caenurgina crassiuscula|[3380]|BLTIB732-08|BL1022|658[0n]bp|Canada.Ontario|BOLD:AAA4171
Caenurgina crassiuscula|[3381]|XAH367-05|2005-ONT-1950|655[0n]bp|Canada.Ontario|BOLD:AAA4171
Caenurgina crassiuscula|[3382]|XAJ810-06|2006-ONT-0810|658[0n]bp|Canada.Ontario|BOLD:AAA4171
Caenurgina crassiuscula|[3383]|XAD283-04|04HBL007283|591[0n]bp|Canada.Ontario|BOLD:AAA4171
Caenurgina crassiuscula|[3384]|RDLQ422-07|DH004506|606[4n]bp|Canada.Quebec|BOLD:AAA4171
Caenurgina crassiuscula|[3385]|TMG95-03|CAEN1.00|639[0n]bp|Canada.Ontario|BOLD:AAA4171
Caenurgina crassiuscula|[3386]|PMG096-03|moth138.01|617[0n]bp|Canada.Ontario|BOLD:AAA4171
Caenurgina crassiuscula|[3387]|TMG94-03|moth144.01|639[0n]bp|Canada.Ontario|BOLD:AAA4171
Caenurgina crassiuscula|[3388]|XAK225-06|2006-ONT-1220|658[0n]bp|Canada.Ontario|BOLD:AAA4171
Caenurgina crassiuscula|[3389]|RDLQ424-07|DH007612|593[3n]bp|Canada.Quebec|BOLD:AAA4171
Caenurgina crassiuscula|[3390]|XAK424-06|2006-ONT-1419|658[0n]bp|Canada.Ontario|BOLD:AAA4171
Caenurgina crassiuscula|[3391]|BLTIB059-08|BL0097|658[0n]bp|Canada.Ontario|BOLD:AAA4171
Caenurgina crassiuscula|[3392]|XAC800-04|04HBL006800|658[0n]bp|Canada.Ontario|BOLD:AAA4171
Caenurgina crassiuscula|[3393]|XAD510-04|04HBL007510|618[0n]bp|Canada.Ontario|BOLD:AAA4171
Caenurgina crassiuscula|[3394]|BBLEC203-09|09BBELE-0203|636[0n]bp|Canada.Nova Scotia|BOLD:AAA4171
Caenurgina crassiuscula|[3395]|BBLPC350-09|09BBELE-1350|655[0n]bp|Canada.New Brunswick|BOLD:AAA4171
Caenurgina crassiuscula|[3396]|KPOEC077-08|08OEC-234|651[0n]bp|Canada.Ontario|BOLD:AAA4171
Caenurgina crassiuscula|[3397]|KPOEC046-08|08OEC-155|646[0n]bp|Canada.Ontario|BOLD:AAA4171
Caenurgina crassiuscula|[3398]|XAJ933-06|2006-ONT-0933|658[0n]bp|Canada.Ontario|BOLD:AAA4171
Caenurgina crassiuscula|[3399]|XAB044-04|04HBL005044|617[2n]bp|Canada.Ontario|BOLD:AAA4171
Caenurgina crassiuscula|[3400]|LPSK038-08|08BBLEP-00741|658[0n]bp|Canada.Saskatchewan|BOLD:AAA4171
Caenurgina crassiuscula|[3401]|MNBB466-05|05-NBSTA-382|658[0n]bp|Canada.New Brunswick|BOLD:AAA4171
Caenurgina crassiuscula|[3402]|MEC131-04|jflandry0131|658[0n]bp|Canada.Quebec|BOLD:AAA4171
Caenurgina crassiuscula|[3403]|KPOEC106-08|08OEC-028|658[0n]bp|Canada.Ontario|BOLD:AAA4171
Caenurgina crassiuscula|[3404]|XAJ839-06|2006-ONT-0839|658[0n]bp|Canada.Ontario|BOLD:AAA4171
Caenurgina crassiuscula|[3405]|LPSK117-08|08BBLEP-01685|658[0n]bp|Canada.Saskatchewan|BOLD:AAA4171
Caenurgina crassiuscula|[3406]|LPMNB472-09|08BBLEP-05510|658[0n]bp|Canada.Manitoba|BOLD:AAA4171
Caenurgina crassiuscula|[3407]|LMIS028-05|05-ONMIS-0028|658[0n]bp|Canada.Ontario|BOLD:AAA4171
Caenurgina crassiuscula|[3408]|LPSK023-08|08BBLEP-00726|658[0n]bp|Canada.Saskatchewan|BOLD:AAA4171
Caenurgina crassiuscula|[3409]|XAK461-06|2006-ONT-1456|658[0n]bp|Canada.Ontario|BOLD:AAA4171
Caenurgina crassiuscula|[3410]|LPSK009-08|08BBLEP-00712|658[0n]bp|Canada.Saskatchewan|BOLD:AAA4171
Caenurgina crassiuscula|[3411]|LPSK045-08|08BBLEP-00748|658[0n]bp|Canada.Saskatchewan|BOLD:AAA4171
Caenurgina crassiuscula|[3412]|XAH449-05|2005-ONT-2032|658[0n]bp|Canada.Ontario|BOLD:AAA4171
Caenurgina crassiuscula|[3413]|LPSK507-08|08BBLEP-02075|658[0n]bp|Canada.Saskatchewan|BOLD:AAA4171
Caenurgina crassiuscula|[3414]|LPSK008-08|08BBLEP-00711|658[0n]bp|Canada.Saskatchewan|BOLD:AAA4171
Caenurgina crassiuscula|[3415]|TTMNB281-06|MNBTT-281|658[0n]bp|Canada.New Brunswick|BOLD:AAA4171
Caenurgina crassiuscula|[3416]|BBLPC384-09|09BBELE-1384|655[0n]bp|Canada.New Brunswick|BOLD:AAA4171
Caenurgina crassiuscula|[3417]|LPSK015-08|08BBLEP-00718|658[0n]bp|Canada.Saskatchewan|BOLD:AAA4171
Caenurgina crassiuscula|[3418]|LPSK114-08|08BBLEP-01682|658[0n]bp|Canada.Saskatchewan|BOLD:AAA4171
Caenurgina crassiuscula|[3419]|LPSK003-08|08BBLEP-00706|569[2n]bp|Canada.Saskatchewan|BOLD:AAA4171
Caenurgina crassiuscula|[3420]|BLTIB661-08|BL942|621[0n]bp|Canada.Ontario|BOLD:AAA4171
Caenurgina crassiuscula|[3421]|LPSK097-08|08BBLEP-01508|658[0n]bp|Canada.Saskatchewan|BOLD:AAA4171
Caenurgina crassiuscula|[3422]|LPSK118-08|08BBLEP-01686|658[0n]bp|Canada.Saskatchewan|BOLD:AAA4171
Caenurgina crassiuscula|[3423]|LPSK035-08|08BBLEP-00738|658[0n]bp|Canada.Saskatchewan|BOLD:AAA4171
Caenurgina crassiuscula|[3424]|LPSK021-08|08BBLEP-00724|658[0n]bp|Canada.Saskatchewan|BOLD:AAA4171
Caenurgina crassiuscula|[3425]|LPSK233-08|08BBLEP-01801|658[0n]bp|Canada.Saskatchewan|BOLD:AAA4171
Caenurgina crassiuscula|[3426]|XAF304-05|HLC-10345|658[0n]bp|Canada.Ontario|BOLD:AAA4171
Caenurgina erechtea|[3427]|LOWCB602-05|CGWC-1542|658[4n]bp|Canada.British Columbia|BOLD:AAB0200
Caenurgina erechtea|[3428]|LOWCB604-05|CGWC-1544|658[3n]bp|Canada.British Columbia|
Caenurgina erechtea|[3429]|LALPA576-10|AVBC 578-10|658[0n]bp|Canada.British Columbia|BOLD:AAB0200
Caenurgina erechtea|[3430]|LPSK202-08|08BBLEP-01770|658[0n]bp|Canada.Saskatchewan|BOLD:AAB0200
Caenurgina erechtea|[3431]|LOWCB595-05|CGWC-1535|658[0n]bp|Canada.British Columbia|BOLD:AAB0200
Caenurgina erechtea|[3432]|LPSK230-08|08BBLEP-01798|658[0n]bp|Canada.Saskatchewan|BOLD:AAB0200
Caenurgina erechtea|[3433]|LALPA546-10|AVBC 548-10|658[0n]bp|Canada.British Columbia|BOLD:AAB0200
Caenurgina erechtea|[3434]|LPSK557-08|08BBLEP-02125|658[0n]bp|Canada.Saskatchewan|BOLD:AAB0200
Caenurgina erechtea|[3435]|LPMNB296-09|08BBLEP-05140|658[0n]bp|Canada.Manitoba|BOLD:AAB0200
Caenurgina erechtea|[3436]|LOWCB599-05|CGWC-1539|590[0n]bp|Canada.British Columbia|BOLD:AAB0200
Caenurgina erechtea|[3437]|LOWCB605-05|CGWC-1545|573[0n]bp|Canada.British Columbia|BOLD:AAB0200
Caenurgina erechtea|[3438]|LOWCB596-05|CGWC-1536|602[0n]bp|Canada.British Columbia|BOLD:AAB0200
Caenurgina erechtea|[3439]|LPSK520-08|08BBLEP-02088|658[0n]bp|Canada.Saskatchewan|BOLD:AAB0200
Caenurgina erechtea|[3440]|LPSK424-08|08BBLEP-01992|658[0n]bp|Canada.Saskatchewan|BOLD:AAB0200
Caenurgina erechtea|[3441]|LPSK209-08|08BBLEP-01777|658[0n]bp|Canada.Saskatchewan|BOLD:AAB0200
Caenurgina erechtea|[3442]|LPSK395-08|08BBLEP-01963|658[0n]bp|Canada.Saskatchewan|BOLD:AAB0200
Caenurgina erechtea|[3443]|LPSK201-08|08BBLEP-01769|658[0n]bp|Canada.Saskatchewan|BOLD:AAB0200
Caenurgina erechtea|[3444]|LOWCE020-06|CGWC-3780|658[0n]bp|Canada.British Columbia|BOLD:AAB0200
Caenurgina erechtea|[3445]|LOWCE021-06|CGWC-3781|658[0n]bp|Canada.British Columbia|BOLD:AAB0200
Caenurgina erechtea|[3446]|LPSK480-08|08BBLEP-02048|658[0n]bp|Canada.Saskatchewan|BOLD:AAB0200
Caenurgina erechtea|[3447]|LALPA1195-11|AVBC 1197-11|658[0n]bp|Canada.British Columbia|BOLD:AAB0200
Caenurgina erechtea|[3448]|LALPA524-10|AVBC 526-10|658[0n]bp|Canada.British Columbia|BOLD:AAB0200
Caenurgina erechtea|[3449]|LOWCB597-05|CGWC-1537|627[0n]bp|Canada.British Columbia|BOLD:AAB0200
Caenurgina erechtea|[3450]|LOWCB606-05|CGWC-1546|599[0n]bp|Canada.British Columbia|BOLD:AAB0200
Caenurgina erechtea|[3451]|LOWCB603-05|CGWC-1543|592[0n]bp|Canada.British Columbia|BOLD:AAB0200
Caenurgina erechtea|[3452]|LOWCB600-05|CGWC-1540|591[2n]bp|Canada.British Columbia|BOLD:AAB0200
Caenurgina erechtea|[3453]|LOWCB601-05|CGWC-1541|603[0n]bp|Canada.British Columbia|BOLD:AAB0200
Caenurgina erechtea|[3454]|LOWCB598-05|CGWC-1538|610[0n]bp|Canada.British Columbia|BOLD:AAB0200
Caenurgina erechtea|[3455]|LPSK523-08|08BBLEP-02091|658[0n]bp|Canada.Saskatchewan|BOLD:AAB0200
Caenurgina erechtea|[3456]|LPSK635-08|08BBLEP-02203|629[0n]bp|Canada.Saskatchewan|BOLD:AAB0200
Caenurgina erechtea|[3457]|LPSK423-08|08BBLEP-01991|658[0n]bp|Canada.Saskatchewan|BOLD:AAB0200
Caenurgina erechtea|[3458]|LOWCE088-06|CGWC-3848|658[0n]bp|Canada.British Columbia|BOLD:AAB0200
Caenurgina erechtea|[3459]|LOWCB594-05|CGWC-1534|658[0n]bp|Canada.British Columbia|BOLD:AAB0200
Doryodes grandipennis|[3460]|MILEQ195-11|11-MISC-670|658[0n]bp|United States.Georgia|BOLD:ACE7232
Doryodes grandipennis|[3461]|MILEP293-10|10-MISC-198|658[0n]bp|United States.Georgia|BOLD:ACE7232
Doryodes grandipennis|[3462]|MILEP294-10|10-MISC-199|658[0n]bp|United States.Georgia|BOLD:ACE7232
Doryodes grandipennis|[3463]|MILEQ196-11|11-MISC-671|658[0n]bp|United States.Georgia|BOLD:ACE7232
Doryodes grandipennis|[3464]|MILEP286-10|10-MISC-191|658[0n]bp|United States.Georgia|BOLD:ACE7232
Doryodes grandipennis|[3465]|MILEP288-10|10-MISC-193|658[0n]bp|United States.Georgia|BOLD:ACE7232
Doryodes grandipennis|[3466]|MILEP290-10|10-MISC-195|658[0n]bp|United States.Georgia|BOLD:ACE7232
Doryodes grandipennis|[3467]|MILEP287-10|10-MISC-192|658[0n]bp|United States.Georgia|BOLD:ACE7232
Doryodes grandipennis|[3468]|MILEP292-10|10-MISC-197|658[0n]bp|United States.Georgia|BOLD:ACE7232
Doryodes grandipennis|[3469]|MILEQ194-11|11-MISC-669|658[0n]bp|United States.Georgia|BOLD:ACE7232
Doryodes grandipennis|[3470]|MILEP289-10|10-MISC-194|658[0n]bp|United States.Georgia|BOLD:ACE7232

Doryodes grandipennis|[3468]|MILEP292-10|10-MISC-197|658[0n]bp|United States.Georgia|BOLD:ACE7232
Doryodes grandipennis|[3469]|MILEQ194-11|11-MISC-669|658[0n]bp|United States.Georgia|BOLD:ACE7232
Doryodes grandipennis|[3470]|MILEP289-10|10-MISC-194|658[0n]bp|United States.Georgia|BOLD:ACE7232
Doryodes grandipennis|[3471]|MILEQ193-11|11-MISC-668|658[0n]bp|United States.Georgia|BOLD:ACE7232
Doryodes grandipennis|[3472]|MILEP291-10|10-MISC-196|658[0n]bp|United States.Georgia|BOLD:ACE7232
Doryodes grandipennis|[3473]|MILEQ192-11|11-MISC-667|658[0n]bp|United States.Georgia|BOLD:ACE7232
Doryodes grandipennis|[3474]|LNC522-06|05-NCCC-522|658[0n]bp|United States.North Carolina|BOLD:ACE7232
Doryodes grandipennis|[3475]|LNC521-06|05-NCCC-521|658[0n]bp|United States.North Carolina|BOLD:ACE7232
Doryodes grandipennis|[3476]|RDNMF524-08|NOC14610|609[0n]bp|United States.Maryland|BOLD:ACE7232
Mocis latipes|[3477]|ABNCC407-07|1407-030603-TX|656[0n]bp|United States.Texas|BOLD:AAA2438
Mocis latipes|[3478]|LNC518-06|05-NCCC-518|585[1n]bp|United States.North Carolina|BOLD:AAA2438
Mocis latipes|[3479]|ABNCC408-07|1408-270503-TX|653[0n]bp|United States.Texas|BOLD:AAA2438
Mocis latipes|[3480]|BBLOD1092-11|BIOUG01822-C05|658[0n]bp|United States.Texas|BOLD:AAA2438
Mocis latipes|[3481]|LNC422-05|05-NCCC-422|658[0n]bp|United States.North Carolina|BOLD:AAA2438
Mocis latipes|[3482]|BBLOD193-11|BIOUG01552-G08|658[0n]bp|United States.Texas|BOLD:AAA2438
Mocis latipes|[3483]|LOFLB491-06|06-FLOR-1431|658[0n]bp|United States.Florida|BOLD:AAA2438
Mocis latipes|[3484]|HKONS039-07|1603-COI-07|658[0n]bp|United States.Florida|BOLD:AAA2438
Mocis latipes|[3485]|HKONS038-07|1602-COI-07|658[0n]bp|United States.Florida|BOLD:AAA2438
Haploa lecontei|[3486]|BBLEC286-09|09BBELE-0286|565[0n]bp|Canada.Nova Scotia|BOLD:AAA8684
Haploa lecontei|[3487]|BBLEC571-09|09BBELE-0571|614[0n]bp|Canada.Nova Scotia|BOLD:AAA8684
Haploa lecontei|[3488]|PHMNB213-04|04HBL007678|609[0n]bp|Canada.New Brunswick|BOLD:AAA8684
Haploa confusa|[3489]|BLTIB770-08|BL1165|658[0n]bp|Canada.Ontario|BOLD:AAA8684
Haploa confusa|[3490]|BLTIB816-08|BL1234|658[0n]bp|Canada.Ontario|BOLD:AAA8684
Haploa confusa|[3491]|TMG75-03|moth1129.01|639[0n]bp|Canada.Ontario|BOLD:AAA8684
Haploa lecontei|[3492]|PHMNB032-03|moth192.02SA|639[0n]bp|Canada.New Brunswick|BOLD:AAA8684
Haploa lecontei|[3493]|BBLPE296-09|09BBELE-2296|648[0n]bp|Canada.Nova Scotia|BOLD:AAA8684
Haploa lecontei|[3494]|BBLEC259-09|09BBELE-0259|658[0n]bp|Canada.Nova Scotia|BOLD:AAA8684
Haploa lecontei|[3495]|BBLEC271-09|09BBELE-0271|638[0n]bp|Canada.Nova Scotia|BOLD:AAA8684
Haploa lecontei|[3496]|BBLEC285-09|09BBELE-0285|658[0n]bp|Canada.Nova Scotia|BOLD:AAA8684
Haploa lecontei|[3497]|RDLQB371-05|DH010457|622[3n]bp|Canada.Quebec|BOLD:AAA8684
Haploa lecontei|[3498]|PHMNB146-04|04HBL007611|658[0n]bp|Canada.New Brunswick|BOLD:AAA8684
Haploa lecontei|[3499]|MNBB254-05|05-NBSTA-170|658[0n]bp|Canada.New Brunswick|BOLD:AAA8684
Haploa lecontei|[3500]|BBLEC590-09|09BBELE-0590|658[0n]bp|Canada.Nova Scotia|BOLD:AAA8684
Haploa lecontei|[3501]|MNBB676-05|05-NBSTA-592|658[0n]bp|Canada.New Brunswick|BOLD:AAA8684
Haploa lecontei|[3502]|BBLPE294-09|09BBELE-2294|658[0n]bp|Canada.Nova Scotia|BOLD:AAA8684
Haploa lecontei|[3503]|BBLEC290-09|09BBELE-0290|658[0n]bp|Canada.Nova Scotia|BOLD:AAA8684
Haploa lecontei|[3504]|BBLPE254-09|09BBELE-2254|658[0n]bp|Canada.Nova Scotia|BOLD:AAA8684
Haploa lecontei|[3505]|PHMNB145-04|04HBL007610|658[0n]bp|Canada.New Brunswick|BOLD:AAA8684
Haploa lecontei|[3506]|BBLEC289-09|09BBELE-0289|658[0n]bp|Canada.Nova Scotia|BOLD:AAA8684
Haploa lecontei|[3507]|MNBB400-05|05-NBSTA-316|658[0n]bp|Canada.New Brunswick|BOLD:AAA8684
Haploa lecontei|[3508]|BBLEC258-09|09BBELE-0258|658[0n]bp|Canada.Nova Scotia|BOLD:AAA8684
Haploa clymene|[3509]|LNCC059-10|10-NCCC-154|658[0n]bp|United States.North Carolina|BOLD:AAA8684
Haploa clymene|[3510]|LNCC1392-11|11-NCCC-917|658[0n]bp|United States.North Carolina|BOLD:AAA8684
Haploa lecontei|[3511]|PHMNB218-04|04HBL007683|658[0n]bp|Canada.New Brunswick|BOLD:AAA8684
Haploa clymene|[3512]|LNCNW033-06|06-NCNW-0033|658[0n]bp|United States.North Carolina|BOLD:AAA8684
Haploa lecontei|[3513]|TMNBD370-07|MNBTT-3171|621[0n]bp|Canada.New Brunswick|BOLD:AAA8684
Haploa lecontei|[3514]|BBLEC340-09|09BBELE-0340|651[0n]bp|Canada.Nova Scotia|BOLD:AAA8684
Haploa lecontei|[3515]|PHMNB144-04|04HBL007609|658[0n]bp|Canada.New Brunswick|BOLD:AAA8684
Haploa lecontei|[3516]|PHMNB232-04|04HBL007697|658[0n]bp|Canada.New Brunswick|BOLD:AAA8684
Haploa confusa|[3517]|TMNBD379-07|MNBTT-3180|658[0n]bp|Canada.New Brunswick|BOLD:AAA8684
Haploa confusa|[3518]|BBLPC253-09|09BBELE-1253|658[0n]bp|Canada.Nova Scotia|BOLD:AAA8684
Haploa confusa|[3519]|XAB119-04|04HBL005119|658[0n]bp|Canada.Ontario|BOLD:AAA8684
Haploa confusa|[3520]|PHMNB074-03|moth75.02SA|639[0n]bp|Canada.New Brunswick|BOLD:AAA8684
Haploa confusa|[3521]|MNBB203-05|05-NBSTA-119|621[0n]bp|Canada.New Brunswick|BOLD:AAA8684
Haploa confusa|[3522]|MNBB626-05|05-NBSTA-542|658[0n]bp|Canada.New Brunswick|BOLD:AAA8684
Haploa lecontei|[3523]|RDLQG446-06|DH012730|658[0n]bp|Canada.Quebec|BOLD:AAA8684
Haploa lecontei|[3524]|RDMAB529-06|UASM58496|622[0n]bp|Canada.Alberta|BOLD:AAA8684
Haploa confusa|[3525]|MNBB253-05|05-NBSTA-169|658[0n]bp|Canada.New Brunswick|BOLD:AAA8684
Haploa confusa|[3526]|MNBB362-05|05-NBSTA-278|658[0n]bp|Canada.New Brunswick|BOLD:AAA8684
Haploa confusa|[3527]|TMNBD375-07|MNBTT-3176|658[0n]bp|Canada.New Brunswick|BOLD:AAA8684
Haploa confusa|[3528]|TMNBD376-07|MNBTT-3177|658[0n]bp|Canada.New Brunswick|BOLD:AAA8684
Haploa lecontei|[3529]|RDMAB528-06|UASM58495|656[0n]bp|Canada.Alberta|BOLD:AAA8684
Haploa lecontei|[3530]|RDNM112-05|CNCNoctuoidea6662|658[0n]bp|Canada.Ontario|BOLD:AAA8684
Haploa confusa|[3531]|TMNBD372-07|MNBTT-3173|656[0n]bp|Canada.New Brunswick|BOLD:AAA8684
Haploa confusa|[3532]|BBLPC262-09|09BBELE-1262|658[0n]bp|Canada.Nova Scotia|BOLD:AAA8684
Haploa lecontei|[3533]|RDLQB697-05|DH010800|658[0n]bp|Canada.Quebec|BOLD:AAA8684
Haploa lecontei|[3534]|TMNBD374-07|MNBTT-3175|658[0n]bp|Canada.New Brunswick|BOLD:AAA8684
Haploa lecontei|[3535]|TMNBD373-07|MNBTT-3174|657[0n]bp|Canada.New Brunswick|BOLD:AAA8684
Haploa lecontei|[3536]|PHMNB767-05|Moth 460.03SA|658[0n]bp|Canada.New Brunswick|BOLD:AAA8684
Haploa lecontei|[3537]|PHMNB765-05|Moth 458.03SA|658[0n]bp|Canada.New Brunswick|BOLD:AAA8684
Haploa lecontei|[3538]|PHMNB441-04|04HBL00667|658[0n]bp|Canada.New Brunswick|BOLD:AAA8684
Haploa confusa|[3539]|XAK591-07|HLC-16144|596[0n]bp|Canada.Ontario|BOLD:AAA8684
Haploa confusa|[3540]|PMG010-03|HAPL1.00|617[0n]bp|Canada.Ontario|BOLD:AAA8684
Haploa confusa|[3541]|MNBB075-05|HBL008685|658[0n]bp|Canada.New Brunswick|BOLD:AAA8684
Haploa confusa|[3542]|XAK511-07|HLC-16064|658[0n]bp|Canada.Ontario|BOLD:AAA8684
Haploa confusa|[3543]|BLTIB771-08|BL1166|658[0n]bp|Canada.Ontario|BOLD:AAA8684
Haploa lecontei|[3544]|RDNM110-05|CNCNoctuoidea6660|658[0n]bp|Canada.Ontario|BOLD:AAA8684
Haploa lecontei|[3545]|RDNM111-05|CNCNoctuoidea6661|658[0n]bp|Canada.Ontario|BOLD:AAA8684
Haploa lecontei|[3546]|RDMAB930-06|UASM7031|658[0n]bp|Canada.Alberta|BOLD:AAA8684
Haploa lecontei|[3547]|XAB196-04|04HBL005196|658[0n]bp|Canada.Ontario|BOLD:AAA8684
Haploa lecontei|[3548]|RDNM113-05|CNCNoctuoidea6663|658[0n]bp|Canada.Ontario|BOLD:AAA8684
Haploa lecontei|[3549]|XAB012-04|04HBL005012|658[0n]bp|Canada.Ontario|BOLD:AAA8684
Haploa confusa|[3550]|XAK008-06|2006-ONT-1003|658[0n]bp|Canada.Ontario|BOLD:AAA8684
Haploa confusa|[3551]|TMNBD378-07|MNBTT-3179|658[0n]bp|Canada.New Brunswick|BOLD:AAA8684
Haploa confusa|[3552]|BBLEC299-09|09BBELE-0299|658[0n]bp|Canada.Nova Scotia|BOLD:AAA8684
Haploa confusa|[3553]|TMNBD377-07|MNBTT-3178|658[0n]bp|Canada.New Brunswick|BOLD:AAA8684
Haploa confusa|[3554]|XAK048-06|2006-ONT-1043|658[0n]bp|Canada.Ontario|BOLD:AAA8684
Haploa confusa|[3555]|XAK047-06|2006-ONT-1042|658[0n]bp|Canada.Ontario|BOLD:AAA8684
Haploa confusa|[3556]|BLTIB918-08|BL1338|658[0n]bp|Canada.Ontario|BOLD:AAA8684
Haploa lecontei|[3557]|PHMO378-03|moth1377.02|639[0n]bp|Canada.Ontario|BOLD:AAA8684
Haploa lecontei|[3558]|TMG74-03|moth1075.01|639[0n]bp|Canada.Ontario|BOLD:AAA8684
Haploa reversa|[3559]|RDNMF055-08|NOC14141|658[0n]bp|United States.New Mexico|BOLD:AAA8684
Haploa lecontei|[3560]|BLTIB662-08|BL943|635[0n]bp|Canada.Ontario|BOLD:AAA8684
Haploa reversa|[3561]|HKONB381-09|3878-COI-08|658[0n]bp|United States.Louisiana|BOLD:AAA8684
Haploa contigua|[3562]|RDMAB969-09|UASM99698|658[0n]bp|Canada.Ontario|BOLD:ABX5655
Haploa contigua|[3563]|RDNMF047-08|NOC14133|658[0n]bp|Canada.Quebec|BOLD:ABX5655
Haploa clymene|[3564]|LNCC1347-11|11-NCCC-872|658[0n]bp|United States.North Carolina|BOLD:ABZ0254
Haploa clymene|[3565]|LNCC1346-11|11-NCCC-871|658[0n]bp|United States.North Carolina|BOLD:ABZ0254
Haploa clymene|[3566]|LILLA595-11|SNS10IL-00762|658[0n]bp|United States.Illinois|BOLD:ABZ0254
Haploa clymene|[3567]|LNCC1393-11|11-NCCC-918|658[0n]bp|United States.North Carolina|BOLD:ABZ0254
Haploa clymene|[3568]|LILLA636-11|SNS10IL-00812|658[0n]bp|United States.Illinois|BOLD:ABZ0254
Haploa clymene|[3569]|LPOKB996-09|MDOK-2038|658[0n]bp|United States.Oklahoma|BOLD:ABZ0254

Haploa clymene|[3567]|LNCC1393-11|11-NCCC-918|658[0n]bp|United States.North Carolina|BOLD:ABZ0254
Haploa clymene|[3568]|LILLA636-11|SNS10IL-00812|658[0n]bp|United States.Illinois|BOLD:ABZ0254
Haploa clymene|[3569]|LPOKB996-09|MDOK-2038|658[0n]bp|United States.Oklahoma|BOLD:ABZ0254
Haploa lecontei|[3570]|BLTIB865-08|BL1284|658[0n]bp|Canada.Ontario|BOLD:ABZ0254
Haploa clymene|[3571]|LNCC058-10|10-NCCC-153|658[0n]bp|United States.North Carolina|BOLD:ABZ0254
Haploa clymene|[3572]|LNCC1390-11|11-NCCC-915|658[0n]bp|United States.North Carolina|BOLD:ABZ0254
Haploa clymene|[3573]|LNCC1345-11|11-NCCC-870|658[0n]bp|United States.North Carolina|BOLD:ABZ0254
Haploa clymene|[3574]|LNCC1391-11|11-NCCC-916|658[0n]bp|United States.North Carolina|BOLD:ABZ0254
Haploa clymene|[3575]|LNC279-05|05-NCCC-279|658[0n]bp|United States.North Carolina|BOLD:ABZ0254
Haploa confusa|[3576]|MNBB564-05|05-NBSTA-480|658[0n]bp|Canada.New Brunswick|BOLD:ABZ0254
Haploa lecontei|[3577]|PHMNB024-03|moth160.02SA|639[0n]bp|Canada.New Brunswick|BOLD:ABZ0254
Haploa lecontei|[3578]|MNBB139-05|05-NBSTA-055|658[0n]bp|Canada.New Brunswick|BOLD:ABZ0254
Haploa clymene|[3579]|LGSMB300-05|DNA-ATBI-1149|583[0n]bp|United States.Tennessee|BOLD:ABZ0257
Haploa reversa|[3580]|LILLA349-11|SNS10IL-00459|658[0n]bp|United States.Illinois|BOLD:ABZ0257
Grammia arge|[3581]|RDMAB762-06|BCSC239|658[0n]bp|Canada.Ontario|BOLD:AAB7789
Grammia arge|[3582]|RDLQH040-06|DH013277|656[0n]bp|Canada.Quebec|BOLD:AAB7789
Grammia arge|[3583]|XAH311-05|2005-ONT-1894|658[0n]bp|Canada.Ontario|BOLD:AAB7789
Grammia arge|[3584]|XAJ402-06|2006-ONT-0402|658[0n]bp|Canada.Ontario|BOLD:AAB7789
Grammia arge|[3585]|XAG083-05|2005-ONT-667|658[0n]bp|Canada.Ontario|BOLD:AAB7789
Grammia arge|[3586]|XAG178-05|2005-ONT-762|658[0n]bp|Canada.Ontario|BOLD:AAB7789
Grammia arge|[3587]|XAK292-06|2006-ONT-1287|658[0n]bp|Canada.Ontario|BOLD:AAB7789
Grammia arge|[3588]|XAG232-05|2005-ONT-816|658[0n]bp|Canada.Ontario|BOLD:AAB7789
Grammia arge|[3589]|XAG233-05|2005-ONT-817|629[1n]bp|Canada.Ontario|BOLD:AAB7789
Grammia arge|[3590]|PMG006-03|APAN1.01|617[0n]bp|Canada.Ontario|BOLD:AAB7789
Grammia arge|[3591]|XAE625-04|Moth4625.03|572[0n]bp|Canada.Ontario|BOLD:AAB7789
Grammia arge|[3592]|XAK266-06|2006-ONT-1261|658[0n]bp|Canada.Ontario|BOLD:AAB7789
Grammia arge|[3593]|XAG252-05|2005-ONT-836|658[0n]bp|Canada.Ontario|BOLD:AAB7789
Grammia arge|[3594]|XAG101-05|2005-ONT-685|658[0n]bp|Canada.Ontario|BOLD:AAB7789
Grammia doris|[3595]|GWNC607-07|CNCLEP00034169|658[0n]bp|Canada.New Brunswick|BOLD:AAE3399
Grammia doris|[3596]|RDMAB760-06|BCSC237|658[0n]bp|Canada.Alberta|BOLD:AAE3399
Grammia doris|[3597]|RDMAB759-06|BCSC236|658[0n]bp|Canada.Alberta|BOLD:AAE3399
Grammia anna|[3598]|RDMAB855-06|BCSC332|658[0n]bp|Canada.Ontario|BOLD:AAD6928
Grammia anna|[3599]|RDLQB557-05|DH010643|658[0n]bp|Canada.Quebec|BOLD:AAD6928
Grammia parthenice|[3600]|XAI125-05|0102-ONT-0125|545[1n]bp|Canada.Ontario|BOLD:AAA8807
Grammia parthenice|[3601]|XAI124-05|0102-ONT-0124|556[0n]bp|Canada.Ontario|BOLD:AAA8807
Grammia parthenice|[3602]|XAG850-05|2005-ONT-1434|658[0n]bp|Canada.Ontario|BOLD:AAA8807
Grammia parthenice|[3603]|XAK296-06|2006-ONT-1291|658[0n]bp|Canada.Ontario|BOLD:AAA8807
Grammia parthenice|[3604]|RDLQB600-05|DH010703|658[0n]bp|Canada.Quebec|BOLD:AAA8807
Grammia figurata|[3605]|RDNM099-05|CNCNoctuoidea6649|658[0n]bp|Canada.Ontario|BOLD:AAA4398
Grammia virguncula|[3606]|LPSOC145-08|PPBP-2144|658[0n]bp|Canada.Ontario|BOLD:AAA4398
Grammia virguncula|[3607]|XAJ721-06|2006-ONT-0721|658[0n]bp|Canada.Ontario|BOLD:AAA4398
Grammia virguncula|[3608]|XAK158-06|2006-ONT-1153|658[0n]bp|Canada.Ontario|BOLD:AAA4398
Grammia virguncula|[3609]|XAJ686-06|2006-ONT-0686|658[0n]bp|Canada.Ontario|BOLD:AAA4398
Grammia virguncula|[3610]|XAB274-04|04HBL005274|658[0n]bp|Canada.Ontario|BOLD:AAA4398
Grammia figurata|[3611]|RDNMB378-05|CNCNoctuoidea10144|658[0n]bp|Canada.Ontario|BOLD:AAA4398
Grammia figurata|[3612]|RDMAB791-06|BCSC268|658[0n]bp|Canada.Ontario|BOLD:AAA4398
Grammia figurata|[3613]|RDNM100-05|CNCNoctuoidea6650|658[0n]bp|Canada.Ontario|BOLD:AAA4398
Grammia virguncula|[3614]|XAB586-04|04HBL005586|606[0n]bp|Canada.Ontario|BOLD:AAA4398
Grammia figurata|[3615]|RDNMB307-05|CNCNoctuoidea10073|613[0n]bp|Canada.Ontario|BOLD:AAA4398
Grammia virguncula|[3616]|XAJ685-06|2006-ONT-0685|658[0n]bp|Canada.Ontario|BOLD:AAA4398
Grammia virguncula|[3617]|LPSOB786-08|PPBP-1785|658[0n]bp|Canada.Ontario|BOLD:AAA4398
Grammia virguncula|[3618]|XAB552-04|04HBL005552|658[0n]bp|Canada.Ontario|BOLD:AAA4398
Grammia virguncula|[3619]|RDMAB817-06|BCSC294|658[0n]bp|Canada.Nova Scotia|BOLD:AAA4398
Grammia virguncula|[3620]|RDMAB851-06|BCSC328|658[0n]bp|Canada.Nova Scotia|BOLD:AAA4398
Grammia virguncula|[3621]|RDMAB845-06|BCSC322|596[0n]bp|Canada.Nova Scotia|BOLD:AAA4398
Grammia virguncula|[3622]|RDMAB818-06|BCSC295|658[0n]bp|Canada.Nova Scotia|BOLD:AAA4398
Grammia virguncula|[3623]|XAF737-05|2005-ONT-386|506[1n]bp|Canada.Ontario|BOLD:AAA4398
Grammia virguncula|[3624]|TMNBB030-06|MNBTT-970|658[0n]bp|Canada.New Brunswick|BOLD:AAA4398
Grammia parthenice|[3625]|LOWCB184-05|CGWC-1124|658[0n]bp|Canada.British Columbia|BOLD:AAA4398
Grammia parthenice|[3626]|RDMAB871-06|BCSC348|658[0n]bp|Canada.Alberta|BOLD:AAA4398
Grammia parthenice|[3627]|LOWCB186-05|CGWC-1126|658[0n]bp|Canada.British Columbia|BOLD:AAA4398
Grammia parthenice|[3628]|LOWCB189-05|CGWC-1129|658[0n]bp|Canada.British Columbia|BOLD:AAA4398
Grammia parthenice|[3629]|LOWCB185-05|CGWC-1125|658[0n]bp|Canada.British Columbia|BOLD:AAA4398
Grammia parthenice|[3630]|LOWCB188-05|CGWC-1128|658[0n]bp|Canada.British Columbia|BOLD:AAA4398
Grammia parthenice|[3631]|LOWCB187-05|CGWC-1127|658[0n]bp|Canada.British Columbia|BOLD:AAA4398
Grammia virguncula|[3632]|RDMAB843-06|BCSC320|658[0n]bp|Canada.Ontario|BOLD:AAA4398
Grammia virguncula|[3633]|RDNMC565-06|CNCNoctuoidea12105|658[0n]bp|Canada.Ontario|BOLD:AAA4398
Grammia quenseli|[3634]|RDNME502-08|LEP037926|648[1n]bp|Canada.Quebec|BOLD:AAA4398
Grammia virguncula|[3635]|LPSOC033-08|PPBP-2032|658[0n]bp|Canada.Ontario|BOLD:AAA4398
Grammia virguncula|[3636]|LPSOC277-08|PPBP-2276|658[0n]bp|Canada.Ontario|BOLD:AAA4398
Grammia virguncula|[3637]|RDLQG144-06|DH012315|614[0n]bp|Canada.Quebec|BOLD:AAA4398
Grammia virguncula|[3638]|LPSOC276-08|PPBP-2275|658[0n]bp|Canada.Ontario|BOLD:AAA4398
Grammia virguncula|[3639]|RDNMB397-05|CNCNoctuoidea10163|658[0n]bp|Canada.Ontario|BOLD:AAA4398
Grammia virguncula|[3640]|LPSOD1044-09|08BBLEP-05971|658[0n]bp|Canada.Ontario|BOLD:AAA4398
Grammia virguncula|[3641]|XAE346-04|Moth4346.03|658[0n]bp|Canada.Ontario|BOLD:AAA4398
Grammia virguncula|[3642]|XAF519-05|2005-ONT-168|658[0n]bp|Canada.Ontario|BOLD:AAA4398
Grammia virguncula|[3643]|RDNMB396-05|CNCNoctuoidea10162|658[0n]bp|Canada.Ontario|BOLD:AAA4398
Grammia virguncula|[3644]|XAB553-04|04HBL005553|658[0n]bp|Canada.Ontario|BOLD:AAA4398
Grammia virguncula|[3645]|LPSOC274-08|PPBP-2273|658[0n]bp|Canada.Ontario|BOLD:AAA4398
Grammia virguncula|[3646]|XAF584-05|2005-ONT-233|658[0n]bp|Canada.Ontario|BOLD:AAA4398
Grammia virguncula|[3647]|XAB585-04|04HBL005585|658[0n]bp|Canada.Ontario|BOLD:AAA4398
Grammia parthenice|[3648]|XAI126-05|0102-ONT-0126|658[0n]bp|Canada.Ontario|BOLD:AAA4398
Grammia parthenice|[3649]|RDMAB816-06|BCSC293|658[0n]bp|Canada.Alberta|BOLD:AAA4398
Grammia parthenice|[3650]|BBLPA337-10|10BBCLP-0337|658[0n]bp|Canada.Alberta|BOLD:AAA4398
Grammia parthenice|[3651]|LPMN168-08|08BBLEP-00967|658[0n]bp|Canada.Manitoba|BOLD:AAA4398
Grammia parthenice|[3652]|BBLPA340-10|10BBCLP-0340|658[0n]bp|Canada.British Columbia|BOLD:AAA4398
Grammia parthenice|[3653]|RDMAB872-06|BCSC349|658[0n]bp|Canada.Ontario|BOLD:AAA4398
Grammia parthenice|[3654]|TMNBD406-07|MNBTT-3207|658[0n]bp|Canada.New Brunswick|BOLD:AAA4398
Grammia parthenice|[3655]|LPSK212-08|08BBLEP-01780|658[0n]bp|Canada.Saskatchewan|BOLD:AAA4398
Grammia parthenice|[3656]|RDNMB298-05|CNCNoctuoidea10064|607[0n]bp|Canada.Ontario|BOLD:AAA4398
Grammia parthenice|[3657]|RDNMB300-05|CNCNoctuoidea10066|566[1n]bp|Canada.Alberta|BOLD:AAA4398
Grammia parthenice|[3658]|TMNBD405-07|MNBTT-3206|658[0n]bp|Canada.New Brunswick|BOLD:AAA4398
Grammia parthenice|[3659]|LPMNB535-09|08BBLEP-05573|658[0n]bp|Canada.Manitoba|BOLD:AAA4398
Grammia parthenice|[3660]|RDLQB599-05|DH010702|658[0n]bp|Canada.Quebec|BOLD:AAA4398
Grammia parthenice|[3661]|BBLPA341-10|10BBCLP-0341|658[0n]bp|Canada.British Columbia|BOLD:AAA4398
Grammia parthenice|[3662]|LPSOD930-09|08BBLEP-05470|658[0n]bp|Canada.Ontario|BOLD:AAA4398
Grammia parthenice|[3663]|LPMN932-08|08BBLEP-02290|658[0n]bp|Canada.Alberta|BOLD:AAA4398
Grammia parthenice|[3664]|BBLPA339-10|10BBCLP-0339|658[0n]bp|Canada.British Columbia|BOLD:AAA4398
Grammia virguncula|[3665]|BBLPA338-10|10BBCLP-0338|658[0n]bp|Canada.Alberta|BOLD:AAA4398
Grammia virguncula|[3666]|RDMAB844-06|BCSC321|658[0n]bp|Canada.Saskatchewan|BOLD:AAA4398
Grammia margo|[3667]|RDMAB792-06|BCSC269|658[0n]bp|Canada.Alberta|BOLD:AAA4398
Grammia parthenice|[3668]|LPSK018-08|08BBLEP-00721|658[0n]bp|Canada.Saskatchewan|BOLD:AAA4398
[illegible]

Grammia virguncula|[3666]|RDMAB844-06|BCSC321|658[0n]bp|Canada.Saskatchewan|BOLD:AAA4398
Grammia margo|[3667]|RDMAB792-06|BCSC269|658[0n]bp|Canada.Alberta|BOLD:AAA4398
Grammia parthenice|[3668]|LPSK018-08|08BBLEP-00721|658[0n]bp|Canada.Saskatchewan|BOLD:AAA4398
Grammia virguncula|[3669]|TMG82-03|moth632.01|639[0n]bp|Canada.Ontario|BOLD:AAA4398
Grammia virguncula|[3670]|PHMO141-03|moth796.01|639[0n]bp|Canada.Ontario|BOLD:AAA4398
Grammia virguncula|[3671]|XAF471-05|2005-ONT-120|658[0n]bp|Canada.Ontario|BOLD:AAA4398
Grammia virguncula|[3672]|PMG008-03|moth691.01|617[0n]bp|Canada.Ontario|BOLD:AAA4398
Grammia virguncula|[3673]|RDLQB818-05|DH010905|617[0n]bp|Canada.Quebec|BOLD:AAA4398
Grammia virguncula|[3674]|XAE476-04|Moth4476.03|574[0n]bp|Canada.Ontario|BOLD:AAA4398
Grammia virguncula|[3675]|RDLQB699-05|DH010802|581[0n]bp|Canada.Quebec|BOLD:AAA4398
Grammia virguncula|[3676]|LPSOC131-08|PPBP-2130|631[0n]bp|Canada.Ontario|BOLD:AAA4398
Grammia virguncula|[3677]|LPSOC275-08|PPBP-2274|637[0n]bp|Canada.Ontario|BOLD:AAA4398
Grammia virguncula|[3678]|XAJ623-06|2006-ONT-0623|636[0n]bp|Canada.Ontario|BOLD:AAA4398
Grammia quenseli|[3679]|RDNME653-08|LEP038077|658[0n]bp|Canada.Newfoundland and Labrador|BOLD:AAA4398
Grammia quenseli|[3680]|MHLEP033-07|CHU06-LEP-033|658[0n]bp|Canada.Manitoba|BOLD:AAA4398
Grammia quenseli|[3681]|LCHP920-07|07PROBE-10682|658[0n]bp|Canada.Manitoba|BOLD:AAA4398
Grammia virguncula|[3682]|TMNBB029-06|MNBTT-969|658[0n]bp|Canada.New Brunswick|BOLD:AAA4398
Grammia virguncula|[3683]|RDLQF827-06|DH011980|658[0n]bp|Canada.Quebec|BOLD:AAA4398
Grammia virguncula|[3684]|RDLQF826-06|DH011979|658[0n]bp|Canada.Quebec|BOLD:AAA4398
Grammia virguncula|[3685]|BLGSM056-09|BL375|632[0n]bp|Canada.Ontario|BOLD:AAA4398
Grammia virguncula|[3686]|RDLQF825-06|DH011978|658[0n]bp|Canada.Quebec|BOLD:AAA4398
Grammia virguncula|[3687]|RDLQB700-05|DH010803|658[0n]bp|Canada.Quebec|BOLD:AAA4398
Grammia virguncula|[3688]|TTMNB027-06|MNBTT-027|658[0n]bp|Canada.New Brunswick|BOLD:AAA4398
Grammia virguncula|[3689]|XAF726-05|2005-ONT-375|658[0n]bp|Canada.Ontario|BOLD:AAA4398
Grammia virguncula|[3690]|RDMAB842-06|BCSC319|658[0n]bp|Canada.Alberta|BOLD:AAA4398
Grammia virguncula|[3691]|RDNMB395-05|CNCNoctuoidea10161|658[0n]bp|Canada.Alberta|BOLD:AAA4398
Grammia virguncula|[3692]|LPMN018-08|08BBLEP-00816|658[0n]bp|Canada.Manitoba|BOLD:AAA4398
Grammia virguncula|[3693]|LPMN021-08|08BBLEP-00819|658[0n]bp|Canada.Manitoba|BOLD:AAA4398
Grammia virguncula|[3694]|LPMN016-08|08BBLEP-00814|658[0n]bp|Canada.Manitoba|BOLD:AAA4398
Grammia virguncula|[3695]|LPMN023-08|08BBLEP-00821|658[0n]bp|Canada.Manitoba|BOLD:AAA4398
Grammia virguncula|[3696]|LPMN014-08|08BBLEP-00812|658[0n]bp|Canada.Manitoba|BOLD:AAA4398
Grammia virguncula|[3697]|LPMN019-08|08BBLEP-00817|658[0n]bp|Canada.Manitoba|BOLD:AAA4398
Grammia virguncula|[3698]|LPMN020-08|08BBLEP-00818|658[0n]bp|Canada.Manitoba|BOLD:AAA4398
Grammia virguncula|[3699]|LPMN167-08|08BBLEP-00966|658[0n]bp|Canada.Manitoba|BOLD:AAA4398
Grammia virguncula|[3700]|RDLQB208-05|DH010294|658[0n]bp|Canada.Quebec|BOLD:AAA4398
Grammia virguncula|[3701]|LPMN017-08|08BBLEP-00815|658[0n]bp|Canada.Manitoba|BOLD:AAA4398
Grammia virguncula|[3702]|LPMN024-08|08BBLEP-00822|658[0n]bp|Canada.Manitoba|BOLD:AAA4398
Grammia virguncula|[3703]|XAF794-05|2005-ONT-443|658[0n]bp|Canada.Ontario|BOLD:AAA4398
Grammia virguncula|[3704]|RDMAB819-06|BCSC296|658[0n]bp|Canada.Nova Scotia|BOLD:AAA4398
Grammia virguncula|[3705]|LPMN015-08|08BBLEP-00813|658[0n]bp|Canada.Manitoba|BOLD:AAA4398
Grammia virguncula|[3706]|LPMN022-08|08BBLEP-00820|658[0n]bp|Canada.Manitoba|BOLD:AAA4398
Grammia williamsii|[3707]|RDMAB891-06|BCSC368|658[0n]bp|Canada.Alberta|BOLD:AAA4398
Grammia williamsii|[3708]|BBLPA336-10|10BBCLP-0336|658[0n]bp|Canada.Alberta|BOLD:AAA4398
Grammia williamsii|[3709]|RDMAB890-06|BCSC367|658[0n]bp|Canada.Alberta|BOLD:AAA4398
Grammia williamsii|[3710]|BBLPA335-10|10BBCLP-0335|658[0n]bp|Canada.Alberta|BOLD:AAA4398
Grammia williamsii|[3711]|RDMAB892-06|BCSC369|658[0n]bp|Canada.Alberta|BOLD:AAA4398
Grammia williamsii|[3712]|RDMAB893-06|BCSC370|658[0n]bp|Canada.Alberta|BOLD:AAA4398
Grammia virguncula|[3713]|JGLL045-10|10PROBE-19309|658[0n]bp|Canada.Manitoba|BOLD:AAA4398
Grammia virguncula|[3714]|RDMAB858-06|BCSC335|658[0n]bp|Canada.Quebec|BOLD:AAA4398
Grammia williamsii|[3715]|RDLQB207-05|DH010293|658[0n]bp|Canada.Quebec|BOLD:AAA4398
Grammia speciosa|[3716]|LCHP190-07|07PROBE-00117|658[0n]bp|Canada.Manitoba|BOLD:AAA4398
Grammia speciosa|[3717]|LCHP111-07|07PROBE-00552|658[0n]bp|Canada.Manitoba|BOLD:AAA4398
Grammia speciosa|[3718]|LCHP189-07|07PROBE-00116|658[0n]bp|Canada.Manitoba|BOLD:AAA4398
Grammia virguncula|[3719]|RDMAB848-06|BCSC325|658[0n]bp|Canada.Alberta|BOLD:AAA4398
Grammia virguncula|[3720]|RDMAB850-06|BCSC327|658[0n]bp|Canada.Alberta|BOLD:AAA4398
Grammia margo|[3721]|RDMAB793-06|BCSC270|658[0n]bp|Canada.Alberta|BOLD:AAA4398
Grammia margo|[3722]|RDMAB833-06|BCSC310|658[0n]bp|Canada.British Columbia|BOLD:AAA4398
Grammia elongata|[3723]|RDMAB918-06|BCSC392|658[0n]bp|Canada.Alberta|BOLD:AAA4398
Grammia margo|[3724]|RDMAB773-06|BCSC250|590[0n]bp|Canada.British Columbia|BOLD:AAA4398
Grammia virguncula|[3725]|RDMAB847-06|BCSC324|573[1n]bp|Canada.Yukon Territory|BOLD:AAA4398
Grammia virguncula|[3726]|RDMAB854-06|BCSC331|572[0n]bp|Canada.Alberta|BOLD:AAA4398
Grammia williamsii|[3727]|RDNMB370-05|CNCNoctuoidea10136|572[0n]bp|Canada.Alberta|BOLD:AAA4398
Grammia williamsii|[3728]|RDMAB879-06|BCSC356|658[0n]bp|Canada.Alberta|BOLD:AAA4398
Grammia williamsii|[3729]|RDMAB909-06|BCSC383|658[0n]bp|Canada.Alberta|BOLD:AAA4398
Grammia quenseli|[3730]|RDNME652-08|LEP038076|648[3n]bp|Canada.Newfoundland and Labrador|BOLD:AAA4398
Grammia franconia|[3731]|RDNMB368-05|CNCNoctuoidea10134|658[0n]bp|Canada.Ontario|BOLD:AAA4398
Grammia franconia|[3732]|RDMAB911-06|BCSC385|658[0n]bp|Canada.Ontario|BOLD:AAA4398
Grammia quenseli|[3733]|RDMAB865-06|BCSC342|658[0n]bp|Canada.Manitoba|BOLD:AAA4398
Grammia quenseli|[3734]|RDNME840-08|LEP041305|639[2n]bp|Canada.Quebec|BOLD:AAA4398
Grammia quenseli|[3735]|RDMAB862-06|BCSC339|658[0n]bp|Canada.British Columbia|BOLD:AAA4398
Grammia virguncula|[3736]|RDLQB796-05|DH010883|617[0n]bp|Canada.Quebec|BOLD:AAA4398
Grammia franconia|[3737]|RDNMB309-05|CNCNoctuoidea10075|615[0n]bp|Canada.Ontario|BOLD:AAA4398
Grammia virguncula|[3738]|RDMAB857-06|BCSC334|658[0n]bp|Canada.Alberta|BOLD:AAA4398
Grammia williamsii|[3739]|RDMAB878-06|BCSC355|658[0n]bp|Canada.Alberta|BOLD:AAA4398
Grammia williamsii|[3740]|RDLQB210-05|DH010296|658[0n]bp|Canada.Quebec|BOLD:AAA4398
Grammia williamsii|[3741]|RDMAB907-06|BCSC381|658[0n]bp|Canada.Alberta|BOLD:AAA4398
Grammia williamsii|[3742]|RDMAB880-06|BCSC357|658[0n]bp|Canada.Alberta|BOLD:AAA4398
Grammia virguncula|[3743]|RDMAB856-06|BCSC333|658[0n]bp|Canada.Alberta|BOLD:AAA4398
Grammia quenseli|[3744]|RDMAB866-06|BCSC343|658[0n]bp|Canada.Manitoba|BOLD:AAA4398
Grammia quenseli|[3745]|LCHQ893-08|07WNP-10785|658[0n]bp|Canada.Manitoba|BOLD:AAA4398
Grammia quenseli|[3746]|RDMAB867-06|BCSC344|658[0n]bp|Canada.Manitoba|BOLD:AAA4398
Grammia franconia|[3747]|RDNMB308-05|CNCNoctuoidea10074|583[0n]bp|Canada.Ontario|BOLD:AAA4398
Grammia quenseli|[3748]|RDMAB863-06|BCSC340|585[0n]bp|Canada.British Columbia|BOLD:AAA4398
Grammia quenseli|[3749]|RDNME839-08|LEP041304|587[0n]bp|Canada.Quebec|BOLD:AAA4398
Grammia williamsii|[3750]|RDMAB908-06|BCSC382|658[0n]bp|Canada.Alberta|BOLD:AAA4398
Grammia franconia|[3751]|RDLQB209-05|DH010295|658[0n]bp|Canada.Quebec|BOLD:AAA4398
Grammia williamsii|[3752]|RDLQB205-05|DH010291|658[0n]bp|Canada.Quebec|BOLD:AAA4398
Grammia williamsii|[3753]|RDLQB206-05|DH010292|658[0n]bp|Canada.Quebec|BOLD:AAA4398
Grammia phyllira|[3754]|RDMAB787-06|BCSC264|658[0n]bp|Canada.Ontario|BOLD:AAC5868
Grammia phyllira|[3755]|RDNMB371-05|CNCNoctuoidea10137|658[0n]bp|Canada.Ontario|BOLD:AAC5868
Grammia phyllira|[3756]|RDMAB790-06|BCSC267|658[0n]bp|Canada.Alberta|BOLD:ABZ5913
Grammia phyllira|[3757]|RDMAB789-06|BCSC266|658[0n]bp|Canada.Alberta|BOLD:ABZ5913
Grammia phyllira|[3758]|RDMAB788-06|BCSC265|658[0n]bp|Canada.Ontario|BOLD:ABX5768
Grammia virgo|[3759]|TMNBB031-06|MNBTT-971|656[0n]bp|Canada.New Brunswick|BOLD:AAA7208
Grammia virgo|[3760]|PHMNB139-04|04HBL007604|658[0n]bp|Canada.New Brunswick|BOLD:AAA7208
Grammia virgo|[3761]|LPMN556-08|08BBLEP-01357|658[0n]bp|Canada.Manitoba|BOLD:AAA7208
Grammia virgo|[3762]|TMNBD408-07|MNBTT-3209|658[0n]bp|Canada.New Brunswick|BOLD:AAA7208
Grammia virgo|[3763]|RDNMB299-05|CNCNoctuoidea10065|599[0n]bp|Canada.Alberta|BOLD:AAA7208
Grammia virgo|[3764]|LPMN707-08|08BBLEP-01510|658[0n]bp|Canada.Manitoba|BOLD:AAA7208
Grammia virgo|[3765]|LPMN288-08|08BBLEP-01087|658[0n]bp|Canada.Manitoba|BOLD:AAA7208
Grammia virgo|[3766]|XAJ938-06|2006-ONT-0938|656[0n]bp|Canada.Ontario|BOLD:AAA7208
Grammia virgo|[3767]|BBLEC208-09|09BBELE-0208|658[0n]bp|Canada.Nova Scotia|BOLD:AAA7208
Grammia virgo|[3768]|BBLEC562-09|09BBELE-0562|658[0n]bp|Canada.Nova Scotia|BOLD:AAA7208

Grammia virgo|[3765]|LPMNB268-08|08BBLEP-01687|658[0n]bp|Canada.Manitoba|BOLD:AAA7208
Grammia virgo|[3766]|XAJ938-06|2006-ONT-0938|656[0n]bp|Canada.Ontario|BOLD:AAA7208
Grammia virgo|[3767]|BBLEC208-09|09BBELE-0208|658[0n]bp|Canada.Nova Scotia|BOLD:AAA7208
Grammia virgo|[3768]|BBLEC563-09|09BBELE-0563|658[0n]bp|Canada.Nova Scotia|BOLD:AAA7208
Grammia virgo|[3769]|RDMAB875-06|BCSC352|658[0n]bp|Canada.Ontario|BOLD:AAA7208
Grammia virgo|[3770]|TMNBD407-07|MNBTT-3208|658[0n]bp|Canada.New Brunswick|BOLD:AAA7208
Grammia virgo|[3771]|MNBB134-05|05-NBSTA-050|658[0n]bp|Canada.New Brunswick|BOLD:AAA7208
Grammia virgo|[3772]|TMNBD409-07|MNBTT-3210|657[0n]bp|Canada.New Brunswick|BOLD:AAA7208
Grammia virgo|[3773]|TMNBD410-07|MNBTT-3211|646[0n]bp|Canada.New Brunswick|BOLD:AAA7208
Grammia virgo|[3774]|PHMNB042-03|moth217.02SA|639[0n]bp|Canada.New Brunswick|BOLD:AAA7208
Grammia virgo|[3775]|TMNBB032-06|MNBTT-972|658[0n]bp|Canada.New Brunswick|BOLD:AAA7208
Grammia virgo|[3776]|TMNBD411-07|MNBTT-3212|658[0n]bp|Canada.New Brunswick|BOLD:AAA7208
Grammia virgo|[3777]|MNBB644-05|05-NBSTA-560|658[0n]bp|Canada.New Brunswick|BOLD:AAA7208
Grammia virgo|[3778]|PHMNB714-05|Moth 407.03SA|658[0n]bp|Canada.New Brunswick|BOLD:AAA7208
Grammia virgo|[3779]|XAI129-05|0102-ONT-0129|603[0n]bp|Canada.Ontario|BOLD:AAA7208
Grammia virgo|[3780]|XAB080-04|04HBL005080|658[0n]bp|Canada.Ontario|BOLD:AAA7208
Grammia virgo|[3781]|PMG007-03|APAN2.01|617[0n]bp|Canada.Ontario|BOLD:AAA7208
Grammia virgo|[3782]|XAI128-05|0102-ONT-0128|616[0n]bp|Canada.Ontario|BOLD:AAA7208
Grammia virgo|[3783]|XAK037-06|2006-ONT-1032|658[0n]bp|Canada.Ontario|BOLD:AAA7208
Grammia virgo|[3784]|XAJ993-06|2006-ONT-0993|658[0n]bp|Canada.Ontario|BOLD:AAA7208
Grammia virgo|[3785]|XAJ936-06|2006-ONT-0936|658[0n]bp|Canada.Ontario|BOLD:AAA7208
Grammia virgo|[3786]|XAG896-05|2005-ONT-1480|658[0n]bp|Canada.Ontario|BOLD:AAA7208
Grammia virgo|[3787]|XAB105-04|04HBL005105|658[0n]bp|Canada.Ontario|BOLD:AAA7208
Grammia virgo|[3788]|XAJ939-06|2006-ONT-0939|658[0n]bp|Canada.Ontario|BOLD:AAA7208
Grammia virgo|[3789]|RDLQB523-05|DH010609|658[0n]bp|Canada.Quebec|BOLD:AAA7208
Grammia virgo|[3790]|XAK038-06|2006-ONT-1033|658[0n]bp|Canada.Ontario|BOLD:AAA7208
Grammia virgo|[3791]|XAB184-04|04HBL005184|658[0n]bp|Canada.Ontario|BOLD:AAA7208
Grammia virgo|[3792]|XAI127-05|0102-ONT-0127|658[0n]bp|Canada.Ontario|BOLD:AAA7208
Grammia virgo|[3793]|RDMAB814-06|BCSC291|658[0n]bp|Canada.Alberta|BOLD:AAA7208
Grammia virgo|[3794]|LPMNB560-09|08BBLEP-05595|658[0n]bp|Canada.Manitoba|BOLD:AAA7208
Grammia elongata|[3795]|RDMAB774-06|BCSC251|519[2n]bp|Canada.British Columbia|BOLD:ABZ6253
Grammia virgo|[3796]|GWORT809-10|BC AB Lep 00049|658[0n]bp|Canada.Ontario|BOLD:AAA7209
Grammia virgo|[3797]|XAJ937-06|2006-ONT-0937|658[0n]bp|Canada.Ontario|BOLD:AAA7209
Grammia quenseli|[3798]|RDMAB868-06|BCSC345|658[0n]bp|Canada.Manitoba|BOLD:ACF3388
Grammia williamsii|[3799]|RDMAB881-06|BCSC358|658[0n]bp|Canada.Alberta|BOLD:AAA7209
Grammia nevadensis|[3800]|LOWCD481-06|CGWC-3301|513[0n]bp|Canada.British Columbia|BOLD:ABZ6253
Grammia nevadensis|[3801]|LOWCD483-06|CGWC-3303|514[0n]bp|Canada.British Columbia|BOLD:ABZ6253
Grammia nevadensis|[3802]|LOWCD480-06|CGWC-3300|512[0n]bp|Canada.British Columbia|BOLD:ABZ6253
Grammia nevadensis|[3803]|RDNMB402-05|CNCNoctuoidea10168|509[0n]bp|Canada.British Columbia|BOLD:ABZ6253
Grammia williamsii|[3804]|RDMAB901-06|BCSC378|511[1n]bp|Canada.British Columbia|BOLD:ABZ6253
Grammia williamsii|[3805]|RDMAB899-06|BCSC376|658[0n]bp|Canada.British Columbia|BOLD:ABZ6253
Grammia williamsii|[3806]|RDMAB900-06|BCSC377|634[0n]bp|Canada.British Columbia|BOLD:ABZ6253
Grammia williamsii|[3807]|RDMAB902-06|BCSC379|658[0n]bp|Canada.British Columbia|BOLD:ABZ6253
Grammia nevadensis|[3808]|RDNMB401-05|CNCNoctuoidea10167|577[4n]bp|Canada.British Columbia|BOLD:ABZ6253
Grammia nevadensis|[3809]|RDMAB764-06|BCSC241|569[1n]bp|Canada.British Columbia|BOLD:ABZ6253
Grammia nevadensis|[3810]|LOWCD466-06|CGWC-3286|658[0n]bp|Canada.British Columbia|BOLD:ABZ6253
Grammia nevadensis|[3811]|LBCG2897-09|08-JDWBC-2897|658[0n]bp|Canada.British Columbia|BOLD:ABZ6253
Grammia nevadensis|[3812]|LBCH7520-10|10-JDWBC-7520|658[0n]bp|Canada.British Columbia|BOLD:ABZ6253
Grammia nevadensis|[3813]|LBCH6332-10|10-JDWBC-6332|658[0n]bp|Canada.British Columbia|BOLD:ABZ6253
Grammia nevadensis|[3814]|LBCH6955-10|10-JDWBC-6955|658[0n]bp|Canada.British Columbia|BOLD:ABZ6253
Grammia nevadensis|[3815]|LBCH6416-10|10-JDWBC-6416|658[0n]bp|Canada.British Columbia|BOLD:ABZ6253
Grammia nevadensis|[3816]|LBCH7570-10|10-JDWBC-7570|658[0n]bp|Canada.British Columbia|BOLD:ABZ6253
Grammia nevadensis|[3817]|LBCH6412-10|10-JDWBC-6412|658[0n]bp|Canada.British Columbia|BOLD:ABZ6253
Grammia nevadensis|[3818]|LBCH7571-10|10-JDWBC-7571|658[0n]bp|Canada.British Columbia|BOLD:ABZ6253
Grammia nevadensis|[3819]|LBCH6836-10|10-JDWBC-6836|658[0n]bp|Canada.British Columbia|BOLD:ABZ6253
Grammia nevadensis|[3820]|LBCH7569-10|10-JDWBC-7569|658[0n]bp|Canada.British Columbia|BOLD:ABZ6253
Grammia nevadensis|[3821]|LBCH6956-10|10-JDWBC-6956|658[0n]bp|Canada.British Columbia|BOLD:ABZ6253
Grammia nevadensis|[3822]|LBCH6961-10|10-JDWBC-6961|658[0n]bp|Canada.British Columbia|BOLD:ABZ6253
Grammia nevadensis|[3823]|LBCH6410-10|10-JDWBC-6410|658[0n]bp|Canada.British Columbia|BOLD:ABZ6253
Grammia nevadensis|[3824]|LBCH7516-10|10-JDWBC-7516|642[0n]bp|Canada.British Columbia|BOLD:ABZ6253
Grammia nevadensis|[3825]|RDNMB420-05|CNCNoctuoidea10186|571[1n]bp|Canada.British Columbia|BOLD:ABZ6253
Grammia blakei|[3826]|RDNMG527-08|CNC LEP00052351|658[0n]bp|Canada.Alberta|BOLD:ABZ6253
Grammia williamsii|[3827]|RDMAB914-06|BCSC388|658[0n]bp|Canada.Alberta|BOLD:ABZ6253
Grammia blakei|[3828]|RDMAB813-06|BCSC290|658[0n]bp|Canada.Alberta|BOLD:ABZ6253
Grammia blakei|[3829]|RDNMG529-08|CNC LEP00052353|658[0n]bp|Canada.Alberta|BOLD:ABZ6253
Grammia blakei|[3830]|RDNMG528-08|CNC LEP00052352|658[0n]bp|Canada.Alberta|BOLD:ABZ6253
Grammia blakei|[3831]|RDMAB753-06|BCSC230|658[0n]bp|Canada.Alberta|BOLD:ABZ6253
Grammia williamsii|[3832]|RDNMB369-05|CNCNoctuoidea10135|589[0n]bp|Canada.Alberta|BOLD:ABZ6253
Grammia williamsii|[3833]|RDMAB904-06|BCSC26|658[0n]bp|Canada.Alberta|BOLD:ABZ6253
Grammia williamsii|[3834]|RDMAB896-06|BCSC373|658[0n]bp|Canada.Alberta|BOLD:ABZ6253
Grammia williamsii|[3835]|LPABB500-08|08BBLEP-03765|631[0n]bp|Canada.Alberta|BOLD:ABZ6253
Grammia williamsii|[3836]|RDMAB903-06|BCSC25|658[0n]bp|Canada.Alberta|BOLD:ABZ6253
Grammia williamsii|[3837]|RDMAB905-06|BCSC27|658[0n]bp|Canada.Alberta|BOLD:ABZ6253
Grammia williamsii|[3838]|RDMAB894-06|BCSC371|658[0n]bp|Canada.Alberta|BOLD:ABZ6253
Grammia williamsii|[3839]|RDMAB913-06|BCSC387|658[0n]bp|Canada.Alberta|BOLD:ABZ6253
Grammia williamsii|[3840]|RDMAB912-06|BCSC386|658[0n]bp|Canada.Alberta|BOLD:ABZ6253
Grammia williamsii|[3841]|RDMAB915-06|BCSC389|658[0n]bp|Canada.Alberta|BOLD:ABZ6253
Grammia complicata|[3842]|RDNMB460-05|CNCNoctuoidea10226|658[0n]bp|Canada.British Columbia|BOLD:ABZ6253
Grammia complicata|[3843]|RDMAB755-06|BCSC232|658[0n]bp|Canada.British Columbia|BOLD:ABZ6253
Grammia complicata|[3844]|RDMAB754-06|BCSC231|658[0n]bp|Canada.British Columbia|BOLD:ABZ6253
Grammia elongata|[3845]|MHLEP084-07|CHU06-LEP-084|658[0n]bp|Canada.Manitoba|BOLD:ABZ6253
Grammia virguncula|[3846]|RDNMB394-05|CNCNoctuoidea10160|522[0n]bp|Canada.Quebec|BOLD:ABZ6253
Grammia ornata|[3847]|RDNMB376-05|CNCNoctuoidea10142|579[0n]bp|Canada.British Columbia|BOLD:ABZ6253
Grammia ornata|[3848]|LBCH5073-10|10-JDWBC-5073|658[0n]bp|Canada.British Columbia|BOLD:ABZ6253
Grammia elongata|[3849]|RDMAB910-06|BCSC384|584[0n]bp|Canada.British Columbia|BOLD:ABZ6253
Grammia ornata|[3850]|LBCH5267-10|10-JDWBC-5267|658[0n]bp|Canada.British Columbia|BOLD:ABZ6253
Grammia ornata|[3851]|LBCH5269-10|10-JDWBC-5269|658[0n]bp|Canada.British Columbia|BOLD:ABZ6253
Grammia ornata|[3852]|LBCH5270-10|10-JDWBC-5270|658[0n]bp|Canada.British Columbia|BOLD:ABZ6253
Grammia ornata|[3853]|LBCH5275-10|10-JDWBC-5275|658[0n]bp|Canada.British Columbia|BOLD:ABZ6253
Grammia ornata|[3854]|LBCH5268-10|10-JDWBC-5268|658[0n]bp|Canada.British Columbia|BOLD:ABZ6253
Grammia ornata|[3855]|LBCH5080-10|10-JDWBC-5080|658[0n]bp|Canada.British Columbia|BOLD:ABZ6253
Grammia ornata|[3856]|LBCH5273-10|10-JDWBC-5273|640[0n]bp|Canada.British Columbia|BOLD:ABZ6253
Grammia ornata|[3857]|LBCH5272-10|10-JDWBC-5272|658[0n]bp|Canada.British Columbia|BOLD:ABZ6253
Grammia ornata|[3858]|LBCH5081-10|10-JDWBC-5081|658[0n]bp|Canada.British Columbia|BOLD:ABZ6253
Grammia ornata|[3859]|LBCG017-08|08-JDWBC-0017|658[0n]bp|Canada.British Columbia|BOLD:ABZ6253
Grammia ornata|[3860]|LBCH5271-10|10-JDWBC-5271|658[0n]bp|Canada.British Columbia|BOLD:ABZ6253
Grammia ornata|[3861]|LBCH5079-10|10-JDWBC-5079|658[0n]bp|Canada.British Columbia|BOLD:ABZ6253
Grammia ornata|[3862]|LBCH5077-10|10-JDWBC-5077|658[0n]bp|Canada.British Columbia|BOLD:ABZ6253
Grammia ornata|[3863]|LBCH5074-10|10-JDWBC-5074|658[0n]bp|Canada.British Columbia|BOLD:ABZ6253
Grammia ornata|[3864]|LBCH5274-10|10-JDWBC-5274|658[0n]bp|Canada.British Columbia|BOLD:ABZ6253
Grammia ornata|[3865]|LBCG018-08|08-JDWBC-0018|658[0n]bp|Canada.British Columbia|BOLD:ABZ6253
Grammia ornata|[3866]|LPVIC108-08|PFC-2006-2684|658[0n]bp|Canada.British Columbia|BOLD:ABZ6253
Grammia ornata|[3867]|LBCH5076-10|10-JDWBC-5076|658[0n]bp|Canada.British Columbia|BOLD:ABZ6253

Grammia ornata|[3864]|LBCH5274-10|10-JDWBC-5274|658[0n]bp|Canada.British Columbia|BOLD:ABZ6253
Grammia ornata|[3865]|LBCG018-08|08-JDWBC-0018|658[0n]bp|Canada.British Columbia|BOLD:ABZ6253
Grammia ornata|[3866]|LPVIC108-08|PFC-2006-2684|658[0n]bp|Canada.British Columbia|BOLD:ABZ6253
Grammia ornata|[3867]|LBCH5076-10|10-JDWBC-5076|658[0n]bp|Canada.British Columbia|BOLD:ABZ6253
Grammia williamsii|[3868]|RDMAB895-06|BCSC372|658[0n]bp|Canada.Alberta|BOLD:ABZ6253
Grammia elongata|[3869]|RDMAB775-06|BCSC252|658[0n]bp|Canada.Alberta|BOLD:ABZ6253
Grammia ornata|[3870]|LBCH5075-10|10-JDWBC-5075|658[0n]bp|Canada.British Columbia|BOLD:ABZ6253
Grammia ornata|[3871]|LBCH5078-10|10-JDWBC-5078|658[0n]bp|Canada.British Columbia|BOLD:ABZ6253
Grammia ornata|[3872]|LBCH5154-10|10-JDWBC-5154|658[0n]bp|Canada.British Columbia|BOLD:ABZ6253
Grammia williamsii|[3873]|RDMAB916-06|BCSC390|658[0n]bp|Canada.Alberta|BOLD:ABZ6253
Grammia williamsii|[3874]|RDMAB898-06|BCSC375|658[0n]bp|Canada.Alberta|BOLD:ABZ6253
Grammia williamsii|[3875]|RDMAB897-06|BCSC374|658[0n]bp|Canada.Alberta|BOLD:ABZ6253
Grammia nevadensis|[3876]|LOWCD471-06|CGWC-3291|561[0n]bp|Canada.British Columbia|BOLD:ABZ6253
Grammia nevadensis|[3877]|LOWCD475-06|CGWC-3295|604[0n]bp|Canada.British Columbia|BOLD:ABZ6253
Grammia nevadensis|[3878]|LBCH7118-10|10-JDWBC-7118|658[0n]bp|Canada.British Columbia|BOLD:ABZ6253
Grammia nevadensis|[3879]|LOWCD469-06|CGWC-3289|590[0n]bp|Canada.British Columbia|BOLD:ABZ6253
Grammia nevadensis|[3880]|RDNMB399-05|CNCNoctuoidea10165|571[1n]bp|Canada.British Columbia|BOLD:ABZ6253
Grammia nevadensis|[3881]|LBCH6862-10|10-JDWBC-6862|658[0n]bp|Canada.British Columbia|BOLD:ABZ6253
Grammia nevadensis|[3882]|LBCH6590-10|10-JDWBC-6590|658[0n]bp|Canada.British Columbia|BOLD:ABZ6253
Grammia nevadensis|[3883]|LBCH6092-10|10-JDWBC-6092|658[0n]bp|Canada.British Columbia|BOLD:ABZ6253
Grammia nevadensis|[3884]|LBCH6857-10|10-JDWBC-6857|658[0n]bp|Canada.British Columbia|BOLD:ABZ6253
Grammia nevadensis|[3885]|LBCH5991-10|10-JDWBC-5991|658[0n]bp|Canada.British Columbia|BOLD:ABZ6253
Grammia nevadensis|[3886]|LBCH6861-10|10-JDWBC-6861|658[0n]bp|Canada.British Columbia|BOLD:ABZ6253
Grammia nevadensis|[3887]|LBCH6337-10|10-JDWBC-6337|658[1n]bp|Canada.British Columbia|BOLD:ABZ6253
Grammia nevadensis|[3888]|LBCH7714-10|10-JDWBC-7714|658[0n]bp|Canada.British Columbia|BOLD:ABZ6253
Grammia nevadensis|[3889]|LBCH7568-10|10-JDWBC-7568|658[0n]bp|Canada.British Columbia|BOLD:ABZ6253
Grammia nevadensis|[3890]|LBCH6863-10|10-JDWBC-6863|658[0n]bp|Canada.British Columbia|BOLD:ABZ6253
Grammia nevadensis|[3891]|LBCH6415-10|10-JDWBC-6415|658[0n]bp|Canada.British Columbia|BOLD:ABZ6253
Grammia nevadensis|[3892]|LBCH7572-10|10-JDWBC-7572|658[0n]bp|Canada.British Columbia|BOLD:ABZ6253
Grammia nevadensis|[3893]|LBCH7573-10|10-JDWBC-7573|658[0n]bp|Canada.British Columbia|BOLD:ABZ6253
Grammia nevadensis|[3894]|LBCH6957-10|10-JDWBC-6957|658[0n]bp|Canada.British Columbia|BOLD:ABZ6253
Grammia nevadensis|[3895]|LBCH6958-10|10-JDWBC-6958|658[0n]bp|Canada.British Columbia|BOLD:ABZ6253
Grammia nevadensis|[3896]|LBCH6334-10|10-JDWBC-6334|658[0n]bp|Canada.British Columbia|BOLD:ABZ6253
Grammia nevadensis|[3897]|LBCH7868-10|10-JDWBC-7868|658[0n]bp|Canada.British Columbia|BOLD:ABZ6253
Grammia nevadensis|[3898]|LBCH6473-10|10-JDWBC-6473|658[0n]bp|Canada.British Columbia|BOLD:ABZ6253
Grammia nevadensis|[3899]|LBCH6954-10|10-JDWBC-6954|658[0n]bp|Canada.British Columbia|BOLD:ABZ6253
Grammia nevadensis|[3900]|LBCG2896-09|08-JDWBC-2896|658[0n]bp|Canada.British Columbia|BOLD:ABZ6253
Grammia nevadensis|[3901]|LBCH6960-10|10-JDWBC-6960|658[0n]bp|Canada.British Columbia|BOLD:ABZ6253
Grammia nevadensis|[3902]|LBCG2898-09|08-JDWBC-2898|658[0n]bp|Canada.British Columbia|BOLD:ABZ6253
Grammia nevadensis|[3903]|LBCH6858-10|10-JDWBC-6858|658[0n]bp|Canada.British Columbia|BOLD:ABZ6253
Grammia nevadensis|[3904]|LBCH7567-10|10-JDWBC-7567|658[0n]bp|Canada.British Columbia|BOLD:ABZ6253
Grammia nevadensis|[3905]|LBCH6594-10|10-JDWBC-6594|658[0n]bp|Canada.British Columbia|BOLD:ABZ6253
Grammia nevadensis|[3906]|LBCH6039-10|10-JDWBC-6039|658[0n]bp|Canada.British Columbia|BOLD:ABZ6253
Grammia nevadensis|[3907]|LOWCD464-06|CGWC-3284|598[0n]bp|Canada.British Columbia|BOLD:ABZ6253
Grammia nevadensis|[3908]|LBCH6591-10|10-JDWBC-6591|658[0n]bp|Canada.British Columbia|BOLD:ABZ6253
Grammia nevadensis|[3909]|LBCH6593-10|10-JDWBC-6593|658[0n]bp|Canada.British Columbia|BOLD:ABZ6253
Grammia nevadensis|[3910]|LOWCD462-06|CGWC-3282|579[0n]bp|Canada.British Columbia|BOLD:ABZ6253
Grammia nevadensis|[3911]|LBCG486-08|08-JDWBC-0486|658[0n]bp|Canada.British Columbia|BOLD:ABZ6253
Grammia nevadensis|[3912]|LOWCB191-05|CGWC-1131|658[0n]bp|Canada.British Columbia|BOLD:ABZ6253
Grammia nevadensis|[3913]|LBCG484-08|08-JDWBC-0484|658[0n]bp|Canada.British Columbia|BOLD:ABZ6253
Grammia nevadensis|[3914]|LBCH7717-10|10-JDWBC-7717|658[0n]bp|Canada.British Columbia|BOLD:ABZ6253
Grammia nevadensis|[3915]|LOWCD461-06|CGWC-3281|656[0n]bp|Canada.British Columbia|BOLD:ABZ6253
Grammia nevadensis|[3916]|LBCH6468-10|10-JDWBC-6468|658[0n]bp|Canada.British Columbia|BOLD:ABZ6253
Grammia nevadensis|[3917]|LBCH6758-10|10-JDWBC-6758|658[0n]bp|Canada.British Columbia|BOLD:ABZ6253
Grammia nevadensis|[3918]|LBCH6588-10|10-JDWBC-6588|658[0n]bp|Canada.British Columbia|BOLD:ABZ6253
Grammia nevadensis|[3919]|LBCH7518-10|10-JDWBC-7518|658[0n]bp|Canada.British Columbia|BOLD:ABZ6253
Grammia nevadensis|[3920]|LOWCD479-06|CGWC-3299|658[0n]bp|Canada.British Columbia|BOLD:ABZ6253
Grammia nevadensis|[3921]|LBCH7521-10|10-JDWBC-7521|658[0n]bp|Canada.British Columbia|BOLD:ABZ6253
Grammia nevadensis|[3922]|LBCG2105-09|08-JDWBC-2105|658[0n]bp|Canada.British Columbia|BOLD:ABZ6253
Grammia nevadensis|[3923]|LBCH7864-10|10-JDWBC-7864|658[0n]bp|Canada.British Columbia|BOLD:ABZ6253
Grammia nevadensis|[3924]|LBCH6336-10|10-JDWBC-6336|658[0n]bp|Canada.British Columbia|BOLD:ABZ6253
Grammia nevadensis|[3925]|LOWCD473-06|CGWC-3293|658[0n]bp|Canada.British Columbia|BOLD:ABZ6253
Grammia nevadensis|[3926]|LBCH6860-10|10-JDWBC-6860|658[0n]bp|Canada.British Columbia|BOLD:ABZ6253
Grammia nevadensis|[3927]|LBCH6333-10|10-JDWBC-6333|658[0n]bp|Canada.British Columbia|BOLD:ABZ6253
Grammia nevadensis|[3928]|RDMAB765-06|BCSC242|658[0n]bp|Canada.Alberta|BOLD:ABZ6253
Grammia nevadensis|[3929]|LOWCD482-06|CGWC-3302|658[0n]bp|Canada.British Columbia|BOLD:ABZ6253
Grammia nevadensis|[3930]|LBCH6587-10|10-JDWBC-6587|658[0n]bp|Canada.British Columbia|BOLD:ABZ6253
Grammia nevadensis|[3931]|LBCH6409-10|10-JDWBC-6409|658[0n]bp|Canada.British Columbia|BOLD:ABZ6253
Grammia nevadensis|[3932]|LBCH7870-10|10-JDWBC-7870|658[0n]bp|Canada.British Columbia|BOLD:ABZ6253
Grammia nevadensis|[3933]|LBCH7863-10|10-JDWBC-7863|658[0n]bp|Canada.British Columbia|BOLD:ABZ6253
Grammia nevadensis|[3934]|LBCH7301-10|10-JDWBC-7301|658[0n]bp|Canada.British Columbia|BOLD:ABZ6253
Grammia nevadensis|[3935]|LBCH7715-10|10-JDWBC-7715|658[0n]bp|Canada.British Columbia|BOLD:ABZ6253
Grammia nevadensis|[3936]|LOWCD477-06|CGWC-3297|658[0n]bp|Canada.British Columbia|BOLD:ABZ6253
Grammia nevadensis|[3937]|LBCH6330-10|10-JDWBC-6330|658[0n]bp|Canada.British Columbia|BOLD:ABZ6253
Grammia nevadensis|[3938]|LBCH7716-10|10-JDWBC-7716|658[0n]bp|Canada.British Columbia|BOLD:ABZ6253
Grammia nevadensis|[3939]|LBCH6469-10|10-JDWBC-6469|658[0n]bp|Canada.British Columbia|BOLD:ABZ6253
Grammia nevadensis|[3940]|LBCH7865-10|10-JDWBC-7865|658[0n]bp|Canada.British Columbia|BOLD:ABZ6253
Grammia nevadensis|[3941]|LOWCD467-06|CGWC-3287|658[0n]bp|Canada.British Columbia|BOLD:ABZ6253
Grammia nevadensis|[3942]|RDMAB763-06|BCSC240|658[0n]bp|Canada.British Columbia|BOLD:ABZ6253
Grammia nevadensis|[3943]|LOWCD478-06|CGWC-3298|608[0n]bp|Canada.British Columbia|BOLD:ABZ6253
Grammia nevadensis|[3944]|LBCH7298-10|10-JDWBC-7298|635[0n]bp|Canada.British Columbia|BOLD:ABZ6253
Grammia nevadensis|[3945]|LBCH7390-10|10-JDWBC-7390|630[0n]bp|Canada.British Columbia|BOLD:ABZ6253
Grammia nevadensis|[3946]|LBCH7296-10|10-JDWBC-7296|627[0n]bp|Canada.British Columbia|BOLD:ABZ6253
Grammia nevadensis|[3947]|LOWCD460-06|CGWC-3280|612[0n]bp|Canada.British Columbia|BOLD:ABZ6253
Grammia nevadensis|[3948]|LBCH7514-10|10-JDWBC-7514|626[0n]bp|Canada.British Columbia|BOLD:ABZ6253
Grammia nevadensis|[3949]|RDNMB398-05|CNCNoctuoidea10164|611[0n]bp|Canada.Alberta|BOLD:ABZ6253
Grammia nevadensis|[3950]|LOWCD468-06|CGWC-3288|613[0n]bp|Canada.British Columbia|BOLD:ABZ6253
Grammia nevadensis|[3951]|LBCH6331-10|10-JDWBC-6331|619[4n]bp|Canada.British Columbia|BOLD:ABZ6253
Grammia nevadensis|[3952]|LOWCD474-06|CGWC-3294|610[0n]bp|Canada.British Columbia|BOLD:ABZ6253
Grammia nevadensis|[3953]|LBCH7517-10|10-JDWBC-7517|632[0n]bp|Canada.British Columbia|BOLD:ABZ6253
Grammia nevadensis|[3954]|LBCH7713-10|10-JDWBC-7713|658[0n]bp|Canada.British Columbia|BOLD:ABZ6253
Grammia nevadensis|[3955]|LBCH7299-10|10-JDWBC-7299|658[0n]bp|Canada.British Columbia|BOLD:ABZ6253
Grammia nevadensis|[3956]|LBCH7303-10|10-JDWBC-7303|658[0n]bp|Canada.British Columbia|BOLD:ABZ6253
Grammia nevadensis|[3957]|LBCH6959-10|10-JDWBC-6959|658[0n]bp|Canada.British Columbia|BOLD:ABZ6253
Grammia nevadensis|[3958]|LOWCB193-05|CGWC-1133|658[0n]bp|Canada.British Columbia|BOLD:ABZ6253
Grammia nevadensis|[3959]|LBCG237-08|08-JDWBC-0237|658[0n]bp|Canada.British Columbia|BOLD:ABZ6253
Grammia nevadensis|[3960]|LBCH6592-10|10-JDWBC-6592|658[0n]bp|Canada.British Columbia|BOLD:ABZ6253
Grammia nevadensis|[3961]|LOWCD472-06|CGWC-3292|658[0n]bp|Canada.British Columbia|BOLD:ABZ6253
Grammia nevadensis|[3962]|LBCH7718-10|10-JDWBC-7718|658[0n]bp|Canada.British Columbia|BOLD:ABZ6253
Grammia nevadensis|[3963]|LBCH6411-10|10-JDWBC-6411|658[0n]bp|Canada.British Columbia|BOLD:ABZ6253
Grammia nevadensis|[3964]|LBCH6856-10|10-JDWBC-6856|658[0n]bp|Canada.British Columbia|BOLD:ABZ6253
Grammia nevadensis|[3965]|LBCH7223-10|10-JDWBC-7223|658[0n]bp|Canada.British Columbia|BOLD:ABZ6253
Grammia nevadensis|[3966]|LOWCB190-05|CGWC-1130|658[0n]bp|Canada.British Columbia|BOLD:ABZ6253

Grammia nevadensis|[3964]|LBCH6856-10|10-JDWBC-6856|658[0n]bp|Canada.British Columbia|BOLD:ABZ6253
Grammia nevadensis|[3965]|LBCH7223-10|10-JDWBC-7223|658[0n]bp|Canada.British Columbia|BOLD:ABZ6253
Grammia nevadensis|[3966]|LOWCB190-05|CGWC-1130|658[0n]bp|Canada.British Columbia|BOLD:ABZ6253
Grammia nevadensis|[3967]|LBCH7566-10|10-JDWBC-7566|658[0n]bp|Canada.British Columbia|BOLD:ABZ6253
Grammia nevadensis|[3968]|LBCH6474-10|10-JDWBC-6474|658[0n]bp|Canada.British Columbia|BOLD:ABZ6253
Grammia nevadensis|[3969]|LOWCD476-06|CGWC-3296|658[0n]bp|Canada.British Columbia|BOLD:ABZ6253
Grammia nevadensis|[3970]|LBCH6589-10|10-JDWBC-6589|658[0n]bp|Canada.British Columbia|BOLD:ABZ6253
Grammia nevadensis|[3971]|LBCH6472-10|10-JDWBC-6472|658[0n]bp|Canada.British Columbia|BOLD:ABZ6253
Grammia nevadensis|[3972]|LBCH7097-10|10-JDWBC-7097|658[0n]bp|Canada.British Columbia|BOLD:ABZ6253
Grammia nevadensis|[3973]|LBCH7464-10|10-JDWBC-7464|658[0n]bp|Canada.British Columbia|BOLD:ABZ6253
Grammia nevadensis|[3974]|LBCH7866-10|10-JDWBC-7866|658[0n]bp|Canada.British Columbia|BOLD:ABZ6253
Grammia nevadensis|[3975]|LBCH7867-10|10-JDWBC-7867|658[0n]bp|Canada.British Columbia|BOLD:ABZ6253
Grammia nevadensis|[3976]|LBCH7300-10|10-JDWBC-7300|658[0n]bp|Canada.British Columbia|BOLD:ABZ6253
Grammia nevadensis|[3977]|LBCH6859-10|10-JDWBC-6859|658[0n]bp|Canada.British Columbia|BOLD:ABZ6253
Grammia nevadensis|[3978]|LBCH7712-10|10-JDWBC-7712|658[0n]bp|Canada.British Columbia|BOLD:ABZ6253
Grammia nevadensis|[3979]|LBCH7869-10|10-JDWBC-7869|658[0n]bp|Canada.British Columbia|BOLD:ABZ6253
Grammia nevadensis|[3980]|LOWCD470-06|CGWC-3290|658[0n]bp|Canada.British Columbia|BOLD:ABZ6253
Grammia nevadensis|[3981]|LBCH7711-10|10-JDWBC-7711|658[0n]bp|Canada.British Columbia|BOLD:ABZ6253
Grammia nevadensis|[3982]|LBCH7297-10|10-JDWBC-7297|658[0n]bp|Canada.British Columbia|BOLD:ABZ6253
Grammia nevadensis|[3983]|LOWCD458-06|CGWC-3278|658[0n]bp|Canada.British Columbia|BOLD:ABZ6253
Grammia nevadensis|[3984]|LOWCD459-06|CGWC-3279|658[0n]bp|Canada.British Columbia|BOLD:ABZ6253
Grammia nevadensis|[3985]|LBCH7519-10|10-JDWBC-7519|658[0n]bp|Canada.British Columbia|BOLD:ABZ6253
Grammia nevadensis|[3986]|LBCG485-08|08-JDWBC-0485|658[0n]bp|Canada.British Columbia|BOLD:ABZ6253
Grammia nevadensis|[3987]|LBCH6475-10|10-JDWBC-6475|658[0n]bp|Canada.British Columbia|BOLD:ABZ6253
Grammia nevadensis|[3988]|LOWCD463-06|CGWC-3283|658[0n]bp|Canada.British Columbia|BOLD:ABZ6253
Grammia nevadensis|[3989]|LOWCB192-05|CGWC-1132|658[0n]bp|Canada.British Columbia|BOLD:ABZ6253
Grammia nevadensis|[3990]|LOWCD465-06|CGWC-3285|658[0n]bp|Canada.British Columbia|BOLD:ABZ6253
Grammia nevadensis|[3991]|LBCH7302-10|10-JDWBC-7302|658[0n]bp|Canada.British Columbia|BOLD:ABZ6253
Grammia nevadensis|[3992]|LBCH6470-10|10-JDWBC-6470|635[0n]bp|Canada.British Columbia|BOLD:ABZ6253
Grammia nevadensis|[3993]|LBCH6471-10|10-JDWBC-6471|658[0n]bp|Canada.British Columbia|BOLD:ABZ6253
Grammia ornata|[3994]|RDMAB757-06|BCSC234|658[0n]bp|Canada.British Columbia|BOLD:ABZ6253
Grammia ornata|[3995]|RDMAB758-06|BCSC235|658[0n]bp|Canada.British Columbia|BOLD:ABZ6253
Grammia nevadensis|[3996]|RDNMB400-05|CNCNoctuoidea10166|533[1n]bp|Canada.Alberta|BOLD:ABZ6253
Grammia nevadensis|[3997]|RDNMB421-05|CNCNoctuoidea10187|658[0n]bp|Canada.Saskatchewan|BOLD:ABZ6253
Grammia nevadensis|[3998]|RDNMB422-05|CNCNoctuoidea10188|658[0n]bp|Canada.Saskatchewan|BOLD:ABZ6253
Grammia nevadensis|[3999]|RDMAB766-06|BCSC243|658[0n]bp|Canada.Alberta|BOLD:ABZ6253
Grammia quenseli|[4000]|RDNME385-08|LEP037809|658[0n]bp|Canada.Yukon Territory|BOLD:ABZ6253
Grammia quenseli|[4001]|RDMAB859-06|BCSC336|599[0n]bp|Canada.Yukon Territory|BOLD:ABZ6253
Grammia quenseli|[4002]|RDMAB749-06|BCSC418|585[0n]bp|Canada.Yukon Territory|BOLD:ABZ6253
Grammia yukona|[4003]|RDMAB783-06|BCSC260|658[0n]bp|Canada.Yukon Territory|BOLD:ABZ6253
Grammia yukona|[4004]|RDMAB782-06|BCSC259|658[0n]bp|Canada.Yukon Territory|BOLD:ABZ6253
Apantesis nais|[4005]|RDNMB382-05|CNCNoctuoidea10148|658[0n]bp|Canada.Ontario|BOLD:AAA8555
Apantesis nais|[4006]|RDNME340-07|CNCNoctuoidea13947|658[1n]bp|Canada.Ontario|BOLD:AAA8555
Apantesis nais|[4007]|PHMO314-03|moth2374.02|639[0n]bp|Canada.Ontario|BOLD:AAA8555
Apantesis phalerata|[4008]|XAK255-06|2006-ONT-1250|658[0n]bp|Canada.Ontario|BOLD:ABY9321
Apantesis phalerata|[4009]|XAK254-06|2006-ONT-1249|658[0n]bp|Canada.Ontario|BOLD:ABY9321
Apantesis phalerata|[4010]|XAJ496-06|2006-ONT-0496|658[0n]bp|Canada.Ontario|BOLD:ABY9321
Apantesis phalerata|[4011]|XAG515-05|2005-ONT-1099|658[0n]bp|Canada.Ontario|BOLD:ABY9321
Apantesis phalerata|[4012]|XAJ340-06|2006-ONT-0340|658[0n]bp|Canada.Ontario|BOLD:ABY9321
Apantesis phalerata|[4013]|XAJ424-06|2006-ONT-0424|658[0n]bp|Canada.Ontario|BOLD:ABY9321
Apantesis phalerata|[4014]|XAG010-05|2005-ONT-594|619[0n]bp|Canada.Ontario|BOLD:ABY9321
Apantesis phalerata|[4015]|XAG251-05|2005-ONT-835|658[0n]bp|Canada.Ontario|BOLD:ABY9321
Apantesis phalerata|[4016]|RDNMB381-05|CNCNoctuoidea10147|658[0n]bp|Canada.Ontario|BOLD:ABY9321
Apantesis phalerata|[4017]|XAF687-05|2005-ONT-336|658[0n]bp|Canada.Ontario|BOLD:ABY9321
Holarctia obliterata|[4018]|RDMAB838-06|BCSC315|658[0n]bp|Canada.Alberta|BOLD:AAE3362
Holarctia obliterata|[4019]|RDMAB836-06|BCSC313|540[0n]bp|Canada.Alberta|BOLD:AAE3362
Holarctia obliterata|[4020]|LPABC813-09|08BBLEP-05032|658[0n]bp|Canada.Alberta|BOLD:AAE3362
Holarctia obliterata|[4021]|RDMAB837-06|BCSC314|658[0n]bp|Canada.Alberta|BOLD:AAE3362
Holoarctia n. sp.|[4022]|RDNME381-08|LEP037805|658[0n]bp|Canada.Yukon Territory|BOLD:ACF1107
Holoarctia n. sp.|[4023]|RDNMK701-11|CNCLEP 81847|658[0n]bp|Canada.Yukon Territory|BOLD:ACF1107
Holoarctia sordida|[4024]|RDMAB933-06|UASM57344|658[0n]bp|Canada.Alberta|BOLD:ABY8300
Holoarctia sordida|[4025]|RDMAB932-06|BCSC399|658[0n]bp|Canada.Alberta|BOLD:ABY8300
Holoarctia sordida|[4026]|RDNMK125-11|CNCLEP 80412|658[0n]bp|Canada.Alberta|BOLD:ABY8300
Neoarctia beanii|[4027]|RDNMG069-08|NOC15010|658[0n]bp|Canada.Alberta|BOLD:AAE4175
Neoarctia beanii|[4028]|LBCH1561-10|10-JDWBC-1561|658[0n]bp|Canada.British Columbia|BOLD:AAE4175
Neoarctia beanii|[4029]|RDNMG070-08|NOC15011|658[0n]bp|Canada.Alberta|BOLD:AAE4175
Neoarctia beanii|[4030]|RDMAB934-06|BCSC400|658[0n]bp|Canada.Alberta|BOLD:AAE4175
Neoarctia beanii|[4031]|LPABC095-09|08BBLEP-04314|658[0n]bp|Canada.Alberta|BOLD:AAE4175
Neoarctia brucei|[4032]|RDMAB919-06|BCSC393|658[0n]bp|Canada.British Columbia|BOLD:AAE4175
Neoarctia lafontainei|[4033]|RDNMF381-08|NOC14467|582[0n]bp|Canada.Yukon Territory|BOLD:ABY7044
Platarctia parthenos|[4034]|LOWCE072-06|CGWC-3832|658[1n]bp|Canada.British Columbia|BOLD:AAB2132
Parasemia plantaginis|[4035]|RDNME164-07|CNCNoctuoidea13692|658[3n]bp|Canada.Alberta|BOLD:AAB6883
Parasemia plantaginis|[4036]|LOWCE082-06|CGWC-3842|658[0n]bp|Canada.British Columbia|BOLD:AAB6883
Parasemia plantaginis|[4037]|RDMAB736-06|BCS_DNA405|632[0n]bp|Canada.Alberta|BOLD:AAB6883
Parasemia plantaginis|[4038]|LPSK104-08|08BBLEP-01672|658[0n]bp|Canada.Saskatchewan|BOLD:AAB6883
Parasemia plantaginis|[4039]|LPSK106-08|08BBLEP-01674|658[0n]bp|Canada.Saskatchewan|BOLD:AAB6883
Parasemia plantaginis|[4040]|LPSK105-08|08BBLEP-01673|658[0n]bp|Canada.Saskatchewan|BOLD:AAB6883
Parasemia plantaginis|[4041]|LOWCE083-06|CGWC-3843|658[0n]bp|Canada.British Columbia|BOLD:AAB6883
Parasemia plantaginis|[4042]|RDNME163-07|CNCNoctuoidea13691|658[0n]bp|Canada.Alberta|BOLD:AAB6883
Parasemia plantaginis|[4043]|BBLPA385-10|10BBCLP-0385|658[0n]bp|Canada.British Columbia|BOLD:AAB6883
Parasemia plantaginis|[4044]|RDNME158-07|CNCNoctuoidea13686|658[0n]bp|Canada.Alberta|BOLD:AAB6883
Parasemia plantaginis|[4045]|LOWCE081-06|CGWC-3841|658[0n]bp|Canada.British Columbia|BOLD:AAB6883
Parasemia plantaginis|[4046]|BBLPA386-10|10BBCLP-0386|658[0n]bp|Canada.British Columbia|BOLD:AAB6883
Parasemia plantaginis|[4047]|RDNME165-07|CNCNoctuoidea13693|617[0n]bp|Canada.Alberta|BOLD:AAB6883
Parasemia plantaginis|[4048]|BBLPA387-10|10BBCLP-0387|634[0n]bp|Canada.British Columbia|BOLD:AAB6883
Arctia caja|[4049]|RDNMB388-05|CNCNoctuoidea10154|616[0n]bp|Canada.Ontario|BOLD:AAA8530
Arctia caja americana|[4050]|RDLQB571-05|DH010674|658[0n]bp|Canada.Quebec|BOLD:AAA8530
Arctia caja|[4051]|XAG615-05|2005-ONT-1199|658[0n]bp|Canada.Ontario|BOLD:AAA8530
Arctia caja|[4052]|XAK250-06|2006-ONT-1245|658[0n]bp|Canada.Ontario|BOLD:AAA8530
Arctia caja|[4053]|XAG617-05|2005-ONT-1201|658[0n]bp|Canada.Ontario|BOLD:AAA8530
Arctia caja|[4054]|XAG579-05|2005-ONT-1163|658[0n]bp|Canada.Ontario|BOLD:AAA8530
Arctia caja|[4055]|XAG404-05|2005-ONT-988|658[0n]bp|Canada.Ontario|BOLD:AAA8530
Arctia caja|[4056]|TMNBD404-07|MNBTT-3205|658[0n]bp|Canada.New Brunswick|BOLD:AAA8530
Arctia caja americana|[4057]|RDLQB836-05|DH010923|621[0n]bp|Canada.Quebec|BOLD:AAA8530
Arctia caja|[4058]|RDNMB386-05|CNCNoctuoidea10152|516[0n]bp|Canada.British Columbia|BOLD:ACE6050
Arctia caja|[4059]|RDMAB386-05|BCSC60|658[1n]bp|Canada.British Columbia|BOLD:ACE6050
Arctia caja|[4060]|RDMAB387-05|BCSC61|658[0n]bp|Canada.Alberta|BOLD:ACE6050
Arctia brachyptera|[4061]|RDNMB383-05|CNCNoctuoidea10149|569[1n]bp|Canada.Yukon Territory|BOLD:ACE6050
Arctia brachyptera|[4062]|RDNMK540-11|CNCLEP 81969|658[0n]bp|Canada.Northwest Territories|BOLD:ACE6050
Arctia caja|[4063]|RDMAB388-05|BCSC62|658[0n]bp|Canada.Alberta|BOLD:ACE6050
Arctia opulenta|[4064]|RDNMB384-05|CNCNoctuoidea10150|585[0n]bp|Canada.Yukon Territory|BOLD:ACE6050
Acerbia alpina|[4065]|RDNMC461-05|CNCNoctuoidea12094|658[0n]bp|Canada.Northwest Territories|BOLD:AAD7179

Arctia caja|[4063]|RDMAB388-05|BCSC62|658[0n]bp|Canada.Alberta|BOLD:ACE6050
Arctia opulenta|[4064]|RDNMB384-05|CNCNoctuoidea10150|585[0n]bp|Canada.Yukon Territory|BOLD:ACE6050
Acerbia alpina|[4065]|RDNMC461-05|CNCNoctuoidea12094|658[0n]bp|Canada.Northwest Territories|BOLD:AAD7179
Pararctia subnebulosa|[4066]|RDNMF382-08|NOC14468|609[0n]bp|Canada.Yukon Territory|BOLD:AAF7314
Pararctia yarrowii|[4067]|RDNMK702-11|CNCLEP 81848|658[0n]bp|Canada.Yukon Territory|BOLD:ACF2201
Pararctia lapponica|[4068]|RDNME836-08|LEP041301|573[0n]bp|Canada.Newfoundland and Labrador|BOLD:AAD4311
Pararctia lapponica|[4069]|RDNME834-08|LEP041299|584[0n]bp|Canada.Newfoundland and Labrador|BOLD:AAD4311
Pararctia lapponica|[4070]|RDMAB392-05|BCSC65|658[0n]bp|Canada.Alberta|BOLD:AAD4311
Pararctia lapponica|[4071]|RDNME835-08|LEP041300|603[0n]bp|Canada.Newfoundland and Labrador|BOLD:AAD4311
Pararctia yarrowii|[4072]|RDMAB385-05|BCSC59|658[0n]bp|Canada.Alberta|BOLD:AAD4311
Pararctia yarrowii|[4073]|RDMAB393-05|BCSC66|658[0n]bp|Canada.Alberta|BOLD:AAD4311
Pararctia yarrowii|[4074]|GWORT781-10|BC AB Lep 00021|658[0n]bp|Canada.Alberta|BOLD:AAD4311
Pararctia yarrowii|[4075]|RDNMB301-05|CNCNoctuoidea10067|527[0n]bp|Canada.Alberta|BOLD:AAD4311
Pararctia yarrowii|[4076]|BBLPA388-10|10BBCLP-0388|658[0n]bp|Canada.Alberta|BOLD:AAD4311
Pararctia yarrowii|[4077]|BBLPA389-10|10BBCLP-0389|658[0n]bp|Canada.Alberta|BOLD:AAD4311
Platarctia parthenos|[4078]|RDNME358-07|CNCNoctuoidea13965|658[0n]bp|Canada.Yukon Territory|BOLD:AAB2131
Platarctia parthenos|[4079]|LCHP700-07|07PROBE-10382|658[0n]bp|Canada.Manitoba|BOLD:AAB2131
Platarctia parthenos|[4080]|LCHP897-07|07PROBE-10659|658[0n]bp|Canada.Manitoba|BOLD:AAB2131
Platarctia parthenos|[4081]|LCHP026-07|07PROBE-00086|658[0n]bp|Canada.Manitoba|BOLD:AAB2131
Platarctia parthenos|[4082]|LPABC922-09|08BBLEP-05333|658[0n]bp|Canada.Alberta|BOLD:AAB2131
Platarctia parthenos|[4083]|LPAB308-08|08BBLEP-02630|658[0n]bp|Canada.Alberta|BOLD:AAB2131
Platarctia parthenos|[4084]|LOWCD148-06|CGWC-2968|648[0n]bp|Canada.British Columbia|BOLD:AAB2131
Platarctia parthenos|[4085]|LPABC843-09|08BBLEP-05062|658[0n]bp|Canada.Alberta|BOLD:AAB2131
Platarctia parthenos|[4086]|LBCG1934-09|08-JDWBC-1934|658[0n]bp|Canada.British Columbia|BOLD:AAB2131
Platarctia parthenos|[4087]|LBCG639-09|08-JDWBC-0639|658[0n]bp|Canada.British Columbia|BOLD:AAB2131
Platarctia parthenos|[4088]|BBLPA313-10|10BBCLP-0313|658[0n]bp|Canada.British Columbia|BOLD:AAB2131
Platarctia parthenos|[4089]|BBLPA315-10|10BBCLP-0315|658[0n]bp|Canada.British Columbia|BOLD:AAB2131
Platarctia parthenos|[4090]|BBLPA316-10|10BBCLP-0316|658[0n]bp|Canada.British Columbia|BOLD:AAB2131
Platarctia parthenos|[4091]|LPMN520-08|08BBLEP-01319|658[0n]bp|Canada.Manitoba|BOLD:AAB2131
Platarctia parthenos|[4092]|RDLQB183-05|DH010269|632[1n]bp|Canada.Quebec|BOLD:AAB2131
Platarctia parthenos|[4093]|DIMC088-09|DS-Test-088|666[0n]bp|Canada.Ontario|
Platarctia parthenos|[4094]|LPMN554-08|08BBLEP-01355|658[0n]bp|Canada.Manitoba|BOLD:AAB2131
Platarctia parthenos|[4095]|LPMN553-08|08BBLEP-01354|658[0n]bp|Canada.Manitoba|BOLD:AAB2131
Platarctia parthenos|[4096]|LPSOD396-09|08BBLEP-00175|658[0n]bp|Canada.Ontario|BOLD:AAB2131
Platarctia parthenos|[4097]|LPMN555-08|08BBLEP-01356|658[0n]bp|Canada.Manitoba|BOLD:AAB2131
Platarctia parthenos|[4098]|RDLQF857-06|DH012018|658[0n]bp|Canada.Quebec|BOLD:AAB2131
Platarctia parthenos|[4099]|RDLQB182-05|DH010268|658[0n]bp|Canada.Quebec|BOLD:AAB2131
Platarctia parthenos|[4100]|LPMN451-08|08BBLEP-01250|658[0n]bp|Canada.Manitoba|BOLD:AAB2131
Platarctia parthenos|[4101]|RDMAB923-06|BCSC396|657[0n]bp|Canada.Alberta|BOLD:AAB2131
Platarctia parthenos|[4102]|BBLPC976-09|09BBELE-1976|658[0n]bp|Canada.Newfoundland and Labrador|BOLD:...
Platarctia parthenos|[4103]|BBLPA317-10|10BBCLP-0317|658[0n]bp|Canada.Alberta|BOLD:AAB2131
Platarctia parthenos|[4104]|RDLQF473-06|DH011622|658[0n]bp|Canada.Quebec|BOLD:AAB2131
Platarctia parthenos|[4105]|RDLQF472-06|DH011621|658[0n]bp|Canada.Quebec|BOLD:AAB2131
Platarctia parthenos|[4106]|BBLPA312-10|10BBCLP-0312|658[0n]bp|Canada.Saskatchewan|BOLD:AAB2131
Platarctia parthenos|[4107]|LPSOD828-09|08BBLEP-00610|658[0n]bp|Canada.Ontario|BOLD:AAB2131
Platarctia parthenos|[4108]|BBLPA318-10|10BBCLP-0318|658[0n]bp|Canada.Ontario|BOLD:AAB2131
Platarctia parthenos|[4109]|MNAC787-07|CNCLEP00027528|658[0n]bp|Canada.Quebec|BOLD:AAB2131
Platarctia parthenos|[4110]|BBLPA314-10|10BBCLP-0314|658[0n]bp|Canada.Ontario|BOLD:AAB2131
Platyprepia virginalis|[4111]|RDMAB935-06|BCSC401|658[0n]bp|Canada.British Columbia|BOLD:AAE4732
Phragmatobia assimilans|[4112]|LMIS016-05|05-ONMIS-0016|658[0n]bp|Canada.Ontario|BOLD:ACF3777
Phragmatobia assimilans|[4113]|LPSOB325-08|PPBP-1324|658[0n]bp|Canada.Ontario|BOLD:ACF3777
Phragmatobia assimilans|[4114]|TMNBB028-06|MNBTT-968|658[0n]bp|Canada.New Brunswick|BOLD:ACF3777
Phragmatobia assimilans|[4115]|LPSOB594-08|PPBP-1593|658[0n]bp|Canada.Ontario|BOLD:ACF3777
Phragmatobia assimilans|[4116]|LPSOB131-08|PPBP-1130|658[0n]bp|Canada.Ontario|BOLD:ACF3777
Phragmatobia assimilans|[4117]|TTMNB067-06|MNBTT-067|658[0n]bp|Canada.New Brunswick|BOLD:ACF3777
Phragmatobia assimilans|[4118]|TTMNB065-06|MNBTT-065|658[0n]bp|Canada.New Brunswick|BOLD:ACF3777
Phragmatobia assimilans|[4119]|RDLQ768-07|DH009494|658[0n]bp|Canada.Quebec|BOLD:ACF3777
Phragmatobia assimilans|[4120]|TTMNB066-06|MNBTT-066|658[0n]bp|Canada.New Brunswick|BOLD:ACF3777
Phragmatobia assimilans|[4121]|TTMNB062-06|MNBTT-062|658[0n]bp|Canada.New Brunswick|BOLD:ACF3777
Phragmatobia assimilans|[4122]|TTMNB064-06|MNBTT-064|658[0n]bp|Canada.New Brunswick|BOLD:ACF3777
Phragmatobia assimilans|[4123]|TMNBB025-06|MNBTT-965|658[0n]bp|Canada.New Brunswick|BOLD:ACF3777
Phragmatobia assimilans|[4124]|RDMAB920-06|UASM24734|658[0n]bp|Canada.Alberta|BOLD:ACF3777
Phragmatobia assimilans|[4125]|TMNBB027-06|MNBTT-967|658[0n]bp|Canada.New Brunswick|BOLD:ACF3777
Phragmatobia assimilans|[4126]|LPSOB138-08|PPBP-1137|658[0n]bp|Canada.Ontario|BOLD:ACF3777
Phragmatobia assimilans|[4127]|RDLQ767-07|DH009273|658[0n]bp|Canada.Quebec|BOLD:ACF3777
Phragmatobia assimilans|[4128]|LPSOB133-08|PPBP-1132|658[0n]bp|Canada.Ontario|BOLD:ACF3777
Phragmatobia assimilans|[4129]|TMNBB024-06|MNBTT-964|658[0n]bp|Canada.New Brunswick|BOLD:ACF3777
Phragmatobia assimilans|[4130]|TMNBB022-06|MNBTT-962|658[0n]bp|Canada.New Brunswick|BOLD:ACF3777
Phragmatobia assimilans|[4131]|TMNBB026-06|MNBTT-966|658[0n]bp|Canada.New Brunswick|BOLD:ACF3777
Phragmatobia assimilans|[4132]|LPSOB118-08|PPBP-1117|658[0n]bp|Canada.Ontario|BOLD:ACF3777
Phragmatobia assimilans|[4133]|TMNBB023-06|MNBTT-963|658[0n]bp|Canada.New Brunswick|BOLD:ACF3777
Phragmatobia assimilans|[4134]|TTMNB063-06|MNBTT-063|658[0n]bp|Canada.New Brunswick|BOLD:ACF3777
Phragmatobia assimilans|[4135]|BBLPB275-10|10BBCLP-1274|658[0n]bp|Canada.Saskatchewan|BOLD:ACF3777
Phragmatobia assimilans|[4136]|LPSOB395-08|PPBP-1394|658[0n]bp|Canada.Ontario|BOLD:ACF3777
Phragmatobia fuliginosa|[4137]|XAG012-05|2005-ONT-596|658[0n]bp|Canada.Ontario|BOLD:AAA6177
Phragmatobia fuliginosa|[4138]|BBLPE566-09|09BBELE-2566|658[0n]bp|Canada.Nova Scotia|BOLD:AAA6177
Phragmatobia fuliginosa|[4139]|BBLPE563-09|09BBELE-2563|638[0n]bp|Canada.Nova Scotia|BOLD:AAA6177
Phragmatobia fuliginosa|[4140]|BBLPE585-09|09BBELE-2585|658[0n]bp|Canada.Nova Scotia|BOLD:AAA6177
Phragmatobia fuliginosa|[4141]|BBLPE598-09|09BBELE-2598|658[0n]bp|Canada.Nova Scotia|BOLD:AAA6177
Phragmatobia fuliginosa|[4142]|XAG231-05|2005-ONT-815|658[0n]bp|Canada.Ontario|BOLD:AAA6177
Phragmatobia fuliginosa|[4143]|RDMAB389-05|BCSC63|658[0n]bp|Canada.Alberta|BOLD:AAA6177
Phragmatobia fuliginosa|[4144]|XAJ924-06|2006-ONT-0924|658[0n]bp|Canada.Ontario|BOLD:AAA6177
Phragmatobia fuliginosa|[4145]|XAJ931-06|2006-ONT-0931|658[0n]bp|Canada.Ontario|BOLD:AAA6177
Phragmatobia fuliginosa|[4146]|TMNBB018-06|MNBTT-958|658[0n]bp|Canada.New Brunswick|BOLD:AAA6177
Phragmatobia fuliginosa|[4147]|XAB035-04|04HBL005035|658[0n]bp|Canada.Ontario|BOLD:AAA6177
Phragmatobia fuliginosa|[4148]|XAD765-05|2005-ONT-564|658[0n]bp|Canada.Ontario|BOLD:AAA6177
Phragmatobia fuliginosa|[4149]|XAB480-04|04HBL005480|658[0n]bp|Canada.Ontario|BOLD:AAA6177
Phragmatobia fuliginosa|[4150]|XAK014-06|2006-ONT-1009|658[0n]bp|Canada.Ontario|BOLD:AAA6177
Phragmatobia fuliginosa|[4151]|BLTIB957-08|BL1386|658[0n]bp|Canada.Ontario|BOLD:AAA6177
Phragmatobia fuliginosa|[4152]|BBLPE584-09|09BBELE-2584|658[0n]bp|Canada.Nova Scotia|BOLD:AAA6177
Phragmatobia fuliginosa|[4153]|RDNME827-08|LEP041292|658[0n]bp|Canada.Alberta|BOLD:AAA6177
Phragmatobia fuliginosa|[4154]|XAG444-05|2005-ONT-1028|658[0n]bp|Canada.Ontario|BOLD:AAA6177
Phragmatobia fuliginosa|[4155]|RDLQ107-05|DH004615|609[0n]bp|Canada.Quebec|BOLD:AAA6177
Phragmatobia fuliginosa|[4156]|RDNM109-05|CNCNoctuoidea6659|658[0n]bp|Canada.Ontario|BOLD:AAA6177
Phragmatobia fuliginosa|[4157]|XAB481-04|04HBL005481|658[0n]bp|Canada.Ontario|BOLD:AAA6177
Phragmatobia fuliginosa rubricosa|[4158]|RDLQ105-05|DH006364|609[0n]bp|Canada.Quebec|BOLD:AAA6177
Phragmatobia fuliginosa|[4159]|TMNBB020-06|MNBTT-960|612[0n]bp|Canada.New Brunswick|BOLD:AAA6177
Phragmatobia fuliginosa|[4160]|TMNBB019-06|MNBTT-959|612[0n]bp|Canada.New Brunswick|BOLD:AAA6177
Phragmatobia fuliginosa|[4161]|PMG015-03|PHRA1.01|617[0n]bp|Canada.Ontario|BOLD:AAA6177
Phragmatobia fuliginosa rubricosa|[4162]|RDLQF686-06|DH011836|601[0n]bp|Canada.Quebec|BOLD:AAA6177
Phragmatobia fuliginosa|[4163]|TMNBB015-06|MNBTT-955|658[0n]bp|Canada.New Brunswick|BOLD:AAA6177
Phragmatobia fuliginosa|[4164]|RDMAB390-05|UASM41320|658[0n]bp|Canada.Alberta|BOLD:AAA6177

Phragmatobia fuliginosa rubricosa|[4162]|RDLQF686-06|DH011836|601[0n]bp|Canada.Quebec|BOLD:AAA6177
Phragmatobia fuliginosa|[4163]|TMNBB015-06|MNBTT-955|658[0n]bp|Canada.New Brunswick|BOLD:AAA6177
Phragmatobia fuliginosa|[4164]|RDMAB390-05|UASM41320|658[0n]bp|Canada.Alberta|BOLD:AAA6177
Phragmatobia fuliginosa|[4165]|XAJ854-06|2006-ONT-0854|658[0n]bp|Canada.Ontario|BOLD:AAA6177
Phragmatobia fuliginosa|[4166]|MNBB399-05|05-NBSTA-315|658[0n]bp|Canada.New Brunswick|BOLD:AAA6177
Phragmatobia fuliginosa|[4167]|XAK064-06|2006-ONT-1059|658[0n]bp|Canada.Ontario|BOLD:AAA6177
Phragmatobia fuliginosa|[4168]|TMNBB016-06|MNBTT-956|658[0n]bp|Canada.New Brunswick|BOLD:AAA6177
Phragmatobia fuliginosa|[4169]|TMNBB021-06|MNBTT-961|658[0n]bp|Canada.New Brunswick|BOLD:AAA6177
Phragmatobia fuliginosa|[4170]|TMNBB017-06|MNBTT-957|658[0n]bp|Canada.New Brunswick|BOLD:AAA6177
Phragmatobia fuliginosa|[4171]|MNBB618-05|05-NBSTA-534|658[0n]bp|Canada.New Brunswick|BOLD:AAA6177
Phragmatobia fuliginosa|[4172]|XAG427-05|2005-ONT-1011|658[0n]bp|Canada.Ontario|BOLD:AAA6177
Phragmatobia fuliginosa|[4173]|RDLQG269-06|DH012477|658[0n]bp|Canada.Quebec|BOLD:AAA6177
Phragmatobia fuliginosa rubricosa|[4174]|RDLQB740-05|DH010655|658[0n]bp|Canada.Quebec|BOLD:AAA6177
Phragmatobia fuliginosa rubricosa|[4175]|RDLQB511-05|DH010597|658[0n]bp|Canada.Quebec|BOLD:AAA6177
Phragmatobia fuliginosa rubricosa|[4176]|RDLQ106-05|DH007139|658[0n]bp|Canada.Quebec|BOLD:AAA6177
Phragmatobia lineata|[4177]|RDNMH377-09|BIRD22041|658[0n]bp|Canada.Alberta|BOLD:AAA6177
Pyrrharctia isabella|[4178]|LHLEP058-06|UBC-2006-0259|577[0n]bp|Canada.British Columbia|BOLD:ACF1163
Pyrrharctia isabella|[4179]|PHMNB712-05|Moth 405.03SA|616[0n]bp|Canada.New Brunswick|BOLD:ACF1163
Pyrrharctia isabella|[4180]|LHLEP295-06|UBC-2006-0930|629[0n]bp|Canada.British Columbia|BOLD:ACF1163
Pyrrharctia isabella|[4181]|LOWCE046-06|CGWC-3806|658[0n]bp|Canada.British Columbia|BOLD:ACF1163
Pyrrharctia isabella|[4182]|TMNBB011-06|MNBTT-951|658[0n]bp|Canada.New Brunswick|BOLD:ACF1163
Pyrrharctia isabella|[4183]|TMNBD386-07|MNBTT-3187|656[0n]bp|Canada.New Brunswick|BOLD:ACF1163
Pyrrharctia isabella|[4184]|LHLEP294-06|UBC-2006-0727|657[0n]bp|Canada.British Columbia|BOLD:ACF1163
Pyrrharctia isabella|[4185]|LPSO230-08|PPBP-0230|658[0n]bp|Canada.Ontario|BOLD:ACF1163
Pyrrharctia isabella|[4186]|LHLEP059-06|UBC-2006-0260|658[0n]bp|Canada.British Columbia|BOLD:ACF1163
Pyrrharctia isabella|[4187]|LHLEP292-06|UBC-2006-0725|657[0n]bp|Canada.British Columbia|BOLD:ACF1163
Pyrrharctia isabella|[4188]|LOWCE039-06|CGWC-3799|658[0n]bp|Canada.British Columbia|BOLD:ACF1163
Pyrrharctia isabella|[4189]|LHLEP293-06|UBC-2006-0726|653[0n]bp|Canada.British Columbia|BOLD:ACF1163
Pyrrharctia isabella|[4190]|LHLEP060-06|UBC-2006-0261|658[0n]bp|Canada.British Columbia|BOLD:ACF1163
Pyrrharctia isabella|[4191]|LHLEP296-06|UBC-2006-1166|657[0n]bp|Canada.British Columbia|BOLD:ACF1163
Pyrrharctia isabella|[4192]|LHLEP057-06|UBC-2006-0258|658[0n]bp|Canada.British Columbia|BOLD:ACF1163
Pyrrharctia isabella|[4193]|LALPA207-10|AVBC 208-10|658[0n]bp|Canada.British Columbia|BOLD:ACF1163
Pyrrharctia isabella|[4194]|LALPA378-10|AVBC 380-10|658[0n]bp|Canada.British Columbia|BOLD:ACF1163
Pyrrharctia isabella|[4195]|LPMN708-08|08BBLEP-01511|658[0n]bp|Canada.Manitoba|BOLD:ACF1163
Pyrrharctia isabella|[4196]|LPSO927-08|PPBP-0927|658[0n]bp|Canada.Ontario|BOLD:ACF1163
Pyrrharctia isabella|[4197]|BBLPC217-09|09BBELE-1217|658[0n]bp|Canada.Nova Scotia|BOLD:ACF1163
Pyrrharctia isabella|[4198]|TMNBD385-07|MNBTT-3186|658[0n]bp|Canada.New Brunswick|BOLD:ACF1163
Pyrrharctia isabella|[4199]|XAK121-06|2006-ONT-1116|658[0n]bp|Canada.Ontario|BOLD:ACF1163
Pyrrharctia isabella|[4200]|BBLEC567-09|09BBELE-0567|641[0n]bp|Canada.Nova Scotia|BOLD:ACF1163
Pyrrharctia isabella|[4201]|BBLEC568-09|09BBELE-0568|658[0n]bp|Canada.Nova Scotia|BOLD:ACF1163
Pyrrharctia isabella|[4202]|BBLEC569-09|09BBELE-0569|658[0n]bp|Canada.Nova Scotia|BOLD:ACF1163
Pyrrharctia isabella|[4203]|RDLQG002-06|DH012122|658[0n]bp|Canada.Quebec|BOLD:ACF1163
Pyrrharctia isabella|[4204]|BBLPC231-09|09BBELE-1231|658[0n]bp|Canada.Nova Scotia|BOLD:ACF1163
Pyrrharctia isabella|[4205]|TMNBD383-07|MNBTT-3184|654[0n]bp|Canada.New Brunswick|BOLD:ACF1163
Pyrrharctia isabella|[4206]|LHLEP291-06|UBC-2006-0724|657[0n]bp|Canada.British Columbia|BOLD:ACF1163
Pyrrharctia isabella|[4207]|RDLQG212-06|DH012388|538[1n]bp|Canada.Quebec|BOLD:ACF1163
Pyrrharctia isabella|[4208]|PMG016-03|moth305.01|617[0n]bp|Canada.Ontario|BOLD:AAA4533
Pyrrharctia isabella|[4209]|BBLPC990-09|09BBELE-1990|633[0n]bp|Canada.Nova Scotia|
Pyrrharctia isabella|[4210]|LPSOB064-08|PPBP-1063|658[0n]bp|Canada.Ontario|BOLD:AAA4533
Pyrrharctia isabella|[4211]|LPSO515-08|PPBP-0515|658[0n]bp|Canada.Ontario|BOLD:AAA4533
Pyrrharctia isabella|[4212]|XAB104-04|04HBL005104|606[2n]bp|Canada.Ontario|BOLD:AAA4533
Pyrrharctia isabella|[4213]|XAB622-04|04HBL005622|658[0n]bp|Canada.Ontario|BOLD:AAA4533
Pyrrharctia isabella|[4214]|LPSOB065-08|PPBP-1064|658[0n]bp|Canada.Ontario|BOLD:AAA4533
Pyrrharctia isabella|[4215]|LPSO872-08|PPBP-0872|658[0n]bp|Canada.Ontario|BOLD:AAA4533
Pyrrharctia isabella|[4216]|XAK157-06|2006-ONT-1152|658[0n]bp|Canada.Ontario|BOLD:AAA4533
Pyrrharctia isabella|[4217]|XAE372-04|Moth4372.03|658[0n]bp|Canada.Ontario|BOLD:AAA4533
Pyrrharctia isabella|[4218]|TMNBD384-07|MNBTT-3185|658[0n]bp|Canada.New Brunswick|BOLD:AAA4533
Pyrrharctia isabella|[4219]|XAB183-04|04HBL005183|658[0n]bp|Canada.Ontario|BOLD:AAA4533
Pyrrharctia isabella|[4220]|XAB082-04|04HBL005082|658[0n]bp|Canada.Ontario|BOLD:AAA4533
Pyrrharctia isabella|[4221]|BLTIB225-08|BL406|658[0n]bp|Canada.Ontario|BOLD:AAA4533
Pyrrharctia isabella|[4222]|LPMN762-08|08BBLEP-01565|658[0n]bp|Canada.Manitoba|BOLD:AAA4533
Pyrrharctia isabella|[4223]|LPSOC140-08|PPBP-2139|658[0n]bp|Canada.Ontario|BOLD:AAA4533
Pyrrharctia isabella|[4224]|TMNBD382-07|MNBTT-3183|658[0n]bp|Canada.New Brunswick|BOLD:AAA4533
Pyrrharctia isabella|[4225]|BLTIB428-08|BL675|658[0n]bp|Canada.Ontario|BOLD:AAA4533
Pyrrharctia isabella|[4226]|XAJ935-06|2006-ONT-0935|658[0n]bp|Canada.Ontario|BOLD:AAA4533
Pyrrharctia isabella|[4227]|LPSOC128-08|PPBP-2127|658[0n]bp|Canada.Ontario|BOLD:AAA4533
Pyrrharctia isabella|[4228]|LPSO325-08|PPBP-0325|658[0n]bp|Canada.Ontario|BOLD:AAA4533
Pyrrharctia isabella|[4229]|XAF693-05|2005-ONT-342|658[0n]bp|Canada.Ontario|BOLD:AAA4533
Pyrrharctia isabella|[4230]|XAE359-04|Moth4359.03|658[0n]bp|Canada.Ontario|BOLD:AAA4533
Pyrrharctia isabella|[4231]|BBLPC251-09|09BBELE-1251|658[0n]bp|Canada.Nova Scotia|BOLD:AAA4533
Pyrrharctia isabella|[4232]|TMNBB010-06|MNBTT-950|658[0n]bp|Canada.New Brunswick|BOLD:AAA4533
Pyrrharctia isabella|[4233]|TMG76-03|moth540.01|639[0n]bp|Canada.Ontario|BOLD:AAA4533
Pyrrharctia isabella|[4234]|BLTIB226-08|BL407|658[0n]bp|Canada.Ontario|BOLD:AAA4533
Pyrrharctia isabella|[4235]|LPSO514-08|PPBP-0514|658[0n]bp|Canada.Ontario|BOLD:AAA4533
Pyrrharctia isabella|[4236]|RDMAB927-06|UASM57202|658[0n]bp|Canada.Alberta|BOLD:AAA4533
Tyria jacobaeae|[4237]|RDMAB921-06|BCSC394|658[0n]bp|Canada.Nova Scotia|BOLD:AAB5189
Tyria jacobaeae|[4238]|LOWCE193-06|CGWC-3953|658[0n]bp|Canada.British Columbia|BOLD:AAB5189
Tyria jacobaeae|[4239]|RDNMG966-08|CNC LEP00053090|642[0n]bp|Canada.Nova Scotia|BOLD:AAB5189
Bruceia pulverina|[4240]|RDNME446-08|LEP037870|549[0n]bp|Canada.British Columbia|BOLD:AAD9797
Bruceia pulverina|[4241]|RDNME227-07|CNCNoctuoidea13834|615[1n]bp|Canada.British Columbia|BOLD:AAD9797
Bruceia pulverina|[4242]|LBCG2899-09|08-JDWBC-2899|658[0n]bp|Canada.British Columbia|BOLD:AAD9797
Bruceia pulverina|[4243]|LBCH7697-10|10-JDWBC-7697|658[0n]bp|Canada.British Columbia|BOLD:AAD9797
Bruceia pulverina|[4244]|LBCH6241-10|10-JDWBC-6241|658[0n]bp|Canada.British Columbia|BOLD:AAD9797
Bruceia pulverina|[4245]|LBCH6133-10|10-JDWBC-6133|658[0n]bp|Canada.British Columbia|BOLD:AAD9797
Cisthene plumbea|[4246]|USLEP543-10|10BBLEP-00543|658[0n]bp|United States.Florida|BOLD:AAC4269
Cisthene plumbea|[4247]|USLEP233-10|10BBLEP-00233|658[0n]bp|United States.Florida|BOLD:AAC4269
Cisthene plumbea|[4248]|USLEP505-10|10BBLEP-00505|658[0n]bp|United States.Florida|BOLD:AAC4269
Cisthene plumbea|[4249]|HKONS218-08|3123-COI-07|658[0n]bp|United States.Florida|BOLD:AAC4269
Cisthene plumbea|[4250]|USLEP542-10|10BBLEP-00542|658[0n]bp|United States.Florida|BOLD:AAC4269
Cisthene plumbea|[4251]|HKONB163-08|3660-COI-08|658[0n]bp|United States.Texas|BOLD:AAC4269
Cisthene plumbea|[4252]|RDNMH350-09|CNCLEP00057759|658[0n]bp|United States.Arkansas|BOLD:AAC4269
Cisthene plumbea|[4253]|LPOKA186-08|MDOK-0186|658[0n]bp|United States.Oklahoma|BOLD:AAC4269
Cisthene plumbea|[4254]|LSEU612-06|06-JKA-0612|658[0n]bp|United States.Georgia|BOLD:AAC4269
Cisthene plumbea|[4255]|HKONS228-08|3133-COI-07|658[1n]bp|United States.Florida|BOLD:AAC4269
Cisthene plumbea|[4256]|LPOKD202-09|MDOK-3281|657[0n]bp|United States.Oklahoma|BOLD:AAC4269
Cisthene plumbea|[4257]|LNCNW103-06|06-NCNW-0103|658[0n]bp|United States.North Carolina|BOLD:AAC4269
Cisthene plumbea|[4258]|LNCNW105-06|06-NCNW-0105|658[0n]bp|United States.North Carolina|BOLD:AAC4269
Cisthene plumbea|[4259]|LPOKC305-09|MDOK-2382|658[0n]bp|United States.Oklahoma|BOLD:AAC4269
Cisthene plumbea|[4260]|LSUSA141-06|06-SUSA-0141|658[0n]bp|United States.Kentucky|BOLD:AAC4269
Cisthene plumbea|[4261]|LSUSA150-06|06-SUSA-0150|656[0n]bp|United States.Kentucky|BOLD:AAC4269
Cisthene plumbea|[4262]|BBLSU024-09|09BBLEP-04393|658[0n]bp|United States.Arkansas|BOLD:AAC4269
Cisthene plumbea|[4263]|RDNMH349-09|CNCLEP00057758|658[0n]bp|United States.Mississippi|BOLD:AAC4269

Cisthene plumbea|[4261]|LSUSA150-06|06-SUSA-0150|656[0n]bp|United States.Kentucky|BOLD:AAC4269
Cisthene plumbea|[4262]|BBLSU024-09|09BBLEP-04393|658[0n]bp|United States.Arkansas|BOLD:AAC4269
Cisthene plumbea|[4263]|RDNMH349-09|CNCLEP00057758|658[0n]bp|United States.Mississippi|BOLD:AAC4269
Cisthene plumbea|[4264]|BBLSU038-09|09BBLEP-04407|658[0n]bp|United States.Arkansas|BOLD:AAC4269
Phytometra ernestinana|[4265]|RDNME723-08|LEP041994|658[0n]bp|United States.Florida|BOLD:AAD6965
Phytometra ernestinana|[4266]|BBLOB648-11|BIOUG01398-G06|658[0n]bp|United States.Florida|BOLD:AAD6965
Phytometra ernestinana|[4267]|HKONS594-08|1782-COI-07|658[0n]bp|United States.Florida|BOLD:AAD6965
Phytometra ernestinana|[4268]|LPOKD343-09|MDOK-3422|658[0n]bp|United States.Oklahoma|BOLD:AAD6965
Phytometra ernestinana|[4269]|LMEM111-09|RBMIS-0111|658[0n]bp|United States.Texas|BOLD:AAD6965
Phytometra ernestinana|[4270]|LPOKB245-09|MDOK-1236|658[0n]bp|United States.Oklahoma|BOLD:AAD6965
Phytometra ernestinana|[4271]|LMEM110-09|RBMIS-0110|633[0n]bp|United States.Texas|BOLD:AAD6965
Phytometra ernestinana|[4272]|ABNCC191-07|1191-150702-MS|583[0n]bp|United States.Mississippi|BOLD:AAD...
Phytometra rhodarialis|[4273]|LMEM114-09|RBMIS-0114|658[0n]bp|United States.Alabama|BOLD:AAC3524
Phytometra rhodarialis|[4274]|LMEM115-09|RBMIS-0115|658[0n]bp|United States.Alabama|BOLD:AAC3524
Phytometra rhodarialis|[4275]|LMEM112-09|RBMIS-0112|658[0n]bp|United States.Alabama|BOLD:AAC3524
Phytometra rhodarialis|[4276]|LMEM113-09|RBMIS-0113|658[0n]bp|United States.Alabama|BOLD:AAC3524
Phytometra rhodarialis|[4277]|LNCB847-09|09-MISC-032|658[0n]bp|United States.Alabama|BOLD:AAC0499
Phytometra rhodarialis|[4278]|LMEM117-09|RBMIS-0117|658[0n]bp|United States.Mississippi|BOLD:AAC0499
Phytometra rhodarialis|[4279]|BBLOB1890-11|BIOUG01425-H01|658[0n]bp|United States.Florida|BOLD:AAC0499
Phytometra rhodarialis|[4280]|LMEM116-09|RBMIS-0116|658[0n]bp|United States.Mississippi|BOLD:AAC0499
Phytometra rhodarialis|[4281]|BBLOC036-11|BIOUG01452-D05|658[0n]bp|United States.Florida|BOLD:AAC0499
Phytometra rhodarialis|[4282]|LNCB845-09|09-MISC-030|658[0n]bp|United States.Alabama|BOLD:AAC0499
Phytometra rhodarialis|[4283]|LNCB844-09|09-MISC-029|658[0n]bp|United States.Alabama|BOLD:AAC0499
Phytometra rhodarialis|[4284]|LNCB374-06|06-NCCC-1330|658[0n]bp|United States.North Carolina|BOLD:AAC...
Phytometra rhodarialis|[4285]|LNC064-05|05-NCCC-064|658[0n]bp|United States.North Carolina|BOLD:AAC0499
Phytometra rhodarialis|[4286]|LNCB846-09|09-MISC-031|658[0n]bp|United States.Alabama|BOLD:AAC0499
Phytometra rhodarialis|[4287]|LMEM118-09|RBMIS-0118|658[0n]bp|United States.Mississippi|BOLD:AAC0499
Phytometra rhodarialis|[4288]|LMEM119-09|RBMIS-0119|658[0n]bp|United States.Mississippi|BOLD:AAC0499
Phytometra rhodarialis|[4289]|LNC063-05|05-NCCC-063|658[0n]bp|United States.North Carolina|BOLD:AAC0499
Anticarsia gemmatalis|[4290]|LSEU045-06|06-JKA-0045|658[0n]bp|United States.Georgia|BOLD:AAA6923
Anticarsia gemmatalis|[4291]|HKONS044-07|1608-COI-07|658[0n]bp|United States.Florida|BOLD:AAA6923
Anticarsia gemmatalis|[4292]|LNC484-06|05-NCCC-484|658[0n]bp|United States.North Carolina|BOLD:AAA6923
Anticarsia gemmatalis|[4293]|HKONS388-08|1858-COI-07|658[0n]bp|United States.Florida|BOLD:AAA6923
Anticarsia gemmatalis|[4294]|LNC447-05|05-NCCC-447|658[0n]bp|United States.North Carolina|BOLD:AAA6923
Anticarsia gemmatalis|[4295]|LPOKE268-11|MDOK-4346|658[0n]bp|United States.Oklahoma|BOLD:AAA6923
Anticarsia gemmatalis|[4296]|HKONS389-08|1859-COI-07|658[0n]bp|United States.Florida|BOLD:AAA6923
Anticarsia gemmatalis|[4297]|LPOKE267-11|MDOK-4345|658[0n]bp|United States.Oklahoma|BOLD:AAA6923
Parahypenodes quadralis|[4298]|RDLQG118-06|DH012275|625[0n]bp|Canada.Quebec|BOLD:AAC8390
Parahypenodes quadralis|[4299]|RDLQG121-06|DH012278|614[0n]bp|Canada.Quebec|BOLD:AAC8390
Parahypenodes quadralis|[4300]|BBLEC117-09|09BBELE-0117|658[0n]bp|Canada.Nova Scotia|BOLD:AAC8390
Parahypenodes quadralis|[4301]|RDLQF663-06|DH011813|637[0n]bp|Canada.Quebec|BOLD:AAC8389
Parahypenodes quadralis|[4302]|RDLQF662-06|DH011812|637[0n]bp|Canada.Quebec|BOLD:AAC8389
Parahypenodes quadralis|[4303]|RDLQG120-06|DH012277|621[2n]bp|Canada.Quebec|BOLD:AAC8389
Parahypenodes quadralis|[4304]|RDLQG119-06|DH012276|632[6n]bp|Canada.Quebec|BOLD:AAC8389
Hypocala andremona|[4305]|LEMMZ379-10|MMZ0379|658[0n]bp|Brazil.Parana|BOLD:AAA6609
Hypocala andremona|[4306]|LOCRB787-08|08-CRBS-411|656[0n]bp|Costa Rica.San Jose|BOLD:AAA6609
Hypocala andremona|[4307]|LTOL837-07|KN-06-1071|658[0n]bp|Costa Rica.Guanacaste|BOLD:AAA6609
Hypocala andremona|[4308]|LEMMZ011-10|MMZ0011|639[0n]bp|Brazil.Parana|BOLD:AAA6609
Hypocala andremona|[4309]|GWOST445-11|BC ZSM Lep 50561|658[0n]bp|Argentina|BOLD:AAA6609
Hypocala andremona|[4310]|GWOST446-11|BC ZSM Lep 50562|658[0n]bp|Argentina|BOLD:AAA6609
Hypocala andremona|[4311]|QUNO479-08|5012-130907-LA|658[0n]bp|United States.Louisiana|BOLD:AAA6609
Hypocala andremona|[4312]|LMEMB196-09|RBMIS-1289|658[0n]bp|United States.Texas|BOLD:AAA6609
Hypocala andremona|[4313]|LTOL836-07|KN-06-1041|658[0n]bp|Costa Rica.Puntarenas|BOLD:AAA6609
Hypocala andremona|[4314]|GWOST444-11|BC ZSM Lep 50560|658[0n]bp|Argentina|BOLD:AAA6609
Hypocala andremona|[4315]|LOCRB786-08|08-CRBS-410|658[0n]bp|Costa Rica.Alajuela|BOLD:AAA6609
Hypocala andremona|[4316]|LOCRB788-08|08-CRBS-412|658[0n]bp|Costa Rica.Alajuela|BOLD:AAA6609
Hypocala andremona|[4317]|LPYPB272-08|MAL-00490|658[0n]bp|Mexico.Quintana Roo|BOLD:AAA6609
Hypocala andremona|[4318]|LGSMG564-07|BGS03927|658[0n]bp|United States.North Carolina|BOLD:AAA6609
Hypocala andremona|[4319]|IAWL149-09|IAWAZ-0148|658[0n]bp|United States.Arizona|BOLD:AAA6609
Hypocala andremona|[4320]|LMEMB197-09|RBMIS-1290|658[0n]bp|Honduras|BOLD:AAA6609
Hypocala andremona|[4321]|LEMMZ120-10|MMZ0120|658[0n]bp|Brazil.Parana|BOLD:AAA6609
Hypocala andremona|[4322]|LEMMZ010-10|MMZ0010|658[0n]bp|Brazil.Parana|BOLD:AAA6609
Hypsoropha hormos|[4323]|LGSMC878-05|DNA-ATBI-2878|616[0n]bp|United States.Tennessee|BOLD:AAB8640
Hypsoropha hormos|[4324]|LOTB339-05|05-TN-00339|658[0n]bp|United States.Tennessee|BOLD:AAB8640
Hypsoropha hormos|[4325]|LMEMB095-09|RBMIS-1188|658[0n]bp|United States.Mississippi|BOLD:AAB8640
Hypsoropha hormos|[4326]|LSUSA165-06|06-SUSA-0165|658[0n]bp|United States.Kentucky|BOLD:AAB8640
Hypsoropha hormos|[4327]|RDNMD352-06|CNCNoctuoidea12684|658[0n]bp|United States.Florida|BOLD:AAB8640
Hypsoropha hormos|[4328]|LOFLA606-06|06-FLOR-0606|658[0n]bp|United States.Florida|BOLD:AAB8640
Hypsoropha hormos|[4329]|LNC922-06|06-NCCC-922|658[0n]bp|United States.North Carolina|BOLD:AAB8640
Hypsoropha hormos|[4330]|LNCB329-06|06-NCCC-1285|658[0n]bp|United States.North Carolina|BOLD:AAB8640
Hypsoropha hormos|[4331]|LNC216-05|05-NCCC-216|658[0n]bp|United States.North Carolina|BOLD:AAB8640
Hypsoropha hormos|[4332]|LMEMB092-09|RBMIS-1185|658[0n]bp|United States.Alabama|BOLD:AAB8640
Hypsoropha hormos|[4333]|LOFLA558-06|06-FLOR-0558|658[0n]bp|United States.Florida|BOLD:AAB8640
Hypsoropha hormos|[4334]|LGSMG560-07|BGS03923|658[0n]bp|United States.Tennessee|BOLD:AAB8640
Hypsoropha hormos|[4335]|LILLA702-11|SNS10IL-00890|658[0n]bp|United States.Illinois|BOLD:AAB8640
Hypsoropha hormos|[4336]|BBLSY869-09|09BBLEP-03796|658[0n]bp|United States.Oklahoma|BOLD:AAB8640
Hypsoropha hormos|[4337]|LMEMB091-09|RBMIS-1184|658[0n]bp|United States.Mississippi|BOLD:AAB8640
Hypsoropha hormos|[4338]|LILLA064-11|SNS10IL-00084|658[0n]bp|United States.Illinois|BOLD:AAB8640
Hypsoropha hormos|[4339]|BBLSY867-09|09BBLEP-03794|658[0n]bp|United States.Oklahoma|BOLD:AAB8640
Hypsoropha hormos|[4340]|ABNCC244-07|1244-020603-TX|644[0n]bp|United States.Texas|BOLD:AAB8640
Hypsoropha hormos|[4341]|BBLSW559-09|09BBLEP-01487|631[0n]bp|United States.Oklahoma|BOLD:AAB8640
Hypsoropha hormos|[4342]|BBLSW491-09|09BBLEP-01419|632[0n]bp|United States.Oklahoma|BOLD:AAB8640
Hypsoropha hormos|[4343]|BBLSW557-09|09BBLEP-01485|643[0n]bp|United States.Oklahoma|BOLD:AAB8640
Hypsoropha hormos|[4344]|ABNCC245-07|1245-080504-TX|569[0n]bp|United States.Texas|BOLD:AAB8640
Hypsoropha hormos|[4345]|LMEMB094-09|RBMIS-1187|658[0n]bp|United States.Mississippi|BOLD:AAB8640
Hypsoropha hormos|[4346]|LPOKB784-09|MDOK-1826|658[0n]bp|United States.Oklahoma|BOLD:AAB8640
Hypsoropha hormos|[4347]|BBLSW564-09|09BBLEP-01492|636[0n]bp|United States.Oklahoma|BOLD:AAB8640
Ctenucha virginica|[4348]|XAB640-04|04HBL005640|558[0n]bp|Canada.Ontario|BOLD:AAA6017
Ctenucha virginica|[4349]|MNBB454-05|05-NBSTA-370|616[0n]bp|Canada.New Brunswick|BOLD:AAA6017
Ctenucha virginica|[4350]|BLTIB988-08|BL1425|658[0n]bp|Canada.Ontario|BOLD:AAA6017
Ctenucha virginica|[4351]|XAD697-05|2005-ONT-496|658[0n]bp|Canada.Ontario|BOLD:AAA6017
Ctenucha virginica|[4352]|BLTIB610-08|BL890|634[0n]bp|Canada.Ontario|BOLD:AAA6017
Ctenucha virginica|[4353]|LPSOB704-08|PPBP-1703|651[0n]bp|Canada.Ontario|BOLD:AAA6017
Ctenucha virginica|[4354]|LPMN294-08|08BBLEP-01093|658[0n]bp|Canada.Manitoba|BOLD:AAA6017
Ctenucha virginica|[4355]|LPMN788-08|08BBLEP-01591|658[0n]bp|Canada.Manitoba|BOLD:AAA6017
Ctenucha virginica|[4356]|LPMN203-08|08BBLEP-01002|658[0n]bp|Canada.Manitoba|BOLD:AAA6017
Ctenucha virginica|[4357]|PHMNB759-05|Moth 452.03SA|658[0n]bp|Canada.New Brunswick|BOLD:AAA6017
Ctenucha virginica|[4358]|TMNBB036-06|MNBTT-976|658[0n]bp|Canada.New Brunswick|BOLD:AAA6017
Ctenucha virginica|[4359]|LPMN292-08|08BBLEP-01091|658[0n]bp|Canada.Manitoba|BOLD:AAA6017
Ctenucha virginica|[4360]|BLTIB221-08|BL400|658[0n]bp|Canada.Ontario|BOLD:AAA6017
Ctenucha virginica|[4361]|BBLPE368-09|09BBELE-2368|658[0n]bp|Canada.Newfoundland and Labrador|BOLD:AA...
Ctenucha virginica|[4362]|BLTIB232-08|BL413|658[0n]bp|Canada.Ontario|BOLD:AAA6017
[illegible]

Ctenucha virginica|[4360]|BLTIB221-08|BL400|658[0n]bp|Canada.Ontario|BOLD:AAA6017
Ctenucha virginica|[4361]|BBLPE368-09|09BBELE-2368|658[0n]bp|Canada.Newfoundland and Labrador|BOLD:AA...
Ctenucha virginica|[4362]|BLTIB232-08|BL413|658[0n]bp|Canada.Ontario|BOLD:AAA6017
Ctenucha virginica|[4363]|TMNBD420-07|MNBTT-3221|657[0n]bp|Canada.New Brunswick|BOLD:AAA6017
Ctenucha virginica|[4364]|BLGSM034-09|BL343|658[0n]bp|Canada.Ontario|BOLD:AAA6017
Ctenucha virginica|[4365]|BLTIB283-08|BL468|658[0n]bp|Canada.Ontario|BOLD:AAA6017
Ctenucha virginica|[4366]|BBLPE303-09|09BBELE-2303|658[0n]bp|Canada.Newfoundland and Labrador|BOLD:AA...
Ctenucha virginica|[4367]|TTMNB256-06|MNBTT-256|658[0n]bp|Canada.New Brunswick|BOLD:AAA6017
Ctenucha virginica|[4368]|LPMN300-08|08BBLEP-01099|658[0n]bp|Canada.Manitoba|BOLD:AAA6017
Ctenucha virginica|[4369]|PHMNB140-04|04HBL007605|658[0n]bp|Canada.New Brunswick|BOLD:AAA6017
Ctenucha virginica|[4370]|BBLPA603-10|10BBCLP-0603|658[0n]bp|Canada.Ontario|BOLD:AAA6017
Ctenucha virginica|[4371]|PHMNB724-05|Moth 417.03SA|658[0n]bp|Canada.New Brunswick|BOLD:AAA6017
Ctenucha virginica|[4372]|LPMN293-08|08BBLEP-01092|658[0n]bp|Canada.Manitoba|BOLD:AAA6017
Ctenucha virginica|[4373]|BBLPE408-09|09BBELE-2408|658[0n]bp|Canada.Newfoundland and Labrador|BOLD:AA...
Ctenucha virginica|[4374]|MNBB200-05|05-NBSTA-116|658[0n]bp|Canada.New Brunswick|BOLD:AAA6017
Ctenucha virginica|[4375]|XAF456-05|2005-ONT-105|658[0n]bp|Canada.Ontario|BOLD:AAA6017
Ctenucha virginica|[4376]|MNBB129-05|05-NBSTA-045|658[0n]bp|Canada.New Brunswick|BOLD:AAA6017
Ctenucha virginica|[4377]|XAB554-04|04HBL005554|658[0n]bp|Canada.Ontario|BOLD:AAA6017
Ctenucha virginica|[4378]|RDMAB924-06|BCSC397|658[0n]bp|Canada.Alberta|BOLD:AAA6017
Ctenucha virginica|[4379]|LPSOB796-08|PPBP-1795|658[0n]bp|Canada.Ontario|BOLD:AAA6017
Ctenucha virginica|[4380]|PHMNB353-04|04HBL00579|658[0n]bp|Canada.New Brunswick|BOLD:AAA6017
Ctenucha virginica|[4381]|BBLPE302-09|09BBELE-2302|658[0n]bp|Canada.Newfoundland and Labrador|BOLD:AA...
Ctenucha virginica|[4382]|BLTIB196-08|BL287|658[0n]bp|Canada.Ontario|BOLD:AAA6017
Ctenucha virginica|[4383]|XAD696-05|2005-ONT-495|658[0n]bp|Canada.Ontario|BOLD:AAA6017
Ctenucha virginica|[4384]|RDLQG445-06|DH012729|658[0n]bp|Canada.Quebec|BOLD:AAA6017
Ctenucha virginica|[4385]|TMNBB035-06|MNBTT-975|658[0n]bp|Canada.New Brunswick|BOLD:AAA6017
Ctenucha virginica|[4386]|XAD698-05|2005-ONT-497|658[0n]bp|Canada.Ontario|BOLD:AAA6017
Ctenucha virginica|[4387]|XAJ658-06|2006-ONT-0658|658[0n]bp|Canada.Ontario|BOLD:AAA6017
Ctenucha virginica|[4388]|TMNBB037-06|MNBTT-977|658[0n]bp|Canada.New Brunswick|BOLD:AAA6017
Ctenucha virginica|[4389]|BLTIB220-08|BL399|658[0n]bp|Canada.Ontario|BOLD:AAA6017
Ctenucha virginica|[4390]|BBLEC090-09|09BBELE-0090|658[0n]bp|Canada.Nova Scotia|BOLD:AAA6017
Ctenucha virginica|[4391]|BLTIB444-08|BL691|658[0n]bp|Canada.Ontario|BOLD:AAA6017
Ctenucha virginica|[4392]|TMNBD419-07|MNBTT-3220|658[0n]bp|Canada.New Brunswick|BOLD:AAA6017
Ctenucha virginica|[4393]|XAF692-05|2005-ONT-341|658[0n]bp|Canada.Ontario|BOLD:AAA6017
Ctenucha virginica|[4394]|BBLEC683-09|09BBELE-0683|658[0n]bp|Canada.Nova Scotia|BOLD:AAA6017
Ctenucha virginica|[4395]|LPSOB797-08|PPBP-1796|658[0n]bp|Canada.Ontario|BOLD:AAA6017
Ctenucha virginica|[4396]|LPSOB771-08|PPBP-1770|658[0n]bp|Canada.Ontario|BOLD:AAA6017
Ctenucha virginica|[4397]|XAK439-06|2006-ONT-1434|658[0n]bp|Canada.Ontario|BOLD:AAA6017
Ctenucha virginica|[4398]|TMNBD423-07|MNBTT-3224|658[0n]bp|Canada.New Brunswick|BOLD:AAA6017
Ctenucha virginica|[4399]|BLTIB284-08|BL469|658[0n]bp|Canada.Ontario|BOLD:AAA6017
Ctenucha virginica|[4400]|PHMNB458-04|04HBL00684|658[0n]bp|Canada.New Brunswick|BOLD:AAA6017
Ctenucha virginica|[4401]|TMNBD421-07|MNBTT-3222|658[0n]bp|Canada.New Brunswick|BOLD:AAA6017
Ctenucha virginica|[4402]|BLTIB231-08|BL412|657[0n]bp|Canada.Ontario|BOLD:AAA6017
Ctenucha virginica|[4403]|LPSOD1047-09|08MZPP-108|658[0n]bp|Canada.Ontario|BOLD:AAA6017
Ctenucha virginica|[4404]|BBLPC230-09|09BBELE-1230|658[0n]bp|Canada.Nova Scotia|BOLD:AAA6017
Ctenucha virginica|[4405]|TMNBD422-07|MNBTT-3223|658[0n]bp|Canada.New Brunswick|BOLD:AAA6017
Ctenucha virginica|[4406]|BLTIB215-08|BL318|658[0n]bp|Canada.Ontario|BOLD:AAA6017
Ctenucha virginica|[4407]|PMG002-03|moth605.01|617[0n]bp|Canada.Ontario|BOLD:AAA6017
Ctenucha virginica|[4408]|PHMNB072-03|moth73.02SA|639[0n]bp|Canada.New Brunswick|BOLD:AAA6017
Ctenucha virginica|[4409]|PHMO088-03|moth563.01|639[0n]bp|Canada.Ontario|BOLD:AAA6017
Ctenucha virginica|[4410]|PHMO003-03|CTEN1.01|639[0n]bp|Canada.Ontario|BOLD:AAA6017
Ctenucha virginica|[4411]|MNBB130-05|05-NBSTA-046|658[0n]bp|Canada.New Brunswick|BOLD:AAA6017
Hypercompe permaculata|[4412]|RDNME138-07|CNCNoctuoidea13666|658[0n]bp|Canada.Alberta|BOLD:AAD0396
Hypercompe permaculata|[4413]|RDMAB926-06|UASM58669|658[0n]bp|Canada.Alberta|BOLD:AAD0396
Hypercompe permaculata|[4414]|RDMAB519-06|UASM58480|658[0n]bp|Canada.Alberta|BOLD:AAD0396
Hypercompe permaculata|[4415]|RDNME143-07|CNCNoctuoidea13671|626[0n]bp|Canada.British Columbia|BOLD:A...
Hypercompe scribonia|[4416]|LPSOD1054-09|08MZPP-122|658[0n]bp|Canada.Ontario|BOLD:ABZ5715
Hypercompe scribonia|[4417]|BBLPA602-10|10BBCLP-0602|658[0n]bp|Canada.Ontario|BOLD:ABZ5715
Hypercompe scribonia|[4418]|LPSOD925-09|08BBLEP-00961|658[0n]bp|Canada.Ontario|BOLD:ABZ5715
Spilosoma congrua|[4419]|TMG77-03|moth289.01|639[0n]bp|Canada.Ontario|BOLD:AAA4294
Spilosoma congrua|[4420]|XAK117-06|2006-ONT-1112|658[0n]bp|Canada.Ontario|BOLD:AAA4294
Spilosoma congrua|[4421]|BLTIB025-08|BL0052|658[0n]bp|Canada.Ontario|BOLD:AAA4294
Spilosoma congrua|[4422]|LPSO241-08|PPBP-0241|658[0n]bp|Canada.Ontario|BOLD:AAA4294
Spilosoma congrua|[4423]|LPSOB136-08|PPBP-1135|652[0n]bp|Canada.Ontario|BOLD:AAA4294
Spilosoma congrua|[4424]|TMNBD390-07|MNBTT-3191|658[0n]bp|Canada.New Brunswick|BOLD:AAA4294
Spilosoma congrua|[4425]|PHMNB580-04|04HBL00806|658[0n]bp|Canada.New Brunswick|BOLD:AAA4294
Spilosoma congrua|[4426]|XAF495-05|2005-ONT-144|658[0n]bp|Canada.Ontario|BOLD:AAA4294
Spilosoma congrua|[4427]|LPSOD871-09|08BBLEP-00653|658[0n]bp|Canada.Ontario|BOLD:AAA4294
Spilosoma congrua|[4428]|LPSOD870-09|08BBLEP-00652|658[0n]bp|Canada.Ontario|BOLD:AAA4294
Spilosoma congrua|[4429]|TTMNB545-06|MNBTT-545|658[0n]bp|Canada.New Brunswick|BOLD:AAA4294
Spilosoma congrua|[4430]|BBLPA376-10|10BBCLP-0376|658[0n]bp|Canada.Saskatchewan|BOLD:AAA4294
Spilosoma congrua|[4431]|BBLPA375-10|10BBCLP-0375|658[0n]bp|Canada.Saskatchewan|BOLD:AAA4294
Spilosoma congrua|[4432]|LPSOD848-09|08BBLEP-00630|658[0n]bp|Canada.Ontario|BOLD:AAA4294
Spilosoma congrua|[4433]|TMNBD393-07|MNBTT-3194|658[0n]bp|Canada.New Brunswick|BOLD:AAA4294
Spilosoma congrua|[4434]|TMNBD388-07|MNBTT-3189|658[0n]bp|Canada.New Brunswick|BOLD:AAA4294
Spilosoma congrua|[4435]|PHMNB579-04|04HBL00805|658[0n]bp|Canada.New Brunswick|BOLD:AAA4294
Spilosoma congrua|[4436]|TMNBD392-07|MNBTT-3193|658[0n]bp|Canada.New Brunswick|BOLD:AAA4294
Spilosoma congrua|[4437]|TMNBB013-06|MNBTT-953|658[0n]bp|Canada.New Brunswick|BOLD:AAA4294
Spilosoma congrua|[4438]|LPSOB134-08|PPBP-1133|658[0n]bp|Canada.Ontario|BOLD:AAA4294
Spilosoma congrua|[4439]|TMNBD389-07|MNBTT-3190|658[0n]bp|Canada.New Brunswick|BOLD:AAA4294
Spilosoma congrua|[4440]|LPSOB135-08|PPBP-1134|658[0n]bp|Canada.Ontario|BOLD:AAA4294
Spilosoma congrua|[4441]|LPMN269-08|08BBLEP-01068|658[0n]bp|Canada.Manitoba|BOLD:AAA4294
Spilosoma congrua|[4442]|LPSOD872-09|08BBLEP-00654|658[0n]bp|Canada.Ontario|BOLD:AAA4294
Spilosoma congrua|[4443]|RDNME342-07|CNCNoctuoidea13949|658[0n]bp|Canada.Ontario|BOLD:AAA4294
Spilosoma congrua|[4444]|RDLQ762-07|DH006622|652[0n]bp|Canada.Quebec|BOLD:AAA4294
Spilosoma congrua|[4445]|LPSOB696-08|PPBP-1695|646[0n]bp|Canada.Ontario|BOLD:AAA4294
Spilosoma congrua|[4446]|TMNBD387-07|MNBTT-3188|646[0n]bp|Canada.New Brunswick|BOLD:AAA4294
Spilosoma congrua|[4447]|TMNBD395-07|MNBTT-3196|646[0n]bp|Canada.New Brunswick|BOLD:AAA4294
Spilosoma congrua|[4448]|PMG017-03|moth306.01|617[0n]bp|Canada.Ontario|BOLD:AAA4294
Spilosoma congrua|[4449]|RDNME191-07|CNCNoctuoidea13798|620[0n]bp|Canada.Alberta|BOLD:AAA4294
Spilosoma congrua|[4450]|RDNME192-07|CNCNoctuoidea13799|617[1n]bp|Canada.Alberta|BOLD:AAA4294
Spilosoma congrua|[4451]|TMNBB012-06|MNBTT-952|658[0n]bp|Canada.New Brunswick|BOLD:AAA4294
Spilosoma congrua|[4452]|LPSOB335-08|PPBP-1334|658[0n]bp|Canada.Ontario|BOLD:AAA4294
Spilosoma dubia|[4453]|RDNME341-07|CNCNoctuoidea13948|658[0n]bp|Canada.Ontario|BOLD:AAC8394
Spilosoma dubia|[4454]|KPOEC113-08|08OEC-058|658[0n]bp|Canada.Ontario|BOLD:AAC8394
Spilosoma dubia|[4455]|RDNME173-07|CNCNoctuoidea13701|638[0n]bp|Canada.Alberta|BOLD:AAC8394
Spilosoma dubia|[4456]|RDNME172-07|CNCNoctuoidea13700|641[0n]bp|Canada.Alberta|BOLD:AAC8394
Spilosoma dubia|[4457]|RDNME174-07|CNCNoctuoidea13702|638[1n]bp|Canada.Alberta|BOLD:AAC8394
Spilosoma latipennis|[4458]|RDNME651-08|LEP038075|658[0n]bp|Canada.Ontario|BOLD:AAB6210
Spilosoma latipennis|[4459]|LPSO136-08|PPBP-0136|658[0n]bp|Canada.Ontario|BOLD:AAB6210
Spilosoma latipennis|[4460]|LPSO353-08|PPBP-0353|658[0n]bp|Canada.Ontario|BOLD:AAB6210
Spilosoma latipennis|[4461]|XAI003-05|0102-ONT-0003|658[0n]bp|Canada.Ontario|BOLD:AAB6210
Spilosoma latipennis|[4462]|XAI002-05|0102-ONT-0002|658[0n]bp|Canada.Ontario|BOLD:AAB6210

Spilosoma latipennis|[4459]|LPSO156-08|PPBP-0156|658[0n]bp|Canada.Ontario|BOLD:AAB6210
Spilosoma latipennis|[4460]|LPSO353-08|PPBP-0353|658[0n]bp|Canada.Ontario|BOLD:AAB6210
Spilosoma latipennis|[4461]|XAI003-05|0102-ONT-0003|658[0n]bp|Canada.Ontario|BOLD:AAB6210
Spilosoma latipennis|[4462]|XAI002-05|0102-ONT-0002|658[0n]bp|Canada.Ontario|BOLD:AAB6210
Spilosoma latipennis|[4463]|XAK168-06|2006-ONT-1163|658[0n]bp|Canada.Ontario|BOLD:AAB6210
Spilosoma latipennis|[4464]|PHMO086-03|moth548.02|639[1n]bp|Canada.Ontario|BOLD:AAB6210
Spilosoma latipennis|[4465]|RDNME650-08|LEP038074|658[0n]bp|Canada.Ontario|BOLD:AAB6210
Spilosoma latipennis|[4466]|XAB304-04|04HBL005304|658[0n]bp|Canada.Ontario|BOLD:AAB6210
Spilosoma latipennis|[4467]|XAK169-06|2006-ONT-1164|658[0n]bp|Canada.Ontario|BOLD:AAB6210
Spilosoma latipennis|[4468]|XAK170-06|2006-ONT-1165|658[0n]bp|Canada.Ontario|BOLD:AAB6210
Spilosoma latipennis|[4469]|XAF672-05|2005-ONT-321|658[0n]bp|Canada.Ontario|BOLD:AAB6210
Spilosoma latipennis|[4470]|LPSOB730-08|PPBP-1729|658[0n]bp|Canada.Ontario|BOLD:AAB6210
Spilosoma latipennis|[4471]|PHMO090-03|moth569.02|639[0n]bp|Canada.Ontario|BOLD:AAB6210
Spilosoma latipennis|[4472]|LPSO952-08|PPBP-0952|646[0n]bp|Canada.Ontario|BOLD:AAB6210
Spilosoma latipennis|[4473]|PHMO123-03|moth735.02|639[1n]bp|Canada.Ontario|BOLD:AAB6210
Spilosoma pteridis|[4474]|RDMAB391-05|BCSC64|561[0n]bp|Canada.British Columbia|BOLD:AAB4578
Spilosoma pteridis|[4475]|RDNMH319-09|CNCLEP00057728|658[0n]bp|Canada.British Columbia|BOLD:AAB4578
Spilosoma pteridis|[4476]|RDNME144-07|CNCNoctuoidea13672|616[0n]bp|Canada.Alberta|BOLD:AAB4578
Spilosoma pteridis|[4477]|LPVIC112-08|PFC-2006-2689|616[0n]bp|Canada.British Columbia|BOLD:AAB4578
Spilosoma pteridis|[4478]|LPVIC113-08|PFC-2006-2690|621[0n]bp|Canada.British Columbia|BOLD:AAB4578
Spilosoma pteridis|[4479]|RDNME146-07|CNCNoctuoidea13674|611[0n]bp|Canada.British Columbia|BOLD:AAB4578
Spilosoma vagans|[4480]|RDNME168-07|CNCNoctuoidea13696|658[0n]bp|Canada.British Columbia|BOLD:AAB4578
Spilosoma danbyi|[4481]|RDNME147-07|CNCNoctuoidea13675|381[0n]bp|Canada.British Columbia|BOLD:AAB4578
Spilosoma vagans|[4482]|RDNME167-07|CNCNoctuoidea13695|658[0n]bp|Canada.British Columbia|BOLD:AAB4578
Spilosoma vagans|[4483]|LPAB261-08|08BBLEP-02583|658[0n]bp|Canada.Alberta|BOLD:AAB4578
Spilosoma vagans|[4484]|RDNME166-07|CNCNoctuoidea13694|658[0n]bp|Canada.British Columbia|BOLD:AAB4578
Spilosoma vagans|[4485]|RDMAB411-05|BCSC84|658[0n]bp|Canada.British Columbia|BOLD:AAB4578
Spilosoma vagans|[4486]|LBCG027-08|08-JDWBC-0027|658[0n]bp|Canada.British Columbia|BOLD:AAB4578
Spilosoma vagans|[4487]|LPABB567-08|08BBLEP-03832|658[0n]bp|Canada.Alberta|BOLD:AAB4578
Spilosoma vagans|[4488]|LPABB428-08|08BBLEP-03693|658[0n]bp|Canada.Alberta|BOLD:AAB4578
Spilosoma vagans|[4489]|RDNMB391-05|CNCNoctuoidea10157|658[0n]bp|Canada.British Columbia|BOLD:AAB4578
Leptarctia californiae|[4490]|RDNMF391-08|NOC14477|658[0n]bp|Canada.British Columbia|BOLD:AAE5275
Leptarctia californiae|[4491]|RDNMF390-08|NOC14476|658[0n]bp|Canada.British Columbia|BOLD:AAE5275
Leptarctia californiae|[4492]|RDNMF389-08|NOC14475|658[0n]bp|Canada.British Columbia|BOLD:AAE5275
Leptarctia californiae|[4493]|RDNMF388-08|NOC14474|658[0n]bp|Canada.British Columbia|BOLD:AAE5275
Estigmene acrea|[4494]|XAB241-04|04HBL005241|573[0n]bp|Canada.Ontario|BOLD:AAB1406
Estigmene acrea|[4495]|XAG234-05|2005-ONT-818|658[0n]bp|Canada.Ontario|BOLD:AAB1406
Estigmene acrea|[4496]|RDLQ757-07|DH011386|658[0n]bp|Canada.Quebec|BOLD:AAB1406
Estigmene acrea|[4497]|XAK174-06|2006-ONT-1169|658[0n]bp|Canada.Ontario|BOLD:AAB1406
Estigmene acrea|[4498]|XAK147-06|2006-ONT-1142|658[0n]bp|Canada.Ontario|BOLD:AAB1406
Estigmene acrea|[4499]|XAB069-04|04HBL005069|624[0n]bp|Canada.Ontario|BOLD:AAB1406
Estigmene acrea|[4500]|LPSOC129-08|PPBP-2128|658[0n]bp|Canada.Ontario|BOLD:AAB1406
Estigmene acrea|[4501]|XAB359-04|04HBL005359|658[0n]bp|Canada.Ontario|BOLD:AAB1406
Estigmene acrea|[4502]|LPMN169-08|08BBLEP-00968|658[0n]bp|Canada.Manitoba|BOLD:AAB1406
Estigmene acrea|[4503]|XAG664-05|2005-ONT-1248|658[1n]bp|Canada.Ontario|BOLD:AAB1406
Estigmene acrea|[4504]|XAJ532-06|2006-ONT-0532|658[0n]bp|Canada.Ontario|BOLD:AAB1406
Estigmene acrea|[4505]|LPMN246-08|08BBLEP-01045|658[0n]bp|Canada.Manitoba|BOLD:AAB1406
Estigmene acrea|[4506]|XAJ463-06|2006-ONT-0463|658[0n]bp|Canada.Ontario|BOLD:AAB1406
Estigmene acrea|[4507]|TZBCA172-06|OMAFRA06-107|658[0n]bp|Canada.Ontario|BOLD:AAB1406
Estigmene acrea|[4508]|DUNLP002-08|Dun-08-002|658[0n]bp|Canada.British Columbia|BOLD:AAB1406
Estigmene acrea|[4509]|RDMAB928-06|UASM57426|658[0n]bp|Canada.Alberta|BOLD:AAB1406
Estigmene acrea|[4510]|LPSOC027-08|PPBP-2026|658[0n]bp|Canada.Ontario|BOLD:AAB1406
Estigmene acrea|[4511]|LPVIC102-08|PFC-2006-2677|658[0n]bp|Canada.British Columbia|BOLD:AAB1406
Estigmene acrea|[4512]|XAF697-05|2005-ONT-346|658[0n]bp|Canada.Ontario|BOLD:AAB1406
Estigmene acrea|[4513]|TMNBD430-07|MNBTT-3231|658[0n]bp|Canada.New Brunswick|BOLD:AAB1406
Estigmene acrea|[4514]|LPSOC130-08|PPBP-2129|658[0n]bp|Canada.Ontario|BOLD:AAB1406
Estigmene acrea|[4515]|XAB001-04|04HBL005001|658[0n]bp|Canada.Ontario|BOLD:AAB1406
Estigmene acrea|[4516]|RDLQB842-05|DH010929|616[0n]bp|Canada.Quebec|BOLD:AAB1406
Estigmene acrea|[4517]|XAJ531-06|2006-ONT-0531|658[0n]bp|Canada.Ontario|BOLD:AAB1406
Estigmene acrea|[4518]|XAG248-05|2005-ONT-832|658[0n]bp|Canada.Ontario|BOLD:AAB1406
Estigmene acrea|[4519]|LPVIC100-08|PFC-2006-2675|658[0n]bp|Canada.British Columbia|BOLD:AAB1406
Estigmene acrea|[4520]|XAJ314-06|2006-ONT-0314|658[0n]bp|Canada.Ontario|BOLD:AAB1406
Hyphantria sp. 1|[4521]|RDNMH936-09|CNCLEP00067935|658[0n]bp|Canada.Ontario|BOLD:ACE3204
Hyphantria sp. 1|[4522]|RDMAB372-05|UASM77838|588[0n]bp|Canada.Alberta|BOLD:ACE3204
Hyphantria sp. 1|[4523]|LHLEP289-06|UBC-2006-0163|649[0n]bp|Canada.British Columbia|BOLD:ACE3204
Hyphantria sp. 1|[4524]|RDNMB426-05|CNCNoctuoidea10192|587[2n]bp|Canada.Saskatchewan|BOLD:ACE3204
Hyphantria sp. 1|[4525]|LALPA258-10|AVBC 259-10|658[0n]bp|Canada.British Columbia|BOLD:ACE3204
Hyphantria sp. 1|[4526]|LHLEP287-06|UBC-2006-0161|657[0n]bp|Canada.British Columbia|BOLD:ACE3204
Hyphantria sp. 1|[4527]|LHLEP290-06|UBC-2006-0803|649[0n]bp|Canada.British Columbia|BOLD:ACE3204
Hyphantria sp. 1|[4528]|RDNMB427-05|CNCNoctuoidea10193|658[0n]bp|Canada.British Columbia|BOLD:ACE3204
Hyphantria sp. 1|[4529]|RDMAB100-05|UASM41980|658[0n]bp|Canada.Alberta|BOLD:ACE3204
Hyphantria sp. 1|[4530]|RDMAB101-05|UASM41981|658[0n]bp|Canada.Alberta|BOLD:ACE3204
Hyphantria sp. 1|[4531]|RDMAB428-05|BCSC101|658[0n]bp|Canada.Alberta|BOLD:ACE3204
Hyphantria sp. 1|[4532]|LHLEP288-06|UBC-2006-0162|657[0n]bp|Canada.British Columbia|BOLD:ACE3204
Hyphantria sp. 1|[4533]|LALPA163-10|AVBC 163-10|658[0n]bp|Canada.British Columbia|BOLD:ACE3204
Hyphantria sp. 1|[4534]|LALPA246-10|AVBC 247-10|658[0n]bp|Canada.British Columbia|BOLD:ACE3204
Hyphantria sp. 1|[4535]|BBLPA630-10|10BBCLP-0630|658[0n]bp|Canada.Ontario|BOLD:AAA2436
Hyphantria sp. 1|[4536]|PHMNB085-03|moth180.02SA|639[4n]bp|Canada.New Brunswick|BOLD:AAA2436
Hyphantria sp. 1|[4537]|XAB628-04|04HBL005628|658[0n]bp|Canada.Ontario|BOLD:AAA2436
Hyphantria sp. 1|[4538]|XAB167-04|04HBL005167|658[0n]bp|Canada.Ontario|BOLD:AAA2436
Hyphantria sp. 1|[4539]|PHMNB019-03|moth153.02SA|639[0n]bp|Canada.New Brunswick|BOLD:AAA2436
Hyphantria sp. 1|[4540]|TMNBD399-07|MNBTT-3200|658[0n]bp|Canada.New Brunswick|BOLD:AAA2436
Hyphantria cunea|[4541]|XAF466-05|2005-ONT-115|658[0n]bp|Canada.Ontario|BOLD:AAA2435
Hyphantria cunea|[4542]|LPSOC049-08|PPBP-2048|658[0n]bp|Canada.Ontario|BOLD:AAA2435
Hyphantria cunea|[4543]|XAB570-04|04HBL005570|658[0n]bp|Canada.Ontario|BOLD:AAA2435
Hyphantria cunea|[4544]|RDNMB423-05|CNCNoctuoidea10189|658[0n]bp|Canada.Ontario|BOLD:AAA2435
Hyphantria cunea|[4545]|XAB152-04|04HBL005152|658[0n]bp|Canada.Ontario|BOLD:AAA2435
Hyphantria cunea|[4546]|XAF561-05|2005-ONT-210|658[0n]bp|Canada.Ontario|BOLD:AAA2435
Hyphantria cunea|[4547]|XAK112-06|2006-ONT-1107|658[0n]bp|Canada.Ontario|BOLD:AAA2435
Hyphantria cunea|[4548]|XAF467-05|2005-ONT-116|658[0n]bp|Canada.Ontario|BOLD:AAA2435
Hyphantria cunea|[4549]|RDNME747-08|LEP042294|658[0n]bp|Canada.Quebec|BOLD:AAA2435
Hyphantria cunea|[4550]|BLTIB255-08|BL437|656[0n]bp|Canada.Ontario|BOLD:AAA2435
Hyphantria cunea|[4551]|BLTIB216-08|BL394|658[0n]bp|Canada.Ontario|BOLD:AAA2435
Hyphantria cunea|[4552]|TMNBD403-07|MNBTT-3204|658[0n]bp|Canada.New Brunswick|BOLD:AAA2435
Hyphantria cunea|[4553]|XAB263-04|04HBL005263|658[0n]bp|Canada.Ontario|BOLD:AAA2435
Hyphantria cunea|[4554]|XAF823-05|2005-ONT-472|658[0n]bp|Canada.Ontario|BOLD:AAA2435
Hyphantria cunea|[4555]|BLTIB330-08|BL521|658[0n]bp|Canada.Ontario|BOLD:AAA2435
Hyphantria cunea|[4556]|XAJ534-06|2006-ONT-0534|658[0n]bp|Canada.Ontario|BOLD:AAA2435
Hyphantria cunea|[4557]|XAK435-06|2006-ONT-1430|658[0n]bp|Canada.Ontario|BOLD:AAA2435
Hyphantria cunea|[4558]|XAB569-04|04HBL005569|658[0n]bp|Canada.Ontario|BOLD:AAA2435
Hyphantria cunea|[4559]|XAK167-06|2006-ONT-1162|658[0n]bp|Canada.Ontario|BOLD:AAA2435
Hyphantria cunea|[4560]|PHMNB376-04|04HBL00602|658[0n]bp|Canada.New Brunswick|BOLD:AAA2435
Hyphantria cunea|[4561]|XAB264-04|04HBL005264|658[0n]bp|Canada.Ontario|BOLD:AAA2435

Hyphantria cunea|[4558]|XAB[illegible]-04|04HBL00[illegible]|658[0n]bp|Canada.Ontario|BOLD:AAA2435
Hyphantria cunea|[4559]|XAK167-06|2006-ONT-1162|658[0n]bp|Canada.Ontario|BOLD:AAA2435
Hyphantria cunea|[4560]|PHMNB376-04|04HBL00602|658[0n]bp|Canada.New Brunswick|BOLD:AAA2435
Hyphantria cunea|[4561]|XAB264-04|04HBL005264|658[0n]bp|Canada.Ontario|BOLD:AAA2435
Hyphantria cunea|[4562]|XAB340-04|04HBL005340|658[0n]bp|Canada.Ontario|BOLD:AAA2435
Hyphantria cunea|[4563]|XAF464-05|2005-ONT-113|658[0n]bp|Canada.Ontario|BOLD:AAA2435
Hyphantria cunea|[4564]|XAB090-04|04HBL005090|658[0n]bp|Canada.Ontario|BOLD:AAA2435
Hyphantria cunea|[4565]|TMNBD402-07|MNBTT-3203|658[0n]bp|Canada.New Brunswick|BOLD:AAA2435
Hyphantria cunea|[4566]|XAB237-04|04HBL005237|658[0n]bp|Canada.Ontario|BOLD:AAA2435
Hyphantria cunea|[4567]|RDNME740-08|LEP042287|658[0n]bp|Canada.New Brunswick|BOLD:AAA2435
Hyphantria cunea|[4568]|TMNBD401-07|MNBTT-3202|658[0n]bp|Canada.New Brunswick|BOLD:AAA2435
Hyphantria cunea|[4569]|XAB163-04|04HBL005163|658[0n]bp|Canada.Ontario|BOLD:AAA2435
Hyphantria cunea|[4570]|TMNBB014-06|MNBTT-954|658[0n]bp|Canada.New Brunswick|BOLD:AAA2435
Hyphantria cunea|[4571]|BLTIB329-08|BL520|658[0n]bp|Canada.Ontario|BOLD:AAA2435
Hyphantria cunea|[4572]|RDNME739-08|LEP042286|658[0n]bp|Canada.New Brunswick|BOLD:AAA2435
Hyphantria cunea|[4573]|BLTIB224-08|BL405|658[0n]bp|Canada.Ontario|BOLD:AAA2435
Hyphantria cunea|[4574]|XAF560-05|2005-ONT-209|658[0n]bp|Canada.Ontario|BOLD:AAA2435
Hyphantria cunea|[4575]|PHMNB374-04|04HBL00600|658[0n]bp|Canada.New Brunswick|BOLD:AAA2435
Hyphantria cunea|[4576]|XAF531-05|2005-ONT-180|658[0n]bp|Canada.Ontario|BOLD:AAA2435
Hyphantria cunea|[4577]|TMNBD400-07|MNBTT-3201|658[0n]bp|Canada.New Brunswick|BOLD:AAA2435
Hyphantria cunea|[4578]|RDNME744-08|LEP042291|658[0n]bp|Canada.New Brunswick|BOLD:AAA2435
Hyphantria cunea|[4579]|BLTIB235-08|BL416|608[2n]bp|Canada.Ontario|BOLD:AAA2435
Hyphantria cunea|[4580]|LPSOD301-09|08BBLEP-00079|634[0n]bp|Canada.Ontario|BOLD:AAA2435
Hyphantria cunea|[4581]|PMG011-03|moth589.01|617[0n]bp|Canada.Ontario|BOLD:AAA2435
Hyphantria cunea|[4582]|TMG81-03|moth661.01|639[0n]bp|Canada.Ontario|BOLD:AAA2435
Hyphantria cunea|[4583]|PHMO458-03|moth560.01|639[0n]bp|Canada.Ontario|BOLD:AAA2435
Hyphantria cunea|[4584]|PHMNB764-05|Moth 457.03SA|658[0n]bp|Canada.New Brunswick|BOLD:AAA2435
Hyphantria cunea|[4585]|BLGSM057-09|BL376|658[0n]bp|Canada.Ontario|BOLD:AAA2435
Hyphantria cunea|[4586]|LPSOC389-08|PPBP-2388|658[0n]bp|Canada.Ontario|BOLD:AAA2435
Hyphantria cunea|[4587]|LPSOC390-08|PPBP-2389|658[0n]bp|Canada.Ontario|BOLD:AAA2435
Hyphantria cunea|[4588]|PHMO369-03|moth432.02|639[0n]bp|Canada.Ontario|BOLD:AAA2435
Hyphantria cunea|[4589]|BLTIB175-08|BL254|658[0n]bp|Canada.Ontario|BOLD:AAA2435
Hyphantria cunea|[4590]|XAF465-05|2005-ONT-114|658[0n]bp|Canada.Ontario|BOLD:AAA2435
Hyphantria cunea|[4591]|XAJ419-06|2006-ONT-0419|658[0n]bp|Canada.Ontario|BOLD:AAA2435
Hyphantria cunea|[4592]|XAB579-04|04HBL005579|658[0n]bp|Canada.Ontario|BOLD:AAA2435
Hyphantria cunea|[4593]|RDNMH935-09|CNCLEP00067934|658[0n]bp|Canada.Ontario|BOLD:AAA2435
Hyphantria cunea|[4594]|LPSO052-08|PPBP-0052|658[0n]bp|Canada.Ontario|BOLD:AAA2435
Hyphantria cunea|[4595]|XAF824-05|2005-ONT-473|658[0n]bp|Canada.Ontario|BOLD:AAA2435
Hyphantria cunea|[4596]|XAF713-05|2005-ONT-362|658[0n]bp|Canada.Ontario|BOLD:AAA2435
Hyphantria cunea|[4597]|XAJ691-06|2006-ONT-0691|658[0n]bp|Canada.Ontario|BOLD:AAA2435
Hyphantria cunea|[4598]|XAB349-04|04HBL005349|658[0n]bp|Canada.Ontario|BOLD:AAA2435
Hyphantria cunea|[4599]|XAK173-06|2006-ONT-1168|658[0n]bp|Canada.Ontario|BOLD:AAA2435
Hyphantria cunea|[4600]|XAF701-05|2005-ONT-350|658[0n]bp|Canada.Ontario|BOLD:AAA2435
Hyphantria cunea|[4601]|BLGSM064-09|BL385|658[0n]bp|Canada.Ontario|BOLD:AAA2435
Hyphantria cunea|[4602]|XAB101-04|04HBL005101|658[0n]bp|Canada.Ontario|BOLD:AAA2435
Hyphantria cunea|[4603]|LPSOB386-08|PPBP-1385|658[0n]bp|Canada.Ontario|BOLD:AAA2435
Hyphantria cunea|[4604]|XAB386-04|04HBL005386|658[0n]bp|Canada.Ontario|BOLD:AAA2435
Hyphantria cunea|[4605]|LPSO489-08|PPBP-0489|658[0n]bp|Canada.Ontario|BOLD:AAA2435
Hyphantria cunea|[4606]|LPSOB387-08|PPBP-1386|658[0n]bp|Canada.Ontario|BOLD:AAA2435
Hyphantria cunea|[4607]|LPSOD270-09|08BBLEP-00048|658[0n]bp|Canada.Ontario|BOLD:AAA2435
Hyphantria cunea|[4608]|LPSOC132-08|PPBP-2131|658[0n]bp|Canada.Ontario|BOLD:AAA2435
Hyphantria cunea|[4609]|XAE356-04|Moth4356.03|658[0n]bp|Canada.Ontario|BOLD:AAA2435
Hyphantria cunea|[4610]|RDNME745-08|LEP042292|658[0n]bp|Canada.New Brunswick|BOLD:AAA2435
Hyphantria cunea|[4611]|XAB155-04|04HBL005155|658[0n]bp|Canada.Ontario|BOLD:AAA2435
Hyphantria cunea|[4612]|XAB265-04|04HBL005265|658[0n]bp|Canada.Ontario|BOLD:AAA2435
Hyphantria cunea|[4613]|XAF563-05|2005-ONT-212|658[0n]bp|Canada.Ontario|BOLD:AAA2435
Hyphantria cunea|[4614]|LPSO400-08|PPBP-0400|658[0n]bp|Canada.Ontario|BOLD:AAA2435
Hyphantria cunea|[4615]|RDLQF858-06|DH012019|658[0n]bp|Canada.Quebec|BOLD:AAA2435
Hyphantria cunea|[4616]|XAE225-04|Moth4225.03|658[0n]bp|Canada.Ontario|BOLD:AAA2435
Hyphantria cunea|[4617]|RDNME748-08|LEP042295|658[0n]bp|Canada.Quebec|BOLD:AAA2435
Hyphantria cunea|[4618]|XAB076-04|04HBL005076|658[0n]bp|Canada.Ontario|BOLD:AAA2435
Hyphantria cunea|[4619]|PHMNB606-04|04HBL00832|658[0n]bp|Canada.New Brunswick|BOLD:AAA2435
Hyphantria cunea|[4620]|PHMNB375-04|04HBL00601|658[0n]bp|Canada.New Brunswick|BOLD:AAA2435
Hyphantria cunea|[4621]|PHMNB377-04|04HBL00603|658[0n]bp|Canada.New Brunswick|BOLD:AAA2435
Hyphantria cunea|[4622]|PHMNB007-03|moth120.02SA|639[0n]bp|Canada.New Brunswick|BOLD:AAA2435
Hyphantria cunea|[4623]|PHMNB086-03|moth231.02SA|639[2n]bp|Canada.New Brunswick|BOLD:AAA2435
Hyphantria cunea|[4624]|BLTIB152-08|BL226|658[0n]bp|Canada.Ontario|BOLD:AAA2435
Hyphantria cunea|[4625]|XAB087-04|04HBL005087|658[0n]bp|Canada.Ontario|BOLD:AAA2435
Hyphantria cunea|[4626]|XAF562-05|2005-ONT-211|658[0n]bp|Canada.Ontario|BOLD:AAA2435
Hyphantria cunea|[4627]|RDLQG447-06|DH012731|658[0n]bp|Canada.Quebec|BOLD:AAA2435
Hyphantria cunea|[4628]|BLTIB151-08|BL225|658[0n]bp|Canada.Ontario|BOLD:AAA2435
Hyphantria cunea|[4629]|XAB091-04|04HBL005091|658[0n]bp|Canada.Ontario|BOLD:AAA2435
Hyphantria cunea|[4630]|LPSOD328-09|08BBLEP-00106|658[0n]bp|Canada.Ontario|BOLD:AAA2435
Hyphantria cunea|[4631]|PHMNB474-04|04HBL00700|658[0n]bp|Canada.New Brunswick|BOLD:AAA2435
Hyphantria cunea|[4632]|XAB334-04|04HBL005334|658[0n]bp|Canada.Ontario|BOLD:AAA2435
Hyphantria cunea|[4633]|XAK453-06|2006-ONT-1448|658[0n]bp|Canada.Ontario|BOLD:AAA2435
Hyphantria cunea|[4634]|XAF659-05|2005-ONT-308|658[0n]bp|Canada.Ontario|BOLD:AAA2435
Hyphantria cunea|[4635]|XAJ535-06|2006-ONT-0535|657[0n]bp|Canada.Ontario|BOLD:AAA2435
Hyphantria cunea|[4636]|XAF660-05|2005-ONT-309|658[0n]bp|Canada.Ontario|BOLD:AAA2435
Hyphantria cunea|[4637]|PHMNB372-04|04HBL00598|658[0n]bp|Canada.New Brunswick|BOLD:AAA2435
Spilosoma congrua|[4638]|RDNMJ800-11|CNCLEP 80328|658[0n]bp|Canada.Quebec|BOLD:AAA2435
Spilosoma virginica|[4639]|MNBB486-05|05-NBSTA-402|658[0n]bp|Canada.New Brunswick|BOLD:AAA3348
Spilosoma virginica|[4640]|PHJUN4075-12|BIOUG02430-G06|610[0n]bp|Canada.Ontario|BOLD:ACE7664
Spilosoma virginica|[4641]|LPSO012-08|PPBP-0012|658[0n]bp|Canada.Ontario|BOLD:ACE7664
Spilosoma virginica|[4642]|LPSO011-08|PPBP-0011|658[0n]bp|Canada.Ontario|BOLD:ACE7664
Spilosoma virginica|[4643]|LPSO243-08|PPBP-0243|658[0n]bp|Canada.Ontario|BOLD:ACE7664
Spilosoma virginica|[4644]|PHJUN4074-12|BIOUG02430-G05|632[0n]bp|Canada.Ontario|BOLD:ACE7664
Spilosoma virginica|[4645]|LPVIA201-08|PFC-2006-0282|513[0n]bp|Canada.British Columbia|BOLD:AAA3347
Spilosoma virginica|[4646]|BLTIB176-08|BL255|658[0n]bp|Canada.Ontario|BOLD:AAA3347
Spilosoma virginica|[4647]|LPMN459-08|08BBLEP-01258|658[0n]bp|Canada.Manitoba|BOLD:AAA3347
Spilosoma virginica|[4648]|RDLQ763-07|DH004867|658[0n]bp|Canada.Quebec|BOLD:AAA3347
Spilosoma virginica|[4649]|TTMNB544-06|MNBTT-544|658[0n]bp|Canada.New Brunswick|BOLD:AAA3347
Spilosoma virginica|[4650]|RDNME170-07|CNCNoctuoidea13698|638[0n]bp|Canada.Alberta|BOLD:AAA3347
Spilosoma virginica|[4651]|XAE468-04|Moth4468.03|658[0n]bp|Canada.Ontario|BOLD:AAA3347
Spilosoma virginica|[4652]|BBLPC250-09|09BBELE-1250|658[0n]bp|Canada.Nova Scotia|BOLD:AAA3347
Spilosoma virginica|[4653]|LPMN820-08|08BBLEP-01623|658[0n]bp|Canada.Manitoba|BOLD:AAA3347
Spilosoma virginica|[4654]|LPSK109-08|08BBLEP-01677|658[0n]bp|Canada.Saskatchewan|BOLD:AAA3347
Spilosoma virginica|[4655]|MNBB329-05|05-NBSTA-245|658[0n]bp|Canada.New Brunswick|BOLD:AAA3347
Spilosoma virginica|[4656]|PHMNB233-04|04HBL007698|658[0n]bp|Canada.New Brunswick|BOLD:AAA3347
Spilosoma virginica|[4657]|TMG78-03|moth355.01|639[0n]bp|Canada.Ontario|BOLD:AAA3347
Spilosoma virginica|[4658]|RDNME171-07|CNCNoctuoidea13699|638[1n]bp|Canada.Alberta|BOLD:AAA3347
Spilosoma virginica|[4659]|XAD608-05|2005-ONT-23|658[0n]bp|Canada.Ontario|BOLD:AAA3347
Spilosoma virginica|[4660]|XAK450-06|2006-ONT-1445|626[0n]bp|Canada.Ontario|BOLD:AAA3347

[illegible]
Spilosoma virginica|[4658]|RDNME171-07|CNCNoctuoidea13699|638[1n]bp|Canada.Alberta|BOLD:AAA3347
Spilosoma virginica|[4659]|XAD608-05|2005-ONT-23|658[0n]bp|Canada.Ontario|BOLD:AAA3347
Spilosoma virginica|[4660]|XAK450-06|2006-ONT-1445|626[0n]bp|Canada.Ontario|BOLD:AAA3347
Spilosoma virginica|[4661]|LHLEP054-06|UBC-2006-0255|589[0n]bp|Canada.British Columbia|BOLD:AAA3347
Spilosoma virginica|[4662]|LHLEP056-06|UBC-2006-0257|658[0n]bp|Canada.British Columbia|BOLD:AAA3347
Spilosoma virginica|[4663]|LHLEP052-06|UBC-2006-0253|658[0n]bp|Canada.British Columbia|BOLD:AAA3347
Spilosoma virginica|[4664]|LPVIB910-08|PFC-2006-2439|645[0n]bp|Canada.British Columbia|BOLD:AAA3347
Spilosoma virginica|[4665]|LMH003-06|PFC-2006-0005|635[0n]bp|Canada.British Columbia|BOLD:AAA3347
Spilosoma virginica|[4666]|LPMN925-08|08BBLEP-02283|654[0n]bp|Canada.Alberta|BOLD:AAA3347
Spilosoma virginica|[4667]|LPMN962-08|08BBLEP-02320|658[0n]bp|Canada.Alberta|BOLD:AAA3347
Spilosoma virginica|[4668]|LBCB142-05|HLC-21082|658[0n]bp|Canada.British Columbia|BOLD:AAA3347
Spilosoma virginica|[4669]|LBCA241-05|HLC-20241|658[0n]bp|Canada.British Columbia|BOLD:AAA3347
Spilosoma virginica|[4670]|BBLPA373-10|10BBCLP-0373|658[0n]bp|Canada.British Columbia|BOLD:AAA3347
Spilosoma virginica|[4671]|LBCB143-05|HLC-21083|658[0n]bp|Canada.British Columbia|BOLD:AAA3347
Spilosoma virginica|[4672]|LBCB145-05|HLC-21085|658[0n]bp|Canada.British Columbia|BOLD:AAA3347
Spilosoma virginica|[4673]|BBLPA372-10|10BBCLP-0372|658[0n]bp|Canada.British Columbia|BOLD:AAA3347
Spilosoma virginica|[4674]|LBCA545-05|HLC-20545|658[0n]bp|Canada.British Columbia|BOLD:AAA3347
Spilosoma virginica|[4675]|LOWCE191-06|CGWC-3951|658[0n]bp|Canada.British Columbia|BOLD:AAA3347
Spilosoma virginica|[4676]|LBCB630-05|HLC-21570|658[0n]bp|Canada.British Columbia|BOLD:AAA3347
Spilosoma virginica|[4677]|LBCA494-05|HLC-20494|658[0n]bp|Canada.British Columbia|BOLD:AAA3347
Spilosoma virginica|[4678]|LBCB144-05|HLC-21084|658[0n]bp|Canada.British Columbia|BOLD:AAA3347
Spilosoma virginica|[4679]|LOWCB547-05|CGWC-1487|582[0n]bp|Canada.British Columbia|BOLD:AAA3347
Spilosoma virginica|[4680]|RDNME183-07|CNCNoctuoidea13711|642[0n]bp|Canada.British Columbia|BOLD:AAA3347
Spilosoma virginica|[4681]|LHLEP055-06|UBC-2006-0256|658[0n]bp|Canada.British Columbia|BOLD:AAA3347
Spilosoma virginica|[4682]|LPSK481-08|08BBLEP-02049|658[0n]bp|Canada.Saskatchewan|BOLD:AAA3347
Spilosoma virginica|[4683]|LPVIA202-08|PFC-2006-0283|658[0n]bp|Canada.British Columbia|BOLD:AAA3347
Spilosoma virginica|[4684]|LBCS030-07|UBC-2007-0053|658[0n]bp|Canada.British Columbia|BOLD:AAA3347
Spilosoma virginica|[4685]|LALPA110-10|AVBC 110-10|658[0n]bp|Canada.British Columbia|BOLD:AAA3347
Spilosoma virginica|[4686]|LALPA109-10|AVBC 109-10|658[0n]bp|Canada.British Columbia|BOLD:AAA3347
Spilosoma virginica|[4687]|LPVIA264-08|PFC-2006-0350|658[0n]bp|Canada.British Columbia|BOLD:AAA3347
Spilosoma virginica|[4688]|LBCS444-07|UBC-2007-0200|658[0n]bp|Canada.British Columbia|BOLD:AAA3347
Spilosoma virginica|[4689]|LPGVA733-08|UBC-2006-2076|658[0n]bp|Canada.British Columbia|BOLD:AAA3347
Spilosoma virginica|[4690]|LBCS443-07|UBC-2007-0199|658[0n]bp|Canada.British Columbia|BOLD:AAA3347
Spilosoma virginica|[4691]|LMH028-06|PFC-2006-0149|658[0n]bp|Canada.British Columbia|BOLD:AAA3347
Spilosoma virginica|[4692]|LPVIB912-08|PFC-2006-2442|646[0n]bp|Canada.British Columbia|BOLD:AAA3347
Spilosoma virginica|[4693]|LPVIB911-08|PFC-2006-2441|647[0n]bp|Canada.British Columbia|BOLD:AAA3347
Spilosoma virginica|[4694]|LBCA544-05|HLC-20544|658[0n]bp|Canada.British Columbia|BOLD:AAA3347
Spilosoma virginica|[4695]|LHLEP053-06|UBC-2006-0254|658[0n]bp|Canada.British Columbia|BOLD:AAA3347
Spilosoma virginica|[4696]|LPVIA200-08|PFC-2006-0274|658[0n]bp|Canada.British Columbia|BOLD:AAA3347
Spilosoma virginica|[4697]|LBCA493-05|HLC-20493|658[0n]bp|Canada.British Columbia|BOLD:AAA3347
Spilosoma virginica|[4698]|LPVIA265-08|PFC-2006-0351|658[0n]bp|Canada.British Columbia|BOLD:AAA3347
Spilosoma virginica|[4699]|LPMN309-08|08BBLEP-01108|658[0n]bp|Canada.Manitoba|BOLD:AAA3347
Spilosoma virginica|[4700]|XAE444-04|Moth4444.03|658[0n]bp|Canada.Ontario|BOLD:AAA3347
Spilosoma virginica|[4701]|PHMNB540-04|04HBL00766|658[1n]bp|Canada.New Brunswick|BOLD:AAA3347
Spilosoma virginica|[4702]|TTMNB254-06|MNBTT-254|658[0n]bp|Canada.New Brunswick|BOLD:AAA3347
Spilosoma virginica|[4703]|TMNBD396-07|MNBTT-3197|649[0n]bp|Canada.New Brunswick|BOLD:AAA3347
Spilosoma virginica|[4704]|TZBCA314-07|234-211|656[0n]bp|Canada.Ontario|BOLD:AAA3347
Spilosoma virginica|[4705]|LPSO475-08|PPBP-0475|656[0n]bp|Canada.Ontario|BOLD:AAA3347
Spilosoma virginica|[4706]|XAK172-06|2006-ONT-1167|658[0n]bp|Canada.Ontario|BOLD:AAA3347
Spilosoma virginica|[4707]|PHMNB726-05|Moth 419.03SA|658[0n]bp|Canada.New Brunswick|BOLD:AAA3347
Spilosoma virginica|[4708]|MNBB502-05|05-NBSTA-418|658[0n]bp|Canada.New Brunswick|BOLD:AAA3347
Spilosoma virginica|[4709]|LPMN598-08|08BBLEP-01399|658[0n]bp|Canada.Manitoba|BOLD:AAA3347
Spilosoma virginica|[4710]|PHMNB529-04|04HBL00755|658[0n]bp|Canada.New Brunswick|BOLD:AAA3347
Spilosoma virginica|[4711]|XAB154-04|04HBL005154|658[0n]bp|Canada.Ontario|BOLD:AAA3347
Spilosoma virginica|[4712]|XAD702-05|2005-ONT-501|658[0n]bp|Canada.Ontario|BOLD:AAA3347
Spilosoma virginica|[4713]|LPSO240-08|PPBP-0240|658[0n]bp|Canada.Ontario|BOLD:AAA3347
Spilosoma virginica|[4714]|XAD703-05|2005-ONT-502|658[0n]bp|Canada.Ontario|BOLD:AAA3347
Spilosoma virginica|[4715]|TMNBD397-07|MNBTT-3198|658[0n]bp|Canada.New Brunswick|BOLD:AAA3347
Spilosoma virginica|[4716]|XAE467-04|Moth4467.03|658[0n]bp|Canada.Ontario|BOLD:AAA3347
Spilosoma virginica|[4717]|TMNBD398-07|MNBTT-3199|658[0n]bp|Canada.New Brunswick|BOLD:AAA3347
Spilosoma virginica|[4718]|XAG230-05|2005-ONT-814|658[0n]bp|Canada.Ontario|BOLD:AAA3347
Spilosoma virginica|[4719]|LPSOD641-09|08BBLEP-00422|658[0n]bp|Canada.Ontario|BOLD:AAA3347
Spilosoma virginica|[4720]|BBLPA374-10|10BBCLP-0374|658[0n]bp|Canada.Saskatchewan|BOLD:AAA3347
Spilosoma virginica|[4721]|XAG710-05|2005-ONT-1294|658[0n]bp|Canada.Ontario|BOLD:AAA3347
Spilosoma virginica|[4722]|XAK346-06|2006-ONT-1341|658[0n]bp|Canada.Ontario|BOLD:AAA3347
Spilosoma virginica|[4723]|XAD701-05|2005-ONT-500|658[0n]bp|Canada.Ontario|BOLD:AAA3347
Spilosoma virginica|[4724]|XAB141-04|04HBL005141|658[0n]bp|Canada.Ontario|BOLD:AAA3347
Spilosoma virginica|[4725]|BLTIB093-08|BL0150|658[0n]bp|Canada.Ontario|BOLD:AAA3347
Spilosoma virginica|[4726]|TMNBD391-07|MNBTT-3192|658[0n]bp|Canada.New Brunswick|BOLD:AAA3347
Spilosoma virginica|[4727]|MNBB281-05|05-NBSTA-197|658[0n]bp|Canada.New Brunswick|BOLD:AAA3347
Spilosoma virginica|[4728]|XAB125-04|04HBL005125|658[0n]bp|Canada.Ontario|BOLD:AAA3347
Spilosoma virginica|[4729]|XAF648-05|2005-ONT-297|658[0n]bp|Canada.Ontario|BOLD:AAA3347
Spilosoma virginica|[4730]|XAK171-06|2006-ONT-1166|658[0n]bp|Canada.Ontario|BOLD:AAA3347
Spilosoma virginica|[4731]|PHMNB436-04|04HBL00662|658[0n]bp|Canada.New Brunswick|BOLD:AAA3347
Spilosoma virginica|[4732]|LPSOB713-08|PPBP-1712|658[0n]bp|Canada.Ontario|BOLD:AAA3347
Spilosoma virginica|[4733]|TMNBD394-07|MNBTT-3195|646[0n]bp|Canada.New Brunswick|BOLD:AAA3347
Spilosoma virginica|[4734]|TZBCA171-06|OMAFRA06-106|622[0n]bp|Canada.Ontario|BOLD:AAA3347
Spilosoma virginica|[4735]|PMG018-03|moth373.01|617[0n]bp|Canada.Ontario|BOLD:AAA3347
Spilosoma virginica|[4736]|PHMNB043-03|moth219.02SA|639[0n]bp|Canada.New Brunswick|BOLD:AAA3347
Spilosoma virginica|[4737]|PHMNB057-03|moth34.02SA|639[0n]bp|Canada.New Brunswick|BOLD:AAA3347
Spilosoma virginica|[4738]|TMG80-03|moth357.01|639[0n]bp|Canada.Ontario|BOLD:AAA3347
Spilosoma virginica|[4739]|TMG79-03|moth547.01|639[0n]bp|Canada.Ontario|BOLD:AAA3347
Spilosoma virginica|[4740]|XAB140-04|04HBL005140|658[0n]bp|Canada.Ontario|BOLD:AAA3347
Spilosoma virginica|[4741]|LPSO354-08|PPBP-0354|658[0n]bp|Canada.Ontario|BOLD:AAA3347
Virbia laeta|[4742]|TTMNB253-06|MNBTT-253|658[0n]bp|Canada.New Brunswick|BOLD:AAA7234
Virbia laeta|[4743]|TMNBD381-07|MNBTT-3182|658[0n]bp|Canada.New Brunswick|BOLD:AAA7234
Virbia laeta|[4744]|RDNME819-08|LEP041284|658[0n]bp|Canada.Ontario|BOLD:AAA7234
Virbia laeta|[4745]|TMNBD380-07|MNBTT-3181|658[0n]bp|Canada.New Brunswick|BOLD:AAA7234
Virbia laeta|[4746]|MNAF437-08|CNCLEP00040422|658[0n]bp|Canada.Manitoba|BOLD:AAA7234
Virbia laeta|[4747]|RDNME818-08|LEP041283|658[0n]bp|Canada.Ontario|BOLD:AAA7234
Virbia laeta|[4748]|PHMO229-03|moth1162.02|639[0n]bp|Canada.Ontario|BOLD:AAA7234
Virbia laeta|[4749]|TMNBD451-07|MNBTT-3252|646[0n]bp|Canada.New Brunswick|BOLD:AAA7234
Virbia laeta|[4750]|MNAF477-08|CNCLEP00040462|658[0n]bp|Canada.Manitoba|BOLD:AAA7234
Virbia laeta|[4751]|LPMN046-08|08BBLEP-00844|658[0n]bp|Canada.Manitoba|BOLD:AAA7234
Virbia laeta|[4752]|LPMNB546-09|08BBLEP-05584|658[0n]bp|Canada.Manitoba|BOLD:AAA7234
Virbia laeta|[4753]|MNAF480-08|CNCLEP00040465|658[0n]bp|Canada.Manitoba|BOLD:AAA7234
Virbia laeta|[4754]|MNAF478-08|CNCLEP00040463|658[0n]bp|Canada.Manitoba|BOLD:AAA7234
Virbia laeta|[4755]|MNAF479-08|CNCLEP00040464|658[0n]bp|Canada.Manitoba|BOLD:AAA7234
Virbia laeta treatii|[4756]|RDLQG443-06|DH012727|658[0n]bp|Canada.Quebec|BOLD:AAA7234
Virbia opella|[4757]|RDNME331-07|CNCNoctuoidea13938|658[0n]bp|Canada.Ontario|BOLD:ACF4452
Virbia opella|[4758]|RDNME332-07|CNCNoctuoidea13939|658[0n]bp|Canada.Ontario|BOLD:ACF4452
Virbia opella|[4759]|RDNME346-07|CNCNoctuoidea13953|658[0n]bp|Canada.Ontario|BOLD:ACF4452

Virbia opella|[4757]|RDNME331-07|CNCNoctuoidea13938|658[0n]bp|Canada.Ontario|BOLD:ACF4452
Virbia opella|[4758]|RDNME332-07|CNCNoctuoidea13939|658[0n]bp|Canada.Ontario|BOLD:ACF4452
Virbia opella|[4759]|RDNME346-07|CNCNoctuoidea13953|658[0n]bp|Canada.Ontario|BOLD:ACF4452
Virbia opella|[4760]|RDNME330-07|CNCNoctuoidea13937|658[0n]bp|Canada.Ontario|BOLD:ACF4452
Virbia opella|[4761]|RDNMB414-05|CNCNoctuoidea10180|658[0n]bp|Canada.Ontario|BOLD:ACF4452
Virbia opella|[4762]|RDNME345-07|CNCNoctuoidea13952|658[1n]bp|Canada.Ontario|BOLD:ACF4452
Virbia opella|[4763]|RDNME333-07|CNCNoctuoidea13940|658[0n]bp|Canada.Ontario|BOLD:ACF4452
Virbia ferruginosa|[4764]|RDLQB386-05|DH010472|658[0n]bp|Canada.Quebec|BOLD:AAA4487
Virbia immaculata|[4765]|RDNMB413-05|CNCNoctuoidea10179|658[0n]bp|Canada.Ontario|BOLD:AAC0912
Virbia ferruginosa|[4766]|RDNME796-08|LEP041261|658[0n]bp|Canada.Ontario|BOLD:AAA4493
Virbia ferruginosa|[4767]|RDNMB412-05|CNCNoctuoidea10178|658[0n]bp|Canada.Ontario|BOLD:AAA4486
Virbia ferruginosa|[4768]|RDNME500-08|LEP037924|658[0n]bp|Canada.Quebec|BOLD:AAA4492
Virbia ferruginosa|[4769]|RDNME499-08|LEP037923|658[0n]bp|Canada.Quebec|BOLD:AAA4491
Virbia aurantiaca|[4770]|BBLEC996-09|09BBELE-0996|658[0n]bp|Canada.Nova Scotia|BOLD:AAA4928
Virbia aurantiaca|[4771]|BBLPA593-10|10BBCLP-0593|658[0n]bp|Canada.Ontario|BOLD:AAA4928
Virbia aurantiaca|[4772]|LPSO819-08|PPBP-0819|658[0n]bp|Canada.Ontario|BOLD:AAA4928
Virbia aurantiaca|[4773]|RDNMB405-05|CNCNoctuoidea10171|658[0n]bp|Canada.Manitoba|BOLD:AAA4928
Virbia aurantiaca|[4774]|MNAG036-08|CNCLEP00041021|658[0n]bp|Canada.Manitoba|BOLD:AAA4928
Virbia aurantiaca|[4775]|MNAG037-08|CNCLEP00041022|658[0n]bp|Canada.Manitoba|BOLD:AAA4928
Virbia aurantiaca|[4776]|RDMAB422-05|BCSC95|658[0n]bp|Canada.Manitoba|BOLD:AAA4928
Virbia aurantiaca|[4777]|MNAG040-08|CNCLEP00041025|658[0n]bp|Canada.Manitoba|BOLD:AAA4928
Virbia aurantiaca|[4778]|RDMAB423-05|BCSC96|658[0n]bp|Canada.Manitoba|BOLD:AAA4928
Virbia aurantiaca|[4779]|MNAG039-08|CNCLEP00041024|658[0n]bp|Canada.Manitoba|BOLD:AAA4928
Virbia aurantiaca|[4780]|MNAG038-08|CNCLEP00041023|658[0n]bp|Canada.Manitoba|BOLD:AAA4928
Virbia aurantiaca|[4781]|RDNMB404-05|CNCNoctuoidea10170|658[0n]bp|Canada.Ontario|BOLD:AAA4928
Virbia aurantiaca|[4782]|RDNMB407-05|CNCNoctuoidea10173|658[0n]bp|Canada.Ontario|BOLD:AAA4928
Virbia aurantiaca|[4783]|LPSK197-08|08BBLEP-01765|658[0n]bp|Canada.Saskatchewan|BOLD:AAA4928
Virbia aurantiaca|[4784]|LPSK632-08|08BBLEP-02200|658[0n]bp|Canada.Saskatchewan|BOLD:AAA4928
Virbia aurantiaca|[4785]|LPSK196-08|08BBLEP-01764|658[0n]bp|Canada.Saskatchewan|BOLD:AAA4928
Virbia aurantiaca|[4786]|LPSK400-08|08BBLEP-01968|658[0n]bp|Canada.Saskatchewan|BOLD:AAA4928
Virbia aurantiaca|[4787]|RDNMB406-05|CNCNoctuoidea10172|658[0n]bp|Canada.Ontario|BOLD:AAA4928
Virbia aurantiaca|[4788]|LPSK200-08|08BBLEP-01768|658[0n]bp|Canada.Saskatchewan|BOLD:AAA4928
Virbia aurantiaca|[4789]|BBLPA591-10|10BBCLP-0591|658[0n]bp|Canada.Ontario|BOLD:AAA4928
Virbia aurantiaca|[4790]|BBLPA590-10|10BBCLP-0590|623[0n]bp|Canada.Ontario|BOLD:AAA4928
Virbia ferruginosa|[4791]|RDLQG147-06|DH012318|658[3n]bp|Canada.Quebec|BOLD:AAA4489
Virbia ferruginosa|[4792]|RDLQG146-06|DH012317|594[0n]bp|Canada.Quebec|BOLD:AAA4489
Virbia ferruginosa|[4793]|RDNMB411-05|CNCNoctuoidea10177|658[0n]bp|Canada.Ontario|BOLD:ABZ2730
Virbia ferruginosa|[4794]|RDLQG149-06|DH012320|592[0n]bp|Canada.Quebec|BOLD:ABY6325
Virbia ferruginosa|[4795]|RDNME486-08|LEP037910|658[0n]bp|Canada.Quebec|BOLD:AAA4490
Virbia ferruginosa|[4796]|XAJ882-06|2006-ONT-0882|658[0n]bp|Canada.Ontario|BOLD:ABZ1841
Virbia ferruginosa|[4797]|XAJ889-06|2006-ONT-0889|658[0n]bp|Canada.Ontario|BOLD:ABZ1841
Virbia ferruginosa|[4798]|RDLQG151-06|DH012322|627[0n]bp|Canada.Quebec|BOLD:ABY5771
Virbia ferruginosa|[4799]|RDNME797-08|LEP041262|658[0n]bp|Canada.Ontario|BOLD:ACE5534
Virbia ferruginosa|[4800]|RDNME495-08|LEP037919|658[0n]bp|Canada.Ontario|BOLD:ACE4285
Virbia ferruginosa|[4801]|RDLQG150-06|DH012321|611[3n]bp|Canada.Quebec|BOLD:ACF1306
Virbia ferruginosa|[4802]|RDNME498-08|LEP037922|658[0n]bp|Canada.Quebec|BOLD:ABZ2731
Virbia ferruginosa|[4803]|RDNME501-08|LEP037925|658[0n]bp|Canada.Quebec|BOLD:ABZ2731
Virbia ferruginosa|[4804]|RDLQB534-05|DH010620|658[0n]bp|Canada.Quebec|BOLD:ABZ2731
Virbia ferruginosa|[4805]|RDNME497-08|LEP037921|658[0n]bp|Canada.Quebec|BOLD:ABZ2731
Virbia ferruginosa|[4806]|RDNME209-07|CNCNoctuoidea13816|658[2n]bp|Canada.Ontario|BOLD:ABZ2731
Virbia immaculata|[4807]|RDNME487-08|LEP037911|658[0n]bp|Canada.Quebec|BOLD:ABZ1843
Virbia ferruginosa|[4808]|XAJ883-06|2006-ONT-0883|658[0n]bp|Canada.Ontario|BOLD:AAA4485
Virbia ferruginosa|[4809]|RDLQG148-06|DH012319|640[0n]bp|Canada.Quebec|BOLD:AAA4485
Virbia ferruginosa|[4810]|TMNBB006-06|MNBTT-946|658[0n]bp|Canada.New Brunswick|BOLD:AAA4485
Virbia ferruginosa|[4811]|TMNBB008-06|MNBTT-948|658[0n]bp|Canada.New Brunswick|BOLD:AAA4485
Virbia ferruginosa|[4812]|TMNBB007-06|MNBTT-947|658[0n]bp|Canada.New Brunswick|BOLD:AAA4485
Virbia ferruginosa|[4813]|RDLQ766-07|DH006360|641[0n]bp|Canada.Quebec|BOLD:AAA4485
Virbia ferruginosa|[4814]|RDLQG152-06|DH012323|621[8n]bp|Canada.Quebec|
Virbia ferruginosa|[4815]|XAJ894-06|2006-ONT-0894|658[0n]bp|Canada.Ontario|BOLD:AAA4485
Virbia ferruginosa|[4816]|TMNBB009-06|MNBTT-949|658[0n]bp|Canada.New Brunswick|BOLD:AAA4485
Virbia ferruginosa|[4817]|RDLQ396-05|DH010079|548[0n]bp|Canada.Quebec|BOLD:AAA4485
Virbia ferruginosa|[4818]|RDNMB409-05|CNCNoctuoidea10175|616[0n]bp|Canada.Alberta|BOLD:AAA4485
Virbia ferruginosa|[4819]|RDLQ398-05|DH010081|572[1n]bp|Canada.Quebec|BOLD:AAA4485
Virbia ferruginosa|[4820]|LPMN618-08|08BBLEP-01419|658[0n]bp|Canada.Manitoba|BOLD:AAA4485
Virbia ferruginosa|[4821]|RDMAB362-05|UASM78177|658[0n]bp|Canada.Alberta|BOLD:AAA4485
Virbia ferruginosa|[4822]|RDMAB377-05|BCSC51|658[0n]bp|Canada.Alberta|BOLD:AAA4485
Virbia ferruginosa|[4823]|RDMAB361-05|UASM78179|601[0n]bp|Canada.Alberta|BOLD:AAA4485
Virbia ferruginosa|[4824]|RDNME496-08|LEP037920|658[0n]bp|Canada.Ontario|BOLD:AAA4485
Virbia ferruginosa|[4825]|RDNME798-08|LEP041263|658[0n]bp|Canada.Ontario|BOLD:AAA4485
Virbia ferruginosa|[4826]|LPMN236-08|08BBLEP-01035|658[0n]bp|Canada.Manitoba|BOLD:AAA4485
Virbia ferruginosa|[4827]|RDNME505-08|LEP037929|658[0n]bp|Canada.Ontario|BOLD:AAA4485
Virbia ferruginosa|[4828]|LPMN234-08|08BBLEP-01033|658[0n]bp|Canada.Manitoba|BOLD:AAA4485
Virbia ferruginosa|[4829]|RDNME795-08|LEP041260|658[0n]bp|Canada.Ontario|BOLD:AAA4485
Virbia ferruginosa|[4830]|RDNME507-08|LEP037931|658[0n]bp|Canada.Ontario|BOLD:AAA4485
Virbia ferruginosa|[4831]|RDNME801-08|LEP041266|658[0n]bp|Canada.Ontario|BOLD:AAA4485
Virbia ferruginosa|[4832]|RDNMB410-05|CNCNoctuoidea10176|658[0n]bp|Canada.Ontario|BOLD:AAA4485
Virbia ferruginosa|[4833]|RDNME492-08|LEP037916|658[0n]bp|Canada.Ontario|BOLD:AAA4485
Virbia ferruginosa|[4834]|RDNME799-08|LEP041264|658[0n]bp|Canada.Ontario|BOLD:AAA4485
Virbia ferruginosa|[4835]|LPMN235-08|08BBLEP-01034|658[0n]bp|Canada.Manitoba|BOLD:AAA4485
Virbia ferruginosa|[4836]|LPMN233-08|08BBLEP-01032|658[0n]bp|Canada.Manitoba|BOLD:AAA4485
Virbia ferruginosa|[4837]|RDNME493-08|LEP037917|658[0n]bp|Canada.Ontario|BOLD:AAA4485
Virbia immaculata|[4838]|XAK314-06|2006-ONT-1309|651[0n]bp|Canada.Ontario|BOLD:AAA4485
Virbia immaculata|[4839]|XAK313-06|2006-ONT-1308|658[0n]bp|Canada.Ontario|BOLD:AAA4485
Virbia immaculata|[4840]|RDNME344-07|CNCNoctuoidea13951|643[2n]bp|Canada.Ontario|BOLD:AAA4485
Virbia immaculata|[4841]|RDNME820-08|LEP041285|658[0n]bp|Canada.Ontario|BOLD:AAA4485
Virbia immaculata|[4842]|MEC419-04|jflandry0419|658[0n]bp|Canada.Quebec|BOLD:AAA4485
Virbia immaculata|[4843]|RDNME354-07|CNCNoctuoidea13961|658[0n]bp|Canada.Ontario|BOLD:AAA4485
Virbia immaculata|[4844]|RDNME494-08|LEP037918|658[0n]bp|Canada.Ontario|BOLD:AAA4485
Virbia ferruginosa|[4845]|RDNMC251-05|CNCNoctuoidea11885|543[0n]bp|Canada.New Brunswick|BOLD:AAA4485
Virbia lamae|[4846]|RDMAB376-05|BCSC50|610[0n]bp|Canada.Nova Scotia|BOLD:AAA4485
Virbia ferruginosa|[4847]|RDLQG145-06|DH012316|630[0n]bp|Canada.Quebec|BOLD:AAA4485
Virbia ferruginosa|[4848]|MNAF854-08|CNCLEP00040839|658[0n]bp|Canada.Manitoba|BOLD:AAA4485
Virbia ferruginosa|[4849]|RDMAB425-05|BCSC98|658[0n]bp|Canada.Alberta|BOLD:AAA4485
Virbia ferruginosa|[4850]|RDNME489-08|LEP037913|658[0n]bp|Canada.British Columbia|BOLD:AAA4485
Virbia ferruginosa|[4851]|RDNME490-08|LEP037914|658[0n]bp|Canada.British Columbia|BOLD:AAA4485
Virbia ferruginosa|[4852]|BBLPA480-10|10BBCLP-0480|632[0n]bp|Canada.British Columbia|BOLD:AAA4485
Virbia ferruginosa|[4853]|BBLPA482-10|10BBCLP-0482|658[0n]bp|Canada.British Columbia|BOLD:AAA4485
Virbia ferruginosa|[4854]|BBLPA481-10|10BBCLP-0481|632[0n]bp|Canada.British Columbia|BOLD:AAA4485
Virbia ferruginosa|[4855]|RDMAB424-05|BCSC97|658[0n]bp|Canada.Alberta|BOLD:AAA4485
Virbia ferruginosa|[4856]|LOWCB561-05|CGWC-1501|658[0n]bp|Canada.British Columbia|BOLD:AAA4485
Virbia ferruginosa|[4857]|RDMAB426-05|BCSC99|658[0n]bp|Canada.Alberta|BOLD:AAA4485
Virbia ferruginosa|[4858]|BBLPA484-10|10BBCLP-0484|658[0n]bp|Canada.British Columbia|BOLD:AAA4485

Virbia ferruginosa|[4856]|LOWCB561-05|CGWC-1501|658[0n]bp|Canada.British Columbia|BOLD:AAA4485
Virbia ferruginosa|[4857]|RDMAB426-05|BCSC99|658[0n]bp|Canada.Alberta|BOLD:AAA4485
Virbia ferruginosa|[4858]|BBLPA484-10|10BBCLP-0484|658[0n]bp|Canada.British Columbia|BOLD:AAA4485
Virbia ferruginosa|[4859]|RDNME491-08|LEP037915|658[0n]bp|Canada.British Columbia|BOLD:AAA4485
Virbia ferruginosa|[4860]|BBLPA483-10|10BBCLP-0483|658[0n]bp|Canada.Ontario|BOLD:AAA4485
Virbia ferruginosa|[4861]|LOWCB556-05|CGWC-1496|658[0n]bp|Canada.British Columbia|BOLD:AAA4485
Virbia ferruginosa|[4862]|LPSOD951-09|08BBLEP-05491|658[0n]bp|Canada.Ontario|BOLD:AAA4485
Virbia ferruginosa|[4863]|LOWCB548-05|CGWC-1488|658[0n]bp|Canada.British Columbia|BOLD:AAA4485
Virbia ferruginosa|[4864]|LOWCB559-05|CGWC-1499|658[0n]bp|Canada.British Columbia|BOLD:AAA4485
Virbia ferruginosa|[4865]|RDLQB384-05|DH010470|658[0n]bp|Canada.Quebec|BOLD:AAA4485
Virbia ferruginosa|[4866]|RDNME488-08|LEP037912|658[0n]bp|Canada.British Columbia|BOLD:AAA4485
Virbia ferruginosa|[4867]|LOWCB558-05|CGWC-1498|658[0n]bp|Canada.British Columbia|BOLD:AAA4485
Virbia ferruginosa|[4868]|LOWCC878-05|CGWC-2758|658[0n]bp|Canada.British Columbia|BOLD:AAA4485
Virbia ferruginosa|[4869]|LOWCB549-05|CGWC-1489|658[0n]bp|Canada.British Columbia|BOLD:AAA4485
Virbia ferruginosa|[4870]|LBCH6104-10|10-JDWBC-6104|658[0n]bp|Canada.British Columbia|BOLD:AAA4485
Virbia ferruginosa|[4871]|LOWCB554-05|CGWC-1494|658[0n]bp|Canada.British Columbia|BOLD:AAA4485
Virbia ferruginosa|[4872]|LOWCB557-05|CGWC-1497|658[0n]bp|Canada.British Columbia|BOLD:AAA4485
Virbia|[4873]|RDMAB429-05|BCSC102|658[1n]bp|Canada.Alberta|BOLD:AAA4485
Virbia ferruginosa|[4874]|LOWCB553-05|CGWC-1493|658[0n]bp|Canada.British Columbia|BOLD:AAA4485
Virbia ferruginosa|[4875]|RDLQB383-05|DH010469|658[0n]bp|Canada.Quebec|BOLD:AAA4485
Virbia ferruginosa|[4876]|RDLQB385-05|DH010471|555[0n]bp|Canada.Quebec|BOLD:AAA4485
Virbia ferruginosa|[4877]|RDLQB381-05|DH010467|541[1n]bp|Canada.Quebec|BOLD:AAA4485
Virbia ferruginosa|[4878]|RDLQB382-05|DH010468|551[0n]bp|Canada.Quebec|BOLD:AAA4485
Virbia ferruginosa|[4879]|RDLQB380-05|DH010466|585[0n]bp|Canada.Quebec|BOLD:AAA4485
Virbia n. sp.|[4880]|RDMAB360-05|UASM78169|611[0n]bp|Canada.Alberta|BOLD:AAA4485
Virbia ferruginosa|[4881]|RDMAB427-05|BCSC100|658[0n]bp|Canada.Alberta|BOLD:AAA4485
Virbia ferruginosa|[4882]|LOWCB552-05|CGWC-1492|578[0n]bp|Canada.British Columbia|BOLD:AAA4485
Virbia ferruginosa|[4883]|LOWCB560-05|CGWC-1500|577[0n]bp|Canada.British Columbia|BOLD:AAA4485
Virbia ferruginosa|[4884]|LOWCB555-05|CGWC-1495|571[0n]bp|Canada.British Columbia|BOLD:AAA4485
Virbia ferruginosa|[4885]|LOWCB550-05|CGWC-1490|540[0n]bp|Canada.British Columbia|BOLD:AAA4485
Virbia ferruginosa|[4886]|LOWCB551-05|CGWC-1491|542[0n]bp|Canada.British Columbia|BOLD:AAA4485
Virbia ferruginosa|[4887]|RDNMB408-05|CNCNoctuoidea10174|616[0n]bp|Canada.British Columbia|BOLD:AAA4485
Virbia sp.|[4888]|RDMAB359-05|UASM78173|541[0n]bp|Canada.Alberta|BOLD:AAA4485
Parascotia fuliginaria|[4889]|GWORK467-09|BC ZSM Lep 21797|658[0n]bp|Germany.Bavaria|BOLD:AAC4237
Parascotia fuliginaria|[4890]|GSCMW1364-10|CCDB-04645 F06|658[0n]bp|Austria.Oberoesterreich|BOLD:AAC4237
Parascotia fuliginaria|[4891]|LEFIA357-10|MM01412|658[0n]bp|Finland.South Karelia|BOLD:AAC4237
Parascotia fuliginaria|[4892]|CGUKC085-09|UKLB23B04|658[0n]bp|United Kingdom.England|BOLD:AAC4237
Parascotia fuliginaria|[4893]|GWORK529-09|BC ZSM Lep 21859|658[0n]bp|Germany.Bavaria|BOLD:AAC4237
Parascotia fuliginaria|[4894]|GWORE2022-09|BC ZSM Lep 22420|632[0n]bp|Germany.Bavaria|BOLD:AAC4237
Parascotia fuliginaria|[4895]|LEFIA1279-10|MM00340|670[1n]bp|Finland.Uusimaa|BOLD:AAC4237
Parascotia fuliginaria|[4896]|LEFIA358-10|MM01413|658[0n]bp|Finland.South Karelia|BOLD:AAC4237
Parascotia fuliginaria|[4897]|FBLMZ414-12|BC ZSM Lep 61170|658[0n]bp|Germany.Bavaria|BOLD:AAC4237
Parascotia fuliginaria|[4898]|TTNFS040-09|FG040|658[0n]bp|Serbia|BOLD:AAC4237
Parascotia fuliginaria|[4899]|FBLMV251-09|BC ZSM Lep 28231|658[0n]bp|Germany.Bavaria|BOLD:AAC4237
Parascotia fuliginaria|[4900]|IBLAO877-12|AOC Lep 00972|658[0n]bp|Spain|BOLD:AAC4237
Parascotia fuliginaria|[4901]|IBLAO762-12|AOC Lep 00857|658[0n]bp|Spain|BOLD:AAC4237
Parascotia fuliginaria|[4902]|CGUKB378-09|UKLB15F03|658[0n]bp|United Kingdom.England|BOLD:AAC4237
Parascotia fuliginaria|[4903]|LENOA012-11|LN-BD0012|658[0n]bp|France.Haute Normandie|BOLD:AAC4237
Parascotia fuliginaria|[4904]|GWOR3941-09|BC ZSM Lep 21225|658[0n]bp|Germany.Bavaria|BOLD:AAC4237
Parascotia fuliginaria|[4905]|FBLMW099-10|BC ZSM Lep 29029|658[0n]bp|Germany.Bavaria|BOLD:AAC4237
Mycterophora inexplicata|[4906]|RDNMF375-08|NOC14461|658[0n]bp|Canada.Alberta|BOLD:ABY9282
Mycterophora inexplicata|[4907]|RDNMF825-08|CNC LEP00053186|658[0n]bp|Canada.Alberta|BOLD:ABY9282
Mycterophora inexplicata|[4908]|RDNMF374-08|NOC14460|658[0n]bp|Canada.Alberta|BOLD:ABY9282
Mycterophora inexplicata|[4909]|LBCH4785-10|10-JDWBC-4785|658[0n]bp|Canada.British Columbia|BOLD:ABY9282
Mycterophora longipalpata|[4910]|LBCG530-08|08-JDWBC-0530|658[0n]bp|Canada.British Columbia|BOLD:AAD9555
Mycterophora longipalpata|[4911]|LBCH7832-10|10-JDWBC-7832|636[0n]bp|Canada.British Columbia|BOLD:AAD...
Mycterophora longipalpata|[4912]|LBCH6687-10|10-JDWBC-6687|658[0n]bp|Canada.British Columbia|BOLD:AAD...
Mycterophora longipalpata|[4913]|RDNMF729-08|NOC14815|576[0n]bp|Canada.British Columbia|BOLD:AAD9555
Mycterophora longipalpata|[4914]|LBCH6723-10|10-JDWBC-6723|658[0n]bp|Canada.British Columbia|BOLD:AAD...
Mycterophora longipalpata|[4915]|LBCG1135-09|08-JDWBC-1135|658[0n]bp|Canada.British Columbia|BOLD:AAD...
Mycterophora longipalpata|[4916]|LBCH6192-10|10-JDWBC-6192|658[0n]bp|Canada.British Columbia|BOLD:AAD...
Mycterophora longipalpata|[4917]|LBCH6257-10|10-JDWBC-6257|658[0n]bp|Canada.British Columbia|BOLD:AAD...
Mycterophora longipalpata|[4918]|LBCH7362-10|10-JDWBC-7362|658[0n]bp|Canada.British Columbia|BOLD:AAD...
Mycterophora longipalpata|[4919]|LOWCC738-05|CGWC-2618|658[0n]bp|Canada.British Columbia|BOLD:AAD9555
Mycterophora sp.|[4920]|BBLPD884-10|10BBCLP-2882|658[0n]bp|Canada.British Columbia|BOLD:ABX5596
Mycterophora sp.|[4921]|LBCC807-05|HLC-22687|658[0n]bp|Canada.British Columbia|BOLD:ABX5596
Mycterophora sp.|[4922]|LBCD437-05|HLC-23257|658[0n]bp|Canada.British Columbia|BOLD:ABX5596
Metalectra discalis|[4923]|RDLQF571-06|DH011721|658[0n]bp|Canada.Quebec|BOLD:AAA8207
Metalectra discalis|[4924]|RDLQG080-06|DH012237|625[0n]bp|Canada.Quebec|BOLD:AAA8207
Metalectra discalis|[4925]|RDLQF568-06|DH011718|658[0n]bp|Canada.Quebec|BOLD:AAA8207
Metalectra discalis|[4926]|RDLQG079-06|DH012236|629[0n]bp|Canada.Quebec|BOLD:AAA8207
Metalectra discalis|[4927]|RDLQF751-06|DH011901|658[0n]bp|Canada.Quebec|BOLD:AAA8207
Metalectra discalis|[4928]|RDLQF750-06|DH011900|658[0n]bp|Canada.Quebec|BOLD:AAA8207
Metalectra discalis|[4929]|RDLQG032-06|DH012163|658[0n]bp|Canada.Quebec|BOLD:AAA8207
Metalectra discalis|[4930]|RDLQF523-06|DH011672|658[0n]bp|Canada.Quebec|BOLD:AAA8207
Metalectra discalis|[4931]|RDLQF524-06|DH011673|658[0n]bp|Canada.Quebec|BOLD:AAA8207
Metalectra discalis|[4932]|RDLQF420-06|DH011527|658[0n]bp|Canada.Quebec|BOLD:AAA8207
Metalectra discalis|[4933]|RDLQF597-06|DH011747|658[0n]bp|Canada.Quebec|BOLD:AAA8207
Metalectra discalis|[4934]|RDLQF599-06|DH011749|658[0n]bp|Canada.Quebec|BOLD:AAA8207
Metalectra discalis|[4935]|RDLQF570-06|DH011720|627[0n]bp|Canada.Quebec|BOLD:AAA8207
Metalectra discalis|[4936]|RDLQF752-06|DH011902|595[1n]bp|Canada.Quebec|BOLD:AAA8207
Metalectra discalis|[4937]|RDNMD224-06|CNCNoctuoidea12560|600[0n]bp|Canada.Quebec|BOLD:AAA8207
Metalectra discalis|[4938]|RDNMD223-06|CNCNoctuoidea12559|611[0n]bp|Canada.Quebec|BOLD:AAA8207
Metalectra discalis|[4939]|RDLQF595-06|DH011745|658[0n]bp|Canada.Quebec|BOLD:AAA8207
Metalectra discalis|[4940]|XAJ849-06|2006-ONT-0849|658[0n]bp|Canada.Ontario|BOLD:AAA8207
Metalectra discalis|[4941]|RDLQF598-06|DH011748|658[0n]bp|Canada.Quebec|BOLD:AAA8207
Metalectra quadrisignata|[4942]|RDLQF569-06|DH011719|658[0n]bp|Canada.Quebec|BOLD:AAB4859
Metalectra quadrisignata|[4943]|RDLQF596-06|DH011746|658[0n]bp|Canada.Quebec|BOLD:AAB4859
Metalectra quadrisignata|[4944]|RDLQF594-06|DH011744|649[0n]bp|Canada.Quebec|BOLD:AAB4859
Metalectra quadrisignata|[4945]|RDLQG078-06|DH012235|633[0n]bp|Canada.Quebec|BOLD:AAB4859
Metalectra quadrisignata|[4946]|RDNMD225-06|CNCNoctuoidea12561|610[0n]bp|Canada.Quebec|BOLD:AAB4859
Metalectra quadrisignata|[4947]|RDLQB079-05|DH010165|658[0n]bp|Canada.Quebec|BOLD:AAB4859
Metalectra quadrisignata|[4948]|RDLQB080-05|DH010166|658[0n]bp|Canada.Quebec|BOLD:AAB4859
Melanomma auricinctaria|[4949]|RDLQE915-06|MDH002918|509[0n]bp|Canada.Quebec|BOLD:AAC4698
Melanomma auricinctaria|[4950]|RDLQE914-06|MDH002917|507[0n]bp|Canada.Quebec|BOLD:AAC4698
Melanomma auricinctaria|[4951]|RDLQE913-06|MDH002916|501[0n]bp|Canada.Quebec|
Ledaea perditalis|[4952]|XAJ866-06|2006-ONT-0866|658[0n]bp|Canada.Ontario|BOLD:AAB5246
Ledaea perditalis|[4953]|XAE608-04|Moth4608.03|658[0n]bp|Canada.Ontario|BOLD:AAB5246
Ledaea perditalis|[4954]|LPSO604-08|PPBP-0604|658[0n]bp|Canada.Ontario|BOLD:AAB5246
Pangrapta decoralis|[4955]|RDNMK272-11|CNCLEP 84181|658[0n]bp|Canada.Ontario|BOLD:AAA3086
Pangrapta decoralis|[4956]|BBLEC647-09|09BBELE-0647|658[0n]bp|Canada.Nova Scotia|BOLD:AAA3086
Pangrapta decoralis|[4957]|BBLPC580-09|09BBELE-1580|658[0n]bp|Canada.Nova Scotia|BOLD:AAA3086

Pangrapta decoralis|[4955]|RDNMK272-11|CNCLEP 84181|658[0n]bp|Canada.Ontario|BOLD:AAA3086
Pangrapta decoralis|[4956]|BBLEC647-09|09BBELE-0647|658[0n]bp|Canada.Nova Scotia|BOLD:AAA3086
Pangrapta decoralis|[4957]|BBLPC580-09|09BBELE-1580|658[0n]bp|Canada.Nova Scotia|BOLD:AAA3086
Pangrapta decoralis|[4958]|BBLPE160-09|09BBELE-2160|658[0n]bp|Canada.Nova Scotia|BOLD:AAA3086
Pangrapta decoralis|[4959]|BBLPC143-09|09BBELE-1143|658[0n]bp|Canada.Nova Scotia|BOLD:AAA3086
Pangrapta decoralis|[4960]|BBLEC623-09|09BBELE-0623|658[0n]bp|Canada.Nova Scotia|BOLD:AAA3086
Pangrapta decoralis|[4961]|BBLPE057-09|09BBELE-2057|658[0n]bp|Canada.Nova Scotia|BOLD:AAA3086
Pangrapta decoralis|[4962]|BBLPE093-09|09BBELE-2093|658[0n]bp|Canada.Nova Scotia|BOLD:AAA3086
Pangrapta decoralis|[4963]|TMNBB065-06|MNBTT-1005|658[0n]bp|Canada.New Brunswick|BOLD:ACF1497
Pangrapta decoralis|[4964]|PHMNB152-04|04HBL007617|658[1n]bp|Canada.New Brunswick|BOLD:ACF1497
Pangrapta decoralis|[4965]|RDNMK307-11|CNCLEP 84216|658[0n]bp|Canada.Ontario|BOLD:ACF1497
Pangrapta decoralis|[4966]|RDLQF494-06|DH011643|658[0n]bp|Canada.Quebec|BOLD:ACF1497
Pangrapta decoralis|[4967]|RDLQB372-05|DH010458|658[0n]bp|Canada.Quebec|BOLD:ACF1497
Pangrapta decoralis|[4968]|BBLPB891-10|10BBCLP-1890|633[0n]bp|Canada.Ontario|BOLD:ACF1497
Pangrapta decoralis|[4969]|RDLQB373-05|DH010459|658[0n]bp|Canada.Quebec|BOLD:ACF1497
Pangrapta decoralis|[4970]|RDLQF493-06|DH011642|658[0n]bp|Canada.Quebec|BOLD:ACF1497
Pangrapta decoralis|[4971]|RDLQF495-06|DH011644|658[0n]bp|Canada.Quebec|BOLD:ACF1497
Pangrapta decoralis|[4972]|RDNMK273-11|CNCLEP 84182|658[0n]bp|Canada.Ontario|BOLD:ACF1497
Pangrapta decoralis|[4973]|RDLQG832-06|DH013125|658[0n]bp|Canada.Quebec|BOLD:ACF1497
Pangrapta decoralis|[4974]|RDNML368-13|CNCLEP 92344|658[0n]bp|Canada.Ontario|BOLD:ACF1497
Pangrapta decoralis|[4975]|BBLPE092-09|09BBELE-2092|658[0n]bp|Canada.Nova Scotia|BOLD:ACF1497
Pangrapta decoralis|[4976]|BBLEC627-09|09BBELE-0627|658[0n]bp|Canada.Nova Scotia|BOLD:ACF1497
Pangrapta decoralis|[4977]|BBLEC633-09|09BBELE-0633|658[0n]bp|Canada.Nova Scotia|BOLD:ACF1497
Pangrapta decoralis|[4978]|BBLPE046-09|09BBELE-2046|658[0n]bp|Canada.Nova Scotia|BOLD:ACF1497
Pangrapta decoralis|[4979]|BBLEC930-09|09BBELE-0930|658[0n]bp|Canada.Nova Scotia|BOLD:ACF1497
Melipotis perpendicularis|[4980]|RDNMF503-08|NOC14589|609[0n]bp|United States.Florida|BOLD:ABZ4611
Melipotis perpendicularis|[4981]|RDNML354-13|CNCLEP 92330|658[0n]bp|United States.Florida|BOLD:ABZ4611
Melipotis perpendicularis|[4982]|RDNML355-13|CNCLEP 92331|658[0n]bp|United States.Florida|BOLD:ABZ4611
Melipotis perpendicularis|[4983]|RDNMF501-08|NOC14587|658[0n]bp|United States.Arizona|BOLD:ABZ4611
Melipotis perpendicularis|[4984]|IAWL093-09|IAWAZ-0092|658[0n]bp|United States.Arizona|BOLD:ABZ4611
Melipotis perpendicularis|[4985]|IAWL077-09|IAWAZ-0076|658[0n]bp|United States.Arizona|BOLD:ABZ4611
Melipotis perpendicularis|[4986]|QUNOD173-10|7172-COI-09|658[0n]bp|United States.Texas|BOLD:ABZ4611
Melipotis perpendicularis|[4987]|IAWL103-09|IAWAZ-0102|658[0n]bp|United States.Arizona|BOLD:ABZ4611
Melipotis perpendicularis|[4988]|IAWL095-09|IAWAZ-0094|658[0n]bp|United States.Arizona|BOLD:ABZ4611
Melipotis perpendicularis|[4989]|IAWL105-09|IAWAZ-0104|658[0n]bp|United States.Arizona|BOLD:ABZ4611
Melipotis perpendicularis|[4990]|ABNCC324-07|1324-230603-AZ|627[1n]bp|United States.Arizona|BOLD:ABZ4611
Melipotis perpendicularis|[4991]|IAWL102-09|IAWAZ-0101|658[0n]bp|United States.Arizona|BOLD:ABZ4611
Melipotis perpendicularis|[4992]|CMAZA885-12|BIOUG02040-C12|658[0n]bp|United States.Arizona|BOLD:ABZ4611
Melipotis perpendicularis|[4993]|CMAZA033-09|CMAZ-0033|658[0n]bp|United States.Arizona|BOLD:ABZ4611
Melipotis perpendicularis|[4994]|IAWL101-09|IAWAZ-0100|658[0n]bp|United States.Arizona|BOLD:ABZ4611
Melipotis perpendicularis|[4995]|RDNMJ519-11|CNCLEP 80047|658[0n]bp|United States.Arizona|BOLD:ABZ4611
Melipotis perpendicularis|[4996]|IAWL094-09|IAWAZ-0093|658[0n]bp|United States.Arizona|BOLD:ABZ4611
Amolita fessa|[4997]|PMG089-03|moth1161.01|617[0n]bp|Canada.Ontario|BOLD:AAC1870
Amolita fessa|[4998]|XAJ901-06|2006-ONT-0901|658[0n]bp|Canada.Ontario|BOLD:AAC1870
Amolita fessa|[4999]|BLTIB471-08|BL719|658[0n]bp|Canada.Ontario|BOLD:AAC1870
Amolita fessa|[5000]|BLTIB438-08|BL685|656[2n]bp|Canada.Ontario|BOLD:AAC1870
Amolita fessa|[5001]|BLTIB502-08|BL758|658[0n]bp|Canada.Ontario|BOLD:AAC1870
Amolita fessa|[5002]|XAK020-06|2006-ONT-1015|658[0n]bp|Canada.Ontario|BOLD:AAC1870
Amolita fessa|[5003]|XAJ897-06|2006-ONT-0897|658[0n]bp|Canada.Ontario|BOLD:AAC1870
Amolita fessa|[5004]|RDLQB424-05|DH010510|658[0n]bp|Canada.Quebec|BOLD:AAC1870
Hyperstrotia pervertens|[5005]|TTMNB297-06|MNBTT-297|602[0n]bp|Canada.New Brunswick|BOLD:ACE3003
Hyperstrotia pervertens|[5006]|RDLQG748-06|DH013041|658[0n]bp|Canada.Quebec|BOLD:ACE3003
Hyperstrotia pervertens|[5007]|RDLQG940-06|DH013237|658[0n]bp|Canada.Quebec|BOLD:ACE3003
Hyperstrotia pervertens|[5008]|BBLPC618-09|09BBELE-1618|658[0n]bp|Canada.Nova Scotia|BOLD:ACE3003
Hyperstrotia pervertens|[5009]|RDLQH004-06|DH013241|658[0n]bp|Canada.Quebec|BOLD:ACE3003
Hyperstrotia pervertens|[5010]|RDLQH008-06|DH013245|658[0n]bp|Canada.Quebec|BOLD:ACE3003
Hyperstrotia pervertens|[5011]|RDLQG786-06|DH013079|658[0n]bp|Canada.Quebec|BOLD:ACE3003
Hyperstrotia pervertens|[5012]|RDLQH011-06|DH013248|658[0n]bp|Canada.Quebec|BOLD:ACE3003
Hyperstrotia pervertens|[5013]|RDLQH006-06|DH013243|658[0n]bp|Canada.Quebec|BOLD:ACE3003
Hyperstrotia pervertens|[5014]|RDLQG939-06|DH013236|658[0n]bp|Canada.Quebec|BOLD:ACE3003
Hyperstrotia pervertens|[5015]|RDLQH012-06|DH013249|658[0n]bp|Canada.Quebec|BOLD:ACE3003
Hyperstrotia pervertens|[5016]|RDLQG712-06|DH013005|658[0n]bp|Canada.Quebec|BOLD:ACE3003
Hyperstrotia pervertens|[5017]|TMNBC519-06|MNBTT-2325|658[0n]bp|Canada.New Brunswick|BOLD:ACE3003
Hyperstrotia pervertens|[5018]|RDLQH005-06|DH013242|658[0n]bp|Canada.Quebec|BOLD:ACE3003
Hyperstrotia pervertens|[5019]|RDLQH007-06|DH013244|658[0n]bp|Canada.Quebec|BOLD:ACE3003
Hyperstrotia pervertens|[5020]|RDLQH003-06|DH013240|658[0n]bp|Canada.Quebec|BOLD:ACE3003
Hyperstrotia pervertens|[5021]|RDLQG582-06|DH012875|658[0n]bp|Canada.Quebec|BOLD:ACE3003
Hyperstrotia pervertens|[5022]|RDLQG927-06|DH013220|658[0n]bp|Canada.Quebec|BOLD:ACE3003
Hyperstrotia pervertens|[5023]|RDLQH009-06|DH013246|658[0n]bp|Canada.Quebec|BOLD:ACE3003
Hyperstrotia pervertens|[5024]|RDLQD890-06|MDH001183|658[0n]bp|Canada.Quebec|BOLD:ACE3003
Hyperstrotia pervertens|[5025]|RDLQD889-06|MDH000345|658[0n]bp|Canada.Quebec|BOLD:ACE3003
Hyperstrotia pervertens|[5026]|RDLQG713-06|DH013006|658[0n]bp|Canada.Quebec|BOLD:ACE3003
Hyperstrotia pervertens|[5027]|RDLQH002-06|DH013239|649[0n]bp|Canada.Quebec|BOLD:ACE3003
Hyperstrotia pervertens|[5028]|TTMNB296-06|MNBTT-296|617[0n]bp|Canada.New Brunswick|BOLD:ACE3003
Hyperstrotia pervertens|[5029]|RDLQH010-06|DH013247|654[0n]bp|Canada.Quebec|BOLD:ACE3003
Hyperstrotia pervertens|[5030]|RDLQG747-06|DH013040|658[0n]bp|Canada.Quebec|BOLD:ACE3003
Hyperstrotia pervertens|[5031]|RDLQH001-06|DH013238|656[0n]bp|Canada.Quebec|BOLD:ACE3003
Hyperstrotia pervertens|[5032]|TMNBC528-06|MNBTT-2334|658[0n]bp|Canada.New Brunswick|BOLD:ACE3003
Hyperstrotia pervertens|[5033]|XAK079-06|2006-ONT-1074|658[0n]bp|Canada.Ontario|BOLD:ACE3003
Hyperstrotia secta|[5034]|LSUSA233-06|06-SUSA-0233|658[0n]bp|United States.Kentucky|BOLD:ACF1580
Hyperstrotia secta|[5035]|LSEU601-06|06-JKA-0601|658[0n]bp|United States.Georgia|BOLD:ACF1580
Hyperstrotia secta|[5036]|LMEMB436-09|RBMIS-1529|658[0n]bp|United States.Alabama|BOLD:ACF1580
Hyperstrotia secta|[5037]|LSEU373-06|06-JKA-0373|658[0n]bp|United States.Georgia|BOLD:ACF1580
Hyperstrotia secta|[5038]|LNCB485-07|07-NCNW-0169|658[0n]bp|United States.North Carolina|BOLD:ACF1580
Hyperstrotia secta|[5039]|LNCB484-07|07-NCNW-0168|658[0n]bp|United States.North Carolina|BOLD:ACF1580
Hyperstrotia secta|[5040]|LSEU371-06|06-JKA-0371|658[0n]bp|United States.Georgia|BOLD:ACF1580
Hyperstrotia secta|[5041]|LGSMG628-07|BGS03991|657[0n]bp|United States.Tennessee|BOLD:ACF1580
Hyperstrotia secta|[5042]|LMEMB435-09|RBMIS-1528|658[0n]bp|United States.Alabama|BOLD:ACF1580
Hyperstrotia secta|[5043]|LGSM736-04|DNA-ATBI-0736|658[0n]bp|United States.Tennessee|BOLD:ACF1580
Hyperstrotia villificans|[5044]|MNAF486-08|CNCLEP00040471|658[0n]bp|Canada.Manitoba|BOLD:ACE6422
Hyperstrotia villificans|[5045]|RDLQC460-06|MDH000518|658[0n]bp|Canada.Quebec|BOLD:ACE6422
Hyperstrotia villificans|[5046]|RDLQG882-06|DH013175|658[0n]bp|Canada.Quebec|BOLD:ACE6422
Hyperstrotia villificans|[5047]|RDNMK653-11|CNCLEP 81830|658[0n]bp|Canada.Ontario|BOLD:ACE6422
Dyspyralis illocata|[5048]|CNSLF315-12|BIOUG03817-D04|616[0n]bp|Canada.Ontario|BOLD:AAB6432
Dyspyralis illocata|[5049]|RDLQF733-06|DH011883|658[0n]bp|Canada.Quebec|BOLD:AAB6432
Dyspyralis illocata|[5050]|RDLQD888-06|MDH001939|658[0n]bp|Canada.Quebec|BOLD:AAB6432
Dyspyralis illocata|[5051]|RDLQF672-06|DH011822|624[0n]bp|Canada.Quebec|BOLD:AAB6432
Dyspyralis illocata|[5052]|RDLQF668-06|DH011818|637[0n]bp|Canada.Quebec|BOLD:AAB6432
Dyspyralis illocata|[5053]|RDLQF667-06|DH011817|637[0n]bp|Canada.Quebec|BOLD:AAB6432
Dyspyralis illocata|[5054]|RDLQF665-06|DH011815|631[0n]bp|Canada.Quebec|BOLD:AAB6432
Dyspyralis illocata|[5055]|RDLQF670-06|DH011820|638[0n]bp|Canada.Quebec|BOLD:AAB6432
Dyspyralis illocata|[5056]|RDLQF671-06|DH011821|638[0n]bp|Canada.Quebec|BOLD:AAB6432
[illegible]

Dyspyralis illocata|[5054]|RDLQF665-06|DH011815|631[0n]bp|Canada.Quebec|BOLD:AAB6432
Dyspyralis illocata|[5055]|RDLQF670-06|DH011820|638[0n]bp|Canada.Quebec|BOLD:AAB6432
Dyspyralis illocata|[5056]|RDLQF671-06|DH011821|638[0n]bp|Canada.Quebec|BOLD:AAB6432
Dyspyralis illocata|[5057]|RDLQF664-06|DH011814|643[1n]bp|Canada.Quebec|BOLD:AAB6432
Dyspyralis illocata|[5058]|RDLQF669-06|DH011819|644[0n]bp|Canada.Quebec|BOLD:AAB6432
Dyspyralis illocata|[5059]|RDLQF666-06|DH011816|644[0n]bp|Canada.Quebec|BOLD:AAB6432
Dyspyralis nigellus|[5060]|RDLQB681-05|DH010784|542[0n]bp|Canada.Quebec|BOLD:AAC3111
Dyspyralis nigellus|[5061]|PMG108-03|TETA1.00|617[0n]bp|Canada.Ontario|BOLD:AAC3111
Dyspyralis nigellus|[5062]|RDLQF786-06|DH011936|658[0n]bp|Canada.Quebec|BOLD:AAC3111
Dyspyralis nigellus|[5063]|RDLQG589-06|DH012882|658[0n]bp|Canada.Quebec|BOLD:AAC3111
Dyspyralis nigellus|[5064]|PHMO265-03|moth1542.02|639[4n]bp|Canada.Ontario|BOLD:AAC3111
Dyspyralis nigellus|[5065]|RDLQF673-06|DH011823|637[0n]bp|Canada.Quebec|BOLD:AAC3111
Dyspyralis nigellus|[5066]|RDLQF674-06|DH011824|637[0n]bp|Canada.Quebec|BOLD:AAC3111
Dyspyralis nigellus|[5067]|RDLQG590-06|DH012883|658[0n]bp|Canada.Quebec|BOLD:AAC3111
Dyspyralis nigellus|[5068]|RDLQG583-06|DH012876|658[0n]bp|Canada.Quebec|BOLD:AAC3111
Dyspyralis puncticosta|[5069]|RDLQH160-07|DH060002|632[0n]bp|Canada.Quebec|BOLD:AAE8238
Dyspyralis puncticosta|[5070]|RDLQF675-06|DH011825|611[0n]bp|Canada.Quebec|BOLD:AAE8238
Oxycilla malaca|[5071]|LGSM773-04|DNA-ATBI-0773|658[0n]bp|United States.North Carolina|BOLD:AAC6000
Oxycilla malaca|[5072]|LGSMC393-05|DNA-ATBI-2393|658[0n]bp|United States.Tennessee|BOLD:AAC6000
Oxycilla malaca|[5073]|QUNOD741-11|9455-250609-MI|658[0n]bp|United States.Mississippi|BOLD:AAC6000
Oxycilla malaca|[5074]|LMEM338-09|RBMIS-0338|658[0n]bp|United States.Mississippi|BOLD:AAC6000
Oxycilla malaca|[5075]|LNCB678-09|09-NCCC-148|658[0n]bp|United States.North Carolina|BOLD:AAC6000
Cisseps fulvicollis|[5076]|LPSOC388-08|PPBP-2387|658[0n]bp|Canada.Ontario|BOLD:AAA4200
Cisseps fulvicollis|[5077]|LBCW013-08|08-JDWWI-0013|658[0n]bp|Canada.British Columbia|BOLD:AAA4200
Cisseps fulvicollis|[5078]|LPAB074-08|08BBLEP-02396|658[0n]bp|Canada.Alberta|BOLD:AAA4200
Cisseps fulvicollis|[5079]|LOWCE036-06|CGWC-3796|618[0n]bp|Canada.British Columbia|BOLD:AAA4200
Cisseps fulvicollis|[5080]|LOWCE074-06|CGWC-3834|658[0n]bp|Canada.British Columbia|BOLD:AAA4200
Cisseps fulvicollis|[5081]|LPABB032-08|08BBLEP-03297|658[0n]bp|Canada.Alberta|BOLD:AAA4200
Cisseps fulvicollis|[5082]|LOWCE035-06|CGWC-3795|658[0n]bp|Canada.British Columbia|BOLD:AAA4200
Cisseps fulvicollis|[5083]|LBCW012-08|08-JDWWI-0012|658[0n]bp|Canada.British Columbia|BOLD:AAA4200
Cisseps fulvicollis|[5084]|LPMN872-08|08BBLEP-02230|658[0n]bp|Canada.Alberta|BOLD:AAA4200
Cisseps fulvicollis|[5085]|LOWCE029-06|CGWC-3789|658[0n]bp|Canada.British Columbia|BOLD:AAA4200
Cisseps fulvicollis|[5086]|LOWCE076-06|CGWC-3836|658[0n]bp|Canada.British Columbia|BOLD:AAA4200
Cisseps fulvicollis|[5087]|LPMN873-08|08BBLEP-02231|658[0n]bp|Canada.Alberta|BOLD:AAA4200
Cisseps fulvicollis|[5088]|LOWCE067-06|CGWC-3827|649[0n]bp|Canada.British Columbia|BOLD:AAA4200
Cisseps fulvicollis|[5089]|LPABB802-09|08BBLEP-04122|658[0n]bp|Canada.Alberta|BOLD:AAA4200
Cisseps fulvicollis|[5090]|LPMN871-08|08BBLEP-02229|658[0n]bp|Canada.Alberta|BOLD:AAA4200
Cisseps fulvicollis|[5091]|TTMNB257-06|MNBTT-257|656[0n]bp|Canada.New Brunswick|BOLD:AAA4200
Cisseps fulvicollis|[5092]|BBLPC360-09|09BBELE-1360|658[0n]bp|Canada.New Brunswick|BOLD:AAA4200
Cisseps fulvicollis|[5093]|BBLEC469-09|09BBELE-0469|658[0n]bp|Canada.New Brunswick|BOLD:AAA4200
Cisseps fulvicollis|[5094]|BBLPC467-09|09BBELE-1467|658[0n]bp|Canada.New Brunswick|BOLD:AAA4200
Cisseps fulvicollis|[5095]|NCCH059-11|BIOUG01573-E11|673[0n]bp|Canada.Ontario|BOLD:AAA4200
Cisseps fulvicollis|[5096]|TMNBD425-07|MNBTT-3226|658[0n]bp|Canada.New Brunswick|BOLD:AAA4200
Cisseps fulvicollis|[5097]|XAK326-06|2006-ONT-1321|658[0n]bp|Canada.Ontario|BOLD:AAA4200
Cisseps fulvicollis|[5098]|XAG915-05|2005-ONT-1499|658[0n]bp|Canada.Ontario|BOLD:AAA4200
Cisseps fulvicollis|[5099]|PHMNB358-04|04HBL00584|658[0n]bp|Canada.New Brunswick|BOLD:AAA4200
Cisseps fulvicollis|[5100]|RDLQ764-07|DH007392|658[0n]bp|Canada.Quebec|BOLD:AAA4200
Cisseps fulvicollis|[5101]|BBLPE511-09|09BBELE-2511|658[0n]bp|Canada.Newfoundland and Labrador|BOLD:A...
Cisseps fulvicollis|[5102]|BBLPC020-09|09BBELE-1020|658[0n]bp|Canada.New Brunswick|BOLD:AAA4200
Cisseps fulvicollis|[5103]|MNBB629-05|05-NBSTA-545|658[0n]bp|Canada.New Brunswick|BOLD:AAA4200
Cisseps fulvicollis|[5104]|BBLPE451-09|09BBELE-2451|658[0n]bp|Canada.Newfoundland and Labrador|BOLD:A...
Cisseps fulvicollis|[5105]|MNBB634-05|05-NBSTA-550|658[0n]bp|Canada.New Brunswick|BOLD:AAA4200
Cisseps fulvicollis|[5106]|MNBB455-05|05-NBSTA-371|658[0n]bp|Canada.New Brunswick|BOLD:AAA4200
Cisseps fulvicollis|[5107]|MNBB633-05|05-NBSTA-549|658[0n]bp|Canada.New Brunswick|BOLD:AAA4200
Cisseps fulvicollis|[5108]|BBLPE540-09|09BBELE-2540|658[0n]bp|Canada.Newfoundland and Labrador|BOLD:A...
Cisseps fulvicollis|[5109]|TMNBD424-07|MNBTT-3225|658[0n]bp|Canada.New Brunswick|BOLD:AAA4200
Cisseps fulvicollis|[5110]|LPSOD166-08|08MZPP-017|656[0n]bp|Canada.Ontario|BOLD:AAA4200
Cisseps fulvicollis|[5111]|XAF630-05|2005-ONT-279|658[0n]bp|Canada.Ontario|BOLD:AAA4200
Cisseps fulvicollis|[5112]|PHMNB369-04|04HBL00595|658[0n]bp|Canada.New Brunswick|BOLD:AAA4200
Cisseps fulvicollis|[5113]|XAF703-05|2005-ONT-352|658[0n]bp|Canada.Ontario|BOLD:AAA4200
Cisseps fulvicollis|[5114]|MECC498-06|jflandry2518|658[0n]bp|Canada.Quebec|BOLD:AAA4200
Cisseps fulvicollis|[5115]|XAJ391-06|2006-ONT-0391|658[0n]bp|Canada.Ontario|BOLD:AAA4200
Cisseps fulvicollis|[5116]|PHMNB462-04|04HBL00688|658[0n]bp|Canada.New Brunswick|BOLD:AAA4200
Cisseps fulvicollis|[5117]|BBLEC494-09|09BBELE-0494|658[0n]bp|Canada.New Brunswick|BOLD:AAA4200
Cisseps fulvicollis|[5118]|TMNBD426-07|MNBTT-3227|658[0n]bp|Canada.New Brunswick|BOLD:AAA4200
Cisseps fulvicollis|[5119]|PHMNB473-04|04HBL00699|658[0n]bp|Canada.New Brunswick|BOLD:AAA4200
Cisseps fulvicollis|[5120]|NCCH061-11|BIOUG01573-F01|673[0n]bp|Canada.Ontario|BOLD:AAA4200
Cisseps fulvicollis|[5121]|BBLPE557-09|09BBELE-2557|658[0n]bp|Canada.Newfoundland and Labrador|BOLD:A...
Cisseps fulvicollis|[5122]|LOWCE077-06|CGWC-3837|658[0n]bp|Canada.British Columbia|BOLD:AAA4200
Cisseps fulvicollis|[5123]|MNBB673-05|05-NBSTA-589|658[0n]bp|Canada.New Brunswick|BOLD:AAA4200
Cisseps fulvicollis|[5124]|BBLPC025-09|09BBELE-1025|658[0n]bp|Canada.New Brunswick|BOLD:AAA4200
Cisseps fulvicollis|[5125]|TMNBD427-07|MNBTT-3228|658[0n]bp|Canada.New Brunswick|BOLD:AAA4200
Cisseps fulvicollis|[5126]|BBLPE500-09|09BBELE-2500|658[0n]bp|Canada.Newfoundland and Labrador|BOLD:A...
Cisseps fulvicollis|[5127]|BBLPC013-09|09BBELE-1013|658[0n]bp|Canada.New Brunswick|BOLD:AAA4200
Cisseps fulvicollis|[5128]|BLTIB1033-08|BL1475|658[0n]bp|Canada.Ontario|BOLD:AAA4200
Cisseps fulvicollis|[5129]|XAJ457-06|2006-ONT-0457|658[0n]bp|Canada.Ontario|BOLD:AAA4200
Cisseps fulvicollis|[5130]|LPABB320-08|08BBLEP-03585|658[0n]bp|Canada.Alberta|BOLD:AAA4200
Cisseps fulvicollis|[5131]|MNBB630-05|05-NBSTA-546|658[0n]bp|Canada.New Brunswick|BOLD:AAA4200
Cisseps fulvicollis|[5132]|BBLPC419-09|09BBELE-1419|658[0n]bp|Canada.New Brunswick|BOLD:AAA4200
Cisseps fulvicollis|[5133]|PHMNB605-04|04HBL00831|658[0n]bp|Canada.New Brunswick|BOLD:AAA4200
Cisseps fulvicollis|[5134]|LOWCE073-06|CGWC-3833|658[0n]bp|Canada.British Columbia|BOLD:AAA4200
Cisseps fulvicollis|[5135]|BBLPA604-10|10BBCLP-0604|658[0n]bp|Canada.Ontario|BOLD:AAA4200
Cisseps fulvicollis|[5136]|LPABB023-08|08BBLEP-03288|658[0n]bp|Canada.Alberta|BOLD:AAA4200
Cisseps fulvicollis|[5137]|XAG388-05|2005-ONT-972|658[0n]bp|Canada.Ontario|BOLD:AAA4200
Cisseps fulvicollis|[5138]|MNBB674-05|05-NBSTA-590|658[0n]bp|Canada.New Brunswick|BOLD:AAA4200
Cisseps fulvicollis|[5139]|MNBB675-05|05-NBSTA-591|658[0n]bp|Canada.New Brunswick|BOLD:AAA4200
Cisseps fulvicollis|[5140]|MNBB631-05|05-NBSTA-547|658[0n]bp|Canada.New Brunswick|BOLD:AAA4200
Cisseps fulvicollis|[5141]|LPABC671-09|08BBLEP-04890|658[0n]bp|Canada.Alberta|BOLD:AAA4200
Cisseps fulvicollis|[5142]|LPMN874-08|08BBLEP-02232|658[0n]bp|Canada.Alberta|BOLD:AAA4200
Cisseps fulvicollis|[5143]|LPMN870-08|08BBLEP-02228|652[0n]bp|Canada.Alberta|BOLD:AAA4200
Cisseps fulvicollis|[5144]|TMG90-03|moth611.01|639[0n]bp|Canada.Ontario|BOLD:AAA4200
Cisseps fulvicollis|[5145]|BBLEC501-09|09BBELE-0501|636[0n]bp|Canada.New Brunswick|BOLD:AAA4200
Cisseps fulvicollis|[5146]|BBLPC981-09|09BBELE-1981|658[0n]bp|Canada.Newfoundland and Labrador|BOLD:A...
Cisseps fulvicollis|[5147]|BBLPC715-09|09BBELE-1715|658[0n]bp|Canada.Newfoundland and Labrador|BOLD:A...
Cisseps fulvicollis|[5148]|BBLPA888-10|10BBCLP-0888|658[0n]bp|Canada.Alberta|BOLD:AAA4200
Cisseps fulvicollis|[5149]|BBLPC785-09|09BBELE-1785|658[0n]bp|Canada.Newfoundland and Labrador|BOLD:A...
Cisseps fulvicollis|[5150]|BBLPE489-09|09BBELE-2489|658[0n]bp|Canada.Newfoundland and Labrador|BOLD:A...
Cisseps fulvicollis|[5151]|BBLPE497-09|09BBELE-2497|635[0n]bp|Canada.Newfoundland and Labrador|BOLD:A...
Cisseps fulvicollis|[5152]|PMG001-03|moth599.01|617[0n]bp|Canada.Ontario|BOLD:AAA4200
Cisseps fulvicollis|[5153]|BBLPC637-09|09BBELE-1637|658[0n]bp|Canada.Newfoundland and Labrador|BOLD:A...
Cisseps fulvicollis|[5154]|TMNBD428-07|MNBTT-3229|658[0n]bp|Canada.New Brunswick|BOLD:AAA4200
Hyperstrotia secta|[5155]|LMEMB437-09|RBMIS-1530|658[0n]bp|United States.Tennessee|BOLD:AAA8828
[illegible]

Cisseps fulvicollis|[5153]|BBLPC637-09|09BBELE-1637|658[0n]bp|Canada.Newfoundland and Labrador|BOLD:A...
Cisseps fulvicollis|[5154]|TMNBD428-07|MNBTT-3229|658[0n]bp|Canada.New Brunswick|BOLD:AAA4200
Hyperstrotia secta|[5155]|LMEMB437-09|RBMIS-1530|658[0n]bp|United States.Tennessee|BOLD:AAA8828
Scoliopteryx libatrix|[5156]|LBCG463-08|08-JDWBC-0463|658[0n]bp|Canada.British Columbia|BOLD:ACE7769
Scoliopteryx libatrix|[5157]|LBCH5277-10|10-JDWBC-5277|658[0n]bp|Canada.British Columbia|BOLD:ACE7769
Scoliopteryx libatrix|[5158]|LBCH5341-10|10-JDWBC-5341|658[0n]bp|Canada.British Columbia|BOLD:ACE7769
Scoliopteryx libatrix|[5159]|LOWCB624-05|CGWC-1564|608[0n]bp|Canada.British Columbia|BOLD:ACE7769
Scoliopteryx libatrix|[5160]|RDLQH065-06|DH013302|658[0n]bp|Canada.Quebec|BOLD:AAB0295
Scoliopteryx libatrix|[5161]|RDLQG268-06|DH012476|658[0n]bp|Canada.Quebec|BOLD:AAB0295
Scoliopteryx libatrix|[5162]|LBCS117-07|UBC-2007-0108|658[0n]bp|Canada.British Columbia|BOLD:AAB0295
Scoliopteryx libatrix|[5163]|XAF435-05|HLC-10476|658[0n]bp|Canada.Ontario|BOLD:AAB0295
Scoliopteryx libatrix|[5164]|BLTIB565-08|BL843|658[0n]bp|Canada.Ontario|BOLD:AAB0295
Scoliopteryx libatrix|[5165]|BBLPB500-10|10BBCLP-1499|658[0n]bp|Canada.Ontario|BOLD:AAB0295
Scoliopteryx libatrix|[5166]|LBCA029-05|HLC-20029|658[0n]bp|Canada.British Columbia|BOLD:AAB0295
Scoliopteryx libatrix|[5167]|BBLPB502-10|10BBCLP-1501|658[0n]bp|Canada.British Columbia|BOLD:AAB0295
Scoliopteryx libatrix|[5168]|LMIS013-05|05-ONMIS-0013|658[0n]bp|Canada.Ontario|BOLD:AAB0295
Scoliopteryx libatrix|[5169]|LOWCE112-06|CGWC-3872|658[0n]bp|Canada.British Columbia|BOLD:AAB0295
Scoliopteryx libatrix|[5170]|BBLPB501-10|10BBCLP-1500|658[0n]bp|Canada.Saskatchewan|BOLD:AAB0295
Scoliopteryx libatrix|[5171]|BBLPC635-09|09BBELE-1635|658[0n]bp|Canada.Newfoundland and Labrador|BOLD ...
Scoliopteryx libatrix|[5172]|RDLQH068-06|DH013305|646[0n]bp|Canada.Quebec|BOLD:AAB0295
Scoliopteryx libatrix|[5173]|RDLQH064-06|DH013301|646[0n]bp|Canada.Quebec|BOLD:AAB0295
Scoliopteryx libatrix|[5174]|RDLQH067-06|DH013304|648[0n]bp|Canada.Quebec|BOLD:AAB0295
Scoliopteryx libatrix|[5175]|LBCS019-07|UBC-2007-0021|658[0n]bp|Canada.British Columbia|BOLD:AAB0295
Scoliopteryx libatrix|[5176]|RDLQH066-06|DH013303|658[0n]bp|Canada.Quebec|BOLD:AAB0295
Nigetia formosalis|[5177]|BLTIB874-08|BL1293|658[0n]bp|Canada.Ontario|BOLD:AAC1716
Phobolosia anfracta|[5178]|LBCH7700-10|10-JDWBC-7700|658[0n]bp|Canada.British Columbia|BOLD:AAL6037
Phobolosia anfracta|[5179]|LBCH6722-10|10-JDWBC-6722|658[0n]bp|Canada.British Columbia|BOLD:AAL6037
Hypena atomaria|[5180]|BLTIB272-08|BL456|658[1n]bp|Canada.Ontario|BOLD:ACF1119
Hypena atomaria|[5181]|RDNMG592-08|CNC LEP00052416|658[0n]bp|Canada.Ontario|BOLD:ACF1119
Hypena atomaria|[5182]|RDMAB960-09|UASM99713|622[1n]bp|Canada.Alberta|BOLD:ACF1119
Hypena atomaria|[5183]|RDMAB958-09|UASM7089|625[0n]bp|Canada.Alberta|BOLD:ACF1119
Hypena atomaria|[5184]|RDMAB959-09|UASM99712|584[0n]bp|Canada.Alberta|BOLD:ACF1119
Hypena atomaria|[5185]|RDLQH126-06|AC000689|600[0n]bp|Canada.Quebec|BOLD:ACF1119
Hypena edictalis|[5186]|RDLQH129-06|DH006286|617[2n]bp|Canada.Quebec|BOLD:ACI5170
Hypena edictalis|[5187]|LPABC680-09|08BBLEP-04899|658[0n]bp|Canada.Alberta|BOLD:AAB3069
Hypena edictalis|[5188]|LPMNB260-09|08BBLEP-05104|613[0n]bp|Canada.Manitoba|BOLD:AAB3069
Hypena edictalis|[5189]|BBLPB742-10|10BBCLP-1741|658[0n]bp|Canada.Alberta|BOLD:AAB3069
Hypena edictalis|[5190]|LPMNB262-09|08BBLEP-05106|658[0n]bp|Canada.Manitoba|BOLD:AAB3069
Hypena edictalis|[5191]|LPMNB261-09|08BBLEP-05105|658[0n]bp|Canada.Manitoba|BOLD:AAB3069
Hypena edictalis|[5192]|RDLQH131-06|DH005429|605[0n]bp|Canada.Quebec|BOLD:AAB3069
Hypena edictalis|[5193]|RDLQH130-06|DH006880|606[5n]bp|Canada.Quebec|BOLD:AAB3069
Hypena edictalis|[5194]|BBLPB437-10|10BBCLP-1436|658[0n]bp|Canada.Alberta|BOLD:AAB3069
Hypena californica|[5195]|LOWCE528-06|CGWC-4288|572[0n]bp|Canada.British Columbia|BOLD:AAB4369
Hypena californica|[5196]|LHLEP343-06|UBC-2006-0593|655[0n]bp|Canada.British Columbia|BOLD:AAB4369
Hypena californica|[5197]|LALPA409-10|AVBC 411-10|658[0n]bp|Canada.British Columbia|BOLD:AAB4369
Hypena californica|[5198]|LOWCE503-06|CGWC-4263|658[0n]bp|Canada.British Columbia|BOLD:AAB4369
Hypena californica|[5199]|LOWCE502-06|CGWC-4262|658[0n]bp|Canada.British Columbia|BOLD:AAB4369
Hypena californica|[5200]|LOWCE481-06|CGWC-4241|658[0n]bp|Canada.British Columbia|BOLD:AAB4369
Hypena californica|[5201]|LOWCE504-06|CGWC-4264|606[0n]bp|Canada.British Columbia|BOLD:AAB4369
Hypena californica|[5202]|LOWCE385-06|CGWC-4145|599[0n]bp|Canada.British Columbia|BOLD:AAB4369
Hypena californica|[5203]|LHLEP654-06|UBC-2006-0794|658[0n]bp|Canada.British Columbia|BOLD:AAB4369
Hypena californica|[5204]|LHLEP655-06|UBC-2006-0951|658[0n]bp|Canada.British Columbia|BOLD:AAB4369
Hypena californica|[5205]|LALPA026-10|AVBC 026-10|658[0n]bp|Canada.British Columbia|BOLD:AAB4369
Hypena californica|[5206]|LHLEP342-06|UBC-2006-0592|657[0n]bp|Canada.British Columbia|BOLD:AAB4369
Hypena californica|[5207]|LOWCE400-06|CGWC-4160|615[0n]bp|Canada.British Columbia|BOLD:AAB4369
Hypena californica|[5208]|LOWCB626-05|CGWC-1566|606[0n]bp|Canada.British Columbia|BOLD:AAB4369
Hypena californica|[5209]|LOWCE509-06|CGWC-4269|610[0n]bp|Canada.British Columbia|BOLD:AAB4369
Hypena californica|[5210]|LOWCE505-06|CGWC-4265|601[0n]bp|Canada.British Columbia|BOLD:AAB4369
Hypena californica|[5211]|LOWCB625-05|CGWC-1565|612[0n]bp|Canada.British Columbia|BOLD:AAB4369
Hypena californica|[5212]|LOWCE399-06|CGWC-4159|617[0n]bp|Canada.British Columbia|BOLD:AAB4369
Hypena californica|[5213]|LOWCE510-06|CGWC-4270|611[0n]bp|Canada.British Columbia|BOLD:AAB4369
Hypena californica|[5214]|LALPA429-10|AVBC 431-10|658[0n]bp|Canada.British Columbia|BOLD:AAB4369
Hypena californica|[5215]|LHLEP344-06|UBC-2006-0594|657[0n]bp|Canada.British Columbia|BOLD:AAB4369
Hypena californica|[5216]|LBCF007-07|07-JDWBC-0018|618[0n]bp|Canada.British Columbia|BOLD:AAB4369
Hypena californica|[5217]|LHLEP345-06|UBC-2006-1410|657[0n]bp|Canada.British Columbia|BOLD:AAB4369
Hypena californica|[5218]|LHLEP341-06|UBC-2006-0591|657[0n]bp|Canada.British Columbia|BOLD:AAB4369
Hypena minualis|[5219]|RDNMD533-06|CNCNoctuoidea12865|658[0n]bp|United States.Florida|BOLD:AAE1423
Hypena minualis|[5220]|ABNCC124-07|1123-270503-TX|642[0n]bp|United States.Texas|BOLD:AAE1423
Hypena minualis|[5221]|LPOKB013-09|MDOK-1526|658[0n]bp|United States.Oklahoma|BOLD:AAE1423
Hypena minualis|[5222]|LPOKB552-09|MDOK-1594|658[0n]bp|United States.Oklahoma|BOLD:AAE1423
Hypena scabra|[5223]|LPSOC325-08|PPBP-2324|648[0n]bp|Canada.Ontario|BOLD:AAA4222
Hypena scabra|[5224]|LPSOC347-08|PPBP-2346|656[0n]bp|Canada.Ontario|BOLD:AAA4222
Hypena scabra|[5225]|BLTIB870-08|BL1289|658[0n]bp|Canada.Ontario|BOLD:AAA4222
Hypena scabra|[5226]|BLTIB842-08|BL1261|658[0n]bp|Canada.Ontario|BOLD:AAA4222
Hypena scabra|[5227]|XAB543-04|04HBL005543|658[0n]bp|Canada.Ontario|BOLD:AAA4222
Hypena scabra|[5228]|XAH877-05|2005-ONT-2460|658[0n]bp|Canada.Ontario|BOLD:AAA4222
Hypena scabra|[5229]|BLGSM007-09|BL1616|658[0n]bp|Canada.Ontario|BOLD:AAA4222
Hypena scabra|[5230]|RDLQH024-06|DH013261|656[0n]bp|Canada.Quebec|BOLD:AAA4222
Hypena scabra|[5231]|LPSOC391-08|PPBP-2390|658[0n]bp|Canada.Ontario|BOLD:AAA4222
Hypena scabra|[5232]|XAH666-05|2005-ONT-2249|658[0n]bp|Canada.Ontario|BOLD:AAA4222
Hypena scabra|[5233]|RDLQF210-06|DH011290|658[0n]bp|Canada.Quebec|BOLD:AAA4222
Hypena scabra|[5234]|LPSOC393-08|PPBP-2392|658[0n]bp|Canada.Ontario|BOLD:AAA4222
Hypena scabra|[5235]|RDLQH027-06|DH013264|656[0n]bp|Canada.Quebec|BOLD:AAA4222
Hypena scabra|[5236]|XAB792-05|04HBL005792|658[0n]bp|Canada.Ontario|BOLD:AAA4222
Hypena scabra|[5237]|XAH846-05|2005-ONT-2429|658[0n]bp|Canada.Ontario|BOLD:AAA4222
Hypena scabra|[5238]|XAH840-05|2005-ONT-2423|658[0n]bp|Canada.Ontario|BOLD:AAA4222
Hypena scabra|[5239]|XAH821-05|2005-ONT-2404|658[0n]bp|Canada.Ontario|BOLD:AAA4222
Hypena scabra|[5240]|RDLQG062-06|DH012198|658[0n]bp|Canada.Quebec|BOLD:AAA4222
Hypena scabra|[5241]|BLTIB871-08|BL1290|658[0n]bp|Canada.Ontario|BOLD:AAA4222
Hypena scabra|[5242]|BLTIB923-08|BL1343|658[0n]bp|Canada.Ontario|BOLD:AAA4222
Hypena scabra|[5243]|RDLQF202-06|DH011259|658[0n]bp|Canada.Quebec|BOLD:AAA4222
Hypena scabra|[5244]|XAK282-06|2006-ONT-1277|658[0n]bp|Canada.Ontario|BOLD:AAA4222
Hypena scabra|[5245]|XAH825-05|2005-ONT-2408|658[0n]bp|Canada.Ontario|BOLD:AAA4222
Hypena scabra|[5246]|PHMNB270-04|04HBL007735|658[0n]bp|Canada.New Brunswick|BOLD:AAA4222
Hypena scabra|[5247]|XAH874-05|2005-ONT-2457|658[0n]bp|Canada.Ontario|BOLD:AAA4222
Hypena scabra|[5248]|LPSOC374-08|PPBP-2373|652[0n]bp|Canada.Ontario|BOLD:AAA4222
Hypena scabra|[5249]|XAB684-04|04HBL005684|642[0n]bp|Canada.Ontario|BOLD:AAA4222
Hypena scabra|[5250]|PHMO206-03|moth1055.01|639[2n]bp|Canada.Ontario|BOLD:AAA4222
Hypena scabra|[5251]|XAD269-04|04HBL007269|595[0n]bp|Canada.Ontario|BOLD:AAA4222
Hypena scabra|[5252]|PHMNB288-04|04HBL007753|605[0n]bp|Canada.New Brunswick|BOLD:AAA4222
Hypena scabra|[5253]|PHMNB277-04|04HBL007742|594[4n]bp|Canada.New Brunswick|BOLD:AAA4222
Hypena scabra|[5254]|XAD005-04|04HBL007005|554[0n]bp|Canada.Ontario|BOLD:AAA4222
Hypena scabra|[5255]|RDLQF554-06|DH011703|658[0n]bp|Canada.Quebec|BOLD:AAA4222

Hypena scabra|[5252]|PHMNB2[illegible]-04|04HBL007[illegible]|[illegible]bp|Canada.New Brunswick|BOLD:AAA4222
Hypena scabra|[5253]|PHMNB277-04|04HBL007742|594[4n]bp|Canada.New Brunswick|BOLD:AAA4222
Hypena scabra|[5254]|XAD005-04|04HBL007005|554[0n]bp|Canada.Ontario|BOLD:AAA4222
Hypena scabra|[5255]|RDLQF554-06|DH011703|658[0n]bp|Canada.Quebec|BOLD:AAA4222
Hypena scabra|[5256]|RDLQF201-06|DH011258|658[0n]bp|Canada.Quebec|BOLD:AAA4222
Hypena scabra|[5257]|LPSOC348-08|PPBP-2347|658[0n]bp|Canada.Ontario|BOLD:AAA4222
Hypena scabra|[5258]|XAD465-04|04HBL007465|658[0n]bp|Canada.Ontario|BOLD:AAA4222
Hypena scabra|[5259]|LPSOC349-08|PPBP-2348|658[0n]bp|Canada.Ontario|BOLD:AAA4222
Hypena scabra|[5260]|XAE151-04|Moth4151.03|658[0n]bp|Canada.Ontario|BOLD:AAA4222
Hypena scabra|[5261]|XAH753-05|2005-ONT-2336|658[0n]bp|Canada.Ontario|BOLD:AAA4222
Hypena scabra|[5262]|RDLQH025-06|DH013262|658[0n]bp|Canada.Quebec|BOLD:AAA4222
Hypena scabra|[5263]|XAB793-05|04HBL005793|658[0n]bp|Canada.Ontario|BOLD:AAA4222
Hypena scabra|[5264]|RDLQH026-06|DH013263|658[0n]bp|Canada.Quebec|BOLD:AAA4222
Hypena scabra|[5265]|XAH737-05|2005-ONT-2320|658[0n]bp|Canada.Ontario|BOLD:AAA4222
Hypena scabra|[5266]|XAH838-05|2005-ONT-2421|658[0n]bp|Canada.Ontario|BOLD:AAA4222
Hypena scabra|[5267]|XAB541-04|04HBL005541|658[0n]bp|Canada.Ontario|BOLD:AAA4222
Hypena scabra|[5268]|PHMNB294-04|04HBL007759|658[0n]bp|Canada.New Brunswick|BOLD:AAA4222
Hypena scabra|[5269]|XAJ160-06|2006-ONT-0160|658[0n]bp|Canada.Ontario|BOLD:AAA4222
Hypena scabra|[5270]|XAH875-05|2005-ONT-2458|658[0n]bp|Canada.Ontario|BOLD:AAA4222
Hypena scabra|[5271]|XAH801-05|2005-ONT-2384|658[0n]bp|Canada.Ontario|BOLD:AAA4222
Hypena scabra|[5272]|LPSOC373-08|PPBP-2372|656[0n]bp|Canada.Ontario|BOLD:AAA4222
Hypena scabra|[5273]|BLTIB1012-08|BL1451|658[0n]bp|Canada.Ontario|BOLD:AAA4222
Hypena scabra|[5274]|LPSOC372-08|PPBP-2371|656[0n]bp|Canada.Ontario|BOLD:AAA4222
Hypena scabra|[5275]|BLTIB512-08|BL771|658[0n]bp|Canada.Ontario|BOLD:AAA4222
Hypena scabra|[5276]|XAH842-05|2005-ONT-2425|658[0n]bp|Canada.Ontario|BOLD:AAA4222
Hypena scabra|[5277]|MNAF372-08|CNCLEP00040357|658[0n]bp|Canada.Manitoba|BOLD:AAA4222
Hypena scabra|[5278]|PHMO075-03|moth495.01|639[0n]bp|Canada.Ontario|BOLD:AAA4222
Hypena scabra|[5279]|PHMNB036-03|moth204.02SA|639[0n]bp|Canada.New Brunswick|BOLD:AAA4222
Hypena scabra|[5280]|PHMNB279-04|04HBL007744|573[0n]bp|Canada.New Brunswick|BOLD:AAA4222
Hypena scabra|[5281]|XAB668-04|04HBL005668|616[0n]bp|Canada.Ontario|BOLD:AAA4222
Hypena scabra|[5282]|XAB524-04|04HBL005524|582[1n]bp|Canada.Ontario|BOLD:AAA4222
Hypena scabra|[5283]|XAH711-05|2005-ONT-2294|622[0n]bp|Canada.Ontario|BOLD:AAA4222
Hypena scabra|[5284]|LPSOC364-08|PPBP-2363|653[0n]bp|Canada.Ontario|BOLD:AAA4222
Hypena baltimoralis|[5285]|RDLQG350-06|DH012567|658[0n]bp|Canada.Quebec|BOLD:AAA2330
Hypena baltimoralis|[5286]|RDLQG356-06|DH012590|658[0n]bp|Canada.Quebec|BOLD:AAA2330
Hypena baltimoralis|[5287]|XAC124-04|04HBL006124|554[2n]bp|Canada.Ontario|BOLD:AAA2330
Hypena baltimoralis|[5288]|RDLQF880-06|DH012052|587[3n]bp|Canada.Quebec|BOLD:AAA2330
Hypena baltimoralis|[5289]|BBLEC641-09|09BBELE-0641|658[0n]bp|Canada.Nova Scotia|BOLD:AAA2330
Hypena baltimoralis|[5290]|PHMNB108-04|04HBL007573|626[1n]bp|Canada.New Brunswick|BOLD:AAA2330
Hypena baltimoralis|[5291]|RDLQF326-06|DH011485|658[0n]bp|Canada.Quebec|BOLD:AAA2330
Hypena baltimoralis|[5292]|PHMNB179-04|04HBL007644|505[0n]bp|Canada.New Brunswick|
Hypena baltimoralis|[5293]|PHMO312-03|moth2326.02|639[0n]bp|Canada.Ontario|BOLD:AAA2330
Hypena baltimoralis|[5294]|RDLQF888-06|DH012063|625[0n]bp|Canada.Quebec|BOLD:AAA2330
Hypena baltimoralis|[5295]|PHMO321-03|moth2439.02|639[0n]bp|Canada.Ontario|BOLD:AAA2330
Hypena baltimoralis|[5296]|RDLQF486-06|DH011635|646[0n]bp|Canada.Quebec|BOLD:AAA2330
Hypena baltimoralis|[5297]|TTMNB088-06|MNBTT-088|656[0n]bp|Canada.New Brunswick|BOLD:AAA2330
Hypena baltimoralis|[5298]|RDLQF195-06|DH011252|658[0n]bp|Canada.Quebec|BOLD:AAA2330
Hypena baltimoralis|[5299]|RDLQF431-06|DH011538|658[0n]bp|Canada.Quebec|BOLD:AAA2330
Hypena baltimoralis|[5300]|RDLQF325-06|DH011484|658[0n]bp|Canada.Quebec|BOLD:AAA2330
Hypena baltimoralis|[5301]|RDLQF396-06|DH011463|658[0n]bp|Canada.Quebec|BOLD:AAA2330
Hypena baltimoralis|[5302]|RDLQF397-06|DH011464|658[0n]bp|Canada.Quebec|BOLD:AAA2330
Hypena baltimoralis|[5303]|RDLQF760-06|DH011910|658[0n]bp|Canada.Quebec|BOLD:AAA2330
Hypena baltimoralis|[5304]|RDLQF490-06|DH011639|658[0n]bp|Canada.Quebec|BOLD:AAA2330
Hypena baltimoralis|[5305]|RDLQF196-06|DH011253|658[0n]bp|Canada.Quebec|BOLD:AAA2330
Hypena baltimoralis|[5306]|RDLQG352-06|DH012569|658[0n]bp|Canada.Quebec|BOLD:AAA2330
Hypena baltimoralis|[5307]|RDLQF316-06|DH011475|658[0n]bp|Canada.Quebec|BOLD:AAA2330
Hypena baltimoralis|[5308]|RDLQF483-06|DH011632|658[0n]bp|Canada.Quebec|BOLD:AAA2330
Hypena baltimoralis|[5309]|RDLQF399-06|DH011466|658[0n]bp|Canada.Quebec|BOLD:AAA2330
Hypena baltimoralis|[5310]|RDLQF393-06|DH011460|658[0n]bp|Canada.Quebec|BOLD:AAA2330
Hypena baltimoralis|[5311]|RDLQG238-06|DH012443|658[0n]bp|Canada.Quebec|BOLD:AAA2330
Hypena baltimoralis|[5312]|RDLQF405-06|DH011472|658[0n]bp|Canada.Quebec|BOLD:AAA2330
Hypena baltimoralis|[5313]|TTMNB274-06|MNBTT-274|658[0n]bp|Canada.New Brunswick|BOLD:AAA2330
Hypena baltimoralis|[5314]|TTMNB089-06|MNBTT-089|657[1n]bp|Canada.New Brunswick|BOLD:AAA2330
Hypena baltimoralis|[5315]|RDLQF484-06|DH011633|658[0n]bp|Canada.Quebec|BOLD:AAA2330
Hypena baltimoralis|[5316]|BBLPC138-09|09BBELE-1138|628[0n]bp|Canada.Nova Scotia|BOLD:AAA2330
Hypena baltimoralis|[5317]|RDLQF758-06|DH011908|621[0n]bp|Canada.Quebec|BOLD:AAA2330
Hypena baltimoralis|[5318]|RDLQF753-06|DH011903|576[2n]bp|Canada.Quebec|BOLD:AAA2330
Hypena baltimoralis|[5319]|RDLQB771-05|DH010858|617[0n]bp|Canada.Quebec|BOLD:AAA2330
Hypena baltimoralis|[5320]|RDLQG244-06|DH012449|582[0n]bp|Canada.Quebec|BOLD:AAA2330
Hypena baltimoralis|[5321]|TTMNB271-06|MNBTT-271|658[0n]bp|Canada.New Brunswick|BOLD:AAA2330
Hypena baltimoralis|[5322]|RDLQF426-06|DH011533|645[0n]bp|Canada.Quebec|BOLD:AAA2330
Hypena baltimoralis|[5323]|RDLQF485-06|DH011634|618[0n]bp|Canada.Quebec|BOLD:AAA2330
Hypena baltimoralis|[5324]|PHMNB048-03|moth233.02SA|639[0n]bp|Canada.New Brunswick|BOLD:AAA2330
Hypena baltimoralis|[5325]|XAD300-04|04HBL007300|567[1n]bp|Canada.Ontario|BOLD:AAA2330
Hypena baltimoralis|[5326]|RDLQF320-06|DH011479|567[0n]bp|Canada.Quebec|BOLD:AAA2330
Hypena baltimoralis|[5327]|RDLQG311-06|DH012523|594[0n]bp|Canada.Quebec|BOLD:AAA2330
Hypena baltimoralis|[5328]|RDLQF402-06|DH011469|637[0n]bp|Canada.Quebec|BOLD:AAA2330
Hypena baltimoralis|[5329]|RDLQF197-06|DH011254|658[0n]bp|Canada.Quebec|BOLD:AAA2330
Hypena baltimoralis|[5330]|RDLQG223-06|DH012406|608[0n]bp|Canada.Quebec|BOLD:AAA2330
Hypena baltimoralis|[5331]|TTMNB272-06|MNBTT-272|608[0n]bp|Canada.New Brunswick|BOLD:AAA2330
Hypena baltimoralis|[5332]|RDLQF889-06|DH012064|593[0n]bp|Canada.Quebec|BOLD:AAA2330
Hypena baltimoralis|[5333]|RDLQG240-06|DH012445|658[0n]bp|Canada.Quebec|BOLD:AAA2330
Hypena baltimoralis|[5334]|RDLQG245-06|DH012450|594[0n]bp|Canada.Quebec|BOLD:AAA2330
Hypena baltimoralis|[5335]|RDLQG061-06|DH012197|655[0n]bp|Canada.Quebec|BOLD:AAA2330
Hypena baltimoralis|[5336]|RDLQG241-06|DH012446|594[0n]bp|Canada.Quebec|BOLD:AAA2330
Hypena baltimoralis|[5337]|RDLQG141-06|DH012312|658[3n]bp|Canada.Quebec|BOLD:AAA2330
Hypena baltimoralis|[5338]|RDLQF754-06|DH011904|659[0n]bp|Canada.Quebec|BOLD:AAA2330
Hypena baltimoralis|[5339]|RDLQF894-06|DH012069|658[0n]bp|Canada.Quebec|BOLD:AAA2330
Hypena baltimoralis|[5340]|BBLPC179-09|09BBELE-1179|658[0n]bp|Canada.Nova Scotia|BOLD:AAA2330
Hypena baltimoralis|[5341]|RDLQF491-06|DH011640|658[0n]bp|Canada.Quebec|BOLD:AAA2330
Hypena baltimoralis|[5342]|TMNBB062-06|MNBTT-1002|658[0n]bp|Canada.New Brunswick|BOLD:AAA2330
Hypena baltimoralis|[5343]|RDLQF327-06|DH011486|658[0n]bp|Canada.Quebec|BOLD:AAA2330
Hypena baltimoralis|[5344]|RDLQF398-06|DH011465|658[0n]bp|Canada.Quebec|BOLD:AAA2330
Hypena baltimoralis|[5345]|RDLQG273-06|DH012485|658[0n]bp|Canada.Quebec|BOLD:AAA2330
Hypena baltimoralis|[5346]|RDLQG309-06|DH012521|658[0n]bp|Canada.Quebec|BOLD:AAA2330
Hypena baltimoralis|[5347]|RDLQG348-06|DH012565|658[0n]bp|Canada.Quebec|BOLD:AAA2330
Hypena baltimoralis|[5348]|TTMNB547-06|MNBTT-547|658[0n]bp|Canada.New Brunswick|BOLD:AAA2330
Hypena baltimoralis|[5349]|RDLQG357-06|DH012591|658[0n]bp|Canada.Quebec|BOLD:AAA2330
Hypena baltimoralis|[5350]|XAG797-05|2005-ONT-1381|658[0n]bp|Canada.Ontario|BOLD:AAA2330
Hypena baltimoralis|[5351]|RDLQF318-06|DH011477|658[0n]bp|Canada.Quebec|BOLD:AAA2330
Hypena baltimoralis|[5352]|RDLQF323-06|DH011482|658[0n]bp|Canada.Quebec|BOLD:AAA2330
Hypena baltimoralis|[5353]|RDLQG239-06|DH012444|658[0n]bp|Canada.Quebec|BOLD:AAA2330
Hypena baltimoralis|[5354]|RDLQF482-06|DH011631|658[0n]bp|Canada.Quebec|BOLD:AAA2330

[illegible]
Hypena baltimoralis|[5352]|RDLQF323-06|DH011482|658[0n]bp|Canada.Quebec|BOLD:AAA2330
Hypena baltimoralis|[5353]|RDLQG239-06|DH012444|658[0n]bp|Canada.Quebec|BOLD:AAA2330
Hypena baltimoralis|[5354]|RDLQF482-06|DH011631|658[0n]bp|Canada.Quebec|BOLD:AAA2330
Hypena baltimoralis|[5355]|RDLQF227-06|DH011307|658[0n]bp|Canada.Quebec|BOLD:AAA2330
Hypena baltimoralis|[5356]|RDLQF394-06|DH011461|658[0n]bp|Canada.Quebec|BOLD:AAA2330
Hypena baltimoralis|[5357]|RDLQF403-06|DH011470|658[0n]bp|Canada.Quebec|BOLD:AAA2330
Hypena baltimoralis|[5358]|RDLQF429-06|DH011536|658[0n]bp|Canada.Quebec|BOLD:AAA2330
Hypena baltimoralis|[5359]|RDLQF897-06|DH012072|658[0n]bp|Canada.Quebec|BOLD:AAA2330
Hypena baltimoralis|[5360]|XAK280-06|2006-ONT-1275|658[0n]bp|Canada.Ontario|BOLD:AAA2330
Hypena baltimoralis|[5361]|RDLQF851-06|DH012004|658[0n]bp|Canada.Quebec|BOLD:AAA2330
Hypena baltimoralis|[5362]|RDLQF489-06|DH011638|658[0n]bp|Canada.Quebec|BOLD:AAA2330
Hypena baltimoralis|[5363]|RDLQF759-06|DH011909|658[0n]bp|Canada.Quebec|BOLD:AAA2330
Hypena baltimoralis|[5364]|BBLPC443-09|09BBELE-1443|658[0n]bp|Canada.New Brunswick|BOLD:AAA2330
Hypena baltimoralis|[5365]|BBLEC103-09|09BBELE-0103|658[0n]bp|Canada.Nova Scotia|BOLD:AAA2330
Hypena baltimoralis|[5366]|RDLQF395-06|DH011462|658[0n]bp|Canada.Quebec|BOLD:AAA2330
Hypena baltimoralis|[5367]|BBLPE257-09|09BBELE-2257|658[0n]bp|Canada.Nova Scotia|BOLD:AAA2330
Hypena baltimoralis|[5368]|RDLQG216-06|DH012392|658[0n]bp|Canada.Quebec|BOLD:AAA2330
Hypena baltimoralis|[5369]|RDLQF194-06|DH011251|658[0n]bp|Canada.Quebec|BOLD:AAA2330
Hypena baltimoralis|[5370]|RDLQF481-06|DH011630|658[0n]bp|Canada.Quebec|BOLD:AAA2330
Hypena baltimoralis|[5371]|BBLEC883-09|09BBELE-0883|658[0n]bp|Canada.Nova Scotia|BOLD:AAA2330
Hypena baltimoralis|[5372]|RDLQF895-06|DH012070|658[0n]bp|Canada.Quebec|BOLD:AAA2330
Hypena baltimoralis|[5373]|RDLQF404-06|DH011471|658[0n]bp|Canada.Quebec|BOLD:AAA2330
Hypena baltimoralis|[5374]|RDLQF315-06|DH011474|658[0n]bp|Canada.Quebec|BOLD:AAA2330
Hypena baltimoralis|[5375]|RDLQF893-06|DH012068|658[0n]bp|Canada.Quebec|BOLD:AAA2330
Hypena baltimoralis|[5376]|RDLQF755-06|DH011905|658[0n]bp|Canada.Quebec|BOLD:AAA2330
Hypena baltimoralis|[5377]|TMNBB063-06|MNBTT-1003|658[0n]bp|Canada.New Brunswick|BOLD:AAA2330
Hypena baltimoralis|[5378]|LPSOC139-08|PPBP-2138|658[0n]bp|Canada.Ontario|BOLD:AAA2330
Hypena baltimoralis|[5379]|RDLQF319-06|DH011478|620[0n]bp|Canada.Quebec|BOLD:AAA2330
Hypena baltimoralis|[5380]|RDLQF762-06|DH011912|605[3n]bp|Canada.Quebec|BOLD:AAA2330
Hypena baltimoralis|[5381]|BBLPE171-09|09BBELE-2171|658[0n]bp|Canada.Nova Scotia|BOLD:AAA2330
Hypena baltimoralis|[5382]|RDLQF314-06|DH011473|658[0n]bp|Canada.Quebec|BOLD:AAA2330
Hypena baltimoralis|[5383]|RDLQF896-06|DH012071|594[0n]bp|Canada.Quebec|BOLD:AAA2330
Hypena baltimoralis|[5384]|PHMNB097-04|04HBL007562|590[0n]bp|Canada.New Brunswick|BOLD:AAA2330
Hypena baltimoralis|[5385]|RDLQG312-06|DH012524|583[0n]bp|Canada.Quebec|BOLD:AAA2330
Hypena baltimoralis|[5386]|TTMNB273-06|MNBTT-273|627[0n]bp|Canada.New Brunswick|BOLD:AAA2330
Hypena baltimoralis|[5387]|RDLQF890-06|DH012065|584[0n]bp|Canada.Quebec|BOLD:AAA2330
Hypena baltimoralis|[5388]|RDLQF891-06|DH012066|582[0n]bp|Canada.Quebec|BOLD:AAA2330
Hypena baltimoralis|[5389]|PHMNB361-04|04HBL00587|658[0n]bp|Canada.New Brunswick|BOLD:AAA2330
Hypena baltimoralis|[5390]|RDLQG351-06|DH012568|658[0n]bp|Canada.Quebec|BOLD:AAA2330
Hypena baltimoralis|[5391]|LPSO660-08|PPBP-0660|656[0n]bp|Canada.Ontario|BOLD:AAA2330
Hypena baltimoralis|[5392]|RDLQF488-06|DH011637|658[0n]bp|Canada.Quebec|BOLD:AAA2330
Hypena baltimoralis|[5393]|XAH297-05|2005-ONT-1880|658[0n]bp|Canada.Ontario|BOLD:AAA2330
Hypena baltimoralis|[5394]|RDLQF430-06|DH011537|658[0n]bp|Canada.Quebec|BOLD:AAA2330
Hypena baltimoralis|[5395]|RDLQF193-06|DH011250|658[0n]bp|Canada.Quebec|BOLD:AAA2330
Hypena baltimoralis|[5396]|RDLQF317-06|DH011476|658[0n]bp|Canada.Quebec|BOLD:AAA2330
Hypena baltimoralis|[5397]|BBLPE151-09|09BBELE-2151|658[0n]bp|Canada.Nova Scotia|BOLD:AAA2330
Hypena baltimoralis|[5398]|RDLQF487-06|DH011636|658[0n]bp|Canada.Quebec|BOLD:AAA2330
Hypena baltimoralis|[5399]|RDLQF761-06|DH011911|594[3n]bp|Canada.Quebec|BOLD:AAA2330
Hypena baltimoralis|[5400]|RDLQF192-06|DH011249|632[0n]bp|Canada.Quebec|BOLD:AAA2330
Hypena baltimoralis|[5401]|RDLQF757-06|DH011907|575[6n]bp|Canada.Quebec|
Hypena baltimoralis|[5402]|RDLQG242-06|DH012447|590[0n]bp|Canada.Quebec|BOLD:AAA2330
Hypena baltimoralis|[5403]|RDLQF401-06|DH011468|616[0n]bp|Canada.Quebec|BOLD:AAA2330
Hypena baltimoralis|[5404]|RDLQF400-06|DH011467|630[0n]bp|Canada.Quebec|BOLD:AAA2330
Hypena baltimoralis|[5405]|XAJ509-06|2006-ONT-0509|643[0n]bp|Canada.Ontario|BOLD:AAA2330
Hypena baltimoralis|[5406]|RDLQG349-06|DH012566|658[0n]bp|Canada.Quebec|BOLD:AAA2330
Hypena baltimoralis|[5407]|BLTIB956-08|BL1385|658[0n]bp|Canada.Ontario|BOLD:AAA2330
Hypena baltimoralis|[5408]|RDLQF324-06|DH011483|658[0n]bp|Canada.Quebec|BOLD:AAA2330
Hypena baltimoralis|[5409]|RDLQG347-06|DH012564|658[0n]bp|Canada.Quebec|BOLD:AAA2330
Hypena baltimoralis|[5410]|RDLQF329-06|DH011488|658[0n]bp|Canada.Quebec|BOLD:AAA2330
Hypena baltimoralis|[5411]|RDLQF756-06|DH011906|658[0n]bp|Canada.Quebec|BOLD:AAA2330
Hypena baltimoralis|[5412]|RDLQF245-06|DH011325|658[0n]bp|Canada.Quebec|BOLD:AAA2330
Hypena baltimoralis|[5413]|BBLEC888-09|09BBELE-0888|658[0n]bp|Canada.Nova Scotia|BOLD:AAA2330
Hypena bijugalis|[5414]|LPSO748-08|PPBP-0748|658[0n]bp|Canada.Ontario|BOLD:AAA8814
Hypena bijugalis|[5415]|LPSO692-08|PPBP-0692|658[0n]bp|Canada.Ontario|BOLD:AAA8814
Hypena bijugalis|[5416]|LPSO890-08|PPBP-0890|658[0n]bp|Canada.Ontario|BOLD:AAA8814
Hypena bijugalis|[5417]|LBCS474-07|UBC-2007-0231|658[0n]bp|Canada.British Columbia|BOLD:AAA8814
Hypena bijugalis|[5418]|RDLQB240-05|DH010326|603[0n]bp|Canada.Quebec|BOLD:AAA8814
Hypena bijugalis|[5419]|XAK488-07|HLC-16041|575[0n]bp|Canada.Ontario|BOLD:AAA8814
Hypena bijugalis|[5420]|XAK489-07|HLC-16042|575[0n]bp|Canada.Ontario|BOLD:AAA8814
Hypena bijugalis|[5421]|LPSO063-08|PPBP-0063|658[0n]bp|Canada.Ontario|BOLD:AAA8814
Hypena bijugalis|[5422]|XAG303-05|2005-ONT-887|658[0n]bp|Canada.Ontario|BOLD:AAA8814
Hypena bijugalis|[5423]|LPSO189-08|PPBP-0189|658[0n]bp|Canada.Ontario|BOLD:AAA8814
Hypena bijugalis|[5424]|LPSO281-08|PPBP-0281|658[0n]bp|Canada.Ontario|BOLD:AAA8814
Hypena bijugalis|[5425]|LPSO470-08|PPBP-0470|656[0n]bp|Canada.Ontario|BOLD:AAA8814
Hypena bijugalis|[5426]|LPSO661-08|PPBP-0661|616[0n]bp|Canada.Ontario|BOLD:AAA8814
Hypena bijugalis|[5427]|XAJ717-06|2006-ONT-0717|658[0n]bp|Canada.Ontario|BOLD:AAA8814
Hypena bijugalis|[5428]|RDLQH124-06|DH007391|588[0n]bp|Canada.Quebec|BOLD:AAA8814
Hypena bijugalis|[5429]|XAJ516-06|2006-ONT-0516|658[0n]bp|Canada.Ontario|BOLD:AAA8814
Hypena bijugalis|[5430]|XAJ394-06|2006-ONT-0394|658[0n]bp|Canada.Ontario|BOLD:AAA8814
Hypena bijugalis|[5431]|JSJUL1658-11|BIOUG01521-D11|658[0n]bp|Canada.Ontario|BOLD:AAA8814
Hypena bijugalis|[5432]|RDLQB255-05|DH010341|658[0n]bp|Canada.Quebec|BOLD:AAA8814
Hypena abalienalis|[5433]|LHLEP035-06|UBC-2006-0108|658[0n]bp|Canada.British Columbia|BOLD:ABY9634
Hypena abalienalis|[5434]|LBCS138-07|UBC-2007-0131|658[0n]bp|Canada.British Columbia|BOLD:ABY9634
Hypena abalienalis|[5435]|LBCS140-07|UBC-2007-0133|658[0n]bp|Canada.British Columbia|BOLD:ABY9634
Hypena abalienalis|[5436]|LPVIA631-08|PFC-2006-0864|658[0n]bp|Canada.British Columbia|BOLD:ABY9634
Hypena abalienalis|[5437]|LPVIA608-08|PFC-2006-0836|658[0n]bp|Canada.British Columbia|BOLD:ABY9634
Hypena abalienalis|[5438]|LBCS139-07|UBC-2007-0132|658[0n]bp|Canada.British Columbia|BOLD:ABY9634
Hypena abalienalis|[5439]|RDLQF198-06|DH011255|658[0n]bp|Canada.Quebec|BOLD:ABY9634
Hypena abalienalis|[5440]|RDLQF284-06|DH011376|658[0n]bp|Canada.Quebec|BOLD:ABY9634
Hypena abalienalis|[5441]|RDLQF779-06|DH011929|658[0n]bp|Canada.Quebec|BOLD:ABY9634
Hypena abalienalis|[5442]|LPMN204-08|08BBLEP-01003|658[0n]bp|Canada.Manitoba|BOLD:ABY9634
Hypena abalienalis|[5443]|RDLQG346-06|DH012563|658[0n]bp|Canada.Quebec|BOLD:ABY9634
Hypena abalienalis|[5444]|RDLQB833-05|DH010920|658[0n]bp|Canada.Quebec|BOLD:ABY9634
Hypena abalienalis|[5445]|RDLQF199-06|DH011256|658[0n]bp|Canada.Quebec|BOLD:ABY9634
Hypena abalienalis|[5446]|RDLQG246-06|DH012451|608[0n]bp|Canada.Quebec|BOLD:ABY9634
Hypena abalienalis|[5447]|XAC595-04|04HBL006595|616[0n]bp|Canada.Ontario|BOLD:ABY9634
Hypena abalienalis|[5448]|XAC622-04|04HBL006622|617[0n]bp|Canada.Ontario|BOLD:ABY9634
Hypena abalienalis|[5449]|LPSO658-08|PPBP-0658|646[0n]bp|Canada.Ontario|BOLD:ABY9634
Hypena manalis|[5450]|RDLQH135-06|DH006943|577[2n]bp|Canada.Quebec|BOLD:AAB5993
Hypena manalis|[5451]|LPSOC375-08|PPBP-2374|653[0n]bp|Canada.Ontario|BOLD:AAB5993
Hypena manalis|[5452]|LPSO834-08|PPBP-0834|658[0n]bp|Canada.Ontario|BOLD:AAB5993
Hypena manalis|[5453]|LPSO573-08|PPBP-0573|658[0n]bp|Canada.Ontario|BOLD:AAB5993

Hypena manalis|[5451]|LPSOC375-08|PPBP-2374|653[0n]bp|Canada.Ontario|BOLD:AAB5993
Hypena manalis|[5452]|LPSO834-08|PPBP-0834|658[0n]bp|Canada.Ontario|BOLD:AAB5993
Hypena manalis|[5453]|LPSO573-08|PPBP-0573|658[0n]bp|Canada.Ontario|BOLD:AAB5993
Hypena manalis|[5454]|BLTIB457-08|BL704|658[0n]bp|Canada.Ontario|BOLD:AAB5993
Hypena manalis|[5455]|LPSO574-08|PPBP-0574|658[0n]bp|Canada.Ontario|BOLD:AAB5993
Hypena manalis|[5456]|LPSOB037-08|PPBP-1036|658[0n]bp|Canada.Ontario|BOLD:AAB5993
Hypena manalis|[5457]|LPSO078-08|PPBP-0078|658[0n]bp|Canada.Ontario|BOLD:AAB5993
Hypena manalis|[5458]|LPSO891-08|PPBP-0891|658[0n]bp|Canada.Ontario|BOLD:AAB5993
Hypena eductalis|[5459]|RDLQF909-06|DH012084|658[0n]bp|Canada.Quebec|BOLD:AAB6003
Hypena eductalis|[5460]|RDLQF496-06|DH011645|658[0n]bp|Canada.Quebec|BOLD:AAB6003
Hypena eductalis|[5461]|RDLQF385-06|DH011452|648[0n]bp|Canada.Quebec|BOLD:AAB6003
Hypena eductalis|[5462]|RDLQG604-06|DH012897|621[0n]bp|Canada.Quebec|BOLD:AAB6003
Hypena eductalis|[5463]|RDLQF386-06|DH011453|658[0n]bp|Canada.Quebec|BOLD:AAB6003
Hypena eductalis|[5464]|RDLQF908-06|DH012083|658[0n]bp|Canada.Quebec|BOLD:AAB6003
Hypena eductalis|[5465]|RDLQF448-06|DH011555|658[0n]bp|Canada.Quebec|BOLD:AAB6003
Hypena eductalis|[5466]|BBLPC923-09|09BBELE-1923|658[0n]bp|Canada.Newfoundland and Labrador|BOLD:AAB6003
Hypena eductalis|[5467]|RDLQG315-06|DH012527|658[0n]bp|Canada.Quebec|BOLD:AAB6003
Hypena eductalis|[5468]|BBLPC783-09|09BBELE-1783|658[0n]bp|Canada.Newfoundland and Labrador|BOLD:AAB6003
Hypena eductalis|[5469]|BBLPE097-09|09BBELE-2097|658[0n]bp|Canada.Nova Scotia|BOLD:AAB6003
Hypena eductalis|[5470]|BBLPC814-09|09BBELE-1814|658[0n]bp|Canada.Newfoundland and Labrador|BOLD:AAB6003
Hypena eductalis|[5471]|BBLPE255-09|09BBELE-2255|635[0n]bp|Canada.Nova Scotia|BOLD:AAB6003
Hypena eductalis|[5472]|BBLPE002-09|09BBELE-2002|622[0n]bp|Canada.Nova Scotia|BOLD:AAB6003
Hypena madefactalis|[5473]|LPSO898-08|PPBP-0898|658[0n]bp|Canada.Ontario|BOLD:ACE2873
Hypena madefactalis|[5474]|XAC851-04|04HBL006851|592[0n]bp|Canada.Ontario|BOLD:ACE2873
Hypena madefactalis|[5475]|RDLQF707-06|DH011857|637[0n]bp|Canada.Quebec|BOLD:ACE2873
Hypena madefactalis|[5476]|LPSO666-08|PPBP-0666|658[0n]bp|Canada.Ontario|BOLD:ACE2873
Hypena madefactalis|[5477]|LPSO546-08|PPBP-0546|658[0n]bp|Canada.Ontario|BOLD:ACE2873
Hypena madefactalis|[5478]|LPSO693-08|PPBP-0693|658[0n]bp|Canada.Ontario|BOLD:ACE2873
Hypena madefactalis|[5479]|LPSO650-08|PPBP-0650|658[0n]bp|Canada.Ontario|BOLD:ACE2873
Hypena madefactalis|[5480]|LPSO678-08|PPBP-0678|658[0n]bp|Canada.Ontario|BOLD:ACE2873
Hypena madefactalis|[5481]|RDLQF200-06|DH011257|658[0n]bp|Canada.Quebec|BOLD:ACE2873
Hypena madefactalis|[5482]|RDLQG020-06|DH012140|658[0n]bp|Canada.Quebec|BOLD:ACE2873
Hypena madefactalis|[5483]|LPSO081-08|PPBP-0081|654[0n]bp|Canada.Ontario|BOLD:ACE2873
Hypena madefactalis|[5484]|BBLPA651-10|10BBCLP-0651|658[0n]bp|Canada.Ontario|BOLD:ACE2873
Hypena madefactalis|[5485]|LPSO652-08|PPBP-0652|646[0n]bp|Canada.Ontario|BOLD:ACE2873
Hypena madefactalis|[5486]|LPSO560-08|PPBP-0560|658[0n]bp|Canada.Ontario|BOLD:ACE2873
Hypena madefactalis|[5487]|LPSO542-08|PPBP-0542|658[0n]bp|Canada.Ontario|BOLD:ACE2873
Hypena madefactalis|[5488]|LPSO261-08|PPBP-0261|658[0n]bp|Canada.Ontario|BOLD:ACE2873
Hypena madefactalis|[5489]|RDLQF907-06|DH012082|658[0n]bp|Canada.Quebec|BOLD:ACE2873
Hypena madefactalis|[5490]|RDLQF903-06|DH012078|658[0n]bp|Canada.Quebec|BOLD:ACE2873
Hypena madefactalis|[5491]|RDLQF905-06|DH012080|658[0n]bp|Canada.Quebec|BOLD:ACE2873
Hypena madefactalis|[5492]|LPSO860-08|PPBP-0860|658[0n]bp|Canada.Ontario|BOLD:ACE2873
Hypena madefactalis|[5493]|LPSO642-08|PPBP-0642|658[0n]bp|Canada.Ontario|BOLD:ACE2873
Hypena madefactalis|[5494]|LPSO357-08|PPBP-0357|658[0n]bp|Canada.Ontario|BOLD:ACE2873
Hypena madefactalis|[5495]|RDLQF906-06|DH012081|658[0n]bp|Canada.Quebec|BOLD:ACE2873
Hypena madefactalis|[5496]|LPSO048-08|PPBP-0048|658[0n]bp|Canada.Ontario|BOLD:ACE2873
Hypena madefactalis|[5497]|RDLQF904-06|DH012079|658[1n]bp|Canada.Quebec|BOLD:ACE2873
Hypena madefactalis|[5498]|LPSO659-08|PPBP-0659|630[0n]bp|Canada.Ontario|BOLD:ACE2873
Hypena madefactalis|[5499]|LPSO665-08|PPBP-0665|632[0n]bp|Canada.Ontario|BOLD:ACE2873
Hypena madefactalis|[5500]|RDLQH121-06|DH005625|618[0n]bp|Canada.Quebec|BOLD:ACE2873
Hypena madefactalis|[5501]|RDLQH122-06|DH009754|582[0n]bp|Canada.Quebec|BOLD:ACE2873
Hypena madefactalis|[5502]|LPSO044-08|PPBP-0044|646[0n]bp|Canada.Ontario|BOLD:ACE2873
Hypena deceptalis|[5503]|XAC847-04|04HBL006847|593[1n]bp|Canada.Ontario|BOLD:AAA8145
Hypena deceptalis|[5504]|XAG975-05|2005-ONT-1559|658[0n]bp|Canada.Ontario|BOLD:AAA8145
Hypena deceptalis|[5505]|XAC155-04|04HBL006155|658[0n]bp|Canada.Ontario|BOLD:AAA8145
Hypena deceptalis|[5506]|RDLQF913-06|DH012088|658[0n]bp|Canada.Quebec|BOLD:AAA8145
Hypena deceptalis|[5507]|RDLQF205-06|DH011262|658[0n]bp|Canada.Quebec|BOLD:AAA8145
Hypena deceptalis|[5508]|RDLQF567-06|DH011716|658[0n]bp|Canada.Quebec|BOLD:AAA8145
Hypena deceptalis|[5509]|RDLQG353-06|DH012570|649[0n]bp|Canada.Quebec|BOLD:AAA8145
Hypena deceptalis|[5510]|RDLQG252-06|DH012457|590[2n]bp|Canada.Quebec|BOLD:AAA8145
Hypena deceptalis|[5511]|RDLQH119-06|DH006877|602[1n]bp|Canada.Quebec|BOLD:AAA8145
Hypena deceptalis|[5512]|RDLQG249-06|DH012454|614[0n]bp|Canada.Quebec|BOLD:AAA8145
Hypena deceptalis|[5513]|XAD296-04|04HBL007296|544[0n]bp|Canada.Ontario|BOLD:AAA8145
Hypena deceptalis|[5514]|XAE546-04|Moth4546.03|565[1n]bp|Canada.Ontario|BOLD:AAA8145
Hypena deceptalis|[5515]|RDLQF910-06|DH012085|658[0n]bp|Canada.Quebec|BOLD:AAA8145
Hypena deceptalis|[5516]|RDLQF529-06|DH011678|658[0n]bp|Canada.Quebec|BOLD:AAA8145
Hypena deceptalis|[5517]|RDLQG253-06|DH012458|658[1n]bp|Canada.Quebec|BOLD:AAA8145
Hypena deceptalis|[5518]|RDLQF912-06|DH012087|658[0n]bp|Canada.Quebec|BOLD:AAA8145
Hypena deceptalis|[5519]|RDLQF914-06|DH012089|658[0n]bp|Canada.Quebec|BOLD:AAA8145
Hypena deceptalis|[5520]|RDLQG036-06|DH012167|658[0n]bp|Canada.Quebec|BOLD:AAA8145
Hypena deceptalis|[5521]|RDLQF740-06|DH011890|658[0n]bp|Canada.Quebec|BOLD:AAA8145
Hypena deceptalis|[5522]|RDLQF911-06|DH012086|658[0n]bp|Canada.Quebec|BOLD:AAA8145
Hypena deceptalis|[5523]|RDLQF425-06|DH011532|658[0n]bp|Canada.Quebec|BOLD:AAA8145
Hypena deceptalis|[5524]|RDLQF742-06|DH011892|658[0n]bp|Canada.Quebec|BOLD:AAA8145
Hypena deceptalis|[5525]|RDLQF741-06|DH011891|658[0n]bp|Canada.Quebec|BOLD:AAA8145
Hypena deceptalis|[5526]|RDLQF739-06|DH011889|658[0n]bp|Canada.Quebec|BOLD:AAA8145
Hypena deceptalis|[5527]|RDLQG354-06|DH012588|658[0n]bp|Canada.Quebec|BOLD:AAA8145
Hypena deceptalis|[5528]|RDLQG355-06|DH012589|658[0n]bp|Canada.Quebec|BOLD:AAA8145
Hypena deceptalis|[5529]|RDLQG251-06|DH012456|658[0n]bp|Canada.Quebec|BOLD:AAA8145
Hypena deceptalis|[5530]|RDLQG140-06|DH012311|631[0n]bp|Canada.Quebec|BOLD:AAA8145
Hypena deceptalis|[5531]|RDLQG250-06|DH012455|658[1n]bp|Canada.Quebec|BOLD:AAA8145
Hypena palparia|[5532]|RDLQF900-06|DH012075|658[0n]bp|Canada.Quebec|BOLD:ABY9635
Hypena palparia|[5533]|RDLQF528-06|DH011677|658[0n]bp|Canada.Quebec|BOLD:ABY9635
Hypena palparia|[5534]|RDLQF902-06|DH012077|658[0n]bp|Canada.Quebec|BOLD:ABY9635
Hypena nr. palparia|[5535]|RDNMJ650-11|CNC LEP 70112|658[0n]bp|Canada.Ontario|BOLD:ABY9635
Hypena nr. palparia|[5536]|RDNMJ651-11|CNC LEP 70113|658[0n]bp|Canada.Ontario|BOLD:ABY9635
Hypena palparia|[5537]|RDLQH125-06|DH004909|611[3n]bp|Canada.Quebec|BOLD:ABY9635
Hypena palparia|[5538]|RDNMJ648-11|CNC LEP 70110|619[0n]bp|Canada.Ontario|BOLD:ABY9635
Hypena palparia|[5539]|RDLQF899-06|DH012074|658[0n]bp|Canada.Quebec|BOLD:ABY9635
Hypena palparia|[5540]|LPSOC344-08|PPBP-2343|658[0n]bp|Canada.Ontario|BOLD:ABY9635
Hypena nr. palparia|[5541]|RDNMJ646-11|CNC LEP 70108|658[0n]bp|Canada.New Brunswick|BOLD:ABY9635
Hypena palparia|[5542]|RDLQF226-06|DH011306|658[0n]bp|Canada.Quebec|BOLD:ABY9635
Hypena palparia|[5543]|LPMN181-08|08BBLEP-00980|658[0n]bp|Canada.Manitoba|BOLD:ABY9635
Hypena palparia|[5544]|RDNMJ649-11|CNC LEP 70111|658[0n]bp|Canada.New Brunswick|BOLD:ABY9635
Hypena nr. palparia|[5545]|RDNMJ645-11|CNC LEP 70107|658[0n]bp|Canada.New Brunswick|BOLD:ABY9635
Hypena palparia|[5546]|RDLQB239-05|DH010325|574[1n]bp|Canada.Quebec|BOLD:ABY9635
Hypena palparia|[5547]|RDNME437-08|LEP037861|658[0n]bp|Canada.Alberta|BOLD:ABY9635
Hypena palparia|[5548]|RDNME436-08|LEP037860|658[0n]bp|Canada.Alberta|BOLD:ABY9635
Hypena palparia|[5549]|RDLQF451-06|DH011558|658[0n]bp|Canada.Quebec|BOLD:ABY9635
Hypena palparia|[5550]|RDLQG435-06|DH012714|658[0n]bp|Canada.Quebec|BOLD:ABY9635
Hypena palparia|[5551]|RDLQF901-06|DH012076|658[0n]bp|Canada.Quebec|BOLD:ABY9635
Hypena palparia|[5552]|RDLQB244-05|DH010330|658[0n]bp|Canada.Quebec|BOLD:ABY9635

Hypena palparia|[5550]|RDLQG435-06|DH012714|658[0n]bp|Canada.Quebec|BOLD:ABY9635
Hypena palparia|[5551]|RDLQF901-06|DH012076|658[0n]bp|Canada.Quebec|BOLD:ABY9635
Hypena palparia|[5552]|RDLQB244-05|DH010330|658[0n]bp|Canada.Quebec|BOLD:ABY9635
Hypena palparia|[5553]|RDLQG360-06|DH012594|658[0n]bp|Canada.Quebec|BOLD:ABY9635
Hypena palparia|[5554]|BBLPE145-09|09BBELE-2145|658[0n]bp|Canada.Nova Scotia|BOLD:ABY9635
Hypena palparia|[5555]|LPSOB823-08|PPBP-1822|658[0n]bp|Canada.Ontario|BOLD:ABY9635
Hypena palparia|[5556]|LBCS213-07|UBC-2007-0594|658[0n]bp|Canada.British Columbia|BOLD:ABY9635
Hypena nr. palparia|[5557]|RDNMJ647-11|CNC LEP 70109|613[0n]bp|Canada.New Brunswick|BOLD:ABY9635
Hypena palparia|[5558]|BBLPD754-10|10BBCLP-2752|628[0n]bp|Canada.British Columbia|BOLD:ABY9635
Hypena palparia|[5559]|RDNME435-08|LEP037859|658[0n]bp|Canada.Alberta|BOLD:ABY9635
Hypena palparia|[5560]|BBLPB208-10|10BBCLP-1207|658[0n]bp|Canada.Ontario|BOLD:ABY9635
Hypena sordidula|[5561]|LPSO950-08|PPBP-0950|658[0n]bp|Canada.Ontario|BOLD:ACF0234
Hypena sordidula|[5562]|RDLQH128-06|DH005336|605[1n]bp|Canada.Quebec|BOLD:ACF0234
Hypena sordidula|[5563]|LPSO657-08|PPBP-0657|564[0n]bp|Canada.Ontario|BOLD:ACF0234
Hypena sordidula|[5564]|LPSO839-08|PPBP-0839|658[0n]bp|Canada.Ontario|BOLD:ACF0234
Hypena sordidula|[5565]|LPSOC331-08|PPBP-2330|658[0n]bp|Canada.Ontario|BOLD:ACF0234
Hypena decorata|[5566]|RDNMF108-08|NOC14194|640[0n]bp|United States.California|BOLD:AAX1808
Hypena humuli|[5567]|KPOEC081-08|08OEC-240|658[0n]bp|Canada.Ontario|BOLD:AAB1349
Hypena humuli|[5568]|KPOEC187-08|08OEC-236|658[0n]bp|Canada.Ontario|BOLD:AAB1349
Hypena humuli|[5569]|RDLQF251-06|DH011331|634[0n]bp|Canada.Quebec|BOLD:AAB1349
Hypena humuli|[5570]|PMG119-03|moth343.01|617[0n]bp|Canada.Ontario|BOLD:AAB1349
Hypena humuli|[5571]|RDLQF830-06|DH011983|658[0n]bp|Canada.Quebec|BOLD:AAB1349
Hypena humuli|[5572]|BBLPB152-10|10BBCLP-1151|658[0n]bp|Canada.Saskatchewan|BOLD:AAB1349
Hypena humuli|[5573]|RDLQF378-06|DH011445|641[0n]bp|Canada.Quebec|BOLD:AAB1353
Hypena humuli|[5574]|LBCG041-08|08-JDWBC-0041|658[0n]bp|Canada.British Columbia|BOLD:AAB1350
Hypena humuli|[5575]|LBCA052-05|HLC-20052|658[0n]bp|Canada.British Columbia|BOLD:AAB1350
Hypena humuli|[5576]|LBCA377-05|HLC-20377|658[0n]bp|Canada.British Columbia|BOLD:AAB1350
Hypena humuli|[5577]|LBCA398-05|HLC-20398|658[0n]bp|Canada.British Columbia|BOLD:AAB1350
Hypena humuli|[5578]|LBCA051-05|HLC-20051|658[0n]bp|Canada.British Columbia|BOLD:AAB1350
Hypena humuli|[5579]|LBCA047-05|HLC-20047|658[0n]bp|Canada.British Columbia|BOLD:AAB1350
Hypena humuli|[5580]|LBCA049-05|HLC-20049|658[0n]bp|Canada.British Columbia|BOLD:AAB1350
Hypena humuli|[5581]|LBCA048-05|HLC-20048|658[0n]bp|Canada.British Columbia|BOLD:AAB1350
Hypena humuli|[5582]|LBCA050-05|HLC-20050|658[0n]bp|Canada.British Columbia|BOLD:AAB1350
Hypena humuli|[5583]|LOWCB178-05|CGWC-1118|658[0n]bp|Canada.British Columbia|BOLD:AAB1350
Hypena humuli|[5584]|LBCA516-05|HLC-20516|658[0n]bp|Canada.British Columbia|BOLD:AAB1350
Hypena humuli|[5585]|LALPA1110-11|AVBC 920-11|658[0n]bp|Canada.British Columbia|BOLD:ABX4983
Hypena humuli|[5586]|LBCA378-05|HLC-20378|658[0n]bp|Canada.British Columbia|BOLD:ABX4983
Hypena humuli|[5587]|LBCS473-07|UBC-2007-0230|658[0n]bp|Canada.British Columbia|BOLD:ABX4983
Hypena humuli|[5588]|LBCH1420-10|10-JDWBC-1420|658[0n]bp|Canada.British Columbia|BOLD:ABX4983
Hypena humuli|[5589]|LOWCE380-06|CGWC-4140|658[0n]bp|Canada.British Columbia|BOLD:ABX4983
Hypena humuli|[5590]|BBLPB153-10|10BBCLP-1152|658[0n]bp|Canada.British Columbia|BOLD:ABX4983
Hypena humuli|[5591]|LBCA400-05|HLC-20400|658[0n]bp|Canada.British Columbia|BOLD:ABX4983
Hypena sp.|[5592]|LMIS026-05|05-ONMIS-0026|658[0n]bp|Canada.Ontario|BOLD:AAB1352
Lesmone detrahens|[5593]|BBLOC918-11|BIOUG01467-D09|658[0n]bp|United States.Arkansas|BOLD:AAC0281
Lesmone detrahens|[5594]|LMEMB203-09|RBMIS-1296|658[0n]bp|United States.Mississippi|BOLD:AAC0281
Lesmone detrahens|[5595]|LSUSA153-06|06-SUSA-0153|658[0n]bp|United States.Kentucky|BOLD:AAC0281
Lesmone detrahens|[5596]|BBLSW303-09|09BBLEP-01231|658[0n]bp|United States.Oklahoma|BOLD:AAC0281
Lesmone detrahens|[5597]|LMEMB201-09|RBMIS-1294|658[0n]bp|United States.Alabama|BOLD:AAC0281
Lesmone detrahens|[5598]|LMEMB199-09|RBMIS-1292|658[0n]bp|United States.Alabama|BOLD:AAC0281
Lesmone detrahens|[5599]|LNCB067-06|06-NCC-1023|658[0n]bp|United States.North Carolina|BOLD:AAC0281
Lesmone detrahens|[5600]|BBLSX577-09|09BBLEP-02505|658[0n]bp|United States.Oklahoma|BOLD:AAC0281
Lesmone detrahens|[5601]|LPOKB1030-09|MDOK-2072|658[0n]bp|United States.Oklahoma|BOLD:AAC0281
Lesmone detrahens|[5602]|LNCB068-06|06-NCC-1024|656[0n]bp|United States.North Carolina|BOLD:AAC0281
Lesmone detrahens|[5603]|LMEMB200-09|RBMIS-1293|627[1n]bp|United States.Alabama|BOLD:AAC0281
Lesmone detrahens|[5604]|BBLOC1046-11|BIOUG01468-G06|658[0n]bp|United States.Arkansas|BOLD:AAC0281
Lesmone detrahens|[5605]|LILLA973-11|SNS10IL-01194|658[0n]bp|United States.Illinois|BOLD:AAC0281
Lesmone detrahens|[5606]|BBLOE1256-12|BIOUG01985-D12|658[0n]bp|United States.Texas|BOLD:AAC0281
Lesmone detrahens|[5607]|LMEMB202-09|RBMIS-1295|658[0n]bp|United States.Mississippi|BOLD:AAC0281
Lesmone detrahens|[5608]|BBLOB1876-11|BIOUG01425-F11|658[0n]bp|United States.Florida|BOLD:AAC0281
Lesmone detrahens|[5609]|LPOKA504-09|MDOK-0504|658[0n]bp|United States.Oklahoma|BOLD:AAC0281
Lesmone detrahens|[5610]|ABNCC311-07|1311-090504-TX|642[0n]bp|United States.Texas|BOLD:AAC0281
Lesmone detrahens|[5611]|LPOKA256-08|MDOK-0256|658[0n]bp|United States.Oklahoma|BOLD:AAC0281
Lesmone detrahens|[5612]|LPOKB1027-09|MDOK-2069|658[0n]bp|United States.Oklahoma|BOLD:AAC0281
Isogona tenuis|[5613]|LGSMC923-05|DNA-ATBI-4003|658[1n]bp|United States.Tennessee|BOLD:AAB5798
Isogona tenuis|[5614]|LMEMB049-09|RBMIS-1142|658[0n]bp|United States.Louisiana|BOLD:AAB5798
Isogona tenuis|[5615]|LMEMB051-09|RBMIS-1144|658[0n]bp|United States.Mississippi|BOLD:AAB5798
Isogona tenuis|[5616]|LILLA323-11|SNS10IL-00425|658[0n]bp|United States.Illinois|BOLD:AAB5798
Isogona tenuis|[5617]|LILLA373-11|SNS10IL-00491|658[0n]bp|United States.Illinois|BOLD:AAB5798
Isogona tenuis|[5618]|LMEMB047-09|RBMIS-1140|658[0n]bp|United States.Alabama|BOLD:AAB5798
Isogona tenuis|[5619]|LMEMB050-09|RBMIS-1143|658[0n]bp|United States.Louisiana|BOLD:AAB5798
Isogona tenuis|[5620]|LGSM543-04|DNA-ATBI-0543|658[0n]bp|United States.Tennessee|BOLD:AAB5798
Isogona tenuis|[5621]|LILLA145-11|SNS10IL-00191|658[0n]bp|United States.Illinois|BOLD:AAB5798
Isogona tenuis|[5622]|ABNCC214-07|1214-210504-TX|656[0n]bp|United States.Texas|BOLD:AAB5798
Isogona tenuis|[5623]|BBLOE1254-12|BIOUG01985-D10|658[0n]bp|United States.Texas|BOLD:AAB5798
Isogona tenuis|[5624]|LMEMB048-09|RBMIS-1141|658[0n]bp|United States.Alabama|BOLD:AAB5798
Isogona tenuis|[5625]|LGSMC922-05|DNA-ATBI-4002|658[0n]bp|United States.Tennessee|BOLD:AAB5798
Isogona tenuis|[5626]|LGSM544-04|DNA-ATBI-0544|658[0n]bp|United States.Tennessee|BOLD:AAB5798
Isogona tenuis|[5627]|HKONS360-08|3265-COI-08|658[0n]bp|United States.Florida|BOLD:AAB5798
Isogona tenuis|[5628]|RDNMK058-11|CNCLEP 81181|658[0n]bp|United States.Florida|BOLD:AAB5798
Isogona tenuis|[5629]|ABNCC209-07|1209-060603-TX|641[0n]bp|United States.Texas|BOLD:AAB5798
Isogona tenuis|[5630]|RDNME665-08|LEP031940|658[0n]bp|United States.Texas|BOLD:AAB5798
Isogona tenuis|[5631]|HKONB047-08|3544-COI-08|658[0n]bp|United States.Texas|BOLD:AAB5798
Isogona tenuis|[5632]|HKONB045-08|3542-COI-08|658[0n]bp|United States.Texas|BOLD:AAB5798
Isogona tenuis|[5633]|HKONB048-08|3545-COI-08|658[0n]bp|United States.Texas|BOLD:AAB5798
Isogona tenuis|[5634]|PSAT152-10|CNCLEP 70036|658[0n]bp|United States.Texas|BOLD:AAB5798
Isogona tenuis|[5635]|HKONB046-08|3543-COI-08|658[0n]bp|United States.Texas|BOLD:AAB5798
Isogona tenuis|[5636]|ABNCC210-07|1210-310503-TX|652[0n]bp|United States.Texas|BOLD:AAB5798
Isogona tenuis|[5637]|LNCC1225-11|11-NCCC-750|658[0n]bp|United States.North Carolina|BOLD:AAB5798
Isogona tenuis|[5638]|BBLOC1177-11|BIOUG01540-B02|658[0n]bp|United States.Texas|BOLD:AAB5798
Isogona tenuis|[5639]|USLEP957-10|10BBLEP-00957|658[0n]bp|United States.Texas|BOLD:AAB5798
Isogona tenuis|[5640]|ABNCC211-07|1211-200504-TX|633[0n]bp|United States.Texas|BOLD:AAB5798
Isogona tenuis|[5641]|ABNCC212-07|1212-240504-TX|620[0n]bp|United States.Texas|BOLD:AAB5798
Alabama argillacea|[5642]|RDNMF609-08|NOC14695|614[0n]bp|Canada.British Columbia|BOLD:AAJ2486
Eudocima apta|[5643]|BLPDV063-11|10-SRNP-110720|658[0n]bp|Costa Rica.Guanacaste|BOLD:AAC6829
Eudocima apta|[5644]|BLPDV062-11|10-SRNP-110719|658[0n]bp|Costa Rica.Guanacaste|BOLD:AAC6829
Eudocima apta|[5645]|BLPDV061-11|10-SRNP-110718|658[0n]bp|Costa Rica.Guanacaste|BOLD:AAC6829
Eudocima apta|[5646]|MHAUB573-05|98-SRNP-8348|658[0n]bp|Costa Rica.Guanacaste|BOLD:AAC6829
Eudocima apta|[5647]|BLPAB410-06|06-SRNP-101349|658[0n]bp|Costa Rica.Guanacaste|BOLD:AAC6829
Eudocima apta|[5648]|BLPEC507-11|11-SRNP-103064|658[0n]bp|Costa Rica.Alajuela|BOLD:AAC6829
Eudocima apta|[5649]|BLPAD073-06|06-SRNP-102892|658[0n]bp|Costa Rica.Guanacaste|BOLD:AAC6829
Eudocima apta|[5650]|BLPDO1010-10|10-SRNP-103592|658[0n]bp|Costa Rica.Alajuela|BOLD:AAC6829
Eudocima apta|[5651]|MHMYL3566-11|10-SRNP-65061|658[0n]bp|Costa Rica|BOLD:AAC6829

Eudocima apta|[5649]|BLPAD073-06|06-SRNP-102892|658[0n]bp|Costa Rica.Guanacaste|BOLD:AAC6829
Eudocima apta|[5650]|BLPDO1010-10|10-SRNP-103592|658[0n]bp|Costa Rica.Alajuela|BOLD:AAC6829
Eudocima apta|[5651]|MHMYL3566-11|10-SRNP-65061|658[0n]bp|Costa Rica|BOLD:AAC6829
Calyptra canadensis|[5652]|BLTIB568-08|BL846|658[0n]bp|Canada.Ontario|BOLD:ABY4439
Calyptra canadensis|[5653]|XAJ831-06|2006-ONT-0831|658[0n]bp|Canada.Ontario|BOLD:ABY4439
Calyptra canadensis|[5654]|XAK005-06|2006-ONT-1000|658[0n]bp|Canada.Ontario|BOLD:AAD2635
Calyptra canadensis|[5655]|BLTIB738-08|BL1028|658[0n]bp|Canada.Ontario|BOLD:AAD2635
Calyptra canadensis|[5656]|RDLQH115-06|DH007313|658[0n]bp|Canada.Quebec|BOLD:AAD2635
Calyptra canadensis|[5657]|BLTIB708-08|BL993|658[0n]bp|Canada.Ontario|BOLD:AAD2635
Calyptra canadensis|[5658]|XAD756-05|2005-ONT-555|658[0n]bp|Canada.Ontario|BOLD:AAD2635
Anomis erosa|[5659]|HKONS382-08|1852-COI-07|658[0n]bp|United States.Florida|BOLD:AAC1739
Anomis erosa|[5660]|RDNMF621-08|NOC14707|609[0n]bp|United States.Missouri|BOLD:AAC1739
Anomis erosa|[5661]|LMEMB116-09|RBMIS-1209|658[0n]bp|United States.Mississippi|BOLD:AAC1739
Anomis erosa|[5662]|RDNMI057-10|CNCLEP 69847|658[0n]bp|United States.Florida|BOLD:AAC1739
Anomis erosa|[5663]|HKONS047-07|1611-COI-07|656[0n]bp|United States.Florida|BOLD:AAC1739
Anomis erosa|[5664]|HKONS381-08|1851-COI-07|658[0n]bp|United States.Florida|BOLD:AAC1739
Anomis erosa|[5665]|HKONS380-08|1850-COI-07|658[0n]bp|United States.Florida|BOLD:AAC1739
Anomis erosa|[5666]|RDNMI058-10|CNCLEP 69848|658[0n]bp|United States.Florida|BOLD:AAC1739
Anomis erosa|[5667]|HKONS384-08|1854-COI-07|658[0n]bp|United States.Florida|BOLD:AAC1739
Anomis erosa|[5668]|RDNMF620-08|NOC14706|640[0n]bp|United States.Missouri|BOLD:AAC1739
Anomis erosa|[5669]|HKONS385-08|1855-COI-07|658[0n]bp|United States.Florida|BOLD:AAC1739
Anomis erosa|[5670]|HKONS383-08|1853-COI-07|658[0n]bp|United States.Florida|BOLD:AAC1739
Anomis erosa|[5671]|HKONS379-08|1849-COI-07|658[0n]bp|United States.Florida|BOLD:AAC1739
Anomis erosa|[5672]|RDNMI059-10|CNCLEP 69849|658[0n]bp|United States.Florida|BOLD:AAC1739
Anomis erosa|[5673]|HKONS386-08|1856-COI-07|658[0n]bp|United States.Florida|BOLD:AAC1739
Anomis flava|[5674]|HKONS377-08|1847-COI-07|658[0n]bp|United States.Florida|BOLD:AAB5675
Anomis flava|[5675]|RDNMI056-10|CNCLEP 69846|658[0n]bp|United States.Florida|BOLD:AAB5675
Lycomorpha pholus|[5676]|XAJ974-06|2006-ONT-0974|658[0n]bp|Canada.Ontario|BOLD:AAC3139
Lycomorpha pholus|[5677]|MNAF323-08|CNCLEP00040308|658[0n]bp|Canada.Manitoba|BOLD:AAC3139
Lycomorpha pholus|[5678]|RDNME512-08|LEP037936|658[0n]bp|Canada.Ontario|BOLD:AAC3139
Lycomorpha pholus|[5679]|LPSOD964-09|08BBLEP-05597|658[0n]bp|Canada.Ontario|BOLD:AAC3139
Lycomorpha pholus|[5680]|RDNME511-08|LEP037935|658[0n]bp|Canada.Ontario|BOLD:AAC3139
Lycomorpha pholus|[5681]|RDLQB533-05|DH010619|658[0n]bp|Canada.Quebec|BOLD:AAC3139
Acsala anomala|[5682]|RDNMF752-08|NOC14838|658[0n]bp|Canada.Yukon Territory|BOLD:AAD5206
Acsala anomala|[5683]|RDNMF751-08|NOC14837|658[0n]bp|Canada.Yukon Territory|BOLD:AAD5206
Acsala anomala|[5684]|RDNME384-08|LEP037808|658[0n]bp|Canada.Yukon Territory|BOLD:AAD5206
Acsala anomala|[5685]|TIPSY572-12|STG98|658[0n]bp|Canada.Yukon Territory|BOLD:AAD5206
Acsala anomala|[5686]|RDNMF754-08|NOC14840|597[0n]bp|Canada.Ontario|BOLD:AAD5206
Acsala anomala|[5687]|RDNMF753-08|NOC14839|658[0n]bp|Canada.Yukon Territory|BOLD:AAD5206
Acsala anomala|[5688]|RDNME382-08|LEP037806|658[0n]bp|Canada.Yukon Territory|BOLD:AAD5206
Acsala anomala|[5689]|RDNME383-08|LEP037807|658[0n]bp|Canada.Yukon Territory|BOLD:AAD5206
Crambidia pura|[5690]|RDNME404-08|LEP037828|649[0n]bp|Canada.Ontario|BOLD:AAD3975
Crambidia cephalica|[5691]|RDMAB394-05|BCSC67|658[0n]bp|Canada.Alberta|BOLD:AAB7304
Crambidia casta|[5692]|BBLPB899-10|10BBCLP-1898|658[0n]bp|Canada.Alberta|BOLD:AAB4937
Crambidia casta|[5693]|BBLPA889-10|10BBCLP-0889|658[0n]bp|Canada.Alberta|BOLD:AAB4937
Crambidia casta|[5694]|LPSK555-08|08BBLEP-02123|658[0n]bp|Canada.Saskatchewan|BOLD:AAB4937
Crambidia casta|[5695]|RDMAB398-05|BCSC71|658[0n]bp|Canada.Alberta|BOLD:AAB4937
Crambidia casta|[5696]|LPSOD942-09|08BBLEP-05482|658[0n]bp|Canada.Ontario|BOLD:AAB4937
Crambidia casta|[5697]|RDMAB680-06|UASM58811|658[0n]bp|Canada.Alberta|BOLD:AAB4937
Crambidia casta|[5698]|RDMAB397-05|BCSC70|658[0n]bp|Canada.Alberta|BOLD:AAB4937
Crambidia casta|[5699]|LPABC153-09|08BBLEP-04372|658[0n]bp|Canada.Alberta|BOLD:AAB4937
Crambidia casta|[5700]|RDMAB604-06|UASM58652|658[0n]bp|Canada.Alberta|BOLD:AAB4937
Crambidia casta|[5701]|LPABC151-09|08BBLEP-04370|658[0n]bp|Canada.Alberta|BOLD:AAB4937
Crambidia casta|[5702]|RDMAB593-06|UASM58903|658[0n]bp|Canada.Alberta|BOLD:AAB4937
Crambidia casta|[5703]|LBCG2647-09|08-JDWBC-2647|658[0n]bp|Canada.British Columbia|BOLD:AAB4937
Crambidia casta|[5704]|LBCG2157-09|08-JDWBC-2157|658[0n]bp|Canada.British Columbia|BOLD:AAB4937
Crambidia casta|[5705]|LBCG2587-09|08-JDWBC-2587|658[0n]bp|Canada.British Columbia|BOLD:AAB4937
Crambidia casta|[5706]|LBCG3240-09|08-JDWBC-3240|658[0n]bp|Canada.British Columbia|BOLD:AAB4937
Crambidia casta|[5707]|RDMAB600-06|UASM58644|658[0n]bp|Canada.Alberta|BOLD:AAB4937
Crambidia casta|[5708]|RDMAB592-06|UASM58902|658[0n]bp|Canada.Alberta|BOLD:AAB4937
Crambidia casta|[5709]|LPABC166-09|08BBLEP-04385|658[6n]bp|Canada.Alberta|
Crambidia pallida|[5710]|BBLEC712-09|09BBELE-0712|658[0n]bp|Canada.Nova Scotia|BOLD:AAA5563
Crambidia pallida|[5711]|TMNBD453-07|MNBTT-3254|658[0n]bp|Canada.New Brunswick|BOLD:AAA5563
Crambidia pallida|[5712]|RDLQB687-05|DH010790|658[0n]bp|Canada.Quebec|BOLD:AAA5563
Crambidia pallida|[5713]|TMNBD455-07|MNBTT-3256|658[0n]bp|Canada.New Brunswick|BOLD:AAA5563
Crambidia pallida|[5714]|MNAF438-08|CNCLEP00040423|658[0n]bp|Canada.Manitoba|BOLD:AAA5563
Crambidia pallida|[5715]|MNAF439-08|CNCLEP00040424|658[0n]bp|Canada.Manitoba|BOLD:AAA5563
Crambidia pallida|[5716]|MNAF858-08|CNCLEP00040843|658[0n]bp|Canada.Manitoba|BOLD:AAA5563
Crambidia pallida|[5717]|BLTIB809-08|BL1227|625[0n]bp|Canada.Ontario|BOLD:AAA5563
Crambidia pallida|[5718]|MNAF440-08|CNCLEP00040425|658[0n]bp|Canada.Manitoba|BOLD:AAA5563
Crambidia pallida|[5719]|MNAF859-08|CNCLEP00040844|658[0n]bp|Canada.Manitoba|BOLD:AAA5563
Crambidia pallida|[5720]|MNAF861-08|CNCLEP00040846|658[0n]bp|Canada.Manitoba|BOLD:AAA5563
Crambidia pallida|[5721]|MNAF441-08|CNCLEP00040426|658[0n]bp|Canada.Manitoba|BOLD:AAA5563
Crambidia pallida|[5722]|MNAF057-08|CNCLEP00038542|658[0n]bp|Canada.Manitoba|BOLD:AAA5563
Crambidia pallida|[5723]|MNAF860-08|CNCLEP00040845|658[0n]bp|Canada.Manitoba|BOLD:AAA5563
Crambidia pallida|[5724]|LPSOD980-09|08BBLEP-05613|658[0n]bp|Canada.Ontario|BOLD:AAA5563
Crambidia pallida|[5725]|XAJ879-06|2006-ONT-0879|636[0n]bp|Canada.Ontario|BOLD:AAA5563
Crambidia pallida|[5726]|TMNBD452-07|MNBTT-3253|658[0n]bp|Canada.New Brunswick|BOLD:AAA5563
Crambidia pallida|[5727]|RDLQG754-06|DH013047|658[0n]bp|Canada.Quebec|BOLD:AAA5563
Crambidia pallida|[5728]|BBLEC741-09|09BBELE-0741|658[0n]bp|Canada.Nova Scotia|BOLD:AAA5563
Crambidia pallida|[5729]|RDLQG883-06|DH013176|658[0n]bp|Canada.Quebec|BOLD:AAA5563
Crambidia pallida|[5730]|RDLQB542-05|DH010628|658[0n]bp|Canada.Quebec|BOLD:AAA5563
Crambidia pallida|[5731]|XAK215-06|2006-ONT-1210|658[0n]bp|Canada.Ontario|BOLD:AAA5563
Crambidia pallida|[5732]|RDLQB890-05|DH006630|658[0n]bp|Canada.Quebec|BOLD:AAA5563
Crambidia pallida|[5733]|RDLQG686-06|DH012979|656[0n]bp|Canada.Quebec|BOLD:AAA5563
Crambidia pallida|[5734]|RDNME406-08|LEP037830|658[0n]bp|Canada.Ontario|BOLD:AAA5563
Crambidia pallida|[5735]|LPMNB238-09|08BBLEP-05082|658[0n]bp|Canada.Manitoba|BOLD:AAA5563
Crambidia pallida|[5736]|RDNME405-08|LEP037829|658[0n]bp|Canada.Ontario|BOLD:AAA5563
Crambidia pallida|[5737]|RDLQB685-05|DH010788|658[0n]bp|Canada.Quebec|BOLD:AAA5563
Crambidia pallida|[5738]|RDLQB686-05|DH010789|658[0n]bp|Canada.Quebec|BOLD:AAA5563
Crambidia pallida|[5739]|RDNME407-08|LEP037831|658[0n]bp|Canada.Ontario|BOLD:AAA5563
Crambidia pallida|[5740]|BBLPE619-09|09BBELE-2619|650[0n]bp|Canada.Nova Scotia|BOLD:AAA5563
Crambidia pallida|[5741]|RDLQB683-05|DH010786|613[0n]bp|Canada.Quebec|BOLD:AAA5563
Crambidia pallida|[5742]|MNAF442-08|CNCLEP00040427|658[0n]bp|Canada.Manitoba|BOLD:AAA5563
Crambidia pallida|[5743]|RDLQB682-05|DH010785|623[0n]bp|Canada.Quebec|BOLD:AAA5563
Crambidia pallida|[5744]|TMNBD454-07|MNBTT-3255|654[0n]bp|Canada.New Brunswick|BOLD:AAA5563
Crambidia pallida|[5745]|RDLQB684-05|DH010787|658[0n]bp|Canada.Quebec|BOLD:AAA5563
Crambidia pallida|[5746]|XAG505-05|2005-ONT-1089|597[2n]bp|Canada.Ontario|BOLD:AAA5563
Crambidia pallida|[5747]|XAG585-05|2005-ONT-1169|606[0n]bp|Canada.Ontario|BOLD:AAA5563
Eilema bicolor|[5748]|LOWCB575-05|CGWC-1515|563[1n]bp|Canada.British Columbia|BOLD:AAA4503
Eilema bicolor|[5749]|LOWCB582-05|CGWC-1522|560[0n]bp|Canada.British Columbia|BOLD:AAA4503
Eilema bicolor|[5750]|LOWCB570-05|CGWC-1510|568[0n]bp|Canada.British Columbia|BOLD:AAA4503
[illegible]

Eilema bicolor|[5748]|LOWCB575-05|CGWC-1515|563[1n]bp|Canada.British Columbia|BOLD:AAA4503
Eilema bicolor|[5749]|LOWCB582-05|CGWC-1522|560[0n]bp|Canada.British Columbia|BOLD:AAA4503
Eilema bicolor|[5750]|LOWCB570-05|CGWC-1510|568[0n]bp|Canada.British Columbia|BOLD:AAA4503
Eilema bicolor|[5751]|RDLQB546-05|DH010632|528[0n]bp|Canada.Quebec|BOLD:AAA4503
Eilema bicolor|[5752]|TMNBD360-07|MNBTT-3161|609[0n]bp|Canada.New Brunswick|BOLD:AAA4503
Eilema bicolor|[5753]|RDLQB532-05|DH010618|565[0n]bp|Canada.Quebec|BOLD:AAA4503
Eilema bicolor|[5754]|MNBB635-05|05-NBSTA-551|658[0n]bp|Canada.New Brunswick|BOLD:AAA4503
Eilema bicolor|[5755]|RDLQG814-06|DH013107|658[0n]bp|Canada.Quebec|BOLD:AAA4503
Eilema bicolor|[5756]|MNAC813-07|CNCLEP00027554|658[0n]bp|Canada.Quebec|BOLD:AAA4503
Eilema bicolor|[5757]|MNAC812-07|CNCLEP00027553|658[0n]bp|Canada.Quebec|BOLD:AAA4503
Eilema bicolor|[5758]|MNBB632-05|05-NBSTA-548|658[0n]bp|Canada.New Brunswick|BOLD:AAA4503
Eilema bicolor|[5759]|MEC809-04|jflandry0809|658[0n]bp|Canada.Quebec|BOLD:AAA4503
Eilema bicolor|[5760]|MECD366-06|jflandry2938|658[0n]bp|Canada.Quebec|BOLD:AAA4503
Eilema bicolor|[5761]|LBCH6339-10|10-JDWBC-6339|658[0n]bp|Canada.British Columbia|BOLD:AAA4503
Eilema bicolor|[5762]|LOWCB574-05|CGWC-1514|575[0n]bp|Canada.British Columbia|BOLD:AAA4503
Eilema bicolor|[5763]|LOWCB584-05|CGWC-1524|573[0n]bp|Canada.British Columbia|BOLD:AAA4503
Eilema bicolor|[5764]|LOWCB580-05|CGWC-1520|587[2n]bp|Canada.British Columbia|BOLD:AAA4503
Eilema bicolor|[5765]|LOWCB583-05|CGWC-1523|567[6n]bp|Canada.British Columbia|
Eilema bicolor|[5766]|LOWCB572-05|CGWC-1512|573[0n]bp|Canada.British Columbia|BOLD:AAA4503
Eilema bicolor|[5767]|LBCD384-05|HLC-23204|617[0n]bp|Canada.British Columbia|BOLD:AAA4503
Eilema bicolor|[5768]|LBCG3241-09|08-JDWBC-3241|635[0n]bp|Canada.British Columbia|BOLD:AAA4503
Eilema bicolor|[5769]|LBCD382-05|HLC-23202|658[1n]bp|Canada.British Columbia|BOLD:AAA4503
Eilema bicolor|[5770]|LOWCB579-05|CGWC-1519|577[0n]bp|Canada.British Columbia|BOLD:AAA4503
Eilema bicolor|[5771]|LOWCB573-05|CGWC-1513|590[0n]bp|Canada.British Columbia|BOLD:AAA4503
Eilema bicolor|[5772]|LOWCB587-05|CGWC-1527|604[3n]bp|Canada.British Columbia|BOLD:AAA4503
Eilema bicolor|[5773]|LBCH7507-10|10-JDWBC-7507|658[0n]bp|Canada.British Columbia|BOLD:AAA4503
Eilema bicolor|[5774]|LOWCB571-05|CGWC-1511|610[2n]bp|Canada.British Columbia|BOLD:AAA4503
Eilema bicolor|[5775]|LBCH507-10|10-JDWBC-0507|658[0n]bp|Canada.British Columbia|BOLD:AAA4503
Eilema bicolor|[5776]|LBCH816-10|10-JDWBC-0816|658[0n]bp|Canada.British Columbia|BOLD:AAA4503
Eilema bicolor|[5777]|LBCH4054-10|10-JDWBC-4054|658[0n]bp|Canada.British Columbia|BOLD:AAA4503
Eilema bicolor|[5778]|LBCH7146-10|10-JDWBC-7146|636[0n]bp|Canada.British Columbia|BOLD:AAA4503
Eilema bicolor|[5779]|LBCG475-08|08-JDWBC-0475|658[0n]bp|Canada.British Columbia|BOLD:AAA4503
Eilema bicolor|[5780]|LOWCB577-05|CGWC-1517|658[0n]bp|Canada.British Columbia|BOLD:AAA4503
Eilema bicolor|[5781]|LBCH7142-10|10-JDWBC-7142|658[0n]bp|Canada.British Columbia|BOLD:AAA4503
Eilema bicolor|[5782]|LBCH7380-10|10-JDWBC-7380|658[0n]bp|Canada.British Columbia|BOLD:AAA4503
Eilema bicolor|[5783]|LPMNB326-09|08BBLEP-05170|658[0n]bp|Canada.Manitoba|BOLD:AAA4503
Eilema bicolor|[5784]|LBCH7397-10|10-JDWBC-7397|658[0n]bp|Canada.British Columbia|BOLD:AAA4503
Eilema bicolor|[5785]|LPMNB447-09|08BBLEP-05447|658[0n]bp|Canada.Manitoba|BOLD:AAA4503
Eilema bicolor|[5786]|LBCD377-05|HLC-23197|658[0n]bp|Canada.British Columbia|BOLD:AAA4503
Eilema bicolor|[5787]|LBCH7385-10|10-JDWBC-7385|658[0n]bp|Canada.British Columbia|BOLD:AAA4503
Eilema bicolor|[5788]|LBCH7379-10|10-JDWBC-7379|658[0n]bp|Canada.British Columbia|BOLD:AAA4503
Eilema bicolor|[5789]|RDMAB404-05|BCSC77|658[0n]bp|Canada.Yukon Territory|BOLD:AAA4503
Eilema bicolor|[5790]|LBCH7145-10|10-JDWBC-7145|642[0n]bp|Canada.British Columbia|BOLD:AAA4503
Eilema bicolor|[5791]|LBCH7148-10|10-JDWBC-7148|658[0n]bp|Canada.British Columbia|BOLD:AAA4503
Eilema bicolor|[5792]|LBCH002-10|10-JDWBC-0002|658[0n]bp|Canada.British Columbia|BOLD:AAA4503
Eilema bicolor|[5793]|LBCH7923-10|10-JDWBC-7923|658[0n]bp|Canada.British Columbia|BOLD:AAA4503
Eilema bicolor|[5794]|LBCH7513-10|10-JDWBC-7513|658[0n]bp|Canada.British Columbia|BOLD:AAA4503
Eilema bicolor|[5795]|LBCH706-10|10-JDWBC-0706|658[0n]bp|Canada.British Columbia|BOLD:AAA4503
Eilema bicolor|[5796]|LBCH7741-10|10-JDWBC-7741|658[0n]bp|Canada.British Columbia|BOLD:AAA4503
Eilema bicolor|[5797]|LBCD383-05|HLC-23203|658[0n]bp|Canada.British Columbia|BOLD:AAA4503
Eilema bicolor|[5798]|LBCH7509-10|10-JDWBC-7509|658[0n]bp|Canada.British Columbia|BOLD:AAA4503
Eilema bicolor|[5799]|LBCH7918-10|10-JDWBC-7918|658[0n]bp|Canada.British Columbia|BOLD:AAA4503
Eilema bicolor|[5800]|LBCH7512-10|10-JDWBC-7512|658[0n]bp|Canada.British Columbia|BOLD:AAA4503
Eilema bicolor|[5801]|LBCH7742-10|10-JDWBC-7742|658[0n]bp|Canada.British Columbia|BOLD:AAA4503
Eilema bicolor|[5802]|LBCH7510-10|10-JDWBC-7510|658[0n]bp|Canada.British Columbia|BOLD:AAA4503
Eilema bicolor|[5803]|LBCD380-05|HLC-23200|658[0n]bp|Canada.British Columbia|BOLD:AAA4503
Eilema bicolor|[5804]|LBCH6345-10|10-JDWBC-6345|658[0n]bp|Canada.British Columbia|BOLD:AAA4503
Eilema bicolor|[5805]|LBCH7738-10|10-JDWBC-7738|658[0n]bp|Canada.British Columbia|BOLD:AAA4503
Eilema bicolor|[5806]|LBCH7696-10|10-JDWBC-7696|658[0n]bp|Canada.British Columbia|BOLD:AAA4503
Eilema bicolor|[5807]|LBCH701-10|10-JDWBC-0701|658[0n]bp|Canada.British Columbia|BOLD:AAA4503
Eilema bicolor|[5808]|LBCH817-10|10-JDWBC-0817|658[0n]bp|Canada.British Columbia|BOLD:AAA4503
Eilema bicolor|[5809]|LBCH822-10|10-JDWBC-0822|658[0n]bp|Canada.British Columbia|BOLD:AAA4503
Eilema bicolor|[5810]|LBCH823-10|10-JDWBC-0823|658[0n]bp|Canada.British Columbia|BOLD:AAA4503
Eilema bicolor|[5811]|LBCH6629-10|10-JDWBC-6629|658[0n]bp|Canada.British Columbia|BOLD:AAA4503
Eilema bicolor|[5812]|LBCH7383-10|10-JDWBC-7383|658[0n]bp|Canada.British Columbia|BOLD:AAA4503
Eilema bicolor|[5813]|LBCH704-10|10-JDWBC-0704|658[0n]bp|Canada.British Columbia|BOLD:AAA4503
Eilema bicolor|[5814]|LBCH7147-10|10-JDWBC-7147|658[0n]bp|Canada.British Columbia|BOLD:AAA4503
Eilema bicolor|[5815]|LBCH6276-10|10-JDWBC-6276|658[0n]bp|Canada.British Columbia|BOLD:AAA4503
Eilema bicolor|[5816]|LBCH7740-10|10-JDWBC-7740|658[0n]bp|Canada.British Columbia|BOLD:AAA4503
Eilema bicolor|[5817]|LBCH703-10|10-JDWBC-0703|658[0n]bp|Canada.British Columbia|BOLD:AAA4503
Eilema bicolor|[5818]|LBCH7465-10|10-JDWBC-7465|658[0n]bp|Canada.British Columbia|BOLD:AAA4503
Eilema bicolor|[5819]|LBCH7511-10|10-JDWBC-7511|658[0n]bp|Canada.British Columbia|BOLD:AAA4503
Eilema bicolor|[5820]|LBCH6483-10|10-JDWBC-6483|658[0n]bp|Canada.British Columbia|BOLD:AAA4503
Eilema bicolor|[5821]|BBLPA893-10|10BBCLP-0893|658[0n]bp|Canada.Alberta|BOLD:AAA4503
Eilema bicolor|[5822]|LBCH6480-10|10-JDWBC-6480|658[0n]bp|Canada.British Columbia|BOLD:AAA4503
Eilema bicolor|[5823]|LBCD386-05|HLC-23206|658[0n]bp|Canada.British Columbia|BOLD:AAA4503
Eilema bicolor|[5824]|LOWCB568-05|CGWC-1508|658[0n]bp|Canada.British Columbia|BOLD:AAA4503
Eilema bicolor|[5825]|LBCH7381-10|10-JDWBC-7381|658[0n]bp|Canada.British Columbia|BOLD:AAA4503
Eilema bicolor|[5826]|LBCG474-08|08-JDWBC-0474|658[0n]bp|Canada.British Columbia|BOLD:AAA4503
Eilema bicolor|[5827]|LBCH227-10|10-JDWBC-0227|658[0n]bp|Canada.British Columbia|BOLD:AAA4503
Eilema bicolor|[5828]|LBCH6476-10|10-JDWBC-6476|658[0n]bp|Canada.British Columbia|BOLD:AAA4503
Eilema bicolor|[5829]|LBCH7143-10|10-JDWBC-7143|658[0n]bp|Canada.British Columbia|BOLD:AAA4503
Eilema bicolor|[5830]|RDMAB379-05|BCSC53|658[0n]bp|Canada.British Columbia|BOLD:AAA4503
Eilema bicolor|[5831]|LBCD376-05|HLC-23196|658[0n]bp|Canada.British Columbia|BOLD:AAA4503
Eilema bicolor|[5832]|LBCH7920-10|10-JDWBC-7920|658[0n]bp|Canada.British Columbia|BOLD:AAA4503
Eilema bicolor|[5833]|LBCH699-10|10-JDWBC-0699|658[0n]bp|Canada.British Columbia|BOLD:AAA4503
Eilema bicolor|[5834]|LBCH6839-10|10-JDWBC-6839|658[0n]bp|Canada.British Columbia|BOLD:AAA4503
Eilema bicolor|[5835]|LBCD379-05|HLC-23199|658[0n]bp|Canada.British Columbia|BOLD:AAA4503
Eilema bicolor|[5836]|LBCG2106-09|08-JDWBC-2106|658[0n]bp|Canada.British Columbia|BOLD:AAA4503
Eilema bicolor|[5837]|LBCH7925-10|10-JDWBC-7925|658[0n]bp|Canada.British Columbia|BOLD:AAA4503
Eilema bicolor|[5838]|LBCH7921-10|10-JDWBC-7921|658[0n]bp|Canada.British Columbia|BOLD:AAA4503
Eilema bicolor|[5839]|BBLPA894-10|10BBCLP-0894|658[0n]bp|Canada.Alberta|BOLD:AAA4503
Eilema bicolor|[5840]|LPABB674-08|08BBLEP-03939|658[0n]bp|Canada.Alberta|BOLD:AAA4503
Eilema bicolor|[5841]|LBCH6338-10|10-JDWBC-6338|658[0n]bp|Canada.British Columbia|BOLD:AAA4503
Eilema bicolor|[5842]|LBCH7743-10|10-JDWBC-7743|658[0n]bp|Canada.British Columbia|BOLD:AAA4503
Eilema bicolor|[5843]|LBCH705-10|10-JDWBC-0705|658[0n]bp|Canada.British Columbia|BOLD:AAA4503
Eilema bicolor|[5844]|LBCH6840-10|10-JDWBC-6840|658[0n]bp|Canada.British Columbia|BOLD:AAA4503
Eilema bicolor|[5845]|DUNLP001-08|Dun-08-001|658[0n]bp|Canada.British Columbia|BOLD:AAA4503
Eilema bicolor|[5846]|LBCD378-05|HLC-23198|658[0n]bp|Canada.British Columbia|BOLD:AAA4503
Eilema bicolor|[5847]|LBCH818-10|10-JDWBC-0818|658[0n]bp|Canada.British Columbia|BOLD:AAA4503
Eilema bicolor|[5848]|LBCH7739-10|10-JDWBC-7739|658[0n]bp|Canada.British Columbia|BOLD:AAA4503
Eilema bicolor|[5849]|LBCH6478-10|10-JDWBC-6478|658[0n]bp|Canada.British Columbia|BOLD:AAA4503
[illegible]

Eilema bicolor|[5847]|LBCH818-10|10-JDWBC-0818|658[0n]bp|Canada.British Columbia|BOLD:AAA4503
Eilema bicolor|[5848]|LBCH7739-10|10-JDWBC-7739|658[0n]bp|Canada.British Columbia|BOLD:AAA4503
Eilema bicolor|[5849]|LBCH6478-10|10-JDWBC-6478|658[0n]bp|Canada.British Columbia|BOLD:AAA4503
Eilema bicolor|[5850]|LBCH7919-10|10-JDWBC-7919|658[0n]bp|Canada.British Columbia|BOLD:AAA4503
Eilema bicolor|[5851]|LBCH6479-10|10-JDWBC-6479|658[0n]bp|Canada.British Columbia|BOLD:AAA4503
Eilema bicolor|[5852]|LOWCB578-05|CGWC-1518|658[0n]bp|Canada.British Columbia|BOLD:AAA4503
Eilema bicolor|[5853]|LBCH7736-10|10-JDWBC-7736|658[0n]bp|Canada.British Columbia|BOLD:AAA4503
Eilema bicolor|[5854]|LBCG2588-09|08-JDWBC-2588|658[0n]bp|Canada.British Columbia|BOLD:AAA4503
Eilema bicolor|[5855]|LBCH6477-10|10-JDWBC-6477|658[0n]bp|Canada.British Columbia|BOLD:AAA4503
Eilema bicolor|[5856]|LBCH700-10|10-JDWBC-0700|658[0n]bp|Canada.British Columbia|BOLD:AAA4503
Eilema bicolor|[5857]|LBCD385-05|HLC-23205|658[0n]bp|Canada.British Columbia|BOLD:AAA4503
Eilema bicolor|[5858]|LBCH6342-10|10-JDWBC-6342|658[0n]bp|Canada.British Columbia|BOLD:AAA4503
Eilema bicolor|[5859]|LBCH7378-10|10-JDWBC-7378|658[0n]bp|Canada.British Columbia|BOLD:AAA4503
Eilema bicolor|[5860]|LBCH702-10|10-JDWBC-0702|658[0n]bp|Canada.British Columbia|BOLD:AAA4503
Eilema bicolor|[5861]|LBCH7737-10|10-JDWBC-7737|658[0n]bp|Canada.British Columbia|BOLD:AAA4503
Eilema bicolor|[5862]|LBCH7508-10|10-JDWBC-7508|658[0n]bp|Canada.British Columbia|BOLD:AAA4503
Eilema bicolor|[5863]|LBCH7922-10|10-JDWBC-7922|658[0n]bp|Canada.British Columbia|BOLD:AAA4503
Eilema bicolor|[5864]|LBCH821-10|10-JDWBC-0821|658[0n]bp|Canada.British Columbia|BOLD:AAA4503
Eilema bicolor|[5865]|LBCH6482-10|10-JDWBC-6482|658[0n]bp|Canada.British Columbia|BOLD:AAA4503
Eilema bicolor|[5866]|LBCH7382-10|10-JDWBC-7382|658[0n]bp|Canada.British Columbia|BOLD:AAA4503
Eilema bicolor|[5867]|BBLPA890-10|10BBCLP-0890|658[0n]bp|Canada.British Columbia|BOLD:AAA4503
Eilema bicolor|[5868]|LBCH7384-10|10-JDWBC-7384|658[0n]bp|Canada.British Columbia|BOLD:AAA4503
Eilema bicolor|[5869]|LBCD381-05|HLC-23201|658[0n]bp|Canada.British Columbia|BOLD:AAA4503
Eilema bicolor|[5870]|LBCH6481-10|10-JDWBC-6481|658[0n]bp|Canada.British Columbia|BOLD:AAA4503
Eilema bicolor|[5871]|LBCH6343-10|10-JDWBC-6343|643[0n]bp|Canada.British Columbia|BOLD:AAA4503
Eilema bicolor|[5872]|LBCH7428-10|10-JDWBC-7428|643[0n]bp|Canada.British Columbia|BOLD:AAA4503
Eilema bicolor|[5873]|LOWCB576-05|CGWC-1516|516[1n]bp|Canada.British Columbia|BOLD:AAA4503
Eilema bicolor|[5874]|LOWCB585-05|CGWC-1525|514[3n]bp|Canada.British Columbia|BOLD:AAA4503
Eilema bicolor|[5875]|LPABB154-08|08BBLEP-03419|643[0n]bp|Canada.Alberta|BOLD:AAA4503
Eilema bicolor|[5876]|LOWCB586-05|CGWC-1526|514[1n]bp|Canada.British Columbia|BOLD:AAA4503
Eilema bicolor|[5877]|LBCH7506-10|10-JDWBC-7506|640[0n]bp|Canada.British Columbia|BOLD:AAA4503
Eilema bicolor|[5878]|LOWCB581-05|CGWC-1521|616[0n]bp|Canada.British Columbia|BOLD:AAA4503
Eilema bicolor|[5879]|LOWCB569-05|CGWC-1509|515[0n]bp|Canada.British Columbia|BOLD:AAA4503
Eilema bicolor|[5880]|LBCH7149-10|10-JDWBC-7149|658[0n]bp|Canada.British Columbia|BOLD:AAA4503
Eilema bicolor|[5881]|BBLPA892-10|10BBCLP-0892|658[0n]bp|Canada.Alberta|BOLD:AAA4503
Eilema bicolor|[5882]|BBLPA891-10|10BBCLP-0891|658[0n]bp|Canada.Alberta|BOLD:AAA4503
Eilema bicolor|[5883]|LBCH7924-10|10-JDWBC-7924|658[0n]bp|Canada.British Columbia|BOLD:AAA4503
Eilema bicolor|[5884]|LBCG240-08|08-JDWBC-0240|658[0n]bp|Canada.British Columbia|BOLD:AAA4503
Eilema bicolor|[5885]|LBCH6963-10|10-JDWBC-6963|658[0n]bp|Canada.British Columbia|BOLD:AAA4503
Eilema bicolor|[5886]|LBCH7144-10|10-JDWBC-7144|658[0n]bp|Canada.British Columbia|BOLD:AAA4503
Eilema bicolor|[5887]|LBCH6341-10|10-JDWBC-6341|658[0n]bp|Canada.British Columbia|BOLD:AAA4503
Eilema bicolor|[5888]|LBCH820-10|10-JDWBC-0820|658[0n]bp|Canada.British Columbia|BOLD:AAA4503
Eilema bicolor|[5889]|LBCH819-10|10-JDWBC-0819|658[0n]bp|Canada.British Columbia|BOLD:AAA4503
Eilema bicolor|[5890]|LBCD388-05|HLC-23208|658[0n]bp|Canada.British Columbia|BOLD:AAA4503
Eilema bicolor|[5891]|LBCH122-10|10-JDWBC-0122|658[0n]bp|Canada.British Columbia|BOLD:AAA4503
Eilema bicolor|[5892]|LBCH6340-10|10-JDWBC-6340|658[0n]bp|Canada.British Columbia|BOLD:AAA4503
Eilema bicolor|[5893]|LBCD387-05|HLC-23207|658[0n]bp|Canada.British Columbia|BOLD:AAA4503
Clemensia albata|[5894]|GWNC614-07|CNCLEP00034176|658[0n]bp|Canada.Ontario|BOLD:ACF3441
Clemensia albata|[5895]|RDNMH799-09|CNCLEP00064127|658[0n]bp|Canada.Ontario|BOLD:ACF3441
Clemensia albata|[5896]|XAK559-07|HLC-16112|593[0n]bp|Canada.Ontario|BOLD:ACF3441
Clemensia albata|[5897]|LPVIA957-08|PFC-2006-1272|581[0n]bp|Canada.British Columbia|BOLD:AAA4333
Clemensia albata|[5898]|RDMAB082-05|UASM57609|633[0n]bp|Canada.Alberta|BOLD:AAA4333
Clemensia albata|[5899]|MNAG089-08|CNCLEP00041074|658[0n]bp|Canada.Manitoba|BOLD:AAA4333
Clemensia albata|[5900]|LPMN398-08|08BBLEP-01197|658[0n]bp|Canada.Manitoba|BOLD:AAA4333
Clemensia albata|[5901]|LBCH203-10|10-JDWBC-0203|658[0n]bp|Canada.British Columbia|BOLD:AAA4333
Clemensia albata|[5902]|LPMN402-08|08BBLEP-01201|658[0n]bp|Canada.Manitoba|BOLD:AAA4333
Clemensia albata|[5903]|LBCH4852-10|10-JDWBC-4852|658[0n]bp|Canada.British Columbia|BOLD:AAA4333
Clemensia albata|[5904]|TMNBD367-07|MNBTT-3168|656[0n]bp|Canada.New Brunswick|BOLD:AAA4333
Clemensia albata|[5905]|LBCH605-10|10-JDWBC-0605|658[0n]bp|Canada.British Columbia|BOLD:AAA4333
Clemensia albata|[5906]|LPSOD999-09|08BBLEP-05632|658[0n]bp|Canada.Ontario|BOLD:AAA4333
Clemensia albata|[5907]|LPMN395-08|08BBLEP-01194|658[0n]bp|Canada.Manitoba|BOLD:AAA4333
Clemensia albata|[5908]|LPVIA194-08|PFC-2006-0262|658[0n]bp|Canada.British Columbia|BOLD:AAA4333
Clemensia albata|[5909]|LBCH3661-10|10-JDWBC-3661|658[0n]bp|Canada.British Columbia|BOLD:AAA4333
Clemensia albata|[5910]|LBCC541-05|HLC-22421|658[0n]bp|Canada.British Columbia|BOLD:AAA4333
Clemensia albata|[5911]|LPMN367-08|08BBLEP-01166|658[0n]bp|Canada.Manitoba|BOLD:AAA4333
Clemensia albata|[5912]|MNBB465-05|05-NBSTA-381|658[5n]bp|Canada.New Brunswick|BOLD:AAA4333
Clemensia albata|[5913]|LPVIA580-08|PFC-2006-0807|636[0n]bp|Canada.British Columbia|BOLD:AAA4333
Clemensia albata|[5914]|XAK581-07|HLC-16134|594[0n]bp|Canada.Ontario|BOLD:AAA4333
Clemensia albata|[5915]|RDLQE456-06|MDH002459|656[0n]bp|Canada.Quebec|BOLD:AAA4333
Clemensia albata|[5916]|LBCH4853-10|10-JDWBC-4853|658[0n]bp|Canada.British Columbia|BOLD:AAA4333
Clemensia albata|[5917]|LBCH085-10|10-JDWBC-0085|658[0n]bp|Canada.British Columbia|BOLD:AAA4333
Clemensia albata|[5918]|LBCH3830-10|10-JDWBC-3830|658[0n]bp|Canada.British Columbia|BOLD:AAA4333
Clemensia albata|[5919]|LBCH322-10|10-JDWBC-0322|658[0n]bp|Canada.British Columbia|BOLD:AAA4333
Clemensia albata|[5920]|LBCH3832-10|10-JDWBC-3832|658[0n]bp|Canada.British Columbia|BOLD:AAA4333
Clemensia albata|[5921]|LBCH204-10|10-JDWBC-0204|658[0n]bp|Canada.British Columbia|BOLD:AAA4333
Clemensia albata|[5922]|LBCH4654-10|10-JDWBC-4654|658[0n]bp|Canada.British Columbia|BOLD:AAA4333
Clemensia albata|[5923]|LBCH3372-10|10-JDWBC-3372|658[0n]bp|Canada.British Columbia|BOLD:AAA4333
Clemensia albata|[5924]|LBCH4488-10|10-JDWBC-4488|658[0n]bp|Canada.British Columbia|BOLD:AAA4333
Clemensia albata|[5925]|LBCG2243-09|08-JDWBC-2243|658[0n]bp|Canada.British Columbia|BOLD:AAA4333
Clemensia albata|[5926]|LBCH745-10|10-JDWBC-0745|658[0n]bp|Canada.British Columbia|BOLD:AAA4333
Clemensia albata|[5927]|LBCH3663-10|10-JDWBC-3663|658[0n]bp|Canada.British Columbia|BOLD:AAA4333
Clemensia albata|[5928]|LBCH4082-10|10-JDWBC-4082|658[0n]bp|Canada.British Columbia|BOLD:AAA4333
Clemensia albata|[5929]|BBLPD739-10|10BBCLP-2737|658[0n]bp|Canada.British Columbia|BOLD:AAA4333
Clemensia albata|[5930]|LBCS242-07|UBC-2007-0623|658[0n]bp|Canada.British Columbia|BOLD:AAA4333
Clemensia albata|[5931]|LBCD575-05|HLC-23395|658[0n]bp|Canada.British Columbia|BOLD:AAA4333
Clemensia albata|[5932]|MNBB497-05|05-NBSTA-413|658[0n]bp|Canada.New Brunswick|BOLD:AAA4333
Clemensia albata|[5933]|LALPA401-10|AVBC 403-10|658[0n]bp|Canada.British Columbia|BOLD:AAA4333
Clemensia albata|[5934]|MNBB689-05|05-NBSTA-605|658[0n]bp|Canada.New Brunswick|BOLD:AAA4333
Clemensia albata|[5935]|TMNBD368-07|MNBTT-3169|658[0n]bp|Canada.New Brunswick|BOLD:AAA4333
Clemensia albata|[5936]|LBCD873-05|HLC-23693|658[0n]bp|Canada.British Columbia|BOLD:AAA4333
Clemensia albata|[5937]|LBCD647-05|HLC-23467|658[0n]bp|Canada.British Columbia|BOLD:AAA4333
Clemensia albata|[5938]|BBLPD741-10|10BBCLP-2739|658[0n]bp|Canada.British Columbia|BOLD:AAA4333
Clemensia albata|[5939]|BBLPD738-10|10BBCLP-2736|658[0n]bp|Canada.British Columbia|BOLD:AAA4333
Clemensia albata|[5940]|LPVIA579-08|PFC-2006-0806|658[0n]bp|Canada.British Columbia|BOLD:AAA4333
Clemensia albata|[5941]|LBCD576-05|HLC-23396|658[0n]bp|Canada.British Columbia|BOLD:AAA4333
Clemensia albata|[5942]|LBCB747-05|HLC-21687|658[0n]bp|Canada.British Columbia|BOLD:AAA4333
Clemensia albata|[5943]|BBLPD663-10|10BBCLP-2661|658[0n]bp|Canada.British Columbia|BOLD:AAA4333
Clemensia albata|[5944]|RDLQG613-06|DH012906|658[0n]bp|Canada.Quebec|BOLD:AAA4333
Clemensia albata|[5945]|LBCD874-05|HLC-23694|658[0n]bp|Canada.British Columbia|BOLD:AAA4333
Clemensia albata|[5946]|LALPA459-10|AVBC 461-10|658[0n]bp|Canada.British Columbia|BOLD:AAA4333
Clemensia albata|[5947]|LALPA499-10|AVBC 501-10|658[0n]bp|Canada.British Columbia|BOLD:AAA4333
Clemensia albata|[5948]|LPGVA732-08|UBC-2006-2061|658[0n]bp|Canada.British Columbia|BOLD:AAA4333
Clemensia albata|[5949]|LBCD646-05|HLC-23466|658[0n]bp|Canada.British Columbia|BOLD:AAA4333

Clemensia albata|[5946]|[illegible]|Canada.British Columbia|[illegible]
Clemensia albata|[5947]|LALPA499-10|AVBC 501-10|658[0n]bp|Canada.British Columbia|BOLD:AAA4333
Clemensia albata|[5948]|LPGVA732-08|UBC-2006-2061|658[0n]bp|Canada.British Columbia|BOLD:AAA4333
Clemensia albata|[5949]|LBCD646-05|HLC-23466|658[0n]bp|Canada.British Columbia|BOLD:AAA4333
Clemensia albata|[5950]|LPVIA577-08|PFC-2006-0804|658[0n]bp|Canada.British Columbia|BOLD:AAA4333
Clemensia albata|[5951]|LPVIA849-08|PFC-2006-1150|658[0n]bp|Canada.British Columbia|BOLD:AAA4333
Clemensia albata|[5952]|LALPA437-10|AVBC 439-10|658[0n]bp|Canada.British Columbia|BOLD:AAA4333
Clemensia albata|[5953]|LPVIA851-08|PFC-2006-1152|658[0n]bp|Canada.British Columbia|BOLD:AAA4333
Clemensia albata|[5954]|TMNBD366-07|MNBTT-3167|631[0n]bp|Canada.New Brunswick|BOLD:AAA4333
Clemensia albata|[5955]|LBCH3088-10|10-JDWBC-3088|642[0n]bp|Canada.British Columbia|BOLD:AAA4333
Clemensia albata|[5956]|TMNBD369-07|MNBTT-3170|641[0n]bp|Canada.New Brunswick|BOLD:AAA4333
Clemensia albata|[5957]|BBLPD740-10|10BBCLP-2738|622[0n]bp|Canada.British Columbia|BOLD:AAA4333
Clemensia albata|[5958]|LBCB331-05|HLC-21271|658[0n]bp|Canada.British Columbia|BOLD:AAA4333
Clemensia albata|[5959]|LPVIA578-08|PFC-2006-0805|658[0n]bp|Canada.British Columbia|BOLD:AAA4333
Gabara subnivosella|[5960]|LOFLA871-06|06-FLOR-0871|658[0n]bp|United States.Florida|BOLD:AAB8349
Gabara subnivosella|[5961]|ABNCC239-07|1239-250503-OK|635[0n]bp|United States.Oklahoma|BOLD:ACE7401
Gabara subnivosella|[5962]|LNCB412-06|06-NCCC-1368|658[0n]bp|United States.North Carolina|BOLD:ABX6126
Gabara subnivosella|[5963]|LNCB415-06|06-NCCC-1371|658[0n]bp|United States.North Carolina|BOLD:AAB6666
Gabara subnivosella|[5964]|LNCC823-11|11-NCCC-348|658[0n]bp|United States.North Carolina|BOLD:AAB6666
Gabara subnivosella|[5965]|LNCB403-06|06-NCCC-1359|658[0n]bp|United States.North Carolina|BOLD:AAB6666
Gabara subnivosella|[5966]|LNCB402-06|06-NCCC-1358|658[0n]bp|United States.North Carolina|BOLD:AAB6666
Gabara subnivosella|[5967]|LNCB411-06|06-NCCC-1367|658[0n]bp|United States.North Carolina|BOLD:AAB6666
Gabara subnivosella|[5968]|LNCB414-06|06-NCCC-1370|658[0n]bp|United States.North Carolina|BOLD:AAB6666
Gabara subnivosella|[5969]|LNCB409-06|06-NCCC-1365|658[0n]bp|United States.North Carolina|BOLD:AAB6666
Gabara subnivosella|[5970]|LNCB405-06|06-NCCC-1361|658[0n]bp|United States.North Carolina|BOLD:AAB6666
Gabara subnivosella|[5971]|LNC368-05|05-NCCC-368|565[2n]bp|United States.North Carolina|BOLD:AAB6666
Gabara subnivosella|[5972]|LNC367-05|05-NCCC-367|556[0n]bp|United States.North Carolina|BOLD:AAB6666
Gabara subnivosella|[5973]|LNCB413-06|06-NCCC-1369|658[0n]bp|United States.North Carolina|BOLD:AAB6666
Gabara subnivosella|[5974]|LNCB406-06|06-NCCC-1362|658[0n]bp|United States.North Carolina|BOLD:AAB6666
Gabara subnivosella|[5975]|LNCB408-06|06-NCCC-1364|658[0n]bp|United States.North Carolina|BOLD:AAB6666
Gabara subnivosella|[5976]|LNCC822-11|11-NCCC-347|658[0n]bp|United States.North Carolina|BOLD:AAB6666
Gabara subnivosella|[5977]|LNCB407-06|06-NCCC-1363|658[0n]bp|United States.North Carolina|BOLD:AAB6666
Gabara subnivosella|[5978]|LNCB410-06|06-NCCC-1366|658[0n]bp|United States.North Carolina|BOLD:AAB6666
Scolecocampa liburna|[5979]|LPOKA193-08|MDOK-0193|658[0n]bp|United States.Oklahoma|BOLD:AAC1567
Scolecocampa liburna|[5980]|LPOKB374-09|MDOK-1411|658[0n]bp|United States.Oklahoma|BOLD:AAC1567
Scolecocampa liburna|[5981]|LOFLA546-06|06-FLOR-0546|658[0n]bp|United States.Florida|BOLD:AAC1567
Scolecocampa liburna|[5982]|BBLOB504-11|BIOUG01397-C05|658[0n]bp|United States.Florida|BOLD:AAC1567
Scolecocampa liburna|[5983]|LTOLB762-11|AM-93-1005|658[0n]bp|United States.Maryland|BOLD:AAC1567
Scolecocampa liburna|[5984]|LGSMG559-07|BGS03922|658[0n]bp|United States.North Carolina|BOLD:AAC1567
Scolecocampa liburna|[5985]|LOFLA534-06|06-FLOR-0534|658[0n]bp|United States.Florida|BOLD:AAC1567
Scolecocampa liburna|[5986]|LILLA674-11|SNS10IL-00853|658[0n]bp|United States.Illinois|BOLD:AAC1567
Scolecocampa liburna|[5987]|LOFLC117-06|06-FLOR-1997|652[0n]bp|United States.Florida|BOLD:AAC1567
Scolecocampa liburna|[5988]|LSUSA226-06|06-SUSA-0226|606[0n]bp|United States.Kentucky|BOLD:AAC1567
Scolecocampa liburna|[5989]|LGSM415-04|DNA-ATBI-0415|658[0n]bp|United States.Tennessee|BOLD:AAC1567
Scolecocampa liburna|[5990]|LNCC1402-11|11-NCCC-927|658[0n]bp|United States.North Carolina|BOLD:AAC1567
Scolecocampa liburna|[5991]|RDNMD787-07|CNCNoctuoidea13119|658[0n]bp|United States.North Carolina|BOLD ...
Scolecocampa liburna|[5992]|LOT187-04|04HBL002187|609[0n]bp|United States.Tennessee|BOLD:AAC1567
Scolecocampa liburna|[5993]|LPOKD243-09|MDOK-3322|657[0n]bp|United States.Oklahoma|BOLD:AAC1567
Scolecocampa liburna|[5994]|ABNCC235-07|1235-090504-TX|633[0n]bp|United States.Texas|BOLD:AAC1567
Scolecocampa liburna|[5995]|BBLSX107-09|09BBLEP-02035|658[0n]bp|United States.Oklahoma|BOLD:AAC1567
Scolecocampa liburna|[5996]|MILEQ259-11|11-MISC-734|658[0n]bp|United States.Alabama|BOLD:AAC1567
Scolecocampa liburna|[5997]|BBLSX106-09|09BBLEP-02034|658[0n]bp|United States.Oklahoma|BOLD:AAC1567
Scolecocampa liburna|[5998]|BBLOB655-11|BIOUG01398-H01|658[0n]bp|United States.Florida|BOLD:AAC1567
Scolecocampa liburna|[5999]|MILEQ257-11|11-MISC-732|631[0n]bp|United States.Alabama|BOLD:AAC1567
Scolecocampa liburna|[6000]|LNC244-05|05-NCCC-244|602[0n]bp|United States.North Carolina|BOLD:AAC1567
Scolecocampa liburna|[6001]|LGSM414-04|DNA-ATBI-0414|658[0n]bp|United States.North Carolina|BOLD:AAC1567
Scolecocampa liburna|[6002]|MILEQ258-11|11-MISC-733|658[0n]bp|United States.Alabama|BOLD:AAC1567
Scolecocampa liburna|[6003]|MILEQ256-11|11-MISC-731|658[0n]bp|United States.Alabama|BOLD:AAC1567
Scolecocampa liburna|[6004]|LNCC1403-11|11-NCCC-928|658[0n]bp|United States.North Carolina|BOLD:AAC1567
Scolecocampa liburna|[6005]|LNC109-05|05-NCCC-109|658[0n]bp|United States.North Carolina|BOLD:AAC1567
Scolecocampa liburna|[6006]|LNCC1253-11|11-NCCC-778|658[0n]bp|United States.North Carolina|BOLD:AAC1567
Scolecocampa liburna|[6007]|LNCC1254-11|11-NCCC-779|658[0n]bp|United States.North Carolina|BOLD:AAC1567
Lophocampa argentata|[6008]|RDNME202-07|CNCNoctuoidea13809|612[0n]bp|Canada.British Columbia|BOLD:AAB...
Lophocampa argentata|[6009]|LHLEP298-06|UBC-2006-1055|657[0n]bp|Canada.British Columbia|BOLD:AAB4184
Lophocampa argentata|[6010]|LHLEP299-06|UBC-2006-1651|657[0n]bp|Canada.British Columbia|BOLD:AAB4184
Lophocampa argentata|[6011]|LMH035-06|PFC-2006-0448|658[0n]bp|Canada.British Columbia|BOLD:AAB4184
Lophocampa argentata|[6012]|LBCS257-07|UBC-2007-0765|658[0n]bp|Canada.British Columbia|BOLD:AAB4184
Lophocampa argentata|[6013]|LALPA385-10|AVBC 387-10|658[0n]bp|Canada.British Columbia|BOLD:AAB4184
Lophocampa argentata|[6014]|LALPA374-10|AVBC 376-10|632[0n]bp|Canada.British Columbia|BOLD:AAB4184
Lophocampa argentata|[6015]|RDNME409-08|LEP037833|658[0n]bp|Canada.British Columbia|BOLD:AAB4184
Lophocampa argentata|[6016]|DUNLP005-08|Dun-08-005|658[0n]bp|Canada.British Columbia|BOLD:AAB4184
Lophocampa argentata|[6017]|DUNLP006-08|Dun-08-006|658[0n]bp|Canada.British Columbia|BOLD:AAB4184
Lophocampa argentata|[6018]|LALPA375-10|AVBC 377-10|658[0n]bp|Canada.British Columbia|BOLD:AAB4184
Lophocampa argentata|[6019]|LHLEP297-06|UBC-2006-0732|657[0n]bp|Canada.British Columbia|BOLD:AAB4184
Lophocampa argentata|[6020]|LBCS258-07|UBC-2007-0766|658[0n]bp|Canada.British Columbia|BOLD:AAB4184
Lophocampa argentata|[6021]|LBCW030-08|08-JDWWI-0030|658[0n]bp|Canada.British Columbia|BOLD:AAB4184
Lophocampa argentata|[6022]|RDNME408-08|LEP037832|658[0n]bp|Canada.British Columbia|BOLD:AAB4184
Lophocampa argentata|[6023]|LBCW031-08|08-JDWWI-0031|658[0n]bp|Canada.British Columbia|BOLD:AAB4184
Lophocampa argentata|[6024]|ARCTA072-07|MILA 0072|658[0n]bp|Canada.British Columbia|BOLD:AAB4184
Lophocampa caryae|[6025]|LPSO879-08|PPBP-0879|658[0n]bp|Canada.Ontario|BOLD:AAB3918
Lophocampa caryae|[6026]|LPSO852-08|PPBP-0852|647[0n]bp|Canada.Ontario|BOLD:AAB3918
Lophocampa caryae|[6027]|LPSO853-08|PPBP-0853|609[0n]bp|Canada.Ontario|BOLD:AAB3918
Lophocampa caryae|[6028]|RDNME456-08|LEP037880|658[0n]bp|Canada.Ontario|BOLD:AAB3918
Lophocampa caryae|[6029]|TMNBD167-07|MNBTT-2968|658[0n]bp|Canada.New Brunswick|BOLD:AAB3918
Lophocampa caryae|[6030]|LPSOB511-08|PPBP-1510|658[0n]bp|Canada.Ontario|BOLD:AAB3918
Lophocampa caryae|[6031]|TMNBD163-07|MNBTT-2964|658[0n]bp|Canada.New Brunswick|BOLD:AAB3918
Lophocampa caryae|[6032]|LPSO639-08|PPBP-0639|658[0n]bp|Canada.Ontario|BOLD:AAB3918
Lophocampa caryae|[6033]|LPSO854-08|PPBP-0854|658[0n]bp|Canada.Ontario|BOLD:AAB3918
Lophocampa caryae|[6034]|TMNBD166-07|MNBTT-2967|658[0n]bp|Canada.New Brunswick|BOLD:AAB3918
Lophocampa caryae|[6035]|RDNME411-08|LEP037835|658[0n]bp|Canada.Ontario|BOLD:AAB3918
Lophocampa caryae|[6036]|TMNBD165-07|MNBTT-2966|658[0n]bp|Canada.New Brunswick|BOLD:AAB3918
Lophocampa caryae|[6037]|TMNBD164-07|MNBTT-2965|658[0n]bp|Canada.New Brunswick|BOLD:AAB3918
Lophocampa caryae|[6038]|LPSO880-08|PPBP-0880|658[0n]bp|Canada.Ontario|BOLD:AAB3918
Lophocampa caryae|[6039]|LPSO640-08|PPBP-0640|658[0n]bp|Canada.Ontario|BOLD:AAB3918
Lophocampa maculata|[6040]|LBCS124-07|UBC-2007-0115|658[0n]bp|Canada.British Columbia|BOLD:AAA3071
Lophocampa maculata|[6041]|LBCS664-07|UBC-2007-0369|658[0n]bp|Canada.British Columbia|BOLD:AAA3071
Lophocampa maculata|[6042]|BBLPA348-10|10BBCLP-0348|658[0n]bp|Canada.British Columbia|BOLD:AAA3071
Lophocampa maculata|[6043]|LPSOD423-09|08BBLEP-00202|658[0n]bp|Canada.Ontario|BOLD:AAA3071
Lophocampa maculata|[6044]|LBCA346-05|HLC-20346|658[0n]bp|Canada.British Columbia|BOLD:AAA3071
Lophocampa maculata|[6045]|LOWCE117-06|CGWC-3877|658[0n]bp|Canada.British Columbia|BOLD:AAA3071
Lophocampa maculata|[6046]|LPSOD237-09|08BBLEP-00015|658[0n]bp|Canada.Ontario|BOLD:AAA3071
Lophocampa maculata|[6047]|LBCA740-05|HLC-20740|658[0n]bp|Canada.British Columbia|BOLD:AAA3071
Lophocampa maculata|[6048]|LBCA345-05|HLC-20345|658[0n]bp|Canada.British Columbia|BOLD:AAA3071

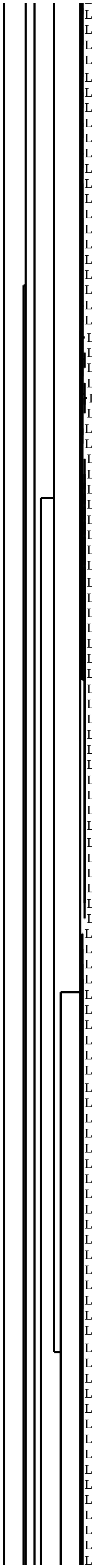

[illegible]
Lophocampa maculata|[6046]|LPSOD237-09|08BBLEP-00015|658[0n]bp|Canada.Ontario|BOLD:AAA3071
Lophocampa maculata|[6047]|LBCA740-05|HLC-20740|658[0n]bp|Canada.British Columbia|BOLD:AAA3071
Lophocampa maculata|[6048]|LBCA345-05|HLC-20345|658[0n]bp|Canada.British Columbia|BOLD:AAA3071
Lophocampa maculata|[6049]|BBLPA347-10|10BBCLP-0347|658[0n]bp|Canada.Ontario|BOLD:AAA3071
Lophocampa maculata|[6050]|LPSOB248-08|PPBP-1247|658[0n]bp|Canada.Ontario|BOLD:AAA3071
Lophocampa maculata|[6051]|LPSOD573-09|08BBLEP-00354|658[0n]bp|Canada.Ontario|BOLD:AAA3071
Lophocampa maculata|[6052]|LBCS123-07|UBC-2007-0114|658[0n]bp|Canada.British Columbia|BOLD:AAA3071
Lophocampa maculata|[6053]|LBCS663-07|UBC-2007-0368|658[0n]bp|Canada.British Columbia|BOLD:AAA3071
Lophocampa maculata|[6054]|LALPA113-10|AVBC 113-10|658[0n]bp|Canada.British Columbia|BOLD:AAA3071
Lophocampa maculata|[6055]|LPSOD422-09|08BBLEP-00201|658[0n]bp|Canada.Ontario|BOLD:AAA3071
Lophocampa maculata|[6056]|LOWCE113-06|CGWC-3873|658[0n]bp|Canada.British Columbia|BOLD:AAA3071
Lophocampa maculata|[6057]|LBCS125-07|UBC-2007-0116|658[0n]bp|Canada.British Columbia|BOLD:AAA3071
Lophocampa maculata|[6058]|LPSOB793-08|PPBP-1792|658[0n]bp|Canada.Ontario|BOLD:AAA3071
Lophocampa maculata|[6059]|LPSOD419-09|08BBLEP-00198|658[0n]bp|Canada.Ontario|BOLD:AAA3071
Lophocampa maculata|[6060]|RDMAB922-06|BCSC395|658[0n]bp|Canada.Alberta|BOLD:AAA3071
Lophocampa maculata|[6061]|LBCA779-05|HLC-20779|658[0n]bp|Canada.British Columbia|BOLD:AAA3071
Lophocampa maculata|[6062]|LALPA146-10|AVBC 146-10|658[0n]bp|Canada.British Columbia|BOLD:AAA3071
Lophocampa maculata|[6063]|XAJ656-06|2006-ONT-0656|658[0n]bp|Canada.Ontario|BOLD:AAA3071
Lophocampa maculata|[6064]|LOWCB667-05|CGWC-1607|658[0n]bp|Canada.British Columbia|BOLD:AAA3071
Lophocampa maculata|[6065]|LPSOD873-09|08BBLEP-00655|658[0n]bp|Canada.Ontario|BOLD:AAA3071
Lophocampa maculata|[6066]|RDLQG444-06|DH012728|658[0n]bp|Canada.Quebec|BOLD:AAA3071
Lophocampa maculata|[6067]|LMH004-06|PFC-2006-0006|658[0n]bp|Canada.British Columbia|BOLD:AAA3071
Lophocampa maculata|[6068]|LOWCE118-06|CGWC-3878|658[0n]bp|Canada.British Columbia|BOLD:AAA3071
Lophocampa maculata|[6069]|LOWCE115-06|CGWC-3875|658[0n]bp|Canada.British Columbia|BOLD:AAA3071
Lophocampa maculata|[6070]|PMG014-03|moth656.01|617[0n]bp|Canada.Ontario|BOLD:AAA3071
Lophocampa maculata|[6071]|TMG85-03|moth655.01|639[0n]bp|Canada.Ontario|BOLD:AAA3071
Lophocampa maculata|[6072]|TMG84-03|moth662.01|639[0n]bp|Canada.Ontario|BOLD:AAA3071
Lophocampa maculata|[6073]|LBCS440-07|UBC-2007-0196|658[0n]bp|Canada.British Columbia|BOLD:AAA3071
Lophocampa maculata|[6074]|LMH026-06|PFC-2006-0147|658[0n]bp|Canada.British Columbia|BOLD:AAA3071
Lophocampa maculata|[6075]|XAJ495-06|2006-ONT-0495|656[0n]bp|Canada.Ontario|BOLD:AAA3071
Lophocampa maculata|[6076]|LBCS666-07|UBC-2007-0371|658[0n]bp|Canada.British Columbia|BOLD:AAA3071
Lophocampa maculata|[6077]|XAB185-04|04HBL005185|658[0n]bp|Canada.Ontario|BOLD:AAA3071
Lophocampa maculata|[6078]|LBCD032-05|HLC-22852|658[0n]bp|Canada.British Columbia|BOLD:AAA3071
Lophocampa maculata|[6079]|LBCC030-05|HLC-21910|658[0n]bp|Canada.British Columbia|BOLD:AAA3071
Lophocampa maculata|[6080]|LBCA492-05|HLC-20492|658[0n]bp|Canada.British Columbia|BOLD:AAA3071
Lophocampa maculata|[6081]|LBCA780-05|HLC-20780|658[0n]bp|Canada.British Columbia|BOLD:AAA3071
Lophocampa maculata|[6082]|LPABB327-08|08BBLEP-03592|658[0n]bp|Canada.Alberta|BOLD:AAA3071
Lophocampa maculata|[6083]|LOWCE116-06|CGWC-3876|658[0n]bp|Canada.British Columbia|BOLD:AAA3071
Lophocampa maculata|[6084]|LBCC033-05|HLC-21913|658[0n]bp|Canada.British Columbia|BOLD:AAA3071
Lophocampa maculata|[6085]|LBCC029-05|HLC-21909|658[0n]bp|Canada.British Columbia|BOLD:AAA3071
Lophocampa maculata|[6086]|BBLPA350-10|10BBCLP-0350|658[0n]bp|Canada.British Columbia|BOLD:AAA3071
Lophocampa maculata|[6087]|LPABB318-08|08BBLEP-03583|658[0n]bp|Canada.Alberta|BOLD:AAA3071
Lophocampa maculata|[6088]|LBCA343-05|HLC-20343|658[0n]bp|Canada.British Columbia|BOLD:AAA3071
Lophocampa maculata|[6089]|LBCA344-05|HLC-20344|658[0n]bp|Canada.British Columbia|BOLD:AAA3071
Lophocampa maculata|[6090]|LBCB137-05|HLC-21077|658[0n]bp|Canada.British Columbia|BOLD:AAA3071
Lophocampa maculata|[6091]|LBCB138-05|HLC-21078|658[0n]bp|Canada.British Columbia|BOLD:AAA3071
Lophocampa maculata|[6092]|LBCC031-05|HLC-21911|658[0n]bp|Canada.British Columbia|BOLD:AAA3071
Lophocampa maculata|[6093]|LBCC032-05|HLC-21912|658[0n]bp|Canada.British Columbia|BOLD:AAA3071
Lophocampa maculata|[6094]|XAE185-04|Moth4185.03|658[0n]bp|Canada.Ontario|BOLD:AAA3071
Lophocampa maculata|[6095]|LBCA280-05|HLC-20280|658[0n]bp|Canada.British Columbia|BOLD:AAA3071
Lophocampa maculata|[6096]|LBCS667-07|UBC-2007-0372|658[0n]bp|Canada.British Columbia|BOLD:AAA3071
Lophocampa maculata|[6097]|XAJ624-06|2006-ONT-0624|658[0n]bp|Canada.Ontario|BOLD:AAA3071
Lophocampa maculata|[6098]|LBCS121-07|UBC-2007-0112|658[0n]bp|Canada.British Columbia|BOLD:AAA3071
Lophocampa maculata|[6099]|LOWCB668-05|CGWC-1608|658[0n]bp|Canada.British Columbia|BOLD:AAA3071
Lophocampa maculata|[6100]|LOWCE114-06|CGWC-3874|658[0n]bp|Canada.British Columbia|BOLD:AAA3071
Lophocampa maculata|[6101]|LBCS665-07|UBC-2007-0370|658[0n]bp|Canada.British Columbia|BOLD:AAA3071
Lophocampa maculata|[6102]|LBCS122-07|UBC-2007-0113|658[0n]bp|Canada.British Columbia|BOLD:AAA3071
Lophocampa maculata|[6103]|XAE389-04|Moth4389.03|658[0n]bp|Canada.Ontario|BOLD:AAA3071
Lophocampa maculata|[6104]|LPABB020-08|08BBLEP-03285|658[0n]bp|Canada.Alberta|BOLD:AAA3071
Lophocampa maculata|[6105]|ARCTA066-07|MILA 0066|658[0n]bp|Canada.British Columbia|BOLD:AAA3071
Lophocampa maculata|[6106]|LPSOB427-08|PPBP-1426|609[0n]bp|Canada.Ontario|BOLD:AAA3071
Lophocampa maculata|[6107]|LPSOC286-08|PPBP-2285|655[0n]bp|Canada.Ontario|BOLD:AAA3071
Lophocampa maculata|[6108]|LPMN296-08|08BBLEP-01095|658[0n]bp|Canada.Manitoba|BOLD:AAA3071
Lophocampa maculata|[6109]|TMNBD417-07|MNBTT-3218|658[0n]bp|Canada.New Brunswick|BOLD:AAA3071
Lophocampa maculata|[6110]|LPSOC168-08|PPBP-2167|658[0n]bp|Canada.Ontario|BOLD:AAA3071
Lophocampa maculata|[6111]|PHMNB450-04|04HBL00676|658[0n]bp|Canada.New Brunswick|BOLD:AAA3071
Lophocampa maculata|[6112]|PHMNB448-04|04HBL00674|658[0n]bp|Canada.New Brunswick|BOLD:AAA3071
Lophocampa maculata|[6113]|PHMNB449-04|04HBL00675|658[0n]bp|Canada.New Brunswick|BOLD:AAA3071
Lophocampa maculata|[6114]|LPSOD658-09|08BBLEP-00439|658[0n]bp|Canada.Ontario|BOLD:AAA3071
Lophocampa maculata|[6115]|LPABB319-08|08BBLEP-03584|658[0n]bp|Canada.Alberta|BOLD:AAA3071
Lophocampa maculata|[6116]|LPMN298-08|08BBLEP-01097|658[0n]bp|Canada.Manitoba|BOLD:AAA3071
Lophocampa maculata|[6117]|LPSOD421-09|08BBLEP-00200|658[0n]bp|Canada.Ontario|BOLD:AAA3071
Lophocampa maculata|[6118]|PHMNB356-04|04HBL00582|658[0n]bp|Canada.New Brunswick|BOLD:AAA3071
Lophocampa maculata|[6119]|LPMN295-08|08BBLEP-01094|658[0n]bp|Canada.Manitoba|BOLD:AAA3071
Lophocampa maculata|[6120]|LPMN709-08|08BBLEP-01512|658[0n]bp|Canada.Manitoba|BOLD:AAA3071
Lophocampa maculata|[6121]|LPSOB314-08|PPBP-1313|658[0n]bp|Canada.Ontario|BOLD:AAA3071
Lophocampa maculata|[6122]|LPSOD417-09|08BBLEP-00196|658[0n]bp|Canada.Ontario|BOLD:AAA3071
Lophocampa maculata|[6123]|TTMNB060-06|MNBTT-060|658[0n]bp|Canada.New Brunswick|BOLD:AAA3071
Lophocampa maculata|[6124]|PHMNB447-04|04HBL00673|658[0n]bp|Canada.New Brunswick|BOLD:AAA3071
Lophocampa maculata|[6125]|LPMN212-08|08BBLEP-01011|658[0n]bp|Canada.Manitoba|BOLD:AAA3071
Lophocampa maculata|[6126]|BBLPE374-09|09BBELE-2374|658[0n]bp|Canada.Newfoundland and Labrador|BOLD:A...
Lophocampa maculata|[6127]|LPABB328-08|08BBLEP-03593|658[0n]bp|Canada.Alberta|BOLD:AAA3071
Lophocampa maculata|[6128]|PHMNB595-04|04HBL00821|658[0n]bp|Canada.New Brunswick|BOLD:AAA3071
Lophocampa maculata|[6129]|LPSOD574-09|08BBLEP-00355|658[0n]bp|Canada.Ontario|BOLD:AAA3071
Lophocampa maculata|[6130]|TTMNB057-06|MNBTT-057|657[0n]bp|Canada.New Brunswick|BOLD:AAA3071
Lophocampa maculata|[6131]|LPMN299-08|08BBLEP-01098|658[0n]bp|Canada.Manitoba|BOLD:AAA3071
Lophocampa maculata|[6132]|LPSOD420-09|08BBLEP-00199|658[0n]bp|Canada.Ontario|BOLD:AAA3071
Lophocampa maculata|[6133]|LPMN301-08|08BBLEP-01100|658[0n]bp|Canada.Manitoba|BOLD:AAA3071
Lophocampa maculata|[6134]|LPSOD330-09|08BBLEP-00108|658[0n]bp|Canada.Ontario|BOLD:AAA3071
Lophocampa maculata|[6135]|LPMN211-08|08BBLEP-01010|658[0n]bp|Canada.Manitoba|BOLD:AAA3071
Lophocampa maculata|[6136]|XAB360-04|04HBL005360|658[0n]bp|Canada.Ontario|BOLD:AAA3071
Lophocampa maculata|[6137]|PHMNB324-04|04HBL00550|658[0n]bp|Canada.New Brunswick|BOLD:AAA3071
Lophocampa maculata|[6138]|PHMNB455-04|04HBL00681|658[0n]bp|Canada.New Brunswick|BOLD:AAA3071
Lophocampa maculata|[6139]|BBLEC371-09|09BBELE-0371|658[0n]bp|Canada.Newfoundland and Labrador|BOLD:A...
Lophocampa maculata|[6140]|BBLPA349-10|10BBCLP-0349|658[0n]bp|Canada.Saskatchewan|BOLD:AAA3071
Lophocampa maculata|[6141]|TMNBB034-06|MNBTT-974|658[0n]bp|Canada.New Brunswick|BOLD:AAA3071
Lophocampa maculata|[6142]|BBLEC275-09|09BBELE-0275|658[0n]bp|Canada.Nova Scotia|BOLD:AAA3071
Lophocampa maculata|[6143]|LPSOD857-09|08BBLEP-00639|658[0n]bp|Canada.Ontario|BOLD:AAA3071
Lophocampa maculata|[6144]|BBLPC894-09|09BBELE-1894|658[0n]bp|Canada.Newfoundland and Labrador|BOLD:A...
Lophocampa maculata|[6145]|LPSOD637-09|08BBLEP-00418|658[0n]bp|Canada.Ontario|BOLD:AAA3071
Lophocampa maculata|[6146]|BBLPA351-10|10BBCLP-0351|658[0n]bp|Canada.Ontario|BOLD:AAA3071
Lophocampa maculata|[6147]|BBLPC966-09|09BBELE-1966|658[0n]bp|Canada.Newfoundland and Labrador|BOLD:A...

Lophocampa maculata|[6145]|LPSOD637-09|08BBLEP-00418|658[0n]bp|Canada.Ontario|BOLD:AAA3071
Lophocampa maculata|[6146]|BBLPA351-10|10BBCLP-0351|658[0n]bp|Canada.Ontario|BOLD:AAA3071
Lophocampa maculata|[6147]|BBLPC966-09|09BBELE-1966|658[0n]bp|Canada.Newfoundland and Labrador|BOLD:A...
Lophocampa maculata|[6148]|TMNBB033-06|MNBTT-973|658[0n]bp|Canada.New Brunswick|BOLD:AAA3071
Lophocampa maculata|[6149]|LPSOB249-08|PPBP-1248|658[0n]bp|Canada.Ontario|BOLD:AAA3071
Lophocampa maculata|[6150]|PHMNB423-04|04HBL00649|658[1n]bp|Canada.New Brunswick|BOLD:AAA3071
Lophocampa maculata|[6151]|BBLPE361-09|09BBELE-2361|639[0n]bp|Canada.Newfoundland and Labrador|BOLD:A...
Lophocampa maculata|[6152]|TMNBD429-07|MNBTT-3230|658[0n]bp|Canada.New Brunswick|BOLD:AAA3071
Lophocampa maculata|[6153]|LPSOD643-09|08BBLEP-00424|634[0n]bp|Canada.Ontario|BOLD:AAA3071
Lophocampa roseata|[6154]|LHLEP305-06|UBC-2006-1475|658[0n]bp|Canada.British Columbia|BOLD:AAC3308
Lophocampa roseata|[6155]|LHLEP304-06|UBC-2006-1221|657[0n]bp|Canada.British Columbia|BOLD:AAC3308
Lophocampa roseata|[6156]|LHLEP300-06|UBC-2006-1217|657[0n]bp|Canada.British Columbia|BOLD:AAC3308
Lophocampa roseata|[6157]|LHLEP302-06|UBC-2006-1219|657[0n]bp|Canada.British Columbia|BOLD:AAC3308
Lophocampa roseata|[6158]|LBCS693-07|UBC-2007-0398|658[0n]bp|Canada.British Columbia|BOLD:AAC3308
Lophocampa roseata|[6159]|LHLEP303-06|UBC-2006-1220|657[0n]bp|Canada.British Columbia|BOLD:AAC3308
Lophocampa roseata|[6160]|LALPA219-10|AVBC 220-10|658[0n]bp|Canada.British Columbia|BOLD:AAC3308
Lophocampa roseata|[6161]|LHLEP301-06|UBC-2006-1218|657[0n]bp|Canada.British Columbia|BOLD:AAC3308
Lophocampa roseata|[6162]|LBCS692-07|UBC-2007-0397|658[0n]bp|Canada.British Columbia|BOLD:AAC3308
Lophocampa roseata|[6163]|LBCS259-07|UBC-2007-0767|658[0n]bp|Canada.British Columbia|BOLD:AAC3308
Cycnia oregonensis|[6164]|XAJ296-06|2006-ONT-0296|658[0n]bp|Canada.Ontario|BOLD:AAB1681
Cycnia oregonensis|[6165]|LBCH5808-10|10-JDWBC-5808|658[0n]bp|Canada.British Columbia|BOLD:AAB1681
Cycnia oregonensis|[6166]|PMG003-03|moth542.01|617[0n]bp|Canada.Ontario|BOLD:AAB1681
Cycnia oregonensis|[6167]|XAK243-06|2006-ONT-1238|658[0n]bp|Canada.Ontario|BOLD:AAB1681
Cycnia oregonensis|[6168]|XAI006-05|0102-ONT-0006|658[0n]bp|Canada.Ontario|BOLD:AAB1681
Cycnia oregonensis|[6169]|XAI007-05|0102-ONT-0007|658[0n]bp|Canada.Ontario|BOLD:AAB1681
Cycnia oregonensis|[6170]|XAB281-04|04HBL005281|658[0n]bp|Canada.Ontario|BOLD:AAB1681
Cycnia oregonensis|[6171]|XAJ601-06|2006-ONT-0601|658[0n]bp|Canada.Ontario|BOLD:AAB1681
Cycnia oregonensis|[6172]|BLTIB063-08|BL0102|658[0n]bp|Canada.Ontario|BOLD:AAB1681
Cycnia oregonensis|[6173]|XAJ657-06|2006-ONT-0657|658[0n]bp|Canada.Ontario|BOLD:AAB1681
Cycnia oregonensis|[6174]|XAJ600-06|2006-ONT-0600|658[0n]bp|Canada.Ontario|BOLD:AAB1681
Cycnia oregonensis|[6175]|TMG87-03|moth732.01|639[0n]bp|Canada.Ontario|BOLD:AAB1681
Cycnia oregonensis|[6176]|XAK224-06|2006-ONT-1219|653[0n]bp|Canada.Ontario|BOLD:AAB1681
Cycnia oregonensis|[6177]|PHMNB141-04|04HBL007606|658[0n]bp|Canada.New Brunswick|BOLD:AAB1681
Cycnia oregonensis|[6178]|XAB063-04|04HBL005063|658[0n]bp|Canada.Ontario|BOLD:AAB1681
Cycnia oregonensis|[6179]|XAI005-05|0102-ONT-0005|658[0n]bp|Canada.Ontario|BOLD:AAB1681
Cycnia oregonensis|[6180]|XAK287-06|2006-ONT-1282|658[0n]bp|Canada.Ontario|BOLD:AAB1681
Cycnia oregonensis|[6181]|LPMN009-08|08BBLEP-00807|649[0n]bp|Canada.Manitoba|BOLD:AAB1681
Cycnia oregonensis|[6182]|RDLQG003-06|DH012123|658[0n]bp|Canada.Quebec|BOLD:AAB1681
Cycnia oregonensis|[6183]|XAG491-05|2005-ONT-1075|658[0n]bp|Canada.Ontario|BOLD:AAB1681
Cycnia oregonensis|[6184]|XAJ354-06|2006-ONT-0354|658[0n]bp|Canada.Ontario|BOLD:AAB1681
Cycnia oregonensis|[6185]|LOWCB664-05|CGWC-1604|658[0n]bp|Canada.British Columbia|BOLD:AAB1681
Cycnia oregonensis|[6186]|XAI004-05|0102-ONT-0004|601[1n]bp|Canada.Ontario|BOLD:AAB1681
Cycnia oregonensis|[6187]|LOWCB663-05|CGWC-1603|658[0n]bp|Canada.British Columbia|BOLD:AAB1681
Cycnia oregonensis|[6188]|LBCH5615-10|10-JDWBC-5615|658[0n]bp|Canada.British Columbia|BOLD:AAB1681
Cycnia oregonensis|[6189]|LBCH5693-10|10-JDWBC-5693|658[0n]bp|Canada.British Columbia|BOLD:AAB1681
Cycnia oregonensis|[6190]|LOWCB666-05|CGWC-1606|658[0n]bp|Canada.British Columbia|BOLD:AAB1681
Cycnia oregonensis|[6191]|XAB243-04|04HBL005243|658[0n]bp|Canada.Ontario|BOLD:AAB1681
Cycnia oregonensis|[6192]|RDMAB925-06|UASM58591|658[0n]bp|Canada.Alberta|BOLD:AAB1681
Cycnia oregonensis|[6193]|LBCH5439-10|10-JDWBC-5439|658[0n]bp|Canada.British Columbia|BOLD:AAB1681
Cycnia oregonensis|[6194]|LOWCB665-05|CGWC-1605|658[0n]bp|Canada.British Columbia|BOLD:AAB1681
Cycnia oregonensis|[6195]|BLTIB051-08|BL0082|582[1n]bp|Canada.Ontario|BOLD:AAB1681
Cycnia tenera|[6196]|BLTIB084-08|BL0126|561[0n]bp|Canada.Ontario|BOLD:AAB0274
Cycnia tenera|[6197]|XAE506-04|Moth4506.03|653[5n]bp|Canada.Ontario|BOLD:AAB0274
Cycnia tenera|[6198]|BLTIB072-08|BL0112|610[0n]bp|Canada.Ontario|BOLD:AAB0274
Cycnia tenera|[6199]|LPABC722-09|08BBLEP-04941|658[0n]bp|Canada.Alberta|BOLD:AAB0274
Cycnia tenera|[6200]|LBCA088-05|HLC-20088|658[0n]bp|Canada.British Columbia|BOLD:AAB0274
Cycnia tenera|[6201]|LBCB208-05|HLC-21148|658[0n]bp|Canada.British Columbia|BOLD:AAB0274
Cycnia tenera|[6202]|XAE459-04|Moth4459.03|658[0n]bp|Canada.Ontario|BOLD:AAB0274
Cycnia tenera|[6203]|TMG86-03|CYCN1.00|639[0n]bp|Canada.Ontario|BOLD:AAB0274
Cycnia tenera|[6204]|BLTIB461-08|BL708|658[0n]bp|Canada.Ontario|BOLD:AAB0274
Cycnia tenera|[6205]|XAJ834-06|2006-ONT-0834|658[0n]bp|Canada.Ontario|BOLD:AAB0274
Cycnia tenera|[6206]|BLTIB885-08|BL1304|658[0n]bp|Canada.Ontario|BOLD:AAB0274
Cycnia tenera|[6207]|XAJ627-06|2006-ONT-0627|658[0n]bp|Canada.Ontario|BOLD:AAB0274
Cycnia tenera|[6208]|LPSOB433-08|PPBP-1432|658[0n]bp|Canada.Ontario|BOLD:AAB0274
Cycnia tenera|[6209]|XAE275-04|Moth4275.03|658[0n]bp|Canada.Ontario|BOLD:AAB0274
Cycnia tenera|[6210]|XAK259-06|2006-ONT-1254|658[0n]bp|Canada.Ontario|BOLD:AAB0274
Cycnia tenera|[6211]|XAJ915-06|2006-ONT-0915|658[0n]bp|Canada.Ontario|BOLD:AAB0274
Cycnia tenera|[6212]|XAE412-04|Moth4412.03|658[0n]bp|Canada.Ontario|BOLD:AAB0274
Cycnia tenera|[6213]|XAB086-04|04HBL005086|658[0n]bp|Canada.Ontario|BOLD:AAB0274
Cycnia tenera|[6214]|LPSOB432-08|PPBP-1431|658[0n]bp|Canada.Ontario|BOLD:AAB0274
Cycnia tenera|[6215]|PMG004-03|moth525.01|617[0n]bp|Canada.Ontario|BOLD:AAB0274
Cycnia tenera|[6216]|XAB244-04|04HBL005244|658[0n]bp|Canada.Ontario|BOLD:AAB0274
Cycnia tenera|[6217]|XAF827-05|2005-ONT-476|658[0n]bp|Canada.Ontario|BOLD:AAB0274
Cycnia tenera|[6218]|XAK449-06|2006-ONT-1444|658[0n]bp|Canada.Ontario|BOLD:AAB0274
Cycnia tenera|[6219]|LPMN050-08|08BBLEP-00848|658[0n]bp|Canada.Manitoba|BOLD:AAB0274
Halysidota harrisii|[6220]|BBLSX102-09|09BBLEP-02030|658[0n]bp|United States.Oklahoma|BOLD:ABZ7359
Halysidota harrisii|[6221]|LGSMC973-05|DNA-ATBI-4053|587[0n]bp|United States.Tennessee|BOLD:ABZ7359
Halysidota harrisii|[6222]|LNCNW039-06|06-NCNW-0039|658[0n]bp|United States.North Carolina|BOLD:ABZ7359
Halysidota harrisii|[6223]|BBLSW528-09|09BBLEP-01456|658[0n]bp|United States.Oklahoma|BOLD:ABZ7359
Halysidota harrisii|[6224]|LGSMG120-07|BGS03441|658[0n]bp|United States.Tennessee|BOLD:ABZ7359
Halysidota harrisii|[6225]|LGSMC721-05|DNA-ATBI-2721|574[0n]bp|United States.Tennessee|BOLD:ABZ7359
Halysidota harrisii|[6226]|LPOKB1009-09|MDOK-2051|658[0n]bp|United States.Oklahoma|BOLD:ABZ7359
Halysidota harrisii|[6227]|UDLEP329-09|v367 Amb|658[0n]bp|United States.Pennsylvania|BOLD:ABZ7359
Halysidota harrisii|[6228]|TMFL002-06|06-TMFL-00002|658[0n]bp|United States.Florida|BOLD:ABZ7359
Halysidota harrisii|[6229]|BBLSX101-09|09BBLEP-02029|658[0n]bp|United States.Oklahoma|BOLD:ABZ7359
Halysidota harrisii|[6230]|BBLSW527-09|09BBLEP-01455|658[0n]bp|United States.Oklahoma|BOLD:ABZ7359
Halysidota harrisii|[6231]|TMFL001-06|06-TMFL-00001|658[0n]bp|United States.Florida|BOLD:ABZ7359
Halysidota harrisii|[6232]|LGSMC934-05|DNA-ATBI-4014|658[0n]bp|United States.Tennessee|BOLD:ABZ7359
Halysidota harrisii|[6233]|LGSMC935-05|DNA-ATBI-4015|658[0n]bp|United States.Tennessee|BOLD:ABZ7359
Halysidota tessellaris|[6234]|BLTIB447-08|BL694|658[0n]bp|Canada.Ontario|BOLD:AAA3425
Halysidota tessellaris|[6235]|PHMNB198-04|04HBL007663|609[0n]bp|Canada.New Brunswick|BOLD:AAA3425
Halysidota tessellaris|[6236]|LPSOC273-08|PPBP-2272|658[0n]bp|Canada.Ontario|BOLD:AAA3425
Halysidota tessellaris|[6237]|BLTIB394-08|BL630|658[0n]bp|Canada.Ontario|BOLD:AAA3425
Halysidota tessellaris|[6238]|MNBB294-05|05-NBSTA-210|658[0n]bp|Canada.New Brunswick|BOLD:AAA3425
Halysidota tessellaris|[6239]|MNBB197-05|05-NBSTA-113|658[0n]bp|Canada.New Brunswick|BOLD:AAA3425
Halysidota tessellaris|[6240]|MNBB235-05|05-NBSTA-151|658[0n]bp|Canada.New Brunswick|BOLD:AAA3425
Halysidota tessellaris|[6241]|MNBB385-05|05-NBSTA-301|658[0n]bp|Canada.New Brunswick|BOLD:AAA3425
Halysidota tessellaris|[6242]|TTMNB255-06|MNBTT-255|658[0n]bp|Canada.New Brunswick|BOLD:AAA3425
Halysidota tessellaris|[6243]|BBLPA574-10|10BBCLP-0574|632[0n]bp|Canada.Ontario|BOLD:AAA3425
Halysidota tessellaris|[6244]|MNBB433-05|05-NBSTA-349|658[0n]bp|Canada.New Brunswick|BOLD:AAA3425
Halysidota tessellaris|[6245]|LPSOC284-08|PPBP-2283|658[0n]bp|Canada.Ontario|BOLD:AAA3425
Halysidota tessellaris|[6246]|BBLPA576-10|10BBCLP-0576|658[0n]bp|Canada.Ontario|BOLD:AAA3425

Halysidota tessellaris|[6244]|MNBB433-05|05-NBSTA-349|658[0n]bp|Canada.New Brunswick|BOLD:AAA3425
Halysidota tessellaris|[6245]|LPSOC284-08|PPBP-2283|658[0n]bp|Canada.Ontario|BOLD:AAA3425
Halysidota tessellaris|[6246]|BBLPA576-10|10BBCLP-0576|658[0n]bp|Canada.Ontario|BOLD:AAA3425
Halysidota tessellaris|[6247]|XAB088-04|04HBL005088|658[0n]bp|Canada.Ontario|BOLD:AAA3425
Halysidota tessellaris|[6248]|MNBB355-05|05-NBSTA-271|658[0n]bp|Canada.New Brunswick|BOLD:AAA3425
Halysidota tessellaris|[6249]|MNBB086-05|05-NBSTA-002|658[0n]bp|Canada.New Brunswick|BOLD:AAA3425
Halysidota tessellaris|[6250]|PMG009-03|moth898.01|617[0n]bp|Canada.Ontario|BOLD:AAA3425
Halysidota tessellaris|[6251]|BBLPA575-10|10BBCLP-0575|658[0n]bp|Canada.Ontario|BOLD:AAA3425
Halysidota tessellaris|[6252]|XAK462-06|2006-ONT-1457|658[0n]bp|Canada.Ontario|BOLD:AAA3425
Halysidota tessellaris|[6253]|MNBB507-05|05-NBSTA-423|658[0n]bp|Canada.New Brunswick|BOLD:AAA3425
Halysidota tessellaris|[6254]|MNBB625-05|05-NBSTA-541|658[0n]bp|Canada.New Brunswick|BOLD:AAA3425
Halysidota tessellaris|[6255]|MNBB085-05|05-NBSTA-001|658[0n]bp|Canada.New Brunswick|BOLD:AAA3425
Halysidota tessellaris|[6256]|MNBB356-05|05-NBSTA-272|658[0n]bp|Canada.New Brunswick|BOLD:AAA3425
Halysidota tessellaris|[6257]|XAB587-04|04HBL005587|658[0n]bp|Canada.Ontario|BOLD:AAA3425
Halysidota tessellaris|[6258]|XAD751-05|2005-ONT-550|658[0n]bp|Canada.Ontario|BOLD:AAA3425
Halysidota tessellaris|[6259]|MNBB556-05|05-NBSTA-472|658[0n]bp|Canada.New Brunswick|BOLD:AAA3425
Halysidota tessellaris|[6260]|BBLPA573-10|10BBCLP-0573|658[0n]bp|Canada.Ontario|BOLD:AAA3425
Halysidota tessellaris|[6261]|MNBB291-05|05-NBSTA-207|658[0n]bp|Canada.New Brunswick|BOLD:AAA3425
Halysidota tessellaris|[6262]|XAJ832-06|2006-ONT-0832|658[0n]bp|Canada.Ontario|BOLD:AAA3425
Halysidota tessellaris|[6263]|XAF544-05|2005-ONT-193|658[0n]bp|Canada.Ontario|BOLD:AAA3425
Halysidota tessellaris|[6264]|BBLEC325-09|09BBELE-0325|658[0n]bp|Canada.Nova Scotia|BOLD:AAA3425
Halysidota tessellaris|[6265]|BLTIB1087-08|BL1096|658[0n]bp|Canada.Ontario|BOLD:AAA3425
Halysidota tessellaris|[6266]|LPSOD1067-09|08MZPP-172|658[0n]bp|Canada.Ontario|BOLD:AAA3425
Halysidota tessellaris|[6267]|MNBB295-05|05-NBSTA-211|658[0n]bp|Canada.New Brunswick|BOLD:AAA3425
Halysidota tessellaris|[6268]|MNBB088-05|05-NBSTA-004|658[0n]bp|Canada.New Brunswick|BOLD:AAA3425
Halysidota tessellaris|[6269]|MNBB508-05|05-NBSTA-424|658[0n]bp|Canada.New Brunswick|BOLD:AAA3425
Halysidota tessellaris|[6270]|MNBB384-05|05-NBSTA-300|658[0n]bp|Canada.New Brunswick|BOLD:AAA3425
Halysidota tessellaris|[6271]|MNBB090-05|05-NBSTA-006|658[0n]bp|Canada.New Brunswick|BOLD:AAA3425
Halysidota tessellaris|[6272]|MNBB509-05|05-NBSTA-425|658[0n]bp|Canada.New Brunswick|BOLD:AAA3425
Halysidota tessellaris|[6273]|MNBB624-05|05-NBSTA-540|658[0n]bp|Canada.New Brunswick|BOLD:AAA3425
Halysidota tessellaris|[6274]|TMNBD415-07|MNBTT-3216|658[0n]bp|Canada.New Brunswick|BOLD:AAA3425
Halysidota tessellaris|[6275]|MNBB434-05|05-NBSTA-350|658[0n]bp|Canada.New Brunswick|BOLD:AAA3425
Halysidota tessellaris|[6276]|TMNBD416-07|MNBTT-3217|658[0n]bp|Canada.New Brunswick|BOLD:AAA3425
Halysidota tessellaris|[6277]|MNBB382-05|05-NBSTA-298|658[0n]bp|Canada.New Brunswick|BOLD:AAA3425
Halysidota tessellaris|[6278]|TMNBD412-07|MNBTT-3213|658[0n]bp|Canada.New Brunswick|BOLD:AAA3425
Halysidota tessellaris|[6279]|MNBB557-05|05-NBSTA-473|658[0n]bp|Canada.New Brunswick|BOLD:AAA3425
Halysidota tessellaris|[6280]|MNBB328-05|05-NBSTA-244|658[0n]bp|Canada.New Brunswick|BOLD:AAA3425
Halysidota tessellaris|[6281]|MNBB510-05|05-NBSTA-426|658[0n]bp|Canada.New Brunswick|BOLD:AAA3425
Halysidota tessellaris|[6282]|MNBB357-05|05-NBSTA-273|658[0n]bp|Canada.New Brunswick|BOLD:AAA3425
Halysidota tessellaris|[6283]|MNBB087-05|05-NBSTA-003|658[0n]bp|Canada.New Brunswick|BOLD:AAA3425
Halysidota tessellaris|[6284]|MNBB091-05|05-NBSTA-007|658[0n]bp|Canada.New Brunswick|BOLD:AAA3425
Halysidota tessellaris|[6285]|MNBB196-05|05-NBSTA-112|658[0n]bp|Canada.New Brunswick|BOLD:AAA3425
Halysidota tessellaris|[6286]|MNBB236-05|05-NBSTA-152|658[0n]bp|Canada.New Brunswick|BOLD:AAA3425
Halysidota tessellaris|[6287]|MNBB089-05|05-NBSTA-005|658[0n]bp|Canada.New Brunswick|BOLD:AAA3425
Halysidota tessellaris|[6288]|TMNBD413-07|MNBTT-3214|658[0n]bp|Canada.New Brunswick|BOLD:AAA3425
Halysidota tessellaris|[6289]|MNBB292-05|05-NBSTA-208|658[0n]bp|Canada.New Brunswick|BOLD:AAA3425
Halysidota tessellaris|[6290]|MNBB358-05|05-NBSTA-274|658[0n]bp|Canada.New Brunswick|BOLD:AAA3425
Halysidota tessellaris|[6291]|MNBB290-05|05-NBSTA-206|658[0n]bp|Canada.New Brunswick|BOLD:AAA3425
Halysidota tessellaris|[6292]|TMNBD414-07|MNBTT-3215|658[0n]bp|Canada.New Brunswick|BOLD:AAA3425
Halysidota tessellaris|[6293]|MNBB623-05|05-NBSTA-539|658[0n]bp|Canada.New Brunswick|BOLD:AAA3425
Halysidota tessellaris|[6294]|MNBB435-05|05-NBSTA-351|658[0n]bp|Canada.New Brunswick|BOLD:AAA3425
Halysidota tessellaris|[6295]|MNBB383-05|05-NBSTA-299|658[0n]bp|Canada.New Brunswick|BOLD:AAA3425
Halysidota tessellaris|[6296]|MNBB293-05|05-NBSTA-209|658[0n]bp|Canada.New Brunswick|BOLD:AAA3425
Halysidota tessellaris|[6297]|PHMNB457-04|04HBL00683|621[1n]bp|Canada.New Brunswick|BOLD:AAA3425
Halysidota tessellaris|[6298]|PHMNB730-05|Moth 423.03SA|621[1n]bp|Canada.New Brunswick|BOLD:AAA3425
Halysidota tessellaris|[6299]|LPSOC285-08|PPBP-2284|642[0n]bp|Canada.Ontario|BOLD:AAA3425
Halysidota tessellaris|[6300]|PHMNB052-03|moth245.02SA|639[0n]bp|Canada.New Brunswick|BOLD:AAA3425
Halysidota tessellaris|[6301]|TMG83-03|HALY1.00|639[0n]bp|Canada.Ontario|BOLD:AAA3425
Halysidota tessellaris|[6302]|PHMNB033-03|moth197.02SA|639[0n]bp|Canada.New Brunswick|BOLD:AAA3425
Halysidota tessellaris|[6303]|XAJ919-06|2006-ONT-0919|658[0n]bp|Canada.Ontario|BOLD:AAA3425
Pygarctia spraguei|[6304]|RDNMF392-08|NOC14478|658[0n]bp|Canada.Manitoba|BOLD:AAD0348
Pygarctia spraguei|[6305]|RDNME450-08|LEP037874|658[0n]bp|Canada.Manitoba|BOLD:AAD0348
Pygarctia spraguei|[6306]|RDNMF393-08|NOC14479|658[0n]bp|Canada.Manitoba|BOLD:AAD0348
Rivula propinqualis|[6307]|BLTIB1026-08|BL1467|658[0n]bp|Canada.Ontario|BOLD:AAA4282
Rivula propinqualis|[6308]|XAF461-05|2005-ONT-110|564[0n]bp|Canada.Ontario|BOLD:AAA4282
Rivula propinqualis|[6309]|PHMO143-03|moth820.01|639[0n]bp|Canada.Ontario|BOLD:AAA4282
Rivula propinqualis|[6310]|PHAUG1522-11|BIOUG01521-A10|658[0n]bp|Canada.Ontario|BOLD:AAA4282
Rivula propinqualis|[6311]|BLGSM010-09|BL1619|658[0n]bp|Canada.Ontario|BOLD:AAA4282
Rivula propinqualis|[6312]|RDLQD886-06|MDH001895|658[0n]bp|Canada.Quebec|BOLD:AAA4282
Rivula propinqualis|[6313]|LPSOD1005-09|08BBLEP-05638|621[0n]bp|Canada.Ontario|BOLD:AAA4282
Rivula propinqualis|[6314]|LPABB173-08|08BBLEP-03438|638[0n]bp|Canada.Alberta|BOLD:AAA4282
Rivula propinqualis|[6315]|XAJ650-06|2006-ONT-0650|656[0n]bp|Canada.Ontario|BOLD:AAA4282
Rivula propinqualis|[6316]|LPSOC253-08|PPBP-2252|656[0n]bp|Canada.Ontario|BOLD:AAA4282
Rivula propinqualis|[6317]|MEC584-04|jflandry0584|655[1n]bp|Canada.Quebec|BOLD:AAA4282
Rivula propinqualis|[6318]|BLTIB1007-08|BL1444|658[0n]bp|Canada.Ontario|BOLD:AAA4282
Rivula propinqualis|[6319]|XAG921-05|2005-ONT-1505|658[0n]bp|Canada.Ontario|BOLD:AAA4282
Rivula propinqualis|[6320]|LPSO762-08|PPBP-0762|658[0n]bp|Canada.Ontario|BOLD:AAA4282
Rivula propinqualis|[6321]|LPMNB253-09|08BBLEP-05097|658[0n]bp|Canada.Manitoba|BOLD:AAA4282
Rivula propinqualis|[6322]|BBLPD974-10|10BBCLP-2972|658[0n]bp|Canada.Alberta|BOLD:AAA4282
Rivula propinqualis|[6323]|BBLPD116-10|10BBCLP-2114|658[0n]bp|Canada.Saskatchewan|BOLD:AAA4282
Rivula propinqualis|[6324]|LPSOC251-08|PPBP-2250|658[0n]bp|Canada.Ontario|BOLD:AAA4282
Rivula propinqualis|[6325]|RDLQB690-05|DH010793|658[0n]bp|Canada.Quebec|BOLD:AAA4282
Rivula propinqualis|[6326]|LPSOC066-08|PPBP-2065|658[0n]bp|Canada.Ontario|BOLD:AAA4282
Rivula propinqualis|[6327]|BBLPD975-10|10BBCLP-2973|658[0n]bp|Canada.Alberta|BOLD:AAA4282
Rivula propinqualis|[6328]|LPABB234-08|08BBLEP-03499|658[0n]bp|Canada.Alberta|BOLD:AAA4282
Rivula propinqualis|[6329]|LPMNB544-09|08BBLEP-05582|658[0n]bp|Canada.Manitoba|BOLD:AAA4282
Rivula propinqualis|[6330]|LPMNB516-09|08BBLEP-05554|658[0n]bp|Canada.Manitoba|BOLD:AAA4282
Rivula propinqualis|[6331]|PHMTV441-10|10PHMAL-2541|658[0n]bp|Canada.Ontario|BOLD:AAA4282
Rivula propinqualis|[6332]|RDLQD887-06|MDH001904|658[0n]bp|Canada.Quebec|BOLD:AAA4282
Rivula propinqualis|[6333]|PHMTV439-10|10PHMAL-2539|658[0n]bp|Canada.Ontario|BOLD:AAA4282
Rivula propinqualis|[6334]|LPMN875-08|08BBLEP-02233|658[0n]bp|Canada.Alberta|BOLD:AAA4282
Rivula propinqualis|[6335]|XAG953-05|2005-ONT-1537|658[0n]bp|Canada.Ontario|BOLD:AAA4282
Rivula propinqualis|[6336]|LPMNB237-09|08BBLEP-05081|658[0n]bp|Canada.Manitoba|BOLD:AAA4282
Rivula propinqualis|[6337]|LPSOB032-08|PPBP-1031|658[0n]bp|Canada.Ontario|BOLD:AAA4282
Rivula propinqualis|[6338]|LPMNB235-09|08BBLEP-05079|658[0n]bp|Canada.Manitoba|BOLD:AAA4282
Rivula propinqualis|[6339]|XAE317-04|Moth4317.03|658[0n]bp|Canada.Ontario|BOLD:AAA4282
Rivula propinqualis|[6340]|LPMNB453-09|08BBLEP-05453|658[0n]bp|Canada.Manitoba|BOLD:AAA4282
Rivula propinqualis|[6341]|XAD389-04|04HBL007389|582[0n]bp|Canada.Ontario|BOLD:AAA4282
Rivula propinqualis|[6342]|XAD730-05|2005-ONT-529|524[2n]bp|Canada.Ontario|BOLD:AAA4282
Rivula propinqualis|[6343]|MEC585-04|jflandry0585|587[0n]bp|Canada.Quebec|BOLD:AAA4282
Rivula propinqualis|[6344]|BLTIB308-08|BL497|658[1n]bp|Canada.Ontario|BOLD:AAA4282
Rivula propinqualis|[6345]|LPMNB410-09|08BBLEP-05254|597[0n]bp|Canada.Manitoba|BOLD:AAA4282

Rivula propinqualis|[6343]|MEC585-04|jflandry0585|587[0n]bp|Canada.Quebec|BOLD:AAA4282
Rivula propinqualis|[6344]|BLTIB308-08|BL497|658[1n]bp|Canada.Ontario|BOLD:AAA4282
Rivula propinqualis|[6345]|LPMNB410-09|08BBLEP-05254|597[0n]bp|Canada.Manitoba|BOLD:AAA4282
Rivula propinqualis|[6346]|BLTIB883-08|BL1302|658[0n]bp|Canada.Ontario|BOLD:AAA4282
Rivula propinqualis|[6347]|BLTIB305-08|BL494|650[0n]bp|Canada.Ontario|BOLD:AAA4282
Rivula propinqualis|[6348]|XAG045-05|2005-ONT-629|658[0n]bp|Canada.Ontario|BOLD:AAA4282
Rivula propinqualis|[6349]|BLTIB172-08|BL251|658[0n]bp|Canada.Ontario|BOLD:AAA4282
Rivula propinqualis|[6350]|LPSOB043-08|PPBP-1042|658[0n]bp|Canada.Ontario|BOLD:AAA4282
Rivula propinqualis|[6351]|PHMTV435-10|10PHMAL-2535|658[0n]bp|Canada.Ontario|BOLD:AAA4282
Rivula propinqualis|[6352]|LPSOC252-08|PPBP-2251|658[0n]bp|Canada.Ontario|BOLD:AAA4282
Rivula propinqualis|[6353]|LPSO593-08|PPBP-0593|658[0n]bp|Canada.Ontario|BOLD:AAA4282
Rivula propinqualis|[6354]|LALPA559-10|AVBC 561-10|658[0n]bp|Canada.British Columbia|BOLD:AAA4282
Rivula propinqualis|[6355]|RDLQE417-06|MDH002420|657[0n]bp|Canada.Quebec|BOLD:AAA4282
Rivula propinqualis|[6356]|RDLQG605-06|DH012898|658[0n]bp|Canada.Quebec|BOLD:AAA4282
Rivula propinqualis|[6357]|TTMNB513-06|MNBTT-513|658[0n]bp|Canada.New Brunswick|BOLD:AAA4282
Rivula propinqualis|[6358]|LMIS048-06|05-ONMIS-0048|658[0n]bp|Canada.Ontario|BOLD:AAA4282
Rivula propinqualis|[6359]|RDLQG493-06|DH012786|654[0n]bp|Canada.Quebec|BOLD:AAA4282
Rivula propinqualis|[6360]|RDLQE342-06|MDH002345|656[0n]bp|Canada.Quebec|BOLD:AAA4282
Rivula propinqualis|[6361]|BBLPC731-09|09BBELE-1731|658[0n]bp|Canada.Newfoundland and Labrador|BOLD:A...
Rivula propinqualis|[6362]|BBLPB973-10|10BBCLP-1972|658[0n]bp|Canada.Alberta|BOLD:AAA4282
Rivula propinqualis|[6363]|RDLQG552-06|DH012845|658[0n]bp|Canada.Quebec|BOLD:AAA4282
Rivula propinqualis|[6364]|RDLQG570-06|DH012863|658[0n]bp|Canada.Quebec|BOLD:AAA4282
Rivula propinqualis|[6365]|BLTIB211-08|BL312|658[0n]bp|Canada.Ontario|BOLD:AAA4282
Rivula propinqualis|[6366]|RDLQG695-06|DH012988|658[0n]bp|Canada.Quebec|BOLD:AAA4282
Rivula propinqualis|[6367]|RDLQG697-06|DH012990|658[0n]bp|Canada.Quebec|BOLD:AAA4282
Rivula propinqualis|[6368]|RDLQG553-06|DH012846|658[0n]bp|Canada.Quebec|BOLD:AAA4282
Rivula propinqualis|[6369]|RDLQG042-06|DH012173|603[0n]bp|Canada.Quebec|BOLD:AAA4282
Rivula propinqualis|[6370]|BLTIB803-08|BL1221|658[0n]bp|Canada.Ontario|BOLD:AAA4282
Rivula propinqualis|[6371]|RDLQG696-06|DH012989|658[0n]bp|Canada.Quebec|BOLD:AAA4282
Rivula propinqualis|[6372]|JSAUG510-11|BIOUG01602-F06|658[0n]bp|Canada.Ontario|BOLD:AAA4282
Rivula propinqualis|[6373]|RDLQG554-06|DH012847|658[0n]bp|Canada.Quebec|BOLD:AAA4282
Rivula propinqualis|[6374]|RDLQG571-06|DH012864|658[0n]bp|Canada.Quebec|BOLD:AAA4282
Rivula propinqualis|[6375]|RDLQG555-06|DH012848|655[0n]bp|Canada.Quebec|BOLD:AAA4282
Rivula propinqualis|[6376]|LPSOB874-08|PPBP-1873|658[1n]bp|Canada.Ontario|BOLD:AAA4282
Rivula propinqualis|[6377]|BLTIB434-08|BL681|658[0n]bp|Canada.Ontario|BOLD:AAA4282
Rivula propinqualis|[6378]|BLGSM053-09|BL372|612[0n]bp|Canada.Ontario|BOLD:AAA4282
Rivula propinqualis|[6379]|LPSOB873-08|PPBP-1872|658[0n]bp|Canada.Ontario|BOLD:AAA4282
Rivula propinqualis|[6380]|JSAUG482-11|BIOUG01602-D02|658[0n]bp|Canada.Ontario|BOLD:AAA4282
Rivula propinqualis|[6381]|LPSOB875-08|PPBP-1874|658[0n]bp|Canada.Ontario|BOLD:AAA4282
Rivula propinqualis|[6382]|PMG159-03|moth647.01|617[0n]bp|Canada.Ontario|BOLD:AAA4282
Rivula propinqualis|[6383]|LPSOB675-08|PPBP-1674|646[0n]bp|Canada.Ontario|BOLD:AAA4282
Euchaetes egle|[6384]|XAI013-05|0102-ONT-0013|611[3n]bp|Canada.Ontario|BOLD:AAC2978
Euchaetes egle|[6385]|XAK451-06|2006-ONT-1446|658[0n]bp|Canada.Ontario|BOLD:AAC2978
Euchaetes egle|[6386]|BLTIB564-08|BL842|658[0n]bp|Canada.Ontario|BOLD:AAC2978
Euchaetes egle|[6387]|PMG005-03|moth706.01|617[0n]bp|Canada.Ontario|BOLD:AAC2978
Euchaetes egle|[6388]|XAI011-05|0102-ONT-0011|658[0n]bp|Canada.Ontario|BOLD:AAC2978
Euchaetes egle|[6389]|XAI014-05|0102-ONT-0014|658[0n]bp|Canada.Ontario|BOLD:AAC2978
Euchaetes egle|[6390]|TMG89-03|EUCHA1.00|639[0n]bp|Canada.Ontario|BOLD:AAC2978
Euchaetes egle|[6391]|XAE390-04|Moth4390.03|658[0n]bp|Canada.Ontario|BOLD:AAC2978
Euchaetes egle|[6392]|XAI012-05|0102-ONT-0012|516[2n]bp|Canada.Ontario|BOLD:AAC2978
Euchaetes egle|[6393]|TMG88-03|moth651.01|639[0n]bp|Canada.Ontario|BOLD:AAC2978
Euchaetes egle|[6394]|XAD752-05|2005-ONT-551|658[0n]bp|Canada.Ontario|BOLD:AAC2978
Colobochyla interpuncta|[6395]|RDLQF383-06|DH011450|659[0n]bp|Canada.Quebec|BOLD:AAB9362
Colobochyla interpuncta|[6396]|RDLQG685-06|DH012978|646[0n]bp|Canada.Quebec|BOLD:AAB9362
Colobochyla interpuncta|[6397]|XAJ633-06|2006-ONT-0633|658[0n]bp|Canada.Ontario|BOLD:AAB9362
Colobochyla interpuncta|[6398]|RDLQG931-06|DH013228|645[0n]bp|Canada.Quebec|BOLD:AAB9362
Tathorhynchus exsiccata|[6399]|RDNMI192-12|CNCLEP00030531|601[0n]bp|United States.Wyoming|BOLD:AAD0612
Tathorhynchus exsiccata|[6400]|RDNMI195-12|CNCLEP00092220|621[0n]bp|Canada.Manitoba|BOLD:AAD0612
Tathorhynchus exsiccata|[6401]|RDNMF623-08|NOC14709|658[0n]bp|Canada.British Columbia|BOLD:AAD0612
Tathorhynchus exsiccata|[6402]|RDNMF625-08|NOC14711|658[0n]bp|Canada.British Columbia|BOLD:AAD0612
Tathorhynchus exsiccata|[6403]|RDNMF624-08|NOC14710|658[0n]bp|Canada.British Columbia|BOLD:AAD0612
Lygephila victoria|[6404]|LOWCB613-05|CGWC-1553|511[0n]bp|Canada.British Columbia|BOLD:AAB4508
Lygephila victoria|[6405]|LOWCB623-05|CGWC-1563|506[0n]bp|Canada.British Columbia|BOLD:AAB4508
Lygephila victoria|[6406]|LBCD318-05|HLC-23138|658[0n]bp|Canada.British Columbia|BOLD:AAB4508
Lygephila victoria|[6407]|LOWCD457-06|CGWC-3277|658[0n]bp|Canada.British Columbia|BOLD:AAB4508
Lygephila victoria|[6408]|BBLPB315-10|10BBCLP-1314|658[0n]bp|Canada.British Columbia|BOLD:AAB4508
Lygephila victoria|[6409]|LBCD323-05|HLC-23143|658[0n]bp|Canada.British Columbia|BOLD:AAB4508
Lygephila victoria|[6410]|LBCH6622-10|10-JDWBC-6622|658[0n]bp|Canada.British Columbia|BOLD:AAB4508
Lygephila victoria|[6411]|LOWCD456-06|CGWC-3276|658[0n]bp|Canada.British Columbia|BOLD:AAB4508
Lygephila victoria|[6412]|LOWCD455-06|CGWC-3275|658[0n]bp|Canada.British Columbia|BOLD:AAB4508
Lygephila victoria|[6413]|LOWCB617-05|CGWC-1557|533[0n]bp|Canada.British Columbia|BOLD:AAB4508
Lygephila victoria|[6414]|LOWCB619-05|CGWC-1559|589[1n]bp|Canada.British Columbia|BOLD:AAB4508
Lygephila victoria|[6415]|LOWCB612-05|CGWC-1552|532[1n]bp|Canada.British Columbia|BOLD:AAB4508
Lygephila victoria|[6416]|LOWCB615-05|CGWC-1555|608[2n]bp|Canada.British Columbia|BOLD:AAB4508
Lygephila victoria|[6417]|LOWCD452-06|CGWC-3272|598[0n]bp|Canada.British Columbia|BOLD:AAB4508
Lygephila victoria|[6418]|LOWCB620-05|CGWC-1560|610[1n]bp|Canada.British Columbia|BOLD:AAB4508
Lygephila victoria|[6419]|LOWCD454-06|CGWC-3274|608[0n]bp|Canada.British Columbia|BOLD:AAB4508
Lygephila victoria|[6420]|LOWCB622-05|CGWC-1562|608[0n]bp|Canada.British Columbia|BOLD:AAB4508
Lygephila victoria|[6421]|LOWCB616-05|CGWC-1556|605[1n]bp|Canada.British Columbia|BOLD:AAB4508
Lygephila victoria|[6422]|LOWCB614-05|CGWC-1554|608[1n]bp|Canada.British Columbia|BOLD:AAB4508
Lygephila victoria|[6423]|LOWCD451-06|CGWC-3271|605[0n]bp|Canada.British Columbia|BOLD:AAB4508
Lygephila victoria|[6424]|LOWCD453-06|CGWC-3273|603[0n]bp|Canada.British Columbia|BOLD:AAB4508
Lygephila victoria|[6425]|LOWCB621-05|CGWC-1561|510[1n]bp|Canada.British Columbia|BOLD:AAB4508
Lygephila victoria|[6426]|LOWCB618-05|CGWC-1558|604[0n]bp|Canada.British Columbia|BOLD:AAB4508
Plusiodonta compressipalpis|[6427]|RDLQH116-06|AC000687|601[0n]bp|Canada.Quebec|BOLD:AAB7156
Plusiodonta compressipalpis|[6428]|BBLPA655-10|10BBCLP-0655|658[0n]bp|Canada.Ontario|BOLD:AAB7156
Leucoma salicis|[6429]|PHMNB083-03|moth96.02SA|639[0n]bp|Canada.New Brunswick|BOLD:AAA5528
Leucoma salicis|[6430]|BBLPE553-09|09BBELE-2553|639[0n]bp|Canada.Newfoundland and Labrador|BOLD:AAA5528
Leucoma salicis|[6431]|LPVIB872-08|PFC-2006-2387|632[0n]bp|Canada.British Columbia|BOLD:AAA5528
Leucoma salicis|[6432]|RDLQF828-06|DH011981|658[0n]bp|Canada.Quebec|BOLD:AAA5528
Leucoma salicis|[6433]|LBCC404-05|HLC-22284|658[0n]bp|Canada.British Columbia|BOLD:AAA5528
Leucoma salicis|[6434]|LBCC555-05|HLC-22435|658[0n]bp|Canada.British Columbia|BOLD:AAA5528
Leucoma salicis|[6435]|DUNLP004-08|Dun-08-004|658[0n]bp|Canada.British Columbia|BOLD:AAA5528
Leucoma salicis|[6436]|DUNLP003-08|Dun-08-003|658[0n]bp|Canada.British Columbia|BOLD:AAA5528
Leucoma salicis|[6437]|LBCC040-05|HLC-21920|658[0n]bp|Canada.British Columbia|BOLD:AAA5528
Leucoma salicis|[6438]|LPABB079-08|08BBLEP-03344|658[0n]bp|Canada.Alberta|BOLD:AAA5528
Leucoma salicis|[6439]|BBLPA368-10|10BBCLP-0368|658[0n]bp|Canada.British Columbia|BOLD:AAA5528
Leucoma salicis|[6440]|LPABB043-08|08BBLEP-03308|658[0n]bp|Canada.Alberta|BOLD:AAA5528
Leucoma salicis|[6441]|BBLPA369-10|10BBCLP-0369|658[0n]bp|Canada.British Columbia|BOLD:AAA5528
Leucoma salicis|[6442]|LPABC911-09|08BBLEP-05322|658[0n]bp|Canada.Alberta|BOLD:AAA5528
Leucoma salicis|[6443]|LBCC039-05|HLC-21919|658[0n]bp|Canada.British Columbia|BOLD:AAA5528
Leucoma salicis|[6444]|BBLPA371-10|10BBCLP-0371|658[0n]bp|Canada.British Columbia|BOLD:AAA5528

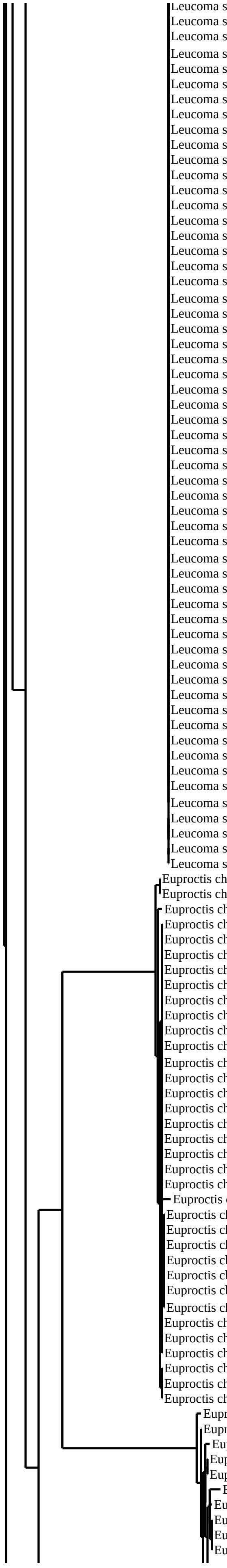

Leucoma salicis|[6442]|LPABC911-09|08BBLEP-05322|658[0n]bp|Canada.Alberta|BOLD:AAA5528
Leucoma salicis|[6443]|LBCC039-05|HLC-21919|658[0n]bp|Canada.British Columbia|BOLD:AAA5528
Leucoma salicis|[6444]|BBLPA371-10|10BBCLP-0371|658[0n]bp|Canada.British Columbia|BOLD:AAA5528
Leucoma salicis|[6445]|LBCC408-05|HLC-22288|658[0n]bp|Canada.British Columbia|BOLD:AAA5528
Leucoma salicis|[6446]|LALPA890-11|AVBC 1063-11|658[0n]bp|Canada.British Columbia|BOLD:AAA5528
Leucoma salicis|[6447]|LBCH3452-10|10-JDWBC-3452|658[0n]bp|Canada.British Columbia|BOLD:AAA5528
Leucoma salicis|[6448]|LBCD034-05|HLC-22854|658[0n]bp|Canada.British Columbia|BOLD:AAA5528
Leucoma salicis|[6449]|LBCC409-05|HLC-22289|658[0n]bp|Canada.British Columbia|BOLD:AAA5528
Leucoma salicis|[6450]|LPABB081-08|08BBLEP-03346|658[0n]bp|Canada.Alberta|BOLD:AAA5528
Leucoma salicis|[6451]|LBCC406-05|HLC-22286|658[0n]bp|Canada.British Columbia|BOLD:AAA5528
Leucoma salicis|[6452]|LBCD035-05|HLC-22855|658[0n]bp|Canada.British Columbia|BOLD:AAA5528
Leucoma salicis|[6453]|LBCD110-05|HLC-22930|658[0n]bp|Canada.British Columbia|BOLD:AAA5528
Leucoma salicis|[6454]|RDLQB430-05|DH010516|658[0n]bp|Canada.Quebec|BOLD:AAA5528
Leucoma salicis|[6455]|LPVIB839-08|PFC-2006-2346|658[0n]bp|Canada.British Columbia|BOLD:AAA5528
Leucoma salicis|[6456]|LBCD033-05|HLC-22853|658[0n]bp|Canada.British Columbia|BOLD:AAA5528
Leucoma salicis|[6457]|LBCH225-10|10-JDWBC-0225|658[0n]bp|Canada.British Columbia|BOLD:AAA5528
Leucoma salicis|[6458]|LBCC034-05|HLC-21914|658[0n]bp|Canada.British Columbia|BOLD:AAA5528
Leucoma salicis|[6459]|LBCD111-05|HLC-22931|658[0n]bp|Canada.British Columbia|BOLD:AAA5528
Leucoma salicis|[6460]|RDLQB423-05|DH010509|658[0n]bp|Canada.Quebec|BOLD:AAA5528
Leucoma salicis|[6461]|LALPA273-10|AVBC 274-10|658[0n]bp|Canada.British Columbia|BOLD:AAA5528
Leucoma salicis|[6462]|LBCB146-05|HLC-21086|658[0n]bp|Canada.British Columbia|BOLD:AAA5528
Leucoma salicis|[6463]|LBCC035-05|HLC-21915|658[0n]bp|Canada.British Columbia|BOLD:AAA5528
Leucoma salicis|[6464]|BLTIB531-08|BL797|658[0n]bp|Canada.Ontario|BOLD:AAA5528
Leucoma salicis|[6465]|LBCD269-05|HLC-23089|658[0n]bp|Canada.British Columbia|BOLD:AAA5528
Leucoma salicis|[6466]|LBCD267-05|HLC-23087|658[0n]bp|Canada.British Columbia|BOLD:AAA5528
Leucoma salicis|[6467]|LBCD109-05|HLC-22929|658[0n]bp|Canada.British Columbia|BOLD:AAA5528
Leucoma salicis|[6468]|LBCC037-05|HLC-21917|658[0n]bp|Canada.British Columbia|BOLD:AAA5528
Leucoma salicis|[6469]|MNBB069-05|HBL008679|658[0n]bp|Canada.New Brunswick|BOLD:AAA5528
Leucoma salicis|[6470]|LBCD037-05|HLC-22857|658[0n]bp|Canada.British Columbia|BOLD:AAA5528
Leucoma salicis|[6471]|LBCC747-05|HLC-22627|658[0n]bp|Canada.British Columbia|BOLD:AAA5528
Leucoma salicis|[6472]|LBCC036-05|HLC-21916|658[0n]bp|Canada.British Columbia|BOLD:AAA5528
Leucoma salicis|[6473]|BBLPA367-10|10BBCLP-0367|658[0n]bp|Canada.British Columbia|BOLD:AAA5528
Leucoma salicis|[6474]|BBLPC689-09|09BBELE-1689|658[0n]bp|Canada.Newfoundland and Labrador|BOLD:AAA5528
Leucoma salicis|[6475]|BBLPC688-09|09BBELE-1688|658[0n]bp|Canada.Newfoundland and Labrador|BOLD:AAA5528
Leucoma salicis|[6476]|LPABB037-08|08BBLEP-03302|658[0n]bp|Canada.Alberta|BOLD:AAA5528
Leucoma salicis|[6477]|LPABC683-09|08BBLEP-04902|658[0n]bp|Canada.Alberta|BOLD:AAA5528
Leucoma salicis|[6478]|LBCD036-05|HLC-22856|658[0n]bp|Canada.British Columbia|BOLD:AAA5528
Leucoma salicis|[6479]|LBCA574-05|HLC-20574|658[0n]bp|Canada.British Columbia|BOLD:AAA5528
Leucoma salicis|[6480]|BBLPC642-09|09BBELE-1642|658[0n]bp|Canada.Newfoundland and Labrador|BOLD:AAA5528
Leucoma salicis|[6481]|RDLQB431-05|DH010517|658[0n]bp|Canada.Quebec|BOLD:AAA5528
Leucoma salicis|[6482]|LBCC407-05|HLC-22287|658[0n]bp|Canada.British Columbia|BOLD:AAA5528
Leucoma salicis|[6483]|LPABB019-08|08BBLEP-03284|658[0n]bp|Canada.Alberta|BOLD:AAA5528
Leucoma salicis|[6484]|LBCD268-05|HLC-23088|658[0n]bp|Canada.British Columbia|BOLD:AAA5528
Leucoma salicis|[6485]|LPVIA002-08|PFC-2006-0004|658[0n]bp|Canada.British Columbia|BOLD:AAA5528
Leucoma salicis|[6486]|BBLPA370-10|10BBCLP-0370|658[0n]bp|Canada.British Columbia|BOLD:AAA5528
Leucoma salicis|[6487]|TMNBD449-07|MNBTT-3250|658[0n]bp|Canada.New Brunswick|BOLD:AAA5528
Leucoma salicis|[6488]|LBCC405-05|HLC-22285|658[0n]bp|Canada.British Columbia|BOLD:AAA5528
Leucoma salicis|[6489]|LBCD265-05|HLC-23085|656[0n]bp|Canada.British Columbia|BOLD:AAA5528
Leucoma salicis|[6490]|LBCD266-05|HLC-23086|637[0n]bp|Canada.British Columbia|BOLD:AAA5528
Leucoma salicis|[6491]|LPVIA094-08|PFC-2006-0151|637[0n]bp|Canada.British Columbia|BOLD:AAA5528
Leucoma salicis|[6492]|TMNBD450-07|MNBTT-3251|646[0n]bp|Canada.New Brunswick|BOLD:AAA5528
Leucoma salicis|[6493]|LPVIB883-08|PFC-2006-2401|635[0n]bp|Canada.British Columbia|BOLD:AAA5528
Leucoma salicis|[6494]|BLTIB547-08|BL819|656[0n]bp|Canada.Ontario|BOLD:AAA5528
Leucoma salicis|[6495]|LPABB044-08|08BBLEP-03309|658[0n]bp|Canada.Alberta|BOLD:AAA5528
Leucoma salicis|[6496]|LBCC038-05|HLC-21918|658[0n]bp|Canada.British Columbia|BOLD:AAA5528
Leucoma salicis|[6497]|LPABB383-08|08BBLEP-03648|658[0n]bp|Canada.Alberta|BOLD:AAA5528
Leucoma salicis|[6498]|LMH002-06|PFC-2006-0002|658[0n]bp|Canada.British Columbia|BOLD:AAA5528
Euproctis chrysorrhoea|[6499]|BTM058-10|EF_D_ROM_N061_N|658[0n]bp|Romania.Moldavia|BOLD:AAD5030
Euproctis chrysorrhoea|[6500]|BTM057-10|EF_D_ROM_N051_L|658[0n]bp|Romania.Moldavia|BOLD:AAD5030
Euproctis chrysorrhoea|[6501]|NLLEA782-12|RMNH.INS.540977|658[0n]bp|Netherlands.South Holland|BOLD:AA...
Euproctis chrysorrhoea|[6502]|BTM011-10|EF_E_GRA_N011_L|658[0n]bp|Spain.Andalusia|BOLD:AAD5030
Euproctis chrysorrhoea|[6503]|FBLMU524-09|BC ZSM Lep 27174|617[0n]bp|Germany.Bavaria|BOLD:AAD5030
Euproctis chrysorrhoea|[6504]|BTM027-10|EF_D_SUS_N023_L|658[0n]bp|Spain.La Rioja|BOLD:AAD5030
Euproctis chrysorrhoea|[6505]|BTM056-10|EF_D_ROM_N042_N|658[0n]bp|Romania.Moldavia|BOLD:AAD5030
Euproctis chrysorrhoea|[6506]|GWOR4215-09|BC ZSM Lep 21499|658[0n]bp|Germany.Bavaria|BOLD:AAD5030
Euproctis chrysorrhoea|[6507]|BTM049-10|EF_D_ROM_N011_L|658[0n]bp|Romania.Moldavia|BOLD:AAD5030
Euproctis chrysorrhoea|[6508]|BTM026-10|EF_D_SUS_N022_L|658[0n]bp|Spain.La Rioja|BOLD:AAD5030
Euproctis chrysorrhoea|[6509]|BTM050-10|EF_D_ROM_N012_N|658[0n]bp|Romania.Moldavia|BOLD:AAD5030
Euproctis chrysorrhoea|[6510]|BTM085-10|EF_E_MEN_N091_L|658[0n]bp|Spain.Balearic Islands|BOLD:AAD5030
Euproctis chrysorrhoea|[6511]|BTM081-10|EF_E_MEN_N051_L|658[0n]bp|Spain.Balearic Islands|BOLD:AAD5030
Euproctis chrysorrhoea|[6512]|BTM082-10|EF_E_MEN_N061_L|658[0n]bp|Spain.Balearic Islands|BOLD:AAD5030
Euproctis chrysorrhoea|[6513]|BTM080-10|EF_E_MEN_N041_L|658[0n]bp|Spain.Balearic Islands|BOLD:AAD5030
Euproctis chrysorrhoea|[6514]|BTM078-10|EF_E_MEN_N021_L|658[0n]bp|Spain.Balearic Islands|BOLD:AAD5030
Euproctis chrysorrhoea|[6515]|BTM076-10|EF_E_MEN_N011_L|658[0n]bp|Spain.Balearic Islands|BOLD:AAD5030
Euproctis chrysorrhoea|[6516]|BTM055-10|EF_D_ROM_N041_L|658[0n]bp|Romania.Moldavia|BOLD:AAD5030
Euproctis chrysorrhoea|[6517]|BTM077-10|EF_E_MEN_N012_L|658[0n]bp|Spain.Balearic Islands|BOLD:AAD5030
Euproctis chrysorrhoea|[6518]|BTM079-10|EF_E_MEN_N031_L|658[0n]bp|Spain.Balearic Islands|BOLD:AAD5030
Euproctis chrysorrhoea|[6519]|GBLGC264-12|BC ZSM Lep R 21499|658[0n]bp|Germany.Bavaria|BOLD:AAD5030
Euproctis chrysorrhoea|[6520]|GWOSP624-11|INRGREF 0244|658[0n]bp|Tunisia|BOLD:AAD5030
Euproctis chrysorrhoea|[6521]|CGUKA293-09|UKLB4A11|658[0n]bp|United Kingdom.England|BOLD:AAD5030
Euproctis chrysorrhoea|[6522]|NLLEA383-12|RMNH.INS.538998|658[0n]bp|Netherlands.South Holland|BOLD:AA...
Euproctis chrysorrhoea|[6523]|NLLEA797-12|RMNH.INS.540992|658[0n]bp|Netherlands.South Holland|BOLD:AA...
Euproctis chrysorrhoea|[6524]|BTM053-10|EF_D_ROM_N031_L|658[0n]bp|Romania.Moldavia|BOLD:AAD5030
Euproctis chrysorrhoea|[6525]|BTM054-10|EF_D_ROM_N032_N|658[0n]bp|Romania.Moldavia|BOLD:AAD5030
Euproctis chrysorrhoea|[6526]|GWOR4168-09|BC ZSM Lep 21452|658[0n]bp|Germany.Bavaria|BOLD:AAD5030
Euproctis chrysorrhoea|[6527]|GBLGC217-12|BC ZSM Lep R 21452|658[0n]bp|Germany.Bavaria|BOLD:AAD5030
Euproctis chrysorrhoea|[6528]|BTM051-10|EF_D_ROM_N021_L|658[0n]bp|Romania.Moldavia|BOLD:AAD5030
Euproctis chrysorrhoea|[6529]|BTM052-10|EF_D_ROM_N022_N|658[0n]bp|Romania.Moldavia|BOLD:AAD5030
Euproctis chrysorrhoea|[6530]|BTM084-10|EF_E_MEN_N081_L|658[0n]bp|Spain.Balearic Islands|BOLD:AAD5030
Euproctis chrysorrhoea|[6531]|GWORU379-10|BC ZSM Lep 31970|658[0n]bp|Italy.Calabria|BOLD:AAD5030
Euproctis chrysorrhoea|[6532]|PHLSA637-11|TLMF Lep 06092|658[0n]bp|Italy|BOLD:AAD5030
Euproctis chrysorrhoea|[6533]|GWORZ097-10|BC ZSM Lep 30738|658[0n]bp|Italy.Basilicata|BOLD:AAD5030
Euproctis similis|[6534]|RFELP034-08|PaA-08-557|658[0n]bp|Russia.Primorsky Krai|BOLD:AAC3712
Euproctis similis|[6535]|LEFIC264-10|MM03695|658[0n]bp|Finland.Uusimaa|BOLD:AAC3712
Euproctis similis|[6536]|LEFIC263-10|MM03694|658[0n]bp|Finland.Uusimaa|BOLD:AAC3712
Euproctis similis|[6537]|FBLMW374-10|BC ZSM Lep 37475|658[0n]bp|Germany.Bavaria|BOLD:AAC3712
Euproctis similis|[6538]|LEFIK943-10|MM18518|658[0n]bp|Finland|BOLD:AAC3712
Euproctis similis|[6539]|CGUKC168-09|UKLB24A04|658[0n]bp|United Kingdom.England|BOLD:AAC3712
Euproctis similis|[6540]|GWORO669-09|BC ZSM Lep 27605|658[0n]bp|Germany.Bavaria|BOLD:AAC3712
Euproctis similis|[6541]|NLLEA759-12|RMNH.INS.540954|658[0n]bp|Netherlands.South Holland|BOLD:AAC3712
Euproctis similis|[6542]|CGUKB617-09|UKLB18B07|658[0n]bp|United Kingdom.England|BOLD:AAC3712
Euproctis similis|[6543]|PHLAA249-09|TLMF Lep 00289|655[0n]bp|Austria.Steiermark|BOLD:AAC3712
[illegible]

Euproctis similis|[6541]|NLLEA759-12|RMNH.INS.540954|658[0n]bp|Netherlands.South Holland|BOLD:AAC3712
Euproctis similis|[6542]|CGUKB617-09|UKLB18B07|658[0n]bp|United Kingdom.England|BOLD:AAC3712
Euproctis similis|[6543]|PHLAA249-09|TLMF Lep 00289|655[0n]bp|Austria.Steiermark|BOLD:AAC3712
Euproctis similis|[6544]|NLLEA813-12|RMNH.INS.541008|658[0n]bp|Netherlands.South Holland|BOLD:AAC3712
Euproctis similis|[6545]|CGUKA283-09|UKLB4A01|637[0n]bp|United Kingdom.England|BOLD:AAC3712
Euproctis similis|[6546]|CGUKD553-09|UKLB38G03|641[0n]bp|United Kingdom.England|BOLD:AAC3712
Euproctis similis|[6547]|CGUKA696-09|UKLB8D04|658[0n]bp|United Kingdom|BOLD:AAC3712
Euproctis similis|[6548]|CGUKA710-09|UKLB8E06|658[0n]bp|United Kingdom|BOLD:AAC3712
Euproctis similis|[6549]|CGUKB318-09|UKLB15A01|658[0n]bp|United Kingdom.England|BOLD:AAC3712
Euproctis similis|[6550]|CGUKB901-09|UKLB21B09|658[0n]bp|United Kingdom.Wales|BOLD:AAC3712
Lymantria dispar|[6551]|LYMMK015-09|LymMk_AA-3|658[0n]bp|Canada.British Columbia|BOLD:AAA2052
Lymantria dispar|[6552]|LBCH7981-10|10-JDWBC-7981|658[0n]bp|Canada.British Columbia|BOLD:AAA2052
Lymantria dispar|[6553]|LYMMK014-09|LymMk_AA-2|658[0n]bp|Canada.British Columbia|BOLD:AAA2052
Lymantria dispar|[6554]|LYMMK013-09|LymMk_AA-1|658[0n]bp|Canada.British Columbia|BOLD:AAA2052
Lymantria dispar|[6555]|LPSOD1058-09|08MZPP-148|632[0n]bp|Canada.Ontario|BOLD:AAA2052
Lymantria dispar dispar|[6556]|TTMNB259-06|MNBTT-259|658[0n]bp|Canada.New Brunswick|BOLD:AAA2052
Lymantria dispar dispar|[6557]|TTMNB260-06|MNBTT-260|658[0n]bp|Canada.New Brunswick|BOLD:AAA2052
Lymantria dispar|[6558]|GBGL1515-06|DQ116098|624[0n]bp|Canada.Ontario|BOLD:AAA2052
Lymantria dispar dispar|[6559]|LMHRG025-06|SPI V-93-1 A|658[0n]bp|Canada.British Columbia|BOLD:AAA2052
Lymantria dispar dispar|[6560]|XAG214-05|2005-ONT-798|658[0n]bp|Canada.Ontario|BOLD:AAA2052
Lymantria dispar dispar|[6561]|LMHRG004-06|SPI C-03-17 A|658[0n]bp|Canada.British Columbia|BOLD:AAA2052
Lymantria dispar dispar|[6562]|XAG177-05|2005-ONT-761|658[0n]bp|Canada.Ontario|BOLD:AAA2052
Lymantria dispar dispar|[6563]|XAG005-05|2005-ONT-589|658[0n]bp|Canada.Ontario|BOLD:AAA2052
Lymantria dispar dispar|[6564]|LMHRG001-06|SPI B-5-2 A|658[0n]bp|Canada.British Columbia|BOLD:AAA2052
Lymantria dispar dispar|[6565]|LMHRG012-06|SPI V-26-5 A|658[0n]bp|Canada.British Columbia|BOLD:AAA2052
Lymantria dispar dispar|[6566]|LMHRG033-06|SPI W-17-1 A|658[0n]bp|Canada.British Columbia|BOLD:AAA2052
Lymantria dispar dispar|[6567]|LMHRG020-06|SPI V-92-1 C|658[0n]bp|Canada.British Columbia|BOLD:AAA2052
Lymantria dispar dispar|[6568]|LMHRG036-06|KAM DAB-9 A|658[0n]bp|Canada.British Columbia|BOLD:AAA2052
Lymantria dispar dispar|[6569]|LMHRG007-06|SPI L-2-5 A|658[0n]bp|Canada.British Columbia|BOLD:AAA2052
Lymantria dispar dispar|[6570]|XAG308-05|2005-ONT-892|658[0n]bp|Canada.Ontario|BOLD:AAA2052
Lymantria dispar dispar|[6571]|XAK268-06|2006-ONT-1263|658[0n]bp|Canada.Ontario|BOLD:AAA2052
Lymantria dispar dispar|[6572]|LMHRG015-06|SPI V-90-2 A|658[0n]bp|Canada.British Columbia|BOLD:AAA2052
Lymantria dispar dispar|[6573]|LMHRG035-06|SPI X-39-1 A|658[0n]bp|Canada.British Columbia|BOLD:AAA2052
Lymantria dispar dispar|[6574]|LMHRG010-06|SPI V-24-3 A|658[0n]bp|Canada.British Columbia|BOLD:AAA2052
Lymantria dispar dispar|[6575]|LMHRG031-06|SPI V-94-1 D|658[0n]bp|Canada.British Columbia|BOLD:AAA2052
Lymantria dispar dispar|[6576]|XAG216-05|2005-ONT-800|658[0n]bp|Canada.Ontario|BOLD:AAA2052
Lymantria dispar dispar|[6577]|LMHRG006-06|SPI I-3-17 A|658[0n]bp|Canada.British Columbia|BOLD:AAA2052
Lymantria dispar dispar|[6578]|LMHRG027-06|SPI V-93-2 B|658[0n]bp|Canada.British Columbia|BOLD:AAA2052
Lymantria dispar dispar|[6579]|BLTIB831-08|BL1249|658[0n]bp|Canada.Ontario|BOLD:AAA2052
Lymantria dispar dispar|[6580]|TMNBD447-07|MNBTT-3248|658[0n]bp|Canada.New Brunswick|BOLD:AAA2052
Lymantria dispar dispar|[6581]|XAG307-05|2005-ONT-891|658[0n]bp|Canada.Ontario|BOLD:AAA2052
Lymantria dispar dispar|[6582]|LMHRG030-06|SPI V-94-1 C|658[0n]bp|Canada.British Columbia|BOLD:AAA2052
Lymantria dispar dispar|[6583]|XAG215-05|2005-ONT-799|658[0n]bp|Canada.Ontario|BOLD:AAA2052
Lymantria dispar dispar|[6584]|LMHRG014-06|SPI V-90-2 B|658[0n]bp|Canada.British Columbia|BOLD:AAA2052
Lymantria dispar dispar|[6585]|LMHRG002-06|SPI C-02-02 A|658[0n]bp|Canada.British Columbia|BOLD:AAA2052
Lymantria dispar dispar|[6586]|LMHRG029-06|SPI V-94-1 B|658[0n]bp|Canada.British Columbia|BOLD:AAA2052
Lymantria dispar dispar|[6587]|XAG277-05|2005-ONT-861|658[0n]bp|Canada.Ontario|BOLD:AAA2052
Lymantria dispar dispar|[6588]|LMHRG021-06|SPI V-92-1 D|658[0n]bp|Canada.British Columbia|BOLD:AAA2052
Lymantria dispar dispar|[6589]|XAG235-05|2005-ONT-819|658[0n]bp|Canada.Ontario|BOLD:AAA2052
Lymantria dispar dispar|[6590]|LMHRG023-06|SPI V-92-3 A|658[0n]bp|Canada.British Columbia|BOLD:AAA2052
Lymantria dispar dispar|[6591]|LMHRG009-06|SPI V-23-8 B|658[0n]bp|Canada.British Columbia|BOLD:AAA2052
Lymantria dispar dispar|[6592]|LMHRG016-06|SPI V-90-3 A|658[0n]bp|Canada.British Columbia|BOLD:AAA2052
Lymantria dispar dispar|[6593]|XAK267-06|2006-ONT-1262|658[0n]bp|Canada.Ontario|BOLD:AAA2052
Lymantria dispar dispar|[6594]|TMNBD445-07|MNBTT-3246|658[0n]bp|Canada.New Brunswick|BOLD:AAA2052
Lymantria dispar dispar|[6595]|LMHRG019-06|SPI V-92-1 B|658[0n]bp|Canada.British Columbia|BOLD:AAA2052
Lymantria dispar dispar|[6596]|BLTIB844-08|BL1263|658[0n]bp|Canada.Ontario|BOLD:AAA2052
Lymantria dispar|[6597]|LPSOD1060-09|08MZPP-150|616[0n]bp|Canada.Ontario|BOLD:AAA2052
Lymantria dispar dispar|[6598]|LMHRG024-06|SPI V-92-3 B|658[0n]bp|Canada.British Columbia|BOLD:AAA2052
Lymantria dispar dispar|[6599]|LMHRG026-06|SPI V-93-2 A|658[0n]bp|Canada.British Columbia|BOLD:AAA2052
Lymantria dispar dispar|[6600]|LMHRG008-06|SPI V-23-8 A|658[0n]bp|Canada.British Columbia|BOLD:AAA2052
Lymantria dispar|[6601]|GBGL1535-06|DQ116118|621[0n]bp|Canada.Ontario|BOLD:AAA2052
Lymantria dispar dispar|[6602]|LMHRG011-06|SPI V-25-5 A|638[0n]bp|Canada.British Columbia|BOLD:AAA2052
Lymantria dispar dispar|[6603]|XAG217-05|2005-ONT-801|626[1n]bp|Canada.Ontario|BOLD:AAA2052
Lymantria dispar|[6604]|GBGL1520-06|DQ116103|624[0n]bp|Canada.Ontario|BOLD:AAA2052
Lymantria dispar dispar|[6605]|BLTIB832-08|BL1250|645[0n]bp|Canada.Ontario|BOLD:AAA2052
Lymantria dispar dispar|[6606]|TMNBD448-07|MNBTT-3249|646[0n]bp|Canada.New Brunswick|BOLD:AAA2052
Lymantria dispar dispar|[6607]|XAG658-05|2005-ONT-1242|658[3n]bp|Canada.Ontario|BOLD:AAA2052
Lymantria dispar dispar|[6608]|XAG348-05|2005-ONT-932|658[0n]bp|Canada.Ontario|BOLD:AAA2052
Lymantria dispar dispar|[6609]|BLTIB901-08|BL1320|658[0n]bp|Canada.Ontario|BOLD:AAA2052
Lymantria dispar dispar|[6610]|LMHRG022-06|SPI V-92-1 E|658[0n]bp|Canada.British Columbia|BOLD:AAA2052
Lymantria dispar dispar|[6611]|TMNBD446-07|MNBTT-3247|658[0n]bp|Canada.New Brunswick|BOLD:AAA2052
Lymantria dispar dispar|[6612]|LMHRG032-06|SPI V-98-2 A|658[0n]bp|Canada.British Columbia|BOLD:AAA2052
Orgyia antiqua|[6613]|LBCH2462-10|10-JDWBC-2462|658[0n]bp|Canada.British Columbia|BOLD:AAA6432
Orgyia antiqua|[6614]|RDNME228-07|CNCNoctuoidea13835|658[0n]bp|Canada.Alberta|BOLD:AAA6432
Orgyia antiqua nova|[6615]|RDLQF824-06|DH011977|658[0n]bp|Canada.Quebec|BOLD:AAA6432
Orgyia antiqua nova|[6616]|RDLQF548-06|DH011697|658[0n]bp|Canada.Quebec|BOLD:AAA6432
Orgyia definita|[6617]|XAG923-05|2005-ONT-1507|658[0n]bp|Canada.Ontario|BOLD:AAA7686
Orgyia definita|[6618]|XAB406-04|04HBL005406|658[0n]bp|Canada.Ontario|BOLD:AAA7686
Orgyia definita|[6619]|XAH749-05|2005-ONT-2332|658[0n]bp|Canada.Ontario|BOLD:AAA7686
Orgyia definita|[6620]|XAH554-05|2005-ONT-2137|658[0n]bp|Canada.Ontario|BOLD:AAA7686
Orgyia definita|[6621]|XAB405-04|04HBL005405|614[0n]bp|Canada.Ontario|BOLD:AAA7686
Orgyia definita|[6622]|XAH717-05|2005-ONT-2300|658[0n]bp|Canada.Ontario|BOLD:AAA7686
Orgyia definita|[6623]|XAH178-05|2005-ONT-1761|658[0n]bp|Canada.Ontario|BOLD:AAA7686
Orgyia definita|[6624]|XAH413-05|2005-ONT-1996|658[0n]bp|Canada.Ontario|BOLD:AAA7686
Orgyia definita|[6625]|XAH038-05|2005-ONT-1621|658[0n]bp|Canada.Ontario|BOLD:AAA7686
Orgyia definita|[6626]|RDLQF887-06|DH012062|658[0n]bp|Canada.Quebec|BOLD:AAA7686
Orgyia definita|[6627]|XAH515-05|2005-ONT-2098|658[0n]bp|Canada.Ontario|BOLD:AAA7686
Orgyia definita|[6628]|XAH298-05|2005-ONT-1881|658[0n]bp|Canada.Ontario|BOLD:AAA7686
Orgyia definita|[6629]|XAB404-04|04HBL005404|658[0n]bp|Canada.Ontario|BOLD:AAA7686
Orgyia definita|[6630]|XAH528-05|2005-ONT-2111|658[0n]bp|Canada.Ontario|BOLD:AAA7686
Orgyia definita|[6631]|XAB451-04|04HBL005451|658[0n]bp|Canada.Ontario|BOLD:AAA7686
Orgyia definita|[6632]|XAB427-04|04HBL005427|658[0n]bp|Canada.Ontario|BOLD:AAA7686
Orgyia definita|[6633]|XAH414-05|2005-ONT-1997|658[0n]bp|Canada.Ontario|BOLD:AAA7686
Orgyia definita|[6634]|XAB644-04|04HBL005644|658[0n]bp|Canada.Ontario|BOLD:AAA7686
Orgyia definita|[6635]|RDLQF250-06|DH011330|658[0n]bp|Canada.Quebec|BOLD:AAA7686
Orgyia definita|[6636]|XAH456-05|2005-ONT-2039|658[0n]bp|Canada.Ontario|BOLD:AAA7686
Orgyia definita|[6637]|XAH527-05|2005-ONT-2110|658[0n]bp|Canada.Ontario|BOLD:AAA7686
Orgyia definita|[6638]|XAH553-05|2005-ONT-2136|658[0n]bp|Canada.Ontario|BOLD:AAA7686
Orgyia definita|[6639]|XAH347-05|2005-ONT-1930|658[0n]bp|Canada.Ontario|BOLD:AAA7686
Orgyia definita|[6640]|XAH598-05|2005-ONT-2181|658[0n]bp|Canada.Ontario|BOLD:AAA7686
Orgyia definita|[6641]|XAB651-04|04HBL005651|658[0n]bp|Canada.Ontario|BOLD:AAA7686
Orgyia definita|[6642]|XAH457-05|2005-ONT-2040|646[0n]bp|Canada.Ontario|BOLD:AAA7686
Orgyia definita|[6643]|XAB430-04|04HBL005430|616[0n]bp|Canada.Ontario|BOLD:AAA7686

Orgyia definita|[6640]|XAH[illegible]-05|2005-ONT-[illegible]|658[0n]bp|Canada.Ontario|BOLD:AAA7686
Orgyia definita|[6641]|XAB651-04|04HBL005651|658[0n]bp|Canada.Ontario|BOLD:AAA7686
Orgyia definita|[6642]|XAH457-05|2005-ONT-2040|646[0n]bp|Canada.Ontario|BOLD:AAA7686
Orgyia definita|[6643]|XAB420-04|04HBL005420|616[0n]bp|Canada.Ontario|BOLD:AAA7686
Orgyia definita|[6644]|PHMO308-03|moth2306.02|639[0n]bp|Canada.Ontario|BOLD:AAA7686
Orgyia definita|[6645]|RDLQB506-05|DH010592|591[0n]bp|Canada.Quebec|BOLD:AAA7686
Orgyia definita|[6646]|XAG972-05|2005-ONT-1556|633[0n]bp|Canada.Ontario|BOLD:AAA7686
Orgyia definita|[6647]|XAH611-05|2005-ONT-2194|658[0n]bp|Canada.Ontario|BOLD:AAA7686
Orgyia leucostigma|[6648]|XAG765-05|2005-ONT-1349|658[0n]bp|Canada.Ontario|BOLD:AAA6431
Orgyia leucostigma|[6649]|TMNBB040-06|MNBTT-980|658[0n]bp|Canada.New Brunswick|BOLD:AAA6431
Orgyia leucostigma|[6650]|XAH579-05|2005-ONT-2162|658[0n]bp|Canada.Ontario|BOLD:AAA6431
Orgyia leucostigma|[6651]|XAG648-05|2005-ONT-1232|657[0n]bp|Canada.Ontario|BOLD:AAA6431
Orgyia leucostigma|[6652]|XAB403-04|04HBL005403|658[0n]bp|Canada.Ontario|BOLD:AAA6431
Orgyia leucostigma|[6653]|XAH597-05|2005-ONT-2180|658[0n]bp|Canada.Ontario|BOLD:AAA6431
Orgyia leucostigma|[6654]|XAG846-05|2005-ONT-1430|658[0n]bp|Canada.Ontario|BOLD:AAA6431
Orgyia leucostigma|[6655]|XAH784-05|2005-ONT-2367|658[0n]bp|Canada.Ontario|BOLD:AAA6431
Orgyia leucostigma|[6656]|MNBB081-05|HBL008691|658[0n]bp|Canada.New Brunswick|BOLD:AAA6431
Orgyia leucostigma|[6657]|XAB214-04|04HBL005214|658[0n]bp|Canada.Ontario|BOLD:AAA6431
Orgyia leucostigma|[6658]|TMNBD444-07|MNBTT-3245|646[0n]bp|Canada.New Brunswick|BOLD:AAA6431
Orgyia leucostigma|[6659]|XAB418-04|04HBL005418|658[0n]bp|Canada.Ontario|BOLD:AAA6431
Orgyia leucostigma|[6660]|XAB449-04|04HBL005449|658[0n]bp|Canada.Ontario|BOLD:AAA6431
Orgyia leucostigma|[6661]|RDNMH871-09|CNCLEP00067179|658[0n]bp|Canada.Nova Scotia|BOLD:AAA6431
Orgyia leucostigma|[6662]|RDNMH870-09|CNCLEP00067178|658[0n]bp|Canada.Nova Scotia|BOLD:AAA6431
Orgyia leucostigma|[6663]|TMNBD441-07|MNBTT-3242|658[0n]bp|Canada.New Brunswick|BOLD:AAA6431
Orgyia leucostigma|[6664]|XAB642-04|04HBL005642|658[0n]bp|Canada.Ontario|BOLD:AAA6431
Orgyia leucostigma|[6665]|TMNBD442-07|MNBTT-3243|658[0n]bp|Canada.New Brunswick|BOLD:AAA6431
Orgyia leucostigma|[6666]|XAG350-05|2005-ONT-934|658[0n]bp|Canada.Ontario|BOLD:AAA6431
Orgyia leucostigma|[6667]|XAB464-04|04HBL005464|658[0n]bp|Canada.Ontario|BOLD:AAA6431
Orgyia leucostigma intermedia|[6668]|RDLQF248-06|DH011328|655[0n]bp|Canada.Quebec|BOLD:AAA6431
Orgyia leucostigma|[6669]|XAG647-05|2005-ONT-1231|658[0n]bp|Canada.Ontario|BOLD:AAA6431
Orgyia leucostigma|[6670]|XAK017-06|2006-ONT-1012|658[0n]bp|Canada.Ontario|BOLD:AAA6431
Orgyia leucostigma|[6671]|XAH085-05|2005-ONT-1668|658[0n]bp|Canada.Ontario|BOLD:AAA6431
Orgyia leucostigma|[6672]|XAH482-05|2005-ONT-2065|658[0n]bp|Canada.Ontario|BOLD:AAA6431
Orgyia leucostigma|[6673]|XAH269-05|2005-ONT-1852|658[0n]bp|Canada.Ontario|BOLD:AAA6431
Orgyia leucostigma|[6674]|TMNBB041-06|MNBTT-981|658[0n]bp|Canada.New Brunswick|BOLD:AAA6431
Orgyia leucostigma|[6675]|XAH599-05|2005-ONT-2182|658[0n]bp|Canada.Ontario|BOLD:AAA6431
Orgyia leucostigma|[6676]|TTMNB258-06|MNBTT-258|658[0n]bp|Canada.New Brunswick|BOLD:AAA6431
Orgyia leucostigma|[6677]|TMNBD440-07|MNBTT-3241|658[0n]bp|Canada.New Brunswick|BOLD:AAA6431
Orgyia leucostigma|[6678]|TMNBB039-06|MNBTT-979|658[0n]bp|Canada.New Brunswick|BOLD:AAA6431
Orgyia leucostigma|[6679]|XAG610-05|2005-ONT-1194|658[0n]bp|Canada.Ontario|BOLD:AAA6431
Orgyia leucostigma|[6680]|XAH578-05|2005-ONT-2161|658[0n]bp|Canada.Ontario|BOLD:AAA6431
Orgyia leucostigma|[6681]|XAH279-05|2005-ONT-1862|658[0n]bp|Canada.Ontario|BOLD:AAA6431
Orgyia leucostigma|[6682]|XAG316-05|2005-ONT-900|658[0n]bp|Canada.Ontario|BOLD:AAA6431
Orgyia leucostigma|[6683]|TMNBD443-07|MNBTT-3244|648[0n]bp|Canada.New Brunswick|BOLD:AAA6431
Orgyia leucostigma|[6684]|XAK615-07|HLC-16168|604[1n]bp|Canada.Ontario|BOLD:AAA6431
Orgyia leucostigma|[6685]|PHMO311-03|moth2318.02|639[0n]bp|Canada.Ontario|BOLD:AAA6431
Orgyia leucostigma|[6686]|PHMO309-03|moth2308.02|639[0n]bp|Canada.Ontario|BOLD:AAA6431
Orgyia leucostigma intermedia|[6687]|RDLQB513-05|DH010599|658[0n]bp|Canada.Quebec|BOLD:AAA6431
Orgyia leucostigma intermedia|[6688]|RDLQF249-06|DH011329|658[0n]bp|Canada.Quebec|BOLD:AAA6431
Orgyia pseudotsugata|[6689]|DUNLP143-08|Dun-08-143|621[6n]bp|Canada.British Columbia|
Orgyia pseudotsugata|[6690]|LBCH7515-10|10-JDWBC-7515|658[0n]bp|Canada.British Columbia|BOLD:AAE5045
Orgyia pseudotsugata|[6691]|LBCH7204-10|10-JDWBC-7204|658[0n]bp|Canada.British Columbia|BOLD:AAE5045
Orgyia pseudotsugata|[6692]|LBCH7082-10|10-JDWBC-7082|658[0n]bp|Canada.British Columbia|BOLD:AAE5045
Orgyia pseudotsugata|[6693]|LBCH7075-10|10-JDWBC-7075|658[0n]bp|Canada.British Columbia|BOLD:AAE5045
Orgyia pseudotsugata|[6694]|LBCH6151-10|10-JDWBC-6151|658[0n]bp|Canada.British Columbia|BOLD:AAE5045
Orgyia pseudotsugata|[6695]|LBCH7080-10|10-JDWBC-7080|658[0n]bp|Canada.British Columbia|BOLD:AAE5045
Orgyia pseudotsugata|[6696]|LBCH6152-10|10-JDWBC-6152|658[0n]bp|Canada.British Columbia|BOLD:AAE5045
Orgyia pseudotsugata|[6697]|LBCH6268-10|10-JDWBC-6268|658[0n]bp|Canada.British Columbia|BOLD:AAE5045
Orgyia pseudotsugata|[6698]|LBCH6263-10|10-JDWBC-6263|658[0n]bp|Canada.British Columbia|BOLD:AAE5045
Orgyia pseudotsugata|[6699]|LBCH6265-10|10-JDWBC-6265|658[0n]bp|Canada.British Columbia|BOLD:AAE5045
Orgyia pseudotsugata|[6700]|LBCH6142-10|10-JDWBC-6142|658[0n]bp|Canada.British Columbia|BOLD:AAE5045
Orgyia pseudotsugata|[6701]|LBCH6143-10|10-JDWBC-6143|658[0n]bp|Canada.British Columbia|BOLD:AAE5045
Orgyia pseudotsugata|[6702]|LBCH7273-10|10-JDWBC-7273|658[0n]bp|Canada.British Columbia|BOLD:AAE5045
Orgyia pseudotsugata|[6703]|LBCH6264-10|10-JDWBC-6264|658[0n]bp|Canada.British Columbia|BOLD:AAE5045
Orgyia pseudotsugata|[6704]|LBCH7427-10|10-JDWBC-7427|658[0n]bp|Canada.British Columbia|BOLD:AAE5045
Orgyia pseudotsugata|[6705]|LBCH7275-10|10-JDWBC-7275|658[0n]bp|Canada.British Columbia|BOLD:AAE5045
Orgyia pseudotsugata|[6706]|LBCH7203-10|10-JDWBC-7203|658[0n]bp|Canada.British Columbia|BOLD:AAE5045
Orgyia pseudotsugata|[6707]|LBCH7166-10|10-JDWBC-7166|658[0n]bp|Canada.British Columbia|BOLD:AAE5045
Orgyia pseudotsugata|[6708]|LBCH7471-10|10-JDWBC-7471|658[0n]bp|Canada.British Columbia|BOLD:AAE5045
Orgyia pseudotsugata|[6709]|LBCH7165-10|10-JDWBC-7165|658[0n]bp|Canada.British Columbia|BOLD:AAE5045
Orgyia pseudotsugata|[6710]|LBCH7206-10|10-JDWBC-7206|658[0n]bp|Canada.British Columbia|BOLD:AAE5045
Orgyia pseudotsugata|[6711]|LBCH6149-10|10-JDWBC-6149|658[0n]bp|Canada.British Columbia|BOLD:AAE5045
Orgyia pseudotsugata|[6712]|LBCH7208-10|10-JDWBC-7208|658[0n]bp|Canada.British Columbia|BOLD:AAE5045
Orgyia pseudotsugata|[6713]|LBCH7278-10|10-JDWBC-7278|658[0n]bp|Canada.British Columbia|BOLD:AAE5045
Orgyia pseudotsugata|[6714]|LBCH7077-10|10-JDWBC-7077|658[0n]bp|Canada.British Columbia|BOLD:AAE5045
Orgyia pseudotsugata|[6715]|LBCH7277-10|10-JDWBC-7277|658[0n]bp|Canada.British Columbia|BOLD:AAE5045
Orgyia pseudotsugata|[6716]|LBCH6267-10|10-JDWBC-6267|658[0n]bp|Canada.British Columbia|BOLD:AAE5045
Orgyia pseudotsugata|[6717]|LBCH7163-10|10-JDWBC-7163|658[0n]bp|Canada.British Columbia|BOLD:AAE5045
Orgyia pseudotsugata|[6718]|LBCH6269-10|10-JDWBC-6269|658[0n]bp|Canada.British Columbia|BOLD:AAE5045
Orgyia pseudotsugata|[6719]|LBCH7276-10|10-JDWBC-7276|658[0n]bp|Canada.British Columbia|BOLD:AAE5045
Orgyia pseudotsugata|[6720]|LBCH7081-10|10-JDWBC-7081|658[0n]bp|Canada.British Columbia|BOLD:AAE5045
Orgyia pseudotsugata|[6721]|LBCH6150-10|10-JDWBC-6150|658[0n]bp|Canada.British Columbia|BOLD:AAE5045
Orgyia pseudotsugata|[6722]|LBCH7167-10|10-JDWBC-7167|658[0n]bp|Canada.British Columbia|BOLD:AAE5045
Orgyia pseudotsugata|[6723]|LBCH7161-10|10-JDWBC-7161|658[0n]bp|Canada.British Columbia|BOLD:AAE5045
Orgyia pseudotsugata|[6724]|LBCH7076-10|10-JDWBC-7076|657[0n]bp|Canada.British Columbia|BOLD:AAE5045
Orgyia pseudotsugata|[6725]|LBCH7202-10|10-JDWBC-7202|658[0n]bp|Canada.British Columbia|BOLD:AAE5045
Orgyia pseudotsugata|[6726]|LBCH6224-10|10-JDWBC-6224|658[0n]bp|Canada.British Columbia|BOLD:AAE5045
Orgyia pseudotsugata|[6727]|LBCH7164-10|10-JDWBC-7164|658[0n]bp|Canada.British Columbia|BOLD:AAE5045
Orgyia pseudotsugata|[6728]|LBCH7272-10|10-JDWBC-7272|658[0n]bp|Canada.British Columbia|BOLD:AAE5045
Orgyia pseudotsugata|[6729]|LBCH7205-10|10-JDWBC-7205|658[0n]bp|Canada.British Columbia|BOLD:AAE5045
Orgyia pseudotsugata|[6730]|LBCH6266-10|10-JDWBC-6266|658[0n]bp|Canada.British Columbia|BOLD:AAE5045
Orgyia pseudotsugata|[6731]|LBCH7391-10|10-JDWBC-7391|658[0n]bp|Canada.British Columbia|BOLD:AAE5045
Orgyia pseudotsugata|[6732]|LBCH7079-10|10-JDWBC-7079|658[0n]bp|Canada.British Columbia|BOLD:AAE5045
Orgyia pseudotsugata|[6733]|LBCH6147-10|10-JDWBC-6147|658[0n]bp|Canada.British Columbia|BOLD:AAE5045
Orgyia pseudotsugata|[6734]|LBCH6270-10|10-JDWBC-6270|658[0n]bp|Canada.British Columbia|BOLD:AAE5045
Orgyia pseudotsugata|[6735]|LALPA1329-12|AVBC 1331-11|601[0n]bp|Canada.British Columbia|BOLD:AAE5045
Orgyia pseudotsugata|[6736]|MNAD418-07|CNCLEP00029327|658[0n]bp|Canada.British Columbia|BOLD:AAE5045
Orgyia pseudotsugata|[6737]|LALPA805-10|AVBC 807-10|658[0n]bp|Canada.British Columbia|BOLD:AAE5045
Orgyia pseudotsugata|[6738]|LBCH7493-10|10-JDWBC-7493|658[0n]bp|Canada.British Columbia|BOLD:AAE5045
Orgyia pseudotsugata|[6739]|LBCH6953-10|10-JDWBC-6953|658[0n]bp|Canada.British Columbia|BOLD:AAE5045
Orgyia pseudotsugata|[6740]|LBCH7840-10|10-JDWBC-7840|658[0n]bp|Canada.British Columbia|BOLD:AAE5045
Orgyia pseudotsugata|[6741]|LBCH7201-10|10-JDWBC-7201|658[0n]bp|Canada.British Columbia|BOLD:AAE5045
Orgyia pseudotsugata|[6742]|LBCH7594-10|10-JDWBC-7594|658[0n]bp|Canada.British Columbia|BOLD:AAE5045

[illegible]
Orgyia pseudotsugata|[6740]|LBCH7840-10|10-JDWBC-7840|658[0n]bp|Canada.British Columbia|BOLD:AAE5045
Orgyia pseudotsugata|[6741]|LBCH7201-10|10-JDWBC-7201|658[0n]bp|Canada.British Columbia|BOLD:AAE5045
Orgyia pseudotsugata|[6742]|LBCH7594-10|10-JDWBC-7594|658[0n]bp|Canada.British Columbia|BOLD:AAE5045
Orgyia pseudotsugata|[6743]|LBCH6145-10|10-JDWBC-6145|658[0n]bp|Canada.British Columbia|BOLD:AAE5045
Orgyia pseudotsugata|[6744]|LBCH6595-10|10-JDWBC-6595|658[0n]bp|Canada.British Columbia|BOLD:AAE5045
Orgyia pseudotsugata|[6745]|LBCH6871-10|10-JDWBC-6871|658[0n]bp|Canada.British Columbia|BOLD:AAE5045
Orgyia pseudotsugata|[6746]|LBCH6404-10|10-JDWBC-6404|658[0n]bp|Canada.British Columbia|BOLD:AAE5045
Orgyia pseudotsugata|[6747]|LBCH6141-10|10-JDWBC-6141|658[0n]bp|Canada.British Columbia|BOLD:AAE5045
Orgyia pseudotsugata|[6748]|LBCH6405-10|10-JDWBC-6405|658[0n]bp|Canada.British Columbia|BOLD:AAE5045
Orgyia pseudotsugata|[6749]|LBCH7499-10|10-JDWBC-7499|658[0n]bp|Canada.British Columbia|BOLD:AAE5045
Orgyia pseudotsugata|[6750]|LBCH7728-10|10-JDWBC-7728|658[0n]bp|Canada.British Columbia|BOLD:AAE5045
Orgyia pseudotsugata|[6751]|LBCH7842-10|10-JDWBC-7842|658[0n]bp|Canada.British Columbia|BOLD:AAE5045
Orgyia pseudotsugata|[6752]|LBCH7492-10|10-JDWBC-7492|658[0n]bp|Canada.British Columbia|BOLD:AAE5045
Orgyia pseudotsugata|[6753]|LBCH6768-10|10-JDWBC-6768|658[0n]bp|Canada.British Columbia|BOLD:AAE5045
Orgyia pseudotsugata|[6754]|LBCH6837-10|10-JDWBC-6837|658[0n]bp|Canada.British Columbia|BOLD:AAE5045
Orgyia pseudotsugata|[6755]|LBCH6402-10|10-JDWBC-6402|658[0n]bp|Canada.British Columbia|BOLD:AAE5045
Orgyia pseudotsugata|[6756]|LBCH6148-10|10-JDWBC-6148|658[0n]bp|Canada.British Columbia|BOLD:AAE5045
Orgyia pseudotsugata|[6757]|LBCH6598-10|10-JDWBC-6598|658[0n]bp|Canada.British Columbia|BOLD:AAE5045
Orgyia pseudotsugata|[6758]|LBCH6520-10|10-JDWBC-6520|658[0n]bp|Canada.British Columbia|BOLD:AAE5045
Orgyia pseudotsugata|[6759]|LBCH7843-10|10-JDWBC-7843|658[0n]bp|Canada.British Columbia|BOLD:AAE5045
Orgyia pseudotsugata|[6760]|LBCH6864-10|10-JDWBC-6864|658[0n]bp|Canada.British Columbia|BOLD:AAE5045
Orgyia pseudotsugata|[6761]|LBCH7734-10|10-JDWBC-7734|658[0n]bp|Canada.British Columbia|BOLD:AAE5045
Orgyia pseudotsugata|[6762]|LBCH7497-10|10-JDWBC-7497|658[0n]bp|Canada.British Columbia|BOLD:AAE5045
Orgyia pseudotsugata|[6763]|LBCH6346-10|10-JDWBC-6346|658[0n]bp|Canada.British Columbia|BOLD:AAE5045
Orgyia pseudotsugata|[6764]|LBCH7023-10|10-JDWBC-7023|658[0n]bp|Canada.British Columbia|BOLD:AAE5045
Orgyia pseudotsugata|[6765]|LBCH7841-10|10-JDWBC-7841|658[0n]bp|Canada.British Columbia|BOLD:AAE5045
Orgyia pseudotsugata|[6766]|LBCH7025-10|10-JDWBC-7025|658[0n]bp|Canada.British Columbia|BOLD:AAE5045
Orgyia pseudotsugata|[6767]|LBCH7590-10|10-JDWBC-7590|658[0n]bp|Canada.British Columbia|BOLD:AAE5045
Orgyia pseudotsugata|[6768]|LBCH6867-10|10-JDWBC-6867|658[0n]bp|Canada.British Columbia|BOLD:AAE5045
Orgyia pseudotsugata|[6769]|LBCH7498-10|10-JDWBC-7498|658[0n]bp|Canada.British Columbia|BOLD:AAE5045
Orgyia pseudotsugata|[6770]|LBCH7839-10|10-JDWBC-7839|658[0n]bp|Canada.British Columbia|BOLD:AAE5045
Orgyia pseudotsugata|[6771]|LBCH7592-10|10-JDWBC-7592|658[0n]bp|Canada.British Columbia|BOLD:AAE5045
Orgyia pseudotsugata|[6772]|LBCH6602-10|10-JDWBC-6602|658[0n]bp|Canada.British Columbia|BOLD:AAE5045
Orgyia pseudotsugata|[6773]|LBCH6521-10|10-JDWBC-6521|658[0n]bp|Canada.British Columbia|BOLD:AAE5045
Orgyia pseudotsugata|[6774]|LBCH6596-10|10-JDWBC-6596|658[0n]bp|Canada.British Columbia|BOLD:AAE5045
Orgyia pseudotsugata|[6775]|LBCH7844-10|10-JDWBC-7844|658[0n]bp|Canada.British Columbia|BOLD:AAE5045
Orgyia pseudotsugata|[6776]|LBCH6773-10|10-JDWBC-6773|658[0n]bp|Canada.British Columbia|BOLD:AAE5045
Orgyia pseudotsugata|[6777]|LBCH7028-10|10-JDWBC-7028|658[0n]bp|Canada.British Columbia|BOLD:AAE5045
Orgyia pseudotsugata|[6778]|LBCH6869-10|10-JDWBC-6869|658[0n]bp|Canada.British Columbia|BOLD:AAE5045
Orgyia pseudotsugata|[6779]|LBCH7593-10|10-JDWBC-7593|658[0n]bp|Canada.British Columbia|BOLD:AAE5045
Orgyia pseudotsugata|[6780]|LBCH6515-10|10-JDWBC-6515|658[0n]bp|Canada.British Columbia|BOLD:AAE5045
Orgyia pseudotsugata|[6781]|LBCH6519-10|10-JDWBC-6519|658[0n]bp|Canada.British Columbia|BOLD:AAE5045
Orgyia pseudotsugata|[6782]|LBCH7279-10|10-JDWBC-7279|658[0n]bp|Canada.British Columbia|BOLD:AAE5045
Orgyia pseudotsugata|[6783]|LBCH7207-10|10-JDWBC-7207|658[0n]bp|Canada.British Columbia|BOLD:AAE5045
Orgyia pseudotsugata|[6784]|LBCH6406-10|10-JDWBC-6406|658[0n]bp|Canada.British Columbia|BOLD:AAE5045
Orgyia pseudotsugata|[6785]|LBCH6600-10|10-JDWBC-6600|658[0n]bp|Canada.British Columbia|BOLD:AAE5045
Orgyia pseudotsugata|[6786]|LBCH6516-10|10-JDWBC-6516|658[0n]bp|Canada.British Columbia|BOLD:AAE5045
Orgyia pseudotsugata|[6787]|LBCH6349-10|10-JDWBC-6349|658[0n]bp|Canada.British Columbia|BOLD:AAE5045
Orgyia pseudotsugata|[6788]|LBCH6146-10|10-JDWBC-6146|658[0n]bp|Canada.British Columbia|BOLD:AAE5045
Orgyia pseudotsugata|[6789]|LBCH6769-10|10-JDWBC-6769|658[0n]bp|Canada.British Columbia|BOLD:AAE5045
Orgyia pseudotsugata|[6790]|LBCH6353-10|10-JDWBC-6353|658[0n]bp|Canada.British Columbia|BOLD:AAE5045
Orgyia pseudotsugata|[6791]|LBCH6771-10|10-JDWBC-6771|658[0n]bp|Canada.British Columbia|BOLD:AAE5045
Orgyia pseudotsugata|[6792]|LBCH6870-10|10-JDWBC-6870|658[0n]bp|Canada.British Columbia|BOLD:AAE5045
Orgyia pseudotsugata|[6793]|LBCH7595-10|10-JDWBC-7595|658[0n]bp|Canada.British Columbia|BOLD:AAE5045
Orgyia pseudotsugata|[6794]|LBCH6770-10|10-JDWBC-6770|658[0n]bp|Canada.British Columbia|BOLD:AAE5045
Orgyia pseudotsugata|[6795]|LBCH7845-10|10-JDWBC-7845|658[0n]bp|Canada.British Columbia|BOLD:AAE5045
Orgyia pseudotsugata|[6796]|LBCH6767-10|10-JDWBC-6767|658[0n]bp|Canada.British Columbia|BOLD:AAE5045
Orgyia pseudotsugata|[6797]|LBCH7596-10|10-JDWBC-7596|658[0n]bp|Canada.British Columbia|BOLD:AAE5045
Orgyia pseudotsugata|[6798]|LBCH7495-10|10-JDWBC-7495|658[0n]bp|Canada.British Columbia|BOLD:AAE5045
Orgyia pseudotsugata|[6799]|LBCH7022-10|10-JDWBC-7022|658[0n]bp|Canada.British Columbia|BOLD:AAE5045
Orgyia pseudotsugata|[6800]|LBCH6518-10|10-JDWBC-6518|658[0n]bp|Canada.British Columbia|BOLD:AAE5045
Orgyia pseudotsugata|[6801]|LBCH7732-10|10-JDWBC-7732|658[0n]bp|Canada.British Columbia|BOLD:AAE5045
Orgyia pseudotsugata|[6802]|LBCH7733-10|10-JDWBC-7733|658[0n]bp|Canada.British Columbia|BOLD:AAE5045
Orgyia pseudotsugata|[6803]|LBCH7735-10|10-JDWBC-7735|658[0n]bp|Canada.British Columbia|BOLD:AAE5045
Orgyia pseudotsugata|[6804]|LBCH7729-10|10-JDWBC-7729|658[0n]bp|Canada.British Columbia|BOLD:AAE5045
Orgyia pseudotsugata|[6805]|LBCH7597-10|10-JDWBC-7597|658[0n]bp|Canada.British Columbia|BOLD:AAE5045
Orgyia pseudotsugata|[6806]|LBCH7026-10|10-JDWBC-7026|658[0n]bp|Canada.British Columbia|BOLD:AAE5045
Orgyia pseudotsugata|[6807]|LBCH6868-10|10-JDWBC-6868|658[0n]bp|Canada.British Columbia|BOLD:AAE5045
Orgyia pseudotsugata|[6808]|LBCH7027-10|10-JDWBC-7027|658[0n]bp|Canada.British Columbia|BOLD:AAE5045
Orgyia pseudotsugata|[6809]|LBCH6401-10|10-JDWBC-6401|658[0n]bp|Canada.British Columbia|BOLD:AAE5045
Orgyia pseudotsugata|[6810]|LBCH6408-10|10-JDWBC-6408|658[0n]bp|Canada.British Columbia|BOLD:AAE5045
Orgyia pseudotsugata|[6811]|LBCH6866-10|10-JDWBC-6866|658[0n]bp|Canada.British Columbia|BOLD:AAE5045
Orgyia pseudotsugata|[6812]|LBCH7494-10|10-JDWBC-7494|658[0n]bp|Canada.British Columbia|BOLD:AAE5045
Orgyia pseudotsugata|[6813]|LBCH6347-10|10-JDWBC-6347|658[0n]bp|Canada.British Columbia|BOLD:AAE5045
Orgyia pseudotsugata|[6814]|LBCH7730-10|10-JDWBC-7730|658[0n]bp|Canada.British Columbia|BOLD:AAE5045
Orgyia pseudotsugata|[6815]|LBCH6517-10|10-JDWBC-6517|658[0n]bp|Canada.British Columbia|BOLD:AAE5045
Orgyia pseudotsugata|[6816]|LBCH7846-10|10-JDWBC-7846|658[0n]bp|Canada.British Columbia|BOLD:AAE5045
Orgyia pseudotsugata|[6817]|LBCH7024-10|10-JDWBC-7024|658[0n]bp|Canada.British Columbia|BOLD:AAE5045
Orgyia pseudotsugata|[6818]|LBCH7078-10|10-JDWBC-7078|658[0n]bp|Canada.British Columbia|BOLD:AAE5045
Orgyia pseudotsugata|[6819]|LBCH6352-10|10-JDWBC-6352|658[0n]bp|Canada.British Columbia|BOLD:AAE5045
Orgyia pseudotsugata|[6820]|LBCH6766-10|10-JDWBC-6766|658[0n]bp|Canada.British Columbia|BOLD:AAE5045
Orgyia pseudotsugata|[6821]|LBCH6403-10|10-JDWBC-6403|658[0n]bp|Canada.British Columbia|BOLD:AAE5045
Orgyia pseudotsugata|[6822]|LBCH7731-10|10-JDWBC-7731|658[0n]bp|Canada.British Columbia|BOLD:AAE5045
Orgyia pseudotsugata|[6823]|LBCH7021-10|10-JDWBC-7021|658[0n]bp|Canada.British Columbia|BOLD:AAE5045
Orgyia pseudotsugata|[6824]|LBCH6601-10|10-JDWBC-6601|658[0n]bp|Canada.British Columbia|BOLD:AAE5045
Orgyia pseudotsugata|[6825]|LBCH6350-10|10-JDWBC-6350|658[0n]bp|Canada.British Columbia|BOLD:AAE5045
Orgyia pseudotsugata|[6826]|LBCH6351-10|10-JDWBC-6351|658[0n]bp|Canada.British Columbia|BOLD:AAE5045
Orgyia pseudotsugata|[6827]|LBCH6407-10|10-JDWBC-6407|658[0n]bp|Canada.British Columbia|BOLD:AAE5045
Orgyia pseudotsugata|[6828]|LBCH6597-10|10-JDWBC-6597|658[0n]bp|Canada.British Columbia|BOLD:AAE5045
Orgyia pseudotsugata|[6829]|LBCH6865-10|10-JDWBC-6865|658[0n]bp|Canada.British Columbia|BOLD:AAE5045
Orgyia pseudotsugata|[6830]|LBCH6514-10|10-JDWBC-6514|658[0n]bp|Canada.British Columbia|BOLD:AAE5045
Orgyia pseudotsugata|[6831]|LBCH7162-10|10-JDWBC-7162|639[0n]bp|Canada.British Columbia|BOLD:AAE5045
Orgyia pseudotsugata|[6832]|LBCH6772-10|10-JDWBC-6772|645[0n]bp|Canada.British Columbia|BOLD:AAE5045
Orgyia pseudotsugata|[6833]|LBCH7274-10|10-JDWBC-7274|629[0n]bp|Canada.British Columbia|BOLD:AAE5045
Orgyia pseudotsugata|[6834]|LBCH7591-10|10-JDWBC-7591|641[0n]bp|Canada.British Columbia|BOLD:AAE5045
Orgyia pseudotsugata|[6835]|LBCH7160-10|10-JDWBC-7160|636[0n]bp|Canada.British Columbia|BOLD:AAE5045
Orgyia pseudotsugata|[6836]|LBCH6599-10|10-JDWBC-6599|658[0n]bp|Canada.British Columbia|BOLD:AAE5045
Orgyia pseudotsugata|[6837]|LBCH7496-10|10-JDWBC-7496|658[0n]bp|Canada.British Columbia|BOLD:AAE5045
Orgyia pseudotsugata|[6838]|LBCH6348-10|10-JDWBC-6348|658[0n]bp|Canada.British Columbia|BOLD:AAE5045
Orgyia pseudotsugata|[6839]|LBCH7371-10|10-JDWBC-7371|658[0n]bp|Canada.British Columbia|BOLD:AAE5045
