# Supplementary material for: A Transcontinental Challenge — A Test of DNA Barcode Performance for 1,541 Species of Canadian Noctuoidea (Lepidoptera)
Source: PLoS One. 2014 Mar 25;9(3):e92797. doi: 10.1371/journal.pone.0092797 (PMC3965468; doi:10.1371/journal.pone.0092797)
Supplement: Tree S5 — NJ tree for Canadian species in the family Noctuidae. (PDF) [file pone.0092797.s009.pdf]

BOLD TaxonID Tree

Title	:	Canadian Noctuoidea, continued [DATASET-CANOC2]
Date	:	28-October-2013
Data Type	:	Nucleotide
Distance Model	:	Kimura 2 Parameter
Marker	:	COI-5P
Codon Positions	:	
Labels	:	Country & Province, SampleID, ProcessID, Sequence Length, BIN URI
Filters	:	Length > 200
Colorization	:	[blue]=Stop Codons [red]=Contamination or misidentification
Attachment	:	Photographs & Spreadsheet

Sequence Count	:	21726
Species count	:	1159
Genus count	:	247
Family count	:	1
Unidentified	:	0

BIN Count	:	1090

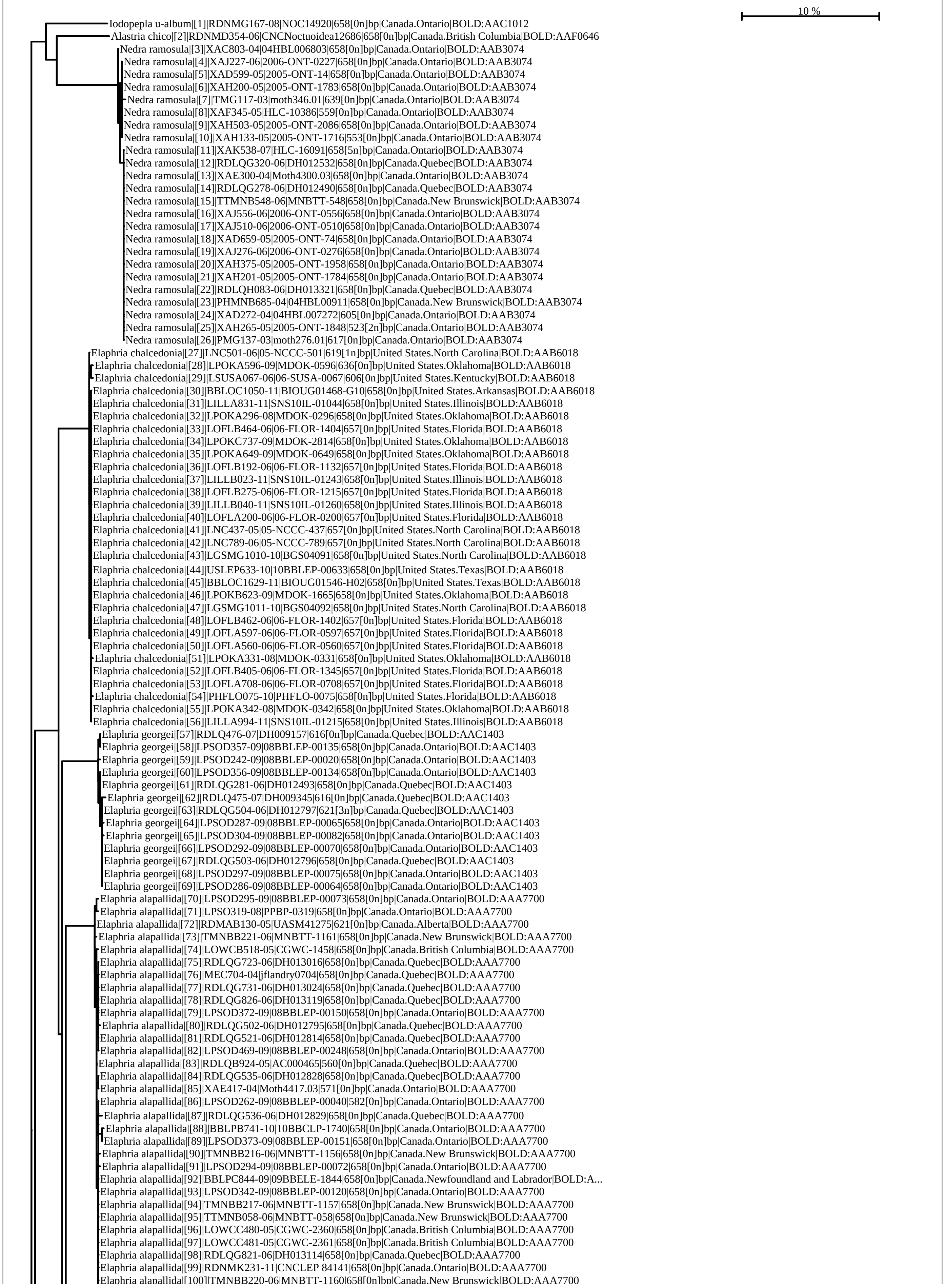
10 %
Iodopepla u-album|[1]|RDNMG167-08|NOC14920|658[0n]bp|Canada.Ontario|BOLD:AAC1012
Alastria chico|[2]|RDNMD354-06|CNCNoctuoidea12686|658[0n]bp|Canada.British Columbia|BOLD:AAF0646
Nedra ramosula|[3]|XAC803-04|04HBL006803|658[0n]bp|Canada.Ontario|BOLD:AAB3074
Nedra ramosula|[4]|XAJ227-06|2006-ONT-0227|658[0n]bp|Canada.Ontario|BOLD:AAB3074
Nedra ramosula|[5]|XAD599-05|2005-ONT-14|658[0n]bp|Canada.Ontario|BOLD:AAB3074
Nedra ramosula|[6]|XAH200-05|2005-ONT-1783|658[0n]bp|Canada.Ontario|BOLD:AAB3074
Nedra ramosula|[7]|TMG117-03|moth346.01|639[0n]bp|Canada.Ontario|BOLD:AAB3074
Nedra ramosula|[8]|XAF345-05|HLC-10386|559[0n]bp|Canada.Ontario|BOLD:AAB3074
Nedra ramosula|[9]|XAH503-05|2005-ONT-2086|658[0n]bp|Canada.Ontario|BOLD:AAB3074
Nedra ramosula|[10]|XAH133-05|2005-ONT-1716|553[0n]bp|Canada.Ontario|BOLD:AAB3074
Nedra ramosula|[11]|XAK538-07|HLC-16091|658[5n]bp|Canada.Ontario|BOLD:AAB3074
Nedra ramosula|[12]|RDLQG320-06|DH012532|658[0n]bp|Canada.Quebec|BOLD:AAB3074
Nedra ramosula|[13]|XAE300-04|Moth4300.03|658[0n]bp|Canada.Ontario|BOLD:AAB3074
Nedra ramosula|[14]|RDLQG278-06|DH012490|658[0n]bp|Canada.Quebec|BOLD:AAB3074
Nedra ramosula|[15]|TTMNB548-06|MNBTT-548|658[0n]bp|Canada.New Brunswick|BOLD:AAB3074
Nedra ramosula|[16]|XAJ556-06|2006-ONT-0556|658[0n]bp|Canada.Ontario|BOLD:AAB3074
Nedra ramosula|[17]|XAJ510-06|2006-ONT-0510|658[0n]bp|Canada.Ontario|BOLD:AAB3074
Nedra ramosula|[18]|XAD659-05|2005-ONT-74|658[0n]bp|Canada.Ontario|BOLD:AAB3074
Nedra ramosula|[19]|XAJ276-06|2006-ONT-0276|658[0n]bp|Canada.Ontario|BOLD:AAB3074
Nedra ramosula|[20]|XAH375-05|2005-ONT-1958|658[0n]bp|Canada.Ontario|BOLD:AAB3074
Nedra ramosula|[21]|XAH201-05|2005-ONT-1784|658[0n]bp|Canada.Ontario|BOLD:AAB3074
Nedra ramosula|[22]|RDLQH083-06|DH013321|658[0n]bp|Canada.Quebec|BOLD:AAB3074
Nedra ramosula|[23]|PHMNB685-04|04HBL00911|658[0n]bp|Canada.New Brunswick|BOLD:AAB3074
Nedra ramosula|[24]|XAD272-04|04HBL007272|605[0n]bp|Canada.Ontario|BOLD:AAB3074
Nedra ramosula|[25]|XAH265-05|2005-ONT-1848|523[2n]bp|Canada.Ontario|BOLD:AAB3074
Nedra ramosula|[26]|PMG137-03|moth276.01|617[0n]bp|Canada.Ontario|BOLD:AAB3074
Elaphria chalcedonia|[27]|LNC501-06|05-NCCC-501|619[1n]bp|United States.North Carolina|BOLD:AAB6018
Elaphria chalcedonia|[28]|LPOKA596-09|MDOK-0596|636[0n]bp|United States.Oklahoma|BOLD:AAB6018
Elaphria chalcedonia|[29]|LSUSA067-06|06-SUSA-0067|606[0n]bp|United States.Kentucky|BOLD:AAB6018
Elaphria chalcedonia|[30]|BBLOC1050-11|BIOUG01468-G10|658[0n]bp|United States.Arkansas|BOLD:AAB6018
Elaphria chalcedonia|[31]|LILLA831-11|SNS10IL-01044|658[0n]bp|United States.Illinois|BOLD:AAB6018
Elaphria chalcedonia|[32]|LPOKA296-08|MDOK-0296|658[0n]bp|United States.Oklahoma|BOLD:AAB6018
Elaphria chalcedonia|[33]|LOFLB464-06|06-FLOR-1404|657[0n]bp|United States.Florida|BOLD:AAB6018
Elaphria chalcedonia|[34]|LPOKC737-09|MDOK-2814|658[0n]bp|United States.Oklahoma|BOLD:AAB6018
Elaphria chalcedonia|[35]|LPOKA649-09|MDOK-0649|658[0n]bp|United States.Oklahoma|BOLD:AAB6018
Elaphria chalcedonia|[36]|LOFLB192-06|06-FLOR-1132|657[0n]bp|United States.Florida|BOLD:AAB6018
Elaphria chalcedonia|[37]|LILLB023-11|SNS10IL-01243|658[0n]bp|United States.Illinois|BOLD:AAB6018
Elaphria chalcedonia|[38]|LOFLB275-06|06-FLOR-1215|657[0n]bp|United States.Florida|BOLD:AAB6018
Elaphria chalcedonia|[39]|LILLB040-11|SNS10IL-01260|658[0n]bp|United States.Illinois|BOLD:AAB6018
Elaphria chalcedonia|[40]|LOFLA200-06|06-FLOR-0200|657[0n]bp|United States.Florida|BOLD:AAB6018
Elaphria chalcedonia|[41]|LNC437-05|05-NCCC-437|657[0n]bp|United States.North Carolina|BOLD:AAB6018
Elaphria chalcedonia|[42]|LNC789-06|05-NCCC-789|657[0n]bp|United States.North Carolina|BOLD:AAB6018
Elaphria chalcedonia|[43]|LGSMG1010-10|BGS04091|658[0n]bp|United States.North Carolina|BOLD:AAB6018
Elaphria chalcedonia|[44]|USLEP633-10|10BBLEP-00633|658[0n]bp|United States.Texas|BOLD:AAB6018
Elaphria chalcedonia|[45]|BBLOC1629-11|BIOUG01546-H02|658[0n]bp|United States.Texas|BOLD:AAB6018
Elaphria chalcedonia|[46]|LPOKB623-09|MDOK-1665|658[0n]bp|United States.Oklahoma|BOLD:AAB6018
Elaphria chalcedonia|[47]|LGSMG1011-10|BGS04092|658[0n]bp|United States.North Carolina|BOLD:AAB6018
Elaphria chalcedonia|[48]|LOFLB462-06|06-FLOR-1402|657[0n]bp|United States.Florida|BOLD:AAB6018
Elaphria chalcedonia|[49]|LOFLA597-06|06-FLOR-0597|657[0n]bp|United States.Florida|BOLD:AAB6018
Elaphria chalcedonia|[50]|LOFLA560-06|06-FLOR-0560|657[0n]bp|United States.Florida|BOLD:AAB6018
Elaphria chalcedonia|[51]|LPOKA331-08|MDOK-0331|658[0n]bp|United States.Oklahoma|BOLD:AAB6018
Elaphria chalcedonia|[52]|LOFLB405-06|06-FLOR-1345|657[0n]bp|United States.Florida|BOLD:AAB6018
Elaphria chalcedonia|[53]|LOFLA708-06|06-FLOR-0708|657[0n]bp|United States.Florida|BOLD:AAB6018
Elaphria chalcedonia|[54]|PHFLO075-10|PHFLO-0075|658[0n]bp|United States.Florida|BOLD:AAB6018
Elaphria chalcedonia|[55]|LPOKA342-08|MDOK-0342|658[0n]bp|United States.Oklahoma|BOLD:AAB6018
Elaphria chalcedonia|[56]|LILLA994-11|SNS10IL-01215|658[0n]bp|United States.Illinois|BOLD:AAB6018
Elaphria georgei|[57]|RDLQ476-07|DH009157|616[0n]bp|Canada.Quebec|BOLD:AAC1403
Elaphria georgei|[58]|LPSOD357-09|08BBLEP-00135|658[0n]bp|Canada.Ontario|BOLD:AAC1403
Elaphria georgei|[59]|LPSOD242-09|08BBLEP-00020|658[0n]bp|Canada.Ontario|BOLD:AAC1403
Elaphria georgei|[60]|LPSOD356-09|08BBLEP-00134|658[0n]bp|Canada.Ontario|BOLD:AAC1403
Elaphria georgei|[61]|RDLQG281-06|DH012493|658[0n]bp|Canada.Quebec|BOLD:AAC1403
Elaphria georgei|[62]|RDLQ475-07|DH009345|616[0n]bp|Canada.Quebec|BOLD:AAC1403
Elaphria georgei|[63]|RDLQG504-06|DH012797|621[3n]bp|Canada.Quebec|BOLD:AAC1403
Elaphria georgei|[64]|LPSOD287-09|08BBLEP-00065|658[0n]bp|Canada.Ontario|BOLD:AAC1403
Elaphria georgei|[65]|LPSOD304-09|08BBLEP-00082|658[0n]bp|Canada.Ontario|BOLD:AAC1403
Elaphria georgei|[66]|LPSOD292-09|08BBLEP-00070|658[0n]bp|Canada.Ontario|BOLD:AAC1403
Elaphria georgei|[67]|RDLQG503-06|DH012796|658[0n]bp|Canada.Quebec|BOLD:AAC1403
Elaphria georgei|[68]|LPSOD297-09|08BBLEP-00075|658[0n]bp|Canada.Ontario|BOLD:AAC1403
Elaphria georgei|[69]|LPSOD286-09|08BBLEP-00064|658[0n]bp|Canada.Ontario|BOLD:AAC1403
Elaphria alapallida|[70]|LPSOD295-09|08BBLEP-00073|658[0n]bp|Canada.Ontario|BOLD:AAA7700
Elaphria alapallida|[71]|LPSO319-08|PPBP-0319|658[0n]bp|Canada.Ontario|BOLD:AAA7700
Elaphria alapallida|[72]|RDMAB130-05|UASM41275|621[0n]bp|Canada.Alberta|BOLD:AAA7700
Elaphria alapallida|[73]|TMNBB221-06|MNBTT-1161|658[0n]bp|Canada.New Brunswick|BOLD:AAA7700
Elaphria alapallida|[74]|LOWCB518-05|CGWC-1458|658[0n]bp|Canada.British Columbia|BOLD:AAA7700
Elaphria alapallida|[75]|RDLQG723-06|DH013016|658[0n]bp|Canada.Quebec|BOLD:AAA7700
Elaphria alapallida|[76]|MEC704-04|jflandry0704|658[0n]bp|Canada.Quebec|BOLD:AAA7700
Elaphria alapallida|[77]|RDLQG731-06|DH013024|658[0n]bp|Canada.Quebec|BOLD:AAA7700
Elaphria alapallida|[78]|RDLQG826-06|DH013119|658[0n]bp|Canada.Quebec|BOLD:AAA7700
Elaphria alapallida|[79]|LPSOD372-09|08BBLEP-00150|658[0n]bp|Canada.Ontario|BOLD:AAA7700
Elaphria alapallida|[80]|RDLQG502-06|DH012795|658[0n]bp|Canada.Quebec|BOLD:AAA7700
Elaphria alapallida|[81]|RDLQG521-06|DH012814|658[0n]bp|Canada.Quebec|BOLD:AAA7700
Elaphria alapallida|[82]|LPSOD469-09|08BBLEP-00248|658[0n]bp|Canada.Ontario|BOLD:AAA7700
Elaphria alapallida|[83]|RDLQB924-05|AC000465|560[0n]bp|Canada.Quebec|BOLD:AAA7700
Elaphria alapallida|[84]|RDLQG535-06|DH012828|658[0n]bp|Canada.Quebec|BOLD:AAA7700
Elaphria alapallida|[85]|XAE417-04|Moth4417.03|571[0n]bp|Canada.Ontario|BOLD:AAA7700
Elaphria alapallida|[86]|LPSOD262-09|08BBLEP-00040|582[0n]bp|Canada.Ontario|BOLD:AAA7700
Elaphria alapallida|[87]|RDLQG536-06|DH012829|658[0n]bp|Canada.Quebec|BOLD:AAA7700
Elaphria alapallida|[88]|BBLPB741-10|10BBCLP-1740|658[0n]bp|Canada.Ontario|BOLD:AAA7700
Elaphria alapallida|[89]|LPSOD373-09|08BBLEP-00151|658[0n]bp|Canada.Ontario|BOLD:AAA7700
Elaphria alapallida|[90]|TMNBB216-06|MNBTT-1156|658[0n]bp|Canada.New Brunswick|BOLD:AAA7700
Elaphria alapallida|[91]|LPSOD294-09|08BBLEP-00072|658[0n]bp|Canada.Ontario|BOLD:AAA7700
Elaphria alapallida|[92]|BBLPC844-09|09BBELE-1844|658[0n]bp|Canada.Newfoundland and Labrador|BOLD:A...
Elaphria alapallida|[93]|LPSOD342-09|08BBLEP-00120|658[0n]bp|Canada.Ontario|BOLD:AAA7700
Elaphria alapallida|[94]|TMNBB217-06|MNBTT-1157|658[0n]bp|Canada.New Brunswick|BOLD:AAA7700
Elaphria alapallida|[95]|TTMNB058-06|MNBTT-058|658[0n]bp|Canada.New Brunswick|BOLD:AAA7700
Elaphria alapallida|[96]|LOWCC480-05|CGWC-2360|658[0n]bp|Canada.British Columbia|BOLD:AAA7700
Elaphria alapallida|[97]|LOWCC481-05|CGWC-2361|658[0n]bp|Canada.British Columbia|BOLD:AAA7700
Elaphria alapallida|[98]|RDLQG821-06|DH013114|658[0n]bp|Canada.Quebec|BOLD:AAA7700
Elaphria alapallida|[99]|RDNMK231-11|CNCLEP 84141|658[0n]bp|Canada.Ontario|BOLD:AAA7700
Elaphria alapallida|[100]|TMNBB220-06|MNBTT-1160|658[0n]bp|Canada.New Brunswick|BOLD:AAA7700

Elaphria alapallida|[98]|RDLQG821-06|DH013114|658[0n]bp|Canada.Quebec|BOLD:AAA7700
Elaphria alapallida|[99]|RDNMK231-11|CNCLEP 84141|658[0n]bp|Canada.Ontario|BOLD:AAA7700
Elaphria alapallida|[100]|TMNBB220-06|MNBTT-1160|658[0n]bp|Canada.New Brunswick|BOLD:AAA7700
Elaphria alapallida|[101]|LALPA289-10|AVBC 291-10|658[0n]bp|Canada.British Columbia|BOLD:AAA7700
Elaphria alapallida|[102]|LPSOB488-08|PPBP-1487|658[0n]bp|Canada.Ontario|BOLD:AAA7700
Elaphria alapallida|[103]|TMNBB214-06|MNBTT-1154|658[0n]bp|Canada.New Brunswick|BOLD:AAA7700
Elaphria alapallida|[104]|LPSOD285-09|08BBLEP-00063|658[0n]bp|Canada.Ontario|BOLD:AAA7700
Elaphria alapallida|[105]|LPSOD377-09|08BBLEP-00156|658[0n]bp|Canada.Ontario|BOLD:AAA7700
Elaphria alapallida|[106]|LPSOD309-09|08BBLEP-00087|658[0n]bp|Canada.Ontario|BOLD:AAA7700
Elaphria alapallida|[107]|TMNBB219-06|MNBTT-1159|658[0n]bp|Canada.New Brunswick|BOLD:AAA7700
Elaphria alapallida|[108]|RDLQB271-05|DH010357|658[0n]bp|Canada.Quebec|BOLD:AAA7700
Elaphria alapallida|[109]|RDLQG500-06|DH012793|658[0n]bp|Canada.Quebec|BOLD:AAA7700
Elaphria alapallida|[110]|LPMN090-08|08BBLEP-00888|658[0n]bp|Canada.Manitoba|BOLD:AAA7700
Elaphria alapallida|[111]|LPSOD374-09|08BBLEP-00152|658[0n]bp|Canada.Ontario|BOLD:AAA7700
Elaphria alapallida|[112]|RDLQG282-06|DH012494|658[0n]bp|Canada.Quebec|BOLD:AAA7700
Elaphria alapallida|[113]|LPSOD655-09|08BBLEP-00436|658[0n]bp|Canada.Ontario|BOLD:AAA7700
Elaphria alapallida|[114]|LPSOD359-09|08BBLEP-00137|658[0n]bp|Canada.Ontario|BOLD:AAA7700
Elaphria alapallida|[115]|RDNMK229-11|CNCLEP 84139|658[0n]bp|Canada.Ontario|BOLD:AAA7700
Elaphria alapallida|[116]|TMNBB215-06|MNBTT-1155|658[0n]bp|Canada.New Brunswick|BOLD:AAA7700
Elaphria alapallida|[117]|BBLPC809-09|09BBELE-1809|658[0n]bp|Canada.Newfoundland and Labrador|BOLD:A...
Elaphria alapallida|[118]|RDLQG501-06|DH012794|647[0n]bp|Canada.Quebec|BOLD:AAA7700
Elaphria alapallida|[119]|LOWCB516-05|CGWC-1456|591[0n]bp|Canada.British Columbia|BOLD:AAA7700
Elaphria alapallida|[120]|RDLQB776-05|DH010863|617[0n]bp|Canada.Quebec|BOLD:AAA7700
Elaphria alapallida|[121]|RDLQH212-07|MDH003998|621[0n]bp|Canada.Quebec|BOLD:AAA7700
Elaphria alapallida|[122]|LPSOD367-09|08BBLEP-00145|658[0n]bp|Canada.Ontario|BOLD:AAA7700
Elaphria alapallida|[123]|LPSOD284-09|08BBLEP-00062|658[0n]bp|Canada.Ontario|BOLD:AAA7700
Elaphria alapallida|[124]|PHMNB641-04|04HBL00867|658[0n]bp|Canada.New Brunswick|BOLD:AAA7700
Elaphria alapallida|[125]|LPMN795-08|08BBLEP-01598|658[0n]bp|Canada.Manitoba|BOLD:AAA7700
Elaphria alapallida|[126]|RDNMK230-11|CNCLEP 84140|658[0n]bp|Canada.Ontario|BOLD:AAA7700
Elaphria grata|[127]|LPSO758-08|PPBP-0758|656[0n]bp|Canada.Ontario|BOLD:AAB4093
Elaphria grata|[128]|LPSO505-08|PPBP-0505|633[0n]bp|Canada.Ontario|BOLD:AAB4093
Elaphria grata|[129]|LPSO485-08|PPBP-0485|658[0n]bp|Canada.Ontario|BOLD:AAB4093
Elaphria grata|[130]|LPSO195-08|PPBP-0195|658[0n]bp|Canada.Ontario|BOLD:AAB4093
Elaphria grata|[131]|LPSO096-08|PPBP-0096|658[0n]bp|Canada.Ontario|BOLD:AAB4093
Elaphria grata|[132]|LPSO106-08|PPBP-0106|658[0n]bp|Canada.Ontario|BOLD:AAB4093
Elaphria grata|[133]|LPSO945-08|PPBP-0945|658[0n]bp|Canada.Ontario|BOLD:AAB4093
Pyrrhia aurantiago|[134]|HELNA518-09|USNM148211|599[0n]bp|United States.Florida|BOLD:AAF5886
Pyrrhia aurantiago|[135]|RDNMF495-08|NOC14581|641[0n]bp|United States.Florida|BOLD:AAF5886
Eutricopis nexilis|[136]|HELNA869-10|64289|658[0n]bp|Canada.British Columbia|BOLD:AAC8108
Eutricopis nexilis|[137]|HELNA867-10|64287|658[0n]bp|Canada.British Columbia|BOLD:AAC8108
Eutricopis nexilis|[138]|HELNA868-10|64288|658[0n]bp|Canada.British Columbia|BOLD:AAC8108
Eutricopis nexilis|[139]|RDNMG538-08|CNC LEP00052362|658[0n]bp|Canada.Alberta|BOLD:AAC8108
Eutricopis nexilis|[140]|RDNMG535-08|CNC LEP00052359|658[0n]bp|Canada.Alberta|BOLD:AAC8108
Eutricopis nexilis|[141]|RDNMF052-08|NOC14138|658[0n]bp|Canada.Alberta|BOLD:AAC8108
Eutricopis nexilis|[142]|RDNMG536-08|CNC LEP00052360|658[0n]bp|Canada.Alberta|BOLD:AAC8108
Eutricopis nexilis|[143]|RDNMG537-08|CNC LEP00052361|658[0n]bp|Canada.Alberta|BOLD:AAC8108
Pyrrhia cilisca|[144]|RDNMG823-08|CNC LEP00052947|658[0n]bp|Canada.Ontario|BOLD:AAC7528
Pyrrhia cilisca|[145]|RDNMC036-05|CNCNoctuoidea10777|658[0n]bp|Canada.Ontario|BOLD:AAC7528
Pyrrhia exprimens|[146]|LHLEP189-06|UBC-2006-1229|658[5n]bp|Canada.British Columbia|BOLD:AAB3280
Pyrrhia exprimens|[147]|RDLQB216-05|DH010302|658[0n]bp|Canada.Quebec|BOLD:AAB3280
Pyrrhia exprimens|[148]|RDLQB214-05|DH010300|658[0n]bp|Canada.Quebec|BOLD:AAB3280
Pyrrhia exprimens|[149]|LPSOC096-08|PPBP-2095|658[0n]bp|Canada.Ontario|BOLD:AAB3280
Pyrrhia exprimens|[150]|BBLPB499-10|10BBCLP-1498|658[0n]bp|Canada.Ontario|BOLD:AAB3280
Pyrrhia exprimens|[151]|RDLQB799-05|DH010886|658[0n]bp|Canada.Quebec|BOLD:AAB3280
Pyrrhia exprimens|[152]|LHLEP271-06|UBC-2006-0164|658[0n]bp|Canada.British Columbia|BOLD:AAB3280
Pyrrhia exprimens|[153]|RDLQB798-05|DH010885|533[0n]bp|Canada.Quebec|BOLD:AAB3280
Pyrrhia exprimens|[154]|RDLQB215-05|DH010301|658[1n]bp|Canada.Quebec|
Pyrrhia exprimens|[155]|BBLPB496-10|10BBCLP-1495|658[0n]bp|Canada.Saskatchewan|BOLD:AAB3280
Pyrrhia exprimens|[156]|LBCH2960-10|10-JDWBC-2960|658[0n]bp|Canada.British Columbia|BOLD:AAB3280
Schinia gaurae|[157]|HELNA045-06|221602|657[5n]bp|United States.Colorado|BOLD:AAD6868
Schinia gaurae|[158]|HELNA046-06|144194|658[5n]bp|United States.Colorado|BOLD:AAD6868
Schinia gaurae|[159]|CMAZA537-10|CMAZ-0537|658[0n]bp|United States.Arizona|BOLD:AAD6868
Schinia gaurae|[160]|CMAZA1074-12|BIOUG02042-D09|658[0n]bp|United States.Arizona|BOLD:AAD6868
Schinia gaurae|[161]|CMAZA404-10|CMAZ-0404|658[0n]bp|United States.Arizona|BOLD:AAD6868
Schinia gaurae|[162]|CMAZA803-10|CMAZ-0803|658[0n]bp|United States.Arizona|BOLD:AAD6868
Schinia gaurae|[163]|LPOKE032-10|MDOK-4110|658[0n]bp|United States.Oklahoma|BOLD:AAD6868
Schinia gaurae|[164]|HELNA284-09|USNM148142|658[0n]bp|United States.New Mexico|BOLD:AAD6868
Schinia gaurae|[165]|LSEU074-06|06-JKA-0074|551[1n]bp|United States.Florida|BOLD:AAD6868
Schinia gaurae|[166]|LSEU073-06|06-JKA-0073|546[2n]bp|United States.Florida|BOLD:AAD6868
Schinia bina group A|[167]|LPOKC819-09|MDOK-2896|658[0n]bp|United States.Oklahoma|BOLD:ACF4964
Schinia bina group A|[168]|RDNMF386-08|NOC14472|641[0n]bp|United States.Texas|BOLD:ACF4964
Schinia bina group A|[169]|HELNA275-09|USNM161855|658[0n]bp|United States.Kansas|BOLD:ACF4964
Schinia bina group A|[170]|HELNA367-09|USNM148051|658[0n]bp|United States.Colorado|BOLD:ACF4964
Schinia florida|[171]|HELNA151-06|USNM00161643|658[0n]bp|Canada.Nova Scotia|BOLD:AAB6844
Schinia florida|[172]|PHMNB722-05|Moth 415.03SA|658[0n]bp|Canada.New Brunswick|BOLD:AAB6844
Schinia florida|[173]|HELNA885-10|65409|658[0n]bp|Canada.New Brunswick|BOLD:AAB6844
Schinia florida|[174]|BBLPC972-09|09BBELE-1972|658[0n]bp|Canada.Newfoundland and Labrador|BOLD:AAB6844
Schinia florida|[175]|HELNA214-06|USNM00157472|634[0n]bp|Canada.Nova Scotia|BOLD:AAB6844
Schinia florida|[176]|HELNA888-10|148290|658[0n]bp|Canada.New Brunswick|BOLD:AAB6844
Schinia florida|[177]|XAD016-04|04HBL007016|520[0n]bp|Canada.Ontario|BOLD:AAB6844
Schinia florida|[178]|HELNA887-10|148289|658[0n]bp|Canada.New Brunswick|BOLD:AAB6844
Schinia florida|[179]|HELNA886-10|65408|658[0n]bp|Canada.New Brunswick|BOLD:AAB6844
Schinia florida|[180]|XAD006-04|04HBL007006|658[0n]bp|Canada.Ontario|BOLD:AAB6844
Schinia florida|[181]|PMG160-03|SCHI1.00|617[0n]bp|Canada.Ontario|BOLD:AAB6844
Schinia florida|[182]|JBAZ159-09|JLB-0159|658[0n]bp|Canada.Ontario|BOLD:AAB6844
Schinia florida|[183]|XAD004-04|04HBL007004|658[0n]bp|Canada.Ontario|BOLD:AAB6844
Schinia florida|[184]|HELNA152-06|USNM00161645|658[0n]bp|Canada.Manitoba|BOLD:AAB6844
Schinia florida|[185]|BLTIB971-08|BL1400|658[0n]bp|Canada.Ontario|BOLD:AAB6844
Schinia florida|[186]|XAI054-05|0102-ONT-0054|658[0n]bp|Canada.Ontario|BOLD:AAB6844
Schinia jaguarina|[187]|LTOLB1301-11|RSP-96-0610|658[0n]bp|United States.Colorado|BOLD:AAC6459
Schinia jaguarina|[188]|HELNA067-06|144248|658[0n]bp|United States.Kansas|BOLD:AAC6459
Schinia jaguarina|[189]|HELNA249-06|USNM00158249|658[0n]bp|United States.Montana|BOLD:AAC6459
Schinia jaguarina|[190]|HELNA855-10|148269|658[0n]bp|United States.Kansas|BOLD:AAC6459
Schinia jaguarina|[191]|HELNA161-06|USNM00161655|658[0n]bp|United States.Montana|BOLD:AAC6459
Schinia jaguarina|[192]|HELNA248-06|USNM00158245|658[0n]bp|United States.Montana|BOLD:AAC6459
Schinia jaguarina|[193]|HELNA247-06|USNM00158246|658[0n]bp|United States.Montana|BOLD:AAC6459
Schinia jaguarina|[194]|HELNA066-06|144247|658[0n]bp|United States.Wyoming|BOLD:AAC6459
Schinia jaguarina|[195]|HELNA162-06|USNM00161656|658[0n]bp|United States.Colorado|BOLD:AAC6459
Schinia jaguarina|[196]|HELNA536-09|USNM148229|658[0n]bp|United States.New Mexico|BOLD:AAC6459
Schinia persimilis|[197]|RDNME247-07|CNCNoctuoidea13854|616[1n]bp|Canada.Alberta|BOLD:AAI0752
Schinia persimilis|[198]|RDNME359-07|CNCNoctuoidea13966|658[0n]bp|Canada.Yukon Territory|BOLD:AAI0752
Schinia lucens|[199]|RDMAB1031-09|USNM Ent 00159792|658[0n]bp|United States.Colorado|BOLD:AAI0692
Schinia lucens|[200]|HELNA235-06|USNM00157985|658[0n]bp|United States.Nebraska|BOLD:AAI0692

Schinia persimilis|[197]|RDNME247-07|CNCNoctuoidea13[illegible]|Canada.Alberta|BOLD:AAI0752
Schinia persimilis|[198]|RDNME359-07|CNCNoctuoidea13966|658[0n]bp|Canada.Yukon Territory|BOLD:AAI0752
Schinia lucens|[199]|RDMAB1031-09|USNM Ent 00159792|658[0n]bp|United States.Colorado|BOLD:AAI0692
Schinia lucens|[200]|HELNA235-06|USNM00157985|658[0n]bp|United States.Nebraska|BOLD:AAI0692
Schinia suetus|[201]|HELNA281-09|USNM161861|658[0n]bp|United States.Utah|BOLD:ACE9042
Schinia suetus|[202]|HELNA374-09|USNM161870|658[0n]bp|United States.Utah|BOLD:ACE9042
Schinia suetus|[203]|NAMUM332-08|RR-89-4002|658[0n]bp|United States.California|BOLD:ACE9042
Schinia suetus|[204]|NAMUM331-08|RR-89-4001|658[0n]bp|United States.California|BOLD:ACE9042
Schinia suetus|[205]|NAMUM329-08|RR-89-0143|658[0n]bp|United States.California|BOLD:ACE9042
Schinia suetus|[206]|HELNA375-09|USNM161871|658[0n]bp|United States.Nevada|BOLD:ACE9042
Schinia mortua|[207]|HELNA072-06|144486|658[0n]bp|United States.Colorado|BOLD:AAE3962
Schinia mortua|[208]|HELNA075-06|221629|658[0n]bp|United States.New Mexico|BOLD:AAE3962
Schinia mortua|[209]|HELNA073-06|144496|658[0n]bp|United States.Colorado|BOLD:AAE3962
Schinia mortua|[210]|HELNA071-06|144488|658[0n]bp|United States.Colorado|BOLD:AAE3962
Schinia mortua|[211]|HELNA076-06|221630|658[0n]bp|United States.New Mexico|BOLD:AAE3962
Schinia mortua|[212]|LTOLB1302-11|CAE-94-0007|658[0n]bp|United States.Kansas|BOLD:AAE3962
Schinia mortua|[213]|LTOLB1303-11|CAE-94-0026|658[0n]bp|United States.Kansas|BOLD:AAE3962
Schinia mortua|[214]|BBLOD849-11|BIOUG01568-F11|658[0n]bp|United States.Arizona|BOLD:AAE3962
Schinia septentrionalis|[215]|LNAUP086-13|SI148368|658[0n]bp|United States.New Mexico|BOLD:AAD6937
Schinia septentrionalis|[216]|HELNA273-09|USNM161853|658[0n]bp|United States.Colorado|BOLD:AAD6937
Schinia septentrionalis|[217]|HELNA099-06|220830|658[0n]bp|United States.Colorado|BOLD:AAD6937
Schinia septentrionalis|[218]|RDNMG626-08|CNC LEP00052450|649[0n]bp|United States.Massachusetts|BOLD ...
Schinia septentrionalis|[219]|RDNMG625-08|CNC LEP00052449|658[0n]bp|United States.Massachusetts|BOLD ...
Schinia septentrionalis|[220]|HELNA895-10|148297|658[0n]bp|United States.Massachusetts|BOLD:AAD6937
Schinia septentrionalis|[221]|HELNA896-10|148298|658[0n]bp|United States.Massachusetts|BOLD:AAD6937
Schinia septentrionalis|[222]|HELNA1014-10|148296|658[0n]bp|United States.Massachusetts|BOLD:AAD6937
Schinia septentrionalis|[223]|HELNA894-10|148295|658[0n]bp|United States.Massachusetts|BOLD:AAD6937
Schinia septentrionalis|[224]|HELNA272-09|USNM161852|526[3n]bp|United States.Kentucky|BOLD:AAD6937
Schinia septentrionalis|[225]|HELNA269-09|USNM161846|658[0n]bp|United States.Connecticut|BOLD:AAD6937
Schinia honesta|[226]|RDMAB1027-09|UASM19604|658[0n]bp|Canada.Alberta|BOLD:AAF2456
Schinia honesta|[227]|RDNMF049-08|NOC14135|658[0n]bp|Canada.British Columbia|BOLD:AAF2456
Schinia verna|[228]|RDNMF777-08|UASM59892|658[0n]bp|Canada.Alberta|BOLD:AAF2456
Schinia verna|[229]|RDNMF776-08|UASM24441|658[0n]bp|Canada.Alberta|BOLD:AAF2456
Schinia avemensis|[230]|RDMAB091-05|UASM58213|602[0n]bp|Canada.Manitoba|BOLD:AAD0775
Schinia avemensis|[231]|HELNA179-06|USNM00161676|658[0n]bp|Canada.Manitoba|BOLD:AAD0775
Schinia avemensis|[232]|RDNMB496-05|CNCNoctuoidea10262|616[0n]bp|Canada.Alberta|BOLD:AAD0775
Schinia avemensis|[233]|RDMAB092-05|UASM58156|639[0n]bp|Canada.Alberta|BOLD:AAD0775
Schinia avemensis|[234]|HELNA180-06|USNM00161678|658[0n]bp|Canada.Alberta|BOLD:AAD0775
Schinia avemensis|[235]|RDNMB115-05|CNCNoctuoidea10267|658[0n]bp|Canada.Saskatchewan|BOLD:AAD0775
Schinia meadi|[236]|RDMAB523-06|UASM58479|658[0n]bp|Canada.Alberta|BOLD:AAF2436
Schinia roseitincta|[237]|LNAUP094-13|SI148376|658[1n]bp|United States.New Mexico|BOLD:AAF2082
Schinia roseitincta|[238]|RDNMF421-08|NOC14507|640[2n]bp|United States.New Mexico|BOLD:AAF2082
Schinia roseitincta|[239]|RDNMF420-08|NOC14506|621[0n]bp|United States.Texas|BOLD:AAF2082
Schinia roseitincta|[240]|RDNMF419-08|NOC14505|620[0n]bp|United States.Texas|BOLD:AAF2082
Schinia lynx|[241]|RDNMF430-08|NOC14516|658[0n]bp|United States.Florida|BOLD:AAB9588
Schinia lynx|[242]|HELNA499-09|USNM148192|658[0n]bp|United States.Maryland|BOLD:AAB9588
Schinia lynx|[243]|RDNMF431-08|NOC14517|658[0n]bp|United States.Florida|BOLD:AAB9588
Schinia lynx|[244]|BBLOE1636-12|BIOUG01989-D12|658[0n]bp|United States.Oklahoma|BOLD:AAB9588
Schinia lynx|[245]|HELNA502-09|USNM148195|658[0n]bp|United States.Maryland|BOLD:AAB9588
Schinia lynx|[246]|HELNA500-09|USNM148193|658[0n]bp|United States.Maryland|BOLD:AAB9588
Schinia lynx|[247]|HELNA501-09|USNM148194|658[0n]bp|United States.Maryland|BOLD:AAB9588
Schinia lynx|[248]|HKONS499-08|3028-COI-08|658[0n]bp|United States.Florida|BOLD:AAB9588
Schinia lynx|[249]|RDNMF427-08|NOC14513|658[0n]bp|United States.Arizona|BOLD:AAB9588
Schinia lynx|[250]|HELNA519-09|USNM148212|658[0n]bp|United States.Maryland|BOLD:AAB9588
Schinia lynx|[251]|HELNA339-09|USNM147991|658[0n]bp|United States.Maryland|BOLD:AAB9588
Schinia lynx|[252]|HELNA939-10|148339|658[0n]bp|United States.Maryland|BOLD:AAB9588
Schinia lynx|[253]|LPOKD275-09|MDOK-3354|641[0n]bp|United States.Oklahoma|BOLD:AAB9588
Schinia lynx|[254]|LPOKA475-09|MDOK-0475|658[0n]bp|United States.Oklahoma|BOLD:AAB9588
Schinia lynx|[255]|HKONS498-08|3027-COI-08|658[0n]bp|United States.Florida|BOLD:AAB9588
Schinia lynx|[256]|RDNMF428-08|NOC14514|658[0n]bp|United States.Texas|BOLD:AAB9588
Schinia lynx|[257]|HELNA293-09|USNM147998|658[0n]bp|United States.Georgia|BOLD:AAB9588
Schinia lynx|[258]|HELNA340-09|USNM147992|603[0n]bp|United States.Maryland|BOLD:AAB9588
Schinia obscurata|[259]|RDLQB415-05|DH010501|658[0n]bp|Canada.Quebec|BOLD:AAB9588
Schinia obscurata|[260]|XAE570-04|Moth4570.03|625[0n]bp|Canada.Ontario|BOLD:AAB9588
Schinia nundina|[261]|HELNA237-06|USNM00159918|658[0n]bp|United States.Arkansas|BOLD:AAD0906
Schinia nundina|[262]|HELNA516-09|USNM148209|658[0n]bp|United States.Maryland|BOLD:AAD0906
Schinia nundina|[263]|HELNA081-06|221610|658[0n]bp|United States.Oklahoma|BOLD:AAD0906
Schinia nundina|[264]|HELNA297-09|USNM148002|658[0n]bp|United States.Georgia|BOLD:AAD0906
Schinia nundina|[265]|HELNA517-09|USNM148210|658[0n]bp|United States.Maryland|BOLD:AAD0906
Schinia nundina|[266]|HELNA236-06|USNM00159917|658[0n]bp|United States.Arkansas|BOLD:AAD0906
Schinia nundina|[267]|HELNA515-09|USNM148208|658[0n]bp|United States.Maryland|BOLD:AAD0906
Schinia acutilinea|[268]|HELNA836-10|148254|658[0n]bp|Canada.Alberta|BOLD:AAB4314
Schinia acutilinea|[269]|RDMAB550-06|UASM58529|657[0n]bp|Canada.Alberta|BOLD:AAB4314
Schinia acutilinea|[270]|HELNA837-10|148255|658[0n]bp|Canada.Alberta|BOLD:AAB4314
Schinia acutilinea|[271]|RDNMC439-05|CNCNoctuoidea12072|592[0n]bp|Canada.Alberta|BOLD:AAB4314
Schinia acutilinea|[272]|RDNMC441-05|CNCNoctuoidea12074|589[0n]bp|Canada.Alberta|BOLD:AAB4314
Schinia acutilinea|[273]|RDNMB217-05|CNCNoctuoidea7963|658[0n]bp|Canada.Alberta|BOLD:AAB4314
Schinia acutilinea|[274]|RDMAB549-06|UASM58528|658[0n]bp|Canada.Alberta|BOLD:AAB4314
Schinia acutilinea|[275]|RDNMB218-05|CNCNoctuoidea7964|658[0n]bp|Canada.Alberta|BOLD:ACF4896
Schinia acutilinea|[276]|RDNMC438-05|CNCNoctuoidea12071|602[0n]bp|Canada.Alberta|BOLD:ACF4896
Schinia acutilinea|[277]|RDNMC440-05|CNCNoctuoidea12073|597[0n]bp|Canada.Alberta|BOLD:ACF4896
Schinia acutilinea|[278]|RDNMC443-05|CNCNoctuoidea12076|599[0n]bp|Canada.Alberta|BOLD:ACF4896
Schinia acutilinea|[279]|RDNMC442-05|CNCNoctuoidea12075|599[0n]bp|Canada.Alberta|BOLD:ACF4896
Schinia thoreaui|[280]|LNAUP167-13|SI148752|658[0n]bp|United States.Maryland|BOLD:AAB4299
Schinia bimatris|[281]|RDNME199-07|CNCNoctuoidea13806|658[0n]bp|Canada.Manitoba|BOLD:AAD3739
Schinia bimatris|[282]|RDNME205-07|CNCNoctuoidea13812|614[0n]bp|Canada.Manitoba|BOLD:AAD3739
Schinia bimatris|[283]|RDNME200-07|CNCNoctuoidea13807|592[0n]bp|Canada.Manitoba|BOLD:AAD3739
Schinia sanguinea|[284]|LNCB808-09|09-NCCC-278|658[0n]bp|United States.North Carolina|BOLD:ABX6641
Schinia sanguinea|[285]|LNCB809-09|09-NCCC-279|658[1n]bp|United States.North Carolina|BOLD:ABX6641
Schinia sanguinea|[286]|HELNA146-06|USNM00161638|658[0n]bp|United States.Montana|BOLD:ABX6641
Schinia sanguinea|[287]|HELNA147-06|USNM00161639|610[0n]bp|United States.Oklahoma|BOLD:ABX6641
Schinia sanguinea|[288]|HELNA094-06|221625|658[0n]bp|United States.Oklahoma|BOLD:ABX6641
Schinia sanguinea|[289]|HELNA148-06|USNM00161640|658[0n]bp|United States.Arkansas|BOLD:ABX6641
Schinia sanguinea|[290]|HELNA093-06|221624|658[0n]bp|United States.Oklahoma|BOLD:ABX6641
Schinia thoreaui|[291]|RDNMF396-08|NOC14482|616[0n]bp|United States.Mississippi|BOLD:AAD3713
Schinia thoreaui|[292]|LPOKD295-09|MDOK-3374|658[0n]bp|United States.Oklahoma|BOLD:AAD3713
Schinia thoreaui|[293]|LPOKD188-09|MDOK-3267|658[0n]bp|United States.Oklahoma|BOLD:AAD3713
Schinia thoreaui|[294]|LPOKA461-09|MDOK-0461|658[0n]bp|United States.Oklahoma|BOLD:AAD3713
Schinia thoreaui|[295]|NAMUM145-08|CAE-94-0009|657[0n]bp|United States.Kansas|BOLD:AAD3713
Schinia thoreaui|[296]|RDNMF397-08|NOC14483|658[0n]bp|United States.Mississippi|BOLD:AAD3713
Schinia thoreaui|[297]|LPOKA041-08|MDOK-0041|658[0n]bp|United States.Oklahoma|BOLD:AAD3713
Schinia thoreaui|[298]|LPOKD020-09|MDOK-3099|658[0n]bp|United States.Oklahoma|BOLD:AAD3713
Schinia thoreaui|[299]|LPOKD192-09|MDOK-3271|658[0n]bp|United States.Oklahoma|BOLD:AAD3713
[illegible]

Schinia thoreaui|[297]|LPOKA041-08|MDOK-0041|658[0n]bp|United States.Oklahoma|BOLD:AAD3713
Schinia thoreaui|[298]|LPOKD020-09|MDOK-3099|658[0n]bp|United States.Oklahoma|BOLD:AAD3713
Schinia thoreaui|[299]|LPOKD192-09|MDOK-3271|658[0n]bp|United States.Oklahoma|BOLD:AAD3713
Schinia thoreaui|[300]|LPOKA137-08|MDOK-0137|658[0n]bp|United States.Oklahoma|BOLD:AAD3713
Schinia thoreaui|[301]|LPOKA474-09|MDOK-0474|658[0n]bp|United States.Oklahoma|BOLD:AAD3713
Schinia arcigera|[302]|XAI046-05|0102-ONT-0046|658[0n]bp|Canada.Ontario|BOLD:AAB0117
Schinia arcigera|[303]|XAG712-05|2005-ONT-1296|658[0n]bp|Canada.Ontario|BOLD:AAB0117
Schinia arcigera|[304]|XAG674-05|2005-ONT-1258|624[3n]bp|Canada.Ontario|BOLD:AAB0117
Schinia arcigera|[305]|XAH086-05|2005-ONT-1669|635[0n]bp|Canada.Ontario|BOLD:AAB0117
Schinia arcigera|[306]|XAH055-05|2005-ONT-1638|658[0n]bp|Canada.Ontario|BOLD:AAB0117
Schinia arcigera|[307]|XAG021-05|2005-ONT-605|658[0n]bp|Canada.Ontario|BOLD:AAB0117
Schinia arcigera|[308]|XAH041-05|2005-ONT-1624|658[2n]bp|Canada.Ontario|BOLD:AAB0117
Schinia arcigera|[309]|PHMO310-03|moth2316.02|639[0n]bp|Canada.Ontario|BOLD:AAB0117
Schinia arcigera|[310]|PHMO318-03|moth2394.02|639[0n]bp|Canada.Ontario|BOLD:AAB0117
Schinia cumatilis|[311]|RDMAB502-06|UASM58467|658[0n]bp|Canada.Alberta|BOLD:ABZ3209
Schinia rivulosa|[312]|RDLQB839-05|DH010926|658[0n]bp|Canada.Quebec|BOLD:AAB5917
Schinia trifascia|[313]|HELNA890-10|148291|658[0n]bp|Canada.Ontario|BOLD:AAC4627
Schinia trifascia|[314]|HELNA891-10|148292|658[0n]bp|Canada.Ontario|BOLD:AAC4627
Schinia trifascia|[315]|HELNA893-10|148294|562[0n]bp|Canada.Ontario|BOLD:AAC4627
Schinia trifascia|[316]|HELNA892-10|148293|658[0n]bp|Canada.Ontario|BOLD:AAC4627
Schinia intermontana|[317]|HELNA172-06|USNM00161667|658[0n]bp|United States.Montana|BOLD:ACE6404
Schinia intermontana|[318]|NAMUM330-08|RR-89-0456|658[0n]bp|United States.California|BOLD:ACE6404
Schinia sexata|[319]|HELNA843-10|148260|658[0n]bp|Canada.Manitoba|BOLD:ACE6404
Schinia sexata|[320]|HELNA1012-10|65805|658[0n]bp|Canada.Manitoba|BOLD:ACE6404
Schinia sexata|[321]|HELNA844-10|148261|658[0n]bp|Canada.Manitoba|BOLD:ACE6404
Schinia villosa|[322]|RDMAB1030-09|USNM Ent 00157330|658[0n]bp|United States.Colorado|BOLD:AAE3854
Schinia villosa|[323]|HELNA173-06|USNM00161668|640[2n]bp|United States.Colorado|BOLD:AAE3854
Schinia villosa|[324]|HELNA207-06|USNM00157332|658[0n]bp|United States.Colorado|BOLD:AAE3854
Schinia villosa|[325]|RDMAB1028-09|USNM Ent 00157352|652[0n]bp|United States.Colorado|BOLD:AAE3854
Schinia villosa|[326]|RDMAB1029-09|USNM Ent 00157343|649[0n]bp|United States.Colorado|BOLD:AAE3854
Schinia walsinghami|[327]|LBCH7626-10|10-JDWBC-7626|658[0n]bp|Canada.British Columbia|BOLD:AAC2308
Schinia walsinghami|[328]|LBCH6390-10|10-JDWBC-6390|658[0n]bp|Canada.British Columbia|BOLD:AAC2308
Schinia walsinghami|[329]|LBCH7625-10|10-JDWBC-7625|658[0n]bp|Canada.British Columbia|BOLD:AAC2308
Schinia walsinghami|[330]|LBCH6426-10|10-JDWBC-6426|658[0n]bp|Canada.British Columbia|BOLD:AAC2308
Schinia walsinghami|[331]|LBCH6428-10|10-JDWBC-6428|658[0n]bp|Canada.British Columbia|BOLD:AAC2308
Schinia walsinghami|[332]|LBCH6432-10|10-JDWBC-6432|658[0n]bp|Canada.British Columbia|BOLD:AAC2308
Schinia walsinghami|[333]|LBCH7773-10|10-JDWBC-7773|658[0n]bp|Canada.British Columbia|BOLD:AAC2308
Schinia walsinghami|[334]|LBCH7047-10|10-JDWBC-7047|658[0n]bp|Canada.British Columbia|BOLD:AAC2308
Schinia walsinghami|[335]|LBCH6555-10|10-JDWBC-6555|658[0n]bp|Canada.British Columbia|BOLD:AAC2308
Schinia walsinghami|[336]|LBCH7620-10|10-JDWBC-7620|658[0n]bp|Canada.British Columbia|BOLD:AAC2308
Schinia walsinghami|[337]|LBCH6388-10|10-JDWBC-6388|658[0n]bp|Canada.British Columbia|BOLD:AAC2308
Schinia walsinghami|[338]|LBCH7778-10|10-JDWBC-7778|658[0n]bp|Canada.British Columbia|BOLD:AAC2308
Schinia walsinghami|[339]|LBCH7772-10|10-JDWBC-7772|658[0n]bp|Canada.British Columbia|BOLD:AAC2308
Schinia walsinghami|[340]|LBCH7776-10|10-JDWBC-7776|658[0n]bp|Canada.British Columbia|BOLD:AAC2308
Schinia walsinghami|[341]|LBCH6430-10|10-JDWBC-6430|658[0n]bp|Canada.British Columbia|BOLD:AAC2308
Schinia walsinghami|[342]|LBCH7624-10|10-JDWBC-7624|658[0n]bp|Canada.British Columbia|BOLD:AAC2308
Schinia walsinghami|[343]|LBCH6631-10|10-JDWBC-6631|658[0n]bp|Canada.British Columbia|BOLD:AAC2308
Schinia walsinghami|[344]|LBCH7775-10|10-JDWBC-7775|658[0n]bp|Canada.British Columbia|BOLD:AAC2308
Schinia walsinghami|[345]|LBCH6429-10|10-JDWBC-6429|658[0n]bp|Canada.British Columbia|BOLD:AAC2308
Schinia walsinghami|[346]|LBCH7777-10|10-JDWBC-7777|658[0n]bp|Canada.British Columbia|BOLD:AAC2308
Schinia walsinghami|[347]|LBCH7619-10|10-JDWBC-7619|658[0n]bp|Canada.British Columbia|BOLD:AAC2308
Schinia walsinghami|[348]|LBCH6427-10|10-JDWBC-6427|658[0n]bp|Canada.British Columbia|BOLD:AAC2308
Schinia walsinghami|[349]|LBCH7621-10|10-JDWBC-7621|658[0n]bp|Canada.British Columbia|BOLD:AAC2308
Schinia walsinghami|[350]|LBCH6425-10|10-JDWBC-6425|658[0n]bp|Canada.British Columbia|BOLD:AAC2308
Schinia walsinghami|[351]|LBCH7779-10|10-JDWBC-7779|658[0n]bp|Canada.British Columbia|BOLD:AAC2308
Schinia walsinghami|[352]|LBCH7622-10|10-JDWBC-7622|658[0n]bp|Canada.British Columbia|BOLD:AAC2308
Schinia walsinghami|[353]|LBCH7623-10|10-JDWBC-7623|658[0n]bp|Canada.British Columbia|BOLD:AAC2308
Schinia walsinghami|[354]|LBCH7774-10|10-JDWBC-7774|658[0n]bp|Canada.British Columbia|BOLD:AAC2308
Schinia walsinghami|[355]|LBCH6431-10|10-JDWBC-6431|658[0n]bp|Canada.British Columbia|BOLD:AAC2308
Callopistria cordata|[356]|BBLPC258-09|09BBELE-1258|658[0n]bp|Canada.Nova Scotia|BOLD:AAA7113
Callopistria cordata|[357]|BBLPE037-09|09BBELE-2037|658[0n]bp|Canada.Nova Scotia|BOLD:AAA7113
Callopistria cordata|[358]|BBLPE183-09|09BBELE-2183|639[0n]bp|Canada.Nova Scotia|BOLD:AAA7113
Callopistria cordata|[359]|BBLPE157-09|09BBELE-2157|658[0n]bp|Canada.Nova Scotia|BOLD:AAA7113
Callopistria cordata|[360]|BBLEC100-09|09BBELE-0100|658[0n]bp|Canada.Nova Scotia|BOLD:AAA7113
Callopistria cordata|[361]|TMNBB201-06|MNBTT-1141|658[0n]bp|Canada.New Brunswick|BOLD:AAA7113
Callopistria cordata|[362]|TMNBB196-06|MNBTT-1136|658[0n]bp|Canada.New Brunswick|BOLD:AAA7113
Callopistria cordata|[363]|RDLQF938-06|DH012118|658[0n]bp|Canada.Quebec|BOLD:AAA7113
Callopistria cordata|[364]|TTMNB036-06|MNBTT-036|658[0n]bp|Canada.New Brunswick|BOLD:AAA7113
Callopistria cordata|[365]|BBLPC195-09|09BBELE-1195|658[0n]bp|Canada.Nova Scotia|BOLD:AAA7113
Callopistria cordata|[366]|BBLEC518-09|09BBELE-0518|658[0n]bp|Canada.New Brunswick|BOLD:AAA7113
Callopistria cordata|[367]|BBLPE042-09|09BBELE-2042|658[0n]bp|Canada.Nova Scotia|BOLD:AAA7113
Callopistria cordata|[368]|BBLPE069-09|09BBELE-2069|658[0n]bp|Canada.Nova Scotia|BOLD:AAA7113
Callopistria cordata|[369]|TMNBB202-06|MNBTT-1142|658[0n]bp|Canada.New Brunswick|BOLD:AAA7113
Callopistria cordata|[370]|TMNBB200-06|MNBTT-1140|658[0n]bp|Canada.New Brunswick|BOLD:AAA7113
Callopistria cordata|[371]|BBLEC643-09|09BBELE-0643|658[0n]bp|Canada.Nova Scotia|BOLD:AAA7113
Callopistria cordata|[372]|BBLPC146-09|09BBELE-1146|658[0n]bp|Canada.Nova Scotia|BOLD:AAA7113
Callopistria cordata|[373]|BBLPC194-09|09BBELE-1194|658[0n]bp|Canada.Nova Scotia|BOLD:AAA7113
Callopistria cordata|[374]|RDLQF521-06|DH011670|658[0n]bp|Canada.Quebec|BOLD:AAA7113
Callopistria cordata|[375]|TMNBB198-06|MNBTT-1138|658[0n]bp|Canada.New Brunswick|BOLD:AAA7113
Callopistria cordata|[376]|MNAC786-07|CNCLEP00027527|658[0n]bp|Canada.Quebec|BOLD:AAA7113
Callopistria cordata|[377]|TMNBB197-06|MNBTT-1137|658[0n]bp|Canada.New Brunswick|BOLD:AAA7113
Callopistria cordata|[378]|BBLPE260-09|09BBELE-2260|658[0n]bp|Canada.Nova Scotia|BOLD:AAA7113
Callopistria cordata|[379]|BBLPE142-09|09BBELE-2142|658[0n]bp|Canada.Nova Scotia|BOLD:AAA7113
Callopistria cordata|[380]|RDLQB264-05|DH010350|658[0n]bp|Canada.Quebec|BOLD:AAA7113
Callopistria cordata|[381]|TMNBB207-06|MNBTT-1147|658[0n]bp|Canada.New Brunswick|BOLD:AAA7113
Callopistria cordata|[382]|TMNBB199-06|MNBTT-1139|658[0n]bp|Canada.New Brunswick|BOLD:AAA7113
Callopistria cordata|[383]|BBLPE034-09|09BBELE-2034|658[0n]bp|Canada.Nova Scotia|BOLD:AAA7113
Callopistria cordata|[384]|BBLPE138-09|09BBELE-2138|658[0n]bp|Canada.Nova Scotia|BOLD:AAA7113
Callopistria cordata|[385]|TMNBB203-06|MNBTT-1143|658[0n]bp|Canada.New Brunswick|BOLD:AAA7113
Callopistria cordata|[386]|TMNBB205-06|MNBTT-1145|658[0n]bp|Canada.New Brunswick|BOLD:AAA7113
Callopistria cordata|[387]|BBLPE064-09|09BBELE-2064|658[0n]bp|Canada.Nova Scotia|BOLD:AAA7113
Callopistria cordata|[388]|BBLEC678-09|09BBELE-0678|658[0n]bp|Canada.Nova Scotia|BOLD:AAA7113
Callopistria cordata|[389]|BBLPE130-09|09BBELE-2130|658[0n]bp|Canada.Nova Scotia|BOLD:AAA7113
Callopistria cordata|[390]|MECD365-06|jflandry2937|656[0n]bp|Canada.Quebec|BOLD:AAA7113
Callopistria cordata|[391]|PHMNB051-03|moth243.02SA|639[0n]bp|Canada.New Brunswick|BOLD:AAA7113
Callopistria cordata|[392]|PHMNB054-03|moth249.02SA|639[0n]bp|Canada.New Brunswick|BOLD:AAA7113
Callopistria cordata|[393]|BBLPE185-09|09BBELE-2185|636[0n]bp|Canada.Nova Scotia|BOLD:AAA7113
Callopistria cordata|[394]|TMNBB204-06|MNBTT-1144|632[0n]bp|Canada.New Brunswick|BOLD:AAA7113
Callopistria cordata|[395]|BBLPE113-09|09BBELE-2113|637[0n]bp|Canada.Nova Scotia|BOLD:AAA7113
Callopistria cordata|[396]|RDLQG522-06|DH012815|621[0n]bp|Canada.Quebec|BOLD:AAA7113
Callopistria cordata|[397]|TTMNB369-06|MNBTT-369|610[0n]bp|Canada.New Brunswick|BOLD:AAA7113
Callopistria cordata|[398]|PHMNB241-04|04HBL007706|609[0n]bp|Canada.New Brunswick|BOLD:AAA7113
Callopistria cordata|[399]|TTMNB032-06|MNBTT-032|656[0n]bp|Canada.New Brunswick|BOLD:AAA7113

Callopistria cordata|[397]|TTMNB369-06|MNBTT-369|610[0n]bp|Canada.New Brunswick|BOLD:AAA7113
Callopistria cordata|[398]|PHMNB241-04|04HBL007706|609[0n]bp|Canada.New Brunswick|BOLD:AAA7113
Callopistria cordata|[399]|TTMNB032-06|MNBTT-032|656[0n]bp|Canada.New Brunswick|BOLD:AAA7113
Callopistria cordata|[400]|TMNBB206-06|MNBTT-1146|658[0n]bp|Canada.New Brunswick|BOLD:AAA7113
Callopistria cordata|[401]|BBLEC115-09|09BBELE-0115|658[0n]bp|Canada.Nova Scotia|BOLD:AAA7113
Callopistria mollissima|[402]|LPSOC183-08|PPBP-2182|656[0n]bp|Canada.Ontario|BOLD:AAB0829
Callopistria mollissima|[403]|PHMNB169-04|04HBL007634|658[0n]bp|Canada.New Brunswick|BOLD:AAB0829
Callopistria mollissima|[404]|TTMNB026-06|MNBTT-026|658[0n]bp|Canada.New Brunswick|BOLD:AAB0829
Callopistria mollissima|[405]|MEC714-04|jflandry0714|658[0n]bp|Canada.Quebec|BOLD:AAB0829
Callopistria mollissima|[406]|MEC811-04|jflandry0811|658[0n]bp|Canada.Quebec|BOLD:AAB0829
Callopistria mollissima|[407]|MNAC729-07|CNCLEP00027463|656[0n]bp|Canada.Quebec|BOLD:AAB0829
Callopistria mollissima|[408]|MNAC853-07|CNCLEP00027594|658[0n]bp|Canada.Quebec|BOLD:AAB0829
Callopistria mollissima|[409]|XAC716-04|04HBL006716|658[0n]bp|Canada.Ontario|BOLD:AAB0829
Callopistria mollissima|[410]|BBLPE041-09|09BBELE-2041|658[0n]bp|Canada.Nova Scotia|BOLD:AAB0829
Callopistria mollissima|[411]|BBLPE074-09|09BBELE-2074|658[0n]bp|Canada.Nova Scotia|BOLD:AAB0829
Callopistria mollissima|[412]|BBLPC594-09|09BBELE-1594|633[0n]bp|Canada.Nova Scotia|BOLD:AAB0829
Callopistria mollissima|[413]|MECD364-06|jflandry2936|656[0n]bp|Canada.Quebec|BOLD:AAB0829
Callopistria mollissima|[414]|PHMNB035-03|moth202.02SA|639[0n]bp|Canada.New Brunswick|BOLD:AAB0829
Callopistria mollissima|[415]|PHMNB164-04|04HBL007629|561[1n]bp|Canada.New Brunswick|BOLD:AAB0829
Callopistria mollissima|[416]|BBLEC260-09|09BBELE-0260|633[0n]bp|Canada.Nova Scotia|BOLD:AAB0829
Callopistria mollissima|[417]|BBLPE127-09|09BBELE-2127|658[0n]bp|Canada.Nova Scotia|BOLD:AAB0829
Callopistria mollissima|[418]|MECD363-06|jflandry2935|658[0n]bp|Canada.Quebec|BOLD:AAB0829
Callopistria mollissima|[419]|PHMNB760-05|Moth 453.03SA|658[0n]bp|Canada.New Brunswick|BOLD:AAB0829
Galgula partita|[420]|XAK306-06|2006-ONT-1301|658[0n]bp|Canada.Ontario|BOLD:AAA6919
Galgula partita|[421]|BBLPD449-10|10BBCLP-2447|658[0n]bp|Canada.Ontario|BOLD:AAA6919
Galgula partita|[422]|BBLPB883-10|10BBCLP-1882|658[0n]bp|Canada.Ontario|BOLD:AAA6919
Galgula partita|[423]|RDLQG800-06|DH013093|658[0n]bp|Canada.Quebec|BOLD:AAA6919
Galgula partita|[424]|XAJ775-06|2006-ONT-0775|658[0n]bp|Canada.Ontario|BOLD:AAA6919
Galgula partita|[425]|RDLQG810-06|DH013103|658[0n]bp|Canada.Quebec|BOLD:AAA6919
Galgula partita|[426]|BBLPB882-10|10BBCLP-1881|658[0n]bp|Canada.Ontario|BOLD:AAA6919
Galgula partita|[427]|XAD568-04|04HBL006983|658[0n]bp|Canada.Ontario|BOLD:AAA6919
Galgula partita|[428]|TMG119-03|moth197.01|639[0n]bp|Canada.Ontario|BOLD:AAA6919
Galgula partita|[429]|PMG117-03|moth255.01|617[0n]bp|Canada.Ontario|BOLD:AAA6919
Galgula partita|[430]|RDLQB778-05|DH010865|617[0n]bp|Canada.Quebec|BOLD:AAA6919
Galgula partita|[431]|TMNBB697-06|MNBTT-1637|643[0n]bp|Canada.New Brunswick|BOLD:AAA6919
Galgula partita|[432]|XAD010-04|04HBL007010|602[0n]bp|Canada.Ontario|BOLD:AAA6919
Spodoptera exigua|[433]|PHMO362-03|moth2759.02|639[0n]bp|Canada.Ontario|BOLD:AAA6644
Spodoptera frugiperda|[434]|XAD239-04|04HBL007239|658[0n]bp|Canada.Ontario|BOLD:ACE4783
Spodoptera frugiperda|[435]|MNBB563-05|05-NBSTA-479|658[0n]bp|Canada.New Brunswick|BOLD:ACE4783
Spodoptera frugiperda|[436]|XAD265-04|04HBL007265|594[0n]bp|Canada.Ontario|BOLD:ACE4783
Spodoptera frugiperda|[437]|RDLQ494-07|DH009868|594[0n]bp|Canada.Quebec|BOLD:ACE4783
Spodoptera frugiperda|[438]|PHMO358-03|moth2745.02|638[4n]bp|Canada.Ontario|BOLD:ACE4783
Spodoptera frugiperda|[439]|XAD490-04|04HBL007490|557[0n]bp|Canada.Ontario|BOLD:AAA4532
Spodoptera frugiperda|[440]|RDLQB738-05|DH010653|593[0n]bp|Canada.Quebec|BOLD:AAA4532
Spodoptera frugiperda|[441]|RDLQB505-05|DH010591|658[0n]bp|Canada.Quebec|BOLD:AAA4532
Spodoptera frugiperda|[442]|TZBCA211-06|OMAFRA06-146|658[0n]bp|Canada.Ontario|BOLD:AAA4532
Spodoptera frugiperda|[443]|TZBCA210-06|OMAFRA06-145|658[0n]bp|Canada.Ontario|BOLD:AAA4532
Spodoptera frugiperda|[444]|XAH550-05|2005-ONT-2133|658[0n]bp|Canada.Ontario|BOLD:AAA4532
Spodoptera frugiperda|[445]|XAD236-04|04HBL007236|658[0n]bp|Canada.Ontario|BOLD:AAA4532
Spodoptera frugiperda|[446]|XAH548-05|2005-ONT-2131|658[0n]bp|Canada.Ontario|BOLD:AAA4532
Spodoptera frugiperda|[447]|PHJUL020-11|BIOUG01133-B03|616[0n]bp|Canada.Ontario|BOLD:AAA4532
Spodoptera frugiperda|[448]|PHJUL079-11|BIOUG01133-G02|607[0n]bp|Canada.Ontario|BOLD:AAA4532
Spodoptera frugiperda|[449]|PHMO299-03|moth2139.02|639[0n]bp|Canada.Ontario|BOLD:AAA4532
Spodoptera frugiperda|[450]|PHMO305-03|moth2243.02|639[0n]bp|Canada.Ontario|BOLD:AAA4532
Spodoptera frugiperda|[451]|XAD509-04|04HBL007509|617[0n]bp|Canada.Ontario|BOLD:AAA4532
Spodoptera ornithogalli|[452]|RDLQ498-07|DH002566|592[0n]bp|Canada.Quebec|BOLD:ABY9250
Spodoptera ornithogalli|[453]|XAB643-04|04HBL005643|658[1n]bp|Canada.Ontario|BOLD:ABY9250
Spodoptera praefica|[454]|LBCH5807-10|10-JDWBC-5807|658[0n]bp|Canada.British Columbia|BOLD:ACE4784
Spodoptera praefica|[455]|RDNMF644-08|NOC14730|658[1n]bp|Canada.Alberta|BOLD:ACE4784
Balsa malana|[456]|RDLQ497-07|DH005173|599[0n]bp|Canada.Quebec|BOLD:AAB2064
Balsa malana|[457]|PHMO368-03|moth279.01|639[0n]bp|Canada.Ontario|BOLD:AAB2064
Balsa malana|[458]|XAK108-06|2006-ONT-1103|658[0n]bp|Canada.Ontario|BOLD:AAB2064
Balsa malana|[459]|KPOEC134-08|08OEC-135|658[0n]bp|Canada.Ontario|BOLD:AAB2064
Balsa malana|[460]|XAI027-05|0102-ONT-0027|658[0n]bp|Canada.Ontario|BOLD:AAB2064
Balsa malana|[461]|XAB313-04|04HBL005313|658[0n]bp|Canada.Ontario|BOLD:AAB2064
Balsa malana|[462]|XAF696-05|2005-ONT-345|658[0n]bp|Canada.Ontario|BOLD:AAB2064
Balsa malana|[463]|XAG211-05|2005-ONT-795|658[0n]bp|Canada.Ontario|BOLD:AAB2064
Balsa malana|[464]|LPSOC400-08|PPBP-2399|630[0n]bp|Canada.Ontario|BOLD:AAB2064
Balsa malana|[465]|LPSOC410-08|PPBP-2409|658[0n]bp|Canada.Ontario|BOLD:AAB2064
Balsa labecula|[466]|XAI026-05|0102-ONT-0026|658[0n]bp|Canada.Ontario|BOLD:AAB2063
Balsa labecula|[467]|RDLQF382-06|DH011449|658[1n]bp|Canada.Quebec|BOLD:AAB2063
Balsa labecula|[468]|MNBB058-05|HBL008668|658[0n]bp|Canada.New Brunswick|BOLD:AAB2063
Balsa labecula|[469]|RDLQG321-06|DH012533|658[0n]bp|Canada.Quebec|BOLD:AAB2063
Balsa labecula|[470]|RDLQF478-06|DH011627|658[0n]bp|Canada.Quebec|BOLD:AAB2063
Balsa labecula|[471]|PHMNB624-04|04HBL00850|658[0n]bp|Canada.New Brunswick|BOLD:AAB2063
Balsa labecula|[472]|XAB308-04|04HBL005308|658[0n]bp|Canada.Ontario|BOLD:AAB2063
Balsa labecula|[473]|RDLQG690-06|DH012983|658[0n]bp|Canada.Quebec|BOLD:AAB2063
Balsa labecula|[474]|MNBB345-05|05-NBSTA-261|658[0n]bp|Canada.New Brunswick|BOLD:AAB2063
Balsa labecula|[475]|RDLQE879-06|MDH002882|658[0n]bp|Canada.Quebec|BOLD:AAB2063
Balsa labecula|[476]|LPSOB347-08|PPBP-1346|658[0n]bp|Canada.Ontario|BOLD:AAB2063
Balsa labecula|[477]|PHMNB194-04|04HBL007659|658[0n]bp|Canada.New Brunswick|BOLD:AAB2063
Balsa labecula|[478]|XAC043-04|04HBL006043|658[0n]bp|Canada.Ontario|BOLD:AAB2063
Balsa labecula|[479]|MNBB064-05|HBL008674|658[0n]bp|Canada.New Brunswick|BOLD:AAB2063
Balsa labecula|[480]|XAC457-04|04HBL006457|658[0n]bp|Canada.Ontario|BOLD:AAB2063
Balsa labecula|[481]|RDLQF480-06|DH011629|658[0n]bp|Canada.Quebec|BOLD:AAB2063
Balsa labecula|[482]|RDLQG782-06|DH013075|658[0n]bp|Canada.Quebec|BOLD:AAB2063
Balsa labecula|[483]|LPSOC060-08|PPBP-2059|658[0n]bp|Canada.Ontario|BOLD:AAB2063
Balsa labecula|[484]|XAE581-04|Moth4581.03|658[0n]bp|Canada.Ontario|BOLD:AAB2063
Balsa labecula|[485]|XAC719-04|04HBL006719|658[0n]bp|Canada.Ontario|BOLD:AAB2063
Balsa labecula|[486]|RDLQG793-06|DH013086|658[0n]bp|Canada.Quebec|BOLD:AAB2063
Balsa labecula|[487]|LPSOC443-08|PPBP-2442|658[0n]bp|Canada.Ontario|BOLD:AAB2063
Balsa labecula|[488]|XAC838-04|04HBL006838|658[0n]bp|Canada.Ontario|BOLD:AAB2063
Balsa labecula|[489]|RDLQG511-06|DH012804|658[0n]bp|Canada.Quebec|BOLD:AAB2063
Balsa labecula|[490]|RDLQF479-06|DH011628|658[0n]bp|Canada.Quebec|BOLD:AAB2063
Balsa labecula|[491]|TMG118-03|moth1152.01|639[0n]bp|Canada.Ontario|BOLD:AAB2063
Balsa labecula|[492]|PMG094-03|moth938.01|617[0n]bp|Canada.Ontario|BOLD:AAB2063
Balsa labecula|[493]|RDLQB394-05|DH010480|658[0n]bp|Canada.Quebec|BOLD:AAB2063
Balsa tristrigella|[494]|LPSOC405-08|PPBP-2404|658[0n]bp|Canada.Ontario|BOLD:AAA6646
Balsa tristrigella|[495]|PHMNB640-04|04HBL00866|658[0n]bp|Canada.New Brunswick|BOLD:AAA6646
Balsa tristrigella|[496]|BLTIB265-08|BL449|627[0n]bp|Canada.Ontario|BOLD:AAA6646
Balsa tristrigella|[497]|LPSOC241-08|PPBP-2240|658[0n]bp|Canada.Ontario|BOLD:AAA6646
Balsa tristrigella|[498]|PHMNB392-04|04HBL00618|658[0n]bp|Canada.New Brunswick|BOLD:AAA6646
Balsa tristrigella|[499]|RDLQG732-06|DH013025|658[0n]bp|Canada.Quebec|BOLD:AAA6646

Balsa tristrigella|[496]|[illegible]|Canada.Ontario|BOLD:[illegible]
Balsa tristrigella|[497]|LPSOC241-08|PPBP-2240|658[0n]bp|Canada.Ontario|BOLD:AAA6646
Balsa tristrigella|[498]|PHMNB392-04|04HBL00618|658[0n]bp|Canada.New Brunswick|BOLD:AAA6646
Balsa tristrigella|[499]|RDLQG732-06|DH013025|658[0n]bp|Canada.Quebec|BOLD:AAA6646
Balsa tristrigella|[500]|MECC002-06|jflandry2022|658[0n]bp|Canada.Quebec|BOLD:AAA6646
Balsa tristrigella|[501]|RDLQG520-06|DH012813|658[0n]bp|Canada.Quebec|BOLD:AAA6646
Balsa tristrigella|[502]|RDLQG724-06|DH013017|658[0n]bp|Canada.Quebec|BOLD:AAA6646
Balsa tristrigella|[503]|MECC186-06|jflandry2206|658[0n]bp|Canada.Quebec|BOLD:AAA6646
Balsa tristrigella|[504]|XAJ700-06|2006-ONT-0700|658[0n]bp|Canada.Ontario|BOLD:AAA6646
Balsa tristrigella|[505]|XAJ630-06|2006-ONT-0630|658[0n]bp|Canada.Ontario|BOLD:AAA6646
Balsa tristrigella|[506]|XAJ699-06|2006-ONT-0699|658[0n]bp|Canada.Ontario|BOLD:AAA6646
Balsa tristrigella|[507]|LPSOC177-08|PPBP-2176|658[0n]bp|Canada.Ontario|BOLD:AAA6646
Balsa tristrigella|[508]|XAK107-06|2006-ONT-1102|658[0n]bp|Canada.Ontario|BOLD:AAA6646
Balsa tristrigella|[509]|LPSOC176-08|PPBP-2175|658[0n]bp|Canada.Ontario|BOLD:AAA6646
Balsa tristrigella|[510]|PMG095-03|moth544.01|617[0n]bp|Canada.Ontario|BOLD:AAA6646
Balsa tristrigella|[511]|PHMO081-03|moth524.01|639[0n]bp|Canada.Ontario|BOLD:AAA6646
Balsa tristrigella|[512]|RDLQG730-06|DH013023|614[0n]bp|Canada.Quebec|BOLD:AAA6646
Balsa tristrigella|[513]|RDLQG691-06|DH012984|658[0n]bp|Canada.Quebec|BOLD:AAA6646
Balsa tristrigella|[514]|PHMNB653-04|04HBL00879|658[0n]bp|Canada.New Brunswick|BOLD:AAA6646
Balsa tristrigella|[515]|XAK192-06|2006-ONT-1187|658[0n]bp|Canada.Ontario|BOLD:AAA6646
Balsa tristrigella|[516]|LPSOB744-08|PPBP-1743|658[0n]bp|Canada.Ontario|BOLD:AAA6646
Balsa tristrigella|[517]|LPSOC407-08|PPBP-2406|658[0n]bp|Canada.Ontario|BOLD:AAA6646
Balsa tristrigella|[518]|PHMNB636-04|04HBL00862|658[0n]bp|Canada.New Brunswick|BOLD:AAA6646
Balsa tristrigella|[519]|PHMNB642-04|04HBL00868|658[0n]bp|Canada.New Brunswick|BOLD:AAA6646
Balsa tristrigella|[520]|LPSOC245-08|PPBP-2244|658[0n]bp|Canada.Ontario|BOLD:AAA6646
Balsa tristrigella|[521]|LPSOC163-08|PPBP-2162|658[0n]bp|Canada.Ontario|BOLD:AAA6646
Balsa tristrigella|[522]|XAB305-04|04HBL005305|658[0n]bp|Canada.Ontario|BOLD:AAA6646
Balsa tristrigella|[523]|RDLQG702-06|DH012995|658[0n]bp|Canada.Quebec|BOLD:AAA6646
Balsa tristrigella|[524]|RDLQG706-06|DH012999|658[0n]bp|Canada.Quebec|BOLD:AAA6646
Balsa tristrigella|[525]|PHMNB394-04|04HBL00620|658[0n]bp|Canada.New Brunswick|BOLD:AAA6646
Balsa tristrigella|[526]|PHMNB637-04|04HBL00863|658[0n]bp|Canada.New Brunswick|BOLD:AAA6646
Balsa tristrigella|[527]|LPSOC244-08|PPBP-2243|658[0n]bp|Canada.Ontario|BOLD:AAA6646
Balsa tristrigella|[528]|PHMNB638-04|04HBL00864|658[0n]bp|Canada.New Brunswick|BOLD:AAA6646
Balsa tristrigella|[529]|PHMNB620-04|04HBL00846|658[0n]bp|Canada.New Brunswick|BOLD:AAA6646
Balsa tristrigella|[530]|LPSOC243-08|PPBP-2242|658[0n]bp|Canada.Ontario|BOLD:AAA6646
Balsa tristrigella|[531]|LPSOC181-08|PPBP-2180|658[0n]bp|Canada.Ontario|BOLD:AAA6646
Balsa tristrigella|[532]|RDLQG519-06|DH012812|658[0n]bp|Canada.Quebec|BOLD:AAA6646
Balsa tristrigella|[533]|LPSOC062-08|PPBP-2061|658[0n]bp|Canada.Ontario|BOLD:AAA6646
Balsa tristrigella|[534]|PHMNB555-04|04HBL00781|658[0n]bp|Canada.New Brunswick|BOLD:AAA6646
Balsa tristrigella|[535]|XAB615-04|04HBL005615|658[0n]bp|Canada.Ontario|BOLD:AAA6646
Balsa tristrigella|[536]|PHMNB646-04|04HBL00872|658[0n]bp|Canada.New Brunswick|BOLD:AAA6646
Balsa tristrigella|[537]|PHMNB175-04|04HBL007640|658[0n]bp|Canada.New Brunswick|BOLD:AAA6646
Balsa tristrigella|[538]|LPSOC242-08|PPBP-2241|656[0n]bp|Canada.Ontario|BOLD:AAA6646
Balsa tristrigella|[539]|XAC027-04|04HBL006027|658[0n]bp|Canada.Ontario|BOLD:AAA6646
Balsa tristrigella|[540]|LPSOC402-08|PPBP-2401|658[0n]bp|Canada.Ontario|BOLD:AAA6646
Balsa tristrigella|[541]|XAK189-06|2006-ONT-1184|658[0n]bp|Canada.Ontario|BOLD:AAA6646
Balsa tristrigella|[542]|XAB583-04|04HBL005583|658[0n]bp|Canada.Ontario|BOLD:AAA6646
Balsa tristrigella|[543]|XAJ705-06|2006-ONT-0705|658[0n]bp|Canada.Ontario|BOLD:AAA6646
Balsa tristrigella|[544]|LPSOC158-08|PPBP-2157|658[0n]bp|Canada.Ontario|BOLD:AAA6646
Balsa tristrigella|[545]|XAJ614-06|2006-ONT-0614|658[0n]bp|Canada.Ontario|BOLD:AAA6646
Balsa tristrigella|[546]|LPSOC159-08|PPBP-2158|658[0n]bp|Canada.Ontario|BOLD:AAA6646
Balsa tristrigella|[547]|XAK181-06|2006-ONT-1176|658[0n]bp|Canada.Ontario|BOLD:AAA6646
Balsa tristrigella|[548]|LPSOC054-08|PPBP-2053|658[0n]bp|Canada.Ontario|BOLD:AAA6646
Balsa tristrigella|[549]|LPSOC160-08|PPBP-2159|658[0n]bp|Canada.Ontario|BOLD:AAA6646
Balsa tristrigella|[550]|XAB357-04|04HBL005357|658[0n]bp|Canada.Ontario|BOLD:AAA6646
Balsa tristrigella|[551]|RDLQG262-06|DH012467|658[0n]bp|Canada.Quebec|BOLD:AAA6646
Balsa tristrigella|[552]|LPSOC161-08|PPBP-2160|658[0n]bp|Canada.Ontario|BOLD:AAA6646
Balsa tristrigella|[553]|RDLQG725-06|DH013018|658[0n]bp|Canada.Quebec|BOLD:AAA6646
Fagitana littera|[554]|RDLQ491-07|DH008707|615[0n]bp|Canada.Quebec|BOLD:AAD1237
Amphipyra pyramidoides|[555]|XAH469-05|2005-ONT-2052|658[0n]bp|Canada.Ontario|BOLD:AAA8525
Amphipyra pyramidoides|[556]|BBLEC482-09|09BBELE-0482|658[0n]bp|Canada.New Brunswick|BOLD:AAA8525
Amphipyra pyramidoides|[557]|JSJUL2388-11|BIOUG01497-E11|658[0n]bp|Canada.Ontario|BOLD:AAA8525
Amphipyra pyramidoides|[558]|XAG175-05|2005-ONT-759|621[0n]bp|Canada.Ontario|BOLD:AAA8525
Amphipyra pyramidoides|[559]|XAH337-05|2005-ONT-1920|658[0n]bp|Canada.Ontario|BOLD:AAA8525
Amphipyra pyramidoides|[560]|XAD097-04|04HBL007097|658[0n]bp|Canada.Ontario|BOLD:AAA8525
Amphipyra pyramidoides|[561]|XAB646-04|04HBL005646|658[0n]bp|Canada.Ontario|BOLD:AAA8525
Amphipyra pyramidoides|[562]|XAB417-04|04HBL005417|658[0n]bp|Canada.Ontario|BOLD:AAA8525
Amphipyra pyramidoides|[563]|TMNBB210-06|MNBTT-1150|658[0n]bp|Canada.New Brunswick|BOLD:AAA8525
Amphipyra pyramidoides|[564]|RDLQB484-05|DH010570|658[0n]bp|Canada.Quebec|BOLD:AAA8525
Amphipyra pyramidoides|[565]|XAK428-06|2006-ONT-1423|658[0n]bp|Canada.Ontario|BOLD:AAA8525
Amphipyra pyramidoides|[566]|TMNBB209-06|MNBTT-1149|656[0n]bp|Canada.New Brunswick|BOLD:AAA8525
Amphipyra pyramidoides|[567]|XAD437-04|04HBL007437|565[0n]bp|Canada.Ontario|BOLD:AAA8525
Amphipyra pyramidoides|[568]|XAH264-05|2005-ONT-1847|604[0n]bp|Canada.Ontario|BOLD:AAA8525
Amphipyra pyramidoides|[569]|BBLEC486-09|09BBELE-0486|639[0n]bp|Canada.New Brunswick|BOLD:AAA8525
Amphipyra pyramidoides|[570]|XAK429-06|2006-ONT-1424|658[0n]bp|Canada.Ontario|BOLD:AAA8525
Amphipyra pyramidoides|[571]|XAB459-04|04HBL005459|640[0n]bp|Canada.Ontario|BOLD:AAA8525
Amphipyra pyramidoides|[572]|XAD102-04|04HBL007102|658[0n]bp|Canada.Ontario|BOLD:AAA8525
Amphipyra pyramidoides|[573]|TMNBB208-06|MNBTT-1148|658[0n]bp|Canada.New Brunswick|BOLD:AAA8525
Amphipyra pyramidoides|[574]|XAK426-06|2006-ONT-1421|658[0n]bp|Canada.Ontario|BOLD:AAA8525
Amphipyra pyramidoides|[575]|XAH518-05|2005-ONT-2101|658[0n]bp|Canada.Ontario|BOLD:AAA8525
Amphipyra pyramidoides|[576]|XAB542-04|04HBL005542|658[0n]bp|Canada.Ontario|BOLD:AAA8525
Amphipyra pyramidoides|[577]|TTMNB512-06|MNBTT-512|656[0n]bp|Canada.New Brunswick|BOLD:AAA8525
Amphipyra pyramidoides|[578]|XAB410-04|04HBL005410|658[0n]bp|Canada.Ontario|BOLD:AAA8525
Amphipyra pyramidoides|[579]|RDNMB966-05|CNCNoctuoidea10741|658[0n]bp|Canada.Ontario|BOLD:AAA8525
Amphipyra pyramidoides|[580]|XAG219-05|2005-ONT-803|658[0n]bp|Canada.Ontario|BOLD:AAA8525
Amphipyra pyramidoides|[581]|XAD466-04|04HBL007466|658[0n]bp|Canada.Ontario|BOLD:AAA8525
Amphipyra pyramidoides|[582]|BBLEC487-09|09BBELE-0487|658[0n]bp|Canada.New Brunswick|BOLD:AAA8525
Amphipyra pyramidoides|[583]|BBLEC505-09|09BBELE-0505|658[0n]bp|Canada.New Brunswick|BOLD:AAA8525
Amphipyra pyramidoides|[584]|XAH090-05|2005-ONT-1673|658[0n]bp|Canada.Ontario|BOLD:AAA8525
Amphipyra pyramidoides|[585]|XAH099-05|2005-ONT-1682|658[0n]bp|Canada.Ontario|BOLD:AAA8525
Amphipyra pyramidoides|[586]|JSJUL2387-11|BIOUG01497-E10|658[0n]bp|Canada.Ontario|BOLD:AAA8525
Amphipyra pyramidoides|[587]|RDLQB845-05|DH010932|658[0n]bp|Canada.Quebec|BOLD:AAA8525
Amphipyra pyramidoides|[588]|TTMNB511-06|MNBTT-511|654[0n]bp|Canada.New Brunswick|BOLD:AAA8525
Amphipyra pyramidoides|[589]|LALPA1230-11|AVBC 1232-11|658[0n]bp|Canada.British Columbia|BOLD:AAA8525
Amphipyra pyramidoides|[590]|LALPA1287-11|AVBC 1289-11|658[0n]bp|Canada.British Columbia|BOLD:AAA8525
Amphipyra pyramidoides|[591]|LOWCC835-05|CGWC-2715|658[0n]bp|Canada.British Columbia|BOLD:AAA8525
Amphipyra tragopoginis|[592]|LBCW059-08|08-JDWWI-0059|658[0n]bp|Canada.British Columbia|BOLD:AAB3277
Amphipyra tragopoginis|[593]|RDLQG219-06|DH012395|658[0n]bp|Canada.Quebec|BOLD:AAB3277
Amphipyra tragopoginis|[594]|PHMO343-03|moth2637.02|639[1n]bp|Canada.Ontario|BOLD:AAB3277
Amphipyra tragopoginis|[595]|XAD156-04|04HBL007156|586[0n]bp|Canada.Ontario|BOLD:AAB3277
Amphipyra tragopoginis|[596]|LALPA1279-11|AVBC 1281-11|658[0n]bp|Canada.British Columbia|BOLD:AAB3277
Amphipyra tragopoginis|[597]|LBCH7951-10|10-JDWBC-7951|658[0n]bp|Canada.British Columbia|BOLD:AAB3277
Amphipyra tragopoginis|[598]|LBCH1503-10|10-JDWBC-1503|658[0n]bp|Canada.British Columbia|BOLD:AAB3277
[illegible]

Amphipyra tragopoginis|[596]|LALPA1279-11|AVBC 1281-11|658[0n]bp|Canada.British Columbia|BOLD:AAB3277
Amphipyra tragopoginis|[597]|LBCH7951-10|10-JDWBC-7951|658[0n]bp|Canada.British Columbia|BOLD:AAB3277
Amphipyra tragopoginis|[598]|LBCH1503-10|10-JDWBC-1503|658[0n]bp|Canada.British Columbia|BOLD:AAB3277
Amphipyra tragopoginis|[599]|LPVIB231-08|PFC-2006-1609|658[0n]bp|Canada.British Columbia|BOLD:AAB3277
Amphipyra tragopoginis|[600]|LOWCB917-05|CGWC-1857|658[0n]bp|Canada.British Columbia|BOLD:AAB3277
Amphipyra tragopoginis|[601]|PHMO276-03|moth1663.02|639[0n]bp|Canada.Ontario|BOLD:AAB3277
Amphipyra tragopoginis|[602]|LALPA600-10|AVBC 602-10|658[0n]bp|Canada.British Columbia|BOLD:AAB3277
Amphipyra tragopoginis|[603]|LALPA760-10|AVBC 762-10|658[0n]bp|Canada.British Columbia|BOLD:AAB3277
Amphipyra tragopoginis|[604]|LPVIB508-08|PFC-2006-1919|658[0n]bp|Canada.British Columbia|BOLD:AAB3277
Amphipyra tragopoginis|[605]|LALPA770-10|AVBC 772-10|658[0n]bp|Canada.British Columbia|BOLD:AAB3277
Amphipyra tragopoginis|[606]|LOWCB919-05|CGWC-1859|658[0n]bp|Canada.British Columbia|BOLD:AAB3277
Amphipyra tragopoginis|[607]|LALPA759-10|AVBC 761-10|658[0n]bp|Canada.British Columbia|BOLD:AAB3277
Amphipyra tragopoginis|[608]|LOWCB916-05|CGWC-1856|658[0n]bp|Canada.British Columbia|BOLD:AAB3277
Amphipyra tragopoginis|[609]|LOWCB918-05|CGWC-1858|658[0n]bp|Canada.British Columbia|BOLD:AAB3277
Amphipyra tragopoginis|[610]|LBCG630-09|08-JDWBC-0630|658[0n]bp|Canada.British Columbia|BOLD:AAB3277
Amphipyra tragopoginis|[611]|LALPA802-10|AVBC 804-10|658[0n]bp|Canada.British Columbia|BOLD:AAB3277
Protoschinia nuchalis|[612]|RDNMF220-08|NOC14306|595[0n]bp|Canada.British Columbia|BOLD:AAD3664
Psaphida rolandi|[613]|MEC130-04|jflandry0130|658[0n]bp|Canada.Quebec|BOLD:AAD4664
Psaphida rolandi|[614]|RDLQ597-07|DH009709|592[0n]bp|Canada.Ontario|BOLD:AAD4664
Psaphida electilis|[615]|RDNMH646-09|CNCLEP00062970|658[0n]bp|Canada.Ontario|BOLD:AAE0827
Psaphida electilis|[616]|RDLQH105-06|DH013343|658[0n]bp|Canada.Quebec|BOLD:AAE0827
Psaphida electilis|[617]|XAF329-05|HLC-10370|572[0n]bp|Canada.Ontario|BOLD:AAE0827
Psaphida resumens|[618]|RDLQ596-07|DH009518|581[0n]bp|Canada.Quebec|BOLD:ACE4774
Psaphida resumens|[619]|RDLQ595-07|DH013453|591[0n]bp|Canada.Ontario|BOLD:ACE4774
Psaphida styracis|[620]|RDLQ594-07|DH009707|593[0n]bp|Canada.Ontario|BOLD:AAD4668
Psaphida styracis|[621]|RDLQ593-07|DH009422|596[0n]bp|Canada.Quebec|BOLD:AAD4668
Psaphida grandis|[622]|LSEU103-06|06-JKA-0103|658[0n]bp|United States.North Carolina|BOLD:AAH7903
Psaphida grandis|[623]|LGSMG695-07|BGS03358|658[0n]bp|United States.Tennessee|BOLD:AAH7903
Psaphida thaxterianus|[624]|RDNMF460-08|NOC14546|658[0n]bp|United States.Alabama|BOLD:AAX8246
Amyna axis|[625]|LPOKE294-11|MDOK-4372|658[0n]bp|United States.Oklahoma|BOLD:AAA8978
Amyna axis|[626]|LPOKA599-09|MDOK-0599|637[0n]bp|United States.Oklahoma|BOLD:AAA8978
Amyna axis|[627]|CMAZA115-09|CMAZ-0115|658[0n]bp|United States.Arizona|BOLD:AAA8978
Amyna axis|[628]|LOFLC228-06|06-FLOR-2108|658[0n]bp|United States.Florida|BOLD:AAA8978
Amyna axis|[629]|LPOKD247-09|MDOK-3326|657[0n]bp|United States.Oklahoma|BOLD:AAA8978
Amyna axis|[630]|LOFLB196-06|06-FLOR-1136|658[0n]bp|United States.Florida|BOLD:AAA8978
Amyna axis|[631]|RDNMD520-06|CNCNoctuoidea12852|658[0n]bp|United States.Florida|BOLD:AAA8978
Amyna axis|[632]|CMAZA1157-12|BIOUG02043-C09|658[0n]bp|United States.Arizona|BOLD:AAA8978
Amyna axis|[633]|LSEU218-06|06-JKA-0218|658[0n]bp|United States.Georgia|BOLD:AAA8978
Amyna axis|[634]|RDNMK089-11|CNCLEP 81212|658[0n]bp|United States.Florida|BOLD:AAA8978
Amyna axis|[635]|LPOKA618-09|MDOK-0618|658[0n]bp|United States.Oklahoma|BOLD:AAA8978
Amyna axis|[636]|LPOKE297-11|MDOK-4375|658[0n]bp|United States.Oklahoma|BOLD:AAA8978
Amyna axis|[637]|LOFLB201-06|06-FLOR-1141|658[0n]bp|United States.Florida|BOLD:AAA8978
Amyna axis|[638]|CMAZA959-12|BIOUG02041-C01|658[0n]bp|United States.Arizona|BOLD:AAA8978
Amyna bullula|[639]|LSEU219-06|06-JKA-0219|658[0n]bp|United States.Georgia|BOLD:AAA8980
Amyna bullula|[640]|LSUSA070-06|06-SUSA-0070|658[0n]bp|United States.Kentucky|BOLD:AAA8980
Annaphila decia|[641]|RDNMF352-08|NOC14438|658[0n]bp|Canada.British Columbia|BOLD:AAR3429
Annaphila diva|[642]|RDNMF787-08|UASM110778|658[0n]bp|Canada.British Columbia|BOLD:AAF0525
Annaphila diva|[643]|LALPA058-10|AVBC 058-10|658[0n]bp|Canada.British Columbia|BOLD:AAF0525
Annaphila diva|[644]|RDNMF786-08|UASM110777|658[0n]bp|Canada.British Columbia|BOLD:AAF0525
Annaphila diva|[645]|LALPA081-10|AVBC 081-10|658[0n]bp|Canada.British Columbia|BOLD:AAF0525
Annaphila diva|[646]|LALPA082-10|AVBC 082-10|658[0n]bp|Canada.British Columbia|BOLD:AAF0525
Cobubatha dividua|[647]|BBLSX815-09|09BBLEP-02743|658[0n]bp|United States.Texas|BOLD:AAE2548
Cobubatha dividua|[648]|LPOKC892-09|MDOK-2969|658[0n]bp|United States.Oklahoma|BOLD:AAE2548
Cobubatha dividua|[649]|BBLOC1452-11|BIOUG01545-A04|658[0n]bp|United States.California|BOLD:AAE2548
Cobubatha dividua|[650]|BBLOC1741-11|BIOUG01548-A08|658[0n]bp|United States.California|BOLD:AAE2548
Cobubatha dividua|[651]|BBLOE1184-12|BIOUG01984-F11|658[0n]bp|United States.Arizona|BOLD:AAE2548
Cobubatha dividua|[652]|LPOKA203-08|MDOK-0203|658[0n]bp|United States.Oklahoma|BOLD:AAE2548
Cobubatha dividua|[653]|BBLOB1950-11|BIOUG01451-E02|658[0n]bp|United States.Arizona|BOLD:AAE2548
Cobubatha dividua|[654]|BBLOB1975-11|BIOUG01451-G03|658[0n]bp|United States.Arizona|BOLD:AAE2548
Cobubatha dividua|[655]|BBLOC1843-11|BIOUG01549-B03|658[0n]bp|United States.California|BOLD:AAE2548
Cobubatha dividua|[656]|BBLSW070-09|09BBLEP-00998|658[0n]bp|United States.Texas|BOLD:AAE2548
Cobubatha dividua|[657]|BBLOC1904-11|BIOUG01549-G04|658[0n]bp|United States.California|BOLD:AAE2548
Cobubatha dividua|[658]|RDNMF745-08|NOC14831|658[0n]bp|United States.California|BOLD:AAE2548
Cobubatha dividua|[659]|BBLOD1947-11|BIOUG01860-C05|658[0n]bp|United States.California|BOLD:AAE2548
Cobubatha dividua|[660]|LPOKD117-09|MDOK-3196|658[0n]bp|United States.Oklahoma|BOLD:AAE2548
Cobubatha dividua|[661]|BBLOC1892-11|BIOUG01549-F04|658[0n]bp|United States.California|BOLD:AAE2548
Cobubatha dividua|[662]|BBLOC1891-11|BIOUG01549-F03|658[0n]bp|United States.California|BOLD:AAE2548
Cobubatha dividua|[663]|BBLOE1191-12|BIOUG01984-G06|658[0n]bp|United States.Arizona|BOLD:AAE2548
Cobubatha dividua|[664]|RDNME923-08|NOC14069|658[0n]bp|United States.Arizona|BOLD:AAE2548
Cobubatha dividua|[665]|CMAZA445-10|CMAZ-0445|658[0n]bp|United States.Arizona|BOLD:AAE2548
Cobubatha dividua|[666]|CMAZA698-10|CMAZ-0698|658[0n]bp|United States.Arizona|BOLD:AAE2548
Cobubatha dividua|[667]|RDNMF743-08|NOC14829|658[0n]bp|United States.California|BOLD:AAE2548
Cobubatha dividua|[668]|RDNMF744-08|NOC14830|652[0n]bp|United States.California|BOLD:AAE2548
Cobubatha dividua|[669]|BBLOC1737-11|BIOUG01548-A04|622[0n]bp|United States.California|BOLD:AAE2548
Cobubatha dividua|[670]|BBLOB040-11|BIOUG01367-D04|604[0n]bp|United States.Arizona|BOLD:AAE2548
Cobubatha dividua|[671]|BBLOC1900-11|BIOUG01549-F12|658[0n]bp|United States.California|BOLD:AAE2548
Ponometia elegantula|[672]|RDNMB267-05|CNCNoctuoidea10033|593[0n]bp|Canada.Alberta|BOLD:AAC6158
Ponometia elegantula|[673]|RDNMB265-05|CNCNoctuoidea10031|658[0n]bp|Canada.Alberta|BOLD:AAC6158
Ponometia fasciatella|[674]|IAWL351-09|IAWAZ-0285|571[1n]bp|United States.Arizona|BOLD:AAE0826
Ponometia fasciatella|[675]|BBLOB1942-11|BIOUG01451-D06|658[0n]bp|United States.Arizona|BOLD:AAE0826
Ponometia fasciatella|[676]|RDNMF710-08|NOC14796|618[1n]bp|United States.Arizona|BOLD:AAE0826
Ponometia fasciatella|[677]|BBLOE1576-12|BIOUG01988-G11|658[0n]bp|United States.Arizona|BOLD:AAE0826
Ponometia fasciatella|[678]|IAWL875-09|IAWAZ-0672|658[0n]bp|United States.Arizona|BOLD:AAE0826
Ponometia fasciatella|[679]|IAWL592-09|IAWAZ-0470|658[0n]bp|United States.Arizona|BOLD:AAE0826
Ponometia fasciatella|[680]|CMAZA746-10|CMAZ-0746|658[0n]bp|United States.Arizona|BOLD:AAE0826
Ponometia fasciatella|[681]|RDNMF709-08|NOC14795|640[0n]bp|United States.Arizona|BOLD:AAE0826
Ponometia fasciatella|[682]|RDNMF711-08|NOC14797|639[0n]bp|United States.Arizona|BOLD:AAE0826
Ponometia fasciatella|[683]|IAWL577-09|IAWAZ-0458|658[0n]bp|United States.Arizona|BOLD:AAE0826
Ponometia fasciatella|[684]|IAWL782-09|IAWAZ-0606|658[0n]bp|United States.Arizona|BOLD:AAE0826
Ponometia fasciatella|[685]|CMAZA470-10|CMAZ-0470|658[0n]bp|United States.Arizona|BOLD:AAE0826
Ponometia fasciatella|[686]|BBLOC797-11|BIOUG01466-B07|658[0n]bp|United States.Arizona|BOLD:AAE0826
Ponometia fasciatella|[687]|BBLOB1940-11|BIOUG01451-D04|658[0n]bp|United States.Arizona|BOLD:AAE0826
Ponometia fasciatella|[688]|RDNMG019-08|NOC14960|658[0n]bp|United States.Arizona|BOLD:AAE0826
Ponometia fasciatella|[689]|RDNMH704-09|CNCLEP00063137|658[0n]bp|United States.Arizona|BOLD:AAE0826
Ponometia tortricina|[690]|RDNMF125-08|NOC14211|658[0n]bp|Canada.Manitoba|BOLD:AAE1800
Ponometia tortricina|[691]|RDNMF129-08|NOC14215|658[1n]bp|Canada.Alberta|BOLD:AAE1800
Ponometia tortricina|[692]|RDNMF126-08|NOC14212|658[0n]bp|Canada.British Columbia|BOLD:AAE1800
Ponometia tortricina|[693]|RDNMF128-08|NOC14214|658[0n]bp|Canada.Alberta|BOLD:AAE1800
Ponometia erastrioides|[694]|XAG460-05|2005-ONT-1044|658[0n]bp|Canada.Ontario|BOLD:AAA8108
Ponometia erastrioides|[695]|TMNBB620-06|MNBTT-1560|658[0n]bp|Canada.New Brunswick|BOLD:AAA8108
Ponometia erastrioides|[696]|RDLQG759-06|DH013052|658[0n]bp|Canada.Quebec|BOLD:AAA8108
Ponometia erastrioides|[697]|RDLQG687-06|DH012980|658[0n]bp|Canada.Quebec|BOLD:AAA8108
Ponometia erastrioides|[698]|RDLQG720-06|DH013013|658[0n]bp|Canada.Quebec|BOLD:AAA8108

Ponometia erastrioides|[696]|RDLQG759-06|DH013052|658[0n]bp|Canada.Quebec|BOLD:AAA8108
Ponometia erastrioides|[697]|RDLQG687-06|DH012980|658[0n]bp|Canada.Quebec|BOLD:AAA8108
Ponometia erastrioides|[698]|RDLQG720-06|DH013013|658[0n]bp|Canada.Quebec|BOLD:AAA8108
Ponometia erastrioides|[699]|BLTIB758-08|BL1048|658[0n]bp|Canada.Ontario|BOLD:AAA8108
Ponometia erastrioides|[700]|XAE582-04|Moth4582.03|658[0n]bp|Canada.Ontario|BOLD:AAA8108
Ponometia erastrioides|[701]|RDLQG558-06|DH012851|658[0n]bp|Canada.Quebec|BOLD:AAA8108
Ponometia erastrioides|[702]|RDLQG539-06|DH012832|658[0n]bp|Canada.Quebec|BOLD:AAA8108
Ponometia erastrioides|[703]|XAD729-05|2005-ONT-528|658[0n]bp|Canada.Ontario|BOLD:AAA8108
Ponometia erastrioides|[704]|XAG568-05|2005-ONT-1152|658[0n]bp|Canada.Ontario|BOLD:AAA8108
Ponometia erastrioides|[705]|XAG959-05|2005-ONT-1543|658[0n]bp|Canada.Ontario|BOLD:AAA8108
Ponometia erastrioides|[706]|XAH025-05|2005-ONT-1608|658[0n]bp|Canada.Ontario|BOLD:AAA8108
Ponometia erastrioides|[707]|RDLQG729-06|DH013022|658[0n]bp|Canada.Quebec|BOLD:AAA8108
Ponometia erastrioides|[708]|BLTIB1009-08|BL1447|658[0n]bp|Canada.Ontario|BOLD:AAA8108
Ponometia erastrioides|[709]|RDLQH022-06|DH013259|658[0n]bp|Canada.Quebec|BOLD:AAA8108
Ponometia erastrioides|[710]|PHMO097-03|moth595.01|658[0n]bp|Canada.Ontario|BOLD:AAA8108
Ponometia erastrioides|[711]|RDLQG757-06|DH013050|658[0n]bp|Canada.Quebec|BOLD:AAA8108
Ponometia erastrioides|[712]|RDLQG767-06|DH013060|658[0n]bp|Canada.Quebec|BOLD:AAA8108
Ponometia erastrioides|[713]|RDLQG801-06|DH013094|658[0n]bp|Canada.Quebec|BOLD:AAA8108
Ponometia erastrioides|[714]|RDLQG850-06|DH013143|656[0n]bp|Canada.Quebec|BOLD:AAA8108
Ponometia erastrioides|[715]|RDLQG770-06|DH013063|658[0n]bp|Canada.Quebec|BOLD:AAA8108
Ponometia erastrioides|[716]|RDLQD892-06|MDH001861|656[0n]bp|Canada.Quebec|BOLD:AAA8108
Ponometia erastrioides|[717]|XAC478-04|04HBL006478|597[0n]bp|Canada.Ontario|BOLD:AAA8108
Ponometia erastrioides|[718]|PMG163-03|moth449.01|617[0n]bp|Canada.Ontario|BOLD:AAA8108
Ponometia erastrioides|[719]|TMG103-03|moth637.01|639[0n]bp|Canada.Ontario|BOLD:AAA8108
Ponometia erastrioides|[720]|XAG904-05|2005-ONT-1488|658[0n]bp|Canada.Ontario|BOLD:AAA8108
Ponometia erastrioides|[721]|XAF775-05|2005-ONT-424|658[0n]bp|Canada.Ontario|BOLD:AAA8108
Ponometia erastrioides|[722]|RDLQD891-06|MDH000869|657[0n]bp|Canada.Quebec|BOLD:AAA8108
Ponometia erastrioides|[723]|XAC024-04|04HBL006024|599[0n]bp|Canada.Ontario|BOLD:AAA8108
Ponometia erastrioides|[724]|XAD015-04|04HBL007015|620[0n]bp|Canada.Ontario|BOLD:AAA8108
Ponometia erastrioides|[725]|BLTIB600-08|BL880|638[0n]bp|Canada.Ontario|BOLD:AAA8108
Ponometia erastrioides|[726]|XAF551-05|2005-ONT-200|658[0n]bp|Canada.Ontario|BOLD:AAA8108
Ponometia erastrioides|[727]|XAC661-04|04HBL006661|658[0n]bp|Canada.Ontario|BOLD:AAA8108
Ponometia erastrioides|[728]|XAG200-05|2005-ONT-784|658[0n]bp|Canada.Ontario|BOLD:AAA8108
Ponometia erastrioides|[729]|XAH258-05|2005-ONT-1841|658[0n]bp|Canada.Ontario|BOLD:AAA8108
Ponometia sutrix|[730]|RDMAB543-06|UASM58075|603[1n]bp|Canada.Alberta|BOLD:AAF0286
Ponometia candefacta|[731]|XAH231-05|2005-ONT-1814|658[0n]bp|Canada.Ontario|BOLD:AAA5642
Ponometia candefacta|[732]|XAK193-06|2006-ONT-1188|642[0n]bp|Canada.Ontario|BOLD:AAA5642
Ponometia candefacta|[733]|RDLQG844-06|DH013137|658[0n]bp|Canada.Quebec|BOLD:AAA5642
Ponometia candefacta|[734]|XAK194-06|2006-ONT-1189|658[0n]bp|Canada.Ontario|BOLD:AAA5642
Ponometia candefacta|[735]|XAH259-05|2005-ONT-1842|614[0n]bp|Canada.Ontario|BOLD:AAA5642
Ponometia candefacta|[736]|XAK077-06|2006-ONT-1072|658[0n]bp|Canada.Ontario|BOLD:AAA5642
Ponometia candefacta|[737]|RDLQE358-06|MDH002361|657[0n]bp|Canada.Quebec|BOLD:AAA5642
Ponometia candefacta|[738]|XAH232-05|2005-ONT-1815|658[0n]bp|Canada.Ontario|BOLD:AAA5642
Ponometia candefacta|[739]|RDLQG888-06|DH013181|658[0n]bp|Canada.Quebec|BOLD:AAA5642
Ponometia candefacta|[740]|RDLQE207-06|MDH002210|657[0n]bp|Canada.Quebec|BOLD:AAA5642
Ponometia candefacta|[741]|RDLQG842-06|DH013135|658[0n]bp|Canada.Quebec|BOLD:AAA5642
Ponometia candefacta|[742]|BLGSM004-09|BL1613|658[0n]bp|Canada.Ontario|BOLD:AAA5642
Ponometia candefacta|[743]|RDLQG887-06|DH013180|658[0n]bp|Canada.Quebec|BOLD:AAA5642
Ponometia candefacta|[744]|RDLQE357-06|MDH002360|658[0n]bp|Canada.Quebec|BOLD:AAA5642
Ponometia candefacta|[745]|RDLQG550-06|DH012843|658[0n]bp|Canada.Quebec|BOLD:AAA5642
Ponometia candefacta|[746]|RDLQG563-06|DH012856|658[0n]bp|Canada.Quebec|BOLD:AAA5642
Ponometia candefacta|[747]|BLTIB1034-08|BL1476|658[0n]bp|Canada.Ontario|BOLD:AAA5642
Ponometia candefacta|[748]|RDLQG562-06|DH012855|658[0n]bp|Canada.Quebec|BOLD:AAA5642
Ponometia candefacta|[749]|XAJ283-06|2006-ONT-0283|658[0n]bp|Canada.Ontario|BOLD:AAA5642
Ponometia candefacta|[750]|RDNMB261-05|CNCNoctuoidea10027|601[0n]bp|Canada.Ontario|BOLD:AAA5642
Ponometia candefacta|[751]|XAG937-05|2005-ONT-1521|617[0n]bp|Canada.Ontario|BOLD:AAA5642
Ponometia candefacta|[752]|XAJ520-06|2006-ONT-0520|657[0n]bp|Canada.Ontario|BOLD:AAA5642
Ponometia candefacta|[753]|PHMO057-03|moth383.02|639[0n]bp|Canada.Ontario|BOLD:AAA5642
Ponometia candefacta|[754]|PHMO062-03|moth427.02|639[0n]bp|Canada.Ontario|BOLD:AAA5642
Ponometia candefacta|[755]|RDLQG560-06|DH012853|658[0n]bp|Canada.Quebec|BOLD:AAA5642
Ponometia candefacta|[756]|XAG792-05|2005-ONT-1376|658[0n]bp|Canada.Ontario|BOLD:AAA5642
Ponometia candefacta|[757]|RDLQD893-06|MDH000747|657[0n]bp|Canada.Quebec|BOLD:AAA5642
Ponometia candefacta|[758]|RDLQG561-06|DH012854|658[0n]bp|Canada.Quebec|BOLD:AAA5642
Ponometia candefacta|[759]|RDLQB751-05|DH010666|658[0n]bp|Canada.Quebec|BOLD:AAA5642
Ponometia candefacta|[760]|RDLQG796-06|DH013089|658[0n]bp|Canada.Quebec|BOLD:AAA5642
Ponometia candefacta|[761]|RDLQG889-06|DH013182|658[0n]bp|Canada.Quebec|BOLD:AAA5642
Ponometia binocula|[762]|RDNMG771-08|CNC LEP00052895|643[1n]bp|United States.Ohio|BOLD:AAE1793
Ponometia binocula|[763]|RDNMG215-08|NOC15067|648[0n]bp|United States.Ohio|BOLD:AAE1793
Ponometia binocula|[764]|RDNMG770-08|CNC LEP00052894|643[1n]bp|United States.Ohio|BOLD:AAE1793
Ponometia binocula|[765]|RDNMG214-08|NOC15066|649[1n]bp|United States.Ohio|BOLD:AAE1793
Ponometia binocula|[766]|LPOKE176-10|MDOK-4254|658[0n]bp|United States.Oklahoma|BOLD:AAE1793
Ponometia binocula|[767]|LPOKE059-10|MDOK-4137|658[0n]bp|United States.Oklahoma|BOLD:AAE1793
Ponometia semiflava|[768]|RDMAB516-06|UASM58481|655[0n]bp|Canada.Alberta|BOLD:AAC8096
Ponometia semiflava|[769]|RDMAB033-05|UASM57516|623[0n]bp|Canada.Alberta|BOLD:AAC8096
Ponometia virginalis|[770]|RDMAB494-06|UASM58429|658[0n]bp|Canada.Alberta|BOLD:AAE1792
Ponometia virginalis|[771]|RDMAB054-05|UASM57537|643[0n]bp|Canada.Alberta|BOLD:AAE1792
Spragueia leo|[772]|BBLPA741-10|10BBCLP-0741|658[0n]bp|Canada.Ontario|BOLD:AAB8175
Tarache augustipennis|[773]|LPABB114-08|08BBLEP-03379|658[0n]bp|Canada.Alberta|BOLD:AAC9149
Tarache augustipennis|[774]|LPABB108-08|08BBLEP-03373|658[0n]bp|Canada.Alberta|BOLD:AAC9149
Tarache augustipennis|[775]|LPABC245-09|08BBLEP-04464|621[2n]bp|Canada.Alberta|BOLD:AAC9149
Tarache augustipennis|[776]|RDNMB259-05|CNCNoctuoidea10025|658[0n]bp|Canada.Alberta|BOLD:AAC9149
Tarache augustipennis|[777]|RDNMB260-05|CNCNoctuoidea10026|658[0n]bp|Canada.Alberta|BOLD:AAC9149
Tarache augustipennis|[778]|LPSK417-08|08BBLEP-01985|658[0n]bp|Canada.Saskatchewan|BOLD:AAC9149
Tarache augustipennis|[779]|LPSK069-08|08BBLEP-00772|658[0n]bp|Canada.Saskatchewan|BOLD:AAC9149
Tarache augustipennis|[780]|RDMAB127-05|UASM41272|658[0n]bp|Canada.Alberta|BOLD:AAC9149
Tarache augustipennis|[781]|LPABB100-08|08BBLEP-03365|658[0n]bp|Canada.Alberta|BOLD:AAC9149
Tarache major|[782]|JMMMB157-11|BIOUG00848-F02|658[0n]bp|United States.California|BOLD:AAD6261
Tarache major|[783]|RDNME089-07|CNCNoctuoidea13386|658[0n]bp|United States.California|BOLD:AAD6261
Tarache major|[784]|RDNME090-07|CNCNoctuoidea13387|658[0n]bp|United States.California|BOLD:AAD6261
Tarache major|[785]|LPABB224-08|08BBLEP-03489|658[0n]bp|Canada.Alberta|BOLD:AAD6261
Tarache major|[786]|JMMMB121-11|BIOUG00848-C02|658[0n]bp|United States.California|BOLD:AAD6261
Tarache major|[787]|LPAB031-08|08BBLEP-02353|658[0n]bp|Canada.Alberta|BOLD:AAD6261
Tarache major|[788]|LPMN881-08|08BBLEP-02239|658[0n]bp|Canada.Alberta|BOLD:AAD6261
Tarache major|[789]|LPABB429-08|08BBLEP-03694|658[0n]bp|Canada.Alberta|BOLD:AAD6261
Tarache areli|[790]|RDNME054-07|CNCNoctuoidea13351|658[0n]bp|United States.California|BOLD:AAF7593
Tarache areli|[791]|RDNMG323-08|NOC15170|658[0n]bp|United States.California|BOLD:AAF7593
Tarache areli|[792]|RDNMG324-08|NOC15171|658[1n]bp|United States.Colorado|BOLD:AAF7593
Tarache areli|[793]|IAWLB309-11|IAWAZ-1217|658[0n]bp|United States.Arizona|BOLD:AAF7593
Tarache areli|[794]|IAWLB310-11|IAWAZ-1218|658[0n]bp|United States.Arizona|BOLD:AAF7593
Tarache aprica|[795]|LPOKC744-09|MDOK-2821|658[0n]bp|United States.Oklahoma|BOLD:AAB8647
Tarache aprica|[796]|HKONB199-09|3696-COI-08|658[0n]bp|United States.Texas|BOLD:AAB8647
Tarache aprica|[797]|LPOKA744-09|MDOK-0744|614[0n]bp|United States.Oklahoma|BOLD:AAB8647
Tarache aprica|[798]|BBLSW934-09|09BBLEP-01862|658[0n]bp|United States.Texas|BOLD:AAB8647

Tarache aprica|[795]|LPOKC744-09|MDOK-2021|658[0n]bp|United States.Oklahoma|BOLD:AAB8647
Tarache aprica|[796]|HKONB199-09|3696-COI-08|658[0n]bp|United States.Texas|BOLD:AAB8647
Tarache aprica|[797]|LPOKA744-09|MDOK-0744|614[0n]bp|United States.Oklahoma|BOLD:AAB8647
Tarache aprica|[798]|BBLSW934-09|09BBLEP-01862|658[0n]bp|United States.Texas|BOLD:AAB8647
Tarache aprica|[799]|LILLA984-11|SNS10IL-01205|658[0n]bp|United States.Illinois|BOLD:AAB8647
Tarache aprica|[800]|BBLSX702-09|09BBLEP-02630|658[0n]bp|United States.Texas|BOLD:AAB8647
Tarache aprica|[801]|LOFLB454-06|06-FLOR-1394|658[0n]bp|United States.Florida|BOLD:AAB8647
Tarache aprica|[802]|LOFLB838-06|06-FLOR-1778|658[0n]bp|United States.Florida|BOLD:AAB8647
Tarache aprica|[803]|USLEP840-10|10BBLEP-00840|658[0n]bp|United States.Texas|BOLD:AAB8647
Tarache aprica|[804]|USLEP841-10|10BBLEP-00841|658[0n]bp|United States.Texas|BOLD:AAB8647
Tarache aprica|[805]|LSUSA084-06|06-SUSA-0084|658[0n]bp|United States.Kentucky|BOLD:AAB8647
Tarache aprica|[806]|LSUSA164-06|06-SUSA-0164|658[0n]bp|United States.Kentucky|BOLD:AAB8647
Tarache aprica|[807]|USLEP839-10|10BBLEP-00839|658[0n]bp|United States.Texas|BOLD:AAB8647
Tarache aprica|[808]|LPOKB731-09|MDOK-1773|658[0n]bp|United States.Oklahoma|BOLD:AAB8647
Tarache aprica|[809]|LTOLB139-08|JKA-97-0011|658[0n]bp|United States.Georgia|BOLD:AAB8647
Tarache aprica|[810]|LPOKD305-09|MDOK-3384|658[0n]bp|United States.Oklahoma|BOLD:AAB8647
Tarache aprica|[811]|BBLOD1308-11|BIOUG01825-E07|658[0n]bp|United States.Texas|BOLD:AAB8647
Tarache aprica|[812]|BBLSW860-09|09BBLEP-01788|658[0n]bp|United States.Texas|BOLD:AAB8647
Tarache aprica|[813]|LOFLB886-06|06-FLOR-1826|658[0n]bp|United States.Florida|BOLD:AAB8647
Tarache aprica|[814]|MNAC699-07|CNCLEP00027149|645[1n]bp|United States.Maryland|BOLD:AAB8647
Tarache aprica|[815]|LSUSA076-06|06-SUSA-0076|594[0n]bp|United States.Kentucky|BOLD:AAB8647
Tarache aprica|[816]|USLEP845-10|10BBLEP-00845|646[0n]bp|United States.Texas|BOLD:AAB8647
Tarache aprica|[817]|BBLSX802-09|09BBLEP-02730|658[0n]bp|United States.Texas|BOLD:AAB8647
Tarache aprica|[818]|LOFLB846-06|06-FLOR-1786|658[0n]bp|United States.Florida|BOLD:AAB8647
Tarache aprica|[819]|HKONB200-09|3697-COI-08|658[0n]bp|United States.Texas|BOLD:AAB8647
Tarache aprica|[820]|BBLSX779-09|09BBLEP-02707|658[0n]bp|United States.Texas|BOLD:AAB8647
Tarache aprica|[821]|LOFLC215-06|06-FLOR-2095|658[0n]bp|United States.Florida|BOLD:AAB8647
Tarache aprica|[822]|LILLB018-11|SNS10IL-01238|658[0n]bp|United States.Illinois|BOLD:AAB8647
Tarache aprica|[823]|BBLSX765-09|09BBLEP-02693|658[0n]bp|United States.Texas|BOLD:AAB8647
Tarache aprica|[824]|USLEP844-10|10BBLEP-00844|658[0n]bp|United States.Texas|BOLD:AAB8647
Tarache aprica|[825]|LOFLB902-06|06-FLOR-1842|658[0n]bp|United States.Florida|BOLD:AAB8647
Tarache aprica|[826]|USLEP842-10|10BBLEP-00842|658[0n]bp|United States.Texas|BOLD:AAB8647
Tarache aprica|[827]|RDNMH786-09|CNCLEP00064114|658[0n]bp|United States.Texas|BOLD:AAB8647
Tarache aprica|[828]|BBLSX822-09|09BBLEP-02750|658[0n]bp|United States.Texas|BOLD:AAB8647
Tarache aprica|[829]|USLEP843-10|10BBLEP-00843|658[0n]bp|United States.Texas|BOLD:AAB8647
Tarache terminimaculata|[830]|LNCB341-06|06-NCCC-1297|658[0n]bp|United States.North Carolina|BOLD:AA...
Tarache terminimaculata|[831]|LNC006-05|05-NCCC-006|658[0n]bp|United States.North Carolina|BOLD:AAE7399
Tarache terminimaculata|[832]|UDLEP186-09|v646 CV|658[0n]bp|United States.Delaware|BOLD:AAE7399
Tarache terminimaculata|[833]|UDLEP125-09|v792 WCC|658[0n]bp|United States.Delaware|BOLD:AAE7399
Tarache terminimaculata|[834]|HKONS496-08|3025-COI-08|658[0n]bp|United States.Florida|BOLD:AAE7399
Tarache terminimaculata|[835]|LNCB340-06|06-NCCC-1296|658[0n]bp|United States.North Carolina|BOLD:AA...
Tarache terminimaculata|[836]|UDLEP075-09|v695 MDT|658[0n]bp|United States.Delaware|BOLD:AAE7399
Tarache terminimaculata|[837]|UDLEP187-09|v647 CV|658[0n]bp|United States.Delaware|BOLD:AAE7399
Cucullia asteroides|[838]|XAE242-04|Moth4242.03|658[0n]bp|Canada.Ontario|BOLD:AAB9406
Cucullia asteroides|[839]|BLGSM012-09|BL320|658[0n]bp|Canada.Ontario|BOLD:AAB9406
Cucullia asteroides|[840]|XAG874-05|2005-ONT-1458|658[0n]bp|Canada.Ontario|BOLD:AAB9406
Cucullia asteroides|[841]|XAD371-04|04HBL007371|658[0n]bp|Canada.Ontario|BOLD:AAB9406
Cucullia asteroides|[842]|XAH373-05|2005-ONT-1956|658[0n]bp|Canada.Ontario|BOLD:AAB9406
Cucullia asteroides|[843]|RDLQ613-07|DH004817|658[0n]bp|Canada.Quebec|BOLD:AAB9406
Cucullia asteroides|[844]|RDLQ611-07|DH009164|658[0n]bp|Canada.Quebec|BOLD:AAB9406
Cucullia asteroides|[845]|XAG015-05|2005-ONT-599|658[0n]bp|Canada.Ontario|BOLD:AAB9406
Cucullia asteroides|[846]|XAG761-05|2005-ONT-1345|658[1n]bp|Canada.Ontario|BOLD:AAB9406
Cucullia asteroides|[847]|XAH221-05|2005-ONT-1804|621[0n]bp|Canada.Ontario|BOLD:AAB9406
Cucullia asteroides|[848]|RDLQ612-07|DH008842|641[0n]bp|Canada.Quebec|BOLD:AAB9406
Cucullia asteroides|[849]|PHMO304-03|moth2240.02|639[1n]bp|Canada.Ontario|BOLD:AAB9406
Cucullia asteroides|[850]|XAE594-04|Moth4594.03|563[0n]bp|Canada.Ontario|BOLD:AAB9406
Cucullia asteroides|[851]|XAD294-04|04HBL007294|583[0n]bp|Canada.Ontario|BOLD:AAB9406
Cucullia asteroides|[852]|XAD395-04|04HBL007395|584[0n]bp|Canada.Ontario|BOLD:AAB9406
Cucullia asteroides|[853]|XAD178-04|04HBL007178|574[0n]bp|Canada.Ontario|BOLD:AAB9406
Cucullia asteroides|[854]|XAH002-05|2005-ONT-1585|620[0n]bp|Canada.Ontario|BOLD:AAB9406
Cucullia omissa|[855]|RDLQB263-05|DH010349|658[0n]bp|Canada.Quebec|BOLD:ABZ8006
Cucullia omissa|[856]|RDNMG483-08|CNC LEP00052307|658[0n]bp|Canada.New Brunswick|BOLD:ABZ8006
Cucullia postera|[857]|RDNMG481-08|CNC LEP00052305|658[0n]bp|Canada.New Brunswick|BOLD:ABZ8007
Cucullia postera|[858]|RDNMG480-08|CNC LEP00052304|658[0n]bp|Canada.New Brunswick|BOLD:ABZ8007
Cucullia postera|[859]|RDLQ608-07|DH003144|616[0n]bp|Canada.Quebec|BOLD:ABZ8007
Cucullia postera|[860]|XAG253-05|2005-ONT-837|658[0n]bp|Canada.Ontario|BOLD:ABZ8007
Cucullia postera|[861]|XAC533-04|04HBL006533|658[0n]bp|Canada.Ontario|BOLD:ABZ8007
Cucullia postera|[862]|XAC046-04|04HBL006046|658[0n]bp|Canada.Ontario|BOLD:ABZ8007
Cucullia postera|[863]|MNBB168-05|05-NBSTA-084|658[0n]bp|Canada.New Brunswick|BOLD:ABZ8007
Cucullia postera|[864]|RDNMG482-08|CNC LEP00052306|658[0n]bp|Canada.New Brunswick|BOLD:ABZ8007
Cucullia florea|[865]|RDNMG484-08|CNC LEP00052308|658[0n]bp|Canada.New Brunswick|BOLD:ABZ8007
Cucullia florea|[866]|BBLPE495-09|09BBELE-2495|658[0n]bp|Canada.Newfoundland and Labrador|BOLD:ABZ8007
Cucullia florea|[867]|PHMNB769-05|Moth 462.03SA|658[0n]bp|Canada.New Brunswick|BOLD:ABZ8007
Cucullia florea|[868]|LOWCC838-05|CGWC-2718|658[0n]bp|Canada.British Columbia|BOLD:ABZ8007
Cucullia florea|[869]|PHMNB185-04|04HBL007650|609[0n]bp|Canada.New Brunswick|BOLD:ABZ8007
Cucullia similaris|[870]|RDNMF143-08|NOC14229|658[0n]bp|Canada.British Columbia|BOLD:ABZ8007
Cucullia similaris|[871]|RDNMF142-08|NOC14228|645[0n]bp|Canada.British Columbia|BOLD:ABZ8007
Cucullia montanae|[872]|RDNMF149-08|NOC14235|658[0n]bp|Canada.British Columbia|BOLD:ABZ8007
Cucullia sp.|[873]|BLGSM023-09|BL332|658[0n]bp|Canada.Ontario|BOLD:ABZ8007
Cucullia sp.|[874]|PHMO168-03|moth891.02|639[0n]bp|Canada.Ontario|BOLD:ABZ8007
Cucullia sp.|[875]|LOWCD760-06|CGWC-3580|657[0n]bp|Canada.British Columbia|BOLD:ABZ8007
Cucullia sp.|[876]|RDNMG485-08|CNC LEP00052309|658[0n]bp|Canada.New Brunswick|BOLD:ABZ8007
Cucullia albida|[877]|RDNMF617-08|NOC14703|658[0n]bp|Canada.Saskatchewan|BOLD:AAE3238
Cucullia albida|[878]|RDNMF618-08|NOC14704|658[0n]bp|Canada.Saskatchewan|BOLD:AAE3238
Cucullia albida|[879]|RDNMF616-08|NOC14702|652[0n]bp|Canada.Saskatchewan|BOLD:AAE3238
Cucullia strigata|[880]|RDNMF144-08|NOC14230|658[0n]bp|Canada.British Columbia|BOLD:AAE3238
Cucullia strigata|[881]|RDNMF148-08|NOC14234|658[0n]bp|Canada.British Columbia|BOLD:AAE3238
Cucullia strigata|[882]|RDNMF145-08|NOC14231|640[0n]bp|Canada.British Columbia|BOLD:AAE3238
Cucullia strigata|[883]|RDNMF147-08|NOC14233|658[0n]bp|Canada.British Columbia|BOLD:AAE3238
Cucullia antipoda group|[884]|RDNMC422-05|CNCNoctuoidea12055|559[0n]bp|Canada.British Columbia|BOLD:...
Cucullia antipoda group|[885]|RDNMC417-05|CNCNoctuoidea12050|557[0n]bp|Canada.British Columbia|BOLD:...
Cucullia antipoda group|[886]|LBCH5547-10|10-JDWBC-5547|658[0n]bp|Canada.British Columbia|BOLD:ABY5329
Cucullia antipoda group|[887]|LBCG339-08|08-JDWBC-0339|658[0n]bp|Canada.British Columbia|BOLD:ABY5329
Cucullia antipoda group|[888]|RDNM904-05|CNCNoctuoidea7744|571[0n]bp|Canada.Alberta|BOLD:AAB2265
Cucullia antipoda group|[889]|RDNMC437-05|CNCNoctuoidea12070|577[1n]bp|Canada.Alberta|BOLD:AAB2265
Cucullia pulla|[890]|RDNMG524-08|CNC LEP00052348|658[0n]bp|Canada.British Columbia|BOLD:AAE3239
Cucullia pulla|[891]|RDNMF054-08|NOC14140|658[0n]bp|Canada.British Columbia|BOLD:AAE3239
Cucullia dorsalis|[892]|RDNMG491-08|CNC LEP00052315|658[0n]bp|Canada.Alberta|BOLD:ABZ3749
Cucullia speyeri|[893]|RDNMG492-08|CNC LEP00052316|658[0n]bp|Canada.Alberta|BOLD:AAD3289
Cucullia speyeri|[894]|RDNMF044-08|NOC14130|658[0n]bp|Canada.Alberta|BOLD:AAD3289
Cucullia speyeri|[895]|RDLQH159-07|DH060001|658[0n]bp|Canada.Quebec|BOLD:AAD3289
Cucullia speyeri|[896]|LPSK535-08|08BBLEP-02103|658[0n]bp|Canada.Saskatchewan|BOLD:AAD3289
Cucullia speyeri|[897]|RDMAB1015-09|UASM34761|658[0n]bp|Canada.Alberta|BOLD:AAD3289
[illegible]

Cucullia speyeri|[895]|RDLQH159-07|DH060001|658[0n]bp|Canada.Quebec|BOLD:AAD3289
Cucullia speyeri|[896]|LPSK535-08|08BBLEP-02103|658[0n]bp|Canada.Saskatchewan|BOLD:AAD3289
Cucullia speyeri|[897]|RDMAB1015-09|UASM34761|658[0n]bp|Canada.Alberta|BOLD:AAD3289
Cucullia speyeri|[898]|XAD580-04|04HBL006995|601[1n]bp|Canada.Ontario|BOLD:AAD3289
Cucullia eulepis|[899]|LBCH6122-10|10-JDWBC-6122|658[0n]bp|Canada.British Columbia|BOLD:AAD2766
Cucullia luna|[900]|RDNM900-05|CNCNoctuoidea7740|540[0n]bp|Canada.Alberta|BOLD:AAE3253
Cucullia luna|[901]|RDNMG486-08|CNC LEP00052310|658[0n]bp|Canada.Saskatchewan|BOLD:AAE3253
Cucullia luna|[902]|RDNMG487-08|CNC LEP00052311|658[0n]bp|Canada.Manitoba|BOLD:AAE3253
Cucullia convexipennis|[903]|MNBB371-05|05-NBSTA-287|658[0n]bp|Canada.New Brunswick|BOLD:AAD2762
Cucullia convexipennis|[904]|RDLQ610-07|AC000594|658[0n]bp|Canada.Quebec|BOLD:AAD2762
Cucullia convexipennis|[905]|RDLQ609-07|DH007999|630[0n]bp|Canada.Quebec|BOLD:AAD2762
Cucullia mcdunnoughi|[906]|RDNMF133-08|NOC14219|658[0n]bp|Canada.British Columbia|BOLD:AAE3252
Cucullia mcdunnoughi|[907]|RDNMF132-08|NOC14218|657[1n]bp|Canada.British Columbia|BOLD:AAE3252
Cucullia intermedia|[908]|LPSK517-08|08BBLEP-02085|658[0n]bp|Canada.Saskatchewan|BOLD:AAB0275
Cucullia intermedia|[909]|PHMNB435-04|04HBL00661|658[0n]bp|Canada.New Brunswick|BOLD:AAB0275
Cucullia intermedia|[910]|XAC183-04|04HBL006183|658[0n]bp|Canada.Ontario|BOLD:AAB0275
Cucullia intermedia|[911]|XAB018-04|04HBL005018|658[0n]bp|Canada.Ontario|BOLD:AAB0275
Cucullia intermedia|[912]|XAJ291-06|2006-ONT-0291|658[0n]bp|Canada.Ontario|BOLD:AAB0275
Cucullia intermedia|[913]|TTMNB392-06|MNBTT-392|658[0n]bp|Canada.New Brunswick|BOLD:AAB0275
Cucullia intermedia|[914]|LALPA891-11|AVBC 1064-11|658[0n]bp|Canada.British Columbia|BOLD:AAB0275
Cucullia intermedia|[915]|LBCG337-08|08-JDWBC-0337|658[0n]bp|Canada.British Columbia|BOLD:AAB0275
Cucullia intermedia|[916]|LPSK543-08|08BBLEP-02111|658[0n]bp|Canada.Saskatchewan|BOLD:AAB0275
Cucullia intermedia|[917]|XAB588-04|04HBL005588|658[0n]bp|Canada.Ontario|BOLD:AAB0275
Cucullia intermedia|[918]|RDLQ616-07|DH008881|658[0n]bp|Canada.Quebec|BOLD:AAB0275
Cucullia intermedia|[919]|XAH049-05|2005-ONT-1632|658[0n]bp|Canada.Ontario|BOLD:AAB0275
Cucullia intermedia|[920]|KPOEC171-08|08OEC-212|658[0n]bp|Canada.Ontario|BOLD:AAB0275
Cucullia intermedia|[921]|XAD639-05|2005-ONT-54|658[0n]bp|Canada.Ontario|BOLD:AAB0275
Cucullia intermedia|[922]|XAB067-04|04HBL005067|658[0n]bp|Canada.Ontario|BOLD:AAB0275
Cucullia intermedia|[923]|XAH196-05|2005-ONT-1779|658[0n]bp|Canada.Ontario|BOLD:AAB0275
Cucullia intermedia|[924]|LBCG2491-09|08-JDWBC-2491|658[0n]bp|Canada.British Columbia|BOLD:AAB0275
Cucullia intermedia|[925]|XAC184-04|04HBL006184|658[0n]bp|Canada.Ontario|BOLD:AAB0275
Cucullia intermedia|[926]|BLGSM074-09|BL1627|658[0n]bp|Canada.Ontario|BOLD:AAB0275
Cucullia intermedia|[927]|LPMN817-08|08BBLEP-01620|658[0n]bp|Canada.Manitoba|BOLD:AAB0275
Cucullia intermedia|[928]|RDLQ615-07|DH002919|658[0n]bp|Canada.Quebec|BOLD:AAB0275
Cucullia intermedia|[929]|BLTIB034-08|BL0061|658[0n]bp|Canada.Ontario|BOLD:AAB0275
Cucullia intermedia|[930]|XAD641-05|2005-ONT-56|658[0n]bp|Canada.Ontario|BOLD:AAB0275
Cucullia intermedia|[931]|RDLQ614-07|DH006210|658[0n]bp|Canada.Quebec|BOLD:AAB0275
Cucullia intermedia|[932]|XAC290-04|04HBL006290|584[0n]bp|Canada.Ontario|BOLD:AAB0275
Cucullia intermedia|[933]|XAH263-05|2005-ONT-1846|658[0n]bp|Canada.Ontario|BOLD:AAB0275
Cucullia intermedia|[934]|XAK154-06|2006-ONT-1149|617[0n]bp|Canada.Ontario|BOLD:AAB0275
Cucullia intermedia|[935]|TMG126-03|CUCU1.00|639[0n]bp|Canada.Ontario|BOLD:AAB0275
Cucullia intermedia|[936]|XAJ490-06|2006-ONT-0490|653[0n]bp|Canada.Ontario|BOLD:AAB0275
Cucullia intermedia|[937]|XAH240-05|2005-ONT-1823|617[0n]bp|Canada.Ontario|BOLD:AAB0275
Cucullia intermedia|[938]|RDLQ617-07|DH004403|623[0n]bp|Canada.Quebec|BOLD:AAB0275
Cucullia intermedia|[939]|PMG106-03|moth181.01|617[0n]bp|Canada.Ontario|BOLD:AAB0275
Cucullia intermedia|[940]|TMG125-03|moth213.01|639[0n]bp|Canada.Ontario|BOLD:AAB0275
Cucullia intermedia|[941]|BLTIB954-08|BL1383|658[0n]bp|Canada.Ontario|BOLD:AAB0275
Cucullia umbratica|[942]|RDNMC456-05|CNCNoctuoidea12089|600[0n]bp|Canada.Quebec|BOLD:AAC0849
Cerma cerintha|[943]|RDLQF934-06|DH012109|658[0n]bp|Canada.Quebec|BOLD:AAB5015
Cerma cerintha|[944]|LPSOC152-08|PPBP-2151|658[0n]bp|Canada.Ontario|BOLD:AAB5015
Cerma cerintha|[945]|XAF568-05|2005-ONT-217|658[0n]bp|Canada.Ontario|BOLD:AAB5015
Cerma cerintha|[946]|BLTIB503-08|BL760|658[0n]bp|Canada.Ontario|BOLD:AAB5015
Cerma cerintha|[947]|XAB635-04|04HBL005635|658[0n]bp|Canada.Ontario|BOLD:AAB5015
Cerma cerintha|[948]|XAK063-06|2006-ONT-1058|658[0n]bp|Canada.Ontario|BOLD:AAB5015
Cerma cerintha|[949]|XAE333-04|Moth4333.03|658[0n]bp|Canada.Ontario|BOLD:AAB5015
Cerma cerintha|[950]|XAK178-06|2006-ONT-1173|658[0n]bp|Canada.Ontario|BOLD:AAB5015
Cerma cerintha|[951]|LPSOC153-08|PPBP-2152|621[0n]bp|Canada.Ontario|BOLD:AAB5015
Cerma cerintha|[952]|BLTIB614-08|BL894|649[0n]bp|Canada.Ontario|BOLD:AAB5015
Cerma cerintha|[953]|TMG101-03|moth866.01|639[0n]bp|Canada.Ontario|BOLD:AAB5015
Cerma cerintha|[954]|PMG100-03|moth631.01|617[0n]bp|Canada.Ontario|BOLD:AAB5015
Cerma cora|[955]|RDLQF843-06|DH011996|658[0n]bp|Canada.Quebec|BOLD:AAD3483
Cerma cora|[956]|RDLQF844-06|DH011997|658[0n]bp|Canada.Quebec|BOLD:AAD3483
Cerma cora|[957]|RDLQF845-06|DH011998|658[0n]bp|Canada.Quebec|BOLD:AAD3483
Cerma cora|[958]|PHMNB069-03|moth59.02SA|639[0n]bp|Canada.New Brunswick|BOLD:AAD3483
Cerma cora|[959]|PHMNB481-04|04HBL00707|617[0n]bp|Canada.New Brunswick|BOLD:AAD3483
Cerma cora|[960]|MNAC056-07|CNCLEP00025117|658[0n]bp|Canada.Ontario|BOLD:AAD3483
Cerma cora|[961]|RDLQF452-06|DH011559|658[0n]bp|Canada.Quebec|BOLD:AAD3483
Polygrammate hebraeicum|[962]|PSAT119-10|CNCLEP 70004|658[0n]bp|Canada.Ontario|BOLD:AAA8249
Crambodes talidiformis|[963]|BLTIB064-08|BL0103|606[6n]bp|Canada.Ontario|
Crambodes talidiformis|[964]|XAC702-04|04HBL006702|658[0n]bp|Canada.Ontario|BOLD:AAD0878
Crambodes talidiformis|[965]|RDLQF886-06|DH012061|611[0n]bp|Canada.Quebec|BOLD:AAD0878
Crambodes talidiformis|[966]|XAC158-04|04HBL006158|658[0n]bp|Canada.Ontario|BOLD:AAD0878
Leuconycta diphteroides|[967]|LPSO668-08|PPBP-0668|577[0n]bp|Canada.Ontario|BOLD:AAA6913
Leuconycta diphteroides|[968]|PHMNB463-04|04HBL00689|658[0n]bp|Canada.New Brunswick|BOLD:AAA6913
Leuconycta diphteroides|[969]|PHMNB739-05|Moth 432.03SA|658[0n]bp|Canada.New Brunswick|BOLD:AAA6913
Leuconycta diphteroides|[970]|PHMNB520-04|04HBL00746|658[0n]bp|Canada.New Brunswick|BOLD:AAA6913
Leuconycta diphteroides|[971]|XAB245-04|04HBL005245|658[0n]bp|Canada.Ontario|BOLD:AAA6913
Leuconycta diphteroides|[972]|LPSOB813-08|PPBP-1812|657[0n]bp|Canada.Ontario|BOLD:AAA6913
Leuconycta diphteroides|[973]|TTMNB310-06|MNBTT-310|658[1n]bp|Canada.New Brunswick|BOLD:AAA6913
Leuconycta diphteroides|[974]|BBLPE079-09|09BBELE-2079|658[0n]bp|Canada.Nova Scotia|BOLD:AAA6913
Leuconycta diphteroides|[975]|XAJ537-06|2006-ONT-0537|658[0n]bp|Canada.Ontario|BOLD:AAA6913
Leuconycta diphteroides|[976]|BLTIB259-08|BL441|658[0n]bp|Canada.Ontario|BOLD:AAA6913
Leuconycta diphteroides|[977]|XAF505-05|2005-ONT-154|658[0n]bp|Canada.Ontario|BOLD:AAA6913
Leuconycta diphteroides|[978]|BLTIB533-08|BL800|658[0n]bp|Canada.Ontario|
Leuconycta diphteroides|[979]|XAH280-05|2005-ONT-1863|658[0n]bp|Canada.Ontario|BOLD:AAA6913
Leuconycta diphteroides|[980]|BLTIB278-08|BL462|658[0n]bp|Canada.Ontario|BOLD:AAA6913
Leuconycta diphteroides|[981]|BLTIB521-08|BL784|658[0n]bp|Canada.Ontario|BOLD:AAA6913
Leuconycta diphteroides|[982]|BLTIB675-08|BL958|658[0n]bp|Canada.Ontario|BOLD:AAA6913
Leuconycta diphteroides|[983]|LPSOC356-08|PPBP-2355|655[0n]bp|Canada.Ontario|BOLD:AAA6913
Leuconycta diphteroides|[984]|BLTIB596-08|BL876|658[0n]bp|Canada.Ontario|BOLD:AAA6913
Leuconycta diphteroides|[985]|RDLQG277-06|DH012489|658[0n]bp|Canada.Quebec|BOLD:AAA6913
Leuconycta diphteroides|[986]|PHMNB625-04|04HBL00851|658[0n]bp|Canada.New Brunswick|BOLD:AAA6913
Leuconycta diphteroides|[987]|PHMNB149-04|04HBL007614|658[0n]bp|Canada.New Brunswick|BOLD:AAA6913
Leuconycta diphteroides|[988]|BLTIB710-08|BL995|658[0n]bp|Canada.Ontario|BOLD:AAA6913
Leuconycta diphteroides|[989]|TMG120-03|moth146.01|617[0n]bp|Canada.Ontario|BOLD:AAA6913
Leuconycta diphteroides|[990]|TMG102-03|LEUC1.00|617[0n]bp|Canada.Ontario|BOLD:AAA6913
Leuconycta diphteroides|[991]|RDLQF428-06|DH011535|658[0n]bp|Canada.Quebec|BOLD:AAA6913
Leuconycta diphteroides|[992]|BLTIB479-08|BL728|658[0n]bp|Canada.Ontario|BOLD:AAA6913
Leuconycta diphteroides|[993]|LPSOD250-09|08BBLEP-00028|658[0n]bp|Canada.Ontario|BOLD:AAA6913
Leuconycta diphteroides|[994]|XAJ343-06|2006-ONT-0343|658[0n]bp|Canada.Ontario|BOLD:AAA6913
Leuconycta diphteroides|[995]|PHMNB214-04|04HBL007679|609[0n]bp|Canada.New Brunswick|BOLD:AAA6913
Leuconycta diphteroides|[996]|PHMNB176-04|04HBL007641|609[0n]bp|Canada.New Brunswick|BOLD:AAA6913
Leuconycta diphteroides|[997]|LPSOB814-08|PPBP-1813|648[0n]bp|Canada.Ontario|BOLD:AAA6913

Leuconycta diphteroides|[995]|PHMNB214-04|04HBL007679|609[0n]bp|Canada.New Brunswick|BOLD:AAA6913
Leuconycta diphteroides|[996]|PHMNB176-04|04HBL007641|609[0n]bp|Canada.New Brunswick|BOLD:AAA6913
Leuconycta diphteroides|[997]|LPSOB814-08|PPBP-1813|648[0n]bp|Canada.Ontario|BOLD:AAA6913
Leuconycta diphteroides|[998]|PHMNB026-03|moth17.02SA|639[0n]bp|Canada.New Brunswick|BOLD:AAA6913
Leuconycta diphteroides|[999]|BLTIB660-08|BL941|634[0n]bp|Canada.Ontario|BOLD:AAA6913
Leuconycta diphteroides|[1000]|TTMNB311-06|MNBTT-311|583[0n]bp|Canada.New Brunswick|BOLD:AAA6913
Leuconycta diphteroides|[1001]|PMG127-03|moth545.01|617[0n]bp|Canada.Ontario|BOLD:AAA6913
Leuconycta diphteroides|[1002]|PHMO139-03|moth793.02|639[0n]bp|Canada.Ontario|BOLD:AAA6913
Leuconycta diphteroides|[1003]|LPSO562-08|PPBP-0562|658[0n]bp|Canada.Ontario|BOLD:AAA6913
Leuconycta diphteroides|[1004]|KPOEC087-08|08OEC-246|658[0n]bp|Canada.Ontario|BOLD:AAA6913
Leuconycta diphteroides|[1005]|XAB172-04|04HBL005172|658[0n]bp|Canada.Ontario|BOLD:AAA6913
Leuconycta diphteroides|[1006]|XAB116-04|04HBL005116|658[0n]bp|Canada.Ontario|BOLD:AAA6913
Leuconycta diphteroides|[1007]|XAF610-05|2005-ONT-259|658[0n]bp|Canada.Ontario|BOLD:AAA6913
Leuconycta diphteroides|[1008]|LPSO553-08|PPBP-0553|658[0n]bp|Canada.Ontario|BOLD:AAA6913
Leuconycta diphteroides|[1009]|LPSO417-08|PPBP-0417|658[0n]bp|Canada.Ontario|BOLD:AAA6913
Leuconycta diphteroides|[1010]|XAB320-04|04HBL005320|658[0n]bp|Canada.Ontario|BOLD:AAA6913
Leuconycta diphteroides|[1011]|LPSO667-08|PPBP-0667|658[0n]bp|Canada.Ontario|BOLD:AAA6913
Leuconycta diphteroides|[1012]|LPSOC035-08|PPBP-2034|658[0n]bp|Canada.Ontario|BOLD:AAA6913
Leuconycta diphteroides|[1013]|LPSOB808-08|PPBP-1807|658[0n]bp|Canada.Ontario|BOLD:AAA6913
Leuconycta diphteroides|[1014]|LPSOC355-08|PPBP-2354|656[1n]bp|Canada.Ontario|BOLD:AAA6913
Leuconycta diphteroides|[1015]|LPSOC354-08|PPBP-2353|647[0n]bp|Canada.Ontario|BOLD:AAA6913
Leuconycta diphteroides|[1016]|LPSO428-08|PPBP-0428|658[0n]bp|Canada.Ontario|BOLD:AAA6913
Leuconycta diphteroides|[1017]|LPSOC353-08|PPBP-2352|657[0n]bp|Canada.Ontario|BOLD:AAA6913
Leuconycta diphteroides|[1018]|LPSO549-08|PPBP-0549|658[0n]bp|Canada.Ontario|BOLD:AAA6913
Magusa divaricata|[1019]|RDLQB627-05|DH010730|658[0n]bp|Canada.Quebec|BOLD:AAA4337
Magusa divaricata|[1020]|XAH035-05|2005-ONT-1618|658[0n]bp|Canada.Ontario|BOLD:AAA4337
Magusa orbifera|[1021]|RDNML321-13|CNCLEP 92297|658[0n]bp|United States.Florida|BOLD:ACD9027
Condica sutor|[1022]|RDNMJ021-10|CNCLEP 73801|658[0n]bp|United States.Florida|BOLD:AAA5860
Condica sutor|[1023]|BBLOD1601-11|BIOUG01829-F03|658[0n]bp|United States.Texas|BOLD:AAA5860
Condica sutor|[1024]|RDNMH496-09|CNCLEP00057815|658[0n]bp|United States.Louisiana|BOLD:AAA5860
Condica sutor|[1025]|LNCNW054-06|06-NCNW-0054|658[0n]bp|United States.North Carolina|BOLD:AAA5860
Condica sutor|[1026]|BBLOC1047-11|BIOUG01468-G07|658[0n]bp|United States.Arkansas|BOLD:AAA5860
Condica sutor|[1027]|RDNMH484-09|CNCLEP00057803|658[0n]bp|United States.Louisiana|BOLD:AAA5860
Condica sutor|[1028]|RDNMH499-09|CNCLEP00057818|658[0n]bp|United States.Louisiana|BOLD:AAA5860
Condica sutor|[1029]|RDNMH501-09|CNCLEP00057820|658[0n]bp|United States.Louisiana|BOLD:AAA5860
Condica sutor|[1030]|RDNMH498-09|CNCLEP00057817|658[0n]bp|United States.Louisiana|BOLD:AAA5860
Condica sutor|[1031]|RDNMH483-09|CNCLEP00057802|658[0n]bp|United States.Louisiana|BOLD:AAA5860
Condica sutor|[1032]|LNC511-06|05-NCCC-511|621[3n]bp|United States.North Carolina|BOLD:AAA5860
Condica sutor|[1033]|RDNMH487-09|CNCLEP00057806|658[0n]bp|United States.Louisiana|BOLD:AAA5860
Condica sutor|[1034]|USLEP299-10|10BBLEP-00299|658[0n]bp|United States.Florida|BOLD:AAA5860
Condica sutor|[1035]|RDNMH497-09|CNCLEP00057816|658[0n]bp|United States.Louisiana|BOLD:AAA5860
Condica sutor|[1036]|RDNMJ016-10|CNCLEP 73796|658[0n]bp|United States.Florida|BOLD:AAA5860
Condica sutor|[1037]|RDNMH485-09|CNCLEP00057804|658[0n]bp|United States.Louisiana|BOLD:AAA5860
Condica sutor|[1038]|RDNMH500-09|CNCLEP00057819|658[0n]bp|United States.Louisiana|BOLD:AAA5860
Condica sutor|[1039]|RDNMJ017-10|CNCLEP 73797|658[0n]bp|United States.Florida|BOLD:AAA5860
Condica sutor|[1040]|RDNMJ023-10|CNCLEP 73803|658[0n]bp|United States.Florida|BOLD:AAA5860
Condica sutor|[1041]|RDNMH488-09|CNCLEP00057807|658[0n]bp|United States.Louisiana|BOLD:AAA5860
Condica sutor|[1042]|RDNMH511-09|CNCLEP00057830|658[0n]bp|United States.Louisiana|BOLD:AAA5860
Condica sutor|[1043]|RDNMH486-09|CNCLEP00057805|658[0n]bp|United States.Louisiana|BOLD:AAA5860
Condica sutor|[1044]|RDNMJ018-10|CNCLEP 73798|658[0n]bp|United States.Florida|BOLD:AAA5860
Condica sutor|[1045]|LNCNW055-06|06-NCNW-0055|658[0n]bp|United States.North Carolina|BOLD:AAA5860
Condica sutor|[1046]|RDNMJ013-10|CNCLEP 73793|658[0n]bp|United States.Florida|BOLD:AAA5860
Condica cupentia|[1047]|RDNMF619-08|NOC14705|615[0n]bp|United States.Florida|BOLD:AAA7985
Condica cupentia|[1048]|BBLOB477-11|BIOUG01397-A02|627[0n]bp|United States.Florida|BOLD:AAA7985
Condica cupentia|[1049]|RDNMJ025-10|CNCLEP 73805|658[0n]bp|United States.Florida|BOLD:AAA7985
Condica cupentia|[1050]|RDNMJ026-10|CNCLEP 73806|658[0n]bp|United States.Florida|BOLD:AAA7985
Condica cupentia|[1051]|RDNMK088-11|CNCLEP 81211|658[0n]bp|United States.Florida|BOLD:AAA7985
Condica cupentia|[1052]|RDNMH495-09|CNCLEP00057814|658[0n]bp|United States.Louisiana|BOLD:AAA7985
Condica cupentia|[1053]|RDNMH494-09|CNCLEP00057813|658[0n]bp|United States.Louisiana|BOLD:AAA7985
Condica vecors|[1054]|RDLQ155-05|DH004398|658[0n]bp|Canada.Quebec|BOLD:AAB5995
Condica sutor|[1055]|LGSMG1021-10|BGS04102|658[0n]bp|United States.Tennessee|BOLD:AAB5995
Condica vecors|[1056]|RDLQG275-06|DH012487|631[0n]bp|Canada.Quebec|BOLD:AAB5995
Condica vecors|[1057]|RDLQF810-06|DH011960|591[1n]bp|Canada.Quebec|BOLD:AAB5995
Condica vecors|[1058]|RDLQF653-06|DH011803|637[0n]bp|Canada.Quebec|BOLD:AAB5995
Condica vecors|[1059]|LPSOC367-08|PPBP-2366|655[0n]bp|Canada.Ontario|BOLD:AAB5995
Condica discistriga|[1060]|LBCH5606-10|10-JDWBC-5606|658[0n]bp|Canada.British Columbia|BOLD:AAC3223
Condica discistriga|[1061]|LBCH5671-10|10-JDWBC-5671|658[0n]bp|Canada.British Columbia|BOLD:AAC3223
Condica discistriga|[1062]|LBCH5722-10|10-JDWBC-5722|658[0n]bp|Canada.British Columbia|BOLD:AAC3223
Condica discistriga|[1063]|LBCH5085-10|10-JDWBC-5085|658[0n]bp|Canada.British Columbia|BOLD:AAC3223
Condica discistriga|[1064]|LBCH5806-10|10-JDWBC-5806|658[0n]bp|Canada.British Columbia|BOLD:AAC3223
Condica discistriga|[1065]|LBCH6453-10|10-JDWBC-6453|658[0n]bp|Canada.British Columbia|BOLD:AAC3223
Condica discistriga|[1066]|LBCH5996-10|10-JDWBC-5996|658[0n]bp|Canada.British Columbia|BOLD:AAC3223
Condica discistriga|[1067]|LBCH5524-10|10-JDWBC-5524|658[0n]bp|Canada.British Columbia|BOLD:AAC3223
Condica discistriga|[1068]|LBCH5495-10|10-JDWBC-5495|658[0n]bp|Canada.British Columbia|BOLD:AAC3223
Condica discistriga|[1069]|LOWCB873-05|CGWC-1813|658[0n]bp|Canada.British Columbia|BOLD:AAC3223
Condica discistriga|[1070]|LOWCB872-05|CGWC-1812|658[0n]bp|Canada.British Columbia|BOLD:AAC3223
Condica discistriga|[1071]|LOWCB864-05|CGWC-1804|658[0n]bp|Canada.British Columbia|BOLD:AAC3223
Condica discistriga|[1072]|LBCH5997-10|10-JDWBC-5997|658[0n]bp|Canada.British Columbia|BOLD:AAC3223
Condica discistriga|[1073]|LBCH5994-10|10-JDWBC-5994|658[0n]bp|Canada.British Columbia|BOLD:AAC3223
Condica discistriga|[1074]|LOWCB868-05|CGWC-1808|658[0n]bp|Canada.British Columbia|BOLD:AAC3223
Condica discistriga|[1075]|LBCH6050-10|10-JDWBC-6050|658[0n]bp|Canada.British Columbia|BOLD:AAC3223
Condica discistriga|[1076]|LBCH6052-10|10-JDWBC-6052|658[0n]bp|Canada.British Columbia|BOLD:AAC3223
Condica discistriga|[1077]|LBCH6048-10|10-JDWBC-6048|658[0n]bp|Canada.British Columbia|BOLD:AAC3223
Condica discistriga|[1078]|LBCH6053-10|10-JDWBC-6053|658[0n]bp|Canada.British Columbia|BOLD:AAC3223
Condica discistriga|[1079]|LOWCB874-05|CGWC-1814|658[0n]bp|Canada.British Columbia|BOLD:AAC3223
Condica discistriga|[1080]|LBCH5992-10|10-JDWBC-5992|658[0n]bp|Canada.British Columbia|BOLD:AAC3223
Condica discistriga|[1081]|LBCH6049-10|10-JDWBC-6049|658[0n]bp|Canada.British Columbia|BOLD:AAC3223
Condica discistriga|[1082]|LBCH5496-10|10-JDWBC-5496|658[0n]bp|Canada.British Columbia|BOLD:AAC3223
Condica discistriga|[1083]|LBCH6047-10|10-JDWBC-6047|658[0n]bp|Canada.British Columbia|BOLD:AAC3223
Condica discistriga|[1084]|LBCH5481-10|10-JDWBC-5481|658[0n]bp|Canada.British Columbia|BOLD:AAC3223
Condica discistriga|[1085]|LOWCB871-05|CGWC-1811|658[0n]bp|Canada.British Columbia|BOLD:AAC3223
Condica discistriga|[1086]|LBCH6051-10|10-JDWBC-6051|658[0n]bp|Canada.British Columbia|BOLD:AAC3223
Condica discistriga|[1087]|LBCH5520-10|10-JDWBC-5520|658[0n]bp|Canada.British Columbia|BOLD:AAC3223
Condica discistriga|[1088]|LBCH5521-10|10-JDWBC-5521|658[0n]bp|Canada.British Columbia|BOLD:AAC3223
Condica discistriga|[1089]|LBCH5995-10|10-JDWBC-5995|658[0n]bp|Canada.British Columbia|BOLD:AAC3223
Condica discistriga|[1090]|LBCH5993-10|10-JDWBC-5993|658[0n]bp|Canada.British Columbia|BOLD:AAC3223
Condica discistriga|[1091]|LBCH5549-10|10-JDWBC-5549|658[0n]bp|Canada.British Columbia|BOLD:AAC3223
Condica discistriga|[1092]|LBCH5519-10|10-JDWBC-5519|658[0n]bp|Canada.British Columbia|BOLD:AAC3223
Condica discistriga|[1093]|LBCH5522-10|10-JDWBC-5522|658[0n]bp|Canada.British Columbia|BOLD:AAC3223
Condica discistriga|[1094]|LBCH5254-10|10-JDWBC-5254|658[0n]bp|Canada.British Columbia|BOLD:AAC3223
Condica discistriga|[1095]|LBCH6046-10|10-JDWBC-6046|658[0n]bp|Canada.British Columbia|BOLD:AAC3223
Condica discistriga|[1096]|LBCH5557-10|10-JDWBC-5557|658[0n]bp|Canada.British Columbia|BOLD:AAC3223
Condica discistriga|[1097]|LBCH5523-10|10-JDWBC-5523|658[0n]bp|Canada.British Columbia|BOLD:AAC3223

Condica discistriga|[1094]|LBCH5254-10|10-JDWBC-5254|658[0n]bp|Canada.British Columbia|BOLD:AAC3223
Condica discistriga|[1095]|LBCH6046-10|10-JDWBC-6046|658[0n]bp|Canada.British Columbia|BOLD:AAC3223
Condica discistriga|[1096]|LBCH5557-10|10-JDWBC-5557|658[0n]bp|Canada.British Columbia|BOLD:AAC3223
Condica discistriga|[1097]|LBCH5523-10|10-JDWBC-5523|658[0n]bp|Canada.British Columbia|BOLD:AAC3223
Condica discistriga|[1098]|LBCH5999-10|10-JDWBC-5999|658[0n]bp|Canada.British Columbia|BOLD:AAC3223
Condica discistriga|[1099]|LBCH5039-10|10-JDWBC-5039|658[0n]bp|Canada.British Columbia|BOLD:AAC3223
Condica discistriga|[1100]|LBCH5574-10|10-JDWBC-5574|658[0n]bp|Canada.British Columbia|BOLD:AAC3223
Condica discistriga|[1101]|LBCH5998-10|10-JDWBC-5998|658[0n]bp|Canada.British Columbia|BOLD:AAC3223
Condica discistriga|[1102]|LBCH6093-10|10-JDWBC-6093|658[0n]bp|Canada.British Columbia|BOLD:AAC3223
Condica discistriga|[1103]|LBCH5525-10|10-JDWBC-5525|658[0n]bp|Canada.British Columbia|BOLD:AAC3223
Condica discistriga|[1104]|LBCH5526-10|10-JDWBC-5526|658[0n]bp|Canada.British Columbia|BOLD:AAC3223
Condica discistriga|[1105]|LBCH5347-10|10-JDWBC-5347|658[0n]bp|Canada.British Columbia|BOLD:AAC3223
Condica discistriga|[1106]|LOWCB869-05|CGWC-1809|648[0n]bp|Canada.British Columbia|BOLD:AAC3223
Condica discistriga|[1107]|LOWCB866-05|CGWC-1806|568[0n]bp|Canada.British Columbia|BOLD:AAC3223
Condica discistriga|[1108]|LOWCD117-06|CGWC-2937|597[0n]bp|Canada.British Columbia|BOLD:AAC3223
Condica discistriga|[1109]|LOWCB865-05|CGWC-1805|587[0n]bp|Canada.British Columbia|BOLD:AAC3223
Condica discistriga|[1110]|LOWCB867-05|CGWC-1807|565[0n]bp|Canada.British Columbia|BOLD:AAC3223
Condica discistriga|[1111]|LOWCB870-05|CGWC-1810|658[0n]bp|Canada.British Columbia|BOLD:AAC3223
Condica videns|[1112]|XAH696-05|2005-ONT-2279|658[0n]bp|Canada.Ontario|BOLD:AAA9166
Condica videns|[1113]|XAD628-05|2005-ONT-43|658[0n]bp|Canada.Ontario|BOLD:AAA9166
Condica videns|[1114]|XAG240-05|2005-ONT-824|658[0n]bp|Canada.Ontario|BOLD:AAA9166
Condica videns|[1115]|PHMO070-03|moth452.01|639[0n]bp|Canada.Ontario|BOLD:AAA9166
Condica videns|[1116]|XAH319-05|2005-ONT-1902|658[0n]bp|Canada.Ontario|BOLD:AAA9166
Condica videns|[1117]|RDLQF368-06|DH011435|658[0n]bp|Canada.Quebec|BOLD:AAA9166
Condica videns|[1118]|XAJ301-06|2006-ONT-0301|658[0n]bp|Canada.Ontario|BOLD:AAA9166
Condica videns|[1119]|RDLQF811-06|DH011961|658[0n]bp|Canada.Quebec|BOLD:AAA9166
Condica videns|[1120]|XAK305-06|2006-ONT-1300|658[0n]bp|Canada.Ontario|BOLD:AAA9166
Condica videns|[1121]|XAH458-05|2005-ONT-2041|658[0n]bp|Canada.Ontario|BOLD:AAA9166
Condica videns|[1122]|LPSO267-08|PPBP-0267|658[0n]bp|Canada.Ontario|BOLD:AAA9166
Condica videns|[1123]|XAB292-04|04HBL005292|658[0n]bp|Canada.Ontario|BOLD:AAA9166
Condica videns|[1124]|KPOEC181-08|08OEC-224|658[0n]bp|Canada.Ontario|BOLD:AAA9166
Condica videns|[1125]|RDLQB673-05|DH010776|658[0n]bp|Canada.Quebec|BOLD:AAA9166
Condica videns|[1126]|KPOEC072-08|08OEC-227|656[0n]bp|Canada.Ontario|BOLD:AAA9166
Condica videns|[1127]|PMG103-03|moth1130.01|617[0n]bp|Canada.Ontario|BOLD:AAA9166
Condica videns|[1128]|PHMO235-03|moth1184.01|639[0n]bp|Canada.Ontario|BOLD:AAA9166
Condica videns|[1129]|PHMO071-03|moth459.01|639[0n]bp|Canada.Ontario|BOLD:AAA9166
Condica videns|[1130]|XAK297-06|2006-ONT-1292|658[0n]bp|Canada.Ontario|BOLD:AAA9166
Condica videns|[1131]|LPSO687-08|PPBP-0687|658[0n]bp|Canada.Ontario|BOLD:AAA9166
Condica videns|[1132]|XAD629-05|2005-ONT-44|658[0n]bp|Canada.Ontario|BOLD:AAA9166
Condica videns|[1133]|XAB271-04|04HBL005271|658[0n]bp|Canada.Ontario|BOLD:AAA9166
Bagisara rectifascia|[1134]|RDNMF527-08|NOC14613|609[0n]bp|United States.Texas|BOLD:AAB2732
Bagisara rectifascia|[1135]|RDNMK481-11|CNCLEP 81912|658[0n]bp|United States.Massachusetts|BOLD:AAB2732
Bagisara rectifascia|[1136]|RDNMF526-08|NOC14612|658[0n]bp|United States.Texas|BOLD:AAB2732
Bagisara rectifascia|[1137]|HKONB417-09|3914-COI-08|658[0n]bp|United States.Indiana|BOLD:AAB2732
Bagisara rectifascia|[1138]|RDNMF525-08|NOC14611|609[0n]bp|United States.Maryland|BOLD:AAB2732
Bagisara rectifascia|[1139]|RDNMK480-11|CNCLEP 81911|602[0n]bp|United States.Connecticut|BOLD:AAB2732
Ogdoconta cinereola|[1140]|RDLQF926-06|DH012101|658[0n]bp|Canada.Quebec|BOLD:AAB2383
Ogdoconta cinereola|[1141]|XAB329-04|04HBL005329|658[0n]bp|Canada.Ontario|BOLD:AAB2383
Ogdoconta cinereola|[1142]|BLTIB153-08|BL227|658[0n]bp|Canada.Ontario|BOLD:AAB2383
Ogdoconta cinereola|[1143]|XAG318-05|2005-ONT-902|658[0n]bp|Canada.Ontario|BOLD:AAB2383
Ogdoconta cinereola|[1144]|XAF762-05|2005-ONT-411|658[0n]bp|Canada.Ontario|BOLD:AAB2383
Ogdoconta cinereola|[1145]|RDLQF558-06|DH011707|658[0n]bp|Canada.Quebec|BOLD:AAB2383
Ogdoconta cinereola|[1146]|XAD298-04|04HBL007298|592[0n]bp|Canada.Ontario|BOLD:AAB2383
Ogdoconta cinereola|[1147]|PHMO348-03|moth2655.02|639[2n]bp|Canada.Ontario|BOLD:AAB2383
Ogdoconta cinereola|[1148]|XAG916-05|2005-ONT-1500|658[0n]bp|Canada.Ontario|BOLD:AAB2383
Ogdoconta cinereola|[1149]|RDLQF927-06|DH012102|658[0n]bp|Canada.Quebec|BOLD:AAB2383
Ogdoconta cinereola|[1150]|XAB515-04|04HBL005515|658[0n]bp|Canada.Ontario|BOLD:AAB2383
Ogdoconta cinereola|[1151]|LPSO322-08|PPBP-0322|658[0n]bp|Canada.Ontario|BOLD:AAB2383
Ogdoconta cinereola|[1152]|XAG820-05|2005-ONT-1404|658[0n]bp|Canada.Ontario|BOLD:AAB2383
Ogdoconta cinereola|[1153]|RDLQB735-05|DH010838|658[0n]bp|Canada.Quebec|BOLD:AAB2383
Perigea xanthioides|[1154]|LGSM679-04|DNA-ATBI-0679|658[0n]bp|United States.Tennessee|BOLD:AAE6318
Perigea xanthioides|[1155]|LNCC1188-11|11-NCCC-713|658[0n]bp|United States.North Carolina|BOLD:AAE6318
Perigea xanthioides|[1156]|LGSMC680-05|DNA-ATBI-2680|658[0n]bp|United States.Tennessee|BOLD:AAE6318
Perigea xanthioides|[1157]|LGSMC939-05|DNA-ATBI-4019|638[0n]bp|United States.Tennessee|BOLD:AAE6318
Perigea xanthioides|[1158]|LPOKA464-09|MDOK-0464|658[0n]bp|United States.Oklahoma|BOLD:AAE6318
Perigea xanthioides|[1159]|LILLA814-11|SNS10IL-01021|658[0n]bp|United States.Illinois|BOLD:AAE6318
Azenia obtusa|[1160]|LPOKC300-09|MDOK-2377|658[0n]bp|United States.Oklahoma|BOLD:AAB6174
Azenia obtusa|[1161]|LSUSA140-06|06-SUSA-0140|658[0n]bp|United States.Kentucky|BOLD:AAB6174
Azenia obtusa|[1162]|LPOKA479-09|MDOK-0479|658[0n]bp|United States.Oklahoma|BOLD:AAB6174
Azenia obtusa|[1163]|LGSM775-04|DNA-ATBI-0775|658[0n]bp|United States.North Carolina|BOLD:AAB6174
Azenia obtusa|[1164]|BBLOC780-11|BIOUG01466-A02|658[0n]bp|United States.Arkansas|BOLD:AAB6174
Azenia obtusa|[1165]|LOFLA470-06|06-FLOR-0470|658[0n]bp|United States.Florida|BOLD:AAB6174
Azenia obtusa|[1166]|LOFLB012-06|06-FLOR-0952|658[0n]bp|United States.Florida|BOLD:AAB6174
Azenia obtusa|[1167]|LOFLB186-06|06-FLOR-1126|658[0n]bp|United States.Florida|BOLD:AAB6174
Azenia obtusa|[1168]|LNC012-05|05-NCCC-012|658[0n]bp|United States.North Carolina|BOLD:AAB6174
Azenia obtusa|[1169]|LNC014-05|05-NCCC-014|658[0n]bp|United States.North Carolina|BOLD:AAB6174
Azenia obtusa|[1170]|LNC013-05|05-NCCC-013|658[0n]bp|United States.North Carolina|BOLD:AAB6174
Azenia obtusa|[1171]|LOFLA700-06|06-FLOR-0700|658[0n]bp|United States.Florida|BOLD:AAB6174
Azenia obtusa|[1172]|LOFLB688-06|06-FLOR-1628|658[0n]bp|United States.Florida|BOLD:AAB6174
Azenia obtusa|[1173]|BBLOB981-11|BIOUG01414-C07|658[0n]bp|United States.Florida|BOLD:AAB6174
Azenia obtusa|[1174]|LNC196-05|05-NCCC-196|658[0n]bp|United States.North Carolina|BOLD:AAB6174
Azenia obtusa|[1175]|BBLOC840-11|BIOUG01466-F02|658[0n]bp|United States.Arkansas|BOLD:AAB6174
Azenia obtusa|[1176]|LPOKB486-09|MDOK-1494|658[0n]bp|United States.Oklahoma|BOLD:AAB6174
Azenia obtusa|[1177]|LOFLA916-06|06-FLOR-0916|658[0n]bp|United States.Florida|BOLD:AAB6174
Azenia obtusa|[1178]|LOFLA915-06|06-FLOR-0915|658[0n]bp|United States.Florida|BOLD:AAB6174
Azenia obtusa|[1179]|LOFLA268-06|06-FLOR-0268|658[0n]bp|United States.Florida|BOLD:AAB6174
Azenia obtusa|[1180]|LNC195-05|05-NCCC-195|658[0n]bp|United States.North Carolina|BOLD:AAB6174
Azenia obtusa|[1181]|LOFLA344-06|06-FLOR-0344|658[0n]bp|United States.Florida|BOLD:AAB6174
Azenia obtusa|[1182]|LILLA353-11|SNS10IL-00464|658[0n]bp|United States.Illinois|BOLD:AAB6174
Azenia obtusa|[1183]|LILLA421-11|SNS10IL-00547|658[0n]bp|United States.Illinois|BOLD:AAB6174
Azenia obtusa|[1184]|MECD345-06|jflandry2917|658[0n]bp|United States.Maryland|BOLD:AAB6174
Azenia obtusa|[1185]|BBLOB1454-11|BIOUG01419-C05|658[0n]bp|United States.Florida|BOLD:AAB6174
Azenia obtusa|[1186]|BBLSX230-09|09BBLEP-02158|658[0n]bp|United States.Texas|BOLD:AAB6174
Azenia obtusa|[1187]|BBLSY250-09|09BBLEP-03177|658[0n]bp|United States.Texas|BOLD:AAB6174
Azenia obtusa|[1188]|BBLSX796-09|09BBLEP-02724|658[0n]bp|United States.Texas|BOLD:AAB6174
Azenia obtusa|[1189]|BBLSX229-09|09BBLEP-02157|658[0n]bp|United States.Texas|BOLD:AAB6174
Micrathetis triplex|[1190]|RDNMJ007-10|CNCLEP 73787|658[0n]bp|Canada.Ontario|BOLD:AAB0866
Abrostola ovalis|[1191]|RDLQB562-05|DH010648|658[0n]bp|Canada.Quebec|BOLD:AAE9121
Abrostola urentis|[1192]|XAJ540-06|2006-ONT-0540|658[0n]bp|Canada.Ontario|BOLD:AAC4871
Abrostola urentis|[1193]|XAG317-05|2005-ONT-901|658[0n]bp|Canada.Ontario|BOLD:AAC4871
Abrostola urentis|[1194]|RDLQF206-06|DH011263|658[0n]bp|Canada.Quebec|BOLD:AAC4871
Abrostola urentis|[1195]|LPSOB817-08|PPBP-1816|658[0n]bp|Canada.Ontario|BOLD:AAC4871
Abrostola urentis|[1196]|RDLQB831-05|DH010918|658[0n]bp|Canada.Quebec|BOLD:AAC4871
[illegible]

Abrostola urentis|[1194]|RDLQF206-06|DH011263|658[0n]bp|Canada.Quebec|BOLD:AAC4871
Abrostola urentis|[1195]|LPSOB817-08|PPBP-1816|658[0n]bp|Canada.Ontario|BOLD:AAC4871
Abrostola urentis|[1196]|RDLQB831-05|DH010918|658[0n]bp|Canada.Quebec|BOLD:AAC4871
Abrostola urentis|[1197]|RDNM840-05|CNCNoctuoidea7680|658[0n]bp|Canada.Ontario|BOLD:AAC4871
Abrostola urentis|[1198]|XAK335-06|2006-ONT-1330|658[0n]bp|Canada.Ontario|BOLD:AAC4871
Abrostola urentis|[1199]|RDLQG055-06|DH012186|608[0n]bp|Canada.Quebec|BOLD:AAC4871
Abrostola urentis|[1200]|RDNM839-05|CNCNoctuoidea7679|588[0n]bp|Canada.Ontario|BOLD:AAC4871
Abrostola urentis|[1201]|RDNM841-05|CNCNoctuoidea7681|568[2n]bp|Canada.British Columbia|BOLD:AAC4871
Maliattha concinnimacula|[1202]|MEC713-04|jflandry0713|658[0n]bp|Canada.Quebec|BOLD:AAB4249
Maliattha concinnimacula|[1203]|LPSOB750-08|PPBP-1749|658[0n]bp|Canada.Ontario|BOLD:AAB4249
Maliattha concinnimacula|[1204]|RDLQG812-06|DH013105|658[0n]bp|Canada.Quebec|BOLD:AAB4249
Maliattha concinnimacula|[1205]|LPSOC196-08|PPBP-2195|658[0n]bp|Canada.Ontario|BOLD:AAB4249
Maliattha concinnimacula|[1206]|LPSOC223-08|PPBP-2222|658[0n]bp|Canada.Ontario|BOLD:AAB4249
Maliattha concinnimacula|[1207]|RDNMB256-05|CNCNoctuoidea10022|658[0n]bp|Canada.Ontario|BOLD:AAB4249
Maliattha concinnimacula|[1208]|LPSOC199-08|PPBP-2198|658[0n]bp|Canada.Ontario|BOLD:AAB4249
Maliattha concinnimacula|[1209]|LPSOC198-08|PPBP-2197|658[0n]bp|Canada.Ontario|BOLD:AAB4249
Maliattha concinnimacula|[1210]|LPSOC267-08|PPBP-2266|658[0n]bp|Canada.Ontario|BOLD:AAB4249
Maliattha concinnimacula|[1211]|PHMNB603-04|04HBL00829|658[0n]bp|Canada.New Brunswick|BOLD:AAB4249
Maliattha concinnimacula|[1212]|PHMNB494-04|04HBL00720|658[0n]bp|Canada.New Brunswick|BOLD:AAB4249
Maliattha concinnimacula|[1213]|TMNBB117-06|MNBTT-1057|658[0n]bp|Canada.New Brunswick|BOLD:AAB4249
Maliattha concinnimacula|[1214]|RDLQG824-06|DH013117|658[0n]bp|Canada.Quebec|BOLD:AAB4249
Maliattha concinnimacula|[1215]|TMNBB115-06|MNBTT-1055|658[0n]bp|Canada.New Brunswick|BOLD:AAB4249
Maliattha concinnimacula|[1216]|PHMNB706-04|04HBL00932|658[0n]bp|Canada.New Brunswick|BOLD:AAB4249
Maliattha concinnimacula|[1217]|RDLQG530-06|DH012823|658[0n]bp|Canada.Quebec|BOLD:AAB4249
Maliattha concinnimacula|[1218]|RDLQG529-06|DH012822|658[0n]bp|Canada.Quebec|BOLD:AAB4249
Maliattha concinnimacula|[1219]|MEC388-04|jflandry0388|658[0n]bp|Canada.Quebec|BOLD:AAB4249
Maliattha concinnimacula|[1220]|RDLQG528-06|DH012821|658[0n]bp|Canada.Quebec|BOLD:AAB4249
Maliattha concinnimacula|[1221]|TMNBB116-06|MNBTT-1056|658[0n]bp|Canada.New Brunswick|BOLD:AAB4249
Maliattha synochitis|[1222]|RDLQG843-06|DH013136|658[0n]bp|Canada.Quebec|BOLD:AAA4972
Maliattha synochitis|[1223]|BLTIB484-08|BL733|656[0n]bp|Canada.Ontario|BOLD:AAA4972
Maliattha synochitis|[1224]|TTMNB305-06|MNBTT-305|658[1n]bp|Canada.New Brunswick|BOLD:AAA4972
Maliattha synochitis|[1225]|BBLEC307-09|09BBELE-0307|658[0n]bp|Canada.Nova Scotia|BOLD:AAA4972
Maliattha synochitis|[1226]|PHMO177-03|moth942.01|639[0n]bp|Canada.Ontario|BOLD:AAA4972
Maliattha synochitis|[1227]|BBLEC607-09|09BBELE-0607|658[0n]bp|Canada.Nova Scotia|BOLD:AAA4972
Maliattha synochitis|[1228]|RDLQG518-06|DH012811|658[0n]bp|Canada.Quebec|BOLD:AAA4972
Maliattha synochitis|[1229]|BBLPC260-09|09BBELE-1260|658[0n]bp|Canada.Nova Scotia|BOLD:AAA4972
Maliattha synochitis|[1230]|BLTIB995-08|BL1432|658[0n]bp|Canada.Ontario|BOLD:AAA4972
Maliattha synochitis|[1231]|BBLPE040-09|09BBELE-2040|658[0n]bp|Canada.Nova Scotia|BOLD:AAA4972
Maliattha synochitis|[1232]|PHMNB344-04|04HBL00570|658[0n]bp|Canada.New Brunswick|BOLD:AAA4972
Maliattha synochitis|[1233]|TTMNB302-06|MNBTT-302|657[0n]bp|Canada.New Brunswick|BOLD:AAA4972
Maliattha synochitis|[1234]|RDLQG688-06|DH012981|658[0n]bp|Canada.Quebec|BOLD:AAA4972
Maliattha synochitis|[1235]|XAG294-05|2005-ONT-878|658[0n]bp|Canada.Ontario|BOLD:AAA4972
Maliattha synochitis|[1236]|LPSO767-08|PPBP-0767|609[0n]bp|Canada.Ontario|BOLD:AAA4972
Maliattha synochitis|[1237]|LPSO716-08|PPBP-0716|658[0n]bp|Canada.Ontario|BOLD:AAA4972
Maliattha synochitis|[1238]|BLTIB250-08|BL432|615[1n]bp|Canada.Ontario|BOLD:AAA4972
Maliattha synochitis|[1239]|LPSO766-08|PPBP-0766|609[0n]bp|Canada.Ontario|BOLD:AAA4972
Maliattha synochitis|[1240]|LPSO768-08|PPBP-0768|609[0n]bp|Canada.Ontario|BOLD:AAA4972
Maliattha synochitis|[1241]|XAE380-04|Moth4380.03|520[1n]bp|Canada.Ontario|BOLD:AAA4972
Maliattha synochitis|[1242]|XAE437-04|Moth4437.03|615[0n]bp|Canada.Ontario|BOLD:AAA4972
Maliattha synochitis|[1243]|PHMNB657-04|04HBL00883|624[0n]bp|Canada.New Brunswick|BOLD:AAA4972
Maliattha synochitis|[1244]|BLTIB605-08|BL885|635[0n]bp|Canada.Ontario|BOLD:AAA4972
Maliattha synochitis|[1245]|BLTIB604-08|BL884|634[0n]bp|Canada.Ontario|BOLD:AAA4972
Maliattha synochitis|[1246]|TTMNB304-06|MNBTT-304|658[4n]bp|Canada.New Brunswick|BOLD:AAA4972
Maliattha synochitis|[1247]|RDLQG517-06|DH012810|658[0n]bp|Canada.Quebec|BOLD:AAA4972
Maliattha synochitis|[1248]|MNBB209-05|05-NBSTA-125|658[0n]bp|Canada.New Brunswick|BOLD:AAA4972
Maliattha synochitis|[1249]|PHMNB059-03|moth37.02SA|639[0n]bp|Canada.New Brunswick|BOLD:AAA4972
Maliattha synochitis|[1250]|LPSO392-08|PPBP-0392|658[0n]bp|Canada.Ontario|BOLD:AAA4972
Maliattha synochitis|[1251]|PHMNB393-04|04HBL00619|658[0n]bp|Canada.New Brunswick|BOLD:AAA4972
Maliattha synochitis|[1252]|PHMNB489-04|04HBL00715|658[0n]bp|Canada.New Brunswick|BOLD:AAA4972
Maliattha synochitis|[1253]|PHMNB397-04|04HBL00623|658[0n]bp|Canada.New Brunswick|BOLD:AAA4972
Maliattha synochitis|[1254]|TMNBB114-06|MNBTT-1054|658[0n]bp|Canada.New Brunswick|BOLD:AAA4972
Maliattha synochitis|[1255]|XAF507-05|2005-ONT-156|658[0n]bp|Canada.Ontario|BOLD:AAA4972
Maliattha synochitis|[1256]|BLGSM048-09|BL367|658[0n]bp|Canada.Ontario|BOLD:AAA4972
Maliattha synochitis|[1257]|BLTIB262-08|BL446|658[0n]bp|Canada.Ontario|BOLD:AAA4972
Maliattha synochitis|[1258]|BLTIB157-08|BL231|658[0n]bp|Canada.Ontario|BOLD:AAA4972
Maliattha synochitis|[1259]|BLTIB493-08|BL746|658[0n]bp|Canada.Ontario|BOLD:AAA4972
Maliattha synochitis|[1260]|BLTIB261-08|BL445|658[0n]bp|Canada.Ontario|BOLD:AAA4972
Maliattha synochitis|[1261]|LPSOD299-09|08BBLEP-00077|658[0n]bp|Canada.Ontario|BOLD:AAA4972
Maliattha synochitis|[1262]|BLTIB274-08|BL458|657[0n]bp|Canada.Ontario|BOLD:AAA4972
Maliattha synochitis|[1263]|XAF792-05|2005-ONT-441|658[0n]bp|Canada.Ontario|BOLD:AAA4972
Maliattha synochitis|[1264]|LPSO626-08|PPBP-0626|658[0n]bp|Canada.Ontario|BOLD:AAA4972
Maliattha synochitis|[1265]|LPSOB915-08|PPBP-1914|658[0n]bp|Canada.Ontario|BOLD:AAA4972
Maliattha synochitis|[1266]|XAB375-04|04HBL005375|658[0n]bp|Canada.Ontario|BOLD:AAA4972
Maliattha synochitis|[1267]|RDLQG831-06|DH013124|658[0n]bp|Canada.Quebec|BOLD:AAA4972
Maliattha synochitis|[1268]|RDLQG728-06|DH013021|658[0n]bp|Canada.Quebec|BOLD:AAA4972
Maliattha synochitis|[1269]|BLTIB930-08|BL1350|658[0n]bp|Canada.Ontario|BOLD:AAA4972
Maliattha synochitis|[1270]|PHMTV434-10|10PHMAL-2534|658[0n]bp|Canada.Ontario|BOLD:AAA4972
Maliattha synochitis|[1271]|XAF706-05|2005-ONT-355|658[0n]bp|Canada.Ontario|BOLD:AAA4972
Maliattha synochitis|[1272]|RDLQG834-06|DH013127|658[0n]bp|Canada.Quebec|BOLD:AAA4972
Maliattha synochitis|[1273]|TMNBB480-06|MNBTT-1420|658[0n]bp|Canada.New Brunswick|BOLD:AAA4972
Maliattha synochitis|[1274]|BLTIB347-08|BL565|658[0n]bp|Canada.Ontario|BOLD:AAA4972
Maliattha synochitis|[1275]|RDLQG835-06|DH013128|658[0n]bp|Canada.Quebec|BOLD:AAA4972
Maliattha synochitis|[1276]|PHMNB550-04|04HBL00776|658[0n]bp|Canada.New Brunswick|BOLD:AAA4972
Maliattha synochitis|[1277]|MEC712-04|jflandry0712|658[0n]bp|Canada.Quebec|BOLD:AAA4972
Maliattha synochitis|[1278]|LPSOB603-08|PPBP-1602|658[0n]bp|Canada.Ontario|BOLD:AAA4972
Maliattha synochitis|[1279]|PHMNB398-04|04HBL00624|658[0n]bp|Canada.New Brunswick|BOLD:AAA4972
Maliattha synochitis|[1280]|LPSO911-08|PPBP-0911|658[0n]bp|Canada.Ontario|BOLD:AAA4972
Maliattha synochitis|[1281]|PHJUN3374-11|BIOUG01486-B06|658[0n]bp|Canada.Ontario|BOLD:AAA4972
Maliattha synochitis|[1282]|BLTIB361-08|BL579|658[0n]bp|Canada.Ontario|BOLD:AAA4972
Maliattha synochitis|[1283]|LPSOB576-08|PPBP-1575|658[0n]bp|Canada.Ontario|BOLD:AAA4972
Maliattha synochitis|[1284]|LPSO931-08|PPBP-0931|658[0n]bp|Canada.Ontario|BOLD:AAA4972
Maliattha synochitis|[1285]|XAD677-05|2005-ONT-92|658[0n]bp|Canada.Ontario|BOLD:AAA4972
Maliattha synochitis|[1286]|LPSO625-08|PPBP-0625|658[0n]bp|Canada.Ontario|BOLD:AAA4972
Maliattha synochitis|[1287]|XAD728-05|2005-ONT-527|658[0n]bp|Canada.Ontario|BOLD:AAA4972
Maliattha synochitis|[1288]|BLTIB487-08|BL736|658[0n]bp|Canada.Ontario|BOLD:AAA4972
Maliattha synochitis|[1289]|LPSOB914-08|PPBP-1913|658[0n]bp|Canada.Ontario|BOLD:AAA4972
Maliattha synochitis|[1290]|BLTIB244-08|BL426|658[0n]bp|Canada.Ontario|BOLD:AAA4972
Maliattha synochitis|[1291]|LPSO453-08|PPBP-0453|658[0n]bp|Canada.Ontario|BOLD:AAA4972
Maliattha synochitis|[1292]|PHMNB664-04|04HBL00890|658[0n]bp|Canada.New Brunswick|BOLD:AAA4972
Maliattha synochitis|[1293]|LPSO205-08|PPBP-0205|658[0n]bp|Canada.Ontario|BOLD:AAA4972
Maliattha synochitis|[1294]|RDLQB777-05|DH010864|658[0n]bp|Canada.Quebec|BOLD:AAA4972
Deltote bellicula|[1295]|RDLQE263-06|MDH002266|656[0n]bp|Canada.Quebec|BOLD:AAB6909
Deltote bellicula|[1296]|RDLQE264-06|MDH002267|657[0n]bp|Canada.Quebec|BOLD:AAB6909

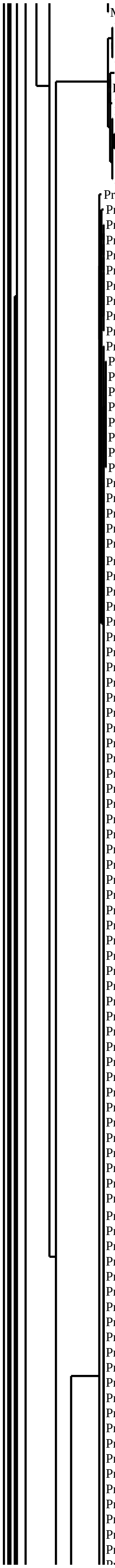

Maliattha synochitis|[1294]|RDLQB777-05|DH010864|658[0n]bp|Canada.Quebec|BOLD:AAA4972
Deltote bellicula|[1295]|RDLQE263-06|MDH002266|656[0n]bp|Canada.Quebec|BOLD:AAB6909
Deltote bellicula|[1296]|RDLQE264-06|MDH002267|657[0n]bp|Canada.Quebec|BOLD:AAB6909
Deltote bellicula|[1297]|RDLQG673-06|DH012966|658[0n]bp|Canada.Quebec|BOLD:AAB6909
Deltote bellicula|[1298]|XAJ873-06|2006-ONT-0873|658[0n]bp|Canada.Ontario|BOLD:AAB6909
Deltote bellicula|[1299]|RDLQG700-06|DH012993|658[0n]bp|Canada.Quebec|BOLD:AAB6909
Deltote bellicula|[1300]|XAD784-05|2005-ONT-583|658[0n]bp|Canada.Ontario|BOLD:AAB6909
Deltote bellicula|[1301]|RDLQG896-06|DH013189|658[0n]bp|Canada.Quebec|BOLD:AAB6909
Deltote bellicula|[1302]|RDLQG898-06|DH013191|658[0n]bp|Canada.Quebec|BOLD:AAB6909
Deltote bellicula|[1303]|RDLQB811-05|DH010898|658[0n]bp|Canada.Quebec|BOLD:AAB6909
Deltote bellicula|[1304]|RDLQE262-06|MDH002265|658[0n]bp|Canada.Quebec|BOLD:AAB6909
Deltote bellicula|[1305]|RDLQG897-06|DH013190|658[0n]bp|Canada.Quebec|BOLD:AAB6909
Protodeltote albidula|[1306]|BBLPE470-09|09BBELE-2470|658[0n]bp|Canada.Newfoundland and Labrador|BOLD ...
Protodeltote albidula|[1307]|BBLPD777-10|10BBCLP-2775|658[0n]bp|Canada.British Columbia|BOLD:AAA2331
Protodeltote albidula|[1308]|BBLPB983-10|10BBCLP-1982|658[0n]bp|Canada.Alberta|BOLD:AAA2331
Protodeltote albidula|[1309]|BBLPB990-10|10BBCLP-1989|658[0n]bp|Canada.Alberta|BOLD:AAA2331
Protodeltote albidula|[1310]|BBLPB989-10|10BBCLP-1988|658[0n]bp|Canada.Alberta|BOLD:AAA2331
Protodeltote albidula|[1311]|BBLPD147-10|10BBCLP-2145|658[0n]bp|Canada.Saskatchewan|BOLD:AAA2331
Protodeltote albidula|[1312]|LPMN657-08|08BBLEP-01458|658[0n]bp|Canada.Manitoba|BOLD:AAA2331
Protodeltote albidula|[1313]|LPMN622-08|08BBLEP-01423|658[0n]bp|Canada.Manitoba|BOLD:AAA2331
Protodeltote albidula|[1314]|BBLPD143-10|10BBCLP-2141|658[0n]bp|Canada.Saskatchewan|BOLD:AAA2331
Protodeltote albidula|[1315]|BBLPD154-10|10BBCLP-2152|658[0n]bp|Canada.Saskatchewan|BOLD:AAA2331
Protodeltote albidula|[1316]|LBCA066-05|HLC-20066|658[0n]bp|Canada.British Columbia|BOLD:AAA2331
Protodeltote albidula|[1317]|LBCA895-05|HLC-20895|658[1n]bp|Canada.British Columbia|BOLD:AAA2331
Protodeltote albidula|[1318]|LBCA518-05|HLC-20518|658[0n]bp|Canada.British Columbia|BOLD:AAA2331
Protodeltote albidula|[1319]|LBCA006-05|HLC-20006|658[0n]bp|Canada.British Columbia|BOLD:AAA2331
Protodeltote albidula|[1320]|LBCA411-05|HLC-20411|658[0n]bp|Canada.British Columbia|BOLD:AAA2331
Protodeltote albidula|[1321]|LBCA409-05|HLC-20409|658[0n]bp|Canada.British Columbia|BOLD:AAA2331
Protodeltote albidula|[1322]|LBCA057-05|HLC-20057|658[0n]bp|Canada.British Columbia|BOLD:AAA2331
Protodeltote albidula|[1323]|LBCA413-05|HLC-20413|658[0n]bp|Canada.British Columbia|BOLD:AAA2331
Protodeltote albidula|[1324]|LBCA521-05|HLC-20521|658[0n]bp|Canada.British Columbia|BOLD:AAA2331
Protodeltote albidula|[1325]|LBCA526-05|HLC-20526|658[0n]bp|Canada.British Columbia|BOLD:AAA2331
Protodeltote albidula|[1326]|LBCW029-08|08-JDWWI-0029|658[0n]bp|Canada.British Columbia|BOLD:AAA2331
Protodeltote albidula|[1327]|BBLPB982-10|10BBCLP-1981|658[0n]bp|Canada.Alberta|BOLD:AAA2331
Protodeltote albidula|[1328]|LBCA134-05|HLC-20134|658[0n]bp|Canada.British Columbia|BOLD:AAA2331
Protodeltote albidula|[1329]|RDLQG434-06|DH012713|658[0n]bp|Canada.Quebec|BOLD:AAA2331
Protodeltote albidula|[1330]|LBCA058-05|HLC-20058|658[0n]bp|Canada.British Columbia|BOLD:AAA2331
Protodeltote albidula|[1331]|LBCA064-05|HLC-20064|658[0n]bp|Canada.British Columbia|BOLD:AAA2331
Protodeltote albidula|[1332]|LBCA414-05|HLC-20414|658[0n]bp|Canada.British Columbia|BOLD:AAA2331
Protodeltote albidula|[1333]|LBCD608-05|HLC-23428|658[0n]bp|Canada.British Columbia|BOLD:AAA2331
Protodeltote albidula|[1334]|LBCA008-05|HLC-20008|658[0n]bp|Canada.British Columbia|BOLD:AAA2331
Protodeltote albidula|[1335]|LHLEP087-06|UBC-2006-0292|658[0n]bp|Canada.British Columbia|BOLD:AAA2331
Protodeltote albidula|[1336]|BBLPD622-10|10BBCLP-2620|658[0n]bp|Canada.British Columbia|BOLD:AAA2331
Protodeltote albidula|[1337]|LBCW028-08|08-JDWWI-0028|658[0n]bp|Canada.British Columbia|BOLD:AAA2331
Protodeltote albidula|[1338]|LBCA419-05|HLC-20419|658[0n]bp|Canada.British Columbia|BOLD:AAA2331
Protodeltote albidula|[1339]|LBCA527-05|HLC-20527|658[0n]bp|Canada.British Columbia|BOLD:AAA2331
Protodeltote albidula|[1340]|RDLQG674-06|DH012967|658[0n]bp|Canada.Quebec|BOLD:AAA2331
Protodeltote albidula|[1341]|LBCA060-05|HLC-20060|658[0n]bp|Canada.British Columbia|BOLD:AAA2331
Protodeltote albidula|[1342]|LHLEP238-06|UBC-2006-0356|658[0n]bp|Canada.British Columbia|BOLD:AAA2331
Protodeltote albidula|[1343]|LBCA007-05|HLC-20007|658[0n]bp|Canada.British Columbia|BOLD:AAA2331
Protodeltote albidula|[1344]|LPSOD618-09|08BBLEP-00399|658[0n]bp|Canada.Ontario|BOLD:AAA2331
Protodeltote albidula|[1345]|RDLQG675-06|DH012968|658[0n]bp|Canada.Quebec|BOLD:AAA2331
Protodeltote albidula|[1346]|LBCA525-05|HLC-20525|658[0n]bp|Canada.British Columbia|BOLD:AAA2331
Protodeltote albidula|[1347]|LPMN524-08|08BBLEP-01323|658[0n]bp|Canada.Manitoba|BOLD:AAA2331
Protodeltote albidula|[1348]|LBCA897-05|HLC-20897|658[0n]bp|Canada.British Columbia|BOLD:AAA2331
Protodeltote albidula|[1349]|LPMN489-08|08BBLEP-01288|658[0n]bp|Canada.Manitoba|BOLD:AAA2331
Protodeltote albidula|[1350]|LBCA068-05|HLC-20068|658[0n]bp|Canada.British Columbia|BOLD:AAA2331
Protodeltote albidula|[1351]|LPMN492-08|08BBLEP-01291|658[0n]bp|Canada.Manitoba|BOLD:AAA2331
Protodeltote albidula|[1352]|LBCA410-05|HLC-20410|658[0n]bp|Canada.British Columbia|BOLD:AAA2331
Protodeltote albidula|[1353]|LBCD605-05|HLC-23425|658[0n]bp|Canada.British Columbia|BOLD:AAA2331
Protodeltote albidula|[1354]|LBCA003-05|HLC-20003|658[0n]bp|Canada.British Columbia|BOLD:AAA2331
Protodeltote albidula|[1355]|BBLPE156-09|09BBELE-2156|658[0n]bp|Canada.Nova Scotia|BOLD:AAA2331
Protodeltote albidula|[1356]|RDLQG432-06|DH012711|658[0n]bp|Canada.Quebec|BOLD:AAA2331
Protodeltote albidula|[1357]|LBCA422-05|HLC-20422|658[0n]bp|Canada.British Columbia|BOLD:AAA2331
Protodeltote albidula|[1358]|LBCA004-05|HLC-20004|658[0n]bp|Canada.British Columbia|BOLD:AAA2331
Protodeltote albidula|[1359]|BBLPD151-10|10BBCLP-2149|658[0n]bp|Canada.Saskatchewan|BOLD:AAA2331
Protodeltote albidula|[1360]|BBLPD197-10|10BBCLP-2195|658[0n]bp|Canada.British Columbia|BOLD:AAA2331
Protodeltote albidula|[1361]|LBCD581-05|HLC-23401|658[0n]bp|Canada.British Columbia|BOLD:AAA2331
Protodeltote albidula|[1362]|BBLPC302-09|09BBELE-1302|658[0n]bp|Canada.Newfoundland and Labrador|BOLD ...
Protodeltote albidula|[1363]|LBCA065-05|HLC-20065|658[0n]bp|Canada.British Columbia|BOLD:AAA2331
Protodeltote albidula|[1364]|LBCA069-05|HLC-20069|658[0n]bp|Canada.British Columbia|BOLD:AAA2331
Protodeltote albidula|[1365]|LOWCB180-05|CGWC-1120|658[0n]bp|Canada.British Columbia|BOLD:AAA2331
Protodeltote albidula|[1366]|BBLPD775-10|10BBCLP-2773|658[0n]bp|Canada.British Columbia|BOLD:AAA2331
Protodeltote albidula|[1367]|LBCA005-05|HLC-20005|658[0n]bp|Canada.British Columbia|BOLD:AAA2331
Protodeltote albidula|[1368]|LBCD602-05|HLC-23422|658[0n]bp|Canada.British Columbia|BOLD:AAA2331
Protodeltote albidula|[1369]|BBLPD146-10|10BBCLP-2144|658[0n]bp|Canada.Saskatchewan|BOLD:AAA2331
Protodeltote albidula|[1370]|LBCD603-05|HLC-23423|658[0n]bp|Canada.British Columbia|BOLD:AAA2331
Protodeltote albidula|[1371]|LBCA529-05|HLC-20529|658[0n]bp|Canada.British Columbia|BOLD:AAA2331
Protodeltote albidula|[1372]|BBLPC659-09|09BBELE-1659|658[0n]bp|Canada.Newfoundland and Labrador|BOLD ...
Protodeltote albidula|[1373]|LBCA071-05|HLC-20071|658[0n]bp|Canada.British Columbia|BOLD:AAA2331
Protodeltote albidula|[1374]|BBLPD628-10|10BBCLP-2626|658[0n]bp|Canada.British Columbia|BOLD:AAA2331
Protodeltote albidula|[1375]|TMNBB111-06|MNBTT-1051|658[0n]bp|Canada.New Brunswick|BOLD:AAA2331
Protodeltote albidula|[1376]|BBLPD432-10|10BBCLP-2430|658[0n]bp|Canada.Ontario|BOLD:AAA2331
Protodeltote albidula|[1377]|BBLPD199-10|10BBCLP-2197|658[0n]bp|Canada.British Columbia|BOLD:AAA2331
Protodeltote albidula|[1378]|LBCA063-05|HLC-20063|658[0n]bp|Canada.British Columbia|BOLD:AAA2331
Protodeltote albidula|[1379]|LBCA421-05|HLC-20421|658[0n]bp|Canada.British Columbia|BOLD:AAA2331
Protodeltote albidula|[1380]|LBCA056-05|HLC-20056|658[0n]bp|Canada.British Columbia|BOLD:AAA2331
Protodeltote albidula|[1381]|BBLPC738-09|09BBELE-1738|658[0n]bp|Canada.Newfoundland and Labrador|BOLD ...
Protodeltote albidula|[1382]|LBCA054-05|HLC-20054|658[0n]bp|Canada.British Columbia|BOLD:AAA2331
Protodeltote albidula|[1383]|LBCA412-05|HLC-20412|658[0n]bp|Canada.British Columbia|BOLD:AAA2331
Protodeltote albidula|[1384]|LPSOD692-09|08BBLEP-00473|658[0n]bp|Canada.Ontario|BOLD:AAA2331
Protodeltote albidula|[1385]|BBLPC614-09|09BBELE-1614|658[0n]bp|Canada.Nova Scotia|BOLD:AAA2331
Protodeltote albidula|[1386]|BBLPC821-09|09BBELE-1821|658[0n]bp|Canada.Newfoundland and Labrador|BOLD ...
Protodeltote albidula|[1387]|LBCA067-05|HLC-20067|658[0n]bp|Canada.British Columbia|BOLD:AAA2331
Protodeltote albidula|[1388]|LBCA417-05|HLC-20417|658[0n]bp|Canada.British Columbia|BOLD:AAA2331
Protodeltote albidula|[1389]|BBLPD198-10|10BBCLP-2196|658[0n]bp|Canada.British Columbia|BOLD:AAA2331
Protodeltote albidula|[1390]|BBLPD153-10|10BBCLP-2151|658[0n]bp|Canada.Saskatchewan|BOLD:AAA2331
Protodeltote albidula|[1391]|LOWCE338-06|CGWC-4098|658[0n]bp|Canada.British Columbia|BOLD:AAA2331
Protodeltote albidula|[1392]|LBCD604-05|HLC-23424|658[0n]bp|Canada.British Columbia|BOLD:AAA2331
Protodeltote albidula|[1393]|BBLPB984-10|10BBCLP-1983|658[0n]bp|Canada.Alberta|BOLD:AAA2331
Protodeltote albidula|[1394]|LBCA010-05|HLC-20010|658[0n]bp|Canada.British Columbia|BOLD:AAA2331
Protodeltote albidula|[1395]|LBCA420-05|HLC-20420|658[0n]bp|Canada.British Columbia|BOLD:AAA2331
Protodeltote albidula|[1396]|LBCA009-05|HLC-20009|658[0n]bp|Canada.British Columbia|BOLD:AAA2331

Protodeltote albidula|[1393]|BBLPD564-10|10BBCLP-1563|658[0n]bp|Canada.Alberta|BOLD:AAA2331
Protodeltote albidula|[1394]|LBCA010-05|HLC-20010|658[0n]bp|Canada.British Columbia|BOLD:AAA2331
Protodeltote albidula|[1395]|LBCA420-05|HLC-20420|658[0n]bp|Canada.British Columbia|BOLD:AAA2331
Protodeltote albidula|[1396]|LBCA009-05|HLC-20009|658[0n]bp|Canada.British Columbia|BOLD:AAA2331
Protodeltote albidula|[1397]|BBLPE473-09|09BBELE-2473|658[0n]bp|Canada.Newfoundland and Labrador|BOLD ...
Protodeltote albidula|[1398]|LBCA416-05|HLC-20416|658[0n]bp|Canada.British Columbia|BOLD:AAA2331
Protodeltote albidula|[1399]|LBCA522-05|HLC-20522|658[0n]bp|Canada.British Columbia|BOLD:AAA2331
Protodeltote albidula|[1400]|LBCA061-05|HLC-20061|658[0n]bp|Canada.British Columbia|BOLD:AAA2331
Protodeltote albidula|[1401]|BBLPD624-10|10BBCLP-2622|658[0n]bp|Canada.British Columbia|BOLD:AAA2331
Protodeltote albidula|[1402]|BBLPC737-09|09BBELE-1737|658[0n]bp|Canada.Newfoundland and Labrador|BOLD ...
Protodeltote albidula|[1403]|RDLQG431-06|DH012710|658[0n]bp|Canada.Quebec|BOLD:AAA2331
Protodeltote albidula|[1404]|LBCA425-05|HLC-20425|658[0n]bp|Canada.British Columbia|BOLD:AAA2331
Protodeltote albidula|[1405]|LOWCB164-05|CGWC-1104|658[0n]bp|Canada.British Columbia|BOLD:AAA2331
Protodeltote albidula|[1406]|BBLPD148-10|10BBCLP-2146|658[0n]bp|Canada.Saskatchewan|BOLD:AAA2331
Protodeltote albidula|[1407]|BBLPC721-09|09BBELE-1721|658[0n]bp|Canada.Newfoundland and Labrador|BOLD ...
Protodeltote albidula|[1408]|BBLPD145-10|10BBCLP-2143|658[0n]bp|Canada.Saskatchewan|BOLD:AAA2331
Protodeltote albidula|[1409]|RDLQG532-06|DH012825|658[0n]bp|Canada.Quebec|BOLD:AAA2331
Protodeltote albidula|[1410]|LPABB279-08|08BBLEP-03544|658[0n]bp|Canada.Alberta|BOLD:AAA2331
Protodeltote albidula|[1411]|RDLQG531-06|DH012824|658[0n]bp|Canada.Quebec|BOLD:AAA2331
Protodeltote albidula|[1412]|LBCD606-05|HLC-23426|658[0n]bp|Canada.British Columbia|BOLD:AAA2331
Protodeltote albidula|[1413]|LBCA062-05|HLC-20062|658[0n]bp|Canada.British Columbia|BOLD:AAA2331
Protodeltote albidula|[1414]|LBCA520-05|HLC-20520|658[0n]bp|Canada.British Columbia|BOLD:AAA2331
Protodeltote albidula|[1415]|RDLQG433-06|DH012712|658[0n]bp|Canada.Quebec|BOLD:AAA2331
Protodeltote albidula|[1416]|BBLPC782-09|09BBELE-1782|658[0n]bp|Canada.Newfoundland and Labrador|BOLD ...
Protodeltote albidula|[1417]|LPABB232-08|08BBLEP-03497|658[0n]bp|Canada.Alberta|BOLD:AAA2331
Protodeltote albidula|[1418]|LBCA523-05|HLC-20523|658[0n]bp|Canada.British Columbia|BOLD:AAA2331
Protodeltote albidula|[1419]|BBLPD438-10|10BBCLP-2436|658[0n]bp|Canada.Ontario|BOLD:AAA2331
Protodeltote albidula|[1420]|LBCA059-05|HLC-20059|658[0n]bp|Canada.British Columbia|BOLD:AAA2331
Protodeltote albidula|[1421]|LBCA055-05|HLC-20055|658[0n]bp|Canada.British Columbia|BOLD:AAA2331
Protodeltote albidula|[1422]|BBLPD152-10|10BBCLP-2150|658[0n]bp|Canada.Saskatchewan|BOLD:AAA2331
Protodeltote albidula|[1423]|LPMN491-08|08BBLEP-01290|658[0n]bp|Canada.Manitoba|BOLD:AAA2331
Protodeltote albidula|[1424]|LPABB272-08|08BBLEP-03537|658[0n]bp|Canada.Alberta|BOLD:AAA2331
Protodeltote albidula|[1425]|BBLPD776-10|10BBCLP-2774|658[0n]bp|Canada.British Columbia|BOLD:AAA2331
Protodeltote albidula|[1426]|LBCA418-05|HLC-20418|658[0n]bp|Canada.British Columbia|BOLD:AAA2331
Protodeltote albidula|[1427]|BBLPE401-09|09BBELE-2401|658[0n]bp|Canada.Newfoundland and Labrador|BOLD ...
Protodeltote albidula|[1428]|LBCD607-05|HLC-23427|658[0n]bp|Canada.British Columbia|BOLD:AAA2331
Protodeltote albidula|[1429]|LHLEP247-06|UBC-2006-1555|658[0n]bp|Canada.British Columbia|BOLD:AAA2331
Protodeltote albidula|[1430]|LPMN616-08|08BBLEP-01417|658[0n]bp|Canada.Manitoba|BOLD:AAA2331
Protodeltote albidula|[1431]|RDLQB258-05|DH010344|658[0n]bp|Canada.Quebec|BOLD:AAA2331
Protodeltote albidula|[1432]|BBLPD200-10|10BBCLP-2198|658[0n]bp|Canada.British Columbia|BOLD:AAA2331
Protodeltote albidula|[1433]|LBCA415-05|HLC-20415|658[0n]bp|Canada.British Columbia|BOLD:AAA2331
Protodeltote albidula|[1434]|LOWCB852-05|CGWC-1792|658[0n]bp|Canada.British Columbia|BOLD:AAA2331
Protodeltote albidula|[1435]|TTMNB300-06|MNBTT-300|658[1n]bp|Canada.New Brunswick|BOLD:AAA2331
Protodeltote albidula|[1436]|LOWCC870-05|CGWC-2750|658[0n]bp|Canada.British Columbia|BOLD:AAA2331
Protodeltote albidula|[1437]|BBLPD626-10|10BBCLP-2624|639[0n]bp|Canada.British Columbia|BOLD:AAA2331
Protodeltote albidula|[1438]|LBCA894-05|HLC-20894|615[0n]bp|Canada.British Columbia|BOLD:AAA2331
Protodeltote albidula|[1439]|LOWCB853-05|CGWC-1793|560[0n]bp|Canada.British Columbia|BOLD:AAA2331
Protodeltote albidula|[1440]|LOWCB851-05|CGWC-1791|592[0n]bp|Canada.British Columbia|BOLD:AAA2331
Protodeltote albidula|[1441]|LOWCB849-05|CGWC-1789|574[0n]bp|Canada.British Columbia|BOLD:AAA2331
Protodeltote albidula|[1442]|LOWCB850-05|CGWC-1790|574[0n]bp|Canada.British Columbia|BOLD:AAA2331
Protodeltote albidula|[1443]|BBLPD440-10|10BBCLP-2438|658[0n]bp|Canada.Ontario|BOLD:AAA2331
Protodeltote albidula|[1444]|TMNBB110-06|MNBTT-1050|658[0n]bp|Canada.New Brunswick|BOLD:AAA2331
Protodeltote albidula|[1445]|TMNBB112-06|MNBTT-1052|658[0n]bp|Canada.New Brunswick|BOLD:AAA2331
Protodeltote albidula|[1446]|RDLQG559-06|DH012852|658[0n]bp|Canada.Quebec|BOLD:AAA2331
Protodeltote albidula|[1447]|XAJ782-06|2006-ONT-0782|658[0n]bp|Canada.Ontario|BOLD:AAA2331
Protodeltote albidula|[1448]|TMNBB113-06|MNBTT-1053|658[0n]bp|Canada.New Brunswick|BOLD:AAA2331
Protodeltote albidula|[1449]|TTMNB303-06|MNBTT-303|658[1n]bp|Canada.New Brunswick|BOLD:AAA2331
Protodeltote albidula|[1450]|TTMNB301-06|MNBTT-301|658[0n]bp|Canada.New Brunswick|BOLD:AAA2331
Protodeltote albidula|[1451]|XAB149-04|04HBL005149|658[0n]bp|Canada.Ontario|BOLD:AAA2331
Protodeltote albidula|[1452]|XAB132-04|04HBL005132|658[0n]bp|Canada.Ontario|BOLD:AAA2331
Protodeltote albidula|[1453]|XAF588-05|2005-ONT-237|658[0n]bp|Canada.Ontario|BOLD:AAA2331
Protodeltote albidula|[1454]|RDLQG595-06|DH012888|658[0n]bp|Canada.Quebec|BOLD:AAA2331
Protodeltote albidula|[1455]|XAG265-05|2005-ONT-849|658[0n]bp|Canada.Ontario|BOLD:AAA2331
Protodeltote albidula|[1456]|RDLQG908-06|DH013201|658[0n]bp|Canada.Quebec|BOLD:AAA2331
Protodeltote albidula|[1457]|RDLQG572-06|DH012865|658[0n]bp|Canada.Quebec|BOLD:AAA2331
Protodeltote albidula|[1458]|RDLQG799-06|DH013092|658[0n]bp|Canada.Quebec|BOLD:AAA2331
Protodeltote albidula|[1459]|LPMN559-08|08BBLEP-01360|658[0n]bp|Canada.Manitoba|BOLD:AAA2331
Protodeltote albidula|[1460]|RDLQG803-06|DH013096|658[0n]bp|Canada.Quebec|BOLD:AAA2331
Protodeltote albidula|[1461]|RDLQG596-06|DH012889|658[0n]bp|Canada.Quebec|BOLD:AAA2331
Protodeltote albidula|[1462]|XAC604-04|04HBL006604|658[0n]bp|Canada.Ontario|BOLD:AAA2331
Protodeltote albidula|[1463]|RDLQG900-06|DH013193|658[0n]bp|Canada.Quebec|BOLD:AAA2331
Protodeltote albidula|[1464]|BLTIB672-08|BL955|658[0n]bp|Canada.Ontario|BOLD:AAA2331
Protodeltote albidula|[1465]|BLGSM049-09|BL368|658[0n]bp|Canada.Ontario|BOLD:AAA2331
Protodeltote albidula|[1466]|BLTIB520-08|BL782|658[0n]bp|Canada.Ontario|BOLD:AAA2331
Protodeltote albidula|[1467]|RDLQG777-06|DH013070|658[0n]bp|Canada.Quebec|BOLD:AAA2331
Protodeltote albidula|[1468]|XAK076-06|2006-ONT-1071|658[0n]bp|Canada.Ontario|BOLD:AAA2331
Protodeltote albidula|[1469]|JSJUN2060-11|BIOUG01690-B07|658[0n]bp|Canada.Ontario|BOLD:AAA2331
Protodeltote albidula|[1470]|RDLQF363-06|DH011430|658[0n]bp|Canada.Quebec|BOLD:AAA2331
Protodeltote albidula|[1471]|XAG119-05|2005-ONT-703|658[0n]bp|Canada.Ontario|BOLD:AAA2331
Protodeltote albidula|[1472]|RDLQG901-06|DH013194|658[0n]bp|Canada.Quebec|BOLD:AAA2331
Protodeltote albidula|[1473]|BLGSM016-09|BL325|632[0n]bp|Canada.Ontario|BOLD:AAA2331
Protodeltote albidula|[1474]|XAF719-05|2005-ONT-368|615[0n]bp|Canada.Ontario|BOLD:AAA2331
Protodeltote albidula|[1475]|LHLEP244-06|UBC-2006-1552|658[0n]bp|Canada.British Columbia|BOLD:AAA2331
Protodeltote albidula|[1476]|RDLQG802-06|DH013095|658[0n]bp|Canada.Quebec|BOLD:AAA2331
Protodeltote albidula|[1477]|LHLEP248-06|UBC-2006-1692|655[0n]bp|Canada.British Columbia|BOLD:AAA2331
Protodeltote albidula|[1478]|PMG128-03|moth685.01|617[0n]bp|Canada.Ontario|BOLD:AAA2331
Protodeltote albidula|[1479]|LHLEP051-06|UBC-2006-0242|649[0n]bp|Canada.British Columbia|BOLD:AAA2331
Protodeltote albidula|[1480]|LHLEP246-06|UBC-2006-1554|658[0n]bp|Canada.British Columbia|BOLD:AAA2331
Protodeltote albidula|[1481]|XAC020-04|04HBL006020|592[0n]bp|Canada.Ontario|BOLD:AAA2331
Protodeltote albidula|[1482]|LALPA224-10|AVBC 225-10|635[0n]bp|Canada.British Columbia|BOLD:AAA2331
Protodeltote albidula|[1483]|BLGSM051-09|BL370|632[0n]bp|Canada.Ontario|BOLD:AAA2331
Protodeltote albidula|[1484]|XAB577-04|04HBL005577|658[0n]bp|Canada.Ontario|BOLD:AAA2331
Protodeltote albidula|[1485]|XAC609-04|04HBL006609|561[0n]bp|Canada.Ontario|BOLD:AAA2331
Protodeltote albidula|[1486]|BLTIB406-08|BL650|658[0n]bp|Canada.Ontario|BOLD:AAA2331
Protodeltote albidula|[1487]|LHLEP237-06|UBC-2006-0196|658[0n]bp|Canada.British Columbia|BOLD:AAA2331
Protodeltote albidula|[1488]|LHLEP240-06|UBC-2006-1083|658[0n]bp|Canada.British Columbia|BOLD:AAA2331
Protodeltote albidula|[1489]|XAB613-04|04HBL005613|658[0n]bp|Canada.Ontario|BOLD:AAA2331
Protodeltote albidula|[1490]|XAB576-04|04HBL005576|658[0n]bp|Canada.Ontario|BOLD:AAA2331
Protodeltote albidula|[1491]|LPMN604-08|08BBLEP-01405|658[0n]bp|Canada.Manitoba|BOLD:AAA2331
Protodeltote albidula|[1492]|RDLQG847-06|DH013140|658[0n]bp|Canada.Quebec|BOLD:AAA2331
Protodeltote albidula|[1493]|BLGSM041-09|BL354|658[0n]bp|Canada.Ontario|BOLD:AAA2331
Protodeltote albidula|[1494]|LHLEP086-06|UBC-2006-0291|658[0n]bp|Canada.British Columbia|BOLD:AAA2331
Protodeltote albidula|[1495]|LHLEP239-06|UBC-2006-0380|658[0n]bp|Canada.British Columbia|BOLD:AAA2331
[illegible]

Protodeltote albidula|[1493]|BLGSM041-09|BL354|658[0n]bp|Canada.Ontario|BOLD:AAA2331
Protodeltote albidula|[1494]|LHLEP086-06|UBC-2006-0291|658[0n]bp|Canada.British Columbia|BOLD:AAA2331
Protodeltote albidula|[1495]|LHLEP239-06|UBC-2006-0380|658[0n]bp|Canada.British Columbia|BOLD:AAA2331
Protodeltote albidula|[1496]|BBLPD144-10|10BBCLP-2142|658[0n]bp|Canada.Saskatchewan|BOLD:AAA2331
Protodeltote albidula|[1497]|LBCS755-07|UBC-2007-0460|658[0n]bp|Canada.British Columbia|BOLD:AAA2331
Protodeltote albidula|[1498]|LHLEP088-06|UBC-2006-0293|658[0n]bp|Canada.British Columbia|BOLD:AAA2331
Protodeltote albidula|[1499]|LHLEP243-06|UBC-2006-1086|658[0n]bp|Canada.British Columbia|BOLD:AAA2331
Protodeltote albidula|[1500]|LALPA225-10|AVBC 226-10|658[0n]bp|Canada.British Columbia|BOLD:AAA2331
Protodeltote albidula|[1501]|LHLEP245-06|UBC-2006-1553|658[0n]bp|Canada.British Columbia|BOLD:AAA2331
Protodeltote albidula|[1502]|LHLEP242-06|UBC-2006-1085|658[0n]bp|Canada.British Columbia|BOLD:AAA2331
Protodeltote albidula|[1503]|BLGSM040-09|BL353|658[0n]bp|Canada.Ontario|BOLD:AAA2331
Protodeltote albidula|[1504]|LHLEP241-06|UBC-2006-1084|658[0n]bp|Canada.British Columbia|BOLD:AAA2331
Protodeltote albidula|[1505]|XAF516-05|2005-ONT-165|658[0n]bp|Canada.Ontario|BOLD:AAA2331
Protodeltote albidula|[1506]|BLTIB741-08|BL1031|658[0n]bp|Canada.Ontario|BOLD:AAA2331
Protodeltote albidula|[1507]|XAB614-04|04HBL005614|658[0n]bp|Canada.Ontario|BOLD:AAA2331
Protodeltote albidula|[1508]|BLTIB248-08|BL430|658[0n]bp|Canada.Ontario|BOLD:AAA2331
Capis archaia|[1509]|RDLQB409-05|DH010495|658[0n]bp|Canada.Quebec|BOLD:AAC6122
Capis archaia|[1510]|RDLQB400-05|DH010486|658[0n]bp|Canada.Quebec|BOLD:AAC6122
Capis archaia|[1511]|RDLQG921-06|DH013214|656[0n]bp|Canada.Quebec|BOLD:AAC6122
Capis archaia|[1512]|RDLQG565-06|DH012858|658[0n]bp|Canada.Quebec|BOLD:AAC6122
Capis archaia|[1513]|RDLQG846-06|DH013139|658[0n]bp|Canada.Quebec|BOLD:AAC6122
Capis archaia|[1514]|RDLQB402-05|DH010488|658[0n]bp|Canada.Quebec|BOLD:AAC6122
Capis archaia|[1515]|RDLQB403-05|DH010489|658[0n]bp|Canada.Quebec|BOLD:AAC6122
Capis archaia|[1516]|RDLQB410-05|DH010496|658[0n]bp|Canada.Quebec|BOLD:AAC6122
Capis archaia|[1517]|RDLQ026-05|DH002752|658[0n]bp|Canada.Quebec|BOLD:AAC6122
Capis archaia|[1518]|RDLQB401-05|DH010487|658[0n]bp|Canada.Quebec|BOLD:AAC6122
Capis curvata|[1519]|RDLQB544-05|DH010630|510[0n]bp|Canada.Quebec|BOLD:AAB7154
Capis curvata|[1520]|BLTIB599-08|BL879|642[0n]bp|Canada.Ontario|BOLD:AAB7154
Capis curvata|[1521]|RDLQG665-06|DH012958|658[0n]bp|Canada.Quebec|BOLD:AAB7154
Capis curvata|[1522]|RDLQG708-06|DH013001|658[0n]bp|Canada.Quebec|BOLD:AAB7154
Capis curvata|[1523]|RDLQG614-06|DH012907|658[0n]bp|Canada.Quebec|BOLD:AAB7154
Capis curvata|[1524]|PHMNB391-04|04HBL00617|658[0n]bp|Canada.New Brunswick|BOLD:AAB7154
Capis curvata|[1525]|RDLQG822-06|DH013115|658[0n]bp|Canada.Quebec|BOLD:AAB7154
Capis curvata|[1526]|MECB076-04|jflandry1020|658[0n]bp|Canada.Quebec|BOLD:AAB7154
Capis curvata|[1527]|RDLQG666-06|DH012959|658[0n]bp|Canada.Quebec|BOLD:AAB7154
Capis curvata|[1528]|LPSOB293-08|PPBP-1292|658[0n]bp|Canada.Ontario|BOLD:AAB7154
Capis curvata|[1529]|BLTIB1061-08|BL1070|658[0n]bp|Canada.Ontario|BOLD:AAB7154
Capis curvata|[1530]|RDLQG701-06|DH012994|658[0n]bp|Canada.Quebec|BOLD:AAB7154
Capis curvata|[1531]|RDLQG744-06|DH013037|658[0n]bp|Canada.Quebec|BOLD:AAB7154
Capis curvata|[1532]|LPMN704-08|08BBLEP-01505|658[0n]bp|Canada.Manitoba|BOLD:AAB7154
Capis curvata|[1533]|RDLQG664-06|DH012957|658[0n]bp|Canada.Quebec|BOLD:AAB7154
Capis curvata|[1534]|RDLQB266-05|DH010352|658[0n]bp|Canada.Quebec|BOLD:AAB7154
Capis curvata|[1535]|RDLQB545-05|DH010631|658[0n]bp|Canada.Quebec|BOLD:AAB7154
Capis curvata|[1536]|RDLQG707-06|DH013000|658[0n]bp|Canada.Quebec|BOLD:AAB7154
Protodeltote muscosula|[1537]|RDLQG710-06|DH013003|658[0n]bp|Canada.Quebec|BOLD:AAA4259
Protodeltote muscosula|[1538]|MNBB050-05|HBL008660|658[0n]bp|Canada.New Brunswick|BOLD:AAA4259
Protodeltote muscosula|[1539]|RDLQH023-06|DH013260|655[0n]bp|Canada.Quebec|BOLD:AAA4259
Protodeltote muscosula|[1540]|XAD153-04|04HBL007153|593[0n]bp|Canada.Ontario|BOLD:AAA4259
Protodeltote muscosula|[1541]|XAB205-04|04HBL005205|658[0n]bp|Canada.Ontario|BOLD:AAA4259
Protodeltote muscosula|[1542]|BLTIB993-08|BL1430|658[0n]bp|Canada.Ontario|BOLD:AAA4259
Protodeltote muscosula|[1543]|BBLPE282-09|09BBELE-2282|647[0n]bp|Canada.Nova Scotia|BOLD:AAA4259
Protodeltote muscosula|[1544]|BLTIB491-08|BL743|658[0n]bp|Canada.Ontario|BOLD:AAA4259
Protodeltote muscosula|[1545]|BLTIB1121-08|BL1134|656[0n]bp|Canada.Ontario|BOLD:AAA4259
Protodeltote muscosula|[1546]|BBLPC169-09|09BBELE-1169|655[0n]bp|Canada.Nova Scotia|BOLD:AAA4259
Protodeltote muscosula|[1547]|BLTIB850-08|BL1269|641[0n]bp|Canada.Ontario|BOLD:AAA4259
Protodeltote muscosula|[1548]|BLTIB1050-08|BL1055|632[0n]bp|Canada.Ontario|BOLD:AAA4259
Protodeltote muscosula|[1549]|BLTIB399-08|BL636|658[0n]bp|Canada.Ontario|BOLD:AAA4259
Protodeltote muscosula|[1550]|RDLQG926-06|DH013219|658[0n]bp|Canada.Quebec|BOLD:AAA4259
Protodeltote muscosula|[1551]|PHMO296-03|moth2115.02|639[0n]bp|Canada.Ontario|BOLD:AAA4259
Protodeltote muscosula|[1552]|XAC598-04|04HBL006598|658[0n]bp|Canada.Ontario|BOLD:AAA4259
Protodeltote muscosula|[1553]|XAG242-05|2005-ONT-826|658[0n]bp|Canada.Ontario|BOLD:AAA4259
Protodeltote muscosula|[1554]|BLTIB1120-08|BL1133|606[0n]bp|Canada.Ontario|BOLD:AAA4259
Protodeltote muscosula|[1555]|TMNBB106-06|MNBTT-1046|658[0n]bp|Canada.New Brunswick|BOLD:AAA4259
Protodeltote muscosula|[1556]|TTMNB028-06|MNBTT-028|658[0n]bp|Canada.New Brunswick|BOLD:AAA4259
Protodeltote muscosula|[1557]|LPSO968-08|PPBP-0968|658[0n]bp|Canada.Ontario|BOLD:AAA4259
Protodeltote muscosula|[1558]|RDLQG709-06|DH013002|658[0n]bp|Canada.Quebec|BOLD:AAA4259
Protodeltote muscosula|[1559]|TTMNB298-06|MNBTT-298|658[0n]bp|Canada.New Brunswick|BOLD:AAA4259
Protodeltote muscosula|[1560]|TMNBB107-06|MNBTT-1047|658[0n]bp|Canada.New Brunswick|BOLD:AAA4259
Protodeltote muscosula|[1561]|BBLPC177-09|09BBELE-1177|658[0n]bp|Canada.Nova Scotia|BOLD:AAA4259
Protodeltote muscosula|[1562]|BLTIB236-08|BL417|658[0n]bp|Canada.Ontario|BOLD:AAA4259
Protodeltote muscosula|[1563]|MNBB414-05|05-NBSTA-330|658[0n]bp|Canada.New Brunswick|BOLD:AAA4259
Protodeltote muscosula|[1564]|XAC605-04|04HBL006605|658[0n]bp|Canada.Ontario|BOLD:AAA4259
Protodeltote muscosula|[1565]|MNBB208-05|05-NBSTA-124|658[0n]bp|Canada.New Brunswick|BOLD:AAA4259
Protodeltote muscosula|[1566]|LPSOC399-08|PPBP-2398|658[0n]bp|Canada.Ontario|BOLD:AAA4259
Protodeltote muscosula|[1567]|PHMNB399-04|04HBL00625|658[0n]bp|Canada.New Brunswick|BOLD:AAA4259
Protodeltote muscosula|[1568]|XAB134-04|04HBL005134|658[0n]bp|Canada.Ontario|BOLD:AAA4259
Protodeltote muscosula|[1569]|LPSOB045-08|PPBP-1044|658[0n]bp|Canada.Ontario|BOLD:AAA4259
Protodeltote muscosula|[1570]|TMNBB108-06|MNBTT-1048|658[0n]bp|Canada.New Brunswick|BOLD:AAA4259
Protodeltote muscosula|[1571]|RDLQG745-06|DH013038|658[0n]bp|Canada.Quebec|BOLD:AAA4259
Protodeltote muscosula|[1572]|LPSOB833-08|PPBP-1832|658[0n]bp|Canada.Ontario|BOLD:AAA4259
Protodeltote muscosula|[1573]|XAC657-04|04HBL006657|658[0n]bp|Canada.Ontario|BOLD:AAA4259
Protodeltote muscosula|[1574]|XAC615-04|04HBL006615|658[0n]bp|Canada.Ontario|BOLD:AAA4259
Protodeltote muscosula|[1575]|TTMNB299-06|MNBTT-299|658[0n]bp|Canada.New Brunswick|BOLD:AAA4259
Protodeltote muscosula|[1576]|PHMNB644-04|04HBL00870|658[0n]bp|Canada.New Brunswick|BOLD:AAA4259
Protodeltote muscosula|[1577]|PHJUN4025-11|BIOUG01497-C12|658[0n]bp|Canada.Ontario|BOLD:AAA4259
Protodeltote muscosula|[1578]|XAC040-04|04HBL006040|658[0n]bp|Canada.Ontario|BOLD:AAA4259
Protodeltote muscosula|[1579]|PHMNB385-04|04HBL00611|658[0n]bp|Canada.New Brunswick|BOLD:AAA4259
Protodeltote muscosula|[1580]|MECB138-04|jflandry1082|658[0n]bp|Canada.Quebec|BOLD:AAA4259
Protodeltote muscosula|[1581]|PHMNB334-04|04HBL00560|658[0n]bp|Canada.New Brunswick|BOLD:AAA4259
Protodeltote muscosula|[1582]|PHMNB147-04|04HBL007612|658[0n]bp|Canada.New Brunswick|BOLD:AAA4259
Protodeltote muscosula|[1583]|RDLQG915-06|DH013208|658[0n]bp|Canada.Quebec|BOLD:AAA4259
Protodeltote muscosula|[1584]|BBLPE050-09|09BBELE-2050|658[0n]bp|Canada.Nova Scotia|BOLD:AAA4259
Protodeltote muscosula|[1585]|RDLQG711-06|DH013004|658[0n]bp|Canada.Quebec|BOLD:AAA4259
Protodeltote muscosula|[1586]|PHMNB521-04|04HBL00747|658[0n]bp|Canada.New Brunswick|BOLD:AAA4259
Protodeltote muscosula|[1587]|PHMNB100-04|04HBL007565|658[0n]bp|Canada.New Brunswick|BOLD:AAA4259
Protodeltote muscosula|[1588]|PHMO134-03|moth779.01|639[0n]bp|Canada.Ontario|BOLD:AAA4259
Protodeltote muscosula|[1589]|PHMNB041-03|moth213.02SA|639[0n]bp|Canada.New Brunswick|BOLD:AAA4259
Protodeltote muscosula|[1590]|PHMO175-03|moth927.01|639[0n]bp|Canada.Ontario|BOLD:AAA4259
Protodeltote muscosula|[1591]|LPSOC401-08|PPBP-2400|634[0n]bp|Canada.Ontario|BOLD:AAA4259
Protodeltote muscosula|[1592]|XAD390-04|04HBL007390|592[0n]bp|Canada.Ontario|BOLD:AAA4259
Protodeltote muscosula|[1593]|XAD281-04|04HBL007281|602[0n]bp|Canada.Ontario|BOLD:AAA4259
Protodeltote muscosula|[1594]|TTMNB031-06|MNBTT-031|622[0n]bp|Canada.New Brunswick|BOLD:AAA4259
Protodeltote muscosula|[1595]|RDLQF650-06|DH011800|595[0n]bp|Canada.Quebec|BOLD:AAA4259

Protodeltote muscosula|[1593]|XAD281-04|04HBL007281|602[0n]bp|Canada.Ontario|BOLD:AAA4259
Protodeltote muscosula|[1594]|TTMNB031-06|MNBTT-031|622[0n]bp|Canada.New Brunswick|BOLD:AAA4259
Protodeltote muscosula|[1595]|RDLQF650-06|DH011800|595[0n]bp|Canada.Quebec|BOLD:AAA4259
Protodeltote muscosula|[1596]|RDLQG792-06|DH013085|655[0n]bp|Canada.Quebec|BOLD:AAA4259
Protodeltote muscosula|[1597]|BLTIB454-08|BL701|658[0n]bp|Canada.Ontario|BOLD:AAA4259
Protodeltote muscosula|[1598]|BBLEC210-09|09BBELE-0210|658[0n]bp|Canada.Nova Scotia|BOLD:AAA4259
Protodeltote muscosula|[1599]|TMNBB109-06|MNBTT-1049|658[0n]bp|Canada.New Brunswick|BOLD:AAA4259
Protodeltote muscosula|[1600]|XAJ864-06|2006-ONT-0864|658[0n]bp|Canada.Ontario|BOLD:AAA4259
Protodeltote muscosula|[1601]|XAK185-06|2006-ONT-1180|658[0n]bp|Canada.Ontario|BOLD:AAA4259
Protodeltote muscosula|[1602]|XAF811-05|2005-ONT-460|658[0n]bp|Canada.Ontario|BOLD:AAA4259
Protodeltote muscosula|[1603]|TMNBB105-06|MNBTT-1045|658[0n]bp|Canada.New Brunswick|BOLD:AAA4259
Colocasia flavicornis|[1604]|RDNMB342-05|CNCNoctuoidea10108|597[0n]bp|Canada.Ontario|BOLD:ACF0763
Colocasia flavicornis|[1605]|RDNMB341-05|CNCNoctuoidea10107|585[0n]bp|Canada.Ontario|BOLD:ACF0763
Colocasia flavicornis|[1606]|RDNMH621-09|CNCLEP00062945|658[0n]bp|Canada.New Brunswick|BOLD:ACF0763
Colocasia flavicornis|[1607]|RDNMH610-09|CNCLEP00062934|658[0n]bp|Canada.Ontario|BOLD:ACF0763
Colocasia flavicornis|[1608]|RDNMH622-09|CNCLEP00062946|658[0n]bp|Canada.New Brunswick|BOLD:ACF0763
Colocasia flavicornis|[1609]|RDNMH611-09|CNCLEP00062935|658[0n]bp|Canada.Ontario|BOLD:ACF0763
Colocasia flavicornis|[1610]|RDNMH612-09|CNCLEP00062936|658[0n]bp|Canada.Ontario|BOLD:ACF0763
Colocasia flavicornis|[1611]|RDNMB339-05|CNCNoctuoidea10105|658[0n]bp|Canada.Ontario|BOLD:ACF0763
Colocasia flavicornis|[1612]|RDNMH623-09|CNCLEP00062947|658[0n]bp|Canada.New Brunswick|BOLD:ACF0763
Colocasia flavicornis|[1613]|BBLPB638-10|10BBCLP-1637|658[0n]bp|Canada.Manitoba|BOLD:ACF0763
Colocasia propinquilinea|[1614]|KPOEC167-08|08OEC-208|658[0n]bp|Canada.Ontario|BOLD:ACF0764
Colocasia propinquilinea|[1615]|RDLQG272-06|DH012484|658[0n]bp|Canada.Quebec|BOLD:ACF0764
Colocasia propinquilinea|[1616]|RDNMH614-09|CNCLEP00062938|658[0n]bp|Canada.Ontario|BOLD:AAA8275
Colocasia propinquilinea|[1617]|RDNMH618-09|CNCLEP00062942|658[0n]bp|Canada.New Brunswick|BOLD:AAA8275
Colocasia propinquilinea|[1618]|RDNMH613-09|CNCLEP00062937|658[0n]bp|Canada.Ontario|BOLD:AAA8275
Colocasia propinquilinea|[1619]|RDNMB340-05|CNCNoctuoidea10106|658[0n]bp|Canada.Quebec|BOLD:AAA8275
Colocasia propinquilinea|[1620]|XAE279-04|Moth4279.03|658[0n]bp|Canada.Ontario|BOLD:AAA8275
Colocasia propinquilinea|[1621]|PMG102-03|moth238.01|617[0n]bp|Canada.Ontario|BOLD:AAA8275
Colocasia propinquilinea|[1622]|XAF550-05|2005-ONT-199|658[0n]bp|Canada.Ontario|BOLD:AAA8275
Colocasia propinquilinea|[1623]|XAB226-04|04HBL005226|658[0n]bp|Canada.Ontario|BOLD:AAA8275
Colocasia propinquilinea|[1624]|RDLQB701-05|DH010804|658[0n]bp|Canada.Quebec|BOLD:AAA8275
Colocasia propinquilinea|[1625]|XAF652-05|2005-ONT-301|658[0n]bp|Canada.Ontario|BOLD:AAA8275
Colocasia propinquilinea|[1626]|XAB276-04|04HBL005276|658[0n]bp|Canada.Ontario|BOLD:AAA8275
Colocasia propinquilinea|[1627]|XAB287-04|04HBL005287|658[0n]bp|Canada.Ontario|BOLD:AAA8275
Colocasia propinquilinea|[1628]|RDLQG271-06|DH012483|658[0n]bp|Canada.Quebec|BOLD:AAA8275
Colocasia propinquilinea|[1629]|TMG107-03|moth381.01|639[0n]bp|Canada.Ontario|BOLD:AAA8275
Colocasia propinquilinea|[1630]|RDMAB211-05|UASM7993|658[0n]bp|Canada.Alberta|BOLD:AAA8275
Colocasia propinquilinea|[1631]|RDMAB212-05|UASM43540|658[0n]bp|Canada.Alberta|BOLD:AAA8275
Colocasia propinquilinea|[1632]|XAB338-04|04HBL005338|658[0n]bp|Canada.Ontario|BOLD:AAA8275
Colocasia propinquilinea|[1633]|XAF651-05|2005-ONT-300|658[0n]bp|Canada.Ontario|BOLD:AAA8275
Colocasia propinquilinea|[1634]|LPSOC032-08|PPBP-2031|658[0n]bp|Canada.Ontario|BOLD:AAA8275
Colocasia propinquilinea|[1635]|RDNMH615-09|CNCLEP00062939|658[0n]bp|Canada.Ontario|BOLD:AAA8275
Colocasia propinquilinea|[1636]|BLTIB070-08|BL0110|658[0n]bp|Canada.Ontario|BOLD:AAA8275
Colocasia propinquilinea|[1637]|XAE312-04|Moth4312.03|658[0n]bp|Canada.Ontario|BOLD:AAA8275
Colocasia propinquilinea|[1638]|LPSOC217-08|PPBP-2216|656[0n]bp|Canada.Ontario|BOLD:AAA8275
Charadra deridens|[1639]|MNBB070-05|HBL008680|658[0n]bp|Canada.New Brunswick|BOLD:AAB0661
Charadra deridens|[1640]|LPSOC084-08|PPBP-2083|658[0n]bp|Canada.Ontario|BOLD:AAB0661
Charadra deridens|[1641]|LPSO337-08|PPBP-0337|658[0n]bp|Canada.Ontario|BOLD:AAB0661
Charadra deridens|[1642]|XAE410-04|Moth4410.03|658[0n]bp|Canada.Ontario|BOLD:AAB0661
Charadra deridens|[1643]|TMG109-03|moth998.01|639[0n]bp|Canada.Ontario|BOLD:AAB0661
Charadra deridens|[1644]|TMG108-03|moth659.01|639[0n]bp|Canada.Ontario|BOLD:AAB0661
Charadra deridens|[1645]|PMG101-03|moth492.01|617[0n]bp|Canada.Ontario|BOLD:AAB0661
Charadra deridens|[1646]|RDLQB560-05|DH010646|658[0n]bp|Canada.Quebec|BOLD:AAB0661
Charadra deridens|[1647]|PHMNB758-05|Moth 451.03SA|658[0n]bp|Canada.New Brunswick|BOLD:AAB0661
Charadra deridens|[1648]|RDLQF852-06|DH012013|658[0n]bp|Canada.Quebec|BOLD:AAB0661
Charadra deridens|[1649]|MNBB283-05|05-NBSTA-199|658[0n]bp|Canada.New Brunswick|BOLD:AAB0661
Charadra deridens|[1650]|LPSOD311-09|08BBLEP-00089|658[0n]bp|Canada.Ontario|BOLD:AAB0661
Charadra deridens|[1651]|RDNMC590-06|CNCNoctuoidea12130|656[0n]bp|Canada.Ontario|BOLD:AAB0661
Charadra deridens|[1652]|LPSO864-08|PPBP-0864|658[0n]bp|Canada.Ontario|BOLD:AAB0661
Charadra deridens|[1653]|XAE353-04|Moth4353.03|658[0n]bp|Canada.Ontario|BOLD:AAB0661
Charadra deridens|[1654]|NCCH111-11|BIOUG01862-F01|658[0n]bp|Canada.Ontario|BOLD:AAB0661
Charadra deridens|[1655]|TMG110-03|moth1157.01|639[0n]bp|Canada.Ontario|BOLD:AAB0661
Raphia frater|[1656]|LPSOC092-08|PPBP-2091|658[2n]bp|Canada.Ontario|BOLD:AAA2370
Raphia frater|[1657]|LPSOB448-08|PPBP-1447|658[0n]bp|Canada.Ontario|BOLD:AAA2370
Raphia frater|[1658]|LPSOB315-08|PPBP-1314|658[0n]bp|Canada.Ontario|BOLD:AAA2370
Raphia frater|[1659]|LPSOB444-08|PPBP-1443|658[0n]bp|Canada.Ontario|BOLD:AAA2370
Raphia frater|[1660]|LPSOB441-08|PPBP-1440|658[0n]bp|Canada.Ontario|BOLD:AAA2370
Raphia frater|[1661]|LPSOB446-08|PPBP-1445|658[0n]bp|Canada.Ontario|BOLD:AAA2370
Raphia frater|[1662]|LPMN062-08|08BBLEP-00860|658[0n]bp|Canada.Manitoba|BOLD:AAA2370
Raphia frater|[1663]|LPSOB380-08|PPBP-1379|647[0n]bp|Canada.Ontario|BOLD:AAA2370
Raphia frater|[1664]|LPMN059-08|08BBLEP-00857|658[0n]bp|Canada.Manitoba|BOLD:AAA2370
Raphia frater|[1665]|LPMN043-08|08BBLEP-00841|658[0n]bp|Canada.Manitoba|BOLD:AAA2370
Raphia frater|[1666]|LPSOB512-08|PPBP-1511|658[0n]bp|Canada.Ontario|BOLD:AAA2370
Raphia frater|[1667]|LPSOB377-08|PPBP-1376|658[0n]bp|Canada.Ontario|BOLD:AAA2370
Raphia frater|[1668]|LPMN066-08|08BBLEP-00864|658[0n]bp|Canada.Manitoba|BOLD:AAA2370
Raphia frater|[1669]|LPSOB447-08|PPBP-1446|658[0n]bp|Canada.Ontario|BOLD:AAA2370
Raphia frater|[1670]|LPSOB321-08|PPBP-1320|658[0n]bp|Canada.Ontario|BOLD:AAA2370
Raphia frater|[1671]|LPSOB430-08|PPBP-1429|658[0n]bp|Canada.Ontario|BOLD:AAA2370
Raphia frater|[1672]|LPMN078-08|08BBLEP-00876|658[0n]bp|Canada.Manitoba|BOLD:AAA2370
Raphia frater|[1673]|LPSOB443-08|PPBP-1442|658[0n]bp|Canada.Ontario|BOLD:AAA2370
Raphia frater|[1674]|BBLPC159-09|09BBELE-1159|658[0n]bp|Canada.Nova Scotia|BOLD:AAA2370
Raphia frater|[1675]|BBLPC134-09|09BBELE-1134|633[0n]bp|Canada.Nova Scotia|BOLD:AAA2370
Raphia frater|[1676]|LPSO846-08|PPBP-0846|658[0n]bp|Canada.Ontario|BOLD:AAA2370
Raphia frater|[1677]|LPSOB605-08|PPBP-1604|658[0n]bp|Canada.Ontario|BOLD:AAA2370
Raphia frater|[1678]|KPOEC168-08|08OEC-209|658[0n]bp|Canada.Ontario|BOLD:AAA2370
Raphia frater|[1679]|TMNBB147-06|MNBTT-1087|658[0n]bp|Canada.New Brunswick|BOLD:AAA2370
Raphia frater|[1680]|LPSOB607-08|PPBP-1606|658[0n]bp|Canada.Ontario|BOLD:AAA2370
Raphia frater|[1681]|LPSOB612-08|PPBP-1611|658[0n]bp|Canada.Ontario|BOLD:AAA2370
Raphia frater|[1682]|XAB153-04|04HBL005153|658[0n]bp|Canada.Ontario|BOLD:AAA2370
Raphia frater|[1683]|XAE435-04|Moth4435.03|658[0n]bp|Canada.Ontario|BOLD:AAA2370
Raphia frater|[1684]|LPSOB442-08|PPBP-1441|658[0n]bp|Canada.Ontario|BOLD:AAA2370
Raphia frater|[1685]|XAB399-04|04HBL005399|658[0n]bp|Canada.Ontario|BOLD:AAA2370
Raphia frater|[1686]|LPSOB312-08|PPBP-1311|658[0n]bp|Canada.Ontario|BOLD:AAA2370
Raphia frater|[1687]|LPSOB445-08|PPBP-1444|658[0n]bp|Canada.Ontario|BOLD:AAA2370
Raphia frater|[1688]|XAE445-04|Moth4445.03|658[0n]bp|Canada.Ontario|BOLD:AAA2370
Raphia frater|[1689]|LPSOB318-08|PPBP-1317|658[0n]bp|Canada.Ontario|BOLD:AAA2370
Raphia frater|[1690]|RDLQB653-05|DH010756|658[0n]bp|Canada.Quebec|BOLD:AAA2370
Raphia frater|[1691]|LPSOB450-08|PPBP-1449|658[0n]bp|Canada.Ontario|BOLD:AAA2370
Raphia frater|[1692]|LPSOB319-08|PPBP-1318|658[0n]bp|Canada.Ontario|BOLD:AAA2370
Raphia frater|[1693]|TMG111-03|moth666.01|639[0n]bp|Canada.Ontario|BOLD:AAA2370
Raphia frater|[1694]|XAE458-04|Moth4458.03|613[0n]bp|Canada.Ontario|BOLD:AAA2370
Raphia frater|[1695]|XAB108-04|04HBL005108|567[0n]bp|Canada.Ontario|BOLD:AAA2370

Raphia frater|[1692]|[illegible]|Canada.Ontario|BOLD:AAA2370
Raphia frater|[1693]|TMG111-03|moth666.01|639[0n]bp|Canada.Ontario|BOLD:AAA2370
Raphia frater|[1694]|XAE458-04|Moth4458.03|613[0n]bp|Canada.Ontario|BOLD:AAA2370
Raphia frater|[1695]|XAB108-04|04HBL005108|567[0n]bp|Canada.Ontario|BOLD:AAA2370
Raphia frater|[1696]|PHMNB015-03|moth138.02SA|639[0n]bp|Canada.New Brunswick|BOLD:AAA2370
Raphia frater|[1697]|MNBB249-05|05-NBSTA-165|576[4n]bp|Canada.New Brunswick|BOLD:AAA2370
Raphia frater|[1698]|BBLPC186-09|09BBELE-1186|658[0n]bp|Canada.Nova Scotia|BOLD:AAA2370
Raphia frater|[1699]|BBLPC228-09|09BBELE-1228|658[0n]bp|Canada.Nova Scotia|BOLD:AAA2370
Raphia frater|[1700]|BBLPC245-09|09BBELE-1245|658[0n]bp|Canada.Nova Scotia|BOLD:AAA2370
Raphia frater|[1701]|TMNBB146-06|MNBTT-1086|658[0n]bp|Canada.New Brunswick|BOLD:AAA2370
Raphia frater|[1702]|BBLEC640-09|09BBELE-0640|658[0n]bp|Canada.Nova Scotia|BOLD:AAA2370
Raphia frater|[1703]|MNBB325-05|05-NBSTA-241|658[0n]bp|Canada.New Brunswick|BOLD:AAA2370
Raphia frater|[1704]|TMNBB150-06|MNBTT-1090|658[0n]bp|Canada.New Brunswick|BOLD:AAA2370
Raphia frater|[1705]|BBLEC626-09|09BBELE-0626|658[0n]bp|Canada.Nova Scotia|BOLD:AAA2370
Raphia frater|[1706]|BBLPC246-09|09BBELE-1246|658[0n]bp|Canada.Nova Scotia|BOLD:AAA2370
Raphia frater|[1707]|TMNBB145-06|MNBTT-1085|658[0n]bp|Canada.New Brunswick|BOLD:AAA2370
Raphia frater|[1708]|LPABB490-08|08BBLEP-03755|658[0n]bp|Canada.Alberta|BOLD:AAA2370
Raphia frater|[1709]|LPAB005-08|08BBLEP-02327|658[0n]bp|Canada.Alberta|BOLD:AAA2370
Raphia frater|[1710]|LPABB606-08|08BBLEP-03871|658[0n]bp|Canada.Alberta|BOLD:AAA2370
Raphia frater|[1711]|LPABB006-08|08BBLEP-03271|658[0n]bp|Canada.Alberta|BOLD:AAA2370
Raphia frater|[1712]|LPABB574-08|08BBLEP-03839|658[0n]bp|Canada.Alberta|BOLD:AAA2370
Raphia frater|[1713]|LBCD284-05|HLC-23104|646[0n]bp|Canada.British Columbia|BOLD:AAA2370
Raphia frater|[1714]|RDMAB252-05|UASM41453|658[0n]bp|Canada.Alberta|BOLD:AAA2370
Raphia frater|[1715]|BBLPB777-10|10BBCLP-1776|658[0n]bp|Canada.British Columbia|BOLD:AAA2370
Raphia frater|[1716]|LBCC428-05|HLC-22308|658[1n]bp|Canada.British Columbia|BOLD:AAA2370
Raphia frater|[1717]|LBCA802-05|HLC-20802|658[0n]bp|Canada.British Columbia|BOLD:AAA2370
Raphia frater|[1718]|LBCA807-05|HLC-20807|658[0n]bp|Canada.British Columbia|BOLD:AAA2370
Raphia frater|[1719]|LBCB200-05|HLC-21140|658[0n]bp|Canada.British Columbia|BOLD:AAA2370
Raphia frater|[1720]|LBCA801-05|HLC-20801|658[1n]bp|Canada.British Columbia|BOLD:AAA2370
Raphia frater|[1721]|LOWCC389-05|CGWC-2269|658[1n]bp|Canada.British Columbia|BOLD:AAA2370
Raphia frater|[1722]|LPVIA593-08|PFC-2006-0821|658[0n]bp|Canada.British Columbia|BOLD:AAA2370
Raphia frater|[1723]|LBCA803-05|HLC-20803|658[0n]bp|Canada.British Columbia|BOLD:AAA2370
Raphia frater|[1724]|LPVIA594-08|PFC-2006-0822|658[0n]bp|Canada.British Columbia|BOLD:AAA2370
Raphia frater|[1725]|LBCA079-05|HLC-20079|658[0n]bp|Canada.British Columbia|BOLD:AAA2370
Raphia frater|[1726]|LOWCC386-05|CGWC-2266|658[0n]bp|Canada.British Columbia|BOLD:AAA2370
Raphia frater|[1727]|LHLEP190-06|UBC-2006-1059|658[0n]bp|Canada.British Columbia|BOLD:AAA2370
Raphia frater|[1728]|LBCC761-05|HLC-22641|658[0n]bp|Canada.British Columbia|BOLD:AAA2370
Raphia frater|[1729]|LBCC005-05|HLC-21885|658[0n]bp|Canada.British Columbia|BOLD:AAA2370
Raphia frater|[1730]|LBCB205-05|HLC-21145|658[0n]bp|Canada.British Columbia|BOLD:AAA2370
Raphia frater|[1731]|LPABB375-08|08BBLEP-03640|658[1n]bp|Canada.Alberta|BOLD:AAA2370
Raphia frater|[1732]|LOWCC383-05|CGWC-2263|658[1n]bp|Canada.British Columbia|BOLD:AAA2370
Raphia frater|[1733]|LBCC453-05|HLC-22333|658[1n]bp|Canada.British Columbia|BOLD:AAA2370
Raphia frater|[1734]|LOWCC387-05|CGWC-2267|658[1n]bp|Canada.British Columbia|BOLD:AAA2370
Raphia frater|[1735]|LOWCC392-05|CGWC-2272|658[1n]bp|Canada.British Columbia|BOLD:AAA2370
Raphia frater|[1736]|RDNMB346-05|CNCNoctuoidea10112|597[0n]bp|Canada.British Columbia|BOLD:AAA2370
Raphia frater|[1737]|RDNMB344-05|CNCNoctuoidea10110|605[0n]bp|Canada.Alberta|BOLD:AAA2370
Raphia frater|[1738]|LOWCC385-05|CGWC-2265|634[1n]bp|Canada.British Columbia|BOLD:AAA2370
Raphia frater|[1739]|LOWCC390-05|CGWC-2270|658[1n]bp|Canada.British Columbia|BOLD:AAA2370
Raphia frater|[1740]|LOWCC393-05|CGWC-2273|658[1n]bp|Canada.British Columbia|BOLD:AAA2370
Raphia frater|[1741]|LALPA881-11|AVBC 1054-11|658[0n]bp|Canada.British Columbia|BOLD:AAA2370
Raphia frater|[1742]|LBCH5603-10|10-JDWBC-5603|658[0n]bp|Canada.British Columbia|BOLD:AAA2370
Raphia frater|[1743]|LBCC760-05|HLC-22640|658[0n]bp|Canada.British Columbia|BOLD:AAA2370
Raphia frater|[1744]|RDNMB349-05|CNCNoctuoidea10115|578[0n]bp|Canada.Alberta|BOLD:AAA2370
Raphia frater|[1745]|BBLPB763-10|10BBCLP-1762|658[0n]bp|Canada.Saskatchewan|BOLD:AAA2370
Raphia frater|[1746]|RDMAB536-06|UASM58513|658[0n]bp|Canada.Alberta|BOLD:AAA2370
Raphia frater|[1747]|BBLPB782-10|10BBCLP-1781|658[0n]bp|Canada.Saskatchewan|BOLD:AAA2370
Raphia frater|[1748]|LPSOD879-09|08BBLEP-00661|658[0n]bp|Canada.Ontario|BOLD:AAA2370
Raphia frater|[1749]|LPMN329-08|08BBLEP-01128|658[0n]bp|Canada.Manitoba|BOLD:AAA2370
Raphia frater|[1750]|LPMN271-08|08BBLEP-01070|658[0n]bp|Canada.Manitoba|BOLD:AAA2370
Raphia frater|[1751]|LPMN273-08|08BBLEP-01072|658[0n]bp|Canada.Manitoba|BOLD:AAA2370
Raphia frater|[1752]|BBLPB780-10|10BBCLP-1779|658[0n]bp|Canada.Saskatchewan|BOLD:AAA2370
Raphia frater|[1753]|BBLPB781-10|10BBCLP-1780|658[0n]bp|Canada.Saskatchewan|BOLD:AAA2370
Raphia frater|[1754]|LPMN317-08|08BBLEP-01116|658[0n]bp|Canada.Manitoba|BOLD:AAA2370
Raphia frater|[1755]|RDMAB262-05|UASM41456|658[0n]bp|Canada.Alberta|BOLD:AAA2370
Raphia frater|[1756]|LPMN038-08|08BBLEP-00836|658[0n]bp|Canada.Manitoba|BOLD:AAA2370
Raphia frater|[1757]|RDMAB268-05|UASM41465|658[0n]bp|Canada.Alberta|BOLD:AAA2370
Raphia frater|[1758]|LPSOD583-09|08BBLEP-00364|658[0n]bp|Canada.Ontario|BOLD:AAA2370
Raphia frater|[1759]|LPMN029-08|08BBLEP-00827|658[0n]bp|Canada.Manitoba|BOLD:AAA2370
Raphia frater|[1760]|LPMN632-08|08BBLEP-01433|658[0n]bp|Canada.Manitoba|BOLD:AAA2370
Raphia frater|[1761]|RDMAB370-05|UASM77836|658[0n]bp|Canada.Alberta|BOLD:AAA2370
Raphia frater|[1762]|BBLPB779-10|10BBCLP-1778|658[0n]bp|Canada.Saskatchewan|BOLD:AAA2370
Raphia frater|[1763]|LPMN324-08|08BBLEP-01123|658[0n]bp|Canada.Manitoba|BOLD:AAA2370
Raphia frater|[1764]|BBLPB776-10|10BBCLP-1775|658[0n]bp|Canada.Alberta|BOLD:AAA2370
Raphia frater|[1765]|LPSOD839-09|08BBLEP-00621|658[0n]bp|Canada.Ontario|BOLD:AAA2370
Raphia frater|[1766]|BBLPB774-10|10BBCLP-1773|658[0n]bp|Canada.Alberta|BOLD:AAA2370
Raphia frater|[1767]|RDMAB257-05|UASM41455|658[0n]bp|Canada.Alberta|BOLD:AAA2370
Raphia frater|[1768]|BBLPB775-10|10BBCLP-1774|658[0n]bp|Canada.Alberta|BOLD:AAA2370
Raphia frater|[1769]|BBLPB783-10|10BBCLP-1782|658[0n]bp|Canada.Saskatchewan|BOLD:AAA2370
Raphia frater|[1770]|LPSOD465-09|08BBLEP-00244|624[0n]bp|Canada.Ontario|BOLD:AAA2370
Raphia frater|[1771]|LPABB612-08|08BBLEP-03877|658[0n]bp|Canada.Alberta|BOLD:AAA2370
Raphia frater|[1772]|LPMN348-08|08BBLEP-01147|658[0n]bp|Canada.Manitoba|BOLD:AAA2370
Raphia frater|[1773]|RDMAB269-05|UASM41454|658[0n]bp|Canada.Alberta|BOLD:AAA2370
Raphia frater|[1774]|LPSOD341-09|08BBLEP-00119|658[0n]bp|Canada.Ontario|BOLD:AAA2370
Raphia frater|[1775]|LPSOB449-08|PPBP-1448|658[0n]bp|Canada.Ontario|BOLD:AAA2370
Raphia frater|[1776]|LPSOD845-09|08BBLEP-00627|658[0n]bp|Canada.Ontario|BOLD:AAA2370
Raphia frater|[1777]|TMNBB151-06|MNBTT-1091|658[0n]bp|Canada.New Brunswick|BOLD:AAA2370
Raphia frater|[1778]|MNBB181-05|05-NBSTA-097|658[0n]bp|Canada.New Brunswick|BOLD:AAA2370
Raphia frater|[1779]|BBLPC236-09|09BBELE-1236|658[0n]bp|Canada.Nova Scotia|BOLD:AAA2370
Raphia frater|[1780]|XAB321-04|04HBL005321|658[0n]bp|Canada.Ontario|BOLD:AAA2370
Raphia frater|[1781]|TTMNB317-06|MNBTT-317|658[0n]bp|Canada.New Brunswick|BOLD:AAA2370
Raphia frater|[1782]|BLTIB716-08|BL1002|658[0n]bp|Canada.Ontario|BOLD:AAA2370
Raphia frater|[1783]|TTMNB315-06|MNBTT-315|658[0n]bp|Canada.New Brunswick|BOLD:AAA2370
Raphia frater|[1784]|XAB567-04|04HBL005567|658[0n]bp|Canada.Ontario|BOLD:AAA2370
Raphia frater|[1785]|TMNBB148-06|MNBTT-1088|658[0n]bp|Canada.New Brunswick|BOLD:AAA2370
Raphia frater|[1786]|PHMNB602-04|04HBL00828|658[0n]bp|Canada.New Brunswick|BOLD:AAA2370
Raphia frater|[1787]|XAB596-04|04HBL005596|658[0n]bp|Canada.Ontario|BOLD:AAA2370
Raphia frater|[1788]|MNBB300-05|05-NBSTA-216|658[0n]bp|Canada.New Brunswick|BOLD:AAA2370
Raphia frater|[1789]|MNBB324-05|05-NBSTA-240|658[0n]bp|Canada.New Brunswick|BOLD:AAA2370
Raphia frater|[1790]|BLTIB332-08|BL523|658[0n]bp|Canada.Ontario|BOLD:AAA2370
Raphia frater|[1791]|BBLEC635-09|09BBELE-0635|658[0n]bp|Canada.Nova Scotia|BOLD:AAA2370
Raphia frater|[1792]|BBLPC232-09|09BBELE-1232|658[0n]bp|Canada.Nova Scotia|BOLD:AAA2370
Raphia frater|[1793]|BBLEC189-09|09BBELE-0189|646[0n]bp|Canada.Nova Scotia|BOLD:AAA2370
Raphia frater|[1794]|TTMNB316-06|MNBTT-316|601[0n]bp|Canada.New Brunswick|BOLD:AAA2370
[illegible]

Raphia frater|[1792]|BBLPC232-09|09BBELE-1232|658[0n]bp|Canada.Nova Scotia|BOLD:AAA2370
Raphia frater|[1793]|BBLEC189-09|09BBELE-0189|646[0n]bp|Canada.Nova Scotia|BOLD:AAA2370
Raphia frater|[1794]|TTMNB316-06|MNBTT-316|601[0n]bp|Canada.New Brunswick|BOLD:AAA2370
Raphia frater|[1795]|PHMNB771-05|Moth 464.03SA|617[0n]bp|Canada.New Brunswick|BOLD:AAA2370
Raphia frater|[1796]|PHMNB045-03|moth221.02SA|639[0n]bp|Canada.New Brunswick|BOLD:AAA2370
Raphia frater|[1797]|XAE086-04|Moth4086.03|606[0n]bp|Canada.Ontario|BOLD:AAA2370
Raphia frater|[1798]|PHMNB153-04|04HBL007618|574[3n]bp|Canada.New Brunswick|BOLD:AAA2370
Raphia frater|[1799]|PHMNB101-04|04HBL007566|570[0n]bp|Canada.New Brunswick|BOLD:AAA2370
Raphia frater|[1800]|XAF655-05|2005-ONT-304|567[0n]bp|Canada.Ontario|BOLD:AAA2370
Raphia frater|[1801]|PHMNB156-04|04HBL007621|655[2n]bp|Canada.New Brunswick|BOLD:AAA2370
Raphia frater|[1802]|MNBB182-05|05-NBSTA-098|577[0n]bp|Canada.New Brunswick|BOLD:AAA2370
Raphia frater|[1803]|XAB256-04|04HBL005256|658[0n]bp|Canada.Ontario|BOLD:AAA2370
Raphia frater|[1804]|PHMNB601-04|04HBL00827|658[0n]bp|Canada.New Brunswick|BOLD:AAA2370
Raphia frater|[1805]|PHMNB752-05|Moth 445.03SA|658[0n]bp|Canada.New Brunswick|BOLD:AAA2370
Raphia frater|[1806]|BBLPC518-09|09BBELE-1518|658[0n]bp|Canada.New Brunswick|BOLD:AAA2370
Raphia frater|[1807]|MNBB617-05|05-NBSTA-533|658[0n]bp|Canada.New Brunswick|BOLD:AAA2370
Raphia frater|[1808]|MNBB396-05|05-NBSTA-312|658[0n]bp|Canada.New Brunswick|BOLD:AAA2370
Raphia frater|[1809]|BBLEC188-09|09BBELE-0188|658[0n]bp|Canada.Nova Scotia|BOLD:AAA2370
Raphia frater|[1810]|XAC288-04|04HBL006288|658[0n]bp|Canada.Ontario|BOLD:AAA2370
Raphia frater|[1811]|XAB356-04|04HBL005356|658[0n]bp|Canada.Ontario|BOLD:AAA2370
Raphia frater|[1812]|XAC187-04|04HBL006187|658[0n]bp|Canada.Ontario|BOLD:AAA2370
Raphia frater|[1813]|KPOEC071-08|08OEC-219|658[0n]bp|Canada.Ontario|BOLD:AAA2370
Raphia frater|[1814]|LPSOD313-09|08BBLEP-00091|658[0n]bp|Canada.Ontario|BOLD:AAA2370
Raphia frater|[1815]|LPSOB378-08|PPBP-1377|658[0n]bp|Canada.Ontario|BOLD:AAA2370
Raphia frater|[1816]|BLTIB363-08|BL581|658[0n]bp|Canada.Ontario|BOLD:AAA2370
Raphia frater|[1817]|TMNBB149-06|MNBTT-1089|658[0n]bp|Canada.New Brunswick|BOLD:AAA2370
Raphia frater|[1818]|LPSOB613-08|PPBP-1612|658[0n]bp|Canada.Ontario|BOLD:AAA2370
Raphia frater|[1819]|LPSOD239-09|08BBLEP-00017|658[0n]bp|Canada.Ontario|BOLD:AAA2370
Raphia frater|[1820]|MNBB301-05|05-NBSTA-217|658[0n]bp|Canada.New Brunswick|BOLD:AAA2370
Raphia frater|[1821]|XAE454-04|Moth4454.03|658[0n]bp|Canada.Ontario|BOLD:AAA2370
Raphia frater|[1822]|XAB142-04|04HBL005142|617[0n]bp|Canada.Ontario|BOLD:AAA2370
Raphia frater|[1823]|PMG157-03|moth583.01|617[0n]bp|Canada.Ontario|BOLD:AAA2370
Raphia frater|[1824]|RDNMB345-05|CNCNoctuoidea10111|605[0n]bp|Canada.Ontario|BOLD:AAA2370
Raphia frater|[1825]|XAD590-05|2005-ONT-5|617[0n]bp|Canada.Ontario|BOLD:AAA2370
Raphia frater|[1826]|XAE456-04|Moth4456.03|649[0n]bp|Canada.Ontario|BOLD:AAA2370
Raphia frater|[1827]|RDLQB702-05|DH010805|658[0n]bp|Canada.Quebec|BOLD:AAA2370
Exyra fax|[1828]|RDLQB267-05|DH010353|658[0n]bp|Canada.Quebec|BOLD:ACF4632
Exyra fax|[1829]|RDNMH150-09|CNCLEP00054368|658[0n]bp|Canada.Quebec|BOLD:ACF4632
Exyra fax|[1830]|RDNMH151-09|CNCLEP00054369|658[0n]bp|Canada.Quebec|BOLD:ACF4632
Euchalcia borealis|[1831]|RDNM847-05|CNCNoctuoidea7687|599[0n]bp|Canada.British Columbia|BOLD:AAX7948
Plusia putnami|[1832]|RDLQB013-05|DH010099|573[0n]bp|Canada.Quebec|BOLD:AAA5394
Plusia putnami|[1833]|RDNM856-05|CNCNoctuoidea7696|504[0n]bp|Canada.New Brunswick|BOLD:AAA5394
Plusia putnami|[1834]|RDLQ293-05|DH007157|622[0n]bp|Canada.Quebec|BOLD:AAA5394
Plusia putnami|[1835]|RDLQF098-06|CB0013|621[5n]bp|Canada.Quebec|BOLD:AAA5394
Plusia putnami|[1836]|RDNM863-05|CNCNoctuoidea7703|577[0n]bp|Canada.British Columbia|BOLD:AAA5394
Plusia putnami|[1837]|RDNM859-05|CNCNoctuoidea7699|658[0n]bp|Canada.Ontario|BOLD:AAA5394
Plusia putnami|[1838]|BBLPC366-09|09BBELE-1366|654[0n]bp|Canada.New Brunswick|BOLD:AAA5394
Plusia putnami|[1839]|RDLQF104-06|CB0019|657[0n]bp|Canada.Quebec|BOLD:AAA5394
Plusia putnami|[1840]|LCHQ716-08|07WNP-10608|658[0n]bp|Canada.Manitoba|BOLD:AAA5394
Plusia putnami|[1841]|RDLQF107-06|CB0022|657[0n]bp|Canada.Quebec|BOLD:AAA5394
Plusia putnami|[1842]|PHMNB683-04|04HBL00909|657[0n]bp|Canada.New Brunswick|BOLD:AAA5394
Plusia putnami|[1843]|PHMNB465-04|04HBL00691|657[0n]bp|Canada.New Brunswick|BOLD:AAA5394
Plusia putnami|[1844]|RDLQF105-06|CB0020|657[0n]bp|Canada.Quebec|BOLD:AAA5394
Plusia putnami|[1845]|BBLPC180-09|09BBELE-1180|658[0n]bp|Canada.Nova Scotia|BOLD:AAA5394
Plusia putnami|[1846]|LCHQ920-08|07WNP-10812|658[0n]bp|Canada.Manitoba|BOLD:AAA5394
Plusia putnami|[1847]|LCHQ312-08|07WNP-10204|658[0n]bp|Canada.Manitoba|BOLD:AAA5394
Plusia putnami|[1848]|LPMN209-08|08BBLEP-01008|658[0n]bp|Canada.Manitoba|BOLD:AAA5394
Plusia putnami|[1849]|RDNM852-05|CNCNoctuoidea7692|658[0n]bp|Canada.British Columbia|BOLD:AAA5394
Plusia putnami|[1850]|LCHP756-07|07PROBE-10441|657[0n]bp|Canada.Manitoba|BOLD:AAA5394
Plusia putnami|[1851]|PHMNB466-04|04HBL00692|657[0n]bp|Canada.New Brunswick|BOLD:AAA5394
Plusia putnami|[1852]|PHMNB320-04|04HBL00546|657[0n]bp|Canada.New Brunswick|BOLD:AAA5394
Plusia putnami|[1853]|RDLQF101-06|CB0016|657[0n]bp|Canada.Quebec|BOLD:AAA5394
Plusia putnami|[1854]|LCHQ715-08|07WNP-10607|657[0n]bp|Canada.Manitoba|BOLD:AAA5394
Plusia putnami|[1855]|RDNM860-05|CNCNoctuoidea7700|658[0n]bp|Canada.Alberta|BOLD:AAA5394
Plusia putnami|[1856]|RDNM850-05|CNCNoctuoidea7690|658[0n]bp|Canada.British Columbia|BOLD:AAA5394
Plusia putnami|[1857]|RDLQF109-06|CB0024|657[0n]bp|Canada.Quebec|BOLD:AAA5394
Plusia putnami|[1858]|RDLQ220-05|DH001801|657[0n]bp|Canada.Quebec|BOLD:AAA5394
Plusia putnami|[1859]|LPABB676-08|08BBLEP-03941|658[0n]bp|Canada.Alberta|BOLD:AAA5394
Plusia putnami|[1860]|RDLQF106-06|CB0021|657[0n]bp|Canada.Quebec|BOLD:AAA5394
Plusia putnami|[1861]|LCHP746-07|07PROBE-10431|655[0n]bp|Canada.Manitoba|BOLD:AAA5394
Plusia putnami|[1862]|RDLQ298-05|DH007312|657[0n]bp|Canada.Quebec|BOLD:AAA5394
Plusia putnami|[1863]|RDLQ119-05|DH001804|657[0n]bp|Canada.Quebec|BOLD:AAA5394
Plusia putnami|[1864]|RDNM857-05|CNCNoctuoidea7697|658[0n]bp|Canada.Ontario|BOLD:AAA5394
Plusia putnami|[1865]|RDLQF110-06|CB0025|619[0n]bp|Canada.Quebec|BOLD:AAA5394
Plusia putnami|[1866]|LCHQ714-08|07WNP-10606|658[0n]bp|Canada.Manitoba|BOLD:AAA5394
Plusia putnami|[1867]|BBLPE082-09|09BBELE-2082|658[0n]bp|Canada.Nova Scotia|BOLD:AAA5394
Plusia putnami|[1868]|RDLQB014-05|DH010100|556[0n]bp|Canada.Quebec|BOLD:AAA5394
Plusia magnimacula|[1869]|RDLQ027-05|DH006704|508[0n]bp|Canada.Quebec|BOLD:AAB2458
Plusia magnimacula|[1870]|LCHP831-07|07PROBE-10588|658[0n]bp|Canada.Manitoba|BOLD:AAB2458
Plusia magnimacula|[1871]|RDLQ285-05|DH006700|573[0n]bp|Canada.Quebec|BOLD:AAB2458
Plusia magnimacula|[1872]|RDLQ284-05|DH006699|600[0n]bp|Canada.Quebec|BOLD:AAB2458
Plusia magnimacula|[1873]|RDLQ295-05|DH007255|603[0n]bp|Canada.Quebec|BOLD:AAB2458
Plusia magnimacula|[1874]|RDNM864-05|CNCNoctuoidea7704|611[0n]bp|Canada.Ontario|BOLD:AAB2458
Plusia magnimacula|[1875]|RDLQB015-05|DH010101|657[0n]bp|Canada.Quebec|BOLD:AAB2458
Plusia magnimacula|[1876]|RDLQB768-05|DH010855|658[0n]bp|Canada.Quebec|BOLD:AAB2458
Plusia magnimacula|[1877]|LPSOB228-08|PPBP-1227|658[0n]bp|Canada.Ontario|BOLD:AAB2458
Plusia magnimacula|[1878]|RDNM867-05|CNCNoctuoidea7707|658[0n]bp|Canada.Ontario|BOLD:AAB2458
Plusia magnimacula|[1879]|RDNM865-05|CNCNoctuoidea7705|658[0n]bp|Canada.Ontario|BOLD:AAB2458
Plusia magnimacula|[1880]|RDLQF096-06|CB0011|657[0n]bp|Canada.Quebec|BOLD:AAB2458
Plusia magnimacula|[1881]|RDLQF111-06|CB0026|657[0n]bp|Canada.Quebec|BOLD:AAB2458
Plusia magnimacula|[1882]|RDLQF103-06|CB0018|657[0n]bp|Canada.Quebec|BOLD:AAB2458
Plusia magnimacula|[1883]|RDLQF102-06|CB0017|657[0n]bp|Canada.Quebec|BOLD:AAB2458
Plusia magnimacula|[1884]|RDNM866-05|CNCNoctuoidea7706|658[0n]bp|Canada.Ontario|BOLD:AAB2458
Plusia magnimacula|[1885]|RDLQF097-06|CB0012|598[0n]bp|Canada.Quebec|BOLD:AAB2458
Plusia magnimacula|[1886]|RDLQF099-06|CB0014|620[0n]bp|Canada.Quebec|BOLD:AAB2458
Plusia magnimacula|[1887]|RDLQF095-06|CB0010|598[0n]bp|Canada.Quebec|BOLD:AAB2458
Plusia magnimacula|[1888]|MECB359-05|jflandry1322|574[0n]bp|Canada.Quebec|BOLD:AAB2458
Plusia magnimacula|[1889]|MECB360-05|jflandry1323|583[0n]bp|Canada.Quebec|BOLD:AAB2458
Plusia magnimacula|[1890]|MECB358-05|jflandry1321|595[0n]bp|Canada.Quebec|BOLD:AAB2458
Plusia magnimacula|[1891]|RDLQ300-05|DH007524|597[0n]bp|Canada.Quebec|BOLD:AAB2458
Plusia magnimacula|[1892]|RDNM858-05|CNCNoctuoidea7698|585[0n]bp|Canada.New Brunswick|BOLD:AAB2458
Plusia magnimacula|[1893]|MECB357-05|jflandry1320|658[0n]bp|Canada.Quebec|BOLD:AAB2458
Plusia magnimacula|[1894]|RDLQB767-05|DH010854|616[0n]bp|Canada.Quebec|BOLD:AAB2458

Plusia magnimacula|[1892]|RDNM858-05|CNCNoctuoidea7698|585[0n]bp|Canada.New Brunswick|BOLD:AAB2458
Plusia magnimacula|[1893]|MECB357-05|jflandry1320|658[0n]bp|Canada.Quebec|BOLD:AAB2458
Plusia magnimacula|[1894]|RDLQB767-05|DH010854|616[0n]bp|Canada.Quebec|BOLD:AAB2458
Plusia magnimacula|[1895]|RDLQ294-05|DH007252|598[0n]bp|Canada.Quebec|BOLD:AAB2458
Plusia contexta|[1896]|RDLQB723-05|DH010826|599[0n]bp|Canada.Quebec|BOLD:AAC3534
Plusia contexta|[1897]|RDLQB762-05|DH010849|617[0n]bp|Canada.Quebec|BOLD:AAC3534
Plusia contexta|[1898]|XAE283-04|Moth4283.03|609[2n]bp|Canada.Ontario|BOLD:AAC3534
Plusia contexta|[1899]|LPSOC120-08|PPBP-2119|654[0n]bp|Canada.Ontario|BOLD:AAC3534
Plusia contexta|[1900]|LPSO882-08|PPBP-0882|658[0n]bp|Canada.Ontario|BOLD:AAC3534
Plusia contexta|[1901]|RDLQB540-05|DH010626|658[0n]bp|Canada.Quebec|BOLD:AAC3534
Plusia contexta|[1902]|RDNMB602-05|CNCNoctuoidea10378|658[0n]bp|Canada.Ontario|BOLD:AAC3534
Plusia contexta|[1903]|RDLQF937-06|DH012117|658[0n]bp|Canada.Quebec|BOLD:AAC3534
Plusia contexta|[1904]|RDNMB600-05|CNCNoctuoidea10376|658[0n]bp|Canada.Ontario|BOLD:AAC3534
Plusia contexta|[1905]|XAJ499-06|2006-ONT-0499|657[0n]bp|Canada.Ontario|BOLD:AAC3534
Plusia contexta|[1906]|RDNMB601-05|CNCNoctuoidea10377|658[0n]bp|Canada.Ontario|BOLD:AAC3534
Plusia contexta|[1907]|LPSOC118-08|PPBP-2117|658[0n]bp|Canada.Ontario|BOLD:AAC3534
Plusia nichollae|[1908]|RDNM855-05|CNCNoctuoidea7695|571[0n]bp|Canada.British Columbia|BOLD:ACJ0937
Plusia nichollae|[1909]|RDNM854-05|CNCNoctuoidea7694|570[0n]bp|Canada.British Columbia|BOLD:ACJ0937
Plusia nichollae|[1910]|RDNM862-05|CNCNoctuoidea7702|554[0n]bp|Canada.British Columbia|BOLD:ACJ0937
Plusia nichollae|[1911]|LBCH488-10|10-JDWBC-0488|658[0n]bp|Canada.British Columbia|BOLD:ACJ0937
Plusia nichollae|[1912]|RDNM853-05|CNCNoctuoidea7693|596[1n]bp|Canada.British Columbia|BOLD:ACJ0937
Plusia nichollae|[1913]|LBCW027-08|08-JDWWI-0027|658[0n]bp|Canada.British Columbia|BOLD:ACJ0937
Plusia nichollae|[1914]|LBCW026-08|08-JDWWI-0026|658[0n]bp|Canada.British Columbia|BOLD:ACJ0937
Plusia nichollae|[1915]|LBCG001-08|08-JDWBC-0001|658[0n]bp|Canada.British Columbia|BOLD:ACJ0937
Plusia venusta|[1916]|XAJ827-06|2006-ONT-0827|657[0n]bp|Canada.Ontario|BOLD:ACJ0936
Plusia venusta|[1917]|LHLEP123-06|UBC-2006-1068|658[0n]bp|Canada.British Columbia|BOLD:ACJ0936
Plusia venusta|[1918]|PHMO319-03|moth2402.02|639[0n]bp|Canada.Ontario|BOLD:ACJ0936
Plusia venusta|[1919]|RDNMB599-05|CNCNoctuoidea10375|658[0n]bp|Canada.Ontario|BOLD:ACJ0936
Plusia venusta|[1920]|RDNMB598-05|CNCNoctuoidea10374|658[0n]bp|Canada.Saskatchewan|BOLD:ACJ0936
Plusia venusta|[1921]|RDNMB597-05|CNCNoctuoidea10373|658[0n]bp|Canada.Ontario|BOLD:ACJ0936
Eosphoropteryx thyatyroides|[1922]|XAG913-05|2005-ONT-1497|658[0n]bp|Canada.Ontario|BOLD:AAD4019
Eosphoropteryx thyatyroides|[1923]|RDLQ425-07|DH006243|645[0n]bp|Canada.Quebec|BOLD:AAD4019
Eosphoropteryx thyatyroides|[1924]|LPMNB327-09|08BBLEP-05171|600[0n]bp|Canada.Manitoba|BOLD:AAD4019
Eosphoropteryx thyatyroides|[1925]|LBCC753-05|HLC-22633|658[0n]bp|Canada.British Columbia|BOLD:AAD4019
Eosphoropteryx thyatyroides|[1926]|LALPA424-10|AVBC 426-10|658[0n]bp|Canada.British Columbia|BOLD:AAD...
Eosphoropteryx thyatyroides|[1927]|LALPA421-10|AVBC 423-10|658[0n]bp|Canada.British Columbia|BOLD:AAD...
Eosphoropteryx thyatyroides|[1928]|LPMNB353-09|08BBLEP-05197|658[0n]bp|Canada.Manitoba|BOLD:AAD4019
Pseudeva palligera|[1929]|RDNMF444-08|NOC14530|658[0n]bp|Canada.British Columbia|BOLD:AAD5210
Pseudeva palligera|[1930]|LPABC877-09|08BBLEP-05288|658[0n]bp|Canada.Alberta|BOLD:AAD5210
Pseudeva palligera|[1931]|LPABC876-09|08BBLEP-05287|658[0n]bp|Canada.Alberta|BOLD:AAD5210
Pseudeva palligera|[1932]|LPABC969-09|08BBLEP-05380|658[0n]bp|Canada.Alberta|BOLD:AAD5210
Pseudeva palligera|[1933]|RDMAB986-09|UASM99700|658[0n]bp|Canada.British Columbia|BOLD:AAD5210
Pseudeva palligera|[1934]|RDMAB985-09|UASM99699|644[0n]bp|Canada.British Columbia|BOLD:AAD5210
Pseudeva palligera|[1935]|RDMAB984-09|UASM99553|627[0n]bp|Canada.Alberta|BOLD:AAD5210
Pseudeva purpurigera|[1936]|RDLQG143-06|DH012314|613[1n]bp|Canada.Quebec|BOLD:AAD7521
Pseudeva purpurigera|[1937]|BLTIB703-08|BL988|658[0n]bp|Canada.Ontario|BOLD:AAD7521
Pseudeva purpurigera|[1938]|LPMNB553-09|08BBLEP-05588|658[0n]bp|Canada.Manitoba|BOLD:AAD7521
Pseudeva purpurigera|[1939]|RDNMG937-08|CNC LEP00053061|658[0n]bp|Canada.Ontario|BOLD:AAD7521
Pseudeva purpurigera|[1940]|RDNMG936-08|CNC LEP00053060|658[0n]bp|Canada.Ontario|BOLD:AAD7521
Pseudeva purpurigera|[1941]|RDLQF815-06|DH011965|658[0n]bp|Canada.Quebec|BOLD:AAD7521
Autographa californica|[1942]|LALPA343-10|AVBC 345-10|658[0n]bp|Canada.British Columbia|BOLD:AAB2628
Autographa californica|[1943]|LBCW053-08|08-JDWWI-0053|658[0n]bp|Canada.British Columbia|BOLD:AAB2628
Autographa californica|[1944]|LALPA453-10|AVBC 455-10|658[0n]bp|Canada.British Columbia|BOLD:AAB2628
Autographa californica|[1945]|LBCA805-05|HLC-20805|658[0n]bp|Canada.British Columbia|BOLD:AAB2628
Autographa californica|[1946]|LPABC039-09|08BBLEP-04258|658[0n]bp|Canada.Alberta|BOLD:AAB2628
Autographa californica|[1947]|LBCS681-07|UBC-2007-0386|658[0n]bp|Canada.British Columbia|BOLD:AAB2628
Autographa californica|[1948]|LHLEP280-06|UBC-2006-1514|658[0n]bp|Canada.British Columbia|BOLD:AAB2628
Autographa californica|[1949]|LOWCE084-06|CGWC-3844|658[0n]bp|Canada.British Columbia|BOLD:AAB2628
Autographa californica|[1950]|LBCH5279-10|10-JDWBC-5279|658[0n]bp|Canada.British Columbia|BOLD:AAB2628
Autographa californica|[1951]|LALPA634-10|AVBC 636-10|658[0n]bp|Canada.British Columbia|BOLD:AAB2628
Autographa californica|[1952]|LHLEP021-06|UBC-2006-0025|658[0n]bp|Canada.British Columbia|BOLD:AAB2628
Autographa californica|[1953]|LBCS186-07|UBC-2007-0567|658[0n]bp|Canada.British Columbia|BOLD:AAB2628
Autographa californica|[1954]|RDNMH183-09|CNCLEP00054400|639[0n]bp|Canada.British Columbia|BOLD:AAB2628
Autographa californica|[1955]|LBCA075-05|HLC-20075|658[0n]bp|Canada.British Columbia|BOLD:AAB2628
Autographa californica|[1956]|LALPA788-10|AVBC 790-10|658[0n]bp|Canada.British Columbia|BOLD:AAB2628
Autographa rubidus|[1957]|LOWCC851-05|CGWC-2731|658[5n]bp|Canada.British Columbia|BOLD:AAD5970
Autographa rubidus|[1958]|LOWCE121-06|CGWC-3881|658[0n]bp|Canada.British Columbia|BOLD:AAD5970
Autographa rubidus|[1959]|LOWCE119-06|CGWC-3879|658[0n]bp|Canada.British Columbia|BOLD:AAD5970
Autographa rubidus|[1960]|BBLPB313-10|10BBCLP-1312|658[0n]bp|Canada.Ontario|BOLD:AAD5970
Autographa rubidus|[1961]|RDLQG264-06|DH012469|658[0n]bp|Canada.Quebec|BOLD:AAD5970
Autographa rubidus|[1962]|LOWCE120-06|CGWC-3880|658[0n]bp|Canada.British Columbia|BOLD:AAD5970
Autographa rubidus|[1963]|BBLPB312-10|10BBCLP-1311|658[0n]bp|Canada.Ontario|BOLD:AAD5970
Autographa rubidus|[1964]|BBLPB311-10|10BBCLP-1310|658[0n]bp|Canada.Ontario|BOLD:AAD5970
Autographa rubidus|[1965]|LPSOD326-09|08BBLEP-00104|658[0n]bp|Canada.Ontario|BOLD:AAD5970
Autographa rubidus|[1966]|BBLPB314-10|10BBCLP-1313|658[0n]bp|Canada.Saskatchewan|BOLD:AAD5970
Autographa precationis|[1967]|XAJ545-06|2006-ONT-0545|656[10n]bp|Canada.Ontario|
Autographa precationis|[1968]|BLTIB1130-08|BL1144|603[1n]bp|Canada.Ontario|BOLD:AAA3836
Autographa precationis|[1969]|XAG875-05|2005-ONT-1459|658[0n]bp|Canada.Ontario|BOLD:AAA3836
Autographa precationis|[1970]|BLTIB847-08|BL1266|658[0n]bp|Canada.Ontario|BOLD:AAA3836
Autographa precationis|[1971]|XAB458-04|04HBL005458|658[0n]bp|Canada.Ontario|BOLD:AAA3836
Autographa precationis|[1972]|XAJ266-06|2006-ONT-0266|658[0n]bp|Canada.Ontario|BOLD:AAA3836
Autographa precationis|[1973]|XAJ427-06|2006-ONT-0427|658[0n]bp|Canada.Ontario|BOLD:AAA3836
Autographa precationis|[1974]|XAH034-05|2005-ONT-1617|658[1n]bp|Canada.Ontario|BOLD:AAA3836
Autographa precationis|[1975]|BLTIB736-08|BL1026|641[4n]bp|Canada.Ontario|BOLD:AAA3836
Autographa precationis|[1976]|XAB674-04|04HBL005674|658[0n]bp|Canada.Ontario|BOLD:AAA3836
Autographa precationis|[1977]|BLTIB785-08|BL1202|658[0n]bp|Canada.Ontario|BOLD:AAA3836
Autographa precationis|[1978]|BLTIB1021-08|BL1462|658[0n]bp|Canada.Ontario|BOLD:AAA3836
Autographa precationis|[1979]|XAH704-05|2005-ONT-2287|658[0n]bp|Canada.Ontario|BOLD:AAA3836
Autographa precationis|[1980]|XAH685-05|2005-ONT-2268|658[0n]bp|Canada.Ontario|BOLD:AAA3836
Autographa precationis|[1981]|XAG613-05|2005-ONT-1197|658[0n]bp|Canada.Ontario|BOLD:AAA3836
Autographa precationis|[1982]|LPSOB324-08|PPBP-1323|658[0n]bp|Canada.Ontario|BOLD:AAA3836
Autographa precationis|[1983]|XAJ494-06|2006-ONT-0494|658[0n]bp|Canada.Ontario|BOLD:AAA3836
Autographa precationis|[1984]|XAG877-05|2005-ONT-1461|658[0n]bp|Canada.Ontario|BOLD:AAA3836
Autographa precationis|[1985]|XAB648-04|04HBL005648|658[0n]bp|Canada.Ontario|BOLD:AAA3836
Autographa precationis|[1986]|XAG085-05|2005-ONT-669|658[0n]bp|Canada.Ontario|BOLD:AAA3836
Autographa precationis|[1987]|XAH317-05|2005-ONT-1900|658[0n]bp|Canada.Ontario|BOLD:AAA3836
Autographa precationis|[1988]|LPSOC360-08|PPBP-2359|655[0n]bp|Canada.Ontario|BOLD:AAA3836
Autographa precationis|[1989]|LPSO918-08|PPBP-0918|658[0n]bp|Canada.Ontario|BOLD:AAA3836
Autographa precationis|[1990]|LPSOB362-08|PPBP-1361|658[0n]bp|Canada.Ontario|BOLD:AAA3836
Autographa precationis|[1991]|PHMNB468-04|04HBL00694|658[0n]bp|Canada.New Brunswick|BOLD:AAA3836
Autographa precationis|[1992]|PHMNB280-04|04HBL007745|658[0n]bp|Canada.New Brunswick|BOLD:AAA3836
Autographa precationis|[1993]|BLTIB861-08|BL1280|658[0n]bp|Canada.Ontario|BOLD:AAA3836
Autographa precationis|[1994]|RDLQB559-05|DH010645|658[0n]bp|Canada.Quebec|BOLD:AAA3836

Autographa precationis|[1991]|PHMNB[illegible]-04|04HBL00[illegible]|658[0n]bp|Canada.New Brunswick|BOLD:AAA3836
Autographa precationis|[1992]|PHMNB280-04|04HBL007745|658[0n]bp|Canada.New Brunswick|BOLD:AAA3836
Autographa precationis|[1993]|BLTIB861-08|BL1280|658[0n]bp|Canada.Ontario|BOLD:AAA3836
Autographa precationis|[1994]|RDLQB559-05|DH010645|658[0n]bp|Canada.Quebec|BOLD:AAA3836
Autographa precationis|[1995]|XAJ544-06|2006-ONT-0544|658[0n]bp|Canada.Ontario|BOLD:AAA3836
Autographa precationis|[1996]|XAG549-05|2005-ONT-1133|658[1n]bp|Canada.Ontario|BOLD:AAA3836
Autographa precationis|[1997]|BLGSM078-09|BL1631|615[0n]bp|Canada.Ontario|BOLD:AAA3836
Autographa precationis|[1998]|PHMO332-03|moth2579.02|639[0n]bp|Canada.Ontario|BOLD:AAA3836
Autographa precationis|[1999]|XAH088-05|2005-ONT-1671|632[1n]bp|Canada.Ontario|BOLD:AAA3836
Autographa precationis|[2000]|XAD289-04|04HBL007289|587[0n]bp|Canada.Ontario|BOLD:AAA3836
Autographa precationis|[2001]|XAD482-04|04HBL007482|586[0n]bp|Canada.Ontario|BOLD:AAA3836
Autographa precationis|[2002]|XAD268-04|04HBL007268|602[0n]bp|Canada.Ontario|BOLD:AAA3836
Autographa precationis|[2003]|XAD266-04|04HBL007266|593[0n]bp|Canada.Ontario|BOLD:AAA3836
Autographa precationis|[2004]|XAD241-04|04HBL007241|592[0n]bp|Canada.Ontario|BOLD:AAA3836
Autographa precationis|[2005]|XAK590-07|HLC-16143|592[1n]bp|Canada.Ontario|BOLD:AAA3836
Autographa precationis|[2006]|XAG313-05|2005-ONT-897|658[0n]bp|Canada.Ontario|BOLD:AAA3836
Autographa precationis|[2007]|PHMNB696-04|04HBL00922|658[0n]bp|Canada.New Brunswick|BOLD:AAA3836
Autographa precationis|[2008]|LPSOB452-08|PPBP-1451|658[0n]bp|Canada.Ontario|BOLD:AAA3836
Autographa precationis|[2009]|BLTIB737-08|BL1027|658[0n]bp|Canada.Ontario|
Autographa precationis|[2010]|XAH628-05|2005-ONT-2211|658[0n]bp|Canada.Ontario|BOLD:AAA3836
Autographa precationis|[2011]|XAH132-05|2005-ONT-1715|658[0n]bp|Canada.Ontario|BOLD:AAA3836
Autographa precationis|[2012]|PHMNB299-04|04HBL007764|658[5n]bp|Canada.New Brunswick|BOLD:AAA3836
Autographa precationis|[2013]|XAD485-04|04HBL007485|589[0n]bp|Canada.Ontario|BOLD:AAA3836
Autographa precationis|[2014]|XAB665-04|04HBL005665|658[0n]bp|Canada.Ontario|BOLD:AAA3836
Autographa precationis|[2015]|XAH408-05|2005-ONT-1991|658[0n]bp|Canada.Ontario|BOLD:AAA3836
Autographa precationis|[2016]|BLTIB902-08|BL1321|658[0n]bp|Canada.Ontario|BOLD:AAA3836
Autographa precationis|[2017]|XAH409-05|2005-ONT-1992|658[0n]bp|Canada.Ontario|BOLD:AAA3836
Autographa precationis|[2018]|LPSO371-08|PPBP-0371|658[0n]bp|Canada.Ontario|BOLD:AAA3836
Autographa precationis|[2019]|BLTIB776-08|BL1172|658[0n]bp|Canada.Ontario|BOLD:AAA3836
Autographa precationis|[2020]|XAD382-04|04HBL007382|658[0n]bp|Canada.Ontario|BOLD:AAA3836
Autographa precationis|[2021]|XAH410-05|2005-ONT-1993|658[0n]bp|Canada.Ontario|BOLD:AAA3836
Autographa precationis|[2022]|LPSO040-08|PPBP-0040|658[0n]bp|Canada.Ontario|BOLD:AAA3836
Autographa precationis|[2023]|XAH552-05|2005-ONT-2135|658[0n]bp|Canada.Ontario|BOLD:AAA3836
Autographa precationis|[2024]|XAJ450-06|2006-ONT-0450|658[0n]bp|Canada.Ontario|BOLD:AAA3836
Autographa precationis|[2025]|XAJ484-06|2006-ONT-0484|658[0n]bp|Canada.Ontario|BOLD:AAA3836
Autographa precationis|[2026]|JSOCT032-11|BIOUG01497-G05|658[0n]bp|Canada.Ontario|BOLD:AAA3836
Autographa precationis|[2027]|LPSOC350-08|PPBP-2349|658[0n]bp|Canada.Ontario|BOLD:AAA3836
Autographa precationis|[2028]|LPSOC359-08|PPBP-2358|658[0n]bp|Canada.Ontario|BOLD:AAA3836
Autographa precationis|[2029]|XAH370-05|2005-ONT-1953|658[0n]bp|Canada.Ontario|BOLD:AAA3836
Autographa precationis|[2030]|XAH027-05|2005-ONT-1610|658[0n]bp|Canada.Ontario|BOLD:AAA3836
Autographa precationis|[2031]|XAJ674-06|2006-ONT-0674|658[0n]bp|Canada.Ontario|BOLD:AAA3836
Autographa precationis|[2032]|XAD431-04|04HBL007431|658[0n]bp|Canada.Ontario|BOLD:AAA3836
Autographa precationis|[2033]|XAD363-04|04HBL007363|658[0n]bp|Canada.Ontario|BOLD:AAA3836
Autographa precationis|[2034]|LPSO039-08|PPBP-0039|658[0n]bp|Canada.Ontario|BOLD:AAA3836
Autographa precationis|[2035]|LPSOB247-08|PPBP-1246|658[0n]bp|Canada.Ontario|BOLD:AAA3836
Autographa precationis|[2036]|BBLPC748-09|09BBELE-1748|658[0n]bp|Canada.Newfoundland and Labrador|BOLD ...
Autographa precationis|[2037]|LPSOB337-08|PPBP-1336|658[0n]bp|Canada.Ontario|BOLD:AAA3836
Autographa precationis|[2038]|RDLQB510-05|DH010596|658[0n]bp|Canada.Quebec|BOLD:AAA3836
Autographa precationis|[2039]|XAG105-05|2005-ONT-689|658[0n]bp|Canada.Ontario|BOLD:AAA3836
Autographa precationis|[2040]|LPSOC329-08|PPBP-2328|658[0n]bp|Canada.Ontario|BOLD:AAA3836
Autographa precationis|[2041]|LMIS046-06|05-ONMIS-0046|658[0n]bp|Canada.Ontario|BOLD:AAA3836
Autographa precationis|[2042]|XAH206-05|2005-ONT-1789|658[0n]bp|Canada.Ontario|BOLD:AAA3836
Autographa precationis|[2043]|XAH371-05|2005-ONT-1954|658[0n]bp|Canada.Ontario|BOLD:AAA3836
Autographa precationis|[2044]|PHMNB365-04|04HBL00591|658[0n]bp|Canada.New Brunswick|BOLD:AAA3836
Autographa precationis|[2045]|XAG204-05|2005-ONT-788|658[1n]bp|Canada.Ontario|BOLD:AAA3836
Autographa precationis|[2046]|XAH300-05|2005-ONT-1883|617[0n]bp|Canada.Ontario|BOLD:AAA3836
Autographa precationis|[2047]|PHMO351-03|moth2671.02|639[0n]bp|Canada.Ontario|BOLD:AAA3836
Autographa precationis|[2048]|XAD464-04|04HBL007464|570[0n]bp|Canada.Ontario|BOLD:AAA3836
Autographa precationis|[2049]|XAH205-05|2005-ONT-1788|658[0n]bp|Canada.Ontario|BOLD:AAA3836
Autographa precationis|[2050]|XAB676-04|04HBL005676|573[0n]bp|Canada.Ontario|BOLD:AAA3836
Autographa precationis|[2051]|XAD263-04|04HBL007263|578[0n]bp|Canada.Ontario|BOLD:AAA3836
Autographa precationis|[2052]|XAG534-05|2005-ONT-1118|566[1n]bp|Canada.Ontario|BOLD:AAA3836
Autographa precationis|[2053]|XAD368-04|04HBL007368|551[0n]bp|Canada.Ontario|BOLD:AAA3836
Autographa precationis|[2054]|XAD432-04|04HBL007432|554[0n]bp|Canada.Ontario|BOLD:AAA3836
Autographa precationis|[2055]|LPSOB233-08|PPBP-1232|658[0n]bp|Canada.Ontario|BOLD:AAA3836
Autographa bimaculata|[2056]|BBLPE117-09|09BBELE-2117|658[0n]bp|Canada.Nova Scotia|BOLD:AAC5751
Autographa bimaculata|[2057]|BBLPB515-10|10BBCLP-1514|658[0n]bp|Canada.Alberta|BOLD:AAC5751
Autographa bimaculata|[2058]|BBLPB514-10|10BBCLP-1513|658[0n]bp|Canada.Alberta|BOLD:AAC5751
Autographa bimaculata|[2059]|LPMNB507-09|08BBLEP-05545|658[0n]bp|Canada.Manitoba|BOLD:AAC5751
Autographa bimaculata|[2060]|RDNMG1004-08|CNC LEP00053128|658[0n]bp|Canada.New Brunswick|BOLD:AAC5751
Autographa bimaculata|[2061]|BBLEC313-09|09BBELE-0313|658[0n]bp|Canada.Nova Scotia|BOLD:AAC5751
Autographa bimaculata|[2062]|BBLPC588-09|09BBELE-1588|658[0n]bp|Canada.Nova Scotia|BOLD:AAC5751
Autographa bimaculata|[2063]|RDNMG1005-08|CNC LEP00053129|658[0n]bp|Canada.New Brunswick|BOLD:AAC5751
Autographa bimaculata|[2064]|LOWCD297-06|CGWC-3117|558[0n]bp|Canada.British Columbia|BOLD:AAC5751
Autographa bimaculata|[2065]|RDLQB624-05|DH010727|584[0n]bp|Canada.Quebec|BOLD:AAC5751
Autographa bimaculata|[2066]|LPMNB512-09|08BBLEP-05550|609[0n]bp|Canada.Manitoba|BOLD:AAC5751
Autographa bimaculata|[2067]|BBLPC991-09|09BBELE-1991|623[0n]bp|Canada.Nova Scotia|
Autographa bimaculata|[2068]|LBCD311-05|HLC-23131|639[0n]bp|Canada.British Columbia|BOLD:AAC5751
Autographa metallica|[2069]|LALPA1325-12|AVBC 1327-11|629[0n]bp|Canada.British Columbia|BOLD:ABX6057
Autographa metallica|[2070]|RDNMF376-08|NOC14462|658[0n]bp|Canada.British Columbia|BOLD:ABX6057
Autographa metallica|[2071]|LALPA1149-11|AVBC 959-11|658[0n]bp|Canada.British Columbia|BOLD:ABX6057
Autographa metallica|[2072]|RDNMF377-08|NOC14463|658[0n]bp|Canada.British Columbia|BOLD:ABX6057
Autographa metallica|[2073]|LALPA1274-11|AVBC 1276-11|658[0n]bp|Canada.British Columbia|BOLD:ABX6057
Autographa metallica|[2074]|LPAB063-08|08BBLEP-02385|658[0n]bp|Canada.Alberta|BOLD:ABX6057
Autographa metallica|[2075]|LBCH1499-10|10-JDWBC-1499|658[0n]bp|Canada.British Columbia|BOLD:ABX6057
Autographa metallica|[2076]|RDNMF378-08|NOC14464|658[0n]bp|Canada.British Columbia|BOLD:ABX6057
Autographa metallica|[2077]|LBCG2840-09|08-JDWBC-2840|658[0n]bp|Canada.British Columbia|BOLD:ABX6057
Autographa flagellum|[2078]|RDLQ755-07|DH013357|658[0n]bp|Canada.Quebec|BOLD:AAC2492
Autographa flagellum|[2079]|PHMO115-03|moth713.01|639[0n]bp|Canada.Ontario|BOLD:AAC2492
Autographa flagellum|[2080]|PHMNB723-05|Moth 416.03SA|658[0n]bp|Canada.New Brunswick|BOLD:AAC2492
Autographa flagellum|[2081]|BBLPE411-09|09BBELE-2411|658[0n]bp|Canada.Newfoundland and Labrador|BOLD:...
Autographa flagellum|[2082]|BBLEC354-09|09BBELE-0354|658[0n]bp|Canada.Newfoundland and Labrador|BOLD:...
Autographa flagellum|[2083]|BBLPE373-09|09BBELE-2373|658[0n]bp|Canada.Newfoundland and Labrador|BOLD:...
Autographa flagellum|[2084]|BBLPC577-09|09BBELE-1577|658[0n]bp|Canada.Nova Scotia|BOLD:AAC2492
Autographa flagellum|[2085]|BBLPC902-09|09BBELE-1902|658[0n]bp|Canada.Newfoundland and Labrador|BOLD:...
Autographa flagellum|[2086]|BBLPE009-09|09BBELE-2009|658[0n]bp|Canada.Nova Scotia|BOLD:AAC2492
Autographa flagellum|[2087]|BBLEC389-09|09BBELE-0389|658[0n]bp|Canada.Newfoundland and Labrador|BOLD:...
Autographa flagellum|[2088]|BBLPC650-09|09BBELE-1650|658[0n]bp|Canada.Newfoundland and Labrador|BOLD:...
Autographa flagellum|[2089]|BBLPC187-09|09BBELE-1187|658[0n]bp|Canada.Nova Scotia|BOLD:AAC2492
Autographa flagellum|[2090]|LPMN758-08|08BBLEP-01561|609[0n]bp|Canada.Manitoba|BOLD:AAC2492
Autographa pseudogamma|[2091]|LPABC086-09|08BBLEP-04305|658[0n]bp|Canada.Alberta|BOLD:AAD2600
Autographa pseudogamma|[2092]|RDNMG611-08|CNC LEP00052435|658[0n]bp|Canada.New Brunswick|BOLD:AAD2600
Autographa pseudogamma|[2093]|LPABC007-09|08BBLEP-04226|636[1n]bp|Canada.Alberta|BOLD:AAD2600
[illegible]

Autographa pseudogamma|[2091]|LPABC086-09|08BBLEP-04305|658[0n]bp|Canada.Alberta|BOLD:AAD2600
Autographa pseudogamma|[2092]|RDNMG611-08|CNC LEP00052435|658[0n]bp|Canada.New Brunswick|BOLD:AAD2600
Autographa pseudogamma|[2093]|LPABC007-09|08BBLEP-04226|636[1n]bp|Canada.Alberta|BOLD:AAD2600
Autographa pseudogamma|[2094]|BBLPE486-09|09BBELE-2486|658[0n]bp|Canada.Newfoundland and Labrador|BOLD ...
Autographa pseudogamma|[2095]|LPABB190-08|08BBLEP-03455|658[0n]bp|Canada.Alberta|BOLD:AAD2600
Autographa pseudogamma|[2096]|RDNMG612-08|CNC LEP00052436|658[0n]bp|Canada.New Brunswick|BOLD:AAD2600
Autographa sansoni|[2097]|LBCH2957-10|10-JDWBC-2957|658[0n]bp|Canada.British Columbia|BOLD:AAC1557
Autographa sansoni|[2098]|LBCA042-05|HLC-20042|658[0n]bp|Canada.British Columbia|BOLD:AAC1557
Autographa sansoni|[2099]|LBCG3026-09|08-JDWBC-3026|658[0n]bp|Canada.British Columbia|BOLD:AAC1557
Autographa sansoni|[2100]|LOWCD134-06|CGWC-2954|658[0n]bp|Canada.British Columbia|BOLD:AAC1557
Autographa sansoni|[2101]|LBCG1885-09|08-JDWBC-1885|658[0n]bp|Canada.British Columbia|BOLD:AAC1557
Autographa sansoni|[2102]|LBCC562-05|HLC-22442|658[0n]bp|Canada.British Columbia|BOLD:AAC1557
Autographa sansoni|[2103]|LBCH1759-10|10-JDWBC-1759|658[0n]bp|Canada.British Columbia|BOLD:AAC1557
Autographa sansoni|[2104]|LOWCE195-06|CGWC-3955|658[0n]bp|Canada.British Columbia|BOLD:AAC1557
Autographa sansoni|[2105]|LBCG2541-09|08-JDWBC-2541|658[0n]bp|Canada.British Columbia|BOLD:AAC1557
Autographa sansoni|[2106]|LBCG3025-09|08-JDWBC-3025|658[0n]bp|Canada.British Columbia|BOLD:AAC1557
Autographa sansoni|[2107]|LBCG3027-09|08-JDWBC-3027|658[0n]bp|Canada.British Columbia|BOLD:AAC1557
Autographa sansoni|[2108]|LBCG2833-09|08-JDWBC-2833|658[0n]bp|Canada.British Columbia|BOLD:AAC1557
Autographa sansoni|[2109]|RDMAB527-06|UASM58487|658[0n]bp|Canada.Alberta|BOLD:AAC1557
Autographa sansoni|[2110]|LBCA036-05|HLC-20036|658[0n]bp|Canada.British Columbia|BOLD:AAC1557
Autographa sansoni|[2111]|LBCG3028-09|08-JDWBC-3028|658[0n]bp|Canada.British Columbia|BOLD:AAC1557
Autographa sansoni|[2112]|LBCH2238-10|10-JDWBC-2238|658[0n]bp|Canada.British Columbia|BOLD:AAC1557
Autographa ampla|[2113]|LOWCD123-06|CGWC-2943|569[0n]bp|Canada.British Columbia|BOLD:AAA8729
Autographa ampla|[2114]|BBLPC227-09|09BBELE-1227|658[0n]bp|Canada.Nova Scotia|BOLD:AAA8729
Autographa ampla|[2115]|BBLPC132-09|09BBELE-1132|621[0n]bp|Canada.Nova Scotia|BOLD:AAA8729
Autographa ampla|[2116]|RDLQB028-05|DH010114|658[0n]bp|Canada.Quebec|BOLD:AAA8729
Autographa ampla|[2117]|BBLPC233-09|09BBELE-1233|658[0n]bp|Canada.Nova Scotia|BOLD:AAA8729
Autographa ampla|[2118]|LHLEP121-06|UBC-2006-0752|617[0n]bp|Canada.British Columbia|BOLD:AAA8729
Autographa ampla|[2119]|RDLQB029-05|DH010115|602[0n]bp|Canada.Quebec|BOLD:AAA8729
Autographa ampla|[2120]|LOWCD171-06|CGWC-2991|599[0n]bp|Canada.British Columbia|BOLD:AAA8729
Autographa ampla|[2121]|LOWCB589-05|CGWC-1529|592[0n]bp|Canada.British Columbia|BOLD:AAA8729
Autographa ampla|[2122]|LOWCB590-05|CGWC-1530|611[0n]bp|Canada.British Columbia|BOLD:AAA8729
Autographa ampla|[2123]|PHMO131-03|moth767.01|639[0n]bp|Canada.Ontario|BOLD:AAA8729
Autographa ampla|[2124]|RDLQB030-05|DH010116|658[0n]bp|Canada.Quebec|BOLD:AAA8729
Autographa ampla|[2125]|RDLQB032-05|DH010118|658[0n]bp|Canada.Quebec|BOLD:AAA8729
Autographa ampla|[2126]|BBLEC773-09|09BBELE-0773|658[0n]bp|Canada.Newfoundland and Labrador|BOLD:AAA8729
Autographa ampla|[2127]|LBCH914-10|10-JDWBC-0914|658[0n]bp|Canada.British Columbia|BOLD:AAA8729
Autographa ampla|[2128]|LBCH118-10|10-JDWBC-0118|658[0n]bp|Canada.British Columbia|BOLD:AAA8729
Autographa ampla|[2129]|BBLPC243-09|09BBELE-1243|658[0n]bp|Canada.Nova Scotia|BOLD:AAA8729
Autographa ampla|[2130]|TMNBB082-06|MNBTT-1022|658[0n]bp|Canada.New Brunswick|BOLD:AAA8729
Autographa ampla|[2131]|LALPA954-11|AVBC 1127-11|658[0n]bp|Canada.British Columbia|BOLD:AAA8729
Autographa ampla|[2132]|LBCH3066-10|10-JDWBC-3066|658[0n]bp|Canada.British Columbia|BOLD:AAA8729
Autographa ampla|[2133]|LBCH809-10|10-JDWBC-0809|658[0n]bp|Canada.British Columbia|BOLD:AAA8729
Autographa ampla|[2134]|LBCA077-05|HLC-20077|658[0n]bp|Canada.British Columbia|BOLD:AAA8729
Autographa ampla|[2135]|LHLEP120-06|UBC-2006-0197|658[0n]bp|Canada.British Columbia|BOLD:AAA8729
Autographa ampla|[2136]|LBCH3332-10|10-JDWBC-3332|658[0n]bp|Canada.British Columbia|BOLD:AAA8729
Autographa ampla|[2137]|LBCH006-10|10-JDWBC-0006|658[0n]bp|Canada.British Columbia|BOLD:AAA8729
Autographa ampla|[2138]|LALPA843-11|AVBC 1016-11|658[0n]bp|Canada.British Columbia|BOLD:AAA8729
Autographa ampla|[2139]|LBCH230-10|10-JDWBC-0230|658[0n]bp|Canada.British Columbia|BOLD:AAA8729
Autographa ampla|[2140]|LHLEP122-06|UBC-2006-0373|658[0n]bp|Canada.British Columbia|BOLD:AAA8729
Autographa ampla|[2141]|LBCH491-10|10-JDWBC-0491|658[0n]bp|Canada.British Columbia|BOLD:AAA8729
Autographa ampla|[2142]|LPVIA612-08|PFC-2006-0840|658[0n]bp|Canada.British Columbia|BOLD:AAA8729
Autographa ampla|[2143]|LBCH4403-10|10-JDWBC-4403|658[0n]bp|Canada.British Columbia|BOLD:AAA8729
Autographa ampla|[2144]|LBCD451-05|HLC-23271|658[0n]bp|Canada.British Columbia|BOLD:AAA8729
Autographa ampla|[2145]|LBCH651-10|10-JDWBC-0651|658[0n]bp|Canada.British Columbia|BOLD:AAA8729
Autographa ampla|[2146]|XAE315-04|Moth4315.03|658[0n]bp|Canada.Ontario|BOLD:AAA8729
Autographa ampla|[2147]|LPABC978-09|08BBLEP-05389|658[0n]bp|Canada.Alberta|BOLD:AAA8729
Autographa ampla|[2148]|LBCC289-05|HLC-22169|658[0n]bp|Canada.British Columbia|BOLD:AAA8729
Autographa ampla|[2149]|LALPA1289-11|AVBC 1291-11|658[0n]bp|Canada.British Columbia|BOLD:AAA8729
Autographa ampla|[2150]|LPABB397-08|08BBLEP-03662|658[0n]bp|Canada.Alberta|BOLD:AAA8729
Autographa ampla|[2151]|RDLQB031-05|DH010117|658[0n]bp|Canada.Quebec|BOLD:AAA8729
Autographa ampla|[2152]|LBCD031-05|HLC-22851|658[0n]bp|Canada.British Columbia|BOLD:AAA8729
Autographa ampla|[2153]|LBCH3781-10|10-JDWBC-3781|658[0n]bp|Canada.British Columbia|BOLD:AAA8729
Autographa ampla|[2154]|LBCH3451-10|10-JDWBC-3451|658[0n]bp|Canada.British Columbia|BOLD:AAA8729
Autographa ampla|[2155]|LBCB904-05|HLC-21844|636[0n]bp|Canada.British Columbia|BOLD:AAA8729
Autographa ampla|[2156]|LBCA610-05|HLC-20610|633[0n]bp|Canada.British Columbia|BOLD:AAA8729
Autographa ampla|[2157]|LBCC641-05|HLC-22521|658[0n]bp|Canada.British Columbia|BOLD:AAA8729
Autographa corusca|[2158]|LALPA661-10|AVBC 663-10|658[0n]bp|Canada.British Columbia|BOLD:ABZ0515
Autographa corusca|[2159]|LALPA687-10|AVBC 689-10|658[0n]bp|Canada.British Columbia|BOLD:ABZ0515
Autographa corusca|[2160]|LBCH120-10|10-JDWBC-0120|658[0n]bp|Canada.British Columbia|BOLD:ABZ0515
Autographa corusca|[2161]|LBCH1302-10|10-JDWBC-1302|658[0n]bp|Canada.British Columbia|BOLD:ABZ0515
Autographa corusca|[2162]|LPVIB068-08|PFC-2006-1416|658[0n]bp|Canada.British Columbia|BOLD:ABZ0515
Autographa corusca|[2163]|RDNMF089-08|NOC14175|658[0n]bp|Canada.British Columbia|BOLD:ABZ0515
Autographa corusca|[2164]|LALPA662-10|AVBC 664-10|658[0n]bp|Canada.British Columbia|BOLD:ABZ0515
Autographa corusca|[2165]|LBCS262-07|UBC-2007-0770|658[0n]bp|Canada.British Columbia|BOLD:ABZ0515
Autographa corusca|[2166]|LHLEP277-06|UBC-2006-1050|658[0n]bp|Canada.British Columbia|BOLD:ABZ0515
Autographa corusca|[2167]|LALPA633-10|AVBC 635-10|658[0n]bp|Canada.British Columbia|BOLD:ABZ0515
Autographa corusca|[2168]|LHLEP278-06|UBC-2006-1659|658[0n]bp|Canada.British Columbia|BOLD:ABZ0515
Autographa corusca|[2169]|LBCH4650-10|10-JDWBC-4650|636[0n]bp|Canada.British Columbia|BOLD:ABZ0515
Autographa corusca|[2170]|LHLEP279-06|UBC-2006-1930|658[0n]bp|Canada.British Columbia|BOLD:ABZ0515
Autographa corusca|[2171]|LBCS261-07|UBC-2007-0769|658[0n]bp|Canada.British Columbia|BOLD:ABZ0515
Autographa corusca|[2172]|LBCH4679-10|10-JDWBC-4679|658[0n]bp|Canada.British Columbia|BOLD:ABZ0515
Autographa buraetica|[2173]|RDNMF084-08|NOC14170|658[0n]bp|Canada.Yukon Territory|BOLD:AAA3990
Autographa mappa|[2174]|BBLPB330-10|10BBCLP-1329|658[0n]bp|Canada.British Columbia|BOLD:AAA9921
Autographa mappa|[2175]|BBLEC274-09|09BBELE-0274|658[0n]bp|Canada.Nova Scotia|BOLD:AAA9921
Autographa mappa|[2176]|BBLPC213-09|09BBELE-1213|658[0n]bp|Canada.Nova Scotia|BOLD:AAA9921
Autographa mappa|[2177]|BBLPB277-10|10BBCLP-1276|658[0n]bp|Canada.British Columbia|BOLD:AAA9921
Autographa mappa|[2178]|RDLQB006-05|DH010092|658[0n]bp|Canada.Quebec|BOLD:AAA9921
Autographa mappa|[2179]|BBLEC372-09|09BBELE-0372|658[0n]bp|Canada.Newfoundland and Labrador|BOLD:AAA9921
Autographa mappa|[2180]|LBCB549-05|HLC-21489|658[0n]bp|Canada.British Columbia|BOLD:AAA9921
Autographa mappa|[2181]|BBLPB278-10|10BBCLP-1277|658[0n]bp|Canada.Ontario|BOLD:AAA9921
Autographa mappa|[2182]|LBCA034-05|HLC-20034|658[0n]bp|Canada.British Columbia|BOLD:AAA9921
Autographa mappa|[2183]|RDLQF855-06|DH012016|658[0n]bp|Canada.Quebec|BOLD:AAA9921
Autographa mappa|[2184]|BBLPC221-09|09BBELE-1221|658[0n]bp|Canada.Nova Scotia|BOLD:AAA9921
Autographa mappa|[2185]|RDLQB008-05|DH010094|658[0n]bp|Canada.Quebec|BOLD:AAA9921
Autographa mappa|[2186]|BBLEC352-09|09BBELE-0352|658[0n]bp|Canada.Newfoundland and Labrador|BOLD:AAA9921
Autographa mappa|[2187]|RDLQB009-05|DH010095|658[0n]bp|Canada.Quebec|BOLD:AAA9921
Autographa mappa|[2188]|RDLQB005-05|DH010091|658[0n]bp|Canada.Quebec|BOLD:AAA9921
Autographa mappa|[2189]|RDLQB007-05|DH010093|658[0n]bp|Canada.Quebec|BOLD:AAA9921
Autographa mappa|[2190]|LPSOC281-08|PPBP-2280|658[0n]bp|Canada.Ontario|BOLD:AAA9921
Autographa mappa|[2191]|RDLQF854-06|DH012015|658[0n]bp|Canada.Quebec|BOLD:AAA9921
Autographa mappa|[2192]|LBCA035-05|HLC-20035|658[0n]bp|Canada.British Columbia|BOLD:AAA9921
Autographa mappa|[2193]|LBCH094-10|10-JDWBC-0094|658[0n]bp|Canada.British Columbia|BOLD:AAA9921

Autographa mappa|[2191]|RDLQF854-06|DH012015|658[0n]bp|Canada.Quebec|BOLD:AAA9921
Autographa mappa|[2192]|LBCA035-05|HLC-20035|658[0n]bp|Canada.British Columbia|BOLD:AAA9921
Autographa mappa|[2193]|LBCH094-10|10-JDWBC-0094|658[0n]bp|Canada.British Columbia|BOLD:AAA9921
Autographa mappa|[2194]|LPABB036-08|08BBLEP-03301|658[0n]bp|Canada.Alberta|BOLD:AAA9921
Autographa mappa|[2195]|LPABC941-09|08BBLEP-05352|658[0n]bp|Canada.Alberta|BOLD:AAA9921
Autographa mappa|[2196]|BBLPE491-09|09BBELE-2491|658[0n]bp|Canada.Newfoundland and Labrador|BOLD:AAA9921
Autographa mappa|[2197]|LBCB921-05|HLC-21861|636[0n]bp|Canada.British Columbia|BOLD:AAA9921
Autographa mappa|[2198]|LOWCD176-06|CGWC-2996|569[0n]bp|Canada.British Columbia|BOLD:AAA9921
Autographa mappa|[2199]|BBLEC102-09|09BBELE-0102|636[0n]bp|Canada.Nova Scotia|BOLD:AAA9921
Autographa mappa|[2200]|BBLEC373-09|09BBELE-0373|637[0n]bp|Canada.Newfoundland and Labrador|BOLD:AAA9921
Autographa mappa|[2201]|BBLEC269-09|09BBELE-0269|633[0n]bp|Canada.Nova Scotia|BOLD:AAA9921
Autographa mappa|[2202]|BBLPE120-09|09BBELE-2120|632[0n]bp|Canada.Nova Scotia|BOLD:AAA9921
Autographa mappa|[2203]|RDLQB010-05|DH010096|595[1n]bp|Canada.Quebec|BOLD:AAA9921
Autographa mappa|[2204]|BBLEC370-09|09BBELE-0370|615[0n]bp|Canada.Newfoundland and Labrador|BOLD:AAA9921
Autographa mappa|[2205]|LBCB920-05|HLC-21860|636[0n]bp|Canada.British Columbia|BOLD:AAA9921
Autographa mappa|[2206]|BBLPE538-09|09BBELE-2538|658[0n]bp|Canada.Newfoundland and Labrador|BOLD:AAA9921
Autographa speciosa|[2207]|RDNMF417-08|NOC14503|658[0n]bp|United States.Oregon|BOLD:ABZ0513
Autographa speciosa|[2208]|RDNMG614-08|CNC LEP00052438|649[0n]bp|United States.Oregon|BOLD:ABZ0513
Autographa speciosa|[2209]|RDNMG613-08|CNC LEP00052437|658[0n]bp|United States.Oregon|BOLD:ABZ0513
Autographa speciosa|[2210]|RDNMF416-08|NOC14502|658[0n]bp|United States.Oregon|BOLD:ABZ0513
Autographa v-alba|[2211]|LPABC073-09|08BBLEP-04292|658[0n]bp|Canada.Alberta|BOLD:ABZ0514
Autographa v-alba|[2212]|LPMN900-08|08BBLEP-02258|658[0n]bp|Canada.Alberta|BOLD:ABZ0514
Megalographa biloba|[2213]|TMG97-03|moth184.01|639[0n]bp|Canada.Ontario|BOLD:AAD7518
Megalographa biloba|[2214]|PMG131-03|moth183.01|617[0n]bp|Canada.Ontario|BOLD:AAD7518
Syngrapha epigaea|[2215]|BBLPB310-10|10BBCLP-1309|658[0n]bp|Canada.British Columbia|BOLD:AAC8825
Syngrapha epigaea|[2216]|RDLQB617-05|DH010720|585[0n]bp|Canada.Quebec|BOLD:AAC8825
Syngrapha epigaea|[2217]|RDLQB620-05|DH010723|643[0n]bp|Canada.Quebec|BOLD:AAC8825
Syngrapha epigaea|[2218]|RDLQB619-05|DH010722|585[0n]bp|Canada.Quebec|BOLD:AAC8825
Syngrapha epigaea|[2219]|RDLQB618-05|DH010721|592[0n]bp|Canada.Quebec|BOLD:AAC8825
Syngrapha epigaea|[2220]|BBLPC995-09|09BBELE-1995|599[0n]bp|Canada.Nova Scotia|BOLD:AAC8825
Syngrapha epigaea|[2221]|LOWCB588-05|CGWC-1528|611[0n]bp|Canada.British Columbia|BOLD:AAC8825
Syngrapha epigaea|[2222]|BBLPE080-09|09BBELE-2080|658[0n]bp|Canada.Nova Scotia|BOLD:AAC8825
Syngrapha epigaea|[2223]|RDLQB841-05|DH010928|658[0n]bp|Canada.Quebec|BOLD:AAC8825
Syngrapha epigaea|[2224]|LBCH3782-10|10-JDWBC-3782|658[0n]bp|Canada.British Columbia|BOLD:AAC8825
Syngrapha epigaea|[2225]|BBLEC524-09|09BBELE-0524|658[0n]bp|Canada.New Brunswick|BOLD:AAC8825
Syngrapha epigaea|[2226]|LALPA695-10|AVBC 697-10|658[0n]bp|Canada.British Columbia|BOLD:AAC8825
Syngrapha epigaea|[2227]|LALPA708-10|AVBC 710-10|658[0n]bp|Canada.British Columbia|BOLD:AAC8825
Syngrapha epigaea|[2228]|LALPA1320-12|AVBC 1322-11|599[0n]bp|Canada.British Columbia|BOLD:AAC8825
Anagrapha falcifera|[2229]|LBCH5211-10|10-JDWBC-5211|658[0n]bp|Canada.British Columbia|BOLD:AAA6337
Anagrapha falcifera|[2230]|LBCH5172-10|10-JDWBC-5172|658[0n]bp|Canada.British Columbia|BOLD:AAA6337
Anagrapha falcifera|[2231]|XAD430-04|04HBL007430|592[0n]bp|Canada.Ontario|BOLD:AAA6337
Anagrapha falcifera|[2232]|LOWCB592-05|CGWC-1532|575[0n]bp|Canada.British Columbia|BOLD:AAA6337
Anagrapha falcifera|[2233]|RDLQB726-05|DH010829|564[0n]bp|Canada.Quebec|BOLD:AAA6337
Anagrapha falcifera|[2234]|PHMO325-03|moth2540.02|639[2n]bp|Canada.Ontario|BOLD:AAA6337
Anagrapha falcifera|[2235]|LOWCB593-05|CGWC-1533|605[0n]bp|Canada.British Columbia|BOLD:AAA6337
Anagrapha falcifera|[2236]|LOWCB591-05|CGWC-1531|585[0n]bp|Canada.British Columbia|BOLD:AAA6337
Anagrapha falcifera|[2237]|XAD303-04|04HBL007303|594[1n]bp|Canada.Ontario|BOLD:AAA6337
Anagrapha falcifera|[2238]|XAD360-04|04HBL007360|582[0n]bp|Canada.Ontario|BOLD:AAA6337
Anagrapha falcifera|[2239]|XAJ487-06|2006-ONT-0487|649[0n]bp|Canada.Ontario|BOLD:AAA6337
Anagrapha falcifera|[2240]|LPSOB390-08|PPBP-1389|658[0n]bp|Canada.Ontario|BOLD:AAA6337
Anagrapha falcifera|[2241]|LPSK534-08|08BBLEP-02102|658[0n]bp|Canada.Saskatchewan|BOLD:AAA6337
Anagrapha falcifera|[2242]|RDLQB730-05|DH010833|592[0n]bp|Canada.Quebec|BOLD:AAA6337
Anagrapha falcifera|[2243]|XAF700-05|2005-ONT-349|658[0n]bp|Canada.Ontario|BOLD:AAA6337
Anagrapha falcifera|[2244]|LPSK415-08|08BBLEP-01983|658[0n]bp|Canada.Saskatchewan|BOLD:AAA6337
Anagrapha falcifera|[2245]|XAJ489-06|2006-ONT-0489|649[0n]bp|Canada.Ontario|BOLD:AAA6337
Anagrapha falcifera|[2246]|XAD591-05|2005-ONT-6|658[0n]bp|Canada.Ontario|BOLD:AAA6337
Anagrapha falcifera|[2247]|LPSOB721-08|PPBP-1720|640[0n]bp|Canada.Ontario|BOLD:AAA6337
Anagrapha falcifera|[2248]|LPSK541-08|08BBLEP-02109|658[0n]bp|Canada.Saskatchewan|BOLD:AAA6337
Anagrapha falcifera|[2249]|LPSOC333-08|PPBP-2332|658[0n]bp|Canada.Ontario|BOLD:AAA6337
Anagrapha falcifera|[2250]|LPSK569-08|08BBLEP-02137|658[0n]bp|Canada.Saskatchewan|BOLD:AAA6337
Anagrapha falcifera|[2251]|TTMNB288-06|MNBTT-288|658[0n]bp|Canada.New Brunswick|BOLD:AAA6337
Anagrapha falcifera|[2252]|LPSOB217-08|PPBP-1216|658[0n]bp|Canada.Ontario|BOLD:AAA6337
Anagrapha falcifera|[2253]|XAK246-06|2006-ONT-1241|658[0n]bp|Canada.Ontario|BOLD:AAA6337
Anagrapha falcifera|[2254]|LPSK538-08|08BBLEP-02106|658[0n]bp|Canada.Saskatchewan|BOLD:AAA6337
Anagrapha falcifera|[2255]|LBCG039-08|08-JDWBC-0039|658[0n]bp|Canada.British Columbia|BOLD:AAA6337
Anagrapha falcifera|[2256]|LPSK591-08|08BBLEP-02159|658[0n]bp|Canada.Saskatchewan|BOLD:AAA6337
Anagrapha falcifera|[2257]|XAJ412-06|2006-ONT-0412|658[0n]bp|Canada.Ontario|BOLD:AAA6337
Anagrapha falcifera|[2258]|XAH835-05|2005-ONT-2418|658[0n]bp|Canada.Ontario|BOLD:AAA6337
Anagrapha falcifera|[2259]|BLTIB752-08|BL1042|658[0n]bp|Canada.Ontario|BOLD:AAA6337
Anagrapha falcifera|[2260]|LPSOB464-08|PPBP-1463|658[0n]bp|Canada.Ontario|BOLD:AAA6337
Anagrapha falcifera|[2261]|XAK247-06|2006-ONT-1242|658[0n]bp|Canada.Ontario|BOLD:AAA6337
Anagrapha falcifera|[2262]|LPSK498-08|08BBLEP-02066|658[0n]bp|Canada.Saskatchewan|BOLD:AAA6337
Anagrapha falcifera|[2263]|LPSOB234-08|PPBP-1233|658[0n]bp|Canada.Ontario|BOLD:AAA6337
Anagrapha falcifera|[2264]|LPSOB371-08|PPBP-1370|657[0n]bp|Canada.Ontario|BOLD:AAA6337
Anagrapha falcifera|[2265]|BLTIB462-08|BL709|658[0n]bp|Canada.Ontario|BOLD:AAA6337
Anagrapha falcifera|[2266]|XAC667-04|04HBL006667|658[0n]bp|Canada.Ontario|BOLD:AAA6337
Anagrapha falcifera|[2267]|LPSK559-08|08BBLEP-02127|658[0n]bp|Canada.Saskatchewan|BOLD:AAA6337
Anagrapha falcifera|[2268]|LPSOB404-08|PPBP-1403|658[0n]bp|Canada.Ontario|BOLD:AAA6337
Anagrapha falcifera|[2269]|LPSK532-08|08BBLEP-02100|658[0n]bp|Canada.Saskatchewan|BOLD:AAA6337
Anagrapha falcifera|[2270]|BLTIB915-08|BL1335|658[0n]bp|Canada.Ontario|BOLD:AAA6337
Anagrapha falcifera|[2271]|LPSOB276-08|PPBP-1275|658[0n]bp|Canada.Ontario|BOLD:AAA6337
Anagrapha falcifera|[2272]|MNBB528-05|05-NBSTA-444|658[0n]bp|Canada.New Brunswick|BOLD:AAA6337
Anagrapha falcifera|[2273]|LPSOD742-09|08BBLEP-00524|658[0n]bp|Canada.Ontario|BOLD:AAA6337
Anagrapha falcifera|[2274]|LPSK048-08|08BBLEP-00751|658[0n]bp|Canada.Saskatchewan|BOLD:AAA6337
Anagrapha falcifera|[2275]|LPSOD636-09|08BBLEP-00417|658[0n]bp|Canada.Ontario|BOLD:AAA6337
Anagrapha falcifera|[2276]|LPSOD1046-09|08MZPP-103|658[0n]bp|Canada.Ontario|BOLD:AAA6337
Anagrapha falcifera|[2277]|LPSK513-08|08BBLEP-02081|658[0n]bp|Canada.Saskatchewan|BOLD:AAA6337
Anagrapha falcifera|[2278]|LPSK526-08|08BBLEP-02094|658[0n]bp|Canada.Saskatchewan|BOLD:AAA6337
Anagrapha falcifera|[2279]|XAJ498-06|2006-ONT-0498|658[0n]bp|Canada.Ontario|BOLD:AAA6337
Anagrapha falcifera|[2280]|XAJ506-06|2006-ONT-0506|658[0n]bp|Canada.Ontario|BOLD:AAA6337
Anagrapha falcifera|[2281]|LPSOC121-08|PPBP-2120|658[0n]bp|Canada.Ontario|BOLD:AAA6337
Anagrapha falcifera|[2282]|LPSK533-08|08BBLEP-02101|658[0n]bp|Canada.Saskatchewan|BOLD:AAA6337
Anagrapha falcifera|[2283]|LPSOB691-08|PPBP-1690|645[0n]bp|Canada.Ontario|BOLD:AAA6337
Anagrapha falcifera|[2284]|BLTIB1003-08|BL1440|658[0n]bp|Canada.Ontario|BOLD:AAA6337
Syngrapha ignea|[2285]|LOWCD145-06|CGWC-2965|603[0n]bp|Canada.British Columbia|BOLD:ABZ2814
Syngrapha ignea|[2286]|LOWCE080-06|CGWC-3840|658[0n]bp|Canada.British Columbia|BOLD:ABZ2814
Syngrapha ignea|[2287]|RDNME527-08|LEP037951|658[0n]bp|Canada.Yukon Territory|BOLD:ABZ2814
Syngrapha ignea|[2288]|LOWCE079-06|CGWC-3839|658[0n]bp|Canada.British Columbia|BOLD:ABZ2814
Syngrapha ignea|[2289]|JSYKA292-10|JSYKB-198|658[0n]bp|Canada.Yukon Territory|BOLD:ABZ2814
Syngrapha alticola|[2290]|LCH523-04|04HBL003523|584[0n]bp|Canada.Manitoba|BOLD:ABY8699
Syngrapha alticola|[2291]|LCHP020-07|07PROBE-00080|644[0n]bp|Canada.Manitoba|BOLD:ABY8699
Syngrapha alticola|[2292]|LCHQ110-07|07PROBE-10879|658[0n]bp|Canada.Manitoba|BOLD:ABY8699
Syngrapha alticola|[2293]|MHLEP114-07|CHU06-LEP-114|658[0n]bp|Canada.Manitoba|BOLD:ABY8699

Syngrapha alticola|[2290]|LCH323-04|04HBL003323|584[0n]bp|Canada.Manitoba|BOLD:ABY8699
Syngrapha alticola|[2291]|LCHP020-07|07PROBE-00080|644[0n]bp|Canada.Manitoba|BOLD:ABY8699
Syngrapha alticola|[2292]|LCHQ110-07|07PROBE-10879|658[0n]bp|Canada.Manitoba|BOLD:ABY8699
Syngrapha alticola|[2293]|MHLEP114-07|CHU06-LEP-114|658[0n]bp|Canada.Manitoba|BOLD:ABY8699
Syngrapha parilis|[2294]|RDNMF739-08|NOC14825|609[0n]bp|Canada.Nunavut|BOLD:AAD7310
Syngrapha parilis|[2295]|RDNME518-08|LEP037942|658[0n]bp|Canada.Yukon Territory|BOLD:AAD7310
Syngrapha parilis|[2296]|RDNMF740-08|NOC14826|658[0n]bp|Canada.Yukon Territory|BOLD:AAD7310
Syngrapha borea|[2297]|RDNMF359-08|NOC14445|658[0n]bp|Canada.Alberta|BOLD:AAE2536
Syngrapha borea|[2298]|RDNMF360-08|NOC14446|658[0n]bp|Canada.Yukon Territory|BOLD:AAE2536
Syngrapha borea|[2299]|RDNMF361-08|NOC14447|658[0n]bp|Canada.British Columbia|BOLD:AAE2536
Syngrapha borea|[2300]|RDNMF362-08|NOC14448|658[0n]bp|Canada.British Columbia|BOLD:AAE2536
Syngrapha borea|[2301]|RDNMF358-08|NOC14444|658[0n]bp|Canada.British Columbia|BOLD:AAE2536
Syngrapha diasema|[2302]|LCHIP055-07|06-PROBE-2524|650[0n]bp|Canada.Manitoba|BOLD:AAA6513
Syngrapha diasema|[2303]|LCH329-04|04HBL003329|658[0n]bp|Canada.Manitoba|BOLD:AAA6513
Syngrapha diasema|[2304]|LCH337-04|04HBL003337|658[0n]bp|Canada.Manitoba|BOLD:AAA6513
Syngrapha diasema|[2305]|LCHP841-07|07PROBE-10598|631[0n]bp|Canada.Manitoba|BOLD:AAA6513
Syngrapha diasema|[2306]|LCH450-04|04HBL003450|658[0n]bp|Canada.Manitoba|BOLD:AAA6513
Syngrapha diasema|[2307]|LCH454-04|04HBL003454|658[0n]bp|Canada.Manitoba|BOLD:AAA6513
Syngrapha diasema|[2308]|LCH333-04|04HBL003333|658[0n]bp|Canada.Manitoba|BOLD:AAA6513
Syngrapha diasema|[2309]|LCH443-04|04HBL003443|658[0n]bp|Canada.Manitoba|BOLD:AAA6513
Syngrapha diasema|[2310]|LCHP578-07|07PROBE-10248|658[0n]bp|Canada.Manitoba|BOLD:AAA6513
Syngrapha diasema|[2311]|LCH338-04|04HBL003338|658[0n]bp|Canada.Manitoba|BOLD:AAA6513
Syngrapha diasema|[2312]|LCHP577-07|07PROBE-10247|654[0n]bp|Canada.Manitoba|BOLD:AAA6513
Syngrapha diasema|[2313]|LCH335-04|04HBL003335|658[0n]bp|Canada.Manitoba|BOLD:AAA6513
Syngrapha diasema|[2314]|LCHP478-07|07PROBE-10146|647[0n]bp|Canada.Manitoba|BOLD:AAA6513
Syngrapha diasema|[2315]|LCHP903-07|07PROBE-10665|644[0n]bp|Canada.Manitoba|BOLD:AAA6513
Syngrapha diasema|[2316]|LCH445-04|04HBL003445|658[0n]bp|Canada.Manitoba|BOLD:AAA6513
Syngrapha diasema|[2317]|LCHP493-07|07PROBE-10161|657[0n]bp|Canada.Manitoba|BOLD:AAA6513
Syngrapha diasema|[2318]|LCHP789-07|07PROBE-10474|658[0n]bp|Canada.Manitoba|BOLD:AAA6513
Syngrapha diasema|[2319]|LCH455-04|04HBL003455|658[0n]bp|Canada.Manitoba|BOLD:AAA6513
Syngrapha diasema|[2320]|LCH453-04|04HBL003453|658[0n]bp|Canada.Manitoba|BOLD:AAA6513
Syngrapha diasema|[2321]|LCHQ191-07|07PROBE-10968|658[0n]bp|Canada.Manitoba|BOLD:AAA6513
Syngrapha diasema|[2322]|LCHP626-07|07PROBE-10307|658[0n]bp|Canada.Manitoba|BOLD:AAA6513
Syngrapha diasema|[2323]|LCHQ482-08|07WNP-10374|657[0n]bp|Canada.Manitoba|BOLD:AAA6513
Syngrapha diasema|[2324]|LCH452-04|04HBL003452|658[0n]bp|Canada.Manitoba|BOLD:AAA6513
Syngrapha diasema|[2325]|LCH451-04|04HBL003451|658[0n]bp|Canada.Manitoba|BOLD:AAA6513
Syngrapha diasema|[2326]|JGLL047-10|10PROBE-19549|658[0n]bp|Canada.Manitoba|BOLD:AAA6513
Syngrapha diasema|[2327]|LCH331-04|04HBL003331|658[0n]bp|Canada.Manitoba|BOLD:AAA6513
Syngrapha diasema|[2328]|LCH336-04|04HBL003336|658[0n]bp|Canada.Manitoba|BOLD:AAA6513
Syngrapha diasema|[2329]|LCH330-04|04HBL003330|658[0n]bp|Canada.Manitoba|BOLD:AAA6513
Syngrapha diasema|[2330]|CHLEP213-09|09PROBE-09508|658[0n]bp|Canada.Manitoba|BOLD:AAA6513
Syngrapha diasema|[2331]|LCH328-04|04HBL003328|658[0n]bp|Canada.Manitoba|BOLD:AAA6513
Syngrapha diasema|[2332]|LCH459-04|04HBL003459|658[0n]bp|Canada.Manitoba|BOLD:AAA6513
Syngrapha diasema|[2333]|LCH524-04|04HBL003524|658[0n]bp|Canada.Manitoba|BOLD:AAA6513
Syngrapha diasema|[2334]|LCHQ909-08|07WNP-10801|658[0n]bp|Canada.Manitoba|BOLD:AAA6513
Syngrapha diasema|[2335]|LCH340-04|04HBL003340|658[0n]bp|Canada.Manitoba|BOLD:AAA6513
Syngrapha diasema|[2336]|LCHQ035-07|07PROBE-10796|657[0n]bp|Canada.Manitoba|BOLD:AAA6513
Syngrapha diasema|[2337]|LCH444-04|04HBL003444|658[0n]bp|Canada.Manitoba|BOLD:AAA6513
Syngrapha diasema|[2338]|LCHP419-07|07PROBE-10043|657[0n]bp|Canada.Manitoba|BOLD:AAA6513
Syngrapha diasema|[2339]|LCH327-04|04HBL003327|658[0n]bp|Canada.Manitoba|BOLD:AAA6513
Syngrapha diasema|[2340]|LCH449-04|04HBL003449|658[0n]bp|Canada.Manitoba|BOLD:AAA6513
Syngrapha diasema|[2341]|LCHP553-07|07PROBE-10223|657[0n]bp|Canada.Manitoba|BOLD:AAA6513
Syngrapha diasema|[2342]|LCHP755-07|07PROBE-10440|658[0n]bp|Canada.Manitoba|BOLD:AAA6513
Syngrapha diasema|[2343]|MHLEP138-07|CHU06-LEP-138|658[0n]bp|Canada.Manitoba|BOLD:AAA6513
Syngrapha diasema|[2344]|LCHP896-07|07PROBE-10658|658[0n]bp|Canada.Manitoba|BOLD:AAA6513
Syngrapha diasema|[2345]|LCHP902-07|07PROBE-10664|658[0n]bp|Canada.Manitoba|BOLD:AAA6513
Syngrapha diasema|[2346]|LCH341-04|04HBL003341|658[1n]bp|Canada.Manitoba|BOLD:AAA6513
Syngrapha diasema|[2347]|LCH446-04|04HBL003446|658[0n]bp|Canada.Manitoba|BOLD:AAA6513
Syngrapha diasema|[2348]|LCH334-04|04HBL003334|658[2n]bp|Canada.Manitoba|BOLD:AAA6513
Syngrapha diasema|[2349]|LCHP901-07|07PROBE-10663|645[0n]bp|Canada.Manitoba|BOLD:AAA6513
Syngrapha diasema|[2350]|LCHP890-07|07PROBE-10647|636[0n]bp|Canada.Manitoba|BOLD:AAA6513
Syngrapha diasema|[2351]|LCHP889-07|07PROBE-10646|636[0n]bp|Canada.Manitoba|BOLD:AAA6513
Syngrapha diasema|[2352]|LCH339-04|04HBL003339|658[0n]bp|Canada.Manitoba|BOLD:AAA6513
Syngrapha diasema|[2353]|LCH332-04|04HBL003332|658[0n]bp|Canada.Manitoba|BOLD:AAA6513
Syngrapha diasema|[2354]|LCHP834-07|07PROBE-10591|657[0n]bp|Canada.Manitoba|BOLD:AAA6513
Syngrapha diasema|[2355]|LCHP539-07|07PROBE-10209|657[0n]bp|Canada.Manitoba|BOLD:AAA6513
Syngrapha diasema|[2356]|CHLEP222-09|09PROBE-09517|658[0n]bp|Canada.Manitoba|BOLD:AAA6513
Syngrapha diasema|[2357]|LCHQ590-08|07WNP-10482|655[0n]bp|Canada.Manitoba|BOLD:AAA6513
Syngrapha orophila|[2358]|LBCG739-09|08-JDWBC-0739|658[0n]bp|Canada.British Columbia|BOLD:AAD2335
Syngrapha orophila|[2359]|RDNMF357-08|NOC14443|658[0n]bp|Canada.Alberta|BOLD:AAD2335
Syngrapha orophila|[2360]|LBCH1093-10|10-JDWBC-1093|658[0n]bp|Canada.British Columbia|BOLD:AAD2335
Syngrapha orophila|[2361]|LBCG764-09|08-JDWBC-0764|658[0n]bp|Canada.British Columbia|BOLD:AAD2335
Syngrapha orophila|[2362]|RDNMF355-08|NOC14441|658[0n]bp|Canada.Alberta|BOLD:AAD2335
Syngrapha orophila|[2363]|RDNMF356-08|NOC14442|658[0n]bp|Canada.Alberta|BOLD:AAD2335
Syngrapha orophila|[2364]|LBCG2837-09|08-JDWBC-2837|658[0n]bp|Canada.British Columbia|BOLD:AAD2335
Syngrapha orophila|[2365]|LBCH1974-10|10-JDWBC-1974|658[0n]bp|Canada.British Columbia|BOLD:AAD2335
Syngrapha orophila|[2366]|LBCH6625-10|10-JDWBC-6625|658[0n]bp|Canada.British Columbia|BOLD:AAD2335
Syngrapha orophila|[2367]|LBCH1161-10|10-JDWBC-1161|658[0n]bp|Canada.British Columbia|BOLD:AAD2335
Syngrapha angulidens|[2368]|LBCG874-09|08-JDWBC-0874|630[0n]bp|Canada.British Columbia|BOLD:ABY6571
Syngrapha angulidens|[2369]|LBCG601-09|08-JDWBC-0601|658[0n]bp|Canada.British Columbia|BOLD:ABY6571
Syngrapha angulidens|[2370]|LBCC559-05|HLC-22439|658[0n]bp|Canada.British Columbia|BOLD:ABY6571
Syngrapha angulidens|[2371]|LBCD453-05|HLC-23273|658[0n]bp|Canada.British Columbia|BOLD:ABY6571
Syngrapha angulidens|[2372]|LBCH1573-10|10-JDWBC-1573|658[0n]bp|Canada.British Columbia|BOLD:ABY6571
Syngrapha angulidens|[2373]|LBCH1507-10|10-JDWBC-1507|658[0n]bp|Canada.British Columbia|BOLD:ABY6571
Syngrapha angulidens|[2374]|LBCC561-05|HLC-22441|658[0n]bp|Canada.British Columbia|BOLD:ABY6571
Syngrapha angulidens|[2375]|LBCH2118-10|10-JDWBC-2118|658[0n]bp|Canada.British Columbia|BOLD:ABY6571
Syngrapha angulidens|[2376]|LBCG884-09|08-JDWBC-0884|658[0n]bp|Canada.British Columbia|BOLD:ABY6571
Syngrapha angulidens|[2377]|LBCG2060-09|08-JDWBC-2060|658[0n]bp|Canada.British Columbia|BOLD:ABY6571
Syngrapha angulidens|[2378]|LBCH1446-10|10-JDWBC-1446|658[0n]bp|Canada.British Columbia|BOLD:ABY6571
Syngrapha angulidens|[2379]|LBCC558-05|HLC-22438|658[0n]bp|Canada.British Columbia|BOLD:ABY6571
Syngrapha angulidens|[2380]|LBCH1512-10|10-JDWBC-1512|658[0n]bp|Canada.British Columbia|BOLD:ABY6571
Syngrapha angulidens|[2381]|LPABB205-08|08BBLEP-03470|658[0n]bp|Canada.Alberta|BOLD:ABY6571
Syngrapha angulidens|[2382]|LBCH1105-10|10-JDWBC-1105|658[0n]bp|Canada.British Columbia|BOLD:ABY6571
Syngrapha angulidens|[2383]|LBCH1513-10|10-JDWBC-1513|658[0n]bp|Canada.British Columbia|BOLD:ABY6571
Syngrapha angulidens|[2384]|LBCH2112-10|10-JDWBC-2112|658[0n]bp|Canada.British Columbia|BOLD:ABY6571
Syngrapha angulidens|[2385]|LBCG738-09|08-JDWBC-0738|658[0n]bp|Canada.British Columbia|BOLD:ABY6571
Syngrapha angulidens|[2386]|LBCH1575-10|10-JDWBC-1575|658[0n]bp|Canada.British Columbia|BOLD:ABY6571
Syngrapha angulidens|[2387]|LBCH1510-10|10-JDWBC-1510|658[0n]bp|Canada.British Columbia|BOLD:ABY6571
Syngrapha angulidens|[2388]|LBCH2116-10|10-JDWBC-2116|658[0n]bp|Canada.British Columbia|BOLD:ABY6571
Syngrapha angulidens|[2389]|LPABC835-09|08BBLEP-05054|658[0n]bp|Canada.Alberta|BOLD:ABY6571
Syngrapha angulidens|[2390]|LBCD140-05|HLC-22960|658[0n]bp|Canada.British Columbia|BOLD:ABY6571
Syngrapha angulidens|[2391]|LBCH1514-10|10-JDWBC-1514|658[0n]bp|Canada.British Columbia|BOLD:ABY6571
Syngrapha angulidens|[2392]|LBCH1515-10|10-JDWBC-1515|658[0n]bp|Canada.British Columbia|BOLD:ABY6571
[illegible]

Syngrapha angulidens|[2390]|LBCD140-05|HLC-22960|658[0n]bp|Canada.British Columbia|BOLD:ABY6571
Syngrapha angulidens|[2391]|LBCH1514-10|10-JDWBC-1514|658[0n]bp|Canada.British Columbia|BOLD:ABY6571
Syngrapha angulidens|[2392]|LBCH1515-10|10-JDWBC-1515|658[0n]bp|Canada.British Columbia|BOLD:ABY6571
Syngrapha angulidens|[2393]|LBCG632-09|08-JDWBC-0632|658[0n]bp|Canada.British Columbia|BOLD:ABY6571
Syngrapha angulidens|[2394]|LBCH1572-10|10-JDWBC-1572|658[0n]bp|Canada.British Columbia|BOLD:ABY6571
Syngrapha angulidens|[2395]|LBCH1576-10|10-JDWBC-1576|658[0n]bp|Canada.British Columbia|BOLD:ABY6571
Syngrapha angulidens|[2396]|LBCC871-05|HLC-22751|658[0n]bp|Canada.British Columbia|BOLD:ABY6571
Syngrapha angulidens|[2397]|LBCC560-05|HLC-22440|658[0n]bp|Canada.British Columbia|BOLD:ABY6571
Syngrapha angulidens|[2398]|LBCD462-05|HLC-23282|658[0n]bp|Canada.British Columbia|BOLD:ABY6571
Syngrapha angulidens|[2399]|LBCH2113-10|10-JDWBC-2113|658[0n]bp|Canada.British Columbia|BOLD:ABY6571
Syngrapha angulidens|[2400]|LBCH2117-10|10-JDWBC-2117|658[0n]bp|Canada.British Columbia|BOLD:ABY6571
Syngrapha angulidens|[2401]|LBCG765-09|08-JDWBC-0765|658[0n]bp|Canada.British Columbia|BOLD:ABY6571
Syngrapha angulidens|[2402]|LBCG2059-09|08-JDWBC-2059|658[0n]bp|Canada.British Columbia|BOLD:ABY6571
Syngrapha angulidens|[2403]|LBCH2119-10|10-JDWBC-2119|658[0n]bp|Canada.British Columbia|BOLD:ABY6571
Syngrapha angulidens|[2404]|LBCH2115-10|10-JDWBC-2115|658[0n]bp|Canada.British Columbia|BOLD:ABY6571
Syngrapha angulidens|[2405]|LBCH1569-10|10-JDWBC-1569|658[0n]bp|Canada.British Columbia|BOLD:ABY6571
Syngrapha angulidens|[2406]|LBCH1516-10|10-JDWBC-1516|658[0n]bp|Canada.British Columbia|BOLD:ABY6571
Syngrapha angulidens|[2407]|LBCC873-05|HLC-22753|658[0n]bp|Canada.British Columbia|BOLD:ABY6571
Syngrapha angulidens|[2408]|LBCC872-05|HLC-22752|658[0n]bp|Canada.British Columbia|BOLD:ABY6571
Syngrapha angulidens|[2409]|LBCH2245-10|10-JDWBC-2245|658[0n]bp|Canada.British Columbia|BOLD:ABY6571
Syngrapha angulidens|[2410]|LBCD141-05|HLC-22961|658[0n]bp|Canada.British Columbia|BOLD:ABY6571
Syngrapha angulidens|[2411]|LBCG1020-09|08-JDWBC-1020|658[0n]bp|Canada.British Columbia|BOLD:ABY6571
Syngrapha angulidens|[2412]|LBCH1447-10|10-JDWBC-1447|658[0n]bp|Canada.British Columbia|BOLD:ABY6571
Syngrapha angulidens|[2413]|LBCH1509-10|10-JDWBC-1509|634[0n]bp|Canada.British Columbia|BOLD:ABY6571
Syngrapha angulidens|[2414]|LBCD215-05|HLC-23035|643[0n]bp|Canada.British Columbia|BOLD:ABY6571
Syngrapha angulidens|[2415]|LBCD423-05|HLC-23243|650[0n]bp|Canada.British Columbia|BOLD:ABY6571
Syngrapha angulidens|[2416]|DUNLP183-08|Dun-08-183|639[0n]bp|Canada.British Columbia|BOLD:ABY6571
Syngrapha angulidens|[2417]|LBCG2067-09|08-JDWBC-2067|658[0n]bp|Canada.British Columbia|BOLD:ABY6571
Syngrapha angulidens|[2418]|LBCH1570-10|10-JDWBC-1570|658[0n]bp|Canada.British Columbia|BOLD:ABY6571
Syngrapha angulidens|[2419]|LBCH1571-10|10-JDWBC-1571|658[0n]bp|Canada.British Columbia|BOLD:ABY6571
Syngrapha angulidens|[2420]|LBCH1511-10|10-JDWBC-1511|658[0n]bp|Canada.British Columbia|BOLD:ABY6571
Syngrapha angulidens|[2421]|LBCH1682-10|10-JDWBC-1682|658[0n]bp|Canada.British Columbia|BOLD:ABY6571
Syngrapha angulidens|[2422]|LBCH1574-10|10-JDWBC-1574|658[0n]bp|Canada.British Columbia|BOLD:ABY6571
Syngrapha angulidens|[2423]|LBCH1508-10|10-JDWBC-1508|639[0n]bp|Canada.British Columbia|BOLD:ABY6571
Syngrapha celsa|[2424]|DUNLP184-08|Dun-08-184|627[0n]bp|Canada.British Columbia|BOLD:ABY6570
Syngrapha celsa|[2425]|LALPA1319-12|AVBC 1321-11|621[0n]bp|Canada.British Columbia|BOLD:ABY6570
Syngrapha celsa|[2426]|LALPA1328-12|AVBC 1330-11|621[0n]bp|Canada.British Columbia|BOLD:ABY6570
Syngrapha celsa|[2427]|LALPA989-11|AVBC 1162-11|658[0n]bp|Canada.British Columbia|BOLD:ABY6570
Syngrapha celsa|[2428]|LBCD139-05|HLC-22959|658[0n]bp|Canada.British Columbia|BOLD:ABY6570
Syngrapha celsa|[2429]|LALPA1298-11|AVBC 1300-11|658[0n]bp|Canada.British Columbia|BOLD:ABY6570
Syngrapha celsa|[2430]|LHLEP266-06|UBC-2006-1658|658[0n]bp|Canada.British Columbia|BOLD:ABY6570
Syngrapha celsa|[2431]|LALPA797-10|AVBC 799-10|658[0n]bp|Canada.British Columbia|BOLD:ABY6570
Syngrapha celsa|[2432]|LBCG861-09|08-JDWBC-0861|658[0n]bp|Canada.British Columbia|BOLD:ABY6570
Syngrapha celsa|[2433]|LBCH3069-10|10-JDWBC-3069|658[0n]bp|Canada.British Columbia|BOLD:ABY6570
Syngrapha celsa|[2434]|LBCS446-07|UBC-2007-0202|658[0n]bp|Canada.British Columbia|BOLD:ABY6570
Syngrapha celsa|[2435]|LBCH804-10|10-JDWBC-0804|658[0n]bp|Canada.British Columbia|BOLD:ABY6570
Syngrapha celsa|[2436]|LBCG2058-09|08-JDWBC-2058|658[0n]bp|Canada.British Columbia|BOLD:ABY6570
Syngrapha celsa|[2437]|LBCH009-10|10-JDWBC-0009|658[0n]bp|Canada.British Columbia|BOLD:ABY6570
Syngrapha celsa|[2438]|LBCD445-05|HLC-23265|658[0n]bp|Canada.British Columbia|BOLD:ABY6570
Syngrapha celsa|[2439]|LALPA1281-11|AVBC 1283-11|658[0n]bp|Canada.British Columbia|BOLD:ABY6570
Syngrapha celsa|[2440]|LBCH2090-10|10-JDWBC-2090|658[0n]bp|Canada.British Columbia|BOLD:ABY6570
Syngrapha celsa|[2441]|LHLEP265-06|UBC-2006-1657|658[0n]bp|Canada.British Columbia|BOLD:ABY6570
Syngrapha celsa|[2442]|LALPA1007-11|AVBC 1180-11|658[1n]bp|Canada.British Columbia|BOLD:ABY6570
Syngrapha celsa|[2443]|LALPA1282-11|AVBC 1284-11|658[0n]bp|Canada.British Columbia|BOLD:ABY6570
Syngrapha celsa|[2444]|LPVIB070-08|PFC-2006-1418|658[0n]bp|Canada.British Columbia|BOLD:ABY6570
Syngrapha celsa|[2445]|LBCH3455-10|10-JDWBC-3455|658[0n]bp|Canada.British Columbia|BOLD:ABY6570
Syngrapha celsa|[2446]|LBCH1518-10|10-JDWBC-1518|658[0n]bp|Canada.British Columbia|BOLD:ABY6570
Syngrapha celsa|[2447]|LBCS680-07|UBC-2007-0385|658[0n]bp|Canada.British Columbia|BOLD:ABY6570
Syngrapha celsa|[2448]|LALPA1295-11|AVBC 1297-11|658[0n]bp|Canada.British Columbia|BOLD:ABY6570
Syngrapha microgamma|[2449]|LCHP832-07|07PROBE-10589|651[0n]bp|Canada.Manitoba|BOLD:ABY6569
Syngrapha microgamma|[2450]|LCH322-04|04HBL003322|658[0n]bp|Canada.Manitoba|BOLD:ABY6569
Syngrapha microgamma|[2451]|LCHP820-07|07PROBE-10577|658[0n]bp|Canada.Manitoba|BOLD:ABY6569
Syngrapha microgamma nearctica|[2452]|RDLQB003-05|DH010089|658[0n]bp|Canada.Quebec|BOLD:ABY6569
Syngrapha microgamma|[2453]|LCH320-04|04HBL003320|658[0n]bp|Canada.Manitoba|BOLD:ABY6569
Syngrapha microgamma|[2454]|LCH321-04|04HBL003321|658[0n]bp|Canada.Manitoba|BOLD:ABY6569
Syngrapha microgamma nearctica|[2455]|RDLQB001-05|DH010087|526[1n]bp|Canada.Quebec|BOLD:ABY6569
Syngrapha microgamma nearctica|[2456]|RDLQB004-05|DH010090|537[0n]bp|Canada.Quebec|BOLD:ABY6569
Syngrapha montana|[2457]|LCH318-04|04HBL003318|658[0n]bp|Canada.Manitoba|BOLD:ABZ6945
Syngrapha montana|[2458]|RDNMG624-08|CNC LEP00052448|658[0n]bp|Canada.New Brunswick|BOLD:ABZ6945
Syngrapha montana|[2459]|RDNMG623-08|CNC LEP00052447|658[0n]bp|Canada.Quebec|BOLD:ABZ6945
Syngrapha montana|[2460]|RDLQG368-06|DH012602|658[0n]bp|Canada.Quebec|BOLD:ABZ6945
Syngrapha rectangula group|[2461]|BBLPB537-10|10BBCLP-1536|658[0n]bp|Canada.British Columbia|BOLD:AAA...
Syngrapha abstrusa|[2462]|BBLPB534-10|10BBCLP-1533|658[0n]bp|Canada.Alberta|BOLD:AAA4309
Syngrapha rectangula|[2463]|BBLPE036-09|09BBELE-2036|658[0n]bp|Canada.Nova Scotia|BOLD:AAA4309
Syngrapha rectangula group|[2464]|LHLEP281-06|UBC-2006-0191|658[0n]bp|Canada.British Columbia|BOLD:AA...
Syngrapha rectangula|[2465]|LHLEP377-06|UBC-2006-1826|658[0n]bp|Canada.British Columbia|BOLD:AAA4309
Syngrapha rectangula|[2466]|LBCS445-07|UBC-2007-0201|658[0n]bp|Canada.British Columbia|BOLD:AAA4309
Syngrapha rectangula|[2467]|LHLEP378-06|UBC-2006-1990|658[0n]bp|Canada.British Columbia|BOLD:AAA4309
Syngrapha rectangula|[2468]|LBCS400-07|UBC-2007-0913|658[0n]bp|Canada.British Columbia|BOLD:AAA4309
Syngrapha cryptica|[2469]|RDNMG621-08|CNC LEP00052445|639[0n]bp|Canada.Ontario|BOLD:AAA4309
Syngrapha cryptica|[2470]|RDNMG622-08|CNC LEP00052446|658[0n]bp|Canada.Ontario|BOLD:AAA4309
Syngrapha rectangula|[2471]|BBLPC082-09|09BBELE-1082|627[0n]bp|Canada.New Brunswick|BOLD:AAA4309
Syngrapha rectangula|[2472]|TTMNB294-06|MNBTT-294|656[0n]bp|Canada.New Brunswick|BOLD:AAA4309
Syngrapha rectangula|[2473]|TTMNB033-06|MNBTT-033|654[0n]bp|Canada.New Brunswick|BOLD:AAA4309
Syngrapha rectangula|[2474]|LALPA624-10|AVBC 626-10|658[0n]bp|Canada.British Columbia|BOLD:AAA4309
Syngrapha rectangula|[2475]|RDLQF366-06|DH011433|658[0n]bp|Canada.Quebec|BOLD:AAA4309
Syngrapha rectangula|[2476]|BBLPC097-09|09BBELE-1097|658[0n]bp|Canada.New Brunswick|BOLD:AAA4309
Syngrapha rectangula|[2477]|LALPA660-10|AVBC 662-10|658[0n]bp|Canada.British Columbia|BOLD:AAA4309
Syngrapha rectangula|[2478]|BBLPE087-09|09BBELE-2087|658[0n]bp|Canada.Nova Scotia|BOLD:AAA4309
Syngrapha rectangula|[2479]|BBLEC071-09|09BBELE-0071|658[0n]bp|Canada.New Brunswick|BOLD:AAA4309
Syngrapha rectangula|[2480]|RDLQB011-05|DH010097|658[0n]bp|Canada.Quebec|BOLD:AAA4309
Syngrapha rectangula|[2481]|LALPA639-10|AVBC 641-10|658[0n]bp|Canada.British Columbia|BOLD:AAA4309
Syngrapha rectangula|[2482]|RDLQB435-05|DH010521|658[0n]bp|Canada.Quebec|BOLD:AAA4309
Syngrapha rectangula|[2483]|RDLQB012-05|DH010098|658[0n]bp|Canada.Quebec|BOLD:AAA4309
Syngrapha rectangula|[2484]|TTMNB295-06|MNBTT-295|658[0n]bp|Canada.New Brunswick|BOLD:AAA4309
Syngrapha rectangula|[2485]|LALPA644-10|AVBC 646-10|658[0n]bp|Canada.British Columbia|BOLD:AAA4309
Syngrapha rectangula group|[2486]|PHMNB366-04|04HBL00592|658[0n]bp|Canada.New Brunswick|BOLD:AAA4309
Syngrapha rectangula group|[2487]|LALPA635-10|AVBC 637-10|658[0n]bp|Canada.British Columbia|BOLD:AAA4309
Syngrapha rectangula group|[2488]|TMNBB084-06|MNBTT-1024|658[0n]bp|Canada.New Brunswick|BOLD:AAA4309
Syngrapha rectangula group|[2489]|BBLPC811-09|09BBELE-1811|658[0n]bp|Canada.Newfoundland and Labrador|BO...
Syngrapha rectangula group|[2490]|BBLPC139-09|09BBELE-1139|658[0n]bp|Canada.Nova Scotia|BOLD:AAA4309
Syngrapha rectangula group|[2491]|BBLPC207-09|09BBELE-1207|658[0n]bp|Canada.Nova Scotia|BOLD:AAA4309
Syngrapha alias|[2492]|BBLPC994-09|09BBELE-1994|658[0n]bp|Canada.Nova Scotia|BOLD:AAA4309

Syngrapha rectangula group|[2490]|BBLPC139-09|09BBELE-1139|658[0n]bp|Canada.Nova Scotia|BOLD:AAA4309
Syngrapha rectangula group|[2491]|BBLPC207-09|09BBELE-1207|658[0n]bp|Canada.Nova Scotia|BOLD:AAA4309
Syngrapha alias|[2492]|BBLPC994-09|09BBELE-1994|658[0n]bp|Canada.Nova Scotia|BOLD:AAA4309
Syngrapha alias|[2493]|XAG212-05|2005-ONT-796|658[0n]bp|Canada.Ontario|BOLD:AAA4309
Syngrapha cryptica|[2494]|RDNMF415-08|NOC14501|658[0n]bp|Canada.Ontario|BOLD:AAA4309
Syngrapha cryptica|[2495]|BBLPE053-09|09BBELE-2053|658[0n]bp|Canada.Nova Scotia|BOLD:AAA4309
Syngrapha cryptica|[2496]|BBLPE261-09|09BBELE-2261|658[0n]bp|Canada.Nova Scotia|BOLD:AAA4309
Syngrapha cryptica|[2497]|BBLPE015-09|09BBELE-2015|658[0n]bp|Canada.Nova Scotia|BOLD:AAA4309
Syngrapha cryptica|[2498]|RDNMF414-08|NOC14500|658[0n]bp|Canada.Ontario|BOLD:AAA4309
Syngrapha rectangula|[2499]|LALPA510-10|AVBC 512-10|658[0n]bp|Canada.British Columbia|BOLD:AAA4309
Syngrapha abstrusa|[2500]|XAK179-06|2006-ONT-1174|658[0n]bp|Canada.Ontario|BOLD:AAA4309
Syngrapha abstrusa|[2501]|RDLQB025-05|DH010111|658[0n]bp|Canada.Quebec|BOLD:AAA4309
Syngrapha abstrusa|[2502]|RDLQB791-05|DH010878|658[0n]bp|Canada.Quebec|BOLD:AAA4309
Syngrapha abstrusa|[2503]|RDLQB020-05|DH010106|658[0n]bp|Canada.Quebec|BOLD:AAA4309
Syngrapha abstrusa|[2504]|RDLQF517-06|DH011666|658[0n]bp|Canada.Quebec|BOLD:AAA4309
Syngrapha abstrusa|[2505]|RDLQB018-05|DH010104|658[0n]bp|Canada.Quebec|BOLD:AAA4309
Syngrapha abstrusa|[2506]|RDLQB021-05|DH010107|658[0n]bp|Canada.Quebec|BOLD:AAA4309
Syngrapha abstrusa|[2507]|RDLQF518-06|DH011667|658[0n]bp|Canada.Quebec|BOLD:AAA4309
Syngrapha abstrusa|[2508]|RDLQB026-05|DH010112|658[0n]bp|Canada.Quebec|BOLD:AAA4309
Syngrapha cryptica|[2509]|BBLPC066-09|09BBELE-1066|643[0n]bp|Canada.New Brunswick|BOLD:AAA4309
Syngrapha rectangula|[2510]|BBLPE083-09|09BBELE-2083|647[0n]bp|Canada.Nova Scotia|BOLD:AAA4309
Syngrapha rectangula|[2511]|BBLEC063-09|09BBELE-0063|625[0n]bp|Canada.New Brunswick|BOLD:AAA4309
Syngrapha abstrusa|[2512]|XAE391-04|Moth4391.03|617[0n]bp|Canada.Ontario|BOLD:AAA4309
Syngrapha abstrusa|[2513]|XAC630-04|04HBL006630|616[0n]bp|Canada.Ontario|BOLD:AAA4309
Syngrapha abstrusa|[2514]|RDLQB792-05|DH010879|617[0n]bp|Canada.Quebec|BOLD:AAA4309
Syngrapha rectangula|[2515]|LPVIA628-08|PFC-2006-0858|635[0n]bp|Canada.British Columbia|BOLD:AAA4309
Syngrapha rectangula|[2516]|RDLQB623-05|DH010726|586[0n]bp|Canada.Quebec|BOLD:AAA4309
Syngrapha rectangula group|[2517]|PHMO116-03|moth714.01|639[0n]bp|Canada.Ontario|BOLD:AAA4309
Syngrapha rectangula group|[2518]|BBLPC578-09|09BBELE-1578|648[0n]bp|Canada.Nova Scotia|BOLD:AAA4309
Syngrapha rectangula group|[2519]|XAB089-04|04HBL005089|658[0n]bp|Canada.Ontario|BOLD:AAA4309
Syngrapha rectangula group|[2520]|XAC600-04|04HBL006600|658[0n]bp|Canada.Ontario|BOLD:AAA4309
Syngrapha rectangula group|[2521]|BBLPC212-09|09BBELE-1212|658[0n]bp|Canada.Nova Scotia|BOLD:AAA4309
Syngrapha abstrusa|[2522]|XAB246-04|04HBL005246|658[1n]bp|Canada.Ontario|BOLD:AAA4309
Syngrapha rectangula group|[2523]|BBLPB541-10|10BBCLP-1540|658[0n]bp|Canada.British Columbia|BOLD:AAA...
Syngrapha rectangula group|[2524]|BBLPB540-10|10BBCLP-1539|658[0n]bp|Canada.British Columbia|BOLD:AAA...
Syngrapha rectangula group|[2525]|PHMNB212-04|04HBL007677|658[0n]bp|Canada.New Brunswick|BOLD:AAA4309
Syngrapha abstrusa|[2526]|PHMNB367-04|04HBL00593|658[0n]bp|Canada.New Brunswick|BOLD:AAA4309
Syngrapha rectangula group|[2527]|TTMNB289-06|MNBTT-289|658[1n]bp|Canada.New Brunswick|BOLD:AAA4309
Syngrapha rectangula group|[2528]|BBLPB536-10|10BBCLP-1535|636[0n]bp|Canada.British Columbia|BOLD:AAA...
Syngrapha alias|[2529]|BBLEC110-09|09BBELE-0110|625[0n]bp|Canada.Nova Scotia|BOLD:AAA4309
Syngrapha alias|[2530]|LBCD446-05|HLC-23266|658[0n]bp|Canada.British Columbia|BOLD:AAA4309
Syngrapha alias|[2531]|LOWCD153-06|CGWC-2973|658[0n]bp|Canada.British Columbia|BOLD:AAA4309
Syngrapha rectangula|[2532]|DUNLP185-08|Dun-08-185|658[0n]bp|Canada.British Columbia|BOLD:AAA4309
Syngrapha rectangula group|[2533]|LBCD447-05|HLC-23267|658[0n]bp|Canada.British Columbia|BOLD:AAA4309
Syngrapha rectangula|[2534]|LOWCD187-06|CGWC-3007|658[0n]bp|Canada.British Columbia|BOLD:AAA4309
Syngrapha rectangula|[2535]|LBCH3331-10|10-JDWBC-3331|658[0n]bp|Canada.British Columbia|BOLD:AAA4309
Syngrapha rectangula|[2536]|LBCG2062-09|08-JDWBC-2062|658[0n]bp|Canada.British Columbia|BOLD:AAA4309
Syngrapha rectangula|[2537]|LBCH3779-10|10-JDWBC-3779|658[0n]bp|Canada.British Columbia|BOLD:AAA4309
Syngrapha rectangula|[2538]|LBCG882-09|08-JDWBC-0882|658[0n]bp|Canada.British Columbia|BOLD:AAA4309
Syngrapha rectangula|[2539]|LBCH010-10|10-JDWBC-0010|658[0n]bp|Canada.British Columbia|BOLD:AAA4309
Syngrapha rectangula|[2540]|LBCH3068-10|10-JDWBC-3068|658[0n]bp|Canada.British Columbia|BOLD:AAA4309
Syngrapha alias|[2541]|LPABB675-08|08BBLEP-03940|658[0n]bp|Canada.Alberta|BOLD:AAA4309
Syngrapha alias|[2542]|LOWCD151-06|CGWC-2971|614[0n]bp|Canada.British Columbia|BOLD:AAA4309
Syngrapha alias|[2543]|DUNLP182-08|Dun-08-182|622[0n]bp|Canada.British Columbia|BOLD:AAA4309
Syngrapha rectangula group|[2544]|BBLPB533-10|10BBCLP-1532|658[0n]bp|Canada.Alberta|BOLD:AAA4309
Syngrapha rectangula group|[2545]|PHMO113-03|moth697.02|639[4n]bp|Canada.Ontario|BOLD:AAA4309
Syngrapha abstrusa|[2546]|RDLQB027-05|DH010113|658[0n]bp|Canada.Quebec|BOLD:AAA4309
Syngrapha rectangula group|[2547]|LBCD291-05|HLC-23111|633[0n]bp|Canada.British Columbia|BOLD:AAA4309
Syngrapha rectangula|[2548]|LALPA842-11|AVBC 1015-11|658[0n]bp|Canada.British Columbia|BOLD:AAA4309
Syngrapha rectangula group|[2549]|LCH585-04|04HBL003585|658[0n]bp|Canada.Manitoba|BOLD:AAA4309
Syngrapha rectangula group|[2550]|LBCH121-10|10-JDWBC-0121|658[0n]bp|Canada.British Columbia|BOLD:AAA...
Syngrapha rectangula group|[2551]|BBLEC634-09|09BBELE-0634|658[0n]bp|Canada.Nova Scotia|BOLD:AAA4309
Syngrapha rectangula group|[2552]|LPMN210-08|08BBLEP-01009|658[0n]bp|Canada.Manitoba|BOLD:AAA4309
Syngrapha rectangula group|[2553]|XAJ724-06|2006-ONT-0724|658[0n]bp|Canada.Ontario|BOLD:AAA4309
Syngrapha rectangula group|[2554]|LBCC439-05|HLC-22319|658[0n]bp|Canada.British Columbia|BOLD:AAA4309
Syngrapha rectangula group|[2555]|PHMNB700-04|04HBL00926|658[0n]bp|Canada.New Brunswick|BOLD:AAA4309
Syngrapha rectangula group|[2556]|LBCH916-10|10-JDWBC-0916|658[0n]bp|Canada.British Columbia|BOLD:AAA...
Syngrapha rectangula group|[2557]|LBCD465-05|HLC-23285|658[0n]bp|Canada.British Columbia|BOLD:AAA4309
Syngrapha rectangula group|[2558]|LBCH231-10|10-JDWBC-0231|658[0n]bp|Canada.British Columbia|BOLD:AAA...
Syngrapha rectangula group|[2559]|BBLPB535-10|10BBCLP-1534|658[0n]bp|Canada.British Columbia|BOLD:AAA...
Syngrapha rectangula|[2560]|LBCH654-10|10-JDWBC-0654|658[0n]bp|Canada.British Columbia|BOLD:AAA4309
Syngrapha rectangula|[2561]|LBCG2061-09|08-JDWBC-2061|658[0n]bp|Canada.British Columbia|BOLD:AAA4309
Syngrapha alias|[2562]|LBCH3780-10|10-JDWBC-3780|658[0n]bp|Canada.British Columbia|BOLD:AAA4309
Syngrapha alias|[2563]|LBCH4046-10|10-JDWBC-4046|658[0n]bp|Canada.British Columbia|BOLD:AAA4309
Syngrapha abstrusa|[2564]|RDLQB016-05|DH010102|658[0n]bp|Canada.Quebec|BOLD:AAA4309
Syngrapha abstrusa|[2565]|RDLQB019-05|DH010105|658[0n]bp|Canada.Quebec|BOLD:AAA4309
Syngrapha abstrusa|[2566]|RDLQF519-06|DH011668|658[0n]bp|Canada.Quebec|BOLD:AAA4309
Syngrapha abstrusa|[2567]|XAK162-06|2006-ONT-1157|658[0n]bp|Canada.Ontario|BOLD:AAA4309
Syngrapha abstrusa|[2568]|RDLQB017-05|DH010103|658[0n]bp|Canada.Quebec|BOLD:AAA4309
Syngrapha abstrusa|[2569]|RDLQB024-05|DH010110|658[0n]bp|Canada.Quebec|BOLD:AAA4309
Syngrapha abstrusa|[2570]|RDLQB022-05|DH010108|658[0n]bp|Canada.Quebec|BOLD:AAA4309
Syngrapha abstrusa|[2571]|RDLQB023-05|DH010109|658[0n]bp|Canada.Quebec|BOLD:AAA4309
Syngrapha rectangula group|[2572]|BBLPC140-09|09BBELE-1140|637[0n]bp|Canada.Nova Scotia|BOLD:AAA4309
Syngrapha rectangula group|[2573]|BBLPB538-10|10BBCLP-1537|658[0n]bp|Canada.British Columbia|BOLD:AAA...
Syngrapha rectangula group|[2574]|RDBBC610-05|CSG23118 C BC|658[1n]bp|Canada.British Columbia|BOLD:AA...
Syngrapha rectangula group|[2575]|LBCD448-05|HLC-23268|658[0n]bp|Canada.British Columbia|BOLD:AAA4309
Syngrapha rectangula group|[2576]|LBCD459-05|HLC-23279|650[0n]bp|Canada.British Columbia|BOLD:AAA4309
Syngrapha rectangula group|[2577]|BBLPE270-09|09BBELE-2270|510[0n]bp|Canada.Nova Scotia|BOLD:AAA4309
Syngrapha rectangula group|[2578]|LBCH802-10|10-JDWBC-0802|639[0n]bp|Canada.British Columbia|BOLD:AAA...
Syngrapha rectangula group|[2579]|BBLPB539-10|10BBCLP-1538|658[0n]bp|Canada.British Columbia|BOLD:AAA...
Syngrapha rectangula group|[2580]|LBCH2111-10|10-JDWBC-2111|658[0n]bp|Canada.British Columbia|BOLD:AA...
Syngrapha rectangula|[2581]|BBLEC273-09|09BBELE-0273|658[0n]bp|Canada.Nova Scotia|BOLD:AAA4309
Syngrapha rectangula group|[2582]|BBLEC493-09|09BBELE-0493|652[0n]bp|Canada.New Brunswick|BOLD:AAA4309
Syngrapha rectangula group|[2583]|BBLPC691-09|09BBELE-1691|658[0n]bp|Canada.Newfoundland and Labrador|BO...
Syngrapha alias|[2584]|RDLQF367-06|DH011434|658[0n]bp|Canada.Quebec|BOLD:AAA4309
Syngrapha alias|[2585]|RDLQB037-05|DH010123|658[0n]bp|Canada.Quebec|BOLD:AAA4309
Syngrapha alias|[2586]|RDLQB622-05|DH010725|642[0n]bp|Canada.Quebec|BOLD:AAA4309
Syngrapha rectangula|[2587]|BBLPE256-09|09BBELE-2256|643[0n]bp|Canada.Nova Scotia|BOLD:AAA4309
Syngrapha rectangula group|[2588]|BBLPC376-09|09BBELE-1376|640[0n]bp|Canada.New Brunswick|BOLD:AAA4309
Syngrapha rectangula group|[2589]|BBLPC318-09|09BBELE-1318|658[0n]bp|Canada.Newfoundland and Labrador|BO...
Syngrapha altera|[2590]|RDLQ432-07|DH011243|658[0n]bp|Canada.Quebec|BOLD:AAB4820
Syngrapha altera|[2591]|BBLEC361-09|09BBELE-0361|658[0n]bp|Canada.Newfoundland and Labrador|BOLD:AAB4820
Syngrapha altera|[2592]|TTMNB290-06|MNBTT-290|658[0n]bp|Canada.New Brunswick|BOLD:AAB4820

Syngrapha rectangula group|[illegible]|Canada.Newfoundland and Labrador|BO...
Syngrapha altera|[2590]|RDLQ432-07|DH011243|658[0n]bp|Canada.Quebec|BOLD:AAB4820
Syngrapha altera|[2591]|BBLEC361-09|09BBELE-0361|658[0n]bp|Canada.Newfoundland and Labrador|BOLD:AAB4820
Syngrapha altera|[2592]|TTMNB290-06|MNBTT-290|658[0n]bp|Canada.New Brunswick|BOLD:AAB4820
Syngrapha altera|[2593]|RDNMD261-06|CNCNoctuoidea12593|658[0n]bp|Canada.New Brunswick|BOLD:AAB4820
Syngrapha altera|[2594]|BBLPE124-09|09BBELE-2124|658[0n]bp|Canada.Nova Scotia|BOLD:AAB4820
Syngrapha altera|[2595]|BBLEC350-09|09BBELE-0350|627[0n]bp|Canada.Newfoundland and Labrador|BOLD:AAB4820
Syngrapha altera|[2596]|RDLQB033-05|DH010119|658[0n]bp|Canada.Quebec|BOLD:AAB4820
Syngrapha altera|[2597]|RDNMD262-06|CNCNoctuoidea12594|658[0n]bp|Canada.New Brunswick|BOLD:AAB4820
Syngrapha altera|[2598]|RDLQ431-07|DH011242|658[0n]bp|Canada.Quebec|BOLD:AAB4820
Syngrapha altera|[2599]|RDNMD260-06|CNCNoctuoidea12592|658[0n]bp|Canada.New Brunswick|BOLD:AAB4820
Syngrapha altera|[2600]|BBLPE344-09|09BBELE-2344|658[0n]bp|Canada.Newfoundland and Labrador|BOLD:AAB4820
Syngrapha altera|[2601]|RDLQB036-05|DH010122|658[0n]bp|Canada.Quebec|BOLD:AAB4820
Syngrapha altera|[2602]|RDLQ430-07|DH011241|636[0n]bp|Canada.Quebec|BOLD:AAB4820
Syngrapha altera|[2603]|RDLQB040-05|DH010126|658[0n]bp|Canada.Quebec|BOLD:AAB4820
Syngrapha altera|[2604]|BBLPE517-09|09BBELE-2517|658[0n]bp|Canada.Newfoundland and Labrador|BOLD:AAB4820
Syngrapha altera|[2605]|RDLQB042-05|DH010128|658[0n]bp|Canada.Quebec|BOLD:AAB4820
Syngrapha altera|[2606]|RDLQB039-05|DH010125|658[0n]bp|Canada.Quebec|BOLD:AAB4820
Syngrapha altera|[2607]|RDLQB041-05|DH010127|658[0n]bp|Canada.Quebec|BOLD:AAB4820
Syngrapha altera|[2608]|RDLQB035-05|DH010121|658[0n]bp|Canada.Quebec|BOLD:AAB4820
Syngrapha altera|[2609]|RDLQB034-05|DH010120|658[0n]bp|Canada.Quebec|BOLD:AAB4820
Syngrapha altera|[2610]|BBLPE536-09|09BBELE-2536|658[0n]bp|Canada.Newfoundland and Labrador|BOLD:AAB4820
Syngrapha altera|[2611]|RDLQB044-05|DH010130|658[0n]bp|Canada.Quebec|BOLD:AAB4820
Syngrapha octoscripta|[2612]|LCHQ706-08|07WNP-10598|658[0n]bp|Canada.Manitoba|BOLD:AAA3949
Syngrapha octoscripta|[2613]|LCHQ597-08|07WNP-10489|658[0n]bp|Canada.Manitoba|BOLD:AAA3949
Syngrapha octoscripta|[2614]|TTMNB292-06|MNBTT-292|621[1n]bp|Canada.New Brunswick|BOLD:AAA3949
Syngrapha octoscripta|[2615]|LCHQ126-07|07PROBE-10895|653[0n]bp|Canada.Manitoba|BOLD:AAA3949
Syngrapha octoscripta|[2616]|TTMNB291-06|MNBTT-291|612[4n]bp|Canada.New Brunswick|BOLD:AAA3949
Syngrapha octoscripta|[2617]|BBLEC437-09|09BBELE-0437|658[0n]bp|Canada.New Brunswick|BOLD:AAA3949
Syngrapha octoscripta|[2618]|LCHQ568-08|07WNP-10460|656[0n]bp|Canada.Manitoba|BOLD:AAA3949
Syngrapha octoscripta|[2619]|BBLPC346-09|09BBELE-1346|656[0n]bp|Canada.New Brunswick|BOLD:AAA3949
Syngrapha octoscripta|[2620]|LCHQ773-08|07WNP-10665|656[0n]bp|Canada.Manitoba|BOLD:AAA3949
Syngrapha octoscripta|[2621]|LCHIP078-07|06-PROBE-2559|651[0n]bp|Canada.Manitoba|BOLD:AAA3949
Syngrapha octoscripta|[2622]|RDLQ428-07|DH007475|587[1n]bp|Canada.Quebec|BOLD:AAA3949
Syngrapha octoscripta|[2623]|RDLQ427-07|DH007369|620[0n]bp|Canada.Quebec|BOLD:AAA3949
Syngrapha octoscripta|[2624]|LCHQ653-08|07WNP-10545|658[0n]bp|Canada.Manitoba|BOLD:AAA3949
Syngrapha octoscripta|[2625]|LCHQ566-08|07WNP-10458|658[0n]bp|Canada.Manitoba|BOLD:AAA3949
Syngrapha octoscripta|[2626]|BBLEC092-09|09BBELE-0092|658[0n]bp|Canada.Nova Scotia|BOLD:AAA3949
Syngrapha octoscripta|[2627]|LCHQ921-08|07WNP-10813|658[0n]bp|Canada.Manitoba|BOLD:AAA3949
Syngrapha octoscripta|[2628]|BBLEC024-09|09BBELE-0024|658[0n]bp|Canada.New Brunswick|BOLD:AAA3949
Syngrapha octoscripta|[2629]|BBLPC349-09|09BBELE-1349|658[0n]bp|Canada.New Brunswick|BOLD:AAA3949
Syngrapha octoscripta|[2630]|TMNBB081-06|MNBTT-1021|658[0n]bp|Canada.New Brunswick|BOLD:AAA3949
Syngrapha octoscripta|[2631]|LCHP666-07|07PROBE-10348|658[0n]bp|Canada.Manitoba|BOLD:AAA3949
Syngrapha octoscripta|[2632]|LBCH1517-10|10-JDWBC-1517|658[0n]bp|Canada.British Columbia|BOLD:AAA3949
Syngrapha octoscripta|[2633]|BBLEC426-09|09BBELE-0426|658[0n]bp|Canada.New Brunswick|BOLD:AAA3949
Syngrapha octoscripta|[2634]|LCHQ646-08|07WNP-10538|658[0n]bp|Canada.Manitoba|BOLD:AAA3949
Syngrapha octoscripta|[2635]|LCHP833-07|07PROBE-10590|658[0n]bp|Canada.Manitoba|BOLD:AAA3949
Syngrapha octoscripta|[2636]|BBLEC499-09|09BBELE-0499|658[0n]bp|Canada.New Brunswick|BOLD:AAA3949
Syngrapha octoscripta|[2637]|BBLPC007-09|09BBELE-1007|658[0n]bp|Canada.New Brunswick|BOLD:AAA3949
Syngrapha octoscripta|[2638]|LCHP824-07|07PROBE-10581|658[0n]bp|Canada.Manitoba|BOLD:AAA3949
Syngrapha octoscripta|[2639]|RDLQB038-05|DH010124|658[0n]bp|Canada.Quebec|BOLD:AAA3949
Syngrapha octoscripta|[2640]|BBLPC362-09|09BBELE-1362|658[0n]bp|Canada.New Brunswick|BOLD:AAA3949
Syngrapha octoscripta|[2641]|BBLEC481-09|09BBELE-0481|658[0n]bp|Canada.New Brunswick|BOLD:AAA3949
Syngrapha octoscripta|[2642]|LCHQ726-08|07WNP-10618|658[0n]bp|Canada.Manitoba|BOLD:AAA3949
Syngrapha octoscripta|[2643]|LCHQ713-08|07WNP-10605|658[0n]bp|Canada.Manitoba|BOLD:AAA3949
Syngrapha octoscripta|[2644]|LCHQ119-07|07PROBE-10888|658[0n]bp|Canada.Manitoba|BOLD:AAA3949
Syngrapha octoscripta|[2645]|BBLPC379-09|09BBELE-1379|658[0n]bp|Canada.New Brunswick|BOLD:AAA3949
Syngrapha octoscripta|[2646]|LCHP957-07|07PROBE-10719|658[0n]bp|Canada.Manitoba|BOLD:AAA3949
Syngrapha octoscripta|[2647]|LCHP958-07|07PROBE-10720|658[0n]bp|Canada.Manitoba|BOLD:AAA3949
Syngrapha octoscripta|[2648]|BBLPC414-09|09BBELE-1414|658[0n]bp|Canada.New Brunswick|BOLD:AAA3949
Syngrapha octoscripta|[2649]|BBLEC480-09|09BBELE-0480|658[0n]bp|Canada.New Brunswick|BOLD:AAA3949
Syngrapha octoscripta|[2650]|LCHP942-07|07PROBE-10704|658[0n]bp|Canada.Manitoba|BOLD:AAA3949
Syngrapha octoscripta|[2651]|LCHQ547-08|07WNP-10439|659[0n]bp|Canada.Manitoba|BOLD:AAA3949
Syngrapha octoscripta|[2652]|BBLPC377-09|09BBELE-1377|658[0n]bp|Canada.New Brunswick|BOLD:AAA3949
Syngrapha octoscripta|[2653]|BBLEC449-09|09BBELE-0449|658[0n]bp|Canada.New Brunswick|BOLD:AAA3949
Syngrapha octoscripta|[2654]|LBCH2114-10|10-JDWBC-2114|658[0n]bp|Canada.British Columbia|BOLD:AAA3949
Syngrapha octoscripta|[2655]|BBLPC361-09|09BBELE-1361|658[0n]bp|Canada.New Brunswick|BOLD:AAA3949
Syngrapha octoscripta|[2656]|BBLEC510-09|09BBELE-0510|658[0n]bp|Canada.New Brunswick|BOLD:AAA3949
Syngrapha octoscripta|[2657]|LCHQ703-08|07WNP-10595|658[0n]bp|Canada.Manitoba|BOLD:AAA3949
Syngrapha octoscripta|[2658]|LCHQ660-08|07WNP-10552|658[0n]bp|Canada.Manitoba|BOLD:AAA3949
Syngrapha octoscripta|[2659]|LCHP817-07|07PROBE-10574|658[0n]bp|Canada.Manitoba|BOLD:AAA3949
Syngrapha octoscripta|[2660]|LCHP869-07|07PROBE-10626|658[0n]bp|Canada.Manitoba|BOLD:AAA3949
Syngrapha octoscripta|[2661]|LCHQ121-07|07PROBE-10890|658[0n]bp|Canada.Manitoba|BOLD:AAA3949
Syngrapha octoscripta|[2662]|BBLPC373-09|09BBELE-1373|658[0n]bp|Canada.New Brunswick|BOLD:AAA3949
Syngrapha octoscripta|[2663]|LCHQ922-08|07WNP-10814|658[0n]bp|Canada.Manitoba|BOLD:AAA3949
Syngrapha octoscripta|[2664]|LCHP952-07|07PROBE-10714|658[0n]bp|Canada.Manitoba|BOLD:AAA3949
Syngrapha octoscripta|[2665]|LCHQ567-08|07WNP-10459|658[0n]bp|Canada.Manitoba|BOLD:AAA3949
Syngrapha octoscripta|[2666]|LBCH4429-10|10-JDWBC-4429|658[0n]bp|Canada.British Columbia|BOLD:AAA3949
Syngrapha octoscripta|[2667]|BBLEC492-09|09BBELE-0492|658[0n]bp|Canada.New Brunswick|BOLD:AAA3949
Syngrapha octoscripta|[2668]|BBLPC380-09|09BBELE-1380|658[0n]bp|Canada.New Brunswick|BOLD:AAA3949
Syngrapha octoscripta|[2669]|LCHQ765-08|07WNP-10657|658[0n]bp|Canada.Manitoba|BOLD:AAA3949
Syngrapha octoscripta|[2670]|BBLEC445-09|09BBELE-0445|658[0n]bp|Canada.New Brunswick|BOLD:AAA3949
Syngrapha octoscripta|[2671]|BBLPC416-09|09BBELE-1416|658[0n]bp|Canada.New Brunswick|BOLD:AAA3949
Syngrapha octoscripta|[2672]|LCHQ564-08|07WNP-10456|658[0n]bp|Canada.Manitoba|BOLD:AAA3949
Syngrapha octoscripta|[2673]|BBLPC364-09|09BBELE-1364|658[0n]bp|Canada.New Brunswick|BOLD:AAA3949
Syngrapha octoscripta|[2674]|BBLPC351-09|09BBELE-1351|658[0n]bp|Canada.New Brunswick|BOLD:AAA3949
Syngrapha octoscripta|[2675]|BBLPC115-09|09BBELE-1115|658[0n]bp|Canada.New Brunswick|BOLD:AAA3949
Syngrapha octoscripta|[2676]|BBLPC354-09|09BBELE-1354|658[0n]bp|Canada.New Brunswick|BOLD:AAA3949
Syngrapha octoscripta|[2677]|BBLEC513-09|09BBELE-0513|658[0n]bp|Canada.New Brunswick|BOLD:AAA3949
Syngrapha octoscripta|[2678]|LCHQ556-08|07WNP-10448|658[0n]bp|Canada.Manitoba|BOLD:AAA3949
Syngrapha octoscripta|[2679]|BBLEC471-09|09BBELE-0471|658[0n]bp|Canada.New Brunswick|BOLD:AAA3949
Syngrapha octoscripta|[2680]|LCHQ722-08|07WNP-10614|658[0n]bp|Canada.Manitoba|BOLD:AAA3949
Syngrapha octoscripta|[2681]|BBLEC525-09|09BBELE-0525|658[0n]bp|Canada.New Brunswick|BOLD:AAA3949
Syngrapha octoscripta|[2682]|LCHQ554-08|07WNP-10446|658[0n]bp|Canada.Manitoba|BOLD:AAA3949
Syngrapha octoscripta|[2683]|BBLEC436-09|09BBELE-0436|658[0n]bp|Canada.New Brunswick|BOLD:AAA3949
Syngrapha octoscripta|[2684]|RDLQB043-05|DH010129|658[0n]bp|Canada.Quebec|BOLD:AAA3949
Syngrapha octoscripta|[2685]|LCHQ430-08|07WNP-10322|658[0n]bp|Canada.Manitoba|BOLD:AAA3949
Syngrapha octoscripta|[2686]|BBLEC442-09|09BBELE-0442|658[0n]bp|Canada.New Brunswick|BOLD:AAA3949
Syngrapha octoscripta|[2687]|LCHP940-07|07PROBE-10702|655[0n]bp|Canada.Manitoba|BOLD:AAA3949
Syngrapha octoscripta|[2688]|BBLPC359-09|09BBELE-1359|658[0n]bp|Canada.New Brunswick|BOLD:AAA3949
Syngrapha octoscripta|[2689]|BBLEC425-09|09BBELE-0425|658[0n]bp|Canada.New Brunswick|BOLD:AAA3949
Syngrapha octoscripta|[2690]|LCHP941-07|07PROBE-10703|658[0n]bp|Canada.Manitoba|BOLD:AAA3949
Syngrapha octoscripta|[2691]|RDNMD259-06|CNCNoctuoidea12591|658[0n]bp|Canada.New Brunswick|BOLD:AAA3949
[illegible]

Syngrapha octoscripta|[2689]|BBLEC425-09|09BBELE-0425|658[0n]bp|Canada.New Brunswick|BOLD:AAA3949
Syngrapha octoscripta|[2690]|LCHP941-07|07PROBE-10703|658[0n]bp|Canada.Manitoba|BOLD:AAA3949
Syngrapha octoscripta|[2691]|RDNMD259-06|CNCNoctuoidea12591|658[0n]bp|Canada.New Brunswick|BOLD:AAA3949
Syngrapha octoscripta|[2692]|BBLEC463-09|09BBELE-0463|658[0n]bp|Canada.New Brunswick|BOLD:AAA3949
Syngrapha octoscripta|[2693]|LPABC373-09|08BBLEP-04592|658[0n]bp|Canada.Alberta|BOLD:AAA3949
Syngrapha octoscripta|[2694]|LCHQ565-08|07WNP-10457|658[0n]bp|Canada.Manitoba|BOLD:AAA3949
Syngrapha octoscripta|[2695]|LCHQ557-08|07WNP-10449|658[0n]bp|Canada.Manitoba|BOLD:AAA3949
Syngrapha octoscripta|[2696]|BBLPB532-10|10BBCLP-1531|658[0n]bp|Canada.Alberta|BOLD:AAA3949
Syngrapha octoscripta|[2697]|BBLPC121-09|09BBELE-1121|658[0n]bp|Canada.New Brunswick|BOLD:AAA3949
Syngrapha octoscripta|[2698]|BBLPC105-09|09BBELE-1105|636[0n]bp|Canada.New Brunswick|BOLD:AAA3949
Syngrapha octoscripta|[2699]|BBLEC039-09|09BBELE-0039|639[0n]bp|Canada.New Brunswick|BOLD:AAA3949
Syngrapha octoscripta|[2700]|BBLPC405-09|09BBELE-1405|632[0n]bp|Canada.New Brunswick|BOLD:AAA3949
Syngrapha octoscripta|[2701]|RDNMD258-06|CNCNoctuoidea12590|632[0n]bp|Canada.New Brunswick|BOLD:AAA3949
Syngrapha octoscripta|[2702]|BBLEC479-09|09BBELE-0479|636[0n]bp|Canada.New Brunswick|BOLD:AAA3949
Syngrapha octoscripta|[2703]|RDLQ429-07|DH007946|606[0n]bp|Canada.Quebec|BOLD:AAA3949
Syngrapha octoscripta|[2704]|BBLEC523-09|09BBELE-0523|638[0n]bp|Canada.New Brunswick|BOLD:AAA3949
Syngrapha octoscripta|[2705]|TTMNB293-06|MNBTT-293|628[0n]bp|Canada.New Brunswick|BOLD:AAA3949
Syngrapha octoscripta|[2706]|LCHQ783-08|07WNP-10675|647[0n]bp|Canada.Manitoba|BOLD:AAA3949
Syngrapha octoscripta|[2707]|BBLEC046-09|09BBELE-0046|658[0n]bp|Canada.New Brunswick|BOLD:AAA3949
Syngrapha octoscripta|[2708]|LCH515-04|04HBL003515|658[0n]bp|Canada.Manitoba|BOLD:AAA3949
Syngrapha selecta|[2709]|LBCH3777-10|10-JDWBC-3777|658[0n]bp|Canada.British Columbia|BOLD:ABZ3678
Syngrapha selecta|[2710]|RDNMD254-06|CNCNoctuoidea12586|627[0n]bp|Canada.New Brunswick|BOLD:ABZ3678
Syngrapha selecta|[2711]|RDNMD255-06|CNCNoctuoidea12587|627[0n]bp|Canada.New Brunswick|BOLD:ABZ3678
Syngrapha selecta|[2712]|DSCNI030-07|06-PROBE-0255|658[0n]bp|Canada.Manitoba|BOLD:ABZ3678
Syngrapha selecta|[2713]|LCHQ634-08|07WNP-10526|658[0n]bp|Canada.Manitoba|BOLD:ABZ3678
Syngrapha selecta|[2714]|LCH520-04|04HBL003520|658[0n]bp|Canada.Manitoba|BOLD:ABZ3678
Syngrapha selecta|[2715]|LCH517-04|04HBL003517|590[0n]bp|Canada.Manitoba|BOLD:ABZ3678
Syngrapha selecta|[2716]|BBLPC039-09|09BBELE-1039|602[0n]bp|Canada.New Brunswick|BOLD:ABZ3678
Syngrapha interrogationis|[2717]|LCHIP137-07|06-PROBE-2675|651[0n]bp|Canada.Manitoba|BOLD:AAB3481
Syngrapha interrogationis|[2718]|LCHIP231-07|06-PROBE-2877|651[0n]bp|Canada.Manitoba|BOLD:AAB3481
Syngrapha interrogationis|[2719]|LCH516-04|04HBL003516|586[0n]bp|Canada.Manitoba|BOLD:AAB3481
Syngrapha interrogationis|[2720]|LCH448-04|04HBL003448|658[0n]bp|Canada.Manitoba|BOLD:AAB3481
Syngrapha interrogationis|[2721]|LBCH2790-10|10-JDWBC-2790|658[0n]bp|Canada.British Columbia|BOLD:AAB...
Syngrapha interrogationis|[2722]|LCH323-04|04HBL003323|658[0n]bp|Canada.Manitoba|BOLD:AAB3481
Syngrapha interrogationis|[2723]|LCH325-04|04HBL003325|658[1n]bp|Canada.Manitoba|BOLD:AAB3481
Syngrapha interrogationis|[2724]|LCHP959-07|07PROBE-10721|658[0n]bp|Canada.Manitoba|BOLD:AAB3481
Syngrapha surena|[2725]|RDNMF413-08|NOC14499|658[0n]bp|Canada.New Brunswick|BOLD:ABX6351
Syngrapha surena|[2726]|LOWCB123-05|CGWC-1063|635[0n]bp|Canada.British Columbia|BOLD:ABX6351
Syngrapha surena|[2727]|RDNMG1008-08|CNC LEP00053132|658[0n]bp|Canada.New Brunswick|BOLD:ABX6351
Syngrapha surena|[2728]|RDNMG1007-08|CNC LEP00053131|658[0n]bp|Canada.New Brunswick|BOLD:ABX6351
Syngrapha surena|[2729]|RDNMF412-08|NOC14498|658[0n]bp|Canada.New Brunswick|BOLD:ABX6351
Syngrapha u-aureum|[2730]|LCH457-04|04HBL003457|617[0n]bp|Canada.Manitoba|BOLD:ABX6351
Syngrapha u-aureum|[2731]|LCHIP141-07|06-PROBE-2683|651[0n]bp|Canada.Manitoba|BOLD:ABX6351
Syngrapha u-aureum|[2732]|LCH590-04|04HBL003590|591[0n]bp|Canada.Manitoba|BOLD:ABX6351
Syngrapha u-aureum|[2733]|MHLEP137-07|CHU06-LEP-137|644[0n]bp|Canada.Manitoba|BOLD:ABX6351
Syngrapha u-aureum|[2734]|LCHQ127-07|07PROBE-10896|657[0n]bp|Canada.Manitoba|BOLD:ABX6351
Syngrapha u-aureum|[2735]|LCH324-04|04HBL003324|658[1n]bp|Canada.Manitoba|BOLD:ABX6351
Syngrapha u-aureum|[2736]|LCHP772-07|07PROBE-10457|658[0n]bp|Canada.Manitoba|BOLD:ABX6351
Syngrapha u-aureum|[2737]|LCHQ381-08|07WNP-10273|658[0n]bp|Canada.Manitoba|BOLD:ABX6351
Syngrapha u-aureum|[2738]|LCH460-04|04HBL003460|658[0n]bp|Canada.Manitoba|BOLD:ABX6351
Syngrapha u-aureum|[2739]|MHLEP136-07|CHU06-LEP-136|658[0n]bp|Canada.Manitoba|BOLD:ABX6351
Syngrapha u-aureum|[2740]|LCH458-04|04HBL003458|658[0n]bp|Canada.Manitoba|BOLD:ABX6351
Syngrapha u-aureum|[2741]|LCHQ029-07|07PROBE-10790|658[0n]bp|Canada.Manitoba|BOLD:ABX6351
Syngrapha viridisigma|[2742]|LOWCC363-05|CGWC-2243|509[1n]bp|Canada.British Columbia|BOLD:AAA8795
Syngrapha viridisigma|[2743]|LOWCC366-05|CGWC-2246|520[1n]bp|Canada.British Columbia|BOLD:AAA8795
Syngrapha viridisigma|[2744]|LOWCC362-05|CGWC-2242|546[0n]bp|Canada.British Columbia|BOLD:AAA8795
Syngrapha viridisigma|[2745]|LCHIP207-07|06-PROBE-2832|651[0n]bp|Canada.Manitoba|BOLD:AAA8795
Syngrapha viridisigma|[2746]|LCHIP105-07|06-PROBE-2591|651[0n]bp|Canada.Manitoba|BOLD:AAA8795
Syngrapha viridisigma|[2747]|BBLPB679-10|10BBCLP-1678|658[0n]bp|Canada.Alberta|BOLD:AAA8795
Syngrapha viridisigma|[2748]|TMNBB083-06|MNBTT-1023|658[0n]bp|Canada.New Brunswick|BOLD:AAA8795
Syngrapha viridisigma|[2749]|LBCH3454-10|10-JDWBC-3454|658[0n]bp|Canada.British Columbia|BOLD:AAA8795
Syngrapha viridisigma|[2750]|LALPA1299-11|AVBC 1301-11|658[0n]bp|Canada.British Columbia|BOLD:AAA8795
Syngrapha viridisigma|[2751]|BBLPB694-10|10BBCLP-1693|658[0n]bp|Canada.Alberta|BOLD:AAA8795
Syngrapha viridisigma|[2752]|LBCH913-10|10-JDWBC-0913|658[0n]bp|Canada.British Columbia|BOLD:AAA8795
Syngrapha viridisigma|[2753]|LALPA753-10|AVBC 755-10|658[0n]bp|Canada.British Columbia|BOLD:AAA8795
Syngrapha viridisigma|[2754]|LBCH4651-10|10-JDWBC-4651|658[0n]bp|Canada.British Columbia|BOLD:AAA8795
Syngrapha viridisigma|[2755]|BBLEC491-09|09BBELE-0491|658[0n]bp|Canada.New Brunswick|BOLD:AAA8795
Syngrapha viridisigma|[2756]|LBCH1504-10|10-JDWBC-1504|658[0n]bp|Canada.British Columbia|BOLD:AAA8795
Syngrapha viridisigma|[2757]|LBCH3453-10|10-JDWBC-3453|658[0n]bp|Canada.British Columbia|BOLD:AAA8795
Syngrapha viridisigma|[2758]|LBCH4118-10|10-JDWBC-4118|658[0n]bp|Canada.British Columbia|BOLD:AAA8795
Syngrapha viridisigma|[2759]|BBLPE027-09|09BBELE-2027|658[0n]bp|Canada.Nova Scotia|BOLD:AAA8795
Syngrapha viridisigma|[2760]|DUNLP186-08|Dun-08-186|658[0n]bp|Canada.British Columbia|BOLD:AAA8795
Syngrapha viridisigma|[2761]|BBLPC375-09|09BBELE-1375|658[0n]bp|Canada.New Brunswick|BOLD:AAA8795
Syngrapha viridisigma|[2762]|LBCH3065-10|10-JDWBC-3065|658[0n]bp|Canada.British Columbia|BOLD:AAA8795
Syngrapha viridisigma|[2763]|LALPA716-10|AVBC 718-10|658[0n]bp|Canada.British Columbia|BOLD:AAA8795
Syngrapha viridisigma|[2764]|RDLQB885-05|DH010962|658[0n]bp|Canada.Quebec|BOLD:AAA8795
Syngrapha viridisigma|[2765]|LBCH4665-10|10-JDWBC-4665|658[0n]bp|Canada.British Columbia|BOLD:AAA8795
Syngrapha viridisigma|[2766]|LPSOD969-09|08BBLEP-05602|658[0n]bp|Canada.Ontario|BOLD:AAA8795
Syngrapha viridisigma|[2767]|LALPA609-10|AVBC 611-10|658[0n]bp|Canada.British Columbia|BOLD:AAA8795
Syngrapha viridisigma|[2768]|BBLEC450-09|09BBELE-0450|658[0n]bp|Canada.New Brunswick|BOLD:AAA8795
Syngrapha viridisigma|[2769]|LALPA689-10|AVBC 691-10|658[0n]bp|Canada.British Columbia|BOLD:AAA8795
Syngrapha viridisigma|[2770]|LOWCD149-06|CGWC-2969|658[0n]bp|Canada.British Columbia|BOLD:AAA8795
Syngrapha viridisigma|[2771]|BBLEC461-09|09BBELE-0461|658[0n]bp|Canada.New Brunswick|BOLD:AAA8795
Syngrapha viridisigma|[2772]|LBCH4117-10|10-JDWBC-4117|658[0n]bp|Canada.British Columbia|BOLD:AAA8795
Syngrapha viridisigma|[2773]|LPSOD1003-09|08BBLEP-05636|658[0n]bp|Canada.Ontario|BOLD:AAA8795
Syngrapha viridisigma|[2774]|LBCH4404-10|10-JDWBC-4404|658[0n]bp|Canada.British Columbia|BOLD:AAA8795
Syngrapha viridisigma|[2775]|LCHQ678-08|07WNP-10570|658[0n]bp|Canada.Manitoba|BOLD:AAA8795
Syngrapha viridisigma|[2776]|BBLPB759-10|10BBCLP-1758|658[0n]bp|Canada.Alberta|BOLD:AAA8795
Syngrapha viridisigma|[2777]|BBLPC439-09|09BBELE-1439|658[0n]bp|Canada.New Brunswick|BOLD:AAA8795
Syngrapha viridisigma|[2778]|LBCH3778-10|10-JDWBC-3778|658[0n]bp|Canada.British Columbia|BOLD:AAA8795
Syngrapha viridisigma|[2779]|LALPA727-10|AVBC 729-10|658[0n]bp|Canada.British Columbia|BOLD:AAA8795
Syngrapha viridisigma|[2780]|BBLPE134-09|09BBELE-2134|658[0n]bp|Canada.Nova Scotia|BOLD:AAA8795
Syngrapha viridisigma|[2781]|BBLPC406-09|09BBELE-1406|658[0n]bp|Canada.New Brunswick|BOLD:AAA8795
Syngrapha viridisigma|[2782]|BBLEC447-09|09BBELE-0447|658[0n]bp|Canada.New Brunswick|BOLD:AAA8795
Syngrapha viridisigma|[2783]|LPSOD977-09|08BBLEP-05610|658[0n]bp|Canada.Ontario|BOLD:AAA8795
Syngrapha viridisigma|[2784]|LOWCC368-05|CGWC-2248|582[0n]bp|Canada.British Columbia|BOLD:AAA8795
Syngrapha viridisigma|[2785]|LOWCC361-05|CGWC-2241|617[0n]bp|Canada.British Columbia|BOLD:AAA8795
Syngrapha viridisigma|[2786]|LOWCC367-05|CGWC-2247|553[0n]bp|Canada.British Columbia|BOLD:AAA8795
Syngrapha viridisigma|[2787]|RDNMD256-06|CNCNoctuoidea12588|627[0n]bp|Canada.New Brunswick|BOLD:AAA8795
Syngrapha viridisigma|[2788]|BBLEC465-09|09BBELE-0465|634[0n]bp|Canada.New Brunswick|BOLD:AAA8795
Syngrapha viridisigma|[2789]|LOWCC369-05|CGWC-2249|555[0n]bp|Canada.British Columbia|BOLD:AAA8795
Syngrapha viridisigma|[2790]|LOWCC370-05|CGWC-2250|579[0n]bp|Canada.British Columbia|BOLD:AAA8795
Syngrapha viridisigma|[2791]|BBLPE165-09|09BBELE-2165|631[0n]bp|Canada.Nova Scotia|BOLD:AAA8795

Syngrapha viridisigma|[2789]|LOWCC369-05|CGWC-2249|555[0n]bp|Canada.British Columbia|BOLD:AAA8795
Syngrapha viridisigma|[2790]|LOWCC370-05|CGWC-2250|579[0n]bp|Canada.British Columbia|BOLD:AAA8795
Syngrapha viridisigma|[2791]|BBLPE165-09|09BBELE-2165|631[0n]bp|Canada.Nova Scotia|BOLD:AAA8795
Syngrapha viridisigma|[2792]|LCH519-04|04HBL003519|584[0n]bp|Canada.Manitoba|BOLD:AAA8795
Syngrapha viridisigma|[2793]|RDNMD257-06|CNCNoctuoidea12589|630[0n]bp|Canada.New Brunswick|BOLD:AAA8795
Syngrapha viridisigma|[2794]|LOWCC365-05|CGWC-2245|584[0n]bp|Canada.British Columbia|BOLD:AAA8795
Syngrapha viridisigma|[2795]|LOWCD127-06|CGWC-2947|616[0n]bp|Canada.British Columbia|BOLD:AAA8795
Syngrapha viridisigma|[2796]|LOWCC364-05|CGWC-2244|568[0n]bp|Canada.British Columbia|BOLD:AAA8795
Syngrapha viridisigma|[2797]|LCHIP201-07|06-PROBE-2826|628[0n]bp|Canada.Manitoba|BOLD:AAA8795
Rachiplusia ou|[2798]|RDNMF443-08|NOC14529|609[0n]bp|Canada.Ontario|BOLD:AAE7504
Rachiplusia ou|[2799]|RDNMF651-08|NOC14737|658[0n]bp|Canada.British Columbia|BOLD:AAE7504
Allagrapha aerea|[2800]|XAE557-04|Moth4557.03|658[4n]bp|Canada.Ontario|BOLD:AAB0752
Allagrapha aerea|[2801]|XAE564-04|Moth4564.03|658[0n]bp|Canada.Ontario|BOLD:AAB0752
Allagrapha aerea|[2802]|XAH418-05|2005-ONT-2001|658[0n]bp|Canada.Ontario|BOLD:AAB0752
Allagrapha aerea|[2803]|XAJ538-06|2006-ONT-0538|658[0n]bp|Canada.Ontario|BOLD:AAB0752
Allagrapha aerea|[2804]|RDLQB558-05|DH010644|658[0n]bp|Canada.Quebec|BOLD:AAB0752
Allagrapha aerea|[2805]|LPSOC314-08|PPBP-2313|658[0n]bp|Canada.Ontario|BOLD:AAB0752
Allagrapha aerea|[2806]|LPSOB099-08|PPBP-1098|658[0n]bp|Canada.Ontario|BOLD:AAB0752
Allagrapha aerea|[2807]|XAK160-06|2006-ONT-1155|658[0n]bp|Canada.Ontario|BOLD:AAB0752
Allagrapha aerea|[2808]|LPSOC361-08|PPBP-2360|656[0n]bp|Canada.Ontario|BOLD:AAB0752
Allagrapha aerea|[2809]|RDLQF273-06|DH011365|658[0n]bp|Canada.Quebec|BOLD:AAB0752
Allagrapha aerea|[2810]|XAC459-04|04HBL006459|658[0n]bp|Canada.Ontario|BOLD:AAB0752
Allagrapha aerea|[2811]|XAB466-04|04HBL005466|658[0n]bp|Canada.Ontario|BOLD:AAB0752
Allagrapha aerea|[2812]|XAD152-04|04HBL007152|658[0n]bp|Canada.Ontario|BOLD:AAB0752
Allagrapha aerea|[2813]|RDLQB720-05|DH010823|658[0n]bp|Canada.Quebec|BOLD:AAB0752
Allagrapha aerea|[2814]|BLTIB218-08|BL396|658[0n]bp|Canada.Ontario|BOLD:AAB0752
Allagrapha aerea|[2815]|XAH278-05|2005-ONT-1861|658[0n]bp|Canada.Ontario|BOLD:AAB0752
Allagrapha aerea|[2816]|XAD388-04|04HBL007388|658[0n]bp|Canada.Ontario|BOLD:AAB0752
Allagrapha aerea|[2817]|LPSOC034-08|PPBP-2033|658[0n]bp|Canada.Ontario|BOLD:AAB0752
Allagrapha aerea|[2818]|PHMO344-03|moth2643.02|640[0n]bp|Canada.Ontario|BOLD:AAB0752
Allagrapha aerea|[2819]|XAD154-04|04HBL007154|576[0n]bp|Canada.Ontario|BOLD:AAB0752
Allagrapha aerea|[2820]|XAD293-04|04HBL007293|597[0n]bp|Canada.Ontario|BOLD:AAB0752
Allagrapha aerea|[2821]|PMG088-03|moth1148.01|617[0n]bp|Canada.Ontario|BOLD:AAB0752
Allagrapha aerea|[2822]|PHMO114-03|moth707.01|639[0n]bp|Canada.Ontario|BOLD:AAB0752
Diachrysia balluca|[2823]|RDLQB422-05|DH010508|658[4n]bp|Canada.Quebec|BOLD:AAD0732
Diachrysia balluca|[2824]|PHMO275-03|moth1661.02|639[2n]bp|Canada.Ontario|BOLD:AAD0732
Diachrysia balluca|[2825]|RDLQB830-05|DH010917|634[0n]bp|Canada.Quebec|BOLD:AAD0732
Diachrysia balluca|[2826]|MECD403-06|jflandry2975|658[0n]bp|Canada.Quebec|BOLD:AAD0732
Diachrysia balluca|[2827]|LPMNB449-09|08BBLEP-05449|658[0n]bp|Canada.Manitoba|BOLD:AAD0732
Diachrysia balluca|[2828]|XAG250-05|2005-ONT-834|658[0n]bp|Canada.Ontario|BOLD:AAD0732
Diachrysia balluca|[2829]|LPMNB568-09|08BBLEP-05646|658[0n]bp|Canada.Manitoba|BOLD:AAD0732
Diachrysia balluca|[2830]|BBLEC957-09|09BBELE-0957|658[3n]bp|Canada.Nova Scotia|BOLD:AAD0732
Chrysanympha formosa|[2831]|RDLQB261-05|DH010347|658[0n]bp|Canada.Quebec|BOLD:AAB6600
Chrysanympha formosa|[2832]|TMNBB079-06|MNBTT-1019|657[0n]bp|Canada.New Brunswick|BOLD:AAB6600
Chrysanympha formosa|[2833]|BBLPE054-09|09BBELE-2054|658[0n]bp|Canada.Nova Scotia|BOLD:AAB6600
Chrysanympha formosa|[2834]|TMNBB077-06|MNBTT-1017|657[1n]bp|Canada.New Brunswick|BOLD:AAB6600
Chrysanympha formosa|[2835]|RDLQB605-05|DH010708|536[0n]bp|Canada.Quebec|BOLD:AAB6600
Chrysanympha formosa|[2836]|TMNBB078-06|MNBTT-1018|657[0n]bp|Canada.New Brunswick|BOLD:AAB6600
Chrysanympha formosa|[2837]|RDLQB260-05|DH010346|658[0n]bp|Canada.Quebec|BOLD:AAB6600
Chrysanympha formosa|[2838]|BLTIB391-08|BL623|657[0n]bp|Canada.Ontario|BOLD:AAB6600
Chrysanympha formosa|[2839]|BBLPE280-09|09BBELE-2280|658[0n]bp|Canada.Nova Scotia|BOLD:AAB6600
Chrysanympha formosa|[2840]|RDLQF933-06|DH012108|657[0n]bp|Canada.Quebec|BOLD:AAB6600
Chrysanympha formosa|[2841]|RDLQB262-05|DH010348|658[0n]bp|Canada.Quebec|BOLD:AAB6600
Chrysanympha formosa|[2842]|TMNBB080-06|MNBTT-1020|657[0n]bp|Canada.New Brunswick|BOLD:AAB6600
Chrysanympha formosa|[2843]|BBLPE115-09|09BBELE-2115|658[0n]bp|Canada.Nova Scotia|BOLD:AAB6600
Chrysanympha formosa|[2844]|BBLPE078-09|09BBELE-2078|658[0n]bp|Canada.Nova Scotia|BOLD:AAB6600
Chrysanympha formosa|[2845]|BBLPE328-09|09BBELE-2328|658[0n]bp|Canada.Newfoundland and Labrador|BOLD:...
Diachrysia aereoides|[2846]|RDLQF813-06|DH011963|658[1n]bp|Canada.Quebec|BOLD:AAB3241
Diachrysia aereoides|[2847]|LOWCE830-06|CGWC-4590|658[0n]bp|Canada.British Columbia|BOLD:AAB3241
Diachrysia aereoides|[2848]|BLTIB843-08|BL1262|658[1n]bp|Canada.Ontario|BOLD:AAB3241
Diachrysia aereoides|[2849]|XAC728-04|04HBL006728|658[0n]bp|Canada.Ontario|BOLD:AAB3241
Diachrysia aereoides|[2850]|XAC793-04|04HBL006793|658[0n]bp|Canada.Ontario|BOLD:AAB3241
Diachrysia aereoides|[2851]|BLTIB724-08|BL1012|658[1n]bp|Canada.Ontario|BOLD:AAB3241
Diachrysia aereoides|[2852]|BBLPE023-09|09BBELE-2023|658[1n]bp|Canada.Nova Scotia|BOLD:AAB3241
Diachrysia aereoides|[2853]|TMG96-03|PLUS1.00|639[0n]bp|Canada.Ontario|BOLD:AAB3241
Diachrysia aereoides|[2854]|BBLPE052-09|09BBELE-2052|658[0n]bp|Canada.Nova Scotia|BOLD:AAB3241
Diachrysia aereoides|[2855]|PHMNB528-04|04HBL00754|658[0n]bp|Canada.New Brunswick|BOLD:AAB3241
Diachrysia aereoides|[2856]|XAC704-04|04HBL006704|658[0n]bp|Canada.Ontario|BOLD:AAB3241
Diachrysia aereoides|[2857]|BBLPE017-09|09BBELE-2017|658[0n]bp|Canada.Nova Scotia|BOLD:AAB3241
Diachrysia aereoides|[2858]|BBLPC604-09|09BBELE-1604|658[0n]bp|Canada.Nova Scotia|BOLD:AAB3241
Diachrysia aereoides|[2859]|XAC458-04|04HBL006458|658[0n]bp|Canada.Ontario|BOLD:AAB3241
Diachrysia aereoides|[2860]|PHJUN3996-11|BIOUG01497-A07|658[0n]bp|Canada.Ontario|BOLD:AAB3241
Diachrysia aereoides|[2861]|XAJ830-06|2006-ONT-0830|658[0n]bp|Canada.Ontario|BOLD:AAB3241
Diachrysia aereoides|[2862]|RDLQF814-06|DH011964|658[0n]bp|Canada.Quebec|BOLD:AAB3241
Diachrysia aereoides|[2863]|XAK223-06|2006-ONT-1218|658[0n]bp|Canada.Ontario|BOLD:AAB3241
Diachrysia aereoides|[2864]|BBLEC321-09|09BBELE-0321|658[0n]bp|Canada.Nova Scotia|BOLD:AAB3241
Diachrysia aereoides|[2865]|BBLEC266-09|09BBELE-0266|658[0n]bp|Canada.Nova Scotia|BOLD:AAB3241
Diachrysia aereoides|[2866]|BBLPC541-09|09BBELE-1541|638[0n]bp|Canada.New Brunswick|BOLD:AAB3241
Diachrysia aereoides|[2867]|RDLQB059-05|DH010145|658[0n]bp|Canada.Quebec|BOLD:AAB3241
Diachrysia aereoides|[2868]|PMG107-03|moth991.01|617[0n]bp|Canada.Ontario|BOLD:AAB3241
Polychrysia esmeralda|[2869]|RDNMD361-06|CNCNoctuoidea12693|658[0n]bp|Canada.Alberta|BOLD:AAF0891
Polychrysia esmeralda|[2870]|RDMAB086-05|UASM57613|639[0n]bp|Canada.Alberta|BOLD:AAF0891
Ctenoplusia oxygramma|[2871]|RDNMF627-08|NOC14713|658[0n]bp|Canada.New Brunswick|BOLD:AAA9797
Ctenoplusia oxygramma|[2872]|RDNMF628-08|NOC14714|658[0n]bp|Canada.New Brunswick|BOLD:AAA9797
Argyrogramma verruca|[2873]|LNC850-06|06-NCCC-850|658[0n]bp|United States.North Carolina|BOLD:AAC5989
Argyrogramma verruca|[2874]|LPOKA597-09|MDOK-0597|658[0n]bp|United States.Oklahoma|BOLD:AAC5989
Argyrogramma verruca|[2875]|LNCB072-06|06-NCC-1028|658[0n]bp|United States.North Carolina|BOLD:AAC5989
Argyrogramma verruca|[2876]|RDNMK050-11|CNCLEP 81173|658[0n]bp|United States.Florida|BOLD:AAC5989
Chrysodeixis chalcites|[2877]|RDNMK154-11|CNCLEP 80441|658[0n]bp|Canada.Ontario|BOLD:AAB3384
Chrysodeixis chalcites|[2878]|RDNMK155-11|CNCLEP 80442|658[0n]bp|Canada.Ontario|BOLD:AAB3384
Chrysodeixis chalcites|[2879]|RDNMH238-09|CNCLEP00054547|658[0n]bp|Canada.Ontario|BOLD:AAB3384
Chrysodeixis chalcites|[2880]|LMHRG044-06|HUM-2006-1394-C|613[0n]bp|Canada.British Columbia|BOLD:AAB3384
Chrysodeixis chalcites|[2881]|LMHRG045-06|HUM-2006-1394-D|635[0n]bp|Canada.British Columbia|BOLD:AAB3384
Chrysodeixis chalcites|[2882]|RDNMK156-11|CNCLEP 80443|658[0n]bp|Canada.Ontario|BOLD:AAB3384
Chrysodeixis chalcites|[2883]|RDNMK153-11|CNCLEP 80440|658[0n]bp|Canada.Ontario|BOLD:AAB3384
Chrysodeixis chalcites|[2884]|TZBCA102-06|OMAFRA06-037|658[0n]bp|Canada.Ontario|BOLD:AAB3384
Chrysodeixis includens|[2885]|LGSMG611-07|BGS03974|658[0n]bp|United States.North Carolina|BOLD:AAA6794
Chrysodeixis includens|[2886]|LNCB071-06|06-NCC-1027|658[0n]bp|United States.North Carolina|BOLD:AAA6794
Chrysodeixis includens|[2887]|LPOKD427-09|MDOK-3506|658[0n]bp|United States.Oklahoma|BOLD:AAA6794
Chrysodeixis includens|[2888]|LGSMG613-07|BGS03976|658[0n]bp|United States.North Carolina|BOLD:AAA6794
Chrysodeixis includens|[2889]|XAH608-05|2005-ONT-2191|658[0n]bp|Canada.Ontario|BOLD:AAA6794
Chrysodeixis includens|[2890]|LOFLB770-06|06-FLOR-1710|658[0n]bp|United States.Florida|BOLD:AAA6794
Chrysodeixis includens|[2891]|LPOKD432-09|MDOK-3511|658[0n]bp|United States.Oklahoma|BOLD:AAA6794

Chrysodeixis includens|[2888]|[illegible]|United States.North Carolina|BOLD:AAA6794
Chrysodeixis includens|[2889]|XAH608-05|2005-ONT-2191|658[0n]bp|Canada.Ontario|BOLD:AAA6794
Chrysodeixis includens|[2890]|LOFLB770-06|06-FLOR-1710|658[0n]bp|United States.Florida|BOLD:AAA6794
Chrysodeixis includens|[2891]|LPOKD432-09|MDOK-3511|658[0n]bp|United States.Oklahoma|BOLD:AAA6794
Chrysodeixis includens|[2892]|LPOKD449-09|MDOK-3528|658[0n]bp|United States.Oklahoma|BOLD:AAA6794
Chrysodeixis includens|[2893]|LPOKA1032-09|MDOK-1505|658[0n]bp|United States.Oklahoma|BOLD:AAA6794
Chrysodeixis includens|[2894]|LGSMG612-07|BGS03975|658[0n]bp|United States.North Carolina|BOLD:AAA6794
Chrysodeixis includens|[2895]|LPOKD407-09|MDOK-3486|658[0n]bp|United States.Oklahoma|BOLD:AAA6794
Chrysodeixis includens|[2896]|XAB463-04|04HBL005463|658[0n]bp|Canada.Ontario|BOLD:AAA6794
Chrysodeixis includens|[2897]|LPOKA567-09|MDOK-0567|658[0n]bp|United States.Oklahoma|BOLD:AAA6794
Chrysodeixis includens|[2898]|LILLB064-11|SNS10IL-01284|658[0n]bp|United States.Illinois|BOLD:AAA6794
Chrysodeixis includens|[2899]|LILLA958-11|SNS10IL-01179|658[0n]bp|United States.Illinois|BOLD:AAA6794
Chrysodeixis includens|[2900]|LNC849-06|06-NCCC-849|658[0n]bp|United States.North Carolina|BOLD:AAA6794
Chrysodeixis includens|[2901]|LPOKA628-09|MDOK-0628|658[0n]bp|United States.Oklahoma|BOLD:AAA6794
Chrysodeixis includens|[2902]|RDLQ426-07|DH009870|643[0n]bp|Canada.Quebec|BOLD:AAA6794
Chrysodeixis includens|[2903]|LPOKA661-09|MDOK-0661|637[0n]bp|United States.Oklahoma|BOLD:AAA6794
Chrysodeixis includens|[2904]|LPOKA343-08|MDOK-0343|658[0n]bp|United States.Oklahoma|BOLD:AAA6794
Chrysodeixis includens|[2905]|LILLA916-11|SNS10IL-01137|658[0n]bp|United States.Illinois|BOLD:AAA6794
Trichoplusia ni|[2906]|RDLQB835-05|DH010922|658[0n]bp|Canada.Quebec|BOLD:AAC3409
Trichoplusia ni|[2907]|LBCA607-05|HLC-20607|618[0n]bp|Canada.British Columbia|BOLD:AAC3409
Trichoplusia ni|[2908]|PHMO357-03|moth2736.02|639[0n]bp|Canada.Ontario|BOLD:AAC3409
Trichoplusia ni|[2909]|RDLQB754-05|DH010841|617[0n]bp|Canada.Quebec|BOLD:AAC3409
Trichoplusia ni|[2910]|RDLQB834-05|DH010921|620[1n]bp|Canada.Quebec|BOLD:AAC3409
Trichoplusia ni|[2911]|BBLPB456-10|10BBCLP-1455|658[0n]bp|Canada.Ontario|BOLD:AAC3409
Trichoplusia ni|[2912]|LBCA806-05|HLC-20806|658[0n]bp|Canada.British Columbia|BOLD:AAC3409
Trichoplusia ni|[2913]|XAD373-04|04HBL007373|658[0n]bp|Canada.Ontario|BOLD:AAC3409
Amphipyra glabella|[2914]|BLTIB1002-08|BL1439|658[0n]bp|Canada.Ontario|BOLD:AAE2518
Amphipyra glabella|[2915]|RDMAB987-09|UASM2460|634[0n]bp|Canada.Alberta|BOLD:AAE2518
Amphipyra glabella|[2916]|RDMAB988-09|UASM59214|634[0n]bp|Canada.Manitoba|BOLD:AAE2518
Amphipyra glabella|[2917]|RDMAB989-09|UASM59215|658[0n]bp|Canada.Alberta|BOLD:AAE2518
Amphipyra glabella|[2918]|RDLQB838-05|DH010925|623[1n]bp|Canada.Quebec|BOLD:AAE2518
Amphipyra glabella|[2919]|BLTIB1043-08|BL1487|658[0n]bp|Canada.Ontario|BOLD:AAE2518
Harrisimemna trisignata|[2920]|TTMNB001-06|MNBTT-001|658[0n]bp|Canada.New Brunswick|BOLD:AAB3137
Harrisimemna trisignata|[2921]|TTMNB029-06|MNBTT-029|658[0n]bp|Canada.New Brunswick|BOLD:AAB3137
Harrisimemna trisignata|[2922]|BBLPC156-09|09BBELE-1156|658[0n]bp|Canada.Nova Scotia|BOLD:AAB3137
Harrisimemna trisignata|[2923]|BBLPC901-09|09BBELE-1901|658[0n]bp|Canada.Newfoundland and Labrador|BOLD...
Harrisimemna trisignata|[2924]|MNBB288-05|05-NBSTA-204|658[0n]bp|Canada.New Brunswick|BOLD:AAB3137
Harrisimemna trisignata|[2925]|BBLPC163-09|09BBELE-1163|658[0n]bp|Canada.Nova Scotia|BOLD:AAB3137
Harrisimemna trisignata|[2926]|LPMN714-08|08BBLEP-01517|658[0n]bp|Canada.Manitoba|BOLD:AAB3137
Harrisimemna trisignata|[2927]|RDLQB067-05|DH010153|658[0n]bp|Canada.Quebec|BOLD:AAB3137
Harrisimemna trisignata|[2928]|RDLQB745-05|DH010660|658[0n]bp|Canada.Quebec|BOLD:AAB3137
Harrisimemna trisignata|[2929]|XAE274-04|Moth4274.03|658[0n]bp|Canada.Ontario|BOLD:AAB3137
Harrisimemna trisignata|[2930]|TTMNB002-06|MNBTT-002|657[0n]bp|Canada.New Brunswick|BOLD:AAB3137
Harrisimemna trisignata|[2931]|RDLQB744-05|DH010659|658[0n]bp|Canada.Quebec|BOLD:AAB3137
Harrisimemna trisignata|[2932]|MECD348-06|jflandry2920|658[0n]bp|Canada.Quebec|BOLD:AAB3137
Harrisimemna trisignata|[2933]|XAK062-06|2006-ONT-1057|658[0n]bp|Canada.Ontario|BOLD:AAB3137
Harrisimemna trisignata|[2934]|LPSOD1064-09|08MZPP-163|658[0n]bp|Canada.Ontario|BOLD:AAB3137
Harrisimemna trisignata|[2935]|RDLQB555-05|DH010641|658[0n]bp|Canada.Quebec|BOLD:AAB3137
Harrisimemna trisignata|[2936]|XAJ927-06|2006-ONT-0927|658[0n]bp|Canada.Ontario|BOLD:AAB3137
Harrisimemna trisignata|[2937]|PHMNB727-05|Moth 420.03SA|658[0n]bp|Canada.New Brunswick|BOLD:AAB3137
Harrisimemna trisignata|[2938]|RDLQB066-05|DH010152|581[0n]bp|Canada.Quebec|BOLD:AAB3137
Behrensia conchiformis|[2939]|LALPA1076-11|AVBC 886-11|627[0n]bp|Canada.British Columbia|BOLD:AAC9584
Behrensia conchiformis|[2940]|LALPA1074-11|AVBC 884-11|658[0n]bp|Canada.British Columbia|BOLD:AAC9584
Behrensia conchiformis|[2941]|LALPA1073-11|AVBC 883-11|658[0n]bp|Canada.British Columbia|BOLD:AAC9584
Calophasia lunula|[2942]|XAJ245-06|2006-ONT-0245|658[0n]bp|Canada.Ontario|BOLD:AAB0052
Calophasia lunula|[2943]|XAG884-05|2005-ONT-1468|658[0n]bp|Canada.Ontario|BOLD:AAB0052
Calophasia lunula|[2944]|RDLQF881-06|DH012056|658[0n]bp|Canada.Quebec|BOLD:AAB0052
Calophasia lunula|[2945]|XAG106-05|2005-ONT-690|658[0n]bp|Canada.Ontario|BOLD:AAB0052
Calophasia lunula|[2946]|XAJ238-06|2006-ONT-0238|658[0n]bp|Canada.Ontario|BOLD:AAB0052
Calophasia lunula|[2947]|XAG260-05|2005-ONT-844|658[0n]bp|Canada.Ontario|BOLD:AAB0052
Calophasia lunula|[2948]|RDLQ607-07|DH012400|658[0n]bp|Canada.Quebec|BOLD:AAB0052
Calophasia lunula|[2949]|XAJ599-06|2006-ONT-0599|658[0n]bp|Canada.Ontario|BOLD:AAB0052
Calophasia lunula|[2950]|XAG945-05|2005-ONT-1529|658[0n]bp|Canada.Ontario|BOLD:AAB0052
Calophasia lunula|[2951]|XAF699-05|2005-ONT-348|658[0n]bp|Canada.Ontario|BOLD:AAB0052
Calophasia lunula|[2952]|XAG020-05|2005-ONT-604|658[0n]bp|Canada.Ontario|BOLD:AAB0052
Calophasia lunula|[2953]|XAG084-05|2005-ONT-668|617[0n]bp|Canada.Ontario|BOLD:AAB0052
Calophasia lunula|[2954]|KPOEC079-08|08OEC-238|640[0n]bp|Canada.Ontario|BOLD:AAB0052
Calophasia lunula|[2955]|XAG763-05|2005-ONT-1347|658[0n]bp|Canada.Ontario|BOLD:AAB0052
Calophasia lunula|[2956]|TMG124-03|moth312.01|639[0n]bp|Canada.Ontario|BOLD:AAB0052
Calophasia lunula|[2957]|PMG097-03|moth281.01|617[0n]bp|Canada.Ontario|BOLD:AAB0052
Calophasia lunula|[2958]|BLTIB069-08|BL0109|547[0n]bp|Canada.Ontario|BOLD:AAB0052
Calophasia lunula|[2959]|XAD013-04|04HBL007013|614[0n]bp|Canada.Ontario|BOLD:AAB0052
Calophasia lunula|[2960]|TTMNB389-06|MNBTT-389|658[0n]bp|Canada.New Brunswick|BOLD:AAB0052
Calophasia lunula|[2961]|XAH602-05|2005-ONT-2185|658[0n]bp|Canada.Ontario|BOLD:AAB0052
Calophasia lunula|[2962]|XAG312-05|2005-ONT-896|658[0n]bp|Canada.Ontario|BOLD:AAB0052
Calophasia lunula|[2963]|TTMNB391-06|MNBTT-391|658[0n]bp|Canada.New Brunswick|BOLD:AAB0052
Calophasia lunula|[2964]|XAJ260-06|2006-ONT-0260|658[0n]bp|Canada.Ontario|BOLD:AAB0052
Calophasia lunula|[2965]|XAJ237-06|2006-ONT-0237|658[0n]bp|Canada.Ontario|BOLD:AAB0052
Calophasia lunula|[2966]|TTMNB388-06|MNBTT-388|658[0n]bp|Canada.New Brunswick|BOLD:AAB0052
Calophasia lunula|[2967]|TTMNB390-06|MNBTT-390|658[0n]bp|Canada.New Brunswick|BOLD:AAB0052
Catabena lineolata|[2968]|RDLQ600-07|DH007746|594[0n]bp|Canada.Quebec|BOLD:AAE4319
Catabena lineolata|[2969]|RDMAB684-06|UASM58812|632[0n]bp|Canada.Alberta|BOLD:AAE4319
Pleromelloida bonuscula|[2970]|RDNMF025-08|NOC14111|658[0n]bp|Canada.British Columbia|BOLD:AAH9194
Pleromelloida bonuscula|[2971]|RDNMG782-08|CNC LEP00052906|643[0n]bp|Canada.British Columbia|BOLD:AAH...
Pleromelloida bonuscula|[2972]|LBCH4988-10|10-JDWBC-4988|658[0n]bp|Canada.British Columbia|BOLD:AAH9194
Pleromelloida bonuscula|[2973]|LBCH4975-10|10-JDWBC-4975|658[0n]bp|Canada.British Columbia|BOLD:AAH9194
Pleromelloida conserta|[2974]|RDNME311-07|CNCNoctuoidea13918|658[0n]bp|Canada.Alberta|BOLD:AAE2130
Pleromelloida conserta|[2975]|RDNME310-07|CNCNoctuoidea13917|657[0n]bp|Canada.Alberta|BOLD:AAE2130
Pleromelloida conserta|[2976]|RDNME312-07|CNCNoctuoidea13919|656[0n]bp|Canada.British Columbia|BOLD:A...
Pleromelloida conserta|[2977]|LBCG306-08|08-JDWBC-0306|658[0n]bp|Canada.British Columbia|BOLD:AAE2130
Pleromelloida conserta|[2978]|LALPA046-10|AVBC 046-10|658[0n]bp|Canada.British Columbia|BOLD:AAE2130
Pleromelloida conserta|[2979]|LALPA024-10|AVBC 024-10|658[0n]bp|Canada.British Columbia|BOLD:AAE2130
Pleromelloida conserta|[2980]|LALPA025-10|AVBC 025-10|658[0n]bp|Canada.British Columbia|BOLD:AAE2130
Pleromelloida conserta|[2981]|LALPA056-10|AVBC 056-10|658[0n]bp|Canada.British Columbia|BOLD:AAE2130
Pleromelloida cinerea|[2982]|LALPA743-10|AVBC 745-10|658[0n]bp|Canada.British Columbia|BOLD:AAD4314
Pleromelloida cinerea|[2983]|LALPA1337-12|AVBC 1339-11|621[0n]bp|Canada.British Columbia|BOLD:AAD4314
Pleromelloida cinerea|[2984]|LALPA769-10|AVBC 771-10|658[0n]bp|Canada.British Columbia|BOLD:AAD4314
Pleromelloida cinerea|[2985]|LOWCB489-05|CGWC-1429|658[0n]bp|Canada.British Columbia|BOLD:AAD4314
Pleromelloida cinerea|[2986]|LOWCB490-05|CGWC-1430|616[2n]bp|Canada.British Columbia|BOLD:AAD4314
Pleromelloida cinerea|[2987]|LOWCC193-05|CGWC-2073|658[0n]bp|Canada.British Columbia|BOLD:AAD4314
Pleromelloida cinerea|[2988]|LOWCB487-05|CGWC-1427|658[0n]bp|Canada.British Columbia|BOLD:AAD4314
Pleromelloida cinerea|[2989]|LOWCB493-05|CGWC-1433|607[6n]bp|Canada.British Columbia|
Pleromelloida cinerea|[2990]|LOWCB492-05|CGWC-1432|611[1n]bp|Canada.British Columbia|BOLD:AAD4314
[illegible]

Pleromelloida cinerea|[2988]|LOWCB487-05|CGWC-1427|658[0n]bp|Canada.British Columbia|BOLD:AAD4314
Pleromelloida cinerea|[2989]|LOWCB493-05|CGWC-1433|607[6n]bp|Canada.British Columbia|
Pleromelloida cinerea|[2990]|LOWCB492-05|CGWC-1432|611[1n]bp|Canada.British Columbia|BOLD:AAD4314
Pleromelloida cinerea|[2991]|LOWCB491-05|CGWC-1431|621[2n]bp|Canada.British Columbia|BOLD:AAD4314
Pleromelloida sp.|[2992]|LBCH7182-10|10-JDWBC-7182|658[0n]bp|Canada.British Columbia|BOLD:ACE3622
Pleromelloida sp.|[2993]|LBCH7121-10|10-JDWBC-7121|658[0n]bp|Canada.British Columbia|BOLD:ACE3622
Pleromelloida sp.|[2994]|LBCH7083-10|10-JDWBC-7083|658[0n]bp|Canada.British Columbia|BOLD:ACE3622
Sympistis lacticollis|[2995]|LBCH6457-10|10-JDWBC-6457|658[0n]bp|Canada.British Columbia|BOLD:AAE1054
Sympistis lacticollis|[2996]|RDNMG956-08|CNC LEP00053080|658[0n]bp|Canada.British Columbia|BOLD:AAE1054
Sympistis dunbari|[2997]|IAWLB523-11|IAWAZ-1431|658[0n]bp|United States.Arizona|BOLD:AAZ0785
Sympistis dunbari|[2998]|LOPN153-06|JCM-OSU-0153|527[0n]bp|United States.Oregon|BOLD:AAE1138
Sympistis dunbari|[2999]|RDNM619-05|CNCNoctuoidea7465|658[0n]bp|United States.Washington|BOLD:AAE1138
Sympistis dunbari|[3000]|LOPN154-06|JCM-OSU-0154|589[1n]bp|United States.Oregon|BOLD:AAE1138
Sympistis dunbari|[3001]|RDNM618-05|CNCNoctuoidea7464|658[0n]bp|United States.Washington|BOLD:AAE1138
Sympistis dunbari|[3002]|LOPN152-06|JCM-OSU-0152|657[1n]bp|United States.Oregon|BOLD:AAE1138
Sympistis extremis|[3003]|RDNMB149-05|CNCNoctuoidea7895|511[0n]bp|Canada.British Columbia|BOLD:AAD8446
Sympistis extremis|[3004]|RDNM627-05|CNCNoctuoidea7473|658[0n]bp|Canada.British Columbia|BOLD:AAD8446
Sympistis extremis|[3005]|RDNMB152-05|CNCNoctuoidea7898|583[0n]bp|Canada.British Columbia|BOLD:AAD8446
Sympistis extremis|[3006]|RDNMB153-05|CNCNoctuoidea7899|605[0n]bp|Canada.British Columbia|BOLD:AAD8446
Sympistis parvanigra|[3007]|RDNMB147-05|CNCNoctuoidea7893|592[0n]bp|Canada.British Columbia|BOLD:AAE5094
Sympistis parvanigra|[3008]|LBCH7229-10|10-JDWBC-7229|658[0n]bp|Canada.British Columbia|BOLD:AAE5094
Sympistis parvanigra|[3009]|LBCH7227-10|10-JDWBC-7227|658[0n]bp|Canada.British Columbia|BOLD:AAE5094
Sympistis parvanigra|[3010]|RDNMG916-08|CNC LEP00053040|658[0n]bp|Canada.British Columbia|BOLD:AAE5094
Sympistis parvanigra|[3011]|RDNMG918-08|CNC LEP00053042|658[0n]bp|Canada.British Columbia|BOLD:AAE5094
Sympistis tenuifascia|[3012]|RDNMF135-08|NOC14221|658[0n]bp|United States.Washington|BOLD:AAD5526
Sympistis tenuifascia|[3013]|RDNMB143-05|CNCNoctuoidea7889|658[0n]bp|United States.Washington|BOLD:AA...
Sympistis tenuifascia|[3014]|RDNMB144-05|CNCNoctuoidea7890|658[0n]bp|United States.Washington|BOLD:AA...
Sympistis tenuifascia|[3015]|RDNMF139-08|NOC14225|658[0n]bp|United States.Washington|BOLD:AAD5526
Sympistis tenuifascia|[3016]|RDNMF138-08|NOC14224|658[0n]bp|United States.Washington|BOLD:AAD5526
Sympistis tenuifascia|[3017]|RDNMF136-08|NOC14222|641[0n]bp|United States.Washington|BOLD:AAD5526
Sympistis tenuifascia|[3018]|RDNMB146-05|CNCNoctuoidea7892|596[0n]bp|United States.Washington|BOLD:AA...
Sympistis tenuifascia|[3019]|RDNMB145-05|CNCNoctuoidea7891|595[0n]bp|United States.Washington|BOLD:AA...
Sympistis tenuifascia|[3020]|RDNMF137-08|NOC14223|658[0n]bp|United States.Washington|BOLD:AAD5526
Sympistis balteata|[3021]|RDNM520-05|CNCNoctuoidea6796|658[0n]bp|United States.Montana|BOLD:AAE1135
Sympistis balteata|[3022]|RDNM519-05|CNCNoctuoidea6795|658[0n]bp|United States.New Mexico|BOLD:AAE1135
Sympistis balteata|[3023]|RDMAB1041-09|UASM99711|649[0n]bp|United States.Wyoming|BOLD:AAE1135
Sympistis balteata|[3024]|RDMAB1040-09|UASM99710|627[0n]bp|United States.Wyoming|BOLD:AAE1135
Sympistis balteata|[3025]|RDMAB1039-09|UASM99709|626[0n]bp|United States.Wyoming|BOLD:AAE1135
Sympistis saundersiana|[3026]|PHMO338-03|moth2610.02|639[2n]bp|Canada.Ontario|BOLD:AAE5097
Sympistis saundersiana|[3027]|XAD433-04|04HBL007433|579[0n]bp|Canada.Ontario|BOLD:AAE5097
Sympistis saundersiana|[3028]|RDNMB192-05|CNCNoctuoidea7938|658[0n]bp|Canada.Ontario|BOLD:AAE5097
Sympistis saundersiana|[3029]|RDLQ606-07|DH009699|658[0n]bp|Canada.Ontario|BOLD:AAE5097
Sympistis umbrifascia|[3030]|RDNMB174-05|CNCNoctuoidea7920|529[0n]bp|Canada.British Columbia|BOLD:AAD...
Sympistis poliochroa|[3031]|LBCH7675-10|10-JDWBC-7675|658[0n]bp|Canada.British Columbia|BOLD:ACE3576
Sympistis poliochroa|[3032]|LOWCC198-05|CGWC-2078|591[1n]bp|Canada.British Columbia|BOLD:AAE1057
Sympistis poliochroa|[3033]|LBCH7224-10|10-JDWBC-7224|658[0n]bp|Canada.British Columbia|BOLD:AAE1057
Sympistis poliochroa|[3034]|RDNMG960-08|CNC LEP00053084|658[0n]bp|Canada.British Columbia|BOLD:AAE1057
Sympistis mackiei|[3035]|RDNMB199-05|CNCNoctuoidea7945|590[0n]bp|Canada.Alberta|BOLD:AAF3800
Sympistis mackiei|[3036]|RDNMB200-05|CNCNoctuoidea7946|597[1n]bp|Canada.Alberta|BOLD:AAF3800
Sympistis mackiei|[3037]|RDNMB198-05|CNCNoctuoidea7944|598[0n]bp|Canada.Alberta|BOLD:AAF3800
Sympistis cibalis|[3038]|RDMAB451-05|BCSC124|658[0n]bp|Canada.Alberta|BOLD:AAC6246
Sympistis cibalis|[3039]|LOWCB488-05|CGWC-1428|658[0n]bp|Canada.British Columbia|BOLD:AAC6246
Sympistis cibalis|[3040]|LOWCB486-05|CGWC-1426|658[0n]bp|Canada.British Columbia|BOLD:AAC6246
Sympistis cibalis|[3041]|RDNMB160-05|CNCNoctuoidea7906|583[0n]bp|Canada.British Columbia|BOLD:AAC6246
Sympistis cibalis|[3042]|LOWCC194-05|CGWC-2074|591[0n]bp|Canada.British Columbia|BOLD:AAC6246
Sympistis cibalis|[3043]|LOWCB485-05|CGWC-1425|589[0n]bp|Canada.British Columbia|BOLD:AAC6246
Sympistis cibalis|[3044]|RDNMB161-05|CNCNoctuoidea7907|598[0n]bp|Canada.Alberta|BOLD:AAC6246
Sympistis cibalis|[3045]|LOWCB484-05|CGWC-1424|615[0n]bp|Canada.British Columbia|BOLD:AAC6246
Sympistis cibalis|[3046]|LOWCB494-05|CGWC-1434|617[0n]bp|Canada.British Columbia|BOLD:AAC6246
Sympistis regina|[3047]|RDNMB871-05|CNCNoctuoidea10646|658[0n]bp|Canada.Alberta|BOLD:ACE3022
Sympistis regina|[3048]|RDNMB872-05|CNCNoctuoidea10647|658[0n]bp|Canada.Alberta|BOLD:ACE3022
Sympistis iricolor|[3049]|RDNM511-05|CNCNoctuoidea6787|658[0n]bp|United States.Montana|BOLD:AAE5105
Sympistis iricolor|[3050]|RDNM513-05|CNCNoctuoidea6789|658[0n]bp|United States.Montana|BOLD:AAE5105
Sympistis iricolor|[3051]|RDNM512-05|CNCNoctuoidea6788|658[0n]bp|United States.Arizona|BOLD:AAE5105
Sympistis iricolor|[3052]|RDNMB142-05|CNCNoctuoidea7888|658[0n]bp|United States.Oregon|BOLD:AAE5105
Sympistis glennyi|[3053]|RDNMC826-06|BOOG74|501[0n]bp|Canada.British Columbia|
Sympistis glennyi|[3054]|RDNMC827-06|BOOG75|598[0n]bp|Canada.British Columbia|BOLD:AAD1770
Sympistis glennyi|[3055]|RDNMC807-06|BOOG55|598[0n]bp|Canada.British Columbia|BOLD:AAD1770
Sympistis glennyi|[3056]|RDNMC828-06|BOOG76|608[0n]bp|Canada.British Columbia|BOLD:AAD1770
Sympistis glennyi|[3057]|RDNMC808-06|BOOG56|610[0n]bp|Canada.British Columbia|BOLD:AAD1770
Sympistis glennyi|[3058]|RDNMC825-06|BOOG73|560[0n]bp|Canada.British Columbia|BOLD:AAD1770
Sympistis glennyi|[3059]|RDNMC806-06|BOOG54|608[0n]bp|Canada.British Columbia|BOLD:AAD1770
Sympistis glennyi|[3060]|RDNMC824-06|BOOG72|560[0n]bp|Canada.British Columbia|BOLD:AAD1770
Sympistis lepipoloides|[3061]|RDNMF735-08|NOC14821|658[0n]bp|Canada.Saskatchewan|BOLD:ACF5739
Sympistis lepipoloides|[3062]|RDNMG915-08|CNC LEP00053039|587[0n]bp|Canada.Saskatchewan|BOLD:ACF5739
Sympistis lepipoloides|[3063]|RDNMG914-08|CNC LEP00053038|588[1n]bp|Canada.Saskatchewan|BOLD:ACF5739
Sympistis levis|[3064]|RDNMB870-05|CNCNoctuoidea10645|658[0n]bp|Canada.Alberta|BOLD:AAE1053
Sympistis sanina|[3065]|RDNMG163-08|NOC14916|658[0n]bp|United States.Utah|BOLD:AAD2911
Sympistis sanina|[3066]|RDNMB558-05|CNCNoctuoidea10334|506[1n]bp|United States.Colorado|BOLD:AAD2911
Sympistis sanina|[3067]|RDNM647-05|CNCNoctuoidea7493|515[1n]bp|United States.Colorado|BOLD:AAD2911
Sympistis sanina|[3068]|RDNM634-05|CNCNoctuoidea7480|525[0n]bp|United States.Colorado|BOLD:AAD2911
Sympistis sanina|[3069]|RDNM533-05|CNCNoctuoidea6809|658[0n]bp|United States.New Mexico|BOLD:AAD2911
Sympistis insanina|[3070]|RDNM615-05|CNCNoctuoidea7461|658[0n]bp|Canada.Alberta|BOLD:AAD2911
Sympistis insanina|[3071]|RDNM617-05|CNCNoctuoidea7463|658[0n]bp|Canada.Saskatchewan|BOLD:AAD2911
Sympistis insanina|[3072]|RDNM616-05|CNCNoctuoidea7462|626[0n]bp|Canada.Alberta|BOLD:AAD2911
Sympistis sanina|[3073]|RDNM532-05|CNCNoctuoidea6808|658[0n]bp|United States.New Mexico|BOLD:AAD2911
Sympistis sanina|[3074]|RDNMB176-05|CNCNoctuoidea7922|658[0n]bp|United States.Wyoming|BOLD:AAD2911
Sympistis sanina|[3075]|RDNMB177-05|CNCNoctuoidea7923|592[0n]bp|United States.Wyoming|BOLD:AAD2911
Sympistis sanina|[3076]|RDNMB178-05|CNCNoctuoidea7924|599[0n]bp|United States.Wyoming|BOLD:AAD2911
Sympistis sanina|[3077]|RDNM610-05|CNCNoctuoidea7456|658[0n]bp|United States.Wyoming|BOLD:AAD2911
Sympistis sanina|[3078]|RDNM648-05|CNCNoctuoidea7494|658[0n]bp|United States.New Mexico|BOLD:AAD2911
Sympistis sanina|[3079]|RDNMB561-05|CNCNoctuoidea10337|658[0n]bp|United States.New Mexico|BOLD:AAD2911
Sympistis sanina|[3080]|RDNMB560-05|CNCNoctuoidea10336|658[0n]bp|United States.New Mexico|BOLD:AAD2911
Sympistis sanina|[3081]|RDNMB559-05|CNCNoctuoidea10335|658[0n]bp|United States.New Mexico|BOLD:AAD2911
Sympistis augustus|[3082]|RDNMB868-05|CNCNoctuoidea10643|658[0n]bp|Canada.Alberta|BOLD:AAE1134
Sympistis augustus|[3083]|RDNMB632-05|CNCNoctuoidea10408|658[0n]bp|Canada.Alberta|BOLD:AAE1134
Sympistis acheron|[3084]|RDNMC809-06|BOOG57|604[1n]bp|Canada.British Columbia|BOLD:AAB6137
Sympistis cocytus|[3085]|RDNMC813-06|BOOG61|557[2n]bp|Canada.British Columbia|BOLD:ABZ6408
Sympistis cocytus|[3086]|RDNMC811-06|BOOG59|563[0n]bp|Canada.British Columbia|BOLD:ABZ6408
Sympistis cocytus|[3087]|RDNMC812-06|BOOG60|564[0n]bp|Canada.British Columbia|BOLD:ABZ6408
Sympistis cocytus|[3088]|RDNMB206-05|CNCNoctuoidea7952|569[0n]bp|Canada.British Columbia|BOLD:ABZ6408
Sympistis cocytus|[3089]|RDNMC810-06|BOOG58|574[0n]bp|Canada.British Columbia|BOLD:ABZ6408
Sympistis cocytus|[3090]|RDNMC814-06|BOOG62|621[0n]bp|Canada.British Columbia|BOLD:ABZ6408

Sympistis cocytus|[3088]|RDNMB206-05|CNCNoctuoidea7952|569[0n]bp|Canada.British Columbia|BOLD:ABZ6408
Sympistis cocytus|[3089]|RDNMC810-06|BOOG58|574[0n]bp|Canada.British Columbia|BOLD:ABZ6408
Sympistis cocytus|[3090]|RDNMC814-06|BOOG62|621[0n]bp|Canada.British Columbia|BOLD:ABZ6408
Sympistis cocytus|[3091]|LBCH7217-10|10-JDWBC-7217|658[0n]bp|Canada.British Columbia|BOLD:ABZ6408
Sympistis pudorata|[3092]|RDNMB124-05|CNCNoctuoidea7870|570[1n]bp|Canada.British Columbia|BOLD:ACE7771
Sympistis pudorata|[3093]|RDMAB1036-09|UASM99706|658[0n]bp|Canada.British Columbia|BOLD:ACE7771
Sympistis pudorata|[3094]|RDMAB1037-09|UASM99707|658[0n]bp|Canada.British Columbia|BOLD:ACE7771
Sympistis pudorata|[3095]|RDMAB1038-09|UASM99708|658[0n]bp|Canada.British Columbia|BOLD:ACE7771
Sympistis sandaraca|[3096]|RDNM598-05|CNCNoctuoidea7444|639[0n]bp|Canada.British Columbia|BOLD:AAD2368
Sympistis viriditincta|[3097]|RDNMF731-08|NOC14817|658[0n]bp|United States.North Dakota|BOLD:AAE5108
Sympistis viriditincta|[3098]|RDNMF730-08|NOC14816|658[1n]bp|United States.Nebraska|BOLD:AAE5108
Sympistis viriditincta|[3099]|RDNMF732-08|NOC14818|658[0n]bp|United States.Nebraska|BOLD:AAE5108
Sympistis viriditincta|[3100]|RDNMF734-08|NOC14820|658[0n]bp|United States.Nebraska|BOLD:AAE5108
Pseudacontia crustaria|[3101]|RDNMF785-08|UASM19655|658[0n]bp|Canada.Alberta|BOLD:AAX8202
Sympistis albifasciata|[3102]|RDNM502-05|CNCNoctuoidea6778|658[0n]bp|United States.California|BOLD:AA...
Sympistis occata|[3103]|RDNMB869-05|CNCNoctuoidea10644|658[0n]bp|Canada.Alberta|BOLD:AAI1668
Sympistis coprocolor|[3104]|RDNM080-05|CNCNoctuoidea6349|622[0n]bp|Canada.British Columbia|BOLD:ACE9017
Sympistis coprocolor|[3105]|RDNMG813-08|CNC LEP00052937|658[0n]bp|Canada.British Columbia|BOLD:ACE9017
Sympistis figurata|[3106]|RDNM493-05|CNCNoctuoidea6769|658[0n]bp|Canada.British Columbia|BOLD:ABY3003
Sympistis figurata|[3107]|RDNM492-05|CNCNoctuoidea6768|658[0n]bp|Canada.British Columbia|BOLD:ABY3003
Sympistis greyi|[3108]|RDNMB165-05|CNCNoctuoidea7911|502[0n]bp|Canada.British Columbia|BOLD:AAC3030
Sympistis greyi|[3109]|LBCH5703-10|10-JDWBC-5703|658[0n]bp|Canada.British Columbia|BOLD:AAC3030
Sympistis greyi|[3110]|LBCH6181-10|10-JDWBC-6181|658[0n]bp|Canada.British Columbia|BOLD:AAC3030
Sympistis greyi|[3111]|LBCH5930-10|10-JDWBC-5930|658[0n]bp|Canada.British Columbia|BOLD:AAC3030
Sympistis greyi|[3112]|LBCH5819-10|10-JDWBC-5819|658[0n]bp|Canada.British Columbia|BOLD:AAC3030
Sympistis greyi|[3113]|LBCH5813-10|10-JDWBC-5813|658[0n]bp|Canada.British Columbia|BOLD:AAC3030
Sympistis greyi|[3114]|LBCH5812-10|10-JDWBC-5812|658[0n]bp|Canada.British Columbia|BOLD:AAC3030
Sympistis greyi|[3115]|LBCH5702-10|10-JDWBC-5702|658[0n]bp|Canada.British Columbia|BOLD:AAC3030
Sympistis greyi|[3116]|LBCH5706-10|10-JDWBC-5706|658[0n]bp|Canada.British Columbia|BOLD:AAC3030
Sympistis greyi|[3117]|LBCH6180-10|10-JDWBC-6180|658[0n]bp|Canada.British Columbia|BOLD:AAC3030
Sympistis greyi|[3118]|LBCH5870-10|10-JDWBC-5870|658[0n]bp|Canada.British Columbia|BOLD:AAC3030
Sympistis greyi|[3119]|LOWCD351-06|CGWC-3171|559[0n]bp|Canada.British Columbia|BOLD:AAC3030
Sympistis greyi|[3120]|LOWCD364-06|CGWC-3184|593[0n]bp|Canada.British Columbia|BOLD:AAC3030
Sympistis greyi|[3121]|LBCG1134-09|08-JDWBC-1134|658[0n]bp|Canada.British Columbia|BOLD:AAC3030
Sympistis greyi|[3122]|LBCH5430-10|10-JDWBC-5430|658[0n]bp|Canada.British Columbia|BOLD:AAC3030
Sympistis greyi|[3123]|LBCH5708-10|10-JDWBC-5708|658[0n]bp|Canada.British Columbia|BOLD:AAC3030
Sympistis greyi|[3124]|LOWCD358-06|CGWC-3178|607[0n]bp|Canada.British Columbia|BOLD:AAC3030
Sympistis greyi|[3125]|LBCH5707-10|10-JDWBC-5707|658[0n]bp|Canada.British Columbia|BOLD:AAC3030
Sympistis greyi|[3126]|LOWCD346-06|CGWC-3166|571[0n]bp|Canada.British Columbia|BOLD:AAC3030
Sympistis greyi|[3127]|LOWCD362-06|CGWC-3182|555[0n]bp|Canada.British Columbia|BOLD:AAC3030
Sympistis greyi|[3128]|LOWCD361-06|CGWC-3181|589[0n]bp|Canada.British Columbia|BOLD:AAC3030
Sympistis greyi|[3129]|LBCH5818-10|10-JDWBC-5818|658[0n]bp|Canada.British Columbia|BOLD:AAC3030
Sympistis greyi|[3130]|LBCH5704-10|10-JDWBC-5704|658[0n]bp|Canada.British Columbia|BOLD:AAC3030
Sympistis greyi|[3131]|LBCH5604-10|10-JDWBC-5604|658[0n]bp|Canada.British Columbia|BOLD:AAC3030
Sympistis greyi|[3132]|LBCH5814-10|10-JDWBC-5814|658[0n]bp|Canada.British Columbia|BOLD:AAC3030
Sympistis greyi|[3133]|LOWCD365-06|CGWC-3185|612[0n]bp|Canada.British Columbia|BOLD:AAC3030
Sympistis pallidior|[3134]|RDMAB450-05|BCSC123|658[0n]bp|Canada.Alberta|BOLD:AAC5159
Sympistis pallidior|[3135]|RDMAB129-05|UASM41274|658[0n]bp|Canada.Alberta|BOLD:AAC5159
Sympistis pallidior|[3136]|RDNM497-05|CNCNoctuoidea6773|658[0n]bp|Canada.Alberta|BOLD:AAC5159
Sympistis pallidior|[3137]|RDNM498-05|CNCNoctuoidea6774|658[0n]bp|Canada.Saskatchewan|BOLD:AAC5159
Sympistis semicollaris|[3138]|LBCH5817-10|10-JDWBC-5817|658[0n]bp|Canada.British Columbia|BOLD:AAB6384
Sympistis semicollaris|[3139]|LBCH5705-10|10-JDWBC-5705|658[0n]bp|Canada.British Columbia|BOLD:AAB6384
Sympistis semicollaris|[3140]|LOWCD352-06|CGWC-3172|658[0n]bp|Canada.British Columbia|BOLD:AAB6384
Sympistis semicollaris|[3141]|LBCH5815-10|10-JDWBC-5815|658[0n]bp|Canada.British Columbia|BOLD:AAB6384
Sympistis semicollaris|[3142]|LBCH5610-10|10-JDWBC-5610|658[0n]bp|Canada.British Columbia|BOLD:AAB6384
Sympistis semicollaris|[3143]|LBCH5816-10|10-JDWBC-5816|658[0n]bp|Canada.British Columbia|BOLD:AAB6384
Sympistis semicollaris|[3144]|RDNM488-05|CNCNoctuoidea6764|658[0n]bp|Canada.British Columbia|BOLD:AAB...
Sympistis semicollaris|[3145]|LALPA918-11|AVBC 1091-11|658[0n]bp|Canada.British Columbia|BOLD:AAB6384
Sympistis semicollaris|[3146]|LOWCD347-06|CGWC-3167|590[0n]bp|Canada.British Columbia|BOLD:AAB6384
Sympistis semicollaris|[3147]|LOWCD348-06|CGWC-3168|587[0n]bp|Canada.British Columbia|BOLD:AAB6384
Sympistis semicollaris|[3148]|LOWCD359-06|CGWC-3179|574[0n]bp|Canada.British Columbia|BOLD:AAB6384
Sympistis semicollaris|[3149]|LOWCD353-06|CGWC-3173|582[0n]bp|Canada.British Columbia|BOLD:AAB6384
Sympistis semicollaris|[3150]|LOWCD355-06|CGWC-3175|565[0n]bp|Canada.British Columbia|BOLD:AAB6384
Sympistis semicollaris|[3151]|LOWCD356-06|CGWC-3176|563[0n]bp|Canada.British Columbia|BOLD:AAB6384
Sympistis semicollaris|[3152]|LOWCD357-06|CGWC-3177|556[0n]bp|Canada.British Columbia|BOLD:AAB6384
Sympistis semicollaris|[3153]|LOWCD368-06|CGWC-3188|600[0n]bp|Canada.British Columbia|BOLD:AAB6384
Sympistis semicollaris|[3154]|LOWCD350-06|CGWC-3170|604[0n]bp|Canada.British Columbia|BOLD:AAB6384
Sympistis semicollaris|[3155]|LOWCD366-06|CGWC-3186|585[0n]bp|Canada.British Columbia|BOLD:AAB6384
Sympistis semicollaris|[3156]|LOWCD363-06|CGWC-3183|612[0n]bp|Canada.British Columbia|BOLD:AAB6384
Sympistis semicollaris|[3157]|LOWCD354-06|CGWC-3174|616[0n]bp|Canada.British Columbia|BOLD:AAB6384
Sympistis semicollaris|[3158]|LOWCD349-06|CGWC-3169|600[0n]bp|Canada.British Columbia|BOLD:AAB6384
Sympistis semicollaris|[3159]|LOWCD367-06|CGWC-3187|581[0n]bp|Canada.British Columbia|BOLD:AAB6384
Sympistis semicollaris|[3160]|LOWCD345-06|CGWC-3165|615[0n]bp|Canada.British Columbia|BOLD:AAB6384
Sympistis semicollaris|[3161]|LOWCD360-06|CGWC-3180|592[0n]bp|Canada.British Columbia|BOLD:AAB6384
Sympistis amun|[3162]|LOWCB731-05|CGWC-1671|658[0n]bp|Canada.British Columbia|BOLD:AAE2626
Sympistis amun|[3163]|RDNM585-05|CNCNoctuoidea7431|658[0n]bp|Canada.British Columbia|BOLD:AAE2626
Sympistis amun|[3164]|LBCH5861-10|10-JDWBC-5861|658[0n]bp|Canada.British Columbia|BOLD:AAE2626
Sympistis chons|[3165]|LBCH5862-10|10-JDWBC-5862|658[0n]bp|Canada.British Columbia|BOLD:AAB4199
Sympistis chons|[3166]|LBCH5709-10|10-JDWBC-5709|658[0n]bp|Canada.British Columbia|BOLD:AAB4199
Sympistis chons|[3167]|RDNM577-05|CNCNoctuoidea7423|645[0n]bp|Canada.British Columbia|BOLD:AAB4199
Sympistis chons|[3168]|RDNM576-05|CNCNoctuoidea7422|578[0n]bp|Canada.British Columbia|BOLD:AAB4199
Sympistis chons|[3169]|RDNM578-05|CNCNoctuoidea7424|658[0n]bp|Canada.British Columbia|BOLD:AAB4199
Sympistis chons|[3170]|LOWCB729-05|CGWC-1669|515[0n]bp|Canada.British Columbia|BOLD:AAB4199
Sympistis chons|[3171]|LBCH5940-10|10-JDWBC-5940|658[0n]bp|Canada.British Columbia|BOLD:AAB4199
Sympistis chons|[3172]|RDMAB453-05|BCSC126|617[0n]bp|Canada.Alberta|BOLD:AAB4199
Sympistis chons|[3173]|LOWCB730-05|CGWC-1670|658[1n]bp|Canada.British Columbia|BOLD:AAB4199
Sympistis chons|[3174]|LBCH6024-10|10-JDWBC-6024|658[0n]bp|Canada.British Columbia|BOLD:AAB4199
Sympistis chons|[3175]|LBCH5670-10|10-JDWBC-5670|658[0n]bp|Canada.British Columbia|BOLD:AAB4199
Sympistis chons|[3176]|LOWCB733-05|CGWC-1673|658[0n]bp|Canada.British Columbia|BOLD:AAB4199
Sympistis chons|[3177]|RDMAB473-06|UASM77898|658[0n]bp|Canada.Alberta|BOLD:AAB4199
Sympistis chons|[3178]|LBCH5607-10|10-JDWBC-5607|658[0n]bp|Canada.British Columbia|BOLD:AAB4199
Sympistis chons|[3179]|LOWCB734-05|CGWC-1674|658[0n]bp|Canada.British Columbia|BOLD:AAB4199
Sympistis chons|[3180]|LBCH5573-10|10-JDWBC-5573|658[0n]bp|Canada.British Columbia|BOLD:AAB4199
Sympistis chons|[3181]|LBCH5804-10|10-JDWBC-5804|658[0n]bp|Canada.British Columbia|BOLD:AAB4199
Sympistis chons|[3182]|RDNM581-05|CNCNoctuoidea7427|658[0n]bp|Canada.British Columbia|BOLD:AAB4199
Sympistis chons|[3183]|RDMAB582-06|UASM58573|658[0n]bp|Canada.Alberta|BOLD:AAB4199
Sympistis chons|[3184]|RDNM580-05|CNCNoctuoidea7426|658[0n]bp|Canada.British Columbia|BOLD:AAB4199
Sympistis chons|[3185]|RDNM583-05|CNCNoctuoidea7429|658[0n]bp|Canada.Alberta|BOLD:AAB4199
Sympistis chons|[3186]|LPABB303-08|08BBLEP-03568|658[0n]bp|Canada.Alberta|BOLD:AAB4199
Sympistis chons|[3187]|LBCH5428-10|10-JDWBC-5428|658[0n]bp|Canada.British Columbia|BOLD:AAB4199
Sympistis chons|[3188]|RDNM582-05|CNCNoctuoidea7428|658[0n]bp|Canada.Alberta|BOLD:AAB4199
Sympistis chons|[3189]|LOWCB738-05|CGWC-1678|658[0n]bp|Canada.British Columbia|BOLD:AAB4199
Sympistis chons|[3190]|LOWCB737-05|CGWC-1677|658[0n]bp|Canada.British Columbia|BOLD:AAB4199

Sympistis chons|[3187]|LBCH5428-10|10-JDWBC-5428|658[0n]bp|Canada.British Columbia|BOLD:AAB4199
Sympistis chons|[3188]|RDNM582-05|CNCNoctuoidea7428|658[0n]bp|Canada.Alberta|BOLD:AAB4199
Sympistis chons|[3189]|LOWCB738-05|CGWC-1678|658[0n]bp|Canada.British Columbia|BOLD:AAB4199
Sympistis chons|[3190]|LOWCB737-05|CGWC-1677|658[0n]bp|Canada.British Columbia|BOLD:AAB4199
Sympistis chons|[3191]|LOWCB736-05|CGWC-1676|559[0n]bp|Canada.British Columbia|BOLD:AAB4199
Sympistis chons|[3192]|LOWCB735-05|CGWC-1675|585[1n]bp|Canada.British Columbia|BOLD:AAB4199
Sympistis chons|[3193]|LBCH5913-10|10-JDWBC-5913|644[0n]bp|Canada.British Columbia|BOLD:AAB4199
Sympistis chons|[3194]|RDNM584-05|CNCNoctuoidea7430|658[0n]bp|Canada.British Columbia|BOLD:AAB4199
Sympistis chons|[3195]|RDNM579-05|CNCNoctuoidea7425|658[0n]bp|Canada.British Columbia|BOLD:AAB4199
Sympistis chons|[3196]|LOWCB732-05|CGWC-1672|658[0n]bp|Canada.British Columbia|BOLD:AAB4199
Sympistis riparia|[3197]|RDNM569-05|CNCNoctuoidea7415|548[0n]bp|Canada.Saskatchewan|BOLD:ACF5261
Sympistis riparia|[3198]|RDMAB490-06|UASM77999|658[0n]bp|Canada.Alberta|BOLD:ACF5261
Sympistis riparia|[3199]|RDNM568-05|CNCNoctuoidea7414|658[0n]bp|Canada.Saskatchewan|BOLD:ACF5261
Sympistis riparia|[3200]|RDNM570-05|CNCNoctuoidea7416|658[0n]bp|Canada.Alberta|BOLD:ACF5261
Sympistis riparia|[3201]|RDNM572-05|CNCNoctuoidea7418|658[0n]bp|Canada.Ontario|BOLD:ACF5261
Sympistis riparia|[3202]|RDNM573-05|CNCNoctuoidea7419|658[0n]bp|Canada.Ontario|BOLD:ACF5261
Sympistis riparia|[3203]|RDMAB452-05|BCSC125|568[3n]bp|Canada.Alberta|BOLD:ACF5261
Sympistis riparia|[3204]|RDNM571-05|CNCNoctuoidea7417|552[0n]bp|Canada.Alberta|BOLD:ACF5261
Sympistis columbia|[3205]|RDNMB183-05|CNCNoctuoidea7929|585[0n]bp|Canada.British Columbia|BOLD:AAE2638
Sympistis columbia|[3206]|RDNMG923-08|CNC LEP00053047|658[0n]bp|Canada.British Columbia|BOLD:AAE1045
Sympistis youngi|[3207]|RDNMB180-05|CNCNoctuoidea7926|654[0n]bp|United States.Washington|BOLD:AAE1045
Sympistis youngi|[3208]|RDNMG922-08|CNC LEP00053046|658[0n]bp|United States.Washington|BOLD:AAE1045
Sympistis youngi|[3209]|RDNMG921-08|CNC LEP00053045|658[0n]bp|United States.Oregon|BOLD:AAE1045
Sympistis youngi|[3210]|RDNMB179-05|CNCNoctuoidea7925|577[1n]bp|United States.Oregon|BOLD:AAE1045
Sympistis dinalda|[3211]|RDNM107-05|CNCNoctuoidea6657|658[0n]bp|Canada.Ontario|BOLD:AAB4182
Sympistis dinalda|[3212]|LPABC938-09|08BBLEP-05349|658[0n]bp|Canada.Alberta|BOLD:AAB4182
Sympistis dinalda|[3213]|LPABC914-09|08BBLEP-05325|658[0n]bp|Canada.Alberta|BOLD:AAB4182
Sympistis dinalda|[3214]|LPABB469-08|08BBLEP-03734|658[0n]bp|Canada.Alberta|BOLD:AAB4182
Sympistis dinalda|[3215]|LPABC980-09|08BBLEP-05391|658[0n]bp|Canada.Alberta|BOLD:AAB4182
Sympistis dinalda|[3216]|LPSK260-08|08BBLEP-01828|658[0n]bp|Canada.Saskatchewan|BOLD:AAB4182
Sympistis dinalda|[3217]|LPABB487-08|08BBLEP-03752|658[0n]bp|Canada.Alberta|BOLD:AAB4182
Sympistis dinalda|[3218]|BBLEC045-09|09BBELE-0045|658[0n]bp|Canada.New Brunswick|BOLD:AAB4182
Sympistis dinalda|[3219]|BBLPE068-09|09BBELE-2068|658[0n]bp|Canada.Nova Scotia|BOLD:AAB4182
Sympistis dinalda|[3220]|LBCD290-05|HLC-23110|658[0n]bp|Canada.British Columbia|BOLD:AAB4182
Sympistis dinalda|[3221]|LPABC899-09|08BBLEP-05310|658[0n]bp|Canada.Alberta|BOLD:AAB4182
Sympistis dinalda|[3222]|LPABC723-09|08BBLEP-04942|658[0n]bp|Canada.Alberta|BOLD:AAB4182
Sympistis dinalda|[3223]|LPABC956-09|08BBLEP-05367|658[0n]bp|Canada.Alberta|BOLD:AAB4182
Sympistis dinalda|[3224]|LPABC952-09|08BBLEP-05363|658[0n]bp|Canada.Alberta|BOLD:AAB4182
Sympistis dinalda|[3225]|LPABC999-09|08BBLEP-05410|658[0n]bp|Canada.Alberta|BOLD:AAB4182
Sympistis dinalda|[3226]|LPABB515-08|08BBLEP-03780|658[0n]bp|Canada.Alberta|BOLD:AAB4182
Sympistis fifia|[3227]|LBCH5720-10|10-JDWBC-5720|658[0n]bp|Canada.British Columbia|BOLD:ACF2135
Sympistis fifia|[3228]|RDNMF716-08|NOC14802|658[0n]bp|Canada.British Columbia|BOLD:ACF2135
Sympistis fifia|[3229]|RDNMF717-08|NOC14803|658[0n]bp|Canada.British Columbia|BOLD:ACF2135
Sympistis fifia|[3230]|LBCH5726-10|10-JDWBC-5726|658[0n]bp|Canada.British Columbia|BOLD:ACF2135
Sympistis fifia|[3231]|LOWCD533-06|CGWC-3353|658[0n]bp|Canada.British Columbia|BOLD:ACF2135
Sympistis fifia|[3232]|LBCD282-05|HLC-23102|658[0n]bp|Canada.British Columbia|BOLD:ACF2135
Sympistis fifia|[3233]|LBCH5941-10|10-JDWBC-5941|658[0n]bp|Canada.British Columbia|BOLD:ACF2135
Sympistis fifia|[3234]|LBCH5803-10|10-JDWBC-5803|658[0n]bp|Canada.British Columbia|BOLD:ACF2135
Sympistis badistriga|[3235]|RDNM108-05|CNCNoctuoidea6658|658[0n]bp|Canada.Ontario|BOLD:AAE4975
Sympistis badistriga|[3236]|LPMNB346-09|08BBLEP-05190|658[0n]bp|Canada.Manitoba|BOLD:AAE4975
Sympistis badistriga|[3237]|LPMNB328-09|08BBLEP-05172|658[0n]bp|Canada.Manitoba|BOLD:AAE4975
Sympistis stabilis|[3238]|RDNMF034-08|NOC14120|658[0n]bp|Canada.Saskatchewan|BOLD:AAF1183
Sympistis stabilis|[3239]|LALPA870-11|AVBC 1043-11|658[0n]bp|Canada.British Columbia|BOLD:AAF1183
Sympistis stabilis|[3240]|RDNMG605-08|CNC LEP00052429|658[0n]bp|Canada.Ontario|BOLD:AAF1183
Sympistis stabilis|[3241]|RDNMG606-08|CNC LEP00052430|658[0n]bp|Canada.British Columbia|BOLD:AAF1183
Sympistis albifasciata|[3242]|NAMUM196-08|RR-96-0222|658[0n]bp|United States.California|BOLD:AAF3869
Sympistis albifasciata|[3243]|RDNMB193-05|CNCNoctuoidea7939|581[0n]bp|United States.Oregon|BOLD:AAF3869
Sympistis albifasciata|[3244]|RDNMB194-05|CNCNoctuoidea7940|580[0n]bp|United States.Oregon|BOLD:AAF3869
Sympistis chionanthi|[3245]|RDNMB214-05|CNCNoctuoidea7960|654[0n]bp|Canada.Alberta|BOLD:AAD7859
Sympistis chionanthi|[3246]|RDNMK771-12|acrorev193|658[0n]bp|Canada.Alberta|BOLD:AAD7859
Sympistis chionanthi|[3247]|RDNMK772-12|acrorev194|658[0n]bp|Canada.Alberta|BOLD:AAD7859
Sympistis chionanthi|[3248]|RDNMB213-05|CNCNoctuoidea7959|658[0n]bp|Canada.Ontario|BOLD:AAD7859
Sympistis chionanthi|[3249]|RDLQ604-07|DH009854|658[0n]bp|Canada.Quebec|BOLD:AAD7859
Sympistis chionanthi|[3250]|XAH314-05|2005-ONT-1897|658[0n]bp|Canada.Ontario|BOLD:AAD7859
Sympistis chionanthi|[3251]|RDLQ605-07|DH007094|658[0n]bp|Canada.Quebec|BOLD:AAD7859
Sympistis chionanthi|[3252]|XAH345-05|2005-ONT-1928|658[0n]bp|Canada.Ontario|BOLD:AAD7859
Sympistis anweileri|[3253]|RDMAB455-05|BCSC128|506[0n]bp|Canada.Alberta|BOLD:AAD4613
Sympistis anweileri|[3254]|RDNMB534-05|CNCNoctuoidea10310|600[0n]bp|Canada.British Columbia|BOLD:AAD4613
Sympistis anweileri|[3255]|RDNMB531-05|CNCNoctuoidea10307|658[0n]bp|Canada.Alberta|BOLD:AAD4613
Sympistis anweileri|[3256]|RDNMB533-05|CNCNoctuoidea10309|549[0n]bp|Canada.Alberta|BOLD:AAD4613
Sympistis anweileri|[3257]|RDMAB454-05|BCSC127|658[0n]bp|Canada.Alberta|BOLD:AAD4613
Sympistis anweileri|[3258]|RDNMB535-05|CNCNoctuoidea10311|658[0n]bp|Canada.British Columbia|BOLD:AAD4613
Sympistis anweileri|[3259]|LBCH4053-10|10-JDWBC-4053|658[0n]bp|Canada.British Columbia|BOLD:AAD4613
Sympistis anweileri|[3260]|RDNMB530-05|CNCNoctuoidea10306|658[0n]bp|Canada.Alberta|BOLD:AAD4613
Sympistis californiae|[3261]|RDNMB536-05|CNCNoctuoidea10312|630[0n]bp|Canada.British Columbia|BOLD:AA...
Sympistis dentata|[3262]|RDLQF364-06|DH011431|658[0n]bp|Canada.Quebec|BOLD:AAB6888
Sympistis dentata|[3263]|BBLPC096-09|09BBELE-1096|658[0n]bp|Canada.New Brunswick|BOLD:AAB6888
Sympistis dentata|[3264]|LBCH6741-10|10-JDWBC-6741|658[0n]bp|Canada.British Columbia|BOLD:AAB6888
Sympistis dentata|[3265]|RDLQB609-05|DH010712|571[2n]bp|Canada.Quebec|BOLD:AAB6888
Sympistis dentata|[3266]|LCHIP138-07|06-PROBE-2680|650[0n]bp|Canada.Manitoba|BOLD:AAB6888
Sympistis dentata|[3267]|TTMNB086-06|MNBTT-086|658[0n]bp|Canada.New Brunswick|BOLD:AAB6888
Sympistis dentata|[3268]|TTMNB087-06|MNBTT-087|658[0n]bp|Canada.New Brunswick|BOLD:AAB6888
Sympistis dentata|[3269]|BBLPE129-09|09BBELE-2129|658[0n]bp|Canada.Nova Scotia|BOLD:AAB6888
Sympistis dentata|[3270]|BBLEC433-09|09BBELE-0433|658[0n]bp|Canada.New Brunswick|BOLD:AAB6888
Sympistis dentata|[3271]|RDLQB616-05|DH010719|578[2n]bp|Canada.Quebec|BOLD:AAB6888
Sympistis dentata|[3272]|RDNMB532-05|CNCNoctuoidea10308|565[1n]bp|Canada.New Brunswick|BOLD:AAB6888
Sympistis dentata|[3273]|RDLQB608-05|DH010711|567[0n]bp|Canada.Quebec|BOLD:AAB6888
Sympistis dentata|[3274]|RDLQB613-05|DH010716|552[0n]bp|Canada.Quebec|BOLD:AAB6888
Sympistis dentata|[3275]|RDLQB612-05|DH010715|602[0n]bp|Canada.Quebec|BOLD:AAB6888
Sympistis dentata|[3276]|RDLQB614-05|DH010717|581[1n]bp|Canada.Quebec|BOLD:AAB6888
Sympistis dentata|[3277]|RDLQB610-05|DH010713|596[1n]bp|Canada.Quebec|BOLD:AAB6888
Sympistis dentata|[3278]|RDLQB611-05|DH010714|569[0n]bp|Canada.Quebec|BOLD:AAB6888
Sympistis dentata|[3279]|RDLQB615-05|DH010718|596[0n]bp|Canada.Quebec|BOLD:AAB6888
Sympistis dentata|[3280]|RDLQB607-05|DH010710|567[1n]bp|Canada.Quebec|BOLD:AAB6888
Sympistis dentata|[3281]|RDLQB084-05|DH010170|658[0n]bp|Canada.Quebec|BOLD:AAB6888
Sympistis funebris|[3282]|RDNMF455-08|NOC14541|618[0n]bp|Canada.Alberta|BOLD:AAD4608
Sympistis funebris|[3283]|CHLEP255-09|09PROBE-09550|658[0n]bp|Canada.Manitoba|BOLD:AAD4608
Sympistis funebris|[3284]|RDNMF456-08|NOC14542|658[0n]bp|Canada.Yukon Territory|BOLD:AAD4608
Sympistis funebris|[3285]|RDNMF458-08|NOC14544|658[0n]bp|Canada.Yukon Territory|BOLD:AAD4608
Sympistis funebris|[3286]|RDNMF457-08|NOC14543|634[0n]bp|Canada.Yukon Territory|BOLD:AAD4608
Sympistis barnesii|[3287]|RDNMB184-05|CNCNoctuoidea7930|598[0n]bp|United States.Washington|BOLD:ABZ4087
Sympistis barnesii|[3288]|RDNMB185-05|CNCNoctuoidea7931|541[0n]bp|United States.Washington|BOLD:ABZ4087
Sympistis chalybdis|[3289]|RDNM484-05|CNCNoctuoidea6760|658[0n]bp|Canada.British Columbia|BOLD:AAE1059
[illegible]

Sympistis barnesii|[3287]|RDNMB184-05|CNCNoctuoidea7930|598[0n]bp|United States.Washington|BOLD:ABZ4087
Sympistis barnesii|[3288]|RDNMB185-05|CNCNoctuoidea7931|541[0n]bp|United States.Washington|BOLD:ABZ4087
Sympistis chalybdis|[3289]|RDNM484-05|CNCNoctuoidea6760|658[0n]bp|Canada.British Columbia|BOLD:AAE1059
Sympistis chalybdis|[3290]|RDNMG878-08|CNC LEP00053002|658[0n]bp|Canada.British Columbia|BOLD:AAE1059
Sympistis piffardi|[3291]|RDLQF241-06|DH011321|636[0n]bp|Canada.Quebec|BOLD:AAE1059
Sympistis piffardi|[3292]|RDNMG808-08|CNC LEP00052932|658[0n]bp|Canada.Alberta|BOLD:AAE1059
Sympistis piffardi|[3293]|RDNM483-05|CNCNoctuoidea6759|658[0n]bp|Canada.New Brunswick|BOLD:AAE1059
Sympistis piffardi|[3294]|RDNMG801-08|CNC LEP00052925|658[0n]bp|Canada.New Brunswick|BOLD:AAE1059
Sympistis piffardi|[3295]|RDNMG807-08|CNC LEP00052931|658[0n]bp|Canada.New Brunswick|BOLD:AAE1059
Sympistis lapponica|[3296]|RDNMG784-08|CNC LEP00052908|658[0n]bp|Canada.Northwest Territories|BOLD:AA...
Sympistis lapponica|[3297]|RDNMG783-08|CNC LEP00052907|590[0n]bp|Canada.Yukon Territory|BOLD:AAC8398
Sympistis lapponica|[3298]|RDNMB921-05|CNCNoctuoidea10696|588[0n]bp|Canada.Nunavut|BOLD:AAC8398
Sympistis lapponica|[3299]|RDNMG785-08|CNC LEP00052909|658[0n]bp|Canada.Yukon Territory|BOLD:AAC8398
Sympistis lapponica|[3300]|RDNME513-08|LEP037937|658[0n]bp|Canada.Yukon Territory|BOLD:AAC8398
Sympistis heliophila|[3301]|RDNME515-08|LEP037939|658[0n]bp|Canada.Yukon Territory|BOLD:AAC8395
Sympistis heliophila|[3302]|RDMAB213-05|UASM78247|658[0n]bp|Canada.Alberta|BOLD:AAC8395
Sympistis heliophila|[3303]|RDMAB214-05|UASM78244|658[0n]bp|Canada.Alberta|BOLD:AAC8395
Sympistis heliophila|[3304]|RDNME524-08|LEP037948|658[0n]bp|Canada.Yukon Territory|BOLD:AAC8395
Sympistis heliophila|[3305]|RDNMB922-05|CNCNoctuoidea10697|570[0n]bp|Canada.Yukon Territory|BOLD:AAC8395
Sympistis wilsoni|[3306]|RDNMF454-08|NOC14540|658[0n]bp|Canada.British Columbia|BOLD:AAC8395
Sympistis wilsoni|[3307]|RDNMF453-08|NOC14539|641[0n]bp|Canada.British Columbia|BOLD:AAC8395
Sympistis zetterstedtii|[3308]|RDNMB926-05|CNCNoctuoidea10701|551[0n]bp|Canada.British Columbia|BOLD:...
Sympistis zetterstedtii|[3309]|RDNMB924-05|CNCNoctuoidea10699|614[0n]bp|Canada.British Columbia|BOLD:...
Sympistis zetterstedtii|[3310]|RDNME523-08|LEP037947|658[0n]bp|Canada.Yukon Territory|BOLD:AAA7102
Sympistis zetterstedtii|[3311]|RDNME522-08|LEP037946|658[0n]bp|Canada.Yukon Territory|BOLD:AAA7102
Sympistis zetterstedtii|[3312]|RDNME514-08|LEP037938|658[0n]bp|Canada.Yukon Territory|BOLD:AAA7102
Sympistis zetterstedtii|[3313]|RDNMI114-10|CNCLEP 69714|658[0n]bp|Canada.Yukon Territory|BOLD:AAA7102
Sympistis zetterstedtii|[3314]|RDNME535-08|LEP037959|658[0n]bp|Canada.Yukon Territory|BOLD:AAA7102
Sympistis zetterstedtii|[3315]|RDNME532-08|LEP037956|658[0n]bp|Canada.Yukon Territory|BOLD:AAA7102
Sympistis zetterstedtii|[3316]|RDNME531-08|LEP037955|646[0n]bp|Canada.Yukon Territory|BOLD:AAA7102
Sympistis zetterstedtii|[3317]|RDNME534-08|LEP037958|658[0n]bp|Canada.Yukon Territory|BOLD:AAA7102
Sympistis zetterstedtii|[3318]|RDNME533-08|LEP037957|658[0n]bp|Canada.Yukon Territory|BOLD:AAA7102
Sympistis zetterstedtii|[3319]|LCHP336-07|07PROBE-03907|658[1n]bp|Canada.Manitoba|BOLD:AAA7102
Sympistis zetterstedtii|[3320]|LCHP023-07|07PROBE-00083|658[0n]bp|Canada.Manitoba|BOLD:AAA7102
Sympistis zetterstedtii|[3321]|LCHP024-07|07PROBE-00084|658[0n]bp|Canada.Manitoba|BOLD:AAA7102
Sympistis zetterstedtii|[3322]|LCHQ925-08|07WNP-10817|658[0n]bp|Canada.Manitoba|BOLD:AAA7102
Sympistis zetterstedtii|[3323]|LCHP052-07|07PROBE-00136|657[0n]bp|Canada.Manitoba|BOLD:AAA7102
Sympistis zetterstedtii|[3324]|LCHP091-07|07PROBE-00527|655[0n]bp|Canada.Manitoba|BOLD:AAA7102
Sympistis zetterstedtii|[3325]|LCHP921-07|07PROBE-10683|658[0n]bp|Canada.Manitoba|BOLD:AAA7102
Sympistis zetterstedtii|[3326]|LCHP335-07|07PROBE-03906|656[0n]bp|Canada.Manitoba|BOLD:AAA7102
Sympistis zetterstedtii|[3327]|RDNMB917-05|CNCNoctuoidea10692|562[0n]bp|Canada.Manitoba|BOLD:AAA7102
Sympistis zetterstedtii|[3328]|RDNMB918-05|CNCNoctuoidea10693|517[0n]bp|Canada.Manitoba|BOLD:AAA7102
Sympistis zetterstedtii|[3329]|RDNMB916-05|CNCNoctuoidea10691|573[2n]bp|Canada.Manitoba|BOLD:AAA7102
Sympistis zetterstedtii|[3330]|MHCOL104-07|CHU06-COL-104|620[0n]bp|Canada.Manitoba|BOLD:AAA7102
Sympistis zetterstedtii|[3331]|LCHP427-07|07PROBE-10051|658[0n]bp|Canada.Manitoba|BOLD:AAA7102
Sympistis zetterstedtii|[3332]|LCHP584-07|07PROBE-10255|658[0n]bp|Canada.Manitoba|BOLD:AAA7102
Sympistis zetterstedtii|[3333]|MHCOL163-07|CHU06-COL-163|658[0n]bp|Canada.Manitoba|BOLD:AAA7102
Sympistis zetterstedtii|[3334]|LCHP113-07|07PROBE-00142|658[0n]bp|Canada.Manitoba|BOLD:AAA7102
Sympistis zetterstedtii|[3335]|LCHP451-07|07PROBE-10114|658[0n]bp|Canada.Manitoba|BOLD:AAA7102
Sympistis zetterstedtii|[3336]|LCHP115-07|07PROBE-00144|658[0n]bp|Canada.Manitoba|BOLD:AAA7102
Sympistis zetterstedtii|[3337]|LCHQ914-08|07WNP-10806|658[0n]bp|Canada.Manitoba|BOLD:AAA7102
Sympistis zetterstedtii|[3338]|LCHP922-07|07PROBE-10684|658[0n]bp|Canada.Manitoba|BOLD:AAA7102
Sympistis zetterstedtii|[3339]|LCHQ924-08|07WNP-10816|658[0n]bp|Canada.Manitoba|BOLD:AAA7102
Sympistis zetterstedtii|[3340]|LCHP022-07|07PROBE-00082|658[0n]bp|Canada.Manitoba|BOLD:AAA7102
Sympistis zetterstedtii|[3341]|LCHP334-07|07PROBE-03905|658[0n]bp|Canada.Manitoba|BOLD:AAA7102
Sympistis zetterstedtii|[3342]|LCHP426-07|07PROBE-10050|658[0n]bp|Canada.Manitoba|BOLD:AAA7102
Sympistis zetterstedtii|[3343]|LCHP114-07|07PROBE-00143|658[0n]bp|Canada.Manitoba|BOLD:AAA7102
Sympistis zetterstedtii|[3344]|LCHP425-07|07PROBE-10049|658[0n]bp|Canada.Manitoba|BOLD:AAA7102
Sympistis zetterstedtii|[3345]|LCHP448-07|07PROBE-10111|658[0n]bp|Canada.Manitoba|BOLD:AAA7102
Sympistis zetterstedtii|[3346]|MHCOL158-07|CHU06-COL-158|658[0n]bp|Canada.Manitoba|BOLD:AAA7102
Sympistis zetterstedtii|[3347]|MHCOL159-07|CHU06-COL-159|658[0n]bp|Canada.Manitoba|BOLD:AAA7102
Sympistis zetterstedtii|[3348]|LCHQ906-08|07WNP-10798|658[0n]bp|Canada.Manitoba|BOLD:AAA7102
Sympistis zetterstedtii|[3349]|LCHP429-07|07PROBE-10053|658[0n]bp|Canada.Manitoba|BOLD:AAA7102
Sympistis zetterstedtii|[3350]|CHLEP278-09|09PROBE-09573|658[0n]bp|Canada.Manitoba|BOLD:AAA7102
Sympistis zetterstedtii|[3351]|MHCOL161-07|CHU06-COL-161|654[0n]bp|Canada.Manitoba|BOLD:AAA7102
Sympistis zetterstedtii|[3352]|LCHP093-07|07PROBE-00529|653[0n]bp|Canada.Manitoba|BOLD:AAA7102
Sympistis zetterstedtii|[3353]|LCHP025-07|07PROBE-00085|656[0n]bp|Canada.Manitoba|BOLD:AAA7102
Sympistis zetterstedtii|[3354]|MHCOL166-07|CHU06-COL-166|645[0n]bp|Canada.Manitoba|BOLD:AAA7102
Sympistis zetterstedtii|[3355]|CHLEP223-09|09PROBE-09518|640[0n]bp|Canada.Manitoba|BOLD:AAA7102
Sympistis zetterstedtii|[3356]|CHLEP279-09|09PROBE-09574|636[0n]bp|Canada.Manitoba|BOLD:AAA7102
Sympistis zetterstedtii|[3357]|LCH326-04|04HBL003326|566[0n]bp|Canada.Manitoba|BOLD:AAA7102
Sympistis zetterstedtii|[3358]|MHLEP027-07|CHU06-LEP-027|658[0n]bp|Canada.Manitoba|BOLD:AAA7102
Euxoa plagigera|[3359]|LBCG3252-09|08-JDWBC-3252|632[0n]bp|Canada.British Columbia|BOLD:ACF2029
Euxoa plagigera|[3360]|LOWCE672-06|CGWC-4432|612[0n]bp|Canada.British Columbia|BOLD:ACF2029
Euxoa plagigera|[3361]|LOWCE655-06|CGWC-4415|654[0n]bp|Canada.British Columbia|BOLD:ACF2029
Euxoa plagigera|[3362]|LOWCE663-06|CGWC-4423|610[0n]bp|Canada.British Columbia|BOLD:ACF2029
Euxoa plagigera|[3363]|LOWCE670-06|CGWC-4430|609[0n]bp|Canada.British Columbia|BOLD:ACF2029
Euxoa plagigera|[3364]|LOWCE668-06|CGWC-4428|607[0n]bp|Canada.British Columbia|BOLD:ACF2029
Euxoa plagigera|[3365]|LOWCE666-06|CGWC-4426|658[0n]bp|Canada.British Columbia|BOLD:ACF2029
Euxoa plagigera|[3366]|LBCH6658-10|10-JDWBC-6658|658[0n]bp|Canada.British Columbia|BOLD:ACF2029
Euxoa plagigera|[3367]|LOWCE671-06|CGWC-4431|627[0n]bp|Canada.British Columbia|BOLD:ACF2029
Euxoa plagigera|[3368]|LBCH6375-10|10-JDWBC-6375|658[0n]bp|Canada.British Columbia|BOLD:ACF2029
Euxoa plagigera|[3369]|LBCH6654-10|10-JDWBC-6654|658[0n]bp|Canada.British Columbia|BOLD:ACF2029
Euxoa plagigera|[3370]|LBCH6655-10|10-JDWBC-6655|658[0n]bp|Canada.British Columbia|BOLD:ACF2029
Euxoa plagigera|[3371]|LOWCE667-06|CGWC-4427|658[0n]bp|Canada.British Columbia|BOLD:ACF2029
Euxoa plagigera|[3372]|LOWCE659-06|CGWC-4419|658[0n]bp|Canada.British Columbia|BOLD:ACF2029
Euxoa plagigera|[3373]|LBCH6652-10|10-JDWBC-6652|658[0n]bp|Canada.British Columbia|BOLD:ACF2029
Euxoa plagigera|[3374]|LBCH6354-10|10-JDWBC-6354|658[0n]bp|Canada.British Columbia|BOLD:ACF2029
Euxoa plagigera|[3375]|BBLPB809-10|10BBCLP-1808|658[0n]bp|Canada.Alberta|BOLD:ACF2029
Euxoa plagigera|[3376]|LOWCE662-06|CGWC-4422|658[0n]bp|Canada.British Columbia|BOLD:ACF2029
Euxoa plagigera|[3377]|LBCH7237-10|10-JDWBC-7237|658[0n]bp|Canada.British Columbia|BOLD:ACF2029
Euxoa plagigera|[3378]|LBCH6498-10|10-JDWBC-6498|658[0n]bp|Canada.British Columbia|BOLD:ACF2029
Euxoa plagigera|[3379]|LBCH6496-10|10-JDWBC-6496|658[0n]bp|Canada.British Columbia|BOLD:ACF2029
Euxoa plagigera|[3380]|LOWCE660-06|CGWC-4420|658[0n]bp|Canada.British Columbia|BOLD:ACF2029
Euxoa plagigera|[3381]|LOWCE658-06|CGWC-4418|658[0n]bp|Canada.British Columbia|BOLD:ACF2029
Euxoa plagigera|[3382]|LOWCE656-06|CGWC-4416|658[0n]bp|Canada.British Columbia|BOLD:ACF2029
Euxoa plagigera|[3383]|LBCH6231-10|10-JDWBC-6231|658[0n]bp|Canada.British Columbia|BOLD:ACF2029
Euxoa plagigera|[3384]|LBCG2875-09|08-JDWBC-2875|658[0n]bp|Canada.British Columbia|BOLD:ACF2029
Euxoa plagigera|[3385]|LBCH6232-10|10-JDWBC-6232|658[0n]bp|Canada.British Columbia|BOLD:ACF2029
Euxoa plagigera|[3386]|LBCH6278-10|10-JDWBC-6278|658[0n]bp|Canada.British Columbia|BOLD:ACF2029
Euxoa plagigera|[3387]|LBCH6503-10|10-JDWBC-6503|658[0n]bp|Canada.British Columbia|BOLD:ACF2029
Euxoa plagigera|[3388]|LBCH7966-10|10-JDWBC-7966|658[0n]bp|Canada.British Columbia|BOLD:ACF2029
Euxoa plagigera|[3389]|LBCH6502-10|10-JDWBC-6502|658[0n]bp|Canada.British Columbia|BOLD:ACF2029

Euxoa plagigera|[3387]|LBCH6503-10|10-JDWBC-6503|658[0n]bp|Canada.British Columbia|BOLD:ACF2029
Euxoa plagigera|[3388]|LBCH7966-10|10-JDWBC-7966|658[0n]bp|Canada.British Columbia|BOLD:ACF2029
Euxoa plagigera|[3389]|LBCH6502-10|10-JDWBC-6502|658[0n]bp|Canada.British Columbia|BOLD:ACF2029
Euxoa plagigera|[3390]|LBCH7312-10|10-JDWBC-7312|658[0n]bp|Canada.British Columbia|BOLD:ACF2029
Euxoa plagigera|[3391]|LBCH7970-10|10-JDWBC-7970|658[0n]bp|Canada.British Columbia|BOLD:ACF2029
Euxoa plagigera|[3392]|LOWCE653-06|CGWC-4413|658[0n]bp|Canada.British Columbia|BOLD:ACF2029
Euxoa plagigera|[3393]|LBCH7877-10|10-JDWBC-7877|658[0n]bp|Canada.British Columbia|BOLD:ACF2029
Euxoa plagigera|[3394]|LOWCE669-06|CGWC-4429|658[0n]bp|Canada.British Columbia|BOLD:ACF2029
Euxoa plagigera|[3395]|LBCH6656-10|10-JDWBC-6656|658[0n]bp|Canada.British Columbia|BOLD:ACF2029
Euxoa plagigera|[3396]|LOWCE673-06|CGWC-4433|658[0n]bp|Canada.British Columbia|BOLD:ACF2029
Euxoa plagigera|[3397]|LBCH6361-10|10-JDWBC-6361|658[0n]bp|Canada.British Columbia|BOLD:ACF2029
Euxoa plagigera|[3398]|LBCH6363-10|10-JDWBC-6363|658[0n]bp|Canada.British Columbia|BOLD:ACF2029
Euxoa plagigera|[3399]|LBCH7876-10|10-JDWBC-7876|658[0n]bp|Canada.British Columbia|BOLD:ACF2029
Euxoa plagigera|[3400]|LBCH7685-10|10-JDWBC-7685|658[0n]bp|Canada.British Columbia|BOLD:ACF2029
Euxoa plagigera|[3401]|LBCH7788-10|10-JDWBC-7788|658[0n]bp|Canada.British Columbia|BOLD:ACF2029
Euxoa plagigera|[3402]|BBLPB810-10|10BBCLP-1809|658[0n]bp|Canada.Alberta|BOLD:ACF2029
Euxoa plagigera|[3403]|LBCH6653-10|10-JDWBC-6653|658[0n]bp|Canada.British Columbia|BOLD:ACF2029
Euxoa plagigera|[3404]|LOWCE652-06|CGWC-4412|658[0n]bp|Canada.British Columbia|BOLD:ACF2029
Euxoa plagigera|[3405]|LBCH6747-10|10-JDWBC-6747|658[0n]bp|Canada.British Columbia|BOLD:ACF2029
Euxoa plagigera|[3406]|LBCH6449-10|10-JDWBC-6449|658[0n]bp|Canada.British Columbia|BOLD:ACF2029
Euxoa plagigera|[3407]|LBCH6373-10|10-JDWBC-6373|658[0n]bp|Canada.British Columbia|BOLD:ACF2029
Euxoa plagigera|[3408]|LOWCE654-06|CGWC-4414|658[0n]bp|Canada.British Columbia|BOLD:ACF2029
Euxoa plagigera|[3409]|BBLPB808-10|10BBCLP-1807|658[0n]bp|Canada.Alberta|BOLD:ACF2029
Euxoa plagigera|[3410]|BBLPB866-10|10BBCLP-1865|658[0n]bp|Canada.British Columbia|BOLD:ACF2029
Euxoa plagigera|[3411]|BBLPB807-10|10BBCLP-1806|658[0n]bp|Canada.Alberta|BOLD:ACF2029
Euxoa plagigera|[3412]|LBCH7688-10|10-JDWBC-7688|658[0n]bp|Canada.British Columbia|BOLD:ACF2029
Euxoa plagigera|[3413]|LBCH6454-10|10-JDWBC-6454|658[0n]bp|Canada.British Columbia|BOLD:ACF2029
Euxoa plagigera|[3414]|LBCH6493-10|10-JDWBC-6493|658[0n]bp|Canada.British Columbia|BOLD:ACF2029
Euxoa plagigera|[3415]|LBCH6096-10|10-JDWBC-6096|658[0n]bp|Canada.British Columbia|BOLD:ACF2029
Euxoa plagigera|[3416]|LBCH6657-10|10-JDWBC-6657|658[0n]bp|Canada.British Columbia|BOLD:ACF2029
Euxoa plagigera|[3417]|LBCG479-08|08-JDWBC-0479|658[0n]bp|Canada.British Columbia|BOLD:ACF2029
Euxoa plagigera|[3418]|LBCH6651-10|10-JDWBC-6651|639[0n]bp|Canada.British Columbia|BOLD:ACF2029
Euxoa plagigera|[3419]|BBLPB811-10|10BBCLP-1810|658[0n]bp|Canada.Alberta|BOLD:ACF2029
Euxoa plagigera|[3420]|LOWCE661-06|CGWC-4421|602[0n]bp|Canada.British Columbia|BOLD:ACF2029
Euxoa plagigera|[3421]|LOWCE657-06|CGWC-4417|617[0n]bp|Canada.British Columbia|BOLD:ACF2029
Euxoa plagigera|[3422]|LBCH7803-10|10-JDWBC-7803|648[0n]bp|Canada.British Columbia|BOLD:ACF2029
Euxoa plagigera|[3423]|LOWCE664-06|CGWC-4424|658[0n]bp|Canada.British Columbia|BOLD:ACF2029
Euxoa plagigera|[3424]|LBCH7366-10|10-JDWBC-7366|658[0n]bp|Canada.British Columbia|BOLD:ACF2029
Euxoa perexcellens|[3425]|RDMAB296-05|UASM77793|539[3n]bp|Canada.Alberta|BOLD:ABZ9415
Euxoa basalis|[3426]|LPSK594-08|08BBLEP-02162|658[0n]bp|Canada.Saskatchewan|BOLD:ABZ9144
Euxoa basalis|[3427]|RDNMF031-08|NOC14117|658[0n]bp|Canada.Alberta|BOLD:ABZ9144
Euxoa basalis|[3428]|RDNMF294-08|NOC14380|658[0n]bp|Canada.Saskatchewan|BOLD:ABZ9144
Euxoa basalis|[3429]|RDNMF295-08|NOC14381|658[0n]bp|Canada.Alberta|BOLD:ABZ9144
Euxoa hollemani|[3430]|RDNMF328-08|NOC14414|658[0n]bp|Canada.British Columbia|BOLD:ABZ9892
Euxoa hollemani|[3431]|RDNMF329-08|NOC14415|658[0n]bp|Canada.British Columbia|BOLD:ABZ9892
Euxoa laetificans|[3432]|RDMAB995-09|UASM24370|645[0n]bp|Canada.Alberta|BOLD:ABZ8980
Euxoa laetificans|[3433]|RDMAB595-06|UASM58619|551[0n]bp|Canada.Alberta|BOLD:ABZ8980
Euxoa laetificans|[3434]|RDMAB994-09|UASM2655|636[0n]bp|Canada.Alberta|BOLD:ABZ8980
Euxoa choris|[3435]|LBCH6377-10|10-JDWBC-6377|513[0n]bp|Canada.British Columbia|
Euxoa choris|[3436]|LBCH6370-10|10-JDWBC-6370|658[0n]bp|Canada.British Columbia|BOLD:ABZ9440
Euxoa choris|[3437]|LBCG2882-09|08-JDWBC-2882|658[0n]bp|Canada.British Columbia|BOLD:ABZ9440
Euxoa choris|[3438]|LBCH6728-10|10-JDWBC-6728|658[0n]bp|Canada.British Columbia|BOLD:ABZ9440
Euxoa choris|[3439]|LBCH5857-10|10-JDWBC-5857|658[0n]bp|Canada.British Columbia|BOLD:ABZ9440
Euxoa choris|[3440]|LBCH6729-10|10-JDWBC-6729|658[0n]bp|Canada.British Columbia|BOLD:ABZ9440
Euxoa choris|[3441]|LBCG3283-09|08-JDWBC-3283|658[0n]bp|Canada.British Columbia|BOLD:ABZ9440
Euxoa choris|[3442]|LBCH6444-10|10-JDWBC-6444|658[0n]bp|Canada.British Columbia|BOLD:ABZ9440
Euxoa choris|[3443]|LBCH6543-10|10-JDWBC-6543|658[0n]bp|Canada.British Columbia|BOLD:ABZ9440
Euxoa choris|[3444]|LBCH6732-10|10-JDWBC-6732|658[0n]bp|Canada.British Columbia|BOLD:ABZ9440
Euxoa choris|[3445]|LBCH6041-10|10-JDWBC-6041|658[0n]bp|Canada.British Columbia|BOLD:ABZ9440
Euxoa choris|[3446]|LBCH6730-10|10-JDWBC-6730|658[0n]bp|Canada.British Columbia|BOLD:ABZ9440
Euxoa choris|[3447]|LBCH6378-10|10-JDWBC-6378|658[0n]bp|Canada.British Columbia|BOLD:ABZ9440
Euxoa choris|[3448]|LBCH6530-10|10-JDWBC-6530|658[0n]bp|Canada.British Columbia|BOLD:ABZ9440
Euxoa choris|[3449]|RDNMG657-08|CNC LEP00052481|658[0n]bp|Canada.Alberta|BOLD:ABZ9440
Euxoa choris|[3450]|LBCH7756-10|10-JDWBC-7756|658[0n]bp|Canada.British Columbia|BOLD:ABZ9440
Euxoa choris|[3451]|LBCH6731-10|10-JDWBC-6731|658[0n]bp|Canada.British Columbia|BOLD:ABZ9440
Euxoa choris|[3452]|LBCG2109-09|08-JDWBC-2109|658[0n]bp|Canada.British Columbia|BOLD:ABZ9440
Euxoa choris|[3453]|LBCH6661-10|10-JDWBC-6661|658[0n]bp|Canada.British Columbia|BOLD:ABZ9440
Euxoa choris|[3454]|LBCH6733-10|10-JDWBC-6733|658[0n]bp|Canada.British Columbia|BOLD:ABZ9440
Euxoa choris|[3455]|LBCH6734-10|10-JDWBC-6734|658[0n]bp|Canada.British Columbia|BOLD:ABZ9440
Euxoa choris|[3456]|LBCH6735-10|10-JDWBC-6735|642[0n]bp|Canada.British Columbia|BOLD:ABZ9440
Euxoa munis|[3457]|RDNMG497-08|CNC LEP00052321|658[0n]bp|Canada.Alberta|BOLD:ABX5442
Euxoa munis|[3458]|RDNMG496-08|CNC LEP00052320|658[0n]bp|Canada.Alberta|BOLD:ABX5442
Euxoa munis|[3459]|RDNMF029-08|NOC14115|658[0n]bp|Canada.Alberta|BOLD:ABX5442
Euxoa nostra|[3460]|RDMAB704-06|UASM24535|658[0n]bp|Canada.Alberta|BOLD:ABZ9893
Euxoa rufula|[3461]|RDNMG638-08|CNC LEP00052462|658[0n]bp|Canada.British Columbia|BOLD:ACE8541
Euxoa rufula|[3462]|RDNMG636-08|CNC LEP00052460|658[0n]bp|Canada.British Columbia|BOLD:ACE8541
Euxoa rufula|[3463]|RDNMG637-08|CNC LEP00052461|658[0n]bp|Canada.British Columbia|BOLD:ACE8541
Euxoa rufula|[3464]|RDNMF038-08|NOC14124|658[0n]bp|Canada.Alberta|BOLD:ACE8541
Euxoa misturata|[3465]|RDMAB687-06|UASM58805|627[0n]bp|Canada.Alberta|BOLD:ACF5206
Euxoa catenula|[3466]|LBCH6897-10|10-JDWBC-6897|658[0n]bp|Canada.British Columbia|BOLD:ABZ8589
Euxoa catenula|[3467]|LBCH6982-10|10-JDWBC-6982|658[0n]bp|Canada.British Columbia|BOLD:ABZ8589
Euxoa catenula|[3468]|LBCH7252-10|10-JDWBC-7252|658[0n]bp|Canada.British Columbia|BOLD:ABZ8589
Euxoa catenula|[3469]|RDMAB555-06|UASM58534|658[0n]bp|Canada.Alberta|BOLD:ABZ8589
Euxoa catenula|[3470]|LBCH6976-10|10-JDWBC-6976|658[0n]bp|Canada.British Columbia|BOLD:ABZ8589
Euxoa catenula|[3471]|LBCH7618-10|10-JDWBC-7618|658[0n]bp|Canada.British Columbia|BOLD:ABZ8589
Euxoa catenula|[3472]|LPVIB798-08|PFC-2006-2272|658[0n]bp|Canada.British Columbia|BOLD:ABZ8589
Euxoa catenula|[3473]|RDNM294-05|CNCNoctuoidea6476|580[0n]bp|Canada.British Columbia|BOLD:ABZ8589
Euxoa catenula|[3474]|RDNM290-05|CNCNoctuoidea6472|658[0n]bp|Canada.Alberta|BOLD:ABZ8589
Euxoa catenula|[3475]|LBCH6826-10|10-JDWBC-6826|658[0n]bp|Canada.British Columbia|BOLD:ABZ8589
Euxoa catenula|[3476]|LBCH6975-10|10-JDWBC-6975|658[0n]bp|Canada.British Columbia|BOLD:ABZ8589
Euxoa catenula|[3477]|LBCH7502-10|10-JDWBC-7502|658[0n]bp|Canada.British Columbia|BOLD:ABZ8589
Euxoa catenula|[3478]|LBCH6979-10|10-JDWBC-6979|658[0n]bp|Canada.British Columbia|BOLD:ABZ8589
Euxoa catenula|[3479]|LBCH6980-10|10-JDWBC-6980|658[0n]bp|Canada.British Columbia|BOLD:ABZ8589
Euxoa catenula|[3480]|LBCH6974-10|10-JDWBC-6974|658[0n]bp|Canada.British Columbia|BOLD:ABZ8589
Euxoa catenula|[3481]|LBCH6977-10|10-JDWBC-6977|658[0n]bp|Canada.British Columbia|BOLD:ABZ8589
Euxoa catenula|[3482]|LBCH6898-10|10-JDWBC-6898|658[0n]bp|Canada.British Columbia|BOLD:ABZ8589
Euxoa catenula|[3483]|LBCH6981-10|10-JDWBC-6981|639[0n]bp|Canada.British Columbia|BOLD:ABZ8589
Euxoa catenula|[3484]|LBCH7351-10|10-JDWBC-7351|640[0n]bp|Canada.British Columbia|BOLD:ABZ8589
Euxoa catenula|[3485]|LBCH6978-10|10-JDWBC-6978|658[0n]bp|Canada.British Columbia|BOLD:ABZ8589
Euxoa catenula|[3486]|LPVIB804-08|PFC-2006-2293|658[0n]bp|Canada.British Columbia|BOLD:ABZ8589
Euxoa catenula|[3487]|LBCH6760-10|10-JDWBC-6760|658[0n]bp|Canada.British Columbia|BOLD:ABZ8589
Euxoa catenula|[3488]|LBCH6761-10|10-JDWBC-6761|658[0n]bp|Canada.British Columbia|BOLD:ABZ8589
Euxoa aequalis|[3489]|RDNM365-05|CNCNoctuoidea6547|523[0n]bp|Canada.Alberta|BOLD:ABZ9912

Euxoa catenula|[3486]|[illegible]|Canada.British Columbia|BOLD:ABZ8589
Euxoa catenula|[3487]|LBCH6760-10|10-JDWBC-6760|658[0n]bp|Canada.British Columbia|BOLD:ABZ8589
Euxoa catenula|[3488]|LBCH6761-10|10-JDWBC-6761|658[0n]bp|Canada.British Columbia|BOLD:ABZ8589
Euxoa aequalis|[3489]|RDNM365-05|CNCNoctuoidea6547|523[0n]bp|Canada.Alberta|BOLD:ABZ9912
Euxoa aequalis|[3490]|RDNM364-05|CNCNoctuoidea6546|658[0n]bp|Canada.Alberta|BOLD:ABZ9912
Euxoa aequalis|[3491]|RDNM363-05|CNCNoctuoidea6545|658[0n]bp|Canada.Alberta|BOLD:ABZ9912
Euxoa aequalis|[3492]|RDNMC083-05|CNCNoctuoidea10824|658[0n]bp|Canada.Alberta|BOLD:ABZ9912
Euxoa messoria|[3493]|LALPA1333-12|AVBC 1335-11|612[0n]bp|Canada.British Columbia|BOLD:AAA2634
Euxoa messoria|[3494]|LBCH7811-10|10-JDWBC-7811|658[0n]bp|Canada.British Columbia|BOLD:AAA2634
Euxoa messoria|[3495]|RDNML017-13|CNCLEP 94247|658[0n]bp|Canada.Nova Scotia|BOLD:AAA2634
Euxoa messoria|[3496]|RDNML016-13|CNCLEP 94246|658[0n]bp|Canada.Nova Scotia|BOLD:AAA2634
Euxoa messoria|[3497]|LOWCD663-06|CGWC-3483|658[0n]bp|Canada.British Columbia|BOLD:AAA2634
Euxoa messoria|[3498]|RDLQ698-07|DH009004|658[0n]bp|Canada.Quebec|BOLD:AAA2634
Euxoa messoria|[3499]|RDLQB269-05|DH010355|658[0n]bp|Canada.Quebec|BOLD:AAA2634
Euxoa messoria|[3500]|LBCH7433-10|10-JDWBC-7433|658[0n]bp|Canada.British Columbia|BOLD:AAA2634
Euxoa messoria|[3501]|LOWCE772-06|CGWC-4532|658[0n]bp|Canada.British Columbia|BOLD:AAA2634
Euxoa messoria|[3502]|XAH007-05|2005-ONT-1590|658[0n]bp|Canada.Ontario|BOLD:AAA2634
Euxoa messoria|[3503]|ABCBF179-12|Cutworm - Agriculture and Agri-Food Canada|658[0n]bp|Canada.British Co...
Euxoa messoria|[3504]|LBCH7816-10|10-JDWBC-7816|658[0n]bp|Canada.British Columbia|BOLD:AAA2634
Euxoa messoria|[3505]|LBCH7555-10|10-JDWBC-7555|658[0n]bp|Canada.British Columbia|BOLD:AAA2634
Euxoa messoria|[3506]|LBCH7971-10|10-JDWBC-7971|658[0n]bp|Canada.British Columbia|BOLD:AAA2634
Euxoa messoria|[3507]|LBCH7374-10|10-JDWBC-7374|658[0n]bp|Canada.British Columbia|BOLD:AAA2634
Euxoa messoria|[3508]|LBCH7817-10|10-JDWBC-7817|658[0n]bp|Canada.British Columbia|BOLD:AAA2634
Euxoa messoria|[3509]|LOWCE322-06|CGWC-4082|658[0n]bp|Canada.British Columbia|BOLD:AAA2634
Euxoa messoria|[3510]|LOWCE773-06|CGWC-4533|658[0n]bp|Canada.British Columbia|BOLD:AAA2634
Euxoa messoria|[3511]|PHMO336-03|moth2606.02|639[0n]bp|Canada.Ontario|BOLD:AAA2634
Euxoa messoria|[3512]|PHMO339-03|moth2611.02|639[0n]bp|Canada.Ontario|BOLD:AAA2634
Euxoa messoria|[3513]|LBCH6755-10|10-JDWBC-6755|615[1n]bp|Canada.British Columbia|BOLD:AAA2634
Euxoa messoria|[3514]|LHLEP443-06|UBC-2006-2116|658[0n]bp|Canada.British Columbia|BOLD:AAA2634
Euxoa messoria|[3515]|LBCH1972-10|10-JDWBC-1972|658[0n]bp|Canada.British Columbia|BOLD:AAA2634
Euxoa dodi|[3516]|RDNMF819-08|CNC LEP00053180|658[0n]bp|Canada.Alberta|BOLD:ACF0932
Euxoa dodi|[3517]|RDNMF890-08|CNC LEP00053251|658[0n]bp|Canada.Alberta|BOLD:ACF0932
Euxoa dodi|[3518]|RDNMF818-08|CNC LEP00053179|658[0n]bp|Canada.Alberta|BOLD:ACF0932
Euxoa infracta|[3519]|LBCH7436-10|10-JDWBC-7436|658[0n]bp|Canada.British Columbia|BOLD:ABZ9442
Euxoa infracta|[3520]|LBCH7960-10|10-JDWBC-7960|658[2n]bp|Canada.British Columbia|BOLD:ABZ9442
Euxoa infracta|[3521]|LBCD458-05|HLC-23278|658[0n]bp|Canada.British Columbia|BOLD:ABZ9442
Euxoa infracta|[3522]|LBCH7820-10|10-JDWBC-7820|658[0n]bp|Canada.British Columbia|BOLD:ABZ9442
Euxoa infracta|[3523]|RDNMG800-08|CNC LEP00052924|590[1n]bp|Canada.British Columbia|BOLD:ABZ9442
Euxoa infracta|[3524]|LBCH7678-10|10-JDWBC-7678|658[0n]bp|Canada.British Columbia|BOLD:ABZ9442
Euxoa infracta|[3525]|LBCH7546-10|10-JDWBC-7546|658[0n]bp|Canada.British Columbia|BOLD:ABZ9442
Euxoa infracta|[3526]|LBCH7547-10|10-JDWBC-7547|658[0n]bp|Canada.British Columbia|BOLD:ABZ9442
Euxoa infracta|[3527]|LBCH6750-10|10-JDWBC-6750|658[0n]bp|Canada.British Columbia|BOLD:ABZ9442
Euxoa infracta|[3528]|RDNMG799-08|CNC LEP00052923|658[0n]bp|Canada.British Columbia|BOLD:ABZ9442
Euxoa infracta|[3529]|LBCH7434-10|10-JDWBC-7434|658[0n]bp|Canada.British Columbia|BOLD:ABZ9442
Euxoa infracta|[3530]|LBCC010-05|HLC-21890|658[0n]bp|Canada.British Columbia|BOLD:ABZ9442
Euxoa infracta|[3531]|RDNMF350-08|NOC14436|658[0n]bp|Canada.British Columbia|BOLD:ABZ9442
Euxoa infracta|[3532]|LBCG1095-09|08-JDWBC-1095|658[0n]bp|Canada.British Columbia|BOLD:ABZ9442
Euxoa pluralis|[3533]|LBCH5698-10|10-JDWBC-5698|658[0n]bp|Canada.British Columbia|BOLD:ABZ9141
Euxoa lillooet|[3534]|RDNMF674-08|NOC14760|636[0n]bp|Canada.British Columbia|BOLD:AAD3167
Euxoa lillooet|[3535]|RDNMF676-08|NOC14762|658[0n]bp|Canada.British Columbia|BOLD:AAD3167
Euxoa lillooet|[3536]|RDNMF675-08|NOC14761|658[0n]bp|Canada.British Columbia|BOLD:AAD3167
Euxoa lillooet|[3537]|RDNMF896-08|CNC LEP00053257|658[0n]bp|Canada.British Columbia|BOLD:AAD3167
Euxoa septentrionalis|[3538]|LBCH7130-10|10-JDWBC-7130|658[0n]bp|Canada.British Columbia|BOLD:AAC8054
Euxoa citricolor|[3539]|RDMAB553-06|UASM58532|658[0n]bp|Canada.Alberta|BOLD:ACE4732
Euxoa citricolor|[3540]|RDMAB590-06|UASM58608|658[0n]bp|Canada.Alberta|BOLD:ACE4732
Euxoa intermontana|[3541]|RDNMG985-08|CNC LEP00053109|658[0n]bp|United States.Colorado|BOLD:AAC3453
Euxoa mimallonis|[3542]|RDNMB649-05|CNCNoctuoidea10425|658[0n]bp|Canada.Alberta|BOLD:AAC3453
Euxoa mimallonis|[3543]|LBCH6660-10|10-JDWBC-6660|658[0n]bp|Canada.British Columbia|BOLD:AAC3453
Euxoa mimallonis|[3544]|LBCH6273-10|10-JDWBC-6273|658[0n]bp|Canada.British Columbia|BOLD:AAC3453
Euxoa biformata|[3545]|RDNMF850-08|CNC LEP00053211|658[0n]bp|United States.Washington|BOLD:AAC3453
Euxoa biformata|[3546]|RDNMB653-05|CNCNoctuoidea10429|587[0n]bp|United States.Washington|BOLD:AAC3453
Euxoa biformata|[3547]|IAWLB154-10|IAWAZ-0963|658[0n]bp|United States.California|BOLD:AAC3453
Euxoa mimallonis|[3548]|RDLQB604-05|DH010707|658[0n]bp|Canada.Quebec|BOLD:AAC3453
Euxoa biformata|[3549]|RDNMF854-08|CNC LEP00053215|658[0n]bp|United States.Washington|BOLD:AAC3453
Euxoa biformata|[3550]|RDNMB655-05|CNCNoctuoidea10431|650[0n]bp|United States.Washington|BOLD:AAC3453
Euxoa biformata|[3551]|RDNMB652-05|CNCNoctuoidea10428|658[1n]bp|United States.Washington|BOLD:AAC3453
Euxoa intermontana|[3552]|RDNMG427-08|CNC LEP00052251|658[0n]bp|United States.Colorado|BOLD:AAC3453
Euxoa mimallonis|[3553]|LBCH6528-10|10-JDWBC-6528|658[0n]bp|Canada.British Columbia|BOLD:AAC3453
Euxoa mimallonis|[3554]|RDNMB647-05|CNCNoctuoidea10423|658[0n]bp|Canada.British Columbia|BOLD:AAC3453
Euxoa mimallonis|[3555]|LBCH6827-10|10-JDWBC-6827|658[0n]bp|Canada.British Columbia|BOLD:AAC3453
Euxoa mimallonis|[3556]|LBCH6759-10|10-JDWBC-6759|658[0n]bp|Canada.British Columbia|BOLD:AAC3453
Euxoa mimallonis|[3557]|LBCH7540-10|10-JDWBC-7540|658[0n]bp|Canada.British Columbia|BOLD:AAC3453
Euxoa mimallonis|[3558]|LBCH6527-10|10-JDWBC-6527|658[0n]bp|Canada.British Columbia|BOLD:AAC3453
Euxoa biformata|[3559]|RDNMF834-08|CNC LEP00053195|658[0n]bp|United States.Washington|BOLD:AAC3453
Euxoa mimallonis|[3560]|RDNMB648-05|CNCNoctuoidea10424|658[0n]bp|Canada.Saskatchewan|BOLD:AAC3453
Euxoa biformata|[3561]|RDNMF851-08|CNC LEP00053212|658[0n]bp|United States.Washington|BOLD:AAC3453
Euxoa intermontana|[3562]|RDNMB654-05|CNCNoctuoidea10430|614[0n]bp|United States.Nevada|BOLD:AAC3453
Euxoa mimallonis|[3563]|RDMAB295-05|UASM77792|658[0n]bp|Canada.Alberta|BOLD:AAC3453
Euxoa shasta|[3564]|RDNMB658-05|CNCNoctuoidea10434|658[0n]bp|United States.California|BOLD:AAC3453
Euxoa shasta|[3565]|IAWLB155-10|IAWAZ-0964|658[0n]bp|United States.California|BOLD:AAC3453
Euxoa shasta|[3566]|RDNMB657-05|CNCNoctuoidea10433|658[0n]bp|United States.Nevada|BOLD:AAC3453
Euxoa shasta|[3567]|RDNMB656-05|CNCNoctuoidea10432|583[1n]bp|United States.California|BOLD:AAC3453
Euxoa mitis|[3568]|RDMAB589-06|UASM58595|658[0n]bp|Canada.Alberta|BOLD:AAE0993
Euxoa mitis|[3569]|RDMAB689-06|UASM58803|561[0n]bp|Canada.Alberta|BOLD:AAE0993
Euxoa mitis|[3570]|RDNMF808-08|CNC LEP00053169|658[0n]bp|Canada.Alberta|BOLD:AAE0993
Euxoa brevipennis|[3571]|RDNMG457-08|CNC LEP00052281|658[0n]bp|Canada.Alberta|BOLD:AAE6690
Euxoa brevipennis|[3572]|RDMAB588-06|UASM58600|658[0n]bp|Canada.Alberta|BOLD:AAE6690
Euxoa obeliscoides|[3573]|LBCH7575-10|10-JDWBC-7575|658[0n]bp|Canada.British Columbia|BOLD:ABZ9441
Euxoa obeliscoides|[3574]|RDLQ703-07|DH009819|658[0n]bp|Canada.Quebec|BOLD:ABZ9441
Euxoa obeliscoides|[3575]|RDNMB867-05|CNCNoctuoidea10642|658[0n]bp|Canada.Alberta|BOLD:ABZ9441
Euxoa obeliscoides|[3576]|RDMAB579-06|UASM58545|658[0n]bp|Canada.Alberta|BOLD:ABZ9441
Euxoa obeliscoides|[3577]|LBCH7580-10|10-JDWBC-7580|658[0n]bp|Canada.British Columbia|BOLD:ABZ9441
Euxoa obeliscoides|[3578]|LBCH7581-10|10-JDWBC-7581|658[0n]bp|Canada.British Columbia|BOLD:ABZ9441
Euxoa obeliscoides|[3579]|LBCH7100-10|10-JDWBC-7100|658[0n]bp|Canada.British Columbia|BOLD:ABZ9441
Euxoa obeliscoides|[3580]|LBCH7577-10|10-JDWBC-7577|658[0n]bp|Canada.British Columbia|BOLD:ABZ9441
Euxoa obeliscoides|[3581]|LBCH7579-10|10-JDWBC-7579|658[0n]bp|Canada.British Columbia|BOLD:ABZ9441
Euxoa obeliscoides|[3582]|LOWCE723-06|CGWC-4483|658[0n]bp|Canada.British Columbia|BOLD:ABZ9441
Euxoa obeliscoides|[3583]|LBCH7128-10|10-JDWBC-7128|658[0n]bp|Canada.British Columbia|BOLD:ABZ9441
Euxoa obeliscoides|[3584]|RDNMB006-05|CNCNoctuoidea7846|658[0n]bp|Canada.Alberta|BOLD:ABZ9441
Euxoa obeliscoides|[3585]|LOWCE725-06|CGWC-4485|616[0n]bp|Canada.British Columbia|BOLD:ABZ9441
Euxoa obeliscoides|[3586]|RDNMB007-05|CNCNoctuoidea7847|594[0n]bp|Canada.Alberta|BOLD:ABZ9441
Euxoa obeliscoides|[3587]|LBCH7578-10|10-JDWBC-7578|658[0n]bp|Canada.British Columbia|BOLD:ABZ9441
Euxoa obeliscoides|[3588]|LBCH6738-10|10-JDWBC-6738|658[0n]bp|Canada.British Columbia|BOLD:ABZ9441
[illegible]

Euxoa obeliscoides|[3586]|RDNMB007-05|CNCNoctuoidea7847|594[0n]bp|Canada.Alberta|BOLD:ABZ9441
Euxoa obeliscoides|[3587]|LBCH7578-10|10-JDWBC-7578|658[0n]bp|Canada.British Columbia|BOLD:ABZ9441
Euxoa obeliscoides|[3588]|LBCH6738-10|10-JDWBC-6738|658[0n]bp|Canada.British Columbia|BOLD:ABZ9441
Euxoa obeliscoides|[3589]|LBCH7782-10|10-JDWBC-7782|658[0n]bp|Canada.British Columbia|BOLD:ABZ9441
Euxoa obeliscoides|[3590]|LBCH7576-10|10-JDWBC-7576|658[0n]bp|Canada.British Columbia|BOLD:ABZ9441
Euxoa obeliscoides|[3591]|LBCH6909-10|10-JDWBC-6909|658[0n]bp|Canada.British Columbia|BOLD:ABZ9441
Euxoa obeliscoides|[3592]|LBCH7534-10|10-JDWBC-7534|658[0n]bp|Canada.British Columbia|BOLD:ABZ9441
Euxoa obeliscoides|[3593]|LBCH7574-10|10-JDWBC-7574|658[0n]bp|Canada.British Columbia|BOLD:ABZ9441
Euxoa obeliscoides|[3594]|LBCH7505-10|10-JDWBC-7505|658[0n]bp|Canada.British Columbia|BOLD:ABZ9441
Euxoa obeliscoides|[3595]|LOWCE724-06|CGWC-4484|658[0n]bp|Canada.British Columbia|BOLD:ABZ9441
Euxoa obeliscoides|[3596]|LBCH7240-10|10-JDWBC-7240|658[0n]bp|Canada.British Columbia|BOLD:ABZ9441
Euxoa oberfoelli|[3597]|RDNMF684-08|NOC14770|658[0n]bp|Canada.Saskatchewan|BOLD:AAF6163
Euxoa punctigera|[3598]|LBCH6274-10|10-JDWBC-6274|658[0n]bp|Canada.British Columbia|BOLD:ABZ9016
Euxoa punctigera|[3599]|LBCH7950-10|10-JDWBC-7950|658[0n]bp|Canada.British Columbia|BOLD:ABZ9016
Euxoa punctigera|[3600]|RDNMG557-08|CNC LEP00052381|658[0n]bp|Canada.British Columbia|BOLD:ABZ9016
Euxoa punctigera|[3601]|LBCH6745-10|10-JDWBC-6745|658[0n]bp|Canada.British Columbia|BOLD:ABZ9016
Euxoa punctigera|[3602]|LBCH6367-10|10-JDWBC-6367|658[0n]bp|Canada.British Columbia|BOLD:ABZ9016
Euxoa punctigera|[3603]|LBCH7791-10|10-JDWBC-7791|658[0n]bp|Canada.British Columbia|BOLD:ABZ9016
Euxoa punctigera|[3604]|LBCH7129-10|10-JDWBC-7129|658[0n]bp|Canada.British Columbia|BOLD:ABZ9016
Euxoa punctigera|[3605]|LBCH7131-10|10-JDWBC-7131|658[0n]bp|Canada.British Columbia|BOLD:ABZ9016
Euxoa punctigera|[3606]|LBCH7541-10|10-JDWBC-7541|658[0n]bp|Canada.British Columbia|BOLD:ABZ9016
Euxoa punctigera|[3607]|LBCH6442-10|10-JDWBC-6442|658[0n]bp|Canada.British Columbia|BOLD:ABZ9016
Euxoa punctigera|[3608]|LBCH6742-10|10-JDWBC-6742|658[0n]bp|Canada.British Columbia|BOLD:ABZ9016
Euxoa punctigera|[3609]|LBCH7550-10|10-JDWBC-7550|658[0n]bp|Canada.British Columbia|BOLD:ABZ9016
Euxoa punctigera|[3610]|LBCH6746-10|10-JDWBC-6746|658[0n]bp|Canada.British Columbia|BOLD:ABZ9016
Euxoa punctigera|[3611]|LBCH7806-10|10-JDWBC-7806|658[0n]bp|Canada.British Columbia|BOLD:ABZ9016
Euxoa spumata|[3612]|RDNMF695-08|NOC14781|609[0n]bp|Canada.Saskatchewan|BOLD:ABZ9016
Euxoa spumata|[3613]|RDNMF694-08|NOC14780|621[0n]bp|Canada.Saskatchewan|BOLD:ABZ9016
Euxoa atristrigata|[3614]|LBCH6847-10|10-JDWBC-6847|634[0n]bp|Canada.British Columbia|BOLD:ACE5990
Euxoa atristrigata|[3615]|LBCH7746-10|10-JDWBC-7746|658[0n]bp|Canada.British Columbia|BOLD:ACE5990
Euxoa atristrigata|[3616]|LBCH6943-10|10-JDWBC-6943|658[0n]bp|Canada.British Columbia|BOLD:ACE5990
Euxoa pallipennis|[3617]|RDNMK361-11|CNCLEP 84270|658[0n]bp|United States.New Mexico|BOLD:ABX6450
Euxoa pallipennis|[3618]|RDNMG672-08|CNC LEP00052496|658[0n]bp|United States.Oregon|BOLD:ABX6450
Euxoa pallipennis|[3619]|RDNMG673-08|CNC LEP00052497|641[0n]bp|United States.Oregon|BOLD:ABX6450
Euxoa pallipennis|[3620]|RDNMG671-08|CNC LEP00052495|658[0n]bp|United States.Oregon|BOLD:ABX6450
Euxoa pallipennis|[3621]|RDNMF023-08|NOC14109|658[0n]bp|United States.Colorado|BOLD:ABX6450
Euxoa detersa|[3622]|RDNMG652-08|CNC LEP00052476|649[4n]bp|Canada.Ontario|BOLD:ABZ9142
Euxoa detersa|[3623]|RDNMG650-08|CNC LEP00052474|658[0n]bp|Canada.Ontario|BOLD:ABZ9142
Euxoa detersa|[3624]|RDNMG651-08|CNC LEP00052475|658[0n]bp|Canada.Ontario|BOLD:ABZ9142
Euxoa detersa|[3625]|BBLEC537-09|09BBELE-0537|658[0n]bp|Canada.New Brunswick|BOLD:ABZ9142
Euxoa cicatricosa|[3626]|RDMAB556-06|UASM58535|658[0n]bp|Canada.Alberta|BOLD:ABZ9142
Euxoa cicatricosa|[3627]|RDNMG795-08|CNC LEP00052919|593[2n]bp|Canada.British Columbia|BOLD:ABZ9142
Euxoa cicatricosa|[3628]|RDNMG796-08|CNC LEP00052920|641[0n]bp|Canada.British Columbia|BOLD:ABZ9142
Euxoa detersa|[3629]|BBLEC503-09|09BBELE-0503|658[0n]bp|Canada.New Brunswick|BOLD:ABZ9142
Euxoa detersa|[3630]|BBLEC502-09|09BBELE-0502|658[0n]bp|Canada.New Brunswick|BOLD:ABZ9142
Euxoa detersa|[3631]|BBLEC490-09|09BBELE-0490|658[0n]bp|Canada.New Brunswick|BOLD:ABZ9142
Euxoa detersa|[3632]|BBLEC512-09|09BBELE-0512|658[0n]bp|Canada.New Brunswick|BOLD:ABZ9142
Euxoa detersa|[3633]|BBLEC498-09|09BBELE-0498|658[0n]bp|Canada.New Brunswick|BOLD:ABZ9142
Euxoa nevada|[3634]|LBCH7053-10|10-JDWBC-7053|658[0n]bp|Canada.British Columbia|BOLD:AAD3157
Euxoa nevada|[3635]|LBCH7056-10|10-JDWBC-7056|658[0n]bp|Canada.British Columbia|BOLD:AAD3157
Euxoa albipennis|[3636]|LBCH7819-10|10-JDWBC-7819|658[0n]bp|Canada.British Columbia|BOLD:AAE6702
Euxoa albipennis|[3637]|LBCH7677-10|10-JDWBC-7677|658[0n]bp|Canada.British Columbia|BOLD:AAE6702
Euxoa albipennis|[3638]|RDLQF240-06|DH011320|630[0n]bp|Canada.Quebec|BOLD:AAE6702
Euxoa albipennis|[3639]|RDLQB770-05|DH010857|658[0n]bp|Canada.Quebec|BOLD:AAE6702
Euxoa albipennis|[3640]|LBCH6448-10|10-JDWBC-6448|658[0n]bp|Canada.British Columbia|BOLD:AAE6702
Euxoa albipennis|[3641]|LOWCE743-06|CGWC-4503|595[0n]bp|Canada.British Columbia|BOLD:AAE6702
Euxoa albipennis|[3642]|LBCH7235-10|10-JDWBC-7235|658[0n]bp|Canada.British Columbia|BOLD:AAE6702
Euxoa albipennis|[3643]|LOWCE745-06|CGWC-4505|595[0n]bp|Canada.British Columbia|BOLD:AAE6702
Euxoa albipennis|[3644]|LBCH7548-10|10-JDWBC-7548|658[0n]bp|Canada.British Columbia|BOLD:AAE6702
Euxoa albipennis|[3645]|LBCH7050-10|10-JDWBC-7050|658[0n]bp|Canada.British Columbia|BOLD:AAE6702
Euxoa albipennis|[3646]|LBCH7689-10|10-JDWBC-7689|658[0n]bp|Canada.British Columbia|BOLD:AAE6702
Euxoa albipennis|[3647]|LBCH7236-10|10-JDWBC-7236|658[0n]bp|Canada.British Columbia|BOLD:AAE6702
Euxoa cinereopallidus|[3648]|RDMAB587-06|UASM58605|658[0n]bp|Canada.Alberta|BOLD:ABZ9422
Euxoa cinereopallidus|[3649]|RDMAB586-06|UASM58592|658[0n]bp|Canada.Alberta|BOLD:ABZ9422
Euxoa teleboa|[3650]|RDMAB562-06|UASM58541|616[1n]bp|Canada.Alberta|BOLD:ABZ8386
Euxoa teleboa|[3651]|RDNMF884-08|CNC LEP00053245|658[0n]bp|Canada.Alberta|BOLD:ABZ8386
Euxoa teleboa|[3652]|RDMAB563-06|UASM58542|658[0n]bp|Canada.Alberta|BOLD:ABZ8386
Euxoa teleboa|[3653]|RDMAB560-06|UASM58539|658[0n]bp|Canada.Alberta|BOLD:ABZ8386
Euxoa teleboa|[3654]|RDNMF883-08|CNC LEP00053244|658[0n]bp|Canada.Alberta|BOLD:ABZ8386
Euxoa teleboa|[3655]|RDMAB561-06|UASM58540|658[0n]bp|Canada.Alberta|BOLD:ABZ8386
Euxoa tronellus|[3656]|RDNMB863-05|CNCNoctuoidea10638|658[0n]bp|Canada.Alberta|BOLD:ABX5591
Euxoa tronellus|[3657]|RDNMB855-05|CNCNoctuoidea10630|658[0n]bp|Canada.Alberta|BOLD:ABX5591
Euxoa tronellus|[3658]|RDNMB852-05|CNCNoctuoidea10627|658[0n]bp|Canada.Alberta|BOLD:ABX5591
Euxoa tronellus|[3659]|RDNMB859-05|CNCNoctuoidea10634|658[0n]bp|Canada.Alberta|BOLD:ABX5591
Euxoa tronellus|[3660]|RDNMB851-05|CNCNoctuoidea10626|658[0n]bp|Canada.Alberta|BOLD:ABX5591
Euxoa tronellus|[3661]|RDNMB854-05|CNCNoctuoidea10629|658[0n]bp|Canada.Alberta|BOLD:ABX5591
Euxoa tronellus|[3662]|RDNMB853-05|CNCNoctuoidea10628|658[0n]bp|Canada.Alberta|BOLD:ABX5591
Euxoa tronellus|[3663]|RDNMB862-05|CNCNoctuoidea10637|658[0n]bp|Canada.Alberta|BOLD:ABX5591
Euxoa tronellus|[3664]|RDNMB860-05|CNCNoctuoidea10635|658[0n]bp|Canada.Alberta|BOLD:ABX5591
Euxoa difformis|[3665]|LOWCE742-06|CGWC-4502|579[1n]bp|Canada.British Columbia|BOLD:ABZ9157
Euxoa difformis|[3666]|LBCH7332-10|10-JDWBC-7332|658[0n]bp|Canada.British Columbia|BOLD:ACE8314
Euxoa difformis|[3667]|LPVIB806-08|PFC-2006-2295|658[0n]bp|Canada.British Columbia|BOLD:ACE8314
Euxoa difformis|[3668]|LPVIB823-08|PFC-2006-2312|658[0n]bp|Canada.British Columbia|BOLD:ACE8314
Euxoa difformis|[3669]|LBCH7055-10|10-JDWBC-7055|658[0n]bp|Canada.British Columbia|BOLD:ACE8314
Euxoa difformis|[3670]|LBCH7261-10|10-JDWBC-7261|658[0n]bp|Canada.British Columbia|BOLD:ACE8314
Euxoa difformis|[3671]|LBCH7264-10|10-JDWBC-7264|658[0n]bp|Canada.British Columbia|BOLD:ACE8314
Euxoa difformis|[3672]|LALPA740-10|AVBC 742-10|658[0n]bp|Canada.British Columbia|BOLD:ACE8314
Euxoa difformis|[3673]|LALPA1296-11|AVBC 1298-11|658[0n]bp|Canada.British Columbia|BOLD:ACE8314
Euxoa difformis|[3674]|LALPA1338-12|AVBC 1340-11|632[0n]bp|Canada.British Columbia|BOLD:ACE8314
Euxoa difformis|[3675]|LALPA1324-12|AVBC 1326-11|630[0n]bp|Canada.British Columbia|BOLD:ACE8314
Euxoa difformis|[3676]|LPVIB810-08|PFC-2006-2299|658[0n]bp|Canada.British Columbia|BOLD:ACE8314
Euxoa difformis|[3677]|LBCH6790-10|10-JDWBC-6790|658[0n]bp|Canada.British Columbia|BOLD:ACE8314
Euxoa difformis|[3678]|LBCH7262-10|10-JDWBC-7262|658[0n]bp|Canada.British Columbia|BOLD:ACE8314
Euxoa difformis|[3679]|LPVIB625-08|PFC-2006-2073|658[0n]bp|Canada.British Columbia|BOLD:ACE8314
Euxoa difformis|[3680]|LBCH7251-10|10-JDWBC-7251|658[0n]bp|Canada.British Columbia|BOLD:ACE8314
Euxoa difformis|[3681]|LBCH7220-10|10-JDWBC-7220|658[0n]bp|Canada.British Columbia|BOLD:ACE8314
Euxoa difformis|[3682]|LBCH7265-10|10-JDWBC-7265|658[0n]bp|Canada.British Columbia|BOLD:ACE8314
Euxoa difformis|[3683]|LBCH7263-10|10-JDWBC-7263|658[0n]bp|Canada.British Columbia|BOLD:ACE8314
Euxoa difformis|[3684]|LPVIB811-08|PFC-2006-2300|658[0n]bp|Canada.British Columbia|BOLD:ACE8314
Euxoa difformis|[3685]|LBCH7959-10|10-JDWBC-7959|658[0n]bp|Canada.British Columbia|BOLD:ACE8314
Euxoa difformis|[3686]|LPVIB822-08|PFC-2006-2311|658[0n]bp|Canada.British Columbia|BOLD:ACE8314
Euxoa difformis|[3687]|LPVIB626-08|PFC-2006-2074|627[0n]bp|Canada.British Columbia|BOLD:ACE8314
Euxoa difformis|[3688]|LPVIB809-08|PFC-2006-2298|658[0n]bp|Canada.British Columbia|BOLD:ACE8314

Euxoa difformis|[3686]|LPVIB822-08|PFC-2006-2311|658[0n]bp|Canada.British Columbia|BOLD:ACE8314
Euxoa difformis|[3687]|LPVIB626-08|PFC-2006-2074|627[0n]bp|Canada.British Columbia|BOLD:ACE8314
Euxoa difformis|[3688]|LPVIB809-08|PFC-2006-2298|658[0n]bp|Canada.British Columbia|BOLD:ACE8314
Euxoa difformis|[3689]|LPVIB745-08|PFC-2006-2207|658[0n]bp|Canada.British Columbia|BOLD:ACE8314
Euxoa difformis|[3690]|LPVIB837-08|PFC-2006-2342|658[0n]bp|Canada.British Columbia|BOLD:ACE8314
Euxoa difformis|[3691]|LPVIB808-08|PFC-2006-2297|658[0n]bp|Canada.British Columbia|BOLD:ACE8314
Euxoa difformis|[3692]|LPVIB807-08|PFC-2006-2296|658[0n]bp|Canada.British Columbia|BOLD:ACE8314
Euxoa difformis|[3693]|LPVIB624-08|PFC-2006-2072|658[0n]bp|Canada.British Columbia|BOLD:ACE8314
Euxoa difformis|[3694]|LPVIB628-08|PFC-2006-2076|629[0n]bp|Canada.British Columbia|BOLD:ACE8314
Euxoa difformis|[3695]|LPVIB631-08|PFC-2006-2079|634[0n]bp|Canada.British Columbia|BOLD:ACE8314
Euxoa difformis|[3696]|LPVIB630-08|PFC-2006-2078|634[0n]bp|Canada.British Columbia|BOLD:ACE8314
Euxoa satiens|[3697]|LBCH7012-10|10-JDWBC-7012|658[0n]bp|Canada.British Columbia|BOLD:AAF6167
Euxoa satiens|[3698]|LBCH6920-10|10-JDWBC-6920|635[0n]bp|Canada.British Columbia|BOLD:AAF6167
Euxoa satiens|[3699]|LBCH6815-10|10-JDWBC-6815|658[0n]bp|Canada.British Columbia|BOLD:AAF6167
Euxoa satiens|[3700]|LBCH7005-10|10-JDWBC-7005|658[0n]bp|Canada.British Columbia|BOLD:AAF6167
Euxoa satiens|[3701]|LBCH6812-10|10-JDWBC-6812|658[0n]bp|Canada.British Columbia|BOLD:AAF6167
Euxoa satiens|[3702]|LBCH6893-10|10-JDWBC-6893|658[0n]bp|Canada.British Columbia|BOLD:AAF6167
Euxoa satiens|[3703]|LBCH6889-10|10-JDWBC-6889|658[0n]bp|Canada.British Columbia|BOLD:AAF6167
Euxoa satiens|[3704]|LBCH6814-10|10-JDWBC-6814|658[0n]bp|Canada.British Columbia|BOLD:AAF6167
Euxoa satiens|[3705]|LBCH6892-10|10-JDWBC-6892|658[0n]bp|Canada.British Columbia|BOLD:AAF6167
Euxoa satiens|[3706]|LBCH7011-10|10-JDWBC-7011|658[0n]bp|Canada.British Columbia|BOLD:AAF6167
Euxoa satiens|[3707]|LBCH6810-10|10-JDWBC-6810|658[0n]bp|Canada.British Columbia|BOLD:AAF6167
Euxoa satiens|[3708]|LBCH7006-10|10-JDWBC-7006|658[0n]bp|Canada.British Columbia|BOLD:AAF6167
Euxoa satiens|[3709]|LBCH6891-10|10-JDWBC-6891|658[0n]bp|Canada.British Columbia|BOLD:AAF6167
Euxoa satiens|[3710]|LBCH6817-10|10-JDWBC-6817|658[0n]bp|Canada.British Columbia|BOLD:AAF6167
Euxoa satiens|[3711]|LBCH7010-10|10-JDWBC-7010|658[0n]bp|Canada.British Columbia|BOLD:AAF6167
Euxoa satiens|[3712]|LBCH7009-10|10-JDWBC-7009|658[0n]bp|Canada.British Columbia|BOLD:AAF6167
Euxoa satiens|[3713]|LBCH6816-10|10-JDWBC-6816|658[0n]bp|Canada.British Columbia|BOLD:AAF6167
Euxoa satiens|[3714]|LBCH6811-10|10-JDWBC-6811|658[0n]bp|Canada.British Columbia|BOLD:AAF6167
Euxoa satiens|[3715]|LBCH6895-10|10-JDWBC-6895|658[0n]bp|Canada.British Columbia|BOLD:AAF6167
Euxoa satiens|[3716]|LBCH6894-10|10-JDWBC-6894|658[0n]bp|Canada.British Columbia|BOLD:AAF6167
Euxoa satiens|[3717]|LBCH7008-10|10-JDWBC-7008|658[0n]bp|Canada.British Columbia|BOLD:AAF6167
Euxoa satiens|[3718]|LBCH7007-10|10-JDWBC-7007|658[0n]bp|Canada.British Columbia|BOLD:AAF6167
Euxoa satiens|[3719]|LBCH6896-10|10-JDWBC-6896|658[0n]bp|Canada.British Columbia|BOLD:AAF6167
Euxoa satiens|[3720]|LBCH6813-10|10-JDWBC-6813|636[0n]bp|Canada.British Columbia|BOLD:AAF6167
Euxoa satiens|[3721]|LBCH6890-10|10-JDWBC-6890|658[0n]bp|Canada.British Columbia|BOLD:AAF6167
Euxoa satiens|[3722]|LBCH6447-10|10-JDWBC-6447|658[0n]bp|Canada.British Columbia|BOLD:AAF6167
Euxoa dargo|[3723]|RDNMG999-08|CNC LEP00053123|658[0n]bp|Canada.Alberta|BOLD:AAE0511
Euxoa moerens|[3724]|RDNMF817-08|CNC LEP00053178|658[0n]bp|United States.Oregon|BOLD:ABZ9003
Euxoa moerens|[3725]|RDNMF815-08|CNC LEP00053176|658[0n]bp|United States.Oregon|BOLD:ABZ9003
Euxoa murdocki|[3726]|RDNMF873-08|CNC LEP00053234|658[0n]bp|Canada.British Columbia|BOLD:ABZ9002
Euxoa subandera|[3727]|RDNMF665-08|NOC14751|618[0n]bp|Canada.British Columbia|BOLD:AAE6682
Euxoa quadridentata|[3728]|RDMAB552-06|UASM58531|658[0n]bp|Canada.Alberta|BOLD:ABZ9158
Euxoa quadridentata|[3729]|LOWCD107-06|CGWC-2927|570[1n]bp|Canada.British Columbia|BOLD:ABZ9158
Euxoa quadridentata|[3730]|LOWCE633-06|CGWC-4393|658[0n]bp|Canada.British Columbia|BOLD:ABZ9158
Euxoa quadridentata|[3731]|LOWCD105-06|CGWC-2925|614[2n]bp|Canada.British Columbia|BOLD:ABZ9158
Euxoa quadridentata|[3732]|LOWCE643-06|CGWC-4403|658[0n]bp|Canada.British Columbia|BOLD:ABZ9158
Euxoa quadridentata|[3733]|LOWCD096-06|CGWC-2916|603[0n]bp|Canada.British Columbia|BOLD:ABZ9158
Euxoa quadridentata|[3734]|LOWCE648-06|CGWC-4408|658[0n]bp|Canada.British Columbia|BOLD:ABZ9158
Euxoa quadridentata|[3735]|LOWCE630-06|CGWC-4390|658[0n]bp|Canada.British Columbia|BOLD:ABZ9158
Euxoa quadridentata|[3736]|LOWCE634-06|CGWC-4394|658[0n]bp|Canada.British Columbia|BOLD:ABZ9158
Euxoa quadridentata|[3737]|LOWCE646-06|CGWC-4406|658[0n]bp|Canada.British Columbia|BOLD:ABZ9158
Euxoa quadridentata|[3738]|LOWCE640-06|CGWC-4400|658[0n]bp|Canada.British Columbia|BOLD:ABZ9158
Euxoa quadridentata|[3739]|LOWCE639-06|CGWC-4399|658[0n]bp|Canada.British Columbia|BOLD:ABZ9158
Euxoa quadridentata|[3740]|LOWCD106-06|CGWC-2926|657[0n]bp|Canada.British Columbia|BOLD:ABZ9158
Euxoa quadridentata|[3741]|LOWCD097-06|CGWC-2917|658[0n]bp|Canada.British Columbia|BOLD:ABZ9158
Euxoa quadridentata|[3742]|LBCH6843-10|10-JDWBC-6843|658[0n]bp|Canada.British Columbia|BOLD:ABZ9158
Euxoa quadridentata|[3743]|LOWCE647-06|CGWC-4407|658[0n]bp|Canada.British Columbia|BOLD:ABZ9158
Euxoa quadridentata|[3744]|LOWCD090-06|CGWC-2910|657[0n]bp|Canada.British Columbia|BOLD:ABZ9158
Euxoa quadridentata|[3745]|LOWCD109-06|CGWC-2929|658[0n]bp|Canada.British Columbia|BOLD:ABZ9158
Euxoa quadridentata|[3746]|LOWCE635-06|CGWC-4395|658[0n]bp|Canada.British Columbia|BOLD:ABZ9158
Euxoa quadridentata|[3747]|LOWCD108-06|CGWC-2928|657[0n]bp|Canada.British Columbia|BOLD:ABZ9158
Euxoa quadridentata|[3748]|LOWCD110-06|CGWC-2930|658[0n]bp|Canada.British Columbia|BOLD:ABZ9158
Euxoa quadridentata|[3749]|LOWCE642-06|CGWC-4402|658[0n]bp|Canada.British Columbia|BOLD:ABZ9158
Euxoa quadridentata|[3750]|LOWCD093-06|CGWC-2913|658[0n]bp|Canada.British Columbia|BOLD:ABZ9158
Euxoa quadridentata|[3751]|LOWCD104-06|CGWC-2924|658[0n]bp|Canada.British Columbia|BOLD:ABZ9158
Euxoa quadridentata|[3752]|LOWCE636-06|CGWC-4396|658[0n]bp|Canada.British Columbia|BOLD:ABZ9158
Euxoa quadridentata|[3753]|LOWCE629-06|CGWC-4389|658[0n]bp|Canada.British Columbia|BOLD:ABZ9158
Euxoa quadridentata|[3754]|LOWCD098-06|CGWC-2918|658[0n]bp|Canada.British Columbia|BOLD:ABZ9158
Euxoa quadridentata|[3755]|LOWCD099-06|CGWC-2919|658[0n]bp|Canada.British Columbia|BOLD:ABZ9158
Euxoa quadridentata|[3756]|LOWCE649-06|CGWC-4409|658[0n]bp|Canada.British Columbia|BOLD:ABZ9158
Euxoa quadridentata|[3757]|LOWCE641-06|CGWC-4401|658[0n]bp|Canada.British Columbia|BOLD:ABZ9158
Euxoa quadridentata|[3758]|LOWCD101-06|CGWC-2921|658[0n]bp|Canada.British Columbia|BOLD:ABZ9158
Euxoa quadridentata|[3759]|LOWCE637-06|CGWC-4397|656[0n]bp|Canada.British Columbia|BOLD:ABZ9158
Euxoa quadridentata|[3760]|LOWCD092-06|CGWC-2912|612[0n]bp|Canada.British Columbia|BOLD:ABZ9158
Euxoa quadridentata|[3761]|LOWCD095-06|CGWC-2915|609[0n]bp|Canada.British Columbia|BOLD:ABZ9158
Euxoa quadridentata|[3762]|LOWCE632-06|CGWC-4392|613[0n]bp|Canada.British Columbia|BOLD:ABZ9158
Euxoa quadridentata|[3763]|LOWCD100-06|CGWC-2920|612[0n]bp|Canada.British Columbia|BOLD:ABZ9158
Euxoa quadridentata|[3764]|LOWCD111-06|CGWC-2931|614[0n]bp|Canada.British Columbia|BOLD:ABZ9158
Euxoa quadridentata|[3765]|LOWCD091-06|CGWC-2911|622[0n]bp|Canada.British Columbia|BOLD:ABZ9158
Euxoa quadridentata|[3766]|LOWCD094-06|CGWC-2914|658[0n]bp|Canada.British Columbia|BOLD:ABZ9158
Euxoa quadridentata|[3767]|LOWCD103-06|CGWC-2923|615[0n]bp|Canada.British Columbia|BOLD:ABZ9158
Euxoa quadridentata|[3768]|LOWCD102-06|CGWC-2922|615[0n]bp|Canada.British Columbia|BOLD:ABZ9158
Euxoa velleripennis|[3769]|XAH222-05|2005-ONT-1805|658[0n]bp|Canada.Ontario|BOLD:ABZ9143
Euxoa velleripennis|[3770]|XAH435-05|2005-ONT-2018|658[0n]bp|Canada.Ontario|BOLD:ABZ9143
Euxoa velleripennis|[3771]|XAH346-05|2005-ONT-1929|658[0n]bp|Canada.Ontario|BOLD:ABZ9143
Euxoa velleripennis|[3772]|XAB454-04|04HBL005454|658[0n]bp|Canada.Ontario|BOLD:ABZ9143
Euxoa velleripennis|[3773]|XAH620-05|2005-ONT-2203|627[0n]bp|Canada.Ontario|BOLD:ABZ9143
Euxoa velleripennis|[3774]|XAH242-05|2005-ONT-1825|658[0n]bp|Canada.Ontario|BOLD:ABZ9143
Euxoa velleripennis|[3775]|XAH272-05|2005-ONT-1855|658[0n]bp|Canada.Ontario|BOLD:ABZ9143
Euxoa velleripennis|[3776]|XAD439-04|04HBL007439|658[0n]bp|Canada.Ontario|BOLD:ABZ9143
Euxoa velleripennis|[3777]|PHMO386-03|moth2533.02|639[0n]bp|Canada.Ontario|BOLD:ABZ9143
Euxoa velleripennis|[3778]|XAH243-05|2005-ONT-1826|658[0n]bp|Canada.Ontario|BOLD:ABZ9143
Euxoa velleripennis|[3779]|XAH131-05|2005-ONT-1714|658[0n]bp|Canada.Ontario|BOLD:ABZ9143
Euxoa scotogrammoides|[3780]|RDNM824-05|CNCNoctuoidea7670|550[1n]bp|United States.California|BOLD:ACE...
Euxoa scotogrammoides|[3781]|RDNM826-05|CNCNoctuoidea7672|658[0n]bp|United States.Oregon|BOLD:ACE8542
Euxoa scotogrammoides|[3782]|RDNM990-05|CNCNoctuoidea7830|658[0n]bp|United States.Oregon|BOLD:ACE8542
Euxoa scotogrammoides|[3783]|RDNM992-05|CNCNoctuoidea7832|601[0n]bp|United States.Washington|BOLD:ACE...
Euxoa scotogrammoides|[3784]|RDNM825-05|CNCNoctuoidea7671|658[0n]bp|United States.Oregon|BOLD:ACE8542
Euxoa scotogrammoides|[3785]|RDNM991-05|CNCNoctuoidea7831|658[0n]bp|United States.Oregon|BOLD:ACE8542
Euxoa edictalis|[3786]|RDNMF682-08|NOC14768|622[0n]bp|Canada.British Columbia|BOLD:ABZ9140
Euxoa edictalis|[3787]|RDNMF681-08|NOC14767|641[0n]bp|Canada.British Columbia|BOLD:ABZ9140
Euxoa tristicula|[3788]|RDNM358-05|CNCNoctuoidea6540|658[0n]bp|Canada.Alberta|BOLD:ABZ9161

Euxoa scotogrammoides|[3785]|[illegible]|United States.Oregon|[illegible]
Euxoa edictalis|[3786]|RDNMF682-08|NOC14768|622[0n]bp|Canada.British Columbia|BOLD:ABZ9140
Euxoa edictalis|[3787]|RDNMF681-08|NOC14767|641[0n]bp|Canada.British Columbia|BOLD:ABZ9140
Euxoa tristicula|[3788]|RDNM358-05|CNCNoctuoidea6540|658[0n]bp|Canada.Alberta|BOLD:ABZ9161
Euxoa tristicula|[3789]|LPSK138-08|08BBLEP-01706|658[0n]bp|Canada.Saskatchewan|BOLD:ABZ9161
Euxoa tristicula|[3790]|LPSK516-08|08BBLEP-02084|658[0n]bp|Canada.Saskatchewan|BOLD:ABZ9161
Euxoa tristicula|[3791]|LPSK130-08|08BBLEP-01698|658[0n]bp|Canada.Saskatchewan|BOLD:ABZ9161
Euxoa tristicula|[3792]|LALPA871-11|AVBC 1044-11|658[0n]bp|Canada.British Columbia|BOLD:ABZ9161
Euxoa tristicula|[3793]|RDNM359-05|CNCNoctuoidea6541|658[0n]bp|Canada.Alberta|BOLD:ABZ9161
Euxoa tristicula|[3794]|RDMAB044-05|UASM57527|634[0n]bp|Canada.Alberta|BOLD:ABZ9161
Euxoa tristicula|[3795]|LPSK122-08|08BBLEP-01690|636[0n]bp|Canada.Saskatchewan|BOLD:ABZ9161
Euxoa tristicula|[3796]|RDMAB043-05|UASM57526|647[0n]bp|Canada.Alberta|BOLD:ABZ9161
Euxoa comosa|[3797]|RDMAB559-06|UASM58538|658[0n]bp|Canada.Alberta|BOLD:ACF2026
Euxoa comosa|[3798]|RDNMB830-05|CNCNoctuoidea10605|658[0n]bp|Canada.Alberta|BOLD:ACF2026
Euxoa comosa|[3799]|RDNMB802-05|CNCNoctuoidea10577|658[0n]bp|Canada.Alberta|BOLD:ACF2026
Euxoa comosa|[3800]|RDNMB837-05|CNCNoctuoidea10612|658[0n]bp|Canada.Alberta|BOLD:ACF2026
Euxoa comosa|[3801]|RDNMB826-05|CNCNoctuoidea10601|658[0n]bp|Canada.Alberta|BOLD:ACF2026
Euxoa comosa|[3802]|RDNMB838-05|CNCNoctuoidea10613|658[0n]bp|Canada.Alberta|BOLD:ACF2026
Euxoa comosa|[3803]|RDNMB827-05|CNCNoctuoidea10602|658[0n]bp|Canada.Alberta|BOLD:ACF2026
Euxoa comosa|[3804]|RDNMB828-05|CNCNoctuoidea10603|658[0n]bp|Canada.Alberta|BOLD:ACF2026
Euxoa comosa|[3805]|RDNMB840-05|CNCNoctuoidea10615|581[0n]bp|Canada.Alberta|BOLD:ACF2026
Euxoa comosa|[3806]|RDNMB823-05|CNCNoctuoidea10598|658[0n]bp|Canada.Alberta|BOLD:ACF2026
Euxoa comosa|[3807]|RDNM302-05|CNCNoctuoidea6484|658[0n]bp|Canada.Alberta|BOLD:ACF2026
Euxoa comosa|[3808]|RDNMB825-05|CNCNoctuoidea10600|658[0n]bp|Canada.Alberta|BOLD:ACF2026
Euxoa comosa|[3809]|RDNMB805-05|CNCNoctuoidea10580|658[0n]bp|Canada.Alberta|BOLD:ACE5080
Euxoa comosa|[3810]|RDNMB842-05|CNCNoctuoidea10617|658[0n]bp|Canada.Alberta|BOLD:ACE5080
Euxoa comosa|[3811]|RDNMB806-05|CNCNoctuoidea10581|658[0n]bp|Canada.Alberta|BOLD:ACE5080
Euxoa comosa|[3812]|RDNMB808-05|CNCNoctuoidea10583|658[0n]bp|Canada.Alberta|BOLD:ACE5080
Euxoa comosa|[3813]|RDMAB316-05|UASM2379|658[0n]bp|Canada.Alberta|BOLD:ACE5080
Euxoa comosa|[3814]|RDNMB803-05|CNCNoctuoidea10578|658[0n]bp|Canada.Alberta|BOLD:ACE5080
Euxoa comosa|[3815]|RDMAB315-05|UASM57920|658[0n]bp|Canada.Alberta|BOLD:ACE5080
Euxoa comosa|[3816]|RDNMB829-05|CNCNoctuoidea10604|658[0n]bp|Canada.Alberta|BOLD:ACE5080
Euxoa comosa|[3817]|RDNMB845-05|CNCNoctuoidea10620|658[0n]bp|Canada.Alberta|BOLD:ACE5080
Euxoa comosa|[3818]|RDNMB804-05|CNCNoctuoidea10579|658[0n]bp|Canada.Alberta|BOLD:ACE5080
Euxoa comosa|[3819]|RDMAB317-05|UASM2380|594[1n]bp|Canada.Alberta|BOLD:ACE5080
Euxoa comosa|[3820]|RDNM322-05|CNCNoctuoidea6504|658[0n]bp|Canada.Alberta|BOLD:ACE5080
Euxoa comosa|[3821]|RDNM324-05|CNCNoctuoidea6506|658[0n]bp|Canada.Alberta|BOLD:ACE5080
Euxoa comosa|[3822]|RDNMB839-05|CNCNoctuoidea10614|658[0n]bp|Canada.Alberta|BOLD:ACE5080
Euxoa comosa|[3823]|RDMAB666-06|UASM58764|573[1n]bp|Canada.Alberta|BOLD:ACE5080
Euxoa comosa|[3824]|RDNM323-05|CNCNoctuoidea6505|658[0n]bp|Canada.Alberta|BOLD:ACE5080
Euxoa comosa|[3825]|RDNMB809-05|CNCNoctuoidea10584|658[0n]bp|Canada.Alberta|BOLD:ACE5080
Euxoa comosa|[3826]|LALPA997-11|AVBC 1170-11|658[0n]bp|Canada.British Columbia|BOLD:ACE5080
Euxoa comosa|[3827]|RDNMB784-05|CNCNoctuoidea10559|616[0n]bp|Canada.British Columbia|BOLD:ACE5080
Euxoa comosa|[3828]|RDNMC085-05|CNCNoctuoidea10826|658[0n]bp|Canada.Alberta|BOLD:ACE5080
Euxoa comosa|[3829]|RDNM325-05|CNCNoctuoidea6507|658[0n]bp|Canada.Ontario|BOLD:ACE5080
Euxoa comosa|[3830]|RDNMC076-05|CNCNoctuoidea10817|658[0n]bp|Canada.New Brunswick|BOLD:ACE5080
Euxoa comosa|[3831]|BBLPC199-09|09BBELE-1199|658[0n]bp|Canada.Nova Scotia|BOLD:ACE5080
Euxoa comosa|[3832]|RDLQF177-06|DH011204|658[0n]bp|Canada.Quebec|BOLD:ACE5080
Euxoa comosa|[3833]|RDNMC212-05|CNCNoctuoideaH024|599[2n]bp|Canada.New Brunswick|BOLD:ACE5080
Euxoa comosa|[3834]|RDNM327-05|CNCNoctuoidea6509|567[0n]bp|Canada.New Brunswick|BOLD:ACE5080
Euxoa comosa|[3835]|RDNMB780-05|CNCNoctuoidea10555|589[0n]bp|Canada.British Columbia|BOLD:ACE5080
Euxoa comosa|[3836]|RDNMB793-05|CNCNoctuoidea10568|546[1n]bp|Canada.British Columbia|BOLD:ACE5080
Euxoa comosa|[3837]|RDNMC059-05|CNCNoctuoidea10800|507[0n]bp|Canada.British Columbia|BOLD:ACE5080
Euxoa comosa|[3838]|RDNMC052-05|CNCNoctuoidea10793|658[1n]bp|Canada.British Columbia|BOLD:ACE5080
Euxoa comosa|[3839]|RDNMB790-05|CNCNoctuoidea10565|658[0n]bp|Canada.British Columbia|BOLD:ACE5080
Euxoa comosa|[3840]|RDNMG660-08|CNC LEP00052484|649[0n]bp|Canada.British Columbia|BOLD:ACE5080
Euxoa comosa|[3841]|LBCH7956-10|10-JDWBC-7956|658[0n]bp|Canada.British Columbia|BOLD:ACE5080
Euxoa comosa|[3842]|RDNMC061-05|CNCNoctuoidea10802|585[2n]bp|Canada.British Columbia|BOLD:ACE5080
Euxoa comosa|[3843]|RDNMB807-05|CNCNoctuoidea10582|658[0n]bp|Canada.Alberta|BOLD:ACE5080
Euxoa comosa|[3844]|BBLPB342-10|10BBCLP-1341|658[0n]bp|Canada.Alberta|BOLD:ACE5080
Euxoa comosa|[3845]|RDNMB801-05|CNCNoctuoidea10576|658[0n]bp|Canada.Alberta|BOLD:ACE5080
Euxoa comosa|[3846]|RDNMB810-05|CNCNoctuoidea10585|658[0n]bp|Canada.Alberta|BOLD:ACE5080
Euxoa comosa|[3847]|RDNMB861-05|CNCNoctuoidea10636|658[0n]bp|Canada.Alberta|BOLD:ACE5080
Euxoa comosa|[3848]|RDNM301-05|CNCNoctuoidea6483|658[0n]bp|Canada.Alberta|BOLD:ACE5080
Euxoa comosa|[3849]|RDNMB850-05|CNCNoctuoidea10625|658[0n]bp|Canada.Alberta|BOLD:ACE5080
Euxoa comosa|[3850]|LPABB867-09|08BBLEP-04187|636[0n]bp|Canada.Alberta|BOLD:ACE5080
Euxoa comosa|[3851]|RDNMB791-05|CNCNoctuoidea10566|563[1n]bp|Canada.British Columbia|BOLD:ACE5080
Euxoa comosa|[3852]|RDNMB800-05|CNCNoctuoidea10575|658[0n]bp|Canada.British Columbia|BOLD:ACE5080
Euxoa comosa|[3853]|RDNMC060-05|CNCNoctuoidea10801|546[0n]bp|Canada.British Columbia|BOLD:ACE5080
Euxoa comosa|[3854]|RDNMB797-05|CNCNoctuoidea10572|658[0n]bp|Canada.British Columbia|BOLD:ACE5080
Euxoa comosa|[3855]|RDNMC053-05|CNCNoctuoidea10794|658[0n]bp|Canada.British Columbia|BOLD:ACE5080
Euxoa comosa|[3856]|RDNMB783-05|CNCNoctuoidea10558|658[0n]bp|Canada.British Columbia|BOLD:ACE5080
Euxoa comosa|[3857]|RDNMB792-05|CNCNoctuoidea10567|658[0n]bp|Canada.British Columbia|BOLD:ACE5080
Euxoa comosa|[3858]|RDNMB813-05|CNCNoctuoidea10588|658[0n]bp|Canada.Alberta|BOLD:ACE5080
Euxoa comosa|[3859]|RDNMC049-05|CNCNoctuoidea10790|658[0n]bp|Canada.British Columbia|BOLD:ACE5080
Euxoa comosa|[3860]|LPABB611-08|08BBLEP-03876|658[0n]bp|Canada.Alberta|BOLD:ACE5080
Euxoa comosa|[3861]|RDNMB814-05|CNCNoctuoidea10589|658[0n]bp|Canada.Alberta|BOLD:ACE5080
Euxoa comosa|[3862]|RDNMB818-05|CNCNoctuoidea10593|658[0n]bp|Canada.Alberta|BOLD:ACE5080
Euxoa comosa|[3863]|RDNMB817-05|CNCNoctuoidea10592|658[0n]bp|Canada.Alberta|BOLD:ACE5080
Euxoa comosa|[3864]|RDNM321-05|CNCNoctuoidea6503|658[0n]bp|Canada.British Columbia|BOLD:ACE5080
Euxoa comosa|[3865]|RDMAB676-06|UASM58792|657[0n]bp|Canada.Alberta|BOLD:ACE5080
Euxoa comosa|[3866]|RDNMB816-05|CNCNoctuoidea10591|658[0n]bp|Canada.British Columbia|BOLD:ACE5080
Euxoa comosa|[3867]|RDNMB815-05|CNCNoctuoidea10590|614[0n]bp|Canada.Alberta|BOLD:ACE5080
Euxoa comosa|[3868]|RDNMC041-05|CNCNoctuoidea10782|551[1n]bp|Canada.British Columbia|BOLD:ACE5080
Euxoa comosa|[3869]|RDNMC043-05|CNCNoctuoidea10784|594[0n]bp|Canada.British Columbia|BOLD:ACE5080
Euxoa comosa|[3870]|RDNMB795-05|CNCNoctuoidea10570|658[0n]bp|Canada.British Columbia|BOLD:ACE5080
Euxoa comosa|[3871]|RDNMC044-05|CNCNoctuoidea10785|520[0n]bp|Canada.British Columbia|BOLD:ACE5080
Euxoa comosa|[3872]|LALPA632-10|AVBC 634-10|658[0n]bp|Canada.British Columbia|BOLD:ACE5080
Euxoa comosa|[3873]|RDNMC084-05|CNCNoctuoidea10825|658[0n]bp|Canada.Alberta|BOLD:ACE5080
Euxoa comosa|[3874]|BBLPB756-10|10BBCLP-1755|658[0n]bp|Canada.Alberta|BOLD:ACE5080
Euxoa comosa|[3875]|LBCH6538-10|10-JDWBC-6538|658[0n]bp|Canada.British Columbia|BOLD:ACE5080
Euxoa comosa|[3876]|LBCH6537-10|10-JDWBC-6537|658[0n]bp|Canada.British Columbia|BOLD:ACE5080
Euxoa comosa|[3877]|RDNM303-05|CNCNoctuoidea6485|658[0n]bp|Canada.Alberta|BOLD:ACF2027
Euxoa comosa|[3878]|RDNMB824-05|CNCNoctuoidea10599|658[0n]bp|Canada.Alberta|BOLD:ACF2027
Euxoa fumalis|[3879]|RDNMB821-05|CNCNoctuoidea10596|658[0n]bp|Canada.Ontario|BOLD:ACE5080
Euxoa fumalis|[3880]|TMNBB367-06|MNBTT-1307|658[0n]bp|Canada.New Brunswick|BOLD:ACE5080
Euxoa fumalis|[3881]|RDNM326-05|CNCNoctuoidea6508|520[3n]bp|Canada.New Brunswick|BOLD:ACE5080
Euxoa fumalis|[3882]|RDNMB819-05|CNCNoctuoidea10594|658[0n]bp|Canada.Ontario|BOLD:ACE5080
Euxoa fumalis|[3883]|RDNM152-05|CNCNoctuoidea6702|658[0n]bp|Canada.Ontario|BOLD:ACE5080
Euxoa fumalis|[3884]|RDNMB820-05|CNCNoctuoidea10595|658[0n]bp|Canada.Ontario|BOLD:ACE5080
Euxoa occidentalis|[3885]|LBCH7957-10|10-JDWBC-7957|658[0n]bp|Canada.British Columbia|BOLD:ACF3236
Euxoa occidentalis|[3886]|LBCH6369-10|10-JDWBC-6369|658[0n]bp|Canada.British Columbia|BOLD:ACF3236
Euxoa occidentalis|[3887]|RDMAB1018-09|UASM112127|651[0n]bp|United States.Oregon|BOLD:ACF3236

Euxoa occidentalis|[3885]|LBCH7957-10|10-JDWBC-7957|658[0n]bp|Canada.British Columbia|BOLD:ACF3236
Euxoa occidentalis|[3886]|LBCH6369-10|10-JDWBC-6369|658[0n]bp|Canada.British Columbia|BOLD:ACF3236
Euxoa occidentalis|[3887]|RDMAB1018-09|UASM112127|651[0n]bp|United States.Oregon|BOLD:ACF3236
Euxoa occidentalis|[3888]|LOWCE775-06|CGWC-4535|658[0n]bp|Canada.British Columbia|BOLD:ACF3236
Euxoa occidentalis|[3889]|LBCG2889-09|08-JDWBC-2889|658[0n]bp|Canada.British Columbia|BOLD:ACF3236
Euxoa occidentalis|[3890]|LBCH7789-10|10-JDWBC-7789|658[0n]bp|Canada.British Columbia|BOLD:ACF3236
Euxoa occidentalis|[3891]|LBCH6460-10|10-JDWBC-6460|658[0n]bp|Canada.British Columbia|BOLD:ACF3236
Euxoa occidentalis|[3892]|LBCH7545-10|10-JDWBC-7545|658[0n]bp|Canada.British Columbia|BOLD:ACF3236
Euxoa occidentalis|[3893]|LBCH7539-10|10-JDWBC-7539|658[0n]bp|Canada.British Columbia|BOLD:ACF3236
Euxoa occidentalis|[3894]|LBCG2887-09|08-JDWBC-2887|658[0n]bp|Canada.British Columbia|BOLD:ACF3236
Euxoa occidentalis|[3895]|LBCH6539-10|10-JDWBC-6539|658[0n]bp|Canada.British Columbia|BOLD:ACF3236
Euxoa occidentalis|[3896]|LBCH6374-10|10-JDWBC-6374|658[0n]bp|Canada.British Columbia|BOLD:ACF3236
Euxoa occidentalis|[3897]|LBCG477-08|08-JDWBC-0477|658[0n]bp|Canada.British Columbia|BOLD:ACF3236
Euxoa occidentalis|[3898]|RDMAB1019-09|UASM112126|635[0n]bp|United States.Oregon|BOLD:ACF3236
Euxoa occidentalis|[3899]|LBCH6828-10|10-JDWBC-6828|658[0n]bp|Canada.British Columbia|BOLD:ACF3236
Euxoa occidentalis|[3900]|LBCH6446-10|10-JDWBC-6446|658[0n]bp|Canada.British Columbia|BOLD:ACF3236
Euxoa divergens|[3901]|RDNMB618-05|CNCNoctuoidea10394|523[0n]bp|Canada.British Columbia|BOLD:ACF2023
Euxoa divergens|[3902]|RDNM774-05|CNCNoctuoidea7620|522[0n]bp|Canada.British Columbia|BOLD:ACF2023
Euxoa divergens|[3903]|LBCH1445-10|10-JDWBC-1445|658[0n]bp|Canada.British Columbia|BOLD:ACF2023
Euxoa divergens|[3904]|LPABB831-09|08BBLEP-04151|658[0n]bp|Canada.Alberta|BOLD:ACF2023
Euxoa divergens|[3905]|LBCH2251-10|10-JDWBC-2251|658[0n]bp|Canada.British Columbia|BOLD:ACF2023
Euxoa divergens|[3906]|LBCH1953-10|10-JDWBC-1953|658[0n]bp|Canada.British Columbia|BOLD:ACF2023
Euxoa divergens|[3907]|LBCH1863-10|10-JDWBC-1863|658[0n]bp|Canada.British Columbia|BOLD:ACF2023
Euxoa divergens|[3908]|RDNMB624-05|CNCNoctuoidea10400|658[1n]bp|Canada.New Brunswick|BOLD:ACE5082
Euxoa divergens|[3909]|RDMAB036-05|UASM57519|611[0n]bp|Canada.Alberta|BOLD:ACE5082
Euxoa divergens|[3910]|LBCG1350-09|08-JDWBC-1350|658[0n]bp|Canada.British Columbia|BOLD:ACE5082
Euxoa divergens|[3911]|RDNMB623-05|CNCNoctuoidea10399|658[0n]bp|Canada.New Brunswick|BOLD:ACE5082
Euxoa divergens|[3912]|LBCH5253-10|10-JDWBC-5253|658[0n]bp|Canada.British Columbia|BOLD:ACE5082
Euxoa divergens|[3913]|RDMAB108-05|UASM41993|658[0n]bp|Canada.Alberta|BOLD:ACE5082
Euxoa divergens|[3914]|RDMAB109-05|UASM41999|658[0n]bp|Canada.Alberta|BOLD:ACE5082
Euxoa divergens|[3915]|LOWCE326-06|CGWC-4086|658[0n]bp|Canada.British Columbia|BOLD:ACE5082
Euxoa divergens|[3916]|RDMAB532-06|UASM58516|658[0n]bp|Canada.Alberta|BOLD:ACE5082
Euxoa divergens|[3917]|LBCG2870-09|08-JDWBC-2870|658[0n]bp|Canada.British Columbia|BOLD:ACE5082
Euxoa divergens|[3918]|LBCG1347-09|08-JDWBC-1347|658[0n]bp|Canada.British Columbia|BOLD:ACE5082
Euxoa divergens|[3919]|LBCH6011-10|10-JDWBC-6011|658[0n]bp|Canada.British Columbia|BOLD:ACE5082
Euxoa divergens|[3920]|LOWCE325-06|CGWC-4085|658[0n]bp|Canada.British Columbia|BOLD:ACE5082
Euxoa divergens|[3921]|BBLPB721-10|10BBCLP-1720|658[0n]bp|Canada.Ontario|BOLD:ACE5082
Euxoa divergens|[3922]|LBCG1342-09|08-JDWBC-1342|658[0n]bp|Canada.British Columbia|BOLD:ACE5082
Euxoa divergens|[3923]|LPSK139-08|08BBLEP-01707|658[0n]bp|Canada.Saskatchewan|BOLD:ACE5082
Euxoa divergens|[3924]|RDNM784-05|CNCNoctuoidea7630|658[0n]bp|Canada.Quebec|BOLD:ACE5082
Euxoa divergens|[3925]|LPMN738-08|08BBLEP-01541|658[0n]bp|Canada.Manitoba|BOLD:ACE5082
Euxoa divergens|[3926]|LPSK234-08|08BBLEP-01802|658[0n]bp|Canada.Saskatchewan|BOLD:ACE5082
Euxoa divergens|[3927]|RDMAB113-05|UASM41286|658[0n]bp|Canada.Alberta|BOLD:ACE5082
Euxoa divergens|[3928]|LBCH5480-10|10-JDWBC-5480|658[0n]bp|Canada.British Columbia|BOLD:ACE5082
Euxoa divergens|[3929]|LOWCD426-06|CGWC-3246|656[0n]bp|Canada.British Columbia|BOLD:ACE5082
Euxoa divergens|[3930]|PHMNB201-04|04HBL007666|574[0n]bp|Canada.New Brunswick|BOLD:ACE5082
Euxoa divergens|[3931]|LOWCD421-06|CGWC-3241|621[0n]bp|Canada.British Columbia|BOLD:ACE5082
Euxoa divergens|[3932]|LOWCD422-06|CGWC-3242|604[0n]bp|Canada.British Columbia|BOLD:ACE5082
Euxoa divergens|[3933]|RDMAB132-05|UASM41277|631[0n]bp|Canada.Alberta|BOLD:ACE5082
Euxoa divergens|[3934]|LOWCD420-06|CGWC-3240|601[0n]bp|Canada.British Columbia|BOLD:ACE5082
Euxoa divergens|[3935]|LOWCD413-06|CGWC-3233|589[0n]bp|Canada.British Columbia|BOLD:ACE5082
Euxoa divergens|[3936]|LOWCD423-06|CGWC-3243|593[0n]bp|Canada.British Columbia|BOLD:ACE5082
Euxoa divergens|[3937]|LOWCD412-06|CGWC-3232|594[0n]bp|Canada.British Columbia|BOLD:ACE5082
Euxoa divergens|[3938]|LOWCD414-06|CGWC-3234|604[0n]bp|Canada.British Columbia|BOLD:ACE5082
Euxoa divergens|[3939]|RDLQF147-06|CB0062|658[0n]bp|Canada.Quebec|BOLD:ACE5082
Euxoa divergens|[3940]|RDNM776-05|CNCNoctuoidea7622|583[0n]bp|Canada.British Columbia|BOLD:ACE5082
Euxoa divergens|[3941]|LPABB125-08|08BBLEP-03390|638[0n]bp|Canada.Alberta|BOLD:ACE5082
Euxoa divergens|[3942]|RDMAB256-05|UASM41467|658[0n]bp|Canada.Alberta|BOLD:ACE5082
Euxoa divergens|[3943]|RDMAB267-05|UASM41466|658[0n]bp|Canada.Alberta|BOLD:ACE5082
Euxoa divergens|[3944]|LPABB616-08|08BBLEP-03881|658[0n]bp|Canada.Alberta|BOLD:ACE5082
Euxoa divergens|[3945]|LPABB380-08|08BBLEP-03645|658[0n]bp|Canada.Alberta|BOLD:ACE5082
Euxoa divergens|[3946]|LOWCE294-06|CGWC-4054|658[0n]bp|Canada.British Columbia|BOLD:ACE5082
Euxoa divergens|[3947]|LPABB124-08|08BBLEP-03389|658[0n]bp|Canada.Alberta|BOLD:ACE5082
Euxoa divergens|[3948]|LPABB343-08|08BBLEP-03608|658[0n]bp|Canada.Alberta|BOLD:ACE5082
Euxoa divergens|[3949]|LBCG2585-09|08-JDWBC-2585|658[0n]bp|Canada.British Columbia|BOLD:ACE5082
Euxoa divergens|[3950]|LPABB352-08|08BBLEP-03617|658[0n]bp|Canada.Alberta|BOLD:ACE5082
Euxoa divergens|[3951]|LPABC590-09|08BBLEP-04809|658[0n]bp|Canada.Alberta|BOLD:ACE5082
Euxoa divergens|[3952]|LPABB856-09|08BBLEP-04176|658[0n]bp|Canada.Alberta|BOLD:ACE5082
Euxoa divergens|[3953]|LPABC573-09|08BBLEP-04792|658[0n]bp|Canada.Alberta|BOLD:ACE5082
Euxoa divergens|[3954]|LPABB421-08|08BBLEP-03686|658[0n]bp|Canada.Alberta|BOLD:ACE5082
Euxoa divergens|[3955]|RDNMB620-05|CNCNoctuoidea10396|509[0n]bp|Canada.Saskatchewan|BOLD:ACE5082
Euxoa divergens|[3956]|RDNM773-05|CNCNoctuoidea7619|507[0n]bp|Canada.Saskatchewan|BOLD:ACE5082
Euxoa divergens|[3957]|RDLQF148-06|CB0063|658[0n]bp|Canada.Quebec|BOLD:ACE5082
Euxoa divergens|[3958]|LOWCD419-06|CGWC-3239|593[0n]bp|Canada.British Columbia|BOLD:ACE5082
Euxoa divergens|[3959]|LOWCD418-06|CGWC-3238|576[0n]bp|Canada.British Columbia|BOLD:ACE5082
Euxoa divergens|[3960]|LOWCD416-06|CGWC-3236|579[0n]bp|Canada.British Columbia|BOLD:ACE5082
Euxoa divergens|[3961]|LOWCD338-06|CGWC-3158|563[2n]bp|Canada.British Columbia|BOLD:ACE5082
Euxoa divergens|[3962]|LOWCD342-06|CGWC-3162|558[1n]bp|Canada.British Columbia|BOLD:ACE5082
Euxoa divergens|[3963]|RDMAB037-05|UASM57520|628[0n]bp|Canada.Alberta|BOLD:ACE5082
Euxoa divergens|[3964]|RDNMB619-05|CNCNoctuoidea10395|658[0n]bp|Canada.British Columbia|BOLD:ACE5082
Euxoa divergens|[3965]|RDLQF849-06|DH012002|658[0n]bp|Canada.Quebec|BOLD:ACE5082
Euxoa divergens|[3966]|LPSOD335-09|08BBLEP-00113|658[0n]bp|Canada.Ontario|BOLD:ACE5082
Euxoa divergens|[3967]|LPSK125-08|08BBLEP-01693|658[0n]bp|Canada.Saskatchewan|BOLD:ACE5082
Euxoa divergens|[3968]|LPMN577-08|08BBLEP-01378|658[0n]bp|Canada.Manitoba|BOLD:ACE5082
Euxoa divergens|[3969]|BBLPB858-10|10BBCLP-1857|658[0n]bp|Canada.British Columbia|BOLD:ACE5082
Euxoa divergens|[3970]|LOWCE330-06|CGWC-4090|658[0n]bp|Canada.British Columbia|BOLD:ACE5082
Euxoa divergens|[3971]|LOWCE329-06|CGWC-4089|658[0n]bp|Canada.British Columbia|BOLD:ACE5082
Euxoa divergens|[3972]|LPABB613-08|08BBLEP-03878|658[0n]bp|Canada.Alberta|BOLD:ACE5082
Euxoa divergens|[3973]|LPSK144-08|08BBLEP-01712|658[0n]bp|Canada.Saskatchewan|BOLD:ACE5082
Euxoa divergens|[3974]|LBCH6009-10|10-JDWBC-6009|658[0n]bp|Canada.British Columbia|BOLD:ACE5082
Euxoa divergens|[3975]|LPSK013-08|08BBLEP-00716|658[0n]bp|Canada.Saskatchewan|BOLD:ACE5082
Euxoa divergens|[3976]|RDNMB617-05|CNCNoctuoidea10393|658[0n]bp|Canada.Alberta|BOLD:ACE5082
Euxoa divergens|[3977]|RDLQB282-05|DH010368|658[0n]bp|Canada.Quebec|BOLD:ACE5082
Euxoa divergens|[3978]|LBCH5945-10|10-JDWBC-5945|658[0n]bp|Canada.British Columbia|BOLD:ACE5082
Euxoa divergens|[3979]|LOWCD425-06|CGWC-3245|657[0n]bp|Canada.British Columbia|BOLD:ACE5082
Euxoa divergens|[3980]|RDNMB621-05|CNCNoctuoidea10397|658[0n]bp|Canada.Saskatchewan|BOLD:ACE5082
Euxoa divergens|[3981]|LOWCE319-06|CGWC-4079|658[0n]bp|Canada.British Columbia|BOLD:ACE5082
Euxoa divergens|[3982]|LPSK565-08|08BBLEP-02133|658[0n]bp|Canada.Saskatchewan|BOLD:ACE5082
Euxoa divergens|[3983]|LPSK140-08|08BBLEP-01708|658[0n]bp|Canada.Saskatchewan|BOLD:ACE5082
Euxoa divergens|[3984]|LOWCE321-06|CGWC-4081|658[0n]bp|Canada.British Columbia|BOLD:ACE5082
Euxoa sinelinea|[3985]|RDLQB283-05|DH010369|658[0n]bp|Canada.Quebec|BOLD:ACE5082
Euxoa sinelinea|[3986]|RDNMH398-09|CNCLEP00054412|658[0n]bp|Canada.Alberta|BOLD:ACE5082
Euxoa sinelinea|[3987]|RDNMH397-09|CNCLEP00054411|658[0n]bp|Canada.Alberta|BOLD:ACE5082

Euxoa sinelinea|[3985]|RDLQB283-05|DH010369|658[0n]bp|Canada.Quebec|BOLD:ACE5082
Euxoa sinelinea|[3986]|RDNMH398-09|CNCLEP00054412|658[0n]bp|Canada.Alberta|BOLD:ACE5082
Euxoa sinelinea|[3987]|RDNMH397-09|CNCLEP00054411|658[0n]bp|Canada.Alberta|BOLD:ACE5082
Euxoa sinelinea|[3988]|RDLQB289-05|DH010375|658[0n]bp|Canada.Quebec|BOLD:ACE5082
Euxoa sinelinea|[3989]|RDLQB288-05|DH010374|658[0n]bp|Canada.Quebec|BOLD:ACE5082
Euxoa divergens|[3990]|LPABB398-08|08BBLEP-03663|658[0n]bp|Canada.Alberta|BOLD:ACE5082
Euxoa divergens|[3991]|RDNMB616-05|CNCNoctuoidea10392|658[0n]bp|Canada.Alberta|BOLD:ACE5082
Euxoa divergens|[3992]|LOWCE328-06|CGWC-4088|658[0n]bp|Canada.British Columbia|BOLD:ACE5082
Euxoa divergens|[3993]|RDNMI116-10|CNCLEP 69716|658[0n]bp|Canada.Yukon Territory|BOLD:ACE5082
Euxoa divergens|[3994]|LPSK232-08|08BBLEP-01800|658[0n]bp|Canada.Saskatchewan|BOLD:ACE5082
Euxoa divergens|[3995]|LBCG1343-09|08-JDWBC-1343|658[0n]bp|Canada.British Columbia|BOLD:ACE5082
Euxoa divergens|[3996]|LPSK574-08|08BBLEP-02142|658[0n]bp|Canada.Saskatchewan|BOLD:ACE5082
Euxoa divergens|[3997]|LPABB620-08|08BBLEP-03885|658[0n]bp|Canada.Alberta|BOLD:ACE5082
Euxoa divergens|[3998]|BBLPB727-10|10BBCLP-1726|658[0n]bp|Canada.Alberta|BOLD:ACE5082
Euxoa divergens|[3999]|RDMAB435-05|BCSC108|658[0n]bp|Canada.Yukon Territory|BOLD:ACE5082
Euxoa divergens|[4000]|LPMN345-08|08BBLEP-01144|658[0n]bp|Canada.Manitoba|BOLD:ACE5082
Euxoa divergens|[4001]|LPSK235-08|08BBLEP-01803|658[0n]bp|Canada.Saskatchewan|BOLD:ACE5082
Euxoa divergens|[4002]|LPSK250-08|08BBLEP-01818|658[0n]bp|Canada.Saskatchewan|BOLD:ACE5082
Euxoa divergens|[4003]|LOWCE327-06|CGWC-4087|658[0n]bp|Canada.British Columbia|BOLD:ACE5082
Euxoa divergens|[4004]|LOWCE320-06|CGWC-4080|658[0n]bp|Canada.British Columbia|BOLD:ACE5082
Euxoa divergens|[4005]|LPMN174-08|08BBLEP-00973|658[0n]bp|Canada.Manitoba|BOLD:ACE5082
Euxoa sinelinea|[4006]|RDLQB285-05|DH010371|658[1n]bp|Canada.Quebec|BOLD:ACE5082
Euxoa divergens|[4007]|BBLPE466-09|09BBELE-2466|654[0n]bp|Canada.Newfoundland and Labrador|BOLD:ACE5082
Euxoa divergens|[4008]|LOWCD415-06|CGWC-3235|599[0n]bp|Canada.British Columbia|BOLD:ACE5082
Euxoa divergens|[4009]|RDLQ687-07|DH006634|597[0n]bp|Canada.Quebec|BOLD:ACE5082
Euxoa divergens|[4010]|LOWCD333-06|CGWC-3153|592[0n]bp|Canada.British Columbia|BOLD:ACE5082
Euxoa divergens|[4011]|LOWCD424-06|CGWC-3244|592[0n]bp|Canada.British Columbia|BOLD:ACE5082
Euxoa divergens|[4012]|LOWCD417-06|CGWC-3237|551[0n]bp|Canada.British Columbia|BOLD:ACE5082
Euxoa sinelinea|[4013]|RDLQB284-05|DH010370|598[0n]bp|Canada.Quebec|BOLD:ACE5082
Euxoa sinelinea|[4014]|RDLQB286-05|DH010372|603[0n]bp|Canada.Quebec|BOLD:ACE5082
Euxoa sinelinea|[4015]|RDLQB287-05|DH010373|658[0n]bp|Canada.Quebec|BOLD:ACE5082
Euxoa atomaris|[4016]|RDNM275-05|CNCNoctuoidea6457|609[0n]bp|Canada.British Columbia|BOLD:ABX5971
Euxoa atomaris|[4017]|LBCH7807-10|10-JDWBC-7807|658[0n]bp|Canada.British Columbia|BOLD:ABX5971
Euxoa atomaris|[4018]|LBCH7234-10|10-JDWBC-7234|658[0n]bp|Canada.British Columbia|BOLD:ABX5971
Euxoa atomaris|[4019]|LBCH7330-10|10-JDWBC-7330|658[0n]bp|Canada.British Columbia|BOLD:ABX5971
Euxoa atomaris|[4020]|LBCH7333-10|10-JDWBC-7333|658[0n]bp|Canada.British Columbia|BOLD:ABX5971
Euxoa extranea|[4021]|RDNMF848-08|CNC LEP00053209|658[0n]bp|United States.Oregon|BOLD:ABZ9891
Euxoa extranea|[4022]|IAWLB015-10|IAWAZ-0828|658[0n]bp|United States.California|BOLD:ABZ9891
Euxoa extranea|[4023]|RDNMF839-08|CNC LEP00053200|658[0n]bp|United States.California|BOLD:ABZ9891
Euxoa extranea|[4024]|RDNMF318-08|NOC14404|658[0n]bp|United States.Washington|BOLD:ABZ9891
Euxoa extranea|[4025]|RDNMF319-08|NOC14405|658[0n]bp|United States.Oregon|BOLD:ABZ9891
Euxoa extranea|[4026]|IAWLB014-10|IAWAZ-0827|658[0n]bp|United States.California|BOLD:ABZ9891
Euxoa extranea|[4027]|IAWLB013-10|IAWAZ-0826|658[0n]bp|United States.California|BOLD:ABZ9891
Euxoa extranea|[4028]|RDNMF320-08|NOC14406|658[0n]bp|United States.Oregon|BOLD:ABZ9891
Euxoa chimoensis|[4029]|RDNMC207-05|CNCNoctuoideaH019|512[0n]bp|Canada.Quebec|BOLD:ACF3481
Euxoa chimoensis|[4030]|MECB945-05|jflandry1977|524[0n]bp|Canada.Quebec|BOLD:ACF3481
Euxoa chimoensis|[4031]|LCHP416-07|07PROBE-10040|658[0n]bp|Canada.Manitoba|BOLD:ACF3481
Euxoa chimoensis|[4032]|LCHP524-07|07PROBE-10192|658[0n]bp|Canada.Manitoba|BOLD:ACF3481
Euxoa chimoensis|[4033]|RDNMF863-08|CNC LEP00053224|658[0n]bp|Canada.Manitoba|BOLD:ACF3481
Euxoa chimoensis|[4034]|LCH261-04|04HBL003261|658[0n]bp|Canada.Manitoba|BOLD:ACF3481
Euxoa chimoensis|[4035]|LCHP852-07|07PROBE-10609|643[0n]bp|Canada.Manitoba|BOLD:ACF3481
Euxoa chimoensis|[4036]|RDNMF864-08|CNC LEP00053225|658[0n]bp|Canada.Manitoba|BOLD:ACF3481
Euxoa chimoensis|[4037]|RDNMF862-08|CNC LEP00053223|658[0n]bp|Canada.Manitoba|BOLD:ACF3481
Euxoa churchillensis|[4038]|RDNMF323-08|NOC14409|658[0n]bp|Canada.Nunavut|BOLD:ACE5992
Euxoa churchillensis|[4039]|RDNMF325-08|NOC14411|658[0n]bp|Canada.Nunavut|BOLD:ACE5992
Euxoa churchillensis|[4040]|RDNMF324-08|NOC14410|658[0n]bp|Canada.Nunavut|BOLD:ACE5992
Euxoa churchillensis|[4041]|RDNMG975-08|CNC LEP00053099|592[0n]bp|Canada.Northwest Territories|BOLD:A...
Euxoa churchillensis|[4042]|RDNMG974-08|CNC LEP00053098|642[0n]bp|Canada.Northwest Territories|BOLD:A...
Euxoa churchillensis|[4043]|RDNMG976-08|CNC LEP00053100|658[0n]bp|Canada.Nunavut|BOLD:ACE5992
Euxoa dissona|[4044]|RDLQ415-05|CB0001|618[0n]bp|Canada.Quebec|BOLD:ACE5992
Euxoa dissona|[4045]|MECB946-05|jflandry1978|578[0n]bp|Canada.Manitoba|BOLD:ACE5992
Euxoa dissona|[4046]|RDNMG1037-08|CNC LEP00053161|641[0n]bp|Canada.Manitoba|BOLD:ACE5992
Euxoa dissona|[4047]|RDNMG1038-08|CNC LEP00053162|658[0n]bp|Canada.Manitoba|BOLD:ACE5992
Euxoa dissona|[4048]|RDNMG1036-08|CNC LEP00053160|658[0n]bp|Canada.Manitoba|BOLD:ACE5992
Euxoa dissona|[4049]|CHLEP208-09|09PROBE-09503|658[0n]bp|Canada.Manitoba|BOLD:ACE5992
Euxoa hyperborea|[4050]|RDNMF322-08|NOC14408|640[0n]bp|United States.Alaska|BOLD:ABZ9739
Euxoa hyperborea|[4051]|RDNMF321-08|NOC14407|658[0n]bp|United States.Alaska|BOLD:ABZ9739
Euxoa muldersi|[4052]|RDNMH1034-09|CNCLEP00068036|658[0n]bp|Canada.Nunavut|BOLD:ABZ9739
Euxoa muldersi|[4053]|RDNMH1035-09|CNCLEP00068037|658[1n]bp|Canada.Nunavut|BOLD:ABZ9739
Euxoa lewisi|[4054]|RDNMF032-08|NOC14118|658[0n]bp|Canada.Alberta|BOLD:ACE5991
Euxoa lewisi|[4055]|RDNMH1031-09|CNCLEP00031854|658[0n]bp|Canada.British Columbia|BOLD:ACE5991
Euxoa lewisi|[4056]|LBCH2200-10|10-JDWBC-2200|658[0n]bp|Canada.British Columbia|BOLD:ACE5991
Euxoa lewisi|[4057]|BBLPB871-10|10BBCLP-1870|658[0n]bp|Canada.British Columbia|BOLD:ACE5991
Euxoa altens|[4058]|LBCH2199-10|10-JDWBC-2199|658[0n]bp|Canada.British Columbia|BOLD:AAF6228
Euxoa westermanni|[4059]|LCH250-04|04HBL003250|658[0n]bp|Canada.Manitoba|BOLD:ABZ8446
Euxoa westermanni|[4060]|LCHP581-07|07PROBE-10251|658[0n]bp|Canada.Manitoba|BOLD:ABZ8446
Euxoa westermanni|[4061]|LCHQ478-08|07WNP-10370|658[0n]bp|Canada.Manitoba|BOLD:ABZ8446
Euxoa westermanni|[4062]|LCH252-04|04HBL003252|658[0n]bp|Canada.Manitoba|BOLD:ABZ8446
Euxoa westermanni|[4063]|LCH255-04|04HBL003255|658[0n]bp|Canada.Manitoba|BOLD:ABZ8446
Euxoa westermanni|[4064]|LCH254-04|04HBL003254|658[0n]bp|Canada.Manitoba|BOLD:ABZ8446
Euxoa westermanni|[4065]|LCHQ702-08|07WNP-10594|658[0n]bp|Canada.Manitoba|BOLD:ABZ8446
Euxoa westermanni|[4066]|LCHP840-07|07PROBE-10597|658[0n]bp|Canada.Manitoba|BOLD:ABZ8446
Euxoa westermanni|[4067]|LCH253-04|04HBL003253|658[0n]bp|Canada.Manitoba|BOLD:ABZ8446
Euxoa westermanni|[4068]|LCHP948-07|07PROBE-10710|639[0n]bp|Canada.Manitoba|BOLD:ABZ8446
Euxoa westermanni|[4069]|LCHP871-07|07PROBE-10628|658[0n]bp|Canada.Manitoba|BOLD:ABZ8446
Euxoa westermanni|[4070]|LCH251-04|04HBL003251|658[0n]bp|Canada.Manitoba|BOLD:ABZ8446
Euxoa westermanni|[4071]|LCHP417-07|07PROBE-10041|658[0n]bp|Canada.Manitoba|BOLD:ABZ8446
Euxoa westermanni|[4072]|LCHP608-07|07PROBE-10289|658[0n]bp|Canada.Manitoba|BOLD:ABZ8446
Euxoa westermanni|[4073]|LCHP873-07|07PROBE-10630|658[0n]bp|Canada.Manitoba|BOLD:ABZ8446
Euxoa nomas|[4074]|RDMAB978-09|UASM78755|590[0n]bp|Canada.Alberta|BOLD:ACF3241
Euxoa nomas|[4075]|RDMAB977-09|UASM78863|632[0n]bp|Canada.Alberta|BOLD:ACF3241
Euxoa nomas|[4076]|RDNMF035-08|NOC14121|658[0n]bp|Canada.Alberta|BOLD:ACF3241
Euxoa nomas|[4077]|RDMAB976-09|UASM59579|658[0n]bp|Canada.Alberta|BOLD:ACF3241
Euxoa flavicollis|[4078]|LBCH7963-10|10-JDWBC-7963|658[0n]bp|Canada.British Columbia|BOLD:ACF3241
Euxoa flavicollis|[4079]|RDNMG494-08|CNC LEP00052318|658[0n]bp|Canada.British Columbia|BOLD:ACF3241
Euxoa flavicollis|[4080]|LPSK137-08|08BBLEP-01705|658[0n]bp|Canada.Saskatchewan|BOLD:ACF3241
Euxoa flavicollis|[4081]|LPSK393-08|08BBLEP-01961|658[0n]bp|Canada.Saskatchewan|BOLD:ACF3241
Euxoa flavicollis|[4082]|RDNMF770-08|UASM107734|658[0n]bp|Canada.Saskatchewan|BOLD:ACF3241
Euxoa flavicollis|[4083]|LBCH6091-10|10-JDWBC-6091|658[0n]bp|Canada.British Columbia|BOLD:ACF3241
Euxoa flavicollis|[4084]|LOWCE714-06|CGWC-4474|658[0n]bp|Canada.British Columbia|BOLD:ACF3241
Euxoa flavicollis|[4085]|RDNMG495-08|CNC LEP00052319|639[0n]bp|Canada.British Columbia|BOLD:ACF3241
Euxoa flavicollis|[4086]|RDNMF037-08|NOC14123|658[0n]bp|Canada.Alberta|BOLD:ACF3241
Euxoa flavicollis|[4087]|LPSK145-08|08BBLEP-01713|658[0n]bp|Canada.Saskatchewan|BOLD:ACF3241

Euxoa flavicollis|[4084]|LOWCE714-06|CGWC-4474|658[0n]bp|Canada.British Columbia|BOLD:ACF3241
Euxoa flavicollis|[4085]|RDNMG495-08|CNC LEP00052319|639[0n]bp|Canada.British Columbia|BOLD:ACF3241
Euxoa flavicollis|[4086]|RDNMF037-08|NOC14123|658[0n]bp|Canada.Alberta|BOLD:ACF3241
Euxoa flavicollis|[4087]|LPSK145-08|08BBLEP-01713|658[0n]bp|Canada.Saskatchewan|BOLD:ACF3241
Euxoa maimes|[4088]|LOWCE715-06|CGWC-4475|658[0n]bp|Canada.British Columbia|BOLD:ACF3241
Euxoa maimes|[4089]|LOWCE718-06|CGWC-4478|604[0n]bp|Canada.British Columbia|BOLD:ACF3241
Euxoa aberrans|[4090]|RDNMG453-08|CNC LEP00052277|658[0n]bp|Canada.British Columbia|BOLD:ACF3241
Euxoa aberrans|[4091]|RDNMG455-08|CNC LEP00052279|658[0n]bp|Canada.British Columbia|BOLD:ACF3241
Euxoa aberrans|[4092]|LBCH7002-10|10-JDWBC-7002|658[0n]bp|Canada.British Columbia|BOLD:ACF3241
Euxoa aberrans|[4093]|LBCH7054-10|10-JDWBC-7054|658[0n]bp|Canada.British Columbia|BOLD:ACF3241
Euxoa aberrans|[4094]|RDNMG454-08|CNC LEP00052278|658[0n]bp|Canada.British Columbia|BOLD:ACF3241
Euxoa maimes|[4095]|RDMAB596-06|UASM58625|658[0n]bp|Canada.Alberta|BOLD:ACF3241
Euxoa maimes|[4096]|LBCH6784-10|10-JDWBC-6784|658[0n]bp|Canada.British Columbia|BOLD:ACF3241
Euxoa maimes|[4097]|RDMAB597-06|UASM58626|658[0n]bp|Canada.Alberta|BOLD:ACF3241
Euxoa maimes|[4098]|LOWCE717-06|CGWC-4477|658[0n]bp|Canada.British Columbia|BOLD:ACF3241
Euxoa macrodentata|[4099]|RDNMF702-08|NOC14788|658[0n]bp|Canada.Yukon Territory|BOLD:ACF3241
Euxoa perpolita|[4100]|LBCH7680-10|10-JDWBC-7680|658[0n]bp|Canada.British Columbia|BOLD:ACF3241
Euxoa perpolita|[4101]|RDLQB498-05|DH010584|658[0n]bp|Canada.Quebec|BOLD:ACF3241
Euxoa perpolita|[4102]|RDLQB497-05|DH010583|658[0n]bp|Canada.Quebec|BOLD:ACF3241
Euxoa perpolita|[4103]|RDLQF176-06|DH011203|658[0n]bp|Canada.Quebec|BOLD:ACF3241
Euxoa perpolita|[4104]|LBCH7681-10|10-JDWBC-7681|658[0n]bp|Canada.British Columbia|BOLD:ACF3241
Euxoa perpolita|[4105]|RDNMG1018-08|CNC LEP00053142|658[0n]bp|Canada.Ontario|BOLD:ACF3241
Euxoa perpolita|[4106]|RDNMG1016-08|CNC LEP00053140|658[0n]bp|Canada.Ontario|BOLD:ACF3241
Euxoa ridingsiana|[4107]|BBLPB726-10|10BBCLP-1725|658[0n]bp|Canada.Alberta|BOLD:ACF3241
Euxoa ridingsiana|[4108]|BBLPB731-10|10BBCLP-1730|658[0n]bp|Canada.Alberta|BOLD:ACF3241
Euxoa ridingsiana|[4109]|BBLPB733-10|10BBCLP-1732|658[0n]bp|Canada.Alberta|BOLD:ACF3241
Euxoa ridingsiana|[4110]|RDNMF768-08|UASM34725|658[0n]bp|Canada.Alberta|BOLD:ACF3241
Euxoa ridingsiana|[4111]|LOWCE722-06|CGWC-4482|658[0n]bp|Canada.British Columbia|BOLD:ACF3241
Euxoa ridingsiana|[4112]|BBLPB722-10|10BBCLP-1721|658[0n]bp|Canada.Alberta|BOLD:ACF3241
Euxoa ridingsiana|[4113]|LBCH7964-10|10-JDWBC-7964|658[0n]bp|Canada.British Columbia|BOLD:ACF3241
Euxoa ridingsiana|[4114]|BBLPB724-10|10BBCLP-1723|658[0n]bp|Canada.Alberta|BOLD:ACF3241
Euxoa ridingsiana|[4115]|BBLPB735-10|10BBCLP-1734|658[0n]bp|Canada.Alberta|BOLD:ACF3241
Euxoa ridingsiana|[4116]|LBCG1103-09|08-JDWBC-1103|658[0n]bp|Canada.British Columbia|BOLD:ACF3241
Euxoa ridingsiana|[4117]|RDNMG560-08|CNC LEP00052384|658[0n]bp|Canada.British Columbia|BOLD:ACF3241
Euxoa ridingsiana|[4118]|RDNMG501-08|CNC LEP00052325|658[0n]bp|Canada.Alberta|BOLD:ACF3241
Euxoa ridingsiana|[4119]|RDNMG500-08|CNC LEP00052324|658[0n]bp|Canada.Alberta|BOLD:ACF3241
Euxoa ridingsiana|[4120]|BBLPB734-10|10BBCLP-1733|658[0n]bp|Canada.Alberta|BOLD:ACF3241
Euxoa ridingsiana|[4121]|RDNMF775-08|UASM34606|658[0n]bp|Canada.Alberta|BOLD:ACF3241
Euxoa ridingsiana|[4122]|RDNMF772-08|UASM34604|658[0n]bp|Canada.Alberta|BOLD:ACF3241
Euxoa ridingsiana|[4123]|RDNMF771-08|UASM107733|658[0n]bp|Canada.Saskatchewan|BOLD:ACF3241
Euxoa ridingsiana|[4124]|BBLPB732-10|10BBCLP-1731|658[0n]bp|Canada.Alberta|BOLD:ACF3241
Euxoa ridingsiana|[4125]|BBLPB723-10|10BBCLP-1722|658[0n]bp|Canada.Alberta|BOLD:ACF3241
Euxoa ridingsiana|[4126]|RDNMG499-08|CNC LEP00052323|658[0n]bp|Canada.British Columbia|BOLD:ACF3241
Euxoa ridingsiana|[4127]|RDNMF769-08|UASM58273|658[0n]bp|Canada.Alberta|BOLD:ACF3241
Euxoa ridingsiana|[4128]|BBLPB737-10|10BBCLP-1736|658[0n]bp|Canada.Alberta|BOLD:ACF3241
Euxoa ridingsiana|[4129]|LBCH6501-10|10-JDWBC-6501|658[0n]bp|Canada.British Columbia|BOLD:ACF3241
Euxoa ridingsiana|[4130]|RDNMF773-08|UASM24203|658[0n]bp|Canada.Alberta|BOLD:ACF3241
Euxoa ridingsiana|[4131]|LBCH7785-10|10-JDWBC-7785|658[0n]bp|Canada.British Columbia|BOLD:ACF3241
Euxoa ridingsiana|[4132]|BBLPB738-10|10BBCLP-1737|658[0n]bp|Canada.Alberta|BOLD:ACF3241
Euxoa ridingsiana|[4133]|RDNMF774-08|UASM24202|658[0n]bp|Canada.Alberta|BOLD:ACF3241
Euxoa ridingsiana|[4134]|BBLPB725-10|10BBCLP-1724|658[0n]bp|Canada.Alberta|BOLD:ACF3241
Euxoa taura|[4135]|RDMAB1022-09|UASM107837|658[0n]bp|Canada.Alberta|BOLD:ACF3241
Euxoa taura|[4136]|RDNMF040-08|NOC14126|658[0n]bp|Canada.Alberta|BOLD:ACF3241
Euxoa manitobana|[4137]|RDMAB488-06|UASM77993|636[0n]bp|Canada.Alberta|BOLD:ACF3241
Euxoa ridingsiana|[4138]|LBCH6783-10|10-JDWBC-6783|645[0n]bp|Canada.British Columbia|BOLD:ACF3241
Euxoa ridingsiana|[4139]|LOWCE719-06|CGWC-4479|602[0n]bp|Canada.British Columbia|BOLD:ACF3241
Euxoa taura|[4140]|RDMAB1023-09|UASM107836|635[0n]bp|Canada.Alberta|BOLD:ACF3241
Euxoa taura|[4141]|RDMAB1021-09|UASM107841|620[0n]bp|Canada.Alberta|BOLD:ACF3241
Euxoa perolivalis|[4142]|LPABB405-08|08BBLEP-03670|658[0n]bp|Canada.Alberta|BOLD:ACF3241
Euxoa perolivalis|[4143]|RDMAB975-09|UASM109019|635[0n]bp|Canada.Alberta|BOLD:ACF3241
Euxoa perolivalis|[4144]|RDMAB974-09|UASM109025|635[0n]bp|Canada.Alberta|BOLD:ACF3241
Euxoa perolivalis|[4145]|RDNMF030-08|NOC14116|658[0n]bp|Canada.Alberta|BOLD:ACF3241
Euxoa perolivalis|[4146]|LPABB424-08|08BBLEP-03689|658[0n]bp|Canada.Alberta|BOLD:ACF3241
Euxoa taura|[4147]|RDNMF027-08|NOC14113|658[0n]bp|Canada.Alberta|BOLD:ACF3241
Euxoa wilsoni|[4148]|RDNMF317-08|NOC14403|658[0n]bp|Canada.British Columbia|BOLD:ACF3241
Euxoa wilsoni|[4149]|RDNMF316-08|NOC14402|658[0n]bp|Canada.British Columbia|BOLD:ACF3241
Euxoa wilsoni|[4150]|RDNMG648-08|CNC LEP00052472|658[0n]bp|Canada.British Columbia|BOLD:ACF3241
Euxoa setonia|[4151]|LOWCE310-06|CGWC-4070|658[0n]bp|Canada.British Columbia|BOLD:ABZ9160
Euxoa setonia|[4152]|BBLPB695-10|10BBCLP-1694|658[0n]bp|Canada.British Columbia|BOLD:ABZ9160
Euxoa setonia|[4153]|LPABB345-08|08BBLEP-03610|658[0n]bp|Canada.Alberta|BOLD:ABZ9160
Euxoa setonia|[4154]|LBCH5700-10|10-JDWBC-5700|647[0n]bp|Canada.British Columbia|BOLD:ABZ9160
Euxoa setonia|[4155]|LPABB423-08|08BBLEP-03688|658[0n]bp|Canada.Alberta|BOLD:ABZ9160
Euxoa setonia|[4156]|LBCH5699-10|10-JDWBC-5699|658[0n]bp|Canada.British Columbia|BOLD:ABZ9160
Euxoa pallidimacula|[4157]|LBCH5512-10|10-JDWBC-5512|658[0n]bp|Canada.British Columbia|BOLD:ABZ9160
Euxoa setonia|[4158]|RDNMB844-05|CNCNoctuoidea10619|658[0n]bp|Canada.Alberta|BOLD:ACE5989
Euxoa setonia|[4159]|RDNMB843-05|CNCNoctuoidea10618|658[0n]bp|Canada.Alberta|BOLD:ACE5989
Euxoa setonia|[4160]|RDMAB104-05|UASM41987|658[0n]bp|Canada.Alberta|BOLD:ACE5989
Euxoa setonia|[4161]|RDNMB841-05|CNCNoctuoidea10616|658[0n]bp|Canada.Alberta|BOLD:ACE5989
Euxoa bicollaris|[4162]|LBCH5942-10|10-JDWBC-5942|658[0n]bp|Canada.British Columbia|BOLD:ACE8317
Euxoa bicollaris|[4163]|LBCH5811-10|10-JDWBC-5811|658[0n]bp|Canada.British Columbia|BOLD:ACE8317
Euxoa satis|[4164]|LBCH7553-10|10-JDWBC-7553|658[0n]bp|Canada.British Columbia|BOLD:ACF3240
Euxoa satis|[4165]|RDMAB1016-09|UASM112124|658[0n]bp|Canada.British Columbia|BOLD:ACF3240
Euxoa satis|[4166]|LBCG1091-09|08-JDWBC-1091|658[0n]bp|Canada.British Columbia|BOLD:ACF3240
Euxoa satis|[4167]|LBCH6752-10|10-JDWBC-6752|658[0n]bp|Canada.British Columbia|BOLD:ACF3240
Euxoa satis|[4168]|LBCH6286-10|10-JDWBC-6286|658[0n]bp|Canada.British Columbia|BOLD:ACF3240
Euxoa satis|[4169]|LBCG1065-09|08-JDWBC-1065|658[0n]bp|Canada.British Columbia|BOLD:ACF3240
Euxoa satis|[4170]|LBCH6155-10|10-JDWBC-6155|658[0n]bp|Canada.British Columbia|BOLD:ACF3240
Euxoa satis|[4171]|LBCH6288-10|10-JDWBC-6288|658[0n]bp|Canada.British Columbia|BOLD:ACF3240
Euxoa satis|[4172]|LBCH6379-10|10-JDWBC-6379|658[0n]bp|Canada.British Columbia|BOLD:ACF3240
Euxoa satis|[4173]|LBCH5876-10|10-JDWBC-5876|658[0n]bp|Canada.British Columbia|BOLD:ACF3240
Euxoa satis|[4174]|RDNMB641-05|CNCNoctuoidea10417|658[0n]bp|Canada.British Columbia|BOLD:ACF3240
Euxoa satis|[4175]|LBCH5944-10|10-JDWBC-5944|658[0n]bp|Canada.British Columbia|BOLD:ACF3240
Euxoa satis|[4176]|LBCH6045-10|10-JDWBC-6045|658[0n]bp|Canada.British Columbia|BOLD:ACF3240
Euxoa satis|[4177]|LOWCD487-06|CGWC-3307|658[0n]bp|Canada.British Columbia|BOLD:ACF3240
Euxoa satis|[4178]|LBCG1090-09|08-JDWBC-1090|658[0n]bp|Canada.British Columbia|BOLD:ACF3240
Euxoa occidentalis|[4179]|RDMAB1017-09|UASM112123|638[0n]bp|Canada.British Columbia|BOLD:ACF3240
Euxoa satis|[4180]|LBCH6289-10|10-JDWBC-6289|634[0n]bp|Canada.British Columbia|BOLD:ACF3240
Euxoa satis|[4181]|RDNM804-05|CNCNoctuoidea7650|605[1n]bp|Canada.British Columbia|BOLD:ACF3240
Euxoa satis|[4182]|LOWCD488-06|CGWC-3308|541[0n]bp|Canada.British Columbia|BOLD:ACF3240
Euxoa satis|[4183]|RDNMB640-05|CNCNoctuoidea10416|509[3n]bp|Canada.British Columbia|BOLD:ACF3240
Euxoa satis|[4184]|LOWCD490-06|CGWC-3310|596[0n]bp|Canada.British Columbia|BOLD:ACF3240
Euxoa satis|[4185]|LBCG876-09|08-JDWBC-0876|658[0n]bp|Canada.British Columbia|BOLD:ACF3240
Euxoa brunneigera|[4186]|LBCH7815-10|10-JDWBC-7815|658[0n]bp|Canada.British Columbia|BOLD:ACF0812
[illegible]

Euxoa satis|[4184]|LOWCD490-06|CGWC-3310|596[0n]bp|Canada.British Columbia|BOLD:ACF3240
Euxoa satis|[4185]|LBCG876-09|08-JDWBC-0876|658[0n]bp|Canada.British Columbia|BOLD:ACF3240
Euxoa brunneigera|[4186]|LBCH7815-10|10-JDWBC-7815|658[0n]bp|Canada.British Columbia|BOLD:ACF0812
Euxoa brunneigera|[4187]|LBCH6529-10|10-JDWBC-6529|658[0n]bp|Canada.British Columbia|BOLD:ACF0812
Euxoa brunneigera|[4188]|LOWCD485-06|CGWC-3305|658[0n]bp|Canada.British Columbia|BOLD:ACF0812
Euxoa brunneigera|[4189]|LBCH6550-10|10-JDWBC-6550|658[0n]bp|Canada.British Columbia|BOLD:ACF0812
Euxoa brunneigera|[4190]|LBCH6027-10|10-JDWBC-6027|658[0n]bp|Canada.British Columbia|BOLD:ACF0812
Euxoa brunneigera|[4191]|LOWCD486-06|CGWC-3306|536[0n]bp|Canada.British Columbia|BOLD:ACF0812
Euxoa brunneigera|[4192]|LOWCD493-06|CGWC-3313|577[0n]bp|Canada.British Columbia|BOLD:ACF0812
Euxoa satis|[4193]|BBLPB698-10|10BBCLP-1697|636[0n]bp|Canada.British Columbia|BOLD:ACF3239
Euxoa satis|[4194]|RDNMB638-05|CNCNoctuoidea10414|658[0n]bp|Canada.British Columbia|BOLD:ACF3238
Euxoa excogita|[4195]|LBCG505-08|08-JDWBC-0505|658[0n]bp|Canada.British Columbia|BOLD:ACF3238
Euxoa excogita|[4196]|LBCH6551-10|10-JDWBC-6551|658[0n]bp|Canada.British Columbia|BOLD:ACF3238
Euxoa excogita|[4197]|LBCH6548-10|10-JDWBC-6548|658[0n]bp|Canada.British Columbia|BOLD:ACF3238
Euxoa excogita|[4198]|LBCH6372-10|10-JDWBC-6372|658[0n]bp|Canada.British Columbia|BOLD:ACF3238
Euxoa excogita|[4199]|RDMAB1006-09|UASM111961|651[0n]bp|Canada.British Columbia|BOLD:ACF3238
Euxoa excogita|[4200]|RDMAB1009-09|UASM111963|635[0n]bp|Canada.British Columbia|BOLD:ACF3238
Euxoa excogita|[4201]|LBCH5793-10|10-JDWBC-5793|658[0n]bp|Canada.British Columbia|BOLD:ACF3238
Euxoa excogita|[4202]|LBCG2879-09|08-JDWBC-2879|658[0n]bp|Canada.British Columbia|BOLD:ACF3238
Euxoa infausta|[4203]|RDNM869-05|CNCNoctuoidea7709|658[0n]bp|Canada.Saskatchewan|BOLD:ACF3238
Euxoa infausta|[4204]|LBCH5864-10|10-JDWBC-5864|658[0n]bp|Canada.British Columbia|BOLD:ACF3238
Euxoa infausta|[4205]|RDMAB318-05|UASM77801|658[0n]bp|Canada.Alberta|BOLD:ACF3238
Euxoa infausta|[4206]|LBCG476-08|08-JDWBC-0476|658[0n]bp|Canada.British Columbia|BOLD:ACF3238
Euxoa infausta|[4207]|RDNM868-05|CNCNoctuoidea7708|658[0n]bp|Canada.British Columbia|BOLD:ACF3238
Euxoa satis|[4208]|LOWCD492-06|CGWC-3312|596[0n]bp|Canada.British Columbia|BOLD:ACF3238
Euxoa satis|[4209]|LBCD455-05|HLC-23275|658[0n]bp|Canada.British Columbia|BOLD:ACF3238
Euxoa satis|[4210]|LOWCD489-06|CGWC-3309|658[0n]bp|Canada.British Columbia|BOLD:ACF3238
Euxoa satis|[4211]|LBCD463-05|HLC-23283|658[0n]bp|Canada.British Columbia|BOLD:ACF3238
Euxoa satis|[4212]|LOWCD491-06|CGWC-3311|658[0n]bp|Canada.British Columbia|BOLD:ACF3238
Euxoa satis|[4213]|LBCH6553-10|10-JDWBC-6553|658[0n]bp|Canada.British Columbia|BOLD:ACF3238
Euxoa satis|[4214]|LOWCD484-06|CGWC-3304|548[0n]bp|Canada.British Columbia|BOLD:ACF3238
Euxoa satis|[4215]|LPSK584-08|08BBLEP-02152|658[0n]bp|Canada.Saskatchewan|BOLD:ACF3238
Euxoa satis|[4216]|LBCH6022-10|10-JDWBC-6022|658[0n]bp|Canada.British Columbia|BOLD:ACF3238
Euxoa satis|[4217]|LBCC012-05|HLC-21892|658[0n]bp|Canada.British Columbia|BOLD:ACF3238
Euxoa satis|[4218]|LPMN930-08|08BBLEP-02288|658[0n]bp|Canada.Alberta|BOLD:ACF3238
Euxoa sp.|[4219]|LBCG1078-09|08-JDWBC-1078|658[0n]bp|Canada.British Columbia|BOLD:ACF3238
Euxoa sp.|[4220]|LBCG1070-09|08-JDWBC-1070|658[0n]bp|Canada.British Columbia|BOLD:ACF3238
Euxoa sp.|[4221]|LBCG1100-09|08-JDWBC-1100|658[0n]bp|Canada.British Columbia|BOLD:ACF3238
Euxoa brunneigera|[4222]|LBCH6753-10|10-JDWBC-6753|658[0n]bp|Canada.British Columbia|BOLD:ACF3238
Euxoa brunneigera|[4223]|LBCH5867-10|10-JDWBC-5867|658[0n]bp|Canada.British Columbia|BOLD:ACF0818
Euxoa agema|[4224]|RDNMF661-08|NOC14747|578[1n]bp|United States.Colorado|BOLD:ACE8316
Euxoa agema|[4225]|RDNMF662-08|NOC14748|609[0n]bp|United States.Colorado|BOLD:ACE8316
Euxoa agema|[4226]|IAWLB072-10|IAWAZ-0885|658[0n]bp|United States.California|BOLD:ACE8316
Euxoa agema|[4227]|RDNMF659-08|NOC14745|640[0n]bp|United States.California|BOLD:ACE8316
Euxoa agema|[4228]|IAWLB071-10|IAWAZ-0884|658[0n]bp|United States.California|BOLD:ACE8316
Euxoa agema|[4229]|RDNMF658-08|NOC14744|658[0n]bp|United States.Colorado|BOLD:ACE8316
Euxoa agema|[4230]|RDNMF660-08|NOC14746|544[0n]bp|United States.Colorado|BOLD:ACE8316
Euxoa oblongistigma|[4231]|RDNMF891-08|CNC LEP00053252|658[0n]bp|Canada.Alberta|BOLD:ACE8316
Euxoa oblongistigma|[4232]|RDMAB554-06|UASM58533|658[0n]bp|Canada.Alberta|BOLD:ACE8316
Euxoa oblongistigma|[4233]|RDMAB998-09|UASM2626|602[0n]bp|Canada.Alberta|BOLD:ACE8316
Euxoa oblongistigma|[4234]|RDMAB996-09|UASM2582|652[0n]bp|Canada.Alberta|BOLD:ACE8316
Euxoa oblongistigma|[4235]|RDMAB997-09|UASM2485|652[0n]bp|Canada.Alberta|BOLD:ACE8316
Euxoa oblongistigma|[4236]|LBCH6846-10|10-JDWBC-6846|658[0n]bp|Canada.British Columbia|BOLD:ACE8316
Euxoa oblongistigma|[4237]|LBCH7873-10|10-JDWBC-7873|658[0n]bp|Canada.British Columbia|BOLD:ACE8316
Euxoa oblongistigma|[4238]|LBCH7723-10|10-JDWBC-7723|658[0n]bp|Canada.British Columbia|BOLD:ACE8316
Euxoa oblongistigma|[4239]|LBCH6787-10|10-JDWBC-6787|658[0n]bp|Canada.British Columbia|BOLD:ACE8316
Euxoa oblongistigma|[4240]|LBCH7968-10|10-JDWBC-7968|658[0n]bp|Canada.British Columbia|BOLD:ACE8316
Euxoa intrita|[4241]|LBCH2088-10|10-JDWBC-2088|658[0n]bp|Canada.British Columbia|BOLD:ACE7161
Euxoa intrita|[4242]|RDNMG377-08|CNC LEP00052201|658[0n]bp|Canada.Saskatchewan|BOLD:ACE7161
Euxoa intrita|[4243]|LOWCD316-06|CGWC-3136|658[0n]bp|Canada.British Columbia|BOLD:ACE7161
Euxoa intrita|[4244]|BBLPB565-10|10BBCLP-1564|658[0n]bp|Canada.Alberta|BOLD:ACE7161
Euxoa intrita|[4245]|LOWCD317-06|CGWC-3137|592[0n]bp|Canada.British Columbia|BOLD:ACE7161
Euxoa intrita|[4246]|LOWCD311-06|CGWC-3131|580[1n]bp|Canada.British Columbia|BOLD:ACE7161
Euxoa intrita|[4247]|LOWCD319-06|CGWC-3139|609[0n]bp|Canada.British Columbia|BOLD:ACE7161
Euxoa intrita|[4248]|LOWCD312-06|CGWC-3132|594[0n]bp|Canada.British Columbia|BOLD:ACE7161
Euxoa intrita|[4249]|LOWCD314-06|CGWC-3134|599[0n]bp|Canada.British Columbia|BOLD:ACE7161
Euxoa intrita|[4250]|LOWCD318-06|CGWC-3138|608[0n]bp|Canada.British Columbia|BOLD:ACE7161
Euxoa intrita|[4251]|RDNMF028-08|NOC14114|658[0n]bp|Canada.Alberta|BOLD:ACE7161
Euxoa intrita|[4252]|LOWCD313-06|CGWC-3133|586[2n]bp|Canada.British Columbia|BOLD:ACE7161
Euxoa intrita|[4253]|LOWCD315-06|CGWC-3135|580[0n]bp|Canada.British Columbia|BOLD:ACE7161
Euxoa intrita|[4254]|LOWCD320-06|CGWC-3140|607[0n]bp|Canada.British Columbia|BOLD:ACE7161
Euxoa intrita|[4255]|RDNMF895-08|CNC LEP00053256|658[0n]bp|Canada.British Columbia|BOLD:ACE7161
Euxoa unica|[4256]|RDNMF698-08|NOC14784|658[0n]bp|Canada.Saskatchewan|BOLD:ACE7161
Euxoa silens|[4257]|LOWCD329-06|CGWC-3149|557[0n]bp|Canada.British Columbia|BOLD:AAC7725
Euxoa silens|[4258]|LOWCD327-06|CGWC-3147|601[0n]bp|Canada.British Columbia|BOLD:AAC7725
Euxoa silens|[4259]|LOWCD330-06|CGWC-3150|612[0n]bp|Canada.British Columbia|BOLD:AAC7725
Euxoa silens|[4260]|RDNMF308-08|NOC14394|658[0n]bp|Canada.British Columbia|BOLD:AAC7725
Euxoa silens|[4261]|LBCH5824-10|10-JDWBC-5824|658[0n]bp|Canada.British Columbia|BOLD:AAC7725
Euxoa silens|[4262]|LOWCD328-06|CGWC-3148|658[0n]bp|Canada.British Columbia|BOLD:AAC7725
Euxoa silens|[4263]|LBCH5718-10|10-JDWBC-5718|658[0n]bp|Canada.British Columbia|BOLD:AAC7725
Euxoa silens|[4264]|LBCH5729-10|10-JDWBC-5729|658[0n]bp|Canada.British Columbia|BOLD:AAC7725
Euxoa silens|[4265]|LBCH6058-10|10-JDWBC-6058|658[0n]bp|Canada.British Columbia|BOLD:AAC7725
Euxoa silens|[4266]|LOWCD326-06|CGWC-3146|658[0n]bp|Canada.British Columbia|BOLD:AAC7725
Euxoa bostoniensis|[4267]|RDMAB970-09|UASM113669|644[0n]bp|Canada.Ontario|BOLD:ACE5950
Euxoa bostoniensis|[4268]|RDMAB972-09|UASM113671|635[0n]bp|Canada.Ontario|BOLD:ACE5950
Euxoa bostoniensis|[4269]|RDMAB971-09|UASM113673|614[0n]bp|Canada.Ontario|BOLD:ACE5950
Euxoa bostoniensis|[4270]|PHMO387-03|moth2640.02|639[0n]bp|Canada.Ontario|BOLD:ACE5950
Euxoa medialis|[4271]|RDMAB503-06|UASM41369|658[0n]bp|Canada.Alberta|BOLD:AAE0848
Euxoa medialis|[4272]|RDMAB504-06|UASM41370|643[0n]bp|Canada.Alberta|BOLD:AAE0848
Euxoa siccata|[4273]|RDMAB706-06|UASM57914|658[0n]bp|Canada.Alberta|BOLD:AAE6721
Euxoa siccata|[4274]|RDMAB707-06|UASM57924|608[0n]bp|Canada.Alberta|BOLD:AAE6721
Euxoa siccata|[4275]|RDMAB708-06|UASM57922|658[0n]bp|Canada.Alberta|BOLD:AAE6721
Euxoa siccata|[4276]|RDMAB705-06|UASM57923|578[0n]bp|Canada.Alberta|BOLD:AAE6721
Euxoa simulata|[4277]|RDNMG641-08|CNC LEP00052465|658[0n]bp|Canada.British Columbia|BOLD:AAD7748
Euxoa tessellata|[4278]|LBCG1101-09|08-JDWBC-1101|658[0n]bp|Canada.British Columbia|BOLD:ACF2025
Euxoa tessellata|[4279]|LBCG1099-09|08-JDWBC-1099|658[0n]bp|Canada.British Columbia|BOLD:ACF2025
Euxoa tessellata|[4280]|LBCH5810-10|10-JDWBC-5810|658[0n]bp|Canada.British Columbia|BOLD:ACF2025
Euxoa tessellata|[4281]|LBCH5856-10|10-JDWBC-5856|658[0n]bp|Canada.British Columbia|BOLD:ACF2025
Euxoa tessellata|[4282]|LBCH7686-10|10-JDWBC-7686|658[0n]bp|Canada.British Columbia|BOLD:ACF2025
Euxoa tessellata|[4283]|LBCG2888-09|08-JDWBC-2888|658[0n]bp|Canada.British Columbia|BOLD:ACF2025
Euxoa tessellata|[4284]|LBCH5914-10|10-JDWBC-5914|658[0n]bp|Canada.British Columbia|BOLD:ACF2025
Euxoa tessellata|[4285]|LOWCE780-06|CGWC-4540|658[0n]bp|Canada.British Columbia|BOLD:ACF2025
Euxoa tessellata|[4286]|LOWCE774-06|CGWC-4534|658[0n]bp|Canada.British Columbia|BOLD:ACF2025

Euxoa tessellata|[4284]|LBCH5914-10|10-JDWBC-5914|658[0n]bp|Canada.British Columbia|BOLD:ACF2025
Euxoa tessellata|[4285]|LOWCE780-06|CGWC-4540|658[0n]bp|Canada.British Columbia|BOLD:ACF2025
Euxoa tessellata|[4286]|LOWCE774-06|CGWC-4534|658[0n]bp|Canada.British Columbia|BOLD:ACF2025
Euxoa tessellata|[4287]|LOWCE783-06|CGWC-4543|658[0n]bp|Canada.British Columbia|BOLD:ACF2025
Euxoa tessellata|[4288]|LOWCE779-06|CGWC-4539|658[0n]bp|Canada.British Columbia|BOLD:ACF2025
Euxoa tessellata|[4289]|LBCH6756-10|10-JDWBC-6756|658[0n]bp|Canada.British Columbia|BOLD:ACF2025
Euxoa tessellata|[4290]|LOWCE777-06|CGWC-4537|575[0n]bp|Canada.British Columbia|BOLD:ACF2025
Euxoa tessellata|[4291]|LBCH6541-10|10-JDWBC-6541|658[0n]bp|Canada.British Columbia|BOLD:ACF2025
Euxoa tessellata|[4292]|RDNMB625-05|CNCNoctuoidea10401|551[0n]bp|Canada.British Columbia|BOLD:ACF2025
Euxoa tessellata|[4293]|LOWCE781-06|CGWC-4541|658[0n]bp|Canada.British Columbia|BOLD:ACF2025
Euxoa tessellata|[4294]|LBCH7804-10|10-JDWBC-7804|658[0n]bp|Canada.British Columbia|BOLD:ACF2025
Euxoa tessellata|[4295]|LBCH6376-10|10-JDWBC-6376|658[0n]bp|Canada.British Columbia|BOLD:ACF2025
Euxoa tessellata|[4296]|LOWCE782-06|CGWC-4542|658[0n]bp|Canada.British Columbia|BOLD:ACF2025
Euxoa tessellata|[4297]|LBCH6156-10|10-JDWBC-6156|658[0n]bp|Canada.British Columbia|BOLD:ACF2025
Euxoa tessellata|[4298]|LOWCE323-06|CGWC-4083|658[0n]bp|Canada.British Columbia|BOLD:ACF2025
Euxoa tessellata|[4299]|BBLPB344-10|10BBCLP-1343|577[0n]bp|Canada.British Columbia|BOLD:ACF2025
Euxoa tessellata|[4300]|LBCH6023-10|10-JDWBC-6023|658[0n]bp|Canada.British Columbia|BOLD:ACF2025
Euxoa tessellata|[4301]|BBLPB349-10|10BBCLP-1348|658[0n]bp|Canada.Alberta|BOLD:ACF2025
Euxoa tessellata|[4302]|LBCG503-08|08-JDWBC-0503|658[0n]bp|Canada.British Columbia|BOLD:ACF2025
Euxoa tessellata|[4303]|LBCH5863-10|10-JDWBC-5863|658[0n]bp|Canada.British Columbia|BOLD:ACF2025
Euxoa tessellata|[4304]|LBCA608-05|HLC-20608|633[0n]bp|Canada.British Columbia|BOLD:ACF2025
Euxoa tessellata|[4305]|LBCH6744-10|10-JDWBC-6744|658[0n]bp|Canada.British Columbia|BOLD:ACF2025
Euxoa tessellata|[4306]|LBCH5597-10|10-JDWBC-5597|658[0n]bp|Canada.British Columbia|BOLD:ACF2025
Euxoa tessellata|[4307]|LOWCE778-06|CGWC-4538|658[0n]bp|Canada.British Columbia|BOLD:ACF2025
Euxoa tessellata|[4308]|LBCG2880-09|08-JDWBC-2880|658[0n]bp|Canada.British Columbia|BOLD:ACF2025
Euxoa tessellata|[4309]|BBLPB863-10|10BBCLP-1862|658[0n]bp|Canada.British Columbia|BOLD:ACF2025
Euxoa tessellata|[4310]|LBCG482-08|08-JDWBC-0482|658[0n]bp|Canada.British Columbia|BOLD:ACF2025
Euxoa tessellata|[4311]|LOWCE776-06|CGWC-4536|658[0n]bp|Canada.British Columbia|BOLD:ACF2025
Euxoa tessellata|[4312]|XAJ847-06|2006-ONT-0847|658[0n]bp|Canada.Ontario|BOLD:ACF2025
Euxoa tessellata|[4313]|XAJ838-06|2006-ONT-0838|658[0n]bp|Canada.Ontario|BOLD:ACF2025
Euxoa tessellata|[4314]|XAJ819-06|2006-ONT-0819|658[0n]bp|Canada.Ontario|BOLD:ACF2025
Euxoa tessellata|[4315]|RDLQF833-06|DH011986|658[0n]bp|Canada.Quebec|BOLD:ACF2025
Euxoa tessellata|[4316]|RDLQB421-05|DH010507|590[0n]bp|Canada.Quebec|BOLD:ACF2025
Euxoa tessellata|[4317]|RDNMB630-05|CNCNoctuoidea10406|601[0n]bp|Canada.New Brunswick|BOLD:ACF2025
Euxoa tessellata|[4318]|RDMAB720-06|UASM41340|658[0n]bp|Canada.Alberta|BOLD:ACF2025
Euxoa tessellata|[4319]|LCHIP091-07|06-PROBE-2572|650[0n]bp|Canada.Manitoba|BOLD:ACF2025
Euxoa tessellata|[4320]|RDNMB628-05|CNCNoctuoidea10404|589[0n]bp|Canada.Alberta|BOLD:ACF2025
Euxoa tessellata|[4321]|LPSK544-08|08BBLEP-02112|658[0n]bp|Canada.Saskatchewan|BOLD:ACF2025
Euxoa tessellata|[4322]|LPSK126-08|08BBLEP-01694|658[0n]bp|Canada.Saskatchewan|BOLD:ACF2025
Euxoa tessellata|[4323]|RDLQF140-06|CB0055|601[0n]bp|Canada.Quebec|BOLD:ACF2025
Euxoa tessellata|[4324]|RDNMB627-05|CNCNoctuoidea10403|595[0n]bp|Canada.Alberta|BOLD:ACF2025
Euxoa tessellata|[4325]|RDMAB721-06|UASM58209|558[0n]bp|Canada.Manitoba|BOLD:ACF2025
Euxoa tessellata|[4326]|XAB174-04|04HBL005174|658[0n]bp|Canada.Ontario|BOLD:ACF2025
Euxoa tessellata|[4327]|LPSK113-08|08BBLEP-01681|658[0n]bp|Canada.Saskatchewan|BOLD:ACF2025
Euxoa tessellata|[4328]|LPSK502-08|08BBLEP-02070|658[0n]bp|Canada.Saskatchewan|BOLD:ACF2025
Euxoa tessellata|[4329]|LPSK542-08|08BBLEP-02110|658[0n]bp|Canada.Saskatchewan|BOLD:ACF2025
Euxoa tessellata|[4330]|LPABB827-09|08BBLEP-04147|636[0n]bp|Canada.Alberta|BOLD:ACF2025
Euxoa tessellata|[4331]|RDMAB722-06|UASM2236|636[0n]bp|Canada.Alberta|BOLD:ACF2025
Euxoa tessellata|[4332]|LPSK512-08|08BBLEP-02080|658[0n]bp|Canada.Saskatchewan|BOLD:ACF2025
Euxoa tessellata|[4333]|BBLPB855-10|10BBCLP-1854|658[0n]bp|Canada.Alberta|BOLD:ACF2025
Euxoa tessellata|[4334]|LPSK033-08|08BBLEP-00736|658[0n]bp|Canada.Saskatchewan|BOLD:ACF2025
Euxoa tessellata|[4335]|LPSK236-08|08BBLEP-01804|658[0n]bp|Canada.Saskatchewan|BOLD:ACF2025
Euxoa tessellata|[4336]|BBLPB347-10|10BBCLP-1346|658[0n]bp|Canada.Alberta|BOLD:ACF2025
Euxoa tessellata|[4337]|BBLPB548-10|10BBCLP-1547|658[0n]bp|Canada.Alberta|BOLD:ACF2025
Euxoa tessellata|[4338]|RDNMH399-09|CNCLEP00054413|658[0n]bp|Canada.Alberta|BOLD:ACF2025
Euxoa tessellata|[4339]|RDLQG052-06|DH012183|658[0n]bp|Canada.Quebec|BOLD:ACF2025
Euxoa tessellata|[4340]|LPSK116-08|08BBLEP-01684|658[0n]bp|Canada.Saskatchewan|BOLD:ACF2025
Euxoa tessellata|[4341]|BBLPB337-10|10BBCLP-1336|658[0n]bp|Canada.Alberta|BOLD:ACF2025
Euxoa tessellata|[4342]|RDLQF141-06|CB0056|658[0n]bp|Canada.Quebec|BOLD:ACF2025
Euxoa tessellata|[4343]|XAB135-04|04HBL005135|658[0n]bp|Canada.Ontario|BOLD:ACF2025
Euxoa tessellata|[4344]|RDLQG189-06|DH012363|658[0n]bp|Canada.Quebec|BOLD:ACF2025
Euxoa tessellata|[4345]|RDNMB631-05|CNCNoctuoidea10407|658[0n]bp|Canada.Saskatchewan|BOLD:ACF2025
Euxoa tessellata|[4346]|RDNMB629-05|CNCNoctuoidea10405|658[0n]bp|Canada.Saskatchewan|BOLD:ACF2025
Euxoa tessellata|[4347]|BBLPB728-10|10BBCLP-1727|658[0n]bp|Canada.Alberta|BOLD:ACF2025
Euxoa tessellata|[4348]|XAJ982-06|2006-ONT-0982|658[0n]bp|Canada.Ontario|BOLD:ACF2025
Euxoa tessellata|[4349]|RDLQB434-05|DH010520|658[0n]bp|Canada.Quebec|BOLD:ACF2025
Euxoa tessellata|[4350]|LPSK576-08|08BBLEP-02144|658[0n]bp|Canada.Saskatchewan|BOLD:ACF2025
Euxoa tessellata|[4351]|RDLQF562-06|DH011711|658[0n]bp|Canada.Quebec|BOLD:ACF2025
Euxoa tessellata|[4352]|XAE585-04|Moth4585.03|567[0n]bp|Canada.Ontario|BOLD:ACF2025
Euxoa tessellata|[4353]|RDNM806-05|CNCNoctuoidea7652|559[1n]bp|Canada.Ontario|BOLD:ACF2025
Euxoa tessellata|[4354]|BLTIB733-08|BL1023|658[0n]bp|Canada.Ontario|BOLD:ACF2025
Euxoa tessellata|[4355]|RDLQB425-05|DH010511|658[0n]bp|Canada.Quebec|BOLD:ACF2025
Euxoa tessellata|[4356]|RDLQG025-06|DH012156|658[0n]bp|Canada.Quebec|BOLD:ACF2025
Euxoa tessellata|[4357]|BLTIB580-08|BL859|658[0n]bp|Canada.Ontario|BOLD:ACF2025
Euxoa tessellata|[4358]|XAJ820-06|2006-ONT-0820|658[0n]bp|Canada.Ontario|BOLD:ACF2025
Euxoa tessellata|[4359]|RDLQB433-05|DH010519|658[0n]bp|Canada.Quebec|BOLD:ACF2025
Euxoa tessellata|[4360]|RDMAB719-06|UASM2484|598[0n]bp|Canada.Alberta|BOLD:ACF2025
Euxoa tessellata|[4361]|XAC052-04|04HBL006052|598[0n]bp|Canada.Ontario|BOLD:ACF2025
Euxoa tessellata|[4362]|RDMAB723-06|UASM34962|570[0n]bp|Canada.Alberta|BOLD:ACF2025
Euxoa tessellata|[4363]|XAC853-04|04HBL006853|591[0n]bp|Canada.Ontario|BOLD:ACF2025
Euxoa pleuritica|[4364]|RDNMF880-08|CNC LEP00053241|658[0n]bp|Canada.Alberta|BOLD:ACE5988
Euxoa pleuritica|[4365]|RDNMF881-08|CNC LEP00053242|658[0n]bp|Canada.Alberta|BOLD:ACE5988
Euxoa pleuritica|[4366]|RDMAB489-06|UASM77998|658[0n]bp|Canada.Alberta|BOLD:ACE5988
Euxoa pleuritica|[4367]|RDNMF882-08|CNC LEP00053243|658[0n]bp|Canada.Alberta|BOLD:ACE5988
Euxoa pestula|[4368]|RDMAB981-09|UASM24563|635[0n]bp|Canada.Alberta|BOLD:ACE5988
Euxoa pestula|[4369]|RDMAB980-09|UASM57917|635[0n]bp|Canada.Alberta|BOLD:ACE5988
Euxoa pestula|[4370]|RDNMF039-08|NOC14125|658[0n]bp|Canada.Alberta|BOLD:ACE5988
Euxoa pestula|[4371]|RDMAB979-09|UASM19975|632[0n]bp|Canada.Alberta|BOLD:ACE5988
Euxoa simona|[4372]|IAWLB124-10|IAWAZ-0914|658[0n]bp|United States.California|BOLD:ACE5988
Euxoa simona|[4373]|RDNMG670-08|CNC LEP00052494|658[0n]bp|United States.Oregon|BOLD:ACE5988
Euxoa simona|[4374]|RDNMG1041-08|CNC LEP00053165|643[0n]bp|United States.Oregon|BOLD:ACE5988
Euxoa simona|[4375]|RDNMF653-08|NOC14739|658[0n]bp|United States.California|BOLD:ACE5988
Euxoa simona|[4376]|RDNMF654-08|NOC14740|658[0n]bp|United States.Oregon|BOLD:ACE5988
Euxoa pleuritica|[4377]|RDNM807-05|CNCNoctuoidea7653|658[0n]bp|Canada.Ontario|BOLD:ACE5988
Euxoa macleani|[4378]|RDNMF856-08|CNC LEP00053217|658[0n]bp|Canada.British Columbia|BOLD:ACE5988
Euxoa apopsis|[4379]|RDNMH1033-09|CNCLEP00068035|658[0n]bp|Canada.British Columbia|BOLD:ACE5988
Euxoa macleani|[4380]|RDNM997-05|CNCNoctuoidea7837|658[0n]bp|Canada.British Columbia|BOLD:ACE5988
Euxoa macleani|[4381]|RDNMF853-08|CNC LEP00053214|658[0n]bp|Canada.British Columbia|BOLD:ACE5988
Euxoa macleani|[4382]|RDNM996-05|CNCNoctuoidea7836|658[0n]bp|Canada.British Columbia|BOLD:ACE5988
Euxoa macleani|[4383]|RDNMH1032-09|CNCLEP00031848|658[0n]bp|Canada.British Columbia|BOLD:ACE5988
Euxoa macleani|[4384]|RDNM999-05|CNCNoctuoidea7839|656[0n]bp|Canada.British Columbia|BOLD:ACE5988
Euxoa macleani|[4385]|RDNMF852-08|CNC LEP00053213|658[0n]bp|Canada.British Columbia|BOLD:ACE5988
Euxoa vallus|[4386]|RDNMB011-05|CNCNoctuoidea7851|658[0n]bp|Canada.British Columbia|BOLD:ACE5988

[illegible]
Euxoa macleani|[4384]|RDNM999-05|CNCNoctuoidea7839|656[0n]bp|Canada.British Columbia|BOLD:ACE5988
Euxoa macleani|[4385]|RDNMF852-08|CNC LEP00053213|658[0n]bp|Canada.British Columbia|BOLD:ACE5988
Euxoa vallus|[4386]|RDNMB011-05|CNCNoctuoidea7851|658[0n]bp|Canada.British Columbia|BOLD:ACE5988
Euxoa costata|[4387]|RDNMF838-08|CNC LEP00053199|658[0n]bp|Canada.British Columbia|BOLD:ACF2028
Euxoa scholastica|[4388]|XAJ818-06|2006-ONT-0818|658[0n]bp|Canada.Ontario|BOLD:ACF3210
Euxoa scholastica|[4389]|RDNMF701-08|NOC14787|658[0n]bp|Canada.Ontario|BOLD:ACF3210
Euxoa furtivus|[4390]|RDNMB663-05|CNCNoctuoidea10439|658[0n]bp|Canada.British Columbia|BOLD:ACF2028
Euxoa sp.|[4391]|LBCG189-08|08-JDWBC-0189|658[0n]bp|Canada.British Columbia|BOLD:ACF2028
Euxoa idahoensis group|[4392]|LBCH7952-10|10-JDWBC-7952|658[0n]bp|Canada.British Columbia|BOLD:ACF2028
Euxoa idahoensis|[4393]|RDNM330-05|CNCNoctuoidea6512|658[0n]bp|Canada.Alberta|BOLD:ACF2028
Euxoa idahoensis|[4394]|RDNM341-05|CNCNoctuoidea6523|658[0n]bp|Canada.Alberta|BOLD:ACF2028
Euxoa idahoensis|[4395]|RDNM328-05|CNCNoctuoidea6510|658[0n]bp|Canada.Alberta|BOLD:ACF2028
Euxoa idahoensis|[4396]|RDNM340-05|CNCNoctuoidea6522|578[0n]bp|Canada.Alberta|BOLD:ACF2028
Euxoa idahoensis|[4397]|BBLPB835-10|10BBCLP-1834|658[0n]bp|Canada.British Columbia|BOLD:ACF2028
Euxoa idahoensis|[4398]|LBCG194-08|08-JDWBC-0194|658[0n]bp|Canada.British Columbia|BOLD:ACF2028
Euxoa idahoensis|[4399]|RDNM331-05|CNCNoctuoidea6513|658[3n]bp|Canada.Alberta|BOLD:ACF2028
Euxoa idahoensis|[4400]|LBCH6505-10|10-JDWBC-6505|658[0n]bp|Canada.British Columbia|BOLD:ACF2028
Euxoa idahoensis|[4401]|LBCG3250-09|08-JDWBC-3250|638[0n]bp|Canada.British Columbia|BOLD:ACF2028
Euxoa idahoensis|[4402]|BBLPB836-10|10BBCLP-1835|658[0n]bp|Canada.British Columbia|BOLD:ACF2028
Euxoa idahoensis|[4403]|LBCG1069-09|08-JDWBC-1069|658[0n]bp|Canada.British Columbia|BOLD:ACF2028
Euxoa idahoensis|[4404]|LBCG2583-09|08-JDWBC-2583|658[0n]bp|Canada.British Columbia|BOLD:ACF2028
Euxoa idahoensis|[4405]|LBCG251-08|08-JDWBC-0251|658[0n]bp|Canada.British Columbia|BOLD:ACF2028
Euxoa idahoensis|[4406]|RDNMB319-05|CNCNoctuoidea10085|658[0n]bp|Canada.British Columbia|BOLD:ACF2028
Euxoa idahoensis|[4407]|BBLPB834-10|10BBCLP-1833|658[0n]bp|Canada.Alberta|BOLD:ACF2028
Euxoa idahoensis|[4408]|LBCH6004-10|10-JDWBC-6004|658[0n]bp|Canada.British Columbia|BOLD:ACF2028
Euxoa idahoensis|[4409]|LBCH6544-10|10-JDWBC-6544|658[0n]bp|Canada.British Columbia|BOLD:ACF2028
Euxoa idahoensis|[4410]|LBCH6230-10|10-JDWBC-6230|658[0n]bp|Canada.British Columbia|BOLD:ACF2028
Euxoa idahoensis|[4411]|LPAB226-08|08BBLEP-02548|658[0n]bp|Canada.Alberta|BOLD:ACF2028
Euxoa idahoensis|[4412]|LBCH6743-10|10-JDWBC-6743|658[0n]bp|Canada.British Columbia|BOLD:ACF2028
Euxoa idahoensis|[4413]|RDNMB659-05|CNCNoctuoidea10435|658[0n]bp|Canada.British Columbia|BOLD:ACF2028
Euxoa idahoensis|[4414]|LOWCD336-06|CGWC-3156|656[0n]bp|Canada.British Columbia|BOLD:ACF2028
Euxoa idahoensis|[4415]|LOWCD339-06|CGWC-3159|602[0n]bp|Canada.British Columbia|BOLD:ACF2028
Euxoa idahoensis|[4416]|LPABB823-09|08BBLEP-04143|658[0n]bp|Canada.Alberta|BOLD:ACF2028
Euxoa idahoensis|[4417]|RDNMB317-05|CNCNoctuoidea10083|597[1n]bp|Canada.British Columbia|BOLD:ACF2028
Euxoa idahoensis|[4418]|LOWCD341-06|CGWC-3161|598[0n]bp|Canada.British Columbia|BOLD:ACF2028
Euxoa idahoensis|[4419]|LOWCD343-06|CGWC-3163|658[0n]bp|Canada.British Columbia|BOLD:ACF2028
Euxoa idahoensis|[4420]|RDNMB679-05|CNCNoctuoidea10455|658[0n]bp|Canada.British Columbia|BOLD:ACF2028
Euxoa idahoensis|[4421]|RDNMB662-05|CNCNoctuoidea10438|658[0n]bp|Canada.British Columbia|BOLD:ACF2028
Euxoa idahoensis|[4422]|LBCG3268-09|08-JDWBC-3268|631[0n]bp|Canada.British Columbia|BOLD:ACF2028
Euxoa idahoensis|[4423]|RDNMB660-05|CNCNoctuoidea10436|616[0n]bp|Canada.British Columbia|BOLD:ACF2028
Euxoa idahoensis|[4424]|RDNM346-05|CNCNoctuoidea6528|658[0n]bp|Canada.British Columbia|BOLD:ACF2028
Euxoa idahoensis|[4425]|RDNMB661-05|CNCNoctuoidea10437|606[0n]bp|Canada.British Columbia|BOLD:ACF2028
Euxoa idahoensis|[4426]|LOWCD344-06|CGWC-3164|638[0n]bp|Canada.British Columbia|BOLD:ACF2028
Euxoa idahoensis|[4427]|LPABB875-09|08BBLEP-04195|658[0n]bp|Canada.Alberta|BOLD:ACF2028
Euxoa idahoensis|[4428]|RDNM329-05|CNCNoctuoidea6511|506[0n]bp|Canada.Alberta|
Euxoa idahoensis group|[4429]|RDNM337-05|CNCNoctuoidea6519|567[0n]bp|Canada.Saskatchewan|BOLD:ACF2028
Euxoa idahoensis group|[4430]|RDNM338-05|CNCNoctuoidea6520|535[0n]bp|Canada.Saskatchewan|BOLD:ACF2028
Euxoa idahoensis group|[4431]|RDNM339-05|CNCNoctuoidea6521|511[0n]bp|Canada.Saskatchewan|BOLD:ACF2028
Euxoa idahoensis group|[4432]|RDNMB315-05|CNCNoctuoidea10081|615[0n]bp|Canada.Alberta|BOLD:ACF2028
Euxoa idahoensis group|[4433]|RDNMB312-05|CNCNoctuoidea10078|658[0n]bp|Canada.Alberta|BOLD:ACF2028
Euxoa idahoensis group|[4434]|RDNMB675-05|CNCNoctuoidea10451|658[0n]bp|Canada.Alberta|BOLD:ACF2028
Euxoa idahoensis group|[4435]|RDNMB311-05|CNCNoctuoidea10077|599[0n]bp|Canada.Alberta|BOLD:ACF2028
Euxoa idahoensis group|[4436]|RDNMB310-05|CNCNoctuoidea10076|658[0n]bp|Canada.Alberta|BOLD:ACF2028
Euxoa castanea|[4437]|RDNMB324-05|CNCNoctuoidea10090|519[3n]bp|Canada.Alberta|BOLD:ACF2028
Euxoa castanea|[4438]|RDNMB329-05|CNCNoctuoidea10095|557[0n]bp|Canada.British Columbia|BOLD:ACF2028
Euxoa castanea|[4439]|RDNMB322-05|CNCNoctuoidea10088|549[0n]bp|Canada.British Columbia|BOLD:ACF2028
Euxoa castanea|[4440]|RDNM351-05|CNCNoctuoidea6533|567[0n]bp|Canada.British Columbia|BOLD:ACF2028
Euxoa castanea|[4441]|LOWCD393-06|CGWC-3213|613[0n]bp|Canada.British Columbia|BOLD:ACF2028
Euxoa castanea|[4442]|LOWCD396-06|CGWC-3216|607[0n]bp|Canada.British Columbia|BOLD:ACF2028
Euxoa castanea|[4443]|LOWCD392-06|CGWC-3212|609[0n]bp|Canada.British Columbia|BOLD:ACF2028
Euxoa castanea|[4444]|LOWCD402-06|CGWC-3222|612[0n]bp|Canada.British Columbia|BOLD:ACF2028
Euxoa castanea|[4445]|LOWCD404-06|CGWC-3224|606[0n]bp|Canada.British Columbia|BOLD:ACF2028
Euxoa castanea|[4446]|LOWCD405-06|CGWC-3225|605[0n]bp|Canada.British Columbia|BOLD:ACF2028
Euxoa castanea|[4447]|BBLPB839-10|10BBCLP-1838|658[1n]bp|Canada.British Columbia|BOLD:ACF2028
Euxoa castanea|[4448]|LOWCD410-06|CGWC-3230|609[0n]bp|Canada.British Columbia|BOLD:ACF2028
Euxoa castanea|[4449]|RDNM352-05|CNCNoctuoidea6534|603[0n]bp|Canada.British Columbia|BOLD:ACF2028
Euxoa castanea|[4450]|LOWCD397-06|CGWC-3217|603[0n]bp|Canada.British Columbia|BOLD:ACF2028
Euxoa castanea|[4451]|RDNMB328-05|CNCNoctuoidea10094|599[0n]bp|Canada.British Columbia|BOLD:ACF2028
Euxoa castanea|[4452]|LOWCD408-06|CGWC-3228|599[0n]bp|Canada.British Columbia|BOLD:ACF2028
Euxoa castanea|[4453]|LOWCD403-06|CGWC-3223|600[0n]bp|Canada.British Columbia|BOLD:ACF2028
Euxoa castanea|[4454]|LOWCD395-06|CGWC-3215|599[0n]bp|Canada.British Columbia|BOLD:ACF2028
Euxoa castanea|[4455]|RDNMB323-05|CNCNoctuoidea10089|597[1n]bp|Canada.British Columbia|BOLD:ACF2028
Euxoa castanea|[4456]|LPABC159-09|08BBLEP-04378|653[2n]bp|Canada.Alberta|BOLD:ACF2028
Euxoa castanea|[4457]|LOWCD398-06|CGWC-3218|595[0n]bp|Canada.British Columbia|BOLD:ACF2028
Euxoa castanea|[4458]|LOWCD411-06|CGWC-3231|595[0n]bp|Canada.British Columbia|BOLD:ACF2028
Euxoa castanea|[4459]|LOWCD394-06|CGWC-3214|595[0n]bp|Canada.British Columbia|BOLD:ACF2028
Euxoa castanea|[4460]|RDNMB330-05|CNCNoctuoidea10096|595[1n]bp|Canada.British Columbia|BOLD:ACF2028
Euxoa castanea|[4461]|RDNMB326-05|CNCNoctuoidea10092|579[0n]bp|Canada.British Columbia|BOLD:ACF2028
Euxoa castanea|[4462]|LOWCD400-06|CGWC-3220|594[0n]bp|Canada.British Columbia|BOLD:ACF2028
Euxoa castanea|[4463]|BBLPB837-10|10BBCLP-1836|626[0n]bp|Canada.British Columbia|BOLD:ACF2028
Euxoa castanea|[4464]|LPABC341-09|08BBLEP-04560|636[0n]bp|Canada.Alberta|BOLD:ACF2028
Euxoa castanea|[4465]|LBCG3249-09|08-JDWBC-3249|638[0n]bp|Canada.British Columbia|BOLD:ACF2028
Euxoa castanea|[4466]|RDNMB325-05|CNCNoctuoidea10091|615[0n]bp|Canada.British Columbia|BOLD:ACF2028
Euxoa castanea|[4467]|LOWCD399-06|CGWC-3219|626[0n]bp|Canada.British Columbia|BOLD:ACF2028
Euxoa castanea|[4468]|BBLPB736-10|10BBCLP-1735|658[0n]bp|Canada.Alberta|BOLD:ACF2028
Euxoa castanea|[4469]|BBLPB840-10|10BBCLP-1839|658[0n]bp|Canada.Alberta|BOLD:ACF2028
Euxoa castanea|[4470]|BBLPB841-10|10BBCLP-1840|658[0n]bp|Canada.Alberta|BOLD:ACF2028
Euxoa castanea|[4471]|LBCH6365-10|10-JDWBC-6365|658[0n]bp|Canada.British Columbia|BOLD:ACF2028
Euxoa castanea|[4472]|LOWCD409-06|CGWC-3229|658[0n]bp|Canada.British Columbia|BOLD:ACF2028
Euxoa castanea|[4473]|LBCH7784-10|10-JDWBC-7784|658[0n]bp|Canada.British Columbia|BOLD:ACF2028
Euxoa castanea|[4474]|BBLPB843-10|10BBCLP-1842|658[0n]bp|Canada.Alberta|BOLD:ACF2028
Euxoa castanea|[4475]|LOWCD407-06|CGWC-3227|658[0n]bp|Canada.British Columbia|BOLD:ACF2028
Euxoa castanea|[4476]|LOWCD406-06|CGWC-3226|658[0n]bp|Canada.British Columbia|BOLD:ACF2028
Euxoa castanea|[4477]|BBLPB842-10|10BBCLP-1841|658[0n]bp|Canada.Alberta|BOLD:ACF2028
Euxoa castanea|[4478]|BBLPB838-10|10BBCLP-1837|658[0n]bp|Canada.British Columbia|BOLD:ACF2028
Euxoa castanea|[4479]|LPABC344-09|08BBLEP-04563|658[0n]bp|Canada.Alberta|BOLD:ACF2028
Euxoa castanea|[4480]|LOWCD401-06|CGWC-3221|619[0n]bp|Canada.British Columbia|BOLD:ACF2028
Euxoa furtivus|[4481]|RDNMB670-05|CNCNoctuoidea10446|658[0n]bp|Canada.British Columbia|BOLD:ACF2028
Euxoa castanea|[4482]|LPSK570-08|08BBLEP-02138|658[0n]bp|Canada.Saskatchewan|BOLD:ACF2028
Euxoa castanea|[4483]|RDNMB676-05|CNCNoctuoidea10452|658[0n]bp|Canada.British Columbia|BOLD:ACF2028
Euxoa castanea|[4484]|LBCG2876-09|08-JDWBC-2876|658[0n]bp|Canada.British Columbia|BOLD:ACF2028
Euxoa castanea|[4485]|RDNMB665-05|CNCNoctuoidea10441|658[0n]bp|Canada.Alberta|BOLD:ACF2028
[illegible]

Euxoa castanea|[4483]|RDNMB676-05|CNCNoctuoidea10452|658[0n]bp|Canada.British Columbia|BOLD:ACF2028
Euxoa castanea|[4484]|LBCG2876-09|08-JDWBC-2876|658[0n]bp|Canada.British Columbia|BOLD:ACF2028
Euxoa castanea|[4485]|RDNMB665-05|CNCNoctuoidea10441|658[0n]bp|Canada.Alberta|BOLD:ACF2028
Euxoa castanea|[4486]|LPSK505-08|08BBLEP-02073|658[0n]bp|Canada.Saskatchewan|BOLD:ACF2028
Euxoa castanea|[4487]|BBLPB832-10|10BBCLP-1831|658[0n]bp|Canada.Alberta|BOLD:ACF2028
Euxoa castanea|[4488]|LBCH7693-10|10-JDWBC-7693|658[0n]bp|Canada.British Columbia|BOLD:ACF2028
Euxoa castanea|[4489]|LOWCD335-06|CGWC-3155|657[0n]bp|Canada.British Columbia|BOLD:ACF2028
Euxoa castanea|[4490]|RDNMC332-05|CNCNoctuoidea11966|658[0n]bp|Canada.Alberta|BOLD:ACF2028
Euxoa castanea|[4491]|RDMAB495-06|UASM58436|658[0n]bp|Canada.Alberta|BOLD:ACF2028
Euxoa castanea|[4492]|LBCH6495-10|10-JDWBC-6495|658[0n]bp|Canada.British Columbia|BOLD:ACF2028
Euxoa castanea|[4493]|RDNMB668-05|CNCNoctuoidea10444|658[0n]bp|Canada.Alberta|BOLD:ACF2028
Euxoa castanea|[4494]|LBCH6492-10|10-JDWBC-6492|658[0n]bp|Canada.British Columbia|BOLD:ACF2028
Euxoa castanea|[4495]|LBCH6371-10|10-JDWBC-6371|658[0n]bp|Canada.British Columbia|BOLD:ACF2028
Euxoa castanea|[4496]|LOWCD334-06|CGWC-3154|606[0n]bp|Canada.British Columbia|BOLD:ACF2028
Euxoa castanea|[4497]|LOWCD331-06|CGWC-3151|610[0n]bp|Canada.British Columbia|BOLD:ACF2028
Euxoa castanea|[4498]|RDNMB680-05|CNCNoctuoidea10456|528[0n]bp|Canada.British Columbia|BOLD:ACF2028
Euxoa castanea|[4499]|BBLPB833-10|10BBCLP-1832|658[0n]bp|Canada.Alberta|BOLD:ACF2028
Euxoa castanea|[4500]|RDNMB669-05|CNCNoctuoidea10445|543[0n]bp|Canada.Alberta|BOLD:ACF2028
Euxoa castanea|[4501]|LOWCD332-06|CGWC-3152|549[0n]bp|Canada.British Columbia|BOLD:ACF2028
Euxoa castanea|[4502]|LOWCD337-06|CGWC-3157|597[0n]bp|Canada.British Columbia|BOLD:ACF2028
Euxoa castanea|[4503]|LOWCD340-06|CGWC-3160|601[0n]bp|Canada.British Columbia|BOLD:ACF2028
Euxoa nr. idahoensis sp. 4BC|[4504]|RDNM350-05|CNCNoctuoidea6532|658[0n]bp|Canada.British Columbia|BOLD...
Euxoa castanea|[4505]|LBCH6494-10|10-JDWBC-6494|658[0n]bp|Canada.British Columbia|BOLD:ACF2028
Euxoa castanea|[4506]|LBCH6020-10|10-JDWBC-6020|658[0n]bp|Canada.British Columbia|BOLD:ACF2028
Euxoa castanea|[4507]|LBCH6749-10|10-JDWBC-6749|658[0n]bp|Canada.British Columbia|BOLD:ACF2028
Euxoa sp.|[4508]|LBCG3312-09|08-JDWBC-3312|658[0n]bp|Canada.British Columbia|BOLD:ACF2028
Euxoa olivalis|[4509]|LBCH6277-10|10-JDWBC-6277|658[0n]bp|Canada.British Columbia|BOLD:ACE9577
Euxoa olivalis|[4510]|LBCG3254-09|08-JDWBC-3254|639[0n]bp|Canada.British Columbia|BOLD:ACE9577
Euxoa olivalis|[4511]|RDNMF898-08|CNC LEP00053259|658[0n]bp|Canada.British Columbia|BOLD:ACE9577
Euxoa olivalis|[4512]|LBCH6364-10|10-JDWBC-6364|658[0n]bp|Canada.British Columbia|BOLD:ACE9577
Euxoa olivalis|[4513]|LBCH6057-10|10-JDWBC-6057|658[0n]bp|Canada.British Columbia|BOLD:ACE9577
Euxoa olivalis|[4514]|LBCH6751-10|10-JDWBC-6751|658[0n]bp|Canada.British Columbia|BOLD:ACE9577
Euxoa olivalis|[4515]|LBCG3313-09|08-JDWBC-3313|658[0n]bp|Canada.British Columbia|BOLD:ACE9577
Euxoa olivalis|[4516]|LBCG2112-09|08-JDWBC-2112|658[0n]bp|Canada.British Columbia|BOLD:ACE9577
Euxoa olivalis|[4517]|LOWCE771-06|CGWC-4531|658[0n]bp|Canada.British Columbia|BOLD:ACE9577
Euxoa olivalis|[4518]|LPSK572-08|08BBLEP-02140|658[0n]bp|Canada.Saskatchewan|BOLD:ACE9577
Euxoa olivalis|[4519]|LPSK482-08|08BBLEP-02050|658[0n]bp|Canada.Saskatchewan|BOLD:ACE9577
Euxoa olivalis|[4520]|LBCH5554-10|10-JDWBC-5554|658[0n]bp|Canada.British Columbia|BOLD:ACE9577
Euxoa olivalis|[4521]|LBCH6455-10|10-JDWBC-6455|658[0n]bp|Canada.British Columbia|BOLD:ACE9577
Euxoa declarata|[4522]|LOWCE738-06|CGWC-4498|656[0n]bp|Canada.British Columbia|BOLD:ACF3242
Euxoa declarata|[4523]|LOWCE733-06|CGWC-4493|612[0n]bp|Canada.British Columbia|BOLD:ACF3242
Euxoa declarata|[4524]|LOWCE731-06|CGWC-4491|617[0n]bp|Canada.British Columbia|BOLD:ACF3242
Euxoa declarata|[4525]|LBCH6789-10|10-JDWBC-6789|658[0n]bp|Canada.British Columbia|BOLD:ACF3242
Euxoa declarata|[4526]|LOWCE737-06|CGWC-4497|613[0n]bp|Canada.British Columbia|BOLD:ACF3242
Euxoa declarata|[4527]|LOWCE730-06|CGWC-4490|608[0n]bp|Canada.British Columbia|BOLD:ACF3242
Euxoa declarata|[4528]|LBCH6842-10|10-JDWBC-6842|658[0n]bp|Canada.British Columbia|BOLD:ACF3242
Euxoa declarata|[4529]|LBCH7962-10|10-JDWBC-7962|658[0n]bp|Canada.British Columbia|BOLD:ACF3242
Euxoa declarata|[4530]|LBCH7544-10|10-JDWBC-7544|658[0n]bp|Canada.British Columbia|BOLD:ACF3242
Euxoa declarata|[4531]|LOWCE735-06|CGWC-4495|658[0n]bp|Canada.British Columbia|BOLD:ACF3242
Euxoa declarata|[4532]|LBCH1624-10|10-JDWBC-1624|658[0n]bp|Canada.British Columbia|BOLD:ACF3242
Euxoa campestris|[4533]|LOWCE740-06|CGWC-4500|618[0n]bp|Canada.British Columbia|BOLD:ACF3242
Euxoa campestris|[4534]|LOWCE736-06|CGWC-4496|619[0n]bp|Canada.British Columbia|BOLD:ACF3242
Euxoa campestris|[4535]|LOWCE741-06|CGWC-4501|617[0n]bp|Canada.British Columbia|BOLD:ACF3242
Euxoa campestris|[4536]|LOWCE734-06|CGWC-4494|614[0n]bp|Canada.British Columbia|BOLD:ACF3242
Euxoa campestris|[4537]|BBLPB865-10|10BBCLP-1864|658[0n]bp|Canada.Alberta|BOLD:ACF3242
Euxoa campestris|[4538]|LOWCE739-06|CGWC-4499|658[0n]bp|Canada.British Columbia|BOLD:ACF3242
Euxoa campestris|[4539]|RDLQF142-06|CB0057|658[0n]bp|Canada.Quebec|BOLD:ACF3242
Euxoa campestris|[4540]|LOWCE729-06|CGWC-4489|658[0n]bp|Canada.British Columbia|BOLD:ACF3242
Euxoa campestris|[4541]|RDLQF143-06|CB0058|658[0n]bp|Canada.Quebec|BOLD:ACF3242
Euxoa campestris|[4542]|LBCD457-05|HLC-23277|657[0n]bp|Canada.British Columbia|BOLD:ACF3242
Euxoa campestris|[4543]|RDLQF144-06|CB0059|658[0n]bp|Canada.Quebec|BOLD:ACF3242
Euxoa campestris|[4544]|BBLPB862-10|10BBCLP-1861|658[0n]bp|Canada.Alberta|BOLD:ACF3242
Euxoa campestris|[4545]|BBLPB730-10|10BBCLP-1729|658[0n]bp|Canada.Alberta|BOLD:ACF3242
Euxoa campestris|[4546]|LBCH7961-10|10-JDWBC-7961|658[0n]bp|Canada.British Columbia|BOLD:ACF3242
Euxoa campestris|[4547]|BBLPB729-10|10BBCLP-1728|658[0n]bp|Canada.Alberta|BOLD:ACF3242
Euxoa campestris|[4548]|BBLPB859-10|10BBCLP-1858|658[0n]bp|Canada.Alberta|BOLD:ACF3242
Euxoa campestris|[4549]|BBLPB864-10|10BBCLP-1863|658[0n]bp|Canada.Alberta|BOLD:ACF3242
Euxoa campestris|[4550]|RDLQ696-07|DH013506|658[0n]bp|Canada.Quebec|BOLD:ACF3242
Euxoa campestris|[4551]|RDLQB442-05|DH010528|658[0n]bp|Canada.Quebec|BOLD:ACF3242
Euxoa campestris|[4552]|BBLPC122-09|09BBELE-1122|649[0n]bp|Canada.New Brunswick|BOLD:ACF3242
Euxoa campestris|[4553]|RDLQ695-07|DH013504|658[3n]bp|Canada.Quebec|BOLD:ACF3242
Euxoa campestris|[4554]|RDLQF283-06|DH011375|658[0n]bp|Canada.Quebec|BOLD:ACF3242
Euxoa campestris|[4555]|RDLQF817-06|DH011970|658[0n]bp|Canada.Quebec|BOLD:ACF3242
Euxoa campestris|[4556]|BBLEC509-09|09BBELE-0509|658[0n]bp|Canada.New Brunswick|BOLD:ACF3242
Euxoa campestris|[4557]|RDLQ697-07|DH007931|658[0n]bp|Canada.Quebec|BOLD:ACF3242
Euxoa campestris|[4558]|LOWCE732-06|CGWC-4492|614[0n]bp|Canada.British Columbia|BOLD:ACF3242
Euxoa rockburnei|[4559]|LPVIC049-08|PFC-2006-2614|617[0n]bp|Canada.British Columbia|BOLD:ACF3242
Euxoa rockburnei|[4560]|RDNMF691-08|NOC14777|612[0n]bp|United States.California|BOLD:ACF3242
Euxoa rockburnei|[4561]|JMMMB382-11|BIOUG00851-A02|658[0n]bp|United States.California|BOLD:ACF3242
Euxoa rockburnei|[4562]|RDNMF689-08|NOC14775|658[0n]bp|United States.Washington|BOLD:ACF3242
Euxoa rockburnei|[4563]|RDNMF690-08|NOC14776|658[0n]bp|United States.Washington|BOLD:ACF3242
Euxoa rockburnei|[4564]|JMMMB376-11|BIOUG00850-H07|658[0n]bp|United States.California|BOLD:ACF3242
Euxoa rockburnei|[4565]|RDNMF692-08|NOC14778|658[0n]bp|United States.California|BOLD:ACF3242
Euxoa rockburnei|[4566]|LBCH7132-10|10-JDWBC-7132|658[0n]bp|Canada.British Columbia|BOLD:ACF3242
Euxoa rockburnei|[4567]|RDNMF688-08|NOC14774|609[0n]bp|United States.Washington|BOLD:ACF3242
Euxoa auripennis|[4568]|LOWCD427-06|CGWC-3247|530[0n]bp|Canada.British Columbia|BOLD:ACF3242
Euxoa auripennis|[4569]|LOWCD435-06|CGWC-3255|568[0n]bp|Canada.British Columbia|BOLD:ACF3242
Euxoa auripennis|[4570]|LOWCD438-06|CGWC-3258|532[0n]bp|Canada.British Columbia|BOLD:ACF3242
Euxoa auripennis|[4571]|LOWCD450-06|CGWC-3270|653[0n]bp|Canada.British Columbia|BOLD:ACF3242
Euxoa auripennis|[4572]|LOWCD432-06|CGWC-3252|617[0n]bp|Canada.British Columbia|BOLD:ACF3242
Euxoa auripennis|[4573]|LOWCD442-06|CGWC-3262|613[0n]bp|Canada.British Columbia|BOLD:ACF3242
Euxoa auripennis|[4574]|LOWCD446-06|CGWC-3266|611[0n]bp|Canada.British Columbia|BOLD:ACF3242
Euxoa auripennis|[4575]|LOWCD441-06|CGWC-3261|612[0n]bp|Canada.British Columbia|BOLD:ACF3242
Euxoa auripennis|[4576]|LOWCD430-06|CGWC-3250|611[0n]bp|Canada.British Columbia|BOLD:ACF3242
Euxoa auripennis|[4577]|LOWCD436-06|CGWC-3256|610[0n]bp|Canada.British Columbia|BOLD:ACF3242
Euxoa auripennis|[4578]|LOWCD440-06|CGWC-3260|608[0n]bp|Canada.British Columbia|BOLD:ACF3242
Euxoa auripennis|[4579]|LOWCD439-06|CGWC-3259|608[0n]bp|Canada.British Columbia|BOLD:ACF3242
Euxoa auripennis|[4580]|LBCH7048-10|10-JDWBC-7048|632[0n]bp|Canada.British Columbia|BOLD:ACF3242
Euxoa auripennis|[4581]|LOWCD431-06|CGWC-3251|602[0n]bp|Canada.British Columbia|BOLD:ACF3242
Euxoa auripennis|[4582]|LOWCD443-06|CGWC-3263|658[1n]bp|Canada.British Columbia|BOLD:ACF3242
Euxoa auripennis|[4583]|LOWCD429-06|CGWC-3249|600[0n]bp|Canada.British Columbia|BOLD:ACF3242
Euxoa auripennis|[4584]|LOWCD437-06|CGWC-3257|599[0n]bp|Canada.British Columbia|BOLD:ACF3242
Euxoa auripennis|[4585]|LBCH6785-10|10-JDWBC-6785|658[0n]bp|Canada.British Columbia|BOLD:ACF3242

Euxoa auripennis|[4583]|LOWCD429-06|CGWC-3249|600[0n]bp|Canada.British Columbia|BOLD:ACF3242
Euxoa auripennis|[4584]|LOWCD437-06|CGWC-3257|599[0n]bp|Canada.British Columbia|BOLD:ACF3242
Euxoa auripennis|[4585]|LBCH6785-10|10-JDWBC-6785|658[0n]bp|Canada.British Columbia|BOLD:ACF3242
Euxoa auripennis|[4586]|LBCH6487-10|10-JDWBC-6487|658[0n]bp|Canada.British Columbia|BOLD:ACF3242
Euxoa auripennis|[4587]|LBCH6500-10|10-JDWBC-6500|658[0n]bp|Canada.British Columbia|BOLD:ACF3242
Euxoa auripennis|[4588]|LBCH7967-10|10-JDWBC-7967|658[0n]bp|Canada.British Columbia|BOLD:ACF3242
Euxoa auripennis|[4589]|LBCH7687-10|10-JDWBC-7687|658[0n]bp|Canada.British Columbia|BOLD:ACF3242
Euxoa auripennis|[4590]|LBCH7549-10|10-JDWBC-7549|658[0n]bp|Canada.British Columbia|BOLD:ACF3242
Euxoa auripennis|[4591]|LBCH7969-10|10-JDWBC-7969|658[0n]bp|Canada.British Columbia|BOLD:ACF3242
Euxoa auripennis|[4592]|LBCH7787-10|10-JDWBC-7787|658[0n]bp|Canada.British Columbia|BOLD:ACF3242
Euxoa auripennis|[4593]|LBCH7965-10|10-JDWBC-7965|658[0n]bp|Canada.British Columbia|BOLD:ACF3242
Euxoa auripennis|[4594]|LBCH6491-10|10-JDWBC-6491|658[0n]bp|Canada.British Columbia|BOLD:ACF3242
Euxoa auripennis|[4595]|LOWCD447-06|CGWC-3267|658[0n]bp|Canada.British Columbia|BOLD:ACF3242
Euxoa auripennis|[4596]|LBCH6229-10|10-JDWBC-6229|658[0n]bp|Canada.British Columbia|BOLD:ACF3242
Euxoa auripennis|[4597]|LOWCD444-06|CGWC-3264|595[0n]bp|Canada.British Columbia|BOLD:ACF3242
Euxoa auripennis|[4598]|LOWCD798-06|CGWC-3618|592[0n]bp|Canada.British Columbia|BOLD:ACF3242
Euxoa auripennis|[4599]|LOWCD445-06|CGWC-3265|598[0n]bp|Canada.British Columbia|BOLD:ACF3242
Euxoa auripennis|[4600]|LOWCD448-06|CGWC-3268|586[0n]bp|Canada.British Columbia|BOLD:ACF3242
Euxoa auripennis|[4601]|LOWCD433-06|CGWC-3253|611[0n]bp|Canada.British Columbia|BOLD:ACF3242
Euxoa auripennis|[4602]|LBCH7783-10|10-JDWBC-7783|658[0n]bp|Canada.British Columbia|BOLD:ACF3242
Euxoa auripennis|[4603]|LOWCD434-06|CGWC-3254|531[0n]bp|Canada.British Columbia|BOLD:ACF3242
Euxoa auripennis|[4604]|LOWCD449-06|CGWC-3269|531[0n]bp|Canada.British Columbia|BOLD:ACF3242
Euxoa redimicula|[4605]|RDNMF867-08|CNC LEP00053228|658[0n]bp|Canada.Ontario|BOLD:ACF3242
Euxoa redimicula|[4606]|RDNMF869-08|CNC LEP00053230|658[0n]bp|Canada.Ontario|BOLD:ACF3242
Euxoa redimicula|[4607]|RDNMF870-08|CNC LEP00053231|658[0n]bp|Canada.Ontario|BOLD:ACF3242
Euxoa redimicula|[4608]|RDNMF868-08|CNC LEP00053229|658[0n]bp|Canada.Ontario|BOLD:ACF3242
Euxoa redimicula|[4609]|RDNMF290-08|NOC14376|658[0n]bp|Canada.Ontario|BOLD:ACF3242
Euxoa redimicula|[4610]|RDNMF291-08|NOC14377|658[0n]bp|Canada.Ontario|BOLD:ACF3242
Euxoa redimicula|[4611]|RDNMF289-08|NOC14375|658[0n]bp|Canada.Ontario|BOLD:ACF3242
Euxoa servitus|[4612]|LPABC084-09|08BBLEP-04303|658[0n]bp|Canada.Alberta|BOLD:ABZ9439
Euxoa servitus|[4613]|LPABC839-09|08BBLEP-05058|658[0n]bp|Canada.Alberta|BOLD:ABZ9439
Euxoa servitus|[4614]|BBLPB813-10|10BBCLP-1812|658[0n]bp|Canada.Alberta|BOLD:ABZ9439
Euxoa servitus|[4615]|LOWCD799-06|CGWC-3619|658[0n]bp|Canada.British Columbia|BOLD:ABZ9439
Euxoa servitus|[4616]|LPABC837-09|08BBLEP-05056|658[0n]bp|Canada.Alberta|BOLD:ABZ9439
Euxoa servitus|[4617]|BBLPB812-10|10BBCLP-1811|658[0n]bp|Canada.Alberta|BOLD:ABZ9439
Euxoa servitus|[4618]|LPABB844-09|08BBLEP-04164|658[0n]bp|Canada.Alberta|BOLD:ABZ9439
Euxoa servitus|[4619]|LPABC814-09|08BBLEP-05033|658[0n]bp|Canada.Alberta|BOLD:ABZ9439
Euxoa servitus|[4620]|LPAB087-08|08BBLEP-02409|658[0n]bp|Canada.Alberta|BOLD:ABZ9439
Euxoa servitus|[4621]|LOWCD855-06|CGWC-3675|607[0n]bp|Canada.British Columbia|BOLD:ABZ9439
Euxoa servitus|[4622]|LOWCD857-06|CGWC-3677|612[0n]bp|Canada.British Columbia|BOLD:ABZ9439
Euxoa servitus|[4623]|BBLPB816-10|10BBCLP-1815|658[0n]bp|Canada.Alberta|BOLD:ABZ9439
Euxoa servitus|[4624]|LPABC754-09|08BBLEP-04973|658[0n]bp|Canada.Alberta|BOLD:ACF3237
Euxoa servitus|[4625]|LPABC810-09|08BBLEP-05029|658[0n]bp|Canada.Alberta|BOLD:ACF3237
Euxoa servitus|[4626]|LPABC794-09|08BBLEP-05013|658[0n]bp|Canada.Alberta|BOLD:ACF3237
Euxoa servitus|[4627]|LPABC836-09|08BBLEP-05055|658[0n]bp|Canada.Alberta|BOLD:ACF3237
Euxoa servitus|[4628]|LPABC838-09|08BBLEP-05057|658[0n]bp|Canada.Alberta|BOLD:ACF3237
Euxoa servitus|[4629]|LPABC949-09|08BBLEP-05360|658[0n]bp|Canada.Alberta|BOLD:ACF3237
Euxoa servitus|[4630]|LBCH7543-10|10-JDWBC-7543|658[0n]bp|Canada.British Columbia|BOLD:ABZ9439
Euxoa servitus|[4631]|LBCG191-08|08-JDWBC-0191|658[0n]bp|Canada.British Columbia|BOLD:ABZ9439
Euxoa servitus|[4632]|LBCG197-08|08-JDWBC-0197|658[0n]bp|Canada.British Columbia|BOLD:ABZ9439
Euxoa servitus|[4633]|LBCG3258-09|08-JDWBC-3258|641[0n]bp|Canada.British Columbia|BOLD:ABZ9439
Euxoa servitus|[4634]|LBCG3267-09|08-JDWBC-3267|641[0n]bp|Canada.British Columbia|BOLD:ABZ9439
Euxoa servitus|[4635]|LBCG3272-09|08-JDWBC-3272|642[0n]bp|Canada.British Columbia|BOLD:ABZ9439
Euxoa servitus|[4636]|LBCG3270-09|08-JDWBC-3270|642[0n]bp|Canada.British Columbia|BOLD:ABZ9439
Euxoa servitus|[4637]|LBCG3263-09|08-JDWBC-3263|641[0n]bp|Canada.British Columbia|BOLD:ABZ9439
Euxoa servitus|[4638]|LBCG198-08|08-JDWBC-0198|658[0n]bp|Canada.British Columbia|BOLD:ABZ9439
Euxoa servitus|[4639]|LBCH6359-10|10-JDWBC-6359|658[0n]bp|Canada.British Columbia|BOLD:ABZ9439
Euxoa servitus|[4640]|LOWCE644-06|CGWC-4404|658[0n]bp|Canada.British Columbia|BOLD:ABZ9439
Euxoa servitus|[4641]|LBCH5958-10|10-JDWBC-5958|658[0n]bp|Canada.British Columbia|BOLD:ABZ9439
Euxoa servitus|[4642]|LBCH6490-10|10-JDWBC-6490|658[0n]bp|Canada.British Columbia|BOLD:ABZ9439
Euxoa servitus|[4643]|LBCG3314-09|08-JDWBC-3314|658[0n]bp|Canada.British Columbia|BOLD:ABZ9439
Euxoa servitus|[4644]|LBCG195-08|08-JDWBC-0195|658[0n]bp|Canada.British Columbia|BOLD:ABZ9439
Euxoa servitus|[4645]|LBCH6488-10|10-JDWBC-6488|658[0n]bp|Canada.British Columbia|BOLD:ABZ9439
Euxoa servitus|[4646]|LOWCD853-06|CGWC-3673|658[0n]bp|Canada.British Columbia|BOLD:ABZ9439
Euxoa servitus|[4647]|LBCH6499-10|10-JDWBC-6499|658[0n]bp|Canada.British Columbia|BOLD:ABZ9439
Euxoa servitus|[4648]|LOWCD800-06|CGWC-3620|658[0n]bp|Canada.British Columbia|BOLD:ABZ9439
Euxoa servitus|[4649]|LBCG3266-09|08-JDWBC-3266|658[0n]bp|Canada.British Columbia|BOLD:ABZ9439
Euxoa servitus|[4650]|LBCG3262-09|08-JDWBC-3262|658[0n]bp|Canada.British Columbia|BOLD:ABZ9439
Euxoa servitus|[4651]|BBLPB815-10|10BBCLP-1814|658[0n]bp|Canada.Alberta|BOLD:ABZ9439
Euxoa servitus|[4652]|LOWCE324-06|CGWC-4084|658[0n]bp|Canada.British Columbia|BOLD:ABZ9439
Euxoa servitus|[4653]|LBCG2894-09|08-JDWBC-2894|658[0n]bp|Canada.British Columbia|BOLD:ABZ9439
Euxoa servitus|[4654]|LBCG3260-09|08-JDWBC-3260|658[0n]bp|Canada.British Columbia|BOLD:ABZ9439
Euxoa servitus|[4655]|LBCH6489-10|10-JDWBC-6489|658[0n]bp|Canada.British Columbia|BOLD:ABZ9439
Euxoa servitus|[4656]|LBCH6360-10|10-JDWBC-6360|658[0n]bp|Canada.British Columbia|BOLD:ABZ9439
Euxoa servitus|[4657]|LBCH5957-10|10-JDWBC-5957|658[0n]bp|Canada.British Columbia|BOLD:ABZ9439
Euxoa servitus|[4658]|LBCH6356-10|10-JDWBC-6356|658[0n]bp|Canada.British Columbia|BOLD:ABZ9439
Euxoa servitus|[4659]|LOWCD852-06|CGWC-3672|658[0n]bp|Canada.British Columbia|BOLD:ABZ9439
Euxoa servitus|[4660]|LOWCE645-06|CGWC-4405|658[0n]bp|Canada.British Columbia|BOLD:ABZ9439
Euxoa servitus|[4661]|LBCH6355-10|10-JDWBC-6355|658[0n]bp|Canada.British Columbia|BOLD:ABZ9439
Euxoa servitus|[4662]|LBCH6484-10|10-JDWBC-6484|658[0n]bp|Canada.British Columbia|BOLD:ABZ9439
Euxoa servitus|[4663]|LBCH6362-10|10-JDWBC-6362|658[0n]bp|Canada.British Columbia|BOLD:ABZ9439
Euxoa servitus|[4664]|LBCG3259-09|08-JDWBC-3259|658[0n]bp|Canada.British Columbia|BOLD:ABZ9439
Euxoa servitus|[4665]|LBCG2893-09|08-JDWBC-2893|658[0n]bp|Canada.British Columbia|BOLD:ABZ9439
Euxoa servitus|[4666]|BBLPB814-10|10BBCLP-1813|658[0n]bp|Canada.Alberta|BOLD:ABZ9439
Euxoa servitus|[4667]|LBCG196-08|08-JDWBC-0196|658[0n]bp|Canada.British Columbia|BOLD:ABZ9439
Euxoa servitus|[4668]|LBCG3253-09|08-JDWBC-3253|658[0n]bp|Canada.British Columbia|BOLD:ABZ9439
Euxoa servitus|[4669]|LBCG3269-09|08-JDWBC-3269|658[0n]bp|Canada.British Columbia|BOLD:ABZ9439
Euxoa servitus|[4670]|LBCH6485-10|10-JDWBC-6485|658[0n]bp|Canada.British Columbia|BOLD:ABZ9439
Euxoa servitus|[4671]|LBCH6358-10|10-JDWBC-6358|658[0n]bp|Canada.British Columbia|BOLD:ABZ9439
Euxoa servitus|[4672]|LBCH5956-10|10-JDWBC-5956|658[0n]bp|Canada.British Columbia|BOLD:ABZ9439
Euxoa servitus|[4673]|LOWCE638-06|CGWC-4398|658[0n]bp|Canada.British Columbia|BOLD:ABZ9439
Euxoa servitus|[4674]|LBCG3256-09|08-JDWBC-3256|638[0n]bp|Canada.British Columbia|BOLD:ABZ9439
Euxoa servitus|[4675]|LBCG3265-09|08-JDWBC-3265|638[0n]bp|Canada.British Columbia|BOLD:ABZ9439
Euxoa servitus|[4676]|LBCG3317-09|08-JDWBC-3317|658[0n]bp|Canada.British Columbia|BOLD:ABZ9439
Euxoa servitus|[4677]|LBCG3255-09|08-JDWBC-3255|658[0n]bp|Canada.British Columbia|BOLD:ABZ9439
Euxoa servitus|[4678]|LBCG2111-09|08-JDWBC-2111|658[0n]bp|Canada.British Columbia|BOLD:ABZ9439
Euxoa servitus|[4679]|LBCG3261-09|08-JDWBC-3261|636[0n]bp|Canada.British Columbia|BOLD:ABZ9439
Euxoa servitus|[4680]|LBCG3264-09|08-JDWBC-3264|632[0n]bp|Canada.British Columbia|BOLD:ABZ9439
Euxoa servitus|[4681]|LBCG3271-09|08-JDWBC-3271|616[0n]bp|Canada.British Columbia|BOLD:ABZ9439
Euxoa servitus|[4682]|LOWCD854-06|CGWC-3674|608[1n]bp|Canada.British Columbia|BOLD:ABZ9439
Euxoa servitus|[4683]|LOWCE631-06|CGWC-4391|607[0n]bp|Canada.British Columbia|BOLD:ABZ9439
Euxoa servitus|[4684]|LOWCD851-06|CGWC-3671|579[0n]bp|Canada.British Columbia|BOLD:ABZ9439
Euxoa servitus|[4685]|LOWCD850-06|CGWC-3670|607[0n]bp|Canada.British Columbia|BOLD:ABZ9439

Euxoa servitus|[4682]|LOWCD854-06|CGWC-3674|[illegible]bp|Canada.British Columbia|BOLD:ABZ9439
Euxoa servitus|[4683]|LOWCE631-06|CGWC-4391|607[0n]bp|Canada.British Columbia|BOLD:ABZ9439
Euxoa servitus|[4684]|LOWCD851-06|CGWC-3671|579[0n]bp|Canada.British Columbia|BOLD:ABZ9439
Euxoa servitus|[4685]|LOWCD850-06|CGWC-3670|607[0n]bp|Canada.British Columbia|BOLD:ABZ9439
Euxoa servitus|[4686]|LBCH6486-10|10-JDWBC-6486|658[0n]bp|Canada.British Columbia|BOLD:ABZ9439
Euxoa servitus|[4687]|LOWCD856-06|CGWC-3676|585[0n]bp|Canada.British Columbia|BOLD:ABZ9439
Euxoa servitus|[4688]|LOWCD858-06|CGWC-3678|610[0n]bp|Canada.British Columbia|BOLD:ABZ9439
Euxoa servitus|[4689]|LBCG192-08|08-JDWBC-0192|658[0n]bp|Canada.British Columbia|BOLD:ABZ9439
Euxoa servitus|[4690]|LOWCD859-06|CGWC-3679|658[0n]bp|Canada.British Columbia|BOLD:ABZ9439
Euxoa servitus|[4691]|LBCG2110-09|08-JDWBC-2110|658[0n]bp|Canada.British Columbia|BOLD:ABZ9439
Euxoa servitus|[4692]|LBCG2892-09|08-JDWBC-2892|658[0n]bp|Canada.British Columbia|BOLD:ABZ9439
Euxoa clausa|[4693]|RDNMF699-08|NOC14785|658[0n]bp|United States.Colorado|BOLD:ACF2028
Euxoa aurulenta|[4694]|RDNM284-05|CNCNoctuoidea6466|658[0n]bp|Canada.Saskatchewan|BOLD:AAC8211
Euxoa aurulenta|[4695]|RDNM285-05|CNCNoctuoidea6467|658[0n]bp|Canada.Saskatchewan|BOLD:AAC8211
Euxoa aurulenta|[4696]|RDNM286-05|CNCNoctuoidea6468|658[0n]bp|Canada.Saskatchewan|BOLD:AAC8211
Euxoa niveilinea|[4697]|RDNMJ121-10|CNCLEP 70149|658[0n]bp|United States.New Mexico|BOLD:ABX5403
Euxoa niveilinea|[4698]|RDNMF088-08|NOC14174|658[0n]bp|United States.Colorado|BOLD:AAE6689
Euxoa niveilinea|[4699]|RDNMF016-08|NOC14102|658[0n]bp|United States.Colorado|BOLD:AAE6689
Euxoa niveilinea|[4700]|RDNMG417-08|CNC LEP00052241|658[0n]bp|United States.Colorado|BOLD:AAE6689
Euxoa niveilinea|[4701]|RDNMG418-08|CNC LEP00052242|658[0n]bp|United States.Colorado|BOLD:AAE6689
Euxoa olivia|[4702]|LBCH7228-10|10-JDWBC-7228|658[0n]bp|Canada.British Columbia|BOLD:AAD3112
Euxoa olivia|[4703]|LBCH7314-10|10-JDWBC-7314|658[0n]bp|Canada.British Columbia|BOLD:AAD3112
Euxoa olivia|[4704]|LOWCE665-06|CGWC-4425|658[0n]bp|Canada.British Columbia|BOLD:AAD3112
Euxoa olivia|[4705]|LALPA816-10|AVBC 818-10|658[0n]bp|Canada.British Columbia|BOLD:AAD3112
Euxoa vetusta|[4706]|LALPA1244-11|AVBC 1246-11|658[0n]bp|Canada.British Columbia|BOLD:ABZ9012
Euxoa vetusta|[4707]|LALPA1269-11|AVBC 1271-11|658[0n]bp|Canada.British Columbia|BOLD:ABZ9012
Euxoa vetusta|[4708]|LALPA1293-11|AVBC 1295-11|658[0n]bp|Canada.British Columbia|BOLD:ABZ9012
Euxoa vetusta|[4709]|LALPA1247-11|AVBC 1249-11|658[0n]bp|Canada.British Columbia|BOLD:ABZ9012
Euxoa vetusta|[4710]|RDNMF301-08|NOC14387|658[0n]bp|Canada.British Columbia|BOLD:ABZ9012
Euxoa vetusta|[4711]|LBCW056-08|08-JDWWI-0056|658[0n]bp|Canada.British Columbia|BOLD:ABZ9012
Euxoa vetusta|[4712]|RDNMF300-08|NOC14386|658[0n]bp|Canada.British Columbia|BOLD:ABZ9012
Euxoa adumbrata|[4713]|LBCH7004-10|10-JDWBC-7004|658[0n]bp|Canada.British Columbia|BOLD:ACF0816
Euxoa adumbrata|[4714]|LBCH6829-10|10-JDWBC-6829|658[0n]bp|Canada.British Columbia|BOLD:ACF0816
Euxoa adumbrata|[4715]|LCHQ094-07|07PROBE-10855|658[0n]bp|Canada.Manitoba|BOLD:ACF0816
Euxoa adumbrata|[4716]|LPABB357-08|08BBLEP-03622|658[0n]bp|Canada.Alberta|BOLD:ACF0816
Euxoa adumbrata|[4717]|LCHP751-07|07PROBE-10436|655[0n]bp|Canada.Manitoba|BOLD:ACF0816
Euxoa adumbrata|[4718]|LCH262-04|04HBL003262|658[0n]bp|Canada.Manitoba|BOLD:ACF0816
Euxoa adumbrata|[4719]|LCHP827-07|07PROBE-10584|658[0n]bp|Canada.Manitoba|BOLD:ACF0816
Euxoa adumbrata|[4720]|BBLPB750-10|10BBCLP-1749|658[0n]bp|Canada.Alberta|BOLD:ACF0816
Euxoa adumbrata|[4721]|LCHQ141-07|07PROBE-10910|658[0n]bp|Canada.Manitoba|BOLD:ACF0816
Euxoa adumbrata|[4722]|LCHP946-07|07PROBE-10708|658[0n]bp|Canada.Manitoba|BOLD:ACF0816
Euxoa adumbrata|[4723]|LBCH7598-10|10-JDWBC-7598|658[0n]bp|Canada.British Columbia|BOLD:ACF0816
Euxoa adumbrata|[4724]|LCHQ589-08|07WNP-10481|655[0n]bp|Canada.Manitoba|BOLD:ACF0816
Euxoa auxiliaris|[4725]|LPABB004-08|08BBLEP-03269|658[0n]bp|Canada.Alberta|BOLD:ABZ9343
Euxoa auxiliaris|[4726]|LPSK110-08|08BBLEP-01678|658[0n]bp|Canada.Saskatchewan|BOLD:ABZ9343
Euxoa auxiliaris|[4727]|LPSK494-08|08BBLEP-02062|658[0n]bp|Canada.Saskatchewan|BOLD:ABZ9343
Euxoa auxiliaris|[4728]|LPSK012-08|08BBLEP-00715|658[0n]bp|Canada.Saskatchewan|BOLD:ABZ9343
Euxoa auxiliaris|[4729]|LPSK112-08|08BBLEP-01680|658[0n]bp|Canada.Saskatchewan|BOLD:ABZ9343
Euxoa auxiliaris|[4730]|LPABC177-09|08BBLEP-04396|658[0n]bp|Canada.Alberta|BOLD:ABZ9343
Euxoa auxiliaris|[4731]|LPSK146-08|08BBLEP-01714|658[0n]bp|Canada.Saskatchewan|BOLD:ABZ9343
Euxoa quebecensis|[4732]|BBLPB411-10|10BBCLP-1410|658[0n]bp|Canada.Ontario|BOLD:ACF0815
Euxoa quebecensis|[4733]|RDLQG364-06|DH012598|658[0n]bp|Canada.Quebec|BOLD:ACF0815
Euxoa quebecensis|[4734]|RDLQG363-06|DH012597|658[0n]bp|Canada.Quebec|BOLD:ACF0815
Euxoa quebecensis|[4735]|RDLQB085-05|DH010171|658[0n]bp|Canada.Quebec|BOLD:ACF0815
Euxoa quebecensis|[4736]|RDLQB086-05|DH010172|658[0n]bp|Canada.Quebec|BOLD:ACF0815
Euxoa quebecensis|[4737]|RDLQB088-05|DH010174|658[0n]bp|Canada.Quebec|BOLD:ACF0815
Euxoa quebecensis|[4738]|LBCD466-05|HLC-23286|616[0n]bp|Canada.British Columbia|BOLD:ACF0815
Euxoa quebecensis|[4739]|BBLPB376-10|10BBCLP-1375|658[0n]bp|Canada.Alberta|BOLD:ACF0815
Euxoa quebecensis|[4740]|BBLPB379-10|10BBCLP-1378|658[0n]bp|Canada.British Columbia|BOLD:ACF0815
Euxoa quebecensis|[4741]|BBLPB378-10|10BBCLP-1377|658[0n]bp|Canada.British Columbia|BOLD:ACF0815
Euxoa quebecensis|[4742]|LPABC713-09|08BBLEP-04932|658[0n]bp|Canada.Alberta|BOLD:ACF0815
Euxoa quebecensis|[4743]|BBLPB381-10|10BBCLP-1380|658[0n]bp|Canada.Alberta|BOLD:ACF0815
Euxoa quebecensis|[4744]|BBLPB382-10|10BBCLP-1381|658[0n]bp|Canada.Alberta|BOLD:ACF0815
Euxoa quebecensis|[4745]|LPABB378-08|08BBLEP-03643|658[0n]bp|Canada.Alberta|BOLD:ACF0815
Euxoa quebecensis|[4746]|RDLQB087-05|DH010173|658[0n]bp|Canada.Quebec|BOLD:ACF0815
Euxoa quebecensis|[4747]|LPABB597-08|08BBLEP-03862|658[0n]bp|Canada.Alberta|BOLD:ACF0815
Euxoa quebecensis|[4748]|LBCD016-05|HLC-22836|658[0n]bp|Canada.British Columbia|BOLD:ACF0815
Euxoa quebecensis|[4749]|BBLPB377-10|10BBCLP-1376|658[0n]bp|Canada.British Columbia|BOLD:ACF0815
Euxoa quebecensis|[4750]|RDMAB434-05|BCSC107|658[0n]bp|Canada.Yukon Territory|BOLD:ACF0815
Euxoa scandens|[4751]|XAE453-04|Moth4453.03|602[0n]bp|Canada.Ontario|BOLD:ABZ9001
Euxoa scandens|[4752]|RDNMF876-08|CNC LEP00053237|658[0n]bp|Canada.Ontario|BOLD:ABZ9001
Euxoa scandens|[4753]|RDMAB095-05|UASM41975|658[0n]bp|Canada.Alberta|BOLD:ABZ9001
Euxoa scandens|[4754]|RDLQF369-06|DH011436|658[0n]bp|Canada.Quebec|BOLD:ABZ9001
Euxoa scandens|[4755]|XAE443-04|Moth4443.03|658[0n]bp|Canada.Ontario|BOLD:ABZ9001
Euxoa scandens|[4756]|RDNMF897-08|CNC LEP00053258|658[0n]bp|Canada.Saskatchewan|BOLD:ABZ9001
Euxoa cursoria|[4757]|RDMAB1002-09|UASM59973|654[0n]bp|Canada.Alberta|BOLD:ACE9573
Euxoa cursoria|[4758]|RDMAB1005-09|UASM59334|633[2n]bp|Canada.Alberta|BOLD:ACE9573
Euxoa cursoria|[4759]|RDNMB013-05|CNCNoctuoidea7853|581[0n]bp|Canada.Alberta|BOLD:ACE9573
Euxoa cursoria|[4760]|RDMAB1004-09|UASM34734|644[0n]bp|Canada.Alberta|BOLD:ACE9573
Euxoa cursoria|[4761]|RDNMG656-08|CNC LEP00052480|658[0n]bp|Canada.Alberta|BOLD:ACE9573
Euxoa cursoria|[4762]|RDMAB1003-09|UASM34987|658[0n]bp|Canada.Alberta|BOLD:ACE9573
Euxoa ochrogaster|[4763]|BBLPB799-10|10BBCLP-1798|658[0n]bp|Canada.Alberta|BOLD:ACE9574
Euxoa ochrogaster|[4764]|LALPA677-10|AVBC 679-10|658[0n]bp|Canada.British Columbia|BOLD:ACE9574
Euxoa ochrogaster|[4765]|LOWCD324-06|CGWC-3144|657[0n]bp|Canada.British Columbia|BOLD:ACE9574
Euxoa ochrogaster|[4766]|BBLPB794-10|10BBCLP-1793|658[0n]bp|Canada.Alberta|BOLD:ACE9574
Euxoa ochrogaster|[4767]|BBLPB559-10|10BBCLP-1558|658[0n]bp|Canada.Alberta|BOLD:ACE9574
Euxoa ochrogaster|[4768]|BBLPB613-10|10BBCLP-1612|658[0n]bp|Canada.British Columbia|BOLD:ACE9574
Euxoa ochrogaster|[4769]|BBLPB795-10|10BBCLP-1794|658[0n]bp|Canada.Alberta|BOLD:ACE9574
Euxoa ochrogaster|[4770]|LALPA691-10|AVBC 693-10|658[0n]bp|Canada.British Columbia|BOLD:ACE9574
Euxoa ochrogaster|[4771]|RDLQ699-07|DH007227|658[0n]bp|Canada.Quebec|BOLD:ACE9574
Euxoa ochrogaster|[4772]|RDLQ700-07|AC000706|658[0n]bp|Canada.Quebec|BOLD:ACE9574
Euxoa ochrogaster|[4773]|BBLPB564-10|10BBCLP-1563|658[0n]bp|Canada.Alberta|BOLD:ACE9574
Euxoa ochrogaster|[4774]|LALPA688-10|AVBC 690-10|658[0n]bp|Canada.British Columbia|BOLD:ACE9574
Euxoa ochrogaster|[4775]|BBLPB798-10|10BBCLP-1797|658[0n]bp|Canada.Alberta|BOLD:ACE9574
Euxoa ochrogaster|[4776]|BBLPB797-10|10BBCLP-1796|658[0n]bp|Canada.Alberta|BOLD:ACE9574
Euxoa ochrogaster|[4777]|BBLPB614-10|10BBCLP-1613|658[0n]bp|Canada.Alberta|BOLD:ACE9574
Euxoa ochrogaster|[4778]|LOWCD322-06|CGWC-3142|614[0n]bp|Canada.British Columbia|BOLD:ACE9574
Euxoa ochrogaster|[4779]|LOWCD323-06|CGWC-3143|598[0n]bp|Canada.British Columbia|BOLD:ACE9574
Euxoa ochrogaster|[4780]|LOWCD321-06|CGWC-3141|554[0n]bp|Canada.British Columbia|BOLD:ACE9574
Euxoa ochrogaster|[4781]|BBLPB796-10|10BBCLP-1795|658[0n]bp|Canada.Alberta|BOLD:ACE9574
Euxoa terrenus|[4782]|LBCG3031-09|08-JDWBC-3031|658[0n]bp|Canada.British Columbia|BOLD:ACF3235
Euxoa terrenus|[4783]|LBCH5943-10|10-JDWBC-5943|658[0n]bp|Canada.British Columbia|BOLD:ACF3235
Euxoa terrenus|[4784]|LBCH5822-10|10-JDWBC-5822|658[0n]bp|Canada.British Columbia|BOLD:ACF3235
[illegible]

Euxoa terrenus|[4782]|LBCG3031-09|08-JDWBC-3031|658[0n]bp|Canada.British Columbia|BOLD:ACF3235
Euxoa terrenus|[4783]|LBCH5943-10|10-JDWBC-5943|658[0n]bp|Canada.British Columbia|BOLD:ACF3235
Euxoa terrenus|[4784]|LBCH5822-10|10-JDWBC-5822|658[0n]bp|Canada.British Columbia|BOLD:ACF3235
Euxoa terrenus|[4785]|LBCH5869-10|10-JDWBC-5869|658[0n]bp|Canada.British Columbia|BOLD:ACF3235
Euxoa terrenus|[4786]|LBCD280-05|HLC-23100|614[0n]bp|Canada.British Columbia|BOLD:ACF3235
Euxoa terrenus|[4787]|LBCH5598-10|10-JDWBC-5598|658[0n]bp|Canada.British Columbia|BOLD:ACF3235
Euxoa terrenus|[4788]|LOWCD494-06|CGWC-3314|583[0n]bp|Canada.British Columbia|BOLD:ACF3235
Euxoa terrenus|[4789]|LOWCD498-06|CGWC-3318|580[0n]bp|Canada.British Columbia|BOLD:ACF3235
Euxoa terrenus|[4790]|LBCH6443-10|10-JDWBC-6443|658[0n]bp|Canada.British Columbia|BOLD:ACF3235
Euxoa terrenus|[4791]|LBCH5730-10|10-JDWBC-5730|658[0n]bp|Canada.British Columbia|BOLD:ACF3235
Euxoa terrenus|[4792]|LOWCD495-06|CGWC-3315|658[0n]bp|Canada.British Columbia|BOLD:ACF3235
Euxoa terrenus|[4793]|LOWCE312-06|CGWC-4072|658[0n]bp|Canada.British Columbia|BOLD:ACF3235
Euxoa terrenus|[4794]|LBCH5855-10|10-JDWBC-5855|658[0n]bp|Canada.British Columbia|BOLD:ACF3235
Euxoa terrenus|[4795]|LBCH5866-10|10-JDWBC-5866|658[0n]bp|Canada.British Columbia|BOLD:ACF3235
Euxoa terrenus|[4796]|LBCH5859-10|10-JDWBC-5859|658[0n]bp|Canada.British Columbia|BOLD:ACF3235
Euxoa terrenus|[4797]|LBCH5724-10|10-JDWBC-5724|658[0n]bp|Canada.British Columbia|BOLD:ACF3235
Euxoa terrenus|[4798]|LBCH5595-10|10-JDWBC-5595|658[0n]bp|Canada.British Columbia|BOLD:ACF3235
Euxoa terrenus|[4799]|LBCH5860-10|10-JDWBC-5860|658[0n]bp|Canada.British Columbia|BOLD:ACF3235
Euxoa terrenus|[4800]|LBCH5858-10|10-JDWBC-5858|658[0n]bp|Canada.British Columbia|BOLD:ACF3235
Euxoa terrenus|[4801]|LOWCE317-06|CGWC-4077|658[0n]bp|Canada.British Columbia|BOLD:ACF3235
Euxoa terrenus|[4802]|LBCH5599-10|10-JDWBC-5599|658[0n]bp|Canada.British Columbia|BOLD:ACF3235
Euxoa terrenus|[4803]|LBCH5591-10|10-JDWBC-5591|658[0n]bp|Canada.British Columbia|BOLD:ACF3235
Euxoa terrenus|[4804]|LOWCD496-06|CGWC-3316|658[0n]bp|Canada.British Columbia|BOLD:ACF3235
Euxoa terrenus|[4805]|LBCH5672-10|10-JDWBC-5672|658[0n]bp|Canada.British Columbia|BOLD:ACF3235
Euxoa terrenus|[4806]|LOWCE311-06|CGWC-4071|658[0n]bp|Canada.British Columbia|BOLD:ACF3235
Euxoa terrenus|[4807]|LBCH5425-10|10-JDWBC-5425|658[0n]bp|Canada.British Columbia|BOLD:ACF3235
Euxoa terrenus|[4808]|LBCH5713-10|10-JDWBC-5713|658[0n]bp|Canada.British Columbia|BOLD:ACF3235
Euxoa terrenus|[4809]|LOWCE297-06|CGWC-4057|658[0n]bp|Canada.British Columbia|BOLD:ACF3235
Euxoa terrenus|[4810]|LBCH5593-10|10-JDWBC-5593|658[0n]bp|Canada.British Columbia|BOLD:ACF3235
Euxoa terrenus|[4811]|LBCH5721-10|10-JDWBC-5721|658[0n]bp|Canada.British Columbia|BOLD:ACF3235
Euxoa terrenus|[4812]|LOWCE316-06|CGWC-4076|658[0n]bp|Canada.British Columbia|BOLD:ACF3235
Euxoa terrenus|[4813]|LOWCE318-06|CGWC-4078|658[0n]bp|Canada.British Columbia|BOLD:ACF3235
Euxoa terrenus|[4814]|LALPA984-11|AVBC 1157-11|658[0n]bp|Canada.British Columbia|BOLD:ACF3235
Euxoa terrenus|[4815]|LOWCE313-06|CGWC-4073|658[0n]bp|Canada.British Columbia|BOLD:ACF3235
Euxoa terrenus|[4816]|LBCH5865-10|10-JDWBC-5865|658[0n]bp|Canada.British Columbia|BOLD:ACF3235
Euxoa terrenus|[4817]|LBCH6003-10|10-JDWBC-6003|658[0n]bp|Canada.British Columbia|BOLD:ACF3235
Euxoa terrenus|[4818]|LBCH6002-10|10-JDWBC-6002|658[0n]bp|Canada.British Columbia|BOLD:ACF3235
Euxoa terrenus|[4819]|LOWCE315-06|CGWC-4075|658[0n]bp|Canada.British Columbia|BOLD:ACF3235
Euxoa terrenus|[4820]|LOWCE314-06|CGWC-4074|658[0n]bp|Canada.British Columbia|BOLD:ACF3235
Euxoa terrenus|[4821]|LBCH6222-10|10-JDWBC-6222|658[0n]bp|Canada.British Columbia|BOLD:ACF3235
Euxoa terrenus|[4822]|LOWCD497-06|CGWC-3317|588[0n]bp|Canada.British Columbia|BOLD:ACF3235
Euxoa terrenus|[4823]|LOWCE309-06|CGWC-4069|615[0n]bp|Canada.British Columbia|BOLD:ACF3235
Euxoa terrenus|[4824]|LOWCE300-06|CGWC-4060|612[0n]bp|Canada.British Columbia|BOLD:ACF3235
Euxoa bochus|[4825]|LBCH6628-10|10-JDWBC-6628|658[0n]bp|Canada.British Columbia|BOLD:AAC8204
Euxoa bochus|[4826]|LBCH7760-10|10-JDWBC-7760|658[0n]bp|Canada.British Columbia|BOLD:AAC8204
Euxoa bochus|[4827]|LOWCB672-05|CGWC-1612|658[0n]bp|Canada.British Columbia|BOLD:AAC8204
Euxoa bochus|[4828]|LOWCB671-05|CGWC-1611|657[0n]bp|Canada.British Columbia|BOLD:AAC8204
Euxoa bochus|[4829]|LOWCB673-05|CGWC-1613|658[0n]bp|Canada.British Columbia|BOLD:AAC8204
Euxoa bochus|[4830]|LOWCB677-05|CGWC-1617|658[0n]bp|Canada.British Columbia|BOLD:AAC8204
Euxoa bochus|[4831]|LOWCB669-05|CGWC-1609|658[0n]bp|Canada.British Columbia|BOLD:AAC8204
Euxoa bochus|[4832]|LOWCB675-05|CGWC-1615|658[0n]bp|Canada.British Columbia|BOLD:AAC8204
Euxoa bochus|[4833]|LBCH7226-10|10-JDWBC-7226|658[0n]bp|Canada.British Columbia|BOLD:AAC8204
Euxoa bochus|[4834]|LBCH6531-10|10-JDWBC-6531|658[0n]bp|Canada.British Columbia|BOLD:AAC8204
Euxoa bochus|[4835]|LOWCB678-05|CGWC-1618|577[0n]bp|Canada.British Columbia|BOLD:AAC8204
Euxoa bochus|[4836]|LOWCB674-05|CGWC-1614|650[0n]bp|Canada.British Columbia|BOLD:AAC8204
Euxoa bochus|[4837]|LOWCB670-05|CGWC-1610|504[0n]bp|Canada.British Columbia|
Euxoa bochus|[4838]|LOWCB676-05|CGWC-1616|658[0n]bp|Canada.British Columbia|BOLD:AAC8204
Diarsia rosaria freemani|[4839]|LPMN794-08|08BBLEP-01597|658[0n]bp|Canada.Manitoba|BOLD:ACE5645
Diarsia rosaria freemani|[4840]|LCH296-04|04HBL003296|658[1n]bp|Canada.Manitoba|BOLD:ACE5645
Diarsia rosaria freemani|[4841]|LPABC404-09|08BBLEP-04623|658[0n]bp|Canada.Alberta|BOLD:ACE5645
Diarsia rosaria freemani|[4842]|LPABC449-09|08BBLEP-04668|658[0n]bp|Canada.Alberta|BOLD:ACE5645
Diarsia rosaria freemani|[4843]|BBLPE316-09|09BBELE-2316|658[0n]bp|Canada.Newfoundland and Labrador|BOLD...
Diarsia rosaria freemani|[4844]|LCH267-04|04HBL003267|658[0n]bp|Canada.Manitoba|BOLD:ACE5645
Diarsia rosaria freemani|[4845]|LOWCC166-05|CGWC-2046|544[0n]bp|Canada.British Columbia|BOLD:ACE5645
Diarsia rosaria freemani|[4846]|LCH275-04|04HBL003275|658[0n]bp|Canada.Manitoba|BOLD:ACE5645
Diarsia rosaria freemani|[4847]|LPABC442-09|08BBLEP-04661|658[0n]bp|Canada.Alberta|BOLD:ACE5645
Diarsia rosaria freemani|[4848]|LCH280-04|04HBL003280|658[0n]bp|Canada.Manitoba|BOLD:ACE5645
Diarsia rosaria freemani|[4849]|LOWCD883-06|CGWC-3703|658[0n]bp|Canada.British Columbia|BOLD:ACE5645
Diarsia rosaria freemani|[4850]|LPABB866-09|08BBLEP-04186|658[0n]bp|Canada.Alberta|BOLD:ACE5645
Diarsia rosaria freemani|[4851]|LPMN336-08|08BBLEP-01135|658[0n]bp|Canada.Manitoba|BOLD:ACE5645
Diarsia rosaria freemani|[4852]|LCH272-04|04HBL003272|652[0n]bp|Canada.Manitoba|BOLD:ACE5645
Diarsia rosaria freemani|[4853]|LCH559-04|04HBL003559|589[0n]bp|Canada.Manitoba|BOLD:ACE5645
Diarsia rosaria rosaria|[4854]|LBCG830-09|08-JDWBC-0830|658[0n]bp|Canada.British Columbia|BOLD:AAA5639
Diarsia rosaria rosaria|[4855]|LBCB492-05|HLC-21432|658[0n]bp|Canada.British Columbia|BOLD:AAA5639
Diarsia rosaria rosaria|[4856]|BBLPB617-10|10BBCLP-1616|614[0n]bp|Canada.British Columbia|BOLD:AAA5639
Diarsia rosaria rosaria|[4857]|LPABC181-09|08BBLEP-04400|640[3n]bp|Canada.Alberta|BOLD:AAA5639
Diarsia rosaria rosaria|[4858]|LALPA1159-11|AVBC 969-11|658[0n]bp|Canada.British Columbia|BOLD:AAA5639
Diarsia rosaria rosaria|[4859]|BBLPB619-10|10BBCLP-1618|658[0n]bp|Canada.British Columbia|BOLD:AAA5639
Diarsia rosaria rosaria|[4860]|LPABC075-09|08BBLEP-04294|658[0n]bp|Canada.Alberta|BOLD:AAA5639
Diarsia rosaria rosaria|[4861]|LBCH1865-10|10-JDWBC-1865|658[0n]bp|Canada.British Columbia|BOLD:AAA5639
Diarsia rosaria rosaria|[4862]|LPABC319-09|08BBLEP-04538|658[0n]bp|Canada.Alberta|BOLD:AAA5639
Diarsia rosaria rosaria|[4863]|BBLPB596-10|10BBCLP-1595|658[0n]bp|Canada.British Columbia|BOLD:AAA5639
Diarsia rosaria rosaria|[4864]|BBLPB595-10|10BBCLP-1594|658[0n]bp|Canada.British Columbia|BOLD:AAA5639
Diarsia rosaria rosaria|[4865]|LBCG2469-09|08-JDWBC-2469|658[0n]bp|Canada.British Columbia|BOLD:AAA5639
Diarsia rosaria rosaria|[4866]|LBCC669-05|HLC-22549|658[0n]bp|Canada.British Columbia|BOLD:AAA5639
Diarsia rosaria rosaria|[4867]|LBCH2202-10|10-JDWBC-2202|658[0n]bp|Canada.British Columbia|BOLD:AAA5639
Diarsia rosaria rosaria|[4868]|BBLPB555-10|10BBCLP-1554|658[0n]bp|Canada.British Columbia|BOLD:AAA5639
Diarsia rosaria rosaria|[4869]|LBCG2472-09|08-JDWBC-2472|658[0n]bp|Canada.British Columbia|BOLD:AAA5639
Diarsia rosaria rosaria|[4870]|BBLPB601-10|10BBCLP-1600|658[0n]bp|Canada.British Columbia|BOLD:AAA5639
Diarsia rosaria rosaria|[4871]|BBLPB554-10|10BBCLP-1553|658[0n]bp|Canada.British Columbia|BOLD:AAA5639
Diarsia rosaria rosaria|[4872]|LBCG3029-09|08-JDWBC-3029|658[0n]bp|Canada.British Columbia|BOLD:AAA5639
Diarsia rosaria rosaria|[4873]|LPABB635-08|08BBLEP-03900|658[0n]bp|Canada.Alberta|BOLD:AAA5639
Diarsia rosaria rosaria|[4874]|BBLPB602-10|10BBCLP-1601|658[0n]bp|Canada.British Columbia|BOLD:AAA5639
Diarsia rosaria rosaria|[4875]|LBCH1775-10|10-JDWBC-1775|658[0n]bp|Canada.British Columbia|BOLD:AAA5639
Diarsia rosaria rosaria|[4876]|BBLPB575-10|10BBCLP-1574|658[0n]bp|Canada.British Columbia|BOLD:AAA5639
Diarsia rosaria rosaria|[4877]|BBLPB576-10|10BBCLP-1575|658[0n]bp|Canada.British Columbia|BOLD:AAA5639
Diarsia rosaria rosaria|[4878]|BBLPB578-10|10BBCLP-1577|658[0n]bp|Canada.British Columbia|BOLD:AAA5639
Diarsia rosaria rosaria|[4879]|BBLPB849-10|10BBCLP-1848|658[0n]bp|Canada.British Columbia|BOLD:AAA5639
Diarsia rosaria rosaria|[4880]|LBCD054-05|HLC-22874|658[0n]bp|Canada.British Columbia|BOLD:AAA5639
Diarsia rosaria rosaria|[4881]|LBCH2250-10|10-JDWBC-2250|658[0n]bp|Canada.British Columbia|BOLD:AAA5639
Diarsia rosaria rosaria|[4882]|BBLPB592-10|10BBCLP-1591|658[0n]bp|Canada.British Columbia|BOLD:AAA5639
Diarsia rosaria rosaria|[4883]|LBCH1683-10|10-JDWBC-1683|658[0n]bp|Canada.British Columbia|BOLD:AAA5639
Diarsia rosaria rosaria|[4884]|LBCB493-05|HLC-21433|658[0n]bp|Canada.British Columbia|BOLD:AAA5639

Diarsia rosaria rosaria|[4882]|BBLPB592-10|10BBCLP-1591|658[0n]bp|Canada.British Columbia|BOLD:AAA5639
Diarsia rosaria rosaria|[4883]|LBCH1683-10|10-JDWBC-1683|658[0n]bp|Canada.British Columbia|BOLD:AAA5639
Diarsia rosaria rosaria|[4884]|LBCB493-05|HLC-21433|658[0n]bp|Canada.British Columbia|BOLD:AAA5639
Diarsia rosaria rosaria|[4885]|BBLPB594-10|10BBCLP-1593|658[0n]bp|Canada.British Columbia|BOLD:AAA5639
Diarsia rosaria rosaria|[4886]|BBLPB577-10|10BBCLP-1576|658[0n]bp|Canada.British Columbia|BOLD:AAA5639
Diarsia rosaria rosaria|[4887]|BBLPB574-10|10BBCLP-1573|658[0n]bp|Canada.British Columbia|BOLD:AAA5639
Diarsia rosaria rosaria|[4888]|BBLPB616-10|10BBCLP-1615|658[0n]bp|Canada.British Columbia|BOLD:AAA5639
Diarsia rosaria rosaria|[4889]|BBLPB618-10|10BBCLP-1617|658[0n]bp|Canada.British Columbia|BOLD:AAA5639
Diarsia rosaria rosaria|[4890]|LPABB826-09|08BBLEP-04146|658[0n]bp|Canada.Alberta|BOLD:AAA5639
Diarsia rosaria rosaria|[4891]|LALPA1323-12|AVBC 1325-11|601[0n]bp|Canada.British Columbia|BOLD:AAA5639
Diarsia rosaria rosaria|[4892]|LOWCC868-05|CGWC-2748|514[1n]bp|Canada.British Columbia|
Diarsia rosaria rosaria|[4893]|LBCH1317-10|10-JDWBC-1317|632[0n]bp|Canada.British Columbia|BOLD:AAA5639
Diarsia rosaria rosaria|[4894]|LOWCD886-06|CGWC-3706|543[0n]bp|Canada.British Columbia|BOLD:AAA5639
Diarsia rosaria rosaria|[4895]|LOWCD884-06|CGWC-3704|598[0n]bp|Canada.British Columbia|BOLD:AAA5639
Diarsia rosaria rosaria|[4896]|LOWCC168-05|CGWC-2048|658[0n]bp|Canada.British Columbia|BOLD:AAA5639
Diarsia rosaria rosaria|[4897]|LALPA1158-11|AVBC 968-11|658[0n]bp|Canada.British Columbia|BOLD:AAA5639
Diarsia rosaria rosaria|[4898]|LOWCC171-05|CGWC-2051|658[0n]bp|Canada.British Columbia|BOLD:AAA5639
Diarsia rosaria rosaria|[4899]|LOWCD885-06|CGWC-3705|658[0n]bp|Canada.British Columbia|BOLD:AAA5639
Diarsia rosaria rosaria|[4900]|LOWCC167-05|CGWC-2047|658[0n]bp|Canada.British Columbia|BOLD:AAA5639
Diarsia rosaria rosaria|[4901]|LHLEP426-06|UBC-2006-1988|658[0n]bp|Canada.British Columbia|BOLD:AAA5639
Diarsia rosaria rosaria|[4902]|LBCH1104-10|10-JDWBC-1104|658[0n]bp|Canada.British Columbia|BOLD:AAA5639
Diarsia rosaria rosaria|[4903]|BBLPB615-10|10BBCLP-1614|658[0n]bp|Canada.British Columbia|BOLD:AAA5639
Diarsia rosaria rosaria|[4904]|LBCG309-08|08-JDWBC-0309|658[0n]bp|Canada.British Columbia|BOLD:AAA5639
Diarsia rosaria rosaria|[4905]|LBCW080-08|08-JDWWI-0080|658[0n]bp|Canada.British Columbia|BOLD:AAA5639
Diarsia rosaria rosaria|[4906]|LBCH2894-10|10-JDWBC-2894|658[0n]bp|Canada.British Columbia|BOLD:AAA5639
Diarsia rosaria rosaria|[4907]|LBCH2903-10|10-JDWBC-2903|658[0n]bp|Canada.British Columbia|BOLD:AAA5639
Diarsia rosaria rosaria|[4908]|LBCH2985-10|10-JDWBC-2985|658[0n]bp|Canada.British Columbia|BOLD:AAA5639
Diarsia rosaria rosaria|[4909]|LOWCD882-06|CGWC-3702|604[0n]bp|Canada.British Columbia|BOLD:AAA5639
Diarsia rosaria rosaria|[4910]|LOWCE761-06|CGWC-4521|604[0n]bp|Canada.British Columbia|BOLD:AAA5639
Diarsia rosaria rosaria|[4911]|LOWCD887-06|CGWC-3707|596[0n]bp|Canada.British Columbia|BOLD:AAA5639
Diarsia rosaria rosaria|[4912]|LOWCE753-06|CGWC-4513|600[0n]bp|Canada.British Columbia|BOLD:AAA5639
Diarsia rosaria rosaria|[4913]|LBCH2897-10|10-JDWBC-2897|658[0n]bp|Canada.British Columbia|BOLD:AAA5639
Diarsia rosaria rosaria|[4914]|LBCH3006-10|10-JDWBC-3006|658[0n]bp|Canada.British Columbia|BOLD:AAA5639
Diarsia rosaria rosaria|[4915]|LBCH2904-10|10-JDWBC-2904|658[0n]bp|Canada.British Columbia|BOLD:AAA5639
Diarsia rosaria rosaria|[4916]|LBCH528-10|10-JDWBC-0528|658[0n]bp|Canada.British Columbia|BOLD:AAA5639
Diarsia rosaria rosaria|[4917]|LOWCE760-06|CGWC-4520|658[0n]bp|Canada.British Columbia|BOLD:AAA5639
Diarsia rosaria rosaria|[4918]|LBCH2087-10|10-JDWBC-2087|658[0n]bp|Canada.British Columbia|BOLD:AAA5639
Diarsia rosaria rosaria|[4919]|LBCG150-08|08-JDWBC-0150|658[0n]bp|Canada.British Columbia|BOLD:AAA5639
Diarsia rosaria rosaria|[4920]|LBCH409-10|10-JDWBC-0409|658[0n]bp|Canada.British Columbia|BOLD:AAA5639
Diarsia rosaria rosaria|[4921]|LBCG315-08|08-JDWBC-0315|658[0n]bp|Canada.British Columbia|BOLD:AAA5639
Diarsia rosaria rosaria|[4922]|LALPA1227-11|AVBC 1229-11|658[0n]bp|Canada.British Columbia|BOLD:AAA5639
Diarsia rosaria rosaria|[4923]|LBCH2896-10|10-JDWBC-2896|658[0n]bp|Canada.British Columbia|BOLD:AAA5639
Diarsia rosaria rosaria|[4924]|LBCH543-10|10-JDWBC-0543|658[0n]bp|Canada.British Columbia|BOLD:AAA5639
Diarsia rosaria rosaria|[4925]|LBCH2900-10|10-JDWBC-2900|658[0n]bp|Canada.British Columbia|BOLD:AAA5639
Diarsia rosaria rosaria|[4926]|LBCH149-10|10-JDWBC-0149|658[0n]bp|Canada.British Columbia|BOLD:AAA5639
Diarsia rosaria rosaria|[4927]|LOWCC170-05|CGWC-2050|658[0n]bp|Canada.British Columbia|BOLD:AAA5639
Diarsia rosaria rosaria|[4928]|BBLPB593-10|10BBCLP-1592|658[0n]bp|Canada.British Columbia|BOLD:AAA5639
Diarsia rosaria rosaria|[4929]|LOWCE750-06|CGWC-4510|658[0n]bp|Canada.British Columbia|BOLD:AAA5639
Diarsia rosaria rosaria|[4930]|LBCG180-08|08-JDWBC-0180|658[0n]bp|Canada.British Columbia|BOLD:AAA5639
Diarsia rosaria rosaria|[4931]|LBCH2895-10|10-JDWBC-2895|658[0n]bp|Canada.British Columbia|BOLD:AAA5639
Diarsia rosaria rosaria|[4932]|LBCH2898-10|10-JDWBC-2898|658[0n]bp|Canada.British Columbia|BOLD:AAA5639
Diarsia rosaria rosaria|[4933]|LOWCE762-06|CGWC-4522|658[0n]bp|Canada.British Columbia|BOLD:AAA5639
Diarsia rosaria rosaria|[4934]|LBCH410-10|10-JDWBC-0410|658[0n]bp|Canada.British Columbia|BOLD:AAA5639
Diarsia rosaria rosaria|[4935]|LBCH2902-10|10-JDWBC-2902|658[0n]bp|Canada.British Columbia|BOLD:AAA5639
Diarsia rosaria rosaria|[4936]|LBCH3008-10|10-JDWBC-3008|658[0n]bp|Canada.British Columbia|BOLD:AAA5639
Diarsia rosaria rosaria|[4937]|LBCH2201-10|10-JDWBC-2201|658[0n]bp|Canada.British Columbia|BOLD:AAA5639
Diarsia rosaria rosaria|[4938]|LOWCC169-05|CGWC-2049|510[0n]bp|Canada.British Columbia|BOLD:AAA5639
Diarsia rosaria rosaria|[4939]|LBCH6005-10|10-JDWBC-6005|658[0n]bp|Canada.British Columbia|BOLD:AAA5639
Diarsia rosaria rosaria|[4940]|LBCG308-08|08-JDWBC-0308|658[0n]bp|Canada.British Columbia|BOLD:AAA5639
Diarsia rosaria rosaria|[4941]|LBCD055-05|HLC-22875|658[0n]bp|Canada.British Columbia|BOLD:AAA5639
Diarsia rosaria rosaria|[4942]|LOWCE751-06|CGWC-4511|658[0n]bp|Canada.British Columbia|BOLD:AAA5639
Diarsia rosaria rosaria|[4943]|LBCH3007-10|10-JDWBC-3007|658[0n]bp|Canada.British Columbia|BOLD:AAA5639
Diarsia rosaria rosaria|[4944]|LBCH2901-10|10-JDWBC-2901|658[0n]bp|Canada.British Columbia|BOLD:AAA5639
Diarsia jucunda|[4945]|BBLPC587-09|09BBELE-1587|658[0n]bp|Canada.Nova Scotia|BOLD:AAB0038
Diarsia jucunda|[4946]|BBLEC700-09|09BBELE-0700|658[0n]bp|Canada.Nova Scotia|BOLD:AAB0038
Diarsia jucunda|[4947]|BBLEC016-09|09BBELE-0016|658[0n]bp|Canada.New Brunswick|BOLD:AAB0038
Diarsia dislocata|[4948]|BBLPB458-10|10BBCLP-1457|658[0n]bp|Canada.Alberta|BOLD:AAA1521
Diarsia dislocata|[4949]|LPABB869-09|08BBLEP-04189|631[0n]bp|Canada.Alberta|BOLD:AAA1521
Diarsia dislocata|[4950]|LCH300-04|04HBL003300|658[0n]bp|Canada.Manitoba|BOLD:AAA1521
Diarsia dislocata|[4951]|LPABC437-09|08BBLEP-04656|658[0n]bp|Canada.Alberta|BOLD:AAA1521
Diarsia dislocata|[4952]|LCHQ028-07|07PROBE-10789|658[0n]bp|Canada.Manitoba|BOLD:AAA1521
Diarsia dislocata|[4953]|RDLQG136-06|DH012303|658[0n]bp|Canada.Quebec|BOLD:AAA1521
Diarsia dislocata|[4954]|LBCH966-10|10-JDWBC-0966|658[0n]bp|Canada.British Columbia|BOLD:AAA1521
Diarsia dislocata|[4955]|LBCH549-10|10-JDWBC-0549|658[0n]bp|Canada.British Columbia|BOLD:AAA1521
Diarsia dislocata|[4956]|LBCH383-10|10-JDWBC-0383|658[0n]bp|Canada.British Columbia|BOLD:AAA1521
Diarsia dislocata|[4957]|LBCH3511-10|10-JDWBC-3511|658[0n]bp|Canada.British Columbia|BOLD:AAA1521
Diarsia dislocata|[4958]|LBCH4745-10|10-JDWBC-4745|658[0n]bp|Canada.British Columbia|BOLD:AAA1521
Diarsia dislocata|[4959]|LCHP482-07|07PROBE-10150|658[0n]bp|Canada.Manitoba|BOLD:AAA1521
Diarsia dislocata|[4960]|LCHP652-07|07PROBE-10333|658[0n]bp|Canada.Manitoba|BOLD:AAA1521
Diarsia dislocata|[4961]|LBCH4030-10|10-JDWBC-4030|658[0n]bp|Canada.British Columbia|BOLD:AAA1521
Diarsia dislocata|[4962]|BBLPE233-09|09BBELE-2233|614[0n]bp|Canada.Newfoundland and Labrador|BOLD:AAA...
Diarsia dislocata|[4963]|LBCH239-10|10-JDWBC-0239|658[0n]bp|Canada.British Columbia|BOLD:AAA1521
Diarsia dislocata|[4964]|LCH314-04|04HBL003314|632[0n]bp|Canada.Manitoba|BOLD:AAA1521
Diarsia dislocata|[4965]|BBLPC938-09|09BBELE-1938|632[0n]bp|Canada.Newfoundland and Labrador|BOLD:AAA...
Diarsia dislocata|[4966]|LBCH839-10|10-JDWBC-0839|636[0n]bp|Canada.British Columbia|BOLD:AAA1521
Diarsia dislocata|[4967]|LCH288-04|04HBL003288|658[0n]bp|Canada.Manitoba|BOLD:AAA1521
Diarsia dislocata|[4968]|BBLPC840-09|09BBELE-1840|658[0n]bp|Canada.Newfoundland and Labrador|BOLD:AAA...
Diarsia dislocata|[4969]|BBLPE114-09|09BBELE-2114|658[0n]bp|Canada.Nova Scotia|BOLD:AAA1521
Diarsia dislocata|[4970]|LPABC074-09|08BBLEP-04293|658[0n]bp|Canada.Alberta|BOLD:AAA1521
Diarsia dislocata|[4971]|LCH299-04|04HBL003299|658[0n]bp|Canada.Manitoba|BOLD:AAA1521
Diarsia dislocata|[4972]|LCH295-04|04HBL003295|658[0n]bp|Canada.Manitoba|BOLD:AAA1521
Diarsia dislocata|[4973]|LCH281-04|04HBL003281|658[0n]bp|Canada.Manitoba|BOLD:AAA1521
Diarsia dislocata|[4974]|LCH289-04|04HBL003289|658[0n]bp|Canada.Manitoba|BOLD:AAA1521
Diarsia dislocata|[4975]|LCH301-04|04HBL003301|658[0n]bp|Canada.Manitoba|BOLD:AAA1521
Diarsia dislocata|[4976]|LCH576-04|04HBL003576|658[1n]bp|Canada.Manitoba|BOLD:AAA1521
Diarsia dislocata|[4977]|LCH309-04|04HBL003309|658[0n]bp|Canada.Manitoba|BOLD:AAA1521
Diarsia dislocata|[4978]|LCHP486-07|07PROBE-10154|658[0n]bp|Canada.Manitoba|BOLD:AAA1521
Diarsia dislocata|[4979]|LCHP874-07|07PROBE-10631|658[0n]bp|Canada.Manitoba|BOLD:AAA1521
Diarsia dislocata|[4980]|BBLPE061-09|09BBELE-2061|658[0n]bp|Canada.Nova Scotia|BOLD:AAA1521
Diarsia dislocata|[4981]|LCHP207-07|07PROBE-00132|658[0n]bp|Canada.Manitoba|BOLD:AAA1521
Diarsia dislocata|[4982]|BBLPC926-09|09BBELE-1926|658[0n]bp|Canada.Newfoundland and Labrador|BOLD:AAA...
Diarsia dislocata|[4983]|LCHP487-07|07PROBE-10155|658[0n]bp|Canada.Manitoba|BOLD:AAA1521
Diarsia dislocata|[4984]|LCH293-04|04HBL003293|658[0n]bp|Canada.Manitoba|BOLD:AAA1521

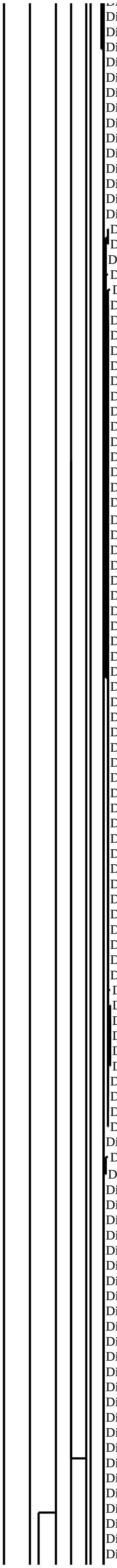

Diarsia dislocata|[4981]|LCHP207-07|07PROBE-00152|658[0n]bp|Canada.Manitoba|BOLD:AAA1521
Diarsia dislocata|[4982]|BBLPC926-09|09BBELE-1926|658[0n]bp|Canada.Newfoundland and Labrador|BOLD:AAA...
Diarsia dislocata|[4983]|LCHP487-07|07PROBE-10155|658[0n]bp|Canada.Manitoba|BOLD:AAA1521
Diarsia dislocata|[4984]|LCH293-04|04HBL003293|658[0n]bp|Canada.Manitoba|BOLD:AAA1521
Diarsia dislocata|[4985]|BBLPC300-09|09BBELE-1300|658[0n]bp|Canada.Newfoundland and Labrador|BOLD:AAA...
Diarsia dislocata|[4986]|LCH577-04|04HBL003577|658[0n]bp|Canada.Manitoba|BOLD:AAA1521
Diarsia dislocata|[4987]|LCH279-04|04HBL003279|658[0n]bp|Canada.Manitoba|BOLD:AAA1521
Diarsia dislocata|[4988]|LCH287-04|04HBL003287|658[0n]bp|Canada.Manitoba|BOLD:AAA1521
Diarsia dislocata|[4989]|LPABB894-09|08BBLEP-04214|658[0n]bp|Canada.Alberta|BOLD:AAA1521
Diarsia dislocata|[4990]|BBLPE221-09|09BBELE-2221|658[0n]bp|Canada.Newfoundland and Labrador|BOLD:AAA...
Diarsia dislocata|[4991]|BBLPE250-09|09BBELE-2250|658[0n]bp|Canada.Newfoundland and Labrador|BOLD:AAA...
Diarsia dislocata|[4992]|LCHP818-07|07PROBE-10575|658[0n]bp|Canada.Manitoba|BOLD:AAA1521
Diarsia dislocata|[4993]|LCHP651-07|07PROBE-10332|658[0n]bp|Canada.Manitoba|BOLD:AAA1521
Diarsia dislocata|[4994]|LCHP648-07|07PROBE-10329|658[0n]bp|Canada.Manitoba|BOLD:AAA1521
Diarsia dislocata|[4995]|LCH270-04|04HBL003270|658[0n]bp|Canada.Manitoba|BOLD:AAA1521
Diarsia dislocata|[4996]|LCHP279-07|07PROBE-03845|658[0n]bp|Canada.Manitoba|BOLD:AAA1521
Diarsia dislocata|[4997]|LCH292-04|04HBL003292|658[0n]bp|Canada.Manitoba|BOLD:AAA1521
Diarsia dislocata|[4998]|LCH271-04|04HBL003271|615[0n]bp|Canada.Manitoba|BOLD:AAA1521
Diarsia dislocata|[4999]|LCHP518-07|07PROBE-10186|658[0n]bp|Canada.Manitoba|BOLD:AAA1521
Diarsia dislocata|[5000]|LBCH3508-10|10-JDWBC-3508|658[0n]bp|Canada.British Columbia|BOLD:AAA1521
Diarsia dislocata|[5001]|LBCH3824-10|10-JDWBC-3824|658[0n]bp|Canada.British Columbia|BOLD:AAA1521
Diarsia dislocata|[5002]|LBCH4740-10|10-JDWBC-4740|658[0n]bp|Canada.British Columbia|BOLD:AAA1521
Diarsia dislocata|[5003]|LCHP255-07|07PROBE-03821|658[0n]bp|Canada.Manitoba|BOLD:AAA1521
Diarsia dislocata|[5004]|LBCG2247-09|08-JDWBC-2247|658[0n]bp|Canada.British Columbia|BOLD:AAA1521
Diarsia dislocata|[5005]|LCHP866-07|07PROBE-10623|658[0n]bp|Canada.Manitoba|BOLD:AAA1521
Diarsia dislocata|[5006]|LBCH3126-10|10-JDWBC-3126|658[0n]bp|Canada.British Columbia|BOLD:AAA1521
Diarsia dislocata|[5007]|LBCG2263-09|08-JDWBC-2263|658[0n]bp|Canada.British Columbia|BOLD:AAA1521
Diarsia dislocata|[5008]|LBCH4747-10|10-JDWBC-4747|658[0n]bp|Canada.British Columbia|BOLD:AAA1521
Diarsia dislocata|[5009]|LCHP900-07|07PROBE-10662|658[0n]bp|Canada.Manitoba|BOLD:AAA1521
Diarsia dislocata|[5010]|LBCH4464-10|10-JDWBC-4464|658[0n]bp|Canada.British Columbia|BOLD:AAA1521
Diarsia dislocata|[5011]|LBCH4044-10|10-JDWBC-4044|658[0n]bp|Canada.British Columbia|BOLD:AAA1521
Diarsia dislocata|[5012]|LBCH4453-10|10-JDWBC-4453|658[0n]bp|Canada.British Columbia|BOLD:AAA1521
Diarsia dislocata|[5013]|LCH282-04|04HBL003282|658[0n]bp|Canada.Manitoba|BOLD:AAA1521
Diarsia dislocata|[5014]|LBCH529-10|10-JDWBC-0529|658[0n]bp|Canada.British Columbia|BOLD:AAA1521
Diarsia dislocata|[5015]|LBCH4017-10|10-JDWBC-4017|658[0n]bp|Canada.British Columbia|BOLD:AAA1521
Diarsia dislocata|[5016]|LCHP650-07|07PROBE-10331|658[0n]bp|Canada.Manitoba|BOLD:AAA1521
Diarsia dislocata|[5017]|LBCH3810-10|10-JDWBC-3810|658[0n]bp|Canada.British Columbia|BOLD:AAA1521
Diarsia dislocata|[5018]|LBCH3141-10|10-JDWBC-3141|658[0n]bp|Canada.British Columbia|BOLD:AAA1521
Diarsia dislocata|[5019]|LCH286-04|04HBL003286|658[0n]bp|Canada.Manitoba|BOLD:AAA1521
Diarsia dislocata|[5020]|LBCH369-10|10-JDWBC-0369|658[0n]bp|Canada.British Columbia|BOLD:AAA1521
Diarsia dislocata|[5021]|LBCH4702-10|10-JDWBC-4702|658[0n]bp|Canada.British Columbia|BOLD:AAA1521
Diarsia dislocata|[5022]|LBCH3802-10|10-JDWBC-3802|658[0n]bp|Canada.British Columbia|BOLD:AAA1521
Diarsia dislocata|[5023]|LBCH373-10|10-JDWBC-0373|658[0n]bp|Canada.British Columbia|BOLD:AAA1521
Diarsia dislocata|[5024]|LBCH4738-10|10-JDWBC-4738|658[0n]bp|Canada.British Columbia|BOLD:AAA1521
Diarsia dislocata|[5025]|LBCH4718-10|10-JDWBC-4718|658[0n]bp|Canada.British Columbia|BOLD:AAA1521
Diarsia dislocata|[5026]|LCH312-04|04HBL003312|658[0n]bp|Canada.Manitoba|BOLD:AAA1521
Diarsia dislocata|[5027]|LBCH3118-10|10-JDWBC-3118|658[0n]bp|Canada.British Columbia|BOLD:AAA1521
Diarsia dislocata|[5028]|LBCH513-10|10-JDWBC-0513|658[0n]bp|Canada.British Columbia|BOLD:AAA1521
Diarsia dislocata|[5029]|LBCH3524-10|10-JDWBC-3524|658[0n]bp|Canada.British Columbia|BOLD:AAA1521
Diarsia dislocata|[5030]|LBCH4025-10|10-JDWBC-4025|658[0n]bp|Canada.British Columbia|BOLD:AAA1521
Diarsia dislocata|[5031]|LBCH3353-10|10-JDWBC-3353|658[0n]bp|Canada.British Columbia|BOLD:AAA1521
Diarsia dislocata|[5032]|LCHP392-07|07PROBE-10016|658[0n]bp|Canada.Manitoba|BOLD:AAA1521
Diarsia dislocata|[5033]|LBCH4443-10|10-JDWBC-4443|658[0n]bp|Canada.British Columbia|BOLD:AAA1521
Diarsia dislocata|[5034]|LCHP583-07|07PROBE-10253|658[0n]bp|Canada.Manitoba|BOLD:AAA1521
Diarsia dislocata|[5035]|LCH305-04|04HBL003305|658[0n]bp|Canada.Manitoba|BOLD:AAA1521
Diarsia dislocata|[5036]|LCH303-04|04HBL003303|658[0n]bp|Canada.Manitoba|BOLD:AAA1521
Diarsia dislocata|[5037]|LCH313-04|04HBL003313|658[0n]bp|Canada.Manitoba|BOLD:AAA1521
Diarsia dislocata|[5038]|LBCH825-10|10-JDWBC-0825|658[0n]bp|Canada.British Columbia|BOLD:AAA1521
Diarsia dislocata|[5039]|LBCH677-10|10-JDWBC-0677|658[0n]bp|Canada.British Columbia|BOLD:AAA1521
Diarsia dislocata|[5040]|LBCH4448-10|10-JDWBC-4448|658[0n]bp|Canada.British Columbia|BOLD:AAA1521
Diarsia dislocata|[5041]|LBCH398-10|10-JDWBC-0398|658[0n]bp|Canada.British Columbia|BOLD:AAA1521
Diarsia dislocata|[5042]|LBCH4463-10|10-JDWBC-4463|658[0n]bp|Canada.British Columbia|BOLD:AAA1521
Diarsia dislocata|[5043]|LBCH3130-10|10-JDWBC-3130|658[0n]bp|Canada.British Columbia|BOLD:AAA1521
Diarsia dislocata|[5044]|LBCH4708-10|10-JDWBC-4708|658[0n]bp|Canada.British Columbia|BOLD:AAA1521
Diarsia dislocata|[5045]|LBCH3800-10|10-JDWBC-3800|658[0n]bp|Canada.British Columbia|BOLD:AAA1521
Diarsia dislocata|[5046]|LCH306-04|04HBL003306|658[0n]bp|Canada.Manitoba|BOLD:AAA1521
Diarsia dislocata|[5047]|LCHP825-07|07PROBE-10582|652[0n]bp|Canada.Manitoba|BOLD:AAA1521
Diarsia dislocata|[5048]|LBCH4442-10|10-JDWBC-4442|643[0n]bp|Canada.British Columbia|BOLD:AAA1521
Diarsia dislocata|[5049]|LBCH3510-10|10-JDWBC-3510|644[0n]bp|Canada.British Columbia|BOLD:AAA1521
Diarsia dislocata|[5050]|LCHQ487-08|07WNP-10379|634[0n]bp|Canada.Manitoba|BOLD:AAA1521
Diarsia dislocata|[5051]|LBCH3497-10|10-JDWBC-3497|635[0n]bp|Canada.British Columbia|BOLD:AAA1521
Diarsia dislocata|[5052]|LBCH4450-10|10-JDWBC-4450|658[0n]bp|Canada.British Columbia|BOLD:AAA1521
Diarsia dislocata|[5053]|LCHP638-07|07PROBE-10319|658[0n]bp|Canada.Manitoba|BOLD:AAA1521
Diarsia dislocata|[5054]|LBCH238-10|10-JDWBC-0238|658[0n]bp|Canada.British Columbia|BOLD:AAA1521
Diarsia dislocata|[5055]|LCH587-04|04HBL003587|658[0n]bp|Canada.Manitoba|BOLD:AAA1521
Diarsia dislocata|[5056]|LPABC263-09|08BBLEP-04482|566[1n]bp|Canada.Alberta|BOLD:AAA1521
Diarsia dislocata|[5057]|LBCH3506-10|10-JDWBC-3506|642[0n]bp|Canada.British Columbia|BOLD:AAA1521
Diarsia dislocata|[5058]|LCH277-04|04HBL003277|617[0n]bp|Canada.Manitoba|BOLD:AAA1521
Diarsia dislocata|[5059]|LBCH4041-10|10-JDWBC-4041|658[0n]bp|Canada.British Columbia|BOLD:AAA1521
Diarsia dislocata|[5060]|LBCH3095-10|10-JDWBC-3095|658[0n]bp|Canada.British Columbia|BOLD:AAA1521
Diarsia dislocata|[5061]|LBCH4461-10|10-JDWBC-4461|658[0n]bp|Canada.British Columbia|BOLD:AAA1521
Diarsia dislocata|[5062]|LBCH3509-10|10-JDWBC-3509|658[0n]bp|Canada.British Columbia|BOLD:AAA1521
Diarsia dislocata|[5063]|LBCH404-10|10-JDWBC-0404|658[0n]bp|Canada.British Columbia|BOLD:AAA1521
Diarsia dislocata|[5064]|LBCH2379-10|10-JDWBC-2379|658[0n]bp|Canada.British Columbia|BOLD:AAA1521
Diarsia dislocata|[5065]|LBCH3507-10|10-JDWBC-3507|658[0n]bp|Canada.British Columbia|BOLD:AAA1521
Diarsia dislocata|[5066]|LBCH4469-10|10-JDWBC-4469|658[0n]bp|Canada.British Columbia|BOLD:AAA1521
Diarsia dislocata|[5067]|LBCH125-10|10-JDWBC-0125|658[0n]bp|Canada.British Columbia|BOLD:AAA1521
Diarsia dislocata|[5068]|LBCH530-10|10-JDWBC-0530|658[0n]bp|Canada.British Columbia|BOLD:AAA1521
Diarsia dislocata|[5069]|LBCH4454-10|10-JDWBC-4454|658[0n]bp|Canada.British Columbia|BOLD:AAA1521
Diarsia dislocata|[5070]|LPAB227-08|08BBLEP-02549|658[0n]bp|Canada.Alberta|BOLD:AAA1521
Diarsia dislocata|[5071]|LBCH940-10|10-JDWBC-0940|658[0n]bp|Canada.British Columbia|BOLD:AAA1521
Diarsia dislocata|[5072]|LBCH387-10|10-JDWBC-0387|658[0n]bp|Canada.British Columbia|BOLD:AAA1521
Diarsia dislocata|[5073]|LPABB152-08|08BBLEP-03417|658[0n]bp|Canada.Alberta|BOLD:AAA1521
Diarsia dislocata|[5074]|LCH294-04|04HBL003294|658[0n]bp|Canada.Manitoba|BOLD:AAA1521
Diarsia dislocata|[5075]|LCH265-04|04HBL003265|658[0n]bp|Canada.Manitoba|BOLD:AAA1521
Diarsia dislocata|[5076]|LCHP899-07|07PROBE-10661|658[0n]bp|Canada.Manitoba|BOLD:AAA1521
Diarsia dislocata|[5077]|LPABC406-09|08BBLEP-04625|658[0n]bp|Canada.Alberta|BOLD:AAA1521
Diarsia dislocata|[5078]|LBCH2385-10|10-JDWBC-2385|658[0n]bp|Canada.British Columbia|BOLD:AAA1521
Diarsia dislocata|[5079]|LBCH3522-10|10-JDWBC-3522|658[0n]bp|Canada.British Columbia|BOLD:AAA1521
Diarsia dislocata|[5080]|LBCH3129-10|10-JDWBC-3129|658[0n]bp|Canada.British Columbia|BOLD:AAA1521
Diarsia dislocata|[5081]|LBCH3516-10|10-JDWBC-3516|658[0n]bp|Canada.British Columbia|BOLD:AAA1521
Diarsia dislocata|[5082]|LCHP479-07|07PROBE-10147|658[0n]bp|Canada.Manitoba|BOLD:AAA1521
Diarsia dislocata|[5083]|LBCH4447-10|10-JDWBC-4447|658[0n]bp|Canada.British Columbia|BOLD:AAA1521
[illegible]

Diarsia dislocata|[5081]|LBCH3516-10|10-JDWBC-3516|658[0n]bp|Canada.British Columbia|BOLD:AAA1521
Diarsia dislocata|[5082]|LCHP479-07|07PROBE-10147|658[0n]bp|Canada.Manitoba|BOLD:AAA1521
Diarsia dislocata|[5083]|LBCH4447-10|10-JDWBC-4447|658[0n]bp|Canada.British Columbia|BOLD:AAA1521
Diarsia dislocata|[5084]|LBCH4697-10|10-JDWBC-4697|658[0n]bp|Canada.British Columbia|BOLD:AAA1521
Diarsia dislocata|[5085]|LBCH408-10|10-JDWBC-0408|658[0n]bp|Canada.British Columbia|BOLD:AAA1521
Diarsia dislocata|[5086]|LBCH3798-10|10-JDWBC-3798|658[0n]bp|Canada.British Columbia|BOLD:AAA1521
Diarsia dislocata|[5087]|LBCH3104-10|10-JDWBC-3104|658[0n]bp|Canada.British Columbia|BOLD:AAA1521
Diarsia dislocata|[5088]|LBCH679-10|10-JDWBC-0679|658[0n]bp|Canada.British Columbia|BOLD:AAA1521
Diarsia dislocata|[5089]|LPABC019-09|08BBLEP-04238|658[0n]bp|Canada.Alberta|BOLD:AAA1521
Diarsia dislocata|[5090]|LBCH683-10|10-JDWBC-0683|658[0n]bp|Canada.British Columbia|BOLD:AAA1521
Diarsia dislocata|[5091]|LBCH3119-10|10-JDWBC-3119|658[0n]bp|Canada.British Columbia|BOLD:AAA1521
Diarsia dislocata|[5092]|LBCH3498-10|10-JDWBC-3498|658[0n]bp|Canada.British Columbia|BOLD:AAA1521
Diarsia dislocata|[5093]|LBCH4720-10|10-JDWBC-4720|658[0n]bp|Canada.British Columbia|BOLD:AAA1521
Diarsia dislocata|[5094]|LBCH240-10|10-JDWBC-0240|658[0n]bp|Canada.British Columbia|BOLD:AAA1521
Diarsia dislocata|[5095]|LBCG2248-09|08-JDWBC-2248|658[0n]bp|Canada.British Columbia|BOLD:AAA1521
Diarsia dislocata|[5096]|LBCH4699-10|10-JDWBC-4699|658[0n]bp|Canada.British Columbia|BOLD:AAA1521
Diarsia dislocata|[5097]|LBCH4043-10|10-JDWBC-4043|658[0n]bp|Canada.British Columbia|BOLD:AAA1521
Diarsia dislocata|[5098]|LBCH382-10|10-JDWBC-0382|658[0n]bp|Canada.British Columbia|BOLD:AAA1521
Diarsia dislocata|[5099]|LBCG2279-09|08-JDWBC-2279|658[0n]bp|Canada.British Columbia|BOLD:AAA1521
Diarsia dislocata|[5100]|LBCH371-10|10-JDWBC-0371|658[0n]bp|Canada.British Columbia|BOLD:AAA1521
Diarsia dislocata|[5101]|LBCH124-10|10-JDWBC-0124|658[0n]bp|Canada.British Columbia|BOLD:AAA1521
Diarsia dislocata|[5102]|LBCH689-10|10-JDWBC-0689|658[0n]bp|Canada.British Columbia|BOLD:AAA1521
Diarsia dislocata|[5103]|LCHP523-07|07PROBE-10191|658[0n]bp|Canada.Manitoba|BOLD:AAA1521
Diarsia dislocata|[5104]|LBCH664-10|10-JDWBC-0664|658[0n]bp|Canada.British Columbia|BOLD:AAA1521
Diarsia dislocata|[5105]|LBCH3801-10|10-JDWBC-3801|658[0n]bp|Canada.British Columbia|BOLD:AAA1521
Diarsia dislocata|[5106]|LBCH4018-10|10-JDWBC-4018|658[0n]bp|Canada.British Columbia|BOLD:AAA1521
Diarsia dislocata|[5107]|LBCH838-10|10-JDWBC-0838|658[0n]bp|Canada.British Columbia|BOLD:AAA1521
Diarsia dislocata|[5108]|LBCH4474-10|10-JDWBC-4474|658[0n]bp|Canada.British Columbia|BOLD:AAA1521
Diarsia dislocata|[5109]|LBCH3132-10|10-JDWBC-3132|658[0n]bp|Canada.British Columbia|BOLD:AAA1521
Diarsia dislocata|[5110]|LCH578-04|04HBL003578|658[0n]bp|Canada.Manitoba|BOLD:AAA1521
Diarsia dislocata|[5111]|LBCH378-10|10-JDWBC-0378|658[0n]bp|Canada.British Columbia|BOLD:AAA1521
Diarsia dislocata|[5112]|LCH276-04|04HBL003276|658[0n]bp|Canada.Manitoba|BOLD:AAA1521
Diarsia dislocata|[5113]|LBCH4457-10|10-JDWBC-4457|658[0n]bp|Canada.British Columbia|BOLD:AAA1521
Diarsia dislocata|[5114]|LBCH4714-10|10-JDWBC-4714|658[0n]bp|Canada.British Columbia|BOLD:AAA1521
Diarsia dislocata|[5115]|LBCH4704-10|10-JDWBC-4704|658[0n]bp|Canada.British Columbia|BOLD:AAA1521
Diarsia dislocata|[5116]|LBCH4716-10|10-JDWBC-4716|658[0n]bp|Canada.British Columbia|BOLD:AAA1521
Diarsia dislocata|[5117]|LBCH3080-10|10-JDWBC-3080|658[0n]bp|Canada.British Columbia|BOLD:AAA1521
Diarsia dislocata|[5118]|LCH298-04|04HBL003298|658[0n]bp|Canada.Manitoba|BOLD:AAA1521
Diarsia dislocata|[5119]|LBCH4723-10|10-JDWBC-4723|658[0n]bp|Canada.British Columbia|BOLD:AAA1521
Diarsia dislocata|[5120]|LBCH4743-10|10-JDWBC-4743|658[0n]bp|Canada.British Columbia|BOLD:AAA1521
Diarsia dislocata|[5121]|LBCH251-10|10-JDWBC-0251|658[0n]bp|Canada.British Columbia|BOLD:AAA1521
Diarsia dislocata|[5122]|LBCH3128-10|10-JDWBC-3128|658[0n]bp|Canada.British Columbia|BOLD:AAA1521
Diarsia dislocata|[5123]|LPABC364-09|08BBLEP-04583|658[0n]bp|Canada.Alberta|BOLD:AAA1521
Diarsia dislocata|[5124]|LBCH4703-10|10-JDWBC-4703|658[0n]bp|Canada.British Columbia|BOLD:AAA1521
Diarsia dislocata|[5125]|LBCH4033-10|10-JDWBC-4033|658[0n]bp|Canada.British Columbia|BOLD:AAA1521
Diarsia dislocata|[5126]|LBCH390-10|10-JDWBC-0390|658[0n]bp|Canada.British Columbia|BOLD:AAA1521
Diarsia dislocata|[5127]|LPABC390-09|08BBLEP-04609|658[0n]bp|Canada.Alberta|BOLD:AAA1521
Diarsia dislocata|[5128]|LBCH3098-10|10-JDWBC-3098|658[0n]bp|Canada.British Columbia|BOLD:AAA1521
Diarsia dislocata|[5129]|LBCH375-10|10-JDWBC-0375|658[0n]bp|Canada.British Columbia|BOLD:AAA1521
Diarsia dislocata|[5130]|LBCH4711-10|10-JDWBC-4711|658[0n]bp|Canada.British Columbia|BOLD:AAA1521
Diarsia dislocata|[5131]|LBCH392-10|10-JDWBC-0392|658[0n]bp|Canada.British Columbia|BOLD:AAA1521
Diarsia dislocata|[5132]|LBCH4477-10|10-JDWBC-4477|658[0n]bp|Canada.British Columbia|BOLD:AAA1521
Diarsia dislocata|[5133]|LBCH3520-10|10-JDWBC-3520|658[0n]bp|Canada.British Columbia|BOLD:AAA1521
Diarsia dislocata|[5134]|LBCH536-10|10-JDWBC-0536|658[0n]bp|Canada.British Columbia|BOLD:AAA1521
Diarsia dislocata|[5135]|LBCG2280-09|08-JDWBC-2280|658[0n]bp|Canada.British Columbia|BOLD:AAA1521
Diarsia dislocata|[5136]|LBCH3121-10|10-JDWBC-3121|658[0n]bp|Canada.British Columbia|BOLD:AAA1521
Diarsia dislocata|[5137]|LBCH368-10|10-JDWBC-0368|658[0n]bp|Canada.British Columbia|BOLD:AAA1521
Diarsia dislocata|[5138]|LCH268-04|04HBL003268|658[0n]bp|Canada.Manitoba|BOLD:AAA1521
Diarsia dislocata|[5139]|LBCH372-10|10-JDWBC-0372|658[0n]bp|Canada.British Columbia|BOLD:AAA1521
Diarsia dislocata|[5140]|LBCH3340-10|10-JDWBC-3340|658[0n]bp|Canada.British Columbia|BOLD:AAA1521
Diarsia dislocata|[5141]|LBCH3805-10|10-JDWBC-3805|658[0n]bp|Canada.British Columbia|BOLD:AAA1521
Diarsia dislocata|[5142]|LBCH3512-10|10-JDWBC-3512|658[0n]bp|Canada.British Columbia|BOLD:AAA1521
Diarsia dislocata|[5143]|LCHP704-07|07PROBE-10386|658[0n]bp|Canada.Manitoba|BOLD:AAA1521
Diarsia dislocata|[5144]|LBCH828-10|10-JDWBC-0828|658[0n]bp|Canada.British Columbia|BOLD:AAA1521
Diarsia dislocata|[5145]|LBCH053-10|10-JDWBC-0053|658[0n]bp|Canada.British Columbia|BOLD:AAA1521
Diarsia dislocata|[5146]|LPABC474-09|08BBLEP-04693|658[0n]bp|Canada.Alberta|BOLD:AAA1521
Diarsia dislocata|[5147]|LCHP786-07|07PROBE-10471|658[0n]bp|Canada.Manitoba|BOLD:AAA1521
Diarsia dislocata|[5148]|LBCH377-10|10-JDWBC-0377|658[0n]bp|Canada.British Columbia|BOLD:AAA1521
Diarsia dislocata|[5149]|LBCH4744-10|10-JDWBC-4744|658[0n]bp|Canada.British Columbia|BOLD:AAA1521
Diarsia dislocata|[5150]|LBCH3818-10|10-JDWBC-3818|658[0n]bp|Canada.British Columbia|BOLD:AAA1521
Diarsia dislocata|[5151]|LBCH031-10|10-JDWBC-0031|658[0n]bp|Canada.British Columbia|BOLD:AAA1521
Diarsia dislocata|[5152]|LBCH4040-10|10-JDWBC-4040|658[0n]bp|Canada.British Columbia|BOLD:AAA1521
Diarsia dislocata|[5153]|LBCH4719-10|10-JDWBC-4719|658[0n]bp|Canada.British Columbia|BOLD:AAA1521
Diarsia dislocata|[5154]|LBCH241-10|10-JDWBC-0241|658[0n]bp|Canada.British Columbia|BOLD:AAA1521
Diarsia dislocata|[5155]|LBCH3137-10|10-JDWBC-3137|658[0n]bp|Canada.British Columbia|BOLD:AAA1521
Diarsia dislocata|[5156]|LBCH4027-10|10-JDWBC-4027|658[0n]bp|Canada.British Columbia|BOLD:AAA1521
Diarsia dislocata|[5157]|LBCH3094-10|10-JDWBC-3094|658[0n]bp|Canada.British Columbia|BOLD:AAA1521
Diarsia dislocata|[5158]|LBCH841-10|10-JDWBC-0841|658[0n]bp|Canada.British Columbia|BOLD:AAA1521
Diarsia dislocata|[5159]|LBCH242-10|10-JDWBC-0242|658[0n]bp|Canada.British Columbia|BOLD:AAA1521
Diarsia dislocata|[5160]|LBCH4709-10|10-JDWBC-4709|658[0n]bp|Canada.British Columbia|BOLD:AAA1521
Diarsia dislocata|[5161]|LBCH3517-10|10-JDWBC-3517|658[0n]bp|Canada.British Columbia|BOLD:AAA1521
Diarsia dislocata|[5162]|LCHP868-07|07PROBE-10625|658[0n]bp|Canada.Manitoba|BOLD:AAA1521
Diarsia dislocata|[5163]|LCH297-04|04HBL003297|658[0n]bp|Canada.Manitoba|BOLD:AAA1521
Diarsia dislocata|[5164]|LBCG2275-09|08-JDWBC-2275|658[0n]bp|Canada.British Columbia|BOLD:AAA1521
Diarsia dislocata|[5165]|BBLPB343-10|10BBCLP-1342|658[0n]bp|Canada.Alberta|BOLD:AAA1521
Diarsia dislocata|[5166]|LBCH029-10|10-JDWBC-0029|658[0n]bp|Canada.British Columbia|BOLD:AAA1521
Diarsia dislocata|[5167]|LBCH366-10|10-JDWBC-0366|658[0n]bp|Canada.British Columbia|BOLD:AAA1521
Diarsia dislocata|[5168]|LBCH3500-10|10-JDWBC-3500|658[0n]bp|Canada.British Columbia|BOLD:AAA1521
Diarsia dislocata|[5169]|LBCH393-10|10-JDWBC-0393|658[0n]bp|Canada.British Columbia|BOLD:AAA1521
Diarsia dislocata|[5170]|LPABC418-09|08BBLEP-04637|658[0n]bp|Canada.Alberta|BOLD:AAA1521
Diarsia dislocata|[5171]|LCH273-04|04HBL003273|658[0n]bp|Canada.Manitoba|BOLD:AAA1521
Diarsia dislocata|[5172]|LBCH3502-10|10-JDWBC-3502|658[0n]bp|Canada.British Columbia|BOLD:AAA1521
Diarsia dislocata|[5173]|LBCH3109-10|10-JDWBC-3109|658[0n]bp|Canada.British Columbia|BOLD:AAA1521
Diarsia dislocata|[5174]|LBCH4471-10|10-JDWBC-4471|658[0n]bp|Canada.British Columbia|BOLD:AAA1521
Diarsia dislocata|[5175]|LBCH2382-10|10-JDWBC-2382|658[0n]bp|Canada.British Columbia|BOLD:AAA1521
Diarsia dislocata|[5176]|LBCG2284-09|08-JDWBC-2284|658[0n]bp|Canada.British Columbia|BOLD:AAA1521
Diarsia dislocata|[5177]|LBCH380-10|10-JDWBC-0380|658[0n]bp|Canada.British Columbia|BOLD:AAA1521
Diarsia dislocata|[5178]|LBCH3117-10|10-JDWBC-3117|658[0n]bp|Canada.British Columbia|BOLD:AAA1521
Diarsia dislocata|[5179]|LPABC017-09|08BBLEP-04236|658[0n]bp|Canada.Alberta|BOLD:AAA1521
Diarsia dislocata|[5180]|LBCH3806-10|10-JDWBC-3806|658[0n]bp|Canada.British Columbia|BOLD:AAA1521
Diarsia dislocata|[5181]|LBCH676-10|10-JDWBC-0676|658[0n]bp|Canada.British Columbia|BOLD:AAA1521
Diarsia dislocata|[5182]|LBCH3124-10|10-JDWBC-3124|658[0n]bp|Canada.British Columbia|BOLD:AAA1521
Diarsia dislocata|[5183]|LBCH126-10|10-JDWBC-0126|658[0n]bp|Canada.British Columbia|BOLD:AAA1521

Diarsia dislocata|[5181]|LBCH676-10|10-JDWBC-0676|658[0n]bp|Canada.British Columbia|BOLD:AAA1521
Diarsia dislocata|[5182]|LBCH3124-10|10-JDWBC-3124|658[0n]bp|Canada.British Columbia|BOLD:AAA1521
Diarsia dislocata|[5183]|LBCH126-10|10-JDWBC-0126|658[0n]bp|Canada.British Columbia|BOLD:AAA1521
Diarsia dislocata|[5184]|LBCH547-10|10-JDWBC-0547|658[0n]bp|Canada.British Columbia|BOLD:AAA1521
Diarsia dislocata|[5185]|LBCH542-10|10-JDWBC-0542|658[0n]bp|Canada.British Columbia|BOLD:AAA1521
Diarsia dislocata|[5186]|LBCH4470-10|10-JDWBC-4470|658[0n]bp|Canada.British Columbia|BOLD:AAA1521
Diarsia dislocata|[5187]|LBCH245-10|10-JDWBC-0245|658[0n]bp|Canada.British Columbia|BOLD:AAA1521
Diarsia dislocata|[5188]|LBCH3103-10|10-JDWBC-3103|658[0n]bp|Canada.British Columbia|BOLD:AAA1521
Diarsia dislocata|[5189]|LBCH4700-10|10-JDWBC-4700|658[0n]bp|Canada.British Columbia|BOLD:AAA1521
Diarsia dislocata|[5190]|LPABC382-09|08BBLEP-04601|658[0n]bp|Canada.Alberta|BOLD:AAA1521
Diarsia dislocata|[5191]|LBCH508-10|10-JDWBC-0508|658[0n]bp|Canada.British Columbia|BOLD:AAA1521
Diarsia dislocata|[5192]|LBCH949-10|10-JDWBC-0949|658[0n]bp|Canada.British Columbia|BOLD:AAA1521
Diarsia dislocata|[5193]|LBCH672-10|10-JDWBC-0672|658[0n]bp|Canada.British Columbia|BOLD:AAA1521
Diarsia dislocata|[5194]|LBCH3515-10|10-JDWBC-3515|658[0n]bp|Canada.British Columbia|BOLD:AAA1521
Diarsia dislocata|[5195]|LCHQ144-07|07PROBE-10913|658[0n]bp|Canada.Manitoba|BOLD:AAA1521
Diarsia dislocata|[5196]|LBCH3496-10|10-JDWBC-3496|658[0n]bp|Canada.British Columbia|BOLD:AAA1521
Diarsia dislocata|[5197]|LBCH526-10|10-JDWBC-0526|658[0n]bp|Canada.British Columbia|BOLD:AAA1521
Diarsia dislocata|[5198]|LBCH4476-10|10-JDWBC-4476|658[0n]bp|Canada.British Columbia|BOLD:AAA1521
Diarsia dislocata|[5199]|LBCH3108-10|10-JDWBC-3108|658[0n]bp|Canada.British Columbia|BOLD:AAA1521
Diarsia dislocata|[5200]|LBCH4715-10|10-JDWBC-4715|658[0n]bp|Canada.British Columbia|BOLD:AAA1521
Diarsia dislocata|[5201]|LBCH3808-10|10-JDWBC-3808|658[0n]bp|Canada.British Columbia|BOLD:AAA1521
Diarsia dislocata|[5202]|LBCH4034-10|10-JDWBC-4034|658[0n]bp|Canada.British Columbia|BOLD:AAA1521
Diarsia dislocata|[5203]|LBCH842-10|10-JDWBC-0842|658[0n]bp|Canada.British Columbia|BOLD:AAA1521
Diarsia dislocata|[5204]|LCHP521-07|07PROBE-10189|658[0n]bp|Canada.Manitoba|BOLD:AAA1521
Diarsia dislocata|[5205]|LBCH3821-10|10-JDWBC-3821|658[0n]bp|Canada.British Columbia|BOLD:AAA1521
Diarsia dislocata|[5206]|LBCH4690-10|10-JDWBC-4690|658[0n]bp|Canada.British Columbia|BOLD:AAA1521
Diarsia dislocata|[5207]|LBCH666-10|10-JDWBC-0666|658[0n]bp|Canada.British Columbia|BOLD:AAA1521
Diarsia dislocata|[5208]|LBCH4020-10|10-JDWBC-4020|658[0n]bp|Canada.British Columbia|BOLD:AAA1521
Diarsia dislocata|[5209]|LBCH3337-10|10-JDWBC-3337|658[0n]bp|Canada.British Columbia|BOLD:AAA1521
Diarsia dislocata|[5210]|LCH304-04|04HBL003304|658[0n]bp|Canada.Manitoba|BOLD:AAA1521
Diarsia dislocata|[5211]|LBCH3131-10|10-JDWBC-3131|658[0n]bp|Canada.British Columbia|BOLD:AAA1521
Diarsia dislocata|[5212]|LBCG2282-09|08-JDWBC-2282|658[0n]bp|Canada.British Columbia|BOLD:AAA1521
Diarsia dislocata|[5213]|LBCH515-10|10-JDWBC-0515|658[0n]bp|Canada.British Columbia|BOLD:AAA1521
Diarsia dislocata|[5214]|LBCH3814-10|10-JDWBC-3814|658[0n]bp|Canada.British Columbia|BOLD:AAA1521
Diarsia dislocata|[5215]|LBCH3107-10|10-JDWBC-3107|658[0n]bp|Canada.British Columbia|BOLD:AAA1521
Diarsia dislocata|[5216]|LBCH395-10|10-JDWBC-0395|658[0n]bp|Canada.British Columbia|BOLD:AAA1521
Diarsia dislocata|[5217]|LBCH832-10|10-JDWBC-0832|658[0n]bp|Canada.British Columbia|BOLD:AAA1521
Diarsia dislocata|[5218]|LBCH535-10|10-JDWBC-0535|658[0n]bp|Canada.British Columbia|BOLD:AAA1521
Diarsia dislocata|[5219]|LBCH682-10|10-JDWBC-0682|658[0n]bp|Canada.British Columbia|BOLD:AAA1521
Diarsia dislocata|[5220]|LBCH3523-10|10-JDWBC-3523|658[0n]bp|Canada.British Columbia|BOLD:AAA1521
Diarsia dislocata|[5221]|LBCH3134-10|10-JDWBC-3134|658[0n]bp|Canada.British Columbia|BOLD:AAA1521
Diarsia dislocata|[5222]|LBCH3339-10|10-JDWBC-3339|658[0n]bp|Canada.British Columbia|BOLD:AAA1521
Diarsia dislocata|[5223]|LBCH3501-10|10-JDWBC-3501|658[0n]bp|Canada.British Columbia|BOLD:AAA1521
Diarsia dislocata|[5224]|LBCH3521-10|10-JDWBC-3521|658[0n]bp|Canada.British Columbia|BOLD:AAA1521
Diarsia dislocata|[5225]|LBCH4468-10|10-JDWBC-4468|658[0n]bp|Canada.British Columbia|BOLD:AAA1521
Diarsia dislocata|[5226]|LBCH674-10|10-JDWBC-0674|658[0n]bp|Canada.British Columbia|BOLD:AAA1521
Diarsia dislocata|[5227]|LBCH548-10|10-JDWBC-0548|658[0n]bp|Canada.British Columbia|BOLD:AAA1521
Diarsia dislocata|[5228]|LBCH669-10|10-JDWBC-0669|658[0n]bp|Canada.British Columbia|BOLD:AAA1521
Diarsia dislocata|[5229]|LBCH4445-10|10-JDWBC-4445|658[0n]bp|Canada.British Columbia|BOLD:AAA1521
Diarsia dislocata|[5230]|LBCH3338-10|10-JDWBC-3338|658[0n]bp|Canada.British Columbia|BOLD:AAA1521
Diarsia dislocata|[5231]|LBCH965-10|10-JDWBC-0965|658[0n]bp|Canada.British Columbia|BOLD:AAA1521
Diarsia dislocata|[5232]|LCH311-04|04HBL003311|658[0n]bp|Canada.Manitoba|BOLD:AAA1521
Diarsia dislocata|[5233]|LBCH2380-10|10-JDWBC-2380|658[0n]bp|Canada.British Columbia|BOLD:AAA1521
Diarsia dislocata|[5234]|LBCH843-10|10-JDWBC-0843|658[0n]bp|Canada.British Columbia|BOLD:AAA1521
Diarsia dislocata|[5235]|LBCH3138-10|10-JDWBC-3138|658[0n]bp|Canada.British Columbia|BOLD:AAA1521
Diarsia dislocata|[5236]|LBCG2273-09|08-JDWBC-2273|658[0n]bp|Canada.British Columbia|BOLD:AAA1521
Diarsia dislocata|[5237]|LBCH3096-10|10-JDWBC-3096|658[0n]bp|Canada.British Columbia|BOLD:AAA1521
Diarsia dislocata|[5238]|LBCH3820-10|10-JDWBC-3820|658[0n]bp|Canada.British Columbia|BOLD:AAA1521
Diarsia dislocata|[5239]|LBCH4462-10|10-JDWBC-4462|658[0n]bp|Canada.British Columbia|BOLD:AAA1521
Diarsia dislocata|[5240]|LBCH388-10|10-JDWBC-0388|658[0n]bp|Canada.British Columbia|BOLD:AAA1521
Diarsia dislocata|[5241]|LBCH4735-10|10-JDWBC-4735|658[0n]bp|Canada.British Columbia|BOLD:AAA1521
Diarsia dislocata|[5242]|BBLPB620-10|10BBCLP-1619|658[0n]bp|Canada.British Columbia|BOLD:AAA1521
Diarsia dislocata|[5243]|LBCG2276-09|08-JDWBC-2276|658[0n]bp|Canada.British Columbia|BOLD:AAA1521
Diarsia dislocata|[5244]|LPABC402-09|08BBLEP-04621|658[0n]bp|Canada.Alberta|BOLD:AAA1521
Diarsia dislocata|[5245]|LBCH4705-10|10-JDWBC-4705|658[0n]bp|Canada.British Columbia|BOLD:AAA1521
Diarsia dislocata|[5246]|LPABC349-09|08BBLEP-04568|658[0n]bp|Canada.Alberta|BOLD:AAA1521
Diarsia dislocata|[5247]|LCH302-04|04HBL003302|658[0n]bp|Canada.Manitoba|BOLD:AAA1521
Diarsia dislocata|[5248]|LBCH3819-10|10-JDWBC-3819|658[0n]bp|Canada.British Columbia|BOLD:AAA1521
Diarsia dislocata|[5249]|LBCH3495-10|10-JDWBC-3495|658[0n]bp|Canada.British Columbia|BOLD:AAA1521
Diarsia dislocata|[5250]|LBCH959-10|10-JDWBC-0959|658[0n]bp|Canada.British Columbia|BOLD:AAA1521
Diarsia dislocata|[5251]|LPABC405-09|08BBLEP-04624|658[0n]bp|Canada.Alberta|BOLD:AAA1521
Diarsia dislocata|[5252]|LPABC401-09|08BBLEP-04620|658[0n]bp|Canada.Alberta|BOLD:AAA1521
Diarsia dislocata|[5253]|LPABC440-09|08BBLEP-04659|658[0n]bp|Canada.Alberta|BOLD:AAA1521
Diarsia dislocata|[5254]|LBCH3114-10|10-JDWBC-3114|658[0n]bp|Canada.British Columbia|BOLD:AAA1521
Diarsia dislocata|[5255]|LBCH3135-10|10-JDWBC-3135|658[0n]bp|Canada.British Columbia|BOLD:AAA1521
Diarsia dislocata|[5256]|LBCH3120-10|10-JDWBC-3120|658[0n]bp|Canada.British Columbia|BOLD:AAA1521
Diarsia dislocata|[5257]|LBCH3097-10|10-JDWBC-3097|658[0n]bp|Canada.British Columbia|BOLD:AAA1521
Diarsia dislocata|[5258]|LBCH4730-10|10-JDWBC-4730|658[0n]bp|Canada.British Columbia|BOLD:AAA1521
Diarsia dislocata|[5259]|LBCH397-10|10-JDWBC-0397|658[0n]bp|Canada.British Columbia|BOLD:AAA1521
Diarsia dislocata|[5260]|LBCH3341-10|10-JDWBC-3341|658[0n]bp|Canada.British Columbia|BOLD:AAA1521
Diarsia dislocata|[5261]|LBCG2296-09|08-JDWBC-2296|658[0n]bp|Canada.British Columbia|BOLD:AAA1521
Diarsia dislocata|[5262]|LCH315-04|04HBL003315|658[0n]bp|Canada.Manitoba|BOLD:AAA1521
Diarsia dislocata|[5263]|LBCH385-10|10-JDWBC-0385|658[0n]bp|Canada.British Columbia|BOLD:AAA1521
Diarsia dislocata|[5264]|LCH285-04|04HBL003285|658[0n]bp|Canada.Manitoba|BOLD:AAA1521
Diarsia dislocata|[5265]|LBCH4736-10|10-JDWBC-4736|658[0n]bp|Canada.British Columbia|BOLD:AAA1521
Diarsia dislocata|[5266]|LBCH249-10|10-JDWBC-0249|658[0n]bp|Canada.British Columbia|BOLD:AAA1521
Diarsia dislocata|[5267]|LBCH539-10|10-JDWBC-0539|658[0n]bp|Canada.British Columbia|BOLD:AAA1521
Diarsia dislocata|[5268]|LBCH964-10|10-JDWBC-0964|658[0n]bp|Canada.British Columbia|BOLD:AAA1521
Diarsia dislocata|[5269]|LBCH4031-10|10-JDWBC-4031|658[0n]bp|Canada.British Columbia|BOLD:AAA1521
Diarsia dislocata|[5270]|LBCH3499-10|10-JDWBC-3499|658[0n]bp|Canada.British Columbia|BOLD:AAA1521
Diarsia dislocata|[5271]|LBCH4446-10|10-JDWBC-4446|658[0n]bp|Canada.British Columbia|BOLD:AAA1521
Diarsia dislocata|[5272]|LBCH4472-10|10-JDWBC-4472|658[0n]bp|Canada.British Columbia|BOLD:AAA1521
Diarsia dislocata|[5273]|LBCH3139-10|10-JDWBC-3139|658[0n]bp|Canada.British Columbia|BOLD:AAA1521
Diarsia dislocata|[5274]|LBCH4444-10|10-JDWBC-4444|658[0n]bp|Canada.British Columbia|BOLD:AAA1521
Diarsia dislocata|[5275]|LPABC338-09|08BBLEP-04557|658[0n]bp|Canada.Alberta|BOLD:AAA1521
Diarsia dislocata|[5276]|LBCH525-10|10-JDWBC-0525|658[0n]bp|Canada.British Columbia|BOLD:AAA1521
Diarsia dislocata|[5277]|LBCH844-10|10-JDWBC-0844|658[0n]bp|Canada.British Columbia|BOLD:AAA1521
Diarsia dislocata|[5278]|LBCH3116-10|10-JDWBC-3116|658[0n]bp|Canada.British Columbia|BOLD:AAA1521
Diarsia dislocata|[5279]|LBCH244-10|10-JDWBC-0244|658[0n]bp|Canada.British Columbia|BOLD:AAA1521
Diarsia dislocata|[5280]|LBCH386-10|10-JDWBC-0386|658[0n]bp|Canada.British Columbia|BOLD:AAA1521
Diarsia dislocata|[5281]|LBCH4737-10|10-JDWBC-4737|658[0n]bp|Canada.British Columbia|BOLD:AAA1521
Diarsia dislocata|[5282]|LBCH3804-10|10-JDWBC-3804|658[0n]bp|Canada.British Columbia|BOLD:AAA1521
Diarsia dislocata|[5283]|LPABC376-09|08BBLEP-04595|658[0n]bp|Canada.Alberta|BOLD:AAA1521

Diarsia dislocata|[5280]|LBCH[illegible]-10|10-JDWBC-[illegible]|658[0n]bp|Canada.British Columbia|BOLD:AAA1521
Diarsia dislocata|[5281]|LBCH4737-10|10-JDWBC-4737|658[0n]bp|Canada.British Columbia|BOLD:AAA1521
Diarsia dislocata|[5282]|LBCH3804-10|10-JDWBC-3804|658[0n]bp|Canada.British Columbia|BOLD:AAA1521
Diarsia dislocata|[5283]|LPABC376-09|08BBLEP-04595|658[0n]bp|Canada.Alberta|BOLD:AAA1521
Diarsia dislocata|[5284]|LCH558-04|04HBL003558|658[0n]bp|Canada.Manitoba|BOLD:AAA1521
Diarsia dislocata|[5285]|LBCH510-10|10-JDWBC-0510|658[0n]bp|Canada.British Columbia|BOLD:AAA1521
Diarsia dislocata|[5286]|LPABC002-09|08BBLEP-04221|658[0n]bp|Canada.Alberta|BOLD:AAA1521
Diarsia dislocata|[5287]|LBCH833-10|10-JDWBC-0833|658[0n]bp|Canada.British Columbia|BOLD:AAA1521
Diarsia dislocata|[5288]|LBCH3822-10|10-JDWBC-3822|658[0n]bp|Canada.British Columbia|BOLD:AAA1521
Diarsia dislocata|[5289]|LCHP522-07|07PROBE-10190|658[0n]bp|Canada.Manitoba|BOLD:AAA1521
Diarsia dislocata|[5290]|LBCH4733-10|10-JDWBC-4733|658[0n]bp|Canada.British Columbia|BOLD:AAA1521
Diarsia dislocata|[5291]|LBCH401-10|10-JDWBC-0401|658[0n]bp|Canada.British Columbia|BOLD:AAA1521
Diarsia dislocata|[5292]|LBCH3133-10|10-JDWBC-3133|658[0n]bp|Canada.British Columbia|BOLD:AAA1521
Diarsia dislocata|[5293]|LBCH4710-10|10-JDWBC-4710|658[0n]bp|Canada.British Columbia|BOLD:AAA1521
Diarsia dislocata|[5294]|LBCH3513-10|10-JDWBC-3513|658[0n]bp|Canada.British Columbia|BOLD:AAA1521
Diarsia dislocata|[5295]|LPABC395-09|08BBLEP-04614|658[0n]bp|Canada.Alberta|BOLD:AAA1521
Diarsia dislocata|[5296]|LBCH243-10|10-JDWBC-0243|658[0n]bp|Canada.British Columbia|BOLD:AAA1521
Diarsia dislocata|[5297]|LBCH365-10|10-JDWBC-0365|658[0n]bp|Canada.British Columbia|BOLD:AAA1521
Diarsia dislocata|[5298]|LBCH831-10|10-JDWBC-0831|658[0n]bp|Canada.British Columbia|BOLD:AAA1521
Diarsia dislocata|[5299]|LBCH4731-10|10-JDWBC-4731|658[0n]bp|Canada.British Columbia|BOLD:AAA1521
Diarsia dislocata|[5300]|LBCH248-10|10-JDWBC-0248|658[0n]bp|Canada.British Columbia|BOLD:AAA1521
Diarsia dislocata|[5301]|LBCH3122-10|10-JDWBC-3122|658[0n]bp|Canada.British Columbia|BOLD:AAA1521
Diarsia dislocata|[5302]|LBCH684-10|10-JDWBC-0684|658[0n]bp|Canada.British Columbia|BOLD:AAA1521
Diarsia dislocata|[5303]|LBCH4038-10|10-JDWBC-4038|658[0n]bp|Canada.British Columbia|BOLD:AAA1521
Diarsia dislocata|[5304]|LBCH686-10|10-JDWBC-0686|658[0n]bp|Canada.British Columbia|BOLD:AAA1521
Diarsia dislocata|[5305]|LBCH4032-10|10-JDWBC-4032|658[0n]bp|Canada.British Columbia|BOLD:AAA1521
Diarsia dislocata|[5306]|LBCH4729-10|10-JDWBC-4729|658[0n]bp|Canada.British Columbia|BOLD:AAA1521
Diarsia dislocata|[5307]|LBCH690-10|10-JDWBC-0690|658[0n]bp|Canada.British Columbia|BOLD:AAA1521
Diarsia dislocata|[5308]|LBCH826-10|10-JDWBC-0826|658[0n]bp|Canada.British Columbia|BOLD:AAA1521
Diarsia dislocata|[5309]|LBCH4029-10|10-JDWBC-4029|658[0n]bp|Canada.British Columbia|BOLD:AAA1521
Diarsia dislocata|[5310]|LBCG2269-09|08-JDWBC-2269|658[0n]bp|Canada.British Columbia|BOLD:AAA1521
Diarsia dislocata|[5311]|LBCH4455-10|10-JDWBC-4455|658[0n]bp|Canada.British Columbia|BOLD:AAA1521
Diarsia dislocata|[5312]|LPABC368-09|08BBLEP-04587|658[0n]bp|Canada.Alberta|BOLD:AAA1521
Diarsia dislocata|[5313]|LBCH544-10|10-JDWBC-0544|658[0n]bp|Canada.British Columbia|BOLD:AAA1521
Diarsia dislocata|[5314]|LBCH3815-10|10-JDWBC-3815|658[0n]bp|Canada.British Columbia|BOLD:AAA1521
Diarsia dislocata|[5315]|LBCH830-10|10-JDWBC-0830|658[0n]bp|Canada.British Columbia|BOLD:AAA1521
Diarsia dislocata|[5316]|LBCH3817-10|10-JDWBC-3817|658[0n]bp|Canada.British Columbia|BOLD:AAA1521
Diarsia dislocata|[5317]|LCHP163-07|07PROBE-00565|658[0n]bp|Canada.Manitoba|BOLD:AAA1521
Diarsia dislocata|[5318]|LPABC375-09|08BBLEP-04594|658[0n]bp|Canada.Alberta|BOLD:AAA1521
Diarsia dislocata|[5319]|LPABB899-09|08BBLEP-04219|658[0n]bp|Canada.Alberta|BOLD:AAA1521
Diarsia dislocata|[5320]|LCH266-04|04HBL003266|658[0n]bp|Canada.Manitoba|BOLD:AAA1521
Diarsia dislocata|[5321]|LPABC422-09|08BBLEP-04641|658[0n]bp|Canada.Alberta|BOLD:AAA1521
Diarsia dislocata|[5322]|LBCH947-10|10-JDWBC-0947|658[0n]bp|Canada.British Columbia|BOLD:AAA1521
Diarsia dislocata|[5323]|LBCH667-10|10-JDWBC-0667|658[0n]bp|Canada.British Columbia|BOLD:AAA1521
Diarsia dislocata|[5324]|LCHP288-07|07PROBE-03854|658[0n]bp|Canada.Manitoba|BOLD:AAA1521
Diarsia dislocata|[5325]|LBCH3809-10|10-JDWBC-3809|658[0n]bp|Canada.British Columbia|BOLD:AAA1521
Diarsia dislocata|[5326]|LCH291-04|04HBL003291|658[0n]bp|Canada.Manitoba|BOLD:AAA1521
Diarsia dislocata|[5327]|LBCH4458-10|10-JDWBC-4458|658[0n]bp|Canada.British Columbia|BOLD:AAA1521
Diarsia dislocata|[5328]|LBCH4707-10|10-JDWBC-4707|658[0n]bp|Canada.British Columbia|BOLD:AAA1521
Diarsia dislocata|[5329]|LBCH3111-10|10-JDWBC-3111|658[0n]bp|Canada.British Columbia|BOLD:AAA1521
Diarsia dislocata|[5330]|LBCH374-10|10-JDWBC-0374|658[0n]bp|Canada.British Columbia|BOLD:AAA1521
Diarsia dislocata|[5331]|LBCH4456-10|10-JDWBC-4456|658[0n]bp|Canada.British Columbia|BOLD:AAA1521
Diarsia dislocata|[5332]|LBCG2278-09|08-JDWBC-2278|658[0n]bp|Canada.British Columbia|BOLD:AAA1521
Diarsia dislocata|[5333]|LCH308-04|04HBL003308|658[0n]bp|Canada.Manitoba|BOLD:AAA1521
Diarsia dislocata|[5334]|LBCH950-10|10-JDWBC-0950|658[0n]bp|Canada.British Columbia|BOLD:AAA1521
Diarsia dislocata|[5335]|LBCH3127-10|10-JDWBC-3127|658[0n]bp|Canada.British Columbia|BOLD:AAA1521
Diarsia dislocata|[5336]|LBCH538-10|10-JDWBC-0538|658[0n]bp|Canada.British Columbia|BOLD:AAA1521
Diarsia dislocata|[5337]|LBCH948-10|10-JDWBC-0948|658[0n]bp|Canada.British Columbia|BOLD:AAA1521
Diarsia dislocata|[5338]|LBCH4045-10|10-JDWBC-4045|658[0n]bp|Canada.British Columbia|BOLD:AAA1521
Diarsia dislocata|[5339]|LCH278-04|04HBL003278|658[0n]bp|Canada.Manitoba|BOLD:AAA1521
Diarsia dislocata|[5340]|LBCH516-10|10-JDWBC-0516|658[0n]bp|Canada.British Columbia|BOLD:AAA1521
Diarsia dislocata|[5341]|LBCH2381-10|10-JDWBC-2381|658[0n]bp|Canada.British Columbia|BOLD:AAA1521
Diarsia dislocata|[5342]|LBCH364-10|10-JDWBC-0364|658[0n]bp|Canada.British Columbia|BOLD:AAA1521
Diarsia dislocata|[5343]|LBCH6748-10|10-JDWBC-6748|658[0n]bp|Canada.British Columbia|BOLD:AAA1521
Diarsia dislocata|[5344]|LCH310-04|04HBL003310|658[0n]bp|Canada.Manitoba|BOLD:AAA1521
Diarsia dislocata|[5345]|LBCH685-10|10-JDWBC-0685|658[0n]bp|Canada.British Columbia|BOLD:AAA1521
Diarsia dislocata|[5346]|LBCH675-10|10-JDWBC-0675|658[0n]bp|Canada.British Columbia|BOLD:AAA1521
Diarsia dislocata|[5347]|LBCH546-10|10-JDWBC-0546|658[0n]bp|Canada.British Columbia|BOLD:AAA1521
Diarsia dislocata|[5348]|LBCH840-10|10-JDWBC-0840|658[0n]bp|Canada.British Columbia|BOLD:AAA1521
Diarsia dislocata|[5349]|LBCH3105-10|10-JDWBC-3105|658[0n]bp|Canada.British Columbia|BOLD:AAA1521
Diarsia dislocata|[5350]|LBCH3816-10|10-JDWBC-3816|658[0n]bp|Canada.British Columbia|BOLD:AAA1521
Diarsia dislocata|[5351]|LBCH2383-10|10-JDWBC-2383|658[0n]bp|Canada.British Columbia|BOLD:AAA1521
Diarsia dislocata|[5352]|LBCH3494-10|10-JDWBC-3494|658[0n]bp|Canada.British Columbia|BOLD:AAA1521
Diarsia dislocata|[5353]|LBCH4467-10|10-JDWBC-4467|658[0n]bp|Canada.British Columbia|BOLD:AAA1521
Diarsia dislocata|[5354]|LBCH367-10|10-JDWBC-0367|658[0n]bp|Canada.British Columbia|BOLD:AAA1521
Diarsia dislocata|[5355]|LBCH3812-10|10-JDWBC-3812|658[0n]bp|Canada.British Columbia|BOLD:AAA1521
Diarsia dislocata|[5356]|LCHP702-07|07PROBE-10384|658[0n]bp|Canada.Manitoba|BOLD:AAA1521
Diarsia dislocata|[5357]|LBCH3807-10|10-JDWBC-3807|658[0n]bp|Canada.British Columbia|BOLD:AAA1521
Diarsia dislocata|[5358]|LBCH3100-10|10-JDWBC-3100|658[0n]bp|Canada.British Columbia|BOLD:AAA1521
Diarsia dislocata|[5359]|LBCH662-10|10-JDWBC-0662|658[0n]bp|Canada.British Columbia|BOLD:AAA1521
Diarsia dislocata|[5360]|LCH316-04|04HBL003316|658[0n]bp|Canada.Manitoba|BOLD:AAA1521
Diarsia dislocata|[5361]|LBCH680-10|10-JDWBC-0680|658[0n]bp|Canada.British Columbia|BOLD:AAA1521
Diarsia dislocata|[5362]|LBCH3102-10|10-JDWBC-3102|658[0n]bp|Canada.British Columbia|BOLD:AAA1521
Diarsia dislocata|[5363]|LBCH370-10|10-JDWBC-0370|658[0n]bp|Canada.British Columbia|BOLD:AAA1521
Diarsia dislocata|[5364]|LBCH381-10|10-JDWBC-0381|658[0n]bp|Canada.British Columbia|BOLD:AAA1521
Diarsia dislocata|[5365]|LBCH4459-10|10-JDWBC-4459|658[0n]bp|Canada.British Columbia|BOLD:AAA1521
Diarsia dislocata|[5366]|LBCH511-10|10-JDWBC-0511|658[0n]bp|Canada.British Columbia|BOLD:AAA1521
Diarsia dislocata|[5367]|LBCH4746-10|10-JDWBC-4746|658[0n]bp|Canada.British Columbia|BOLD:AAA1521
Diarsia dislocata|[5368]|LBCH128-10|10-JDWBC-0128|658[0n]bp|Canada.British Columbia|BOLD:AAA1521
Diarsia dislocata|[5369]|LCH290-04|04HBL003290|658[0n]bp|Canada.Manitoba|BOLD:AAA1521
Diarsia dislocata|[5370]|LBCH4021-10|10-JDWBC-4021|658[0n]bp|Canada.British Columbia|BOLD:AAA1521
Diarsia dislocata|[5371]|LBCH3803-10|10-JDWBC-3803|658[0n]bp|Canada.British Columbia|BOLD:AAA1521
Diarsia dislocata|[5372]|LBCH4035-10|10-JDWBC-4035|658[0n]bp|Canada.British Columbia|BOLD:AAA1521
Diarsia dislocata|[5373]|LCHP525-07|07PROBE-10193|658[0n]bp|Canada.Manitoba|BOLD:AAA1521
Diarsia dislocata|[5374]|LBCH4742-10|10-JDWBC-4742|658[0n]bp|Canada.British Columbia|BOLD:AAA1521
Diarsia dislocata|[5375]|LBCH3125-10|10-JDWBC-3125|658[0n]bp|Canada.British Columbia|BOLD:AAA1521
Diarsia dislocata|[5376]|LBCH4451-10|10-JDWBC-4451|658[0n]bp|Canada.British Columbia|BOLD:AAA1521
Diarsia dislocata|[5377]|LBCH3813-10|10-JDWBC-3813|658[0n]bp|Canada.British Columbia|BOLD:AAA1521
Diarsia dislocata|[5378]|LBCH4022-10|10-JDWBC-4022|658[0n]bp|Canada.British Columbia|BOLD:AAA1521
Diarsia dislocata|[5379]|LPABC018-09|08BBLEP-04237|658[0n]bp|Canada.Alberta|BOLD:AAA1521
Diarsia dislocata|[5380]|LBCH3519-10|10-JDWBC-3519|658[0n]bp|Canada.British Columbia|BOLD:AAA1521
Diarsia dislocata|[5381]|LBCH4725-10|10-JDWBC-4725|658[0n]bp|Canada.British Columbia|BOLD:AAA1521
Diarsia dislocata|[5382]|LBCH127-10|10-JDWBC-0127|658[0n]bp|Canada.British Columbia|BOLD:AAA1521
[illegible]

Diarsia dislocata|[5380]|LBCH3519-10|10-JDWBC-3519|658[0n]bp|Canada.British Columbia|BOLD:AAA1521
Diarsia dislocata|[5381]|LBCH4725-10|10-JDWBC-4725|658[0n]bp|Canada.British Columbia|BOLD:AAA1521
Diarsia dislocata|[5382]|LBCH127-10|10-JDWBC-0127|658[0n]bp|Canada.British Columbia|BOLD:AAA1521
Diarsia dislocata|[5383]|LBCH835-10|10-JDWBC-0835|658[0n]bp|Canada.British Columbia|BOLD:AAA1521
Diarsia dislocata|[5384]|LBCH246-10|10-JDWBC-0246|658[0n]bp|Canada.British Columbia|BOLD:AAA1521
Diarsia dislocata|[5385]|LBCH4732-10|10-JDWBC-4732|658[0n]bp|Canada.British Columbia|BOLD:AAA1521
Diarsia dislocata|[5386]|LBCH824-10|10-JDWBC-0824|658[0n]bp|Canada.British Columbia|BOLD:AAA1521
Diarsia dislocata|[5387]|LBCH687-10|10-JDWBC-0687|658[0n]bp|Canada.British Columbia|BOLD:AAA1521
Diarsia dislocata|[5388]|LBCH3113-10|10-JDWBC-3113|658[0n]bp|Canada.British Columbia|BOLD:AAA1521
Diarsia dislocata|[5389]|LBCH4712-10|10-JDWBC-4712|658[0n]bp|Canada.British Columbia|BOLD:AAA1521
Diarsia dislocata|[5390]|LBCH509-10|10-JDWBC-0509|658[0n]bp|Canada.British Columbia|BOLD:AAA1521
Diarsia dislocata|[5391]|LBCH827-10|10-JDWBC-0827|658[0n]bp|Canada.British Columbia|BOLD:AAA1521
Diarsia dislocata|[5392]|LBCH4727-10|10-JDWBC-4727|658[0n]bp|Canada.British Columbia|BOLD:AAA1521
Diarsia dislocata|[5393]|LBCG2271-09|08-JDWBC-2271|658[0n]bp|Canada.British Columbia|BOLD:AAA1521
Diarsia dislocata|[5394]|LBCH688-10|10-JDWBC-0688|658[0n]bp|Canada.British Columbia|BOLD:AAA1521
Diarsia dislocata|[5395]|LBCH678-10|10-JDWBC-0678|658[0n]bp|Canada.British Columbia|BOLD:AAA1521
Diarsia dislocata|[5396]|LBCH030-10|10-JDWBC-0030|658[0n]bp|Canada.British Columbia|BOLD:AAA1521
Diarsia dislocata|[5397]|LBCH3514-10|10-JDWBC-3514|658[0n]bp|Canada.British Columbia|BOLD:AAA1521
Diarsia dislocata|[5398]|LBCH3099-10|10-JDWBC-3099|658[0n]bp|Canada.British Columbia|BOLD:AAA1521
Diarsia dislocata|[5399]|LBCH052-10|10-JDWBC-0052|658[0n]bp|Canada.British Columbia|BOLD:AAA1521
Diarsia dislocata|[5400]|LBCH3115-10|10-JDWBC-3115|658[0n]bp|Canada.British Columbia|BOLD:AAA1521
Diarsia dislocata|[5401]|LBCH032-10|10-JDWBC-0032|658[0n]bp|Canada.British Columbia|BOLD:AAA1521
Diarsia dislocata|[5402]|LBCH692-10|10-JDWBC-0692|658[0n]bp|Canada.British Columbia|BOLD:AAA1521
Diarsia dislocata|[5403]|LBCH3336-10|10-JDWBC-3336|658[0n]bp|Canada.British Columbia|BOLD:AAA1521
Diarsia dislocata|[5404]|LBCH4441-10|10-JDWBC-4441|644[0n]bp|Canada.British Columbia|BOLD:AAA1521
Diarsia dislocata|[5405]|LBCH551-10|10-JDWBC-0551|658[0n]bp|Canada.British Columbia|BOLD:AAA1521
Diarsia dislocata|[5406]|LBCH512-10|10-JDWBC-0512|658[0n]bp|Canada.British Columbia|BOLD:AAA1521
Diarsia dislocata|[5407]|LBCH384-10|10-JDWBC-0384|658[0n]bp|Canada.British Columbia|BOLD:AAA1521
Diarsia dislocata|[5408]|LBCH3112-10|10-JDWBC-3112|658[0n]bp|Canada.British Columbia|BOLD:AAA1521
Diarsia dislocata|[5409]|LBCH540-10|10-JDWBC-0540|658[0n]bp|Canada.British Columbia|BOLD:AAA1521
Diarsia dislocata|[5410]|LBCH3140-10|10-JDWBC-3140|658[0n]bp|Canada.British Columbia|BOLD:AAA1521
Diarsia dislocata|[5411]|LBCH4721-10|10-JDWBC-4721|658[0n]bp|Canada.British Columbia|BOLD:AAA1521
Diarsia dislocata|[5412]|LBCH3504-10|10-JDWBC-3504|658[0n]bp|Canada.British Columbia|BOLD:AAA1521
Diarsia dislocata|[5413]|LBCH394-10|10-JDWBC-0394|658[0n]bp|Canada.British Columbia|BOLD:AAA1521
Diarsia dislocata|[5414]|LBCH129-10|10-JDWBC-0129|658[0n]bp|Canada.British Columbia|BOLD:AAA1521
Diarsia dislocata|[5415]|LBCH3123-10|10-JDWBC-3123|658[0n]bp|Canada.British Columbia|BOLD:AAA1521
Diarsia dislocata|[5416]|LBCH3110-10|10-JDWBC-3110|658[0n]bp|Canada.British Columbia|BOLD:AAA1521
Diarsia dislocata|[5417]|LBCH376-10|10-JDWBC-0376|658[0n]bp|Canada.British Columbia|BOLD:AAA1521
Diarsia dislocata|[5418]|LCH283-04|04HBL003283|654[0n]bp|Canada.Manitoba|BOLD:AAA1521
Diarsia dislocata|[5419]|LCH579-04|04HBL003579|594[0n]bp|Canada.Manitoba|BOLD:AAA1521
Diarsia dislocata|[5420]|LBCH4713-10|10-JDWBC-4713|643[0n]bp|Canada.British Columbia|BOLD:AAA1521
Diarsia dislocata|[5421]|BBLPE244-09|09BBELE-2244|622[0n]bp|Canada.Newfoundland and Labrador|BOLD:AAA...
Diarsia dislocata|[5422]|BBLPE230-09|09BBELE-2230|634[0n]bp|Canada.Newfoundland and Labrador|BOLD:AAA...
Diarsia dislocata|[5423]|LCH274-04|04HBL003274|645[0n]bp|Canada.Manitoba|BOLD:AAA1521
Diarsia dislocata|[5424]|LBCH4728-10|10-JDWBC-4728|649[0n]bp|Canada.British Columbia|BOLD:AAA1521
Diarsia dislocata|[5425]|LBCH4452-10|10-JDWBC-4452|647[0n]bp|Canada.British Columbia|BOLD:AAA1521
Diarsia dislocata|[5426]|TTCFW642-08|08BBCOL-0415|631[0n]bp|Canada.Alberta|
Diarsia dislocata|[5427]|LCHP515-07|07PROBE-10183|634[0n]bp|Canada.Manitoba|BOLD:AAA1521
Diarsia dislocata|[5428]|LBCH3797-10|10-JDWBC-3797|644[0n]bp|Canada.British Columbia|BOLD:AAA1521
Diarsia dislocata|[5429]|LBCH4465-10|10-JDWBC-4465|650[0n]bp|Canada.British Columbia|BOLD:AAA1521
Diarsia dislocata|[5430]|LCHQ142-07|07PROBE-10911|658[1n]bp|Canada.Manitoba|BOLD:AAA1521
Diarsia dislocata|[5431]|LBCH4460-10|10-JDWBC-4460|658[0n]bp|Canada.British Columbia|BOLD:AAA1521
Diarsia dislocata|[5432]|LBCH4028-10|10-JDWBC-4028|658[0n]bp|Canada.British Columbia|BOLD:AAA1521
Diarsia dislocata|[5433]|LBCH2384-10|10-JDWBC-2384|658[0n]bp|Canada.British Columbia|BOLD:AAA1521
Diarsia dislocata|[5434]|LBCH389-10|10-JDWBC-0389|658[0n]bp|Canada.British Columbia|BOLD:AAA1521
Diarsia dislocata|[5435]|LBCG2283-09|08-JDWBC-2283|658[0n]bp|Canada.British Columbia|BOLD:AAA1521
Diarsia dislocata|[5436]|LBCH4701-10|10-JDWBC-4701|658[0n]bp|Canada.British Columbia|BOLD:AAA1521
Diarsia dislocata|[5437]|LBCG2297-09|08-JDWBC-2297|658[0n]bp|Canada.British Columbia|BOLD:AAA1521
Diarsia dislocata|[5438]|LBCH4726-10|10-JDWBC-4726|658[0n]bp|Canada.British Columbia|BOLD:AAA1521
Diarsia dislocata|[5439]|LBCH3503-10|10-JDWBC-3503|658[0n]bp|Canada.British Columbia|BOLD:AAA1521
Diarsia dislocata|[5440]|LBCH4698-10|10-JDWBC-4698|658[0n]bp|Canada.British Columbia|BOLD:AAA1521
Diarsia dislocata|[5441]|LBCH2386-10|10-JDWBC-2386|658[0n]bp|Canada.British Columbia|BOLD:AAA1521
Diarsia dislocata|[5442]|LBCH3136-10|10-JDWBC-3136|658[0n]bp|Canada.British Columbia|BOLD:AAA1521
Diarsia dislocata|[5443]|LBCH4042-10|10-JDWBC-4042|658[0n]bp|Canada.British Columbia|BOLD:AAA1521
Diarsia dislocata|[5444]|LBCH4449-10|10-JDWBC-4449|658[0n]bp|Canada.British Columbia|BOLD:AAA1521
Diarsia dislocata|[5445]|LBCH537-10|10-JDWBC-0537|658[0n]bp|Canada.British Columbia|BOLD:AAA1521
Diarsia dislocata|[5446]|LBCH670-10|10-JDWBC-0670|658[0n]bp|Canada.British Columbia|BOLD:AAA1521
Diarsia dislocata|[5447]|LBCH4706-10|10-JDWBC-4706|658[0n]bp|Canada.British Columbia|BOLD:AAA1521
Diarsia dislocata|[5448]|LBCH4741-10|10-JDWBC-4741|658[0n]bp|Canada.British Columbia|BOLD:AAA1521
Diarsia dislocata|[5449]|LBCH514-10|10-JDWBC-0514|658[0n]bp|Canada.British Columbia|BOLD:AAA1521
Diarsia dislocata|[5450]|LBCH379-10|10-JDWBC-0379|658[0n]bp|Canada.British Columbia|BOLD:AAA1521
Diarsia dislocata|[5451]|LBCH4724-10|10-JDWBC-4724|658[0n]bp|Canada.British Columbia|BOLD:AAA1521
Diarsia dislocata|[5452]|LCH269-04|04HBL003269|658[0n]bp|Canada.Manitoba|BOLD:AAA1521
Diarsia dislocata|[5453]|LBCH3101-10|10-JDWBC-3101|658[0n]bp|Canada.British Columbia|BOLD:AAA1521
Diarsia dislocata|[5454]|LBCH3823-10|10-JDWBC-3823|658[0n]bp|Canada.British Columbia|BOLD:AAA1521
Diarsia dislocata|[5455]|LCH307-04|04HBL003307|658[0n]bp|Canada.Manitoba|BOLD:AAA1521
Diarsia jucunda|[5456]|RDLQ721-07|DH008275|562[0n]bp|Canada.Quebec|BOLD:AAA1521
Diarsia jucunda|[5457]|RDLQ710-07|DH007295|617[0n]bp|Canada.Quebec|BOLD:AAA1521
Diarsia jucunda|[5458]|MNBB611-05|05-NBSTA-527|658[0n]bp|Canada.New Brunswick|BOLD:AAA1521
Diarsia jucunda|[5459]|BBLEC330-09|09BBELE-0330|658[0n]bp|Canada.Nova Scotia|BOLD:AAA1521
Diarsia jucunda|[5460]|BBLPE026-09|09BBELE-2026|658[0n]bp|Canada.Nova Scotia|BOLD:AAA1521
Diarsia jucunda|[5461]|BBLPE024-09|09BBELE-2024|621[0n]bp|Canada.Nova Scotia|BOLD:AAA1521
Diarsia jucunda|[5462]|RDLQ712-07|DH007311|646[0n]bp|Canada.Quebec|BOLD:AAA1521
Diarsia jucunda|[5463]|BBLPE123-09|09BBELE-2123|658[0n]bp|Canada.Nova Scotia|BOLD:AAA1521
Diarsia jucunda|[5464]|RDLQB908-05|DH009356|658[0n]bp|Canada.Quebec|BOLD:AAA1521
Diarsia jucunda|[5465]|BBLEC280-09|09BBELE-0280|658[0n]bp|Canada.Nova Scotia|BOLD:AAA1521
Diarsia jucunda|[5466]|BBLPC448-09|09BBELE-1448|658[0n]bp|Canada.New Brunswick|BOLD:AAA1521
Diarsia jucunda|[5467]|BBLEC322-09|09BBELE-0322|658[0n]bp|Canada.Nova Scotia|BOLD:AAA1521
Diarsia jucunda|[5468]|BBLPC094-09|09BBELE-1094|658[0n]bp|Canada.New Brunswick|BOLD:AAA1521
Diarsia jucunda|[5469]|BBLEC303-09|09BBELE-0303|645[0n]bp|Canada.Nova Scotia|BOLD:AAA1521
Diarsia jucunda|[5470]|BBLEC338-09|09BBELE-0338|622[0n]bp|Canada.Nova Scotia|BOLD:AAA1521
Diarsia jucunda|[5471]|BBLPE182-09|09BBELE-2182|636[0n]bp|Canada.Nova Scotia|BOLD:AAA1521
Diarsia jucunda|[5472]|BBLEC276-09|09BBELE-0276|629[0n]bp|Canada.Nova Scotia|BOLD:AAA1521
Diarsia jucunda|[5473]|BBLEC331-09|09BBELE-0331|658[0n]bp|Canada.Nova Scotia|BOLD:AAA1521
Diarsia jucunda|[5474]|BBLPE085-09|09BBELE-2085|658[0n]bp|Canada.Nova Scotia|BOLD:AAA1521
Diarsia jucunda|[5475]|BBLPE031-09|09BBELE-2031|658[0n]bp|Canada.Nova Scotia|BOLD:AAA1521
Diarsia jucunda|[5476]|RDLQB254-05|DH010340|658[0n]bp|Canada.Quebec|BOLD:AAA1521
Diarsia jucunda|[5477]|BBLEC106-09|09BBELE-0106|658[0n]bp|Canada.Nova Scotia|BOLD:AAA1521
Diarsia jucunda|[5478]|BBLPE039-09|09BBELE-2039|658[0n]bp|Canada.Nova Scotia|BOLD:AAA1521
Diarsia jucunda|[5479]|BBLEC270-09|09BBELE-0270|658[0n]bp|Canada.Nova Scotia|BOLD:AAA1521
Diarsia jucunda|[5480]|BBLPC589-09|09BBELE-1589|658[0n]bp|Canada.Nova Scotia|BOLD:AAA1521
Diarsia jucunda|[5481]|BBLEC261-09|09BBELE-0261|658[0n]bp|Canada.Nova Scotia|BOLD:AAA1521
Diarsia calgary|[5482]|BBLPB443-10|10BBCLP-1442|658[0n]bp|Canada.Alberta|BOLD:ABX6134

Diarsia jucunda|[5480]|BBLPC589-09|09BBELE-1589|658[0n]bp|Canada.Nova Scotia|BOLD:AAA1521
Diarsia jucunda|[5481]|BBLEC261-09|09BBELE-0261|658[0n]bp|Canada.Nova Scotia|BOLD:AAA1521
Diarsia calgary|[5482]|BBLPB443-10|10BBCLP-1442|658[0n]bp|Canada.Alberta|BOLD:ABX6134
Diarsia calgary|[5483]|RDMAB255-05|UASM41463|658[0n]bp|Canada.Alberta|BOLD:ABX6134
Diarsia calgary|[5484]|LOWCD294-06|CGWC-3114|658[0n]bp|Canada.British Columbia|BOLD:ABX6134
Diarsia calgary|[5485]|LOWCE752-06|CGWC-4512|658[0n]bp|Canada.British Columbia|BOLD:ABX6134
Diarsia calgary|[5486]|BBLPB452-10|10BBCLP-1451|658[0n]bp|Canada.British Columbia|BOLD:ABX6134
Diarsia calgary|[5487]|RDMAB264-05|UASM41464|658[0n]bp|Canada.Alberta|BOLD:ABX6134
Diarsia calgary|[5488]|LBCH521-10|10-JDWBC-0521|658[0n]bp|Canada.British Columbia|BOLD:ACE3480
Diarsia calgary|[5489]|LBCH049-10|10-JDWBC-0049|658[0n]bp|Canada.British Columbia|BOLD:ACE3480
Diarsia calgary|[5490]|LBCH041-10|10-JDWBC-0041|658[0n]bp|Canada.British Columbia|BOLD:ACE3480
Diarsia calgary|[5491]|LBCH671-10|10-JDWBC-0671|658[0n]bp|Canada.British Columbia|BOLD:ACE3480
Diarsia calgary|[5492]|LBCH150-10|10-JDWBC-0150|658[0n]bp|Canada.British Columbia|BOLD:ACE3480
Diarsia calgary|[5493]|LBCH2899-10|10-JDWBC-2899|658[0n]bp|Canada.British Columbia|BOLD:ACE3480
Diarsia calgary|[5494]|LBCH253-10|10-JDWBC-0253|658[0n]bp|Canada.British Columbia|BOLD:ACE3480
Diarsia calgary|[5495]|LBCH138-10|10-JDWBC-0138|658[0n]bp|Canada.British Columbia|BOLD:ACE3480
Diarsia esurialis|[5496]|LBCH523-10|10-JDWBC-0523|658[0n]bp|Canada.British Columbia|BOLD:ACF1489
Diarsia esurialis|[5497]|LBCH131-10|10-JDWBC-0131|658[0n]bp|Canada.British Columbia|BOLD:ACF1489
Diarsia esurialis|[5498]|LBCH657-10|10-JDWBC-0657|658[0n]bp|Canada.British Columbia|BOLD:ACF1489
Diarsia esurialis|[5499]|LBCH264-10|10-JDWBC-0264|658[0n]bp|Canada.British Columbia|BOLD:ACF1489
Diarsia esurialis|[5500]|LBCH668-10|10-JDWBC-0668|658[0n]bp|Canada.British Columbia|BOLD:ACF1489
Diarsia esurialis|[5501]|LBCH2906-10|10-JDWBC-2906|658[0n]bp|Canada.British Columbia|BOLD:ACF1489
Diarsia esurialis|[5502]|LBCH261-10|10-JDWBC-0261|658[0n]bp|Canada.British Columbia|BOLD:ACF1489
Diarsia esurialis|[5503]|LBCH2387-10|10-JDWBC-2387|658[0n]bp|Canada.British Columbia|BOLD:ACF1489
Diarsia esurialis|[5504]|LBCH3041-10|10-JDWBC-3041|658[0n]bp|Canada.British Columbia|BOLD:ACF1489
Diarsia esurialis|[5505]|LBCS136-07|UBC-2007-0129|658[0n]bp|Canada.British Columbia|BOLD:ACF1489
Diarsia esurialis|[5506]|LBCH681-10|10-JDWBC-0681|658[0n]bp|Canada.British Columbia|BOLD:ACF1489
Diarsia esurialis|[5507]|LBCS135-07|UBC-2007-0128|658[0n]bp|Canada.British Columbia|BOLD:ACF1489
Diarsia esurialis|[5508]|LBCH2392-10|10-JDWBC-2392|658[0n]bp|Canada.British Columbia|BOLD:ACF1489
Diarsia esurialis|[5509]|LBCH407-10|10-JDWBC-0407|658[0n]bp|Canada.British Columbia|BOLD:ACF1489
Diarsia esurialis|[5510]|LBCH3047-10|10-JDWBC-3047|658[0n]bp|Canada.British Columbia|BOLD:ACF1489
Diarsia esurialis|[5511]|LBCH1937-10|10-JDWBC-1937|636[0n]bp|Canada.British Columbia|BOLD:ACF1489
Diarsia esurialis|[5512]|LBCH849-10|10-JDWBC-0849|658[0n]bp|Canada.British Columbia|BOLD:ACF1489
Diarsia esurialis|[5513]|LBCH399-10|10-JDWBC-0399|658[0n]bp|Canada.British Columbia|BOLD:ACF1489
Diarsia esurialis|[5514]|LBCH829-10|10-JDWBC-0829|658[0n]bp|Canada.British Columbia|BOLD:ACF1489
Diarsia esurialis|[5515]|LBCH517-10|10-JDWBC-0517|658[0n]bp|Canada.British Columbia|BOLD:ACF1489
Diarsia esurialis|[5516]|LBCH043-10|10-JDWBC-0043|658[0n]bp|Canada.British Columbia|BOLD:ACF1489
Diarsia esurialis|[5517]|LBCH846-10|10-JDWBC-0846|658[0n]bp|Canada.British Columbia|BOLD:ACF1489
Diarsia esurialis|[5518]|LBCH2961-10|10-JDWBC-2961|658[0n]bp|Canada.British Columbia|BOLD:ACF1489
Diarsia esurialis|[5519]|LBCH968-10|10-JDWBC-0968|658[0n]bp|Canada.British Columbia|BOLD:ABX6710
Diarsia esurialis|[5520]|LBCH265-10|10-JDWBC-0265|658[0n]bp|Canada.British Columbia|BOLD:ABX6710
Diarsia esurialis|[5521]|LBCH2393-10|10-JDWBC-2393|658[0n]bp|Canada.British Columbia|BOLD:ABX6710
Diarsia esurialis|[5522]|LBCH156-10|10-JDWBC-0156|658[0n]bp|Canada.British Columbia|BOLD:ABX6710
Diarsia esurialis|[5523]|LBCH2391-10|10-JDWBC-2391|658[0n]bp|Canada.British Columbia|BOLD:ABX6710
Diarsia esurialis|[5524]|LBCH145-10|10-JDWBC-0145|658[0n]bp|Canada.British Columbia|BOLD:ABX6710
Diarsia esurialis|[5525]|LBCH519-10|10-JDWBC-0519|658[0n]bp|Canada.British Columbia|BOLD:ABX6710
Diarsia esurialis|[5526]|LBCH2938-10|10-JDWBC-2938|658[0n]bp|Canada.British Columbia|BOLD:ABX6710
Diarsia esurialis|[5527]|LBCH412-10|10-JDWBC-0412|658[0n]bp|Canada.British Columbia|BOLD:ABX6710
Diarsia esurialis|[5528]|LBCH673-10|10-JDWBC-0673|658[0n]bp|Canada.British Columbia|BOLD:ABX6710
Diarsia esurialis|[5529]|LBCH259-10|10-JDWBC-0259|658[0n]bp|Canada.British Columbia|BOLD:ABX6710
Diarsia esurialis|[5530]|LBCH406-10|10-JDWBC-0406|658[0n]bp|Canada.British Columbia|BOLD:ABX6710
Diarsia esurialis|[5531]|LBCH845-10|10-JDWBC-0845|658[0n]bp|Canada.British Columbia|BOLD:ABX6710
Diarsia esurialis|[5532]|LBCH2916-10|10-JDWBC-2916|658[0n]bp|Canada.British Columbia|BOLD:ABX6710
Diarsia esurialis|[5533]|LBCH534-10|10-JDWBC-0534|658[0n]bp|Canada.British Columbia|BOLD:ABX6710
Diarsia esurialis|[5534]|LBCH391-10|10-JDWBC-0391|658[0n]bp|Canada.British Columbia|BOLD:ABX6710
Diarsia esurialis|[5535]|LBCH3046-10|10-JDWBC-3046|658[0n]bp|Canada.British Columbia|BOLD:ABX6710
Diarsia esurialis|[5536]|LBCH851-10|10-JDWBC-0851|658[0n]bp|Canada.British Columbia|BOLD:ABX6710
Diarsia esurialis|[5537]|LBCH658-10|10-JDWBC-0658|658[0n]bp|Canada.British Columbia|BOLD:ABX6710
Diarsia esurialis|[5538]|LBCH524-10|10-JDWBC-0524|658[0n]bp|Canada.British Columbia|BOLD:ABX6710
Diarsia esurialis|[5539]|LBCH051-10|10-JDWBC-0051|658[0n]bp|Canada.British Columbia|BOLD:ABX6710
Diarsia esurialis|[5540]|LBCH139-10|10-JDWBC-0139|658[0n]bp|Canada.British Columbia|BOLD:ABX6710
Diarsia esurialis|[5541]|LBCH3043-10|10-JDWBC-3043|658[0n]bp|Canada.British Columbia|BOLD:ABX6710
Diarsia esurialis|[5542]|LBCH3005-10|10-JDWBC-3005|658[0n]bp|Canada.British Columbia|BOLD:ABX6710
Diarsia esurialis|[5543]|LBCH260-10|10-JDWBC-0260|658[0n]bp|Canada.British Columbia|BOLD:ABX6710
Diarsia esurialis|[5544]|LBCH2963-10|10-JDWBC-2963|658[0n]bp|Canada.British Columbia|BOLD:ABX6710
Diarsia esurialis|[5545]|LBCH837-10|10-JDWBC-0837|658[0n]bp|Canada.British Columbia|BOLD:ABX6710
Diarsia esurialis|[5546]|LBCH142-10|10-JDWBC-0142|658[0n]bp|Canada.British Columbia|BOLD:ABX6710
Diarsia esurialis|[5547]|LBCH044-10|10-JDWBC-0044|658[0n]bp|Canada.British Columbia|BOLD:ABX6710
Diarsia esurialis|[5548]|LBCH665-10|10-JDWBC-0665|658[0n]bp|Canada.British Columbia|BOLD:ABX6710
Diarsia esurialis|[5549]|LBCH255-10|10-JDWBC-0255|658[0n]bp|Canada.British Columbia|BOLD:ABX6710
Diarsia esurialis|[5550]|LBCH2394-10|10-JDWBC-2394|658[0n]bp|Canada.British Columbia|BOLD:ABX6710
Diarsia esurialis|[5551]|LBCH157-10|10-JDWBC-0157|658[0n]bp|Canada.British Columbia|BOLD:ABX6710
Diarsia esurialis|[5552]|LBCH405-10|10-JDWBC-0405|658[0n]bp|Canada.British Columbia|BOLD:ABX6710
Diarsia esurialis|[5553]|LBCH2914-10|10-JDWBC-2914|658[0n]bp|Canada.British Columbia|BOLD:ABX6710
Diarsia esurialis|[5554]|LBCH2936-10|10-JDWBC-2936|658[0n]bp|Canada.British Columbia|BOLD:ABX6710
Diarsia esurialis|[5555]|LBCH527-10|10-JDWBC-0527|658[0n]bp|Canada.British Columbia|BOLD:ABX6710
Diarsia esurialis|[5556]|LBCH2389-10|10-JDWBC-2389|658[0n]bp|Canada.British Columbia|BOLD:ABX6710
Diarsia esurialis|[5557]|LBCH3050-10|10-JDWBC-3050|658[0n]bp|Canada.British Columbia|BOLD:ABX6710
Diarsia esurialis|[5558]|LBCH130-10|10-JDWBC-0130|658[0n]bp|Canada.British Columbia|BOLD:ABX6710
Diarsia esurialis|[5559]|LBCH250-10|10-JDWBC-0250|658[0n]bp|Canada.British Columbia|BOLD:ABX6710
Diarsia esurialis|[5560]|LBCH267-10|10-JDWBC-0267|658[0n]bp|Canada.British Columbia|BOLD:ABX6710
Diarsia esurialis|[5561]|LBCH257-10|10-JDWBC-0257|658[0n]bp|Canada.British Columbia|BOLD:ABX6710
Diarsia esurialis|[5562]|LBCH541-10|10-JDWBC-0541|658[0n]bp|Canada.British Columbia|BOLD:ABX6710
Diarsia esurialis|[5563]|LBCH140-10|10-JDWBC-0140|658[0n]bp|Canada.British Columbia|BOLD:ABX6710
Diarsia esurialis|[5564]|LBCH263-10|10-JDWBC-0263|658[0n]bp|Canada.British Columbia|BOLD:ABX6710
Diarsia esurialis|[5565]|LBCH2905-10|10-JDWBC-2905|658[0n]bp|Canada.British Columbia|BOLD:ABX6710
Diarsia esurialis|[5566]|LBCH2964-10|10-JDWBC-2964|658[0n]bp|Canada.British Columbia|BOLD:ABX6710
Diarsia esurialis|[5567]|LBCH132-10|10-JDWBC-0132|658[0n]bp|Canada.British Columbia|BOLD:ABX6710
Diarsia esurialis|[5568]|LBCH047-10|10-JDWBC-0047|658[0n]bp|Canada.British Columbia|BOLD:ABX6710
Diarsia esurialis|[5569]|LBCH2918-10|10-JDWBC-2918|658[0n]bp|Canada.British Columbia|BOLD:ABX6710
Diarsia esurialis|[5570]|LBCH048-10|10-JDWBC-0048|658[0n]bp|Canada.British Columbia|BOLD:ABX6710
Diarsia esurialis|[5571]|LBCH2893-10|10-JDWBC-2893|658[0n]bp|Canada.British Columbia|BOLD:ABX6710
Diarsia esurialis|[5572]|LBCH133-10|10-JDWBC-0133|658[0n]bp|Canada.British Columbia|BOLD:ABX6710
Diarsia esurialis|[5573]|LBCH247-10|10-JDWBC-0247|658[0n]bp|Canada.British Columbia|BOLD:ABX6710
Diarsia esurialis|[5574]|LBCH2395-10|10-JDWBC-2395|658[0n]bp|Canada.British Columbia|BOLD:ABX6710
Diarsia esurialis|[5575]|LBCH834-10|10-JDWBC-0834|658[0n]bp|Canada.British Columbia|BOLD:ABX6710
Diarsia esurialis|[5576]|LALPA962-11|AVBC 1135-11|658[0n]bp|Canada.British Columbia|BOLD:ABX6710
Diarsia esurialis|[5577]|LALPA963-11|AVBC 1136-11|658[0n]bp|Canada.British Columbia|BOLD:ABX6710
Diarsia esurialis|[5578]|LALPA311-10|AVBC 313-10|658[0n]bp|Canada.British Columbia|BOLD:ABX6710
Diarsia esurialis|[5579]|LBCH2937-10|10-JDWBC-2937|658[0n]bp|Canada.British Columbia|BOLD:ABX6710
Diarsia esurialis|[5580]|LBCH272-10|10-JDWBC-0272|658[0n]bp|Canada.British Columbia|BOLD:ABX6710
Diarsia esurialis|[5581]|LBCH545-10|10-JDWBC-0545|658[0n]bp|Canada.British Columbia|BOLD:ABX6710
Diarsia esurialis|[5582]|LBCH850-10|10-JDWBC-0850|658[0n]bp|Canada.British Columbia|BOLD:ABX6710

Diarsia esurialis|[5579]|LBCH2957-10|10-JDWBC-2957|658[0n]bp|Canada.British Columbia|BOLD:ABX6710
Diarsia esurialis|[5580]|LBCH272-10|10-JDWBC-0272|658[0n]bp|Canada.British Columbia|BOLD:ABX6710
Diarsia esurialis|[5581]|LBCH545-10|10-JDWBC-0545|658[0n]bp|Canada.British Columbia|BOLD:ABX6710
Diarsia esurialis|[5582]|LBCH850-10|10-JDWBC-0850|658[0n]bp|Canada.British Columbia|BOLD:ABX6710
Diarsia esurialis|[5583]|LBCH036-10|10-JDWBC-0036|658[0n]bp|Canada.British Columbia|BOLD:ABX6710
Diarsia esurialis|[5584]|LBCH161-10|10-JDWBC-0161|658[0n]bp|Canada.British Columbia|BOLD:ABX6710
Diarsia esurialis|[5585]|LBCH045-10|10-JDWBC-0045|658[0n]bp|Canada.British Columbia|BOLD:ABX6710
Diarsia esurialis|[5586]|LBCH159-10|10-JDWBC-0159|658[0n]bp|Canada.British Columbia|BOLD:ABX6710
Diarsia esurialis|[5587]|LBCH254-10|10-JDWBC-0254|658[0n]bp|Canada.British Columbia|BOLD:ABX6710
Diarsia esurialis|[5588]|LBCH262-10|10-JDWBC-0262|658[0n]bp|Canada.British Columbia|BOLD:ABX6710
Diarsia esurialis|[5589]|LBCH2390-10|10-JDWBC-2390|658[0n]bp|Canada.British Columbia|BOLD:ABX6710
Diarsia esurialis|[5590]|LBCH258-10|10-JDWBC-0258|658[0n]bp|Canada.British Columbia|BOLD:ABX6710
Diarsia esurialis|[5591]|LBCH2388-10|10-JDWBC-2388|658[0n]bp|Canada.British Columbia|BOLD:ABX6710
Diarsia esurialis|[5592]|LBCH054-10|10-JDWBC-0054|658[0n]bp|Canada.British Columbia|BOLD:ABX6710
Diarsia esurialis|[5593]|LBCG442-08|08-JDWBC-0442|658[0n]bp|Canada.British Columbia|BOLD:ABX6710
Diarsia esurialis|[5594]|LBCH836-10|10-JDWBC-0836|658[0n]bp|Canada.British Columbia|BOLD:ABX6710
Diarsia esurialis|[5595]|LBCH273-10|10-JDWBC-0273|658[0n]bp|Canada.British Columbia|BOLD:ABX6710
Diarsia esurialis|[5596]|LBCH040-10|10-JDWBC-0040|658[0n]bp|Canada.British Columbia|BOLD:ABX6710
Diarsia esurialis|[5597]|LBCH252-10|10-JDWBC-0252|658[0n]bp|Canada.British Columbia|BOLD:ABX6710
Diarsia esurialis|[5598]|LBCH522-10|10-JDWBC-0522|658[0n]bp|Canada.British Columbia|BOLD:ABX6710
Diarsia esurialis|[5599]|LBCH3784-10|10-JDWBC-3784|658[0n]bp|Canada.British Columbia|BOLD:ABX6710
Diarsia esurialis|[5600]|LBCH151-10|10-JDWBC-0151|658[0n]bp|Canada.British Columbia|BOLD:ABX6710
Diarsia esurialis|[5601]|LBCH663-10|10-JDWBC-0663|658[0n]bp|Canada.British Columbia|BOLD:ABX6710
Diarsia esurialis|[5602]|LBCH256-10|10-JDWBC-0256|658[0n]bp|Canada.British Columbia|BOLD:ABX6710
Diarsia esurialis|[5603]|LBCH035-10|10-JDWBC-0035|658[0n]bp|Canada.British Columbia|BOLD:ABX6710
Diarsia esurialis|[5604]|LBCH143-10|10-JDWBC-0143|658[0n]bp|Canada.British Columbia|BOLD:ABX6710
Diarsia esurialis|[5605]|LBCH3048-10|10-JDWBC-3048|658[0n]bp|Canada.British Columbia|BOLD:ABX6710
Diarsia esurialis|[5606]|LBCH037-10|10-JDWBC-0037|658[0n]bp|Canada.British Columbia|BOLD:ABX6710
Diarsia esurialis|[5607]|LBCH656-10|10-JDWBC-0656|658[0n]bp|Canada.British Columbia|BOLD:ABX6710
Diarsia esurialis|[5608]|LBCH532-10|10-JDWBC-0532|658[0n]bp|Canada.British Columbia|BOLD:ABX6710
Diarsia esurialis|[5609]|LBCH3042-10|10-JDWBC-3042|658[0n]bp|Canada.British Columbia|BOLD:ABX6710
Diarsia esurialis|[5610]|LBCH038-10|10-JDWBC-0038|658[0n]bp|Canada.British Columbia|BOLD:ABX6710
Diarsia esurialis|[5611]|LBCH039-10|10-JDWBC-0039|658[0n]bp|Canada.British Columbia|BOLD:ABX6710
Diarsia esurialis|[5612]|LBCH134-10|10-JDWBC-0134|658[0n]bp|Canada.British Columbia|BOLD:ABX6710
Diarsia esurialis|[5613]|LBCH3045-10|10-JDWBC-3045|658[0n]bp|Canada.British Columbia|BOLD:ABX6710
Diarsia esurialis|[5614]|LBCH3049-10|10-JDWBC-3049|658[0n]bp|Canada.British Columbia|BOLD:ABX6710
Diarsia esurialis|[5615]|LBCH271-10|10-JDWBC-0271|658[0n]bp|Canada.British Columbia|BOLD:ABX6710
Diarsia esurialis|[5616]|LBCH533-10|10-JDWBC-0533|658[0n]bp|Canada.British Columbia|BOLD:ABX6710
Diarsia esurialis|[5617]|LBCH2917-10|10-JDWBC-2917|658[0n]bp|Canada.British Columbia|BOLD:ABX6710
Diarsia esurialis|[5618]|LBCH531-10|10-JDWBC-0531|658[0n]bp|Canada.British Columbia|BOLD:ABX6710
Diarsia esurialis|[5619]|LBCH661-10|10-JDWBC-0661|658[0n]bp|Canada.British Columbia|BOLD:ABX6710
Diarsia esurialis|[5620]|LBCH148-10|10-JDWBC-0148|658[0n]bp|Canada.British Columbia|BOLD:ABX6710
Diarsia esurialis|[5621]|LBCH270-10|10-JDWBC-0270|658[0n]bp|Canada.British Columbia|BOLD:ABX6710
Diarsia esurialis|[5622]|LBCH158-10|10-JDWBC-0158|658[0n]bp|Canada.British Columbia|BOLD:ABX6710
Diarsia esurialis|[5623]|LBCH034-10|10-JDWBC-0034|658[0n]bp|Canada.British Columbia|BOLD:ABX6710
Diarsia esurialis|[5624]|LBCH403-10|10-JDWBC-0403|658[0n]bp|Canada.British Columbia|BOLD:ABX6710
Diarsia esurialis|[5625]|LBCH660-10|10-JDWBC-0660|658[0n]bp|Canada.British Columbia|BOLD:ABX6710
Diarsia esurialis|[5626]|LBCH402-10|10-JDWBC-0402|658[0n]bp|Canada.British Columbia|BOLD:ABX6710
Diarsia esurialis|[5627]|LBCH033-10|10-JDWBC-0033|658[0n]bp|Canada.British Columbia|BOLD:ABX6710
Diarsia esurialis|[5628]|LBCH042-10|10-JDWBC-0042|658[0n]bp|Canada.British Columbia|BOLD:ABX6710
Diarsia esurialis|[5629]|LBCH141-10|10-JDWBC-0141|658[0n]bp|Canada.British Columbia|BOLD:ABX6710
Diarsia esurialis|[5630]|LBCH518-10|10-JDWBC-0518|658[0n]bp|Canada.British Columbia|BOLD:ABX6710
Diarsia esurialis|[5631]|LBCH550-10|10-JDWBC-0550|658[0n]bp|Canada.British Columbia|BOLD:ABX6710
Diarsia esurialis|[5632]|LBCH146-10|10-JDWBC-0146|658[0n]bp|Canada.British Columbia|BOLD:ABX6710
Diarsia esurialis|[5633]|LBCH3044-10|10-JDWBC-3044|658[0n]bp|Canada.British Columbia|BOLD:ABX6710
Diarsia esurialis|[5634]|LBCH274-10|10-JDWBC-0274|658[0n]bp|Canada.British Columbia|BOLD:ABX6710
Diarsia esurialis|[5635]|LBCH400-10|10-JDWBC-0400|658[0n]bp|Canada.British Columbia|BOLD:ABX6710
Diarsia esurialis|[5636]|LBCH2915-10|10-JDWBC-2915|658[0n]bp|Canada.British Columbia|BOLD:ABX6710
Diarsia esurialis|[5637]|LBCH266-10|10-JDWBC-0266|658[0n]bp|Canada.British Columbia|BOLD:ABX6710
Diarsia esurialis|[5638]|LBCH2935-10|10-JDWBC-2935|658[0n]bp|Canada.British Columbia|BOLD:ABX6710
Diarsia esurialis|[5639]|LBCH2987-10|10-JDWBC-2987|658[0n]bp|Canada.British Columbia|BOLD:ABX6710
Diarsia esurialis|[5640]|LBCH155-10|10-JDWBC-0155|658[0n]bp|Canada.British Columbia|BOLD:ABX6710
Diarsia esurialis|[5641]|LBCH411-10|10-JDWBC-0411|658[0n]bp|Canada.British Columbia|BOLD:ABX6710
Diarsia esurialis|[5642]|LBCH135-10|10-JDWBC-0135|658[0n]bp|Canada.British Columbia|BOLD:ABX6710
Diarsia esurialis|[5643]|LBCH655-10|10-JDWBC-0655|658[0n]bp|Canada.British Columbia|BOLD:ABX6710
Diarsia esurialis|[5644]|LBCH691-10|10-JDWBC-0691|658[0n]bp|Canada.British Columbia|BOLD:ABX6710
Diarsia esurialis|[5645]|LBCH847-10|10-JDWBC-0847|658[0n]bp|Canada.British Columbia|BOLD:ABX6710
Diarsia esurialis|[5646]|LBCH268-10|10-JDWBC-0268|658[0n]bp|Canada.British Columbia|BOLD:ABX6710
Diarsia esurialis|[5647]|LBCH050-10|10-JDWBC-0050|658[0n]bp|Canada.British Columbia|BOLD:ABX6710
Diarsia esurialis|[5648]|LBCH396-10|10-JDWBC-0396|658[0n]bp|Canada.British Columbia|BOLD:ABX6710
Diarsia esurialis|[5649]|LBCH046-10|10-JDWBC-0046|658[0n]bp|Canada.British Columbia|BOLD:ABX6710
Diarsia esurialis|[5650]|LBCH2984-10|10-JDWBC-2984|658[0n]bp|Canada.British Columbia|BOLD:ABX6710
Diarsia esurialis|[5651]|LBCH520-10|10-JDWBC-0520|658[0n]bp|Canada.British Columbia|BOLD:ABX6710
Diarsia esurialis|[5652]|LBCH2962-10|10-JDWBC-2962|658[0n]bp|Canada.British Columbia|BOLD:ABX6710
Diarsia esurialis|[5653]|LBCH136-10|10-JDWBC-0136|658[0n]bp|Canada.British Columbia|BOLD:ABX6710
Diarsia esurialis|[5654]|LBCH269-10|10-JDWBC-0269|658[0n]bp|Canada.British Columbia|BOLD:ABX6710
Diarsia esurialis|[5655]|LBCH144-10|10-JDWBC-0144|658[0n]bp|Canada.British Columbia|BOLD:ABX6710
Diarsia esurialis|[5656]|LBCH160-10|10-JDWBC-0160|658[0n]bp|Canada.British Columbia|BOLD:ABX6710
Diarsia esurialis|[5657]|LBCH147-10|10-JDWBC-0147|658[0n]bp|Canada.British Columbia|BOLD:ABX6710
Diarsia esurialis|[5658]|LBCH659-10|10-JDWBC-0659|658[0n]bp|Canada.British Columbia|BOLD:ABX6710
Diarsia esurialis|[5659]|LBCH848-10|10-JDWBC-0848|658[0n]bp|Canada.British Columbia|BOLD:ABX6710
Diarsia rubifera|[5660]|RDLQ716-07|DH008017|627[0n]bp|Canada.Quebec|BOLD:ABX5264
Diarsia rubifera|[5661]|BBLEC008-09|09BBELE-0008|621[0n]bp|Canada.New Brunswick|BOLD:ABX5264
Diarsia rubifera|[5662]|RDLQB651-05|DH010754|587[1n]bp|Canada.Quebec|BOLD:ABX5264
Diarsia rubifera|[5663]|RDLQ719-07|DH007869|658[0n]bp|Canada.Quebec|BOLD:ABX5264
Diarsia rubifera|[5664]|RDLQ720-07|DH006307|632[0n]bp|Canada.Quebec|BOLD:ABX5264
Diarsia rubifera|[5665]|BBLEC017-09|09BBELE-0017|626[0n]bp|Canada.New Brunswick|BOLD:ABX5264
Diarsia rubifera|[5666]|LPSOD961-09|08BBLEP-05501|658[0n]bp|Canada.Ontario|BOLD:ABX5264
Diarsia rubifera|[5667]|RDLQ715-07|DH007451|658[0n]bp|Canada.Quebec|BOLD:ABX5264
Diarsia rubifera|[5668]|LHLEP424-06|UBC-2006-1523|658[0n]bp|Canada.British Columbia|BOLD:ABX5264
Diarsia rubifera|[5669]|RDLQF175-06|DH011202|658[0n]bp|Canada.Quebec|BOLD:ABX5264
Diarsia rubifera|[5670]|RDLQF371-06|DH011438|658[0n]bp|Canada.Quebec|BOLD:ABX5264
Diarsia rubifera|[5671]|LPGVA608-08|UBC-2006-1766|658[0n]bp|Canada.British Columbia|BOLD:ABX5264
Diarsia rubifera|[5672]|BBLEC516-09|09BBELE-0516|658[0n]bp|Canada.New Brunswick|BOLD:ABX5264
Diarsia rubifera|[5673]|RDLQF372-06|DH011439|658[0n]bp|Canada.Quebec|BOLD:ABX5264
Diarsia rubifera|[5674]|RDLQB832-05|DH010919|620[0n]bp|Canada.Quebec|BOLD:ABX5264
Diarsia rubifera|[5675]|BBLPC037-09|09BBELE-1037|634[0n]bp|Canada.New Brunswick|BOLD:ABX5264
Diarsia rubifera|[5676]|LPMNB468-09|08BBLEP-05506|658[0n]bp|Canada.Manitoba|BOLD:ABX5264
Diarsia rubifera|[5677]|LPSOD938-09|08BBLEP-05478|658[0n]bp|Canada.Ontario|BOLD:ABX5264
Diarsia rubifera|[5678]|LPMNB508-09|08BBLEP-05546|658[0n]bp|Canada.Manitoba|BOLD:ABX5264
Diarsia rubifera|[5679]|BBLPB566-10|10BBCLP-1565|658[0n]bp|Canada.Alberta|BOLD:ABX5264
Diarsia rubifera|[5680]|BBLPB553-10|10BBCLP-1552|658[0n]bp|Canada.Alberta|BOLD:ABX5264
Diarsia rubifera|[5681]|LPSOD941-09|08BBLEP-05481|658[0n]bp|Canada.Ontario|BOLD:ABX5264
[illegible]

Diarsia rubifera|[5679]|BBLPB566-10|10BBCLP-1565|658[0n]bp|Canada.Alberta|BOLD:ABX5264
Diarsia rubifera|[5680]|BBLPB553-10|10BBCLP-1552|658[0n]bp|Canada.Alberta|BOLD:ABX5264
Diarsia rubifera|[5681]|LPSOD941-09|08BBLEP-05481|658[0n]bp|Canada.Ontario|BOLD:ABX5264
Diarsia rubifera|[5682]|LPMNB470-09|08BBLEP-05508|658[0n]bp|Canada.Manitoba|BOLD:ABX5264
Diarsia rubifera|[5683]|BBLEC004-09|09BBELE-0004|621[0n]bp|Canada.New Brunswick|BOLD:ABX5264
Diarsia rubifera|[5684]|RDLQF799-06|DH011949|609[0n]bp|Canada.Quebec|BOLD:ABX5264
Diarsia rubifera|[5685]|LBCG2259-09|08-JDWBC-2259|658[0n]bp|Canada.British Columbia|BOLD:ABX5264
Diarsia rubifera|[5686]|LBCH3355-10|10-JDWBC-3355|658[0n]bp|Canada.British Columbia|BOLD:ABX5264
Diarsia rubifera|[5687]|BBLPB359-10|10BBCLP-1358|658[0n]bp|Canada.Alberta|BOLD:ABX5264
Diarsia rubifera|[5688]|LBCG2264-09|08-JDWBC-2264|658[0n]bp|Canada.British Columbia|BOLD:ABX5264
Diarsia rubifera|[5689]|LBCH4717-10|10-JDWBC-4717|658[0n]bp|Canada.British Columbia|BOLD:ABX5264
Diarsia rubifera|[5690]|BBLPB552-10|10BBCLP-1551|658[0n]bp|Canada.Alberta|BOLD:ABX5264
Diarsia rubifera|[5691]|BBLPB568-10|10BBCLP-1567|658[0n]bp|Canada.Alberta|BOLD:ABX5264
Diarsia rubifera|[5692]|BBLPB450-10|10BBCLP-1449|658[0n]bp|Canada.Alberta|BOLD:ABX5264
Diarsia rubifera|[5693]|BBLPB354-10|10BBCLP-1353|645[0n]bp|Canada.Alberta|BOLD:ABX5264
Diarsia rubifera|[5694]|LBCH4019-10|10-JDWBC-4019|658[0n]bp|Canada.British Columbia|BOLD:ABX5264
Diarsia rubifera|[5695]|BBLPB353-10|10BBCLP-1352|658[0n]bp|Canada.Alberta|BOLD:ABX5264
Diarsia rubifera|[5696]|BBLPB567-10|10BBCLP-1566|658[0n]bp|Canada.Alberta|BOLD:ABX5264
Diarsia rubifera|[5697]|LBCG2258-09|08-JDWBC-2258|658[0n]bp|Canada.British Columbia|BOLD:ABX5264
Diarsia rubifera|[5698]|LBCH3357-10|10-JDWBC-3357|658[0n]bp|Canada.British Columbia|BOLD:ABX5264
Diarsia rubifera|[5699]|BBLPB355-10|10BBCLP-1354|658[0n]bp|Canada.Alberta|BOLD:ABX5264
Diarsia rubifera|[5700]|LBCH3359-10|10-JDWBC-3359|658[0n]bp|Canada.British Columbia|BOLD:ABX5264
Diarsia rubifera|[5701]|LBCH3356-10|10-JDWBC-3356|658[0n]bp|Canada.British Columbia|BOLD:ABX5264
Diarsia rubifera|[5702]|LBCG2266-09|08-JDWBC-2266|658[0n]bp|Canada.British Columbia|BOLD:ABX5264
Diarsia rubifera|[5703]|LBCH4015-10|10-JDWBC-4015|658[0n]bp|Canada.British Columbia|BOLD:ABX5264
Diarsia rubifera|[5704]|LBCG2270-09|08-JDWBC-2270|658[0n]bp|Canada.British Columbia|BOLD:ABX5264
Diarsia rubifera|[5705]|LBCG2268-09|08-JDWBC-2268|658[0n]bp|Canada.British Columbia|BOLD:ABX5264
Diarsia rubifera|[5706]|LOWCE788-06|CGWC-4548|658[0n]bp|Canada.British Columbia|BOLD:ABX5264
Diarsia rubifera|[5707]|LBCH3106-10|10-JDWBC-3106|658[0n]bp|Canada.British Columbia|BOLD:ABX5264
Diarsia rubifera|[5708]|LBCH3342-10|10-JDWBC-3342|658[0n]bp|Canada.British Columbia|BOLD:ABX5264
Diarsia rubifera|[5709]|LBCH962-10|10-JDWBC-0962|658[0n]bp|Canada.British Columbia|BOLD:ABX5264
Diarsia rubifera|[5710]|LBCG2250-09|08-JDWBC-2250|658[0n]bp|Canada.British Columbia|BOLD:ABX5264
Diarsia rubifera|[5711]|BBLPB551-10|10BBCLP-1550|658[0n]bp|Canada.Alberta|BOLD:ABX5264
Diarsia rubifera|[5712]|LBCG2262-09|08-JDWBC-2262|658[0n]bp|Canada.British Columbia|BOLD:ABX5264
Diarsia rubifera|[5713]|LOWCE789-06|CGWC-4549|658[0n]bp|Canada.British Columbia|BOLD:ABX5264
Diarsia rubifera|[5714]|LALPA728-10|AVBC 730-10|658[0n]bp|Canada.British Columbia|BOLD:ABX5264
Diarsia rubifera|[5715]|LBCH4750-10|10-JDWBC-4750|658[0n]bp|Canada.British Columbia|BOLD:ABX5264
Diarsia rubifera|[5716]|LBCG2257-09|08-JDWBC-2257|658[0n]bp|Canada.British Columbia|BOLD:ABX5264
Diarsia rubifera|[5717]|LBCH3358-10|10-JDWBC-3358|658[0n]bp|Canada.British Columbia|BOLD:ABX5264
Diarsia rubifera|[5718]|BBLPB571-10|10BBCLP-1570|658[0n]bp|Canada.Alberta|BOLD:ABX5264
Diarsia rubifera|[5719]|LBCH946-10|10-JDWBC-0946|658[0n]bp|Canada.British Columbia|BOLD:ABX5264
Diarsia rubifera|[5720]|LBCH3505-10|10-JDWBC-3505|658[0n]bp|Canada.British Columbia|BOLD:ABX5264
Diarsia rubifera|[5721]|BBLPB745-10|10BBCLP-1744|658[0n]bp|Canada.Alberta|BOLD:ABX5264
Diarsia rubifera|[5722]|LBCH3360-10|10-JDWBC-3360|658[0n]bp|Canada.British Columbia|BOLD:ABX5264
Diarsia rubifera|[5723]|BBLPB461-10|10BBCLP-1460|658[0n]bp|Canada.Alberta|BOLD:ABX5264
Diarsia rubifera|[5724]|LHLEP425-06|UBC-2006-1524|658[0n]bp|Canada.British Columbia|BOLD:ABX5264
Diarsia rubifera|[5725]|LBCH961-10|10-JDWBC-0961|658[0n]bp|Canada.British Columbia|BOLD:ABX5264
Diarsia rubifera|[5726]|LBCG2267-09|08-JDWBC-2267|658[0n]bp|Canada.British Columbia|BOLD:ABX5264
Diarsia rubifera|[5727]|LBCH4734-10|10-JDWBC-4734|658[0n]bp|Canada.British Columbia|BOLD:ABX5264
Diarsia rubifera|[5728]|LBCH3142-10|10-JDWBC-3142|658[0n]bp|Canada.British Columbia|BOLD:ABX5264
Diarsia rubifera|[5729]|LBCG2265-09|08-JDWBC-2265|658[0n]bp|Canada.British Columbia|BOLD:ABX5264
Diarsia rubifera|[5730]|BBLPB582-10|10BBCLP-1581|658[0n]bp|Canada.Alberta|BOLD:ABX5264
Diarsia rubifera|[5731]|LBCH4722-10|10-JDWBC-4722|644[0n]bp|Canada.British Columbia|BOLD:ABX5264
Diarsia rubifera|[5732]|LBCH3491-10|10-JDWBC-3491|636[0n]bp|Canada.British Columbia|BOLD:ABX5264
Diarsia rubifera|[5733]|LBCH4748-10|10-JDWBC-4748|658[0n]bp|Canada.British Columbia|BOLD:ABX5264
Diarsia rubifera|[5734]|LBCG2277-09|08-JDWBC-2277|658[0n]bp|Canada.British Columbia|BOLD:ABX5264
Diarsia rubifera|[5735]|LBCH3354-10|10-JDWBC-3354|658[0n]bp|Canada.British Columbia|BOLD:ABX5264
Diarsia rubifera|[5736]|LBCH3799-10|10-JDWBC-3799|658[0n]bp|Canada.British Columbia|BOLD:ABX5264
Diarsia rubifera|[5737]|LBCH3518-10|10-JDWBC-3518|658[0n]bp|Canada.British Columbia|BOLD:ABX5264
Diarsia rubifera|[5738]|LBCG2260-09|08-JDWBC-2260|658[0n]bp|Canada.British Columbia|BOLD:ABX5264
Diarsia rubifera|[5739]|LBCH4749-10|10-JDWBC-4749|658[0n]bp|Canada.British Columbia|BOLD:ABX5264
Diarsia rubifera|[5740]|LBCG2261-09|08-JDWBC-2261|658[0n]bp|Canada.British Columbia|BOLD:ABX5264
Diarsia rubifera|[5741]|LOWCE787-06|CGWC-4547|658[0n]bp|Canada.British Columbia|BOLD:ABX5264
Diarsia rubifera|[5742]|LBCH960-10|10-JDWBC-0960|658[0n]bp|Canada.British Columbia|BOLD:ABX5264
Diarsia rubifera|[5743]|BBLPB550-10|10BBCLP-1549|658[0n]bp|Canada.Alberta|BOLD:ABX5264
Diarsia rubifera|[5744]|BBLPB356-10|10BBCLP-1355|658[0n]bp|Canada.Alberta|BOLD:ABX5264
Diarsia rubifera|[5745]|LBCG2251-09|08-JDWBC-2251|658[0n]bp|Canada.British Columbia|BOLD:ABX5264
Diarsia rubifera|[5746]|BBLPB549-10|10BBCLP-1548|658[0n]bp|Canada.Alberta|BOLD:ABX5264
Abagrotis apposita|[5747]|LPVIC032-08|PFC-2006-2587|621[0n]bp|Canada.British Columbia|BOLD:AAE9047
Abagrotis apposita|[5748]|LALPA1285-11|AVBC 1287-11|658[0n]bp|Canada.British Columbia|BOLD:AAE9047
Abagrotis apposita|[5749]|RDNMC101-05|CNCNoctuoidea10842|658[0n]bp|Canada.British Columbia|BOLD:AAE9047
Abagrotis apposita|[5750]|LALPA1018-11|AVBC 1191-11|658[0n]bp|Canada.British Columbia|BOLD:AAE9047
Abagrotis apposita|[5751]|LPVIB274-08|PFC-2006-1664|658[0n]bp|Canada.British Columbia|BOLD:AAE9047
Abagrotis apposita|[5752]|LBCH6285-10|10-JDWBC-6285|658[0n]bp|Canada.British Columbia|BOLD:AAE9047
Abagrotis apposita|[5753]|LPVIB447-08|PFC-2006-1854|658[0n]bp|Canada.British Columbia|BOLD:AAE9047
Abagrotis apposita|[5754]|LPVIA968-08|PFC-2006-1294|635[0n]bp|Canada.British Columbia|BOLD:AAE9047
Abagrotis apposita|[5755]|RDNMC099-05|CNCNoctuoidea10840|658[0n]bp|Canada.British Columbia|BOLD:AAE9047
Abagrotis apposita|[5756]|LBCC293-05|HLC-22173|658[0n]bp|Canada.British Columbia|BOLD:AAE9047
Graphiphora augur|[5757]|LOWCC860-05|CGWC-2740|658[0n]bp|Canada.British Columbia|BOLD:ACF0936
Graphiphora augur|[5758]|LBCH1170-10|10-JDWBC-1170|658[0n]bp|Canada.British Columbia|BOLD:ACF0936
Graphiphora augur|[5759]|LPABC270-09|08BBLEP-04489|613[0n]bp|Canada.Alberta|BOLD:ACF0936
Graphiphora augur|[5760]|LBCG2306-09|08-JDWBC-2306|658[0n]bp|Canada.British Columbia|BOLD:ACF0936
Graphiphora augur|[5761]|LOWCC892-05|CGWC-2772|586[0n]bp|Canada.British Columbia|BOLD:ACF0936
Graphiphora augur|[5762]|LOWCD893-06|CGWC-3713|618[0n]bp|Canada.British Columbia|BOLD:ACF0936
Graphiphora augur|[5763]|LOWCD891-06|CGWC-3711|606[1n]bp|Canada.British Columbia|BOLD:ACF0936
Graphiphora augur|[5764]|LBCH358-10|10-JDWBC-0358|552[0n]bp|Canada.British Columbia|BOLD:ACF0936
Graphiphora augur|[5765]|LOWCD804-06|CGWC-3624|596[2n]bp|Canada.British Columbia|BOLD:ACF0936
Graphiphora augur|[5766]|RDNM846-05|CNCNoctuoidea7686|594[1n]bp|Canada.British Columbia|BOLD:ACF0936
Graphiphora augur|[5767]|LBCH016-10|10-JDWBC-0016|643[0n]bp|Canada.British Columbia|BOLD:ACF0936
Graphiphora augur|[5768]|LOWCD138-06|CGWC-2958|580[0n]bp|Canada.British Columbia|BOLD:ACF0936
Graphiphora augur|[5769]|LBCH3468-10|10-JDWBC-3468|641[0n]bp|Canada.British Columbia|BOLD:ACF0936
Graphiphora augur|[5770]|LBCH925-10|10-JDWBC-0925|658[0n]bp|Canada.British Columbia|BOLD:ACF0936
Graphiphora augur|[5771]|LBCD041-05|HLC-22861|658[0n]bp|Canada.British Columbia|BOLD:ACF0936
Graphiphora augur|[5772]|RDNMB900-05|CNCNoctuoidea10675|658[2n]bp|Canada.British Columbia|BOLD:ACF0936
Graphiphora augur|[5773]|LOWCD892-06|CGWC-3712|658[0n]bp|Canada.British Columbia|BOLD:ACF0936
Graphiphora augur|[5774]|LALPA1190-11|AVBC 1192-11|658[0n]bp|Canada.British Columbia|BOLD:ACF0936
Graphiphora augur|[5775]|RDMAB469-05|BCSC134|658[0n]bp|Canada.Yukon Territory|BOLD:ACF0936
Graphiphora augur|[5776]|LBCH4696-10|10-JDWBC-4696|643[0n]bp|Canada.British Columbia|BOLD:ACF0936
Graphiphora augur|[5777]|LOWCD888-06|CGWC-3708|610[0n]bp|Canada.British Columbia|BOLD:ACF0936
Graphiphora augur|[5778]|LOWCD805-06|CGWC-3625|595[0n]bp|Canada.British Columbia|BOLD:ACF0936
Graphiphora augur|[5779]|LOWC833-05|CGWC-0833|658[0n]bp|Canada.British Columbia|BOLD:ACF0936
Graphiphora augur|[5780]|LPABC412-09|08BBLEP-04631|658[0n]bp|Canada.Alberta|BOLD:ACF0936
Graphiphora augur|[5781]|LBCH2027-10|10-JDWBC-2027|658[0n]bp|Canada.British Columbia|BOLD:ACF0936

Graphiphora augur|[5779]|LOWC833-05|CGWC-0833|658[0n]bp|Canada.British Columbia|BOLD:ACF0936
Graphiphora augur|[5780]|LPABC412-09|08BBLEP-04631|658[0n]bp|Canada.Alberta|BOLD:ACF0936
Graphiphora augur|[5781]|LBCH2027-10|10-JDWBC-2027|658[0n]bp|Canada.British Columbia|BOLD:ACF0936
Graphiphora augur|[5782]|LALPA652-10|AVBC 654-10|636[0n]bp|Canada.British Columbia|BOLD:ACF0936
Graphiphora augur|[5783]|LPABC264-09|08BBLEP-04483|631[0n]bp|Canada.Alberta|BOLD:ACF0936
Graphiphora augur|[5784]|LBCD101-05|HLC-22921|658[0n]bp|Canada.British Columbia|BOLD:ACF0936
Graphiphora augur|[5785]|LOWC832-05|CGWC-0832|658[0n]bp|Canada.British Columbia|BOLD:ACF0936
Graphiphora augur|[5786]|BBLPB706-10|10BBCLP-1705|658[0n]bp|Canada.British Columbia|BOLD:ACF0936
Graphiphora augur|[5787]|LPABC394-09|08BBLEP-04613|658[0n]bp|Canada.Alberta|BOLD:ACF0936
Graphiphora augur|[5788]|LPABC456-09|08BBLEP-04675|658[0n]bp|Canada.Alberta|BOLD:ACF0936
Graphiphora augur|[5789]|LBCH4431-10|10-JDWBC-4431|658[0n]bp|Canada.British Columbia|BOLD:ACF0936
Graphiphora augur|[5790]|LPABC473-09|08BBLEP-04692|658[0n]bp|Canada.Alberta|BOLD:ACF0936
Graphiphora augur|[5791]|LBCC836-05|HLC-22716|658[0n]bp|Canada.British Columbia|BOLD:ACF0936
Graphiphora augur|[5792]|LPABC430-09|08BBLEP-04649|658[0n]bp|Canada.Alberta|BOLD:ACF0936
Graphiphora augur|[5793]|LPABB822-09|08BBLEP-04142|658[0n]bp|Canada.Alberta|BOLD:ACF0936
Graphiphora augur|[5794]|LBCC837-05|HLC-22717|658[0n]bp|Canada.British Columbia|BOLD:ACF0936
Graphiphora augur|[5795]|LBCC835-05|HLC-22715|658[0n]bp|Canada.British Columbia|BOLD:ACF0936
Graphiphora augur|[5796]|LBCD325-05|HLC-23145|658[0n]bp|Canada.British Columbia|BOLD:ACF0936
Graphiphora augur|[5797]|LPABC425-09|08BBLEP-04644|658[0n]bp|Canada.Alberta|BOLD:ACF0936
Graphiphora augur|[5798]|LBCH3366-10|10-JDWBC-3366|658[0n]bp|Canada.British Columbia|BOLD:ACF0936
Graphiphora augur|[5799]|LBCH3086-10|10-JDWBC-3086|658[0n]bp|Canada.British Columbia|BOLD:ACF0936
Graphiphora augur|[5800]|LBCH362-10|10-JDWBC-0362|658[0n]bp|Canada.British Columbia|BOLD:ACF0936
Graphiphora augur|[5801]|LBCH4682-10|10-JDWBC-4682|658[0n]bp|Canada.British Columbia|BOLD:ACF0936
Graphiphora augur|[5802]|LBCG766-09|08-JDWBC-0766|658[0n]bp|Canada.British Columbia|BOLD:ACF0936
Graphiphora augur|[5803]|LBCH357-10|10-JDWBC-0357|658[0n]bp|Canada.British Columbia|BOLD:ACF0936
Graphiphora augur|[5804]|LBCG2305-09|08-JDWBC-2305|658[0n]bp|Canada.British Columbia|BOLD:ACF0936
Graphiphora augur|[5805]|LPABC438-09|08BBLEP-04657|658[0n]bp|Canada.Alberta|BOLD:ACF0936
Graphiphora augur|[5806]|RDNMB901-05|CNCNoctuoidea10676|658[0n]bp|Canada.British Columbia|BOLD:ACF0936
Graphiphora augur|[5807]|LBCH919-10|10-JDWBC-0919|658[0n]bp|Canada.British Columbia|BOLD:ACF0936
Graphiphora augur|[5808]|LBCH3364-10|10-JDWBC-3364|658[0n]bp|Canada.British Columbia|BOLD:ACF0936
Graphiphora augur|[5809]|LBCD425-05|HLC-23245|658[0n]bp|Canada.British Columbia|BOLD:ACF0936
Graphiphora augur|[5810]|LBCH360-10|10-JDWBC-0360|658[0n]bp|Canada.British Columbia|BOLD:ACF0936
Graphiphora augur|[5811]|LOWC834-05|CGWC-0834|658[0n]bp|Canada.British Columbia|BOLD:ACF0936
Graphiphora augur|[5812]|LBCH114-10|10-JDWBC-0114|658[0n]bp|Canada.British Columbia|BOLD:ACF0936
Graphiphora augur|[5813]|LBCH941-10|10-JDWBC-0941|658[0n]bp|Canada.British Columbia|BOLD:ACF0936
Graphiphora augur|[5814]|LBCH359-10|10-JDWBC-0359|658[0n]bp|Canada.British Columbia|BOLD:ACF0936
Graphiphora augur|[5815]|LOWC835-05|CGWC-0835|658[0n]bp|Canada.British Columbia|BOLD:ACF0936
Graphiphora augur|[5816]|LBCG2311-09|08-JDWBC-2311|658[0n]bp|Canada.British Columbia|BOLD:ACF0936
Graphiphora augur|[5817]|LBCH493-10|10-JDWBC-0493|658[0n]bp|Canada.British Columbia|BOLD:ACF0936
Graphiphora augur|[5818]|LBCH3362-10|10-JDWBC-3362|658[0n]bp|Canada.British Columbia|BOLD:ACF0936
Graphiphora augur|[5819]|LOWCC185-05|CGWC-2065|658[0n]bp|Canada.British Columbia|BOLD:ACF0936
Graphiphora augur|[5820]|LBCH361-10|10-JDWBC-0361|658[0n]bp|Canada.British Columbia|BOLD:ACF0936
Graphiphora augur|[5821]|LBCH4688-10|10-JDWBC-4688|658[0n]bp|Canada.British Columbia|BOLD:ACF0936
Graphiphora augur|[5822]|LBCH3371-10|10-JDWBC-3371|658[0n]bp|Canada.British Columbia|BOLD:ACF0936
Graphiphora augur|[5823]|LBCH3072-10|10-JDWBC-3072|658[0n]bp|Canada.British Columbia|BOLD:ACF0936
Graphiphora augur|[5824]|LBCH922-10|10-JDWBC-0922|658[0n]bp|Canada.British Columbia|BOLD:ACF0936
Graphiphora augur|[5825]|LALPA587-10|AVBC 589-10|658[0n]bp|Canada.British Columbia|BOLD:ACF0936
Graphiphora augur|[5826]|LBCG2312-09|08-JDWBC-2312|658[0n]bp|Canada.British Columbia|BOLD:ACF0936
Graphiphora augur|[5827]|LBCH942-10|10-JDWBC-0942|658[0n]bp|Canada.British Columbia|BOLD:ACF0936
Graphiphora augur|[5828]|LPABC272-09|08BBLEP-04491|658[0n]bp|Canada.Alberta|BOLD:ACF0936
Graphiphora augur|[5829]|LOWCE784-06|CGWC-4544|658[0n]bp|Canada.British Columbia|BOLD:ACF0936
Graphiphora augur|[5830]|LOWC839-05|CGWC-0839|658[0n]bp|Canada.British Columbia|BOLD:ACF0936
Graphiphora augur|[5831]|LOWCC184-05|CGWC-2064|658[0n]bp|Canada.British Columbia|BOLD:ACF0936
Graphiphora augur|[5832]|LPABC351-09|08BBLEP-04570|658[0n]bp|Canada.Alberta|BOLD:ACF0936
Graphiphora augur|[5833]|LBCH926-10|10-JDWBC-0926|658[0n]bp|Canada.British Columbia|BOLD:ACF0936
Graphiphora augur|[5834]|LBCH4680-10|10-JDWBC-4680|658[0n]bp|Canada.British Columbia|BOLD:ACF0936
Graphiphora augur|[5835]|LBCH3368-10|10-JDWBC-3368|658[0n]bp|Canada.British Columbia|BOLD:ACF0936
Graphiphora augur|[5836]|LBCH4686-10|10-JDWBC-4686|658[0n]bp|Canada.British Columbia|BOLD:ACF0936
Graphiphora augur|[5837]|LBCH3363-10|10-JDWBC-3363|658[0n]bp|Canada.British Columbia|BOLD:ACF0936
Graphiphora augur|[5838]|LBCH808-10|10-JDWBC-0808|658[0n]bp|Canada.British Columbia|BOLD:ACF0936
Graphiphora augur|[5839]|LOWC837-05|CGWC-0837|658[0n]bp|Canada.British Columbia|BOLD:ACF0936
Graphiphora augur|[5840]|LBCH921-10|10-JDWBC-0921|658[0n]bp|Canada.British Columbia|BOLD:ACF0936
Graphiphora augur|[5841]|LBCH4423-10|10-JDWBC-4423|658[0n]bp|Canada.British Columbia|BOLD:ACF0936
Graphiphora augur|[5842]|LBCH363-10|10-JDWBC-0363|658[0n]bp|Canada.British Columbia|BOLD:ACF0936
Graphiphora augur|[5843]|LBCH3369-10|10-JDWBC-3369|658[0n]bp|Canada.British Columbia|BOLD:ACF0936
Graphiphora augur|[5844]|LOWC836-05|CGWC-0836|658[0n]bp|Canada.British Columbia|BOLD:ACF0936
Graphiphora augur|[5845]|LBCH3776-10|10-JDWBC-3776|658[0n]bp|Canada.British Columbia|BOLD:ACF0936
Graphiphora augur|[5846]|LPABC381-09|08BBLEP-04600|658[0n]bp|Canada.Alberta|BOLD:ACF0936
Graphiphora augur|[5847]|LBCH2378-10|10-JDWBC-2378|658[0n]bp|Canada.British Columbia|BOLD:ACF0936
Graphiphora augur|[5848]|LBCD043-05|HLC-22863|658[0n]bp|Canada.British Columbia|BOLD:ACF0936
Graphiphora augur|[5849]|LBCH923-10|10-JDWBC-0923|658[0n]bp|Canada.British Columbia|BOLD:ACF0936
Graphiphora augur|[5850]|LBCH924-10|10-JDWBC-0924|658[0n]bp|Canada.British Columbia|BOLD:ACF0936
Graphiphora augur|[5851]|LOWCC186-05|CGWC-2066|658[0n]bp|Canada.British Columbia|BOLD:ACF0936
Graphiphora augur|[5852]|LBCH4424-10|10-JDWBC-4424|658[0n]bp|Canada.British Columbia|BOLD:ACF0936
Graphiphora augur|[5853]|LBCG2310-09|08-JDWBC-2310|658[0n]bp|Canada.British Columbia|BOLD:ACF0936
Graphiphora augur|[5854]|LBCH3365-10|10-JDWBC-3365|658[0n]bp|Canada.British Columbia|BOLD:ACF0936
Graphiphora augur|[5855]|LPABB603-08|08BBLEP-03868|658[0n]bp|Canada.Alberta|BOLD:ACF0936
Graphiphora augur|[5856]|LOWCC187-05|CGWC-2067|658[0n]bp|Canada.British Columbia|BOLD:ACF0936
Graphiphora augur|[5857]|LBCH356-10|10-JDWBC-0356|658[0n]bp|Canada.British Columbia|BOLD:ACF0936
Graphiphora augur|[5858]|LBCG1878-09|08-JDWBC-1878|658[0n]bp|Canada.British Columbia|BOLD:ACF0936
Graphiphora augur|[5859]|LBCH920-10|10-JDWBC-0920|658[0n]bp|Canada.British Columbia|BOLD:ACF0936
Graphiphora augur|[5860]|LBCH4689-10|10-JDWBC-4689|658[0n]bp|Canada.British Columbia|BOLD:ACF0936
Graphiphora augur|[5861]|LOWCD890-06|CGWC-3710|658[0n]bp|Canada.British Columbia|BOLD:ACF0936
Graphiphora augur|[5862]|LPABC334-09|08BBLEP-04553|658[0n]bp|Canada.Alberta|BOLD:ACF0936
Graphiphora augur|[5863]|LOWC838-05|CGWC-0838|658[0n]bp|Canada.British Columbia|BOLD:ACF0936
Graphiphora augur|[5864]|LOWC840-05|CGWC-0840|658[0n]bp|Canada.British Columbia|BOLD:ACF0936
Graphiphora augur|[5865]|LBCD042-05|HLC-22862|658[0n]bp|Canada.British Columbia|BOLD:ACF0936
Graphiphora augur|[5866]|LBCH1305-10|10-JDWBC-1305|658[0n]bp|Canada.British Columbia|BOLD:ACF0936
Graphiphora augur|[5867]|LBCH4683-10|10-JDWBC-4683|658[0n]bp|Canada.British Columbia|BOLD:ACF0936
Graphiphora augur|[5868]|LPABC403-09|08BBLEP-04622|658[0n]bp|Canada.Alberta|BOLD:ACF0936
Graphiphora augur|[5869]|LBCH3370-10|10-JDWBC-3370|658[0n]bp|Canada.British Columbia|BOLD:ACF0936
Graphiphora augur|[5870]|LBCH693-10|10-JDWBC-0693|658[0n]bp|Canada.British Columbia|BOLD:ACF0936
Graphiphora augur|[5871]|LBCH3367-10|10-JDWBC-3367|658[0n]bp|Canada.British Columbia|BOLD:ACF0936
Graphiphora augur|[5872]|LOWCC188-05|CGWC-2068|658[0n]bp|Canada.British Columbia|BOLD:ACF0936
Graphiphora augur|[5873]|LBCH4687-10|10-JDWBC-4687|658[0n]bp|Canada.British Columbia|BOLD:ACF0936
Graphiphora augur|[5874]|LOWCD889-06|CGWC-3709|658[0n]bp|Canada.British Columbia|BOLD:ACF0936
Graphiphora augur|[5875]|LOWCC852-05|CGWC-2732|658[0n]bp|Canada.British Columbia|BOLD:ACF0936
Graphiphora augur|[5876]|LBCH4684-10|10-JDWBC-4684|658[0n]bp|Canada.British Columbia|BOLD:ACF0936
Graphiphora augur|[5877]|LBCD316-05|HLC-23136|658[0n]bp|Canada.British Columbia|BOLD:ACF0936
Graphiphora augur|[5878]|LBCC834-05|HLC-22714|658[0n]bp|Canada.British Columbia|BOLD:ACF0936
Graphiphora augur|[5879]|LBCH3361-10|10-JDWBC-3361|658[0n]bp|Canada.British Columbia|BOLD:ACF0936
Graphiphora augur|[5880]|LBCH4685-10|10-JDWBC-4685|658[0n]bp|Canada.British Columbia|BOLD:ACF0936
Graphiphora augur|[5881]|LBCH4119-10|10-JDWBC-4119|658[0n]bp|Canada.British Columbia|BOLD:ACF0936

[illegible]
Graphiphora augur|[5879]|LBCH3361-10|10-JDWBC-3361|658[0n]bp|Canada.British Columbia|BOLD:ACF0936
Graphiphora augur|[5880]|LBCH4685-10|10-JDWBC-4685|658[0n]bp|Canada.British Columbia|BOLD:ACF0936
Graphiphora augur|[5881]|LBCH4119-10|10-JDWBC-4119|658[0n]bp|Canada.British Columbia|BOLD:ACF0936
Graphiphora augur|[5882]|LBCH232-10|10-JDWBC-0232|658[0n]bp|Canada.British Columbia|BOLD:ACF0936
Graphiphora augur|[5883]|RDNM845-05|CNCNoctuoidea7685|658[0n]bp|Canada.British Columbia|BOLD:ACF0936
Graphiphora augur|[5884]|LPSOD983-09|08BBLEP-05616|658[0n]bp|Canada.Ontario|BOLD:ABZ5642
Graphiphora augur|[5885]|RDNMC075-05|CNCNoctuoidea10816|542[0n]bp|Canada.New Brunswick|BOLD:ABZ5642
Graphiphora augur|[5886]|XAG543-05|2005-ONT-1127|592[0n]bp|Canada.Ontario|BOLD:ABZ5642
Graphiphora augur|[5887]|LPMNB467-09|08BBLEP-05505|658[0n]bp|Canada.Manitoba|BOLD:ABZ5642
Graphiphora augur|[5888]|MNBB378-05|05-NBSTA-294|658[0n]bp|Canada.New Brunswick|BOLD:ABZ5642
Graphiphora augur|[5889]|BLTIB784-08|BL1201|658[0n]bp|Canada.Ontario|BOLD:AAA2707
Graphiphora augur|[5890]|RDNMB904-05|CNCNoctuoidea10679|658[0n]bp|Canada.Manitoba|BOLD:AAA2707
Graphiphora augur|[5891]|XAD573-04|04HBL006988|658[0n]bp|Canada.Ontario|BOLD:AAA2707
Graphiphora augur|[5892]|XAG205-05|2005-ONT-789|658[2n]bp|Canada.Ontario|BOLD:AAA2707
Graphiphora augur|[5893]|XAG704-05|2005-ONT-1288|658[0n]bp|Canada.Ontario|BOLD:AAA2707
Graphiphora augur|[5894]|MNBB605-05|05-NBSTA-521|658[0n]bp|Canada.New Brunswick|BOLD:AAA2707
Graphiphora augur|[5895]|RDNMB903-05|CNCNoctuoidea10678|658[0n]bp|Canada.Alberta|BOLD:AAA2707
Graphiphora augur|[5896]|LPABC407-09|08BBLEP-04626|658[0n]bp|Canada.Alberta|BOLD:AAA2707
Graphiphora augur|[5897]|XAB002-04|04HBL005002|567[0n]bp|Canada.Ontario|BOLD:AAA2707
Graphiphora augur|[5898]|XAG479-05|2005-ONT-1063|587[0n]bp|Canada.Ontario|BOLD:AAA2707
Graphiphora augur|[5899]|LPSOD936-09|08BBLEP-05476|658[0n]bp|Canada.Ontario|BOLD:AAA2707
Graphiphora augur|[5900]|LCHP659-07|07PROBE-10341|626[0n]bp|Canada.Manitoba|BOLD:AAA2707
Graphiphora augur|[5901]|JSJUL2389-11|BIOUG01497-E12|658[0n]bp|Canada.Ontario|BOLD:AAA2707
Graphiphora augur|[5902]|LCHIP246-07|06-PROBE-2896|650[0n]bp|Canada.Manitoba|BOLD:AAA2707
Graphiphora augur|[5903]|PHMNB064-03|moth50.02SA|639[0n]bp|Canada.New Brunswick|BOLD:AAA2707
Graphiphora augur|[5904]|RDNMD051-06|CNCNoctuoidea12437|609[0n]bp|Canada|BOLD:AAA2707
Graphiphora augur|[5905]|RDNM844-05|CNCNoctuoidea7684|658[0n]bp|Canada.Ontario|BOLD:AAA2707
Graphiphora augur|[5906]|RDLQ706-07|DH002869|658[0n]bp|Canada.Quebec|BOLD:AAA2707
Graphiphora augur|[5907]|RDNMB905-05|CNCNoctuoidea10680|658[0n]bp|Canada.Alberta|BOLD:AAA2707
Choephora fungorum|[5908]|NAMUM317-08|JKA-97-0033|658[0n]bp|United States.Georgia|BOLD:AAC7430
Choephora fungorum|[5909]|NAMUM318-08|JKA-97-0034|658[0n]bp|United States.Georgia|BOLD:AAC7430
Choephora fungorum|[5910]|LPOKA371-08|MDOK-0371|658[0n]bp|United States.Oklahoma|BOLD:AAC7430
Choephora fungorum|[5911]|LNC479-06|05-NCCC-479|658[0n]bp|United States.North Carolina|BOLD:AAC7430
Choephora fungorum|[5912]|LNC478-06|05-NCCC-478|658[0n]bp|United States.North Carolina|BOLD:AAC7430
Choephora fungorum|[5913]|LPOKA375-08|MDOK-0375|658[0n]bp|United States.Oklahoma|BOLD:AAC7430
Choephora fungorum|[5914]|RDNMG1032-08|CNC LEP00053156|658[0n]bp|United States.Maryland|BOLD:AAC7430
Choephora fungorum|[5915]|LPOKA610-09|MDOK-0610|639[0n]bp|United States.Oklahoma|BOLD:AAC7430
Choephora fungorum|[5916]|LPOKA624-09|MDOK-0624|658[0n]bp|United States.Oklahoma|BOLD:AAC7430
Choephora fungorum|[5917]|LPOKD450-09|MDOK-3529|658[0n]bp|United States.Oklahoma|BOLD:AAC7430
Choephora fungorum|[5918]|LPOKA613-09|MDOK-0613|617[0n]bp|United States.Oklahoma|BOLD:AAC7430
Hemipachnobia monochromatea|[5919]|RDLQG138-06|DH012305|640[0n]bp|Canada.Quebec|BOLD:AAE5284
Hemipachnobia monochromatea|[5920]|RDLQF460-06|DH011567|658[0n]bp|Canada.Quebec|BOLD:AAE5284
Hemipachnobia monochromatea|[5921]|TMNBB391-06|MNBTT-1331|658[0n]bp|Canada.New Brunswick|BOLD:AAE5284
Hemipachnobia monochromatea|[5922]|TMNBB390-06|MNBTT-1330|658[0n]bp|Canada.New Brunswick|BOLD:AAE5284
Pseudohermonassa flavotincta|[5923]|RDNMF648-08|NOC14734|640[0n]bp|Canada.British Columbia|BOLD:AAB9841
Pseudohermonassa flavotincta|[5924]|LBCH1309-10|10-JDWBC-1309|658[0n]bp|Canada.British Columbia|BOLD:...
Pseudohermonassa flavotincta|[5925]|LBCH1313-10|10-JDWBC-1313|658[0n]bp|Canada.British Columbia|BOLD:...
Pseudohermonassa flavotincta|[5926]|LBCH1312-10|10-JDWBC-1312|658[0n]bp|Canada.British Columbia|BOLD:...
Pseudohermonassa flavotincta|[5927]|LBCH1314-10|10-JDWBC-1314|658[0n]bp|Canada.British Columbia|BOLD:...
Pseudohermonassa flavotincta|[5928]|LBCH1307-10|10-JDWBC-1307|658[0n]bp|Canada.British Columbia|BOLD:...
Pseudohermonassa flavotincta|[5929]|RDNMF649-08|NOC14735|640[0n]bp|Canada.British Columbia|BOLD:AAB9841
Pseudohermonassa flavotincta|[5930]|RDNMF646-08|NOC14732|652[0n]bp|Canada.British Columbia|BOLD:AAB9841
Pseudohermonassa flavotincta|[5931]|RDNMF647-08|NOC14733|658[0n]bp|Canada.British Columbia|BOLD:AAB9841
Pseudohermonassa flavotincta|[5932]|LBCH1308-10|10-JDWBC-1308|632[0n]bp|Canada.British Columbia|BOLD:...
Pseudohermonassa flavotincta|[5933]|LBCH1310-10|10-JDWBC-1310|658[0n]bp|Canada.British Columbia|BOLD:...
Pseudohermonassa flavotincta|[5934]|LBCH1306-10|10-JDWBC-1306|658[0n]bp|Canada.British Columbia|BOLD:...
Pseudohermonassa flavotincta|[5935]|LBCH1315-10|10-JDWBC-1315|636[0n]bp|Canada.British Columbia|BOLD:...
Pseudohermonassa flavotincta|[5936]|LBCH1311-10|10-JDWBC-1311|658[0n]bp|Canada.British Columbia|BOLD:...
Prognorisma substrigata|[5937]|LOWC933-05|CGWC-0933|658[0n]bp|Canada.British Columbia|BOLD:AAB2661
Prognorisma substrigata|[5938]|LOWCD374-06|CGWC-3194|581[0n]bp|Canada.British Columbia|BOLD:AAB2661
Prognorisma substrigata|[5939]|LOWCD381-06|CGWC-3201|590[0n]bp|Canada.British Columbia|BOLD:AAB2661
Prognorisma substrigata|[5940]|LOWCD380-06|CGWC-3200|589[0n]bp|Canada.British Columbia|BOLD:AAB2661
Prognorisma substrigata|[5941]|LOWCD383-06|CGWC-3203|582[0n]bp|Canada.British Columbia|BOLD:AAB2661
Prognorisma substrigata|[5942]|LOWCD375-06|CGWC-3195|544[0n]bp|Canada.British Columbia|BOLD:AAB2661
Prognorisma substrigata|[5943]|LOWCD378-06|CGWC-3198|577[0n]bp|Canada.British Columbia|BOLD:AAB2661
Prognorisma substrigata|[5944]|LOWCD372-06|CGWC-3192|582[0n]bp|Canada.British Columbia|BOLD:AAB2661
Prognorisma substrigata|[5945]|LOWCD371-06|CGWC-3191|590[0n]bp|Canada.British Columbia|BOLD:AAB2661
Prognorisma substrigata|[5946]|LOWC928-05|CGWC-0928|658[0n]bp|Canada.British Columbia|BOLD:AAB2661
Prognorisma substrigata|[5947]|LOWCC163-05|CGWC-2043|658[0n]bp|Canada.British Columbia|BOLD:AAB2661
Prognorisma substrigata|[5948]|LOWC926-05|CGWC-0926|658[0n]bp|Canada.British Columbia|BOLD:AAB2661
Prognorisma substrigata|[5949]|LOWC934-05|CGWC-0934|658[0n]bp|Canada.British Columbia|BOLD:AAB2661
Prognorisma substrigata|[5950]|LOWC929-05|CGWC-0929|658[0n]bp|Canada.British Columbia|BOLD:AAB2661
Prognorisma substrigata|[5951]|LOWC930-05|CGWC-0930|658[0n]bp|Canada.British Columbia|BOLD:AAB2661
Prognorisma substrigata|[5952]|LOWCD377-06|CGWC-3197|658[0n]bp|Canada.British Columbia|BOLD:AAB2661
Prognorisma substrigata|[5953]|LOWC932-05|CGWC-0932|658[0n]bp|Canada.British Columbia|BOLD:AAB2661
Prognorisma substrigata|[5954]|LOWC927-05|CGWC-0927|658[0n]bp|Canada.British Columbia|BOLD:AAB2661
Prognorisma substrigata|[5955]|LOWCD384-06|CGWC-3204|583[0n]bp|Canada.British Columbia|BOLD:AAB2661
Prognorisma substrigata|[5956]|LOWCD385-06|CGWC-3205|551[0n]bp|Canada.British Columbia|BOLD:AAB2661
Prognorisma substrigata|[5957]|LOWCD376-06|CGWC-3196|658[0n]bp|Canada.British Columbia|BOLD:AAB2661
Prognorisma substrigata|[5958]|LOWCD370-06|CGWC-3190|610[0n]bp|Canada.British Columbia|BOLD:AAB2661
Prognorisma substrigata|[5959]|LOWCD373-06|CGWC-3193|609[0n]bp|Canada.British Columbia|BOLD:AAB2661
Prognorisma substrigata|[5960]|LOWC931-05|CGWC-0931|511[0n]bp|Canada.British Columbia|BOLD:AAB2661
Prognorisma substrigata|[5961]|LOWCD382-06|CGWC-3202|610[0n]bp|Canada.British Columbia|BOLD:AAB2661
Prognorisma substrigata|[5962]|LOWCD379-06|CGWC-3199|546[0n]bp|Canada.British Columbia|BOLD:AAB2661
Prognorisma substrigata|[5963]|LOWC925-05|CGWC-0925|658[0n]bp|Canada.British Columbia|BOLD:AAB2661
Pseudohermonassa bicarnea|[5964]|BBLPC023-09|09BBELE-1023|657[0n]bp|Canada.New Brunswick|BOLD:AAB1024
Pseudohermonassa bicarnea|[5965]|BBLEC429-09|09BBELE-0429|658[0n]bp|Canada.New Brunswick|BOLD:AAB1024
Pseudohermonassa bicarnea|[5966]|XAI052-05|0102-ONT-0052|658[0n]bp|Canada.Ontario|BOLD:AAB1024
Pseudohermonassa bicarnea|[5967]|XAD171-04|04HBL007171|658[0n]bp|Canada.Ontario|BOLD:AAB1024
Pseudohermonassa bicarnea|[5968]|XAG709-05|2005-ONT-1293|658[0n]bp|Canada.Ontario|BOLD:AAB1024
Pseudohermonassa bicarnea|[5969]|XAG878-05|2005-ONT-1462|658[0n]bp|Canada.Ontario|BOLD:AAB1024
Pseudohermonassa bicarnea|[5970]|PHAUG1796-11|BIOUG01497-E06|658[0n]bp|Canada.Ontario|BOLD:AAB1024
Pseudohermonassa bicarnea|[5971]|XAH220-05|2005-ONT-1803|658[0n]bp|Canada.Ontario|BOLD:AAB1024
Pseudohermonassa bicarnea|[5972]|XAK421-06|2006-ONT-1416|658[0n]bp|Canada.Ontario|BOLD:AAB1024
Pseudohermonassa bicarnea|[5973]|XAD181-04|04HBL007181|593[0n]bp|Canada.Ontario|BOLD:AAB1024
Pseudohermonassa bicarnea|[5974]|XAD243-04|04HBL007243|658[0n]bp|Canada.Ontario|BOLD:AAB1024
Pseudohermonassa bicarnea|[5975]|RDLQF812-06|DH011962|658[0n]bp|Canada.Quebec|BOLD:AAB1024
Pseudohermonassa bicarnea|[5976]|MNBB478-05|05-NBSTA-394|658[0n]bp|Canada.New Brunswick|BOLD:AAB1024
Pseudohermonassa bicarnea|[5977]|MNBB591-05|05-NBSTA-507|658[0n]bp|Canada.New Brunswick|BOLD:AAB1024
Pseudohermonassa bicarnea|[5978]|XAK420-06|2006-ONT-1415|658[0n]bp|Canada.Ontario|BOLD:AAB1024
Pseudohermonassa bicarnea|[5979]|MNBB592-05|05-NBSTA-508|658[0n]bp|Canada.New Brunswick|BOLD:AAB1024
Pseudohermonassa bicarnea|[5980]|XAD176-04|04HBL007176|658[0n]bp|Canada.Ontario|BOLD:AAB1024
[illegible]

Pseudohermonassa bicarnea|[5978]|XAK420-06|2006-ONT-1415|658[0n]bp|Canada.Ontario|BOLD:AAB1024
Pseudohermonassa bicarnea|[5979]|MNBB592-05|05-NBSTA-508|658[0n]bp|Canada.New Brunswick|BOLD:AAB1024
Pseudohermonassa bicarnea|[5980]|XAD176-04|04HBL007176|658[0n]bp|Canada.Ontario|BOLD:AAB1024
Pseudohermonassa bicarnea|[5981]|MNBB596-05|05-NBSTA-512|658[0n]bp|Canada.New Brunswick|BOLD:AAB1024
Pseudohermonassa bicarnea|[5982]|TTMNB452-06|MNBTT-452|658[0n]bp|Canada.New Brunswick|BOLD:AAB1024
Pseudohermonassa bicarnea|[5983]|RDLQB843-05|DH010930|658[0n]bp|Canada.Quebec|BOLD:AAB1024
Pseudohermonassa bicarnea|[5984]|TTMNB450-06|MNBTT-450|658[0n]bp|Canada.New Brunswick|BOLD:AAB1024
Pseudohermonassa bicarnea|[5985]|MNBB595-05|05-NBSTA-511|658[0n]bp|Canada.New Brunswick|BOLD:AAB1024
Pseudohermonassa bicarnea|[5986]|XAD341-04|04HBL007341|658[0n]bp|Canada.Ontario|BOLD:AAB1024
Pseudohermonassa bicarnea|[5987]|MNBB426-05|05-NBSTA-342|658[0n]bp|Canada.New Brunswick|BOLD:AAB1024
Pseudohermonassa bicarnea|[5988]|MNBB594-05|05-NBSTA-510|658[0n]bp|Canada.New Brunswick|BOLD:AAB1024
Pseudohermonassa tenuicula|[5989]|TTMNB451-06|MNBTT-451|658[0n]bp|Canada.New Brunswick|BOLD:ACE5721
Pseudohermonassa tenuicula|[5990]|BBLPC418-09|09BBELE-1418|658[0n]bp|Canada.New Brunswick|BOLD:ACE5721
Pseudohermonassa tenuicula|[5991]|MNBB597-05|05-NBSTA-513|658[0n]bp|Canada.New Brunswick|BOLD:ACE5721
Pseudohermonassa tenuicula|[5992]|BBLPC390-09|09BBELE-1390|658[0n]bp|Canada.New Brunswick|BOLD:ACE5721
Pseudohermonassa tenuicula|[5993]|MNBB598-05|05-NBSTA-514|658[0n]bp|Canada.New Brunswick|BOLD:ACE5721
Pseudohermonassa tenuicula|[5994]|RDLQ730-07|DH007448|658[0n]bp|Canada.Quebec|BOLD:ACE5721
Pseudohermonassa tenuicula|[5995]|BBLPC413-09|09BBELE-1413|658[0n]bp|Canada.New Brunswick|BOLD:ACE5721
Pseudohermonassa tenuicula|[5996]|RDLQ729-07|DH011283|658[0n]bp|Canada.Quebec|BOLD:ACE5721
Pseudohermonassa tenuicula|[5997]|XAD383-04|04HBL007383|658[0n]bp|Canada.Ontario|BOLD:ACE5721
Pseudohermonassa tenuicula|[5998]|RDLQB766-05|DH010853|658[0n]bp|Canada.Quebec|BOLD:ACE5721
Pseudohermonassa tenuicula|[5999]|MNBB667-05|05-NBSTA-583|658[0n]bp|Canada.New Brunswick|BOLD:ACE5721
Pseudohermonassa tenuicula|[6000]|BBLPC369-09|09BBELE-1369|658[0n]bp|Canada.New Brunswick|BOLD:ACE5721
Pseudohermonassa tenuicula|[6001]|MNBB482-05|05-NBSTA-398|658[0n]bp|Canada.New Brunswick|BOLD:ACE5721
Pseudohermonassa tenuicula|[6002]|BBLPC417-09|09BBELE-1417|658[0n]bp|Canada.New Brunswick|BOLD:ACE5721
Pseudohermonassa tenuicula|[6003]|BBLPC353-09|09BBELE-1353|658[0n]bp|Canada.New Brunswick|BOLD:ACE5721
Pseudohermonassa tenuicula|[6004]|LPMNB232-09|08BBLEP-05076|658[0n]bp|Canada.Manitoba|BOLD:ACE5721
Pseudohermonassa tenuicula|[6005]|BBLPB860-10|10BBCLP-1859|658[0n]bp|Canada.Alberta|BOLD:ACE5721
Pseudohermonassa tenuicula|[6006]|LOWCD369-06|CGWC-3189|586[0n]bp|Canada.British Columbia|BOLD:ACE5721
Pseudohermonassa tenuicula|[6007]|LOWCD500-06|CGWC-3320|587[0n]bp|Canada.British Columbia|BOLD:ACE5721
Pseudohermonassa tenuicula|[6008]|BBLPB861-10|10BBCLP-1860|658[0n]bp|Canada.Alberta|BOLD:ACE5721
Pseudohermonassa tenuicula|[6009]|XAD246-04|04HBL007246|658[0n]bp|Canada.Ontario|BOLD:ACE5721
Agnorisma badinodis|[6010]|LPOKD634-09|MDOK-3713|658[0n]bp|United States.Oklahoma|BOLD:AAD4960
Agnorisma badinodis|[6011]|LPOKA620-09|MDOK-0620|630[0n]bp|United States.Oklahoma|BOLD:AAD4960
Agnorisma badinodis|[6012]|LPOKA671-09|MDOK-0671|602[0n]bp|United States.Oklahoma|BOLD:AAD4960
Agnorisma badinodis|[6013]|LPOKA1040-09|MDOK-1520|658[0n]bp|United States.Oklahoma|BOLD:AAD4960
Agnorisma badinodis|[6014]|LPOKA651-09|MDOK-0651|630[0n]bp|United States.Oklahoma|BOLD:AAD4960
Agnorisma badinodis|[6015]|LNC515-06|05-NCCC-515|658[0n]bp|United States.North Carolina|BOLD:AAD4960
Agnorisma badinodis|[6016]|JRLAA024-09|JRLAA-024|621[0n]bp|United States.Alabama|BOLD:AAD4960
Agnorisma badinodis|[6017]|LNCC400-10|10-NCCC-495|658[0n]bp|United States.North Carolina|BOLD:AAD4960
Agnorisma badinodis|[6018]|LNCC401-10|10-NCCC-496|658[0n]bp|United States.North Carolina|BOLD:AAD4960
Agnorisma badinodis|[6019]|RDNMG752-08|CNC LEP00052876|658[0n]bp|United States.Georgia|BOLD:AAD4960
Agnorisma badinodis|[6020]|LSEU165-06|06-JKA-0165|658[0n]bp|United States.Georgia|BOLD:AAD4960
Agnorisma badinodis|[6021]|MJMSL147-10|TDWG-0064|658[0n]bp|United States.Massachusetts|BOLD:AAD4960
Agnorisma badinodis|[6022]|MJMSL019-10|10MA-0019|658[0n]bp|United States.Massachusetts|BOLD:AAD4960
Agnorisma bugrai|[6023]|LOWCC130-05|CGWC-2010|595[0n]bp|Canada.British Columbia|BOLD:AAB0245
Agnorisma bugrai|[6024]|LOWCD809-06|CGWC-3629|603[0n]bp|Canada.British Columbia|BOLD:AAB0245
Agnorisma bugrai|[6025]|LOWCD801-06|CGWC-3621|658[0n]bp|Canada.British Columbia|BOLD:AAB0245
Agnorisma bugrai|[6026]|LOWCD802-06|CGWC-3622|658[0n]bp|Canada.British Columbia|BOLD:AAB0245
Agnorisma bugrai|[6027]|LOWCC135-05|CGWC-2015|658[0n]bp|Canada.British Columbia|BOLD:AAB0245
Agnorisma bugrai|[6028]|LOWCC131-05|CGWC-2011|569[0n]bp|Canada.British Columbia|BOLD:AAB0245
Agnorisma bugrai|[6029]|LOWCC127-05|CGWC-2007|596[0n]bp|Canada.British Columbia|BOLD:AAB0245
Agnorisma bugrai|[6030]|LOWCC126-05|CGWC-2006|658[0n]bp|Canada.British Columbia|BOLD:AAB0245
Agnorisma bugrai|[6031]|LOWCC145-05|CGWC-2025|658[0n]bp|Canada.British Columbia|BOLD:AAB0245
Agnorisma bugrai|[6032]|LOWCC137-05|CGWC-2017|658[0n]bp|Canada.British Columbia|BOLD:AAB0245
Agnorisma bugrai|[6033]|LOWCC132-05|CGWC-2012|658[0n]bp|Canada.British Columbia|BOLD:AAB0245
Agnorisma bugrai|[6034]|LOWCC141-05|CGWC-2021|658[0n]bp|Canada.British Columbia|BOLD:AAB0245
Agnorisma bugrai|[6035]|LOWCC134-05|CGWC-2014|560[0n]bp|Canada.British Columbia|BOLD:AAB0245
Agnorisma bugrai|[6036]|LOWCC146-05|CGWC-2026|658[0n]bp|Canada.British Columbia|BOLD:AAB0245
Agnorisma bugrai|[6037]|XAD384-04|04HBL007384|594[0n]bp|Canada.Ontario|BOLD:AAB0245
Agnorisma bugrai|[6038]|LOWCC138-05|CGWC-2018|585[0n]bp|Canada.British Columbia|BOLD:AAB0245
Agnorisma bugrai|[6039]|LOWCC136-05|CGWC-2016|658[0n]bp|Canada.British Columbia|BOLD:AAB0245
Agnorisma bugrai|[6040]|LOWCC129-05|CGWC-2009|592[0n]bp|Canada.British Columbia|BOLD:AAB0245
Agnorisma bugrai|[6041]|LOWCD807-06|CGWC-3627|605[0n]bp|Canada.British Columbia|BOLD:AAB0245
Agnorisma bugrai|[6042]|LOWCD803-06|CGWC-3623|549[0n]bp|Canada.British Columbia|BOLD:AAB0245
Agnorisma bugrai|[6043]|LOWCD806-06|CGWC-3626|589[0n]bp|Canada.British Columbia|BOLD:AAB0245
Agnorisma bugrai|[6044]|LOWCC144-05|CGWC-2024|596[0n]bp|Canada.British Columbia|BOLD:AAB0245
Agnorisma bugrai|[6045]|PHMO394-03|moth2654.02|639[0n]bp|Canada.Ontario|BOLD:AAB0245
Agnorisma bugrai|[6046]|PHMO393-03|moth2583.02|639[0n]bp|Canada.Ontario|BOLD:AAB0245
Agnorisma bugrai|[6047]|LOWCD499-06|CGWC-3319|613[0n]bp|Canada.British Columbia|BOLD:AAB0245
Agnorisma bugrai|[6048]|LOWCC142-05|CGWC-2022|596[0n]bp|Canada.British Columbia|BOLD:AAB0245
Agnorisma bugrai|[6049]|LOWCD808-06|CGWC-3628|603[0n]bp|Canada.British Columbia|BOLD:AAB0245
Agnorisma bugrai|[6050]|LOWCC133-05|CGWC-2013|658[0n]bp|Canada.British Columbia|BOLD:AAB0245
Agnorisma bugrai|[6051]|XAI050-05|0102-ONT-0050|658[0n]bp|Canada.Ontario|BOLD:AAB0245
Agnorisma bugrai|[6052]|LOWCC140-05|CGWC-2020|658[0n]bp|Canada.British Columbia|BOLD:AAB0245
Agnorisma bugrai|[6053]|LOWCC139-05|CGWC-2019|658[0n]bp|Canada.British Columbia|BOLD:AAB0245
Agnorisma bugrai|[6054]|LOWCC143-05|CGWC-2023|658[0n]bp|Canada.British Columbia|BOLD:AAB0245
Agnorisma bugrai|[6055]|XAI051-05|0102-ONT-0051|658[0n]bp|Canada.Ontario|BOLD:AAB0245
Agnorisma bugrai|[6056]|LOWCC128-05|CGWC-2008|594[0n]bp|Canada.British Columbia|BOLD:AAB0245
Adelphagrotis indeterminata|[6057]|LPVIB558-08|PFC-2006-1984|658[0n]bp|Canada.British Columbia|BOLD:A...
Adelphagrotis indeterminata|[6058]|LALPA1270-11|AVBC 1272-11|658[0n]bp|Canada.British Columbia|BOLD:A...
Adelphagrotis indeterminata|[6059]|LALPA1272-11|AVBC 1274-11|658[0n]bp|Canada.British Columbia|BOLD:A...
Adelphagrotis indeterminata|[6060]|LALPA741-10|AVBC 743-10|658[0n]bp|Canada.British Columbia|BOLD:ABZ...
Adelphagrotis indeterminata|[6061]|LBCH6275-10|10-JDWBC-6275|658[0n]bp|Canada.British Columbia|BOLD:A...
Adelphagrotis indeterminata|[6062]|LALPA1209-11|AVBC 1211-11|658[0n]bp|Canada.British Columbia|BOLD:A...
Adelphagrotis stellaris|[6063]|LHLEP390-06|UBC-2006-1518|658[0n]bp|Canada.British Columbia|BOLD:AAC2434
Adelphagrotis stellaris|[6064]|LPVIB256-08|PFC-2006-1646|658[0n]bp|Canada.British Columbia|BOLD:AAC2434
Adelphagrotis stellaris|[6065]|LPVIA611-08|PFC-2006-0839|658[0n]bp|Canada.British Columbia|BOLD:AAC2434
Adelphagrotis stellaris|[6066]|LPVIB085-08|PFC-2006-1439|658[1n]bp|Canada.British Columbia|BOLD:AAC2434
Adelphagrotis stellaris|[6067]|LPVIB237-08|PFC-2006-1616|658[0n]bp|Canada.British Columbia|BOLD:AAC2434
Adelphagrotis stellaris|[6068]|LALPA606-10|AVBC 608-10|658[0n]bp|Canada.British Columbia|BOLD:AAC2434
Adelphagrotis stellaris|[6069]|LBCS265-07|UBC-2007-0773|658[0n]bp|Canada.British Columbia|BOLD:AAC2434
Adelphagrotis stellaris|[6070]|RDNMF615-08|NOC14701|658[0n]bp|Canada.British Columbia|BOLD:AAC2434
Adelphagrotis stellaris|[6071]|LPVIA099-08|PFC-2006-0164|658[0n]bp|Canada.British Columbia|BOLD:AAC2434
Adelphagrotis stellaris|[6072]|LPVIA835-08|PFC-2006-1130|623[0n]bp|Canada.British Columbia|BOLD:AAC2434
Adelphagrotis stellaris|[6073]|LALPA671-10|AVBC 673-10|658[1n]bp|Canada.British Columbia|BOLD:AAC2434
Adelphagrotis stellaris|[6074]|LPVIB072-08|PFC-2006-1420|658[0n]bp|Canada.British Columbia|BOLD:AAC2434
Adelphagrotis stellaris|[6075]|RDNMF614-08|NOC14700|658[0n]bp|Canada.British Columbia|BOLD:AAC2434
Adelphagrotis stellaris|[6076]|RDNMF613-08|NOC14699|658[0n]bp|Canada.British Columbia|BOLD:AAC2434
Adelphagrotis stellaris|[6077]|LPVIB067-08|PFC-2006-1415|658[0n]bp|Canada.British Columbia|BOLD:AAC2434
Adelphagrotis stellaris|[6078]|LHLEP356-06|UBC-2006-1517|657[0n]bp|Canada.British Columbia|BOLD:AAC2434
Adelphagrotis stellaris|[6079]|LHLEP435-06|UBC-2006-1656|658[0n]bp|Canada.British Columbia|BOLD:AAC2434
Adelphagrotis stellaris|[6080]|RDNMF612-08|NOC14698|658[0n]bp|Canada.British Columbia|BOLD:AAC2434

Adelphagrotis stellaris|[6078]|LHLEP356-06|UBC-2006-1517|657[0n]bp|Canada.British Columbia|BOLD:AAC2434
Adelphagrotis stellaris|[6079]|LHLEP435-06|UBC-2006-1656|658[0n]bp|Canada.British Columbia|BOLD:AAC2434
Adelphagrotis stellaris|[6080]|RDNMF612-08|NOC14698|658[0n]bp|Canada.British Columbia|BOLD:AAC2434
Adelphagrotis stellaris|[6081]|LHLEP357-06|UBC-2006-1655|657[0n]bp|Canada.British Columbia|BOLD:AAC2434
Eurois astricta|[6082]|LBCH1860-10|10-JDWBC-1860|658[0n]bp|Canada.British Columbia|BOLD:AAA2221
Eurois astricta|[6083]|LALPA712-10|AVBC 714-10|658[0n]bp|Canada.British Columbia|BOLD:AAA2221
Eurois astricta|[6084]|LALPA1280-11|AVBC 1282-11|658[0n]bp|Canada.British Columbia|BOLD:AAA2221
Eurois astricta|[6085]|LALPA756-10|AVBC 758-10|658[0n]bp|Canada.British Columbia|BOLD:AAA2221
Eurois astricta|[6086]|LALPA1318-12|AVBC 1320-11|632[0n]bp|Canada.British Columbia|BOLD:AAA2221
Eurois astricta|[6087]|LBCG2891-09|08-JDWBC-2891|658[0n]bp|Canada.British Columbia|BOLD:AAA2221
Eurois astricta|[6088]|LBCG2883-09|08-JDWBC-2883|658[0n]bp|Canada.British Columbia|BOLD:AAA2221
Eurois astricta|[6089]|LBCB042-05|HLC-20982|658[0n]bp|Canada.British Columbia|BOLD:AAA2221
Eurois astricta|[6090]|LBCG816-09|08-JDWBC-0816|658[0n]bp|Canada.British Columbia|BOLD:AAA2221
Eurois astricta|[6091]|LOWCC173-05|CGWC-2053|658[0n]bp|Canada.British Columbia|BOLD:AAA2221
Eurois astricta|[6092]|LBCH1857-10|10-JDWBC-1857|658[0n]bp|Canada.British Columbia|BOLD:AAA2221
Eurois astricta|[6093]|LBCD232-05|HLC-23052|632[0n]bp|Canada.British Columbia|BOLD:AAA2221
Eurois astricta|[6094]|LBCD242-05|HLC-23062|632[0n]bp|Canada.British Columbia|BOLD:AAA2221
Eurois astricta|[6095]|LBCH1675-10|10-JDWBC-1675|658[0n]bp|Canada.British Columbia|BOLD:AAA2221
Eurois astricta|[6096]|LBCD243-05|HLC-23063|658[0n]bp|Canada.British Columbia|BOLD:AAA2221
Eurois astricta|[6097]|LBCH2056-10|10-JDWBC-2056|658[0n]bp|Canada.British Columbia|BOLD:AAA2221
Eurois astricta|[6098]|LBCD246-05|HLC-23066|616[0n]bp|Canada.British Columbia|BOLD:AAA2221
Eurois astricta|[6099]|LPABC493-09|08BBLEP-04712|658[0n]bp|Canada.Alberta|BOLD:AAA2221
Eurois astricta|[6100]|LBCD438-05|HLC-23258|658[0n]bp|Canada.British Columbia|BOLD:AAA2221
Eurois astricta|[6101]|LBCG638-09|08-JDWBC-0638|658[0n]bp|Canada.British Columbia|BOLD:AAA2221
Eurois astricta|[6102]|LBCC325-05|HLC-22205|658[0n]bp|Canada.British Columbia|BOLD:AAA2221
Eurois astricta|[6103]|LBCD271-05|HLC-23091|631[0n]bp|Canada.British Columbia|BOLD:AAA2221
Eurois astricta|[6104]|LBCD241-05|HLC-23061|638[0n]bp|Canada.British Columbia|BOLD:AAA2221
Eurois astricta|[6105]|LBCD419-05|HLC-23239|636[0n]bp|Canada.British Columbia|BOLD:AAA2221
Eurois astricta|[6106]|LBCD244-05|HLC-23064|636[0n]bp|Canada.British Columbia|BOLD:AAA2221
Eurois astricta|[6107]|LBCD237-05|HLC-23057|616[0n]bp|Canada.British Columbia|BOLD:AAA2221
Eurois astricta|[6108]|LBCD216-05|HLC-23036|616[0n]bp|Canada.British Columbia|BOLD:AAA2221
Eurois astricta|[6109]|LBCD420-05|HLC-23240|616[0n]bp|Canada.British Columbia|BOLD:AAA2221
Eurois astricta|[6110]|LBCB037-05|HLC-20977|658[0n]bp|Canada.British Columbia|BOLD:AAA2221
Eurois astricta|[6111]|LBCB045-05|HLC-20985|658[0n]bp|Canada.British Columbia|BOLD:AAA2221
Eurois astricta|[6112]|BBLPB528-10|10BBCLP-1527|658[0n]bp|Canada.British Columbia|BOLD:AAA2221
Eurois astricta|[6113]|LBCH2094-10|10-JDWBC-2094|658[0n]bp|Canada.British Columbia|BOLD:AAA2221
Eurois astricta|[6114]|LBCD222-05|HLC-23042|658[0n]bp|Canada.British Columbia|BOLD:AAA2221
Eurois astricta|[6115]|LBCH1091-10|10-JDWBC-1091|658[0n]bp|Canada.British Columbia|BOLD:AAA2221
Eurois astricta|[6116]|LBCH1566-10|10-JDWBC-1566|658[0n]bp|Canada.British Columbia|BOLD:AAA2221
Eurois astricta|[6117]|LBCD238-05|HLC-23058|658[0n]bp|Canada.British Columbia|BOLD:AAA2221
Eurois astricta|[6118]|LBCH1502-10|10-JDWBC-1502|658[0n]bp|Canada.British Columbia|BOLD:AAA2221
Eurois astricta|[6119]|LBCH1428-10|10-JDWBC-1428|658[0n]bp|Canada.British Columbia|BOLD:AAA2221
Eurois astricta|[6120]|LBCB043-05|HLC-20983|658[0n]bp|Canada.British Columbia|BOLD:AAA2221
Eurois astricta|[6121]|LBCB044-05|HLC-20984|658[0n]bp|Canada.British Columbia|BOLD:AAA2221
Eurois astricta|[6122]|LBCA756-05|HLC-20756|658[0n]bp|Canada.British Columbia|BOLD:AAA2221
Eurois astricta|[6123]|LBCH1859-10|10-JDWBC-1859|658[0n]bp|Canada.British Columbia|BOLD:AAA2221
Eurois astricta|[6124]|LBCD250-05|HLC-23070|658[0n]bp|Canada.British Columbia|BOLD:AAA2221
Eurois astricta|[6125]|LBCH1856-10|10-JDWBC-1856|658[0n]bp|Canada.British Columbia|BOLD:AAA2221
Eurois astricta|[6126]|LBCH2248-10|10-JDWBC-2248|658[0n]bp|Canada.British Columbia|BOLD:AAA2221
Eurois astricta|[6127]|LBCC324-05|HLC-22204|658[0n]bp|Canada.British Columbia|BOLD:AAA2221
Eurois astricta|[6128]|LBCD044-05|HLC-22864|658[0n]bp|Canada.British Columbia|BOLD:AAA2221
Eurois astricta|[6129]|LHLEP389-06|UBC-2006-2056|658[0n]bp|Canada.British Columbia|BOLD:AAA2221
Eurois astricta|[6130]|LPABC400-09|08BBLEP-04619|658[0n]bp|Canada.Alberta|BOLD:AAA2221
Eurois astricta|[6131]|LBCC323-05|HLC-22203|658[0n]bp|Canada.British Columbia|BOLD:AAA2221
Eurois astricta|[6132]|LPAB210-08|08BBLEP-02532|658[0n]bp|Canada.Alberta|BOLD:AAA2221
Eurois astricta|[6133]|LBCG685-09|08-JDWBC-0685|658[0n]bp|Canada.British Columbia|BOLD:AAA2221
Eurois astricta|[6134]|LBCH2093-10|10-JDWBC-2093|658[0n]bp|Canada.British Columbia|BOLD:AAA2221
Eurois astricta|[6135]|BBLPB675-10|10BBCLP-1674|658[0n]bp|Canada.British Columbia|BOLD:AAA2221
Eurois astricta|[6136]|GWOSN429-11|BC ZSM Lep 48930|658[0n]bp|Canada.British Columbia|BOLD:AAA2221
Eurois astricta|[6137]|LBCB038-05|HLC-20978|658[0n]bp|Canada.British Columbia|BOLD:AAA2221
Eurois astricta|[6138]|BBLPB531-10|10BBCLP-1530|658[0n]bp|Canada.British Columbia|BOLD:AAA2221
Eurois astricta|[6139]|LBCB615-05|HLC-21555|658[0n]bp|Canada.British Columbia|BOLD:AAA2221
Eurois astricta|[6140]|LBCH1772-10|10-JDWBC-1772|658[0n]bp|Canada.British Columbia|BOLD:AAA2221
Eurois astricta|[6141]|LBCH1858-10|10-JDWBC-1858|658[0n]bp|Canada.British Columbia|BOLD:AAA2221
Eurois astricta|[6142]|LBCG637-09|08-JDWBC-0637|658[0n]bp|Canada.British Columbia|BOLD:AAA2221
Eurois astricta|[6143]|LBCG693-09|08-JDWBC-0693|658[0n]bp|Canada.British Columbia|BOLD:AAA2221
Eurois astricta|[6144]|LBCC827-05|HLC-22707|658[0n]bp|Canada.British Columbia|BOLD:AAA2221
Eurois astricta|[6145]|BBLPB530-10|10BBCLP-1529|658[0n]bp|Canada.British Columbia|BOLD:AAA2221
Eurois astricta|[6146]|BBLPB529-10|10BBCLP-1528|658[0n]bp|Canada.British Columbia|BOLD:AAA2221
Eurois astricta|[6147]|LBCG3321-09|08-JDWBC-3321|658[0n]bp|Canada.British Columbia|BOLD:AAA2221
Eurois astricta|[6148]|LBCC750-05|HLC-22630|658[0n]bp|Canada.British Columbia|BOLD:AAA2221
Eurois astricta|[6149]|LALPA811-10|AVBC 813-10|658[0n]bp|Canada.British Columbia|BOLD:AAA2221
Eurois astricta|[6150]|LBCH1236-10|10-JDWBC-1236|658[0n]bp|Canada.British Columbia|BOLD:AAA2221
Eurois astricta|[6151]|LBCD253-05|HLC-23073|658[0n]bp|Canada.British Columbia|BOLD:AAA2221
Eurois astricta|[6152]|LBCB040-05|HLC-20980|658[0n]bp|Canada.British Columbia|BOLD:AAA2221
Eurois astricta|[6153]|LBCH1165-10|10-JDWBC-1165|658[0n]bp|Canada.British Columbia|BOLD:AAA2221
Eurois astricta|[6154]|LBCH1861-10|10-JDWBC-1861|658[0n]bp|Canada.British Columbia|BOLD:AAA2221
Eurois astricta|[6155]|LPABB480-08|08BBLEP-03745|658[0n]bp|Canada.Alberta|BOLD:AAA2221
Eurois astricta|[6156]|LBCB039-05|HLC-20979|658[0n]bp|Canada.British Columbia|BOLD:AAA2221
Eurois astricta|[6157]|LBCC826-05|HLC-22706|658[0n]bp|Canada.British Columbia|BOLD:AAA2221
Eurois astricta|[6158]|LBCH1996-10|10-JDWBC-1996|658[0n]bp|Canada.British Columbia|BOLD:AAA2221
Eurois astricta|[6159]|LBCH1090-10|10-JDWBC-1090|658[0n]bp|Canada.British Columbia|BOLD:AAA2221
Eurois astricta|[6160]|LBCD240-05|HLC-23060|658[0n]bp|Canada.British Columbia|BOLD:AAA2221
Eurois astricta|[6161]|LBCH1855-10|10-JDWBC-1855|658[0n]bp|Canada.British Columbia|BOLD:AAA2221
Eurois astricta|[6162]|LBCH2101-10|10-JDWBC-2101|658[0n]bp|Canada.British Columbia|BOLD:AAA2221
Eurois astricta|[6163]|LBCH1565-10|10-JDWBC-1565|658[0n]bp|Canada.British Columbia|BOLD:AAA2221
Eurois astricta|[6164]|LBCD249-05|HLC-23069|658[0n]bp|Canada.British Columbia|BOLD:AAA2221
Eurois astricta|[6165]|LBCC024-05|HLC-21904|658[0n]bp|Canada.British Columbia|BOLD:AAA2221
Eurois astricta|[6166]|LBCC671-05|HLC-22551|658[0n]bp|Canada.British Columbia|BOLD:AAA2221
Eurois astricta|[6167]|LBCD223-05|HLC-23043|658[0n]bp|Canada.British Columbia|BOLD:AAA2221
Eurois astricta|[6168]|LBCB046-05|HLC-20986|658[0n]bp|Canada.British Columbia|BOLD:AAA2221
Eurois astricta|[6169]|LBCD247-05|HLC-23067|655[0n]bp|Canada.British Columbia|BOLD:AAA2221
Eurois astricta|[6170]|LPAB233-08|08BBLEP-02555|658[0n]bp|Canada.Alberta|BOLD:AAA2221
Eurois astricta|[6171]|LBCH1854-10|10-JDWBC-1854|658[0n]bp|Canada.British Columbia|BOLD:AAA2221
Eurois astricta|[6172]|LBCG849-09|08-JDWBC-0849|658[0n]bp|Canada.British Columbia|BOLD:AAA2221
Eurois astricta|[6173]|BBLPB527-10|10BBCLP-1526|658[0n]bp|Canada.British Columbia|BOLD:AAA2221
Eurois astricta|[6174]|LBCG684-09|08-JDWBC-0684|658[0n]bp|Canada.British Columbia|BOLD:AAA2221
Eurois astricta|[6175]|LBCD329-05|HLC-23149|658[1n]bp|Canada.British Columbia|BOLD:AAA2221
Eurois astricta|[6176]|LBCA753-05|HLC-20753|646[0n]bp|Canada.British Columbia|BOLD:AAA2221
Eurois astricta|[6177]|LBCD245-05|HLC-23065|648[0n]bp|Canada.British Columbia|BOLD:AAA2221
Eurois astricta|[6178]|LBCD328-05|HLC-23148|623[0n]bp|Canada.British Columbia|BOLD:AAA2221
Eurois astricta|[6179]|LPABC492-09|08BBLEP-04711|605[0n]bp|Canada.Alberta|BOLD:AAA2221
Eurois astricta|[6180]|LBCB041-05|HLC-20981|658[0n]bp|Canada.British Columbia|BOLD:AAA2221

Eurois astricta|[6177]|LBCD243-05|HLC-23063|648[0n]bp|Canada.British Columbia|BOLD:AAA2221
Eurois astricta|[6178]|LBCD328-05|HLC-23148|623[0n]bp|Canada.British Columbia|BOLD:AAA2221
Eurois astricta|[6179]|LPABC492-09|08BBLEP-04711|605[0n]bp|Canada.Alberta|BOLD:AAA2221
Eurois astricta|[6180]|LBCB041-05|HLC-20981|658[0n]bp|Canada.British Columbia|BOLD:AAA2221
Eurois astricta|[6181]|LBCD440-05|HLC-23260|651[0n]bp|Canada.British Columbia|BOLD:AAA2221
Eurois astricta|[6182]|LBCD234-05|HLC-23054|648[1n]bp|Canada.British Columbia|BOLD:AAA2221
Eurois astricta|[6183]|LBCG629-09|08-JDWBC-0629|658[0n]bp|Canada.British Columbia|BOLD:AAA2221
Eurois astricta|[6184]|LPABC494-09|08BBLEP-04713|658[0n]bp|Canada.Alberta|BOLD:AAA2221
Eurois astricta|[6185]|LBCD239-05|HLC-23059|658[0n]bp|Canada.British Columbia|BOLD:AAA2221
Eurois astricta|[6186]|LPAB209-08|08BBLEP-02531|658[0n]bp|Canada.Alberta|BOLD:AAA2220
Eurois astricta|[6187]|TTMNB439-06|MNBTT-439|658[0n]bp|Canada.New Brunswick|BOLD:AAA2220
Eurois astricta|[6188]|LOWC819-05|CGWC-0819|658[0n]bp|Canada.British Columbia|BOLD:AAA2220
Eurois astricta|[6189]|LALPA849-11|AVBC 1022-11|658[0n]bp|Canada.British Columbia|BOLD:AAA2220
Eurois astricta|[6190]|LBCH489-10|10-JDWBC-0489|658[0n]bp|Canada.British Columbia|BOLD:AAA2220
Eurois astricta|[6191]|LBCH4102-10|10-JDWBC-4102|658[0n]bp|Canada.British Columbia|BOLD:AAA2220
Eurois astricta|[6192]|LBCH3764-10|10-JDWBC-3764|658[0n]bp|Canada.British Columbia|BOLD:AAA2220
Eurois astricta|[6193]|LPABB486-08|08BBLEP-03751|658[0n]bp|Canada.Alberta|BOLD:AAA2220
Eurois astricta|[6194]|LBCH4108-10|10-JDWBC-4108|658[0n]bp|Canada.British Columbia|BOLD:AAA2220
Eurois astricta|[6195]|LPABB874-09|08BBLEP-04194|658[0n]bp|Canada.Alberta|BOLD:AAA2220
Eurois astricta|[6196]|LOWCC865-05|CGWC-2745|606[0n]bp|Canada.British Columbia|BOLD:AAA2220
Eurois astricta|[6197]|LPABC199-09|08BBLEP-04418|658[0n]bp|Canada.Alberta|BOLD:AAA2220
Eurois astricta|[6198]|LBCH3152-10|10-JDWBC-3152|658[0n]bp|Canada.British Columbia|BOLD:AAA2220
Eurois astricta|[6199]|RDLQF348-06|DH011415|658[0n]bp|Canada.Quebec|BOLD:AAA2220
Eurois astricta|[6200]|LBCH4414-10|10-JDWBC-4414|658[0n]bp|Canada.British Columbia|BOLD:AAA2220
Eurois astricta|[6201]|LBCG957-09|08-JDWBC-0957|658[0n]bp|Canada.British Columbia|BOLD:AAA2220
Eurois astricta|[6202]|LBCG955-09|08-JDWBC-0955|658[0n]bp|Canada.British Columbia|BOLD:AAA2220
Eurois astricta|[6203]|LBCH929-10|10-JDWBC-0929|658[0n]bp|Canada.British Columbia|BOLD:AAA2220
Eurois astricta|[6204]|LCHQ422-08|07WNP-10314|658[0n]bp|Canada.Manitoba|BOLD:AAA2220
Eurois astricta|[6205]|MNBB663-05|05-NBSTA-579|658[0n]bp|Canada.New Brunswick|BOLD:AAA2220
Eurois astricta|[6206]|LBCH3765-10|10-JDWBC-3765|658[0n]bp|Canada.British Columbia|BOLD:AAA2220
Eurois astricta|[6207]|LCH552-04|04HBL003552|658[0n]bp|Canada.Manitoba|BOLD:AAA2220
Eurois astricta|[6208]|LBCH931-10|10-JDWBC-0931|658[0n]bp|Canada.British Columbia|BOLD:AAA2220
Eurois astricta|[6209]|LCH553-04|04HBL003553|658[0n]bp|Canada.Manitoba|BOLD:AAA2220
Eurois astricta|[6210]|LOWC820-05|CGWC-0820|658[0n]bp|Canada.British Columbia|BOLD:AAA2220
Eurois astricta|[6211]|LCHQ621-08|07WNP-10513|658[0n]bp|Canada.Manitoba|BOLD:AAA2220
Eurois astricta|[6212]|LBCH3327-10|10-JDWBC-3327|658[0n]bp|Canada.British Columbia|BOLD:AAA2220
Eurois astricta|[6213]|LOWC823-05|CGWC-0823|658[0n]bp|Canada.British Columbia|BOLD:AAA2220
Eurois astricta|[6214]|LCHQ668-08|07WNP-10560|658[0n]bp|Canada.Manitoba|BOLD:AAA2220
Eurois astricta|[6215]|LBCH3480-10|10-JDWBC-3480|658[0n]bp|Canada.British Columbia|BOLD:AAA2220
Eurois astricta|[6216]|LCH554-04|04HBL003554|658[0n]bp|Canada.Manitoba|BOLD:AAA2220
Eurois astricta|[6217]|TTMNB438-06|MNBTT-438|658[0n]bp|Canada.New Brunswick|BOLD:AAA2220
Eurois astricta|[6218]|LOWCE851-06|CGWC-4611|658[0n]bp|Canada.British Columbia|BOLD:AAA2220
Eurois astricta|[6219]|LCH555-04|04HBL003555|658[0n]bp|Canada.Manitoba|BOLD:AAA2220
Eurois astricta|[6220]|LCHP928-07|07PROBE-10690|658[0n]bp|Canada.Manitoba|BOLD:AAA2220
Eurois astricta|[6221]|BBLPB668-10|10BBCLP-1667|658[0n]bp|Canada.Saskatchewan|BOLD:AAA2220
Eurois astricta|[6222]|BBLPB526-10|10BBCLP-1525|658[0n]bp|Canada.Alberta|BOLD:AAA2220
Eurois astricta|[6223]|LBCH3153-10|10-JDWBC-3153|658[0n]bp|Canada.British Columbia|BOLD:AAA2220
Eurois astricta|[6224]|LCH546-04|04HBL003546|658[0n]bp|Canada.Manitoba|BOLD:AAA2220
Eurois astricta|[6225]|LBCH3758-10|10-JDWBC-3758|658[0n]bp|Canada.British Columbia|BOLD:AAA2220
Eurois astricta|[6226]|LBCH930-10|10-JDWBC-0930|658[0n]bp|Canada.British Columbia|BOLD:AAA2220
Eurois astricta|[6227]|TTMNB010-06|MNBTT-010|658[0n]bp|Canada.New Brunswick|BOLD:AAA2220
Eurois astricta|[6228]|LPMNB503-09|08BBLEP-05541|658[0n]bp|Canada.Manitoba|BOLD:AAA2220
Eurois astricta|[6229]|LCHP926-07|07PROBE-10688|658[0n]bp|Canada.Manitoba|BOLD:AAA2220
Eurois astricta|[6230]|LBCH3762-10|10-JDWBC-3762|658[0n]bp|Canada.British Columbia|BOLD:AAA2220
Eurois astricta|[6231]|BBLPC029-09|09BBELE-1029|658[0n]bp|Canada.New Brunswick|BOLD:AAA2220
Eurois astricta|[6232]|LCHQ269-08|07WNP-10161|658[0n]bp|Canada.Manitoba|BOLD:AAA2220
Eurois astricta|[6233]|LOWCD520-06|CGWC-3340|658[0n]bp|Canada.British Columbia|BOLD:AAA2220
Eurois astricta|[6234]|LBCH928-10|10-JDWBC-0928|658[0n]bp|Canada.British Columbia|BOLD:AAA2220
Eurois astricta|[6235]|LBCH4409-10|10-JDWBC-4409|658[0n]bp|Canada.British Columbia|BOLD:AAA2220
Eurois astricta|[6236]|LBCH4410-10|10-JDWBC-4410|658[0n]bp|Canada.British Columbia|BOLD:AAA2220
Eurois astricta|[6237]|LOWC818-05|CGWC-0818|658[0n]bp|Canada.British Columbia|BOLD:AAA2220
Eurois astricta|[6238]|LOWC824-05|CGWC-0824|658[0n]bp|Canada.British Columbia|BOLD:AAA2220
Eurois astricta|[6239]|LBCH4664-10|10-JDWBC-4664|658[0n]bp|Canada.British Columbia|BOLD:AAA2220
Eurois astricta|[6240]|LCHQ133-07|07PROBE-10902|658[0n]bp|Canada.Manitoba|BOLD:AAA2220
Eurois astricta|[6241]|LPABC667-09|08BBLEP-04886|658[0n]bp|Canada.Alberta|BOLD:AAA2220
Eurois astricta|[6242]|LBCH6621-10|10-JDWBC-6621|658[0n]bp|Canada.British Columbia|BOLD:AAA2220
Eurois astricta|[6243]|LBCH3481-10|10-JDWBC-3481|658[0n]bp|Canada.British Columbia|BOLD:AAA2220
Eurois astricta|[6244]|LOWCC933-05|CGWC-2813|658[0n]bp|Canada.British Columbia|BOLD:AAA2220
Eurois astricta|[6245]|LCHQ471-08|07WNP-10363|658[0n]bp|Canada.Manitoba|BOLD:AAA2220
Eurois astricta|[6246]|LBCH4408-10|10-JDWBC-4408|658[0n]bp|Canada.British Columbia|BOLD:AAA2220
Eurois astricta|[6247]|LBCH7682-10|10-JDWBC-7682|658[0n]bp|Canada.British Columbia|BOLD:AAA2220
Eurois astricta|[6248]|LCHQ683-08|07WNP-10575|656[0n]bp|Canada.Manitoba|BOLD:AAA2220
Eurois astricta|[6249]|LCHQ513-08|07WNP-10405|658[0n]bp|Canada.Manitoba|BOLD:AAA2220
Eurois astricta|[6250]|TTMNB437-06|MNBTT-437|658[0n]bp|Canada.New Brunswick|BOLD:AAA2220
Eurois astricta|[6251]|LBCH4106-10|10-JDWBC-4106|658[0n]bp|Canada.British Columbia|BOLD:AAA2220
Eurois astricta|[6252]|LBCH3759-10|10-JDWBC-3759|658[0n]bp|Canada.British Columbia|BOLD:AAA2220
Eurois astricta|[6253]|LBCH3150-10|10-JDWBC-3150|658[0n]bp|Canada.British Columbia|BOLD:AAA2220
Eurois astricta|[6254]|LBCH4107-10|10-JDWBC-4107|658[0n]bp|Canada.British Columbia|BOLD:AAA2220
Eurois astricta|[6255]|LBCH4411-10|10-JDWBC-4411|658[0n]bp|Canada.British Columbia|BOLD:AAA2220
Eurois astricta|[6256]|LBCH3760-10|10-JDWBC-3760|658[0n]bp|Canada.British Columbia|BOLD:AAA2220
Eurois astricta|[6257]|LOWCD519-06|CGWC-3339|658[0n]bp|Canada.British Columbia|BOLD:AAA2220
Eurois astricta|[6258]|LBCH3151-10|10-JDWBC-3151|658[0n]bp|Canada.British Columbia|BOLD:AAA2220
Eurois astricta|[6259]|BBLPB524-10|10BBCLP-1523|658[0n]bp|Canada.Alberta|BOLD:AAA2220
Eurois astricta|[6260]|TMNBB409-06|MNBTT-1349|658[0n]bp|Canada.New Brunswick|BOLD:AAA2220
Eurois astricta|[6261]|RDLQB573-05|DH010676|658[0n]bp|Canada.Quebec|BOLD:AAA2220
Eurois astricta|[6262]|TTMNB016-06|MNBTT-016|657[0n]bp|Canada.New Brunswick|BOLD:AAA2220
Eurois astricta|[6263]|LBCH4413-10|10-JDWBC-4413|658[0n]bp|Canada.British Columbia|BOLD:AAA2220
Eurois astricta|[6264]|LBCH4667-10|10-JDWBC-4667|658[0n]bp|Canada.British Columbia|BOLD:AAA2220
Eurois astricta|[6265]|LPABC025-09|08BBLEP-04244|658[0n]bp|Canada.Alberta|BOLD:AAA2220
Eurois astricta|[6266]|LBCH4412-10|10-JDWBC-4412|658[0n]bp|Canada.British Columbia|BOLD:AAA2220
Eurois astricta|[6267]|LBCH3487-10|10-JDWBC-3487|658[0n]bp|Canada.British Columbia|BOLD:AAA2220
Eurois astricta|[6268]|LCHP754-07|07PROBE-10439|658[0n]bp|Canada.Manitoba|BOLD:AAA2220
Eurois astricta|[6269]|LOWC821-05|CGWC-0821|658[0n]bp|Canada.British Columbia|BOLD:AAA2220
Eurois astricta|[6270]|LPABB842-09|08BBLEP-04162|658[0n]bp|Canada.Alberta|BOLD:AAA2220
Eurois astricta|[6271]|LBCH4407-10|10-JDWBC-4407|658[0n]bp|Canada.British Columbia|BOLD:AAA2220
Eurois astricta|[6272]|LBCH3146-10|10-JDWBC-3146|658[0n]bp|Canada.British Columbia|BOLD:AAA2220
Eurois astricta|[6273]|LCHQ559-08|07WNP-10451|658[0n]bp|Canada.Manitoba|BOLD:AAA2220
Eurois astricta|[6274]|LCHQ245-08|07WNP-10137|658[0n]bp|Canada.Manitoba|BOLD:AAA2220
Eurois astricta|[6275]|LBCH3148-10|10-JDWBC-3148|658[0n]bp|Canada.British Columbia|BOLD:AAA2220
Eurois astricta|[6276]|LBCH3147-10|10-JDWBC-3147|658[0n]bp|Canada.British Columbia|BOLD:AAA2220
Eurois astricta|[6277]|LBCH3761-10|10-JDWBC-3761|658[0n]bp|Canada.British Columbia|BOLD:AAA2220
Eurois astricta|[6278]|LOWCD259-06|CGWC-3079|658[0n]bp|Canada.British Columbia|BOLD:AAA2220
Eurois astricta|[6279]|LBCH3763-10|10-JDWBC-3763|658[0n]bp|Canada.British Columbia|BOLD:AAA2220
[illegible]

Eurois astricta|[6277]|LBCH3761-10|10-JDWBC-3761|658[0n]bp|Canada.British Columbia|BOLD:AAA2220
Eurois astricta|[6278]|LOWCD259-06|CGWC-3079|658[0n]bp|Canada.British Columbia|BOLD:AAA2220
Eurois astricta|[6279]|LBCH3763-10|10-JDWBC-3763|658[0n]bp|Canada.British Columbia|BOLD:AAA2220
Eurois astricta|[6280]|LBCH4105-10|10-JDWBC-4105|658[0n]bp|Canada.British Columbia|BOLD:AAA2220
Eurois astricta|[6281]|LBCH4104-10|10-JDWBC-4104|658[0n]bp|Canada.British Columbia|BOLD:AAA2220
Eurois astricta|[6282]|LBCH3145-10|10-JDWBC-3145|658[0n]bp|Canada.British Columbia|BOLD:AAA2220
Eurois astricta|[6283]|LBCH4103-10|10-JDWBC-4103|658[0n]bp|Canada.British Columbia|BOLD:AAA2220
Eurois astricta|[6284]|LBCH3149-10|10-JDWBC-3149|658[0n]bp|Canada.British Columbia|BOLD:AAA2220
Eurois astricta|[6285]|LCHQ470-08|07WNP-10362|658[1n]bp|Canada.Manitoba|BOLD:AAA2220
Eurois astricta|[6286]|LCH547-04|04HBL003547|596[0n]bp|Canada.Manitoba|BOLD:AAA2220
Eurois astricta|[6287]|LBCH932-10|10-JDWBC-0932|640[0n]bp|Canada.British Columbia|BOLD:AAA2220
Eurois astricta|[6288]|LCH543-04|04HBL003543|585[1n]bp|Canada.Manitoba|BOLD:AAA2220
Eurois astricta|[6289]|LBCH3482-10|10-JDWBC-3482|632[0n]bp|Canada.British Columbia|BOLD:AAA2220
Eurois astricta|[6290]|LBCH3469-10|10-JDWBC-3469|641[0n]bp|Canada.British Columbia|BOLD:AAA2220
Eurois astricta|[6291]|LCH539-04|04HBL003539|650[0n]bp|Canada.Manitoba|BOLD:AAA2220
Eurois astricta|[6292]|LCHP753-07|07PROBE-10438|650[0n]bp|Canada.Manitoba|BOLD:AAA2220
Eurois astricta|[6293]|LBCH3486-10|10-JDWBC-3486|635[0n]bp|Canada.British Columbia|BOLD:AAA2220
Eurois astricta|[6294]|LPABC026-09|08BBLEP-04245|621[0n]bp|Canada.Alberta|BOLD:AAA2220
Eurois astricta|[6295]|LBCH3485-10|10-JDWBC-3485|639[0n]bp|Canada.British Columbia|BOLD:AAA2220
Eurois astricta|[6296]|LCH544-04|04HBL003544|627[0n]bp|Canada.Manitoba|BOLD:AAA2220
Eurois astricta|[6297]|TTMNB015-06|MNBTT-015|635[0n]bp|Canada.New Brunswick|BOLD:AAA2220
Eurois astricta|[6298]|LBCH3483-10|10-JDWBC-3483|635[0n]bp|Canada.British Columbia|BOLD:AAA2220
Eurois astricta|[6299]|LCH548-04|04HBL003548|625[0n]bp|Canada.Manitoba|BOLD:AAA2220
Eurois astricta|[6300]|LCH551-04|04HBL003551|615[0n]bp|Canada.Manitoba|BOLD:AAA2220
Eurois astricta|[6301]|LOWCD518-06|CGWC-3338|609[0n]bp|Canada.British Columbia|BOLD:AAA2220
Eurois astricta|[6302]|LOWC822-05|CGWC-0822|585[0n]bp|Canada.British Columbia|BOLD:AAA2220
Eurois astricta|[6303]|LCH549-04|04HBL003549|578[0n]bp|Canada.Manitoba|BOLD:AAA2220
Eurois astricta|[6304]|LOWCD517-06|CGWC-3337|581[0n]bp|Canada.British Columbia|BOLD:AAA2220
Eurois astricta|[6305]|LOWCD516-06|CGWC-3336|580[0n]bp|Canada.British Columbia|BOLD:AAA2220
Eurois astricta|[6306]|LOWCD514-06|CGWC-3334|602[0n]bp|Canada.British Columbia|BOLD:AAA2220
Eurois astricta|[6307]|LOWCD140-06|CGWC-2960|572[0n]bp|Canada.British Columbia|BOLD:AAA2220
Eurois astricta|[6308]|RDLQB572-05|DH010675|519[1n]bp|Canada.Quebec|BOLD:AAA2220
Eurois astricta|[6309]|LCH542-04|04HBL003542|595[0n]bp|Canada.Manitoba|BOLD:AAA2220
Eurois astricta|[6310]|LCH441-04|04HBL003441|561[1n]bp|Canada.Manitoba|BOLD:AAA2220
Eurois astricta|[6311]|LCH541-04|04HBL003541|560[0n]bp|Canada.Manitoba|BOLD:AAA2220
Eurois astricta|[6312]|LCH550-04|04HBL003550|520[4n]bp|Canada.Manitoba|BOLD:AAA2220
Eurois astricta|[6313]|LBCH3484-10|10-JDWBC-3484|632[0n]bp|Canada.British Columbia|BOLD:AAA2220
Eurois astricta|[6314]|LOWCD515-06|CGWC-3335|569[0n]bp|Canada.British Columbia|BOLD:AAA2220
Eurois astricta|[6315]|LCH545-04|04HBL003545|586[0n]bp|Canada.Manitoba|BOLD:AAA2220
Eurois astricta|[6316]|LCH540-04|04HBL003540|605[0n]bp|Canada.Manitoba|BOLD:AAA2220
Eurois astricta|[6317]|LCHP752-07|07PROBE-10437|657[0n]bp|Canada.Manitoba|BOLD:AAA2220
Eurois astricta|[6318]|TTMNB436-06|MNBTT-436|658[0n]bp|Canada.New Brunswick|BOLD:AAA2220
Eurois nigra|[6319]|LOWC810-05|CGWC-0810|658[0n]bp|Canada.British Columbia|BOLD:AAB0434
Eurois nigra|[6320]|LOWC815-05|CGWC-0815|658[0n]bp|Canada.British Columbia|BOLD:AAB0434
Eurois nigra|[6321]|LOWCC832-05|CGWC-2712|590[0n]bp|Canada.British Columbia|BOLD:AAB0434
Eurois nigra|[6322]|BBLPA333-10|10BBCLP-0333|658[0n]bp|Canada.Alberta|BOLD:AAB0434
Eurois nigra|[6323]|LOWC811-05|CGWC-0811|658[0n]bp|Canada.British Columbia|BOLD:AAB0434
Eurois nigra|[6324]|LOWC805-05|CGWC-0805|658[0n]bp|Canada.British Columbia|BOLD:AAB0434
Eurois nigra|[6325]|LOWC813-05|CGWC-0813|658[0n]bp|Canada.British Columbia|BOLD:AAB0434
Eurois nigra|[6326]|LPABC495-09|08BBLEP-04714|634[0n]bp|Canada.Alberta|BOLD:AAB0434
Eurois nigra|[6327]|LOWCD785-06|CGWC-3605|657[0n]bp|Canada.British Columbia|BOLD:AAB0434
Eurois nigra|[6328]|LOWC814-05|CGWC-0814|658[0n]bp|Canada.British Columbia|BOLD:AAB0434
Eurois nigra|[6329]|LPABC029-09|08BBLEP-04248|658[1n]bp|Canada.Alberta|BOLD:AAB0434
Eurois nigra|[6330]|LOWC816-05|CGWC-0816|658[0n]bp|Canada.British Columbia|BOLD:AAB0434
Eurois nigra|[6331]|LALPA812-10|AVBC 814-10|658[0n]bp|Canada.British Columbia|BOLD:AAB0434
Eurois nigra|[6332]|LPABC499-09|08BBLEP-04718|658[0n]bp|Canada.Alberta|BOLD:AAB0434
Eurois nigra|[6333]|LOWC807-05|CGWC-0807|658[0n]bp|Canada.British Columbia|BOLD:AAB0434
Eurois nigra|[6334]|LOWCD786-06|CGWC-3606|657[0n]bp|Canada.British Columbia|BOLD:AAB0434
Eurois nigra|[6335]|LPABB876-09|08BBLEP-04196|658[0n]bp|Canada.Alberta|BOLD:AAB0434
Eurois nigra|[6336]|LOWC806-05|CGWC-0806|658[0n]bp|Canada.British Columbia|BOLD:AAB0434
Eurois nigra|[6337]|LOWC812-05|CGWC-0812|658[0n]bp|Canada.British Columbia|BOLD:AAB0434
Eurois nigra|[6338]|LOWC808-05|CGWC-0808|658[0n]bp|Canada.British Columbia|BOLD:AAB0434
Eurois nigra|[6339]|LOWCD788-06|CGWC-3608|657[0n]bp|Canada.British Columbia|BOLD:AAB0434
Eurois nigra|[6340]|LOWC817-05|CGWC-0817|658[0n]bp|Canada.British Columbia|BOLD:AAB0434
Eurois nigra|[6341]|LOWCD787-06|CGWC-3607|656[0n]bp|Canada.British Columbia|BOLD:AAB0434
Eurois nigra|[6342]|LPABC439-09|08BBLEP-04658|658[0n]bp|Canada.Alberta|BOLD:AAB0434
Eurois nigra|[6343]|LPABB718-08|08BBLEP-03983|658[0n]bp|Canada.Alberta|BOLD:AAB0434
Eurois nigra|[6344]|LPABB881-09|08BBLEP-04201|658[0n]bp|Canada.Alberta|BOLD:AAB0434
Eurois nigra|[6345]|LOWCD784-06|CGWC-3604|658[0n]bp|Canada.British Columbia|BOLD:AAB0434
Eurois nigra|[6346]|LPABC496-09|08BBLEP-04715|634[0n]bp|Canada.Alberta|BOLD:AAB0434
Eurois nigra|[6347]|LPABC432-09|08BBLEP-04651|658[0n]bp|Canada.Alberta|BOLD:AAB0434
Eurois nigra|[6348]|LPABC038-09|08BBLEP-04257|658[0n]bp|Canada.Alberta|BOLD:AAB0434
Eurois nigra|[6349]|LPABC809-09|08BBLEP-05028|658[0n]bp|Canada.Alberta|BOLD:AAB0434
Eurois nigra|[6350]|LPABC481-09|08BBLEP-04700|658[0n]bp|Canada.Alberta|BOLD:AAB0434
Eurois nigra|[6351]|LOWC809-05|CGWC-0809|658[0n]bp|Canada.British Columbia|BOLD:AAB0434
Eurois occulta|[6352]|LCHQ538-08|07WNP-10430|658[0n]bp|Canada.Manitoba|BOLD:AAA3312
Eurois occulta|[6353]|LOWC829-05|CGWC-0829|658[0n]bp|Canada.British Columbia|BOLD:AAA3312
Eurois occulta|[6354]|LBCH7683-10|10-JDWBC-7683|658[0n]bp|Canada.British Columbia|BOLD:AAA3312
Eurois occulta|[6355]|LOWC825-05|CGWC-0825|658[0n]bp|Canada.British Columbia|BOLD:AAA3312
Eurois occulta|[6356]|LBCG2003-09|08-JDWBC-2003|658[0n]bp|Canada.British Columbia|BOLD:AAA3312
Eurois occulta|[6357]|LBCG706-09|08-JDWBC-0706|658[0n]bp|Canada.British Columbia|BOLD:AAA3312
Eurois occulta|[6358]|LBCH7364-10|10-JDWBC-7364|633[0n]bp|Canada.British Columbia|BOLD:AAA3312
Eurois occulta|[6359]|BBLEC460-09|09BBELE-0460|658[0n]bp|Canada.New Brunswick|BOLD:AAA3312
Eurois occulta|[6360]|LBCH107-10|10-JDWBC-0107|658[0n]bp|Canada.British Columbia|BOLD:AAA3312
Eurois occulta|[6361]|LBCH1163-10|10-JDWBC-1163|658[0n]bp|Canada.British Columbia|BOLD:AAA3312
Eurois occulta|[6362]|LCHQ026-07|07PROBE-10787|658[0n]bp|Canada.Manitoba|BOLD:AAA3312
Eurois occulta|[6363]|LOWC831-05|CGWC-0831|658[0n]bp|Canada.British Columbia|BOLD:AAA3312
Eurois occulta|[6364]|LBCH4093-10|10-JDWBC-4093|658[0n]bp|Canada.British Columbia|BOLD:AAA3312
Eurois occulta|[6365]|TMNBB369-06|MNBTT-1309|658[0n]bp|Canada.New Brunswick|BOLD:AAA3312
Eurois occulta|[6366]|LCHQ111-07|07PROBE-10880|658[0n]bp|Canada.Manitoba|BOLD:AAA3312
Eurois occulta|[6367]|LBCH4662-10|10-JDWBC-4662|658[0n]bp|Canada.British Columbia|BOLD:AAA3312
Eurois occulta|[6368]|BBLPA327-10|10BBCLP-0327|658[0n]bp|Canada.Alberta|BOLD:AAA3312
Eurois occulta|[6369]|TTMNB432-06|MNBTT-432|658[0n]bp|Canada.New Brunswick|BOLD:AAA3312
Eurois occulta|[6370]|LBCH4658-10|10-JDWBC-4658|658[0n]bp|Canada.British Columbia|BOLD:AAA3312
Eurois occulta|[6371]|LBCC825-05|HLC-22705|658[0n]bp|Canada.British Columbia|BOLD:AAA3312
Eurois occulta|[6372]|BBLEC452-09|09BBELE-0452|658[0n]bp|Canada.New Brunswick|BOLD:AAA3312
Eurois occulta|[6373]|LBCH644-10|10-JDWBC-0644|658[0n]bp|Canada.British Columbia|BOLD:AAA3312
Eurois occulta|[6374]|TTMNB434-06|MNBTT-434|658[0n]bp|Canada.New Brunswick|BOLD:AAA3312
Eurois occulta|[6375]|LBCH3757-10|10-JDWBC-3757|658[0n]bp|Canada.British Columbia|BOLD:AAA3312
Eurois occulta|[6376]|LBCH2716-10|10-JDWBC-2716|658[0n]bp|Canada.British Columbia|BOLD:AAA3312
Eurois occulta|[6377]|LBCH2091-10|10-JDWBC-2091|658[0n]bp|Canada.British Columbia|BOLD:AAA3312
Eurois occulta|[6378]|LBCH485-10|10-JDWBC-0485|658[0n]bp|Canada.British Columbia|BOLD:AAA3312
Eurois occulta|[6379]|LBCH4391-10|10-JDWBC-4391|658[0n]bp|Canada.British Columbia|BOLD:AAA3312

Eurois occulta|[6377]|LBCH2091-10|10-JDWBC-2091|658[0n]bp|Canada.British Columbia|BOLD:AAA3312
Eurois occulta|[6378]|LBCH485-10|10-JDWBC-0485|658[0n]bp|Canada.British Columbia|BOLD:AAA3312
Eurois occulta|[6379]|LBCH4391-10|10-JDWBC-4391|658[0n]bp|Canada.British Columbia|BOLD:AAA3312
Eurois occulta|[6380]|LBCH4087-10|10-JDWBC-4087|658[0n]bp|Canada.British Columbia|BOLD:AAA3312
Eurois occulta|[6381]|LOWCD524-06|CGWC-3344|658[0n]bp|Canada.British Columbia|BOLD:AAA3312
Eurois occulta|[6382]|LBCH4092-10|10-JDWBC-4092|658[0n]bp|Canada.British Columbia|BOLD:AAA3312
Eurois occulta|[6383]|LBCH1162-10|10-JDWBC-1162|658[0n]bp|Canada.British Columbia|BOLD:AAA3312
Eurois occulta|[6384]|LBCH4661-10|10-JDWBC-4661|658[0n]bp|Canada.British Columbia|BOLD:AAA3312
Eurois occulta|[6385]|LCHP925-07|07PROBE-10687|658[0n]bp|Canada.Manitoba|BOLD:AAA3312
Eurois occulta|[6386]|BBLEC484-09|09BBELE-0484|658[0n]bp|Canada.New Brunswick|BOLD:AAA3312
Eurois occulta|[6387]|LBCH7836-10|10-JDWBC-7836|658[0n]bp|Canada.British Columbia|BOLD:AAA3312
Eurois occulta|[6388]|LBCG735-09|08-JDWBC-0735|658[0n]bp|Canada.British Columbia|BOLD:AAA3312
Eurois occulta|[6389]|LOWCD523-06|CGWC-3343|609[0n]bp|Canada.British Columbia|BOLD:AAA3312
Eurois occulta|[6390]|LCHP621-07|07PROBE-10302|658[0n]bp|Canada.Manitoba|BOLD:AAA3312
Eurois occulta|[6391]|LCHP929-07|07PROBE-10691|658[0n]bp|Canada.Manitoba|BOLD:AAA3312
Eurois occulta|[6392]|LBCW034-08|08-JDWWI-0034|658[0n]bp|Canada.British Columbia|BOLD:AAA3312
Eurois occulta|[6393]|LBCH4387-10|10-JDWBC-4387|658[0n]bp|Canada.British Columbia|BOLD:AAA3312
Eurois occulta|[6394]|LBCH4090-10|10-JDWBC-4090|658[0n]bp|Canada.British Columbia|BOLD:AAA3312
Eurois occulta|[6395]|TTMNB435-06|MNBTT-435|658[0n]bp|Canada.New Brunswick|BOLD:AAA3312
Eurois occulta|[6396]|LCHP924-07|07PROBE-10686|636[0n]bp|Canada.Manitoba|BOLD:AAA3312
Eurois occulta|[6397]|LCHIP122-07|06-PROBE-2643|650[0n]bp|Canada.Manitoba|BOLD:AAA3312
Eurois occulta|[6398]|LCHIP052-07|06-PROBE-2521|650[0n]bp|Canada.Manitoba|BOLD:AAA3312
Eurois occulta|[6399]|XAE496-04|Moth4496.03|647[0n]bp|Canada.Ontario|BOLD:AAA3312
Eurois occulta|[6400]|LBCH005-10|10-JDWBC-0005|658[0n]bp|Canada.British Columbia|BOLD:AAA3312
Eurois occulta|[6401]|BBLEC459-09|09BBELE-0459|658[0n]bp|Canada.New Brunswick|BOLD:AAA3312
Eurois occulta|[6402]|LBCH7116-10|10-JDWBC-7116|658[0n]bp|Canada.British Columbia|BOLD:AAA3312
Eurois occulta|[6403]|LBCH4392-10|10-JDWBC-4392|634[0n]bp|Canada.British Columbia|BOLD:AAA3312
Eurois occulta|[6404]|LCHQ113-07|07PROBE-10882|658[0n]bp|Canada.Manitoba|BOLD:AAA3312
Eurois occulta|[6405]|LBCD439-05|HLC-23259|658[0n]bp|Canada.British Columbia|BOLD:AAA3312
Eurois occulta|[6406]|LOWCD525-06|CGWC-3345|593[0n]bp|Canada.British Columbia|BOLD:AAA3312
Eurois occulta|[6407]|LOWCD522-06|CGWC-3342|618[0n]bp|Canada.British Columbia|BOLD:AAA3312
Eurois occulta|[6408]|LPABC189-09|08BBLEP-04408|647[1n]bp|Canada.Alberta|BOLD:AAA3312
Eurois occulta|[6409]|LALPA1336-12|AVBC 1338-11|632[0n]bp|Canada.British Columbia|BOLD:AAA3312
Eurois occulta|[6410]|LBCH3754-10|10-JDWBC-3754|658[0n]bp|Canada.British Columbia|BOLD:AAA3312
Eurois occulta|[6411]|LBCH4659-10|10-JDWBC-4659|658[0n]bp|Canada.British Columbia|BOLD:AAA3312
Eurois occulta|[6412]|LPABC497-09|08BBLEP-04716|658[0n]bp|Canada.Alberta|BOLD:AAA3312
Eurois occulta|[6413]|LBCH7425-10|10-JDWBC-7425|658[0n]bp|Canada.British Columbia|BOLD:AAA3312
Eurois occulta|[6414]|LBCG2827-09|08-JDWBC-2827|658[0n]bp|Canada.British Columbia|BOLD:AAA3312
Eurois occulta|[6415]|LCHQ919-08|07WNP-10811|658[0n]bp|Canada.Manitoba|BOLD:AAA3312
Eurois occulta|[6416]|LCHQ539-08|07WNP-10431|658[0n]bp|Canada.Manitoba|BOLD:AAA3312
Eurois occulta|[6417]|LALPA621-10|AVBC 623-10|658[0n]bp|Canada.British Columbia|BOLD:AAA3312
Eurois occulta|[6418]|LBCG1880-09|08-JDWBC-1880|658[0n]bp|Canada.British Columbia|BOLD:AAA3312
Eurois occulta|[6419]|PHMNB422-04|04HBL00648|658[0n]bp|Canada.New Brunswick|BOLD:AAA3312
Eurois occulta|[6420]|LBCB047-05|HLC-20987|658[0n]bp|Canada.British Columbia|BOLD:AAA3312
Eurois occulta|[6421]|BBLEC451-09|09BBELE-0451|658[0n]bp|Canada.New Brunswick|BOLD:AAA3312
Eurois occulta|[6422]|LBCG2004-09|08-JDWBC-2004|658[0n]bp|Canada.British Columbia|BOLD:AAA3312
Eurois occulta|[6423]|LPABC223-09|08BBLEP-04442|658[0n]bp|Canada.Alberta|BOLD:AAA3312
Eurois occulta|[6424]|LPABC194-09|08BBLEP-04413|658[0n]bp|Canada.Alberta|BOLD:AAA3312
Eurois occulta|[6425]|LBCH1950-10|10-JDWBC-1950|658[0n]bp|Canada.British Columbia|BOLD:AAA3312
Eurois occulta|[6426]|LBCH3750-10|10-JDWBC-3750|658[0n]bp|Canada.British Columbia|BOLD:AAA3312
Eurois occulta|[6427]|TTMNB431-06|MNBTT-431|658[0n]bp|Canada.New Brunswick|BOLD:AAA3312
Eurois occulta|[6428]|LBCH800-10|10-JDWBC-0800|658[0n]bp|Canada.British Columbia|BOLD:AAA3312
Eurois occulta|[6429]|XAE347-04|Moth4347.03|658[0n]bp|Canada.Ontario|BOLD:AAA3312
Eurois occulta|[6430]|LBCG850-09|08-JDWBC-0850|658[0n]bp|Canada.British Columbia|BOLD:AAA3312
Eurois occulta|[6431]|LBCH7758-10|10-JDWBC-7758|658[0n]bp|Canada.British Columbia|BOLD:AAA3312
Eurois occulta|[6432]|LBCG2826-09|08-JDWBC-2826|658[0n]bp|Canada.British Columbia|BOLD:AAA3312
Eurois occulta|[6433]|LBCH3756-10|10-JDWBC-3756|658[0n]bp|Canada.British Columbia|BOLD:AAA3312
Eurois occulta|[6434]|LBCH912-10|10-JDWBC-0912|658[0n]bp|Canada.British Columbia|BOLD:AAA3312
Eurois occulta|[6435]|LBCH1827-10|10-JDWBC-1827|658[0n]bp|Canada.British Columbia|BOLD:AAA3312
Eurois occulta|[6436]|LBCH4091-10|10-JDWBC-4091|658[0n]bp|Canada.British Columbia|BOLD:AAA3312
Eurois occulta|[6437]|LBCH3462-10|10-JDWBC-3462|658[0n]bp|Canada.British Columbia|BOLD:AAA3312
Eurois occulta|[6438]|BBLPA330-10|10BBCLP-0330|658[0n]bp|Canada.British Columbia|BOLD:AAA3312
Eurois occulta|[6439]|LCHP792-07|07PROBE-10477|658[0n]bp|Canada.Manitoba|BOLD:AAA3312
Eurois occulta|[6440]|LCHP927-07|07PROBE-10689|658[0n]bp|Canada.Manitoba|BOLD:AAA3312
Eurois occulta|[6441]|LALPA622-10|AVBC 624-10|658[0n]bp|Canada.British Columbia|BOLD:AAA3312
Eurois occulta|[6442]|LBCH1092-10|10-JDWBC-1092|658[0n]bp|Canada.British Columbia|BOLD:AAA3312
Eurois occulta|[6443]|LALPA715-10|AVBC 717-10|658[0n]bp|Canada.British Columbia|BOLD:AAA3312
Eurois occulta|[6444]|LBCG1935-09|08-JDWBC-1935|658[0n]bp|Canada.British Columbia|BOLD:AAA3312
Eurois occulta|[6445]|LALPA608-10|AVBC 610-10|658[0n]bp|Canada.British Columbia|BOLD:AAA3312
Eurois occulta|[6446]|LBCH1995-10|10-JDWBC-1995|658[0n]bp|Canada.British Columbia|BOLD:AAA3312
Eurois occulta|[6447]|LCHQ659-08|07WNP-10551|658[0n]bp|Canada.Manitoba|BOLD:AAA3312
Eurois occulta|[6448]|LBCH3751-10|10-JDWBC-3751|658[0n]bp|Canada.British Columbia|BOLD:AAA3312
Eurois occulta|[6449]|BBLPC397-09|09BBELE-1397|658[0n]bp|Canada.New Brunswick|BOLD:AAA3312
Eurois occulta|[6450]|MNBB558-05|05-NBSTA-474|658[0n]bp|Canada.New Brunswick|BOLD:AAA3312
Eurois occulta|[6451]|TMNBB368-06|MNBTT-1308|658[0n]bp|Canada.New Brunswick|BOLD:AAA3312
Eurois occulta|[6452]|LOWC830-05|CGWC-0830|658[0n]bp|Canada.British Columbia|BOLD:AAA3312
Eurois occulta|[6453]|BBLEC458-09|09BBELE-0458|658[0n]bp|Canada.New Brunswick|BOLD:AAA3312
Eurois occulta|[6454]|LBCH4089-10|10-JDWBC-4089|658[0n]bp|Canada.British Columbia|BOLD:AAA3312
Eurois occulta|[6455]|LBCH3062-10|10-JDWBC-3062|658[0n]bp|Canada.British Columbia|BOLD:AAA3312
Eurois occulta|[6456]|LBCH2636-10|10-JDWBC-2636|658[0n]bp|Canada.British Columbia|BOLD:AAA3312
Eurois occulta|[6457]|LBCH3456-10|10-JDWBC-3456|658[0n]bp|Canada.British Columbia|BOLD:AAA3312
Eurois occulta|[6458]|LBCH2243-10|10-JDWBC-2243|658[0n]bp|Canada.British Columbia|BOLD:AAA3312
Eurois occulta|[6459]|LBCW035-08|08-JDWWI-0035|658[0n]bp|Canada.British Columbia|BOLD:AAA3312
Eurois occulta|[6460]|BBLPC393-09|09BBELE-1393|658[0n]bp|Canada.New Brunswick|BOLD:AAA3312
Eurois occulta|[6461]|LBCH4663-10|10-JDWBC-4663|658[0n]bp|Canada.British Columbia|BOLD:AAA3312
Eurois occulta|[6462]|BBLPC403-09|09BBELE-1403|658[0n]bp|Canada.New Brunswick|BOLD:AAA3312
Eurois occulta|[6463]|LALPA631-10|AVBC 633-10|658[0n]bp|Canada.British Columbia|BOLD:AAA3312
Eurois occulta|[6464]|LBCH3752-10|10-JDWBC-3752|658[0n]bp|Canada.British Columbia|BOLD:AAA3312
Eurois occulta|[6465]|LBCH3461-10|10-JDWBC-3461|658[0n]bp|Canada.British Columbia|BOLD:AAA3312
Eurois occulta|[6466]|BBLPB669-10|10BBCLP-1668|658[0n]bp|Canada.Alberta|BOLD:AAA3312
Eurois occulta|[6467]|LBCH1495-10|10-JDWBC-1495|658[0n]bp|Canada.British Columbia|BOLD:AAA3312
Eurois occulta|[6468]|TTMNB011-06|MNBTT-011|658[0n]bp|Canada.New Brunswick|BOLD:AAA3312
Eurois occulta|[6469]|LPAB206-08|08BBLEP-02528|658[0n]bp|Canada.Alberta|BOLD:AAA3312
Eurois occulta|[6470]|LALPA1294-11|AVBC 1296-11|658[0n]bp|Canada.British Columbia|BOLD:AAA3312
Eurois occulta|[6471]|BBLPC102-09|09BBELE-1102|658[0n]bp|Canada.New Brunswick|BOLD:AAA3312
Eurois occulta|[6472]|LBCH2092-10|10-JDWBC-2092|658[0n]bp|Canada.British Columbia|BOLD:AAA3312
Eurois occulta|[6473]|LBCH2913-10|10-JDWBC-2913|658[0n]bp|Canada.British Columbia|BOLD:AAA3312
Eurois occulta|[6474]|LBCG2828-09|08-JDWBC-2828|658[0n]bp|Canada.British Columbia|BOLD:AAA3312
Eurois occulta|[6475]|BBLPC385-09|09BBELE-1385|658[0n]bp|Canada.New Brunswick|BOLD:AAA3312
Eurois occulta|[6476]|LBCG848-09|08-JDWBC-0848|658[0n]bp|Canada.British Columbia|BOLD:AAA3312
Eurois occulta|[6477]|TTMNB433-06|MNBTT-433|658[0n]bp|Canada.New Brunswick|BOLD:AAA3312
Eurois occulta|[6478]|LBCH3753-10|10-JDWBC-3753|658[0n]bp|Canada.British Columbia|BOLD:AAA3312
Eurois occulta|[6479]|LBCH3457-10|10-JDWBC-3457|658[0n]bp|Canada.British Columbia|BOLD:AAA3312

[illegible]
Eurois occulta|[6477]|TTMNB433-06|MNBTT-433|658[0n]bp|Canada.New Brunswick|BOLD:AAA3312
Eurois occulta|[6478]|LBCH3753-10|10-JDWBC-3753|658[0n]bp|Canada.British Columbia|BOLD:AAA3312
Eurois occulta|[6479]|LBCH3457-10|10-JDWBC-3457|658[0n]bp|Canada.British Columbia|BOLD:AAA3312
Eurois occulta|[6480]|LBCH4088-10|10-JDWBC-4088|658[0n]bp|Canada.British Columbia|BOLD:AAA3312
Eurois occulta|[6481]|LOWC826-05|CGWC-0826|658[0n]bp|Canada.British Columbia|BOLD:AAA3312
Eurois occulta|[6482]|LOWC828-05|CGWC-0828|658[0n]bp|Canada.British Columbia|BOLD:AAA3312
Eurois occulta|[6483]|LCHP826-07|07PROBE-10583|658[0n]bp|Canada.Manitoba|BOLD:AAA3312
Eurois occulta|[6484]|LBCH3326-10|10-JDWBC-3326|658[0n]bp|Canada.British Columbia|BOLD:AAA3312
Eurois occulta|[6485]|LBCH4389-10|10-JDWBC-4389|658[0n]bp|Canada.British Columbia|BOLD:AAA3312
Eurois occulta|[6486]|LBCH4660-10|10-JDWBC-4660|658[0n]bp|Canada.British Columbia|BOLD:AAA3312
Eurois occulta|[6487]|LALPA623-10|AVBC 625-10|658[0n]bp|Canada.British Columbia|BOLD:AAA3312
Eurois occulta|[6488]|LPMN177-08|08BBLEP-00976|658[0n]bp|Canada.Manitoba|BOLD:AAA3312
Eurois occulta|[6489]|LBCH3463-10|10-JDWBC-3463|658[0n]bp|Canada.British Columbia|BOLD:AAA3312
Eurois occulta|[6490]|LBCH4657-10|10-JDWBC-4657|641[0n]bp|Canada.British Columbia|BOLD:AAA3312
Eurois occulta|[6491]|BBLEC454-09|09BBELE-0454|638[0n]bp|Canada.New Brunswick|BOLD:AAA3312
Eurois occulta|[6492]|LCHQ112-07|07PROBE-10881|639[0n]bp|Canada.Manitoba|BOLD:AAA3312
Eurois occulta|[6493]|LPABC210-09|08BBLEP-04429|649[0n]bp|Canada.Alberta|BOLD:AAA3312
Eurois occulta|[6494]|LBCH3460-10|10-JDWBC-3460|641[0n]bp|Canada.British Columbia|BOLD:AAA3312
Eurois occulta|[6495]|LBCH3458-10|10-JDWBC-3458|658[0n]bp|Canada.British Columbia|BOLD:AAA3312
Eurois occulta|[6496]|LBCH3459-10|10-JDWBC-3459|591[0n]bp|Canada.British Columbia|BOLD:AAA3312
Eurois occulta|[6497]|LPABC480-09|08BBLEP-04699|612[0n]bp|Canada.Alberta|BOLD:AAA3312
Eurois occulta|[6498]|LBCD258-05|HLC-23078|658[0n]bp|Canada.British Columbia|BOLD:AAA3312
Eurois occulta|[6499]|RDLQB575-05|DH010678|519[0n]bp|Canada.Quebec|BOLD:AAA3312
Eurois occulta|[6500]|LCHQ512-08|07WNP-10404|658[0n]bp|Canada.Manitoba|BOLD:AAA3312
Eurois occulta|[6501]|LOWCD526-06|CGWC-3346|658[0n]bp|Canada.British Columbia|BOLD:AAA3312
Eurois occulta|[6502]|LCHQ537-08|07WNP-10429|658[0n]bp|Canada.Manitoba|BOLD:AAA3312
Eurois occulta|[6503]|LOWCD521-06|CGWC-3341|658[0n]bp|Canada.British Columbia|BOLD:AAA3312
Eurois occulta|[6504]|LBCH4393-10|10-JDWBC-4393|658[0n]bp|Canada.British Columbia|BOLD:AAA3312
Eurois occulta|[6505]|LBCH4086-10|10-JDWBC-4086|658[0n]bp|Canada.British Columbia|BOLD:AAA3312
Eurois occulta|[6506]|LBCH2859-10|10-JDWBC-2859|658[0n]bp|Canada.British Columbia|BOLD:AAA3312
Eurois occulta|[6507]|LBCH4388-10|10-JDWBC-4388|658[0n]bp|Canada.British Columbia|BOLD:AAA3312
Eurois occulta|[6508]|LBCG2005-09|08-JDWBC-2005|658[0n]bp|Canada.British Columbia|BOLD:AAA3312
Eurois occulta|[6509]|LBCH3755-10|10-JDWBC-3755|658[0n]bp|Canada.British Columbia|BOLD:AAA3312
Eurois occulta|[6510]|BBLPC394-09|09BBELE-1394|654[0n]bp|Canada.New Brunswick|BOLD:AAA3312
Eurois occulta|[6511]|LBCH4394-10|10-JDWBC-4394|639[0n]bp|Canada.British Columbia|BOLD:AAA3312
Eurois occulta|[6512]|LBCH3464-10|10-JDWBC-3464|636[0n]bp|Canada.British Columbia|BOLD:AAA3312
Eurois occulta|[6513]|LBCH4390-10|10-JDWBC-4390|658[0n]bp|Canada.British Columbia|BOLD:AAA3312
Eurois occulta|[6514]|LOWC827-05|CGWC-0827|658[0n]bp|Canada.British Columbia|BOLD:AAA3312
Eurois occulta|[6515]|LOWCE819-06|CGWC-4579|658[0n]bp|Canada.British Columbia|BOLD:AAA3312
Xestia plebeia|[6516]|LPVIB799-08|PFC-2006-2273|658[0n]bp|Canada.British Columbia|BOLD:AAC5794
Xestia plebeia|[6517]|LBCW022-08|08-JDWWI-0022|658[0n]bp|Canada.British Columbia|BOLD:AAC5794
Xestia plebeia|[6518]|LBCW023-08|08-JDWWI-0023|658[0n]bp|Canada.British Columbia|BOLD:AAC5794
Xestia plebeia|[6519]|RDNMG753-08|CNC LEP00052877|658[0n]bp|Canada.British Columbia|BOLD:AAC5794
Xestia plebeia|[6520]|RDNMF010-08|NOC14096|658[0n]bp|Canada.British Columbia|BOLD:AAC5794
Xestia plebeia|[6521]|LALPA1304-11|AVBC 1306-11|658[0n]bp|Canada.British Columbia|BOLD:AAC5794
Xestia plebeia|[6522]|RDNMG754-08|CNC LEP00052878|658[0n]bp|Canada.British Columbia|BOLD:AAC5794
Chersotis juncta|[6523]|LOWCD502-06|CGWC-3322|525[0n]bp|Canada.British Columbia|BOLD:AAB1122
Chersotis juncta|[6524]|LPSK463-08|08BBLEP-02031|633[0n]bp|Canada.Saskatchewan|BOLD:AAB1122
Chersotis juncta|[6525]|LOWC874-05|CGWC-0874|658[0n]bp|Canada.British Columbia|BOLD:AAB1122
Chersotis juncta|[6526]|LPABC011-09|08BBLEP-04230|658[0n]bp|Canada.Alberta|BOLD:AAB1122
Chersotis juncta|[6527]|LOWCD696-06|CGWC-3516|658[0n]bp|Canada.British Columbia|BOLD:AAB1122
Chersotis juncta|[6528]|LOWC866-05|CGWC-0866|658[0n]bp|Canada.British Columbia|BOLD:AAB1122
Chersotis juncta|[6529]|LOWC870-05|CGWC-0870|658[0n]bp|Canada.British Columbia|BOLD:AAB1122
Chersotis juncta|[6530]|LOWC869-05|CGWC-0869|658[0n]bp|Canada.British Columbia|BOLD:AAB1122
Chersotis juncta|[6531]|LOWCD505-06|CGWC-3325|658[0n]bp|Canada.British Columbia|BOLD:AAB1122
Chersotis juncta|[6532]|LOWC864-05|CGWC-0864|658[0n]bp|Canada.British Columbia|BOLD:AAB1122
Chersotis juncta|[6533]|LOWC868-05|CGWC-0868|658[0n]bp|Canada.British Columbia|BOLD:AAB1122
Chersotis juncta|[6534]|LPABB861-09|08BBLEP-04181|658[0n]bp|Canada.Alberta|BOLD:AAB1122
Chersotis juncta|[6535]|LOWC872-05|CGWC-0872|658[0n]bp|Canada.British Columbia|BOLD:AAB1122
Chersotis juncta|[6536]|LPABC175-09|08BBLEP-04394|658[0n]bp|Canada.Alberta|BOLD:AAB1122
Chersotis juncta|[6537]|LOWC867-05|CGWC-0867|658[0n]bp|Canada.British Columbia|BOLD:AAB1122
Chersotis juncta|[6538]|LPABC015-09|08BBLEP-04234|658[0n]bp|Canada.Alberta|BOLD:AAB1122
Chersotis juncta|[6539]|LOWCD504-06|CGWC-3324|658[0n]bp|Canada.British Columbia|BOLD:AAB1122
Chersotis juncta|[6540]|LPABB863-09|08BBLEP-04183|658[0n]bp|Canada.Alberta|BOLD:AAB1122
Chersotis juncta|[6541]|LOWC875-05|CGWC-0875|658[0n]bp|Canada.British Columbia|BOLD:AAB1122
Chersotis juncta|[6542]|LOWCD698-06|CGWC-3518|658[0n]bp|Canada.British Columbia|BOLD:AAB1122
Chersotis juncta|[6543]|LOWC865-05|CGWC-0865|658[0n]bp|Canada.British Columbia|BOLD:AAB1122
Chersotis juncta|[6544]|LBCG1351-09|08-JDWBC-1351|658[0n]bp|Canada.British Columbia|BOLD:AAB1122
Chersotis juncta|[6545]|LOWC877-05|CGWC-0877|658[0n]bp|Canada.British Columbia|BOLD:AAB1122
Chersotis juncta|[6546]|LOWCD503-06|CGWC-3323|658[0n]bp|Canada.British Columbia|BOLD:AAB1122
Chersotis juncta|[6547]|LOWC876-05|CGWC-0876|658[0n]bp|Canada.British Columbia|BOLD:AAB1122
Chersotis juncta|[6548]|LOWC863-05|CGWC-0863|658[0n]bp|Canada.British Columbia|BOLD:AAB1122
Chersotis juncta|[6549]|LOWC871-05|CGWC-0871|658[0n]bp|Canada.British Columbia|BOLD:AAB1122
Chersotis juncta|[6550]|LOWC873-05|CGWC-0873|658[0n]bp|Canada.British Columbia|BOLD:AAB1122
Chersotis juncta|[6551]|LOWCD697-06|CGWC-3517|589[0n]bp|Canada.British Columbia|BOLD:AAB1122
Chersotis juncta|[6552]|LOWCD695-06|CGWC-3515|595[0n]bp|Canada.British Columbia|BOLD:AAB1122
Chersotis juncta|[6553]|LOWC878-05|CGWC-0878|658[0n]bp|Canada.British Columbia|BOLD:AAB1122
Aplectoides condita|[6554]|RDLQB101-05|DH010187|658[0n]bp|Canada.Quebec|BOLD:AAA6359
Aplectoides condita|[6555]|BBLPB771-10|10BBCLP-1770|658[0n]bp|Canada.Saskatchewan|BOLD:AAA6359
Aplectoides condita|[6556]|RDLQB099-05|DH010185|658[0n]bp|Canada.Quebec|BOLD:AAA6359
Aplectoides condita|[6557]|RDLQB102-05|DH010188|658[0n]bp|Canada.Quebec|BOLD:AAA6359
Aplectoides condita|[6558]|RDLQF850-06|DH012003|658[0n]bp|Canada.Quebec|BOLD:AAA6359
Aplectoides condita|[6559]|BBLPB765-10|10BBCLP-1764|658[0n]bp|Canada.Saskatchewan|BOLD:AAA6359
Aplectoides condita|[6560]|TMNBB396-06|MNBTT-1336|658[0n]bp|Canada.New Brunswick|BOLD:AAA6359
Aplectoides condita|[6561]|RDLQB100-05|DH010186|658[0n]bp|Canada.Quebec|BOLD:AAA6359
Aplectoides condita|[6562]|RDLQ736-07|DH008446|636[0n]bp|Canada.Quebec|BOLD:AAA6359
Aplectoides condita|[6563]|LPSOD657-09|08BBLEP-00438|658[0n]bp|Canada.Ontario|BOLD:AAA6359
Aplectoides condita|[6564]|RDNM981-05|CNCNoctuoidea7821|658[0n]bp|Canada.Ontario|BOLD:AAA6359
Aplectoides condita|[6565]|RDNM982-05|CNCNoctuoidea7822|658[0n]bp|Canada.Ontario|BOLD:AAA6359
Aplectoides condita|[6566]|TMNBB395-06|MNBTT-1335|658[0n]bp|Canada.New Brunswick|BOLD:AAA6359
Aplectoides condita|[6567]|TTMNB565-06|MNBTT-565|658[0n]bp|Canada.New Brunswick|BOLD:AAA6359
Aplectoides condita|[6568]|RDLQF459-06|DH011566|658[0n]bp|Canada.Quebec|BOLD:AAA6359
Aplectoides condita|[6569]|TTMNB563-06|MNBTT-563|658[0n]bp|Canada.New Brunswick|BOLD:AAA6359
Aplectoides condita|[6570]|TTMNB562-06|MNBTT-562|658[0n]bp|Canada.New Brunswick|BOLD:AAA6359
Aplectoides condita|[6571]|TTMNB564-06|MNBTT-564|658[0n]bp|Canada.New Brunswick|BOLD:AAA6359
Aplectoides condita|[6572]|RDLQF506-06|DH011655|658[0n]bp|Canada.Quebec|BOLD:AAA6359
Aplectoides condita|[6573]|RDLQG379-06|DH012647|658[0n]bp|Canada.Quebec|BOLD:AAA6359
Aplectoides condita|[6574]|LBCA099-05|HLC-20099|655[0n]bp|Canada.British Columbia|BOLD:AAA6359
Aplectoides condita|[6575]|LBCA825-05|HLC-20825|658[0n]bp|Canada.British Columbia|BOLD:AAA6359
Aplectoides condita|[6576]|LOWCD599-06|CGWC-3419|573[0n]bp|Canada.British Columbia|BOLD:AAA6359
Aplectoides condita|[6577]|RDLQG377-06|DH012645|658[0n]bp|Canada.Quebec|BOLD:AAA6359
Aplectoides condita|[6578]|BBLPB410-10|10BBCLP-1409|658[0n]bp|Canada.Ontario|BOLD:AAA6359
[illegible]

Aplectoides condita|[6576]|LOWCD599-06|CGWC-3419|573[0n]bp|Canada.British Columbia|BOLD:AAA6359
Aplectoides condita|[6577]|RDLQG377-06|DH012645|658[0n]bp|Canada.Quebec|BOLD:AAA6359
Aplectoides condita|[6578]|BBLPB410-10|10BBCLP-1409|658[0n]bp|Canada.Ontario|BOLD:AAA6359
Aplectoides condita|[6579]|RDLQG380-06|DH012648|658[0n]bp|Canada.Quebec|BOLD:AAA6359
Aplectoides condita|[6580]|LOWCD597-06|CGWC-3417|658[0n]bp|Canada.British Columbia|BOLD:AAA6359
Aplectoides condita|[6581]|LOWCC175-05|CGWC-2055|658[0n]bp|Canada.British Columbia|BOLD:AAA6359
Aplectoides condita|[6582]|RDLQG376-06|DH012644|658[0n]bp|Canada.Quebec|BOLD:AAA6359
Aplectoides condita|[6583]|LPSOD457-09|08BBLEP-00236|658[0n]bp|Canada.Ontario|BOLD:AAA6359
Aplectoides condita|[6584]|LPSOD895-09|08BBLEP-00677|658[0n]bp|Canada.Ontario|BOLD:AAA6359
Aplectoides condita|[6585]|LOWC919-05|CGWC-0919|658[0n]bp|Canada.British Columbia|BOLD:AAA6359
Aplectoides condita|[6586]|LALPA196-10|AVBC 197-10|658[0n]bp|Canada.British Columbia|BOLD:AAA6359
Aplectoides condita|[6587]|BBLEC400-09|09BBELE-0400|658[0n]bp|Canada.Newfoundland and Labrador|BOLD:A...
Aplectoides condita|[6588]|LBCB637-05|HLC-21577|658[0n]bp|Canada.British Columbia|BOLD:AAA6359
Aplectoides condita|[6589]|LPSOD850-09|08BBLEP-00632|658[0n]bp|Canada.Ontario|BOLD:AAA6359
Aplectoides condita|[6590]|LOWCD598-06|CGWC-3418|658[0n]bp|Canada.British Columbia|BOLD:AAA6359
Aplectoides condita|[6591]|LBCB083-05|HLC-21023|658[0n]bp|Canada.British Columbia|BOLD:AAA6359
Aplectoides condita|[6592]|LPSOD740-09|08BBLEP-00522|658[0n]bp|Canada.Ontario|BOLD:AAA6359
Aplectoides condita|[6593]|RDLQG378-06|DH012646|658[0n]bp|Canada.Quebec|BOLD:AAA6359
Aplectoides condita|[6594]|CDIBC003-07|06-CDI-0003|658[0n]bp|Canada.British Columbia|BOLD:AAA6359
Aplectoides condita|[6595]|LBCA881-05|HLC-20881|658[0n]bp|Canada.British Columbia|BOLD:AAA6359
Aplectoides condita|[6596]|LBCB213-05|HLC-21153|658[0n]bp|Canada.British Columbia|BOLD:AAA6359
Aplectoides condita|[6597]|LPSOB317-08|PPBP-1316|658[0n]bp|Canada.Ontario|BOLD:AAA6359
Aplectoides condita|[6598]|LOWCD595-06|CGWC-3415|658[0n]bp|Canada.British Columbia|BOLD:AAA6359
Aplectoides condita|[6599]|LBCA081-05|HLC-20081|658[0n]bp|Canada.British Columbia|BOLD:AAA6359
Aplectoides condita|[6600]|LBCA835-05|HLC-20835|658[0n]bp|Canada.British Columbia|BOLD:AAA6359
Aplectoides condita|[6601]|LBCA082-05|HLC-20082|658[0n]bp|Canada.British Columbia|BOLD:AAA6359
Aplectoides condita|[6602]|LBCA826-05|HLC-20826|658[0n]bp|Canada.British Columbia|BOLD:AAA6359
Aplectoides condita|[6603]|LOWCD596-06|CGWC-3416|523[0n]bp|Canada.British Columbia|BOLD:AAA6359
Aplectoides condita|[6604]|RDLQG375-06|DH012643|649[0n]bp|Canada.Quebec|BOLD:AAA6359
Aplectoides condita|[6605]|RDMAB079-05|UASM57606|598[0n]bp|Canada.Alberta|BOLD:AAA6359
Aplectoides condita|[6606]|LOWCD593-06|CGWC-3413|585[0n]bp|Canada.British Columbia|BOLD:AAA6359
Aplectoides condita|[6607]|LOWC920-05|CGWC-0920|559[1n]bp|Canada.British Columbia|BOLD:AAA6359
Aplectoides condita|[6608]|LOWCD594-06|CGWC-3414|582[0n]bp|Canada.British Columbia|BOLD:AAA6359
Aplectoides condita|[6609]|RDNM984-05|CNCNoctuoidea7824|560[0n]bp|Canada.British Columbia|BOLD:AAA6359
Aplectoides condita|[6610]|LPMN759-08|08BBLEP-01562|609[0n]bp|Canada.Manitoba|BOLD:AAA6359
Aplectoides condita|[6611]|BBLPB463-10|10BBCLP-1462|658[1n]bp|Canada.British Columbia|BOLD:AAA6359
Aplectoides condita|[6612]|LOWC917-05|CGWC-0917|658[0n]bp|Canada.British Columbia|BOLD:AAA6359
Aplectoides condita|[6613]|BBLPC686-09|09BBELE-1686|658[0n]bp|Canada.Newfoundland and Labrador|BOLD:A...
Aplectoides condita|[6614]|LPSOD851-09|08BBLEP-00633|658[0n]bp|Canada.Ontario|BOLD:AAA6359
Protolampra rufipectus|[6615]|LBCH6736-10|10-JDWBC-6736|658[0n]bp|Canada.British Columbia|BOLD:ACE6699
Protolampra rufipectus|[6616]|BBLPB412-10|10BBCLP-1411|658[0n]bp|Canada.Saskatchewan|BOLD:ACE6699
Protolampra rufipectus|[6617]|LBCH7805-10|10-JDWBC-7805|658[0n]bp|Canada.British Columbia|BOLD:ACE6699
Protolampra rufipectus|[6618]|LOWCD665-06|CGWC-3485|599[0n]bp|Canada.British Columbia|BOLD:ACE6699
Protolampra rufipectus|[6619]|LOWCD672-06|CGWC-3492|590[0n]bp|Canada.British Columbia|BOLD:ACE6699
Protolampra rufipectus|[6620]|LPMN956-08|08BBLEP-02314|658[0n]bp|Canada.Alberta|BOLD:ACE6699
Protolampra rufipectus|[6621]|LOWC886-05|CGWC-0886|658[0n]bp|Canada.British Columbia|BOLD:ACE6699
Protolampra rufipectus|[6622]|LOWCD669-06|CGWC-3489|658[0n]bp|Canada.British Columbia|BOLD:ACE6699
Protolampra rufipectus|[6623]|LPABC796-09|08BBLEP-05015|658[0n]bp|Canada.Alberta|BOLD:ACE6699
Protolampra rufipectus|[6624]|LOWC889-05|CGWC-0889|658[0n]bp|Canada.British Columbia|BOLD:ACE6699
Protolampra rufipectus|[6625]|LOWC884-05|CGWC-0884|658[0n]bp|Canada.British Columbia|BOLD:ACE6699
Protolampra rufipectus|[6626]|LOWC885-05|CGWC-0885|658[0n]bp|Canada.British Columbia|BOLD:ACE6699
Protolampra rufipectus|[6627]|LPABB496-08|08BBLEP-03761|658[0n]bp|Canada.Alberta|BOLD:ACE6699
Protolampra rufipectus|[6628]|LBCH1864-10|10-JDWBC-1864|658[0n]bp|Canada.British Columbia|BOLD:ACE6699
Protolampra rufipectus|[6629]|LOWCD668-06|CGWC-3488|610[0n]bp|Canada.British Columbia|BOLD:ACE6699
Protolampra rufipectus|[6630]|RDLQB636-05|DH010739|658[0n]bp|Canada.Quebec|BOLD:ACE6699
Protolampra rufipectus|[6631]|BBLPB373-10|10BBCLP-1372|658[0n]bp|Canada.Alberta|BOLD:AAA6777
Protolampra rufipectus|[6632]|LOWC879-05|CGWC-0879|658[0n]bp|Canada.British Columbia|BOLD:AAA6777
Protolampra rufipectus|[6633]|LOWC883-05|CGWC-0883|658[0n]bp|Canada.British Columbia|BOLD:AAA6777
Protolampra rufipectus|[6634]|BBLPB372-10|10BBCLP-1371|658[0n]bp|Canada.Alberta|BOLD:AAA6777
Protolampra rufipectus|[6635]|LOWCD670-06|CGWC-3490|601[0n]bp|Canada.British Columbia|BOLD:AAA6777
Protolampra rufipectus|[6636]|LOWCD673-06|CGWC-3493|658[0n]bp|Canada.British Columbia|BOLD:AAA6777
Protolampra rufipectus|[6637]|LBCH3793-10|10-JDWBC-3793|658[0n]bp|Canada.British Columbia|BOLD:AAA6777
Protolampra rufipectus|[6638]|LOWC887-05|CGWC-0887|658[0n]bp|Canada.British Columbia|BOLD:AAA6777
Protolampra rufipectus|[6639]|LBCH7818-10|10-JDWBC-7818|658[0n]bp|Canada.British Columbia|BOLD:AAA6777
Protolampra rufipectus|[6640]|LOWCD677-06|CGWC-3497|658[0n]bp|Canada.British Columbia|BOLD:AAA6777
Protolampra rufipectus|[6641]|LOWC881-05|CGWC-0881|658[7n]bp|Canada.British Columbia|
Protolampra rufipectus|[6642]|LBCH3085-10|10-JDWBC-3085|658[0n]bp|Canada.British Columbia|BOLD:AAA6777
Protolampra rufipectus|[6643]|LBCH4048-10|10-JDWBC-4048|658[0n]bp|Canada.British Columbia|BOLD:AAA6777
Protolampra rufipectus|[6644]|LPABB369-08|08BBLEP-03634|658[0n]bp|Canada.Alberta|BOLD:AAA6777
Protolampra rufipectus|[6645]|LBCD288-05|HLC-23108|618[0n]bp|Canada.British Columbia|BOLD:AAA6777
Protolampra rufipectus|[6646]|MNBB530-05|05-NBSTA-446|658[0n]bp|Canada.New Brunswick|BOLD:AAA6777
Protolampra rufipectus|[6647]|RDLQB633-05|DH010736|599[0n]bp|Canada.Quebec|BOLD:AAA6777
Protolampra rufipectus|[6648]|LOWCD674-06|CGWC-3494|609[0n]bp|Canada.British Columbia|BOLD:AAA6777
Protolampra rufipectus|[6649]|LOWC882-05|CGWC-0882|658[0n]bp|Canada.British Columbia|BOLD:AAA6777
Protolampra rufipectus|[6650]|LOWC888-05|CGWC-0888|658[0n]bp|Canada.British Columbia|BOLD:AAA6777
Protolampra rufipectus|[6651]|LOWCD675-06|CGWC-3495|658[0n]bp|Canada.British Columbia|BOLD:AAA6777
Protolampra rufipectus|[6652]|LOWCD678-06|CGWC-3498|658[0n]bp|Canada.British Columbia|BOLD:AAA6777
Protolampra rufipectus|[6653]|LOWCD671-06|CGWC-3491|658[0n]bp|Canada.British Columbia|BOLD:AAA6777
Protolampra rufipectus|[6654]|LPABC429-09|08BBLEP-04648|658[0n]bp|Canada.Alberta|BOLD:AAA6777
Protolampra rufipectus|[6655]|LPABC003-09|08BBLEP-04222|658[0n]bp|Canada.Alberta|BOLD:AAA6777
Protolampra rufipectus|[6656]|LOWCD666-06|CGWC-3486|658[0n]bp|Canada.British Columbia|BOLD:AAA6777
Protolampra rufipectus|[6657]|LOWCD664-06|CGWC-3484|657[0n]bp|Canada.British Columbia|BOLD:AAA6777
Protolampra rufipectus|[6658]|RDLQF186-06|DH011213|658[0n]bp|Canada.Quebec|BOLD:AAA6777
Protolampra rufipectus|[6659]|BBLPB375-10|10BBCLP-1374|658[0n]bp|Canada.Alberta|BOLD:AAA6777
Protolampra rufipectus|[6660]|BBLPB371-10|10BBCLP-1370|658[0n]bp|Canada.British Columbia|BOLD:AAA6777
Protolampra rufipectus|[6661]|LOWCD660-06|CGWC-3480|592[0n]bp|Canada.British Columbia|BOLD:ABY5873
Protolampra rufipectus|[6662]|LOWCD662-06|CGWC-3482|592[0n]bp|Canada.British Columbia|BOLD:ABY5873
Protolampra rufipectus|[6663]|RDLQB637-05|DH010740|540[0n]bp|Canada.Quebec|BOLD:ABY5873
Protolampra rufipectus|[6664]|LOWCD128-06|CGWC-2948|602[1n]bp|Canada.British Columbia|BOLD:ABY5873
Protolampra rufipectus|[6665]|RDLQB635-05|DH010738|600[0n]bp|Canada.Quebec|BOLD:ABY5873
Protolampra rufipectus|[6666]|LOWCD667-06|CGWC-3487|610[0n]bp|Canada.British Columbia|BOLD:ABY5873
Protolampra rufipectus|[6667]|BBLPB449-10|10BBCLP-1448|658[0n]bp|Canada.Saskatchewan|BOLD:ABY5873
Protolampra rufipectus|[6668]|LOWCD659-06|CGWC-3479|658[0n]bp|Canada.British Columbia|BOLD:ABY5873
Protolampra rufipectus|[6669]|LOWC890-05|CGWC-0890|658[0n]bp|Canada.British Columbia|BOLD:ABY5873
Protolampra rufipectus|[6670]|LBCH7552-10|10-JDWBC-7552|658[0n]bp|Canada.British Columbia|BOLD:ABY5873
Protolampra rufipectus|[6671]|TTMNB490-06|MNBTT-490|658[0n]bp|Canada.New Brunswick|BOLD:ABY5873
Protolampra rufipectus|[6672]|LOWC891-05|CGWC-0891|658[0n]bp|Canada.British Columbia|BOLD:ABY5873
Protolampra rufipectus|[6673]|BBLPB374-10|10BBCLP-1373|658[0n]bp|Canada.Alberta|BOLD:ABY5873
Protolampra rufipectus|[6674]|LOWC880-05|CGWC-0880|658[0n]bp|Canada.British Columbia|BOLD:ABY5873
Protolampra rufipectus|[6675]|LBCH6287-10|10-JDWBC-6287|658[0n]bp|Canada.British Columbia|BOLD:ABY5873
Protolampra rufipectus|[6676]|RDLQB634-05|DH010737|540[0n]bp|Canada.Quebec|BOLD:ABY5873
Protolampra rufipectus|[6677]|LOWCD661-06|CGWC-3481|604[0n]bp|Canada.British Columbia|BOLD:ABY5873
Protolampra rufipectus|[6678]|LBCH927-10|10-JDWBC-0927|658[0n]bp|Canada.British Columbia|BOLD:ABY5873

Protolampra rufipectus|[6676]|RDLQB634-05|DH010737|540[0n]bp|Canada.Quebec|BOLD:ABY5873
Protolampra rufipectus|[6677]|LOWCD661-06|CGWC-3481|604[0n]bp|Canada.British Columbia|BOLD:ABY5873
Protolampra rufipectus|[6678]|LBCH927-10|10-JDWBC-0927|658[0n]bp|Canada.British Columbia|BOLD:ABY5873
Protolampra rufipectus|[6679]|LOWCD658-06|CGWC-3478|658[0n]bp|Canada.British Columbia|BOLD:ABY5873
Rhyacia quadrangula|[6680]|LCHP960-07|07PROBE-10722|658[0n]bp|Canada.Manitoba|BOLD:AAA4280
Rhyacia clemens|[6681]|RDMAB281-05|UASM41500|658[0n]bp|Canada.Alberta|BOLD:AAA4280
Rhyacia quadrangula|[6682]|LCHQ480-08|07WNP-10372|656[0n]bp|Canada.Manitoba|BOLD:AAA4280
Rhyacia quadrangula|[6683]|LCHIP023-07|06-PROBE-0043|650[0n]bp|Canada.Manitoba|BOLD:AAA4280
Rhyacia quadrangula|[6684]|LCHP585-07|07PROBE-10256|658[0n]bp|Canada.Manitoba|BOLD:AAA4280
Rhyacia quadrangula|[6685]|LCHP951-07|07PROBE-10713|658[0n]bp|Canada.Manitoba|BOLD:AAA4280
Rhyacia quadrangula|[6686]|LCH222-04|04HBL003222|658[0n]bp|Canada.Manitoba|BOLD:AAA4280
Rhyacia quadrangula|[6687]|LCH223-04|04HBL003223|658[0n]bp|Canada.Manitoba|BOLD:AAA4280
Rhyacia quadrangula|[6688]|LCHP851-07|07PROBE-10608|630[0n]bp|Canada.Manitoba|BOLD:AAA4280
Rhyacia quadrangula|[6689]|LCH225-04|04HBL003225|654[0n]bp|Canada.Manitoba|BOLD:AAA4280
Rhyacia quadrangula|[6690]|LCH226-04|04HBL003226|632[2n]bp|Canada.Manitoba|BOLD:AAA4280
Rhyacia quadrangula|[6691]|LCHP367-07|07PROBE-03942|640[0n]bp|Canada.Manitoba|BOLD:AAA4280
Rhyacia quadrangula|[6692]|LCH228-04|04HBL003228|658[0n]bp|Canada.Manitoba|BOLD:AAA4280
Rhyacia quadrangula|[6693]|LCHP870-07|07PROBE-10627|658[0n]bp|Canada.Manitoba|BOLD:AAA4280
Rhyacia quadrangula|[6694]|LCH230-04|04HBL003230|658[0n]bp|Canada.Manitoba|BOLD:AAA4280
Rhyacia quadrangula|[6695]|LCHP622-07|07PROBE-10303|658[0n]bp|Canada.Manitoba|BOLD:AAA4280
Rhyacia quadrangula|[6696]|LCHQ432-08|07WNP-10324|658[0n]bp|Canada.Manitoba|BOLD:AAA4280
Rhyacia quadrangula|[6697]|LCHQ503-08|07WNP-10395|658[0n]bp|Canada.Manitoba|BOLD:AAA4280
Rhyacia quadrangula|[6698]|LCHQ122-07|07PROBE-10891|658[0n]bp|Canada.Manitoba|BOLD:AAA4280
Rhyacia quadrangula|[6699]|LCH224-04|04HBL003224|658[0n]bp|Canada.Manitoba|BOLD:AAA4280
Rhyacia quadrangula|[6700]|LCHQ751-08|07WNP-10643|658[0n]bp|Canada.Manitoba|BOLD:AAA4280
Rhyacia quadrangula|[6701]|LCH232-04|04HBL003232|658[0n]bp|Canada.Manitoba|BOLD:AAA4280
Rhyacia quadrangula|[6702]|LCHP836-07|07PROBE-10593|658[0n]bp|Canada.Manitoba|BOLD:AAA4280
Rhyacia quadrangula|[6703]|LCHQ135-07|07PROBE-10904|658[0n]bp|Canada.Manitoba|BOLD:AAA4280
Rhyacia quadrangula|[6704]|LCHP633-07|07PROBE-10314|658[0n]bp|Canada.Manitoba|BOLD:AAA4280
Rhyacia quadrangula|[6705]|LCHQ474-08|07WNP-10366|658[0n]bp|Canada.Manitoba|BOLD:AAA4280
Rhyacia quadrangula|[6706]|LCH227-04|04HBL003227|658[0n]bp|Canada.Manitoba|BOLD:AAA4280
Rhyacia quadrangula|[6707]|LCHP894-07|07PROBE-10651|658[0n]bp|Canada.Manitoba|BOLD:AAA4280
Rhyacia quadrangula|[6708]|LCHQ395-08|07WNP-10287|658[0n]bp|Canada.Manitoba|BOLD:AAA4280
Rhyacia quadrangula|[6709]|LCHQ670-08|07WNP-10562|658[0n]bp|Canada.Manitoba|BOLD:AAA4280
Rhyacia quadrangula|[6710]|LCHQ648-08|07WNP-10540|658[0n]bp|Canada.Manitoba|BOLD:AAA4280
Rhyacia quadrangula|[6711]|LCHQ136-07|07PROBE-10905|658[0n]bp|Canada.Manitoba|BOLD:AAA4280
Rhyacia quadrangula|[6712]|LCHP892-07|07PROBE-10649|658[0n]bp|Canada.Manitoba|BOLD:AAA4280
Rhyacia quadrangula|[6713]|LCHQ526-08|07WNP-10418|658[0n]bp|Canada.Manitoba|BOLD:AAA4280
Rhyacia quadrangula|[6714]|LCH221-04|04HBL003221|658[0n]bp|Canada.Manitoba|BOLD:AAA4280
Rhyacia quadrangula|[6715]|LCHP504-07|07PROBE-10172|658[0n]bp|Canada.Manitoba|BOLD:AAA4280
Rhyacia quadrangula|[6716]|LCHQ116-07|07PROBE-10885|658[0n]bp|Canada.Manitoba|BOLD:AAA4280
Rhyacia quadrangula|[6717]|LCHP278-07|07PROBE-03844|658[0n]bp|Canada.Manitoba|BOLD:AAA4280
Rhyacia quadrangula|[6718]|LCHQ428-08|07WNP-10320|658[0n]bp|Canada.Manitoba|BOLD:AAA4280
Rhyacia quadrangula|[6719]|LCHQ225-08|07WNP-10117|658[0n]bp|Canada.Manitoba|BOLD:AAA4280
Rhyacia quadrangula|[6720]|LCHQ572-08|07WNP-10464|658[0n]bp|Canada.Manitoba|BOLD:AAA4280
Rhyacia quadrangula|[6721]|LCHP485-07|07PROBE-10153|658[0n]bp|Canada.Manitoba|BOLD:AAA4280
Rhyacia quadrangula|[6722]|LCHP774-07|07PROBE-10459|658[0n]bp|Canada.Manitoba|BOLD:AAA4280
Rhyacia quadrangula|[6723]|LCHP785-07|07PROBE-10470|658[0n]bp|Canada.Manitoba|BOLD:AAA4280
Rhyacia quadrangula|[6724]|LCHP947-07|07PROBE-10709|658[0n]bp|Canada.Manitoba|BOLD:AAA4280
Rhyacia quadrangula|[6725]|LCHQ120-07|07PROBE-10889|658[0n]bp|Canada.Manitoba|BOLD:AAA4280
Rhyacia quadrangula|[6726]|LCHQ306-08|07WNP-10198|658[0n]bp|Canada.Manitoba|BOLD:AAA4280
Rhyacia quadrangula|[6727]|LCHQ593-08|07WNP-10485|658[0n]bp|Canada.Manitoba|BOLD:AAA4280
Rhyacia quadrangula|[6728]|LCHP848-07|07PROBE-10605|658[0n]bp|Canada.Manitoba|BOLD:AAA4280
Rhyacia quadrangula|[6729]|LCH220-04|04HBL003220|658[0n]bp|Canada.Manitoba|BOLD:AAA4280
Rhyacia quadrangula|[6730]|LCHP893-07|07PROBE-10650|658[0n]bp|Canada.Manitoba|BOLD:AAA4280
Rhyacia quadrangula|[6731]|LCHQ186-07|07PROBE-10963|658[0n]bp|Canada.Manitoba|BOLD:AAA4280
Rhyacia quadrangula|[6732]|LCHP862-07|07PROBE-10619|658[0n]bp|Canada.Manitoba|BOLD:AAA4280
Rhyacia quadrangula|[6733]|LCHQ483-08|07WNP-10375|657[0n]bp|Canada.Manitoba|BOLD:AAA4280
Rhyacia quadrangula|[6734]|LCHQ661-08|07WNP-10553|658[0n]bp|Canada.Manitoba|BOLD:AAA4280
Rhyacia quadrangula|[6735]|LCHQ520-08|07WNP-10412|658[0n]bp|Canada.Manitoba|BOLD:AAA4280
Rhyacia quadrangula|[6736]|MHLEP124-07|CHU06-LEP-124|658[0n]bp|Canada.Manitoba|BOLD:AAA4280
Rhyacia quadrangula|[6737]|LCHQ705-08|07WNP-10597|657[0n]bp|Canada.Manitoba|BOLD:AAA4280
Rhyacia quadrangula|[6738]|LCHP576-07|07PROBE-10246|658[0n]bp|Canada.Manitoba|BOLD:AAA4280
Rhyacia quadrangula|[6739]|LCH218-04|04HBL003218|658[0n]bp|Canada.Manitoba|BOLD:AAA4280
Rhyacia quadrangula|[6740]|LCHQ704-08|07WNP-10596|658[0n]bp|Canada.Manitoba|BOLD:AAA4280
Rhyacia quadrangula|[6741]|LCHP962-07|07PROBE-10724|658[0n]bp|Canada.Manitoba|BOLD:AAA4280
Rhyacia quadrangula|[6742]|LCHP310-07|07PROBE-03876|658[0n]bp|Canada.Manitoba|BOLD:AAA4280
Rhyacia quadrangula|[6743]|LCHP961-07|07PROBE-10723|658[0n]bp|Canada.Manitoba|BOLD:AAA4280
Rhyacia quadrangula|[6744]|LCHP845-07|07PROBE-10602|658[0n]bp|Canada.Manitoba|BOLD:AAA4280
Rhyacia quadrangula|[6745]|LCHP663-07|07PROBE-10345|658[0n]bp|Canada.Manitoba|BOLD:AAA4280
Rhyacia quadrangula|[6746]|LCHQ770-08|07WNP-10662|658[0n]bp|Canada.Manitoba|BOLD:AAA4280
Rhyacia quadrangula|[6747]|LCHQ427-08|07WNP-10319|658[0n]bp|Canada.Manitoba|BOLD:AAA4280
Rhyacia quadrangula|[6748]|LCHP955-07|07PROBE-10717|658[0n]bp|Canada.Manitoba|BOLD:AAA4280
Rhyacia quadrangula|[6749]|LCHQ602-08|07WNP-10494|658[0n]bp|Canada.Manitoba|BOLD:AAA4280
Rhyacia quadrangula|[6750]|LCHP354-07|07PROBE-03929|658[0n]bp|Canada.Manitoba|BOLD:AAA4280
Rhyacia quadrangula|[6751]|LCHQ804-08|07WNP-10696|658[0n]bp|Canada.Manitoba|BOLD:AAA4280
Rhyacia quadrangula|[6752]|LCHP963-07|07PROBE-10725|658[0n]bp|Canada.Manitoba|BOLD:AAA4280
Rhyacia quadrangula|[6753]|LCHQ819-08|07WNP-10711|658[0n]bp|Canada.Manitoba|BOLD:AAA4280
Rhyacia quadrangula|[6754]|LCHQ708-08|07WNP-10600|658[0n]bp|Canada.Manitoba|BOLD:AAA4280
Rhyacia quadrangula|[6755]|LCHQ645-08|07WNP-10537|656[0n]bp|Canada.Manitoba|BOLD:AAA4280
Rhyacia quadrangula|[6756]|LCHQ429-08|07WNP-10321|656[0n]bp|Canada.Manitoba|BOLD:AAA4280
Rhyacia quadrangula|[6757]|LCH233-04|04HBL003233|654[0n]bp|Canada.Manitoba|BOLD:AAA4280
Rhyacia quadrangula|[6758]|LCHQ898-08|07WNP-10790|656[0n]bp|Canada.Manitoba|BOLD:AAA4280
Rhyacia quadrangula|[6759]|LCHQ720-08|07WNP-10612|656[0n]bp|Canada.Manitoba|BOLD:AAA4280
Rhyacia quadrangula|[6760]|LCHP843-07|07PROBE-10600|655[0n]bp|Canada.Manitoba|BOLD:AAA4280
Rhyacia quadrangula|[6761]|LCHP241-07|07PROBE-03809|634[0n]bp|Canada.Manitoba|BOLD:AAA4280
Rhyacia quadrangula|[6762]|LCHIP022-07|06-PROBE-0042|650[0n]bp|Canada.Manitoba|BOLD:AAA4280
Rhyacia quadrangula|[6763]|LCHQ304-08|07WNP-10196|626[0n]bp|Canada.Manitoba|BOLD:AAA4280
Rhyacia quadrangula|[6764]|LCH229-04|04HBL003229|658[0n]bp|Canada.Manitoba|BOLD:AAA4280
Rhyacia quadrangula|[6765]|LCH219-04|04HBL003219|658[0n]bp|Canada.Manitoba|BOLD:AAA4280
Rhyacia quadrangula|[6766]|LCH231-04|04HBL003231|658[0n]bp|Canada.Manitoba|BOLD:AAA4280
Rhyacia quadrangula|[6767]|LCHQ820-08|07WNP-10712|658[0n]bp|Canada.Manitoba|BOLD:AAA4280
Lycophotia phyllophora|[6768]|BBLPE239-09|09BBELE-2239|658[0n]bp|Canada.Newfoundland and Labrador|BOLD ...
Lycophotia phyllophora|[6769]|TTMNB503-06|MNBTT-503|658[0n]bp|Canada.New Brunswick|BOLD:AAA7117
Lycophotia phyllophora|[6770]|PHMNB177-04|04HBL007642|609[0n]bp|Canada.New Brunswick|BOLD:AAA7117
Lycophotia phyllophora|[6771]|BBLPC936-09|09BBELE-1936|618[0n]bp|Canada.Newfoundland and Labrador|BOLD ...
Lycophotia phyllophora|[6772]|BBLEC375-09|09BBELE-0375|658[0n]bp|Canada.Newfoundland and Labrador|BOLD ...
Lycophotia phyllophora|[6773]|BBLPE308-09|09BBELE-2308|658[0n]bp|Canada.Newfoundland and Labrador|BOLD ...
Lycophotia phyllophora|[6774]|BBLPE086-09|09BBELE-2086|658[0n]bp|Canada.Nova Scotia|BOLD:AAA7117
Lycophotia phyllophora|[6775]|BBLPC796-09|09BBELE-1796|658[0n]bp|Canada.Newfoundland and Labrador|BOLD ...
Lycophotia phyllophora|[6776]|BBLPC895-09|09BBELE-1895|658[0n]bp|Canada.Newfoundland and Labrador|BOLD ...
Lycophotia phyllophora|[6777]|BBLEC095-09|09BBELE-0095|658[0n]bp|Canada.Nova Scotia|BOLD:AAA7117
Lycophotia phyllophora|[6778]|BBLPE335-09|09BBELE-2335|658[0n]bp|Canada.Newfoundland and Labrador|BOLD [illegible]

Lycophotia phyllophora|[6775]|BBLPC790-09|09BBELE-1790|658[0n]bp|Canada.Newfoundland and Labrador|BOLD ...
Lycophotia phyllophora|[6776]|BBLPC895-09|09BBELE-1895|658[0n]bp|Canada.Newfoundland and Labrador|BOLD ...
Lycophotia phyllophora|[6777]|BBLEC095-09|09BBELE-0095|658[0n]bp|Canada.Nova Scotia|BOLD:AAA7117
Lycophotia phyllophora|[6778]|BBLPE335-09|09BBELE-2335|658[0n]bp|Canada.Newfoundland and Labrador|BOLD ...
Lycophotia phyllophora|[6779]|BBLPE188-09|09BBELE-2188|658[0n]bp|Canada.Newfoundland and Labrador|BOLD ...
Lycophotia phyllophora|[6780]|BBLPE020-09|09BBELE-2020|658[0n]bp|Canada.Nova Scotia|BOLD:AAA7117
Lycophotia phyllophora|[6781]|BBLPE197-09|09BBELE-2197|658[0n]bp|Canada.Newfoundland and Labrador|BOLD ...
Lycophotia phyllophora|[6782]|BBLPC717-09|09BBELE-1717|658[0n]bp|Canada.Newfoundland and Labrador|BOLD ...
Lycophotia phyllophora|[6783]|BBLPC692-09|09BBELE-1692|658[0n]bp|Canada.Newfoundland and Labrador|BOLD ...
Lycophotia phyllophora|[6784]|BBLEC107-09|09BBELE-0107|658[0n]bp|Canada.Nova Scotia|BOLD:AAA7117
Lycophotia phyllophora|[6785]|BBLPE236-09|09BBELE-2236|658[0n]bp|Canada.Newfoundland and Labrador|BOLD ...
Lycophotia phyllophora|[6786]|BBLPC234-09|09BBELE-1234|658[0n]bp|Canada.Nova Scotia|BOLD:AAA7117
Lycophotia phyllophora|[6787]|BBLPE348-09|09BBELE-2348|658[0n]bp|Canada.Newfoundland and Labrador|BOLD ...
Lycophotia phyllophora|[6788]|BBLPC971-09|09BBELE-1971|658[0n]bp|Canada.Newfoundland and Labrador|BOLD ...
Lycophotia phyllophora|[6789]|BBLPE243-09|09BBELE-2243|658[0n]bp|Canada.Newfoundland and Labrador|BOLD ...
Lycophotia phyllophora|[6790]|BBLPE025-09|09BBELE-2025|658[0n]bp|Canada.Nova Scotia|BOLD:AAA7117
Lycophotia phyllophora|[6791]|BBLPE321-09|09BBELE-2321|658[0n]bp|Canada.Newfoundland and Labrador|BOLD ...
Lycophotia phyllophora|[6792]|BBLEC097-09|09BBELE-0097|658[0n]bp|Canada.Nova Scotia|BOLD:AAA7117
Lycophotia phyllophora|[6793]|BBLPE384-09|09BBELE-2384|658[0n]bp|Canada.Newfoundland and Labrador|BOLD ...
Lycophotia phyllophora|[6794]|BBLPE212-09|09BBELE-2212|658[0n]bp|Canada.Newfoundland and Labrador|BOLD ...
Lycophotia phyllophora|[6795]|BBLPE392-09|09BBELE-2392|658[0n]bp|Canada.Newfoundland and Labrador|BOLD ...
Lycophotia phyllophora|[6796]|BBLPC660-09|09BBELE-1660|658[0n]bp|Canada.Newfoundland and Labrador|BOLD ...
Lycophotia phyllophora|[6797]|BBLPE317-09|09BBELE-2317|658[0n]bp|Canada.Newfoundland and Labrador|BOLD ...
Lycophotia phyllophora|[6798]|BBLEC365-09|09BBELE-0365|658[0n]bp|Canada.Newfoundland and Labrador|BOLD ...
Lycophotia phyllophora|[6799]|BBLEC639-09|09BBELE-0639|658[0n]bp|Canada.Nova Scotia|BOLD:AAA7117
Lycophotia phyllophora|[6800]|BBLPE161-09|09BBELE-2161|658[0n]bp|Canada.Nova Scotia|BOLD:AAA7117
Lycophotia phyllophora|[6801]|BBLEC642-09|09BBELE-0642|658[0n]bp|Canada.Nova Scotia|BOLD:AAA7117
Lycophotia phyllophora|[6802]|PHMNB248-04|04HBL007713|551[0n]bp|Canada.New Brunswick|BOLD:AAA7117
Lycophotia phyllophora|[6803]|BBLPC751-09|09BBELE-1751|658[0n]bp|Canada.Newfoundland and Labrador|BOLD ...
Lycophotia phyllophora|[6804]|BBLPC775-09|09BBELE-1775|658[0n]bp|Canada.Newfoundland and Labrador|BOLD ...
Lycophotia phyllophora|[6805]|PHMNB202-04|04HBL007667|519[0n]bp|Canada.New Brunswick|BOLD:AAA7117
Lycophotia phyllophora|[6806]|BBLPE143-09|09BBELE-2143|635[0n]bp|Canada.Nova Scotia|BOLD:AAA7117
Lycophotia phyllophora|[6807]|PHMNB195-04|04HBL007660|527[0n]bp|Canada.New Brunswick|BOLD:AAA7117
Lycophotia phyllophora|[6808]|BBLPE187-09|09BBELE-2187|621[0n]bp|Canada.Newfoundland and Labrador|BOLD ...
Lycophotia phyllophora|[6809]|BBLPE007-09|09BBELE-2007|622[0n]bp|Canada.Nova Scotia|BOLD:AAA7117
Lycophotia phyllophora|[6810]|BBLPE249-09|09BBELE-2249|620[0n]bp|Canada.Newfoundland and Labrador|BOLD ...
Lycophotia phyllophora|[6811]|BBLPE224-09|09BBELE-2224|632[0n]bp|Canada.Newfoundland and Labrador|BOLD ...
Lycophotia phyllophora|[6812]|PHMNB204-04|04HBL007669|526[0n]bp|Canada.New Brunswick|BOLD:AAA7117
Lycophotia phyllophora|[6813]|RDLQG130-06|DH012297|658[1n]bp|Canada.Quebec|BOLD:AAA7117
Lycophotia phyllophora|[6814]|TTMNB505-06|MNBTT-505|658[0n]bp|Canada.New Brunswick|BOLD:AAA7117
Lycophotia phyllophora|[6815]|TTMNB504-06|MNBTT-504|658[0n]bp|Canada.New Brunswick|BOLD:AAA7117
Lycophotia phyllophora|[6816]|XAD778-05|2005-ONT-577|658[0n]bp|Canada.Ontario|BOLD:AAA7117
Lycophotia phyllophora|[6817]|BBLPC201-09|09BBELE-1201|658[0n]bp|Canada.Nova Scotia|BOLD:AAA7117
Lycophotia phyllophora|[6818]|RDLQB300-05|DH010386|658[0n]bp|Canada.Quebec|BOLD:AAA7117
Lycophotia phyllophora|[6819]|RDLQG131-06|DH012298|658[3n]bp|Canada.Quebec|BOLD:AAA7117
Lycophotia phyllophora|[6820]|XAB479-04|04HBL005479|658[0n]bp|Canada.Ontario|BOLD:AAA7117
Lycophotia phyllophora|[6821]|PHMNB148-04|04HBL007613|658[0n]bp|Canada.New Brunswick|BOLD:AAA7117
Lycophotia phyllophora|[6822]|TTMNB500-06|MNBTT-500|656[0n]bp|Canada.New Brunswick|BOLD:AAA7117
Lycophotia phyllophora|[6823]|TTMNB501-06|MNBTT-501|658[0n]bp|Canada.New Brunswick|BOLD:AAA7117
Lycophotia phyllophora|[6824]|TTMNB502-06|MNBTT-502|658[0n]bp|Canada.New Brunswick|BOLD:AAA7117
Cerastis enigmatica|[6825]|LALPA037-10|AVBC 037-10|637[1n]bp|Canada.British Columbia|BOLD:AAE1521
Cerastis enigmatica|[6826]|RDNMG1028-08|CNC LEP00053152|658[0n]bp|Canada.British Columbia|BOLD:AAE1521
Cerastis enigmatica|[6827]|LALPA075-10|AVBC 075-10|658[0n]bp|Canada.British Columbia|BOLD:AAE1521
Cerastis enigmatica|[6828]|LALPA053-10|AVBC 053-10|658[0n]bp|Canada.British Columbia|BOLD:AAE1521
Cerastis enigmatica|[6829]|LALPA1056-11|AVBC 866-11|658[0n]bp|Canada.British Columbia|BOLD:AAE1521
Cerastis enigmatica|[6830]|RDNMG1027-08|CNC LEP00053151|658[0n]bp|Canada.British Columbia|BOLD:AAE1521
Cerastis enigmatica|[6831]|RDNMF353-08|NOC14439|658[0n]bp|Canada.British Columbia|BOLD:AAE1521
Cerastis enigmatica|[6832]|RDNMF354-08|NOC14440|658[0n]bp|Canada.British Columbia|BOLD:AAE1521
Cerastis enigmatica|[6833]|RDNMG1026-08|CNC LEP00053150|658[0n]bp|Canada.British Columbia|BOLD:AAE1521
Cerastis salicarum|[6834]|LOWCE028-06|CGWC-3788|658[1n]bp|Canada.British Columbia|BOLD:AAC4727
Cerastis salicarum|[6835]|LOWCE500-06|CGWC-4260|616[0n]bp|Canada.British Columbia|BOLD:AAC4727
Cerastis salicarum|[6836]|LOWCE344-06|CGWC-4104|658[0n]bp|Canada.British Columbia|BOLD:AAC4727
Cerastis salicarum|[6837]|LOWC923-05|CGWC-0923|658[0n]bp|Canada.British Columbia|BOLD:AAC4727
Cerastis salicarum|[6838]|RDLQH098-06|DH013336|626[0n]bp|Canada.Quebec|BOLD:AAC4727
Cerastis salicarum|[6839]|LOWCE246-06|CGWC-4006|658[0n]bp|Canada.British Columbia|BOLD:AAC4727
Cerastis salicarum|[6840]|RDLQH097-06|DH013335|639[0n]bp|Canada.Quebec|BOLD:AAC4727
Cerastis salicarum|[6841]|LOWCE027-06|CGWC-3787|658[0n]bp|Canada.British Columbia|BOLD:AAC4727
Cerastis salicarum|[6842]|RDLQH096-06|DH013334|606[0n]bp|Canada.Quebec|BOLD:AAC4727
Cerastis salicarum|[6843]|LOWC922-05|CGWC-0922|658[0n]bp|Canada.British Columbia|BOLD:AAC4727
Cerastis salicarum|[6844]|LOWCE463-06|CGWC-4223|621[0n]bp|Canada.British Columbia|BOLD:AAC4727
Cerastis salicarum|[6845]|LOWCE508-06|CGWC-4268|617[0n]bp|Canada.British Columbia|BOLD:AAC4727
Cerastis fishii|[6846]|TMNBB393-06|MNBTT-1333|658[0n]bp|Canada.New Brunswick|BOLD:AAE1522
Cerastis fishii|[6847]|RDLQ732-07|DH004488|632[0n]bp|Canada.Quebec|BOLD:AAE1522
Cerastis tenebrifera|[6848]|TMNBB392-06|MNBTT-1332|658[1n]bp|Canada.New Brunswick|BOLD:AAC1487
Cerastis tenebrifera|[6849]|RDLQ734-07|DH009331|658[0n]bp|Canada.Quebec|BOLD:AAC1487
Cerastis tenebrifera|[6850]|TMNBB394-06|MNBTT-1334|658[0n]bp|Canada.New Brunswick|BOLD:AAC1487
Cerastis tenebrifera|[6851]|RDLQ733-07|DH009677|646[0n]bp|Canada.Quebec|BOLD:AAC1487
Cerastis tenebrifera|[6852]|XAJ210-06|2006-ONT-0210|658[0n]bp|Canada.Ontario|BOLD:AAC1487
Cerastis tenebrifera|[6853]|TTMNB485-06|MNBTT-485|658[0n]bp|Canada.New Brunswick|BOLD:AAC1487
Anaplectoides prasina|[6854]|LBCS367-07|UBC-2007-0880|658[0n]bp|Canada.British Columbia|BOLD:AAA2948
Anaplectoides prasina|[6855]|BBLPB661-10|10BBCLP-1660|658[0n]bp|Canada.Saskatchewan|BOLD:AAA2948
Anaplectoides prasina|[6856]|LPABC023-09|08BBLEP-04242|658[0n]bp|Canada.Alberta|BOLD:AAA2948
Anaplectoides prasina|[6857]|PHMNB740-05|Moth 433.03SA|658[0n]bp|Canada.New Brunswick|BOLD:AAA2948
Anaplectoides prasina|[6858]|BBLPC620-09|09BBELE-1620|658[0n]bp|Canada.Nova Scotia|BOLD:AAA2948
Anaplectoides prasina|[6859]|MNBB103-05|05-NBSTA-019|658[0n]bp|Canada.New Brunswick|BOLD:AAA2948
Anaplectoides prasina|[6860]|MNBB514-05|05-NBSTA-430|658[0n]bp|Canada.New Brunswick|BOLD:AAA2948
Anaplectoides prasina|[6861]|MNBB102-05|05-NBSTA-018|658[0n]bp|Canada.New Brunswick|BOLD:AAA2948
Anaplectoides prasina|[6862]|BBLPC099-09|09BBELE-1099|658[0n]bp|Canada.New Brunswick|BOLD:AAA2948
Anaplectoides prasina|[6863]|TTMNB487-06|MNBTT-487|658[0n]bp|Canada.New Brunswick|BOLD:AAA2948
Anaplectoides prasina|[6864]|MNBB101-05|05-NBSTA-017|658[0n]bp|Canada.New Brunswick|BOLD:AAA2948
Anaplectoides prasina|[6865]|MNBB105-05|05-NBSTA-021|658[0n]bp|Canada.New Brunswick|BOLD:AAA2948
Anaplectoides prasina|[6866]|BBLPC598-09|09BBELE-1598|658[0n]bp|Canada.Nova Scotia|BOLD:AAA2948
Anaplectoides prasina|[6867]|TTMNB486-06|MNBTT-486|658[0n]bp|Canada.New Brunswick|BOLD:AAA2948
Anaplectoides prasina|[6868]|XAC482-04|04HBL006482|658[0n]bp|Canada.Ontario|BOLD:AAA2948
Anaplectoides prasina|[6869]|MNBB438-05|05-NBSTA-354|658[0n]bp|Canada.New Brunswick|BOLD:AAA2948
Anaplectoides prasina|[6870]|MNBB437-05|05-NBSTA-353|658[0n]bp|Canada.New Brunswick|BOLD:AAA2948
Anaplectoides prasina|[6871]|MNBB651-05|05-NBSTA-567|658[0n]bp|Canada.New Brunswick|BOLD:AAA2948
Anaplectoides prasina|[6872]|MNBB096-05|05-NBSTA-012|658[0n]bp|Canada.New Brunswick|BOLD:AAA2948
Anaplectoides prasina|[6873]|LPMNB419-09|08BBLEP-05419|658[0n]bp|Canada.Manitoba|BOLD:AAA2948
Anaplectoides prasina|[6874]|RDNMC001-05|CNCNoctuoidea10742|658[0n]bp|Canada.Ontario|BOLD:AAA2948
Anaplectoides prasina|[6875]|MNBB174-05|05-NBSTA-090|658[0n]bp|Canada.New Brunswick|BOLD:AAA2948
Anaplectoides prasina|[6876]|MNBB100-05|05-NBSTA-016|658[0n]bp|Canada.New Brunswick|BOLD:AAA2948
Anaplectoides prasina|[6877]|MNBB612-05|05-NBSTA-528|658[0n]bp|Canada.New Brunswick|BOLD:AAA2948
[illegible]

Anaplectoides prasina|[6875]|MNBB174-05|05-NBSTA-090|658[0n]bp|Canada.New Brunswick|BOLD:AAA2948
Anaplectoides prasina|[6876]|MNBB100-05|05-NBSTA-016|658[0n]bp|Canada.New Brunswick|BOLD:AAA2948
Anaplectoides prasina|[6877]|MNBB612-05|05-NBSTA-528|658[0n]bp|Canada.New Brunswick|BOLD:AAA2948
Anaplectoides prasina|[6878]|MNBB359-05|05-NBSTA-275|658[0n]bp|Canada.New Brunswick|BOLD:AAA2948
Anaplectoides prasina|[6879]|MNBB242-05|05-NBSTA-158|658[0n]bp|Canada.New Brunswick|BOLD:AAA2948
Anaplectoides prasina|[6880]|RDLQG373-06|DH012607|658[0n]bp|Canada.Quebec|BOLD:AAA2948
Anaplectoides prasina|[6881]|TMNBB397-06|MNBTT-1337|658[0n]bp|Canada.New Brunswick|BOLD:AAA2948
Anaplectoides prasina|[6882]|RDLQG031-06|DH012162|658[0n]bp|Canada.Quebec|BOLD:AAA2948
Anaplectoides prasina|[6883]|MNBB099-05|05-NBSTA-015|658[0n]bp|Canada.New Brunswick|BOLD:AAA2948
Anaplectoides prasina|[6884]|BBLEC422-09|09BBELE-0422|658[0n]bp|Canada.New Brunswick|BOLD:AAA2948
Anaplectoides prasina|[6885]|XAI053-05|0102-ONT-0053|658[0n]bp|Canada.Ontario|BOLD:AAA2948
Anaplectoides prasina|[6886]|BBLEC790-09|09BBELE-0790|658[0n]bp|Canada.Newfoundland and Labrador|BOLD ...
Anaplectoides prasina|[6887]|MNBB481-05|05-NBSTA-397|658[0n]bp|Canada.New Brunswick|BOLD:AAA2948
Anaplectoides prasina|[6888]|MNBB118-05|05-NBSTA-034|658[0n]bp|Canada.New Brunswick|BOLD:AAA2948
Anaplectoides prasina|[6889]|MNBB098-05|05-NBSTA-014|658[0n]bp|Canada.New Brunswick|BOLD:AAA2948
Anaplectoides prasina|[6890]|TMNBB410-06|MNBTT-1350|658[0n]bp|Canada.New Brunswick|BOLD:AAA2948
Anaplectoides prasina|[6891]|PHMNB102-04|04HBL007567|658[0n]bp|Canada.New Brunswick|BOLD:AAA2948
Anaplectoides prasina|[6892]|BBLPC113-09|09BBELE-1113|658[0n]bp|Canada.New Brunswick|BOLD:AAA2948
Anaplectoides prasina|[6893]|MNBB350-05|05-NBSTA-266|658[0n]bp|Canada.New Brunswick|BOLD:AAA2948
Anaplectoides prasina|[6894]|BBLEC272-09|09BBELE-0272|658[0n]bp|Canada.Nova Scotia|BOLD:AAA2948
Anaplectoides prasina|[6895]|MNBB173-05|05-NBSTA-089|658[0n]bp|Canada.New Brunswick|BOLD:AAA2948
Anaplectoides prasina|[6896]|XAC611-04|04HBL006611|658[0n]bp|Canada.Ontario|BOLD:AAA2948
Anaplectoides prasina|[6897]|MNBB349-05|05-NBSTA-265|658[0n]bp|Canada.New Brunswick|BOLD:AAA2948
Anaplectoides prasina|[6898]|MNBB323-05|05-NBSTA-239|658[0n]bp|Canada.New Brunswick|BOLD:AAA2948
Anaplectoides prasina|[6899]|MNBB351-05|05-NBSTA-267|658[0n]bp|Canada.New Brunswick|BOLD:AAA2948
Anaplectoides prasina|[6900]|MNBB175-05|05-NBSTA-091|658[0n]bp|Canada.New Brunswick|BOLD:AAA2948
Anaplectoides prasina|[6901]|MNBB097-05|05-NBSTA-013|658[0n]bp|Canada.New Brunswick|BOLD:AAA2948
Anaplectoides prasina|[6902]|MNBB176-05|05-NBSTA-092|658[0n]bp|Canada.New Brunswick|BOLD:AAA2948
Anaplectoides prasina|[6903]|BBLPC026-09|09BBELE-1026|656[0n]bp|Canada.New Brunswick|BOLD:AAA2948
Anaplectoides prasina|[6904]|PMG090-03|moth995.01|617[0n]bp|Canada.Ontario|BOLD:AAA2948
Anaplectoides prasina|[6905]|PHMNB065-03|moth52.02SA|639[0n]bp|Canada.New Brunswick|BOLD:AAA2948
Anaplectoides prasina|[6906]|LOWCD583-06|CGWC-3403|586[0n]bp|Canada.British Columbia|BOLD:AAA2948
Anaplectoides prasina|[6907]|LOWCD589-06|CGWC-3409|600[0n]bp|Canada.British Columbia|BOLD:AAA2948
Anaplectoides prasina|[6908]|LPVIB632-08|PFC-2006-2080|634[0n]bp|Canada.British Columbia|BOLD:AAA2948
Anaplectoides prasina|[6909]|MNBB244-05|05-NBSTA-160|575[0n]bp|Canada.New Brunswick|BOLD:AAA2948
Anaplectoides prasina|[6910]|XAC568-04|04HBL006568|596[0n]bp|Canada.Ontario|BOLD:AAA2948
Anaplectoides prasina|[6911]|MNBB095-05|05-NBSTA-011|554[0n]bp|Canada.New Brunswick|BOLD:AAA2948
Anaplectoides prasina|[6912]|MNBB287-05|05-NBSTA-203|591[0n]bp|Canada.New Brunswick|BOLD:AAA2948
Anaplectoides prasina|[6913]|LOWCD590-06|CGWC-3410|575[0n]bp|Canada.British Columbia|BOLD:AAA2948
Anaplectoides prasina|[6914]|MNBB215-05|05-NBSTA-131|540[0n]bp|Canada.New Brunswick|BOLD:AAA2948
Anaplectoides prasina|[6915]|MNBB104-05|05-NBSTA-020|658[0n]bp|Canada.New Brunswick|BOLD:AAA2948
Anaplectoides prasina|[6916]|RDNMC002-05|CNCNoctuoidea10743|658[0n]bp|Canada.British Columbia|BOLD:AA...
Anaplectoides prasina|[6917]|LBCH4678-10|10-JDWBC-4678|658[0n]bp|Canada.British Columbia|BOLD:AAA2948
Anaplectoides prasina|[6918]|LOWC853-05|CGWC-0853|658[0n]bp|Canada.British Columbia|BOLD:AAA2948
Anaplectoides prasina|[6919]|BBLPB662-10|10BBCLP-1661|658[0n]bp|Canada.Alberta|BOLD:AAA2948
Anaplectoides prasina|[6920]|LOWCD585-06|CGWC-3405|586[0n]bp|Canada.British Columbia|BOLD:AAA2948
Anaplectoides prasina|[6921]|LBCH3143-10|10-JDWBC-3143|658[0n]bp|Canada.British Columbia|BOLD:AAA2948
Anaplectoides prasina|[6922]|LBCB888-05|HLC-21828|640[1n]bp|Canada.British Columbia|BOLD:AAA2948
Anaplectoides prasina|[6923]|LPABC278-09|08BBLEP-04497|545[0n]bp|Canada.Alberta|BOLD:AAA2948
Anaplectoides prasina|[6924]|LBCG007-08|08-JDWBC-0007|658[0n]bp|Canada.British Columbia|BOLD:AAA2948
Anaplectoides prasina|[6925]|LBCC751-05|HLC-22631|658[1n]bp|Canada.British Columbia|BOLD:AAA2948
Anaplectoides prasina|[6926]|LBCS654-07|UBC-2007-0357|658[0n]bp|Canada.British Columbia|BOLD:AAA2948
Anaplectoides prasina|[6927]|LBCH490-10|10-JDWBC-0490|658[0n]bp|Canada.British Columbia|BOLD:AAA2948
Anaplectoides prasina|[6928]|LHLEP404-06|UBC-2006-2081|658[0n]bp|Canada.British Columbia|BOLD:AAA2948
Anaplectoides prasina|[6929]|LBCB614-05|HLC-21554|658[0n]bp|Canada.British Columbia|BOLD:AAA2948
Anaplectoides prasina|[6930]|LHLEP403-06|UBC-2006-1253|658[0n]bp|Canada.British Columbia|BOLD:AAA2948
Anaplectoides prasina|[6931]|LPGVA599-08|UBC-2006-1757|658[0n]bp|Canada.British Columbia|BOLD:AAA2948
Anaplectoides prasina|[6932]|LBCH4421-10|10-JDWBC-4421|658[0n]bp|Canada.British Columbia|BOLD:AAA2948
Anaplectoides prasina|[6933]|LBCH4677-10|10-JDWBC-4677|658[0n]bp|Canada.British Columbia|BOLD:AAA2948
Anaplectoides prasina|[6934]|LOWC852-05|CGWC-0852|658[0n]bp|Canada.British Columbia|BOLD:AAA2948
Anaplectoides prasina|[6935]|LBCH933-10|10-JDWBC-0933|658[0n]bp|Canada.British Columbia|BOLD:AAA2948
Anaplectoides prasina|[6936]|LBCH2242-10|10-JDWBC-2242|658[0n]bp|Canada.British Columbia|BOLD:AAA2948
Anaplectoides prasina|[6937]|LALPA804-10|AVBC 806-10|658[0n]bp|Canada.British Columbia|BOLD:AAA2948
Anaplectoides prasina|[6938]|LBCG2315-09|08-JDWBC-2315|658[0n]bp|Canada.British Columbia|BOLD:AAA2948
Anaplectoides prasina|[6939]|LHLEP402-06|UBC-2006-1042|658[0n]bp|Canada.British Columbia|BOLD:AAA2948
Anaplectoides prasina|[6940]|LOWC855-05|CGWC-0855|658[0n]bp|Canada.British Columbia|BOLD:AAA2948
Anaplectoides prasina|[6941]|LBCH4415-10|10-JDWBC-4415|658[0n]bp|Canada.British Columbia|BOLD:AAA2948
Anaplectoides prasina|[6942]|LBCH4420-10|10-JDWBC-4420|658[0n]bp|Canada.British Columbia|BOLD:AAA2948
Anaplectoides prasina|[6943]|LBCD094-05|HLC-22914|658[0n]bp|Canada.British Columbia|BOLD:AAA2948
Anaplectoides prasina|[6944]|LOWC848-05|CGWC-0848|658[0n]bp|Canada.British Columbia|BOLD:AAA2948
Anaplectoides prasina|[6945]|LBCD093-05|HLC-22913|658[0n]bp|Canada.British Columbia|BOLD:AAA2948
Anaplectoides prasina|[6946]|LBCH3328-10|10-JDWBC-3328|658[0n]bp|Canada.British Columbia|BOLD:AAA2948
Anaplectoides prasina|[6947]|LBCD092-05|HLC-22912|658[0n]bp|Canada.British Columbia|BOLD:AAA2948
Anaplectoides prasina|[6948]|LALPA801-10|AVBC 803-10|658[0n]bp|Canada.British Columbia|BOLD:AAA2948
Anaplectoides prasina|[6949]|LBCC326-05|HLC-22206|658[0n]bp|Canada.British Columbia|BOLD:AAA2948
Anaplectoides prasina|[6950]|LOWCD584-06|CGWC-3404|658[0n]bp|Canada.British Columbia|BOLD:AAA2948
Anaplectoides prasina|[6951]|LBCH4418-10|10-JDWBC-4418|658[0n]bp|Canada.British Columbia|BOLD:AAA2948
Anaplectoides prasina|[6952]|LBCH345-10|10-JDWBC-0345|658[0n]bp|Canada.British Columbia|BOLD:AAA2948
Anaplectoides prasina|[6953]|LBCH4419-10|10-JDWBC-4419|658[0n]bp|Canada.British Columbia|BOLD:AAA2948
Anaplectoides prasina|[6954]|LBCH4674-10|10-JDWBC-4674|658[0n]bp|Canada.British Columbia|BOLD:AAA2948
Anaplectoides prasina|[6955]|LBCH3144-10|10-JDWBC-3144|658[0n]bp|Canada.British Columbia|BOLD:AAA2948
Anaplectoides prasina|[6956]|LBCG818-09|08-JDWBC-0818|658[0n]bp|Canada.British Columbia|BOLD:AAA2948
Anaplectoides prasina|[6957]|LBCH4671-10|10-JDWBC-4671|658[0n]bp|Canada.British Columbia|BOLD:AAA2948
Anaplectoides prasina|[6958]|LBCH013-10|10-JDWBC-0013|658[0n]bp|Canada.British Columbia|BOLD:AAA2948
Anaplectoides prasina|[6959]|LBCH224-10|10-JDWBC-0224|658[0n]bp|Canada.British Columbia|BOLD:AAA2948
Anaplectoides prasina|[6960]|LBCH1563-10|10-JDWBC-1563|658[0n]bp|Canada.British Columbia|BOLD:AAA2948
Anaplectoides prasina|[6961]|LBCH1426-10|10-JDWBC-1426|658[0n]bp|Canada.British Columbia|BOLD:AAA2948
Anaplectoides prasina|[6962]|LALPA803-10|AVBC 805-10|658[0n]bp|Canada.British Columbia|BOLD:AAA2948
Anaplectoides prasina|[6963]|LBCC752-05|HLC-22632|658[0n]bp|Canada.British Columbia|BOLD:AAA2948
Anaplectoides prasina|[6964]|LBCH4675-10|10-JDWBC-4675|658[0n]bp|Canada.British Columbia|BOLD:AAA2948
Anaplectoides prasina|[6965]|LOWC854-05|CGWC-0854|658[0n]bp|Canada.British Columbia|BOLD:AAA2948
Anaplectoides prasina|[6966]|LBCH934-10|10-JDWBC-0934|658[0n]bp|Canada.British Columbia|BOLD:AAA2948
Anaplectoides prasina|[6967]|LBCH803-10|10-JDWBC-0803|658[0n]bp|Canada.British Columbia|BOLD:AAA2948
Anaplectoides prasina|[6968]|LOWCD588-06|CGWC-3408|658[0n]bp|Canada.British Columbia|BOLD:AAA2948
Anaplectoides prasina|[6969]|LBCH3766-10|10-JDWBC-3766|658[0n]bp|Canada.British Columbia|BOLD:AAA2948
Anaplectoides prasina|[6970]|LALPA764-10|AVBC 766-10|658[0n]bp|Canada.British Columbia|BOLD:AAA2948
Anaplectoides prasina|[6971]|LBCH110-10|10-JDWBC-0110|658[0n]bp|Canada.British Columbia|BOLD:AAA2948
Anaplectoides prasina|[6972]|LBCB613-05|HLC-21553|658[0n]bp|Canada.British Columbia|BOLD:AAA2948
Anaplectoides prasina|[6973]|LBCH3333-10|10-JDWBC-3333|658[0n]bp|Canada.British Columbia|BOLD:AAA2948
Anaplectoides prasina|[6974]|LOWCD586-06|CGWC-3406|658[0n]bp|Canada.British Columbia|BOLD:AAA2948
Anaplectoides prasina|[6975]|LBCH4672-10|10-JDWBC-4672|658[0n]bp|Canada.British Columbia|BOLD:AAA2948
Anaplectoides prasina|[6976]|LBCH4111-10|10-JDWBC-4111|658[0n]bp|Canada.British Columbia|BOLD:AAA2948
Anaplectoides prasina|[6977]|LBCH4422-10|10-JDWBC-4422|658[0n]bp|Canada.British Columbia|BOLD:AAA2948

Anaplectoides prasina|[6975]|LBCH4672-10|10-JDWBC-4672|658[0n]bp|Canada.British Columbia|BOLD:AAA2948
Anaplectoides prasina|[6976]|LBCH4111-10|10-JDWBC-4111|658[0n]bp|Canada.British Columbia|BOLD:AAA2948
Anaplectoides prasina|[6977]|LBCH4422-10|10-JDWBC-4422|658[0n]bp|Canada.British Columbia|BOLD:AAA2948
Anaplectoides prasina|[6978]|LPABC339-09|08BBLEP-04558|658[0n]bp|Canada.Alberta|BOLD:AAA2948
Anaplectoides prasina|[6979]|LBCH6524-10|10-JDWBC-6524|658[0n]bp|Canada.British Columbia|BOLD:AAA2948
Anaplectoides prasina|[6980]|LBCW054-08|08-JDWWI-0054|658[0n]bp|Canada.British Columbia|BOLD:AAA2948
Anaplectoides prasina|[6981]|LOWCD587-06|CGWC-3407|658[0n]bp|Canada.British Columbia|BOLD:AAA2948
Anaplectoides prasina|[6982]|LOWC847-05|CGWC-0847|658[0n]bp|Canada.British Columbia|BOLD:AAA2948
Anaplectoides prasina|[6983]|LOWC849-05|CGWC-0849|658[0n]bp|Canada.British Columbia|BOLD:AAA2948
Anaplectoides prasina|[6984]|LBCH4416-10|10-JDWBC-4416|658[0n]bp|Canada.British Columbia|BOLD:AAA2948
Anaplectoides prasina|[6985]|LBCH4417-10|10-JDWBC-4417|658[0n]bp|Canada.British Columbia|BOLD:AAA2948
Anaplectoides prasina|[6986]|LBCH4676-10|10-JDWBC-4676|658[0n]bp|Canada.British Columbia|BOLD:AAA2948
Anaplectoides prasina|[6987]|LBCC014-05|HLC-21894|658[0n]bp|Canada.British Columbia|BOLD:AAA2948
Anaplectoides prasina|[6988]|LOWCD591-06|CGWC-3411|658[0n]bp|Canada.British Columbia|BOLD:AAA2948
Anaplectoides prasina|[6989]|LBCH4673-10|10-JDWBC-4673|658[0n]bp|Canada.British Columbia|BOLD:AAA2948
Anaplectoides prasina|[6990]|LOWCD592-06|CGWC-3412|658[0n]bp|Canada.British Columbia|BOLD:AAA2948
Anaplectoides prasina|[6991]|LBCC829-05|HLC-22709|658[0n]bp|Canada.British Columbia|BOLD:AAA2948
Anaplectoides prasina|[6992]|LALPA720-10|AVBC 722-10|658[0n]bp|Canada.British Columbia|BOLD:AAA2948
Anaplectoides prasina|[6993]|LOWC851-05|CGWC-0851|658[0n]bp|Canada.British Columbia|BOLD:AAA2948
Anaplectoides prasina|[6994]|LOWC850-05|CGWC-0850|658[0n]bp|Canada.British Columbia|BOLD:AAA2948
Anaplectoides prasina|[6995]|LBCD330-05|HLC-23150|650[0n]bp|Canada.British Columbia|BOLD:AAA2948
Anaplectoides prasina|[6996]|LBCH3465-10|10-JDWBC-3465|658[0n]bp|Canada.British Columbia|BOLD:AAA2948
Anaplectoides pressus|[6997]|LBCH025-10|10-JDWBC-0025|658[0n]bp|Canada.British Columbia|BOLD:AAA3756
Anaplectoides pressus|[6998]|LPABC489-09|08BBLEP-04708|658[0n]bp|Canada.Alberta|BOLD:AAA3756
Anaplectoides pressus|[6999]|LPABC452-09|08BBLEP-04671|658[0n]bp|Canada.Alberta|BOLD:AAA3756
Anaplectoides pressus|[7000]|BBLPE363-09|09BBELE-2363|658[0n]bp|Canada.Newfoundland and Labrador|BOLD ...
Anaplectoides pressus|[7001]|LPABC459-09|08BBLEP-04678|658[0n]bp|Canada.Alberta|BOLD:AAA3756
Anaplectoides pressus|[7002]|LPABC271-09|08BBLEP-04490|641[0n]bp|Canada.Alberta|BOLD:AAA3756
Anaplectoides pressus|[7003]|RDLQB291-05|DH010377|658[0n]bp|Canada.Quebec|BOLD:AAA3756
Anaplectoides pressus|[7004]|LOWC894-05|CGWC-0894|658[0n]bp|Canada.British Columbia|BOLD:AAA3756
Anaplectoides pressus|[7005]|LBCH237-10|10-JDWBC-0237|658[0n]bp|Canada.British Columbia|BOLD:AAA3756
Anaplectoides pressus|[7006]|LBCH4114-10|10-JDWBC-4114|658[0n]bp|Canada.British Columbia|BOLD:AAA3756
Anaplectoides pressus|[7007]|LBCH4435-10|10-JDWBC-4435|658[0n]bp|Canada.British Columbia|BOLD:AAA3756
Anaplectoides pressus|[7008]|BBLPC645-09|09BBELE-1645|658[0n]bp|Canada.Newfoundland and Labrador|BOLD ...
Anaplectoides pressus|[7009]|LPABC441-09|08BBLEP-04660|658[0n]bp|Canada.Alberta|BOLD:AAA3756
Anaplectoides pressus|[7010]|LOWC862-05|CGWC-0862|658[0n]bp|Canada.British Columbia|BOLD:AAA3756
Anaplectoides pressus|[7011]|LBCH022-10|10-JDWBC-0022|658[0n]bp|Canada.British Columbia|BOLD:AAA3756
Anaplectoides pressus|[7012]|LBCH4693-10|10-JDWBC-4693|658[0n]bp|Canada.British Columbia|BOLD:AAA3756
Anaplectoides pressus|[7013]|TTMNB488-06|MNBTT-488|658[0n]bp|Canada.New Brunswick|BOLD:AAA3756
Anaplectoides pressus|[7014]|LPABC478-09|08BBLEP-04697|658[0n]bp|Canada.Alberta|BOLD:AAA3756
Anaplectoides pressus|[7015]|BBLPB856-10|10BBCLP-1855|658[0n]bp|Canada.British Columbia|BOLD:AAA3756
Anaplectoides pressus|[7016]|LALPA874-11|AVBC 1047-11|658[0n]bp|Canada.British Columbia|BOLD:AAA3756
Anaplectoides pressus|[7017]|LBCH055-10|10-JDWBC-0055|658[0n]bp|Canada.British Columbia|BOLD:AAA3756
Anaplectoides pressus|[7018]|BBLPC244-09|09BBELE-1244|658[0n]bp|Canada.Nova Scotia|BOLD:AAA3756
Anaplectoides pressus|[7019]|LOWC893-05|CGWC-0893|658[0n]bp|Canada.British Columbia|BOLD:AAA3756
Anaplectoides pressus|[7020]|LALPA1256-11|AVBC 1258-11|658[0n]bp|Canada.British Columbia|BOLD:AAA3756
Anaplectoides pressus|[7021]|LBCH812-10|10-JDWBC-0812|658[0n]bp|Canada.British Columbia|BOLD:AAA3756
Anaplectoides pressus|[7022]|LOWCD579-06|CGWC-3399|658[0n]bp|Canada.British Columbia|BOLD:AAA3756
Anaplectoides pressus|[7023]|BBLPC897-09|09BBELE-1897|658[0n]bp|Canada.Newfoundland and Labrador|BOLD ...
Anaplectoides pressus|[7024]|LPABC462-09|08BBLEP-04681|658[0n]bp|Canada.Alberta|BOLD:AAA3756
Anaplectoides pressus|[7025]|LBCH3467-10|10-JDWBC-3467|658[0n]bp|Canada.British Columbia|BOLD:AAA3756
Anaplectoides pressus|[7026]|LOWCE786-06|CGWC-4546|658[0n]bp|Canada.British Columbia|BOLD:AAA3756
Anaplectoides pressus|[7027]|LOWCD576-06|CGWC-3396|658[0n]bp|Canada.British Columbia|BOLD:AAA3756
Anaplectoides pressus|[7028]|LPABC461-09|08BBLEP-04680|658[0n]bp|Canada.Alberta|BOLD:AAA3756
Anaplectoides pressus|[7029]|LPABC372-09|08BBLEP-04591|658[0n]bp|Canada.Alberta|BOLD:AAA3756
Anaplectoides pressus|[7030]|LBCH3093-10|10-JDWBC-3093|658[0n]bp|Canada.British Columbia|BOLD:AAA3756
Anaplectoides pressus|[7031]|RDLQB293-05|DH010379|658[0n]bp|Canada.Quebec|BOLD:AAA3756
Anaplectoides pressus|[7032]|LPAB244-08|08BBLEP-02566|658[0n]bp|Canada.Alberta|BOLD:AAA3756
Anaplectoides pressus|[7033]|LBCH4115-10|10-JDWBC-4115|658[0n]bp|Canada.British Columbia|BOLD:AAA3756
Anaplectoides pressus|[7034]|LBCH697-10|10-JDWBC-0697|658[0n]bp|Canada.British Columbia|BOLD:AAA3756
Anaplectoides pressus|[7035]|LOWCD581-06|CGWC-3401|658[0n]bp|Canada.British Columbia|BOLD:AAA3756
Anaplectoides pressus|[7036]|LBCH3090-10|10-JDWBC-3090|658[0n]bp|Canada.British Columbia|BOLD:AAA3756
Anaplectoides pressus|[7037]|LOWCD577-06|CGWC-3397|658[0n]bp|Canada.British Columbia|BOLD:AAA3756
Anaplectoides pressus|[7038]|LPABC391-09|08BBLEP-04610|658[0n]bp|Canada.Alberta|BOLD:AAA3756
Anaplectoides pressus|[7039]|LPABC465-09|08BBLEP-04684|658[0n]bp|Canada.Alberta|BOLD:AAA3756
Anaplectoides pressus|[7040]|LBCH235-10|10-JDWBC-0235|658[0n]bp|Canada.British Columbia|BOLD:AAA3756
Anaplectoides pressus|[7041]|BBLPB869-10|10BBCLP-1868|658[0n]bp|Canada.British Columbia|BOLD:AAA3756
Anaplectoides pressus|[7042]|LBCH236-10|10-JDWBC-0236|658[0n]bp|Canada.British Columbia|BOLD:AAA3756
Anaplectoides pressus|[7043]|TTMNB489-06|MNBTT-489|658[0n]bp|Canada.New Brunswick|BOLD:AAA3756
Anaplectoides pressus|[7044]|LPABC332-09|08BBLEP-04551|658[0n]bp|Canada.Alberta|BOLD:AAA3756
Anaplectoides pressus|[7045]|LBCH967-10|10-JDWBC-0967|658[0n]bp|Canada.British Columbia|BOLD:AAA3756
Anaplectoides pressus|[7046]|LOWCD580-06|CGWC-3400|658[0n]bp|Canada.British Columbia|BOLD:AAA3756
Anaplectoides pressus|[7047]|LBCH3091-10|10-JDWBC-3091|658[0n]bp|Canada.British Columbia|BOLD:AAA3756
Anaplectoides pressus|[7048]|LBCH495-10|10-JDWBC-0495|658[0n]bp|Canada.British Columbia|BOLD:AAA3756
Anaplectoides pressus|[7049]|LPABC450-09|08BBLEP-04669|658[0n]bp|Canada.Alberta|BOLD:AAA3756
Anaplectoides pressus|[7050]|LOWCD582-06|CGWC-3402|658[0n]bp|Canada.British Columbia|BOLD:AAA3756
Anaplectoides pressus|[7051]|LPABC421-09|08BBLEP-04640|658[0n]bp|Canada.Alberta|BOLD:AAA3756
Anaplectoides pressus|[7052]|LBCG845-09|08-JDWBC-0845|658[0n]bp|Canada.British Columbia|BOLD:AAA3756
Anaplectoides pressus|[7053]|LPABC371-09|08BBLEP-04590|658[0n]bp|Canada.Alberta|BOLD:AAA3756
Anaplectoides pressus|[7054]|BBLPB704-10|10BBCLP-1703|658[0n]bp|Canada.British Columbia|BOLD:AAA3756
Anaplectoides pressus|[7055]|LBCH026-10|10-JDWBC-0026|658[0n]bp|Canada.British Columbia|BOLD:AAA3756
Anaplectoides pressus|[7056]|LBCH021-10|10-JDWBC-0021|658[0n]bp|Canada.British Columbia|BOLD:AAA3756
Anaplectoides pressus|[7057]|RDLQB290-05|DH010376|658[0n]bp|Canada.Quebec|BOLD:AAA3756
Anaplectoides pressus|[7058]|RDNM977-05|CNCNoctuoidea7817|658[0n]bp|Canada.Ontario|BOLD:AAA3756
Anaplectoides pressus|[7059]|LPABC451-09|08BBLEP-04670|658[0n]bp|Canada.Alberta|BOLD:AAA3756
Anaplectoides pressus|[7060]|LPABC424-09|08BBLEP-04643|658[0n]bp|Canada.Alberta|BOLD:AAA3756
Anaplectoides pressus|[7061]|LPABC393-09|08BBLEP-04612|658[0n]bp|Canada.Alberta|BOLD:AAA3756
Anaplectoides pressus|[7062]|LOWC918-05|CGWC-0918|658[0n]bp|Canada.British Columbia|BOLD:AAA3756
Anaplectoides pressus|[7063]|LPABC455-09|08BBLEP-04674|658[0n]bp|Canada.Alberta|BOLD:AAA3756
Anaplectoides pressus|[7064]|BBLPC241-09|09BBELE-1241|658[0n]bp|Canada.Nova Scotia|BOLD:AAA3756
Anaplectoides pressus|[7065]|LBCH4432-10|10-JDWBC-4432|658[0n]bp|Canada.British Columbia|BOLD:AAA3756
Anaplectoides pressus|[7066]|LBCH027-10|10-JDWBC-0027|658[0n]bp|Canada.British Columbia|BOLD:AAA3756
Anaplectoides pressus|[7067]|LBCH813-10|10-JDWBC-0813|658[0n]bp|Canada.British Columbia|BOLD:AAA3756
Anaplectoides pressus|[7068]|RDLQB292-05|DH010378|658[0n]bp|Canada.Quebec|BOLD:AAA3756
Anaplectoides pressus|[7069]|LOWCE785-06|CGWC-4545|658[0n]bp|Canada.British Columbia|BOLD:AAA3756
Anaplectoides pressus|[7070]|LPABC377-09|08BBLEP-04596|658[0n]bp|Canada.Alberta|BOLD:AAA3756
Anaplectoides pressus|[7071]|LPABC417-09|08BBLEP-04636|658[0n]bp|Canada.Alberta|BOLD:AAA3756
Anaplectoides pressus|[7072]|LOWC856-05|CGWC-0856|658[0n]bp|Canada.British Columbia|BOLD:AAA3756
Anaplectoides pressus|[7073]|LPABC350-09|08BBLEP-04569|658[0n]bp|Canada.Alberta|BOLD:AAA3756
Anaplectoides pressus|[7074]|BBLPC155-09|09BBELE-1155|658[0n]bp|Canada.Nova Scotia|BOLD:AAA3756
Anaplectoides pressus|[7075]|BBLPB408-10|10BBCLP-1407|658[0n]bp|Canada.Saskatchewan|BOLD:AAA3756
Anaplectoides pressus|[7076]|LOWCD575-06|CGWC-3395|658[0n]bp|Canada.British Columbia|BOLD:AAA3756
Anaplectoides pressus|[7077]|LOWC860-05|CGWC-0860|658[0n]bp|Canada.British Columbia|BOLD:AAA3756

Anaplectoides pressus|[7074]|BBLPC153-09|09BBELE-1153|658[0n]bp|Canada.Nova Scotia|BOLD:AAA3756
Anaplectoides pressus|[7075]|BBLPB408-10|10BBCLP-1407|658[0n]bp|Canada.Saskatchewan|BOLD:AAA3756
Anaplectoides pressus|[7076]|LOWCD575-06|CGWC-3395|658[0n]bp|Canada.British Columbia|BOLD:AAA3756
Anaplectoides pressus|[7077]|LOWC860-05|CGWC-0860|658[0n]bp|Canada.British Columbia|BOLD:AAA3756
Anaplectoides pressus|[7078]|LPABC470-09|08BBLEP-04689|658[0n]bp|Canada.Alberta|BOLD:AAA3756
Anaplectoides pressus|[7079]|LOWC892-05|CGWC-0892|658[0n]bp|Canada.British Columbia|BOLD:AAA3756
Anaplectoides pressus|[7080]|LBCH352-10|10-JDWBC-0352|658[0n]bp|Canada.British Columbia|BOLD:AAA3756
Anaplectoides pressus|[7081]|LOWC859-05|CGWC-0859|658[0n]bp|Canada.British Columbia|BOLD:AAA3756
Anaplectoides pressus|[7082]|RDNM979-05|CNCNoctuoidea7819|658[0n]bp|Canada.British Columbia|BOLD:AAA3756
Anaplectoides pressus|[7083]|LBCH4692-10|10-JDWBC-4692|658[0n]bp|Canada.British Columbia|BOLD:AAA3756
Anaplectoides pressus|[7084]|LBCH028-10|10-JDWBC-0028|658[0n]bp|Canada.British Columbia|BOLD:AAA3756
Anaplectoides pressus|[7085]|LBCH024-10|10-JDWBC-0024|658[0n]bp|Canada.British Columbia|BOLD:AAA3756
Anaplectoides pressus|[7086]|LBCH2396-10|10-JDWBC-2396|658[0n]bp|Canada.British Columbia|BOLD:AAA3756
Anaplectoides pressus|[7087]|LBCG571-09|08-JDWBC-0571|658[0n]bp|Canada.British Columbia|BOLD:AAA3756
Anaplectoides pressus|[7088]|BBLPC656-09|09BBELE-1656|658[0n]bp|Canada.Newfoundland and Labrador|BOLD ...
Anaplectoides pressus|[7089]|BBLEC855-09|09BBELE-0855|658[0n]bp|Canada.Newfoundland and Labrador|BOLD ...
Anaplectoides pressus|[7090]|LBCG2771-09|08-JDWBC-2771|658[0n]bp|Canada.British Columbia|BOLD:AAA3756
Anaplectoides pressus|[7091]|LOWC857-05|CGWC-0857|608[0n]bp|Canada.British Columbia|BOLD:AAA3756
Anaplectoides pressus|[7092]|LOWC921-05|CGWC-0921|601[0n]bp|Canada.British Columbia|BOLD:AAA3756
Anaplectoides pressus|[7093]|LOWC861-05|CGWC-0861|658[0n]bp|Canada.British Columbia|BOLD:AAA3756
Anaplectoides pressus|[7094]|LOWCD578-06|CGWC-3398|546[0n]bp|Canada.British Columbia|BOLD:AAA3756
Anaplectoides pressus|[7095]|LOWC858-05|CGWC-0858|578[0n]bp|Canada.British Columbia|BOLD:AAA3756
Anaplectoides pressus|[7096]|LPABC488-09|08BBLEP-04707|635[0n]bp|Canada.Alberta|BOLD:AAA3756
Anaplectoides pressus|[7097]|LPABC482-09|08BBLEP-04701|623[0n]bp|Canada.Alberta|BOLD:AAA3756
Anaplectoides pressus|[7098]|BBLPB703-10|10BBCLP-1702|621[0n]bp|Canada.British Columbia|BOLD:AAA3756
Anaplectoides pressus|[7099]|LPABC490-09|08BBLEP-04709|612[0n]bp|Canada.Alberta|BOLD:AAA3756
Anaplectoides pressus|[7100]|LPABC193-09|08BBLEP-04412|619[0n]bp|Canada.Alberta|BOLD:AAA3756
Anaplectoides pressus|[7101]|LBCH023-10|10-JDWBC-0023|658[0n]bp|Canada.British Columbia|BOLD:AAA3756
Anaplectoides pressus|[7102]|LBCH811-10|10-JDWBC-0811|658[0n]bp|Canada.British Columbia|BOLD:AAA3756
Cryptocala acadiensis|[7103]|LPSK240-08|08BBLEP-01808|658[0n]bp|Canada.Saskatchewan|BOLD:AAA4464
Cryptocala acadiensis|[7104]|LPABC275-09|08BBLEP-04494|613[0n]bp|Canada.Alberta|BOLD:AAA4464
Cryptocala acadiensis|[7105]|BBLPB507-10|10BBCLP-1506|658[0n]bp|Canada.Alberta|BOLD:AAA4464
Cryptocala acadiensis|[7106]|PHMNB046-03|moth229.02SA|639[0n]bp|Canada.New Brunswick|BOLD:AAA4464
Cryptocala acadiensis|[7107]|BBLEC099-09|09BBELE-0099|631[0n]bp|Canada.Nova Scotia|BOLD:AAA4464
Cryptocala acadiensis|[7108]|BBLEC019-09|09BBELE-0019|650[0n]bp|Canada.New Brunswick|BOLD:AAA4464
Cryptocala acadiensis|[7109]|LPSK239-08|08BBLEP-01807|658[0n]bp|Canada.Saskatchewan|BOLD:AAA4464
Cryptocala acadiensis|[7110]|BBLPC681-09|09BBELE-1681|658[0n]bp|Canada.Newfoundland and Labrador|BOLD ...
Cryptocala acadiensis|[7111]|BBLPE394-09|09BBELE-2394|658[0n]bp|Canada.Newfoundland and Labrador|BOLD ...
Cryptocala acadiensis|[7112]|BBLPE337-09|09BBELE-2337|658[0n]bp|Canada.Newfoundland and Labrador|BOLD ...
Cryptocala acadiensis|[7113]|BBLPE356-09|09BBELE-2356|658[0n]bp|Canada.Newfoundland and Labrador|BOLD ...
Cryptocala acadiensis|[7114]|LPMNB534-09|08BBLEP-05572|658[0n]bp|Canada.Manitoba|BOLD:AAA4464
Cryptocala acadiensis|[7115]|LPABB216-08|08BBLEP-03481|658[0n]bp|Canada.Alberta|BOLD:AAA4464
Cryptocala acadiensis|[7116]|BBLPB510-10|10BBCLP-1509|658[0n]bp|Canada.British Columbia|BOLD:AAA4464
Cryptocala acadiensis|[7117]|BBLPC114-09|09BBELE-1114|658[0n]bp|Canada.New Brunswick|BOLD:AAA4464
Cryptocala acadiensis|[7118]|MNBB450-05|05-NBSTA-366|658[0n]bp|Canada.New Brunswick|BOLD:AAA4464
Cryptocala acadiensis|[7119]|BBLPC042-09|09BBELE-1042|621[0n]bp|Canada.New Brunswick|BOLD:AAA4464
Cryptocala acadiensis|[7120]|BBLPB504-10|10BBCLP-1503|658[0n]bp|Canada.Alberta|BOLD:AAA4464
Cryptocala acadiensis|[7121]|BBLPB508-10|10BBCLP-1507|658[0n]bp|Canada.British Columbia|BOLD:AAA4464
Cryptocala acadiensis|[7122]|LPABC140-09|08BBLEP-04359|658[0n]bp|Canada.Alberta|BOLD:AAA4464
Cryptocala acadiensis|[7123]|BBLPB401-10|10BBCLP-1400|658[0n]bp|Canada.Alberta|BOLD:AAA4464
Cryptocala acadiensis|[7124]|BBLPB402-10|10BBCLP-1401|658[0n]bp|Canada.Alberta|BOLD:AAA4464
Cryptocala acadiensis|[7125]|LPABC526-09|08BBLEP-04745|629[0n]bp|Canada.Alberta|BOLD:AAA4464
Cryptocala acadiensis|[7126]|LPSK237-08|08BBLEP-01805|658[0n]bp|Canada.Saskatchewan|BOLD:AAA4464
Cryptocala acadiensis|[7127]|LOWC906-05|CGWC-0906|658[0n]bp|Canada.British Columbia|BOLD:AAA4464
Cryptocala acadiensis|[7128]|LPABC517-09|08BBLEP-04736|658[0n]bp|Canada.Alberta|BOLD:AAA4464
Cryptocala acadiensis|[7129]|BBLPE331-09|09BBELE-2331|658[0n]bp|Canada.Newfoundland and Labrador|BOLD ...
Cryptocala acadiensis|[7130]|BBLPB397-10|10BBCLP-1396|658[0n]bp|Canada.British Columbia|BOLD:AAA4464
Cryptocala acadiensis|[7131]|LOWCD693-06|CGWC-3513|658[0n]bp|Canada.British Columbia|BOLD:AAA4464
Cryptocala acadiensis|[7132]|LPABC317-09|08BBLEP-04536|658[0n]bp|Canada.Alberta|BOLD:AAA4464
Cryptocala acadiensis|[7133]|LOWC901-05|CGWC-0901|658[0n]bp|Canada.British Columbia|BOLD:AAA4464
Cryptocala acadiensis|[7134]|LPABB253-08|08BBLEP-03518|658[0n]bp|Canada.Alberta|BOLD:AAA4464
Cryptocala acadiensis|[7135]|LBCH3084-10|10-JDWBC-3084|658[0n]bp|Canada.British Columbia|BOLD:AAA4464
Cryptocala acadiensis|[7136]|LOWCD689-06|CGWC-3509|658[0n]bp|Canada.British Columbia|BOLD:AAA4464
Cryptocala acadiensis|[7137]|LBCH4023-10|10-JDWBC-4023|658[0n]bp|Canada.British Columbia|BOLD:AAA4464
Cryptocala acadiensis|[7138]|LBCH3335-10|10-JDWBC-3335|658[0n]bp|Canada.British Columbia|BOLD:AAA4464
Cryptocala acadiensis|[7139]|LPABC472-09|08BBLEP-04691|658[0n]bp|Canada.Alberta|BOLD:AAA4464
Cryptocala acadiensis|[7140]|LOWC897-05|CGWC-0897|658[0n]bp|Canada.British Columbia|BOLD:AAA4464
Cryptocala acadiensis|[7141]|BBLPC481-09|09BBELE-1481|658[0n]bp|Canada.New Brunswick|BOLD:AAA4464
Cryptocala acadiensis|[7142]|LPABC512-09|08BBLEP-04731|611[0n]bp|Canada.Alberta|BOLD:AAA4464
Cryptocala acadiensis|[7143]|LBCH3493-10|10-JDWBC-3493|658[0n]bp|Canada.British Columbia|BOLD:AAA4464
Cryptocala acadiensis|[7144]|LBCH937-10|10-JDWBC-0937|658[0n]bp|Canada.British Columbia|BOLD:AAA4464
Cryptocala acadiensis|[7145]|LPABB491-08|08BBLEP-03756|658[0n]bp|Canada.Alberta|BOLD:AAA4464
Cryptocala acadiensis|[7146]|BBLPB404-10|10BBCLP-1403|658[0n]bp|Canada.Alberta|BOLD:AAA4464
Cryptocala acadiensis|[7147]|LOWC903-05|CGWC-0903|658[0n]bp|Canada.British Columbia|BOLD:AAA4464
Cryptocala acadiensis|[7148]|LPABC524-09|08BBLEP-04743|658[0n]bp|Canada.Alberta|BOLD:AAA4464
Cryptocala acadiensis|[7149]|LPABC006-09|08BBLEP-04225|658[0n]bp|Canada.Alberta|BOLD:AAA4464
Cryptocala acadiensis|[7150]|LOWCE078-06|CGWC-3838|658[0n]bp|Canada.British Columbia|BOLD:AAA4464
Cryptocala acadiensis|[7151]|GWOSN431-11|BC ZSM Lep 48932|658[0n]bp|Canada.British Columbia|BOLD:AAA4464
Cryptocala acadiensis|[7152]|LOWCD691-06|CGWC-3511|658[0n]bp|Canada.British Columbia|BOLD:AAA4464
Cryptocala acadiensis|[7153]|LOWCD690-06|CGWC-3510|584[0n]bp|Canada.British Columbia|BOLD:AAA4464
Cryptocala acadiensis|[7154]|BBLPC062-09|09BBELE-1062|658[0n]bp|Canada.New Brunswick|BOLD:AAA4464
Cryptocala acadiensis|[7155]|LBCH3783-10|10-JDWBC-3783|658[0n]bp|Canada.British Columbia|BOLD:AAA4464
Cryptocala acadiensis|[7156]|LOWCD692-06|CGWC-3512|584[0n]bp|Canada.British Columbia|BOLD:AAA4464
Cryptocala acadiensis|[7157]|RDLQF695-06|DH011845|609[0n]bp|Canada.Quebec|BOLD:AAA4464
Cryptocala acadiensis|[7158]|LOWC899-05|CGWC-0899|555[1n]bp|Canada.British Columbia|BOLD:AAA4464
Cryptocala acadiensis|[7159]|LPABC511-09|08BBLEP-04730|634[0n]bp|Canada.Alberta|BOLD:AAA4464
Cryptocala acadiensis|[7160]|LPABC471-09|08BBLEP-04690|634[0n]bp|Canada.Alberta|BOLD:AAA4464
Cryptocala acadiensis|[7161]|LPABC247-09|08BBLEP-04466|621[0n]bp|Canada.Alberta|BOLD:AAA4464
Cryptocala acadiensis|[7162]|LPABC257-09|08BBLEP-04476|624[0n]bp|Canada.Alberta|BOLD:AAA4464
Cryptocala acadiensis|[7163]|LPABC324-09|08BBLEP-04543|608[0n]bp|Canada.Alberta|BOLD:AAA4464
Cryptocala acadiensis|[7164]|BBLPB399-10|10BBCLP-1398|658[0n]bp|Canada.Alberta|BOLD:AAA4464
Cryptocala acadiensis|[7165]|MNBB133-05|05-NBSTA-049|658[0n]bp|Canada.New Brunswick|BOLD:AAA4464
Cryptocala acadiensis|[7166]|LOWC909-05|CGWC-0909|658[0n]bp|Canada.British Columbia|BOLD:AAA4464
Cryptocala acadiensis|[7167]|BBLPB509-10|10BBCLP-1508|658[0n]bp|Canada.British Columbia|BOLD:AAA4464
Cryptocala acadiensis|[7168]|BBLPB398-10|10BBCLP-1397|658[0n]bp|Canada.Alberta|BOLD:AAA4464
Cryptocala acadiensis|[7169]|LPMNB499-09|08BBLEP-05537|658[0n]bp|Canada.Manitoba|BOLD:AAA4464
Cryptocala acadiensis|[7170]|LPABB144-08|08BBLEP-03409|658[0n]bp|Canada.Alberta|BOLD:AAA4464
Cryptocala acadiensis|[7171]|LOWC896-05|CGWC-0896|658[0n]bp|Canada.British Columbia|BOLD:AAA4464
Cryptocala acadiensis|[7172]|LPABC009-09|08BBLEP-04228|658[0n]bp|Canada.Alberta|BOLD:AAA4464
Cryptocala acadiensis|[7173]|LOWCD688-06|CGWC-3508|658[0n]bp|Canada.British Columbia|BOLD:AAA4464
Cryptocala acadiensis|[7174]|LPABC328-09|08BBLEP-04547|658[0n]bp|Canada.Alberta|BOLD:AAA4464
Cryptocala acadiensis|[7175]|LOWCD694-06|CGWC-3514|658[0n]bp|Canada.British Columbia|BOLD:AAA4464
Cryptocala acadiensis|[7176]|LPMNB244-09|08BBLEP-05088|658[0n]bp|Canada.Manitoba|BOLD:AAA4464
[illegible]

Cryptocala acadiensis|[7174]|LPABC328-09|08BBLEP-04547|658[0n]bp|Canada.Alberta|BOLD:AAA4464
Cryptocala acadiensis|[7175]|LOWCD694-06|CGWC-3514|658[0n]bp|Canada.British Columbia|BOLD:AAA4464
Cryptocala acadiensis|[7176]|LPMNB244-09|08BBLEP-05088|658[0n]bp|Canada.Manitoba|BOLD:AAA4464
Cryptocala acadiensis|[7177]|BBLPB513-10|10BBCLP-1512|658[0n]bp|Canada.British Columbia|BOLD:AAA4464
Cryptocala acadiensis|[7178]|LPMNB277-09|08BBLEP-05121|658[0n]bp|Canada.Manitoba|BOLD:AAA4464
Cryptocala acadiensis|[7179]|BBLPB403-10|10BBCLP-1402|658[0n]bp|Canada.Alberta|BOLD:AAA4464
Cryptocala acadiensis|[7180]|LBCG2252-09|08-JDWBC-2252|658[0n]bp|Canada.British Columbia|BOLD:AAA4464
Cryptocala acadiensis|[7181]|LPABC447-09|08BBLEP-04666|658[0n]bp|Canada.Alberta|BOLD:AAA4464
Cryptocala acadiensis|[7182]|LOWC900-05|CGWC-0900|658[0n]bp|Canada.British Columbia|BOLD:AAA4464
Cryptocala acadiensis|[7183]|LPABC409-09|08BBLEP-04628|658[0n]bp|Canada.Alberta|BOLD:AAA4464
Cryptocala acadiensis|[7184]|BBLPE359-09|09BBELE-2359|658[0n]bp|Canada.Newfoundland and Labrador|BOLD ...
Cryptocala acadiensis|[7185]|LPABC316-09|08BBLEP-04535|658[0n]bp|Canada.Alberta|BOLD:AAA4464
Cryptocala acadiensis|[7186]|LBCH3488-10|10-JDWBC-3488|658[0n]bp|Canada.British Columbia|BOLD:AAA4464
Cryptocala acadiensis|[7187]|LBCG2254-09|08-JDWBC-2254|658[0n]bp|Canada.British Columbia|BOLD:AAA4464
Cryptocala acadiensis|[7188]|LOWC895-05|CGWC-0895|658[0n]bp|Canada.British Columbia|BOLD:AAA4464
Cryptocala acadiensis|[7189]|LOWC907-05|CGWC-0907|658[0n]bp|Canada.British Columbia|BOLD:AAA4464
Cryptocala acadiensis|[7190]|BBLPC104-09|09BBELE-1104|658[0n]bp|Canada.New Brunswick|BOLD:AAA4464
Cryptocala acadiensis|[7191]|LOWC904-05|CGWC-0904|658[0n]bp|Canada.British Columbia|BOLD:AAA4464
Cryptocala acadiensis|[7192]|LOWC898-05|CGWC-0898|658[0n]bp|Canada.British Columbia|BOLD:AAA4464
Cryptocala acadiensis|[7193]|LOWC902-05|CGWC-0902|658[0n]bp|Canada.British Columbia|BOLD:AAA4464
Cryptocala acadiensis|[7194]|LPMNB259-09|08BBLEP-05103|658[0n]bp|Canada.Manitoba|BOLD:AAA4464
Cryptocala acadiensis|[7195]|BBLPB512-10|10BBCLP-1511|658[0n]bp|Canada.British Columbia|BOLD:AAA4464
Cryptocala acadiensis|[7196]|LBCH3490-10|10-JDWBC-3490|658[0n]bp|Canada.British Columbia|BOLD:AAA4464
Cryptocala acadiensis|[7197]|LPMNB257-09|08BBLEP-05101|658[0n]bp|Canada.Manitoba|BOLD:AAA4464
Cryptocala acadiensis|[7198]|LBCG2253-09|08-JDWBC-2253|658[0n]bp|Canada.British Columbia|BOLD:AAA4464
Cryptocala acadiensis|[7199]|BBLPC214-09|09BBELE-1214|658[0n]bp|Canada.Nova Scotia|BOLD:AAA4464
Cryptocala acadiensis|[7200]|LBCH3786-10|10-JDWBC-3786|658[0n]bp|Canada.British Columbia|BOLD:AAA4464
Cryptocala acadiensis|[7201]|LOWC911-05|CGWC-0911|658[0n]bp|Canada.British Columbia|BOLD:AAA4464
Cryptocala acadiensis|[7202]|LOWC905-05|CGWC-0905|658[0n]bp|Canada.British Columbia|BOLD:AAA4464
Cryptocala acadiensis|[7203]|LBCG2255-09|08-JDWBC-2255|658[0n]bp|Canada.British Columbia|BOLD:AAA4464
Cryptocala acadiensis|[7204]|LOWC910-05|CGWC-0910|658[0n]bp|Canada.British Columbia|BOLD:AAA4464
Cryptocala acadiensis|[7205]|LOWC908-05|CGWC-0908|658[0n]bp|Canada.British Columbia|BOLD:AAA4464
Cryptocala acadiensis|[7206]|LBCH3492-10|10-JDWBC-3492|658[0n]bp|Canada.British Columbia|BOLD:AAA4464
Cryptocala acadiensis|[7207]|BBLPB506-10|10BBCLP-1505|658[0n]bp|Canada.Alberta|BOLD:AAA4464
Cryptocala acadiensis|[7208]|BBLPB396-10|10BBCLP-1395|658[0n]bp|Canada.British Columbia|BOLD:AAA4464
Cryptocala acadiensis|[7209]|LBCG2272-09|08-JDWBC-2272|658[0n]bp|Canada.British Columbia|BOLD:AAA4464
Cryptocala acadiensis|[7210]|LPMNB258-09|08BBLEP-05102|658[0n]bp|Canada.Manitoba|BOLD:AAA4464
Cryptocala acadiensis|[7211]|LPABC446-09|08BBLEP-04665|658[0n]bp|Canada.Alberta|BOLD:AAA4464
Noctua comes|[7212]|LBCW046-08|08-JDWWI-0046|658[0n]bp|Canada.British Columbia|BOLD:AAA2633
Noctua comes|[7213]|LMH033-06|PFC-2006-0715|658[0n]bp|Canada.British Columbia|BOLD:AAA2633
Noctua comes|[7214]|LALPA761-10|AVBC 763-10|658[0n]bp|Canada.British Columbia|BOLD:AAA2633
Noctua comes|[7215]|LALPA799-10|AVBC 801-10|658[0n]bp|Canada.British Columbia|BOLD:AAA2633
Noctua comes|[7216]|LHLEP127-06|UBC-2006-2083|658[0n]bp|Canada.British Columbia|BOLD:AAA2633
Noctua comes|[7217]|LHLEP124-06|UBC-2006-2124|658[0n]bp|Canada.British Columbia|BOLD:AAA2633
Noctua comes|[7218]|LALPA767-10|AVBC 769-10|658[0n]bp|Canada.British Columbia|BOLD:AAA2633
Noctua comes|[7219]|LHLEP126-06|UBC-2006-2084|658[0n]bp|Canada.British Columbia|BOLD:AAA2633
Noctua comes|[7220]|LALPA789-10|AVBC 791-10|658[0n]bp|Canada.British Columbia|BOLD:AAA2633
Noctua comes|[7221]|LHLEP129-06|UBC-2006-1056|658[0n]bp|Canada.British Columbia|BOLD:AAA2633
Noctua comes|[7222]|LALPA1194-11|AVBC 1196-11|658[0n]bp|Canada.British Columbia|BOLD:AAA2633
Noctua comes|[7223]|DUNLP177-08|Dun-08-177|658[0n]bp|Canada.British Columbia|BOLD:AAA2633
Noctua comes|[7224]|LALPA1302-11|AVBC 1304-11|658[0n]bp|Canada.British Columbia|BOLD:AAA2633
Noctua comes|[7225]|LPVIB825-08|PFC-2006-2314|658[0n]bp|Canada.British Columbia|BOLD:AAA2633
Noctua comes|[7226]|LHLEP128-06|UBC-2006-2082|658[0n]bp|Canada.British Columbia|BOLD:AAA2633
Noctua comes|[7227]|LHLEP125-06|UBC-2006-2123|658[0n]bp|Canada.British Columbia|BOLD:AAA2633
Noctua pronuba|[7228]|LCHP814-07|07PROBE-10571|658[0n]bp|Canada.Manitoba|BOLD:AAA2632
Noctua pronuba|[7229]|LALPA121-10|AVBC 121-10|658[0n]bp|Canada.British Columbia|BOLD:AAA2632
Noctua pronuba|[7230]|LBCS246-07|UBC-2007-0754|658[0n]bp|Canada.British Columbia|BOLD:AAA2632
Noctua pronuba|[7231]|LBCS181-07|UBC-2007-0562|658[0n]bp|Canada.British Columbia|BOLD:AAA2632
Noctua pronuba|[7232]|LBCS639-07|UBC-2007-0339|647[0n]bp|Canada.British Columbia|BOLD:AAA2632
Noctua pronuba|[7233]|LBCS104-07|UBC-2007-0094|658[0n]bp|Canada.British Columbia|BOLD:AAA2632
Noctua pronuba|[7234]|BBLEC455-09|09BBELE-0455|658[0n]bp|Canada.New Brunswick|BOLD:AAA2632
Noctua pronuba|[7235]|LPSO732-08|PPBP-0732|658[0n]bp|Canada.Ontario|BOLD:AAA2632
Noctua pronuba|[7236]|BBLPC035-09|09BBELE-1035|632[0n]bp|Canada.New Brunswick|BOLD:AAA2632
Noctua pronuba|[7237]|LBCH7424-10|10-JDWBC-7424|658[0n]bp|Canada.British Columbia|BOLD:AAA2632
Noctua pronuba|[7238]|MNBB475-05|05-NBSTA-391|658[0n]bp|Canada.New Brunswick|BOLD:AAA2632
Noctua pronuba|[7239]|XAF573-05|2005-ONT-222|658[0n]bp|Canada.Ontario|BOLD:AAA2632
Noctua pronuba|[7240]|LBCS245-07|UBC-2007-0753|658[0n]bp|Canada.British Columbia|BOLD:AAA2632
Noctua pronuba|[7241]|MNBB576-05|05-NBSTA-492|658[0n]bp|Canada.New Brunswick|BOLD:AAA2632
Noctua pronuba|[7242]|BBLPC402-09|09BBELE-1402|658[0n]bp|Canada.New Brunswick|BOLD:AAA2632
Noctua pronuba|[7243]|RDLQF474-06|DH011623|658[0n]bp|Canada.Quebec|BOLD:AAA2632
Noctua pronuba|[7244]|LBCS247-07|UBC-2007-0755|658[0n]bp|Canada.British Columbia|BOLD:AAA2632
Noctua pronuba|[7245]|BBLPC396-09|09BBELE-1396|658[0n]bp|Canada.New Brunswick|BOLD:AAA2632
Noctua pronuba|[7246]|LCHQ658-08|07WNP-10550|658[0n]bp|Canada.Manitoba|BOLD:AAA2632
Noctua pronuba|[7247]|XAK448-06|2006-ONT-1443|658[0n]bp|Canada.Ontario|BOLD:AAA2632
Noctua pronuba|[7248]|LBCW045-08|08-JDWWI-0045|658[0n]bp|Canada.British Columbia|BOLD:AAA2632
Noctua pronuba|[7249]|BBLEC423-09|09BBELE-0423|658[0n]bp|Canada.New Brunswick|BOLD:AAA2632
Noctua pronuba|[7250]|BBLPC438-09|09BBELE-1438|658[0n]bp|Canada.New Brunswick|BOLD:AAA2632
Noctua pronuba|[7251]|MNBB575-05|05-NBSTA-491|658[0n]bp|Canada.New Brunswick|BOLD:AAA2632
Noctua pronuba|[7252]|LPMNB466-09|08BBLEP-05504|658[0n]bp|Canada.Manitoba|BOLD:AAA2632
Noctua pronuba|[7253]|LBCS183-07|UBC-2007-0564|658[0n]bp|Canada.British Columbia|BOLD:AAA2632
Noctua pronuba|[7254]|LBCH7326-10|10-JDWBC-7326|658[0n]bp|Canada.British Columbia|BOLD:AAA2632
Noctua pronuba|[7255]|LBCS107-07|UBC-2007-0097|658[0n]bp|Canada.British Columbia|BOLD:AAA2632
Noctua pronuba|[7256]|LPSOB798-08|PPBP-1797|658[0n]bp|Canada.Ontario|BOLD:AAA2632
Noctua pronuba|[7257]|LHLEP024-06|UBC-2006-0028|658[0n]bp|Canada.British Columbia|BOLD:AAA2632
Noctua pronuba|[7258]|LHLEP061-06|UBC-2006-0266|658[0n]bp|Canada.British Columbia|BOLD:AAA2632
Noctua pronuba|[7259]|MNBB380-05|05-NBSTA-296|658[0n]bp|Canada.New Brunswick|BOLD:AAA2632
Noctua pronuba|[7260]|LBCS027-07|UBC-2007-0050|658[0n]bp|Canada.British Columbia|BOLD:AAA2632
Noctua pronuba|[7261]|BBLEC419-09|09BBELE-0419|658[0n]bp|Canada.New Brunswick|BOLD:AAA2632
Noctua pronuba|[7262]|LBCG3745-09|08-JDWBC-3745|658[0n]bp|Canada.British Columbia|BOLD:AAA2632
Noctua pronuba|[7263]|BLTIB704-08|BL989|658[0n]bp|Canada.Ontario|BOLD:AAA2632
Noctua pronuba|[7264]|BBLEC416-09|09BBELE-0416|658[0n]bp|Canada.New Brunswick|BOLD:AAA2632
Noctua pronuba|[7265]|LBCS243-07|UBC-2007-0751|658[0n]bp|Canada.British Columbia|BOLD:AAA2632
Noctua pronuba|[7266]|LBCG3749-09|08-JDWBC-3749|658[0n]bp|Canada.British Columbia|BOLD:AAA2632
Noctua pronuba|[7267]|LBCS365-07|UBC-2007-0878|658[0n]bp|Canada.British Columbia|BOLD:AAA2632
Noctua pronuba|[7268]|BBLEC441-09|09BBELE-0441|658[0n]bp|Canada.New Brunswick|BOLD:AAA2632
Noctua pronuba|[7269]|BBLPC392-09|09BBELE-1392|658[0n]bp|Canada.New Brunswick|BOLD:AAA2632
Noctua pronuba|[7270]|LBCG3748-09|08-JDWBC-3748|658[0n]bp|Canada.British Columbia|BOLD:AAA2632
Noctua pronuba|[7271]|LBCS001-07|UBC-2007-0001|658[0n]bp|Canada.British Columbia|BOLD:AAA2632
Noctua pronuba|[7272]|BLTIB1000-08|BL1437|658[0n]bp|Canada.Ontario|BOLD:AAA2632
Noctua pronuba|[7273]|LBCS106-07|UBC-2007-0096|658[0n]bp|Canada.British Columbia|BOLD:AAA2632
Noctua pronuba|[7274]|LALPA122-10|AVBC 122-10|658[0n]bp|Canada.British Columbia|BOLD:AAA2632
Noctua pronuba|[7275]|MNBB381-05|05-NBSTA-297|658[0n]bp|Canada.New Brunswick|BOLD:AAA2632
Noctua pronuba|[7276]|LPSOB946-08|PPBP-1945|658[0n]bp|Canada.Ontario|BOLD:AAA2632

Noctua pronuba|[7274]|LALPA122-10|AVBC 122-10|658[0n]bp|Canada.British Columbia|BOLD:AAA2632
Noctua pronuba|[7275]|MNBB381-05|05-NBSTA-297|658[0n]bp|Canada.New Brunswick|BOLD:AAA2632
Noctua pronuba|[7276]|LPSOB946-08|PPBP-1945|658[0n]bp|Canada.Ontario|BOLD:AAA2632
Noctua pronuba|[7277]|LPSOB079-08|PPBP-1078|658[0n]bp|Canada.Ontario|BOLD:AAA2632
Noctua pronuba|[7278]|LCHP771-07|07PROBE-10456|658[0n]bp|Canada.Manitoba|BOLD:AAA2632
Noctua pronuba|[7279]|BBLEC420-09|09BBELE-0420|658[0n]bp|Canada.New Brunswick|BOLD:AAA2632
Noctua pronuba|[7280]|BLTIB783-08|BL1200|658[0n]bp|Canada.Ontario|BOLD:AAA2632
Noctua pronuba|[7281]|XAH336-05|2005-ONT-1919|658[0n]bp|Canada.Ontario|BOLD:AAA2632
Noctua pronuba|[7282]|LBCS638-07|UBC-2007-0338|658[0n]bp|Canada.British Columbia|BOLD:AAA2632
Noctua pronuba|[7283]|LBCS244-07|UBC-2007-0752|658[0n]bp|Canada.British Columbia|BOLD:AAA2632
Noctua pronuba|[7284]|XAG911-05|2005-ONT-1495|658[0n]bp|Canada.Ontario|BOLD:AAA2632
Noctua pronuba|[7285]|LBCG3747-09|08-JDWBC-3747|658[0n]bp|Canada.British Columbia|BOLD:AAA2632
Noctua pronuba|[7286]|XAH450-05|2005-ONT-2033|658[0n]bp|Canada.Ontario|BOLD:AAA2632
Noctua pronuba|[7287]|XAJ684-06|2006-ONT-0684|658[0n]bp|Canada.Ontario|BOLD:AAA2632
Noctua pronuba|[7288]|LBCS362-07|UBC-2007-0875|658[0n]bp|Canada.British Columbia|BOLD:AAA2632
Noctua pronuba|[7289]|LBCS637-07|UBC-2007-0337|658[0n]bp|Canada.British Columbia|BOLD:AAA2632
Noctua pronuba|[7290]|LBCS105-07|UBC-2007-0095|658[0n]bp|Canada.British Columbia|BOLD:AAA2632
Noctua pronuba|[7291]|XAD098-04|04HBL007098|658[0n]bp|Canada.Ontario|BOLD:AAA2632
Noctua pronuba|[7292]|LBCS025-07|UBC-2007-0048|658[0n]bp|Canada.British Columbia|BOLD:AAA2632
Noctua pronuba|[7293]|LBCS439-07|UBC-2007-0195|658[0n]bp|Canada.British Columbia|BOLD:AAA2632
Noctua pronuba|[7294]|MNBB131-05|05-NBSTA-047|658[0n]bp|Canada.New Brunswick|BOLD:AAA2632
Noctua pronuba|[7295]|BBLEC456-09|09BBELE-0456|658[0n]bp|Canada.New Brunswick|BOLD:AAA2632
Noctua pronuba|[7296]|LBCS438-07|UBC-2007-0194|658[0n]bp|Canada.British Columbia|BOLD:AAA2632
Noctua pronuba|[7297]|PHJUN3992-11|BIOUG01497-A03|658[0n]bp|Canada.Ontario|BOLD:AAA2632
Noctua pronuba|[7298]|BBLPC100-09|09BBELE-1100|658[0n]bp|Canada.New Brunswick|BOLD:AAA2632
Noctua pronuba|[7299]|LPSO731-08|PPBP-0731|658[0n]bp|Canada.Ontario|BOLD:AAA2632
Noctua pronuba|[7300]|LBCS108-07|UBC-2007-0098|658[0n]bp|Canada.British Columbia|BOLD:AAA2632
Noctua pronuba|[7301]|LBCS026-07|UBC-2007-0049|658[0n]bp|Canada.British Columbia|BOLD:AAA2632
Noctua pronuba|[7302]|TTMNB430-06|MNBTT-430|658[0n]bp|Canada.New Brunswick|BOLD:AAA2632
Noctua pronuba|[7303]|MNBB132-05|05-NBSTA-048|658[0n]bp|Canada.New Brunswick|BOLD:AAA2632
Noctua pronuba|[7304]|LPSO933-08|PPBP-0933|658[0n]bp|Canada.Ontario|BOLD:AAA2632
Noctua pronuba|[7305]|LBCH7117-10|10-JDWBC-7117|658[0n]bp|Canada.British Columbia|BOLD:AAA2632
Noctua pronuba|[7306]|XAJ812-06|2006-ONT-0812|658[0n]bp|Canada.Ontario|BOLD:AAA2632
Noctua pronuba|[7307]|BBLEC132-09|09BBELE-0132|658[0n]bp|Canada.New Brunswick|BOLD:AAA2632
Noctua pronuba|[7308]|BBLEC564-09|09BBELE-0564|658[0n]bp|Canada.Nova Scotia|BOLD:AAA2632
Noctua pronuba|[7309]|PHMNB182-04|04HBL007647|533[0n]bp|Canada.New Brunswick|BOLD:AAA2632
Noctua pronuba|[7310]|DUNLP176-08|Dun-08-176|654[0n]bp|Canada.British Columbia|BOLD:AAA2632
Noctua pronuba|[7311]|LHLEP023-06|UBC-2006-0027|654[0n]bp|Canada.British Columbia|BOLD:AAA2632
Noctua pronuba|[7312]|PHMNB084-03|moth97.02SA|639[0n]bp|Canada.New Brunswick|BOLD:AAA2632
Noctua pronuba|[7313]|BBLEC418-09|09BBELE-0418|658[0n]bp|Canada.New Brunswick|BOLD:AAA2632
Noctua pronuba|[7314]|LBCS366-07|UBC-2007-0879|658[0n]bp|Canada.British Columbia|BOLD:AAA2632
Noctua pronuba|[7315]|PPGB342-12|BIOUG03353-E06|658[0n]bp|Canada.Ontario|
Noctua pronuba|[7316]|BBLEC049-09|09BBELE-0049|647[0n]bp|Canada.New Brunswick|BOLD:AAA2632
Noctua pronuba|[7317]|BBLPC101-09|09BBELE-1101|634[0n]bp|Canada.New Brunswick|BOLD:AAA2632
Noctua pronuba|[7318]|XAD173-04|04HBL007173|616[0n]bp|Canada.Ontario|BOLD:AAA2632
Noctua pronuba|[7319]|PHMO100-03|moth606.01|639[0n]bp|Canada.Ontario|BOLD:AAA2632
Noctua pronuba|[7320]|XAC570-04|04HBL006570|602[0n]bp|Canada.Ontario|BOLD:AAA2632
Noctua pronuba|[7321]|XAH097-05|2005-ONT-1680|638[0n]bp|Canada.Ontario|BOLD:AAA2632
Noctua pronuba|[7322]|BBLEC682-09|09BBELE-0682|642[0n]bp|Canada.Nova Scotia|BOLD:AAA2632
Noctua pronuba|[7323]|LPSO631-08|PPBP-0631|658[0n]bp|Canada.Ontario|BOLD:AAA2632
Noctua pronuba|[7324]|BBLPC395-09|09BBELE-1395|658[0n]bp|Canada.New Brunswick|BOLD:AAA2632
Noctua pronuba|[7325]|LALPA706-10|AVBC 708-10|658[0n]bp|Canada.British Columbia|BOLD:AAA2632
Noctua pronuba|[7326]|BLTIB311-08|BL500|658[0n]bp|Canada.Ontario|BOLD:AAA2632
Noctua pronuba|[7327]|BBLEC485-09|09BBELE-0485|658[0n]bp|Canada.New Brunswick|BOLD:AAA2632
Noctua pronuba|[7328]|LBCW044-08|08-JDWWI-0044|658[0n]bp|Canada.British Columbia|BOLD:AAA2632
Noctua pronuba|[7329]|MNBB643-05|05-NBSTA-559|658[0n]bp|Canada.New Brunswick|BOLD:AAA2632
Noctua pronuba|[7330]|LCHQ320-08|07WNP-10212|658[0n]bp|Canada.Manitoba|BOLD:AAA2632
Noctua pronuba|[7331]|LBCS182-07|UBC-2007-0563|658[0n]bp|Canada.British Columbia|BOLD:AAA2632
Noctua pronuba|[7332]|LBCS363-07|UBC-2007-0876|658[0n]bp|Canada.British Columbia|BOLD:AAA2632
Noctua pronuba|[7333]|PHMNB229-04|04HBL007694|609[0n]bp|Canada.New Brunswick|BOLD:AAA2632
Noctua pronuba|[7334]|LPSOB705-08|PPBP-1704|609[0n]bp|Canada.Ontario|BOLD:AAA2632
Noctua pronuba|[7335]|PMG138-03|NOCTU1.00|617[0n]bp|Canada.Ontario|BOLD:AAA2632
Noctua pronuba|[7336]|LBCS364-07|UBC-2007-0877|658[0n]bp|Canada.British Columbia|BOLD:AAA2632
Noctua pronuba|[7337]|PHMO004-03|NOCTU2|639[0n]bp|Canada.Ontario|BOLD:AAA2632
Noctua pronuba|[7338]|XAJ948-06|2006-ONT-0948|658[0n]bp|Canada.Ontario|BOLD:AAA2632
Noctua pronuba|[7339]|BBLEC453-09|09BBELE-0453|658[0n]bp|Canada.New Brunswick|BOLD:AAA2632
Eueretagrotis perattentus|[7340]|TTMNB498-06|MNBTT-498|658[0n]bp|Canada.New Brunswick|BOLD:AAA8152
Eueretagrotis perattentus|[7341]|JSJUN2103-11|BIOUG01690-F02|618[0n]bp|Canada.Ontario|BOLD:AAA8151
Eueretagrotis perattentus|[7342]|RDMAB072-05|UASM57599|611[0n]bp|Canada.Alberta|BOLD:AAA8151
Eueretagrotis perattentus|[7343]|RDLQB305-05|DH010391|658[0n]bp|Canada.Quebec|BOLD:AAA8151
Eueretagrotis perattentus|[7344]|RDLQB304-05|DH010390|658[0n]bp|Canada.Quebec|BOLD:AAA8151
Eueretagrotis perattentus|[7345]|BBLPE354-09|09BBELE-2354|658[0n]bp|Canada.Newfoundland and Labrador|BOL...
Eueretagrotis perattentus|[7346]|BBLPE006-09|09BBELE-2006|658[0n]bp|Canada.Nova Scotia|BOLD:AAA8151
Eueretagrotis perattentus|[7347]|RDLQB306-05|DH010392|658[0n]bp|Canada.Quebec|BOLD:AAA8151
Eueretagrotis perattentus|[7348]|BBLPE028-09|09BBELE-2028|658[0n]bp|Canada.Nova Scotia|BOLD:AAA8151
Eueretagrotis perattentus|[7349]|BBLPC168-09|09BBELE-1168|656[0n]bp|Canada.Nova Scotia|BOLD:AAA8151
Eueretagrotis perattentus|[7350]|BBLPB718-10|10BBCLP-1717|658[0n]bp|Canada.Ontario|BOLD:AAA8151
Eueretagrotis perattentus|[7351]|BLTIB443-08|BL690|658[0n]bp|Canada.Ontario|BOLD:AAA8151
Eueretagrotis perattentus|[7352]|XAD777-05|2005-ONT-576|658[0n]bp|Canada.Ontario|BOLD:AAA8151
Eueretagrotis perattentus|[7353]|RDLQB303-05|DH010389|658[0n]bp|Canada.Quebec|BOLD:AAA8151
Eueretagrotis perattentus|[7354]|BBLPE136-09|09BBELE-2136|658[0n]bp|Canada.Nova Scotia|BOLD:AAA8151
Eueretagrotis perattentus|[7355]|RDLQB301-05|DH010387|658[0n]bp|Canada.Quebec|BOLD:AAA8151
Eueretagrotis perattentus|[7356]|RDLQB302-05|DH010388|658[0n]bp|Canada.Quebec|BOLD:AAA8151
Eueretagrotis perattentus|[7357]|BBLPE071-09|09BBELE-2071|607[0n]bp|Canada.Nova Scotia|BOLD:AAA8151
Eueretagrotis perattentus|[7358]|BBLPC680-09|09BBELE-1680|658[0n]bp|Canada.Newfoundland and Labrador|BOL...
Eueretagrotis perattentus|[7359]|TTMNB496-06|MNBTT-496|658[0n]bp|Canada.New Brunswick|BOLD:AAA8151
Eueretagrotis perattentus|[7360]|BBLPB853-10|10BBCLP-1852|658[0n]bp|Canada.British Columbia|BOLD:AAA8151
Eueretagrotis perattentus|[7361]|XAB093-04|04HBL005093|658[0n]bp|Canada.Ontario|BOLD:AAA8151
Eueretagrotis perattentus|[7362]|BLTIB270-08|BL454|658[1n]bp|Canada.Ontario|BOLD:AAA8151
Eueretagrotis perattentus|[7363]|LBCH152-10|10-JDWBC-0152|658[0n]bp|Canada.British Columbia|BOLD:AAA8151
Eueretagrotis perattentus|[7364]|LHLEP394-06|UBC-2006-1016|658[0n]bp|Canada.British Columbia|BOLD:AAA...
Eueretagrotis perattentus|[7365]|LHLEP393-06|UBC-2006-1012|658[0n]bp|Canada.British Columbia|BOLD:AAA...
Eueretagrotis perattentus|[7366]|LBCH852-10|10-JDWBC-0852|658[0n]bp|Canada.British Columbia|BOLD:AAA8151
Eueretagrotis perattentus|[7367]|LHLEP434-06|UBC-2006-1525|658[0n]bp|Canada.British Columbia|BOLD:AAA...
Eueretagrotis perattentus|[7368]|LBCH154-10|10-JDWBC-0154|658[0n]bp|Canada.British Columbia|BOLD:AAA8151
Eueretagrotis perattentus|[7369]|LBCB623-05|HLC-21563|658[0n]bp|Canada.British Columbia|BOLD:AAA8151
Eueretagrotis perattentus|[7370]|LBCH153-10|10-JDWBC-0153|658[0n]bp|Canada.British Columbia|BOLD:AAA8151
Eueretagrotis perattentus|[7371]|RDLQG139-06|DH012310|646[1n]bp|Canada.Quebec|BOLD:AAA8151
Eueretagrotis perattentus|[7372]|PHMNB424-04|04HBL00650|658[2n]bp|Canada.New Brunswick|BOLD:AAA8151
Eueretagrotis perattentus|[7373]|TTMNB495-06|MNBTT-495|658[0n]bp|Canada.New Brunswick|BOLD:AAA8151
Eueretagrotis perattentus|[7374]|PHMNB467-04|04HBL00693|658[0n]bp|Canada.New Brunswick|BOLD:AAA8151
Eueretagrotis perattentus|[7375]|PHMNB684-04|04HBL00910|658[0n]bp|Canada.New Brunswick|BOLD:AAA8151
Eueretagrotis perattentus|[7376]|RDLQB307-05|DH010393|658[0n]bp|Canada.Quebec|BOLD:AAA8151

Eueretagrotis perattentus|[7373]|TTMNB495-06|MNBTT-495|658[0n]bp|Canada.New Brunswick|BOLD:AAA8151
Eueretagrotis perattentus|[7374]|PHMNB467-04|04HBL00693|658[0n]bp|Canada.New Brunswick|BOLD:AAA8151
Eueretagrotis perattentus|[7375]|PHMNB684-04|04HBL00910|658[0n]bp|Canada.New Brunswick|BOLD:AAA8151
Eueretagrotis perattentus|[7376]|RDLQB307-05|DH010393|658[0n]bp|Canada.Quebec|BOLD:AAA8151
Eueretagrotis perattentus|[7377]|PHMNB368-04|04HBL00594|658[0n]bp|Canada.New Brunswick|BOLD:AAA8151
Eueretagrotis perattentus|[7378]|PHMNB001-03|moth1.02SA|639[0n]bp|Canada.New Brunswick|BOLD:AAA8151
Eueretagrotis perattentus|[7379]|TTMNB497-06|MNBTT-497|655[0n]bp|Canada.New Brunswick|BOLD:AAA8151
Eueretagrotis perattentus|[7380]|RDMAB071-05|UASM57598|615[0n]bp|Canada.Alberta|BOLD:AAA8151
Eueretagrotis attentus|[7381]|BBLEC969-09|09BBELE-0969|658[0n]bp|Canada.Nova Scotia|BOLD:AAB3386
Eueretagrotis attentus|[7382]|BBLEC191-09|09BBELE-0191|577[0n]bp|Canada.Nova Scotia|BOLD:AAB3386
Eueretagrotis attentus|[7383]|TMNBB402-06|MNBTT-1342|658[0n]bp|Canada.New Brunswick|BOLD:AAB3386
Eueretagrotis attentus|[7384]|BBLEC549-09|09BBELE-0549|658[0n]bp|Canada.Nova Scotia|BOLD:AAB3386
Eueretagrotis attentus|[7385]|RDLQB299-05|DH010385|658[0n]bp|Canada.Quebec|BOLD:AAB3386
Eueretagrotis attentus|[7386]|RDLQB296-05|DH010382|597[0n]bp|Canada.Quebec|BOLD:AAB3386
Eueretagrotis attentus|[7387]|RDLQB297-05|DH010383|658[0n]bp|Canada.Quebec|BOLD:AAB3386
Eueretagrotis attentus|[7388]|TTMNB013-06|MNBTT-013|657[0n]bp|Canada.New Brunswick|BOLD:AAB3386
Eueretagrotis attentus|[7389]|TTMNB020-06|MNBTT-020|658[0n]bp|Canada.New Brunswick|BOLD:AAB3386
Eueretagrotis attentus|[7390]|TMNBB399-06|MNBTT-1339|658[0n]bp|Canada.New Brunswick|BOLD:AAB3386
Eueretagrotis attentus|[7391]|BBLEC116-09|09BBELE-0116|658[0n]bp|Canada.Nova Scotia|BOLD:AAB3386
Eueretagrotis attentus|[7392]|TTMNB019-06|MNBTT-019|658[0n]bp|Canada.New Brunswick|BOLD:AAB3386
Eueretagrotis attentus|[7393]|TMNBB401-06|MNBTT-1341|658[0n]bp|Canada.New Brunswick|BOLD:AAB3386
Eueretagrotis attentus|[7394]|TMNBB400-06|MNBTT-1340|658[0n]bp|Canada.New Brunswick|BOLD:AAB3386
Eueretagrotis attentus|[7395]|RDLQB298-05|DH010384|658[0n]bp|Canada.Quebec|BOLD:AAB3386
Eueretagrotis attentus|[7396]|RDLQB294-05|DH010380|658[0n]bp|Canada.Quebec|BOLD:AAB3386
Eueretagrotis attentus|[7397]|TTMNB012-06|MNBTT-012|658[0n]bp|Canada.New Brunswick|BOLD:AAB3386
Eueretagrotis attentus|[7398]|BBLPE617-09|09BBELE-2617|658[0n]bp|Canada.Nova Scotia|BOLD:AAB3386
Eueretagrotis attentus|[7399]|TTMNB078-06|MNBTT-078|657[0n]bp|Canada.New Brunswick|BOLD:AAB3386
Eueretagrotis attentus|[7400]|TTMNB046-06|MNBTT-046|618[0n]bp|Canada.New Brunswick|BOLD:AAB3386
Eueretagrotis attentus|[7401]|RDLQB295-05|DH010381|658[0n]bp|Canada.Quebec|BOLD:AAB3386
Eueretagrotis attentus|[7402]|TMNBB398-06|MNBTT-1338|658[0n]bp|Canada.New Brunswick|BOLD:AAB3386
Eueretagrotis sigmoides|[7403]|RDNMG1035-08|CNC LEP00053159|642[0n]bp|Canada.Ontario|BOLD:AAE4603
Eueretagrotis sigmoides|[7404]|RDLQ738-07|DH009805|658[0n]bp|Canada.Quebec|BOLD:AAE4603
Eueretagrotis sigmoides|[7405]|RDNMG1034-08|CNC LEP00053158|643[0n]bp|Canada.Ontario|BOLD:AAE4603
Eueretagrotis sigmoides|[7406]|RDLQ739-07|DH005452|658[0n]bp|Canada.Quebec|BOLD:AAE4603
Paradiarsia littoralis|[7407]|LOWCD603-06|CGWC-3423|587[0n]bp|Canada.British Columbia|BOLD:AAA7373
Paradiarsia littoralis|[7408]|LOWCD617-06|CGWC-3437|658[0n]bp|Canada.British Columbia|BOLD:AAA7373
Paradiarsia littoralis|[7409]|LOWCD607-06|CGWC-3427|559[0n]bp|Canada.British Columbia|BOLD:AAA7373
Paradiarsia littoralis|[7410]|LOWCD618-06|CGWC-3438|608[0n]bp|Canada.British Columbia|BOLD:AAA7373
Paradiarsia littoralis|[7411]|LOWCD602-06|CGWC-3422|581[0n]bp|Canada.British Columbia|BOLD:AAA7373
Paradiarsia littoralis|[7412]|LOWCD611-06|CGWC-3431|596[0n]bp|Canada.British Columbia|BOLD:AAA7373
Paradiarsia littoralis|[7413]|LOWCD601-06|CGWC-3421|658[0n]bp|Canada.British Columbia|BOLD:AAA7373
Paradiarsia littoralis|[7414]|LBCG357-08|08-JDWBC-0357|658[0n]bp|Canada.British Columbia|BOLD:AAA7373
Paradiarsia littoralis|[7415]|LOWCD615-06|CGWC-3435|658[0n]bp|Canada.British Columbia|BOLD:AAA7373
Paradiarsia littoralis|[7416]|LOWCD608-06|CGWC-3428|658[0n]bp|Canada.British Columbia|BOLD:AAA7373
Paradiarsia littoralis|[7417]|LOWCD610-06|CGWC-3430|658[0n]bp|Canada.British Columbia|BOLD:AAA7373
Paradiarsia littoralis|[7418]|LOWCD609-06|CGWC-3429|658[0n]bp|Canada.British Columbia|BOLD:AAA7373
Paradiarsia littoralis|[7419]|LOWCD612-06|CGWC-3432|658[0n]bp|Canada.British Columbia|BOLD:AAA7373
Paradiarsia littoralis|[7420]|LOWCD616-06|CGWC-3436|658[0n]bp|Canada.British Columbia|BOLD:AAA7373
Paradiarsia littoralis|[7421]|LBCG364-08|08-JDWBC-0364|658[0n]bp|Canada.British Columbia|BOLD:AAA7373
Paradiarsia littoralis|[7422]|LOWCD613-06|CGWC-3433|658[0n]bp|Canada.British Columbia|BOLD:AAA7373
Paradiarsia littoralis|[7423]|LBCG389-08|08-JDWBC-0389|658[0n]bp|Canada.British Columbia|BOLD:AAA7373
Paradiarsia littoralis|[7424]|LOWCD600-06|CGWC-3420|658[0n]bp|Canada.British Columbia|BOLD:AAA7373
Paradiarsia littoralis|[7425]|LOWCD605-06|CGWC-3425|657[0n]bp|Canada.British Columbia|BOLD:AAA7373
Paradiarsia littoralis|[7426]|LOWCD619-06|CGWC-3439|600[0n]bp|Canada.British Columbia|BOLD:AAA7373
Paradiarsia littoralis|[7427]|LOWCD606-06|CGWC-3426|558[1n]bp|Canada.British Columbia|BOLD:AAA7373
Paradiarsia littoralis|[7428]|LOWCD614-06|CGWC-3434|558[0n]bp|Canada.British Columbia|BOLD:AAA7373
Paradiarsia littoralis|[7429]|LOWCD604-06|CGWC-3424|658[0n]bp|Canada.British Columbia|BOLD:AAA7373
Paradiarsia littoralis|[7430]|RDLQB393-05|DH010479|589[0n]bp|Canada.Quebec|BOLD:AAA7373
Paradiarsia littoralis|[7431]|BBLPB455-10|10BBCLP-1454|658[0n]bp|Canada.Alberta|BOLD:AAA7373
Paradiarsia littoralis|[7432]|LPABB351-08|08BBLEP-03616|658[0n]bp|Canada.Alberta|BOLD:AAA7373
Paradiarsia littoralis|[7433]|LPSK456-08|08BBLEP-02024|658[0n]bp|Canada.Saskatchewan|BOLD:AAA7373
Paradiarsia littoralis|[7434]|BBLPB587-10|10BBCLP-1586|658[0n]bp|Canada.Alberta|BOLD:AAA7373
Paradiarsia littoralis|[7435]|BBLPB370-10|10BBCLP-1369|658[0n]bp|Canada.Alberta|BOLD:AAA7373
Paradiarsia littoralis|[7436]|LPABB872-09|08BBLEP-04192|657[0n]bp|Canada.Alberta|BOLD:AAA7373
Paradiarsia littoralis|[7437]|LPSK441-08|08BBLEP-02009|658[0n]bp|Canada.Saskatchewan|BOLD:AAA7373
Paradiarsia littoralis|[7438]|LPAB229-08|08BBLEP-02551|658[0n]bp|Canada.Alberta|BOLD:AAA7373
Paradiarsia littoralis|[7439]|LPMN717-08|08BBLEP-01520|658[0n]bp|Canada.Manitoba|BOLD:AAA7373
Paradiarsia littoralis|[7440]|LPMN711-08|08BBLEP-01514|658[0n]bp|Canada.Manitoba|BOLD:AAA7373
Paradiarsia littoralis|[7441]|RDMAB076-05|UASM57603|658[0n]bp|Canada.Alberta|BOLD:AAA7373
Paradiarsia littoralis|[7442]|LPMN330-08|08BBLEP-01129|658[0n]bp|Canada.Manitoba|BOLD:AAA7373
Paradiarsia littoralis|[7443]|LPABB366-08|08BBLEP-03631|658[0n]bp|Canada.Alberta|BOLD:AAA7373
Paradiarsia littoralis|[7444]|LPSK134-08|08BBLEP-01702|658[0n]bp|Canada.Saskatchewan|BOLD:AAA7373
Paradiarsia littoralis|[7445]|LPABB181-08|08BBLEP-03446|658[0n]bp|Canada.Alberta|BOLD:AAA7373
Paradiarsia littoralis|[7446]|LPSK601-08|08BBLEP-02169|658[0n]bp|Canada.Saskatchewan|BOLD:AAA7373
Paradiarsia littoralis|[7447]|LPSK316-08|08BBLEP-01884|658[0n]bp|Canada.Saskatchewan|BOLD:AAA7373
Paradiarsia littoralis|[7448]|LPMN880-08|08BBLEP-02238|658[0n]bp|Canada.Alberta|BOLD:AAA7373
Paradiarsia littoralis|[7449]|LPABB195-08|08BBLEP-03460|658[0n]bp|Canada.Alberta|BOLD:AAA7373
Paradiarsia littoralis|[7450]|LPABB346-08|08BBLEP-03611|658[0n]bp|Canada.Alberta|BOLD:AAA7373
Paradiarsia littoralis|[7451]|LPABC703-09|08BBLEP-04922|658[0n]bp|Canada.Alberta|BOLD:AAA7373
Paradiarsia littoralis|[7452]|LPMN879-08|08BBLEP-02237|658[0n]bp|Canada.Alberta|BOLD:AAA7373
Paradiarsia littoralis|[7453]|LPABB386-08|08BBLEP-03651|658[0n]bp|Canada.Alberta|BOLD:AAA7373
Paradiarsia littoralis|[7454]|LPABB420-08|08BBLEP-03685|658[0n]bp|Canada.Alberta|BOLD:AAA7373
Paradiarsia littoralis|[7455]|LPABB192-08|08BBLEP-03457|658[0n]bp|Canada.Alberta|BOLD:AAA7373
Paradiarsia littoralis|[7456]|LPABB395-08|08BBLEP-03660|658[0n]bp|Canada.Alberta|BOLD:AAA7373
Paradiarsia littoralis|[7457]|LPSK467-08|08BBLEP-02035|658[0n]bp|Canada.Saskatchewan|BOLD:AAA7373
Tesagrotis corrodera|[7458]|RDNMF338-08|NOC14424|658[0n]bp|Canada.British Columbia|BOLD:AAE9272
Tesagrotis corrodera|[7459]|LBCH7125-10|10-JDWBC-7125|658[0n]bp|Canada.British Columbia|BOLD:AAE9272
Tesagrotis atrifrons|[7460]|NAMUM147-08|RR-95-0168|658[0n]bp|United States.California|BOLD:ACF3460
Tesagrotis atrifrons|[7461]|IAWLB025-10|IAWAZ-0838|658[0n]bp|United States.California|BOLD:ACF3460
Tesagrotis atrifrons|[7462]|IAWLB026-10|IAWAZ-0839|658[0n]bp|United States.California|BOLD:ACF3460
Tesagrotis piscipellis|[7463]|LPABB497-08|08BBLEP-03762|658[0n]bp|Canada.Alberta|BOLD:AAE9266
Tesagrotis piscipellis|[7464]|LBCA599-05|HLC-20599|658[0n]bp|Canada.British Columbia|BOLD:AAE9266
Tesagrotis piscipellis|[7465]|LBCH7103-10|10-JDWBC-7103|658[0n]bp|Canada.British Columbia|BOLD:AAE9266
Tesagrotis piscipellis|[7466]|LBCD322-05|HLC-23142|658[0n]bp|Canada.British Columbia|BOLD:AAE9266
Tesagrotis piscipellis|[7467]|LBCH5794-10|10-JDWBC-5794|658[0n]bp|Canada.British Columbia|BOLD:AAE9266
Abagrotis alternata|[7468]|TMNBB404-06|MNBTT-1344|658[1n]bp|Canada.New Brunswick|BOLD:ABZ1830
Abagrotis alternata|[7469]|RDLQ744-07|DH007395|658[0n]bp|Canada.Quebec|BOLD:ABZ1830
Abagrotis alternata|[7470]|XAJ815-06|2006-ONT-0815|639[0n]bp|Canada.Ontario|BOLD:ABZ1830
Abagrotis alternata|[7471]|RDLQ004-05|DH008023|656[0n]bp|Canada.Quebec|BOLD:ABZ1830
Abagrotis alternata|[7472]|TMNBB403-06|MNBTT-1343|658[0n]bp|Canada.New Brunswick|BOLD:ABZ1830
Abagrotis alternata|[7473]|RDLQF269-06|DH011361|658[0n]bp|Canada.Quebec|BOLD:ABZ1830
Abagrotis alternata|[7474]|TTMNB570-06|MNBTT-570|658[0n]bp|Canada.New Brunswick|BOLD:ABZ1830
Abagrotis alternata|[7475]|RDLQ743-07|DH007396|658[0n]bp|Canada.Quebec|BOLD:ABZ1830
[illegible]

Abagrotis alternata|[7473]|RDLQF269-06|DH011361|658[0n]bp|Canada.Quebec|BOLD:ABZ1830
Abagrotis alternata|[7474]|TTMNB570-06|MNBTT-570|658[0n]bp|Canada.New Brunswick|BOLD:ABZ1830
Abagrotis alternata|[7475]|RDLQ743-07|DH007396|658[0n]bp|Canada.Quebec|BOLD:ABZ1830
Abagrotis alternata|[7476]|TMNBB405-06|MNBTT-1345|658[0n]bp|Canada.New Brunswick|BOLD:ABZ1830
Abagrotis trigona|[7477]|LBCH7426-10|10-JDWBC-7426|658[0n]bp|Canada.British Columbia|BOLD:ABY3181
Abagrotis trigona|[7478]|LBCH6236-10|10-JDWBC-6236|658[0n]bp|Canada.British Columbia|BOLD:ABY3181
Abagrotis trigona|[7479]|LBCH7337-10|10-JDWBC-7337|658[0n]bp|Canada.British Columbia|BOLD:ABY3181
Abagrotis trigona|[7480]|RDNMC155-05|CNCNoctuoidea10896|658[0n]bp|Canada.British Columbia|BOLD:ABY3181
Abagrotis trigona|[7481]|LPVIA607-08|PFC-2006-0835|658[0n]bp|Canada.British Columbia|BOLD:ABY3181
Abagrotis trigona|[7482]|LBCH7114-10|10-JDWBC-7114|658[0n]bp|Canada.British Columbia|BOLD:ABY3181
Abagrotis trigona|[7483]|LBCH6125-10|10-JDWBC-6125|658[0n]bp|Canada.British Columbia|BOLD:ABY3181
Abagrotis trigona|[7484]|LBCG481-08|08-JDWBC-0481|658[0n]bp|Canada.British Columbia|BOLD:ABY3181
Abagrotis trigona|[7485]|LBCH6299-10|10-JDWBC-6299|658[0n]bp|Canada.British Columbia|BOLD:ABY3181
Abagrotis trigona|[7486]|LBCH7809-10|10-JDWBC-7809|658[0n]bp|Canada.British Columbia|BOLD:ABY3181
Abagrotis trigona|[7487]|LBCG3248-09|08-JDWBC-3248|632[0n]bp|Canada.British Columbia|BOLD:ABY3181
Abagrotis trigona|[7488]|LBCH6272-10|10-JDWBC-6272|642[0n]bp|Canada.British Columbia|BOLD:ABY3181
Abagrotis trigona|[7489]|LBCH7472-10|10-JDWBC-7472|658[0n]bp|Canada.British Columbia|BOLD:ABY3181
Abagrotis trigona|[7490]|LBCH7676-10|10-JDWBC-7676|658[0n]bp|Canada.British Columbia|BOLD:ABY3181
Abagrotis trigona|[7491]|LOWCE758-06|CGWC-4518|658[0n]bp|Canada.British Columbia|BOLD:ABY3181
Abagrotis trigona|[7492]|RDNMC157-05|CNCNoctuoidea10898|658[0n]bp|Canada.British Columbia|BOLD:ABY3181
Abagrotis trigona|[7493]|LBCH6450-10|10-JDWBC-6450|658[0n]bp|Canada.British Columbia|BOLD:ABY3181
Abagrotis trigona|[7494]|LBCH6309-10|10-JDWBC-6309|658[0n]bp|Canada.British Columbia|BOLD:ABY3181
Abagrotis trigona|[7495]|LPVIB271-08|PFC-2006-1661|658[0n]bp|Canada.British Columbia|BOLD:ABY3181
Abagrotis trigona|[7496]|RDNMC158-05|CNCNoctuoidea10899|658[0n]bp|Canada.Alberta|BOLD:ABY3181
Abagrotis trigona|[7497]|LBCG478-08|08-JDWBC-0478|658[0n]bp|Canada.British Columbia|BOLD:ABY3181
Abagrotis trigona|[7498]|LBCC340-05|HLC-22220|658[0n]bp|Canada.British Columbia|BOLD:ABY3181
Abagrotis duanca|[7499]|RDNMC108-05|CNCNoctuoidea10849|658[0n]bp|Canada.British Columbia|BOLD:ACF4293
Abagrotis duanca|[7500]|LBCG1353-09|08-JDWBC-1353|658[0n]bp|Canada.British Columbia|BOLD:ACF4293
Abagrotis duanca|[7501]|LOWCE759-06|CGWC-4519|658[0n]bp|Canada.British Columbia|BOLD:ACF4293
Abagrotis duanca|[7502]|LBCG193-08|08-JDWBC-0193|658[0n]bp|Canada.British Columbia|BOLD:ACF4293
Abagrotis duanca|[7503]|LBCG2872-09|08-JDWBC-2872|658[0n]bp|Canada.British Columbia|BOLD:ACF4293
Abagrotis duanca|[7504]|RDMAB656-06|UASM58763|658[0n]bp|Canada.Alberta|BOLD:ACF4293
Abagrotis duanca|[7505]|LBCG1349-09|08-JDWBC-1349|658[0n]bp|Canada.British Columbia|BOLD:ACF4293
Abagrotis scopeops|[7506]|LALPA1315-11|AVBC 1317-11|658[0n]bp|Canada.British Columbia|BOLD:ACF0138
Abagrotis scopeops|[7507]|LBCH7814-10|10-JDWBC-7814|658[0n]bp|Canada.British Columbia|BOLD:ACF0138
Abagrotis scopeops|[7508]|LALPA1228-11|AVBC 1230-11|658[0n]bp|Canada.British Columbia|BOLD:ACF0138
Abagrotis scopeops|[7509]|RDNMG392-08|CNC LEP00052216|658[0n]bp|Canada.British Columbia|BOLD:ACF0138
Abagrotis scopeops|[7510]|RDNMG391-08|CNC LEP00052215|658[0n]bp|Canada.British Columbia|BOLD:ACF0138
Abagrotis scopeops|[7511]|LBCH7393-10|10-JDWBC-7393|658[0n]bp|Canada.British Columbia|BOLD:ACF0138
Abagrotis scopeops|[7512]|LBCH7331-10|10-JDWBC-7331|658[0n]bp|Canada.British Columbia|BOLD:ACF0138
Abagrotis scopeops|[7513]|LALPA1334-12|AVBC 1336-11|632[0n]bp|Canada.British Columbia|BOLD:ACF0138
Abagrotis scopeops|[7514]|LALPA703-10|AVBC 705-10|658[0n]bp|Canada.British Columbia|BOLD:ACF0138
Abagrotis scopeops|[7515]|LBCH1776-10|10-JDWBC-1776|658[0n]bp|Canada.British Columbia|BOLD:ACF0138
Abagrotis pulchrata|[7516]|DUNLP147-08|Dun-08-147|658[0n]bp|Canada.British Columbia|BOLD:ABZ7253
Abagrotis baueri|[7517]|LPVIB660-08|PFC-2006-2109|634[0n]bp|Canada.British Columbia|BOLD:ACF3416
Abagrotis baueri|[7518]|LALPA729-10|AVBC 731-10|658[0n]bp|Canada.British Columbia|BOLD:ACF3416
Abagrotis baueri|[7519]|LALPA1243-11|AVBC 1245-11|658[0n]bp|Canada.British Columbia|BOLD:ACF3416
Abagrotis baueri|[7520]|RDNM674-05|CNCNoctuoidea7520|658[0n]bp|Canada.British Columbia|BOLD:ACF3416
Abagrotis baueri|[7521]|RDNM673-05|CNCNoctuoidea7519|658[0n]bp|Canada.British Columbia|BOLD:ACF3416
Abagrotis baueri|[7522]|LALPA931-11|AVBC 1104-11|658[0n]bp|Canada.British Columbia|BOLD:ACF3416
Abagrotis orbis|[7523]|RDNMG385-08|CNC LEP00052209|658[0n]bp|Canada.Alberta|BOLD:ACF3347
Abagrotis orbis|[7524]|RDNMC129-05|CNCNoctuoidea10870|658[0n]bp|Canada.Alberta|BOLD:ACF3347
Abagrotis orbis|[7525]|RDNMG387-08|CNC LEP00052211|658[0n]bp|Canada.Ontario|BOLD:ACF3347
Abagrotis glenni|[7526]|NAMUM068-08|RR-98-2104|657[0n]bp|United States.Arizona|BOLD:ACE4051
Abagrotis mirabilis|[7527]|LBCH7837-10|10-JDWBC-7837|658[0n]bp|Canada.British Columbia|BOLD:ACF3643
Abagrotis mirabilis|[7528]|LBCH6153-10|10-JDWBC-6153|658[0n]bp|Canada.British Columbia|BOLD:ACF3643
Abagrotis mirabilis|[7529]|LOWCD391-06|CGWC-3211|658[0n]bp|Canada.British Columbia|BOLD:ACF3643
Abagrotis mirabilis|[7530]|BBLPB755-10|10BBCLP-1754|658[0n]bp|Canada.British Columbia|BOLD:ACF3643
Abagrotis mirabilis|[7531]|RDNMC164-05|CNCNoctuoidea10905|658[0n]bp|Canada.British Columbia|BOLD:ACF3643
Abagrotis mirabilis|[7532]|LBCH7325-10|10-JDWBC-7325|658[0n]bp|Canada.British Columbia|BOLD:ACF3643
Abagrotis mirabilis|[7533]|RDNM659-05|CNCNoctuoidea7505|658[0n]bp|Canada.British Columbia|BOLD:ACF3643
Abagrotis mirabilis|[7534]|RDNM658-05|CNCNoctuoidea7504|658[0n]bp|Canada.British Columbia|BOLD:ACF3643
Abagrotis mirabilis|[7535]|RDNMC163-05|CNCNoctuoidea10904|658[0n]bp|Canada.British Columbia|BOLD:ACF3643
Abagrotis mirabilis|[7536]|LBCH7239-10|10-JDWBC-7239|658[0n]bp|Canada.British Columbia|BOLD:ACF3643
Abagrotis mirabilis|[7537]|RDNM661-05|CNCNoctuoidea7507|616[0n]bp|Canada.British Columbia|BOLD:ACF3643
Abagrotis mirabilis|[7538]|LOWCD390-06|CGWC-3210|543[0n]bp|Canada.British Columbia|BOLD:ACF3643
Abagrotis mirabilis|[7539]|LBCH6763-10|10-JDWBC-6763|658[0n]bp|Canada.British Columbia|BOLD:ACF3643
Abagrotis mirabilis|[7540]|LBCH7101-10|10-JDWBC-7101|658[0n]bp|Canada.British Columbia|BOLD:ACF3643
Abagrotis glenni|[7541]|RDNM656-05|CNCNoctuoidea7502|658[0n]bp|United States.Oregon|BOLD:ACF3643
Abagrotis glenni|[7542]|RDNMC159-05|CNCNoctuoidea10900|658[0n]bp|United States.Oregon|BOLD:ACF3643
Abagrotis glenni|[7543]|RDNMC160-05|CNCNoctuoidea10901|658[0n]bp|United States.Oregon|BOLD:ACF3643
Abagrotis glenni|[7544]|RDNM657-05|CNCNoctuoidea7503|530[0n]bp|United States.Oregon|BOLD:ACF3643
Abagrotis mirabilis|[7545]|RDNMC140-05|CNCNoctuoidea10881|658[0n]bp|Canada.British Columbia|BOLD:ACF3643
Abagrotis mirabilis|[7546]|DUNLP145-08|Dun-08-145|625[1n]bp|Canada.British Columbia|BOLD:ACF3643
Abagrotis reedi|[7547]|RDNM686-05|CNCNoctuoidea7532|579[0n]bp|Canada.British Columbia|BOLD:ACF3347
Abagrotis reedi|[7548]|LBCH6552-10|10-JDWBC-6552|658[0n]bp|Canada.British Columbia|BOLD:ACF3347
Abagrotis reedi|[7549]|LBCH7808-10|10-JDWBC-7808|658[0n]bp|Canada.British Columbia|BOLD:ACF3347
Abagrotis reedi|[7550]|RDNMC148-05|CNCNoctuoidea10889|658[0n]bp|Canada.British Columbia|BOLD:ACF3347
Abagrotis reedi|[7551]|LBCH7813-10|10-JDWBC-7813|658[0n]bp|Canada.British Columbia|BOLD:ACF3347
Abagrotis reedi|[7552]|RDNMC151-05|CNCNoctuoidea10892|658[0n]bp|Canada.British Columbia|BOLD:ACF3347
Abagrotis reedi|[7553]|LBCH7375-10|10-JDWBC-7375|658[0n]bp|Canada.British Columbia|BOLD:ACF3347
Abagrotis reedi|[7554]|RDMAB240-05|UASM2428|658[0n]bp|Canada.Alberta|BOLD:ACF3347
Abagrotis reedi|[7555]|RDMAB652-06|UASM58435|658[0n]bp|Canada.Alberta|BOLD:ACF3347
Abagrotis reedi|[7556]|RDMAB518-06|UASM58459|658[0n]bp|Canada.Alberta|BOLD:ACF3347
Abagrotis reedi|[7557]|RDNMB292-05|CNCNoctuoidea10058|595[0n]bp|Canada.Manitoba|BOLD:ACF3347
Abagrotis reedi|[7558]|RDNMC143-05|CNCNoctuoidea10884|658[0n]bp|Canada.Saskatchewan|BOLD:ACF3347
Abagrotis reedi|[7559]|RDNMB290-05|CNCNoctuoidea10056|658[0n]bp|Canada.Manitoba|BOLD:ACF3347
Abagrotis variata|[7560]|LPSK436-08|08BBLEP-02004|658[0n]bp|Canada.Saskatchewan|BOLD:ACF5352
Abagrotis variata|[7561]|LALPA1275-11|AVBC 1277-11|658[0n]bp|Canada.British Columbia|BOLD:ACF5346
Abagrotis variata|[7562]|LALPA766-10|AVBC 768-10|658[0n]bp|Canada.British Columbia|BOLD:ACF5346
Abagrotis variata|[7563]|LALPA724-10|AVBC 726-10|658[0n]bp|Canada.British Columbia|BOLD:ACF5346
Abagrotis variata|[7564]|RDNMF340-08|NOC14426|658[0n]bp|Canada.British Columbia|BOLD:ABZ7251
Abagrotis variata|[7565]|RDNMF341-08|NOC14427|658[0n]bp|Canada.British Columbia|BOLD:ABZ7251
Abagrotis duanca|[7566]|RDNMC109-05|CNCNoctuoidea10850|658[0n]bp|Canada.British Columbia|BOLD:ACF3564
Abagrotis forbesi|[7567]|LBCH7958-10|10-JDWBC-7958|658[0n]bp|Canada.British Columbia|BOLD:ACF3351
Abagrotis forbesi|[7568]|RDNMC095-05|CNCNoctuoidea10836|658[0n]bp|Canada.British Columbia|BOLD:ACF3351
Abagrotis nanalis|[7569]|LBCG1370-09|08-JDWBC-1370|658[0n]bp|Canada.British Columbia|BOLD:ABZ7250
Abagrotis nanalis|[7570]|LBCG1369-09|08-JDWBC-1369|658[0n]bp|Canada.British Columbia|BOLD:ABZ7250
Abagrotis nanalis|[7571]|LBCH6000-10|10-JDWBC-6000|658[0n]bp|Canada.British Columbia|BOLD:ABZ7250
Abagrotis nanalis|[7572]|LBCH7000-10|10-JDWBC-7000|658[0n]bp|Canada.British Columbia|BOLD:ABZ7250
Abagrotis nanalis|[7573]|LBCH6765-10|10-JDWBC-6765|658[0n]bp|Canada.British Columbia|BOLD:ABZ7250
Abagrotis nanalis|[7574]|LBCH7882-10|10-JDWBC-7882|658[0n]bp|Canada.British Columbia|BOLD:ABZ7250
Abagrotis nanalis|[7575]|LBCH6850-10|10-JDWBC-6850|658[0n]bp|Canada.British Columbia|BOLD:ABZ7250

Abagrotis nanalis|[7573]|LBCH6765-10|10-JDWBC-6765|658[0n]bp|Canada.British Columbia|BOLD:ABZ7250
Abagrotis nanalis|[7574]|LBCH7882-10|10-JDWBC-7882|658[0n]bp|Canada.British Columbia|BOLD:ABZ7250
Abagrotis nanalis|[7575]|LBCH6850-10|10-JDWBC-6850|658[0n]bp|Canada.British Columbia|BOLD:ABZ7250
Abagrotis nanalis|[7576]|LBCH6534-10|10-JDWBC-6534|658[0n]bp|Canada.British Columbia|BOLD:ABZ7250
Abagrotis nanalis|[7577]|LBCH6947-10|10-JDWBC-6947|658[0n]bp|Canada.British Columbia|BOLD:ABZ7250
Abagrotis nanalis|[7578]|LBCG2869-09|08-JDWBC-2869|658[0n]bp|Canada.British Columbia|BOLD:ABZ7250
Abagrotis nanalis|[7579]|LBCH7780-10|10-JDWBC-7780|658[0n]bp|Canada.British Columbia|BOLD:ABZ7250
Abagrotis nanalis|[7580]|LBCH6834-10|10-JDWBC-6834|658[0n]bp|Canada.British Columbia|BOLD:ABZ7250
Abagrotis nanalis|[7581]|LBCH6919-10|10-JDWBC-6919|634[0n]bp|Canada.British Columbia|BOLD:ABZ7250
Abagrotis nanalis|[7582]|LBCH7538-10|10-JDWBC-7538|634[0n]bp|Canada.British Columbia|BOLD:ABZ7250
Abagrotis nanalis|[7583]|LBCH7694-10|10-JDWBC-7694|643[0n]bp|Canada.British Columbia|BOLD:ABZ7250
Abagrotis nanalis|[7584]|RDNMC092-05|CNCNoctuoidea10833|658[0n]bp|Canada.Alberta|BOLD:ABZ7250
Abagrotis placida|[7585]|RDNMC146-05|CNCNoctuoidea10887|577[7n]bp|Canada.Alberta|
Abagrotis placida|[7586]|RDNMC119-05|CNCNoctuoidea10860|658[0n]bp|Canada.British Columbia|BOLD:ACE7993
Abagrotis placida|[7587]|RDNMC111-05|CNCNoctuoidea10852|658[0n]bp|Canada.British Columbia|BOLD:ACE7993
Abagrotis placida|[7588]|LOWCE765-06|CGWC-4525|658[0n]bp|Canada.British Columbia|BOLD:ACE7993
Abagrotis placida|[7589]|RDMAB627-06|UASM58745|658[0n]bp|Canada.Alberta|BOLD:ACE7993
Abagrotis placida|[7590]|RDMAB628-06|UASM58749|658[0n]bp|Canada.Alberta|BOLD:ACE7993
Abagrotis placida|[7591]|LBCG2584-09|08-JDWBC-2584|658[0n]bp|Canada.British Columbia|BOLD:ACE7993
Abagrotis placida|[7592]|LBCH6659-10|10-JDWBC-6659|658[0n]bp|Canada.British Columbia|BOLD:ACE7993
Abagrotis placida|[7593]|RDMAB233-05|UASM58302|658[0n]bp|Canada.Alberta|BOLD:ACE7993
Abagrotis placida|[7594]|LBCH7792-10|10-JDWBC-7792|658[0n]bp|Canada.British Columbia|BOLD:ACE7993
Abagrotis placida|[7595]|RDMAB624-06|UASM58748|658[0n]bp|Canada.Alberta|BOLD:ACE7993
Abagrotis placida|[7596]|RDNMC170-05|CNCNoctuoidea10911|658[0n]bp|Canada.British Columbia|BOLD:ACE7993
Abagrotis placida|[7597]|RDNMC128-05|CNCNoctuoidea10869|658[0n]bp|Canada.British Columbia|BOLD:ACE7993
Abagrotis placida|[7598]|LBCH6549-10|10-JDWBC-6549|658[0n]bp|Canada.British Columbia|BOLD:ACE7993
Abagrotis placida|[7599]|RDMAB231-05|UASM58191|658[0n]bp|Canada.Alberta|BOLD:ACE7993
Abagrotis placida|[7600]|RDMAB598-06|UASM58634|658[0n]bp|Canada.Alberta|BOLD:ACE7993
Abagrotis placida|[7601]|RDMAB234-05|UASM2634|658[0n]bp|Canada.Alberta|BOLD:ACE7993
Abagrotis placida|[7602]|RDMAB626-06|UASM58750|658[0n]bp|Canada.Alberta|BOLD:ACE7993
Abagrotis placida|[7603]|LBCH6223-10|10-JDWBC-6223|658[0n]bp|Canada.British Columbia|BOLD:ACE7993
Abagrotis placida|[7604]|LBCH7431-10|10-JDWBC-7431|658[0n]bp|Canada.British Columbia|BOLD:ACE7993
Abagrotis placida|[7605]|BBLPB556-10|10BBCLP-1555|658[0n]bp|Canada.British Columbia|BOLD:ACE7993
Abagrotis placida|[7606]|LOWCE764-06|CGWC-4524|658[0n]bp|Canada.British Columbia|BOLD:ACE7993
Abagrotis placida|[7607]|RDMAB232-05|UASM58186|658[0n]bp|Canada.Alberta|BOLD:ACE7993
Abagrotis placida|[7608]|RDMAB625-06|UASM58752|621[0n]bp|Canada.Alberta|BOLD:ACE7993
Abagrotis placida|[7609]|RDNMC116-05|CNCNoctuoidea10857|558[0n]bp|Canada.Alberta|BOLD:ACE7993
Abagrotis placida|[7610]|RDNMC118-05|CNCNoctuoidea10859|574[0n]bp|Canada.Alberta|BOLD:ACE7993
Abagrotis placida|[7611]|RDNMC112-05|CNCNoctuoidea10853|570[0n]bp|Canada.Alberta|BOLD:ACE7993
Abagrotis placida|[7612]|RDNMC117-05|CNCNoctuoidea10858|594[0n]bp|Canada.Alberta|BOLD:ACE7993
Abagrotis placida|[7613]|RDNM694-05|CNCNoctuoidea7540|585[0n]bp|Canada.British Columbia|BOLD:ACE7993
Abagrotis placida|[7614]|LBCH4037-10|10-JDWBC-4037|658[0n]bp|Canada.British Columbia|BOLD:AAA5965
Abagrotis placida|[7615]|LBCH936-10|10-JDWBC-0936|658[0n]bp|Canada.British Columbia|BOLD:AAA5965
Abagrotis placida|[7616]|RDNMC123-05|CNCNoctuoidea10864|658[0n]bp|Canada.Alberta|BOLD:AAA5965
Abagrotis placida|[7617]|RDNMC122-05|CNCNoctuoidea10863|658[0n]bp|Canada.Alberta|BOLD:AAA5965
Abagrotis placida|[7618]|RDNM689-05|CNCNoctuoidea7535|658[0n]bp|Canada.British Columbia|BOLD:AAA5965
Abagrotis placida|[7619]|RDNMC120-05|CNCNoctuoidea10861|658[0n]bp|Canada.British Columbia|BOLD:AAA5965
Abagrotis placida|[7620]|RDNM688-05|CNCNoctuoidea7534|658[0n]bp|Canada.British Columbia|BOLD:AAA5965
Abagrotis placida|[7621]|RDNM690-05|CNCNoctuoidea7536|599[0n]bp|Canada.British Columbia|BOLD:AAA5965
Abagrotis placida|[7622]|RDNM696-05|CNCNoctuoidea7542|599[0n]bp|Canada.British Columbia|BOLD:AAA5965
Abagrotis placida|[7623]|BBLPB744-10|10BBCLP-1743|658[0n]bp|Canada.British Columbia|BOLD:AAA5965
Abagrotis placida|[7624]|DUNLP190-08|Dun-08-190|649[1n]bp|Canada.British Columbia|BOLD:AAA5965
Abagrotis placida|[7625]|RDMAB651-06|UASM58126|658[0n]bp|Canada.Alberta|BOLD:AAA5965
Abagrotis placida|[7626]|LOWCE766-06|CGWC-4526|658[0n]bp|Canada.British Columbia|BOLD:AAA5965
Abagrotis placida|[7627]|RDNMC121-05|CNCNoctuoidea10862|658[0n]bp|Canada.Alberta|BOLD:AAA5965
Abagrotis placida|[7628]|RDMAB236-05|UASM24113|658[0n]bp|Canada.Alberta|BOLD:AAA5965
Abagrotis placida|[7629]|RDMAB650-06|UASM58125|658[0n]bp|Canada.Alberta|BOLD:AAA5965
Abagrotis placida|[7630]|RDNMC171-05|CNCNoctuoidea10912|658[0n]bp|Canada.Alberta|BOLD:AAA5965
Abagrotis placida|[7631]|LOWCE763-06|CGWC-4523|658[0n]bp|Canada.British Columbia|BOLD:AAA5965
Abagrotis placida|[7632]|RDMAB655-06|UASM58762|658[0n]bp|Canada.Alberta|BOLD:AAA5965
Abagrotis placida|[7633]|RDMAB235-05|UASM24093|658[0n]bp|Canada.Alberta|BOLD:AAA5965
Abagrotis placida|[7634]|RDMAB654-06|UASM58761|658[0n]bp|Canada.Alberta|BOLD:AAA5965
Abagrotis placida|[7635]|RDNMC342-05|CNCNoctuoidea11976|631[0n]bp|Canada.Manitoba|BOLD:AAA5965
Abagrotis placida|[7636]|RDNMB287-05|CNCNoctuoidea10053|602[0n]bp|Canada.Manitoba|BOLD:AAA5965
Abagrotis placida|[7637]|RDNMB286-05|CNCNoctuoidea10052|601[0n]bp|Canada.Manitoba|BOLD:AAA5965
Abagrotis placida|[7638]|RDMAB373-05|UASM77839|605[0n]bp|Canada.Alberta|BOLD:AAA5965
Abagrotis cupida|[7639]|BBLEC446-09|09BBELE-0446|658[0n]bp|Canada.New Brunswick|BOLD:ACF4293
Abagrotis cupida|[7640]|BBLPC425-09|09BBELE-1425|658[0n]bp|Canada.New Brunswick|BOLD:ACF4293
Abagrotis cupida|[7641]|RDMAB230-05|UASM58303|600[1n]bp|Canada.Alberta|BOLD:ACF4293
Abagrotis cupida|[7642]|RDNMB291-05|CNCNoctuoidea10057|566[0n]bp|Canada.Manitoba|BOLD:ACF4293
Abagrotis cupida|[7643]|RDNM723-05|CNCNoctuoidea7569|618[0n]bp|Canada.Ontario|BOLD:ACF4293
Abagrotis cupida|[7644]|RDNM721-05|CNCNoctuoidea7567|618[0n]bp|Canada.Ontario|BOLD:ACF4293
Abagrotis cupida|[7645]|RDNMC730-06|CNCNoctuoidea12270|658[0n]bp|Canada.Ontario|BOLD:ACF4293
Abagrotis cupida|[7646]|RDMAB238-05|UASM34589|658[0n]bp|Canada.Alberta|BOLD:ACF4293
Abagrotis cupida|[7647]|RDMAB243-05|UASM2403|617[0n]bp|Canada.Alberta|BOLD:ACF4293
Abagrotis cupida|[7648]|RDNMC729-06|CNCNoctuoidea12269|658[0n]bp|Canada.Ontario|BOLD:ACF4293
Abagrotis cupida|[7649]|RDMAB682-06|UASM58794|658[0n]bp|Canada.Alberta|BOLD:ACF4293
Abagrotis cupida|[7650]|RDNM718-05|CNCNoctuoidea7564|609[0n]bp|Canada.Ontario|BOLD:ACF4293
Abagrotis cupida|[7651]|RDNM720-05|CNCNoctuoidea7566|607[0n]bp|Canada.Ontario|BOLD:ACF4293
Abagrotis brunneipennis|[7652]|RDNM682-05|CNCNoctuoidea7528|611[0n]bp|Canada.British Columbia|BOLD:AC...
Abagrotis cupida|[7653]|RDNM719-05|CNCNoctuoidea7565|607[0n]bp|Canada.Ontario|BOLD:ACF4293
Abagrotis brunneipennis|[7654]|RDNM669-05|CNCNoctuoidea7515|658[0n]bp|Canada.British Columbia|BOLD:AC...
Abagrotis cupida|[7655]|RDNMB288-05|CNCNoctuoidea10054|601[0n]bp|Canada.Manitoba|BOLD:ACF4293
Abagrotis cupida|[7656]|RDNM722-05|CNCNoctuoidea7568|658[0n]bp|Canada.Ontario|BOLD:ACF4293
Abagrotis cupida|[7657]|RDNMB297-05|CNCNoctuoidea10063|603[0n]bp|Canada.Manitoba|BOLD:ACF4293
Abagrotis cupida|[7658]|RDNMB270-05|CNCNoctuoidea10036|599[0n]bp|Canada.Manitoba|BOLD:ACF4293
Abagrotis brunneipennis|[7659]|RDNM680-05|CNCNoctuoidea7526|658[0n]bp|Canada.British Columbia|BOLD:AC...
Abagrotis brunneipennis|[7660]|LBCH7334-10|10-JDWBC-7334|658[0n]bp|Canada.British Columbia|BOLD:ACF4293
Abagrotis brunneipennis|[7661]|RDMAB653-06|UASM58760|658[0n]bp|Canada.Alberta|BOLD:ACF4293
Abagrotis brunneipennis|[7662]|RDNM681-05|CNCNoctuoidea7527|658[0n]bp|Canada.Ontario|BOLD:ACF4293
Abagrotis brunneipennis|[7663]|RDMAB242-05|UASM34754|658[0n]bp|Canada.Alberta|BOLD:ACF4293
Abagrotis brunneipennis|[7664]|RDMAB247-05|UASM41491|658[0n]bp|Canada.Alberta|BOLD:ACF4293
Abagrotis brunneipennis|[7665]|RDMAB245-05|UASM2386|658[0n]bp|Canada.Alberta|BOLD:ACF4293
Abagrotis brunneipennis|[7666]|TTMNB492-06|MNBTT-492|658[0n]bp|Canada.New Brunswick|BOLD:ACF4293
Abagrotis brunneipennis|[7667]|TTMNB494-06|MNBTT-494|658[0n]bp|Canada.New Brunswick|BOLD:ACF4293
Abagrotis brunneipennis|[7668]|RDMAB248-05|UASM41492|634[0n]bp|Canada.Alberta|BOLD:ACF4293
Abagrotis brunneipennis|[7669]|RDNM679-05|CNCNoctuoidea7525|658[0n]bp|Canada.Ontario|BOLD:ACF4293
Abagrotis brunneipennis|[7670]|TTMNB493-06|MNBTT-493|658[0n]bp|Canada.New Brunswick|BOLD:ACF4293
Abagrotis brunneipennis|[7671]|RDMAB246-05|UASM2387|658[0n]bp|Canada.Alberta|BOLD:ACF4293
Abagrotis cupida|[7672]|RDNMC726-06|CNCNoctuoidea12266|658[0n]bp|Canada.Manitoba|BOLD:ACF4293
Abagrotis cupida|[7673]|RDNMC731-06|CNCNoctuoidea12271|658[0n]bp|Canada.Ontario|BOLD:ACF4293
Abagrotis cupida|[7674]|RDMAB239-05|UASM34593|658[0n]bp|Canada.Alberta|BOLD:ACF4293
Abagrotis cupida|[7675]|TMNBB406-06|MNBTT-1346|658[0n]bp|Canada.New Brunswick|BOLD:ACF4293

Abagrotis cupida|[7672]|RDNMC720-06|CNCNoctuoidea12266|658[0n]bp|Canada.Manitoba|BOLD:ACF4293
Abagrotis cupida|[7673]|RDNMC731-06|CNCNoctuoidea12271|658[0n]bp|Canada.Ontario|BOLD:ACF4293
Abagrotis cupida|[7674]|RDMAB239-05|UASM34593|658[0n]bp|Canada.Alberta|BOLD:ACF4293
Abagrotis cupida|[7675]|TMNBB406-06|MNBTT-1346|658[0n]bp|Canada.New Brunswick|BOLD:ACF4293
Abagrotis cupida|[7676]|RDNM717-05|CNCNoctuoidea7563|658[0n]bp|Canada.Ontario|BOLD:ACF4293
Abagrotis cupida|[7677]|RDNMB293-05|CNCNoctuoidea10059|658[0n]bp|Canada.Manitoba|BOLD:ACF4293
Abagrotis cupida|[7678]|RDNMB449-05|CNCNoctuoidea10215|658[0n]bp|Canada.Manitoba|BOLD:ACF4293
Abagrotis cupida|[7679]|TMNBB407-06|MNBTT-1347|658[0n]bp|Canada.New Brunswick|BOLD:ACF4293
Abagrotis cupida|[7680]|RDNMB295-05|CNCNoctuoidea10061|658[0n]bp|Canada.Manitoba|BOLD:ACF4293
Abagrotis cupida|[7681]|RDNMB271-05|CNCNoctuoidea10037|658[0n]bp|Canada.Manitoba|BOLD:ACF4293
Abagrotis cupida|[7682]|XAJ855-06|2006-ONT-0855|658[0n]bp|Canada.Ontario|BOLD:ACF4293
Abagrotis cupida|[7683]|TMNBB408-06|MNBTT-1348|658[0n]bp|Canada.New Brunswick|BOLD:ACF4293
Abagrotis cupida|[7684]|RDNMB269-05|CNCNoctuoidea10035|658[0n]bp|Canada.Manitoba|BOLD:ACF4293
Abagrotis cupida|[7685]|RDNMB289-05|CNCNoctuoidea10055|658[0n]bp|Canada.Manitoba|BOLD:ACF4293
Abagrotis cupida|[7686]|RDMAB241-05|UASM2154|658[0n]bp|Canada.Alberta|BOLD:ACF4293
Abagrotis cupida|[7687]|RDLQ006-05|DH008353|658[0n]bp|Canada.Quebec|BOLD:ACF4293
Abagrotis cupida|[7688]|RDNMB296-05|CNCNoctuoidea10062|658[0n]bp|Canada.Manitoba|BOLD:ACF4293
Abagrotis cupida|[7689]|RDNMC725-06|CNCNoctuoidea12265|658[0n]bp|Canada.Ontario|BOLD:ACF4293
Abagrotis cupida|[7690]|RDMAB244-05|UASM58189|658[0n]bp|Canada.Alberta|BOLD:ACF4293
Abagrotis cupida|[7691]|RDNMB294-05|CNCNoctuoidea10060|658[0n]bp|Canada.Manitoba|BOLD:ACF4293
Abagrotis discoidalis|[7692]|RDNMC107-05|CNCNoctuoidea10848|658[0n]bp|Canada.Alberta|BOLD:ACF4293
Abagrotis nefascia|[7693]|RDNM652-05|CNCNoctuoidea7498|658[0n]bp|Canada.British Columbia|BOLD:ACF4293
Abagrotis nefascia|[7694]|RDNMC137-05|CNCNoctuoidea10878|658[0n]bp|Canada.British Columbia|BOLD:ACF4293
Abagrotis hermina|[7695]|LBCD456-05|HLC-23276|658[0n]bp|Canada.British Columbia|BOLD:ACF3428
Abagrotis hermina|[7696]|LBCH7885-10|10-JDWBC-7885|644[0n]bp|Canada.British Columbia|BOLD:ACF0135
Abagrotis hermina|[7697]|RDNMC167-05|CNCNoctuoidea10908|658[0n]bp|Canada.Alberta|BOLD:ACF0135
Abagrotis hermina|[7698]|RDMAB237-05|UASM34594|658[0n]bp|Canada.Alberta|BOLD:ACF0135
Abagrotis hermina|[7699]|RDMAB681-06|UASM58793|622[0n]bp|Canada.Alberta|BOLD:ACF0135
Abagrotis hermina|[7700]|RDNMC165-05|CNCNoctuoidea10906|655[0n]bp|Canada.Saskatchewan|BOLD:ACF0135
Abagrotis hermina|[7701]|RDNMC126-05|CNCNoctuoidea10867|658[0n]bp|Canada.British Columbia|BOLD:ACF0135
Abagrotis hermina|[7702]|BBLPB746-10|10BBCLP-1745|658[0n]bp|Canada.Alberta|BOLD:ACF0135
Abagrotis hermina|[7703]|RDNM692-05|CNCNoctuoidea7538|612[0n]bp|Canada.British Columbia|BOLD:ACF0135
Abagrotis hermina|[7704]|RDNMC166-05|CNCNoctuoidea10907|658[0n]bp|Canada.Alberta|BOLD:ACE7022
Abagrotis hermina|[7705]|RDNMB942-05|CNCNoctuoidea10717|658[0n]bp|Canada.Alberta|BOLD:ACE7022
Abagrotis hermina|[7706]|RDNMC153-05|CNCNoctuoidea10894|658[0n]bp|Canada.Alberta|BOLD:ACE7022
Abagrotis hermina|[7707]|RDMAB599-06|UASM58635|658[0n]bp|Canada.Alberta|BOLD:ACE7022
Abagrotis dickeli|[7708]|RDNMC082-05|CNCNoctuoidea10823|658[0n]bp|Canada.British Columbia|BOLD:ACF3422
Abagrotis dickeli|[7709]|RDNMC115-05|CNCNoctuoidea10856|658[0n]bp|Canada.British Columbia|BOLD:ACF3422
Abagrotis dickeli|[7710]|RDNM695-05|CNCNoctuoidea7541|610[0n]bp|Canada.British Columbia|BOLD:ACF3422
Abagrotis dickeli|[7711]|RDNMB273-05|CNCNoctuoidea10039|602[0n]bp|Canada.Manitoba|BOLD:ACE9894
Abagrotis dickeli|[7712]|RDNMB272-05|CNCNoctuoidea10038|658[0n]bp|Canada.Manitoba|BOLD:ACE9894
Abagrotis dickeli|[7713]|RDNMC127-05|CNCNoctuoidea10868|658[0n]bp|Canada.Saskatchewan|BOLD:ACE9894
Abagrotis dickeli|[7714]|RDNMC343-05|CNCNoctuoidea11977|658[0n]bp|Canada.Manitoba|BOLD:ACE9894
Abagrotis dickeli|[7715]|RDNMC727-06|CNCNoctuoidea12267|658[0n]bp|Canada.Manitoba|BOLD:ACE9894
Abagrotis turbulenta|[7716]|RDNMC161-05|CNCNoctuoidea10902|658[0n]bp|Canada.British Columbia|BOLD:ACE...
Abagrotis turbulenta|[7717]|RDNMG679-08|CNC LEP00052803|658[0n]bp|Canada.British Columbia|BOLD:ACE9894
Abagrotis turbulenta|[7718]|RDNMG680-08|CNC LEP00052804|658[0n]bp|Canada.British Columbia|BOLD:ACE9894
Abagrotis anchocelioides|[7719]|RDLQ740-07|DH008024|658[0n]bp|Canada.Quebec|BOLD:ACE6487
Abagrotis anchocelioides|[7720]|RDLQ005-05|DH008337|573[0n]bp|Canada.Quebec|BOLD:ACE6487
Abagrotis anchocelioides|[7721]|RDLQ742-07|DH007093|658[0n]bp|Canada.Quebec|BOLD:ACE6487
Abagrotis anchocelioides|[7722]|RDLQ741-07|DH007394|658[0n]bp|Canada.Quebec|BOLD:ACE6487
Abagrotis nefascia|[7723]|LBCH7600-10|10-JDWBC-7600|658[0n]bp|Canada.British Columbia|BOLD:ABZ7252
Abagrotis nefascia|[7724]|RDNMC138-05|CNCNoctuoidea10879|658[0n]bp|Canada.British Columbia|BOLD:ABZ7252
Abagrotis nefascia|[7725]|RDNM653-05|CNCNoctuoidea7499|636[0n]bp|Canada.British Columbia|BOLD:ABZ7252
Abagrotis nefascia|[7726]|LBCH7169-10|10-JDWBC-7169|658[0n]bp|Canada.British Columbia|BOLD:ABZ7252
Abagrotis vittifrons|[7727]|LBCH6619-10|10-JDWBC-6619|658[0n]bp|Canada.British Columbia|BOLD:ACF3351
Abagrotis vittifrons|[7728]|LBCH7727-10|10-JDWBC-7727|658[0n]bp|Canada.British Columbia|BOLD:ACF3351
Abagrotis vittifrons|[7729]|LBCH7119-10|10-JDWBC-7119|658[0n]bp|Canada.British Columbia|BOLD:ACF3351
Abagrotis vittifrons|[7730]|RDNMC093-05|CNCNoctuoidea10834|658[0n]bp|Canada.British Columbia|BOLD:ACF...
Abagrotis vittifrons|[7731]|LOWC915-05|CGWC-0915|658[0n]bp|Canada.British Columbia|BOLD:ACF3351
Abagrotis vittifrons|[7732]|LOWC916-05|CGWC-0916|658[0n]bp|Canada.British Columbia|BOLD:ACF3351
Abagrotis vittifrons|[7733]|LOWC914-05|CGWC-0914|658[0n]bp|Canada.British Columbia|BOLD:ACF3351
Abagrotis vittifrons|[7734]|LOWC913-05|CGWC-0913|658[0n]bp|Canada.British Columbia|BOLD:ACF3351
Abagrotis vittifrons|[7735]|LOWCD687-06|CGWC-3507|609[0n]bp|Canada.British Columbia|BOLD:ACF3351
Abagrotis vittifrons|[7736]|LBCH7329-10|10-JDWBC-7329|658[0n]bp|Canada.British Columbia|BOLD:ACF3351
Abagrotis vittifrons|[7737]|LOWCD685-06|CGWC-3505|658[0n]bp|Canada.British Columbia|BOLD:ACF3351
Abagrotis vittifrons|[7738]|LBCH7504-10|10-JDWBC-7504|658[0n]bp|Canada.British Columbia|BOLD:ACF3351
Abagrotis vittifrons|[7739]|LBCH6835-10|10-JDWBC-6835|658[0n]bp|Canada.British Columbia|BOLD:ACF3351
Abagrotis vittifrons|[7740]|LOWCD686-06|CGWC-3506|658[0n]bp|Canada.British Columbia|BOLD:ACF3351
Abagrotis vittifrons|[7741]|LBCH7692-10|10-JDWBC-7692|658[0n]bp|Canada.British Columbia|BOLD:ACF3351
Abagrotis vittifrons|[7742]|LBCH7881-10|10-JDWBC-7881|658[0n]bp|Canada.British Columbia|BOLD:ACF3351
Abagrotis erratica|[7743]|RDNMC131-05|CNCNoctuoidea10872|635[0n]bp|Canada.British Columbia|BOLD:AAE2248
Abagrotis erratica|[7744]|RDNMC132-05|CNCNoctuoidea10873|581[0n]bp|Canada.British Columbia|BOLD:AAE2248
Pronoctua craboi|[7745]|RDMAB342-05|UASM34869|658[0n]bp|Canada.Alberta|BOLD:AAD8045
Pronoctua craboi|[7746]|RDNMF433-08|NOC14519|658[0n]bp|Canada.British Columbia|BOLD:AAD8045
Pronoctua craboi|[7747]|RDNMF432-08|NOC14518|658[0n]bp|Canada.British Columbia|BOLD:AAD8045
Pronoctua craboi|[7748]|RDMAB340-05|UASM24066|658[0n]bp|Canada.Alberta|BOLD:AAD8045
Pronoctua peabodyae|[7749]|RDMAB341-05|UASM58178|658[0n]bp|Canada.Alberta|BOLD:AAD8042
Pronoctua peabodyae|[7750]|RDMAB629-06|UASM2404|658[0n]bp|Canada.Alberta|BOLD:AAD8042
Abagrotis dodi|[7751]|LBCH7557-10|10-JDWBC-7557|658[0n]bp|Canada.British Columbia|BOLD:AAF3346
Abagrotis dodi|[7752]|RDNMC105-05|CNCNoctuoidea10846|658[0n]bp|Canada.British Columbia|BOLD:AAF3346
Abagrotis dodi|[7753]|LBCH7887-10|10-JDWBC-7887|658[0n]bp|Canada.British Columbia|BOLD:AAF3346
Abagrotis dodi|[7754]|LBCH6545-10|10-JDWBC-6545|658[0n]bp|Canada.British Columbia|BOLD:AAF3346
Abagrotis dodi|[7755]|LBCH7554-10|10-JDWBC-7554|658[0n]bp|Canada.British Columbia|BOLD:AAF3346
Pronoctua typica|[7756]|RDMAB293-05|UASM77789|658[0n]bp|Canada.Alberta|BOLD:AAD9535
Pronoctua typica|[7757]|RDNME777-08|LEP041242|658[0n]bp|Canada.Alberta|BOLD:AAD9535
Pronoctua typica|[7758]|LBCH6218-10|10-JDWBC-6218|658[0n]bp|Canada.British Columbia|BOLD:AAD9535
Pronoctua typica|[7759]|LBCH5719-10|10-JDWBC-5719|658[0n]bp|Canada.British Columbia|BOLD:AAD9535
Pronoctua typica|[7760]|LBCH7466-10|10-JDWBC-7466|658[0n]bp|Canada.British Columbia|BOLD:AAD9535
Pronoctua typica|[7761]|RDNME776-08|LEP041241|658[0n]bp|Canada.Alberta|BOLD:AAD9535
Parabagrotis exsertistigma|[7762]|LPABC602-09|08BBLEP-04821|632[0n]bp|Canada.Alberta|BOLD:AAA3352
Parabagrotis exsertistigma|[7763]|LPABC569-09|08BBLEP-04788|658[0n]bp|Canada.Alberta|BOLD:AAA3352
Parabagrotis exsertistigma|[7764]|LPABC572-09|08BBLEP-04791|658[0n]bp|Canada.Alberta|BOLD:AAA3352
Parabagrotis exsertistigma|[7765]|LPABC576-09|08BBLEP-04795|658[0n]bp|Canada.Alberta|BOLD:AAA3352
Parabagrotis exsertistigma|[7766]|LPABB465-08|08BBLEP-03730|658[0n]bp|Canada.Alberta|BOLD:AAA3352
Parabagrotis exsertistigma|[7767]|LBCH7943-10|10-JDWBC-7943|658[0n]bp|Canada.British Columbia|BOLD:AA...
Parabagrotis exsertistigma|[7768]|LPABC614-09|08BBLEP-04833|658[0n]bp|Canada.Alberta|BOLD:AAA3352
Parabagrotis exsertistigma|[7769]|LPABC651-09|08BBLEP-04870|658[1n]bp|Canada.Alberta|
Parabagrotis exsertistigma|[7770]|LBCH7949-10|10-JDWBC-7949|658[0n]bp|Canada.British Columbia|BOLD:AA...
Parabagrotis exsertistigma|[7771]|LPABC581-09|08BBLEP-04800|658[0n]bp|Canada.Alberta|BOLD:AAA3352
Parabagrotis exsertistigma|[7772]|LPABB461-08|08BBLEP-03726|658[0n]bp|Canada.Alberta|BOLD:AAA3352
Parabagrotis exsertistigma|[7773]|LPABC575-09|08BBLEP-04794|658[0n]bp|Canada.Alberta|BOLD:AAA3352
Parabagrotis exsertistigma|[7774]|LPABC595-09|08BBLEP-04814|658[0n]bp|Canada.Alberta|BOLD:AAA3352
[illegible]

Parabagrotis exsertistigma|[7772]|LPABB461-08|08BBLEP-03726|658[0n]bp|Canada.Alberta|BOLD:AAA3352
Parabagrotis exsertistigma|[7773]|LPABC575-09|08BBLEP-04794|658[0n]bp|Canada.Alberta|BOLD:AAA3352
Parabagrotis exsertistigma|[7774]|LPABC595-09|08BBLEP-04814|658[0n]bp|Canada.Alberta|BOLD:AAA3352
Parabagrotis exsertistigma|[7775]|LBCH6946-10|10-JDWBC-6946|658[0n]bp|Canada.British Columbia|BOLD:AA...
Parabagrotis exsertistigma|[7776]|LPABB456-08|08BBLEP-03721|658[0n]bp|Canada.Alberta|BOLD:AAA3352
Parabagrotis exsertistigma|[7777]|LPABC589-09|08BBLEP-04808|658[0n]bp|Canada.Alberta|BOLD:AAA3352
Parabagrotis exsertistigma|[7778]|LPABB464-08|08BBLEP-03729|658[0n]bp|Canada.Alberta|BOLD:AAA3352
Parabagrotis exsertistigma|[7779]|LPABC621-09|08BBLEP-04840|658[0n]bp|Canada.Alberta|BOLD:AAA3352
Parabagrotis exsertistigma|[7780]|LPABC588-09|08BBLEP-04807|658[0n]bp|Canada.Alberta|BOLD:AAA3352
Parabagrotis exsertistigma|[7781]|LPAB016-08|08BBLEP-02338|658[0n]bp|Canada.Alberta|BOLD:AAA3352
Parabagrotis exsertistigma|[7782]|LPABC635-09|08BBLEP-04854|658[0n]bp|Canada.Alberta|BOLD:AAA3352
Parabagrotis exsertistigma|[7783]|LPABC591-09|08BBLEP-04810|658[0n]bp|Canada.Alberta|BOLD:AAA3352
Parabagrotis exsertistigma|[7784]|LPAB077-08|08BBLEP-02399|658[0n]bp|Canada.Alberta|BOLD:AAA3352
Parabagrotis exsertistigma|[7785]|LPABC615-09|08BBLEP-04834|658[0n]bp|Canada.Alberta|BOLD:AAA3352
Parabagrotis exsertistigma|[7786]|LPABC598-09|08BBLEP-04817|658[0n]bp|Canada.Alberta|BOLD:AAA3352
Parabagrotis exsertistigma|[7787]|LPABC639-09|08BBLEP-04858|658[0n]bp|Canada.Alberta|BOLD:AAA3352
Parabagrotis exsertistigma|[7788]|LPABC620-09|08BBLEP-04839|658[0n]bp|Canada.Alberta|BOLD:AAA3352
Parabagrotis exsertistigma|[7789]|LBCH7133-10|10-JDWBC-7133|658[0n]bp|Canada.British Columbia|BOLD:AA...
Parabagrotis exsertistigma|[7790]|LPABC607-09|08BBLEP-04826|658[0n]bp|Canada.Alberta|BOLD:AAA3352
Parabagrotis exsertistigma|[7791]|LBCH6819-10|10-JDWBC-6819|658[0n]bp|Canada.British Columbia|BOLD:AA...
Parabagrotis exsertistigma|[7792]|LBCH7313-10|10-JDWBC-7313|658[0n]bp|Canada.British Columbia|BOLD:AA...
Parabagrotis exsertistigma|[7793]|LPAB009-08|08BBLEP-02331|642[0n]bp|Canada.Alberta|BOLD:AAA3352
Parabagrotis exsertistigma|[7794]|LPABC638-09|08BBLEP-04857|632[0n]bp|Canada.Alberta|BOLD:AAA3352
Parabagrotis exsertistigma|[7795]|LBCH6913-10|10-JDWBC-6913|635[0n]bp|Canada.British Columbia|BOLD:AA...
Parabagrotis exsertistigma|[7796]|RDMAB047-05|UASM57530|635[0n]bp|Canada.Alberta|BOLD:AAA3352
Parabagrotis exsertistigma|[7797]|LBCH5107-10|10-JDWBC-5107|658[0n]bp|Canada.British Columbia|BOLD:AA...
Parabagrotis exsertistigma|[7798]|LPABC583-09|08BBLEP-04802|658[0n]bp|Canada.Alberta|BOLD:AAA3352
Parabagrotis exsertistigma|[7799]|LBCH6144-10|10-JDWBC-6144|658[0n]bp|Canada.British Columbia|BOLD:AA...
Parabagrotis exsertistigma|[7800]|LPABC586-09|08BBLEP-04805|658[0n]bp|Canada.Alberta|BOLD:AAA3352
Parabagrotis exsertistigma|[7801]|LPABC604-09|08BBLEP-04823|658[0n]bp|Canada.Alberta|BOLD:AAA3352
Parabagrotis exsertistigma|[7802]|LPAB019-08|08BBLEP-02341|658[0n]bp|Canada.Alberta|BOLD:AAA3352
Parabagrotis exsertistigma|[7803]|LPABC568-09|08BBLEP-04787|605[0n]bp|Canada.Alberta|BOLD:AAA3352
Parabagrotis exsertistigma|[7804]|LPABC605-09|08BBLEP-04824|600[0n]bp|Canada.Alberta|BOLD:AAA3352
Parabagrotis exsertistigma|[7805]|LPABC567-09|08BBLEP-04786|658[0n]bp|Canada.Alberta|BOLD:AAA3352
Parabagrotis exsertistigma|[7806]|LPABC585-09|08BBLEP-04804|658[0n]bp|Canada.Alberta|BOLD:AAA3352
Parabagrotis exsertistigma|[7807]|LPABC628-09|08BBLEP-04847|658[0n]bp|Canada.Alberta|BOLD:AAA3352
Parabagrotis exsertistigma|[7808]|LPABC610-09|08BBLEP-04829|658[0n]bp|Canada.Alberta|BOLD:AAA3352
Parabagrotis exsertistigma|[7809]|LPABC632-09|08BBLEP-04851|658[0n]bp|Canada.Alberta|BOLD:AAA3352
Parabagrotis exsertistigma|[7810]|LPABC570-09|08BBLEP-04789|658[0n]bp|Canada.Alberta|BOLD:AAA3352
Parabagrotis exsertistigma|[7811]|LPABC579-09|08BBLEP-04798|658[0n]bp|Canada.Alberta|BOLD:AAA3352
Parabagrotis exsertistigma|[7812]|LBCH6825-10|10-JDWBC-6825|658[0n]bp|Canada.British Columbia|BOLD:AA...
Parabagrotis exsertistigma|[7813]|LPABC599-09|08BBLEP-04818|658[0n]bp|Canada.Alberta|BOLD:AAA3352
Parabagrotis exsertistigma|[7814]|LPABC603-09|08BBLEP-04822|658[0n]bp|Canada.Alberta|BOLD:AAA3352
Parabagrotis exsertistigma|[7815]|LPABC616-09|08BBLEP-04835|658[0n]bp|Canada.Alberta|BOLD:AAA3352
Parabagrotis exsertistigma|[7816]|LPABC629-09|08BBLEP-04848|658[0n]bp|Canada.Alberta|BOLD:AAA3352
Parabagrotis exsertistigma|[7817]|LPABC566-09|08BBLEP-04785|658[0n]bp|Canada.Alberta|BOLD:AAA3352
Parabagrotis exsertistigma|[7818]|LPABC594-09|08BBLEP-04813|658[0n]bp|Canada.Alberta|BOLD:AAA3352
Parabagrotis exsertistigma|[7819]|LPABC592-09|08BBLEP-04811|658[0n]bp|Canada.Alberta|BOLD:AAA3352
Parabagrotis exsertistigma|[7820]|LBCH7503-10|10-JDWBC-7503|658[0n]bp|Canada.British Columbia|BOLD:AA...
Parabagrotis exsertistigma|[7821]|LPABC619-09|08BBLEP-04838|658[0n]bp|Canada.Alberta|BOLD:AAA3352
Parabagrotis exsertistigma|[7822]|LBCH6822-10|10-JDWBC-6822|658[0n]bp|Canada.British Columbia|BOLD:AA...
Parabagrotis exsertistigma|[7823]|LPABB462-08|08BBLEP-03727|658[0n]bp|Canada.Alberta|BOLD:AAA3352
Parabagrotis exsertistigma|[7824]|LPABC571-09|08BBLEP-04790|658[0n]bp|Canada.Alberta|BOLD:AAA3352
Parabagrotis exsertistigma|[7825]|LPABC625-09|08BBLEP-04844|658[0n]bp|Canada.Alberta|BOLD:AAA3352
Parabagrotis exsertistigma|[7826]|LBCG418-08|08-JDWBC-0418|658[0n]bp|Canada.British Columbia|BOLD:AAA...
Parabagrotis exsertistigma|[7827]|LPABC574-09|08BBLEP-04793|658[0n]bp|Canada.Alberta|BOLD:AAA3352
Parabagrotis exsertistigma|[7828]|LPAB011-08|08BBLEP-02333|658[0n]bp|Canada.Alberta|BOLD:AAA3352
Parabagrotis exsertistigma|[7829]|LOWCC182-05|CGWC-2062|658[0n]bp|Canada.British Columbia|BOLD:AAA3352
Parabagrotis exsertistigma|[7830]|LPABB007-08|08BBLEP-03272|658[0n]bp|Canada.Alberta|BOLD:AAA3352
Parabagrotis exsertistigma|[7831]|LPVIC116-08|PFC-2006-2693|658[0n]bp|Canada.British Columbia|BOLD:AA...
Parabagrotis exsertistigma|[7832]|LPABC596-09|08BBLEP-04815|658[0n]bp|Canada.Alberta|BOLD:AAA3352
Parabagrotis exsertistigma|[7833]|LPVIC115-08|PFC-2006-2692|658[0n]bp|Canada.British Columbia|BOLD:AA...
Parabagrotis exsertistigma|[7834]|LPABC578-09|08BBLEP-04797|658[0n]bp|Canada.Alberta|BOLD:AAA3352
Parabagrotis exsertistigma|[7835]|LPABC608-09|08BBLEP-04827|658[0n]bp|Canada.Alberta|BOLD:AAA3352
Parabagrotis exsertistigma|[7836]|LPABC611-09|08BBLEP-04830|658[0n]bp|Canada.Alberta|BOLD:AAA3352
Parabagrotis exsertistigma|[7837]|LBCH6935-10|10-JDWBC-6935|658[0n]bp|Canada.British Columbia|BOLD:AA...
Parabagrotis exsertistigma|[7838]|LPABC909-09|08BBLEP-05320|658[0n]bp|Canada.Alberta|BOLD:AAA3352
Parabagrotis exsertistigma|[7839]|LPABC593-09|08BBLEP-04812|658[0n]bp|Canada.Alberta|BOLD:AAA3352
Parabagrotis exsertistigma|[7840]|LPABC926-09|08BBLEP-05337|658[0n]bp|Canada.Alberta|BOLD:AAA3352
Parabagrotis exsertistigma|[7841]|LPABC634-09|08BBLEP-04853|658[0n]bp|Canada.Alberta|BOLD:AAA3352
Parabagrotis exsertistigma|[7842]|LPABB463-08|08BBLEP-03728|658[0n]bp|Canada.Alberta|BOLD:AAA3352
Parabagrotis exsertistigma|[7843]|LBCH6820-10|10-JDWBC-6820|658[0n]bp|Canada.British Columbia|BOLD:AA...
Parabagrotis exsertistigma|[7844]|LBCH7029-10|10-JDWBC-7029|658[0n]bp|Canada.British Columbia|BOLD:AA...
Parabagrotis exsertistigma|[7845]|LPABC630-09|08BBLEP-04849|658[0n]bp|Canada.Alberta|BOLD:AAA3352
Parabagrotis exsertistigma|[7846]|LBCH7942-10|10-JDWBC-7942|658[0n]bp|Canada.British Columbia|BOLD:AA...
Parabagrotis exsertistigma|[7847]|LOWCC181-05|CGWC-2061|658[0n]bp|Canada.British Columbia|BOLD:AAA3352
Parabagrotis exsertistigma|[7848]|LPABC617-09|08BBLEP-04836|658[0n]bp|Canada.Alberta|BOLD:AAA3352
Parabagrotis exsertistigma|[7849]|LBCH7944-10|10-JDWBC-7944|658[0n]bp|Canada.British Columbia|BOLD:AA...
Parabagrotis exsertistigma|[7850]|LPABC584-09|08BBLEP-04803|658[0n]bp|Canada.Alberta|BOLD:AAA3352
Parabagrotis exsertistigma|[7851]|LBCH6823-10|10-JDWBC-6823|658[0n]bp|Canada.British Columbia|BOLD:AA...
Parabagrotis exsertistigma|[7852]|LPABC622-09|08BBLEP-04841|658[0n]bp|Canada.Alberta|BOLD:AAA3352
Parabagrotis exsertistigma|[7853]|LBCH6824-10|10-JDWBC-6824|658[0n]bp|Canada.British Columbia|BOLD:AA...
Parabagrotis exsertistigma|[7854]|LBCH6788-10|10-JDWBC-6788|658[0n]bp|Canada.British Columbia|BOLD:AA...
Parabagrotis exsertistigma|[7855]|LPABC587-09|08BBLEP-04806|658[0n]bp|Canada.Alberta|BOLD:AAA3352
Parabagrotis exsertistigma|[7856]|LPABC624-09|08BBLEP-04843|658[0n]bp|Canada.Alberta|BOLD:AAA3352
Parabagrotis exsertistigma|[7857]|LPABB333-08|08BBLEP-03598|658[0n]bp|Canada.Alberta|BOLD:AAA3352
Parabagrotis exsertistigma|[7858]|LPABB332-08|08BBLEP-03597|658[0n]bp|Canada.Alberta|BOLD:AAA3352
Parabagrotis exsertistigma|[7859]|LPABB460-08|08BBLEP-03725|658[0n]bp|Canada.Alberta|BOLD:AAA3352
Parabagrotis exsertistigma|[7860]|LBCH7238-10|10-JDWBC-7238|658[0n]bp|Canada.British Columbia|BOLD:AA...
Parabagrotis exsertistigma|[7861]|LPABB457-08|08BBLEP-03722|658[0n]bp|Canada.Alberta|BOLD:AAA3352
Parabagrotis exsertistigma|[7862]|LPABB458-08|08BBLEP-03723|658[0n]bp|Canada.Alberta|BOLD:AAA3352
Parabagrotis exsertistigma|[7863]|LPABC631-09|08BBLEP-04850|658[0n]bp|Canada.Alberta|BOLD:AAA3352
Parabagrotis exsertistigma|[7864]|LBCG1348-09|08-JDWBC-1348|658[0n]bp|Canada.British Columbia|BOLD:AA...
Parabagrotis exsertistigma|[7865]|LPABC565-09|08BBLEP-04784|658[0n]bp|Canada.Alberta|BOLD:AAA3352
Parabagrotis exsertistigma|[7866]|LPABC577-09|08BBLEP-04796|658[0n]bp|Canada.Alberta|BOLD:AAA3352
Parabagrotis exsertistigma|[7867]|LPABC975-09|08BBLEP-05386|658[0n]bp|Canada.Alberta|BOLD:AAA3352
Parabagrotis exsertistigma|[7868]|LPAB007-08|08BBLEP-02329|658[0n]bp|Canada.Alberta|BOLD:AAA3352
Parabagrotis exsertistigma|[7869]|LPABC597-09|08BBLEP-04816|658[0n]bp|Canada.Alberta|BOLD:AAA3352
Parabagrotis exsertistigma|[7870]|LPABC618-09|08BBLEP-04837|658[0n]bp|Canada.Alberta|BOLD:AAA3352
Parabagrotis exsertistigma|[7871]|LBCH7948-10|10-JDWBC-7948|658[0n]bp|Canada.British Columbia|BOLD:AA...
Parabagrotis exsertistigma|[7872]|LPAB017-08|08BBLEP-02339|658[0n]bp|Canada.Alberta|BOLD:AAA3352
Parabagrotis exsertistigma|[7873]|LBCH6818-10|10-JDWBC-6818|658[0n]bp|Canada.British Columbia|BOLD:AA...
Parabagrotis exsertistigma|[7874]|LPABC633-09|08BBLEP-04852|658[0n]bp|Canada.Alberta|BOLD:AAA3352

Parabagrotis exsertistigma|[7872]|LPAB017-08|08BBLEP-02339|658[0n]bp|Canada.Alberta|BOLD:AAA3352
Parabagrotis exsertistigma|[7873]|LBCH6818-10|10-JDWBC-6818|658[0n]bp|Canada.British Columbia|BOLD:AA...
Parabagrotis exsertistigma|[7874]|LPABC633-09|08BBLEP-04852|658[0n]bp|Canada.Alberta|BOLD:AAA3352
Parabagrotis exsertistigma|[7875]|LPABB459-08|08BBLEP-03724|658[0n]bp|Canada.Alberta|BOLD:AAA3352
Parabagrotis exsertistigma|[7876]|LPABC580-09|08BBLEP-04799|658[0n]bp|Canada.Alberta|BOLD:AAA3352
Parabagrotis exsertistigma|[7877]|LPABC612-09|08BBLEP-04831|658[0n]bp|Canada.Alberta|BOLD:AAA3352
Parabagrotis exsertistigma|[7878]|LBCH7945-10|10-JDWBC-7945|658[0n]bp|Canada.British Columbia|BOLD:AA...
Parabagrotis exsertistigma|[7879]|LPAB018-08|08BBLEP-02340|658[0n]bp|Canada.Alberta|BOLD:AAA3352
Parabagrotis exsertistigma|[7880]|LPABC609-09|08BBLEP-04828|658[0n]bp|Canada.Alberta|BOLD:AAA3352
Parabagrotis exsertistigma|[7881]|LPABB358-08|08BBLEP-03623|658[0n]bp|Canada.Alberta|BOLD:AAA3352
Parabagrotis exsertistigma|[7882]|LPABC627-09|08BBLEP-04846|658[0n]bp|Canada.Alberta|BOLD:AAA3352
Parabagrotis exsertistigma|[7883]|LPABC640-09|08BBLEP-04859|658[0n]bp|Canada.Alberta|BOLD:AAA3352
Parabagrotis exsertistigma|[7884]|LPAB006-08|08BBLEP-02328|640[0n]bp|Canada.Alberta|BOLD:AAA3352
Parabagrotis exsertistigma|[7885]|LPABC626-09|08BBLEP-04845|632[0n]bp|Canada.Alberta|BOLD:AAA3352
Parabagrotis exsertistigma|[7886]|LPABC606-09|08BBLEP-04825|632[0n]bp|Canada.Alberta|BOLD:AAA3352
Parabagrotis exsertistigma|[7887]|LPABC637-09|08BBLEP-04856|632[0n]bp|Canada.Alberta|BOLD:AAA3352
Parabagrotis exsertistigma|[7888]|LPABC601-09|08BBLEP-04820|632[0n]bp|Canada.Alberta|BOLD:AAA3352
Parabagrotis exsertistigma|[7889]|LPABC613-09|08BBLEP-04832|632[0n]bp|Canada.Alberta|BOLD:AAA3352
Parabagrotis exsertistigma|[7890]|LPABC636-09|08BBLEP-04855|632[0n]bp|Canada.Alberta|BOLD:AAA3352
Parabagrotis exsertistigma|[7891]|LPABC582-09|08BBLEP-04801|632[0n]bp|Canada.Alberta|BOLD:AAA3352
Parabagrotis exsertistigma|[7892]|LPABC623-09|08BBLEP-04842|630[0n]bp|Canada.Alberta|BOLD:AAA3352
Parabagrotis exsertistigma|[7893]|LBCH7233-10|10-JDWBC-7233|637[0n]bp|Canada.British Columbia|BOLD:AA...
Parabagrotis exsertistigma|[7894]|LBCH6915-10|10-JDWBC-6915|609[0n]bp|Canada.British Columbia|BOLD:AA...
Parabagrotis exsertistigma|[7895]|LPVIB640-08|PFC-2006-2088|600[0n]bp|Canada.British Columbia|BOLD:AA...
Parabagrotis exsertistigma|[7896]|LBCH6914-10|10-JDWBC-6914|639[0n]bp|Canada.British Columbia|BOLD:AA...
Parabagrotis exsertistigma|[7897]|LBCH6821-10|10-JDWBC-6821|639[0n]bp|Canada.British Columbia|BOLD:AA...
Parabagrotis exsertistigma|[7898]|LPABC600-09|08BBLEP-04819|635[0n]bp|Canada.Alberta|BOLD:AAA3352
Parabagrotis exsertistigma|[7899]|LPABC933-09|08BBLEP-05344|658[0n]bp|Canada.Alberta|BOLD:AAA3352
Parabagrotis exsertistigma|[7900]|LBCH6912-10|10-JDWBC-6912|658[0n]bp|Canada.British Columbia|BOLD:AA...
Parabagrotis cupidissima|[7901]|RDNMC088-05|CNCNoctuoidea10829|658[0n]bp|Canada.British Columbia|BOLD ...
Parabagrotis formalis|[7902]|LPVIB641-08|PFC-2006-2089|630[0n]bp|Canada.British Columbia|BOLD:ACE7476
Parabagrotis insularis|[7903]|IAWLB456-11|IAWAZ-1364|658[0n]bp|United States.California|BOLD:ABZ2364
Parabagrotis insularis|[7904]|GMLC814-12|2011GM-0510|658[0n]bp|United States.California|BOLD:ABZ2364
Parabagrotis insularis|[7905]|IAWLB455-11|IAWAZ-1363|658[0n]bp|United States.California|BOLD:ABZ2364
Parabagrotis insularis|[7906]|LOCBC462-06|06-BLLOC-2342|658[0n]bp|United States.California|BOLD:ABZ2364
Parabagrotis insularis|[7907]|LOCBD285-06|06-BLLOC-3105|658[0n]bp|United States.California|BOLD:ABZ2364
Parabagrotis insularis|[7908]|GMLC709-11|2011GM-0405|658[0n]bp|United States.California|BOLD:ABZ2364
Parabagrotis insularis|[7909]|RDNMF008-08|NOC14094|658[0n]bp|United States.California|BOLD:ABZ2364
Parabagrotis insularis|[7910]|GMLC569-11|2011GM-0265|658[0n]bp|United States.California|BOLD:ABZ2364
Parabagrotis insularis|[7911]|LOCBE090-06|06-BLLOC-3909|658[0n]bp|United States.California|BOLD:ABZ2364
Parabagrotis insularis|[7912]|GMLC873-12|2011GM-0569|658[0n]bp|United States.California|BOLD:ABZ2364
Parabagrotis insularis|[7913]|IAWLB446-11|IAWAZ-1354|658[0n]bp|United States.California|BOLD:ABZ2364
Parabagrotis insularis|[7914]|NAMUM413-09|RR-96-0718|658[0n]bp|United States.California|BOLD:ABZ2364
Parabagrotis insularis|[7915]|GMLC1198-12|2011GM-0894|658[0n]bp|United States.California|BOLD:ABZ2364
Parabagrotis insularis|[7916]|BBLOE1775-12|BIOUG01990-H08|658[0n]bp|United States.California|BOLD:ABZ...
Parabagrotis insularis|[7917]|GMLC1157-12|2011GM-0853|658[0n]bp|United States.California|BOLD:ABZ2364
Parabagrotis insularis|[7918]|IAWLB453-11|IAWAZ-1361|658[0n]bp|United States.California|BOLD:ABZ2364
Parabagrotis insularis|[7919]|JBAZ028-09|JLB-0028|658[4n]bp|United States.California|BOLD:ABZ2364
Parabagrotis insularis|[7920]|BBLOE1774-12|BIOUG01990-H07|658[0n]bp|United States.California|BOLD:ABZ...
Parabagrotis insularis|[7921]|BBLOC936-11|BIOUG01467-F03|658[0n]bp|United States.California|BOLD:ABZ2364
Parabagrotis insularis|[7922]|LOCBB322-06|06-BLLOC-1262|658[0n]bp|United States.California|BOLD:ABZ2364
Parabagrotis insularis|[7923]|IAWLB457-11|IAWAZ-1365|658[0n]bp|United States.California|BOLD:ABZ2364
Parabagrotis insularis|[7924]|GMLC717-11|2011GM-0413|632[0n]bp|United States.California|BOLD:ABZ2364
Parabagrotis insularis|[7925]|RWWC074-10|RWWA-2051|658[0n]bp|United States.Washington|BOLD:ABZ2364
Parabagrotis insularis|[7926]|GMLC735-12|2011GM-0431|658[0n]bp|United States.California|BOLD:ABZ2364
Parabagrotis insularis|[7927]|LOCBC464-06|06-BLLOC-2344|658[0n]bp|United States.California|BOLD:ABZ2364
Parabagrotis insularis|[7928]|GMLC616-11|2011GM-0312|658[0n]bp|United States.California|BOLD:ABZ2364
Parabagrotis insularis|[7929]|LOCB529-06|06-BLLOC-529|658[0n]bp|United States.California|BOLD:ABZ2364
Parabagrotis insularis|[7930]|BBLOD576-11|BIOUG01565-G11|658[0n]bp|United States.California|BOLD:ABZ2364
Parabagrotis insularis|[7931]|LOCBB323-06|06-BLLOC-1263|658[0n]bp|United States.California|BOLD:ABZ2364
Parabagrotis insularis|[7932]|IAWLB454-11|IAWAZ-1362|658[0n]bp|United States.California|BOLD:ABZ2364
Parabagrotis insularis|[7933]|LOCBE086-06|06-BLLOC-3905|658[0n]bp|United States.California|BOLD:ABZ2364
Parabagrotis insularis|[7934]|LOCBB325-06|06-BLLOC-1265|658[0n]bp|United States.California|BOLD:ABZ2364
Parabagrotis insularis|[7935]|LOCBB324-06|06-BLLOC-1264|658[0n]bp|United States.California|BOLD:ABZ2364
Parabagrotis sulinaris|[7936]|LHLEP212-06|UBC-2006-2077|658[0n]bp|Canada.British Columbia|BOLD:ABZ1410
Parabagrotis sulinaris|[7937]|LBCH7947-10|10-JDWBC-7947|658[0n]bp|Canada.British Columbia|BOLD:ABZ1410
Parabagrotis sulinaris|[7938]|LALPA1253-11|AVBC 1255-11|658[0n]bp|Canada.British Columbia|BOLD:ABZ1410
Parabagrotis sulinaris|[7939]|LBCG2065-09|08-JDWBC-2065|658[0n]bp|Canada.British Columbia|BOLD:ABZ1410
Parabagrotis sulinaris|[7940]|LBCH1102-10|10-JDWBC-1102|658[0n]bp|Canada.British Columbia|BOLD:ABZ1410
Parabagrotis sulinaris|[7941]|LBCG577-09|08-JDWBC-0577|658[0n]bp|Canada.British Columbia|BOLD:ABZ1410
Parabagrotis sulinaris|[7942]|RDMAB294-05|UASM77791|658[0n]bp|Canada.Alberta|BOLD:ABZ1410
Parabagrotis sulinaris|[7943]|LBCH7946-10|10-JDWBC-7946|658[0n]bp|Canada.British Columbia|BOLD:ABZ1410
Parabagrotis sulinaris|[7944]|LHLEP225-06|UBC-2006-1991|658[0n]bp|Canada.British Columbia|BOLD:ABZ1410
Parabagrotis sulinaris|[7945]|LALPA1303-11|AVBC 1305-11|658[0n]bp|Canada.British Columbia|BOLD:ABZ1410
Parabagrotis sulinaris|[7946]|LPVIB260-08|PFC-2006-1650|658[0n]bp|Canada.British Columbia|BOLD:ABZ1410
Parabagrotis sulinaris|[7947]|LBCH6845-10|10-JDWBC-6845|630[0n]bp|Canada.British Columbia|BOLD:ABZ1410
Parabagrotis sulinaris|[7948]|LPVIB639-08|PFC-2006-2087|637[0n]bp|Canada.British Columbia|BOLD:ABZ1410
Parabagrotis sulinaris|[7949]|LALPA1322-12|AVBC 1324-11|633[0n]bp|Canada.British Columbia|BOLD:ABZ1410
Parabagrotis sulinaris|[7950]|LBCH7127-10|10-JDWBC-7127|658[0n]bp|Canada.British Columbia|BOLD:ABZ1410
Parabagrotis sulinaris|[7951]|LALPA798-10|AVBC 800-10|658[0n]bp|Canada.British Columbia|BOLD:ABZ1410
Parabagrotis sulinaris|[7952]|LHLEP215-06|UBC-2006-2114|658[0n]bp|Canada.British Columbia|BOLD:ABZ1410
Parabagrotis sulinaris|[7953]|LHLEP213-06|UBC-2006-2078|658[0n]bp|Canada.British Columbia|BOLD:ABZ1410
Spaelotis clandestina|[7954]|LOWC843-05|CGWC-0843|658[0n]bp|Canada.British Columbia|BOLD:ABZ2365
Spaelotis clandestina|[7955]|LOWCD903-06|CGWC-3723|658[0n]bp|Canada.British Columbia|BOLD:ABZ2365
Spaelotis clandestina|[7956]|LOWCD904-06|CGWC-3724|601[0n]bp|Canada.British Columbia|BOLD:ABZ2365
Spaelotis clandestina|[7957]|LOWCD894-06|CGWC-3714|553[0n]bp|Canada.British Columbia|BOLD:ABZ2365
Spaelotis clandestina|[7958]|LOWCD911-06|CGWC-3731|566[0n]bp|Canada.British Columbia|BOLD:ABZ2365
Spaelotis clandestina|[7959]|LOWCC176-05|CGWC-2056|658[0n]bp|Canada.British Columbia|BOLD:ABZ2365
Spaelotis clandestina|[7960]|LOWCD898-06|CGWC-3718|658[0n]bp|Canada.British Columbia|BOLD:ABZ2365
Spaelotis clandestina|[7961]|LBCH5723-10|10-JDWBC-5723|658[0n]bp|Canada.British Columbia|BOLD:ABZ2365
Spaelotis clandestina|[7962]|LBCH7955-10|10-JDWBC-7955|658[0n]bp|Canada.British Columbia|BOLD:ABZ2365
Spaelotis clandestina|[7963]|LBCH7003-10|10-JDWBC-7003|658[0n]bp|Canada.British Columbia|BOLD:ABZ2365
Spaelotis clandestina|[7964]|LPSK129-08|08BBLEP-01697|658[0n]bp|Canada.Saskatchewan|BOLD:ABZ2365
Spaelotis clandestina|[7965]|LOWC842-05|CGWC-0842|658[0n]bp|Canada.British Columbia|BOLD:ABZ2365
Spaelotis clandestina|[7966]|LOWCC177-05|CGWC-2057|658[0n]bp|Canada.British Columbia|BOLD:ABZ2365
Spaelotis clandestina|[7967]|LOWCD895-06|CGWC-3715|658[0n]bp|Canada.British Columbia|BOLD:ABZ2365
Spaelotis clandestina|[7968]|LOWCD900-06|CGWC-3720|599[0n]bp|Canada.British Columbia|BOLD:ABZ2365
Spaelotis clandestina|[7969]|LOWCD908-06|CGWC-3728|604[0n]bp|Canada.British Columbia|BOLD:ABZ2365
Spaelotis clandestina|[7970]|XAD271-04|04HBL007271|594[0n]bp|Canada.Ontario|BOLD:ABZ2365
Spaelotis clandestina|[7971]|LOWCD901-06|CGWC-3721|593[0n]bp|Canada.British Columbia|BOLD:ABZ2365
Spaelotis clandestina|[7972]|LBCB071-05|HLC-21011|658[0n]bp|Canada.British Columbia|BOLD:ABZ2365
Spaelotis clandestina|[7973]|RDNMC539-06|CNCNoctuoidea12361|628[0n]bp|Canada.Saskatchewan|BOLD:ABZ2365
Spaelotis clandestina|[7974]|LOWC845-05|CGWC-0845|658[0n]bp|Canada.British Columbia|BOLD:ABZ2365

[illegible]
Spaelotis clandestina|[7972]|LBCB071-05|HLC-21011|658[0n]bp|Canada.British Columbia|BOLD:ABZ2365
Spaelotis clandestina|[7973]|RDNMC539-06|CNCNoctuoidea12361|628[0n]bp|Canada.Saskatchewan|BOLD:ABZ2365
Spaelotis clandestina|[7974]|LOWC845-05|CGWC-0845|658[0n]bp|Canada.British Columbia|BOLD:ABZ2365
Spaelotis clandestina|[7975]|LOWCC179-05|CGWC-2059|658[0n]bp|Canada.British Columbia|BOLD:ABZ2365
Spaelotis clandestina|[7976]|XAH601-05|2005-ONT-2184|658[0n]bp|Canada.Ontario|BOLD:ABZ2365
Spaelotis clandestina|[7977]|LOWC846-05|CGWC-0846|658[0n]bp|Canada.British Columbia|BOLD:ABZ2365
Spaelotis clandestina|[7978]|RDNMC540-06|CNCNoctuoidea12362|658[0n]bp|Canada.Saskatchewan|BOLD:ABZ2365
Spaelotis clandestina|[7979]|BBLPB753-10|10BBCLP-1752|658[0n]bp|Canada.Alberta|BOLD:ABZ2365
Spaelotis clandestina|[7980]|RDLQG133-06|DH012300|593[0n]bp|Canada.Quebec|BOLD:ABZ2365
Spaelotis clandestina|[7981]|LOWCD907-06|CGWC-3727|589[0n]bp|Canada.British Columbia|BOLD:ABZ2365
Spaelotis clandestina|[7982]|LOWCD902-06|CGWC-3722|595[0n]bp|Canada.British Columbia|BOLD:ABZ2365
Spaelotis clandestina|[7983]|BLTIB312-08|BL501|658[0n]bp|Canada.Ontario|BOLD:ABZ2365
Spaelotis clandestina|[7984]|MNBB645-05|05-NBSTA-561|658[0n]bp|Canada.New Brunswick|BOLD:ABZ2365
Spaelotis clandestina|[7985]|LOWC844-05|CGWC-0844|658[0n]bp|Canada.British Columbia|BOLD:ABZ2365
Spaelotis clandestina|[7986]|RDLQF542-06|DH011691|658[0n]bp|Canada.Quebec|BOLD:ABZ2365
Spaelotis clandestina|[7987]|LBCH7218-10|10-JDWBC-7218|658[0n]bp|Canada.British Columbia|BOLD:ABZ2365
Spaelotis clandestina|[7988]|RDLQG362-06|DH012596|658[0n]bp|Canada.Quebec|BOLD:ABZ2365
Spaelotis clandestina|[7989]|RDMAB116-05|UASM41256|658[0n]bp|Canada.Alberta|BOLD:ABZ2365
Spaelotis clandestina|[7990]|LPSK119-08|08BBLEP-01687|658[0n]bp|Canada.Saskatchewan|BOLD:ABZ2365
Spaelotis clandestina|[7991]|LOWC841-05|CGWC-0841|658[0n]bp|Canada.British Columbia|BOLD:ABZ2365
Spaelotis clandestina|[7992]|XAB011-04|04HBL005011|573[1n]bp|Canada.Ontario|BOLD:ABZ2365
Spaelotis clandestina|[7993]|MNBB529-05|05-NBSTA-445|658[0n]bp|Canada.New Brunswick|BOLD:ABZ2365
Spaelotis clandestina|[7994]|PMG162-03|moth1169.01|617[0n]bp|Canada.Ontario|BOLD:ABZ2365
Spaelotis clandestina|[7995]|RDLQF261-06|DH011341|658[0n]bp|Canada.Quebec|BOLD:ABZ2365
Spaelotis clandestina|[7996]|LOWCD899-06|CGWC-3719|658[0n]bp|Canada.British Columbia|BOLD:ABZ2365
Spaelotis clandestina|[7997]|XAD267-04|04HBL007267|658[0n]bp|Canada.Ontario|BOLD:ABZ2365
Spaelotis clandestina|[7998]|PHMNB209-04|04HBL007674|609[0n]bp|Canada.New Brunswick|BOLD:ABZ2365
Spaelotis clandestina|[7999]|RDNMC541-06|CNCNoctuoidea12363|658[0n]bp|Canada.Saskatchewan|BOLD:ABZ2365
Spaelotis clandestina|[8000]|RDLQG030-06|DH012161|658[0n]bp|Canada.Quebec|BOLD:ABZ2365
Spaelotis clandestina|[8001]|PHMO182-03|moth955.02|639[0n]bp|Canada.Ontario|BOLD:ABZ2365
Spaelotis bicava|[8002]|RDMAB135-05|UASM41279|658[0n]bp|Canada.Alberta|BOLD:ABZ2365
Spaelotis bicava|[8003]|RDMAB677-06|UASM77976|658[0n]bp|Canada.Alberta|BOLD:ABZ2365
Spaelotis bicava|[8004]|RDMAB678-06|UASM77983|658[0n]bp|Canada.Alberta|BOLD:ABZ2365
Spaelotis bicava|[8005]|LBCH7192-10|10-JDWBC-7192|658[0n]bp|Canada.British Columbia|BOLD:ABZ2365
Spaelotis bicava|[8006]|LPABB331-08|08BBLEP-03596|658[0n]bp|Canada.Alberta|BOLD:ABZ2365
Spaelotis sp.|[8007]|LBCH7181-10|10-JDWBC-7181|658[0n]bp|Canada.British Columbia|BOLD:ABZ2365
Spaelotis sp.|[8008]|LBCH5474-10|10-JDWBC-5474|658[0n]bp|Canada.British Columbia|BOLD:ABZ2365
Spaelotis sp.|[8009]|LBCG096-08|08-JDWBC-0096|658[0n]bp|Canada.British Columbia|BOLD:ABZ2365
Spaelotis sp.|[8010]|LBCH5714-10|10-JDWBC-5714|658[0n]bp|Canada.British Columbia|BOLD:ABZ2365
Spaelotis sp.|[8011]|LALPA817-10|AVBC 819-10|658[0n]bp|Canada.British Columbia|BOLD:ABZ2365
Spaelotis sp.|[8012]|LOWCC178-05|CGWC-2058|564[0n]bp|Canada.British Columbia|BOLD:ABZ2365
Spaelotis sp.|[8013]|LOWCD906-06|CGWC-3726|608[0n]bp|Canada.British Columbia|BOLD:ABZ2365
Setagrotis radiola|[8014]|RDMAB603-06|UASM58649|658[0n]bp|Canada.Alberta|BOLD:ABZ0944
Setagrotis pallidicollis|[8015]|LBCG1341-09|08-JDWBC-1341|658[4n]bp|Canada.British Columbia|BOLD:AAC3592
Setagrotis pallidicollis|[8016]|LBCH5725-10|10-JDWBC-5725|658[0n]bp|Canada.British Columbia|BOLD:AAC3592
Setagrotis pallidicollis|[8017]|LOWCD676-06|CGWC-3496|658[0n]bp|Canada.British Columbia|BOLD:AAC3592
Setagrotis pallidicollis|[8018]|LBCH6019-10|10-JDWBC-6019|658[0n]bp|Canada.British Columbia|BOLD:AAC3592
Setagrotis pallidicollis|[8019]|LBCH6119-10|10-JDWBC-6119|658[0n]bp|Canada.British Columbia|BOLD:AAC3592
Setagrotis pallidicollis|[8020]|RDNMC094-05|CNCNoctuoidea10835|658[0n]bp|Canada.Alberta|BOLD:AAC3592
Setagrotis pallidicollis|[8021]|LALPA1257-11|AVBC 1259-11|658[0n]bp|Canada.British Columbia|BOLD:AAC3592
Setagrotis pallidicollis|[8022]|LBCB068-05|HLC-21008|658[0n]bp|Canada.British Columbia|BOLD:AAC3592
Setagrotis pallidicollis|[8023]|LBCG2881-09|08-JDWBC-2881|658[0n]bp|Canada.British Columbia|BOLD:AAC3592
Setagrotis pallidicollis|[8024]|LBCH6306-10|10-JDWBC-6306|658[0n]bp|Canada.British Columbia|BOLD:AAC3592
Setagrotis pallidicollis|[8025]|LPABB639-08|08BBLEP-03904|658[0n]bp|Canada.Alberta|BOLD:AAC3592
Setagrotis pallidicollis|[8026]|LBCH5716-10|10-JDWBC-5716|658[0n]bp|Canada.British Columbia|BOLD:AAC3592
Setagrotis pallidicollis|[8027]|LOWC924-05|CGWC-0924|658[0n]bp|Canada.British Columbia|BOLD:AAC3592
Setagrotis pallidicollis|[8028]|LPABB879-09|08BBLEP-04199|658[0n]bp|Canada.Alberta|BOLD:AAC3592
Setagrotis pallidicollis|[8029]|LBCH5800-10|10-JDWBC-5800|658[0n]bp|Canada.British Columbia|BOLD:AAC3592
Setagrotis pallidicollis|[8030]|LBCH6021-10|10-JDWBC-6021|648[0n]bp|Canada.British Columbia|BOLD:AAC3592
Setagrotis pallidicollis|[8031]|LOWCD386-06|CGWC-3206|579[0n]bp|Canada.British Columbia|BOLD:AAC3592
Setagrotis vocalis|[8032]|RDNMK108-11|CNCLEP 80395|508[0n]bp|United States.Utah|BOLD:AAC3592
Setagrotis vocalis|[8033]|BBLOC132-11|BIOUG01453-D06|658[0n]bp|United States.Texas|BOLD:ACF4384
Setagrotis vocalis|[8034]|BBLOC145-11|BIOUG01453-E07|658[0n]bp|United States.Texas|BOLD:ACF4384
Setagrotis vocalis|[8035]|IAWLB022-10|IAWAZ-0835|658[0n]bp|United States.California|BOLD:ACF4384
Setagrotis vocalis|[8036]|BBLOC143-11|BIOUG01453-E05|658[0n]bp|United States.Texas|BOLD:ACF4384
Setagrotis vocalis|[8037]|NAMUM365-09|RR-98-1206|658[0n]bp|United States.California|BOLD:ACF4384
Xestia mustelina|[8038]|LBCD289-05|HLC-23109|652[0n]bp|Canada.British Columbia|BOLD:AAB7040
Xestia mustelina|[8039]|LBCD279-05|HLC-23099|658[0n]bp|Canada.British Columbia|BOLD:AAB7040
Xestia mustelina|[8040]|LBCD287-05|HLC-23107|616[0n]bp|Canada.British Columbia|BOLD:AAB7040
Xestia mustelina|[8041]|LALPA629-10|AVBC 631-10|658[0n]bp|Canada.British Columbia|BOLD:AAB7040
Xestia mustelina|[8042]|LALPA1306-11|AVBC 1308-11|658[0n]bp|Canada.British Columbia|BOLD:AAB7040
Xestia mustelina|[8043]|LALPA668-10|AVBC 670-10|658[0n]bp|Canada.British Columbia|BOLD:AAB7040
Xestia mustelina|[8044]|LALPA749-10|AVBC 751-10|658[0n]bp|Canada.British Columbia|BOLD:AAB7040
Xestia mustelina|[8045]|DUNLP187-08|Dun-08-187|643[0n]bp|Canada.British Columbia|BOLD:AAB7040
Xestia mustelina|[8046]|LBCH4691-10|10-JDWBC-4691|658[0n]bp|Canada.British Columbia|BOLD:AAB7040
Xestia mustelina|[8047]|LBCH3787-10|10-JDWBC-3787|658[0n]bp|Canada.British Columbia|BOLD:AAB7040
Xestia mustelina|[8048]|LBCG2290-09|08-JDWBC-2290|658[0n]bp|Canada.British Columbia|BOLD:AAB7040
Xestia mustelina|[8049]|LBCH939-10|10-JDWBC-0939|658[0n]bp|Canada.British Columbia|BOLD:AAB7040
Xestia mustelina|[8050]|LBCG2281-09|08-JDWBC-2281|658[0n]bp|Canada.British Columbia|BOLD:AAB7040
Xestia mustelina|[8051]|LBCG2288-09|08-JDWBC-2288|658[0n]bp|Canada.British Columbia|BOLD:AAB7040
Xestia mustelina|[8052]|LBCG2289-09|08-JDWBC-2289|658[0n]bp|Canada.British Columbia|BOLD:AAB7040
Xestia mustelina|[8053]|LBCH943-10|10-JDWBC-0943|658[0n]bp|Canada.British Columbia|BOLD:AAB7040
Xestia mustelina|[8054]|LBCH4436-10|10-JDWBC-4436|658[0n]bp|Canada.British Columbia|BOLD:AAB7040
Xestia mustelina|[8055]|LBCH3471-10|10-JDWBC-3471|658[0n]bp|Canada.British Columbia|BOLD:AAB7040
Xestia mustelina|[8056]|LBCH4116-10|10-JDWBC-4116|658[0n]bp|Canada.British Columbia|BOLD:AAB7040
Xestia mustelina|[8057]|LBCG2287-09|08-JDWBC-2287|658[0n]bp|Canada.British Columbia|BOLD:AAB7040
Coenophila opacifrons|[8058]|RDLQB816-05|DH010903|622[0n]bp|Canada.Quebec|BOLD:AAD2661
Coenophila opacifrons|[8059]|RDLQB650-05|DH010753|658[0n]bp|Canada.Quebec|BOLD:AAD2661
Coenophila opacifrons|[8060]|LBCH4036-10|10-JDWBC-4036|658[0n]bp|Canada.British Columbia|BOLD:AAD2661
Coenophila opacifrons|[8061]|LBCH4475-10|10-JDWBC-4475|658[0n]bp|Canada.British Columbia|BOLD:AAD2661
Coenophila opacifrons|[8062]|RDLQF311-06|DH011411|658[0n]bp|Canada.Quebec|BOLD:AAD2661
Coenophila opacifrons|[8063]|RDLQF879-06|DH012051|658[0n]bp|Canada.Quebec|BOLD:AAD2661
Coenophila opacifrons|[8064]|RDLQB814-05|DH010901|621[0n]bp|Canada.Quebec|BOLD:AAD2661
Coenophila opacifrons|[8065]|RDLQB815-05|DH010902|658[0n]bp|Canada.Quebec|BOLD:AAD2661
Coenophila opacifrons|[8066]|RDLQB821-05|DH010908|658[0n]bp|Canada.Quebec|BOLD:AAD2661
Protolampra brunneicollis|[8067]|XAD350-04|04HBL007350|604[1n]bp|Canada.Ontario|BOLD:AAC5112
Protolampra brunneicollis|[8068]|XAD248-04|04HBL007248|649[0n]bp|Canada.Ontario|BOLD:AAC5112
Protolampra brunneicollis|[8069]|XAD286-04|04HBL007286|572[0n]bp|Canada.Ontario|BOLD:AAC5112
Protolampra brunneicollis|[8070]|XAH040-05|2005-ONT-1623|636[0n]bp|Canada.Ontario|BOLD:AAC5112
Protolampra brunneicollis|[8071]|XAH197-05|2005-ONT-1780|658[0n]bp|Canada.Ontario|BOLD:AAC5112
Protolampra brunneicollis|[8072]|RDLQB819-05|DH010906|658[0n]bp|Canada.Quebec|BOLD:AAC5112
Protolampra brunneicollis|[8073]|XAJ814-06|2006-ONT-0814|658[0n]bp|Canada.Ontario|BOLD:AAC5112
[illegible]

Protolampra brunneicollis|[8071]|XAH197-05|2005-ONT-1780|658[0n]bp|Canada.Ontario|BOLD:AAC5112
Protolampra brunneicollis|[8072]|RDLQB819-05|DH010906|658[0n]bp|Canada.Quebec|BOLD:AAC5112
Protolampra brunneicollis|[8073]|XAJ814-06|2006-ONT-0814|658[0n]bp|Canada.Ontario|BOLD:AAC5112
Protolampra brunneicollis|[8074]|XAH275-05|2005-ONT-1858|658[0n]bp|Canada.Ontario|BOLD:AAC5112
Protolampra brunneicollis|[8075]|LBCH7392-10|10-JDWBC-7392|658[0n]bp|Canada.British Columbia|BOLD:AAC...
Xestia badicollis|[8076]|TMNBB370-06|MNBTT-1310|658[8n]bp|Canada.New Brunswick|
Xestia badicollis|[8077]|TTMNB471-06|MNBTT-471|658[3n]bp|Canada.New Brunswick|BOLD:AAA5962
Xestia dilucida|[8078]|RDNML149-13|13-NCCC-054|612[0n]bp|United States.North Carolina|BOLD:AAA5962
Xestia dilucida|[8079]|LNC357-05|05-NCCC-357|503[0n]bp|United States.North Carolina|BOLD:AAA5962
Xestia dilucida|[8080]|LNC355-05|05-NCCC-355|527[1n]bp|United States.North Carolina|BOLD:AAA5962
Xestia dilucida|[8081]|QUNOD667-11|9381-MI|658[0n]bp|United States.Michigan|BOLD:AAA5962
Xestia dilucida|[8082]|QUNOD666-11|9380-MI|658[0n]bp|United States.Michigan|BOLD:AAA5962
Xestia dilucida|[8083]|RDNMB110-05|USNM219557|658[0n]bp|United States.Tennessee|BOLD:AAA5962
Xestia dilucida|[8084]|QUNOD668-11|9382-MI|658[0n]bp|United States.Michigan|BOLD:AAA5962
Xestia dilucida|[8085]|RDLQB647-05|DH010750|549[0n]bp|Canada.Quebec|BOLD:AAA5962
Xestia dilucida|[8086]|TMNBB387-06|MNBTT-1327|656[2n]bp|Canada.New Brunswick|BOLD:AAA5962
Xestia dilucida|[8087]|TTMNB484-06|MNBTT-484|658[0n]bp|Canada.New Brunswick|BOLD:AAA5962
Xestia dilucida|[8088]|RDLQF877-06|DH012049|658[0n]bp|Canada.Quebec|BOLD:AAA5962
Xestia dilucida|[8089]|TTMNB477-06|MNBTT-477|658[0n]bp|Canada.New Brunswick|BOLD:AAA5962
Xestia dilucida|[8090]|TTMNB480-06|MNBTT-480|658[0n]bp|Canada.New Brunswick|BOLD:AAA5962
Xestia dilucida|[8091]|TTMNB475-06|MNBTT-475|658[0n]bp|Canada.New Brunswick|BOLD:AAA5962
Xestia dilucida|[8092]|RDLQB775-05|DH010862|658[0n]bp|Canada.Quebec|BOLD:AAA5962
Xestia dilucida|[8093]|MJMSL146-10|TDWG-0063|658[0n]bp|United States.Massachusetts|BOLD:AAA5962
Xestia dilucida|[8094]|RDLQF876-06|DH012048|658[0n]bp|Canada.Quebec|BOLD:AAA5962
Xestia dilucida|[8095]|RDLQF310-06|DH011410|658[0n]bp|Canada.Quebec|BOLD:AAA5962
Xestia dilucida|[8096]|RDLQF874-06|DH012046|658[0n]bp|Canada.Quebec|BOLD:AAA5962
Xestia dilucida|[8097]|TTMNB483-06|MNBTT-483|658[0n]bp|Canada.New Brunswick|BOLD:AAA5962
Xestia dilucida|[8098]|LNCC403-10|10-NCCC-498|658[0n]bp|United States.North Carolina|BOLD:AAA5962
Xestia dilucida|[8099]|MJMSL052-10|10MA-0052|658[0n]bp|United States.Massachusetts|BOLD:AAA5962
Xestia dilucida|[8100]|TMNBB381-06|MNBTT-1321|658[0n]bp|Canada.New Brunswick|BOLD:AAA5962
Xestia dilucida|[8101]|RDLQF293-06|DH011393|658[0n]bp|Canada.Quebec|BOLD:AAA5962
Xestia dilucida|[8102]|TMNBB382-06|MNBTT-1322|658[0n]bp|Canada.New Brunswick|BOLD:AAA5962
Xestia dilucida|[8103]|RDLQF295-06|DH011395|658[0n]bp|Canada.Quebec|BOLD:AAA5962
Xestia dilucida|[8104]|TTMNB481-06|MNBTT-481|658[0n]bp|Canada.New Brunswick|BOLD:AAA5962
Xestia dilucida|[8105]|TMNBB389-06|MNBTT-1329|658[0n]bp|Canada.New Brunswick|BOLD:AAA5962
Xestia dilucida|[8106]|TMNBB377-06|MNBTT-1317|658[0n]bp|Canada.New Brunswick|BOLD:AAA5962
Xestia dilucida|[8107]|TMNBB385-06|MNBTT-1325|658[0n]bp|Canada.New Brunswick|BOLD:AAA5962
Xestia dilucida|[8108]|RDLQB764-05|DH010851|658[0n]bp|Canada.Quebec|BOLD:AAA5962
Xestia dilucida|[8109]|RDLQF285-06|DH011377|658[0n]bp|Canada.Quebec|BOLD:AAA5962
Xestia dilucida|[8110]|TMNBB378-06|MNBTT-1318|658[0n]bp|Canada.New Brunswick|BOLD:AAA5962
Xestia dilucida|[8111]|MJMSL034-10|10MA-0034|658[0n]bp|United States.Massachusetts|BOLD:AAA5962
Xestia dilucida|[8112]|RDLQF290-06|DH011390|658[0n]bp|Canada.Quebec|BOLD:AAA5962
Xestia dilucida|[8113]|TTMNB478-06|MNBTT-478|658[0n]bp|Canada.New Brunswick|BOLD:AAA5962
Xestia dilucida|[8114]|RDLQF286-06|DH011378|658[0n]bp|Canada.Quebec|BOLD:AAA5962
Xestia dilucida|[8115]|RDLQF301-06|DH011401|658[0n]bp|Canada.Quebec|BOLD:AAA5962
Xestia dilucida|[8116]|TMNBB384-06|MNBTT-1324|658[0n]bp|Canada.New Brunswick|BOLD:AAA5962
Xestia dilucida|[8117]|RDLQF875-06|DH012047|658[0n]bp|Canada.Quebec|BOLD:AAA5962
Xestia dilucida|[8118]|RDLQF298-06|DH011398|658[0n]bp|Canada.Quebec|BOLD:AAA5962
Xestia dilucida|[8119]|RDLQF292-06|DH011392|658[0n]bp|Canada.Quebec|BOLD:AAA5962
Xestia dilucida|[8120]|TTMNB479-06|MNBTT-479|658[0n]bp|Canada.New Brunswick|BOLD:AAA5962
Xestia dilucida|[8121]|RDLQF304-06|DH011404|658[0n]bp|Canada.Quebec|BOLD:AAA5962
Xestia dilucida|[8122]|TTMNB482-06|MNBTT-482|658[0n]bp|Canada.New Brunswick|BOLD:AAA5962
Xestia dilucida|[8123]|RDLQF291-06|DH011391|658[0n]bp|Canada.Quebec|BOLD:AAA5962
Xestia dilucida|[8124]|MJMSL033-10|10MA-0033|658[0n]bp|United States.Massachusetts|BOLD:AAA5962
Xestia dilucida|[8125]|RDLQF299-06|DH011399|658[0n]bp|Canada.Quebec|BOLD:AAA5962
Xestia dilucida|[8126]|TMNBB374-06|MNBTT-1314|658[0n]bp|Canada.New Brunswick|BOLD:AAA5962
Xestia dilucida|[8127]|RDLQF300-06|DH011400|658[0n]bp|Canada.Quebec|BOLD:AAA5962
Xestia dilucida|[8128]|RDLQF305-06|DH011405|658[0n]bp|Canada.Quebec|BOLD:AAA5962
Xestia dilucida|[8129]|TMNBB375-06|MNBTT-1315|658[0n]bp|Canada.New Brunswick|BOLD:AAA5962
Xestia dilucida|[8130]|RDLQF873-06|DH012045|658[0n]bp|Canada.Quebec|BOLD:AAA5962
Xestia dilucida|[8131]|RDLQF289-06|DH011389|658[0n]bp|Canada.Quebec|BOLD:AAA5962
Xestia dilucida|[8132]|MJMSL054-10|10MA-0054|658[0n]bp|United States.Massachusetts|BOLD:AAA5962
Xestia dilucida|[8133]|TMNBB386-06|MNBTT-1326|658[0n]bp|Canada.New Brunswick|BOLD:AAA5962
Xestia dilucida|[8134]|TTMNB476-06|MNBTT-476|658[0n]bp|Canada.New Brunswick|BOLD:AAA5962
Xestia dilucida|[8135]|RDNMI048-10|CNCLEP 69838|658[0n]bp|United States.Florida|BOLD:AAA5962
Xestia dilucida|[8136]|LNCC402-10|10-NCCC-497|658[1n]bp|United States.North Carolina|BOLD:AAA5962
Xestia dilucida|[8137]|RDLQF306-06|DH011406|658[1n]bp|Canada.Quebec|BOLD:AAA5962
Xestia dilucida|[8138]|TMNBB383-06|MNBTT-1323|658[0n]bp|Canada.New Brunswick|BOLD:AAA5962
Xestia dilucida|[8139]|RDLQF302-06|DH011402|658[1n]bp|Canada.Quebec|BOLD:AAA5962
Xestia dilucida|[8140]|TMNBB380-06|MNBTT-1320|658[0n]bp|Canada.New Brunswick|BOLD:AAA5962
Xestia dilucida|[8141]|GWOTA071-12|BC ZSM Lep 58547|658[0n]bp|United States.Massachusetts|BOLD:AAA5962
Xestia dilucida|[8142]|RDLQF303-06|DH011403|658[2n]bp|Canada.Quebec|BOLD:AAA5962
Xestia dilucida|[8143]|RDLQF297-06|DH011397|643[0n]bp|Canada.Quebec|BOLD:AAA5962
Xestia dilucida|[8144]|RDLQF309-06|DH011409|632[0n]bp|Canada.Quebec|BOLD:AAA5962
Xestia dilucida|[8145]|RDLQF308-06|DH011408|654[2n]bp|Canada.Quebec|BOLD:AAA5962
Xestia dilucida|[8146]|RDLQF294-06|DH011394|630[0n]bp|Canada.Quebec|BOLD:AAA5962
Xestia dilucida|[8147]|RDLQF307-06|DH011407|632[0n]bp|Canada.Quebec|BOLD:AAA5962
Xestia dilucida|[8148]|TMNBB376-06|MNBTT-1316|658[0n]bp|Canada.New Brunswick|BOLD:AAA5962
Xestia dilucida|[8149]|TMNBB388-06|MNBTT-1328|658[0n]bp|Canada.New Brunswick|BOLD:AAA5962
Xestia dilucida|[8150]|RDLQF296-06|DH011396|638[2n]bp|Canada.Quebec|BOLD:AAA5962
Xestia dilucida|[8151]|MJMSL053-10|10MA-0053|658[0n]bp|United States.Massachusetts|BOLD:AAA5962
Xestia dilucida|[8152]|TMNBB379-06|MNBTT-1319|658[0n]bp|Canada.New Brunswick|BOLD:AAA5962
Xestia dilucida|[8153]|MJMSL032-10|10MA-0032|658[0n]bp|United States.Massachusetts|BOLD:AAA5962
Xestia badicollis|[8154]|RDLQF288-06|DH011380|658[2n]bp|Canada.Quebec|BOLD:AAA5962
Xestia badicollis|[8155]|BBLEC517-09|09BBELE-0517|613[0n]bp|Canada.New Brunswick|BOLD:AAA5962
Xestia dilucida|[8156]|LNC450-05|05-NCCC-450|658[0n]bp|United States.North Carolina|BOLD:AAA5962
Xestia dilucida|[8157]|LNC451-05|05-NCCC-451|658[0n]bp|United States.North Carolina|BOLD:AAA5962
Xestia dilucida|[8158]|LNC477-06|05-NCCC-477|658[0n]bp|United States.North Carolina|BOLD:AAA5962
Xestia dilucida|[8159]|LNC449-05|05-NCCC-449|658[0n]bp|United States.North Carolina|BOLD:AAA5962
Xestia badicollis|[8160]|RDLQB675-05|DH010778|501[0n]bp|Canada.Quebec|
Xestia badicollis|[8161]|RDLQF180-06|DH011207|658[0n]bp|Canada.Quebec|BOLD:AAA5962
Xestia badicollis|[8162]|LPSOD985-09|08BBLEP-05618|658[0n]bp|Canada.Ontario|BOLD:AAA5962
Xestia badicollis|[8163]|RDLQF182-06|DH011209|658[0n]bp|Canada.Quebec|BOLD:AAA5962
Xestia badicollis|[8164]|LPSOD971-09|08BBLEP-05604|658[0n]bp|Canada.Ontario|BOLD:AAA5962
Xestia badicollis|[8165]|RDLQF181-06|DH011208|658[0n]bp|Canada.Quebec|BOLD:AAA5962
Xestia badicollis|[8166]|RDLQF184-06|DH011211|658[0n]bp|Canada.Quebec|BOLD:AAA5962
Xestia badicollis|[8167]|LPSOD993-09|08BBLEP-05626|658[0n]bp|Canada.Ontario|BOLD:AAA5962
Xestia badicollis|[8168]|RDLQF178-06|DH011205|658[0n]bp|Canada.Quebec|BOLD:AAA5962
Xestia badicollis|[8169]|LPSOD963-09|08BBLEP-05503|658[0n]bp|Canada.Ontario|BOLD:AAA5962
Xestia badicollis|[8170]|TTMNB472-06|MNBTT-472|658[4n]bp|Canada.New Brunswick|BOLD:AAA5962
Xestia praevia|[8171]|RDNMC383-05|CNCNoctuoidea12017|658[1n]bp|Canada.Ontario|BOLD:AAA5962
Xestia badicollis|[8172]|RDLQF183-06|DH011210|658[1n]bp|Canada.Quebec|BOLD:AAA5962
Xestia badicollis|[8173]|BBLEC504-09|09BBELE-0504|658[0n]bp|Canada.New Brunswick|BOLD:AAA5962

Xestia praevia|[8171]|RDNMC383-05|CNCNoctuoidea12017|658[1n]bp|Canada.Ontario|BOLD:AAA5962
Xestia badicollis|[8172]|RDLQF183-06|DH011210|658[1n]bp|Canada.Quebec|BOLD:AAA5962
Xestia badicollis|[8173]|BBLEC504-09|09BBELE-0504|658[0n]bp|Canada.New Brunswick|BOLD:AAA5962
Xestia badicollis|[8174]|TMNBB373-06|MNBTT-1313|658[0n]bp|Canada.New Brunswick|BOLD:AAA5962
Xestia badicollis|[8175]|RDNMC386-05|CNCNoctuoidea12020|658[0n]bp|Canada.Ontario|BOLD:AAA5962
Xestia badicollis|[8176]|TTMNB473-06|MNBTT-473|658[0n]bp|Canada.New Brunswick|BOLD:AAA5962
Xestia badicollis|[8177]|BBLEC432-09|09BBELE-0432|658[0n]bp|Canada.New Brunswick|BOLD:AAA5962
Xestia badicollis|[8178]|RDNMC385-05|CNCNoctuoidea12019|658[0n]bp|Canada.Ontario|BOLD:AAA5962
Xestia badicollis|[8179]|BBLEC527-09|09BBELE-0527|594[0n]bp|Canada.New Brunswick|BOLD:AAA5962
Xestia badicollis|[8180]|TMNBB372-06|MNBTT-1312|658[0n]bp|Canada.New Brunswick|BOLD:AAA5962
Xestia badicollis|[8181]|BBLPC036-09|09BBELE-1036|621[0n]bp|Canada.New Brunswick|BOLD:AAA5962
Xestia badicollis|[8182]|BBLPC378-09|09BBELE-1378|632[0n]bp|Canada.New Brunswick|BOLD:AAA5962
Xestia badicollis|[8183]|TTMNB470-06|MNBTT-470|658[0n]bp|Canada.New Brunswick|BOLD:AAA5962
Xestia badicollis|[8184]|BBLPC547-09|09BBELE-1547|658[0n]bp|Canada.New Brunswick|BOLD:AAA5962
Xestia badicollis|[8185]|RDLQF185-06|DH011212|658[0n]bp|Canada.Quebec|BOLD:AAA5962
Xestia badicollis|[8186]|RDLQF287-06|DH011379|658[0n]bp|Canada.Quebec|BOLD:AAA5962
Xestia badicollis|[8187]|RDLQF179-06|DH011206|658[0n]bp|Canada.Quebec|BOLD:AAA5962
Xestia badicollis|[8188]|BBLEC514-09|09BBELE-0514|658[0n]bp|Canada.New Brunswick|BOLD:AAA5962
Xestia badicollis|[8189]|TTMNB474-06|MNBTT-474|658[0n]bp|Canada.New Brunswick|BOLD:AAA5962
Xestia badicollis|[8190]|BBLPC019-09|09BBELE-1019|658[0n]bp|Canada.New Brunswick|BOLD:AAA5962
Xestia badicollis|[8191]|TTMNB469-06|MNBTT-469|658[0n]bp|Canada.New Brunswick|BOLD:AAA5962
Xestia badicollis|[8192]|BBLPC018-09|09BBELE-1018|658[0n]bp|Canada.New Brunswick|BOLD:AAA5962
Xestia praevia|[8193]|RDLQB525-05|DH010611|658[1n]bp|Canada.Quebec|BOLD:AAA5962
Xestia praevia|[8194]|RDLQF376-06|DH011443|658[0n]bp|Canada.Quebec|BOLD:AAA5962
Xestia praevia|[8195]|BBLPC152-09|09BBELE-1152|658[0n]bp|Canada.Nova Scotia|BOLD:AAA5962
Xestia praevia|[8196]|RDLQF377-06|DH011444|658[0n]bp|Canada.Quebec|BOLD:AAA5962
Xestia praevia|[8197]|BBLEC687-09|09BBELE-0687|658[0n]bp|Canada.Nova Scotia|BOLD:AAA5962
Xestia praevia|[8198]|RDNMC384-05|CNCNoctuoidea12018|658[0n]bp|Canada.Ontario|BOLD:AAA5962
Xestia praevia|[8199]|RDLQF798-06|DH011948|658[0n]bp|Canada.Quebec|BOLD:AAA5962
Xestia praevia|[8200]|BBLEC917-09|09BBELE-0917|658[0n]bp|Canada.Nova Scotia|BOLD:AAA5962
Xestia praevia|[8201]|BBLPC461-09|09BBELE-1461|658[0n]bp|Canada.New Brunswick|BOLD:AAA5962
Xestia praevia|[8202]|BBLEC697-09|09BBELE-0697|658[0n]bp|Canada.Nova Scotia|BOLD:AAA5962
Xestia praevia|[8203]|BBLEC977-09|09BBELE-0977|658[0n]bp|Canada.Nova Scotia|BOLD:AAA5962
Xestia praevia|[8204]|RDLQB739-05|DH010654|658[0n]bp|Canada.Quebec|BOLD:AAA5962
Xestia infimatis|[8205]|LALPA731-10|AVBC 733-10|658[1n]bp|Canada.British Columbia|BOLD:ABY8915
Xestia infimatis|[8206]|LPVIB658-08|PFC-2006-2107|588[0n]bp|Canada.British Columbia|BOLD:ABY8915
Xestia infimatis|[8207]|LPVIB670-08|PFC-2006-2119|632[0n]bp|Canada.British Columbia|BOLD:ABY8915
Xestia finatimis|[8208]|RDNMF424-08|NOC14510|658[0n]bp|Canada.British Columbia|BOLD:ABY8915
Xestia vernilis|[8209]|LPABC217-09|08BBLEP-04436|658[0n]bp|Canada.Alberta|BOLD:ABY8915
Xestia finatimis|[8210]|LOWCB001-05|CGWC-0941|658[0n]bp|Canada.British Columbia|BOLD:ABY8915
Xestia finatimis|[8211]|RDNMF423-08|NOC14509|658[0n]bp|Canada.British Columbia|BOLD:ABY8915
Xestia finatimis|[8212]|LOWCB009-05|CGWC-0949|658[0n]bp|Canada.British Columbia|BOLD:ABY8915
Xestia finatimis|[8213]|LOWC937-05|CGWC-0937|658[0n]bp|Canada.British Columbia|BOLD:ABY8915
Xestia vernilis|[8214]|RDNMG756-08|CNC LEP00052880|642[0n]bp|Canada.Alberta|BOLD:ABY8915
Xestia vernilis|[8215]|RDNMF053-08|NOC14139|658[0n]bp|Canada.Alberta|BOLD:ABY8915
Xestia vernilis|[8216]|LPABC008-09|08BBLEP-04227|658[0n]bp|Canada.Alberta|BOLD:ABY8915
Xestia vernilis|[8217]|LPABC209-09|08BBLEP-04428|658[1n]bp|Canada.Alberta|BOLD:ABY8915
Xestia finatimis|[8218]|LOWCB005-05|CGWC-0945|658[3n]bp|Canada.British Columbia|BOLD:AAB0107
Xestia vernilis|[8219]|LPABC184-09|08BBLEP-04403|658[0n]bp|Canada.Alberta|BOLD:AAB0107
Xestia vernilis|[8220]|LPABC216-09|08BBLEP-04435|632[1n]bp|Canada.Alberta|BOLD:AAB0107
Xestia vernilis|[8221]|LPABB864-09|08BBLEP-04184|646[0n]bp|Canada.Alberta|BOLD:AAB0107
Xestia vernilis|[8222]|LPABC219-09|08BBLEP-04438|632[1n]bp|Canada.Alberta|BOLD:AAB0107
Xestia vernilis|[8223]|LPABB720-08|08BBLEP-03985|642[0n]bp|Canada.Alberta|BOLD:AAB0107
Xestia vernilis|[8224]|LPABC192-09|08BBLEP-04411|612[0n]bp|Canada.Alberta|BOLD:AAB0107
Xestia finatimis|[8225]|LOWCD389-06|CGWC-3209|570[0n]bp|Canada.British Columbia|BOLD:AAB0107
Xestia finatimis|[8226]|LALPA1317-11|AVBC 1319-11|658[0n]bp|Canada.British Columbia|BOLD:AAB0107
Xestia verniloides|[8227]|RDNMF083-08|NOC14169|650[0n]bp|Canada.British Columbia|BOLD:AAB0107
Xestia vernilis|[8228]|LPABB818-09|08BBLEP-04138|658[0n]bp|Canada.Alberta|BOLD:AAB0107
Xestia vernilis|[8229]|LPABC214-09|08BBLEP-04433|658[0n]bp|Canada.Alberta|BOLD:AAB0107
Xestia finatimis|[8230]|LOWCB002-05|CGWC-0942|658[0n]bp|Canada.British Columbia|BOLD:AAB0107
Xestia finatimis|[8231]|LOWC939-05|CGWC-0939|658[0n]bp|Canada.British Columbia|BOLD:AAB0107
Xestia finatimis|[8232]|LOWCB007-05|CGWC-0947|658[0n]bp|Canada.British Columbia|BOLD:AAB0107
Xestia finatimis|[8233]|LOWCB011-05|CGWC-0951|658[0n]bp|Canada.British Columbia|BOLD:AAB0107
Xestia finatimis|[8234]|LOWC938-05|CGWC-0938|658[0n]bp|Canada.British Columbia|BOLD:AAB0107
Xestia finatimis|[8235]|LALPA672-10|AVBC 674-10|658[0n]bp|Canada.British Columbia|BOLD:AAB0107
Xestia finatimis|[8236]|RDNMF425-08|NOC14511|658[0n]bp|Canada.British Columbia|BOLD:AAB0107
Xestia finatimis|[8237]|LOWCB004-05|CGWC-0944|658[0n]bp|Canada.British Columbia|BOLD:AAB0107
Xestia finatimis|[8238]|LOWCB003-05|CGWC-0943|658[0n]bp|Canada.British Columbia|BOLD:AAB0107
Xestia finatimis|[8239]|LOWCB008-05|CGWC-0948|658[0n]bp|Canada.British Columbia|BOLD:AAB0107
Xestia finatimis|[8240]|LOWC935-05|CGWC-0935|658[0n]bp|Canada.British Columbia|BOLD:AAB0107
Xestia finatimis|[8241]|LOWCB006-05|CGWC-0946|658[0n]bp|Canada.British Columbia|BOLD:AAB0107
Xestia finatimis|[8242]|LOWC936-05|CGWC-0936|658[0n]bp|Canada.British Columbia|BOLD:AAB0107
Xestia finatimis|[8243]|LOWC940-05|CGWC-0940|658[0n]bp|Canada.British Columbia|BOLD:AAB0107
Xestia finatimis|[8244]|LOWCB010-05|CGWC-0950|658[0n]bp|Canada.British Columbia|BOLD:AAB0107
Xestia finatimis|[8245]|LOWCD387-06|CGWC-3207|580[0n]bp|Canada.British Columbia|BOLD:AAB0107
Xestia verniloides|[8246]|RDNMG758-08|CNC LEP00052882|642[0n]bp|Canada.British Columbia|BOLD:AAB0107
Xestia finatimis|[8247]|LOWCD388-06|CGWC-3208|587[0n]bp|Canada.British Columbia|BOLD:AAB0107
Xestia vernilis|[8248]|LPABC196-09|08BBLEP-04415|633[0n]bp|Canada.Alberta|BOLD:AAB0107
Xestia verniloides|[8249]|RDNMG759-08|CNC LEP00052883|642[0n]bp|Canada.British Columbia|BOLD:AAB0107
Xestia c-nigrum|[8250]|MNBB425-05|05-NBSTA-341|658[0n]bp|Canada.New Brunswick|BOLD:AAA2144
Xestia c-nigrum|[8251]|LOWC795-05|CGWC-0795|658[0n]bp|Canada.British Columbia|BOLD:AAA2144
Xestia c-nigrum|[8252]|XAH087-05|2005-ONT-1670|597[13n]bp|Canada.Ontario|
Xestia c-nigrum|[8253]|LOWC803-05|CGWC-0803|658[1n]bp|Canada.British Columbia|BOLD:AAA2144
Xestia c-nigrum|[8254]|BBLPB824-10|10BBCLP-1823|658[0n]bp|Canada.Alberta|BOLD:AAA2144
Xestia c-nigrum|[8255]|LPSOB715-08|PPBP-1714|609[0n]bp|Canada.Ontario|BOLD:AAA2144
Xestia c-nigrum|[8256]|BBLEC849-09|09BBELE-0849|658[0n]bp|Canada.Newfoundland and Labrador|BOLD:AAA2144
Xestia c-nigrum|[8257]|LBCB892-05|HLC-21832|616[0n]bp|Canada.British Columbia|BOLD:AAA2144
Xestia c-nigrum|[8258]|LBCB890-05|HLC-21830|598[0n]bp|Canada.British Columbia|BOLD:AAA2144
Xestia c-nigrum|[8259]|LBCB893-05|HLC-21833|598[0n]bp|Canada.British Columbia|BOLD:AAA2144
Xestia c-nigrum|[8260]|LBCB891-05|HLC-21831|615[1n]bp|Canada.British Columbia|BOLD:AAA2144
Xestia c-nigrum|[8261]|RDLQB639-05|DH010742|573[2n]bp|Canada.Quebec|BOLD:AAA2144
Xestia c-nigrum|[8262]|MNBB523-05|05-NBSTA-439|658[0n]bp|Canada.New Brunswick|BOLD:AAA2144
Xestia c-nigrum|[8263]|PHMNB247-04|04HBL007712|537[0n]bp|Canada.New Brunswick|BOLD:AAA2144
Xestia c-nigrum|[8264]|PHMNB178-04|04HBL007643|500[0n]bp|Canada.New Brunswick|
Xestia c-nigrum|[8265]|LOWCD783-06|CGWC-3603|657[0n]bp|Canada.British Columbia|BOLD:AAA2144
Xestia c-nigrum|[8266]|TTMNB447-06|MNBTT-447|658[0n]bp|Canada.New Brunswick|BOLD:AAA2144
Xestia c-nigrum|[8267]|LPSOB716-08|PPBP-1715|658[0n]bp|Canada.Ontario|BOLD:AAA2144
Xestia c-nigrum|[8268]|MNBB424-05|05-NBSTA-340|658[0n]bp|Canada.New Brunswick|BOLD:AAA2144
Xestia c-nigrum|[8269]|XAK337-06|2006-ONT-1332|658[0n]bp|Canada.Ontario|BOLD:AAA2144
Xestia c-nigrum|[8270]|TTMNB443-06|MNBTT-443|658[0n]bp|Canada.New Brunswick|BOLD:AAA2144
Xestia c-nigrum|[8271]|LPSOC332-08|PPBP-2331|658[0n]bp|Canada.Ontario|BOLD:AAA2144
Xestia c-nigrum|[8272]|LOWC802-05|CGWC-0802|658[0n]bp|Canada.British Columbia|BOLD:AAA2144
Xestia c-nigrum|[8273]|LPSOB979-08|PPBP-1978|658[0n]bp|Canada.Ontario|BOLD:AAA2144

[illegible]
Xestia c-nigrum|[8271]|LPSOC332-08|PPBP-2331|658[0n]bp|Canada.Ontario|BOLD:AAA2144
Xestia c-nigrum|[8272]|LOWC802-05|CGWC-0802|658[0n]bp|Canada.British Columbia|BOLD:AAA2144
Xestia c-nigrum|[8273]|LPSOB979-08|PPBP-1978|658[0n]bp|Canada.Ontario|BOLD:AAA2144
Xestia c-nigrum|[8274]|LOWC800-05|CGWC-0800|658[0n]bp|Canada.British Columbia|BOLD:AAA2144
Xestia c-nigrum|[8275]|LPSOC363-08|PPBP-2362|655[0n]bp|Canada.Ontario|BOLD:AAA2144
Xestia c-nigrum|[8276]|XAB150-04|04HBL005150|658[0n]bp|Canada.Ontario|BOLD:AAA2144
Xestia c-nigrum|[8277]|XAD244-04|04HBL007244|658[0n]bp|Canada.Ontario|BOLD:AAA2144
Xestia c-nigrum|[8278]|LOWCE812-06|CGWC-4572|658[0n]bp|Canada.British Columbia|BOLD:AAA2144
Xestia c-nigrum|[8279]|MNBB427-05|05-NBSTA-343|658[0n]bp|Canada.New Brunswick|BOLD:AAA2144
Xestia c-nigrum|[8280]|RDLQF885-06|DH012060|658[0n]bp|Canada.Quebec|BOLD:AAA2144
Xestia c-nigrum|[8281]|BBLPC063-09|09BBELE-1063|658[0n]bp|Canada.New Brunswick|BOLD:AAA2144
Xestia c-nigrum|[8282]|MNBB387-05|05-NBSTA-303|658[0n]bp|Canada.New Brunswick|BOLD:AAA2144
Xestia c-nigrum|[8283]|LHLEP427-06|UBC-2006-0351|658[0n]bp|Canada.British Columbia|BOLD:AAA2144
Xestia c-nigrum|[8284]|LPSOC299-08|PPBP-2298|658[0n]bp|Canada.Ontario|BOLD:AAA2144
Xestia c-nigrum|[8285]|MNBB171-05|05-NBSTA-087|658[0n]bp|Canada.New Brunswick|BOLD:AAA2144
Xestia c-nigrum|[8286]|LOWCE840-06|CGWC-4600|658[0n]bp|Canada.British Columbia|BOLD:AAA2144
Xestia c-nigrum|[8287]|TTMNB444-06|MNBTT-444|658[0n]bp|Canada.New Brunswick|BOLD:AAA2144
Xestia c-nigrum|[8288]|LPSOC346-08|PPBP-2345|655[0n]bp|Canada.Ontario|BOLD:AAA2144
Xestia c-nigrum|[8289]|BBLPC430-09|09BBELE-1430|658[0n]bp|Canada.New Brunswick|BOLD:AAA2144
Xestia c-nigrum|[8290]|XAH223-05|2005-ONT-1806|658[0n]bp|Canada.Ontario|BOLD:AAA2144
Xestia c-nigrum|[8291]|MNBB522-05|05-NBSTA-438|658[0n]bp|Canada.New Brunswick|BOLD:AAA2144
Xestia c-nigrum|[8292]|LPSOC108-08|PPBP-2107|658[0n]bp|Canada.Ontario|BOLD:AAA2144
Xestia c-nigrum|[8293]|TTMNB446-06|MNBTT-446|658[0n]bp|Canada.New Brunswick|BOLD:AAA2144
Xestia c-nigrum|[8294]|XAD434-04|04HBL007434|658[0n]bp|Canada.Ontario|BOLD:AAA2144
Xestia c-nigrum|[8295]|BBLPC093-09|09BBELE-1093|658[0n]bp|Canada.New Brunswick|BOLD:AAA2144
Xestia c-nigrum|[8296]|LPSOB978-08|PPBP-1977|658[0n]bp|Canada.Ontario|BOLD:AAA2144
Xestia c-nigrum|[8297]|MNBB608-05|05-NBSTA-524|658[0n]bp|Canada.New Brunswick|BOLD:AAA2144
Xestia c-nigrum|[8298]|LPSOC298-08|PPBP-2297|658[0n]bp|Canada.Ontario|BOLD:AAA2144
Xestia c-nigrum|[8299]|BBLPC407-09|09BBELE-1407|658[0n]bp|Canada.New Brunswick|BOLD:AAA2144
Xestia c-nigrum|[8300]|LBCD029-05|HLC-22849|658[0n]bp|Canada.British Columbia|BOLD:AAA2144
Xestia c-nigrum|[8301]|XAH549-05|2005-ONT-2132|658[0n]bp|Canada.Ontario|BOLD:AAA2144
Xestia c-nigrum|[8302]|XAD500-04|04HBL007500|658[0n]bp|Canada.Ontario|BOLD:AAA2144
Xestia c-nigrum|[8303]|LPSOC301-08|PPBP-2300|658[0n]bp|Canada.Ontario|BOLD:AAA2144
Xestia c-nigrum|[8304]|LPMNB443-09|08BBLEP-05443|658[0n]bp|Canada.Manitoba|BOLD:AAA2144
Xestia c-nigrum|[8305]|PHMNB745-05|Moth 438.03SA|658[0n]bp|Canada.New Brunswick|BOLD:AAA2144
Xestia c-nigrum|[8306]|LOWC799-05|CGWC-0799|658[0n]bp|Canada.British Columbia|BOLD:AAA2144
Xestia c-nigrum|[8307]|LPSOC345-08|PPBP-2344|658[0n]bp|Canada.Ontario|BOLD:AAA2144
Xestia c-nigrum|[8308]|LOWC794-05|CGWC-0794|658[0n]bp|Canada.British Columbia|BOLD:AAA2144
Xestia c-nigrum|[8309]|LPSOC305-08|PPBP-2304|658[0n]bp|Canada.Ontario|BOLD:AAA2144
Xestia c-nigrum|[8310]|BBLEC424-09|09BBELE-0424|658[0n]bp|Canada.New Brunswick|BOLD:AAA2144
Xestia c-nigrum|[8311]|TTMNB448-06|MNBTT-448|658[0n]bp|Canada.New Brunswick|BOLD:AAA2144
Xestia c-nigrum|[8312]|MNBB593-05|05-NBSTA-509|658[0n]bp|Canada.New Brunswick|BOLD:AAA2144
Xestia c-nigrum|[8313]|LPSOC115-08|PPBP-2114|658[0n]bp|Canada.Ontario|BOLD:AAA2144
Xestia c-nigrum|[8314]|XAK434-06|2006-ONT-1429|658[0n]bp|Canada.Ontario|BOLD:AAA2144
Xestia c-nigrum|[8315]|BBLEC444-09|09BBELE-0444|658[0n]bp|Canada.New Brunswick|BOLD:AAA2144
Xestia c-nigrum|[8316]|MNBB423-05|05-NBSTA-339|658[0n]bp|Canada.New Brunswick|BOLD:AAA2144
Xestia c-nigrum|[8317]|MNBB560-05|05-NBSTA-476|658[0n]bp|Canada.New Brunswick|BOLD:AAA2144
Xestia c-nigrum|[8318]|LPSOC293-08|PPBP-2292|658[0n]bp|Canada.Ontario|BOLD:AAA2144
Xestia c-nigrum|[8319]|TTMNB441-06|MNBTT-441|658[0n]bp|Canada.New Brunswick|BOLD:AAA2144
Xestia c-nigrum|[8320]|XAB363-04|04HBL005363|658[0n]bp|Canada.Ontario|BOLD:AAA2144
Xestia c-nigrum|[8321]|LALPA1249-11|AVBC 1251-11|658[0n]bp|Canada.British Columbia|BOLD:AAA2144
Xestia c-nigrum|[8322]|LPSOC292-08|PPBP-2291|658[0n]bp|Canada.Ontario|BOLD:AAA2144
Xestia c-nigrum|[8323]|RDLQ726-07|DH007161|658[0n]bp|Canada.Quebec|BOLD:AAA2144
Xestia c-nigrum|[8324]|LALPA638-10|AVBC 640-10|658[0n]bp|Canada.British Columbia|BOLD:AAA2144
Xestia c-nigrum|[8325]|LPSOC095-08|PPBP-2094|658[0n]bp|Canada.Ontario|BOLD:AAA2144
Xestia c-nigrum|[8326]|LPSOC294-08|PPBP-2293|658[0n]bp|Canada.Ontario|BOLD:AAA2144
Xestia c-nigrum|[8327]|LOWC798-05|CGWC-0798|658[0n]bp|Canada.British Columbia|BOLD:AAA2144
Xestia c-nigrum|[8328]|MNBB524-05|05-NBSTA-440|658[0n]bp|Canada.New Brunswick|BOLD:AAA2144
Xestia c-nigrum|[8329]|BBLPB827-10|10BBCLP-1826|658[0n]bp|Canada.British Columbia|BOLD:AAA2144
Xestia c-nigrum|[8330]|BLTIB269-08|BL453|658[0n]bp|Canada.Ontario|BOLD:AAA2144
Xestia c-nigrum|[8331]|LPSOC291-08|PPBP-2290|658[0n]bp|Canada.Ontario|BOLD:AAA2144
Xestia c-nigrum|[8332]|LPSOC302-08|PPBP-2301|658[0n]bp|Canada.Ontario|BOLD:AAA2144
Xestia c-nigrum|[8333]|XAH530-05|2005-ONT-2113|658[0n]bp|Canada.Ontario|BOLD:AAA2144
Xestia c-nigrum|[8334]|LALPA714-10|AVBC 716-10|658[0n]bp|Canada.British Columbia|BOLD:AAA2144
Xestia c-nigrum|[8335]|LOWC804-05|CGWC-0804|658[0n]bp|Canada.British Columbia|BOLD:AAA2144
Xestia c-nigrum|[8336]|XAF549-05|2005-ONT-198|658[0n]bp|Canada.Ontario|BOLD:AAA2144
Xestia c-nigrum|[8337]|TTMNB445-06|MNBTT-445|658[0n]bp|Canada.New Brunswick|BOLD:AAA2144
Xestia c-nigrum|[8338]|LOWC797-05|CGWC-0797|658[0n]bp|Canada.British Columbia|BOLD:AAA2144
Xestia c-nigrum|[8339]|LPSOC105-08|PPBP-2104|658[0n]bp|Canada.Ontario|BOLD:AAA2144
Xestia c-nigrum|[8340]|LPSOC296-08|PPBP-2295|658[0n]bp|Canada.Ontario|BOLD:AAA2144
Xestia c-nigrum|[8341]|LOWC912-05|CGWC-0912|658[0n]bp|Canada.British Columbia|BOLD:AAA2144
Xestia c-nigrum|[8342]|LPSOC112-08|PPBP-2111|658[0n]bp|Canada.Ontario|BOLD:AAA2144
Xestia c-nigrum|[8343]|BBLPB825-10|10BBCLP-1824|658[0n]bp|Canada.Alberta|BOLD:AAA2144
Xestia c-nigrum|[8344]|LOWC801-05|CGWC-0801|658[0n]bp|Canada.British Columbia|BOLD:AAA2144
Xestia c-nigrum|[8345]|MNBB600-05|05-NBSTA-516|658[0n]bp|Canada.New Brunswick|BOLD:AAA2144
Xestia c-nigrum|[8346]|LOWCE806-06|CGWC-4566|658[0n]bp|Canada.British Columbia|BOLD:AAA2144
Xestia c-nigrum|[8347]|MNBB666-05|05-NBSTA-582|658[0n]bp|Canada.New Brunswick|BOLD:AAA2144
Xestia c-nigrum|[8348]|BBLEC684-09|09BBELE-0684|658[0n]bp|Canada.Nova Scotia|BOLD:AAA2144
Xestia c-nigrum|[8349]|BBLPC050-09|09BBELE-1050|658[0n]bp|Canada.New Brunswick|BOLD:AAA2144
Xestia c-nigrum|[8350]|LOWCE858-06|CGWC-4618|658[0n]bp|Canada.British Columbia|BOLD:AAA2144
Xestia c-nigrum|[8351]|BBLEC430-09|09BBELE-0430|658[0n]bp|Canada.New Brunswick|BOLD:AAA2144
Xestia c-nigrum|[8352]|LPMNB442-09|08BBLEP-05442|658[0n]bp|Canada.Manitoba|BOLD:AAA2144
Xestia c-nigrum|[8353]|LPSOC114-08|PPBP-2113|658[0n]bp|Canada.Ontario|BOLD:AAA2144
Xestia c-nigrum|[8354]|LPSOC303-08|PPBP-2302|658[0n]bp|Canada.Ontario|BOLD:AAA2144
Xestia c-nigrum|[8355]|LBCC288-05|HLC-22168|658[0n]bp|Canada.British Columbia|BOLD:AAA2144
Xestia c-nigrum|[8356]|BBLPE424-09|09BBELE-2424|658[0n]bp|Canada.Newfoundland and Labrador|BOLD:AAA2144
Xestia c-nigrum|[8357]|LOWCE810-06|CGWC-4570|658[0n]bp|Canada.British Columbia|BOLD:AAA2144
Xestia c-nigrum|[8358]|LPSOD970-09|08BBLEP-05603|658[0n]bp|Canada.Ontario|BOLD:AAA2144
Xestia c-nigrum|[8359]|LPSOC304-08|PPBP-2303|658[0n]bp|Canada.Ontario|BOLD:AAA2144
Xestia c-nigrum|[8360]|MNBB668-05|05-NBSTA-584|658[0n]bp|Canada.New Brunswick|BOLD:AAA2144
Xestia c-nigrum|[8361]|MNBB521-05|05-NBSTA-437|658[0n]bp|Canada.New Brunswick|BOLD:AAA2144
Xestia c-nigrum|[8362]|BBLEC448-09|09BBELE-0448|658[0n]bp|Canada.New Brunswick|BOLD:AAA2144
Xestia c-nigrum|[8363]|RDLQ727-07|DH008432|658[0n]bp|Canada.Quebec|BOLD:AAA2144
Xestia c-nigrum|[8364]|TTMNB442-06|MNBTT-442|658[0n]bp|Canada.New Brunswick|BOLD:AAA2144
Xestia c-nigrum|[8365]|LPSOC116-08|PPBP-2115|658[0n]bp|Canada.Ontario|BOLD:AAA2144
Xestia c-nigrum|[8366]|LBCD030-05|HLC-22850|658[0n]bp|Canada.British Columbia|BOLD:AAA2144
Xestia c-nigrum|[8367]|BBLPB829-10|10BBCLP-1828|658[0n]bp|Canada.British Columbia|BOLD:AAA2144
Xestia c-nigrum|[8368]|BBLPC061-09|09BBELE-1061|658[0n]bp|Canada.New Brunswick|BOLD:AAA2144
Xestia c-nigrum|[8369]|LPSOC297-08|PPBP-2296|658[0n]bp|Canada.Ontario|BOLD:AAA2144
Xestia c-nigrum|[8370]|TTMNB566-06|MNBTT-566|658[0n]bp|Canada.New Brunswick|BOLD:AAA2144
Xestia c-nigrum|[8371]|BBLPC058-09|09BBELE-1058|658[0n]bp|Canada.New Brunswick|BOLD:AAA2144
Xestia c-nigrum|[8372]|MNBB169-05|05-NBSTA-085|658[0n]bp|Canada.New Brunswick|BOLD:AAA2144
[illegible]

Xestia c-nigrum|[8370]|TTMNB566-06|MNBTT-566|658[0n]bp|Canada.New Brunswick|BOLD:AAA2144
Xestia c-nigrum|[8371]|BBLPC058-09|09BBELE-1058|658[0n]bp|Canada.New Brunswick|BOLD:AAA2144
Xestia c-nigrum|[8372]|MNBB169-05|05-NBSTA-085|658[0n]bp|Canada.New Brunswick|BOLD:AAA2144
Xestia c-nigrum|[8373]|LPVIC114-08|PFC-2006-2691|658[0n]bp|Canada.British Columbia|BOLD:AAA2144
Xestia c-nigrum|[8374]|XAH605-05|2005-ONT-2188|658[0n]bp|Canada.Ontario|BOLD:AAA2144
Xestia c-nigrum|[8375]|MNBB599-05|05-NBSTA-515|658[0n]bp|Canada.New Brunswick|BOLD:AAA2144
Xestia c-nigrum|[8376]|BBLPB826-10|10BBCLP-1825|658[0n]bp|Canada.British Columbia|BOLD:AAA2144
Xestia c-nigrum|[8377]|LPABC910-09|08BBLEP-05321|658[1n]bp|Canada.Alberta|BOLD:AAA2144
Xestia c-nigrum|[8378]|BBLEC466-09|09BBELE-0466|658[0n]bp|Canada.New Brunswick|BOLD:AAA2144
Xestia c-nigrum|[8379]|BBLPC060-09|09BBELE-1060|644[0n]bp|Canada.New Brunswick|BOLD:AAA2144
Xestia c-nigrum|[8380]|LPSOC109-08|PPBP-2108|656[0n]bp|Canada.Ontario|BOLD:AAA2144
Xestia c-nigrum|[8381]|BBLPB828-10|10BBCLP-1827|632[1n]bp|Canada.British Columbia|BOLD:AAA2144
Xestia c-nigrum|[8382]|XAF780-05|2005-ONT-429|579[4n]bp|Canada.Ontario|BOLD:AAA2144
Xestia c-nigrum|[8383]|BBLPC030-09|09BBELE-1030|632[0n]bp|Canada.New Brunswick|BOLD:AAA2144
Xestia c-nigrum|[8384]|PHMNB222-04|04HBL007687|538[1n]bp|Canada.New Brunswick|BOLD:AAA2144
Xestia c-nigrum|[8385]|MNBB422-05|05-NBSTA-338|540[0n]bp|Canada.New Brunswick|BOLD:AAA2144
Xestia c-nigrum|[8386]|MNBB170-05|05-NBSTA-086|537[0n]bp|Canada.New Brunswick|BOLD:AAA2144
Xestia c-nigrum|[8387]|TTMNB440-06|MNBTT-440|605[0n]bp|Canada.New Brunswick|BOLD:AAA2144
Xestia c-nigrum|[8388]|RDLQB707-05|DH010810|596[0n]bp|Canada.Quebec|BOLD:AAA2144
Xestia c-nigrum|[8389]|PHMNB003-03|moth104.02SA|639[0n]bp|Canada.New Brunswick|BOLD:AAA2144
Xestia c-nigrum|[8390]|PHMO085-03|moth543.01|639[0n]bp|Canada.Ontario|BOLD:AAA2144
Xestia c-nigrum|[8391]|LPSOC295-08|PPBP-2294|658[0n]bp|Canada.Ontario|BOLD:AAA2144
Xestia c-nigrum|[8392]|XAH562-05|2005-ONT-2145|658[0n]bp|Canada.Ontario|BOLD:AAA2144
Xestia c-nigrum|[8393]|BBLPB830-10|10BBCLP-1829|658[0n]bp|Canada.British Columbia|BOLD:AAA2144
Xestia c-nigrum|[8394]|LPSOC104-08|PPBP-2103|658[0n]bp|Canada.Ontario|BOLD:AAA2144
Xestia c-nigrum|[8395]|LBCC287-05|HLC-22167|658[0n]bp|Canada.British Columbia|BOLD:AAA2144
Xestia c-nigrum|[8396]|BLTIB339-08|BL535|658[0n]bp|Canada.Ontario|BOLD:AAA2144
Xestia c-nigrum|[8397]|BBLPC347-09|09BBELE-1347|658[0n]bp|Canada.New Brunswick|BOLD:AAA2144
Xestia c-nigrum|[8398]|BLTIB315-08|BL504|658[0n]bp|Canada.Ontario|BOLD:AAA2144
Xestia c-nigrum|[8399]|LPSOC300-08|PPBP-2299|658[0n]bp|Canada.Ontario|BOLD:AAA2144
Xestia c-nigrum|[8400]|XAJ549-06|2006-ONT-0549|658[0n]bp|Canada.Ontario|BOLD:AAA2144
Xestia c-nigrum|[8401]|LBCD493-05|HLC-23313|658[0n]bp|Canada.British Columbia|BOLD:AAA2144
Xestia c-nigrum|[8402]|MNBB119-05|05-NBSTA-035|658[0n]bp|Canada.New Brunswick|BOLD:AAA2144
Xestia c-nigrum|[8403]|LOWC796-05|CGWC-0796|658[0n]bp|Canada.British Columbia|BOLD:AAA2144
Xestia c-nigrum|[8404]|LPSOC326-08|PPBP-2325|658[0n]bp|Canada.Ontario|BOLD:AAA2144
Xestia c-nigrum|[8405]|LPSOB972-08|PPBP-1971|658[0n]bp|Canada.Ontario|BOLD:AAA2144
Xestia c-nigrum|[8406]|MNBB590-05|05-NBSTA-506|658[0n]bp|Canada.New Brunswick|BOLD:AAA2144
Xestia c-nigrum|[8407]|LPSOC103-08|PPBP-2102|658[0n]bp|Canada.Ontario|BOLD:AAA2144
Xestia c-nigrum|[8408]|TTMNB449-06|MNBTT-449|658[0n]bp|Canada.New Brunswick|BOLD:AAA2144
Xestia c-nigrum|[8409]|BBLPC085-09|09BBELE-1085|658[0n]bp|Canada.New Brunswick|BOLD:AAA2144
Xestia c-nigrum|[8410]|MNBB386-05|05-NBSTA-302|658[0n]bp|Canada.New Brunswick|BOLD:AAA2144
Xestia dolosa|[8411]|XAH621-05|2005-ONT-2204|658[0n]bp|Canada.Ontario|BOLD:AAA2144
Xestia dolosa|[8412]|PHMNB278-04|04HBL007743|658[0n]bp|Canada.New Brunswick|BOLD:AAA2144
Xestia dolosa|[8413]|LPSOC290-08|PPBP-2289|658[0n]bp|Canada.Ontario|BOLD:AAA2144
Xestia dolosa|[8414]|PHMNB598-04|04HBL00824|658[0n]bp|Canada.New Brunswick|BOLD:AAA2144
Xestia dolosa|[8415]|XAB550-04|04HBL005550|658[0n]bp|Canada.Ontario|BOLD:AAA2144
Xestia dolosa|[8416]|XAH702-05|2005-ONT-2285|658[0n]bp|Canada.Ontario|BOLD:AAA2144
Xestia dolosa|[8417]|XAH619-05|2005-ONT-2202|658[0n]bp|Canada.Ontario|BOLD:AAA2144
Xestia maculata|[8418]|RDMAB1035-09|UASM78759|658[0n]bp|Canada.Alberta|BOLD:ABY8431
Xestia maculata|[8419]|RDMAB1033-09|UASM78865|644[0n]bp|Canada.Alberta|BOLD:ABY8431
Xestia maculata|[8420]|RDMAB1034-09|UASM78864|537[0n]bp|Canada.Alberta|BOLD:ABY8431
Xestia maculata|[8421]|RDMAB1032-09|UASM78757|644[0n]bp|Canada.Alberta|BOLD:ABY8431
Xestia scropulana|[8422]|LCHQ883-08|07WNP-10775|658[0n]bp|Canada.Manitoba|BOLD:ABY8431
Xestia inuitica|[8423]|RDNMG708-08|CNC LEP00052832|658[1n]bp|Canada.Nunavut|BOLD:ABY8431
Xestia inuitica|[8424]|RDNMG709-08|CNC LEP00052833|658[2n]bp|Canada.Nunavut|BOLD:ABY8431
Xestia scropulana|[8425]|LCHP867-07|07PROBE-10624|658[0n]bp|Canada.Manitoba|BOLD:ABY8431
Xestia scropulana|[8426]|LCHP157-07|07PROBE-00559|658[0n]bp|Canada.Manitoba|BOLD:ABY8431
Xestia scropulana|[8427]|LCHP176-07|07PROBE-00578|658[0n]bp|Canada.Manitoba|BOLD:ABY8431
Xestia scropulana|[8428]|LCHP629-07|07PROBE-10310|658[0n]bp|Canada.Manitoba|BOLD:ABY8431
Xestia scropulana|[8429]|LCHQ876-08|07WNP-10768|658[0n]bp|Canada.Manitoba|BOLD:ABY8431
Xestia scropulana|[8430]|LCHQ871-08|07WNP-10763|658[0n]bp|Canada.Manitoba|BOLD:ABY8431
Xestia scropulana|[8431]|LCHP174-07|07PROBE-00576|658[0n]bp|Canada.Manitoba|BOLD:ABY8431
Xestia scropulana|[8432]|LCHQ874-08|07WNP-10766|658[0n]bp|Canada.Manitoba|BOLD:ABY8431
Xestia scropulana|[8433]|LCHQ880-08|07WNP-10772|658[0n]bp|Canada.Manitoba|BOLD:ABY8431
Xestia scropulana|[8434]|LCHQ870-08|07WNP-10762|658[0n]bp|Canada.Manitoba|BOLD:ABY8431
Xestia scropulana|[8435]|LCHP161-07|07PROBE-00563|658[0n]bp|Canada.Manitoba|BOLD:ABY8431
Xestia scropulana|[8436]|LCHQ879-08|07WNP-10771|658[0n]bp|Canada.Manitoba|BOLD:ABY8431
Xestia scropulana|[8437]|LCHQ885-08|07WNP-10777|658[0n]bp|Canada.Manitoba|BOLD:ABY8431
Xestia scropulana|[8438]|LCHQ882-08|07WNP-10774|658[0n]bp|Canada.Manitoba|BOLD:ABY8431
Xestia scropulana|[8439]|LCH256-04|04HBL003256|658[0n]bp|Canada.Manitoba|BOLD:ABY8431
Xestia scropulana|[8440]|LCHQ896-08|07WNP-10788|658[0n]bp|Canada.Manitoba|BOLD:ABY8431
Xestia scropulana|[8441]|LCHQ873-08|07WNP-10765|658[0n]bp|Canada.Manitoba|BOLD:ABY8431
Xestia scropulana|[8442]|LCHQ872-08|07WNP-10764|657[0n]bp|Canada.Manitoba|BOLD:ABY8431
Xestia scropulana|[8443]|LCHP162-07|07PROBE-00564|658[0n]bp|Canada.Manitoba|BOLD:ABY8431
Xestia scropulana|[8444]|LCHQ913-08|07WNP-10805|658[0n]bp|Canada.Manitoba|BOLD:ABY8431
Xestia scropulana|[8445]|LCHP214-07|07PROBE-03784|658[0n]bp|Canada.Manitoba|BOLD:ABY8431
Xestia scropulana|[8446]|LCHP206-07|07PROBE-00131|658[0n]bp|Canada.Manitoba|BOLD:ABY8431
Xestia scropulana|[8447]|LCHP158-07|07PROBE-00560|658[0n]bp|Canada.Manitoba|BOLD:ABY8431
Xestia scropulana|[8448]|LCHQ878-08|07WNP-10770|658[0n]bp|Canada.Manitoba|BOLD:ABY8431
Xestia scropulana|[8449]|LCHP173-07|07PROBE-00575|658[0n]bp|Canada.Manitoba|BOLD:ABY8431
Xestia scropulana|[8450]|MHLEP090-07|CHU06-LEP-090|658[0n]bp|Canada.Manitoba|BOLD:ABY8431
Xestia scropulana|[8451]|LCHP007-07|07PROBE-00065|655[0n]bp|Canada.Manitoba|BOLD:ABY8431
Xestia scropulana|[8452]|LCHQ887-08|07WNP-10779|658[0n]bp|Canada.Manitoba|BOLD:ABY8431
Xestia scropulana|[8453]|LCHQ892-08|07WNP-10784|658[0n]bp|Canada.Manitoba|BOLD:ABY8431
Xestia inuitica|[8454]|RDNMG707-08|CNC LEP00052831|658[0n]bp|Canada.Nunavut|BOLD:ABY8431
Xestia scropulana|[8455]|LCHP160-07|07PROBE-00562|656[0n]bp|Canada.Manitoba|BOLD:ABY8431
Xestia scropulana|[8456]|LCHQ875-08|07WNP-10767|656[0n]bp|Canada.Manitoba|BOLD:ABY8431
Xestia scropulana|[8457]|LCHP159-07|07PROBE-00561|656[0n]bp|Canada.Manitoba|BOLD:ABY8431
Xestia scropulana|[8458]|LCHP390-07|07PROBE-10014|638[0n]bp|Canada.Manitoba|BOLD:ABY8431
Xestia inuitica|[8459]|RDNMF075-08|NOC14161|551[7n]bp|Canada.Nunavut|
Xestia scropulana|[8460]|LCHQ923-08|07WNP-10815|658[0n]bp|Canada.Manitoba|BOLD:ABY8431
Xestia scropulana|[8461]|LCHQ881-08|07WNP-10773|658[0n]bp|Canada.Manitoba|BOLD:ABY8431
Xestia scropulana|[8462]|LCHQ884-08|07WNP-10776|658[0n]bp|Canada.Manitoba|BOLD:ABY8431
Xestia wockei|[8463]|RDNMG733-08|CNC LEP00052857|609[0n]bp|Canada.Yukon Territory|BOLD:ABY8431
Xestia wockei|[8464]|RDNMG732-08|CNC LEP00052856|641[0n]bp|Canada.Yukon Territory|BOLD:ABY8431
Xestia liquidaria|[8465]|RDNMG728-08|CNC LEP00052852|658[5n]bp|Canada.Yukon Territory|BOLD:AAH9449
Xestia lyngei|[8466]|RDNMG722-08|CNC LEP00052846|642[0n]bp|Canada.Yukon Territory|BOLD:AAD0055
Xestia lyngei|[8467]|RDNMG697-08|CNC LEP00052821|658[0n]bp|Canada.Yukon Territory|BOLD:AAD0055
Xestia lyngei|[8468]|RDNMF069-08|NOC14155|658[0n]bp|Canada.Yukon Territory|BOLD:AAD0055
Xestia lyngei|[8469]|RDNMG696-08|CNC LEP00052820|658[0n]bp|Canada.Yukon Territory|BOLD:AAD0055
Xestia lyngei|[8470]|RDNMG695-08|CNC LEP00052819|642[0n]bp|Canada.Yukon Territory|BOLD:AAD0055
Xestia liquidaria|[8471]|RDNMG729-08|CNC LEP00052853|618[4n]bp|Canada.Yukon Territory|BOLD:ABZ1295
Xestia woodi|[8472]|RDNMF065-08|NOC14151|658[0n]bp|Canada.Yukon Territory|BOLD:ABZ1295

Xestia lyngei|[8470]|RDNMG695-08|CNC LEP00052819|642[0n]bp|Canada.Yukon Territory|BOLD:AAD0055
Xestia liquidaria|[8471]|RDNMG729-08|CNC LEP00052853|618[4n]bp|Canada.Yukon Territory|BOLD:ABZ1295
Xestia woodi|[8472]|RDNMF065-08|NOC14151|658[0n]bp|Canada.Yukon Territory|BOLD:ABZ1295
Xestia woodi|[8473]|RDNMG724-08|CNC LEP00052848|658[0n]bp|Canada.Yukon Territory|BOLD:ABZ1295
Xestia kolymae|[8474]|RDNMG743-08|CNC LEP00052867|642[0n]bp|Canada.Yukon Territory|BOLD:AAF1130
Xestia kolymae|[8475]|RDNMG742-08|CNC LEP00052866|641[0n]bp|Canada.Yukon Territory|BOLD:AAF1130
Xestia kolymae|[8476]|RDNMF070-08|NOC14156|658[0n]bp|Canada.Yukon Territory|BOLD:AAF1130
Xestia fabulosa|[8477]|LBCG2490-09|08-JDWBC-2490|638[0n]bp|Canada.British Columbia|BOLD:AAA9525
Xestia fabulosa|[8478]|LBCH2981-10|10-JDWBC-2981|658[0n]bp|Canada.British Columbia|BOLD:AAA9525
Xestia fabulosa|[8479]|LBCH3027-10|10-JDWBC-3027|658[0n]bp|Canada.British Columbia|BOLD:AAA9525
Xestia fabulosa|[8480]|LBCH2955-10|10-JDWBC-2955|658[0n]bp|Canada.British Columbia|BOLD:AAA9525
Xestia fabulosa|[8481]|LBCH2941-10|10-JDWBC-2941|658[0n]bp|Canada.British Columbia|BOLD:AAA9525
Xestia fabulosa|[8482]|LBCG2539-09|08-JDWBC-2539|658[0n]bp|Canada.British Columbia|BOLD:AAA9525
Xestia fabulosa|[8483]|LCHP538-07|07PROBE-10208|658[0n]bp|Canada.Manitoba|BOLD:AAA9525
Xestia fabulosa|[8484]|LBCG2757-09|08-JDWBC-2757|658[0n]bp|Canada.British Columbia|BOLD:AAA9525
Xestia fabulosa|[8485]|LBCG2385-09|08-JDWBC-2385|658[0n]bp|Canada.British Columbia|BOLD:AAA9525
Xestia fabulosa|[8486]|LBCG2387-09|08-JDWBC-2387|658[0n]bp|Canada.British Columbia|BOLD:AAA9525
Xestia fabulosa|[8487]|LBCG3054-09|08-JDWBC-3054|658[0n]bp|Canada.British Columbia|BOLD:AAA9525
Xestia fabulosa|[8488]|LBCG2823-09|08-JDWBC-2823|658[0n]bp|Canada.British Columbia|BOLD:AAA9525
Xestia fabulosa|[8489]|LBCG2759-09|08-JDWBC-2759|658[0n]bp|Canada.British Columbia|BOLD:AAA9525
Xestia fabulosa|[8490]|LBCG1903-09|08-JDWBC-1903|658[0n]bp|Canada.British Columbia|BOLD:AAA9525
Xestia fabulosa|[8491]|LBCG3055-09|08-JDWBC-3055|658[0n]bp|Canada.British Columbia|BOLD:AAA9525
Xestia fabulosa|[8492]|LBCH2249-10|10-JDWBC-2249|658[0n]bp|Canada.British Columbia|BOLD:AAA9525
Xestia fabulosa|[8493]|LCHP588-07|07PROBE-10269|658[0n]bp|Canada.Manitoba|BOLD:AAA9525
Xestia fabulosa|[8494]|LBCG2390-09|08-JDWBC-2390|658[0n]bp|Canada.British Columbia|BOLD:AAA9525
Xestia fabulosa|[8495]|LBCG2383-09|08-JDWBC-2383|658[0n]bp|Canada.British Columbia|BOLD:AAA9525
Xestia fabulosa|[8496]|LBCG2384-09|08-JDWBC-2384|658[0n]bp|Canada.British Columbia|BOLD:AAA9525
Xestia fabulosa|[8497]|LBCH1933-10|10-JDWBC-1933|658[0n]bp|Canada.British Columbia|BOLD:AAA9525
Xestia fabulosa|[8498]|LBCG1904-09|08-JDWBC-1904|658[0n]bp|Canada.British Columbia|BOLD:AAA9525
Xestia fabulosa|[8499]|LBCG1906-09|08-JDWBC-1906|658[0n]bp|Canada.British Columbia|BOLD:AAA9525
Xestia fabulosa|[8500]|LBCG3056-09|08-JDWBC-3056|658[0n]bp|Canada.British Columbia|BOLD:AAA9525
Xestia fabulosa|[8501]|LBCB804-05|HLC-21744|658[0n]bp|Canada.British Columbia|BOLD:AAA9525
Xestia fabulosa|[8502]|LBCG2761-09|08-JDWBC-2761|658[0n]bp|Canada.British Columbia|BOLD:AAA9525
Xestia fabulosa|[8503]|LBCG2758-09|08-JDWBC-2758|658[0n]bp|Canada.British Columbia|BOLD:AAA9525
Xestia fabulosa|[8504]|LBCG2825-09|08-JDWBC-2825|658[0n]bp|Canada.British Columbia|BOLD:AAA9525
Xestia fabulosa|[8505]|LBCG2824-09|08-JDWBC-2824|658[0n]bp|Canada.British Columbia|BOLD:AAA9525
Xestia fabulosa|[8506]|LBCG2386-09|08-JDWBC-2386|658[0n]bp|Canada.British Columbia|BOLD:AAA9525
Xestia fabulosa|[8507]|LBCG2382-09|08-JDWBC-2382|658[0n]bp|Canada.British Columbia|BOLD:AAA9525
Xestia fabulosa|[8508]|LBCG2756-09|08-JDWBC-2756|658[0n]bp|Canada.British Columbia|BOLD:AAA9525
Xestia fabulosa|[8509]|LBCG2392-09|08-JDWBC-2392|658[0n]bp|Canada.British Columbia|BOLD:AAA9525
Xestia fabulosa|[8510]|LBCG2389-09|08-JDWBC-2389|658[0n]bp|Canada.British Columbia|BOLD:AAA9525
Xestia fabulosa|[8511]|LBCG2762-09|08-JDWBC-2762|658[0n]bp|Canada.British Columbia|BOLD:AAA9525
Xestia fabulosa|[8512]|LBCG2763-09|08-JDWBC-2763|658[0n]bp|Canada.British Columbia|BOLD:AAA9525
Xestia fabulosa|[8513]|LBCG2388-09|08-JDWBC-2388|658[0n]bp|Canada.British Columbia|BOLD:AAA9525
Xestia fabulosa|[8514]|LCHP311-07|07PROBE-03877|658[0n]bp|Canada.Manitoba|BOLD:AAA9525
Xestia fabulosa|[8515]|LBCG3057-09|08-JDWBC-3057|658[0n]bp|Canada.British Columbia|BOLD:AAA9525
Xestia fabulosa|[8516]|LBCG2391-09|08-JDWBC-2391|658[0n]bp|Canada.British Columbia|BOLD:AAA9525
Xestia fabulosa|[8517]|LBCG2760-09|08-JDWBC-2760|658[0n]bp|Canada.British Columbia|BOLD:AAA9525
Xestia albuncula|[8518]|RDNMG749-08|CNC LEP00052873|658[0n]bp|Canada.Yukon Territory|BOLD:AAA2274
Xestia albuncula|[8519]|RDNMF005-08|NOC14091|658[0n]bp|Canada.Yukon Territory|BOLD:AAA2274
Xestia albuncula|[8520]|RDNMG751-08|CNC LEP00052875|658[0n]bp|Canada.Yukon Territory|BOLD:AAA2274
Xestia imperita|[8521]|LCHP788-07|07PROBE-10473|658[0n]bp|Canada.Manitoba|BOLD:ABZ1722
Xestia imperita|[8522]|LCHQ895-08|07WNP-10787|632[2n]bp|Canada.Manitoba|BOLD:ABZ1722
Xestia imperita|[8523]|LCHQ894-08|07WNP-10786|658[0n]bp|Canada.Manitoba|BOLD:ABZ1722
Xestia imperita|[8524]|LCHP956-07|07PROBE-10718|658[0n]bp|Canada.Manitoba|BOLD:ABZ1722
Xestia imperita|[8525]|LCHQ724-08|07WNP-10616|658[0n]bp|Canada.Manitoba|BOLD:ABZ1722
Xestia imperita|[8526]|RDLQF165-06|DH011192|658[0n]bp|Canada.Quebec|BOLD:ABZ1722
Xestia imperita|[8527]|LOWCE102-06|CGWC-3862|658[0n]bp|Canada.British Columbia|BOLD:ABZ1722
Xestia imperita|[8528]|LCHQ546-08|07WNP-10438|658[0n]bp|Canada.Manitoba|BOLD:ABZ1722
Xestia imperita|[8529]|LCHP617-07|07PROBE-10298|658[0n]bp|Canada.Manitoba|BOLD:ABZ1722
Xestia imperita|[8530]|LCHP615-07|07PROBE-10296|658[0n]bp|Canada.Manitoba|BOLD:ABZ1722
Xestia imperita|[8531]|LCHP964-07|07PROBE-10726|658[0n]bp|Canada.Manitoba|BOLD:ABZ1722
Xestia imperita|[8532]|LCHQ586-08|07WNP-10478|658[0n]bp|Canada.Manitoba|BOLD:ABZ1722
Xestia imperita|[8533]|LCHP807-07|07PROBE-10564|658[0n]bp|Canada.Manitoba|BOLD:ABZ1722
Xestia imperita|[8534]|LCHQ911-08|07WNP-10803|658[0n]bp|Canada.Manitoba|BOLD:ABZ1722
Xestia imperita|[8535]|LCHP612-07|07PROBE-10293|658[0n]bp|Canada.Manitoba|BOLD:ABZ1722
Xestia imperita|[8536]|LCHP812-07|07PROBE-10569|658[0n]bp|Canada.Manitoba|BOLD:ABZ1722
Xestia imperita|[8537]|LCHQ138-07|07PROBE-10907|658[0n]bp|Canada.Manitoba|BOLD:ABZ1722
Xestia imperita|[8538]|LCHP809-07|07PROBE-10566|658[0n]bp|Canada.Manitoba|BOLD:ABZ1722
Xestia imperita|[8539]|LCHQ192-07|07PROBE-10969|658[0n]bp|Canada.Manitoba|BOLD:ABZ1722
Xestia imperita|[8540]|LCHP701-07|07PROBE-10383|658[0n]bp|Canada.Manitoba|BOLD:ABZ1722
Xestia imperita|[8541]|LCHP614-07|07PROBE-10295|658[0n]bp|Canada.Manitoba|BOLD:ABZ1722
Xestia imperita|[8542]|LCHP606-07|07PROBE-10287|658[0n]bp|Canada.Manitoba|BOLD:ABZ1722
Xestia imperita|[8543]|LCHP536-07|07PROBE-10206|658[0n]bp|Canada.Manitoba|BOLD:ABZ1722
Xestia imperita|[8544]|LCHQ527-08|07WNP-10419|658[0n]bp|Canada.Manitoba|BOLD:ABZ1722
Xestia imperita|[8545]|LCHP598-07|07PROBE-10279|658[0n]bp|Canada.Manitoba|BOLD:ABZ1722
Xestia imperita|[8546]|LCHQ542-08|07WNP-10434|658[0n]bp|Canada.Manitoba|BOLD:ABZ1722
Xestia imperita|[8547]|LCHQ489-08|07WNP-10381|658[0n]bp|Canada.Manitoba|BOLD:ABZ1722
Xestia imperita|[8548]|LCHQ479-08|07WNP-10371|658[0n]bp|Canada.Manitoba|BOLD:ABZ1722
Xestia imperita|[8549]|LCHQ595-08|07WNP-10487|658[0n]bp|Canada.Manitoba|BOLD:ABZ1722
Xestia imperita|[8550]|LCHP601-07|07PROBE-10282|658[0n]bp|Canada.Manitoba|BOLD:ABZ1722
Xestia imperita|[8551]|LCHP671-07|07PROBE-10353|658[0n]bp|Canada.Manitoba|BOLD:ABZ1722
Xestia imperita|[8552]|LCHQ196-07|07PROBE-10973|658[0n]bp|Canada.Manitoba|BOLD:ABZ1722
Xestia imperita|[8553]|LCHP749-07|07PROBE-10434|655[0n]bp|Canada.Manitoba|BOLD:ABZ1722
Xestia imperita|[8554]|LCHP945-07|07PROBE-10707|658[0n]bp|Canada.Manitoba|BOLD:ABZ1722
Xestia imperita|[8555]|LCHP645-07|07PROBE-10326|658[0n]bp|Canada.Manitoba|BOLD:ABZ1722
Xestia imperita|[8556]|LCHQ481-08|07WNP-10373|658[0n]bp|Canada.Manitoba|BOLD:ABZ1722
Xestia imperita|[8557]|LCHQ183-07|07PROBE-10960|658[0n]bp|Canada.Manitoba|BOLD:ABZ1722
Xestia imperita|[8558]|LCHP872-07|07PROBE-10629|658[0n]bp|Canada.Manitoba|BOLD:ABZ1722
Xestia imperita|[8559]|LPABC218-09|08BBLEP-04437|658[1n]bp|Canada.Alberta|BOLD:ABZ1722
Xestia imperita|[8560]|LCHP863-07|07PROBE-10620|658[0n]bp|Canada.Manitoba|BOLD:ABZ1722
Xestia imperita|[8561]|LPABC205-09|08BBLEP-04424|632[1n]bp|Canada.Alberta|BOLD:ABZ1722
Xestia imperita|[8562]|LCHQ778-08|07WNP-10670|656[0n]bp|Canada.Manitoba|BOLD:ABZ1722
Xestia imperita|[8563]|MHCOL160-07|CHU06-COL-160|653[0n]bp|Canada.Manitoba|BOLD:ABZ1722
Xestia imperita|[8564]|LPABC220-09|08BBLEP-04439|622[0n]bp|Canada.Alberta|BOLD:ABZ1722
Xestia imperita|[8565]|LPABC195-09|08BBLEP-04414|597[1n]bp|Canada.Alberta|BOLD:ABZ1722
Xestia imperita|[8566]|LCHQ573-08|07WNP-10465|658[0n]bp|Canada.Manitoba|BOLD:ABZ1722
Xestia imperita|[8567]|LCHQ303-08|07WNP-10195|656[0n]bp|Canada.Manitoba|BOLD:ABZ1722
Xestia imperita|[8568]|LCHQ185-07|07PROBE-10962|656[0n]bp|Canada.Manitoba|BOLD:ABZ1722
Xestia imperita|[8569]|LCHP611-07|07PROBE-10292|658[0n]bp|Canada.Manitoba|BOLD:ABZ1722
Xestia imperita|[8570]|LCHQ776-08|07WNP-10668|658[0n]bp|Canada.Manitoba|BOLD:ABZ1722
Xestia imperita|[8571]|LCHQ143-07|07PROBE-10912|658[0n]bp|Canada.Manitoba|BOLD:ABZ1722
Xestia imperita|[8572]|LCHQ132-07|07PROBE-10901|658[0n]bp|Canada.Manitoba|BOLD:ABZ1722

[illegible]
Xestia imperita|[8570]|LCHQ776-08|07WNP-10668|658[0n]bp|Canada.Manitoba|BOLD:ABZ1722
Xestia imperita|[8571]|LCHQ143-07|07PROBE-10912|658[0n]bp|Canada.Manitoba|BOLD:ABZ1722
Xestia imperita|[8572]|LCHQ132-07|07PROBE-10901|658[0n]bp|Canada.Manitoba|BOLD:ABZ1722
Xestia imperita|[8573]|LCHP661-07|07PROBE-10343|658[0n]bp|Canada.Manitoba|BOLD:ABZ1722
Xestia imperita|[8574]|LPABC174-09|08BBLEP-04393|658[1n]bp|Canada.Alberta|BOLD:ABZ1722
Xestia imperita|[8575]|LCHQ383-08|07WNP-10275|658[0n]bp|Canada.Manitoba|BOLD:ABZ1722
Xestia imperita|[8576]|LCHQ195-07|07PROBE-10972|658[0n]bp|Canada.Manitoba|BOLD:ABZ1722
Xestia imperita|[8577]|LCHQ354-08|07WNP-10246|658[0n]bp|Canada.Manitoba|BOLD:ABZ1722
Xestia imperita|[8578]|LCHP949-07|07PROBE-10711|658[0n]bp|Canada.Manitoba|BOLD:ABZ1722
Xestia imperita|[8579]|LCHP619-07|07PROBE-10300|658[0n]bp|Canada.Manitoba|BOLD:ABZ1722
Xestia imperita|[8580]|LOWCC174-05|CGWC-2054|658[0n]bp|Canada.British Columbia|BOLD:ABZ1722
Xestia imperita|[8581]|LCHQ769-08|07WNP-10661|658[0n]bp|Canada.Manitoba|BOLD:ABZ1722
Xestia imperita|[8582]|LOWCC183-05|CGWC-2063|658[0n]bp|Canada.British Columbia|BOLD:ABZ1722
Xestia imperita|[8583]|LBCG3318-09|08-JDWBC-3318|625[0n]bp|Canada.British Columbia|BOLD:ABZ1722
Xestia imperita|[8584]|LPABC256-09|08BBLEP-04475|658[1n]bp|Canada.Alberta|BOLD:ABZ1722
Xestia imperita|[8585]|LPABC213-09|08BBLEP-04432|658[0n]bp|Canada.Alberta|BOLD:ABZ1722
Xestia imperita|[8586]|LPABB729-08|08BBLEP-03994|658[0n]bp|Canada.Alberta|BOLD:ABZ1722
Xestia imperita|[8587]|LCHQ777-08|07WNP-10669|658[0n]bp|Canada.Manitoba|BOLD:ABZ1722
Xestia imperita|[8588]|LCHQ555-08|07WNP-10447|658[0n]bp|Canada.Manitoba|BOLD:ABZ1722
Xestia imperita|[8589]|LCHP662-07|07PROBE-10344|658[0n]bp|Canada.Manitoba|BOLD:ABZ1722
Xestia imperita|[8590]|RDLQF163-06|DH011190|658[0n]bp|Canada.Quebec|BOLD:ABZ1722
Xestia imperita|[8591]|LCHIP223-07|06-PROBE-2868|650[0n]bp|Canada.Manitoba|BOLD:ABZ1722
Xestia imperita|[8592]|LCHQ169-07|07PROBE-10938|658[0n]bp|Canada.Manitoba|BOLD:ABZ1722
Xestia imperita|[8593]|LCHQ588-08|07WNP-10480|658[0n]bp|Canada.Manitoba|BOLD:ABZ1722
Xestia imperita|[8594]|LCHP808-07|07PROBE-10565|658[0n]bp|Canada.Manitoba|BOLD:ABZ1722
Xestia imperita|[8595]|LCHQ182-07|07PROBE-10959|658[0n]bp|Canada.Manitoba|BOLD:ABZ1722
Xestia imperita|[8596]|LCHP798-07|07PROBE-10555|658[0n]bp|Canada.Manitoba|BOLD:ABZ1722
Xestia imperita|[8597]|LCHQ440-08|07WNP-10332|658[0n]bp|Canada.Manitoba|BOLD:ABZ1722
Xestia imperita|[8598]|LCHQ905-08|07WNP-10797|658[0n]bp|Canada.Manitoba|BOLD:ABZ1722
Xestia imperita|[8599]|LCHQ552-08|07WNP-10444|658[0n]bp|Canada.Manitoba|BOLD:ABZ1722
Xestia imperita|[8600]|LCHP646-07|07PROBE-10327|657[0n]bp|Canada.Manitoba|BOLD:ABZ1722
Xestia imperita|[8601]|LCHP610-07|07PROBE-10291|658[0n]bp|Canada.Manitoba|BOLD:ABZ1722
Xestia imperita|[8602]|LCHQ486-08|07WNP-10378|633[0n]bp|Canada.Manitoba|BOLD:ABZ1722
Xestia imperita|[8603]|LCHP616-07|07PROBE-10297|658[0n]bp|Canada.Manitoba|BOLD:ABZ1722
Xestia imperita|[8604]|LCHP672-07|07PROBE-10354|658[0n]bp|Canada.Manitoba|BOLD:ABZ1722
Xestia imperita|[8605]|LCHQ194-07|07PROBE-10971|656[0n]bp|Canada.Manitoba|BOLD:ABZ1722
Xestia imperita|[8606]|LCHP658-07|07PROBE-10339|658[0n]bp|Canada.Manitoba|BOLD:ABZ1722
Xestia imperita|[8607]|LCHQ101-07|07PROBE-10862|658[0n]bp|Canada.Manitoba|BOLD:ABZ1722
Xestia imperita|[8608]|RDLQF162-06|DH011189|658[0n]bp|Canada.Quebec|BOLD:ABZ1722
Xestia imperita|[8609]|LCHQ193-07|07PROBE-10970|658[0n]bp|Canada.Manitoba|BOLD:ABZ1722
Xestia imperita|[8610]|LCHP668-07|07PROBE-10350|658[0n]bp|Canada.Manitoba|BOLD:ABZ1722
Xestia imperita|[8611]|LCHP810-07|07PROBE-10567|658[0n]bp|Canada.Manitoba|BOLD:ABZ1722
Xestia imperita|[8612]|LCHQ091-07|07PROBE-10852|658[0n]bp|Canada.Manitoba|BOLD:ABZ1722
Xestia imperita|[8613]|LCHQ504-08|07WNP-10396|658[0n]bp|Canada.Manitoba|BOLD:ABZ1722
Xestia imperita|[8614]|LCHQ131-07|07PROBE-10900|658[0n]bp|Canada.Manitoba|BOLD:ABZ1722
Xestia imperita|[8615]|LCHQ485-08|07WNP-10377|658[0n]bp|Canada.Manitoba|BOLD:ABZ1722
Xestia imperita|[8616]|RDLQF170-06|DH011197|658[0n]bp|Canada.Quebec|BOLD:ABZ1722
Xestia imperita|[8617]|LCHP600-07|07PROBE-10281|658[0n]bp|Canada.Manitoba|BOLD:ABZ1722
Xestia imperita|[8618]|LCHP670-07|07PROBE-10352|658[0n]bp|Canada.Manitoba|BOLD:ABZ1722
Xestia imperita|[8619]|LCHQ355-08|07WNP-10247|658[0n]bp|Canada.Manitoba|BOLD:ABZ1722
Xestia imperita|[8620]|LCHP602-07|07PROBE-10283|658[0n]bp|Canada.Manitoba|BOLD:ABZ1722
Xestia imperita|[8621]|RDLQF160-06|DH011187|658[0n]bp|Canada.Quebec|BOLD:ABZ1722
Xestia imperita|[8622]|LCH575-04|04HBL003575|658[0n]bp|Canada.Manitoba|BOLD:ABZ1722
Xestia imperita|[8623]|LCHP813-07|07PROBE-10570|658[0n]bp|Canada.Manitoba|BOLD:ABZ1722
Xestia imperita|[8624]|LCHQ592-08|07WNP-10484|657[0n]bp|Canada.Manitoba|BOLD:ABZ1722
Xestia imperita|[8625]|LCHQ476-08|07WNP-10368|657[0n]bp|Canada.Manitoba|BOLD:ABZ1722
Xestia imperita|[8626]|LCHP660-07|07PROBE-10342|658[0n]bp|Canada.Manitoba|BOLD:ABZ1722
Xestia imperita|[8627]|LCHP535-07|07PROBE-10205|658[0n]bp|Canada.Manitoba|BOLD:ABZ1722
Xestia imperita|[8628]|LCHQ226-08|07WNP-10118|658[0n]bp|Canada.Manitoba|BOLD:ABZ1722
Xestia imperita|[8629]|LCHP982-07|07PROBE-10744|658[0n]bp|Canada.Manitoba|BOLD:ABZ1722
Xestia imperita|[8630]|LCHP667-07|07PROBE-10349|658[0n]bp|Canada.Manitoba|BOLD:ABZ1722
Xestia imperita|[8631]|LCHP819-07|07PROBE-10576|658[0n]bp|Canada.Manitoba|BOLD:ABZ1722
Xestia imperita|[8632]|LCHQ145-07|07PROBE-10914|658[0n]bp|Canada.Manitoba|BOLD:ABZ1722
Xestia imperita|[8633]|LCHP618-07|07PROBE-10299|658[0n]bp|Canada.Manitoba|BOLD:ABZ1722
Xestia imperita|[8634]|RDLQF167-06|DH011194|658[0n]bp|Canada.Quebec|BOLD:ABZ1722
Xestia imperita|[8635]|LCHP594-07|07PROBE-10275|658[0n]bp|Canada.Manitoba|BOLD:ABZ1722
Xestia imperita|[8636]|RDLQF166-06|DH011193|658[0n]bp|Canada.Quebec|BOLD:ABZ1722
Xestia imperita|[8637]|LCHQ098-07|07PROBE-10859|658[0n]bp|Canada.Manitoba|BOLD:ABZ1722
Xestia imperita|[8638]|LCHQ775-08|07WNP-10667|658[0n]bp|Canada.Manitoba|BOLD:ABZ1722
Xestia imperita|[8639]|RDLQF169-06|DH011196|658[0n]bp|Canada.Quebec|BOLD:ABZ1722
Xestia imperita|[8640]|RDLQF168-06|DH011195|658[0n]bp|Canada.Quebec|BOLD:ABZ1722
Xestia imperita|[8641]|MHLEP092-07|CHU06-LEP-092|658[0n]bp|Canada.Manitoba|BOLD:ABZ1722
Xestia imperita|[8642]|LCHQ184-07|07PROBE-10961|658[0n]bp|Canada.Manitoba|BOLD:ABZ1722
Xestia imperita|[8643]|LCHP620-07|07PROBE-10301|658[0n]bp|Canada.Manitoba|BOLD:ABZ1722
Xestia imperita|[8644]|LCHQ197-07|07PROBE-10974|658[0n]bp|Canada.Manitoba|BOLD:ABZ1722
Xestia imperita|[8645]|LCHP944-07|07PROBE-10706|658[0n]bp|Canada.Manitoba|BOLD:ABZ1722
Xestia imperita|[8646]|LCHP797-07|07PROBE-10554|658[0n]bp|Canada.Manitoba|BOLD:ABZ1722
Xestia imperita|[8647]|LCHP953-07|07PROBE-10715|658[0n]bp|Canada.Manitoba|BOLD:ABZ1722
Xestia imperita|[8648]|LCHP599-07|07PROBE-10280|658[0n]bp|Canada.Manitoba|BOLD:ABZ1722
Xestia imperita|[8649]|RDLQF171-06|DH011198|658[0n]bp|Canada.Quebec|BOLD:ABZ1722
Xestia imperita|[8650]|RDLQF161-06|DH011188|658[0n]bp|Canada.Quebec|BOLD:ABZ1722
Xestia imperita|[8651]|LCHP842-07|07PROBE-10599|651[0n]bp|Canada.Manitoba|BOLD:ABZ1722
Xestia imperita|[8652]|LCHQ731-08|07WNP-10623|636[0n]bp|Canada.Manitoba|BOLD:ABZ1722
Xestia imperita|[8653]|LCHQ763-08|07WNP-10655|636[0n]bp|Canada.Manitoba|BOLD:ABZ1722
Xestia imperita|[8654]|LCHQ100-07|07PROBE-10861|637[0n]bp|Canada.Manitoba|BOLD:ABZ1722
Xestia imperita|[8655]|LCHQ584-08|07WNP-10476|636[0n]bp|Canada.Manitoba|BOLD:ABZ1722
Xestia imperita|[8656]|LCHQ188-07|07PROBE-10965|658[0n]bp|Canada.Manitoba|BOLD:ABZ1722
Xestia imperita|[8657]|LCHP950-07|07PROBE-10712|658[0n]bp|Canada.Manitoba|BOLD:ABZ1722
Xestia imperita|[8658]|LCHP806-07|07PROBE-10563|658[0n]bp|Canada.Manitoba|BOLD:ABZ1722
Xestia imperita|[8659]|LCHQ198-07|07PROBE-10975|658[0n]bp|Canada.Manitoba|BOLD:ABZ1722
Xestia imperita|[8660]|LCHQ097-07|07PROBE-10858|658[0n]bp|Canada.Manitoba|BOLD:ABZ1722
Xestia imperita|[8661]|LCHQ090-07|07PROBE-10851|658[0n]bp|Canada.Manitoba|BOLD:ABZ1722
Xestia imperita|[8662]|LCHQ583-08|07WNP-10475|658[0n]bp|Canada.Manitoba|BOLD:ABZ1722
Xestia imperita|[8663]|RDLQF164-06|DH011191|658[0n]bp|Canada.Quebec|BOLD:ABZ1722
Xestia imperita|[8664]|LCHQ262-08|07WNP-10154|658[0n]bp|Canada.Manitoba|BOLD:ABZ1722
Xestia imperita|[8665]|LCHP603-07|07PROBE-10284|658[0n]bp|Canada.Manitoba|BOLD:ABZ1722
Xestia imperita|[8666]|LCHQ719-08|07WNP-10611|658[0n]bp|Canada.Manitoba|BOLD:ABZ1722
Xestia imperita|[8667]|MHCOL157-07|CHU06-COL-157|658[0n]bp|Canada.Manitoba|BOLD:ABZ1722
Xestia bryanti|[8668]|RDNMF046-08|NOC14132|658[0n]bp|Canada.British Columbia|BOLD:AAE4633
Xestia bryanti|[8669]|RDNMG531-08|CNC LEP00052355|658[0n]bp|Canada.British Columbia|BOLD:AAE4633
Xestia bryanti|[8670]|RDNMG532-08|CNC LEP00052356|658[0n]bp|Canada.British Columbia|BOLD:AAE4633
Xestia bryanti|[8671]|RDNMG533-08|CNC LEP00052357|658[0n]bp|Canada.British Columbia|BOLD:AAE4633
[illegible]

Xestia bryanti|[8669]|RDNMG531-08|CNC LEP00052355|658[0n]bp|Canada.British Columbia|BOLD:AAE4633
Xestia bryanti|[8670]|RDNMG532-08|CNC LEP00052356|658[0n]bp|Canada.British Columbia|BOLD:AAE4633
Xestia bryanti|[8671]|RDNMG533-08|CNC LEP00052357|658[0n]bp|Canada.British Columbia|BOLD:AAE4633
Xestia laxa|[8672]|RDNMG727-08|CNC LEP00052851|642[0n]bp|Canada.Yukon Territory|BOLD:AAH9453
Xestia laxa|[8673]|RDNMF071-08|NOC14157|658[0n]bp|Canada.Yukon Territory|BOLD:AAH9453
Xestia mixta|[8674]|LCHQ030-07|07PROBE-10791|642[0n]bp|Canada.Manitoba|BOLD:ABY7781
Xestia mixta|[8675]|LCHP234-07|07PROBE-03804|658[0n]bp|Canada.Manitoba|BOLD:ABY7781
Xestia mixta|[8676]|LCHP748-07|07PROBE-10433|655[0n]bp|Canada.Manitoba|BOLD:ABY7781
Xestia mixta|[8677]|LCHP624-07|07PROBE-10305|658[0n]bp|Canada.Manitoba|BOLD:ABY7781
Xestia mixta|[8678]|LCHQ027-07|07PROBE-10788|658[0n]bp|Canada.Manitoba|BOLD:ABY7781
Xestia mixta|[8679]|LCHQ092-07|07PROBE-10853|658[0n]bp|Canada.Manitoba|BOLD:ABY7781
Xestia mixta|[8680]|LCHP604-07|07PROBE-10285|658[0n]bp|Canada.Manitoba|BOLD:ABY7781
Xestia mixta|[8681]|LCHP757-07|07PROBE-10442|658[0n]bp|Canada.Manitoba|BOLD:ABY7781
Xestia mixta|[8682]|LCHQ095-07|07PROBE-10856|658[0n]bp|Canada.Manitoba|BOLD:ABY7781
Xestia mixta|[8683]|LCHP494-07|07PROBE-10162|658[0n]bp|Canada.Manitoba|BOLD:ABY7781
Xestia mixta|[8684]|LCHP596-07|07PROBE-10277|658[0n]bp|Canada.Manitoba|BOLD:ABY7781
Xestia mixta|[8685]|LCHP665-07|07PROBE-10347|658[0n]bp|Canada.Manitoba|BOLD:ABY7781
Xestia mixta|[8686]|LCHQ031-07|07PROBE-10792|658[0n]bp|Canada.Manitoba|BOLD:ABY7781
Xestia mixta|[8687]|LCHP309-07|07PROBE-03875|658[0n]bp|Canada.Manitoba|BOLD:ABY7781
Xestia mixta|[8688]|LCHP480-07|07PROBE-10148|658[0n]bp|Canada.Manitoba|BOLD:ABY7781
Xestia mixta|[8689]|LCHP516-07|07PROBE-10184|658[0n]bp|Canada.Manitoba|BOLD:ABY7781
Xestia mixta|[8690]|LCHP699-07|07PROBE-10381|658[0n]bp|Canada.Manitoba|BOLD:ABY7781
Xestia mixta|[8691]|LCHP669-07|07PROBE-10351|658[0n]bp|Canada.Manitoba|BOLD:ABY7781
Xestia mixta|[8692]|LCHQ034-07|07PROBE-10795|658[0n]bp|Canada.Manitoba|BOLD:ABY7781
Xestia mixta|[8693]|LCHP647-07|07PROBE-10328|658[0n]bp|Canada.Manitoba|BOLD:ABY7781
Xestia mixta|[8694]|LCHP517-07|07PROBE-10185|658[0n]bp|Canada.Manitoba|BOLD:ABY7781
Xestia mixta|[8695]|LCHQ025-07|07PROBE-10786|658[0n]bp|Canada.Manitoba|BOLD:ABY7781
Xestia mixta|[8696]|LCHP767-07|07PROBE-10452|658[0n]bp|Canada.Manitoba|BOLD:ABY7781
Xestia mixta|[8697]|LCHP484-07|07PROBE-10152|658[0n]bp|Canada.Manitoba|BOLD:ABY7781
Xestia mixta|[8698]|LCHP513-07|07PROBE-10181|658[0n]bp|Canada.Manitoba|BOLD:ABY7781
Xestia speciosa|[8699]|LCHQ475-08|07WNP-10367|657[0n]bp|Canada.Manitoba|BOLD:ACF2639
Xestia speciosa|[8700]|LCHP773-07|07PROBE-10458|658[0n]bp|Canada.Manitoba|BOLD:ACF2639
Xestia speciosa|[8701]|LCHQ700-08|07WNP-10592|658[0n]bp|Canada.Manitoba|BOLD:ACF2639
Xestia speciosa|[8702]|LCHQ701-08|07WNP-10593|658[0n]bp|Canada.Manitoba|BOLD:ACF2639
Xestia speciosa|[8703]|LCHP865-07|07PROBE-10622|658[0n]bp|Canada.Manitoba|BOLD:ACF2639
Xestia speciosa|[8704]|LBCG873-09|08-JDWBC-0873|658[0n]bp|Canada.British Columbia|BOLD:AAA2273
Xestia speciosa|[8705]|LBCG2012-09|08-JDWBC-2012|636[0n]bp|Canada.British Columbia|BOLD:AAA2273
Xestia speciosa|[8706]|LBCG2045-09|08-JDWBC-2045|658[0n]bp|Canada.British Columbia|BOLD:AAA2273
Xestia speciosa|[8707]|LBCG2028-09|08-JDWBC-2028|658[0n]bp|Canada.British Columbia|BOLD:AAA2273
Xestia speciosa|[8708]|LBCG2016-09|08-JDWBC-2016|658[0n]bp|Canada.British Columbia|BOLD:AAA2273
Xestia speciosa|[8709]|LBCG2037-09|08-JDWBC-2037|658[0n]bp|Canada.British Columbia|BOLD:AAA2273
Xestia speciosa|[8710]|LBCH1431-10|10-JDWBC-1431|658[0n]bp|Canada.British Columbia|BOLD:AAA2273
Xestia speciosa|[8711]|LBCG2015-09|08-JDWBC-2015|658[0n]bp|Canada.British Columbia|BOLD:AAA2273
Xestia speciosa|[8712]|LBCG2031-09|08-JDWBC-2031|658[0n]bp|Canada.British Columbia|BOLD:AAA2273
Xestia speciosa|[8713]|LBCH1436-10|10-JDWBC-1436|638[0n]bp|Canada.British Columbia|BOLD:AAA2273
Xestia speciosa|[8714]|LBCG580-09|08-JDWBC-0580|658[0n]bp|Canada.British Columbia|BOLD:AAA2273
Xestia speciosa|[8715]|LBCG1857-09|08-JDWBC-1857|658[0n]bp|Canada.British Columbia|BOLD:AAA2273
Xestia speciosa|[8716]|LBCG999-09|08-JDWBC-0999|658[0n]bp|Canada.British Columbia|BOLD:AAA2273
Xestia speciosa|[8717]|LBCH4425-10|10-JDWBC-4425|658[0n]bp|Canada.British Columbia|BOLD:AAA2273
Xestia speciosa|[8718]|LBCG1015-09|08-JDWBC-1015|658[0n]bp|Canada.British Columbia|BOLD:AAA2273
Xestia speciosa|[8719]|LBCG761-09|08-JDWBC-0761|658[0n]bp|Canada.British Columbia|BOLD:AAA2273
Xestia speciosa|[8720]|BBLPB659-10|10BBCLP-1658|658[0n]bp|Canada.British Columbia|BOLD:AAA2273
Xestia speciosa|[8721]|LBCG2021-09|08-JDWBC-2021|658[0n]bp|Canada.British Columbia|BOLD:AAA2273
Xestia speciosa|[8722]|LBCH4110-10|10-JDWBC-4110|658[0n]bp|Canada.British Columbia|BOLD:AAA2273
Xestia speciosa|[8723]|LBCH4109-10|10-JDWBC-4109|658[0n]bp|Canada.British Columbia|BOLD:AAA2273
Xestia speciosa|[8724]|LBCG2032-09|08-JDWBC-2032|658[0n]bp|Canada.British Columbia|BOLD:AAA2273
Xestia speciosa|[8725]|LBCG2025-09|08-JDWBC-2025|658[0n]bp|Canada.British Columbia|BOLD:AAA2273
Xestia speciosa|[8726]|LBCD452-05|HLC-23272|658[0n]bp|Canada.British Columbia|BOLD:AAA2273
Xestia speciosa|[8727]|LBCH1676-10|10-JDWBC-1676|658[0n]bp|Canada.British Columbia|BOLD:AAA2273
Xestia speciosa|[8728]|LBCH1527-10|10-JDWBC-1527|658[0n]bp|Canada.British Columbia|BOLD:AAA2273
Xestia speciosa|[8729]|LBCG2033-09|08-JDWBC-2033|658[0n]bp|Canada.British Columbia|BOLD:AAA2273
Xestia speciosa|[8730]|LBCH3466-10|10-JDWBC-3466|658[0n]bp|Canada.British Columbia|BOLD:AAA2273
Xestia speciosa|[8731]|LBCG575-09|08-JDWBC-0575|658[0n]bp|Canada.British Columbia|BOLD:AAA2273
Xestia speciosa|[8732]|LBCG823-09|08-JDWBC-0823|658[0n]bp|Canada.British Columbia|BOLD:AAA2273
Xestia speciosa|[8733]|LBCG2049-09|08-JDWBC-2049|658[0n]bp|Canada.British Columbia|BOLD:AAA2273
Xestia speciosa|[8734]|LBCG969-09|08-JDWBC-0969|658[0n]bp|Canada.British Columbia|BOLD:AAA2273
Xestia speciosa|[8735]|LBCH1442-10|10-JDWBC-1442|658[0n]bp|Canada.British Columbia|BOLD:AAA2273
Xestia speciosa|[8736]|LBCG708-09|08-JDWBC-0708|658[0n]bp|Canada.British Columbia|BOLD:AAA2273
Xestia speciosa|[8737]|LBCH496-10|10-JDWBC-0496|658[0n]bp|Canada.British Columbia|BOLD:AAA2273
Xestia speciosa|[8738]|LBCG2017-09|08-JDWBC-2017|658[0n]bp|Canada.British Columbia|BOLD:AAA2273
Xestia speciosa|[8739]|LBCH1434-10|10-JDWBC-1434|658[0n]bp|Canada.British Columbia|BOLD:AAA2273
Xestia speciosa|[8740]|LBCH4426-10|10-JDWBC-4426|658[0n]bp|Canada.British Columbia|BOLD:AAA2273
Xestia speciosa|[8741]|LBCG968-09|08-JDWBC-0968|658[0n]bp|Canada.British Columbia|BOLD:AAA2273
Xestia speciosa|[8742]|LBCG2024-09|08-JDWBC-2024|658[0n]bp|Canada.British Columbia|BOLD:AAA2273
Xestia speciosa|[8743]|LBCH1677-10|10-JDWBC-1677|658[0n]bp|Canada.British Columbia|BOLD:AAA2273
Xestia speciosa|[8744]|LBCG692-09|08-JDWBC-0692|658[0n]bp|Canada.British Columbia|BOLD:AAA2273
Xestia speciosa|[8745]|LBCH1529-10|10-JDWBC-1529|658[0n]bp|Canada.British Columbia|BOLD:AAA2273
Xestia speciosa|[8746]|LBCG710-09|08-JDWBC-0710|658[0n]bp|Canada.British Columbia|BOLD:AAA2273
Xestia speciosa|[8747]|LBCH503-10|10-JDWBC-0503|658[0n]bp|Canada.British Columbia|BOLD:AAA2273
Xestia speciosa|[8748]|LBCG854-09|08-JDWBC-0854|658[0n]bp|Canada.British Columbia|BOLD:AAA2273
Xestia speciosa|[8749]|LBCG996-09|08-JDWBC-0996|658[0n]bp|Canada.British Columbia|BOLD:AAA2273
Xestia speciosa|[8750]|LBCG578-09|08-JDWBC-0578|658[0n]bp|Canada.British Columbia|BOLD:AAA2273
Xestia speciosa|[8751]|LBCG851-09|08-JDWBC-0851|658[0n]bp|Canada.British Columbia|BOLD:AAA2273
Xestia speciosa|[8752]|LBCG829-09|08-JDWBC-0829|658[0n]bp|Canada.British Columbia|BOLD:AAA2273
Xestia speciosa|[8753]|LBCH1523-10|10-JDWBC-1523|658[0n]bp|Canada.British Columbia|BOLD:AAA2273
Xestia speciosa|[8754]|LBCG1001-09|08-JDWBC-1001|658[0n]bp|Canada.British Columbia|BOLD:AAA2273
Xestia speciosa|[8755]|LBCG592-09|08-JDWBC-0592|658[0n]bp|Canada.British Columbia|BOLD:AAA2273
Xestia speciosa|[8756]|LBCG2043-09|08-JDWBC-2043|658[0n]bp|Canada.British Columbia|BOLD:AAA2273
Xestia speciosa|[8757]|BBLPB652-10|10BBCLP-1651|658[0n]bp|Canada.British Columbia|BOLD:AAA2273
Xestia speciosa|[8758]|LBCG820-09|08-JDWBC-0820|658[0n]bp|Canada.British Columbia|BOLD:AAA2273
Xestia speciosa|[8759]|LPABC392-09|08BBLEP-04611|658[0n]bp|Canada.Alberta|BOLD:AAA2273
Xestia speciosa|[8760]|LBCG2035-09|08-JDWBC-2035|658[0n]bp|Canada.British Columbia|BOLD:AAA2273
Xestia speciosa|[8761]|LBCG839-09|08-JDWBC-0839|658[0n]bp|Canada.British Columbia|BOLD:AAA2273
Xestia speciosa|[8762]|LBCH1433-10|10-JDWBC-1433|658[0n]bp|Canada.British Columbia|BOLD:AAA2273
Xestia speciosa|[8763]|LBCG586-09|08-JDWBC-0586|658[0n]bp|Canada.British Columbia|BOLD:AAA2273
Xestia speciosa|[8764]|LBCH1525-10|10-JDWBC-1525|658[0n]bp|Canada.British Columbia|BOLD:AAA2273
Xestia speciosa|[8765]|LBCH1545-10|10-JDWBC-1545|658[0n]bp|Canada.British Columbia|BOLD:AAA2273
Xestia speciosa|[8766]|LBCG2007-09|08-JDWBC-2007|658[0n]bp|Canada.British Columbia|BOLD:AAA2273
Xestia speciosa|[8767]|LBCG2011-09|08-JDWBC-2011|658[0n]bp|Canada.British Columbia|BOLD:AAA2273
Xestia speciosa|[8768]|LBCH1427-10|10-JDWBC-1427|658[0n]bp|Canada.British Columbia|BOLD:AAA2273
Xestia speciosa|[8769]|LBCG855-09|08-JDWBC-0855|658[0n]bp|Canada.British Columbia|BOLD:AAA2273
Xestia speciosa|[8770]|LBCH3087-10|10-JDWBC-3087|658[0n]bp|Canada.British Columbia|BOLD:AAA2273
Xestia speciosa|[8771]|LBCG827-09|08-JDWBC-0827|658[0n]bp|Canada.British Columbia|BOLD:AAA2273

Xestia speciosa|[8769]|LBCG855-09|08-JDWBC-0855|658[0n]bp|Canada.British Columbia|BOLD:AAA2273
Xestia speciosa|[8770]|LBCH3087-10|10-JDWBC-3087|658[0n]bp|Canada.British Columbia|BOLD:AAA2273
Xestia speciosa|[8771]|LBCG827-09|08-JDWBC-0827|658[0n]bp|Canada.British Columbia|BOLD:AAA2273
Xestia speciosa|[8772]|BBLPB654-10|10BBCLP-1653|658[0n]bp|Canada.British Columbia|BOLD:AAA2273
Xestia speciosa|[8773]|LBCG1877-09|08-JDWBC-1877|658[0n]bp|Canada.British Columbia|BOLD:AAA2273
Xestia speciosa|[8774]|LBCG960-09|08-JDWBC-0960|658[0n]bp|Canada.British Columbia|BOLD:AAA2273
Xestia speciosa|[8775]|LBCH1166-10|10-JDWBC-1166|658[0n]bp|Canada.British Columbia|BOLD:AAA2273
Xestia speciosa|[8776]|LBCH648-10|10-JDWBC-0648|658[0n]bp|Canada.British Columbia|BOLD:AAA2273
Xestia speciosa|[8777]|LBCG2013-09|08-JDWBC-2013|658[0n]bp|Canada.British Columbia|BOLD:AAA2273
Xestia speciosa|[8778]|LBCG966-09|08-JDWBC-0966|658[0n]bp|Canada.British Columbia|BOLD:AAA2273
Xestia speciosa|[8779]|LBCG2034-09|08-JDWBC-2034|658[0n]bp|Canada.British Columbia|BOLD:AAA2273
Xestia speciosa|[8780]|BBLPB651-10|10BBCLP-1650|658[0n]bp|Canada.British Columbia|BOLD:AAA2273
Xestia speciosa|[8781]|LBCH501-10|10-JDWBC-0501|658[0n]bp|Canada.British Columbia|BOLD:AAA2273
Xestia speciosa|[8782]|LBCH1238-10|10-JDWBC-1238|658[0n]bp|Canada.British Columbia|BOLD:AAA2273
Xestia speciosa|[8783]|LBCG733-09|08-JDWBC-0733|658[0n]bp|Canada.British Columbia|BOLD:AAA2273
Xestia speciosa|[8784]|LBCG581-09|08-JDWBC-0581|658[0n]bp|Canada.British Columbia|BOLD:AAA2273
Xestia speciosa|[8785]|LBCH499-10|10-JDWBC-0499|658[0n]bp|Canada.British Columbia|BOLD:AAA2273
Xestia speciosa|[8786]|LBCG1876-09|08-JDWBC-1876|658[0n]bp|Canada.British Columbia|BOLD:AAA2273
Xestia speciosa|[8787]|LBCH4681-10|10-JDWBC-4681|658[0n]bp|Canada.British Columbia|BOLD:AAA2273
Xestia speciosa|[8788]|LBCH1528-10|10-JDWBC-1528|658[0n]bp|Canada.British Columbia|BOLD:AAA2273
Xestia speciosa|[8789]|LBCG2020-09|08-JDWBC-2020|658[0n]bp|Canada.British Columbia|BOLD:AAA2273
Xestia speciosa|[8790]|LBCH500-10|10-JDWBC-0500|658[0n]bp|Canada.British Columbia|BOLD:AAA2273
Xestia speciosa|[8791]|LBCH1107-10|10-JDWBC-1107|658[0n]bp|Canada.British Columbia|BOLD:AAA2273
Xestia speciosa|[8792]|LBCG2023-09|08-JDWBC-2023|658[0n]bp|Canada.British Columbia|BOLD:AAA2273
Xestia speciosa|[8793]|LBCG574-09|08-JDWBC-0574|658[0n]bp|Canada.British Columbia|BOLD:AAA2273
Xestia speciosa|[8794]|LBCG1872-09|08-JDWBC-1872|658[0n]bp|Canada.British Columbia|BOLD:AAA2273
Xestia speciosa|[8795]|LBCH218-10|10-JDWBC-0218|658[0n]bp|Canada.British Columbia|BOLD:AAA2273
Xestia speciosa|[8796]|LBCG1002-09|08-JDWBC-1002|658[0n]bp|Canada.British Columbia|BOLD:AAA2273
Xestia speciosa|[8797]|LBCH1430-10|10-JDWBC-1430|658[0n]bp|Canada.British Columbia|BOLD:AAA2273
Xestia speciosa|[8798]|LBCG2038-09|08-JDWBC-2038|658[0n]bp|Canada.British Columbia|BOLD:AAA2273
Xestia speciosa|[8799]|LBCG1867-09|08-JDWBC-1867|658[0n]bp|Canada.British Columbia|BOLD:AAA2273
Xestia speciosa|[8800]|LBCG870-09|08-JDWBC-0870|658[0n]bp|Canada.British Columbia|BOLD:AAA2273
Xestia speciosa|[8801]|LBCG736-09|08-JDWBC-0736|658[0n]bp|Canada.British Columbia|BOLD:AAA2273
Xestia speciosa|[8802]|LBCH815-10|10-JDWBC-0815|658[0n]bp|Canada.British Columbia|BOLD:AAA2273
Xestia speciosa|[8803]|BBLPB653-10|10BBCLP-1652|658[0n]bp|Canada.British Columbia|BOLD:AAA2273
Xestia speciosa|[8804]|LBCG2022-09|08-JDWBC-2022|658[0n]bp|Canada.British Columbia|BOLD:AAA2273
Xestia speciosa|[8805]|LPABC279-09|08BBLEP-04498|658[0n]bp|Canada.Alberta|BOLD:AAA2273
Xestia speciosa|[8806]|LBCH3795-10|10-JDWBC-3795|658[0n]bp|Canada.British Columbia|BOLD:AAA2273
Xestia speciosa|[8807]|LBCG1863-09|08-JDWBC-1863|658[0n]bp|Canada.British Columbia|BOLD:AAA2273
Xestia speciosa|[8808]|LBCG651-09|08-JDWBC-0651|658[0n]bp|Canada.British Columbia|BOLD:AAA2273
Xestia speciosa|[8809]|LBCG822-09|08-JDWBC-0822|658[0n]bp|Canada.British Columbia|BOLD:AAA2273
Xestia speciosa|[8810]|LBCG641-09|08-JDWBC-0641|658[0n]bp|Canada.British Columbia|BOLD:AAA2273
Xestia speciosa|[8811]|BBLPB655-10|10BBCLP-1654|658[0n]bp|Canada.British Columbia|BOLD:AAA2273
Xestia speciosa|[8812]|LBCG584-09|08-JDWBC-0584|658[0n]bp|Canada.British Columbia|BOLD:AAA2273
Xestia speciosa|[8813]|LBCG2030-09|08-JDWBC-2030|658[0n]bp|Canada.British Columbia|BOLD:AAA2273
Xestia speciosa|[8814]|LBCH1544-10|10-JDWBC-1544|658[0n]bp|Canada.British Columbia|BOLD:AAA2273
Xestia speciosa|[8815]|LBCG2036-09|08-JDWBC-2036|658[0n]bp|Canada.British Columbia|BOLD:AAA2273
Xestia speciosa|[8816]|LBCH1971-10|10-JDWBC-1971|658[0n]bp|Canada.British Columbia|BOLD:AAA2273
Xestia speciosa|[8817]|BBLPB657-10|10BBCLP-1656|658[0n]bp|Canada.British Columbia|BOLD:AAA2273
Xestia speciosa|[8818]|BBLPB658-10|10BBCLP-1657|658[0n]bp|Canada.British Columbia|BOLD:AAA2273
Xestia speciosa|[8819]|LBCH109-10|10-JDWBC-0109|658[0n]bp|Canada.British Columbia|BOLD:AAA2273
Xestia speciosa|[8820]|LBCG2026-09|08-JDWBC-2026|658[0n]bp|Canada.British Columbia|BOLD:AAA2273
Xestia speciosa|[8821]|LBCG2008-09|08-JDWBC-2008|658[0n]bp|Canada.British Columbia|BOLD:AAA2273
Xestia speciosa|[8822]|LBCH1526-10|10-JDWBC-1526|658[0n]bp|Canada.British Columbia|BOLD:AAA2273
Xestia speciosa|[8823]|LBCH498-10|10-JDWBC-0498|658[0n]bp|Canada.British Columbia|BOLD:AAA2273
Xestia speciosa|[8824]|LBCG2019-09|08-JDWBC-2019|658[0n]bp|Canada.British Columbia|BOLD:AAA2273
Xestia speciosa|[8825]|LBCG2053-09|08-JDWBC-2053|658[0n]bp|Canada.British Columbia|BOLD:AAA2273
Xestia speciosa|[8826]|LBCG1868-09|08-JDWBC-1868|658[0n]bp|Canada.British Columbia|BOLD:AAA2273
Xestia speciosa|[8827]|LBCG852-09|08-JDWBC-0852|658[0n]bp|Canada.British Columbia|BOLD:AAA2273
Xestia speciosa|[8828]|LBCH1524-10|10-JDWBC-1524|658[0n]bp|Canada.British Columbia|BOLD:AAA2273
Xestia speciosa|[8829]|LBCH2130-10|10-JDWBC-2130|658[0n]bp|Canada.British Columbia|BOLD:AAA2273
Xestia speciosa|[8830]|LBCH1932-10|10-JDWBC-1932|658[0n]bp|Canada.British Columbia|BOLD:AAA2273
Xestia speciosa|[8831]|LBCG2010-09|08-JDWBC-2010|658[0n]bp|Canada.British Columbia|BOLD:AAA2273
Xestia speciosa|[8832]|LBCG853-09|08-JDWBC-0853|658[0n]bp|Canada.British Columbia|BOLD:AAA2273
Xestia speciosa|[8833]|LBCH935-10|10-JDWBC-0935|658[0n]bp|Canada.British Columbia|BOLD:AAA2273
Xestia speciosa|[8834]|LBCG572-09|08-JDWBC-0572|658[0n]bp|Canada.British Columbia|BOLD:AAA2273
Xestia speciosa|[8835]|LBCH3089-10|10-JDWBC-3089|658[0n]bp|Canada.British Columbia|BOLD:AAA2273
Xestia speciosa|[8836]|LBCH502-10|10-JDWBC-0502|658[0n]bp|Canada.British Columbia|BOLD:AAA2273
Xestia speciosa|[8837]|LBCH3788-10|10-JDWBC-3788|658[0n]bp|Canada.British Columbia|BOLD:AAA2273
Xestia speciosa|[8838]|LBCG954-09|08-JDWBC-0954|658[0n]bp|Canada.British Columbia|BOLD:AAA2273
Xestia speciosa|[8839]|LBCG2027-09|08-JDWBC-2027|658[0n]bp|Canada.British Columbia|BOLD:AAA2273
Xestia speciosa|[8840]|LBCG997-09|08-JDWBC-0997|658[0n]bp|Canada.British Columbia|BOLD:AAA2273
Xestia speciosa|[8841]|LBCG2314-09|08-JDWBC-2314|658[0n]bp|Canada.British Columbia|BOLD:AAA2273
Xestia speciosa|[8842]|BBLPB656-10|10BBCLP-1655|658[0n]bp|Canada.British Columbia|BOLD:AAA2273
Xestia speciosa|[8843]|LBCG2009-09|08-JDWBC-2009|658[0n]bp|Canada.British Columbia|BOLD:AAA2273
Xestia speciosa|[8844]|LBCH497-10|10-JDWBC-0497|658[0n]bp|Canada.British Columbia|BOLD:AAA2273
Xestia speciosa|[8845]|LBCG2041-09|08-JDWBC-2041|658[0n]bp|Canada.British Columbia|BOLD:AAA2273
Xestia speciosa|[8846]|LBCG1019-09|08-JDWBC-1019|658[0n]bp|Canada.British Columbia|BOLD:AAA2273
Xestia speciosa|[8847]|LBCG953-09|08-JDWBC-0953|658[0n]bp|Canada.British Columbia|BOLD:AAA2273
Xestia speciosa|[8848]|LBCG2006-09|08-JDWBC-2006|658[0n]bp|Canada.British Columbia|BOLD:AAA2273
Xestia speciosa|[8849]|LBCH4427-10|10-JDWBC-4427|658[0n]bp|Canada.British Columbia|BOLD:AAA2273
Xestia speciosa|[8850]|LBCH915-10|10-JDWBC-0915|658[0n]bp|Canada.British Columbia|BOLD:AAA2273
Xestia speciosa|[8851]|LBCG965-09|08-JDWBC-0965|658[0n]bp|Canada.British Columbia|BOLD:AAA2273
Xestia speciosa|[8852]|LBCG1873-09|08-JDWBC-1873|658[0n]bp|Canada.British Columbia|BOLD:AAA2273
Xestia speciosa|[8853]|LBCG2029-09|08-JDWBC-2029|658[0n]bp|Canada.British Columbia|BOLD:AAA2273
Xestia speciosa|[8854]|LBCG2372-09|08-JDWBC-2372|658[0n]bp|Canada.British Columbia|BOLD:AAA2273
Xestia speciosa|[8855]|LBCG587-09|08-JDWBC-0587|658[0n]bp|Canada.British Columbia|BOLD:AAA2273
Xestia speciosa|[8856]|LBCG709-09|08-JDWBC-0709|658[0n]bp|Canada.British Columbia|BOLD:AAA2273
Xestia speciosa|[8857]|LPABC197-09|08BBLEP-04416|658[0n]bp|Canada.Alberta|BOLD:AAA2273
Xestia speciosa|[8858]|LBCG826-09|08-JDWBC-0826|658[0n]bp|Canada.British Columbia|BOLD:AAA2273
Xestia speciosa|[8859]|LBCH018-10|10-JDWBC-0018|658[0n]bp|Canada.British Columbia|BOLD:AAA2273
Xestia speciosa|[8860]|LBCG1870-09|08-JDWBC-1870|658[0n]bp|Canada.British Columbia|BOLD:AAA2273
Xestia speciosa|[8861]|LBCG2040-09|08-JDWBC-2040|658[0n]bp|Canada.British Columbia|BOLD:AAA2273
Xestia speciosa|[8862]|LBCH1437-10|10-JDWBC-1437|643[0n]bp|Canada.British Columbia|BOLD:AAA2273
Xestia speciosa|[8863]|LBCH347-10|10-JDWBC-0347|625[0n]bp|Canada.British Columbia|BOLD:AAA2273
Xestia speciosa|[8864]|LBCH1522-10|10-JDWBC-1522|636[0n]bp|Canada.British Columbia|BOLD:AAA2273
Xestia speciosa|[8865]|LBCG819-09|08-JDWBC-0819|658[0n]bp|Canada.British Columbia|BOLD:AAA2273
Xestia quieta|[8866]|RDNMF060-08|NOC14146|658[0n]bp|Canada.Northwest Territories|BOLD:AAF0710
Xestia quieta|[8867]|RDNMG725-08|CNC LEP00052849|658[0n]bp|Canada.Nunavut|BOLD:AAF0710
Xestia quieta|[8868]|RDNMG726-08|CNC LEP00052850|658[0n]bp|Canada.Nunavut|BOLD:AAF0710
Xestia perquiritata|[8869]|LOWCC153-05|CGWC-2033|569[0n]bp|Canada.British Columbia|BOLD:ACE9300
Xestia perquiritata|[8870]|LOWCC155-05|CGWC-2035|633[1n]bp|Canada.British Columbia|BOLD:ACE9300
Xestia perquiritata|[8871]|LBCG763-09|08-JDWBC-0763|658[0n]bp|Canada.British Columbia|BOLD:ACE9300

[illegible]
Xestia perquiritata|[8869]|LOWCC153-05|CGWC-2033|569[0n]bp|Canada.British Columbia|BOLD:ACE9300
Xestia perquiritata|[8870]|LOWCC155-05|CGWC-2035|633[1n]bp|Canada.British Columbia|BOLD:ACE9300
Xestia perquiritata|[8871]|LBCG763-09|08-JDWBC-0763|658[0n]bp|Canada.British Columbia|BOLD:ACE9300
Xestia perquiritata|[8872]|LOWCC156-05|CGWC-2036|658[0n]bp|Canada.British Columbia|BOLD:ACE9300
Xestia perquiritata|[8873]|LOWCC152-05|CGWC-2032|658[0n]bp|Canada.British Columbia|BOLD:ACE9300
Xestia perquiritata|[8874]|LBCG1874-09|08-JDWBC-1874|658[0n]bp|Canada.British Columbia|BOLD:ACE9300
Xestia perquiritata|[8875]|LBCG1849-09|08-JDWBC-1849|658[0n]bp|Canada.British Columbia|BOLD:ACE9300
Xestia perquiritata|[8876]|LBCG1859-09|08-JDWBC-1859|658[0n]bp|Canada.British Columbia|BOLD:ACE9300
Xestia perquiritata|[8877]|LBCG959-09|08-JDWBC-0959|658[0n]bp|Canada.British Columbia|BOLD:ACE9300
Xestia perquiritata|[8878]|LBCG1869-09|08-JDWBC-1869|658[0n]bp|Canada.British Columbia|BOLD:ACE9300
Xestia perquiritata|[8879]|LOWCC147-05|CGWC-2027|658[0n]bp|Canada.British Columbia|BOLD:ACE9300
Xestia perquiritata|[8880]|LOWCC151-05|CGWC-2031|658[0n]bp|Canada.British Columbia|BOLD:ACE9300
Xestia perquiritata|[8881]|LBCG1851-09|08-JDWBC-1851|658[0n]bp|Canada.British Columbia|BOLD:ACE9300
Xestia perquiritata|[8882]|LBCG732-09|08-JDWBC-0732|658[0n]bp|Canada.British Columbia|BOLD:ACE9300
Xestia perquiritata|[8883]|LBCG745-09|08-JDWBC-0745|658[0n]bp|Canada.British Columbia|BOLD:ACE9300
Xestia perquiritata|[8884]|LOWCC148-05|CGWC-2028|658[0n]bp|Canada.British Columbia|BOLD:ACE9300
Xestia perquiritata|[8885]|LOWCC149-05|CGWC-2029|658[0n]bp|Canada.British Columbia|BOLD:ACE9300
Xestia perquiritata|[8886]|LOWCE823-06|CGWC-4583|624[0n]bp|Canada.British Columbia|BOLD:ACE9300
Xestia perquiritata|[8887]|LOWCC154-05|CGWC-2034|540[0n]bp|Canada.British Columbia|BOLD:ACE9300
Xestia perquiritata|[8888]|LBCH3092-10|10-JDWBC-3092|658[0n]bp|Canada.British Columbia|BOLD:ACE9300
Xestia perquiritata|[8889]|LBCG1858-09|08-JDWBC-1858|658[0n]bp|Canada.British Columbia|BOLD:ACE9300
Xestia perquiritata|[8890]|LBCG998-09|08-JDWBC-0998|658[0n]bp|Canada.British Columbia|BOLD:ACE9300
Xestia perquiritata|[8891]|LBCG762-09|08-JDWBC-0762|658[0n]bp|Canada.British Columbia|BOLD:ACE9300
Xestia perquiritata|[8892]|LOWCC150-05|CGWC-2030|658[0n]bp|Canada.British Columbia|BOLD:ACE9300
Xestia perquiritata|[8893]|LBCH3796-10|10-JDWBC-3796|658[0n]bp|Canada.British Columbia|BOLD:ACE9300
Xestia perquiritata|[8894]|LCHP587-07|07PROBE-10268|658[0n]bp|Canada.Manitoba|BOLD:AAA4307
Xestia perquiritata|[8895]|BBLPB521-10|10BBCLP-1520|658[0n]bp|Canada.Ontario|BOLD:AAA4307
Xestia perquiritata|[8896]|BBLPB522-10|10BBCLP-1521|658[0n]bp|Canada.Ontario|BOLD:AAA4307
Xestia perquiritata|[8897]|RDLQB640-05|DH010743|584[0n]bp|Canada.Quebec|BOLD:AAA4307
Xestia perquiritata|[8898]|RDLQB641-05|DH010744|566[0n]bp|Canada.Quebec|BOLD:AAA4307
Xestia perquiritata|[8899]|LCHP534-07|07PROBE-10204|658[0n]bp|Canada.Manitoba|BOLD:AAA4307
Xestia perquiritata|[8900]|LCHP906-07|07PROBE-10668|658[0n]bp|Canada.Manitoba|BOLD:AAA4307
Xestia perquiritata|[8901]|LCH241-04|04HBL003241|658[0n]bp|Canada.Manitoba|BOLD:AAA4307
Xestia perquiritata|[8902]|LBCG2838-09|08-JDWBC-2838|658[0n]bp|Canada.British Columbia|BOLD:ACE9312
Xestia perquiritata|[8903]|LBCG2765-09|08-JDWBC-2765|658[0n]bp|Canada.British Columbia|BOLD:ACE9312
Xestia perquiritata|[8904]|LBCG2368-09|08-JDWBC-2368|658[0n]bp|Canada.British Columbia|BOLD:ACE9312
Xestia perquiritata|[8905]|LBCD187-05|HLC-23007|658[0n]bp|Canada.British Columbia|BOLD:ACE9312
Xestia perquiritata|[8906]|LBCB359-05|HLC-21299|658[0n]bp|Canada.British Columbia|BOLD:ACE9312
Xestia perquiritata|[8907]|LBCB756-05|HLC-21696|658[0n]bp|Canada.British Columbia|BOLD:ACE9312
Xestia perquiritata|[8908]|LBCB360-05|HLC-21300|658[0n]bp|Canada.British Columbia|BOLD:ACE9312
Xestia perquiritata|[8909]|LBCG2371-09|08-JDWBC-2371|658[0n]bp|Canada.British Columbia|BOLD:ACE9312
Xestia perquiritata|[8910]|LBCG2376-09|08-JDWBC-2376|658[0n]bp|Canada.British Columbia|BOLD:ACE9312
Xestia perquiritata|[8911]|LBCG2381-09|08-JDWBC-2381|658[0n]bp|Canada.British Columbia|BOLD:ACE9312
Xestia perquiritata|[8912]|BBLPB660-10|10BBCLP-1659|658[0n]bp|Canada.British Columbia|BOLD:ACE9312
Xestia perquiritata|[8913]|LBCG1886-09|08-JDWBC-1886|658[0n]bp|Canada.British Columbia|BOLD:ACE9312
Xestia perquiritata|[8914]|LBCH2270-10|10-JDWBC-2270|658[0n]bp|Canada.British Columbia|BOLD:ACE9312
Xestia perquiritata|[8915]|LBCH2267-10|10-JDWBC-2267|658[0n]bp|Canada.British Columbia|BOLD:ACE9312
Xestia perquiritata|[8916]|LBCG1902-09|08-JDWBC-1902|658[0n]bp|Canada.British Columbia|BOLD:ACE9312
Xestia perquiritata|[8917]|LBCH1444-10|10-JDWBC-1444|658[0n]bp|Canada.British Columbia|BOLD:ACE9312
Xestia perquiritata|[8918]|LBCD149-05|HLC-22969|658[0n]bp|Canada.British Columbia|BOLD:ACE9312
Xestia perquiritata|[8919]|LBCG3048-09|08-JDWBC-3048|658[0n]bp|Canada.British Columbia|BOLD:ACE9312
Xestia perquiritata|[8920]|LBCG2378-09|08-JDWBC-2378|658[0n]bp|Canada.British Columbia|BOLD:ACE9312
Xestia perquiritata|[8921]|LBCG2375-09|08-JDWBC-2375|658[0n]bp|Canada.British Columbia|BOLD:ACE9312
Xestia perquiritata|[8922]|LBCH1429-10|10-JDWBC-1429|658[0n]bp|Canada.British Columbia|BOLD:ACE9312
Xestia perquiritata|[8923]|LBCH2266-10|10-JDWBC-2266|658[0n]bp|Canada.British Columbia|BOLD:ACE9312
Xestia perquiritata|[8924]|LBCG2369-09|08-JDWBC-2369|658[0n]bp|Canada.British Columbia|BOLD:ACE9312
Xestia perquiritata|[8925]|LBCH2271-10|10-JDWBC-2271|658[0n]bp|Canada.British Columbia|BOLD:ACE9312
Xestia perquiritata|[8926]|LBCG2764-09|08-JDWBC-2764|658[0n]bp|Canada.British Columbia|BOLD:ACE9312
Xestia perquiritata|[8927]|LBCG1900-09|08-JDWBC-1900|658[0n]bp|Canada.British Columbia|BOLD:ACE9312
Xestia perquiritata|[8928]|LBCH2272-10|10-JDWBC-2272|658[0n]bp|Canada.British Columbia|BOLD:ACE9312
Xestia perquiritata|[8929]|LBCG1901-09|08-JDWBC-1901|658[0n]bp|Canada.British Columbia|BOLD:ACE9312
Xestia perquiritata|[8930]|LBCH1543-10|10-JDWBC-1543|658[0n]bp|Canada.British Columbia|BOLD:ACE9312
Xestia perquiritata|[8931]|LBCG2370-09|08-JDWBC-2370|658[0n]bp|Canada.British Columbia|BOLD:ACE9312
Xestia perquiritata|[8932]|LBCG2766-09|08-JDWBC-2766|658[0n]bp|Canada.British Columbia|BOLD:ACE9312
Xestia perquiritata|[8933]|LBCH2268-10|10-JDWBC-2268|658[0n]bp|Canada.British Columbia|BOLD:ACE9312
Xestia perquiritata|[8934]|LBCG2377-09|08-JDWBC-2377|658[0n]bp|Canada.British Columbia|BOLD:ACE9312
Xestia perquiritata|[8935]|LBCC674-05|HLC-22554|658[0n]bp|Canada.British Columbia|BOLD:ACE9312
Xestia perquiritata|[8936]|LBCA737-05|HLC-20737|655[0n]bp|Canada.British Columbia|BOLD:ACE9312
Xestia perquiritata|[8937]|LBCH1108-10|10-JDWBC-1108|658[0n]bp|Canada.British Columbia|BOLD:ACE9312
Xestia perquiritata|[8938]|LBCB485-05|HLC-21425|658[0n]bp|Canada.British Columbia|BOLD:ACE9312
Xestia perquiritata|[8939]|LBCH2269-10|10-JDWBC-2269|658[0n]bp|Canada.British Columbia|BOLD:ACE9312
Xestia perquiritata|[8940]|LBCG595-09|08-JDWBC-0595|658[0n]bp|Canada.British Columbia|BOLD:ACE9312
Xestia perquiritata|[8941]|LBCG2373-09|08-JDWBC-2373|658[0n]bp|Canada.British Columbia|BOLD:ACE9312
Xestia perquiritata|[8942]|LBCH2099-10|10-JDWBC-2099|658[0n]bp|Canada.British Columbia|BOLD:ACE9312
Xestia perquiritata|[8943]|LBCG2380-09|08-JDWBC-2380|658[0n]bp|Canada.British Columbia|BOLD:ACE9312
Xestia perquiritata|[8944]|LBCG1892-09|08-JDWBC-1892|658[0n]bp|Canada.British Columbia|BOLD:ACE9312
Xestia perquiritata|[8945]|LBCB358-05|HLC-21298|658[0n]bp|Canada.British Columbia|BOLD:ACE9312
Xestia perquiritata|[8946]|LBCH1568-10|10-JDWBC-1568|658[0n]bp|Canada.British Columbia|BOLD:ACE9312
Xestia perquiritata|[8947]|LBCH1460-10|10-JDWBC-1460|658[0n]bp|Canada.British Columbia|BOLD:ACE9312
Xestia perquiritata|[8948]|LBCG3036-09|08-JDWBC-3036|658[0n]bp|Canada.British Columbia|BOLD:ACE9312
Xestia perquiritata|[8949]|LBCG2379-09|08-JDWBC-2379|658[0n]bp|Canada.British Columbia|BOLD:ACE9312
Xestia perquiritata|[8950]|LBCG2767-09|08-JDWBC-2767|658[0n]bp|Canada.British Columbia|BOLD:ACE9312
Xestia perquiritata|[8951]|LBCH1541-10|10-JDWBC-1541|658[0n]bp|Canada.British Columbia|BOLD:ACE9312
Xestia perquiritata|[8952]|LBCH1580-10|10-JDWBC-1580|658[0n]bp|Canada.British Columbia|BOLD:ACE9312
Xestia perquiritata|[8953]|LBCG2014-09|08-JDWBC-2014|658[0n]bp|Canada.British Columbia|BOLD:ACE9312
Xestia perquiritata|[8954]|LBCA305-05|HLC-20305|658[0n]bp|Canada.British Columbia|BOLD:ACE9312
Xestia perquiritata|[8955]|LBCB582-05|HLC-21522|652[0n]bp|Canada.British Columbia|BOLD:ACE9312
Xestia perquiritata|[8956]|LBCH1438-10|10-JDWBC-1438|658[0n]bp|Canada.British Columbia|BOLD:ACE9312
Xestia perquiritata|[8957]|LBCH2128-10|10-JDWBC-2128|636[0n]bp|Canada.British Columbia|BOLD:ACE9312
Xestia perquiritata|[8958]|LBCH1542-10|10-JDWBC-1542|640[0n]bp|Canada.British Columbia|BOLD:ACE9312
Xestia perquiritata|[8959]|LBCG2374-09|08-JDWBC-2374|658[0n]bp|Canada.British Columbia|BOLD:ACE9312
Xestia perquiritata|[8960]|LBCH1439-10|10-JDWBC-1439|658[0n]bp|Canada.British Columbia|BOLD:ACE9312
Xestia perquiritata|[8961]|LBCB803-05|HLC-21743|658[0n]bp|Canada.British Columbia|BOLD:ACE9312
Xestia perquiritata|[8962]|LBCB757-05|HLC-21697|658[0n]bp|Canada.British Columbia|BOLD:ACE9312
Xestia perquiritata|[8963]|LBCA736-05|HLC-20736|658[0n]bp|Canada.British Columbia|BOLD:ACE9312
Xestia perquiritata|[8964]|LBCG2039-09|08-JDWBC-2039|658[0n]bp|Canada.British Columbia|BOLD:ACE9312
Xestia perquiritata|[8965]|LBCH1519-10|10-JDWBC-1519|658[0n]bp|Canada.British Columbia|BOLD:ACE9312
Xestia perquiritata|[8966]|LBCG2839-09|08-JDWBC-2839|658[0n]bp|Canada.British Columbia|BOLD:ACE9312
Xestia tecta|[8967]|LCHQ912-08|07WNP-10804|658[0n]bp|Canada.Manitoba|BOLD:AAC5747
Xestia tecta|[8968]|LCHQ901-08|07WNP-10793|658[0n]bp|Canada.Manitoba|BOLD:AAC5747
Xestia tecta|[8969]|LCHQ908-08|07WNP-10800|658[0n]bp|Canada.Manitoba|BOLD:AAC5747
Xestia tecta|[8970]|LCHQ907-08|07WNP-10799|658[0n]bp|Canada.Manitoba|BOLD:AAC5747
[illegible]

Xestia tecta|[8968]|LCHQ901-08|07WNP-10793|658[0n]bp|Canada.Manitoba|BOLD:AAC5747
Xestia tecta|[8969]|LCHQ908-08|07WNP-10800|658[0n]bp|Canada.Manitoba|BOLD:AAC5747
Xestia tecta|[8970]|LCHQ907-08|07WNP-10799|658[0n]bp|Canada.Manitoba|BOLD:AAC5747
Xestia tecta|[8971]|LCHQ899-08|07WNP-10791|658[0n]bp|Canada.Manitoba|BOLD:AAC5747
Xestia tecta|[8972]|LCHQ900-08|07WNP-10792|658[0n]bp|Canada.Manitoba|BOLD:AAC5747
Xestia tecta|[8973]|LCHQ897-08|07WNP-10789|658[0n]bp|Canada.Manitoba|BOLD:AAC5747
Xestia tecta|[8974]|LCHP188-07|07PROBE-00115|621[0n]bp|Canada.Manitoba|BOLD:AAC5747
Xestia atrata|[8975]|RDNMG745-08|CNC LEP00052869|658[0n]bp|Canada.Yukon Territory|BOLD:ACF5082
Xestia atrata|[8976]|LCHP256-07|07PROBE-03822|658[0n]bp|Canada.Manitoba|BOLD:AAC1499
Xestia atrata|[8977]|LCHP276-07|07PROBE-03842|657[0n]bp|Canada.Manitoba|BOLD:AAC1499
Xestia atrata|[8978]|RDNMF607-08|NOC14693|658[0n]bp|Canada.Manitoba|BOLD:AAC1499
Xestia atrata|[8979]|LCHQ625-08|07WNP-10517|658[0n]bp|Canada.Manitoba|BOLD:AAC1499
Xestia atrata|[8980]|LCHP408-07|07PROBE-10032|658[0n]bp|Canada.Manitoba|BOLD:AAC1499
Xestia atrata|[8981]|LCHP636-07|07PROBE-10317|658[0n]bp|Canada.Manitoba|BOLD:AAC1499
Xestia atrata|[8982]|LCHP519-07|07PROBE-10187|658[0n]bp|Canada.Manitoba|BOLD:AAC1499
Xestia atrata|[8983]|LCHP386-07|07PROBE-10010|649[0n]bp|Canada.Manitoba|BOLD:AAC1499
Xestia atrata|[8984]|LCHQ096-07|07PROBE-10857|658[0n]bp|Canada.Manitoba|BOLD:AAC1499
Xestia atrata|[8985]|LCHP631-07|07PROBE-10312|658[0n]bp|Canada.Manitoba|BOLD:AAC1499
Xestia atrata|[8986]|LCHP388-07|07PROBE-10012|658[0n]bp|Canada.Manitoba|BOLD:AAC1499
Xestia atrata|[8987]|LCHP257-07|07PROBE-03823|658[0n]bp|Canada.Manitoba|BOLD:AAC1499
Xestia atrata|[8988]|CHLEP309-09|09PROBE-09604|658[0n]bp|Canada.Manitoba|BOLD:AAC1499
Xestia atrata|[8989]|LCHQ046-07|07PROBE-10807|658[0n]bp|Canada.Manitoba|BOLD:AAE1738
Xestia atrata|[8990]|LCHP258-07|07PROBE-03824|656[0n]bp|Canada.Manitoba|BOLD:AAE1738
Xestia atrata|[8991]|LCHP628-07|07PROBE-10309|658[0n]bp|Canada.Manitoba|BOLD:AAE1738
Xestia atrata|[8992]|LCHP275-07|07PROBE-03841|658[0n]bp|Canada.Manitoba|BOLD:AAE1738
Xestia atrata|[8993]|LCHP505-07|07PROBE-10173|658[0n]bp|Canada.Manitoba|BOLD:AAE1738
Xestia okakensis|[8994]|RDNMG690-08|CNC LEP00052814|658[0n]bp|Canada.Alberta|BOLD:AAB9912
Xestia okakensis|[8995]|RDNMG689-08|CNC LEP00052813|642[0n]bp|Canada.Alberta|BOLD:AAB9912
Xestia okakensis|[8996]|CHLEP155-09|09PROBE-09450|658[0n]bp|Canada.Manitoba|BOLD:AAB9912
Xestia okakensis|[8997]|LCHP210-07|07PROBE-00135|658[0n]bp|Canada.Manitoba|BOLD:AAB9912
Xestia okakensis|[8998]|LCHP411-07|07PROBE-10035|658[0n]bp|Canada.Manitoba|BOLD:AAB9912
Xestia okakensis|[8999]|LCHP008-07|07PROBE-00066|656[0n]bp|Canada.Manitoba|BOLD:AAB9912
Xestia okakensis|[9000]|CHLEP211-09|09PROBE-09506|642[0n]bp|Canada.Manitoba|BOLD:AAB9912
Xestia okakensis|[9001]|LCHP259-07|07PROBE-03825|644[0n]bp|Canada.Manitoba|BOLD:AAB9912
Xestia okakensis|[9002]|CHLEP203-09|09PROBE-09498|637[0n]bp|Canada.Manitoba|BOLD:AAB9912
Xestia okakensis|[9003]|CHLEP210-09|09PROBE-09505|636[0n]bp|Canada.Manitoba|BOLD:AAB9912
Xestia okakensis|[9004]|CHLEP087-09|09PROBE-09382|635[0n]bp|Canada.Manitoba|BOLD:AAB9912
Xestia okakensis|[9005]|LCHP165-07|07PROBE-00567|634[0n]bp|Canada.Manitoba|BOLD:AAB9912
Xestia okakensis|[9006]|LCHP006-07|07PROBE-00064|635[0n]bp|Canada.Manitoba|BOLD:AAB9912
Xestia okakensis|[9007]|CHLEP216-09|09PROBE-09511|634[0n]bp|Canada.Manitoba|BOLD:AAB9912
Xestia okakensis|[9008]|CHLEP209-09|09PROBE-09504|658[0n]bp|Canada.Manitoba|BOLD:AAB9912
Xestia intermedia|[9009]|RDNME519-08|LEP037943|658[0n]bp|Canada.Yukon Territory|BOLD:AAE4575
Xestia intermedia|[9010]|RDNMF048-08|NOC14134|658[0n]bp|Canada.British Columbia|BOLD:AAE4575
Xestia intermedia|[9011]|RDNMG692-08|CNC LEP00052816|642[0n]bp|Canada.Yukon Territory|BOLD:AAE4575
Xestia intermedia|[9012]|RDNMG691-08|CNC LEP00052815|642[0n]bp|Canada.Yukon Territory|BOLD:AAE4575
Xestia lupa|[9013]|RDNMG747-08|CNC LEP00052871|642[0n]bp|Canada.Yukon Territory|BOLD:ACE6805
Xestia lupa|[9014]|RDNMG746-08|CNC LEP00052870|642[0n]bp|Canada.Alberta|BOLD:ACE6805
Xestia lupa|[9015]|RDNMF063-08|NOC14149|658[0n]bp|Canada.Yukon Territory|BOLD:ACE6805
Xestia lupa|[9016]|RDNMG748-08|CNC LEP00052872|658[0n]bp|Canada.Yukon Territory|BOLD:ACE6805
Xestia aequaeva|[9017]|RDNMG686-08|CNC LEP00052810|652[0n]bp|Canada.Yukon Territory|BOLD:ABZ6386
Xestia aequaeva|[9018]|RDNMG687-08|CNC LEP00052811|640[0n]bp|Canada.Yukon Territory|BOLD:AAD9297
Xestia aequaeva|[9019]|RDNMF068-08|NOC14154|658[0n]bp|Canada.Yukon Territory|BOLD:AAD9297
Xestia alaskae|[9020]|RDNMF061-08|NOC14147|658[0n]bp|Canada.Yukon Territory|BOLD:AAD9296
Xestia thula|[9021]|RDNMF067-08|NOC14153|641[0n]bp|Canada.Nunavut|BOLD:AAD9296
Parabarrovia keelei|[9022]|RDNMG704-08|CNC LEP00052828|641[0n]bp|Canada.Yukon Territory|BOLD:ACE9951
Parabarrovia keelei|[9023]|RDNMF073-08|NOC14159|658[0n]bp|Canada.Yukon Territory|BOLD:ABZ2143
Parabarrovia keelei|[9024]|RDNMG705-08|CNC LEP00052829|658[0n]bp|Canada.Yukon Territory|BOLD:ABZ2143
Parabarrovia ogilviensis|[9025]|RDNMG703-08|CNC LEP00052827|641[2n]bp|Canada.Yukon Territory|BOLD:AAD...
Parabarrovia ogilviensis|[9026]|RDNMG701-08|CNC LEP00052825|642[0n]bp|Canada.Yukon Territory|BOLD:AAD...
Parabarrovia ogilviensis|[9027]|RDNMG700-08|CNC LEP00052824|658[0n]bp|Canada.Yukon Territory|BOLD:AAD...
Parabarrovia ogilviensis|[9028]|RDNMG699-08|CNC LEP00052823|658[0n]bp|Canada.Yukon Territory|BOLD:AAD...
Parabarrovia ogilviensis|[9029]|RDNMG702-08|CNC LEP00052826|658[0n]bp|Canada.Yukon Territory|BOLD:AAD...
Parabarrovia ogilviensis|[9030]|RDNMF074-08|NOC14160|658[0n]bp|Canada.Yukon Territory|BOLD:AAD5961
Xestia homogena|[9031]|LCHP805-07|07PROBE-10562|658[0n]bp|Canada.Manitoba|BOLD:AAA3385
Xestia homogena|[9032]|LCH593-04|04HBL003593|595[1n]bp|Canada.Manitoba|BOLD:AAA3385
Xestia homogena|[9033]|LCHQ488-08|07WNP-10380|632[0n]bp|Canada.Manitoba|BOLD:AAA3385
Xestia homogena|[9034]|RDNMD037-06|CNCNoctuoidea12423|658[0n]bp|Canada|BOLD:AAA3385
Xestia homogena|[9035]|LCHQ190-07|07PROBE-10967|658[0n]bp|Canada.Manitoba|BOLD:AAA3385
Xestia homogena|[9036]|LCH319-04|04HBL003319|658[0n]bp|Canada.Manitoba|BOLD:AAA3385
Xestia homogena|[9037]|LCH447-04|04HBL003447|658[0n]bp|Canada.Manitoba|BOLD:AAA3385
Xestia homogena|[9038]|RDNMD038-06|CNCNoctuoidea12424|614[0n]bp|Canada|BOLD:AAA3385
Xestia homogena|[9039]|LCH246-04|04HBL003246|658[0n]bp|Canada.Manitoba|BOLD:AAA3385
Xestia homogena|[9040]|LCHQ099-07|07PROBE-10860|658[0n]bp|Canada.Manitoba|BOLD:AAA3385
Xestia homogena|[9041]|LBCH1448-10|10-JDWBC-1448|658[0n]bp|Canada.British Columbia|BOLD:AAA3385
Xestia homogena|[9042]|LBCG872-09|08-JDWBC-0872|658[0n]bp|Canada.British Columbia|BOLD:AAA3385
Xestia homogena|[9043]|LBCG963-09|08-JDWBC-0963|658[0n]bp|Canada.British Columbia|BOLD:AAA3385
Xestia homogena|[9044]|LBCG1853-09|08-JDWBC-1853|658[0n]bp|Canada.British Columbia|BOLD:AAA3385
Xestia homogena|[9045]|LBCG1862-09|08-JDWBC-1862|658[0n]bp|Canada.British Columbia|BOLD:AAA3385
Xestia homogena|[9046]|LBCG767-09|08-JDWBC-0767|658[0n]bp|Canada.British Columbia|BOLD:AAA3385
Xestia homogena|[9047]|LBCG1856-09|08-JDWBC-1856|658[0n]bp|Canada.British Columbia|BOLD:AAA3385
Xestia homogena|[9048]|LBCG1016-09|08-JDWBC-1016|658[0n]bp|Canada.British Columbia|BOLD:AAA3385
Xestia homogena|[9049]|LBCG967-09|08-JDWBC-0967|658[0n]bp|Canada.British Columbia|BOLD:AAA3385
Xestia homogena|[9050]|LBCG734-09|08-JDWBC-0734|658[0n]bp|Canada.British Columbia|BOLD:AAA3385
Xestia homogena|[9051]|LBCG962-09|08-JDWBC-0962|658[0n]bp|Canada.British Columbia|BOLD:AAA3385
Xestia homogena|[9052]|LBCH3470-10|10-JDWBC-3470|658[0n]bp|Canada.British Columbia|BOLD:AAA3385
Xestia homogena|[9053]|LBCG964-09|08-JDWBC-0964|658[0n]bp|Canada.British Columbia|BOLD:AAA3385
Xestia homogena|[9054]|LBCG1018-09|08-JDWBC-1018|658[0n]bp|Canada.British Columbia|BOLD:AAA3385
Xestia homogena|[9055]|LBCG768-09|08-JDWBC-0768|658[0n]bp|Canada.British Columbia|BOLD:AAA3385
Xestia homogena|[9056]|LBCG1875-09|08-JDWBC-1875|658[0n]bp|Canada.British Columbia|BOLD:AAA3385
Xestia homogena|[9057]|LBCG1861-09|08-JDWBC-1861|658[0n]bp|Canada.British Columbia|BOLD:AAA3385
Xestia homogena|[9058]|LBCH1094-10|10-JDWBC-1094|658[0n]bp|Canada.British Columbia|BOLD:AAA3385
Xestia homogena|[9059]|LBCG2055-09|08-JDWBC-2055|658[0n]bp|Canada.British Columbia|BOLD:AAA3385
Xestia homogena|[9060]|LBCG1014-09|08-JDWBC-1014|658[0n]bp|Canada.British Columbia|BOLD:AAA3385
Xestia homogena|[9061]|LBCH1536-10|10-JDWBC-1536|658[0n]bp|Canada.British Columbia|BOLD:AAA3385
Xestia homogena|[9062]|LBCH2107-10|10-JDWBC-2107|658[0n]bp|Canada.British Columbia|BOLD:AAA3385
Xestia homogena|[9063]|LBCH1535-10|10-JDWBC-1535|658[0n]bp|Canada.British Columbia|BOLD:AAA3385
Xestia homogena|[9064]|LBCG2050-09|08-JDWBC-2050|658[0n]bp|Canada.British Columbia|BOLD:AAA3385
Xestia homogena|[9065]|LBCC670-05|HLC-22550|658[0n]bp|Canada.British Columbia|BOLD:AAA3385
Xestia homogena|[9066]|LBCD183-05|HLC-23003|658[0n]bp|Canada.British Columbia|BOLD:AAA3385
Xestia homogena|[9067]|LBCH1453-10|10-JDWBC-1453|658[0n]bp|Canada.British Columbia|BOLD:AAA3385
Xestia homogena|[9068]|LBCH1235-10|10-JDWBC-1235|658[0n]bp|Canada.British Columbia|BOLD:AAA3385
Xestia homogena|[9069]|LBCG593-09|08-JDWBC-0593|658[0n]bp|Canada.British Columbia|BOLD:AAA3385
Xestia homogena|[9070]|LBCH2109-10|10-JDWBC-2109|658[0n]bp|Canada.British Columbia|BOLD:AAA3385

Xestia homogena|[9068]|LBCH1235-10|10-JDWBC-1235|658[0n]bp|Canada.British Columbia|BOLD:AAA3385
Xestia homogena|[9069]|LBCG593-09|08-JDWBC-0593|658[0n]bp|Canada.British Columbia|BOLD:AAA3385
Xestia homogena|[9070]|LBCH2109-10|10-JDWBC-2109|658[0n]bp|Canada.British Columbia|BOLD:AAA3385
Xestia homogena|[9071]|BBLPB287-10|10BBCLP-1286|658[0n]bp|Canada.British Columbia|BOLD:AAA3385
Xestia homogena|[9072]|LBCH1584-10|10-JDWBC-1584|658[0n]bp|Canada.British Columbia|BOLD:AAA3385
Xestia homogena|[9073]|BBLPB289-10|10BBCLP-1288|658[0n]bp|Canada.British Columbia|BOLD:AAA3385
Xestia homogena|[9074]|LBCG2048-09|08-JDWBC-2048|658[0n]bp|Canada.British Columbia|BOLD:AAA3385
Xestia homogena|[9075]|LBCH1521-10|10-JDWBC-1521|658[0n]bp|Canada.British Columbia|BOLD:AAA3385
Xestia homogena|[9076]|LBCG1850-09|08-JDWBC-1850|658[0n]bp|Canada.British Columbia|BOLD:AAA3385
Xestia homogena|[9077]|LBCG2046-09|08-JDWBC-2046|658[0n]bp|Canada.British Columbia|BOLD:AAA3385
Xestia homogena|[9078]|LBCG2018-09|08-JDWBC-2018|658[0n]bp|Canada.British Columbia|BOLD:AAA3385
Xestia homogena|[9079]|LBCG2052-09|08-JDWBC-2052|658[0n]bp|Canada.British Columbia|BOLD:AAA3385
Xestia homogena|[9080]|LBCH1451-10|10-JDWBC-1451|658[0n]bp|Canada.British Columbia|BOLD:AAA3385
Xestia homogena|[9081]|LBCG1871-09|08-JDWBC-1871|658[0n]bp|Canada.British Columbia|BOLD:AAA3385
Xestia homogena|[9082]|LBCH1951-10|10-JDWBC-1951|658[0n]bp|Canada.British Columbia|BOLD:AAA3385
Xestia homogena|[9083]|LBCH2106-10|10-JDWBC-2106|658[0n]bp|Canada.British Columbia|BOLD:AAA3385
Xestia homogena|[9084]|LBCG570-09|08-JDWBC-0570|658[0n]bp|Canada.British Columbia|BOLD:AAA3385
Xestia homogena|[9085]|LBCH1679-10|10-JDWBC-1679|658[0n]bp|Canada.British Columbia|BOLD:AAA3385
Xestia homogena|[9086]|LBCD185-05|HLC-23005|658[0n]bp|Canada.British Columbia|BOLD:AAA3385
Xestia homogena|[9087]|LBCH1537-10|10-JDWBC-1537|658[0n]bp|Canada.British Columbia|BOLD:AAA3385
Xestia homogena|[9088]|LBCG994-09|08-JDWBC-0994|658[0n]bp|Canada.British Columbia|BOLD:AAA3385
Xestia homogena|[9089]|LBCH2103-10|10-JDWBC-2103|658[0n]bp|Canada.British Columbia|BOLD:AAA3385
Xestia homogena|[9090]|LBCD144-05|HLC-22964|658[0n]bp|Canada.British Columbia|BOLD:AAA3385
Xestia homogena|[9091]|LBCH1533-10|10-JDWBC-1533|658[0n]bp|Canada.British Columbia|BOLD:AAA3385
Xestia homogena|[9092]|LBCH1455-10|10-JDWBC-1455|658[0n]bp|Canada.British Columbia|BOLD:AAA3385
Xestia homogena|[9093]|LBCC563-05|HLC-22443|658[0n]bp|Canada.British Columbia|BOLD:AAA3385
Xestia homogena|[9094]|BBLPB290-10|10BBCLP-1289|658[0n]bp|Canada.British Columbia|BOLD:AAA3385
Xestia homogena|[9095]|LBCH1532-10|10-JDWBC-1532|658[0n]bp|Canada.British Columbia|BOLD:AAA3385
Xestia homogena|[9096]|LBCG2047-09|08-JDWBC-2047|658[0n]bp|Canada.British Columbia|BOLD:AAA3385
Xestia homogena|[9097]|LBCG591-09|08-JDWBC-0591|658[0n]bp|Canada.British Columbia|BOLD:AAA3385
Xestia homogena|[9098]|LBCH1096-10|10-JDWBC-1096|658[0n]bp|Canada.British Columbia|BOLD:AAA3385
Xestia homogena|[9099]|LBCG949-09|08-JDWBC-0949|658[0n]bp|Canada.British Columbia|BOLD:AAA3385
Xestia homogena|[9100]|LBCG2044-09|08-JDWBC-2044|658[0n]bp|Canada.British Columbia|BOLD:AAA3385
Xestia homogena|[9101]|LBCG650-09|08-JDWBC-0650|658[0n]bp|Canada.British Columbia|BOLD:AAA3385
Xestia homogena|[9102]|LBCH1452-10|10-JDWBC-1452|658[0n]bp|Canada.British Columbia|BOLD:AAA3385
Xestia homogena|[9103]|LBCG860-09|08-JDWBC-0860|658[0n]bp|Canada.British Columbia|BOLD:AAA3385
Xestia homogena|[9104]|LPABC428-09|08BBLEP-04647|658[0n]bp|Canada.Alberta|BOLD:AAA3385
Xestia homogena|[9105]|LBCD184-05|HLC-23004|658[0n]bp|Canada.British Columbia|BOLD:AAA3385
Xestia homogena|[9106]|LBCG956-09|08-JDWBC-0956|658[0n]bp|Canada.British Columbia|BOLD:AAA3385
Xestia homogena|[9107]|LBCG958-09|08-JDWBC-0958|658[0n]bp|Canada.British Columbia|BOLD:AAA3385
Xestia homogena|[9108]|LBCG972-09|08-JDWBC-0972|658[0n]bp|Canada.British Columbia|BOLD:AAA3385
Xestia homogena|[9109]|LBCG2056-09|08-JDWBC-2056|658[0n]bp|Canada.British Columbia|BOLD:AAA3385
Xestia homogena|[9110]|LBCG2057-09|08-JDWBC-2057|658[0n]bp|Canada.British Columbia|BOLD:AAA3385
Xestia homogena|[9111]|LBCG579-09|08-JDWBC-0579|658[0n]bp|Canada.British Columbia|BOLD:AAA3385
Xestia homogena|[9112]|LBCG1855-09|08-JDWBC-1855|658[0n]bp|Canada.British Columbia|BOLD:AAA3385
Xestia homogena|[9113]|LBCG1854-09|08-JDWBC-1854|658[0n]bp|Canada.British Columbia|BOLD:AAA3385
Xestia homogena|[9114]|LBCG971-09|08-JDWBC-0971|658[0n]bp|Canada.British Columbia|BOLD:AAA3385
Xestia homogena|[9115]|LBCC874-05|HLC-22754|658[0n]bp|Canada.British Columbia|BOLD:AAA3385
Xestia homogena|[9116]|LBCH1440-10|10-JDWBC-1440|658[0n]bp|Canada.British Columbia|BOLD:AAA3385
Xestia homogena|[9117]|LBCH1680-10|10-JDWBC-1680|658[0n]bp|Canada.British Columbia|BOLD:AAA3385
Xestia homogena|[9118]|LBCG737-09|08-JDWBC-0737|658[0n]bp|Canada.British Columbia|BOLD:AAA3385
Xestia homogena|[9119]|LBCD102-05|HLC-22922|658[0n]bp|Canada.British Columbia|BOLD:AAA3385
Xestia homogena|[9120]|LBCG594-09|08-JDWBC-0594|658[0n]bp|Canada.British Columbia|BOLD:AAA3385
Xestia homogena|[9121]|LBCH1530-10|10-JDWBC-1530|658[0n]bp|Canada.British Columbia|BOLD:AAA3385
Xestia homogena|[9122]|LBCG2051-09|08-JDWBC-2051|658[0n]bp|Canada.British Columbia|BOLD:AAA3385
Xestia homogena|[9123]|LBCH1100-10|10-JDWBC-1100|658[0n]bp|Canada.British Columbia|BOLD:AAA3385
Xestia homogena|[9124]|LBCG858-09|08-JDWBC-0858|658[0n]bp|Canada.British Columbia|BOLD:AAA3385
Xestia homogena|[9125]|LBCG2042-09|08-JDWBC-2042|658[0n]bp|Canada.British Columbia|BOLD:AAA3385
Xestia homogena|[9126]|LBCH1441-10|10-JDWBC-1441|658[0n]bp|Canada.British Columbia|BOLD:AAA3385
Xestia homogena|[9127]|LBCC875-05|HLC-22755|658[2n]bp|Canada.British Columbia|BOLD:AAA3385
Xestia homogena|[9128]|LBCH1449-10|10-JDWBC-1449|621[0n]bp|Canada.British Columbia|BOLD:AAA3385
Xestia homogena|[9129]|LBCH1454-10|10-JDWBC-1454|658[0n]bp|Canada.British Columbia|BOLD:AAA3385
Xestia homogena|[9130]|LBCH2110-10|10-JDWBC-2110|658[0n]bp|Canada.British Columbia|BOLD:AAA3385
Xestia homogena|[9131]|LBCH1101-10|10-JDWBC-1101|658[0n]bp|Canada.British Columbia|BOLD:AAA3385
Xestia homogena|[9132]|LBCH1099-10|10-JDWBC-1099|658[0n]bp|Canada.British Columbia|BOLD:AAA3385
Xestia homogena|[9133]|LBCH2105-10|10-JDWBC-2105|658[0n]bp|Canada.British Columbia|BOLD:AAA3385
Xestia homogena|[9134]|LBCH1095-10|10-JDWBC-1095|658[0n]bp|Canada.British Columbia|BOLD:AAA3385
Xestia homogena|[9135]|LBCH1534-10|10-JDWBC-1534|658[0n]bp|Canada.British Columbia|BOLD:AAA3385
Xestia homogena|[9136]|LBCG590-09|08-JDWBC-0590|658[0n]bp|Canada.British Columbia|BOLD:AAA3385
Xestia homogena|[9137]|LBCH1443-10|10-JDWBC-1443|658[0n]bp|Canada.British Columbia|BOLD:AAA3385
Xestia homogena|[9138]|LBCH1998-10|10-JDWBC-1998|658[0n]bp|Canada.British Columbia|BOLD:AAA3385
Xestia homogena|[9139]|LBCG2054-09|08-JDWBC-2054|658[0n]bp|Canada.British Columbia|BOLD:AAA3385
Xestia homogena|[9140]|LBCH2104-10|10-JDWBC-2104|658[0n]bp|Canada.British Columbia|BOLD:AAA3385
Xestia homogena|[9141]|LBCG833-09|08-JDWBC-0833|658[0n]bp|Canada.British Columbia|BOLD:AAA3385
Xestia homogena|[9142]|LBCG995-09|08-JDWBC-0995|658[0n]bp|Canada.British Columbia|BOLD:AAA3385
Xestia homogena|[9143]|LBCD186-05|HLC-23006|658[0n]bp|Canada.British Columbia|BOLD:AAA3385
Xestia homogena|[9144]|BBLPB288-10|10BBCLP-1287|658[0n]bp|Canada.British Columbia|BOLD:AAA3385
Xestia homogena|[9145]|LBCD143-05|HLC-22963|658[0n]bp|Canada.British Columbia|BOLD:AAA3385
Xestia homogena|[9146]|LBCG1860-09|08-JDWBC-1860|658[0n]bp|Canada.British Columbia|BOLD:AAA3385
Xestia homogena|[9147]|LBCH1531-10|10-JDWBC-1531|658[0n]bp|Canada.British Columbia|BOLD:AAA3385
Xestia homogena|[9148]|LBCH1450-10|10-JDWBC-1450|658[0n]bp|Canada.British Columbia|BOLD:AAA3385
Xestia homogena|[9149]|LBCH1098-10|10-JDWBC-1098|658[0n]bp|Canada.British Columbia|BOLD:AAA3385
Xestia homogena|[9150]|LBCG1017-09|08-JDWBC-1017|658[0n]bp|Canada.British Columbia|BOLD:AAA3385
Xestia homogena|[9151]|LBCH2108-10|10-JDWBC-2108|658[0n]bp|Canada.British Columbia|BOLD:AAA3385
Xestia homogena|[9152]|LBCH1435-10|10-JDWBC-1435|658[0n]bp|Canada.British Columbia|BOLD:AAA3385
Xestia homogena|[9153]|LBCH1097-10|10-JDWBC-1097|658[0n]bp|Canada.British Columbia|BOLD:AAA3385
Xestia ursae|[9154]|LCHQ910-08|07WNP-10802|658[0n]bp|Canada.Manitoba|BOLD:AAB0146
Xestia ursae|[9155]|LCHP582-07|07PROBE-10252|658[0n]bp|Canada.Manitoba|BOLD:AAB0146
Xestia ursae|[9156]|LCHP166-07|07PROBE-00568|658[0n]bp|Canada.Manitoba|BOLD:AAB0146
Xestia ursae|[9157]|LCHP274-07|07PROBE-03840|658[0n]bp|Canada.Manitoba|BOLD:AAB0146
Xestia ursae|[9158]|LCHP461-07|07PROBE-10129|658[0n]bp|Canada.Manitoba|BOLD:AAB0146
Xestia ursae|[9159]|LCHP389-07|07PROBE-10013|658[0n]bp|Canada.Manitoba|BOLD:AAB0146
Xestia ursae|[9160]|LCHP421-07|07PROBE-10045|658[0n]bp|Canada.Manitoba|BOLD:AAB0146
Xestia ursae|[9161]|LCHP954-07|07PROBE-10716|658[0n]bp|Canada.Manitoba|BOLD:AAB0146
Xestia ursae|[9162]|LCHP273-07|07PROBE-03839|658[0n]bp|Canada.Manitoba|BOLD:AAB0146
Xestia ursae|[9163]|LCHP750-07|07PROBE-10435|655[0n]bp|Canada.Manitoba|BOLD:AAB0146
Xestia ursae|[9164]|LCHQ189-07|07PROBE-10966|658[0n]bp|Canada.Manitoba|BOLD:AAB0146
Xestia ursae|[9165]|LCHP463-07|07PROBE-10131|658[0n]bp|Canada.Manitoba|BOLD:AAB0146
Xestia ursae|[9166]|LCHP222-07|07PROBE-03792|658[0n]bp|Canada.Manitoba|BOLD:AAB0146
Xestia ursae|[9167]|LCHP703-07|07PROBE-10385|658[0n]bp|Canada.Manitoba|BOLD:AAB0146
Xestia ursae|[9168]|LCHP409-07|07PROBE-10033|638[0n]bp|Canada.Manitoba|BOLD:AAB0146
Xestia ursae|[9169]|LCHP464-07|07PROBE-10132|658[0n]bp|Canada.Manitoba|BOLD:AAB0146
Xestia ursae|[9170]|LCHQ903-08|07WNP-10795|658[0n]bp|Canada.Manitoba|BOLD:AAB0146

[illegible]
Xestia ursae|[9168]|LCHP409-07|07PROBE-10033|638[0n]bp|Canada.Manitoba|BOLD:AAB0146
Xestia ursae|[9169]|LCHP464-07|07PROBE-10132|658[0n]bp|Canada.Manitoba|BOLD:AAB0146
Xestia ursae|[9170]|LCHQ903-08|07WNP-10795|658[0n]bp|Canada.Manitoba|BOLD:AAB0146
Xestia ursae|[9171]|LCHQ904-08|07WNP-10796|658[0n]bp|Canada.Manitoba|BOLD:AAB0146
Xestia ursae|[9172]|LCHP713-07|07PROBE-10395|658[0n]bp|Canada.Manitoba|BOLD:AAB0146
Xestia ursae|[9173]|LCHP607-07|07PROBE-10288|658[0n]bp|Canada.Manitoba|BOLD:AAB0146
Xestia ursae|[9174]|LCHP586-07|07PROBE-10257|658[0n]bp|Canada.Manitoba|BOLD:AAB0146
Xestia ursae|[9175]|LCHP164-07|07PROBE-00566|658[0n]bp|Canada.Manitoba|BOLD:AAB0146
Xestia ursae|[9176]|LCHP595-07|07PROBE-10276|658[0n]bp|Canada.Manitoba|BOLD:AAB0146
Xestia ursae|[9177]|LCHQ877-08|07WNP-10769|658[0n]bp|Canada.Manitoba|BOLD:AAB0146
Xestia ursae|[9178]|LCHP413-07|07PROBE-10037|658[0n]bp|Canada.Manitoba|BOLD:AAB0146
Xestia ursae|[9179]|LCHP537-07|07PROBE-10207|658[0n]bp|Canada.Manitoba|BOLD:AAB0146
Xestia ursae|[9180]|LCHP514-07|07PROBE-10182|658[0n]bp|Canada.Manitoba|BOLD:AAB0146
Xestia ursae|[9181]|LCHP387-07|07PROBE-10011|658[0n]bp|Canada.Manitoba|BOLD:AAB0146
Xestia ursae|[9182]|LCHP634-07|07PROBE-10315|658[0n]bp|Canada.Manitoba|BOLD:AAB0146
Xestia ursae|[9183]|LCHP630-07|07PROBE-10311|658[0n]bp|Canada.Manitoba|BOLD:AAB0146
Xestia ursae|[9184]|LCHQ128-07|07PROBE-10897|658[0n]bp|Canada.Manitoba|BOLD:AAB0146
Xestia ursae|[9185]|LCHP905-07|07PROBE-10667|658[0n]bp|Canada.Manitoba|BOLD:AAB0146
Xestia ursae|[9186]|LCHP856-07|07PROBE-10613|658[0n]bp|Canada.Manitoba|BOLD:AAB0146
Xestia ursae|[9187]|LCHP649-07|07PROBE-10330|658[0n]bp|Canada.Manitoba|BOLD:AAB0146
Xestia normanianus|[9188]|XAH135-05|2005-ONT-1718|658[1n]bp|Canada.Ontario|BOLD:ABY7124
Xestia normanianus|[9189]|BBLEC943-09|09BBELE-0943|621[4n]bp|Canada.Nova Scotia|BOLD:ABY7124
Xestia normanianus|[9190]|BBLEC186-09|09BBELE-0186|638[0n]bp|Canada.Nova Scotia|BOLD:ABY7124
Xestia normanianus|[9191]|BBLEC935-09|09BBELE-0935|658[0n]bp|Canada.Nova Scotia|BOLD:ABY7124
Xestia normanianus|[9192]|XAG898-05|2005-ONT-1482|658[0n]bp|Canada.Ontario|BOLD:AAA8650
Xestia normanianus|[9193]|LPSOD972-09|08BBLEP-05605|658[0n]bp|Canada.Ontario|BOLD:AAA8650
Xestia normanianus|[9194]|XAD359-04|04HBL007359|572[0n]bp|Canada.Ontario|BOLD:AAA8650
Xestia normanianus|[9195]|XAD174-04|04HBL007174|658[0n]bp|Canada.Ontario|BOLD:AAA8650
Xestia normanianus|[9196]|BBLEC947-09|09BBELE-0947|614[0n]bp|Canada.Nova Scotia|BOLD:AAA8650
Xestia normanianus|[9197]|TTMNB464-06|MNBTT-464|658[0n]bp|Canada.New Brunswick|BOLD:AAA8650
Xestia normanianus|[9198]|BBLEC040-09|09BBELE-0040|658[0n]bp|Canada.New Brunswick|BOLD:AAA8650
Xestia normanianus|[9199]|XAG446-05|2005-ONT-1030|658[1n]bp|Canada.Ontario|BOLD:AAA8650
Xestia normanianus|[9200]|XAH050-05|2005-ONT-1633|658[1n]bp|Canada.Ontario|BOLD:AAA8650
Xestia normanianus|[9201]|RDLQB643-05|DH010746|577[0n]bp|Canada.Quebec|BOLD:AAA8650
Xestia normanianus|[9202]|XAD375-04|04HBL007375|658[0n]bp|Canada.Ontario|BOLD:AAA8650
Xestia normanianus|[9203]|BBLEC898-09|09BBELE-0898|658[0n]bp|Canada.Nova Scotia|BOLD:AAA8650
Xestia normanianus|[9204]|BBLEC920-09|09BBELE-0920|658[0n]bp|Canada.Nova Scotia|BOLD:AAA8650
Xestia normanianus|[9205]|PHAUG1795-11|BIOUG01497-E05|658[0n]bp|Canada.Ontario|BOLD:AAA8650
Xestia normanianus|[9206]|XAH136-05|2005-ONT-1719|658[0n]bp|Canada.Ontario|BOLD:AAA8650
Xestia normanianus|[9207]|BBLEC036-09|09BBELE-0036|658[0n]bp|Canada.New Brunswick|BOLD:AAA8650
Xestia normanianus|[9208]|BBLPC086-09|09BBELE-1086|658[0n]bp|Canada.New Brunswick|BOLD:AAA8650
Xestia normanianus|[9209]|TTMNB462-06|MNBTT-462|658[0n]bp|Canada.New Brunswick|BOLD:AAA8650
Xestia normanianus|[9210]|BBLEC495-09|09BBELE-0495|658[0n]bp|Canada.New Brunswick|BOLD:AAA8650
Xestia normanianus|[9211]|LPMNB440-09|08BBLEP-05440|658[0n]bp|Canada.Manitoba|BOLD:AAA8650
Xestia normanianus|[9212]|XAG804-05|2005-ONT-1388|658[0n]bp|Canada.Ontario|BOLD:AAA8650
Xestia normanianus|[9213]|BBLEC014-09|09BBELE-0014|658[0n]bp|Canada.New Brunswick|BOLD:AAA8650
Xestia normanianus|[9214]|BBLEC044-09|09BBELE-0044|658[0n]bp|Canada.New Brunswick|BOLD:AAA8650
Xestia normanianus|[9215]|BBLEC515-09|09BBELE-0515|633[0n]bp|Canada.New Brunswick|BOLD:AAA8650
Xestia normanianus|[9216]|PHAUG1788-11|BIOUG01497-D10|611[0n]bp|Canada.Ontario|BOLD:AAA8650
Xestia normanianus|[9217]|BBLPC108-09|09BBELE-1108|633[0n]bp|Canada.New Brunswick|BOLD:AAA8650
Xestia normanianus|[9218]|TMNBB411-06|MNBTT-1351|658[0n]bp|Canada.New Brunswick|BOLD:AAA8650
Xestia normanianus|[9219]|RDLQB644-05|DH010747|658[0n]bp|Canada.Quebec|BOLD:AAA8650
Xestia normanianus|[9220]|TTMNB463-06|MNBTT-463|658[0n]bp|Canada.New Brunswick|BOLD:AAA8650
Xestia normanianus|[9221]|XAG802-05|2005-ONT-1386|658[0n]bp|Canada.Ontario|BOLD:AAA8650
Xestia normanianus|[9222]|BBLEC047-09|09BBELE-0047|658[0n]bp|Canada.New Brunswick|BOLD:AAA8650
Xestia normanianus|[9223]|XAD376-04|04HBL007376|658[0n]bp|Canada.Ontario|BOLD:AAA8650
Xestia normanianus|[9224]|BBLEC942-09|09BBELE-0942|607[0n]bp|Canada.Nova Scotia|BOLD:AAA8650
Xestia oblata|[9225]|LPABC076-09|08BBLEP-04295|658[1n]bp|Canada.Alberta|BOLD:AAA5964
Xestia oblata|[9226]|LOWCD794-06|CGWC-3614|570[0n]bp|Canada.British Columbia|BOLD:AAA5964
Xestia oblata|[9227]|LOWC124-05|CGWC-0124|591[0n]bp|Canada.British Columbia|BOLD:AAA5964
Xestia oblata|[9228]|LOWCD792-06|CGWC-3612|658[0n]bp|Canada.British Columbia|BOLD:AAA5964
Xestia oblata|[9229]|LOWC123-05|CGWC-0123|658[0n]bp|Canada.British Columbia|BOLD:AAA5964
Xestia oblata|[9230]|LALPA507-10|AVBC 509-10|658[0n]bp|Canada.British Columbia|BOLD:AAA5964
Xestia oblata|[9231]|LPMN903-08|08BBLEP-02261|658[0n]bp|Canada.Alberta|BOLD:AAA5964
Xestia oblata|[9232]|BBLPB606-10|10BBCLP-1605|658[0n]bp|Canada.British Columbia|BOLD:AAA5964
Xestia oblata|[9233]|LOWC120-05|CGWC-0120|658[0n]bp|Canada.British Columbia|BOLD:AAA5964
Xestia oblata|[9234]|LBCC341-05|HLC-22221|658[0n]bp|Canada.British Columbia|BOLD:AAA5964
Xestia oblata|[9235]|LBCB550-05|HLC-21490|658[0n]bp|Canada.British Columbia|BOLD:AAA5964
Xestia oblata|[9236]|LOWCD791-06|CGWC-3611|658[0n]bp|Canada.British Columbia|BOLD:AAA5964
Xestia oblata|[9237]|LOWCD795-06|CGWC-3615|657[0n]bp|Canada.British Columbia|BOLD:AAA5964
Xestia oblata|[9238]|LPABC803-09|08BBLEP-05022|658[0n]bp|Canada.Alberta|BOLD:AAA5964
Xestia oblata|[9239]|LOWC122-05|CGWC-0122|658[0n]bp|Canada.British Columbia|BOLD:AAA5964
Xestia oblata|[9240]|LPABC929-09|08BBLEP-05340|658[0n]bp|Canada.Alberta|BOLD:AAA5964
Xestia oblata|[9241]|LALPA612-10|AVBC 614-10|658[0n]bp|Canada.British Columbia|BOLD:AAA5964
Xestia oblata|[9242]|LBCH1567-10|10-JDWBC-1567|658[0n]bp|Canada.British Columbia|BOLD:AAA5964
Xestia oblata|[9243]|LOWC127-05|CGWC-0127|658[0n]bp|Canada.British Columbia|BOLD:AAA5964
Xestia oblata|[9244]|LBCH1867-10|10-JDWBC-1867|658[0n]bp|Canada.British Columbia|BOLD:AAA5964
Xestia oblata|[9245]|LOWCD797-06|CGWC-3617|657[0n]bp|Canada.British Columbia|BOLD:AAA5964
Xestia oblata|[9246]|LBCD313-05|HLC-23133|658[0n]bp|Canada.British Columbia|BOLD:AAA5964
Xestia oblata|[9247]|LOWCD796-06|CGWC-3616|657[0n]bp|Canada.British Columbia|BOLD:AAA5964
Xestia oblata|[9248]|LBCH2029-10|10-JDWBC-2029|658[0n]bp|Canada.British Columbia|BOLD:AAA5964
Xestia oblata|[9249]|LOWC125-05|CGWC-0125|582[0n]bp|Canada.British Columbia|BOLD:AAA5964
Xestia oblata|[9250]|LOWC126-05|CGWC-0126|578[0n]bp|Canada.British Columbia|BOLD:AAA5964
Xestia oblata|[9251]|LPABB367-08|08BBLEP-03632|658[0n]bp|Canada.Alberta|BOLD:AAA5964
Xestia oblata|[9252]|LPAB088-08|08BBLEP-02410|658[0n]bp|Canada.Alberta|BOLD:AAA5964
Xestia oblata|[9253]|LALPA896-11|AVBC 1069-11|658[0n]bp|Canada.British Columbia|BOLD:AAA5964
Xestia oblata|[9254]|LPABB474-08|08BBLEP-03739|658[0n]bp|Canada.Alberta|BOLD:AAA5964
Xestia oblata|[9255]|LOWCD789-06|CGWC-3609|657[0n]bp|Canada.British Columbia|BOLD:AAA5964
Xestia oblata|[9256]|LALPA1234-11|AVBC 1236-11|658[0n]bp|Canada.British Columbia|BOLD:AAA5964
Xestia oblata|[9257]|LOWCD790-06|CGWC-3610|658[0n]bp|Canada.British Columbia|BOLD:AAA5964
Xestia oblata|[9258]|LBCH6220-10|10-JDWBC-6220|658[0n]bp|Canada.British Columbia|BOLD:AAA5964
Xestia oblata|[9259]|LOWC121-05|CGWC-0121|658[0n]bp|Canada.British Columbia|BOLD:AAA5964
Xestia oblata|[9260]|LOWC128-05|CGWC-0128|658[0n]bp|Canada.British Columbia|BOLD:AAA5964
Xestia oblata|[9261]|LCH522-04|04HBL003522|658[0n]bp|Canada.Manitoba|BOLD:AAA5964
Xestia oblata|[9262]|LCH521-04|04HBL003521|658[1n]bp|Canada.Manitoba|BOLD:AAA5964
Xestia oblata|[9263]|RDLQB094-05|DH010180|658[0n]bp|Canada.Quebec|BOLD:AAA5964
Xestia oblata|[9264]|RDLQB097-05|DH010183|658[0n]bp|Canada.Quebec|BOLD:AAA5964
Xestia oblata|[9265]|RDLQB095-05|DH010181|658[0n]bp|Canada.Quebec|BOLD:AAA5964
Xestia oblata|[9266]|RDLQB093-05|DH010179|589[0n]bp|Canada.Quebec|BOLD:AAA5964
Xestia oblata|[9267]|RDLQB098-05|DH010184|658[0n]bp|Canada.Quebec|BOLD:AAA5964
Xestia oblata|[9268]|RDLQB096-05|DH010182|658[0n]bp|Canada.Quebec|BOLD:AAA5964
Xestia oblata|[9269]|BBLEC096-09|09BBELE-0096|658[0n]bp|Canada.Nova Scotia|BOLD:AAA5964
[illegible]

Xestia oblata|[9267]|RDLQB098-05|DH010184|658[0n]bp|Canada.Quebec|BOLD:AAA5964
Xestia oblata|[9268]|RDLQB096-05|DH010182|658[0n]bp|Canada.Quebec|BOLD:AAA5964
Xestia oblata|[9269]|BBLEC096-09|09BBELE-0096|658[0n]bp|Canada.Nova Scotia|BOLD:AAA5964
Xestia oblata|[9270]|BBLPC935-09|09BBELE-1935|658[0n]bp|Canada.Newfoundland and Labrador|BOLD:AAA5964
Xestia oblata|[9271]|BBLPC584-09|09BBELE-1584|658[0n]bp|Canada.Nova Scotia|BOLD:AAA5964
Xestia oblata|[9272]|BBLPC225-09|09BBELE-1225|658[0n]bp|Canada.Nova Scotia|BOLD:AAA5964
Xestia oblata|[9273]|BBLPC896-09|09BBELE-1896|658[0n]bp|Canada.Newfoundland and Labrador|BOLD:AAA5964
Xestia oblata|[9274]|BBLPE383-09|09BBELE-2383|658[0n]bp|Canada.Newfoundland and Labrador|BOLD:AAA5964
Xestia oblata|[9275]|BBLPC224-09|09BBELE-1224|638[0n]bp|Canada.Nova Scotia|BOLD:AAA5964
Xestia oblata|[9276]|BBLPC606-09|09BBELE-1606|639[0n]bp|Canada.Nova Scotia|BOLD:AAA5964
Xestia oblata|[9277]|BBLPC211-09|09BBELE-1211|658[0n]bp|Canada.Nova Scotia|BOLD:AAA5964
Xestia oblata|[9278]|BBLPE350-09|09BBELE-2350|658[0n]bp|Canada.Newfoundland and Labrador|BOLD:AAA5964
Xestia oblata|[9279]|BBLPE018-09|09BBELE-2018|658[0n]bp|Canada.Nova Scotia|BOLD:AAA5964
Xestia oblata|[9280]|LOWC132-05|CGWC-0132|590[0n]bp|Canada.British Columbia|BOLD:AAA5964
Xestia oblata|[9281]|LOWCC885-05|CGWC-2765|588[0n]bp|Canada.British Columbia|BOLD:AAA5964
Xestia oblata|[9282]|LOWC131-05|CGWC-0131|658[0n]bp|Canada.British Columbia|BOLD:AAA5964
Xestia oblata|[9283]|LBCH348-10|10-JDWBC-0348|658[0n]bp|Canada.British Columbia|BOLD:AAA5964
Xestia oblata|[9284]|LOWC129-05|CGWC-0129|658[0n]bp|Canada.British Columbia|BOLD:AAA5964
Xestia oblata|[9285]|LOWCD793-06|CGWC-3613|657[0n]bp|Canada.British Columbia|BOLD:AAA5964
Xestia oblata|[9286]|LOWC130-05|CGWC-0130|658[0n]bp|Canada.British Columbia|BOLD:AAA5964
Xestia oblata|[9287]|LPAB010-08|08BBLEP-02332|658[0n]bp|Canada.Alberta|BOLD:AAA5964
Xestia oblata|[9288]|LPMN581-08|08BBLEP-01382|658[0n]bp|Canada.Manitoba|BOLD:AAA5964
Xestia oblata|[9289]|BBLPB610-10|10BBCLP-1609|658[0n]bp|Canada.British Columbia|BOLD:AAA5964
Xestia oblata|[9290]|LPABB072-08|08BBLEP-03337|658[0n]bp|Canada.Alberta|BOLD:AAA5964
Xestia oblata|[9291]|LBCC640-05|HLC-22520|658[0n]bp|Canada.British Columbia|BOLD:AAA5964
Xestia oblata|[9292]|BBLPB611-10|10BBCLP-1610|658[0n]bp|Canada.Alberta|BOLD:AAA5964
Xestia oblata|[9293]|LPABB350-08|08BBLEP-03615|658[0n]bp|Canada.Alberta|BOLD:AAA5964
Xestia oblata|[9294]|LCH284-04|04HBL003284|658[0n]bp|Canada.Manitoba|BOLD:AAA5964
Xestia oblata|[9295]|LOWCE843-06|CGWC-4603|658[0n]bp|Canada.British Columbia|BOLD:AAA5964
Xestia oblata|[9296]|BBLPB503-10|10BBCLP-1502|658[0n]bp|Canada.British Columbia|BOLD:AAA5964
Xestia oblata|[9297]|BBLPB607-10|10BBCLP-1606|658[0n]bp|Canada.British Columbia|BOLD:AAA5964
Xestia oblata|[9298]|LBCD317-05|HLC-23137|658[0n]bp|Canada.British Columbia|BOLD:AAA5964
Xestia oblata|[9299]|BBLPB609-10|10BBCLP-1608|658[0n]bp|Canada.British Columbia|BOLD:AAA5964
Xestia oblata|[9300]|BBLPB608-10|10BBCLP-1607|658[0n]bp|Canada.British Columbia|BOLD:AAA5964
Xestia smithii|[9301]|JSAUG1680-11|BIOUG01497-F08|658[0n]bp|Canada.Ontario|BOLD:AAA2590
Xestia smithii|[9302]|BBLPC374-09|09BBELE-1374|658[0n]bp|Canada.New Brunswick|BOLD:AAA2590
Xestia smithii|[9303]|XAH126-05|2005-ONT-1709|658[0n]bp|Canada.Ontario|BOLD:AAA2590
Xestia smithii|[9304]|TMNBB371-06|MNBTT-1311|658[0n]bp|Canada.New Brunswick|BOLD:AAA2590
Xestia smithii|[9305]|RDLQG063-06|DH012199|658[0n]bp|Canada.Quebec|BOLD:AAA2590
Xestia smithii|[9306]|PHAUG1794-11|BIOUG01497-E04|658[0n]bp|Canada.Ontario|BOLD:AAA2590
Xestia smithii|[9307]|LPMNB482-09|08BBLEP-05520|658[0n]bp|Canada.Manitoba|BOLD:AAA2590
Xestia smithii|[9308]|BBLEC007-09|09BBELE-0007|658[0n]bp|Canada.New Brunswick|BOLD:AAA2590
Xestia smithii|[9309]|PHAUG1792-11|BIOUG01497-E02|658[0n]bp|Canada.Ontario|BOLD:AAA2590
Xestia smithii|[9310]|XAG994-05|2005-ONT-1578|637[0n]bp|Canada.Ontario|BOLD:AAA2590
Xestia smithii|[9311]|LPMNB329-09|08BBLEP-05173|658[0n]bp|Canada.Manitoba|BOLD:AAA2590
Xestia smithii|[9312]|PHAUG1785-11|BIOUG01497-D07|658[0n]bp|Canada.Ontario|BOLD:AAA2590
Xestia smithii|[9313]|PHAUG1783-11|BIOUG01497-D05|658[0n]bp|Canada.Ontario|BOLD:AAA2590
Xestia smithii|[9314]|LBCH951-10|10-JDWBC-0951|658[0n]bp|Canada.British Columbia|BOLD:AAA2590
Xestia smithii|[9315]|LHLEP569-06|UBC-2006-2055|658[0n]bp|Canada.British Columbia|BOLD:AAA2590
Xestia smithii|[9316]|LPABC014-09|08BBLEP-04233|634[1n]bp|Canada.Alberta|BOLD:AAA2590
Xestia smithii|[9317]|LBCH1103-10|10-JDWBC-1103|658[0n]bp|Canada.British Columbia|BOLD:AAA2590
Xestia smithii|[9318]|LOWC785-05|CGWC-0785|658[0n]bp|Canada.British Columbia|BOLD:AAA2590
Xestia smithii|[9319]|LOWCD819-06|CGWC-3639|657[0n]bp|Canada.British Columbia|BOLD:AAA2590
Xestia smithii|[9320]|LOWCD834-06|CGWC-3654|658[0n]bp|Canada.British Columbia|BOLD:AAA2590
Xestia smithii|[9321]|LPABB516-08|08BBLEP-03781|658[0n]bp|Canada.Alberta|BOLD:AAA2590
Xestia smithii|[9322]|LOWC789-05|CGWC-0789|658[0n]bp|Canada.British Columbia|BOLD:AAA2590
Xestia smithii|[9323]|LOWCD821-06|CGWC-3641|657[0n]bp|Canada.British Columbia|BOLD:AAA2590
Xestia smithii|[9324]|LOWC788-05|CGWC-0788|658[0n]bp|Canada.British Columbia|BOLD:AAA2590
Xestia smithii|[9325]|LBCH3345-10|10-JDWBC-3345|658[0n]bp|Canada.British Columbia|BOLD:AAA2590
Xestia smithii|[9326]|LBCH4352-10|10-JDWBC-4352|658[0n]bp|Canada.British Columbia|BOLD:AAA2590
Xestia smithii|[9327]|LBCD426-05|HLC-23246|658[0n]bp|Canada.British Columbia|BOLD:AAA2590
Xestia smithii|[9328]|LHLEP440-06|UBC-2006-1989|658[0n]bp|Canada.British Columbia|BOLD:AAA2590
Xestia smithii|[9329]|LALPA686-10|AVBC 688-10|658[0n]bp|Canada.British Columbia|BOLD:AAA2590
Xestia smithii|[9330]|LBCG2285-09|08-JDWBC-2285|658[0n]bp|Canada.British Columbia|BOLD:AAA2590
Xestia smithii|[9331]|LBCH4024-10|10-JDWBC-4024|658[0n]bp|Canada.British Columbia|BOLD:AAA2590
Xestia smithii|[9332]|LALPA738-10|AVBC 740-10|658[0n]bp|Canada.British Columbia|BOLD:AAA2590
Xestia smithii|[9333]|LALPA1237-11|AVBC 1239-11|658[0n]bp|Canada.British Columbia|BOLD:AAA2590
Xestia smithii|[9334]|LBCH3073-10|10-JDWBC-3073|658[0n]bp|Canada.British Columbia|BOLD:AAA2590
Xestia smithii|[9335]|LBCH4647-10|10-JDWBC-4647|658[0n]bp|Canada.British Columbia|BOLD:AAA2590
Xestia smithii|[9336]|LOWCD823-06|CGWC-3643|658[0n]bp|Canada.British Columbia|BOLD:AAA2590
Xestia smithii|[9337]|LBCH957-10|10-JDWBC-0957|658[0n]bp|Canada.British Columbia|BOLD:AAA2590
Xestia smithii|[9338]|LPVIB571-08|PFC-2006-1997|658[0n]bp|Canada.British Columbia|BOLD:AAA2590
Xestia smithii|[9339]|LBCH963-10|10-JDWBC-0963|658[0n]bp|Canada.British Columbia|BOLD:AAA2590
Xestia smithii|[9340]|LBCH4644-10|10-JDWBC-4644|658[0n]bp|Canada.British Columbia|BOLD:AAA2590
Xestia smithii|[9341]|LBCG2313-09|08-JDWBC-2313|658[0n]bp|Canada.British Columbia|BOLD:AAA2590
Xestia smithii|[9342]|LPVIB261-08|PFC-2006-1651|658[0n]bp|Canada.British Columbia|BOLD:AAA2590
Xestia smithii|[9343]|LBCH4346-10|10-JDWBC-4346|658[0n]bp|Canada.British Columbia|BOLD:AAA2590
Xestia smithii|[9344]|LBCH4026-10|10-JDWBC-4026|658[0n]bp|Canada.British Columbia|BOLD:AAA2590
Xestia smithii|[9345]|LBCH3532-10|10-JDWBC-3532|658[0n]bp|Canada.British Columbia|BOLD:AAA2590
Xestia smithii|[9346]|LHLEP415-06|UBC-2006-1935|658[0n]bp|Canada.British Columbia|BOLD:AAA2590
Xestia smithii|[9347]|LALPA725-10|AVBC 727-10|658[0n]bp|Canada.British Columbia|BOLD:AAA2590
Xestia smithii|[9348]|LPVIB505-08|PFC-2006-1916|658[0n]bp|Canada.British Columbia|BOLD:AAA2590
Xestia smithii|[9349]|LBCH956-10|10-JDWBC-0956|658[0n]bp|Canada.British Columbia|BOLD:AAA2590
Xestia smithii|[9350]|LALPA656-10|AVBC 658-10|658[0n]bp|Canada.British Columbia|BOLD:AAA2590
Xestia smithii|[9351]|LBCH3525-10|10-JDWBC-3525|658[0n]bp|Canada.British Columbia|BOLD:AAA2590
Xestia smithii|[9352]|LBCH4099-10|10-JDWBC-4099|658[0n]bp|Canada.British Columbia|BOLD:AAA2590
Xestia smithii|[9353]|LBCH3074-10|10-JDWBC-3074|658[0n]bp|Canada.British Columbia|BOLD:AAA2590
Xestia smithii|[9354]|LBCH3529-10|10-JDWBC-3529|658[0n]bp|Canada.British Columbia|BOLD:AAA2590
Xestia smithii|[9355]|LBCH3792-10|10-JDWBC-3792|658[0n]bp|Canada.British Columbia|BOLD:AAA2590
Xestia smithii|[9356]|LBCH3352-10|10-JDWBC-3352|658[0n]bp|Canada.British Columbia|BOLD:AAA2590
Xestia smithii|[9357]|LALPA1248-11|AVBC 1250-11|658[0n]bp|Canada.British Columbia|BOLD:AAA2590
Xestia smithii|[9358]|LBCH4643-10|10-JDWBC-4643|658[0n]bp|Canada.British Columbia|BOLD:AAA2590
Xestia smithii|[9359]|LBCH3347-10|10-JDWBC-3347|658[0n]bp|Canada.British Columbia|BOLD:AAA2590
Xestia smithii|[9360]|LBCH3076-10|10-JDWBC-3076|658[0n]bp|Canada.British Columbia|BOLD:AAA2590
Xestia smithii|[9361]|LPVIB569-08|PFC-2006-1995|658[0n]bp|Canada.British Columbia|BOLD:AAA2590
Xestia smithii|[9362]|LBCH3075-10|10-JDWBC-3075|658[0n]bp|Canada.British Columbia|BOLD:AAA2590
Xestia smithii|[9363]|LBCH4649-10|10-JDWBC-4649|658[0n]bp|Canada.British Columbia|BOLD:AAA2590
Xestia smithii|[9364]|LPVIB506-08|PFC-2006-1917|658[0n]bp|Canada.British Columbia|BOLD:AAA2590
Xestia smithii|[9365]|LOWCD830-06|CGWC-3650|657[0n]bp|Canada.British Columbia|BOLD:AAA2590
Xestia smithii|[9366]|LALPA544-10|AVBC 546-10|637[0n]bp|Canada.British Columbia|BOLD:AAA2590
Xestia smithii|[9367]|LBCH4645-10|10-JDWBC-4645|643[0n]bp|Canada.British Columbia|BOLD:AAA2590
Xestia smithii|[9368]|LBCG2299-09|08-JDWBC-2299|621[0n]bp|Canada.British Columbia|BOLD:AAA2590
Xestia smithii|[9369]|LBCH2705-10|10-JDWBC-2705|658[0n]bp|Canada.British Columbia|BOLD:AAA2590

Xestia smithii|[9367]|LBCH4645-10|10-JDWBC-4645|643[0n]bp|Canada.British Columbia|BOLD:AAA2590
Xestia smithii|[9368]|LBCG2299-09|08-JDWBC-2299|621[0n]bp|Canada.British Columbia|BOLD:AAA2590
Xestia smithii|[9369]|LBCH2705-10|10-JDWBC-2705|658[0n]bp|Canada.British Columbia|BOLD:AAA2590
Xestia smithii|[9370]|LBCH4100-10|10-JDWBC-4100|658[0n]bp|Canada.British Columbia|BOLD:AAA2590
Xestia smithii|[9371]|LBCG2303-09|08-JDWBC-2303|658[0n]bp|Canada.British Columbia|BOLD:AAA2590
Xestia smithii|[9372]|LBCH4350-10|10-JDWBC-4350|658[0n]bp|Canada.British Columbia|BOLD:AAA2590
Xestia smithii|[9373]|LBCH3531-10|10-JDWBC-3531|658[0n]bp|Canada.British Columbia|BOLD:AAA2590
Xestia smithii|[9374]|LBCH3077-10|10-JDWBC-3077|658[0n]bp|Canada.British Columbia|BOLD:AAA2590
Xestia smithii|[9375]|LBCH3079-10|10-JDWBC-3079|658[0n]bp|Canada.British Columbia|BOLD:AAA2590
Xestia smithii|[9376]|LALPA1258-11|AVBC 1260-11|658[0n]bp|Canada.British Columbia|BOLD:AAA2590
Xestia smithii|[9377]|LBCH3794-10|10-JDWBC-3794|658[0n]bp|Canada.British Columbia|BOLD:AAA2590
Xestia smithii|[9378]|PHAUG1791-11|BIOUG01497-E01|658[0n]bp|Canada.Ontario|BOLD:AAA2590
Xestia smithii|[9379]|XAG973-05|2005-ONT-1557|638[0n]bp|Canada.Ontario|BOLD:AAA2590
Xestia smithii|[9380]|XAH124-05|2005-ONT-1707|658[0n]bp|Canada.Ontario|BOLD:AAA2590
Xestia smithii|[9381]|PHAUG1781-11|BIOUG01497-D03|658[1n]bp|Canada.Ontario|BOLD:AAA2590
Xestia smithii|[9382]|BBLPB417-10|10BBCLP-1416|658[0n]bp|Canada.Alberta|BOLD:AAA2590
Xestia smithii|[9383]|LPABC013-09|08BBLEP-04232|658[1n]bp|Canada.Alberta|BOLD:AAA2590
Xestia smithii|[9384]|JSAUG1682-11|BIOUG01497-F10|658[0n]bp|Canada.Ontario|BOLD:AAA2590
Xestia smithii|[9385]|LALPA1235-11|AVBC 1237-11|658[0n]bp|Canada.British Columbia|BOLD:AAA2590
Xestia smithii|[9386]|XAH125-05|2005-ONT-1708|658[0n]bp|Canada.Ontario|BOLD:AAA2590
Xestia smithii|[9387]|LPSOD967-09|08BBLEP-05600|658[0n]bp|Canada.Ontario|BOLD:AAA2590
Xestia smithii|[9388]|XAH194-05|2005-ONT-1777|658[0n]bp|Canada.Ontario|BOLD:AAA2590
Xestia smithii|[9389]|LOWC787-05|CGWC-0787|658[0n]bp|Canada.British Columbia|BOLD:AAA2590
Xestia smithii|[9390]|JSAUG1679-11|BIOUG01497-F07|658[0n]bp|Canada.Ontario|BOLD:AAA2590
Xestia smithii|[9391]|XAH276-05|2005-ONT-1859|658[0n]bp|Canada.Ontario|BOLD:AAA2590
Xestia smithii|[9392]|TTMNB468-06|MNBTT-468|658[0n]bp|Canada.New Brunswick|BOLD:AAA2590
Xestia smithii|[9393]|BBLPB584-10|10BBCLP-1583|658[0n]bp|Canada.Alberta|BOLD:AAA2590
Xestia smithii|[9394]|RDLQB628-05|DH010731|580[0n]bp|Canada.Quebec|BOLD:AAA2590
Xestia smithii|[9395]|BBLEC507-09|09BBELE-0507|634[0n]bp|Canada.New Brunswick|BOLD:AAA2590
Xestia smithii|[9396]|BBLPB416-10|10BBCLP-1415|620[0n]bp|Canada.Alberta|BOLD:AAA2590
Xestia smithii|[9397]|RDLQ723-07|DH008022|652[0n]bp|Canada.Quebec|BOLD:AAA2590
Xestia smithii|[9398]|PHAUG1790-11|BIOUG01497-D12|658[0n]bp|Canada.Ontario|BOLD:AAA2590
Xestia smithii|[9399]|XAH464-05|2005-ONT-2047|658[0n]bp|Canada.Ontario|BOLD:AAA2590
Xestia smithii|[9400]|XAH130-05|2005-ONT-1713|658[0n]bp|Canada.Ontario|BOLD:AAA2590
Xestia smithii|[9401]|LPMNB504-09|08BBLEP-05542|658[0n]bp|Canada.Manitoba|BOLD:AAA2590
Xestia smithii|[9402]|LPSOD960-09|08BBLEP-05500|658[0n]bp|Canada.Ontario|BOLD:AAA2590
Xestia smithii|[9403]|XAG734-05|2005-ONT-1318|658[0n]bp|Canada.Ontario|BOLD:AAA2590
Xestia smithii|[9404]|XAH273-05|2005-ONT-1856|658[0n]bp|Canada.Ontario|BOLD:AAA2590
Xestia smithii|[9405]|RDLQ722-07|DH011284|658[0n]bp|Canada.Quebec|BOLD:AAA2590
Xestia smithii|[9406]|PHAUG1789-11|BIOUG01497-D11|658[0n]bp|Canada.Ontario|BOLD:AAA2590
Xestia smithii|[9407]|BBLEC439-09|09BBELE-0439|658[0n]bp|Canada.New Brunswick|BOLD:AAA2590
Xestia smithii|[9408]|LBCG2300-09|08-JDWBC-2300|658[0n]bp|Canada.British Columbia|BOLD:AAA2590
Xestia smithii|[9409]|LOWC783-05|CGWC-0783|658[0n]bp|Canada.British Columbia|BOLD:AAA2590
Xestia smithii|[9410]|LOWCD822-06|CGWC-3642|658[0n]bp|Canada.British Columbia|BOLD:AAA2590
Xestia smithii|[9411]|PHAUG1782-11|BIOUG01497-D04|658[1n]bp|Canada.Ontario|BOLD:AAA2590
Xestia smithii|[9412]|LBCH4646-10|10-JDWBC-4646|636[0n]bp|Canada.British Columbia|BOLD:AAA2590
Xestia smithii|[9413]|LBCG2274-09|08-JDWBC-2274|658[0n]bp|Canada.British Columbia|BOLD:AAA2590
Xestia smithii|[9414]|LOWC790-05|CGWC-0790|658[0n]bp|Canada.British Columbia|BOLD:AAA2590
Xestia smithii|[9415]|LOWCD812-06|CGWC-3632|657[0n]bp|Canada.British Columbia|BOLD:AAA2590
Xestia smithii|[9416]|XAH008-05|2005-ONT-1591|616[0n]bp|Canada.Ontario|BOLD:AAA2590
Xestia smithii|[9417]|LBCG2295-09|08-JDWBC-2295|658[0n]bp|Canada.British Columbia|BOLD:AAA2590
Xestia smithii|[9418]|LBCG2291-09|08-JDWBC-2291|658[0n]bp|Canada.British Columbia|BOLD:AAA2590
Xestia smithii|[9419]|LOWCD828-06|CGWC-3648|657[0n]bp|Canada.British Columbia|BOLD:AAA2590
Xestia smithii|[9420]|LBCH4351-10|10-JDWBC-4351|658[0n]bp|Canada.British Columbia|BOLD:AAA2590
Xestia smithii|[9421]|LPMNB506-09|08BBLEP-05544|658[0n]bp|Canada.Manitoba|BOLD:AAA2590
Xestia smithii|[9422]|LOWCD831-06|CGWC-3651|658[0n]bp|Canada.British Columbia|BOLD:AAA2590
Xestia smithii|[9423]|LOWCD835-06|CGWC-3655|658[0n]bp|Canada.British Columbia|BOLD:AAA2590
Xestia smithii|[9424]|LPSOD937-09|08BBLEP-05477|658[0n]bp|Canada.Ontario|BOLD:AAA2590
Xestia smithii|[9425]|LOWCD827-06|CGWC-3647|658[0n]bp|Canada.British Columbia|BOLD:AAA2590
Xestia smithii|[9426]|LBCG2292-09|08-JDWBC-2292|658[0n]bp|Canada.British Columbia|BOLD:AAA2590
Xestia smithii|[9427]|LALPA1259-11|AVBC 1261-11|658[0n]bp|Canada.British Columbia|BOLD:AAA2590
Xestia smithii|[9428]|BBLPB415-10|10BBCLP-1414|658[0n]bp|Canada.Alberta|BOLD:AAA2590
Xestia smithii|[9429]|LOWCD816-06|CGWC-3636|657[0n]bp|Canada.British Columbia|BOLD:AAA2590
Xestia smithii|[9430]|LBCH3528-10|10-JDWBC-3528|658[0n]bp|Canada.British Columbia|BOLD:AAA2590
Xestia smithii|[9431]|LBCH2863-10|10-JDWBC-2863|658[0n]bp|Canada.British Columbia|BOLD:AAA2590
Xestia smithii|[9432]|LBCH1498-10|10-JDWBC-1498|658[0n]bp|Canada.British Columbia|BOLD:AAA2590
Xestia smithii|[9433]|BBLPA829-10|10BBCLP-0829|658[0n]bp|Canada.Saskatchewan|BOLD:AAA2590
Xestia smithii|[9434]|LBCH4349-10|10-JDWBC-4349|658[0n]bp|Canada.British Columbia|BOLD:AAA2590
Xestia smithii|[9435]|XAH195-05|2005-ONT-1778|658[0n]bp|Canada.Ontario|BOLD:AAA2590
Xestia smithii|[9436]|LBCH952-10|10-JDWBC-0952|658[0n]bp|Canada.British Columbia|BOLD:AAA2590
Xestia smithii|[9437]|LBCH3530-10|10-JDWBC-3530|658[0n]bp|Canada.British Columbia|BOLD:AAA2590
Xestia smithii|[9438]|BBLPB579-10|10BBCLP-1578|658[0n]bp|Canada.Alberta|BOLD:AAA2590
Xestia smithii|[9439]|LBCG631-09|08-JDWBC-0631|658[0n]bp|Canada.British Columbia|BOLD:AAA2590
Xestia smithii|[9440]|BBLPB573-10|10BBCLP-1572|658[0n]bp|Canada.Saskatchewan|BOLD:AAA2590
Xestia smithii|[9441]|BBLPB414-10|10BBCLP-1413|658[0n]bp|Canada.Alberta|BOLD:AAA2590
Xestia smithii|[9442]|LBCH1774-10|10-JDWBC-1774|658[0n]bp|Canada.British Columbia|BOLD:AAA2590
Xestia smithii|[9443]|BBLPB558-10|10BBCLP-1557|658[0n]bp|Canada.Alberta|BOLD:AAA2590
Xestia smithii|[9444]|LBCH2718-10|10-JDWBC-2718|658[0n]bp|Canada.British Columbia|BOLD:AAA2590
Xestia smithii|[9445]|LOWCD813-06|CGWC-3633|657[0n]bp|Canada.British Columbia|BOLD:AAA2590
Xestia smithii|[9446]|BBLPB384-10|10BBCLP-1383|658[0n]bp|Canada.Saskatchewan|BOLD:AAA2590
Xestia smithii|[9447]|LBCH4095-10|10-JDWBC-4095|658[0n]bp|Canada.British Columbia|BOLD:AAA2590
Xestia smithii|[9448]|LBCH955-10|10-JDWBC-0955|658[0n]bp|Canada.British Columbia|BOLD:AAA2590
Xestia smithii|[9449]|LBCH2822-10|10-JDWBC-2822|658[0n]bp|Canada.British Columbia|BOLD:AAA2590
Xestia smithii|[9450]|LOWCD832-06|CGWC-3652|658[0n]bp|Canada.British Columbia|BOLD:AAA2590
Xestia smithii|[9451]|LOWCD829-06|CGWC-3649|658[0n]bp|Canada.British Columbia|BOLD:AAA2590
Xestia smithii|[9452]|LBCH953-10|10-JDWBC-0953|658[0n]bp|Canada.British Columbia|BOLD:AAA2590
Xestia smithii|[9453]|LBCH4098-10|10-JDWBC-4098|658[0n]bp|Canada.British Columbia|BOLD:AAA2590
Xestia smithii|[9454]|LPABC398-09|08BBLEP-04617|658[0n]bp|Canada.Alberta|BOLD:AAA2590
Xestia smithii|[9455]|LOWCD836-06|CGWC-3656|658[0n]bp|Canada.British Columbia|BOLD:AAA2590
Xestia smithii|[9456]|BBLPB561-10|10BBCLP-1560|658[0n]bp|Canada.Saskatchewan|BOLD:AAA2590
Xestia smithii|[9457]|TTMNB465-06|MNBTT-465|658[0n]bp|Canada.New Brunswick|BOLD:AAA2590
Xestia smithii|[9458]|LOWCD824-06|CGWC-3644|658[0n]bp|Canada.British Columbia|BOLD:AAA2590
Xestia smithii|[9459]|LBCH3348-10|10-JDWBC-3348|658[0n]bp|Canada.British Columbia|BOLD:AAA2590
Xestia smithii|[9460]|LOWC792-05|CGWC-0792|658[0n]bp|Canada.British Columbia|BOLD:AAA2590
Xestia smithii|[9461]|BBLPB395-10|10BBCLP-1394|658[0n]bp|Canada.Saskatchewan|BOLD:AAA2590
Xestia smithii|[9462]|LBCG2286-09|08-JDWBC-2286|658[0n]bp|Canada.British Columbia|BOLD:AAA2590
Xestia smithii|[9463]|LBCH4094-10|10-JDWBC-4094|658[0n]bp|Canada.British Columbia|BOLD:AAA2590
Xestia smithii|[9464]|LBCH3343-10|10-JDWBC-3343|658[0n]bp|Canada.British Columbia|BOLD:AAA2590
Xestia smithii|[9465]|LBCH4039-10|10-JDWBC-4039|658[0n]bp|Canada.British Columbia|BOLD:AAA2590
Xestia smithii|[9466]|LBCG2309-09|08-JDWBC-2309|658[0n]bp|Canada.British Columbia|BOLD:AAA2590
Xestia smithii|[9467]|LBCG2304-09|08-JDWBC-2304|658[0n]bp|Canada.British Columbia|BOLD:AAA2590
Xestia smithii|[9468]|BBLPB581-10|10BBCLP-1580|658[0n]bp|Canada.Alberta|BOLD:AAA2590
Xestia smithii|[9469]|LOWCD826-06|CGWC-3646|657[0n]bp|Canada.British Columbia|BOLD:AAA2590

[illegible]
Xestia smithii|[9467]|LBCG2304-09|08-JDWBC-2304|658[0n]bp|Canada.British Columbia|BOLD:AAA2590
Xestia smithii|[9468]|BBLPB581-10|10BBCLP-1580|658[0n]bp|Canada.Alberta|BOLD:AAA2590
Xestia smithii|[9469]|LOWCD826-06|CGWC-3646|657[0n]bp|Canada.British Columbia|BOLD:AAA2590
Xestia smithii|[9470]|LBCG2298-09|08-JDWBC-2298|658[0n]bp|Canada.British Columbia|BOLD:AAA2590
Xestia smithii|[9471]|BBLPB444-10|10BBCLP-1443|658[0n]bp|Canada.Saskatchewan|BOLD:AAA2590
Xestia smithii|[9472]|LBCH2840-10|10-JDWBC-2840|658[0n]bp|Canada.British Columbia|BOLD:AAA2590
Xestia smithii|[9473]|LBCH123-10|10-JDWBC-0123|658[0n]bp|Canada.British Columbia|BOLD:AAA2590
Xestia smithii|[9474]|LPABC021-09|08BBLEP-04240|658[0n]bp|Canada.Alberta|BOLD:AAA2590
Xestia smithii|[9475]|LBCD106-05|HLC-22926|658[0n]bp|Canada.British Columbia|BOLD:AAA2590
Xestia smithii|[9476]|LBCH954-10|10-JDWBC-0954|658[0n]bp|Canada.British Columbia|BOLD:AAA2590
Xestia smithii|[9477]|LBCH3350-10|10-JDWBC-3350|658[0n]bp|Canada.British Columbia|BOLD:AAA2590
Xestia smithii|[9478]|LBCH2791-10|10-JDWBC-2791|658[0n]bp|Canada.British Columbia|BOLD:AAA2590
Xestia smithii|[9479]|LOWC791-05|CGWC-0791|658[0n]bp|Canada.British Columbia|BOLD:AAA2590
Xestia smithii|[9480]|BBLPB560-10|10BBCLP-1559|658[0n]bp|Canada.Saskatchewan|BOLD:AAA2590
Xestia smithii|[9481]|LBCH4648-10|10-JDWBC-4648|658[0n]bp|Canada.British Columbia|BOLD:AAA2590
Xestia smithii|[9482]|LBCG2308-09|08-JDWBC-2308|658[0n]bp|Canada.British Columbia|BOLD:AAA2590
Xestia smithii|[9483]|LOWCC160-05|CGWC-2040|658[0n]bp|Canada.British Columbia|BOLD:AAA2590
Xestia smithii|[9484]|BBLPB394-10|10BBCLP-1393|658[0n]bp|Canada.Saskatchewan|BOLD:AAA2590
Xestia smithii|[9485]|BBLPB583-10|10BBCLP-1582|658[0n]bp|Canada.Saskatchewan|BOLD:AAA2590
Xestia smithii|[9486]|LBCH3346-10|10-JDWBC-3346|658[0n]bp|Canada.British Columbia|BOLD:AAA2590
Xestia smithii|[9487]|LOWC793-05|CGWC-0793|658[0n]bp|Canada.British Columbia|BOLD:AAA2590
Xestia smithii|[9488]|LOWCD825-06|CGWC-3645|658[0n]bp|Canada.British Columbia|BOLD:AAA2590
Xestia smithii|[9489]|LBCH4642-10|10-JDWBC-4642|658[0n]bp|Canada.British Columbia|BOLD:AAA2590
Xestia smithii|[9490]|LBCG2302-09|08-JDWBC-2302|658[0n]bp|Canada.British Columbia|BOLD:AAA2590
Xestia smithii|[9491]|LOWCD817-06|CGWC-3637|657[0n]bp|Canada.British Columbia|BOLD:AAA2590
Xestia smithii|[9492]|LOWC784-05|CGWC-0784|658[0n]bp|Canada.British Columbia|BOLD:AAA2590
Xestia smithii|[9493]|BBLPB462-10|10BBCLP-1461|658[0n]bp|Canada.Alberta|BOLD:AAA2590
Xestia smithii|[9494]|TTMNB466-06|MNBTT-466|658[0n]bp|Canada.New Brunswick|BOLD:AAA2590
Xestia smithii|[9495]|LBCH4347-10|10-JDWBC-4347|658[0n]bp|Canada.British Columbia|BOLD:AAA2590
Xestia smithii|[9496]|LBCH4096-10|10-JDWBC-4096|658[0n]bp|Canada.British Columbia|BOLD:AAA2590
Xestia smithii|[9497]|LOWCD811-06|CGWC-3631|657[0n]bp|Canada.British Columbia|BOLD:AAA2590
Xestia smithii|[9498]|LBCD312-05|HLC-23132|658[0n]bp|Canada.British Columbia|BOLD:AAA2590
Xestia smithii|[9499]|LBCH3790-10|10-JDWBC-3790|658[0n]bp|Canada.British Columbia|BOLD:AAA2590
Xestia smithii|[9500]|LBCH3526-10|10-JDWBC-3526|658[0n]bp|Canada.British Columbia|BOLD:AAA2590
Xestia smithii|[9501]|JSAUG1681-11|BIOUG01497-F09|658[0n]bp|Canada.Ontario|BOLD:AAA2590
Xestia smithii|[9502]|LBCH3078-10|10-JDWBC-3078|658[0n]bp|Canada.British Columbia|BOLD:AAA2590
Xestia smithii|[9503]|BBLPB413-10|10BBCLP-1412|658[0n]bp|Canada.Saskatchewan|BOLD:AAA2590
Xestia smithii|[9504]|TTMNB467-06|MNBTT-467|658[0n]bp|Canada.New Brunswick|BOLD:AAA2590
Xestia smithii|[9505]|LBCH3527-10|10-JDWBC-3527|658[0n]bp|Canada.British Columbia|BOLD:AAA2590
Xestia smithii|[9506]|RDLQB652-05|DH010755|658[0n]bp|Canada.Quebec|BOLD:AAA2590
Xestia smithii|[9507]|LOWCC161-05|CGWC-2041|658[0n]bp|Canada.British Columbia|BOLD:AAA2590
Xestia smithii|[9508]|LBCH3791-10|10-JDWBC-3791|658[0n]bp|Canada.British Columbia|BOLD:AAA2590
Xestia smithii|[9509]|LBCH4641-10|10-JDWBC-4641|658[0n]bp|Canada.British Columbia|BOLD:AAA2590
Xestia smithii|[9510]|LOWCD810-06|CGWC-3630|657[0n]bp|Canada.British Columbia|BOLD:AAA2590
Xestia smithii|[9511]|LOWCD818-06|CGWC-3638|656[0n]bp|Canada.British Columbia|BOLD:AAA2590
Xestia smithii|[9512]|LOWCD820-06|CGWC-3640|656[0n]bp|Canada.British Columbia|BOLD:AAA2590
Xestia smithii|[9513]|LOWCD814-06|CGWC-3634|656[0n]bp|Canada.British Columbia|BOLD:AAA2590
Xestia smithii|[9514]|LOWCD815-06|CGWC-3635|656[0n]bp|Canada.British Columbia|BOLD:AAA2590
Xestia smithii|[9515]|BBLPB563-10|10BBCLP-1562|658[0n]bp|Canada.Saskatchewan|BOLD:AAA2590
Xestia smithii|[9516]|LBCH958-10|10-JDWBC-0958|643[0n]bp|Canada.British Columbia|BOLD:AAA2590
Xestia smithii|[9517]|LBCH7793-10|10-JDWBC-7793|658[0n]bp|Canada.British Columbia|BOLD:AAA2590
Xestia smithii|[9518]|LOWCD833-06|CGWC-3653|564[0n]bp|Canada.British Columbia|BOLD:AAA2590
Xestia smithii|[9519]|LBCH3351-10|10-JDWBC-3351|658[0n]bp|Canada.British Columbia|BOLD:AAA2590
Xestia smithii|[9520]|LBCG2294-09|08-JDWBC-2294|658[0n]bp|Canada.British Columbia|BOLD:AAA2590
Xestia smithii|[9521]|LOWC786-05|CGWC-0786|658[0n]bp|Canada.British Columbia|BOLD:AAA2590
Xestia smithii|[9522]|LBCH3349-10|10-JDWBC-3349|658[0n]bp|Canada.British Columbia|BOLD:AAA2590
Xestia smithii|[9523]|BBLPB586-10|10BBCLP-1585|658[0n]bp|Canada.British Columbia|BOLD:AAA2590
Xestia smithii|[9524]|BBLPB580-10|10BBCLP-1579|658[0n]bp|Canada.Alberta|BOLD:AAA2590
Xestia smithii|[9525]|LBCH1674-10|10-JDWBC-1674|658[0n]bp|Canada.British Columbia|BOLD:AAA2590
Xestia smithii|[9526]|LBCH4101-10|10-JDWBC-4101|658[0n]bp|Canada.British Columbia|BOLD:AAA2590
Xestia smithii|[9527]|LBCH4345-10|10-JDWBC-4345|658[0n]bp|Canada.British Columbia|BOLD:AAA2590
Xestia smithii|[9528]|LBCG2307-09|08-JDWBC-2307|658[0n]bp|Canada.British Columbia|BOLD:AAA2590
Xestia smithii|[9529]|LBCH4097-10|10-JDWBC-4097|658[0n]bp|Canada.British Columbia|BOLD:AAA2590
Xestia smithii|[9530]|LBCG2293-09|08-JDWBC-2293|658[0n]bp|Canada.British Columbia|BOLD:AAA2590
Xestia lorezi|[9531]|RDNMF062-08|NOC14148|658[0n]bp|Canada.Yukon Territory|BOLD:AAD2545
Xestia lorezi|[9532]|RDNMG685-08|CNC LEP00052809|642[0n]bp|Canada.Yukon Territory|BOLD:AAD2545
Xestia xanthographa|[9533]|LHLEP198-06|UBC-2006-1984|658[0n]bp|Canada.British Columbia|BOLD:AAA6806
Xestia xanthographa|[9534]|LPVIB556-08|PFC-2006-1982|658[0n]bp|Canada.British Columbia|BOLD:AAA6806
Xestia xanthographa|[9535]|LHLEP199-06|UBC-2006-1985|658[0n]bp|Canada.British Columbia|BOLD:AAA6806
Xestia xanthographa|[9536]|LPVIB805-08|PFC-2006-2294|658[0n]bp|Canada.British Columbia|BOLD:AAA6806
Xestia xanthographa|[9537]|LHLEP391-06|UBC-2006-2073|658[0n]bp|Canada.British Columbia|BOLD:AAA6806
Xestia xanthographa|[9538]|LHLEP202-06|UBC-2006-2047|658[0n]bp|Canada.British Columbia|BOLD:AAA6806
Xestia xanthographa|[9539]|LHLEP195-06|UBC-2006-1927|658[0n]bp|Canada.British Columbia|BOLD:AAA6806
Xestia xanthographa|[9540]|LPVIB744-08|PFC-2006-2206|658[0n]bp|Canada.British Columbia|BOLD:AAA6806
Xestia xanthographa|[9541]|LPVIB504-08|PFC-2006-1915|658[0n]bp|Canada.British Columbia|BOLD:AAA6806
Xestia xanthographa|[9542]|LHLEP207-06|UBC-2006-2054|658[0n]bp|Canada.British Columbia|BOLD:AAA6806
Xestia xanthographa|[9543]|LPVIB702-08|PFC-2006-2151|658[0n]bp|Canada.British Columbia|BOLD:AAA6806
Xestia xanthographa|[9544]|LHLEP197-06|UBC-2006-1929|658[0n]bp|Canada.British Columbia|BOLD:AAA6806
Xestia xanthographa|[9545]|LHLEP206-06|UBC-2006-2051|658[0n]bp|Canada.British Columbia|BOLD:AAA6806
Xestia xanthographa|[9546]|LALPA607-10|AVBC 609-10|658[0n]bp|Canada.British Columbia|BOLD:AAA6806
Xestia xanthographa|[9547]|LBCW042-08|08-JDWWI-0042|658[0n]bp|Canada.British Columbia|BOLD:AAA6806
Xestia xanthographa|[9548]|LALPA640-10|AVBC 642-10|658[0n]bp|Canada.British Columbia|BOLD:AAA6806
Xestia xanthographa|[9549]|LPVIB572-08|PFC-2006-2001|658[0n]bp|Canada.British Columbia|BOLD:AAA6806
Xestia xanthographa|[9550]|LHLEP204-06|UBC-2006-2049|658[0n]bp|Canada.British Columbia|BOLD:AAA6806
Xestia xanthographa|[9551]|LHLEP194-06|UBC-2006-1926|658[0n]bp|Canada.British Columbia|BOLD:AAA6806
Xestia xanthographa|[9552]|LBCW043-08|08-JDWWI-0043|658[0n]bp|Canada.British Columbia|BOLD:AAA6806
Xestia xanthographa|[9553]|RDNMF011-08|NOC14097|658[0n]bp|Canada.British Columbia|BOLD:AAA6806
Xestia xanthographa|[9554]|LPVIB746-08|PFC-2006-2208|658[0n]bp|Canada.British Columbia|BOLD:AAA6806
Xestia xanthographa|[9555]|LPVIB824-08|PFC-2006-2313|658[0n]bp|Canada.British Columbia|BOLD:AAA6806
Xestia xanthographa|[9556]|LPVIB557-08|PFC-2006-1983|658[0n]bp|Canada.British Columbia|BOLD:AAA6806
Xestia xanthographa|[9557]|LALPA704-10|AVBC 706-10|658[0n]bp|Canada.British Columbia|BOLD:AAA6806
Xestia xanthographa|[9558]|LHLEP441-06|UBC-2006-2072|658[0n]bp|Canada.British Columbia|BOLD:AAA6806
Xestia xanthographa|[9559]|LHLEP205-06|UBC-2006-2050|658[0n]bp|Canada.British Columbia|BOLD:AAA6806
Xestia xanthographa|[9560]|LPVIB004-08|PFC-2006-1914|658[0n]bp|Canada.British Columbia|BOLD:AAA6806
Xestia xanthographa|[9561]|LHLEP200-06|UBC-2006-1986|658[0n]bp|Canada.British Columbia|BOLD:AAA6806
Xestia xanthographa|[9562]|LHLEP196-06|UBC-2006-1928|658[0n]bp|Canada.British Columbia|BOLD:AAA6806
Xestia xanthographa|[9563]|LPVIB668-08|PFC-2006-2117|633[0n]bp|Canada.British Columbia|BOLD:AAA6806
Xestia xanthographa|[9564]|LPVIB659-08|PFC-2006-2108|632[0n]bp|Canada.British Columbia|BOLD:AAA6806
Xestia xanthographa|[9565]|LPVIB655-08|PFC-2006-2103|573[0n]bp|Canada.British Columbia|BOLD:AAA6806
Xestia xanthographa|[9566]|LPVIB651-08|PFC-2006-2099|610[0n]bp|Canada.British Columbia|BOLD:AAA6806
Xestia xanthographa|[9567]|LPVIB647-08|PFC-2006-2095|636[0n]bp|Canada.British Columbia|BOLD:AAA6806
Xestia xanthographa|[9568]|LPVIB597-08|PFC-2006-2038|636[0n]bp|Canada.British Columbia|BOLD:AAA6806
[illegible]

Xestia xanthographa|[9566]|LPVIB651-08|PFC-2006-2099|610[0n]bp|Canada.British Columbia|BOLD:AAA6806
Xestia xanthographa|[9567]|LPVIB647-08|PFC-2006-2095|636[0n]bp|Canada.British Columbia|BOLD:AAA6806
Xestia xanthographa|[9568]|LPVIB597-08|PFC-2006-2038|636[0n]bp|Canada.British Columbia|BOLD:AAA6806
Xestia xanthographa|[9569]|LPVIB653-08|PFC-2006-2101|636[0n]bp|Canada.British Columbia|BOLD:AAA6806
Xestia xanthographa|[9570]|LPVIB648-08|PFC-2006-2096|634[0n]bp|Canada.British Columbia|BOLD:AAA6806
Xestia xanthographa|[9571]|LPVIB649-08|PFC-2006-2097|623[0n]bp|Canada.British Columbia|BOLD:AAA6806
Xestia xanthographa|[9572]|LPVIB652-08|PFC-2006-2100|623[0n]bp|Canada.British Columbia|BOLD:AAA6806
Xestia xanthographa|[9573]|LPVIB656-08|PFC-2006-2104|623[0n]bp|Canada.British Columbia|BOLD:AAA6806
Xestia xanthographa|[9574]|LPVIB650-08|PFC-2006-2098|572[0n]bp|Canada.British Columbia|BOLD:AAA6806
Xestia xanthographa|[9575]|LHLEP392-06|UBC-2006-2075|658[0n]bp|Canada.British Columbia|BOLD:AAA6806
Xestia xanthographa|[9576]|LHLEP201-06|UBC-2006-1987|658[0n]bp|Canada.British Columbia|BOLD:AAA6806
Xestia xanthographa|[9577]|LALPA1245-11|AVBC 1247-11|658[0n]bp|Canada.British Columbia|BOLD:AAA6806
Xestia xanthographa|[9578]|LALPA682-10|AVBC 684-10|658[0n]bp|Canada.British Columbia|BOLD:AAA6806
Xestia xanthographa|[9579]|LHLEP203-06|UBC-2006-2048|658[0n]bp|Canada.British Columbia|BOLD:AAA6806
Xestia xanthographa|[9580]|LHLEP193-06|UBC-2006-1925|658[0n]bp|Canada.British Columbia|BOLD:AAA6806
Agrotis ipsilon|[9581]|XAB709-04|04HBL005709|658[0n]bp|Canada.Ontario|BOLD:AAA3364
Agrotis ipsilon|[9582]|PHMNB276-04|04HBL007741|569[0n]bp|Canada.New Brunswick|BOLD:AAA3364
Agrotis ipsilon|[9583]|XAB677-04|04HBL005677|571[0n]bp|Canada.Ontario|BOLD:AAA3364
Agrotis ipsilon|[9584]|XAD508-04|04HBL007508|577[0n]bp|Canada.Ontario|BOLD:AAA3364
Agrotis ipsilon|[9585]|MHCOL167-07|CHU06-COL-167|625[0n]bp|Canada.Manitoba|BOLD:AAA3364
Agrotis ipsilon|[9586]|MHLEP085-07|CHU06-LEP-085|658[0n]bp|Canada.Manitoba|BOLD:AAA3364
Agrotis ipsilon|[9587]|MNBB550-05|05-NBSTA-466|658[0n]bp|Canada.New Brunswick|BOLD:AAA3364
Agrotis ipsilon|[9588]|JGLL060-10|10PROBE-25723|658[0n]bp|Canada.Manitoba|BOLD:AAA3364
Agrotis ipsilon|[9589]|MHCOL156-07|CHU06-COL-156|658[0n]bp|Canada.Manitoba|BOLD:AAA3364
Agrotis ipsilon|[9590]|XAH638-05|2005-ONT-2221|658[0n]bp|Canada.Ontario|BOLD:AAA3364
Agrotis ipsilon|[9591]|MNBB477-05|05-NBSTA-393|658[0n]bp|Canada.New Brunswick|BOLD:AAA3364
Agrotis ipsilon|[9592]|MHLEP078-07|CHU06-LEP-078|658[0n]bp|Canada.Manitoba|BOLD:AAA3364
Agrotis ipsilon|[9593]|XAH615-05|2005-ONT-2198|658[0n]bp|Canada.Ontario|BOLD:AAA3364
Agrotis ipsilon|[9594]|XAB774-04|04HBL005774|658[0n]bp|Canada.Ontario|BOLD:AAA3364
Agrotis ipsilon|[9595]|XAB750-04|04HBL005750|658[0n]bp|Canada.Ontario|BOLD:AAA3364
Agrotis ipsilon|[9596]|XAD374-04|04HBL007374|658[0n]bp|Canada.Ontario|BOLD:AAA3364
Agrotis ipsilon|[9597]|BLTIB545-08|BL817|658[0n]bp|Canada.Ontario|BOLD:AAA3364
Agrotis ipsilon|[9598]|XAC035-04|04HBL006035|658[0n]bp|Canada.Ontario|BOLD:AAA3364
Agrotis ipsilon|[9599]|BBLEC543-09|09BBELE-0543|658[0n]bp|Canada.Nova Scotia|BOLD:AAA3364
Agrotis ipsilon|[9600]|MNBB179-05|05-NBSTA-095|658[0n]bp|Canada.New Brunswick|BOLD:AAA3364
Agrotis ipsilon|[9601]|BLTIB830-08|BL1248|658[0n]bp|Canada.Ontario|BOLD:AAA3364
Agrotis ipsilon|[9602]|MHCOL360-07|CHU06-COL-360|658[0n]bp|Canada.Manitoba|BOLD:AAA3364
Agrotis ipsilon|[9603]|JGLL061-10|10PROBE-25724|658[0n]bp|Canada.Manitoba|BOLD:AAA3364
Agrotis ipsilon|[9604]|TTMNB420-06|MNBTT-420|658[0n]bp|Canada.New Brunswick|BOLD:AAA3364
Agrotis ipsilon|[9605]|LPSO022-08|PPBP-0022|658[0n]bp|Canada.Ontario|BOLD:AAA3364
Agrotis ipsilon|[9606]|MHLEP093-07|CHU06-LEP-093|658[0n]bp|Canada.Manitoba|BOLD:AAA3364
Agrotis ipsilon|[9607]|BBLPC508-09|09BBELE-1508|658[0n]bp|Canada.New Brunswick|BOLD:AAA3364
Agrotis ipsilon|[9608]|MHLEP015-07|CHU06-LEP-015|658[0n]bp|Canada.Manitoba|BOLD:AAA3364
Agrotis ipsilon|[9609]|MNBB474-05|05-NBSTA-390|658[0n]bp|Canada.New Brunswick|BOLD:AAA3364
Agrotis ipsilon|[9610]|MNBB428-05|05-NBSTA-344|658[0n]bp|Canada.New Brunswick|BOLD:AAA3364
Agrotis ipsilon|[9611]|XAH667-05|2005-ONT-2250|658[0n]bp|Canada.Ontario|BOLD:AAA3364
Agrotis ipsilon|[9612]|XAB667-04|04HBL005667|658[0n]bp|Canada.Ontario|BOLD:AAA3364
Agrotis ipsilon|[9613]|PHMNB286-04|04HBL007751|658[0n]bp|Canada.New Brunswick|BOLD:AAA3364
Agrotis ipsilon|[9614]|XAB775-04|04HBL005775|658[0n]bp|Canada.Ontario|BOLD:AAA3364
Agrotis ipsilon|[9615]|BBLPC033-09|09BBELE-1033|658[0n]bp|Canada.New Brunswick|BOLD:AAA3364
Agrotis ipsilon|[9616]|LPSOD1045-09|08MZPP-102|658[0n]bp|Canada.Ontario|BOLD:AAA3364
Agrotis ipsilon|[9617]|MHCOL114-07|CHU06-COL-114|658[0n]bp|Canada.Manitoba|BOLD:AAA3364
Agrotis ipsilon|[9618]|MHCOL366-07|CHU06-COL-366|658[0n]bp|Canada.Manitoba|BOLD:AAA3364
Agrotis ipsilon|[9619]|JGLL062-10|10PROBE-25725|658[0n]bp|Canada.Manitoba|BOLD:AAA3364
Agrotis ipsilon|[9620]|LALPA175-10|AVBC 175-10|658[0n]bp|Canada.British Columbia|BOLD:AAA3364
Agrotis ipsilon|[9621]|TTMNB421-06|MNBTT-421|658[0n]bp|Canada.New Brunswick|BOLD:AAA3364
Agrotis ipsilon|[9622]|LBCS028-07|UBC-2007-0051|658[0n]bp|Canada.British Columbia|BOLD:AAA3364
Agrotis ipsilon|[9623]|MNBB476-05|05-NBSTA-392|658[0n]bp|Canada.New Brunswick|BOLD:AAA3364
Agrotis ipsilon|[9624]|MHCOL284-07|CHU06-COL-284|658[0n]bp|Canada.Manitoba|BOLD:AAA3364
Agrotis ipsilon|[9625]|MNBB374-05|05-NBSTA-290|658[0n]bp|Canada.New Brunswick|BOLD:AAA3364
Agrotis ipsilon|[9626]|LBCS250-07|UBC-2007-0758|658[0n]bp|Canada.British Columbia|BOLD:AAA3364
Agrotis ipsilon|[9627]|LBCS184-07|UBC-2007-0565|658[0n]bp|Canada.British Columbia|BOLD:AAA3364
Agrotis ipsilon|[9628]|XAB690-04|04HBL005690|658[0n]bp|Canada.Ontario|BOLD:AAA3364
Agrotis ipsilon|[9629]|XAJ813-06|2006-ONT-0813|658[0n]bp|Canada.Ontario|BOLD:AAA3364
Agrotis ipsilon|[9630]|MHCOL368-07|CHU06-COL-368|650[0n]bp|Canada.Manitoba|BOLD:AAA3364
Agrotis ipsilon|[9631]|PHLCH800-11|10PROBE-21912|632[0n]bp|Canada.Manitoba|BOLD:AAA3364
Agrotis ipsilon|[9632]|MHCOL007-07|CHU06-COL-007|646[0n]bp|Canada.Manitoba|BOLD:AAA3364
Agrotis ipsilon|[9633]|MHCOL113-07|CHU06-COL-113|628[0n]bp|Canada.Manitoba|BOLD:AAA3364
Agrotis ipsilon|[9634]|RDLQF818-06|DH011971|551[0n]bp|Canada.Quebec|BOLD:AAA3364
Agrotis ipsilon|[9635]|PMG086-03|moth252.01|617[0n]bp|Canada.Ontario|BOLD:AAA3364
Agrotis ipsilon|[9636]|XAB710-04|04HBL005710|589[0n]bp|Canada.Ontario|BOLD:AAA3364
Agrotis ipsilon|[9637]|PHMO170-03|moth899.01|639[0n]bp|Canada.Ontario|BOLD:AAA3364
Agrotis ipsilon|[9638]|MHCOL107-07|CHU06-COL-107|658[0n]bp|Canada.Manitoba|BOLD:AAA3364
Agrotis gladiaria|[9639]|XAD381-04|04HBL007381|658[0n]bp|Canada.Ontario|BOLD:ACF0067
Agrotis gladiaria|[9640]|XAD435-04|04HBL007435|575[0n]bp|Canada.Ontario|BOLD:ACF0067
Agrotis gladiaria|[9641]|XAD385-04|04HBL007385|595[0n]bp|Canada.Ontario|BOLD:ACF0067
Agrotis gladiaria|[9642]|XAD377-04|04HBL007377|595[0n]bp|Canada.Ontario|BOLD:ACF0067
Agrotis gladiaria|[9643]|XAD380-04|04HBL007380|548[0n]bp|Canada.Ontario|BOLD:ACF0067
Agrotis gladiaria|[9644]|XAD369-04|04HBL007369|593[0n]bp|Canada.Ontario|BOLD:ACF0067
Agrotis gladiaria|[9645]|XAD378-04|04HBL007378|658[0n]bp|Canada.Ontario|BOLD:ACF0067
Agrotis gladiaria|[9646]|XAD387-04|04HBL007387|658[0n]bp|Canada.Ontario|BOLD:ACF0067
Agrotis gladiaria|[9647]|XAH459-05|2005-ONT-2042|658[0n]bp|Canada.Ontario|BOLD:ACF0067
Agrotis gladiaria|[9648]|XAH354-05|2005-ONT-1937|658[0n]bp|Canada.Ontario|BOLD:ACF0067
Agrotis gladiaria|[9649]|PHMO331-03|moth2570.02|639[0n]bp|Canada.Ontario|BOLD:ACF0067
Agrotis gladiaria|[9650]|PHMO340-03|moth2615.02|639[0n]bp|Canada.Ontario|BOLD:ACF0067
Agrotis gladiaria|[9651]|XAH361-05|2005-ONT-1944|658[0n]bp|Canada.Ontario|BOLD:ACF0067
Agrotis gladiaria|[9652]|XAH391-05|2005-ONT-1974|658[0n]bp|Canada.Ontario|BOLD:ACF0067
Agrotis gladiaria|[9653]|XAH412-05|2005-ONT-1995|658[0n]bp|Canada.Ontario|BOLD:ACF0067
Agrotis gladiaria|[9654]|XAH460-05|2005-ONT-2043|658[0n]bp|Canada.Ontario|BOLD:ACF0067
Agrotis venerabilis|[9655]|TTMNB419-06|MNBTT-419|658[0n]bp|Canada.New Brunswick|BOLD:ABZ1938
Agrotis venerabilis|[9656]|TTMNB415-06|MNBTT-415|658[0n]bp|Canada.New Brunswick|BOLD:ABZ1938
Agrotis venerabilis|[9657]|TTMNB418-06|MNBTT-418|658[0n]bp|Canada.New Brunswick|BOLD:ABZ1938
Agrotis venerabilis|[9658]|XAB412-04|04HBL005412|580[0n]bp|Canada.Ontario|BOLD:ABZ1938
Agrotis venerabilis|[9659]|XAH363-05|2005-ONT-1946|658[0n]bp|Canada.Ontario|BOLD:ABZ1938
Agrotis venerabilis|[9660]|XAH313-05|2005-ONT-1896|658[1n]bp|Canada.Ontario|BOLD:ABZ1938
Agrotis venerabilis|[9661]|XAH411-05|2005-ONT-1994|658[0n]bp|Canada.Ontario|BOLD:ABZ1938
Agrotis venerabilis|[9662]|XAH529-05|2005-ONT-2112|658[0n]bp|Canada.Ontario|BOLD:ABZ1938
Agrotis venerabilis|[9663]|XAH531-05|2005-ONT-2114|658[0n]bp|Canada.Ontario|BOLD:ABZ1938
Agrotis venerabilis|[9664]|XAH316-05|2005-ONT-1899|658[0n]bp|Canada.Ontario|BOLD:ABZ1938
Agrotis venerabilis|[9665]|XAH448-05|2005-ONT-2031|658[0n]bp|Canada.Ontario|BOLD:ABZ1938
Agrotis venerabilis|[9666]|XAH387-05|2005-ONT-1970|658[0n]bp|Canada.Ontario|BOLD:ABZ1938
Agrotis venerabilis|[9667]|XAB428-04|04HBL005428|658[0n]bp|Canada.Ontario|BOLD:ABZ1938
Agrotis venerabilis|[9668]|XAB441-04|04HBL005441|658[0n]bp|Canada.Ontario|BOLD:ABZ1938

Agrotis venerabilis|[9666]|XAH387-05|2005-ONT-1970|658[0n]bp|Canada.Ontario|BOLD:ABZ1938
Agrotis venerabilis|[9667]|XAB428-04|04HBL005428|658[0n]bp|Canada.Ontario|BOLD:ABZ1938
Agrotis venerabilis|[9668]|XAB441-04|04HBL005441|658[0n]bp|Canada.Ontario|BOLD:ABZ1938
Agrotis venerabilis|[9669]|XAD386-04|04HBL007386|658[0n]bp|Canada.Ontario|BOLD:ABZ1938
Agrotis venerabilis|[9670]|XAH566-05|2005-ONT-2149|658[0n]bp|Canada.Ontario|BOLD:ABZ1938
Agrotis venerabilis|[9671]|XAH436-05|2005-ONT-2019|658[0n]bp|Canada.Ontario|BOLD:ABZ1938
Agrotis venerabilis|[9672]|PHMO347-03|moth2652.02|639[0n]bp|Canada.Ontario|BOLD:ABZ1938
Agrotis venerabilis|[9673]|PHMO341-03|moth2617.02|639[0n]bp|Canada.Ontario|BOLD:ABZ1938
Agrotis venerabilis|[9674]|XAH390-05|2005-ONT-1973|658[0n]bp|Canada.Ontario|BOLD:ABZ1938
Agrotis venerabilis|[9675]|LOWC108-05|CGWC-0108|598[1n]bp|Canada.British Columbia|BOLD:ABZ1938
Agrotis venerabilis|[9676]|LOWC106-05|CGWC-0106|658[0n]bp|Canada.British Columbia|BOLD:ABZ1938
Agrotis venerabilis|[9677]|LOWC117-05|CGWC-0117|590[3n]bp|Canada.British Columbia|BOLD:ABZ1938
Agrotis venerabilis|[9678]|LOWC109-05|CGWC-0109|590[4n]bp|Canada.British Columbia|BOLD:ABZ1938
Agrotis venerabilis|[9679]|LOWC118-05|CGWC-0118|604[1n]bp|Canada.British Columbia|BOLD:ABZ1938
Agrotis venerabilis|[9680]|LBCH6805-10|10-JDWBC-6805|658[0n]bp|Canada.British Columbia|BOLD:ABZ1938
Agrotis venerabilis|[9681]|LBCH7014-10|10-JDWBC-7014|658[0n]bp|Canada.British Columbia|BOLD:ABZ1938
Agrotis venerabilis|[9682]|TTMNB416-06|MNBTT-416|658[0n]bp|Canada.New Brunswick|BOLD:ABZ1938
Agrotis venerabilis|[9683]|LBCH7013-10|10-JDWBC-7013|658[0n]bp|Canada.British Columbia|BOLD:ABZ1938
Agrotis venerabilis|[9684]|LBCH6902-10|10-JDWBC-6902|658[0n]bp|Canada.British Columbia|BOLD:ABZ1938
Agrotis venerabilis|[9685]|LOWCD879-06|CGWC-3699|658[0n]bp|Canada.British Columbia|BOLD:ABZ1938
Agrotis venerabilis|[9686]|LBCH6941-10|10-JDWBC-6941|658[0n]bp|Canada.British Columbia|BOLD:ABZ1938
Agrotis venerabilis|[9687]|LBCH7015-10|10-JDWBC-7015|658[0n]bp|Canada.British Columbia|BOLD:ABZ1938
Agrotis venerabilis|[9688]|LOWC119-05|CGWC-0119|658[0n]bp|Canada.British Columbia|BOLD:ABZ1938
Agrotis venerabilis|[9689]|TMNBB364-06|MNBTT-1304|658[0n]bp|Canada.New Brunswick|BOLD:ABZ1938
Agrotis venerabilis|[9690]|LBCH6904-10|10-JDWBC-6904|658[0n]bp|Canada.British Columbia|BOLD:ABZ1938
Agrotis venerabilis|[9691]|LBCH6906-10|10-JDWBC-6906|658[0n]bp|Canada.British Columbia|BOLD:ABZ1938
Agrotis venerabilis|[9692]|LBCH6899-10|10-JDWBC-6899|658[0n]bp|Canada.British Columbia|BOLD:ABZ1938
Agrotis venerabilis|[9693]|LBCH6905-10|10-JDWBC-6905|658[0n]bp|Canada.British Columbia|BOLD:ABZ1938
Agrotis venerabilis|[9694]|LBCH6774-10|10-JDWBC-6774|658[0n]bp|Canada.British Columbia|BOLD:ABZ1938
Agrotis venerabilis|[9695]|LBCH6931-10|10-JDWBC-6931|658[0n]bp|Canada.British Columbia|BOLD:ABZ1938
Agrotis venerabilis|[9696]|LBCH7016-10|10-JDWBC-7016|658[0n]bp|Canada.British Columbia|BOLD:ABZ1938
Agrotis venerabilis|[9697]|RDLQF267-06|DH011359|658[2n]bp|Canada.Quebec|BOLD:ABZ1938
Agrotis venerabilis|[9698]|RDLQF279-06|DH011371|658[0n]bp|Canada.Quebec|BOLD:ABZ1938
Agrotis venerabilis|[9699]|TTMNB417-06|MNBTT-417|658[0n]bp|Canada.New Brunswick|BOLD:ABZ1938
Agrotis venerabilis|[9700]|LBCH6936-10|10-JDWBC-6936|636[0n]bp|Canada.British Columbia|BOLD:ABZ1938
Agrotis venerabilis|[9701]|LBCH7020-10|10-JDWBC-7020|646[0n]bp|Canada.British Columbia|BOLD:ABZ1938
Agrotis venerabilis|[9702]|LBCH6900-10|10-JDWBC-6900|639[0n]bp|Canada.British Columbia|BOLD:ABZ1938
Agrotis venerabilis|[9703]|LBCH6903-10|10-JDWBC-6903|658[0n]bp|Canada.British Columbia|BOLD:ABZ1938
Agrotis venerabilis|[9704]|LBCH7017-10|10-JDWBC-7017|658[0n]bp|Canada.British Columbia|BOLD:ABZ1938
Agrotis venerabilis|[9705]|LBCH7019-10|10-JDWBC-7019|658[0n]bp|Canada.British Columbia|BOLD:ABZ1938
Agrotis venerabilis|[9706]|LBCH6804-10|10-JDWBC-6804|658[0n]bp|Canada.British Columbia|BOLD:ABZ1938
Agrotis venerabilis|[9707]|LBCH6901-10|10-JDWBC-6901|658[0n]bp|Canada.British Columbia|BOLD:ABZ1938
Agrotis venerabilis|[9708]|LBCH6779-10|10-JDWBC-6779|658[0n]bp|Canada.British Columbia|BOLD:ABZ1938
Agrotis venerabilis|[9709]|LBCH6808-10|10-JDWBC-6808|658[0n]bp|Canada.British Columbia|BOLD:ABZ1938
Agrotis venerabilis|[9710]|LBCH7066-10|10-JDWBC-7066|658[0n]bp|Canada.British Columbia|BOLD:ABZ1938
Agrotis venerabilis|[9711]|LBCH6841-10|10-JDWBC-6841|658[0n]bp|Canada.British Columbia|BOLD:ABZ1938
Agrotis venerabilis|[9712]|LBCH7018-10|10-JDWBC-7018|658[0n]bp|Canada.British Columbia|BOLD:ABZ1938
Agrotis venerabilis|[9713]|LBCH6776-10|10-JDWBC-6776|658[0n]bp|Canada.British Columbia|BOLD:ABZ1938
Agrotis venerabilis|[9714]|LBCH6937-10|10-JDWBC-6937|658[0n]bp|Canada.British Columbia|BOLD:ABZ1938
Agrotis venerabilis|[9715]|LBCH6940-10|10-JDWBC-6940|658[0n]bp|Canada.British Columbia|BOLD:ABZ1938
Agrotis venerabilis|[9716]|LBCH6775-10|10-JDWBC-6775|658[0n]bp|Canada.British Columbia|BOLD:ABZ1938
Agrotis venerabilis|[9717]|LBCH7243-10|10-JDWBC-7243|658[0n]bp|Canada.British Columbia|BOLD:ABZ1938
Agrotis venerabilis|[9718]|LBCH7138-10|10-JDWBC-7138|658[0n]bp|Canada.British Columbia|BOLD:ABZ1938
Agrotis venerabilis|[9719]|LBCH6778-10|10-JDWBC-6778|658[0n]bp|Canada.British Columbia|BOLD:ABZ1938
Agrotis venerabilis|[9720]|LBCH6777-10|10-JDWBC-6777|658[0n]bp|Canada.British Columbia|BOLD:ABZ1938
Agrotis venerabilis|[9721]|LBCH7242-10|10-JDWBC-7242|658[0n]bp|Canada.British Columbia|BOLD:ABZ1938
Agrotis venerabilis|[9722]|LBCH7248-10|10-JDWBC-7248|658[0n]bp|Canada.British Columbia|BOLD:ABZ1938
Agrotis venerabilis|[9723]|LOWC111-05|CGWC-0111|658[0n]bp|Canada.British Columbia|BOLD:ABZ1938
Agrotis venerabilis|[9724]|LBCH7304-10|10-JDWBC-7304|658[0n]bp|Canada.British Columbia|BOLD:ABZ1938
Agrotis venerabilis|[9725]|LBCH7690-10|10-JDWBC-7690|658[0n]bp|Canada.British Columbia|BOLD:ABZ1938
Agrotis venerabilis|[9726]|LBCH7139-10|10-JDWBC-7139|658[0n]bp|Canada.British Columbia|BOLD:ABZ1938
Agrotis venerabilis|[9727]|LBCH6939-10|10-JDWBC-6939|658[0n]bp|Canada.British Columbia|BOLD:ABZ1938
Agrotis venerabilis|[9728]|LBCH7134-10|10-JDWBC-7134|658[0n]bp|Canada.British Columbia|BOLD:ABZ1938
Agrotis venerabilis|[9729]|LBCH7141-10|10-JDWBC-7141|658[0n]bp|Canada.British Columbia|BOLD:ABZ1938
Agrotis venerabilis|[9730]|LBCH7306-10|10-JDWBC-7306|658[0n]bp|Canada.British Columbia|BOLD:ABZ1938
Agrotis venerabilis|[9731]|LBCH7249-10|10-JDWBC-7249|658[0n]bp|Canada.British Columbia|BOLD:ABZ1938
Agrotis venerabilis|[9732]|LBCH6809-10|10-JDWBC-6809|658[0n]bp|Canada.British Columbia|BOLD:ABZ1938
Agrotis venerabilis|[9733]|LBCH7310-10|10-JDWBC-7310|658[0n]bp|Canada.British Columbia|BOLD:ABZ1938
Agrotis venerabilis|[9734]|LBCH7247-10|10-JDWBC-7247|658[0n]bp|Canada.British Columbia|BOLD:ABZ1938
Agrotis venerabilis|[9735]|LBCH6942-10|10-JDWBC-6942|658[0n]bp|Canada.British Columbia|BOLD:ABZ1938
Agrotis venerabilis|[9736]|LBCH6848-10|10-JDWBC-6848|658[0n]bp|Canada.British Columbia|BOLD:ABZ1938
Agrotis venerabilis|[9737]|LBCH7307-10|10-JDWBC-7307|658[0n]bp|Canada.British Columbia|BOLD:ABZ1938
Agrotis venerabilis|[9738]|LBCH6781-10|10-JDWBC-6781|658[0n]bp|Canada.British Columbia|BOLD:ABZ1938
Agrotis venerabilis|[9739]|LOWC104-05|CGWC-0104|658[0n]bp|Canada.British Columbia|BOLD:ABZ1938
Agrotis venerabilis|[9740]|LBCH7305-10|10-JDWBC-7305|658[0n]bp|Canada.British Columbia|BOLD:ABZ1938
Agrotis venerabilis|[9741]|LBCH7245-10|10-JDWBC-7245|658[0n]bp|Canada.British Columbia|BOLD:ABZ1938
Agrotis venerabilis|[9742]|LBCH7135-10|10-JDWBC-7135|658[0n]bp|Canada.British Columbia|BOLD:ABZ1938
Agrotis venerabilis|[9743]|LBCH7311-10|10-JDWBC-7311|658[0n]bp|Canada.British Columbia|BOLD:ABZ1938
Agrotis venerabilis|[9744]|LBCH7137-10|10-JDWBC-7137|658[0n]bp|Canada.British Columbia|BOLD:ABZ1938
Agrotis venerabilis|[9745]|LBCH6807-10|10-JDWBC-6807|658[0n]bp|Canada.British Columbia|BOLD:ABZ1938
Agrotis venerabilis|[9746]|LBCH6780-10|10-JDWBC-6780|658[0n]bp|Canada.British Columbia|BOLD:ABZ1938
Agrotis venerabilis|[9747]|LBCH7246-10|10-JDWBC-7246|658[0n]bp|Canada.British Columbia|BOLD:ABZ1938
Agrotis venerabilis|[9748]|LBCH7309-10|10-JDWBC-7309|658[0n]bp|Canada.British Columbia|BOLD:ABZ1938
Agrotis venerabilis|[9749]|LBCH7140-10|10-JDWBC-7140|658[0n]bp|Canada.British Columbia|BOLD:ABZ1938
Agrotis venerabilis|[9750]|LBCH6938-10|10-JDWBC-6938|658[0n]bp|Canada.British Columbia|BOLD:ABZ1938
Agrotis venerabilis|[9751]|LOWCD881-06|CGWC-3701|601[0n]bp|Canada.British Columbia|BOLD:ABZ1938
Agrotis venerabilis|[9752]|LBCH7244-10|10-JDWBC-7244|634[0n]bp|Canada.British Columbia|BOLD:ABZ1938
Agrotis venerabilis|[9753]|LOWC116-05|CGWC-0116|590[2n]bp|Canada.British Columbia|BOLD:ABZ1938
Agrotis venerabilis|[9754]|LBCH6803-10|10-JDWBC-6803|641[0n]bp|Canada.British Columbia|BOLD:ABZ1938
Agrotis venerabilis|[9755]|LBCH6802-10|10-JDWBC-6802|641[0n]bp|Canada.British Columbia|BOLD:ABZ1938
Agrotis venerabilis|[9756]|LBCH6806-10|10-JDWBC-6806|641[0n]bp|Canada.British Columbia|BOLD:ABZ1938
Agrotis venerabilis|[9757]|LBCH7308-10|10-JDWBC-7308|630[0n]bp|Canada.British Columbia|BOLD:ABZ1938
Agrotis venerabilis|[9758]|LOWC115-05|CGWC-0115|599[1n]bp|Canada.British Columbia|BOLD:ABZ1938
Agrotis venerabilis|[9759]|LOWCD878-06|CGWC-3698|574[1n]bp|Canada.British Columbia|BOLD:ABZ1938
Agrotis venerabilis|[9760]|BBLPB845-10|10BBCLP-1844|658[0n]bp|Canada.Saskatchewan|BOLD:ABZ1938
Agrotis venerabilis|[9761]|LBCH7136-10|10-JDWBC-7136|658[0n]bp|Canada.British Columbia|BOLD:ABZ1938
Agrotis venerabilis|[9762]|LOWCD877-06|CGWC-3697|658[1n]bp|Canada.British Columbia|BOLD:ABZ1938
Agrotis venerabilis|[9763]|LOWC112-05|CGWC-0112|609[0n]bp|Canada.British Columbia|BOLD:ABZ1938
Agrotis venerabilis|[9764]|LOWCD876-06|CGWC-3696|658[0n]bp|Canada.British Columbia|BOLD:ABZ1938
Agrotis venerabilis|[9765]|LOWCD880-06|CGWC-3700|658[0n]bp|Canada.British Columbia|BOLD:ABZ1938
Agrotis venerabilis|[9766]|LOWC105-05|CGWC-0105|658[0n]bp|Canada.British Columbia|BOLD:ABZ1938
Agrotis venerabilis|[9767]|LOWC107-05|CGWC-0107|658[0n]bp|Canada.British Columbia|BOLD:ABZ1938
Agrotis venerabilis|[9768]|LOWC110-05|CGWC-0110|590[2n]bp|Canada.British Columbia|BOLD:ABZ1938

[illegible]
Agrotis venerabilis|[9766]|LOWC105-05|CGWC-0105|658[0n]bp|Canada.British Columbia|BOLD:ABZ1938
Agrotis venerabilis|[9767]|LOWC107-05|CGWC-0107|658[0n]bp|Canada.British Columbia|BOLD:ABZ1938
Agrotis venerabilis|[9768]|LOWC110-05|CGWC-0110|590[2n]bp|Canada.British Columbia|BOLD:ABZ1938
Agrotis venerabilis|[9769]|LOWC113-05|CGWC-0113|658[1n]bp|Canada.British Columbia|BOLD:ABZ1938
Agrotis daedalus|[9770]|RDNMF041-08|NOC14127|658[0n]bp|Canada.Alberta|BOLD:AAF1591
Agrotis rileyana|[9771]|RDNMG879-08|CNC LEP00053003|658[0n]bp|Canada.Alberta|BOLD:ACF3230
Agrotis rileyana|[9772]|RDNMG880-08|CNC LEP00053004|658[0n]bp|Canada.Alberta|BOLD:ACF3230
Agrotis rileyana|[9773]|RDMAB594-06|UASM58615|658[0n]bp|Canada.Alberta|BOLD:ACF3230
Agrotis rileyana|[9774]|RDMAB306-05|UASM77794|658[0n]bp|Canada.Alberta|BOLD:ACF3230
Agrotis orthogonia|[9775]|RDNMG401-08|CNC LEP00052225|658[0n]bp|Canada.Alberta|BOLD:ABZ7032
Agrotis orthogonia|[9776]|RDNMG402-08|CNC LEP00052226|658[0n]bp|Canada.Alberta|BOLD:ABZ7032
Agrotis robustior|[9777]|RDNMF330-08|NOC14416|658[0n]bp|Canada.Alberta|BOLD:ACF3665
Agrotis robustior|[9778]|RDNMF331-08|NOC14417|658[0n]bp|Canada.Alberta|BOLD:ACF3665
Agrotis robustior|[9779]|RDNMF203-08|NOC14289|658[0n]bp|Canada.Alberta|BOLD:ACF3665
Agrotis robustior|[9780]|RDNMF202-08|NOC14288|658[0n]bp|Canada.Alberta|BOLD:ACF3665
Agrotis ruta|[9781]|LCHQ631-08|07WNP-10523|658[0n]bp|Canada.Manitoba|BOLD:AAA1629
Agrotis ruta|[9782]|LCHQ622-08|07WNP-10514|658[0n]bp|Canada.Manitoba|BOLD:AAA1629
Agrotis ruta|[9783]|LCHQ562-08|07WNP-10454|658[0n]bp|Canada.Manitoba|BOLD:AAA1629
Agrotis ruta|[9784]|LCHQ325-08|07WNP-10217|658[0n]bp|Canada.Manitoba|BOLD:AAA1629
Agrotis ruta|[9785]|LCHP762-07|07PROBE-10447|658[2n]bp|Canada.Manitoba|BOLD:AAA1629
Agrotis ruta|[9786]|LCHQ423-08|07WNP-10315|616[0n]bp|Canada.Manitoba|BOLD:AAA1629
Agrotis ruta|[9787]|LCHQ221-08|07WNP-10113|658[0n]bp|Canada.Manitoba|BOLD:AAA1629
Agrotis ruta|[9788]|LCHIP095-07|06-PROBE-2576|650[0n]bp|Canada.Manitoba|BOLD:AAA1629
Agrotis ruta|[9789]|LCHQ416-08|07WNP-10308|658[1n]bp|Canada.Manitoba|BOLD:AAA1629
Agrotis ruta|[9790]|LCHQ540-08|07WNP-10432|656[1n]bp|Canada.Manitoba|BOLD:AAA1629
Agrotis ruta|[9791]|LCHQ813-08|07WNP-10705|658[0n]bp|Canada.Manitoba|BOLD:AAA1629
Agrotis ruta|[9792]|LCHQ817-08|07WNP-10709|632[0n]bp|Canada.Manitoba|BOLD:AAA1629
Agrotis ruta|[9793]|LCHQ654-08|07WNP-10546|658[0n]bp|Canada.Manitoba|BOLD:AAA1629
Agrotis ruta|[9794]|LCHQ514-08|07WNP-10406|632[0n]bp|Canada.Manitoba|BOLD:AAA1629
Agrotis ruta|[9795]|LCHQ519-08|07WNP-10411|658[0n]bp|Canada.Manitoba|BOLD:AAA1629
Agrotis ruta|[9796]|LCHQ332-08|07WNP-10224|656[0n]bp|Canada.Manitoba|BOLD:AAA1629
Agrotis ruta|[9797]|LCHIP024-07|06-PROBE-0044|650[0n]bp|Canada.Manitoba|BOLD:AAA1629
Agrotis ruta|[9798]|LCHQ339-08|07WNP-10231|658[0n]bp|Canada.Manitoba|BOLD:AAA1629
Agrotis ruta|[9799]|LCHQ425-08|07WNP-10317|658[0n]bp|Canada.Manitoba|BOLD:AAA1629
Agrotis ruta|[9800]|LCHQ812-08|07WNP-10704|658[0n]bp|Canada.Manitoba|BOLD:AAA1629
Agrotis ruta|[9801]|LCHQ419-08|07WNP-10311|658[0n]bp|Canada.Manitoba|BOLD:AAA1629
Agrotis ruta|[9802]|LCHQ502-08|07WNP-10394|658[0n]bp|Canada.Manitoba|BOLD:AAA1629
Agrotis ruta|[9803]|LCHQ576-08|07WNP-10468|657[0n]bp|Canada.Manitoba|BOLD:AAA1629
Agrotis ruta|[9804]|LCHQ627-08|07WNP-10519|658[0n]bp|Canada.Manitoba|BOLD:AAA1629
Agrotis ruta|[9805]|LCHQ676-08|07WNP-10568|657[0n]bp|Canada.Manitoba|BOLD:AAA1629
Agrotis ruta|[9806]|LCHQ677-08|07WNP-10569|658[0n]bp|Canada.Manitoba|BOLD:AAA1629
Agrotis ruta|[9807]|LCHQ807-08|07WNP-10699|658[0n]bp|Canada.Manitoba|BOLD:AAA1629
Agrotis ruta|[9808]|LCHQ266-08|07WNP-10158|658[0n]bp|Canada.Manitoba|BOLD:AAA1629
Agrotis ruta|[9809]|LCHQ349-08|07WNP-10241|658[0n]bp|Canada.Manitoba|BOLD:AAA1629
Agrotis ruta|[9810]|LCHQ210-08|07WNP-10102|658[0n]bp|Canada.Manitoba|BOLD:AAA1629
Agrotis ruta|[9811]|LCHQ249-08|07WNP-10141|658[0n]bp|Canada.Manitoba|BOLD:AAA1629
Agrotis ruta|[9812]|LCHQ237-08|07WNP-10129|658[0n]bp|Canada.Manitoba|BOLD:AAA1629
Agrotis ruta|[9813]|LCHQ264-08|07WNP-10156|658[0n]bp|Canada.Manitoba|BOLD:AAA1629
Agrotis ruta|[9814]|LCHQ329-08|07WNP-10221|658[0n]bp|Canada.Manitoba|BOLD:AAA1629
Agrotis ruta|[9815]|LCHQ637-08|07WNP-10529|658[0n]bp|Canada.Manitoba|BOLD:AAA1629
Agrotis ruta|[9816]|LCHQ574-08|07WNP-10466|658[0n]bp|Canada.Manitoba|BOLD:AAA1629
Agrotis ruta|[9817]|LCHQ550-08|07WNP-10442|658[0n]bp|Canada.Manitoba|BOLD:AAA1629
Agrotis ruta|[9818]|LCHQ601-08|07WNP-10493|658[0n]bp|Canada.Manitoba|BOLD:AAA1629
Agrotis ruta|[9819]|LCHQ247-08|07WNP-10139|658[0n]bp|Canada.Manitoba|BOLD:AAA1629
Agrotis ruta|[9820]|LCHQ682-08|07WNP-10574|658[0n]bp|Canada.Manitoba|BOLD:AAA1629
Agrotis ruta|[9821]|LCHQ250-08|07WNP-10142|658[0n]bp|Canada.Manitoba|BOLD:AAA1629
Agrotis ruta|[9822]|LCHQ672-08|07WNP-10564|658[0n]bp|Canada.Manitoba|BOLD:AAA1629
Agrotis ruta|[9823]|LCHQ675-08|07WNP-10567|658[0n]bp|Canada.Manitoba|BOLD:AAA1629
Agrotis ruta|[9824]|LCHQ680-08|07WNP-10572|658[0n]bp|Canada.Manitoba|BOLD:AAA1629
Agrotis ruta|[9825]|LCHQ468-08|07WNP-10360|657[0n]bp|Canada.Manitoba|BOLD:AAA1629
Agrotis ruta|[9826]|LCHQ624-08|07WNP-10516|658[0n]bp|Canada.Manitoba|BOLD:AAA1629
Agrotis ruta|[9827]|LCHQ638-08|07WNP-10530|657[0n]bp|Canada.Manitoba|BOLD:AAA1629
Agrotis ruta|[9828]|LCHQ635-08|07WNP-10527|658[0n]bp|Canada.Manitoba|BOLD:AAA1629
Agrotis ruta|[9829]|LCHQ239-08|07WNP-10131|658[0n]bp|Canada.Manitoba|BOLD:AAA1629
Agrotis ruta|[9830]|LCHQ208-08|07WNP-10100|658[0n]bp|Canada.Manitoba|BOLD:AAA1629
Agrotis ruta|[9831]|LCHQ640-08|07WNP-10532|658[0n]bp|Canada.Manitoba|BOLD:AAA1629
Agrotis ruta|[9832]|LCHQ649-08|07WNP-10541|658[0n]bp|Canada.Manitoba|BOLD:AAA1629
Agrotis ruta|[9833]|MHLEP089-07|CHU06-LEP-089|658[0n]bp|Canada.Manitoba|BOLD:AAA1629
Agrotis ruta|[9834]|LCHQ334-08|07WNP-10226|658[0n]bp|Canada.Manitoba|BOLD:AAA1629
Agrotis ruta|[9835]|LCHQ333-08|07WNP-10225|658[0n]bp|Canada.Manitoba|BOLD:AAA1629
Agrotis ruta|[9836]|LCHQ352-08|07WNP-10244|658[0n]bp|Canada.Manitoba|BOLD:AAA1629
Agrotis ruta|[9837]|LCHQ664-08|07WNP-10556|658[0n]bp|Canada.Manitoba|BOLD:AAA1629
Agrotis ruta|[9838]|LCHQ472-08|07WNP-10364|658[0n]bp|Canada.Manitoba|BOLD:AAA1629
Agrotis ruta|[9839]|LCHQ342-08|07WNP-10234|658[0n]bp|Canada.Manitoba|BOLD:AAA1629
Agrotis ruta|[9840]|LCHQ560-08|07WNP-10452|658[0n]bp|Canada.Manitoba|BOLD:AAA1629
Agrotis ruta|[9841]|LCHQ623-08|07WNP-10515|658[0n]bp|Canada.Manitoba|BOLD:AAA1629
Agrotis ruta|[9842]|LCHQ216-08|07WNP-10108|658[0n]bp|Canada.Manitoba|BOLD:AAA1629
Agrotis ruta|[9843]|LCHQ330-08|07WNP-10222|658[0n]bp|Canada.Manitoba|BOLD:AAA1629
Agrotis ruta|[9844]|LCHQ393-08|07WNP-10285|658[0n]bp|Canada.Manitoba|BOLD:AAA1629
Agrotis ruta|[9845]|LCHQ431-08|07WNP-10323|658[0n]bp|Canada.Manitoba|BOLD:AAA1629
Agrotis ruta|[9846]|LCHQ219-08|07WNP-10111|658[0n]bp|Canada.Manitoba|BOLD:AAA1629
Agrotis ruta|[9847]|LCHQ630-08|07WNP-10522|658[0n]bp|Canada.Manitoba|BOLD:AAA1629
Agrotis ruta|[9848]|LCHQ558-08|07WNP-10450|658[0n]bp|Canada.Manitoba|BOLD:AAA1629
Agrotis ruta|[9849]|LCHQ327-08|07WNP-10219|658[0n]bp|Canada.Manitoba|BOLD:AAA1629
Agrotis ruta|[9850]|LCHQ642-08|07WNP-10534|658[0n]bp|Canada.Manitoba|BOLD:AAA1629
Agrotis ruta|[9851]|LCHQ343-08|07WNP-10235|658[0n]bp|Canada.Manitoba|BOLD:AAA1629
Agrotis ruta|[9852]|LCHQ251-08|07WNP-10143|658[0n]bp|Canada.Manitoba|BOLD:AAA1629
Agrotis ruta|[9853]|LCHQ699-08|07WNP-10591|658[0n]bp|Canada.Manitoba|BOLD:AAA1629
Agrotis ruta|[9854]|LCHQ541-08|07WNP-10433|658[0n]bp|Canada.Manitoba|BOLD:AAA1629
Agrotis ruta|[9855]|LCHQ815-08|07WNP-10707|658[0n]bp|Canada.Manitoba|BOLD:AAA1629
Agrotis ruta|[9856]|LCHQ614-08|07WNP-10506|658[0n]bp|Canada.Manitoba|BOLD:AAA1629
Agrotis ruta|[9857]|LCHQ655-08|07WNP-10547|658[0n]bp|Canada.Manitoba|BOLD:AAA1629
Agrotis ruta|[9858]|LCHQ466-08|07WNP-10358|658[0n]bp|Canada.Manitoba|BOLD:AAA1629
Agrotis ruta|[9859]|LCHQ241-08|07WNP-10133|658[0n]bp|Canada.Manitoba|BOLD:AAA1629
Agrotis ruta|[9860]|LCHQ695-08|07WNP-10587|658[0n]bp|Canada.Manitoba|BOLD:AAA1629
Agrotis ruta|[9861]|LCHQ570-08|07WNP-10462|657[0n]bp|Canada.Manitoba|BOLD:AAA1629
Agrotis ruta|[9862]|LCHQ673-08|07WNP-10565|657[0n]bp|Canada.Manitoba|BOLD:AAA1629
Agrotis ruta|[9863]|LCHQ543-08|07WNP-10435|658[0n]bp|Canada.Manitoba|BOLD:AAA1629
Agrotis ruta|[9864]|LCHQ222-08|07WNP-10114|658[0n]bp|Canada.Manitoba|BOLD:AAA1629
Agrotis ruta|[9865]|LCHQ689-08|07WNP-10581|658[0n]bp|Canada.Manitoba|BOLD:AAA1629
Agrotis ruta|[9866]|LCHQ615-08|07WNP-10507|658[0n]bp|Canada.Manitoba|BOLD:AAA1629
Agrotis ruta|[9867]|LCHQ669-08|07WNP-10561|657[0n]bp|Canada.Manitoba|BOLD:AAA1629
[illegible]

Agrotis ruta|[9865]|LCHQ689-08|07WNP-10581|658[0n]bp|Canada.Manitoba|BOLD:AAA1629
Agrotis ruta|[9866]|LCHQ615-08|07WNP-10507|658[0n]bp|Canada.Manitoba|BOLD:AAA1629
Agrotis ruta|[9867]|LCHQ669-08|07WNP-10561|657[0n]bp|Canada.Manitoba|BOLD:AAA1629
Agrotis ruta|[9868]|LCHQ394-08|07WNP-10286|658[0n]bp|Canada.Manitoba|BOLD:AAA1629
Agrotis ruta|[9869]|LCHQ270-08|07WNP-10162|658[0n]bp|Canada.Manitoba|BOLD:AAA1629
Agrotis ruta|[9870]|LCHQ212-08|07WNP-10104|658[0n]bp|Canada.Manitoba|BOLD:AAA1629
Agrotis ruta|[9871]|LCHQ392-08|07WNP-10284|658[0n]bp|Canada.Manitoba|BOLD:AAA1629
Agrotis ruta|[9872]|LCHQ220-08|07WNP-10112|658[0n]bp|Canada.Manitoba|BOLD:AAA1629
Agrotis ruta|[9873]|LCHQ579-08|07WNP-10471|658[0n]bp|Canada.Manitoba|BOLD:AAA1629
Agrotis ruta|[9874]|LCHQ501-08|07WNP-10393|658[0n]bp|Canada.Manitoba|BOLD:AAA1629
Agrotis ruta|[9875]|LCHQ679-08|07WNP-10571|658[0n]bp|Canada.Manitoba|BOLD:AAA1629
Agrotis ruta|[9876]|LCHQ548-08|07WNP-10440|658[0n]bp|Canada.Manitoba|BOLD:AAA1629
Agrotis ruta|[9877]|LCHQ666-08|07WNP-10558|658[0n]bp|Canada.Manitoba|BOLD:AAA1629
Agrotis ruta|[9878]|LCHQ258-08|07WNP-10150|658[0n]bp|Canada.Manitoba|BOLD:AAA1629
Agrotis ruta|[9879]|LCHQ692-08|07WNP-10584|658[0n]bp|Canada.Manitoba|BOLD:AAA1629
Agrotis ruta|[9880]|LCHQ521-08|07WNP-10413|658[0n]bp|Canada.Manitoba|BOLD:AAA1629
Agrotis ruta|[9881]|LCHQ549-08|07WNP-10441|658[0n]bp|Canada.Manitoba|BOLD:AAA1629
Agrotis ruta|[9882]|LCHQ382-08|07WNP-10274|658[0n]bp|Canada.Manitoba|BOLD:AAA1629
Agrotis ruta|[9883]|LCHQ340-08|07WNP-10232|658[0n]bp|Canada.Manitoba|BOLD:AAA1629
Agrotis ruta|[9884]|LCHQ246-08|07WNP-10138|658[0n]bp|Canada.Manitoba|BOLD:AAA1629
Agrotis ruta|[9885]|LCHQ633-08|07WNP-10525|658[0n]bp|Canada.Manitoba|BOLD:AAA1629
Agrotis ruta|[9886]|LCHQ257-08|07WNP-10149|658[0n]bp|Canada.Manitoba|BOLD:AAA1629
Agrotis ruta|[9887]|LCHQ657-08|07WNP-10549|658[0n]bp|Canada.Manitoba|BOLD:AAA1629
Agrotis ruta|[9888]|LCHQ575-08|07WNP-10467|658[0n]bp|Canada.Manitoba|BOLD:AAA1629
Agrotis ruta|[9889]|LCHQ613-08|07WNP-10505|658[0n]bp|Canada.Manitoba|BOLD:AAA1629
Agrotis ruta|[9890]|LCHQ500-08|07WNP-10392|658[0n]bp|Canada.Manitoba|BOLD:AAA1629
Agrotis ruta|[9891]|LCHQ545-08|07WNP-10437|658[0n]bp|Canada.Manitoba|BOLD:AAA1629
Agrotis ruta|[9892]|LCHQ561-08|07WNP-10453|657[0n]bp|Canada.Manitoba|BOLD:AAA1629
Agrotis ruta|[9893]|LCHQ252-08|07WNP-10144|658[0n]bp|Canada.Manitoba|BOLD:AAA1629
Agrotis ruta|[9894]|LCHQ345-08|07WNP-10237|658[0n]bp|Canada.Manitoba|BOLD:AAA1629
Agrotis ruta|[9895]|LCHQ663-08|07WNP-10555|658[0n]bp|Canada.Manitoba|BOLD:AAA1629
Agrotis ruta|[9896]|LCHQ240-08|07WNP-10132|658[0n]bp|Canada.Manitoba|BOLD:AAA1629
Agrotis ruta|[9897]|LCHQ691-08|07WNP-10583|658[0n]bp|Canada.Manitoba|BOLD:AAA1629
Agrotis ruta|[9898]|LCHQ244-08|07WNP-10136|658[0n]bp|Canada.Manitoba|BOLD:AAA1629
Agrotis ruta|[9899]|LCHQ518-08|07WNP-10410|658[0n]bp|Canada.Manitoba|BOLD:AAA1629
Agrotis ruta|[9900]|LCHQ571-08|07WNP-10463|658[0n]bp|Canada.Manitoba|BOLD:AAA1629
Agrotis ruta|[9901]|LCHQ811-08|07WNP-10703|658[0n]bp|Canada.Manitoba|BOLD:AAA1629
Agrotis ruta|[9902]|LCHQ267-08|07WNP-10159|658[0n]bp|Canada.Manitoba|BOLD:AAA1629
Agrotis ruta|[9903]|LCHQ641-08|07WNP-10533|658[0n]bp|Canada.Manitoba|BOLD:AAA1629
Agrotis ruta|[9904]|LCHQ223-08|07WNP-10115|658[0n]bp|Canada.Manitoba|BOLD:AAA1629
Agrotis ruta|[9905]|LCHQ620-08|07WNP-10512|657[0n]bp|Canada.Manitoba|BOLD:AAA1629
Agrotis ruta|[9906]|LCHQ322-08|07WNP-10214|658[0n]bp|Canada.Manitoba|BOLD:AAA1629
Agrotis ruta|[9907]|LCHQ693-08|07WNP-10585|658[0n]bp|Canada.Manitoba|BOLD:AAA1629
Agrotis ruta|[9908]|LCHQ261-08|07WNP-10153|658[0n]bp|Canada.Manitoba|BOLD:AAA1629
Agrotis ruta|[9909]|LCHQ467-08|07WNP-10359|658[0n]bp|Canada.Manitoba|BOLD:AAA1629
Agrotis ruta|[9910]|LCHQ516-08|07WNP-10408|658[0n]bp|Canada.Manitoba|BOLD:AAA1629
Agrotis ruta|[9911]|LCHQ338-08|07WNP-10230|658[0n]bp|Canada.Manitoba|BOLD:AAA1629
Agrotis ruta|[9912]|LCHQ651-08|07WNP-10543|658[0n]bp|Canada.Manitoba|BOLD:AAA1629
Agrotis ruta|[9913]|LCHQ331-08|07WNP-10223|658[0n]bp|Canada.Manitoba|BOLD:AAA1629
Agrotis ruta|[9914]|LCHQ619-08|07WNP-10511|658[0n]bp|Canada.Manitoba|BOLD:AAA1629
Agrotis ruta|[9915]|LCHQ697-08|07WNP-10589|658[0n]bp|Canada.Manitoba|BOLD:AAA1629
Agrotis ruta|[9916]|LCHQ211-08|07WNP-10103|658[0n]bp|Canada.Manitoba|BOLD:AAA1629
Agrotis ruta|[9917]|LCHQ424-08|07WNP-10316|658[0n]bp|Canada.Manitoba|BOLD:AAA1629
Agrotis ruta|[9918]|LCHQ809-08|07WNP-10701|658[0n]bp|Canada.Manitoba|BOLD:AAA1629
Agrotis ruta|[9919]|LCHQ259-08|07WNP-10151|658[0n]bp|Canada.Manitoba|BOLD:AAA1629
Agrotis ruta|[9920]|LCHQ629-08|07WNP-10521|658[0n]bp|Canada.Manitoba|BOLD:AAA1629
Agrotis ruta|[9921]|LCHQ688-08|07WNP-10580|658[0n]bp|Canada.Manitoba|BOLD:AAA1629
Agrotis ruta|[9922]|LCHQ662-08|07WNP-10554|658[0n]bp|Canada.Manitoba|BOLD:AAA1629
Agrotis ruta|[9923]|LCHQ600-08|07WNP-10492|658[0n]bp|Canada.Manitoba|BOLD:AAA1629
Agrotis ruta|[9924]|LCHQ215-08|07WNP-10107|658[0n]bp|Canada.Manitoba|BOLD:AAA1629
Agrotis ruta|[9925]|LCHQ587-08|07WNP-10479|658[0n]bp|Canada.Manitoba|BOLD:AAA1629
Agrotis ruta|[9926]|LCHP747-07|07PROBE-10432|655[0n]bp|Canada.Manitoba|BOLD:AAA1629
Agrotis ruta|[9927]|LCHQ469-08|07WNP-10361|658[0n]bp|Canada.Manitoba|BOLD:AAA1629
Agrotis ruta|[9928]|LCHQ551-08|07WNP-10443|658[0n]bp|Canada.Manitoba|BOLD:AAA1629
Agrotis ruta|[9929]|LCHQ391-08|07WNP-10283|658[0n]bp|Canada.Manitoba|BOLD:AAA1629
Agrotis ruta|[9930]|LCHQ256-08|07WNP-10148|658[0n]bp|Canada.Manitoba|BOLD:AAA1629
Agrotis ruta|[9931]|LCHQ248-08|07WNP-10140|658[0n]bp|Canada.Manitoba|BOLD:AAA1629
Agrotis ruta|[9932]|LCHQ242-08|07WNP-10134|658[0n]bp|Canada.Manitoba|BOLD:AAA1629
Agrotis ruta|[9933]|LCHQ810-08|07WNP-10702|658[0n]bp|Canada.Manitoba|BOLD:AAA1629
Agrotis ruta|[9934]|LCHQ698-08|07WNP-10590|658[0n]bp|Canada.Manitoba|BOLD:AAA1629
Agrotis ruta|[9935]|LCHQ324-08|07WNP-10216|658[0n]bp|Canada.Manitoba|BOLD:AAA1629
Agrotis ruta|[9936]|LCHQ616-08|07WNP-10508|658[0n]bp|Canada.Manitoba|BOLD:AAA1629
Agrotis ruta|[9937]|LCHQ238-08|07WNP-10130|658[0n]bp|Canada.Manitoba|BOLD:AAA1629
Agrotis ruta|[9938]|LCHQ632-08|07WNP-10524|658[0n]bp|Canada.Manitoba|BOLD:AAA1629
Agrotis ruta|[9939]|LCHQ323-08|07WNP-10215|658[0n]bp|Canada.Manitoba|BOLD:AAA1629
Agrotis ruta|[9940]|LCHQ681-08|07WNP-10573|657[0n]bp|Canada.Manitoba|BOLD:AAA1629
Agrotis ruta|[9941]|LCHQ350-08|07WNP-10242|658[0n]bp|Canada.Manitoba|BOLD:AAA1629
Agrotis ruta|[9942]|MHLEP094-07|CHU06-LEP-094|658[0n]bp|Canada.Manitoba|BOLD:AAA1629
Agrotis ruta|[9943]|LCHQ346-08|07WNP-10238|658[0n]bp|Canada.Manitoba|BOLD:AAA1629
Agrotis ruta|[9944]|LCHQ553-08|07WNP-10445|658[0n]bp|Canada.Manitoba|BOLD:AAA1629
Agrotis ruta|[9945]|LCHQ599-08|07WNP-10491|658[0n]bp|Canada.Manitoba|BOLD:AAA1629
Agrotis ruta|[9946]|LCHQ674-08|07WNP-10566|658[0n]bp|Canada.Manitoba|BOLD:AAA1629
Agrotis ruta|[9947]|LCHQ581-08|07WNP-10473|658[0n]bp|Canada.Manitoba|BOLD:AAA1629
Agrotis ruta|[9948]|LCHQ243-08|07WNP-10135|658[0n]bp|Canada.Manitoba|BOLD:AAA1629
Agrotis ruta|[9949]|LCHQ335-08|07WNP-10227|658[0n]bp|Canada.Manitoba|BOLD:AAA1629
Agrotis ruta|[9950]|LCHQ209-08|07WNP-10101|657[0n]bp|Canada.Manitoba|BOLD:AAA1629
Agrotis ruta|[9951]|LCHQ351-08|07WNP-10243|658[0n]bp|Canada.Manitoba|BOLD:AAA1629
Agrotis ruta|[9952]|LCHQ218-08|07WNP-10110|658[0n]bp|Canada.Manitoba|BOLD:AAA1629
Agrotis ruta|[9953]|LCHQ696-08|07WNP-10588|657[0n]bp|Canada.Manitoba|BOLD:AAA1629
Agrotis ruta|[9954]|LCHQ639-08|07WNP-10531|658[0n]bp|Canada.Manitoba|BOLD:AAA1629
Agrotis ruta|[9955]|LCHQ336-08|07WNP-10228|658[0n]bp|Canada.Manitoba|BOLD:AAA1629
Agrotis ruta|[9956]|LCHQ265-08|07WNP-10157|658[0n]bp|Canada.Manitoba|BOLD:AAA1629
Agrotis ruta|[9957]|LCHQ254-08|07WNP-10146|658[0n]bp|Canada.Manitoba|BOLD:AAA1629
Agrotis ruta|[9958]|LCHQ326-08|07WNP-10218|658[0n]bp|Canada.Manitoba|BOLD:AAA1629
Agrotis ruta|[9959]|LCHQ260-08|07WNP-10152|658[0n]bp|Canada.Manitoba|BOLD:AAA1629
Agrotis ruta|[9960]|LCHQ690-08|07WNP-10582|658[0n]bp|Canada.Manitoba|BOLD:AAA1629
Agrotis ruta|[9961]|LCHQ421-08|07WNP-10313|657[0n]bp|Canada.Manitoba|BOLD:AAA1629
Agrotis ruta|[9962]|LCHQ544-08|07WNP-10436|658[0n]bp|Canada.Manitoba|BOLD:AAA1629
Agrotis ruta|[9963]|LCHQ522-08|07WNP-10414|658[0n]bp|Canada.Manitoba|BOLD:AAA1629
Agrotis ruta|[9964]|LCHQ353-08|07WNP-10245|658[0n]bp|Canada.Manitoba|BOLD:AAA1629
Agrotis ruta|[9965]|LCHQ307-08|07WNP-10199|657[0n]bp|Canada.Manitoba|BOLD:AAA1629
Agrotis ruta|[9966]|LCHQ337-08|07WNP-10229|658[0n]bp|Canada.Manitoba|BOLD:AAA1629
Agrotis ruta|[9967]|LCHQ686-08|07WNP-10578|658[0n]bp|Canada.Manitoba|BOLD:AAA1629

Agrotis ruta|[9965]|LCHQ307-08|07WNP-10199|657[0n]bp|Canada.Manitoba|BOLD:AAA1629
Agrotis ruta|[9966]|LCHQ337-08|07WNP-10229|658[0n]bp|Canada.Manitoba|BOLD:AAA1629
Agrotis ruta|[9967]|LCHQ686-08|07WNP-10578|658[0n]bp|Canada.Manitoba|BOLD:AAA1629
Agrotis ruta|[9968]|LCHQ347-08|07WNP-10239|658[0n]bp|Canada.Manitoba|BOLD:AAA1629
Agrotis ruta|[9969]|LCHQ515-08|07WNP-10407|658[0n]bp|Canada.Manitoba|BOLD:AAA1629
Agrotis ruta|[9970]|LCHQ569-08|07WNP-10461|658[0n]bp|Canada.Manitoba|BOLD:AAA1629
Agrotis ruta|[9971]|LCHQ563-08|07WNP-10455|658[0n]bp|Canada.Manitoba|BOLD:AAA1629
Agrotis ruta|[9972]|LCHQ268-08|07WNP-10160|658[0n]bp|Canada.Manitoba|BOLD:AAA1629
Agrotis ruta|[9973]|LCHQ255-08|07WNP-10147|658[0n]bp|Canada.Manitoba|BOLD:AAA1629
Agrotis ruta|[9974]|LCHQ213-08|07WNP-10105|658[0n]bp|Canada.Manitoba|BOLD:AAA1629
Agrotis ruta|[9975]|LCHQ253-08|07WNP-10145|658[0n]bp|Canada.Manitoba|BOLD:AAA1629
Agrotis ruta|[9976]|LCHQ687-08|07WNP-10579|658[0n]bp|Canada.Manitoba|BOLD:AAA1629
Agrotis ruta|[9977]|LCHQ816-08|07WNP-10708|658[0n]bp|Canada.Manitoba|BOLD:AAA1629
Agrotis ruta|[9978]|LCHQ224-08|07WNP-10116|658[0n]bp|Canada.Manitoba|BOLD:AAA1629
Agrotis ruta|[9979]|LCHQ214-08|07WNP-10106|658[0n]bp|Canada.Manitoba|BOLD:AAA1629
Agrotis ruta|[9980]|LCHQ628-08|07WNP-10520|658[0n]bp|Canada.Manitoba|BOLD:AAA1629
Agrotis ruta|[9981]|LCHQ217-08|07WNP-10109|658[0n]bp|Canada.Manitoba|BOLD:AAA1629
Agrotis ruta|[9982]|LCHQ618-08|07WNP-10510|658[0n]bp|Canada.Manitoba|BOLD:AAA1629
Agrotis ruta|[9983]|LCHQ803-08|07WNP-10695|645[0n]bp|Canada.Manitoba|BOLD:AAA1629
Agrotis ruta|[9984]|LCHQ328-08|07WNP-10220|658[0n]bp|Canada.Manitoba|BOLD:AAA1629
Agrotis ruta|[9985]|LCHQ321-08|07WNP-10213|658[0n]bp|Canada.Manitoba|BOLD:AAA1629
Agrotis ruta|[9986]|LCHQ473-08|07WNP-10365|655[0n]bp|Canada.Manitoba|BOLD:AAA1629
Agrotis ruta|[9987]|LCHQ650-08|07WNP-10542|655[0n]bp|Canada.Manitoba|BOLD:AAA1629
Agrotis ruta|[9988]|LCHQ585-08|07WNP-10477|655[0n]bp|Canada.Manitoba|BOLD:AAA1629
Agrotis ruta|[9989]|LCHQ665-08|07WNP-10557|656[0n]bp|Canada.Manitoba|BOLD:AAA1629
Agrotis ruta|[9990]|LCHQ626-08|07WNP-10518|656[0n]bp|Canada.Manitoba|BOLD:AAA1629
Agrotis ruta|[9991]|LCHQ685-08|07WNP-10577|656[0n]bp|Canada.Manitoba|BOLD:AAA1629
Agrotis ruta|[9992]|LCHQ656-08|07WNP-10548|655[0n]bp|Canada.Manitoba|BOLD:AAA1629
Agrotis ruta|[9993]|LCHIP128-07|06-PROBE-2659|650[0n]bp|Canada.Manitoba|BOLD:AAA1629
Agrotis ruta|[9994]|LCHIP112-07|06-PROBE-2609|650[0n]bp|Canada.Manitoba|BOLD:AAA1629
Agrotis ruta|[9995]|LCHQ420-08|07WNP-10312|621[0n]bp|Canada.Manitoba|BOLD:AAA1629
Agrotis ruta|[9996]|LCHQ418-08|07WNP-10310|632[0n]bp|Canada.Manitoba|BOLD:AAA1629
Agrotis ruta|[9997]|LCHQ667-08|07WNP-10559|632[0n]bp|Canada.Manitoba|BOLD:AAA1629
Agrotis ruta|[9998]|LCHIP096-07|06-PROBE-2577|639[0n]bp|Canada.Manitoba|BOLD:AAA1629
Agrotis ruta|[9999]|LCHQ414-08|07WNP-10306|635[0n]bp|Canada.Manitoba|BOLD:AAA1629
Agrotis ruta|[10000]|LCHQ263-08|07WNP-10155|658[0n]bp|Canada.Manitoba|BOLD:AAA1629
Agrotis ruta|[10001]|LCHQ598-08|07WNP-10490|658[0n]bp|Canada.Manitoba|BOLD:AAA1629
Agrotis ruta|[10002]|LCHIP097-07|06-PROBE-2578|650[0n]bp|Canada.Manitoba|BOLD:AAA1629
Agrotis ruta|[10003]|LCHQ814-08|07WNP-10706|648[0n]bp|Canada.Manitoba|BOLD:AAA1629
Agrotis ruta|[10004]|LCHQ805-08|07WNP-10697|646[0n]bp|Canada.Manitoba|BOLD:AAA1629
Agrotis ruta|[10005]|LCHQ643-08|07WNP-10535|643[0n]bp|Canada.Manitoba|BOLD:AAA1629
Agrotis ruta|[10006]|LCHQ415-08|07WNP-10307|649[0n]bp|Canada.Manitoba|BOLD:AAA1629
Agrotis ruta|[10007]|LCHQ808-08|07WNP-10700|656[0n]bp|Canada.Manitoba|BOLD:AAA1629
Agrotis ruta|[10008]|LCHQ617-08|07WNP-10509|658[0n]bp|Canada.Manitoba|BOLD:AAA1629
Agrotis ruta|[10009]|LCHQ578-08|07WNP-10470|658[0n]bp|Canada.Manitoba|BOLD:AAA1629
Agrotis ruta|[10010]|LCHQ684-08|07WNP-10576|657[0n]bp|Canada.Manitoba|BOLD:AAA1629
Agrotis ruta|[10011]|LCHQ582-08|07WNP-10474|658[0n]bp|Canada.Manitoba|BOLD:AAA1629
Agrotis ruta|[10012]|LCHQ671-08|07WNP-10563|658[0n]bp|Canada.Manitoba|BOLD:AAA1629
Agrotis ruta|[10013]|LCHQ636-08|07WNP-10528|658[0n]bp|Canada.Manitoba|BOLD:AAA1629
Agrotis ruta|[10014]|LCHQ341-08|07WNP-10233|656[0n]bp|Canada.Manitoba|BOLD:AAA1629
Agrotis ruta|[10015]|LCHQ577-08|07WNP-10469|658[0n]bp|Canada.Manitoba|BOLD:AAA1629
Agrotis ruta|[10016]|LCHQ348-08|07WNP-10240|658[0n]bp|Canada.Manitoba|BOLD:AAA1629
Agrotis ruta|[10017]|LCHQ644-08|07WNP-10536|658[0n]bp|Canada.Manitoba|BOLD:AAA1629
Agrotis ruta|[10018]|LCHQ694-08|07WNP-10586|658[0n]bp|Canada.Manitoba|BOLD:AAA1629
Agrotis ruta|[10019]|LCHQ647-08|07WNP-10539|658[0n]bp|Canada.Manitoba|BOLD:AAA1629
Agrotis ruta|[10020]|LCHQ344-08|07WNP-10236|658[0n]bp|Canada.Manitoba|BOLD:AAA1629
Agrotis ruta|[10021]|LCHQ426-08|07WNP-10318|658[0n]bp|Canada.Manitoba|BOLD:AAA1629
Agrotis vetusta|[10022]|LOWC103-05|CGWC-0103|658[0n]bp|Canada.British Columbia|BOLD:ACF0067
Agrotis vetusta|[10023]|BBLPB318-10|10BBCLP-1317|658[0n]bp|Canada.Saskatchewan|BOLD:ACF0067
Agrotis vetusta|[10024]|RDLQB603-05|DH010706|658[0n]bp|Canada.Quebec|BOLD:ACF0067
Agrotis vetusta|[10025]|BBLPB317-10|10BBCLP-1316|622[0n]bp|Canada.Alberta|BOLD:ACF0067
Agrotis vetusta|[10026]|BBLEC530-09|09BBELE-0530|658[0n]bp|Canada.New Brunswick|BOLD:ACF0067
Agrotis vetusta|[10027]|RDLQF276-06|DH011368|658[0n]bp|Canada.Quebec|BOLD:ACF0067
Agrotis vetusta|[10028]|RDLQB602-05|DH010705|658[0n]bp|Canada.Quebec|BOLD:ACF0067
Agrotis vetusta|[10029]|BBLEC489-09|09BBELE-0489|658[0n]bp|Canada.New Brunswick|BOLD:ACF0067
Agrotis antica|[10030]|LBCG378-08|08-JDWBC-0378|658[0n]bp|Canada.British Columbia|BOLD:ACE9952
Agrotis obliqua|[10031]|LBCG3030-09|08-JDWBC-3030|658[0n]bp|Canada.British Columbia|BOLD:ACE9952
Agrotis obliqua|[10032]|RDNME438-08|LEP037862|658[0n]bp|Canada.Alberta|BOLD:ACE9952
Agrotis obliqua|[10033]|LPABB373-08|08BBLEP-03638|658[0n]bp|Canada.Alberta|BOLD:ACE9952
Agrotis obliqua|[10034]|RDNME439-08|LEP037863|658[0n]bp|Canada.Alberta|BOLD:ACE9952
Agrotis obliqua|[10035]|BBLPB612-10|10BBCLP-1611|658[0n]bp|Canada.British Columbia|BOLD:ACE9952
Agrotis obliqua|[10036]|LBCD053-05|HLC-22873|658[0n]bp|Canada.British Columbia|BOLD:ACE9952
Agrotis obliqua|[10037]|BBLPB719-10|10BBCLP-1718|658[0n]bp|Canada.Alberta|BOLD:ACE9952
Agrotis obliqua|[10038]|BBLPB844-10|10BBCLP-1843|658[0n]bp|Canada.Alberta|BOLD:ACE9952
Agrotis sp.|[10039]|LBCH5207-10|10-JDWBC-5207|658[0n]bp|Canada.British Columbia|BOLD:ABZ0371
Agrotis sp.|[10040]|LBCH5278-10|10-JDWBC-5278|658[0n]bp|Canada.British Columbia|BOLD:ABZ0371
Agrotis gravis|[10041]|RDNMG405-08|CNC LEP00052229|658[0n]bp|Canada.British Columbia|BOLD:AAE4279
Agrotis obliqua|[10042]|LCH258-04|04HBL003258|658[0n]bp|Canada.Manitoba|BOLD:ABZ7027
Agrotis obliqua|[10043]|RDMAB1000-09|UASM106934|658[0n]bp|Canada.Alberta|BOLD:ABZ7027
Agrotis obliqua|[10044]|RDMAB355-05|UASM41684|538[1n]bp|Canada.Alberta|BOLD:ABZ7027
Agrotis obliqua|[10045]|RDMAB352-05|UASM56943|604[0n]bp|Canada.Alberta|BOLD:ABZ7027
Agrotis obliqua|[10046]|RDMAB349-05|UASM57445|603[0n]bp|Canada.Alberta|BOLD:ABZ7027
Agrotis obliqua|[10047]|RDNMF200-08|NOC14286|658[0n]bp|Canada.Alberta|BOLD:ABZ7027
Agrotis obliqua|[10048]|RDMAB1001-09|UASM106935|658[0n]bp|Canada.Alberta|BOLD:ABZ7027
Agrotis obliqua|[10049]|RDLQG394-06|DH012662|658[0n]bp|Canada.Quebec|BOLD:ABZ7027
Agrotis obliqua|[10050]|RDLQ685-07|DH005366|591[0n]bp|Canada.Quebec|BOLD:ABZ7027
Agrotis obliqua|[10051]|RDLQG393-06|DH012661|658[0n]bp|Canada.Quebec|BOLD:ABZ7027
Agrotis obliqua|[10052]|LPSOD392-09|08BBLEP-00171|658[0n]bp|Canada.Ontario|BOLD:ABZ7027
Agrotis obliqua|[10053]|RDLQG395-06|DH012663|658[0n]bp|Canada.Quebec|BOLD:ABZ7027
Agrotis arenarius|[10054]|RDNMF077-08|NOC14163|618[1n]bp|Canada.Nova Scotia|BOLD:ACF2766
Agrotis arenarius|[10055]|RDNMG400-08|CNC LEP00052224|640[1n]bp|Canada.Nova Scotia|BOLD:ACF2766
Agrotis arenarius|[10056]|RDNMG399-08|CNC LEP00052223|640[0n]bp|Canada.Nova Scotia|BOLD:ACF2766
Agrotis stigmosa|[10057]|RDMAB999-09|UASM106929|639[0n]bp|Canada.Alberta|BOLD:ABZ7028
Agrotis vancouverensis|[10058]|LOWCD919-06|CGWC-3739|575[0n]bp|Canada.British Columbia|BOLD:ABZ7030
Agrotis vancouverensis|[10059]|LHLEP381-06|UBC-2006-0763|582[0n]bp|Canada.British Columbia|BOLD:ABZ7030
Agrotis vancouverensis|[10060]|RDMAB348-05|UASM56946|578[0n]bp|Canada.Alberta|BOLD:ABZ7030
Agrotis volubilis|[10061]|XAE217-04|Moth4217.03|617[0n]bp|Canada.Ontario|BOLD:ABZ7030
Agrotis vancouverensis|[10062]|LMH006-06|PFC-2006-0008|658[1n]bp|Canada.British Columbia|BOLD:ABZ7030
Agrotis vancouverensis|[10063]|LBCG037-08|08-JDWBC-0037|658[0n]bp|Canada.British Columbia|BOLD:ABZ7030
Agrotis vancouverensis|[10064]|LPABB403-08|08BBLEP-03668|658[0n]bp|Canada.Alberta|BOLD:ABZ7030
Agrotis vancouverensis|[10065]|LPVIA095-08|PFC-2006-0154|658[0n]bp|Canada.British Columbia|BOLD:ABZ7030
Agrotis vancouverensis|[10066]|LHLEP398-06|UBC-2006-0203|658[0n]bp|Canada.British Columbia|BOLD:ABZ7030
Agrotis vancouverensis|[10067]|LHLEP396-06|UBC-2006-0201|658[0n]bp|Canada.British Columbia|BOLD:ABZ7030

[illegible]
Agrotis vancouverensis|[10065]|LPVIA095-08|PFC-2006-0154|658[0n]bp|Canada.British Columbia|BOLD:ABZ7030
Agrotis vancouverensis|[10066]|LHLEP398-06|UBC-2006-0203|658[0n]bp|Canada.British Columbia|BOLD:ABZ7030
Agrotis vancouverensis|[10067]|LHLEP396-06|UBC-2006-0201|658[0n]bp|Canada.British Columbia|BOLD:ABZ7030
Agrotis vancouverensis|[10068]|RDMAB105-05|UASM41989|658[0n]bp|Canada.Alberta|BOLD:ABZ7030
Agrotis vancouverensis|[10069]|LHLEP380-06|UBC-2006-0762|658[0n]bp|Canada.British Columbia|BOLD:ABZ7030
Agrotis vancouverensis|[10070]|LPVIA096-08|PFC-2006-0155|658[0n]bp|Canada.British Columbia|BOLD:ABZ7030
Agrotis vancouverensis|[10071]|LPVIA097-08|PFC-2006-0156|658[0n]bp|Canada.British Columbia|BOLD:ABZ7030
Agrotis vancouverensis|[10072]|LPVIC024-08|PFC-2006-2579|658[0n]bp|Canada.British Columbia|BOLD:ABZ7030
Agrotis vancouverensis|[10073]|LHLEP400-06|UBC-2006-0760|658[0n]bp|Canada.British Columbia|BOLD:ABZ7030
Agrotis vancouverensis|[10074]|LPAB014-08|08BBLEP-02336|658[0n]bp|Canada.Alberta|BOLD:ABZ7030
Agrotis vancouverensis|[10075]|LHLEP410-06|UBC-2006-0198|658[0n]bp|Canada.British Columbia|BOLD:ABZ7030
Agrotis vancouverensis|[10076]|BBLPB851-10|10BBCLP-1850|658[0n]bp|Canada.British Columbia|BOLD:ABZ7030
Agrotis vancouverensis|[10077]|BBLPB716-10|10BBCLP-1715|658[0n]bp|Canada.Alberta|BOLD:ABZ7030
Agrotis vancouverensis|[10078]|RDNM726-05|CNCNoctuoidea7572|658[0n]bp|Canada.Alberta|BOLD:ABZ7030
Agrotis vancouverensis|[10079]|LHLEP395-06|UBC-2006-0200|658[0n]bp|Canada.British Columbia|BOLD:ABZ7030
Agrotis vancouverensis|[10080]|LOWCD918-06|CGWC-3738|658[0n]bp|Canada.British Columbia|BOLD:ABZ7030
Agrotis vancouverensis|[10081]|RDMAB350-05|UASM41962|658[0n]bp|Canada.Alberta|BOLD:ABZ7030
Agrotis vancouverensis|[10082]|RDMAB102-05|UASM41984|658[0n]bp|Canada.Alberta|BOLD:ABZ7030
Agrotis vancouverensis|[10083]|LHLEP406-06|UBC-2006-0199|658[0n]bp|Canada.British Columbia|BOLD:ABZ7030
Agrotis volubilis|[10084]|LPSOB693-08|PPBP-1692|652[0n]bp|Canada.Ontario|BOLD:ABZ7030
Agrotis vancouverensis|[10085]|LPVIB928-08|PFC-2006-2462|637[0n]bp|Canada.British Columbia|BOLD:ABZ7030
Agrotis vancouverensis|[10086]|RDMAB354-05|UASM57249|608[0n]bp|Canada.Alberta|BOLD:ABZ7030
Agrotis vancouverensis|[10087]|LOWCC199-05|CGWC-2079|602[0n]bp|Canada.British Columbia|BOLD:ABZ7030
Agrotis vancouverensis|[10088]|LPVIC096-08|PFC-2006-2668|658[0n]bp|Canada.British Columbia|BOLD:ABZ7030
Agrotis vancouverensis|[10089]|RDMAB356-05|UASM24515|609[0n]bp|Canada.Alberta|BOLD:ABZ7030
Agrotis vancouverensis|[10090]|LPVIA266-08|PFC-2006-0352|643[0n]bp|Canada.British Columbia|BOLD:ABZ7030
Agrotis vancouverensis|[10091]|LMH016-06|PFC-2006-0086|649[1n]bp|Canada.British Columbia|BOLD:ABZ7030
Agrotis vancouverensis|[10092]|LPVIB908-08|PFC-2006-2434|647[0n]bp|Canada.British Columbia|BOLD:ABZ7030
Agrotis volubilis|[10093]|TMG146-03|moth388.01|639[0n]bp|Canada.Ontario|BOLD:ABZ7030
Agrotis volubilis|[10094]|PMG087-03|moth356.01|617[0n]bp|Canada.Ontario|BOLD:ABZ7030
Agrotis volubilis|[10095]|LPSOB214-08|PPBP-1213|658[0n]bp|Canada.Ontario|BOLD:ABZ7030
Agrotis volubilis|[10096]|LPSOC094-08|PPBP-2093|658[0n]bp|Canada.Ontario|BOLD:ABZ7030
Agrotis vancouverensis|[10097]|LALPA425-10|AVBC 427-10|658[0n]bp|Canada.British Columbia|BOLD:ABZ7030
Agrotis vancouverensis|[10098]|BBLPB691-10|10BBCLP-1690|658[0n]bp|Canada.Alberta|BOLD:ABZ7030
Agrotis vancouverensis|[10099]|LPABB005-08|08BBLEP-03270|658[0n]bp|Canada.Alberta|BOLD:ABZ7030
Agrotis vancouverensis|[10100]|LPVIA278-08|PFC-2006-0366|657[0n]bp|Canada.British Columbia|BOLD:ABZ7030
Agrotis vancouverensis|[10101]|LPVIA290-08|PFC-2006-0381|658[0n]bp|Canada.British Columbia|BOLD:ABZ7030
Agrotis vancouverensis|[10102]|LHLEP401-06|UBC-2006-0761|658[0n]bp|Canada.British Columbia|BOLD:ABZ7030
Agrotis vancouverensis|[10103]|RDMAB122-05|UASM41268|658[0n]bp|Canada.Alberta|BOLD:ABZ7030
Agrotis vancouverensis|[10104]|LPVIA289-08|PFC-2006-0380|658[0n]bp|Canada.British Columbia|BOLD:ABZ7030
Agrotis vancouverensis|[10105]|BBLPB717-10|10BBCLP-1716|658[0n]bp|Canada.Alberta|BOLD:ABZ7030
Agrotis vancouverensis|[10106]|LALPA161-10|AVBC 161-10|658[0n]bp|Canada.British Columbia|BOLD:ABZ7030
Agrotis vancouverensis|[10107]|LALPA1128-11|AVBC 938-11|658[0n]bp|Canada.British Columbia|BOLD:ABZ7030
Agrotis vancouverensis|[10108]|RDNM727-05|CNCNoctuoidea7573|658[0n]bp|Canada.Alberta|BOLD:ABZ7030
Agrotis vancouverensis|[10109]|LHLEP397-06|UBC-2006-0202|658[0n]bp|Canada.British Columbia|BOLD:ABZ7030
Agrotis vancouverensis|[10110]|LHLEP399-06|UBC-2006-0204|658[0n]bp|Canada.British Columbia|BOLD:ABZ7030
Agrotis vancouverensis|[10111]|LALPA1165-11|AVBC 975-11|658[0n]bp|Canada.British Columbia|BOLD:ABZ7030
Agrotis vancouverensis|[10112]|LALPA214-10|AVBC 215-10|658[0n]bp|Canada.British Columbia|BOLD:ABZ7030
Agrotis vancouverensis|[10113]|LHLEP382-06|UBC-2006-0764|658[0n]bp|Canada.British Columbia|BOLD:ABZ7030
Agrotis vancouverensis|[10114]|RDMAB357-05|UASM7243|658[0n]bp|Canada.Alberta|BOLD:ABZ7030
Agrotis vancouverensis|[10115]|LPABB454-08|08BBLEP-03719|658[0n]bp|Canada.Alberta|BOLD:ABZ7030
Agrotis vancouverensis|[10116]|LPABB819-09|08BBLEP-04139|658[0n]bp|Canada.Alberta|BOLD:ABZ7030
Agrotis vancouverensis|[10117]|LPVIB961-08|PFC-2006-2503|658[0n]bp|Canada.British Columbia|BOLD:ABZ7030
Agrotis vancouverensis|[10118]|BBLPB720-10|10BBCLP-1719|658[0n]bp|Canada.Alberta|BOLD:ABZ7030
Agrotis vancouverensis|[10119]|LALPA406-10|AVBC 408-10|658[0n]bp|Canada.British Columbia|BOLD:ABZ7030
Agrotis vancouverensis|[10120]|LPVIB838-08|PFC-2006-2344|658[0n]bp|Canada.British Columbia|BOLD:ABZ7030
Agrotis volubilis|[10121]|RDNMF201-08|NOC14287|658[0n]bp|Canada.Ontario|BOLD:ABZ7030
Agrotis volubilis|[10122]|LPSOB218-08|PPBP-1217|658[0n]bp|Canada.Ontario|BOLD:ABZ7030
Agrotis volubilis|[10123]|LPSOB231-08|PPBP-1230|658[0n]bp|Canada.Ontario|BOLD:ABZ7030
Agrotis volubilis|[10124]|XAB231-04|04HBL005231|658[0n]bp|Canada.Ontario|BOLD:ABZ7030
Agrotis volubilis|[10125]|KPOEC039-08|08OEC-147|658[0n]bp|Canada.Ontario|BOLD:ABZ7030
Agrotis volubilis|[10126]|LPSOD333-09|08BBLEP-00111|658[0n]bp|Canada.Ontario|BOLD:ABZ7030
Agrotis volubilis|[10127]|XAE097-04|Moth4097.03|658[0n]bp|Canada.Ontario|BOLD:ABZ7030
Agrotis volubilis|[10128]|XAB219-04|04HBL005219|658[0n]bp|Canada.Ontario|BOLD:ABZ7030
Agrotis volubilis|[10129]|BLTIB111-08|BL176|658[0n]bp|Canada.Ontario|BOLD:ABZ7030
Agrotis volubilis|[10130]|LPSO134-08|PPBP-0134|658[0n]bp|Canada.Ontario|BOLD:ABZ7030
Agrotis volubilis|[10131]|BLTIB121-08|BL188|658[0n]bp|Canada.Ontario|BOLD:ABZ7030
Agrotis volubilis|[10132]|BLTIB041-08|BL0068|658[0n]bp|Canada.Ontario|BOLD:ABZ7030
Agrotis volubilis|[10133]|LPSOB339-08|PPBP-1338|658[0n]bp|Canada.Ontario|BOLD:ABZ7030
Agrotis volubilis|[10134]|LPSOB216-08|PPBP-1215|658[0n]bp|Canada.Ontario|BOLD:ABZ7030
Agrotis volubilis|[10135]|XAD640-05|2005-ONT-55|658[0n]bp|Canada.Ontario|BOLD:ABZ7030
Agrotis volubilis|[10136]|XAB254-04|04HBL005254|658[0n]bp|Canada.Ontario|BOLD:ABZ7030
Actebia fennica|[10137]|RDLQG022-06|DH012153|658[0n]bp|Canada.Quebec|BOLD:AAD5720
Actebia fennica|[10138]|BBLPB831-10|10BBCLP-1830|658[0n]bp|Canada.Alberta|BOLD:AAD5720
Actebia fennica|[10139]|LPVIB629-08|PFC-2006-2077|636[0n]bp|Canada.British Columbia|BOLD:AAD5720
Actebia fennica|[10140]|RDLQF247-06|DH011327|641[0n]bp|Canada.Quebec|BOLD:AAD5720
Actebia fennica|[10141]|LCHP943-07|07PROBE-10705|658[0n]bp|Canada.Manitoba|BOLD:AAD5720
Actebia fennica|[10142]|LBCH4405-10|10-JDWBC-4405|658[0n]bp|Canada.British Columbia|BOLD:AAD5720
Actebia fennica|[10143]|RDLQG059-06|DH012195|658[0n]bp|Canada.Quebec|BOLD:AAD5720
Actebia fennica|[10144]|LALPA868-11|AVBC 1041-11|658[0n]bp|Canada.British Columbia|BOLD:AAD5720
Copablepharon longipenne|[10145]|RDNM248-05|CNCNoctuoidea6430|658[0n]bp|Canada.Saskatchewan|BOLD:ACE5221
Copablepharon longipenne|[10146]|RDNM250-05|CNCNoctuoidea6432|658[0n]bp|Canada.Saskatchewan|BOLD:ACE5221
Copablepharon longipenne|[10147]|RDNM249-05|CNCNoctuoidea6431|658[0n]bp|Canada.Saskatchewan|BOLD:ACE5221
Copablepharon absidum|[10148]|RDNM232-05|CNCNoctuoidea6414|658[0n]bp|United States.Washington|BOLD:ABY...
Copablepharon absidum|[10149]|RDNM229-05|CNCNoctuoidea6411|544[0n]bp|United States.Washington|BOLD:ABY...
Copablepharon absidum|[10150]|RDNM231-05|CNCNoctuoidea6413|580[0n]bp|United States.Washington|BOLD:ABY...
Copablepharon fuscum|[10151]|RDNMB572-05|CNCNoctuoidea10348|609[0n]bp|United States.Washington|BOLD:AB...
Copablepharon fuscum|[10152]|RDNMB571-05|CNCNoctuoidea10347|658[0n]bp|United States.Washington|BOLD:AB...
Copablepharon fuscum|[10153]|RDNM711-05|CNCNoctuoidea7557|526[0n]bp|United States.Washington|
Copablepharon grandis|[10154]|RDNME985-08|CNC LEP 00047460|658[7n]bp|United States.New Mexico|
Copablepharon grandis|[10155]|RDNMF007-08|NOC14093|658[0n]bp|United States.Colorado|BOLD:ABY9003
Copablepharon grandis|[10156]|RDNMG424-08|CNC LEP00052248|658[0n]bp|United States.Colorado|BOLD:ABY9003
Copablepharon grandis|[10157]|RDNMF094-08|NOC14180|658[0n]bp|United States.Colorado|BOLD:ABY9003
Copablepharon grandis|[10158]|RDNMG425-08|CNC LEP00052249|658[0n]bp|United States.Colorado|BOLD:ABY9003
Copablepharon viridisparsa|[10159]|RDNM222-05|CNCNoctuoidea6404|658[0n]bp|Canada.Alberta|BOLD:AAC4953
Copablepharon viridisparsa|[10160]|RDNM223-05|CNCNoctuoidea6405|658[0n]bp|Canada.Alberta|BOLD:AAC4953
Copablepharon viridisparsa|[10161]|RDNMJ624-11|acrorev gga54|658[1n]bp|Canada.Saskatchewan|BOLD:AAC4953
Copablepharon viridisparsa|[10162]|RDNM221-05|CNCNoctuoidea6403|658[0n]bp|Canada.Alberta|BOLD:AAC4953
Dichagyris reliqua|[10163]|RDNMI194-12|CNCLEP00092219|601[0n]bp|United States.Michigan|BOLD:ACD0448
Dichagyris reliqua|[10164]|RDNMI193-12|CNCLEP00092218|627[0n]bp|Canada.Manitoba|BOLD:ACD0448
Dichagyris variabilis|[10165]|LBCH7221-10|10-JDWBC-7221|658[0n]bp|Canada.British Columbia|BOLD:AAD0257
Dichagyris variabilis|[10166]|LBCH7470-10|10-JDWBC-7470|658[0n]bp|Canada.British Columbia|BOLD:AAD0257
[illegible]

Dichagyris reliqua|[10164]|RDNMI193-12|CNCLEP00092218|627[0n]bp|Canada.Manitoba|BOLD:ACD0448
Dichagyris variabilis|[10165]|LBCH7221-10|10-JDWBC-7221|658[0n]bp|Canada.British Columbia|BOLD:AAD0257
Dichagyris variabilis|[10166]|LBCH7470-10|10-JDWBC-7470|658[0n]bp|Canada.British Columbia|BOLD:AAD0257
Dichagyris variabilis|[10167]|LOWCB682-05|CGWC-1622|658[0n]bp|Canada.British Columbia|BOLD:AAD0257
Dichagyris variabilis|[10168]|LBCH7928-10|10-JDWBC-7928|658[0n]bp|Canada.British Columbia|BOLD:AAD0257
Dichagyris variabilis|[10169]|LOWCB679-05|CGWC-1619|658[0n]bp|Canada.British Columbia|BOLD:AAD0257
Dichagyris variabilis|[10170]|LBCH7932-10|10-JDWBC-7932|658[0n]bp|Canada.British Columbia|BOLD:AAD0257
Dichagyris variabilis|[10171]|LALPA1252-11|AVBC 1254-11|658[0n]bp|Canada.British Columbia|BOLD:AAD0257
Dichagyris variabilis|[10172]|LBCH7927-10|10-JDWBC-7927|658[0n]bp|Canada.British Columbia|BOLD:AAD0257
Dichagyris variabilis|[10173]|LBCH7930-10|10-JDWBC-7930|658[0n]bp|Canada.British Columbia|BOLD:AAD0257
Dichagyris variabilis|[10174]|LOWCB685-05|CGWC-1625|658[0n]bp|Canada.British Columbia|BOLD:AAD0257
Dichagyris variabilis|[10175]|LBCH7926-10|10-JDWBC-7926|658[0n]bp|Canada.British Columbia|BOLD:AAD0257
Dichagyris variabilis|[10176]|LBCH7500-10|10-JDWBC-7500|658[0n]bp|Canada.British Columbia|BOLD:AAD0257
Dichagyris variabilis|[10177]|LOWCB683-05|CGWC-1623|658[0n]bp|Canada.British Columbia|BOLD:AAD0257
Dichagyris variabilis|[10178]|LOWCB680-05|CGWC-1620|658[0n]bp|Canada.British Columbia|BOLD:AAD0257
Dichagyris variabilis|[10179]|LBCH7092-10|10-JDWBC-7092|644[0n]bp|Canada.British Columbia|BOLD:AAD0257
Dichagyris variabilis|[10180]|LOWCD917-06|CGWC-3737|608[0n]bp|Canada.British Columbia|BOLD:AAD0257
Dichagyris variabilis|[10181]|LOWCB681-05|CGWC-1621|658[0n]bp|Canada.British Columbia|BOLD:AAD0257
Dichagyris variabilis|[10182]|LBCH7931-10|10-JDWBC-7931|658[0n]bp|Canada.British Columbia|BOLD:AAD0257
Dichagyris variabilis|[10183]|LOWCB684-05|CGWC-1624|658[0n]bp|Canada.British Columbia|BOLD:AAD0257
Dichagyris variabilis|[10184]|LPVIB445-08|PFC-2006-1851|658[0n]bp|Canada.British Columbia|BOLD:AAD0257
Dichagyris variabilis|[10185]|LBCH7929-10|10-JDWBC-7929|658[0n]bp|Canada.British Columbia|BOLD:AAD0257
Dichagyris variabilis|[10186]|LPVIB573-08|PFC-2006-2002|658[0n]bp|Canada.British Columbia|BOLD:AAD0257
Dichagyris variabilis|[10187]|LPVIB268-08|PFC-2006-1658|658[0n]bp|Canada.British Columbia|BOLD:AAD0257
Dichagyris variabilis|[10188]|LBCH7744-10|10-JDWBC-7744|658[0n]bp|Canada.British Columbia|BOLD:AAD0257
Dichagyris variabilis|[10189]|LPVIB446-08|PFC-2006-1852|658[0n]bp|Canada.British Columbia|BOLD:AAD0257
Dichagyris variabilis|[10190]|LPVIB267-08|PFC-2006-1657|658[0n]bp|Canada.British Columbia|BOLD:AAD0257
Dichagyris variabilis|[10191]|LPVIB266-08|PFC-2006-1656|658[0n]bp|Canada.British Columbia|BOLD:AAD0257
Dichagyris variabilis|[10192]|LBCH7933-10|10-JDWBC-7933|658[0n]bp|Canada.British Columbia|BOLD:AAD0257
Dichagyris variabilis|[10193]|LBCH7150-10|10-JDWBC-7150|658[0n]bp|Canada.British Columbia|BOLD:AAD0257
Dichagyris acclivis|[10194]|RDNMG213-08|NOC15065|658[0n]bp|United States.Maryland|BOLD:AAE0890
Dichagyris acclivis|[10195]|RDNMF600-08|NOC14686|658[0n]bp|United States.Kansas|BOLD:AAE0890
Dichagyris acclivis|[10196]|RDNMF599-08|NOC14685|658[0n]bp|United States.Texas|BOLD:AAE0890
Dichagyris acclivis|[10197]|RDNMG212-08|NOC15064|658[0n]bp|United States.Maryland|BOLD:AAE0890
Dichagyris acclivis|[10198]|RDNMF596-08|NOC14682|658[0n]bp|United States.Pennsylvania|BOLD:AAE0890
Dichagyris acclivis|[10199]|LILLA796-11|SNS10IL-01001|658[0n]bp|United States.Illinois|BOLD:AAE0890
Dichagyris acclivis|[10200]|NAMUM326-08|RWP-87-0869-2|658[0n]bp|United States.Maryland|BOLD:AAE0890
Dichagyris acclivis|[10201]|NAMUM325-08|RWP-87-0869-1|658[0n]bp|United States.Maryland|BOLD:AAE0890
Dichagyris grotei|[10202]|RDNMD863-07|CNCNoctuoidea13194|655[0n]bp|United States.New Mexico|BOLD:AAE0889
Dichagyris grotei|[10203]|RDNMD862-07|CNCNoctuoidea13193|655[0n]bp|United States.New Mexico|BOLD:AAE0889
Dichagyris grotei|[10204]|NAMUM058-08|RR-98-2154|656[1n]bp|United States.Arizona|BOLD:AAE0889
Feltia subterranea|[10205]|CMAZA271-09|CMAZ-0271|658[0n]bp|United States.Arizona|BOLD:AAB6793
Feltia subterranea|[10206]|CMAZA1022-12|BIOUG02041-H04|658[0n]bp|United States.Arizona|BOLD:AAB6793
Feltia subterranea|[10207]|RDNMD885-07|CNCNoctuoidea13216|655[0n]bp|United States.Arizona|BOLD:AAB6793
Feltia subterranea|[10208]|BBLSW342-09|09BBLEP-01270|658[0n]bp|United States.Arizona|BOLD:AAB6793
Feltia subterranea|[10209]|BBLOD953-11|BIOUG01569-G08|658[0n]bp|United States.Texas|BOLD:AAB6793
Feltia subterranea|[10210]|LOCBE089-06|06-BLLOC-3908|658[0n]bp|United States.California|BOLD:AAB6793
Feltia subterranea|[10211]|GMLC1377-12|2012GM-0108|636[0n]bp|United States.California|BOLD:AAB6793
Feltia subterranea|[10212]|GMLC1072-12|2011GM-0768|658[0n]bp|United States.California|BOLD:AAB6793
Feltia subterranea|[10213]|BBLSW334-09|09BBLEP-01262|647[0n]bp|United States.Arizona|BOLD:AAB6793
Feltia subterranea|[10214]|BBLOB450-11|BIOUG01396-F10|658[0n]bp|United States.Florida|BOLD:AAB6793
Feltia subterranea|[10215]|LPOKA886-09|MDOK-0981|658[0n]bp|United States.Oklahoma|BOLD:AAB6793
Feltia subterranea|[10216]|BBLOC1049-11|BIOUG01468-G09|658[0n]bp|United States.Arkansas|BOLD:AAB6793
Feltia subterranea|[10217]|BBLOC876-11|BIOUG01467-A03|658[0n]bp|United States.Arkansas|BOLD:AAB6793
Feltia subterranea|[10218]|BBLOC654-11|BIOUG01458-H05|621[0n]bp|United States.Arkansas|BOLD:AAB6793
Feltia subterranea|[10219]|LNC482-06|05-NCCC-482|586[0n]bp|United States.North Carolina|BOLD:AAB6793
Feltia subterranea|[10220]|LGSM681-04|DNA-ATBI-0681|527[1n]bp|United States.Tennessee|BOLD:AAB6793
Feltia subterranea|[10221]|BBLOC652-11|BIOUG01458-H03|622[0n]bp|United States.Arkansas|BOLD:AAB6793
Feltia subterranea|[10222]|BBLOC651-11|BIOUG01458-H02|622[0n]bp|United States.Arkansas|BOLD:AAB6793
Feltia subterranea|[10223]|BBLOC881-11|BIOUG01467-A08|658[0n]bp|United States.Arkansas|BOLD:AAB6793
Feltia subterranea|[10224]|BBLSW847-09|09BBLEP-01775|658[0n]bp|United States.Texas|BOLD:AAB6793
Feltia subterranea|[10225]|BBLOB501-11|BIOUG01397-C02|658[0n]bp|United States.Florida|BOLD:AAB6793
Feltia subterranea|[10226]|LOFLA735-06|06-FLOR-0735|658[0n]bp|United States.Florida|BOLD:AAB6793
Feltia subterranea|[10227]|BBLSX698-09|09BBLEP-02626|658[0n]bp|United States.Texas|BOLD:AAB6793
Feltia subterranea|[10228]|LOFLA687-06|06-FLOR-0687|658[0n]bp|United States.Florida|BOLD:AAB6793
Feltia subterranea|[10229]|BBLOC1041-11|BIOUG01468-G01|658[0n]bp|United States.Arkansas|BOLD:AAB6793
Feltia subterranea|[10230]|LOFLB115-06|06-FLOR-1055|658[0n]bp|United States.Florida|BOLD:AAB6793
Feltia subterranea|[10231]|BBLOB1689-11|BIOUG01421-G02|658[0n]bp|United States.Florida|BOLD:AAB6793
Feltia subterranea|[10232]|BBLOE1361-12|BIOUG01986-E10|658[0n]bp|United States.Florida|BOLD:AAB6793
Feltia subterranea|[10233]|BBLOC1045-11|BIOUG01468-G05|658[0n]bp|United States.Arkansas|BOLD:AAB6793
Feltia subterranea|[10234]|BBLOC1034-11|BIOUG01468-F06|658[0n]bp|United States.Texas|BOLD:AAB6793
Feltia subterranea|[10235]|LOFLB479-06|06-FLOR-1419|658[0n]bp|United States.Florida|BOLD:AAB6793
Feltia subterranea|[10236]|BBLSW841-09|09BBLEP-01769|658[0n]bp|United States.Texas|BOLD:AAB6793
Feltia subterranea|[10237]|BBLOB1674-11|BIOUG01421-E11|658[0n]bp|United States.Florida|BOLD:AAB6793
Feltia subterranea|[10238]|LNC481-06|05-NCCC-481|658[0n]bp|United States.North Carolina|BOLD:AAB6793
Feltia subterranea|[10239]|LPOKB141-09|MDOK-1284|658[0n]bp|United States.Oklahoma|BOLD:AAB6793
Feltia subterranea|[10240]|BBLOC1043-11|BIOUG01468-G03|658[0n]bp|United States.Arkansas|BOLD:AAB6793
Feltia subterranea|[10241]|BBLOC1060-11|BIOUG01468-H08|658[0n]bp|United States.Arkansas|BOLD:AAB6793
Feltia subterranea|[10242]|BBLOC1036-11|BIOUG01468-F08|658[0n]bp|United States.Texas|BOLD:AAB6793
Feltia subterranea|[10243]|LOFLB512-06|06-FLOR-1452|658[0n]bp|United States.Florida|BOLD:AAB6793
Feltia subterranea|[10244]|LOFLB269-06|06-FLOR-1209|658[0n]bp|United States.Florida|BOLD:AAB6793
Feltia subterranea|[10245]|BBLOC1042-11|BIOUG01468-G02|658[0n]bp|United States.Arkansas|BOLD:AAB6793
Feltia subterranea|[10246]|LOCBC661-06|06-BLLOC-2541|630[0n]bp|United States.California|BOLD:AAB6793
Feltia subterranea|[10247]|BBLSW335-09|09BBLEP-01263|658[0n]bp|United States.Arizona|BOLD:AAB6793
Feltia subterranea|[10248]|GMLC1178-12|2011GM-0874|658[0n]bp|United States.California|BOLD:AAB6793
Feltia subterranea|[10249]|GMLC1279-12|2012GM-0010|658[0n]bp|United States.California|BOLD:AAB6793
Feltia subterranea|[10250]|GMLC1112-12|2011GM-0808|658[0n]bp|United States.California|BOLD:AAB6793
Feltia subterranea|[10251]|RDNMD383-06|CNCNoctuoidea12715|658[0n]bp|United States.California|BOLD:AAB6793
Feltia subterranea|[10252]|CMAZA1064-12|BIOUG02042-C11|658[0n]bp|United States.Arizona|BOLD:AAB6793
Feltia subterranea|[10253]|CMAZA002-09|CMAZ-0002|658[0n]bp|United States.Arizona|BOLD:AAB6793
Feltia subterranea|[10254]|LOCBC451-06|06-BLLOC-2331|658[0n]bp|United States.California|BOLD:AAB6793
Feltia subterranea|[10255]|BBLOD950-11|BIOUG01569-G05|658[0n]bp|United States.California|BOLD:AAB6793
Feltia subterranea|[10256]|BBLSW777-09|09BBLEP-01705|658[0n]bp|United States.Arizona|BOLD:AAB6793
Feltia subterranea|[10257]|BBLSX684-09|09BBLEP-02612|658[0n]bp|United States.Arizona|BOLD:AAB6793
Feltia subterranea|[10258]|BBLOD587-11|BIOUG01565-H10|658[0n]bp|United States.California|BOLD:AAB6793
Feltia subterranea|[10259]|AWCLB416-10|AWC-08095|658[0n]bp|United States.Arizona|BOLD:AAB6793
Feltia subterranea|[10260]|AWCLB273-10|AWC-07361|658[0n]bp|United States.Arizona|BOLD:AAB6793
Feltia subterranea|[10261]|BBLOD956-11|BIOUG01569-G11|658[0n]bp|United States.Texas|BOLD:AAB6793
Feltia subterranea|[10262]|GMLC951-12|2011GM-0647|658[0n]bp|United States.California|BOLD:AAB6793
Feltia subterranea|[10263]|BBLSW322-09|09BBLEP-01250|658[0n]bp|United States.Arizona|BOLD:AAB6793
Feltia jaculifera|[10264]|LPMNB356-09|08BBLEP-05200|658[0n]bp|Canada.Manitoba|BOLD:ACE9864
Feltia jaculifera|[10265]|RDNM698-05|CNCNoctuoidea7544|617[0n]bp|Canada.Ontario|BOLD:ACE9864
Feltia jaculifera|[10266]|LPABC840-09|08BBLEP-05059|658[0n]bp|Canada.Alberta|BOLD:ACE9866

Feltia jaculifera|[10264]|LPMNB356-09|08BBLEP-05200|658[0n]bp|Canada.Manitoba|BOLD:ACE9864
Feltia jaculifera|[10265]|RDNM698-05|CNCNoctuoidea7544|617[0n]bp|Canada.Ontario|BOLD:ACE9864
Feltia jaculifera|[10266]|LPABC840-09|08BBLEP-05059|658[0n]bp|Canada.Alberta|BOLD:ACE9866
Feltia jaculifera|[10267]|LBCH7318-10|10-JDWBC-7318|658[0n]bp|Canada.British Columbia|BOLD:ACE9866
Feltia jaculifera|[10268]|RDNM700-05|CNCNoctuoidea7546|571[2n]bp|Canada.British Columbia|BOLD:ACE9866
Feltia jaculifera|[10269]|LPMNB437-09|08BBLEP-05437|658[0n]bp|Canada.Manitoba|BOLD:ACE9866
Feltia jaculifera|[10270]|RDNM703-05|CNCNoctuoidea7549|519[0n]bp|Canada.Alberta|BOLD:AAA3351
Feltia jaculifera|[10271]|XAD357-04|04HBL007357|513[0n]bp|Canada.Ontario|BOLD:AAA3351
Feltia jaculifera|[10272]|RDLQB727-05|DH010830|525[0n]bp|Canada.Quebec|BOLD:AAA3351
Feltia jaculifera|[10273]|RDNM699-05|CNCNoctuoidea7545|513[2n]bp|Canada.Alberta|BOLD:AAA3351
Feltia jaculifera|[10274]|RDNM701-05|CNCNoctuoidea7547|514[0n]bp|Canada.British Columbia|BOLD:AAA3351
Feltia jaculifera|[10275]|RDNM705-05|CNCNoctuoidea7551|506[0n]bp|Canada.British Columbia|BOLD:AAA3351
Feltia jaculifera|[10276]|LOWCD574-06|CGWC-3394|524[0n]bp|Canada.British Columbia|BOLD:AAA3351
Feltia jaculifera|[10277]|LOWCD564-06|CGWC-3384|658[0n]bp|Canada.British Columbia|BOLD:AAA3351
Feltia jaculifera|[10278]|XAH551-05|2005-ONT-2134|658[2n]bp|Canada.Ontario|BOLD:AAA3351
Feltia jaculifera|[10279]|TTMNB424-06|MNBTT-424|658[0n]bp|Canada.New Brunswick|BOLD:AAA3351
Feltia jaculifera|[10280]|XAD175-04|04HBL007175|658[0n]bp|Canada.Ontario|BOLD:AAA3351
Feltia jaculifera|[10281]|XAH149-05|2005-ONT-1732|658[0n]bp|Canada.Ontario|BOLD:AAA3351
Feltia jaculifera|[10282]|LPABC918-09|08BBLEP-05329|658[0n]bp|Canada.Alberta|BOLD:AAA3351
Feltia jaculifera|[10283]|BBLEC467-09|09BBELE-0467|658[0n]bp|Canada.New Brunswick|BOLD:AAA3351
Feltia jaculifera|[10284]|TTMNB423-06|MNBTT-423|658[0n]bp|Canada.New Brunswick|BOLD:AAA3351
Feltia jaculifera|[10285]|BBLEC131-09|09BBELE-0131|658[0n]bp|Canada.New Brunswick|BOLD:AAA3351
Feltia jaculifera|[10286]|XAH461-05|2005-ONT-2044|658[0n]bp|Canada.Ontario|BOLD:AAA3351
Feltia jaculifera|[10287]|XAD170-04|04HBL007170|578[0n]bp|Canada.Ontario|BOLD:AAA3351
Feltia jaculifera|[10288]|XAD290-04|04HBL007290|518[0n]bp|Canada.Ontario|BOLD:AAA3351
Feltia jaculifera|[10289]|LBCH7724-10|10-JDWBC-7724|658[0n]bp|Canada.British Columbia|BOLD:AAA3351
Feltia jaculifera|[10290]|XAH376-05|2005-ONT-1959|658[0n]bp|Canada.Ontario|BOLD:AAA3351
Feltia jaculifera|[10291]|XAG881-05|2005-ONT-1465|564[0n]bp|Canada.Ontario|BOLD:AAA3351
Feltia jaculifera|[10292]|XAH587-05|2005-ONT-2170|658[0n]bp|Canada.Ontario|BOLD:AAA3351
Feltia jaculifera|[10293]|XAB678-04|04HBL005678|658[0n]bp|Canada.Ontario|BOLD:AAA3351
Feltia jaculifera|[10294]|BBLPB819-10|10BBCLP-1818|658[0n]bp|Canada.Alberta|BOLD:AAA3351
Feltia jaculifera|[10295]|LPABB274-08|08BBLEP-03539|658[0n]bp|Canada.Alberta|BOLD:AAA3351
Feltia jaculifera|[10296]|XAH299-05|2005-ONT-1882|658[0n]bp|Canada.Ontario|BOLD:AAA3351
Feltia jaculifera|[10297]|XAH139-05|2005-ONT-1722|658[0n]bp|Canada.Ontario|BOLD:AAA3351
Feltia jaculifera|[10298]|LPABB624-08|08BBLEP-03889|658[0n]bp|Canada.Alberta|BOLD:AAA3351
Feltia jaculifera|[10299]|RDLQF207-06|DH011287|658[0n]bp|Canada.Quebec|BOLD:AAA3351
Feltia jaculifera|[10300]|LPMNB439-09|08BBLEP-05439|658[0n]bp|Canada.Manitoba|BOLD:AAA3351
Feltia jaculifera|[10301]|LPABC931-09|08BBLEP-05342|658[0n]bp|Canada.Alberta|BOLD:AAA3351
Feltia jaculifera|[10302]|XAD470-04|04HBL007470|658[0n]bp|Canada.Ontario|BOLD:AAA3351
Feltia jaculifera|[10303]|BBLPB821-10|10BBCLP-1820|658[0n]bp|Canada.Alberta|BOLD:AAA3351
Feltia jaculifera|[10304]|XAH431-05|2005-ONT-2014|658[0n]bp|Canada.Ontario|BOLD:AAA3351
Feltia jaculifera|[10305]|RDLQB759-05|DH010846|658[0n]bp|Canada.Quebec|BOLD:AAA3351
Feltia jaculifera|[10306]|XAH282-05|2005-ONT-1865|658[0n]bp|Canada.Ontario|BOLD:AAA3351
Feltia jaculifera|[10307]|TTMNB422-06|MNBTT-422|658[0n]bp|Canada.New Brunswick|BOLD:AAA3351
Feltia jaculifera|[10308]|LPMN955-08|08BBLEP-02313|658[0n]bp|Canada.Alberta|BOLD:AAA3351
Feltia jaculifera|[10309]|TTMNB426-06|MNBTT-426|658[0n]bp|Canada.New Brunswick|BOLD:AAA3351
Feltia jaculifera|[10310]|TTMNB425-06|MNBTT-425|658[0n]bp|Canada.New Brunswick|BOLD:AAA3351
Feltia jaculifera|[10311]|XAH138-05|2005-ONT-1721|658[0n]bp|Canada.Ontario|BOLD:AAA3351
Feltia jaculifera|[10312]|XAH089-05|2005-ONT-1672|658[2n]bp|Canada.Ontario|BOLD:AAA3351
Feltia jaculifera|[10313]|XAH315-05|2005-ONT-1898|607[0n]bp|Canada.Ontario|BOLD:AAA3351
Feltia jaculifera|[10314]|XAB429-04|04HBL005429|616[0n]bp|Canada.Ontario|BOLD:AAA3351
Feltia jaculifera|[10315]|XAH417-05|2005-ONT-2000|658[0n]bp|Canada.Ontario|BOLD:AAA3351
Feltia jaculifera|[10316]|LPABB115-08|08BBLEP-03380|650[0n]bp|Canada.Alberta|BOLD:AAA3351
Feltia jaculifera|[10317]|XAH462-05|2005-ONT-2045|603[0n]bp|Canada.Ontario|BOLD:AAA3351
Feltia jaculifera|[10318]|XAH463-05|2005-ONT-2046|658[0n]bp|Canada.Ontario|BOLD:AAA3351
Feltia jaculifera|[10319]|LPABB625-08|08BBLEP-03890|658[0n]bp|Canada.Alberta|BOLD:AAA3351
Feltia jaculifera|[10320]|XAH416-05|2005-ONT-1999|658[0n]bp|Canada.Ontario|BOLD:AAA3351
Feltia jaculifera|[10321]|XAB430-04|04HBL005430|658[0n]bp|Canada.Ontario|BOLD:AAA3351
Feltia jaculifera|[10322]|XAH360-05|2005-ONT-1943|658[0n]bp|Canada.Ontario|BOLD:AAA3351
Feltia jaculifera|[10323]|LBCH7198-10|10-JDWBC-7198|658[0n]bp|Canada.British Columbia|BOLD:AAA3351
Feltia jaculifera|[10324]|LBCH7200-10|10-JDWBC-7200|658[0n]bp|Canada.British Columbia|BOLD:AAA3351
Feltia jaculifera|[10325]|LBCH7589-10|10-JDWBC-7589|658[0n]bp|Canada.British Columbia|BOLD:AAA3351
Feltia jaculifera|[10326]|LOWCB704-05|CGWC-1644|658[0n]bp|Canada.British Columbia|BOLD:AAA3351
Feltia jaculifera|[10327]|LBCH6120-10|10-JDWBC-6120|658[0n]bp|Canada.British Columbia|BOLD:AAA3351
Feltia jaculifera|[10328]|LBCH7033-10|10-JDWBC-7033|658[0n]bp|Canada.British Columbia|BOLD:AAA3351
Feltia jaculifera|[10329]|LOWCB703-05|CGWC-1643|593[0n]bp|Canada.British Columbia|BOLD:AAA3351
Feltia jaculifera|[10330]|LOWCD561-06|CGWC-3381|658[0n]bp|Canada.British Columbia|BOLD:AAA3351
Feltia jaculifera|[10331]|LBCH7456-10|10-JDWBC-7456|658[0n]bp|Canada.British Columbia|BOLD:AAA3351
Feltia jaculifera|[10332]|LOWCB705-05|CGWC-1645|658[0n]bp|Canada.British Columbia|BOLD:AAA3351
Feltia jaculifera|[10333]|LBCH7532-10|10-JDWBC-7532|658[0n]bp|Canada.British Columbia|BOLD:AAA3351
Feltia jaculifera|[10334]|LBCG3316-09|08-JDWBC-3316|658[0n]bp|Canada.British Columbia|BOLD:AAA3351
Feltia jaculifera|[10335]|XAH532-05|2005-ONT-2115|658[0n]bp|Canada.Ontario|BOLD:AAA3351
Feltia jaculifera|[10336]|LBCH7438-10|10-JDWBC-7438|658[0n]bp|Canada.British Columbia|BOLD:AAA3351
Feltia jaculifera|[10337]|LBCH7444-10|10-JDWBC-7444|658[0n]bp|Canada.British Columbia|BOLD:AAA3351
Feltia jaculifera|[10338]|LBCH7872-10|10-JDWBC-7872|658[0n]bp|Canada.British Columbia|BOLD:AAA3351
Feltia jaculifera|[10339]|LOWCB699-05|CGWC-1639|658[0n]bp|Canada.British Columbia|BOLD:AAA3351
Feltia jaculifera|[10340]|LBCH6786-10|10-JDWBC-6786|658[0n]bp|Canada.British Columbia|BOLD:AAA3351
Feltia jaculifera|[10341]|LOWCB706-05|CGWC-1646|658[0n]bp|Canada.British Columbia|BOLD:AAA3351
Feltia jaculifera|[10342]|LBCH7722-10|10-JDWBC-7722|658[0n]bp|Canada.British Columbia|BOLD:AAA3351
Feltia jaculifera|[10343]|LBCH7582-10|10-JDWBC-7582|658[0n]bp|Canada.British Columbia|BOLD:AAA3351
Feltia jaculifera|[10344]|LBCG190-08|08-JDWBC-0190|658[0n]bp|Canada.British Columbia|BOLD:AAA3351
Feltia jaculifera|[10345]|XAD284-04|04HBL007284|658[0n]bp|Canada.Ontario|BOLD:AAA3351
Feltia jaculifera|[10346]|LOWCD569-06|CGWC-3389|657[0n]bp|Canada.British Columbia|BOLD:AAA3351
Feltia jaculifera|[10347]|LBCH7878-10|10-JDWBC-7878|658[0n]bp|Canada.British Columbia|BOLD:AAA3351
Feltia jaculifera|[10348]|LBCH7535-10|10-JDWBC-7535|658[0n]bp|Canada.British Columbia|BOLD:AAA3351
Feltia jaculifera|[10349]|LOWCD571-06|CGWC-3391|658[0n]bp|Canada.British Columbia|BOLD:AAA3351
Feltia jaculifera|[10350]|LBCH7126-10|10-JDWBC-7126|658[0n]bp|Canada.British Columbia|BOLD:AAA3351
Feltia jaculifera|[10351]|LBCH7031-10|10-JDWBC-7031|658[0n]bp|Canada.British Columbia|BOLD:AAA3351
Feltia jaculifera|[10352]|LBCH7871-10|10-JDWBC-7871|658[0n]bp|Canada.British Columbia|BOLD:AAA3351
Feltia jaculifera|[10353]|LBCH7586-10|10-JDWBC-7586|658[0n]bp|Canada.British Columbia|BOLD:AAA3351
Feltia jaculifera|[10354]|LOWCD558-06|CGWC-3378|658[0n]bp|Canada.British Columbia|BOLD:AAA3351
Feltia jaculifera|[10355]|LBCH7320-10|10-JDWBC-7320|658[0n]bp|Canada.British Columbia|BOLD:AAA3351
Feltia jaculifera|[10356]|LBCH6908-10|10-JDWBC-6908|658[0n]bp|Canada.British Columbia|BOLD:AAA3351
Feltia jaculifera|[10357]|LOWCB702-05|CGWC-1642|658[0n]bp|Canada.British Columbia|BOLD:AAA3351
Feltia jaculifera|[10358]|LBCH7195-10|10-JDWBC-7195|658[0n]bp|Canada.British Columbia|BOLD:AAA3351
Feltia jaculifera|[10359]|LBCH7317-10|10-JDWBC-7317|658[0n]bp|Canada.British Columbia|BOLD:AAA3351
Feltia jaculifera|[10360]|LBCH7720-10|10-JDWBC-7720|658[0n]bp|Canada.British Columbia|BOLD:AAA3351
Feltia jaculifera|[10361]|LOWCD563-06|CGWC-3383|658[0n]bp|Canada.British Columbia|BOLD:AAA3351
Feltia jaculifera|[10362]|LBCH7457-10|10-JDWBC-7457|658[0n]bp|Canada.British Columbia|BOLD:AAA3351
Feltia jaculifera|[10363]|LBCH7458-10|10-JDWBC-7458|658[0n]bp|Canada.British Columbia|BOLD:AAA3351
Feltia jaculifera|[10364]|LBCH7537-10|10-JDWBC-7537|658[0n]bp|Canada.British Columbia|BOLD:AAA3351
Feltia jaculifera|[10365]|LBCH6445-10|10-JDWBC-6445|658[0n]bp|Canada.British Columbia|BOLD:AAA3351
Feltia jaculifera|[10366]|LOWCD113-06|CGWC-2933|658[0n]bp|Canada.British Columbia|BOLD:AAA3351

Feltia jaculifera|[10364]|LBCH7537-10|10-JDWBC-7537|658[0n]bp|Canada.British Columbia|BOLD:AAA3351
Feltia jaculifera|[10365]|LBCH6445-10|10-JDWBC-6445|658[0n]bp|Canada.British Columbia|BOLD:AAA3351
Feltia jaculifera|[10366]|LOWCD113-06|CGWC-2933|658[0n]bp|Canada.British Columbia|BOLD:AAA3351
Feltia jaculifera|[10367]|LBCH7437-10|10-JDWBC-7437|658[0n]bp|Canada.British Columbia|BOLD:AAA3351
Feltia jaculifera|[10368]|LBCG1104-09|08-JDWBC-1104|658[0n]bp|Canada.British Columbia|BOLD:AAA3351
Feltia jaculifera|[10369]|LBCG3315-09|08-JDWBC-3315|658[0n]bp|Canada.British Columbia|BOLD:AAA3351
Feltia jaculifera|[10370]|LOWCB701-05|CGWC-1641|658[0n]bp|Canada.British Columbia|BOLD:AAA3351
Feltia jaculifera|[10371]|LPABB575-08|08BBLEP-03840|658[0n]bp|Canada.Alberta|BOLD:AAA3351
Feltia jaculifera|[10372]|LBCH7442-10|10-JDWBC-7442|658[0n]bp|Canada.British Columbia|BOLD:AAA3351
Feltia jaculifera|[10373]|LBCH6121-10|10-JDWBC-6121|658[0n]bp|Canada.British Columbia|BOLD:AAA3351
Feltia jaculifera|[10374]|LOWCD570-06|CGWC-3390|658[0n]bp|Canada.British Columbia|BOLD:AAA3351
Feltia jaculifera|[10375]|LBCH7461-10|10-JDWBC-7461|658[0n]bp|Canada.British Columbia|BOLD:AAA3351
Feltia jaculifera|[10376]|LOWCB698-05|CGWC-1638|658[0n]bp|Canada.British Columbia|BOLD:AAA3351
Feltia jaculifera|[10377]|LOWCD557-06|CGWC-3377|658[0n]bp|Canada.British Columbia|BOLD:AAA3351
Feltia jaculifera|[10378]|LOWCD559-06|CGWC-3379|658[0n]bp|Canada.British Columbia|BOLD:AAA3351
Feltia jaculifera|[10379]|LBCH7440-10|10-JDWBC-7440|658[0n]bp|Canada.British Columbia|BOLD:AAA3351
Feltia jaculifera|[10380]|XAH128-05|2005-ONT-1711|658[0n]bp|Canada.Ontario|BOLD:AAA3351
Feltia jaculifera|[10381]|LBCH7196-10|10-JDWBC-7196|658[0n]bp|Canada.British Columbia|BOLD:AAA3351
Feltia jaculifera|[10382]|LOWCC165-05|CGWC-2045|658[0n]bp|Canada.British Columbia|BOLD:AAA3351
Feltia jaculifera|[10383]|LBCH7367-10|10-JDWBC-7367|658[0n]bp|Canada.British Columbia|BOLD:AAA3351
Feltia jaculifera|[10384]|LBCH7463-10|10-JDWBC-7463|658[0n]bp|Canada.British Columbia|BOLD:AAA3351
Feltia jaculifera|[10385]|LBCH7719-10|10-JDWBC-7719|658[0n]bp|Canada.British Columbia|BOLD:AAA3351
Feltia jaculifera|[10386]|LBCH7585-10|10-JDWBC-7585|658[0n]bp|Canada.British Columbia|BOLD:AAA3351
Feltia jaculifera|[10387]|LBCH7460-10|10-JDWBC-7460|658[0n]bp|Canada.British Columbia|BOLD:AAA3351
Feltia jaculifera|[10388]|LBCH7786-10|10-JDWBC-7786|658[0n]bp|Canada.British Columbia|BOLD:AAA3351
Feltia jaculifera|[10389]|LBCH7439-10|10-JDWBC-7439|658[0n]bp|Canada.British Columbia|BOLD:AAA3351
Feltia jaculifera|[10390]|LBCH7194-10|10-JDWBC-7194|658[0n]bp|Canada.British Columbia|BOLD:AAA3351
Feltia jaculifera|[10391]|LBCH7321-10|10-JDWBC-7321|658[0n]bp|Canada.British Columbia|BOLD:AAA3351
Feltia jaculifera|[10392]|LOWCD572-06|CGWC-3392|658[0n]bp|Canada.British Columbia|BOLD:AAA3351
Feltia jaculifera|[10393]|LBCH7530-10|10-JDWBC-7530|658[0n]bp|Canada.British Columbia|BOLD:AAA3351
Feltia jaculifera|[10394]|LBCH7032-10|10-JDWBC-7032|658[0n]bp|Canada.British Columbia|BOLD:AAA3351
Feltia jaculifera|[10395]|LBCH7443-10|10-JDWBC-7443|658[0n]bp|Canada.British Columbia|BOLD:AAA3351
Feltia jaculifera|[10396]|LBCH7533-10|10-JDWBC-7533|658[0n]bp|Canada.British Columbia|BOLD:AAA3351
Feltia jaculifera|[10397]|LBCH7536-10|10-JDWBC-7536|658[0n]bp|Canada.British Columbia|BOLD:AAA3351
Feltia jaculifera|[10398]|LBCH7036-10|10-JDWBC-7036|658[0n]bp|Canada.British Columbia|BOLD:AAA3351
Feltia jaculifera|[10399]|LOWCD560-06|CGWC-3380|657[0n]bp|Canada.British Columbia|BOLD:AAA3351
Feltia jaculifera|[10400]|LOWCC164-05|CGWC-2044|658[0n]bp|Canada.British Columbia|BOLD:AAA3351
Feltia jaculifera|[10401]|LBCH7034-10|10-JDWBC-7034|658[0n]bp|Canada.British Columbia|BOLD:AAA3351
Feltia jaculifera|[10402]|LBCH7038-10|10-JDWBC-7038|658[0n]bp|Canada.British Columbia|BOLD:AAA3351
Feltia jaculifera|[10403]|LBCH7721-10|10-JDWBC-7721|658[0n]bp|Canada.British Columbia|BOLD:AAA3351
Feltia jaculifera|[10404]|LBCH7102-10|10-JDWBC-7102|658[0n]bp|Canada.British Columbia|BOLD:AAA3351
Feltia jaculifera|[10405]|LBCH6504-10|10-JDWBC-6504|658[0n]bp|Canada.British Columbia|BOLD:AAA3351
Feltia jaculifera|[10406]|LOWCD562-06|CGWC-3382|658[0n]bp|Canada.British Columbia|BOLD:AAA3351
Feltia jaculifera|[10407]|LBCH7197-10|10-JDWBC-7197|658[0n]bp|Canada.British Columbia|BOLD:AAA3351
Feltia jaculifera|[10408]|LBCH7099-10|10-JDWBC-7099|658[0n]bp|Canada.British Columbia|BOLD:AAA3351
Feltia jaculifera|[10409]|LOWCD566-06|CGWC-3386|658[0n]bp|Canada.British Columbia|BOLD:AAA3351
Feltia jaculifera|[10410]|LBCH7584-10|10-JDWBC-7584|658[0n]bp|Canada.British Columbia|BOLD:AAA3351
Feltia jaculifera|[10411]|XAH039-05|2005-ONT-1622|658[0n]bp|Canada.Ontario|BOLD:AAA3351
Feltia jaculifera|[10412]|LBCH7199-10|10-JDWBC-7199|658[0n]bp|Canada.British Columbia|BOLD:AAA3351
Feltia jaculifera|[10413]|LBCH7588-10|10-JDWBC-7588|658[0n]bp|Canada.British Columbia|BOLD:AAA3351
Feltia jaculifera|[10414]|LBCH7193-10|10-JDWBC-7193|658[0n]bp|Canada.British Columbia|BOLD:AAA3351
Feltia jaculifera|[10415]|LBCG2895-09|08-JDWBC-2895|658[0n]bp|Canada.British Columbia|BOLD:AAA3351
Feltia jaculifera|[10416]|LBCH7874-10|10-JDWBC-7874|658[0n]bp|Canada.British Columbia|BOLD:AAA3351
Feltia jaculifera|[10417]|LOWCD565-06|CGWC-3385|658[0n]bp|Canada.British Columbia|BOLD:AAA3351
Feltia jaculifera|[10418]|LOWCB700-05|CGWC-1640|658[0n]bp|Canada.British Columbia|BOLD:AAA3351
Feltia jaculifera|[10419]|LBCH7875-10|10-JDWBC-7875|658[0n]bp|Canada.British Columbia|BOLD:AAA3351
Feltia jaculifera|[10420]|LBCH7587-10|10-JDWBC-7587|658[0n]bp|Canada.British Columbia|BOLD:AAA3351
Feltia jaculifera|[10421]|XAH129-05|2005-ONT-1712|658[1n]bp|Canada.Ontario|BOLD:AAA3351
Feltia jaculifera|[10422]|LBCH7531-10|10-JDWBC-7531|658[0n]bp|Canada.British Columbia|BOLD:AAA3351
Feltia jaculifera|[10423]|LOWCD567-06|CGWC-3387|598[2n]bp|Canada.British Columbia|BOLD:AAA3351
Feltia jaculifera|[10424]|XAH140-05|2005-ONT-1723|658[0n]bp|Canada.Ontario|BOLD:AAA3351
Feltia jaculifera|[10425]|LOWCD501-06|CGWC-3321|599[0n]bp|Canada.British Columbia|BOLD:AAA3351
Feltia jaculifera|[10426]|LBCH7316-10|10-JDWBC-7316|636[0n]bp|Canada.British Columbia|BOLD:AAA3351
Feltia jaculifera|[10427]|RDNM706-05|CNCNoctuoidea7552|609[0n]bp|Canada.British Columbia|BOLD:AAA3351
Feltia jaculifera|[10428]|LBCH6911-10|10-JDWBC-6911|633[0n]bp|Canada.British Columbia|BOLD:AAA3351
Feltia jaculifera|[10429]|LBCH7583-10|10-JDWBC-7583|643[0n]bp|Canada.British Columbia|BOLD:AAA3351
Feltia jaculifera|[10430]|XAD177-04|04HBL007177|592[0n]bp|Canada.Ontario|BOLD:AAA3351
Feltia jaculifera|[10431]|LOWCD573-06|CGWC-3393|612[0n]bp|Canada.British Columbia|BOLD:AAA3351
Feltia jaculifera|[10432]|LOWCD568-06|CGWC-3388|605[0n]bp|Canada.British Columbia|BOLD:AAA3351
Feltia jaculifera|[10433]|LBCH7037-10|10-JDWBC-7037|639[0n]bp|Canada.British Columbia|BOLD:AAA3351
Feltia jaculifera|[10434]|LOWCD556-06|CGWC-3376|658[0n]bp|Canada.British Columbia|BOLD:AAA3351
Feltia jaculifera|[10435]|LBCH7035-10|10-JDWBC-7035|658[0n]bp|Canada.British Columbia|BOLD:AAA3351
Feltia jaculifera|[10436]|LBCH7726-10|10-JDWBC-7726|658[0n]bp|Canada.British Columbia|BOLD:AAA3351
Feltia jaculifera|[10437]|LBCH6910-10|10-JDWBC-6910|632[0n]bp|Canada.British Columbia|BOLD:AAA3351
Feltia jaculifera|[10438]|LBCH7441-10|10-JDWBC-7441|658[0n]bp|Canada.British Columbia|BOLD:AAA3351
Feltia jaculifera|[10439]|LBCH7323-10|10-JDWBC-7323|658[0n]bp|Canada.British Columbia|BOLD:AAA3351
Feltia jaculifera|[10440]|LBCH7319-10|10-JDWBC-7319|658[0n]bp|Canada.British Columbia|BOLD:AAA3351
Feltia jaculifera|[10441]|LBCH7725-10|10-JDWBC-7725|658[0n]bp|Canada.British Columbia|BOLD:AAA3351
Feltia jaculifera|[10442]|LBCH7459-10|10-JDWBC-7459|640[0n]bp|Canada.British Columbia|BOLD:AAA3351
Feltia jaculifera|[10443]|LBCH7462-10|10-JDWBC-7462|636[0n]bp|Canada.British Columbia|BOLD:AAA3351
Feltia jaculifera|[10444]|LBCH7322-10|10-JDWBC-7322|658[0n]bp|Canada.British Columbia|BOLD:AAA3351
Feltia herilis|[10445]|LPMNB566-09|08BBLEP-05644|658[0n]bp|Canada.Manitoba|BOLD:ACE9865
Feltia herilis|[10446]|XAG880-05|2005-ONT-1464|658[0n]bp|Canada.Ontario|BOLD:ACE9865
Feltia herilis|[10447]|LPMN991-09|08BBLEP-04032|658[0n]bp|Canada.Manitoba|BOLD:ACE9865
Feltia herilis|[10448]|XAG858-05|2005-ONT-1442|617[0n]bp|Canada.Ontario|BOLD:ACE9865
Feltia herilis|[10449]|BLTIB751-08|BL1041|658[0n]bp|Canada.Ontario|BOLD:ACE9865
Feltia herilis|[10450]|LPMNB565-09|08BBLEP-05643|658[0n]bp|Canada.Manitoba|BOLD:ACE9865
Feltia herilis|[10451]|RDLQB541-05|DH010627|658[0n]bp|Canada.Quebec|BOLD:ACE9865
Feltia herilis|[10452]|LPMN963-08|08BBLEP-02321|658[0n]bp|Canada.Alberta|BOLD:ACE9865
Feltia herilis|[10453]|XAC794-04|04HBL006794|658[0n]bp|Canada.Ontario|BOLD:ACE9865
Feltia herilis|[10454]|RDLQ690-07|DH012033|656[0n]bp|Canada.Quebec|BOLD:ACE9865
Feltia herilis|[10455]|TTMNB428-06|MNBTT-428|658[0n]bp|Canada.New Brunswick|BOLD:ACE9865
Feltia herilis|[10456]|XAD274-04|04HBL007274|658[0n]bp|Canada.Ontario|BOLD:ACE9865
Feltia herilis|[10457]|TMNBB366-06|MNBTT-1306|658[0n]bp|Canada.New Brunswick|BOLD:ACE9865
Feltia herilis|[10458]|LPMNB434-09|08BBLEP-05434|658[0n]bp|Canada.Manitoba|BOLD:ACE9865
Feltia herilis|[10459]|LPMNB433-09|08BBLEP-05433|658[0n]bp|Canada.Manitoba|BOLD:ACE9865
Feltia herilis|[10460]|XAK418-06|2006-ONT-1413|658[0n]bp|Canada.Ontario|BOLD:ACE9865
Feltia herilis|[10461]|BBLPB823-10|10BBCLP-1822|658[0n]bp|Canada.Alberta|BOLD:ACE9865
Feltia herilis|[10462]|BBLPB820-10|10BBCLP-1819|658[0n]bp|Canada.Alberta|BOLD:ACE9865
Feltia herilis|[10463]|LPMNB241-09|08BBLEP-05085|658[0n]bp|Canada.Manitoba|BOLD:ACE9865
Feltia herilis|[10464]|BLTIB903-08|BL1322|658[0n]bp|Canada.Ontario|BOLD:ACE9865
Feltia herilis|[10465]|TTMNB427-06|MNBTT-427|658[0n]bp|Canada.New Brunswick|BOLD:ACE9865

Feltia herilis|[10463]|LPMNB241-09|08BBLEP-05085|658[0n]bp|Canada.Manitoba|BOLD:ACE9865
Feltia herilis|[10464]|BLTIB903-08|BL1322|658[0n]bp|Canada.Ontario|BOLD:ACE9865
Feltia herilis|[10465]|TTMNB427-06|MNBTT-427|658[0n]bp|Canada.New Brunswick|BOLD:ACE9865
Feltia herilis|[10466]|BBLPB822-10|10BBCLP-1821|658[0n]bp|Canada.British Columbia|BOLD:ACE9865
Feltia herilis|[10467]|LPMNB438-09|08BBLEP-05438|636[0n]bp|Canada.Manitoba|BOLD:ACE9865
Feltia herilis|[10468]|PHMO306-03|moth2260.02|639[0n]bp|Canada.Ontario|BOLD:ACE9865
Feltia herilis|[10469]|BLTIB581-08|BL860|621[0n]bp|Canada.Ontario|BOLD:ACE9865
Feltia herilis|[10470]|BBLPC352-09|09BBELE-1352|658[0n]bp|Canada.New Brunswick|BOLD:ACE9865
Feltia herilis|[10471]|XAH123-05|2005-ONT-1706|658[0n]bp|Canada.Ontario|BOLD:ACE9865
Feltia herilis|[10472]|LALPA1242-11|AVBC 1244-11|658[0n]bp|Canada.British Columbia|BOLD:ACE9865
Feltia herilis|[10473]|LPMNB435-09|08BBLEP-05435|658[0n]bp|Canada.Manitoba|BOLD:ACE9865
Feltia herilis|[10474]|XAH368-05|2005-ONT-1951|658[0n]bp|Canada.Ontario|BOLD:ACE9865
Feltia herilis|[10475]|TMNBB365-06|MNBTT-1305|658[0n]bp|Canada.New Brunswick|BOLD:ACE9865
Feltia herilis|[10476]|LPMN989-09|08BBLEP-04030|658[0n]bp|Canada.Manitoba|BOLD:ACE9865
Feltia herilis|[10477]|LPMN999-09|08BBLEP-04040|658[0n]bp|Canada.Manitoba|BOLD:ACE9865
Feltia herilis|[10478]|XAH244-05|2005-ONT-1827|658[0n]bp|Canada.Ontario|BOLD:ACE9865
Feltia herilis|[10479]|LPMNB236-09|08BBLEP-05080|658[0n]bp|Canada.Manitoba|BOLD:ACE9865
Feltia herilis|[10480]|XAD172-04|04HBL007172|658[0n]bp|Canada.Ontario|BOLD:ACE9865
Feltia herilis|[10481]|LPMNB505-09|08BBLEP-05543|658[0n]bp|Canada.Manitoba|BOLD:ACE9865
Feltia herilis|[10482]|LPABC928-09|08BBLEP-05339|658[0n]bp|Canada.Alberta|BOLD:ACE9865
Feltia herilis|[10483]|RDLQB509-05|DH010595|658[0n]bp|Canada.Quebec|BOLD:ACE9865
Feltia tricosa|[10484]|RDLQB438-05|DH010524|593[0n]bp|Canada.Quebec|BOLD:ACE9865
Feltia subgothica|[10485]|RDLQ692-07|DH012206|658[0n]bp|Canada.Quebec|BOLD:ACE9865
Feltia subgothica|[10486]|XAD579-04|04HBL006994|528[0n]bp|Canada.Ontario|BOLD:ACE9865
Feltia subgothica|[10487]|XAG096-05|2005-ONT-680|658[0n]bp|Canada.Ontario|BOLD:ACE9865
Feltia subgothica|[10488]|XAG017-05|2005-ONT-601|658[0n]bp|Canada.Ontario|BOLD:ACE9865
Feltia subgothica|[10489]|XAK422-06|2006-ONT-1417|658[0n]bp|Canada.Ontario|BOLD:ACE9865
Feltia subgothica|[10490]|XAG851-05|2005-ONT-1435|658[0n]bp|Canada.Ontario|BOLD:ACE9865
Feltia subgothica|[10491]|XAK347-06|2006-ONT-1342|658[0n]bp|Canada.Ontario|BOLD:ACE9865
Feltia subgothica|[10492]|XAG651-05|2005-ONT-1235|658[0n]bp|Canada.Ontario|BOLD:ACE9865
Feltia subgothica|[10493]|RDLQ688-07|DH008173|607[0n]bp|Canada.Quebec|BOLD:ACE9865
Feltia subgothica|[10494]|XAG241-05|2005-ONT-825|636[0n]bp|Canada.Ontario|BOLD:ACE9865
Feltia subgothica|[10495]|PHMO260-03|moth1513.02|639[0n]bp|Canada.Ontario|BOLD:ACE9865
Feltia subgothica|[10496]|PHMO382-03|moth2127.02|639[0n]bp|Canada.Ontario|BOLD:ACE9865
Feltia tricosa|[10497]|RDLQB760-05|DH010847|620[0n]bp|Canada.Quebec|BOLD:ACE9865
Feltia tricosa|[10498]|RDLQ689-07|DH012360|632[0n]bp|Canada.Quebec|BOLD:ACE9867
Feltia tricosa|[10499]|PHMO233-03|moth1176.01|639[0n]bp|Canada.Ontario|BOLD:ACE9865
Feltia tricosa|[10500]|RDLQ691-07|DH012203|642[0n]bp|Canada.Quebec|BOLD:ACE9865
Feltia tricosa|[10501]|XAC724-04|04HBL006724|658[0n]bp|Canada.Ontario|BOLD:ACE9865
Feltia tricosa|[10502]|PHMO279-03|moth1839.02|639[0n]bp|Canada.Ontario|BOLD:ACE9865
Feltia tricosa|[10503]|RDLQB756-05|DH010843|617[0n]bp|Canada.Quebec|BOLD:ACE9865
Feltia tricosa|[10504]|BLTIB750-08|BL1040|658[0n]bp|Canada.Ontario|BOLD:ACE9865
Feltia tricosa|[10505]|BLTIB797-08|BL1215|658[0n]bp|Canada.Ontario|BOLD:ACE9865
Feltia tricosa|[10506]|XAG109-05|2005-ONT-693|637[0n]bp|Canada.Ontario|BOLD:ACE9865
Feltia tricosa|[10507]|RDLQB757-05|DH010844|658[0n]bp|Canada.Quebec|BOLD:ACE9865
Feltia tricosa|[10508]|PMG113-03|moth1109.01|617[0n]bp|Canada.Ontario|BOLD:ACE9865
Feltia tricosa|[10509]|RDLQB725-05|DH010828|515[3n]bp|Canada.Quebec|BOLD:ACE9865
Feltia tricosa|[10510]|BLTIB1129-08|BL1143|656[0n]bp|Canada.Ontario|BOLD:ACE9865
Feltia tricosa|[10511]|BLTIB1013-08|BL1452|658[1n]bp|Canada.Ontario|BOLD:ACE9865
Feltia tricosa|[10512]|XAK482-07|HLC-16035|603[0n]bp|Canada.Ontario|BOLD:ACE9865
Feltia tricosa|[10513]|BLTIB749-08|BL1039|658[0n]bp|Canada.Ontario|BOLD:ACE9865
Feltia tricosa|[10514]|BLTIB798-08|BL1216|658[0n]bp|Canada.Ontario|BOLD:ACE9865
Feltia tricosa|[10515]|XAG309-05|2005-ONT-893|658[0n]bp|Canada.Ontario|BOLD:ACE9865
Feltia tricosa|[10516]|XAG095-05|2005-ONT-679|658[0n]bp|Canada.Ontario|BOLD:ACE9865
Feltia tricosa|[10517]|BLTIB775-08|BL1171|658[0n]bp|Canada.Ontario|BOLD:ACE9865
Feltia tricosa|[10518]|XAJ817-06|2006-ONT-0817|658[0n]bp|Canada.Ontario|BOLD:ACE9865
Feltia tricosa|[10519]|JSAUG1676-11|BIOUG01497-F04|658[0n]bp|Canada.Ontario|BOLD:ACE9865
Feltia manifesta|[10520]|LSEU522-06|06-JKA-0522|658[0n]bp|United States.Georgia|BOLD:AAE6661
Feltia manifesta|[10521]|LSEU523-06|06-JKA-0523|658[0n]bp|United States.Georgia|BOLD:AAE6661
Feltia manifesta|[10522]|LSEU520-06|06-JKA-0520|658[0n]bp|United States.Georgia|BOLD:AAE6661
Feltia manifesta|[10523]|LSEU521-06|06-JKA-0521|658[0n]bp|United States.Georgia|BOLD:AAE6661
Feltia geniculata|[10524]|RDLQF282-06|DH011374|658[0n]bp|Canada.Quebec|BOLD:AAE6660
Feltia geniculata|[10525]|RDLQB772-05|DH010859|658[0n]bp|Canada.Quebec|BOLD:AAE6660
Feltia geniculata|[10526]|TTMNB429-06|MNBTT-429|658[0n]bp|Canada.New Brunswick|BOLD:AAE6660
Feltia mollis|[10527]|LPABC022-09|08BBLEP-04241|658[0n]bp|Canada.Alberta|BOLD:ABY6006
Feltia mollis|[10528]|LCH628-05|111CH|658[0n]bp|Canada.Manitoba|BOLD:ABY6006
Feltia mollis|[10529]|LBCH5955-10|10-JDWBC-5955|658[0n]bp|Canada.British Columbia|BOLD:ABY6006
Feltia mollis|[10530]|BBLPB867-10|10BBCLP-1866|658[0n]bp|Canada.Alberta|BOLD:ABY6006
Feltia mollis|[10531]|LOWCB692-05|CGWC-1632|658[0n]bp|Canada.British Columbia|BOLD:ABY6006
Feltia mollis|[10532]|LOWCB689-05|CGWC-1629|564[0n]bp|Canada.British Columbia|BOLD:ABY6006
Feltia mollis|[10533]|LOWCB688-05|CGWC-1628|649[0n]bp|Canada.British Columbia|BOLD:ABY6006
Feltia mollis|[10534]|LOWCB686-05|CGWC-1626|562[1n]bp|Canada.British Columbia|BOLD:ABY6006
Feltia mollis|[10535]|LOWCB696-05|CGWC-1636|658[0n]bp|Canada.British Columbia|BOLD:ABY6006
Feltia mollis|[10536]|LOWCD916-06|CGWC-3736|658[0n]bp|Canada.British Columbia|BOLD:ABY6006
Feltia mollis|[10537]|LOWCB694-05|CGWC-1634|658[0n]bp|Canada.British Columbia|BOLD:ABY6006
Feltia mollis|[10538]|LOWCD913-06|CGWC-3733|658[0n]bp|Canada.British Columbia|BOLD:ABY6006
Feltia mollis|[10539]|LOWCB697-05|CGWC-1637|658[0n]bp|Canada.British Columbia|BOLD:ABY6006
Feltia mollis|[10540]|LOWCB690-05|CGWC-1630|658[0n]bp|Canada.British Columbia|BOLD:ABY6006
Feltia mollis|[10541]|LOWCB693-05|CGWC-1633|658[0n]bp|Canada.British Columbia|BOLD:ABY6006
Feltia mollis|[10542]|LBCH5954-10|10-JDWBC-5954|658[0n]bp|Canada.British Columbia|BOLD:ABY6006
Feltia mollis|[10543]|LOWCB691-05|CGWC-1631|658[0n]bp|Canada.British Columbia|BOLD:ABY6006
Feltia mollis|[10544]|LOWCD912-06|CGWC-3732|609[0n]bp|Canada.British Columbia|BOLD:ABY6006
Feltia mollis|[10545]|LOWCD914-06|CGWC-3734|585[0n]bp|Canada.British Columbia|BOLD:ABY6006
Feltia mollis|[10546]|LOWCD915-06|CGWC-3735|589[0n]bp|Canada.British Columbia|BOLD:ABY6006
Feltia mollis|[10547]|LOWCB695-05|CGWC-1635|658[0n]bp|Canada.British Columbia|BOLD:ABY6006
Feltia mollis|[10548]|LOWCB687-05|CGWC-1627|658[0n]bp|Canada.British Columbia|BOLD:ABY6006
Feltia nigrita|[10549]|RDNMG713-08|CNC LEP00052837|642[0n]bp|Canada.Yukon Territory|BOLD:ABY6005
Feltia nigrita|[10550]|RDNMG712-08|CNC LEP00052836|658[0n]bp|Canada.Yukon Territory|BOLD:ABY6005
Feltia nigrita|[10551]|RDNMG711-08|CNC LEP00052835|658[0n]bp|Canada.Yukon Territory|BOLD:ABY6005
Feltia nigrita|[10552]|RDNMF791-08|UASM2053|658[0n]bp|Canada.Alberta|BOLD:ABY6005
Feltia nigrita|[10553]|RDNMF793-08|UASM107561|658[0n]bp|Canada.Alberta|BOLD:ABY6005
Feltia nigrita|[10554]|RDNMF790-08|UASM56912|658[0n]bp|Canada.Alberta|BOLD:ABY6005
Feltia nigrita|[10555]|RDNMG710-08|CNC LEP00052834|658[0n]bp|Canada.Saskatchewan|BOLD:ABY6005
Feltia nigrita|[10556]|BBLPB754-10|10BBCLP-1753|658[0n]bp|Canada.Alberta|BOLD:ABY6005
Feltia nigrita|[10557]|RDNMF789-08|UASM107564|658[0n]bp|Canada.Alberta|BOLD:ABY6005
Feltia nigrita|[10558]|RDNMF788-08|UASM41655|658[0n]bp|Canada.Alberta|BOLD:ABY6005
Feltia nigrita|[10559]|RDNMG714-08|CNC LEP00052838|658[0n]bp|Canada.Alberta|BOLD:ABY6005
Feltia nigrita|[10560]|RDNMF792-08|UASM107560|658[0n]bp|Canada.Alberta|BOLD:ABY6005
Feltia nigrita|[10561]|RDNMF794-08|UASM107565|658[0n]bp|Canada.British Columbia|BOLD:ABY6005
Feltia boreana|[10562]|RDNMF066-08|NOC14152|658[0n]bp|Canada.Yukon Territory|BOLD:AAD9414
Feltia boreana|[10563]|RDNMG734-08|CNC LEP00052858|642[0n]bp|Canada.Yukon Territory|BOLD:AAD9414
Feltia boreana|[10564]|RDNMG721-08|CNC LEP00052845|642[0n]bp|Canada.Yukon Territory|BOLD:AAD9414
Feltia boreana|[10565]|RDNMG720-08|CNC LEP00052844|633[2n]bp|Canada.Northwest Territories|BOLD:AAD9414

Feltia boreana|[10563]|RDNMG734-08|CNC LEP00052858|642[0n]bp|Canada.Yukon Territory|BOLD:AAD9414
Feltia boreana|[10564]|RDNMG721-08|CNC LEP00052845|642[0n]bp|Canada.Yukon Territory|BOLD:AAD9414
Feltia boreana|[10565]|RDNMG720-08|CNC LEP00052844|633[2n]bp|Canada.Northwest Territories|BOLD:AAD9414
Feltia boreana|[10566]|RDNMG719-08|CNC LEP00052843|642[1n]bp|Canada.Northwest Territories|BOLD:AAD9414
Feltia boreana|[10567]|RDNMG718-08|CNC LEP00052842|645[0n]bp|Canada.Northwest Territories|BOLD:AAD9414
Feltia beringiana|[10568]|RDNMG736-08|CNC LEP00052860|642[0n]bp|Canada.Yukon Territory|BOLD:ABY9079
Feltia beringiana|[10569]|RDNMF059-08|NOC14145|658[0n]bp|Canada.Yukon Territory|BOLD:ABY9079
Feltia troubridgei|[10570]|RDNMI191-12|CNCLEP00019235|614[0n]bp|Canada.Yukon Territory|BOLD:ACE9137
Feltia woodiana|[10571]|RDNMG717-08|CNC LEP00052841|658[0n]bp|Canada.Yukon Territory|BOLD:ACE9137
Feltia woodiana|[10572]|RDNMG715-08|CNC LEP00052839|641[0n]bp|Canada.Yukon Territory|BOLD:ACE9137
Feltia woodiana|[10573]|RDNMG716-08|CNC LEP00052840|642[0n]bp|Canada.Yukon Territory|BOLD:ACE9137
Feltia woodiana|[10574]|RDNMF058-08|NOC14144|658[0n]bp|Canada.Yukon Territory|BOLD:ACE9137
Actebia balanitis|[10575]|LOWCE307-06|CGWC-4067|658[0n]bp|Canada.British Columbia|BOLD:AAB2919
Actebia balanitis|[10576]|LOWCD897-06|CGWC-3717|658[0n]bp|Canada.British Columbia|BOLD:AAB2919
Actebia balanitis|[10577]|LOWCE304-06|CGWC-4064|658[0n]bp|Canada.British Columbia|BOLD:AAB2919
Actebia balanitis|[10578]|LOWCD511-06|CGWC-3331|658[0n]bp|Canada.British Columbia|BOLD:AAB2919
Actebia balanitis|[10579]|LBCH6844-10|10-JDWBC-6844|658[0n]bp|Canada.British Columbia|BOLD:AAB2919
Actebia balanitis|[10580]|LPABB417-08|08BBLEP-03682|658[0n]bp|Canada.Alberta|BOLD:AAB2919
Actebia balanitis|[10581]|LPABB334-08|08BBLEP-03599|658[0n]bp|Canada.Alberta|BOLD:AAB2919
Actebia balanitis|[10582]|LOWCD910-06|CGWC-3730|658[0n]bp|Canada.British Columbia|BOLD:AAB2919
Actebia balanitis|[10583]|LOWCD508-06|CGWC-3328|658[0n]bp|Canada.British Columbia|BOLD:AAB2919
Actebia balanitis|[10584]|LOWCE302-06|CGWC-4062|658[0n]bp|Canada.British Columbia|BOLD:AAB2919
Actebia balanitis|[10585]|LPABB415-08|08BBLEP-03680|658[0n]bp|Canada.Alberta|BOLD:AAB2919
Actebia balanitis|[10586]|LPSK127-08|08BBLEP-01695|658[0n]bp|Canada.Saskatchewan|BOLD:AAB2919
Actebia balanitis|[10587]|LOWCE303-06|CGWC-4063|658[0n]bp|Canada.British Columbia|BOLD:AAB2919
Actebia balanitis|[10588]|LOWCD512-06|CGWC-3332|658[0n]bp|Canada.British Columbia|BOLD:AAB2919
Actebia balanitis|[10589]|LPABB406-08|08BBLEP-03671|658[0n]bp|Canada.Alberta|BOLD:AAB2919
Actebia balanitis|[10590]|LOWCE301-06|CGWC-4061|658[0n]bp|Canada.British Columbia|BOLD:AAB2919
Actebia balanitis|[10591]|LPABB337-08|08BBLEP-03602|658[0n]bp|Canada.Alberta|BOLD:AAB2919
Actebia balanitis|[10592]|LOWCD507-06|CGWC-3327|656[0n]bp|Canada.British Columbia|BOLD:AAB2919
Actebia balanitis|[10593]|LOWCD513-06|CGWC-3333|594[0n]bp|Canada.British Columbia|BOLD:AAB2919
Actebia balanitis|[10594]|LOWCD506-06|CGWC-3326|578[0n]bp|Canada.British Columbia|BOLD:AAB2919
Actebia balanitis|[10595]|LPABC221-09|08BBLEP-04440|632[0n]bp|Canada.Alberta|BOLD:AAB2919
Actebia balanitis|[10596]|LOWCD905-06|CGWC-3725|611[0n]bp|Canada.British Columbia|BOLD:AAB2919
Actebia balanitis|[10597]|LOWCD510-06|CGWC-3330|607[0n]bp|Canada.British Columbia|BOLD:AAB2919
Actebia balanitis|[10598]|LBCH6921-10|10-JDWBC-6921|601[0n]bp|Canada.British Columbia|BOLD:AAB2919
Actebia balanitis|[10599]|LOWCD509-06|CGWC-3329|597[0n]bp|Canada.British Columbia|BOLD:AAB2919
Actebia balanitis|[10600]|LPABB385-08|08BBLEP-03650|658[0n]bp|Canada.Alberta|BOLD:AAB2919
Actebia balanitis|[10601]|LOWCE308-06|CGWC-4068|658[0n]bp|Canada.British Columbia|BOLD:AAB2919
Actebia balanitis|[10602]|LOWCE306-06|CGWC-4066|658[0n]bp|Canada.British Columbia|BOLD:AAB2919
Eucoptocnemis fimbriaris|[10603]|LNC474-06|05-NCCC-474|658[1n]bp|United States.North Carolina|BOLD:AAE...
Eucoptocnemis fimbriaris|[10604]|LNC631-06|06-NCCC-631|658[0n]bp|United States.North Carolina|BOLD:AAE...
Eucoptocnemis fimbriaris|[10605]|GWOTA063-12|BC ZSM Lep 58539|658[0n]bp|United States.Massachusetts|BOLD...
Eucoptocnemis fimbriaris|[10606]|LNC473-06|05-NCCC-473|658[0n]bp|United States.North Carolina|BOLD:AAE...
Eucoptocnemis fimbriaris|[10607]|LNC630-06|06-NCCC-630|658[0n]bp|United States.North Carolina|BOLD:AAE...
Protogygia alberta|[10608]|RDNMB592-05|CNCNoctuoidea10368|658[0n]bp|Canada.Alberta|BOLD:ACE7812
Protogygia enalaga|[10609]|RDMAB137-05|UASM41284|658[0n]bp|Canada.Alberta|BOLD:AAE8189
Protogygia enalaga|[10610]|RDNMC226-05|CNCNoctuoidea11860|658[0n]bp|Canada.Alberta|BOLD:AAE8189
Protogygia postera|[10611]|RDNMC228-05|CNCNoctuoidea11862|582[0n]bp|Canada.Alberta|BOLD:AAF6326
Protogygia querula|[10612]|RDNMF045-08|NOC14131|658[0n]bp|Canada.Alberta|BOLD:AAC5097
Protogygia querula|[10613]|RDNMG739-08|CNC LEP00052863|658[0n]bp|Canada.Alberta|BOLD:AAC5097
Homorthodes hanhami|[10614]|LALPA240-10|AVBC 241-10|658[0n]bp|Canada.British Columbia|BOLD:AAC8664
Homorthodes hanhami|[10615]|LPVIA234-08|PFC-2006-0316|658[0n]bp|Canada.British Columbia|BOLD:AAC8664
Homorthodes hanhami|[10616]|LALPA245-10|AVBC 246-10|658[0n]bp|Canada.British Columbia|BOLD:AAC8664
Homorthodes hanhami|[10617]|LPVIB042-08|PFC-2006-1367|658[0n]bp|Canada.British Columbia|BOLD:AAC8664
Homorthodes hanhami|[10618]|LPVIA269-08|PFC-2006-0355|658[0n]bp|Canada.British Columbia|BOLD:AAC8664
Homorthodes hanhami|[10619]|LALPA223-10|AVBC 224-10|658[0n]bp|Canada.British Columbia|BOLD:AAC8664
Homorthodes hanhami|[10620]|LPVIA102-08|PFC-2006-0167|658[0n]bp|Canada.British Columbia|BOLD:AAC8664
Homorthodes hanhami|[10621]|LPVIA287-08|PFC-2006-0378|658[0n]bp|Canada.British Columbia|BOLD:AAC8664
Homorthodes hanhami|[10622]|LALPA218-10|AVBC 219-10|658[0n]bp|Canada.British Columbia|BOLD:AAC8664
Homorthodes hanhami|[10623]|LPVIA273-08|PFC-2006-0361|644[0n]bp|Canada.British Columbia|BOLD:AAC8664
Homorthodes hanhami|[10624]|LPVIA606-08|PFC-2006-0834|658[0n]bp|Canada.British Columbia|BOLD:AAC8664
Homorthodes hanhami|[10625]|LPVIB056-08|PFC-2006-1384|658[0n]bp|Canada.British Columbia|BOLD:AAC8664
Homorthodes hanhami|[10626]|LPVIA272-08|PFC-2006-0360|658[0n]bp|Canada.British Columbia|BOLD:AAC8664
Homorthodes hanhami|[10627]|LPVIA605-08|PFC-2006-0833|658[0n]bp|Canada.British Columbia|BOLD:AAC8664
Homorthodes hanhami|[10628]|LPVIA842-08|PFC-2006-1142|658[0n]bp|Canada.British Columbia|BOLD:AAC8664
Homorthodes hanhami|[10629]|LPVIB044-08|PFC-2006-1369|658[0n]bp|Canada.British Columbia|BOLD:AAC8664
Homorthodes hanhami|[10630]|LPVIA274-08|PFC-2006-0362|658[0n]bp|Canada.British Columbia|BOLD:AAC8664
Homorthodes hanhami|[10631]|LPVIA571-08|PFC-2006-0798|658[0n]bp|Canada.British Columbia|BOLD:AAC8664
Homorthodes hanhami|[10632]|LPVIA101-08|PFC-2006-0166|658[0n]bp|Canada.British Columbia|BOLD:AAC8664
Homorthodes hanhami|[10633]|LPVIA268-08|PFC-2006-0354|658[0n]bp|Canada.British Columbia|BOLD:AAC8664
Homorthodes hanhami|[10634]|LPVIA275-08|PFC-2006-0363|658[0n]bp|Canada.British Columbia|BOLD:AAC8664
Homorthodes hanhami|[10635]|LPVIA277-08|PFC-2006-0365|658[0n]bp|Canada.British Columbia|BOLD:AAC8664
Homorthodes hanhami|[10636]|LPVIA276-08|PFC-2006-0364|658[2n]bp|Canada.British Columbia|BOLD:AAC8664
Homorthodes hanhami|[10637]|LPVIA973-08|PFC-2006-1300|613[0n]bp|Canada.British Columbia|BOLD:AAC8664
Homorthodes hanhami|[10638]|LPVIB043-08|PFC-2006-1368|619[0n]bp|Canada.British Columbia|BOLD:AAC8664
Homorthodes hanhami|[10639]|LPVIA969-08|PFC-2006-1295|635[0n]bp|Canada.British Columbia|BOLD:AAC8664
Homorthodes hanhami|[10640]|LPVIA971-08|PFC-2006-1297|635[0n]bp|Canada.British Columbia|BOLD:AAC8664
Anicla infecta|[10641]|LOFLC186-06|06-FLOR-2066|658[0n]bp|United States.Florida|BOLD:ACE9898
Anicla infecta|[10642]|LOFLB487-06|06-FLOR-1427|658[0n]bp|United States.Florida|BOLD:ACE9898
Anicla infecta|[10643]|USLEP1192-10|10BBLEP-01192|658[0n]bp|United States.Florida|BOLD:ACE9898
Anicla infecta|[10644]|BBLOE1435-12|BIOUG01987-D01|658[0n]bp|United States.Arkansas|BOLD:ACE9898
Anicla infecta|[10645]|USLEP292-10|10BBLEP-00292|658[0n]bp|United States.Florida|BOLD:ACE9898
Anicla infecta|[10646]|USLEP1148-10|10BBLEP-01148|658[0n]bp|United States.Florida|BOLD:ACE9898
Anicla infecta|[10647]|LPOKA668-09|MDOK-0668|658[0n]bp|United States.Oklahoma|BOLD:ACE9898
Anicla infecta|[10648]|LGSMG895-10|BGS04164|658[0n]bp|United States.North Carolina|BOLD:ACE9898
Anicla infecta|[10649]|LOFLB126-06|06-FLOR-1066|658[0n]bp|United States.Florida|BOLD:ACE9898
Anicla infecta|[10650]|USLEP642-10|10BBLEP-00642|658[0n]bp|United States.Florida|BOLD:ACE9898
Anicla infecta|[10651]|LGSMG894-10|BGS04163|658[0n]bp|United States.Tennessee|BOLD:ACE9898
Anicla infecta|[10652]|USLEP632-10|10BBLEP-00632|658[0n]bp|United States.Florida|BOLD:ACE9898
Anicla infecta|[10653]|LILLA797-11|SNS10IL-01002|658[0n]bp|United States.Illinois|BOLD:ACE9898
Anicla infecta|[10654]|LPOKA961-09|MDOK-1056|658[0n]bp|United States.Oklahoma|BOLD:ACE9898
Anicla infecta|[10655]|BBLOC1037-11|BIOUG01468-F09|658[0n]bp|United States.Texas|BOLD:ACE9898
Anicla infecta|[10656]|BBLOB1307-11|BIOUG01417-F12|658[0n]bp|United States.Florida|BOLD:ACE9898
Anicla infecta|[10657]|LNC485-06|05-NCCC-485|658[0n]bp|United States.North Carolina|BOLD:ACE9898
Anicla infecta|[10658]|LPOKA616-09|MDOK-0616|639[0n]bp|United States.Oklahoma|BOLD:ACE9898
Anicla infecta|[10659]|LPOKA518-09|MDOK-0518|632[0n]bp|United States.Oklahoma|BOLD:ACE9898
Anicla infecta|[10660]|LNC486-06|05-NCCC-486|650[0n]bp|United States.North Carolina|BOLD:ACE9898
Anicla infecta|[10661]|BBLSW846-09|09BBLEP-01774|632[0n]bp|United States.Texas|BOLD:ACE9898
Anicla infecta|[10662]|USLEP300-10|10BBLEP-00300|658[0n]bp|United States.Florida|BOLD:ACE9898
Anicla illapsa|[10663]|NCCH102-11|BIOUG01862-E04|658[0n]bp|Canada.Ontario|BOLD:ACF5764
Anicla illapsa|[10664]|MEC404-04|jflandry0404|658[0n]bp|Canada.Quebec|BOLD:ACF5764
Anicla illapsa|[10665]|XAC188-04|04HBL006188|658[0n]bp|Canada.Ontario|BOLD:ACF5764

[illegible]
Anicla illapsa|[10663]|NCCH102-11|BIOUG01862-E04|658[0n]bp|Canada.Ontario|BOLD:ACF5764
Anicla illapsa|[10664]|MEC404-04|jflandry0404|658[0n]bp|Canada.Quebec|BOLD:ACF5764
Anicla illapsa|[10665]|XAC188-04|04HBL006188|658[0n]bp|Canada.Ontario|BOLD:ACF5764
Anicla illapsa|[10666]|XAC627-04|04HBL006627|658[0n]bp|Canada.Ontario|BOLD:ACF5764
Anicla illapsa|[10667]|XAC178-04|04HBL006178|658[0n]bp|Canada.Ontario|BOLD:ACF5764
Anicla illapsa|[10668]|XAJ617-06|2006-ONT-0617|658[0n]bp|Canada.Ontario|BOLD:ACF5764
Anicla illapsa|[10669]|BLTIB379-08|BL607|638[0n]bp|Canada.Ontario|BOLD:ACF5764
Anicla tenuescens|[10670]|RDNMF593-08|NOC14679|658[0n]bp|United States.Nebraska|BOLD:AAE5009
Anicla tenuescens|[10671]|RDNMF595-08|NOC14681|658[0n]bp|United States.Wyoming|BOLD:AAE5009
Anicla tenuescens|[10672]|RDNMF592-08|NOC14678|658[0n]bp|United States.Colorado|BOLD:AAE5009
Anicla tenuescens|[10673]|RDNMF594-08|NOC14680|522[2n]bp|United States.Nebraska|
Anicla exuberans|[10674]|LOWCE767-06|CGWC-4527|658[1n]bp|Canada.British Columbia|BOLD:AAC5892
Anicla exuberans|[10675]|LBCG413-08|08-JDWBC-0413|658[0n]bp|Canada.British Columbia|BOLD:AAC5892
Anicla exuberans|[10676]|LBCH6040-10|10-JDWBC-6040|658[0n]bp|Canada.British Columbia|BOLD:AAC5892
Anicla exuberans|[10677]|LOWCE296-06|CGWC-4056|658[0n]bp|Canada.British Columbia|BOLD:AAC5892
Anicla exuberans|[10678]|LBCH6095-10|10-JDWBC-6095|658[0n]bp|Canada.British Columbia|BOLD:AAC5892
Anicla exuberans|[10679]|LBCH5511-10|10-JDWBC-5511|658[0n]bp|Canada.British Columbia|BOLD:AAC5892
Anicla exuberans|[10680]|LBCH6042-10|10-JDWBC-6042|658[0n]bp|Canada.British Columbia|BOLD:AAC5892
Anicla exuberans|[10681]|LBCH6043-10|10-JDWBC-6043|658[0n]bp|Canada.British Columbia|BOLD:AAC5892
Anicla exuberans|[10682]|LOWCE298-06|CGWC-4058|658[0n]bp|Canada.British Columbia|BOLD:AAC5892
Anicla exuberans|[10683]|LBCG414-08|08-JDWBC-0414|658[0n]bp|Canada.British Columbia|BOLD:AAC5892
Anicla exuberans|[10684]|LBCH5570-10|10-JDWBC-5570|658[0n]bp|Canada.British Columbia|BOLD:AAC5892
Anicla exuberans|[10685]|LOWCE769-06|CGWC-4529|623[0n]bp|Canada.British Columbia|BOLD:AAC5892
Anicla exuberans|[10686]|LOWCE768-06|CGWC-4528|658[0n]bp|Canada.British Columbia|BOLD:AAC5892
Anicla exuberans|[10687]|LBCH5571-10|10-JDWBC-5571|658[0n]bp|Canada.British Columbia|BOLD:AAC5892
Anicla exuberans|[10688]|LBCH5513-10|10-JDWBC-5513|658[0n]bp|Canada.British Columbia|BOLD:AAC5892
Anicla exuberans|[10689]|LOWCE295-06|CGWC-4055|658[0n]bp|Canada.British Columbia|BOLD:AAC5892
Anicla exuberans|[10690]|LBCG412-08|08-JDWBC-0412|658[0n]bp|Canada.British Columbia|BOLD:AAC5892
Anicla forbesi|[10691]|RDLQB679-05|DH010782|658[0n]bp|Canada.Quebec|BOLD:ACF5752
Anicla forbesi|[10692]|RDNMG941-08|CNC LEP00053065|658[0n]bp|Canada.Ontario|BOLD:ACF5752
Anicla forbesi|[10693]|RDNMG942-08|CNC LEP00053066|658[0n]bp|Canada.Ontario|BOLD:ACF5752
Anicla forbesi|[10694]|RDLQ705-07|DH007930|658[0n]bp|Canada.Quebec|BOLD:ACF5752
Anicla forbesi|[10695]|RDNMG943-08|CNC LEP00053067|658[0n]bp|Canada.Ontario|BOLD:ACF5752
Anicla tepperi|[10696]|LPSK131-08|08BBLEP-01699|658[0n]bp|Canada.Saskatchewan|BOLD:AAC5355
Anicla tepperi|[10697]|LPSK545-08|08BBLEP-02113|658[0n]bp|Canada.Saskatchewan|BOLD:AAC5355
Anicla tepperi|[10698]|LPSK398-08|08BBLEP-01966|658[0n]bp|Canada.Saskatchewan|
Anicla tepperi|[10699]|RDNMF095-08|NOC14181|658[0n]bp|Canada.Alberta|BOLD:AAC5355
Anicla tepperi|[10700]|LPSK504-08|08BBLEP-02072|658[0n]bp|Canada.Saskatchewan|BOLD:AAC5355
Anicla tepperi|[10701]|LPSK248-08|08BBLEP-01816|658[0n]bp|Canada.Saskatchewan|BOLD:AAC5355
Anicla tepperi|[10702]|LPSK141-08|08BBLEP-01709|658[0n]bp|Canada.Saskatchewan|BOLD:AAC5355
Peridroma saucia|[10703]|XAB693-04|04HBL005693|611[0n]bp|Canada.Ontario|BOLD:AAA6377
Peridroma saucia|[10704]|RDLQ707-07|DH006206|658[3n]bp|Canada.Quebec|BOLD:AAA6377
Peridroma saucia|[10705]|LALPA1124-11|AVBC 934-11|658[0n]bp|Canada.British Columbia|BOLD:AAA6377
Peridroma saucia|[10706]|BBLPB692-10|10BBCLP-1691|658[0n]bp|Canada.Ontario|BOLD:AAA6377
Peridroma saucia|[10707]|XAB689-04|04HBL005689|658[0n]bp|Canada.Ontario|BOLD:AAA6377
Peridroma saucia|[10708]|XAH834-05|2005-ONT-2417|658[0n]bp|Canada.Ontario|BOLD:AAA6377
Peridroma saucia|[10709]|LBCW055-08|08-JDWWI-0055|658[0n]bp|Canada.British Columbia|BOLD:AAA6377
Peridroma saucia|[10710]|XAC150-04|04HBL006150|626[0n]bp|Canada.Ontario|BOLD:AAA6377
Peridroma saucia|[10711]|MHCOL120-07|CHU06-COL-120|603[0n]bp|Canada.Manitoba|BOLD:AAA6377
Peridroma saucia|[10712]|JGLL050-10|10PROBE-19713|658[0n]bp|Canada.Manitoba|BOLD:AAA6377
Peridroma saucia|[10713]|LBCG856-09|08-JDWBC-0856|658[0n]bp|Canada.British Columbia|BOLD:AAA6377
Peridroma saucia|[10714]|LCHP394-07|07PROBE-10018|658[0n]bp|Canada.Manitoba|BOLD:AAA6377
Ochropleura implecta|[10715]|MNBB128-05|05-NBSTA-044|606[2n]bp|Canada.New Brunswick|BOLD:AAA3073
Ochropleura implecta|[10716]|LHLEP069-06|UBC-2006-0274|658[0n]bp|Canada.British Columbia|BOLD:AAA3073
Ochropleura implecta|[10717]|PHMNB607-04|04HBL00833|658[0n]bp|Canada.New Brunswick|BOLD:AAA3073
Ochropleura implecta|[10718]|BBLPE274-09|09BBELE-2274|658[0n]bp|Canada.Nova Scotia|BOLD:AAA3073
Ochropleura implecta|[10719]|BBLPE407-09|09BBELE-2407|633[0n]bp|Canada.Newfoundland and Labrador|BOLD:...
Ochropleura implecta|[10720]|LPGVA605-08|UBC-2006-1763|632[0n]bp|Canada.British Columbia|BOLD:AAA3073
Ochropleura implecta|[10721]|PHMNB608-04|04HBL00834|658[0n]bp|Canada.New Brunswick|BOLD:AAA3073
Ochropleura implecta|[10722]|RDLQF225-06|DH011305|658[0n]bp|Canada.Quebec|BOLD:AAA3073
Ochropleura implecta|[10723]|PHMNB383-04|04HBL00609|658[0n]bp|Canada.New Brunswick|BOLD:AAA3073
Ochropleura implecta|[10724]|LHLEP218-06|UBC-2006-0166|658[0n]bp|Canada.British Columbia|BOLD:AAA3073
Ochropleura implecta|[10725]|RDLQF270-06|DH011362|658[0n]bp|Canada.Quebec|BOLD:AAA3073
Ochropleura implecta|[10726]|RDLQB536-05|DH010622|658[0n]bp|Canada.Quebec|BOLD:AAA3073
Ochropleura implecta|[10727]|BBLPC694-09|09BBELE-1694|658[0n]bp|Canada.Newfoundland and Labrador|BOLD:...
Ochropleura implecta|[10728]|LHLEP227-06|UBC-2006-2068|658[0n]bp|Canada.British Columbia|BOLD:AAA3073
Ochropleura implecta|[10729]|LPMN805-08|08BBLEP-01608|658[0n]bp|Canada.Manitoba|BOLD:AAA3073
Ochropleura implecta|[10730]|LHLEP070-06|UBC-2006-0275|658[0n]bp|Canada.British Columbia|BOLD:AAA3073
Ochropleura implecta|[10731]|RDLQF456-06|DH011563|658[0n]bp|Canada.Quebec|BOLD:AAA3073
Ochropleura implecta|[10732]|LALPA251-10|AVBC 252-10|658[0n]bp|Canada.British Columbia|BOLD:AAA3073
Ochropleura implecta|[10733]|LHLEP231-06|UBC-2006-2110|658[0n]bp|Canada.British Columbia|BOLD:AAA3073
Ochropleura implecta|[10734]|LHLEP219-06|UBC-2006-0167|658[0n]bp|Canada.British Columbia|BOLD:AAA3073
Ochropleura implecta|[10735]|LPGVA603-08|UBC-2006-1761|658[0n]bp|Canada.British Columbia|BOLD:AAA3073
Ochropleura implecta|[10736]|LBCH2986-10|10-JDWBC-2986|658[0n]bp|Canada.British Columbia|BOLD:AAA3073
Ochropleura implecta|[10737]|LBCA812-05|HLC-20812|658[0n]bp|Canada.British Columbia|BOLD:AAA3073
Ochropleura implecta|[10738]|BBLPE277-09|09BBELE-2277|658[0n]bp|Canada.Nova Scotia|BOLD:AAA3073
Ochropleura implecta|[10739]|BBLEC603-09|09BBELE-0603|658[0n]bp|Canada.Nova Scotia|BOLD:AAA3073
Ochropleura implecta|[10740]|LHLEP235-06|UBC-2006-2136|658[0n]bp|Canada.British Columbia|BOLD:AAA3073
Ochropleura implecta|[10741]|MNBB451-05|05-NBSTA-367|658[0n]bp|Canada.New Brunswick|BOLD:AAA3073
Ochropleura implecta|[10742]|BBLPB846-10|10BBCLP-1845|658[0n]bp|Canada.British Columbia|BOLD:AAA3073
Ochropleura implecta|[10743]|LPABB056-08|08BBLEP-03321|658[0n]bp|Canada.Alberta|BOLD:AAA3073
Ochropleura implecta|[10744]|LPABC815-09|08BBLEP-05034|658[0n]bp|Canada.Alberta|BOLD:AAA3073
Ochropleura implecta|[10745]|BBLPB847-10|10BBCLP-1846|658[0n]bp|Canada.British Columbia|BOLD:AAA3073
Ochropleura implecta|[10746]|LHLEP223-06|UBC-2006-1049|658[0n]bp|Canada.British Columbia|BOLD:AAA3073
Ochropleura implecta|[10747]|BBLPB850-10|10BBCLP-1849|658[0n]bp|Canada.Ontario|BOLD:AAA3073
Ochropleura implecta|[10748]|BBLPE116-09|09BBELE-2116|658[0n]bp|Canada.Nova Scotia|BOLD:AAA3073
Ochropleura implecta|[10749]|BBLPE081-09|09BBELE-2081|658[0n]bp|Canada.Nova Scotia|BOLD:AAA3073
Ochropleura implecta|[10750]|BBLPC726-09|09BBELE-1726|658[0n]bp|Canada.Newfoundland and Labrador|BOLD:...
Ochropleura implecta|[10751]|LHLEP233-06|UBC-2006-2112|658[0n]bp|Canada.British Columbia|BOLD:AAA3073
Ochropleura implecta|[10752]|LHLEP234-06|UBC-2006-2135|658[0n]bp|Canada.British Columbia|BOLD:AAA3073
Ochropleura implecta|[10753]|BBLPC600-09|09BBELE-1600|658[0n]bp|Canada.Nova Scotia|BOLD:AAA3073
Ochropleura implecta|[10754]|LHLEP232-06|UBC-2006-2111|658[0n]bp|Canada.British Columbia|BOLD:AAA3073
Ochropleura implecta|[10755]|LHLEP071-06|UBC-2006-0276|658[0n]bp|Canada.British Columbia|BOLD:AAA3073
Ochropleura implecta|[10756]|LHLEP226-06|UBC-2006-1931|658[0n]bp|Canada.British Columbia|BOLD:AAA3073
Ochropleura implecta|[10757]|LBCA933-05|HLC-20933|658[0n]bp|Canada.British Columbia|BOLD:AAA3073
Ochropleura implecta|[10758]|BBLPB848-10|10BBCLP-1847|658[0n]bp|Canada.British Columbia|BOLD:AAA3073
Ochropleura implecta|[10759]|BBLPC695-09|09BBELE-1695|658[0n]bp|Canada.Newfoundland and Labrador|BOLD:...
Ochropleura implecta|[10760]|LCH264-04|04HBL003264|658[0n]bp|Canada.Manitoba|BOLD:AAA3073
Ochropleura implecta|[10761]|BBLPC257-09|09BBELE-1257|658[0n]bp|Canada.Nova Scotia|BOLD:AAA3073
Ochropleura implecta|[10762]|LALPA185-10|AVBC 185-10|658[0n]bp|Canada.British Columbia|BOLD:AAA3073
Ochropleura implecta|[10763]|BBLPC593-09|09BBELE-1593|658[0n]bp|Canada.Nova Scotia|BOLD:AAA3073
Ochropleura implecta|[10764]|TTMNB044-06|MNBTT-044|658[0n]bp|Canada.New Brunswick|BOLD:AAA3073
[illegible]

Ochropleura implecta|[10762]|LALPA185-10|AVBC 185-10|658[0n]bp|Canada.British Columbia|BOLD:AAA3073
Ochropleura implecta|[10763]|BBLPC593-09|09BBELE-1593|658[0n]bp|Canada.Nova Scotia|BOLD:AAA3073
Ochropleura implecta|[10764]|TTMNB044-06|MNBTT-044|658[0n]bp|Canada.New Brunswick|BOLD:AAA3073
Ochropleura implecta|[10765]|LHLEP222-06|UBC-2006-1048|658[0n]bp|Canada.British Columbia|BOLD:AAA3073
Ochropleura implecta|[10766]|RDLQB537-05|DH010623|658[0n]bp|Canada.Quebec|BOLD:AAA3073
Ochropleura implecta|[10767]|LHLEP224-06|UBC-2006-1522|658[0n]bp|Canada.British Columbia|BOLD:AAA3073
Ochropleura implecta|[10768]|RDLQF546-06|DH011695|658[0n]bp|Canada.Quebec|BOLD:AAA3073
Ochropleura implecta|[10769]|LHLEP230-06|UBC-2006-2071|658[0n]bp|Canada.British Columbia|BOLD:AAA3073
Ochropleura implecta|[10770]|BBLEC666-09|09BBELE-0666|658[0n]bp|Canada.Nova Scotia|BOLD:AAA3073
Ochropleura implecta|[10771]|LHLEP220-06|UBC-2006-0765|658[0n]bp|Canada.British Columbia|BOLD:AAA3073
Ochropleura implecta|[10772]|BBLPC662-09|09BBELE-1662|658[0n]bp|Canada.Newfoundland and Labrador|BOLD:...
Ochropleura implecta|[10773]|BBLPE390-09|09BBELE-2390|658[0n]bp|Canada.Newfoundland and Labrador|BOLD:...
Ochropleura implecta|[10774]|BBLPB457-10|10BBCLP-1456|658[0n]bp|Canada.British Columbia|BOLD:AAA3073
Ochropleura implecta|[10775]|LHLEP229-06|UBC-2006-2070|658[0n]bp|Canada.British Columbia|BOLD:AAA3073
Ochropleura implecta|[10776]|LBCH3004-10|10-JDWBC-3004|658[0n]bp|Canada.British Columbia|BOLD:AAA3073
Ochropleura implecta|[10777]|PHMNB378-04|04HBL00604|658[0n]bp|Canada.New Brunswick|BOLD:AAA3073
Ochropleura implecta|[10778]|LALPA405-10|AVBC 407-10|658[0n]bp|Canada.British Columbia|BOLD:AAA3073
Ochropleura implecta|[10779]|LBCC294-05|HLC-22174|658[0n]bp|Canada.British Columbia|BOLD:AAA3073
Ochropleura implecta|[10780]|XAD775-05|2005-ONT-574|658[0n]bp|Canada.Ontario|BOLD:AAA3073
Ochropleura implecta|[10781]|LPGVA607-08|UBC-2006-1765|658[0n]bp|Canada.British Columbia|BOLD:AAA3073
Ochropleura implecta|[10782]|LPMN727-08|08BBLEP-01530|658[0n]bp|Canada.Manitoba|BOLD:AAA3073
Ochropleura implecta|[10783]|LHLEP221-06|UBC-2006-1017|658[0n]bp|Canada.British Columbia|BOLD:AAA3073
Ochropleura implecta|[10784]|LPVIA114-08|PFC-2006-0180|658[0n]bp|Canada.British Columbia|BOLD:AAA3073
Ochropleura implecta|[10785]|BBLPC254-09|09BBELE-1254|658[0n]bp|Canada.Nova Scotia|BOLD:AAA3073
Ochropleura implecta|[10786]|BBLPC599-09|09BBELE-1599|658[0n]bp|Canada.Nova Scotia|BOLD:AAA3073
Ochropleura implecta|[10787]|BBLEC624-09|09BBELE-0624|658[0n]bp|Canada.Nova Scotia|BOLD:AAA3073
Ochropleura implecta|[10788]|LHLEP228-06|UBC-2006-2069|658[0n]bp|Canada.British Columbia|BOLD:AAA3073
Ochropleura implecta|[10789]|LHLEP217-06|UBC-2006-0165|658[0n]bp|Canada.British Columbia|BOLD:AAA3073
Ochropleura implecta|[10790]|LBCH1501-10|10-JDWBC-1501|658[0n]bp|Canada.British Columbia|BOLD:AAA3073
Ochropleura implecta|[10791]|LPMN741-08|08BBLEP-01544|609[0n]bp|Canada.Manitoba|BOLD:AAA3073
Ochropleura implecta|[10792]|LPGVA606-08|UBC-2006-1764|627[0n]bp|Canada.British Columbia|BOLD:AAA3073
Ochropleura implecta|[10793]|BBLEC387-09|09BBELE-0387|658[0n]bp|Canada.Newfoundland and Labrador|BOLD:...
Ochropleura implecta|[10794]|RDLQB703-05|DH010806|579[2n]bp|Canada.Quebec|BOLD:AAA3073
Ochropleura implecta|[10795]|BBLEC864-09|09BBELE-0864|654[0n]bp|Canada.Newfoundland and Labrador|BOLD:...
Ochropleura implecta|[10796]|RDLQF224-06|DH011304|655[0n]bp|Canada.Quebec|BOLD:AAA3073
Ochropleura implecta|[10797]|TTMNB090-06|MNBTT-090|656[0n]bp|Canada.New Brunswick|BOLD:AAA3073
Ochropleura implecta|[10798]|RDLQG214-06|DH012390|654[0n]bp|Canada.Quebec|BOLD:AAA3073
Ochropleura implecta|[10799]|LHLEP072-06|UBC-2006-0277|652[0n]bp|Canada.British Columbia|BOLD:AAA3073
Ochropleura implecta|[10800]|LBCA375-05|HLC-20375|649[0n]bp|Canada.British Columbia|BOLD:AAA3073
Ochropleura implecta|[10801]|PHMNB753-05|Moth 446.03SA|650[0n]bp|Canada.New Brunswick|BOLD:AAA3073
Ochropleura implecta|[10802]|BBLPC771-09|09BBELE-1771|620[0n]bp|Canada.Newfoundland and Labrador|BOLD:...
Ochropleura implecta|[10803]|LPGVA635-08|UBC-2006-1803|649[0n]bp|Canada.British Columbia|BOLD:AAA3073
Ochropleura implecta|[10804]|PHMNB674-04|04HBL00900|624[0n]bp|Canada.New Brunswick|BOLD:AAA3073
Ochropleura implecta|[10805]|LPGVA604-08|UBC-2006-1762|626[0n]bp|Canada.British Columbia|BOLD:AAA3073
Ochropleura implecta|[10806]|BBLPC145-09|09BBELE-1145|658[0n]bp|Canada.Nova Scotia|BOLD:AAA3073
Ochropleura implecta|[10807]|BBLEC842-09|09BBELE-0842|634[0n]bp|Canada.Newfoundland and Labrador|BOLD:...
Ochropleura implecta|[10808]|BBLPC664-09|09BBELE-1664|634[0n]bp|Canada.Newfoundland and Labrador|BOLD:...
Ochropleura implecta|[10809]|PHMNB025-03|moth163.02SA|639[0n]bp|Canada.New Brunswick|BOLD:AAA3073
Ochropleura implecta|[10810]|XAF781-05|2005-ONT-430|503[3n]bp|Canada.Ontario|
Ochropleura implecta|[10811]|RDLQB731-05|DH010834|593[1n]bp|Canada.Quebec|BOLD:AAA3073
Ochropleura implecta|[10812]|XAJ840-06|2006-ONT-0840|619[0n]bp|Canada.Ontario|BOLD:AAA3073
Ochropleura implecta|[10813]|LPGVA634-08|UBC-2006-1802|633[0n]bp|Canada.British Columbia|BOLD:AAA3073
Ochropleura implecta|[10814]|LBCA507-05|HLC-20507|658[0n]bp|Canada.British Columbia|BOLD:AAA3073
Ochropleura implecta|[10815]|BBLPE353-09|09BBELE-2353|658[0n]bp|Canada.Newfoundland and Labrador|BOLD:...
Ochropleura implecta|[10816]|LPSOB996-08|PPBP-1995|658[0n]bp|Canada.Ontario|BOLD:AAA3073
Ochropleura implecta|[10817]|LPSOB080-08|PPBP-1079|658[0n]bp|Canada.Ontario|BOLD:AAA3073
Ochropleura implecta|[10818]|LPSOB821-08|PPBP-1820|658[0n]bp|Canada.Ontario|BOLD:AAA3073
Ochropleura implecta|[10819]|XAB071-04|04HBL005071|562[0n]bp|Canada.Ontario|BOLD:AAA3073
Ochropleura implecta|[10820]|BLTIB110-08|BL175|658[1n]bp|Canada.Ontario|BOLD:AAA3073
Ochropleura implecta|[10821]|LPSOB822-08|PPBP-1821|658[0n]bp|Canada.Ontario|BOLD:AAA3073
Ochropleura implecta|[10822]|BLTIB506-08|BL763|658[0n]bp|Canada.Ontario|BOLD:AAA3073
Ochropleura implecta|[10823]|BLGSM050-09|BL369|658[0n]bp|Canada.Ontario|BOLD:AAA3073
Ochropleura implecta|[10824]|LPSOB986-08|PPBP-1985|658[0n]bp|Canada.Ontario|BOLD:AAA3073
Ochropleura implecta|[10825]|BLTIB1023-08|BL1464|658[0n]bp|Canada.Ontario|BOLD:AAA3073
Ochropleura implecta|[10826]|BLTIB247-08|BL429|658[0n]bp|Canada.Ontario|BOLD:AAA3073
Ochropleura implecta|[10827]|LPSOC110-08|PPBP-2109|658[0n]bp|Canada.Ontario|BOLD:AAA3073
Ochropleura implecta|[10828]|LPSO841-08|PPBP-0841|658[0n]bp|Canada.Ontario|BOLD:AAA3073
Ochropleura implecta|[10829]|PMG139-03|moth308.01|617[0n]bp|Canada.Ontario|BOLD:AAA3073
Ochropleura implecta|[10830]|RDLQG224-06|DH012407|632[0n]bp|Canada.Quebec|BOLD:AAA3073
Ochropleura implecta|[10831]|BLTIB222-08|BL402|658[0n]bp|Canada.Ontario|BOLD:AAA3073
Ochropleura implecta|[10832]|XAK318-06|2006-ONT-1313|658[0n]bp|Canada.Ontario|BOLD:AAA3073
Ochropleura implecta|[10833]|BLGSM043-09|BL356|658[0n]bp|Canada.Ontario|BOLD:AAA3073
Ochropleura implecta|[10834]|LPSO436-08|PPBP-0436|658[0n]bp|Canada.Ontario|BOLD:AAA3073
Ochropleura implecta|[10835]|XAH267-05|2005-ONT-1850|617[0n]bp|Canada.Ontario|BOLD:AAA3073
Ochropleura implecta|[10836]|XAK300-06|2006-ONT-1295|656[0n]bp|Canada.Ontario|BOLD:AAA3073
Ochropleura implecta|[10837]|XAD776-05|2005-ONT-575|658[0n]bp|Canada.Ontario|BOLD:AAA3073
Ochropleura implecta|[10838]|BLTIB280-08|BL464|658[0n]bp|Canada.Ontario|BOLD:AAA3073
Ochropleura implecta|[10839]|BLGSM063-09|BL382|658[0n]bp|Canada.Ontario|BOLD:AAA3073
Ochropleura implecta|[10840]|BLTIB213-08|BL316|658[0n]bp|Canada.Ontario|BOLD:AAA3073
Ochropleura implecta|[10841]|LPSOC117-08|PPBP-2116|658[0n]bp|Canada.Ontario|BOLD:AAA3073
Ochropleura implecta|[10842]|BLTIB098-08|BL157|658[0n]bp|Canada.Ontario|BOLD:AAA3073
Ochropleura implecta|[10843]|BLGSM044-09|BL357|658[0n]bp|Canada.Ontario|BOLD:AAA3073
Ochropleura implecta|[10844]|LPSOC387-08|PPBP-2386|658[0n]bp|Canada.Ontario|BOLD:AAA3073
Ochropleura implecta|[10845]|LPSOC119-08|PPBP-2118|658[0n]bp|Canada.Ontario|BOLD:AAA3073
Ochropleura implecta|[10846]|BLGSM037-09|BL348|658[0n]bp|Canada.Ontario|BOLD:AAA3073
Ochropleura implecta|[10847]|LPSOB989-08|PPBP-1988|658[0n]bp|Canada.Ontario|BOLD:AAA3073
Ochropleura implecta|[10848]|XAE184-04|Moth4184.03|658[0n]bp|Canada.Ontario|BOLD:AAA3073
Ochropleura implecta|[10849]|BLTIB251-08|BL433|658[0n]bp|Canada.Ontario|BOLD:AAA3073
Ochropleura implecta|[10850]|LPSO498-08|PPBP-0498|658[0n]bp|Canada.Ontario|BOLD:AAA3073
Ochropleura implecta|[10851]|LPSOC334-08|PPBP-2333|658[0n]bp|Canada.Ontario|BOLD:AAA3073
Ochropleura implecta|[10852]|BLTIB135-08|BL203|658[0n]bp|Canada.Ontario|BOLD:AAA3073
Ochropleura implecta|[10853]|XAG735-05|2005-ONT-1319|658[3n]bp|Canada.Ontario|BOLD:AAA3073
Homorthodes furfurata|[10854]|RDLQ681-07|DH009421|594[0n]bp|Canada.Quebec|BOLD:AAA3454
Homorthodes furfurata|[10855]|RDLQB309-05|DH010395|658[0n]bp|Canada.Quebec|BOLD:AAA3454
Homorthodes furfurata|[10856]|RDLQ680-07|DH009418|591[0n]bp|Canada.Quebec|BOLD:AAA3454
Homorthodes furfurata|[10857]|LOWCD554-06|CGWC-3374|658[0n]bp|Canada.British Columbia|BOLD:AAA3454
Homorthodes furfurata|[10858]|LBCH6100-10|10-JDWBC-6100|658[0n]bp|Canada.British Columbia|BOLD:AAA3454
Homorthodes furfurata|[10859]|LBCH5959-10|10-JDWBC-5959|658[0n]bp|Canada.British Columbia|BOLD:AAA3454
Homorthodes furfurata|[10860]|LBCC456-05|HLC-22336|658[0n]bp|Canada.British Columbia|BOLD:AAA3454
Homorthodes furfurata|[10861]|RDMAB292-05|UASM77787|614[1n]bp|Canada.Alberta|BOLD:AAA3454
Homorthodes furfurata|[10862]|LBCA618-05|HLC-20618|633[0n]bp|Canada.British Columbia|BOLD:AAA3454
Homorthodes furfurata|[10863]|LBCA614-05|HLC-20614|634[0n]bp|Canada.British Columbia|BOLD:AAA3454
Homorthodes furfurata|[10864]|LBCA617-05|HLC-20617|634[0n]bp|Canada.British Columbia|BOLD:AAA3454

Homorthodes furfurata|[10862]|LBCA618-05|HLC-20618|633[0n]bp|Canada.British Columbia|BOLD:AAA3454
Homorthodes furfurata|[10863]|LBCA614-05|HLC-20614|634[0n]bp|Canada.British Columbia|BOLD:AAA3454
Homorthodes furfurata|[10864]|LBCA617-05|HLC-20617|634[0n]bp|Canada.British Columbia|BOLD:AAA3454
Homorthodes furfurata|[10865]|LBCA616-05|HLC-20616|634[0n]bp|Canada.British Columbia|BOLD:AAA3454
Homorthodes furfurata|[10866]|LBCA698-05|HLC-20698|658[0n]bp|Canada.British Columbia|BOLD:AAA3454
Homorthodes furfurata|[10867]|LBCA701-05|HLC-20701|658[0n]bp|Canada.British Columbia|BOLD:AAA3454
Homorthodes furfurata|[10868]|LBCB624-05|HLC-21564|658[0n]bp|Canada.British Columbia|BOLD:AAA3454
Homorthodes furfurata|[10869]|LBCA696-05|HLC-20696|658[0n]bp|Canada.British Columbia|BOLD:AAA3454
Homorthodes furfurata|[10870]|LPABB529-08|08BBLEP-03794|658[0n]bp|Canada.Alberta|BOLD:AAA3454
Homorthodes furfurata|[10871]|LBCA697-05|HLC-20697|658[0n]bp|Canada.British Columbia|BOLD:AAA3454
Homorthodes furfurata|[10872]|LBCA699-05|HLC-20699|658[0n]bp|Canada.British Columbia|BOLD:AAA3454
Homorthodes furfurata|[10873]|LBCA613-05|HLC-20613|653[0n]bp|Canada.British Columbia|BOLD:AAA3454
Homorthodes furfurata|[10874]|LBCA700-05|HLC-20700|647[0n]bp|Canada.British Columbia|BOLD:AAA3454
Homorthodes furfurata|[10875]|RDNME245-07|CNCNoctuoidea13852|616[0n]bp|Canada.Alberta|BOLD:AAA3454
Homorthodes furfurata|[10876]|LBCB625-05|HLC-21565|658[0n]bp|Canada.British Columbia|BOLD:AAA3454
Homorthodes furfurata|[10877]|LOWCD649-06|CGWC-3469|658[0n]bp|Canada.British Columbia|BOLD:AAA3454
Homorthodes furfurata|[10878]|LBCA615-05|HLC-20615|658[0n]bp|Canada.British Columbia|BOLD:AAA3454
Homorthodes furfurata|[10879]|LBCH5961-10|10-JDWBC-5961|658[0n]bp|Canada.British Columbia|BOLD:AAA3454
Homorthodes furfurata|[10880]|LBCG1360-09|08-JDWBC-1360|658[0n]bp|Canada.British Columbia|BOLD:AAA3454
Homorthodes furfurata|[10881]|LBCH5963-10|10-JDWBC-5963|644[0n]bp|Canada.British Columbia|BOLD:AAA3454
Homorthodes furfurata|[10882]|LOWCD547-06|CGWC-3367|658[0n]bp|Canada.British Columbia|BOLD:AAA3454
Homorthodes furfurata|[10883]|LOWC102-05|CGWC-0102|610[1n]bp|Canada.British Columbia|BOLD:AAA3454
Homorthodes furfurata|[10884]|LOWCD650-06|CGWC-3470|600[1n]bp|Canada.British Columbia|BOLD:AAA3454
Homorthodes furfurata|[10885]|LBCG425-08|08-JDWBC-0425|658[0n]bp|Canada.British Columbia|BOLD:AAA3454
Homorthodes furfurata|[10886]|LBCA827-05|HLC-20827|658[0n]bp|Canada.British Columbia|BOLD:AAA3454
Homorthodes furfurata|[10887]|LBCG1354-09|08-JDWBC-1354|658[0n]bp|Canada.British Columbia|BOLD:AAA3454
Homorthodes furfurata|[10888]|LBCC350-05|HLC-22230|658[0n]bp|Canada.British Columbia|BOLD:AAA3454
Homorthodes furfurata|[10889]|LBCH6167-10|10-JDWBC-6167|658[0n]bp|Canada.British Columbia|BOLD:AAA3454
Homorthodes furfurata|[10890]|LBCH6291-10|10-JDWBC-6291|658[0n]bp|Canada.British Columbia|BOLD:AAA3454
Homorthodes furfurata|[10891]|LBCC346-05|HLC-22226|658[0n]bp|Canada.British Columbia|BOLD:AAA3454
Homorthodes furfurata|[10892]|LBCH5960-10|10-JDWBC-5960|658[0n]bp|Canada.British Columbia|BOLD:AAA3454
Homorthodes furfurata|[10893]|LBCH5875-10|10-JDWBC-5875|658[0n]bp|Canada.British Columbia|BOLD:AAA3454
Homorthodes furfurata|[10894]|LBCC458-05|HLC-22338|658[0n]bp|Canada.British Columbia|BOLD:AAA3454
Homorthodes furfurata|[10895]|LBCH5966-10|10-JDWBC-5966|658[0n]bp|Canada.British Columbia|BOLD:AAA3454
Homorthodes furfurata|[10896]|LBCG1359-09|08-JDWBC-1359|658[0n]bp|Canada.British Columbia|BOLD:AAA3454
Homorthodes furfurata|[10897]|LBCH6025-10|10-JDWBC-6025|658[0n]bp|Canada.British Columbia|BOLD:AAA3454
Homorthodes furfurata|[10898]|LBCG1364-09|08-JDWBC-1364|658[0n]bp|Canada.British Columbia|BOLD:AAA3454
Homorthodes furfurata|[10899]|LOWCD551-06|CGWC-3371|658[0n]bp|Canada.British Columbia|BOLD:AAA3454
Homorthodes furfurata|[10900]|LBCG1366-09|08-JDWBC-1366|658[0n]bp|Canada.British Columbia|BOLD:AAA3454
Homorthodes furfurata|[10901]|LOWCD546-06|CGWC-3366|658[0n]bp|Canada.British Columbia|BOLD:AAA3454
Homorthodes furfurata|[10902]|LBCH5965-10|10-JDWBC-5965|658[0n]bp|Canada.British Columbia|BOLD:AAA3454
Homorthodes furfurata|[10903]|LBCH6298-10|10-JDWBC-6298|658[0n]bp|Canada.British Columbia|BOLD:AAA3454
Homorthodes furfurata|[10904]|LBCG1368-09|08-JDWBC-1368|658[0n]bp|Canada.British Columbia|BOLD:AAA3454
Homorthodes furfurata|[10905]|LOWCD654-06|CGWC-3474|658[0n]bp|Canada.British Columbia|BOLD:AAA3454
Homorthodes furfurata|[10906]|LBCG1365-09|08-JDWBC-1365|658[0n]bp|Canada.British Columbia|BOLD:AAA3454
Homorthodes furfurata|[10907]|LBCH6754-10|10-JDWBC-6754|658[0n]bp|Canada.British Columbia|BOLD:AAA3454
Homorthodes furfurata|[10908]|LOWCD539-06|CGWC-3359|658[0n]bp|Canada.British Columbia|BOLD:AAA3454
Homorthodes furfurata|[10909]|BBLPB357-10|10BBCLP-1356|658[0n]bp|Canada.British Columbia|BOLD:AAA3454
Homorthodes furfurata|[10910]|LBCC349-05|HLC-22229|658[0n]bp|Canada.British Columbia|BOLD:AAA3454
Homorthodes furfurata|[10911]|LBCH6294-10|10-JDWBC-6294|658[0n]bp|Canada.British Columbia|BOLD:AAA3454
Homorthodes furfurata|[10912]|LBCB114-05|HLC-21054|658[0n]bp|Canada.British Columbia|BOLD:AAA3454
Homorthodes furfurata|[10913]|LBCB627-05|HLC-21567|658[0n]bp|Canada.British Columbia|BOLD:AAA3454
Homorthodes furfurata|[10914]|LOWC087-05|CGWC-0087|658[0n]bp|Canada.British Columbia|BOLD:AAA3454
Homorthodes furfurata|[10915]|LOWC088-05|CGWC-0088|658[0n]bp|Canada.British Columbia|BOLD:AAA3454
Homorthodes furfurata|[10916]|LBCC351-05|HLC-22231|658[0n]bp|Canada.British Columbia|BOLD:AAA3454
Homorthodes furfurata|[10917]|LBCC347-05|HLC-22227|658[0n]bp|Canada.British Columbia|BOLD:AAA3454
Homorthodes furfurata|[10918]|LOWCD543-06|CGWC-3363|658[0n]bp|Canada.British Columbia|BOLD:AAA3454
Homorthodes furfurata|[10919]|LBCH5968-10|10-JDWBC-5968|658[0n]bp|Canada.British Columbia|BOLD:AAA3454
Homorthodes furfurata|[10920]|LOWC099-05|CGWC-0099|658[0n]bp|Canada.British Columbia|BOLD:AAA3454
Homorthodes furfurata|[10921]|LBCH5967-10|10-JDWBC-5967|658[0n]bp|Canada.British Columbia|BOLD:AAA3454
Homorthodes furfurata|[10922]|LOWCD653-06|CGWC-3473|658[0n]bp|Canada.British Columbia|BOLD:AAA3454
Homorthodes furfurata|[10923]|LOWCD652-06|CGWC-3472|658[0n]bp|Canada.British Columbia|BOLD:AAA3454
Homorthodes furfurata|[10924]|LOWC101-05|CGWC-0101|658[0n]bp|Canada.British Columbia|BOLD:AAA3454
Homorthodes furfurata|[10925]|LBCH5560-10|10-JDWBC-5560|658[0n]bp|Canada.British Columbia|BOLD:AAA3454
Homorthodes furfurata|[10926]|LBCC454-05|HLC-22334|658[0n]bp|Canada.British Columbia|BOLD:AAA3454
Homorthodes furfurata|[10927]|LBCB080-05|HLC-21020|658[0n]bp|Canada.British Columbia|BOLD:AAA3454
Homorthodes furfurata|[10928]|LBCH5613-10|10-JDWBC-5613|658[0n]bp|Canada.British Columbia|BOLD:AAA3454
Homorthodes furfurata|[10929]|LBCC455-05|HLC-22335|658[0n]bp|Canada.British Columbia|BOLD:AAA3454
Homorthodes furfurata|[10930]|LBCH5969-10|10-JDWBC-5969|658[0n]bp|Canada.British Columbia|BOLD:AAA3454
Homorthodes furfurata|[10931]|LBCH5550-10|10-JDWBC-5550|658[0n]bp|Canada.British Columbia|BOLD:AAA3454
Homorthodes furfurata|[10932]|LBCH5795-10|10-JDWBC-5795|658[0n]bp|Canada.British Columbia|BOLD:AAA3454
Homorthodes furfurata|[10933]|LBCH5962-10|10-JDWBC-5962|658[0n]bp|Canada.British Columbia|BOLD:AAA3454
Homorthodes furfurata|[10934]|LBCB079-05|HLC-21019|658[0n]bp|Canada.British Columbia|BOLD:AAA3454
Homorthodes furfurata|[10935]|LBCC345-05|HLC-22225|658[0n]bp|Canada.British Columbia|BOLD:AAA3454
Homorthodes furfurata|[10936]|BBLPB358-10|10BBCLP-1357|658[0n]bp|Canada.British Columbia|BOLD:AAA3454
Homorthodes furfurata|[10937]|LOWCD552-06|CGWC-3372|658[0n]bp|Canada.British Columbia|BOLD:AAA3454
Homorthodes furfurata|[10938]|LOWCD548-06|CGWC-3368|658[0n]bp|Canada.British Columbia|BOLD:AAA3454
Homorthodes furfurata|[10939]|LOWC092-05|CGWC-0092|658[0n]bp|Canada.British Columbia|BOLD:AAA3454
Homorthodes furfurata|[10940]|LBCG1367-09|08-JDWBC-1367|658[0n]bp|Canada.British Columbia|BOLD:AAA3454
Homorthodes furfurata|[10941]|LOWC096-05|CGWC-0096|658[0n]bp|Canada.British Columbia|BOLD:AAA3454
Homorthodes furfurata|[10942]|LOWCD544-06|CGWC-3364|658[0n]bp|Canada.British Columbia|BOLD:AAA3454
Homorthodes furfurata|[10943]|LBCB626-05|HLC-21566|658[0n]bp|Canada.British Columbia|BOLD:AAA3454
Homorthodes furfurata|[10944]|LOWCD651-06|CGWC-3471|658[0n]bp|Canada.British Columbia|BOLD:AAA3454
Homorthodes furfurata|[10945]|LBCG1362-09|08-JDWBC-1362|658[0n]bp|Canada.British Columbia|BOLD:AAA3454
Homorthodes furfurata|[10946]|LOWC091-05|CGWC-0091|658[0n]bp|Canada.British Columbia|BOLD:AAA3454
Homorthodes furfurata|[10947]|LOWCD540-06|CGWC-3360|658[0n]bp|Canada.British Columbia|BOLD:AAA3454
Homorthodes furfurata|[10948]|LOWC098-05|CGWC-0098|658[0n]bp|Canada.British Columbia|BOLD:AAA3454
Homorthodes furfurata|[10949]|LBCC457-05|HLC-22337|658[0n]bp|Canada.British Columbia|BOLD:AAA3454
Homorthodes furfurata|[10950]|LBCC646-05|HLC-22526|658[0n]bp|Canada.British Columbia|BOLD:AAA3454
Homorthodes furfurata|[10951]|LBCB116-05|HLC-21056|658[0n]bp|Canada.British Columbia|BOLD:AAA3454
Homorthodes furfurata|[10952]|LBCB354-05|HLC-21294|658[0n]bp|Canada.British Columbia|BOLD:AAA3454
Homorthodes furfurata|[10953]|LOWC089-05|CGWC-0089|658[0n]bp|Canada.British Columbia|BOLD:AAA3454
Homorthodes furfurata|[10954]|LBCH5874-10|10-JDWBC-5874|658[0n]bp|Canada.British Columbia|BOLD:AAA3454
Homorthodes furfurata|[10955]|LBCG1358-09|08-JDWBC-1358|658[0n]bp|Canada.British Columbia|BOLD:AAA3454
Homorthodes furfurata|[10956]|LOWCD542-06|CGWC-3362|658[0n]bp|Canada.British Columbia|BOLD:AAA3454
Homorthodes furfurata|[10957]|LBCG2865-09|08-JDWBC-2865|658[0n]bp|Canada.British Columbia|BOLD:AAA3454
Homorthodes furfurata|[10958]|LOWCD550-06|CGWC-3370|658[0n]bp|Canada.British Columbia|BOLD:AAA3454
Homorthodes furfurata|[10959]|LOWCD555-06|CGWC-3375|658[0n]bp|Canada.British Columbia|BOLD:AAA3454
Homorthodes furfurata|[10960]|LOWC093-05|CGWC-0093|658[0n]bp|Canada.British Columbia|BOLD:AAA3454
Homorthodes furfurata|[10961]|LOWCD549-06|CGWC-3369|658[0n]bp|Canada.British Columbia|BOLD:AAA3454
Homorthodes furfurata|[10962]|LBCG1361-09|08-JDWBC-1361|658[0n]bp|Canada.British Columbia|BOLD:AAA3454
Homorthodes furfurata|[10963]|LBCH5873-10|10-JDWBC-5873|658[0n]bp|Canada.British Columbia|BOLD:AAA3454
Homorthodes furfurata|[10964]|LBCG1357-09|08-JDWBC-1357|658[0n]bp|Canada.British Columbia|BOLD:AAA3454

[illegible]
Homorthodes furfurata|[10962]|LBCG1361-09|08-JDWBC-1361|658[0n]bp|Canada.British Columbia|BOLD:AAA3454
Homorthodes furfurata|[10963]|LBCH5873-10|10-JDWBC-5873|658[0n]bp|Canada.British Columbia|BOLD:AAA3454
Homorthodes furfurata|[10964]|LBCG1357-09|08-JDWBC-1357|658[0n]bp|Canada.British Columbia|BOLD:AAA3454
Homorthodes furfurata|[10965]|LOWCD553-06|CGWC-3373|658[0n]bp|Canada.British Columbia|BOLD:AAA3454
Homorthodes furfurata|[10966]|LOWCD655-06|CGWC-3475|658[0n]bp|Canada.British Columbia|BOLD:AAA3454
Homorthodes furfurata|[10967]|LOWCD656-06|CGWC-3476|658[0n]bp|Canada.British Columbia|BOLD:AAA3454
Homorthodes furfurata|[10968]|LBCC348-05|HLC-22228|658[0n]bp|Canada.British Columbia|BOLD:AAA3454
Homorthodes furfurata|[10969]|LBCH6099-10|10-JDWBC-6099|658[0n]bp|Canada.British Columbia|BOLD:AAA3454
Homorthodes furfurata|[10970]|LOWC094-05|CGWC-0094|658[0n]bp|Canada.British Columbia|BOLD:AAA3454
Homorthodes furfurata|[10971]|LBCB119-05|HLC-21059|658[0n]bp|Canada.British Columbia|BOLD:AAA3454
Homorthodes furfurata|[10972]|LBCG2256-09|08-JDWBC-2256|658[0n]bp|Canada.British Columbia|BOLD:AAA3454
Homorthodes furfurata|[10973]|LOWC090-05|CGWC-0090|658[0n]bp|Canada.British Columbia|BOLD:AAA3454
Homorthodes furfurata|[10974]|LOWCD545-06|CGWC-3365|658[0n]bp|Canada.British Columbia|BOLD:AAA3454
Homorthodes furfurata|[10975]|LOWCD657-06|CGWC-3477|658[0n]bp|Canada.British Columbia|BOLD:AAA3454
Homorthodes furfurata|[10976]|LALPA887-11|AVBC 1060-11|640[0n]bp|Canada.British Columbia|BOLD:AAA3454
Homorthodes furfurata|[10977]|LBCG1356-09|08-JDWBC-1356|641[0n]bp|Canada.British Columbia|BOLD:AAA3454
Homorthodes furfurata|[10978]|LOWC095-05|CGWC-0095|617[0n]bp|Canada.British Columbia|BOLD:AAA3454
Homorthodes furfurata|[10979]|LOWC100-05|CGWC-0100|604[0n]bp|Canada.British Columbia|BOLD:AAA3454
Homorthodes furfurata|[10980]|LBCH6060-10|10-JDWBC-6060|646[0n]bp|Canada.British Columbia|BOLD:AAA3454
Homorthodes furfurata|[10981]|LBCH6059-10|10-JDWBC-6059|646[0n]bp|Canada.British Columbia|BOLD:AAA3454
Homorthodes furfurata|[10982]|LBCG1355-09|08-JDWBC-1355|643[0n]bp|Canada.British Columbia|BOLD:AAA3454
Homorthodes furfurata|[10983]|LBCH5964-10|10-JDWBC-5964|644[0n]bp|Canada.British Columbia|BOLD:AAA3454
Homorthodes furfurata|[10984]|LOWC097-05|CGWC-0097|658[0n]bp|Canada.British Columbia|BOLD:AAA3454
Homorthodes furfurata|[10985]|LBCG1363-09|08-JDWBC-1363|658[0n]bp|Canada.British Columbia|BOLD:AAA3454
Pseudorthodes irrorata|[10986]|LBCG2249-09|08-JDWBC-2249|658[0n]bp|Canada.British Columbia|BOLD:ABZ1590
Pseudorthodes irrorata|[10987]|DUNLP189-08|Dun-08-189|650[0n]bp|Canada.British Columbia|BOLD:ABZ1590
Pseudorthodes irrorata|[10988]|LPVIA844-08|PFC-2006-1144|658[0n]bp|Canada.British Columbia|BOLD:ABZ1590
Pseudorthodes irrorata|[10989]|LBCS487-07|UBC-2007-0244|658[0n]bp|Canada.British Columbia|BOLD:ABZ1590
Pseudorthodes irrorata|[10990]|LBCS137-07|UBC-2007-0130|658[0n]bp|Canada.British Columbia|BOLD:ABZ1590
Pseudorthodes irrorata|[10991]|LBCH4473-10|10-JDWBC-4473|658[0n]bp|Canada.British Columbia|BOLD:ABZ1590
Pseudorthodes irrorata|[10992]|LBCS486-07|UBC-2007-0243|658[0n]bp|Canada.British Columbia|BOLD:ABZ1590
Pseudorthodes irrorata|[10993]|LALPA932-11|AVBC 1105-11|658[0n]bp|Canada.British Columbia|BOLD:ABZ1590
Pseudorthodes irrorata|[10994]|LBCH4016-10|10-JDWBC-4016|658[0n]bp|Canada.British Columbia|BOLD:ABZ1590
Pseudorthodes irrorata|[10995]|LBCS676-07|UBC-2007-0381|658[0n]bp|Canada.British Columbia|BOLD:ABZ1590
Pseudorthodes irrorata|[10996]|LBCS678-07|UBC-2007-0383|658[0n]bp|Canada.British Columbia|BOLD:ABZ1590
Pseudorthodes irrorata|[10997]|LBCS679-07|UBC-2007-0384|658[0n]bp|Canada.British Columbia|BOLD:ABZ1590
Pseudorthodes irrorata|[10998]|LBCS677-07|UBC-2007-0382|658[0n]bp|Canada.British Columbia|BOLD:ABZ1590
Pseudorthodes irrorata|[10999]|LBCS675-07|UBC-2007-0380|658[0n]bp|Canada.British Columbia|BOLD:ABZ1590
Pseudorthodes irrorata|[11000]|LHLEP431-06|UBC-2006-1232|658[0n]bp|Canada.British Columbia|BOLD:ABZ1590
Pseudorthodes irrorata|[11001]|LBCH4739-10|10-JDWBC-4739|658[0n]bp|Canada.British Columbia|BOLD:ABZ1590
Pseudorthodes vecors|[11002]|PHMNB681-04|04HBL00907|658[0n]bp|Canada.New Brunswick|BOLD:AAA8885
Pseudorthodes vecors|[11003]|PMG155-03|moth950.01|617[1n]bp|Canada.Ontario|BOLD:AAA8885
Pseudorthodes vecors|[11004]|RDLQF035-06|DH007671|658[0n]bp|Canada.Quebec|BOLD:AAA8885
Pseudorthodes vecors|[11005]|PHJUN4015-11|BIOUG01497-C02|658[0n]bp|Canada.Ontario|BOLD:AAA8885
Pseudorthodes vecors|[11006]|PHJUN4007-11|BIOUG01497-B06|658[0n]bp|Canada.Ontario|BOLD:AAA8885
Pseudorthodes vecors|[11007]|RDLQ674-07|DH007574|658[0n]bp|Canada.Quebec|BOLD:AAA8885
Pseudorthodes vecors|[11008]|RDLQB069-05|DH010155|627[0n]bp|Canada.Quebec|BOLD:AAA8885
Pseudorthodes vecors|[11009]|RDLQ306-05|DH007977|658[0n]bp|Canada.Quebec|BOLD:AAA8885
Pseudorthodes vecors|[11010]|RDLQ676-07|DH009355|632[0n]bp|Canada.Quebec|BOLD:ACF5054
Pseudorthodes vecors|[11011]|XAJ843-06|2006-ONT-0843|658[0n]bp|Canada.Ontario|BOLD:ACF5054
Pseudorthodes vecors|[11012]|RDLQF455-06|DH011562|658[0n]bp|Canada.Quebec|BOLD:ACF5054
Pseudorthodes vecors|[11013]|RDLQ675-07|DH009354|658[0n]bp|Canada.Quebec|BOLD:ACF5054
Pseudorthodes vecors|[11014]|RDLQ677-07|DH007893|658[0n]bp|Canada.Quebec|BOLD:ACF5054
Pseudorthodes vecors|[11015]|RDLQF034-06|DH006710|658[0n]bp|Canada.Quebec|BOLD:ACF5054
Pseudorthodes vecors|[11016]|RDLQ673-07|DH007843|617[0n]bp|Canada.Quebec|BOLD:ACF5054
Pseudorthodes vecors|[11017]|PHMO185-03|moth961.01|639[0n]bp|Canada.Ontario|BOLD:ACF5054
Pseudorthodes vecors|[11018]|RDLQ304-05|DH007786|608[0n]bp|Canada.Quebec|BOLD:ACF5054
Pseudorthodes vecors|[11019]|RDLQ672-07|DH005666|608[0n]bp|Canada.Quebec|BOLD:ACF5054
Pseudorthodes vecors|[11020]|RDLQG382-06|DH012650|658[0n]bp|Canada.Quebec|BOLD:ACF5054
Pseudorthodes vecors|[11021]|RDLQG365-06|DH012599|658[0n]bp|Canada.Quebec|BOLD:ACF5054
Pseudorthodes vecors|[11022]|RDLQG381-06|DH012649|658[0n]bp|Canada.Quebec|BOLD:ACF5054
Pseudorthodes vecors|[11023]|RDLQ678-07|DH006640|625[0n]bp|Canada.Quebec|BOLD:ACF5054
Pseudorthodes vecors|[11024]|RDLQ679-07|DH007907|658[0n]bp|Canada.Quebec|BOLD:ACF5054
Polia imbrifera|[11025]|RDLQ627-07|DH007846|631[0n]bp|Canada.Quebec|BOLD:AAB3022
Polia imbrifera|[11026]|XAK460-06|2006-ONT-1455|658[0n]bp|Canada.Ontario|BOLD:AAB3022
Polia imbrifera|[11027]|XAK457-06|2006-ONT-1452|658[0n]bp|Canada.Ontario|BOLD:AAB3022
Polia imbrifera|[11028]|BBLPE217-09|09BBELE-2217|634[0n]bp|Canada.Newfoundland and Labrador|BOLD:AAB3022
Polia imbrifera|[11029]|RDLQ625-07|AC000447|658[0n]bp|Canada.Quebec|BOLD:AAB3022
Polia imbrifera|[11030]|RDLQB220-05|DH010306|658[0n]bp|Canada.Quebec|BOLD:AAB3022
Polia imbrifera|[11031]|XAK459-06|2006-ONT-1454|658[0n]bp|Canada.Ontario|BOLD:AAB3022
Polia imbrifera|[11032]|LBCA582-05|HLC-20582|655[0n]bp|Canada.British Columbia|BOLD:AAB3022
Polia imbrifera|[11033]|RDLQB217-05|DH010303|658[0n]bp|Canada.Quebec|BOLD:AAB3022
Polia imbrifera|[11034]|BLTIB567-08|BL845|658[0n]bp|Canada.Ontario|BOLD:AAB3022
Polia imbrifera|[11035]|LPMN915-08|08BBLEP-02273|658[0n]bp|Canada.Alberta|BOLD:AAB3022
Polia imbrifera|[11036]|BBLPC992-09|09BBELE-1992|658[0n]bp|Canada.Nova Scotia|BOLD:AAB3022
Polia imbrifera|[11037]|RDLQ626-07|DH009922|658[0n]bp|Canada.Quebec|BOLD:AAB3022
Polia imbrifera|[11038]|XAK455-06|2006-ONT-1450|658[0n]bp|Canada.Ontario|BOLD:AAB3022
Polia imbrifera|[11039]|TMNBB306-06|MNBTT-1246|658[0n]bp|Canada.New Brunswick|BOLD:AAB3022
Polia imbrifera|[11040]|XAC709-04|04HBL006709|658[0n]bp|Canada.Ontario|BOLD:AAB3022
Polia imbrifera|[11041]|BBLEC091-09|09BBELE-0091|658[0n]bp|Canada.Nova Scotia|BOLD:AAB3022
Polia imbrifera|[11042]|RDLQG126-06|DH012293|658[0n]bp|Canada.Quebec|BOLD:AAB3022
Polia imbrifera|[11043]|XAK040-06|2006-ONT-1035|658[0n]bp|Canada.Ontario|BOLD:AAB3022
Polia imbrifera|[11044]|PMG150-03|EURO1.00|617[0n]bp|Canada.Ontario|BOLD:AAB3022
Polia imbrifera|[11045]|TTMNB393-06|MNBTT-393|658[0n]bp|Canada.New Brunswick|BOLD:AAB3022
Polia imbrifera|[11046]|XAK458-06|2006-ONT-1453|658[0n]bp|Canada.Ontario|BOLD:AAB3022
Polia nimbosa|[11047]|PHMNB267-04|04HBL007732|658[0n]bp|Canada.New Brunswick|BOLD:AAA4058
Polia nimbosa|[11048]|BBLPC252-09|09BBELE-1252|614[0n]bp|Canada.Nova Scotia|BOLD:AAA4058
Polia nimbosa|[11049]|LOWCC195-05|CGWC-2075|579[0n]bp|Canada.British Columbia|BOLD:AAA4058
Polia nimbosa|[11050]|LBCA357-05|HLC-20357|614[0n]bp|Canada.British Columbia|BOLD:AAA4058
Polia nimbosa|[11051]|LBCA351-05|HLC-20351|658[0n]bp|Canada.British Columbia|BOLD:AAA4058
Polia nimbosa|[11052]|RDLQ621-07|DH007717|594[0n]bp|Canada.Quebec|BOLD:AAA4058
Polia nimbosa|[11053]|RDMAB017-05|UASM57562|641[0n]bp|Canada.Alberta|BOLD:AAA4058
Polia nimbosa|[11054]|LBCA349-05|HLC-20349|640[0n]bp|Canada.British Columbia|BOLD:AAA4058
Polia nimbosa|[11055]|LBCA350-05|HLC-20350|615[0n]bp|Canada.British Columbia|BOLD:AAA4058
Polia nimbosa|[11056]|LBCA356-05|HLC-20356|646[0n]bp|Canada.British Columbia|BOLD:AAA4058
Polia nimbosa|[11057]|LBCA355-05|HLC-20355|610[0n]bp|Canada.British Columbia|BOLD:AAA4058
Polia nimbosa|[11058]|LBCA348-05|HLC-20348|628[0n]bp|Canada.British Columbia|BOLD:AAA4058
Polia nimbosa|[11059]|RDLQ622-07|DH007922|645[0n]bp|Canada.Quebec|BOLD:AAA4058
Polia nimbosa|[11060]|BBLPC987-09|09BBELE-1987|658[0n]bp|Canada.Nova Scotia|BOLD:AAA4058
Polia nimbosa|[11061]|BBLEC093-09|09BBELE-0093|658[0n]bp|Canada.Nova Scotia|BOLD:AAA4058
Polia nimbosa|[11062]|LBCA577-05|HLC-20577|655[0n]bp|Canada.British Columbia|BOLD:AAA4058
Polia nimbosa|[11063]|LOWCC196-05|CGWC-2076|658[0n]bp|Canada.British Columbia|BOLD:AAA4058
[illegible]

Polia nimbosa|[11061]|BBLEC093-09|09BBELE-0093|658[0n]bp|Canada.Nova Scotia|BOLD:AAA4058
Polia nimbosa|[11062]|LBCA577-05|HLC-20577|655[0n]bp|Canada.British Columbia|BOLD:AAA4058
Polia nimbosa|[11063]|LOWCC196-05|CGWC-2076|658[0n]bp|Canada.British Columbia|BOLD:AAA4058
Polia nimbosa|[11064]|LPMN123-08|08BBLEP-00921|658[0n]bp|Canada.Manitoba|BOLD:AAA4058
Polia nimbosa|[11065]|LBCA469-05|HLC-20469|658[0n]bp|Canada.British Columbia|BOLD:AAA4058
Polia nimbosa|[11066]|LBCH3071-10|10-JDWBC-3071|658[0n]bp|Canada.British Columbia|BOLD:AAA4058
Polia nimbosa|[11067]|BBLPC998-09|09BBELE-1998|658[0n]bp|Canada.Nova Scotia|BOLD:AAA4058
Polia nimbosa|[11068]|LOWCB335-05|CGWC-1275|658[0n]bp|Canada.British Columbia|BOLD:AAA4058
Polia nimbosa|[11069]|LBCH2377-10|10-JDWBC-2377|658[0n]bp|Canada.British Columbia|BOLD:AAA4058
Polia nimbosa|[11070]|LBCH019-10|10-JDWBC-0019|658[0n]bp|Canada.British Columbia|BOLD:AAA4058
Polia nimbosa|[11071]|LBCA490-05|HLC-20490|658[0n]bp|Canada.British Columbia|BOLD:AAA4058
Polia nimbosa|[11072]|LBCA488-05|HLC-20488|658[0n]bp|Canada.British Columbia|BOLD:AAA4058
Polia nimbosa|[11073]|MNBB152-05|05-NBSTA-068|658[0n]bp|Canada.New Brunswick|BOLD:AAA4058
Polia nimbosa|[11074]|LHLEP249-06|UBC-2006-0931|658[0n]bp|Canada.British Columbia|BOLD:AAA4058
Polia nimbosa|[11075]|MNBB421-05|05-NBSTA-337|658[0n]bp|Canada.New Brunswick|BOLD:AAA4058
Polia nimbosa|[11076]|LBCA475-05|HLC-20475|658[0n]bp|Canada.British Columbia|BOLD:AAA4058
Polia nimbosa|[11077]|LPMN331-08|08BBLEP-01130|658[0n]bp|Canada.Manitoba|BOLD:AAA4058
Polia nimbosa|[11078]|LBCA471-05|HLC-20471|658[0n]bp|Canada.British Columbia|BOLD:AAA4058
Polia nimbosa|[11079]|LBCA484-05|HLC-20484|658[0n]bp|Canada.British Columbia|BOLD:AAA4058
Polia nimbosa|[11080]|LBCH108-10|10-JDWBC-0108|658[0n]bp|Canada.British Columbia|BOLD:AAA4058
Polia nimbosa|[11081]|LBCH504-10|10-JDWBC-0504|658[0n]bp|Canada.British Columbia|BOLD:AAA4058
Polia nimbosa|[11082]|MNBB156-05|05-NBSTA-072|658[0n]bp|Canada.New Brunswick|BOLD:AAA4058
Polia nimbosa|[11083]|LBCA477-05|HLC-20477|658[0n]bp|Canada.British Columbia|BOLD:AAA4058
Polia nimbosa|[11084]|LBCA489-05|HLC-20489|658[0n]bp|Canada.British Columbia|BOLD:AAA4058
Polia nimbosa|[11085]|LOWCD255-06|CGWC-3075|658[0n]bp|Canada.British Columbia|BOLD:AAA4058
Polia nimbosa|[11086]|LBCA481-05|HLC-20481|658[0n]bp|Canada.British Columbia|BOLD:AAA4058
Polia nimbosa|[11087]|LPABB362-08|08BBLEP-03627|658[0n]bp|Canada.Alberta|BOLD:AAA4058
Polia nimbosa|[11088]|LBCH219-10|10-JDWBC-0219|658[0n]bp|Canada.British Columbia|BOLD:AAA4058
Polia nimbosa|[11089]|LOWCE814-06|CGWC-4574|658[0n]bp|Canada.British Columbia|BOLD:AAA4058
Polia nimbosa|[11090]|LBCH814-10|10-JDWBC-0814|658[0n]bp|Canada.British Columbia|BOLD:AAA4058
Polia nimbosa|[11091]|LBCA478-05|HLC-20478|658[0n]bp|Canada.British Columbia|BOLD:AAA4058
Polia nimbosa|[11092]|LOWCD250-06|CGWC-3070|658[0n]bp|Canada.British Columbia|BOLD:AAA4058
Polia nimbosa|[11093]|LBCA476-05|HLC-20476|658[0n]bp|Canada.British Columbia|BOLD:AAA4058
Polia nimbosa|[11094]|LPABB355-08|08BBLEP-03620|658[0n]bp|Canada.Alberta|BOLD:AAA4058
Polia nimbosa|[11095]|LBCA472-05|HLC-20472|658[0n]bp|Canada.British Columbia|BOLD:AAA4058
Polia nimbosa|[11096]|BBLPC999-09|09BBELE-1999|658[0n]bp|Canada.Nova Scotia|BOLD:AAA4058
Polia nimbosa|[11097]|LBCA479-05|HLC-20479|658[0n]bp|Canada.British Columbia|BOLD:AAA4058
Polia nimbosa|[11098]|BBLPB673-10|10BBCLP-1672|658[0n]bp|Canada.British Columbia|BOLD:AAA4058
Polia nimbosa|[11099]|LBCA486-05|HLC-20486|658[0n]bp|Canada.British Columbia|BOLD:AAA4058
Polia nimbosa|[11100]|LBCA470-05|HLC-20470|658[0n]bp|Canada.British Columbia|BOLD:AAA4058
Polia nimbosa|[11101]|LBCA347-05|HLC-20347|658[0n]bp|Canada.British Columbia|BOLD:AAA4058
Polia nimbosa|[11102]|LOWCD180-06|CGWC-3000|658[0n]bp|Canada.British Columbia|BOLD:AAA4058
Polia nimbosa|[11103]|LBCH111-10|10-JDWBC-0111|658[0n]bp|Canada.British Columbia|BOLD:AAA4058
Polia nimbosa|[11104]|LBCA485-05|HLC-20485|658[0n]bp|Canada.British Columbia|BOLD:AAA4058
Polia nimbosa|[11105]|LBCA353-05|HLC-20353|658[0n]bp|Canada.British Columbia|BOLD:AAA4058
Polia nimbosa|[11106]|BBLPC986-09|09BBELE-1986|658[0n]bp|Canada.Nova Scotia|BOLD:AAA4058
Polia nimbosa|[11107]|LBCH346-10|10-JDWBC-0346|658[0n]bp|Canada.British Columbia|BOLD:AAA4058
Polia nimbosa|[11108]|LBCH020-10|10-JDWBC-0020|658[0n]bp|Canada.British Columbia|BOLD:AAA4058
Polia nimbosa|[11109]|LALPA913-11|AVBC 1086-11|658[0n]bp|Canada.British Columbia|BOLD:AAA4058
Polia nimbosa|[11110]|LBCA482-05|HLC-20482|658[0n]bp|Canada.British Columbia|BOLD:AAA4058
Polia nimbosa|[11111]|LOWCE852-06|CGWC-4612|658[0n]bp|Canada.British Columbia|BOLD:AAA4058
Polia nimbosa|[11112]|BBLPB674-10|10BBCLP-1673|658[0n]bp|Canada.British Columbia|BOLD:AAA4058
Polia nimbosa|[11113]|LBCA474-05|HLC-20474|658[0n]bp|Canada.British Columbia|BOLD:AAA4058
Polia nimbosa|[11114]|LOWCC197-05|CGWC-2077|658[0n]bp|Canada.British Columbia|BOLD:AAA4058
Polia nimbosa|[11115]|LBCH649-10|10-JDWBC-0649|658[0n]bp|Canada.British Columbia|BOLD:AAA4058
Polia nimbosa|[11116]|LBCA360-05|HLC-20360|658[0n]bp|Canada.British Columbia|BOLD:AAA4058
Polia nimbosa|[11117]|LBCA359-05|HLC-20359|636[0n]bp|Canada.British Columbia|BOLD:AAA4058
Polia nimbosa|[11118]|LOWCC873-05|CGWC-2753|561[0n]bp|Canada.British Columbia|BOLD:AAA4058
Polia nimbosa|[11119]|LBCA354-05|HLC-20354|658[0n]bp|Canada.British Columbia|BOLD:AAA4058
Polia nimbosa|[11120]|LOWCE829-06|CGWC-4589|603[0n]bp|Canada.British Columbia|BOLD:AAA4058
Polia nimbosa|[11121]|LBCA358-05|HLC-20358|608[0n]bp|Canada.British Columbia|BOLD:AAA4058
Polia nimbosa|[11122]|RDMAB016-05|UASM57561|566[0n]bp|Canada.Alberta|BOLD:AAA4058
Polia nimbosa|[11123]|LBCA487-05|HLC-20487|658[0n]bp|Canada.British Columbia|BOLD:AAA4058
Polia nimbosa|[11124]|LBCA480-05|HLC-20480|658[0n]bp|Canada.British Columbia|BOLD:AAA4058
Polia nimbosa|[11125]|PHMNB228-04|04HBL007693|586[0n]bp|Canada.New Brunswick|BOLD:AAA4058
Polia nimbosa|[11126]|LOWCC853-05|CGWC-2733|586[1n]bp|Canada.British Columbia|BOLD:AAA4058
Polia nimbosa|[11127]|LOWCD252-06|CGWC-3072|582[0n]bp|Canada.British Columbia|BOLD:AAA4058
Polia nimbosa|[11128]|PHMO230-03|moth1165.01|639[0n]bp|Canada.Ontario|BOLD:AAA4058
Polia nimbosa|[11129]|LBCA473-05|HLC-20473|658[0n]bp|Canada.British Columbia|BOLD:AAA4058
Polia nimbosa|[11130]|LBCA361-05|HLC-20361|633[0n]bp|Canada.British Columbia|BOLD:AAA4058
Polia discalis|[11131]|LPABC102-09|08BBLEP-04321|658[0n]bp|Canada.Alberta|BOLD:ACE7737
Polia discalis|[11132]|RDNMB245-05|CNCNoctuoidea10011|599[0n]bp|Canada.Saskatchewan|BOLD:ACE7737
Polia discalis|[11133]|RDMAB019-05|UASM57564|617[0n]bp|Canada.Alberta|BOLD:ACE7737
Polia piniae|[11134]|LOWCD848-06|CGWC-3668|576[0n]bp|Canada.British Columbia|BOLD:AAA6561
Polia piniae|[11135]|BBLPB670-10|10BBCLP-1669|658[0n]bp|Canada.Alberta|BOLD:AAA6561
Polia piniae|[11136]|BBLPB321-10|10BBCLP-1320|658[0n]bp|Canada.Alberta|BOLD:AAA6561
Polia piniae|[11137]|LPABB836-09|08BBLEP-04156|658[0n]bp|Canada.Alberta|BOLD:AAA6561
Polia piniae|[11138]|BBLPB323-10|10BBCLP-1322|658[0n]bp|Canada.Alberta|BOLD:AAA6561
Polia piniae|[11139]|LPABB325-08|08BBLEP-03590|658[0n]bp|Canada.Alberta|BOLD:AAA6561
Polia piniae|[11140]|LOWCD844-06|CGWC-3664|658[0n]bp|Canada.British Columbia|BOLD:AAA6561
Polia piniae|[11141]|LPABB065-08|08BBLEP-03330|615[0n]bp|Canada.Alberta|BOLD:AAA6561
Polia piniae|[11142]|LPABB501-08|08BBLEP-03766|658[0n]bp|Canada.Alberta|BOLD:AAA6561
Polia piniae|[11143]|LBCD229-05|HLC-23049|658[0n]bp|Canada.British Columbia|BOLD:AAA6561
Polia piniae|[11144]|LPABB084-08|08BBLEP-03349|641[0n]bp|Canada.Alberta|BOLD:AAA6561
Polia piniae|[11145]|LBCH6624-10|10-JDWBC-6624|658[0n]bp|Canada.British Columbia|BOLD:AAA6561
Polia piniae|[11146]|LBCH5797-10|10-JDWBC-5797|658[0n]bp|Canada.British Columbia|BOLD:AAA6561
Polia piniae|[11147]|LPABB082-08|08BBLEP-03347|658[0n]bp|Canada.Alberta|BOLD:AAA6561
Polia piniae|[11148]|LOWCD842-06|CGWC-3662|577[7n]bp|Canada.British Columbia|
Polia piniae|[11149]|LOWCD840-06|CGWC-3660|658[0n]bp|Canada.British Columbia|BOLD:AAA6561
Polia piniae|[11150]|LBCB889-05|HLC-21829|615[1n]bp|Canada.British Columbia|BOLD:AAA6561
Polia piniae|[11151]|LBCD235-05|HLC-23055|658[0n]bp|Canada.British Columbia|BOLD:AAA6561
Polia piniae|[11152]|LBCH7365-10|10-JDWBC-7365|658[0n]bp|Canada.British Columbia|BOLD:AAA6561
Polia piniae|[11153]|LBCD225-05|HLC-23045|658[0n]bp|Canada.British Columbia|BOLD:AAA6561
Polia piniae|[11154]|LBCH5798-10|10-JDWBC-5798|658[0n]bp|Canada.British Columbia|BOLD:AAA6561
Polia piniae|[11155]|LBCD230-05|HLC-23050|658[0n]bp|Canada.British Columbia|BOLD:AAA6561
Polia piniae|[11156]|LBCD228-05|HLC-23048|658[0n]bp|Canada.British Columbia|BOLD:AAA6561
Polia piniae|[11157]|LOWCD841-06|CGWC-3661|658[0n]bp|Canada.British Columbia|BOLD:AAA6561
Polia piniae|[11158]|LBCH1931-10|10-JDWBC-1931|658[0n]bp|Canada.British Columbia|BOLD:AAA6561
Polia piniae|[11159]|LBCH2244-10|10-JDWBC-2244|658[0n]bp|Canada.British Columbia|BOLD:AAA6561
Polia piniae|[11160]|LBCH2085-10|10-JDWBC-2085|658[0n]bp|Canada.British Columbia|BOLD:AAA6561
Polia piniae|[11161]|LALPA665-10|AVBC 667-10|658[0n]bp|Canada.British Columbia|BOLD:AAA6561
Polia piniae|[11162]|LPABB519-08|08BBLEP-03784|658[0n]bp|Canada.Alberta|BOLD:AAA6561
Polia piniae|[11163]|LOWCD839-06|CGWC-3659|658[0n]bp|Canada.British Columbia|BOLD:AAA6561

Polia piniae|[11161]|LALPA665-10|AVBC 667-10|658[0n]bp|Canada.British Columbia|BOLD:AAA6561
Polia piniae|[11162]|LPABB519-08|08BBLEP-03784|658[0n]bp|Canada.Alberta|BOLD:AAA6561
Polia piniae|[11163]|LOWCD839-06|CGWC-3659|658[0n]bp|Canada.British Columbia|BOLD:AAA6561
Polia piniae|[11164]|RDMAB472-06|UASM77896|658[0n]bp|Canada.Alberta|BOLD:AAA6561
Polia piniae|[11165]|LBCD236-05|HLC-23056|657[0n]bp|Canada.British Columbia|BOLD:AAA6561
Polia piniae|[11166]|LBCD224-05|HLC-23044|657[0n]bp|Canada.British Columbia|BOLD:AAA6561
Polia piniae|[11167]|LBCG2841-09|08-JDWBC-2841|658[0n]bp|Canada.British Columbia|BOLD:AAA6561
Polia piniae|[11168]|LBCH5691-10|10-JDWBC-5691|658[0n]bp|Canada.British Columbia|BOLD:AAA6561
Polia piniae|[11169]|LPABB024-08|08BBLEP-03289|658[0n]bp|Canada.Alberta|BOLD:AAA6561
Polia piniae|[11170]|LBCG817-09|08-JDWBC-0817|658[0n]bp|Canada.British Columbia|BOLD:AAA6561
Polia piniae|[11171]|LALPA578-10|AVBC 580-10|658[0n]bp|Canada.British Columbia|BOLD:AAA6561
Polia piniae|[11172]|BBLPB672-10|10BBCLP-1671|658[0n]bp|Canada.British Columbia|BOLD:AAA6561
Polia piniae|[11173]|LALPA592-10|AVBC 594-10|658[0n]bp|Canada.British Columbia|BOLD:AAA6561
Polia piniae|[11174]|LPABB324-08|08BBLEP-03589|658[0n]bp|Canada.Alberta|BOLD:AAA6561
Polia piniae|[11175]|LPABB038-08|08BBLEP-03303|658[0n]bp|Canada.Alberta|BOLD:AAA6561
Polia piniae|[11176]|LBCH5417-10|10-JDWBC-5417|658[0n]bp|Canada.British Columbia|BOLD:AAA6561
Polia piniae|[11177]|LPABB200-08|08BBLEP-03465|658[0n]bp|Canada.Alberta|BOLD:AAA6561
Polia piniae|[11178]|LOWCD843-06|CGWC-3663|658[0n]bp|Canada.British Columbia|BOLD:AAA6561
Polia piniae|[11179]|LBCH1520-10|10-JDWBC-1520|658[0n]bp|Canada.British Columbia|BOLD:AAA6561
Polia piniae|[11180]|LBCD220-05|HLC-23040|658[0n]bp|Canada.British Columbia|BOLD:AAA6561
Polia piniae|[11181]|LPABB025-08|08BBLEP-03290|658[0n]bp|Canada.Alberta|BOLD:AAA6561
Polia piniae|[11182]|LPABB039-08|08BBLEP-03304|658[0n]bp|Canada.Alberta|BOLD:AAA6561
Polia piniae|[11183]|LBCD227-05|HLC-23047|655[0n]bp|Canada.British Columbia|BOLD:AAA6561
Polia piniae|[11184]|LBCH1564-10|10-JDWBC-1564|658[0n]bp|Canada.British Columbia|BOLD:AAA6561
Polia piniae|[11185]|LBCA585-05|HLC-20585|638[0n]bp|Canada.British Columbia|BOLD:AAA6561
Polia piniae|[11186]|LBCD226-05|HLC-23046|651[0n]bp|Canada.British Columbia|BOLD:AAA6561
Polia piniae|[11187]|LOWCE833-06|CGWC-4593|656[0n]bp|Canada.British Columbia|BOLD:AAA6561
Polia piniae|[11188]|LBCA593-05|HLC-20593|650[0n]bp|Canada.British Columbia|BOLD:AAA6561
Polia piniae|[11189]|LBCD231-05|HLC-23051|638[0n]bp|Canada.British Columbia|BOLD:AAA6561
Polia piniae|[11190]|LPABB187-08|08BBLEP-03452|648[0n]bp|Canada.Alberta|BOLD:AAA6561
Polia piniae|[11191]|LOWCD847-06|CGWC-3667|583[0n]bp|Canada.British Columbia|BOLD:AAA6561
Polia piniae|[11192]|LPABB085-08|08BBLEP-03350|636[0n]bp|Canada.Alberta|BOLD:AAA6561
Polia piniae|[11193]|LPABC959-09|08BBLEP-05370|560[0n]bp|Canada.Alberta|BOLD:AAA6561
Polia piniae|[11194]|LPABC408-09|08BBLEP-04627|634[0n]bp|Canada.Alberta|BOLD:AAA6561
Polia piniae|[11195]|LOWCD838-06|CGWC-3658|600[0n]bp|Canada.British Columbia|BOLD:AAA6561
Polia piniae|[11196]|LOWCB714-05|CGWC-1654|586[0n]bp|Canada.British Columbia|BOLD:AAA6561
Polia piniae|[11197]|LOWCD849-06|CGWC-3669|596[0n]bp|Canada.British Columbia|BOLD:AAA6561
Polia piniae|[11198]|LOWCD846-06|CGWC-3666|596[0n]bp|Canada.British Columbia|BOLD:AAA6561
Polia piniae|[11199]|RDNMB244-05|CNCNoctuoidea10010|595[0n]bp|Canada.British Columbia|BOLD:AAA6561
Polia piniae|[11200]|LOWCB717-05|CGWC-1657|587[0n]bp|Canada.British Columbia|BOLD:AAA6561
Polia piniae|[11201]|LOWCB716-05|CGWC-1656|587[0n]bp|Canada.British Columbia|BOLD:AAA6561
Polia piniae|[11202]|LBCC643-05|HLC-22523|632[0n]bp|Canada.British Columbia|BOLD:AAA6561
Polia piniae|[11203]|LPABC831-09|08BBLEP-05050|658[0n]bp|Canada.Alberta|BOLD:AAA6561
Polia purpurissata|[11204]|LOWCE679-06|CGWC-4439|631[0n]bp|Canada.British Columbia|BOLD:AAA6697
Polia nugatis|[11205]|LBCH6932-10|10-JDWBC-6932|658[0n]bp|Canada.British Columbia|BOLD:AAD3359
Polia nugatis|[11206]|LBCH6881-10|10-JDWBC-6881|658[0n]bp|Canada.British Columbia|BOLD:AAD3359
Polia nugatis|[11207]|LBCH6882-10|10-JDWBC-6882|658[0n]bp|Canada.British Columbia|BOLD:AAD3359
Polia nugatis|[11208]|LBCH6887-10|10-JDWBC-6887|658[0n]bp|Canada.British Columbia|BOLD:AAD3359
Polia nugatis|[11209]|LBCH6542-10|10-JDWBC-6542|658[0n]bp|Canada.British Columbia|BOLD:AAD3359
Polia nugatis|[11210]|LBCH6526-10|10-JDWBC-6526|658[0n]bp|Canada.British Columbia|BOLD:AAD3359
Polia nugatis|[11211]|LBCH6885-10|10-JDWBC-6885|658[0n]bp|Canada.British Columbia|BOLD:AAD3359
Polia nugatis|[11212]|LBCH7853-10|10-JDWBC-7853|658[0n]bp|Canada.British Columbia|BOLD:AAD3359
Polia nugatis|[11213]|LBCH7852-10|10-JDWBC-7852|658[0n]bp|Canada.British Columbia|BOLD:AAD3359
Polia nugatis|[11214]|LBCH7849-10|10-JDWBC-7849|658[0n]bp|Canada.British Columbia|BOLD:AAD3359
Polia nugatis|[11215]|LBCH7850-10|10-JDWBC-7850|658[0n]bp|Canada.British Columbia|BOLD:AAD3359
Polia nugatis|[11216]|LBCH6884-10|10-JDWBC-6884|658[0n]bp|Canada.British Columbia|BOLD:AAD3359
Polia nugatis|[11217]|LBCH7753-10|10-JDWBC-7753|658[0n]bp|Canada.British Columbia|BOLD:AAD3359
Polia nugatis|[11218]|LBCH7748-10|10-JDWBC-7748|658[0n]bp|Canada.British Columbia|BOLD:AAD3359
Polia nugatis|[11219]|LBCH7752-10|10-JDWBC-7752|658[0n]bp|Canada.British Columbia|BOLD:AAD3359
Polia nugatis|[11220]|LBCH7854-10|10-JDWBC-7854|658[0n]bp|Canada.British Columbia|BOLD:AAD3359
Polia nugatis|[11221]|LBCH6883-10|10-JDWBC-6883|658[0n]bp|Canada.British Columbia|BOLD:AAD3359
Polia nugatis|[11222]|LBCH7751-10|10-JDWBC-7751|658[0n]bp|Canada.British Columbia|BOLD:AAD3359
Polia nugatis|[11223]|LBCH6888-10|10-JDWBC-6888|658[0n]bp|Canada.British Columbia|BOLD:AAD3359
Polia nugatis|[11224]|LBCH6886-10|10-JDWBC-6886|658[0n]bp|Canada.British Columbia|BOLD:AAD3359
Polia nugatis|[11225]|LBCH6525-10|10-JDWBC-6525|658[0n]bp|Canada.British Columbia|BOLD:AAD3359
Polia nugatis|[11226]|LBCH6907-10|10-JDWBC-6907|658[0n]bp|Canada.British Columbia|BOLD:AAD3359
Polia nugatis|[11227]|LBCH7848-10|10-JDWBC-7848|658[0n]bp|Canada.British Columbia|BOLD:AAD3359
Polia nugatis|[11228]|LBCH7754-10|10-JDWBC-7754|658[0n]bp|Canada.British Columbia|BOLD:AAD3359
Polia nugatis|[11229]|LBCH7599-10|10-JDWBC-7599|658[0n]bp|Canada.British Columbia|BOLD:AAD3359
Polia nugatis|[11230]|LBCH7750-10|10-JDWBC-7750|658[0n]bp|Canada.British Columbia|BOLD:AAD3359
Polia nugatis|[11231]|LBCH6880-10|10-JDWBC-6880|658[0n]bp|Canada.British Columbia|BOLD:AAD3359
Polia nugatis|[11232]|LOWCB323-05|CGWC-1263|658[0n]bp|Canada.British Columbia|BOLD:AAD3359
Polia nugatis|[11233]|LBCH6962-10|10-JDWBC-6962|658[0n]bp|Canada.British Columbia|BOLD:AAD3359
Polia nugatis|[11234]|LOWCB326-05|CGWC-1266|658[0n]bp|Canada.British Columbia|BOLD:AAD3359
Polia nugatis|[11235]|LBCH6368-10|10-JDWBC-6368|658[0n]bp|Canada.British Columbia|BOLD:AAD3359
Polia nugatis|[11236]|LBCH7847-10|10-JDWBC-7847|658[0n]bp|Canada.British Columbia|BOLD:AAD3359
Polia nugatis|[11237]|LBCH7747-10|10-JDWBC-7747|646[0n]bp|Canada.British Columbia|BOLD:AAD3359
Polia nugatis|[11238]|LBCH6922-10|10-JDWBC-6922|624[0n]bp|Canada.British Columbia|BOLD:AAD3359
Polia nugatis|[11239]|LBCH7851-10|10-JDWBC-7851|658[0n]bp|Canada.British Columbia|BOLD:AAD3359
Polia purpurissata|[11240]|LOWCE811-06|CGWC-4571|658[0n]bp|Canada.British Columbia|BOLD:AAA6697
Polia purpurissata|[11241]|LPABC467-09|08BBLEP-04686|658[0n]bp|Canada.Alberta|BOLD:AAA6697
Polia purpurissata|[11242]|TMNBB307-06|MNBTT-1247|658[0n]bp|Canada.New Brunswick|BOLD:AAA6697
Polia purpurissata|[11243]|RDLQ623-07|DH006322|632[0n]bp|Canada.Quebec|BOLD:AAA6697
Polia purpurissata|[11244]|TTMNB507-06|MNBTT-507|658[0n]bp|Canada.New Brunswick|BOLD:AAA6697
Polia purpurissata|[11245]|LPSK231-08|08BBLEP-01799|658[0n]bp|Canada.Saskatchewan|BOLD:AAA6697
Polia nugatis|[11246]|XAD577-04|04HBL006992|589[0n]bp|Canada.Ontario|BOLD:AAA6697
Polia nugatis|[11247]|RDNMB242-05|CNCNoctuoidea10008|594[0n]bp|Canada.Alberta|BOLD:AAA6697
Polia purpurissata|[11248]|LPABC027-09|08BBLEP-04246|658[0n]bp|Canada.Alberta|BOLD:AAA6697
Polia purpurissata|[11249]|LPSK448-08|08BBLEP-02016|658[0n]bp|Canada.Saskatchewan|BOLD:AAA6697
Polia purpurissata|[11250]|LBCG3320-09|08-JDWBC-3320|658[0n]bp|Canada.British Columbia|BOLD:AAA6697
Polia purpurissata|[11251]|BBLPB665-10|10BBCLP-1664|658[0n]bp|Canada.British Columbia|BOLD:AAA6697
Polia purpurissata|[11252]|LOWCE684-06|CGWC-4444|658[0n]bp|Canada.British Columbia|BOLD:AAA6697
Polia purpurissata|[11253]|LOWCE677-06|CGWC-4437|658[0n]bp|Canada.British Columbia|BOLD:AAA6697
Polia purpurissata|[11254]|LOWCE675-06|CGWC-4435|658[0n]bp|Canada.British Columbia|BOLD:AAA6697
Polia purpurissata|[11255]|BBLPB666-10|10BBCLP-1665|658[0n]bp|Canada.British Columbia|BOLD:AAA6697
Polia purpurissata|[11256]|LBCH6623-10|10-JDWBC-6623|658[0n]bp|Canada.British Columbia|BOLD:AAA6697
Polia purpurissata|[11257]|LOWCC172-05|CGWC-2052|658[0n]bp|Canada.British Columbia|BOLD:AAA6697
Polia purpurissata|[11258]|LBCH5952-10|10-JDWBC-5952|634[0n]bp|Canada.British Columbia|BOLD:AAA6697
Polia purpurissata|[11259]|LOWCE676-06|CGWC-4436|596[0n]bp|Canada.British Columbia|BOLD:AAA6697
Polia purpurissata|[11260]|LOWCB334-05|CGWC-1274|558[0n]bp|Canada.British Columbia|BOLD:AAA6697
Polia purpurissata|[11261]|LOWCE674-06|CGWC-4434|616[0n]bp|Canada.British Columbia|BOLD:AAA6697
Polia purpurissata|[11262]|LOWCB328-05|CGWC-1268|515[0n]bp|Canada.British Columbia|BOLD:AAA6697
Polia purpurissata|[11263]|TMNBB308-06|MNBTT-1248|658[0n]bp|Canada.New Brunswick|BOLD:AAA6697

[illegible]
Polia purpurissata|[11261]|LOWCE674-06|CGWC-4434|616[0n]bp|Canada.British Columbia|BOLD:AAA6697
Polia purpurissata|[11262]|LOWCB328-05|CGWC-1268|515[0n]bp|Canada.British Columbia|BOLD:AAA6697
Polia purpurissata|[11263]|TMNBB308-06|MNBTT-1248|658[0n]bp|Canada.New Brunswick|BOLD:AAA6697
Polia purpurissata|[11264]|LBCC828-05|HLC-22708|658[0n]bp|Canada.British Columbia|BOLD:AAA6697
Polia purpurissata|[11265]|LBCA592-05|HLC-20592|617[0n]bp|Canada.British Columbia|BOLD:AAA6697
Polia purpurissata|[11266]|LPMNB436-09|08BBLEP-05436|658[0n]bp|Canada.Manitoba|BOLD:AAA6697
Polia purpurissata|[11267]|LBCA594-05|HLC-20594|632[0n]bp|Canada.British Columbia|BOLD:AAA6697
Polia purpurissata|[11268]|BBLPB700-10|10BBCLP-1699|632[0n]bp|Canada.British Columbia|BOLD:AAA6697
Polia purpurissata|[11269]|LPABC114-09|08BBLEP-04333|658[1n]bp|Canada.Alberta|BOLD:AAA6697
Polia purpurissata|[11270]|RDLQ624-07|DH007443|624[0n]bp|Canada.Quebec|BOLD:AAA6697
Polia purpurissata|[11271]|LALPA884-11|AVBC 1057-11|658[0n]bp|Canada.British Columbia|BOLD:AAA6697
Polia purpurissata|[11272]|LOWCE682-06|CGWC-4442|658[0n]bp|Canada.British Columbia|BOLD:AAA6697
Polia purpurissata|[11273]|LPABC336-09|08BBLEP-04555|658[0n]bp|Canada.Alberta|BOLD:AAA6697
Polia purpurissata|[11274]|LOWCB331-05|CGWC-1271|658[0n]bp|Canada.British Columbia|BOLD:AAA6697
Polia purpurissata|[11275]|BBLPB320-10|10BBCLP-1319|658[0n]bp|Canada.British Columbia|BOLD:AAA6697
Polia purpurissata|[11276]|LPABB040-08|08BBLEP-03305|658[0n]bp|Canada.Alberta|BOLD:AAA6697
Polia purpurissata|[11277]|LHLEP256-06|UBC-2006-1507|658[0n]bp|Canada.British Columbia|BOLD:AAA6697
Polia purpurissata|[11278]|LBCH4666-10|10-JDWBC-4666|658[0n]bp|Canada.British Columbia|BOLD:AAA6697
Polia purpurissata|[11279]|LOWCB325-05|CGWC-1265|658[0n]bp|Canada.British Columbia|BOLD:AAA6697
Polia purpurissata|[11280]|BBLPB671-10|10BBCLP-1670|658[0n]bp|Canada.British Columbia|BOLD:AAA6697
Polia purpurissata|[11281]|XAD354-04|04HBL007354|658[0n]bp|Canada.Ontario|BOLD:AAA6697
Polia purpurissata|[11282]|LALPA878-11|AVBC 1051-11|658[0n]bp|Canada.British Columbia|BOLD:AAA6697
Polia purpurissata|[11283]|BBLPB319-10|10BBCLP-1318|658[0n]bp|Canada.British Columbia|BOLD:AAA6697
Polia purpurissata|[11284]|LPABC397-09|08BBLEP-04616|658[0n]bp|Canada.Alberta|BOLD:AAA6697
Polia purpurissata|[11285]|LPABB189-08|08BBLEP-03454|658[0n]bp|Canada.Alberta|BOLD:AAA6697
Polia purpurissata|[11286]|LOWCE678-06|CGWC-4438|658[0n]bp|Canada.British Columbia|BOLD:AAA6697
Polia purpurissata|[11287]|LPABC927-09|08BBLEP-05338|658[0n]bp|Canada.Alberta|BOLD:AAA6697
Polia purpurissata|[11288]|TTMNB506-06|MNBTT-506|658[0n]bp|Canada.New Brunswick|BOLD:AAA6697
Polia purpurissata|[11289]|TTMNB510-06|MNBTT-510|658[0n]bp|Canada.New Brunswick|BOLD:AAA6697
Polia purpurissata|[11290]|LPABC094-09|08BBLEP-04313|658[0n]bp|Canada.Alberta|BOLD:AAA6697
Polia purpurissata|[11291]|TTMNB508-06|MNBTT-508|658[0n]bp|Canada.New Brunswick|BOLD:AAA6697
Polia purpurissata|[11292]|LOWCE683-06|CGWC-4443|658[0n]bp|Canada.British Columbia|BOLD:AAA6697
Polia purpurissata|[11293]|TTMNB509-06|MNBTT-509|658[0n]bp|Canada.New Brunswick|BOLD:AAA6697
Polia purpurissata|[11294]|XAH051-05|2005-ONT-1634|645[0n]bp|Canada.Ontario|BOLD:AAA6697
Polia nugatis|[11295]|RDMAB546-06|UASM2603|622[2n]bp|Canada.Alberta|BOLD:AAA6697
Polia purpurissata|[11296]|LOWCB330-05|CGWC-1270|582[0n]bp|Canada.British Columbia|BOLD:AAA6697
Polia purpurissata|[11297]|LBCD221-05|HLC-23041|657[0n]bp|Canada.British Columbia|BOLD:AAA6697
Polia nugatis|[11298]|RDMAB545-06|UASM2554|629[0n]bp|Canada.Alberta|BOLD:AAA6697
Polia purpurissata|[11299]|LOWCB324-05|CGWC-1264|594[0n]bp|Canada.British Columbia|BOLD:AAA6697
Polia purpurissata|[11300]|LOWCB333-05|CGWC-1273|594[0n]bp|Canada.British Columbia|BOLD:AAA6697
Polia purpurissata|[11301]|LOWCB327-05|CGWC-1267|591[0n]bp|Canada.British Columbia|BOLD:AAA6697
Polia purpurissata|[11302]|LOWCB322-05|CGWC-1262|596[1n]bp|Canada.British Columbia|BOLD:AAA6697
Polia purpurissata|[11303]|PMG151-03|ORTH1.00|617[0n]bp|Canada.Ontario|BOLD:AAA6697
Polia purpurissata|[11304]|LOWCB329-05|CGWC-1269|557[0n]bp|Canada.British Columbia|BOLD:AAA6697
Polia purpurissata|[11305]|LOWCB332-05|CGWC-1272|557[0n]bp|Canada.British Columbia|BOLD:AAA6697
Polia purpurissata|[11306]|XAG311-05|2005-ONT-895|658[1n]bp|Canada.Ontario|BOLD:AAA6697
Polia purpurissata|[11307]|XAG801-05|2005-ONT-1385|658[0n]bp|Canada.Ontario|BOLD:AAA6697
Polia propodea|[11308]|LCHP520-07|07PROBE-10188|658[0n]bp|Canada.Manitoba|BOLD:AAA9949
Polia propodea|[11309]|LCH239-04|04HBL003239|658[0n]bp|Canada.Manitoba|BOLD:AAA9949
Polia propodea|[11310]|LCH240-04|04HBL003240|658[0n]bp|Canada.Manitoba|BOLD:AAA9949
Polia propodea|[11311]|RDNME711-08|LEP041982|658[0n]bp|Canada.Quebec|BOLD:AAA9949
Polia propodea|[11312]|GWNC609-07|CNCLEP00034171|658[0n]bp|Canada.Alberta|BOLD:AAA9949
Polia propodea|[11313]|RDNME646-08|LEP038070|658[0n]bp|Canada.Alberta|BOLD:AAA9949
Polia propodea|[11314]|RDNME672-08|LEP031947|658[0n]bp|Canada.Quebec|BOLD:AAA9949
Polia propodea|[11315]|LOWCC834-05|CGWC-2714|658[0n]bp|Canada.British Columbia|BOLD:AAA9949
Polia propodea|[11316]|LBCG952-09|08-JDWBC-0952|658[2n]bp|Canada.British Columbia|BOLD:AAA9949
Polia propodea|[11317]|LOWCD247-06|CGWC-3067|658[2n]bp|Canada.British Columbia|BOLD:AAA9949
Polia propodea|[11318]|LOWCD260-06|CGWC-3080|658[0n]bp|Canada.British Columbia|BOLD:AAA9949
Polia propodea|[11319]|LOWCC831-05|CGWC-2711|658[0n]bp|Canada.British Columbia|BOLD:AAA9949
Polia propodea|[11320]|RDNME647-08|LEP038071|658[0n]bp|Canada.Alberta|BOLD:AAA9949
Polia propodea|[11321]|LOWCC829-05|CGWC-2709|658[0n]bp|Canada.British Columbia|BOLD:AAA9949
Polia propodea|[11322]|LBCH1496-10|10-JDWBC-1496|658[0n]bp|Canada.British Columbia|BOLD:AAA9949
Polia propodea|[11323]|LBCH1432-10|10-JDWBC-1432|658[0n]bp|Canada.British Columbia|BOLD:AAA9949
Polia propodea|[11324]|GWNC615-07|CNCLEP00034177|658[0n]bp|Canada.Alberta|BOLD:AAA9949
Polia propodea|[11325]|LOWCD254-06|CGWC-3074|658[0n]bp|Canada.British Columbia|BOLD:AAA9949
Polia propodea|[11326]|LBCH2102-10|10-JDWBC-2102|658[0n]bp|Canada.British Columbia|BOLD:AAA9949
Polia propodea|[11327]|LOWCD248-06|CGWC-3068|658[0n]bp|Canada.British Columbia|BOLD:AAA9949
Polia propodea|[11328]|LBCH1164-10|10-JDWBC-1164|658[0n]bp|Canada.British Columbia|BOLD:AAA9949
Polia propodea|[11329]|LOWCD256-06|CGWC-3076|658[0n]bp|Canada.British Columbia|BOLD:AAA9949
Polia propodea|[11330]|LBCH2057-10|10-JDWBC-2057|658[0n]bp|Canada.British Columbia|BOLD:AAA9949
Polia propodea|[11331]|LBCH1237-10|10-JDWBC-1237|658[0n]bp|Canada.British Columbia|BOLD:AAA9949
Polia propodea|[11332]|LOWCC833-05|CGWC-2713|658[0n]bp|Canada.British Columbia|BOLD:AAA9949
Polia propodea|[11333]|LBCG1866-09|08-JDWBC-1866|658[0n]bp|Canada.British Columbia|BOLD:AAA9949
Polia propodea|[11334]|LBCG948-09|08-JDWBC-0948|658[0n]bp|Canada.British Columbia|BOLD:AAA9949
Polia propodea|[11335]|LBCG707-09|08-JDWBC-0707|658[0n]bp|Canada.British Columbia|BOLD:AAA9949
Polia propodea|[11336]|LOWCD253-06|CGWC-3073|654[0n]bp|Canada.British Columbia|BOLD:AAA9949
Polia propodea|[11337]|LBCH2100-10|10-JDWBC-2100|642[0n]bp|Canada.British Columbia|BOLD:AAA9949
Polia propodea|[11338]|LOWCD257-06|CGWC-3077|658[0n]bp|Canada.British Columbia|BOLD:AAA9949
Polia propodea|[11339]|LOWCD249-06|CGWC-3069|658[0n]bp|Canada.British Columbia|BOLD:AAA9949
Polia propodea|[11340]|LOWCD245-06|CGWC-3065|546[0n]bp|Canada.British Columbia|BOLD:AAA9949
Polia propodea|[11341]|LOWCD258-06|CGWC-3078|591[0n]bp|Canada.British Columbia|BOLD:AAA9949
Polia propodea|[11342]|LOWCD251-06|CGWC-3071|608[0n]bp|Canada.British Columbia|BOLD:AAA9949
Polia propodea|[11343]|LOWCD246-06|CGWC-3066|599[0n]bp|Canada.British Columbia|BOLD:AAA9949
Polia propodea|[11344]|LOWCC830-05|CGWC-2710|593[0n]bp|Canada.British Columbia|BOLD:AAA9949
Polia propodea|[11345]|LBCG1865-09|08-JDWBC-1865|621[0n]bp|Canada.British Columbia|BOLD:AAA9949
Polia rogenhoferi|[11346]|RDLQB219-05|DH010305|658[0n]bp|Canada.Quebec|BOLD:AAA9583
Polia rogenhoferi|[11347]|RDLQB218-05|DH010304|658[0n]bp|Canada.Quebec|BOLD:AAA9583
Polia rogenhoferi|[11348]|BBLPB525-10|10BBCLP-1524|658[0n]bp|Canada.British Columbia|BOLD:AAA9583
Polia rogenhoferi|[11349]|RDLQB221-05|DH010307|658[1n]bp|Canada.Quebec|BOLD:AAA9583
Polia rogenhoferi|[11350]|RDLQF038-06|DH001481|573[1n]bp|Canada.Quebec|BOLD:AAA9583
Polia rogenhoferi|[11351]|RDLQF037-06|DH001480|658[0n]bp|Canada.Quebec|BOLD:AAA9583
Polia rogenhoferi|[11352]|RDLQF036-06|DH001479|658[0n]bp|Canada.Quebec|BOLD:AAA9583
Polia rogenhoferi|[11353]|LBCG797-09|08-JDWBC-0797|658[0n]bp|Canada.British Columbia|BOLD:AAA9583
Polia rogenhoferi|[11354]|RDNME649-08|LEP038073|658[0n]bp|Canada.British Columbia|BOLD:AAA9583
Polia rogenhoferi|[11355]|GWNC608-07|CNCLEP00034170|658[0n]bp|Canada.Alberta|BOLD:AAA9583
Polia richardsoni|[11356]|RDNME517-08|LEP037941|658[0n]bp|Canada.Yukon Territory|BOLD:AAA9583
Polia richardsoni|[11357]|RDNME525-08|LEP037949|658[0n]bp|Canada.Yukon Territory|BOLD:AAA9583
Polia richardsoni|[11358]|RDNMF051-08|NOC14137|658[0n]bp|Canada.Yukon Territory|BOLD:AAA9583
Polia rogenhoferi|[11359]|LCHP828-07|07PROBE-10585|658[0n]bp|Canada.Manitoba|BOLD:AAA9583
Polia rogenhoferi|[11360]|LCHP201-07|07PROBE-00126|658[1n]bp|Canada.Manitoba|BOLD:AAA9583
Polia rogenhoferi|[11361]|LCHQ140-07|07PROBE-10909|658[0n]bp|Canada.Manitoba|BOLD:AAA9583
Polia rogenhoferi|[11362]|LCHP202-07|07PROBE-00127|658[0n]bp|Canada.Manitoba|BOLD:AAA9583
[illegible]

Polia rogenhoferi|[11360]|LCHP201-07|07PROBE-00126|658[1n]bp|Canada.Manitoba|BOLD:AAA9583
Polia rogenhoferi|[11361]|LCHQ140-07|07PROBE-10909|658[0n]bp|Canada.Manitoba|BOLD:AAA9583
Polia rogenhoferi|[11362]|LCHP202-07|07PROBE-00127|658[0n]bp|Canada.Manitoba|BOLD:AAA9583
Polia rogenhoferi|[11363]|LCHP623-07|07PROBE-10304|658[0n]bp|Canada.Manitoba|BOLD:AAA9583
Polia rogenhoferi|[11364]|LCHP272-07|07PROBE-03838|657[0n]bp|Canada.Manitoba|BOLD:AAA9583
Polia rogenhoferi|[11365]|LCHP705-07|07PROBE-10387|658[0n]bp|Canada.Manitoba|BOLD:AAA9583
Polia rogenhoferi|[11366]|LCHP155-07|07PROBE-00557|658[0n]bp|Canada.Manitoba|BOLD:AAA9583
Polia rogenhoferi|[11367]|LCHP154-07|07PROBE-00556|658[0n]bp|Canada.Manitoba|BOLD:AAA9583
Polia rogenhoferi|[11368]|LCHQ888-08|07WNP-10780|658[0n]bp|Canada.Manitoba|BOLD:AAA9583
Polia rogenhoferi|[11369]|CHLEP154-09|09PROBE-09449|658[0n]bp|Canada.Manitoba|BOLD:AAA9583
Polia rogenhoferi|[11370]|LCHP233-07|07PROBE-03803|658[0n]bp|Canada.Manitoba|BOLD:AAA9583
Polia rogenhoferi|[11371]|LCHP199-07|07PROBE-00124|658[0n]bp|Canada.Manitoba|BOLD:AAA9583
Polia rogenhoferi|[11372]|GWNC610-07|CNCLEP00034172|658[0n]bp|Canada.Alberta|BOLD:AAA9583
Polia rogenhoferi|[11373]|LCHP152-07|07PROBE-00554|656[0n]bp|Canada.Manitoba|BOLD:AAA9583
Polia rogenhoferi|[11374]|LCHP198-07|07PROBE-00123|632[0n]bp|Canada.Manitoba|BOLD:AAA9583
Polia rogenhoferi|[11375]|LCHP811-07|07PROBE-10568|658[0n]bp|Canada.Manitoba|BOLD:AAA9583
Polia rogenhoferi|[11376]|LCHP232-07|07PROBE-03802|658[0n]bp|Canada.Manitoba|BOLD:AAA9583
Polia rogenhoferi|[11377]|LCHQ139-07|07PROBE-10908|658[0n]bp|Canada.Manitoba|BOLD:AAA9583
Polia rogenhoferi|[11378]|LCHP153-07|07PROBE-00555|658[0n]bp|Canada.Manitoba|BOLD:AAA9583
Polia rogenhoferi|[11379]|LCHP930-07|07PROBE-10692|658[0n]bp|Canada.Manitoba|BOLD:AAA9583
Polia rogenhoferi|[11380]|LCHQ045-07|07PROBE-10806|658[0n]bp|Canada.Manitoba|BOLD:AAA9583
Polia rogenhoferi|[11381]|LCHQ044-07|07PROBE-10805|658[0n]bp|Canada.Manitoba|BOLD:AAA9583
Polia rogenhoferi|[11382]|LCHP205-07|07PROBE-00130|658[0n]bp|Canada.Manitoba|BOLD:AAA9583
Polia rogenhoferi|[11383]|LCHP203-07|07PROBE-00128|658[0n]bp|Canada.Manitoba|BOLD:AAA9583
Polia rogenhoferi|[11384]|LCHP156-07|07PROBE-00558|658[0n]bp|Canada.Manitoba|BOLD:AAA9583
Polia rogenhoferi|[11385]|LCHP637-07|07PROBE-10318|658[0n]bp|Canada.Manitoba|BOLD:AAA9583
Polia rogenhoferi|[11386]|LCHP200-07|07PROBE-00125|658[0n]bp|Canada.Manitoba|BOLD:AAA9583
Polia rogenhoferi|[11387]|LCHQ889-08|07WNP-10781|658[0n]bp|Canada.Manitoba|BOLD:AAA9583
Polia rogenhoferi|[11388]|LCHP204-07|07PROBE-00129|658[0n]bp|Canada.Manitoba|BOLD:AAA9583
Properigea costa|[11389]|CMAZA1068-12|BIOUG02042-D03|658[0n]bp|United States.Arizona|BOLD:ABW6337
Properigea costa|[11390]|RDNMF528-08|NOC14614|658[0n]bp|United States.Arizona|BOLD:AAH7705
Properigea costa|[11391]|RDNMF529-08|NOC14615|609[0n]bp|United States.Arizona|BOLD:AAH7705
Properigea albimacula|[11392]|LBCH6018-10|10-JDWBC-6018|658[0n]bp|Canada.British Columbia|BOLD:AAC7834
Properigea albimacula|[11393]|LBCH5935-10|10-JDWBC-5935|658[0n]bp|Canada.British Columbia|BOLD:AAC7834
Properigea albimacula|[11394]|LPVIA572-08|PFC-2006-0799|658[0n]bp|Canada.British Columbia|BOLD:AAC7834
Properigea albimacula|[11395]|LBCH5932-10|10-JDWBC-5932|658[0n]bp|Canada.British Columbia|BOLD:AAC7834
Properigea albimacula|[11396]|LBCH6233-10|10-JDWBC-6233|658[0n]bp|Canada.British Columbia|BOLD:AAC7834
Properigea albimacula|[11397]|LBCH5933-10|10-JDWBC-5933|658[0n]bp|Canada.British Columbia|BOLD:AAC7834
Properigea albimacula|[11398]|LBCH5936-10|10-JDWBC-5936|658[0n]bp|Canada.British Columbia|BOLD:AAC7834
Properigea albimacula|[11399]|LBCH5934-10|10-JDWBC-5934|658[0n]bp|Canada.British Columbia|BOLD:AAC7834
Properigea albimacula|[11400]|LBCH5938-10|10-JDWBC-5938|658[0n]bp|Canada.British Columbia|BOLD:AAC7834
Properigea albimacula|[11401]|LBCH5937-10|10-JDWBC-5937|658[0n]bp|Canada.British Columbia|BOLD:AAC7834
Properigea niveirena|[11402]|LALPA911-11|AVBC 1084-11|658[0n]bp|Canada.British Columbia|BOLD:AAF0135
Pseudobryomima muscosa|[11403]|LALPA813-10|AVBC 815-10|658[0n]bp|Canada.British Columbia|BOLD:AAD7262
Pseudobryomima muscosa|[11404]|RDNMF159-08|NOC14245|658[0n]bp|Canada.British Columbia|BOLD:AAD7262
Euplexia benesimilis|[11405]|LPSO842-08|PPBP-0842|658[0n]bp|Canada.Ontario|BOLD:AAA4098
Euplexia benesimilis|[11406]|BBLPE099-09|09BBELE-2099|658[0n]bp|Canada.Nova Scotia|BOLD:AAA4097
Euplexia benesimilis|[11407]|LPSO743-08|PPBP-0743|658[0n]bp|Canada.Ontario|BOLD:AAA4097
Euplexia benesimilis|[11408]|PHMNB058-03|moth36.02SA|639[0n]bp|Canada.New Brunswick|BOLD:AAA4097
Euplexia benesimilis|[11409]|LPVIA237-08|PFC-2006-0319|658[0n]bp|Canada.British Columbia|BOLD:AAA4097
Euplexia benesimilis|[11410]|LPABB581-08|08BBLEP-03846|658[0n]bp|Canada.Alberta|BOLD:AAA4097
Euplexia benesimilis|[11411]|LOWCB437-05|CGWC-1377|590[0n]bp|Canada.British Columbia|BOLD:AAA4097
Euplexia benesimilis|[11412]|LOWCB458-05|CGWC-1398|617[0n]bp|Canada.British Columbia|BOLD:AAA4097
Euplexia benesimilis|[11413]|BBLPC222-09|09BBELE-1222|633[0n]bp|Canada.Nova Scotia|BOLD:AAA4097
Euplexia benesimilis|[11414]|LBCC335-05|HLC-22215|658[0n]bp|Canada.British Columbia|BOLD:AAA4097
Euplexia benesimilis|[11415]|LPVIA236-08|PFC-2006-0318|658[0n]bp|Canada.British Columbia|BOLD:AAA4097
Euplexia benesimilis|[11416]|LHLEP428-06|UBC-2006-0709|658[0n]bp|Canada.British Columbia|BOLD:AAA4097
Euplexia benesimilis|[11417]|LBCC296-05|HLC-22176|658[0n]bp|Canada.British Columbia|BOLD:AAA4097
Euplexia benesimilis|[11418]|LBCD052-05|HLC-22872|658[0n]bp|Canada.British Columbia|BOLD:AAA4097
Euplexia benesimilis|[11419]|BBLPA814-10|10BBCLP-0814|658[0n]bp|Canada.British Columbia|BOLD:AAA4097
Euplexia benesimilis|[11420]|LBCC440-05|HLC-22320|658[0n]bp|Canada.British Columbia|BOLD:AAA4097
Euplexia benesimilis|[11421]|LBCC303-05|HLC-22183|658[0n]bp|Canada.British Columbia|BOLD:AAA4097
Euplexia benesimilis|[11422]|LBCH2983-10|10-JDWBC-2983|658[0n]bp|Canada.British Columbia|BOLD:AAA4097
Euplexia benesimilis|[11423]|LALPA1122-11|AVBC 932-11|658[0n]bp|Canada.British Columbia|BOLD:AAA4097
Euplexia benesimilis|[11424]|LBCC334-05|HLC-22214|658[0n]bp|Canada.British Columbia|BOLD:AAA4097
Euplexia benesimilis|[11425]|LBCC006-05|HLC-21886|658[0n]bp|Canada.British Columbia|BOLD:AAA4097
Euplexia benesimilis|[11426]|LOWCB456-05|CGWC-1396|658[0n]bp|Canada.British Columbia|BOLD:AAA4097
Euplexia benesimilis|[11427]|LBCB206-05|HLC-21146|658[0n]bp|Canada.British Columbia|BOLD:AAA4097
Euplexia benesimilis|[11428]|LBCC042-05|HLC-21922|658[0n]bp|Canada.British Columbia|BOLD:AAA4097
Euplexia benesimilis|[11429]|LBCC008-05|HLC-21888|658[0n]bp|Canada.British Columbia|BOLD:AAA4097
Euplexia benesimilis|[11430]|LBCS749-07|UBC-2007-0454|658[0n]bp|Canada.British Columbia|BOLD:AAA4097
Euplexia benesimilis|[11431]|LOWCB457-05|CGWC-1397|658[0n]bp|Canada.British Columbia|BOLD:AAA4097
Euplexia benesimilis|[11432]|LBCH2958-10|10-JDWBC-2958|658[0n]bp|Canada.British Columbia|BOLD:AAA4097
Euplexia benesimilis|[11433]|LBCG2542-09|08-JDWBC-2542|658[0n]bp|Canada.British Columbia|BOLD:AAA4097
Euplexia benesimilis|[11434]|LBCD103-05|HLC-22923|658[0n]bp|Canada.British Columbia|BOLD:AAA4097
Euplexia benesimilis|[11435]|LALPA601-10|AVBC 603-10|658[0n]bp|Canada.British Columbia|BOLD:AAA4097
Euplexia benesimilis|[11436]|LBCB552-05|HLC-21492|658[0n]bp|Canada.British Columbia|BOLD:AAA4097
Euplexia benesimilis|[11437]|LBCB407-05|HLC-21347|658[0n]bp|Canada.British Columbia|BOLD:AAA4097
Euplexia benesimilis|[11438]|LBCB622-05|HLC-21562|658[0n]bp|Canada.British Columbia|BOLD:AAA4097
Euplexia benesimilis|[11439]|LBCB075-05|HLC-21015|658[0n]bp|Canada.British Columbia|BOLD:AAA4097
Euplexia benesimilis|[11440]|LBCD056-05|HLC-22876|658[0n]bp|Canada.British Columbia|BOLD:AAA4097
Euplexia benesimilis|[11441]|BBLPA812-10|10BBCLP-0812|658[0n]bp|Canada.British Columbia|BOLD:AAA4097
Euplexia benesimilis|[11442]|LBCC007-05|HLC-21887|658[0n]bp|Canada.British Columbia|BOLD:AAA4097
Euplexia benesimilis|[11443]|LBCB073-05|HLC-21013|658[0n]bp|Canada.British Columbia|BOLD:AAA4097
Euplexia benesimilis|[11444]|BBLPA813-10|10BBCLP-0813|658[0n]bp|Canada.British Columbia|BOLD:AAA4097
Euplexia benesimilis|[11445]|BBLPB852-10|10BBCLP-1851|658[0n]bp|Canada.British Columbia|BOLD:AAA4097
Euplexia benesimilis|[11446]|BBLPA815-10|10BBCLP-0815|658[0n]bp|Canada.British Columbia|BOLD:AAA4097
Euplexia benesimilis|[11447]|LALPA141-10|AVBC 141-10|658[0n]bp|Canada.British Columbia|BOLD:AAA4097
Euplexia benesimilis|[11448]|LBCD489-05|HLC-23309|654[0n]bp|Canada.British Columbia|BOLD:AAA4097
Euplexia benesimilis|[11449]|LBCD460-05|HLC-23280|645[0n]bp|Canada.British Columbia|BOLD:AAA4097
Euplexia benesimilis|[11450]|LCH556-04|04HBL003556|585[0n]bp|Canada.Manitoba|BOLD:AAA4097
Euplexia benesimilis|[11451]|LOWCB438-05|CGWC-1378|626[1n]bp|Canada.British Columbia|BOLD:AAA4097
Euplexia benesimilis|[11452]|LBCD104-05|HLC-22924|636[0n]bp|Canada.British Columbia|BOLD:AAA4097
Euplexia benesimilis|[11453]|RDMAB075-05|UASM57602|611[0n]bp|Canada.Alberta|BOLD:AAA4097
Euplexia benesimilis|[11454]|LPMN724-08|08BBLEP-01527|658[0n]bp|Canada.Manitoba|BOLD:AAA4097
Euplexia benesimilis|[11455]|BBLPC973-09|09BBELE-1973|658[0n]bp|Canada.Newfoundland and Labrador|BOLD:...
Euplexia benesimilis|[11456]|BBLPB542-10|10BBCLP-1541|658[0n]bp|Canada.Ontario|BOLD:AAA4097
Euplexia benesimilis|[11457]|BBLPC629-09|09BBELE-1629|658[0n]bp|Canada.Newfoundland and Labrador|BOLD:...
Euplexia benesimilis|[11458]|BBLEC804-09|09BBELE-0804|658[0n]bp|Canada.Newfoundland and Labrador|BOLD:...
Euplexia benesimilis|[11459]|BBLPE347-09|09BBELE-2347|658[0n]bp|Canada.Newfoundland and Labrador|BOLD:...
Euplexia benesimilis|[11460]|BBLPE100-09|09BBELE-2100|658[0n]bp|Canada.Nova Scotia|BOLD:AAA4097
Euplexia benesimilis|[11461]|BBLPE266-09|09BBELE-2266|658[0n]bp|Canada.Nova Scotia|BOLD:AAA4097
Euplexia benesimilis|[11462]|BBLEC357-09|09BBELE-0357|658[0n]bp|Canada.Newfoundland and Labrador|BOLD:...

Euplexia benesimilis|[11460]|BBLPE100-09|09BBELE-2100|658[0n]bp|Canada.Nova Scotia|BOLD:AAA4097
Euplexia benesimilis|[11461]|BBLPE266-09|09BBELE-2266|658[0n]bp|Canada.Nova Scotia|BOLD:AAA4097
Euplexia benesimilis|[11462]|BBLEC357-09|09BBELE-0357|658[0n]bp|Canada.Newfoundland and Labrador|BOLD:...
Euplexia benesimilis|[11463]|BBLEC383-09|09BBELE-0383|658[0n]bp|Canada.Newfoundland and Labrador|BOLD:...
Euplexia benesimilis|[11464]|BBLPB749-10|10BBCLP-1748|658[0n]bp|Canada.Ontario|BOLD:AAA4097
Euplexia benesimilis|[11465]|BBLPA811-10|10BBCLP-0811|658[0n]bp|Canada.Ontario|BOLD:AAA4097
Euplexia benesimilis|[11466]|BBLPC266-09|09BBELE-1266|658[0n]bp|Canada.Nova Scotia|BOLD:AAA4097
Euplexia benesimilis|[11467]|TTMNB034-06|MNBTT-034|658[0n]bp|Canada.New Brunswick|BOLD:AAA4097
Euplexia benesimilis|[11468]|LBCC766-05|HLC-22646|658[0n]bp|Canada.British Columbia|BOLD:AAA4097
Euplexia benesimilis|[11469]|BBLPA810-10|10BBCLP-0810|658[0n]bp|Canada.Ontario|BOLD:AAA4097
Euplexia benesimilis|[11470]|BBLPA808-10|10BBCLP-0808|658[0n]bp|Canada.Ontario|BOLD:AAA4097
Euplexia benesimilis|[11471]|BBLPE126-09|09BBELE-2126|658[0n]bp|Canada.Nova Scotia|BOLD:AAA4097
Euplexia benesimilis|[11472]|BBLPA809-10|10BBCLP-0809|658[0n]bp|Canada.Ontario|BOLD:AAA4097
Euplexia benesimilis|[11473]|BBLPE357-09|09BBELE-2357|658[0n]bp|Canada.Newfoundland and Labrador|BOLD:...
Euplexia benesimilis|[11474]|BBLPE014-09|09BBELE-2014|658[0n]bp|Canada.Nova Scotia|BOLD:AAA4097
Euplexia benesimilis|[11475]|BBLPE128-09|09BBELE-2128|658[0n]bp|Canada.Nova Scotia|BOLD:AAA4097
Euplexia benesimilis|[11476]|LPSO384-08|PPBP-0384|658[0n]bp|Canada.Ontario|BOLD:AAA4097
Euplexia benesimilis|[11477]|BLTIB113-08|BL180|609[1n]bp|Canada.Ontario|BOLD:AAA4097
Euplexia benesimilis|[11478]|LPSO359-08|PPBP-0359|658[0n]bp|Canada.Ontario|BOLD:AAA4097
Euplexia benesimilis|[11479]|XAC799-04|04HBL006799|658[0n]bp|Canada.Ontario|BOLD:AAA4097
Euplexia benesimilis|[11480]|RDLQG202-06|DH012378|658[0n]bp|Canada.Quebec|BOLD:AAA4097
Euplexia benesimilis|[11481]|BLTIB459-08|BL706|658[0n]bp|Canada.Ontario|BOLD:AAA4097
Euplexia benesimilis|[11482]|XAC601-04|04HBL006601|658[0n]bp|Canada.Ontario|BOLD:AAA4097
Euplexia benesimilis|[11483]|BLTIB145-08|BL213|658[0n]bp|Canada.Ontario|BOLD:AAA4097
Euplexia benesimilis|[11484]|LPSOB093-08|PPBP-1092|658[0n]bp|Canada.Ontario|BOLD:AAA4097
Euplexia benesimilis|[11485]|BLTIB090-08|BL0146|658[0n]bp|Canada.Ontario|BOLD:AAA4097
Euplexia benesimilis|[11486]|RDLQF566-06|DH011715|658[0n]bp|Canada.Quebec|BOLD:AAA4097
Euplexia benesimilis|[11487]|LPSO496-08|PPBP-0496|658[0n]bp|Canada.Ontario|BOLD:AAA4097
Euplexia benesimilis|[11488]|LPSOB818-08|PPBP-1817|658[0n]bp|Canada.Ontario|BOLD:AAA4097
Euplexia benesimilis|[11489]|PHMO049-03|moth268.02|639[0n]bp|Canada.Ontario|BOLD:AAA4097
Euplexia benesimilis|[11490]|BLGSM059-09|BL378|621[0n]bp|Canada.Ontario|BOLD:AAA4097
Euplexia benesimilis|[11491]|LPSOC111-08|PPBP-2110|658[0n]bp|Canada.Ontario|BOLD:AAA4097
Euplexia benesimilis|[11492]|TTMNB361-06|MNBTT-361|658[0n]bp|Canada.New Brunswick|BOLD:AAA4097
Euplexia benesimilis|[11493]|LPSO495-08|PPBP-0495|658[0n]bp|Canada.Ontario|BOLD:AAA4097
Euplexia benesimilis|[11494]|LPSO364-08|PPBP-0364|658[0n]bp|Canada.Ontario|BOLD:AAA4097
Euplexia benesimilis|[11495]|LPSOB816-08|PPBP-1815|658[0n]bp|Canada.Ontario|BOLD:AAA4097
Conservula anodonta|[11496]|RDLQF545-06|DH011694|658[0n]bp|Canada.Quebec|BOLD:AAF1231
Conservula anodonta|[11497]|RDLQG132-06|DH012299|574[0n]bp|Canada.Quebec|BOLD:AAF1231
Conservula anodonta|[11498]|RDLQB674-05|DH010777|658[0n]bp|Canada.Quebec|BOLD:AAF1231
Homorthodes discreta|[11499]|LBCH6634-10|10-JDWBC-6634|658[0n]bp|Canada.British Columbia|BOLD:AAD9145
Homorthodes discreta|[11500]|LBCG1102-09|08-JDWBC-1102|658[0n]bp|Canada.British Columbia|BOLD:AAD9145
Homorthodes discreta|[11501]|LBCH7474-10|10-JDWBC-7474|658[0n]bp|Canada.British Columbia|BOLD:AAD9145
Homorthodes discreta|[11502]|LBCH6238-10|10-JDWBC-6238|658[0n]bp|Canada.British Columbia|BOLD:AAD9145
Homorthodes discreta|[11503]|LBCH6296-10|10-JDWBC-6296|658[0n]bp|Canada.British Columbia|BOLD:AAD9145
Homorthodes discreta|[11504]|LBCH6239-10|10-JDWBC-6239|658[0n]bp|Canada.British Columbia|BOLD:AAD9145
Homorthodes discreta|[11505]|LBCH7341-10|10-JDWBC-7341|658[0n]bp|Canada.British Columbia|BOLD:AAD9145
Homorthodes discreta|[11506]|LBCH6166-10|10-JDWBC-6166|658[0n]bp|Canada.British Columbia|BOLD:AAD9145
Homorthodes discreta|[11507]|LOWCB899-05|CGWC-1839|658[0n]bp|Canada.British Columbia|BOLD:AAD9145
Homorthodes discreta|[11508]|LBCH7376-10|10-JDWBC-7376|636[0n]bp|Canada.British Columbia|BOLD:AAD9145
Homorthodes discreta|[11509]|LBCH6295-10|10-JDWBC-6295|658[0n]bp|Canada.British Columbia|BOLD:AAD9145
Phlogophora iris|[11510]|PHMNB151-04|04HBL007616|565[5n]bp|Canada.New Brunswick|BOLD:AAA9019
Phlogophora iris|[11511]|BBLEC364-09|09BBELE-0364|658[0n]bp|Canada.Newfoundland and Labrador|BOLD:AAA9019
Phlogophora iris|[11512]|TMNBB188-06|MNBTT-1128|658[1n]bp|Canada.New Brunswick|BOLD:AAA9019
Phlogophora iris|[11513]|PHMNB235-04|04HBL007700|568[1n]bp|Canada.New Brunswick|BOLD:AAA9019
Phlogophora iris|[11514]|RDLQ486-07|DH012440|599[0n]bp|Canada.Quebec|BOLD:AAA9019
Phlogophora iris|[11515]|PHMNB318-04|04HBL00544|658[0n]bp|Canada.New Brunswick|BOLD:AAA9019
Phlogophora iris|[11516]|PHMNB563-04|04HBL00789|658[1n]bp|Canada.New Brunswick|BOLD:AAA9019
Phlogophora iris|[11517]|TMNBB187-06|MNBTT-1127|658[0n]bp|Canada.New Brunswick|BOLD:AAA9019
Phlogophora iris|[11518]|TMNBB189-06|MNBTT-1129|658[0n]bp|Canada.New Brunswick|BOLD:AAA9019
Phlogophora iris|[11519]|PHMNB691-04|04HBL00917|658[0n]bp|Canada.New Brunswick|BOLD:AAA9019
Phlogophora iris|[11520]|XAE455-04|Moth4455.03|658[0n]bp|Canada.Ontario|BOLD:AAA9019
Phlogophora iris|[11521]|LPSOB784-08|PPBP-1783|658[0n]bp|Canada.Ontario|BOLD:AAA9019
Phlogophora iris|[11522]|PHMNB461-04|04HBL00687|658[0n]bp|Canada.New Brunswick|BOLD:AAA9019
Phlogophora iris|[11523]|LPMN814-08|08BBLEP-01617|658[0n]bp|Canada.Manitoba|BOLD:AAA9019
Phlogophora iris|[11524]|BBLPB520-10|10BBCLP-1519|658[0n]bp|Canada.Ontario|BOLD:AAA9019
Phlogophora iris|[11525]|LPSOB785-08|PPBP-1784|658[0n]bp|Canada.Ontario|BOLD:AAA9019
Phlogophora iris|[11526]|BBLEC358-09|09BBELE-0358|658[0n]bp|Canada.Newfoundland and Labrador|BOLD:AAA9019
Phlogophora iris|[11527]|PHMNB319-04|04HBL00545|658[0n]bp|Canada.New Brunswick|BOLD:AAA9019
Phlogophora iris|[11528]|LPSOB008-08|PPBP-1007|658[0n]bp|Canada.Ontario|BOLD:AAA9019
Phlogophora iris|[11529]|BBLPE501-09|09BBELE-2501|658[0n]bp|Canada.Newfoundland and Labrador|BOLD:AAA9019
Phlogophora iris|[11530]|BBLEC348-09|09BBELE-0348|658[0n]bp|Canada.Newfoundland and Labrador|BOLD:AAA9019
Phlogophora iris|[11531]|LPSOC311-08|PPBP-2310|658[0n]bp|Canada.Ontario|BOLD:AAA9019
Phlogophora iris|[11532]|LPSOB313-08|PPBP-1312|658[0n]bp|Canada.Ontario|BOLD:AAA9019
Phlogophora iris|[11533]|BBLPE492-09|09BBELE-2492|658[0n]bp|Canada.Newfoundland and Labrador|BOLD:AAA9019
Phlogophora iris|[11534]|XAE276-04|Moth4276.03|658[0n]bp|Canada.Ontario|BOLD:AAA9019
Phlogophora iris|[11535]|LPMN340-08|08BBLEP-01139|658[0n]bp|Canada.Manitoba|BOLD:AAA9019
Phlogophora iris|[11536]|BBLPC321-09|09BBELE-1321|658[0n]bp|Canada.Newfoundland and Labrador|BOLD:AAA9019
Phlogophora iris|[11537]|XAB551-04|04HBL005551|658[0n]bp|Canada.Ontario|BOLD:AAA9019
Phlogophora iris|[11538]|BBLPB516-10|10BBCLP-1515|658[0n]bp|Canada.Ontario|BOLD:AAA9019
Phlogophora iris|[11539]|BBLPB519-10|10BBCLP-1518|658[0n]bp|Canada.Ontario|BOLD:AAA9019
Phlogophora iris|[11540]|BBLPB518-10|10BBCLP-1517|658[0n]bp|Canada.Ontario|BOLD:AAA9019
Phlogophora iris|[11541]|TTMNB362-06|MNBTT-362|658[0n]bp|Canada.New Brunswick|BOLD:AAA9019
Phlogophora iris|[11542]|BBLPB517-10|10BBCLP-1516|658[0n]bp|Canada.Ontario|BOLD:AAA9019
Phlogophora iris|[11543]|PHMNB464-04|04HBL00690|658[0n]bp|Canada.New Brunswick|BOLD:AAA9019
Phlogophora iris|[11544]|BBLPE494-09|09BBELE-2494|658[0n]bp|Canada.Newfoundland and Labrador|BOLD:AAA9019
Phlogophora iris|[11545]|PHMNB469-04|04HBL00695|658[0n]bp|Canada.New Brunswick|BOLD:AAA9019
Phlogophora iris|[11546]|PHMNB380-04|04HBL00606|658[0n]bp|Canada.New Brunswick|BOLD:AAA9019
Phlogophora iris|[11547]|PHMNB679-04|04HBL00905|616[0n]bp|Canada.New Brunswick|BOLD:AAA9019
Phlogophora iris|[11548]|PHMNB720-05|Moth 413.03SA|616[0n]bp|Canada.New Brunswick|BOLD:AAA9019
Phlogophora iris|[11549]|XAB397-04|04HBL005397|658[0n]bp|Canada.Ontario|BOLD:AAA9019
Phlogophora iris|[11550]|PHMNB188-04|04HBL007653|609[0n]bp|Canada.New Brunswick|BOLD:AAA9019
Phlogophora iris|[11551]|PHMNB066-03|moth53.02SA|639[0n]bp|Canada.New Brunswick|BOLD:AAA9019
Phlogophora iris|[11552]|PHMO083-03|moth541.01|639[0n]bp|Canada.Ontario|BOLD:AAA9019
Phlogophora iris|[11553]|PHMO084-03|moth542.02|639[0n]bp|Canada.Ontario|BOLD:AAA9019
Phlogophora periculosa|[11554]|XAD087-04|04HBL007087|576[0n]bp|Canada.Ontario|BOLD:AAA7228
Phlogophora periculosa|[11555]|XAD351-04|04HBL007351|576[1n]bp|Canada.Ontario|BOLD:AAA7228
Phlogophora periculosa|[11556]|XAD305-04|04HBL007305|592[0n]bp|Canada.Ontario|BOLD:AAA7228
Phlogophora periculosa|[11557]|LPSOD1001-09|08BBLEP-05634|658[0n]bp|Canada.Ontario|BOLD:AAA7228
Phlogophora periculosa|[11558]|BBLEC009-09|09BBELE-0009|658[0n]bp|Canada.New Brunswick|BOLD:AAA7228
Phlogophora periculosa|[11559]|BBLPB633-10|10BBCLP-1632|658[0n]bp|Canada.Alberta|BOLD:AAA7228
Phlogophora periculosa|[11560]|BBLEC010-09|09BBELE-0010|658[0n]bp|Canada.New Brunswick|BOLD:AAA7228
Phlogophora periculosa|[11561]|LOWCC008-05|CGWC-1888|620[0n]bp|Canada.British Columbia|BOLD:AAA7228
Phlogophora periculosa|[11562]|TMNBB190-06|MNBTT-1130|658[0n]bp|Canada.New Brunswick|BOLD:AAA7228

[illegible]
Phlogophora periculosa|[11560]|BBLEC010-09|09BBELE-0010|658[0n]bp|Canada.New Brunswick|BOLD:AAA7228
Phlogophora periculosa|[11561]|LOWCC008-05|CGWC-1888|620[0n]bp|Canada.British Columbia|BOLD:AAA7228
Phlogophora periculosa|[11562]|TMNBB190-06|MNBTT-1130|658[0n]bp|Canada.New Brunswick|BOLD:AAA7228
Phlogophora periculosa|[11563]|DUNLP181-08|Dun-08-181|658[0n]bp|Canada.British Columbia|BOLD:AAA7228
Phlogophora periculosa|[11564]|XAH001-05|2005-ONT-1584|614[0n]bp|Canada.Ontario|BOLD:AAA7228
Phlogophora periculosa|[11565]|BBLPC106-09|09BBELE-1106|633[0n]bp|Canada.New Brunswick|BOLD:AAA7228
Phlogophora periculosa|[11566]|BBLPC103-09|09BBELE-1103|631[0n]bp|Canada.New Brunswick|BOLD:AAA7228
Phlogophora periculosa|[11567]|TTMNB363-06|MNBTT-363|658[1n]bp|Canada.New Brunswick|BOLD:AAA7228
Phlogophora periculosa|[11568]|LBCH4397-10|10-JDWBC-4397|658[0n]bp|Canada.British Columbia|BOLD:AAA7228
Phlogophora periculosa|[11569]|LBCH944-10|10-JDWBC-0944|658[0n]bp|Canada.British Columbia|BOLD:AAA7228
Phlogophora periculosa|[11570]|LOWCC017-05|CGWC-1897|658[0n]bp|Canada.British Columbia|BOLD:AAA7228
Phlogophora periculosa|[11571]|LHLEP260-06|UBC-2006-1499|658[0n]bp|Canada.British Columbia|BOLD:AAA7228
Phlogophora periculosa|[11572]|LBCH4400-10|10-JDWBC-4400|658[0n]bp|Canada.British Columbia|BOLD:AAA7228
Phlogophora periculosa|[11573]|LBCH3773-10|10-JDWBC-3773|658[0n]bp|Canada.British Columbia|BOLD:AAA7228
Phlogophora periculosa|[11574]|LBCH3769-10|10-JDWBC-3769|658[0n]bp|Canada.British Columbia|BOLD:AAA7228
Phlogophora periculosa|[11575]|BBLEC528-09|09BBELE-0528|658[0n]bp|Canada.New Brunswick|BOLD:AAA7228
Phlogophora periculosa|[11576]|LOWCC009-05|CGWC-1889|658[0n]bp|Canada.British Columbia|BOLD:AAA7228
Phlogophora periculosa|[11577]|LBCH3329-10|10-JDWBC-3329|658[0n]bp|Canada.British Columbia|BOLD:AAA7228
Phlogophora periculosa|[11578]|LBCH4402-10|10-JDWBC-4402|658[0n]bp|Canada.British Columbia|BOLD:AAA7228
Phlogophora periculosa|[11579]|LBCH3770-10|10-JDWBC-3770|658[0n]bp|Canada.British Columbia|BOLD:AAA7228
Phlogophora periculosa|[11580]|BBLEC043-09|09BBELE-0043|658[0n]bp|Canada.New Brunswick|BOLD:AAA7228
Phlogophora periculosa|[11581]|LBCH945-10|10-JDWBC-0945|658[0n]bp|Canada.British Columbia|BOLD:AAA7228
Phlogophora periculosa|[11582]|LBCH3472-10|10-JDWBC-3472|658[0n]bp|Canada.British Columbia|BOLD:AAA7228
Phlogophora periculosa|[11583]|LBCH3775-10|10-JDWBC-3775|658[0n]bp|Canada.British Columbia|BOLD:AAA7228
Phlogophora periculosa|[11584]|RDLQB483-05|DH010569|658[0n]bp|Canada.Quebec|BOLD:AAA7228
Phlogophora periculosa|[11585]|LHLEP261-06|UBC-2006-1500|658[0n]bp|Canada.British Columbia|BOLD:AAA7228
Phlogophora periculosa|[11586]|LBCH3473-10|10-JDWBC-3473|658[0n]bp|Canada.British Columbia|BOLD:AAA7228
Phlogophora periculosa|[11587]|LOWCC015-05|CGWC-1895|658[0n]bp|Canada.British Columbia|BOLD:AAA7228
Phlogophora periculosa|[11588]|TMNBB191-06|MNBTT-1131|658[0n]bp|Canada.New Brunswick|BOLD:AAA7228
Phlogophora periculosa|[11589]|LBCH3772-10|10-JDWBC-3772|658[0n]bp|Canada.British Columbia|BOLD:AAA7228
Phlogophora periculosa|[11590]|LALPA555-10|AVBC 557-10|658[0n]bp|Canada.British Columbia|BOLD:AAA7228
Phlogophora periculosa|[11591]|LBCH3767-10|10-JDWBC-3767|658[0n]bp|Canada.British Columbia|BOLD:AAA7228
Phlogophora periculosa|[11592]|LHLEP264-06|UBC-2006-1650|658[0n]bp|Canada.British Columbia|BOLD:AAA7228
Phlogophora periculosa|[11593]|BBLPC123-09|09BBELE-1123|658[0n]bp|Canada.New Brunswick|BOLD:AAA7228
Phlogophora periculosa|[11594]|LBCH1500-10|10-JDWBC-1500|658[0n]bp|Canada.British Columbia|BOLD:AAA7228
Phlogophora periculosa|[11595]|LPMNB531-09|08BBLEP-05569|658[0n]bp|Canada.Manitoba|BOLD:AAA7228
Phlogophora periculosa|[11596]|LHLEP258-06|UBC-2006-1409|658[0n]bp|Canada.British Columbia|BOLD:AAA7228
Phlogophora periculosa|[11597]|LOWCD156-06|CGWC-2976|658[0n]bp|Canada.British Columbia|BOLD:AAA7228
Phlogophora periculosa|[11598]|LBCH3477-10|10-JDWBC-3477|658[0n]bp|Canada.British Columbia|BOLD:AAA7228
Phlogophora periculosa|[11599]|LBCH3476-10|10-JDWBC-3476|658[0n]bp|Canada.British Columbia|BOLD:AAA7228
Phlogophora periculosa|[11600]|LBCH4395-10|10-JDWBC-4395|658[0n]bp|Canada.British Columbia|BOLD:AAA7228
Phlogophora periculosa|[11601]|LOWCC010-05|CGWC-1890|658[0n]bp|Canada.British Columbia|BOLD:AAA7228
Phlogophora periculosa|[11602]|LBCH2084-10|10-JDWBC-2084|658[0n]bp|Canada.British Columbia|BOLD:AAA7228
Phlogophora periculosa|[11603]|LOWCC013-05|CGWC-1893|658[0n]bp|Canada.British Columbia|BOLD:AAA7228
Phlogophora periculosa|[11604]|LBCH3771-10|10-JDWBC-3771|658[0n]bp|Canada.British Columbia|BOLD:AAA7228
Phlogophora periculosa|[11605]|LHLEP257-06|UBC-2006-1408|658[0n]bp|Canada.British Columbia|BOLD:AAA7228
Phlogophora periculosa|[11606]|LBCH3475-10|10-JDWBC-3475|658[0n]bp|Canada.British Columbia|BOLD:AAA7228
Phlogophora periculosa|[11607]|LPMNB248-09|08BBLEP-05092|658[0n]bp|Canada.Manitoba|BOLD:AAA7228
Phlogophora periculosa|[11608]|LBCH4120-10|10-JDWBC-4120|658[0n]bp|Canada.British Columbia|BOLD:AAA7228
Phlogophora periculosa|[11609]|LOWCC007-05|CGWC-1887|658[0n]bp|Canada.British Columbia|BOLD:AAA7228
Phlogophora periculosa|[11610]|LBCC443-05|HLC-22323|658[0n]bp|Canada.British Columbia|BOLD:AAA7228
Phlogophora periculosa|[11611]|LBCH3474-10|10-JDWBC-3474|658[0n]bp|Canada.British Columbia|BOLD:AAA7228
Phlogophora periculosa|[11612]|LBCH4399-10|10-JDWBC-4399|658[0n]bp|Canada.British Columbia|BOLD:AAA7228
Phlogophora periculosa|[11613]|LALPA572-10|AVBC 574-10|658[0n]bp|Canada.British Columbia|BOLD:AAA7228
Phlogophora periculosa|[11614]|LBCH3774-10|10-JDWBC-3774|658[0n]bp|Canada.British Columbia|BOLD:AAA7228
Phlogophora periculosa|[11615]|LHLEP259-06|UBC-2006-1498|658[0n]bp|Canada.British Columbia|BOLD:AAA7228
Phlogophora periculosa|[11616]|LBCH3478-10|10-JDWBC-3478|658[0n]bp|Canada.British Columbia|BOLD:AAA7228
Phlogophora periculosa|[11617]|BBLPC107-09|09BBELE-1107|658[0n]bp|Canada.New Brunswick|BOLD:AAA7228
Phlogophora periculosa|[11618]|LALPA577-10|AVBC 579-10|658[0n]bp|Canada.British Columbia|BOLD:AAA7228
Phlogophora periculosa|[11619]|LBCH4396-10|10-JDWBC-4396|658[0n]bp|Canada.British Columbia|BOLD:AAA7228
Phlogophora periculosa|[11620]|LOWCC012-05|CGWC-1892|658[0n]bp|Canada.British Columbia|BOLD:AAA7228
Phlogophora periculosa|[11621]|LBCH4121-10|10-JDWBC-4121|658[0n]bp|Canada.British Columbia|BOLD:AAA7228
Phlogophora periculosa|[11622]|XAG942-05|2005-ONT-1526|658[0n]bp|Canada.Ontario|BOLD:AAA7228
Phlogophora periculosa|[11623]|LBCH3479-10|10-JDWBC-3479|658[0n]bp|Canada.British Columbia|BOLD:AAA7228
Phlogophora periculosa|[11624]|LOWCC014-05|CGWC-1894|658[0n]bp|Canada.British Columbia|BOLD:AAA7228
Phlogophora periculosa|[11625]|LBCH4401-10|10-JDWBC-4401|658[0n]bp|Canada.British Columbia|BOLD:AAA7228
Phlogophora periculosa|[11626]|LBCH3768-10|10-JDWBC-3768|658[0n]bp|Canada.British Columbia|BOLD:AAA7228
Phlogophora periculosa|[11627]|LBCH4398-10|10-JDWBC-4398|658[0n]bp|Canada.British Columbia|BOLD:AAA7228
Phlogophora periculosa|[11628]|LOWCC016-05|CGWC-1896|653[0n]bp|Canada.British Columbia|BOLD:AAA7228
Phlogophora periculosa|[11629]|PHAUG1779-11|BIOUG01497-D01|658[0n]bp|Canada.Ontario|BOLD:AAA7228
Phlogophora periculosa|[11630]|LOWCC011-05|CGWC-1891|658[0n]bp|Canada.British Columbia|BOLD:AAA7228
Phlogophora periculosa|[11631]|LHLEP263-06|UBC-2006-1502|658[0n]bp|Canada.British Columbia|BOLD:AAA7228
Phlogophora periculosa|[11632]|LBCH3067-10|10-JDWBC-3067|658[0n]bp|Canada.British Columbia|BOLD:AAA7228
Phlogophora periculosa|[11633]|LHLEP262-06|UBC-2006-1501|658[0n]bp|Canada.British Columbia|BOLD:AAA7228
Acopa perpallida|[11634]|RDNMF636-08|NOC14722|658[0n]bp|United States.New Mexico|BOLD:ACE4350
Acopa perpallida|[11635]|RDNMJ180-10|CNCLEP 70208|658[0n]bp|United States.New Mexico|BOLD:ACE4350
Acopa perpallida|[11636]|RDNMJ181-10|CNCLEP 70209|658[0n]bp|United States.New Mexico|BOLD:ACE4350
Acopa perpallida|[11637]|RDNMF639-08|NOC14725|658[0n]bp|United States.Arizona|BOLD:AAD5594
Acopa perpallida|[11638]|RDNMF635-08|NOC14721|658[5n]bp|United States.Arizona|BOLD:AAD5594
Acopa perpallida|[11639]|RDNMF640-08|NOC14726|658[0n]bp|United States.Arizona|BOLD:AAD5594
Acopa perpallida|[11640]|RDNMF638-08|NOC14724|658[0n]bp|United States.Arizona|BOLD:AAD5594
Acopa perpallida|[11641]|RDNMF637-08|NOC14723|658[0n]bp|United States.Arizona|BOLD:AAD5594
Cryphia cuerva|[11642]|LBCG2858-09|08-JDWBC-2858|658[0n]bp|Canada.British Columbia|BOLD:AAC1995
Cryphia cuerva|[11643]|LOWCC358-05|CGWC-2238|658[0n]bp|Canada.British Columbia|BOLD:AAC1995
Cryphia cuerva|[11644]|LOWCB181-05|CGWC-1121|658[0n]bp|Canada.British Columbia|BOLD:AAC1995
Cryphia cuerva|[11645]|LOWCC359-05|CGWC-2239|658[0n]bp|Canada.British Columbia|BOLD:AAC1995
Cryphia cuerva|[11646]|LOWCC355-05|CGWC-2235|658[0n]bp|Canada.British Columbia|BOLD:AAC1995
Cryphia cuerva|[11647]|LOWCC360-05|CGWC-2240|658[0n]bp|Canada.British Columbia|BOLD:AAC1995
Cryphia cuerva|[11648]|LOWCC357-05|CGWC-2237|520[0n]bp|Canada.British Columbia|
Cryphia cuerva|[11649]|LOWCC356-05|CGWC-2236|580[0n]bp|Canada.British Columbia|BOLD:AAC1995
Cryphia cuerva|[11650]|LBCH6964-10|10-JDWBC-6964|658[0n]bp|Canada.British Columbia|BOLD:AAC1995
Cryphia cuerva|[11651]|LBCH7606-10|10-JDWBC-7606|658[0n]bp|Canada.British Columbia|BOLD:AAC1995
Cryphia cuerva|[11652]|LBCH7601-10|10-JDWBC-7601|658[0n]bp|Canada.British Columbia|BOLD:AAC1995
Cryphia cuerva|[11653]|LBCH7603-10|10-JDWBC-7603|658[0n]bp|Canada.British Columbia|BOLD:AAC1995
Cryphia cuerva|[11654]|LBCH7766-10|10-JDWBC-7766|658[0n]bp|Canada.British Columbia|BOLD:AAC1995
Cryphia cuerva|[11655]|LBCH7825-10|10-JDWBC-7825|658[0n]bp|Canada.British Columbia|BOLD:AAC1995
Cryphia cuerva|[11656]|LBCH7764-10|10-JDWBC-7764|658[0n]bp|Canada.British Columbia|BOLD:AAC1995
Cryphia cuerva|[11657]|LBCH7608-10|10-JDWBC-7608|658[0n]bp|Canada.British Columbia|BOLD:AAC1995
Cryphia cuerva|[11658]|LBCH7602-10|10-JDWBC-7602|658[0n]bp|Canada.British Columbia|BOLD:AAC1995
Cryphia cuerva|[11659]|LBCH7604-10|10-JDWBC-7604|658[0n]bp|Canada.British Columbia|BOLD:AAC1995
Cryphia cuerva|[11660]|LBCG2857-09|08-JDWBC-2857|658[0n]bp|Canada.British Columbia|BOLD:AAC1995
Cryphia cuerva|[11661]|LBCH7768-10|10-JDWBC-7768|658[0n]bp|Canada.British Columbia|BOLD:AAC1995
[illegible]

Cryphia cuerva|[11659]|LBCH7604-10|10-JDWBC-7604|658[0n]bp|Canada.British Columbia|BOLD:AAC1995
Cryphia cuerva|[11660]|LBCG2857-09|08-JDWBC-2857|658[0n]bp|Canada.British Columbia|BOLD:AAC1995
Cryphia cuerva|[11661]|LBCH7768-10|10-JDWBC-7768|658[0n]bp|Canada.British Columbia|BOLD:AAC1995
Cryphia cuerva|[11662]|LBCH6726-10|10-JDWBC-6726|658[0n]bp|Canada.British Columbia|BOLD:AAC1995
Cryphia cuerva|[11663]|LBCG2860-09|08-JDWBC-2860|658[0n]bp|Canada.British Columbia|BOLD:AAC1995
Cryphia cuerva|[11664]|LBCH7605-10|10-JDWBC-7605|658[0n]bp|Canada.British Columbia|BOLD:AAC1995
Cryphia cuerva|[11665]|LBCH7765-10|10-JDWBC-7765|658[0n]bp|Canada.British Columbia|BOLD:AAC1995
Cryphia cuerva|[11666]|LBCG2859-09|08-JDWBC-2859|658[0n]bp|Canada.British Columbia|BOLD:AAC1995
Cryphia cuerva|[11667]|LBCH6925-10|10-JDWBC-6925|658[0n]bp|Canada.British Columbia|BOLD:AAC1995
Cryphia cuerva|[11668]|LBCH7607-10|10-JDWBC-7607|658[0n]bp|Canada.British Columbia|BOLD:AAC1995
Cryphia cuerva|[11669]|LBCH7763-10|10-JDWBC-7763|658[0n]bp|Canada.British Columbia|BOLD:AAC1995
Cryphia cuerva|[11670]|LBCH7762-10|10-JDWBC-7762|658[0n]bp|Canada.British Columbia|BOLD:AAC1995
Cryphia cuerva|[11671]|LOWCB182-05|CGWC-1122|613[0n]bp|Canada.British Columbia|BOLD:AAC1995
Cryphia cuerva|[11672]|RDNME775-08|LEP041240|658[0n]bp|Canada.Alberta|BOLD:AAC1995
Cryphia cuerva|[11673]|LBCG2861-09|08-JDWBC-2861|658[0n]bp|Canada.British Columbia|BOLD:AAC1995
Cryphia cuerva|[11674]|LBCH6458-10|10-JDWBC-6458|658[0n]bp|Canada.British Columbia|BOLD:AAC1995
Cryphia cuerva|[11675]|LBCG2856-09|08-JDWBC-2856|658[0n]bp|Canada.British Columbia|BOLD:AAC1995
Cryphia cuerva|[11676]|LBCH6462-10|10-JDWBC-6462|658[1n]bp|Canada.British Columbia|BOLD:AAC1995
Cryphia olivacea|[11677]|LBCH6532-10|10-JDWBC-6532|658[0n]bp|Canada.British Columbia|BOLD:AAE9413
Cryphia olivacea|[11678]|LBCH7346-10|10-JDWBC-7346|658[0n]bp|Canada.British Columbia|BOLD:ACE9980
Cryphia olivacea|[11679]|LBCH7348-10|10-JDWBC-7348|658[0n]bp|Canada.British Columbia|BOLD:ACE9980
Cryphia olivacea|[11680]|LBCH7338-10|10-JDWBC-7338|658[0n]bp|Canada.British Columbia|BOLD:ACE9980
Cryphia olivacea|[11681]|LBCH7344-10|10-JDWBC-7344|658[0n]bp|Canada.British Columbia|BOLD:ACE9980
Cryphia olivacea|[11682]|LBCH7347-10|10-JDWBC-7347|658[0n]bp|Canada.British Columbia|BOLD:ACE9980
Cryphia olivacea|[11683]|LBCH7345-10|10-JDWBC-7345|658[0n]bp|Canada.British Columbia|BOLD:ACE9980
Cryphia olivacea|[11684]|LBCH7446-10|10-JDWBC-7446|658[0n]bp|Canada.British Columbia|BOLD:ACE9980
Cryphia olivacea|[11685]|LBCH7093-10|10-JDWBC-7093|658[0n]bp|Canada.British Columbia|BOLD:ACE9980
Cryphia olivacea|[11686]|LBCH7349-10|10-JDWBC-7349|628[0n]bp|Canada.British Columbia|BOLD:ACE9980
Cryphia olivacea|[11687]|LBCH7343-10|10-JDWBC-7343|658[0n]bp|Canada.British Columbia|BOLD:ACE9980
Cryphia olivacea|[11688]|LBCH7373-10|10-JDWBC-7373|658[0n]bp|Canada.British Columbia|BOLD:ACE9980
Cryphia olivacea|[11689]|LBCH6137-10|10-JDWBC-6137|658[0n]bp|Canada.British Columbia|BOLD:ACE9980
Cryphia olivacea|[11690]|LBCH7350-10|10-JDWBC-7350|658[0n]bp|Canada.British Columbia|BOLD:ACE9980
Cryphia olivacea|[11691]|LBCH6237-10|10-JDWBC-6237|658[0n]bp|Canada.British Columbia|BOLD:ACE9980
Cryphia olivacea|[11692]|LBCH7478-10|10-JDWBC-7478|658[0n]bp|Canada.British Columbia|BOLD:ACE9980
Mythimna unipuncta|[11693]|XAB623-04|04HBL005623|658[0n]bp|Canada.Ontario|BOLD:AAA2482
Mythimna unipuncta|[11694]|LPSOD448-09|08BBLEP-00227|658[0n]bp|Canada.Ontario|BOLD:AAA2482
Mythimna unipuncta|[11695]|LPSOD424-09|08BBLEP-00203|658[0n]bp|Canada.Ontario|BOLD:AAA2482
Mythimna unipuncta|[11696]|LPSOB146-08|PPBP-1145|658[0n]bp|Canada.Ontario|BOLD:AAA2482
Mythimna unipuncta|[11697]|LBCS134-07|UBC-2007-0127|658[0n]bp|Canada.British Columbia|BOLD:AAA2482
Mythimna unipuncta|[11698]|LPSOD449-09|08BBLEP-00228|658[0n]bp|Canada.Ontario|BOLD:AAA2482
Mythimna unipuncta|[11699]|LPSOD519-09|08BBLEP-00298|658[0n]bp|Canada.Ontario|BOLD:AAA2482
Mythimna unipuncta|[11700]|PHMNB350-04|04HBL00576|658[0n]bp|Canada.New Brunswick|BOLD:AAA2482
Mythimna unipuncta|[11701]|BBLPA828-10|10BBCLP-0828|658[0n]bp|Canada.Saskatchewan|BOLD:AAA2482
Mythimna unipuncta|[11702]|LPSOD570-09|08BBLEP-00351|658[0n]bp|Canada.Ontario|BOLD:AAA2482
Mythimna unipuncta|[11703]|LPSOD331-09|08BBLEP-00109|658[0n]bp|Canada.Ontario|BOLD:AAA2482
Mythimna unipuncta|[11704]|XAH004-05|2005-ONT-1587|658[0n]bp|Canada.Ontario|BOLD:AAA2482
Mythimna unipuncta|[11705]|LPSOC321-08|PPBP-2320|658[0n]bp|Canada.Ontario|BOLD:AAA2482
Mythimna unipuncta|[11706]|LPSOB215-08|PPBP-1214|658[0n]bp|Canada.Ontario|BOLD:AAA2482
Mythimna unipuncta|[11707]|LPSOD799-09|08BBLEP-00581|658[0n]bp|Canada.Ontario|BOLD:AAA2482
Mythimna unipuncta|[11708]|LPSOC098-08|PPBP-2097|658[0n]bp|Canada.Ontario|BOLD:AAA2482
Mythimna unipuncta|[11709]|LPSO334-08|PPBP-0334|658[0n]bp|Canada.Ontario|BOLD:AAA2482
Mythimna unipuncta|[11710]|LPSOB110-08|PPBP-1109|658[0n]bp|Canada.Ontario|BOLD:AAA2482
Mythimna unipuncta|[11711]|LPSOD802-09|08BBLEP-00584|658[0n]bp|Canada.Ontario|BOLD:AAA2482
Mythimna unipuncta|[11712]|LPSOD517-09|08BBLEP-00296|658[0n]bp|Canada.Ontario|BOLD:AAA2482
Mythimna unipuncta|[11713]|LPSO328-08|PPBP-0328|658[0n]bp|Canada.Ontario|BOLD:AAA2482
Mythimna unipuncta|[11714]|LPSOD509-09|08BBLEP-00288|658[0n]bp|Canada.Ontario|BOLD:AAA2482
Mythimna unipuncta|[11715]|BLTIB975-08|BL1408|658[0n]bp|Canada.Ontario|BOLD:AAA2482
Mythimna unipuncta|[11716]|LPSOD324-09|08BBLEP-00102|658[0n]bp|Canada.Ontario|BOLD:AAA2482
Mythimna unipuncta|[11717]|BLTIB548-08|BL820|658[0n]bp|Canada.Ontario|BOLD:AAA2482
Mythimna unipuncta|[11718]|BLTIB109-08|BL174|658[0n]bp|Canada.Ontario|BOLD:AAA2482
Mythimna unipuncta|[11719]|XAD438-04|04HBL007438|658[0n]bp|Canada.Ontario|BOLD:AAA2482
Mythimna unipuncta|[11720]|LPSOC288-08|PPBP-2287|658[0n]bp|Canada.Ontario|BOLD:AAA2482
Mythimna unipuncta|[11721]|LPSO029-08|PPBP-0029|658[0n]bp|Canada.Ontario|BOLD:AAA2482
Mythimna unipuncta|[11722]|BBLPA825-10|10BBCLP-0825|658[0n]bp|Canada.Ontario|BOLD:AAA2482
Mythimna unipuncta|[11723]|PHMNB355-04|04HBL00581|658[0n]bp|Canada.New Brunswick|BOLD:AAA2482
Mythimna unipuncta|[11724]|LPSOB587-08|PPBP-1586|658[0n]bp|Canada.Ontario|BOLD:AAA2482
Mythimna unipuncta|[11725]|BLTIB912-08|BL1332|658[0n]bp|Canada.Ontario|BOLD:AAA2482
Mythimna unipuncta|[11726]|LPSOD866-09|08BBLEP-00648|658[0n]bp|Canada.Ontario|BOLD:AAA2482
Mythimna unipuncta|[11727]|LPSOC322-08|PPBP-2321|658[0n]bp|Canada.Ontario|BOLD:AAA2482
Mythimna unipuncta|[11728]|BLGSM027-09|BL336|658[0n]bp|Canada.Ontario|BOLD:AAA2482
Mythimna unipuncta|[11729]|PHMNB315-04|04HBL00541|658[0n]bp|Canada.New Brunswick|BOLD:AAA2482
Mythimna unipuncta|[11730]|BLTIB972-08|BL1402|658[0n]bp|Canada.Ontario|BOLD:AAA2482
Mythimna unipuncta|[11731]|LPSOB459-08|PPBP-1458|658[0n]bp|Canada.Ontario|BOLD:AAA2482
Mythimna unipuncta|[11732]|LPSOB111-08|PPBP-1110|658[0n]bp|Canada.Ontario|BOLD:AAA2482
Mythimna unipuncta|[11733]|RDLQF871-06|DH012043|658[0n]bp|Canada.Quebec|BOLD:AAA2482
Mythimna unipuncta|[11734]|LPSOD518-09|08BBLEP-00297|658[0n]bp|Canada.Ontario|BOLD:AAA2482
Mythimna unipuncta|[11735]|BLTIB509-08|BL767|658[0n]bp|Canada.Ontario|BOLD:AAA2482
Mythimna unipuncta|[11736]|LPSOD1050-09|08MZPP-116|658[0n]bp|Canada.Ontario|BOLD:AAA2482
Mythimna unipuncta|[11737]|MNBB445-05|05-NBSTA-361|658[0n]bp|Canada.New Brunswick|BOLD:AAA2482
Mythimna unipuncta|[11738]|BLTIB735-08|BL1025|658[0n]bp|Canada.Ontario|BOLD:AAA2482
Mythimna unipuncta|[11739]|LPSO144-08|PPBP-0144|658[0n]bp|Canada.Ontario|BOLD:AAA2482
Mythimna unipuncta|[11740]|LPSOC205-08|PPBP-2204|658[0n]bp|Canada.Ontario|BOLD:AAA2482
Mythimna unipuncta|[11741]|LPSOD743-09|08BBLEP-00525|658[0n]bp|Canada.Ontario|BOLD:AAA2482
Mythimna unipuncta|[11742]|LPSOD273-09|08BBLEP-00051|658[0n]bp|Canada.Ontario|BOLD:AAA2482
Mythimna unipuncta|[11743]|LPSOC318-08|PPBP-2317|658[0n]bp|Canada.Ontario|BOLD:AAA2482
Mythimna unipuncta|[11744]|LPSOB213-08|PPBP-1212|658[0n]bp|Canada.Ontario|BOLD:AAA2482
Mythimna unipuncta|[11745]|MHCOL287-07|CHU06-COL-287|658[0n]bp|Canada.Manitoba|BOLD:AAA2482
Mythimna unipuncta|[11746]|LPSOD487-09|08BBLEP-00266|658[0n]bp|Canada.Ontario|BOLD:AAA2482
Mythimna unipuncta|[11747]|PHMNB308-04|04HBL007773|658[0n]bp|Canada.New Brunswick|BOLD:AAA2482
Mythimna unipuncta|[11748]|LPSOD640-09|08BBLEP-00421|658[0n]bp|Canada.Ontario|BOLD:AAA2482
Mythimna unipuncta|[11749]|LPSO935-08|PPBP-0935|658[0n]bp|Canada.Ontario|BOLD:AAA2482
Mythimna unipuncta|[11750]|LPSOB291-08|PPBP-1290|658[0n]bp|Canada.Ontario|BOLD:AAA2482
Mythimna unipuncta|[11751]|LPSOD733-09|08BBLEP-00515|658[0n]bp|Canada.Ontario|BOLD:AAA2482
Mythimna unipuncta|[11752]|LPSOD338-09|08BBLEP-00116|658[0n]bp|Canada.Ontario|BOLD:AAA2482
Mythimna unipuncta|[11753]|XAH651-05|2005-ONT-2234|658[0n]bp|Canada.Ontario|BOLD:AAA2482
Mythimna unipuncta|[11754]|LPSOD571-09|08BBLEP-00352|658[0n]bp|Canada.Ontario|BOLD:AAA2482
Mythimna unipuncta|[11755]|KPOEC153-08|08OEC-194|658[0n]bp|Canada.Ontario|BOLD:AAA2482
Mythimna unipuncta|[11756]|LPSOD315-09|08BBLEP-00093|658[0n]bp|Canada.Ontario|BOLD:AAA2482
Mythimna unipuncta|[11757]|LPSOD806-09|08BBLEP-00588|658[0n]bp|Canada.Ontario|BOLD:AAA2482
Mythimna unipuncta|[11758]|LPSOB697-08|PPBP-1696|655[0n]bp|Canada.Ontario|BOLD:AAA2482
Mythimna unipuncta|[11759]|LPSOD567-09|08BBLEP-00348|658[0n]bp|Canada.Ontario|BOLD:AAA2482
Mythimna unipuncta|[11760]|LPSOD569-09|08BBLEP-00350|658[0n]bp|Canada.Ontario|BOLD:AAA2482
Mythimna unipuncta|[11761]|BBLPA826-10|10BBCLP-0826|658[0n]bp|Canada.Ontario|BOLD:AAA2482

Mythimna unipuncta|[11759]|LPSOD567-09|08BBLEP-00348|658[0n]bp|Canada.Ontario|BOLD:AAA2482
Mythimna unipuncta|[11760]|LPSOD569-09|08BBLEP-00350|658[0n]bp|Canada.Ontario|BOLD:AAA2482
Mythimna unipuncta|[11761]|BBLPA826-10|10BBCLP-0826|658[0n]bp|Canada.Ontario|BOLD:AAA2482
Mythimna unipuncta|[11762]|LPSOD566-09|08BBLEP-00347|658[0n]bp|Canada.Ontario|BOLD:AAA2482
Mythimna unipuncta|[11763]|BBLPA827-10|10BBCLP-0827|658[0n]bp|Canada.Ontario|BOLD:AAA2482
Mythimna unipuncta|[11764]|BLTIB081-08|BL0123|658[0n]bp|Canada.Ontario|BOLD:AAA2482
Mythimna unipuncta|[11765]|LPSOB153-08|PPBP-1152|658[0n]bp|Canada.Ontario|BOLD:AAA2482
Mythimna unipuncta|[11766]|LPSOC320-08|PPBP-2319|658[0n]bp|Canada.Ontario|BOLD:AAA2482
Mythimna unipuncta|[11767]|LPSOD568-09|08BBLEP-00349|658[0n]bp|Canada.Ontario|BOLD:AAA2482
Mythimna unipuncta|[11768]|KPOEC139-08|08OEC-143|658[0n]bp|Canada.Ontario|BOLD:AAA2482
Mythimna unipuncta|[11769]|BBLPC065-09|09BBELE-1065|658[0n]bp|Canada.New Brunswick|BOLD:AAA2482
Mythimna unipuncta|[11770]|LPSO028-08|PPBP-0028|658[0n]bp|Canada.Ontario|BOLD:AAA2482
Mythimna unipuncta|[11771]|BLTIB505-08|BL762|658[0n]bp|Canada.Ontario|BOLD:AAA2482
Mythimna unipuncta|[11772]|BLTIB510-08|BL768|658[0n]bp|Canada.Ontario|BOLD:AAA2482
Mythimna unipuncta|[11773]|LPSOB074-08|PPBP-1073|658[0n]bp|Canada.Ontario|BOLD:AAA2482
Mythimna unipuncta|[11774]|BLTIB214-08|BL317|658[0n]bp|Canada.Ontario|BOLD:AAA2482
Mythimna unipuncta|[11775]|LPSOD425-09|08BBLEP-00204|658[0n]bp|Canada.Ontario|BOLD:AAA2482
Mythimna unipuncta|[11776]|LPSO886-08|PPBP-0886|658[0n]bp|Canada.Ontario|BOLD:AAA2482
Mythimna unipuncta|[11777]|LPSOB703-08|PPBP-1702|657[0n]bp|Canada.Ontario|BOLD:AAA2482
Mythimna unipuncta|[11778]|JSJUL2390-11|BIOUG01497-F01|658[0n]bp|Canada.Ontario|BOLD:AAA2482
Mythimna unipuncta|[11779]|BBLPC056-09|09BBELE-1056|658[0n]bp|Canada.New Brunswick|BOLD:AAA2482
Mythimna unipuncta|[11780]|BLTIB734-08|BL1024|658[0n]bp|Canada.Ontario|BOLD:AAA2482
Mythimna unipuncta|[11781]|BBLPE191-09|09BBELE-2191|614[0n]bp|Canada.Newfoundland and Labrador|BOLD:AA...
Mythimna unipuncta|[11782]|XAH176-05|2005-ONT-1759|658[1n]bp|Canada.Ontario|BOLD:AAA2482
Mythimna unipuncta|[11783]|BLTIB689-08|BL972|658[1n]bp|Canada.Ontario|BOLD:AAA2482
Mythimna unipuncta|[11784]|BLTIB875-08|BL1294|658[0n]bp|Canada.Ontario|BOLD:AAA2482
Mythimna unipuncta|[11785]|LPSOB698-08|PPBP-1697|653[0n]bp|Canada.Ontario|BOLD:AAA2482
Mythimna unipuncta|[11786]|LPSOB711-08|PPBP-1710|653[0n]bp|Canada.Ontario|BOLD:AAA2482
Mythimna unipuncta|[11787]|BLTIB478-08|BL727|656[0n]bp|Canada.Ontario|BOLD:AAA2482
Mythimna unipuncta|[11788]|XAD347-04|04HBL007347|658[0n]bp|Canada.Ontario|BOLD:AAA2482
Mythimna unipuncta|[11789]|LPSOC093-08|PPBP-2092|658[0n]bp|Canada.Ontario|BOLD:AAA2482
Mythimna unipuncta|[11790]|XAH003-05|2005-ONT-1586|621[0n]bp|Canada.Ontario|BOLD:AAA2482
Mythimna unipuncta|[11791]|BLTIB291-08|BL478|649[0n]bp|Canada.Ontario|BOLD:AAA2482
Mythimna unipuncta|[11792]|LPSOB694-08|PPBP-1693|645[0n]bp|Canada.Ontario|BOLD:AAA2482
Mythimna unipuncta|[11793]|LPSOB619-08|PPBP-1618|650[0n]bp|Canada.Ontario|BOLD:AAA2482
Mythimna unipuncta|[11794]|BLTIB613-08|BL893|643[1n]bp|Canada.Ontario|BOLD:AAA2482
Mythimna unipuncta|[11795]|LPSOB706-08|PPBP-1705|648[0n]bp|Canada.Ontario|BOLD:AAA2482
Mythimna unipuncta|[11796]|LPSOB670-08|PPBP-1669|646[0n]bp|Canada.Ontario|BOLD:AAA2482
Mythimna unipuncta|[11797]|LPSOB663-08|PPBP-1662|646[0n]bp|Canada.Ontario|BOLD:AAA2482
Mythimna unipuncta|[11798]|LPSOD651-09|08BBLEP-00432|582[0n]bp|Canada.Ontario|BOLD:AAA2482
Mythimna unipuncta|[11799]|BLTIB108-08|BL173|519[0n]bp|Canada.Ontario|BOLD:AAA2482
Mythimna unipuncta|[11800]|BLTIB586-08|BL866|638[0n]bp|Canada.Ontario|BOLD:AAA2482
Mythimna unipuncta|[11801]|XAD480-04|04HBL007480|583[0n]bp|Canada.Ontario|BOLD:AAA2482
Mythimna unipuncta|[11802]|TMG132-03|moth174.01|639[0n]bp|Canada.Ontario|BOLD:AAA2482
Mythimna unipuncta|[11803]|PMG154-03|moth155.01|617[0n]bp|Canada.Ontario|BOLD:AAA2482
Mythimna unipuncta|[11804]|XAG895-05|2005-ONT-1479|643[0n]bp|Canada.Ontario|BOLD:AAA2482
Mythimna unipuncta|[11805]|LPSO324-08|PPBP-0324|658[0n]bp|Canada.Ontario|BOLD:AAA2482
Mythimna unipuncta|[11806]|LPSO027-08|PPBP-0027|658[0n]bp|Canada.Ontario|BOLD:AAA2482
Leucania linita|[11807]|LPSO840-08|PPBP-0840|658[1n]bp|Canada.Ontario|BOLD:AAC3156
Leucania linita|[11808]|LPSO572-08|PPBP-0572|658[0n]bp|Canada.Ontario|BOLD:AAC3156
Leucania linita|[11809]|PHMO171-03|moth906.01|639[0n]bp|Canada.Ontario|BOLD:AAC3156
Leucania linita|[11810]|LPMN804-08|08BBLEP-01607|658[0n]bp|Canada.Manitoba|BOLD:AAC3156
Leucania linita|[11811]|LPMN796-08|08BBLEP-01599|658[0n]bp|Canada.Manitoba|BOLD:AAC3156
Leucania linita|[11812]|LPMN797-08|08BBLEP-01600|658[0n]bp|Canada.Manitoba|BOLD:AAC3156
Leucania linita|[11813]|RDLQ647-07|DH004810|646[0n]bp|Canada.Quebec|BOLD:AAC3156
Leucania linita|[11814]|RDLQ646-07|DH003859|621[0n]bp|Canada.Quebec|BOLD:AAC3156
Leucania adjuta|[11815]|LGSM529-04|DNA-ATBI-0529|657[2n]bp|United States.Tennessee|BOLD:AAC0179
Leucania adjuta|[11816]|LGSMC682-05|DNA-ATBI-2682|658[0n]bp|United States.Tennessee|BOLD:AAC0179
Leucania adjuta|[11817]|LNC430-05|05-NCCC-430|616[1n]bp|United States.North Carolina|BOLD:AAC0179
Leucania adjuta|[11818]|LGSMD680-05|DNA-ATBI-4137|658[1n]bp|United States.North Carolina|BOLD:AAC0179
Leucania adjuta|[11819]|JRLAA011-09|JRLAA-011|658[0n]bp|United States.Alabama|BOLD:AAC0179
Leucania adjuta|[11820]|LOFLA274-06|06-FLOR-0274|657[0n]bp|United States.Florida|BOLD:AAC0179
Leucania adjuta|[11821]|LNC640-06|06-NCCC-640|657[0n]bp|United States.North Carolina|BOLD:AAC0179
Leucania adjuta|[11822]|LPOKA284-08|MDOK-0284|658[0n]bp|United States.Oklahoma|BOLD:AAC0179
Leucania adjuta|[11823]|LGSMC380-05|DNA-ATBI-2380|658[0n]bp|United States.Tennessee|BOLD:AAC0179
Leucania adjuta|[11824]|LGSMG862-10|BGS04131|658[0n]bp|United States.North Carolina|BOLD:AAC0179
Leucania adjuta|[11825]|LOFLA170-06|06-FLOR-0170|657[0n]bp|United States.Florida|BOLD:AAC0179
Leucania adjuta|[11826]|USLEP1193-10|10BBLEP-01193|658[0n]bp|United States.Florida|BOLD:AAC0179
Leucania adjuta|[11827]|LPOKD610-09|MDOK-3689|658[0n]bp|United States.Oklahoma|BOLD:AAC0179
Leucania adjuta|[11828]|LILLA948-11|SNS10IL-01169|658[0n]bp|United States.Illinois|BOLD:AAC0179
Leucania adjuta|[11829]|BBLSW298-09|09BBLEP-01226|658[0n]bp|United States.Oklahoma|BOLD:AAC0179
Leucania adjuta|[11830]|RDNMD571-06|CNCNoctuoidea12903|657[0n]bp|United States.Florida|BOLD:AAC0179
Leucania adjuta|[11831]|LPOKC782-09|MDOK-2859|658[0n]bp|United States.Oklahoma|BOLD:AAC0179
Leucania adjuta|[11832]|RDNMD565-06|CNCNoctuoidea12897|657[0n]bp|United States.Florida|BOLD:AAC0179
Leucania adjuta|[11833]|LGSM530-04|DNA-ATBI-0530|658[0n]bp|United States.Tennessee|BOLD:AAC0179
Leucania adjuta|[11834]|BBLSW454-09|09BBLEP-01382|658[0n]bp|United States.Oklahoma|BOLD:AAC0179
Leucania adjuta|[11835]|BBLSX285-09|09BBLEP-02213|657[0n]bp|United States.Oklahoma|BOLD:AAC0179
Leucania adjuta|[11836]|BBLSU088-09|09BBLEP-04457|658[0n]bp|United States.Mississippi|BOLD:AAC0179
Leucania adjuta|[11837]|RDNMD572-06|CNCNoctuoidea12904|657[0n]bp|United States.Florida|BOLD:AAC0179
Leucania adjuta|[11838]|LGSMC685-05|DNA-ATBI-2685|658[1n]bp|United States.Tennessee|BOLD:AAC0179
Leucania oregona|[11839]|RDNMF576-08|NOC14662|609[0n]bp|Canada.British Columbia|BOLD:AAC7175
Leucania oregona|[11840]|RDNMF577-08|NOC14663|609[0n]bp|Canada.British Columbia|BOLD:AAC7175
Leucania commoides|[11841]|MNBB444-05|05-NBSTA-360|658[0n]bp|Canada.New Brunswick|BOLD:AAA8386
Leucania commoides|[11842]|PHMNB748-05|Moth 441.03SA|658[0n]bp|Canada.New Brunswick|BOLD:AAA8386
Leucania commoides|[11843]|XAB175-04|04HBL005175|658[0n]bp|Canada.Ontario|BOLD:AAA8386
Leucania commoides|[11844]|MNBB113-05|05-NBSTA-029|658[0n]bp|Canada.New Brunswick|BOLD:AAA8386
Leucania commoides|[11845]|XAC714-04|04HBL006714|658[0n]bp|Canada.Ontario|BOLD:AAA8386
Leucania commoides|[11846]|MNBB442-05|05-NBSTA-358|658[0n]bp|Canada.New Brunswick|BOLD:AAA8386
Leucania commoides|[11847]|BBLEC696-09|09BBELE-0696|658[0n]bp|Canada.Nova Scotia|BOLD:AAA8386
Leucania commoides|[11848]|PHMNB096-04|04HBL007561|658[0n]bp|Canada.New Brunswick|BOLD:AAA8386
Leucania commoides|[11849]|MNBB162-05|05-NBSTA-078|658[0n]bp|Canada.New Brunswick|BOLD:AAA8386
Leucania commoides|[11850]|MNBB650-05|05-NBSTA-566|658[0n]bp|Canada.New Brunswick|BOLD:AAA8386
Leucania commoides|[11851]|MNBB109-05|05-NBSTA-025|658[0n]bp|Canada.New Brunswick|BOLD:AAA8386
Leucania commoides|[11852]|MNBB609-05|05-NBSTA-525|658[0n]bp|Canada.New Brunswick|BOLD:AAA8386
Leucania commoides|[11853]|XAC461-04|04HBL006461|613[0n]bp|Canada.Ontario|BOLD:AAA8386
Leucania commoides|[11854]|MNBB610-05|05-NBSTA-526|658[0n]bp|Canada.New Brunswick|BOLD:AAA8386
Leucania commoides|[11855]|MNBB164-05|05-NBSTA-080|534[2n]bp|Canada.New Brunswick|BOLD:AAA8386
Leucania commoides|[11856]|XAC073-04|04HBL006073|582[0n]bp|Canada.Ontario|BOLD:AAA8386
Leucania commoides|[11857]|MNBB107-05|05-NBSTA-023|548[0n]bp|Canada.New Brunswick|BOLD:AAA8386
Leucania commoides|[11858]|MNBB163-05|05-NBSTA-079|547[1n]bp|Canada.New Brunswick|BOLD:AAA8386
Leucania commoides|[11859]|RDLQG128-06|DH012295|603[0n]bp|Canada.Quebec|BOLD:AAA8386
Leucania commoides|[11860]|MNBB106-05|05-NBSTA-022|658[0n]bp|Canada.New Brunswick|BOLD:AAA8386
Leucania commoides|[11861]|MNBB239-05|05-NBSTA-155|538[0n]bp|Canada.New Brunswick|BOLD:AAA8386

Leucania commoides|[11859]|RDLQG128-06|DH012295|603[0n]bp|Canada.Quebec|BOLD:AAA8386
Leucania commoides|[11860]|MNBB106-05|05-NBSTA-022|658[0n]bp|Canada.New Brunswick|BOLD:AAA8386
Leucania commoides|[11861]|MNBB239-05|05-NBSTA-155|538[0n]bp|Canada.New Brunswick|BOLD:AAA8386
Leucania commoides|[11862]|XAC715-04|04HBL006715|658[0n]bp|Canada.Ontario|BOLD:AAA8386
Leucania commoides|[11863]|BBLEC708-09|09BBELE-0708|656[0n]bp|Canada.Nova Scotia|BOLD:AAA8386
Leucania commoides|[11864]|TTMNB413-06|MNBTT-413|658[0n]bp|Canada.New Brunswick|BOLD:AAA8386
Leucania commoides|[11865]|MNBB112-05|05-NBSTA-028|658[0n]bp|Canada.New Brunswick|BOLD:AAA8386
Leucania commoides|[11866]|MNBB111-05|05-NBSTA-027|658[0n]bp|Canada.New Brunswick|BOLD:AAA8386
Leucania commoides|[11867]|LPSK007-08|08BBLEP-00710|658[0n]bp|Canada.Saskatchewan|BOLD:AAA8386
Leucania commoides|[11868]|MNBB110-05|05-NBSTA-026|658[0n]bp|Canada.New Brunswick|BOLD:AAA8386
Leucania commoides|[11869]|LPMNB418-09|08BBLEP-05418|658[0n]bp|Canada.Manitoba|BOLD:AAA8386
Leucania commoides|[11870]|MNBB327-05|05-NBSTA-243|658[0n]bp|Canada.New Brunswick|BOLD:AAA8386
Leucania commoides|[11871]|MNBB108-05|05-NBSTA-024|658[0n]bp|Canada.New Brunswick|BOLD:AAA8386
Leucania commoides|[11872]|XAC613-04|04HBL006613|658[0n]bp|Canada.Ontario|BOLD:AAA8386
Leucania commoides|[11873]|XAJ841-06|2006-ONT-0841|658[0n]bp|Canada.Ontario|BOLD:AAA8386
Leucania commoides|[11874]|XAJ917-06|2006-ONT-0917|658[0n]bp|Canada.Ontario|BOLD:AAA8386
Leucania commoides|[11875]|LPMN085-08|08BBLEP-00883|658[0n]bp|Canada.Manitoba|BOLD:AAA8386
Leucania commoides|[11876]|XAB496-04|04HBL005496|658[0n]bp|Canada.Ontario|BOLD:AAA8386
Leucania commoides|[11877]|XAJ844-06|2006-ONT-0844|658[0n]bp|Canada.Ontario|BOLD:AAA8386
Leucania commoides|[11878]|BLTIB786-08|BL1203|658[0n]bp|Canada.Ontario|BOLD:AAA8386
Leucania commoides|[11879]|RDLQB913-05|DH002849|549[0n]bp|Canada.Quebec|BOLD:AAA8386
Leucania commoides|[11880]|RDLQG129-06|DH012296|607[2n]bp|Canada.Quebec|BOLD:AAA8386
Leucania commoides|[11881]|XAJ826-06|2006-ONT-0826|596[0n]bp|Canada.Ontario|BOLD:AAA8386
Leucania linda|[11882]|BBLPA582-10|10BBCLP-0582|658[0n]bp|Canada.Ontario|BOLD:ABY6338
Leucania linda|[11883]|LPSO992-08|PPBP-0992|658[0n]bp|Canada.Ontario|BOLD:ABY6338
Leucania linda|[11884]|XAH504-05|2005-ONT-2087|658[0n]bp|Canada.Ontario|BOLD:ABY6338
Leucania linda|[11885]|LPSOB970-08|PPBP-1969|658[0n]bp|Canada.Ontario|BOLD:ABY6338
Leucania phragmitidicola|[11886]|LPSOB227-08|PPBP-1226|658[0n]bp|Canada.Ontario|BOLD:ABX6101
Leucania phragmitidicola|[11887]|LPSOB212-08|PPBP-1211|658[0n]bp|Canada.Ontario|BOLD:ABX6101
Leucania phragmitidicola|[11888]|XAB257-04|04HBL005257|658[4n]bp|Canada.Ontario|BOLD:ABX6101
Leucania phragmitidicola|[11889]|XAB595-04|04HBL005595|590[0n]bp|Canada.Ontario|BOLD:ABX6101
Leucania phragmitidicola|[11890]|LPSOB385-08|PPBP-1384|658[0n]bp|Canada.Ontario|BOLD:ABX6101
Leucania phragmitidicola|[11891]|XAD578-04|04HBL006993|588[3n]bp|Canada.Ontario|BOLD:ABX6101
Leucania phragmitidicola|[11892]|XAD720-05|2005-ONT-519|658[0n]bp|Canada.Ontario|BOLD:ABX6101
Leucania phragmitidicola|[11893]|XAD288-04|04HBL007288|591[0n]bp|Canada.Ontario|BOLD:ABX6101
Leucania phragmitidicola|[11894]|XAH134-05|2005-ONT-1717|658[1n]bp|Canada.Ontario|BOLD:ABX6101
Leucania lapidaria|[11895]|XAG976-05|2005-ONT-1560|585[0n]bp|Canada.Ontario|BOLD:AAB8995
Leucania lapidaria|[11896]|RDLQ655-07|DH004821|623[0n]bp|Canada.Quebec|BOLD:AAB8995
Leucania lapidaria|[11897]|RDLQ653-07|DH004837|658[0n]bp|Canada.Quebec|BOLD:AAB8995
Leucania lapidaria|[11898]|XAJ689-06|2006-ONT-0689|656[0n]bp|Canada.Ontario|BOLD:AAB8995
Leucania lapidaria|[11899]|XAJ690-06|2006-ONT-0690|656[0n]bp|Canada.Ontario|BOLD:AAB8995
Leucania lapidaria|[11900]|RDLQ656-07|DH005175|625[0n]bp|Canada.Quebec|BOLD:AAB8995
Leucania lapidaria|[11901]|XAD302-04|04HBL007302|600[1n]bp|Canada.Ontario|BOLD:AAB8995
Leucania lapidaria|[11902]|XAB260-04|04HBL005260|658[0n]bp|Canada.Ontario|BOLD:AAB8995
Leucania lapidaria|[11903]|XAK164-06|2006-ONT-1159|657[0n]bp|Canada.Ontario|BOLD:AAB8995
Leucania lapidaria|[11904]|BLGSM066-09|BL388|658[0n]bp|Canada.Ontario|BOLD:AAB8995
Leucania lapidaria|[11905]|XAJ449-06|2006-ONT-0449|657[1n]bp|Canada.Ontario|BOLD:AAB8995
Leucania lapidaria|[11906]|XAJ451-06|2006-ONT-0451|657[1n]bp|Canada.Ontario|BOLD:AAB8995
Leucania anteroclara|[11907]|LOWC048-05|CGWC-0048|658[1n]bp|Canada.British Columbia|BOLD:ACE5750
Leucania anteroclara|[11908]|LOWC061-05|CGWC-0061|658[2n]bp|Canada.British Columbia|BOLD:ACE5750
Leucania anteroclara|[11909]|LOWC054-05|CGWC-0054|658[0n]bp|Canada.British Columbia|BOLD:ACE5750
Leucania anteroclara|[11910]|LOWCD229-06|CGWC-3049|605[0n]bp|Canada.British Columbia|BOLD:ACE5750
Leucania anteroclara|[11911]|LOWC047-05|CGWC-0047|658[0n]bp|Canada.British Columbia|BOLD:ACE5750
Leucania anteroclara|[11912]|LPABB393-08|08BBLEP-03658|658[0n]bp|Canada.Alberta|BOLD:ACE5750
Leucania anteroclara|[11913]|LPABB304-08|08BBLEP-03569|658[0n]bp|Canada.Alberta|BOLD:ACE5750
Leucania anteroclara|[11914]|LPABC004-09|08BBLEP-04223|658[1n]bp|Canada.Alberta|BOLD:ACE5750
Leucania anteroclara|[11915]|LPABC431-09|08BBLEP-04650|658[0n]bp|Canada.Alberta|BOLD:ACE5750
Leucania anteroclara|[11916]|LPABB118-08|08BBLEP-03383|647[0n]bp|Canada.Alberta|BOLD:ACE5750
Leucania anteroclara|[11917]|LPABC005-09|08BBLEP-04224|658[2n]bp|Canada.Alberta|BOLD:ACE5750
Leucania anteroclara|[11918]|LOWC026-05|CGWC-0026|658[0n]bp|Canada.British Columbia|BOLD:ACE5750
Leucania anteroclara|[11919]|LOWCD230-06|CGWC-3050|583[0n]bp|Canada.British Columbia|BOLD:ACE5750
Leucania anteroclara|[11920]|LOWC051-05|CGWC-0051|658[0n]bp|Canada.British Columbia|BOLD:ACE5750
Leucania anteroclara|[11921]|RDMAB128-05|UASM41273|658[0n]bp|Canada.Alberta|BOLD:ACE5750
Leucania anteroclara|[11922]|LPABB402-08|08BBLEP-03667|658[0n]bp|Canada.Alberta|BOLD:ACE5750
Leucania anteroclara|[11923]|LOWC056-05|CGWC-0056|658[0n]bp|Canada.British Columbia|BOLD:ACE5750
Leucania anteroclara|[11924]|LPABB450-08|08BBLEP-03715|658[0n]bp|Canada.Alberta|BOLD:ACE5750
Leucania anteroclara|[11925]|LPABB843-09|08BBLEP-04163|658[0n]bp|Canada.Alberta|BOLD:ACE5750
Leucania anteroclara|[11926]|LPSK040-08|08BBLEP-00743|658[0n]bp|Canada.Saskatchewan|BOLD:ACE5750
Leucania anteroclara|[11927]|LPABC321-09|08BBLEP-04540|658[0n]bp|Canada.Alberta|BOLD:ACE5750
Leucania anteroclara|[11928]|LOWCD773-06|CGWC-3593|657[0n]bp|Canada.British Columbia|BOLD:ACE5750
Leucania anteroclara|[11929]|LPABC853-09|08BBLEP-05264|658[0n]bp|Canada.Alberta|BOLD:ACE5750
Leucania anteroclara|[11930]|LPABC824-09|08BBLEP-05043|658[0n]bp|Canada.Alberta|BOLD:ACE5750
Leucania anteroclara|[11931]|BBLPB453-10|10BBCLP-1452|658[0n]bp|Canada.Alberta|BOLD:ACE5750
Leucania anteroclara|[11932]|LOWC062-05|CGWC-0062|623[0n]bp|Canada.British Columbia|BOLD:ACE5750
Leucania anteroclara|[11933]|LPABB573-08|08BBLEP-03838|658[0n]bp|Canada.Alberta|BOLD:ACE5750
Leucania anteroclara|[11934]|LBCH6523-10|10-JDWBC-6523|658[0n]bp|Canada.British Columbia|BOLD:ACE5750
Leucania anteroclara|[11935]|LPABB193-08|08BBLEP-03458|658[0n]bp|Canada.Alberta|BOLD:ACE5750
Leucania anteroclara|[11936]|LBCH6094-10|10-JDWBC-6094|658[0n]bp|Canada.British Columbia|BOLD:ACE5750
Leucania anteroclara|[11937]|LOWCD771-06|CGWC-3591|657[0n]bp|Canada.British Columbia|BOLD:ACE5750
Leucania anteroclara|[11938]|LPABB145-08|08BBLEP-03410|658[0n]bp|Canada.Alberta|BOLD:ACE5750
Leucania anteroclara|[11939]|LPABB723-08|08BBLEP-03988|658[0n]bp|Canada.Alberta|BOLD:ACE5750
Leucania anteroclara|[11940]|LPABC411-09|08BBLEP-04630|658[0n]bp|Canada.Alberta|BOLD:ACE5750
Leucania anteroclara|[11941]|LPMN898-08|08BBLEP-02256|658[0n]bp|Canada.Alberta|BOLD:ACE5750
Leucania anteroclara|[11942]|LPABB194-08|08BBLEP-03459|658[0n]bp|Canada.Alberta|BOLD:ACE5750
Leucania anteroclara|[11943]|LPAB239-08|08BBLEP-02561|658[0n]bp|Canada.Alberta|BOLD:ACE5750
Leucania anteroclara|[11944]|LPSK452-08|08BBLEP-02020|658[0n]bp|Canada.Saskatchewan|BOLD:ACE5750
Leucania anteroclara|[11945]|LOWCD777-06|CGWC-3597|657[0n]bp|Canada.British Columbia|BOLD:ACE5750
Leucania anteroclara|[11946]|LPABC466-09|08BBLEP-04685|658[0n]bp|Canada.Alberta|BOLD:ACE5750
Leucania anteroclara|[11947]|LBCH6627-10|10-JDWBC-6627|658[0n]bp|Canada.British Columbia|BOLD:ACE5750
Leucania anteroclara|[11948]|LBCH6044-10|10-JDWBC-6044|658[0n]bp|Canada.British Columbia|BOLD:ACE5750
Leucania anteroclara|[11949]|LPSK585-08|08BBLEP-02153|658[0n]bp|Canada.Saskatchewan|BOLD:ACE5750
Leucania anteroclara|[11950]|LPABB848-09|08BBLEP-04168|658[0n]bp|Canada.Alberta|BOLD:ACE5750
Leucania anteroclara|[11951]|LPABB630-08|08BBLEP-03895|658[0n]bp|Canada.Alberta|BOLD:ACE5750
Leucania anteroclara|[11952]|LBCH6441-10|10-JDWBC-6441|658[0n]bp|Canada.British Columbia|BOLD:ACE5750
Leucania anteroclara|[11953]|LPABB868-09|08BBLEP-04188|658[0n]bp|Canada.Alberta|BOLD:ACE5750
Leucania anteroclara|[11954]|LPABC065-09|08BBLEP-04284|658[0n]bp|Canada.Alberta|BOLD:ACE5750
Leucania anteroclara|[11955]|LPABB579-08|08BBLEP-03844|658[0n]bp|Canada.Alberta|BOLD:ACE5750
Leucania anteroclara|[11956]|LOWCD775-06|CGWC-3595|657[0n]bp|Canada.British Columbia|BOLD:ACE5750
Leucania anteroclara|[11957]|LPABC946-09|08BBLEP-05357|658[0n]bp|Canada.Alberta|BOLD:ACE5750
Leucania anteroclara|[11958]|LBCH5559-10|10-JDWBC-5559|658[0n]bp|Canada.British Columbia|BOLD:ACE5750
Leucania anteroclara|[11959]|LOWCD228-06|CGWC-3048|658[0n]bp|Canada.British Columbia|BOLD:ACE5750
Leucania anteroclara|[11960]|LOWC049-05|CGWC-0049|658[0n]bp|Canada.British Columbia|BOLD:ACE5750

Leucania anteroclara|[11958]|LBCH5559-10|10-JDWBC-5559|658[0n]bp|Canada.British Columbia|BOLD:ACE5750
Leucania anteroclara|[11959]|LOWCD228-06|CGWC-3048|658[0n]bp|Canada.British Columbia|BOLD:ACE5750
Leucania anteroclara|[11960]|LOWC049-05|CGWC-0049|658[0n]bp|Canada.British Columbia|BOLD:ACE5750
Leucania anteroclara|[11961]|LPABB849-09|08BBLEP-04169|658[0n]bp|Canada.Alberta|BOLD:ACE5750
Leucania anteroclara|[11962]|LOWCD772-06|CGWC-3592|656[0n]bp|Canada.British Columbia|BOLD:ACE5750
Leucania anteroclara|[11963]|LPABB359-08|08BBLEP-03624|639[0n]bp|Canada.Alberta|BOLD:ACE5750
Leucania anteroclara|[11964]|LPABB365-08|08BBLEP-03630|638[0n]bp|Canada.Alberta|BOLD:ACE5750
Leucania anteroclara|[11965]|LBCH5953-10|10-JDWBC-5953|641[0n]bp|Canada.British Columbia|BOLD:ACE5750
Leucania anteroclara|[11966]|LOWCD227-06|CGWC-3047|610[0n]bp|Canada.British Columbia|BOLD:ACE5750
Leucania anteroclara|[11967]|LOWCD226-06|CGWC-3046|607[0n]bp|Canada.British Columbia|BOLD:ACE5750
Leucania anteroclara|[11968]|LOWCD769-06|CGWC-3589|609[0n]bp|Canada.British Columbia|BOLD:ACE5750
Leucania anteroclara|[11969]|LOWCD774-06|CGWC-3594|604[1n]bp|Canada.British Columbia|BOLD:ACE5750
Leucania anteroclara|[11970]|LOWC050-05|CGWC-0050|583[0n]bp|Canada.British Columbia|BOLD:ACE5750
Leucania farcta|[11971]|LPVIC031-08|PFC-2006-2586|658[1n]bp|Canada.British Columbia|BOLD:AAA2483
Leucania farcta roseola|[11972]|LBCA208-05|HLC-20208|658[0n]bp|Canada.British Columbia|BOLD:AAA2483
Leucania farcta|[11973]|LALPA888-11|AVBC 1061-11|658[0n]bp|Canada.British Columbia|BOLD:AAA2483
Leucania farcta roseola|[11974]|LOWCE813-06|CGWC-4573|657[0n]bp|Canada.British Columbia|BOLD:AAA2483
Leucania farcta roseola|[11975]|LOWCE832-06|CGWC-4592|657[0n]bp|Canada.British Columbia|BOLD:AAA2483
Leucania farcta roseola|[11976]|LHLEP104-06|UBC-2006-1531|658[2n]bp|Canada.British Columbia|BOLD:AAA2483
Leucania farcta|[11977]|LPVIA839-08|PFC-2006-1139|658[1n]bp|Canada.British Columbia|BOLD:AAA2483
Leucania farcta|[11978]|LALPA895-11|AVBC 1068-11|658[0n]bp|Canada.British Columbia|BOLD:AAA2483
Leucania farcta|[11979]|BBLPA819-10|10BBCLP-0819|658[0n]bp|Canada.British Columbia|BOLD:AAA2483
Leucania farcta|[11980]|LALPA1172-11|AVBC 982-11|658[0n]bp|Canada.British Columbia|BOLD:AAA2483
Leucania farcta roseola|[11981]|BBLPA822-10|10BBCLP-0822|658[0n]bp|Canada.British Columbia|BOLD:AAA2483
Leucania farcta roseola|[11982]|LHLEP108-06|UBC-2006-1528|658[0n]bp|Canada.British Columbia|BOLD:AAA2483
Leucania farcta|[11983]|LALPA368-10|AVBC 370-10|658[0n]bp|Canada.British Columbia|BOLD:AAA2483
Leucania farcta|[11984]|LALPA474-10|AVBC 476-10|658[0n]bp|Canada.British Columbia|BOLD:AAA2483
Leucania farcta roseola|[11985]|LHLEP111-06|UBC-2006-1062|658[0n]bp|Canada.British Columbia|BOLD:AAA2483
Leucania farcta roseola|[11986]|LMH040-06|PFC-2006-0460|658[0n]bp|Canada.British Columbia|BOLD:AAA2483
Leucania farcta|[11987]|LPVIA627-08|PFC-2006-0857|623[0n]bp|Canada.British Columbia|BOLD:AAA2483
Leucania farcta roseola|[11988]|LOWCE825-06|CGWC-4585|625[0n]bp|Canada.British Columbia|BOLD:AAA2483
Leucania farcta roseola|[11989]|RDNMB439-05|CNCNoctuoidea10205|608[1n]bp|Canada.British Columbia|BOLD:...
Leucania farcta roseola|[11990]|LPVIA410-08|PFC-2006-0596|658[0n]bp|Canada.British Columbia|BOLD:AAA2483
Leucania farcta roseola|[11991]|LPGVA713-08|UBC-2006-1982|658[0n]bp|Canada.British Columbia|BOLD:AAA2483
Leucania farcta roseola|[11992]|LHLEP109-06|UBC-2006-1653|658[0n]bp|Canada.British Columbia|BOLD:AAA2483
Leucania farcta roseola|[11993]|LHLEP112-06|UBC-2006-1061|658[0n]bp|Canada.British Columbia|BOLD:AAA2483
Leucania farcta roseola|[11994]|LHLEP101-06|UBC-2006-0480|658[0n]bp|Canada.British Columbia|BOLD:AAA2483
Leucania farcta roseola|[11995]|LHLEP106-06|UBC-2006-1530|658[0n]bp|Canada.British Columbia|BOLD:AAA2483
Leucania farcta|[11996]|LALPA339-10|AVBC 341-10|658[0n]bp|Canada.British Columbia|BOLD:AAA2483
Leucania farcta|[11997]|LPVIB980-08|PFC-2006-2530|658[0n]bp|Canada.British Columbia|BOLD:AAA2483
Leucania farcta|[11998]|LALPA496-10|AVBC 498-10|658[0n]bp|Canada.British Columbia|BOLD:AAA2483
Leucania farcta|[11999]|LPVIA303-08|PFC-2006-0462|658[0n]bp|Canada.British Columbia|BOLD:AAA2483
Leucania farcta|[12000]|LPVIB061-08|PFC-2006-1398|658[0n]bp|Canada.British Columbia|BOLD:AAA2483
Leucania farcta|[12001]|LALPA552-10|AVBC 554-10|658[0n]bp|Canada.British Columbia|BOLD:AAA2483
Leucania farcta roseola|[12002]|LPGVA714-08|UBC-2006-1983|638[0n]bp|Canada.British Columbia|BOLD:AAA2483
Leucania farcta roseola|[12003]|LHLEP103-06|UBC-2006-0767|658[0n]bp|Canada.British Columbia|BOLD:AAA2483
Leucania farcta roseola|[12004]|LHLEP113-06|UBC-2006-1060|658[0n]bp|Canada.British Columbia|BOLD:AAA2483
Leucania farcta roseola|[12005]|LHLEP102-06|UBC-2006-0766|658[0n]bp|Canada.British Columbia|BOLD:AAA2483
Leucania farcta roseola|[12006]|LHLEP105-06|UBC-2006-1529|658[2n]bp|Canada.British Columbia|BOLD:AAA2483
Leucania farcta roseola|[12007]|LBCD474-05|HLC-23294|646[0n]bp|Canada.British Columbia|BOLD:AAA2483
Leucania farcta roseola|[12008]|LBCD320-05|HLC-23140|658[0n]bp|Canada.British Columbia|BOLD:AAA2483
Leucania farcta|[12009]|LALPA494-10|AVBC 496-10|658[0n]bp|Canada.British Columbia|BOLD:AAA2483
Leucania farcta|[12010]|BBLPA823-10|10BBCLP-0823|658[0n]bp|Canada.British Columbia|BOLD:AAA2483
Leucania farcta|[12011]|LALPA397-10|AVBC 399-10|658[0n]bp|Canada.British Columbia|BOLD:AAA2483
Leucania farcta|[12012]|BBLPA821-10|10BBCLP-0821|658[0n]bp|Canada.British Columbia|BOLD:AAA2483
Leucania farcta|[12013]|LBCH5605-10|10-JDWBC-5605|658[0n]bp|Canada.British Columbia|BOLD:AAA2483
Leucania farcta|[12014]|BBLPA816-10|10BBCLP-0816|658[0n]bp|Canada.British Columbia|BOLD:AAA2483
Leucania farcta|[12015]|BBLPA817-10|10BBCLP-0817|658[0n]bp|Canada.British Columbia|BOLD:AAA2483
Leucania farcta|[12016]|BBLPA818-10|10BBCLP-0818|658[0n]bp|Canada.British Columbia|BOLD:AAA2483
Leucania farcta|[12017]|LPVIB520-08|PFC-2006-1935|658[0n]bp|Canada.British Columbia|BOLD:AAA2483
Leucania farcta|[12018]|BBLPA820-10|10BBCLP-0820|658[0n]bp|Canada.British Columbia|BOLD:AAA2483
Leucania farcta|[12019]|LALPA498-10|AVBC 500-10|658[0n]bp|Canada.British Columbia|BOLD:AAA2483
Leucania farcta|[12020]|LBCH7880-10|10-JDWBC-7880|658[0n]bp|Canada.British Columbia|BOLD:AAA2483
Leucania farcta roseola|[12021]|LBCD475-05|HLC-23295|658[0n]bp|Canada.British Columbia|BOLD:AAA2483
Leucania farcta roseola|[12022]|LBCD476-05|HLC-23296|658[0n]bp|Canada.British Columbia|BOLD:AAA2483
Leucania farcta roseola|[12023]|LBCD428-05|HLC-23248|658[0n]bp|Canada.British Columbia|BOLD:AAA2483
Leucania farcta roseola|[12024]|LHLEP110-06|UBC-2006-1127|658[0n]bp|Canada.British Columbia|BOLD:AAA2483
Leucania farcta roseola|[12025]|LBCC301-05|HLC-22181|658[0n]bp|Canada.British Columbia|BOLD:AAA2483
Leucania farcta roseola|[12026]|LHLEP107-06|UBC-2006-1654|658[0n]bp|Canada.British Columbia|BOLD:AAA2483
Leucania farcta roseola|[12027]|LBCD321-05|HLC-23141|658[0n]bp|Canada.British Columbia|BOLD:AAA2483
Leucania multilinea|[12028]|LHLEP114-06|UBC-2006-1065|658[0n]bp|Canada.British Columbia|BOLD:ACF1540
Leucania multilinea|[12029]|LHLEP116-06|UBC-2006-1063|658[0n]bp|Canada.British Columbia|BOLD:ACF1540
Leucania multilinea|[12030]|LHLEP115-06|UBC-2006-1064|658[0n]bp|Canada.British Columbia|BOLD:ACF1540
Leucania multilinea|[12031]|LPSK444-08|08BBLEP-02012|658[0n]bp|Canada.Saskatchewan|BOLD:ACF1540
Leucania multilinea|[12032]|LBCC027-05|HLC-21907|658[0n]bp|Canada.British Columbia|BOLD:ACF1540
Leucania multilinea|[12033]|LPABC157-09|08BBLEP-04376|645[1n]bp|Canada.Alberta|BOLD:ACF1540
Leucania multilinea|[12034]|LPSK443-08|08BBLEP-02011|658[0n]bp|Canada.Saskatchewan|BOLD:ACF1540
Leucania multilinea|[12035]|LPSK010-08|08BBLEP-00713|658[0n]bp|Canada.Saskatchewan|BOLD:ACF1540
Leucania multilinea|[12036]|LPSK253-08|08BBLEP-01821|658[0n]bp|Canada.Saskatchewan|BOLD:ACF1540
Leucania multilinea|[12037]|BBLPA824-10|10BBCLP-0824|658[0n]bp|Canada.British Columbia|BOLD:ACF1540
Leucania multilinea|[12038]|LPMN819-08|08BBLEP-01622|658[0n]bp|Canada.Manitoba|BOLD:ACF1540
Leucania multilinea|[12039]|LPSK508-08|08BBLEP-02076|658[0n]bp|Canada.Saskatchewan|BOLD:ACF1540
Leucania multilinea|[12040]|RDLQ650-07|DH006298|608[1n]bp|Canada.Quebec|BOLD:ACF1540
Leucania multilinea|[12041]|LPABC158-09|08BBLEP-04377|658[0n]bp|Canada.Alberta|BOLD:ACF1540
Leucania multilinea|[12042]|LOWC055-05|CGWC-0055|658[0n]bp|Canada.British Columbia|BOLD:ACF1540
Leucania multilinea|[12043]|LBCH6366-10|10-JDWBC-6366|658[0n]bp|Canada.British Columbia|BOLD:ACF1540
Leucania multilinea|[12044]|LHLEP119-06|UBC-2006-1222|658[0n]bp|Canada.British Columbia|BOLD:ACF1540
Leucania multilinea|[12045]|XAB107-04|04HBL005107|658[0n]bp|Canada.Ontario|BOLD:ACF1540
Leucania multilinea|[12046]|LPSK252-08|08BBLEP-01820|658[0n]bp|Canada.Saskatchewan|BOLD:ACF1540
Leucania multilinea|[12047]|RDLQ654-07|DH003862|623[0n]bp|Canada.Quebec|BOLD:ACF1540
Leucania multilinea|[12048]|BBLPE343-09|09BBELE-2343|658[0n]bp|Canada.Newfoundland and Labrador|BOLD:A...
Leucania multilinea|[12049]|BBLPE141-09|09BBELE-2141|658[0n]bp|Canada.Nova Scotia|BOLD:ACF1540
Leucania multilinea|[12050]|BBLPE122-09|09BBELE-2122|636[0n]bp|Canada.Nova Scotia|BOLD:ACF1540
Leucania multilinea|[12051]|BBLPC601-09|09BBELE-1601|658[0n]bp|Canada.Nova Scotia|BOLD:ACF1540
Leucania multilinea|[12052]|XAB156-04|04HBL005156|658[1n]bp|Canada.Ontario|BOLD:ACF1540
Leucania multilinea|[12053]|XAK051-06|2006-ONT-1046|658[0n]bp|Canada.Ontario|BOLD:ACF1540
Leucania multilinea|[12054]|BBLPE339-09|09BBELE-2339|658[0n]bp|Canada.Newfoundland and Labrador|BOLD:A...
Leucania multilinea|[12055]|BBLPE360-09|09BBELE-2360|658[0n]bp|Canada.Newfoundland and Labrador|BOLD:A...
Leucania multilinea|[12056]|RDLQ652-07|DH003860|658[0n]bp|Canada.Quebec|BOLD:ACF1540
Leucania multilinea|[12057]|BBLEC268-09|09BBELE-0268|658[0n]bp|Canada.Nova Scotia|BOLD:ACF1540
Leucania multilinea|[12058]|MNBB443-05|05-NBSTA-359|658[0n]bp|Canada.New Brunswick|BOLD:ACF1540
Leucania multilinea|[12059]|BBLPE338-09|09BBELE-2338|658[0n]bp|Canada.Newfoundland and Labrador|BOLD:A...
Leucania multilinea|[12060]|BBLPC699-09|09BBELE-1699|658[0n]bp|Canada.Newfoundland and Labrador|BOLD:A...

Leucania multilinea|[12058]|MNBB443-05|05-NBSTA-359|658[0n]bp|Canada.New Brunswick|BOLD:ACF1540
Leucania multilinea|[12059]|BBLPE338-09|09BBELE-2338|658[0n]bp|Canada.Newfoundland and Labrador|BOLD:A...
Leucania multilinea|[12060]|BBLPC699-09|09BBELE-1699|658[0n]bp|Canada.Newfoundland and Labrador|BOLD:A...
Leucania multilinea|[12061]|RDLQ651-07|DH003864|658[0n]bp|Canada.Quebec|BOLD:ACF1540
Leucania multilinea|[12062]|MNBB023-05|HBL008633|658[0n]bp|Canada.New Brunswick|BOLD:ACF1540
Leucania multilinea|[12063]|BBLEC347-09|09BBELE-0347|658[0n]bp|Canada.Newfoundland and Labrador|BOLD:A...
Leucania multilinea|[12064]|BBLPE340-09|09BBELE-2340|658[0n]bp|Canada.Newfoundland and Labrador|BOLD:A...
Leucania multilinea|[12065]|BBLPE320-09|09BBELE-2320|658[0n]bp|Canada.Newfoundland and Labrador|BOLD:A...
Leucania multilinea|[12066]|BBLEC414-09|09BBELE-0414|658[0n]bp|Canada.Newfoundland and Labrador|BOLD:A...
Leucania multilinea|[12067]|BBLPC655-09|09BBELE-1655|658[0n]bp|Canada.Newfoundland and Labrador|BOLD:A...
Leucania multilinea|[12068]|BBLPC943-09|09BBELE-1943|658[0n]bp|Canada.Newfoundland and Labrador|BOLD:A...
Leucania multilinea|[12069]|BLTIB393-08|BL629|658[0n]bp|Canada.Ontario|BOLD:ACF1540
Leucania multilinea|[12070]|RDLQ649-07|DH009713|658[0n]bp|Canada.Ontario|BOLD:ACF1540
Leucania multilinea|[12071]|BBLPE333-09|09BBELE-2333|658[0n]bp|Canada.Newfoundland and Labrador|BOLD:A...
Leucania multilinea|[12072]|LOWCE827-06|CGWC-4587|609[2n]bp|Canada.British Columbia|BOLD:ACF1540
Leucania multilinea|[12073]|RDLQ657-07|DH003861|601[0n]bp|Canada.Quebec|BOLD:ACF1540
Leucania multilinea|[12074]|RDLQ648-07|DH009712|624[0n]bp|Canada.Ontario|BOLD:ACF1540
Leucania multilinea|[12075]|PHMO156-03|moth860.01|639[0n]bp|Canada.Ontario|BOLD:ACF1540
Leucania multilinea|[12076]|LHLEP117-06|UBC-2006-1066|658[0n]bp|Canada.British Columbia|BOLD:ACF1540
Leucania multilinea|[12077]|XAB195-04|04HBL005195|658[0n]bp|Canada.Ontario|BOLD:ACF1540
Leucania multilinea|[12078]|LHLEP118-06|UBC-2006-1067|658[0n]bp|Canada.British Columbia|BOLD:ACF1540
Leucania multilinea|[12079]|LPSK446-08|08BBLEP-02014|658[0n]bp|Canada.Saskatchewan|BOLD:ACF1540
Leucania multilinea|[12080]|LOWCD225-06|CGWC-3045|658[0n]bp|Canada.British Columbia|BOLD:ACF1540
Leucania multilinea|[12081]|BLTIB789-08|BL1206|658[0n]bp|Canada.Ontario|BOLD:ACF1540
Leucania multilinea|[12082]|LPSK445-08|08BBLEP-02013|658[0n]bp|Canada.Saskatchewan|BOLD:ACF1540
Leucania multilinea|[12083]|XAC668-04|04HBL006668|658[0n]bp|Canada.Ontario|BOLD:ACF1540
Leucania multilinea|[12084]|XAC713-04|04HBL006713|658[0n]bp|Canada.Ontario|BOLD:ACF1540
Leucania multilinea|[12085]|PHMNB761-05|Moth 454.03SA|658[0n]bp|Canada.New Brunswick|BOLD:ACF1540
Leucania multilinea|[12086]|LPSK017-08|08BBLEP-00720|658[0n]bp|Canada.Saskatchewan|BOLD:ACF1540
Leucania multilinea|[12087]|LPSK005-08|08BBLEP-00708|658[0n]bp|Canada.Saskatchewan|BOLD:ACF1540
Leucania multilinea|[12088]|LPMNB567-09|08BBLEP-05645|658[0n]bp|Canada.Manitoba|BOLD:ACF1540
Leucania multilinea|[12089]|LPSK600-08|08BBLEP-02168|658[0n]bp|Canada.Saskatchewan|BOLD:ACF1540
Leucania multilinea|[12090]|BBLPA835-10|10BBCLP-0835|658[0n]bp|Canada.Alberta|BOLD:ACF1540
Leucania multilinea|[12091]|LPSK025-08|08BBLEP-00728|658[0n]bp|Canada.Saskatchewan|BOLD:ACF1540
Leucania multilinea|[12092]|LPSK495-08|08BBLEP-02063|658[0n]bp|Canada.Saskatchewan|BOLD:ACF1540
Leucania multilinea|[12093]|LPSK120-08|08BBLEP-01688|658[0n]bp|Canada.Saskatchewan|BOLD:ACF1540
Leucania multilinea|[12094]|LPSK589-08|08BBLEP-02157|658[0n]bp|Canada.Saskatchewan|BOLD:ACF1540
Leucania multilinea|[12095]|LPSK486-08|08BBLEP-02054|658[0n]bp|Canada.Saskatchewan|BOLD:ACF1540
Leucania multilinea|[12096]|LPSK046-08|08BBLEP-00749|658[0n]bp|Canada.Saskatchewan|BOLD:ACF1540
Leucania multilinea|[12097]|LPSK548-08|08BBLEP-02116|658[0n]bp|Canada.Saskatchewan|BOLD:ACF1540
Leucania multilinea|[12098]|LPSK568-08|08BBLEP-02136|658[0n]bp|Canada.Saskatchewan|BOLD:ACF1540
Leucania multilinea|[12099]|LPSK558-08|08BBLEP-02126|658[0n]bp|Canada.Saskatchewan|BOLD:ACF1540
Leucania multilinea|[12100]|LPSK471-08|08BBLEP-02039|658[0n]bp|Canada.Saskatchewan|BOLD:ACF1540
Leucania multilinea|[12101]|LPSK032-08|08BBLEP-00735|658[0n]bp|Canada.Saskatchewan|BOLD:ACF1540
Leucania multilinea|[12102]|LPSK598-08|08BBLEP-02166|658[0n]bp|Canada.Saskatchewan|BOLD:ACF1540
Leucania multilinea|[12103]|LPSK509-08|08BBLEP-02077|658[0n]bp|Canada.Saskatchewan|BOLD:ACF1540
Leucania multilinea|[12104]|LPSK510-08|08BBLEP-02078|658[0n]bp|Canada.Saskatchewan|BOLD:ACF1540
Leucania multilinea|[12105]|LPSK530-08|08BBLEP-02098|658[0n]bp|Canada.Saskatchewan|BOLD:ACF1540
Leucania multilinea|[12106]|LPMNB563-09|08BBLEP-05641|658[0n]bp|Canada.Manitoba|BOLD:ACF1540
Leucania multilinea|[12107]|LPSK006-08|08BBLEP-00709|658[0n]bp|Canada.Saskatchewan|BOLD:ACF1540
Leucania multilinea|[12108]|LPSK440-08|08BBLEP-02008|658[0n]bp|Canada.Saskatchewan|BOLD:ACF1540
Leucania multilinea|[12109]|LPSK049-08|08BBLEP-00752|658[0n]bp|Canada.Saskatchewan|BOLD:ACF1540
Leucania multilinea|[12110]|LPSK531-08|08BBLEP-02099|658[0n]bp|Canada.Saskatchewan|BOLD:ACF1540
Leucania pseudargyria|[12111]|LPMN988-09|08BBLEP-04029|658[0n]bp|Canada.Manitoba|BOLD:AAB6246
Leucania pseudargyria|[12112]|LPMN996-09|08BBLEP-04037|658[0n]bp|Canada.Manitoba|BOLD:AAB6246
Leucania pseudargyria|[12113]|RDLQG039-06|DH012170|658[0n]bp|Canada.Quebec|BOLD:AAB6246
Leucania pseudargyria|[12114]|LPMN987-09|08BBLEP-04028|658[0n]bp|Canada.Manitoba|BOLD:AAB6246
Leucania pseudargyria|[12115]|MNBB165-05|05-NBSTA-081|658[0n]bp|Canada.New Brunswick|BOLD:AAB6246
Leucania pseudargyria|[12116]|XAJ828-06|2006-ONT-0828|657[0n]bp|Canada.Ontario|BOLD:AAB6246
Leucania pseudargyria|[12117]|BBLEC561-09|09BBELE-0561|658[0n]bp|Canada.Nova Scotia|BOLD:AAB6246
Leucania pseudargyria|[12118]|LPMNB319-09|08BBLEP-05163|658[0n]bp|Canada.Manitoba|BOLD:AAB6246
Leucania pseudargyria|[12119]|BBLEC559-09|09BBELE-0559|643[0n]bp|Canada.Nova Scotia|BOLD:AAB6246
Leucania pseudargyria|[12120]|MNBB116-05|05-NBSTA-032|658[0n]bp|Canada.New Brunswick|BOLD:AAB6246
Leucania pseudargyria|[12121]|TTMNB414-06|MNBTT-414|657[0n]bp|Canada.New Brunswick|BOLD:AAB6246
Leucania pseudargyria|[12122]|XAK052-06|2006-ONT-1047|657[0n]bp|Canada.Ontario|BOLD:AAB6246
Leucania pseudargyria|[12123]|PHJUN3995-11|BIOUG01497-A06|658[0n]bp|Canada.Ontario|BOLD:AAB6246
Leucania pseudargyria|[12124]|PHJUN3994-11|BIOUG01497-A05|658[0n]bp|Canada.Ontario|BOLD:AAB6246
Leucania pseudargyria|[12125]|MNBB166-05|05-NBSTA-082|658[0n]bp|Canada.New Brunswick|BOLD:AAB6246
Leucania pseudargyria|[12126]|BLTIB504-08|BL761|657[0n]bp|Canada.Ontario|BOLD:AAB6246
Leucania pseudargyria|[12127]|PHJUN3998-11|BIOUG01497-A09|658[0n]bp|Canada.Ontario|BOLD:AAB6246
Leucania pseudargyria|[12128]|MNBB021-05|HBL008631|658[0n]bp|Canada.New Brunswick|BOLD:AAB6246
Leucania pseudargyria|[12129]|XAE524-04|Moth4524.03|592[0n]bp|Canada.Ontario|BOLD:AAB6246
Leucania pseudargyria|[12130]|PMG126-03|moth1166.01|617[0n]bp|Canada.Ontario|BOLD:AAB6246
Leucania inermis|[12131]|LPMN072-08|08BBLEP-00870|658[0n]bp|Canada.Manitoba|BOLD:ACE5714
Leucania inermis|[12132]|PHMO056-03|moth375.02|639[0n]bp|Canada.Ontario|BOLD:ACE5714
Leucania inermis|[12133]|RDLQ660-07|DH005416|644[0n]bp|Canada.Quebec|BOLD:ACE5714
Leucania inermis|[12134]|RDLQF935-06|DH012115|658[0n]bp|Canada.Quebec|BOLD:ACE5714
Leucania inermis|[12135]|RDLQ659-07|DH005446|611[1n]bp|Canada.Quebec|BOLD:ACE5714
Leucania ursula|[12136]|LPSO656-08|PPBP-0656|658[0n]bp|Canada.Ontario|BOLD:ACE7380
Leucania ursula|[12137]|LPSO429-08|PPBP-0429|658[0n]bp|Canada.Ontario|BOLD:ACE7380
Leucania ursula|[12138]|LPSO257-08|PPBP-0257|658[0n]bp|Canada.Ontario|BOLD:ACE7380
Leucania ursula|[12139]|LPSO920-08|PPBP-0920|658[0n]bp|Canada.Ontario|BOLD:ACE7380
Leucania ursula|[12140]|LPSO561-08|PPBP-0561|658[0n]bp|Canada.Ontario|BOLD:ACE7380
Leucania ursula|[12141]|LPSO919-08|PPBP-0919|658[0n]bp|Canada.Ontario|BOLD:ACE7380
Leucania ursula|[12142]|LPSO741-08|PPBP-0741|658[0n]bp|Canada.Ontario|BOLD:ACE7380
Leucania ursula|[12143]|LPSO238-08|PPBP-0238|656[0n]bp|Canada.Ontario|BOLD:ACE7380
Leucania ursula|[12144]|LPSO430-08|PPBP-0430|638[0n]bp|Canada.Ontario|BOLD:ACE7380
Leucania insueta|[12145]|RDLQB910-05|DH009412|658[0n]bp|Canada.Quebec|BOLD:AAA3150
Leucania insueta|[12146]|RDLQB909-05|AC001025|504[0n]bp|Canada.Quebec|BOLD:AAA3150
Leucania insueta|[12147]|XAE556-04|Moth4556.03|658[0n]bp|Canada.Ontario|BOLD:AAA3150
Leucania insueta|[12148]|XAE428-04|Moth4428.03|658[0n]bp|Canada.Ontario|BOLD:AAA3150
Leucania insueta|[12149]|LOWCD244-06|CGWC-3064|567[0n]bp|Canada.British Columbia|BOLD:AAA3150
Leucania insueta|[12150]|LOWCD231-06|CGWC-3051|563[0n]bp|Canada.British Columbia|BOLD:AAA3150
Leucania insueta|[12151]|RDNMB428-05|CNCNoctuoidea10194|541[1n]bp|Canada.British Columbia|BOLD:AAA3150
Leucania insueta|[12152]|LOWCD240-06|CGWC-3060|596[0n]bp|Canada.British Columbia|BOLD:AAA3150
Leucania insueta|[12153]|LOWC059-05|CGWC-0059|513[0n]bp|Canada.British Columbia|BOLD:AAA3150
Leucania insueta|[12154]|LOWC058-05|CGWC-0058|658[0n]bp|Canada.British Columbia|BOLD:AAA3150
Leucania insueta|[12155]|LOWCD233-06|CGWC-3053|569[0n]bp|Canada.British Columbia|BOLD:AAA3150
Leucania insueta|[12156]|LPSK599-08|08BBLEP-02167|658[0n]bp|Canada.Saskatchewan|BOLD:AAA3150
Leucania insueta|[12157]|PHMNB610-04|04HBL00836|658[0n]bp|Canada.New Brunswick|BOLD:AAA3150
Leucania insueta|[12158]|RDLQF840-06|DH011993|658[0n]bp|Canada.Quebec|BOLD:AAA3150
Leucania insueta|[12159]|LPSK447-08|08BBLEP-02015|658[0n]bp|Canada.Saskatchewan|BOLD:AAA3150
Leucania insueta|[12160]|RDNMB429-05|CNCNoctuoidea10195|658[0n]bp|Canada.Alberta|BOLD:AAA3150

[illegible]
Leucania insueta|[12158]|RDLQF840-06|DH011993|658[0n]bp|Canada.Quebec|BOLD:AAA3150
Leucania insueta|[12159]|LPSK447-08|08BBLEP-02015|658[0n]bp|Canada.Saskatchewan|BOLD:AAA3150
Leucania insueta|[12160]|RDNMB429-05|CNCNoctuoidea10195|658[0n]bp|Canada.Alberta|BOLD:AAA3150
Leucania insueta|[12161]|BBLPE306-09|09BBELE-2306|658[0n]bp|Canada.Newfoundland and Labrador|BOLD:AAA3150
Leucania insueta|[12162]|LOWC052-05|CGWC-0052|658[0n]bp|Canada.British Columbia|BOLD:AAA3150
Leucania insueta|[12163]|PHMNB328-04|04HBL00554|658[0n]bp|Canada.New Brunswick|BOLD:AAA3150
Leucania insueta|[12164]|RDLQF842-06|DH011995|658[0n]bp|Canada.Quebec|BOLD:AAA3150
Leucania insueta|[12165]|LOWC065-05|CGWC-0065|658[0n]bp|Canada.British Columbia|BOLD:AAA3150
Leucania insueta|[12166]|BBLPC698-09|09BBELE-1698|658[0n]bp|Canada.Newfoundland and Labrador|BOLD:AAA3150
Leucania insueta|[12167]|LOWCD239-06|CGWC-3059|658[0n]bp|Canada.British Columbia|BOLD:AAA3150
Leucania insueta|[12168]|LOWC060-05|CGWC-0060|658[0n]bp|Canada.British Columbia|BOLD:AAA3150
Leucania insueta|[12169]|RDLQB911-05|AC000239|658[0n]bp|Canada.Quebec|BOLD:AAA3150
Leucania insueta|[12170]|LOWCD243-06|CGWC-3063|658[0n]bp|Canada.British Columbia|BOLD:AAA3150
Leucania insueta|[12171]|LOWC064-05|CGWC-0064|658[0n]bp|Canada.British Columbia|BOLD:AAA3150
Leucania insueta|[12172]|RDLQF026-06|DH011156|658[0n]bp|Canada.Quebec|BOLD:AAA3150
Leucania insueta|[12173]|LOWCD241-06|CGWC-3061|658[0n]bp|Canada.British Columbia|BOLD:AAA3150
Leucania insueta|[12174]|RDLQF839-06|DH011992|658[0n]bp|Canada.Quebec|BOLD:AAA3150
Leucania insueta|[12175]|LOWCD242-06|CGWC-3062|658[0n]bp|Canada.British Columbia|BOLD:AAA3150
Leucania insueta|[12176]|LOWC057-05|CGWC-0057|658[0n]bp|Canada.British Columbia|BOLD:AAA3150
Leucania insueta|[12177]|BBLPC718-09|09BBELE-1718|658[0n]bp|Canada.Newfoundland and Labrador|BOLD:AAA3150
Leucania insueta|[12178]|RDLQF841-06|DH011994|658[0n]bp|Canada.Quebec|BOLD:AAA3150
Leucania insueta|[12179]|LOWCD238-06|CGWC-3058|658[0n]bp|Canada.British Columbia|BOLD:AAA3150
Leucania insueta|[12180]|LOWC025-05|CGWC-0025|658[0n]bp|Canada.British Columbia|BOLD:AAA3150
Leucania insueta|[12181]|XAJ836-06|2006-ONT-0836|658[0n]bp|Canada.Ontario|BOLD:AAA3150
Leucania insueta|[12182]|LPSK560-08|08BBLEP-02128|658[0n]bp|Canada.Saskatchewan|BOLD:AAA3150
Leucania insueta|[12183]|RDMAB125-05|UASM41287|658[0n]bp|Canada.Alberta|BOLD:AAA3150
Leucania insueta|[12184]|RDLQF838-06|DH011991|658[0n]bp|Canada.Quebec|BOLD:AAA3150
Leucania insueta|[12185]|LOWC063-05|CGWC-0063|658[0n]bp|Canada.British Columbia|BOLD:AAA3150
Leucania dia|[12186]|LBCH5805-10|10-JDWBC-5805|658[0n]bp|Canada.British Columbia|BOLD:AAA3150
Leucania dia|[12187]|LBCH5165-10|10-JDWBC-5165|658[0n]bp|Canada.British Columbia|BOLD:AAA3150
Leucania dia|[12188]|RDNME363-07|CNCNoctuoidea13970|656[0n]bp|Canada.Yukon Territory|BOLD:AAA3150
Leucania dia|[12189]|BBLPA830-10|10BBCLP-0830|658[0n]bp|Canada.Alberta|BOLD:AAA3150
Leucania dia|[12190]|BBLPA833-10|10BBCLP-0833|658[0n]bp|Canada.Alberta|BOLD:AAA3150
Leucania dia|[12191]|LBCH5166-10|10-JDWBC-5166|658[0n]bp|Canada.British Columbia|BOLD:AAA3150
Leucania dia|[12192]|LBCG103-08|08-JDWBC-0103|658[0n]bp|Canada.British Columbia|BOLD:AAA3150
Leucania dia|[12193]|LBCG356-08|08-JDWBC-0356|658[0n]bp|Canada.British Columbia|BOLD:AAA3150
Leucania dia|[12194]|LBCG377-08|08-JDWBC-0377|658[0n]bp|Canada.British Columbia|BOLD:AAA3150
Leucania dia|[12195]|LBCH5006-10|10-JDWBC-5006|658[0n]bp|Canada.British Columbia|BOLD:AAA3150
Leucania dia|[12196]|LBCG338-08|08-JDWBC-0338|658[0n]bp|Canada.British Columbia|BOLD:AAA3150
Leucania dia|[12197]|LBCH5086-10|10-JDWBC-5086|658[0n]bp|Canada.British Columbia|BOLD:AAA3150
Leucania dia|[12198]|LBCG466-08|08-JDWBC-0466|658[0n]bp|Canada.British Columbia|BOLD:AAA3150
Leucania dia|[12199]|LBCH5358-10|10-JDWBC-5358|658[0n]bp|Canada.British Columbia|BOLD:AAA3150
Leucania dia|[12200]|LBCG120-08|08-JDWBC-0120|658[0n]bp|Canada.British Columbia|BOLD:AAA3150
Leucania dia|[12201]|LBCH5469-10|10-JDWBC-5469|658[0n]bp|Canada.British Columbia|BOLD:AAA3150
Leucania dia|[12202]|LBCH4984-10|10-JDWBC-4984|658[0n]bp|Canada.British Columbia|BOLD:AAA3150
Leucania dia|[12203]|LBCH5558-10|10-JDWBC-5558|658[0n]bp|Canada.British Columbia|BOLD:AAA3150
Leucania dia|[12204]|LBCG334-08|08-JDWBC-0334|658[0n]bp|Canada.British Columbia|BOLD:AAA3150
Leucania dia|[12205]|LBCH5485-10|10-JDWBC-5485|658[0n]bp|Canada.British Columbia|BOLD:AAA3150
Leucania dia|[12206]|LBCH5518-10|10-JDWBC-5518|658[0n]bp|Canada.British Columbia|BOLD:AAA3150
Leucania dia|[12207]|LBCG336-08|08-JDWBC-0336|658[0n]bp|Canada.British Columbia|BOLD:AAA3150
Leucania dia|[12208]|LBCG126-08|08-JDWBC-0126|658[0n]bp|Canada.British Columbia|BOLD:AAA3150
Leucania dia|[12209]|LBCG043-08|08-JDWBC-0043|658[0n]bp|Canada.British Columbia|BOLD:AAA3150
Leucania dia|[12210]|LBCG360-08|08-JDWBC-0360|658[0n]bp|Canada.British Columbia|BOLD:AAA3150
Leucania dia|[12211]|LBCH5285-10|10-JDWBC-5285|658[0n]bp|Canada.British Columbia|BOLD:AAA3150
Leucania dia|[12212]|LBCH5008-10|10-JDWBC-5008|658[0n]bp|Canada.British Columbia|BOLD:AAA3150
Leucania dia|[12213]|LBCG346-08|08-JDWBC-0346|658[0n]bp|Canada.British Columbia|BOLD:AAA3150
Leucania dia|[12214]|LBCG341-08|08-JDWBC-0341|658[0n]bp|Canada.British Columbia|BOLD:AAA3150
Leucania dia|[12215]|LBCH5007-10|10-JDWBC-5007|658[0n]bp|Canada.British Columbia|BOLD:AAA3150
Leucania dia|[12216]|LBCG464-08|08-JDWBC-0464|658[0n]bp|Canada.British Columbia|BOLD:AAA3150
Leucania dia|[12217]|LBCG374-08|08-JDWBC-0374|658[0n]bp|Canada.British Columbia|BOLD:AAA3150
Leucania dia|[12218]|LBCH5264-10|10-JDWBC-5264|658[0n]bp|Canada.British Columbia|BOLD:AAA3150
Leucania dia|[12219]|LBCG354-08|08-JDWBC-0354|658[0n]bp|Canada.British Columbia|BOLD:AAA3150
Leucania dia|[12220]|LBCH5361-10|10-JDWBC-5361|658[0n]bp|Canada.British Columbia|BOLD:AAA3150
Leucania dia|[12221]|LBCG042-08|08-JDWBC-0042|657[0n]bp|Canada.British Columbia|BOLD:AAA3150
Leucania dia|[12222]|LBCH5360-10|10-JDWBC-5360|658[0n]bp|Canada.British Columbia|BOLD:AAA3150
Leucania dia|[12223]|LBCG344-08|08-JDWBC-0344|658[0n]bp|Canada.British Columbia|BOLD:AAA3150
Leucania dia|[12224]|LBCG115-08|08-JDWBC-0115|658[0n]bp|Canada.British Columbia|BOLD:AAA3150
Leucania dia|[12225]|LBCH5608-10|10-JDWBC-5608|658[0n]bp|Canada.British Columbia|BOLD:AAA3150
Leucania dia|[12226]|LBCH5362-10|10-JDWBC-5362|658[0n]bp|Canada.British Columbia|BOLD:AAA3150
Leucania dia|[12227]|LBCH5363-10|10-JDWBC-5363|658[0n]bp|Canada.British Columbia|BOLD:AAA3150
Leucania dia|[12228]|LBCH5169-10|10-JDWBC-5169|658[0n]bp|Canada.British Columbia|BOLD:AAA3150
Leucania dia|[12229]|LBCG415-08|08-JDWBC-0415|658[0n]bp|Canada.British Columbia|BOLD:AAA3150
Leucania dia|[12230]|LBCH5089-10|10-JDWBC-5089|658[0n]bp|Canada.British Columbia|BOLD:AAA3150
Leucania dia|[12231]|LBCH5091-10|10-JDWBC-5091|658[0n]bp|Canada.British Columbia|BOLD:AAA3150
Leucania dia|[12232]|LBCH5357-10|10-JDWBC-5357|644[0n]bp|Canada.British Columbia|BOLD:AAA3150
Leucania dia|[12233]|RDNMF573-08|NOC14659|640[0n]bp|Canada.British Columbia|BOLD:AAA3150
Leucania dia|[12234]|LHLEP096-06|UBC-2006-0481|658[0n]bp|Canada.British Columbia|BOLD:AAA3150
Leucania dia|[12235]|LALPA1278-11|AVBC 1280-11|658[0n]bp|Canada.British Columbia|BOLD:AAA3150
Leucania dia|[12236]|LALPA213-10|AVBC 214-10|658[0n]bp|Canada.British Columbia|BOLD:AAA3150
Leucania dia|[12237]|LBCG365-08|08-JDWBC-0365|658[0n]bp|Canada.British Columbia|BOLD:AAA3150
Leucania dia|[12238]|LHLEP097-06|UBC-2006-0622|658[0n]bp|Canada.British Columbia|BOLD:AAA3150
Leucania dia|[12239]|LHLEP100-06|UBC-2006-0186|658[0n]bp|Canada.British Columbia|BOLD:AAA3150
Leucania dia|[12240]|LALPA228-10|AVBC 229-10|658[0n]bp|Canada.British Columbia|BOLD:AAA3150
Leucania dia|[12241]|LPVIA298-08|PFC-2006-0389|658[0n]bp|Canada.British Columbia|BOLD:AAA3150
Leucania dia|[12242]|LALPA103-10|AVBC 103-10|658[0n]bp|Canada.British Columbia|BOLD:AAA3150
Leucania dia|[12243]|LALPA403-10|AVBC 405-10|658[0n]bp|Canada.British Columbia|BOLD:AAA3150
Leucania dia|[12244]|LPVIB852-08|PFC-2006-2361|658[0n]bp|Canada.British Columbia|BOLD:AAA3150
Leucania dia|[12245]|LALPA192-10|AVBC 192-10|658[0n]bp|Canada.British Columbia|BOLD:AAA3150
Leucania dia|[12246]|LPVIB881-08|PFC-2006-2398|639[0n]bp|Canada.British Columbia|BOLD:AAA3150
Leucania dia|[12247]|LPVIB907-08|PFC-2006-2433|638[0n]bp|Canada.British Columbia|BOLD:AAA3150
Leucania dia|[12248]|LPVIC099-08|PFC-2006-2672|637[0n]bp|Canada.British Columbia|BOLD:AAA3150
Leucania dia|[12249]|LHLEP098-06|UBC-2006-0184|658[0n]bp|Canada.British Columbia|BOLD:AAA3150
Leucania dia|[12250]|LBCG376-08|08-JDWBC-0376|658[0n]bp|Canada.British Columbia|BOLD:AAA3150
Leucania dia|[12251]|LPABB151-08|08BBLEP-03416|658[0n]bp|Canada.Alberta|BOLD:AAA3150
Leucania dia|[12252]|RDLQB912-05|DH008660|658[0n]bp|Canada.Quebec|BOLD:AAA3150
Leucania dia|[12253]|BBLPA834-10|10BBCLP-0834|658[0n]bp|Canada.Alberta|BOLD:AAA3150
Leucania dia|[12254]|BBLPA832-10|10BBCLP-0832|658[0n]bp|Canada.Alberta|BOLD:AAA3150
Leucania dia|[12255]|BBLPA831-10|10BBCLP-0831|658[0n]bp|Canada.Alberta|BOLD:AAA3150
Leucania dia|[12256]|LBCG106-08|08-JDWBC-0106|658[0n]bp|Canada.British Columbia|BOLD:AAA3150
Leucania dia|[12257]|LHLEP095-06|UBC-2006-1533|658[0n]bp|Canada.British Columbia|BOLD:AAA3150
Leucania dia|[12258]|LHLEP099-06|UBC-2006-0185|658[0n]bp|Canada.British Columbia|BOLD:AAA3150
Leucania dia|[12259]|LPABC016-09|08BBLEP-04235|633[0n]bp|Canada.Alberta|BOLD:AAA3150
[illegible]

Leucania dia|[12257]|LHLEP095-06|UBC-2006-1533|658[0n]bp|Canada.British Columbia|BOLD:AAA3150
Leucania dia|[12258]|LHLEP099-06|UBC-2006-0185|658[0n]bp|Canada.British Columbia|BOLD:AAA3150
Leucania dia|[12259]|LPABC016-09|08BBLEP-04235|633[0n]bp|Canada.Alberta|BOLD:AAA3150
Leucania dia|[12260]|LPABB137-08|08BBLEP-03402|638[0n]bp|Canada.Alberta|BOLD:AAA3150
Leucania dia|[12261]|LPVIB013-08|PFC-2006-2461|641[0n]bp|Canada.British Columbia|BOLD:AAA3150
Leucania dia|[12262]|LOWCD237-06|CGWC-3057|614[0n]bp|Canada.British Columbia|BOLD:AAA3150
Leucania dia|[12263]|RDLQF025-06|DH011155|658[0n]bp|Canada.Quebec|BOLD:AAA3150
Leucania dia|[12264]|LBCH5092-10|10-JDWBC-5092|658[0n]bp|Canada.British Columbia|BOLD:AAA3150
Leucania dia|[12265]|LBCG369-08|08-JDWBC-0369|658[0n]bp|Canada.British Columbia|BOLD:AAA3150
Leucania dia|[12266]|RDNME362-07|CNCNoctuoidea13969|658[0n]bp|Canada.Yukon Territory|BOLD:AAA3150
Leucania dia|[12267]|LBCH5162-10|10-JDWBC-5162|658[0n]bp|Canada.British Columbia|BOLD:AAA3150
Leucania dia|[12268]|LBCH5168-10|10-JDWBC-5168|658[0n]bp|Canada.British Columbia|BOLD:AAA3150
Leucania dia|[12269]|LBCH5009-10|10-JDWBC-5009|658[0n]bp|Canada.British Columbia|BOLD:AAA3150
Leucania dia|[12270]|LBCG379-08|08-JDWBC-0379|658[0n]bp|Canada.British Columbia|BOLD:AAA3150
Leucania dia|[12271]|LBCH5088-10|10-JDWBC-5088|658[0n]bp|Canada.British Columbia|BOLD:AAA3150
Leucania dia|[12272]|LBCG105-08|08-JDWBC-0105|658[0n]bp|Canada.British Columbia|BOLD:AAA3150
Leucania dia|[12273]|RDNMF572-08|NOC14658|658[0n]bp|Canada.British Columbia|BOLD:AAA3150
Leucania dia|[12274]|LBCG122-08|08-JDWBC-0122|658[0n]bp|Canada.British Columbia|BOLD:AAA3150
Leucania dia|[12275]|LBCH5005-10|10-JDWBC-5005|658[0n]bp|Canada.British Columbia|BOLD:AAA3150
Leucania dia|[12276]|LBCH5090-10|10-JDWBC-5090|658[0n]bp|Canada.British Columbia|BOLD:AAA3150
Leucania dia|[12277]|LBCG363-08|08-JDWBC-0363|658[0n]bp|Canada.British Columbia|BOLD:AAA3150
Leucania dia|[12278]|LBCH5572-10|10-JDWBC-5572|658[0n]bp|Canada.British Columbia|BOLD:AAA3150
Leucania dia|[12279]|LBCH5164-10|10-JDWBC-5164|658[0n]bp|Canada.British Columbia|BOLD:AAA3150
Leucania dia|[12280]|LBCH5052-10|10-JDWBC-5052|658[0n]bp|Canada.British Columbia|BOLD:AAA3150
Leucania dia|[12281]|LOWCD236-06|CGWC-3056|658[0n]bp|Canada.British Columbia|BOLD:AAA3150
Leucania dia|[12282]|LBCH5484-10|10-JDWBC-5484|658[0n]bp|Canada.British Columbia|BOLD:AAA3150
Leucania dia|[12283]|LBCG392-08|08-JDWBC-0392|658[0n]bp|Canada.British Columbia|BOLD:AAA3150
Leucania dia|[12284]|LBCG345-08|08-JDWBC-0345|658[0n]bp|Canada.British Columbia|BOLD:AAA3150
Leucania dia|[12285]|LBCG370-08|08-JDWBC-0370|658[0n]bp|Canada.British Columbia|BOLD:AAA3150
Leucania dia|[12286]|RDNMF575-08|NOC14661|658[0n]bp|Canada.British Columbia|BOLD:AAA3150
Leucania dia|[12287]|LBCH5010-10|10-JDWBC-5010|658[0n]bp|Canada.British Columbia|BOLD:AAA3150
Leucania dia|[12288]|LOWCE111-06|CGWC-3871|658[0n]bp|Canada.British Columbia|BOLD:AAA3150
Leucania dia|[12289]|LBCG372-08|08-JDWBC-0372|658[0n]bp|Canada.British Columbia|BOLD:AAA3150
Leucania dia|[12290]|LBCH5004-10|10-JDWBC-5004|658[0n]bp|Canada.British Columbia|BOLD:AAA3150
Leucania dia|[12291]|LBCH5167-10|10-JDWBC-5167|658[0n]bp|Canada.British Columbia|BOLD:AAA3150
Leucania dia|[12292]|LBCG375-08|08-JDWBC-0375|658[0n]bp|Canada.British Columbia|BOLD:AAA3150
Leucania dia|[12293]|LOWCD234-06|CGWC-3054|658[0n]bp|Canada.British Columbia|BOLD:AAA3150
Leucania dia|[12294]|LOWCD235-06|CGWC-3055|658[0n]bp|Canada.British Columbia|BOLD:AAA3150
Leucania dia|[12295]|LBCG426-08|08-JDWBC-0426|658[0n]bp|Canada.British Columbia|BOLD:AAA3150
Leucania dia|[12296]|LBCH5420-10|10-JDWBC-5420|658[0n]bp|Canada.British Columbia|BOLD:AAA3150
Leucania dia|[12297]|LBCH5359-10|10-JDWBC-5359|658[0n]bp|Canada.British Columbia|BOLD:AAA3150
Leucania dia|[12298]|LBCH5003-10|10-JDWBC-5003|658[0n]bp|Canada.British Columbia|BOLD:AAA3150
Leucania dia|[12299]|LBCH5087-10|10-JDWBC-5087|658[0n]bp|Canada.British Columbia|BOLD:AAA3150
Leucania dia|[12300]|LBCG117-08|08-JDWBC-0117|658[0n]bp|Canada.British Columbia|BOLD:AAA3150
Leucania dia|[12301]|LBCH5163-10|10-JDWBC-5163|658[0n]bp|Canada.British Columbia|BOLD:AAA3150
Leucania dia|[12302]|LBCG416-08|08-JDWBC-0416|658[0n]bp|Canada.British Columbia|BOLD:AAA3150
Leucania dia|[12303]|LBCG127-08|08-JDWBC-0127|658[0n]bp|Canada.British Columbia|BOLD:AAA3150
Leucania dia|[12304]|LBCG110-08|08-JDWBC-0110|658[0n]bp|Canada.British Columbia|BOLD:AAA3150
Leucania dia|[12305]|LBCH5093-10|10-JDWBC-5093|658[0n]bp|Canada.British Columbia|BOLD:AAA3150
Leucania dia|[12306]|LBCH5364-10|10-JDWBC-5364|658[0n]bp|Canada.British Columbia|BOLD:AAA3150
Leucania dia|[12307]|LBCG114-08|08-JDWBC-0114|658[0n]bp|Canada.British Columbia|BOLD:AAA3150
Leucania dia|[12308]|LBCH5419-10|10-JDWBC-5419|658[0n]bp|Canada.British Columbia|BOLD:AAA3150
Leucania dia|[12309]|LBCG373-08|08-JDWBC-0373|658[0n]bp|Canada.British Columbia|BOLD:AAA3150
Leucania dia|[12310]|LBCG358-08|08-JDWBC-0358|658[0n]bp|Canada.British Columbia|BOLD:AAA3150
Leucania dia|[12311]|RDNMF574-08|NOC14660|652[0n]bp|Canada.British Columbia|BOLD:AAA3150
Leucania dia|[12312]|LOWCD232-06|CGWC-3052|591[0n]bp|Canada.British Columbia|BOLD:AAA3150
Leucania dia|[12313]|RDNME364-07|CNCNoctuoidea13971|608[0n]bp|Canada.Yukon Territory|BOLD:AAA3150
Leucania dia|[12314]|LBCG1352-09|08-JDWBC-1352|636[0n]bp|Canada.British Columbia|BOLD:AAA3150
Leucania dia|[12315]|LBCG382-08|08-JDWBC-0382|658[0n]bp|Canada.British Columbia|BOLD:AAA3150
Mythimna yukonensis|[12316]|UAMIC522-13|UAM Insects 131237|648[0n]bp|United States.Alaska|BOLD:AAA3150
Mythimna oxygala|[12317]|BLTIB237-08|BL419|658[0n]bp|Canada.Ontario|BOLD:AAA2815
Mythimna oxygala|[12318]|LHLEP134-06|UBC-2006-2063|658[0n]bp|Canada.British Columbia|BOLD:AAA2815
Mythimna oxygala|[12319]|LOWCD142-06|CGWC-2962|658[0n]bp|Canada.British Columbia|BOLD:AAA2815
Mythimna oxygala|[12320]|LPSK462-08|08BBLEP-02030|658[0n]bp|Canada.Saskatchewan|BOLD:AAA2815
Mythimna oxygala|[12321]|LHLEP141-06|UBC-2006-2134|658[0n]bp|Canada.British Columbia|BOLD:AAA2815
Mythimna oxygala|[12322]|LHLEP133-06|UBC-2006-2062|658[0n]bp|Canada.British Columbia|BOLD:AAA2815
Mythimna oxygala|[12323]|LHLEP144-06|UBC-2006-2105|658[0n]bp|Canada.British Columbia|BOLD:AAA2815
Mythimna oxygala|[12324]|BBLPA838-10|10BBCLP-0838|658[0n]bp|Canada.Alberta|BOLD:AAA2815
Mythimna oxygala|[12325]|LBCC830-05|HLC-22710|658[0n]bp|Canada.British Columbia|BOLD:AAA2815
Mythimna oxygala|[12326]|LPABB382-08|08BBLEP-03647|658[0n]bp|Canada.Alberta|BOLD:AAA2815
Mythimna oxygala|[12327]|LHLEP145-06|UBC-2006-2106|658[0n]bp|Canada.British Columbia|BOLD:AAA2815
Mythimna oxygala|[12328]|LPGVA636-08|UBC-2006-1804|632[0n]bp|Canada.British Columbia|BOLD:AAA2815
Mythimna oxygala|[12329]|LPGVA609-08|UBC-2006-1767|636[0n]bp|Canada.British Columbia|BOLD:AAA2815
Mythimna oxygala|[12330]|LHLEP080-06|UBC-2006-0285|624[0n]bp|Canada.British Columbia|BOLD:AAA2815
Mythimna oxygala|[12331]|LPGVA637-08|UBC-2006-1805|622[0n]bp|Canada.British Columbia|BOLD:AAA2815
Mythimna oxygala|[12332]|LOWC045-05|CGWC-0045|658[0n]bp|Canada.British Columbia|BOLD:AAA2815
Mythimna oxygala|[12333]|LBCA206-05|HLC-20206|658[0n]bp|Canada.British Columbia|BOLD:AAA2815
Mythimna oxygala|[12334]|LPVIB006-08|PFC-2006-2343|658[0n]bp|Canada.British Columbia|BOLD:AAA2815
Mythimna oxygala|[12335]|LPVIB062-08|PFC-2006-1399|658[0n]bp|Canada.British Columbia|BOLD:AAA2815
Mythimna oxygala|[12336]|LOWC040-05|CGWC-0040|575[0n]bp|Canada.British Columbia|BOLD:AAA2815
Mythimna oxygala|[12337]|LOWC041-05|CGWC-0041|658[0n]bp|Canada.British Columbia|BOLD:AAA2815
Mythimna oxygala|[12338]|LOWC036-05|CGWC-0036|658[0n]bp|Canada.British Columbia|BOLD:AAA2815
Mythimna oxygala|[12339]|LPABC454-09|08BBLEP-04673|658[0n]bp|Canada.Alberta|BOLD:AAA2815
Mythimna oxygala|[12340]|LOWC046-05|CGWC-0046|658[0n]bp|Canada.British Columbia|BOLD:AAA2815
Mythimna oxygala|[12341]|LOWC044-05|CGWC-0044|658[0n]bp|Canada.British Columbia|BOLD:AAA2815
Mythimna oxygala|[12342]|LOWCD162-06|CGWC-2982|658[0n]bp|Canada.British Columbia|BOLD:AAA2815
Mythimna oxygala|[12343]|LOWC037-05|CGWC-0037|658[0n]bp|Canada.British Columbia|BOLD:AAA2815
Mythimna oxygala|[12344]|LHLEP148-06|UBC-2006-2109|658[0n]bp|Canada.British Columbia|BOLD:AAA2815
Mythimna oxygala|[12345]|LHLEP137-06|UBC-2006-2066|658[0n]bp|Canada.British Columbia|BOLD:AAA2815
Mythimna oxygala|[12346]|LPSK254-08|08BBLEP-01822|658[0n]bp|Canada.Saskatchewan|BOLD:AAA2815
Mythimna oxygala|[12347]|LPABC363-09|08BBLEP-04582|658[0n]bp|Canada.Alberta|BOLD:AAA2815
Mythimna oxygala|[12348]|LOWCD779-06|CGWC-3599|658[0n]bp|Canada.British Columbia|BOLD:AAA2815
Mythimna oxygala|[12349]|LBCH1316-10|10-JDWBC-1316|658[0n]bp|Canada.British Columbia|BOLD:AAA2815
Mythimna oxygala|[12350]|LHLEP131-06|UBC-2006-0382|658[0n]bp|Canada.British Columbia|BOLD:AAA2815
Mythimna oxygala|[12351]|LOWC042-05|CGWC-0042|658[0n]bp|Canada.British Columbia|BOLD:AAA2815
Mythimna oxygala|[12352]|LHLEP130-06|UBC-2006-0381|658[0n]bp|Canada.British Columbia|BOLD:AAA2815
Mythimna oxygala|[12353]|LHLEP147-06|UBC-2006-2108|658[0n]bp|Canada.British Columbia|BOLD:AAA2815
Mythimna oxygala|[12354]|LBCC832-05|HLC-22712|658[0n]bp|Canada.British Columbia|BOLD:AAA2815
Mythimna oxygala|[12355]|LPABC378-09|08BBLEP-04597|658[0n]bp|Canada.Alberta|BOLD:AAA2815
Mythimna oxygala|[12356]|LALPA1204-11|AVBC 1206-11|658[0n]bp|Canada.British Columbia|BOLD:AAA2815
Mythimna oxygala|[12357]|LOWC043-05|CGWC-0043|658[0n]bp|Canada.British Columbia|BOLD:AAA2815
Mythimna oxygala|[12358]|LOWC038-05|CGWC-0038|658[0n]bp|Canada.British Columbia|BOLD:AAA2815
Mythimna oxygala|[12359]|LOWCD188-06|CGWC-3008|658[0n]bp|Canada.British Columbia|BOLD:AAA2815

Mythimna oxygala|[12357]|LOWC043-05|CGWC-0043|658[0n]bp|Canada.British Columbia|BOLD:AAA2815
Mythimna oxygala|[12358]|LOWC038-05|CGWC-0038|658[0n]bp|Canada.British Columbia|BOLD:AAA2815
Mythimna oxygala|[12359]|LOWCD188-06|CGWC-3008|658[0n]bp|Canada.British Columbia|BOLD:AAA2815
Mythimna oxygala|[12360]|LOWC039-05|CGWC-0039|658[0n]bp|Canada.British Columbia|BOLD:AAA2815
Mythimna oxygala|[12361]|LPGVA611-08|UBC-2006-1769|658[0n]bp|Canada.British Columbia|BOLD:AAA2815
Mythimna oxygala|[12362]|RDNMC034-05|CNCNoctuoidea10775|657[0n]bp|Canada.British Columbia|BOLD:AAA2815
Mythimna oxygala|[12363]|LMH041-06|PFC-2006-0461|658[0n]bp|Canada.British Columbia|BOLD:AAA2815
Mythimna oxygala|[12364]|BBLPA837-10|10BBCLP-0837|658[0n]bp|Canada.Alberta|BOLD:AAA2815
Mythimna oxygala|[12365]|LHLEP135-06|UBC-2006-2064|658[0n]bp|Canada.British Columbia|BOLD:AAA2815
Mythimna oxygala|[12366]|LOWCD778-06|CGWC-3598|658[0n]bp|Canada.British Columbia|BOLD:AAA2815
Mythimna oxygala|[12367]|LPABC420-09|08BBLEP-04639|658[0n]bp|Canada.Alberta|BOLD:AAA2815
Mythimna oxygala|[12368]|LBCH350-10|10-JDWBC-0350|658[0n]bp|Canada.British Columbia|BOLD:AAA2815
Mythimna oxygala|[12369]|RDNMC033-05|CNCNoctuoidea10774|658[0n]bp|Canada.British Columbia|BOLD:AAA2815
Mythimna oxygala|[12370]|LHLEP142-06|UBC-2006-2139|658[0n]bp|Canada.British Columbia|BOLD:AAA2815
Mythimna oxygala|[12371]|LHLEP079-06|UBC-2006-0284|658[0n]bp|Canada.British Columbia|BOLD:AAA2815
Mythimna oxygala|[12372]|LHLEP146-06|UBC-2006-2107|658[0n]bp|Canada.British Columbia|BOLD:AAA2815
Mythimna oxygala|[12373]|LHLEP143-06|UBC-2006-2104|658[0n]bp|Canada.British Columbia|BOLD:AAA2815
Mythimna oxygala|[12374]|LBCA207-05|HLC-20207|653[0n]bp|Canada.British Columbia|BOLD:AAA2815
Mythimna oxygala|[12375]|LHLEP140-06|UBC-2006-2133|652[0n]bp|Canada.British Columbia|BOLD:AAA2815
Mythimna oxygala|[12376]|LHLEP136-06|UBC-2006-2065|646[0n]bp|Canada.British Columbia|BOLD:AAA2815
Mythimna oxygala|[12377]|LPGVA610-08|UBC-2006-1768|618[0n]bp|Canada.British Columbia|BOLD:AAA2815
Mythimna oxygala|[12378]|LBCD319-05|HLC-23139|643[0n]bp|Canada.British Columbia|BOLD:AAA2815
Mythimna oxygala|[12379]|LOWCC899-05|CGWC-2779|583[1n]bp|Canada.British Columbia|BOLD:AAA2815
Mythimna oxygala|[12380]|LHLEP138-06|UBC-2006-2067|624[0n]bp|Canada.British Columbia|BOLD:AAA2815
Mythimna oxygala|[12381]|LBCA374-05|HLC-20374|622[0n]bp|Canada.British Columbia|BOLD:AAA2815
Mythimna oxygala|[12382]|LOWCD770-06|CGWC-3590|591[0n]bp|Canada.British Columbia|BOLD:AAA2815
Mythimna oxygala|[12383]|LPABC171-09|08BBLEP-04390|613[0n]bp|Canada.Alberta|BOLD:AAA2815
Mythimna oxygala|[12384]|LHLEP139-06|UBC-2006-1128|612[0n]bp|Canada.British Columbia|BOLD:AAA2815
Mythimna oxygala|[12385]|LBCC831-05|HLC-22711|658[0n]bp|Canada.British Columbia|BOLD:AAA2815
Mythimna oxygala|[12386]|LHLEP132-06|UBC-2006-0383|658[0n]bp|Canada.British Columbia|BOLD:AAA2815
Mythimna oxygala|[12387]|BBLPA842-10|10BBCLP-0842|658[0n]bp|Canada.British Columbia|BOLD:AAA2815
Mythimna oxygala|[12388]|BBLPE352-09|09BBELE-2352|658[0n]bp|Canada.Newfoundland and Labrador|BOLD:AAA2815
Mythimna oxygala|[12389]|BBLPC057-09|09BBELE-1057|658[0n]bp|Canada.New Brunswick|BOLD:AAA2815
Mythimna oxygala|[12390]|XAB516-04|04HBL005516|658[0n]bp|Canada.Ontario|BOLD:AAA2815
Mythimna oxygala|[12391]|BLTIB338-08|BL534|658[0n]bp|Canada.Ontario|BOLD:AAA2815
Mythimna oxygala|[12392]|RDLQF884-06|DH012059|658[0n]bp|Canada.Quebec|BOLD:AAA2815
Mythimna oxygala|[12393]|XAB366-04|04HBL005366|658[0n]bp|Canada.Ontario|BOLD:AAA2815
Mythimna oxygala|[12394]|BLTIB268-08|BL452|658[0n]bp|Canada.Ontario|BOLD:AAA2815
Mythimna oxygala|[12395]|XAD719-05|2005-ONT-518|658[0n]bp|Canada.Ontario|BOLD:AAA2815
Mythimna oxygala|[12396]|LPMNB225-09|08BBLEP-05069|658[0n]bp|Canada.Manitoba|BOLD:AAA2815
Mythimna oxygala|[12397]|LPABC367-09|08BBLEP-04586|638[0n]bp|Canada.Alberta|BOLD:AAA2815
Mythimna oxygala|[12398]|LPSOC133-08|PPBP-2132|658[0n]bp|Canada.Ontario|BOLD:AAA2815
Mythimna oxygala|[12399]|LCH557-04|04HBL003557|658[0n]bp|Canada.Manitoba|BOLD:AAA2815
Mythimna oxygala|[12400]|LPABC246-09|08BBLEP-04465|658[0n]bp|Canada.Alberta|BOLD:AAA2815
Mythimna oxygala|[12401]|BBLPA839-10|10BBCLP-0839|658[0n]bp|Canada.Saskatchewan|BOLD:AAA2815
Mythimna oxygala|[12402]|BBLPA847-10|10BBCLP-0847|658[0n]bp|Canada.Alberta|BOLD:AAA2815
Mythimna oxygala|[12403]|BLTIB273-08|BL457|658[0n]bp|Canada.Ontario|BOLD:AAA2815
Mythimna oxygala|[12404]|XAH171-05|2005-ONT-1754|658[0n]bp|Canada.Ontario|BOLD:AAA2815
Mythimna oxygala|[12405]|RDMAB062-05|UASM57589|645[1n]bp|Canada.Alberta|BOLD:AAA2815
Mythimna oxygala|[12406]|LPABC315-09|08BBLEP-04534|633[0n]bp|Canada.Alberta|BOLD:AAA2815
Mythimna oxygala|[12407]|BLGSM028-09|BL337|646[0n]bp|Canada.Ontario|BOLD:AAA2815
Mythimna oxygala|[12408]|LPSOC338-08|PPBP-2337|609[0n]bp|Canada.Ontario|BOLD:AAA2815
Mythimna oxygala|[12409]|PHMO117-03|moth724.01|639[0n]bp|Canada.Ontario|BOLD:AAA2815
Mythimna oxygala|[12410]|LPSOC340-08|PPBP-2339|658[0n]bp|Canada.Ontario|BOLD:AAA2815
Mythimna oxygala|[12411]|BBLPA846-10|10BBCLP-0846|658[0n]bp|Canada.Alberta|BOLD:AAA2815
Mythimna oxygala|[12412]|LPSOB618-08|PPBP-1617|646[0n]bp|Canada.Ontario|BOLD:AAA2815
Mythimna oxygala|[12413]|BLTIB511-08|BL769|606[0n]bp|Canada.Ontario|BOLD:AAA2815
Mythimna oxygala|[12414]|XAD717-05|2005-ONT-516|650[0n]bp|Canada.Ontario|BOLD:AAA2815
Mythimna oxygala|[12415]|XAJ668-06|2006-ONT-0668|658[0n]bp|Canada.Ontario|BOLD:AAA2815
Mythimna oxygala|[12416]|RDNMC035-05|CNCNoctuoidea10776|658[0n]bp|Canada.Alberta|BOLD:AAA2815
Mythimna oxygala|[12417]|XAF825-05|2005-ONT-474|658[0n]bp|Canada.Ontario|BOLD:AAA2815
Mythimna oxygala|[12418]|BBLPA844-10|10BBCLP-0844|658[0n]bp|Canada.Alberta|BOLD:AAA2815
Mythimna oxygala|[12419]|XAH380-05|2005-ONT-1963|658[0n]bp|Canada.Ontario|BOLD:AAA2815
Mythimna oxygala|[12420]|LPSOC328-08|PPBP-2327|658[0n]bp|Canada.Ontario|BOLD:AAA2815
Mythimna oxygala|[12421]|BLGSM076-09|BL1629|658[0n]bp|Canada.Ontario|BOLD:AAA2815
Mythimna oxygala|[12422]|XAJ628-06|2006-ONT-0628|658[0n]bp|Canada.Ontario|BOLD:AAA2815
Mythimna oxygala|[12423]|LPSOC339-08|PPBP-2338|658[0n]bp|Canada.Ontario|BOLD:AAA2815
Mythimna oxygala|[12424]|LPSOC337-08|PPBP-2336|657[0n]bp|Canada.Ontario|BOLD:AAA2815
Mythimna oxygala|[12425]|BLTIB790-08|BL1207|658[0n]bp|Canada.Ontario|BOLD:AAA2815
Mythimna oxygala|[12426]|BBLPA841-10|10BBCLP-0841|658[0n]bp|Canada.British Columbia|BOLD:AAA2815
Mythimna oxygala|[12427]|BBLPA845-10|10BBCLP-0845|658[0n]bp|Canada.Alberta|BOLD:AAA2815
Mythimna oxygala|[12428]|XAH454-05|2005-ONT-2037|658[0n]bp|Canada.Ontario|BOLD:AAA2815
Mythimna oxygala|[12429]|XAJ452-06|2006-ONT-0452|658[0n]bp|Canada.Ontario|BOLD:AAA2815
Mythimna yukonensis|[12430]|ABKWR093-07|KENWR 6646d|658[0n]bp|United States.Alaska|BOLD:AAA2815
Mythimna oxygala|[12431]|BLTIB047-08|BL0077|658[0n]bp|Canada.Ontario|BOLD:AAA2815
Mythimna oxygala|[12432]|XAJ316-06|2006-ONT-0316|658[0n]bp|Canada.Ontario|BOLD:AAA2815
Mythimna oxygala|[12433]|BBLEC277-09|09BBELE-0277|658[0n]bp|Canada.Nova Scotia|BOLD:AAA2815
Mythimna oxygala|[12434]|LPABC337-09|08BBLEP-04556|658[0n]bp|Canada.Alberta|BOLD:AAA2815
Mythimna oxygala|[12435]|LBCC028-05|HLC-21908|658[0n]bp|Canada.British Columbia|BOLD:AAA2815
Mythimna oxygala|[12436]|XAD361-04|04HBL007361|658[0n]bp|Canada.Ontario|BOLD:AAA2815
Mythimna oxygala|[12437]|LPSOC229-08|PPBP-2228|658[0n]bp|Canada.Ontario|BOLD:AAA2815
Mythimna oxygala|[12438]|LPSOC342-08|PPBP-2341|658[0n]bp|Canada.Ontario|BOLD:AAA2815
Mythimna oxygala|[12439]|XAB612-04|04HBL005612|658[0n]bp|Canada.Ontario|BOLD:AAA2815
Mythimna oxygala|[12440]|BLGSM067-09|BL389|658[0n]bp|Canada.Ontario|BOLD:AAA2815
Mythimna oxygala|[12441]|XAD718-05|2005-ONT-517|658[0n]bp|Canada.Ontario|BOLD:AAA2815
Mythimna oxygala|[12442]|BLGSM094-09|BL1647|658[0n]bp|Canada.Ontario|BOLD:AAA2815
Mythimna oxygala|[12443]|XAK166-06|2006-ONT-1161|658[0n]bp|Canada.Ontario|BOLD:AAA2815
Mythimna oxygala|[12444]|BLTIB678-08|BL961|658[1n]bp|Canada.Ontario|BOLD:AAA2815
Mythimna oxygala|[12445]|BLGSM065-09|BL387|658[0n]bp|Canada.Ontario|BOLD:AAA2815
Mythimna oxygala|[12446]|LPSOC341-08|PPBP-2340|646[0n]bp|Canada.Ontario|BOLD:AAA2815
Mythimna oxygala|[12447]|LPSOB724-08|PPBP-1723|603[0n]bp|Canada.Ontario|BOLD:AAA2815
Mythimna oxygala|[12448]|PHMO065-03|moth437.02|639[0n]bp|Canada.Ontario|BOLD:AAA2815
Mythimna oxygala|[12449]|LPABC477-09|08BBLEP-04696|609[0n]bp|Canada.Alberta|BOLD:AAA2815
Mythimna oxygala|[12450]|RDLQ658-07|DH007059|552[0n]bp|Canada.Quebec|BOLD:AAA2815
Mythimna oxygala|[12451]|RDNMC193-05|CNCNoctuoideaH005|585[0n]bp|Canada.Quebec|BOLD:AAA2815
Mythimna oxygala|[12452]|XAD365-04|04HBL007365|572[0n]bp|Canada.Ontario|BOLD:AAA2815
Mythimna oxygala|[12453]|RDMAB063-05|UASM57590|573[0n]bp|Canada.Alberta|BOLD:AAA2815
Mythimna oxygala|[12454]|XAD366-04|04HBL007366|587[0n]bp|Canada.Ontario|BOLD:AAA2815
Mythimna yukonensis|[12455]|ABKWR055-07|KENWR 6606|658[0n]bp|United States.Alaska|BOLD:AAA2815
Mythimna oxygala|[12456]|LPSOC336-08|PPBP-2335|658[0n]bp|Canada.Ontario|BOLD:AAA2815
Mythimna yukonensis|[12457]|UAMIC523-13|UAM Insects 131240|599[5n]bp|United States.Alaska|BOLD:AAA2815
Homorthodes communis|[12458]|LBCW058-08|08-JDWWI-0058|658[0n]bp|Canada.British Columbia|BOLD:AAC1982
Homorthodes communis|[12459]|LPVIB667-08|PFC-2006-2116|631[0n]bp|Canada.British Columbia|BOLD:AAC1982

[illegible]
Mythimna yukonensis|[12457]|UAMIC523-13|UAM Insects 131240|599[5n]bp|United States.Alaska|BOLD:AAA2815
Homorthodes communis|[12458]|LBCW058-08|08-JDWWI-0058|658[0n]bp|Canada.British Columbia|BOLD:AAC1982
Homorthodes communis|[12459]|LPVIB667-08|PFC-2006-2116|631[0n]bp|Canada.British Columbia|BOLD:AAC1982
Homorthodes communis|[12460]|LPVIB679-08|PFC-2006-2128|637[1n]bp|Canada.British Columbia|BOLD:AAC1982
Homorthodes communis|[12461]|RDNMG907-08|CNC LEP00053031|658[0n]bp|Canada.British Columbia|BOLD:AAC1982
Homorthodes communis|[12462]|LPVIB765-08|PFC-2006-2230|658[0n]bp|Canada.British Columbia|BOLD:AAC1982
Homorthodes communis|[12463]|LPVIB427-08|PFC-2006-1830|658[0n]bp|Canada.British Columbia|BOLD:AAC1982
Homorthodes communis|[12464]|LPVIB666-08|PFC-2006-2115|575[0n]bp|Canada.British Columbia|BOLD:AAC1982
Homorthodes communis|[12465]|LPVIB673-08|PFC-2006-2122|606[0n]bp|Canada.British Columbia|BOLD:AAC1982
Homorthodes communis|[12466]|LPVIB665-08|PFC-2006-2114|634[0n]bp|Canada.British Columbia|BOLD:AAC1982
Homorthodes communis|[12467]|LPVIB664-08|PFC-2006-2113|601[0n]bp|Canada.British Columbia|BOLD:AAC1982
Homorthodes communis|[12468]|RDNMG908-08|CNC LEP00053032|658[0n]bp|Canada.British Columbia|BOLD:AAC1982
Homorthodes communis|[12469]|LPVIB663-08|PFC-2006-2112|634[0n]bp|Canada.British Columbia|BOLD:AAC1982
Homorthodes communis|[12470]|LPVIB273-08|PFC-2006-1663|658[0n]bp|Canada.British Columbia|BOLD:AAC1982
Homorthodes communis|[12471]|LPVIB495-08|PFC-2006-1905|658[0n]bp|Canada.British Columbia|BOLD:AAC1982
Homorthodes communis|[12472]|LPVIB764-08|PFC-2006-2229|658[0n]bp|Canada.British Columbia|BOLD:AAC1982
Homorthodes communis|[12473]|LPVIB426-08|PFC-2006-1829|658[0n]bp|Canada.British Columbia|BOLD:AAC1982
Homorthodes communis|[12474]|LPVIB672-08|PFC-2006-2121|639[0n]bp|Canada.British Columbia|BOLD:AAC1982
Homorthodes communis|[12475]|LALPA1201-11|AVBC 1203-11|658[0n]bp|Canada.British Columbia|BOLD:AAC1982
Homorthodes communis|[12476]|LPVIB315-08|PFC-2006-1708|658[0n]bp|Canada.British Columbia|BOLD:AAC1982
Homorthodes communis|[12477]|LPVIB272-08|PFC-2006-1662|658[0n]bp|Canada.British Columbia|BOLD:AAC1982
Homorthodes fractura mecrona|[12478]|LALPA1017-11|AVBC 1190-11|658[0n]bp|Canada.British Columbia|BOLD:...
Homorthodes fractura mecrona|[12479]|LALPA979-11|AVBC 1152-11|658[0n]bp|Canada.British Columbia|BOLD:A...
Homorthodes fractura mecrona|[12480]|LALPA934-11|AVBC 1107-11|658[0n]bp|Canada.British Columbia|BOLD:A...
Homorthodes fractura mecrona|[12481]|LALPA504-10|AVBC 506-11|658[0n]bp|Canada.British Columbia|BOLD:AA...
Homorthodes fractura mecrona|[12482]|LALPA287-10|AVBC 288-10|658[0n]bp|Canada.British Columbia|BOLD:AA...
Homorthodes fractura mecrona|[12483]|BBLPB447-10|10BBCLP-1446|658[0n]bp|Canada.British Columbia|BOLD:A...
Homorthodes fractura mecrona|[12484]|LHLEP429-06|UBC-2006-0795|658[0n]bp|Canada.British Columbia|BOLD:...
Homorthodes fractura mecrona|[12485]|LALPA183-10|AVBC 183-10|658[0n]bp|Canada.British Columbia|BOLD:AA...
Homorthodes fractura mecrona|[12486]|LPVIB058-08|PFC-2006-1386|658[0n]bp|Canada.British Columbia|BOLD:...
Homorthodes fractura mecrona|[12487]|LHLEP430-06|UBC-2006-0796|658[0n]bp|Canada.British Columbia|BOLD:...
Homorthodes fractura mecrona|[12488]|RDNMF408-08|NOC14494|652[0n]bp|Canada.British Columbia|BOLD:AAB4377
Homorthodes fractura mecrona|[12489]|LPVIA626-08|PFC-2006-0855|638[0n]bp|Canada.British Columbia|BOLD:...
Homorthodes fractura mecrona|[12490]|LALPA539-10|AVBC 541-10|658[0n]bp|Canada.British Columbia|BOLD:AA...
Homorthodes fractura mecrona|[12491]|LALPA538-10|AVBC 540-10|658[0n]bp|Canada.British Columbia|BOLD:AA...
Homorthodes fractura mecrona|[12492]|LALPA540-10|AVBC 542-10|658[0n]bp|Canada.British Columbia|BOLD:AA...
Homorthodes fractura mecrona|[12493]|LHLEP075-06|UBC-2006-0280|658[0n]bp|Canada.British Columbia|BOLD:...
Pseudanarta crocea|[12494]|LBCH7098-10|10-JDWBC-7098|658[0n]bp|Canada.British Columbia|BOLD:AAD4581
Pseudanarta crocea|[12495]|LBCH7232-10|10-JDWBC-7232|658[0n]bp|Canada.British Columbia|BOLD:AAD4581
Pseudanarta crocea|[12496]|LBCH7352-10|10-JDWBC-7352|658[0n]bp|Canada.British Columbia|BOLD:AAD4581
Pseudanarta crocea|[12497]|LBCH7635-10|10-JDWBC-7635|658[0n]bp|Canada.British Columbia|BOLD:AAD4581
Pseudanarta crocea|[12498]|LBCH7642-10|10-JDWBC-7642|658[1n]bp|Canada.British Columbia|BOLD:AAD4581
Pseudanarta crocea|[12499]|LBCH6386-10|10-JDWBC-6386|658[0n]bp|Canada.British Columbia|BOLD:AAD4581
Pseudanarta crocea|[12500]|RDNMF157-08|NOC14243|658[0n]bp|Canada.British Columbia|BOLD:AAD4581
Pseudanarta crocea|[12501]|LBCH7558-10|10-JDWBC-7558|658[0n]bp|Canada.British Columbia|BOLD:AAD4581
Pseudanarta crocea|[12502]|LBCH7636-10|10-JDWBC-7636|658[0n]bp|Canada.British Columbia|BOLD:AAD4581
Pseudanarta crocea|[12503]|LBCH7640-10|10-JDWBC-7640|658[0n]bp|Canada.British Columbia|BOLD:AAD4581
Pseudanarta crocea|[12504]|LBCH6971-10|10-JDWBC-6971|658[0n]bp|Canada.British Columbia|BOLD:AAD4581
Pseudanarta crocea|[12505]|LBCH6970-10|10-JDWBC-6970|658[0n]bp|Canada.British Columbia|BOLD:AAD4581
Pseudanarta crocea|[12506]|RDNMF154-08|NOC14240|658[0n]bp|Canada.British Columbia|BOLD:AAD4581
Pseudanarta crocea|[12507]|LBCH6965-10|10-JDWBC-6965|658[0n]bp|Canada.British Columbia|BOLD:AAD4581
Pseudanarta crocea|[12508]|LBCH6968-10|10-JDWBC-6968|658[0n]bp|Canada.British Columbia|BOLD:AAD4581
Pseudanarta crocea|[12509]|LBCH6972-10|10-JDWBC-6972|658[0n]bp|Canada.British Columbia|BOLD:AAD4581
Pseudanarta crocea|[12510]|LBCH7637-10|10-JDWBC-7637|658[0n]bp|Canada.British Columbia|BOLD:AAD4581
Pseudanarta crocea|[12511]|LBCH6966-10|10-JDWBC-6966|658[0n]bp|Canada.British Columbia|BOLD:AAD4581
Pseudanarta crocea|[12512]|LBCH7638-10|10-JDWBC-7638|658[0n]bp|Canada.British Columbia|BOLD:AAD4581
Pseudanarta crocea|[12513]|LBCH7639-10|10-JDWBC-7639|658[0n]bp|Canada.British Columbia|BOLD:AAD4581
Pseudanarta crocea|[12514]|RDNMF153-08|NOC14239|658[0n]bp|Canada.British Columbia|BOLD:AAD4581
Pseudanarta crocea|[12515]|LBCH6969-10|10-JDWBC-6969|658[0n]bp|Canada.British Columbia|BOLD:AAD4581
Pseudanarta crocea|[12516]|LBCH7641-10|10-JDWBC-7641|658[0n]bp|Canada.British Columbia|BOLD:AAD4581
Pseudanarta crocea|[12517]|LBCH6967-10|10-JDWBC-6967|658[0n]bp|Canada.British Columbia|BOLD:AAD4581
Pseudanarta flava|[12518]|LALPA818-10|AVBC 820-10|658[0n]bp|Canada.British Columbia|BOLD:AAE0933
Pseudanarta flava|[12519]|LBCH6973-10|10-JDWBC-6973|658[0n]bp|Canada.British Columbia|BOLD:AAE0933
Acronicta funeralis|[12520]|PHMNB187-04|04HBL007652|524[0n]bp|Canada.New Brunswick|BOLD:AAB7001
Acronicta funeralis|[12521]|PHMNB155-04|04HBL007620|653[1n]bp|Canada.New Brunswick|BOLD:AAB7001
Acronicta funeralis|[12522]|PHMNB002-03|moth102.02SA|639[0n]bp|Canada.New Brunswick|BOLD:AAB7001
Acronicta funeralis|[12523]|PHMNB070-03|moth62.02SA|639[0n]bp|Canada.New Brunswick|BOLD:AAB7001
Acronicta funeralis|[12524]|PHMNB246-04|04HBL007711|658[0n]bp|Canada.New Brunswick|BOLD:AAB7001
Acronicta funeralis|[12525]|RDLQB159-05|DH010245|658[0n]bp|Canada.Quebec|BOLD:AAB7001
Acronicta funeralis|[12526]|MNBB245-05|05-NBSTA-161|658[0n]bp|Canada.New Brunswick|BOLD:AAB7001
Acronicta funeralis|[12527]|RDNMI017-10|CNCLEP 69807|658[0n]bp|United States.California|BOLD:AAB7001
Acronicta funeralis|[12528]|RDLQF512-06|DH011661|658[0n]bp|Canada.Quebec|BOLD:AAB7001
Acronicta funeralis|[12529]|TMNBB155-06|MNBTT-1095|658[0n]bp|Canada.New Brunswick|BOLD:AAB7001
Acronicta funeralis|[12530]|TMNBB156-06|MNBTT-1096|658[0n]bp|Canada.New Brunswick|BOLD:AAB7001
Acronicta funeralis|[12531]|PHMNB770-05|Moth 463.03SA|658[0n]bp|Canada.New Brunswick|BOLD:AAB7001
Acronicta funeralis|[12532]|LPABC804-09|08BBLEP-05023|658[0n]bp|Canada.Alberta|BOLD:AAB7001
Acronicta funeralis|[12533]|RDLQF510-06|DH011659|658[0n]bp|Canada.Quebec|BOLD:AAB7001
Acronicta funeralis|[12534]|LOTB311-05|05-TN-00311|658[0n]bp|United States.Tennessee|BOLD:AAB7001
Acronicta funeralis|[12535]|PHMNB695-04|04HBL00921|658[0n]bp|Canada.New Brunswick|BOLD:AAB7001
Acronicta funeralis|[12536]|RDLQF511-06|DH011660|658[0n]bp|Canada.Quebec|BOLD:AAB7001
Acronicta funeralis|[12537]|RDNMJ083-10|CNCLEP 73862|658[0n]bp|United States.Texas|BOLD:AAB7001
Acronicta funeralis|[12538]|LPABB388-08|08BBLEP-03653|658[0n]bp|Canada.Alberta|BOLD:AAB7001
Acronicta funeralis|[12539]|PHMNB578-04|04HBL00804|658[0n]bp|Canada.New Brunswick|BOLD:AAB7001
Acronicta funeralis|[12540]|RDNMJ622-11|acrorev gga52|622[0n]bp|United States.Texas|BOLD:AAB7001
Acronicta funeralis|[12541]|RDNMJ621-11|acrorev gga51|623[0n]bp|United States.Texas|BOLD:AAB7001
Acronicta funeralis|[12542]|UDLEP252-09|v212 TY|658[0n]bp|United States.Pennsylvania|BOLD:AAB7001
Acronicta funeralis|[12543]|RDLQB158-05|DH010244|658[0n]bp|Canada.Quebec|BOLD:AAB7001
Chytonix sensilis|[12544]|PSAT117-10|CNCLEP 70002|658[0n]bp|Canada.Ontario|BOLD:AAD8379
Chytonix sensilis|[12545]|RDLQF375-06|DH011442|658[0n]bp|Canada.Quebec|BOLD:AAD8379
Chytonix sensilis|[12546]|RDLQF374-06|DH011441|658[0n]bp|Canada.Quebec|BOLD:AAD8379
Dypterygia rozmani|[12547]|RDLQF563-06|DH011712|658[0n]bp|Canada.Quebec|BOLD:AAE8028
Dypterygia rozmani|[12548]|RDLQB561-05|DH010647|658[0n]bp|Canada.Quebec|BOLD:AAE8028
Dypterygia rozmani|[12549]|RDLQG058-06|DH012189|658[0n]bp|Canada.Quebec|BOLD:AAE8028
Acronicta americana|[12550]|MNBB149-05|05-NBSTA-065|658[0n]bp|Canada.New Brunswick|BOLD:AAA5139
Acronicta americana|[12551]|MNBB150-05|05-NBSTA-066|658[0n]bp|Canada.New Brunswick|BOLD:AAA5139
Acronicta americana|[12552]|MNBB553-05|05-NBSTA-469|658[0n]bp|Canada.New Brunswick|BOLD:AAA5139
Acronicta americana|[12553]|BLTIB1062-08|BL1071|658[0n]bp|Canada.Ontario|BOLD:AAA5139
Acronicta americana|[12554]|MNBB320-05|05-NBSTA-236|658[0n]bp|Canada.New Brunswick|BOLD:AAA5139
Acronicta americana|[12555]|BBLPE314-09|09BBELE-2314|658[0n]bp|Canada.Newfoundland and Labrador|BOLD:A...
Acronicta americana|[12556]|BBLPE520-09|09BBELE-2520|658[0n]bp|Canada.Newfoundland and Labrador|BOLD:A...
Acronicta americana|[12557]|BBLPE522-09|09BBELE-2522|658[0n]bp|Canada.Newfoundland and Labrador|BOLD:A...
Acronicta americana|[12558]|MNBB147-05|05-NBSTA-063|658[0n]bp|Canada.New Brunswick|BOLD:AAA5139
[illegible]

Acronicta americana|[12556]|BBLPE520-09|09BBELE-2520|658[0n]bp|Canada.Newfoundland and Labrador|BOLD:A...
Acronicta americana|[12557]|BBLPE522-09|09BBELE-2522|658[0n]bp|Canada.Newfoundland and Labrador|BOLD:A...
Acronicta americana|[12558]|MNBB147-05|05-NBSTA-063|658[0n]bp|Canada.New Brunswick|BOLD:AAA5139
Acronicta americana|[12559]|BBLPE531-09|09BBELE-2531|658[0n]bp|Canada.Newfoundland and Labrador|BOLD:A...
Acronicta americana|[12560]|XAC618-04|04HBL006618|658[0n]bp|Canada.Ontario|BOLD:AAA5139
Acronicta americana|[12561]|MNBB266-05|05-NBSTA-182|658[0n]bp|Canada.New Brunswick|BOLD:AAA5139
Acronicta americana|[12562]|BBLPE523-09|09BBELE-2523|658[0n]bp|Canada.Newfoundland and Labrador|BOLD:A...
Acronicta americana|[12563]|XAE547-04|Moth4547.03|617[0n]bp|Canada.Ontario|BOLD:AAA5139
Acronicta americana|[12564]|BBLPE482-09|09BBELE-2482|632[0n]bp|Canada.Newfoundland and Labrador|BOLD:A...
Acronicta americana|[12565]|PHMO390-03|moth971.01|639[0n]bp|Canada.Ontario|BOLD:AAA5139
Acronicta americana|[12566]|PHMNB076-03|moth80.02SA|639[0n]bp|Canada.New Brunswick|BOLD:AAA5139
Acronicta americana|[12567]|LPSOC283-08|PPBP-2282|658[0n]bp|Canada.Ontario|BOLD:AAA5139
Acronicta americana|[12568]|BBLPE521-09|09BBELE-2521|658[0n]bp|Canada.Newfoundland and Labrador|BOLD:A...
Acronicta americana|[12569]|XAK152-06|2006-ONT-1147|658[0n]bp|Canada.Ontario|BOLD:AAA5139
Acronicta americana|[12570]|BBLEC409-09|09BBELE-0409|658[0n]bp|Canada.Newfoundland and Labrador|BOLD:A...
Acronicta americana|[12571]|XAB120-04|04HBL005120|658[0n]bp|Canada.Ontario|BOLD:AAA5139
Acronicta americana|[12572]|MNBB420-05|05-NBSTA-336|658[0n]bp|Canada.New Brunswick|BOLD:AAA5139
Acronicta americana|[12573]|MNBB588-05|05-NBSTA-504|658[0n]bp|Canada.New Brunswick|BOLD:AAA5139
Acronicta americana|[12574]|MNBB155-05|05-NBSTA-071|658[0n]bp|Canada.New Brunswick|BOLD:AAA5139
Acronicta americana|[12575]|MNBB178-05|05-NBSTA-094|658[0n]bp|Canada.New Brunswick|BOLD:AAA5139
Acronicta americana|[12576]|BBLPE371-09|09BBELE-2371|658[0n]bp|Canada.Newfoundland and Labrador|BOLD:A...
Acronicta americana|[12577]|MNBB270-05|05-NBSTA-186|658[0n]bp|Canada.New Brunswick|BOLD:AAA5139
Acronicta americana|[12578]|PHMNB586-04|04HBL00812|658[0n]bp|Canada.New Brunswick|BOLD:AAA5139
Acronicta americana|[12579]|MNBB354-05|05-NBSTA-270|658[0n]bp|Canada.New Brunswick|BOLD:AAA5139
Acronicta americana|[12580]|MNBB221-05|05-NBSTA-137|658[0n]bp|Canada.New Brunswick|BOLD:AAA5139
Acronicta americana|[12581]|MNBB375-05|05-NBSTA-291|658[0n]bp|Canada.New Brunswick|BOLD:AAA5139
Acronicta americana|[12582]|RDLQ442-07|DH008864|658[0n]bp|Canada.Quebec|BOLD:AAA5139
Acronicta americana|[12583]|MNBB224-05|05-NBSTA-140|658[0n]bp|Canada.New Brunswick|BOLD:AAA5139
Acronicta americana|[12584]|MNBB222-05|05-NBSTA-138|658[0n]bp|Canada.New Brunswick|BOLD:AAA5139
Acronicta americana|[12585]|MNBB223-05|05-NBSTA-139|658[0n]bp|Canada.New Brunswick|BOLD:AAA5139
Acronicta americana|[12586]|MNBB151-05|05-NBSTA-067|658[0n]bp|Canada.New Brunswick|BOLD:AAA5139
Acronicta americana|[12587]|BBLPE299-09|09BBELE-2299|658[0n]bp|Canada.Newfoundland and Labrador|BOLD:A...
Acronicta americana|[12588]|BBLEC042-09|09BBELE-0042|658[0n]bp|Canada.New Brunswick|BOLD:AAA5139
Acronicta americana|[12589]|MNBB219-05|05-NBSTA-135|658[0n]bp|Canada.New Brunswick|BOLD:AAA5139
Acronicta americana|[12590]|MNBB589-05|05-NBSTA-505|658[0n]bp|Canada.New Brunswick|BOLD:AAA5139
Acronicta americana|[12591]|MNBB269-05|05-NBSTA-185|658[0n]bp|Canada.New Brunswick|BOLD:AAA5139
Acronicta americana|[12592]|MNBB092-05|05-NBSTA-008|658[0n]bp|Canada.New Brunswick|BOLD:AAA5139
Acronicta americana|[12593]|BBLPE532-09|09BBELE-2532|658[0n]bp|Canada.Newfoundland and Labrador|BOLD:A...
Acronicta americana|[12594]|PHMNB351-04|04HBL00577|654[0n]bp|Canada.New Brunswick|BOLD:AAA5139
Acronicta americana|[12595]|BBLPE300-09|09BBELE-2300|648[0n]bp|Canada.Newfoundland and Labrador|BOLD:A...
Acronicta americana|[12596]|XAE550-04|Moth4550.03|625[0n]bp|Canada.Ontario|BOLD:AAA5139
Acronicta americana|[12597]|XAB179-04|04HBL005179|658[0n]bp|Canada.Ontario|BOLD:AAA5139
Acronicta americana|[12598]|XAC571-04|04HBL006571|658[0n]bp|Canada.Ontario|BOLD:AAA5139
Acronicta americana|[12599]|MNBB218-05|05-NBSTA-134|658[0n]bp|Canada.New Brunswick|BOLD:AAA5139
Acronicta americana|[12600]|PHMNB741-05|Moth 434.03SA|658[0n]bp|Canada.New Brunswick|BOLD:AAA5139
Acronicta americana|[12601]|RDLQ441-07|DH012151|658[0n]bp|Canada.Quebec|BOLD:AAA5139
Acronicta americana|[12602]|BBLPE483-09|09BBELE-2483|658[0n]bp|Canada.Newfoundland and Labrador|BOLD:A...
Acronicta dactylina|[12603]|RDNMK507-11|CNCLEP 70898|658[0n]bp|Canada.Ontario|BOLD:AAJ1616
Acronicta dactylina|[12604]|RDNMJ473-11|CNCLEP 80001|658[0n]bp|Canada.Ontario|BOLD:AAJ1616
Acronicta dactylina|[12605]|PHMO124-03|moth737.01|639[0n]bp|Canada.Ontario|BOLD:AAJ1616
Acronicta dactylina|[12606]|PMG081-03|moth682.01|617[0n]bp|Canada.Ontario|BOLD:AAJ1616
Acronicta dactylina|[12607]|RDNMK663-11|CNCLEP 81840|658[0n]bp|Canada.Ontario|BOLD:AAJ1616
Acronicta dactylina|[12608]|MNBB226-05|05-NBSTA-142|658[0n]bp|Canada.New Brunswick|BOLD:AAA2802
Acronicta dactylina|[12609]|MNBB268-05|05-NBSTA-184|658[0n]bp|Canada.New Brunswick|BOLD:AAA2802
Acronicta dactylina|[12610]|LHLEP367-06|UBC-2006-0748|657[0n]bp|Canada.British Columbia|BOLD:AAA2802
Acronicta dactylina|[12611]|MNBB513-05|05-NBSTA-429|658[0n]bp|Canada.New Brunswick|BOLD:AAA2802
Acronicta dactylina|[12612]|MNBB148-05|05-NBSTA-064|658[0n]bp|Canada.New Brunswick|BOLD:AAA2802
Acronicta dactylina|[12613]|RDLQB151-05|DH010237|658[0n]bp|Canada.Quebec|BOLD:AAA2802
Acronicta dactylina|[12614]|XAC177-04|04HBL006177|656[0n]bp|Canada.Ontario|BOLD:AAA2802
Acronicta dactylina|[12615]|MNBB158-05|05-NBSTA-074|658[0n]bp|Canada.New Brunswick|BOLD:AAA2802
Acronicta dactylina|[12616]|LALPA909-11|AVBC 1082-11|658[0n]bp|Canada.British Columbia|BOLD:AAA2802
Acronicta dactylina|[12617]|LALPA1011-11|AVBC 1184-11|658[0n]bp|Canada.British Columbia|BOLD:AAA2802
Acronicta dactylina|[12618]|LOWCB855-05|CGWC-1795|658[0n]bp|Canada.British Columbia|BOLD:AAA2802
Acronicta dactylina|[12619]|TMNBB152-06|MNBTT-1092|656[0n]bp|Canada.New Brunswick|BOLD:AAA2802
Acronicta dactylina|[12620]|LBCA467-05|HLC-20467|627[0n]bp|Canada.British Columbia|BOLD:AAA2802
Acronicta dactylina|[12621]|LBCA483-05|HLC-20483|632[0n]bp|Canada.British Columbia|BOLD:AAA2802
Acronicta dactylina|[12622]|LBCA352-05|HLC-20352|632[0n]bp|Canada.British Columbia|BOLD:AAA2802
Acronicta dactylina|[12623]|LBCS640-07|UBC-2007-0340|658[0n]bp|Canada.British Columbia|BOLD:AAA2802
Acronicta dactylina|[12624]|TTMNB320-06|MNBTT-320|658[3n]bp|Canada.New Brunswick|BOLD:AAA2802
Acronicta dactylina|[12625]|LBCB177-05|HLC-21117|658[0n]bp|Canada.British Columbia|BOLD:AAA2802
Acronicta dactylina|[12626]|RDNMK661-11|CNCLEP 81838|658[0n]bp|Canada.British Columbia|BOLD:AAA2802
Acronicta dactylina|[12627]|BBLPB667-10|10BBCLP-1666|658[0n]bp|Canada.British Columbia|BOLD:AAA2802
Acronicta dactylina|[12628]|LBCA466-05|HLC-20466|651[0n]bp|Canada.British Columbia|BOLD:AAA2802
Acronicta dactylina|[12629]|MNBB661-05|05-NBSTA-577|633[0n]bp|Canada.New Brunswick|BOLD:AAA2802
Acronicta dactylina|[12630]|LBCA746-05|HLC-20746|631[0n]bp|Canada.British Columbia|BOLD:AAA2802
Acronicta dactylina|[12631]|LBCS641-07|UBC-2007-0341|658[0n]bp|Canada.British Columbia|BOLD:AAA2802
Acronicta dactylina|[12632]|MNBB353-05|05-NBSTA-269|658[0n]bp|Canada.New Brunswick|BOLD:AAA2802
Acronicta dactylina|[12633]|PHMNB317-04|04HBL00543|658[0n]bp|Canada.New Brunswick|BOLD:AAA2802
Acronicta dactylina|[12634]|LALPA666-10|AVBC 668-10|658[0n]bp|Canada.British Columbia|BOLD:AAA2802
Acronicta dactylina|[12635]|MNBB473-05|05-NBSTA-389|658[0n]bp|Canada.New Brunswick|BOLD:AAA2802
Acronicta dactylina|[12636]|LHLEP370-06|UBC-2006-0932|657[0n]bp|Canada.British Columbia|BOLD:AAA2802
Acronicta dactylina|[12637]|LMH010-06|PFC-2006-0012|658[1n]bp|Canada.British Columbia|BOLD:AAA2802
Acronicta dactylina|[12638]|XAE367-04|Moth4367.03|658[0n]bp|Canada.Ontario|BOLD:AAA2802
Acronicta dactylina|[12639]|RDLQB152-05|DH010238|658[0n]bp|Canada.Quebec|BOLD:AAA2802
Acronicta dactylina|[12640]|MNBB555-05|05-NBSTA-471|658[0n]bp|Canada.New Brunswick|BOLD:AAA2802
Acronicta dactylina|[12641]|TMNBB154-06|MNBTT-1094|658[0n]bp|Canada.New Brunswick|BOLD:AAA2802
Acronicta dactylina|[12642]|MNBB160-05|05-NBSTA-076|658[0n]bp|Canada.New Brunswick|BOLD:AAA2802
Acronicta dactylina|[12643]|PHMNB742-05|Moth 435.03SA|658[0n]bp|Canada.New Brunswick|BOLD:AAA2802
Acronicta dactylina|[12644]|MNBB154-05|05-NBSTA-070|658[0n]bp|Canada.New Brunswick|BOLD:AAA2802
Acronicta dactylina|[12645]|RDLQB797-05|DH010884|658[0n]bp|Canada.Quebec|BOLD:AAA2802
Acronicta dactylina|[12646]|XAI057-05|0102-ONT-0057|658[0n]bp|Canada.Ontario|BOLD:AAA2802
Acronicta dactylina|[12647]|MNBB273-05|05-NBSTA-189|658[0n]bp|Canada.New Brunswick|BOLD:AAA2802
Acronicta dactylina|[12648]|RDLQG137-06|DH012304|658[3n]bp|Canada.Quebec|BOLD:AAA2802
Acronicta dactylina|[12649]|MNBB227-05|05-NBSTA-143|658[0n]bp|Canada.New Brunswick|BOLD:AAA2802
Acronicta dactylina|[12650]|XAC654-04|04HBL006654|658[0n]bp|Canada.Ontario|BOLD:AAA2802
Acronicta dactylina|[12651]|LPMN666-08|08BBLEP-01467|658[0n]bp|Canada.Manitoba|BOLD:AAA2802
Acronicta dactylina|[12652]|XAF572-05|2005-ONT-221|658[0n]bp|Canada.Ontario|BOLD:AAA2802
Acronicta dactylina|[12653]|LHLEP364-06|UBC-2006-0620|657[0n]bp|Canada.British Columbia|BOLD:AAA2802
Acronicta dactylina|[12654]|MNBB267-05|05-NBSTA-183|658[0n]bp|Canada.New Brunswick|BOLD:AAA2802
Acronicta dactylina|[12655]|MNBB587-05|05-NBSTA-503|658[0n]bp|Canada.New Brunswick|BOLD:AAA2802
Acronicta dactylina|[12656]|MNBB376-05|05-NBSTA-292|658[0n]bp|Canada.New Brunswick|BOLD:AAA2802
Acronicta dactylina|[12657]|LHLEP365-06|UBC-2006-0746|657[0n]bp|Canada.British Columbia|BOLD:AAA2802
Acronicta dactylina|[12658]|BBLPC670-09|09BBELE-1670|658[0n]bp|Canada.Newfoundland and Labrador|BOLD:A...

Acronicta dactylina|[12656]|MNBB376-05|05-NBSTA-292|658[0n]bp|Canada.New Brunswick|BOLD:AAA2802
Acronicta dactylina|[12657]|LHLEP365-06|UBC-2006-0746|657[0n]bp|Canada.British Columbia|BOLD:AAA2802
Acronicta dactylina|[12658]|BBLPC670-09|09BBELE-1670|658[0n]bp|Canada.Newfoundland and Labrador|BOLD:A...
Acronicta dactylina|[12659]|LBCS642-07|UBC-2007-0342|658[0n]bp|Canada.British Columbia|BOLD:AAA2802
Acronicta dactylina|[12660]|BBLEC839-09|09BBELE-0839|658[0n]bp|Canada.Newfoundland and Labrador|BOLD:A...
Acronicta dactylina|[12661]|LHLEP308-06|UBC-2006-1355|657[0n]bp|Canada.British Columbia|BOLD:AAA2802
Acronicta dactylina|[12662]|TTMNB318-06|MNBTT-318|658[0n]bp|Canada.New Brunswick|BOLD:AAA2802
Acronicta dactylina|[12663]|LPSOC082-08|PPBP-2081|658[0n]bp|Canada.Ontario|BOLD:AAA2802
Acronicta dactylina|[12664]|MNBB662-05|05-NBSTA-578|658[0n]bp|Canada.New Brunswick|BOLD:AAA2802
Acronicta dactylina|[12665]|MNBB554-05|05-NBSTA-470|658[0n]bp|Canada.New Brunswick|BOLD:AAA2802
Acronicta dactylina|[12666]|MNBB272-05|05-NBSTA-188|658[0n]bp|Canada.New Brunswick|BOLD:AAA2802
Acronicta dactylina|[12667]|LPMN667-08|08BBLEP-01468|658[0n]bp|Canada.Manitoba|BOLD:AAA2802
Acronicta dactylina|[12668]|LHLEP362-06|UBC-2006-0465|657[0n]bp|Canada.British Columbia|BOLD:AAA2802
Acronicta dactylina|[12669]|PHMNB763-05|Moth 456.03SA|658[0n]bp|Canada.New Brunswick|BOLD:AAA2802
Acronicta dactylina|[12670]|LPSOC081-08|PPBP-2080|658[0n]bp|Canada.Ontario|BOLD:AAA2802
Acronicta dactylina|[12671]|LALPA1199-11|AVBC 1201-11|658[0n]bp|Canada.British Columbia|BOLD:AAA2802
Acronicta dactylina|[12672]|MNBB271-05|05-NBSTA-187|658[0n]bp|Canada.New Brunswick|BOLD:AAA2802
Acronicta dactylina|[12673]|RDNMB877-05|CNCNoctuoidea10652|658[0n]bp|Canada.New Brunswick|BOLD:AAA2802
Acronicta dactylina|[12674]|MNBB093-05|05-NBSTA-009|658[0n]bp|Canada.New Brunswick|BOLD:AAA2802
Acronicta dactylina|[12675]|XAJ580-06|2006-ONT-0580|658[0n]bp|Canada.Ontario|BOLD:AAA2802
Acronicta dactylina|[12676]|MNBB512-05|05-NBSTA-428|658[0n]bp|Canada.New Brunswick|BOLD:AAA2802
Acronicta dactylina|[12677]|MNBB217-05|05-NBSTA-133|658[0n]bp|Canada.New Brunswick|BOLD:AAA2802
Acronicta dactylina|[12678]|MNBB296-05|05-NBSTA-212|658[0n]bp|Canada.New Brunswick|BOLD:AAA2802
Acronicta dactylina|[12679]|XAF455-05|2005-ONT-104|658[0n]bp|Canada.Ontario|BOLD:AAA2802
Acronicta dactylina|[12680]|XAB476-04|04HBL005476|658[0n]bp|Canada.Ontario|BOLD:AAA2802
Acronicta dactylina|[12681]|LHLEP358-06|UBC-2006-0169|657[0n]bp|Canada.British Columbia|BOLD:AAA2802
Acronicta dactylina|[12682]|PHMNB768-05|Moth 461.03SA|658[0n]bp|Canada.New Brunswick|BOLD:AAA2802
Acronicta dactylina|[12683]|LHLEP366-06|UBC-2006-0747|657[0n]bp|Canada.British Columbia|BOLD:AAA2802
Acronicta dactylina|[12684]|LALPA1121-11|AVBC 931-11|658[0n]bp|Canada.British Columbia|BOLD:AAA2802
Acronicta dactylina|[12685]|LPVIA508-08|PFC-2006-0719|658[0n]bp|Canada.British Columbia|BOLD:AAA2802
Acronicta dactylina|[12686]|RDLQ122-05|DH007134|658[0n]bp|Canada.Quebec|BOLD:AAA2802
Acronicta dactylina|[12687]|MNBB660-05|05-NBSTA-576|658[0n]bp|Canada.New Brunswick|BOLD:AAA2802
Acronicta dactylina|[12688]|PHMNB584-04|04HBL00810|658[0n]bp|Canada.New Brunswick|BOLD:AAA2802
Acronicta dactylina|[12689]|MNBB322-05|05-NBSTA-238|658[0n]bp|Canada.New Brunswick|BOLD:AAA2802
Acronicta dactylina|[12690]|TTMNB321-06|MNBTT-321|658[0n]bp|Canada.New Brunswick|BOLD:AAA2802
Acronicta dactylina|[12691]|TMNBB153-06|MNBTT-1093|658[0n]bp|Canada.New Brunswick|BOLD:AAA2802
Acronicta dactylina|[12692]|BBLEC356-09|09BBELE-0356|658[0n]bp|Canada.Newfoundland and Labrador|BOLD:A...
Acronicta dactylina|[12693]|MNBB153-05|05-NBSTA-069|658[0n]bp|Canada.New Brunswick|BOLD:AAA2802
Acronicta dactylina|[12694]|BBLPC632-09|09BBELE-1632|658[0n]bp|Canada.Newfoundland and Labrador|BOLD:A...
Acronicta dactylina|[12695]|MNBB647-05|05-NBSTA-563|658[0n]bp|Canada.New Brunswick|BOLD:AAA2802
Acronicta dactylina|[12696]|BBLPC639-09|09BBELE-1639|658[0n]bp|Canada.Newfoundland and Labrador|BOLD:A...
Acronicta dactylina|[12697]|MNBB071-05|HBL008681|658[0n]bp|Canada.New Brunswick|BOLD:AAA2802
Acronicta dactylina|[12698]|LPMN565-08|08BBLEP-01366|658[0n]bp|Canada.Manitoba|BOLD:AAA2802
Acronicta dactylina|[12699]|MNBB157-05|05-NBSTA-073|658[0n]bp|Canada.New Brunswick|BOLD:AAA2802
Acronicta dactylina|[12700]|PHMNB438-04|04HBL00664|658[0n]bp|Canada.New Brunswick|BOLD:AAA2802
Acronicta dactylina|[12701]|LHLEP360-06|UBC-2006-0463|657[0n]bp|Canada.British Columbia|BOLD:AAA2802
Acronicta dactylina|[12702]|MNBB511-05|05-NBSTA-427|658[0n]bp|Canada.New Brunswick|BOLD:AAA2802
Acronicta dactylina|[12703]|PHMNB587-04|04HBL00813|658[0n]bp|Canada.New Brunswick|BOLD:AAA2802
Acronicta dactylina|[12704]|RDNMJ474-11|CNCLEP 80002|658[0n]bp|Canada.Ontario|BOLD:AAA2802
Acronicta dactylina|[12705]|XAC617-04|04HBL006617|658[0n]bp|Canada.Ontario|BOLD:AAA2802
Acronicta dactylina|[12706]|LHLEP368-06|UBC-2006-0749|657[0n]bp|Canada.British Columbia|BOLD:AAA2802
Acronicta dactylina|[12707]|TTMNB319-06|MNBTT-319|658[0n]bp|Canada.New Brunswick|BOLD:AAA2802
Acronicta dactylina|[12708]|LHLEP363-06|UBC-2006-0466|657[0n]bp|Canada.British Columbia|BOLD:AAA2802
Acronicta dactylina|[12709]|MNBB377-05|05-NBSTA-293|622[0n]bp|Canada.New Brunswick|BOLD:AAA2802
Acronicta dactylina|[12710]|LALPA1015-11|AVBC 1188-11|658[0n]bp|Canada.British Columbia|BOLD:AAA2802
Acronicta dactylina|[12711]|LPVIA838-08|PFC-2006-1136|658[0n]bp|Canada.British Columbia|BOLD:AAA2802
Acronicta dactylina|[12712]|MNBB159-05|05-NBSTA-075|658[0n]bp|Canada.New Brunswick|BOLD:AAA2802
Acronicta dactylina|[12713]|LHLEP369-06|UBC-2006-0750|649[0n]bp|Canada.British Columbia|BOLD:AAA2802
Acronicta dactylina|[12714]|LHLEP361-06|UBC-2006-0464|657[0n]bp|Canada.British Columbia|BOLD:AAA2802
Acronicta dactylina|[12715]|PHMNB701-04|04HBL00927|658[0n]bp|Canada.New Brunswick|BOLD:AAA2802
Acronicta dactylina|[12716]|RDLQB793-05|DH010880|658[0n]bp|Canada.Quebec|BOLD:AAA2802
Acronicta dactylina|[12717]|RDLQ120-05|DH002078|658[0n]bp|Canada.Quebec|BOLD:AAA2802
Acronicta dactylina|[12718]|PHMNB316-04|04HBL00542|658[0n]bp|Canada.New Brunswick|BOLD:AAA2802
Acronicta dactylina|[12719]|RDLQB150-05|DH010236|658[0n]bp|Canada.Quebec|BOLD:AAA2802
Acronicta dactylina|[12720]|MNBB602-05|05-NBSTA-518|658[0n]bp|Canada.New Brunswick|BOLD:AAA2802
Acronicta dactylina|[12721]|PHMNB266-04|04HBL007731|609[0n]bp|Canada.New Brunswick|BOLD:AAA2802
Acronicta dactylina|[12722]|LPVIB874-08|PFC-2006-2391|647[0n]bp|Canada.British Columbia|BOLD:AAA2802
Acronicta dactylina|[12723]|RDLQ123-05|DH007508|648[0n]bp|Canada.Quebec|BOLD:AAA2802
Acronicta dactylina|[12724]|LHLEP371-06|UBC-2006-1011|648[0n]bp|Canada.British Columbia|BOLD:AAA2802
Acronicta dactylina|[12725]|LHLEP372-06|UBC-2006-1471|631[0n]bp|Canada.British Columbia|BOLD:AAA2802
Acronicta dactylina|[12726]|LPMN313-08|08BBLEP-01112|636[0n]bp|Canada.Manitoba|BOLD:AAA2802
Acronicta dactylina|[12727]|PHMNB013-03|moth136.02SA|639[0n]bp|Canada.New Brunswick|BOLD:AAA2802
Acronicta dactylina|[12728]|LHLEP359-06|UBC-2006-0462|623[0n]bp|Canada.British Columbia|BOLD:AAA2802
Acronicta dactylina|[12729]|LPVIA629-08|PFC-2006-0861|634[0n]bp|Canada.British Columbia|BOLD:AAA2802
Acronicta dactylina|[12730]|LPVIA967-08|PFC-2006-1293|627[0n]bp|Canada.British Columbia|BOLD:AAA2802
Acronicta dactylina|[12731]|MNBB220-05|05-NBSTA-136|539[0n]bp|Canada.New Brunswick|BOLD:AAA2802
Acronicta dactylina|[12732]|MNBB228-05|05-NBSTA-144|543[0n]bp|Canada.New Brunswick|BOLD:AAA2802
Acronicta dactylina|[12733]|MNBB321-05|05-NBSTA-237|576[2n]bp|Canada.New Brunswick|BOLD:AAA2802
Acronicta dactylina|[12734]|MNBB225-05|05-NBSTA-141|585[0n]bp|Canada.New Brunswick|BOLD:AAA2802
Acronicta dactylina|[12735]|RDLQ121-05|DH007748|589[0n]bp|Canada.Quebec|BOLD:AAA2802
Acronicta dactylina|[12736]|PHMNB762-05|Moth 455.03SA|608[0n]bp|Canada.New Brunswick|BOLD:AAA2802
Acronicta rubricoma|[12737]|LPSO870-08|PPBP-0870|611[2n]bp|Canada.Ontario|BOLD:AAB6117
Acronicta rubricoma|[12738]|LPSO139-08|PPBP-0139|658[0n]bp|Canada.Ontario|BOLD:AAB6117
Acronicta rubricoma|[12739]|LPSO954-08|PPBP-0954|658[0n]bp|Canada.Ontario|BOLD:AAB6117
Acronicta rubricoma|[12740]|LPSO349-08|PPBP-0349|658[0n]bp|Canada.Ontario|BOLD:AAB6117
Acronicta rubricoma|[12741]|LPSO450-08|PPBP-0450|658[0n]bp|Canada.Ontario|BOLD:AAB6117
Acronicta rubricoma|[12742]|LPSO739-08|PPBP-0739|658[0n]bp|Canada.Ontario|BOLD:AAB6117
Acronicta rubricoma|[12743]|LPSO649-08|PPBP-0649|645[0n]bp|Canada.Ontario|BOLD:AAB6117
Acronicta rubricoma|[12744]|LPSO843-08|PPBP-0843|609[1n]bp|Canada.Ontario|BOLD:AAB6117
Acronicta rubricoma|[12745]|LPSO845-08|PPBP-0845|658[0n]bp|Canada.Ontario|BOLD:AAB6117
Acronicta rubricoma|[12746]|LPSO363-08|PPBP-0363|658[0n]bp|Canada.Ontario|BOLD:AAB6117
Acronicta rubricoma|[12747]|LPSO869-08|PPBP-0869|658[0n]bp|Canada.Ontario|BOLD:AAB6117
Acronicta rubricoma|[12748]|LPSO342-08|PPBP-0342|658[0n]bp|Canada.Ontario|BOLD:AAB6117
Acronicta rubricoma|[12749]|LPSO738-08|PPBP-0738|658[0n]bp|Canada.Ontario|BOLD:AAB6117
Acronicta impressa|[12750]|LBCC003-05|HLC-21883|658[0n]bp|Canada.British Columbia|BOLD:ACF2279
Acronicta impressa|[12751]|LBCC762-05|HLC-22642|658[0n]bp|Canada.British Columbia|BOLD:ACF2279
Acronicta impressa|[12752]|RDNMK838-12|CNCLEP73163|658[0n]bp|Canada.British Columbia|BOLD:ACF2279
Acronicta impressa|[12753]|RDNMK767-12|acrorev189|658[0n]bp|Canada.Alberta|BOLD:ACF2279
Acronicta impressa|[12754]|LBCC291-05|HLC-22171|658[0n]bp|Canada.British Columbia|BOLD:ACF2279
Acronicta impressa|[12755]|RDLQ192-05|05HBLT0192|553[0n]bp|Canada.Quebec|BOLD:AAA7955
Acronicta impressa|[12756]|RDNMK765-12|acrorev187|658[0n]bp|Canada.Alberta|BOLD:AAA7955
Acronicta impressa|[12757]|XAF406-05|HLC-10447|658[0n]bp|Canada.Ontario|BOLD:AAA7955
Acronicta impressa|[12758]|RDLQ143-05|DH007401|658[0n]bp|Canada.Quebec|BOLD:AAA7955

[illegible]
Acronicta impressa|[12756]|RDNMK765-12|acrorev187|658[0n]bp|Canada.Alberta|BOLD:AAA7955
Acronicta impressa|[12757]|XAF406-05|HLC-10447|658[0n]bp|Canada.Ontario|BOLD:AAA7955
Acronicta impressa|[12758]|RDLQ143-05|DH007401|658[0n]bp|Canada.Quebec|BOLD:AAA7955
Acronicta impressa|[12759]|RDNMB608-05|CNCNoctuoidea10384|658[0n]bp|Canada.Quebec|BOLD:AAA7955
Acronicta impressa|[12760]|RDNMB610-05|CNCNoctuoidea10386|658[0n]bp|Canada.Quebec|BOLD:AAA7955
Acronicta impressa|[12761]|TTMNB009-06|MNBTT-009|657[0n]bp|Canada.New Brunswick|BOLD:AAA7955
Acronicta impressa|[12762]|RDLQB167-05|DH010253|658[0n]bp|Canada.Quebec|BOLD:AAA7955
Acronicta impressa|[12763]|RDLQ299-05|DH007442|593[0n]bp|Canada.Quebec|BOLD:AAA7955
Acronicta impressa|[12764]|TTMNB007-06|MNBTT-007|656[0n]bp|Canada.New Brunswick|BOLD:AAA7955
Acronicta impressa|[12765]|TTMNB005-06|MNBTT-005|656[0n]bp|Canada.New Brunswick|BOLD:AAA7955
Acronicta impressa|[12766]|RDLQF690-06|DH011840|637[0n]bp|Canada.Quebec|BOLD:AAA7955
Acronicta impressa|[12767]|TMG114-03|moth364.01|639[0n]bp|Canada.Ontario|BOLD:AAA7955
Acronicta impressa|[12768]|RDLQB166-05|DH010252|658[0n]bp|Canada.Quebec|BOLD:AAA7955
Acronicta impressa|[12769]|LMIS024-05|05-ONMIS-0024|658[0n]bp|Canada.Ontario|BOLD:AAA7955
Acronicta impressa|[12770]|XAD665-05|2005-ONT-80|658[0n]bp|Canada.Ontario|BOLD:AAA7955
Acronicta impressa|[12771]|RDLQ404-05|DH050274|658[0n]bp|Canada.Quebec|BOLD:AAA7955
Acronicta impressa|[12772]|RDLQ453-07|DH013377|613[0n]bp|Canada.Quebec|BOLD:AAA7955
Acronicta impressa|[12773]|MNBB449-05|05-NBSTA-365|658[0n]bp|Canada.New Brunswick|BOLD:AAA7955
Acronicta impressa|[12774]|BBLPC486-09|09BBELE-1486|658[0n]bp|Canada.New Brunswick|BOLD:AAA7955
Acronicta impressa|[12775]|LPSOD524-09|08BBLEP-00303|658[0n]bp|Canada.Ontario|BOLD:AAA7955
Acronicta impressa|[12776]|RDNMK766-12|acrorev188|658[0n]bp|Canada.Alberta|BOLD:AAA7955
Acronicta impressa|[12777]|RDNMK689-11|acrorev gga-131|658[0n]bp|Canada.Alberta|BOLD:AAA7955
Acronicta impressa|[12778]|BBLPC381-09|09BBELE-1381|658[0n]bp|Canada.New Brunswick|BOLD:AAA7955
Acronicta impressa|[12779]|CHLEP082-09|09PROBE-09377|658[0n]bp|Canada.Manitoba|BOLD:AAA7955
Acronicta impressa|[12780]|RDNMK768-12|acrorev190|658[0n]bp|Canada.Alberta|BOLD:AAA7955
Acronicta impressa|[12781]|BBLPC511-09|09BBELE-1511|658[0n]bp|Canada.New Brunswick|BOLD:AAA7955
Acronicta impressa|[12782]|XAK011-06|2006-ONT-1006|658[0n]bp|Canada.Ontario|BOLD:AAA7955
Acronicta impressa|[12783]|TTMNB008-06|MNBTT-008|656[0n]bp|Canada.New Brunswick|BOLD:AAA7955
Acronicta impressa|[12784]|RDNMK764-12|acrorev186|658[0n]bp|Canada.Alberta|BOLD:AAA7955
Acronicta impressa|[12785]|RDNMK687-11|acrorev gga-129|658[0n]bp|Canada.Alberta|BOLD:AAA7955
Acronicta impressa|[12786]|XAF423-05|HLC-10464|658[0n]bp|Canada.Ontario|BOLD:AAA7955
Acronicta impressa|[12787]|RDLQ405-05|DH050275|658[0n]bp|Canada.Quebec|BOLD:AAA7955
Acronicta impressa|[12788]|RDLQB736-05|DH010839|658[0n]bp|Canada.Quebec|BOLD:AAA7955
Acronicta impressa|[12789]|RDNMB607-05|CNCNoctuoidea10383|658[0n]bp|Canada.Quebec|BOLD:AAA7955
Acronicta impressa|[12790]|RDNMK763-12|acrorev185|658[0n]bp|Canada.Alberta|BOLD:AAA7955
Acronicta impressa|[12791]|XAK284-06|2006-ONT-1279|658[0n]bp|Canada.Ontario|BOLD:AAA7955
Acronicta impressa|[12792]|RDNMB609-05|CNCNoctuoidea10385|535[0n]bp|Canada.Quebec|BOLD:AAA7955
Acronicta impressa|[12793]|RDLQB524-05|DH010610|658[0n]bp|Canada.Quebec|BOLD:AAA7955
Acronicta impressa|[12794]|XAG707-05|2005-ONT-1291|658[0n]bp|Canada.Ontario|BOLD:AAA7955
Acronicta impressa|[12795]|RDLQ305-05|DH007882|656[0n]bp|Canada.Quebec|BOLD:AAA7955
Acronicta impressa|[12796]|XAJ136-06|2006-ONT-0136|634[0n]bp|Canada.Ontario|BOLD:AAA7955
Acronicta impressa|[12797]|PMG083-03|moth248.01|617[0n]bp|Canada.Ontario|BOLD:AAA7955
Acronicta impressa|[12798]|RDLQ291-05|DH007124|658[0n]bp|Canada.Quebec|BOLD:AAA7955
Acronicta retardata|[12799]|RDLQ449-07|AC000628|658[0n]bp|Canada.Quebec|BOLD:ACF1696
Acronicta retardata|[12800]|BBLEC112-09|09BBELE-0112|632[0n]bp|Canada.Nova Scotia|BOLD:ACF1696
Acronicta retardata|[12801]|MNBB138-05|05-NBSTA-054|658[0n]bp|Canada.New Brunswick|BOLD:ACF1696
Acronicta retardata|[12802]|RDNM103-05|CNCNoctuoidea6653|658[0n]bp|Canada.Ontario|BOLD:ACF1696
Acronicta retardata|[12803]|BBLPC264-09|09BBELE-1264|658[0n]bp|Canada.Nova Scotia|BOLD:ACF1696
Acronicta retardata|[12804]|PSAT156-10|CNCLEP 70040|658[0n]bp|Canada.Ontario|BOLD:ACF1696
Acronicta retardata|[12805]|XAE351-04|Moth4351.03|658[0n]bp|Canada.Ontario|BOLD:ACF1696
Acronicta retardata|[12806]|BBLPE146-09|09BBELE-2146|658[0n]bp|Canada.Nova Scotia|BOLD:ACF1696
Acronicta retardata|[12807]|BBLEC575-09|09BBELE-0575|658[0n]bp|Canada.Nova Scotia|BOLD:ACF1696
Acronicta retardata|[12808]|RDLQ317-05|DH008870|566[0n]bp|Canada.Quebec|BOLD:ACF1696
Acronicta retardata|[12809]|BBLPC238-09|09BBELE-1238|658[0n]bp|Canada.Nova Scotia|BOLD:ACF1696
Acronicta retardata|[12810]|RDNM104-05|CNCNoctuoidea6654|658[0n]bp|Canada.Ontario|BOLD:ACF1696
Acronicta retardata|[12811]|MNBB009-05|HBL008619|658[0n]bp|Canada.New Brunswick|BOLD:ACF1696
Acronicta retardata|[12812]|XAE352-04|Moth4352.03|658[0n]bp|Canada.Ontario|BOLD:ACF1696
Acronicta afflicta|[12813]|PHMNB452-04|04HBL00678|629[0n]bp|Canada.New Brunswick|BOLD:AAB4198
Acronicta marmorata|[12814]|LMH009-06|PFC-2006-0011|658[0n]bp|Canada.British Columbia|BOLD:AAD4321
Acronicta marmorata|[12815]|RDNMF119-08|NOC14205|658[0n]bp|Canada.British Columbia|BOLD:AAD4321
Acronicta marmorata|[12816]|RDNMF121-08|NOC14207|658[0n]bp|Canada.British Columbia|BOLD:AAD4321
Acronicta marmorata|[12817]|RDNMF122-08|NOC14208|658[0n]bp|Canada.British Columbia|BOLD:AAD4321
Acronicta n sp.|[12818]|RDNMJ072-10|CNCLEP 73851|658[0n]bp|Canada.Ontario|BOLD:ACE9903
Acronicta n sp.|[12819]|RDNMJ629-11|CNC LEP 70091|658[0n]bp|Canada.Ontario|BOLD:ACE9903
Acronicta haesitata|[12820]|RDNMI063-10|CNCLEP 69853|658[0n]bp|Canada.Ontario|BOLD:ACF1195
Acronicta haesitata|[12821]|RDNMI065-10|CNCLEP 69855|612[0n]bp|Canada.Ontario|BOLD:ACF1195
Acronicta albarufa|[12822]|RDNMG216-08|NOC15068|658[0n]bp|United States.New Jersey|BOLD:ACF1195
Acronicta haesitata|[12823]|RDLQ126-05|DH002235|658[0n]bp|Canada.Quebec|BOLD:ACF1195
Acronicta haesitata|[12824]|RDNMI071-10|CNCLEP 69861|621[0n]bp|Canada.Ontario|BOLD:ACF1195
Acronicta haesitata|[12825]|RDLQB564-05|DH010650|658[0n]bp|Canada.Quebec|BOLD:ACF1195
Acronicta haesitata|[12826]|RDNM101-05|CNCNoctuoidea6651|658[0n]bp|Canada.Ontario|BOLD:ACF1195
Acronicta haesitata|[12827]|RDNMI066-10|CNCLEP 69856|658[0n]bp|Canada.Ontario|BOLD:ACF1195
Acronicta haesitata|[12828]|RDNMI060-10|CNCLEP 69850|658[0n]bp|Canada.Ontario|BOLD:ACF1195
Acronicta modica|[12829]|RDNMK247-11|CNCLEP 84156|658[0n]bp|Canada.Ontario|BOLD:ACF1195
Acronicta modica|[12830]|RDNMK248-11|CNCLEP 84157|658[0n]bp|Canada.Ontario|BOLD:ACF1195
Acronicta modica|[12831]|RDNMI069-10|CNCLEP 69859|658[0n]bp|Canada.Ontario|BOLD:ACF1195
Acronicta ovata|[12832]|RDNMI092-10|CNCLEP 69882|658[0n]bp|Canada.Ontario|BOLD:ACF1195
Acronicta ovata|[12833]|RDNMI190-10|CNCLEP 69885|658[0n]bp|Canada|BOLD:ACF1195
Acronicta ovata|[12834]|RDNMI091-10|CNCLEP 69881|658[0n]bp|Canada.Ontario|BOLD:ACF1195
Acronicta exilis|[12835]|RDNMJ664-11|NOC 10224|658[0n]bp|United States.Maryland|BOLD:AAA7126
Acronicta exilis|[12836]|LGSMG943-10|BGS04024|658[0n]bp|United States.Tennessee|BOLD:AAA7126
Acronicta exilis|[12837]|LGSM562-04|DNA-ATBI-0562|658[0n]bp|United States.Tennessee|BOLD:AAA7126
Acronicta exilis|[12838]|RDNMJ075-10|CNCLEP 73854|658[0n]bp|United States.Texas|BOLD:AAA7126
Acronicta exilis|[12839]|QUNOD738-11|9452-220509-TX|658[0n]bp|United States.Texas|BOLD:AAA7126
Acronicta exilis|[12840]|RDNMJ644-11|CNC LEP 70106|632[0n]bp|United States.Florida|BOLD:AAA7126
Acronicta exilis|[12841]|BBLOC1192-11|BIOUG01540-C05|634[0n]bp|United States.Texas|BOLD:AAA7126
Acronicta exilis|[12842]|RDNMI072-10|CNCLEP 69862|658[0n]bp|United States.Mississippi|BOLD:AAA7126
Acronicta increta|[12843]|RDNMJ630-11|CNC LEP 70092|658[0n]bp|Canada.Ontario|BOLD:AAA7126
Acronicta increta|[12844]|RDLQB552-05|DH010638|658[0n]bp|Canada.Quebec|BOLD:AAA7126
Acronicta increta|[12845]|PSAT157-10|CNCLEP 70041|658[0n]bp|Canada.Ontario|BOLD:AAA7126
Acronicta increta|[12846]|PHMNB593-04|04HBL00819|658[0n]bp|Canada.New Brunswick|BOLD:AAA7126
Acronicta increta|[12847]|BBLPE262-09|09BBELE-2262|658[0n]bp|Canada.Nova Scotia|BOLD:AAA7126
Acronicta increta|[12848]|XAC663-04|04HBL006663|658[0n]bp|Canada.Ontario|BOLD:AAA7126
Acronicta increta|[12849]|RDLQ134-05|DH005071|658[0n]bp|Canada.Quebec|BOLD:AAA7126
Acronicta increta|[12850]|RDLQ130-05|DH002216|658[0n]bp|Canada.Quebec|BOLD:AAA7126
Acronicta increta|[12851]|RDLQ139-05|DH002211|658[0n]bp|Canada.Quebec|BOLD:AAA7126
Acronicta increta|[12852]|RDNMI080-10|CNCLEP 69870|658[0n]bp|Canada.Ontario|BOLD:AAA7126
Acronicta increta|[12853]|LPMNB322-09|08BBLEP-05166|658[0n]bp|Canada.Manitoba|BOLD:AAA7126
Acronicta increta|[12854]|RDNMI082-10|CNCLEP 69872|658[0n]bp|Canada.Ontario|BOLD:AAA7126
Acronicta increta|[12855]|RDNMI189-10|CNCLEP 69884|658[0n]bp|Canada|BOLD:AAA7126
Acronicta increta|[12856]|RDNMI062-10|CNCLEP 69852|658[0n]bp|Canada.Ontario|BOLD:AAA7126
Acronicta increta|[12857]|RDNM102-05|CNCNoctuoidea6652|658[0n]bp|Canada.Ontario|BOLD:AAA7126
[illegible]

Acronicta increta|[12855]|RDNMI189-10|CNCLEP 69884|658[0n]bp|Canada|BOLD:AAA7126
Acronicta increta|[12856]|RDNMI062-10|CNCLEP 69852|658[0n]bp|Canada.Ontario|BOLD:AAA7126
Acronicta increta|[12857]|RDNM102-05|CNCNoctuoidea6652|658[0n]bp|Canada.Ontario|BOLD:AAA7126
Acronicta increta|[12858]|RDNMI089-10|CNCLEP 69879|658[0n]bp|Canada.New Brunswick|BOLD:AAA7126
Acronicta increta|[12859]|RDLQ133-05|DH002194|594[0n]bp|Canada.Quebec|BOLD:AAA7126
Acronicta increta|[12860]|RDNMI061-10|CNCLEP 69851|541[0n]bp|Canada.Manitoba|BOLD:AAA7126
Acronicta increta|[12861]|TTMNB327-06|MNBTT-327|649[3n]bp|Canada.New Brunswick|BOLD:AAA7126
Acronicta increta|[12862]|RDNMI085-10|CNCLEP 69875|658[0n]bp|Canada.New Brunswick|BOLD:AAA7126
Acronicta increta|[12863]|RDNMJ336-11|CNCLEP 80244|658[0n]bp|Canada.Ontario|BOLD:AAA7126
Acronicta increta|[12864]|RDLQB554-05|DH010640|658[0n]bp|Canada.Quebec|BOLD:AAA7126
Acronicta increta|[12865]|RDLQG038-06|DH012169|658[0n]bp|Canada.Quebec|BOLD:AAA7126
Acronicta increta|[12866]|RDNMI083-10|CNCLEP 69873|658[0n]bp|Canada.Ontario|BOLD:AAA7126
Acronicta increta|[12867]|RDNMI064-10|CNCLEP 69854|658[0n]bp|Canada.New Brunswick|BOLD:AAA7126
Acronicta increta|[12868]|RDLQ129-05|DH005526|658[0n]bp|Canada.Quebec|BOLD:AAA7126
Acronicta increta|[12869]|RDLQ135-05|DH002203|658[0n]bp|Canada.Quebec|BOLD:AAA7126
Acronicta increta|[12870]|RDNMJ658-11|CNC LEP 70120|658[0n]bp|Canada.Ontario|BOLD:AAA7126
Acronicta increta|[12871]|RDNMK245-11|CNCLEP 84154|658[0n]bp|Canada.Ontario|BOLD:AAA7126
Acronicta increta|[12872]|RDNMJ659-11|CNC LEP 70121|658[0n]bp|Canada.Ontario|BOLD:AAA7126
Acronicta increta|[12873]|RDLQ138-05|DH002230|658[0n]bp|Canada.Quebec|BOLD:AAA7126
Acronicta increta|[12874]|RDNMK246-11|CNCLEP 84155|658[0n]bp|Canada.Ontario|BOLD:AAA7126
Acronicta increta|[12875]|XAF738-05|2005-ONT-387|658[0n]bp|Canada.Ontario|BOLD:AAA7126
Acronicta increta|[12876]|RDNMI081-10|CNCLEP 69871|658[0n]bp|Canada.Ontario|BOLD:AAA7126
Acronicta increta|[12877]|RDLQ131-05|DH005295|658[0n]bp|Canada.Quebec|BOLD:AAA7126
Acronicta increta|[12878]|RDLQ136-05|DH002198|658[0n]bp|Canada.Quebec|BOLD:AAA7126
Acronicta increta|[12879]|RDLQ140-05|DH007585|658[0n]bp|Canada.Quebec|BOLD:AAA7126
Acronicta increta|[12880]|XAC664-04|04HBL006664|658[0n]bp|Canada.Ontario|BOLD:AAA7126
Acronicta increta|[12881]|XAE560-04|Moth4560.03|568[0n]bp|Canada.Ontario|BOLD:AAA7126
Acronicta tristis|[12882]|PHMNB227-04|04HBL007692|609[0n]bp|Canada.New Brunswick|BOLD:ACE9904
Acronicta tristis|[12883]|PHMNB217-04|04HBL007682|609[0n]bp|Canada.New Brunswick|BOLD:ACE9904
Acronicta tristis|[12884]|MNBB616-05|05-NBSTA-532|658[0n]bp|Canada.New Brunswick|BOLD:ACE9904
Acronicta tristis|[12885]|TTMNB326-06|MNBTT-326|658[0n]bp|Canada.New Brunswick|BOLD:ACE9904
Acronicta tristis|[12886]|RDNMI093-10|CNCLEP 69883|658[0n]bp|Canada.New Brunswick|BOLD:ACE9904
Acronicta tristis|[12887]|RDLQ128-05|DH002215|658[0n]bp|Canada.Quebec|BOLD:ACE9904
Acronicta tristis|[12888]|TTMNB004-06|MNBTT-004|657[0n]bp|Canada.New Brunswick|BOLD:ACE9904
Acronicta tristis|[12889]|MNBB394-05|05-NBSTA-310|658[0n]bp|Canada.New Brunswick|BOLD:ACE9904
Acronicta tristis|[12890]|MNBB392-05|05-NBSTA-308|658[0n]bp|Canada.New Brunswick|BOLD:ACE9904
Acronicta tristis|[12891]|PHMNB750-05|Moth 443.03SA|658[0n]bp|Canada.New Brunswick|BOLD:ACE9904
Acronicta tristis|[12892]|MNBB195-05|05-NBSTA-111|658[0n]bp|Canada.New Brunswick|BOLD:ACE9904
Acronicta tristis|[12893]|RDNMI088-10|CNCLEP 69878|658[0n]bp|Canada.New Brunswick|BOLD:ACE9904
Acronicta tristis|[12894]|TMNBB162-06|MNBTT-1102|658[0n]bp|Canada.New Brunswick|BOLD:ACE9904
Acronicta tristis|[12895]|PHMNB589-04|04HBL00815|658[0n]bp|Canada.New Brunswick|BOLD:ACE9904
Acronicta tristis|[12896]|PHMNB725-05|Moth 418.03SA|658[0n]bp|Canada.New Brunswick|BOLD:ACE9904
Acronicta tristis|[12897]|TTMNB003-06|MNBTT-003|657[0n]bp|Canada.New Brunswick|BOLD:ACE9904
Acronicta tristis|[12898]|RDLQB553-05|DH010639|658[0n]bp|Canada.Quebec|BOLD:ACE9904
Acronicta tristis|[12899]|MNBB505-05|05-NBSTA-421|658[0n]bp|Canada.New Brunswick|BOLD:ACE9904
Acronicta tristis|[12900]|PHMNB454-04|04HBL00680|658[0n]bp|Canada.New Brunswick|BOLD:ACE9904
Acronicta tristis|[12901]|TTMNB325-06|MNBTT-325|658[0n]bp|Canada.New Brunswick|BOLD:ACE9904
Acronicta tristis|[12902]|MNBB393-05|05-NBSTA-309|658[0n]bp|Canada.New Brunswick|BOLD:ACE9904
Acronicta tristis|[12903]|MNBB395-05|05-NBSTA-311|658[0n]bp|Canada.New Brunswick|BOLD:ACE9904
Acronicta tristis|[12904]|RDNMI090-10|CNCLEP 69880|658[0n]bp|Canada.New Brunswick|BOLD:ACE9904
Acronicta tristis|[12905]|TMNBB160-06|MNBTT-1100|658[2n]bp|Canada.New Brunswick|BOLD:ACE9904
Acronicta tristis|[12906]|PHMNB038-03|moth21.02SA|639[0n]bp|Canada.New Brunswick|BOLD:ACE9904
Acronicta tristis|[12907]|PHMNB252-04|04HBL007717|616[0n]bp|Canada.New Brunswick|BOLD:ACE9904
Acronicta tristis|[12908]|RDNMI087-10|CNCLEP 69877|620[0n]bp|Canada.New Brunswick|BOLD:ACE9904
Acronicta tristis|[12909]|RDNMI086-10|CNCLEP 69876|620[0n]bp|Canada.Ontario|BOLD:ACE9904
Acronicta tristis|[12910]|PHMNB159-04|04HBL007624|658[0n]bp|Canada.New Brunswick|BOLD:ACE9904
Acronicta tristis|[12911]|TMNBB161-06|MNBTT-1101|658[0n]bp|Canada.New Brunswick|BOLD:ACE9904
Acronicta tristis|[12912]|MNBB506-05|05-NBSTA-422|658[0n]bp|Canada.New Brunswick|BOLD:ACE9904
Acronicta tristis|[12913]|RDNMI084-10|CNCLEP 69874|658[0n]bp|Canada.Ontario|BOLD:ACE9904
Acronicta tristis|[12914]|MNBB615-05|05-NBSTA-531|658[0n]bp|Canada.New Brunswick|BOLD:ACE9904
Acronicta tristis|[12915]|PHMNB591-04|04HBL00817|658[0n]bp|Canada.New Brunswick|BOLD:ACE9904
Acronicta tristis|[12916]|RDLQ132-05|DH002213|658[0n]bp|Canada.Quebec|BOLD:ACE9904
Acronicta vulpina|[12917]|XAJ530-06|2006-ONT-0530|658[0n]bp|Canada.Ontario|BOLD:AAB3897
Acronicta vulpina|[12918]|RDNMD365-06|CNCNoctuoidea12697|656[0n]bp|Canada.New Brunswick|BOLD:AAB3897
Acronicta vulpina|[12919]|XAF454-05|2005-ONT-103|658[0n]bp|Canada.Ontario|BOLD:AAB3897
Acronicta vulpina|[12920]|LPABB330-08|08BBLEP-03595|658[0n]bp|Canada.Alberta|BOLD:AAB3897
Acronicta vulpina|[12921]|BBLEC398-09|09BBELE-0398|658[0n]bp|Canada.Newfoundland and Labrador|BOLD:AAB...
Acronicta vulpina|[12922]|RDLQ444-07|DH012006|650[0n]bp|Canada.Quebec|BOLD:AAB3897
Acronicta vulpina|[12923]|LPMN924-08|08BBLEP-02282|648[0n]bp|Canada.Alberta|BOLD:AAB3897
Acronicta vulpina|[12924]|LPMN563-08|08BBLEP-01364|658[0n]bp|Canada.Manitoba|BOLD:AAB3897
Acronicta vulpina|[12925]|RDNMD269-06|CNCNoctuoidea12601|658[0n]bp|Canada.New Brunswick|BOLD:AAB3897
Acronicta vulpina|[12926]|LPMN320-08|08BBLEP-01119|658[0n]bp|Canada.Manitoba|BOLD:AAB3897
Acronicta vulpina|[12927]|RDNMD267-06|CNCNoctuoidea12599|658[0n]bp|Canada.New Brunswick|BOLD:AAB3897
Acronicta vulpina|[12928]|LPMN916-08|08BBLEP-02274|658[0n]bp|Canada.Alberta|BOLD:AAB3897
Acronicta vulpina|[12929]|RDNMD268-06|CNCNoctuoidea12600|658[0n]bp|Canada.New Brunswick|BOLD:AAB3897
Acronicta vulpina|[12930]|LBCA576-05|HLC-20576|658[0n]bp|Canada.British Columbia|BOLD:AAB3897
Acronicta vulpina|[12931]|BBLPA381-10|10BBCLP-0381|658[0n]bp|Canada.Ontario|BOLD:AAB3897
Acronicta vulpina|[12932]|RDLQ445-07|DH012005|658[0n]bp|Canada.Quebec|BOLD:AAB3897
Acronicta vulpina|[12933]|LPMN312-08|08BBLEP-01111|658[0n]bp|Canada.Manitoba|BOLD:AAB3897
Acronicta vulpina|[12934]|RDNMD364-06|CNCNoctuoidea12696|658[0n]bp|Canada.New Brunswick|BOLD:AAB3897
Acronicta vulpina|[12935]|LPSOB436-08|PPBP-1435|658[0n]bp|Canada.Ontario|BOLD:AAB3897
Acronicta vulpina|[12936]|LPMN321-08|08BBLEP-01120|658[0n]bp|Canada.Manitoba|BOLD:AAB3897
Acronicta vulpina|[12937]|XAB216-04|04HBL005216|658[0n]bp|Canada.Ontario|BOLD:AAB3897
Acronicta vulpina|[12938]|PHMNB583-04|04HBL00809|658[0n]bp|Canada.New Brunswick|BOLD:AAB3897
Acronicta vulpina|[12939]|LPMN308-08|08BBLEP-01107|658[0n]bp|Canada.Manitoba|BOLD:AAB3897
Acronicta vulpina|[12940]|LBCD045-05|HLC-22865|596[1n]bp|Canada.British Columbia|BOLD:AAB3897
Acronicta vulpina|[12941]|LPMN172-08|08BBLEP-00971|658[0n]bp|Canada.Manitoba|BOLD:AAB3897
Acronicta fragilis|[12942]|PHMNB221-04|04HBL007686|609[0n]bp|Canada.New Brunswick|BOLD:ACF0048
Acronicta fragilis|[12943]|MNBB490-05|05-NBSTA-406|658[0n]bp|Canada.New Brunswick|BOLD:ACF0048
Acronicta fragilis|[12944]|RDLQ303-05|DH007781|656[0n]bp|Canada.Quebec|BOLD:ACF0048
Acronicta fragilis|[12945]|PHMNB329-04|04HBL00555|658[0n]bp|Canada.New Brunswick|BOLD:ACF0048
Acronicta fragilis|[12946]|BBLPB207-10|10BBCLP-1206|658[0n]bp|Canada.Ontario|BOLD:ACF0048
Acronicta fragilis|[12947]|TTMNB324-06|MNBTT-324|658[0n]bp|Canada.New Brunswick|BOLD:ACF0048
Acronicta fragilis|[12948]|BBLPC798-09|09BBELE-1798|658[0n]bp|Canada.Newfoundland and Labrador|BOLD:AA...
Acronicta fragilis|[12949]|LBCG2546-09|08-JDWBC-2546|658[0n]bp|Canada.British Columbia|BOLD:AAA9541
Acronicta fragilis|[12950]|LBCG3221-09|08-JDWBC-3221|658[0n]bp|Canada.British Columbia|BOLD:AAA9541
Acronicta fragilis|[12951]|BBLPE400-09|09BBELE-2400|658[0n]bp|Canada.Newfoundland and Labrador|BOLD:AA...
Acronicta fragilis|[12952]|TTMNB323-06|MNBTT-323|658[0n]bp|Canada.New Brunswick|BOLD:AAA9541
Acronicta fragilis|[12953]|LBCG2545-09|08-JDWBC-2545|658[0n]bp|Canada.British Columbia|BOLD:AAA9541
Acronicta fragilis|[12954]|LBCG2544-09|08-JDWBC-2544|658[0n]bp|Canada.British Columbia|BOLD:AAA9541
Acronicta fragilis|[12955]|TMNBB157-06|MNBTT-1097|658[0n]bp|Canada.New Brunswick|BOLD:AAA9541
Acronicta fragilis|[12956]|RDNMH339-09|CNCLEP00057748|658[0n]bp|Canada.New Brunswick|BOLD:AAA9541
Acronicta fragilis|[12957]|LBCG177-08|08-JDWBC-0177|658[0n]bp|Canada.British Columbia|BOLD:AAA9541

Acronicta fragilis|[12955]|TMNBB157-06|MNBTT-1097|658[0n]bp|Canada.New Brunswick|BOLD:AAA9541
Acronicta fragilis|[12956]|RDNMH339-09|CNCLEP00057748|658[0n]bp|Canada.New Brunswick|BOLD:AAA9541
Acronicta fragilis|[12957]|LBCG177-08|08-JDWBC-0177|658[0n]bp|Canada.British Columbia|BOLD:AAA9541
Acronicta fragilis|[12958]|LPMN397-08|08BBLEP-01196|658[0n]bp|Canada.Manitoba|BOLD:AAA9541
Acronicta fragilis|[12959]|LBCH3031-10|10-JDWBC-3031|658[0n]bp|Canada.British Columbia|BOLD:AAA9541
Acronicta fragilis|[12960]|BBLPB523-10|10BBCLP-1522|658[0n]bp|Canada.Ontario|BOLD:AAA9541
Acronicta fragilis|[12961]|RDLQG322-06|DH012537|658[0n]bp|Canada.Quebec|BOLD:AAA9541
Acronicta fragilis|[12962]|LBCH228-10|10-JDWBC-0228|658[0n]bp|Canada.British Columbia|BOLD:AAA9541
Acronicta fragilis|[12963]|RDNMH338-09|CNCLEP00057747|658[0n]bp|Canada.New Brunswick|BOLD:AAA9541
Acronicta fragilis|[12964]|LBCA505-05|HLC-20505|658[0n]bp|Canada.British Columbia|BOLD:AAA9541
Acronicta fragilis|[12965]|BBLPE530-09|09BBELE-2530|658[0n]bp|Canada.Newfoundland and Labrador|BOLD:AA...
Acronicta fragilis|[12966]|RDLQF513-06|DH011662|658[0n]bp|Canada.Quebec|BOLD:AAA9541
Acronicta fragilis|[12967]|LBCG2543-09|08-JDWBC-2543|658[0n]bp|Canada.British Columbia|BOLD:AAA9541
Acronicta fragilis|[12968]|BBLEC336-09|09BBELE-0336|658[0n]bp|Canada.Nova Scotia|BOLD:AAA9541
Acronicta fragilis|[12969]|LBCA830-05|HLC-20830|658[0n]bp|Canada.British Columbia|BOLD:AAA9541
Acronicta fragilis|[12970]|LALPA1008-11|AVBC 1181-11|658[0n]bp|Canada.British Columbia|BOLD:AAA9541
Acronicta fragilis|[12971]|BBLEC819-09|09BBELE-0819|658[0n]bp|Canada.Newfoundland and Labrador|BOLD:AA...
Acronicta fragilis|[12972]|LALPA156-10|AVBC 156-10|658[0n]bp|Canada.British Columbia|BOLD:AAA9541
Acronicta fragilis|[12973]|LALPA921-11|AVBC 1094-11|634[0n]bp|Canada.British Columbia|BOLD:AAA9541
Acronicta fragilis|[12974]|RDLQF514-06|DH011663|658[0n]bp|Canada.Quebec|BOLD:AAA9541
Acronicta fragilis|[12975]|LALPA988-11|AVBC 1161-11|658[0n]bp|Canada.British Columbia|BOLD:AAA9541
Acronicta fragilis|[12976]|LBCC442-05|HLC-22322|658[0n]bp|Canada.British Columbia|BOLD:AAA9541
Acronicta fragilis|[12977]|PHMNB261-04|04HBL007726|597[0n]bp|Canada.New Brunswick|BOLD:ACF4239
Acronicta heitzmani|[12978]|RDNMK841-12|CNCLEP83454|658[0n]bp|Canada.Ontario|BOLD:ACF4251
Acronicta heitzmani|[12979]|RDNMK842-12|CNCLEP83455|658[0n]bp|Canada.Ontario|BOLD:ACF4251
Acronicta connecta|[12980]|PHMO161-03|moth878.02|639[0n]bp|Canada.Ontario|BOLD:AAF0028
Acronicta connecta|[12981]|XAJ595-06|2006-ONT-0595|656[0n]bp|Canada.Ontario|BOLD:AAF0028
Acronicta connecta|[12982]|BLTIB281-08|BL465|658[0n]bp|Canada.Ontario|BOLD:AAF0028
Acronicta interrupta|[12983]|XAJ505-06|2006-ONT-0505|643[0n]bp|Canada.Ontario|BOLD:AAB7005
Acronicta interrupta|[12984]|LPSOB805-08|PPBP-1804|658[0n]bp|Canada.Ontario|BOLD:AAB7005
Acronicta interrupta|[12985]|XAJ455-06|2006-ONT-0455|658[0n]bp|Canada.Ontario|BOLD:AAB7005
Acronicta interrupta|[12986]|XAB303-04|04HBL005303|658[0n]bp|Canada.Ontario|BOLD:AAB7005
Acronicta interrupta|[12987]|RDLQ264-05|DH005074|658[0n]bp|Canada.Quebec|BOLD:AAB7005
Acronicta interrupta|[12988]|LPSOD276-09|08BBLEP-00054|658[0n]bp|Canada.Ontario|BOLD:AAB7005
Acronicta interrupta|[12989]|MNBB240-05|05-NBSTA-156|524[0n]bp|Canada.New Brunswick|BOLD:AAB7005
Acronicta interrupta|[12990]|PHMNB585-04|04HBL00811|658[0n]bp|Canada.New Brunswick|BOLD:AAB7005
Acronicta interrupta|[12991]|MNBB649-05|05-NBSTA-565|658[0n]bp|Canada.New Brunswick|BOLD:AAB7005
Acronicta interrupta|[12992]|MNBB480-05|05-NBSTA-396|658[0n]bp|Canada.New Brunswick|BOLD:AAB7005
Acronicta interrupta|[12993]|MNBB190-05|05-NBSTA-106|658[0n]bp|Canada.New Brunswick|BOLD:AAB7005
Acronicta hasta|[12994]|RDNM106-05|CNCNoctuoidea6656|658[0n]bp|Canada.Ontario|BOLD:AAA6038
Acronicta hasta|[12995]|LPSOB440-08|PPBP-1439|658[0n]bp|Canada.Ontario|BOLD:AAA6038
Acronicta hasta|[12996]|RDNMB481-05|CNCNoctuoidea10247|658[0n]bp|Canada.Ontario|BOLD:AAA6038
Acronicta hasta|[12997]|LPSO362-08|PPBP-0362|658[0n]bp|Canada.Ontario|BOLD:AAA6038
Acronicta hasta|[12998]|XAD099-04|04HBL007099|658[0n]bp|Canada.Ontario|BOLD:AAA6038
Acronicta hasta|[12999]|XAE473-04|Moth4473.03|658[0n]bp|Canada.Ontario|BOLD:AAA6038
Acronicta hasta|[13000]|RDNMB474-05|CNCNoctuoidea10240|658[0n]bp|Canada.British Columbia|BOLD:AAA6038
Acronicta hasta|[13001]|RDNMB475-05|CNCNoctuoidea10241|528[0n]bp|Canada.British Columbia|BOLD:AAA6038
Acronicta hasta|[13002]|RDNMB477-05|CNCNoctuoidea10243|658[0n]bp|Canada.Ontario|BOLD:AAA6038
Acronicta hasta|[13003]|BLTIB293-08|BL480|658[0n]bp|Canada.Ontario|BOLD:AAA6038
Acronicta hasta|[13004]|RDLQ301-05|DH007554|608[0n]bp|Canada.Quebec|BOLD:AAA6038
Acronicta hasta|[13005]|RDNMD278-06|CNCNoctuoidea12610|658[0n]bp|Canada.New Brunswick|BOLD:AAA6038
Acronicta hasta|[13006]|RDLQ451-07|DH012415|658[0n]bp|Canada.Quebec|BOLD:AAA6038
Acronicta hasta|[13007]|RDNMB483-05|CNCNoctuoidea10249|658[0n]bp|Canada.Alberta|BOLD:AAA6038
Acronicta hasta|[13008]|RDLQ269-05|DH005214|658[0n]bp|Canada.Quebec|BOLD:AAA6038
Acronicta hasta|[13009]|XAJ454-06|2006-ONT-0454|658[0n]bp|Canada.Ontario|BOLD:AAA6038
Acronicta hasta|[13010]|XAE621-04|Moth4621.03|657[0n]bp|Canada.Ontario|BOLD:AAA6038
Acronicta hasta|[13011]|XAJ582-06|2006-ONT-0582|658[0n]bp|Canada.Ontario|BOLD:AAA6038
Acronicta hasta|[13012]|XAJ289-06|2006-ONT-0289|658[0n]bp|Canada.Ontario|BOLD:AAA6038
Acronicta hasta|[13013]|XAE583-04|Moth4583.03|658[0n]bp|Canada.Ontario|BOLD:AAA6038
Acronicta hasta|[13014]|RDNMD275-06|CNCNoctuoidea12607|658[0n]bp|Canada.New Brunswick|BOLD:AAA6038
Acronicta hasta|[13015]|RDNMB476-05|CNCNoctuoidea10242|658[0n]bp|Canada.Ontario|BOLD:AAA6038
Acronicta hasta|[13016]|XAJ979-06|2006-ONT-0979|658[0n]bp|Canada.Ontario|BOLD:AAA6038
Acronicta hasta|[13017]|RDNMD280-06|CNCNoctuoidea12612|658[0n]bp|Canada.New Brunswick|BOLD:AAA6038
Acronicta hasta|[13018]|LPSOC087-08|PPBP-2086|658[0n]bp|Canada.Ontario|BOLD:AAA6038
Acronicta hasta|[13019]|RDLQ452-07|DH009401|658[0n]bp|Canada.Quebec|BOLD:AAA6038
Acronicta hasta|[13020]|XAF567-05|2005-ONT-216|658[0n]bp|Canada.Ontario|BOLD:AAA6038
Acronicta hasta|[13021]|PHMNB588-04|04HBL00814|658[0n]bp|Canada.New Brunswick|BOLD:AAA6038
Acronicta hasta|[13022]|LPSOB789-08|PPBP-1788|658[0n]bp|Canada.Ontario|BOLD:AAA6038
Acronicta hasta|[13023]|BBLPE263-09|09BBELE-2263|658[0n]bp|Canada.Nova Scotia|BOLD:AAA6038
Acronicta hasta|[13024]|XAJ399-06|2006-ONT-0399|658[0n]bp|Canada.Ontario|BOLD:AAA6038
Acronicta hasta|[13025]|XAE618-04|Moth4618.03|658[0n]bp|Canada.Ontario|BOLD:AAA6038
Acronicta hasta|[13026]|XAF643-05|2005-ONT-292|658[0n]bp|Canada.Ontario|BOLD:AAA6038
Acronicta hasta|[13027]|LPSOC289-08|PPBP-2288|658[0n]bp|Canada.Ontario|BOLD:AAA6038
Acronicta hasta|[13028]|LPSOC030-08|PPBP-2029|658[0n]bp|Canada.Ontario|BOLD:AAA6038
Acronicta hasta|[13029]|LPSOC088-08|PPBP-2087|658[0n]bp|Canada.Ontario|BOLD:AAA6038
Acronicta hasta|[13030]|BBLPB644-10|10BBCLP-1643|658[0n]bp|Canada.Saskatchewan|BOLD:AAA6038
Acronicta hasta|[13031]|RDNMB480-05|CNCNoctuoidea10246|658[0n]bp|Canada.Alberta|BOLD:AAA6038
Acronicta hasta|[13032]|RDLQ256-05|DH004659|658[0n]bp|Canada.Quebec|BOLD:AAA6038
Acronicta hasta|[13033]|RDNMD279-06|CNCNoctuoidea12611|658[0n]bp|Canada.New Brunswick|BOLD:AAA6038
Acronicta hasta|[13034]|XAF543-05|2005-ONT-192|658[0n]bp|Canada.Ontario|BOLD:AAA6038
Acronicta hasta|[13035]|XAB164-04|04HBL005164|658[0n]bp|Canada.Ontario|BOLD:AAA6038
Acronicta hasta|[13036]|RDLQB160-05|DH010246|658[0n]bp|Canada.Quebec|BOLD:AAA6038
Acronicta hasta|[13037]|XAG405-05|2005-ONT-989|658[0n]bp|Canada.Ontario|BOLD:AAA6038
Acronicta hasta|[13038]|XAJ409-06|2006-ONT-0409|658[0n]bp|Canada.Ontario|BOLD:AAA6038
Acronicta hasta|[13039]|XAE314-04|Moth4314.03|658[0n]bp|Canada.Ontario|BOLD:AAA6038
Acronicta hasta|[13040]|XAJ488-06|2006-ONT-0488|656[0n]bp|Canada.Ontario|BOLD:AAA6038
Acronicta hasta|[13041]|LPMN572-08|08BBLEP-01373|651[0n]bp|Canada.Manitoba|BOLD:AAA6038
Acronicta hasta|[13042]|XAC626-04|04HBL006626|615[0n]bp|Canada.Ontario|BOLD:AAA6038
Acronicta hasta|[13043]|TMG112-03|moth413.01|639[0n]bp|Canada.Ontario|BOLD:AAA6038
Acronicta hasta|[13044]|PMG082-03|moth378.01|617[0n]bp|Canada.Ontario|BOLD:AAA6038
Acronicta hasta|[13045]|RDNMB482-05|CNCNoctuoidea10248|658[0n]bp|Canada.New Brunswick|BOLD:AAA6038
Acronicta innotata|[13046]|LBCB147-05|HLC-21087|658[0n]bp|Canada.British Columbia|BOLD:AAA3813
Acronicta innotata|[13047]|RDNMK658-11|CNCLEP 81835|658[0n]bp|Canada.British Columbia|BOLD:AAA3813
Acronicta innotata|[13048]|LHLEP373-06|UBC-2006-0753|657[0n]bp|Canada.British Columbia|BOLD:AAA3813
Acronicta innotata|[13049]|LOWCC352-05|CGWC-2232|658[0n]bp|Canada.British Columbia|BOLD:AAA3813
Acronicta innotata|[13050]|LALPA875-11|AVBC 1048-11|658[0n]bp|Canada.British Columbia|BOLD:AAA3813
Acronicta innotata|[13051]|LOWCE808-06|CGWC-4568|658[0n]bp|Canada.British Columbia|BOLD:AAA3813
Acronicta innotata|[13052]|LBCB629-05|HLC-21569|658[0n]bp|Canada.British Columbia|BOLD:AAA3813
Acronicta innotata|[13053]|LBCC041-05|HLC-21921|658[0n]bp|Canada.British Columbia|BOLD:AAA3813
Acronicta innotata|[13054]|LOWCC351-05|CGWC-2231|658[0n]bp|Canada.British Columbia|BOLD:AAA3813
Acronicta innotata|[13055]|LBCC410-05|HLC-22290|658[0n]bp|Canada.British Columbia|BOLD:AAA3813
Acronicta innotata|[13056]|RDNMK654-11|CNCLEP 81831|658[0n]bp|Canada.British Columbia|BOLD:AAA3813
Acronicta innotata|[13057]|BBLPA378-10|10BBCLP-0378|658[0n]bp|Canada.British Columbia|BOLD:AAA3813

Acronicta innotata|[13055]|LBCC410-05|HLC-22290|658[0n]bp|Canada.British Columbia|BOLD:AAA3813
Acronicta innotata|[13056]|RDNMK654-11|CNCLEP 81831|658[0n]bp|Canada.British Columbia|BOLD:AAA3813
Acronicta innotata|[13057]|BBLPA378-10|10BBCLP-0378|658[0n]bp|Canada.British Columbia|BOLD:AAA3813
Acronicta innotata|[13058]|BBLPA379-10|10BBCLP-0379|658[0n]bp|Canada.British Columbia|BOLD:AAA3813
Acronicta innotata|[13059]|LALPA872-11|AVBC 1045-11|658[0n]bp|Canada.British Columbia|BOLD:AAA3813
Acronicta innotata|[13060]|LPABB392-08|08BBLEP-03657|658[0n]bp|Canada.Alberta|BOLD:AAA3813
Acronicta innotata|[13061]|LBCD422-05|HLC-23242|658[0n]bp|Canada.British Columbia|BOLD:AAA3813
Acronicta innotata|[13062]|LOWCE196-06|CGWC-3956|658[0n]bp|Canada.British Columbia|BOLD:AAA3813
Acronicta innotata|[13063]|LOWCC862-05|CGWC-2742|575[1n]bp|Canada.British Columbia|BOLD:AAA3813
Acronicta innotata|[13064]|LOWCC354-05|CGWC-2234|529[0n]bp|Canada.British Columbia|BOLD:AAA3813
Acronicta innotata|[13065]|LOWCC349-05|CGWC-2229|582[0n]bp|Canada.British Columbia|BOLD:AAA3813
Acronicta innotata|[13066]|LOWCC353-05|CGWC-2233|658[0n]bp|Canada.British Columbia|BOLD:AAA3813
Acronicta innotata|[13067]|LOWCC350-05|CGWC-2230|658[0n]bp|Canada.British Columbia|BOLD:AAA3813
Acronicta innotata|[13068]|XAB253-04|04HBL005253|658[2n]bp|Canada.Ontario|BOLD:AAA3813
Acronicta innotata|[13069]|BBLPC671-09|09BBELE-1671|658[1n]bp|Canada.Newfoundland and Labrador|BOLD:AA...
Acronicta innotata|[13070]|PHMNB590-04|04HBL00816|658[0n]bp|Canada.New Brunswick|BOLD:AAA3813
Acronicta innotata|[13071]|XAE465-04|Moth4465.03|613[0n]bp|Canada.Ontario|BOLD:AAA3813
Acronicta innotata|[13072]|MNBB278-05|05-NBSTA-194|658[0n]bp|Canada.New Brunswick|BOLD:AAA3813
Acronicta innotata|[13073]|MNBB483-05|05-NBSTA-399|658[1n]bp|Canada.New Brunswick|BOLD:AAA3813
Acronicta innotata|[13074]|MNBB280-05|05-NBSTA-196|658[0n]bp|Canada.New Brunswick|BOLD:AAA3813
Acronicta innotata|[13075]|BBLEC005-09|09BBELE-0005|632[0n]bp|Canada.New Brunswick|BOLD:AAA3813
Acronicta innotata|[13076]|MNBB192-05|05-NBSTA-108|658[1n]bp|Canada.New Brunswick|BOLD:AAA3813
Acronicta innotata|[13077]|BBLPE175-09|09BBELE-2175|639[0n]bp|Canada.Nova Scotia|BOLD:AAA3813
Acronicta innotata|[13078]|BBLPC950-09|09BBELE-1950|658[0n]bp|Canada.Newfoundland and Labrador|BOLD:AA...
Acronicta innotata|[13079]|BBLPE487-09|09BBELE-2487|658[0n]bp|Canada.Newfoundland and Labrador|BOLD:AA...
Acronicta innotata|[13080]|BBLPC851-09|09BBELE-1851|658[0n]bp|Canada.Newfoundland and Labrador|BOLD:AA...
Acronicta innotata|[13081]|BBLEC366-09|09BBELE-0366|658[0n]bp|Canada.Newfoundland and Labrador|BOLD:AA...
Acronicta innotata|[13082]|BBLPE301-09|09BBELE-2301|658[0n]bp|Canada.Newfoundland and Labrador|BOLD:AA...
Acronicta innotata|[13083]|PHMNB360-04|04HBL00586|658[0n]bp|Canada.New Brunswick|BOLD:AAA3813
Acronicta innotata|[13084]|MNBB241-05|05-NBSTA-157|658[0n]bp|Canada.New Brunswick|BOLD:AAA3813
Acronicta innotata|[13085]|LPSOB383-08|PPBP-1382|658[0n]bp|Canada.Ontario|BOLD:AAA3813
Acronicta innotata|[13086]|BBLPC576-09|09BBELE-1576|658[0n]bp|Canada.Nova Scotia|BOLD:AAA3813
Acronicta innotata|[13087]|BBLEC783-09|09BBELE-0783|658[0n]bp|Canada.Newfoundland and Labrador|BOLD:AA...
Acronicta innotata|[13088]|BBLPE490-09|09BBELE-2490|658[0n]bp|Canada.Newfoundland and Labrador|BOLD:AA...
Acronicta innotata|[13089]|BBLEC882-09|09BBELE-0882|658[0n]bp|Canada.Newfoundland and Labrador|BOLD:AA...
Acronicta innotata|[13090]|MNBB448-05|05-NBSTA-364|658[0n]bp|Canada.New Brunswick|BOLD:AAA3813
Acronicta innotata|[13091]|MNBB484-05|05-NBSTA-400|658[0n]bp|Canada.New Brunswick|BOLD:AAA3813
Acronicta innotata|[13092]|MNBB191-05|05-NBSTA-107|658[0n]bp|Canada.New Brunswick|BOLD:AAA3813
Acronicta innotata|[13093]|MNBB504-05|05-NBSTA-420|658[0n]bp|Canada.New Brunswick|BOLD:AAA3813
Acronicta innotata|[13094]|MNBB275-05|05-NBSTA-191|658[0n]bp|Canada.New Brunswick|BOLD:AAA3813
Acronicta innotata|[13095]|MNBB194-05|05-NBSTA-110|658[0n]bp|Canada.New Brunswick|BOLD:AAA3813
Acronicta innotata|[13096]|MNBB391-05|05-NBSTA-307|658[0n]bp|Canada.New Brunswick|BOLD:AAA3813
Acronicta innotata|[13097]|XAE358-04|Moth4358.03|658[0n]bp|Canada.Ontario|BOLD:AAA3813
Acronicta innotata|[13098]|BBLPE484-09|09BBELE-2484|658[0n]bp|Canada.Newfoundland and Labrador|BOLD:AA...
Acronicta innotata|[13099]|BBLPE035-09|09BBELE-2035|658[0n]bp|Canada.Nova Scotia|BOLD:AAA3813
Acronicta innotata|[13100]|XAJ407-06|2006-ONT-0407|658[0n]bp|Canada.Ontario|BOLD:AAA3813
Acronicta innotata|[13101]|BBLPE514-09|09BBELE-2514|658[0n]bp|Canada.Newfoundland and Labrador|BOLD:AA...
Acronicta innotata|[13102]|MNBB485-05|05-NBSTA-401|658[0n]bp|Canada.New Brunswick|BOLD:AAA3813
Acronicta innotata|[13103]|MNBB043-05|HBL008653|658[0n]bp|Canada.New Brunswick|BOLD:AAA3813
Acronicta innotata|[13104]|MNBB094-05|05-NBSTA-010|658[0n]bp|Canada.New Brunswick|BOLD:AAA3813
Acronicta innotata|[13105]|LPMN267-08|08BBLEP-01066|658[0n]bp|Canada.Manitoba|BOLD:AAA3813
Acronicta innotata|[13106]|BBLPA377-10|10BBCLP-0377|658[0n]bp|Canada.Ontario|BOLD:AAA3813
Acronicta innotata|[13107]|MNBB604-05|05-NBSTA-520|658[0n]bp|Canada.New Brunswick|BOLD:AAA3813
Acronicta innotata|[13108]|XAF517-05|2005-ONT-166|658[0n]bp|Canada.Ontario|BOLD:AAA3813
Acronicta innotata|[13109]|MNBB045-05|HBL008655|658[0n]bp|Canada.New Brunswick|BOLD:AAA3813
Acronicta innotata|[13110]|MNBB277-05|05-NBSTA-193|658[0n]bp|Canada.New Brunswick|BOLD:AAA3813
Acronicta innotata|[13111]|MNBB276-05|05-NBSTA-192|658[0n]bp|Canada.New Brunswick|BOLD:AAA3813
Acronicta innotata|[13112]|BBLPE488-09|09BBELE-2488|658[0n]bp|Canada.Newfoundland and Labrador|BOLD:AA...
Acronicta innotata|[13113]|MNBB330-05|05-NBSTA-246|658[0n]bp|Canada.New Brunswick|BOLD:AAA3813
Acronicta innotata|[13114]|XAE461-04|Moth4461.03|658[0n]bp|Canada.Ontario|BOLD:AAA3813
Acronicta innotata|[13115]|BBLPC571-09|09BBELE-1571|658[0n]bp|Canada.Nova Scotia|BOLD:AAA3813
Acronicta innotata|[13116]|BBLEC359-09|09BBELE-0359|658[0n]bp|Canada.Newfoundland and Labrador|BOLD:AA...
Acronicta innotata|[13117]|MNBB279-05|05-NBSTA-195|658[0n]bp|Canada.New Brunswick|BOLD:AAA3813
Acronicta innotata|[13118]|BBLPA380-10|10BBCLP-0380|658[0n]bp|Canada.Ontario|BOLD:AAA3813
Acronicta innotata|[13119]|XAK050-06|2006-ONT-1045|658[0n]bp|Canada.Ontario|BOLD:AAA3813
Acronicta innotata|[13120]|BBLPA382-10|10BBCLP-0382|658[0n]bp|Canada.Ontario|BOLD:AAA3813
Acronicta innotata|[13121]|MNBB430-05|05-NBSTA-346|658[0n]bp|Canada.New Brunswick|BOLD:AAA3813
Acronicta innotata|[13122]|MNBB390-05|05-NBSTA-306|658[0n]bp|Canada.New Brunswick|BOLD:AAA3813
Acronicta innotata|[13123]|BBLPE409-09|09BBELE-2409|658[0n]bp|Canada.Newfoundland and Labrador|BOLD:AA...
Acronicta innotata|[13124]|XAK053-06|2006-ONT-1048|658[0n]bp|Canada.Ontario|BOLD:AAA3813
Acronicta innotata|[13125]|RDLQB162-05|DH010248|658[0n]bp|Canada.Quebec|BOLD:AAA3813
Acronicta innotata|[13126]|MNBB503-05|05-NBSTA-419|658[0n]bp|Canada.New Brunswick|BOLD:AAA3813
Acronicta innotata|[13127]|MNBB648-05|05-NBSTA-564|658[1n]bp|Canada.New Brunswick|BOLD:AAA3813
Acronicta innotata|[13128]|MNBB389-05|05-NBSTA-305|658[0n]bp|Canada.New Brunswick|BOLD:AAA3813
Acronicta innotata|[13129]|MNBB388-05|05-NBSTA-304|658[0n]bp|Canada.New Brunswick|BOLD:AAA3813
Acronicta innotata|[13130]|BBLEC075-09|09BBELE-0075|656[0n]bp|Canada.New Brunswick|BOLD:AAA3813
Acronicta innotata|[13131]|MNBB331-05|05-NBSTA-247|570[0n]bp|Canada.New Brunswick|BOLD:AAA3813
Acronicta innotata|[13132]|XAK479-07|HLC-16032|594[0n]bp|Canada.Ontario|BOLD:AAA3813
Acronicta innotata|[13133]|BBLEC346-09|09BBELE-0346|633[0n]bp|Canada.Newfoundland and Labrador|BOLD:AA...
Acronicta innotata|[13134]|BBLEC382-09|09BBELE-0382|648[0n]bp|Canada.Newfoundland and Labrador|BOLD:AA...
Acronicta innotata|[13135]|PHMO215-03|moth1098.01|639[0n]bp|Canada.Ontario|BOLD:AAA3813
Acronicta innotata|[13136]|BBLPE541-09|09BBELE-2541|627[0n]bp|Canada.Newfoundland and Labrador|BOLD:AA...
Acronicta innotata|[13137]|TTMNB322-06|MNBTT-322|608[0n]bp|Canada.New Brunswick|BOLD:AAA3813
Acronicta innotata|[13138]|XAE284-04|Moth4284.03|658[0n]bp|Canada.Ontario|BOLD:AAA3813
Acronicta innotata|[13139]|XAE470-04|Moth4470.03|615[0n]bp|Canada.Ontario|BOLD:AAA3813
Acronicta innotata|[13140]|BBLEC388-09|09BBELE-0388|658[0n]bp|Canada.Newfoundland and Labrador|BOLD:AA...
Acronicta quadrata|[13141]|BBLPB705-10|10BBCLP-1704|658[0n]bp|Canada.Ontario|BOLD:AAF6773
Acronicta quadrata|[13142]|RDNMB880-05|CNCNoctuoidea10655|511[0n]bp|Canada.Saskatchewan|BOLD:AAF6773
Acronicta quadrata|[13143]|RDLQB161-05|DH010247|658[0n]bp|Canada.Quebec|BOLD:AAF6773
Acronicta quadrata|[13144]|RDNMB881-05|CNCNoctuoidea10656|658[0n]bp|Canada.Manitoba|BOLD:AAF6773
Acronicta quadrata|[13145]|BBLPB251-10|10BBCLP-1250|658[0n]bp|Canada.Ontario|BOLD:AAF6773
Acronicta radcliffei|[13146]|LPVIA405-08|PFC-2006-0591|658[0n]bp|Canada.British Columbia|BOLD:AAC4642
Acronicta radcliffei|[13147]|RDNMB485-05|CNCNoctuoidea10251|658[0n]bp|Canada.British Columbia|BOLD:AAC...
Acronicta radcliffei|[13148]|LALPA967-11|AVBC 1140-11|634[0n]bp|Canada.British Columbia|BOLD:AAC4642
Acronicta radcliffei|[13149]|RDNMB486-05|CNCNoctuoidea10252|658[0n]bp|Canada.British Columbia|BOLD:AAC...
Acronicta radcliffei|[13150]|LPABB502-08|08BBLEP-03767|658[0n]bp|Canada.Alberta|BOLD:AAC4642
Acronicta radcliffei|[13151]|RDNMB889-05|CNCNoctuoidea10664|658[0n]bp|Canada.British Columbia|BOLD:AAC...
Acronicta radcliffei|[13152]|RDLQB177-05|DH010263|658[0n]bp|Canada.Quebec|BOLD:AAC4642
Acronicta radcliffei|[13153]|LPVIA109-08|PFC-2006-0175|658[0n]bp|Canada.British Columbia|BOLD:AAC4642
Acronicta radcliffei|[13154]|LPVIA230-08|PFC-2006-0312|658[0n]bp|Canada.British Columbia|BOLD:AAC4642
Acronicta radcliffei|[13155]|LBCA598-05|HLC-20598|658[0n]bp|Canada.British Columbia|BOLD:AAC4642
Acronicta radcliffei|[13156]|DUNLP148-08|Dun-08-148|650[0n]bp|Canada.British Columbia|BOLD:AAC4642

Acronicta radcliffei|[13154]|LPVIA230-08|PFC-2006-0312|658[0n]bp|Canada.British Columbia|BOLD:AAC4642
Acronicta radcliffei|[13155]|LBCA598-05|HLC-20598|658[0n]bp|Canada.British Columbia|BOLD:AAC4642
Acronicta radcliffei|[13156]|DUNLP148-08|Dun-08-148|650[0n]bp|Canada.British Columbia|BOLD:AAC4642
Acronicta laetifica|[13157]|RDLQ412-05|AC000896|601[0n]bp|Canada.Quebec|BOLD:AAB3866
Acronicta laetifica|[13158]|RDNMD277-06|CNCNoctuoidea12609|658[0n]bp|Canada.New Brunswick|BOLD:AAB3866
Acronicta laetifica|[13159]|RDLQ450-07|AC000627|658[0n]bp|Canada.Quebec|BOLD:AAB3866
Acronicta laetifica|[13160]|RDNMD276-06|CNCNoctuoidea12608|658[0n]bp|Canada.New Brunswick|BOLD:AAB3866
Acronicta laetifica|[13161]|XAB207-04|04HBL005207|658[0n]bp|Canada.Ontario|BOLD:AAB3866
Acronicta laetifica|[13162]|RDNMB479-05|CNCNoctuoidea10245|658[0n]bp|Canada.Ontario|BOLD:AAB3866
Acronicta laetifica|[13163]|RDNM105-05|CNCNoctuoidea6655|658[0n]bp|Canada.Ontario|BOLD:AAB3866
Acronicta laetifica|[13164]|RDNMB478-05|CNCNoctuoidea10244|614[0n]bp|Canada.New Brunswick|BOLD:AAB3866
Acronicta laetifica|[13165]|RDLQ124-05|DH002117|658[0n]bp|Canada.Quebec|BOLD:AAB3866
Acronicta laetifica|[13166]|RDNMD274-06|CNCNoctuoidea12606|658[0n]bp|Canada.New Brunswick|BOLD:AAB3866
Acronicta laetifica|[13167]|RDLQ125-05|DH002148|658[0n]bp|Canada.Quebec|BOLD:AAB3866
Acronicta laetifica|[13168]|RDLQB708-05|DH010811|658[0n]bp|Canada.Quebec|BOLD:AAB3866
Acronicta spinigera|[13169]|LPSOC080-08|PPBP-2079|658[0n]bp|Canada.Ontario|BOLD:ABY8980
Acronicta spinigera|[13170]|RDLQ302-05|DH007587|621[0n]bp|Canada.Quebec|BOLD:ABY8980
Acronicta spinigera|[13171]|XAJ618-06|2006-ONT-0618|633[0n]bp|Canada.Ontario|BOLD:ABY8980
Acronicta spinigera|[13172]|RDNMB883-05|CNCNoctuoidea10658|658[0n]bp|Canada.Ontario|BOLD:ABY8980
Acronicta spinigera|[13173]|RDNMB882-05|CNCNoctuoidea10657|658[0n]bp|Canada.Ontario|BOLD:ABY8980
Acronicta spinigera|[13174]|LPSOC085-08|PPBP-2084|658[0n]bp|Canada.Ontario|BOLD:ABY8980
Acronicta lobeliae|[13175]|RDLQ316-05|DH008837|658[0n]bp|Canada.Quebec|BOLD:AAC7189
Acronicta lobeliae|[13176]|LPSO005-08|PPBP-0005|658[0n]bp|Canada.Ontario|BOLD:AAC7189
Acronicta lobeliae|[13177]|RDLQ406-05|DH050726|658[0n]bp|Canada.Quebec|BOLD:AAC7189
Acronicta clarescens|[13178]|LNC941-06|06-NCCC-941|658[0n]bp|United States.North Carolina|BOLD:AAH4715
Acronicta clarescens|[13179]|LNC940-06|06-NCCC-940|658[0n]bp|United States.North Carolina|BOLD:AAH4715
Acronicta clarescens|[13180]|RDNMJ586-11|acrorev gga16|619[0n]bp|United States.Texas|BOLD:AAH4715
Acronicta clarescens|[13181]|UDLEP074-09|v691 MDT|658[0n]bp|United States.Delaware|BOLD:AAH4715
Acronicta strigulata|[13182]|LBCH5609-10|10-JDWBC-5609|658[0n]bp|Canada.British Columbia|BOLD:AAF5220
Acronicta strigulata|[13183]|RDNMB887-05|CNCNoctuoidea10662|658[0n]bp|Canada.British Columbia|BOLD:AAF...
Acronicta strigulata|[13184]|LOWCB176-05|CGWC-1116|658[0n]bp|Canada.British Columbia|BOLD:AAF5220
Acronicta morula|[13185]|XAJ401-06|2006-ONT-0401|658[0n]bp|Canada.Ontario|BOLD:AAB3303
Acronicta morula|[13186]|XAJ616-06|2006-ONT-0616|658[0n]bp|Canada.Ontario|BOLD:AAB3303
Acronicta morula|[13187]|PHMNB231-04|04HBL007696|609[0n]bp|Canada.New Brunswick|BOLD:AAB3303
Acronicta morula|[13188]|RDLQ224-05|DH002065|658[0n]bp|Canada.Quebec|BOLD:AAB3303
Acronicta morula|[13189]|MNBB561-05|05-NBSTA-477|658[0n]bp|Canada.New Brunswick|BOLD:AAB3303
Acronicta morula|[13190]|XAB110-04|04HBL005110|658[0n]bp|Canada.Ontario|BOLD:AAB3303
Acronicta morula|[13191]|XAC729-04|04HBL006729|658[0n]bp|Canada.Ontario|BOLD:AAB3303
Acronicta morula|[13192]|XAF649-05|2005-ONT-298|658[0n]bp|Canada.Ontario|BOLD:AAB3303
Acronicta morula|[13193]|MNBB562-05|05-NBSTA-478|658[0n]bp|Canada.New Brunswick|BOLD:AAB3303
Acronicta morula|[13194]|BLTIB174-08|BL253|658[0n]bp|Canada.Ontario|BOLD:AAB3303
Acronicta morula|[13195]|MNBB603-05|05-NBSTA-519|658[0n]bp|Canada.New Brunswick|BOLD:AAB3303
Acronicta morula|[13196]|XAB161-04|04HBL005161|658[0n]bp|Canada.Ontario|BOLD:AAB3303
Acronicta morula|[13197]|PHMNB352-04|04HBL00578|658[0n]bp|Canada.New Brunswick|BOLD:AAB3303
Acronicta morula|[13198]|XAF545-05|2005-ONT-194|658[0n]bp|Canada.Ontario|BOLD:AAB3303
Acronicta morula|[13199]|MNBB601-05|05-NBSTA-517|658[0n]bp|Canada.New Brunswick|BOLD:AAB3303
Acronicta morula|[13200]|XAE549-04|Moth4549.03|582[0n]bp|Canada.Ontario|BOLD:AAB3303
Acronicta morula|[13201]|XAC572-04|04HBL006572|594[0n]bp|Canada.Ontario|BOLD:AAB3303
Acronicta morula|[13202]|PHMNB029-03|moth181.02SA|639[0n]bp|Canada.New Brunswick|BOLD:AAB3303
Acronicta morula|[13203]|TMG113-03|moth1108.01|639[0n]bp|Canada.Ontario|BOLD:AAB3303
Acronicta morula|[13204]|PMG085-03|ACRO1.00|617[0n]bp|Canada.Ontario|BOLD:AAB3303
Acronicta morula|[13205]|PHMNB056-03|moth29.02SA|639[0n]bp|Canada.New Brunswick|BOLD:AAB3303
Acronicta superans|[13206]|PHMNB236-04|04HBL007701|526[0n]bp|Canada.New Brunswick|BOLD:AAB7444
Acronicta superans|[13207]|PHMNB223-04|04HBL007688|572[0n]bp|Canada.New Brunswick|BOLD:AAB7444
Acronicta superans|[13208]|LPSOC365-08|PPBP-2364|655[0n]bp|Canada.Ontario|BOLD:AAB7444
Acronicta superans|[13209]|XAE396-04|Moth4396.03|658[0n]bp|Canada.Ontario|BOLD:AAB7444
Acronicta superans|[13210]|PHMNB444-04|04HBL00670|658[3n]bp|Canada.New Brunswick|BOLD:AAB7444
Acronicta superans|[13211]|MNBB515-05|05-NBSTA-431|658[0n]bp|Canada.New Brunswick|BOLD:AAB7444
Acronicta superans|[13212]|XAC730-04|04HBL006730|658[0n]bp|Canada.Ontario|BOLD:AAB7444
Acronicta superans|[13213]|BBLPB648-10|10BBCLP-1647|658[0n]bp|Canada.Ontario|BOLD:AAB7444
Acronicta superans|[13214]|XAF327-05|HLC-10368|658[0n]bp|Canada.Ontario|BOLD:AAB7444
Acronicta superans|[13215]|XAK156-06|2006-ONT-1151|658[0n]bp|Canada.Ontario|BOLD:AAB7444
Acronicta superans|[13216]|LMIS030-05|05-ONMIS-0030|658[0n]bp|Canada.Ontario|BOLD:AAB7444
Acronicta superans|[13217]|RDLQ454-07|DH012733|617[1n]bp|Canada.Quebec|BOLD:AAB7444
Acronicta superans|[13218]|LPSOD337-09|08BBLEP-00115|658[0n]bp|Canada.Ontario|BOLD:AAB7444
Acronicta superans|[13219]|MNBB282-05|05-NBSTA-198|658[0n]bp|Canada.New Brunswick|BOLD:AAB7444
Acronicta superans|[13220]|LPSOD366-09|08BBLEP-00144|658[0n]bp|Canada.Ontario|BOLD:AAB7444
Acronicta superans|[13221]|LPSOC312-08|PPBP-2311|658[0n]bp|Canada.Ontario|BOLD:AAB7444
Acronicta mansueta|[13222]|RDNMB466-05|CNCNoctuoidea10232|658[0n]bp|Canada.Alberta|BOLD:AAD2021
Acronicta mansueta|[13223]|LBCH5210-10|10-JDWBC-5210|658[0n]bp|Canada.British Columbia|BOLD:AAD2021
Acronicta mansueta|[13224]|LBCG502-08|08-JDWBC-0502|658[0n]bp|Canada.British Columbia|BOLD:AAD2021
Acronicta mansueta|[13225]|RDNMB467-05|CNCNoctuoidea10233|583[1n]bp|Canada.Alberta|BOLD:AAD2021
Acronicta mansueta|[13226]|RDMAB118-05|UASM41259|658[0n]bp|Canada.Alberta|BOLD:AAD2021
Acronicta mansueta|[13227]|RDNMB461-05|CNCNoctuoidea10227|658[0n]bp|Canada.Alberta|BOLD:AAD2021
Acronicta mansueta|[13228]|RDNMB462-05|CNCNoctuoidea10228|658[0n]bp|Canada.British Columbia|BOLD:AAD2021
Acronicta mansueta|[13229]|LPABC904-09|08BBLEP-05315|658[0n]bp|Canada.Alberta|BOLD:AAD2021
Acronicta vinnula|[13230]|RDLQH215-07|MDH004121|644[0n]bp|Canada.Quebec|BOLD:AAC1018
Acronicta vinnula|[13231]|XAK333-06|2006-ONT-1328|658[0n]bp|Canada.Ontario|BOLD:AAC1018
Acronicta vinnula|[13232]|XAK319-06|2006-ONT-1314|658[0n]bp|Canada.Ontario|BOLD:AAC1018
Acronicta vinnula|[13233]|LPSOC357-08|PPBP-2356|658[0n]bp|Canada.Ontario|BOLD:AAC1018
Acronicta vinnula|[13234]|XAK332-06|2006-ONT-1327|658[0n]bp|Canada.Ontario|BOLD:AAC1018
Acronicta vinnula|[13235]|LPSOB825-08|PPBP-1824|658[0n]bp|Canada.Ontario|BOLD:AAC1018
Acronicta vinnula|[13236]|XAJ553-06|2006-ONT-0553|658[0n]bp|Canada.Ontario|BOLD:AAC1018
Acronicta grisea|[13237]|RDLQB168-05|DH010254|513[0n]bp|Canada.Quebec|BOLD:AAA7688
Acronicta grisea|[13238]|LALPA541-10|AVBC 543-10|658[0n]bp|Canada.British Columbia|BOLD:AAA7688
Acronicta grisea|[13239]|PHMNB592-04|04HBL00818|658[0n]bp|Canada.New Brunswick|BOLD:AAA7688
Acronicta grisea|[13240]|LPSOD336-09|08BBLEP-00114|658[0n]bp|Canada.Ontario|BOLD:AAA7688
Acronicta grisea|[13241]|LPVIA229-08|PFC-2006-0311|658[0n]bp|Canada.British Columbia|BOLD:AAA7688
Acronicta grisea|[13242]|LBCA362-05|HLC-20362|658[0n]bp|Canada.British Columbia|BOLD:AAA7688
Acronicta grisea|[13243]|RDLQ292-05|DH007128|600[0n]bp|Canada.Quebec|BOLD:AAA7688
Acronicta grisea|[13244]|BBLPB645-10|10BBCLP-1644|658[0n]bp|Canada.Alberta|BOLD:AAA7688
Acronicta grisea|[13245]|PHMNB363-04|04HBL00589|658[0n]bp|Canada.New Brunswick|BOLD:AAA7688
Acronicta grisea|[13246]|BBLPB778-10|10BBCLP-1777|658[0n]bp|Canada.Ontario|BOLD:AAA7688
Acronicta grisea|[13247]|PHMNB250-04|04HBL007715|609[0n]bp|Canada.New Brunswick|BOLD:AAA7688
Acronicta grisea|[13248]|LPABB488-08|08BBLEP-03753|658[0n]bp|Canada.Alberta|BOLD:AAA7688
Acronicta grisea|[13249]|PHMNB027-03|moth172.02SA|639[0n]bp|Canada.New Brunswick|BOLD:AAA7688
Acronicta grisea|[13250]|LPMN347-08|08BBLEP-01146|658[0n]bp|Canada.Manitoba|BOLD:AAA7688
Acronicta grisea|[13251]|RDNMK655-11|CNCLEP 81832|658[0n]bp|Canada.British Columbia|BOLD:AAA7688
Acronicta grisea|[13252]|LPABB448-08|08BBLEP-03713|658[0n]bp|Canada.Alberta|BOLD:AAA7688
Acronicta grisea|[13253]|LBCD142-05|HLC-22962|658[0n]bp|Canada.British Columbia|BOLD:AAA7688
Acronicta grisea|[13254]|LPAB012-08|08BBLEP-02334|658[0n]bp|Canada.Alberta|BOLD:AAA7688
Acronicta grisea|[13255]|LBCA363-05|HLC-20363|636[0n]bp|Canada.British Columbia|BOLD:AAA7688
Acronicta grisea|[13256]|LOWCE803-06|CGWC-4563|658[1n]bp|Canada.British Columbia|BOLD:AAA7688

Acronicta grisea|[13254]|LPAB012-08|08BBLEP-02334|658[0n]bp|Canada.Alberta|BOLD:AAA7688
Acronicta grisea|[13255]|LBCA363-05|HLC-20363|636[0n]bp|Canada.British Columbia|BOLD:AAA7688
Acronicta grisea|[13256]|LOWCE803-06|CGWC-4563|658[1n]bp|Canada.British Columbia|BOLD:AAA7688
Acronicta grisea|[13257]|LPMN322-08|08BBLEP-01121|658[0n]bp|Canada.Manitoba|BOLD:AAA7688
Acronicta grisea|[13258]|RDLQB175-05|DH010261|658[0n]bp|Canada.Quebec|BOLD:AAA7688
Acronicta grisea|[13259]|RDLQB171-05|DH010257|658[0n]bp|Canada.Quebec|BOLD:AAA7688
Acronicta grisea|[13260]|LALPA920-11|AVBC 1093-11|658[0n]bp|Canada.British Columbia|BOLD:AAA7688
Acronicta grisea|[13261]|RDLQB173-05|DH010259|658[0n]bp|Canada.Quebec|BOLD:AAA7688
Acronicta grisea|[13262]|RDLQB169-05|DH010255|658[0n]bp|Canada.Quebec|BOLD:AAA7688
Acronicta grisea|[13263]|RDLQF515-06|DH011664|658[0n]bp|Canada.Quebec|BOLD:AAA7688
Acronicta grisea|[13264]|RDLQB170-05|DH010256|658[0n]bp|Canada.Quebec|BOLD:AAA7688
Acronicta grisea|[13265]|LPABB371-08|08BBLEP-03636|658[0n]bp|Canada.Alberta|BOLD:AAA7688
Acronicta grisea|[13266]|BBLPE275-09|09BBELE-2275|658[0n]bp|Canada.Nova Scotia|BOLD:AAA7688
Acronicta grisea|[13267]|LPABC974-09|08BBLEP-05385|658[0n]bp|Canada.Alberta|BOLD:AAA7688
Acronicta grisea|[13268]|PHMNB678-04|04HBL00904|658[0n]bp|Canada.New Brunswick|BOLD:AAA7688
Acronicta grisea|[13269]|RDLQB174-05|DH010260|658[0n]bp|Canada.Quebec|BOLD:AAA7688
Acronicta grisea|[13270]|LCHP361-07|07PROBE-03936|658[0n]bp|Canada.Manitoba|BOLD:AAA7688
Acronicta grisea|[13271]|LPMN635-08|08BBLEP-01436|658[0n]bp|Canada.Manitoba|BOLD:AAA7688
Acronicta grisea|[13272]|LBCA391-05|HLC-20391|658[0n]bp|Canada.British Columbia|BOLD:AAA7688
Acronicta grisea|[13273]|RDLQB800-05|DH010887|617[0n]bp|Canada.Quebec|BOLD:AAA7688
Acronicta grisea|[13274]|LPVIA999-08|PFC-2006-1334|635[0n]bp|Canada.British Columbia|BOLD:AAA7688
Acronicta grisea|[13275]|LOWCB854-05|CGWC-1794|547[0n]bp|Canada.British Columbia|BOLD:AAA7688
Acronicta grisea|[13276]|LOWCE809-06|CGWC-4569|658[0n]bp|Canada.British Columbia|BOLD:AAA7688
Acronicta grisea|[13277]|LPMN573-08|08BBLEP-01374|658[0n]bp|Canada.Manitoba|BOLD:AAA7688
Acronicta grisea|[13278]|LOWCC914-05|CGWC-2794|658[0n]bp|Canada.British Columbia|BOLD:AAA7688
Acronicta grisea|[13279]|PHMNB680-04|04HBL00906|658[0n]bp|Canada.New Brunswick|BOLD:AAA7688
Acronicta grisea|[13280]|BBLPC746-09|09BBELE-1746|658[0n]bp|Canada.Newfoundland and Labrador|BOLD:AAA7688
Acronicta grisea|[13281]|LBCA497-05|HLC-20497|658[0n]bp|Canada.British Columbia|BOLD:AAA7688
Acronicta grisea|[13282]|LALPA943-11|AVBC 1116-11|658[0n]bp|Canada.British Columbia|BOLD:AAA7688
Acronicta grisea|[13283]|RDNMB890-05|CNCNoctuoidea10665|658[0n]bp|Canada.New Brunswick|BOLD:AAA7688
Acronicta grisea|[13284]|BBLPB646-10|10BBCLP-1645|658[0n]bp|Canada.British Columbia|BOLD:AAA7688
Acronicta grisea|[13285]|PHMNB535-04|04HBL00761|658[0n]bp|Canada.New Brunswick|BOLD:AAA7688
Acronicta grisea|[13286]|BBLPE544-09|09BBELE-2544|658[0n]bp|Canada.Newfoundland and Labrador|BOLD:AAA7688
Acronicta grisea|[13287]|RDLQB172-05|DH010258|658[0n]bp|Canada.Quebec|BOLD:AAA7688
Acronicta grisea|[13288]|PHMNB527-04|04HBL00753|658[0n]bp|Canada.New Brunswick|BOLD:AAA7688
Acronicta grisea|[13289]|RDLQB176-05|DH010262|658[0n]bp|Canada.Quebec|BOLD:AAA7688
Acronicta grisea|[13290]|RDNMB891-05|CNCNoctuoidea10666|658[0n]bp|Canada.British Columbia|BOLD:AAA7688
Acronicta grisea|[13291]|LPSOD586-09|08BBLEP-00367|658[0n]bp|Canada.Ontario|BOLD:AAA7688
Acronicta hamamelis|[13292]|QUNOC037-09|5868-070506-LA|658[0n]bp|United States.Louisiana|BOLD:AAJ1606
Acronicta hamamelis|[13293]|MILEQ293-11|11-MISC-768|658[0n]bp|United States.Alabama|BOLD:AAJ1606
Acronicta hamamelis|[13294]|LNCB069-06|06-NCC-1025|658[0n]bp|United States.North Carolina|BOLD:AAJ1606
Acronicta lithospila|[13295]|LPOKB1035-09|MDOK-2077|658[0n]bp|United States.Oklahoma|BOLD:AAF0152
Acronicta lithospila|[13296]|LNCC657-11|11-NCCC-182|658[0n]bp|United States.North Carolina|BOLD:AAF0152
Acronicta lithospila|[13297]|LSEU392-06|06-JKA-0392|658[0n]bp|United States.Georgia|BOLD:AAF0152
Acronicta lithospila|[13298]|LSEU334-06|06-JKA-0334|658[0n]bp|United States.Georgia|BOLD:AAF0152
Acronicta lithospila|[13299]|LOTB298-05|05-TN-00298|658[0n]bp|United States.Tennessee|BOLD:AAF0152
Acronicta tritona|[13300]|MMNA003-05|HH001|658[0n]bp|Canada.New Brunswick|BOLD:AAC1000
Acronicta tritona|[13301]|LCH263-04|04HBL003263|658[0n]bp|Canada.Manitoba|BOLD:AAC1000
Acronicta tritona|[13302]|RDLQF711-06|DH011861|637[0n]bp|Canada.Quebec|BOLD:AAC1000
Agriopodes fallax|[13303]|PHMNB596-04|04HBL00822|658[0n]bp|Canada.New Brunswick|BOLD:AAB6889
Agriopodes fallax|[13304]|RDLQB064-05|DH010150|658[0n]bp|Canada.Quebec|BOLD:AAB6889
Agriopodes fallax|[13305]|BBLPE272-09|09BBELE-2272|658[0n]bp|Canada.Nova Scotia|BOLD:AAB6889
Agriopodes fallax|[13306]|RDLQB065-05|DH010151|553[0n]bp|Canada.Quebec|BOLD:AAB6889
Agriopodes fallax|[13307]|BBLPE029-09|09BBELE-2029|658[0n]bp|Canada.Nova Scotia|BOLD:AAB6889
Agriopodes fallax|[13308]|XAB606-04|04HBL005606|658[0n]bp|Canada.Ontario|BOLD:AAB6889
Agriopodes fallax|[13309]|XAE561-04|Moth4561.03|658[0n]bp|Canada.Ontario|BOLD:AAB6889
Agriopodes fallax|[13310]|XAF497-05|2005-ONT-146|658[0n]bp|Canada.Ontario|BOLD:AAB6889
Agriopodes fallax|[13311]|LPSOC352-08|PPBP-2351|658[0n]bp|Canada.Ontario|BOLD:AAB6889
Agriopodes fallax|[13312]|XAI039-05|0102-ONT-0039|658[0n]bp|Canada.Ontario|BOLD:AAB6889
Agriopodes fallax|[13313]|LPSO355-08|PPBP-0355|658[0n]bp|Canada.Ontario|BOLD:AAB6889
Agriopodes fallax|[13314]|XAF754-05|2005-ONT-403|578[0n]bp|Canada.Ontario|BOLD:AAB6889
Agriopodes fallax|[13315]|PHMO133-03|moth772.01|639[0n]bp|Canada.Ontario|BOLD:AAB6889
Agriopodes fallax|[13316]|XAC797-04|04HBL006797|615[0n]bp|Canada.Ontario|BOLD:AAB6889
Agriopodes fallax|[13317]|PHMO147-03|moth829.01|639[0n]bp|Canada.Ontario|BOLD:AAB6889
Agriopodes geminata|[13318]|RDNMF449-08|NOC14535|641[1n]bp|Canada.Manitoba|BOLD:AAB6889
Acronicta lepusculina|[13319]|RDMAB614-06|UASM58661B|658[0n]bp|Canada.Alberta|BOLD:AAB0891
Acronicta cyanescens|[13320]|RDNME193-07|CNCNoctuoidea13800|658[0n]bp|Canada.Yukon Territory|BOLD:AAB0891
Acronicta cyanescens|[13321]|LPABB609-08|08BBLEP-03874|658[0n]bp|Canada.Alberta|BOLD:AAB0891
Acronicta cyanescens|[13322]|RDNMJ599-11|acrorev gga29|658[0n]bp|Canada.British Columbia|BOLD:AAB0891
Acronicta cyanescens|[13323]|RDNMJ598-11|acrorev gga28|658[0n]bp|Canada.British Columbia|BOLD:AAB0891
Acronicta cyanescens|[13324]|RDNMB874-05|CNCNoctuoidea10649|658[0n]bp|Canada.British Columbia|BOLD:AAB...
Acronicta cyanescens|[13325]|RDMAB572-06|UASM58571|658[0n]bp|Canada.Alberta|BOLD:AAB0891
Acronicta cyanescens|[13326]|LPABC976-09|08BBLEP-05387|658[0n]bp|Canada.Alberta|BOLD:AAB0891
Acronicta cyanescens|[13327]|LPABC812-09|08BBLEP-05031|658[0n]bp|Canada.Alberta|BOLD:AAB0891
Acronicta cyanescens|[13328]|RDNMJ595-11|acrorev gga25|658[0n]bp|Canada.British Columbia|BOLD:AAB0891
Acronicta cyanescens|[13329]|LPABB608-08|08BBLEP-03873|658[0n]bp|Canada.Alberta|BOLD:AAB0891
Acronicta cyanescens|[13330]|RDNMJ623-11|acrorev gga53|658[0n]bp|Canada.Alberta|BOLD:AAB0891
Acronicta cyanescens|[13331]|RDMAB573-06|UASM58572|629[0n]bp|Canada.Alberta|BOLD:AAB0891
Acronicta cyanescens|[13332]|RDNMB873-05|CNCNoctuoidea10648|550[0n]bp|Canada.British Columbia|BOLD:AAB...
Acronicta lepusculina|[13333]|RDMAB615-06|UASM24156|658[0n]bp|Canada.Alberta|BOLD:AAB0891
Acronicta lepusculina|[13334]|RDMAB612-06|UASM57541|658[0n]bp|Canada.Alberta|BOLD:AAB0891
Acronicta lepusculina|[13335]|RDMAB605-06|UASM58661A|658[0n]bp|Canada.Alberta|BOLD:AAB0891
Acronicta lepusculina|[13336]|RDMAB613-06|UASM2704|658[0n]bp|Canada.Alberta|BOLD:AAB0891
Acronicta lepusculina|[13337]|RDNMJ605-11|acrorev gga35|658[0n]bp|Canada.New Brunswick|BOLD:AAB0891
Acronicta lepusculina|[13338]|LPSOB322-08|PPBP-1321|658[0n]bp|Canada.Ontario|BOLD:AAB0891
Acronicta lepusculina|[13339]|RDNMD272-06|CNCNoctuoidea12604|658[0n]bp|Canada.New Brunswick|BOLD:AAB0891
Acronicta lepusculina|[13340]|KPOEC129-08|08OEC-130|658[0n]bp|Canada.Ontario|BOLD:AAB0891
Acronicta lepusculina|[13341]|RDNMJ607-11|acrorev gga37|658[0n]bp|Canada.Alberta|BOLD:AAB0891
Acronicta lepusculina|[13342]|RDMAB606-06|UASM58662|658[0n]bp|Canada.Alberta|BOLD:AAB0891
Acronicta lepusculina|[13343]|RDNMD270-06|CNCNoctuoidea12602|658[0n]bp|Canada.New Brunswick|BOLD:AAB0891
Acronicta lepusculina|[13344]|RDLQB153-05|DH010239|658[0n]bp|Canada.Quebec|BOLD:AAB0891
Acronicta lepusculina|[13345]|XAK537-07|HLC-16090|658[0n]bp|Canada.Ontario|BOLD:AAB0891
Acronicta lepusculina|[13346]|RDLQB154-05|DH010240|658[0n]bp|Canada.Quebec|BOLD:AAB0891
Acronicta lepusculina|[13347]|RDNMD271-06|CNCNoctuoidea12603|658[0n]bp|Canada.New Brunswick|BOLD:AAB0891
Acronicta lepusculina|[13348]|RDNMD273-06|CNCNoctuoidea12605|658[0n]bp|Canada.New Brunswick|BOLD:AAB0891
Acronicta lepusculina|[13349]|RDNMJ597-11|acrorev gga27|658[0n]bp|Canada.Alberta|BOLD:AAB0891
Acronicta lepusculina|[13350]|KPOEC166-08|08OEC-207|658[0n]bp|Canada.Ontario|BOLD:AAB0891
Acronicta lepusculina|[13351]|RDLQB156-05|DH010242|658[0n]bp|Canada.Quebec|BOLD:AAB0891
Acronicta lepusculina|[13352]|XAK509-07|HLC-16062|658[0n]bp|Canada.Ontario|BOLD:AAB0891
Acronicta lepusculina|[13353]|XAK159-06|2006-ONT-1154|658[0n]bp|Canada.Ontario|BOLD:AAB0891
Acronicta lepusculina|[13354]|XAJ597-06|2006-ONT-0597|656[0n]bp|Canada.Ontario|BOLD:AAB0891
Acronicta lepusculina|[13355]|PMG084-03|moth239.01|617[0n]bp|Canada.Ontario|BOLD:AAB0891
Acronicta lepusculina|[13356]|RDNMB879-05|CNCNoctuoidea10654|638[0n]bp|Canada.New Brunswick|BOLD:AAB0891

[illegible]
Acronicta lepusculina|[13354]|XAJ597-06|2006-ONT-0597|656[0n]bp|Canada.Ontario|BOLD:AAB0891
Acronicta lepusculina|[13355]|PMG084-03|moth239.01|617[0n]bp|Canada.Ontario|BOLD:AAB0891
Acronicta lepusculina|[13356]|RDNMB879-05|CNCNoctuoidea10654|638[0n]bp|Canada.New Brunswick|BOLD:AAB0891
Acronicta lepusculina|[13357]|RDLQ443-07|DH012630|658[0n]bp|Canada.Quebec|BOLD:AAB0891
Acronicta lepusculina|[13358]|RDLQB157-05|DH010243|658[0n]bp|Canada.Quebec|BOLD:AAB0891
Acronicta lepusculina|[13359]|RDLQB155-05|DH010241|658[0n]bp|Canada.Quebec|BOLD:AAB0891
Acronicta lepusculina|[13360]|XAF793-05|2005-ONT-442|658[0n]bp|Canada.Ontario|BOLD:AAB0891
Acronicta lepusculina|[13361]|RDNMB878-05|CNCNoctuoidea10653|658[0n]bp|Canada.New Brunswick|BOLD:AAB0891
Acronicta lanceolaria|[13362]|RDNMB892-05|CNCNoctuoidea10667|658[0n]bp|Canada.Alberta|BOLD:AAJ1615
Acronicta lanceolaria|[13363]|RDNMG539-08|CNC LEP00052363|658[0n]bp|Canada.Alberta|BOLD:AAJ1615
Acronicta impleta|[13364]|PHMNB017-03|moth146.02SA|639[0n]bp|Canada.New Brunswick|BOLD:AAB0826
Acronicta impleta|[13365]|LPSOB376-08|PPBP-1375|657[0n]bp|Canada.Ontario|BOLD:AAB0826
Acronicta impleta|[13366]|RDNMB885-05|CNCNoctuoidea10660|658[0n]bp|Canada.Ontario|BOLD:AAB0826
Acronicta impleta|[13367]|LPSOD238-09|08BBLEP-00016|658[1n]bp|Canada.Ontario|BOLD:AAB0826
Acronicta impleta|[13368]|RDLQ446-07|DH009482|645[0n]bp|Canada.Quebec|BOLD:AAB0826
Acronicta impleta|[13369]|RDLQ447-07|DH012008|645[0n]bp|Canada.Quebec|BOLD:AAB0826
Acronicta impleta|[13370]|RDLQ448-07|DH009406|658[0n]bp|Canada.Quebec|BOLD:AAB0826
Acronicta impleta|[13371]|PSAT135-10|CNCLEP 70019|658[0n]bp|Canada.British Columbia|BOLD:AAB0826
Acronicta impleta|[13372]|LPVIA110-08|PFC-2006-0176|658[0n]bp|Canada.British Columbia|BOLD:AAB0826
Acronicta impleta|[13373]|RDMAB123-05|UASM41269|658[0n]bp|Canada.Alberta|BOLD:AAB0826
Acronicta impleta|[13374]|BBLPB650-10|10BBCLP-1649|658[0n]bp|Canada.Ontario|BOLD:AAB0826
Acronicta impleta|[13375]|RDNMB884-05|CNCNoctuoidea10659|658[0n]bp|Canada.Ontario|BOLD:AAB0826
Acronicta impleta|[13376]|LPSOB590-08|PPBP-1589|658[0n]bp|Canada.Ontario|BOLD:AAB0826
Acronicta perdita|[13377]|RDNMF116-08|NOC14202|658[0n]bp|Canada.British Columbia|BOLD:ABZ6712
Acronicta perdita|[13378]|RDNMF114-08|NOC14200|647[0n]bp|Canada.British Columbia|BOLD:ABZ6712
Acronicta perdita|[13379]|LPVIB875-08|PFC-2006-2392|630[0n]bp|Canada.British Columbia|BOLD:ABZ6712
Acronicta sperata|[13380]|RDNMB453-05|CNCNoctuoidea10219|658[0n]bp|Canada.Alberta|BOLD:AAD7893
Acronicta sperata|[13381]|RDNMB452-05|CNCNoctuoidea10218|658[0n]bp|Canada.Ontario|BOLD:AAD7893
Acronicta sperata|[13382]|KPOEC142-08|08OEC-146|658[0n]bp|Canada.Ontario|BOLD:AAD7893
Acronicta sperata|[13383]|RDNMB454-05|CNCNoctuoidea10220|658[0n]bp|Canada.Alberta|BOLD:AAD7893
Acronicta sperata|[13384]|RDNMB450-05|CNCNoctuoidea10216|658[0n]bp|Canada.Ontario|BOLD:AAD7893
Acronicta sperata|[13385]|RDNMB451-05|CNCNoctuoidea10217|658[0n]bp|Canada.Ontario|BOLD:AAD7893
Acronicta lupini|[13386]|RDNMB455-05|CNCNoctuoidea10221|658[0n]bp|Canada.British Columbia|BOLD:AAC4122
Acronicta lupini|[13387]|LBCB937-05|HLC-21877|624[0n]bp|Canada.British Columbia|BOLD:AAC4122
Acronicta lupini|[13388]|LPABB404-08|08BBLEP-03669|658[0n]bp|Canada.Alberta|BOLD:AAC4122
Acronicta lupini|[13389]|RDNMD281-06|CNCNoctuoidea12613|608[0n]bp|Canada.British Columbia|BOLD:AAC4122
Acronicta lupini|[13390]|GWNC612-07|CNCLEP00034174|658[0n]bp|Canada.Yukon Territory|BOLD:AAC4122
Acronicta lupini|[13391]|LPMN928-08|08BBLEP-02286|658[0n]bp|Canada.Alberta|BOLD:AAC4122
Acronicta lupini|[13392]|RDNMD282-06|CNCNoctuoidea12614|658[0n]bp|Canada.British Columbia|BOLD:AAC4122
Acronicta lupini|[13393]|CHLEP068-09|09PROBE-09363|627[0n]bp|Canada.Manitoba|BOLD:AAC4122
Acronicta lupini|[13394]|RDLQ455-07|DH008732|658[0n]bp|Canada.Newfoundland and Labrador|BOLD:AAC4122
Acronicta lupini|[13395]|GWNC611-07|CNCLEP00034173|658[0n]bp|Canada.Yukon Territory|BOLD:AAC4122
Acronicta lupini|[13396]|RDLQF112-06|CB0027|656[0n]bp|Canada.Quebec|BOLD:AAC4122
Acronicta longa|[13397]|RDNMK683-11|acrorev gga-125|658[0n]bp|Canada.Alberta|BOLD:AAD2547
Acronicta longa|[13398]|RDNMJ051-10|CNCLEP 73831|658[0n]bp|Canada.Alberta|BOLD:AAD2547
Acronicta longa|[13399]|TTMNB035-06|MNBTT-035|656[0n]bp|Canada.New Brunswick|BOLD:AAD2547
Acronicta longa|[13400]|RDLQ289-05|DH007121|656[0n]bp|Canada.Quebec|BOLD:AAD2547
Acronicta noctivaga|[13401]|PHMNB251-04|04HBL007716|567[0n]bp|Canada.New Brunswick|BOLD:AAC0015
Acronicta noctivaga|[13402]|LPSOD874-09|08BBLEP-00656|658[0n]bp|Canada.Ontario|BOLD:AAC0015
Acronicta noctivaga|[13403]|XAJ352-06|2006-ONT-0352|658[0n]bp|Canada.Ontario|BOLD:AAC0015
Acronicta noctivaga|[13404]|TMNBB159-06|MNBTT-1099|658[0n]bp|Canada.New Brunswick|BOLD:AAC0015
Acronicta noctivaga|[13405]|RDLQF516-06|DH011665|658[0n]bp|Canada.Quebec|BOLD:AAC0015
Acronicta noctivaga|[13406]|TMNBB158-06|MNBTT-1098|658[0n]bp|Canada.New Brunswick|BOLD:AAC0015
Acronicta noctivaga|[13407]|RDLQB165-05|DH010251|658[0n]bp|Canada.Quebec|BOLD:AAC0015
Acronicta noctivaga|[13408]|RDLQB795-05|DH010882|658[0n]bp|Canada.Quebec|BOLD:AAC0015
Acronicta noctivaga|[13409]|RDLQB164-05|DH010250|658[0n]bp|Canada.Quebec|BOLD:AAC0015
Acronicta noctivaga|[13410]|KPOEC131-08|08OEC-132|658[0n]bp|Canada.Ontario|BOLD:AAC0015
Acronicta noctivaga|[13411]|LPSOD244-09|08BBLEP-00022|658[0n]bp|Canada.Ontario|BOLD:AAC0015
Acronicta noctivaga|[13412]|RDLQ141-05|DH002142|614[0n]bp|Canada.Quebec|BOLD:AAC0015
Acronicta oblinita|[13413]|RDLQB802-05|DH010889|563[1n]bp|Canada.Quebec|BOLD:AAB1100
Acronicta oblinita|[13414]|LPSOB600-08|PPBP-1599|658[0n]bp|Canada.Ontario|BOLD:AAB1100
Acronicta oblinita|[13415]|XAE309-04|Moth4309.03|658[0n]bp|Canada.Ontario|BOLD:AAB1100
Acronicta oblinita|[13416]|RDLQB804-05|DH010891|615[0n]bp|Canada.Quebec|BOLD:AAB1100
Acronicta oblinita|[13417]|RDLQB163-05|DH010249|658[0n]bp|Canada.Quebec|BOLD:AAB1100
Acronicta oblinita|[13418]|XAF755-05|2005-ONT-404|587[4n]bp|Canada.Ontario|BOLD:AAB1100
Acronicta oblinita|[13419]|RDLQB801-05|DH010888|658[0n]bp|Canada.Quebec|BOLD:AAB1100
Acronicta oblinita|[13420]|BBLPE033-09|09BBELE-2033|658[0n]bp|Canada.Nova Scotia|BOLD:AAB1100
Acronicta oblinita|[13421]|RDLQB803-05|DH010890|658[0n]bp|Canada.Quebec|BOLD:AAB1100
Acronicta oblinita|[13422]|XAI059-05|0102-ONT-0059|658[0n]bp|Canada.Ontario|BOLD:AAB1100
Acronicta oblinita|[13423]|XAJ594-06|2006-ONT-0594|656[0n]bp|Canada.Ontario|BOLD:AAB1100
Acronicta oblinita|[13424]|XAJ400-06|2006-ONT-0400|658[0n]bp|Canada.Ontario|BOLD:AAB1100
Acronicta oblinita|[13425]|PHMNB211-04|04HBL007676|589[0n]bp|Canada.New Brunswick|BOLD:AAB1100
Acronicta oblinita|[13426]|TTMNB030-06|MNBTT-030|658[0n]bp|Canada.New Brunswick|BOLD:AAB1100
Acronicta oblinita|[13427]|RDLQ290-05|DH007122|597[0n]bp|Canada.Quebec|BOLD:AAB1100
Acronicta oblinita|[13428]|LPSO525-08|PPBP-0525|656[0n]bp|Canada.Ontario|BOLD:AAB1100
Acronicta oblinita|[13429]|XAC625-04|04HBL006625|615[2n]bp|Canada.Ontario|BOLD:AAB1100
Acronicta oblinita|[13430]|XAE206-04|Moth4206.03|658[0n]bp|Canada.Ontario|BOLD:AAB1100
Acronicta oblinita|[13431]|LPSO544-08|PPBP-0544|658[0n]bp|Canada.Ontario|BOLD:AAB1100
Acronicta oblinita|[13432]|LPSO847-08|PPBP-0847|658[0n]bp|Canada.Ontario|BOLD:AAB1100
Simyra insularis|[13433]|RDMAB709-06|UASM57362|566[0n]bp|Canada.Alberta|BOLD:AAA7111
Simyra insularis|[13434]|XAC154-04|04HBL006154|658[0n]bp|Canada.Ontario|BOLD:AAA7111
Simyra insularis|[13435]|RDMAB710-06|UASM24544|636[0n]bp|Canada.Alberta|BOLD:AAA7111
Simyra insularis|[13436]|RDLQB922-05|DH002289|580[3n]bp|Canada.Quebec|BOLD:AAA7111
Simyra insularis|[13437]|XAK600-07|HLC-16153|592[0n]bp|Canada.Ontario|BOLD:AAA7111
Simyra insularis|[13438]|PMG161-03|moth253.01|617[0n]bp|Canada.Ontario|BOLD:AAA7111
Simyra insularis|[13439]|TMG115-03|moth361.01|639[0n]bp|Canada.Ontario|BOLD:AAA7111
Simyra insularis|[13440]|RDLQG221-06|DH012404|658[0n]bp|Canada.Quebec|BOLD:AAA7111
Simyra insularis|[13441]|RDLQB499-05|DH010585|658[0n]bp|Canada.Quebec|BOLD:AAA7111
Simyra insularis|[13442]|RDLQB706-05|DH010809|658[0n]bp|Canada.Quebec|BOLD:AAA7111
Simyra insularis|[13443]|XAC153-04|04HBL006153|658[0n]bp|Canada.Ontario|BOLD:AAA7111
Simyra insularis|[13444]|RDLQB921-05|DH009282|658[0n]bp|Canada.Quebec|BOLD:AAA7111
Simyra insularis|[13445]|XAG014-05|2005-ONT-598|658[0n]bp|Canada.Ontario|BOLD:AAA7111
Simyra insularis|[13446]|XAK531-07|HLC-16084|658[0n]bp|Canada.Ontario|BOLD:AAA7111
Simyra insularis|[13447]|RDLQG222-06|DH012405|658[0n]bp|Canada.Quebec|BOLD:AAA7111
Simyra insularis|[13448]|XAK260-06|2006-ONT-1255|658[0n]bp|Canada.Ontario|BOLD:AAA7111
Simyra insularis|[13449]|XAC181-04|04HBL006181|658[0n]bp|Canada.Ontario|BOLD:AAA7111
Simyra insularis|[13450]|RDLQH082-06|DH013320|658[0n]bp|Canada.Quebec|BOLD:AAA7111
Simyra insularis|[13451]|XAD660-05|2005-ONT-75|658[0n]bp|Canada.Ontario|BOLD:AAA7111
Simyra insularis|[13452]|XAK532-07|HLC-16085|658[0n]bp|Canada.Ontario|BOLD:AAA7111
Simyra insularis|[13453]|BLTIB057-08|BL0090|658[0n]bp|Canada.Ontario|BOLD:AAA7111
Simyra insularis|[13454]|LPMN297-08|08BBLEP-01096|634[1n]bp|Canada.Manitoba|BOLD:AAA7111
Simyra insularis|[13455]|RDMAB718-06|UASM7054|658[0n]bp|Canada.Alberta|BOLD:AAA7111
[illegible]

Simyra insularis|[13453]|BLTIB057-08|BL0090|658[0n]bp|Canada.Ontario|BOLD:AAA7111
Simyra insularis|[13454]|LPMN297-08|08BBLEP-01096|634[1n]bp|Canada.Manitoba|BOLD:AAA7111
Simyra insularis|[13455]|RDMAB718-06|UASM7054|658[0n]bp|Canada.Alberta|BOLD:AAA7111
Simyra insularis|[13456]|XAG013-05|2005-ONT-597|658[0n]bp|Canada.Ontario|BOLD:AAA7111
Simyra insularis|[13457]|XAE638-04|Moth4638.03|658[0n]bp|Canada.Ontario|BOLD:AAA7111
Simyra insularis|[13458]|RDLQB429-05|DH010515|658[0n]bp|Canada.Quebec|BOLD:AAA7111
Simyra insularis|[13459]|XAJ453-06|2006-ONT-0453|658[0n]bp|Canada.Ontario|BOLD:AAA7111
Simyra insularis sp. A|[13460]|NCCH103-11|BIOUG01862-E05|658[0n]bp|Canada.Ontario|BOLD:AAA7111
Hydroeciodes serrata|[13461]|RDNMF442-08|NOC14528|658[0n]bp|Canada.Alberta|BOLD:AAE5159
Hydroeciodes serrata|[13462]|LPABC693-09|08BBLEP-04912|658[0n]bp|Canada.Alberta|BOLD:AAE5159
Protorthodes curtica|[13463]|LBCH7340-10|10-JDWBC-7340|658[0n]bp|Canada.British Columbia|BOLD:ABZ7207
Protorthodes curtica|[13464]|LBCH7185-10|10-JDWBC-7185|658[0n]bp|Canada.British Columbia|BOLD:ABZ7207
Protorthodes curtica|[13465]|LBCH7288-10|10-JDWBC-7288|658[0n]bp|Canada.British Columbia|BOLD:ABZ7207
Protorthodes curtica|[13466]|LBCH7614-10|10-JDWBC-7614|658[0n]bp|Canada.British Columbia|BOLD:ABZ7207
Protorthodes curtica|[13467]|LBCH7679-10|10-JDWBC-7679|658[0n]bp|Canada.British Columbia|BOLD:ABZ7207
Protorthodes curtica|[13468]|LBCH7112-10|10-JDWBC-7112|658[0n]bp|Canada.British Columbia|BOLD:ABZ7207
Protorthodes curtica|[13469]|LBCH6995-10|10-JDWBC-6995|658[0n]bp|Canada.British Columbia|BOLD:ABZ7207
Protorthodes curtica|[13470]|LBCH7794-10|10-JDWBC-7794|658[0n]bp|Canada.British Columbia|BOLD:ABZ7207
Protorthodes curtica|[13471]|LBCH7186-10|10-JDWBC-7186|658[0n]bp|Canada.British Columbia|BOLD:ABZ7207
Protorthodes curtica|[13472]|LBCH7886-10|10-JDWBC-7886|658[0n]bp|Canada.British Columbia|BOLD:ABZ7207
Protorthodes curtica|[13473]|LBCH7293-10|10-JDWBC-7293|658[0n]bp|Canada.British Columbia|BOLD:ABZ7207
Protorthodes curtica|[13474]|LBCH6830-10|10-JDWBC-6830|658[0n]bp|Canada.British Columbia|BOLD:ABZ7207
Protorthodes curtica|[13475]|LBCH6547-10|10-JDWBC-6547|658[0n]bp|Canada.British Columbia|BOLD:ABZ7207
Protorthodes curtica|[13476]|LBCH7291-10|10-JDWBC-7291|658[0n]bp|Canada.British Columbia|BOLD:ABZ7207
Protorthodes curtica|[13477]|LBCH7889-10|10-JDWBC-7889|658[0n]bp|Canada.British Columbia|BOLD:ABZ7207
Protorthodes curtica|[13478]|LBCH7184-10|10-JDWBC-7184|658[0n]bp|Canada.British Columbia|BOLD:ABZ7207
Protorthodes curtica|[13479]|LBCH7294-10|10-JDWBC-7294|658[0n]bp|Canada.British Columbia|BOLD:ABZ7207
Protorthodes curtica|[13480]|LBCH7890-10|10-JDWBC-7890|658[0n]bp|Canada.British Columbia|BOLD:ABZ7207
Protorthodes curtica|[13481]|LBCH7556-10|10-JDWBC-7556|658[0n]bp|Canada.British Columbia|BOLD:ABZ7207
Protorthodes curtica|[13482]|LBCH7892-10|10-JDWBC-7892|658[0n]bp|Canada.British Columbia|BOLD:ABZ7207
Protorthodes curtica|[13483]|LBCH7616-10|10-JDWBC-7616|658[0n]bp|Canada.British Columbia|BOLD:ABZ7207
Protorthodes curtica|[13484]|LBCH7111-10|10-JDWBC-7111|658[0n]bp|Canada.British Columbia|BOLD:ABZ7207
Protorthodes curtica|[13485]|LBCH6831-10|10-JDWBC-6831|658[0n]bp|Canada.British Columbia|BOLD:ABZ7207
Protorthodes curtica|[13486]|LBCH7110-10|10-JDWBC-7110|658[0n]bp|Canada.British Columbia|BOLD:ABZ7207
Protorthodes curtica|[13487]|LBCH7113-10|10-JDWBC-7113|658[0n]bp|Canada.British Columbia|BOLD:ABZ7207
Protorthodes curtica|[13488]|LBCH7289-10|10-JDWBC-7289|658[0n]bp|Canada.British Columbia|BOLD:ABZ7207
Protorthodes curtica|[13489]|LBCH7189-10|10-JDWBC-7189|658[0n]bp|Canada.British Columbia|BOLD:ABZ7207
Protorthodes curtica|[13490]|LBCH7430-10|10-JDWBC-7430|658[0n]bp|Canada.British Columbia|BOLD:ABZ7207
Protorthodes curtica|[13491]|LBCH7109-10|10-JDWBC-7109|658[0n]bp|Canada.British Columbia|BOLD:ABZ7207
Protorthodes curtica|[13492]|LBCH6998-10|10-JDWBC-6998|658[0n]bp|Canada.British Columbia|BOLD:ABZ7207
Protorthodes curtica|[13493]|LBCH7260-10|10-JDWBC-7260|658[0n]bp|Canada.British Columbia|BOLD:ABZ7207
Protorthodes curtica|[13494]|LBCH7610-10|10-JDWBC-7610|658[0n]bp|Canada.British Columbia|BOLD:ABZ7207
Protorthodes curtica|[13495]|LBCH6764-10|10-JDWBC-6764|658[0n]bp|Canada.British Columbia|BOLD:ABZ7207
Protorthodes curtica|[13496]|LBCH7615-10|10-JDWBC-7615|658[0n]bp|Canada.British Columbia|BOLD:ABZ7207
Protorthodes curtica|[13497]|LBCH7477-10|10-JDWBC-7477|658[0n]bp|Canada.British Columbia|BOLD:ABZ7207
Protorthodes curtica|[13498]|LBCH7295-10|10-JDWBC-7295|658[0n]bp|Canada.British Columbia|BOLD:ABZ7207
Protorthodes curtica|[13499]|LBCH7183-10|10-JDWBC-7183|658[0n]bp|Canada.British Columbia|BOLD:ABZ7207
Protorthodes curtica|[13500]|LBCH6791-10|10-JDWBC-6791|658[0n]bp|Canada.British Columbia|BOLD:ABZ7207
Protorthodes curtica|[13501]|LBCH7613-10|10-JDWBC-7613|658[0n]bp|Canada.British Columbia|BOLD:ABZ7207
Protorthodes curtica|[13502]|LBCH6996-10|10-JDWBC-6996|658[0n]bp|Canada.British Columbia|BOLD:ABZ7207
Protorthodes curtica|[13503]|LBCH7888-10|10-JDWBC-7888|658[0n]bp|Canada.British Columbia|BOLD:ABZ7207
Protorthodes curtica|[13504]|LBCH7609-10|10-JDWBC-7609|658[0n]bp|Canada.British Columbia|BOLD:ABZ7207
Protorthodes curtica|[13505]|LBCH7106-10|10-JDWBC-7106|658[0n]bp|Canada.British Columbia|BOLD:ABZ7207
Protorthodes curtica|[13506]|LBCH7891-10|10-JDWBC-7891|658[0n]bp|Canada.British Columbia|BOLD:ABZ7207
Protorthodes curtica|[13507]|LBCH7812-10|10-JDWBC-7812|658[0n]bp|Canada.British Columbia|BOLD:ABZ7207
Protorthodes curtica|[13508]|LBCH7104-10|10-JDWBC-7104|658[0n]bp|Canada.British Columbia|BOLD:ABZ7207
Protorthodes curtica|[13509]|LBCH7188-10|10-JDWBC-7188|658[0n]bp|Canada.British Columbia|BOLD:ABZ7207
Protorthodes curtica|[13510]|LBCH7191-10|10-JDWBC-7191|658[0n]bp|Canada.British Columbia|BOLD:ABZ7207
Protorthodes curtica|[13511]|LBCH7335-10|10-JDWBC-7335|658[0n]bp|Canada.British Columbia|BOLD:ABZ7207
Protorthodes curtica|[13512]|LBCH7254-10|10-JDWBC-7254|658[0n]bp|Canada.British Columbia|BOLD:ABZ7207
Protorthodes curtica|[13513]|LBCH7107-10|10-JDWBC-7107|658[0n]bp|Canada.British Columbia|BOLD:ABZ7207
Protorthodes curtica|[13514]|LBCH6928-10|10-JDWBC-6928|658[0n]bp|Canada.British Columbia|BOLD:ABZ7207
Protorthodes curtica|[13515]|LBCH6934-10|10-JDWBC-6934|658[0n]bp|Canada.British Columbia|BOLD:ABZ7207
Protorthodes curtica|[13516]|LBCH7108-10|10-JDWBC-7108|658[0n]bp|Canada.British Columbia|BOLD:ABZ7207
Protorthodes curtica|[13517]|LBCH7290-10|10-JDWBC-7290|658[0n]bp|Canada.British Columbia|BOLD:ABZ7207
Protorthodes curtica|[13518]|LBCH6992-10|10-JDWBC-6992|658[0n]bp|Canada.British Columbia|BOLD:ABZ7207
Protorthodes curtica|[13519]|LBCH6997-10|10-JDWBC-6997|658[0n]bp|Canada.British Columbia|BOLD:ABZ7207
Protorthodes curtica|[13520]|LBCH6994-10|10-JDWBC-6994|658[0n]bp|Canada.British Columbia|BOLD:ABZ7207
Protorthodes curtica|[13521]|LBCH7893-10|10-JDWBC-7893|658[0n]bp|Canada.British Columbia|BOLD:ABZ7207
Protorthodes curtica|[13522]|LBCH6999-10|10-JDWBC-6999|658[0n]bp|Canada.British Columbia|BOLD:ABZ7207
Protorthodes curtica|[13523]|LBCH6993-10|10-JDWBC-6993|658[0n]bp|Canada.British Columbia|BOLD:ABZ7207
Protorthodes curtica|[13524]|LBCH7258-10|10-JDWBC-7258|626[0n]bp|Canada.British Columbia|BOLD:ABZ7207
Protorthodes curtica|[13525]|LBCH7883-10|10-JDWBC-7883|658[0n]bp|Canada.British Columbia|BOLD:ABZ7207
Protorthodes curtica|[13526]|LBCH6792-10|10-JDWBC-6792|658[0n]bp|Canada.British Columbia|BOLD:ABZ7207
Protorthodes curtica|[13527]|LBCH7255-10|10-JDWBC-7255|658[0n]bp|Canada.British Columbia|BOLD:ABZ7207
Protorthodes curtica|[13528]|LBCH7611-10|10-JDWBC-7611|642[0n]bp|Canada.British Columbia|BOLD:ABZ7207
Protorthodes curtica|[13529]|LBCH7612-10|10-JDWBC-7612|644[0n]bp|Canada.British Columbia|BOLD:ABZ7207
Protorthodes curtica|[13530]|LBCH7256-10|10-JDWBC-7256|641[0n]bp|Canada.British Columbia|BOLD:ABZ7207
Protorthodes curtica|[13531]|LBCH6929-10|10-JDWBC-6929|634[0n]bp|Canada.British Columbia|BOLD:ABZ7207
Protorthodes curtica|[13532]|LBCH6916-10|10-JDWBC-6916|629[0n]bp|Canada.British Columbia|BOLD:ABZ7207
Protorthodes curtica|[13533]|RDNMB274-05|CNCNoctuoidea10040|538[0n]bp|Canada.British Columbia|BOLD:ABZ...
Protorthodes curtica|[13534]|LBCH7257-10|10-JDWBC-7257|622[0n]bp|Canada.British Columbia|BOLD:ABZ7207
Protorthodes curtica|[13535]|LBCH7292-10|10-JDWBC-7292|626[0n]bp|Canada.British Columbia|BOLD:ABZ7207
Protorthodes curtica|[13536]|LBCH7259-10|10-JDWBC-7259|626[0n]bp|Canada.British Columbia|BOLD:ABZ7207
Protorthodes curtica|[13537]|LBCH7336-10|10-JDWBC-7336|636[0n]bp|Canada.British Columbia|BOLD:ABZ7207
Protorthodes curtica|[13538]|LBCH7253-10|10-JDWBC-7253|658[0n]bp|Canada.British Columbia|BOLD:ABZ7207
Protorthodes eureka|[13539]|RDMAB714-06|UASM34977|658[0n]bp|Canada.Alberta|BOLD:AAD9514
Protorthodes eureka|[13540]|RDMAB713-06|UASM24256|607[0n]bp|Canada.Alberta|BOLD:AAD9514
Protorthodes incincta|[13541]|RDNMB284-05|CNCNoctuoidea10050|658[0n]bp|Canada.Manitoba|BOLD:AAB5164
Protorthodes incincta|[13542]|RDNMF758-08|UASM99408|658[0n]bp|Canada.Alberta|BOLD:AAB5164
Protorthodes incincta|[13543]|RDNMB283-05|CNCNoctuoidea10049|598[0n]bp|Canada.Ontario|BOLD:AAB5164
Protorthodes incincta|[13544]|RDMAB711-06|UASM24353|576[1n]bp|Canada.Alberta|BOLD:AAB5164
Protorthodes incincta|[13545]|RDNMB282-05|CNCNoctuoidea10048|658[0n]bp|Canada.Ontario|BOLD:AAB5164
Protorthodes incincta|[13546]|RDNMF765-08|UASM99475|658[0n]bp|Canada.Alberta|BOLD:AAB5164
Protorthodes incincta|[13547]|RDNMF763-08|UASM99473|658[0n]bp|Canada.Alberta|BOLD:AAB5164
Protorthodes incincta|[13548]|RDNMF764-08|UASM99474|658[0n]bp|Canada.Alberta|BOLD:AAB5164
Protorthodes incincta|[13549]|RDNMF591-08|NOC14677|658[0n]bp|Canada.Saskatchewan|BOLD:AAB5164
Protorthodes incincta|[13550]|RDNMF767-08|UASM99477|658[0n]bp|Canada.Alberta|BOLD:AAB5164
Protorthodes incincta|[13551]|RDNMB277-05|CNCNoctuoidea10043|658[0n]bp|Canada.Alberta|BOLD:AAB5164
Protorthodes incincta|[13552]|RDNMB279-05|CNCNoctuoidea10045|658[0n]bp|Canada.Alberta|BOLD:AAB5164
Protorthodes incincta|[13553]|RDNMB281-05|CNCNoctuoidea10047|658[0n]bp|Canada.Alberta|BOLD:AAB5164
Protorthodes incincta|[13554]|RDNMB278-05|CNCNoctuoidea10044|573[0n]bp|Canada.Alberta|BOLD:AAB5164
Protorthodes incincta|[13555]|RDNMF766-08|UASM99476|658[0n]bp|Canada.Alberta|BOLD:AAB5164

Protorthodes incincta|[13553]|RDNMB281-05|CNCNoctuoidea10047|658[0n]bp|Canada.Alberta|BOLD:AAB5164
Protorthodes incincta|[13554]|RDNMB278-05|CNCNoctuoidea10044|573[0n]bp|Canada.Alberta|BOLD:AAB5164
Protorthodes incincta|[13555]|RDNMF766-08|UASM99476|658[0n]bp|Canada.Alberta|BOLD:AAB5164
Protorthodes incincta|[13556]|RDNMB280-05|CNCNoctuoidea10046|658[0n]bp|Canada.Alberta|BOLD:AAB5164
Protorthodes incincta|[13557]|RDMAB712-06|UASM24248|658[0n]bp|Canada.Alberta|BOLD:AAB5164
Protorthodes oviduca|[13558]|LOWCE754-06|CGWC-4514|658[0n]bp|Canada.British Columbia|BOLD:AAB0331
Protorthodes oviduca|[13559]|LOWCE756-06|CGWC-4516|658[0n]bp|Canada.British Columbia|BOLD:AAB0331
Protorthodes oviduca|[13560]|LOWCE755-06|CGWC-4515|658[0n]bp|Canada.British Columbia|BOLD:AAB0331
Protorthodes oviduca|[13561]|LOWCD541-06|CGWC-3361|658[0n]bp|Canada.British Columbia|BOLD:AAB0331
Protorthodes oviduca|[13562]|XAD647-05|2005-ONT-62|639[0n]bp|Canada.Ontario|BOLD:AAB0331
Protorthodes oviduca|[13563]|XAC152-04|04HBL006152|658[0n]bp|Canada.Ontario|BOLD:AAB0331
Protorthodes oviduca|[13564]|PMG152-03|moth283.01|617[0n]bp|Canada.Ontario|BOLD:AAB0331
Protorthodes oviduca|[13565]|PHMO079-03|moth511.01|639[0n]bp|Canada.Ontario|BOLD:AAB0331
Protorthodes oviduca|[13566]|XAF556-05|2005-ONT-205|658[0n]bp|Canada.Ontario|BOLD:AAB0331
Protorthodes oviduca|[13567]|RDNMG380-08|CNC LEP00052204|658[0n]bp|Canada.Newfoundland and Labrador|BOLD...
Protorthodes oviduca|[13568]|LPSOD834-09|08BBLEP-00616|658[0n]bp|Canada.Ontario|BOLD:AAB0331
Protorthodes oviduca|[13569]|RDNMG379-08|CNC LEP00052203|658[0n]bp|Canada.Newfoundland and Labrador|BOLD...
Protorthodes oviduca|[13570]|RDLQG383-06|DH012651|658[0n]bp|Canada.Quebec|BOLD:AAB0331
Protorthodes oviduca|[13571]|XAJ392-06|2006-ONT-0392|658[0n]bp|Canada.Ontario|BOLD:AAB0331
Protorthodes oviduca|[13572]|BBLPB440-10|10BBCLP-1439|658[0n]bp|Canada.Saskatchewan|BOLD:AAB0331
Protorthodes oviduca|[13573]|RDLQG385-06|DH012653|658[0n]bp|Canada.Quebec|BOLD:AAB0331
Protorthodes oviduca|[13574]|RDLQG384-06|DH012652|658[0n]bp|Canada.Quebec|BOLD:AAB0331
Protorthodes oviduca|[13575]|RDLQG265-06|DH012470|658[0n]bp|Canada.Quebec|BOLD:AAB0331
Protorthodes oviduca|[13576]|LPSOD887-09|08BBLEP-00669|658[0n]bp|Canada.Ontario|BOLD:AAB0331
Protorthodes oviduca|[13577]|LPSOD256-09|08BBLEP-00034|658[0n]bp|Canada.Ontario|BOLD:AAB0331
Protorthodes oviduca|[13578]|XAF581-05|2005-ONT-230|658[0n]bp|Canada.Ontario|BOLD:AAB0331
Protorthodes oviduca|[13579]|XAB353-04|04HBL005353|658[0n]bp|Canada.Ontario|BOLD:AAB0331
Protorthodes oviduca|[13580]|RDLQG386-06|DH012654|658[0n]bp|Canada.Quebec|BOLD:AAB0331
Protorthodes oviduca|[13581]|BBLPB368-10|10BBCLP-1367|658[0n]bp|Canada.Saskatchewan|BOLD:AAB0331
Protorthodes oviduca|[13582]|XAC123-04|04HBL006123|658[0n]bp|Canada.Ontario|BOLD:AAB0331
Protorthodes oviduca|[13583]|BBLPB445-10|10BBCLP-1444|658[0n]bp|Canada.Saskatchewan|BOLD:AAB0331
Protorthodes oviduca|[13584]|RDLQG387-06|DH012655|658[0n]bp|Canada.Quebec|BOLD:AAB0331
Protorthodes oviduca|[13585]|LPSOD395-09|08BBLEP-00174|658[0n]bp|Canada.Ontario|BOLD:AAB0331
Protorthodes oviduca|[13586]|LPSOD624-09|08BBLEP-00405|658[0n]bp|Canada.Ontario|BOLD:AAB0331
Protorthodes oviduca|[13587]|LPSOD725-09|08BBLEP-00507|658[0n]bp|Canada.Ontario|BOLD:AAB0331
Protorthodes oviduca|[13588]|RDLQF507-06|DH011656|658[0n]bp|Canada.Quebec|BOLD:AAB0331
Protorthodes oviduca|[13589]|TMG142-03|moth340.01|639[0n]bp|Canada.Ontario|BOLD:AAB0331
Protorthodes oviduca|[13590]|PHMO073-03|moth473.01|639[0n]bp|Canada.Ontario|BOLD:AAB0331
Protorthodes oviduca|[13591]|RDLQ288-05|DH007119|656[0n]bp|Canada.Quebec|BOLD:AAB0331
Protorthodes oviduca|[13592]|LPSOB242-08|PPBP-1241|658[0n]bp|Canada.Ontario|BOLD:AAB0331
Protorthodes oviduca|[13593]|LPSOD652-09|08BBLEP-00433|658[0n]bp|Canada.Ontario|BOLD:AAB0331
Protorthodes oviduca|[13594]|BBLPB367-10|10BBCLP-1366|658[0n]bp|Canada.Saskatchewan|BOLD:AAB0331
Protorthodes oviduca|[13595]|BBLPB366-10|10BBCLP-1365|658[0n]bp|Canada.Ontario|BOLD:AAB0331
Protorthodes rufula|[13596]|LOCBC012-06|06-BLLOC-1892|658[0n]bp|United States.California|BOLD:AAB0747
Protorthodes rufula|[13597]|LOCB686-06|06-BLLOC-686|658[0n]bp|United States.California|BOLD:AAB0747
Protorthodes rufula|[13598]|GMLC1204-12|2011GM-0900|658[0n]bp|United States.California|BOLD:AAB0747
Protorthodes rufula|[13599]|GMLC1018-12|2011GM-0714|658[0n]bp|United States.California|BOLD:AAB0747
Protorthodes rufula|[13600]|GMLC1450-12|2012GM-0181|600[0n]bp|United States.California|BOLD:AAB0747
Protorthodes rufula|[13601]|GMLC477-11|2011GM-0173|658[0n]bp|United States.California|BOLD:AAB0747
Protorthodes rufula|[13602]|GMLC1449-12|2012GM-0180|600[0n]bp|United States.California|BOLD:AAB0747
Protorthodes rufula|[13603]|GMLC462-11|2011GM-0158|658[0n]bp|United States.California|BOLD:AAB0747
Protorthodes rufula|[13604]|GMLC1017-12|2011GM-0713|658[0n]bp|United States.California|BOLD:AAB0747
Protorthodes rufula|[13605]|GMLC465-11|2011GM-0161|658[0n]bp|United States.California|BOLD:AAB0747
Protorthodes rufula|[13606]|GMLC500-11|2011GM-0196|658[0n]bp|United States.California|BOLD:AAB0747
Protorthodes rufula|[13607]|GMLC1095-12|2011GM-0791|658[0n]bp|United States.California|BOLD:AAB0747
Protorthodes rufula|[13608]|GMLC229-11|2009GM-0098|658[0n]bp|United States.California|BOLD:AAB0747
Protorthodes rufula|[13609]|GMLC514-11|2011GM-0210|658[0n]bp|United States.California|BOLD:AAB0747
Protorthodes rufula|[13610]|GMLC369-11|2011GM-0065|658[0n]bp|United States.California|BOLD:AAB0747
Protorthodes rufula|[13611]|GMLC368-11|2011GM-0064|658[0n]bp|United States.California|BOLD:AAB0747
Protorthodes rufula|[13612]|GMLC450-11|2011GM-0146|658[0n]bp|United States.California|BOLD:AAB0747
Protorthodes rufula|[13613]|GMLC424-11|2011GM-0120|658[0n]bp|United States.California|BOLD:AAB0747
Protorthodes rufula|[13614]|GMLC448-11|2011GM-0144|658[0n]bp|United States.California|BOLD:AAB0747
Protorthodes rufula|[13615]|GMLC352-11|2011GM-0048|658[0n]bp|United States.California|BOLD:AAB0747
Protorthodes rufula|[13616]|GMLC453-11|2011GM-0149|658[0n]bp|United States.California|BOLD:AAB0747
Protorthodes rufula|[13617]|GMLC452-11|2011GM-0148|658[0n]bp|United States.California|BOLD:AAB0747
Protorthodes rufula|[13618]|GMLC386-11|2011GM-0082|658[0n]bp|United States.California|BOLD:AAB0747
Protorthodes rufula|[13619]|GMLC511-11|2011GM-0207|658[0n]bp|United States.California|BOLD:AAB0747
Protorthodes rufula|[13620]|GMLC1126-12|2011GM-0822|658[0n]bp|United States.California|BOLD:AAB0747
Protorthodes rufula|[13621]|GMLC451-11|2011GM-0147|658[0n]bp|United States.California|BOLD:AAB0747
Protorthodes rufula|[13622]|GMLC1020-12|2011GM-0716|658[0n]bp|United States.California|BOLD:AAB0747
Protorthodes rufula|[13623]|GMLC1175-12|2011GM-0871|658[0n]bp|United States.California|BOLD:AAB0747
Protorthodes rufula|[13624]|RDNMD423-06|CNCNoctuoidea12755|658[0n]bp|United States.California|BOLD:AAB...
Protorthodes rufula|[13625]|RWWB073-09|RWWA-1072|658[0n]bp|United States.Washington|BOLD:AAB0747
Protorthodes rufula|[13626]|RWWB635-10|RWWA-1634|639[0n]bp|United States.Washington|BOLD:AAB0747
Protorthodes rufula|[13627]|LOCB688-06|06-BLLOC-688|658[0n]bp|United States.California|BOLD:AAB0747
Protorthodes rufula|[13628]|LOCB699-06|06-BLLOC-699|658[0n]bp|United States.California|BOLD:AAB0747
Protorthodes rufula|[13629]|RDNMC592-06|CNCNoctuoidea12132|519[0n]bp|United States.Oregon|BOLD:AAB0747
Protorthodes rufula|[13630]|RWWA977-09|RWWA-0977|658[0n]bp|United States.Washington|BOLD:AAB0747
Protorthodes rufula|[13631]|GMLC510-11|2011GM-0206|658[0n]bp|United States.California|BOLD:AAB0747
Protorthodes rufula|[13632]|LOCBB298-06|06-BLLOC-1238|658[0n]bp|United States.California|BOLD:AAB0747
Protorthodes rufula|[13633]|LOCB707-06|06-BLLOC-707|658[0n]bp|United States.California|BOLD:AAB0747
Protorthodes rufula|[13634]|LOCBB302-06|06-BLLOC-1242|627[0n]bp|United States.California|BOLD:AAB0747
Protorthodes rufula|[13635]|RDNMD426-06|CNCNoctuoidea12758|658[0n]bp|United States.California|BOLD:AAB...
Protorthodes rufula|[13636]|LOCBB651-06|06-BLLOC-1591|658[0n]bp|United States.California|BOLD:AAB0747
Protorthodes rufula|[13637]|LOCB831-06|06-BLLOC-831|658[0n]bp|United States.California|BOLD:AAB0747
Protorthodes rufula|[13638]|LOCB634-06|06-BLLOC-634|658[0n]bp|United States.California|BOLD:AAB0747
Protorthodes rufula|[13639]|LOCB716-06|06-BLLOC-716|658[0n]bp|United States.California|BOLD:AAB0747
Protorthodes rufula|[13640]|LOCB706-06|06-BLLOC-706|658[0n]bp|United States.California|BOLD:AAB0747
Protorthodes rufula|[13641]|LOCBB300-06|06-BLLOC-1240|658[0n]bp|United States.California|BOLD:AAB0747
Protorthodes rufula|[13642]|LOCBB301-06|06-BLLOC-1241|658[0n]bp|United States.California|BOLD:AAB0747
Protorthodes rufula|[13643]|LOCB704-06|06-BLLOC-704|658[0n]bp|United States.California|BOLD:AAB0747
Protorthodes rufula|[13644]|RDNMD425-06|CNCNoctuoidea12757|658[0n]bp|United States.California|BOLD:AAB...
Protorthodes rufula|[13645]|LOCB773-06|06-BLLOC-773|658[0n]bp|United States.California|BOLD:AAB0747
Protorthodes rufula|[13646]|LOCB702-06|06-BLLOC-702|658[0n]bp|United States.California|BOLD:AAB0747
Protorthodes rufula|[13647]|LOCB687-06|06-BLLOC-687|658[0n]bp|United States.California|BOLD:AAB0747
Protorthodes rufula|[13648]|LOCB701-06|06-BLLOC-701|658[0n]bp|United States.California|BOLD:AAB0747
Protorthodes rufula|[13649]|LOCB494-06|06-BLLOC-494|658[0n]bp|United States.California|BOLD:AAB0747
Protorthodes rufula|[13650]|LOCBB299-06|06-BLLOC-1239|658[0n]bp|United States.California|BOLD:AAB0747
Protorthodes rufula|[13651]|RDNMD427-06|CNCNoctuoidea12759|658[0n]bp|United States.California|BOLD:AAB...
Protorthodes rufula|[13652]|RDNMG860-08|CNC LEP00052984|658[0n]bp|United States.California|BOLD:AAB0747
Protorthodes rufula|[13653]|LOCB689-06|06-BLLOC-689|658[0n]bp|United States.California|BOLD:AAB0747
Protorthodes rufula|[13654]|RDNMG859-08|CNC LEP00052983|658[0n]bp|United States.California|BOLD:AAB0747
Aphimella contrahens|[13655]|RDNMG945-08|CNC LEP00053069|658[0n]bp|Canada.Ontario|BOLD:AAE5019

[illegible]
Protorthodes rufula|[13653]|LOCB689-06|06-BLLOC-689|658[0n]bp|United States.California|BOLD:AAB0747
Protorthodes rufula|[13654]|RDNMG859-08|CNC LEP00052983|658[0n]bp|United States.California|BOLD:AAB0747
Anhimella contrahens|[13655]|RDNMG945-08|CNC LEP00053069|658[0n]bp|Canada.Ontario|BOLD:AAE5019
Anhimella contrahens|[13656]|RDNMG946-08|CNC LEP00053070|658[0n]bp|Canada.Alberta|BOLD:AAE5017
Anhimella contrahens|[13657]|XAG420-05|2005-ONT-1004|658[0n]bp|Canada.Ontario|BOLD:AAE5017
Anhimella pacifica|[13658]|RDNMF587-08|NOC14673|658[0n]bp|United States.California|BOLD:AAJ2704
Anhimella pacifica|[13659]|RDNMG944-08|CNC LEP00053068|658[0n]bp|Canada.British Columbia|BOLD:AAE5018
Anhimella pacifica|[13660]|AWCLB271-10|AWC-07359|658[0n]bp|United States.Arizona|BOLD:AAJ2705
Anhimella pacifica|[13661]|RDNMG973-08|CNC LEP00053097|658[0n]bp|United States.California|BOLD:AAJ2705
Anhimella pacifica|[13662]|AWCLB272-10|AWC-07360|658[0n]bp|United States.Arizona|BOLD:AAJ2705
Anhimella perbrunnea|[13663]|RDNMF586-08|NOC14672|658[2n]bp|United States.Washington|BOLD:AAF1328
Anhimella perbrunnea|[13664]|GMLC1223-12|2011GM-0919|658[0n]bp|United States.California|BOLD:AAF1328
Anhimella perbrunnea|[13665]|RDNMF585-08|NOC14671|658[0n]bp|United States.Oregon|BOLD:AAF1328
Anhimella perbrunnea|[13666]|GMLC1185-12|2011GM-0881|658[0n]bp|United States.California|BOLD:AAF1328
Anhimella perbrunnea|[13667]|GMLC1447-12|2012GM-0178|614[0n]bp|United States.California|BOLD:AAF1328
Anhimella perbrunnea|[13668]|RWWC625-11|RWWA-2602|658[0n]bp|United States.Washington|BOLD:ACE4177
Anhimella perbrunnea|[13669]|RWWC008-10|RWWA-1985|634[0n]bp|United States.Washington|BOLD:ACE4177
Anhimella perbrunnea|[13670]|RWWB150-09|RWWA-1149|658[0n]bp|United States.Washington|BOLD:ACE4177
Ulolonche disticha|[13671]|RDNMD914-07|CNCNoctuoidea13245|655[0n]bp|Canada.Alberta|BOLD:ABZ0724
Ulolonche culea|[13672]|BBLCU214-09|09BBLEP-04701|658[0n]bp|United States.Michigan|BOLD:AAE0713
Ulolonche culea|[13673]|BBLCU243-09|09BBLEP-04730|658[0n]bp|United States.Michigan|BOLD:AAE0713
Ulolonche culea|[13674]|BBLCU126-09|09BBLEP-04613|658[0n]bp|United States.Michigan|BOLD:AAE0713
Ulolonche culea|[13675]|BBLCU246-09|09BBLEP-04733|614[0n]bp|United States.Michigan|BOLD:AAE0713
Ulolonche culea|[13676]|BBLSX953-09|09BBLEP-02881|644[0n]bp|United States.Oklahoma|BOLD:AAE0713
Ulolonche culea|[13677]|LOCT051-05|05-CTATBI-0051|633[0n]bp|United States.Connecticut|BOLD:AAE0713
Ulolonche culea|[13678]|BBLCU193-09|09BBLEP-04680|658[0n]bp|United States.Michigan|BOLD:AAE0713
Ulolonche culea|[13679]|LNCC161-10|10-NCCC-256|658[0n]bp|United States.North Carolina|BOLD:AAE0713
Ulolonche culea|[13680]|LSEU346-06|06-JKA-0346|658[0n]bp|United States.Georgia|BOLD:AAE0713
Ulolonche culea|[13681]|LOCT052-05|05-CTATBI-0052|658[0n]bp|United States.Connecticut|BOLD:AAE0713
Ulolonche culea|[13682]|LGSMC419-05|DNA-ATBI-2419|658[0n]bp|United States.Tennessee|BOLD:AAE0713
Ulolonche culea|[13683]|LNCB951-10|10-NCCC-041|658[0n]bp|United States.North Carolina|BOLD:AAE0713
Ulolonche culea|[13684]|LSEU347-06|06-JKA-0347|658[0n]bp|United States.Georgia|BOLD:AAE0713
Ulolonche modesta|[13685]|LPSO552-08|PPBP-0552|657[0n]bp|Canada.Ontario|BOLD:AAD9680
Ulolonche modesta|[13686]|RDLQ682-07|DH005083|631[0n]bp|Canada.Quebec|BOLD:AAD9680
Ulolonche orbiculata|[13687]|RDNMF370-08|NOC14456|609[0n]bp|Canada.Alberta|BOLD:AAD7571
Ulolonche orbiculata|[13688]|RDNMF372-08|NOC14458|658[0n]bp|Canada.Alberta|BOLD:AAD7571
Ulolonche orbiculata|[13689]|RDNMF371-08|NOC14457|658[0n]bp|Canada.Alberta|BOLD:AAD7571
Ulolonche orbiculata|[13690]|RDNMF373-08|NOC14459|658[0n]bp|Canada.Alberta|BOLD:AAD7571
Ulolonche orbiculata|[13691]|RDNMF369-08|NOC14455|658[0n]bp|Canada.Alberta|BOLD:AAD7571
Lacinipolia anguina|[13692]|LPSOD902-09|08BBLEP-00684|658[0n]bp|Canada.Ontario|BOLD:AAB8456
Lacinipolia anguina|[13693]|RDLQB074-05|DH010160|658[0n]bp|Canada.Quebec|BOLD:AAB8456
Lacinipolia anguina|[13694]|TTMNB555-06|MNBTT-555|656[0n]bp|Canada.New Brunswick|BOLD:AAB8456
Lacinipolia anguina|[13695]|TMNBB325-06|MNBTT-1265|656[0n]bp|Canada.New Brunswick|BOLD:AAB8456
Lacinipolia anguina|[13696]|BBLPB335-10|10BBCLP-1334|658[0n]bp|Canada.Ontario|BOLD:AAB8456
Lacinipolia anguina|[13697]|TMNBB324-06|MNBTT-1264|658[0n]bp|Canada.New Brunswick|BOLD:AAB8456
Lacinipolia anguina|[13698]|RDLQB073-05|DH010159|658[0n]bp|Canada.Quebec|BOLD:AAB8456
Lacinipolia anguina|[13699]|BBLPB481-10|10BBCLP-1480|658[0n]bp|Canada.Alberta|BOLD:AAB8456
Lacinipolia anguina|[13700]|BBLPB491-10|10BBCLP-1490|658[0n]bp|Canada.Alberta|BOLD:AAB8456
Lacinipolia anguina|[13701]|BBLPB482-10|10BBCLP-1481|658[0n]bp|Canada.Alberta|BOLD:AAB8456
Lacinipolia anguina|[13702]|BBLPB493-10|10BBCLP-1492|658[0n]bp|Canada.Alberta|BOLD:AAB8456
Lacinipolia anguina|[13703]|LBCG045-08|08-JDWBC-0045|658[0n]bp|Canada.British Columbia|BOLD:AAB8456
Lacinipolia anguina|[13704]|LOWCC083-05|CGWC-1963|658[0n]bp|Canada.British Columbia|BOLD:AAB8456
Lacinipolia anguina|[13705]|RDMAB640-06|UASM58759|658[0n]bp|Canada.Alberta|BOLD:AAB8456
Lacinipolia anguina|[13706]|LBCH5339-10|10-JDWBC-5339|658[0n]bp|Canada.British Columbia|BOLD:AAB8456
Lacinipolia anguina|[13707]|BBLPB492-10|10BBCLP-1491|658[0n]bp|Canada.Alberta|BOLD:AAB8456
Lacinipolia anguina|[13708]|BBLPB477-10|10BBCLP-1476|658[0n]bp|Canada.British Columbia|BOLD:AAB8456
Lacinipolia anguina|[13709]|LOWCE549-06|CGWC-4309|616[0n]bp|Canada.British Columbia|BOLD:AAB8456
Lacinipolia anguina|[13710]|LOWCE553-06|CGWC-4313|627[0n]bp|Canada.British Columbia|BOLD:AAB8456
Lacinipolia anguina|[13711]|RDMAB369-05|UASM77835|617[0n]bp|Canada.Alberta|BOLD:AAB8456
Lacinipolia anguina|[13712]|LOWCE550-06|CGWC-4310|607[0n]bp|Canada.British Columbia|BOLD:AAB8456
Lacinipolia anguina|[13713]|RDMAB261-05|UASM41470|570[0n]bp|Canada.Alberta|BOLD:AAB8456
Lacinipolia anguina|[13714]|BBLPB479-10|10BBCLP-1478|522[1n]bp|Canada.Alberta|BOLD:AAB8456
Lacinipolia anguina|[13715]|LBCH5515-10|10-JDWBC-5515|658[0n]bp|Canada.British Columbia|BOLD:AAB8456
Lacinipolia anguina|[13716]|RDMAB309-05|UASM57064|658[0n]bp|Canada.Alberta|BOLD:AAB8456
Lacinipolia anguina|[13717]|BBLPB483-10|10BBCLP-1482|658[0n]bp|Canada.Alberta|BOLD:AAB8456
Lacinipolia longiclava|[13718]|RDMAB307-05|UASM57154|658[0n]bp|Canada.Alberta|BOLD:ACE5163
Lacinipolia longiclava|[13719]|RDMAB308-05|UASM57066|658[0n]bp|Canada.Alberta|BOLD:ACE5163
Lacinipolia longiclava|[13720]|RDMAB310-05|UASM19837|658[0n]bp|Canada.Alberta|BOLD:ACE5163
Lacinipolia longiclava|[13721]|RDMAB107-05|UASM41992|658[1n]bp|Canada.Alberta|BOLD:ACE5163
Lacinipolia patalis|[13722]|LHLEP267-06|UBC-2006-0187|658[0n]bp|Canada.British Columbia|BOLD:AAB1769
Lacinipolia patalis|[13723]|BBLPB407-10|10BBCLP-1406|658[0n]bp|Canada.British Columbia|BOLD:AAB1769
Lacinipolia patalis|[13724]|LPVIA238-08|PFC-2006-0322|658[0n]bp|Canada.British Columbia|BOLD:AAB1769
Lacinipolia patalis|[13725]|BBLPB465-10|10BBCLP-1464|658[0n]bp|Canada.British Columbia|BOLD:AAB1769
Lacinipolia patalis|[13726]|LBCS103-07|UBC-2007-0077|658[0n]bp|Canada.British Columbia|BOLD:AAB1769
Lacinipolia patalis|[13727]|LPVIA104-08|PFC-2006-0169|658[0n]bp|Canada.British Columbia|BOLD:AAB1769
Lacinipolia patalis|[13728]|LMH008-06|PFC-2006-0010|658[0n]bp|Canada.British Columbia|BOLD:AAB1769
Lacinipolia patalis|[13729]|LALPA117-10|AVBC 117-10|658[0n]bp|Canada.British Columbia|BOLD:AAB1769
Lacinipolia patalis|[13730]|LPVIA294-08|PFC-2006-0385|658[0n]bp|Canada.British Columbia|BOLD:AAB1769
Lacinipolia patalis|[13731]|LHLEP268-06|UBC-2006-0188|658[0n]bp|Canada.British Columbia|BOLD:AAB1769
Lacinipolia patalis|[13732]|LALPA204-10|AVBC 205-10|658[0n]bp|Canada.British Columbia|BOLD:AAB1769
Lacinipolia patalis|[13733]|LALPA230-10|AVBC 231-10|658[0n]bp|Canada.British Columbia|BOLD:AAB1769
Lacinipolia patalis|[13734]|LPVIA267-08|PFC-2006-0353|644[0n]bp|Canada.British Columbia|BOLD:AAB1769
Lacinipolia patalis|[13735]|RDNMC388-05|CNCNoctuoidea12022|579[0n]bp|Canada.British Columbia|BOLD:AAB1769
Lacinipolia patalis|[13736]|RDNMC280-05|CNCNoctuoidea11914|602[0n]bp|Canada.British Columbia|BOLD:AAB1769
Lacinipolia patalis|[13737]|LPVIA103-08|PFC-2006-0168|658[0n]bp|Canada.British Columbia|BOLD:AAB1769
Lacinipolia olivacea|[13738]|BBLPC520-09|09BBELE-1520|658[0n]bp|Canada.New Brunswick|BOLD:AAA2596
Lacinipolia olivacea|[13739]|BBLPE334-09|09BBELE-2334|658[0n]bp|Canada.Newfoundland and Labrador|BOLD:...
Lacinipolia olivacea|[13740]|BBLEC573-09|09BBELE-0573|658[0n]bp|Canada.Nova Scotia|BOLD:AAA2596
Lacinipolia olivacea|[13741]|XAK322-06|2006-ONT-1317|658[0n]bp|Canada.Ontario|BOLD:AAA2596
Lacinipolia olivacea|[13742]|XAG798-05|2005-ONT-1382|658[0n]bp|Canada.Ontario|BOLD:AAA2596
Lacinipolia olivacea|[13743]|MNBB670-05|05-NBSTA-586|658[0n]bp|Canada.New Brunswick|BOLD:AAA2596
Lacinipolia olivacea|[13744]|XAK334-06|2006-ONT-1329|658[0n]bp|Canada.Ontario|BOLD:AAA2596
Lacinipolia olivacea|[13745]|MNBB672-05|05-NBSTA-588|658[0n]bp|Canada.New Brunswick|BOLD:AAA2596
Lacinipolia olivacea|[13746]|BBLEC576-09|09BBELE-0576|658[0n]bp|Canada.Nova Scotia|BOLD:AAA2596
Lacinipolia olivacea|[13747]|BBLPC412-09|09BBELE-1412|632[0n]bp|Canada.New Brunswick|BOLD:AAA2596
Lacinipolia olivacea|[13748]|BBLPC067-09|09BBELE-1067|658[0n]bp|Canada.New Brunswick|BOLD:AAA2596
Lacinipolia olivacea|[13749]|BBLPC064-09|09BBELE-1064|658[0n]bp|Canada.New Brunswick|BOLD:AAA2596
Lacinipolia olivacea|[13750]|XAI037-05|0102-ONT-0037|658[0n]bp|Canada.Ontario|BOLD:AAA2596
Lacinipolia olivacea|[13751]|RDLQB747-05|DH010662|658[0n]bp|Canada.Quebec|BOLD:AAA2596
Lacinipolia olivacea|[13752]|XAK321-06|2006-ONT-1316|658[0n]bp|Canada.Ontario|BOLD:AAA2596
Lacinipolia olivacea|[13753]|MNBB620-05|05-NBSTA-536|658[0n]bp|Canada.New Brunswick|BOLD:AAA2596
Lacinipolia olivacea|[13754]|BLTIB944-08|BL1371|658[0n]bp|Canada.Ontario|BOLD:AAA2596
[illegible]

Lacinipolia olivacea|[13752]|XAK321-06|2006-ONT-1316|658[0n]bp|Canada.Ontario|BOLD:AAA2596
Lacinipolia olivacea|[13753]|MNBB620-05|05-NBSTA-536|658[0n]bp|Canada.New Brunswick|BOLD:AAA2596
Lacinipolia olivacea|[13754]|BLTIB944-08|BL1371|658[0n]bp|Canada.Ontario|BOLD:AAA2596
Lacinipolia olivacea|[13755]|XAG210-05|2005-ONT-794|658[0n]bp|Canada.Ontario|BOLD:AAA2596
Lacinipolia olivacea|[13756]|XAI035-05|0102-ONT-0035|658[0n]bp|Canada.Ontario|BOLD:AAA2596
Lacinipolia olivacea|[13757]|BBLPC059-09|09BBELE-1059|658[0n]bp|Canada.New Brunswick|BOLD:AAA2596
Lacinipolia olivacea|[13758]|XAK323-06|2006-ONT-1318|658[0n]bp|Canada.Ontario|BOLD:AAA2596
Lacinipolia olivacea|[13759]|XAI036-05|0102-ONT-0036|658[0n]bp|Canada.Ontario|BOLD:AAA2596
Lacinipolia olivacea|[13760]|MNBB452-05|05-NBSTA-368|658[0n]bp|Canada.New Brunswick|BOLD:AAA2596
Lacinipolia olivacea|[13761]|XAG711-05|2005-ONT-1295|658[0n]bp|Canada.Ontario|BOLD:AAA2596
Lacinipolia olivacea|[13762]|BLTIB800-08|BL1218|658[0n]bp|Canada.Ontario|BOLD:AAA2596
Lacinipolia olivacea|[13763]|BLTIB1063-08|BL1072|658[0n]bp|Canada.Ontario|BOLD:AAA2596
Lacinipolia olivacea|[13764]|TTMNB403-06|MNBTT-403|658[0n]bp|Canada.New Brunswick|BOLD:AAA2596
Lacinipolia olivacea|[13765]|MNBB621-05|05-NBSTA-537|658[0n]bp|Canada.New Brunswick|BOLD:AAA2596
Lacinipolia olivacea|[13766]|RDLQB632-05|DH010735|658[0n]bp|Canada.Quebec|BOLD:AAA2596
Lacinipolia olivacea|[13767]|BLTIB839-08|BL1258|658[0n]bp|Canada.Ontario|BOLD:AAA2596
Lacinipolia olivacea|[13768]|XAI034-05|0102-ONT-0034|658[0n]bp|Canada.Ontario|BOLD:AAA2596
Lacinipolia olivacea|[13769]|XAG815-05|2005-ONT-1399|658[1n]bp|Canada.Ontario|BOLD:AAA2596
Lacinipolia olivacea|[13770]|XAD249-04|04HBL007249|593[0n]bp|Canada.Ontario|BOLD:AAA2596
Lacinipolia olivacea|[13771]|PHMO222-03|moth1131.01|639[0n]bp|Canada.Ontario|BOLD:AAA2596
Lacinipolia olivacea|[13772]|XAK485-07|HLC-16038|598[0n]bp|Canada.Ontario|BOLD:AAA2596
Lacinipolia olivacea|[13773]|XAK560-07|HLC-16113|595[0n]bp|Canada.Ontario|BOLD:AAA2596
Lacinipolia olivacea|[13774]|XAK561-07|HLC-16114|604[0n]bp|Canada.Ontario|BOLD:AAA2596
Lacinipolia olivacea|[13775]|XAD008-04|04HBL007008|599[0n]bp|Canada.Ontario|BOLD:AAA2596
Lacinipolia olivacea|[13776]|XAC854-04|04HBL006854|581[0n]bp|Canada.Ontario|BOLD:AAA2596
Lacinipolia olivacea|[13777]|RDLQB638-05|DH010741|614[1n]bp|Canada.Quebec|BOLD:AAA2596
Lacinipolia olivacea|[13778]|BBLPC529-09|09BBELE-1529|658[0n]bp|Canada.New Brunswick|BOLD:AAA2596
Lacinipolia olivacea|[13779]|BBLPC070-09|09BBELE-1070|658[0n]bp|Canada.New Brunswick|BOLD:AAA2596
Lacinipolia olivacea|[13780]|MNBB622-05|05-NBSTA-538|658[0n]bp|Canada.New Brunswick|BOLD:AAA2596
Lacinipolia olivacea|[13781]|BBLPC389-09|09BBELE-1389|658[0n]bp|Canada.New Brunswick|BOLD:AAA2596
Lacinipolia olivacea|[13782]|TTMNB402-06|MNBTT-402|658[0n]bp|Canada.New Brunswick|BOLD:AAA2596
Lacinipolia olivacea|[13783]|BBLPC109-09|09BBELE-1109|658[0n]bp|Canada.New Brunswick|BOLD:AAA2596
Lacinipolia olivacea|[13784]|TMNBB327-06|MNBTT-1267|658[0n]bp|Canada.New Brunswick|BOLD:AAA2596
Lacinipolia olivacea|[13785]|TMNBB328-06|MNBTT-1268|658[0n]bp|Canada.New Brunswick|BOLD:AAA2596
Lacinipolia olivacea|[13786]|BBLPC410-09|09BBELE-1410|658[0n]bp|Canada.New Brunswick|BOLD:AAA2596
Lacinipolia olivacea|[13787]|BBLPC040-09|09BBELE-1040|658[0n]bp|Canada.New Brunswick|BOLD:AAA2596
Lacinipolia olivacea|[13788]|BBLPC043-09|09BBELE-1043|656[0n]bp|Canada.New Brunswick|BOLD:AAA2596
Lacinipolia olivacea|[13789]|BBLEC748-09|09BBELE-0748|632[0n]bp|Canada.Nova Scotia|BOLD:AAA2596
Lacinipolia olivacea|[13790]|PHMNB006-03|moth118.02SA|639[0n]bp|Canada.New Brunswick|BOLD:AAA2596
Lacinipolia olivacea|[13791]|BBLPC422-09|09BBELE-1422|632[0n]bp|Canada.New Brunswick|BOLD:AAA2596
Lacinipolia olivacea|[13792]|BBLEC431-09|09BBELE-0431|658[0n]bp|Canada.New Brunswick|BOLD:AAA2596
Lacinipolia olivacea|[13793]|BBLPC555-09|09BBELE-1555|658[0n]bp|Canada.New Brunswick|BOLD:AAA2596
Lacinipolia olivacea|[13794]|BBLPC424-09|09BBELE-1424|658[0n]bp|Canada.New Brunswick|BOLD:AAA2596
Lacinipolia olivacea|[13795]|BBLPC488-09|09BBELE-1488|658[0n]bp|Canada.New Brunswick|BOLD:AAA2596
Lacinipolia olivacea|[13796]|BBLEC745-09|09BBELE-0745|658[0n]bp|Canada.Nova Scotia|BOLD:AAA2596
Lacinipolia olivacea|[13797]|BBLEC470-09|09BBELE-0470|658[0n]bp|Canada.New Brunswick|BOLD:AAA2596
Lacinipolia olivacea|[13798]|BBLEC031-09|09BBELE-0031|658[0n]bp|Canada.New Brunswick|BOLD:AAA2596
Lacinipolia olivacea|[13799]|BBLPC388-09|09BBELE-1388|658[0n]bp|Canada.New Brunswick|BOLD:AAA2596
Lacinipolia olivacea|[13800]|MNBB671-05|05-NBSTA-587|658[0n]bp|Canada.New Brunswick|BOLD:AAA2596
Lacinipolia olivacea|[13801]|MNBB619-05|05-NBSTA-535|658[0n]bp|Canada.New Brunswick|BOLD:AAA2596
Lacinipolia olivacea|[13802]|BBLPC401-09|09BBELE-1401|658[0n]bp|Canada.New Brunswick|BOLD:AAA2596
Lacinipolia olivacea|[13803]|LPMNB359-09|08BBLEP-05203|658[0n]bp|Canada.Manitoba|BOLD:ACF0280
Lacinipolia olivacea|[13804]|LPMNB456-09|08BBLEP-05456|658[0n]bp|Canada.Manitoba|BOLD:ACF0280
Lacinipolia olivacea|[13805]|LPMNB380-09|08BBLEP-05224|658[0n]bp|Canada.Manitoba|BOLD:ACF0280
Lacinipolia olivacea|[13806]|LPMNB448-09|08BBLEP-05448|658[0n]bp|Canada.Manitoba|BOLD:ACF0280
Lacinipolia olivacea|[13807]|RDLQG064-06|DH012200|656[0n]bp|Canada.Quebec|BOLD:ACF0280
Lacinipolia olivacea|[13808]|LPMNB330-09|08BBLEP-05174|658[0n]bp|Canada.Manitoba|BOLD:ACF0280
Lacinipolia olivacea|[13809]|LPMNB411-09|08BBLEP-05255|658[0n]bp|Canada.Manitoba|BOLD:ACF0280
Lacinipolia olivacea|[13810]|LPMNB445-09|08BBLEP-05445|658[0n]bp|Canada.Manitoba|BOLD:ACF0280
Lacinipolia olivacea|[13811]|LPMNB474-09|08BBLEP-05512|658[0n]bp|Canada.Manitoba|BOLD:ACF0280
Lacinipolia olivacea|[13812]|LPMNB354-09|08BBLEP-05198|658[0n]bp|Canada.Manitoba|BOLD:ACF0280
Lacinipolia olivacea|[13813]|LPMNB417-09|08BBLEP-05417|658[0n]bp|Canada.Manitoba|BOLD:ACF0280
Lacinipolia olivacea|[13814]|LPMNB245-09|08BBLEP-05089|658[0n]bp|Canada.Manitoba|BOLD:ACF0280
Lacinipolia olivacea|[13815]|LALPA511-10|AVBC 513-10|658[0n]bp|Canada.British Columbia|BOLD:ACE9063
Lacinipolia olivacea|[13816]|LPAB251-08|08BBLEP-02573|658[0n]bp|Canada.Alberta|BOLD:ACE9063
Lacinipolia olivacea|[13817]|LPABC821-09|08BBLEP-05040|658[0n]bp|Canada.Alberta|BOLD:ACE9063
Lacinipolia olivacea|[13818]|LOWCE541-06|CGWC-4301|598[0n]bp|Canada.British Columbia|BOLD:ACE9063
Lacinipolia olivacea|[13819]|LPAB236-08|08BBLEP-02558|658[0n]bp|Canada.Alberta|BOLD:ACE9063
Lacinipolia olivacea|[13820]|LPABC935-09|08BBLEP-05346|658[0n]bp|Canada.Alberta|BOLD:ACE9063
Lacinipolia olivacea|[13821]|LPABC820-09|08BBLEP-05039|658[0n]bp|Canada.Alberta|BOLD:ACE9063
Lacinipolia olivacea|[13822]|BBLPB476-10|10BBCLP-1475|658[0n]bp|Canada.British Columbia|BOLD:ACE9063
Lacinipolia olivacea|[13823]|LPABB618-08|08BBLEP-03883|658[0n]bp|Canada.Alberta|BOLD:ACE9063
Lacinipolia olivacea|[13824]|BBLPB484-10|10BBCLP-1483|658[0n]bp|Canada.Alberta|BOLD:ACE9063
Lacinipolia olivacea|[13825]|LALPA860-11|AVBC 1033-11|658[0n]bp|Canada.British Columbia|BOLD:ACE9063
Lacinipolia olivacea|[13826]|LOWCC180-05|CGWC-2060|658[0n]bp|Canada.British Columbia|BOLD:ACE9063
Lacinipolia olivacea|[13827]|LOWC014-05|CGWC-0014|658[0n]bp|Canada.British Columbia|BOLD:ACE9063
Lacinipolia olivacea|[13828]|LOWC021-05|CGWC-0021|658[0n]bp|Canada.British Columbia|BOLD:ACE9063
Lacinipolia olivacea|[13829]|LOWCD179-06|CGWC-2999|658[0n]bp|Canada.British Columbia|BOLD:ACE9063
Lacinipolia olivacea|[13830]|LPABB175-08|08BBLEP-03440|658[0n]bp|Canada.Alberta|BOLD:ACE9063
Lacinipolia olivacea|[13831]|LPABB276-08|08BBLEP-03541|658[0n]bp|Canada.Alberta|BOLD:ACE9063
Lacinipolia olivacea|[13832]|LALPA869-11|AVBC 1042-11|658[0n]bp|Canada.British Columbia|BOLD:ACE9063
Lacinipolia olivacea|[13833]|LOWC017-05|CGWC-0017|658[0n]bp|Canada.British Columbia|BOLD:ACE9063
Lacinipolia olivacea|[13834]|BBLPB485-10|10BBCLP-1484|658[0n]bp|Canada.Alberta|BOLD:ACE9063
Lacinipolia olivacea|[13835]|BBLPB489-10|10BBCLP-1488|658[0n]bp|Canada.Alberta|BOLD:ACE9063
Lacinipolia olivacea|[13836]|LPABB222-08|08BBLEP-03487|658[0n]bp|Canada.Alberta|BOLD:ACE9063
Lacinipolia olivacea|[13837]|LPSOD935-09|08BBLEP-05475|658[0n]bp|Canada.Ontario|BOLD:ACE9063
Lacinipolia olivacea|[13838]|LOWC016-05|CGWC-0016|658[0n]bp|Canada.British Columbia|BOLD:ACE9063
Lacinipolia olivacea|[13839]|LPABB632-08|08BBLEP-03897|658[0n]bp|Canada.Alberta|BOLD:ACE9063
Lacinipolia olivacea|[13840]|LPABC655-09|08BBLEP-04874|658[0n]bp|Canada.Alberta|BOLD:ACE9063
Lacinipolia olivacea|[13841]|LBCC302-05|HLC-22182|658[0n]bp|Canada.British Columbia|BOLD:ACE9063
Lacinipolia olivacea|[13842]|BBLPC906-09|09BBELE-1906|658[0n]bp|Canada.Newfoundland and Labrador|BOLD:...
Lacinipolia olivacea|[13843]|BBLPB473-10|10BBCLP-1472|658[0n]bp|Canada.British Columbia|BOLD:ACE9063
Lacinipolia olivacea|[13844]|BBLPB854-10|10BBCLP-1853|658[0n]bp|Canada.Alberta|BOLD:ACE9063
Lacinipolia olivacea|[13845]|LPABC136-09|08BBLEP-04355|658[0n]bp|Canada.Alberta|BOLD:ACE9063
Lacinipolia olivacea|[13846]|LPABC654-09|08BBLEP-04873|658[0n]bp|Canada.Alberta|BOLD:ACE9063
Lacinipolia olivacea|[13847]|LALPA873-11|AVBC 1046-11|658[0n]bp|Canada.British Columbia|BOLD:ACE9063
Lacinipolia olivacea|[13848]|BBLPB475-10|10BBCLP-1474|658[0n]bp|Canada.British Columbia|BOLD:ACE9063
Lacinipolia olivacea|[13849]|LPABB528-08|08BBLEP-03793|658[0n]bp|Canada.Alberta|BOLD:ACE9063
Lacinipolia olivacea|[13850]|LOWC019-05|CGWC-0019|658[0n]bp|Canada.British Columbia|BOLD:ACE9063
Lacinipolia olivacea|[13851]|LPABC770-09|08BBLEP-04989|658[0n]bp|Canada.Alberta|BOLD:ACE9063
Lacinipolia olivacea|[13852]|LPABC132-09|08BBLEP-04351|658[0n]bp|Canada.Alberta|BOLD:ACE9063
Lacinipolia olivacea|[13853]|BBLPB488-10|10BBCLP-1487|658[0n]bp|Canada.Saskatchewan|BOLD:ACE9063
Lacinipolia olivacea|[13854]|LPABC138-09|08BBLEP-04357|658[0n]bp|Canada.Alberta|BOLD:ACE9063

Lacinipolia olivacea|[13852]|LPABC132-09|08BBLEP-04351|658[0n]bp|Canada.Alberta|BOLD:ACE9063
Lacinipolia olivacea|[13853]|BBLPB488-10|10BBCLP-1487|658[0n]bp|Canada.Saskatchewan|BOLD:ACE9063
Lacinipolia olivacea|[13854]|LPABC138-09|08BBLEP-04357|658[0n]bp|Canada.Alberta|BOLD:ACE9063
Lacinipolia olivacea|[13855]|BBLPB472-10|10BBCLP-1471|658[0n]bp|Canada.British Columbia|BOLD:ACE9063
Lacinipolia olivacea|[13856]|LPABB504-08|08BBLEP-03769|658[0n]bp|Canada.Alberta|BOLD:ACE9063
Lacinipolia olivacea|[13857]|LPABB248-08|08BBLEP-03513|658[0n]bp|Canada.Alberta|BOLD:ACE9063
Lacinipolia olivacea|[13858]|BBLPB494-10|10BBCLP-1493|658[0n]bp|Canada.Alberta|BOLD:ACE9063
Lacinipolia olivacea|[13859]|LOWC023-05|CGWC-0023|658[0n]bp|Canada.British Columbia|BOLD:ACE9063
Lacinipolia olivacea|[13860]|RDMAB647-06|UASM58319|658[0n]bp|Canada.Alberta|BOLD:ACE9063
Lacinipolia olivacea|[13861]|LPABC769-09|08BBLEP-04988|658[0n]bp|Canada.Alberta|BOLD:ACE9063
Lacinipolia olivacea|[13862]|LOWC024-05|CGWC-0024|658[0n]bp|Canada.British Columbia|BOLD:ACE9063
Lacinipolia olivacea|[13863]|LBCH2206-10|10-JDWBC-2206|658[0n]bp|Canada.British Columbia|BOLD:ACE9063
Lacinipolia olivacea|[13864]|LOWC020-05|CGWC-0020|658[0n]bp|Canada.British Columbia|BOLD:ACE9063
Lacinipolia olivacea|[13865]|LOWC018-05|CGWC-0018|658[0n]bp|Canada.British Columbia|BOLD:ACE9063
Lacinipolia olivacea|[13866]|BBLPB490-10|10BBCLP-1489|658[0n]bp|Canada.Alberta|BOLD:ACE9063
Lacinipolia olivacea|[13867]|BBLPB474-10|10BBCLP-1473|658[0n]bp|Canada.British Columbia|BOLD:ACE9063
Lacinipolia olivacea|[13868]|LPABB127-08|08BBLEP-03392|658[0n]bp|Canada.Alberta|BOLD:ACE9063
Lacinipolia olivacea|[13869]|LPABB249-08|08BBLEP-03514|658[0n]bp|Canada.Alberta|BOLD:ACE9063
Lacinipolia olivacea|[13870]|LBCB553-05|HLC-21493|658[0n]bp|Canada.British Columbia|BOLD:ACE9063
Lacinipolia olivacea|[13871]|BBLPB487-10|10BBCLP-1486|658[0n]bp|Canada.Saskatchewan|BOLD:ACE9063
Lacinipolia olivacea|[13872]|LOWC022-05|CGWC-0022|658[0n]bp|Canada.British Columbia|BOLD:ACE9063
Lacinipolia olivacea|[13873]|BBLPB486-10|10BBCLP-1485|658[0n]bp|Canada.Alberta|BOLD:ACE9063
Lacinipolia olivacea|[13874]|LPABC133-09|08BBLEP-04352|654[0n]bp|Canada.Alberta|BOLD:ACE9063
Lacinipolia olivacea|[13875]|LPABC134-09|08BBLEP-04353|633[0n]bp|Canada.Alberta|BOLD:ACE9063
Lacinipolia olivacea|[13876]|LOWCE543-06|CGWC-4303|617[1n]bp|Canada.British Columbia|BOLD:ACE9063
Lacinipolia olivacea|[13877]|BBLPB478-10|10BBCLP-1477|609[0n]bp|Canada.British Columbia|BOLD:ACE9063
Lacinipolia olivacea|[13878]|LPABC276-09|08BBLEP-04495|630[1n]bp|Canada.Alberta|BOLD:ACE9063
Lacinipolia olivacea|[13879]|LPABC129-09|08BBLEP-04348|623[1n]bp|Canada.Alberta|BOLD:ACE9063
Lacinipolia olivacea|[13880]|LOWCE539-06|CGWC-4299|617[0n]bp|Canada.British Columbia|BOLD:ACE9063
Lacinipolia olivacea|[13881]|LOWCE536-06|CGWC-4296|617[0n]bp|Canada.British Columbia|BOLD:ACE9063
Lacinipolia olivacea|[13882]|LOWCE535-06|CGWC-4295|627[0n]bp|Canada.British Columbia|BOLD:ACE9063
Lacinipolia olivacea|[13883]|RDNMC391-05|CNCNoctuoidea12025|610[0n]bp|Canada.Alberta|BOLD:ACE9063
Lacinipolia olivacea|[13884]|LOWCE538-06|CGWC-4298|610[0n]bp|Canada.British Columbia|BOLD:ACE9063
Lacinipolia olivacea|[13885]|LOWCE540-06|CGWC-4300|610[0n]bp|Canada.British Columbia|BOLD:ACE9063
Lacinipolia olivacea|[13886]|LPABC135-09|08BBLEP-04354|568[0n]bp|Canada.Alberta|BOLD:ACE9063
Lacinipolia olivacea|[13887]|LOWCE542-06|CGWC-4302|606[0n]bp|Canada.British Columbia|BOLD:ACE9063
Lacinipolia olivacea|[13888]|LOWCE537-06|CGWC-4297|608[0n]bp|Canada.British Columbia|BOLD:ACE9063
Lacinipolia olivacea|[13889]|LOWCE533-06|CGWC-4293|610[0n]bp|Canada.British Columbia|BOLD:ACE9063
Lacinipolia olivacea|[13890]|LOWCE534-06|CGWC-4294|610[0n]bp|Canada.British Columbia|BOLD:ACE9063
Lacinipolia olivacea|[13891]|RDMAB646-06|UASM7231|658[0n]bp|Canada.Alberta|BOLD:ACE9063
Lacinipolia comis|[13892]|LBCH6739-10|10-JDWBC-6739|658[0n]bp|Canada.British Columbia|BOLD:AAC2984
Lacinipolia comis|[13893]|LBCB074-05|HLC-21014|658[0n]bp|Canada.British Columbia|BOLD:AAC2984
Lacinipolia comis|[13894]|LBCH1952-10|10-JDWBC-1952|658[0n]bp|Canada.British Columbia|BOLD:AAC2984
Lacinipolia comis|[13895]|LALPA617-10|AVBC 619-10|658[0n]bp|Canada.British Columbia|BOLD:AAC2984
Lacinipolia comis|[13896]|LBCD464-05|HLC-23284|658[0n]bp|Canada.British Columbia|BOLD:AAC2984
Lacinipolia comis|[13897]|LBCH2086-10|10-JDWBC-2086|658[0n]bp|Canada.British Columbia|BOLD:AAC2984
Lacinipolia comis|[13898]|LBCH1973-10|10-JDWBC-1973|642[0n]bp|Canada.British Columbia|BOLD:AAC2984
Lacinipolia comis|[13899]|LBCH4052-10|10-JDWBC-4052|658[0n]bp|Canada.British Columbia|BOLD:AAC2984
Lacinipolia comis|[13900]|LALPA942-11|AVBC 1115-11|658[0n]bp|Canada.British Columbia|BOLD:AAC2984
Lacinipolia comis|[13901]|LBCH355-10|10-JDWBC-0355|658[0n]bp|Canada.British Columbia|BOLD:AAC2984
Lacinipolia comis|[13902]|BBLPB470-10|10BBCLP-1469|658[0n]bp|Canada.British Columbia|BOLD:AAC2984
Lacinipolia comis|[13903]|LALPA1314-11|AVBC 1316-11|658[0n]bp|Canada.British Columbia|BOLD:AAC2984
Lacinipolia comis|[13904]|LPABC958-09|08BBLEP-05369|658[0n]bp|Canada.Alberta|BOLD:AAC2984
Lacinipolia comis|[13905]|LALPA982-11|AVBC 1155-11|658[0n]bp|Canada.British Columbia|BOLD:AAC2984
Lacinipolia comis|[13906]|LBCH4050-10|10-JDWBC-4050|658[0n]bp|Canada.British Columbia|BOLD:AAC2984
Lacinipolia comis|[13907]|LBCH4438-10|10-JDWBC-4438|658[0n]bp|Canada.British Columbia|BOLD:AAC2984
Lacinipolia comis|[13908]|LBCH3785-10|10-JDWBC-3785|658[0n]bp|Canada.British Columbia|BOLD:AAC2984
Lacinipolia comis|[13909]|LBCH938-10|10-JDWBC-0938|658[0n]bp|Canada.British Columbia|BOLD:AAC2984
Lacinipolia comis|[13910]|LBCH4440-10|10-JDWBC-4440|658[0n]bp|Canada.British Columbia|BOLD:AAC2984
Lacinipolia davena|[13911]|LHLEP567-06|UBC-2006-0189|658[0n]bp|Canada.British Columbia|BOLD:AAB3076
Lacinipolia davena|[13912]|LOWCE279-06|CGWC-4039|658[1n]bp|Canada.British Columbia|BOLD:AAB3076
Lacinipolia davena|[13913]|LBCA371-05|HLC-20371|610[1n]bp|Canada.British Columbia|BOLD:AAB3076
Lacinipolia davena|[13914]|LBCD146-05|HLC-22966|658[0n]bp|Canada.British Columbia|BOLD:AAB3076
Lacinipolia davena|[13915]|RDNMK660-11|CNCLEP 81837|658[0n]bp|Canada.British Columbia|BOLD:AAB3076
Lacinipolia davena|[13916]|LOWCE278-06|CGWC-4038|658[0n]bp|Canada.British Columbia|BOLD:AAB3076
Lacinipolia davena|[13917]|LPABC905-09|08BBLEP-05316|658[0n]bp|Canada.Alberta|BOLD:AAB3076
Lacinipolia davena|[13918]|LALPA253-10|AVBC 254-10|658[0n]bp|Canada.British Columbia|BOLD:AAB3076
Lacinipolia davena|[13919]|LBCA369-05|HLC-20369|658[0n]bp|Canada.British Columbia|BOLD:AAB3076
Lacinipolia davena|[13920]|RDMAB253-05|UASM41469|655[0n]bp|Canada.Alberta|BOLD:AAB3076
Lacinipolia davena|[13921]|LBCD147-05|HLC-22967|596[0n]bp|Canada.British Columbia|BOLD:AAB3076
Lacinipolia davena|[13922]|LALPA341-10|AVBC 343-10|658[0n]bp|Canada.British Columbia|BOLD:AAB3076
Lacinipolia davena|[13923]|RDMAB251-05|UASM41459|658[0n]bp|Canada.Alberta|BOLD:AAB3076
Lacinipolia davena|[13924]|LBCA370-05|HLC-20370|658[0n]bp|Canada.British Columbia|BOLD:AAB3076
Lacinipolia davena|[13925]|LPABB267-08|08BBLEP-03532|658[0n]bp|Canada.Alberta|BOLD:AAB3076
Lacinipolia davena|[13926]|LBCA506-05|HLC-20506|658[0n]bp|Canada.British Columbia|BOLD:AAB3076
Lacinipolia davena|[13927]|LOWCE280-06|CGWC-4040|658[0n]bp|Canada.British Columbia|BOLD:AAB3076
Lacinipolia davena|[13928]|LPABB631-08|08BBLEP-03896|658[0n]bp|Canada.Alberta|BOLD:AAB3076
Lacinipolia davena|[13929]|LALPA216-10|AVBC 217-10|658[0n]bp|Canada.British Columbia|BOLD:AAB3076
Lacinipolia davena|[13930]|LPABB376-08|08BBLEP-03641|658[0n]bp|Canada.Alberta|BOLD:AAB3076
Lacinipolia davena|[13931]|LBCD145-05|HLC-22965|658[0n]bp|Canada.British Columbia|BOLD:AAB3076
Lacinipolia davena|[13932]|LPABB411-08|08BBLEP-03676|658[0n]bp|Canada.Alberta|BOLD:AAB3076
Lacinipolia davena|[13933]|LPAB020-08|08BBLEP-02342|658[0n]bp|Canada.Alberta|BOLD:AAB3076
Lacinipolia davena|[13934]|LALPA237-10|AVBC 238-10|658[0n]bp|Canada.British Columbia|BOLD:AAB3076
Lacinipolia davena|[13935]|RDNMK659-11|CNCLEP 81836|658[0n]bp|Canada.British Columbia|BOLD:AAB3076
Lacinipolia davena|[13936]|LPABB394-08|08BBLEP-03659|658[0n]bp|Canada.Alberta|BOLD:AAB3076
Lacinipolia davena|[13937]|LPABB122-08|08BBLEP-03387|658[0n]bp|Canada.Alberta|BOLD:AAB3076
Lacinipolia davena|[13938]|LPABB123-08|08BBLEP-03388|658[0n]bp|Canada.Alberta|BOLD:AAB3076
Lacinipolia davena|[13939]|LALPA610-10|AVBC 612-10|658[0n]bp|Canada.British Columbia|BOLD:AAB3076
Lacinipolia davena|[13940]|LHLEP568-06|UBC-2006-0190|658[0n]bp|Canada.British Columbia|BOLD:AAB3076
Lacinipolia rectilinea|[13941]|RDNMF584-08|NOC14670|658[0n]bp|United States.Washington|BOLD:AAJ2964
Lacinipolia rectilinea|[13942]|RDNMF583-08|NOC14669|609[1n]bp|United States.Washington|BOLD:AAJ2964
Lacinipolia sp.|[13943]|LOWC033-05|CGWC-0033|570[0n]bp|Canada.British Columbia|BOLD:ACF0246
Lacinipolia sp.|[13944]|LBCG3245-09|08-JDWBC-3245|632[0n]bp|Canada.British Columbia|BOLD:ACF0246
Lacinipolia sp.|[13945]|LBCG2850-09|08-JDWBC-2850|658[0n]bp|Canada.British Columbia|BOLD:ACF4075
Lacinipolia sp.|[13946]|LBCG2867-09|08-JDWBC-2867|658[0n]bp|Canada.British Columbia|BOLD:ACF4075
Lacinipolia sp.|[13947]|LBCG248-08|08-JDWBC-0248|658[0n]bp|Canada.British Columbia|BOLD:ACF4075
Lacinipolia sp.|[13948]|LBCG3246-09|08-JDWBC-3246|630[0n]bp|Canada.British Columbia|BOLD:ACF4075
Lacinipolia sp.|[13949]|LBCH6461-10|10-JDWBC-6461|658[0n]bp|Canada.British Columbia|BOLD:ACF4075
Lacinipolia sp.|[13950]|LBCH6164-10|10-JDWBC-6164|658[0n]bp|Canada.British Columbia|BOLD:ACF4075
Lacinipolia sp.|[13951]|LBCG2853-09|08-JDWBC-2853|658[0n]bp|Canada.British Columbia|BOLD:ACF4075
Lacinipolia sp.|[13952]|LBCG2846-09|08-JDWBC-2846|658[0n]bp|Canada.British Columbia|BOLD:ACF4075
Lacinipolia sp.|[13953]|LBCH6608-10|10-JDWBC-6608|658[0n]bp|Canada.British Columbia|BOLD:ACF4075
Lacinipolia sp.|[13954]|LBCG2847-09|08-JDWBC-2847|658[0n]bp|Canada.British Columbia|BOLD:ACF4075

[illegible]
Lacinipolia sp.|[13952]|LBCG2846-09|08-JDWBC-2846|658[0n]bp|Canada.British Columbia|BOLD:ACF4075
Lacinipolia sp.|[13953]|LBCH6608-10|10-JDWBC-6608|658[0n]bp|Canada.British Columbia|BOLD:ACF4075
Lacinipolia sp.|[13954]|LBCG2847-09|08-JDWBC-2847|658[0n]bp|Canada.British Columbia|BOLD:ACF4075
Lacinipolia sp.|[13955]|LBCH6158-10|10-JDWBC-6158|658[0n]bp|Canada.British Columbia|BOLD:ACF4075
Lacinipolia sp.|[13956]|LBCG2849-09|08-JDWBC-2849|658[0n]bp|Canada.British Columbia|BOLD:ACF4075
Lacinipolia sp.|[13957]|LOWC029-05|CGWC-0029|658[0n]bp|Canada.British Columbia|BOLD:ACF4075
Lacinipolia sp.|[13958]|LBCH6226-10|10-JDWBC-6226|658[0n]bp|Canada.British Columbia|BOLD:ACF4075
Lacinipolia sp.|[13959]|LOWC034-05|CGWC-0034|658[0n]bp|Canada.British Columbia|BOLD:ACF4075
Lacinipolia sp.|[13960]|LBCG483-08|08-JDWBC-0483|658[0n]bp|Canada.British Columbia|BOLD:ACF4075
Lacinipolia sp.|[13961]|LBCG500-08|08-JDWBC-0500|658[0n]bp|Canada.British Columbia|BOLD:ACF4075
Lacinipolia sp.|[13962]|LBCH7342-10|10-JDWBC-7342|658[0n]bp|Canada.British Columbia|BOLD:ACF4075
Lacinipolia sp.|[13963]|LBCG495-08|08-JDWBC-0495|658[0n]bp|Canada.British Columbia|BOLD:ACF4075
Lacinipolia sp.|[13964]|LBCH7562-10|10-JDWBC-7562|658[0n]bp|Canada.British Columbia|BOLD:ACF4075
Lacinipolia sp.|[13965]|LBCH6557-10|10-JDWBC-6557|658[0n]bp|Canada.British Columbia|BOLD:ACF4075
Lacinipolia sp.|[13966]|LBCG2848-09|08-JDWBC-2848|658[0n]bp|Canada.British Columbia|BOLD:ACF4075
Lacinipolia sp.|[13967]|LBCG2844-09|08-JDWBC-2844|658[0n]bp|Canada.British Columbia|BOLD:ACF4075
Lacinipolia sp.|[13968]|LOWC035-05|CGWC-0035|658[0n]bp|Canada.British Columbia|BOLD:ACF4075
Lacinipolia sp.|[13969]|LOWC031-05|CGWC-0031|658[0n]bp|Canada.British Columbia|BOLD:ACF4075
Lacinipolia sp.|[13970]|LOWC028-05|CGWC-0028|658[0n]bp|Canada.British Columbia|BOLD:ACF4075
Lacinipolia sp.|[13971]|LOWC030-05|CGWC-0030|658[0n]bp|Canada.British Columbia|BOLD:ACF4075
Lacinipolia sp.|[13972]|LOWC032-05|CGWC-0032|658[0n]bp|Canada.British Columbia|BOLD:ACF4075
Lacinipolia sp.|[13973]|LBCH7769-10|10-JDWBC-7769|658[0n]bp|Canada.British Columbia|BOLD:ACF4075
Lacinipolia sp.|[13974]|LOWC027-05|CGWC-0027|658[0n]bp|Canada.British Columbia|BOLD:ACF4075
Lacinipolia sp.|[13975]|LBCH6605-10|10-JDWBC-6605|658[0n]bp|Canada.British Columbia|BOLD:ACF4075
Lacinipolia sp.|[13976]|LBCH7372-10|10-JDWBC-7372|658[0n]bp|Canada.British Columbia|BOLD:ACF4075
Lacinipolia sp.|[13977]|LBCH6632-10|10-JDWBC-6632|658[0n]bp|Canada.British Columbia|BOLD:ACF4075
Lacinipolia sp.|[13978]|LBCH6604-10|10-JDWBC-6604|658[0n]bp|Canada.British Columbia|BOLD:ACF4075
Lacinipolia sp.|[13979]|LBCH6606-10|10-JDWBC-6606|658[0n]bp|Canada.British Columbia|BOLD:ACF4075
Lacinipolia sp.|[13980]|LBCG2854-09|08-JDWBC-2854|658[0n]bp|Canada.British Columbia|BOLD:ACF4075
Lacinipolia sp.|[13981]|LBCH6384-10|10-JDWBC-6384|658[0n]bp|Canada.British Columbia|BOLD:ACF4075
Lacinipolia sp.|[13982]|LBCG250-08|08-JDWBC-0250|658[0n]bp|Canada.British Columbia|BOLD:ACF4075
Lacinipolia sp.|[13983]|LOWCE532-06|CGWC-4292|658[0n]bp|Canada.British Columbia|BOLD:ACF4075
Lacinipolia strigicollis|[13984]|RDNMC281-05|CNCNoctuoidea11915|602[0n]bp|Canada.British Columbia|BOLD ...
Lacinipolia strigicollis|[13985]|LBCH6162-10|10-JDWBC-6162|658[0n]bp|Canada.British Columbia|BOLD:AAB7768
Lacinipolia strigicollis|[13986]|LBCH5948-10|10-JDWBC-5948|658[0n]bp|Canada.British Columbia|BOLD:AAB7768
Lacinipolia strigicollis|[13987]|LBCH6130-10|10-JDWBC-6130|658[0n]bp|Canada.British Columbia|BOLD:AAB7768
Lacinipolia strigicollis|[13988]|LBCH5947-10|10-JDWBC-5947|658[0n]bp|Canada.British Columbia|BOLD:AAB7768
Lacinipolia strigicollis|[13989]|LBCH6282-10|10-JDWBC-6282|658[0n]bp|Canada.British Columbia|BOLD:AAB7768
Lacinipolia strigicollis|[13990]|LBCH6163-10|10-JDWBC-6163|658[0n]bp|Canada.British Columbia|BOLD:AAB7768
Lacinipolia strigicollis|[13991]|LBCH6159-10|10-JDWBC-6159|658[0n]bp|Canada.British Columbia|BOLD:AAB7768
Lacinipolia strigicollis|[13992]|LBCH6227-10|10-JDWBC-6227|658[0n]bp|Canada.British Columbia|BOLD:AAB7768
Lacinipolia strigicollis|[13993]|LBCH6161-10|10-JDWBC-6161|658[0n]bp|Canada.British Columbia|BOLD:AAB7768
Lacinipolia strigicollis|[13994]|LBCH6157-10|10-JDWBC-6157|658[0n]bp|Canada.British Columbia|BOLD:AAB7768
Lacinipolia strigicollis|[13995]|LBCH6129-10|10-JDWBC-6129|658[0n]bp|Canada.British Columbia|BOLD:AAB7768
Lacinipolia strigicollis|[13996]|LBCH6228-10|10-JDWBC-6228|658[0n]bp|Canada.British Columbia|BOLD:AAB7768
Lacinipolia strigicollis|[13997]|LBCH6160-10|10-JDWBC-6160|658[0n]bp|Canada.British Columbia|BOLD:AAB7768
Lacinipolia strigicollis|[13998]|LBCG1106-09|08-JDWBC-1106|658[0n]bp|Canada.British Columbia|BOLD:AAB7768
Lacinipolia strigicollis|[13999]|LBCH6281-10|10-JDWBC-6281|658[0n]bp|Canada.British Columbia|BOLD:AAB7768
Lacinipolia strigicollis|[14000]|LBCH6225-10|10-JDWBC-6225|658[0n]bp|Canada.British Columbia|BOLD:AAB7768
Lacinipolia strigicollis|[14001]|RDNMC392-05|CNCNoctuoidea12026|591[0n]bp|Canada.British Columbia|BOLD ...
Lacinipolia strigicollis|[14002]|LALPA889-11|AVBC 1062-11|658[0n]bp|Canada.British Columbia|BOLD:AAB7768
Lacinipolia strigicollis|[14003]|LBCG2843-09|08-JDWBC-2843|658[0n]bp|Canada.British Columbia|BOLD:AAB7768
Lacinipolia strigicollis|[14004]|LBCH7781-10|10-JDWBC-7781|658[0n]bp|Canada.British Columbia|BOLD:AAB7768
Lacinipolia strigicollis|[14005]|LBCH6609-10|10-JDWBC-6609|658[0n]bp|Canada.British Columbia|BOLD:AAB7768
Lacinipolia strigicollis|[14006]|LBCH6382-10|10-JDWBC-6382|658[0n]bp|Canada.British Columbia|BOLD:AAB7768
Lacinipolia strigicollis|[14007]|LBCH6610-10|10-JDWBC-6610|658[0n]bp|Canada.British Columbia|BOLD:AAB7768
Lacinipolia strigicollis|[14008]|LBCH6603-10|10-JDWBC-6603|658[0n]bp|Canada.British Columbia|BOLD:AAB7768
Lacinipolia strigicollis|[14009]|LBCG2851-09|08-JDWBC-2851|658[0n]bp|Canada.British Columbia|BOLD:AAB7768
Lacinipolia strigicollis|[14010]|LBCG2845-09|08-JDWBC-2845|658[0n]bp|Canada.British Columbia|BOLD:AAB7768
Lacinipolia strigicollis|[14011]|LBCG2852-09|08-JDWBC-2852|658[0n]bp|Canada.British Columbia|BOLD:AAB7768
Lacinipolia strigicollis|[14012]|LBCH7767-10|10-JDWBC-7767|658[0n]bp|Canada.British Columbia|BOLD:AAB7768
Lacinipolia strigicollis|[14013]|LBCH6056-10|10-JDWBC-6056|658[0n]bp|Canada.British Columbia|BOLD:AAB7768
Lacinipolia strigicollis|[14014]|LBCH6607-10|10-JDWBC-6607|658[0n]bp|Canada.British Columbia|BOLD:AAB7768
Lacinipolia strigicollis|[14015]|LBCG2855-09|08-JDWBC-2855|658[0n]bp|Canada.British Columbia|BOLD:AAB7768
Lacinipolia implicata|[14016]|RDLQ661-07|DH013486|644[0n]bp|Canada.Quebec|BOLD:AAE5535
Lacinipolia implicata|[14017]|RDLQ662-07|DH013485|658[0n]bp|Canada.Quebec|BOLD:AAE5535
Lacinipolia meditata|[14018]|RDLQF209-06|DH011289|658[0n]bp|Canada.Quebec|BOLD:AAA8562
Lacinipolia meditata|[14019]|XAD242-04|04HBL007242|658[0n]bp|Canada.Ontario|BOLD:AAA8562
Lacinipolia meditata|[14020]|XAD256-04|04HBL007256|599[0n]bp|Canada.Ontario|BOLD:AAA8562
Lacinipolia meditata|[14021]|XAD253-04|04HBL007253|646[0n]bp|Canada.Ontario|BOLD:AAA8562
Lacinipolia meditata|[14022]|XAD275-04|04HBL007275|589[0n]bp|Canada.Ontario|BOLD:AAA8562
Lacinipolia meditata|[14023]|XAG845-05|2005-ONT-1429|658[0n]bp|Canada.Ontario|BOLD:AAA8562
Lacinipolia meditata|[14024]|LPABC942-09|08BBLEP-05353|658[0n]bp|Canada.Alberta|BOLD:AAA8562
Lacinipolia meditata|[14025]|LPABB184-08|08BBLEP-03449|658[0n]bp|Canada.Alberta|BOLD:AAA8562
Lacinipolia meditata|[14026]|RDNMB248-05|CNCNoctuoidea10014|658[0n]bp|Canada.Alberta|BOLD:AAA8562
Lacinipolia meditata|[14027]|LPMN902-08|08BBLEP-02260|658[0n]bp|Canada.Alberta|BOLD:AAA8562
Lacinipolia meditata|[14028]|XAD364-04|04HBL007364|569[1n]bp|Canada.Ontario|BOLD:AAA8562
Lacinipolia meditata|[14029]|XAH208-05|2005-ONT-1791|658[0n]bp|Canada.Ontario|BOLD:AAA8562
Lacinipolia meditata|[14030]|XAG646-05|2005-ONT-1230|658[0n]bp|Canada.Ontario|BOLD:AAA8562
Lacinipolia meditata|[14031]|XAG733-05|2005-ONT-1317|658[0n]bp|Canada.Ontario|BOLD:AAA8562
Lacinipolia meditata|[14032]|XAD362-04|04HBL007362|658[0n]bp|Canada.Ontario|BOLD:AAA8562
Lacinipolia meditata|[14033]|XAD346-04|04HBL007346|658[0n]bp|Canada.Ontario|BOLD:AAA8562
Lacinipolia meditata|[14034]|RDNMB247-05|CNCNoctuoidea10013|658[0n]bp|Canada.Ontario|BOLD:AAA8562
Lacinipolia meditata|[14035]|XAG995-05|2005-ONT-1579|658[0n]bp|Canada.Ontario|BOLD:AAA8562
Lacinipolia meditata|[14036]|XAG944-05|2005-ONT-1528|658[0n]bp|Canada.Ontario|BOLD:AAA8562
Lacinipolia meditata|[14037]|PHMO392-03|moth2467.02|639[0n]bp|Canada.Ontario|BOLD:AAA8562
Lacinipolia meditata|[14038]|XAD254-04|04HBL007254|607[0n]bp|Canada.Ontario|BOLD:AAA8562
Lacinipolia meditata|[14039]|XAK271-06|2006-ONT-1266|658[0n]bp|Canada.Ontario|BOLD:AAA8562
Lacinipolia meditata|[14040]|XAK291-06|2006-ONT-1286|658[0n]bp|Canada.Ontario|BOLD:AAA8562
Lacinipolia meditata|[14041]|XAH036-05|2005-ONT-1619|658[0n]bp|Canada.Ontario|BOLD:AAA8562
Lacinipolia meditata|[14042]|XAK245-06|2006-ONT-1240|658[0n]bp|Canada.Ontario|BOLD:AAA8562
Lacinipolia meditata|[14043]|XAG852-05|2005-ONT-1436|658[0n]bp|Canada.Ontario|BOLD:AAA8562
Lacinipolia meditata|[14044]|XAG978-05|2005-ONT-1562|633[0n]bp|Canada.Ontario|BOLD:AAA8562
Lacinipolia meditata|[14045]|XAG671-05|2005-ONT-1255|658[1n]bp|Canada.Ontario|BOLD:AAA8562
Lacinipolia meditata|[14046]|XAH127-05|2005-ONT-1710|658[0n]bp|Canada.Ontario|BOLD:AAA8562
Lacinipolia meditata|[14047]|XAG914-05|2005-ONT-1498|658[0n]bp|Canada.Ontario|BOLD:AAA8562
Lacinipolia meditata|[14048]|XAH281-05|2005-ONT-1864|658[0n]bp|Canada.Ontario|BOLD:AAA8562
Lacinipolia meditata|[14049]|XAH270-05|2005-ONT-1853|658[0n]bp|Canada.Ontario|BOLD:AAA8562
Lacinipolia meditata|[14050]|XAH028-05|2005-ONT-1611|658[0n]bp|Canada.Ontario|BOLD:AAA8562
Lacinipolia meditata|[14051]|XAH153-05|2005-ONT-1736|658[0n]bp|Canada.Ontario|BOLD:AAA8562
Lacinipolia meditata|[14052]|XAH152-05|2005-ONT-1735|658[0n]bp|Canada.Ontario|BOLD:AAA8562
Lacinipolia meditata|[14053]|XAH151-05|2005-ONT-1734|658[1n]bp|Canada.Ontario|BOLD:AAA8562
[illegible]

Lacinipolia meditata|[14051]|XAH153-05|2005-ONT-1736|658[0n]bp|Canada.Ontario|BOLD:AAA8562
Lacinipolia meditata|[14052]|XAH152-05|2005-ONT-1735|658[0n]bp|Canada.Ontario|BOLD:AAA8562
Lacinipolia meditata|[14053]|XAH151-05|2005-ONT-1734|658[1n]bp|Canada.Ontario|BOLD:AAA8562
Lacinipolia meditata|[14054]|XAD273-04|04HBL007273|604[0n]bp|Canada.Ontario|BOLD:AAA8562
Lacinipolia meditata|[14055]|XAD003-04|04HBL007003|580[0n]bp|Canada.Ontario|BOLD:AAA8562
Lacinipolia lustralis|[14056]|RDMAB522-06|UASM58483|658[0n]bp|Canada.Alberta|BOLD:AAA7130
Lacinipolia lustralis|[14057]|RDMAB368-05|UASM77833|587[0n]bp|Canada.Alberta|BOLD:AAA7130
Lacinipolia lustralis|[14058]|RDMAB034-05|UASM57517|628[0n]bp|Canada.Alberta|BOLD:AAA7130
Lacinipolia lustralis|[14059]|LPMN013-08|08BBLEP-00811|658[0n]bp|Canada.Manitoba|BOLD:AAA7130
Lacinipolia lustralis|[14060]|LOWCC092-05|CGWC-1972|542[0n]bp|Canada.British Columbia|BOLD:AAA7130
Lacinipolia lustralis|[14061]|PHMNB138-04|04HBL007603|658[0n]bp|Canada.New Brunswick|BOLD:AAA7130
Lacinipolia lustralis|[14062]|PHMNB483-04|04HBL00709|658[0n]bp|Canada.New Brunswick|BOLD:AAA7130
Lacinipolia lustralis|[14063]|RDLQB063-05|DH010149|658[0n]bp|Canada.Quebec|BOLD:AAA7130
Lacinipolia lustralis|[14064]|LPSOD252-09|08BBLEP-00030|658[0n]bp|Canada.Ontario|BOLD:AAA7130
Lacinipolia lustralis|[14065]|PHMNB477-04|04HBL00703|658[0n]bp|Canada.New Brunswick|BOLD:AAA7130
Lacinipolia lustralis|[14066]|RDLQB061-05|DH010147|658[0n]bp|Canada.Quebec|BOLD:AAA7130
Lacinipolia lustralis|[14067]|RDLQB060-05|DH010146|658[0n]bp|Canada.Quebec|BOLD:AAA7130
Lacinipolia lustralis|[14068]|RDLQB062-05|DH010148|658[0n]bp|Canada.Quebec|BOLD:AAA7130
Lacinipolia lustralis|[14069]|XAC621-04|04HBL006621|617[0n]bp|Canada.Ontario|BOLD:AAA7130
Lacinipolia lustralis|[14070]|RDNMC401-05|CNCNoctuoidea12035|613[0n]bp|Canada.Ontario|BOLD:AAA7130
Lacinipolia lustralis|[14071]|RDMAB258-05|UASM41457|658[0n]bp|Canada.Alberta|BOLD:AAA7130
Lacinipolia lustralis|[14072]|LPMN633-08|08BBLEP-01434|658[0n]bp|Canada.Manitoba|BOLD:AAA7130
Lacinipolia lustralis|[14073]|LOWCE568-06|CGWC-4328|658[0n]bp|Canada.British Columbia|BOLD:AAA7130
Lacinipolia lustralis|[14074]|LOWCE277-06|CGWC-4037|563[0n]bp|Canada.British Columbia|BOLD:AAA7130
Lacinipolia lustralis|[14075]|LPMN264-08|08BBLEP-01063|658[0n]bp|Canada.Manitoba|BOLD:AAA7130
Lacinipolia lustralis|[14076]|LOWCE580-06|CGWC-4340|658[0n]bp|Canada.British Columbia|BOLD:AAA7130
Lacinipolia lustralis|[14077]|LOWCC095-05|CGWC-1975|658[0n]bp|Canada.British Columbia|BOLD:AAA7130
Lacinipolia lustralis|[14078]|LPSK466-08|08BBLEP-02034|658[0n]bp|Canada.Saskatchewan|BOLD:AAA7130
Lacinipolia lustralis|[14079]|LPSK251-08|08BBLEP-01819|658[0n]bp|Canada.Saskatchewan|BOLD:AAA7130
Lacinipolia lustralis|[14080]|LOWCC093-05|CGWC-1973|658[0n]bp|Canada.British Columbia|BOLD:AAA7130
Lacinipolia lustralis|[14081]|LOWCE575-06|CGWC-4335|658[0n]bp|Canada.British Columbia|BOLD:AAA7130
Lacinipolia lustralis|[14082]|LOWCE565-06|CGWC-4325|658[0n]bp|Canada.British Columbia|BOLD:AAA7130
Lacinipolia lustralis|[14083]|LPMN323-08|08BBLEP-01122|658[0n]bp|Canada.Manitoba|BOLD:AAA7130
Lacinipolia lustralis|[14084]|LOWCE567-06|CGWC-4327|658[0n]bp|Canada.British Columbia|BOLD:AAA7130
Lacinipolia lustralis|[14085]|LOWCE566-06|CGWC-4326|658[0n]bp|Canada.British Columbia|BOLD:AAA7130
Lacinipolia lustralis|[14086]|LOWCE564-06|CGWC-4324|658[0n]bp|Canada.British Columbia|BOLD:AAA7130
Lacinipolia lustralis|[14087]|LOWCE571-06|CGWC-4331|658[0n]bp|Canada.British Columbia|BOLD:AAA7130
Lacinipolia lustralis|[14088]|LOWCC096-05|CGWC-1976|658[0n]bp|Canada.British Columbia|BOLD:AAA7130
Lacinipolia lustralis|[14089]|LOWCC094-05|CGWC-1974|658[0n]bp|Canada.British Columbia|BOLD:AAA7130
Lacinipolia lustralis|[14090]|LOWCE573-06|CGWC-4333|658[0n]bp|Canada.British Columbia|BOLD:AAA7130
Lacinipolia lustralis|[14091]|LOWCE578-06|CGWC-4338|658[0n]bp|Canada.British Columbia|BOLD:AAA7130
Lacinipolia lustralis|[14092]|RDMAB575-06|UASM58565|658[0n]bp|Canada.Alberta|BOLD:AAA7130
Lacinipolia lustralis|[14093]|RDMAB576-06|UASM58566|658[0n]bp|Canada.Alberta|BOLD:AAA7130
Lacinipolia lustralis|[14094]|LOWCE579-06|CGWC-4339|658[0n]bp|Canada.British Columbia|BOLD:AAA7130
Lacinipolia lustralis|[14095]|LOWCC090-05|CGWC-1970|658[0n]bp|Canada.British Columbia|BOLD:AAA7130
Lacinipolia lustralis|[14096]|LOWCE577-06|CGWC-4337|658[0n]bp|Canada.British Columbia|BOLD:AAA7130
Lacinipolia lustralis|[14097]|LPMN165-08|08BBLEP-00964|658[0n]bp|Canada.Manitoba|BOLD:AAA7130
Lacinipolia lustralis|[14098]|LOWCE563-06|CGWC-4323|658[0n]bp|Canada.British Columbia|BOLD:AAA7130
Lacinipolia lustralis|[14099]|LOWCE561-06|CGWC-4321|658[0n]bp|Canada.British Columbia|BOLD:AAA7130
Lacinipolia lustralis|[14100]|LOWCE562-06|CGWC-4322|654[0n]bp|Canada.British Columbia|BOLD:AAA7130
Lacinipolia lustralis|[14101]|RDMAB289-05|UASM77784|612[1n]bp|Canada.Alberta|BOLD:AAA7130
Lacinipolia lustralis|[14102]|LOWCE576-06|CGWC-4336|649[0n]bp|Canada.British Columbia|BOLD:AAA7130
Lacinipolia lustralis|[14103]|RDNMC387-05|CNCNoctuoidea12021|578[0n]bp|Canada.Alberta|BOLD:AAA7130
Lacinipolia lustralis|[14104]|LOWCE862-06|CGWC-4622|617[0n]bp|Canada.British Columbia|BOLD:AAA7130
Lacinipolia lustralis|[14105]|LOWCD759-06|CGWC-3579|583[1n]bp|Canada.British Columbia|BOLD:AAA7130
Lacinipolia lustralis|[14106]|RDMAB312-05|UASM19846|611[0n]bp|Canada.Alberta|BOLD:AAA7130
Lacinipolia lustralis|[14107]|RDMAB313-05|UASM24107|612[0n]bp|Canada.Alberta|BOLD:AAA7130
Lacinipolia lustralis|[14108]|LPSK442-08|08BBLEP-02010|658[0n]bp|Canada.Saskatchewan|BOLD:AAA7130
Lacinipolia lustralis|[14109]|RDMAB099-05|UASM41979|658[0n]bp|Canada.Alberta|BOLD:AAA7130
Lacinipolia lustralis|[14110]|LOWCE581-06|CGWC-4341|658[0n]bp|Canada.British Columbia|BOLD:AAA7130
Lacinipolia lustralis|[14111]|LPMN643-08|08BBLEP-01444|658[0n]bp|Canada.Manitoba|BOLD:AAA7130
Lacinipolia lustralis|[14112]|LOWCE569-06|CGWC-4329|658[0n]bp|Canada.British Columbia|BOLD:AAA7130
Lacinipolia lustralis|[14113]|LOWCE570-06|CGWC-4330|658[0n]bp|Canada.British Columbia|BOLD:AAA7130
Lacinipolia lustralis|[14114]|LPMN527-08|08BBLEP-01326|658[0n]bp|Canada.Manitoba|BOLD:AAA7130
Lacinipolia lustralis|[14115]|LOWCC097-05|CGWC-1977|658[0n]bp|Canada.British Columbia|BOLD:AAA7130
Lacinipolia lustralis|[14116]|RDMAB111-05|UASM41289|658[0n]bp|Canada.Alberta|BOLD:AAA7130
Lacinipolia naevia|[14117]|RDMAB374-05|UASM77840|575[1n]bp|Canada.Alberta|BOLD:AAH5399
Lacinipolia pensilis|[14118]|LOWCE525-06|CGWC-4285|570[1n]bp|Canada.British Columbia|BOLD:ABZ1332
Lacinipolia pensilis|[14119]|RDMAB304-05|UASM2652|658[0n]bp|Canada.Alberta|BOLD:ABZ7094
Lacinipolia pensilis|[14120]|RDMAB602-06|UASM58647|658[0n]bp|Canada.Alberta|BOLD:ABZ7094
Lacinipolia pensilis|[14121]|RDMAB305-05|UASM2532|658[0n]bp|Canada.Alberta|BOLD:ABZ7094
Lacinipolia pensilis|[14122]|RDMAB601-06|UASM58646|658[0n]bp|Canada.Alberta|BOLD:ABZ7094
Lacinipolia pensilis|[14123]|LBCH7673-10|10-JDWBC-7673|658[0n]bp|Canada.British Columbia|BOLD:ABZ7094
Lacinipolia pensilis|[14124]|LOWCD766-06|CGWC-3586|657[0n]bp|Canada.British Columbia|BOLD:ABZ7094
Lacinipolia pensilis|[14125]|LBCH7671-10|10-JDWBC-7671|658[0n]bp|Canada.British Columbia|BOLD:ABZ7094
Lacinipolia pensilis|[14126]|LOWCE555-06|CGWC-4315|658[0n]bp|Canada.British Columbia|BOLD:ABZ7094
Lacinipolia pensilis|[14127]|RDNMB232-05|CNCNoctuoidea7978|615[0n]bp|Canada.British Columbia|BOLD:ABZ7094
Lacinipolia pensilis|[14128]|LOWCE554-06|CGWC-4314|610[0n]bp|Canada.British Columbia|BOLD:ABZ7094
Lacinipolia pensilis|[14129]|LOWCD768-06|CGWC-3588|591[0n]bp|Canada.British Columbia|BOLD:ABZ7094
Lacinipolia pensilis|[14130]|RDNMB224-05|CNCNoctuoidea7970|594[0n]bp|Canada.British Columbia|BOLD:ABZ7094
Lacinipolia pensilis|[14131]|LBCH7435-10|10-JDWBC-7435|658[0n]bp|Canada.British Columbia|BOLD:ABZ7094
Lacinipolia pensilis|[14132]|LBCH6456-10|10-JDWBC-6456|658[0n]bp|Canada.British Columbia|BOLD:ABZ7094
Lacinipolia pensilis|[14133]|LBCH7884-10|10-JDWBC-7884|658[0n]bp|Canada.British Columbia|BOLD:ABZ7094
Lacinipolia pensilis|[14134]|LBCH7670-10|10-JDWBC-7670|658[0n]bp|Canada.British Columbia|BOLD:ABZ7094
Lacinipolia pensilis|[14135]|LBCH7669-10|10-JDWBC-7669|658[0n]bp|Canada.British Columbia|BOLD:ABZ7094
Lacinipolia pensilis|[14136]|LBCH7001-10|10-JDWBC-7001|658[0n]bp|Canada.British Columbia|BOLD:ABZ7094
Lacinipolia pensilis|[14137]|LBCH7810-10|10-JDWBC-7810|658[0n]bp|Canada.British Columbia|BOLD:ABZ7094
Lacinipolia pensilis|[14138]|LBCH7667-10|10-JDWBC-7667|658[0n]bp|Canada.British Columbia|BOLD:ABZ7094
Lacinipolia pensilis|[14139]|LBCH7674-10|10-JDWBC-7674|658[0n]bp|Canada.British Columbia|BOLD:ABZ7094
Lacinipolia pensilis|[14140]|LBCH6724-10|10-JDWBC-6724|640[0n]bp|Canada.British Columbia|BOLD:ABZ7094
Lacinipolia pensilis|[14141]|LOWCE552-06|CGWC-4312|610[0n]bp|Canada.British Columbia|BOLD:ABZ7094
Lacinipolia pensilis|[14142]|LBCH7672-10|10-JDWBC-7672|636[0n]bp|Canada.British Columbia|BOLD:ABZ7094
Lacinipolia pensilis|[14143]|LBCG3324-09|08-JDWBC-3324|658[0n]bp|Canada.British Columbia|BOLD:ABZ7094
Lacinipolia pensilis|[14144]|LBCH7668-10|10-JDWBC-7668|658[0n]bp|Canada.British Columbia|BOLD:ABZ7094
Lacinipolia pensilis|[14145]|LBCH7122-10|10-JDWBC-7122|658[0n]bp|Canada.British Columbia|BOLD:ABZ7094
Lacinipolia pensilis|[14146]|LBCH6381-10|10-JDWBC-6381|658[0n]bp|Canada.British Columbia|BOLD:ABZ7094
Lacinipolia pensilis|[14147]|LOWCE526-06|CGWC-4286|548[2n]bp|Canada.British Columbia|BOLD:ABZ1332
Lacinipolia pensilis|[14148]|LOWCE557-06|CGWC-4317|617[0n]bp|Canada.British Columbia|BOLD:AAA5195
Lacinipolia pensilis|[14149]|LOWCE544-06|CGWC-4304|605[0n]bp|Canada.British Columbia|BOLD:AAA5195
Lacinipolia pensilis|[14150]|RDNMC081-05|CNCNoctuoidea10822|658[0n]bp|Canada.Alberta|BOLD:AAA5195
Lacinipolia pensilis|[14151]|LBCH6740-10|10-JDWBC-6740|658[0n]bp|Canada.British Columbia|BOLD:AAA5195
Lacinipolia pensilis|[14152]|LBCG3273-09|08-JDWBC-3273|641[0n]bp|Canada.British Columbia|BOLD:AAA5195
Lacinipolia pensilis|[14153]|LBCG2863-09|08-JDWBC-2863|658[0n]bp|Canada.British Columbia|BOLD:AAA5195

Lacinipolia pensilis|[14151]|LBCH6740-10|10-JDWBC-6740|658[0n]bp|Canada.British Columbia|BOLD:AAA5195
Lacinipolia pensilis|[14152]|LBCG3273-09|08-JDWBC-3273|641[0n]bp|Canada.British Columbia|BOLD:AAA5195
Lacinipolia pensilis|[14153]|LBCG2863-09|08-JDWBC-2863|658[0n]bp|Canada.British Columbia|BOLD:AAA5195
Lacinipolia pensilis|[14154]|LBCH7394-10|10-JDWBC-7394|658[0n]bp|Canada.British Columbia|BOLD:AAA5195
Lacinipolia pensilis|[14155]|RDNMB233-05|CNCNoctuoidea7979|592[0n]bp|Canada.British Columbia|BOLD:AAA5195
Lacinipolia pensilis|[14156]|LPVIB634-08|PFC-2006-2082|597[0n]bp|Canada.British Columbia|BOLD:AAA5195
Lacinipolia pensilis|[14157]|LPVIB253-08|PFC-2006-1643|658[0n]bp|Canada.British Columbia|BOLD:AAA5195
Lacinipolia pensilis|[14158]|LOWCE556-06|CGWC-4316|617[0n]bp|Canada.British Columbia|BOLD:AAA5195
Lacinipolia pensilis|[14159]|LOWCE551-06|CGWC-4311|617[0n]bp|Canada.British Columbia|BOLD:AAA5195
Lacinipolia pensilis|[14160]|LBCG3247-09|08-JDWBC-3247|643[0n]bp|Canada.British Columbia|BOLD:AAA5195
Lacinipolia pensilis|[14161]|LBCH7231-10|10-JDWBC-7231|643[0n]bp|Canada.British Columbia|BOLD:AAA5195
Lacinipolia pensilis|[14162]|RDMAB623-06|UASM58742|658[0n]bp|Canada.Alberta|BOLD:AAA5195
Lacinipolia pensilis|[14163]|LPVIB637-08|PFC-2006-2085|634[0n]bp|Canada.British Columbia|BOLD:AAA5195
Lacinipolia pensilis|[14164]|LPVIB258-08|PFC-2006-1648|658[0n]bp|Canada.British Columbia|BOLD:AAA5195
Lacinipolia pensilis|[14165]|LOWCC086-05|CGWC-1966|658[0n]bp|Canada.British Columbia|BOLD:AAA5195
Lacinipolia pensilis|[14166]|LBCG2862-09|08-JDWBC-2862|658[0n]bp|Canada.British Columbia|BOLD:AAA5195
Lacinipolia pensilis|[14167]|LBCD293-05|HLC-23113|633[0n]bp|Canada.British Columbia|BOLD:AAA5195
Lacinipolia pensilis|[14168]|LBCG3257-09|08-JDWBC-3257|642[0n]bp|Canada.British Columbia|BOLD:AAA5195
Lacinipolia pensilis|[14169]|LPABC387-09|08BBLEP-04606|658[0n]bp|Canada.Alberta|BOLD:AAA5195
Lacinipolia pensilis|[14170]|LBCG480-08|08-JDWBC-0480|658[0n]bp|Canada.British Columbia|BOLD:AAA5195
Lacinipolia pensilis|[14171]|LPABB011-08|08BBLEP-03276|658[0n]bp|Canada.Alberta|BOLD:AAA5195
Lacinipolia pensilis|[14172]|LBCH6540-10|10-JDWBC-6540|658[0n]bp|Canada.British Columbia|BOLD:AAA5195
Lacinipolia pensilis|[14173]|LBCG1346-09|08-JDWBC-1346|658[0n]bp|Canada.British Columbia|BOLD:AAA5195
Lacinipolia pensilis|[14174]|LOWCC088-05|CGWC-1968|658[0n]bp|Canada.British Columbia|BOLD:AAA5195
Lacinipolia pensilis|[14175]|LBCG2868-09|08-JDWBC-2868|658[0n]bp|Canada.British Columbia|BOLD:AAA5195
Lacinipolia pensilis|[14176]|LBCG1344-09|08-JDWBC-1344|658[0n]bp|Canada.British Columbia|BOLD:AAA5195
Lacinipolia pensilis|[14177]|LBCH7476-10|10-JDWBC-7476|658[0n]bp|Canada.British Columbia|BOLD:AAA5195
Lacinipolia pensilis|[14178]|LOWCE574-06|CGWC-4334|658[0n]bp|Canada.British Columbia|BOLD:AAA5195
Lacinipolia pensilis|[14179]|LPABC389-09|08BBLEP-04608|658[0n]bp|Canada.Alberta|BOLD:AAA5195
Lacinipolia pensilis|[14180]|LPVIB636-08|PFC-2006-2084|658[0n]bp|Canada.British Columbia|BOLD:AAA5195
Lacinipolia pensilis|[14181]|RDMAB622-06|UASM58743|658[0n]bp|Canada.Alberta|BOLD:AAA5195
Lacinipolia pensilis|[14182]|LOWCE559-06|CGWC-4319|658[0n]bp|Canada.British Columbia|BOLD:AAA5195
Lacinipolia pensilis|[14183]|LBCG2873-09|08-JDWBC-2873|658[0n]bp|Canada.British Columbia|BOLD:AAA5195
Lacinipolia pensilis|[14184]|LBCG2871-09|08-JDWBC-2871|658[0n]bp|Canada.British Columbia|BOLD:AAA5195
Lacinipolia pensilis|[14185]|LBCD461-05|HLC-23281|658[0n]bp|Canada.British Columbia|BOLD:AAA5195
Lacinipolia pensilis|[14186]|LOWCC087-05|CGWC-1967|658[0n]bp|Canada.British Columbia|BOLD:AAA5195
Lacinipolia pensilis|[14187]|LALPA1276-11|AVBC 1278-11|658[0n]bp|Canada.British Columbia|BOLD:AAA5195
Lacinipolia pensilis|[14188]|LBCG3251-09|08-JDWBC-3251|638[0n]bp|Canada.British Columbia|BOLD:AAA5195
Lacinipolia pensilis|[14189]|LBCH6497-10|10-JDWBC-6497|658[0n]bp|Canada.British Columbia|BOLD:AAA5195
Lacinipolia pensilis|[14190]|LBCG252-08|08-JDWBC-0252|658[0n]bp|Canada.British Columbia|BOLD:AAA5195
Lacinipolia pensilis|[14191]|LBCG2108-09|08-JDWBC-2108|658[0n]bp|Canada.British Columbia|BOLD:AAA5195
Lacinipolia vicina|[14192]|LOWCC089-05|CGWC-1969|658[0n]bp|Canada.British Columbia|BOLD:AAA6056
Lacinipolia vicina|[14193]|BBLPB469-10|10BBCLP-1468|658[0n]bp|Canada.Ontario|BOLD:AAA6056
Lacinipolia vicina|[14194]|BBLPB480-10|10BBCLP-1479|658[0n]bp|Canada.Alberta|BOLD:AAA6056
Lacinipolia vicina|[14195]|LPABC370-09|08BBLEP-04589|658[0n]bp|Canada.Alberta|BOLD:AAA6056
Lacinipolia vicina|[14196]|LPABB015-08|08BBLEP-03280|633[0n]bp|Canada.Alberta|BOLD:AAA6056
Lacinipolia vicina|[14197]|RDMAB619-06|UASM58741|658[0n]bp|Canada.Alberta|BOLD:AAA6056
Lacinipolia vicina|[14198]|RDMAB263-05|UASM41460|658[0n]bp|Canada.Alberta|BOLD:AAA6056
Lacinipolia vicina|[14199]|LPABB031-08|08BBLEP-03296|658[0n]bp|Canada.Alberta|BOLD:AAA6056
Lacinipolia vicina|[14200]|RDMAB632-06|CBCC1030|658[0n]bp|Canada.Alberta|BOLD:AAA6056
Lacinipolia vicina|[14201]|RDMAB288-05|UASM77783|634[0n]bp|Canada.Alberta|BOLD:AAA6056
Lacinipolia vicina|[14202]|LPAB249-08|08BBLEP-02571|658[0n]bp|Canada.Alberta|BOLD:AAA6056
Lacinipolia vicina|[14203]|RDMAB635-06|UASM58756|658[0n]bp|Canada.Alberta|BOLD:AAA6056
Lacinipolia vicina|[14204]|RDMAB620-06|UASM77865|658[0n]bp|Canada.Alberta|BOLD:AAA6056
Lacinipolia vicina|[14205]|RDMAB254-05|UASM41468|658[0n]bp|Canada.Alberta|BOLD:AAA6056
Lacinipolia vicina|[14206]|LPABB440-08|08BBLEP-03705|658[0n]bp|Canada.Alberta|BOLD:AAA6056
Lacinipolia vicina|[14207]|LPABB582-08|08BBLEP-03847|658[0n]bp|Canada.Alberta|BOLD:AAA6056
Lacinipolia vicina|[14208]|LPABB068-08|08BBLEP-03333|658[0n]bp|Canada.Alberta|BOLD:AAA6056
Lacinipolia vicina|[14209]|LPABB418-08|08BBLEP-03683|658[0n]bp|Canada.Alberta|BOLD:AAA6056
Lacinipolia vicina|[14210]|LPABB353-08|08BBLEP-03618|658[0n]bp|Canada.Alberta|BOLD:AAA6056
Lacinipolia vicina|[14211]|LPABC986-09|08BBLEP-05397|658[0n]bp|Canada.Alberta|BOLD:AAA6056
Lacinipolia vicina|[14212]|LPMN885-08|08BBLEP-02243|658[0n]bp|Canada.Alberta|BOLD:AAA6056
Lacinipolia vicina|[14213]|LPABC139-09|08BBLEP-04358|658[0n]bp|Canada.Alberta|BOLD:AAA6056
Lacinipolia vicina|[14214]|LPABB431-08|08BBLEP-03696|658[0n]bp|Canada.Alberta|BOLD:AAA6056
Lacinipolia vicina|[14215]|RDMAB639-06|UASM58758|658[0n]bp|Canada.Alberta|BOLD:AAA6056
Lacinipolia vicina|[14216]|RDMAB638-06|UASM57936|658[0n]bp|Canada.Alberta|BOLD:AAA6056
Lacinipolia vicina|[14217]|BBLPB467-10|10BBCLP-1466|658[0n]bp|Canada.British Columbia|BOLD:AAA6056
Lacinipolia vicina|[14218]|LBCH5820-10|10-JDWBC-5820|658[0n]bp|Canada.British Columbia|BOLD:AAA6056
Lacinipolia vicina|[14219]|LBCH5717-10|10-JDWBC-5717|658[0n]bp|Canada.British Columbia|BOLD:AAA6056
Lacinipolia vicina|[14220]|LBCH5602-10|10-JDWBC-5602|658[0n]bp|Canada.British Columbia|BOLD:AAA6056
Lacinipolia vicina|[14221]|LBCH5715-10|10-JDWBC-5715|658[0n]bp|Canada.British Columbia|BOLD:AAA6056
Lacinipolia vicina|[14222]|BBLPB336-10|10BBCLP-1335|658[0n]bp|Canada.British Columbia|BOLD:AAA6056
Lacinipolia vicina|[14223]|LBCH5821-10|10-JDWBC-5821|658[0n]bp|Canada.British Columbia|BOLD:AAA6056
Lacinipolia vicina|[14224]|LBCH5946-10|10-JDWBC-5946|658[0n]bp|Canada.British Columbia|BOLD:AAA6056
Lacinipolia vicina|[14225]|LBCH5429-10|10-JDWBC-5429|658[0n]bp|Canada.British Columbia|BOLD:AAA6056
Lacinipolia vicina|[14226]|BBLPB569-10|10BBCLP-1568|658[0n]bp|Canada.British Columbia|BOLD:AAA6056
Lacinipolia vicina|[14227]|LBCH6098-10|10-JDWBC-6098|658[0n]bp|Canada.British Columbia|BOLD:AAA6056
Lacinipolia vicina|[14228]|LBCG132-08|08-JDWBC-0132|658[0n]bp|Canada.British Columbia|BOLD:AAA6056
Lacinipolia vicina|[14229]|LOWCD763-06|CGWC-3583|656[0n]bp|Canada.British Columbia|BOLD:AAA6056
Lacinipolia vicina|[14230]|LOWCD765-06|CGWC-3585|657[1n]bp|Canada.British Columbia|BOLD:AAA6056
Lacinipolia vicina|[14231]|LOWCC085-05|CGWC-1965|658[0n]bp|Canada.British Columbia|BOLD:AAA6056
Lacinipolia vicina|[14232]|LBCH5575-10|10-JDWBC-5575|658[0n]bp|Canada.British Columbia|BOLD:AAA6056
Lacinipolia vicina|[14233]|LOWCD762-06|CGWC-3582|657[0n]bp|Canada.British Columbia|BOLD:AAA6056
Lacinipolia vicina|[14234]|LBCH5514-10|10-JDWBC-5514|658[0n]bp|Canada.British Columbia|BOLD:AAA6056
Lacinipolia vicina|[14235]|LBCH5548-10|10-JDWBC-5548|658[0n]bp|Canada.British Columbia|BOLD:AAA6056
Lacinipolia vicina|[14236]|LBCG128-08|08-JDWBC-0128|658[0n]bp|Canada.British Columbia|BOLD:AAA6056
Lacinipolia vicina|[14237]|LOWCD764-06|CGWC-3584|658[0n]bp|Canada.British Columbia|BOLD:AAA6056
Lacinipolia vicina|[14238]|LOWCE545-06|CGWC-4305|626[0n]bp|Canada.British Columbia|BOLD:AAA6056
Lacinipolia vicina|[14239]|LOWCE548-06|CGWC-4308|626[0n]bp|Canada.British Columbia|BOLD:AAA6056
Lacinipolia vicina|[14240]|LOWCE547-06|CGWC-4307|615[0n]bp|Canada.British Columbia|BOLD:AAA6056
Lacinipolia vicina|[14241]|RDNMB237-05|CNCNoctuoidea10003|588[0n]bp|Canada.British Columbia|BOLD:AAA6056
Lacinipolia vicina|[14242]|LOWCE546-06|CGWC-4306|598[0n]bp|Canada.British Columbia|BOLD:AAA6056
Lacinipolia vicina|[14243]|LBCG119-08|08-JDWBC-0119|658[0n]bp|Canada.British Columbia|BOLD:AAA6056
Lacinipolia vicina|[14244]|LOWCE558-06|CGWC-4318|658[0n]bp|Canada.British Columbia|BOLD:AAA6056
Lacinipolia vicina|[14245]|LOWCD767-06|CGWC-3587|658[0n]bp|Canada.British Columbia|BOLD:AAA6056
Lacinipolia vicina group|[14246]|RDMAB110-05|UASM41996|658[0n]bp|Canada.Alberta|BOLD:AAA6058
Lacinipolia vicina group|[14247]|LPSK563-08|08BBLEP-02131|658[0n]bp|Canada.Saskatchewan|BOLD:AAA6058
Lacinipolia vicina group|[14248]|LPSK249-08|08BBLEP-01817|658[0n]bp|Canada.Saskatchewan|BOLD:AAA6058
Lacinipolia vicina group|[14249]|RDMAB641-06|UASM24141|658[0n]bp|Canada.Alberta|BOLD:AAA6058
Lacinipolia vicina group|[14250]|LPSK043-08|08BBLEP-00746|658[0n]bp|Canada.Saskatchewan|BOLD:AAA6058
Lacinipolia vicina group|[14251]|RDMAB273-05|UASM41401|658[0n]bp|Canada.Saskatchewan|BOLD:AAA6058
Lacinipolia vicina group|[14252]|RDMAB491-06|UASM78000|658[0n]bp|Canada.Alberta|BOLD:AAA6058
Lacinipolia vicina group|[14253]|RDMAB630-06|UASM58753|658[0n]bp|Canada.Alberta|BOLD:AAA6058

[illegible]
Lacinipolia vicina group|[14251]|RDMAB273-05|UASM41401|658[0n]bp|Canada.Saskatchewan|BOLD:AAA6058
Lacinipolia vicina group|[14252]|RDMAB491-06|UASM78000|658[0n]bp|Canada.Alberta|BOLD:AAA6058
Lacinipolia vicina group|[14253]|RDMAB630-06|UASM58753|658[0n]bp|Canada.Alberta|BOLD:AAA6058
Lacinipolia vicina group|[14254]|RDMAB272-05|UASM41403|658[0n]bp|Canada.Alberta|BOLD:AAA6058
Lacinipolia vicina group|[14255]|LPSK525-08|08BBLEP-02093|658[0n]bp|Canada.Saskatchewan|BOLD:AAA6058
Lacinipolia vicina group|[14256]|RDMAB275-05|UASM41494|658[0n]bp|Canada.Alberta|BOLD:AAA6058
Lacinipolia vicina group|[14257]|RDMAB631-06|UASM57244|658[0n]bp|Canada.Alberta|BOLD:AAA6058
Lacinipolia vicina group|[14258]|RDMAB634-06|UASM57279|658[0n]bp|Canada.Alberta|BOLD:AAA6058
Lacinipolia vicina group|[14259]|RDMAB271-05|UASM41402|658[0n]bp|Canada.Saskatchewan|BOLD:AAA6058
Lacinipolia vicina group|[14260]|LPSK492-08|08BBLEP-02060|658[0n]bp|Canada.Saskatchewan|BOLD:AAA6058
Lacinipolia vicina group|[14261]|LPSK529-08|08BBLEP-02097|658[0n]bp|Canada.Saskatchewan|BOLD:AAA6058
Lacinipolia vicina group|[14262]|RDMAB643-06|UASM58460|658[0n]bp|Canada.Alberta|BOLD:AAA6058
Lacinipolia vicina group|[14263]|LPSK593-08|08BBLEP-02161|658[0n]bp|Canada.Saskatchewan|BOLD:AAA6058
Lacinipolia vicina group|[14264]|LPSK553-08|08BBLEP-02121|658[0n]bp|Canada.Saskatchewan|BOLD:AAA6058
Lacinipolia vicina group|[14265]|RDMAB277-05|UASM41495|587[0n]bp|Canada.Alberta|BOLD:AAA6058
Lacinipolia vicina group|[14266]|LPSK602-08|08BBLEP-02170|658[0n]bp|Canada.Saskatchewan|BOLD:AAA6058
Lacinipolia vicina group|[14267]|RDMAB366-05|UASM77831|658[0n]bp|Canada.Alberta|BOLD:AAA6058
Lacinipolia vicina group|[14268]|LPSK577-08|08BBLEP-02145|658[0n]bp|Canada.Saskatchewan|BOLD:AAA6058
Lacinipolia vicina group|[14269]|LPSK454-08|08BBLEP-02022|658[0n]bp|Canada.Saskatchewan|BOLD:AAA6058
Lacinipolia vicina group|[14270]|LPSK464-08|08BBLEP-02032|658[0n]bp|Canada.Saskatchewan|BOLD:AAA6058
Lacinipolia vicina group|[14271]|RDMAB270-05|UASM41400|658[0n]bp|Canada.Saskatchewan|BOLD:AAA6058
Lacinipolia vicina group|[14272]|RDMAB276-05|UASM41496|658[0n]bp|Canada.Alberta|BOLD:AAA6058
Lacinipolia vicina group|[14273]|LPSK561-08|08BBLEP-02129|658[0n]bp|Canada.Saskatchewan|BOLD:AAA6058
Lacinipolia vicina group|[14274]|LPSK473-08|08BBLEP-02041|658[0n]bp|Canada.Saskatchewan|BOLD:AAA6058
Lacinipolia vicina group|[14275]|RDMAB644-06|UASM24517|658[0n]bp|Canada.Alberta|BOLD:AAA6058
Lacinipolia vicina group|[14276]|LPSK044-08|08BBLEP-00747|658[0n]bp|Canada.Saskatchewan|BOLD:AAA6058
Lacinipolia vicina group|[14277]|LPSK587-08|08BBLEP-02155|658[0n]bp|Canada.Saskatchewan|BOLD:AAA6058
Lacinipolia vicina group|[14278]|LPMN337-08|08BBLEP-01136|658[0n]bp|Canada.Manitoba|BOLD:AAA6058
Lacinipolia vicina group|[14279]|RDMAB642-06|UASM56978|658[0n]bp|Canada.Alberta|BOLD:AAA6058
Lacinipolia vicina group|[14280]|LPSK491-08|08BBLEP-02059|658[0n]bp|Canada.Saskatchewan|BOLD:AAA6058
Lacinipolia vicina group|[14281]|LPSK496-08|08BBLEP-02064|658[0n]bp|Canada.Saskatchewan|BOLD:AAA6058
Lacinipolia vicina group|[14282]|LPSK041-08|08BBLEP-00744|658[0n]bp|Canada.Saskatchewan|BOLD:AAA6058
Lacinipolia vicina group|[14283]|LPSK515-08|08BBLEP-02083|658[0n]bp|Canada.Saskatchewan|BOLD:AAA6058
Lacinipolia vicina group|[14284]|LPSK540-08|08BBLEP-02108|658[0n]bp|Canada.Saskatchewan|BOLD:AAA6058
Lacinipolia vicina group|[14285]|LPSK566-08|08BBLEP-02134|658[0n]bp|Canada.Saskatchewan|BOLD:AAA6058
Lacinipolia vicina group|[14286]|LPSK042-08|08BBLEP-00745|658[0n]bp|Canada.Saskatchewan|BOLD:AAA6058
Lacinipolia vicina group|[14287]|RDMAB633-06|UASM57243|658[0n]bp|Canada.Alberta|BOLD:AAA6058
Lacinipolia vicina group|[14288]|LPSK501-08|08BBLEP-02069|658[0n]bp|Canada.Saskatchewan|BOLD:AAA6058
Lacinipolia vicina group|[14289]|RDMAB637-06|UASM58757|658[0n]bp|Canada.Alberta|BOLD:AAA6058
Lacinipolia vicina group|[14290]|LPSK469-08|08BBLEP-02037|658[0n]bp|Canada.Saskatchewan|BOLD:AAA6058
Lacinipolia vicina group|[14291]|RDMAB645-06|UASM24522|658[0n]bp|Canada.Alberta|BOLD:AAA6058
Lacinipolia vicina group|[14292]|LPSK503-08|08BBLEP-02071|658[0n]bp|Canada.Saskatchewan|BOLD:AAA6058
Lacinipolia vicina group|[14293]|LPSK573-08|08BBLEP-02141|658[0n]bp|Canada.Saskatchewan|BOLD:AAA6058
Lacinipolia vicina group|[14294]|LPSK588-08|08BBLEP-02156|658[0n]bp|Canada.Saskatchewan|BOLD:AAA6058
Lacinipolia vicina group|[14295]|LPSK247-08|08BBLEP-01815|658[0n]bp|Canada.Saskatchewan|BOLD:AAA6058
Lacinipolia vicina group|[14296]|RDNMB236-05|CNCNoctuoidea10002|658[0n]bp|Canada.Alberta|BOLD:AAA6058
Lacinipolia vicina group|[14297]|RDMAB367-05|UASM77832|603[0n]bp|Canada.Alberta|BOLD:AAA6058
Lacinipolia vicina group|[14298]|RDMAB311-05|UASM77799|605[0n]bp|Canada.Alberta|BOLD:AAA6058
Lacinipolia vicina group|[14299]|RDNMB238-05|CNCNoctuoidea10004|602[0n]bp|Canada.Alberta|BOLD:AAA6058
Lacinipolia vicina group|[14300]|RDMAB274-05|UASM41493|658[0n]bp|Canada.Alberta|BOLD:AAA6058
Lacinipolia vicina group|[14301]|RDMAB497-06|UASM58450|658[0n]bp|Canada.Alberta|BOLD:AAA6058
Lacinipolia vicina group|[14302]|LPSK539-08|08BBLEP-02107|658[0n]bp|Canada.Saskatchewan|BOLD:AAA6058
Lacinipolia vicina group|[14303]|LPSK475-08|08BBLEP-02043|658[0n]bp|Canada.Saskatchewan|BOLD:AAA6058
Lacinipolia vicina group|[14304]|RDMAB521-06|UASM58484|658[0n]bp|Canada.Alberta|BOLD:AAA6058
Lacinipolia vicina group|[14305]|RDMAB636-06|UASM34842|658[0n]bp|Canada.Alberta|BOLD:AAA6058
Lacinipolia vicina pensilis complex|[14306]|RDLQB448-05|DH010534|658[0n]bp|Canada.Quebec|BOLD:AAA6057
Lacinipolia vicina pensilis complex|[14307]|RDMAB621-06|UASM58744|658[0n]bp|Canada.Alberta|BOLD:AAD4273
Lacinipolia vicina pensilis complex|[14308]|RDMAB303-05|UASM19841|658[0n]bp|Canada.Alberta|BOLD:AAD4273
Lacinipolia vicina pensilis complex|[14309]|RDMAB683-06|UASM58808|574[0n]bp|Canada.Alberta|BOLD:AAD4273
Trichocerapoda oblita|[14310]|NAMUM188-08|RR-96-0203|658[0n]bp|United States.California|BOLD:AAF4923
Trichocerapoda oblita|[14311]|RDNMC404-05|CNCNoctuoidea12104|595[0n]bp|Canada.Saskatchewan|BOLD:AAF4923
Trichocerapoda oblita|[14312]|NAMUM187-08|RR-96-0202|658[0n]bp|United States.California|BOLD:AAF4923
Lasionycta fergusoni|[14313]|LBCG2486-09|08-JDWBC-2486|621[0n]bp|Canada.British Columbia|BOLD:ACF5159
Lasionycta fergusoni|[14314]|RDNM266-05|CNCNoctuoidea6448|658[0n]bp|Canada.British Columbia|BOLD:ACF5159
Lasionycta fergusoni|[14315]|RDNM264-05|CNCNoctuoidea6446|658[0n]bp|Canada.Alberta|BOLD:ACF5159
Lasionycta fergusoni|[14316]|RDNM265-05|CNCNoctuoidea6447|513[0n]bp|Canada.British Columbia|BOLD:ACF5159
Lasionycta fergusoni|[14317]|LBCG3045-09|08-JDWBC-3045|658[0n]bp|Canada.British Columbia|BOLD:ACF5159
Lasionycta subdita|[14318]|RDLQ664-07|DH008667|611[0n]bp|Canada.Newfoundland and Labrador|BOLD:AAC3208
Lasionycta subdita|[14319]|LCHP377-07|07PROBE-10001|641[0n]bp|Canada.Manitoba|BOLD:AAC3208
Lasionycta subdita|[14320]|LCHP242-07|07PROBE-03810|658[0n]bp|Canada.Manitoba|BOLD:AAC3208
Lasionycta subdita|[14321]|LCHP418-07|07PROBE-10042|658[0n]bp|Canada.Manitoba|BOLD:AAC3208
Lasionycta subdita|[14322]|LCHP796-07|07PROBE-10553|658[0n]bp|Canada.Manitoba|BOLD:AAC3208
Lasionycta subdita|[14323]|LCHP151-07|07PROBE-00179|656[0n]bp|Canada.Manitoba|BOLD:AAC3208
Lasionycta subdita|[14324]|LCHP150-07|07PROBE-00178|656[0n]bp|Canada.Manitoba|BOLD:AAC3208
Lasionycta subdita|[14325]|CHLEP214-09|09PROBE-09509|658[0n]bp|Canada.Manitoba|BOLD:AAC3208
Lasionycta subdita|[14326]|LCHP010-07|07PROBE-00068|658[0n]bp|Canada.Manitoba|BOLD:AAC3208
Lasionycta subdita|[14327]|RDNMB711-05|CNCNoctuoidea10487|658[0n]bp|Canada.Manitoba|BOLD:AAC3208
Lasionycta subdita|[14328]|RDNMB712-05|CNCNoctuoidea10488|658[0n]bp|Canada.Manitoba|BOLD:AAC3208
Lasionycta subdita|[14329]|LCHP380-07|07PROBE-10004|645[0n]bp|Canada.Manitoba|BOLD:AAC3208
Lasionycta taigata|[14330]|RDNMF580-08|NOC14666|658[0n]bp|Canada.Quebec|BOLD:AAB7616
Lasionycta taigata|[14331]|RDLQB245-05|DH010331|658[0n]bp|Canada.Quebec|BOLD:AAB7616
Lasionycta taigata|[14332]|RDLQB232-05|DH010318|658[0n]bp|Canada.Quebec|BOLD:AAB7616
Lasionycta taigata|[14333]|RDNMF582-08|NOC14668|658[0n]bp|Canada.Quebec|BOLD:AAB7616
Lasionycta taigata|[14334]|RDLQB247-05|DH010333|658[0n]bp|Canada.Quebec|BOLD:AAB7616
Lasionycta taigata|[14335]|RDNMB713-05|CNCNoctuoidea10489|658[0n]bp|Canada.Quebec|BOLD:AAB7616
Lasionycta taigata|[14336]|LCHP609-07|07PROBE-10290|658[0n]bp|Canada.Manitoba|BOLD:AAB7616
Lasionycta taigata|[14337]|RDNMF581-08|NOC14667|658[0n]bp|Canada.Alberta|BOLD:AAB7616
Lasionycta taigata|[14338]|LCHP171-07|07PROBE-00573|658[0n]bp|Canada.Manitoba|BOLD:AAB7616
Lasionycta taigata|[14339]|LCHP375-07|07PROBE-03950|654[0n]bp|Canada.Manitoba|BOLD:AAB7616
Lasionycta taigata|[14340]|LCHP363-07|07PROBE-03938|632[0n]bp|Canada.Manitoba|BOLD:AAB7616
Lasionycta taigata|[14341]|LCHP378-07|07PROBE-10002|655[0n]bp|Canada.Manitoba|BOLD:AAB7616
Lasionycta taigata|[14342]|LPMN713-08|08BBLEP-01516|658[0n]bp|Canada.Manitoba|BOLD:AAB7616
Lasionycta taigata|[14343]|LCHP483-07|07PROBE-10151|658[0n]bp|Canada.Manitoba|BOLD:AAB7616
Lasionycta taigata|[14344]|RDLQB246-05|DH010332|658[0n]bp|Canada.Quebec|BOLD:AAB7616
Lasionycta taigata|[14345]|RDLQB233-05|DH010319|658[0n]bp|Canada.Quebec|BOLD:AAB7616
Lasionycta skraelingia|[14346]|RDNMF579-08|NOC14665|658[0n]bp|Canada.Yukon Territory|BOLD:AAB7616
Lasionycta skraelingia|[14347]|RDNME679-08|LEP031954|658[0n]bp|Canada.Yukon Territory|BOLD:AAB7616
Lasionycta taigata|[14348]|RDNMH187-09|CNC LEP 00054404|637[0n]bp|Canada.Newfoundland and Labrador|BOLD ...
Lasionycta taigata|[14349]|RDNMB714-05|CNCNoctuoidea10490|658[0n]bp|Canada.Manitoba|BOLD:AAB7616
Lasionycta luteola|[14350]|RDNMB499-05|CNCNoctuoidea10275|534[0n]bp|Canada.British Columbia|BOLD:AAE5146
Lasionycta luteola|[14351]|RDNM403-05|CNCNoctuoidea6585|658[0n]bp|Canada.British Columbia|BOLD:AAE5146
Lasionycta luteola|[14352]|LPABB810-09|08BBLEP-04130|658[0n]bp|Canada.Alberta|BOLD:AAE5146
[illegible]

Lasionycta luteola|[14350]|RDNMB499-05|CNCNoctuoidea10273|534[0n]bp|Canada.British Columbia|BOLD:AAE5146
Lasionycta luteola|[14351]|RDNM403-05|CNCNoctuoidea6585|658[0n]bp|Canada.British Columbia|BOLD:AAE5146
Lasionycta luteola|[14352]|LPABB810-09|08BBLEP-04130|658[0n]bp|Canada.Alberta|BOLD:AAE5146
Lasionycta luteola|[14353]|RDNM402-05|CNCNoctuoidea6584|581[0n]bp|Canada.British Columbia|BOLD:AAE5146
Lasionycta phaea|[14354]|RDNMB710-05|CNCNoctuoidea10486|594[0n]bp|Canada.Nunavut|BOLD:AAX0117
Lasionycta secedens|[14355]|LOWCD266-06|CGWC-3086|658[0n]bp|Canada.British Columbia|BOLD:AAB0756
Lasionycta secedens|[14356]|LCHQ134-07|07PROBE-10903|658[4n]bp|Canada.Manitoba|BOLD:AAB0756
Lasionycta secedens|[14357]|RDNME516-08|LEP037940|658[1n]bp|Canada.Yukon Territory|BOLD:AAB0756
Lasionycta secedens|[14358]|LOWCC102-05|CGWC-1982|596[0n]bp|Canada.British Columbia|BOLD:AAB0756
Lasionycta secedens|[14359]|LOWCD267-06|CGWC-3087|658[0n]bp|Canada.British Columbia|BOLD:AAB0756
Lasionycta secedens|[14360]|LOWCC099-05|CGWC-1979|658[0n]bp|Canada.British Columbia|BOLD:AAB0756
Lasionycta secedens|[14361]|LOWCC103-05|CGWC-1983|658[0n]bp|Canada.British Columbia|BOLD:AAB0756
Lasionycta secedens|[14362]|LOWCC098-05|CGWC-1978|658[0n]bp|Canada.British Columbia|BOLD:AAB0756
Lasionycta secedens|[14363]|LOWCC100-05|CGWC-1980|658[0n]bp|Canada.British Columbia|BOLD:AAB0756
Lasionycta secedens|[14364]|RDNMB729-05|CNCNoctuoidea10505|658[0n]bp|Canada.Quebec|BOLD:AAB0756
Lasionycta secedens|[14365]|RDNMB730-05|CNCNoctuoidea10506|658[0n]bp|Canada.Quebec|BOLD:AAB0756
Lasionycta secedens|[14366]|LOWCC106-05|CGWC-1986|658[0n]bp|Canada.British Columbia|BOLD:AAB0756
Lasionycta secedens|[14367]|LOWCC107-05|CGWC-1987|658[0n]bp|Canada.British Columbia|BOLD:AAB0756
Lasionycta secedens|[14368]|LOWCC101-05|CGWC-1981|658[0n]bp|Canada.British Columbia|BOLD:AAB0756
Lasionycta secedens|[14369]|LOWCD265-06|CGWC-3085|658[0n]bp|Canada.British Columbia|BOLD:AAB0756
Lasionycta secedens|[14370]|LOWCC104-05|CGWC-1984|658[0n]bp|Canada.British Columbia|BOLD:AAB0756
Lasionycta secedens|[14371]|LOWCC105-05|CGWC-1985|658[0n]bp|Canada.British Columbia|BOLD:AAB0756
Lasionycta secedens|[14372]|RDLQ644-07|DH001484|632[0n]bp|Canada.Quebec|BOLD:AAB0756
Lasionycta secedens|[14373]|RDLQ643-07|DH008642|514[8n]bp|Canada.Quebec|
Lasionycta secedens|[14374]|LCHP307-07|07PROBE-03873|658[0n]bp|Canada.Manitoba|BOLD:AAB0756
Lasionycta secedens|[14375]|LOWCD268-06|CGWC-3088|658[0n]bp|Canada.British Columbia|BOLD:AAB0756
Lasionycta secedens|[14376]|LCHP306-07|07PROBE-03872|656[0n]bp|Canada.Manitoba|BOLD:AAB0756
Lasionycta secedens|[14377]|LOWCD269-06|CGWC-3089|658[0n]bp|Canada.British Columbia|BOLD:AAB0756
Lasionycta secedens|[14378]|LOWCC159-05|CGWC-2039|658[0n]bp|Canada.British Columbia|BOLD:AAB0756
Lasionycta secedens|[14379]|LCH317-04|04HBL003317|658[0n]bp|Canada.Manitoba|BOLD:AAB0756
Lasionycta secedens|[14380]|LCHQ037-07|07PROBE-10798|658[0n]bp|Canada.Manitoba|BOLD:AAB0756
Lasionycta secedens|[14381]|LCHQ033-07|07PROBE-10794|658[0n]bp|Canada.Manitoba|BOLD:AAB0756
Lasionycta secedens|[14382]|LOWCC158-05|CGWC-2038|658[0n]bp|Canada.British Columbia|BOLD:AAB0756
Lasionycta secedens|[14383]|LCHP412-07|07PROBE-10036|658[1n]bp|Canada.Manitoba|BOLD:AAB0756
Lasionycta secedens|[14384]|LPMN760-08|08BBLEP-01563|567[0n]bp|Canada.Manitoba|BOLD:AAB0756
Lasionycta secedens|[14385]|LCHP376-07|07PROBE-03951|622[0n]bp|Canada.Manitoba|BOLD:AAB0756
Lasionycta leucocycla|[14386]|RDNMB753-05|CNCNoctuoidea10529|568[1n]bp|Canada.Ontario|BOLD:ACF3546
Lasionycta anthracina|[14387]|RDLQB075-05|DH010161|658[0n]bp|Canada.Quebec|BOLD:ACF3546
Lasionycta anthracina|[14388]|RDLQB076-05|DH010162|658[0n]bp|Canada.Quebec|BOLD:ACF3546
Lasionycta anthracina|[14389]|RDNM272-05|CNCNoctuoidea6454|658[0n]bp|Canada.Quebec|BOLD:ACF3546
Lasionycta flanda|[14390]|RDNMH188-09|CNC LEP 00054405|637[0n]bp|Canada.Newfoundland and Labrador|BOLD ...
Lasionycta leucocycla|[14391]|RDNMC351-05|CNCNoctuoidea11985|564[0n]bp|Canada.Quebec|BOLD:ACF3546
Lasionycta leucocycla|[14392]|RDNMB683-05|CNCNoctuoidea10459|537[2n]bp|Canada.Manitoba|BOLD:ACF3546
Lasionycta leucocycla|[14393]|RDNMB758-05|CNCNoctuoidea10534|572[0n]bp|Canada.Nunavut|BOLD:ACF3546
Lasionycta leucocycla|[14394]|RDNM444-05|CNCNoctuoidea6626|658[0n]bp|Canada.Northwest Territories|BOLD ...
Lasionycta leucocycla|[14395]|RDNMB699-05|CNCNoctuoidea10475|557[0n]bp|Canada.Yukon Territory|BOLD:ACF...
Lasionycta leucocycla|[14396]|RDNMB700-05|CNCNoctuoidea10476|557[0n]bp|Canada.Yukon Territory|BOLD:ACF...
Lasionycta frigida|[14397]|RDNM445-05|CNCNoctuoidea6627|658[0n]bp|Canada.Alberta|BOLD:ACF3546
Lasionycta frigida|[14398]|RDNM426-05|CNCNoctuoidea6608|658[1n]bp|Canada.Alberta|BOLD:ACF3546
Lasionycta leucocycla|[14399]|RDNM405-05|CNCNoctuoidea6587|572[0n]bp|Canada.British Columbia|BOLD:ACF3546
Lasionycta leucocycla|[14400]|RDNMB493-05|CNCNoctuoidea10259|658[0n]bp|Canada.British Columbia|BOLD:AC...
Lasionycta leucocycla|[14401]|RDNMB681-05|CNCNoctuoidea10457|658[0n]bp|Canada.Yukon Territory|BOLD:ACF...
Lasionycta leucocycla|[14402]|RDNMB494-05|CNCNoctuoidea10260|658[0n]bp|Canada.British Columbia|BOLD:AC...
Lasionycta leucocycla|[14403]|RDNM404-05|CNCNoctuoidea6586|564[0n]bp|Canada.Yukon Territory|BOLD:ACF3546
Lasionycta leucocycla|[14404]|RDNMB692-05|CNCNoctuoidea10468|573[0n]bp|Canada.Nunavut|BOLD:ACF3546
Lasionycta leucocycla|[14405]|RDNMB703-05|CNCNoctuoidea10479|603[0n]bp|Canada.British Columbia|BOLD:AC...
Lasionycta leucocycla|[14406]|RDNMB491-05|CNCNoctuoidea10257|658[0n]bp|Canada.British Columbia|BOLD:AC...
Lasionycta leucocycla|[14407]|RDNMB682-05|CNCNoctuoidea10458|658[0n]bp|Canada.Yukon Territory|BOLD:ACF...
Lasionycta anthracina|[14408]|RDNMB506-05|CNCNoctuoidea10282|568[1n]bp|Canada.Alberta|BOLD:ACF3546
Lasionycta leucocycla|[14409]|RDNMB508-05|CNCNoctuoidea10284|558[0n]bp|Canada.Manitoba|BOLD:ACF3546
Lasionycta leucocycla|[14410]|RDNMB701-05|CNCNoctuoidea10477|577[0n]bp|Canada.Yukon Territory|BOLD:ACF...
Lasionycta leucocycla|[14411]|RDNMB694-05|CNCNoctuoidea10470|570[0n]bp|Canada.Nunavut|BOLD:ACF3546
Lasionycta leucocycla|[14412]|RDNMB695-05|CNCNoctuoidea10471|544[0n]bp|Canada.Nunavut|BOLD:ACF3546
Lasionycta leucocycla|[14413]|RDNMB756-05|CNCNoctuoidea10532|573[0n]bp|Canada.Nunavut|BOLD:ACF3546
Lasionycta leucocycla|[14414]|RDNMB697-05|CNCNoctuoidea10473|544[0n]bp|Canada.Yukon Territory|BOLD:ACF...
Lasionycta leucocycla|[14415]|RDNMB693-05|CNCNoctuoidea10469|576[0n]bp|Canada.Nunavut|BOLD:ACF3546
Lasionycta leucocycla|[14416]|RDNMB698-05|CNCNoctuoidea10474|530[0n]bp|Canada.Yukon Territory|BOLD:ACF...
Lasionycta leucocycla|[14417]|RDNMB705-05|CNCNoctuoidea10481|567[2n]bp|Canada.British Columbia|BOLD:AC...
Lasionycta leucocycla|[14418]|RDNM407-05|CNCNoctuoidea6589|535[0n]bp|Canada.Nunavut|BOLD:ACF3546
Lasionycta leucocycla|[14419]|RDNMB509-05|CNCNoctuoidea10285|596[0n]bp|Canada.Manitoba|BOLD:ACF3546
Lasionycta leucocycla|[14420]|RDNM451-05|CNCNoctuoidea6633|658[0n]bp|Canada.Manitoba|BOLD:ACF3546
Lasionycta leucocycla|[14421]|RDNMB688-05|CNCNoctuoidea10464|614[0n]bp|Canada.Manitoba|BOLD:ACF3546
Lasionycta leucocycla|[14422]|RDNMB691-05|CNCNoctuoidea10467|658[0n]bp|Canada.Manitoba|BOLD:ACF3546
Lasionycta leucocycla|[14423]|RDNM406-05|CNCNoctuoidea6588|593[0n]bp|Canada.British Columbia|BOLD:ACF3546
Lasionycta leucocycla|[14424]|RDNMB704-05|CNCNoctuoidea10480|605[0n]bp|Canada.British Columbia|BOLD:AC...
Lasionycta leucocycla|[14425]|RDNMB757-05|CNCNoctuoidea10533|605[0n]bp|Canada.Nunavut|BOLD:ACF3546
Lasionycta leucocycla|[14426]|LARC047-10|08WOLVES-00522|658[0n]bp|Canada.Nunavut|BOLD:ACF3546
Lasionycta leucocycla|[14427]|RDNMB492-05|CNCNoctuoidea10258|658[0n]bp|Canada.British Columbia|BOLD:AC...
Lasionycta leucocycla|[14428]|RDNMB490-05|CNCNoctuoidea10256|658[0n]bp|Canada.British Columbia|BOLD:AC...
Lasionycta leucocycla|[14429]|RDNMB702-05|CNCNoctuoidea10478|615[2n]bp|Canada.British Columbia|BOLD:AC...
Lasionycta leucocycla|[14430]|RDNMB489-05|CNCNoctuoidea10255|658[0n]bp|Canada.Yukon Territory|BOLD:ACF...
Lasionycta leucocycla|[14431]|MNAG254-08|CNCLEP00043300|658[0n]bp|Canada.Nunavut|BOLD:ACF3546
Lasionycta leucocycla|[14432]|RDNMB690-05|CNCNoctuoidea10466|658[0n]bp|Canada.Manitoba|BOLD:ACF3546
Lasionycta leucocycla|[14433]|RDNMB689-05|CNCNoctuoidea10465|657[0n]bp|Canada.Manitoba|BOLD:ACF3546
Lasionycta leucocycla|[14434]|RDNMB687-05|CNCNoctuoidea10463|655[0n]bp|Canada.Manitoba|BOLD:ACF3546
Lasionycta leucocycla|[14435]|RDNMB696-05|CNCNoctuoidea10472|540[1n]bp|Canada.Nunavut|BOLD:ACF3546
Lasionycta leucocycla|[14436]|RDNMB685-05|CNCNoctuoidea10461|616[1n]bp|Canada.Manitoba|BOLD:ACF3546
Lasionycta leucocycla|[14437]|RDNMB507-05|CNCNoctuoidea10283|576[0n]bp|Canada.Manitoba|BOLD:ACF3546
Lasionycta coracina|[14438]|RDNMB752-05|CNCNoctuoidea10528|546[0n]bp|Canada.Yukon Territory|BOLD:ACF3546
Lasionycta coracina|[14439]|RDNMB510-05|CNCNoctuoidea10286|613[4n]bp|Canada.Yukon Territory|BOLD:ACF3546
Lasionycta leucocycla|[14440]|RDNM450-05|CNCNoctuoidea6632|658[0n]bp|Canada.Manitoba|BOLD:ACF3546
Lasionycta leucocycla|[14441]|LCHP069-07|07PROBE-00505|658[0n]bp|Canada.Manitoba|BOLD:ACF3546
Lasionycta leucocycla|[14442]|RDNMB686-05|CNCNoctuoidea10462|591[1n]bp|Canada.Manitoba|BOLD:ACF3546
Lasionycta staudingeri|[14443]|RDNM419-05|CNCNoctuoidea6601|544[0n]bp|Canada.Nunavut|BOLD:ACF3546
Lasionycta staudingeri|[14444]|RDNMK712-11|CNCLEP 81858|658[0n]bp|Canada.Northwest Territories|BOLD:AC...
Lasionycta staudingeri|[14445]|RDNMB755-05|CNCNoctuoidea10531|658[0n]bp|Canada.Yukon Territory|BOLD:AC...
Lasionycta staudingeri|[14446]|RDNMB754-05|CNCNoctuoidea10530|657[1n]bp|Canada.Yukon Territory|BOLD:AC...
Lasionycta staudingeri|[14447]|RDNM420-05|CNCNoctuoidea6602|546[0n]bp|Canada.Nunavut|BOLD:ACF3546
Lasionycta subfumosa|[14448]|LEFII274-11|MM19924|658[0n]bp|Canada|BOLD:ACF3546
Lasionycta subfumosa|[14449]|LEFII275-11|MM19925|658[0n]bp|Canada|BOLD:ACF3546
Lasionycta perplexella|[14450]|RDNMB728-05|CNCNoctuoidea10504|563[0n]bp|Canada.British Columbia|BOLD:A...
Lasionycta perplexella|[14451]|RDNM392-05|CNCNoctuoidea6574|597[0n]bp|Canada.British Columbia|BOLD:ACF...
Lasionycta perplexella|[14452]|RDNMB725-05|CNCNoctuoidea10501|658[0n]bp|Canada.British Columbia|BOLD:A...

Lasionycta perplexella|[14450]|RDNMB728-05|CNCNoctuoidea10504|563[0n]bp|Canada.British Columbia|BOLD:A...
Lasionycta perplexella|[14451]|RDNM392-05|CNCNoctuoidea6574|597[0n]bp|Canada.British Columbia|BOLD:ACF...
Lasionycta perplexella|[14452]|RDNMB725-05|CNCNoctuoidea10501|658[0n]bp|Canada.British Columbia|BOLD:A...
Lasionycta perplexella|[14453]|RDNMB726-05|CNCNoctuoidea10502|658[0n]bp|Canada.British Columbia|BOLD:A...
Lasionycta perplexella|[14454]|RDNMB520-05|CNCNoctuoidea10296|658[0n]bp|Canada.British Columbia|BOLD:A...
Lasionycta perplexella|[14455]|RDNMB724-05|CNCNoctuoidea10500|658[1n]bp|Canada.British Columbia|BOLD:A...
Lasionycta perplexa|[14456]|RDMAB464-05|UASM58286|585[4n]bp|Canada.Alberta|BOLD:ACF3546
Lasionycta perplexa|[14457]|RDNM396-05|CNCNoctuoidea6578|658[0n]bp|Canada.British Columbia|BOLD:ACF3546
Lasionycta perplexa|[14458]|LPAB228-08|08BBLEP-02550|656[1n]bp|Canada.Alberta|BOLD:ACF3546
Lasionycta perplexa|[14459]|LPMN954-08|08BBLEP-02312|631[0n]bp|Canada.Alberta|BOLD:ACF3546
Lasionycta perplexa|[14460]|LPABB120-08|08BBLEP-03385|635[0n]bp|Canada.Alberta|BOLD:ACF3546
Lasionycta perplexa|[14461]|LPABB391-08|08BBLEP-03656|658[0n]bp|Canada.Alberta|BOLD:ACF3546
Lasionycta perplexa|[14462]|BBLPB339-10|10BBCLP-1338|658[0n]bp|Canada.British Columbia|BOLD:ACF3546
Lasionycta perplexa|[14463]|LPABB354-08|08BBLEP-03619|658[0n]bp|Canada.Alberta|BOLD:ACF3546
Lasionycta perplexa|[14464]|LPABB408-08|08BBLEP-03673|658[0n]bp|Canada.Alberta|BOLD:ACF3546
Lasionycta perplexa|[14465]|LPABB484-08|08BBLEP-03749|658[0n]bp|Canada.Alberta|BOLD:ACF3546
Lasionycta perplexa|[14466]|BBLPB333-10|10BBCLP-1332|658[0n]bp|Canada.British Columbia|BOLD:ACF3546
Lasionycta perplexa|[14467]|RDNMB723-05|CNCNoctuoidea10499|658[0n]bp|Canada.British Columbia|BOLD:ACF3546
Lasionycta perplexa|[14468]|RDMAB250-05|UASM41452|658[0n]bp|Canada.Alberta|BOLD:ACF3546
Lasionycta perplexa|[14469]|LPABB339-08|08BBLEP-03604|658[0n]bp|Canada.Alberta|BOLD:ACF3546
Lasionycta perplexa|[14470]|LPAB064-08|08BBLEP-02386|658[0n]bp|Canada.Alberta|BOLD:ACF3546
Lasionycta perplexa|[14471]|RDMAB467-05|BCSC132|658[0n]bp|Canada.Yukon Territory|BOLD:ACF3546
Lasionycta perplexa|[14472]|BBLPB757-10|10BBCLP-1756|658[0n]bp|Canada.Alberta|BOLD:ACF3546
Lasionycta perplexa|[14473]|RDMAB468-05|BCSC133|658[0n]bp|Canada.Alberta|BOLD:ACF3546
Lasionycta perplexa|[14474]|LPAB021-08|08BBLEP-02343|658[0n]bp|Canada.Alberta|BOLD:ACF3546
Lasionycta perplexa|[14475]|LPABB453-08|08BBLEP-03718|658[0n]bp|Canada.Alberta|BOLD:ACF3546
Lasionycta perplexa|[14476]|RDMAB249-05|UASM41451|658[0n]bp|Canada.Alberta|BOLD:ACF3546
Lasionycta perplexa|[14477]|LPABB296-08|08BBLEP-03561|658[0n]bp|Canada.Alberta|BOLD:ACF3546
Lasionycta perplexa|[14478]|LPABB374-08|08BBLEP-03639|658[0n]bp|Canada.Alberta|BOLD:ACF3546
Lasionycta perplexa|[14479]|LPABB387-08|08BBLEP-03652|658[0n]bp|Canada.Alberta|BOLD:ACF3546
Lasionycta perplexa|[14480]|LPABB441-08|08BBLEP-03706|658[0n]bp|Canada.Alberta|BOLD:ACF3546
Lasionycta perplexa|[14481]|LPABB414-08|08BBLEP-03679|658[0n]bp|Canada.Alberta|BOLD:ACF3546
Lasionycta perplexa|[14482]|LPABB341-08|08BBLEP-03606|658[0n]bp|Canada.Alberta|BOLD:ACF3546
Lasionycta perplexa|[14483]|LPAB008-08|08BBLEP-02330|658[0n]bp|Canada.Alberta|BOLD:ACF3546
Lasionycta perplexa|[14484]|LPABB379-08|08BBLEP-03644|658[0n]bp|Canada.Alberta|BOLD:ACF3546
Lasionycta perplexa|[14485]|LPABB455-08|08BBLEP-03720|658[0n]bp|Canada.Alberta|BOLD:ACF3546
Lasionycta perplexa|[14486]|RDMAB461-05|UASM24134|658[0n]bp|Canada.Alberta|BOLD:ACF3546
Lasionycta perplexa|[14487]|RDNM395-05|CNCNoctuoidea6577|658[0n]bp|Canada.British Columbia|BOLD:ACF3546
Lasionycta perplexa|[14488]|RDMAB462-05|UASM24133|658[0n]bp|Canada.Alberta|BOLD:ACF3546
Lasionycta perplexa|[14489]|RDMAB463-05|UASM24132|658[0n]bp|Canada.Alberta|BOLD:ACF3546
Lasionycta perplexa|[14490]|LPABB407-08|08BBLEP-03672|658[0n]bp|Canada.Alberta|BOLD:ACF3546
Lasionycta perplexa|[14491]|RDMAB465-05|UASM58285|604[0n]bp|Canada.Alberta|BOLD:ACF3546
Lasionycta perplexa|[14492]|RDMAB466-05|UASM58284|604[0n]bp|Canada.Alberta|BOLD:ACF3546
Lasionycta perplexa|[14493]|RDNMB727-05|CNCNoctuoidea10503|571[0n]bp|Canada.Alberta|BOLD:ACF3546
Lasionycta perplexa|[14494]|LPABB338-08|08BBLEP-03603|658[0n]bp|Canada.Alberta|BOLD:ACF3546
Lasionycta perplexa|[14495]|LPABB610-08|08BBLEP-03875|658[0n]bp|Canada.Alberta|BOLD:ACF3546
Lasionycta perplexa|[14496]|LPABB400-08|08BBLEP-03665|658[0n]bp|Canada.Alberta|BOLD:ACF3546
Lasionycta perplexa|[14497]|BBLPB764-10|10BBCLP-1763|658[0n]bp|Canada.British Columbia|BOLD:ACF3546
Lasionycta perplexa|[14498]|LPABB485-08|08BBLEP-03750|658[0n]bp|Canada.Alberta|BOLD:ACF3546
Lasionycta perplexa|[14499]|LALPA993-11|AVBC 1166-11|658[0n]bp|Canada.British Columbia|BOLD:ACF3546
Lasionycta perplexa|[14500]|LPABB492-08|08BBLEP-03757|658[0n]bp|Canada.Alberta|BOLD:ACF3546
Lasionycta perplexa|[14501]|LPABB121-08|08BBLEP-03386|658[0n]bp|Canada.Alberta|BOLD:ACF3546
Lasionycta perplexa|[14502]|LPABB016-08|08BBLEP-03281|658[0n]bp|Canada.Alberta|BOLD:ACF3546
Lasionycta perplexa|[14503]|LPABB268-08|08BBLEP-03533|658[0n]bp|Canada.Alberta|BOLD:ACF3546
Lasionycta perplexa|[14504]|LPABB586-08|08BBLEP-03851|658[0n]bp|Canada.Alberta|BOLD:ACF3546
Lasionycta perplexa|[14505]|LPABB627-08|08BBLEP-03892|658[0n]bp|Canada.Alberta|BOLD:ACF3546
Lasionycta perplexa|[14506]|LPABB340-08|08BBLEP-03605|658[0n]bp|Canada.Alberta|BOLD:ACF3546
Lasionycta poca|[14507]|RDNM188-05|CNCNoctuoidea6738|564[0n]bp|Canada.Alberta|BOLD:ACF3546
Lasionycta poca|[14508]|RDNM187-05|CNCNoctuoidea6737|658[0n]bp|Canada.British Columbia|BOLD:ACF3546
Lasionycta poca|[14509]|RDNMB484-05|CNCNoctuoidea10250|658[0n]bp|Canada.Yukon Territory|BOLD:ACF3546
Lasionycta poca|[14510]|RDNM186-05|CNCNoctuoidea6736|658[0n]bp|Canada.British Columbia|BOLD:ACF3546
Lasionycta poca|[14511]|RDNM408-05|CNCNoctuoidea6590|658[0n]bp|Canada.British Columbia|BOLD:ACF3546
Lasionycta subfuscula|[14512]|RDNMB518-05|CNCNoctuoidea10294|576[0n]bp|Canada.British Columbia|BOLD:AC...
Lasionycta subfuscula|[14513]|RDNMB762-05|CNCNoctuoidea10538|572[0n]bp|Canada.British Columbia|BOLD:AC...
Lasionycta subfuscula|[14514]|RDNMB760-05|CNCNoctuoidea10536|571[0n]bp|Canada.British Columbia|BOLD:AC...
Lasionycta subfuscula|[14515]|RDNMB514-05|CNCNoctuoidea10290|658[0n]bp|Canada.British Columbia|BOLD:AC...
Lasionycta subfuscula|[14516]|RDNMB516-05|CNCNoctuoidea10292|658[0n]bp|Canada.British Columbia|BOLD:AC...
Lasionycta subfuscula|[14517]|RDNM397-05|CNCNoctuoidea6579|658[0n]bp|Canada.British Columbia|BOLD:ACF3546
Lasionycta subfuscula|[14518]|RDNMB771-05|CNCNoctuoidea10547|658[0n]bp|Canada.British Columbia|BOLD:AC...
Lasionycta subfuscula|[14519]|RDNMB519-05|CNCNoctuoidea10295|539[1n]bp|Canada.British Columbia|BOLD:AC...
Lasionycta subfuscula|[14520]|RDNMB761-05|CNCNoctuoidea10537|579[0n]bp|Canada.British Columbia|BOLD:AC...
Lasionycta subfuscula|[14521]|RDNMB517-05|CNCNoctuoidea10293|658[0n]bp|Canada.British Columbia|BOLD:AC...
Lasionycta subfuscula|[14522]|BBLPB758-10|10BBCLP-1757|634[0n]bp|Canada.Alberta|BOLD:ACF3546
Lasionycta subfuscula|[14523]|RDNMB759-05|CNCNoctuoidea10535|617[0n]bp|Canada.British Columbia|BOLD:AC...
Lasionycta subfuscula|[14524]|LPABB116-08|08BBLEP-03381|658[0n]bp|Canada.Alberta|BOLD:ACF3546
Lasionycta subfuscula|[14525]|RDNMB773-05|CNCNoctuoidea10549|658[0n]bp|Canada.British Columbia|BOLD:AC...
Lasionycta subfuscula|[14526]|RDNMG097-08|NOC14850|658[0n]bp|Canada.British Columbia|BOLD:ACF3546
Lasionycta subfuscula|[14527]|RDNMB513-05|CNCNoctuoidea10289|658[0n]bp|Canada.British Columbia|BOLD:AC...
Lasionycta subfuscula|[14528]|RDNM398-05|CNCNoctuoidea6580|658[0n]bp|Canada.British Columbia|BOLD:ACF3546
Lasionycta subfuscula|[14529]|RDNMG101-08|NOC14854|658[0n]bp|Canada.British Columbia|BOLD:ACF3546
Lasionycta impingens|[14530]|RDNM415-05|CNCNoctuoidea6597|592[0n]bp|Canada.British Columbia|BOLD:AAA8091
Lasionycta impingens|[14531]|RDNMB717-05|CNCNoctuoidea10493|658[0n]bp|Canada.British Columbia|BOLD:AAA...
Lasionycta impingens|[14532]|LOWCD114-06|CGWC-2934|658[0n]bp|Canada.British Columbia|BOLD:AAA8091
Lasionycta impingens|[14533]|BBLPB466-10|10BBCLP-1465|658[0n]bp|Canada.Alberta|BOLD:AAA8091
Lasionycta impingens|[14534]|RDNMB718-05|CNCNoctuoidea10494|563[2n]bp|Canada.Alberta|BOLD:AAA8091
Lasionycta promulsa|[14535]|RDNMB734-05|CNCNoctuoidea10510|598[0n]bp|Canada.Alberta|BOLD:AAA8091
Lasionycta promulsa|[14536]|RDMAB673-06|UASM24169|621[1n]bp|Canada.Alberta|BOLD:AAA8091
Lasionycta promulsa|[14537]|RDNM434-05|CNCNoctuoidea6616|658[0n]bp|Canada.British Columbia|BOLD:AAA8091
Lasionycta promulsa|[14538]|RDNM433-05|CNCNoctuoidea6615|658[0n]bp|Canada.British Columbia|BOLD:AAA8091
Lasionycta promulsa|[14539]|BBLPB380-10|10BBCLP-1379|658[0n]bp|Canada.British Columbia|BOLD:AAA8091
Lasionycta promulsa|[14540]|RDNMB743-05|CNCNoctuoidea10519|658[0n]bp|Canada.Alberta|BOLD:AAA8091
Lasionycta promulsa|[14541]|RDNMB735-05|CNCNoctuoidea10511|658[0n]bp|Canada.Alberta|BOLD:AAA8091
Lasionycta promulsa|[14542]|RDNMB736-05|CNCNoctuoidea10512|658[0n]bp|Canada.Alberta|BOLD:AAA8091
Lasionycta promulsa|[14543]|RDNM430-05|CNCNoctuoidea6612|658[0n]bp|Canada.British Columbia|BOLD:AAA8091
Lasionycta promulsa|[14544]|RDNMB741-05|CNCNoctuoidea10517|658[0n]bp|Canada.Alberta|BOLD:AAA8091
Lasionycta promulsa|[14545]|RDNM431-05|CNCNoctuoidea6613|658[0n]bp|Canada.British Columbia|BOLD:AAA8091
Lasionycta promulsa|[14546]|RDNMB739-05|CNCNoctuoidea10515|658[0n]bp|Canada.Alberta|BOLD:AAA8091
Lasionycta promulsa|[14547]|BBLPB464-10|10BBCLP-1463|658[0n]bp|Canada.British Columbia|BOLD:AAA8091
Lasionycta promulsa|[14548]|RDNM429-05|CNCNoctuoidea6611|658[0n]bp|Canada.British Columbia|BOLD:AAA8091
Lasionycta promulsa|[14549]|RDNMB732-05|CNCNoctuoidea10508|570[0n]bp|Canada.British Columbia|BOLD:AAA8091
Lasionycta promulsa|[14550]|RDMAB675-06|UASM2182|658[0n]bp|Canada.Alberta|BOLD:AAA8091
Lasionycta promulsa|[14551]|RDNMB744-05|CNCNoctuoidea10520|615[0n]bp|Canada.Alberta|BOLD:AAA8091
Lasionycta promulsa|[14552]|RDNM432-05|CNCNoctuoidea6614|581[0n]bp|Canada.British Columbia|BOLD:AAA8091

[illegible]
Lasionycta promulsa|[14550]|RDMAB675-06|UASM2182|658[0n]bp|Canada.Alberta|BOLD:AAA8091
Lasionycta promulsa|[14551]|RDNMB744-05|CNCNoctuoidea10520|615[0n]bp|Canada.Alberta|BOLD:AAA8091
Lasionycta promulsa|[14552]|RDNM432-05|CNCNoctuoidea6614|581[0n]bp|Canada.British Columbia|BOLD:AAA8091
Lasionycta promulsa|[14553]|RDNM454-05|CNCNoctuoidea6636|524[0n]bp|Canada.Alberta|BOLD:AAA8091
Lasionycta promulsa|[14554]|RDNM435-05|CNCNoctuoidea6617|658[0n]bp|Canada.British Columbia|BOLD:AAA8091
Lasionycta promulsa|[14555]|RDNMB733-05|CNCNoctuoidea10509|658[0n]bp|Canada.British Columbia|BOLD:AAA8091
Lasionycta promulsa|[14556]|RDNMB740-05|CNCNoctuoidea10516|658[0n]bp|Canada.Alberta|BOLD:AAA8091
Lasionycta promulsa|[14557]|RDNMB738-05|CNCNoctuoidea10514|658[0n]bp|Canada.Alberta|BOLD:AAA8091
Lasionycta promulsa|[14558]|RDNMB737-05|CNCNoctuoidea10513|658[0n]bp|Canada.Alberta|BOLD:AAA8091
Lasionycta promulsa|[14559]|RDNMB742-05|CNCNoctuoidea10518|658[0n]bp|Canada.Alberta|BOLD:AAA8091
Lasionycta promulsa|[14560]|RDNM427-05|CNCNoctuoidea6609|658[0n]bp|Canada.Alberta|BOLD:AAA8091
Lasionycta promulsa|[14561]|RDMAB672-06|UASM2181|562[0n]bp|Canada.Alberta|BOLD:AAA8091
Lasionycta promulsa|[14562]|RDMAB674-06|UASM2180|561[0n]bp|Canada.Alberta|BOLD:AAA8091
Lasionycta pulverea|[14563]|RDNMB749-05|CNCNoctuoidea10525|658[0n]bp|Canada.Alberta|BOLD:AAA8091
Lasionycta pulverea|[14564]|RDNMB748-05|CNCNoctuoidea10524|560[0n]bp|Canada.Alberta|BOLD:AAA8091
Lasionycta pulverea|[14565]|RDNM452-05|CNCNoctuoidea6634|601[0n]bp|Canada.Alberta|BOLD:AAA8091
Lasionycta pulverea|[14566]|RDNM453-05|CNCNoctuoidea6635|542[0n]bp|Canada.Alberta|BOLD:AAA8091
Lasionycta pulverea|[14567]|RDMAB460-05|UASM34425|596[0n]bp|Canada.Alberta|BOLD:AAA8091
Lasionycta pulverea|[14568]|RDNMB750-05|CNCNoctuoidea10526|554[0n]bp|Canada.Alberta|BOLD:AAA8091
Lasionycta silacea|[14569]|RDNM176-05|CNCNoctuoidea6726|658[0n]bp|Canada.British Columbia|BOLD:AAA8091
Lasionycta silacea|[14570]|RDNM436-05|CNCNoctuoidea6618|658[0n]bp|Canada.British Columbia|BOLD:AAA8091
Lasionycta silacea|[14571]|RDNM178-05|CNCNoctuoidea6728|658[0n]bp|Canada.British Columbia|BOLD:AAA8091
Lasionycta silacea|[14572]|RDMAB290-05|UASM77790|596[0n]bp|Canada.Alberta|BOLD:AAA8091
Lasionycta caesia|[14573]|RDNM390-05|CNCNoctuoidea6572|658[0n]bp|Canada.British Columbia|BOLD:AAA8091
Lasionycta caesia|[14574]|RDNM389-05|CNCNoctuoidea6571|579[0n]bp|Canada.British Columbia|BOLD:AAA8091
Lasionycta caesia|[14575]|RDNM239-05|CNCNoctuoidea6421|562[0n]bp|Canada.British Columbia|BOLD:AAA8091
Lasionycta quadrilunata yukona|[14576]|RDNMI115-10|CNCLEP 69715|658[0n]bp|Canada.Yukon Territory|BOLD:...
Lasionycta quadrilunata|[14577]|RDNMK520-11|CNCLEP 81949|658[0n]bp|Canada.Yukon Territory|BOLD:AAA8091
Lasionycta quadrilunata|[14578]|RDNMK521-11|CNCLEP 81950|658[0n]bp|Canada.Yukon Territory|BOLD:AAA8091
Lasionycta quadrilunata|[14579]|RDNMK519-11|CNCLEP 81948|658[0n]bp|Canada.Yukon Territory|BOLD:AAA8091
Lasionycta quadrilunata|[14580]|RDNMK517-11|CNCLEP 81946|658[0n]bp|Canada.Yukon Territory|BOLD:AAA8091
Lasionycta quadrilunata|[14581]|RDNMK518-11|CNCLEP 81947|658[0n]bp|Canada.Yukon Territory|BOLD:AAA8091
Lasionycta quadrilunata yukona|[14582]|RDNM461-05|CNCNoctuoidea6643|658[0n]bp|Canada.Alberta|BOLD:AAA8091
Lasionycta brunnea|[14583]|RDNM377-05|CNCNoctuoidea6559|601[0n]bp|Canada.British Columbia|BOLD:AAA8091
Lasionycta brunnea|[14584]|RDNM180-05|CNCNoctuoidea6730|658[0n]bp|Canada.British Columbia|BOLD:AAA8091
Lasionycta brunnea|[14585]|RDNM369-05|CNCNoctuoidea6551|658[0n]bp|Canada.British Columbia|BOLD:AAA8091
Lasionycta brunnea|[14586]|LPABB349-08|08BBLEP-03614|658[0n]bp|Canada.Alberta|BOLD:AAA8091
Lasionycta brunnea|[14587]|RDNM374-05|CNCNoctuoidea6556|658[0n]bp|Canada.British Columbia|BOLD:AAA8091
Lasionycta brunnea|[14588]|RDNM181-05|CNCNoctuoidea6731|567[0n]bp|Canada.British Columbia|BOLD:AAA8091
Lasionycta brunnea|[14589]|RDNM179-05|CNCNoctuoidea6729|534[0n]bp|Canada.British Columbia|BOLD:AAA8091
Lasionycta brunnea|[14590]|RDNM380-05|CNCNoctuoidea6562|568[0n]bp|Canada.British Columbia|BOLD:AAA8091
Lasionycta brunnea|[14591]|RDNMB542-05|CNCNoctuoidea10318|658[0n]bp|Canada.British Columbia|BOLD:AAA8091
Lasionycta carolynae|[14592]|RDNMH920-09|CNCLEP00067919|658[0n]bp|Canada|BOLD:AAA8091
Lasionycta carolynae|[14593]|RDNMH922-09|CNCLEP00067921|658[0n]bp|Canada|BOLD:AAA8091
Lasionycta carolynae|[14594]|RDNMH921-09|CNCLEP00067920|658[0n]bp|Canada|BOLD:AAA8091
Lasionycta lagganata|[14595]|RDNMG843-08|CNC LEP00052967|595[0n]bp|Canada.British Columbia|BOLD:AAA8091
Lasionycta lagganata|[14596]|RDNMG844-08|CNC LEP00052968|643[0n]bp|Canada.British Columbia|BOLD:AAA8091
Lasionycta lagganata|[14597]|RDNMG842-08|CNC LEP00052966|643[1n]bp|Canada.British Columbia|BOLD:AAA8091
Lasionycta lagganata|[14598]|RDNM442-05|CNCNoctuoidea6624|605[0n]bp|Canada.British Columbia|BOLD:AAA8091
Lasionycta uniformis|[14599]|RDNM388-05|CNCNoctuoidea6570|658[0n]bp|Canada.British Columbia|BOLD:AAA8091
Lasionycta uniformis|[14600]|RDNM459-05|CNCNoctuoidea6641|658[0n]bp|Canada.British Columbia|BOLD:AAA8091
Lasionycta uniformis|[14601]|RDNM462-05|CNCNoctuoidea6644|658[0n]bp|Canada.British Columbia|BOLD:AAA8091
Lasionycta uniformis|[14602]|RDNMB502-05|CNCNoctuoidea10278|658[0n]bp|Canada.British Columbia|BOLD:AAA...
Lasionycta uniformis|[14603]|RDNM387-05|CNCNoctuoidea6569|658[0n]bp|Canada.British Columbia|BOLD:AAA8091
Lasionycta uniformis|[14604]|RDNMB709-05|CNCNoctuoidea10485|658[0n]bp|Canada.British Columbia|BOLD:AAA...
Lasionycta uniformis|[14605]|RDNM458-05|CNCNoctuoidea6640|658[0n]bp|Canada.British Columbia|BOLD:AAA8091
Lasionycta uniformis|[14606]|RDNMB511-05|CNCNoctuoidea10287|658[0n]bp|Canada.British Columbia|BOLD:AAA...
Lasionycta uniformis|[14607]|RDNM455-05|CNCNoctuoidea6637|564[0n]bp|Canada.British Columbia|BOLD:AAA8091
Lasionycta uniformis|[14608]|RDNM456-05|CNCNoctuoidea6638|558[0n]bp|Canada.British Columbia|BOLD:AAA8091
Lasionycta uniformis|[14609]|RDNMB504-05|CNCNoctuoidea10280|559[0n]bp|Canada.British Columbia|BOLD:AAA...
Lasionycta uniformis|[14610]|RDNM449-05|CNCNoctuoidea6631|658[0n]bp|Canada.British Columbia|BOLD:AAA8091
Lasionycta uniformis|[14611]|RDNMB500-05|CNCNoctuoidea10276|658[0n]bp|Canada.British Columbia|BOLD:AAA...
Lasionycta gelida|[14612]|RDNMG095-08|NOC14848|609[0n]bp|Canada.British Columbia|BOLD:AAA8091
Lasionycta gelida|[14613]|RDNMG096-08|NOC14849|658[0n]bp|Canada.British Columbia|BOLD:AAA8091
Lasionycta phoca|[14614]|RDNMB770-05|CNCNoctuoidea10546|534[0n]bp|Canada.Quebec|BOLD:AAA8091
Lasionycta phoca|[14615]|LCHP235-07|07PROBE-03805|656[0n]bp|Canada.Manitoba|BOLD:AAA8091
Lasionycta phoca|[14616]|RDLQ663-07|DH008666|648[0n]bp|Canada.Newfoundland and Labrador|BOLD:AAA8091
Lasionycta phoca|[14617]|LCHP385-07|07PROBE-10009|632[0n]bp|Canada.Manitoba|BOLD:AAA8091
Lasionycta phoca|[14618]|RDNM185-05|CNCNoctuoidea6735|658[0n]bp|Canada.Manitoba|BOLD:AAA8091
Lasionycta phoca|[14619]|LCHP308-07|07PROBE-03874|658[0n]bp|Canada.Manitoba|BOLD:AAA8091
Lasionycta phoca|[14620]|LCHP218-07|07PROBE-03788|636[0n]bp|Canada.Manitoba|BOLD:AAA8091
Lasionycta phoca|[14621]|LCHP823-07|07PROBE-10580|631[0n]bp|Canada.Manitoba|BOLD:AAA8091
Lasionycta phoca|[14622]|LCHP219-07|07PROBE-03789|658[0n]bp|Canada.Manitoba|BOLD:AAA8091
Lasionycta phoca|[14623]|RDNM184-05|CNCNoctuoidea6734|658[0n]bp|Canada.Manitoba|BOLD:AAA8091
Lasionycta phoca|[14624]|LCHP393-07|07PROBE-10017|658[0n]bp|Canada.Manitoba|BOLD:AAA8091
Lasionycta phoca|[14625]|RDNM271-05|CNCNoctuoidea6453|658[0n]bp|Canada.Manitoba|BOLD:AAA8091
Lasionycta uniformis|[14626]|RDNM386-05|CNCNoctuoidea6568|658[0n]bp|Canada.British Columbia|BOLD:AAA8091
Lasionycta uniformis|[14627]|RDNMB498-05|CNCNoctuoidea10274|658[0n]bp|Canada.British Columbia|BOLD:AAA...
Lasionycta uniformis|[14628]|RDNMB505-05|CNCNoctuoidea10281|658[0n]bp|Canada.British Columbia|BOLD:AAA...
Lasionycta uniformis|[14629]|RDNMB587-05|CNCNoctuoidea10363|658[0n]bp|Canada.British Columbia|BOLD:AAA...
Lasionycta uniformis|[14630]|RDNM370-05|CNCNoctuoidea6552|567[0n]bp|Canada.British Columbia|BOLD:AAA8091
Lasionycta uniformis|[14631]|RDNMB539-05|CNCNoctuoidea10315|581[1n]bp|Canada.Alberta|BOLD:AAA8091
Lasionycta uniformis|[14632]|RDNM375-05|CNCNoctuoidea6557|590[0n]bp|Canada.British Columbia|BOLD:AAA8091
Lasionycta uniformis|[14633]|RDNM379-05|CNCNoctuoidea6561|567[0n]bp|Canada.British Columbia|BOLD:AAA8091
Lasionycta uniformis|[14634]|RDNMB540-05|CNCNoctuoidea10316|559[0n]bp|Canada.Alberta|BOLD:AAA8091
Lasionycta uniformis|[14635]|RDNMB497-05|CNCNoctuoidea10273|613[0n]bp|Canada.British Columbia|BOLD:AAA...
Lasionycta uniformis|[14636]|RDNMB503-05|CNCNoctuoidea10279|614[0n]bp|Canada.British Columbia|BOLD:AAA...
Lasionycta uniformis|[14637]|RDNM378-05|CNCNoctuoidea6560|567[0n]bp|Canada.British Columbia|BOLD:AAA8091
Lasionycta uniformis|[14638]|RDNM372-05|CNCNoctuoidea6554|563[0n]bp|Canada.British Columbia|BOLD:AAA8091
Lasionycta uniformis|[14639]|RDNM371-05|CNCNoctuoidea6553|612[0n]bp|Canada.British Columbia|BOLD:AAA8091
Lasionycta uniformis|[14640]|RDNM376-05|CNCNoctuoidea6558|658[0n]bp|Canada.British Columbia|BOLD:AAA8091
Lasionycta uniformis|[14641]|RDNM373-05|CNCNoctuoidea6555|658[0n]bp|Canada.British Columbia|BOLD:AAA8091
Lasionycta uniformis|[14642]|RDNMB541-05|CNCNoctuoidea10317|658[0n]bp|Canada.Alberta|BOLD:AAA8091
Lasionycta uniformis|[14643]|RDNM448-05|CNCNoctuoidea6630|571[0n]bp|Canada.British Columbia|BOLD:AAA8091
Lasionycta uniformis|[14644]|RDNM447-05|CNCNoctuoidea6629|658[0n]bp|Canada.British Columbia|BOLD:AAA8091
Lasionycta uniformis|[14645]|RDNM446-05|CNCNoctuoidea6628|658[0n]bp|Canada.British Columbia|BOLD:AAA8091
Lasionycta haida|[14646]|RDNM258-05|CNCNoctuoidea6440|658[0n]bp|Canada.British Columbia|BOLD:ACE5758
Lasionycta haida|[14647]|RDNM256-05|CNCNoctuoidea6438|658[0n]bp|Canada.British Columbia|BOLD:ACE5758
Lasionycta haida|[14648]|RDNM257-05|CNCNoctuoidea6439|658[0n]bp|Canada.British Columbia|BOLD:ACE5758
Lasionycta haida|[14649]|RDNM255-05|CNCNoctuoidea6437|570[0n]bp|Canada.British Columbia|BOLD:ACE5758
Lasionycta haida|[14650]|RDNM259-05|CNCNoctuoidea6441|540[0n]bp|Canada.British Columbia|BOLD:ACE5758
Lasionycta haida|[14651]|RDNM260-05|CNCNoctuoidea6442|565[0n]bp|Canada.British Columbia|BOLD:ACE5758
[illegible]

Lasionycta haida|[14649]|RDNM255-05|CNCNoctuoidea6437|570[0n]bp|Canada.British Columbia|BOLD:ACE5758
Lasionycta haida|[14650]|RDNM259-05|CNCNoctuoidea6441|540[0n]bp|Canada.British Columbia|BOLD:ACE5758
Lasionycta haida|[14651]|RDNM260-05|CNCNoctuoidea6442|565[0n]bp|Canada.British Columbia|BOLD:ACE5758
Lasionycta mutilata|[14652]|RDNM261-05|CNCNoctuoidea6443|658[0n]bp|Canada.British Columbia|BOLD:AAA5423
Lasionycta mutilata|[14653]|RDNM263-05|CNCNoctuoidea6445|658[0n]bp|Canada.British Columbia|BOLD:AAA5423
Lasionycta mutilata|[14654]|RDNM262-05|CNCNoctuoidea6444|658[0n]bp|Canada.British Columbia|BOLD:AAA5423
Lasionycta mutilata|[14655]|LPAB075-08|08BBLEP-02397|658[0n]bp|Canada.Alberta|BOLD:AAA5423
Lasionycta mutilata|[14656]|RDMAB459-05|UASM24708|658[0n]bp|Canada.Alberta|BOLD:AAA5423
Lasionycta mutilata|[14657]|LBCH2189-10|10-JDWBC-2189|658[0n]bp|Canada.British Columbia|BOLD:AAA5423
Lasionycta mutilata|[14658]|LBCH2259-10|10-JDWBC-2259|658[0n]bp|Canada.British Columbia|BOLD:AAA5423
Lasionycta mutilata|[14659]|LBCH1538-10|10-JDWBC-1538|658[0n]bp|Canada.British Columbia|BOLD:AAA5423
Lasionycta mutilata|[14660]|LBCG1889-09|08-JDWBC-1889|658[0n]bp|Canada.British Columbia|BOLD:AAA5423
Lasionycta mutilata|[14661]|LBCH1678-10|10-JDWBC-1678|658[0n]bp|Canada.British Columbia|BOLD:AAA5423
Lasionycta mutilata|[14662]|LBCG2481-09|08-JDWBC-2481|658[0n]bp|Canada.British Columbia|BOLD:AAA5423
Lasionycta mutilata|[14663]|LBCH2263-10|10-JDWBC-2263|658[0n]bp|Canada.British Columbia|BOLD:AAA5423
Lasionycta mutilata|[14664]|LBCH1111-10|10-JDWBC-1111|658[0n]bp|Canada.British Columbia|BOLD:AAA5423
Lasionycta mutilata|[14665]|LBCH2120-10|10-JDWBC-2120|658[0n]bp|Canada.British Columbia|BOLD:AAA5423
Lasionycta mutilata|[14666]|LBCG3042-09|08-JDWBC-3042|658[0n]bp|Canada.British Columbia|BOLD:AAA5423
Lasionycta mutilata|[14667]|LBCG2475-09|08-JDWBC-2475|631[1n]bp|Canada.British Columbia|BOLD:AAA5423
Lasionycta mutilata|[14668]|LBCH2193-10|10-JDWBC-2193|647[0n]bp|Canada.British Columbia|BOLD:AAA5423
Lasionycta mutilata|[14669]|LBCH1868-10|10-JDWBC-1868|642[0n]bp|Canada.British Columbia|BOLD:AAA5423
Lasionycta mutilata|[14670]|LBCG3052-09|08-JDWBC-3052|631[0n]bp|Canada.British Columbia|BOLD:AAA5423
Lasionycta mutilata|[14671]|LBCH1852-10|10-JDWBC-1852|641[0n]bp|Canada.British Columbia|BOLD:AAA5423
Lasionycta mutilata|[14672]|LBCH2190-10|10-JDWBC-2190|658[0n]bp|Canada.British Columbia|BOLD:AAA5423
Lasionycta mutilata|[14673]|LBCH1577-10|10-JDWBC-1577|658[0n]bp|Canada.British Columbia|BOLD:AAA5423
Lasionycta mutilata|[14674]|LBCH2192-10|10-JDWBC-2192|658[0n]bp|Canada.British Columbia|BOLD:AAA5423
Lasionycta mutilata|[14675]|LBCG2768-09|08-JDWBC-2768|658[0n]bp|Canada.British Columbia|BOLD:AAA5423
Lasionycta mutilata|[14676]|LBCH1506-10|10-JDWBC-1506|658[0n]bp|Canada.British Columbia|BOLD:AAA5423
Lasionycta mutilata|[14677]|LBCH2188-10|10-JDWBC-2188|658[0n]bp|Canada.British Columbia|BOLD:AAA5423
Lasionycta mutilata|[14678]|LBCH2260-10|10-JDWBC-2260|658[0n]bp|Canada.British Columbia|BOLD:AAA5423
Lasionycta mutilata|[14679]|LBCH1773-10|10-JDWBC-1773|658[0n]bp|Canada.British Columbia|BOLD:AAA5423
Lasionycta mutilata|[14680]|LBCG1890-09|08-JDWBC-1890|658[0n]bp|Canada.British Columbia|BOLD:AAA5423
Lasionycta mutilata|[14681]|LBCG2477-09|08-JDWBC-2477|658[0n]bp|Canada.British Columbia|BOLD:AAA5423
Lasionycta mutilata|[14682]|LBCH1114-10|10-JDWBC-1114|658[0n]bp|Canada.British Columbia|BOLD:AAA5423
Lasionycta mutilata|[14683]|LBCG2830-09|08-JDWBC-2830|658[0n]bp|Canada.British Columbia|BOLD:AAA5423
Lasionycta mutilata|[14684]|LBCH1716-10|10-JDWBC-1716|658[0n]bp|Canada.British Columbia|BOLD:AAA5423
Lasionycta mutilata|[14685]|LBCG3038-09|08-JDWBC-3038|658[0n]bp|Canada.British Columbia|BOLD:AAA5423
Lasionycta mutilata|[14686]|LBCH1760-10|10-JDWBC-1760|658[0n]bp|Canada.British Columbia|BOLD:AAA5423
Lasionycta mutilata|[14687]|LBCG3039-09|08-JDWBC-3039|658[0n]bp|Canada.British Columbia|BOLD:AAA5423
Lasionycta mutilata|[14688]|LBCH2191-10|10-JDWBC-2191|658[0n]bp|Canada.British Columbia|BOLD:AAA5423
Lasionycta mutilata|[14689]|LBCG2769-09|08-JDWBC-2769|658[0n]bp|Canada.British Columbia|BOLD:AAA5423
Lasionycta mutilata|[14690]|LBCH2257-10|10-JDWBC-2257|658[0n]bp|Canada.British Columbia|BOLD:AAA5423
Lasionycta mutilata|[14691]|LBCG2770-09|08-JDWBC-2770|658[0n]bp|Canada.British Columbia|BOLD:AAA5423
Lasionycta mutilata|[14692]|LBCH2261-10|10-JDWBC-2261|658[0n]bp|Canada.British Columbia|BOLD:AAA5423
Lasionycta mutilata|[14693]|LBCH2126-10|10-JDWBC-2126|658[0n]bp|Canada.British Columbia|BOLD:AAA5423
Lasionycta mutilata|[14694]|LBCG1888-09|08-JDWBC-1888|658[0n]bp|Canada.British Columbia|BOLD:AAA5423
Lasionycta mutilata|[14695]|LBCG2484-09|08-JDWBC-2484|658[0n]bp|Canada.British Columbia|BOLD:AAA5423
Lasionycta mutilata|[14696]|LBCG1895-09|08-JDWBC-1895|658[0n]bp|Canada.British Columbia|BOLD:AAA5423
Lasionycta mutilata|[14697]|LBCH2123-10|10-JDWBC-2123|658[0n]bp|Canada.British Columbia|BOLD:AAA5423
Lasionycta mutilata|[14698]|LBCH2122-10|10-JDWBC-2122|658[0n]bp|Canada.British Columbia|BOLD:AAA5423
Lasionycta mutilata|[14699]|LBCG2832-09|08-JDWBC-2832|658[0n]bp|Canada.British Columbia|BOLD:AAA5423
Lasionycta mutilata|[14700]|LBCG2829-09|08-JDWBC-2829|658[0n]bp|Canada.British Columbia|BOLD:AAA5423
Lasionycta mutilata|[14701]|LBCG1896-09|08-JDWBC-1896|658[0n]bp|Canada.British Columbia|BOLD:AAA5423
Lasionycta mutilata|[14702]|LBCG3041-09|08-JDWBC-3041|658[0n]bp|Canada.British Columbia|BOLD:AAA5423
Lasionycta mutilata|[14703]|LBCG2476-09|08-JDWBC-2476|658[0n]bp|Canada.British Columbia|BOLD:AAA5423
Lasionycta mutilata|[14704]|LBCH2089-10|10-JDWBC-2089|658[0n]bp|Canada.British Columbia|BOLD:AAA5423
Lasionycta mutilata|[14705]|LBCG3037-09|08-JDWBC-3037|658[0n]bp|Canada.British Columbia|BOLD:AAA5423
Lasionycta mutilata|[14706]|LBCG1898-09|08-JDWBC-1898|658[0n]bp|Canada.British Columbia|BOLD:AAA5423
Lasionycta mutilata|[14707]|LBCH1112-10|10-JDWBC-1112|658[0n]bp|Canada.British Columbia|BOLD:AAA5423
Lasionycta mutilata|[14708]|LBCH2127-10|10-JDWBC-2127|658[0n]bp|Canada.British Columbia|BOLD:AAA5423
Lasionycta mutilata|[14709]|LBCH2124-10|10-JDWBC-2124|658[0n]bp|Canada.British Columbia|BOLD:AAA5423
Lasionycta mutilata|[14710]|LBCG1893-09|08-JDWBC-1893|658[0n]bp|Canada.British Columbia|BOLD:AAA5423
Lasionycta mutilata|[14711]|LBCH2121-10|10-JDWBC-2121|658[0n]bp|Canada.British Columbia|BOLD:AAA5423
Lasionycta mutilata|[14712]|LBCH1109-10|10-JDWBC-1109|658[0n]bp|Canada.British Columbia|BOLD:AAA5423
Lasionycta mutilata|[14713]|LBCH1828-10|10-JDWBC-1828|658[0n]bp|Canada.British Columbia|BOLD:AAA5423
Lasionycta mutilata|[14714]|LBCH2028-10|10-JDWBC-2028|658[0n]bp|Canada.British Columbia|BOLD:AAA5423
Lasionycta mutilata|[14715]|LBCG3049-09|08-JDWBC-3049|658[0n]bp|Canada.British Columbia|BOLD:AAA5423
Lasionycta mutilata|[14716]|LBCG3050-09|08-JDWBC-3050|658[0n]bp|Canada.British Columbia|BOLD:AAA5423
Lasionycta mutilata|[14717]|LBCH2195-10|10-JDWBC-2195|658[0n]bp|Canada.British Columbia|BOLD:AAA5423
Lasionycta mutilata|[14718]|LBCG3044-09|08-JDWBC-3044|658[0n]bp|Canada.British Columbia|BOLD:AAA5423
Lasionycta mutilata|[14719]|LBCH1115-10|10-JDWBC-1115|658[0n]bp|Canada.British Columbia|BOLD:AAA5423
Lasionycta mutilata|[14720]|LBCH1113-10|10-JDWBC-1113|658[0n]bp|Canada.British Columbia|BOLD:AAA5423
Lasionycta mutilata|[14721]|LBCH1920-10|10-JDWBC-1920|658[0n]bp|Canada.British Columbia|BOLD:AAA5423
Lasionycta mutilata|[14722]|LBCG1899-09|08-JDWBC-1899|658[0n]bp|Canada.British Columbia|BOLD:AAA5423
Lasionycta mutilata|[14723]|LBCG1887-09|08-JDWBC-1887|658[0n]bp|Canada.British Columbia|BOLD:AAA5423
Lasionycta mutilata|[14724]|LBCH2125-10|10-JDWBC-2125|658[0n]bp|Canada.British Columbia|BOLD:AAA5423
Lasionycta mutilata|[14725]|LBCH2194-10|10-JDWBC-2194|658[0n]bp|Canada.British Columbia|BOLD:AAA5423
Lasionycta mutilata|[14726]|LBCH1110-10|10-JDWBC-1110|658[0n]bp|Canada.British Columbia|BOLD:AAA5423
Lasionycta mutilata|[14727]|LBCH1168-10|10-JDWBC-1168|658[0n]bp|Canada.British Columbia|BOLD:AAA5423
Lasionycta mutilata|[14728]|LBCG2478-09|08-JDWBC-2478|658[0n]bp|Canada.British Columbia|BOLD:AAA5423
Lasionycta mutilata|[14729]|LBCG3051-09|08-JDWBC-3051|658[0n]bp|Canada.British Columbia|BOLD:AAA5423
Lasionycta mutilata|[14730]|LBCG3034-09|08-JDWBC-3034|658[0n]bp|Canada.British Columbia|BOLD:AAA5423
Lasionycta mutilata|[14731]|LBCG3035-09|08-JDWBC-3035|658[0n]bp|Canada.British Columbia|BOLD:AAA5423
Lasionycta mutilata|[14732]|LBCG1894-09|08-JDWBC-1894|658[0n]bp|Canada.British Columbia|BOLD:AAA5423
Lasionycta mutilata|[14733]|LBCG1897-09|08-JDWBC-1897|658[0n]bp|Canada.British Columbia|BOLD:AAA5423
Lasionycta mutilata|[14734]|LBCG1891-09|08-JDWBC-1891|658[0n]bp|Canada.British Columbia|BOLD:AAA5423
Lasionycta mutilata|[14735]|LBCG2480-09|08-JDWBC-2480|658[0n]bp|Canada.British Columbia|BOLD:AAA5423
Lasionycta mutilata|[14736]|LBCG3047-09|08-JDWBC-3047|658[0n]bp|Canada.British Columbia|BOLD:AAA5423
Lasionycta mutilata|[14737]|LBCH2258-10|10-JDWBC-2258|658[0n]bp|Canada.British Columbia|BOLD:AAA5423
Lasionycta mutilata|[14738]|LBCH1539-10|10-JDWBC-1539|658[0n]bp|Canada.British Columbia|BOLD:AAA5423
Lasionycta mutilata|[14739]|LBCG2482-09|08-JDWBC-2482|658[0n]bp|Canada.British Columbia|BOLD:AAA5423
Lasionycta mutilata|[14740]|LBCH1579-10|10-JDWBC-1579|658[0n]bp|Canada.British Columbia|BOLD:AAA5423
Lasionycta mutilata|[14741]|LBCH2262-10|10-JDWBC-2262|658[0n]bp|Canada.British Columbia|BOLD:AAA5423
Lasionycta mutilata|[14742]|LBCG2479-09|08-JDWBC-2479|658[0n]bp|Canada.British Columbia|BOLD:AAA5423
Lasionycta mutilata|[14743]|LBCG3040-09|08-JDWBC-3040|658[0n]bp|Canada.British Columbia|BOLD:AAA5423
Lasionycta mutilata|[14744]|LBCH1749-10|10-JDWBC-1749|658[0n]bp|Canada.British Columbia|BOLD:AAA5423
Lasionycta mutilata|[14745]|LBCG3046-09|08-JDWBC-3046|658[0n]bp|Canada.British Columbia|BOLD:AAA5423
Lasionycta mutilata|[14746]|LBCG2485-09|08-JDWBC-2485|658[0n]bp|Canada.British Columbia|BOLD:AAA5423
Lasionycta mutilata|[14747]|LBCH2264-10|10-JDWBC-2264|658[0n]bp|Canada.British Columbia|BOLD:AAA5423
Lasionycta mutilata|[14748]|LBCH1934-10|10-JDWBC-1934|658[0n]bp|Canada.British Columbia|BOLD:AAA5423
Orthodes detracta|[14749]|RDLQG191-06|DH012367|658[0n]bp|Canada.Quebec|BOLD:AAA6122
Orthodes detracta|[14750]|BBLPA649-10|10BBCLP-0649|658[0n]bp|Canada.Ontario|BOLD:AAA6122
Orthodes detracta|[14751]|LPMN343-08|08BBLEP-01142|658[0n]bp|Canada.Manitoba|BOLD:AAA6122

Orthodes detracta|[14749]|RDLQG191-06|DH012367|658[0n]bp|Canada.Quebec|BOLD:AAA6122
Orthodes detracta|[14750]|BBLPA649-10|10BBCLP-0649|658[0n]bp|Canada.Ontario|BOLD:AAA6122
Orthodes detracta|[14751]|LPMN343-08|08BBLEP-01142|658[0n]bp|Canada.Manitoba|BOLD:AAA6122
Orthodes detracta|[14752]|LOWCD747-06|CGWC-3567|657[0n]bp|Canada.British Columbia|BOLD:AAA6122
Orthodes detracta|[14753]|LOWCD741-06|CGWC-3561|658[0n]bp|Canada.British Columbia|BOLD:AAA6122
Orthodes detracta|[14754]|LPMN250-08|08BBLEP-01049|658[0n]bp|Canada.Manitoba|BOLD:AAA6122
Orthodes detracta|[14755]|LPMN089-08|08BBLEP-00887|658[0n]bp|Canada.Manitoba|BOLD:AAA6122
Orthodes detracta|[14756]|RDLQB058-05|DH010144|657[0n]bp|Canada.Quebec|BOLD:AAA6122
Orthodes detracta|[14757]|LOWCD748-06|CGWC-3568|658[0n]bp|Canada.British Columbia|BOLD:AAA6122
Orthodes detracta|[14758]|LBCH354-10|10-JDWBC-0354|658[0n]bp|Canada.British Columbia|BOLD:AAA6122
Orthodes detracta|[14759]|LOWCD738-06|CGWC-3558|658[0n]bp|Canada.British Columbia|BOLD:AAA6122
Orthodes detracta|[14760]|LOWCD744-06|CGWC-3564|658[0n]bp|Canada.British Columbia|BOLD:AAA6122
Orthodes detracta|[14761]|RDLQB052-05|DH010138|658[0n]bp|Canada.Quebec|BOLD:AAA6122
Orthodes detracta|[14762]|LOWCD742-06|CGWC-3562|658[0n]bp|Canada.British Columbia|BOLD:AAA6122
Orthodes detracta|[14763]|XAJ850-06|2006-ONT-0850|658[0n]bp|Canada.Ontario|BOLD:AAA6122
Orthodes detracta|[14764]|LOWCB315-05|CGWC-1255|658[0n]bp|Canada.British Columbia|BOLD:AAA6122
Orthodes detracta|[14765]|LOWCD743-06|CGWC-3563|658[0n]bp|Canada.British Columbia|BOLD:AAA6122
Orthodes detracta|[14766]|RDLQB048-05|DH010134|658[0n]bp|Canada.Quebec|BOLD:AAA6122
Orthodes detracta|[14767]|LOWCB319-05|CGWC-1259|574[0n]bp|Canada.British Columbia|BOLD:AAA6122
Orthodes detracta|[14768]|LBCA601-05|HLC-20601|638[0n]bp|Canada.British Columbia|BOLD:AAA6122
Orthodes detracta|[14769]|RDNME246-07|CNCNoctuoidea13853|620[1n]bp|Canada.Alberta|BOLD:AAA6122
Orthodes detracta|[14770]|LOWCD746-06|CGWC-3566|582[0n]bp|Canada.British Columbia|BOLD:AAA6122
Orthodes detracta|[14771]|LOWCD736-06|CGWC-3556|595[0n]bp|Canada.British Columbia|BOLD:AAA6122
Orthodes detracta|[14772]|LOWCD740-06|CGWC-3560|610[0n]bp|Canada.British Columbia|BOLD:AAA6122
Orthodes detracta|[14773]|LOWCD745-06|CGWC-3565|610[0n]bp|Canada.British Columbia|BOLD:AAA6122
Orthodes detracta|[14774]|LOWCB320-05|CGWC-1260|577[1n]bp|Canada.British Columbia|BOLD:AAA6122
Orthodes detracta|[14775]|LOWCC091-05|CGWC-1971|587[0n]bp|Canada.British Columbia|BOLD:AAA6122
Orthodes detracta|[14776]|RDNMB251-05|CNCNoctuoidea10017|588[0n]bp|Canada.Quebec|BOLD:AAA6122
Orthodes detracta|[14777]|LOWCB309-05|CGWC-1249|579[0n]bp|Canada.British Columbia|BOLD:AAA6122
Orthodes detracta|[14778]|RDNMB249-05|CNCNoctuoidea10015|554[1n]bp|Canada.British Columbia|BOLD:AAA6122
Orthodes detracta|[14779]|LOWCB321-05|CGWC-1261|606[0n]bp|Canada.British Columbia|BOLD:AAA6122
Orthodes detracta|[14780]|LOWCE275-06|CGWC-4035|605[0n]bp|Canada.British Columbia|BOLD:AAA6122
Orthodes detracta|[14781]|LOWCB311-05|CGWC-1251|606[0n]bp|Canada.British Columbia|BOLD:AAA6122
Orthodes detracta|[14782]|LOWCB312-05|CGWC-1252|606[0n]bp|Canada.British Columbia|BOLD:AAA6122
Orthodes detracta|[14783]|LOWCB318-05|CGWC-1258|606[0n]bp|Canada.British Columbia|BOLD:AAA6122
Orthodes detracta|[14784]|RDLQB046-05|DH010132|524[1n]bp|Canada.Quebec|BOLD:AAA6122
Orthodes detracta|[14785]|LOWCD735-06|CGWC-3555|576[0n]bp|Canada.British Columbia|BOLD:AAA6122
Orthodes detracta|[14786]|LOWCB310-05|CGWC-1250|591[0n]bp|Canada.British Columbia|BOLD:AAA6122
Orthodes detracta|[14787]|LOWCD739-06|CGWC-3559|564[0n]bp|Canada.British Columbia|BOLD:AAA6122
Orthodes detracta|[14788]|LOWCC867-05|CGWC-2747|599[0n]bp|Canada.British Columbia|BOLD:AAA6122
Orthodes detracta|[14789]|LOWCB313-05|CGWC-1253|595[0n]bp|Canada.British Columbia|BOLD:AAA6122
Orthodes detracta|[14790]|RDLQB056-05|DH010142|658[0n]bp|Canada.Quebec|BOLD:AAA6122
Orthodes detracta|[14791]|RDLQB927-05|DH005482|658[0n]bp|Canada.Quebec|BOLD:AAA6122
Orthodes detracta|[14792]|RDLQB047-05|DH010133|658[0n]bp|Canada.Quebec|BOLD:AAA6122
Orthodes detracta|[14793]|RDLQB049-05|DH010135|658[0n]bp|Canada.Quebec|BOLD:AAA6122
Orthodes detracta|[14794]|RDLQB045-05|DH010131|658[0n]bp|Canada.Quebec|BOLD:AAA6122
Orthodes detracta|[14795]|RDLQB054-05|DH010140|658[0n]bp|Canada.Quebec|BOLD:AAA6122
Orthodes detracta|[14796]|RDLQB051-05|DH010137|658[0n]bp|Canada.Quebec|BOLD:AAA6122
Orthodes detracta|[14797]|RDLQB055-05|DH010141|602[0n]bp|Canada.Quebec|BOLD:AAA6122
Orthodes detracta|[14798]|TMNBB310-06|MNBTT-1250|656[0n]bp|Canada.New Brunswick|BOLD:AAA6122
Orthodes detracta|[14799]|TMNBB412-06|MNBTT-1352|658[0n]bp|Canada.New Brunswick|BOLD:AAA6122
Orthodes detracta|[14800]|RDLQB057-05|DH010143|658[0n]bp|Canada.Quebec|BOLD:AAA6122
Orthodes detracta|[14801]|RDLQB926-05|DH005409|658[0n]bp|Canada.Quebec|BOLD:AAA6122
Orthodes detracta|[14802]|RDLQB053-05|DH010139|658[0n]bp|Canada.Quebec|BOLD:AAA6122
Orthodes detracta|[14803]|TMNBB309-06|MNBTT-1249|658[0n]bp|Canada.New Brunswick|BOLD:AAA6122
Orthodes goodelli|[14804]|RDLQB308-05|DH010394|559[0n]bp|Canada.Quebec|BOLD:AAB4023
Orthodes goodelli|[14805]|RDMAB046-05|UASM57529|630[0n]bp|Canada.Alberta|BOLD:AAB4023
Orthodes goodelli|[14806]|BLTIB400-08|BL640|658[0n]bp|Canada.Ontario|BOLD:AAB4023
Orthodes goodelli|[14807]|RDNMB254-05|CNCNoctuoidea10020|658[0n]bp|Canada.Ontario|BOLD:AAB4023
Orthodes goodelli|[14808]|LPSOC107-08|PPBP-2106|658[0n]bp|Canada.Ontario|BOLD:AAB4023
Orthodes goodelli|[14809]|RDNMB253-05|CNCNoctuoidea10019|658[0n]bp|Canada.Ontario|BOLD:AAB4023
Orthodes goodelli|[14810]|RDNMB252-05|CNCNoctuoidea10018|658[0n]bp|Canada.Alberta|BOLD:AAB4023
Orthodes goodelli|[14811]|LPMN654-08|08BBLEP-01455|658[0n]bp|Canada.Manitoba|BOLD:AAB4023
Orthodes goodelli|[14812]|LPMN798-08|08BBLEP-01601|658[0n]bp|Canada.Manitoba|BOLD:AAB4023
Orthodes noverca|[14813]|RDNMG831-08|CNC LEP00052955|658[0n]bp|United States.Washington|BOLD:AAE1343
Orthodes noverca|[14814]|RDNMJ835-11|CNCLEP 80363|658[0n]bp|United States.Utah|BOLD:AAE1343
Orthodes noverca|[14815]|LOWCD625-06|CGWC-3445|658[0n]bp|Canada.British Columbia|BOLD:AAE1343
Orthodes noverca|[14816]|RDNMB250-05|CNCNoctuoidea10016|565[0n]bp|Canada.British Columbia|BOLD:AAE1343
Orthodes noverca|[14817]|RDNMG832-08|CNC LEP00052956|658[0n]bp|United States.New Mexico|BOLD:AAE1343
Orthodes noverca|[14818]|RDNMG634-08|CNC LEP00052458|658[0n]bp|United States.New Mexico|BOLD:AAE1343
Orthodes noverca|[14819]|RDMAB951-09|UASM121361|568[1n]bp|Canada.British Columbia|BOLD:AAE1343
Orthodes noverca|[14820]|RDMAB950-09|UASM121375|621[0n]bp|Canada.British Columbia|BOLD:AAE1343
Orthodes noverca|[14821]|LBCH5823-10|10-JDWBC-5823|658[0n]bp|Canada.British Columbia|BOLD:AAE1343
Orthodes noverca|[14822]|LBCH5108-10|10-JDWBC-5108|658[0n]bp|Canada.British Columbia|BOLD:AAE1343
Orthodes noverca|[14823]|LBCH5422-10|10-JDWBC-5422|658[0n]bp|Canada.British Columbia|BOLD:AAE1343
Orthodes noverca|[14824]|LBCH5829-10|10-JDWBC-5829|658[0n]bp|Canada.British Columbia|BOLD:AAE1343
Orthodes noverca|[14825]|LBCH6097-10|10-JDWBC-6097|658[0n]bp|Canada.British Columbia|BOLD:AAE1343
Orthodes noverca|[14826]|LBCH5828-10|10-JDWBC-5828|658[0n]bp|Canada.British Columbia|BOLD:AAE1343
Orthodes noverca|[14827]|LBCH5452-10|10-JDWBC-5452|658[0n]bp|Canada.British Columbia|BOLD:AAE1343
Orthodes noverca|[14828]|LBCH5450-10|10-JDWBC-5450|658[0n]bp|Canada.British Columbia|BOLD:AAE1343
Orthodes noverca|[14829]|LBCH5594-10|10-JDWBC-5594|658[0n]bp|Canada.British Columbia|BOLD:AAE1343
Orthodes noverca|[14830]|LBCH5596-10|10-JDWBC-5596|658[0n]bp|Canada.British Columbia|BOLD:AAE1343
Orthodes noverca|[14831]|LBCH7370-10|10-JDWBC-7370|658[0n]bp|Canada.British Columbia|BOLD:AAE1343
Orthodes noverca|[14832]|LBCH5939-10|10-JDWBC-5939|658[0n]bp|Canada.British Columbia|BOLD:AAE1343
Orthodes noverca|[14833]|LBCH5451-10|10-JDWBC-5451|658[0n]bp|Canada.British Columbia|BOLD:AAE1343
Orthodes noverca|[14834]|RDMAB949-09|UASM121382|578[0n]bp|United States.Oregon|BOLD:AAE1343
Orthodes noverca|[14835]|CGLCA152-10|CCGBOLD00152|658[0n]bp|United States.California|BOLD:AAE1343
Orthodes noverca|[14836]|RDNMK271-11|CNCLEP 84180|658[0n]bp|United States.California|BOLD:AAE1343
Orthodes noverca|[14837]|BBLOE1237-12|BIOUG01985-C05|658[0n]bp|United States.California|BOLD:AAE1343
Orthodes noverca|[14838]|RDNMK301-11|CNCLEP 84210|658[0n]bp|United States.California|BOLD:AAE1343
Orthodes noverca|[14839]|BBLOE1406-12|BIOUG01987-A08|658[0n]bp|United States.California|BOLD:AAE1343
Orthodes obscura|[14840]|BBLPB345-10|10BBCLP-1344|658[0n]bp|Canada.British Columbia|BOLD:AAD1496
Orthodes obscura|[14841]|LBCG355-08|08-JDWBC-0355|658[0n]bp|Canada.British Columbia|BOLD:AAD1496
Orthodes obscura|[14842]|LOWCD734-06|CGWC-3554|658[0n]bp|Canada.British Columbia|BOLD:AAD1496
Orthodes obscura|[14843]|LBCG381-08|08-JDWBC-0381|658[0n]bp|Canada.British Columbia|BOLD:AAD1496
Orthodes obscura|[14844]|LPABB425-08|08BBLEP-03690|658[0n]bp|Canada.Alberta|BOLD:AAD1496
Orthodes obscura|[14845]|LPABB419-08|08BBLEP-03684|658[0n]bp|Canada.Alberta|BOLD:AAD1496
Orthodes obscura|[14846]|KPOEC044-08|08OEC-153|658[0n]bp|Canada.Ontario|BOLD:AAD1496
Orthodes obscura|[14847]|RDLQ618-07|AC000635|636[0n]bp|Canada.Quebec|BOLD:AAD1496
Orthodes obscura|[14848]|RDNMB255-05|CNCNoctuoidea10021|658[0n]bp|Canada.Ontario|BOLD:AAD1496
Lacinipolia renigera|[14849]|LPSOB238-08|PPBP-1237|658[0n]bp|Canada.Ontario|BOLD:ABZ4680
Lacinipolia renigera|[14850]|LPSOB235-08|PPBP-1234|658[0n]bp|Canada.Ontario|BOLD:ABZ4680
Lacinipolia renigera|[14851]|LPSOB236-08|PPBP-1235|658[0n]bp|Canada.Ontario|BOLD:ABZ4680

[illegible]
Lacinipolia renigera|[14849]|LPSOB238-08|PPBP-1237|658[0n]bp|Canada.Ontario|BOLD:ABZ4680
Lacinipolia renigera|[14850]|LPSOB235-08|PPBP-1234|658[0n]bp|Canada.Ontario|BOLD:ABZ4680
Lacinipolia renigera|[14851]|LPSOB236-08|PPBP-1235|658[0n]bp|Canada.Ontario|BOLD:ABZ4680
Lacinipolia renigera|[14852]|LPSO685-08|PPBP-0685|658[0n]bp|Canada.Ontario|BOLD:ABZ4680
Lacinipolia renigera|[14853]|LPSO835-08|PPBP-0835|658[0n]bp|Canada.Ontario|BOLD:ABZ4680
Lacinipolia renigera|[14854]|LPSOB246-08|PPBP-1245|658[0n]bp|Canada.Ontario|BOLD:ABZ4680
Lacinipolia renigera|[14855]|BBLPA650-10|10BBCLP-0650|658[0n]bp|Canada.Ontario|BOLD:ABZ4680
Lacinipolia renigera|[14856]|LPSOB222-08|PPBP-1221|658[0n]bp|Canada.Ontario|BOLD:ABZ4680
Lacinipolia renigera|[14857]|XAJ692-06|2006-ONT-0692|658[0n]bp|Canada.Ontario|BOLD:ACE7010
Lacinipolia renigera|[14858]|RDMAB011-05|UASM57556|627[0n]bp|Canada.Alberta|BOLD:ACE7010
Lacinipolia renigera|[14859]|RDNMC409-05|CNCNoctuoidea12042|658[0n]bp|Canada.Alberta|BOLD:ACE7010
Lacinipolia renigera|[14860]|RDMAB649-06|UASM24140|658[0n]bp|Canada.Alberta|BOLD:ACE7010
Lacinipolia renigera|[14861]|BBLPB426-10|10BBCLP-1425|658[0n]bp|Canada.Alberta|BOLD:ACE7010
Lacinipolia renigera|[14862]|BBLPB739-10|10BBCLP-1738|658[0n]bp|Canada.Alberta|BOLD:ACE7010
Lacinipolia renigera|[14863]|BBLPB428-10|10BBCLP-1427|658[0n]bp|Canada.Alberta|BOLD:ACE7010
Lacinipolia renigera|[14864]|LPABB499-08|08BBLEP-03764|636[0n]bp|Canada.Alberta|BOLD:ACE7010
Lacinipolia renigera|[14865]|RDLQB920-05|DH010986|658[0n]bp|Canada.Quebec|BOLD:ACE7010
Lacinipolia renigera|[14866]|BBLPE309-09|09BBELE-2309|658[0n]bp|Canada.Newfoundland and Labrador|BOLD:...
Lacinipolia renigera|[14867]|BBLPC265-09|09BBELE-1265|658[0n]bp|Canada.Nova Scotia|BOLD:ACE7010
Lacinipolia renigera|[14868]|BBLEC265-09|09BBELE-0265|658[0n]bp|Canada.Nova Scotia|BOLD:ACE7010
Lacinipolia renigera|[14869]|BBLPE271-09|09BBELE-2271|622[0n]bp|Canada.Nova Scotia|BOLD:ACE7010
Lacinipolia renigera|[14870]|RDLQB919-05|DH010985|658[0n]bp|Canada.Quebec|BOLD:ACE7010
Lacinipolia renigera|[14871]|RDLQB918-05|DH010984|658[0n]bp|Canada.Quebec|BOLD:ACE7010
Lacinipolia renigera|[14872]|XAK301-06|2006-ONT-1296|658[0n]bp|Canada.Ontario|BOLD:ACE7010
Lacinipolia renigera|[14873]|XAD716-05|2005-ONT-515|658[0n]bp|Canada.Ontario|BOLD:ACE7010
Lacinipolia renigera|[14874]|RDLQF072-06|DH007433|658[0n]bp|Canada.Quebec|BOLD:ACE7010
Lacinipolia renigera|[14875]|BBLPC398-09|09BBELE-1398|658[0n]bp|Canada.New Brunswick|BOLD:ACE7010
Lacinipolia renigera|[14876]|RDLQF071-06|DH007084|658[0n]bp|Canada.Quebec|BOLD:ACE7010
Lacinipolia renigera|[14877]|BBLPC421-09|09BBELE-1421|658[0n]bp|Canada.New Brunswick|BOLD:ACE7010
Lacinipolia renigera|[14878]|TTMNB401-06|MNBTT-401|658[0n]bp|Canada.New Brunswick|BOLD:ACE7010
Lacinipolia renigera|[14879]|RDMAB266-05|UASM41458|658[0n]bp|Canada.Alberta|BOLD:ACE7010
Lacinipolia renigera|[14880]|XAD481-04|04HBL007481|600[0n]bp|Canada.Ontario|BOLD:ACE7010
Lacinipolia renigera|[14881]|XAD091-04|04HBL007091|580[0n]bp|Canada.Ontario|BOLD:ACE7010
Lacinipolia renigera|[14882]|RDLQF699-06|DH011849|637[0n]bp|Canada.Quebec|BOLD:ACE7010
Lacinipolia renigera|[14883]|XAD478-04|04HBL007478|658[0n]bp|Canada.Ontario|BOLD:ACE7010
Lacinipolia renigera|[14884]|LPSOB237-08|PPBP-1236|632[0n]bp|Canada.Ontario|BOLD:ACE7008
Lacinipolia renigera|[14885]|BBLPC391-09|09BBELE-1391|658[0n]bp|Canada.New Brunswick|BOLD:ACE7008
Lacinipolia renigera|[14886]|XAK283-06|2006-ONT-1278|658[0n]bp|Canada.Ontario|BOLD:ACE7008
Lacinipolia renigera|[14887]|LPSOB245-08|PPBP-1244|658[0n]bp|Canada.Ontario|BOLD:ACE7008
Lacinipolia renigera|[14888]|LPSOB209-08|PPBP-1208|658[0n]bp|Canada.Ontario|BOLD:AAA2636
Lacinipolia renigera|[14889]|LPSOB177-08|PPBP-1176|658[0n]bp|Canada.Ontario|BOLD:AAA2636
Lacinipolia renigera|[14890]|LPMN742-08|08BBLEP-01545|621[0n]bp|Canada.Manitoba|BOLD:AAA2636
Lacinipolia renigera|[14891]|BBLPB740-10|10BBCLP-1739|658[0n]bp|Canada.Alberta|BOLD:AAA2636
Lacinipolia renigera|[14892]|LPMNB391-09|08BBLEP-05235|658[0n]bp|Canada.Manitoba|BOLD:AAA2636
Lacinipolia renigera|[14893]|LPMNB334-09|08BBLEP-05178|658[0n]bp|Canada.Manitoba|BOLD:AAA2636
Lacinipolia renigera|[14894]|LPMN403-08|08BBLEP-01202|658[0n]bp|Canada.Manitoba|BOLD:AAA2636
Lacinipolia renigera|[14895]|BLTIB896-08|BL1315|638[0n]bp|Canada.Ontario|BOLD:AAA2636
Lacinipolia renigera|[14896]|TMG131-03|moth816.01|639[0n]bp|Canada.Ontario|BOLD:AAA2636
Lacinipolia renigera|[14897]|PMG124-03|LACI1.00|617[0n]bp|Canada.Ontario|BOLD:AAA2636
Lacinipolia renigera|[14898]|XAK486-07|HLC-16039|583[0n]bp|Canada.Ontario|BOLD:AAA2636
Lacinipolia renigera|[14899]|XAH377-05|2005-ONT-1960|643[0n]bp|Canada.Ontario|BOLD:AAA2636
Lacinipolia renigera|[14900]|LPSOD1000-09|08BBLEP-05633|658[0n]bp|Canada.Ontario|BOLD:AAA2636
Lacinipolia renigera|[14901]|LPMN334-08|08BBLEP-01133|658[0n]bp|Canada.Manitoba|BOLD:AAA2636
Lacinipolia renigera|[14902]|BBLPB429-10|10BBCLP-1428|658[0n]bp|Canada.Saskatchewan|BOLD:AAA2636
Lacinipolia renigera|[14903]|XAH037-05|2005-ONT-1620|658[1n]bp|Canada.Ontario|BOLD:AAA2636
Lacinipolia renigera|[14904]|XAG261-05|2005-ONT-845|655[0n]bp|Canada.Ontario|BOLD:AAA2636
Lacinipolia renigera|[14905]|XAB117-04|04HBL005117|658[0n]bp|Canada.Ontario|BOLD:AAA2636
Lacinipolia renigera|[14906]|XAB641-04|04HBL005641|658[0n]bp|Canada.Ontario|BOLD:AAA2636
Lacinipolia renigera|[14907]|XAK184-06|2006-ONT-1179|658[0n]bp|Canada.Ontario|BOLD:AAA2636
Lacinipolia renigera|[14908]|XAB456-04|04HBL005456|658[0n]bp|Canada.Ontario|BOLD:AAA2636
Lacinipolia renigera|[14909]|XAH665-05|2005-ONT-2248|658[0n]bp|Canada.Ontario|BOLD:AAA2636
Lacinipolia renigera|[14910]|XAH613-05|2005-ONT-2196|658[0n]bp|Canada.Ontario|BOLD:AAA2636
Lacinipolia renigera|[14911]|RDLQF067-06|AC000322|658[0n]bp|Canada.Quebec|BOLD:AAA2636
Lacinipolia renigera|[14912]|RDLQF069-06|DH003832|658[0n]bp|Canada.Quebec|BOLD:AAA2636
Lacinipolia renigera|[14913]|XAK145-06|2006-ONT-1140|658[0n]bp|Canada.Ontario|BOLD:AAA2636
Lacinipolia renigera|[14914]|XAC801-04|04HBL006801|658[0n]bp|Canada.Ontario|BOLD:AAA2636
Lacinipolia renigera|[14915]|XAH198-05|2005-ONT-1781|658[0n]bp|Canada.Ontario|BOLD:AAA2636
Lacinipolia renigera|[14916]|XAB354-04|04HBL005354|658[0n]bp|Canada.Ontario|BOLD:AAA2636
Lacinipolia renigera|[14917]|XAB446-04|04HBL005446|658[0n]bp|Canada.Ontario|BOLD:AAA2636
Lacinipolia renigera|[14918]|BBLPC404-09|09BBELE-1404|658[0n]bp|Canada.New Brunswick|BOLD:AAA2636
Lacinipolia renigera|[14919]|BLTIB570-08|BL848|658[0n]bp|Canada.Ontario|BOLD:AAA2636
Lacinipolia renigera|[14920]|XAB383-04|04HBL005383|658[0n]bp|Canada.Ontario|BOLD:AAA2636
Lacinipolia renigera|[14921]|XAJ846-06|2006-ONT-0846|658[0n]bp|Canada.Ontario|BOLD:AAA2636
Lacinipolia renigera|[14922]|BLTIB897-08|BL1316|658[0n]bp|Canada.Ontario|BOLD:AAA2636
Lacinipolia renigera|[14923]|XAJ858-06|2006-ONT-0858|658[0n]bp|Canada.Ontario|BOLD:AAA2636
Lacinipolia renigera|[14924]|BLTIB1132-08|BL1146|658[0n]bp|Canada.Ontario|BOLD:AAA2636
Lacinipolia renigera|[14925]|TMNBB326-06|MNBTT-1266|658[0n]bp|Canada.New Brunswick|BOLD:AAA2636
Lacinipolia renigera|[14926]|XAB124-04|04HBL005124|658[0n]bp|Canada.Ontario|BOLD:AAA2636
Lacinipolia renigera|[14927]|XAI029-05|0102-ONT-0029|658[0n]bp|Canada.Ontario|BOLD:AAA2636
Lacinipolia renigera|[14928]|XAK109-06|2006-ONT-1104|658[0n]bp|Canada.Ontario|BOLD:AAA2636
Lacinipolia renigera|[14929]|XAC839-04|04HBL006839|658[0n]bp|Canada.Ontario|BOLD:AAA2636
Lacinipolia renigera|[14930]|BLTIB712-08|BL997|658[0n]bp|Canada.Ontario|BOLD:AAA2636
Lacinipolia renigera|[14931]|BBLEC252-09|09BBELE-0252|658[0n]bp|Canada.Nova Scotia|BOLD:AAA2636
Lacinipolia renigera|[14932]|BBLEC550-09|09BBELE-0550|658[0n]bp|Canada.Nova Scotia|BOLD:AAA2636
Lacinipolia renigera|[14933]|BBLEC547-09|09BBELE-0547|654[0n]bp|Canada.Nova Scotia|BOLD:AAA2636
Lacinipolia renigera|[14934]|BBLEC180-09|09BBELE-0180|647[0n]bp|Canada.Nova Scotia|BOLD:AAA2636
Lacinipolia renigera|[14935]|MNBB127-05|05-NBSTA-043|584[20n]bp|Canada.New Brunswick|
Lacinipolia renigera|[14936]|LPSK246-08|08BBLEP-01814|658[0n]bp|Canada.Saskatchewan|BOLD:AAA2636
Lacinipolia renigera|[14937]|BBLPE572-09|09BBELE-2572|658[0n]bp|Canada.Nova Scotia|BOLD:AAA2636
Lacinipolia renigera|[14938]|PHMNB379-04|04HBL00605|658[0n]bp|Canada.New Brunswick|BOLD:AAA2636
Lacinipolia renigera|[14939]|BLTIB829-08|BL1247|658[0n]bp|Canada.Ontario|BOLD:ACE7009
Lacinipolia renigera|[14940]|BBLPB468-10|10BBCLP-1467|658[0n]bp|Canada.Alberta|BOLD:ACE7009
Lacinipolia renigera|[14941]|BLTIB711-08|BL996|658[0n]bp|Canada.Ontario|BOLD:ACE7009
Lacinipolia renigera|[14942]|BBLPC507-09|09BBELE-1507|614[0n]bp|Canada.New Brunswick|BOLD:ACE7009
Lacinipolia renigera|[14943]|LPABC137-09|08BBLEP-04356|658[2n]bp|Canada.Alberta|BOLD:ACE7009
Lacinipolia renigera|[14944]|RDMAB010-05|UASM57555|658[0n]bp|Canada.Alberta|BOLD:ACE7009
Lacinipolia renigera|[14945]|LPMNB347-09|08BBLEP-05191|658[0n]bp|Canada.Manitoba|BOLD:ACE7009
Lacinipolia renigera|[14946]|BBLEC688-09|09BBELE-0688|658[0n]bp|Canada.Nova Scotia|BOLD:ACE7009
Lacinipolia renigera|[14947]|LPMNB246-09|08BBLEP-05090|658[0n]bp|Canada.Manitoba|BOLD:ACE7009
Lacinipolia renigera|[14948]|RDLQF208-06|DH011288|658[0n]bp|Canada.Quebec|BOLD:ACE7009
Lacinipolia renigera|[14949]|BBLEC545-09|09BBELE-0545|658[0n]bp|Canada.Nova Scotia|BOLD:ACE7009
Lacinipolia renigera|[14950]|LPABB206-08|08BBLEP-03471|658[0n]bp|Canada.Alberta|BOLD:ACE7009
[illegible]

Lacinipolia renigera|[14948]|RDLQF208-06|DH011288|658[0n]bp|Canada.Quebec|BOLD:ACE7009
Lacinipolia renigera|[14949]|BBLEC545-09|09BBELE-0545|658[0n]bp|Canada.Nova Scotia|BOLD:ACE7009
Lacinipolia renigera|[14950]|LPABB206-08|08BBLEP-03471|658[0n]bp|Canada.Alberta|BOLD:ACE7009
Lacinipolia renigera|[14951]|RDLQF073-06|DH007935|658[0n]bp|Canada.Quebec|BOLD:ACE7009
Lacinipolia renigera|[14952]|BBLEC707-09|09BBELE-0707|658[0n]bp|Canada.Nova Scotia|BOLD:ACE7009
Lacinipolia renigera|[14953]|RDLQF370-06|DH011437|658[0n]bp|Canada.Quebec|BOLD:ACE7009
Lacinipolia renigera|[14954]|BBLPC420-09|09BBELE-1420|658[0n]bp|Canada.New Brunswick|BOLD:ACE7009
Lacinipolia renigera|[14955]|PHMNB749-05|Moth 442.03SA|658[0n]bp|Canada.New Brunswick|BOLD:ACE7009
Lacinipolia renigera|[14956]|BBLEC968-09|09BBELE-0968|658[0n]bp|Canada.Nova Scotia|BOLD:ACE7009
Lacinipolia renigera|[14957]|RDMAB648-06|UASM24109|658[0n]bp|Canada.Alberta|BOLD:ACE7009
Lacinipolia renigera|[14958]|LPMN800-08|08BBLEP-01603|658[0n]bp|Canada.Manitoba|BOLD:ACE7009
Lacinipolia renigera|[14959]|BBLPC387-09|09BBELE-1387|658[0n]bp|Canada.New Brunswick|BOLD:ACE7009
Lacinipolia renigera|[14960]|RDLQB538-05|DH010624|658[0n]bp|Canada.Quebec|BOLD:ACE7009
Lacinipolia renigera|[14961]|BBLEC767-09|09BBELE-0767|658[0n]bp|Canada.Nova Scotia|BOLD:ACE7009
Lacinipolia renigera|[14962]|RDLQF070-06|DH005441|658[0n]bp|Canada.Quebec|BOLD:ACE7009
Lacinipolia renigera|[14963]|RDMAB499-06|UASM58453|658[0n]bp|Canada.Alberta|BOLD:ACE7009
Lacinipolia renigera|[14964]|BLTIB1102-08|BL1114|658[0n]bp|Canada.Ontario|BOLD:ACE7009
Lacinipolia renigera|[14965]|BBLEC706-09|09BBELE-0706|658[0n]bp|Canada.Nova Scotia|BOLD:ACE7009
Lacinipolia renigera|[14966]|BBLEC742-09|09BBELE-0742|633[0n]bp|Canada.Nova Scotia|BOLD:ACE7009
Lacinipolia renigera|[14967]|BBLEC253-09|09BBELE-0253|635[0n]bp|Canada.Nova Scotia|BOLD:ACE7009
Lacinipolia renigera|[14968]|BBLEC716-09|09BBELE-0716|641[0n]bp|Canada.Nova Scotia|BOLD:ACE7009
Lacinipolia renigera|[14969]|BBLEC768-09|09BBELE-0768|650[0n]bp|Canada.Nova Scotia|BOLD:ACE7009
Lacinipolia renigera|[14970]|BBLEC710-09|09BBELE-0710|655[0n]bp|Canada.Nova Scotia|BOLD:ACE7009
Lacinipolia renigera|[14971]|LPMNB509-09|08BBLEP-05547|658[0n]bp|Canada.Manitoba|BOLD:ACE7009
Lacinipolia renigera|[14972]|LPSK245-08|08BBLEP-01813|658[0n]bp|Canada.Saskatchewan|BOLD:ACE7009
Lacinipolia stenotis|[14973]|CMAZA1210-12|BIOUG02043-H02|658[0n]bp|United States.Arizona|BOLD:AAF2596
Lacinipolia stenotis|[14974]|NAMUM132-08|RR-99-1261|658[0n]bp|United States.California|BOLD:AAF2596
Lacinipolia stenotis|[14975]|NAMUM143-08|RR-99-1261-2|656[0n]bp|United States.California|BOLD:AAF2596
Lacinipolia stenotis|[14976]|RDNMC400-05|CNCNoctuoidea12034|579[0n]bp|United States.Oregon|BOLD:AAF2596
Lacinipolia stricta|[14977]|RDMAB314-05|UASM77800|658[0n]bp|Canada.Alberta|BOLD:AAB2883
Lacinipolia stricta|[14978]|LOWCE523-06|CGWC-4283|546[0n]bp|Canada.British Columbia|BOLD:AAI2180
Lacinipolia stricta|[14979]|RDNMB220-05|CNCNoctuoidea7966|599[0n]bp|Canada.British Columbia|BOLD:AAB2880
Lacinipolia stricta|[14980]|LBCH6536-10|10-JDWBC-6536|658[0n]bp|Canada.British Columbia|BOLD:AAB2880
Lacinipolia stricta|[14981]|LBCH7475-10|10-JDWBC-7475|658[0n]bp|Canada.British Columbia|BOLD:AAB2880
Lacinipolia stricta|[14982]|LOWCC078-05|CGWC-1958|658[0n]bp|Canada.British Columbia|BOLD:AAB2880
Lacinipolia stricta|[14983]|LOWCE531-06|CGWC-4291|658[0n]bp|Canada.British Columbia|BOLD:AAB2880
Lacinipolia stricta|[14984]|LOWCD190-06|CGWC-3010|658[0n]bp|Canada.British Columbia|BOLD:AAB2880
Lacinipolia stricta|[14985]|LOWCC073-05|CGWC-1953|658[0n]bp|Canada.British Columbia|BOLD:AAB2880
Lacinipolia stricta|[14986]|LOWCC081-05|CGWC-1961|658[0n]bp|Canada.British Columbia|BOLD:AAB2880
Lacinipolia stricta|[14987]|LOWCC074-05|CGWC-1954|658[0n]bp|Canada.British Columbia|BOLD:AAB2880
Lacinipolia stricta|[14988]|LOWC005-05|CGWC-0005|658[0n]bp|Canada.British Columbia|BOLD:AAB2880
Lacinipolia stricta|[14989]|LOWCC075-05|CGWC-1955|658[0n]bp|Canada.British Columbia|BOLD:AAB2880
Lacinipolia stricta|[14990]|LBCH7354-10|10-JDWBC-7354|658[0n]bp|Canada.British Columbia|BOLD:AAB2880
Lacinipolia stricta|[14991]|LOWCC080-05|CGWC-1960|658[0n]bp|Canada.British Columbia|BOLD:AAB2880
Lacinipolia stricta|[14992]|LOWCC082-05|CGWC-1962|658[0n]bp|Canada.British Columbia|BOLD:AAB2880
Lacinipolia stricta|[14993]|LBCH7123-10|10-JDWBC-7123|658[0n]bp|Canada.British Columbia|BOLD:AAB2880
Lacinipolia stricta|[14994]|LALPA745-10|AVBC 747-10|658[0n]bp|Canada.British Columbia|BOLD:AAB2880
Lacinipolia stricta|[14995]|LOWCC072-05|CGWC-1952|658[0n]bp|Canada.British Columbia|BOLD:AAB2880
Lacinipolia stricta|[14996]|LOWCC071-05|CGWC-1951|658[0n]bp|Canada.British Columbia|BOLD:AAB2880
Lacinipolia stricta|[14997]|LBCH7222-10|10-JDWBC-7222|642[0n]bp|Canada.British Columbia|BOLD:AAB2880
Lacinipolia stricta|[14998]|LOWCE530-06|CGWC-4290|614[0n]bp|Canada.British Columbia|BOLD:AAB2880
Lacinipolia stricta|[14999]|LOWCC079-05|CGWC-1959|658[0n]bp|Canada.British Columbia|BOLD:AAB2880
Lacinipolia stricta|[15000]|LOWCE529-06|CGWC-4289|658[0n]bp|Canada.British Columbia|BOLD:AAB2880
Lacinipolia stricta|[15001]|LOWCC077-05|CGWC-1957|658[0n]bp|Canada.British Columbia|BOLD:AAB2880
Lacinipolia stricta|[15002]|LOWCC076-05|CGWC-1956|658[0n]bp|Canada.British Columbia|BOLD:AAB2880
Psammopolia arietis|[15003]|BBLPB769-10|10BBCLP-1768|658[0n]bp|Canada.British Columbia|BOLD:AAP8033
Psammopolia wyatti|[15004]|RDNMB720-05|CNCNoctuoidea10496|562[1n]bp|Canada.British Columbia|BOLD:AAB0902
Psammopolia wyatti|[15005]|RDNMB719-05|CNCNoctuoidea10495|538[1n]bp|Canada.British Columbia|BOLD:AAB0902
Tricholita notata|[15006]|RDNMF738-08|NOC14824|640[0n]bp|United States.Oklahoma|BOLD:AAJ8475
Tricholita signata|[15007]|LPMNB227-09|08BBLEP-05071|658[0n]bp|Canada.Manitoba|BOLD:AAA6002
Tricholita signata|[15008]|XAG550-05|2005-ONT-1134|597[1n]bp|Canada.Ontario|BOLD:AAA6002
Tricholita signata|[15009]|XAD582-04|04HBL006997|658[0n]bp|Canada.Ontario|BOLD:AAA6002
Tricholita signata|[15010]|XAG547-05|2005-ONT-1131|604[1n]bp|Canada.Ontario|BOLD:AAA6002
Tricholita signata|[15011]|XAG471-05|2005-ONT-1055|546[2n]bp|Canada.Ontario|BOLD:AAA6002
Tricholita signata|[15012]|XAG473-05|2005-ONT-1057|580[1n]bp|Canada.Ontario|BOLD:AAA6002
Tricholita signata|[15013]|XAK257-06|2006-ONT-1252|658[0n]bp|Canada.Ontario|BOLD:AAA6002
Tricholita signata|[15014]|XAG103-05|2005-ONT-687|658[0n]bp|Canada.Ontario|BOLD:AAA6002
Tricholita signata|[15015]|XAG862-05|2005-ONT-1446|658[0n]bp|Canada.Ontario|BOLD:AAA6002
Tricholita signata|[15016]|XAK604-07|HLC-16157|658[0n]bp|Canada.Ontario|BOLD:AAA6002
Tricholita signata|[15017]|XAI049-05|0102-ONT-0049|658[0n]bp|Canada.Ontario|BOLD:AAA6002
Tricholita signata|[15018]|XAK416-06|2006-ONT-1411|656[0n]bp|Canada.Ontario|BOLD:AAA6002
Tricholita signata|[15019]|BLTIB845-08|BL1264|658[0n]bp|Canada.Ontario|BOLD:AAA6002
Tricholita signata|[15020]|BBLPC095-09|09BBELE-1095|658[0n]bp|Canada.New Brunswick|BOLD:AAA6002
Tricholita signata|[15021]|BLTIB989-08|BL1426|658[0n]bp|Canada.Ontario|BOLD:AAA6002
Tricholita signata|[15022]|BLTIB773-08|BL1169|658[0n]bp|Canada.Ontario|BOLD:AAA6002
Tricholita signata|[15023]|BLTIB799-08|BL1217|658[0n]bp|Canada.Ontario|BOLD:AAA6002
Tricholita signata|[15024]|XAC837-04|04HBL006837|658[0n]bp|Canada.Ontario|BOLD:AAA6002
Tricholita signata|[15025]|RDLQF373-06|DH011440|658[0n]bp|Canada.Quebec|BOLD:AAA6002
Tricholita signata|[15026]|XAB013-04|04HBL005013|658[0n]bp|Canada.Ontario|BOLD:AAA6002
Tricholita signata|[15027]|XAG650-05|2005-ONT-1234|658[0n]bp|Canada.Ontario|BOLD:AAA6002
Tricholita signata|[15028]|XAK228-06|2006-ONT-1223|658[0n]bp|Canada.Ontario|BOLD:AAA6002
Tricholita signata|[15029]|BLTIB863-08|BL1282|658[0n]bp|Canada.Ontario|BOLD:AAA6002
Tricholita signata|[15030]|XAG472-05|2005-ONT-1056|658[0n]bp|Canada.Ontario|BOLD:AAA6002
Tricholita signata|[15031]|XAC042-04|04HBL006042|592[0n]bp|Canada.Ontario|BOLD:AAA6002
Tricholita signata|[15032]|RDLQB649-05|DH010752|569[0n]bp|Canada.Quebec|BOLD:AAA6002
Tricholita signata|[15033]|BLTIB1019-08|BL1459|658[0n]bp|Canada.Ontario|BOLD:AAA6002
Tricholita signata|[15034]|BLTIB848-08|BL1267|658[0n]bp|Canada.Ontario|BOLD:AAA6002
Tricholita signata|[15035]|BBLPC543-09|09BBELE-1543|639[0n]bp|Canada.New Brunswick|BOLD:AAA6002
Tricholita signata|[15036]|BBLPE560-09|09BBELE-2560|658[0n]bp|Canada.Nova Scotia|BOLD:AAA6002
Tricholita signata|[15037]|XAG649-05|2005-ONT-1233|658[0n]bp|Canada.Ontario|BOLD:AAA6002
Tricholita signata|[15038]|XAC723-04|04HBL006723|658[0n]bp|Canada.Ontario|BOLD:AAA6002
Tricholita signata|[15039]|XAC834-04|04HBL006834|658[0n]bp|Canada.Ontario|BOLD:AAA6002
Tricholita signata|[15040]|XAG107-05|2005-ONT-691|658[0n]bp|Canada.Ontario|BOLD:AAA6002
Tricholita signata|[15041]|XAG853-05|2005-ONT-1437|658[0n]bp|Canada.Ontario|BOLD:AAA6002
Tricholita signata|[15042]|BLTIB1031-08|BL1473|658[0n]bp|Canada.Ontario|BOLD:AAA6002
Tricholita signata|[15043]|XAI047-05|0102-ONT-0047|658[0n]bp|Canada.Ontario|BOLD:AAA6002
Tricholita signata|[15044]|XAG764-05|2005-ONT-1348|658[0n]bp|Canada.Ontario|BOLD:AAA6002
Tricholita signata|[15045]|XAG528-05|2005-ONT-1112|658[0n]bp|Canada.Ontario|BOLD:AAA6002
Tricholita signata|[15046]|XAG345-05|2005-ONT-929|658[0n]bp|Canada.Ontario|BOLD:AAA6002
Tricholita signata|[15047]|BLTIB862-08|BL1281|658[0n]bp|Canada.Ontario|BOLD:AAA6002
Tricholita signata|[15048]|XAI048-05|0102-ONT-0048|658[0n]bp|Canada.Ontario|BOLD:AAA6002
Tricholita signata|[15049]|BLTIB914-08|BL1334|658[0n]bp|Canada.Ontario|BOLD:AAA6002
Tricholita signata|[15050]|XAC462-04|04HBL006462|658[0n]bp|Canada.Ontario|BOLD:AAA6002

Tricholita signata|[15048]|XAI048-05|0102-ONT-0048|658[0n]bp|Canada.Ontario|BOLD:AAA6002
Tricholita signata|[15049]|BLTIB914-08|BL1334|658[0n]bp|Canada.Ontario|BOLD:AAA6002
Tricholita signata|[15050]|XAC462-04|04HBL006462|658[0n]bp|Canada.Ontario|BOLD:AAA6002
Tricholita signata|[15051]|XAK605-07|HLC-16158|658[0n]bp|Canada.Ontario|BOLD:AAA6002
Tricholita signata|[15052]|XAG672-05|2005-ONT-1256|658[1n]bp|Canada.Ontario|BOLD:AAA6002
Tricholita signata|[15053]|XAG670-05|2005-ONT-1254|658[0n]bp|Canada.Ontario|BOLD:AAA6002
Tricholita signata|[15054]|XAG673-05|2005-ONT-1257|614[0n]bp|Canada.Ontario|BOLD:AAA6002
Tricholita signata|[15055]|XAG654-05|2005-ONT-1238|657[1n]bp|Canada.Ontario|BOLD:AAA6002
Tricholita signata|[15056]|PMG164-03|TRICHO1.00|617[0n]bp|Canada.Ontario|BOLD:AAA6002
Tricholita signata|[15057]|TMG145-03|moth1104.01|639[0n]bp|Canada.Ontario|BOLD:AAA6002
Tricholita signata|[15058]|XAC850-04|04HBL006850|592[0n]bp|Canada.Ontario|BOLD:AAA6002
Tricholita signata|[15059]|XAD585-04|04HBL007000|557[0n]bp|Canada.Ontario|BOLD:AAA6002
Tricholita signata|[15060]|XAD101-04|04HBL007101|593[0n]bp|Canada.Ontario|BOLD:AAA6002
Tricholita signata|[15061]|XAG885-05|2005-ONT-1469|632[0n]bp|Canada.Ontario|BOLD:AAA6002
Orthodes cynica|[15062]|LPSOB983-08|PPBP-1982|658[0n]bp|Canada.Ontario|BOLD:AAA2411
Orthodes cynica|[15063]|LPSOB994-08|PPBP-1993|658[0n]bp|Canada.Ontario|BOLD:AAA2411
Orthodes cynica|[15064]|XAE251-04|Moth4251.03|616[0n]bp|Canada.Ontario|BOLD:AAA2411
Orthodes cynica|[15065]|LPSO263-08|PPBP-0263|657[0n]bp|Canada.Ontario|BOLD:AAA2411
Orthodes cynica|[15066]|XAB592-04|04HBL005592|658[0n]bp|Canada.Ontario|BOLD:AAA2411
Orthodes cynica|[15067]|LPSO035-08|PPBP-0035|658[0n]bp|Canada.Ontario|BOLD:AAA2411
Orthodes cynica|[15068]|TTMNB557-06|MNBTT-557|658[1n]bp|Canada.New Brunswick|BOLD:AAA2411
Orthodes cynica|[15069]|XAC180-04|04HBL006180|658[0n]bp|Canada.Ontario|BOLD:AAA2411
Orthodes cynica|[15070]|BLTIB184-08|BL264|658[0n]bp|Canada.Ontario|BOLD:AAA2411
Orthodes cynica|[15071]|LPSOD886-09|08BBLEP-00668|658[0n]bp|Canada.Ontario|BOLD:AAA2411
Orthodes cynica|[15072]|LPSOB993-08|PPBP-1992|658[0n]bp|Canada.Ontario|BOLD:AAA2411
Orthodes cynica|[15073]|TTMNB559-06|MNBTT-559|658[0n]bp|Canada.New Brunswick|BOLD:AAA2411
Orthodes cynica|[15074]|RDLQG388-06|DH012656|658[0n]bp|Canada.Quebec|BOLD:AAA2411
Orthodes cynica|[15075]|XAB251-04|04HBL005251|658[0n]bp|Canada.Ontario|BOLD:AAA2411
Orthodes cynica|[15076]|LPSO926-08|PPBP-0926|658[0n]bp|Canada.Ontario|BOLD:AAA2411
Orthodes cynica|[15077]|LPSO050-08|PPBP-0050|658[0n]bp|Canada.Ontario|BOLD:AAA2411
Orthodes cynica|[15078]|LPSOD521-09|08BBLEP-00300|658[0n]bp|Canada.Ontario|BOLD:AAA2411
Orthodes cynica|[15079]|RDLQG391-06|DH012659|658[0n]bp|Canada.Quebec|BOLD:AAA2411
Orthodes cynica|[15080]|LPSO493-08|PPBP-0493|658[0n]bp|Canada.Ontario|BOLD:AAA2411
Orthodes cynica|[15081]|LPSOC106-08|PPBP-2105|658[0n]bp|Canada.Ontario|BOLD:AAA2411
Orthodes cynica|[15082]|LPSOB998-08|PPBP-1997|658[0n]bp|Canada.Ontario|BOLD:AAA2411
Orthodes cynica|[15083]|XAB266-04|04HBL005266|658[0n]bp|Canada.Ontario|BOLD:AAA2411
Orthodes cynica|[15084]|RDLQG397-06|DH012665|658[0n]bp|Canada.Quebec|BOLD:AAA2411
Orthodes cynica|[15085]|KPOEC084-08|08OEC-243|658[0n]bp|Canada.Ontario|BOLD:AAA2411
Orthodes cynica|[15086]|LPSOB997-08|PPBP-1996|658[0n]bp|Canada.Ontario|BOLD:AAA2411
Orthodes cynica|[15087]|LPSO036-08|PPBP-0036|658[0n]bp|Canada.Ontario|BOLD:AAA2411
Orthodes cynica|[15088]|XAC296-04|04HBL006296|658[0n]bp|Canada.Ontario|BOLD:AAA2411
Orthodes cynica|[15089]|RDLQG255-06|DH012460|658[0n]bp|Canada.Quebec|BOLD:AAA2411
Orthodes cynica|[15090]|LPSO250-08|PPBP-0250|658[0n]bp|Canada.Ontario|BOLD:AAA2411
Orthodes cynica|[15091]|LPSOB809-08|PPBP-1808|658[0n]bp|Canada.Ontario|BOLD:AAA2411
Orthodes cynica|[15092]|LPSO254-08|PPBP-0254|658[0n]bp|Canada.Ontario|BOLD:AAA2411
Orthodes cynica|[15093]|LPSOC371-08|PPBP-2370|655[0n]bp|Canada.Ontario|BOLD:AAA2411
Orthodes cynica|[15094]|XAF586-05|2005-ONT-235|658[0n]bp|Canada.Ontario|BOLD:AAA2411
Orthodes cynica|[15095]|LPSOD885-09|08BBLEP-00667|658[0n]bp|Canada.Ontario|BOLD:AAA2411
Orthodes cynica|[15096]|LPSOB119-08|PPBP-1118|658[0n]bp|Canada.Ontario|BOLD:AAA2411
Orthodes cynica|[15097]|LPSOD434-09|08BBLEP-00213|658[0n]bp|Canada.Ontario|BOLD:AAA2411
Orthodes cynica|[15098]|RDLQ261-05|DH004970|658[0n]bp|Canada.Quebec|BOLD:AAA2411
Orthodes cynica|[15099]|LPSOC385-08|PPBP-2384|658[0n]bp|Canada.Ontario|BOLD:AAA2411
Orthodes cynica|[15100]|LPSO051-08|PPBP-0051|658[0n]bp|Canada.Ontario|BOLD:AAA2411
Orthodes cynica|[15101]|LPSOB804-08|PPBP-1803|658[0n]bp|Canada.Ontario|BOLD:AAA2411
Orthodes cynica|[15102]|PHMNB382-04|04HBL00608|658[0n]bp|Canada.New Brunswick|BOLD:AAA2411
Orthodes cynica|[15103]|RDLQG390-06|DH012658|658[0n]bp|Canada.Quebec|BOLD:AAA2411
Orthodes cynica|[15104]|XAK180-06|2006-ONT-1175|658[0n]bp|Canada.Ontario|BOLD:AAA2411
Orthodes cynica|[15105]|LPSO669-08|PPBP-0669|658[0n]bp|Canada.Ontario|BOLD:AAA2411
Orthodes cynica|[15106]|LPMN528-08|08BBLEP-01327|658[0n]bp|Canada.Manitoba|BOLD:AAA2411
Orthodes cynica|[15107]|LPSOB232-08|PPBP-1231|658[0n]bp|Canada.Ontario|BOLD:AAA2411
Orthodes cynica|[15108]|XAJ712-06|2006-ONT-0712|658[0n]bp|Canada.Ontario|BOLD:AAA2411
Orthodes cynica|[15109]|RDLQB068-05|DH010154|658[0n]bp|Canada.Quebec|BOLD:AAA2411
Orthodes cynica|[15110]|RDLQG274-06|DH012486|658[0n]bp|Canada.Quebec|BOLD:AAA2411
Orthodes cynica|[15111]|LPSOB120-08|PPBP-1119|658[0n]bp|Canada.Ontario|BOLD:AAA2411
Orthodes cynica|[15112]|LPSO499-08|PPBP-0499|658[0n]bp|Canada.Ontario|BOLD:AAA2411
Orthodes cynica|[15113]|XAJ713-06|2006-ONT-0713|658[0n]bp|Canada.Ontario|BOLD:AAA2411
Orthodes cynica|[15114]|LPSOB987-08|PPBP-1986|658[0n]bp|Canada.Ontario|BOLD:AAA2411
Orthodes cynica|[15115]|RDLQG396-06|DH012664|658[0n]bp|Canada.Quebec|BOLD:AAA2411
Orthodes cynica|[15116]|LPSO360-08|PPBP-0360|658[0n]bp|Canada.Ontario|BOLD:AAA2411
Orthodes cynica|[15117]|TTMNB556-06|MNBTT-556|658[0n]bp|Canada.New Brunswick|BOLD:AAA2411
Orthodes cynica|[15118]|LPSO547-08|PPBP-0547|658[0n]bp|Canada.Ontario|BOLD:AAA2411
Orthodes cynica|[15119]|RDLQG367-06|DH012601|658[0n]bp|Canada.Quebec|BOLD:AAA2411
Orthodes cynica|[15120]|RDLQG389-06|DH012657|658[0n]bp|Canada.Quebec|BOLD:AAA2411
Orthodes cynica|[15121]|LPSO742-08|PPBP-0742|658[0n]bp|Canada.Ontario|BOLD:AAA2411
Orthodes cynica|[15122]|LPSOD440-09|08BBLEP-00219|658[0n]bp|Canada.Ontario|BOLD:AAA2411
Orthodes cynica|[15123]|LPSOC044-08|PPBP-2043|658[0n]bp|Canada.Ontario|BOLD:AAA2411
Orthodes cynica|[15124]|LPSOB094-08|PPBP-1093|658[0n]bp|Canada.Ontario|BOLD:AAA2411
Orthodes cynica|[15125]|KPOEC176-08|08OEC-217|658[0n]bp|Canada.Ontario|BOLD:AAA2411
Orthodes cynica|[15126]|BLTIB252-08|BL434|658[0n]bp|Canada.Ontario|BOLD:AAA2411
Orthodes cynica|[15127]|XAC292-04|04HBL006292|658[0n]bp|Canada.Ontario|BOLD:AAA2411
Orthodes cynica|[15128]|PHMTV426-10|10PHMAL-2526|658[0n]bp|Canada.Ontario|BOLD:AAA2411
Orthodes cynica|[15129]|RDLQF846-06|DH011999|658[0n]bp|Canada.Quebec|BOLD:AAA2411
Orthodes cynica|[15130]|XAB259-04|04HBL005259|658[0n]bp|Canada.Ontario|BOLD:AAA2411
Orthodes cynica|[15131]|LPSOB720-08|PPBP-1719|658[0n]bp|Canada.Ontario|BOLD:AAA2411
Orthodes cynica|[15132]|LPSO277-08|PPBP-0277|658[0n]bp|Canada.Ontario|BOLD:AAA2411
Orthodes cynica|[15133]|BBLPB454-10|10BBCLP-1453|658[0n]bp|Canada.Ontario|BOLD:AAA2411
Orthodes cynica|[15134]|XAJ615-06|2006-ONT-0615|658[0n]bp|Canada.Ontario|BOLD:AAA2411
Orthodes cynica|[15135]|XAB217-04|04HBL005217|658[0n]bp|Canada.Ontario|BOLD:AAA2411
Orthodes cynica|[15136]|LPSOC097-08|PPBP-2096|658[0n]bp|Canada.Ontario|BOLD:AAA2411
Orthodes cynica|[15137]|LPSOB829-08|PPBP-1828|658[0n]bp|Canada.Ontario|BOLD:AAA2411
Orthodes cynica|[15138]|PHMNB413-04|04HBL00639|658[0n]bp|Canada.New Brunswick|BOLD:AAA2411
Orthodes cynica|[15139]|LPSO889-08|PPBP-0889|658[0n]bp|Canada.Ontario|BOLD:AAA2411
Orthodes cynica|[15140]|LPSOD771-09|08BBLEP-00553|658[0n]bp|Canada.Ontario|BOLD:AAA2411
Orthodes cynica|[15141]|LPSO266-08|PPBP-0266|658[0n]bp|Canada.Ontario|BOLD:AAA2411
Orthodes cynica|[15142]|LPSO348-08|PPBP-0348|658[0n]bp|Canada.Ontario|BOLD:AAA2411
Orthodes cynica|[15143]|XAC597-04|04HBL006597|658[0n]bp|Canada.Ontario|BOLD:AAA2411
Orthodes cynica|[15144]|LPSO055-08|PPBP-0055|658[0n]bp|Canada.Ontario|BOLD:AAA2411
Orthodes cynica|[15145]|LPSOB995-08|PPBP-1994|658[0n]bp|Canada.Ontario|BOLD:AAA2411
Orthodes cynica|[15146]|LPSO747-08|PPBP-0747|658[0n]bp|Canada.Ontario|BOLD:AAA2411
Orthodes cynica|[15147]|RDLQG366-06|DH012600|658[0n]bp|Canada.Quebec|BOLD:AAA2411
Orthodes cynica|[15148]|LPSOB803-08|PPBP-1802|658[0n]bp|Canada.Ontario|BOLD:AAA2411
Orthodes cynica|[15149]|RDLQF936-06|DH012116|658[0n]bp|Canada.Quebec|BOLD:AAA2411
Orthodes cynica|[15150]|TTMNB561-06|MNBTT-561|658[0n]bp|Canada.New Brunswick|BOLD:AAA2411

[illegible]
Orthodes cynica|[15148]|LPSOB803-08|PPBP-1802|658[0n]bp|Canada.Ontario|BOLD:AAA2411
Orthodes cynica|[15149]|RDLQF936-06|DH012116|658[0n]bp|Canada.Quebec|BOLD:AAA2411
Orthodes cynica|[15150]|TTMNB561-06|MNBTT-561|658[0n]bp|Canada.New Brunswick|BOLD:AAA2411
Orthodes cynica|[15151]|XAJ653-06|2006-ONT-0653|658[0n]bp|Canada.Ontario|BOLD:AAA2411
Orthodes cynica|[15152]|LPSOD894-09|08BBLEP-00676|658[0n]bp|Canada.Ontario|BOLD:AAA2411
Orthodes cynica|[15153]|LPSO492-08|PPBP-0492|658[0n]bp|Canada.Ontario|BOLD:AAA2411
Orthodes cynica|[15154]|TTMNB558-06|MNBTT-558|658[0n]bp|Canada.New Brunswick|BOLD:AAA2411
Orthodes cynica|[15155]|LPSO409-08|PPBP-0409|658[0n]bp|Canada.Ontario|BOLD:AAA2411
Orthodes cynica|[15156]|LPSOB819-08|PPBP-1818|658[0n]bp|Canada.Ontario|BOLD:AAA2411
Orthodes cynica|[15157]|LPSOB999-08|PPBP-1998|658[0n]bp|Canada.Ontario|BOLD:AAA2411
Orthodes cynica|[15158]|BBLPB585-10|10BBCLP-1584|658[0n]bp|Canada.Ontario|BOLD:AAA2411
Orthodes cynica|[15159]|XAF470-05|2005-ONT-119|658[0n]bp|Canada.Ontario|BOLD:AAA2411
Orthodes cynica|[15160]|XAF821-05|2005-ONT-470|658[0n]bp|Canada.Ontario|BOLD:AAA2411
Orthodes cynica|[15161]|LPSO645-08|PPBP-0645|658[0n]bp|Canada.Ontario|BOLD:AAA2411
Orthodes cynica|[15162]|LPMN578-08|08BBLEP-01379|658[0n]bp|Canada.Manitoba|BOLD:AAA2411
Orthodes cynica|[15163]|LPSO249-08|PPBP-0249|658[0n]bp|Canada.Ontario|BOLD:AAA2411
Orthodes cynica|[15164]|LPMN333-08|08BBLEP-01132|658[0n]bp|Canada.Manitoba|BOLD:AAA2411
Orthodes cynica|[15165]|LPSOB991-08|PPBP-1990|658[0n]bp|Canada.Ontario|BOLD:AAA2411
Orthodes cynica|[15166]|LPSO502-08|PPBP-0502|658[0n]bp|Canada.Ontario|BOLD:AAA2411
Orthodes cynica|[15167]|LPSOD572-09|08BBLEP-00353|658[0n]bp|Canada.Ontario|BOLD:AAA2411
Orthodes cynica|[15168]|LPSO037-08|PPBP-0037|658[0n]bp|Canada.Ontario|BOLD:AAA2411
Orthodes cynica|[15169]|LPSO543-08|PPBP-0543|657[0n]bp|Canada.Ontario|BOLD:AAA2411
Orthodes cynica|[15170]|LPSOD694-09|08BBLEP-00475|658[0n]bp|Canada.Ontario|BOLD:AAA2411
Orthodes cynica|[15171]|RDLQ409-05|AC000566|658[1n]bp|Canada.Quebec|BOLD:AAA2411
Orthodes cynica|[15172]|LPSOD707-09|08BBLEP-00488|658[0n]bp|Canada.Ontario|BOLD:AAA2411
Orthodes cynica|[15173]|TTMNB560-06|MNBTT-560|656[0n]bp|Canada.New Brunswick|BOLD:AAA2411
Orthodes cynica|[15174]|LPSOB158-08|PPBP-1157|656[0n]bp|Canada.Ontario|BOLD:AAA2411
Orthodes cynica|[15175]|LPSOC369-08|PPBP-2368|643[0n]bp|Canada.Ontario|BOLD:AAA2411
Orthodes cynica|[15176]|LPSOB664-08|PPBP-1663|646[0n]bp|Canada.Ontario|BOLD:AAA2411
Orthodes cynica|[15177]|LPSOB692-08|PPBP-1691|646[0n]bp|Canada.Ontario|BOLD:AAA2411
Orthodes cynica|[15178]|LPSOB992-08|PPBP-1991|646[0n]bp|Canada.Ontario|BOLD:AAA2411
Orthodes cynica|[15179]|RDLQB050-05|DH010136|658[0n]bp|Canada.Quebec|BOLD:AAA2411
Orthodes cynica|[15180]|XAB078-04|04HBL005078|569[0n]bp|Canada.Ontario|BOLD:AAA2411
Orthodes cynica|[15181]|RDLQ312-05|DH008150|582[0n]bp|Canada.Quebec|BOLD:AAA2411
Orthodes cynica|[15182]|RDLQB812-05|DH010899|617[0n]bp|Canada.Quebec|BOLD:AAA2411
Orthodes cynica|[15183]|PMG141-03|moth359.01|617[0n]bp|Canada.Ontario|BOLD:AAA2411
Orthodes cynica|[15184]|XAE299-04|Moth4299.03|617[0n]bp|Canada.Ontario|BOLD:AAA2411
Orthodes cynica|[15185]|TMG144-03|moth342.01|639[0n]bp|Canada.Ontario|BOLD:AAA2411
Orthodes cynica|[15186]|PHMO145-03|moth824.01|639[0n]bp|Canada.Ontario|BOLD:AAA2411
Orthodes cynica|[15187]|PHMO059-03|moth395.01|639[0n]bp|Canada.Ontario|BOLD:AAA2411
Orthodes cynica|[15188]|TMG143-03|moth385.01|639[0n]bp|Canada.Ontario|BOLD:AAA2411
Orthodes cynica|[15189]|MEC587-04|jflandry0587|583[0n]bp|Canada.Quebec|BOLD:AAA2411
Orthodes cynica|[15190]|RDLQ313-05|DH008151|578[0n]bp|Canada.Quebec|BOLD:AAA2411
Orthodes cynica|[15191]|RDLQ309-05|DH008144|571[1n]bp|Canada.Quebec|BOLD:AAA2411
Orthodes cynica|[15192]|XAJ554-06|2006-ONT-0554|658[0n]bp|Canada.Ontario|BOLD:AAA2411
Orthodes cynica|[15193]|RDLQ307-05|DH008142|557[0n]bp|Canada.Quebec|BOLD:AAA2411
Orthodes cynica|[15194]|RDLQ308-05|DH008143|591[0n]bp|Canada.Quebec|BOLD:AAA2411
Orthodes cynica|[15195]|RDLQ310-05|DH008147|600[1n]bp|Canada.Quebec|BOLD:AAA2411
Orthodes cynica|[15196]|LPSO282-08|PPBP-0282|643[0n]bp|Canada.Ontario|BOLD:AAA2411
Orthodes cynica|[15197]|RDLQ287-05|DH007083|656[0n]bp|Canada.Quebec|BOLD:AAA2411
Orthodes cynica|[15198]|BLTIB254-08|BL436|658[0n]bp|Canada.Ontario|BOLD:AAA2411
Orthodes cynica|[15199]|LPSO740-08|PPBP-0740|658[0n]bp|Canada.Ontario|BOLD:AAA2411
Orthodes cynica|[15200]|RDLQG392-06|DH012660|658[0n]bp|Canada.Quebec|BOLD:AAA2411
Orthodes cynica|[15201]|KPOEC162-08|08OEC-203|658[0n]bp|Canada.Ontario|BOLD:AAA2411
Orthodes majuscula|[15202]|RDLQG188-06|DH012362|655[0n]bp|Canada.Quebec|BOLD:AAB4054
Orthodes majuscula|[15203]|LPMNB335-09|08BBLEP-05179|640[0n]bp|Canada.Manitoba|BOLD:AAB4054
Orthodes majuscula|[15204]|LPSO487-08|PPBP-0487|658[0n]bp|Canada.Ontario|BOLD:AAB4054
Orthodes majuscula|[15205]|RDLQF544-06|DH011693|658[0n]bp|Canada.Quebec|BOLD:AAB4054
Orthodes majuscula|[15206]|TTMNB037-06|MNBTT-037|658[0n]bp|Canada.New Brunswick|BOLD:AAB4054
Orthodes majuscula|[15207]|LPSO500-08|PPBP-0500|658[0n]bp|Canada.Ontario|BOLD:AAB4054
Orthodes majuscula|[15208]|LPSOB082-08|PPBP-1081|658[0n]bp|Canada.Ontario|BOLD:AAB4054
Orthodes majuscula|[15209]|RDLQG135-06|DH012302|658[0n]bp|Canada.Quebec|BOLD:AAB4054
Neleucania praegracilis|[15210]|RDNMG265-08|NOC15117|658[0n]bp|United States.Texas|BOLD:AAE6946
Neleucania praegracilis|[15211]|RDNMF229-08|NOC14315|658[0n]bp|United States.Wyoming|BOLD:AAE6946
Neleucania praegracilis|[15212]|RDNMF230-08|NOC14316|658[0n]bp|United States.Wyoming|BOLD:AAE6946
Neleucania praegracilis|[15213]|RDNMF231-08|NOC14317|658[0n]bp|United States.Wyoming|BOLD:AAE6946
Zosteropoda hirtipes|[15214]|LPABB506-08|08BBLEP-03771|658[0n]bp|Canada.Alberta|BOLD:AAB4289
Zosteropoda hirtipes|[15215]|LALPA286-10|AVBC 287-10|658[0n]bp|Canada.British Columbia|BOLD:AAB4289
Zosteropoda hirtipes|[15216]|LBCH5576-10|10-JDWBC-5576|658[0n]bp|Canada.British Columbia|BOLD:AAB4289
Zosteropoda hirtipes|[15217]|LALPA272-10|AVBC 273-10|658[0n]bp|Canada.British Columbia|BOLD:AAB4289
Zosteropoda hirtipes|[15218]|LBCH5516-10|10-JDWBC-5516|658[0n]bp|Canada.British Columbia|BOLD:AAB4289
Zosteropoda hirtipes|[15219]|LALPA315-10|AVBC 317-10|658[0n]bp|Canada.British Columbia|BOLD:AAB4289
Lacinipolia cuneata|[15220]|LPVIA098-08|PFC-2006-0163|658[0n]bp|Canada.British Columbia|BOLD:AAB1084
Lacinipolia cuneata|[15221]|LPVIA235-08|PFC-2006-0317|637[0n]bp|Canada.British Columbia|BOLD:AAB1084
Lacinipolia cuneata|[15222]|LALPA479-10|AVBC 481-10|658[0n]bp|Canada.British Columbia|BOLD:AAB1084
Lacinipolia cuneata|[15223]|LALPA1104-11|AVBC 914-11|658[0n]bp|Canada.British Columbia|BOLD:AAB1084
Lacinipolia cuneata|[15224]|LALPA320-10|AVBC 322-10|658[0n]bp|Canada.British Columbia|BOLD:AAB1084
Lacinipolia cuneata|[15225]|LALPA186-10|AVBC 186-10|658[0n]bp|Canada.British Columbia|BOLD:AAB1084
Lacinipolia cuneata|[15226]|LALPA227-10|AVBC 228-10|658[0n]bp|Canada.British Columbia|BOLD:AAB1084
Lacinipolia cuneata|[15227]|LALPA290-10|AVBC 292-10|658[0n]bp|Canada.British Columbia|BOLD:AAB1084
Lacinipolia cuneata|[15228]|LALPA293-10|AVBC 295-10|658[0n]bp|Canada.British Columbia|BOLD:AAB1084
Lacinipolia cuneata|[15229]|LALPA321-10|AVBC 323-10|658[0n]bp|Canada.British Columbia|BOLD:AAB1084
Lacinipolia cuneata|[15230]|LALPA1188-11|AVBC 998-11|658[0n]bp|Canada.British Columbia|BOLD:AAB1084
Lacinipolia cuneata|[15231]|LPVIA100-08|PFC-2006-0165|658[0n]bp|Canada.British Columbia|BOLD:AAB1084
Lacinipolia cuneata|[15232]|LPVIA293-08|PFC-2006-0384|658[0n]bp|Canada.British Columbia|BOLD:AAB1084
Lacinipolia cuneata|[15233]|LALPA423-10|AVBC 425-10|658[0n]bp|Canada.British Columbia|BOLD:AAB1084
Lacinipolia cuneata|[15234]|LPVIB929-08|PFC-2006-2463|648[0n]bp|Canada.British Columbia|BOLD:AAB1084
Lacinipolia cuneata|[15235]|LPVIA292-08|PFC-2006-0383|658[0n]bp|Canada.British Columbia|BOLD:AAB1084
Athetis tarda|[15236]|LPSO671-08|PPBP-0671|658[0n]bp|Canada.Ontario|BOLD:AAA8313
Athetis tarda|[15237]|LPSO437-08|PPBP-0437|658[0n]bp|Canada.Ontario|BOLD:AAA8313
Athetis tarda|[15238]|LPSO275-08|PPBP-0275|658[0n]bp|Canada.Ontario|BOLD:AAA8313
Athetis tarda|[15239]|LPSO551-08|PPBP-0551|658[0n]bp|Canada.Ontario|BOLD:AAA8313
Athetis tarda|[15240]|LPSO749-08|PPBP-0749|658[0n]bp|Canada.Ontario|BOLD:AAA8313
Athetis tarda|[15241]|LPSO684-08|PPBP-0684|658[0n]bp|Canada.Ontario|BOLD:AAA8313
Athetis tarda|[15242]|LPSO751-08|PPBP-0751|658[0n]bp|Canada.Ontario|BOLD:AAA8313
Caradrina morpheus|[15243]|LBCH2252-10|10-JDWBC-2252|658[0n]bp|Canada.British Columbia|BOLD:AAA6694
Caradrina morpheus|[15244]|LHLEP252-06|UBC-2006-1075|658[0n]bp|Canada.British Columbia|BOLD:AAA6694
Caradrina morpheus|[15245]|BBLPB393-10|10BBCLP-1392|658[0n]bp|Canada.British Columbia|BOLD:AAA6694
Caradrina morpheus|[15246]|BBLPC623-09|09BBELE-1623|658[0n]bp|Canada.Newfoundland and Labrador|BOLD:AA...
Caradrina morpheus|[15247]|BBLPB389-10|10BBCLP-1388|658[0n]bp|Canada.British Columbia|BOLD:AAA6694
Caradrina morpheus|[15248]|LBCG008-08|08-JDWBC-0008|658[0n]bp|Canada.British Columbia|BOLD:AAA6694
Caradrina morpheus|[15249]|BBLPB391-10|10BBCLP-1390|658[0n]bp|Canada.British Columbia|BOLD:AAA6694
[illegible]

Caradrina morpheus|[15247]|BBLPB389-10|10BBCLP-1388|658[0n]bp|Canada.British Columbia|BOLD:AAA6694
Caradrina morpheus|[15248]|LBCG008-08|08-JDWBC-0008|658[0n]bp|Canada.British Columbia|BOLD:AAA6694
Caradrina morpheus|[15249]|BBLPB391-10|10BBCLP-1390|658[0n]bp|Canada.British Columbia|BOLD:AAA6694
Caradrina morpheus|[15250]|LHLEP076-06|UBC-2006-0281|658[0n]bp|Canada.British Columbia|BOLD:AAA6694
Caradrina morpheus|[15251]|LOWCB904-05|CGWC-1844|658[0n]bp|Canada.British Columbia|BOLD:AAA6694
Caradrina morpheus|[15252]|BBLPE416-09|09BBELE-2416|658[0n]bp|Canada.Newfoundland and Labrador|BOLD:AA...
Caradrina morpheus|[15253]|LPVIB007-08|PFC-2006-2363|658[0n]bp|Canada.British Columbia|BOLD:AAA6694
Caradrina morpheus|[15254]|LHLEP254-06|UBC-2006-1077|658[0n]bp|Canada.British Columbia|BOLD:AAA6694
Caradrina morpheus|[15255]|LBCA083-05|HLC-20083|658[0n]bp|Canada.British Columbia|BOLD:AAA6694
Caradrina morpheus|[15256]|BBLPB390-10|10BBCLP-1389|658[0n]bp|Canada.British Columbia|BOLD:AAA6694
Caradrina morpheus|[15257]|LHLEP250-06|UBC-2006-0352|658[0n]bp|Canada.British Columbia|BOLD:AAA6694
Caradrina morpheus|[15258]|LHLEP077-06|UBC-2006-0282|658[0n]bp|Canada.British Columbia|BOLD:AAA6694
Caradrina morpheus|[15259]|LBCH5614-10|10-JDWBC-5614|658[0n]bp|Canada.British Columbia|BOLD:AAA6694
Caradrina morpheus|[15260]|LHLEP078-06|UBC-2006-0283|658[0n]bp|Canada.British Columbia|BOLD:AAA6694
Caradrina morpheus|[15261]|LALPA420-10|AVBC 422-10|658[0n]bp|Canada.British Columbia|BOLD:AAA6694
Caradrina morpheus|[15262]|BBLPB747-10|10BBCLP-1746|658[0n]bp|Canada.British Columbia|BOLD:AAA6694
Caradrina morpheus|[15263]|LHLEP255-06|UBC-2006-1129|658[0n]bp|Canada.British Columbia|BOLD:AAA6694
Caradrina morpheus|[15264]|LALPA234-10|AVBC 235-10|658[0n]bp|Canada.British Columbia|BOLD:AAA6694
Caradrina morpheus|[15265]|LPVIB841-08|PFC-2006-2348|658[0n]bp|Canada.British Columbia|BOLD:AAA6694
Caradrina morpheus|[15266]|LOWCB903-05|CGWC-1843|658[0n]bp|Canada.British Columbia|BOLD:AAA6694
Caradrina morpheus|[15267]|LALPA359-10|AVBC 361-10|658[0n]bp|Canada.British Columbia|BOLD:AAA6694
Caradrina morpheus|[15268]|BBLPB748-10|10BBCLP-1747|658[0n]bp|Canada.British Columbia|BOLD:AAA6694
Caradrina morpheus|[15269]|LHLEP253-06|UBC-2006-1076|658[0n]bp|Canada.British Columbia|BOLD:AAA6694
Caradrina morpheus|[15270]|LALPA393-10|AVBC 395-10|658[0n]bp|Canada.British Columbia|BOLD:AAA6694
Caradrina morpheus|[15271]|LOWCB901-05|CGWC-1841|658[0n]bp|Canada.British Columbia|BOLD:AAA6694
Caradrina morpheus|[15272]|PHMNB180-04|04HBL007645|658[0n]bp|Canada.New Brunswick|BOLD:AAA6694
Caradrina morpheus|[15273]|LBCS485-07|UBC-2007-0242|658[0n]bp|Canada.British Columbia|BOLD:AAA6694
Caradrina morpheus|[15274]|LHLEP251-06|UBC-2006-1074|658[0n]bp|Canada.British Columbia|BOLD:AAA6694
Caradrina morpheus|[15275]|LPVIA409-08|PFC-2006-0595|658[0n]bp|Canada.British Columbia|BOLD:AAA6694
Caradrina morpheus|[15276]|LBCG1905-09|08-JDWBC-1905|658[0n]bp|Canada.British Columbia|BOLD:AAA6694
Caradrina morpheus|[15277]|LPVIA613-08|PFC-2006-0841|658[0n]bp|Canada.British Columbia|BOLD:AAA6694
Caradrina morpheus|[15278]|BBLPB392-10|10BBCLP-1391|658[0n]bp|Canada.British Columbia|BOLD:AAA6694
Caradrina morpheus|[15279]|LPVIB915-08|PFC-2006-2446|634[0n]bp|Canada.British Columbia|BOLD:AAA6694
Caradrina morpheus|[15280]|RDMAB015-05|UASM57560|622[0n]bp|Canada.Alberta|BOLD:AAA6694
Caradrina morpheus|[15281]|RDMAB014-05|UASM57559|567[0n]bp|Canada.Alberta|BOLD:AAA6694
Caradrina morpheus|[15282]|LPVIB949-08|PFC-2006-2487|647[0n]bp|Canada.British Columbia|BOLD:AAA6694
Caradrina morpheus|[15283]|LOWCB900-05|CGWC-1840|606[0n]bp|Canada.British Columbia|BOLD:AAA6694
Caradrina morpheus|[15284]|RDLQ495-07|DH002900|601[2n]bp|Canada.Quebec|BOLD:AAA6694
Caradrina morpheus|[15285]|LOWCB905-05|CGWC-1845|580[0n]bp|Canada.British Columbia|BOLD:AAA6694
Caradrina morpheus|[15286]|LOWCB902-05|CGWC-1842|587[0n]bp|Canada.British Columbia|BOLD:AAA6694
Caradrina morpheus|[15287]|RDLQ496-07|DH005660|607[0n]bp|Canada.Quebec|BOLD:AAA6694
Caradrina morpheus|[15288]|LPVIB873-08|PFC-2006-2390|634[0n]bp|Canada.British Columbia|BOLD:AAA6694
Caradrina morpheus|[15289]|LPVIB880-08|PFC-2006-2397|634[0n]bp|Canada.British Columbia|BOLD:AAA6694
Caradrina montana|[15290]|LCHQ718-08|07WNP-10610|657[0n]bp|Canada.Manitoba|BOLD:AAA8639
Caradrina montana|[15291]|LCHQ760-08|07WNP-10652|658[0n]bp|Canada.Manitoba|BOLD:AAA8639
Caradrina montana|[15292]|LBCH7797-10|10-JDWBC-7797|658[0n]bp|Canada.British Columbia|BOLD:AAA8639
Caradrina montana|[15293]|LBCC838-05|HLC-22718|658[0n]bp|Canada.British Columbia|BOLD:AAA8639
Caradrina montana|[15294]|LPSK571-08|08BBLEP-02139|658[0n]bp|Canada.Saskatchewan|BOLD:AAA8639
Caradrina montana|[15295]|LBCH6001-10|10-JDWBC-6001|658[0n]bp|Canada.British Columbia|BOLD:AAA8639
Caradrina montana|[15296]|LOWCE847-06|CGWC-4607|658[0n]bp|Canada.British Columbia|BOLD:AAA8639
Caradrina montana|[15297]|LBCH7856-10|10-JDWBC-7856|658[0n]bp|Canada.British Columbia|BOLD:AAA8639
Caradrina montana|[15298]|LBCG2107-09|08-JDWBC-2107|658[0n]bp|Canada.British Columbia|BOLD:AAA8639
Caradrina montana|[15299]|LPSK147-08|08BBLEP-01715|658[0n]bp|Canada.Saskatchewan|BOLD:AAA8639
Caradrina montana|[15300]|BBLPB423-10|10BBCLP-1422|658[0n]bp|Canada.Alberta|BOLD:AAA8639
Caradrina montana|[15301]|LBCH6459-10|10-JDWBC-6459|658[0n]bp|Canada.British Columbia|BOLD:AAA8639
Caradrina montana|[15302]|LBCH6128-10|10-JDWBC-6128|658[0n]bp|Canada.British Columbia|BOLD:AAA8639
Caradrina montana|[15303]|LBCH7858-10|10-JDWBC-7858|658[0n]bp|Canada.British Columbia|BOLD:AAA8639
Caradrina montana|[15304]|LOWCB875-05|CGWC-1815|658[0n]bp|Canada.British Columbia|BOLD:AAA8639
Caradrina montana|[15305]|LOWCB883-05|CGWC-1823|658[0n]bp|Canada.British Columbia|BOLD:AAA8639
Caradrina montana|[15306]|LOWCB889-05|CGWC-1829|658[0n]bp|Canada.British Columbia|BOLD:AAA8639
Caradrina montana|[15307]|LPSK154-08|08BBLEP-01722|658[0n]bp|Canada.Saskatchewan|BOLD:AAA8639
Caradrina montana|[15308]|RDMAB136-05|UASM41283|658[0n]bp|Canada.Alberta|BOLD:AAA8639
Caradrina montana|[15309]|LOWCB895-05|CGWC-1835|658[0n]bp|Canada.British Columbia|BOLD:AAA8639
Caradrina montana|[15310]|LOWCB879-05|CGWC-1819|658[0n]bp|Canada.British Columbia|BOLD:AAA8639
Caradrina montana|[15311]|LALPA815-10|AVBC 817-10|658[0n]bp|Canada.British Columbia|BOLD:AAA8639
Caradrina montana|[15312]|BBLPB439-10|10BBCLP-1438|658[0n]bp|Canada.British Columbia|BOLD:AAA8639
Caradrina montana|[15313]|LPSK386-08|08BBLEP-01954|658[0n]bp|Canada.Saskatchewan|BOLD:AAA8639
Caradrina montana|[15314]|LBCH6646-10|10-JDWBC-6646|658[0n]bp|Canada.British Columbia|BOLD:AAA8639
Caradrina montana|[15315]|LOWCB885-05|CGWC-1825|658[0n]bp|Canada.British Columbia|BOLD:AAA8639
Caradrina montana|[15316]|LBCH6645-10|10-JDWBC-6645|658[0n]bp|Canada.British Columbia|BOLD:AAA8639
Caradrina montana|[15317]|LPSK155-08|08BBLEP-01723|658[0n]bp|Canada.Saskatchewan|BOLD:AAA8639
Caradrina montana|[15318]|LBCH7855-10|10-JDWBC-7855|658[0n]bp|Canada.British Columbia|BOLD:AAA8639
Caradrina montana|[15319]|LBCH7857-10|10-JDWBC-7857|658[0n]bp|Canada.British Columbia|BOLD:AAA8639
Caradrina montana|[15320]|LOWCB888-05|CGWC-1828|658[0n]bp|Canada.British Columbia|BOLD:AAA8639
Caradrina montana|[15321]|LBCH6983-10|10-JDWBC-6983|658[0n]bp|Canada.British Columbia|BOLD:AAA8639
Caradrina montana|[15322]|LOWCB896-05|CGWC-1836|658[0n]bp|Canada.British Columbia|BOLD:AAA8639
Caradrina montana|[15323]|LOWCB892-05|CGWC-1832|658[0n]bp|Canada.British Columbia|BOLD:AAA8639
Caradrina montana|[15324]|BBLPB427-10|10BBCLP-1426|658[0n]bp|Canada.Alberta|BOLD:AAA8639
Caradrina montana|[15325]|LBCH7801-10|10-JDWBC-7801|658[0n]bp|Canada.British Columbia|BOLD:AAA8639
Caradrina montana|[15326]|LBCH7862-10|10-JDWBC-7862|658[0n]bp|Canada.British Columbia|BOLD:AAA8639
Caradrina montana|[15327]|LPSK152-08|08BBLEP-01720|658[0n]bp|Canada.Saskatchewan|BOLD:AAA8639
Caradrina montana|[15328]|LBCH7796-10|10-JDWBC-7796|658[0n]bp|Canada.British Columbia|BOLD:AAA8639
Caradrina montana|[15329]|LBCH5431-10|10-JDWBC-5431|658[0n]bp|Canada.British Columbia|BOLD:AAA8639
Caradrina montana|[15330]|LBCH6546-10|10-JDWBC-6546|658[0n]bp|Canada.British Columbia|BOLD:AAA8639
Caradrina montana|[15331]|BBLPB424-10|10BBCLP-1423|658[0n]bp|Canada.Alberta|BOLD:AAA8639
Caradrina montana|[15332]|RDMAB040-05|UASM57523|631[0n]bp|Canada.Alberta|BOLD:AAA8639
Caradrina montana|[15333]|LOWCB897-05|CGWC-1837|589[0n]bp|Canada.British Columbia|BOLD:AAA8639
Caradrina montana|[15334]|LOWCB882-05|CGWC-1822|595[0n]bp|Canada.British Columbia|BOLD:AAA8639
Caradrina montana|[15335]|LOWCB890-05|CGWC-1830|563[0n]bp|Canada.British Columbia|BOLD:AAA8639
Caradrina montana|[15336]|LOWCB876-05|CGWC-1816|582[0n]bp|Canada.British Columbia|BOLD:AAA8639
Caradrina montana|[15337]|LOWCB893-05|CGWC-1833|566[0n]bp|Canada.British Columbia|BOLD:AAA8639
Caradrina montana|[15338]|LOWCB898-05|CGWC-1838|503[0n]bp|Canada.British Columbia|
Caradrina montana|[15339]|LOWCB887-05|CGWC-1827|501[0n]bp|Canada.British Columbia|
Caradrina montana|[15340]|LBCH7860-10|10-JDWBC-7860|642[0n]bp|Canada.British Columbia|BOLD:AAA8639
Caradrina montana|[15341]|RDNMI196-12|CNCLEP00094160|614[0n]bp|Canada.Ontario|BOLD:AAA8639
Caradrina montana|[15342]|LPSK123-08|08BBLEP-01691|658[0n]bp|Canada.Saskatchewan|BOLD:AAA8639
Caradrina montana|[15343]|LCHQ313-08|07WNP-10205|656[0n]bp|Canada.Manitoba|BOLD:AAA8639
Caradrina montana|[15344]|BBLPB425-10|10BBCLP-1424|658[0n]bp|Canada.Alberta|BOLD:AAA8639
Caradrina montana|[15345]|LBCH6648-10|10-JDWBC-6648|658[0n]bp|Canada.British Columbia|BOLD:AAA8639
Caradrina montana|[15346]|LALPA505-10|AVBC 507-11|658[0n]bp|Canada.British Columbia|BOLD:AAA8639
Caradrina montana|[15347]|LOWCB884-05|CGWC-1824|658[0n]bp|Canada.British Columbia|BOLD:AAA8639
Caradrina montana|[15348]|LBCH6643-10|10-JDWBC-6643|658[0n]bp|Canada.British Columbia|BOLD:AAA8639
Caradrina montana|[15349]|LBCH7617-10|10-JDWBC-7617|658[0n]bp|Canada.British Columbia|BOLD:AAA8639

Caradrina montana|[15347]|LOWCB884-05|CGWC-1824|658[0n]bp|Canada.British Columbia|BOLD:AAA8639
Caradrina montana|[15348]|LBCH6643-10|10-JDWBC-6643|658[0n]bp|Canada.British Columbia|BOLD:AAA8639
Caradrina montana|[15349]|LBCH7617-10|10-JDWBC-7617|658[0n]bp|Canada.British Columbia|BOLD:AAA8639
Caradrina montana|[15350]|LALPA625-10|AVBC 627-10|658[0n]bp|Canada.British Columbia|BOLD:AAA8639
Caradrina montana|[15351]|LALPA1239-11|AVBC 1241-11|658[0n]bp|Canada.British Columbia|BOLD:AAA8639
Caradrina montana|[15352]|LBCG501-08|08-JDWBC-0501|658[0n]bp|Canada.British Columbia|BOLD:AAA8639
Caradrina montana|[15353]|LALPA953-11|AVBC 1126-11|658[0n]bp|Canada.British Columbia|BOLD:AAA8639
Caradrina montana|[15354]|LBCG2582-09|08-JDWBC-2582|658[0n]bp|Canada.British Columbia|BOLD:AAA8639
Caradrina montana|[15355]|LOWCB881-05|CGWC-1821|658[0n]bp|Canada.British Columbia|BOLD:AAA8639
Caradrina montana|[15356]|LPVIB763-08|PFC-2006-2228|658[0n]bp|Canada.British Columbia|BOLD:AAA8639
Caradrina montana|[15357]|LBCH6644-10|10-JDWBC-6644|658[0n]bp|Canada.British Columbia|BOLD:AAA8639
Caradrina montana|[15358]|LBCH7377-10|10-JDWBC-7377|658[0n]bp|Canada.British Columbia|BOLD:AAA8639
Caradrina montana|[15359]|LOWCB891-05|CGWC-1831|656[0n]bp|Canada.British Columbia|BOLD:AAA8639
Caradrina montana|[15360]|LOWCB880-05|CGWC-1820|573[0n]bp|Canada.British Columbia|BOLD:AAA8639
Caradrina montana|[15361]|LOWCB877-05|CGWC-1817|566[0n]bp|Canada.British Columbia|BOLD:AAA8639
Caradrina montana|[15362]|LOWCB894-05|CGWC-1834|565[0n]bp|Canada.British Columbia|BOLD:AAA8639
Caradrina montana|[15363]|LOWCB878-05|CGWC-1818|564[0n]bp|Canada.British Columbia|BOLD:AAA8639
Caradrina montana|[15364]|LOWCB886-05|CGWC-1826|588[0n]bp|Canada.British Columbia|BOLD:AAA8639
Caradrina multifera|[15365]|TMNBB211-06|MNBTT-1151|658[0n]bp|Canada.New Brunswick|BOLD:AAA8639
Caradrina multifera|[15366]|TMNBB212-06|MNBTT-1152|658[0n]bp|Canada.New Brunswick|BOLD:AAA8639
Caradrina multifera|[15367]|RDNMB216-05|CNCNoctuoidea7962|588[0n]bp|Canada.Ontario|BOLD:AAA8639
Caradrina multifera|[15368]|RDLQ492-07|DH007165|604[1n]bp|Canada.Quebec|BOLD:AAA8639
Caradrina multifera|[15369]|RDLQ493-07|DH009077|608[0n]bp|Canada.Quebec|BOLD:AAA8639
Caradrina multifera|[15370]|TMNBB213-06|MNBTT-1153|658[0n]bp|Canada.New Brunswick|BOLD:AAA8639
Protoperigea anotha|[15371]|RDNMF601-08|NOC14687|658[0n]bp|Canada.Alberta|BOLD:AAF6358
Protoperigea anotha|[15372]|RDNMF603-08|NOC14689|652[0n]bp|Canada.Alberta|BOLD:AAF6358
Protoperigea anotha|[15373]|RDNMF602-08|NOC14688|658[0n]bp|Canada.Alberta|BOLD:AAF6358
Protoperigea anotha|[15374]|BBLPB421-10|10BBCLP-1420|658[0n]bp|Canada.Alberta|BOLD:AAF6358
Caradrina camina|[15375]|RDNMC707-06|CNCNoctuoidea12247|591[0n]bp|Canada.British Columbia|BOLD:ABY7042
Caradrina camina|[15376]|LBCH7908-10|10-JDWBC-7908|658[0n]bp|Canada.British Columbia|BOLD:ABY7042
Caradrina camina|[15377]|LBCH7905-10|10-JDWBC-7905|658[0n]bp|Canada.British Columbia|BOLD:ABY7042
Caradrina camina|[15378]|LBCH7909-10|10-JDWBC-7909|658[0n]bp|Canada.British Columbia|BOLD:ABY7042
Caradrina camina|[15379]|LBCH6533-10|10-JDWBC-6533|658[0n]bp|Canada.British Columbia|BOLD:ABY7042
Caradrina camina|[15380]|LBCH7561-10|10-JDWBC-7561|658[0n]bp|Canada.British Columbia|BOLD:ABY7042
Caradrina camina|[15381]|LBCH7904-10|10-JDWBC-7904|658[0n]bp|Canada.British Columbia|BOLD:ABY7042
Caradrina camina|[15382]|LBCH7065-10|10-JDWBC-7065|658[0n]bp|Canada.British Columbia|BOLD:ABY7042
Caradrina camina|[15383]|LBCH7973-10|10-JDWBC-7973|658[0n]bp|Canada.British Columbia|BOLD:ABY7042
Caradrina camina|[15384]|LBCH7906-10|10-JDWBC-7906|658[0n]bp|Canada.British Columbia|BOLD:ABY7042
Caradrina camina|[15385]|LBCH6926-10|10-JDWBC-6926|658[0n]bp|Canada.British Columbia|BOLD:ABY7042
Caradrina camina|[15386]|LBCH7903-10|10-JDWBC-7903|658[0n]bp|Canada.British Columbia|BOLD:ABY7042
Caradrina camina|[15387]|LBCH7822-10|10-JDWBC-7822|658[0n]bp|Canada.British Columbia|BOLD:ABY7042
Caradrina camina|[15388]|LBCH7907-10|10-JDWBC-7907|658[0n]bp|Canada.British Columbia|BOLD:ABY7042
Caradrina camina|[15389]|LBCH7902-10|10-JDWBC-7902|658[0n]bp|Canada.British Columbia|BOLD:ABY7042
Caradrina meralis|[15390]|LBCH7190-10|10-JDWBC-7190|658[0n]bp|Canada.British Columbia|BOLD:AAB9848
Caradrina meralis|[15391]|LBCH7501-10|10-JDWBC-7501|658[0n]bp|Canada.British Columbia|BOLD:AAB9848
Caradrina meralis|[15392]|LBCH6650-10|10-JDWBC-6650|658[0n]bp|Canada.British Columbia|BOLD:AAB9848
Caradrina meralis|[15393]|LBCH6292-10|10-JDWBC-6292|658[0n]bp|Canada.British Columbia|BOLD:AAB9848
Caradrina meralis|[15394]|LBCH7473-10|10-JDWBC-7473|658[0n]bp|Canada.British Columbia|BOLD:AAB9848
Caradrina meralis|[15395]|LBCH6174-10|10-JDWBC-6174|658[0n]bp|Canada.British Columbia|BOLD:AAB9848
Caradrina meralis|[15396]|LBCG2874-09|08-JDWBC-2874|658[0n]bp|Canada.British Columbia|BOLD:AAB9848
Caradrina meralis|[15397]|LBCH6297-10|10-JDWBC-6297|658[0n]bp|Canada.British Columbia|BOLD:AAB9848
Caradrina meralis|[15398]|LBCH6307-10|10-JDWBC-6307|658[0n]bp|Canada.British Columbia|BOLD:AAB9848
Caradrina meralis|[15399]|LBCH6169-10|10-JDWBC-6169|658[0n]bp|Canada.British Columbia|BOLD:AAB9848
Caradrina meralis|[15400]|LPVIB671-08|PFC-2006-2120|633[0n]bp|Canada.British Columbia|BOLD:AAB9848
Caradrina meralis|[15401]|LBCH7396-10|10-JDWBC-7396|658[0n]bp|Canada.British Columbia|BOLD:AAB9848
Caradrina meralis|[15402]|LBCH6303-10|10-JDWBC-6303|658[0n]bp|Canada.British Columbia|BOLD:AAB9848
Caradrina meralis|[15403]|LBCH6649-10|10-JDWBC-6649|658[0n]bp|Canada.British Columbia|BOLD:AAB9848
Caradrina meralis|[15404]|LBCG492-08|08-JDWBC-0492|658[0n]bp|Canada.British Columbia|BOLD:AAB9848
Caradrina meralis|[15405]|LBCH6172-10|10-JDWBC-6172|658[0n]bp|Canada.British Columbia|BOLD:AAB9848
Caradrina meralis|[15406]|LBCH6235-10|10-JDWBC-6235|658[0n]bp|Canada.British Columbia|BOLD:AAB9848
Caradrina meralis|[15407]|LBCH6240-10|10-JDWBC-6240|658[0n]bp|Canada.British Columbia|BOLD:AAB9848
Caradrina meralis|[15408]|LBCH7821-10|10-JDWBC-7821|658[0n]bp|Canada.British Columbia|BOLD:AAB9848
Caradrina meralis|[15409]|LBCH6170-10|10-JDWBC-6170|658[0n]bp|Canada.British Columbia|BOLD:AAB9848
Caradrina meralis|[15410]|LBCH7799-10|10-JDWBC-7799|658[0n]bp|Canada.British Columbia|BOLD:AAB9848
Caradrina meralis|[15411]|LBCH6300-10|10-JDWBC-6300|658[0n]bp|Canada.British Columbia|BOLD:AAB9848
Caradrina meralis|[15412]|LBCG504-08|08-JDWBC-0504|658[0n]bp|Canada.British Columbia|BOLD:AAB9848
Caradrina meralis|[15413]|LBCH7403-10|10-JDWBC-7403|658[0n]bp|Canada.British Columbia|BOLD:AAB9848
Caradrina meralis|[15414]|LBCH7105-10|10-JDWBC-7105|658[0n]bp|Canada.British Columbia|BOLD:AAB9848
Caradrina meralis|[15415]|LBCH7402-10|10-JDWBC-7402|658[0n]bp|Canada.British Columbia|BOLD:AAB9848
Caradrina meralis|[15416]|LBCH7432-10|10-JDWBC-7432|658[0n]bp|Canada.British Columbia|BOLD:AAB9848
Caradrina meralis|[15417]|LBCH7404-10|10-JDWBC-7404|658[0n]bp|Canada.British Columbia|BOLD:AAB9848
Caradrina meralis|[15418]|LBCG2864-09|08-JDWBC-2864|658[0n]bp|Canada.British Columbia|BOLD:AAB9848
Caradrina meralis|[15419]|LBCH7400-10|10-JDWBC-7400|658[0n]bp|Canada.British Columbia|BOLD:AAB9848
Caradrina meralis|[15420]|LBCH7096-10|10-JDWBC-7096|658[0n]bp|Canada.British Columbia|BOLD:AAB9848
Caradrina meralis|[15421]|LBCH6127-10|10-JDWBC-6127|658[0n]bp|Canada.British Columbia|BOLD:AAB9848
Caradrina meralis|[15422]|LPVIB270-08|PFC-2006-1660|658[0n]bp|Canada.British Columbia|BOLD:AAB9848
Caradrina meralis|[15423]|LBCH6305-10|10-JDWBC-6305|658[0n]bp|Canada.British Columbia|BOLD:AAB9848
Caradrina meralis|[15424]|LBCH6301-10|10-JDWBC-6301|658[0n]bp|Canada.British Columbia|BOLD:AAB9848
Caradrina meralis|[15425]|LOWCD863-06|CGWC-3683|658[0n]bp|Canada.British Columbia|BOLD:AAB9848
Caradrina meralis|[15426]|LBCH7124-10|10-JDWBC-7124|636[0n]bp|Canada.British Columbia|BOLD:AAB9848
Caradrina meralis|[15427]|LPVIB269-08|PFC-2006-1659|646[0n]bp|Canada.British Columbia|BOLD:AAB9848
Caradrina meralis|[15428]|LOWCD862-06|CGWC-3682|596[0n]bp|Canada.British Columbia|BOLD:AAB9848
Caradrina meralis|[15429]|LBCH6310-10|10-JDWBC-6310|658[0n]bp|Canada.British Columbia|BOLD:AAB9848
Caradrina meralis|[15430]|LBCH6171-10|10-JDWBC-6171|658[0n]bp|Canada.British Columbia|BOLD:AAB9848
Caradrina meralis|[15431]|LBCH6173-10|10-JDWBC-6173|658[0n]bp|Canada.British Columbia|BOLD:AAB9848
Caradrina meralis|[15432]|LBCH7405-10|10-JDWBC-7405|658[0n]bp|Canada.British Columbia|BOLD:AAB9848
Caradrina meralis|[15433]|LBCH7219-10|10-JDWBC-7219|658[0n]bp|Canada.British Columbia|BOLD:AAB9848
Caradrina meralis|[15434]|LBCH6168-10|10-JDWBC-6168|658[0n]bp|Canada.British Columbia|BOLD:AAB9848
Caradrina meralis|[15435]|LBCH6175-10|10-JDWBC-6175|658[0n]bp|Canada.British Columbia|BOLD:AAB9848
Caradrina meralis|[15436]|LOWCD861-06|CGWC-3681|658[0n]bp|Canada.British Columbia|BOLD:AAB9848
Caradrina meralis|[15437]|LBCG2113-09|08-JDWBC-2113|658[0n]bp|Canada.British Columbia|BOLD:AAB9848
Caradrina meralis|[15438]|LBCH7800-10|10-JDWBC-7800|658[0n]bp|Canada.British Columbia|BOLD:AAB9848
Caradrina meralis|[15439]|LBCH7339-10|10-JDWBC-7339|658[0n]bp|Canada.British Columbia|BOLD:AAB9848
Caradrina meralis|[15440]|LBCG2114-09|08-JDWBC-2114|658[0n]bp|Canada.British Columbia|BOLD:AAB9848
Caradrina meralis|[15441]|LBCH7795-10|10-JDWBC-7795|658[0n]bp|Canada.British Columbia|BOLD:AAB9848
Caradrina meralis|[15442]|LBCH6308-10|10-JDWBC-6308|658[0n]bp|Canada.British Columbia|BOLD:AAB9848
Caradrina meralis|[15443]|LBCH7399-10|10-JDWBC-7399|658[0n]bp|Canada.British Columbia|BOLD:AAB9848
Caradrina meralis|[15444]|LBCH6302-10|10-JDWBC-6302|658[0n]bp|Canada.British Columbia|BOLD:AAB9848
Caradrina meralis|[15445]|LBCH7861-10|10-JDWBC-7861|658[0n]bp|Canada.British Columbia|BOLD:AAB9848
Caradrina meralis|[15446]|LBCH6126-10|10-JDWBC-6126|658[0n]bp|Canada.British Columbia|BOLD:AAB9848
Caradrina meralis|[15447]|LBCG498-08|08-JDWBC-0498|658[0n]bp|Canada.British Columbia|BOLD:AAB9848
Caradrina meralis|[15448]|LBCH7401-10|10-JDWBC-7401|658[0n]bp|Canada.British Columbia|BOLD:AAB9848
Caradrina meralis|[15449]|LBCH7859-10|10-JDWBC-7859|658[0n]bp|Canada.British Columbia|BOLD:AAB9848

[illegible]
Caradrina meralis|[15447]|LBCG498-08|08-JDWBC-0498|658[0n]bp|Canada.British Columbia|BOLD:AAB9848
Caradrina meralis|[15448]|LBCH7401-10|10-JDWBC-7401|658[0n]bp|Canada.British Columbia|BOLD:AAB9848
Caradrina meralis|[15449]|LBCH7859-10|10-JDWBC-7859|658[0n]bp|Canada.British Columbia|BOLD:AAB9848
Caradrina meralis|[15450]|LBCG249-08|08-JDWBC-0249|658[0n]bp|Canada.British Columbia|BOLD:AAB9848
Caradrina meralis|[15451]|LBCG2115-09|08-JDWBC-2115|658[0n]bp|Canada.British Columbia|BOLD:AAB9848
Caradrina meralis|[15452]|LBCH6304-10|10-JDWBC-6304|658[0n]bp|Canada.British Columbia|BOLD:AAB9848
Caradrina meralis|[15453]|LBCH7798-10|10-JDWBC-7798|658[0n]bp|Canada.British Columbia|BOLD:AAB9848
Caradrina meralis|[15454]|LBCH7802-10|10-JDWBC-7802|658[0n]bp|Canada.British Columbia|BOLD:AAB9848
Caradrina meralis|[15455]|LOWCD860-06|CGWC-3680|658[0n]bp|Canada.British Columbia|BOLD:AAB9848
Caradrina meralis|[15456]|LBCG2866-09|08-JDWBC-2866|658[0n]bp|Canada.British Columbia|BOLD:AAB9848
Caradrina meralis|[15457]|LBCH7398-10|10-JDWBC-7398|658[0n]bp|Canada.British Columbia|BOLD:AAB9848
Protoperigea posticata|[15458]|RDMAB591-06|UASM58597|615[0n]bp|Canada.Alberta|BOLD:AAF6359
Protoperigea posticata|[15459]|RDNMC583-06|CNCNoctuoidea12123|658[0n]bp|Canada.Alberta|BOLD:AAF6359
Proxenus mendosa|[15460]|BBLPB451-10|10BBCLP-1450|658[0n]bp|Canada.Alberta|BOLD:AAC9107
Proxenus mendosa|[15461]|BBLPF010-10|10BBCLP-3007|658[0n]bp|Canada.Alberta|BOLD:AAC9107
Proxenus mendosa|[15462]|RDMAB331-05|UASM77817|505[0n]bp|Canada.Alberta|BOLD:AAC9107
Proxenus mendosa|[15463]|LBCC284-05|HLC-22164|658[0n]bp|Canada.British Columbia|BOLD:AAC9107
Proxenus mendosa|[15464]|LBCC285-05|HLC-22165|658[0n]bp|Canada.British Columbia|BOLD:AAC9107
Proxenus mendosa|[15465]|BBLPF009-10|10BBCLP-3006|658[0n]bp|Canada.Alberta|BOLD:AAC9107
Proxenus mendosa|[15466]|RDMAB501-06|UASM58466|658[0n]bp|Canada.Alberta|BOLD:AAC9107
Proxenus mendosa|[15467]|RDMAB333-05|UASM77815|561[1n]bp|Canada.Alberta|BOLD:AAC9107
Proxenus mendosa|[15468]|LPABB434-08|08BBLEP-03699|658[0n]bp|Canada.Alberta|BOLD:AAC9107
Proxenus mendosa|[15469]|LPABB051-08|08BBLEP-03316|658[0n]bp|Canada.Alberta|BOLD:AAC9107
Proxenus mendosa|[15470]|LPABB412-08|08BBLEP-03677|658[0n]bp|Canada.Alberta|BOLD:AAC9107
Proxenus mendosa|[15471]|RDNMC735-06|CNCNoctuoidea12275|658[0n]bp|Canada.Quebec|BOLD:AAC9107
Proxenus mindara|[15472]|LOCBB257-06|06-BLLOC-1197|658[0n]bp|United States.California|BOLD:AAB9054
Proxenus mindara|[15473]|LOCBC580-06|06-BLLOC-2460|658[0n]bp|United States.California|BOLD:AAB9054
Proxenus mindara|[15474]|LOCBB256-06|06-BLLOC-1196|658[0n]bp|United States.California|BOLD:AAB9054
Proxenus mindara|[15475]|LOCBC195-06|06-BLLOC-2075|656[0n]bp|United States.California|BOLD:AAB9054
Proxenus mindara|[15476]|RDMAB325-05|UASM77823|517[1n]bp|United States.Washington|BOLD:AAB9054
Proxenus mindara|[15477]|RDNMC750-06|CNCNoctuoidea12290|658[0n]bp|United States.California|BOLD:AAB9054
Proxenus mindara|[15478]|LOCBB254-06|06-BLLOC-1194|658[0n]bp|United States.California|BOLD:AAB9054
Proxenus mindara|[15479]|LOCBD926-06|06-BLLOC-3747|658[0n]bp|United States.California|BOLD:AAB9054
Proxenus mindara|[15480]|LOCBB253-06|06-BLLOC-1193|658[0n]bp|United States.California|BOLD:AAB9054
Proxenus mindara|[15481]|LOCBC671-06|06-BLLOC-2551|658[0n]bp|United States.California|BOLD:AAB9054
Proxenus mindara|[15482]|RWWA578-09|RWWA-0596|658[0n]bp|United States.Washington|BOLD:AAB9054
Proxenus mindara|[15483]|RWWC324-11|RWWA-2301|658[0n]bp|United States.Washington|BOLD:AAB9054
Proxenus mindara|[15484]|RWWB935-10|RWWA-1934|658[0n]bp|United States.Washington|BOLD:AAB9054
Proxenus mindara|[15485]|RWWA613-09|RWWA-0631|658[0n]bp|United States.Washington|BOLD:AAB9054
Proxenus mindara|[15486]|RWWA605-09|RWWA-0623|658[0n]bp|United States.Washington|BOLD:AAB9054
Proxenus mindara|[15487]|RWWC456-11|RWWA-2433|658[0n]bp|United States.Washington|BOLD:AAB9054
Proxenus mindara|[15488]|RWWA938-09|RWWA-0938|658[0n]bp|United States.Washington|BOLD:AAB9054
Proxenus mindara|[15489]|RWWB856-10|RWWA-1855|632[0n]bp|United States.Washington|BOLD:AAB9054
Proxenus mindara|[15490]|RWWA633-09|RWWA-0651|658[0n]bp|United States.Washington|BOLD:AAB9054
Proxenus mindara|[15491]|LOCBB255-06|06-BLLOC-1195|658[0n]bp|United States.California|BOLD:AAB9054
Proxenus miranda|[15492]|LPSK468-08|08BBLEP-02036|658[0n]bp|Canada.Saskatchewan|BOLD:AAA5599
Proxenus miranda|[15493]|RDLQB072-05|DH010158|658[0n]bp|Canada.Quebec|BOLD:AAA5599
Proxenus miranda|[15494]|LPSO763-08|PPBP-0763|658[0n]bp|Canada.Ontario|BOLD:AAA5599
Proxenus miranda|[15495]|LPSO999-08|PPBP-0999|658[0n]bp|Canada.Ontario|BOLD:AAA5599
Proxenus miranda|[15496]|RDLQG314-06|DH012526|658[0n]bp|Canada.Quebec|BOLD:AAA5599
Proxenus miranda|[15497]|KPOEC048-08|08OEC-158|658[0n]bp|Canada.Ontario|BOLD:AAA5599
Proxenus miranda|[15498]|RDLQG557-06|DH012850|658[0n]bp|Canada.Quebec|BOLD:AAA5599
Proxenus miranda|[15499]|LPSO753-08|PPBP-0753|658[0n]bp|Canada.Ontario|BOLD:AAA5599
Proxenus miranda|[15500]|LPSO921-08|PPBP-0921|658[0n]bp|Canada.Ontario|BOLD:AAA5599
Proxenus miranda|[15501]|LPSOB211-08|PPBP-1210|658[0n]bp|Canada.Ontario|BOLD:AAA5599
Proxenus miranda|[15502]|LPSO770-08|PPBP-0770|658[0n]bp|Canada.Ontario|BOLD:AAA5599
Proxenus miranda|[15503]|LPSO303-08|PPBP-0303|658[0n]bp|Canada.Ontario|BOLD:AAA5599
Proxenus miranda|[15504]|LPSO690-08|PPBP-0690|658[0n]bp|Canada.Ontario|BOLD:AAA5599
Proxenus miranda|[15505]|LPSO833-08|PPBP-0833|609[0n]bp|Canada.Ontario|BOLD:AAA5599
Proxenus miranda|[15506]|LPSO302-08|PPBP-0302|658[0n]bp|Canada.Ontario|BOLD:AAA5599
Proxenus miranda|[15507]|PMG153-03|moth447.01|617[0n]bp|Canada.Ontario|BOLD:AAA5599
Proxenus miranda|[15508]|XAK274-06|2006-ONT-1269|658[0n]bp|Canada.Ontario|BOLD:AAA5599
Proxenus miranda|[15509]|RDMAB345-05|UASM77816|658[0n]bp|Canada.Alberta|BOLD:AAA5599
Proxenus miranda|[15510]|XAG079-05|2005-ONT-663|658[0n]bp|Canada.Ontario|BOLD:AAA5599
Proxenus miranda|[15511]|KPOEC144-08|08OEC-156|658[0n]bp|Canada.Ontario|BOLD:AAA5599
Proxenus miranda|[15512]|XAD282-04|04HBL007282|593[0n]bp|Canada.Ontario|BOLD:AAA5599
Proxenus miranda|[15513]|XAK540-07|HLC-16093|594[0n]bp|Canada.Ontario|BOLD:AAA5599
Proxenus miranda|[15514]|KPOEC118-08|08OEC-064|621[2n]bp|Canada.Ontario|BOLD:AAA5599
Proxenus miranda|[15515]|LPSO755-08|PPBP-0755|658[0n]bp|Canada.Ontario|BOLD:AAA5599
Proxenus miranda|[15516]|LOWCB912-05|CGWC-1852|658[0n]bp|Canada.British Columbia|BOLD:AAA5599
Proxenus miranda|[15517]|LPSO459-08|PPBP-0459|658[0n]bp|Canada.Ontario|BOLD:AAA5599
Proxenus miranda|[15518]|LPMN393-08|08BBLEP-01192|658[0n]bp|Canada.Manitoba|BOLD:AAA5599
Proxenus miranda|[15519]|LPABB536-08|08BBLEP-03801|658[0n]bp|Canada.Alberta|BOLD:AAA5599
Proxenus miranda|[15520]|LPABC880-09|08BBLEP-05291|658[0n]bp|Canada.Alberta|BOLD:AAA5599
Proxenus miranda|[15521]|LOWCB910-05|CGWC-1850|658[0n]bp|Canada.British Columbia|BOLD:AAA5599
Proxenus miranda|[15522]|LBCG3059-09|08-JDWBC-3059|658[0n]bp|Canada.British Columbia|BOLD:AAA5599
Proxenus miranda|[15523]|MECC102-06|jflandry2122|658[0n]bp|Canada.Ontario|BOLD:AAA5599
Proxenus miranda|[15524]|XAG699-05|2005-ONT-1283|658[0n]bp|Canada.Ontario|BOLD:AAA5599
Proxenus miranda|[15525]|RDLQG313-06|DH012525|658[0n]bp|Canada.Quebec|BOLD:AAA5599
Proxenus miranda|[15526]|LPABC757-09|08BBLEP-04976|658[0n]bp|Canada.Alberta|BOLD:AAA5599
Proxenus miranda|[15527]|LOWCB909-05|CGWC-1849|658[0n]bp|Canada.British Columbia|BOLD:AAA5599
Proxenus miranda|[15528]|LBCG393-08|08-JDWBC-0393|658[0n]bp|Canada.British Columbia|BOLD:AAA5599
Proxenus miranda|[15529]|LPSO301-08|PPBP-0301|658[0n]bp|Canada.Ontario|BOLD:AAA5599
Proxenus miranda|[15530]|RDMAB326-05|UASM77822|658[0n]bp|Canada.Alberta|BOLD:AAA5599
Proxenus miranda|[15531]|LBCG1881-09|08-JDWBC-1881|658[0n]bp|Canada.British Columbia|BOLD:AAA5599
Proxenus miranda|[15532]|LPMN944-08|08BBLEP-02302|658[0n]bp|Canada.Alberta|BOLD:AAA5599
Proxenus miranda|[15533]|LBCG2470-09|08-JDWBC-2470|658[0n]bp|Canada.British Columbia|BOLD:AAA5599
Proxenus miranda|[15534]|LPMN335-08|08BBLEP-01134|658[0n]bp|Canada.Manitoba|BOLD:AAA5599
Proxenus miranda|[15535]|LOWCB911-05|CGWC-1851|658[0n]bp|Canada.British Columbia|BOLD:AAA5599
Proxenus miranda|[15536]|RDLQB071-05|DH010157|658[0n]bp|Canada.Quebec|BOLD:AAA5599
Proxenus miranda|[15537]|RDMAB115-05|UASM41255|658[0n]bp|Canada.Alberta|BOLD:AAA5599
Proxenus miranda|[15538]|KPOEC095-08|08OEC-256|658[0n]bp|Canada.Ontario|BOLD:AAA5599
Proxenus miranda|[15539]|BBLPB442-10|10BBCLP-1441|658[0n]bp|Canada.Ontario|BOLD:AAA5599
Proxenus miranda|[15540]|LPAB105-08|08BBLEP-02427|658[1n]bp|Canada.Alberta|BOLD:AAA5599
Proxenus miranda|[15541]|LPMN949-08|08BBLEP-02307|658[0n]bp|Canada.Alberta|BOLD:AAA5599
Proxenus miranda|[15542]|LOWCB914-05|CGWC-1854|585[0n]bp|Canada.British Columbia|BOLD:AAA5599
Proxenus miranda|[15543]|RDLQB070-05|DH010156|571[0n]bp|Canada.Quebec|BOLD:AAA5599
Proxenus miranda|[15544]|XAK564-07|HLC-16117|584[0n]bp|Canada.Ontario|BOLD:AAA5599
Proxenus miranda|[15545]|RDMAB330-05|UASM77819|558[0n]bp|Canada.Alberta|BOLD:AAA5599
Proxenus miranda|[15546]|LOWCB907-05|CGWC-1847|589[0n]bp|Canada.British Columbia|BOLD:AAA5599
Proxenus miranda|[15547]|RDMAB320-05|UASM77825|517[0n]bp|Canada.Alberta|BOLD:AAA5599
Proxenus miranda|[15548]|LOWCB913-05|CGWC-1853|585[0n]bp|Canada.British Columbia|BOLD:AAA5599
[illegible]

Proxenus miranda|[15546]|LOWCB907-05|CGWC-1847|589[0n]bp|Canada.British Columbia|BOLD:AAA5599
Proxenus miranda|[15547]|RDMAB320-05|UASM77825|517[0n]bp|Canada.Alberta|BOLD:AAA5599
Proxenus miranda|[15548]|LOWCB913-05|CGWC-1853|585[0n]bp|Canada.British Columbia|BOLD:AAA5599
Proxenus miranda|[15549]|LOWCD300-06|CGWC-3120|654[0n]bp|Canada.British Columbia|BOLD:AAA5599
Proxenus miranda|[15550]|LOWCB915-05|CGWC-1855|658[0n]bp|Canada.British Columbia|BOLD:AAA5599
Proxenus miranda|[15551]|LBCG391-08|08-JDWBC-0391|658[0n]bp|Canada.British Columbia|BOLD:AAA5599
Proxenus miranda|[15552]|RDLQG556-06|DH012849|658[0n]bp|Canada.Quebec|BOLD:AAA5599
Proxenus miranda|[15553]|XAH557-05|2005-ONT-2140|658[0n]bp|Canada.Ontario|BOLD:AAA5599
Proxenus miranda|[15554]|LPAB095-08|08BBLEP-02417|658[0n]bp|Canada.Alberta|BOLD:AAA5599
Proxenus miranda|[15555]|LOWCB906-05|CGWC-1846|658[0n]bp|Canada.British Columbia|BOLD:AAA5599
Phosphila miselioides|[15556]|LPSO745-08|PPBP-0745|658[0n]bp|Canada.Ontario|BOLD:AAB2662
Phosphila miselioides|[15557]|LPSO883-08|PPBP-0883|658[0n]bp|Canada.Ontario|BOLD:AAB2662
Phosphila miselioides|[15558]|LPSOB081-08|PPBP-1080|658[0n]bp|Canada.Ontario|BOLD:AAB2662
Phosphila miselioides|[15559]|LPSO343-08|PPBP-0343|658[0n]bp|Canada.Ontario|BOLD:AAB2662
Psectraglaea carnosa|[15560]|RDNMF223-08|NOC14309|658[0n]bp|Canada.Ontario|BOLD:AAE0651
Psectraglaea carnosa|[15561]|RDNMF225-08|NOC14311|658[0n]bp|Canada.Ontario|BOLD:AAE0651
Psectraglaea carnosa|[15562]|RDNMF221-08|NOC14307|658[0n]bp|Canada.Ontario|BOLD:AAE0651
Psectraglaea carnosa|[15563]|RDNMF224-08|NOC14310|658[0n]bp|Canada.Ontario|BOLD:AAE0651
Psectraglaea carnosa|[15564]|RDNMF222-08|NOC14308|658[0n]bp|Canada.Ontario|BOLD:AAE0651
Apamea cinefacta|[15565]|RDNM962-05|CNCNoctuoidea7802|558[0n]bp|Canada.British Columbia|BOLD:AAD3412
Apamea inordinata|[15566]|LBCH5257-10|10-JDWBC-5257|658[0n]bp|Canada.British Columbia|BOLD:AAC7156
Apamea inordinata|[15567]|LBCH5351-10|10-JDWBC-5351|658[0n]bp|Canada.British Columbia|BOLD:AAC7156
Apamea inordinata|[15568]|LBCH5256-10|10-JDWBC-5256|658[0n]bp|Canada.British Columbia|BOLD:AAC7156
Apamea inordinata|[15569]|RDNMD240-06|CNCNoctuoidea12572|658[0n]bp|Canada.Ontario|BOLD:AAC7156
Apamea inordinata|[15570]|LBCH5353-10|10-JDWBC-5353|658[0n]bp|Canada.British Columbia|BOLD:AAC7156
Apamea inordinata|[15571]|LBCG342-08|08-JDWBC-0342|658[0n]bp|Canada.British Columbia|BOLD:AAC7156
Apamea inordinata|[15572]|LBCG340-08|08-JDWBC-0340|658[0n]bp|Canada.British Columbia|BOLD:AAC7156
Apamea inordinata|[15573]|LBCH5356-10|10-JDWBC-5356|658[0n]bp|Canada.British Columbia|BOLD:AAC7156
Apamea inordinata|[15574]|LBCH5050-10|10-JDWBC-5050|658[0n]bp|Canada.British Columbia|BOLD:AAC7156
Apamea inordinata|[15575]|LBCH5261-10|10-JDWBC-5261|658[0n]bp|Canada.British Columbia|BOLD:AAC7156
Apamea inordinata|[15576]|RDMAB446-05|BCSC119|658[0n]bp|Canada.Alberta|BOLD:AAC7156
Apamea inordinata|[15577]|LBCH5349-10|10-JDWBC-5349|658[0n]bp|Canada.British Columbia|BOLD:AAC7156
Apamea inordinata|[15578]|LBCH5352-10|10-JDWBC-5352|658[0n]bp|Canada.British Columbia|BOLD:AAC7156
Apamea inordinata|[15579]|LBCH5350-10|10-JDWBC-5350|658[0n]bp|Canada.British Columbia|BOLD:AAC7156
Apamea inordinata|[15580]|LBCH5049-10|10-JDWBC-5049|658[0n]bp|Canada.British Columbia|BOLD:AAC7156
Apamea inordinata|[15581]|LBCH5354-10|10-JDWBC-5354|658[0n]bp|Canada.British Columbia|BOLD:AAC7156
Apamea inordinata|[15582]|LBCH5260-10|10-JDWBC-5260|658[0n]bp|Canada.British Columbia|BOLD:AAC7156
Apamea inordinata|[15583]|LBCH5355-10|10-JDWBC-5355|658[0n]bp|Canada.British Columbia|BOLD:AAC7156
Apamea inordinata|[15584]|LBCH5258-10|10-JDWBC-5258|642[0n]bp|Canada.British Columbia|BOLD:AAC7156
Apamea inordinata|[15585]|LBCH5259-10|10-JDWBC-5259|641[0n]bp|Canada.British Columbia|BOLD:AAC7156
Apamea inordinata|[15586]|LBCH5048-10|10-JDWBC-5048|658[0n]bp|Canada.British Columbia|BOLD:AAC7156
Apamea inordinata|[15587]|LBCH5263-10|10-JDWBC-5263|658[0n]bp|Canada.British Columbia|BOLD:AAC7156
Apamea inordinata|[15588]|RDNMD241-06|CNCNoctuoidea12573|658[0n]bp|Canada.Ontario|BOLD:AAC7156
Apamea inordinata|[15589]|LBCH5262-10|10-JDWBC-5262|658[0n]bp|Canada.British Columbia|BOLD:AAC7156
Apamea spaldingi|[15590]|LBCH5283-10|10-JDWBC-5283|658[0n]bp|Canada.British Columbia|BOLD:AAD3063
Apamea spaldingi|[15591]|LBCH5046-10|10-JDWBC-5046|658[0n]bp|Canada.British Columbia|BOLD:AAD3063
Apamea spaldingi|[15592]|RDMAB487-06|UASM77906|634[0n]bp|Canada.Alberta|BOLD:AAD3063
Apamea spaldingi|[15593]|LBCH5517-10|10-JDWBC-5517|658[0n]bp|Canada.British Columbia|BOLD:AAD3063
Apamea spaldingi|[15594]|LBCH5045-10|10-JDWBC-5045|658[0n]bp|Canada.British Columbia|BOLD:AAD3063
Apamea spaldingi|[15595]|LBCH5042-10|10-JDWBC-5042|658[0n]bp|Canada.British Columbia|BOLD:AAD3063
Apamea spaldingi|[15596]|LBCH5348-10|10-JDWBC-5348|658[0n]bp|Canada.British Columbia|BOLD:AAD3063
Apamea spaldingi|[15597]|LBCH5041-10|10-JDWBC-5041|658[0n]bp|Canada.British Columbia|BOLD:AAD3063
Apamea spaldingi|[15598]|LBCH5047-10|10-JDWBC-5047|658[0n]bp|Canada.British Columbia|BOLD:AAD3063
Apamea spaldingi|[15599]|LBCH5043-10|10-JDWBC-5043|658[0n]bp|Canada.British Columbia|BOLD:AAD3063
Apamea spaldingi|[15600]|LBCH5044-10|10-JDWBC-5044|658[0n]bp|Canada.British Columbia|BOLD:AAD3063
Apamea spaldingi|[15601]|LBCH5421-10|10-JDWBC-5421|658[0n]bp|Canada.British Columbia|BOLD:AAD3063
Apamea spaldingi|[15602]|LBCH5040-10|10-JDWBC-5040|658[0n]bp|Canada.British Columbia|BOLD:AAD3063
Capsula subflava|[15603]|RDMAB558-06|UASM58537|658[0n]bp|Canada.Alberta|BOLD:AAC6808
Capsula subflava|[15604]|RDNMB023-05|CNCNoctuoidea7863|658[0n]bp|Canada.Ontario|BOLD:AAC6807
Capsula subflava|[15605]|LBCH6522-10|10-JDWBC-6522|658[0n]bp|Canada.British Columbia|BOLD:AAC6807
Capsula subflava|[15606]|RDMAB557-06|UASM58536|658[0n]bp|Canada.Alberta|BOLD:AAC6807
Capsula subflava|[15607]|RDNMB021-05|CNCNoctuoidea7861|658[0n]bp|Canada.Ontario|BOLD:AAC6807
Capsula subflava|[15608]|RDNMC028-05|CNCNoctuoidea10769|658[0n]bp|Canada.Ontario|BOLD:AAC6807
Capsula subflava|[15609]|RDNMB022-05|CNCNoctuoidea7862|658[0n]bp|Canada.Ontario|BOLD:AAC6807
Capsula subflava|[15610]|RDNMC029-05|CNCNoctuoidea10770|658[0n]bp|Canada.Ontario|BOLD:AAC6807
Capsula subflava|[15611]|RDNMB024-05|CNCNoctuoidea7864|658[0n]bp|Canada.Ontario|BOLD:AAC6807
Litholomia napaea|[15612]|LOWCE030-06|CGWC-3790|596[2n]bp|Canada.British Columbia|BOLD:AAB0068
Litholomia napaea|[15613]|TMNBB232-06|MNBTT-1172|658[0n]bp|Canada.New Brunswick|BOLD:AAB0068
Litholomia napaea|[15614]|RDLQ505-07|DH006187|608[0n]bp|Canada.Quebec|BOLD:AAB0068
Litholomia napaea|[15615]|TMNBB231-06|MNBTT-1171|658[0n]bp|Canada.New Brunswick|BOLD:AAB0068
Litholomia napaea|[15616]|TMNBB236-06|MNBTT-1176|658[0n]bp|Canada.New Brunswick|BOLD:AAB0068
Litholomia napaea|[15617]|TMNBB233-06|MNBTT-1173|631[0n]bp|Canada.New Brunswick|BOLD:AAB0068
Litholomia napaea|[15618]|TMNBB235-06|MNBTT-1175|658[0n]bp|Canada.New Brunswick|BOLD:AAB0068
Litholomia napaea|[15619]|TMNBB230-06|MNBTT-1170|658[0n]bp|Canada.New Brunswick|BOLD:AAB0068
Litholomia napaea|[15620]|TMNBB237-06|MNBTT-1177|658[0n]bp|Canada.New Brunswick|BOLD:AAB0068
Litholomia napaea|[15621]|TMNBB234-06|MNBTT-1174|656[0n]bp|Canada.New Brunswick|BOLD:AAB0068
Litholomia napaea|[15622]|LOWCD939-06|CGWC-3759|658[0n]bp|Canada.British Columbia|BOLD:AAB0068
Litholomia napaea|[15623]|LOWCE001-06|CGWC-3761|609[0n]bp|Canada.British Columbia|BOLD:AAB0068
Litholomia napaea|[15624]|LOWCE032-06|CGWC-3792|658[0n]bp|Canada.British Columbia|BOLD:AAB0068
Litholomia napaea|[15625]|LBCG271-08|08-JDWBC-0271|658[0n]bp|Canada.British Columbia|BOLD:AAB0068
Litholomia napaea|[15626]|LALPA013-10|AVBC 013-10|658[0n]bp|Canada.British Columbia|BOLD:AAB0068
Litholomia napaea|[15627]|LOWCE006-06|CGWC-3766|658[0n]bp|Canada.British Columbia|BOLD:AAB0068
Litholomia napaea|[15628]|LOWCE375-06|CGWC-4135|658[0n]bp|Canada.British Columbia|BOLD:AAB0068
Litholomia napaea|[15629]|LOWCE007-06|CGWC-3767|658[0n]bp|Canada.British Columbia|BOLD:AAB0068
Litholomia napaea|[15630]|LOWCD937-06|CGWC-3757|658[0n]bp|Canada.British Columbia|BOLD:AAB0068
Litholomia napaea|[15631]|LOWCE033-06|CGWC-3793|658[0n]bp|Canada.British Columbia|BOLD:AAB0068
Litholomia napaea|[15632]|LALPA076-10|AVBC 076-10|658[0n]bp|Canada.British Columbia|BOLD:AAB0068
Litholomia napaea|[15633]|LOWCD938-06|CGWC-3758|658[0n]bp|Canada.British Columbia|BOLD:AAB0068
Litholomia napaea|[15634]|LOWCE003-06|CGWC-3763|658[0n]bp|Canada.British Columbia|BOLD:AAB0068
Litholomia napaea|[15635]|LOWCE244-06|CGWC-4004|658[0n]bp|Canada.British Columbia|BOLD:AAB0068
Litholomia napaea|[15636]|LOWCE241-06|CGWC-4001|658[0n]bp|Canada.British Columbia|BOLD:AAB0068
Litholomia napaea|[15637]|LBCH1241-10|10-JDWBC-1241|658[0n]bp|Canada.British Columbia|BOLD:AAB0068
Litholomia napaea|[15638]|LOWCE004-06|CGWC-3764|658[0n]bp|Canada.British Columbia|BOLD:AAB0068
Litholomia napaea|[15639]|LOWCE506-06|CGWC-4266|620[0n]bp|Canada.British Columbia|BOLD:AAB0068
Litholomia napaea|[15640]|LOWCE034-06|CGWC-3794|658[0n]bp|Canada.British Columbia|BOLD:AAB0068
Litholomia napaea|[15641]|LOWCE002-06|CGWC-3762|658[0n]bp|Canada.British Columbia|BOLD:AAB0068
Litholomia napaea|[15642]|LOWCE373-06|CGWC-4133|658[0n]bp|Canada.British Columbia|BOLD:AAB0068
Litholomia napaea|[15643]|LALPA032-10|AVBC 032-10|658[0n]bp|Canada.British Columbia|BOLD:AAB0068
Litholomia napaea|[15644]|LOWCE031-06|CGWC-3791|658[0n]bp|Canada.British Columbia|BOLD:AAB0068
Litholomia napaea|[15645]|LOWCB862-05|CGWC-1802|658[0n]bp|Canada.British Columbia|BOLD:AAB0068
Litholomia napaea|[15646]|LOWCE480-06|CGWC-4240|588[0n]bp|Canada.British Columbia|BOLD:AAB0068
Oligia minuscula|[15647]|TTMNB343-06|MNBTT-343|526[1n]bp|Canada.New Brunswick|BOLD:AAB2890
Oligia minuscula|[15648]|TTMNB346-06|MNBTT-346|558[3n]bp|Canada.New Brunswick|BOLD:AAB2890

Litholomia napaea|[15646]|LOWCE480-06|CGWC-4240|588[0n]bp|Canada.British Columbia|BOLD:AAB0068
Oligia minuscula|[15647]|TTMNB343-06|MNBTT-343|526[1n]bp|Canada.New Brunswick|BOLD:AAB2890
Oligia minuscula|[15648]|TTMNB346-06|MNBTT-346|558[3n]bp|Canada.New Brunswick|BOLD:AAB2890
Oligia minuscula|[15649]|TMNBB183-06|MNBTT-1123|658[0n]bp|Canada.New Brunswick|BOLD:AAB2890
Oligia minuscula|[15650]|TMNBB179-06|MNBTT-1119|658[0n]bp|Canada.New Brunswick|BOLD:AAB2890
Oligia minuscula|[15651]|TTMNB341-06|MNBTT-341|658[0n]bp|Canada.New Brunswick|BOLD:AAB2890
Oligia minuscula|[15652]|TTMNB345-06|MNBTT-345|604[0n]bp|Canada.New Brunswick|BOLD:AAB2890
Oligia minuscula|[15653]|TTMNB352-06|MNBTT-352|658[1n]bp|Canada.New Brunswick|BOLD:AAB2890
Oligia minuscula|[15654]|TMNBB178-06|MNBTT-1118|658[0n]bp|Canada.New Brunswick|BOLD:AAB2890
Oligia minuscula|[15655]|TTMNB348-06|MNBTT-348|658[1n]bp|Canada.New Brunswick|BOLD:AAB2890
Oligia minuscula|[15656]|TTMNB350-06|MNBTT-350|613[1n]bp|Canada.New Brunswick|BOLD:AAB2890
Oligia minuscula|[15657]|TTMNB349-06|MNBTT-349|658[5n]bp|Canada.New Brunswick|BOLD:AAB2890
Oligia minuscula|[15658]|RDLQB773-05|DH010860|618[0n]bp|Canada.Quebec|BOLD:AAB2890
Oligia minuscula|[15659]|TMNBB181-06|MNBTT-1121|658[0n]bp|Canada.New Brunswick|BOLD:AAB2890
Oligia minuscula|[15660]|RDLQB769-05|DH010856|617[0n]bp|Canada.Quebec|BOLD:AAB2890
Oligia minuscula|[15661]|TMNBB182-06|MNBTT-1122|658[0n]bp|Canada.New Brunswick|BOLD:AAB2890
Oligia minuscula|[15662]|TTMNB344-06|MNBTT-344|614[1n]bp|Canada.New Brunswick|BOLD:AAB2890
Oligia minuscula|[15663]|TMNBB180-06|MNBTT-1120|658[0n]bp|Canada.New Brunswick|BOLD:AAB2890
Oligia minuscula|[15664]|TTMNB347-06|MNBTT-347|658[6n]bp|Canada.New Brunswick|BOLD:AAB2890
Oligia minuscula|[15665]|TTMNB351-06|MNBTT-351|658[2n]bp|Canada.New Brunswick|BOLD:AAB2890
Oligia minuscula|[15666]|TTMNB342-06|MNBTT-342|658[1n]bp|Canada.New Brunswick|BOLD:AAB2890
Oligia minuscula|[15667]|RDLQB516-05|DH010602|658[0n]bp|Canada.Quebec|BOLD:AAB2890
Oligia minuscula|[15668]|RDLQF312-06|DH011412|658[0n]bp|Canada.Quebec|BOLD:AAB2890
Oligia minuscula|[15669]|RDLQB817-05|DH010904|658[0n]bp|Canada.Quebec|BOLD:AAB2890
Oligia minuscula|[15670]|RDLQB774-05|DH010861|658[0n]bp|Canada.Quebec|BOLD:AAB2890
Oligia minuscula|[15671]|RDLQB763-05|DH010850|658[0n]bp|Canada.Quebec|BOLD:AAB2890
Oligia minuscula|[15672]|TMNBB177-06|MNBTT-1117|658[0n]bp|Canada.New Brunswick|BOLD:AAB2890
Apamea occidens|[15673]|LBCH6626-10|10-JDWBC-6626|658[0n]bp|Canada.British Columbia|BOLD:AAE2925
Apamea occidens|[15674]|LBCH6006-10|10-JDWBC-6006|658[0n]bp|Canada.British Columbia|BOLD:AAE2925
Apamea occidens|[15675]|RDNMC219-05|CNCNoctuoidea11853|658[0n]bp|Canada.British Columbia|BOLD:AAE2925
Capsula laeta|[15676]|RDNMC031-05|CNCNoctuoidea10772|658[0n]bp|Canada.Ontario|BOLD:ABY6766
Capsula laeta|[15677]|RDNMG906-08|CNC LEP00053030|658[0n]bp|Canada.Ontario|BOLD:ABY6766
Capsula laeta|[15678]|RDNMG905-08|CNC LEP00053029|658[0n]bp|Canada.Ontario|BOLD:ABY6766
Capsula oblonga|[15679]|TTMNB354-06|MNBTT-354|658[1n]bp|Canada.New Brunswick|BOLD:AAB3171
Capsula oblonga|[15680]|RDLQB840-05|DH010927|617[0n]bp|Canada.Quebec|BOLD:AAB3171
Capsula oblonga|[15681]|RDLQB837-05|DH010924|658[0n]bp|Canada.Quebec|BOLD:AAB3171
Capsula oblonga|[15682]|RDLQF555-06|DH011704|658[0n]bp|Canada.Quebec|BOLD:AAB3171
Capsula oblonga|[15683]|RDLQF556-06|DH011705|658[0n]bp|Canada.Quebec|BOLD:AAB3171
Capsula oblonga|[15684]|RDLQF557-06|DH011706|658[0n]bp|Canada.Quebec|BOLD:AAB3171
Capsula oblonga|[15685]|RDLQF550-06|DH011699|658[0n]bp|Canada.Quebec|BOLD:AAB3171
Capsula oblonga|[15686]|RDNMC030-05|CNCNoctuoidea10771|658[0n]bp|Canada.Ontario|BOLD:AAB3171
Capsula oblonga|[15687]|XAD100-04|04HBL007100|579[0n]bp|Canada.Ontario|BOLD:AAB3171
Capsula oblonga|[15688]|LBCW041-08|08-JDWWI-0041|658[0n]bp|Canada.British Columbia|BOLD:AAB3171
Capsula oblonga|[15689]|XAD356-04|04HBL007356|658[0n]bp|Canada.Ontario|BOLD:AAB3171
Capsula oblonga|[15690]|LBCW040-08|08-JDWWI-0040|658[0n]bp|Canada.British Columbia|BOLD:AAB3171
Capsula oblonga|[15691]|LPGVA633-08|UBC-2006-1801|658[0n]bp|Canada.British Columbia|BOLD:AAB3171
Capsula oblonga|[15692]|LPGVA632-08|UBC-2006-1800|658[0n]bp|Canada.British Columbia|BOLD:AAB3171
Capsula oblonga|[15693]|RDLQB500-05|DH010586|658[0n]bp|Canada.Quebec|BOLD:AAB3171
Apamea alia|[15694]|RDLQF049-06|DH002853|658[2n]bp|Canada.Quebec|BOLD:AAB7612
Apamea alia|[15695]|LPSK506-08|08BBLEP-02074|658[0n]bp|Canada.Saskatchewan|BOLD:AAB7612
Apamea alia|[15696]|LPVIB864-08|PFC-2006-2378|658[0n]bp|Canada.British Columbia|BOLD:AAB7612
Apamea alia|[15697]|LALPA384-10|AVBC 386-10|658[0n]bp|Canada.British Columbia|BOLD:AAB7612
Apamea alia|[15698]|LALPA200-10|AVBC 201-10|658[0n]bp|Canada.British Columbia|BOLD:AAB7612
Apamea alia|[15699]|LPVIB851-08|PFC-2006-2360|658[0n]bp|Canada.British Columbia|BOLD:AAB7612
Apamea alia|[15700]|LALPA369-10|AVBC 371-10|658[0n]bp|Canada.British Columbia|BOLD:AAB7612
Apamea alia|[15701]|LALPA201-10|AVBC 202-10|658[0n]bp|Canada.British Columbia|BOLD:AAB7612
Apamea alia|[15702]|LPVIB847-08|PFC-2006-2354|658[0n]bp|Canada.British Columbia|BOLD:AAB7612
Apamea alia|[15703]|LPVIA297-08|PFC-2006-0388|658[0n]bp|Canada.British Columbia|BOLD:AAB7612
Apamea alia|[15704]|LPMN793-08|08BBLEP-01596|658[0n]bp|Canada.Manitoba|BOLD:AAB7612
Apamea alia|[15705]|LPMN579-08|08BBLEP-01380|658[0n]bp|Canada.Manitoba|BOLD:AAB7612
Apamea alia|[15706]|LPSK034-08|08BBLEP-00737|658[0n]bp|Canada.Saskatchewan|BOLD:AAB7612
Apamea alia|[15707]|XAB636-04|04HBL005636|658[0n]bp|Canada.Ontario|BOLD:AAB7612
Apamea alia|[15708]|RDLQF048-06|DH005122|658[0n]bp|Canada.Quebec|BOLD:AAB7612
Apamea alia|[15709]|XAC297-04|04HBL006297|658[0n]bp|Canada.Ontario|BOLD:AAB7612
Apamea alia|[15710]|XAF583-05|2005-ONT-232|658[0n]bp|Canada.Ontario|BOLD:AAB7612
Apamea alia|[15711]|LOWCD279-06|CGWC-3099|578[0n]bp|Canada.British Columbia|BOLD:AAB7612
Apamea alia|[15712]|PHMO112-03|moth686.02|639[1n]bp|Canada.Ontario|BOLD:AAB7612
Apamea alia|[15713]|PHMO210-03|moth1068.01|639[0n]bp|Canada.Ontario|BOLD:AAB7612
Apamea alia|[15714]|BBLPB635-10|10BBCLP-1634|658[0n]bp|Canada.British Columbia|BOLD:AAB7612
Apamea alia|[15715]|LPABC330-09|08BBLEP-04549|650[0n]bp|Canada.Alberta|BOLD:AAB7612
Apamea alia|[15716]|RDLQB693-05|DH010796|658[0n]bp|Canada.Quebec|BOLD:AAB7612
Apamea alia|[15717]|LBCA508-05|HLC-20508|658[0n]bp|Canada.British Columbia|BOLD:AAB7612
Apamea alia|[15718]|LOWCD280-06|CGWC-3100|658[0n]bp|Canada.British Columbia|BOLD:AAB7612
Apamea alia|[15719]|LOWCC850-05|CGWC-2730|580[3n]bp|Canada.British Columbia|BOLD:AAB7612
Macronoctua onusta|[15720]|XAD469-04|04HBL007469|658[0n]bp|Canada.Ontario|BOLD:AAD1618
Macronoctua onusta|[15721]|PHMO247-03|moth1400.01|639[0n]bp|Canada.Ontario|BOLD:AAD1618
Macronoctua onusta|[15722]|RDLQF243-06|DH011323|658[0n]bp|Canada.Quebec|BOLD:AAD1618
Macronoctua onusta|[15723]|XAH730-05|2005-ONT-2313|658[0n]bp|Canada.Ontario|BOLD:AAD1618
Macronoctua onusta|[15724]|RDLQF242-06|DH011322|658[0n]bp|Canada.Quebec|BOLD:AAD1618
Macronoctua onusta|[15725]|PHMO335-03|moth2605.02|639[0n]bp|Canada.Ontario|BOLD:AAD1618
Macronoctua onusta|[15726]|XAH563-05|2005-ONT-2146|580[0n]bp|Canada.Ontario|BOLD:AAD1618
Apamea indocilis|[15727]|RDNMD265-06|CNCNoctuoidea12597|658[0n]bp|Canada.New Brunswick|BOLD:ACE8841
Apamea indocilis|[15728]|LBCH234-10|10-JDWBC-0234|658[0n]bp|Canada.British Columbia|BOLD:ACE8841
Apamea indocilis|[15729]|LBCA495-05|HLC-20495|658[0n]bp|Canada.British Columbia|BOLD:ACE8841
Apamea indocilis|[15730]|RDNMD266-06|CNCNoctuoidea12598|654[0n]bp|Canada.British Columbia|BOLD:ACE8841
Apamea indocilis|[15731]|RDNMC507-06|CNCNoctuoidea12329|532[0n]bp|Canada.Ontario|BOLD:ACE8841
Apamea indocilis|[15732]|TTMNB329-06|MNBTT-329|622[0n]bp|Canada.New Brunswick|BOLD:ACE8841
Apamea indocilis|[15733]|RDNMC246-05|CNCNoctuoidea11880|603[0n]bp|Canada.British Columbia|BOLD:ACE8841
Apamea indocilis|[15734]|RDNM947-05|CNCNoctuoidea7787|543[0n]bp|Canada.British Columbia|BOLD:ACE8841
Apamea nr. indocilis|[15735]|RDNMC245-05|CNCNoctuoidea11879|658[0n]bp|Canada.Ontario|BOLD:ACE8841
Apamea nr. indocilis|[15736]|LCHQ652-08|07WNP-10544|658[0n]bp|Canada.Manitoba|BOLD:ACE8841
Apamea nr. indocilis|[15737]|RDMAB1046-09|UASM130020|619[0n]bp|Canada.British Columbia|BOLD:ACE8841
Apamea nr. indocilis|[15738]|LALPA235-10|AVBC 236-10|658[0n]bp|Canada.British Columbia|BOLD:ACE8841
Apamea nr. indocilis|[15739]|LALPA291-10|AVBC 293-10|658[0n]bp|Canada.British Columbia|BOLD:ACE8841
Apamea nr. indocilis|[15740]|RDMAB1047-09|UASM130018|632[0n]bp|Canada.Alberta|BOLD:ACE8841
Apamea remissa|[15741]|LCHQ187-07|07PROBE-10964|658[0n]bp|Canada.Manitoba|BOLD:AAB1790
Apamea impulsa|[15742]|RDLQB077-05|DH010163|658[0n]bp|Canada.Quebec|BOLD:AAD5601
Apamea impulsa|[15743]|LPABC415-09|08BBLEP-04634|658[0n]bp|Canada.Alberta|BOLD:AAD5601
Apamea impulsa|[15744]|PHMNB190-04|04HBL007655|616[0n]bp|Canada.New Brunswick|BOLD:AAD5601
Apamea impulsa|[15745]|RDLQB078-05|DH010164|658[0n]bp|Canada.Quebec|BOLD:AAD5601
Apamea impulsa|[15746]|BBLPC672-09|09BBELE-1672|658[0n]bp|Canada.Newfoundland and Labrador|BOLD:AAD5601
Apamea impulsa|[15747]|LBCH6737-10|10-JDWBC-6737|658[0n]bp|Canada.British Columbia|BOLD:AAD5601
Apamea impulsa|[15748]|BBLPC673-09|09BBELE-1673|658[0n]bp|Canada.Newfoundland and Labrador|BOLD:AAD5601

[illegible]
Apamea impulsa|[15746]|BBLPC672-09|09BBELE-1672|658[0n]bp|Canada.Newfoundland and Labrador|BOLD:AAD5601
Apamea impulsa|[15747]|LBCH6737-10|10-JDWBC-6737|658[0n]bp|Canada.British Columbia|BOLD:AAD5601
Apamea impulsa|[15748]|BBLPC673-09|09BBELE-1673|658[0n]bp|Canada.Newfoundland and Labrador|BOLD:AAD5601
Apamea unita|[15749]|RDNMF078-08|NOC14164|658[0n]bp|United States.Colorado|BOLD:AAJ1441
Apamea unita|[15750]|RDNMG971-08|CNC LEP00053095|592[0n]bp|United States.Colorado|BOLD:AAJ1441
Apamea cuculliformis|[15751]|LOCBC287-06|06-BLLOC-2167|658[0n]bp|United States.California|BOLD:AAF1821
Apamea cuculliformis|[15752]|NAMUM153-08|RR-95-0101|658[0n]bp|United States.California|BOLD:AAF1821
Apamea cuculliformis|[15753]|GMLC649-11|2011GM-0345|658[0n]bp|United States.California|BOLD:AAF1821
Apamea cuculliformis|[15754]|RDNMC677-06|CNCNoctuoidea12217|658[0n]bp|United States.California|BOLD:AA...
Apamea cuculliformis|[15755]|GMLC1081-12|2011GM-0777|658[0n]bp|United States.California|BOLD:AAF1821
Apamea cuculliformis|[15756]|GMLC1427-12|2012GM-0158|614[0n]bp|United States.California|BOLD:AAF1821
Apamea cuculliformis|[15757]|GMLC1423-12|2012GM-0154|614[0n]bp|United States.California|BOLD:AAF1821
Apamea cuculliformis|[15758]|GMLC1119-12|2011GM-0815|658[0n]bp|United States.California|BOLD:AAF1821
Apamea sordens|[15759]|RDNMB017-05|CNCNoctuoidea7857|524[0n]bp|Canada.British Columbia|BOLD:AAA4201
Apamea sordens|[15760]|LBCA503-05|HLC-20503|658[0n]bp|Canada.British Columbia|BOLD:AAA4201
Apamea sordens|[15761]|LALPA217-10|AVBC 218-10|658[0n]bp|Canada.British Columbia|BOLD:AAA4201
Apamea sordens|[15762]|LALPA301-10|AVBC 303-10|658[0n]bp|Canada.British Columbia|BOLD:AAA4201
Apamea sordens|[15763]|RDMAB018-05|UASM57563|576[0n]bp|Canada.Alberta|BOLD:AAA4201
Apamea sordens|[15764]|LPVIB914-08|PFC-2006-2445|642[0n]bp|Canada.British Columbia|BOLD:AAA4201
Apamea sordens|[15765]|RDNM964-05|CNCNoctuoidea7804|557[0n]bp|Canada.British Columbia|BOLD:AAA4201
Apamea sordens|[15766]|LBCG104-08|08-JDWBC-0104|658[0n]bp|Canada.British Columbia|BOLD:AAA4201
Apamea sordens|[15767]|RDMAB437-05|BCSC110|658[0n]bp|Canada.Yukon Territory|BOLD:AAA4201
Apamea sordens|[15768]|RDNMC010-05|CNCNoctuoidea10751|658[0n]bp|Canada.Alberta|BOLD:AAA4201
Apamea sordens|[15769]|RDNMC008-05|CNCNoctuoidea10749|658[0n]bp|Canada.Alberta|BOLD:AAA4201
Apamea sordens|[15770]|BBLPB690-10|10BBCLP-1689|658[0n]bp|Canada.Alberta|BOLD:AAA4201
Apamea sordens|[15771]|RDNMC007-05|CNCNoctuoidea10748|658[0n]bp|Canada.Alberta|BOLD:AAA4201
Apamea sordens|[15772]|RDLQF016-06|DH004811|658[0n]bp|Canada.Quebec|BOLD:AAA4201
Apamea sordens|[15773]|RDNM965-05|CNCNoctuoidea7805|658[0n]bp|Canada.British Columbia|BOLD:AAA4201
Apamea sordens|[15774]|RDLQF011-06|DH008130|579[3n]bp|Canada.Quebec|BOLD:AAA4201
Apamea sordens|[15775]|LPSOB973-08|PPBP-1972|658[0n]bp|Canada.Ontario|BOLD:AAA4201
Apamea sordens|[15776]|XAI062-05|0102-ONT-0062|508[1n]bp|Canada.Ontario|BOLD:AAA4201
Apamea sordens|[15777]|XAI064-05|0102-ONT-0064|658[0n]bp|Canada.Ontario|BOLD:AAA4201
Apamea sordens|[15778]|XAJ673-06|2006-ONT-0673|658[0n]bp|Canada.Ontario|BOLD:AAA4201
Apamea sordens|[15779]|XAC176-04|04HBL006176|658[0n]bp|Canada.Ontario|BOLD:AAA4201
Apamea sordens|[15780]|RDLQF002-06|DH004653|658[0n]bp|Canada.Quebec|BOLD:AAA4201
Apamea sordens|[15781]|BBLPB693-10|10BBCLP-1692|658[0n]bp|Canada.Ontario|BOLD:AAA4201
Apamea sordens|[15782]|XAB350-04|04HBL005350|658[0n]bp|Canada.Ontario|BOLD:AAA4201
Apamea sordens|[15783]|RDNMB016-05|CNCNoctuoidea7856|658[0n]bp|Canada.Ontario|BOLD:AAA4201
Apamea sordens|[15784]|XAK163-06|2006-ONT-1158|658[0n]bp|Canada.Ontario|BOLD:AAA4201
Apamea sordens|[15785]|XAF802-05|2005-ONT-451|658[0n]bp|Canada.Ontario|BOLD:AAA4201
Apamea sordens|[15786]|RDLQF022-06|DH011152|658[0n]bp|Canada.Quebec|BOLD:AAA4201
Apamea sordens|[15787]|RDLQF006-06|AC000251|658[0n]bp|Canada.Quebec|BOLD:AAA4201
Apamea sordens|[15788]|XAB365-04|04HBL005365|658[0n]bp|Canada.Ontario|BOLD:AAA4201
Apamea sordens|[15789]|RDLQF001-06|DH004818|658[0n]bp|Canada.Quebec|BOLD:AAA4201
Apamea sordens|[15790]|XAF518-05|2005-ONT-167|617[0n]bp|Canada.Ontario|BOLD:AAA4201
Apamea sordens|[15791]|PHMO122-03|moth729.01|639[0n]bp|Canada.Ontario|BOLD:AAA4201
Apamea sordens|[15792]|PHMO078-03|moth510.01|639[0n]bp|Canada.Ontario|BOLD:AAA4201
Apamea sordens|[15793]|BLTIB282-08|BL466|657[0n]bp|Canada.Ontario|BOLD:AAA4201
Apamea sordens|[15794]|LPSOC127-08|PPBP-2126|658[0n]bp|Canada.Ontario|BOLD:AAA4201
Apamea sordens|[15795]|RDLQB917-05|DH010983|658[0n]bp|Canada.Quebec|BOLD:AAA4201
Apamea sordens|[15796]|RDNMB018-05|CNCNoctuoidea7858|658[0n]bp|Canada.Ontario|BOLD:AAA4201
Apamea sordens|[15797]|XAI065-05|0102-ONT-0065|658[0n]bp|Canada.Ontario|BOLD:AAA4201
Apamea sordens|[15798]|LPSOC330-08|PPBP-2329|658[0n]bp|Canada.Ontario|BOLD:AAA4201
Apamea sordens|[15799]|RDLQF020-06|DH011150|658[3n]bp|Canada.Quebec|BOLD:AAA4201
Apamea sordens|[15800]|RDNM966-05|CNCNoctuoidea7806|658[0n]bp|Canada.Ontario|BOLD:AAA4201
Apamea sordens|[15801]|LPSOB699-08|PPBP-1698|658[0n]bp|Canada.Ontario|BOLD:AAA4201
Apamea sordens|[15802]|XAF468-05|2005-ONT-117|658[0n]bp|Canada.Ontario|BOLD:AAA4201
Apamea sordens|[15803]|RDLQF004-06|AC000632|631[0n]bp|Canada.Quebec|BOLD:AAA4201
Apamea sordens|[15804]|LPSOC362-08|PPBP-2361|653[0n]bp|Canada.Ontario|BOLD:AAA4201
Apamea sordens|[15805]|RDLQF014-06|DH004822|656[0n]bp|Canada.Quebec|BOLD:AAA4201
Apamea sordens|[15806]|PHMO099-03|moth604.01|639[0n]bp|Canada.Ontario|BOLD:AAA4201
Apamea sordens|[15807]|RDLQB806-05|DH010893|617[0n]bp|Canada.Quebec|BOLD:AAA4201
Apamea sordens|[15808]|LPSOC316-08|PPBP-2315|658[0n]bp|Canada.Ontario|BOLD:AAA4201
Apamea sordens|[15809]|RDLQF012-06|DH008131|658[0n]bp|Canada.Quebec|BOLD:AAA4201
Apamea sordens|[15810]|RDLQF017-06|DH004812|658[0n]bp|Canada.Quebec|BOLD:AAA4201
Apamea sordens|[15811]|XAI061-05|0102-ONT-0061|658[0n]bp|Canada.Ontario|BOLD:AAA4201
Apamea sordens|[15812]|RDNMB020-05|CNCNoctuoidea7860|658[0n]bp|Canada.New Brunswick|BOLD:AAA4201
Apamea sordens|[15813]|BBLPE346-09|09BBELE-2346|658[0n]bp|Canada.Newfoundland and Labrador|BOLD:AAA4201
Apamea sordens|[15814]|RDLQF021-06|DH011151|658[0n]bp|Canada.Quebec|BOLD:AAA4201
Apamea sordens|[15815]|LPSOC031-08|PPBP-2030|658[0n]bp|Canada.Ontario|BOLD:AAA4201
Apamea sordens|[15816]|LPSOC091-08|PPBP-2090|658[0n]bp|Canada.Ontario|BOLD:AAA4201
Apamea sordens|[15817]|RDLQF005-06|AC000245|658[1n]bp|Canada.Quebec|BOLD:AAA4201
Apamea sordens|[15818]|RDLQF023-06|DH011153|658[0n]bp|Canada.Quebec|BOLD:AAA4201
Apamea sordens|[15819]|RDLQF003-06|DH004820|658[0n]bp|Canada.Quebec|BOLD:AAA4201
Apamea sordens|[15820]|RDLQF015-06|DH004923|632[1n]bp|Canada.Quebec|BOLD:AAA4201
Apamea sordens|[15821]|LPSOC327-08|PPBP-2326|658[0n]bp|Canada.Ontario|BOLD:AAA4201
Apamea sordens|[15822]|XAB506-04|04HBL005506|622[0n]bp|Canada.Ontario|BOLD:AAA4201
Apamea sordens|[15823]|RDLQF018-06|DH011148|658[0n]bp|Canada.Quebec|BOLD:AAA4201
Apamea sordens|[15824]|PHMNB327-04|04HBL00553|658[0n]bp|Canada.New Brunswick|BOLD:AAA4201
Apamea sordens|[15825]|RDLQF019-06|DH011149|658[0n]bp|Canada.Quebec|BOLD:AAA4201
Apamea sordens|[15826]|PHMNB126-04|04HBL007591|658[0n]bp|Canada.New Brunswick|BOLD:AAA4201
Apamea sordens|[15827]|RDLQB916-05|DH010982|658[0n]bp|Canada.Quebec|BOLD:AAA4201
Apamea cariosa|[15828]|RDNM202-05|CNCNoctuoidea6384|658[0n]bp|Canada.Ontario|BOLD:AAE3183
Apamea cariosa|[15829]|RDNM201-05|CNCNoctuoidea6383|658[0n]bp|Canada.Ontario|BOLD:AAE3183
Apamea cariosa|[15830]|XAE254-04|Moth4254.03|658[0n]bp|Canada.Ontario|BOLD:AAE3183
Apamea cariosa|[15831]|RDNM200-05|CNCNoctuoidea6382|658[0n]bp|Canada.Ontario|BOLD:AAE3183
Apamea cristata|[15832]|RDLQF042-06|DH005339|658[0n]bp|Canada.Quebec|BOLD:AAF1717
Apamea cristata|[15833]|RDLQF041-06|DH005622|658[0n]bp|Canada.Quebec|BOLD:AAF1717
Apamea cristata|[15834]|RDLQF043-06|DH005341|658[0n]bp|Canada.Quebec|BOLD:AAF1717
Apamea inebriata|[15835]|RDNMG209-08|NOC15061|609[0n]bp|United States.New Jersey|BOLD:AAW5920
Apamea verbascoides|[15836]|RDLQF543-06|DH011692|658[0n]bp|Canada.Quebec|BOLD:AAD6273
Apamea verbascoides|[15837]|RDNM199-05|CNCNoctuoidea6381|658[0n]bp|Canada.Ontario|BOLD:AAD6273
Apamea verbascoides|[15838]|BBLPC683-09|09BBELE-1683|658[0n]bp|Canada.Newfoundland and Labrador|BOLD:A...
Apamea verbascoides|[15839]|RDLQF045-06|DH005497|658[0n]bp|Canada.Quebec|BOLD:AAD6273
Apamea verbascoides|[15840]|RDLQG127-06|DH012294|643[0n]bp|Canada.Quebec|BOLD:AAD6273
Apamea verbascoides|[15841]|RDLQF047-06|DH002306|658[0n]bp|Canada.Quebec|BOLD:AAD6273
Apamea verbascoides|[15842]|PHMTV424-10|10PHMAL-2524|658[0n]bp|Canada.Ontario|BOLD:AAD6273
Apamea nigrior|[15843]|PHMNB321-04|04HBL00547|658[0n]bp|Canada.New Brunswick|BOLD:AAE7006
Apamea nigrior|[15844]|RDNM197-05|CNCNoctuoidea6379|638[0n]bp|Canada.Ontario|BOLD:AAE7006
Apamea nigrior|[15845]|RDNM198-05|CNCNoctuoidea6380|658[0n]bp|Canada.Ontario|BOLD:AAE7006
Apamea nigrior|[15846]|PHMNB582-04|04HBL00808|658[0n]bp|Canada.New Brunswick|BOLD:AAE7006
Apamea vultuosa|[15847]|RDNMG855-08|CNC LEP00052979|658[0n]bp|Canada.Ontario|BOLD:AAB7575
[illegible]

Apamea nigrior|[15845]|RDNM198-05|CNCNoctuoidea6380|658[0n]bp|Canada.Ontario|BOLD:AAE7006
Apamea nigrior|[15846]|PHMNB582-04|04HBL00808|658[0n]bp|Canada.New Brunswick|BOLD:AAE7006
Apamea vultuosa|[15847]|RDNMG855-08|CNC LEP00052979|658[0n]bp|Canada.Ontario|BOLD:AAB7575
Apamea vulgaris|[15848]|RDNMG210-08|NOC15062|658[0n]bp|United States.Maryland|BOLD:AAB7575
Apamea vulgaris|[15849]|RDNMG211-08|NOC15063|649[0n]bp|United States.Maryland|BOLD:AAB7575
Apamea vultuosa|[15850]|LPSO641-08|PPBP-0641|624[0n]bp|Canada.Ontario|BOLD:AAB7575
Apamea unanimis|[15851]|XAC289-04|04HBL006289|658[0n]bp|Canada.Ontario|BOLD:AAA8789
Apamea unanimis|[15852]|LPSOB722-08|PPBP-1721|513[0n]bp|Canada.Ontario|BOLD:AAA8789
Apamea unanimis|[15853]|PHMNB531-04|04HBL00757|658[0n]bp|Canada.New Brunswick|BOLD:AAA8789
Apamea unanimis|[15854]|LPSOB820-08|PPBP-1819|658[0n]bp|Canada.Ontario|BOLD:AAA8789
Apamea unanimis|[15855]|BBLPC330-09|09BBELE-1330|658[0n]bp|Canada.Newfoundland and Labrador|BOLD:AAA8789
Apamea unanimis|[15856]|LPSOC392-08|PPBP-2391|658[0n]bp|Canada.Ontario|BOLD:AAA8789
Apamea unanimis|[15857]|LALPA1173-11|AVBC 983-11|658[0n]bp|Canada.British Columbia|BOLD:AAA8789
Apamea unanimis|[15858]|LPSOC099-08|PPBP-2098|658[0n]bp|Canada.Ontario|BOLD:AAA8789
Apamea unanimis|[15859]|LPSOD283-09|08BBLEP-00061|658[0n]bp|Canada.Ontario|BOLD:AAA8789
Apamea unanimis|[15860]|RDLQF057-06|DH005082|658[0n]bp|Canada.Quebec|BOLD:AAA8789
Apamea unanimis|[15861]|RDLQF058-06|DH005119|658[0n]bp|Canada.Quebec|BOLD:AAA8789
Apamea unanimis|[15862]|XAB590-04|04HBL005590|658[0n]bp|Canada.Ontario|BOLD:AAA8789
Apamea unanimis|[15863]|BLTIB188-08|BL269|658[0n]bp|Canada.Ontario|BOLD:AAA8789
Apamea unanimis|[15864]|PHMNB364-04|04HBL00590|658[0n]bp|Canada.New Brunswick|BOLD:AAA8789
Apamea unanimis|[15865]|RDNMG464-08|CNC LEP00052288|658[0n]bp|Canada.New Brunswick|BOLD:AAA8789
Apamea unanimis|[15866]|RDNMG465-08|CNC LEP00052289|658[0n]bp|Canada.Ontario|BOLD:AAA8789
Apamea unanimis|[15867]|LALPA1171-11|AVBC 981-11|658[0n]bp|Canada.British Columbia|BOLD:AAA8789
Apamea unanimis|[15868]|XAB507-04|04HBL005507|658[0n]bp|Canada.Ontario|BOLD:AAA8789
Apamea unanimis|[15869]|XAE290-04|Moth4290.03|658[0n]bp|Canada.Ontario|BOLD:AAA8789
Apamea unanimis|[15870]|RDLQF056-06|DH009120|658[0n]bp|Canada.Quebec|BOLD:AAA8789
Apamea unanimis|[15871]|RDLQF454-06|DH011561|658[0n]bp|Canada.Quebec|BOLD:AAA8789
Apamea unanimis|[15872]|XAJ626-06|2006-ONT-0626|658[0n]bp|Canada.Ontario|BOLD:AAA8789
Apamea unanimis|[15873]|BBLPC284-09|09BBELE-1284|658[0n]bp|Canada.Newfoundland and Labrador|BOLD:AAA8789
Apamea unanimis|[15874]|BLTIB017-08|BL0018|658[0n]bp|Canada.Ontario|BOLD:AAA8789
Apamea unanimis|[15875]|LPSOB718-08|PPBP-1717|658[0n]bp|Canada.Ontario|BOLD:AAA8789
Apamea unanimis|[15876]|RDNMG463-08|CNC LEP00052287|658[0n]bp|Canada.New Brunswick|BOLD:AAA8789
Apamea unanimis|[15877]|LPSOC123-08|PPBP-2122|658[0n]bp|Canada.Ontario|BOLD:AAA8789
Apamea unanimis|[15878]|XAB564-04|04HBL005564|658[0n]bp|Canada.Ontario|BOLD:AAA8789
Apamea unanimis|[15879]|LPSOB806-08|PPBP-1805|658[0n]bp|Canada.Ontario|BOLD:AAA8789
Apamea unanimis|[15880]|LPSOB665-08|PPBP-1664|644[0n]bp|Canada.Ontario|BOLD:AAA8789
Apamea unanimis|[15881]|PHMO166-03|moth888.01|639[0n]bp|Canada.Ontario|BOLD:AAA8789
Apamea unanimis|[15882]|RDLQB794-05|DH010881|627[0n]bp|Canada.Quebec|BOLD:AAA8789
Apamea unanimis|[15883]|LPSOC366-08|PPBP-2365|658[0n]bp|Canada.Ontario|BOLD:AAA8789
Apamea unanimis|[15884]|XAC186-04|04HBL006186|658[0n]bp|Canada.Ontario|BOLD:AAA8789
Apamea unanimis|[15885]|BLTIB089-08|BL0145|658[0n]bp|Canada.Ontario|BOLD:AAA8789
Apamea vultuosa|[15886]|BBLPB688-10|10BBCLP-1687|658[0n]bp|Canada.British Columbia|BOLD:AAB7574
Apamea vultuosa|[15887]|XAD715-05|2005-ONT-514|658[0n]bp|Canada.Ontario|BOLD:AAB7574
Apamea vultuosa|[15888]|PHMTV412-10|10PHMAL-2512|658[0n]bp|Canada.Ontario|BOLD:AAB7574
Apamea vultuosa|[15889]|PHJUN4003-11|BIOUG01497-B02|658[0n]bp|Canada.Ontario|BOLD:AAB7574
Apamea vultuosa|[15890]|PHMTV410-10|10PHMAL-2510|658[0n]bp|Canada.Ontario|BOLD:AAB7574
Apamea vultuosa|[15891]|RDNM145-05|CNCNoctuoidea6695|658[0n]bp|Canada.Ontario|BOLD:AAB7574
Apamea vultuosa|[15892]|RDLQB082-05|DH010168|658[0n]bp|Canada.Quebec|BOLD:AAB7574
Apamea vultuosa|[15893]|LOWCD126-06|CGWC-2946|600[0n]bp|Canada.British Columbia|BOLD:AAB7574
Apamea vultuosa|[15894]|BLTIB275-08|BL459|658[0n]bp|Canada.Ontario|BOLD:AAB7574
Apamea burgessi|[15895]|RDNMD464-06|CNCNoctuoidea12796|597[0n]bp|United States.Colorado|BOLD:AAC5458
Apamea burgessi|[15896]|RDNMD154-06|CNCNoctuoidea11171|605[0n]bp|United States.Colorado|BOLD:AAC5458
Apamea burgessi|[15897]|RDNMD465-06|CNCNoctuoidea12797|658[0n]bp|United States.Colorado|BOLD:AAC5458
Apamea burgessi|[15898]|RDNMD251-06|CNCNoctuoidea12583|617[0n]bp|United States.Oregon|BOLD:AAC5458
Apamea burgessi|[15899]|RDNMD250-06|CNCNoctuoidea12582|617[0n]bp|United States.Oregon|BOLD:AAC5458
Apamea burgessi|[15900]|RDNMD153-06|CNCNoctuoidea11170|658[0n]bp|United States.Colorado|BOLD:AAC5457
Apamea burgessi|[15901]|RDNMD336-06|CNCNoctuoidea12668|658[0n]bp|United States.Massachusetts|BOLD:AAC5459
Apamea burgessi|[15902]|GWOTA073-12|BC ZSM Lep 58549|658[0n]bp|United States.Massachusetts|BOLD:AAC5459
Apamea burgessi|[15903]|RDNMD253-06|CNCNoctuoidea12585|617[0n]bp|United States.Wyoming|BOLD:AAC5459
Apamea burgessi|[15904]|RDNMG899-08|CNC LEP00053023|658[0n]bp|United States.Colorado|BOLD:AAC5459
Apamea burgessi|[15905]|RDNMD252-06|CNCNoctuoidea12584|591[0n]bp|United States.Arizona|BOLD:AAC5459
Apamea burgessi|[15906]|RDNMD249-06|CNCNoctuoidea12581|617[0n]bp|United States.Montana|BOLD:AAC5459
Apamea scoparia|[15907]|LOWCC426-05|CGWC-2306|584[0n]bp|Canada.British Columbia|BOLD:AAA8064
Apamea scoparia|[15908]|LPSK438-08|08BBLEP-02006|609[0n]bp|Canada.Saskatchewan|BOLD:AAA8064
Apamea scoparia|[15909]|LCHQ524-08|07WNP-10416|643[1n]bp|Canada.Manitoba|BOLD:AAA8064
Apamea scoparia|[15910]|LOWCC214-05|CGWC-2094|608[0n]bp|Canada.British Columbia|BOLD:AAA8064
Apamea scoparia|[15911]|LPABB029-08|08BBLEP-03294|658[0n]bp|Canada.Alberta|BOLD:AAA8064
Apamea scoparia|[15912]|LOWCD282-06|CGWC-3102|657[0n]bp|Canada.British Columbia|BOLD:AAA8064
Apamea scoparia|[15913]|LPABC072-09|08BBLEP-04291|658[0n]bp|Canada.Alberta|BOLD:AAA8064
Apamea scoparia|[15914]|LPSK528-08|08BBLEP-02096|658[0n]bp|Canada.Saskatchewan|BOLD:AAA8064
Apamea scoparia|[15915]|LALPA1216-11|AVBC 1218-11|658[0n]bp|Canada.British Columbia|BOLD:AAA8064
Apamea scoparia|[15916]|LPSK493-08|08BBLEP-02061|658[0n]bp|Canada.Saskatchewan|BOLD:AAA8064
Apamea scoparia|[15917]|LPSK579-08|08BBLEP-02147|658[0n]bp|Canada.Saskatchewan|BOLD:AAA8064
Apamea scoparia|[15918]|LPSK519-08|08BBLEP-02087|658[0n]bp|Canada.Saskatchewan|BOLD:AAA8064
Apamea scoparia|[15919]|DSCNI039-07|06-PROBE-0264|658[0n]bp|Canada.Manitoba|BOLD:AAA8064
Apamea scoparia|[15920]|LPSK527-08|08BBLEP-02095|658[0n]bp|Canada.Saskatchewan|BOLD:AAA8064
Apamea scoparia|[15921]|LPABB722-08|08BBLEP-03987|658[0n]bp|Canada.Alberta|BOLD:AAA8064
Apamea scoparia|[15922]|LPSK521-08|08BBLEP-02089|658[0n]bp|Canada.Saskatchewan|BOLD:AAA8064
Apamea scoparia|[15923]|LPSK485-08|08BBLEP-02053|658[0n]bp|Canada.Saskatchewan|BOLD:AAA8064
Apamea scoparia|[15924]|LBCB406-05|HLC-21346|658[0n]bp|Canada.British Columbia|BOLD:AAA8064
Apamea scoparia|[15925]|LPSK111-08|08BBLEP-01679|658[0n]bp|Canada.Saskatchewan|BOLD:AAA8064
Apamea scoparia|[15926]|RDNMC483-06|CNCNoctuoidea12305|658[0n]bp|Canada.Quebec|BOLD:AAA8064
Apamea scoparia|[15927]|LOWCC423-05|CGWC-2303|658[0n]bp|Canada.British Columbia|BOLD:AAA8064
Apamea scoparia|[15928]|LBCB620-05|HLC-21560|658[0n]bp|Canada.British Columbia|BOLD:AAA8064
Apamea scoparia|[15929]|LPSK474-08|08BBLEP-02042|658[0n]bp|Canada.Saskatchewan|BOLD:AAA8064
Apamea scoparia|[15930]|LPSK551-08|08BBLEP-02119|658[0n]bp|Canada.Saskatchewan|BOLD:AAA8064
Apamea scoparia|[15931]|LALPA886-11|AVBC 1059-11|658[0n]bp|Canada.British Columbia|BOLD:AAA8064
Apamea scoparia|[15932]|LPSK549-08|08BBLEP-02117|658[0n]bp|Canada.Saskatchewan|BOLD:AAA8064
Apamea scoparia|[15933]|LALPA864-11|AVBC 1037-11|658[0n]bp|Canada.British Columbia|BOLD:AAA8064
Apamea scoparia|[15934]|LPSK582-08|08BBLEP-02150|658[0n]bp|Canada.Saskatchewan|BOLD:AAA8064
Apamea scoparia|[15935]|LPSK583-08|08BBLEP-02151|658[0n]bp|Canada.Saskatchewan|BOLD:AAA8064
Apamea scoparia|[15936]|LPABB721-08|08BBLEP-03986|658[0n]bp|Canada.Alberta|BOLD:AAA8064
Apamea scoparia|[15937]|LPSK213-08|08BBLEP-01781|658[0n]bp|Canada.Saskatchewan|BOLD:AAA8064
Apamea scoparia|[15938]|LPSK592-08|08BBLEP-02160|658[0n]bp|Canada.Saskatchewan|BOLD:AAA8064
Apamea scoparia|[15939]|LPSK451-08|08BBLEP-02019|658[0n]bp|Canada.Saskatchewan|BOLD:AAA8064
Apamea scoparia|[15940]|LPSK580-08|08BBLEP-02148|658[0n]bp|Canada.Saskatchewan|BOLD:AAA8064
Apamea scoparia|[15941]|LOWCC424-05|CGWC-2304|658[0n]bp|Canada.British Columbia|BOLD:AAA8064
Apamea scoparia|[15942]|BBLPB793-10|10BBCLP-1792|658[0n]bp|Canada.British Columbia|BOLD:AAA8064
Apamea scoparia|[15943]|LHLEP062-06|UBC-2006-0267|658[0n]bp|Canada.British Columbia|BOLD:AAA8064
Apamea scoparia|[15944]|LPSK596-08|08BBLEP-02164|658[0n]bp|Canada.Saskatchewan|BOLD:AAA8064
Apamea scoparia|[15945]|LPSK586-08|08BBLEP-02154|658[0n]bp|Canada.Saskatchewan|BOLD:AAA8064
Apamea scoparia|[15946]|LCHQ517-08|07WNP-10409|658[0n]bp|Canada.Manitoba|BOLD:AAA8064
Apamea scoparia|[15947]|LOWCC425-05|CGWC-2305|658[0n]bp|Canada.British Columbia|BOLD:AAA8064

Apamea scoparia|[15945]|LPSK586-08|08BBLEP-02154|658[0n]bp|Canada.Saskatchewan|BOLD:AAA8064
Apamea scoparia|[15946]|LCHQ517-08|07WNP-10409|658[0n]bp|Canada.Manitoba|BOLD:AAA8064
Apamea scoparia|[15947]|LOWCC425-05|CGWC-2305|658[0n]bp|Canada.British Columbia|BOLD:AAA8064
Apamea scoparia|[15948]|LPSK108-08|08BBLEP-01676|658[0n]bp|Canada.Saskatchewan|BOLD:AAA8064
Apamea scoparia|[15949]|LPABB728-08|08BBLEP-03993|658[0n]bp|Canada.Alberta|BOLD:AAA8064
Apamea scoparia|[15950]|LPSK220-08|08BBLEP-01788|658[0n]bp|Canada.Saskatchewan|BOLD:AAA8064
Apamea scoparia|[15951]|LPABB336-08|08BBLEP-03601|658[0n]bp|Canada.Alberta|BOLD:AAA8064
Apamea scoparia|[15952]|LPSK518-08|08BBLEP-02086|658[0n]bp|Canada.Saskatchewan|BOLD:AAA8064
Apamea lutosa|[15953]|RDNMG809-08|CNC LEP00052933|658[0n]bp|Canada.Ontario|BOLD:AAE3213
Apamea lutosa|[15954]|RDNMG811-08|CNC LEP00052935|658[0n]bp|Canada.Ontario|BOLD:AAE3213
Apamea lutosa|[15955]|RDNM203-05|CNCNoctuoidea6385|658[0n]bp|Canada.Ontario|BOLD:AAE3213
Apamea lutosa|[15956]|RDNM204-05|CNCNoctuoidea6386|653[0n]bp|Canada.Ontario|BOLD:AAE3213
Apamea lutosa|[15957]|RDNMG810-08|CNC LEP00052934|658[0n]bp|Canada.Ontario|BOLD:AAE3213
Apamea helva|[15958]|RDLQF061-06|DH008083|658[0n]bp|Canada.Quebec|BOLD:AAC5412
Apamea helva|[15959]|RDNM972-05|CNCNoctuoidea7812|658[0n]bp|Canada.Ontario|BOLD:AAC5412
Apamea helva|[15960]|RDLQF553-06|DH011702|658[0n]bp|Canada.Quebec|BOLD:AAC5412
Apamea helva|[15961]|RDLQF059-06|DH008080|658[0n]bp|Canada.Quebec|BOLD:AAC5412
Apamea helva|[15962]|RDLQF060-06|DH008081|658[0n]bp|Canada.Quebec|BOLD:AAC5412
Apamea centralis|[15963]|LOWCB462-05|CGWC-1402|658[0n]bp|Canada.British Columbia|BOLD:ACE3024
Apamea centralis|[15964]|RDNMC025-05|CNCNoctuoidea10766|541[0n]bp|Canada.British Columbia|BOLD:ACE3024
Apamea centralis|[15965]|RDNMD244-06|CNCNoctuoidea12576|658[0n]bp|Canada.British Columbia|BOLD:ACE3024
Apamea centralis|[15966]|RDMAB476-06|UASM77902|658[0n]bp|Canada.Alberta|BOLD:ACE3024
Apamea centralis|[15967]|LOWCD298-06|CGWC-3118|658[0n]bp|Canada.British Columbia|BOLD:ACE3024
Apamea centralis|[15968]|RDMAB447-05|BCSC120|658[0n]bp|Canada.Alberta|BOLD:ACE3024
Apamea centralis|[15969]|RDNMD242-06|CNCNoctuoidea12574|658[0n]bp|Canada.British Columbia|BOLD:ACE3024
Apamea centralis|[15970]|LOWCE749-06|CGWC-4509|658[0n]bp|Canada.British Columbia|BOLD:ACE3024
Apamea centralis|[15971]|LOWCC428-05|CGWC-2308|658[0n]bp|Canada.British Columbia|BOLD:ACE3024
Apamea centralis|[15972]|RDNMD246-06|CNCNoctuoidea12578|596[0n]bp|Canada.British Columbia|BOLD:ACE3024
Apamea centralis|[15973]|LOWCC431-05|CGWC-2311|658[0n]bp|Canada.British Columbia|BOLD:ACE3024
Apamea centralis|[15974]|LOWCC429-05|CGWC-2309|658[0n]bp|Canada.British Columbia|BOLD:ACE3024
Apamea commoda|[15975]|RDNM939-05|CNCNoctuoidea7779|605[0n]bp|Canada.British Columbia|BOLD:ACE3024
Apamea commoda|[15976]|RDNM937-05|CNCNoctuoidea7777|658[0n]bp|Canada.British Columbia|BOLD:ACE3024
Apamea commoda|[15977]|RDNMD247-06|CNCNoctuoidea12579|617[0n]bp|Canada.British Columbia|BOLD:ACE3024
Apamea commoda|[15978]|RDNMC368-05|CNCNoctuoidea12002|616[0n]bp|Canada.British Columbia|BOLD:ACE3024
Apamea commoda|[15979]|RDNMD248-06|CNCNoctuoidea12580|603[0n]bp|Canada.British Columbia|BOLD:ACE3024
Apamea commoda|[15980]|RDNM938-05|CNCNoctuoidea7778|658[0n]bp|Canada.British Columbia|BOLD:ACE3024
Apamea commoda|[15981]|RDNM063-05|CNCNoctuoidea6332|658[0n]bp|Canada.Alberta|BOLD:ACE3024
Apamea commoda|[15982]|BBLPB770-10|10BBCLP-1769|658[0n]bp|Canada.British Columbia|BOLD:ACE3024
Apamea commoda|[15983]|LPSK142-08|08BBLEP-01710|658[0n]bp|Canada.Saskatchewan|BOLD:ACE3024
Apamea commoda|[15984]|RDNM945-05|CNCNoctuoidea7785|658[0n]bp|Canada.Alberta|BOLD:ACE3024
Apamea commoda|[15985]|RDNMC065-05|CNCNoctuoidea10806|658[0n]bp|Canada.Alberta|BOLD:ACE3024
Apamea commoda|[15986]|RDNM146-05|CNCNoctuoidea6696|658[0n]bp|Canada.Saskatchewan|BOLD:ACE3024
Apamea commoda|[15987]|RDNM942-05|CNCNoctuoidea7782|658[0n]bp|Canada.Alberta|BOLD:ACE3024
Apamea commoda|[15988]|RDNM064-05|CNCNoctuoidea6333|658[0n]bp|Canada.Alberta|BOLD:ACE3024
Apamea commoda|[15989]|RDMAB097-05|UASM41977|658[0n]bp|Canada.Alberta|BOLD:ACE3024
Apamea commoda|[15990]|RDNMC063-05|CNCNoctuoidea10804|658[0n]bp|Canada.Saskatchewan|BOLD:ACE3024
Apamea commoda|[15991]|BBLPB699-10|10BBCLP-1698|634[0n]bp|Canada.British Columbia|BOLD:ACE3024
Apamea commoda|[15992]|RDNM946-05|CNCNoctuoidea7786|567[0n]bp|Canada.Saskatchewan|BOLD:ACE3024
Apamea commoda|[15993]|LPSK128-08|08BBLEP-01696|658[0n]bp|Canada.Saskatchewan|BOLD:ACE3024
Apamea commoda|[15994]|LPABC463-09|08BBLEP-04682|658[0n]bp|Canada.Alberta|BOLD:AAA6405
Apamea commoda|[15995]|RDNMC356-05|CNCNoctuoidea11990|565[0n]bp|Canada.Newfoundland and Labrador|BOLD:...
Apamea commoda|[15996]|RDNMC200-05|CNCNoctuoideaH012|557[0n]bp|Canada.Quebec|BOLD:AAA6405
Apamea commoda|[15997]|RDMAB445-05|BCSC118|658[0n]bp|Canada.Yukon Territory|BOLD:AAA6405
Apamea commoda|[15998]|RDNM060-05|CNCNoctuoidea6329|600[0n]bp|Canada.Alberta|BOLD:AAA6405
Apamea commoda|[15999]|RDLQ456-07|DH008696|594[0n]bp|Canada.Quebec|BOLD:AAA6405
Apamea commoda|[16000]|RDNMC201-05|CNCNoctuoideaH013|603[1n]bp|Canada.Quebec|BOLD:AAA6405
Apamea commoda|[16001]|LPAB212-08|08BBLEP-02534|658[0n]bp|Canada.Alberta|BOLD:AAA6405
Apamea commoda|[16002]|RDMAB443-05|BCSC116|658[0n]bp|Canada.Alberta|BOLD:AAA6405
Apamea commoda|[16003]|RDNM944-05|CNCNoctuoidea7784|658[0n]bp|Canada.Saskatchewan|BOLD:AAA6405
Apamea commoda|[16004]|LPSK556-08|08BBLEP-02124|658[0n]bp|Canada.Saskatchewan|BOLD:AAA6405
Apamea commoda|[16005]|RDNM941-05|CNCNoctuoidea7781|658[0n]bp|Canada.Alberta|BOLD:AAA6405
Apamea commoda|[16006]|RDMAB121-05|UASM41267|658[0n]bp|Canada.Alberta|BOLD:AAA6405
Apamea commoda|[16007]|RDNMC064-05|CNCNoctuoidea10805|658[0n]bp|Canada.Alberta|BOLD:AAA6405
Apamea commoda|[16008]|RDNM062-05|CNCNoctuoidea6331|577[0n]bp|Canada.Alberta|BOLD:AAA6405
Apamea commoda|[16009]|RDNM061-05|CNCNoctuoidea6330|614[0n]bp|Canada.British Columbia|BOLD:AAA6405
Apamea commoda|[16010]|BBLPB792-10|10BBCLP-1791|658[0n]bp|Canada.Alberta|BOLD:AAA6405
Apamea commoda|[16011]|RDMAB444-05|BCSC117|658[0n]bp|Canada.Alberta|BOLD:AAA6405
Apamea commoda|[16012]|LPABC180-09|08BBLEP-04399|658[0n]bp|Canada.Alberta|BOLD:AAA6405
Apamea commoda|[16013]|RDMAB480-06|UASM77916|658[0n]bp|Canada.Alberta|BOLD:AAA6405
Apamea commoda|[16014]|LOWCC206-05|CGWC-2086|658[0n]bp|Canada.British Columbia|BOLD:AAA6405
Apamea commoda|[16015]|LPAB211-08|08BBLEP-02533|658[0n]bp|Canada.Alberta|BOLD:AAA6405
Apamea commoda|[16016]|LPABB859-09|08BBLEP-04179|658[0n]bp|Canada.Alberta|BOLD:AAA6405
Apamea commoda|[16017]|LPABC320-09|08BBLEP-04539|658[0n]bp|Canada.Alberta|BOLD:AAA6405
Apamea commoda|[16018]|RDNM147-05|CNCNoctuoidea6697|658[0n]bp|Canada.Alberta|BOLD:AAA6405
Apamea commoda|[16019]|LPABB329-08|08BBLEP-03594|658[0n]bp|Canada.Alberta|BOLD:AAA6405
Apamea commoda|[16020]|LPABB857-09|08BBLEP-04177|658[0n]bp|Canada.Alberta|BOLD:AAA6405
Apamea commoda|[16021]|RDMAB436-05|BCSC109|658[0n]bp|Canada.Yukon Territory|BOLD:AAA6405
Apamea commoda|[16022]|RDMAB479-06|UASM77910|658[0n]bp|Canada.Alberta|BOLD:AAA6405
Apamea commoda|[16023]|RDNMC062-05|CNCNoctuoidea10803|658[0n]bp|Canada.Alberta|BOLD:AAA6405
Apamea commoda|[16024]|BBLPB752-10|10BBCLP-1751|658[0n]bp|Canada.Alberta|BOLD:AAA6405
Apamea commoda|[16025]|LPABB846-09|08BBLEP-04166|658[0n]bp|Canada.Alberta|BOLD:AAA6405
Apamea commoda|[16026]|LOWCC427-05|CGWC-2307|658[0n]bp|Canada.British Columbia|BOLD:AAA6405
Apamea commoda|[16027]|RDNM943-05|CNCNoctuoidea7783|658[0n]bp|Canada.Alberta|BOLD:AAA6405
Apamea commoda|[16028]|LPABB871-09|08BBLEP-04191|658[0n]bp|Canada.Alberta|BOLD:AAA6405
Apamea commoda|[16029]|RDMAB481-06|UASM77914|658[0n]bp|Canada.Alberta|BOLD:AAA6405
Apamea commoda|[16030]|LPABC273-09|08BBLEP-04492|658[0n]bp|Canada.Alberta|BOLD:AAA6405
Apamea commoda|[16031]|LPABC274-09|08BBLEP-04493|658[0n]bp|Canada.Alberta|BOLD:AAA6405
Apamea commoda|[16032]|RDNMC365-05|CNCNoctuoidea11999|601[0n]bp|Canada.British Columbia|BOLD:AAA6405
Apamea commoda|[16033]|BBLPE355-09|09BBELE-2355|643[0n]bp|Canada.Newfoundland and Labrador|BOLD:AAA6405
Apamea commoda|[16034]|RDMAB483-06|UASM77918|638[0n]bp|Canada.Alberta|BOLD:AAA6405
Apamea commoda|[16035]|RDNMC366-05|CNCNoctuoidea12000|599[0n]bp|Canada.British Columbia|BOLD:AAA6405
Apamea commoda|[16036]|RDMAB482-06|UASM77915|627[0n]bp|Canada.Alberta|BOLD:AAA6405
Apamea commoda|[16037]|LPABB854-09|08BBLEP-04174|634[0n]bp|Canada.Alberta|BOLD:AAA6405
Apamea commoda|[16038]|RDMAB332-05|UASM77814|603[0n]bp|Canada.Alberta|BOLD:AAA6405
Apamea commoda|[16039]|RDMAB346-05|UASM41485|604[0n]bp|Canada.Alberta|BOLD:AAA6405
Apamea commoda|[16040]|RDNM069-05|CNCNoctuoidea6338|622[0n]bp|Canada.Alberta|BOLD:AAA6405
Apamea commoda|[16041]|RDMAB327-05|UASM77821|606[0n]bp|Canada.Alberta|BOLD:AAA6405
Apamea commoda|[16042]|LPABB530-08|08BBLEP-03795|658[0n]bp|Canada.Alberta|BOLD:AAA6405
Apamea commoda|[16043]|RDMAB475-06|UASM77903|658[0n]bp|Canada.Alberta|BOLD:AAA6405
Apamea commoda|[16044]|RDNM933-05|CNCNoctuoidea7773|616[0n]bp|Canada.British Columbia|BOLD:AAA6405
Apamea antennata|[16045]|LOWCC401-05|CGWC-2281|658[0n]bp|Canada.British Columbia|BOLD:AAB3665
Apamea antennata|[16046]|LOWCC397-05|CGWC-2277|658[0n]bp|Canada.British Columbia|BOLD:AAB3665
Apamea antennata|[16047]|LBCH5711-10|10-JDWBC-5711|658[0n]bp|Canada.British Columbia|BOLD:AAB3665

[illegible]
Apamea antennata|[16045]|LOWCC401-05|CGWC-2281|658[0n]bp|Canada.British Columbia|BOLD:AAB3665
Apamea antennata|[16046]|LOWCC397-05|CGWC-2277|658[0n]bp|Canada.British Columbia|BOLD:AAB3665
Apamea antennata|[16047]|LBCH5711-10|10-JDWBC-5711|658[0n]bp|Canada.British Columbia|BOLD:AAB3665
Apamea antennata|[16048]|LBCH5710-10|10-JDWBC-5710|658[0n]bp|Canada.British Columbia|BOLD:AAB3665
Apamea antennata|[16049]|LPABB033-08|08BBLEP-03298|658[0n]bp|Canada.Alberta|BOLD:AAB3665
Apamea antennata|[16050]|LOWCC394-05|CGWC-2274|658[0n]bp|Canada.British Columbia|BOLD:AAB3665
Apamea antennata|[16051]|RDNM957-05|CNCNoctuoidea7797|658[0n]bp|Canada.British Columbia|BOLD:AAB3665
Apamea antennata|[16052]|LPABB572-08|08BBLEP-03837|658[0n]bp|Canada.Alberta|BOLD:AAB3665
Apamea antennata|[16053]|LPABB473-08|08BBLEP-03738|658[0n]bp|Canada.Alberta|BOLD:AAB3665
Apamea antennata|[16054]|RDNM958-05|CNCNoctuoidea7798|658[0n]bp|Canada.British Columbia|BOLD:AAB3665
Apamea antennata|[16055]|LPABB619-08|08BBLEP-03884|658[0n]bp|Canada.Alberta|BOLD:AAB3665
Apamea antennata|[16056]|LALPA957-11|AVBC 1130-11|640[0n]bp|Canada.British Columbia|BOLD:AAB3665
Apamea antennata|[16057]|LBCH5796-10|10-JDWBC-5796|649[0n]bp|Canada.British Columbia|BOLD:AAB3665
Apamea antennata|[16058]|LOWCC400-05|CGWC-2280|658[0n]bp|Canada.British Columbia|BOLD:AAB3665
Apamea antennata|[16059]|LOWCC395-05|CGWC-2275|658[0n]bp|Canada.British Columbia|BOLD:AAB3665
Apamea antennata|[16060]|LBCH5854-10|10-JDWBC-5854|658[0n]bp|Canada.British Columbia|BOLD:AAB3665
Apamea antennata|[16061]|RDNM956-05|CNCNoctuoidea7796|658[0n]bp|Canada.British Columbia|BOLD:AAB3665
Apamea antennata|[16062]|LALPA999-11|AVBC 1172-11|658[0n]bp|Canada.British Columbia|BOLD:AAB3665
Apamea antennata|[16063]|LBCH5569-10|10-JDWBC-5569|658[0n]bp|Canada.British Columbia|BOLD:AAB3665
Apamea antennata|[16064]|LBCG1074-09|08-JDWBC-1074|658[0n]bp|Canada.British Columbia|BOLD:AAB3665
Apamea antennata|[16065]|LBCH5612-10|10-JDWBC-5612|658[0n]bp|Canada.British Columbia|BOLD:AAB3665
Apamea antennata|[16066]|LOWCC399-05|CGWC-2279|658[0n]bp|Canada.British Columbia|BOLD:AAB3665
Apamea antennata|[16067]|LBCG1340-09|08-JDWBC-1340|658[0n]bp|Canada.British Columbia|BOLD:AAB3665
Apamea antennata|[16068]|LALPA355-10|AVBC 357-10|658[0n]bp|Canada.British Columbia|BOLD:AAB3665
Apamea antennata|[16069]|LBCA580-05|HLC-20580|658[0n]bp|Canada.British Columbia|BOLD:AAB3665
Apamea antennata|[16070]|LPABB045-08|08BBLEP-03310|658[0n]bp|Canada.Alberta|BOLD:AAB3665
Apamea antennata|[16071]|LBCH6015-10|10-JDWBC-6015|658[0n]bp|Canada.British Columbia|BOLD:AAB3665
Apamea antennata|[16072]|LBCH5853-10|10-JDWBC-5853|658[0n]bp|Canada.British Columbia|BOLD:AAB3665
Apamea antennata|[16073]|LOWCC398-05|CGWC-2278|658[0n]bp|Canada.British Columbia|BOLD:AAB3665
Apamea antennata|[16074]|LBCG1073-09|08-JDWBC-1073|658[0n]bp|Canada.British Columbia|BOLD:AAB3665
Apamea antennata|[16075]|LOWCC396-05|CGWC-2276|658[0n]bp|Canada.British Columbia|BOLD:AAB3665
Apamea antennata|[16076]|LBCH6090-10|10-JDWBC-6090|658[0n]bp|Canada.British Columbia|BOLD:AAB3665
Apamea antennata|[16077]|RDMAB375-05|UASM77841|631[0n]bp|Canada.Alberta|BOLD:AAB3665
Apamea lignicolora|[16078]|LPSK546-08|08BBLEP-02114|658[0n]bp|Canada.Saskatchewan|BOLD:AAB0880
Apamea lignicolora|[16079]|LPSK490-08|08BBLEP-02058|658[0n]bp|Canada.Saskatchewan|BOLD:AAB0880
Apamea lignicolora|[16080]|LPSK478-08|08BBLEP-02046|609[0n]bp|Canada.Saskatchewan|BOLD:AAB0880
Apamea lignicolora|[16081]|LPSK524-08|08BBLEP-02092|658[0n]bp|Canada.Saskatchewan|BOLD:AAB0880
Apamea lignicolora|[16082]|LPSK477-08|08BBLEP-02045|658[0n]bp|Canada.Saskatchewan|BOLD:AAB0880
Apamea lignicolora|[16083]|PHJUN3999-11|BIOUG01497-A10|658[0n]bp|Canada.Ontario|BOLD:AAB0880
Apamea lignicolora|[16084]|RDLQF054-06|DH007582|658[0n]bp|Canada.Quebec|BOLD:AAB0880
Apamea lignicolora|[16085]|LPSK547-08|08BBLEP-02115|658[0n]bp|Canada.Saskatchewan|BOLD:AAB0880
Apamea lignicolora|[16086]|LPSK488-08|08BBLEP-02056|658[0n]bp|Canada.Saskatchewan|BOLD:AAB0880
Apamea lignicolora|[16087]|TTMNB568-06|MNBTT-568|658[0n]bp|Canada.New Brunswick|BOLD:AAB0880
Apamea lignicolora|[16088]|LPSK019-08|08BBLEP-00722|658[0n]bp|Canada.Saskatchewan|BOLD:AAB0880
Apamea lignicolora|[16089]|LPSK489-08|08BBLEP-02057|658[0n]bp|Canada.Saskatchewan|BOLD:AAB0880
Apamea lignicolora|[16090]|TMNBB163-06|MNBTT-1103|658[0n]bp|Canada.New Brunswick|BOLD:AAB0880
Apamea lignicolora|[16091]|RDLQF052-06|AC000448|658[0n]bp|Canada.Quebec|BOLD:AAB0880
Apamea lignicolora|[16092]|RDMAB478-06|UASM77907|658[0n]bp|Canada.Alberta|BOLD:AAB0880
Apamea lignicolora|[16093]|LPSK022-08|08BBLEP-00725|658[0n]bp|Canada.Saskatchewan|BOLD:AAB0880
Apamea lignicolora|[16094]|PHMNB451-04|04HBL00677|658[0n]bp|Canada.New Brunswick|BOLD:AAB0880
Apamea lignicolora|[16095]|XAC072-04|04HBL006072|658[0n]bp|Canada.Ontario|BOLD:AAB0880
Apamea lignicolora|[16096]|LPSK595-08|08BBLEP-02163|658[0n]bp|Canada.Saskatchewan|BOLD:AAB0880
Apamea lignicolora|[16097]|LPSK121-08|08BBLEP-01689|658[0n]bp|Canada.Saskatchewan|BOLD:AAB0880
Apamea lignicolora|[16098]|LPSK479-08|08BBLEP-02047|658[0n]bp|Canada.Saskatchewan|BOLD:AAB0880
Apamea lignicolora|[16099]|RDLQF051-06|DH009350|658[0n]bp|Canada.Quebec|BOLD:AAB0880
Apamea lignicolora|[16100]|LPSK487-08|08BBLEP-02055|658[0n]bp|Canada.Saskatchewan|BOLD:AAB0880
Apamea lignicolora|[16101]|PHMNB088-03|moth10.02SA|639[0n]bp|Canada.New Brunswick|BOLD:AAB0880
Apamea lignicolora|[16102]|PHMO176-03|moth931.02|639[0n]bp|Canada.Ontario|BOLD:AAB0880
Apamea lignicolora|[16103]|PHMO194-03|moth987.01|639[0n]bp|Canada.Ontario|BOLD:AAB0880
Apamea lignicolora|[16104]|PMG092-03|moth985.01|617[0n]bp|Canada.Ontario|BOLD:AAB0880
Apamea lignicolora|[16105]|XAC669-04|04HBL006669|658[0n]bp|Canada.Ontario|BOLD:AAB0880
Apamea lignicolora|[16106]|XAI171-06|0102-ONT-0171|658[0n]bp|Canada.Ontario|BOLD:AAB0880
Apamea atriclava|[16107]|RDNMD263-06|CNCNoctuoidea12595|608[0n]bp|Canada.British Columbia|BOLD:AAD5818
Apamea atriclava|[16108]|RDNM955-05|CNCNoctuoidea7795|658[0n]bp|Canada.British Columbia|BOLD:AAD5818
Apamea atriclava|[16109]|LPVIA270-08|PFC-2006-0356|658[0n]bp|Canada.British Columbia|BOLD:AAD5818
Apamea sora|[16110]|LOWCC404-05|CGWC-2284|658[1n]bp|Canada.British Columbia|BOLD:AAA8200
Apamea sora|[16111]|LPABC419-09|08BBLEP-04638|658[0n]bp|Canada.Alberta|BOLD:AAA8200
Apamea sora|[16112]|LOWCC406-05|CGWC-2286|658[0n]bp|Canada.British Columbia|BOLD:AAA8200
Apamea sora|[16113]|BBLPB707-10|10BBCLP-1706|658[0n]bp|Canada.British Columbia|BOLD:AAA8200
Apamea sora|[16114]|RDMAB485-06|UASM41415|658[0n]bp|Canada.Alberta|BOLD:AAA8200
Apamea sora|[16115]|LPABC458-09|08BBLEP-04677|658[0n]bp|Canada.Alberta|BOLD:AAA8200
Apamea sora|[16116]|LPABB191-08|08BBLEP-03456|658[0n]bp|Canada.Alberta|BOLD:AAA8200
Apamea sora|[16117]|RDNM076-05|CNCNoctuoidea6345|658[0n]bp|Canada.British Columbia|BOLD:AAA8200
Apamea sora|[16118]|LOWCC402-05|CGWC-2282|658[0n]bp|Canada.British Columbia|BOLD:AAA8200
Apamea sora|[16119]|BBLPB689-10|10BBCLP-1688|658[0n]bp|Canada.British Columbia|BOLD:AAA8200
Apamea sora|[16120]|LOWCC409-05|CGWC-2289|658[0n]bp|Canada.British Columbia|BOLD:AAA8200
Apamea sora|[16121]|LPABC183-09|08BBLEP-04402|658[0n]bp|Canada.Alberta|BOLD:AAA8200
Apamea sora|[16122]|LOWCC413-05|CGWC-2293|658[0n]bp|Canada.British Columbia|BOLD:AAA8200
Apamea sora|[16123]|LBCD327-05|HLC-23147|658[0n]bp|Canada.British Columbia|BOLD:AAA8200
Apamea sora|[16124]|RDMAB449-05|BCSC122|658[0n]bp|Canada.Yukon Territory|BOLD:AAA8200
Apamea sora|[16125]|LOWCB183-05|CGWC-1123|658[0n]bp|Canada.British Columbia|BOLD:AAA8200
Apamea sora|[16126]|LOWCC405-05|CGWC-2285|658[0n]bp|Canada.British Columbia|BOLD:AAA8200
Apamea sora|[16127]|LOWCC430-05|CGWC-2310|658[0n]bp|Canada.British Columbia|BOLD:AAA8200
Apamea sora|[16128]|BBLPB634-10|10BBCLP-1633|658[0n]bp|Canada.Alberta|BOLD:AAA8200
Apamea sora|[16129]|LOWCC411-05|CGWC-2291|658[0n]bp|Canada.British Columbia|BOLD:AAA8200
Apamea sora|[16130]|LPAB202-08|08BBLEP-02524|658[0n]bp|Canada.Alberta|BOLD:AAA8200
Apamea sora|[16131]|LPABB814-09|08BBLEP-04134|658[0n]bp|Canada.Alberta|BOLD:AAA8200
Apamea sora|[16132]|LPABB642-08|08BBLEP-03907|658[0n]bp|Canada.Alberta|BOLD:AAA8200
Apamea sora|[16133]|LOWCC410-05|CGWC-2290|658[0n]bp|Canada.British Columbia|BOLD:AAA8200
Apamea sora|[16134]|RDNMC078-05|CNCNoctuoidea10819|658[0n]bp|Canada.British Columbia|BOLD:AAA8200
Apamea sora|[16135]|LPABC895-09|08BBLEP-05306|658[0n]bp|Canada.Alberta|BOLD:AAA8200
Apamea sora|[16136]|LOWCC403-05|CGWC-2283|658[0n]bp|Canada.British Columbia|BOLD:AAA8200
Apamea sora|[16137]|LPABC201-09|08BBLEP-04420|658[0n]bp|Canada.Alberta|BOLD:AAA8200
Apamea sora|[16138]|LPABB730-08|08BBLEP-03995|658[0n]bp|Canada.Alberta|BOLD:AAA8200
Apamea sora|[16139]|LOWCC412-05|CGWC-2292|658[0n]bp|Canada.British Columbia|BOLD:AAA8200
Apamea sora|[16140]|LPABC070-09|08BBLEP-04289|658[0n]bp|Canada.Alberta|BOLD:AAA8200
Apamea sora|[16141]|LOWCC408-05|CGWC-2288|658[0n]bp|Canada.British Columbia|BOLD:AAA8200
Apamea sora|[16142]|RDMAB477-06|UASM77901|658[0n]bp|Canada.Alberta|BOLD:AAA8200
Apamea sora|[16143]|RDNM936-05|CNCNoctuoidea7776|658[0n]bp|Canada.British Columbia|BOLD:AAA8200
Apamea sora|[16144]|LOWCC407-05|CGWC-2287|658[0n]bp|Canada.British Columbia|BOLD:AAA8200
Apamea sora|[16145]|LPABB717-08|08BBLEP-03982|658[0n]bp|Canada.Alberta|BOLD:AAA8200
Apamea sora|[16146]|RDNM077-05|CNCNoctuoidea6346|658[0n]bp|Canada.British Columbia|BOLD:AAA8200
[illegible]

Apamea sora|[16144]|LOWCC407-05|CGWC-2287|658[0n]bp|Canada.British Columbia|BOLD:AAA8200
Apamea sora|[16145]|LPABB717-08|08BBLEP-03982|658[0n]bp|Canada.Alberta|BOLD:AAA8200
Apamea sora|[16146]|RDNM077-05|CNCNoctuoidea6346|658[0n]bp|Canada.British Columbia|BOLD:AAA8200
Apamea sora|[16147]|LPABC030-09|08BBLEP-04249|658[1n]bp|Canada.Alberta|BOLD:AAA8200
Apamea sora|[16148]|LPABC200-09|08BBLEP-04419|632[0n]bp|Canada.Alberta|BOLD:AAA8200
Apamea sora|[16149]|RDMAB486-06|UASM41414|646[0n]bp|Canada.Alberta|BOLD:AAA8200
Apamea sora|[16150]|LPABB815-09|08BBLEP-04135|637[0n]bp|Canada.Alberta|BOLD:AAA8200
Apamea sora|[16151]|RDNM024-05|CNCNoctuoidea6293|598[0n]bp|Canada.British Columbia|BOLD:AAA8200
Apamea sora|[16152]|BBLPB696-10|10BBCLP-1695|658[0n]bp|Canada.British Columbia|BOLD:AAA8200
Apamea sora|[16153]|LPAB207-08|08BBLEP-02529|655[0n]bp|Canada.Alberta|BOLD:AAA8200
Apamea sora|[16154]|RDMAB484-06|UASM77917|615[0n]bp|Canada.Alberta|BOLD:AAA8200
Apamea sora|[16155]|RDNM078-05|CNCNoctuoidea6347|623[0n]bp|Canada.Alberta|BOLD:AAA8200
Apamea sora|[16156]|RDNM079-05|CNCNoctuoidea6348|622[0n]bp|Canada.British Columbia|BOLD:AAA8200
Apamea longula|[16157]|RDNMC376-05|CNCNoctuoidea12010|571[0n]bp|Canada.British Columbia|BOLD:ABZ5701
Apamea longula|[16158]|RDNMC371-05|CNCNoctuoidea12005|598[1n]bp|Canada.British Columbia|BOLD:ABZ5701
Apamea longula|[16159]|RDMAB440-05|BCSC113|658[0n]bp|Canada.Yukon Territory|BOLD:ABZ5701
Apamea longula|[16160]|RDMAB439-05|BCSC112|658[0n]bp|Canada.Yukon Territory|BOLD:ABZ5701
Apamea longula|[16161]|RDNMC374-05|CNCNoctuoidea12008|597[0n]bp|Canada.British Columbia|BOLD:ABZ5701
Apamea longula|[16162]|RDNMC370-05|CNCNoctuoidea12004|598[0n]bp|Canada.British Columbia|BOLD:ABZ5701
Apamea longula|[16163]|LOWCB475-05|CGWC-1415|658[0n]bp|Canada.British Columbia|BOLD:ABZ5701
Apamea longula|[16164]|LOWCB469-05|CGWC-1409|658[0n]bp|Canada.British Columbia|BOLD:ABZ5701
Apamea longula|[16165]|LOWCB473-05|CGWC-1413|658[0n]bp|Canada.British Columbia|BOLD:ABZ5701
Apamea longula|[16166]|LOWCE688-06|CGWC-4448|658[0n]bp|Canada.British Columbia|BOLD:ABZ5701
Apamea longula|[16167]|LOWCE689-06|CGWC-4449|658[0n]bp|Canada.British Columbia|BOLD:ABZ5701
Apamea longula|[16168]|RDMAB442-05|BCSC115|658[0n]bp|Canada.Alberta|BOLD:ABZ5701
Apamea longula|[16169]|LOWCB471-05|CGWC-1411|658[0n]bp|Canada.British Columbia|BOLD:ABZ5701
Apamea longula|[16170]|LBCH7468-10|10-JDWBC-7468|658[0n]bp|Canada.British Columbia|BOLD:ABZ5701
Apamea longula|[16171]|LOWCB470-05|CGWC-1410|658[0n]bp|Canada.British Columbia|BOLD:ABZ5701
Apamea longula|[16172]|LBCH6757-10|10-JDWBC-6757|658[0n]bp|Canada.British Columbia|BOLD:ABZ5701
Apamea longula|[16173]|LOWCB476-05|CGWC-1416|658[0n]bp|Canada.British Columbia|BOLD:ABZ5701
Apamea longula|[16174]|LOWCE685-06|CGWC-4445|658[0n]bp|Canada.British Columbia|BOLD:ABZ5701
Apamea longula|[16175]|LBCH7757-10|10-JDWBC-7757|658[0n]bp|Canada.British Columbia|BOLD:ABZ5701
Apamea longula|[16176]|LOWCB466-05|CGWC-1406|658[0n]bp|Canada.British Columbia|BOLD:ABZ5701
Apamea longula|[16177]|LOWCB474-05|CGWC-1414|658[0n]bp|Canada.British Columbia|BOLD:ABZ5701
Apamea longula|[16178]|LOWCB467-05|CGWC-1407|658[0n]bp|Canada.British Columbia|BOLD:ABZ5701
Apamea longula|[16179]|LOWCB464-05|CGWC-1404|658[0n]bp|Canada.British Columbia|BOLD:ABZ5701
Apamea longula|[16180]|LOWCB463-05|CGWC-1403|658[0n]bp|Canada.British Columbia|BOLD:ABZ5701
Apamea longula|[16181]|LOWCB468-05|CGWC-1408|658[0n]bp|Canada.British Columbia|BOLD:ABZ5701
Apamea longula|[16182]|RDNMC373-05|CNCNoctuoidea12007|617[0n]bp|Canada.British Columbia|BOLD:ABZ5701
Apamea longula|[16183]|LBCG2885-09|08-JDWBC-2885|638[0n]bp|Canada.British Columbia|BOLD:ABZ5701
Apamea longula|[16184]|RDNM970-05|CNCNoctuoidea7810|590[0n]bp|Canada.British Columbia|BOLD:ABZ5701
Apamea longula|[16185]|RDNMC372-05|CNCNoctuoidea12006|552[1n]bp|Canada.British Columbia|BOLD:ABZ5701
Apamea longula|[16186]|LOWCE686-06|CGWC-4446|605[1n]bp|Canada.British Columbia|BOLD:ABZ5701
Apamea longula|[16187]|LOWCB472-05|CGWC-1412|615[0n]bp|Canada.British Columbia|BOLD:ABZ5701
Apamea longula|[16188]|RDMAB441-05|BCSC114|578[0n]bp|Canada.Alberta|BOLD:ABZ5701
Apamea longula|[16189]|LOWCE687-06|CGWC-4447|605[1n]bp|Canada.British Columbia|BOLD:ABZ5701
Apamea longula|[16190]|LOWCB465-05|CGWC-1405|658[0n]bp|Canada.British Columbia|BOLD:ABZ5701
Apamea longula|[16191]|RDNMC017-05|CNCNoctuoidea10758|658[0n]bp|Canada.Alberta|BOLD:ABZ5701
Apamea longula|[16192]|RDNMC012-05|CNCNoctuoidea10753|583[1n]bp|Canada.Alberta|BOLD:ABZ5701
Apamea inficita|[16193]|LOWCE706-06|CGWC-4466|576[1n]bp|Canada.British Columbia|BOLD:ABZ5959
Apamea inficita|[16194]|LOWCE711-06|CGWC-4471|584[1n]bp|Canada.British Columbia|BOLD:ABZ5959
Apamea inficita|[16195]|LOWCB860-05|CGWC-1800|658[0n]bp|Canada.British Columbia|BOLD:ABZ5959
Apamea inficita|[16196]|LOWCE708-06|CGWC-4468|658[0n]bp|Canada.British Columbia|BOLD:ABZ5959
Apamea inficita|[16197]|LOWCB857-05|CGWC-1797|581[0n]bp|Canada.British Columbia|BOLD:ABZ5959
Apamea inficita|[16198]|LOWCE710-06|CGWC-4470|658[0n]bp|Canada.British Columbia|BOLD:ABZ5959
Apamea inficita|[16199]|LBCG188-08|08-JDWBC-0188|658[0n]bp|Canada.British Columbia|BOLD:ABZ5959
Apamea inficita|[16200]|LBCG3311-09|08-JDWBC-3311|658[0n]bp|Canada.British Columbia|BOLD:ABZ5959
Apamea inficita|[16201]|LOWCB861-05|CGWC-1801|557[0n]bp|Canada.British Columbia|BOLD:ABZ5959
Apamea inficita|[16202]|RDMAB448-05|BCSC121|658[0n]bp|Canada.Yukon Territory|BOLD:ABZ5959
Apamea inficita|[16203]|RDMAB458-05|BCSC131|658[0n]bp|Canada.Yukon Territory|BOLD:ABZ5959
Apamea inficita|[16204]|RDNM968-05|CNCNoctuoidea7808|534[0n]bp|Canada.Alberta|BOLD:ABZ5959
Apamea inficita|[16205]|RDNM967-05|CNCNoctuoidea7807|658[0n]bp|Canada.British Columbia|BOLD:ABZ5959
Apamea inficita|[16206]|LPABC826-09|08BBLEP-05045|658[0n]bp|Canada.Alberta|BOLD:ABZ5959
Apamea inficita|[16207]|LPABC206-09|08BBLEP-04425|658[0n]bp|Canada.Alberta|BOLD:ABZ5959
Apamea inficita|[16208]|LOWCB858-05|CGWC-1798|593[0n]bp|Canada.British Columbia|BOLD:ABZ5959
Apamea inficita|[16209]|LPAB247-08|08BBLEP-02569|658[0n]bp|Canada.Alberta|BOLD:ABZ5959
Apamea inficita|[16210]|LPABB724-08|08BBLEP-03989|658[0n]bp|Canada.Alberta|BOLD:ABZ5959
Apamea inficita|[16211]|LPABB858-09|08BBLEP-04178|658[0n]bp|Canada.Alberta|BOLD:ABZ5959
Apamea inficita|[16212]|LPABB847-09|08BBLEP-04167|658[0n]bp|Canada.Alberta|BOLD:ABZ5959
Apamea inficita|[16213]|RDMAB457-05|BCSC130|658[0n]bp|Canada.Alberta|BOLD:ABZ5959
Apamea inficita|[16214]|RDMAB456-05|BCSC129|658[0n]bp|Canada.Alberta|BOLD:ABZ5959
Apamea inficita|[16215]|LPABC827-09|08BBLEP-05046|658[0n]bp|Canada.Alberta|BOLD:ABZ5959
Apamea inficita|[16216]|LBCG187-08|08-JDWBC-0187|658[0n]bp|Canada.British Columbia|BOLD:ABZ5959
Apamea inficita|[16217]|LPABB860-09|08BBLEP-04180|658[0n]bp|Canada.Alberta|BOLD:ABZ5959
Apamea inficita|[16218]|LPABB710-08|08BBLEP-03975|658[0n]bp|Canada.Alberta|BOLD:ABZ5959
Apamea inficita|[16219]|LPABB017-08|08BBLEP-03282|658[0n]bp|Canada.Alberta|BOLD:ABZ5959
Apamea inficita|[16220]|LOWC053-05|CGWC-0053|658[0n]bp|Canada.British Columbia|BOLD:ABZ5959
Apamea inficita|[16221]|LPABB880-09|08BBLEP-04200|636[0n]bp|Canada.Alberta|BOLD:ABZ5959
Apamea inficita|[16222]|LPABC811-09|08BBLEP-05030|658[0n]bp|Canada.Alberta|BOLD:ABZ5959
Apamea inficita|[16223]|LPABB712-08|08BBLEP-03977|658[0n]bp|Canada.Alberta|BOLD:ABZ5959
Apamea inficita|[16224]|LPABB714-08|08BBLEP-03979|631[0n]bp|Canada.Alberta|BOLD:ABZ5959
Apamea inficita|[16225]|LPABB711-08|08BBLEP-03976|637[0n]bp|Canada.Alberta|BOLD:ABZ5959
Apamea inficita|[16226]|LPABC224-09|08BBLEP-04443|658[0n]bp|Canada.Alberta|BOLD:ABZ5959
Apamea inficita|[16227]|LOWCE707-06|CGWC-4467|658[0n]bp|Canada.British Columbia|BOLD:ABZ5959
Apamea inficita|[16228]|LOWCB856-05|CGWC-1796|573[0n]bp|Canada.British Columbia|BOLD:ABZ5959
Apamea inficita|[16229]|BBLEC464-09|09BBELE-0464|658[0n]bp|Canada.New Brunswick|BOLD:ABZ5959
Apamea inficita|[16230]|BBLPB438-10|10BBCLP-1437|658[0n]bp|Canada.Alberta|BOLD:ABZ5959
Apamea inficita|[16231]|LOWCE713-06|CGWC-4473|658[0n]bp|Canada.British Columbia|BOLD:ABZ5959
Apamea cogitata|[16232]|LPABB726-08|08BBLEP-03991|658[0n]bp|Canada.Alberta|BOLD:AAA2865
Apamea cogitata|[16233]|RDLQF063-06|DH008085|658[0n]bp|Canada.Quebec|BOLD:AAA2865
Apamea cogitata|[16234]|LPABC254-09|08BBLEP-04473|633[1n]bp|Canada.Alberta|BOLD:AAA2865
Apamea cogitata|[16235]|LPABC399-09|08BBLEP-04618|658[0n]bp|Canada.Alberta|BOLD:AAA2865
Apamea cogitata|[16236]|LPABB716-08|08BBLEP-03981|658[0n]bp|Canada.Alberta|BOLD:AAA2865
Apamea cogitata|[16237]|LCH627-05|108CH|658[0n]bp|Canada.Manitoba|BOLD:AAA2865
Apamea cogitata|[16238]|BBLPB788-10|10BBCLP-1787|658[0n]bp|Canada.Alberta|BOLD:AAA2865
Apamea cogitata|[16239]|RDLQF064-06|DH008086|658[0n]bp|Canada.Quebec|BOLD:AAA2865
Apamea cogitata|[16240]|RDLQF062-06|DH007553|658[0n]bp|Canada.Quebec|BOLD:AAA2865
Apamea cogitata|[16241]|LPABC460-09|08BBLEP-04679|658[0n]bp|Canada.Alberta|BOLD:AAA2865
Apamea cogitata|[16242]|LPABC208-09|08BBLEP-04427|658[0n]bp|Canada.Alberta|BOLD:AAA2865
Apamea cogitata|[16243]|LPABB715-08|08BBLEP-03980|658[0n]bp|Canada.Alberta|BOLD:AAA2865
Apamea cogitata|[16244]|LPABC342-09|08BBLEP-04561|638[0n]bp|Canada.Alberta|BOLD:AAA2865
Apamea cogitata|[16245]|LBCH1304-10|10-JDWBC-1304|633[0n]bp|Canada.British Columbia|BOLD:AAA2865
Apamea cogitata|[16246]|TTMNB328-06|MNBTT-328|626[0n]bp|Canada.New Brunswick|BOLD:AAA2865

Apamea cogitata|[16244]|LPABC342-09|08BBLEP-04561|638[0n]bp|Canada.Alberta|BOLD:AAA2865
Apamea cogitata|[16245]|LBCH1304-10|10-JDWBC-1304|633[0n]bp|Canada.British Columbia|BOLD:AAA2865
Apamea cogitata|[16246]|TTMNB328-06|MNBTT-328|626[0n]bp|Canada.New Brunswick|BOLD:AAA2865
Apamea cogitata|[16247]|BBLPB787-10|10BBCLP-1786|658[0n]bp|Canada.Alberta|BOLD:AAA2865
Apamea cogitata|[16248]|LPABC204-09|08BBLEP-04423|634[0n]bp|Canada.Alberta|BOLD:AAA2865
Apamea cogitata|[16249]|LBCH1681-10|10-JDWBC-1681|658[0n]bp|Canada.British Columbia|BOLD:AAA2865
Apamea cogitata|[16250]|LBCH7755-10|10-JDWBC-7755|658[0n]bp|Canada.British Columbia|BOLD:AAA2865
Apamea cogitata|[16251]|LBCH917-10|10-JDWBC-0917|658[0n]bp|Canada.British Columbia|BOLD:AAA2865
Apamea cogitata|[16252]|LBCH017-10|10-JDWBC-0017|658[0n]bp|Canada.British Columbia|BOLD:AAA2865
Apamea cogitata|[16253]|LOWCD305-06|CGWC-3125|658[0n]bp|Canada.British Columbia|BOLD:AAA2865
Apamea cogitata|[16254]|LOWCC935-05|CGWC-2815|658[0n]bp|Canada.British Columbia|BOLD:AAA2865
Apamea cogitata|[16255]|LHLEP064-06|UBC-2006-0269|646[0n]bp|Canada.British Columbia|BOLD:AAA2865
Apamea cogitata|[16256]|LOWCD310-06|CGWC-3130|658[0n]bp|Canada.British Columbia|BOLD:AAA2865
Apamea cogitata|[16257]|LBCB069-05|HLC-21009|658[0n]bp|Canada.British Columbia|BOLD:AAA2865
Apamea cogitata|[16258]|LBCH2203-10|10-JDWBC-2203|658[0n]bp|Canada.British Columbia|BOLD:AAA2865
Apamea cogitata|[16259]|LBCH1581-10|10-JDWBC-1581|658[0n]bp|Canada.British Columbia|BOLD:AAA2865
Apamea cogitata|[16260]|LBCH4695-10|10-JDWBC-4695|658[0n]bp|Canada.British Columbia|BOLD:AAA2865
Apamea cogitata|[16261]|LBCG2884-09|08-JDWBC-2884|658[0n]bp|Canada.British Columbia|BOLD:AAA2865
Apamea cogitata|[16262]|LBCG582-09|08-JDWBC-0582|658[0n]bp|Canada.British Columbia|BOLD:AAA2865
Apamea cogitata|[16263]|LBCG2366-09|08-JDWBC-2366|658[0n]bp|Canada.British Columbia|BOLD:AAA2865
Apamea cogitata|[16264]|LOWCC435-05|CGWC-2315|658[0n]bp|Canada.British Columbia|BOLD:AAA2865
Apamea cogitata|[16265]|LOWCC872-05|CGWC-2752|658[0n]bp|Canada.British Columbia|BOLD:AAA2865
Apamea cogitata|[16266]|LOWCC415-05|CGWC-2295|658[0n]bp|Canada.British Columbia|BOLD:AAA2865
Apamea cogitata|[16267]|RDMAB498-06|UASM58433|608[0n]bp|Canada.Alberta|BOLD:AAA2865
Apamea cogitata|[16268]|LBCH2096-10|10-JDWBC-2096|658[0n]bp|Canada.British Columbia|BOLD:AAA2865
Apamea cogitata|[16269]|LHLEP417-06|UBC-2006-1171|658[0n]bp|Canada.British Columbia|BOLD:AAA2865
Apamea cogitata|[16270]|LOWCC433-05|CGWC-2313|589[0n]bp|Canada.British Columbia|BOLD:AAA2865
Apamea cogitata|[16271]|LOWCC421-05|CGWC-2301|604[0n]bp|Canada.British Columbia|BOLD:AAA2865
Apamea cogitata|[16272]|LOWCD303-06|CGWC-3123|585[0n]bp|Canada.British Columbia|BOLD:AAA2865
Apamea cogitata|[16273]|LPGVA631-08|UBC-2006-1799|658[0n]bp|Canada.British Columbia|BOLD:AAA2865
Apamea cogitata|[16274]|RDMAB073-05|UASM57600|633[0n]bp|Canada.Alberta|BOLD:AAA2865
Apamea cogitata|[16275]|BBLPB784-10|10BBCLP-1783|658[0n]bp|Canada.Alberta|BOLD:AAA2865
Apamea cogitata|[16276]|LPABB725-08|08BBLEP-03990|658[0n]bp|Canada.Alberta|BOLD:AAA2865
Apamea cogitata|[16277]|BBLPB791-10|10BBCLP-1790|658[0n]bp|Canada.Alberta|BOLD:AAA2865
Apamea cogitata|[16278]|LBCH1169-10|10-JDWBC-1169|658[0n]bp|Canada.British Columbia|BOLD:AAA2865
Apamea cogitata|[16279]|LPABC908-09|08BBLEP-05319|658[0n]bp|Canada.Alberta|BOLD:AAA2865
Apamea cogitata|[16280]|LBCD449-05|HLC-23269|658[0n]bp|Canada.British Columbia|BOLD:AAA2865
Apamea cogitata|[16281]|LBCH1582-10|10-JDWBC-1582|658[0n]bp|Canada.British Columbia|BOLD:AAA2865
Apamea cogitata|[16282]|LBCD273-05|HLC-23093|658[0n]bp|Canada.British Columbia|BOLD:AAA2865
Apamea cogitata|[16283]|LBCH1497-10|10-JDWBC-1497|658[0n]bp|Canada.British Columbia|BOLD:AAA2865
Apamea cogitata|[16284]|LPSK459-08|08BBLEP-02027|658[0n]bp|Canada.Saskatchewan|BOLD:AAA2865
Apamea cogitata|[16285]|LBCH1583-10|10-JDWBC-1583|658[0n]bp|Canada.British Columbia|BOLD:AAA2865
Apamea cogitata|[16286]|LBCD450-05|HLC-23270|658[0n]bp|Canada.British Columbia|BOLD:AAA2865
Apamea cogitata|[16287]|LBCH796-10|10-JDWBC-0796|658[0n]bp|Canada.British Columbia|BOLD:AAA2865
Apamea cogitata|[16288]|LPABB882-09|08BBLEP-04202|658[0n]bp|Canada.Alberta|BOLD:AAA2865
Apamea cogitata|[16289]|LBCC556-05|HLC-22436|658[0n]bp|Canada.British Columbia|BOLD:AAA2865
Apamea cogitata|[16290]|LBCG3281-09|08-JDWBC-3281|658[0n]bp|Canada.British Columbia|BOLD:AAA2865
Apamea cogitata|[16291]|LBCH113-10|10-JDWBC-0113|658[0n]bp|Canada.British Columbia|BOLD:AAA2865
Apamea cogitata|[16292]|LBCD443-05|HLC-23263|658[0n]bp|Canada.British Columbia|BOLD:AAA2865
Apamea cogitata|[16293]|LPSK453-08|08BBLEP-02021|658[0n]bp|Canada.Saskatchewan|BOLD:AAA2865
Apamea cogitata|[16294]|LBCH3344-10|10-JDWBC-3344|658[0n]bp|Canada.British Columbia|BOLD:AAA2865
Apamea cogitata|[16295]|LHLEP416-06|UBC-2006-1046|658[0n]bp|Canada.British Columbia|BOLD:AAA2865
Apamea cogitata|[16296]|LOWCC416-05|CGWC-2296|658[0n]bp|Canada.British Columbia|BOLD:AAA2865
Apamea cogitata|[16297]|LBCD442-05|HLC-23262|658[0n]bp|Canada.British Columbia|BOLD:AAA2865
Apamea cogitata|[16298]|LBCC824-05|HLC-22704|658[0n]bp|Canada.British Columbia|BOLD:AAA2865
Apamea cogitata|[16299]|LPABB629-08|08BBLEP-03894|658[0n]bp|Canada.Alberta|BOLD:AAA2865
Apamea cogitata|[16300]|LHLEP442-06|UBC-2006-2074|658[0n]bp|Canada.British Columbia|BOLD:AAA2865
Apamea cogitata|[16301]|LOWCC419-05|CGWC-2299|658[0n]bp|Canada.British Columbia|BOLD:AAA2865
Apamea cogitata|[16302]|LBCH2241-10|10-JDWBC-2241|658[0n]bp|Canada.British Columbia|BOLD:AAA2865
Apamea cogitata|[16303]|LBCH3070-10|10-JDWBC-3070|658[0n]bp|Canada.British Columbia|BOLD:AAA2865
Apamea cogitata|[16304]|LBCC869-05|HLC-22749|658[0n]bp|Canada.British Columbia|BOLD:AAA2865
Apamea cogitata|[16305]|LBCH4434-10|10-JDWBC-4434|658[0n]bp|Canada.British Columbia|BOLD:AAA2865
Apamea cogitata|[16306]|LOWCC437-05|CGWC-2317|658[0n]bp|Canada.British Columbia|BOLD:AAA2865
Apamea cogitata|[16307]|LPABB615-08|08BBLEP-03880|658[0n]bp|Canada.Alberta|BOLD:AAA2865
Apamea cogitata|[16308]|LPABC436-09|08BBLEP-04655|658[0n]bp|Canada.Alberta|BOLD:AAA2865
Apamea cogitata|[16309]|LOWCC418-05|CGWC-2298|658[0n]bp|Canada.British Columbia|BOLD:AAA2865
Apamea cogitata|[16310]|LPSK431-08|08BBLEP-01999|658[0n]bp|Canada.Saskatchewan|BOLD:AAA2865
Apamea cogitata|[16311]|LBCG2364-09|08-JDWBC-2364|658[0n]bp|Canada.British Columbia|BOLD:AAA2865
Apamea cogitata|[16312]|LPSK437-08|08BBLEP-02005|658[0n]bp|Canada.Saskatchewan|BOLD:AAA2865
Apamea cogitata|[16313]|LBCG2365-09|08-JDWBC-2365|658[0n]bp|Canada.British Columbia|BOLD:AAA2865
Apamea cogitata|[16314]|LBCG2776-09|08-JDWBC-2776|658[0n]bp|Canada.British Columbia|BOLD:AAA2865
Apamea cogitata|[16315]|LPABB604-08|08BBLEP-03869|658[0n]bp|Canada.Alberta|BOLD:AAA2865
Apamea cogitata|[16316]|LBCH2097-10|10-JDWBC-2097|658[0n]bp|Canada.British Columbia|BOLD:AAA2865
Apamea cogitata|[16317]|BBLPB786-10|10BBCLP-1785|658[0n]bp|Canada.British Columbia|BOLD:AAA2865
Apamea cogitata|[16318]|LPABC255-09|08BBLEP-04474|658[0n]bp|Canada.Alberta|BOLD:AAA2865
Apamea cogitata|[16319]|LBCH2240-10|10-JDWBC-2240|658[0n]bp|Canada.British Columbia|BOLD:AAA2865
Apamea cogitata|[16320]|LOWCC434-05|CGWC-2314|658[0n]bp|Canada.British Columbia|BOLD:AAA2865
Apamea cogitata|[16321]|LPSK434-08|08BBLEP-02002|658[0n]bp|Canada.Saskatchewan|BOLD:AAA2865
Apamea cogitata|[16322]|LBCB802-05|HLC-21742|658[0n]bp|Canada.British Columbia|BOLD:AAA2865
Apamea cogitata|[16323]|LBCH694-10|10-JDWBC-0694|658[0n]bp|Canada.British Columbia|BOLD:AAA2865
Apamea cogitata|[16324]|LBCG2066-09|08-JDWBC-2066|658[0n]bp|Canada.British Columbia|BOLD:AAA2865
Apamea cogitata|[16325]|LOWCD307-06|CGWC-3127|658[0n]bp|Canada.British Columbia|BOLD:AAA2865
Apamea cogitata|[16326]|LPABC207-09|08BBLEP-04426|658[0n]bp|Canada.Alberta|BOLD:AAA2865
Apamea cogitata|[16327]|LBCG3322-09|08-JDWBC-3322|658[0n]bp|Canada.British Columbia|BOLD:AAA2865
Apamea cogitata|[16328]|LPSK433-08|08BBLEP-02001|658[0n]bp|Canada.Saskatchewan|BOLD:AAA2865
Apamea cogitata|[16329]|LPABC212-09|08BBLEP-04431|658[0n]bp|Canada.Alberta|BOLD:AAA2865
Apamea cogitata|[16330]|LOWCD304-06|CGWC-3124|658[0n]bp|Canada.British Columbia|BOLD:AAA2865
Apamea cogitata|[16331]|BBLPB785-10|10BBCLP-1784|658[0n]bp|Canada.British Columbia|BOLD:AAA2865
Apamea cogitata|[16332]|LPABB873-09|08BBLEP-04193|658[0n]bp|Canada.Alberta|BOLD:AAA2865
Apamea cogitata|[16333]|LHLEP063-06|UBC-2006-0268|658[0n]bp|Canada.British Columbia|BOLD:AAA2865
Apamea cogitata|[16334]|LPSK432-08|08BBLEP-02000|658[0n]bp|Canada.Saskatchewan|BOLD:AAA2865
Apamea cogitata|[16335]|LBCG472-08|08-JDWBC-0472|658[0n]bp|Canada.British Columbia|BOLD:AAA2865
Apamea cogitata|[16336]|LBCH4694-10|10-JDWBC-4694|658[0n]bp|Canada.British Columbia|BOLD:AAA2865
Apamea cogitata|[16337]|LBCB753-05|HLC-21693|658[0n]bp|Canada.British Columbia|BOLD:AAA2865
Apamea cogitata|[16338]|LBCH1997-10|10-JDWBC-1997|658[0n]bp|Canada.British Columbia|BOLD:AAA2865
Apamea cogitata|[16339]|LOWCC417-05|CGWC-2297|658[0n]bp|Canada.British Columbia|BOLD:AAA2865
Apamea cogitata|[16340]|LOWCC420-05|CGWC-2300|658[0n]bp|Canada.British Columbia|BOLD:AAA2865
Apamea cogitata|[16341]|LBCD048-05|HLC-22868|658[0n]bp|Canada.British Columbia|BOLD:AAA2865
Apamea cogitata|[16342]|LPABB713-08|08BBLEP-03978|658[0n]bp|Canada.Alberta|BOLD:AAA2865
Apamea cogitata|[16343]|LOWCC422-05|CGWC-2302|658[0n]bp|Canada.British Columbia|BOLD:AAA2865
Apamea cogitata|[16344]|LBCD310-05|HLC-23130|656[0n]bp|Canada.British Columbia|BOLD:AAA2865
Apamea cogitata|[16345]|LBCS187-07|UBC-2007-0568|658[0n]bp|Canada.British Columbia|BOLD:AAA2865
Apamea cogitata|[16346]|LPSK460-08|08BBLEP-02028|658[0n]bp|Canada.Saskatchewan|BOLD:AAA2865

Apamea cogitata|[16343]|[illegible]
Apamea cogitata|[16344]|LBCD310-05|HLC-23130|656[0n]bp|Canada.British Columbia|BOLD:AAA2865
Apamea cogitata|[16345]|LBCS187-07|UBC-2007-0568|658[0n]bp|Canada.British Columbia|BOLD:AAA2865
Apamea cogitata|[16346]|LPSK460-08|08BBLEP-02028|658[0n]bp|Canada.Saskatchewan|BOLD:AAA2865
Apamea cogitata|[16347]|LBCH223-10|10-JDWBC-0223|658[0n]bp|Canada.British Columbia|BOLD:AAA2865
Apamea cogitata|[16348]|LBCB752-05|HLC-21692|658[0n]bp|Canada.British Columbia|BOLD:AAA2865
Apamea cogitata|[16349]|LBCC338-05|HLC-22218|658[0n]bp|Canada.British Columbia|BOLD:AAA2865
Apamea cogitata|[16350]|LBCH1862-10|10-JDWBC-1862|658[0n]bp|Canada.British Columbia|BOLD:AAA2865
Apamea cogitata|[16351]|BBLPB790-10|10BBCLP-1789|658[0n]bp|Canada.British Columbia|BOLD:AAA2865
Apamea cogitata|[16352]|LBCD444-05|HLC-23264|658[0n]bp|Canada.British Columbia|BOLD:AAA2865
Apamea cogitata|[16353]|LBCH4113-10|10-JDWBC-4113|658[0n]bp|Canada.British Columbia|BOLD:AAA2865
Apamea cogitata|[16354]|LPABC202-09|08BBLEP-04421|658[0n]bp|Canada.Alberta|BOLD:AAA2865
Apamea cogitata|[16355]|LBCB581-05|HLC-21521|658[0n]bp|Canada.British Columbia|BOLD:AAA2865
Apamea cogitata|[16356]|LPSK430-08|08BBLEP-01998|609[0n]bp|Canada.Saskatchewan|BOLD:AAA2865
Apamea cogitata|[16357]|LOWCD306-06|CGWC-3126|593[0n]bp|Canada.British Columbia|BOLD:AAA2865
Apamea cogitata|[16358]|LOWCD309-06|CGWC-3129|610[0n]bp|Canada.British Columbia|BOLD:AAA2865
Apamea cogitata|[16359]|LOWCD308-06|CGWC-3128|608[0n]bp|Canada.British Columbia|BOLD:AAA2865
Apamea cogitata|[16360]|LPABB719-08|08BBLEP-03984|644[0n]bp|Canada.Alberta|BOLD:AAA2865
Apamea cogitata|[16361]|LBCD326-05|HLC-23146|650[0n]bp|Canada.British Columbia|BOLD:AAA2865
Apamea cogitata|[16362]|LPSK455-08|08BBLEP-02023|643[0n]bp|Canada.Saskatchewan|BOLD:AAA2865
Apamea cogitata|[16363]|LPABB816-09|08BBLEP-04136|644[0n]bp|Canada.Alberta|BOLD:AAA2865
Apamea cogitata|[16364]|LBCH1935-10|10-JDWBC-1935|658[0n]bp|Canada.British Columbia|BOLD:AAA2865
Apamea cogitata|[16365]|LPSK429-08|08BBLEP-01997|658[0n]bp|Canada.Saskatchewan|BOLD:AAA2865
Apamea cogitata|[16366]|LOWCC436-05|CGWC-2316|658[0n]bp|Canada.British Columbia|BOLD:AAA2865
Apamea cogitata|[16367]|LBCB070-05|HLC-21010|658[0n]bp|Canada.British Columbia|BOLD:AAA2865
Apamea cogitata|[16368]|LOWCC432-05|CGWC-2312|658[0n]bp|Canada.British Columbia|BOLD:AAA2865
Apamea cogitata|[16369]|LPABB577-08|08BBLEP-03842|658[0n]bp|Canada.Alberta|BOLD:AAA2865
Apamea dubitans|[16370]|XAH170-05|2005-ONT-1753|579[4n]bp|Canada.Ontario|BOLD:ACF4111
Apamea dubitans|[16371]|RDLQF552-06|DH011701|658[0n]bp|Canada.Quebec|BOLD:ACF4111
Apamea dubitans|[16372]|RDLQF066-06|DH007552|658[0n]bp|Canada.Quebec|BOLD:ACF4111
Apamea dubitans|[16373]|RDLQF065-06|DH007403|658[0n]bp|Canada.Quebec|BOLD:ACF4111
Apamea dubitans|[16374]|LPMNB240-09|08BBLEP-05084|658[0n]bp|Canada.Manitoba|BOLD:ACF4111
Apamea dubitans|[16375]|LPSOD973-09|08BBLEP-05606|658[0n]bp|Canada.Ontario|BOLD:ACF4111
Apamea dubitans|[16376]|BLTIB955-08|BL1384|658[0n]bp|Canada.Ontario|BOLD:ACF4111
Apamea dubitans|[16377]|BBLEC473-09|09BBELE-0473|638[0n]bp|Canada.New Brunswick|BOLD:ACF4111
Apamea dubitans|[16378]|PHMO298-03|moth2123.02|639[0n]bp|Canada.Ontario|BOLD:ACF4111
Apamea devastator|[16379]|LPABC069-09|08BBLEP-04288|658[0n]bp|Canada.Alberta|BOLD:ABY5257
Apamea devastator|[16380]|LOWCB478-05|CGWC-1418|658[0n]bp|Canada.British Columbia|BOLD:ABY5257
Apamea devastator|[16381]|LPABB833-09|08BBLEP-04153|658[0n]bp|Canada.Alberta|BOLD:ABY5257
Apamea devastator|[16382]|LBCG2886-09|08-JDWBC-2886|658[0n]bp|Canada.British Columbia|BOLD:ABY5257
Apamea devastator|[16383]|LBCH2098-10|10-JDWBC-2098|658[0n]bp|Canada.British Columbia|BOLD:ABY5257
Apamea devastator|[16384]|LPSK564-08|08BBLEP-02132|658[0n]bp|Canada.Saskatchewan|BOLD:ABY5257
Apamea devastator|[16385]|BBLPB702-10|10BBCLP-1701|658[0n]bp|Canada.Alberta|BOLD:ABY5257
Apamea devastator|[16386]|LOWCB169-05|CGWC-1109|658[0n]bp|Canada.British Columbia|BOLD:ABY5257
Apamea devastator|[16387]|LOWCB481-05|CGWC-1421|658[0n]bp|Canada.British Columbia|BOLD:ABY5257
Apamea devastator|[16388]|LOWCB482-05|CGWC-1422|658[0n]bp|Canada.British Columbia|BOLD:ABY5257
Apamea devastator|[16389]|LOWCB171-05|CGWC-1111|658[0n]bp|Canada.British Columbia|BOLD:ABY5257
Apamea devastator|[16390]|BBLPB767-10|10BBCLP-1766|658[0n]bp|Canada.Alberta|BOLD:ABY5257
Apamea devastator|[16391]|LBCH1106-10|10-JDWBC-1106|658[0n]bp|Canada.British Columbia|BOLD:ABY5257
Apamea devastator|[16392]|LPABB196-08|08BBLEP-03461|658[0n]bp|Canada.Alberta|BOLD:ABY5257
Apamea devastator|[16393]|LPABB845-09|08BBLEP-04165|658[0n]bp|Canada.Alberta|BOLD:ABY5257
Apamea devastator|[16394]|LPABC042-09|08BBLEP-04261|658[0n]bp|Canada.Alberta|BOLD:ABY5257
Apamea devastator|[16395]|BBLPB676-10|10BBCLP-1675|658[0n]bp|Canada.Alberta|BOLD:ABY5257
Apamea devastator|[16396]|LPABC704-09|08BBLEP-04923|658[0n]bp|Canada.Alberta|BOLD:ABY5257
Apamea devastator|[16397]|LBCD441-05|HLC-23261|658[0n]bp|Canada.British Columbia|BOLD:ABY5257
Apamea devastator|[16398]|LOWCE699-06|CGWC-4459|658[0n]bp|Canada.British Columbia|BOLD:ABY5257
Apamea devastator|[16399]|LPABB117-08|08BBLEP-03382|658[0n]bp|Canada.Alberta|BOLD:ABY5257
Apamea devastator|[16400]|LOWCB479-05|CGWC-1419|658[0n]bp|Canada.British Columbia|BOLD:ABY5257
Apamea devastator|[16401]|BBLPB711-10|10BBCLP-1710|612[0n]bp|Canada.Alberta|BOLD:ABY5257
Apamea devastator|[16402]|LOWCB483-05|CGWC-1423|611[0n]bp|Canada.British Columbia|BOLD:ABY5257
Apamea devastator|[16403]|LPSK458-08|08BBLEP-02026|658[0n]bp|Canada.Saskatchewan|BOLD:ABY5257
Apamea devastator|[16404]|TTMNB333-06|MNBTT-333|658[0n]bp|Canada.New Brunswick|BOLD:ABY5257
Apamea devastator|[16405]|LPSK428-08|08BBLEP-01996|658[0n]bp|Canada.Saskatchewan|BOLD:ABY5257
Apamea devastator|[16406]|LPSK457-08|08BBLEP-02025|658[0n]bp|Canada.Saskatchewan|BOLD:ABY5257
Apamea devastator|[16407]|LPSK552-08|08BBLEP-02120|658[0n]bp|Canada.Saskatchewan|BOLD:ABY5257
Apamea devastator|[16408]|LPABB614-08|08BBLEP-03879|658[0n]bp|Canada.Alberta|BOLD:ABY5257
Apamea devastator|[16409]|LPSK597-08|08BBLEP-02165|658[0n]bp|Canada.Saskatchewan|BOLD:ABY5257
Apamea devastator|[16410]|LPABC099-09|08BBLEP-04318|658[0n]bp|Canada.Alberta|BOLD:ABY5257
Apamea devastator|[16411]|RDNMJ657-11|CNC LEP 70119|658[0n]bp|Canada.Alberta|BOLD:ABY5257
Apamea devastator|[16412]|LPABB119-08|08BBLEP-03384|658[0n]bp|Canada.Alberta|BOLD:ABY5257
Apamea devastator|[16413]|LOWCB477-05|CGWC-1417|658[1n]bp|Canada.British Columbia|BOLD:ABY5257
Apamea devastator|[16414]|XAG848-05|2005-ONT-1432|658[0n]bp|Canada.Ontario|BOLD:ABY5257
Apamea devastator|[16415]|TTMNB331-06|MNBTT-331|658[1n]bp|Canada.New Brunswick|BOLD:ABY5257
Apamea devastator|[16416]|TTMNB334-06|MNBTT-334|658[1n]bp|Canada.New Brunswick|BOLD:ABY5257
Apamea devastator|[16417]|LPVIC029-08|PFC-2006-2584|592[0n]bp|Canada.British Columbia|BOLD:ABY5257
Apamea devastator|[16418]|PHMNB240-04|04HBL007705|609[0n]bp|Canada.New Brunswick|BOLD:ABY5257
Apamea devastator|[16419]|LPVIB507-08|PFC-2006-1918|658[0n]bp|Canada.British Columbia|BOLD:ABY5257
Apamea devastator|[16420]|LPSK514-08|08BBLEP-02082|658[0n]bp|Canada.Saskatchewan|BOLD:ABY5257
Apamea devastator|[16421]|LBCH1167-10|10-JDWBC-1167|658[0n]bp|Canada.British Columbia|BOLD:ABY5257
Apamea devastator|[16422]|XAB162-04|04HBL005162|658[0n]bp|Canada.Ontario|BOLD:ABY5257
Apamea devastator|[16423]|LOWCE698-06|CGWC-4458|658[0n]bp|Canada.British Columbia|BOLD:ABY5257
Apamea devastator|[16424]|LBCC299-05|HLC-22179|658[0n]bp|Canada.British Columbia|BOLD:ABY5257
Apamea devastator|[16425]|LBCH7684-10|10-JDWBC-7684|658[0n]bp|Canada.British Columbia|BOLD:ABY5257
Apamea devastator|[16426]|LOWCE701-06|CGWC-4461|658[0n]bp|Canada.British Columbia|BOLD:ABY5257
Apamea devastator|[16427]|LBCH6038-10|10-JDWBC-6038|658[0n]bp|Canada.British Columbia|BOLD:ABY5257
Apamea devastator|[16428]|LOWCB480-05|CGWC-1420|658[0n]bp|Canada.British Columbia|BOLD:ABY5257
Apamea devastator|[16429]|LOWCE703-06|CGWC-4463|658[0n]bp|Canada.British Columbia|BOLD:ABY5257
Apamea devastator|[16430]|TTMNB569-06|MNBTT-569|658[0n]bp|Canada.New Brunswick|BOLD:ABY5257
Apamea devastator|[16431]|RDLQB825-05|DH010912|658[0n]bp|Canada.Quebec|BOLD:ABY5257
Apamea devastator|[16432]|LPSK590-08|08BBLEP-02158|658[0n]bp|Canada.Saskatchewan|BOLD:ABY5257
Apamea devastator|[16433]|LOWCB170-05|CGWC-1110|658[0n]bp|Canada.British Columbia|BOLD:ABY5257
Apamea devastator|[16434]|LPSK426-08|08BBLEP-01994|658[0n]bp|Canada.Saskatchewan|BOLD:ABY5257
Apamea devastator|[16435]|LPSK450-08|08BBLEP-02018|658[0n]bp|Canada.Saskatchewan|BOLD:ABY5257
Apamea devastator|[16436]|XAD014-04|04HBL007014|658[0n]bp|Canada.Ontario|BOLD:ABY5257
Apamea devastator|[16437]|LBCG471-08|08-JDWBC-0471|658[0n]bp|Canada.British Columbia|BOLD:ABY5257
Apamea devastator|[16438]|LOWCB168-05|CGWC-1108|658[0n]bp|Canada.British Columbia|BOLD:ABY5257
Apamea devastator|[16439]|LHLEP418-06|UBC-2006-1045|658[0n]bp|Canada.British Columbia|BOLD:ABY5257
Apamea devastator|[16440]|LPABB727-08|08BBLEP-03992|658[0n]bp|Canada.Alberta|BOLD:ABY5257
Apamea devastator|[16441]|TTMNB335-06|MNBTT-335|658[0n]bp|Canada.New Brunswick|BOLD:ABY5257
Apamea devastator|[16442]|LOWCE692-06|CGWC-4452|658[0n]bp|Canada.British Columbia|BOLD:ABY5257
Apamea devastator|[16443]|LPSK020-08|08BBLEP-00723|658[0n]bp|Canada.Saskatchewan|BOLD:ABY5257
Apamea devastator|[16444]|LPSK483-08|08BBLEP-02051|658[0n]bp|Canada.Saskatchewan|BOLD:ABY5257
Apamea devastator|[16445]|BLTIB864-08|BL1283|658[0n]bp|Canada.Ontario|BOLD:ABY5257
Apamea devastator|[16446]|[illegible]

Apamea devastator|[16443]|LPSK020-08|08BBLEP-00723|658[0n]bp|Canada.Saskatchewan|BOLD:ABY5257
Apamea devastator|[16444]|LPSK483-08|08BBLEP-02051|658[0n]bp|Canada.Saskatchewan|BOLD:ABY5257
Apamea devastator|[16445]|BLTIB864-08|BL1283|658[0n]bp|Canada.Ontario|BOLD:ABY5257
Apamea devastator|[16446]|LPSK435-08|08BBLEP-02003|658[0n]bp|Canada.Saskatchewan|BOLD:ABY5257
Apamea devastator|[16447]|LOWCB172-05|CGWC-1112|658[0n]bp|Canada.British Columbia|BOLD:ABY5257
Apamea devastator|[16448]|LOWCE690-06|CGWC-4450|658[0n]bp|Canada.British Columbia|BOLD:ABY5257
Apamea devastator|[16449]|LPSK500-08|08BBLEP-02068|658[0n]bp|Canada.Saskatchewan|BOLD:ABY5257
Apamea devastator|[16450]|LPSK449-08|08BBLEP-02017|658[0n]bp|Canada.Saskatchewan|BOLD:ABY5257
Apamea devastator|[16451]|BBLPB360-10|10BBCLP-1359|658[0n]bp|Canada.Alberta|BOLD:ABY5257
Apamea devastator|[16452]|LPSK461-08|08BBLEP-02029|658[0n]bp|Canada.Saskatchewan|BOLD:ABY5257
Apamea devastator|[16453]|LOWCE702-06|CGWC-4462|658[0n]bp|Canada.British Columbia|BOLD:ABY5257
Apamea devastator|[16454]|TTMNB567-06|MNBTT-567|658[0n]bp|Canada.New Brunswick|BOLD:ABY5257
Apamea devastator|[16455]|LBCH7120-10|10-JDWBC-7120|658[0n]bp|Canada.British Columbia|BOLD:ABY5257
Apamea devastator|[16456]|LOWCE700-06|CGWC-4460|658[0n]bp|Canada.British Columbia|BOLD:ABY5257
Apamea devastator|[16457]|LBCH6026-10|10-JDWBC-6026|658[0n]bp|Canada.British Columbia|BOLD:ABY5257
Apamea devastator|[16458]|LPSK427-08|08BBLEP-01995|658[0n]bp|Canada.Saskatchewan|BOLD:ABY5257
Apamea devastator|[16459]|LPVIC028-08|PFC-2006-2583|658[0n]bp|Canada.British Columbia|BOLD:ABY5257
Apamea devastator|[16460]|LOWCE694-06|CGWC-4454|658[0n]bp|Canada.British Columbia|BOLD:ABY5257
Apamea devastator|[16461]|LOWCC440-05|CGWC-2320|658[0n]bp|Canada.British Columbia|BOLD:ABY5257
Apamea devastator|[16462]|LOWCE705-06|CGWC-4465|658[0n]bp|Canada.British Columbia|BOLD:ABY5257
Apamea devastator|[16463]|LBCH7328-10|10-JDWBC-7328|658[0n]bp|Canada.British Columbia|BOLD:ABY5257
Apamea devastator|[16464]|LBCG2580-09|08-JDWBC-2580|658[0n]bp|Canada.British Columbia|BOLD:ABY5257
Apamea devastator|[16465]|LPABB636-08|08BBLEP-03901|658[0n]bp|Canada.Alberta|BOLD:ABY5257
Apamea devastator|[16466]|XAG016-05|2005-ONT-600|658[0n]bp|Canada.Ontario|BOLD:ABY5257
Apamea devastator|[16467]|LBCG3280-09|08-JDWBC-3280|658[0n]bp|Canada.British Columbia|BOLD:ABY5257
Apamea devastator|[16468]|XAC039-04|04HBL006039|658[0n]bp|Canada.Ontario|BOLD:ABY5257
Apamea devastator|[16469]|LPSK536-08|08BBLEP-02104|658[0n]bp|Canada.Saskatchewan|BOLD:ABY5257
Apamea devastator|[16470]|LPSK115-08|08BBLEP-01683|658[0n]bp|Canada.Saskatchewan|BOLD:ABY5257
Apamea devastator|[16471]|LPVIB069-08|PFC-2006-1417|658[0n]bp|Canada.British Columbia|BOLD:ABY5257
Apamea devastator|[16472]|LPVIB238-08|PFC-2006-1617|658[0n]bp|Canada.British Columbia|BOLD:ABY5257
Apamea devastator|[16473]|LBCH2095-10|10-JDWBC-2095|658[0n]bp|Canada.British Columbia|BOLD:ABY5257
Apamea devastator|[16474]|LPSK550-08|08BBLEP-02118|658[0n]bp|Canada.Saskatchewan|BOLD:ABY5257
Apamea devastator|[16475]|LOWCE691-06|CGWC-4451|658[0n]bp|Canada.British Columbia|BOLD:ABY5257
Apamea devastator|[16476]|LBCG3323-09|08-JDWBC-3323|658[0n]bp|Canada.British Columbia|BOLD:ABY5257
Apamea devastator|[16477]|LBCG3282-09|08-JDWBC-3282|658[0n]bp|Canada.British Columbia|BOLD:ABY5257
Apamea devastator|[16478]|LPABC414-09|08BBLEP-04633|658[0n]bp|Canada.Alberta|BOLD:ABY5257
Apamea devastator|[16479]|LBCG2890-09|08-JDWBC-2890|658[0n]bp|Canada.British Columbia|BOLD:ABY5257
Apamea devastator|[16480]|LPABB390-08|08BBLEP-03655|658[0n]bp|Canada.Alberta|BOLD:ABY5257
Apamea devastator|[16481]|XAJ980-06|2006-ONT-0980|658[0n]bp|Canada.Ontario|BOLD:ABY5257
Apamea devastator|[16482]|BBLPB802-10|10BBCLP-1801|658[0n]bp|Canada.British Columbia|BOLD:ABY5257
Apamea devastator|[16483]|XAC804-04|04HBL006804|658[0n]bp|Canada.Ontario|BOLD:ABY5257
Apamea devastator|[16484]|BBLPB664-10|10BBCLP-1663|636[0n]bp|Canada.British Columbia|BOLD:ABY5257
Apamea devastator|[16485]|TTMNB336-06|MNBTT-336|658[1n]bp|Canada.New Brunswick|BOLD:ABY5257
Apamea devastator|[16486]|XAG732-05|2005-ONT-1316|638[0n]bp|Canada.Ontario|BOLD:ABY5257
Apamea devastator|[16487]|LPABC068-09|08BBLEP-04287|637[0n]bp|Canada.Alberta|BOLD:ABY5257
Apamea devastator|[16488]|XAC036-04|04HBL006036|583[0n]bp|Canada.Ontario|BOLD:ABY5257
Apamea devastator|[16489]|LBCB621-05|HLC-21561|597[0n]bp|Canada.British Columbia|BOLD:ABY5257
Apamea devastator|[16490]|PMG091-03|moth1175.01|617[0n]bp|Canada.Ontario|BOLD:ABY5257
Apamea devastator|[16491]|BBLPB663-10|10BBCLP-1662|658[0n]bp|Canada.British Columbia|BOLD:ABY5257
Apamea devastator|[16492]|BBLPB678-10|10BBCLP-1677|658[0n]bp|Canada.Alberta|BOLD:ABY5257
Apamea devastator|[16493]|LOWCE693-06|CGWC-4453|608[0n]bp|Canada.British Columbia|BOLD:ABY5257
Apamea devastator|[16494]|LOWCE695-06|CGWC-4455|608[0n]bp|Canada.British Columbia|BOLD:ABY5257
Apamea devastator|[16495]|RDLQB758-05|DH010845|631[0n]bp|Canada.Quebec|BOLD:ABY5257
Apamea devastator|[16496]|LOWCE697-06|CGWC-4457|605[0n]bp|Canada.British Columbia|BOLD:ABY5257
Apamea devastator|[16497]|LOWCE696-06|CGWC-4456|605[0n]bp|Canada.British Columbia|BOLD:ABY5257
Apamea devastator|[16498]|RDLQ144-05|DH008076|531[0n]bp|Canada.Quebec|BOLD:ABY5257
Apamea devastator|[16499]|TTMNB332-06|MNBTT-332|611[0n]bp|Canada.New Brunswick|BOLD:ABY5257
Apamea devastator|[16500]|LBCH1866-10|10-JDWBC-1866|658[0n]bp|Canada.British Columbia|BOLD:ABY5257
Apamea devastator|[16501]|BLTIB569-08|BL847|658[0n]bp|Canada.Ontario|BOLD:ABY5257
Apamea devastator|[16502]|TMNBB164-06|MNBTT-1104|658[0n]bp|Canada.New Brunswick|BOLD:ABY5257
Apamea devastator|[16503]|LPVIB570-08|PFC-2006-1996|658[0n]bp|Canada.British Columbia|BOLD:ABY5257
Apamea devastator|[16504]|XAE626-04|Moth4626.03|658[0n]bp|Canada.Ontario|BOLD:ABY5257
Apamea devastator|[16505]|LBCH1970-10|10-JDWBC-1970|658[0n]bp|Canada.British Columbia|BOLD:ABY5257
Apamea amputatrix|[16506]|LBCS119-07|UBC-2007-0110|658[0n]bp|Canada.British Columbia|BOLD:AAA3867
Apamea amputatrix|[16507]|LPMN935-08|08BBLEP-02293|658[0n]bp|Canada.Alberta|BOLD:AAA3867
Apamea amputatrix|[16508]|LPABB188-08|08BBLEP-03453|658[0n]bp|Canada.Alberta|BOLD:AAA3867
Apamea amputatrix|[16509]|RDMAB126-05|UASM41271|658[0n]bp|Canada.Alberta|BOLD:AAA3867
Apamea amputatrix|[16510]|LPABC117-09|08BBLEP-04336|636[0n]bp|Canada.Alberta|BOLD:AAA3867
Apamea amputatrix|[16511]|MNBB429-05|05-NBSTA-345|658[0n]bp|Canada.New Brunswick|BOLD:AAA3867
Apamea amputatrix|[16512]|BLTIB241-08|BL423|658[0n]bp|Canada.Ontario|BOLD:AAA3867
Apamea amputatrix|[16513]|BBLPB293-10|10BBCLP-1292|658[0n]bp|Canada.Alberta|BOLD:AAA3867
Apamea amputatrix|[16514]|LBCS248-07|UBC-2007-0756|658[0n]bp|Canada.British Columbia|BOLD:AAA3867
Apamea amputatrix|[16515]|LOWCC414-05|CGWC-2294|658[0n]bp|Canada.British Columbia|BOLD:AAA3867
Apamea amputatrix|[16516]|LPSK004-08|08BBLEP-00707|658[0n]bp|Canada.Saskatchewan|BOLD:AAA3867
Apamea amputatrix|[16517]|LALPA542-10|AVBC 544-10|658[0n]bp|Canada.British Columbia|BOLD:AAA3867
Apamea amputatrix|[16518]|BBLEC440-09|09BBELE-0440|658[0n]bp|Canada.New Brunswick|BOLD:AAA3867
Apamea amputatrix|[16519]|LPABB568-08|08BBLEP-03833|658[0n]bp|Canada.Alberta|BOLD:AAA3867
Apamea amputatrix|[16520]|LPMN913-08|08BBLEP-02271|658[0n]bp|Canada.Alberta|BOLD:AAA3867
Apamea amputatrix|[16521]|LPABB481-08|08BBLEP-03746|658[0n]bp|Canada.Alberta|BOLD:AAA3867
Apamea amputatrix|[16522]|MNBB216-05|05-NBSTA-132|658[0n]bp|Canada.New Brunswick|BOLD:AAA3867
Apamea amputatrix|[16523]|MNBB652-05|05-NBSTA-568|658[0n]bp|Canada.New Brunswick|BOLD:AAA3867
Apamea amputatrix|[16524]|LPABB028-08|08BBLEP-03293|658[0n]bp|Canada.Alberta|BOLD:AAA3867
Apamea amputatrix|[16525]|BBLPB292-10|10BBCLP-1291|658[0n]bp|Canada.Alberta|BOLD:AAA3867
Apamea amputatrix|[16526]|LPMN901-08|08BBLEP-02259|658[0n]bp|Canada.Alberta|BOLD:AAA3867
Apamea amputatrix|[16527]|PHMNB150-04|04HBL007615|658[0n]bp|Canada.New Brunswick|BOLD:AAA3867
Apamea amputatrix|[16528]|LPABC847-09|08BBLEP-05066|658[0n]bp|Canada.Alberta|BOLD:AAA3867
Apamea amputatrix|[16529]|LBCS185-07|UBC-2007-0566|658[0n]bp|Canada.British Columbia|BOLD:AAA3867
Apamea amputatrix|[16530]|MNBB285-05|05-NBSTA-201|658[0n]bp|Canada.New Brunswick|BOLD:AAA3867
Apamea amputatrix|[16531]|BBLPB291-10|10BBCLP-1290|658[0n]bp|Canada.Alberta|BOLD:AAA3867
Apamea amputatrix|[16532]|LBCC432-05|HLC-22312|658[0n]bp|Canada.British Columbia|BOLD:AAA3867
Apamea amputatrix|[16533]|XAD581-04|04HBL006996|658[0n]bp|Canada.Ontario|BOLD:AAA3867
Apamea amputatrix|[16534]|LPABB344-08|08BBLEP-03609|658[0n]bp|Canada.Alberta|BOLD:AAA3867
Apamea amputatrix|[16535]|LBCS120-07|UBC-2007-0111|658[0n]bp|Canada.British Columbia|BOLD:AAA3867
Apamea amputatrix|[16536]|LHLEP572-06|UBC-2006-1123|658[0n]bp|Canada.British Columbia|BOLD:AAA3867
Apamea amputatrix|[16537]|LHLEP405-06|UBC-2006-1044|658[0n]bp|Canada.British Columbia|BOLD:AAA3867
Apamea amputatrix|[16538]|LPABC121-09|08BBLEP-04340|658[0n]bp|Canada.Alberta|BOLD:AAA3867
Apamea amputatrix|[16539]|LPABC846-09|08BBLEP-05065|658[0n]bp|Canada.Alberta|BOLD:AAA3867
Apamea amputatrix|[16540]|LPABB335-08|08BBLEP-03600|658[0n]bp|Canada.Alberta|BOLD:AAA3867
Apamea amputatrix|[16541]|LPABC842-09|08BBLEP-05061|658[0n]bp|Canada.Alberta|BOLD:AAA3867
Apamea amputatrix|[16542]|LBCS651-07|UBC-2007-0354|658[0n]bp|Canada.British Columbia|BOLD:AAA3867
Apamea amputatrix|[16543]|LPMN936-08|08BBLEP-02294|658[0n]bp|Canada.Alberta|BOLD:AAA3867
Apamea amputatrix|[16544]|LPABC182-09|08BBLEP-04401|603[0n]bp|Canada.Alberta|BOLD:AAA3867
Apamea amputatrix|[16545]|LPABC128-09|08BBLEP-04347|620[2n]bp|Canada.Alberta|BOLD:AAA3867

Apamea amputatrix|[16543]|LPMN936-08|08BBLEP-02294|658[0n]bp|Canada.Alberta|BOLD:AAA3867
Apamea amputatrix|[16544]|LPABC182-09|08BBLEP-04401|603[0n]bp|Canada.Alberta|BOLD:AAA3867
Apamea amputatrix|[16545]|LPABC128-09|08BBLEP-04347|620[2n]bp|Canada.Alberta|BOLD:AAA3867
Apamea amputatrix|[16546]|LPABC097-09|08BBLEP-04316|636[1n]bp|Canada.Alberta|BOLD:AAA3867
Apamea amputatrix|[16547]|PHMNB077-03|moth84.02SA|639[0n]bp|Canada.New Brunswick|BOLD:AAA3867
Apamea amputatrix|[16548]|LPABC124-09|08BBLEP-04343|616[0n]bp|Canada.Alberta|BOLD:AAA3867
Apamea amputatrix|[16549]|LPABC498-09|08BBLEP-04717|611[0n]bp|Canada.Alberta|BOLD:AAA3867
Apamea amputatrix|[16550]|LPABC127-09|08BBLEP-04346|612[0n]bp|Canada.Alberta|BOLD:AAA3867
Apamea amputatrix|[16551]|PHMNB230-04|04HBL007695|609[0n]bp|Canada.New Brunswick|BOLD:AAA3867
Apamea amputatrix|[16552]|LPVIB015-08|PFC-2006-2502|658[0n]bp|Canada.British Columbia|BOLD:AAA3867
Apamea amputatrix|[16553]|LPABC119-09|08BBLEP-04338|608[0n]bp|Canada.Alberta|BOLD:AAA3867
Apamea amputatrix|[16554]|LPVIC030-08|PFC-2006-2585|658[0n]bp|Canada.British Columbia|BOLD:AAA3867
Apamea amputatrix|[16555]|LBCD477-05|HLC-23297|658[0n]bp|Canada.British Columbia|BOLD:AAA3867
Apamea amputatrix|[16556]|LBCS652-07|UBC-2007-0355|658[0n]bp|Canada.British Columbia|BOLD:AAA3867
Apamea amputatrix|[16557]|XAC852-04|04HBL006852|590[1n]bp|Canada.Ontario|BOLD:AAA3867
Apamea amputatrix|[16558]|XAI044-05|0102-ONT-0044|658[0n]bp|Canada.Ontario|BOLD:AAA3867
Apamea amputatrix|[16559]|XAC037-04|04HBL006037|658[0n]bp|Canada.Ontario|BOLD:AAA3867
Apamea amputatrix|[16560]|BBLEC483-09|09BBELE-0483|658[0n]bp|Canada.New Brunswick|BOLD:AAA3867
Apamea amputatrix|[16561]|LALPA581-10|AVBC 583-10|658[0n]bp|Canada.British Columbia|BOLD:AAA3867
Apamea amputatrix|[16562]|XAC832-04|04HBL006832|658[0n]bp|Canada.Ontario|BOLD:AAA3867
Apamea amputatrix|[16563]|LMH043-06|PFC-2006-0464|656[0n]bp|Canada.British Columbia|BOLD:AAA3867
Apamea amputatrix|[16564]|BLTIB494-08|BL748|658[0n]bp|Canada.Ontario|BOLD:AAA3867
Apamea amputatrix|[16565]|RDLQF039-06|DH002270|658[0n]bp|Canada.Quebec|BOLD:AAA3867
Apamea amputatrix|[16566]|LPMN914-08|08BBLEP-02272|658[0n]bp|Canada.Alberta|BOLD:AAA3867
Apamea amputatrix|[16567]|BLTIB349-08|BL567|658[0n]bp|Canada.Ontario|BOLD:AAA3867
Apamea amputatrix|[16568]|RDNM949-05|CNCNoctuoidea7789|658[0n]bp|Canada.Ontario|BOLD:AAA3867
Apamea amputatrix|[16569]|LPSK578-08|08BBLEP-02146|658[0n]bp|Canada.Saskatchewan|BOLD:AAA3867
Apamea amputatrix|[16570]|BLTIB439-08|BL686|658[0n]bp|Canada.Ontario|BOLD:AAA3867
Apamea amputatrix|[16571]|LBCS249-07|UBC-2007-0757|658[0n]bp|Canada.British Columbia|BOLD:AAA3867
Apamea amputatrix|[16572]|RDLQF040-06|DH002272|658[0n]bp|Canada.Quebec|BOLD:AAA3867
Apamea amputatrix|[16573]|LHLEP421-06|UBC-2006-1043|658[0n]bp|Canada.British Columbia|BOLD:AAA3867
Apamea amputatrix|[16574]|LPAB001-08|08BBLEP-02323|658[0n]bp|Canada.Alberta|BOLD:AAA3867
Apamea amputatrix|[16575]|LBCS653-07|UBC-2007-0356|658[0n]bp|Canada.British Columbia|BOLD:AAA3867
Apamea amputatrix|[16576]|BBLEC690-09|09BBELE-0690|658[0n]bp|Canada.Nova Scotia|BOLD:AAA3867
Apamea amputatrix|[16577]|LALPA522-10|AVBC 524-10|658[0n]bp|Canada.British Columbia|BOLD:AAA3867
Apamea amputatrix|[16578]|PHMNB756-05|Moth 449.03SA|658[0n]bp|Canada.New Brunswick|BOLD:AAA3867
Apamea amputatrix|[16579]|LHLEP422-06|UBC-2006-1649|658[0n]bp|Canada.British Columbia|BOLD:AAA3867
Apamea amputatrix|[16580]|LMH044-06|PFC-2006-0465|658[0n]bp|Canada.British Columbia|BOLD:AAA3867
Apamea amputatrix|[16581]|LHLEP573-06|UBC-2006-1508|658[0n]bp|Canada.British Columbia|BOLD:AAA3867
Apamea amputatrix|[16582]|LPABB621-08|08BBLEP-03886|658[0n]bp|Canada.Alberta|BOLD:AAA3867
Apamea amputatrix|[16583]|LPVIB052-08|PFC-2006-1380|658[0n]bp|Canada.British Columbia|BOLD:AAA3867
Apamea amputatrix|[16584]|LALPA487-10|AVBC 489-10|658[0n]bp|Canada.British Columbia|BOLD:AAA3867
Apamea amputatrix|[16585]|BBLPC165-09|09BBELE-1165|658[0n]bp|Canada.Nova Scotia|BOLD:AAA3867
Apamea amputatrix|[16586]|LMH042-06|PFC-2006-0463|658[0n]bp|Canada.British Columbia|BOLD:AAA3867
Apamea amputatrix|[16587]|MNBB117-05|05-NBSTA-033|658[0n]bp|Canada.New Brunswick|BOLD:AAA3867
Apamea amputatrix|[16588]|XAC835-04|04HBL006835|658[0n]bp|Canada.Ontario|BOLD:AAA3867
Apamea amputatrix|[16589]|LPSK581-08|08BBLEP-02149|658[0n]bp|Canada.Saskatchewan|BOLD:AAA3867
Apamea amputatrix|[16590]|BBLEC353-09|09BBELE-0353|658[0n]bp|Canada.Newfoundland and Labrador|BOLD:AAA...
Apamea amputatrix|[16591]|LPVIA414-08|PFC-2006-0600|658[0n]bp|Canada.British Columbia|BOLD:AAA3867
Apamea amputatrix|[16592]|LALPA533-10|AVBC 535-10|658[0n]bp|Canada.British Columbia|BOLD:AAA3867
Apamea amputatrix|[16593]|LPVIA573-08|PFC-2006-0800|658[0n]bp|Canada.British Columbia|BOLD:AAA3867
Apamea amputatrix|[16594]|LHLEP423-06|UBC-2006-1122|658[0n]bp|Canada.British Columbia|BOLD:AAA3867
Apamea amputatrix|[16595]|BBLPC607-09|09BBELE-1607|658[0n]bp|Canada.Nova Scotia|BOLD:AAA3867
Apamea amputatrix|[16596]|LPABC829-09|08BBLEP-05048|658[0n]bp|Canada.Alberta|BOLD:AAA3867
Apamea amputatrix|[16597]|LPABC845-09|08BBLEP-05064|658[0n]bp|Canada.Alberta|BOLD:AAA3867
Apamea amputatrix|[16598]|LPABC098-09|08BBLEP-04317|658[3n]bp|Canada.Alberta|BOLD:AAA3867
Apamea amputatrix|[16599]|BLTIB242-08|BL424|658[0n]bp|Canada.Ontario|BOLD:AAA3867
Apamea amputatrix|[16600]|LPVIB885-08|PFC-2006-2403|639[0n]bp|Canada.British Columbia|BOLD:AAA3867
Apamea amputatrix|[16601]|BLTIB663-08|BL944|648[0n]bp|Canada.Ontario|BOLD:AAA3867
Apamea amputatrix|[16602]|LPVIB909-08|PFC-2006-2435|647[0n]bp|Canada.British Columbia|BOLD:AAA3867
Apamea amputatrix|[16603]|LPGVA601-08|UBC-2006-1759|623[0n]bp|Canada.British Columbia|BOLD:AAA3867
Apamea amputatrix|[16604]|LPVIA966-08|PFC-2006-1292|631[0n]bp|Canada.British Columbia|BOLD:AAA3867
Apamea amputatrix|[16605]|LPGVA600-08|UBC-2006-1758|632[0n]bp|Canada.British Columbia|BOLD:AAA3867
Apamea amputatrix|[16606]|LPGVA602-08|UBC-2006-1760|627[0n]bp|Canada.British Columbia|BOLD:AAA3867
Apamea amputatrix|[16607]|PHMNB234-04|04HBL007699|573[2n]bp|Canada.New Brunswick|BOLD:AAA3867
Apamea amputatrix|[16608]|RDNM950-05|CNCNoctuoidea7790|540[0n]bp|Canada.British Columbia|BOLD:AAA3867
Apamea amputatrix|[16609]|LBCC431-05|HLC-22311|658[0n]bp|Canada.British Columbia|BOLD:AAA3867
Apamea amputatrix|[16610]|LPABC118-09|08BBLEP-04337|641[1n]bp|Canada.Alberta|BOLD:AAA3867
Apamea amputatrix|[16611]|LOWCD278-06|CGWC-3098|599[0n]bp|Canada.British Columbia|BOLD:AAA3867
Apamea amputatrix|[16612]|LPVIA507-08|PFC-2006-0718|658[0n]bp|Canada.British Columbia|BOLD:AAA3867
Apamea lintneri|[16613]|RDNMF742-08|NOC14828|614[0n]bp|Canada.Quebec|BOLD:AAE5928
Apamea lintneri|[16614]|RDNMC266-05|CNCNoctuoidea11900|658[0n]bp|Canada.Quebec|BOLD:AAE5928
Apamea lintneri|[16615]|RDNMG924-08|CNC LEP00053048|643[0n]bp|Canada.Quebec|BOLD:AAE5928
Apamea lintneri|[16616]|RDNMF741-08|NOC14827|658[0n]bp|Canada.Quebec|BOLD:AAE5928
Apamea acera|[16617]|LBCH6217-10|10-JDWBC-6217|658[0n]bp|Canada.British Columbia|BOLD:AAE6994
Apamea acera|[16618]|RDNMF003-08|NOC14089|658[0n]bp|Canada.British Columbia|BOLD:AAE6994
Apamea niveivenosa|[16619]|LPABC111-09|08BBLEP-04330|658[0n]bp|Canada.Alberta|BOLD:AAB8174
Apamea niveivenosa|[16620]|RDNM059-05|CNCNoctuoidea6328|601[0n]bp|Canada.Alberta|BOLD:AAB8174
Apamea niveivenosa|[16621]|RDNM058-05|CNCNoctuoidea6327|600[0n]bp|Canada.Alberta|BOLD:AAB8174
Apamea niveivenosa|[16622]|RDNM057-05|CNCNoctuoidea6326|611[0n]bp|Canada.Alberta|BOLD:AAB8174
Apamea niveivenosa|[16623]|LPABC112-09|08BBLEP-04331|632[2n]bp|Canada.Alberta|BOLD:AAB8174
Apamea niveivenosa|[16624]|LPABB647-08|08BBLEP-03912|658[0n]bp|Canada.Alberta|BOLD:AAB8174
Apamea niveivenosa|[16625]|LPMNB471-09|08BBLEP-05509|658[0n]bp|Canada.Manitoba|BOLD:AAB8174
Apamea niveivenosa|[16626]|LPABB077-08|08BBLEP-03342|658[0n]bp|Canada.Alberta|BOLD:AAB8174
Apamea niveivenosa|[16627]|LPABB384-08|08BBLEP-03649|658[0n]bp|Canada.Alberta|BOLD:AAB8174
Apamea contradicta|[16628]|LPMN716-08|08BBLEP-01519|658[0n]bp|Canada.Manitoba|BOLD:ACE6002
Apamea contradicta|[16629]|RDLQB891-05|DH008729|658[0n]bp|Canada.Newfoundland and Labrador|BOLD:ACE6002
Apamea contradicta|[16630]|LPMN636-08|08BBLEP-01437|658[0n]bp|Canada.Manitoba|BOLD:ACE6002
Apamea contradicta|[16631]|LOWCD302-06|CGWC-3122|585[0n]bp|Canada.British Columbia|BOLD:ACE6002
Apamea contradicta|[16632]|RDNMC211-05|CNCNoctuoideaH023|576[0n]bp|Canada.Quebec|BOLD:ACE6002
Apamea contradicta|[16633]|LPABB850-09|08BBLEP-04170|658[0n]bp|Canada.Alberta|BOLD:ACE6002
Apamea contradicta|[16634]|RDNMC209-05|CNCNoctuoideaH021|658[0n]bp|Canada.Quebec|BOLD:ACE6002
Apamea contradicta|[16635]|RDNMC369-05|CNCNoctuoidea12003|600[0n]bp|Canada.British Columbia|BOLD:ACE6002
Apamea zeta|[16636]|RDNMB605-05|CNCNoctuoidea10381|658[0n]bp|Canada.Quebec|BOLD:ACE6001
Apamea zeta|[16637]|RDNMB606-05|CNCNoctuoidea10382|658[0n]bp|Canada.Quebec|BOLD:ACE6001
Apamea zeta|[16638]|LCH236-04|04HBL003236|658[0n]bp|Canada.Manitoba|BOLD:ACE6001
Apamea zeta|[16639]|RDLQB856-05|DH010939|516[0n]bp|Canada.Quebec|BOLD:AAA5797
Apamea zeta|[16640]|RDNMD232-06|CNCNoctuoidea12564|658[0n]bp|Canada.British Columbia|BOLD:AAA5797
Apamea zeta|[16641]|RDNMD230-06|CNCNoctuoidea12562|658[0n]bp|Canada.British Columbia|BOLD:AAA5797
Apamea zeta|[16642]|RDNMD229-06|CNCNoctuoidea6807b|658[0n]bp|Canada.British Columbia|BOLD:AAA5797
Apamea zeta|[16643]|RDNM531-05|CNCNoctuoidea6807|583[0n]bp|Canada.British Columbia|BOLD:AAA5797
Apamea zeta|[16644]|RDNMB604-05|CNCNoctuoidea10380|658[0n]bp|Canada.Nunavut|BOLD:AAA5797
Apamea zeta|[16645]|RDNMB603-05|CNCNoctuoidea10379|658[0n]bp|Canada.Nunavut|BOLD:AAA5797

[illegible]
Apamea zeta|[16643]|RDNM531-05|CNCNoctuoidea6807|583[0n]bp|Canada.British Columbia|BOLD:AAA5797
Apamea zeta|[16644]|RDNMB604-05|CNCNoctuoidea10380|658[0n]bp|Canada.Nunavut|BOLD:AAA5797
Apamea zeta|[16645]|RDNMB603-05|CNCNoctuoidea10379|658[0n]bp|Canada.Nunavut|BOLD:AAA5797
Apamea zeta|[16646]|RDNM536-05|CNCNoctuoidea6812|658[0n]bp|Canada.Manitoba|BOLD:AAA5797
Apamea zeta|[16647]|LCHQ115-07|07PROBE-10884|658[0n]bp|Canada.Manitoba|BOLD:AAA5797
Apamea zeta|[16648]|LCHP816-07|07PROBE-10573|658[0n]bp|Canada.Manitoba|BOLD:AAA5797
Apamea zeta|[16649]|LCHP860-07|07PROBE-10617|658[0n]bp|Canada.Manitoba|BOLD:AAA5797
Apamea zeta|[16650]|RDNMD236-06|CNCNoctuoidea12568|591[0n]bp|Canada.Manitoba|BOLD:AAA5797
Apamea zeta|[16651]|LCHP579-07|07PROBE-10249|658[0n]bp|Canada.Manitoba|BOLD:AAA5797
Apamea zeta|[16652]|LCHP784-07|07PROBE-10469|658[0n]bp|Canada.Manitoba|BOLD:AAA5797
Apamea zeta|[16653]|LCHP783-07|07PROBE-10468|658[0n]bp|Canada.Manitoba|BOLD:AAA5797
Apamea zeta|[16654]|LCHP898-07|07PROBE-10660|658[0n]bp|Canada.Manitoba|BOLD:AAA5797
Apamea zeta|[16655]|LCHP821-07|07PROBE-10578|658[0n]bp|Canada.Manitoba|BOLD:AAA5797
Apamea zeta|[16656]|LCHP627-07|07PROBE-10308|658[0n]bp|Canada.Manitoba|BOLD:AAA5797
Apamea zeta|[16657]|RDNM149-05|CNCNoctuoidea6699|534[0n]bp|Canada.British Columbia|BOLD:AAA5797
Apamea zeta|[16658]|RDNM148-05|CNCNoctuoidea6698|569[0n]bp|Canada.British Columbia|BOLD:AAA5797
Apamea zeta|[16659]|RDMAB438-05|BCSC111|658[0n]bp|Canada.Yukon Territory|BOLD:AAA5797
Apamea zeta|[16660]|RDNMD235-06|CNCNoctuoidea12567|596[0n]bp|Canada.British Columbia|BOLD:AAA5797
Apamea zeta|[16661]|RDNMD227-06|CNCNoctuoidea9930|658[0n]bp|Canada.British Columbia|BOLD:AAA5797
Apamea zeta|[16662]|RDNM530-05|CNCNoctuoidea6806|658[0n]bp|Canada.British Columbia|BOLD:AAA5797
Apamea zeta|[16663]|RDNMD234-06|CNCNoctuoidea12566|658[0n]bp|Canada.British Columbia|BOLD:AAA5797
Apamea zeta|[16664]|RDNMD233-06|CNCNoctuoidea12565|619[0n]bp|Canada.British Columbia|BOLD:AAA5797
Apamea zeta|[16665]|RDNM527-05|CNCNoctuoidea6803|582[0n]bp|Canada.Manitoba|BOLD:AAA5797
Apamea zeta|[16666]|RDNM529-05|CNCNoctuoidea6805|579[0n]bp|Canada.Manitoba|BOLD:AAA5797
Apamea zeta|[16667]|RDNM537-05|CNCNoctuoidea6813|584[0n]bp|Canada.Manitoba|BOLD:AAA5797
Apamea zeta|[16668]|LCHP854-07|07PROBE-10611|658[0n]bp|Canada.Manitoba|BOLD:AAA5797
Apamea zeta|[16669]|RDNMD237-06|CNCNoctuoidea12569|583[0n]bp|Canada.Manitoba|BOLD:AAA5797
Apamea zeta|[16670]|LCHP855-07|07PROBE-10612|658[0n]bp|Canada.Manitoba|BOLD:AAA5797
Apamea zeta|[16671]|LCHQ168-07|07PROBE-10937|658[0n]bp|Canada.Manitoba|BOLD:AAA5797
Apamea zeta|[16672]|LCHP837-07|07PROBE-10594|658[0n]bp|Canada.Manitoba|BOLD:AAA5797
Apamea zeta|[16673]|LCHP208-07|07PROBE-00133|658[0n]bp|Canada.Manitoba|BOLD:AAA5797
Apamea zeta|[16674]|LCHP887-07|07PROBE-10644|658[0n]bp|Canada.Manitoba|BOLD:AAA5797
Apamea zeta|[16675]|LCHP847-07|07PROBE-10604|658[0n]bp|Canada.Manitoba|BOLD:AAA5797
Apamea zeta|[16676]|LCHP005-07|07PROBE-00063|658[0n]bp|Canada.Manitoba|BOLD:AAA5797
Apamea zeta|[16677]|LCHP526-07|07PROBE-10196|658[0n]bp|Canada.Manitoba|BOLD:AAA5797
Apamea zeta|[16678]|LCHP849-07|07PROBE-10606|658[0n]bp|Canada.Manitoba|BOLD:AAA5797
Apamea zeta|[16679]|RDNM524-05|CNCNoctuoidea6800|658[0n]bp|Canada.Manitoba|BOLD:AAA5797
Apamea zeta|[16680]|RDNMD226-06|CNCNoctuoidea9801|658[0n]bp|Canada.Manitoba|BOLD:AAA5797
Apamea zeta|[16681]|LCHP420-07|07PROBE-10044|658[0n]bp|Canada.Manitoba|BOLD:AAA5797
Apamea zeta|[16682]|LCHP853-07|07PROBE-10610|658[0n]bp|Canada.Manitoba|BOLD:AAA5797
Apamea zeta|[16683]|LCH234-04|04HBL003234|658[0n]bp|Canada.Manitoba|BOLD:AAA5797
Apamea zeta|[16684]|LCHP216-07|07PROBE-03786|658[0n]bp|Canada.Manitoba|BOLD:AAA5797
Apamea zeta|[16685]|LCHP861-07|07PROBE-10618|658[0n]bp|Canada.Manitoba|BOLD:AAA5797
Apamea zeta|[16686]|LCHP859-07|07PROBE-10616|658[0n]bp|Canada.Manitoba|BOLD:AAA5797
Apamea zeta|[16687]|LCHP395-07|07PROBE-10019|658[0n]bp|Canada.Manitoba|BOLD:AAA5797
Apamea zeta|[16688]|LCHP844-07|07PROBE-10601|657[0n]bp|Canada.Manitoba|BOLD:AAA5797
Apamea zeta|[16689]|LCHP858-07|07PROBE-10615|658[0n]bp|Canada.Manitoba|BOLD:AAA5797
Apamea zeta|[16690]|LCHP414-07|07PROBE-10038|658[0n]bp|Canada.Manitoba|BOLD:AAA5797
Apamea zeta|[16691]|LCHP850-07|07PROBE-10607|658[0n]bp|Canada.Manitoba|BOLD:AAA5797
Apamea zeta|[16692]|LCHP580-07|07PROBE-10250|658[0n]bp|Canada.Manitoba|BOLD:AAA5797
Apamea zeta|[16693]|LCHP277-07|07PROBE-03843|656[0n]bp|Canada.Manitoba|BOLD:AAA5797
Apamea zeta|[16694]|RDNM523-05|CNCNoctuoidea6799|658[0n]bp|Canada.Nunavut|BOLD:AAA5797
Apamea zeta|[16695]|RDNM525-05|CNCNoctuoidea6801|658[0n]bp|Canada.Manitoba|BOLD:AAA5797
Apamea zeta|[16696]|LCHP838-07|07PROBE-10595|658[0n]bp|Canada.Manitoba|BOLD:AAA5797
Apamea zeta|[16697]|LCH235-04|04HBL003235|658[0n]bp|Canada.Manitoba|BOLD:AAA5797
Apamea zeta|[16698]|LCHP415-07|07PROBE-10039|658[0n]bp|Canada.Manitoba|BOLD:AAA5797
Apamea zeta|[16699]|LCHP410-07|07PROBE-10034|658[0n]bp|Canada.Manitoba|BOLD:AAA5797
Apamea zeta|[16700]|RDNM538-05|CNCNoctuoidea6814|658[0n]bp|Canada.Manitoba|BOLD:AAA5797
Apamea zeta|[16701]|LCHP384-07|07PROBE-10008|658[0n]bp|Canada.Manitoba|BOLD:AAA5797
Apamea zeta|[16702]|LCHP632-07|07PROBE-10313|658[0n]bp|Canada.Manitoba|BOLD:AAA5797
Apamea zeta|[16703]|LCHP217-07|07PROBE-03787|658[0n]bp|Canada.Manitoba|BOLD:AAA5797
Apamea zeta|[16704]|LCHP215-07|07PROBE-03785|658[0n]bp|Canada.Manitoba|BOLD:AAA5797
Apamea zeta|[16705]|LCHP260-07|07PROBE-03826|658[0n]bp|Canada.Manitoba|BOLD:AAA5797
Apamea zeta|[16706]|LCHP209-07|07PROBE-00134|658[0n]bp|Canada.Manitoba|BOLD:AAA5797
Apamea zeta|[16707]|LCHQ117-07|07PROBE-10886|658[0n]bp|Canada.Manitoba|BOLD:AAA5797
Apamea zeta|[16708]|LCHP625-07|07PROBE-10306|658[0n]bp|Canada.Manitoba|BOLD:AAA5797
Apamea zeta|[16709]|CHLEP158-09|09PROBE-09453|658[0n]bp|Canada.Manitoba|BOLD:AAA5797
Apamea zeta|[16710]|LCHP635-07|07PROBE-10316|658[0n]bp|Canada.Manitoba|BOLD:AAA5797
Apamea zeta|[16711]|RDNMD228-06|CNCNoctuoidea9805|603[0n]bp|Canada.Manitoba|BOLD:AAA5797
Apamea zeta|[16712]|LCHP004-07|07PROBE-00062|655[0n]bp|Canada.Manitoba|BOLD:AAA5797
Apamea zeta|[16713]|LCHQ170-07|07PROBE-10939|656[0n]bp|Canada.Manitoba|BOLD:AAA5797
Apamea zeta|[16714]|LCHP857-07|07PROBE-10614|634[0n]bp|Canada.Manitoba|BOLD:AAA5797
Apamea zeta|[16715]|LCHP846-07|07PROBE-10603|655[0n]bp|Canada.Manitoba|BOLD:AAA5797
Apamea maxima|[16716]|BBLPB250-10|10BBCLP-1249|658[0n]bp|Canada.British Columbia|BOLD:AAC7976
Apamea maxima|[16717]|RDNMG459-08|CNC LEP00052283|658[0n]bp|Canada.British Columbia|BOLD:AAC7976
Apamea maxima|[16718]|RDNMC253-05|CNCNoctuoidea11887|658[0n]bp|Canada.British Columbia|BOLD:AAC7976
Apamea maxima|[16719]|BBLPB249-10|10BBCLP-1248|658[0n]bp|Canada.British Columbia|BOLD:AAC7976
Apamea maxima|[16720]|RDNMC252-05|CNCNoctuoidea11886|597[0n]bp|Canada.British Columbia|BOLD:AAC7976
Apamea maxima|[16721]|RDNMG458-08|CNC LEP00052282|658[0n]bp|Canada.British Columbia|BOLD:AAC7976
Apamea maxima|[16722]|RDNMG460-08|CNC LEP00052284|658[0n]bp|Canada.British Columbia|BOLD:AAC7976
Apamea maxima|[16723]|LBCH1303-10|10-JDWBC-1303|658[0n]bp|Canada.British Columbia|BOLD:AAC7976
Loscopia velata|[16724]|XAJ852-06|2006-ONT-0852|658[0n]bp|Canada.Ontario|BOLD:AAB5969
Loscopia velata|[16725]|PHSEP340-11|BIOUG01292-A11|657[0n]bp|Canada.Ontario|BOLD:AAB5969
Loscopia velata|[16726]|MNBB076-05|HBL008686|658[0n]bp|Canada.New Brunswick|BOLD:AAB5969
Loscopia velata|[16727]|MNBB525-05|05-NBSTA-441|658[0n]bp|Canada.New Brunswick|BOLD:AAB5969
Loscopia velata|[16728]|BBLEC544-09|09BBELE-0544|658[0n]bp|Canada.Nova Scotia|BOLD:AAB5969
Loscopia velata|[16729]|BBLPC112-09|09BBELE-1112|658[0n]bp|Canada.New Brunswick|BOLD:AAB5969
Loscopia velata|[16730]|HEJUL2101-12|BIOUG02170-G12|658[0n]bp|Canada.Ontario|BOLD:AAB5969
Loscopia velata|[16731]|RDLQB648-05|DH010751|601[0n]bp|Canada.Quebec|BOLD:AAB5969
Loscopia velata|[16732]|RDLQ472-07|DH005582|585[0n]bp|Canada.Quebec|BOLD:AAB5969
Loscopia velata|[16733]|BBLPB572-10|10BBCLP-1571|658[0n]bp|Canada.Saskatchewan|BOLD:AAB5969
Loscopia velata|[16734]|BLTIB1125-08|BL1138|658[0n]bp|Canada.Ontario|BOLD:AAB5969
Loscopia velata|[16735]|MNBB526-05|05-NBSTA-442|658[0n]bp|Canada.New Brunswick|BOLD:AAB5969
Loscopia velata|[16736]|XAC849-04|04HBL006849|658[0n]bp|Canada.Ontario|BOLD:AAB5969
Loscopia velata|[16737]|BLTIB913-08|BL1333|658[0n]bp|Canada.Ontario|BOLD:AAB5969
Loscopia velata|[16738]|RDLQ465-07|DH007610|585[0n]bp|Canada.Quebec|BOLD:AAB5969
Loscopia velata|[16739]|BLTIB532-08|BL798|658[0n]bp|Canada.Ontario|BOLD:AAB5969
Apamea apamiformis|[16740]|RDNM971-05|CNCNoctuoidea7811|658[0n]bp|Canada.Ontario|BOLD:AAJ1428
Apamea apamiformis|[16741]|RDLQF050-06|DH002308|658[0n]bp|Canada.Quebec|BOLD:AAJ1428
Apamea plutonia|[16742]|PHMTV423-10|10PHMAL-2523|658[0n]bp|Canada.Ontario|BOLD:AAD3061
Apamea plutonia|[16743]|XAE566-04|Moth4566.03|658[0n]bp|Canada.Ontario|BOLD:AAD3061
Apamea plutonia|[16744]|PHMTV420-10|10PHMAL-2520|658[0n]bp|Canada.Ontario|BOLD:AAD3061
[illegible]

Apamea plutonia|[16742]|PHMTV423-10|10PHMAL-2523|658[0n]bp|Canada.Ontario|BOLD:AAD3061
Apamea plutonia|[16743]|XAE566-04|Moth4566.03|658[0n]bp|Canada.Ontario|BOLD:AAD3061
Apamea plutonia|[16744]|PHMTV420-10|10PHMAL-2520|658[0n]bp|Canada.Ontario|BOLD:AAD3061
Apamea plutonia|[16745]|RDNM196-05|CNCNoctuoidea6378|658[0n]bp|Canada.Ontario|BOLD:AAD3061
Apamea plutonia|[16746]|LOWCE804-06|CGWC-4564|658[0n]bp|Canada.British Columbia|BOLD:AAD3061
Apamea plutonia|[16747]|RDMAB291-05|UASM77786|600[0n]bp|Canada.Alberta|BOLD:AAD3061
Oligia bridghamii|[16748]|RDNM738-05|CNCNoctuoidea7584|658[0n]bp|Canada.Ontario|BOLD:AAE1433
Oligia bridghamii|[16749]|RDNMG510-08|CNC LEP00052334|658[0n]bp|Canada.Quebec|BOLD:AAE1433
Oligia bridghamii|[16750]|RDNM739-05|CNCNoctuoidea7585|525[1n]bp|Canada.New Brunswick|BOLD:AAE1433
Oligia bridghamii|[16751]|RDNMG513-08|CNC LEP00052337|658[0n]bp|Canada.New Brunswick|BOLD:AAE1433
Oligia bridghamii|[16752]|RDNMG514-08|CNC LEP00052338|658[0n]bp|Canada.British Columbia|BOLD:AAE1433
Oligia egens|[16753]|RDNM735-05|CNCNoctuoidea7581|658[0n]bp|Canada.Alberta|BOLD:ACE9969
Oligia egens|[16754]|RDNM737-05|CNCNoctuoidea7583|567[2n]bp|Canada.Alberta|BOLD:ACE9969
Oligia egens|[16755]|RDLQ460-07|DH007224|658[0n]bp|Canada.Quebec|BOLD:ACE9969
Oligia egens|[16756]|RDNM736-05|CNCNoctuoidea7582|658[0n]bp|Canada.Alberta|BOLD:ACE9969
Oligia rampartensis|[16757]|RDNMB525-05|CNCNoctuoidea10301|601[0n]bp|Canada.British Columbia|BOLD:AAE1470
Oligia violacea|[16758]|RDNMB524-05|CNCNoctuoidea10300|658[0n]bp|Canada.British Columbia|BOLD:AAB1458
Ufeus hulstii|[16759]|RDNMG853-08|CNC LEP00052977|592[0n]bp|Canada.British Columbia|BOLD:ACF0561
Ufeus hulstii|[16760]|RDNMG854-08|CNC LEP00052978|658[0n]bp|Canada.British Columbia|BOLD:ACF0561
Ufeus plicatus|[16761]|RDLQ750-07|DH008754|658[0n]bp|Canada.Quebec|BOLD:AAD7193
Ufeus plicatus|[16762]|RDLQ749-07|DH002857|635[0n]bp|Canada.Quebec|BOLD:AAD7193
Ufeus satyricus|[16763]|RDLQ751-07|DH008515|635[0n]bp|Canada.Quebec|BOLD:AAB8639
Ufeus satyricus|[16764]|RDNME260-07|CNCNoctuoidea13867|617[0n]bp|Canada.New Brunswick|BOLD:AAB8639
Ufeus satyricus|[16765]|RDNME261-07|CNCNoctuoidea13868|619[1n]bp|Canada.New Brunswick|BOLD:AAB8639
Ufeus satyricus|[16766]|LOWCE274-06|CGWC-4034|658[0n]bp|Canada.British Columbia|BOLD:AAB8639
Ufeus satyricus|[16767]|LOWCD682-06|CGWC-3502|570[0n]bp|Canada.British Columbia|BOLD:AAB8639
Ufeus satyricus|[16768]|LOWCD681-06|CGWC-3501|658[0n]bp|Canada.British Columbia|BOLD:AAB8639
Ufeus satyricus|[16769]|RDNME258-07|CNCNoctuoidea13865|658[0n]bp|Canada.New Brunswick|BOLD:AAB8639
Ufeus satyricus|[16770]|LOWCD680-06|CGWC-3500|565[0n]bp|Canada.British Columbia|BOLD:AAB8639
Ufeus satyricus|[16771]|LOWCD684-06|CGWC-3504|589[0n]bp|Canada.British Columbia|BOLD:AAB8639
Ufeus satyricus|[16772]|PHMNB268-04|04HBL007733|658[0n]bp|Canada.New Brunswick|BOLD:AAB8639
Ufeus satyricus|[16773]|LOWCD679-06|CGWC-3499|585[1n]bp|Canada.British Columbia|BOLD:AAB8639
Ufeus satyricus|[16774]|LOWCD683-06|CGWC-3503|592[0n]bp|Canada.British Columbia|BOLD:AAB8639
Ufeus satyricus|[16775]|PHMO363-03|moth2760.02|639[0n]bp|Canada.Ontario|BOLD:AAB8639
Ufeus satyricus|[16776]|XAB671-04|04HBL005671|658[0n]bp|Canada.Ontario|BOLD:AAB8639
Ufeus satyricus|[16777]|XAB647-04|04HBL005647|658[0n]bp|Canada.Ontario|BOLD:AAB8639
Achatodes zeae|[16778]|BBLPC554-09|09BBELE-1554|658[0n]bp|Canada.New Brunswick|BOLD:AAD5924
Achatodes zeae|[16779]|RDLQB445-05|DH010531|658[0n]bp|Canada.Quebec|BOLD:AAD5924
Achatodes zeae|[16780]|RDLQB576-05|DH010679|598[0n]bp|Canada.Quebec|BOLD:AAD5924
Oligia chlorostigma|[16781]|RDNMF379-08|NOC14465|658[0n]bp|Canada.Quebec|BOLD:AAE5953
Oligia chlorostigma|[16782]|RDNMG512-08|CNC LEP00052336|658[0n]bp|Canada.New Brunswick|BOLD:AAE5953
Oligia chlorostigma|[16783]|RDNMF380-08|NOC14466|658[0n]bp|Canada.Quebec|BOLD:AAE5953
Eremobina claudens|[16784]|RDNMB469-05|CNCNoctuoidea10235|658[0n]bp|Canada.Alberta|BOLD:AAC2614
Eremobina claudens|[16785]|RDLQ461-07|DH007225|608[0n]bp|Canada.Quebec|BOLD:AAC2614
Eremobina claudens|[16786]|LBCH4433-10|10-JDWBC-4433|658[0n]bp|Canada.British Columbia|BOLD:AAC2614
Eremobina claudens|[16787]|LALPA1286-11|AVBC 1288-11|658[0n]bp|Canada.British Columbia|BOLD:AAC2614
Eremobina claudens|[16788]|LBCH1239-10|10-JDWBC-1239|658[0n]bp|Canada.British Columbia|BOLD:AAC2614
Eremobina claudens|[16789]|LBCH1553-10|10-JDWBC-1553|658[0n]bp|Canada.British Columbia|BOLD:AAC2614
Eremobina claudens|[16790]|LBCH2058-10|10-JDWBC-2058|658[0n]bp|Canada.British Columbia|BOLD:AAC2614
Eremobina leucoscelis|[16791]|RDNMB471-05|CNCNoctuoidea10237|658[0n]bp|Canada.Alberta|BOLD:AAC2614
Eremobina leucoscelis|[16792]|BBLPB773-10|10BBCLP-1772|658[0n]bp|Canada.Alberta|BOLD:AAC2614
Eremobina leucoscelis|[16793]|RDNMB472-05|CNCNoctuoidea10238|658[0n]bp|Canada.Alberta|BOLD:AAC2614
Eremobina leucoscelis|[16794]|BBLPB768-10|10BBCLP-1767|658[0n]bp|Canada.Alberta|BOLD:AAC2614
Eremobina leucoscelis|[16795]|XAD379-04|04HBL007379|658[0n]bp|Canada.Ontario|BOLD:AAC2614
Eremobina leucoscelis|[16796]|RDLQ468-07|DH008801|601[0n]bp|Canada.Quebec|BOLD:AAC2614
Eremobina leucoscelis|[16797]|RDNMI168-10|CNCLEP 69768|658[0n]bp|Canada.New Brunswick|BOLD:AAC2614
Eremobina leucoscelis|[16798]|RDLQB642-05|DH010745|578[0n]bp|Canada.Quebec|BOLD:AAC2614
Lemmeria digitalis|[16799]|RDNMD791-07|CNCNoctuoidea13123|658[0n]bp|Canada.Ontario|BOLD:AAF0679
Lemmeria digitalis|[16800]|RDLQF259-06|DH011339|658[0n]bp|Canada.Quebec|BOLD:AAF0679
Lemmeria digitalis|[16801]|RDLQF260-06|DH011340|658[0n]bp|Canada.Quebec|BOLD:AAF0679
Papaipema appassionata|[16802]|RDNMG776-08|CNC LEP00052900|658[0n]bp|Canada.New Brunswick|BOLD:AAF1834
Papaipema appassionata|[16803]|RDNMG775-08|CNC LEP00052899|658[0n]bp|Canada.New Brunswick|BOLD:AAF1834
Papaipema appassionata|[16804]|RDLQ296-05|DH007259|656[0n]bp|Canada.Quebec|BOLD:AAF1834
Papaipema cerussata|[16805]|LSEU207-06|06-JKA-0207|658[0n]bp|United States.Georgia|BOLD:AAE7004
Papaipema cerussata|[16806]|LSEU208-06|06-JKA-0208|658[0n]bp|United States.Georgia|BOLD:AAE7004
Papaipema cerussata|[16807]|LNCC395-10|10-NCCC-490|658[0n]bp|United States.North Carolina|BOLD:AAE7004
Papaipema cerussata|[16808]|LSEU209-06|06-JKA-0209|658[0n]bp|United States.Georgia|BOLD:AAE7004
Papaipema cerussata|[16809]|LNCC394-10|10-NCCC-489|658[0n]bp|United States.North Carolina|BOLD:AAE7004
Papaipema cerussata|[16810]|LSUSA017-06|06-SUSA-0017|658[0n]bp|United States.Kentucky|BOLD:AAE7004
Papaipema cerussata|[16811]|LNCC397-10|10-NCCC-492|658[0n]bp|United States.North Carolina|BOLD:AAE7004
Papaipema cerussata|[16812]|LNCC396-10|10-NCCC-491|658[0n]bp|United States.North Carolina|BOLD:AAE7004
Papaipema furcata|[16813]|RDNMG777-08|CNC LEP00052901|658[0n]bp|Canada.Ontario|BOLD:AAF1779
Papaipema nelita|[16814]|RDLQB464-05|DH010550|658[0n]bp|Canada.Quebec|BOLD:AAB8711
Papaipema nelita|[16815]|XAB213-04|04HBL005213|658[0n]bp|Canada.Ontario|BOLD:AAB8711
Papaipema nelita|[16816]|RDLQ148-05|DH007263|658[0n]bp|Canada.Quebec|BOLD:AAB8711
Papaipema nelita|[16817]|RDLQ151-05|DH002372|658[0n]bp|Canada.Quebec|BOLD:AAB8711
Papaipema nelita|[16818]|JSAUG1675-11|BIOUG01497-F03|658[0n]bp|Canada.Ontario|BOLD:AAB8711
Papaipema nelita|[16819]|RDLQB470-05|DH010556|658[0n]bp|Canada.Quebec|BOLD:AAB8711
Papaipema nelita|[16820]|XAB215-04|04HBL005215|658[0n]bp|Canada.Ontario|BOLD:AAB8711
Papaipema nelita|[16821]|RDLQ149-05|DH002371|597[0n]bp|Canada.Quebec|BOLD:AAB8711
Papaipema nelita|[16822]|RDLQ485-07|DH009862|619[0n]bp|Canada.Quebec|BOLD:AAB8711
Papaipema nelita|[16823]|RDLQB846-05|DH010933|620[0n]bp|Canada.Quebec|BOLD:AAB8711
Papaipema nelita|[16824]|RDLQ150-05|DH002370|573[0n]bp|Canada.Quebec|BOLD:AAB8711
Papaipema nelita|[16825]|RDLQ154-05|DH002373|569[0n]bp|Canada.Quebec|BOLD:AAB8711
Papaipema nelita|[16826]|RDNMH869-09|CNCLEP00067177|658[0n]bp|Canada.Nova Scotia|BOLD:AAB8711
Papaipema nelita|[16827]|RDNMH868-09|CNCLEP00067176|658[0n]bp|Canada.Nova Scotia|BOLD:AAB8711
Papaipema nr. pterisii|[16828]|RDNM007-05|CNCNoctuoidea6276|658[0n]bp|Canada.Ontario|BOLD:AAB8711
Papaipema nr. pterisii|[16829]|XAH453-05|2005-ONT-2036|658[0n]bp|Canada.Ontario|BOLD:AAB8711
Papaipema nr. pterisii|[16830]|RDNM823-05|CNCNoctuoidea7669|658[0n]bp|Canada.Ontario|BOLD:AAB8711
Papaipema nr. pterisii|[16831]|RDNM008-05|CNCNoctuoidea6277|627[0n]bp|Canada.New Brunswick|BOLD:AAB8711
Papaipema nr. pterisii|[16832]|RDNM009-05|CNCNoctuoidea6278|625[0n]bp|Canada.New Brunswick|BOLD:AAB8711
Papaipema impecuniosa|[16833]|PHMO356-03|moth2728.02|639[0n]bp|Canada.Ontario|BOLD:AAB8711
Papaipema impecuniosa|[16834]|RDLQH038-06|DH013275|658[0n]bp|Canada.Quebec|BOLD:AAB8711
Papaipema impecuniosa|[16835]|RDLQF268-06|DH011360|658[0n]bp|Canada.Quebec|BOLD:AAB8711
Papaipema impecuniosa|[16836]|XAD270-04|04HBL007270|658[0n]bp|Canada.Ontario|BOLD:AAB8711
Papaipema impecuniosa|[16837]|XAH560-05|2005-ONT-2143|658[0n]bp|Canada.Ontario|BOLD:AAB8711
Papaipema harrisii|[16838]|RDNMD846-07|CNCNoctuoidea13178|658[0n]bp|Canada.Quebec|BOLD:AAB8711
Papaipema harrisii|[16839]|RDLQ025-05|DH006253|658[0n]bp|Canada.Quebec|BOLD:AAB8711
Papaipema harrisii|[16840]|PHMNB300-04|04HBL007765|658[0n]bp|Canada.New Brunswick|BOLD:AAB8711
Papaipema harrisii|[16841]|RDLQ390-05|DH010073|658[0n]bp|Canada.New Brunswick|BOLD:AAB8711
Papaipema harrisii|[16842]|RDLQ392-05|DH010075|658[0n]bp|Canada.New Brunswick|BOLD:AAB8711
Papaipema harrisii|[16843]|RDLQ391-05|DH010074|658[0n]bp|Canada.New Brunswick|BOLD:AAB8711
Papaipema harrisii|[16844]|RDLQ389-05|DH010072|658[0n]bp|Canada.New Brunswick|BOLD:AAB8711

Papaipema harrisii|[16842]|RDLQ392-05|DH010075|658[0n]bp|Canada.New Brunswick|BOLD:AAB8711
Papaipema harrisii|[16843]|RDLQ391-05|DH010074|658[0n]bp|Canada.New Brunswick|BOLD:AAB8711
Papaipema harrisii|[16844]|RDLQ389-05|DH010072|658[0n]bp|Canada.New Brunswick|BOLD:AAB8711
Papaipema harrisii|[16845]|RDNMB120-05|CNCNoctuoidea10272|614[0n]bp|Canada.Alberta|BOLD:AAB8711
Papaipema harrisii|[16846]|RDNMB119-05|CNCNoctuoidea10271|614[0n]bp|Canada.Alberta|BOLD:AAB8711
Papaipema nr. pterisii|[16847]|RDNM820-05|CNCNoctuoidea7666|658[0n]bp|Canada.Ontario|BOLD:AAB8711
Papaipema nr. pterisii|[16848]|XAH559-05|2005-ONT-2142|658[0n]bp|Canada.Ontario|BOLD:AAB8711
Papaipema nr. pterisii|[16849]|RDNM770-05|CNCNoctuoidea7616|603[0n]bp|Canada.New Brunswick|BOLD:AAB8711
Papaipema nr. pterisii|[16850]|XAB453-04|04HBL005453|658[0n]bp|Canada.Ontario|BOLD:AAB8711
Papaipema nr. pterisii|[16851]|RDNM005-05|CNCNoctuoidea6274|658[0n]bp|Canada.Ontario|BOLD:AAB8711
Papaipema nr. pterisii|[16852]|RDLQ297-05|DH007264|607[0n]bp|Canada.Quebec|BOLD:AAB8711
Papaipema nr. pterisii|[16853]|RDNM769-05|CNCNoctuoidea7615|658[0n]bp|Canada.Ontario|BOLD:AAB8711
Papaipema nr. pterisii|[16854]|RDLQF266-06|DH011358|658[0n]bp|Canada.Quebec|BOLD:AAB8711
Papaipema nr. pterisii|[16855]|RDNM006-05|CNCNoctuoidea6275|658[0n]bp|Canada.Ontario|BOLD:AAB8711
Papaipema nr. pterisii|[16856]|RDNM832-05|CNCNoctuoidea7678|617[0n]bp|Canada.Ontario|BOLD:AAB8711
Papaipema nr. pterisii|[16857]|RDNM003-05|CNCNoctuoidea6272|658[0n]bp|Canada.Ontario|BOLD:AAB8711
Papaipema nr. pterisii|[16858]|RDLQ314-05|DH008771|658[0n]bp|Canada.Quebec|BOLD:AAB8711
Papaipema nr. pterisii|[16859]|RDNMB589-05|CNCNoctuoidea10365|617[0n]bp|Canada.Ontario|BOLD:AAB8711
Papaipema nr. pterisii|[16860]|RDNM831-05|CNCNoctuoidea7677|617[0n]bp|Canada.Ontario|BOLD:AAB8711
Papaipema cataphracta|[16861]|RDNMG929-08|CNC LEP00053053|658[0n]bp|Canada.Ontario|BOLD:ABZ6984
Papaipema lysimachiae|[16862]|RDMAB1049-09|UASM128614|656[0n]bp|Canada.Ontario|BOLD:ACF1518
Papaipema lysimachiae|[16863]|RDLQ147-05|DH006673|658[0n]bp|Canada.Quebec|BOLD:ACF1518
Papaipema leucostigma|[16864]|RDNM828-05|CNCNoctuoidea7674|658[0n]bp|Canada.Ontario|BOLD:AAB8711
Papaipema leucostigma|[16865]|RDNM818-05|CNCNoctuoidea7664|658[0n]bp|Canada.Ontario|BOLD:AAB8711
Papaipema leucostigma|[16866]|RDNM759-05|CNCNoctuoidea7605|658[0n]bp|Canada.Ontario|BOLD:AAB8711
Papaipema leucostigma|[16867]|RDNM763-05|CNCNoctuoidea7609|658[0n]bp|Canada.Ontario|BOLD:AAB8711
Papaipema leucostigma|[16868]|RDNM771-05|CNCNoctuoidea7617|658[0n]bp|Canada.Ontario|BOLD:AAB8711
Papaipema leucostigma|[16869]|RDNM819-05|CNCNoctuoidea7665|658[0n]bp|Canada.Ontario|BOLD:AAB8711
Papaipema leucostigma|[16870]|RDNM760-05|CNCNoctuoidea7606|658[0n]bp|Canada.Ontario|BOLD:AAB8711
Papaipema leucostigma|[16871]|RDMAB1050-09|UASM128613|649[0n]bp|Canada.Ontario|BOLD:AAB8711
Papaipema leucostigma|[16872]|RDNM762-05|CNCNoctuoidea7608|658[0n]bp|Canada.Ontario|BOLD:AAB8711
Papaipema leucostigma|[16873]|RDNM772-05|CNCNoctuoidea7618|658[0n]bp|Canada.Ontario|BOLD:AAB8711
Papaipema leucostigma|[16874]|RDNM010-05|CNCNoctuoidea6279|658[0n]bp|Canada.Ontario|BOLD:AAB8711
Papaipema leucostigma|[16875]|RDNM761-05|CNCNoctuoidea7607|658[0n]bp|Canada.Ontario|BOLD:AAB8711
Papaipema circumlucens|[16876]|RDNMF475-08|NOC14561|609[0n]bp|Canada.Saskatchewan|BOLD:AAB8711
Papaipema pterisii|[16877]|RDNM827-05|CNCNoctuoidea7673|658[0n]bp|Canada.Ontario|BOLD:AAB8711
Papaipema pterisii|[16878]|RDNM766-05|CNCNoctuoidea7612|658[0n]bp|Canada.Ontario|BOLD:AAB8711
Papaipema pterisii|[16879]|RDNMB590-05|CNCNoctuoidea10366|658[0n]bp|Canada.Ontario|BOLD:AAB8711
Papaipema pterisii|[16880]|RDNM767-05|CNCNoctuoidea7613|658[0n]bp|Canada.Ontario|BOLD:AAB8711
Papaipema pterisii|[16881]|RDNM821-05|CNCNoctuoidea7667|658[0n]bp|Canada.Ontario|BOLD:AAB8711
Papaipema pterisii|[16882]|RDNM822-05|CNCNoctuoidea7668|658[0n]bp|Canada.Ontario|BOLD:AAB8711
Papaipema pterisii|[16883]|RDNM830-05|CNCNoctuoidea7676|658[0n]bp|Canada.Ontario|BOLD:AAB8711
Papaipema pterisii|[16884]|RDNM004-05|CNCNoctuoidea6273|658[0n]bp|Canada.Ontario|BOLD:AAB8711
Papaipema pterisii|[16885]|RDNM765-05|CNCNoctuoidea7611|658[0n]bp|Canada.Ontario|BOLD:AAB8711
Papaipema pterisii|[16886]|RDLQ286-05|DH007050|576[0n]bp|Canada.Quebec|BOLD:AAB8711
Papaipema pterisii|[16887]|RDNM829-05|CNCNoctuoidea7675|658[0n]bp|Canada.Ontario|BOLD:AAB8711
Papaipema pterisii|[16888]|RDNM002-05|CNCNoctuoidea6271|658[0n]bp|Canada.Ontario|BOLD:AAB8711
Papaipema pterisii|[16889]|RDNM708-05|CNCNoctuoidea7554|601[0n]bp|Canada.Ontario|BOLD:AAB8711
Papaipema insulidens|[16890]|RDNM707-05|CNCNoctuoidea7553|598[0n]bp|United States.Oregon|BOLD:ABZ0884
Papaipema birdi|[16891]|RDMAB1057-09|UASM128722|658[0n]bp|Canada.British Columbia|BOLD:ABZ0884
Papaipema birdi|[16892]|RDLQF264-06|DH011356|658[0n]bp|Canada.Quebec|BOLD:ABZ0884
Papaipema birdi|[16893]|RDNME420-08|LEP037844|658[0n]bp|Canada.Ontario|BOLD:ABZ0884
Papaipema birdi|[16894]|RDNMF474-08|NOC14560|658[0n]bp|Canada.British Columbia|BOLD:ABZ0884
Papaipema birdi|[16895]|RDMAB1056-09|UASM128723|658[0n]bp|Canada.British Columbia|BOLD:ABZ0884
Papaipema birdi|[16896]|XAB433-04|04HBL005433|658[0n]bp|Canada.Ontario|BOLD:ABZ0884
Papaipema insulidens|[16897]|RDMAB1052-09|UASM128729|651[0n]bp|United States.Oregon|BOLD:ABZ0884
Papaipema insulidens|[16898]|RDMAB1053-09|UASM128728|656[0n]bp|United States.Oregon|BOLD:ABZ0884
Papaipema birdi|[16899]|RDMAB1055-09|UASM128724|635[0n]bp|Canada.British Columbia|BOLD:ABZ0884
Papaipema insulidens|[16900]|RDMAB1054-09|UASM128725|630[0n]bp|United States.Oregon|BOLD:ABZ0884
Papaipema necopina|[16901]|RDNMF476-08|NOC14562|587[0n]bp|Canada.Ontario|BOLD:ACE4041
Papaipema aweme|[16902]|RDNMD635-06|CNCNoctuoidea12967|658[1n]bp|Canada.Ontario|BOLD:ABU6937
Papaipema cerina|[16903]|RDNMF473-08|NOC14559|658[0n]bp|Canada.Ontario|BOLD:ABZ5369
Papaipema eupatorii|[16904]|RDLQF263-06|DH011355|658[0n]bp|Canada.Quebec|BOLD:ACF3647
Papaipema eupatorii|[16905]|RDNMG793-08|CNC LEP00052917|658[0n]bp|Canada.New Brunswick|BOLD:ACF3647
Papaipema eupatorii|[16906]|RDNMG794-08|CNC LEP00052918|592[0n]bp|Canada.New Brunswick|BOLD:ACF3647
Papaipema eupatorii|[16907]|RDNME421-08|LEP037845|658[0n]bp|Canada.Ontario|BOLD:ACF3647
Papaipema nepheleptena|[16908]|RDNMG779-08|CNC LEP00052903|643[0n]bp|Canada.Ontario|BOLD:ACF3647
Papaipema nepheleptena|[16909]|XAH622-05|2005-ONT-2205|658[0n]bp|Canada.Ontario|BOLD:ACF3647
Papaipema nepheleptena|[16910]|XAH604-05|2005-ONT-2187|658[0n]bp|Canada.Ontario|BOLD:ACF3647
Papaipema nepheleptena|[16911]|RDNMG778-08|CNC LEP00052902|658[0n]bp|Canada.Ontario|BOLD:ACF3647
Papaipema arctivorens|[16912]|XAH246-05|2005-ONT-1829|658[0n]bp|Canada.Ontario|BOLD:ACF1624
Papaipema arctivorens|[16913]|XAD237-04|04HBL007237|658[0n]bp|Canada.Ontario|BOLD:ACF1624
Papaipema arctivorens|[16914]|RDNM764-05|CNCNoctuoidea7610|658[0n]bp|Canada.Ontario|BOLD:ACF1624
Papaipema arctivorens|[16915]|XAH150-05|2005-ONT-1733|658[0n]bp|Canada.Ontario|BOLD:ACF1624
Papaipema arctivorens|[16916]|XAB411-04|04HBL005411|658[0n]bp|Canada.Ontario|BOLD:ACF1624
Papaipema arctivorens|[16917]|RDNM768-05|CNCNoctuoidea7614|658[0n]bp|Canada.Ontario|BOLD:ACF1624
Papaipema arctivorens|[16918]|XAI170-06|0102-ONT-0170|656[0n]bp|Canada.Ontario|BOLD:ACF1624
Papaipema arctivorens|[16919]|RDLQB824-05|DH010911|617[0n]bp|Canada.Quebec|BOLD:ACF1624
Papaipema arctivorens|[16920]|RDLQB844-05|DH010931|627[0n]bp|Canada.Quebec|BOLD:ACF1624
Papaipema arctivorens|[16921]|PHMO301-03|moth2204.02|639[0n]bp|Canada.Ontario|BOLD:ACF1624
Papaipema arctivorens|[16922]|PHMO330-03|moth2563.02|639[0n]bp|Canada.Ontario|BOLD:ACF1624
Papaipema arctivorens|[16923]|XAH147-05|2005-ONT-1730|658[0n]bp|Canada.Ontario|BOLD:ACF1624
Papaipema arctivorens|[16924]|RDLQG060-06|DH012196|658[0n]bp|Canada.Quebec|BOLD:ACF1624
Papaipema baptisiae|[16925]|PHMO248-03|moth1402.01|639[1n]bp|Canada.Ontario|BOLD:ACF3649
Papaipema baptisiae|[16926]|XAD467-04|04HBL007467|588[0n]bp|Canada.Ontario|BOLD:ACF3649
Papaipema baptisiae|[16927]|RDLQ251-05|DH002389|614[0n]bp|Canada.Quebec|BOLD:ACF3649
Papaipema baptisiae|[16928]|RDMAB1051-09|UASM128703|653[0n]bp|Canada.Ontario|BOLD:ACF3649
Papaipema baptisiae|[16929]|XAB439-04|04HBL005439|658[0n]bp|Canada.Ontario|BOLD:ACF3649
Papaipema nebris|[16930]|RDNML186-13|13-NCCC-091|658[0n]bp|United States.North Carolina|BOLD:ACF1624
Papaipema nebris|[16931]|RDNML179-13|13-NCCC-084|658[0n]bp|United States.North Carolina|BOLD:ACF1624
Papaipema nebris|[16932]|RDNML180-13|13-NCCC-085|658[0n]bp|United States.North Carolina|BOLD:ACF1624
Papaipema nebris|[16933]|LPOKA322-08|MDOK-0322|658[0n]bp|United States.Oklahoma|BOLD:ACF1624
Papaipema nebris|[16934]|LPOKD617-09|MDOK-3696|658[0n]bp|United States.Oklahoma|BOLD:ACF1624
Papaipema nebris|[16935]|LPOKD409-09|MDOK-3488|658[0n]bp|United States.Oklahoma|BOLD:ACF1624
Papaipema nebris|[16936]|LPOKA072-08|MDOK-0072|658[0n]bp|United States.Oklahoma|BOLD:ACF1624
Papaipema nebris|[16937]|LPOKE287-11|MDOK-4365|658[0n]bp|United States.Oklahoma|BOLD:ACF1624
Papaipema nebris|[16938]|LPOKD603-09|MDOK-3682|658[0n]bp|United States.Oklahoma|BOLD:ACF1624
Papaipema nebris|[16939]|LSUSA016-06|06-SUSA-0016|658[0n]bp|United States.Kentucky|BOLD:ACF1624
Papaipema nebris|[16940]|LSUSA014-06|06-SUSA-0014|658[0n]bp|United States.Kentucky|BOLD:ACF1624
Papaipema nebris|[16941]|LILLA862-11|SNS10IL-01083|658[0n]bp|United States.Illinois|BOLD:ACF1624
Papaipema nebris|[16942]|LSUSA074-06|06-SUSA-0074|658[0n]bp|United States.Kentucky|BOLD:ACF1624
Papaipema nebris|[16943]|LILLA943-11|SNS10IL-01164|658[0n]bp|United States.Illinois|BOLD:ACF1624
Papaipema nebris|[16944]|RDNML178-13|13-NCCC-083|658[0n]bp|United States.North Carolina|BOLD:ACF1624

[illegible]
Papaipema nebris|[16942]|LSUSA074-06|06-SUSA-0074|658[0n]bp|United States.Kentucky|BOLD:ACF1624
Papaipema nebris|[16943]|LILLA943-11|SNS10IL-01164|658[0n]bp|United States.Illinois|BOLD:ACF1624
Papaipema nebris|[16944]|RDNML178-13|13-NCCC-083|658[0n]bp|United States.North Carolina|BOLD:ACF1624
Papaipema nebris|[16945]|LPOKA366-08|MDOK-0366|658[0n]bp|United States.Oklahoma|BOLD:ACF1624
Papaipema rigida|[16946]|RDLQ152-05|DH002392|658[0n]bp|Canada.Quebec|BOLD:ACF1228
Papaipema rigida|[16947]|RDNMG802-08|CNC LEP00052926|658[0n]bp|Canada.New Brunswick|BOLD:ACF1228
Papaipema rigida|[16948]|RDNMG803-08|CNC LEP00052927|658[0n]bp|Canada.New Brunswick|BOLD:ACF1228
Papaipema inquaesita|[16949]|XAH733-05|2005-ONT-2316|658[0n]bp|Canada.Ontario|BOLD:ACF3648
Papaipema inquaesita|[16950]|XAH707-05|2005-ONT-2290|658[0n]bp|Canada.Ontario|BOLD:ACF3648
Papaipema inquaesita|[16951]|XAH581-05|2005-ONT-2164|658[0n]bp|Canada.Ontario|BOLD:ACF3648
Papaipema inquaesita|[16952]|RDLQF265-06|DH011357|658[0n]bp|Canada.Quebec|BOLD:ACF3648
Papaipema inquaesita|[16953]|XAH705-05|2005-ONT-2288|658[0n]bp|Canada.Ontario|BOLD:ACF3648
Papaipema inquaesita|[16954]|XAH558-05|2005-ONT-2141|658[0n]bp|Canada.Ontario|BOLD:ACF3648
Papaipema inquaesita|[16955]|XAH695-05|2005-ONT-2278|658[0n]bp|Canada.Ontario|BOLD:ACF3648
Papaipema inquaesita|[16956]|XAD471-04|04HBL007471|566[0n]bp|Canada.Ontario|BOLD:ACF3648
Papaipema inquaesita|[16957]|XAH706-05|2005-ONT-2289|612[0n]bp|Canada.Ontario|BOLD:ACF3648
Papaipema inquaesita|[16958]|PHMO327-03|moth2546.02|639[0n]bp|Canada.Ontario|BOLD:ACF3648
Papaipema inquaesita|[16959]|XAH606-05|2005-ONT-2189|658[0n]bp|Canada.Ontario|BOLD:ACF3648
Papaipema inquaesita|[16960]|XAH546-05|2005-ONT-2129|658[0n]bp|Canada.Ontario|BOLD:ACF3648
Papaipema inquaesita|[16961]|PHMO355-03|moth2724.02|639[0n]bp|Canada.Ontario|BOLD:ACF3648
Papaipema speciosissima|[16962]|RDNMG774-08|CNC LEP00052898|658[0n]bp|Canada.Ontario|BOLD:AAF1797
Papaipema unimoda|[16963]|RDNMC581-06|CNCNoctuoidea12121|658[0n]bp|Canada.Alberta|BOLD:AAC2395
Papaipema unimoda|[16964]|XAH564-05|2005-ONT-2147|658[0n]bp|Canada.Ontario|BOLD:AAC2395
Papaipema unimoda|[16965]|RDNMC580-06|CNCNoctuoidea12120|658[0n]bp|Canada.New Brunswick|BOLD:AAC2395
Papaipema unimoda|[16966]|RDLQ482-07|AC000537|627[0n]bp|Canada.Quebec|BOLD:AAC2395
Papaipema unimoda|[16967]|RDLQ484-07|AC000528|619[0n]bp|Canada.Quebec|BOLD:AAC2395
Papaipema unimoda|[16968]|RDLQ481-07|AC000526|622[0n]bp|Canada.Quebec|BOLD:AAC2395
Papaipema unimoda|[16969]|RDLQ477-07|DH011351|622[0n]bp|Canada.Quebec|BOLD:AAC2395
Papaipema unimoda|[16970]|RDLQ479-07|DH011352|627[0n]bp|Canada.Quebec|BOLD:AAC2395
Papaipema unimoda|[16971]|RDLQ483-07|DH011350|630[0n]bp|Canada.Quebec|BOLD:AAC2395
Papaipema unimoda|[16972]|RDLQ480-07|DH011353|623[0n]bp|Canada.Quebec|BOLD:AAC2395
Papaipema unimoda|[16973]|RDLQ478-07|DH011354|613[0n]bp|Canada.Quebec|BOLD:AAC2395
Papaipema unimoda|[16974]|RDNMC579-06|CNCNoctuoidea12119|658[0n]bp|Canada.New Brunswick|BOLD:AAC2395
Photedes defecta|[16975]|BLTIB1030-08|BL1471|658[0n]bp|Canada.Ontario|BOLD:AAC8594
Photedes defecta|[16976]|BLTIB1006-08|BL1443|658[0n]bp|Canada.Ontario|BOLD:AAC8594
Photedes defecta|[16977]|BLTIB963-08|BL1392|658[0n]bp|Canada.Ontario|BOLD:AAC8594
Photedes defecta|[16978]|BLTIB794-08|BL1212|658[0n]bp|Canada.Ontario|BOLD:AAC8594
Photedes defecta|[16979]|XAJ880-06|2006-ONT-0880|658[0n]bp|Canada.Ontario|BOLD:AAC8594
Photedes defecta|[16980]|BLTIB998-08|BL1435|658[0n]bp|Canada.Ontario|BOLD:AAC8594
Photedes defecta|[16981]|XAJ990-06|2006-ONT-0990|658[0n]bp|Canada.Ontario|BOLD:AAC8594
Photedes defecta|[16982]|RDLQ471-07|DH007386|547[2n]bp|Canada.Quebec|BOLD:AAC8594
Photedes defecta|[16983]|BLTIB946-08|BL1374|658[0n]bp|Canada.Ontario|BOLD:AAC8594
Photedes enervata|[16984]|RDNMF470-08|NOC14556|658[0n]bp|Canada.Nova Scotia|BOLD:AAR9607
Melanapamea mixta|[16985]|RDNM194-05|CNCNoctuoidea6376|658[0n]bp|Canada.Ontario|BOLD:AAE7007
Photedes includens|[16986]|BBLPB994-10|10BBCLP-1993|658[0n]bp|Canada.Alberta|BOLD:AAE2566
Photedes includens|[16987]|BBLPB406-10|10BBCLP-1405|658[0n]bp|Canada.Alberta|BOLD:AAE2566
Photedes includens|[16988]|BBLPB995-10|10BBCLP-1994|658[0n]bp|Canada.Alberta|BOLD:AAE2566
Photedes includens|[16989]|BBLPB405-10|10BBCLP-1404|658[0n]bp|Canada.Alberta|BOLD:AAE2566
Photedes includens|[16990]|RDMAB942-09|UASM99289|585[0n]bp|Canada.Alberta|BOLD:AAE2566
Photedes includens|[16991]|RDMAB940-09|UASM7142|610[0n]bp|Canada.Alberta|BOLD:AAE2566
Photedes includens|[16992]|RDMAB941-09|UASM129298|619[0n]bp|Canada.Ontario|BOLD:AAE2566
Photedes includens|[16993]|RDNMG848-08|CNC LEP00052972|658[0n]bp|Canada.Ontario|BOLD:AAE2566
Photedes includens|[16994]|RDLQB404-05|DH010490|658[0n]bp|Canada.Quebec|BOLD:AAE2566
Neoligia atlantica|[16995]|BBLPC712-09|09BBELE-1712|658[0n]bp|Canada.Newfoundland and Labrador|BOLD:AA...
Neoligia atlantica|[16996]|RDNMF168-08|NOC14254|632[0n]bp|Canada.Nova Scotia|BOLD:AAK7888
Neoligia canadensis|[16997]|RDNM097-05|CNCNoctuoidea6647|658[0n]bp|Canada.Ontario|BOLD:AAE1829
Neoligia canadensis|[16998]|RDNME639-08|LEP038063|658[0n]bp|Canada.Quebec|BOLD:AAE1829
Neoligia canadensis|[16999]|RDNMG950-08|CNC LEP00053074|658[0n]bp|Canada.Ontario|BOLD:AAE1829
Neoligia canadensis|[17000]|RDNM096-05|CNCNoctuoidea6646|658[0n]bp|Canada.Ontario|BOLD:AAE1829
Neoligia canadensis|[17001]|RDLQB414-05|DH010500|658[0n]bp|Canada.Quebec|BOLD:AAE1829
Neoligia semicana|[17002]|RDNMG949-08|CNC LEP00053073|658[0n]bp|Canada.Ontario|BOLD:AAG4393
Neoligia semicana|[17003]|RDNMG948-08|CNC LEP00053072|658[0n]bp|Canada.Ontario|BOLD:AAG4393
Neoligia lancea|[17004]|RDNMF174-08|NOC14260|658[0n]bp|Canada.British Columbia|BOLD:AAD9222
Neoligia lancea|[17005]|LBCH7353-10|10-JDWBC-7353|658[0n]bp|Canada.British Columbia|BOLD:AAD9222
Neoligia invenusta|[17006]|LBCH7560-10|10-JDWBC-7560|658[0n]bp|Canada.British Columbia|BOLD:AAD8668
Neoligia invenusta|[17007]|RDNMF173-08|NOC14259|658[0n]bp|Canada.British Columbia|BOLD:AAD8668
Neoligia invenusta|[17008]|LBCH6725-10|10-JDWBC-6725|658[0n]bp|Canada.British Columbia|BOLD:AAD8668
Neoligia invenusta|[17009]|LBCH7664-10|10-JDWBC-7664|658[0n]bp|Canada.British Columbia|BOLD:AAD8668
Neoligia invenusta|[17010]|LBCH6383-10|10-JDWBC-6383|658[0n]bp|Canada.British Columbia|BOLD:AAD8668
Neoligia invenusta|[17011]|LBCG1388-09|08-JDWBC-1388|658[0n]bp|Canada.British Columbia|BOLD:AAD8668
Neoligia invenusta|[17012]|RDNMF169-08|NOC14255|658[0n]bp|Canada.British Columbia|BOLD:AAD8668
Neoligia invenusta|[17013]|RDNMF171-08|NOC14257|658[0n]bp|Canada.British Columbia|BOLD:AAD8668
Neoligia invenusta|[17014]|LALPA755-10|AVBC 757-10|658[0n]bp|Canada.British Columbia|BOLD:AAD8668
Neoligia invenusta|[17015]|LBCH6165-10|10-JDWBC-6165|658[0n]bp|Canada.British Columbia|BOLD:AAD8668
Neoligia invenusta|[17016]|LBCH6385-10|10-JDWBC-6385|658[0n]bp|Canada.British Columbia|BOLD:AAD8668
Neoligia invenusta|[17017]|LALPA730-10|AVBC 732-10|658[0n]bp|Canada.British Columbia|BOLD:AAD8668
Neoligia invenusta|[17018]|RDNMF170-08|NOC14256|658[0n]bp|Canada.British Columbia|BOLD:AAD8668
Neoligia invenusta|[17019]|LALPA675-10|AVBC 677-10|658[0n]bp|Canada.British Columbia|BOLD:AAD8668
Neoligia albirena|[17020]|RDNMF163-08|NOC14249|658[0n]bp|Canada.British Columbia|BOLD:ABX4938
Neoligia albirena|[17021]|RDNMF165-08|NOC14251|658[0n]bp|Canada.British Columbia|BOLD:ABX4938
Neoligia albirena|[17022]|RDNMF166-08|NOC14252|647[0n]bp|Canada.British Columbia|BOLD:ABX4938
Neoligia lillooet|[17023]|LBCH7559-10|10-JDWBC-7559|658[0n]bp|Canada.British Columbia|BOLD:AAD8644
Neoligia lillooet|[17024]|RDNMF179-08|NOC14265|645[0n]bp|Canada.British Columbia|BOLD:AAD8644
Neoligia lillooet|[17025]|RDNMF367-08|NOC14453|658[0n]bp|Canada.Alberta|BOLD:AAD8644
Neoligia lillooet|[17026]|RDNMF366-08|NOC14452|657[0n]bp|Canada.Alberta|BOLD:AAD8644
Neoligia lillooet|[17027]|LOWCC626-05|CGWC-2506|658[0n]bp|Canada.British Columbia|BOLD:AAD8644
Neoligia lillooet|[17028]|LOWCC627-05|CGWC-2507|596[0n]bp|Canada.British Columbia|BOLD:AAD8644
Neoligia lillooet|[17029]|LBCH7953-10|10-JDWBC-7953|658[0n]bp|Canada.British Columbia|BOLD:AAD8644
Neoligia lillooet|[17030]|RDNMF368-08|NOC14454|658[0n]bp|Canada.Alberta|BOLD:AAD8644
Neoligia tonsa|[17031]|RDNMF183-08|NOC14269|658[0n]bp|Canada.British Columbia|BOLD:AAE1791
Neoligia tonsa|[17032]|LBCH7662-10|10-JDWBC-7662|658[0n]bp|Canada.British Columbia|BOLD:AAE1791
Neoligia tonsa|[17033]|LBCH6387-10|10-JDWBC-6387|658[0n]bp|Canada.British Columbia|BOLD:AAE1791
Neoligia tonsa|[17034]|LBCH6558-10|10-JDWBC-6558|658[0n]bp|Canada.British Columbia|BOLD:AAE1791
Neoligia tonsa|[17035]|LBCH7824-10|10-JDWBC-7824|658[0n]bp|Canada.British Columbia|BOLD:AAE1791
Neoligia tonsa|[17036]|LBCH7665-10|10-JDWBC-7665|658[0n]bp|Canada.British Columbia|BOLD:AAE1791
Neoligia tonsa|[17037]|RDNMF181-08|NOC14267|658[0n]bp|Canada.British Columbia|BOLD:AAE1791
Neoligia tonsa|[17038]|LBCH7660-10|10-JDWBC-7660|658[0n]bp|Canada.British Columbia|BOLD:AAE1791
Neoligia tonsa|[17039]|LBCG1401-09|08-JDWBC-1401|658[0n]bp|Canada.British Columbia|BOLD:AAE1791
Neoligia tonsa|[17040]|RDNMF182-08|NOC14268|658[0n]bp|Canada.British Columbia|BOLD:AAE1791
Neoligia tonsa|[17041]|LBCH7661-10|10-JDWBC-7661|636[0n]bp|Canada.British Columbia|BOLD:AAE1791
Neoligia tonsa|[17042]|RDNMF184-08|NOC14270|632[0n]bp|Canada.British Columbia|BOLD:AAE1791
Neoligia tonsa|[17043]|LBCH7659-10|10-JDWBC-7659|658[0n]bp|Canada.British Columbia|BOLD:AAE1791
[illegible]

Neoligia tonsa|[17041]|LBCH7661-10|10-JDWBC-7661|658[0n]bp|Canada.British Columbia|BOLD:AAE1791
Neoligia tonsa|[17042]|RDNMF184-08|NOC14270|632[0n]bp|Canada.British Columbia|BOLD:AAE1791
Neoligia tonsa|[17043]|LBCH7659-10|10-JDWBC-7659|658[0n]bp|Canada.British Columbia|BOLD:AAE1791
Neoligia tonsa|[17044]|LBCH6727-10|10-JDWBC-6727|658[0n]bp|Canada.British Columbia|BOLD:AAE1791
Neoligia tonsa|[17045]|LBCH7663-10|10-JDWBC-7663|658[0n]bp|Canada.British Columbia|BOLD:AAE1791
Neoligia tonsa|[17046]|LBCH7666-10|10-JDWBC-7666|658[0n]bp|Canada.British Columbia|BOLD:AAE1791
Neoligia tonsa|[17047]|LBCH6556-10|10-JDWBC-6556|658[0n]bp|Canada.British Columbia|BOLD:AAE1791
Neoligia tonsa|[17048]|LBCH7823-10|10-JDWBC-7823|658[0n]bp|Canada.British Columbia|BOLD:AAE1791
Neoligia subjuncta|[17049]|LPABC155-09|08BBLEP-04374|627[0n]bp|Canada.Alberta|BOLD:AAA8279
Neoligia subjuncta|[17050]|LOWCB519-05|CGWC-1459|658[0n]bp|Canada.British Columbia|BOLD:AAA8279
Neoligia subjuncta|[17051]|LOWCB522-05|CGWC-1462|605[0n]bp|Canada.British Columbia|BOLD:AAA8279
Neoligia subjuncta|[17052]|LPABB537-08|08BBLEP-03802|658[0n]bp|Canada.Alberta|BOLD:AAA8279
Neoligia subjuncta|[17053]|LOWCB520-05|CGWC-1460|658[0n]bp|Canada.British Columbia|BOLD:AAA8279
Neoligia subjuncta|[17054]|LPABC896-09|08BBLEP-05307|658[0n]bp|Canada.Alberta|BOLD:AAA8279
Neoligia subjuncta|[17055]|LOWCD531-06|CGWC-3351|658[0n]bp|Canada.British Columbia|BOLD:AAA8279
Neoligia subjuncta|[17056]|LBCH1335-10|10-JDWBC-1335|658[0n]bp|Canada.British Columbia|BOLD:AAA8279
Neoligia subjuncta|[17057]|BBLPD546-10|10BBCLP-2544|658[0n]bp|Canada.British Columbia|BOLD:AAA8279
Neoligia subjuncta|[17058]|LOWCD530-06|CGWC-3350|658[0n]bp|Canada.British Columbia|BOLD:AAA8279
Neoligia subjuncta|[17059]|MNBB083-05|HBL008693|658[0n]bp|Canada.New Brunswick|BOLD:AAA8279
Neoligia subjuncta|[17060]|LBCC241-05|HLC-22121|658[0n]bp|Canada.British Columbia|BOLD:AAA8279
Neoligia subjuncta|[17061]|XAB131-04|04HBL005131|658[0n]bp|Canada.Ontario|BOLD:AAA8279
Neoligia subjuncta|[17062]|BBLEC875-09|09BBELE-0875|658[0n]bp|Canada.Newfoundland and Labrador|BOLD:AA...
Neoligia subjuncta|[17063]|LALPA938-11|AVBC 1111-11|658[0n]bp|Canada.British Columbia|BOLD:AAA8279
Neoligia subjuncta|[17064]|RDLQB398-05|DH010484|658[0n]bp|Canada.Quebec|BOLD:AAA8279
Neoligia subjuncta|[17065]|BBLPB190-10|10BBCLP-1189|658[0n]bp|Canada.Alberta|BOLD:AAA8279
Neoligia subjuncta|[17066]|BLTIB542-08|BL813|658[0n]bp|Canada.Ontario|BOLD:AAA8279
Neoligia subjuncta|[17067]|BBLPC716-09|09BBELE-1716|658[0n]bp|Canada.Newfoundland and Labrador|BOLD:AA...
Neoligia subjuncta|[17068]|BLTIB455-08|BL702|658[0n]bp|Canada.Ontario|BOLD:AAA8279
Neoligia subjuncta|[17069]|RDLQB396-05|DH010482|658[0n]bp|Canada.Quebec|BOLD:AAA8279
Neoligia subjuncta|[17070]|RDLQG806-06|DH013099|658[0n]bp|Canada.Quebec|BOLD:AAA8279
Neoligia subjuncta|[17071]|PMG140-03|moth954.01|617[0n]bp|Canada.Ontario|BOLD:AAA8279
Neoligia subjuncta|[17072]|LOWCB515-05|CGWC-1455|610[0n]bp|Canada.British Columbia|BOLD:AAA8279
Neoligia subjuncta|[17073]|LOWCB517-05|CGWC-1457|608[0n]bp|Canada.British Columbia|BOLD:AAA8279
Neoligia subjuncta|[17074]|LOWCB514-05|CGWC-1454|615[0n]bp|Canada.British Columbia|BOLD:AAA8279
Neoligia subjuncta|[17075]|LOWCB513-05|CGWC-1453|605[0n]bp|Canada.British Columbia|BOLD:AAA8279
Neoligia subjuncta|[17076]|BLTIB615-08|BL895|631[0n]bp|Canada.Ontario|BOLD:AAA8279
Neoligia subjuncta|[17077]|RDMAB078-05|UASM57605|622[0n]bp|Canada.Alberta|BOLD:AAA8279
Neoligia subjuncta|[17078]|RDLQG773-06|DH013066|658[0n]bp|Canada.Quebec|BOLD:AAA8279
Neoligia subjuncta|[17079]|BBLPD856-10|10BBCLP-2854|658[0n]bp|Canada.British Columbia|BOLD:AAA8279
Neoligia subjuncta|[17080]|XAC710-04|04HBL006710|658[0n]bp|Canada.Ontario|BOLD:AAA8279
Neoligia subjuncta|[17081]|LPABC435-09|08BBLEP-04654|658[0n]bp|Canada.Alberta|BOLD:AAA8279
Neoligia subjuncta|[17082]|RDLQB397-05|DH010483|658[0n]bp|Canada.Quebec|BOLD:AAA8279
Neoligia subjuncta|[17083]|BLTIB498-08|BL754|658[0n]bp|Canada.Ontario|BOLD:AAA8279
Neoligia subjuncta|[17084]|LPMN070-08|08BBLEP-00868|658[0n]bp|Canada.Manitoba|BOLD:AAA8279
Neoligia subjuncta|[17085]|XAB122-04|04HBL005122|658[0n]bp|Canada.Ontario|BOLD:AAA8279
Neoligia subjuncta|[17086]|LPABC359-09|08BBLEP-04578|658[0n]bp|Canada.Alberta|BOLD:AAA8279
Neoligia subjuncta|[17087]|BBLPE389-09|09BBELE-2389|658[0n]bp|Canada.Newfoundland and Labrador|BOLD:AA...
Neoligia subjuncta|[17088]|LPMNB420-09|08BBLEP-05420|658[0n]bp|Canada.Manitoba|BOLD:AAA8279
Neoligia subjuncta|[17089]|LOWCB521-05|CGWC-1461|658[0n]bp|Canada.British Columbia|BOLD:AAA8279
Neoligia subjuncta|[17090]|RDLQG788-06|DH013081|658[0n]bp|Canada.Quebec|BOLD:AAA8279
Neoligia subjuncta|[17091]|RDLQG795-06|DH013088|658[0n]bp|Canada.Quebec|BOLD:AAA8279
Neoligia subjuncta|[17092]|LPABC361-09|08BBLEP-04580|658[0n]bp|Canada.Alberta|BOLD:AAA8279
Neoligia subjuncta|[17093]|BBLPB471-10|10BBCLP-1470|658[0n]bp|Canada.British Columbia|BOLD:AAA8279
Neoligia subjuncta|[17094]|RDLQG807-06|DH013100|658[0n]bp|Canada.Quebec|BOLD:AAA8279
Neoligia subjuncta|[17095]|LPSK262-08|08BBLEP-01830|658[0n]bp|Canada.Saskatchewan|BOLD:AAA8279
Neoligia subjuncta|[17096]|RDLQB411-05|DH010497|658[0n]bp|Canada.Quebec|BOLD:AAA8279
Neoligia subjuncta|[17097]|LPABC469-09|08BBLEP-04688|658[0n]bp|Canada.Alberta|BOLD:AAA8279
Hypocoena rufostrigata|[17098]|RDNMF271-08|NOC14357|658[0n]bp|Canada.Saskatchewan|BOLD:AAD8056
Hypocoena rufostrigata|[17099]|LPMN733-08|08BBLEP-01536|658[0n]bp|Canada.Manitoba|BOLD:AAD8056
Hypocoena rufostrigata|[17100]|RDNMF272-08|NOC14358|658[1n]bp|Canada.Saskatchewan|BOLD:AAD8056
Hypocoena rufostrigata|[17101]|RDNMF274-08|NOC14360|658[0n]bp|Canada.Alberta|BOLD:AAD8056
Hypocoena rufostrigata|[17102]|RDNMF273-08|NOC14359|658[0n]bp|Canada.Saskatchewan|BOLD:AAD8056
Hypocoena rufostrigata|[17103]|RDNMF270-08|NOC14356|658[0n]bp|Canada.Saskatchewan|BOLD:AAD8056
Lateroligia ophiogramma|[17104]|LPVIA299-08|PFC-2006-0455|658[0n]bp|Canada.British Columbia|BOLD:AAB0872
Lateroligia ophiogramma|[17105]|XAG548-05|2005-ONT-1132|605[1n]bp|Canada.Ontario|BOLD:AAB0872
Lateroligia ophiogramma|[17106]|LPVIB977-08|PFC-2006-2527|658[0n]bp|Canada.British Columbia|BOLD:AAB0872
Lateroligia ophiogramma|[17107]|RDLQB436-05|DH010522|576[0n]bp|Canada.Quebec|BOLD:AAB0872
Lateroligia ophiogramma|[17108]|LHLEP574-06|UBC-2006-1069|622[0n]bp|Canada.British Columbia|BOLD:AAB0872
Lateroligia ophiogramma|[17109]|LPVIB001-08|PFC-2006-1335|622[0n]bp|Canada.British Columbia|BOLD:AAB0872
Lateroligia ophiogramma|[17110]|LMH038-06|PFC-2006-0454|658[0n]bp|Canada.British Columbia|BOLD:AAB0872
Lateroligia ophiogramma|[17111]|LPVIA300-08|PFC-2006-0456|658[0n]bp|Canada.British Columbia|BOLD:AAB0872
Lateroligia ophiogramma|[17112]|LHLEP074-06|UBC-2006-0279|658[0n]bp|Canada.British Columbia|BOLD:AAB0872
Lateroligia ophiogramma|[17113]|LPVIB075-08|PFC-2006-1423|658[0n]bp|Canada.British Columbia|BOLD:AAB0872
Lateroligia ophiogramma|[17114]|LHLEP575-06|UBC-2006-1070|658[0n]bp|Canada.British Columbia|BOLD:AAB0872
Lateroligia ophiogramma|[17115]|LALPA683-10|AVBC 685-10|658[0n]bp|Canada.British Columbia|BOLD:AAB0872
Lateroligia ophiogramma|[17116]|LPMN1002-09|08BBLEP-04043|658[0n]bp|Canada.Manitoba|BOLD:AAB0872
Lateroligia ophiogramma|[17117]|LALPA312-10|AVBC 314-10|658[0n]bp|Canada.British Columbia|BOLD:AAB0872
Lateroligia ophiogramma|[17118]|LPABB067-08|08BBLEP-03332|658[0n]bp|Canada.Alberta|BOLD:AAB0872
Lateroligia ophiogramma|[17119]|BLTIB507-08|BL764|658[0n]bp|Canada.Ontario|BOLD:AAB0872
Lateroligia ophiogramma|[17120]|BBLPC409-09|09BBELE-1409|658[0n]bp|Canada.New Brunswick|BOLD:AAB0872
Lateroligia ophiogramma|[17121]|LHLEP576-06|UBC-2006-1172|658[0n]bp|Canada.British Columbia|BOLD:AAB0872
Lateroligia ophiogramma|[17122]|LPVIA610-08|PFC-2006-0838|658[0n]bp|Canada.British Columbia|BOLD:AAB0872
Lateroligia ophiogramma|[17123]|RDLQG054-06|DH012185|658[0n]bp|Canada.Quebec|BOLD:AAB0872
Lateroligia ophiogramma|[17124]|LPVIB087-08|PFC-2006-1441|658[0n]bp|Canada.British Columbia|BOLD:AAB0872
Lateroligia ophiogramma|[17125]|LPVIB978-08|PFC-2006-2528|658[0n]bp|Canada.British Columbia|BOLD:AAB0872
Lateroligia ophiogramma|[17126]|RDLQB722-05|DH010825|658[0n]bp|Canada.Quebec|BOLD:AAB0872
Lateroligia ophiogramma|[17127]|LPVIA301-08|PFC-2006-0457|658[0n]bp|Canada.British Columbia|BOLD:AAB0872
Lateroligia ophiogramma|[17128]|LPVIA302-08|PFC-2006-0458|658[0n]bp|Canada.British Columbia|BOLD:AAB0872
Lateroligia ophiogramma|[17129]|LALPA575-10|AVBC 577-10|658[0n]bp|Canada.British Columbia|BOLD:AAB0872
Lateroligia ophiogramma|[17130]|BBLPB346-10|10BBCLP-1345|658[0n]bp|Canada.Alberta|BOLD:AAB0872
Lateroligia ophiogramma|[17131]|RDLQB729-05|DH010832|658[0n]bp|Canada.Quebec|BOLD:AAB0872
Lateroligia ophiogramma|[17132]|BLTIB658-08|BL939|658[0n]bp|Canada.Ontario|BOLD:AAB0872
Lateroligia ophiogramma|[17133]|MNBB125-05|05-NBSTA-041|658[0n]bp|Canada.New Brunswick|BOLD:AAB0872
Lateroligia ophiogramma|[17134]|LALPA455-10|AVBC 457-10|658[0n]bp|Canada.British Columbia|BOLD:AAB0872
Lateroligia ophiogramma|[17135]|LHLEP733-06|UBC-2006-0278|644[0n]bp|Canada.British Columbia|BOLD:AAB0872
Lateroligia ophiogramma|[17136]|PHMO264-03|moth1536.02|639[0n]bp|Canada.Ontario|BOLD:AAB0872
Lateroligia ophiogramma|[17137]|LHLEP577-06|UBC-2006-1230|634[0n]bp|Canada.British Columbia|BOLD:AAB0872
Lateroligia ophiogramma|[17138]|BBLPC068-09|09BBELE-1068|658[0n]bp|Canada.New Brunswick|BOLD:AAB0872
Oligia modica|[17139]|TTMNB330-06|MNBTT-330|585[0n]bp|Canada.New Brunswick|BOLD:ABZ2233
Oligia modica|[17140]|RDLQF835-06|DH011988|658[0n]bp|Canada.Quebec|BOLD:ABZ2233
Oligia modica|[17141]|RDLQB427-05|DH010513|658[0n]bp|Canada.Quebec|BOLD:AAE1462
Mesapamea fractilinea|[17142]|RDLQB734-05|DH010837|658[0n]bp|Canada.Quebec|BOLD:AAC5476
Mesapamea fractilinea|[17143]|RDLQB813-05|DH010900|658[0n]bp|Canada.Quebec|BOLD:AAC5476

Oligia modica|[17141]|RDLQB427-05|DH010513|658[0n]bp|Canada.Quebec|BOLD:AAE1462
Mesapamea fractilinea|[17142]|RDLQB734-05|DH010837|658[0n]bp|Canada.Quebec|BOLD:AAC5476
Mesapamea fractilinea|[17143]|RDLQB813-05|DH010900|658[0n]bp|Canada.Quebec|BOLD:AAC5476
Mesapamea fractilinea|[17144]|RDLQ462-07|DH007618|608[0n]bp|Canada.Quebec|BOLD:AAC5476
Mesapamea fractilinea|[17145]|XAD342-04|04HBL007342|658[0n]bp|Canada.Ontario|BOLD:AAC5476
Mesapamea fractilinea|[17146]|XAD297-04|04HBL007297|579[0n]bp|Canada.Ontario|BOLD:AAC5476
Mesapamea fractilinea|[17147]|HEAUG1529-12|BIOUG02430-E11|618[0n]bp|Canada.Ontario|BOLD:AAC5476
Mesapamea fractilinea|[17148]|RDLQ156-05|DH007889|609[0n]bp|Canada.Quebec|BOLD:AAC5476
Mesapamea fractilinea|[17149]|PHSEP346-11|BIOUG01292-B05|652[0n]bp|Canada.Ontario|BOLD:AAC5476
Mesapamea fractilinea|[17150]|PHAUG1513-11|BIOUG01521-A01|658[0n]bp|Canada.Ontario|BOLD:AAC5476
Hypocoena inquinata|[17151]|RDNMG938-08|CNC LEP00053062|658[0n]bp|Canada.Alberta|BOLD:AAD0213
Hypocoena inquinata|[17152]|BBLPB388-10|10BBCLP-1387|658[0n]bp|Canada.Alberta|BOLD:AAD0213
Hypocoena inquinata|[17153]|RDNMG939-08|CNC LEP00053063|658[0n]bp|Canada.Ontario|BOLD:AAD0213
Hypocoena inquinata|[17154]|LPABC290-09|08BBLEP-04509|658[0n]bp|Canada.Alberta|BOLD:AAD0213
Hypocoena inquinata|[17155]|BBLPB385-10|10BBCLP-1384|658[0n]bp|Canada.Alberta|BOLD:AAD0213
Hypocoena inquinata|[17156]|BBLPB386-10|10BBCLP-1385|658[0n]bp|Canada.Alberta|BOLD:AAD0213
Hypocoena inquinata|[17157]|BBLPA843-10|10BBCLP-0843|658[0n]bp|Canada.Alberta|BOLD:AAD0213
Hypocoena inquinata|[17158]|BBLPB387-10|10BBCLP-1386|658[0n]bp|Canada.Alberta|BOLD:AAD0213
Hypocoena inquinata|[17159]|LPABC266-09|08BBLEP-04485|658[0n]bp|Canada.Alberta|BOLD:AAD0213
Hypocoena inquinata|[17160]|RDNMG940-08|CNC LEP00053064|658[0n]bp|Canada.Alberta|BOLD:AAD0213
Hypocoena inquinata|[17161]|RDNMG504-08|CNC LEP00052328|658[0n]bp|Canada.Ontario|BOLD:ACE4044
Hypocoena inquinata|[17162]|BBLPE618-09|09BBELE-2618|632[0n]bp|Canada.Nova Scotia|BOLD:ACE4044
Hypocoena inquinata|[17163]|RDNMG503-08|CNC LEP00052327|658[0n]bp|Canada.Manitoba|BOLD:AAD0213
Neoligia exhausta|[17164]|BLTIB1122-08|BL1135|658[0n]bp|Canada.Ontario|BOLD:AAB6095
Neoligia exhausta|[17165]|RDNM095-05|CNCNoctuoidea6645|658[0n]bp|Canada.Ontario|BOLD:AAB6095
Neoligia exhausta|[17166]|RDLQG591-06|DH012884|658[0n]bp|Canada.Quebec|BOLD:AAB6095
Neoligia exhausta|[17167]|XAC602-04|04HBL006602|658[0n]bp|Canada.Ontario|BOLD:AAB6095
Neoligia exhausta|[17168]|RDLQG917-06|DH013210|658[0n]bp|Canada.Quebec|BOLD:AAB6095
Neoligia exhausta|[17169]|RDLQG753-06|DH013046|658[0n]bp|Canada.Quebec|BOLD:AAB6095
Neoligia exhausta|[17170]|PHMO188-03|moth966.01|639[2n]bp|Canada.Ontario|BOLD:AAB6095
Neoligia exhausta|[17171]|BLTIB577-08|BL856|619[0n]bp|Canada.Ontario|BOLD:AAB6095
Neoligia exhausta|[17172]|RDLQG805-06|DH013098|653[0n]bp|Canada.Quebec|BOLD:AAB6095
Neoligia exhausta|[17173]|HEJUL255-12|BIOUG02385-F03|604[0n]bp|Canada.Ontario|BOLD:AAB6095
Neoligia exhausta|[17174]|PHMO278-03|moth1710.02|638[0n]bp|Canada.Ontario|BOLD:AAB6095
Neoligia exhausta|[17175]|BLTIB602-08|BL882|634[0n]bp|Canada.Ontario|BOLD:AAB6095
Neoligia exhausta|[17176]|XAJ802-06|2006-ONT-0802|632[0n]bp|Canada.Ontario|BOLD:AAB6095
Neoligia exhausta|[17177]|XAG058-05|2005-ONT-642|658[0n]bp|Canada.Ontario|BOLD:AAB6095
Neoligia exhausta|[17178]|RDLQG776-06|DH013069|658[0n]bp|Canada.Quebec|BOLD:AAB6095
Neoligia exhausta|[17179]|RDNME669-08|LEP031944|658[0n]bp|Canada.New Brunswick|BOLD:AAB6095
Neoligia exhausta|[17180]|XAJ863-06|2006-ONT-0863|658[0n]bp|Canada.Ontario|BOLD:AAB6095
Neoligia exhausta|[17181]|RDLQB395-05|DH010481|658[0n]bp|Canada.Quebec|BOLD:AAB6095
Neoligia exhausta|[17182]|XAJ865-06|2006-ONT-0865|658[0n]bp|Canada.Ontario|BOLD:AAB6095
Neoligia exhausta|[17183]|XAB144-04|04HBL005144|658[0n]bp|Canada.Ontario|BOLD:AAB6095
Neoligia exhausta|[17184]|XAC733-04|04HBL006733|658[0n]bp|Canada.Ontario|BOLD:AAB6095
Oligia tusa|[17185]|RDNMF726-08|NOC14812|658[0n]bp|Canada.British Columbia|BOLD:AAB5984
Oligia tusa|[17186]|RDNMF725-08|NOC14811|658[0n]bp|Canada.British Columbia|BOLD:AAB5984
Oligia tusa|[17187]|RDNMF724-08|NOC14810|658[0n]bp|Canada.British Columbia|BOLD:AAB5984
Neoligia crytora|[17188]|RDLQG751-06|DH013044|658[0n]bp|Canada.Quebec|BOLD:AAF0862
Neoligia crytora|[17189]|RDLQG750-06|DH013043|658[0n]bp|Canada.Quebec|BOLD:AAF0862
Neoligia crytora|[17190]|RDLQG749-06|DH013042|658[0n]bp|Canada.Quebec|BOLD:AAF0862
Benjaminiola colorada|[17191]|RDNMF283-08|NOC14369|658[0n]bp|Canada.British Columbia|BOLD:ACF2615
Photedes didonea|[17192]|RDNMD792-07|CNCNoctuoidea13124|658[0n]bp|United States.Oregon|BOLD:AAF1495
Photedes didonea|[17193]|RDMAB957-09|UASM129132|638[0n]bp|United States.Oregon|BOLD:AAF1495
Photedes didonea|[17194]|RDMAB955-09|UASM129127|621[0n]bp|United States.Oregon|BOLD:AAF1495
Photedes didonea|[17195]|RDMAB956-09|UASM129126|642[0n]bp|United States.Washington|BOLD:AAF1495
Photedes panatela|[17196]|LPSOB827-08|PPBP-1826|658[0n]bp|Canada.Ontario|BOLD:AAC7821
Photedes panatela|[17197]|LPSOB852-08|PPBP-1851|658[0n]bp|Canada.Ontario|BOLD:AAC7821
Photedes panatela|[17198]|TMNBB470-06|MNBTT-1410|658[0n]bp|Canada.New Brunswick|BOLD:AAC7821
Photedes panatela|[17199]|RDNMF280-08|NOC14366|609[0n]bp|Canada.New Brunswick|BOLD:AAC7821
Photedes panatela|[17200]|LPSOB837-08|PPBP-1836|658[0n]bp|Canada.Ontario|BOLD:AAC7821
Photedes panatela|[17201]|RDNMF282-08|NOC14368|658[0n]bp|Canada.Ontario|BOLD:AAC7821
Photedes panatela|[17202]|RDLQB270-05|DH010356|658[0n]bp|Canada.Quebec|BOLD:AAC7821
Photedes panatela|[17203]|LPSOC195-08|PPBP-2194|658[0n]bp|Canada.Ontario|BOLD:AAC7821
Photedes panatela|[17204]|BLGSM046-09|BL359|658[0n]bp|Canada.Ontario|BOLD:AAC7821
Photedes panatela|[17205]|LPSOD296-09|08BBLEP-00074|658[0n]bp|Canada.Ontario|BOLD:AAC7821
Photedes panatela|[17206]|RDNMF281-08|NOC14367|658[0n]bp|Canada.New Brunswick|BOLD:AAC7821
Brachylomia cascadia|[17207]|RDNMG619-08|CNC LEP00052443|658[0n]bp|Canada.British Columbia|BOLD:ABX6835
Brachylomia cascadia|[17208]|RDNMG620-08|CNC LEP00052444|658[0n]bp|Canada.British Columbia|BOLD:ABX6835
Brachylomia cascadia|[17209]|LALPA923-11|AVBC 1096-11|658[0n]bp|Canada.British Columbia|BOLD:ABX6835
Brachylomia cascadia|[17210]|RDNM163-05|CNCNoctuoidea6713|658[0n]bp|Canada.British Columbia|BOLD:ABX6835
Brachylomia cascadia|[17211]|RDNM162-05|CNCNoctuoidea6712|580[0n]bp|Canada.British Columbia|BOLD:ABX6835
Brachylomia rectifascia|[17212]|RDNM160-05|CNCNoctuoidea6710|658[0n]bp|United States.California|BOLD:A...
Brachylomia rectifascia|[17213]|RDNM161-05|CNCNoctuoidea6711|658[0n]bp|United States.California|BOLD:A...
Brachylomia algens|[17214]|RDLQF172-06|DH011199|658[0n]bp|Canada.Quebec|BOLD:AAD7480
Brachylomia algens|[17215]|BBLPB766-10|10BBCLP-1765|658[0n]bp|Canada.Saskatchewan|BOLD:AAD7480
Brachylomia algens|[17216]|RDNMG931-08|CNC LEP00053055|658[0n]bp|Canada.Ontario|BOLD:AAD7480
Brachylomia algens|[17217]|RDNMG933-08|CNC LEP00053057|658[0n]bp|Canada.Ontario|BOLD:ABY6308
Brachylomia algens|[17218]|BBLPB334-10|10BBCLP-1333|658[0n]bp|Canada.Saskatchewan|BOLD:ABY6308
Brachylomia algens|[17219]|RDLQF652-06|DH011802|637[0n]bp|Canada.Quebec|BOLD:ABY6308
Brachylomia algens|[17220]|RDNMG932-08|CNC LEP00053056|658[0n]bp|Canada.Alberta|BOLD:ABY6308
Brachylomia discinigra|[17221]|RDLQF173-06|DH011200|658[0n]bp|Canada.Quebec|BOLD:AAD7477
Brachylomia discinigra|[17222]|RDNMB945-05|CNCNoctuoidea10720|658[0n]bp|Canada.Ontario|BOLD:AAD7477
Brachylomia discinigra|[17223]|RDNMB944-05|CNCNoctuoidea10719|658[0n]bp|Canada.Ontario|BOLD:AAD7477
Brachylomia thula|[17224]|RDMAB1020-09|UASM125964|658[0n]bp|United States.Oregon|BOLD:AAD7482
Brachylomia thula|[17225]|RDNM174-05|CNCNoctuoidea6724|658[0n]bp|United States.Washington|BOLD:AAD7482
Brachylomia thula|[17226]|RDNMG618-08|CNC LEP00052442|658[0n]bp|United States.Washington|BOLD:AAD7482
Oligia obtusa|[17227]|RDNMG952-08|CNC LEP00053076|658[0n]bp|Canada.Ontario|BOLD:AAD7847
Oligia obtusa|[17228]|RDLQB822-05|DH010909|621[0n]bp|Canada.Quebec|BOLD:AAD7847
Oligia obtusa|[17229]|RDNMG951-08|CNC LEP00053075|658[0n]bp|Canada.British Columbia|BOLD:AAD7847
Oligia obtusa|[17230]|PHMO328-03|moth2559.02|639[0n]bp|Canada.Ontario|BOLD:AAD7847
Oligia obtusa|[17231]|PHMO324-03|moth2522.02|639[1n]bp|Canada.Ontario|BOLD:AAD7847
Hydraecia columbia|[17232]|RDNM273-05|CNCNoctuoidea6455|571[0n]bp|Canada.British Columbia|BOLD:ABY5447
Hydraecia columbia|[17233]|RDNM274-05|CNCNoctuoidea6456|507[0n]bp|Canada.British Columbia|BOLD:ABY5447
Hydraecia micacea|[17234]|RDNMB933-05|CNCNoctuoidea10708|658[0n]bp|Canada.Ontario|BOLD:AAB1631
Hydraecia micacea|[17235]|XAK510-07|HLC-16063|658[0n]bp|Canada.Ontario|BOLD:AAB1631
Hydraecia micacea|[17236]|BLTIB935-08|BL1355|658[0n]bp|Canada.Ontario|BOLD:AAB1631
Hydraecia micacea|[17237]|XAG382-05|2005-ONT-966|658[0n]bp|Canada.Ontario|BOLD:AAB1631
Hydraecia micacea|[17238]|RDLQB820-05|DH010907|618[0n]bp|Canada.Quebec|BOLD:AAB1631
Hydraecia micacea|[17239]|PMG118-03|moth1181.01|617[0n]bp|Canada.Ontario|BOLD:AAB1631
Hydraecia micacea|[17240]|XAK348-06|2006-ONT-1343|658[0n]bp|Canada.Ontario|BOLD:AAB1631
Hydraecia micacea|[17241]|XAK609-07|HLC-16162|566[0n]bp|Canada.Ontario|BOLD:AAB1631
Hydraecia pallescens|[17242]|RDNM039-05|CNCNoctuoidea6308|596[1n]bp|Canada.British Columbia|BOLD:ABZ6168
Hydraecia pallescens|[17243]|RDNM040-05|CNCNoctuoidea6309|614[1n]bp|Canada.British Columbia|BOLD:ABZ6168

[illegible]
Hydraecia micacea|[17241]|XAK609-07|HLC-16162|566[0n]bp|Canada.Ontario|BOLD:AAB1631
Hydraecia pallescens|[17242]|RDNM039-05|CNCNoctuoidea6308|596[1n]bp|Canada.British Columbia|BOLD:ABZ6168
Hydraecia pallescens|[17243]|RDNM040-05|CNCNoctuoidea6309|614[1n]bp|Canada.British Columbia|BOLD:ABZ6168
Hydraecia immanis|[17244]|RDNMB934-05|CNCNoctuoidea10709|658[0n]bp|Canada.Ontario|BOLD:AAF0898
Hydraecia stramentosa|[17245]|RDNMC080-05|CNCNoctuoidea10821|658[0n]bp|Canada.Ontario|BOLD:AAI8720
Hydraecia stramentosa|[17246]|XAD299-04|04HBL007299|579[0n]bp|Canada.Ontario|BOLD:AAI8720
Amphipoea americana|[17247]|RWWB168-09|RWWA-1167|658[0n]bp|United States.Washington|BOLD:ABZ0147
Amphipoea interoceanica|[17248]|RDNML375-13|CNCLEP 92351|658[0n]bp|Canada.Ontario|BOLD:ABZ0147
Amphipoea interoceanica|[17249]|RDNML376-13|CNCLEP 92352|658[0n]bp|Canada.Alberta|BOLD:ABZ0147
Amphipoea sp. 1|[17250]|BBLEC699-09|09BBELE-0699|658[0n]bp|Canada.Nova Scotia|BOLD:ABZ0147
Amphipoea sp. 1|[17251]|TTMNB355-06|MNBTT-355|658[0n]bp|Canada.New Brunswick|BOLD:ABZ0147
Amphipoea sp. 2|[17252]|RDLQ469-07|AC000763|596[3n]bp|Canada.Quebec|BOLD:AAC0644
Amphipoea americana|[17253]|LGSMG976-10|BGS04057|658[0n]bp|United States.North Carolina|BOLD:AAC0644
Amphipoea americana|[17254]|LGSMG972-10|BGS04053|658[0n]bp|United States.North Carolina|BOLD:AAC0644
Amphipoea sp. 2|[17255]|BLTIB1044-08|BL1488|658[0n]bp|Canada.Ontario|BOLD:AAC0644
Amphipoea sp. 2|[17256]|BBLEC709-09|09BBELE-0709|658[0n]bp|Canada.Nova Scotia|BOLD:AAC0644
Amphipoea sp. 2|[17257]|TTMNB360-06|MNBTT-360|658[1n]bp|Canada.New Brunswick|BOLD:AAC0644
Amphipoea sp. 2|[17258]|BBLPB632-10|10BBCLP-1631|658[0n]bp|Canada.Saskatchewan|BOLD:AAC0644
Amphipoea sp. 2|[17259]|LOWCD178-06|CGWC-2998|658[0n]bp|Canada.British Columbia|BOLD:AAC0644
Amphipoea sp. 2|[17260]|XAH323-05|2005-ONT-1906|658[0n]bp|Canada.Ontario|BOLD:AAC0644
Amphipoea sp. 2|[17261]|RDLQB724-05|DH010827|658[0n]bp|Canada.Quebec|BOLD:AAC0644
Amphipoea sp. 2|[17262]|MNBB664-05|05-NBSTA-580|658[0n]bp|Canada.New Brunswick|BOLD:AAC0644
Amphipoea sp. 2|[17263]|XAK339-06|2006-ONT-1334|658[0n]bp|Canada.Ontario|BOLD:AAC0644
Amphipoea sp. 2|[17264]|TTMNB356-06|MNBTT-356|658[0n]bp|Canada.New Brunswick|BOLD:AAC0644
Amphipoea sp. 2|[17265]|BBLPB627-10|10BBCLP-1626|658[0n]bp|Canada.Alberta|BOLD:AAC0644
Amphipoea sp. 2|[17266]|BBLEC692-09|09BBELE-0692|658[0n]bp|Canada.Nova Scotia|BOLD:AAC0644
Amphipoea sp. 2|[17267]|TMNBB186-06|MNBTT-1126|658[0n]bp|Canada.New Brunswick|BOLD:AAC0644
Amphipoea sp. 2|[17268]|XAJ984-06|2006-ONT-0984|657[0n]bp|Canada.Ontario|BOLD:AAC0644
Amphipoea sp. 2|[17269]|BBLPB626-10|10BBCLP-1625|658[0n]bp|Canada.Alberta|BOLD:AAC0644
Amphipoea sp. 2|[17270]|TTMNB359-06|MNBTT-359|658[0n]bp|Canada.New Brunswick|BOLD:AAC0644
Amphipoea sp. 2|[17271]|BBLPB629-10|10BBCLP-1628|658[0n]bp|Canada.Saskatchewan|BOLD:AAC0644
Amphipoea sp. 2|[17272]|BBLPB630-10|10BBCLP-1629|658[0n]bp|Canada.Saskatchewan|BOLD:AAC0644
Amphipoea americana|[17273]|LGSMG978-10|BGS04059|658[0n]bp|United States.North Carolina|BOLD:AAC0644
Amphipoea sp. 2|[17274]|BBLPB625-10|10BBCLP-1624|658[0n]bp|Canada.Alberta|BOLD:AAC0644
Amphipoea sp. 2|[17275]|BBLPB624-10|10BBCLP-1623|658[0n]bp|Canada.Alberta|BOLD:AAC0644
Amphipoea sp. 2|[17276]|XAK415-06|2006-ONT-1410|658[0n]bp|Canada.Ontario|BOLD:AAC0644
Amphipoea americana|[17277]|LSEU750-06|06-JKA-0750|658[0n]bp|United States.Georgia|BOLD:AAC0644
Amphipoea americana|[17278]|LNCC348-10|10-NCCC-348|658[0n]bp|United States.North Carolina|BOLD:AAC0644
Amphipoea americana|[17279]|LGSMG973-10|BGS04054|658[0n]bp|United States.North Carolina|BOLD:AAC0644
Amphipoea americana|[17280]|LGSMG981-10|BGS04062|658[0n]bp|United States.North Carolina|BOLD:AAC0644
Amphipoea americana|[17281]|LGSMG974-10|BGS04055|658[0n]bp|United States.North Carolina|BOLD:AAC0644
Amphipoea americana|[17282]|LGSMG975-10|BGS04056|658[0n]bp|United States.North Carolina|BOLD:AAC0644
Amphipoea americana|[17283]|LGSMG979-10|BGS04060|658[0n]bp|United States.North Carolina|BOLD:AAC0644
Amphipoea americana|[17284]|LGSMG980-10|BGS04061|658[0n]bp|United States.North Carolina|BOLD:AAC0644
Amphipoea americana|[17285]|LGSMG977-10|BGS04058|658[0n]bp|United States.North Carolina|BOLD:AAC0644
Amphipoea americana|[17286]|LNCC330-10|10-NCCC-330|658[0n]bp|United States.North Carolina|BOLD:AAC0644
Amphipoea sp. 2|[17287]|BBLEC137-09|09BBELE-0137|658[0n]bp|Canada.Nova Scotia|BOLD:AAC0644
Amphipoea sp. 2|[17288]|BBLPB631-10|10BBCLP-1630|658[0n]bp|Canada.Saskatchewan|BOLD:AAC0644
Amphipoea sp. 2|[17289]|XAD304-04|04HBL007304|584[0n]bp|Canada.Ontario|BOLD:AAC0644
Amphipoea sp. 2|[17290]|TTMNB357-06|MNBTT-357|599[0n]bp|Canada.New Brunswick|BOLD:AAC0644
Amphipoea sp. 2|[17291]|RDLQ470-07|DH006295|608[0n]bp|Canada.Quebec|BOLD:AAC0644
Amphipoea sp. 2|[17292]|TTMNB358-06|MNBTT-358|611[0n]bp|Canada.New Brunswick|BOLD:AAC0644
Amphipoea sp. 2|[17293]|BBLPB628-10|10BBCLP-1627|658[0n]bp|Canada.Alberta|BOLD:AAC0644
Helotropha reniformis|[17294]|BBLPB751-10|10BBCLP-1750|638[0n]bp|Canada.Alberta|BOLD:AAB8703
Helotropha reniformis|[17295]|RDNMB937-05|CNCNoctuoidea10712|658[0n]bp|Canada.British Columbia|BOLD:AA...
Helotropha reniformis|[17296]|BBLPB872-10|10BBCLP-1871|658[0n]bp|Canada.Alberta|BOLD:AAB8703
Helotropha reniformis|[17297]|XAH219-05|2005-ONT-1802|658[0n]bp|Canada.Ontario|BOLD:AAB8703
Helotropha reniformis|[17298]|LHLEP407-06|UBC-2006-2137|658[0n]bp|Canada.British Columbia|BOLD:AAB8703
Helotropha reniformis|[17299]|RDNMB938-05|CNCNoctuoidea10713|655[0n]bp|Canada.Alberta|BOLD:AAB8703
Helotropha reniformis|[17300]|LALPA690-10|AVBC 692-10|658[0n]bp|Canada.British Columbia|BOLD:AAB8703
Helotropha reniformis|[17301]|RDLQB601-05|DH010704|658[0n]bp|Canada.Quebec|BOLD:AAB8703
Helotropha reniformis|[17302]|LALPA786-10|AVBC 788-10|658[0n]bp|Canada.British Columbia|BOLD:AAB8703
Helotropha reniformis|[17303]|LBCH4430-10|10-JDWBC-4430|658[0n]bp|Canada.British Columbia|BOLD:AAB8703
Helotropha reniformis|[17304]|BBLPB804-10|10BBCLP-1803|658[0n]bp|Canada.Alberta|BOLD:AAB8703
Helotropha reniformis|[17305]|BBLPB803-10|10BBCLP-1802|658[0n]bp|Canada.Alberta|BOLD:AAB8703
Hypocoena basistriga|[17306]|DSCNI065-07|06-PROBE-0290|658[0n]bp|Canada.Manitoba|BOLD:AAE2019
Hypocoena basistriga|[17307]|DSCNI027-07|06-PROBE-0252|658[1n]bp|Canada.Manitoba|BOLD:AAE2019
Hypocoena basistriga|[17308]|LCHIP030-07|06-PROBE-0116|650[0n]bp|Canada.Manitoba|BOLD:AAE2019
Hypocoena basistriga|[17309]|DSCNI064-07|06-PROBE-0289|658[1n]bp|Canada.Manitoba|BOLD:AAE2019
Hypocoena basistriga|[17310]|DSCNI037-07|06-PROBE-0262|658[0n]bp|Canada.Manitoba|BOLD:AAE2019
Oligia divesta|[17311]|LOWCB935-05|CGWC-1875|578[1n]bp|Canada.British Columbia|BOLD:AAC8262
Oligia divesta|[17312]|LOWCB940-05|CGWC-1880|658[0n]bp|Canada.British Columbia|BOLD:AAC8262
Oligia divesta|[17313]|LOWCB937-05|CGWC-1877|658[0n]bp|Canada.British Columbia|BOLD:AAC8262
Oligia divesta|[17314]|LOWCB938-05|CGWC-1878|658[1n]bp|Canada.British Columbia|BOLD:AAC8262
Oligia divesta|[17315]|LPVIB512-08|PFC-2006-1927|658[0n]bp|Canada.British Columbia|BOLD:AAC8262
Oligia divesta|[17316]|LPVIB257-08|PFC-2006-1647|658[0n]bp|Canada.British Columbia|BOLD:AAC8262
Oligia divesta|[17317]|LPVIA834-08|PFC-2006-1129|658[0n]bp|Canada.British Columbia|BOLD:AAC8262
Oligia divesta|[17318]|LBCH6280-10|10-JDWBC-6280|658[0n]bp|Canada.British Columbia|BOLD:AAC8262
Oligia divesta|[17319]|LBCH7972-10|10-JDWBC-7972|658[0n]bp|Canada.British Columbia|BOLD:AAC8262
Oligia divesta|[17320]|LALPA650-10|AVBC 652-10|658[0n]bp|Canada.British Columbia|BOLD:AAC8262
Oligia divesta|[17321]|LALPA1251-11|AVBC 1253-11|658[0n]bp|Canada.British Columbia|BOLD:AAC8262
Oligia divesta|[17322]|LALPA694-10|AVBC 696-10|658[0n]bp|Canada.British Columbia|BOLD:AAC8262
Oligia divesta|[17323]|LPVIB259-08|PFC-2006-1649|658[0n]bp|Canada.British Columbia|BOLD:AAC8262
Oligia divesta|[17324]|LPVIB638-08|PFC-2006-2086|644[0n]bp|Canada.British Columbia|BOLD:AAC8262
Oligia divesta|[17325]|LALPA1326-12|AVBC 1328-11|621[0n]bp|Canada.British Columbia|BOLD:AAC8262
Oligia divesta|[17326]|LOWCB939-05|CGWC-1879|658[0n]bp|Canada.British Columbia|BOLD:AAC8262
Oligia divesta|[17327]|LBCH6452-10|10-JDWBC-6452|658[0n]bp|Canada.British Columbia|BOLD:AAC8262
Oligia divesta|[17328]|LOWCB936-05|CGWC-1876|658[0n]bp|Canada.British Columbia|BOLD:AAC8262
Oligia divesta|[17329]|LPVIB499-08|PFC-2006-1909|658[0n]bp|Canada.British Columbia|BOLD:AAC8262
Oligia divesta|[17330]|LBCH6290-10|10-JDWBC-6290|658[0n]bp|Canada.British Columbia|BOLD:AAC8262
Oligia divesta|[17331]|LBCH6124-10|10-JDWBC-6124|658[0n]bp|Canada.British Columbia|BOLD:AAC8262
Oligia divesta|[17332]|LBCH6832-10|10-JDWBC-6832|658[0n]bp|Canada.British Columbia|BOLD:AAC8262
Oligia divesta|[17333]|LBCH6123-10|10-JDWBC-6123|658[0n]bp|Canada.British Columbia|BOLD:AAC8262
Oligia divesta|[17334]|LBCG2877-09|08-JDWBC-2877|658[0n]bp|Canada.British Columbia|BOLD:AAC8262
Oligia divesta|[17335]|LBCH6279-10|10-JDWBC-6279|658[0n]bp|Canada.British Columbia|BOLD:AAC8262
Hydraecia perobliqua|[17336]|RDNMG607-08|CNC LEP00052431|658[0n]bp|Canada.Manitoba|BOLD:AAF0892
Hydraecia perobliqua|[17337]|RDNMB932-05|CNCNoctuoidea10707|658[0n]bp|Canada.Manitoba|BOLD:AAF0892
Resapamea stipata|[17338]|TMNBB168-06|MNBTT-1108|658[0n]bp|Canada.New Brunswick|BOLD:AAB8481
Resapamea stipata|[17339]|TMNBB176-06|MNBTT-1116|658[0n]bp|Canada.New Brunswick|BOLD:AAB8481
Resapamea stipata|[17340]|TTMNB337-06|MNBTT-337|658[1n]bp|Canada.New Brunswick|BOLD:AAB8481
Resapamea stipata|[17341]|TTMNB338-06|MNBTT-338|658[0n]bp|Canada.New Brunswick|BOLD:AAB8481
Resapamea stipata|[17342]|TMNBB172-06|MNBTT-1112|658[0n]bp|Canada.New Brunswick|BOLD:AAB8481
[illegible]

Resapamea stipata|[17340]|TTMNB337-06|MNBTT-337|658[1n]bp|Canada.New Brunswick|BOLD:AAB8481
Resapamea stipata|[17341]|TTMNB338-06|MNBTT-338|658[0n]bp|Canada.New Brunswick|BOLD:AAB8481
Resapamea stipata|[17342]|TMNBB172-06|MNBTT-1112|658[0n]bp|Canada.New Brunswick|BOLD:AAB8481
Resapamea stipata|[17343]|TTMNB339-06|MNBTT-339|658[0n]bp|Canada.New Brunswick|BOLD:AAB8481
Resapamea stipata|[17344]|TMNBB169-06|MNBTT-1109|658[0n]bp|Canada.New Brunswick|BOLD:AAB8481
Resapamea stipata|[17345]|TMNBB173-06|MNBTT-1113|658[0n]bp|Canada.New Brunswick|BOLD:AAB8481
Resapamea stipata|[17346]|TMNBB165-06|MNBTT-1105|658[0n]bp|Canada.New Brunswick|BOLD:AAB8481
Resapamea stipata|[17347]|TTMNB340-06|MNBTT-340|658[0n]bp|Canada.New Brunswick|BOLD:AAB8481
Resapamea stipata|[17348]|TMNBB175-06|MNBTT-1115|658[0n]bp|Canada.New Brunswick|BOLD:AAB8481
Resapamea stipata|[17349]|TMNBB174-06|MNBTT-1114|658[0n]bp|Canada.New Brunswick|BOLD:AAB8481
Resapamea stipata|[17350]|TMNBB170-06|MNBTT-1110|594[0n]bp|Canada.New Brunswick|BOLD:AAB8481
Resapamea stipata|[17351]|TMNBB167-06|MNBTT-1107|658[0n]bp|Canada.New Brunswick|BOLD:AAB8481
Resapamea stipata|[17352]|TMNBB171-06|MNBTT-1111|658[0n]bp|Canada.New Brunswick|BOLD:AAB8481
Resapamea stipata|[17353]|TMNBB166-06|MNBTT-1106|658[0n]bp|Canada.New Brunswick|BOLD:AAB8481
Photedes inops|[17354]|RDNMF277-08|NOC14363|658[0n]bp|Canada.Alberta|BOLD:AAD7226
Photedes inops|[17355]|RDNMF275-08|NOC14361|658[0n]bp|Canada.Alberta|BOLD:AAD7226
Photedes inops|[17356]|RDNMF276-08|NOC14362|658[0n]bp|Canada.Alberta|BOLD:AAD7226
Photedes inops|[17357]|RDNMD790-07|CNCNoctuoidea13122|658[0n]bp|Canada.Alberta|BOLD:AAD7226
Photedes inops|[17358]|RDNMF279-08|NOC14365|658[0n]bp|Canada.Alberta|BOLD:AAD7226
Photedes inops|[17359]|RDNMF278-08|NOC14364|658[0n]bp|Canada.Alberta|BOLD:AAD7226
Epidemas cinerea|[17360]|LOWCB503-05|CGWC-1443|658[0n]bp|Canada.British Columbia|BOLD:ABY4336
Epidemas cinerea|[17361]|LOWCB502-05|CGWC-1442|615[0n]bp|Canada.British Columbia|BOLD:ABY4336
Epidemas cinerea|[17362]|LOWCB499-05|CGWC-1439|598[2n]bp|Canada.British Columbia|BOLD:ABY4336
Epidemas cinerea|[17363]|LOWCB496-05|CGWC-1436|658[0n]bp|Canada.British Columbia|BOLD:ABY4336
Epidemas cinerea|[17364]|LOWCB511-05|CGWC-1451|658[0n]bp|Canada.British Columbia|BOLD:ABY4336
Epidemas cinerea|[17365]|LOWCB507-05|CGWC-1447|616[1n]bp|Canada.British Columbia|BOLD:ABY4336
Epidemas cinerea|[17366]|LOWCB500-05|CGWC-1440|615[1n]bp|Canada.British Columbia|BOLD:ABY4336
Epidemas cinerea|[17367]|LOWCB506-05|CGWC-1446|658[0n]bp|Canada.British Columbia|BOLD:ABY4336
Epidemas cinerea|[17368]|LOWCB509-05|CGWC-1449|658[0n]bp|Canada.British Columbia|BOLD:ABY4336
Epidemas cinerea|[17369]|LOWCB508-05|CGWC-1448|658[0n]bp|Canada.British Columbia|BOLD:ABY4336
Epidemas cinerea|[17370]|LOWCB498-05|CGWC-1438|658[0n]bp|Canada.British Columbia|BOLD:ABY4336
Epidemas cinerea|[17371]|LOWCB505-05|CGWC-1445|658[0n]bp|Canada.British Columbia|BOLD:ABY4336
Epidemas cinerea|[17372]|LOWCB497-05|CGWC-1437|658[0n]bp|Canada.British Columbia|BOLD:ABY4336
Epidemas cinerea|[17373]|RDNM125-05|CNCNoctuoidea6675|658[0n]bp|Canada.Alberta|BOLD:ACF0588
Epidemas cinerea|[17374]|RDNM127-05|CNCNoctuoidea6677|658[0n]bp|Canada.Alberta|BOLD:ACF0588
Epidemas cinerea|[17375]|RDNM126-05|CNCNoctuoidea6676|658[0n]bp|Canada.Alberta|BOLD:ACF0588
Epidemas cinerea|[17376]|LOWCC191-05|CGWC-2071|605[0n]bp|Canada.British Columbia|BOLD:AAB1675
Epidemas cinerea|[17377]|LOWCB504-05|CGWC-1444|658[0n]bp|Canada.British Columbia|BOLD:AAB1675
Epidemas cinerea|[17378]|LOWCB510-05|CGWC-1450|658[0n]bp|Canada.British Columbia|BOLD:AAB1675
Epidemas cinerea|[17379]|LOWCB495-05|CGWC-1435|658[0n]bp|Canada.British Columbia|BOLD:AAB1675
Epidemas cinerea|[17380]|LOWCB501-05|CGWC-1441|658[0n]bp|Canada.British Columbia|BOLD:AAB1675
Epidemas cinerea|[17381]|LOWCB512-05|CGWC-1452|658[0n]bp|Canada.British Columbia|BOLD:AAB1675
Epidemas obscurus|[17382]|RDNMF500-08|NOC14586|609[0n]bp|Canada.British Columbia|BOLD:AAB1674
Epidemas obscurus|[17383]|RDNMF499-08|NOC14585|658[0n]bp|Canada.British Columbia|BOLD:AAB1674
Epidemas obscurus|[17384]|LBCH6849-10|10-JDWBC-6849|658[0n]bp|Canada.British Columbia|BOLD:AAB1674
Epidemas obscurus|[17385]|LBCH6782-10|10-JDWBC-6782|658[0n]bp|Canada.British Columbia|BOLD:AAB1674
Epidemas cinerea|[17386]|LBCH7230-10|10-JDWBC-7230|658[0n]bp|Canada.British Columbia|BOLD:AAB1674
Epidemas obscurus|[17387]|LBCH7052-10|10-JDWBC-7052|658[0n]bp|Canada.British Columbia|BOLD:AAB1674
Epidemas obscurus|[17388]|LBCH6927-10|10-JDWBC-6927|658[0n]bp|Canada.British Columbia|BOLD:AAB1674
Epidemas obscurus|[17389]|LBCH6833-10|10-JDWBC-6833|658[0n]bp|Canada.British Columbia|BOLD:AAB1674
Epidemas obscurus|[17390]|LBCH7051-10|10-JDWBC-7051|658[0n]bp|Canada.British Columbia|BOLD:AAB1674
Oligia strigilis|[17391]|RDNMG511-08|CNC LEP00052335|658[0n]bp|Canada.Ontario|BOLD:AAB4833
Oligia strigilis|[17392]|RDLQ467-07|DH002359|606[0n]bp|Canada.Quebec|BOLD:AAB4833
Resapamea mammuthus|[17393]|CNCLA568-13|CNCLEP00094163|658[0n]bp|Canada.Yukon Territory|BOLD:ABX0034
Resapamea passer|[17394]|LBCA373-05|HLC-20373|582[0n]bp|Canada.British Columbia|BOLD:AAB5260
Resapamea passer|[17395]|RDNM541-05|CNCNoctuoidea6817|581[0n]bp|Canada.British Columbia|BOLD:AAB5260
Resapamea passer|[17396]|BBLPB870-10|10BBCLP-1869|658[0n]bp|Canada.Alberta|BOLD:AAB5260
Resapamea passer|[17397]|LALPA340-10|AVBC 342-10|658[0n]bp|Canada.British Columbia|BOLD:AAB5260
Resapamea passer|[17398]|LCHQ171-07|07PROBE-10940|658[0n]bp|Canada.Manitoba|BOLD:AAB5260
Resapamea passer|[17399]|RDNM973-05|CNCNoctuoidea7813|658[0n]bp|Canada.Alberta|BOLD:AAB5260
Resapamea passer|[17400]|LBCA032-05|HLC-20032|658[0n]bp|Canada.British Columbia|BOLD:AAB5260
Resapamea passer|[17401]|RDNM032-05|CNCNoctuoidea6301|658[0n]bp|Canada.Alberta|BOLD:AAB5260
Resapamea passer|[17402]|LBCA038-05|HLC-20038|658[0n]bp|Canada.British Columbia|BOLD:AAB5260
Resapamea passer|[17403]|LPVIB840-08|PFC-2006-2347|658[0n]bp|Canada.British Columbia|BOLD:AAB5260
Resapamea passer|[17404]|LBCA031-05|HLC-20031|658[0n]bp|Canada.British Columbia|BOLD:AAB5260
Resapamea passer|[17405]|LBCA037-05|HLC-20037|658[0n]bp|Canada.British Columbia|BOLD:AAB5260
Resapamea passer|[17406]|LBCA033-05|HLC-20033|658[0n]bp|Canada.British Columbia|BOLD:AAB5260
Resapamea passer|[17407]|LBCA041-05|HLC-20041|658[0n]bp|Canada.British Columbia|BOLD:AAB5260
Resapamea passer|[17408]|LPVIA232-08|PFC-2006-0314|631[0n]bp|Canada.British Columbia|BOLD:AAB5260
Resapamea passer|[17409]|RDNM031-05|CNCNoctuoidea6300|624[0n]bp|Canada.Alberta|BOLD:AAB5260
Resapamea passer|[17410]|RDNM542-05|CNCNoctuoidea6818|567[0n]bp|Canada.British Columbia|BOLD:AAB5260
Resapamea passer|[17411]|RDNM034-05|CNCNoctuoidea6303|658[0n]bp|Canada.Alberta|BOLD:AAB5260
Resapamea passer|[17412]|LPSO877-08|PPBP-0877|658[0n]bp|Canada.Ontario|BOLD:AAB5260
Resapamea passer|[17413]|RDNM033-05|CNCNoctuoidea6302|658[0n]bp|Canada.Alberta|BOLD:AAB5260
Resapamea venosa|[17414]|RDNMF723-08|NOC14809|658[0n]bp|Canada.British Columbia|BOLD:AAB5260
Meropleon ambifusca|[17415]|LPOKA407-09|MDOK-0407|658[0n]bp|United States.Oklahoma|BOLD:AAD8634
Meropleon ambifusca|[17416]|LSEU114-06|06-JKA-0114|658[0n]bp|United States.Georgia|BOLD:AAD8634
Meropleon ambifusca|[17417]|RDNMC709-06|CNCNoctuoidea12249|658[0n]bp|United States.Oklahoma|BOLD:AAD8634
Meropleon ambifusca|[17418]|LSEU777-06|06-JKA-0777|658[0n]bp|United States.Missouri|BOLD:AAD8634
Meropleon ambifusca|[17419]|LSEU778-06|06-JKA-0778|656[0n]bp|United States.Missouri|BOLD:AAD8634
Meropleon ambifusca|[17420]|LILLA726-11|SNS10IL-00917|658[0n]bp|United States.Illinois|BOLD:AAD8634
Meropleon ambifusca|[17421]|LSEU776-06|06-JKA-0776|658[0n]bp|United States.Missouri|BOLD:AAD8634
Meropleon diversicolor|[17422]|RDLQ466-07|DH007158|608[0n]bp|Canada.Quebec|BOLD:AAF0922
Meropleon diversicolor|[17423]|XAD399-04|04HBL007399|577[0n]bp|Canada.Ontario|BOLD:AAF0922
Pyreferra hesperidago|[17424]|RDLQ570-07|DH002734|575[0n]bp|Canada.Quebec|BOLD:AAF5736
Pyreferra citrombra|[17425]|RDNMH866-09|CNCLEP00062948|658[0n]bp|Canada.Ontario|BOLD:AAD2863
Pyreferra citrombra|[17426]|MEC126-04|jflandry0126|658[0n]bp|Canada.Quebec|BOLD:AAD2862
Pyreferra citrombra|[17427]|RDLQH112-06|DH013350|621[0n]bp|Canada.Quebec|BOLD:AAD2862
Pyreferra citrombra|[17428]|RDNMH625-09|CNCLEP00062949|658[0n]bp|Canada.Ontario|BOLD:AAD2862
Pyreferra citrombra|[17429]|RDLQH113-06|DH013351|658[0n]bp|Canada.Quebec|BOLD:AAD2862
Pyreferra pettiti|[17430]|XAF279-05|HLC-10320|658[0n]bp|Canada.Ontario|BOLD:AAD2999
Pyreferra pettiti|[17431]|RDLQ573-07|DH009526|556[0n]bp|Canada.Quebec|BOLD:AAD2999
Pyreferra pettiti|[17432]|XAF051-05|HBL008867|658[0n]bp|Canada.Ontario|BOLD:AAD2999
Pyreferra pettiti|[17433]|RDLQ571-07|DH009310|566[0n]bp|Canada.Quebec|BOLD:AAD2999
Pyreferra pettiti|[17434]|MECB882-05|jflandry1914|658[0n]bp|Canada.Quebec|BOLD:AAD2999
Pyreferra pettiti|[17435]|MECB881-05|jflandry1913|658[0n]bp|Canada.Quebec|BOLD:AAD2999
Pyreferra pettiti|[17436]|MEC186-04|jflandry0186|658[0n]bp|Canada.Quebec|BOLD:AAD2999
Rhizedra lutosa|[17437]|RDLQB761-05|DH010848|589[7n]bp|Canada.Quebec|
Rhizedra lutosa|[17438]|RDLQH037-06|DH013274|658[0n]bp|Canada.Quebec|BOLD:AAB8633
Rhizedra lutosa|[17439]|RDLQF882-06|DH012057|658[0n]bp|Canada.Quebec|BOLD:AAB8633
Rhizedra lutosa|[17440]|RDLQH035-06|DH013272|658[0n]bp|Canada.Quebec|BOLD:AAB8633
Rhizedra lutosa|[17441]|RDLQH036-06|DH013273|658[0n]bp|Canada.Quebec|BOLD:AAB8633
Rhizedra lutosa|[17442]|RDLQB459-05|DH010545|658[0n]bp|Canada.Quebec|BOLD:AAB8633

Rhizedra lutosa|[17440]|RDLQH035-06|DH013272|658[0n]bp|Canada.Quebec|BOLD:AAB8633
Rhizedra lutosa|[17441]|RDLQH036-06|DH013273|658[0n]bp|Canada.Quebec|BOLD:AAB8633
Rhizedra lutosa|[17442]|RDLQB459-05|DH010545|658[0n]bp|Canada.Quebec|BOLD:AAB8633
Rhizedra lutosa|[17443]|RDLQG217-06|DH012393|658[0n]bp|Canada.Quebec|BOLD:AAB8633
Brachylomia populi|[17444]|RDNMF206-08|NOC14292|658[0n]bp|United States.Washington|BOLD:AAE0555
Brachylomia populi|[17445]|RDNMF205-08|NOC14291|658[0n]bp|United States.Washington|BOLD:AAE0555
Brachylomia populi|[17446]|RDNMF207-08|NOC14293|658[0n]bp|United States.Washington|BOLD:AAE0555
Brachylomia populi|[17447]|RDNMF204-08|NOC14290|658[0n]bp|United States.Washington|BOLD:AAE0555
Brachylomia populi|[17448]|RDNMF208-08|NOC14294|658[0n]bp|United States.Washington|BOLD:AAE0555
Xylomoia chagnoni|[17449]|BLTIB367-08|BL586|642[0n]bp|Canada.Ontario|BOLD:AAE4227
Xylomoia chagnoni|[17450]|RDNMG582-08|CNC LEP00052406|658[0n]bp|Canada.Ontario|BOLD:AAE4227
Xylomoia chagnoni|[17451]|RDNMG581-08|CNC LEP00052405|658[0n]bp|Canada.Ontario|BOLD:AAE4227
Xylomoia chagnoni|[17452]|RDNMG580-08|CNC LEP00052404|658[0n]bp|Canada.Ontario|BOLD:AAE4227
Xylomoia indirecta|[17453]|LALPA468-10|AVBC 470-10|658[0n]bp|Canada.British Columbia|BOLD:AAB1776
Xylomoia indirecta|[17454]|LALPA418-10|AVBC 420-10|658[0n]bp|Canada.British Columbia|BOLD:AAB1776
Xylomoia indirecta|[17455]|LPVIA407-08|PFC-2006-0593|658[0n]bp|Canada.British Columbia|BOLD:AAB1776
Xylomoia indirecta|[17456]|LALPA480-10|AVBC 482-10|658[0n]bp|Canada.British Columbia|BOLD:AAB1776
Xylomoia indirecta|[17457]|LALPA414-10|AVBC 416-10|658[0n]bp|Canada.British Columbia|BOLD:AAB1776
Xylomoia indirecta|[17458]|LALPA532-10|AVBC 534-10|658[0n]bp|Canada.British Columbia|BOLD:AAB1776
Xylomoia indirecta|[17459]|LPVIA517-08|PFC-2006-0733|658[0n]bp|Canada.British Columbia|BOLD:AAB1776
Xylomoia indirecta|[17460]|LALPA410-10|AVBC 412-10|658[0n]bp|Canada.British Columbia|BOLD:AAB1776
Xylomoia indirecta|[17461]|LHLEP384-06|UBC-2006-1534|658[0n]bp|Canada.British Columbia|BOLD:AAB1776
Xylomoia indirecta|[17462]|LHLEP409-06|UBC-2006-1661|658[0n]bp|Canada.British Columbia|BOLD:AAB1776
Xylomoia indirecta|[17463]|LOWCD281-06|CGWC-3101|658[0n]bp|Canada.British Columbia|BOLD:AAB1776
Xylomoia indirecta|[17464]|LHLEP408-06|UBC-2006-1660|658[0n]bp|Canada.British Columbia|BOLD:AAB1776
Xylomoia indirecta|[17465]|LHLEP436-06|UBC-2006-1819|658[0n]bp|Canada.British Columbia|BOLD:AAB1776
Xylomoia indirecta|[17466]|LHLEP438-06|UBC-2006-1933|658[0n]bp|Canada.British Columbia|BOLD:AAB1776
Xylomoia indirecta|[17467]|LHLEP386-06|UBC-2006-1536|658[0n]bp|Canada.British Columbia|BOLD:AAB1776
Xylomoia indirecta|[17468]|LHLEP420-06|UBC-2006-1072|658[0n]bp|Canada.British Columbia|BOLD:AAB1776
Xylomoia indirecta|[17469]|RDNMB527-05|CNCNoctuoidea10303|565[1n]bp|Canada.British Columbia|BOLD:AAB1776
Xylomoia indirecta|[17470]|LHLEP385-06|UBC-2006-1535|658[0n]bp|Canada.British Columbia|BOLD:AAB1776
Xylomoia indirecta|[17471]|LHLEP387-06|UBC-2006-1537|658[0n]bp|Canada.British Columbia|BOLD:AAB1776
Xylomoia indirecta|[17472]|LBCS267-07|UBC-2007-0775|658[0n]bp|Canada.British Columbia|BOLD:AAB1776
Xylomoia indirecta|[17473]|LPVIA609-08|PFC-2006-0837|658[0n]bp|Canada.British Columbia|BOLD:AAB1776
Xylomoia indirecta|[17474]|LHLEP388-06|UBC-2006-1538|658[0n]bp|Canada.British Columbia|BOLD:AAB1776
Xylomoia indirecta|[17475]|LHLEP419-06|UBC-2006-1071|658[0n]bp|Canada.British Columbia|BOLD:AAB1776
Eupsilia devia|[17476]|PHMO036-03|moth136.02|639[0n]bp|Canada.Ontario|BOLD:AAD9847
Eupsilia devia|[17477]|RDLQH111-06|DH013349|652[0n]bp|Canada.Quebec|BOLD:AAD9847
Eupsilia devia|[17478]|RDLQH093-06|DH013331|658[0n]bp|Canada.Quebec|BOLD:AAD9847
Eupsilia devia|[17479]|XAF032-05|HBL008848|658[0n]bp|Canada.Ontario|BOLD:AAD9847
Eupsilia devia|[17480]|TMNBB251-06|MNBTT-1191|658[0n]bp|Canada.New Brunswick|BOLD:AAD9847
Eupsilia tristigmata|[17481]|LALPA807-10|AVBC 809-10|658[0n]bp|Canada.British Columbia|BOLD:ABY3690
Eupsilia tristigmata|[17482]|TMNBB246-06|MNBTT-1186|658[0n]bp|Canada.New Brunswick|BOLD:ABY3690
Eupsilia tristigmata|[17483]|LOWCE041-06|CGWC-3801|658[0n]bp|Canada.British Columbia|BOLD:ABY3690
Eupsilia tristigmata|[17484]|TMNBB248-06|MNBTT-1188|657[0n]bp|Canada.New Brunswick|BOLD:ABY3690
Eupsilia tristigmata|[17485]|LALPA022-10|AVBC 022-10|658[0n]bp|Canada.British Columbia|BOLD:ABY3690
Eupsilia tristigmata|[17486]|LOWCC019-05|CGWC-1899|658[0n]bp|Canada.British Columbia|BOLD:ABY3690
Eupsilia tristigmata|[17487]|LOWCE042-06|CGWC-3802|658[0n]bp|Canada.British Columbia|BOLD:ABY3690
Eupsilia tristigmata|[17488]|LALPA004-10|AVBC 004-10|658[0n]bp|Canada.British Columbia|BOLD:ABY3690
Eupsilia tristigmata|[17489]|RDLQ565-07|DH009633|647[0n]bp|Canada.Quebec|BOLD:ABY3690
Eupsilia tristigmata|[17490]|PHMO018-03|moth50.02|639[0n]bp|Canada.Ontario|BOLD:ABY3690
Eupsilia tristigmata|[17491]|RDLQ562-07|DH009689|596[0n]bp|Canada.Quebec|BOLD:ABY3690
Eupsilia tristigmata|[17492]|RDLQ563-07|DH009690|658[0n]bp|Canada.Quebec|BOLD:ABY3690
Eupsilia tristigmata|[17493]|RDMAB577-06|UASM58547|593[0n]bp|Canada.Alberta|BOLD:ABY3690
Eupsilia sidus|[17494]|LNCB970-10|10-NCCC-060|658[0n]bp|United States.North Carolina|BOLD:ABZ6254
Eupsilia sidus|[17495]|RDNML119-13|13-NCCC-024|658[0n]bp|United States.New Jersey|BOLD:ABZ6254
Eupsilia sidus|[17496]|RDNMF479-08|NOC14565|658[0n]bp|United States.New York|BOLD:ABZ6254
Eupsilia sidus|[17497]|LNCC498-11|11-NCCC-0023|658[0n]bp|United States.North Carolina|BOLD:ABZ6254
Eupsilia sidus|[17498]|LNCC485-11|11-NCCC-0010|658[0n]bp|United States.North Carolina|BOLD:ABZ6254
Eupsilia sidus|[17499]|RDNML116-13|13-NCCC-021|658[0n]bp|United States.New Jersey|BOLD:ABZ6254
Eupsilia sidus|[17500]|RDNML117-13|13-NCCC-022|658[0n]bp|United States.New Jersey|BOLD:ABZ6254
Eupsilia sidus|[17501]|LNCC504-11|11-NCCC-0029|658[0n]bp|United States.North Carolina|BOLD:ABZ6254
Eupsilia sidus|[17502]|RDNML118-13|13-NCCC-023|658[0n]bp|United States.New Jersey|BOLD:ABZ6254
Eupsilia tristigmata|[17503]|XAJ176-06|2006-ONT-0176|656[0n]bp|Canada.Ontario|BOLD:ABZ6254
Eupsilia morrisoni|[17504]|XAH672-05|2005-ONT-2255|658[0n]bp|Canada.Ontario|BOLD:AAB4640
Eupsilia morrisoni|[17505]|XAJ096-06|2006-ONT-0096|658[0n]bp|Canada.Ontario|BOLD:AAB4640
Eupsilia morrisoni|[17506]|PMG111-03|moth126.01|617[0n]bp|Canada.Ontario|BOLD:AAB4640
Eupsilia vinulenta|[17507]|TMNBB250-06|MNBTT-1190|658[0n]bp|Canada.New Brunswick|BOLD:AAB4640
Eupsilia vinulenta|[17508]|TTMNB372-06|MNBTT-372|658[0n]bp|Canada.New Brunswick|BOLD:AAB4640
Eupsilia morrisoni|[17509]|XAI028-05|0102-ONT-0028|658[0n]bp|Canada.Ontario|BOLD:AAB4640
Eupsilia morrisoni|[17510]|XAJ135-06|2006-ONT-0135|658[0n]bp|Canada.Ontario|BOLD:AAB4640
Eupsilia morrisoni|[17511]|XAJ068-06|2006-ONT-0068|658[0n]bp|Canada.Ontario|BOLD:AAB4640
Eupsilia morrisoni|[17512]|XAJ175-06|2006-ONT-0175|658[0n]bp|Canada.Ontario|BOLD:AAB4640
Eupsilia morrisoni|[17513]|XAF238-05|HLC-10279|658[0n]bp|Canada.Ontario|BOLD:AAB4640
Eupsilia morrisoni|[17514]|PHAPR1313-11|BIOUG01497-A01|658[0n]bp|Canada.Ontario|BOLD:AAB4640
Eupsilia morrisoni|[17515]|XAF202-05|HLC-10243|658[0n]bp|Canada.Ontario|BOLD:AAB4640
Eupsilia morrisoni|[17516]|XAJ107-06|2006-ONT-0107|658[0n]bp|Canada.Ontario|BOLD:AAB4640
Eupsilia morrisoni|[17517]|UOFTL004-11|J1X3LB|658[0n]bp|Canada.Ontario|
Eupsilia morrisoni|[17518]|PHNOV551-11|BIOUG01497-E08|658[0n]bp|Canada.Ontario|BOLD:AAB4640
Eupsilia morrisoni|[17519]|XAJ052-06|2006-ONT-0052|658[0n]bp|Canada.Ontario|BOLD:AAB4640
Eupsilia morrisoni|[17520]|XAJ024-06|2006-ONT-0024|658[0n]bp|Canada.Ontario|BOLD:AAB4640
Eupsilia morrisoni|[17521]|XAF235-05|HLC-10276|658[0n]bp|Canada.Ontario|BOLD:AAB4640
Eupsilia morrisoni|[17522]|XAJ112-06|2006-ONT-0112|658[0n]bp|Canada.Ontario|BOLD:AAB4640
Eupsilia morrisoni|[17523]|MEC136-04|jflandry0136|658[0n]bp|Canada.Quebec|BOLD:AAB4640
Eupsilia morrisoni|[17524]|RDLQ569-07|DH009665|575[0n]bp|Canada.Quebec|BOLD:AAB4640
Eupsilia morrisoni|[17525]|XAJ174-06|2006-ONT-0174|626[0n]bp|Canada.Ontario|BOLD:AAB4640
Eupsilia morrisoni|[17526]|RDLQ568-07|DH009638|586[0n]bp|Canada.Quebec|BOLD:AAB4640
Eupsilia morrisoni|[17527]|XAJ066-06|2006-ONT-0066|643[0n]bp|Canada.Ontario|BOLD:AAB4640
Eupsilia vinulenta|[17528]|RDLQ561-07|DH009643|536[0n]bp|Canada.Quebec|BOLD:AAB4640
Eupsilia morrisoni|[17529]|TMNBB249-06|MNBTT-1189|632[0n]bp|Canada.New Brunswick|BOLD:AAB4640
Eupsilia vinulenta|[17530]|TMNBB247-06|MNBTT-1187|658[0n]bp|Canada.New Brunswick|BOLD:AAB4640
Lithophane contenta|[17531]|RDNMF209-08|NOC14295|658[0n]bp|United States.California|BOLD:AAK6635
Lithophane contenta|[17532]|RDNMJ136-10|CNCLEP 70164|658[0n]bp|United States.New Mexico|BOLD:AAV8098
Lithophane atara|[17533]|DUNLP164-08|Dun-08-164|600[3n]bp|Canada.British Columbia|BOLD:ABY9616
Lithophane atara|[17534]|RDNMF191-08|NOC14277|609[0n]bp|Canada.British Columbia|BOLD:ABY9616
Lithophane atara|[17535]|RDNMF193-08|NOC14279|609[0n]bp|Canada.British Columbia|BOLD:ABY9616
Lithophane atara|[17536]|RDNMF192-08|NOC14278|658[0n]bp|Canada.British Columbia|BOLD:ABY9616
Lithophane atara|[17537]|LBCH5156-10|10-JDWBC-5156|658[0n]bp|Canada.British Columbia|BOLD:ABY9616
Lithophane adipel|[17538]|RDMAB702-06|UASM19663|658[0n]bp|Canada.Alberta|BOLD:AAC7746
Lithophane lepida|[17539]|RDNMK477-11|CNCLEP 81908|633[1n]bp|United States.New Hampshire|BOLD:AAC7746
Lithophane ponderosa|[17540]|DUNLP172-08|Dun-08-172|650[2n]bp|Canada.British Columbia|BOLD:AAC7746
Lithophane ponderosa|[17541]|LBCH5106-10|10-JDWBC-5106|658[0n]bp|Canada.British Columbia|BOLD:AAC7746
Lithophane ponderosa|[17542]|DUNLP171-08|Dun-08-171|658[0n]bp|Canada.British Columbia|BOLD:AAC7746

Lithophane ponderosa|[17540]|DUNLP172-08|Dun-08-172|650[2n]bp|Canada.British Columbia|BOLD:AAC7746
Lithophane ponderosa|[17541]|LBCH5106-10|10-JDWBC-5106|658[0n]bp|Canada.British Columbia|BOLD:AAC7746
Lithophane ponderosa|[17542]|DUNLP171-08|Dun-08-171|658[0n]bp|Canada.British Columbia|BOLD:AAC7746
Lithophane ponderosa|[17543]|DUNLP170-08|Dun-08-170|658[0n]bp|Canada.British Columbia|BOLD:AAC7746
Lithophane ponderosa|[17544]|DUNLP173-08|Dun-08-173|658[0n]bp|Canada.British Columbia|BOLD:AAC7746
Lithophane ponderosa|[17545]|DUNLP169-08|Dun-08-169|644[5n]bp|Canada.British Columbia|BOLD:AAC7746
Lithophane ponderosa|[17546]|RDNMF189-08|NOC14275|640[23n]bp|Canada.British Columbia|
Lithophane itata|[17547]|RDNMG857-08|CNC LEP00052981|592[0n]bp|Canada.British Columbia|BOLD:ABZ4930
Lithophane itata|[17548]|RDNMD629-06|CNCNoctuoidea12961|631[1n]bp|Canada.British Columbia|BOLD:ABZ4930
Lithophane itata|[17549]|DUNLP167-08|Dun-08-167|658[0n]bp|Canada.British Columbia|BOLD:ABZ4930
Lithophane itata|[17550]|RDNMG856-08|CNC LEP00052980|658[0n]bp|Canada.British Columbia|BOLD:ABZ4930
Lithophane thujae|[17551]|RDNMF822-08|CNC LEP00053183|658[0n]bp|Canada.New Brunswick|BOLD:ABZ4926
Lithophane thujae|[17552]|RDNMF820-08|CNC LEP00053181|658[0n]bp|Canada.New Brunswick|BOLD:ABZ4926
Lithophane thujae|[17553]|RDNMD633-06|CNCNoctuoidea12965|658[0n]bp|Canada.New Brunswick|BOLD:ABZ4926
Lithophane thujae|[17554]|RDNMF821-08|CNC LEP00053182|658[0n]bp|Canada.New Brunswick|BOLD:ABZ4926
Lithophane fagina|[17555]|XAB692-04|04HBL005692|645[0n]bp|Canada.Ontario|BOLD:AAB3686
Lithophane fagina|[17556]|RDLQF254-06|DH011334|658[0n]bp|Canada.Quebec|BOLD:AAB3686
Lithophane fagina|[17557]|RDLQG466-06|DH012759|658[0n]bp|Canada.Quebec|BOLD:AAB3686
Lithophane fagina|[17558]|LOWCE023-06|CGWC-3783|658[0n]bp|Canada.British Columbia|BOLD:AAB3686
Lithophane fagina|[17559]|XAF154-05|HBL008970|658[0n]bp|Canada.Ontario|BOLD:AAB3686
Lithophane fagina|[17560]|XAF201-05|HLC-10242|658[0n]bp|Canada.Ontario|BOLD:AAB3686
Lithophane fagina|[17561]|XAE039-04|Moth4039.03|658[0n]bp|Canada.Ontario|BOLD:AAB3686
Lithophane fagina|[17562]|PHMNB778-05|Moth 471.02SA|658[0n]bp|Canada.New Brunswick|BOLD:AAB3686
Lithophane fagina|[17563]|XAH701-05|2005-ONT-2284|658[0n]bp|Canada.Ontario|BOLD:AAB3686
Lithophane fagina|[17564]|XAJ086-06|2006-ONT-0086|658[0n]bp|Canada.Ontario|BOLD:AAB3686
Lithophane fagina|[17565]|XAF026-05|HBL008842|658[0n]bp|Canada.Ontario|BOLD:AAB3686
Lithophane fagina|[17566]|XAF027-05|HBL008843|658[0n]bp|Canada.Ontario|BOLD:AAB3686
Lithophane fagina|[17567]|XAF153-05|HBL008969|658[0n]bp|Canada.Ontario|BOLD:AAB3686
Lithophane fagina|[17568]|XAE149-04|Moth4149.03|658[0n]bp|Canada.Ontario|BOLD:AAB3686
Lithophane fagina|[17569]|XAB539-04|04HBL005539|658[0n]bp|Canada.Ontario|BOLD:AAB3686
Lithophane fagina|[17570]|XAF241-05|HLC-10282|658[0n]bp|Canada.Ontario|BOLD:AAB3686
Lithophane fagina|[17571]|XAF240-05|HLC-10281|658[0n]bp|Canada.Ontario|BOLD:AAB3686
Lithophane fagina|[17572]|XAH746-05|2005-ONT-2329|658[0n]bp|Canada.Ontario|BOLD:AAB3686
Lithophane fagina|[17573]|PHMO013-03|moth29.02|639[0n]bp|Canada.Ontario|BOLD:AAB3686
Lithophane fagina|[17574]|PHMO016-03|moth38.02|639[0n]bp|Canada.Ontario|BOLD:AAB3686
Lithophane fagina|[17575]|PHMO388-03|moth40.02|639[0n]bp|Canada.Ontario|BOLD:AAB3686
Lithophane fagina|[17576]|LOWCB546-05|CGWC-1486|523[2n]bp|Canada.British Columbia|
Lithophane fagina|[17577]|XAJ083-06|2006-ONT-0083|574[0n]bp|Canada.Ontario|BOLD:AAB3686
Lithophane fagina|[17578]|LOWCE466-06|CGWC-4226|656[0n]bp|Canada.British Columbia|BOLD:AAB3686
Lithophane amanda|[17579]|RDNMG562-08|CNC LEP00052386|658[0n]bp|Canada.New Brunswick|BOLD:AAE0248
Lithophane amanda|[17580]|RDNMG561-08|CNC LEP00052385|658[0n]bp|Canada.New Brunswick|BOLD:AAE0248
Lithophane amanda|[17581]|XAE168-04|Moth4168.03|658[0n]bp|Canada.Ontario|BOLD:AAE0248
Lithophane amanda|[17582]|RDNMG567-08|CNC LEP00052391|658[0n]bp|Canada.New Brunswick|BOLD:AAE0248
Lithophane amanda|[17583]|RDLQ509-07|DH002603|576[1n]bp|Canada.Quebec|BOLD:AAE0248
Lithophane dilatocula|[17584]|LALPA1105-11|AVBC 915-11|642[0n]bp|Canada.British Columbia|BOLD:AAF7039
Lithophane pexata|[17585]|RDLQ518-07|DH009467|658[0n]bp|Canada.Quebec|BOLD:AAD9908
Lithophane pexata|[17586]|XAJ089-06|2006-ONT-0089|658[0n]bp|Canada.Ontario|BOLD:AAD9908
Lithophane pexata|[17587]|LOWCC018-05|CGWC-1898|658[0n]bp|Canada.British Columbia|BOLD:AAD9908
Lithophane pexata|[17588]|RDLQ519-07|DH009452|565[0n]bp|Canada.Quebec|BOLD:AAD9908
Lithophane pexata|[17589]|RDLQG462-06|DH012755|648[1n]bp|Canada.Quebec|BOLD:AAD9908
Lithophane thaxteri|[17590]|RDNMF195-08|NOC14281|658[0n]bp|Canada.New Brunswick|BOLD:AAE0044
Lithophane thaxteri|[17591]|RDNMF194-08|NOC14280|658[0n]bp|Canada.Ontario|BOLD:AAE0044
Lithophane thaxteri|[17592]|DUNLP174-08|Dun-08-174|576[0n]bp|Canada.British Columbia|BOLD:AAE0044
Lithophane thaxteri|[17593]|RDLQ506-07|AC000548|609[0n]bp|Canada.Quebec|BOLD:AAE0044
Lithophane thaxteri|[17594]|RDNMF196-08|NOC14282|658[0n]bp|Canada.British Columbia|BOLD:AAE0044
Lithophane baileyi|[17595]|DUNLP175-08|Dun-08-175|658[0n]bp|Canada.British Columbia|BOLD:AAD9328
Lithophane baileyi|[17596]|RDNM118-05|CNCNoctuoidea6668|658[0n]bp|Canada.British Columbia|BOLD:AAD9328
Lithophane baileyi|[17597]|LALPA768-10|AVBC 770-10|658[0n]bp|Canada.British Columbia|BOLD:AAD9328
Lithophane baileyi|[17598]|LALPA736-10|AVBC 738-10|658[0n]bp|Canada.British Columbia|BOLD:AAD9328
Lithophane baileyi|[17599]|LALPA1305-11|AVBC 1307-11|658[0n]bp|Canada.British Columbia|BOLD:AAD9328
Lithophane baileyi|[17600]|RDNM117-05|CNCNoctuoidea6667|658[0n]bp|Canada.British Columbia|BOLD:AAD9328
Lithophane baileyi|[17601]|RDNM116-05|CNCNoctuoidea6666|658[0n]bp|Canada.Ontario|BOLD:AAD9328
Lithophane baileyi|[17602]|RDNM115-05|CNCNoctuoidea6665|658[0n]bp|Canada.Ontario|BOLD:AAD9328
Lithophane baileyi|[17603]|TMNBB244-06|MNBTT-1184|658[0n]bp|Canada.New Brunswick|BOLD:AAD9328
Lithophane baileyi|[17604]|RDLQ520-07|DH009653|658[0n]bp|Canada.Quebec|BOLD:AAD9328
Lithophane baileyi|[17605]|RDNM114-05|CNCNoctuoidea6664|658[0n]bp|Canada.Ontario|BOLD:AAD9328
Lithophane baileyi|[17606]|RDLQ521-07|DH009649|632[0n]bp|Canada.Quebec|BOLD:AAD9328
Lithophane querquera|[17607]|RDNMC562-06|CNCNoctuoidea12384|559[0n]bp|United States.Georgia|BOLD:ABZ0521
Lithophane querquera|[17608]|RDNMG570-08|CNC LEP00052394|658[0n]bp|United States.New Jersey|BOLD:ABZ0521
Lithophane querquera|[17609]|RDNMG572-08|CNC LEP00052396|658[0n]bp|United States.New Jersey|BOLD:ABZ0521
Lithophane querquera|[17610]|RDNMG571-08|CNC LEP00052395|658[0n]bp|United States.New Jersey|BOLD:ABZ0521
Lithophane querquera|[17611]|RDNM011-05|CNCNoctuoidea6280|593[0n]bp|United States.Georgia|BOLD:ABZ0521
Lithophane scottae|[17612]|RDNM012-05|CNCNoctuoidea6281|658[0n]bp|Canada.Ontario|BOLD:ABZ0521
Lithophane tepida|[17613]|RDNM120-05|CNCNoctuoidea6670|658[0n]bp|Canada.New Brunswick|BOLD:ABZ0523
Lithophane tepida|[17614]|RDLQF255-06|DH011335|658[0n]bp|Canada.Quebec|BOLD:ABZ0523
Lithophane tepida|[17615]|RDNM121-05|CNCNoctuoidea6671|658[0n]bp|Canada.New Brunswick|BOLD:ABZ0523
Lithophane patefacta|[17616]|XAF207-05|HLC-10248|658[0n]bp|Canada.Ontario|BOLD:AAB1070
Lithophane oriunda|[17617]|RDLQ511-07|DH004055|577[0n]bp|Canada.Quebec|BOLD:AAB1070
Lithophane oriunda|[17618]|RDLQ510-07|AC000028|553[0n]bp|Canada.Quebec|BOLD:AAB1070
Lithophane oriunda|[17619]|PHMO047-03|moth238.02|639[0n]bp|Canada.Ontario|BOLD:AAB1070
Lithophane oriunda|[17620]|XAJ010-06|2006-ONT-0010|610[0n]bp|Canada.Ontario|BOLD:AAB1070
Lithophane oriunda|[17621]|RDLQ512-07|DH009655|604[0n]bp|Canada.Quebec|BOLD:AAB1070
Lithophane oriunda|[17622]|XAE169-04|Moth4169.03|658[0n]bp|Canada.Ontario|BOLD:AAB1070
Lithophane oriunda|[17623]|XAF294-05|HLC-10335|658[0n]bp|Canada.Ontario|BOLD:AAB1070
Lithophane oriunda|[17624]|XAJ212-06|2006-ONT-0212|658[0n]bp|Canada.Ontario|BOLD:AAB1070
Lithophane patefacta|[17625]|XAF197-05|HLC-10238|658[0n]bp|Canada.Ontario|BOLD:AAB1070
Lithophane bethunei|[17626]|XAJ133-06|2006-ONT-0133|658[0n]bp|Canada.Ontario|BOLD:AAB1070
Lithophane bethunei|[17627]|XAF209-05|HLC-10250|658[0n]bp|Canada.Ontario|BOLD:AAB1070
Lithophane bethunei|[17628]|RDLQ160-05|DH002600|581[0n]bp|Canada.Quebec|BOLD:AAB1070
Lithophane bethunei|[17629]|XAF117-05|HBL008933|658[0n]bp|Canada.Ontario|BOLD:AAB1070
Lithophane bethunei|[17630]|XAF268-05|HLC-10309|658[0n]bp|Canada.Ontario|BOLD:AAB1070
Lithophane bethunei|[17631]|RDLQ515-07|DH009687|658[0n]bp|Canada.Quebec|BOLD:AAB1070
Lithophane bethunei|[17632]|XAF211-05|HLC-10252|658[0n]bp|Canada.Ontario|BOLD:AAB1070
Lithophane bethunei|[17633]|XAF198-05|HLC-10239|658[0n]bp|Canada.Ontario|BOLD:AAB1070
Lithophane bethunei|[17634]|XAF212-05|HLC-10253|658[0n]bp|Canada.Ontario|BOLD:AAB1070
Lithophane patefacta|[17635]|RDLQ546-07|DH009470|658[0n]bp|Canada.Quebec|BOLD:AAB1070
Lithophane bethunei|[17636]|XAF269-05|HLC-10310|658[0n]bp|Canada.Ontario|BOLD:AAB1070
Lithophane bethunei|[17637]|XAF210-05|HLC-10251|658[0n]bp|Canada.Ontario|BOLD:AAB1070
Lithophane bethunei|[17638]|RDLQ517-07|DH009608|658[0n]bp|Canada.Quebec|BOLD:AAB1070
Lithophane hemina|[17639]|RDLQ171-05|DH004388|561[0n]bp|Canada.Quebec|BOLD:AAB1070
Lithophane hemina|[17640]|RDLQ172-05|DH002672|658[0n]bp|Canada.Quebec|BOLD:AAB1070
Lithophane hemina|[17641]|RDLQ535-07|DH004151|658[0n]bp|Canada.Quebec|BOLD:AAB1070

Lithophane hemina|[17639]|RDLQ171-05|DH004388|561[0n]bp|Canada.Quebec|BOLD:AAB1070
Lithophane hemina|[17640]|RDLQ172-05|DH002672|658[0n]bp|Canada.Quebec|BOLD:AAB1070
Lithophane hemina|[17641]|RDLQ535-07|DH004151|658[0n]bp|Canada.Quebec|BOLD:AAB1070
Lithophane hemina|[17642]|RDLQ551-07|DH009673|658[0n]bp|Canada.Quebec|BOLD:AAB1070
Lithophane hemina|[17643]|RDLQ537-07|DH009659|658[0n]bp|Canada.Quebec|BOLD:AAB1070
Lithophane hemina|[17644]|RDLQ534-07|DH009671|658[0n]bp|Canada.Quebec|BOLD:AAB1070
Lithophane hemina|[17645]|RDLQ168-05|DH004145|658[0n]bp|Canada.Quebec|BOLD:AAB1070
Lithophane hemina|[17646]|RDLQH029-06|DH013266|655[0n]bp|Canada.Quebec|BOLD:AAB1070
Lithophane hemina|[17647]|RDLQ170-05|DH004130|658[0n]bp|Canada.Quebec|BOLD:AAB1070
Lithophane hemina|[17648]|RDNM074-05|CNCNoctuoidea6343|658[0n]bp|Canada.Ontario|BOLD:AAB1070
Lithophane hemina|[17649]|RDLQH031-06|DH013268|658[0n]bp|Canada.Quebec|BOLD:AAB1070
Lithophane hemina|[17650]|RDLQ539-07|DH009658|658[0n]bp|Canada.Quebec|BOLD:AAB1070
Lithophane hemina|[17651]|XAF150-05|HBL008966|658[0n]bp|Canada.Ontario|BOLD:AAB1070
Lithophane hemina|[17652]|RDLQ536-07|DH009657|658[0n]bp|Canada.Quebec|BOLD:AAB1070
Lithophane hemina|[17653]|RDNM070-05|CNCNoctuoidea6339|658[0n]bp|Canada.Ontario|BOLD:AAB1070
Lithophane hemina|[17654]|RDLQH028-06|DH013265|658[0n]bp|Canada.Quebec|BOLD:AAB1070
Lithophane hemina|[17655]|RDNM071-05|CNCNoctuoidea6340|658[0n]bp|Canada.Ontario|BOLD:AAB1070
Lithophane hemina|[17656]|RDNM051-05|CNCNoctuoidea6320|658[0n]bp|Canada.Ontario|BOLD:AAB1070
Lithophane hemina|[17657]|TMNBB242-06|MNBTT-1182|658[0n]bp|Canada.New Brunswick|BOLD:AAB1070
Lithophane hemina|[17658]|RDLQ531-07|DH004020|658[0n]bp|Canada.Quebec|BOLD:AAB1070
Lithophane hemina|[17659]|RDLQ538-07|DH002639|595[0n]bp|Canada.Quebec|BOLD:AAB1070
Lithophane hemina|[17660]|RDLQ552-07|DH013433|595[0n]bp|Canada.Quebec|BOLD:AAB1070
Lithophane hemina|[17661]|RDLQ532-07|DH004097|601[0n]bp|Canada.Quebec|BOLD:AAB1070
Lithophane hemina|[17662]|RDLQ533-07|DH004014|601[0n]bp|Canada.Quebec|BOLD:AAB1070
Lithophane hemina|[17663]|RDNM073-05|CNCNoctuoidea6342|658[0n]bp|Canada.Ontario|BOLD:AAB1070
Lithophane patefacta|[17664]|XAF206-05|HLC-10247|658[0n]bp|Canada.Ontario|BOLD:AAB1070
Lithophane disposita|[17665]|XAJ120-06|2006-ONT-0120|658[0n]bp|Canada.Ontario|BOLD:AAB1070
Lithophane bethunei|[17666]|XAF260-05|HLC-10301|658[0n]bp|Canada.Ontario|BOLD:AAB1070
Lithophane bethunei|[17667]|XAF298-05|HLC-10339|658[0n]bp|Canada.Ontario|BOLD:AAB1070
Lithophane bethunei|[17668]|RDLQ516-07|DH009609|658[0n]bp|Canada.Quebec|BOLD:AAB1070
Lithophane bethunei|[17669]|XAJ015-06|2006-ONT-0015|622[0n]bp|Canada.Ontario|BOLD:AAB1070
Lithophane disposita|[17670]|RDLQ529-07|DH013435|595[0n]bp|Canada.Quebec|BOLD:AAB1070
Lithophane disposita|[17671]|XAJ016-06|2006-ONT-0016|618[0n]bp|Canada.Ontario|BOLD:AAB1070
Lithophane patefacta|[17672]|XAD643-05|2005-ONT-58|658[0n]bp|Canada.Ontario|BOLD:AAB1070
Lithophane patefacta|[17673]|RDNM848-05|CNCNoctuoidea7688|658[0n]bp|Canada.Ontario|BOLD:AAB1070
Lithophane patefacta|[17674]|RDLQ547-07|DH009660|658[0n]bp|Canada.Quebec|BOLD:AAB1070
Lithophane patefacta|[17675]|RDLQ548-07|DH009472|658[0n]bp|Canada.Quebec|BOLD:AAB1070
Lithophane patefacta|[17676]|XAF295-05|HLC-10336|658[0n]bp|Canada.Ontario|BOLD:AAB1070
Lithophane patefacta|[17677]|XAF448-05|HLC-10489|658[0n]bp|Canada.Ontario|BOLD:AAB1070
Lithophane patefacta|[17678]|RDNM849-05|CNCNoctuoidea7689|616[0n]bp|Canada.Ontario|BOLD:AAB1070
Lithophane innominata|[17679]|RDLQ530-07|DH009629|658[0n]bp|Canada.Quebec|BOLD:AAB1070
Lithophane innominata|[17680]|RDLQ162-05|DH004144|625[0n]bp|Canada.Quebec|BOLD:AAB1070
Lithophane lanei|[17681]|RDNM050-05|CNCNoctuoidea6319|658[0n]bp|Canada.Ontario|BOLD:AAB1070
Lithophane lanei|[17682]|RDNM047-05|CNCNoctuoidea6316|658[0n]bp|Canada.Ontario|BOLD:AAB1070
Lithophane lanei|[17683]|RDLQ549-07|DH009672|596[0n]bp|Canada.Quebec|BOLD:AAB1070
Lithophane lanei|[17684]|XAF151-05|HBL008967|658[0n]bp|Canada.Ontario|BOLD:AAB1070
Lithophane lanei|[17685]|XAF262-05|HLC-10303|658[0n]bp|Canada.Ontario|BOLD:AAB1070
Lithophane lanei|[17686]|XAF204-05|HLC-10245|658[0n]bp|Canada.Ontario|BOLD:AAB1070
Lithophane lanei|[17687]|RDNM049-05|CNCNoctuoidea6318|658[0n]bp|Canada.Ontario|BOLD:AAB1070
Lithophane lanei|[17688]|RDNM048-05|CNCNoctuoidea6317|658[0n]bp|Canada.Ontario|BOLD:AAB1070
Lithophane lanei|[17689]|RDLQ550-07|DH009693|658[0n]bp|Canada.Quebec|BOLD:AAB1070
Lithophane semiusta|[17690]|RDLQ157-05|DH004183|575[0n]bp|Canada.Quebec|BOLD:AAB1070
Lithophane semiusta|[17691]|RDLQ540-07|DH009436|658[0n]bp|Canada.Quebec|BOLD:AAB1070
Lithophane innominata|[17692]|XAJ177-06|2006-ONT-0177|658[0n]bp|Canada.Ontario|BOLD:ACE4172
Lithophane innominata|[17693]|LOWCE250-06|CGWC-4010|658[0n]bp|Canada.British Columbia|BOLD:ACE4172
Lithophane innominata|[17694]|PMG129-03|moth185.01|617[0n]bp|Canada.Ontario|BOLD:ACE4172
Lithophane innominata|[17695]|RDLQ161-05|DH002671|658[0n]bp|Canada.Quebec|BOLD:ACE4172
Lithophane petulca|[17696]|RDNM044-05|CNCNoctuoidea6313|658[0n]bp|Canada.Ontario|BOLD:ACE4172
Lithophane innominata|[17697]|RDMAB581-06|UASM58543|658[0n]bp|Canada.Alberta|BOLD:ACE4172
Lithophane innominata|[17698]|XAF208-05|HLC-10249|658[0n]bp|Canada.Ontario|BOLD:ACE4172
Lithophane innominata|[17699]|LOWCE043-06|CGWC-3803|658[0n]bp|Canada.British Columbia|BOLD:ACE4172
Lithophane innominata|[17700]|LOWCE474-06|CGWC-4234|658[0n]bp|Canada.British Columbia|BOLD:ACE4172
Lithophane innominata|[17701]|RDLQH032-06|DH013269|658[0n]bp|Canada.Quebec|BOLD:ACE4172
Lithophane innominata|[17702]|TMNBB239-06|MNBTT-1179|658[0n]bp|Canada.New Brunswick|BOLD:ACE4172
Lithophane innominata|[17703]|RDLQ544-07|DH009664|658[0n]bp|Canada.Quebec|BOLD:ACE4172
Lithophane innominata|[17704]|RDLQ543-07|DH006199|658[0n]bp|Canada.Quebec|BOLD:ACE4172
Lithophane innominata|[17705]|XAJ056-06|2006-ONT-0056|658[0n]bp|Canada.Ontario|BOLD:ACE4172
Lithophane innominata|[17706]|LMIS031-05|05-ONMIS-0031|658[0n]bp|Canada.Ontario|BOLD:ACE4172
Lithophane innominata|[17707]|XAJ014-06|2006-ONT-0014|658[0n]bp|Canada.Ontario|BOLD:ACE4172
Lithophane innominata|[17708]|LALPA054-10|AVBC 054-10|658[0n]bp|Canada.British Columbia|BOLD:ACE4172
Lithophane innominata|[17709]|DUNLP166-08|Dun-08-166|658[0n]bp|Canada.British Columbia|BOLD:ACE4172
Lithophane innominata|[17710]|TMNBB240-06|MNBTT-1180|658[0n]bp|Canada.New Brunswick|BOLD:ACE4172
Lithophane innominata|[17711]|LALPA033-10|AVBC 033-10|658[0n]bp|Canada.British Columbia|BOLD:ACE4172
Lithophane innominata|[17712]|XAF412-05|HLC-10453|658[0n]bp|Canada.Ontario|BOLD:ACE4172
Lithophane innominata|[17713]|LOWCC446-05|CGWC-2326|658[0n]bp|Canada.British Columbia|BOLD:ACE4172
Lithophane innominata|[17714]|XAJ023-06|2006-ONT-0023|658[0n]bp|Canada.Ontario|BOLD:ACE4172
Lithophane innominata|[17715]|TMNBB238-06|MNBTT-1178|656[0n]bp|Canada.New Brunswick|BOLD:ACE4172
Lithophane innominata|[17716]|LOWCC439-05|CGWC-2319|594[0n]bp|Canada.British Columbia|BOLD:ACE4172
Lithophane innominata|[17717]|RDNM046-05|CNCNoctuoidea6315|580[0n]bp|Canada.British Columbia|BOLD:ACE4172
Lithophane innominata|[17718]|TMNBB241-06|MNBTT-1181|658[0n]bp|Canada.New Brunswick|BOLD:ACE4172
Lithophane innominata|[17719]|RDLQ545-07|DH004239|595[0n]bp|Canada.Quebec|BOLD:ACE4172
Lithophane innominata|[17720]|LOWCD170-06|CGWC-2990|552[0n]bp|Canada.British Columbia|BOLD:ACE4172
Lithophane innominata|[17721]|LOWCC438-05|CGWC-2318|564[0n]bp|Canada.British Columbia|BOLD:ACE4172
Lithophane innominata|[17722]|RDLQ542-07|DH002623|599[0n]bp|Canada.Quebec|BOLD:ACE4172
Lithophane innominata|[17723]|RDLQ541-07|DH006151|598[0n]bp|Canada.Quebec|BOLD:ACE4172
Lithophane petulca|[17724]|RDLQ159-05|DH002590|658[0n]bp|Canada.Quebec|BOLD:ACE4172
Lithophane petulca|[17725]|XAJ259-06|2006-ONT-0259|658[0n]bp|Canada.Ontario|BOLD:ACE4172
Lithophane petulca|[17726]|XAF296-05|HLC-10337|658[0n]bp|Canada.Ontario|BOLD:ACE4172
Lithophane petulca|[17727]|LOWCE026-06|CGWC-3786|658[0n]bp|Canada.British Columbia|BOLD:ACE4172
Lithophane petulca|[17728]|LOWCE025-06|CGWC-3785|658[0n]bp|Canada.British Columbia|BOLD:ACE4172
Lithophane petulca|[17729]|XAJ093-06|2006-ONT-0093|658[0n]bp|Canada.Ontario|BOLD:ACE4172
Lithophane petulca|[17730]|XAJ009-06|2006-ONT-0009|658[0n]bp|Canada.Ontario|BOLD:ACE4172
Lithophane petulca|[17731]|RDNM045-05|CNCNoctuoidea6314|658[0n]bp|Canada.Ontario|BOLD:ACE4172
Lithophane petulca|[17732]|LALPA1143-11|AVBC 953-11|658[0n]bp|Canada.British Columbia|BOLD:ACE4172
Lithophane petulca|[17733]|TMNBB243-06|MNBTT-1183|657[0n]bp|Canada.New Brunswick|BOLD:ACE4172
Lithophane petulca|[17734]|RDNM043-05|CNCNoctuoidea6312|658[0n]bp|Canada.Ontario|BOLD:ACE4172
Lithophane petulca|[17735]|RDNM072-05|CNCNoctuoidea6341|658[0n]bp|Canada.Ontario|BOLD:ACE4172
Lithophane petulca|[17736]|RDLQ167-05|DH002643|658[0n]bp|Canada.Quebec|BOLD:ACE4172
Lithophane signosa|[17737]|RDNMF477-08|NOC14563|609[0n]bp|United States.New York|BOLD:ACF1025
Lithophane signosa|[17738]|LNCB977-10|10-NCCC-067|658[0n]bp|United States.North Carolina|BOLD:ACF1025
Lithophane signosa|[17739]|RDNMJ100-10|CNCLEP 70128|658[0n]bp|United States.Indiana|BOLD:ACF1025
Lithophane signosa|[17740]|LNCC477-11|11-NCCC-0002|658[0n]bp|United States.North Carolina|BOLD:ACF1025
Lithophane signosa|[17741]|RDNML109-13|13-NCCC-014|658[0n]bp|United States.New Jersey|BOLD:ACF1025

Lithophane signosa|[17739]|RDNMJ100-10|CNCLEP 70128|658[0n]bp|United States.Indiana|BOLD:ACF1025
Lithophane signosa|[17740]|LNCC477-11|11-NCCC-0002|658[0n]bp|United States.North Carolina|BOLD:ACF1025
Lithophane signosa|[17741]|RDNML109-13|13-NCCC-014|658[0n]bp|United States.New Jersey|BOLD:ACF1025
Lithophane georgii|[17742]|LALPA1028-11|AVBC 838-11|658[0n]bp|Canada.British Columbia|BOLD:AAC2621
Lithophane georgii|[17743]|LALPA1118-11|AVBC 928-11|658[0n]bp|Canada.British Columbia|BOLD:AAC2621
Lithophane georgii|[17744]|LALPA1066-11|AVBC 876-11|634[0n]bp|Canada.British Columbia|BOLD:AAC2621
Lithophane georgii|[17745]|LOWCE022-06|CGWC-3782|658[0n]bp|Canada.British Columbia|BOLD:AAC2621
Lithophane georgii|[17746]|LOWCB545-05|CGWC-1485|615[1n]bp|Canada.British Columbia|BOLD:AAC2621
Lithophane georgii|[17747]|LOWCB544-05|CGWC-1484|658[0n]bp|Canada.British Columbia|BOLD:AAC2621
Lithophane georgii|[17748]|LOWCE208-06|CGWC-3968|658[0n]bp|Canada.British Columbia|BOLD:AAC2621
Lithophane georgii|[17749]|LALPA1030-11|AVBC 840-11|658[0n]bp|Canada.British Columbia|BOLD:AAC2621
Lithophane georgii|[17750]|LALPA1029-11|AVBC 839-11|658[0n]bp|Canada.British Columbia|BOLD:AAC2621
Lithophane grotei|[17751]|XAF007-05|HBL008823|658[0n]bp|Canada.Ontario|BOLD:AAB5821
Lithophane grotei|[17752]|XAF001-05|HBL008817|614[0n]bp|Canada.Ontario|BOLD:AAB5821
Lithophane grotei|[17753]|RDLQ526-07|DH009695|658[0n]bp|Canada.Quebec|BOLD:AAB5821
Lithophane grotei|[17754]|RDNMG595-08|CNC LEP00052419|658[0n]bp|Canada.New Brunswick|BOLD:AAB5821
Lithophane grotei|[17755]|XAE013-04|Moth4013.03|658[0n]bp|Canada.Ontario|BOLD:AAB5821
Lithophane grotei|[17756]|XAH783-05|2005-ONT-2366|658[0n]bp|Canada.Ontario|BOLD:AAB5821
Lithophane grotei|[17757]|RDLQ527-07|DH009579|658[0n]bp|Canada.Quebec|BOLD:AAB5821
Lithophane grotei|[17758]|RDLQH094-06|DH013332|632[0n]bp|Canada.Quebec|BOLD:AAB5821
Lithophane grotei|[17759]|PHMO014-03|moth30.02|639[0n]bp|Canada.Ontario|BOLD:AAB5821
Lithophane grotei|[17760]|RDLQH034-06|DH013271|658[0n]bp|Canada.Quebec|BOLD:AAB5821
Lithophane grotei|[17761]|XAB546-04|04HBL005546|658[0n]bp|Canada.Ontario|BOLD:AAB5821
Lithophane grotei|[17762]|RDLQ525-07|DH009842|658[0n]bp|Canada.Quebec|BOLD:AAB5821
Lithophane grotei|[17763]|TMNBB245-06|MNBTT-1185|658[0n]bp|Canada.New Brunswick|BOLD:AAB5821
Lithophane grotei|[17764]|PHMNB269-04|04HBL007734|577[0n]bp|Canada.New Brunswick|BOLD:AAB5821
Lithophane grotei|[17765]|RDLQ524-07|DH009886|638[0n]bp|Canada.Quebec|BOLD:AAB5821
Lithophane grotei|[17766]|RDLQH033-06|DH013270|658[0n]bp|Canada.Quebec|BOLD:AAB5821
Lithophane antennata|[17767]|RDLQ502-07|DH004045|608[0n]bp|Canada.Quebec|BOLD:AAB5821
Lithophane antennata|[17768]|XAF052-05|HBL008868|658[0n]bp|Canada.Ontario|BOLD:AAB5821
Lithophane antennata|[17769]|XAF152-05|HBL008968|658[0n]bp|Canada.Ontario|BOLD:AAB5821
Lithophane antennata|[17770]|XAF205-05|HLC-10246|658[0n]bp|Canada.Ontario|BOLD:AAB5821
Lithophane antennata|[17771]|RDLQ503-07|DH013440|563[0n]bp|Canada.Quebec|BOLD:AAB5821
Lithophane antennata|[17772]|XAJ019-06|2006-ONT-0019|658[0n]bp|Canada.Ontario|BOLD:AAB5821
Lithophane antennata|[17773]|XAF258-05|HLC-10299|658[0n]bp|Canada.Ontario|BOLD:AAB5821
Lithophane antennata|[17774]|XAH858-05|2005-ONT-2441|658[0n]bp|Canada.Ontario|BOLD:AAB5821
Lithophane laticinerea|[17775]|XAF004-05|HBL008820|658[0n]bp|Canada.Ontario|BOLD:AAB5821
Lithophane laticinerea|[17776]|XAJ204-06|2006-ONT-0204|658[0n]bp|Canada.Ontario|BOLD:AAB5821
Lithophane laticinerea|[17777]|RDNMG594-08|CNC LEP00052418|658[0n]bp|Canada.New Brunswick|BOLD:AAB5821
Lithophane laticinerea|[17778]|XAJ197-06|2006-ONT-0197|658[0n]bp|Canada.Ontario|BOLD:AAB5821
Lithophane laticinerea|[17779]|XAB699-04|04HBL005699|658[0n]bp|Canada.Ontario|BOLD:AAB5821
Lithophane laticinerea|[17780]|RDNMG593-08|CNC LEP00052417|658[0n]bp|Canada.New Brunswick|BOLD:AAB5821
Lithophane laticinerea|[17781]|XAJ203-06|2006-ONT-0203|596[0n]bp|Canada.Ontario|BOLD:AAB5821
Lithophane laticinerea|[17782]|PHMO006-03|moth6.02|639[0n]bp|Canada.Ontario|BOLD:AAB5821
Lithophane laticinerea|[17783]|XAJ246-06|2006-ONT-0246|658[0n]bp|Canada.Ontario|BOLD:AAB5821
Lithophane laticinerea|[17784]|RDLQ528-07|DH004044|658[0n]bp|Canada.Quebec|BOLD:AAB5821
Lithophane laticinerea|[17785]|XAF050-05|HBL008866|658[0n]bp|Canada.Ontario|BOLD:AAB5821
Lithophane laticinerea|[17786]|XAJ020-06|2006-ONT-0020|658[0n]bp|Canada.Ontario|BOLD:AAB5821
Lithophane laticinerea|[17787]|XAH752-05|2005-ONT-2335|658[0n]bp|Canada.Ontario|BOLD:AAB5821
Lithophane pertorrida|[17788]|LALPA1041-11|AVBC 851-11|658[0n]bp|Canada.British Columbia|BOLD:AAB5821
Lithophane pertorrida|[17789]|LALPA1057-11|AVBC 867-11|658[0n]bp|Canada.British Columbia|BOLD:AAB5821
Lithophane pertorrida|[17790]|LALPA010-10|AVBC 010-10|658[0n]bp|Canada.British Columbia|BOLD:AAB5821
Lithophane pertorrida|[17791]|RDNMF215-08|NOC14301|658[0n]bp|Canada.British Columbia|BOLD:AAB5821
Lithophane pertorrida|[17792]|RDNMF213-08|NOC14299|658[0n]bp|Canada.British Columbia|BOLD:AAB5821
Lithophane pertorrida|[17793]|LALPA1047-11|AVBC 857-11|658[0n]bp|Canada.British Columbia|BOLD:AAB5821
Lithophane pertorrida|[17794]|RDNMF212-08|NOC14298|658[0n]bp|Canada.British Columbia|BOLD:AAB5821
Lithophane pertorrida|[17795]|LALPA763-10|AVBC 765-10|658[0n]bp|Canada.British Columbia|BOLD:AAB5821
Lithophane pertorrida|[17796]|RDNMF211-08|NOC14297|658[0n]bp|Canada.British Columbia|BOLD:AAB5821
Lithophane unimoda|[17797]|XAH770-05|2005-ONT-2353|658[0n]bp|Canada.Ontario|BOLD:AAB5821
Lithophane unimoda|[17798]|RDMAB696-06|UASM56924|616[0n]bp|Canada.Alberta|BOLD:AAB5821
Lithophane unimoda|[17799]|RDMAB699-06|UASM56920|658[0n]bp|Canada.Alberta|BOLD:AAB5821
Lithophane unimoda|[17800]|RDMAB697-06|UASM34667|574[1n]bp|Canada.Alberta|BOLD:AAB5821
Lithophane unimoda|[17801]|RDMAB700-06|UASM56927|658[0n]bp|Canada.Alberta|BOLD:AAB5821
Lithophane unimoda|[17802]|XAJ171-06|2006-ONT-0171|601[0n]bp|Canada.Ontario|BOLD:AAB5821
Lithophane unimoda|[17803]|RDLQH084-06|DH013322|639[0n]bp|Canada.Quebec|BOLD:AAB5821
Lithophane unimoda|[17804]|XAJ258-06|2006-ONT-0258|658[0n]bp|Canada.Ontario|BOLD:AAB5821
Lithophane unimoda|[17805]|RDNMG568-08|CNC LEP00052392|658[0n]bp|Canada.New Brunswick|BOLD:AAB5821
Lithophane unimoda|[17806]|PHMO025-03|moth77.02|639[0n]bp|Canada.Ontario|BOLD:AAB5821
Lithophane unimoda|[17807]|RDMAB701-06|UASM24050|563[1n]bp|Canada.Alberta|BOLD:AAB5821
Lithophane unimoda|[17808]|XAJ069-06|2006-ONT-0069|658[0n]bp|Canada.Ontario|BOLD:AAB5821
Lithophane unimoda|[17809]|XAC125-04|04HBL006125|658[0n]bp|Canada.Ontario|BOLD:AAB5821
Lithophane unimoda|[17810]|XAC098-04|04HBL006098|658[0n]bp|Canada.Ontario|BOLD:AAB5821
Lithophane unimoda|[17811]|RDNMG569-08|CNC LEP00052393|658[0n]bp|Canada.New Brunswick|BOLD:AAB5821
Lithophane unimoda|[17812]|RDMAB698-06|UASM56921|658[0n]bp|Canada.Alberta|BOLD:AAB5821
Lithophane unimoda|[17813]|XAJ172-06|2006-ONT-0172|658[0n]bp|Canada.Ontario|BOLD:AAB5821
Lithophane unimoda|[17814]|XAF411-05|HLC-10452|658[0n]bp|Canada.Ontario|BOLD:AAB5821
Lithophane unimoda|[17815]|RDLQ523-07|DH009619|593[0n]bp|Canada.Quebec|BOLD:AAB5821
Lithophane unimoda|[17816]|XAH860-05|2005-ONT-2443|658[0n]bp|Canada.Ontario|BOLD:AAB5821
Lithophane unimoda|[17817]|RDLQ522-07|DH004287|593[0n]bp|Canada.Quebec|BOLD:AAB5821
Lithophane unimoda|[17818]|XAH822-05|2005-ONT-2405|607[0n]bp|Canada.Ontario|BOLD:AAB5821
Lithomoia germana|[17819]|LBCH2775-10|10-JDWBC-2775|658[0n]bp|Canada.British Columbia|BOLD:ABZ6598
Lithomoia germana|[17820]|XAH625-05|2005-ONT-2208|658[0n]bp|Canada.Ontario|BOLD:ABZ6598
Lithomoia germana|[17821]|LBCH2777-10|10-JDWBC-2777|658[0n]bp|Canada.British Columbia|BOLD:ABZ6598
Lithomoia germana|[17822]|LBCH2609-10|10-JDWBC-2609|658[0n]bp|Canada.British Columbia|BOLD:ABZ6598
Lithomoia germana|[17823]|LBCH2570-10|10-JDWBC-2570|658[0n]bp|Canada.British Columbia|BOLD:ABZ6598
Lithomoia germana|[17824]|LOWCD181-06|CGWC-3001|658[0n]bp|Canada.British Columbia|BOLD:ABZ6598
Lithomoia germana|[17825]|LOWCD177-06|CGWC-2997|658[0n]bp|Canada.British Columbia|BOLD:ABZ6598
Lithomoia germana|[17826]|LBCH2778-10|10-JDWBC-2778|658[0n]bp|Canada.British Columbia|BOLD:ABZ6598
Lithomoia germana|[17827]|LBCH2773-10|10-JDWBC-2773|658[0n]bp|Canada.British Columbia|BOLD:ABZ6598
Lithomoia germana|[17828]|RDLQ508-07|DH006137|601[0n]bp|Canada.Quebec|BOLD:AAB3483
Lithomoia germana|[17829]|XAH333-05|2005-ONT-1916|658[0n]bp|Canada.Ontario|BOLD:AAB3483
Lithomoia germana|[17830]|XAH332-05|2005-ONT-1915|658[0n]bp|Canada.Ontario|BOLD:AAB3483
Lithomoia germana|[17831]|TTMNB371-06|MNBTT-371|658[0n]bp|Canada.New Brunswick|BOLD:AAB3483
Lithomoia germana|[17832]|RDLQ507-07|DH007212|601[0n]bp|Canada.Quebec|BOLD:AAB3483
Lithomoia germana|[17833]|LBCH2779-10|10-JDWBC-2779|658[0n]bp|Canada.British Columbia|BOLD:AAB3483
Lithomoia germana|[17834]|TMNBB227-06|MNBTT-1167|658[0n]bp|Canada.New Brunswick|BOLD:AAB3483
Lithomoia germana|[17835]|XAH238-05|2005-ONT-1821|658[0n]bp|Canada.Ontario|BOLD:AAB3483
Lithomoia germana|[17836]|LPVIB669-08|PFC-2006-2118|633[0n]bp|Canada.British Columbia|BOLD:AAB3483
Lithomoia germana|[17837]|LPVIB657-08|PFC-2006-2106|614[0n]bp|Canada.British Columbia|BOLD:AAB3483
Lithomoia germana|[17838]|LALPA710-10|AVBC 712-10|658[0n]bp|Canada.British Columbia|BOLD:AAB3483
Lithomoia germana|[17839]|LBCH2717-10|10-JDWBC-2717|658[0n]bp|Canada.British Columbia|BOLD:AAB3483
Lithomoia germana|[17840]|XAH362-05|2005-ONT-1945|658[0n]bp|Canada.Ontario|BOLD:AAB3483
Lithomoia germana|[17841]|TMNBB225-06|MNBTT-1165|658[0n]bp|Canada.New Brunswick|BOLD:AAB3483

Lithomoia germana|[17839]|LBCH2717-10|10-JDWBC-2717|658[0n]bp|Canada.British Columbia|BOLD:AAB3483
Lithomoia germana|[17840]|XAH362-05|2005-ONT-1945|658[0n]bp|Canada.Ontario|BOLD:AAB3483
Lithomoia germana|[17841]|TMNBB225-06|MNBTT-1165|658[0n]bp|Canada.New Brunswick|BOLD:AAB3483
Lithomoia germana|[17842]|TMNBB226-06|MNBTT-1166|658[0n]bp|Canada.New Brunswick|BOLD:AAB3483
Lithomoia germana|[17843]|LBCH2772-10|10-JDWBC-2772|658[0n]bp|Canada.British Columbia|BOLD:AAB3483
Lithomoia germana|[17844]|LBCH2838-10|10-JDWBC-2838|658[0n]bp|Canada.British Columbia|BOLD:AAB3483
Lithomoia germana|[17845]|XAB413-04|04HBL005413|658[0n]bp|Canada.Ontario|BOLD:AAB3483
Lithomoia germana|[17846]|LBCH2681-10|10-JDWBC-2681|658[0n]bp|Canada.British Columbia|BOLD:AAB3483
Lithomoia germana|[17847]|LBCH2506-10|10-JDWBC-2506|658[0n]bp|Canada.British Columbia|BOLD:AAB3483
Lithomoia germana|[17848]|LBCH2776-10|10-JDWBC-2776|658[0n]bp|Canada.British Columbia|BOLD:AAB3483
Lithomoia germana|[17849]|LALPA762-10|AVBC 764-10|658[0n]bp|Canada.British Columbia|BOLD:AAB3483
Lithomoia germana|[17850]|XAH374-05|2005-ONT-1957|658[0n]bp|Canada.Ontario|BOLD:AAB3483
Lithomoia germana|[17851]|LBCH2860-10|10-JDWBC-2860|658[0n]bp|Canada.British Columbia|BOLD:AAB3483
Lithomoia germana|[17852]|LBCH2774-10|10-JDWBC-2774|658[0n]bp|Canada.British Columbia|BOLD:AAB3483
Xylena nupera|[17853]|RDLQH092-06|DH013330|658[0n]bp|Canada.Quebec|BOLD:AAD3567
Xylena nupera|[17854]|LCHIP182-07|06-PROBE-2807|641[0n]bp|Canada.Manitoba|BOLD:AAD3567
Xylena nupera|[17855]|RDLQH089-06|DH013327|658[0n]bp|Canada.Quebec|BOLD:AAD3567
Xylena nupera|[17856]|RDLQH091-06|DH013329|658[0n]bp|Canada.Quebec|BOLD:AAD3567
Xylena nupera-sp. n.|[17857]|LOWCC212-05|CGWC-2092|658[0n]bp|Canada.British Columbia|BOLD:AAD3566
Xylena nupera-sp. n.|[17858]|LALPA1050-11|AVBC 860-11|658[0n]bp|Canada.British Columbia|BOLD:AAD3566
Xylena nupera-sp. n.|[17859]|LOWCC040-05|CGWC-1920|658[0n]bp|Canada.British Columbia|BOLD:AAD3566
Xylena nupera-sp. n.|[17860]|LALPA062-10|AVBC 062-10|658[0n]bp|Canada.British Columbia|BOLD:AAD3566
Xylena brucei|[17861]|LOWCC213-05|CGWC-2093|658[0n]bp|Canada.British Columbia|BOLD:AAE4681
Xylena brucei|[17862]|LOWCD925-06|CGWC-3745|576[0n]bp|Canada.British Columbia|BOLD:AAE4681
Xylena cineritia|[17863]|LOWCE412-06|CGWC-4172|587[1n]bp|Canada.British Columbia|BOLD:ABZ7519
Xylena cineritia|[17864]|LOWCD922-06|CGWC-3742|658[0n]bp|Canada.British Columbia|BOLD:ABZ7519
Xylena cineritia|[17865]|LOWCE059-06|CGWC-3819|658[0n]bp|Canada.British Columbia|BOLD:ABZ7519
Xylena cineritia|[17866]|LOWCD924-06|CGWC-3744|658[0n]bp|Canada.British Columbia|BOLD:ABZ7519
Xylena cineritia|[17867]|LOWCE058-06|CGWC-3818|658[0n]bp|Canada.British Columbia|BOLD:ABZ7519
Xylena cineritia|[17868]|LOWCE465-06|CGWC-4225|658[0n]bp|Canada.British Columbia|BOLD:ABZ7519
Xylena cineritia|[17869]|LOWCB541-05|CGWC-1481|658[0n]bp|Canada.British Columbia|BOLD:ABZ7519
Xylena cineritia|[17870]|LOWCD920-06|CGWC-3740|658[0n]bp|Canada.British Columbia|BOLD:ABZ7519
Xylena cineritia|[17871]|LOWCB542-05|CGWC-1482|658[0n]bp|Canada.British Columbia|BOLD:ABZ7519
Xylena cineritia|[17872]|LOWCC210-05|CGWC-2090|658[0n]bp|Canada.British Columbia|BOLD:ABZ7519
Xylena cineritia|[17873]|LOWCE057-06|CGWC-3817|658[0n]bp|Canada.British Columbia|BOLD:ABZ7519
Xylena cineritia|[17874]|LOWCE207-06|CGWC-3967|632[0n]bp|Canada.British Columbia|BOLD:ABZ7519
Xylena cineritia|[17875]|LBCH4991-10|10-JDWBC-4991|658[0n]bp|Canada.British Columbia|BOLD:ABZ7519
Xylena cineritia|[17876]|LOWCD923-06|CGWC-3743|658[0n]bp|Canada.British Columbia|BOLD:ABZ7519
Xylena cineritia|[17877]|LOWCE205-06|CGWC-3965|658[0n]bp|Canada.British Columbia|BOLD:ABZ7519
Xylena cineritia|[17878]|LOWCE411-06|CGWC-4171|608[0n]bp|Canada.British Columbia|BOLD:ABZ7519
Xylena cineritia|[17879]|LOWCE356-06|CGWC-4116|577[0n]bp|Canada.British Columbia|BOLD:ABZ7519
Xylena cineritia|[17880]|LOWCE360-06|CGWC-4120|604[0n]bp|Canada.British Columbia|BOLD:ABZ7519
Xylena cineritia|[17881]|LOWCE209-06|CGWC-3969|658[0n]bp|Canada.British Columbia|BOLD:ABZ7519
Xylena cineritia|[17882]|LOWCD921-06|CGWC-3741|658[0n]bp|Canada.British Columbia|BOLD:ABZ7519
Xylena curvimacula|[17883]|LALPA1024-11|AVBC 834-11|658[0n]bp|Canada.British Columbia|BOLD:ABZ7519
Xylena curvimacula|[17884]|LALPA1043-11|AVBC 853-11|658[0n]bp|Canada.British Columbia|BOLD:ABZ7519
Xylena curvimacula|[17885]|LOWCC211-05|CGWC-2091|618[0n]bp|Canada.British Columbia|BOLD:ABZ7519
Xylena curvimacula|[17886]|XAF293-05|HLC-10334|658[0n]bp|Canada.Ontario|BOLD:ABZ7519
Xylena curvimacula|[17887]|LMIS014-05|05-ONMIS-0014|658[0n]bp|Canada.Ontario|BOLD:ABZ7519
Xylena curvimacula|[17888]|LOWCD296-06|CGWC-3116|658[0n]bp|Canada.British Columbia|BOLD:ABZ7519
Xylena curvimacula|[17889]|LOWCC024-05|CGWC-1904|658[0n]bp|Canada.British Columbia|BOLD:ABZ7519
Xylena curvimacula|[17890]|LALPA031-10|AVBC 031-10|658[0n]bp|Canada.British Columbia|BOLD:ABZ7519
Xylena curvimacula|[17891]|LOWCE061-06|CGWC-3821|658[0n]bp|Canada.British Columbia|BOLD:ABZ7519
Xylena curvimacula|[17892]|RDLQ499-07|DH009719|621[0n]bp|Canada.Quebec|BOLD:ABZ7519
Xylena curvimacula|[17893]|RDLQH085-06|DH013323|621[0n]bp|Canada.Quebec|BOLD:ABZ7519
Xylena curvimacula|[17894]|RDLQ500-07|DH009721|601[0n]bp|Canada.Quebec|BOLD:ABZ7519
Xylena curvimacula|[17895]|LOWCC020-05|CGWC-1900|555[0n]bp|Canada.British Columbia|BOLD:ABZ7519
Xylena curvimacula|[17896]|PMG165-03|moth244.01|617[0n]bp|Canada.Ontario|BOLD:ABZ7519
Xylena curvimacula|[17897]|LOWCE060-06|CGWC-3820|658[0n]bp|Canada.British Columbia|BOLD:ABZ7519
Xylena curvimacula|[17898]|LOWCE064-06|CGWC-3824|658[0n]bp|Canada.British Columbia|BOLD:ABZ7519
Xylena curvimacula|[17899]|LOWCC022-05|CGWC-1902|658[0n]bp|Canada.British Columbia|BOLD:ABZ7519
Xylena curvimacula|[17900]|LOWCE065-06|CGWC-3825|658[0n]bp|Canada.British Columbia|BOLD:ABZ7519
Xylena curvimacula|[17901]|LOWCC021-05|CGWC-1901|658[0n]bp|Canada.British Columbia|BOLD:ABZ7519
Xylena thoracica|[17902]|DSCNI032-07|06-PROBE-0257|658[0n]bp|Canada.Manitoba|BOLD:ACF2663
Xylena thoracica|[17903]|LALPA001-10|AVBC 001-10|658[0n]bp|Canada.British Columbia|BOLD:ACF2663
Xylena thoracica|[17904]|LBCG467-08|08-JDWBC-0467|658[0n]bp|Canada.British Columbia|BOLD:ACF2663
Xylena thoracica|[17905]|LALPA030-10|AVBC 030-10|658[0n]bp|Canada.British Columbia|BOLD:ACF2663
Xylena thoracica|[17906]|LOWCD926-06|CGWC-3746|658[0n]bp|Canada.British Columbia|BOLD:ACF2663
Xylena thoracica|[17907]|LBCH5342-10|10-JDWBC-5342|658[0n]bp|Canada.British Columbia|BOLD:ACF2663
Xylena thoracica|[17908]|DSCNI033-07|06-PROBE-0258|658[0n]bp|Canada.Manitoba|BOLD:ACF2663
Xylena thoracica|[17909]|LOWCD708-06|CGWC-3528|658[0n]bp|Canada.British Columbia|BOLD:ACF2663
Xylena thoracica|[17910]|LBCH4992-10|10-JDWBC-4992|658[0n]bp|Canada.British Columbia|BOLD:ACF2663
Xylena thoracica|[17911]|RDNME237-07|CNCNoctuoidea13844|618[0n]bp|Canada.Alberta|BOLD:ACF2663
Xylena thoracica|[17912]|TMNBB223-06|MNBTT-1163|658[0n]bp|Canada.New Brunswick|BOLD:AAB5195
Xylena thoracica|[17913]|TTMNB581-06|MNBTT-581|658[0n]bp|Canada.New Brunswick|BOLD:AAB5195
Xylena thoracica|[17914]|LOWCD705-06|CGWC-3525|658[0n]bp|Canada.British Columbia|BOLD:AAB5195
Xylena thoracica|[17915]|LOWCD710-06|CGWC-3530|658[0n]bp|Canada.British Columbia|BOLD:AAB5195
Xylena thoracica|[17916]|TMNBB224-06|MNBTT-1164|658[0n]bp|Canada.New Brunswick|BOLD:AAB5195
Xylena thoracica|[17917]|LOWCD707-06|CGWC-3527|658[0n]bp|Canada.British Columbia|BOLD:AAB5195
Xylena thoracica|[17918]|LOWCD709-06|CGWC-3529|658[0n]bp|Canada.British Columbia|BOLD:AAB5195
Xylena thoracica|[17919]|LOWCD712-06|CGWC-3532|658[0n]bp|Canada.British Columbia|BOLD:AAB5195
Xylena thoracica|[17920]|LOWCD711-06|CGWC-3531|654[0n]bp|Canada.British Columbia|BOLD:AAB5195
Xylena thoracica|[17921]|LOWCB543-05|CGWC-1483|511[1n]bp|Canada.British Columbia|BOLD:AAB5195
Xylena thoracica|[17922]|TMNBB222-06|MNBTT-1162|658[0n]bp|Canada.New Brunswick|BOLD:AAB5195
Xylena thoracica|[17923]|RDLQ501-07|DH009706|597[0n]bp|Canada.Ontario|BOLD:AAB5195
Xylena thoracica|[17924]|RDNME238-07|CNCNoctuoidea13845|658[0n]bp|Canada.Alberta|BOLD:AAB5195
Xylena thoracica|[17925]|LOWCD706-06|CGWC-3526|658[0n]bp|Canada.British Columbia|BOLD:AAB5195
Enargia decolor|[17926]|RDMAB287-05|UASM77884|517[0n]bp|Canada.Alberta|BOLD:AAA7455
Enargia decolor|[17927]|RDMAB286-05|UASM77909|548[0n]bp|Canada.Alberta|BOLD:AAA7455
Enargia decolor|[17928]|LOWCC002-05|CGWC-1882|658[0n]bp|Canada.British Columbia|BOLD:AAA7455
Enargia decolor|[17929]|BBLPB242-10|10BBCLP-1241|658[0n]bp|Canada.Saskatchewan|BOLD:AAA7455
Enargia decolor|[17930]|XAG971-05|2005-ONT-1555|629[0n]bp|Canada.Ontario|BOLD:AAA7455
Enargia decolor|[17931]|XAG758-05|2005-ONT-1342|658[0n]bp|Canada.Ontario|BOLD:AAA7455
Enargia decolor|[17932]|BBLPB243-10|10BBCLP-1242|658[0n]bp|Canada.Saskatchewan|BOLD:AAA7455
Enargia decolor|[17933]|XAG614-05|2005-ONT-1198|658[0n]bp|Canada.Ontario|BOLD:AAA7455
Enargia decolor|[17934]|BBLPB245-10|10BBCLP-1244|658[0n]bp|Canada.Saskatchewan|BOLD:AAA7455
Enargia decolor|[17935]|LOWCC006-05|CGWC-1886|658[0n]bp|Canada.British Columbia|BOLD:AAA7455
Enargia decolor|[17936]|RDMAB663-06|UASM34750|639[2n]bp|Canada.Alberta|BOLD:AAA7455
Enargia decolor|[17937]|LOWCC001-05|CGWC-1881|658[0n]bp|Canada.British Columbia|BOLD:AAA7455
Enargia decolor|[17938]|XAD353-04|04HBL007353|549[0n]bp|Canada.Ontario|BOLD:AAA7455
Enargia decolor|[17939]|RDLQB597-05|DH010700|537[0n]bp|Canada.Quebec|BOLD:AAA7455
Enargia decolor|[17940]|LPMNB320-09|08BBLEP-05164|658[0n]bp|Canada.Manitoba|BOLD:AAA7455

Enargia decolor|[17938]|XAD333-04|04HBL007333|549[0n]bp|Canada.Ontario|BOLD:AAA7455
Enargia decolor|[17939]|RDLQB597-05|DH010700|537[0n]bp|Canada.Quebec|BOLD:AAA7455
Enargia decolor|[17940]|LPMNB320-09|08BBLEP-05164|658[0n]bp|Canada.Manitoba|BOLD:AAA7455
Enargia decolor|[17941]|RDMAB665-06|UASM7188|658[0n]bp|Canada.Alberta|BOLD:AAA7455
Enargia decolor|[17942]|XAG969-05|2005-ONT-1553|636[0n]bp|Canada.Ontario|BOLD:AAA7455
Enargia decolor|[17943]|RDNMH998-09|CNC LEP 68006|658[0n]bp|Canada.New Brunswick|BOLD:AAA7455
Enargia decolor|[17944]|XAH052-05|2005-ONT-1635|658[0n]bp|Canada.Ontario|BOLD:AAA7455
Enargia decolor|[17945]|XAD391-04|04HBL007391|658[0n]bp|Canada.Ontario|BOLD:AAA7455
Enargia decolor|[17946]|LBCH2784-10|10-JDWBC-2784|658[0n]bp|Canada.British Columbia|BOLD:AAA7455
Enargia decolor|[17947]|PHMO337-03|moth2609.02|639[1n]bp|Canada.Ontario|BOLD:AAA7455
Enargia decolor|[17948]|TTMNB364-06|MNBTT-364|658[0n]bp|Canada.New Brunswick|BOLD:AAA7455
Enargia decolor|[17949]|LPMN994-09|08BBLEP-04035|658[0n]bp|Canada.Manitoba|BOLD:AAA7455
Enargia decolor|[17950]|RDNMH996-09|CNC LEP 68004|658[0n]bp|Canada.New Brunswick|BOLD:AAA7455
Enargia decolor|[17951]|LBCH2837-10|10-JDWBC-2837|658[0n]bp|Canada.British Columbia|BOLD:AAA7455
Enargia decolor|[17952]|LBCH2783-10|10-JDWBC-2783|658[0n]bp|Canada.British Columbia|BOLD:AAA7455
Enargia decolor|[17953]|LBCH2788-10|10-JDWBC-2788|658[0n]bp|Canada.British Columbia|BOLD:AAA7455
Enargia decolor|[17954]|LBCH2683-10|10-JDWBC-2683|658[0n]bp|Canada.British Columbia|BOLD:AAA7455
Enargia decolor|[17955]|TTMNB365-06|MNBTT-365|658[0n]bp|Canada.New Brunswick|BOLD:AAA7455
Enargia decolor|[17956]|XAH172-05|2005-ONT-1755|658[0n]bp|Canada.Ontario|BOLD:AAA7455
Enargia decolor|[17957]|LBCH2820-10|10-JDWBC-2820|658[0n]bp|Canada.British Columbia|BOLD:AAA7455
Enargia decolor|[17958]|BBLPB248-10|10BBCLP-1247|658[0n]bp|Canada.Saskatchewan|BOLD:AAA7455
Enargia decolor|[17959]|BBLPB244-10|10BBCLP-1243|658[0n]bp|Canada.Saskatchewan|BOLD:AAA7455
Enargia decolor|[17960]|LBCH2543-10|10-JDWBC-2543|658[0n]bp|Canada.British Columbia|BOLD:AAA7455
Enargia decolor|[17961]|RDMAB664-06|UASM58333|658[0n]bp|Canada.Alberta|BOLD:AAA7455
Enargia decolor|[17962]|LPMN995-09|08BBLEP-04036|658[0n]bp|Canada.Manitoba|BOLD:AAA7455
Enargia decolor|[17963]|XAH173-05|2005-ONT-1756|658[0n]bp|Canada.Ontario|BOLD:AAA7455
Enargia decolor|[17964]|XAG943-05|2005-ONT-1527|658[0n]bp|Canada.Ontario|BOLD:AAA7455
Enargia decolor|[17965]|LBCH2787-10|10-JDWBC-2787|658[0n]bp|Canada.British Columbia|BOLD:AAA7455
Enargia decolor|[17966]|LBCH2786-10|10-JDWBC-2786|658[0n]bp|Canada.British Columbia|BOLD:AAA7455
Enargia decolor|[17967]|XAH467-05|2005-ONT-2050|658[0n]bp|Canada.Ontario|BOLD:AAA7455
Enargia decolor|[17968]|LBCH2782-10|10-JDWBC-2782|658[0n]bp|Canada.British Columbia|BOLD:AAA7455
Enargia decolor|[17969]|LBCH2785-10|10-JDWBC-2785|658[0n]bp|Canada.British Columbia|BOLD:AAA7455
Enargia decolor|[17970]|LOWCD174-06|CGWC-2994|658[0n]bp|Canada.British Columbia|BOLD:AAA7455
Enargia decolor|[17971]|LBCH2507-10|10-JDWBC-2507|658[0n]bp|Canada.British Columbia|BOLD:AAA7455
Enargia decolor|[17972]|LBCH2781-10|10-JDWBC-2781|658[0n]bp|Canada.British Columbia|BOLD:AAA7455
Enargia decolor|[17973]|BBLPB241-10|10BBCLP-1240|658[0n]bp|Canada.Saskatchewan|BOLD:AAA7455
Enargia decolor|[17974]|LOWCC005-05|CGWC-1885|658[0n]bp|Canada.British Columbia|BOLD:AAA7455
Enargia decolor|[17975]|LOWCD125-06|CGWC-2945|614[0n]bp|Canada.British Columbia|BOLD:AAA7455
Enargia decolor|[17976]|PHMO350-03|moth2670.02|639[0n]bp|Canada.Ontario|BOLD:AAA7455
Enargia decolor|[17977]|DSCNI038-07|06-PROBE-0263|655[0n]bp|Canada.Manitoba|BOLD:AAA7455
Enargia decolor|[17978]|LOWCC004-05|CGWC-1884|658[0n]bp|Canada.British Columbia|BOLD:AAA7455
Enargia decolor|[17979]|LOWCC003-05|CGWC-1883|658[0n]bp|Canada.British Columbia|BOLD:AAA7455
Enargia decolor|[17980]|RDLQB596-05|DH010699|658[0n]bp|Canada.Quebec|BOLD:AAA7455
Enargia decolor|[17981]|XAG849-05|2005-ONT-1433|658[0n]bp|Canada.Ontario|BOLD:AAA7455
Enargia decolor|[17982]|BBLPB246-10|10BBCLP-1245|658[0n]bp|Canada.Saskatchewan|BOLD:AAA7455
Enargia decolor|[17983]|LBCH2610-10|10-JDWBC-2610|658[0n]bp|Canada.British Columbia|BOLD:AAA7455
Enargia fausta|[17984]|BBLPB247-10|10BBCLP-1246|658[0n]bp|Canada.Saskatchewan|BOLD:AAA7455
Enargia fausta|[17985]|LPMNB339-09|08BBLEP-05183|658[0n]bp|Canada.Manitoba|BOLD:AAA7455
Enargia fausta|[17986]|BBLPB237-10|10BBCLP-1236|658[0n]bp|Canada.Saskatchewan|BOLD:AAA7455
Enargia fausta|[17987]|LPMNB430-09|08BBLEP-05430|658[0n]bp|Canada.Manitoba|BOLD:AAA7455
Enargia fausta|[17988]|BBLPB239-10|10BBCLP-1238|658[0n]bp|Canada.Saskatchewan|BOLD:AAA7455
Enargia fausta|[17989]|RDNMH997-09|CNC LEP 68005|658[0n]bp|Canada.New Brunswick|BOLD:AAA7455
Enargia fausta|[17990]|TMNBB192-06|MNBTT-1132|658[0n]bp|Canada.New Brunswick|BOLD:AAA7455
Enargia infumata|[17991]|RDMAB659-06|UASM41852|547[0n]bp|Canada.Alberta|BOLD:AAA7455
Enargia infumata|[17992]|RDNME190-07|CNCNoctuoidea13797|604[1n]bp|Canada.Ontario|BOLD:AAA7455
Enargia infumata|[17993]|XAB147-04|04HBL005147|658[0n]bp|Canada.Ontario|BOLD:AAA7455
Enargia infumata|[17994]|RDMAB284-05|UASM77834|509[0n]bp|Canada.Alberta|BOLD:AAA7455
Enargia infumata|[17995]|LBCD304-05|HLC-23124|618[0n]bp|Canada.British Columbia|BOLD:AAA7455
Enargia infumata|[17996]|LBCD300-05|HLC-23120|618[0n]bp|Canada.British Columbia|BOLD:AAA7455
Enargia infumata|[17997]|RDMAB662-06|UASM58312|606[2n]bp|Canada.Alberta|BOLD:AAA7455
Enargia infumata|[17998]|LBCD309-05|HLC-23129|639[0n]bp|Canada.British Columbia|BOLD:AAA7455
Enargia infumata|[17999]|LBCD305-05|HLC-23125|618[0n]bp|Canada.British Columbia|BOLD:AAA7455
Enargia infumata|[18000]|RDMAB285-05|UASM77859|502[1n]bp|Canada.Alberta|
Enargia infumata|[18001]|LBCD302-05|HLC-23122|618[0n]bp|Canada.British Columbia|BOLD:AAA7455
Enargia infumata|[18002]|LPMNB323-09|08BBLEP-05167|658[0n]bp|Canada.Manitoba|BOLD:AAA7455
Enargia infumata|[18003]|BBLPB240-10|10BBCLP-1239|658[0n]bp|Canada.Saskatchewan|BOLD:AAA7455
Enargia infumata|[18004]|BBLPB238-10|10BBCLP-1237|658[0n]bp|Canada.Saskatchewan|BOLD:AAA7455
Enargia infumata|[18005]|LPMN993-09|08BBLEP-04034|658[0n]bp|Canada.Manitoba|BOLD:AAA7455
Enargia infumata|[18006]|LBCD306-05|HLC-23126|658[0n]bp|Canada.British Columbia|BOLD:AAA7455
Enargia infumata|[18007]|XAB198-04|04HBL005198|658[0n]bp|Canada.Ontario|BOLD:AAA7455
Enargia infumata|[18008]|LBCA596-05|HLC-20596|647[0n]bp|Canada.British Columbia|BOLD:AAA7455
Enargia infumata|[18009]|RDMAB661-06|UASM58131|601[0n]bp|Canada.Alberta|BOLD:AAA7455
Enargia infumata|[18010]|RDNME188-07|CNCNoctuoidea13716|642[0n]bp|Canada.New Brunswick|BOLD:AAA7455
Enargia infumata|[18011]|RDLQB598-05|DH010701|519[0n]bp|Canada.Quebec|BOLD:AAA7455
Enargia infumata|[18012]|RDNME185-07|CNCNoctuoidea13713|642[0n]bp|Canada.New Brunswick|BOLD:AAA7455
Enargia infumata|[18013]|RDNME189-07|CNCNoctuoidea13796|619[0n]bp|Canada.Ontario|BOLD:AAA7455
Enargia infumata|[18014]|LBCD303-05|HLC-23123|618[0n]bp|Canada.British Columbia|BOLD:AAA7455
Enargia infumata|[18015]|LBCA595-05|HLC-20595|633[0n]bp|Canada.British Columbia|BOLD:AAA7455
Enargia infumata|[18016]|LBCA597-05|HLC-20597|641[0n]bp|Canada.British Columbia|BOLD:AAA7455
Enargia infumata|[18017]|LBCB036-05|HLC-20976|658[0n]bp|Canada.British Columbia|BOLD:AAA7455
Enargia infumata|[18018]|LBCD427-05|HLC-23247|658[0n]bp|Canada.British Columbia|BOLD:AAA7455
Enargia infumata|[18019]|RDLQF541-06|DH011690|658[0n]bp|Canada.Quebec|BOLD:AAA7455
Enargia infumata|[18020]|RDNMH1005-09|CNCLEP00068013|658[0n]bp|Canada.British Columbia|BOLD:AAA7455
Enargia infumata|[18021]|RDNMH999-09|CNCLEP00068007|658[0n]bp|Canada.British Columbia|BOLD:AAA7455
Enargia infumata|[18022]|LPVIC003-08|PFC-2006-2556|658[0n]bp|Canada.British Columbia|BOLD:AAA7455
Enargia infumata|[18023]|RDMAB657-06|UASM58133|658[0n]bp|Canada.Alberta|BOLD:AAA7455
Enargia infumata|[18024]|RDMAB658-06|UASM58130|658[0n]bp|Canada.Alberta|BOLD:AAA7455
Enargia infumata|[18025]|XAB194-04|04HBL005194|658[0n]bp|Canada.Ontario|BOLD:AAA7455
Enargia infumata|[18026]|LBCD307-05|HLC-23127|658[0n]bp|Canada.British Columbia|BOLD:AAA7455
Enargia infumata|[18027]|XAK054-06|2006-ONT-1049|658[0n]bp|Canada.Ontario|BOLD:AAA7455
Enargia infumata|[18028]|LPVIB233-08|PFC-2006-1612|658[0n]bp|Canada.British Columbia|BOLD:AAA7455
Enargia infumata|[18029]|LBCB199-05|HLC-21139|658[0n]bp|Canada.British Columbia|BOLD:AAA7455
Enargia infumata|[18030]|LBCD467-05|HLC-23287|658[0n]bp|Canada.British Columbia|BOLD:AAA7455
Enargia infumata|[18031]|RDNMH1003-09|CNC LEP 68011|658[0n]bp|Canada.British Columbia|BOLD:AAA7455
Enargia infumata|[18032]|XAB493-04|04HBL005493|658[0n]bp|Canada.Ontario|BOLD:AAA7455
Enargia infumata|[18033]|XAC594-04|04HBL006594|658[0n]bp|Canada.Ontario|BOLD:AAA7455
Enargia infumata|[18034]|LBCD308-05|HLC-23128|658[0n]bp|Canada.British Columbia|BOLD:AAA7455
Enargia infumata|[18035]|XAC566-04|04HBL006566|658[0n]bp|Canada.Ontario|BOLD:AAA7455
Enargia infumata|[18036]|XAC038-04|04HBL006038|658[0n]bp|Canada.Ontario|BOLD:AAA7455
Enargia infumata|[18037]|RDNMH1001-09|CNC LEP 68009|658[0n]bp|Canada.British Columbia|BOLD:AAA7455
Enargia infumata|[18038]|RDNME187-07|CNCNoctuoidea13715|641[0n]bp|Canada.New Brunswick|BOLD:AAA7455
Enargia infumata|[18039]|RDNME186-07|CNCNoctuoidea13714|640[0n]bp|Canada.New Brunswick|BOLD:AAA7455
Enargia infumata|[18040]|RDMAB660-06|UASM58132|658[0n]bp|Canada.Alberta|BOLD:AAA7455

Enargia infumata|[18038]|RDNME187-07|CNCNoctuoidea13715|641[0n]bp|Canada.New Brunswick|BOLD:AAA7455
Enargia infumata|[18039]|RDNME186-07|CNCNoctuoidea13714|640[0n]bp|Canada.New Brunswick|BOLD:AAA7455
Enargia infumata|[18040]|RDMAB660-06|UASM58132|658[0n]bp|Canada.Alberta|BOLD:AAA7455
Enargia infumata|[18041]|LBCD301-05|HLC-23121|618[0n]bp|Canada.British Columbia|BOLD:AAA7455
Homoglaea californica|[18042]|RDNMG890-08|CNC LEP00053014|658[0n]bp|Canada.British Columbia|BOLD:AAI8247
Andropolia diversilineata|[18043]|RDNMF315-08|NOC14401|658[0n]bp|United States.Wyoming|BOLD:AAE1573
Andropolia diversilineata|[18044]|JMMMB362-11|BIOUG00850-G05|658[0n]bp|United States.California|BOLD:A...
Andropolia diversilineata|[18045]|RDNMF313-08|NOC14399|658[0n]bp|United States.Nevada|BOLD:AAE1573
Andropolia diversilineata|[18046]|RDNMF312-08|NOC14398|658[0n]bp|United States.Nevada|BOLD:AAE1573
Andropolia diversilineata|[18047]|BBLOC1215-11|BIOUG01540-E04|658[0n]bp|United States.California|BOLD:...
Andropolia diversilineata|[18048]|BBLOC994-11|BIOUG01468-C02|658[0n]bp|United States.California|BOLD:A...
Andropolia diversilineata|[18049]|BBLOC1369-11|BIOUG01542-B04|658[0n]bp|United States.California|BOLD:...
Andropolia diversilineata|[18050]|RDNMF314-08|NOC14400|658[0n]bp|United States.California|BOLD:AAE1573
Andropolia diversilineata|[18051]|BBLOC1381-11|BIOUG01542-C04|658[0n]bp|United States.California|BOLD:...
Andropolia diversilineata|[18052]|BBLOC1214-11|BIOUG01540-E03|658[0n]bp|United States.California|BOLD:...
Andropolia diversilineata|[18053]|BBLOC1375-11|BIOUG01542-B10|658[0n]bp|United States.California|BOLD:...
Andropolia diversilineata|[18054]|NAMUM165-08|RR-95-0130|658[0n]bp|United States.California|BOLD:AAE1573
Andropolia diversilineata|[18055]|BBLOC1377-11|BIOUG01542-B12|628[0n]bp|United States.California|BOLD:...
Andropolia contacta|[18056]|RDLQ487-07|DH011172|618[0n]bp|Canada.Quebec|BOLD:AAC6243
Andropolia contacta|[18057]|RDLQ488-07|DH011170|618[0n]bp|Canada.Quebec|BOLD:AAC6243
Andropolia contacta|[18058]|RDLQ489-07|DH011171|594[0n]bp|Canada.Quebec|BOLD:AAC6243
Andropolia contacta|[18059]|LPABC957-09|08BBLEP-05368|658[0n]bp|Canada.Alberta|BOLD:AAC6243
Andropolia contacta|[18060]|RDLQB574-05|DH010677|658[0n]bp|Canada.Quebec|BOLD:AAC6243
Andropolia contacta|[18061]|RDMAB507-06|UASM41374|658[0n]bp|Canada.Alberta|BOLD:AAC6243
Andropolia contacta|[18062]|DSCNI034-07|06-PROBE-0259|658[0n]bp|Canada.Manitoba|BOLD:AAC6243
Andropolia contacta|[18063]|LCHIP104-07|06-PROBE-2590|649[0n]bp|Canada.Manitoba|BOLD:AAC6243
Andropolia contacta|[18064]|LBCD248-05|HLC-23068|616[0n]bp|Canada.British Columbia|BOLD:AAC6243
Andropolia contacta|[18065]|LBCD421-05|HLC-23241|632[0n]bp|Canada.British Columbia|BOLD:AAC6243
Andropolia aedon|[18066]|LBCH7327-10|10-JDWBC-7327|658[0n]bp|Canada.British Columbia|BOLD:ACE5322
Andropolia aedon|[18067]|LALPA618-10|AVBC 620-10|658[0n]bp|Canada.British Columbia|BOLD:ACE5322
Andropolia aedon|[18068]|RDMAB506-06|UASM41373|631[0n]bp|Canada.Alberta|BOLD:ACE5322
Andropolia aedon|[18069]|LALPA1202-11|AVBC 1204-11|658[0n]bp|Canada.British Columbia|BOLD:ACE5322
Andropolia aedon|[18070]|LALPA637-10|AVBC 639-10|658[0n]bp|Canada.British Columbia|BOLD:ACE5322
Andropolia theodori|[18071]|RDMAB505-06|UASM41372|658[0n]bp|Canada.Alberta|BOLD:ACE5842
Andropolia theodori|[18072]|LALPA667-10|AVBC 669-10|658[0n]bp|Canada.British Columbia|BOLD:ACE5842
Andropolia theodori|[18073]|LBCH7749-10|10-JDWBC-7749|658[0n]bp|Canada.British Columbia|BOLD:ACE5842
Andropolia theodori|[18074]|LBCH7551-10|10-JDWBC-7551|634[0n]bp|Canada.British Columbia|BOLD:ACE5842
Rhizagrotis albalis|[18075]|BBLSX617-09|09BBLEP-02545|658[0n]bp|United States.Arizona|BOLD:AAF7150
Rhizagrotis albalis|[18076]|RDNMG441-08|CNC LEP00052265|658[0n]bp|United States.Colorado|BOLD:AAF7150
Rhizagrotis albalis|[18077]|BBLSW413-09|09BBLEP-01341|658[0n]bp|United States.New Mexico|BOLD:AAF7150
Rhizagrotis albalis|[18078]|RDNMD645-06|CNCNoctuoidea12977|658[0n]bp|United States.New Mexico|BOLD:AAF...
Rhizagrotis albalis|[18079]|BBLOC146-11|BIOUG01453-E08|658[0n]bp|United States.Texas|BOLD:AAF7150
Rhizagrotis albalis|[18080]|BBLSX618-09|09BBLEP-02546|658[0n]bp|United States.Arizona|BOLD:AAF7150
Rhizagrotis cloanthoides|[18081]|RDNMG468-08|CNC LEP00052292|658[0n]bp|United States.Oregon|BOLD:AAE3645
Rhizagrotis cloanthoides|[18082]|RDNMD648-06|CNCNoctuoidea12980|658[0n]bp|United States.New Mexico|BOLD ...
Rhizagrotis cloanthoides|[18083]|RDNMG469-08|CNC LEP00052293|658[0n]bp|United States.Oregon|BOLD:AAE3645
Rhizagrotis cloanthoides|[18084]|RDNMD647-06|CNCNoctuoidea12979|658[0n]bp|United States.New Mexico|BOLD ...
Rhizagrotis cloanthoides|[18085]|RDNMG470-08|CNC LEP00052294|658[0n]bp|United States.Oregon|BOLD:AAE3645
Rhizagrotis stylata|[18086]|RDNMF092-08|NOC14178|658[0n]bp|United States.Colorado|BOLD:AAE1530
Rhizagrotis stylata|[18087]|RDNMF004-08|NOC14090|658[0n]bp|United States.Colorado|BOLD:AAE1530
Rhizagrotis stylata|[18088]|RDNMG442-08|CNC LEP00052266|636[0n]bp|United States.Utah|BOLD:AAE1530
Rhizagrotis stylata|[18089]|NAMUM275-08|RR-98-1109|658[0n]bp|United States.California|BOLD:AAE1530
Rhizagrotis stylata|[18090]|RDNMG440-08|CNC LEP00052264|658[0n]bp|United States.Utah|BOLD:AAE1530
Rhizagrotis stylata|[18091]|RDNME601-08|LEP038025|658[0n]bp|United States.Colorado|BOLD:AAE1530
Aseptis adnixa|[18092]|LBCS671-07|UBC-2007-0376|658[0n]bp|Canada.British Columbia|BOLD:AAA7134
Aseptis adnixa|[18093]|LHLEP411-06|UBC-2006-0757|658[0n]bp|Canada.British Columbia|BOLD:AAA7134
Aseptis adnixa|[18094]|LBCS673-07|UBC-2007-0378|658[0n]bp|Canada.British Columbia|BOLD:AAA7134
Aseptis adnixa|[18095]|LBCS672-07|UBC-2007-0377|658[0n]bp|Canada.British Columbia|BOLD:AAA7134
Aseptis adnixa|[18096]|LHLEP413-06|UBC-2006-0759|658[0n]bp|Canada.British Columbia|BOLD:AAA7134
Aseptis adnixa|[18097]|RDNMC666-06|CNCNoctuoidea12206|658[0n]bp|Canada.British Columbia|BOLD:AAA7134
Aseptis adnixa|[18098]|LBCS670-07|UBC-2007-0375|658[0n]bp|Canada.British Columbia|BOLD:AAA7134
Aseptis adnixa|[18099]|RDNMC661-06|CNCNoctuoidea12201|658[0n]bp|Canada.British Columbia|BOLD:AAA7134
Aseptis adnixa|[18100]|LHLEP412-06|UBC-2006-0758|658[0n]bp|Canada.British Columbia|BOLD:AAA7134
Aseptis adnixa|[18101]|LPVIA291-08|PFC-2006-0382|658[0n]bp|Canada.British Columbia|BOLD:AAA7134
Aseptis adnixa|[18102]|LBCS674-07|UBC-2007-0379|658[0n]bp|Canada.British Columbia|BOLD:AAA7134
Aseptis adnixa|[18103]|LMHRG037-06|SPI H-2-17 A|658[0n]bp|Canada.British Columbia|BOLD:AAA7134
Aseptis binotata|[18104]|LOWCB445-05|CGWC-1385|508[1n]bp|Canada.British Columbia|
Aseptis binotata|[18105]|RDNMC645-06|CNCNoctuoidea12185|535[0n]bp|Canada.British Columbia|BOLD:AAA4142
Aseptis binotata|[18106]|LALPA512-10|AVBC 514-10|658[0n]bp|Canada.British Columbia|BOLD:AAA4141
Aseptis binotata|[18107]|LPVIB073-08|PFC-2006-1421|658[0n]bp|Canada.British Columbia|BOLD:AAA4141
Aseptis binotata|[18108]|NAGEO261-09|09-JDWGEO-261|658[0n]bp|Canada.British Columbia|
Aseptis binotata|[18109]|LALPA528-10|AVBC 530-10|633[0n]bp|Canada.British Columbia|BOLD:AAA4141
Aseptis binotata|[18110]|RDNMC644-06|CNCNoctuoidea12184|519[0n]bp|Canada.British Columbia|BOLD:AAA4141
Aseptis binotata|[18111]|LPVIB016-08|PFC-2006-2507|658[0n]bp|Canada.British Columbia|BOLD:AAA4141
Aseptis binotata|[18112]|LPVIA561-08|PFC-2006-0788|658[0n]bp|Canada.British Columbia|BOLD:AAA4141
Aseptis binotata|[18113]|LHLEP437-06|UBC-2006-1820|658[0n]bp|Canada.British Columbia|BOLD:AAA4141
Aseptis binotata|[18114]|LALPA1313-11|AVBC 1315-11|658[0n]bp|Canada.British Columbia|BOLD:AAA4141
Aseptis binotata|[18115]|LALPA1196-11|AVBC 1198-11|658[0n]bp|Canada.British Columbia|BOLD:AAA4141
Aseptis binotata|[18116]|LPVIA563-08|PFC-2006-0790|658[0n]bp|Canada.British Columbia|BOLD:AAA4141
Aseptis binotata|[18117]|LBCS266-07|UBC-2007-0774|658[0n]bp|Canada.British Columbia|BOLD:AAA4141
Aseptis binotata|[18118]|LPVIB509-08|PFC-2006-1920|658[0n]bp|Canada.British Columbia|BOLD:AAA4141
Aseptis binotata|[18119]|LALPA530-10|AVBC 532-10|658[0n]bp|Canada.British Columbia|BOLD:AAA4141
Aseptis binotata|[18120]|LPVIB232-08|PFC-2006-1610|658[0n]bp|Canada.British Columbia|BOLD:AAA4141
Aseptis binotata|[18121]|LPVIB262-08|PFC-2006-1652|658[0n]bp|Canada.British Columbia|BOLD:AAA4141
Aseptis binotata|[18122]|LPVIB074-08|PFC-2006-1422|658[0n]bp|Canada.British Columbia|BOLD:AAA4141
Aseptis binotata|[18123]|LPVIB263-08|PFC-2006-1653|658[0n]bp|Canada.British Columbia|BOLD:AAA4141
Aseptis binotata|[18124]|LALPA1010-11|AVBC 1183-11|658[0n]bp|Canada.British Columbia|BOLD:AAA4141
Aseptis binotata|[18125]|LPVIA837-08|PFC-2006-1134|658[0n]bp|Canada.British Columbia|BOLD:AAA4141
Aseptis binotata|[18126]|LPVIA562-08|PFC-2006-0789|658[0n]bp|Canada.British Columbia|BOLD:AAA4141
Aseptis binotata|[18127]|LPVIB251-08|PFC-2006-1641|658[0n]bp|Canada.British Columbia|BOLD:AAA4141
Aseptis binotata|[18128]|RDNME031-07|TMustelin#314|609[0n]bp|Canada.British Columbia|BOLD:AAA4141
Aseptis binotata|[18129]|LBCD294-05|HLC-23114|617[0n]bp|Canada.British Columbia|BOLD:AAA4141
Aseptis binotata|[18130]|LBCD283-05|HLC-23103|616[0n]bp|Canada.British Columbia|BOLD:AAA4141
Aseptis binotata|[18131]|LBCB061-05|HLC-21001|658[0n]bp|Canada.British Columbia|BOLD:AAA4141
Aseptis binotata|[18132]|LBCC328-05|HLC-22208|658[0n]bp|Canada.British Columbia|BOLD:AAA4141
Aseptis binotata|[18133]|LBCC645-05|HLC-22525|658[0n]bp|Canada.British Columbia|BOLD:AAA4141
Aseptis binotata|[18134]|LOWCB442-05|CGWC-1382|658[0n]bp|Canada.British Columbia|BOLD:AAA4141
Aseptis binotata|[18135]|LOWCB451-05|CGWC-1391|658[0n]bp|Canada.British Columbia|BOLD:AAA4141
Aseptis binotata|[18136]|LPMN950-08|08BBLEP-02308|658[0n]bp|Canada.Alberta|BOLD:AAA4141
Aseptis binotata|[18137]|LBCD286-05|HLC-23106|658[0n]bp|Canada.British Columbia|BOLD:AAA4141
Aseptis binotata|[18138]|LBCC330-05|HLC-22210|658[0n]bp|Canada.British Columbia|BOLD:AAA4141
Aseptis binotata|[18139]|LOWCB448-05|CGWC-1388|658[0n]bp|Canada.British Columbia|BOLD:AAA4141
Aseptis binotata|[18140]|LBCC438-05|HLC-22318|658[0n]bp|Canada.British Columbia|BOLD:AAA4141

[illegible]
Aseptis binotata|[18138]|LBCC330-05|HLC-22210|658[0n]bp|Canada.British Columbia|BOLD:AAA4141
Aseptis binotata|[18139]|LOWCB448-05|CGWC-1388|658[0n]bp|Canada.British Columbia|BOLD:AAA4141
Aseptis binotata|[18140]|LBCC438-05|HLC-22318|658[0n]bp|Canada.British Columbia|BOLD:AAA4141
Aseptis binotata|[18141]|LBCC332-05|HLC-22212|658[0n]bp|Canada.British Columbia|BOLD:AAA4141
Aseptis binotata|[18142]|LBCC437-05|HLC-22317|658[0n]bp|Canada.British Columbia|BOLD:AAA4141
Aseptis binotata|[18143]|LOWCB449-05|CGWC-1389|658[0n]bp|Canada.British Columbia|BOLD:AAA4141
Aseptis binotata|[18144]|LPAB085-08|08BBLEP-02407|658[0n]bp|Canada.Alberta|BOLD:AAA4141
Aseptis binotata|[18145]|LALPA1193-11|AVBC 1195-11|658[0n]bp|Canada.British Columbia|BOLD:AAA4141
Aseptis binotata|[18146]|LOWCB447-05|CGWC-1387|658[0n]bp|Canada.British Columbia|BOLD:AAA4141
Aseptis binotata|[18147]|LBCD295-05|HLC-23115|658[0n]bp|Canada.British Columbia|BOLD:AAA4141
Aseptis binotata|[18148]|LPABC851-09|08BBLEP-05262|658[0n]bp|Canada.Alberta|BOLD:AAA4141
Aseptis binotata|[18149]|LBCD285-05|HLC-23105|658[0n]bp|Canada.British Columbia|BOLD:AAA4141
Aseptis binotata|[18150]|LBCC337-05|HLC-22217|658[0n]bp|Canada.British Columbia|BOLD:AAA4141
Aseptis binotata|[18151]|LBCD278-05|HLC-23098|658[0n]bp|Canada.British Columbia|BOLD:AAA4141
Aseptis binotata|[18152]|LPABB628-08|08BBLEP-03893|658[0n]bp|Canada.Alberta|BOLD:AAA4141
Aseptis binotata|[18153]|LBCC764-05|HLC-22644|658[0n]bp|Canada.British Columbia|BOLD:AAA4141
Aseptis binotata|[18154]|LPAB086-08|08BBLEP-02408|658[0n]bp|Canada.Alberta|BOLD:AAA4141
Aseptis binotata|[18155]|LOWCB444-05|CGWC-1384|658[0n]bp|Canada.British Columbia|BOLD:AAA4141
Aseptis binotata|[18156]|LHLEP432-06|UBC-2006-1521|658[0n]bp|Canada.British Columbia|BOLD:AAA4141
Aseptis binotata|[18157]|LBCB062-05|HLC-21002|658[0n]bp|Canada.British Columbia|BOLD:AAA4141
Aseptis binotata|[18158]|LPABC830-09|08BBLEP-05049|658[0n]bp|Canada.Alberta|BOLD:AAA4141
Aseptis binotata|[18159]|LPABC924-09|08BBLEP-05335|658[0n]bp|Canada.Alberta|BOLD:AAA4141
Aseptis binotata|[18160]|LOWCB446-05|CGWC-1386|658[0n]bp|Canada.British Columbia|BOLD:AAA4141
Aseptis binotata|[18161]|LBCC441-05|HLC-22321|658[0n]bp|Canada.British Columbia|BOLD:AAA4141
Aseptis binotata|[18162]|LBCD424-05|HLC-23244|658[0n]bp|Canada.British Columbia|BOLD:AAA4141
Aseptis binotata|[18163]|LBCC333-05|HLC-22213|658[0n]bp|Canada.British Columbia|BOLD:AAA4141
Aseptis binotata|[18164]|LBCD276-05|HLC-23096|658[0n]bp|Canada.British Columbia|BOLD:AAA4141
Aseptis binotata|[18165]|LBCD274-05|HLC-23094|658[0n]bp|Canada.British Columbia|BOLD:AAA4141
Aseptis binotata|[18166]|LPABC992-09|08BBLEP-05403|658[0n]bp|Canada.Alberta|BOLD:AAA4141
Aseptis binotata|[18167]|LOWCB443-05|CGWC-1383|658[0n]bp|Canada.British Columbia|BOLD:AAA4141
Aseptis binotata|[18168]|LBCD277-05|HLC-23097|658[1n]bp|Canada.British Columbia|BOLD:AAA4141
Aseptis binotata|[18169]|LOWCD527-06|CGWC-3347|623[0n]bp|Canada.British Columbia|BOLD:AAA4141
Aseptis binotata|[18170]|LOWCB440-05|CGWC-1380|590[1n]bp|Canada.British Columbia|BOLD:AAA4141
Aseptis binotata|[18171]|LOWCB441-05|CGWC-1381|587[0n]bp|Canada.British Columbia|BOLD:AAA4141
Aseptis binotata|[18172]|LHLEP570-06|UBC-2006-0756|658[0n]bp|Canada.British Columbia|BOLD:AAA4141
Aseptis binotata|[18173]|LHLEP433-06|UBC-2006-1527|658[0n]bp|Canada.British Columbia|BOLD:AAA4141
Aseptis binotata|[18174]|LALPA514-10|AVBC 516-10|658[0n]bp|Canada.British Columbia|BOLD:AAA4141
Aseptis binotata|[18175]|LOWCC205-05|CGWC-2085|658[0n]bp|Canada.British Columbia|BOLD:AAA4141
Aseptis binotata|[18176]|LBCD296-05|HLC-23116|658[0n]bp|Canada.British Columbia|BOLD:AAA4141
Aseptis binotata|[18177]|LOWCB450-05|CGWC-1390|658[0n]bp|Canada.British Columbia|BOLD:AAA4141
Aseptis binotata|[18178]|LBCC329-05|HLC-22209|658[0n]bp|Canada.British Columbia|BOLD:AAA4141
Aseptis binotata|[18179]|LOWCE859-06|CGWC-4619|658[0n]bp|Canada.British Columbia|BOLD:AAA4141
Aseptis binotata|[18180]|LOWCB439-05|CGWC-1379|658[0n]bp|Canada.British Columbia|BOLD:AAA4141
Aseptis characta|[18181]|RDNMC669-06|CNCNoctuoidea12209|658[0n]bp|Canada.British Columbia|BOLD:AAA6683
Aseptis characta|[18182]|RDMAB134-05|UASM41278|658[0n]bp|Canada.Alberta|BOLD:AAA6683
Aseptis characta|[18183]|RDNMC611-06|CNCNoctuoidea12151|658[0n]bp|Canada.Alberta|BOLD:AAA6683
Aseptis characta|[18184]|RDNMC617-06|CNCNoctuoidea12157|658[0n]bp|Canada.British Columbia|BOLD:AAA6683
Aseptis characta|[18185]|RDMAB039-05|UASM57522|558[0n]bp|Canada.Alberta|BOLD:AAA6683
Aseptis fumosa|[18186]|LOPN160-06|JCM-OSU-0160|526[0n]bp|United States.Oregon|BOLD:AAC3839
Aseptis fumosa|[18187]|LOPN158-06|JCM-OSU-0158|581[0n]bp|United States.Oregon|BOLD:AAC3839
Aseptis fumosa|[18188]|RDNME004-07|TMustelin#33|609[0n]bp|United States.California|BOLD:AAC3839
Aseptis fumosa|[18189]|RDNME014-07|TMustelin#172|609[0n]bp|United States.California|BOLD:AAC3839
Aseptis fumosa|[18190]|RDNME025-07|TMustelin#306|609[0n]bp|United States.California|BOLD:AAC3839
Aseptis fumosa|[18191]|LOPN159-06|JCM-OSU-0159|589[0n]bp|United States.Oregon|BOLD:AAC3839
Aseptis fumosa|[18192]|RDNMC619-06|CNCNoctuoidea12159|593[0n]bp|United States.Nevada|BOLD:AAC3839
Aseptis fumosa|[18193]|LOPN161-06|JCM-OSU-0161|592[1n]bp|United States.Oregon|BOLD:AAC3839
Aseptis fumosa|[18194]|BBLOC1212-11|BIOUG01540-E01|658[0n]bp|United States.California|BOLD:AAC3839
Aseptis fumosa|[18195]|RDNMC621-06|CNCNoctuoidea12161|658[0n]bp|United States.California|BOLD:AAC3839
Aseptis fumosa|[18196]|BBLOC207-11|BIOUG01454-B10|658[0n]bp|United States.Texas|BOLD:AAC3839
Aseptis fumosa|[18197]|BBLOC942-11|BIOUG01467-F09|658[0n]bp|United States.Texas|BOLD:AAC3839
Aseptis fumosa|[18198]|RDNMC623-06|CNCNoctuoidea12163|658[0n]bp|United States.California|BOLD:AAC3839
Aseptis fumosa|[18199]|BBLOC215-11|BIOUG01454-C06|658[0n]bp|United States.Texas|BOLD:AAC3839
Aseptis fumosa|[18200]|LOCBD211-06|06-BLLOC-3031|658[0n]bp|United States.California|BOLD:AAC3839
Aseptis fumosa|[18201]|RDNMC622-06|CNCNoctuoidea12162|658[0n]bp|United States.Nevada|BOLD:AAC3839
Aseptis fumosa|[18202]|BBLOC1269-11|BIOUG01541-A11|658[0n]bp|United States.California|BOLD:AAC3839
Cosmia calami|[18203]|XAK298-06|2006-ONT-1293|658[0n]bp|Canada.Ontario|BOLD:AAA4468
Cosmia calami|[18204]|RDLQB742-05|DH010657|658[0n]bp|Canada.Quebec|BOLD:AAA4468
Cosmia calami|[18205]|RDLQB526-05|DH010612|658[0n]bp|Canada.Quebec|BOLD:AAA4468
Cosmia calami|[18206]|LPMNB396-09|08BBLEP-05240|658[0n]bp|Canada.Manitoba|BOLD:AAA4468
Cosmia calami|[18207]|LPMNB349-09|08BBLEP-05193|658[0n]bp|Canada.Manitoba|BOLD:AAA4468
Cosmia calami|[18208]|LPMNB344-09|08BBLEP-05188|658[0n]bp|Canada.Manitoba|BOLD:AAA4468
Cosmia calami|[18209]|LPMNB399-09|08BBLEP-05243|658[0n]bp|Canada.Manitoba|BOLD:AAA4468
Cosmia calami|[18210]|LPMNB385-09|08BBLEP-05229|658[0n]bp|Canada.Manitoba|BOLD:AAA4468
Cosmia calami|[18211]|LPMNB383-09|08BBLEP-05227|658[0n]bp|Canada.Manitoba|BOLD:AAA4468
Cosmia calami|[18212]|LPMNB367-09|08BBLEP-05211|658[0n]bp|Canada.Manitoba|BOLD:AAA4468
Cosmia calami|[18213]|LPMNB371-09|08BBLEP-05215|658[0n]bp|Canada.Manitoba|BOLD:AAA4468
Cosmia calami|[18214]|LPMNB379-09|08BBLEP-05223|658[0n]bp|Canada.Manitoba|BOLD:AAA4468
Cosmia calami|[18215]|LPMNB408-09|08BBLEP-05252|658[0n]bp|Canada.Manitoba|BOLD:AAA4468
Cosmia calami|[18216]|LPMNB388-09|08BBLEP-05232|658[0n]bp|Canada.Manitoba|BOLD:AAA4468
Cosmia calami|[18217]|LPMNB480-09|08BBLEP-05518|658[0n]bp|Canada.Manitoba|BOLD:AAA4468
Cosmia calami|[18218]|LPMNB475-09|08BBLEP-05513|658[0n]bp|Canada.Manitoba|BOLD:AAA4468
Cosmia calami|[18219]|LPMNB414-09|08BBLEP-05258|658[0n]bp|Canada.Manitoba|BOLD:AAA4468
Cosmia calami|[18220]|LPMNB361-09|08BBLEP-05205|658[0n]bp|Canada.Manitoba|BOLD:AAA4468
Cosmia calami|[18221]|LPMNB345-09|08BBLEP-05189|658[0n]bp|Canada.Manitoba|BOLD:AAA4468
Cosmia calami|[18222]|LPMNB401-09|08BBLEP-05245|658[0n]bp|Canada.Manitoba|BOLD:AAA4468
Cosmia calami|[18223]|LPMNB366-09|08BBLEP-05210|658[0n]bp|Canada.Manitoba|BOLD:AAA4468
Cosmia calami|[18224]|LPMNB393-09|08BBLEP-05237|658[0n]bp|Canada.Manitoba|BOLD:AAA4468
Cosmia calami|[18225]|LPMNB333-09|08BBLEP-05177|658[0n]bp|Canada.Manitoba|BOLD:AAA4468
Cosmia calami|[18226]|LPMNB481-09|08BBLEP-05519|658[0n]bp|Canada.Manitoba|BOLD:AAA4468
Cosmia calami|[18227]|LPMNB381-09|08BBLEP-05225|658[0n]bp|Canada.Manitoba|BOLD:AAA4468
Cosmia calami|[18228]|LPMNB387-09|08BBLEP-05231|658[0n]bp|Canada.Manitoba|BOLD:AAA4468
Cosmia calami|[18229]|LPMNB373-09|08BBLEP-05217|658[0n]bp|Canada.Manitoba|BOLD:AAA4468
Cosmia calami|[18230]|LPMNB390-09|08BBLEP-05234|637[0n]bp|Canada.Manitoba|BOLD:AAA4468
Cosmia calami|[18231]|LPMNB374-09|08BBLEP-05218|609[0n]bp|Canada.Manitoba|BOLD:AAA4468
Cosmia calami|[18232]|LPMNB394-09|08BBLEP-05238|609[0n]bp|Canada.Manitoba|BOLD:AAA4468
Cosmia calami|[18233]|LPMNB409-09|08BBLEP-05253|658[0n]bp|Canada.Manitoba|BOLD:AAA4468
Cosmia calami|[18234]|LPMNB365-09|08BBLEP-05209|658[0n]bp|Canada.Manitoba|BOLD:AAA4468
Cosmia calami|[18235]|LPMNB402-09|08BBLEP-05246|658[0n]bp|Canada.Manitoba|BOLD:AAA4468
Cosmia calami|[18236]|LPMNB369-09|08BBLEP-05213|658[0n]bp|Canada.Manitoba|BOLD:AAA4468
Cosmia calami|[18237]|LPMNB357-09|08BBLEP-05201|658[0n]bp|Canada.Manitoba|BOLD:AAA4468
Cosmia calami|[18238]|LPMNB476-09|08BBLEP-05514|658[0n]bp|Canada.Manitoba|BOLD:AAA4468
Cosmia calami|[18239]|LPMNB407-09|08BBLEP-05251|658[0n]bp|Canada.Manitoba|BOLD:AAA4468
[illegible]

Cosmia calami|[18237]|LPMNB537-09|08BBLEP-05201|658[0n]bp|Canada.Manitoba|BOLD:AAA4468
Cosmia calami|[18238]|LPMNB476-09|08BBLEP-05514|658[0n]bp|Canada.Manitoba|BOLD:AAA4468
Cosmia calami|[18239]|LPMNB407-09|08BBLEP-05251|658[0n]bp|Canada.Manitoba|BOLD:AAA4468
Cosmia calami|[18240]|BBLEC140-09|09BBELE-0140|658[0n]bp|Canada.Nova Scotia|BOLD:AAA4468
Cosmia calami|[18241]|BBLEC908-09|09BBELE-0908|658[0n]bp|Canada.Nova Scotia|BOLD:AAA4468
Cosmia calami|[18242]|BBLEC916-09|09BBELE-0916|658[0n]bp|Canada.Nova Scotia|BOLD:AAA4468
Cosmia calami|[18243]|BBLPE562-09|09BBELE-2562|658[0n]bp|Canada.Nova Scotia|BOLD:AAA4468
Cosmia calami|[18244]|BBLPE571-09|09BBELE-2571|658[0n]bp|Canada.Nova Scotia|BOLD:AAA4468
Cosmia calami|[18245]|BBLEC907-09|09BBELE-0907|658[0n]bp|Canada.Nova Scotia|BOLD:AAA4468
Cosmia calami|[18246]|BBLPE620-09|09BBELE-2620|658[0n]bp|Canada.Nova Scotia|BOLD:AAA4468
Cosmia calami|[18247]|BBLPE586-09|09BBELE-2586|658[0n]bp|Canada.Nova Scotia|BOLD:AAA4468
Cosmia calami|[18248]|BBLEC945-09|09BBELE-0945|658[0n]bp|Canada.Nova Scotia|BOLD:AAA4468
Cosmia calami|[18249]|BBLEC934-09|09BBELE-0934|658[0n]bp|Canada.Nova Scotia|BOLD:AAA4468
Cosmia calami|[18250]|BBLEC155-09|09BBELE-0155|658[0n]bp|Canada.Nova Scotia|BOLD:AAA4468
Cosmia calami|[18251]|BBLPE607-09|09BBELE-2607|658[0n]bp|Canada.Nova Scotia|BOLD:AAA4468
Cosmia calami|[18252]|BBLPE600-09|09BBELE-2600|658[0n]bp|Canada.Nova Scotia|BOLD:AAA4468
Cosmia calami|[18253]|BBLPE595-09|09BBELE-2595|658[0n]bp|Canada.Nova Scotia|BOLD:AAA4468
Cosmia calami|[18254]|BBLPE615-09|09BBELE-2615|658[0n]bp|Canada.Nova Scotia|BOLD:AAA4468
Cosmia calami|[18255]|BBLEC221-09|09BBELE-0221|636[0n]bp|Canada.Nova Scotia|BOLD:AAA4468
Cosmia calami|[18256]|BBLEC730-09|09BBELE-0730|632[0n]bp|Canada.Nova Scotia|BOLD:AAA4468
Cosmia calami|[18257]|BBLEC142-09|09BBELE-0142|658[0n]bp|Canada.Nova Scotia|BOLD:AAA4468
Cosmia elisae|[18258]|LBCH4669-10|10-JDWBC-4669|658[0n]bp|Canada.British Columbia|BOLD:AAB0874
Cosmia elisae|[18259]|LBCH4049-10|10-JDWBC-4049|620[0n]bp|Canada.British Columbia|BOLD:AAB0874
Cosmia elisae|[18260]|LBCH4437-10|10-JDWBC-4437|658[0n]bp|Canada.British Columbia|BOLD:AAB0874
Cosmia elisae|[18261]|LBCD297-05|HLC-23117|616[0n]bp|Canada.British Columbia|BOLD:AAB0874
Cosmia elisae|[18262]|LOWCC474-05|CGWC-2354|580[0n]bp|Canada.British Columbia|BOLD:AAB0874
Cosmia elisae|[18263]|LOWCC048-05|CGWC-1928|658[0n]bp|Canada.British Columbia|BOLD:AAB0874
Cosmia elisae|[18264]|LOWCC050-05|CGWC-1930|658[0n]bp|Canada.British Columbia|BOLD:AAB0874
Cosmia elisae|[18265]|LOWCC052-05|CGWC-1932|658[0n]bp|Canada.British Columbia|BOLD:AAB0874
Cosmia elisae|[18266]|LBCD275-05|HLC-23095|658[0n]bp|Canada.British Columbia|BOLD:AAB0874
Cosmia elisae|[18267]|LOWCC043-05|CGWC-1923|658[0n]bp|Canada.British Columbia|BOLD:AAB0874
Cosmia elisae|[18268]|LOWCC045-05|CGWC-1925|658[0n]bp|Canada.British Columbia|BOLD:AAB0874
Cosmia elisae|[18269]|DUNLP149-08|Dun-08-149|658[0n]bp|Canada.British Columbia|BOLD:AAB0874
Cosmia elisae|[18270]|LBCH1240-10|10-JDWBC-1240|658[0n]bp|Canada.British Columbia|BOLD:AAB0874
Cosmia elisae|[18271]|LBCH6055-10|10-JDWBC-6055|658[0n]bp|Canada.British Columbia|BOLD:AAB0874
Cosmia elisae|[18272]|LOWCC055-05|CGWC-1935|658[0n]bp|Canada.British Columbia|BOLD:AAB0874
Cosmia elisae|[18273]|LBCH6008-10|10-JDWBC-6008|658[0n]bp|Canada.British Columbia|BOLD:AAB0874
Cosmia elisae|[18274]|LOWCC053-05|CGWC-1933|658[0n]bp|Canada.British Columbia|BOLD:AAB0874
Cosmia elisae|[18275]|LOWCB173-05|CGWC-1113|658[0n]bp|Canada.British Columbia|BOLD:AAB0874
Cosmia elisae|[18276]|LOWCC044-05|CGWC-1924|658[0n]bp|Canada.British Columbia|BOLD:AAB0874
Cosmia elisae|[18277]|LOWCB174-05|CGWC-1114|658[0n]bp|Canada.British Columbia|BOLD:AAB0874
Cosmia elisae|[18278]|LOWCC047-05|CGWC-1927|658[0n]bp|Canada.British Columbia|BOLD:AAB0874
Cosmia elisae|[18279]|LOWCC042-05|CGWC-1922|658[0n]bp|Canada.British Columbia|BOLD:AAB0874
Cosmia elisae|[18280]|LOWCC051-05|CGWC-1931|658[0n]bp|Canada.British Columbia|BOLD:AAB0874
Cosmia elisae|[18281]|RDMAB347-05|UASM477803|605[0n]bp|Canada.Alberta|BOLD:AAB0874
Cosmia elisae|[18282]|LBCH6007-10|10-JDWBC-6007|643[0n]bp|Canada.British Columbia|BOLD:AAB0874
Cosmia elisae|[18283]|LOWCC041-05|CGWC-1921|657[0n]bp|Canada.British Columbia|BOLD:AAB0874
Cosmia elisae|[18284]|LOWCC049-05|CGWC-1929|658[0n]bp|Canada.British Columbia|BOLD:AAB0874
Cosmia elisae|[18285]|LOWCC069-05|CGWC-1949|658[0n]bp|Canada.British Columbia|BOLD:AAB0874
Cosmia elisae|[18286]|LOWCC054-05|CGWC-1934|658[0n]bp|Canada.British Columbia|BOLD:AAB0874
Cosmia praeacuta|[18287]|LPVIA828-08|PFC-2006-1118|618[1n]bp|Canada.British Columbia|BOLD:AAB0874
Cosmia praeacuta|[18288]|LPVIB250-08|PFC-2006-1640|658[0n]bp|Canada.British Columbia|BOLD:AAB0874
Cosmia praeacuta|[18289]|LOWCC057-05|CGWC-1937|658[0n]bp|Canada.British Columbia|BOLD:AAB0874
Cosmia praeacuta|[18290]|LPVIB169-08|PFC-2006-1531|658[0n]bp|Canada.British Columbia|BOLD:AAB0874
Cosmia praeacuta|[18291]|LPVIB493-08|PFC-2006-1903|658[0n]bp|Canada.British Columbia|BOLD:AAB0874
Cosmia praeacuta|[18292]|LPVIA565-08|PFC-2006-0792|658[0n]bp|Canada.British Columbia|BOLD:AAB0874
Cosmia praeacuta|[18293]|LOWCC056-05|CGWC-1936|658[0n]bp|Canada.British Columbia|BOLD:AAB0874
Cosmia praeacuta|[18294]|LOWCC063-05|CGWC-1943|658[0n]bp|Canada.British Columbia|BOLD:AAB0874
Cosmia praeacuta|[18295]|LOWCC061-05|CGWC-1941|658[0n]bp|Canada.British Columbia|BOLD:AAB0874
Cosmia praeacuta|[18296]|LOWCC064-05|CGWC-1944|658[0n]bp|Canada.British Columbia|BOLD:AAB0874
Cosmia praeacuta|[18297]|LOWCC066-05|CGWC-1946|658[0n]bp|Canada.British Columbia|BOLD:AAB0874
Cosmia praeacuta|[18298]|LOWCC065-05|CGWC-1945|658[0n]bp|Canada.British Columbia|BOLD:AAB0874
Cosmia praeacuta|[18299]|LOWCC046-05|CGWC-1926|658[0n]bp|Canada.British Columbia|BOLD:AAB0874
Cosmia praeacuta|[18300]|LOWCC060-05|CGWC-1940|658[0n]bp|Canada.British Columbia|BOLD:AAB0874
Cosmia praeacuta|[18301]|LOWCD295-06|CGWC-3115|658[0n]bp|Canada.British Columbia|BOLD:AAB0874
Cosmia praeacuta|[18302]|LOWCC062-05|CGWC-1942|658[0n]bp|Canada.British Columbia|BOLD:AAB0874
Cosmia praeacuta|[18303]|LOWCC059-05|CGWC-1939|658[0n]bp|Canada.British Columbia|BOLD:AAB0874
Cosmia praeacuta|[18304]|LOWCC067-05|CGWC-1947|658[0n]bp|Canada.British Columbia|BOLD:AAB0874
Cosmia praeacuta|[18305]|LOWCC068-05|CGWC-1948|658[0n]bp|Canada.British Columbia|BOLD:AAB0874
Cosmia praeacuta|[18306]|LOWCC058-05|CGWC-1938|658[0n]bp|Canada.British Columbia|BOLD:AAB0874
Cosmia praeacuta|[18307]|LOWCC070-05|CGWC-1950|658[0n]bp|Canada.British Columbia|BOLD:AAB0874
Cosmia praeacuta|[18308]|LALPA1332-12|AVBC 1334-11|614[0n]bp|Canada.British Columbia|BOLD:AAB0874
Cosmia praeacuta|[18309]|LPVIA406-08|PFC-2006-0592|658[0n]bp|Canada.British Columbia|BOLD:AAB0874
Cosmia praeacuta|[18310]|LPVIB170-08|PFC-2006-1532|658[0n]bp|Canada.British Columbia|BOLD:AAB0874
Cosmia praeacuta|[18311]|LHLEP211-06|UBC-2006-1824|658[0n]bp|Canada.British Columbia|BOLD:AAB0874
Cosmia praeacuta|[18312]|LPVIA560-08|PFC-2006-0787|658[0n]bp|Canada.British Columbia|BOLD:AAB0874
Cosmia praeacuta|[18313]|LPVIA564-08|PFC-2006-0791|658[0n]bp|Canada.British Columbia|BOLD:AAB0874
Cosmia praeacuta|[18314]|LHLEP208-06|UBC-2006-1821|658[0n]bp|Canada.British Columbia|BOLD:AAB0874
Cosmia praeacuta|[18315]|LHLEP374-06|UBC-2006-1932|657[0n]bp|Canada.British Columbia|BOLD:AAB0874
Cosmia praeacuta|[18316]|LPVIC027-08|PFC-2006-2582|658[0n]bp|Canada.British Columbia|BOLD:AAB0874
Cosmia praeacuta|[18317]|LALPA647-10|AVBC 649-10|658[0n]bp|Canada.British Columbia|BOLD:AAB0874
Cosmia praeacuta|[18318]|LPVIB071-08|PFC-2006-1419|658[0n]bp|Canada.British Columbia|BOLD:AAB0874
Cosmia praeacuta|[18319]|LALPA664-10|AVBC 666-10|658[0n]bp|Canada.British Columbia|BOLD:AAB0874
Cosmia praeacuta|[18320]|LHLEP210-06|UBC-2006-1823|658[0n]bp|Canada.British Columbia|BOLD:AAB0874
Cosmia praeacuta|[18321]|LPVIA568-08|PFC-2006-0795|658[0n]bp|Canada.British Columbia|BOLD:AAB0874
Cosmia praeacuta|[18322]|LPVIA570-08|PFC-2006-0797|658[0n]bp|Canada.British Columbia|BOLD:AAB0874
Cosmia praeacuta|[18323]|LPVIA827-08|PFC-2006-1117|658[0n]bp|Canada.British Columbia|BOLD:AAB0874
Cosmia praeacuta|[18324]|LPVIB235-08|PFC-2006-1614|658[0n]bp|Canada.British Columbia|BOLD:AAB0874
Cosmia praeacuta|[18325]|LPVIB252-08|PFC-2006-1642|658[0n]bp|Canada.British Columbia|BOLD:AAB0874
Cosmia praeacuta|[18326]|LPVIC026-08|PFC-2006-2581|658[0n]bp|Canada.British Columbia|BOLD:AAB0874
Cosmia praeacuta|[18327]|LBCS264-07|UBC-2007-0772|658[0n]bp|Canada.British Columbia|BOLD:AAB0874
Cosmia praeacuta|[18328]|LHLEP439-06|UBC-2006-1936|658[0n]bp|Canada.British Columbia|BOLD:AAB0874
Cosmia praeacuta|[18329]|LALPA659-10|AVBC 661-10|658[0n]bp|Canada.British Columbia|BOLD:AAB0874
Cosmia praeacuta|[18330]|LHLEP571-06|UBC-2006-1389|658[0n]bp|Canada.British Columbia|BOLD:AAB0874
Cosmia praeacuta|[18331]|LBCS192-07|UBC-2007-0573|658[0n]bp|Canada.British Columbia|BOLD:AAB0874
Cosmia praeacuta|[18332]|LPVIA566-08|PFC-2006-0793|658[0n]bp|Canada.British Columbia|BOLD:AAB0874
Cosmia praeacuta|[18333]|LPVIB255-08|PFC-2006-1645|658[0n]bp|Canada.British Columbia|BOLD:AAB0874
Cosmia praeacuta|[18334]|LHLEP209-06|UBC-2006-1822|658[0n]bp|Canada.British Columbia|BOLD:AAB0874
Cosmia praeacuta|[18335]|LPVIA569-08|PFC-2006-0796|658[0n]bp|Canada.British Columbia|BOLD:AAB0874
Cosmia praeacuta|[18336]|LPVIA829-08|PFC-2006-1119|658[0n]bp|Canada.British Columbia|BOLD:AAB0874
Cosmia praeacuta|[18337]|LPVIB254-08|PFC-2006-1644|658[0n]bp|Canada.British Columbia|BOLD:AAB0874
Cosmia praeacuta|[18338]|LPVIB167-08|PFC-2006-1529|658[0n]bp|Canada.British Columbia|BOLD:AAB0874
Cosmia praeacuta|[18339]|LPVIA567-08|PFC-2006-0794|658[0n]bp|Canada.British Columbia|BOLD:AAB0874

Cosmia praeacuta|[18337]|LPVIB254-08|PFC-2006-1644|658[0n]bp|Canada.British Columbia|BOLD:AAB0874
Cosmia praeacuta|[18338]|LPVIB167-08|PFC-2006-1529|658[0n]bp|Canada.British Columbia|BOLD:AAB0874
Cosmia praeacuta|[18339]|LPVIA567-08|PFC-2006-0794|658[0n]bp|Canada.British Columbia|BOLD:AAB0874
Cosmia praeacuta|[18340]|LPVIB236-08|PFC-2006-1615|658[0n]bp|Canada.British Columbia|BOLD:AAB0874
Cosmia praeacuta|[18341]|LPVIB076-08|PFC-2006-1424|658[0n]bp|Canada.British Columbia|BOLD:AAB0874
Cosmia praeacuta|[18342]|LALPA599-10|AVBC 601-10|658[0n]bp|Canada.British Columbia|BOLD:AAB0874
Cosmia praeacuta|[18343]|LPVIA408-08|PFC-2006-0594|658[0n]bp|Canada.British Columbia|BOLD:AAB0874
Cosmia praeacuta|[18344]|LPVIB240-08|PFC-2006-1623|658[0n]bp|Canada.British Columbia|BOLD:AAB0874
Cosmia praeacuta|[18345]|LPVIB249-08|PFC-2006-1639|658[0n]bp|Canada.British Columbia|BOLD:AAB0874
Cosmia praeacuta|[18346]|LPVIB168-08|PFC-2006-1530|658[0n]bp|Canada.British Columbia|BOLD:AAB0874
Cosmia praeacuta|[18347]|LPVIC025-08|PFC-2006-2580|658[0n]bp|Canada.British Columbia|BOLD:AAB0874
Cosmia praeacuta|[18348]|DUNLP150-08|Dun-08-150|643[0n]bp|Canada.British Columbia|BOLD:AAB0874
Cosmia praeacuta|[18349]|LALPA658-10|AVBC 660-10|658[0n]bp|Canada.British Columbia|BOLD:AAB0874
Cosmia praeacuta|[18350]|LALPA645-10|AVBC 647-10|658[0n]bp|Canada.British Columbia|BOLD:AAB0874
Cosmia praeacuta|[18351]|LALPA556-10|AVBC 558-10|658[0n]bp|Canada.British Columbia|BOLD:AAB0874
Cosmia praeacuta|[18352]|LHLEP376-06|UBC-2006-1520|656[0n]bp|Canada.British Columbia|BOLD:AAB0874
Cosmia praeacuta|[18353]|LHLEP375-06|UBC-2006-1519|656[0n]bp|Canada.British Columbia|BOLD:AAB0874
Cosmia praeacuta|[18354]|LPVIA830-08|PFC-2006-1120|633[0n]bp|Canada.British Columbia|BOLD:AAB0874
Cosmia praeacuta|[18355]|LPVIA831-08|PFC-2006-1121|633[0n]bp|Canada.British Columbia|BOLD:AAB0874
Eucirroedia pampina|[18356]|LOWCD115-06|CGWC-2935|614[0n]bp|Canada.British Columbia|BOLD:AAB8057
Eucirroedia pampina|[18357]|TMNBB262-06|MNBTT-1202|658[0n]bp|Canada.New Brunswick|BOLD:AAB8057
Eucirroedia pampina|[18358]|TTMNB374-06|MNBTT-374|658[0n]bp|Canada.New Brunswick|BOLD:AAB8057
Eucirroedia pampina|[18359]|TMNBB264-06|MNBTT-1204|658[0n]bp|Canada.New Brunswick|BOLD:AAB8057
Eucirroedia pampina|[18360]|TMNBB266-06|MNBTT-1206|658[0n]bp|Canada.New Brunswick|BOLD:AAB8057
Eucirroedia pampina|[18361]|TMNBB265-06|MNBTT-1205|658[0n]bp|Canada.New Brunswick|BOLD:AAB8057
Eucirroedia pampina|[18362]|TMNBB268-06|MNBTT-1208|658[0n]bp|Canada.New Brunswick|BOLD:AAB8057
Eucirroedia pampina|[18363]|TMNBB263-06|MNBTT-1203|658[0n]bp|Canada.New Brunswick|BOLD:AAB8057
Eucirroedia pampina|[18364]|TMNBB270-06|MNBTT-1210|658[0n]bp|Canada.New Brunswick|BOLD:AAB8057
Eucirroedia pampina|[18365]|TMNBB261-06|MNBTT-1201|656[0n]bp|Canada.New Brunswick|BOLD:AAB8057
Eucirroedia pampina|[18366]|TMNBB267-06|MNBTT-1207|658[0n]bp|Canada.New Brunswick|BOLD:AAB8057
Eucirroedia pampina|[18367]|RDLQF262-06|DH011342|658[0n]bp|Canada.Quebec|BOLD:AAB8057
Eucirroedia pampina|[18368]|TMNBB271-06|MNBTT-1211|658[0n]bp|Canada.New Brunswick|BOLD:AAB8057
Eucirroedia pampina|[18369]|TMNBB269-06|MNBTT-1209|658[0n]bp|Canada.New Brunswick|BOLD:AAB8057
Chaetaglaea cerata|[18370]|RDNMG521-08|CNC LEP00052345|658[0n]bp|Canada.Ontario|BOLD:AAF4072
Chaetaglaea cerata|[18371]|RDNMF718-08|NOC14804|658[0n]bp|Canada.Ontario|BOLD:AAF4072
Chaetaglaea sericea|[18372]|RDNMG542-08|CNC LEP00052366|658[0n]bp|Canada.Ontario|BOLD:AAE1199
Chaetaglaea sericea|[18373]|RDNMG544-08|CNC LEP00052368|658[0n]bp|Canada.Ontario|BOLD:AAE1199
Chaetaglaea sericea|[18374]|RDNMG543-08|CNC LEP00052367|658[0n]bp|Canada.Ontario|BOLD:AAE1199
Chaetaglaea tremula|[18375]|RDNMG1031-08|CNC LEP00053155|658[0n]bp|Canada.Ontario|BOLD:AAE1201
Chaetaglaea tremula|[18376]|RDNMG1030-08|CNC LEP00053154|658[0n]bp|Canada.Ontario|BOLD:AAE1201
Chaetaglaea tremula|[18377]|RDNMG1029-08|CNC LEP00053153|658[0n]bp|Canada.Ontario|BOLD:AAE1201
Mesogona olivata|[18378]|LALPA1297-11|AVBC 1299-11|658[0n]bp|Canada.British Columbia|BOLD:ACE4866
Mesogona olivata|[18379]|LBCH7151-10|10-JDWBC-7151|658[0n]bp|Canada.British Columbia|BOLD:ACE4866
Mesogona olivata|[18380]|LBCH7250-10|10-JDWBC-7250|658[0n]bp|Canada.British Columbia|BOLD:ACE4866
Mesogona olivata|[18381]|LBCH7315-10|10-JDWBC-7315|642[0n]bp|Canada.British Columbia|BOLD:ACE4866
Mesogona olivata|[18382]|LPVIB643-08|PFC-2006-2091|631[0n]bp|Canada.British Columbia|BOLD:ACE4866
Mesogona olivata|[18383]|LPVIB642-08|PFC-2006-2090|641[0n]bp|Canada.British Columbia|BOLD:ACE4866
Mesogona subcuprea|[18384]|RDNMF483-08|NOC14569|658[0n]bp|United States.Nevada|BOLD:AAC4482
Mesogona subcuprea|[18385]|RDNMF484-08|NOC14570|658[0n]bp|United States.Oregon|BOLD:AAC4482
Mesogona subcuprea|[18386]|RDNMF482-08|NOC14568|658[0n]bp|United States.Washington|BOLD:AAC4482
Mesogona subcuprea|[18387]|RDNMF485-08|NOC14571|658[0n]bp|United States.California|BOLD:AAC4482
Mesogona subcuprea|[18388]|RDNMF481-08|NOC14567|658[0n]bp|United States.Washington|BOLD:AAC4482
Metaxaglaea inulta|[18389]|RDLQB504-05|DH010590|658[0n]bp|Canada.Quebec|BOLD:AAC1629
Metaxaglaea inulta|[18390]|RDLQF238-06|DH011318|658[0n]bp|Canada.Quebec|BOLD:AAC1629
Metaxaglaea inulta|[18391]|RDLQF239-06|DH011319|656[0n]bp|Canada.Quebec|BOLD:AAC1629
Metaxaglaea inulta|[18392]|RDLQF235-06|DH011315|658[0n]bp|Canada.Quebec|BOLD:AAC1629
Metaxaglaea inulta|[18393]|PHMO360-03|moth2751.02|639[0n]bp|Canada.Ontario|BOLD:AAC1629
Metaxaglaea inulta|[18394]|XAH700-05|2005-ONT-2283|658[0n]bp|Canada.Ontario|BOLD:AAC1629
Metaxaglaea inulta|[18395]|PHMO190-03|moth970.02|639[1n]bp|Canada.Ontario|BOLD:AAC1629
Metaxaglaea inulta|[18396]|PHMO329-03|moth2560.02|639[2n]bp|Canada.Ontario|BOLD:AAC1629
Metaxaglaea inulta|[18397]|TMNBB252-06|MNBTT-1192|658[0n]bp|Canada.New Brunswick|BOLD:AAC1629
Metaxaglaea inulta|[18398]|TTMNB373-06|MNBTT-373|658[0n]bp|Canada.New Brunswick|BOLD:AAC1629
Metaxaglaea inulta|[18399]|TMNBB254-06|MNBTT-1194|656[0n]bp|Canada.New Brunswick|BOLD:AAC1629
Metaxaglaea inulta|[18400]|TMNBB253-06|MNBTT-1193|658[0n]bp|Canada.New Brunswick|BOLD:AAC1629
Hillia iris|[18401]|LCHQ823-08|07WNP-10715|658[0n]bp|Canada.Manitoba|BOLD:AAA1264
Hillia iris|[18402]|LCHQ717-08|07WNP-10609|658[0n]bp|Canada.Manitoba|BOLD:AAA1264
Hillia iris|[18403]|LCHQ744-08|07WNP-10636|656[0n]bp|Canada.Manitoba|BOLD:AAA1264
Hillia iris|[18404]|LCHIP092-07|06-PROBE-2573|650[0n]bp|Canada.Manitoba|BOLD:AAA1264
Hillia iris|[18405]|LCHIP123-07|06-PROBE-2644|650[0n]bp|Canada.Manitoba|BOLD:AAA1264
Hillia iris|[18406]|LCHIP212-07|06-PROBE-2837|650[0n]bp|Canada.Manitoba|BOLD:AAA1264
Hillia iris|[18407]|LCHIP198-07|06-PROBE-2823|650[0n]bp|Canada.Manitoba|BOLD:AAA1264
Hillia iris|[18408]|LCHIP085-07|06-PROBE-2566|650[0n]bp|Canada.Manitoba|BOLD:AAA1264
Hillia iris|[18409]|LCHQ294-08|07WNP-10186|658[0n]bp|Canada.Manitoba|BOLD:AAA1264
Hillia iris|[18410]|LCHQ806-08|07WNP-10698|657[0n]bp|Canada.Manitoba|BOLD:AAA1264
Hillia iris|[18411]|LCHQ228-08|07WNP-10120|658[0n]bp|Canada.Manitoba|BOLD:AAA1264
Hillia iris|[18412]|LCHIP039-07|06-PROBE-0163|650[1n]bp|Canada.Manitoba|BOLD:AAA1264
Hillia iris|[18413]|LCHQ308-08|07WNP-10200|657[0n]bp|Canada.Manitoba|BOLD:AAA1264
Hillia iris|[18414]|LCHQ272-08|07WNP-10164|656[0n]bp|Canada.Manitoba|BOLD:AAA1264
Hillia iris|[18415]|LCHQ838-08|07WNP-10730|658[0n]bp|Canada.Manitoba|BOLD:AAA1264
Hillia iris|[18416]|LCHIP254-07|06-PROBE-2918|650[0n]bp|Canada.Manitoba|BOLD:AAA1264
Hillia iris|[18417]|MHLEP122-07|CHU06-LEP-122|658[0n]bp|Canada.Manitoba|BOLD:AAA1264
Hillia iris|[18418]|LCHIP121-07|06-PROBE-2641|650[0n]bp|Canada.Manitoba|BOLD:AAA1264
Hillia iris|[18419]|LCHQ379-08|07WNP-10271|658[0n]bp|Canada.Manitoba|BOLD:AAA1264
Hillia iris|[18420]|LCHQ821-08|07WNP-10713|658[0n]bp|Canada.Manitoba|BOLD:AAA1264
Hillia iris|[18421]|LCHQ374-08|07WNP-10266|658[0n]bp|Canada.Manitoba|BOLD:AAA1264
Hillia iris|[18422]|LCHIP034-07|06-PROBE-0158|650[0n]bp|Canada.Manitoba|BOLD:AAA1264
Hillia iris|[18423]|MHLEP120-07|CHU06-LEP-120|658[0n]bp|Canada.Manitoba|BOLD:AAA1264
Hillia iris|[18424]|LCHQ836-08|07WNP-10728|658[0n]bp|Canada.Manitoba|BOLD:AAA1264
Hillia iris|[18425]|LCHQ728-08|07WNP-10620|658[0n]bp|Canada.Manitoba|BOLD:AAA1264
Hillia iris|[18426]|LCHIP090-07|06-PROBE-2571|650[0n]bp|Canada.Manitoba|BOLD:AAA1264
Hillia iris|[18427]|LCHQ410-08|07WNP-10302|658[0n]bp|Canada.Manitoba|BOLD:AAA1264
Hillia iris|[18428]|LCHQ443-08|07WNP-10335|658[0n]bp|Canada.Manitoba|BOLD:AAA1264
Hillia iris|[18429]|LCHQ438-08|07WNP-10330|658[0n]bp|Canada.Manitoba|BOLD:AAA1264
Hillia iris|[18430]|LCHQ398-08|07WNP-10290|657[0n]bp|Canada.Manitoba|BOLD:AAA1264
Hillia iris|[18431]|LCHIP027-07|06-PROBE-0109|650[0n]bp|Canada.Manitoba|BOLD:AAA1264
Hillia iris|[18432]|LCHQ764-08|07WNP-10656|621[0n]bp|Canada.Manitoba|BOLD:AAA1264
Hillia iris|[18433]|LCHIP213-07|06-PROBE-2844|650[0n]bp|Canada.Manitoba|BOLD:AAA1264
Hillia iris|[18434]|LCHQ787-08|07WNP-10679|656[0n]bp|Canada.Manitoba|BOLD:AAA1264
Hillia iris|[18435]|LCHQ275-08|07WNP-10167|658[0n]bp|Canada.Manitoba|BOLD:AAA1264
Hillia iris|[18436]|LCHQ710-08|07WNP-10602|621[0n]bp|Canada.Manitoba|BOLD:AAA1264
Hillia iris|[18437]|LCHIP235-07|06-PROBE-2881|650[0n]bp|Canada.Manitoba|BOLD:AAA1264
Hillia iris|[18438]|LCHQ594-08|07WNP-10486|658[0n]bp|Canada.Manitoba|BOLD:AAA1264
Hillia iris|[18439]|LCHQ711-08|07WNP-10603|658[0n]bp|Canada.Manitoba|BOLD:AAA1264

[illegible]
Hillia iris|[18437]|LCHIP235-07|06-PROBE-2881|650[0n]bp|Canada.Manitoba|BOLD:AAA1264
Hillia iris|[18438]|LCHQ594-08|07WNP-10486|658[0n]bp|Canada.Manitoba|BOLD:AAA1264
Hillia iris|[18439]|LCHQ711-08|07WNP-10603|658[0n]bp|Canada.Manitoba|BOLD:AAA1264
Hillia iris|[18440]|LCHQ723-08|07WNP-10615|658[0n]bp|Canada.Manitoba|BOLD:AAA1264
Hillia iris|[18441]|LCHQ281-08|07WNP-10173|658[0n]bp|Canada.Manitoba|BOLD:AAA1264
Hillia iris|[18442]|LCHQ439-08|07WNP-10331|658[0n]bp|Canada.Manitoba|BOLD:AAA1264
Hillia iris|[18443]|MHLEP125-07|CHU06-LEP-125|658[0n]bp|Canada.Manitoba|BOLD:AAA1264
Hillia iris|[18444]|LCHIP258-07|06-PROBE-2922|650[0n]bp|Canada.Manitoba|BOLD:AAA1264
Hillia iris|[18445]|LCHQ286-08|07WNP-10178|658[0n]bp|Canada.Manitoba|BOLD:AAA1264
Hillia iris|[18446]|LCHQ748-08|07WNP-10640|632[0n]bp|Canada.Manitoba|BOLD:AAA1264
Hillia iris|[18447]|LCHIP037-07|06-PROBE-0161|650[0n]bp|Canada.Manitoba|BOLD:AAA1264
Hillia iris|[18448]|LCHIP020-07|06-PROBE-0040|650[0n]bp|Canada.Manitoba|BOLD:AAA1264
Hillia iris|[18449]|LCHQ609-08|07WNP-10501|657[0n]bp|Canada.Manitoba|BOLD:AAA1264
Hillia iris|[18450]|LCHIP149-07|06-PROBE-2691|650[1n]bp|Canada.Manitoba|BOLD:AAA1264
Hillia iris|[18451]|LCHQ833-08|07WNP-10725|658[0n]bp|Canada.Manitoba|BOLD:AAA1264
Hillia iris|[18452]|LCHQ271-08|07WNP-10163|658[0n]bp|Canada.Manitoba|BOLD:AAA1264
Hillia iris|[18453]|LCHQ828-08|07WNP-10720|658[0n]bp|Canada.Manitoba|BOLD:AAA1264
Hillia iris|[18454]|LCHQ741-08|07WNP-10633|658[0n]bp|Canada.Manitoba|BOLD:AAA1264
Hillia iris|[18455]|LCHQ835-08|07WNP-10727|656[0n]bp|Canada.Manitoba|BOLD:AAA1264
Hillia iris|[18456]|LCHIP059-07|06-PROBE-2528|650[0n]bp|Canada.Manitoba|BOLD:AAA1264
Hillia iris|[18457]|LCHIP107-07|06-PROBE-2593|650[0n]bp|Canada.Manitoba|BOLD:AAA1264
Hillia iris|[18458]|LCHIP012-07|06-PROBE-0032|650[0n]bp|Canada.Manitoba|BOLD:AAA1264
Hillia iris|[18459]|LCHIP064-07|06-PROBE-2533|650[0n]bp|Canada.Manitoba|BOLD:AAA1264
Hillia iris|[18460]|LCHIP066-07|06-PROBE-2535|650[0n]bp|Canada.Manitoba|BOLD:AAA1264
Hillia iris|[18461]|LCHQ232-08|07WNP-10124|658[0n]bp|Canada.Manitoba|BOLD:AAA1264
Hillia iris|[18462]|LCHQ596-08|07WNP-10488|657[0n]bp|Canada.Manitoba|BOLD:AAA1264
Hillia iris|[18463]|LCHQ402-08|07WNP-10294|657[0n]bp|Canada.Manitoba|BOLD:AAA1264
Hillia iris|[18464]|LCHQ406-08|07WNP-10298|639[1n]bp|Canada.Manitoba|BOLD:AAA1264
Hillia iris|[18465]|LCHQ434-08|07WNP-10326|656[0n]bp|Canada.Manitoba|BOLD:AAA1264
Hillia iris|[18466]|LCHQ844-08|07WNP-10736|658[0n]bp|Canada.Manitoba|BOLD:AAA1264
Hillia iris|[18467]|LCHQ822-08|07WNP-10714|658[0n]bp|Canada.Manitoba|BOLD:AAA1264
Hillia iris|[18468]|LCHIP111-07|06-PROBE-2598|650[1n]bp|Canada.Manitoba|BOLD:AAA1264
Hillia iris|[18469]|LCHQ721-08|07WNP-10613|658[0n]bp|Canada.Manitoba|BOLD:AAA1264
Hillia iris|[18470]|LCHQ284-08|07WNP-10176|658[0n]bp|Canada.Manitoba|BOLD:AAA1264
Hillia iris|[18471]|LCHQ301-08|07WNP-10193|658[0n]bp|Canada.Manitoba|BOLD:AAA1264
Hillia iris|[18472]|LCHQ405-08|07WNP-10297|658[1n]bp|Canada.Manitoba|BOLD:AAA1264
Hillia iris|[18473]|LCHIP047-07|06-PROBE-0171|650[0n]bp|Canada.Manitoba|BOLD:AAA1264
Hillia iris|[18474]|LCHQ445-08|07WNP-10337|657[0n]bp|Canada.Manitoba|BOLD:AAA1264
Hillia iris|[18475]|LCHQ368-08|07WNP-10260|658[0n]bp|Canada.Manitoba|BOLD:AAA1264
Hillia iris|[18476]|LCHQ603-08|07WNP-10495|658[0n]bp|Canada.Manitoba|BOLD:AAA1264
Hillia iris|[18477]|LCHQ274-08|07WNP-10166|658[0n]bp|Canada.Manitoba|BOLD:AAA1264
Hillia iris|[18478]|LCHIP234-07|06-PROBE-2880|650[0n]bp|Canada.Manitoba|BOLD:AAA1264
Hillia iris|[18479]|LCHIP261-07|06-PROBE-2926|650[0n]bp|Canada.Manitoba|BOLD:AAA1264
Hillia iris|[18480]|ENLCW091-09|09PROBE-01376|658[0n]bp|Canada.Manitoba|BOLD:AAA1264
Hillia iris|[18481]|LCHQ785-08|07WNP-10677|658[0n]bp|Canada.Manitoba|BOLD:AAA1264
Hillia iris|[18482]|LCHQ276-08|07WNP-10168|658[0n]bp|Canada.Manitoba|BOLD:AAA1264
Hillia iris|[18483]|LCHQ755-08|07WNP-10647|658[0n]bp|Canada.Manitoba|BOLD:AAA1264
Hillia iris|[18484]|LCHQ797-08|07WNP-10689|658[0n]bp|Canada.Manitoba|BOLD:AAA1264
Hillia iris|[18485]|LCHQ361-08|07WNP-10253|658[0n]bp|Canada.Manitoba|BOLD:AAA1264
Hillia iris|[18486]|LCHQ477-08|07WNP-10369|658[0n]bp|Canada.Manitoba|BOLD:AAA1264
Hillia iris|[18487]|LCHQ709-08|07WNP-10601|656[0n]bp|Canada.Manitoba|BOLD:AAA1264
Hillia iris|[18488]|LCHQ795-08|07WNP-10687|658[0n]bp|Canada.Manitoba|BOLD:AAA1264
Hillia iris|[18489]|LCHQ404-08|07WNP-10296|656[0n]bp|Canada.Manitoba|BOLD:AAA1264
Hillia iris|[18490]|LCHQ279-08|07WNP-10171|656[0n]bp|Canada.Manitoba|BOLD:AAA1264
Hillia iris|[18491]|LCHQ525-08|07WNP-10417|655[0n]bp|Canada.Manitoba|BOLD:AAA1264
Hillia iris|[18492]|LCHQ380-08|07WNP-10272|656[0n]bp|Canada.Manitoba|BOLD:AAA1264
Hillia iris|[18493]|LCHQ846-08|07WNP-10738|656[0n]bp|Canada.Manitoba|BOLD:AAA1264
Hillia iris|[18494]|LCHQ604-08|07WNP-10496|656[0n]bp|Canada.Manitoba|BOLD:AAA1264
Hillia iris|[18495]|LCHQ827-08|07WNP-10719|655[0n]bp|Canada.Manitoba|BOLD:AAA1264
Hillia iris|[18496]|LCHQ847-08|07WNP-10739|656[0n]bp|Canada.Manitoba|BOLD:AAA1264
Hillia iris|[18497]|LCHQ839-08|07WNP-10731|656[0n]bp|Canada.Manitoba|BOLD:AAA1264
Hillia iris|[18498]|LCHQ830-08|07WNP-10722|655[0n]bp|Canada.Manitoba|BOLD:AAA1264
Hillia iris|[18499]|LCHQ712-08|07WNP-10604|655[0n]bp|Canada.Manitoba|BOLD:AAA1264
Hillia iris|[18500]|LCHQ735-08|07WNP-10627|656[0n]bp|Canada.Manitoba|BOLD:AAA1264
Hillia iris|[18501]|LCHQ273-08|07WNP-10165|656[0n]bp|Canada.Manitoba|BOLD:AAA1264
Hillia iris|[18502]|LCHQ280-08|07WNP-10172|656[0n]bp|Canada.Manitoba|BOLD:AAA1264
Hillia iris|[18503]|LCHIP221-07|06-PROBE-2866|650[0n]bp|Canada.Manitoba|BOLD:AAA1264
Hillia iris|[18504]|LCHQ727-08|07WNP-10619|656[0n]bp|Canada.Manitoba|BOLD:AAA1264
Hillia iris|[18505]|LCHIP263-07|06-PROBE-2928|650[0n]bp|Canada.Manitoba|BOLD:AAA1264
Hillia iris|[18506]|LCHQ399-08|07WNP-10291|637[0n]bp|Canada.Manitoba|BOLD:AAA1264
Hillia iris|[18507]|LCHIP002-07|06-PROBE-0022|628[0n]bp|Canada.Manitoba|BOLD:AAA1264
Hillia iris|[18508]|LCHQ412-08|07WNP-10304|657[0n]bp|Canada.Manitoba|BOLD:AAA1264
Hillia iris|[18509]|LCHIP013-07|06-PROBE-0033|618[0n]bp|Canada.Manitoba|BOLD:AAA1264
Hillia iris|[18510]|LCHIP041-07|06-PROBE-0165|619[0n]bp|Canada.Manitoba|BOLD:AAA1264
Hillia iris|[18511]|LCHIP251-07|06-PROBE-2915|650[1n]bp|Canada.Manitoba|BOLD:AAA1264
Hillia iris|[18512]|LCHIP073-07|06-PROBE-2542|650[0n]bp|Canada.Manitoba|BOLD:AAA1264
Hillia iris|[18513]|LCHIP208-07|06-PROBE-2833|650[0n]bp|Canada.Manitoba|BOLD:AAA1264
Hillia iris|[18514]|LCHIP209-07|06-PROBE-2834|650[0n]bp|Canada.Manitoba|BOLD:AAA1264
Hillia iris|[18515]|LCHIP065-07|06-PROBE-2534|650[0n]bp|Canada.Manitoba|BOLD:AAA1264
Hillia iris|[18516]|LCHIP100-07|06-PROBE-2581|650[0n]bp|Canada.Manitoba|BOLD:AAA1264
Hillia iris|[18517]|LCHIP032-07|06-PROBE-0156|621[0n]bp|Canada.Manitoba|BOLD:AAA1264
Hillia iris|[18518]|LCHQ449-08|07WNP-10341|634[0n]bp|Canada.Manitoba|BOLD:AAA1264
Hillia iris|[18519]|LCHIP005-07|06-PROBE-0025|650[0n]bp|Canada.Manitoba|BOLD:AAA1264
Hillia iris|[18520]|LCHIP156-07|06-PROBE-2781|650[0n]bp|Canada.Manitoba|BOLD:AAA1264
Hillia iris|[18521]|LCHIP130-07|06-PROBE-2661|650[0n]bp|Canada.Manitoba|BOLD:AAA1264
Hillia iris|[18522]|LCHIP219-07|06-PROBE-2864|650[0n]bp|Canada.Manitoba|BOLD:AAA1264
Hillia iris|[18523]|LCHIP233-07|06-PROBE-2879|650[0n]bp|Canada.Manitoba|BOLD:AAA1264
Hillia iris|[18524]|LCHIP225-07|06-PROBE-2870|650[0n]bp|Canada.Manitoba|BOLD:AAA1264
Hillia iris|[18525]|LCHIP094-07|06-PROBE-2575|650[0n]bp|Canada.Manitoba|BOLD:AAA1264
Hillia iris|[18526]|LCHIP183-07|06-PROBE-2808|650[0n]bp|Canada.Manitoba|BOLD:AAA1264
Hillia iris|[18527]|LCHIP262-07|06-PROBE-2927|650[0n]bp|Canada.Manitoba|BOLD:AAA1264
Hillia iris|[18528]|LCHIP057-07|06-PROBE-2526|650[0n]bp|Canada.Manitoba|BOLD:AAA1264
Hillia iris|[18529]|LCHIP080-07|06-PROBE-2561|650[0n]bp|Canada.Manitoba|BOLD:AAA1264
Hillia iris|[18530]|LCHIP036-07|06-PROBE-0160|650[0n]bp|Canada.Manitoba|BOLD:AAA1264
Hillia iris|[18531]|LCHIP079-07|06-PROBE-2560|650[0n]bp|Canada.Manitoba|BOLD:AAA1264
Hillia iris|[18532]|LCHIP050-07|06-PROBE-0174|650[0n]bp|Canada.Manitoba|BOLD:AAA1264
Hillia iris|[18533]|LCHIP011-07|06-PROBE-0031|650[0n]bp|Canada.Manitoba|BOLD:AAA1264
Hillia iris|[18534]|LCHIP230-07|06-PROBE-2875|650[0n]bp|Canada.Manitoba|BOLD:AAA1264
Hillia iris|[18535]|LCHIP025-07|06-PROBE-0106|650[0n]bp|Canada.Manitoba|BOLD:AAA1264
Hillia iris|[18536]|LCHIP136-07|06-PROBE-2674|650[0n]bp|Canada.Manitoba|BOLD:AAA1264
Hillia iris|[18537]|LCHIP054-07|06-PROBE-2523|650[0n]bp|Canada.Manitoba|BOLD:AAA1264
Hillia iris|[18538]|LCHIP040-07|06-PROBE-0164|650[0n]bp|Canada.Manitoba|BOLD:AAA1264
[illegible]

Hillia iris|[18536]|LCHIP136-07|06-PROBE-2674|650[0n]bp|Canada.Manitoba|BOLD:AAA1264
Hillia iris|[18537]|LCHIP054-07|06-PROBE-2523|650[0n]bp|Canada.Manitoba|BOLD:AAA1264
Hillia iris|[18538]|LCHIP040-07|06-PROBE-0164|650[0n]bp|Canada.Manitoba|BOLD:AAA1264
Hillia iris|[18539]|LCHIP265-07|06-PROBE-2930|650[0n]bp|Canada.Manitoba|BOLD:AAA1264
Hillia iris|[18540]|LCHIP224-07|06-PROBE-2869|650[0n]bp|Canada.Manitoba|BOLD:AAA1264
Hillia iris|[18541]|LCHIP103-07|06-PROBE-2584|650[0n]bp|Canada.Manitoba|BOLD:AAA1264
Hillia iris|[18542]|LCHIP260-07|06-PROBE-2924|650[0n]bp|Canada.Manitoba|BOLD:AAA1264
Hillia iris|[18543]|LCHIP119-07|06-PROBE-2616|650[0n]bp|Canada.Manitoba|BOLD:AAA1264
Hillia iris|[18544]|LCHIP144-07|06-PROBE-2686|650[0n]bp|Canada.Manitoba|BOLD:AAA1264
Hillia iris|[18545]|LCHIP093-07|06-PROBE-2574|650[0n]bp|Canada.Manitoba|BOLD:AAA1264
Hillia iris|[18546]|LCHIP274-07|06-PROBE-2959|650[0n]bp|Canada.Manitoba|BOLD:AAA1264
Hillia iris|[18547]|LCHIP015-07|06-PROBE-0035|650[0n]bp|Canada.Manitoba|BOLD:AAA1264
Hillia iris|[18548]|LCHIP142-07|06-PROBE-2684|650[0n]bp|Canada.Manitoba|BOLD:AAA1264
Hillia iris|[18549]|LCHIP267-07|06-PROBE-2932|650[0n]bp|Canada.Manitoba|BOLD:AAA1264
Hillia iris|[18550]|LCHIP249-07|06-PROBE-2913|650[0n]bp|Canada.Manitoba|BOLD:AAA1264
Hillia iris|[18551]|LCHIP226-07|06-PROBE-2871|650[0n]bp|Canada.Manitoba|BOLD:AAA1264
Hillia iris|[18552]|LCHIP222-07|06-PROBE-2867|650[0n]bp|Canada.Manitoba|BOLD:AAA1264
Hillia iris|[18553]|LCHIP018-07|06-PROBE-0038|650[0n]bp|Canada.Manitoba|BOLD:AAA1264
Hillia iris|[18554]|LCHIP009-07|06-PROBE-0029|650[0n]bp|Canada.Manitoba|BOLD:AAA1264
Hillia iris|[18555]|LCHIP115-07|06-PROBE-2612|650[0n]bp|Canada.Manitoba|BOLD:AAA1264
Hillia iris|[18556]|LCHIP127-07|06-PROBE-2658|650[0n]bp|Canada.Manitoba|BOLD:AAA1264
Hillia iris|[18557]|LCHIP256-07|06-PROBE-2920|650[0n]bp|Canada.Manitoba|BOLD:AAA1264
Hillia iris|[18558]|LCHIP277-07|06-PROBE-2967|650[0n]bp|Canada.Manitoba|BOLD:AAA1264
Hillia iris|[18559]|LCHIP232-07|06-PROBE-2878|650[0n]bp|Canada.Manitoba|BOLD:AAA1264
Hillia iris|[18560]|LCHIP269-07|06-PROBE-2934|650[0n]bp|Canada.Manitoba|BOLD:AAA1264
Hillia iris|[18561]|LCHIP281-07|06-PROBE-2971|650[0n]bp|Canada.Manitoba|BOLD:AAA1264
Hillia iris|[18562]|LCHIP268-07|06-PROBE-2933|650[0n]bp|Canada.Manitoba|BOLD:AAA1264
Hillia iris|[18563]|LCHIP010-07|06-PROBE-0030|650[0n]bp|Canada.Manitoba|BOLD:AAA1264
Hillia iris|[18564]|LCHIP211-07|06-PROBE-2836|650[0n]bp|Canada.Manitoba|BOLD:AAA1264
Hillia iris|[18565]|LCHIP028-07|06-PROBE-0110|650[0n]bp|Canada.Manitoba|BOLD:AAA1264
Hillia iris|[18566]|LCHIP255-07|06-PROBE-2919|650[0n]bp|Canada.Manitoba|BOLD:AAA1264
Hillia iris|[18567]|LCHIP038-07|06-PROBE-0162|650[0n]bp|Canada.Manitoba|BOLD:AAA1264
Hillia iris|[18568]|LCHIP155-07|06-PROBE-2780|650[0n]bp|Canada.Manitoba|BOLD:AAA1264
Hillia iris|[18569]|LCHIP081-07|06-PROBE-2562|650[0n]bp|Canada.Manitoba|BOLD:AAA1264
Hillia iris|[18570]|LCHIP086-07|06-PROBE-2567|650[0n]bp|Canada.Manitoba|BOLD:AAA1264
Hillia iris|[18571]|LCHQ408-08|07WNP-10300|652[0n]bp|Canada.Manitoba|BOLD:AAA1264
Hillia iris|[18572]|LCHIP210-07|06-PROBE-2835|650[0n]bp|Canada.Manitoba|BOLD:AAA1264
Hillia iris|[18573]|LCHIP004-07|06-PROBE-0024|650[0n]bp|Canada.Manitoba|BOLD:AAA1264
Hillia iris|[18574]|LCHIP264-07|06-PROBE-2929|650[0n]bp|Canada.Manitoba|BOLD:AAA1264
Hillia iris|[18575]|LCHIP098-07|06-PROBE-2579|650[0n]bp|Canada.Manitoba|BOLD:AAA1264
Hillia iris|[18576]|LCHIP001-07|06-PROBE-0021|650[0n]bp|Canada.Manitoba|BOLD:AAA1264
Hillia iris|[18577]|LCHIP035-07|06-PROBE-0159|650[0n]bp|Canada.Manitoba|BOLD:AAA1264
Hillia iris|[18578]|LCHIP033-07|06-PROBE-0157|650[0n]bp|Canada.Manitoba|BOLD:AAA1264
Hillia iris|[18579]|LCHIP068-07|06-PROBE-2537|650[0n]bp|Canada.Manitoba|BOLD:AAA1264
Hillia iris|[18580]|LCHIP279-07|06-PROBE-2969|650[0n]bp|Canada.Manitoba|BOLD:AAA1264
Hillia iris|[18581]|LCHIP026-07|06-PROBE-0108|650[0n]bp|Canada.Manitoba|BOLD:AAA1264
Hillia iris|[18582]|LCHIP131-07|06-PROBE-2662|650[0n]bp|Canada.Manitoba|BOLD:AAA1264
Hillia iris|[18583]|LCHIP140-07|06-PROBE-2682|650[0n]bp|Canada.Manitoba|BOLD:AAA1264
Hillia iris|[18584]|LCHIP101-07|06-PROBE-2582|650[0n]bp|Canada.Manitoba|BOLD:AAA1264
Hillia iris|[18585]|LCHIP214-07|06-PROBE-2845|650[0n]bp|Canada.Manitoba|BOLD:AAA1264
Hillia iris|[18586]|LCHIP061-07|06-PROBE-2530|650[0n]bp|Canada.Manitoba|BOLD:AAA1264
Hillia iris|[18587]|LCHIP072-07|06-PROBE-2541|650[0n]bp|Canada.Manitoba|BOLD:AAA1264
Hillia iris|[18588]|LCHIP056-07|06-PROBE-2525|650[0n]bp|Canada.Manitoba|BOLD:AAA1264
Hillia iris|[18589]|LCHIP008-07|06-PROBE-0028|650[0n]bp|Canada.Manitoba|BOLD:AAA1264
Hillia iris|[18590]|LCHIP049-07|06-PROBE-0173|650[0n]bp|Canada.Manitoba|BOLD:AAA1264
Hillia iris|[18591]|LCHIP006-07|06-PROBE-0026|650[0n]bp|Canada.Manitoba|BOLD:AAA1264
Hillia iris|[18592]|LCHIP069-07|06-PROBE-2538|650[0n]bp|Canada.Manitoba|BOLD:AAA1264
Hillia iris|[18593]|LCHIP067-07|06-PROBE-2536|650[0n]bp|Canada.Manitoba|BOLD:AAA1264
Hillia iris|[18594]|LCHIP029-07|06-PROBE-0111|650[0n]bp|Canada.Manitoba|BOLD:AAA1264
Hillia iris|[18595]|LCHIP046-07|06-PROBE-0170|650[0n]bp|Canada.Manitoba|BOLD:AAA1264
Hillia iris|[18596]|LCHIP275-07|06-PROBE-2965|650[0n]bp|Canada.Manitoba|BOLD:AAA1264
Hillia iris|[18597]|LCHIP045-07|06-PROBE-0169|650[0n]bp|Canada.Manitoba|BOLD:AAA1264
Hillia iris|[18598]|LCHIP218-07|06-PROBE-2863|650[0n]bp|Canada.Manitoba|BOLD:AAA1264
Hillia iris|[18599]|LCHIP129-07|06-PROBE-2660|650[0n]bp|Canada.Manitoba|BOLD:AAA1264
Hillia iris|[18600]|LCHIP102-07|06-PROBE-2583|650[0n]bp|Canada.Manitoba|BOLD:AAA1264
Hillia iris|[18601]|LCHIP014-07|06-PROBE-0034|650[0n]bp|Canada.Manitoba|BOLD:AAA1264
Hillia iris|[18602]|LCHIP017-07|06-PROBE-0037|650[0n]bp|Canada.Manitoba|BOLD:AAA1264
Hillia iris|[18603]|LCHIP053-07|06-PROBE-2522|650[0n]bp|Canada.Manitoba|BOLD:AAA1264
Hillia iris|[18604]|LCHIP160-07|06-PROBE-2785|650[0n]bp|Canada.Manitoba|BOLD:AAA1264
Hillia iris|[18605]|LCHIP043-07|06-PROBE-0167|650[0n]bp|Canada.Manitoba|BOLD:AAA1264
Hillia iris|[18606]|LCHIP139-07|06-PROBE-2681|650[0n]bp|Canada.Manitoba|BOLD:AAA1264
Hillia iris|[18607]|LCHIP070-07|06-PROBE-2539|650[0n]bp|Canada.Manitoba|BOLD:AAA1264
Hillia iris|[18608]|LCHIP071-07|06-PROBE-2540|650[0n]bp|Canada.Manitoba|BOLD:AAA1264
Hillia iris|[18609]|LCHIP044-07|06-PROBE-0168|650[0n]bp|Canada.Manitoba|BOLD:AAA1264
Hillia iris|[18610]|LCHIP125-07|06-PROBE-2646|650[0n]bp|Canada.Manitoba|BOLD:AAA1264
Hillia iris|[18611]|LCHIP109-07|06-PROBE-2595|650[0n]bp|Canada.Manitoba|BOLD:AAA1264
Hillia iris|[18612]|LCHIP273-07|06-PROBE-2958|650[0n]bp|Canada.Manitoba|BOLD:AAA1264
Hillia iris|[18613]|LCHIP124-07|06-PROBE-2645|650[0n]bp|Canada.Manitoba|BOLD:AAA1264
Hillia iris|[18614]|LCHIP252-07|06-PROBE-2916|650[0n]bp|Canada.Manitoba|BOLD:AAA1264
Hillia iris|[18615]|LCHIP099-07|06-PROBE-2580|650[0n]bp|Canada.Manitoba|BOLD:AAA1264
Hillia iris|[18616]|LCHIP228-07|06-PROBE-2873|650[0n]bp|Canada.Manitoba|BOLD:AAA1264
Hillia iris|[18617]|LCHIP253-07|06-PROBE-2917|650[0n]bp|Canada.Manitoba|BOLD:AAA1264
Hillia iris|[18618]|LCHIP007-07|06-PROBE-0027|650[0n]bp|Canada.Manitoba|BOLD:AAA1264
Hillia iris|[18619]|LCHIP247-07|06-PROBE-2897|650[0n]bp|Canada.Manitoba|BOLD:AAA1264
Hillia iris|[18620]|LCHIP089-07|06-PROBE-2570|650[0n]bp|Canada.Manitoba|BOLD:AAA1264
Hillia iris|[18621]|LCHIP146-07|06-PROBE-2688|650[0n]bp|Canada.Manitoba|BOLD:AAA1264
Hillia iris|[18622]|LCHIP220-07|06-PROBE-2865|650[0n]bp|Canada.Manitoba|BOLD:AAA1264
Hillia iris|[18623]|LCHIP143-07|06-PROBE-2685|650[0n]bp|Canada.Manitoba|BOLD:AAA1264
Hillia iris|[18624]|LCHIP248-07|06-PROBE-2898|650[0n]bp|Canada.Manitoba|BOLD:AAA1264
Hillia iris|[18625]|LCHIP227-07|06-PROBE-2872|650[1n]bp|Canada.Manitoba|BOLD:AAA1264
Hillia iris|[18626]|LCHIP259-07|06-PROBE-2923|650[1n]bp|Canada.Manitoba|BOLD:AAA1264
Hillia iris|[18627]|LCHIP278-07|06-PROBE-2968|650[0n]bp|Canada.Manitoba|BOLD:AAA1264
Hillia iris|[18628]|LCHQ793-08|07WNP-10685|646[0n]bp|Canada.Manitoba|BOLD:AAA1264
Hillia iris|[18629]|LCHQ293-08|07WNP-10185|646[0n]bp|Canada.Manitoba|BOLD:AAA1264
Hillia iris|[18630]|LCHIP250-07|06-PROBE-2914|650[0n]bp|Canada.Manitoba|BOLD:AAA1264
Hillia iris|[18631]|DSCNI062-07|06-PROBE-0287|658[0n]bp|Canada.Manitoba|BOLD:AAA1264
Hillia iris|[18632]|LCHQ358-08|07WNP-10250|657[0n]bp|Canada.Manitoba|BOLD:AAA1264
Hillia iris|[18633]|LCHQ377-08|07WNP-10269|658[0n]bp|Canada.Manitoba|BOLD:AAA1264
Hillia iris|[18634]|LCHQ825-08|07WNP-10717|658[0n]bp|Canada.Manitoba|BOLD:AAA1264
Hillia iris|[18635]|DSCNI029-07|06-PROBE-0254|658[0n]bp|Canada.Manitoba|BOLD:AAA1264
Hillia iris|[18636]|LCHQ299-08|07WNP-10191|658[0n]bp|Canada.Manitoba|BOLD:AAA1264
Hillia iris|[18637]|LCHQ433-08|07WNP-10325|658[0n]bp|Canada.Manitoba|BOLD:AAA1264
Hillia iris|[18638]|LCHQ227-08|07WNP-10119|657[0n]bp|Canada.Manitoba|BOLD:AAA1264

Hillia iris|[18636]|LCHQ299-08|07WNP-10191|658[0n]bp|Canada.Manitoba|BOLD:AAA1264
Hillia iris|[18637]|LCHQ433-08|07WNP-10325|658[0n]bp|Canada.Manitoba|BOLD:AAA1264
Hillia iris|[18638]|LCHQ227-08|07WNP-10119|657[0n]bp|Canada.Manitoba|BOLD:AAA1264
Hillia iris|[18639]|LCHQ781-08|07WNP-10673|658[0n]bp|Canada.Manitoba|BOLD:AAA1264
Hillia iris|[18640]|LCHQ857-08|07WNP-10749|658[0n]bp|Canada.Manitoba|BOLD:AAA1264
Hillia iris|[18641]|MHLEP129-07|CHU06-LEP-129|658[0n]bp|Canada.Manitoba|BOLD:AAA1264
Hillia iris|[18642]|LCHQ771-08|07WNP-10663|658[0n]bp|Canada.Manitoba|BOLD:AAA1264
Hillia iris|[18643]|LCHQ436-08|07WNP-10328|657[0n]bp|Canada.Manitoba|BOLD:AAA1264
Hillia iris|[18644]|LCHQ442-08|07WNP-10334|657[0n]bp|Canada.Manitoba|BOLD:AAA1264
Hillia iris|[18645]|LCHQ745-08|07WNP-10637|658[0n]bp|Canada.Manitoba|BOLD:AAA1264
Hillia iris|[18646]|LCHQ707-08|07WNP-10599|658[0n]bp|Canada.Manitoba|BOLD:AAA1264
Hillia iris|[18647]|LCHQ375-08|07WNP-10267|657[0n]bp|Canada.Manitoba|BOLD:AAA1264
Hillia iris|[18648]|ENLCW083-09|09PROBE-01368|658[0n]bp|Canada.Manitoba|BOLD:AAA1264
Hillia iris|[18649]|LCHQ608-08|07WNP-10500|658[0n]bp|Canada.Manitoba|BOLD:AAA1264
Hillia iris|[18650]|LCHQ505-08|07WNP-10397|658[0n]bp|Canada.Manitoba|BOLD:AAA1264
Hillia iris|[18651]|LCHQ372-08|07WNP-10264|658[0n]bp|Canada.Manitoba|BOLD:AAA1264
Hillia iris|[18652]|LCHQ295-08|07WNP-10187|658[0n]bp|Canada.Manitoba|BOLD:AAA1264
Hillia iris|[18653]|LCHQ289-08|07WNP-10181|658[0n]bp|Canada.Manitoba|BOLD:AAA1264
Hillia iris|[18654]|ENLCW008-09|09PROBE-01293|658[0n]bp|Canada.Manitoba|BOLD:AAA1264
Hillia iris|[18655]|LCHQ291-08|07WNP-10183|658[0n]bp|Canada.Manitoba|BOLD:AAA1264
Hillia iris|[18656]|LCHQ411-08|07WNP-10303|658[0n]bp|Canada.Manitoba|BOLD:AAA1264
Hillia iris|[18657]|LCHQ441-08|07WNP-10333|658[0n]bp|Canada.Manitoba|BOLD:AAA1264
Hillia iris|[18658]|LCHQ855-08|07WNP-10747|658[0n]bp|Canada.Manitoba|BOLD:AAA1264
Hillia iris|[18659]|LCHQ753-08|07WNP-10645|658[0n]bp|Canada.Manitoba|BOLD:AAA1264
Hillia iris|[18660]|LCHQ365-08|07WNP-10257|658[0n]bp|Canada.Manitoba|BOLD:AAA1264
Hillia iris|[18661]|DSCNI025-07|06-PROBE-0250|658[0n]bp|Canada.Manitoba|BOLD:AAA1264
Hillia iris|[18662]|LCHQ435-08|07WNP-10327|658[0n]bp|Canada.Manitoba|BOLD:AAA1264
Hillia iris|[18663]|LCHQ756-08|07WNP-10648|658[0n]bp|Canada.Manitoba|BOLD:AAA1264
Hillia iris|[18664]|MHLEP127-07|CHU06-LEP-127|658[0n]bp|Canada.Manitoba|BOLD:AAA1264
Hillia iris|[18665]|LCHQ747-08|07WNP-10639|657[0n]bp|Canada.Manitoba|BOLD:AAA1264
Hillia iris|[18666]|LCH583-04|04HBL003583|658[0n]bp|Canada.Manitoba|BOLD:AAA1264
Hillia iris|[18667]|LCHQ230-08|07WNP-10122|658[0n]bp|Canada.Manitoba|BOLD:AAA1264
Hillia iris|[18668]|LCHQ840-08|07WNP-10732|658[0n]bp|Canada.Manitoba|BOLD:AAA1264
Hillia iris|[18669]|LCHQ591-08|07WNP-10483|658[0n]bp|Canada.Manitoba|BOLD:AAA1264
Hillia iris|[18670]|DSCNI026-07|06-PROBE-0251|658[0n]bp|Canada.Manitoba|BOLD:AAA1264
Hillia iris|[18671]|LCHQ300-08|07WNP-10192|658[0n]bp|Canada.Manitoba|BOLD:AAA1264
Hillia iris|[18672]|LCHQ730-08|07WNP-10622|658[0n]bp|Canada.Manitoba|BOLD:AAA1264
Hillia iris|[18673]|LCHQ852-08|07WNP-10744|658[0n]bp|Canada.Manitoba|BOLD:AAA1264
Hillia iris|[18674]|LCHQ834-08|07WNP-10726|657[0n]bp|Canada.Manitoba|BOLD:AAA1264
Hillia iris|[18675]|LCHQ396-08|07WNP-10288|658[0n]bp|Canada.Manitoba|BOLD:AAA1264
Hillia iris|[18676]|LCHQ233-08|07WNP-10125|658[0n]bp|Canada.Manitoba|BOLD:AAA1264
Hillia iris|[18677]|MHLEP130-07|CHU06-LEP-130|658[0n]bp|Canada.Manitoba|BOLD:AAA1264
Hillia iris|[18678]|LCHQ370-08|07WNP-10262|658[0n]bp|Canada.Manitoba|BOLD:AAA1264
Hillia iris|[18679]|LCHQ786-08|07WNP-10678|658[0n]bp|Canada.Manitoba|BOLD:AAA1264
Hillia iris|[18680]|LCHQ371-08|07WNP-10263|658[0n]bp|Canada.Manitoba|BOLD:AAA1264
Hillia iris|[18681]|LCHQ287-08|07WNP-10179|658[0n]bp|Canada.Manitoba|BOLD:AAA1264
Hillia iris|[18682]|LCHQ366-08|07WNP-10258|658[0n]bp|Canada.Manitoba|BOLD:AAA1264
Hillia iris|[18683]|LCHQ853-08|07WNP-10745|658[0n]bp|Canada.Manitoba|BOLD:AAA1264
Hillia iris|[18684]|LCHQ444-08|07WNP-10336|657[0n]bp|Canada.Manitoba|BOLD:AAA1264
Hillia iris|[18685]|LCHQ506-08|07WNP-10398|658[0n]bp|Canada.Manitoba|BOLD:AAA1264
Hillia iris|[18686]|LCHQ738-08|07WNP-10630|658[0n]bp|Canada.Manitoba|BOLD:AAA1264
Hillia iris|[18687]|LCHQ401-08|07WNP-10293|658[0n]bp|Canada.Manitoba|BOLD:AAA1264
Hillia iris|[18688]|LCHQ446-08|07WNP-10338|658[0n]bp|Canada.Manitoba|BOLD:AAA1264
Hillia iris|[18689]|LCHQ403-08|07WNP-10295|658[0n]bp|Canada.Manitoba|BOLD:AAA1264
Hillia iris|[18690]|LCHQ282-08|07WNP-10174|658[0n]bp|Canada.Manitoba|BOLD:AAA1264
Hillia iris|[18691]|LCHQ292-08|07WNP-10184|657[0n]bp|Canada.Manitoba|BOLD:AAA1264
Hillia iris|[18692]|LCHQ737-08|07WNP-10629|658[0n]bp|Canada.Manitoba|BOLD:AAA1264
Hillia iris|[18693]|LCHQ831-08|07WNP-10723|658[0n]bp|Canada.Manitoba|BOLD:AAA1264
Hillia iris|[18694]|LCHQ448-08|07WNP-10340|658[0n]bp|Canada.Manitoba|BOLD:AAA1264
Hillia iris|[18695]|LCHQ829-08|07WNP-10721|658[0n]bp|Canada.Manitoba|BOLD:AAA1264
Hillia iris|[18696]|LCHQ356-08|07WNP-10248|658[0n]bp|Canada.Manitoba|BOLD:AAA1264
Hillia iris|[18697]|LCHQ508-08|07WNP-10400|658[0n]bp|Canada.Manitoba|BOLD:AAA1264
Hillia iris|[18698]|LCHQ229-08|07WNP-10121|658[0n]bp|Canada.Manitoba|BOLD:AAA1264
Hillia iris|[18699]|LCHQ409-08|07WNP-10301|658[0n]bp|Canada.Manitoba|BOLD:AAA1264
Hillia iris|[18700]|LCHQ367-08|07WNP-10259|658[0n]bp|Canada.Manitoba|BOLD:AAA1264
Hillia iris|[18701]|LCHQ841-08|07WNP-10733|658[0n]bp|Canada.Manitoba|BOLD:AAA1264
Hillia iris|[18702]|LCHQ447-08|07WNP-10339|657[0n]bp|Canada.Manitoba|BOLD:AAA1264
Hillia iris|[18703]|LCHQ788-08|07WNP-10680|658[0n]bp|Canada.Manitoba|BOLD:AAA1264
Hillia iris|[18704]|LCHQ796-08|07WNP-10688|658[0n]bp|Canada.Manitoba|BOLD:AAA1264
Hillia iris|[18705]|LCHQ854-08|07WNP-10746|658[0n]bp|Canada.Manitoba|BOLD:AAA1264
Hillia iris|[18706]|LCHQ363-08|07WNP-10255|658[0n]bp|Canada.Manitoba|BOLD:AAA1264
Hillia iris|[18707]|MHLEP123-07|CHU06-LEP-123|658[0n]bp|Canada.Manitoba|BOLD:AAA1264
Hillia iris|[18708]|LCHQ364-08|07WNP-10256|658[0n]bp|Canada.Manitoba|BOLD:AAA1264
Hillia iris|[18709]|DSCNI028-07|06-PROBE-0253|658[0n]bp|Canada.Manitoba|BOLD:AAA1264
Hillia iris|[18710]|LOWCC837-05|CGWC-2717|658[0n]bp|Canada.British Columbia|BOLD:AAA1264
Hillia iris|[18711]|LCHQ484-08|07WNP-10376|657[0n]bp|Canada.Manitoba|BOLD:AAA1264
Hillia iris|[18712]|LCHQ437-08|07WNP-10329|658[0n]bp|Canada.Manitoba|BOLD:AAA1264
Hillia iris|[18713]|LCHQ369-08|07WNP-10261|658[0n]bp|Canada.Manitoba|BOLD:AAA1264
Hillia iris|[18714]|LCHQ826-08|07WNP-10718|658[0n]bp|Canada.Manitoba|BOLD:AAA1264
Hillia iris|[18715]|LCHQ848-08|07WNP-10740|658[0n]bp|Canada.Manitoba|BOLD:AAA1264
Hillia iris|[18716]|LCHQ607-08|07WNP-10499|658[0n]bp|Canada.Manitoba|BOLD:AAA1264
Hillia iris|[18717]|MHLEP126-07|CHU06-LEP-126|658[0n]bp|Canada.Manitoba|BOLD:AAA1264
Hillia iris|[18718]|MHLEP121-07|CHU06-LEP-121|658[0n]bp|Canada.Manitoba|BOLD:AAA1264
Hillia iris|[18719]|LCHQ523-08|07WNP-10415|658[0n]bp|Canada.Manitoba|BOLD:AAA1264
Hillia iris|[18720]|LCHQ290-08|07WNP-10182|658[0n]bp|Canada.Manitoba|BOLD:AAA1264
Hillia iris|[18721]|LCHQ288-08|07WNP-10180|658[0n]bp|Canada.Manitoba|BOLD:AAA1264
Hillia iris|[18722]|LCHQ611-08|07WNP-10503|658[0n]bp|Canada.Manitoba|BOLD:AAA1264
Hillia iris|[18723]|LCHQ790-08|07WNP-10682|658[0n]bp|Canada.Manitoba|BOLD:AAA1264
Hillia iris|[18724]|LCHQ843-08|07WNP-10735|658[0n]bp|Canada.Manitoba|BOLD:AAA1264
Hillia iris|[18725]|LCHQ734-08|07WNP-10626|658[0n]bp|Canada.Manitoba|BOLD:AAA1264
Hillia iris|[18726]|LCHQ850-08|07WNP-10742|658[0n]bp|Canada.Manitoba|BOLD:AAA1264
Hillia iris|[18727]|LCHQ782-08|07WNP-10674|657[0n]bp|Canada.Manitoba|BOLD:AAA1264
Hillia iris|[18728]|ENLCW002-09|09PROBE-01287|658[0n]bp|Canada.Manitoba|BOLD:AAA1264
Hillia iris|[18729]|LCHQ837-08|07WNP-10729|658[0n]bp|Canada.Manitoba|BOLD:AAA1264
Hillia iris|[18730]|LCHQ314-08|07WNP-10206|658[0n]bp|Canada.Manitoba|BOLD:AAA1264
Hillia iris|[18731]|LCHQ610-08|07WNP-10502|658[0n]bp|Canada.Manitoba|BOLD:AAA1264
Hillia iris|[18732]|LCHQ758-08|07WNP-10650|658[0n]bp|Canada.Manitoba|BOLD:AAA1264
Hillia iris|[18733]|LCHQ378-08|07WNP-10270|658[0n]bp|Canada.Manitoba|BOLD:AAA1264
Hillia iris|[18734]|LCHQ359-08|07WNP-10251|658[0n]bp|Canada.Manitoba|BOLD:AAA1264
Hillia iris|[18735]|DSCNI036-07|06-PROBE-0261|658[0n]bp|Canada.Manitoba|BOLD:AAA1264
Hillia iris|[18736]|LCHQ605-08|07WNP-10497|658[0n]bp|Canada.Manitoba|BOLD:AAA1264
Hillia iris|[18737]|LCHQ742-08|07WNP-10634|658[0n]bp|Canada.Manitoba|BOLD:AAA1264
Hillia iris|[18738]|LCHQ278-08|07WNP-10170|658[0n]bp|Canada.Manitoba|BOLD:AAA1264

[illegible]
Hillia iris|[18736]|LCHQ605-08|07WNP-10497|658[0n]bp|Canada.Manitoba|BOLD:AAA1264
Hillia iris|[18737]|LCHQ742-08|07WNP-10634|658[0n]bp|Canada.Manitoba|BOLD:AAA1264
Hillia iris|[18738]|LCHQ278-08|07WNP-10170|658[0n]bp|Canada.Manitoba|BOLD:AAA1264
Hillia iris|[18739]|LCHQ845-08|07WNP-10737|658[0n]bp|Canada.Manitoba|BOLD:AAA1264
Hillia iris|[18740]|LCHQ397-08|07WNP-10289|658[0n]bp|Canada.Manitoba|BOLD:AAA1264
Hillia iris|[18741]|LCHQ302-08|07WNP-10194|657[0n]bp|Canada.Manitoba|BOLD:AAA1264
Hillia iris|[18742]|ENLCW093-09|09PROBE-01378|658[0n]bp|Canada.Manitoba|BOLD:AAA1264
Hillia iris|[18743]|LCHQ305-08|07WNP-10197|658[0n]bp|Canada.Manitoba|BOLD:AAA1264
Hillia iris|[18744]|LCHQ376-08|07WNP-10268|658[0n]bp|Canada.Manitoba|BOLD:AAA1264
Hillia iris|[18745]|LCHQ759-08|07WNP-10651|658[0n]bp|Canada.Manitoba|BOLD:AAA1264
Hillia iris|[18746]|LCHQ732-08|07WNP-10624|658[0n]bp|Canada.Manitoba|BOLD:AAA1264
Hillia iris|[18747]|LCHQ296-08|07WNP-10188|658[0n]bp|Canada.Manitoba|BOLD:AAA1264
Hillia iris|[18748]|LCHQ507-08|07WNP-10399|622[0n]bp|Canada.Manitoba|BOLD:AAA1264
Hillia iris|[18749]|RDLQ592-07|DH008781|596[0n]bp|Canada.Quebec|BOLD:AAA1264
Hillia iris|[18750]|PHMNB284-04|04HBL007749|571[1n]bp|Canada.New Brunswick|BOLD:AAA1264
Hillia iris|[18751]|LCHIP113-07|06-PROBE-2610|650[0n]bp|Canada.Manitoba|BOLD:AAA1264
Hillia iris|[18752]|LCHQ725-08|07WNP-10617|658[0n]bp|Canada.Manitoba|BOLD:AAA1264
Hillia iris|[18753]|LCHQ285-08|07WNP-10177|658[0n]bp|Canada.Manitoba|BOLD:AAA1264
Hillia iris|[18754]|LCHQ752-08|07WNP-10644|658[0n]bp|Canada.Manitoba|BOLD:AAA1264
Hillia iris|[18755]|LCHQ580-08|07WNP-10472|656[0n]bp|Canada.Manitoba|BOLD:AAA1264
Hillia iris|[18756]|LCHIP048-07|06-PROBE-0172|650[0n]bp|Canada.Manitoba|BOLD:AAA1264
Hillia iris|[18757]|LCHIP276-07|06-PROBE-2966|650[0n]bp|Canada.Manitoba|BOLD:AAA1264
Hillia iris|[18758]|LCHIP063-07|06-PROBE-2532|650[0n]bp|Canada.Manitoba|BOLD:AAA1264
Hillia iris|[18759]|LCHQ746-08|07WNP-10638|632[0n]bp|Canada.Manitoba|BOLD:AAA1264
Hillia iris|[18760]|LCHQ818-08|07WNP-10710|631[0n]bp|Canada.Manitoba|BOLD:AAA1264
Hillia iris|[18761]|LCHQ749-08|07WNP-10641|632[0n]bp|Canada.Manitoba|BOLD:AAA1264
Hillia iris|[18762]|LCHQ733-08|07WNP-10625|632[0n]bp|Canada.Manitoba|BOLD:AAA1264
Hillia iris|[18763]|LCHQ851-08|07WNP-10743|632[0n]bp|Canada.Manitoba|BOLD:AAA1264
Hillia iris|[18764]|LCHQ362-08|07WNP-10254|658[0n]bp|Canada.Manitoba|BOLD:AAA1264
Hillia iris|[18765]|LCHIP003-07|06-PROBE-0023|650[0n]bp|Canada.Manitoba|BOLD:AAA1264
Hillia iris|[18766]|LCHQ231-08|07WNP-10123|656[0n]bp|Canada.Manitoba|BOLD:AAA1264
Hillia iris|[18767]|LCHQ757-08|07WNP-10649|658[0n]bp|Canada.Manitoba|BOLD:AAA1264
Hillia iris|[18768]|LCHQ772-08|07WNP-10664|658[0n]bp|Canada.Manitoba|BOLD:AAA1264
Hillia iris|[18769]|LCHQ400-08|07WNP-10292|658[0n]bp|Canada.Manitoba|BOLD:AAA1264
Hillia iris|[18770]|LCHQ824-08|07WNP-10716|658[0n]bp|Canada.Manitoba|BOLD:AAA1264
Hillia iris|[18771]|LCHQ360-08|07WNP-10252|658[0n]bp|Canada.Manitoba|BOLD:AAA1264
Hillia iris|[18772]|LCHQ780-08|07WNP-10672|655[0n]bp|Canada.Manitoba|BOLD:AAA1264
Hillia iris|[18773]|LCHQ791-08|07WNP-10683|658[0n]bp|Canada.Manitoba|BOLD:AAA1264
Hillia iris|[18774]|LCHQ298-08|07WNP-10190|657[0n]bp|Canada.Manitoba|BOLD:AAA1264
Hillia iris|[18775]|LCHQ739-08|07WNP-10631|658[0n]bp|Canada.Manitoba|BOLD:AAA1264
Hillia iris|[18776]|LCHQ373-08|07WNP-10265|658[0n]bp|Canada.Manitoba|BOLD:AAA1264
Hillia iris|[18777]|LCHQ277-08|07WNP-10169|658[0n]bp|Canada.Manitoba|BOLD:AAA1264
Hillia iris|[18778]|LCHQ856-08|07WNP-10748|658[0n]bp|Canada.Manitoba|BOLD:AAA1264
Hillia iris|[18779]|MHLEP128-07|CHU06-LEP-128|658[0n]bp|Canada.Manitoba|BOLD:AAA1264
Hillia iris|[18780]|LCHQ784-08|07WNP-10676|658[0n]bp|Canada.Manitoba|BOLD:AAA1264
Hillia iris|[18781]|LCHQ832-08|07WNP-10724|657[0n]bp|Canada.Manitoba|BOLD:AAA1264
Hillia iris|[18782]|LCHQ779-08|07WNP-10671|656[0n]bp|Canada.Manitoba|BOLD:AAA1264
Hillia iris|[18783]|LCHQ762-08|07WNP-10654|658[0n]bp|Canada.Manitoba|BOLD:AAA1264
Hillia iris|[18784]|LCHQ740-08|07WNP-10632|658[0n]bp|Canada.Manitoba|BOLD:AAA1264
Hillia iris|[18785]|LCHQ754-08|07WNP-10646|658[0n]bp|Canada.Manitoba|BOLD:AAA1264
Hillia iris|[18786]|LCHQ849-08|07WNP-10741|658[0n]bp|Canada.Manitoba|BOLD:AAA1264
Hillia iris|[18787]|LCHQ794-08|07WNP-10686|658[0n]bp|Canada.Manitoba|BOLD:AAA1264
Hillia iris|[18788]|LCHQ842-08|07WNP-10734|658[0n]bp|Canada.Manitoba|BOLD:AAA1264
Hillia iris|[18789]|LCHQ297-08|07WNP-10189|657[0n]bp|Canada.Manitoba|BOLD:AAA1264
Hillia iris|[18790]|LCHQ761-08|07WNP-10653|658[0n]bp|Canada.Manitoba|BOLD:AAA1264
Hillia iris|[18791]|LCHQ750-08|07WNP-10642|658[0n]bp|Canada.Manitoba|BOLD:AAA1264
Hillia iris|[18792]|LCHQ606-08|07WNP-10498|658[0n]bp|Canada.Manitoba|BOLD:AAA1264
Hillia iris|[18793]|LCHQ736-08|07WNP-10628|658[0n]bp|Canada.Manitoba|BOLD:AAA1264
Hillia iris|[18794]|LCHQ357-08|07WNP-10249|656[0n]bp|Canada.Manitoba|BOLD:AAA1264
Hillia iris|[18795]|LCHQ283-08|07WNP-10175|655[0n]bp|Canada.Manitoba|BOLD:AAA1264
Hillia iris|[18796]|LCHQ743-08|07WNP-10635|655[0n]bp|Canada.Manitoba|BOLD:AAA1264
Hillia iris|[18797]|LCHQ729-08|07WNP-10621|656[0n]bp|Canada.Manitoba|BOLD:AAA1264
Hillia iris|[18798]|LCHIP108-07|06-PROBE-2594|650[0n]bp|Canada.Manitoba|BOLD:AAA1264
Hillia iris|[18799]|LCHIP217-07|06-PROBE-2862|650[0n]bp|Canada.Manitoba|BOLD:AAA1264
Hillia iris|[18800]|LCHIP151-07|06-PROBE-2696|650[0n]bp|Canada.Manitoba|BOLD:AAA1264
Hillia iris|[18801]|LCHIP019-07|06-PROBE-0039|650[0n]bp|Canada.Manitoba|BOLD:AAA1264
Hillia iris|[18802]|LCHIP280-07|06-PROBE-2970|650[0n]bp|Canada.Manitoba|BOLD:AAA1264
Hillia iris|[18803]|LCHIP266-07|06-PROBE-2931|650[0n]bp|Canada.Manitoba|BOLD:AAA1264
Hillia iris|[18804]|LCHQ774-08|07WNP-10666|646[0n]bp|Canada.Manitoba|BOLD:AAA1264
Hillia iris|[18805]|LCHQ407-08|07WNP-10299|633[0n]bp|Canada.Manitoba|BOLD:AAA1264
Hillia iris|[18806]|LCHIP135-07|06-PROBE-2673|650[0n]bp|Canada.Manitoba|BOLD:AAA1264
Hillia iris|[18807]|LCHIP016-07|06-PROBE-0036|650[0n]bp|Canada.Manitoba|BOLD:AAA1264
Hillia iris|[18808]|LCHIP106-07|06-PROBE-2592|650[0n]bp|Canada.Manitoba|BOLD:AAA1264
Hillia maida|[18809]|NAMUM280-08|RR-98-1163|542[0n]bp|United States.California|BOLD:AAI8475
Hillia maida|[18810]|RDNMF497-08|NOC14583|658[0n]bp|United States.Colorado|BOLD:AAI8474
Epiglaea apiata|[18811]|RDLQF272-06|DH011364|658[0n]bp|Canada.Quebec|BOLD:AAC2729
Epiglaea apiata|[18812]|TMNBB255-06|MNBTT-1195|658[0n]bp|Canada.New Brunswick|BOLD:AAC2729
Epiglaea apiata|[18813]|TMNBB258-06|MNBTT-1198|658[0n]bp|Canada.New Brunswick|BOLD:AAC2729
Epiglaea apiata|[18814]|TMNBB259-06|MNBTT-1199|658[0n]bp|Canada.New Brunswick|BOLD:AAC2729
Epiglaea apiata|[18815]|TMNBB257-06|MNBTT-1197|658[0n]bp|Canada.New Brunswick|BOLD:AAC2729
Epiglaea apiata|[18816]|RDLQF236-06|DH011316|658[0n]bp|Canada.Quebec|BOLD:AAC2729
Epiglaea apiata|[18817]|RDLQF234-06|DH011314|658[0n]bp|Canada.Quebec|BOLD:AAC2729
Epiglaea apiata|[18818]|RDLQF237-06|DH011317|658[0n]bp|Canada.Quebec|BOLD:AAC2729
Epiglaea apiata|[18819]|TMNBB260-06|MNBTT-1200|658[0n]bp|Canada.New Brunswick|BOLD:AAC2729
Epiglaea apiata|[18820]|TMNBB256-06|MNBTT-1196|658[0n]bp|Canada.New Brunswick|BOLD:AAC2729
Parastichtis suspecta|[18821]|LOWCB131-05|CGWC-1071|582[0n]bp|Canada.British Columbia|BOLD:ABY7871
Parastichtis suspecta|[18822]|BBLPB352-10|10BBCLP-1351|658[0n]bp|Canada.Alberta|BOLD:ABY7871
Parastichtis suspecta|[18823]|RDNMB935-05|CNCNoctuoidea10710|658[0n]bp|Canada.British Columbia|BOLD:AB...
Parastichtis suspecta|[18824]|LOWCB130-05|CGWC-1070|577[0n]bp|Canada.British Columbia|BOLD:ABY7871
Parastichtis suspecta|[18825]|RDNMC278-05|CNCNoctuoidea11912|579[0n]bp|Canada.British Columbia|BOLD:AB...
Parastichtis suspecta|[18826]|BBLPB570-10|10BBCLP-1569|658[0n]bp|Canada.British Columbia|BOLD:ABY7871
Parastichtis suspecta|[18827]|BBLPB743-10|10BBCLP-1742|658[0n]bp|Canada.Alberta|BOLD:ABY7871
Parastichtis suspecta|[18828]|BBLPB422-10|10BBCLP-1421|658[0n]bp|Canada.Alberta|BOLD:ABY7871
Parastichtis suspecta|[18829]|BBLPB351-10|10BBCLP-1350|658[0n]bp|Canada.Alberta|BOLD:ABY7871
Parastichtis suspecta|[18830]|MHLEP132-07|CHU06-LEP-132|658[0n]bp|Canada.Manitoba|BOLD:ABY7871
Parastichtis suspecta|[18831]|LOWCD299-06|CGWC-3119|658[0n]bp|Canada.British Columbia|BOLD:ABY7871
Parastichtis suspecta|[18832]|LOWCD534-06|CGWC-3354|658[0n]bp|Canada.British Columbia|BOLD:ABY7871
Parastichtis suspecta|[18833]|LOWCB129-05|CGWC-1069|658[0n]bp|Canada.British Columbia|BOLD:ABY7871
Parastichtis suspecta|[18834]|BBLPB350-10|10BBCLP-1349|658[0n]bp|Canada.Alberta|BOLD:ABY7871
Parastichtis suspecta|[18835]|BBLPB459-10|10BBCLP-1458|658[0n]bp|Canada.Alberta|BOLD:ABY7871
Parastichtis suspecta|[18836]|LCHIP257-07|06-PROBE-2921|650[0n]bp|Canada.Manitoba|BOLD:ABY7871
Parastichtis suspecta|[18837]|BBLPB419-10|10BBCLP-1418|658[0n]bp|Canada.Alberta|BOLD:AAB4551
[illegible]

Parastichtis suspecta|[18835]|BBLPB439-10|10BBCLP-1438|658[0n]bp|Canada.Alberta|BOLD:ABY7871
Parastichtis suspecta|[18836]|LCHIP257-07|06-PROBE-2921|650[0n]bp|Canada.Manitoba|BOLD:ABY7871
Parastichtis suspecta|[18837]|BBLPB419-10|10BBCLP-1418|658[0n]bp|Canada.Alberta|BOLD:AAB4551
Parastichtis suspecta|[18838]|RDNMC277-05|CNCNoctuoidea11911|616[0n]bp|Canada.Alberta|BOLD:AAB4551
Parastichtis suspecta|[18839]|RDNMC279-05|CNCNoctuoidea11913|658[0n]bp|Canada.British Columbia|BOLD:AA...
Parastichtis suspecta|[18840]|RDNMB936-05|CNCNoctuoidea10711|658[0n]bp|Canada.Alberta|BOLD:AAB4551
Parastichtis suspecta|[18841]|BBLPB460-10|10BBCLP-1459|658[0n]bp|Canada.Alberta|BOLD:AAB4551
Parastichtis suspecta|[18842]|LBCH3334-10|10-JDWBC-3334|658[0n]bp|Canada.British Columbia|BOLD:AAB4551
Parastichtis suspecta|[18843]|LBCD416-05|HLC-23236|658[0n]bp|Canada.British Columbia|BOLD:AAB4551
Epiglaea decliva|[18844]|RDLQ574-07|DH009877|658[0n]bp|Canada.Quebec|BOLD:AAX7020
Homoglaea dives|[18845]|GMLC1266-12|2011GM-0962|658[0n]bp|United States.California|BOLD:AAM3001
Homoglaea dives|[18846]|RWWB484-10|RWWA-1483|658[0n]bp|United States.Washington|BOLD:AAM3001
Homoglaea dives|[18847]|RWWC111-10|RWWA-2088|658[0n]bp|United States.Washington|BOLD:AAM3001
Homoglaea dives|[18848]|RWWB529-10|RWWA-1528|658[0n]bp|United States.Washington|BOLD:AAM3001
Homoglaea dives|[18849]|RWWB491-10|RWWA-1490|658[0n]bp|United States.Washington|BOLD:AAM3001
Homoglaea carbonaria|[18850]|LALPA1023-11|AVBC 833-11|658[0n]bp|Canada.British Columbia|BOLD:AAE4974
Homoglaea carbonaria|[18851]|LOWCD928-06|CGWC-3748|521[1n]bp|Canada.British Columbia|BOLD:AAE4974
Homoglaea carbonaria|[18852]|LOWCE593-06|CGWC-4353|658[0n]bp|Canada.British Columbia|BOLD:AAE4974
Homoglaea carbonaria|[18853]|LOWCE592-06|CGWC-4352|658[0n]bp|Canada.British Columbia|BOLD:AAE4974
Homoglaea carbonaria|[18854]|LOWCD927-06|CGWC-3747|658[0n]bp|Canada.British Columbia|BOLD:AAE4974
Homoglaea carbonaria|[18855]|LALPA814-10|AVBC 816-10|658[0n]bp|Canada.British Columbia|BOLD:AAE4974
Homoglaea carbonaria|[18856]|LOWCE590-06|CGWC-4350|658[0n]bp|Canada.British Columbia|BOLD:AAE4974
Homoglaea carbonaria|[18857]|LOWCE591-06|CGWC-4351|658[0n]bp|Canada.British Columbia|BOLD:AAE4974
Homoglaea hircina|[18858]|LOWCE589-06|CGWC-4349|658[0n]bp|Canada.British Columbia|BOLD:AAE1607
Homoglaea hircina|[18859]|XAE639-04|Moth4639.03|610[0n]bp|Canada.Ontario|BOLD:AAE1607
Homoglaea hircina|[18860]|TMNBB228-06|MNBTT-1168|658[0n]bp|Canada.New Brunswick|BOLD:AAE1607
Homoglaea hircina|[18861]|TMNBB229-06|MNBTT-1169|658[0n]bp|Canada.New Brunswick|BOLD:AAE1607
Homoglaea hircina|[18862]|LOWCE588-06|CGWC-4348|656[0n]bp|Canada.British Columbia|BOLD:AAE1607
Agrochola lota|[18863]|RDNMF487-08|NOC14573|609[0n]bp|Canada.Newfoundland and Labrador|BOLD:AAC0283
Xanthia tatago|[18864]|PHMNB296-04|04HBL007761|657[0n]bp|Canada.New Brunswick|BOLD:AAE4535
Xanthia tatago|[18865]|LOWCD167-06|CGWC-2987|614[0n]bp|Canada.British Columbia|BOLD:AAE4535
Xanthia tatago|[18866]|PHMNB272-04|04HBL007737|658[0n]bp|Canada.New Brunswick|BOLD:AAE4535
Xanthia tatago|[18867]|PHMNB283-04|04HBL007748|658[0n]bp|Canada.New Brunswick|BOLD:AAE4535
Xanthia tatago|[18868]|LBCH2864-10|10-JDWBC-2864|658[0n]bp|Canada.British Columbia|BOLD:AAE4535
Xanthia tatago|[18869]|LALPA809-10|AVBC 811-10|658[0n]bp|Canada.British Columbia|BOLD:AAE4535
Bellura obliqua|[18870]|LPVIA285-08|PFC-2006-0376|658[0n]bp|Canada.British Columbia|BOLD:AAA9614
Bellura obliqua|[18871]|LALPA203-10|AVBC 204-10|658[0n]bp|Canada.British Columbia|BOLD:AAA9614
Bellura obliqua|[18872]|LALPA244-10|AVBC 245-10|658[0n]bp|Canada.British Columbia|BOLD:AAA9614
Bellura obliqua|[18873]|BBLPA442-10|10BBCLP-0442|658[0n]bp|Canada.British Columbia|BOLD:AAA9614
Bellura obliqua|[18874]|BBLPA441-10|10BBCLP-0441|658[0n]bp|Canada.British Columbia|BOLD:AAA9614
Bellura obliqua|[18875]|RDNMB927-05|CNCNoctuoidea10702|658[0n]bp|Canada.British Columbia|BOLD:AAA9614
Bellura obliqua|[18876]|BBLPA443-10|10BBCLP-0443|658[0n]bp|Canada.British Columbia|BOLD:AAA9614
Bellura obliqua|[18877]|BBLPA440-10|10BBCLP-0440|642[0n]bp|Canada.British Columbia|BOLD:AAA9614
Bellura obliqua|[18878]|BBLPA439-10|10BBCLP-0439|641[0n]bp|Canada.British Columbia|BOLD:AAA9614
Bellura obliqua|[18879]|LALPA188-10|AVBC 188-10|658[0n]bp|Canada.British Columbia|BOLD:AAA9614
Bellura obliqua|[18880]|LPSOB425-08|PPBP-1424|658[0n]bp|Canada.Ontario|BOLD:AAA9614
Bellura obliqua|[18881]|XAJ420-06|2006-ONT-0420|658[0n]bp|Canada.Ontario|BOLD:AAA9614
Bellura obliqua|[18882]|RDNMB118-05|CNCNoctuoidea10270|658[0n]bp|Canada.Alberta|BOLD:AAA9614
Bellura obliqua|[18883]|RDNMB931-05|CNCNoctuoidea10706|599[0n]bp|Canada.Alberta|BOLD:AAA9614
Bellura obliqua|[18884]|RDNMJ149-10|CNCLEP 70177|658[0n]bp|Canada.Alberta|BOLD:AAA9614
Bellura obliqua|[18885]|RDNMJ150-10|CNCLEP 70178|658[0n]bp|Canada.Alberta|BOLD:AAA9614
Bellura obliqua|[18886]|BBLPA095-10|10BBCLP-0095|658[0n]bp|Canada.Manitoba|BOLD:AAA9614
Bellura obliqua|[18887]|LPMN310-08|08BBLEP-01109|658[0n]bp|Canada.Manitoba|BOLD:AAA9614
Bellura obliqua|[18888]|XAI055-05|0102-ONT-0055|658[0n]bp|Canada.Ontario|BOLD:AAA9614
Bellura obliqua|[18889]|RDNMJ173-10|CNCLEP 70201|658[0n]bp|Canada.Ontario|BOLD:AAA9614
Bellura obliqua|[18890]|LPSO848-08|PPBP-0848|658[0n]bp|Canada.Ontario|BOLD:AAA9614
Bellura obliqua|[18891]|LPSOB424-08|PPBP-1423|658[0n]bp|Canada.Ontario|BOLD:AAA9614
Bellura obliqua|[18892]|XAJ671-06|2006-ONT-0671|658[0n]bp|Canada.Ontario|BOLD:AAA9614
Bellura obliqua|[18893]|BLTIB200-08|BL291|658[0n]bp|Canada.Ontario|BOLD:AAA9614
Bellura obliqua|[18894]|XAE366-04|Moth4366.03|658[0n]bp|Canada.Ontario|BOLD:AAA9614
Bellura obliqua|[18895]|XAB275-04|04HBL005275|658[0n]bp|Canada.Ontario|BOLD:AAA9614
Bellura obliqua|[18896]|XAK338-06|2006-ONT-1333|658[0n]bp|Canada.Ontario|BOLD:AAA9614
Bellura obliqua|[18897]|BLTIB198-08|BL289|658[0n]bp|Canada.Ontario|BOLD:AAA9614
Bellura obliqua|[18898]|RDNM975-05|CNCNoctuoidea7815|658[0n]bp|Canada.Ontario|BOLD:AAA9614
Bellura obliqua|[18899]|RDNMG058-08|NOC14999|658[0n]bp|Canada.Ontario|BOLD:AAA9614
Bellura obliqua|[18900]|BBLPA094-10|10BBCLP-0094|658[0n]bp|Canada.Saskatchewan|BOLD:AAA9614
Bellura obliqua|[18901]|XAI056-05|0102-ONT-0056|658[0n]bp|Canada.Ontario|BOLD:AAA9614
Bellura obliqua|[18902]|RDNMB928-05|CNCNoctuoidea10703|658[0n]bp|Canada.Ontario|BOLD:AAA9614
Bellura sp.|[18903]|RDNML025-13|CNCLEP 94255|658[0n]bp|Canada.Ontario|BOLD:AAA9614
Bellura obliqua|[18904]|RDLQB746-05|DH010661|658[0n]bp|Canada.Quebec|BOLD:AAA9614
Bellura obliqua|[18905]|XAE464-04|Moth4464.03|613[1n]bp|Canada.Ontario|BOLD:AAA9614
Bellura obliqua|[18906]|XAJ539-06|2006-ONT-0539|622[0n]bp|Canada.Ontario|BOLD:AAA9614
Bellura obliqua|[18907]|PHMNB581-04|04HBL00807|658[0n]bp|Canada.New Brunswick|BOLD:AAA9614
Bellura obliqua|[18908]|RDNM976-05|CNCNoctuoidea7816|658[0n]bp|Canada.Ontario|BOLD:AAA9614
Bellura obliqua|[18909]|XAK155-06|2006-ONT-1150|658[0n]bp|Canada.Ontario|BOLD:AAA9614
Bellura obliqua|[18910]|XAG470-05|2005-ONT-1054|658[0n]bp|Canada.Ontario|BOLD:AAA9614
Bellura obliqua|[18911]|RDLQG374-06|DH012608|658[0n]bp|Canada.Quebec|BOLD:AAA9614
Bellura sp.|[18912]|RDNML026-13|CNCLEP 94256|658[0n]bp|Canada.Ontario|BOLD:AAA9614
Bellura vulnifica|[18913]|XAJ829-06|2006-ONT-0829|658[0n]bp|Canada.Ontario|BOLD:AAD3485
Bellura sp.|[18914]|LPSO851-08|PPBP-0851|658[0n]bp|Canada.Ontario|BOLD:AAD3485
Bellura gortynoides|[18915]|RDNMC578-06|CNCNoctuoidea12118|658[0n]bp|Canada.Ontario|BOLD:AAD3485
Bellura vulnifica|[18916]|RDNML371-13|CNCLEP 92347|658[0n]bp|Canada.Ontario|BOLD:AAD3485
Bellura vulnifica|[18917]|RDNML372-13|CNCLEP 92348|658[0n]bp|Canada.Ontario|BOLD:AAD3485
Bellura vulnifica|[18918]|RDNMB930-05|CNCNoctuoidea10705|658[0n]bp|Canada.Quebec|BOLD:AAD3485
Bellura vulnifica|[18919]|XAK423-06|2006-ONT-1418|658[0n]bp|Canada.Ontario|BOLD:AAD3485
Hyppa brunneicrista|[18920]|RDMAB279-05|UASM41498|509[0n]bp|Canada.Alberta|BOLD:ABZ2288
Hyppa brunneicrista|[18921]|LCHP379-07|07PROBE-10003|632[0n]bp|Canada.Manitoba|BOLD:ABZ2288
Hyppa brunneicrista|[18922]|LCHP462-07|07PROBE-10130|658[0n]bp|Canada.Manitoba|BOLD:ABZ2288
Hyppa brunneicrista|[18923]|LCHP383-07|07PROBE-10007|658[0n]bp|Canada.Manitoba|BOLD:ABZ2288
Hyppa brunneicrista|[18924]|LCHP481-07|07PROBE-10149|658[0n]bp|Canada.Manitoba|BOLD:ABZ2288
Hyppa brunneicrista|[18925]|LCH245-04|04HBL003245|658[0n]bp|Canada.Manitoba|BOLD:ABZ2288
Hyppa brunneicrista|[18926]|CHLEP083-09|09PROBE-09378|658[0n]bp|Canada.Manitoba|BOLD:ABZ2288
Hyppa brunneicrista|[18927]|LCHP891-07|07PROBE-10648|658[0n]bp|Canada.Manitoba|BOLD:ABZ2288
Hyppa brunneicrista|[18928]|CHLEP078-09|09PROBE-09373|658[0n]bp|Canada.Manitoba|BOLD:ABZ2288
Hyppa brunneicrista|[18929]|LCHP895-07|07PROBE-10657|658[0n]bp|Canada.Manitoba|BOLD:ABZ2288
Hyppa brunneicrista|[18930]|CHLEP074-09|09PROBE-09369|658[0n]bp|Canada.Manitoba|BOLD:ABZ2288
Hyppa brunneicrista|[18931]|LCH244-04|04HBL003244|658[0n]bp|Canada.Manitoba|BOLD:ABZ2288
Hyppa brunneicrista|[18932]|LCHP184-07|07PROBE-00111|658[0n]bp|Canada.Manitoba|BOLD:ABZ2288
Hyppa brunneicrista|[18933]|LCH243-04|04HBL003243|658[0n]bp|Canada.Manitoba|BOLD:ABZ2288
Hyppa brunneicrista|[18934]|BBLPA447-10|10BBCLP-0447|658[0n]bp|Canada.British Columbia|BOLD:ABZ2288
Hyppa indistincta|[18935]|LBCA367-05|HLC-20367|588[1n]bp|Canada.British Columbia|BOLD:ABX5989
Hyppa indistincta|[18936]|BBLPA454-10|10BBCLP-0454|658[0n]bp|Canada.British Columbia|BOLD:ABX5989
Hyppa indistincta|[18937]|LBCH1919-10|10-JDWBC-1919|640[0n]bp|Canada.British Columbia|BOLD:ABX5989

Hyppa indistincta|[18935]|LBCA367-05|HLC-20367|588[1n]bp|Canada.British Columbia|BOLD:ABX5989
Hyppa indistincta|[18936]|BBLPA454-10|10BBCLP-0454|658[0n]bp|Canada.British Columbia|BOLD:ABX5989
Hyppa indistincta|[18937]|LBCH1919-10|10-JDWBC-1919|640[0n]bp|Canada.British Columbia|BOLD:ABX5989
Hyppa indistincta|[18938]|LBCD095-05|HLC-22915|658[0n]bp|Canada.British Columbia|BOLD:ABX5989
Hyppa indistincta|[18939]|LBCG2393-09|08-JDWBC-2393|658[0n]bp|Canada.British Columbia|BOLD:ABX5989
Hyppa indistincta|[18940]|LBCG2836-09|08-JDWBC-2836|658[0n]bp|Canada.British Columbia|BOLD:ABX5989
Hyppa indistincta|[18941]|LBCH2256-10|10-JDWBC-2256|658[0n]bp|Canada.British Columbia|BOLD:ABX5989
Hyppa indistincta|[18942]|LBCC833-05|HLC-22713|658[0n]bp|Canada.British Columbia|BOLD:ABX5989
Hyppa indistincta|[18943]|LBCC429-05|HLC-22309|658[0n]bp|Canada.British Columbia|BOLD:ABX5989
Hyppa indistincta|[18944]|LPMN918-08|08BBLEP-02276|658[0n]bp|Canada.Alberta|BOLD:ABX5989
Hyppa indistincta|[18945]|LBCG2394-09|08-JDWBC-2394|658[0n]bp|Canada.British Columbia|BOLD:ABX5989
Hyppa indistincta|[18946]|LBCG3053-09|08-JDWBC-3053|658[0n]bp|Canada.British Columbia|BOLD:ABX5989
Hyppa indistincta|[18947]|BBLPA445-10|10BBCLP-0445|658[0n]bp|Canada.Alberta|BOLD:ABX5989
Hyppa indistincta|[18948]|RDMAB548-06|CBCC1043|658[0n]bp|Canada.Alberta|BOLD:ABX5989
Hyppa indistincta|[18949]|BBLPA453-10|10BBCLP-0453|658[0n]bp|Canada.British Columbia|BOLD:ABX5989
Hyppa indistincta|[18950]|BBLPA451-10|10BBCLP-0451|658[0n]bp|Canada.British Columbia|BOLD:ABX5989
Hyppa indistincta|[18951]|LBCA364-05|HLC-20364|658[0n]bp|Canada.British Columbia|BOLD:ABX5989
Hyppa indistincta|[18952]|LBCG2772-09|08-JDWBC-2772|658[0n]bp|Canada.British Columbia|BOLD:ABX5989
Hyppa indistincta|[18953]|LALPA508-10|AVBC 510-10|658[0n]bp|Canada.British Columbia|BOLD:ABX5989
Hyppa indistincta|[18954]|LALPA737-10|AVBC 739-10|658[0n]bp|Canada.British Columbia|BOLD:ABX5989
Hyppa contrasta|[18955]|LOWCB933-05|CGWC-1873|535[0n]bp|Canada.British Columbia|BOLD:AAA5659
Hyppa contrasta|[18956]|LOWCB934-05|CGWC-1874|535[0n]bp|Canada.British Columbia|BOLD:AAA5659
Hyppa contrasta|[18957]|LOWCB922-05|CGWC-1862|536[0n]bp|Canada.British Columbia|BOLD:AAA5659
Hyppa contrasta|[18958]|LOWCE839-06|CGWC-4599|617[0n]bp|Canada.British Columbia|BOLD:AAA5659
Hyppa contrasta|[18959]|LBCB938-05|HLC-21878|637[0n]bp|Canada.British Columbia|BOLD:AAA5659
Hyppa contrasta|[18960]|LBCC430-05|HLC-22310|596[0n]bp|Canada.British Columbia|BOLD:AAA5659
Hyppa contrasta|[18961]|LBCG2835-09|08-JDWBC-2835|658[0n]bp|Canada.British Columbia|BOLD:AAA5659
Hyppa contrasta|[18962]|LBCB408-05|HLC-21348|658[0n]bp|Canada.British Columbia|BOLD:AAA5659
Hyppa contrasta|[18963]|BBLPB685-10|10BBCLP-1684|658[0n]bp|Canada.British Columbia|BOLD:AAA5659
Hyppa contrasta|[18964]|LBCC673-05|HLC-22553|658[0n]bp|Canada.British Columbia|BOLD:AAA5659
Hyppa contrasta|[18965]|LBCA496-05|HLC-20496|658[0n]bp|Canada.British Columbia|BOLD:AAA5659
Hyppa contrasta|[18966]|BBLPA450-10|10BBCLP-0450|658[0n]bp|Canada.British Columbia|BOLD:AAA5659
Hyppa contrasta|[18967]|RDMAB278-05|UASM41497|658[0n]bp|Canada.Alberta|BOLD:AAA5659
Hyppa contrasta|[18968]|LBCA931-05|HLC-20931|658[0n]bp|Canada.British Columbia|BOLD:AAA5659
Hyppa contrasta|[18969]|BBLPA452-10|10BBCLP-0452|658[0n]bp|Canada.British Columbia|BOLD:AAA5659
Hyppa contrasta|[18970]|LBCD028-05|HLC-22848|658[0n]bp|Canada.British Columbia|BOLD:AAA5659
Hyppa contrasta|[18971]|BBLPA449-10|10BBCLP-0449|658[0n]bp|Canada.British Columbia|BOLD:AAA5659
Hyppa contrasta|[18972]|BBLPA448-10|10BBCLP-0448|658[0n]bp|Canada.British Columbia|BOLD:AAA5659
Hyppa contrasta|[18973]|LBCA271-05|HLC-20271|658[0n]bp|Canada.British Columbia|BOLD:AAA5659
Hyppa contrasta|[18974]|LBCD025-05|HLC-22845|658[0n]bp|Canada.British Columbia|BOLD:AAA5659
Hyppa contrasta|[18975]|PHMNB143-04|04HBL007608|658[0n]bp|Canada.New Brunswick|BOLD:AAA5659
Hyppa contrasta|[18976]|BBLPA446-10|10BBCLP-0446|658[0n]bp|Canada.British Columbia|BOLD:AAA5659
Hyppa contrasta|[18977]|LOWCB930-05|CGWC-1870|658[0n]bp|Canada.British Columbia|BOLD:AAA5659
Hyppa contrasta|[18978]|LPMN158-08|08BBLEP-00956|658[0n]bp|Canada.Manitoba|BOLD:AAA5659
Hyppa contrasta|[18979]|RDLQB083-05|DH010169|658[0n]bp|Canada.Quebec|BOLD:AAA5659
Hyppa contrasta|[18980]|BBLPE313-09|09BBELE-2313|658[0n]bp|Canada.Newfoundland and Labrador|BOLD:AAA5659
Hyppa contrasta|[18981]|PHMNB718-05|Moth 411.03SA|658[0n]bp|Canada.New Brunswick|BOLD:AAA5659
Hyppa contrasta|[18982]|LOWCB927-05|CGWC-1867|658[0n]bp|Canada.British Columbia|BOLD:AAA5659
Hyppa contrasta|[18983]|BBLPC939-09|09BBELE-1939|658[0n]bp|Canada.Newfoundland and Labrador|BOLD:AAA5659
Hyppa contrasta|[18984]|BBLPC283-09|09BBELE-1283|658[0n]bp|Canada.Newfoundland and Labrador|BOLD:AAA5659
Hyppa contrasta|[18985]|LBCH2982-10|10-JDWBC-2982|658[0n]bp|Canada.British Columbia|BOLD:AAA5659
Hyppa potamus|[18986]|CNCLA569-13|CNCLEP00019491|658[0n]bp|Canada.Yukon Territory|BOLD:AAA5659
Hyppa contrasta|[18987]|LOWCB926-05|CGWC-1866|639[0n]bp|Canada.British Columbia|BOLD:AAA5659
Hyppa contrasta|[18988]|PHMNB254-04|04HBL007719|561[0n]bp|Canada.New Brunswick|BOLD:AAA5659
Hyppa contrasta|[18989]|LOWCB931-05|CGWC-1871|658[0n]bp|Canada.British Columbia|BOLD:AAA5659
Hyppa contrasta|[18990]|LALPA655-10|AVBC 657-10|658[0n]bp|Canada.British Columbia|BOLD:AAA5659
Hyppa contrasta|[18991]|LALPA1261-11|AVBC 1263-11|658[0n]bp|Canada.British Columbia|BOLD:AAA5659
Hyppa contrasta|[18992]|BBLPB684-10|10BBCLP-1683|658[0n]bp|Canada.British Columbia|BOLD:AAA5659
Hyppa contrasta|[18993]|LALPA910-11|AVBC 1083-11|658[0n]bp|Canada.British Columbia|BOLD:AAA5659
Hyppa contrasta|[18994]|LOWCB932-05|CGWC-1872|658[0n]bp|Canada.British Columbia|BOLD:AAA5659
Hyppa contrasta|[18995]|LOWCB925-05|CGWC-1865|658[0n]bp|Canada.British Columbia|BOLD:AAA5659
Hyppa contrasta|[18996]|LPVIB053-08|PFC-2006-1381|658[0n]bp|Canada.British Columbia|BOLD:AAA5659
Hyppa contrasta|[18997]|BBLPE150-09|09BBELE-2150|658[0n]bp|Canada.Nova Scotia|BOLD:AAA5659
Hyppa contrasta|[18998]|BBLPA444-10|10BBCLP-0444|658[0n]bp|Canada.Alberta|BOLD:AAA5659
Hyppa contrasta|[18999]|BBLPE364-09|09BBELE-2364|658[0n]bp|Canada.Newfoundland and Labrador|BOLD:AAA5659
Hyppa contrasta|[19000]|LOWCB924-05|CGWC-1864|658[0n]bp|Canada.British Columbia|BOLD:AAA5659
Hyppa contrasta|[19001]|LPMN564-08|08BBLEP-01365|655[0n]bp|Canada.Manitoba|BOLD:AAA5659
Hyppa contrasta|[19002]|LOWCB920-05|CGWC-1860|658[0n]bp|Canada.British Columbia|BOLD:AAA5659
Hyppa contrasta|[19003]|BBLEC349-09|09BBELE-0349|658[0n]bp|Canada.Newfoundland and Labrador|BOLD:AAA5659
Hyppa contrasta|[19004]|LPMN818-08|08BBLEP-01621|658[0n]bp|Canada.Manitoba|BOLD:AAA5659
Hyppa contrasta|[19005]|BBLPE056-09|09BBELE-2056|658[0n]bp|Canada.Nova Scotia|BOLD:AAA5659
Hyppa contrasta|[19006]|PHMNB757-05|Moth 450.03SA|658[0n]bp|Canada.New Brunswick|BOLD:AAA5659
Hyppa contrasta|[19007]|BBLPE366-09|09BBELE-2366|658[0n]bp|Canada.Newfoundland and Labrador|BOLD:AAA5659
Hyppa contrasta|[19008]|LOWCB929-05|CGWC-1869|658[0n]bp|Canada.British Columbia|BOLD:AAA5659
Hyppa contrasta|[19009]|BBLPB686-10|10BBCLP-1685|658[0n]bp|Canada.British Columbia|BOLD:AAA5659
Hyppa contrasta|[19010]|LBCC013-05|HLC-21893|658[0n]bp|Canada.British Columbia|BOLD:AAA5659
Hyppa contrasta|[19011]|LCH242-04|04HBL003242|658[0n]bp|Canada.Manitoba|BOLD:AAA5659
Hyppa contrasta|[19012]|PHMNB174-04|04HBL007639|658[0n]bp|Canada.New Brunswick|BOLD:AAA5659
Hyppa contrasta|[19013]|LOWCB921-05|CGWC-1861|658[0n]bp|Canada.British Columbia|BOLD:AAA5659
Hyppa contrasta|[19014]|BBLPE412-09|09BBELE-2412|658[0n]bp|Canada.Newfoundland and Labrador|BOLD:AAA5659
Hyppa contrasta|[19015]|LALPA1170-11|AVBC 980-11|658[0n]bp|Canada.British Columbia|BOLD:AAA5659
Hyppa contrasta|[19016]|LBCH2246-10|10-JDWBC-2246|658[0n]bp|Canada.British Columbia|BOLD:AAA5659
Hyppa contrasta|[19017]|LOWCB928-05|CGWC-1868|658[0n]bp|Canada.British Columbia|BOLD:AAA5659
Hyppa contrasta|[19018]|LPAB215-08|08BBLEP-02537|658[0n]bp|Canada.Alberta|BOLD:AAA5659
Hyppa contrasta|[19019]|LBCB405-05|HLC-21345|658[0n]bp|Canada.British Columbia|BOLD:AAA5659
Hyppa contrasta|[19020]|RDMAB280-05|UASM41499|658[0n]bp|Canada.Alberta|BOLD:AAA5659
Hyppa contrasta|[19021]|LOWCB923-05|CGWC-1863|649[0n]bp|Canada.British Columbia|BOLD:AAA5659
Hyppa contrasta|[19022]|LCHP830-07|07PROBE-10587|643[0n]bp|Canada.Manitoba|BOLD:AAA5659
Hyppa contrasta|[19023]|BBLPE279-09|09BBELE-2279|638[0n]bp|Canada.Nova Scotia|BOLD:AAA5659
Hyppa contrasta|[19024]|BBLPE088-09|09BBELE-2088|634[0n]bp|Canada.Nova Scotia|BOLD:AAA5659
Hyppa contrasta|[19025]|PHMNB034-03|moth20.02SA|639[0n]bp|Canada.New Brunswick|BOLD:AAA5659
Hyppa contrasta|[19026]|LBCH494-10|10-JDWBC-0494|658[0n]bp|Canada.British Columbia|BOLD:AAA5659
Hyppa contrasta|[19027]|BBLPE351-09|09BBELE-2351|658[0n]bp|Canada.Newfoundland and Labrador|BOLD:AAA5659
Hyppa contrasta|[19028]|BBLPE269-09|09BBELE-2269|658[0n]bp|Canada.Nova Scotia|BOLD:AAA5659
Hyppa potamus|[19029]|CNCLA565-13|CNCLEP00098997|658[0n]bp|Canada.Yukon Territory|BOLD:AAA5659
Hyppa xylinoides|[19030]|LPSOB714-08|PPBP-1713|621[0n]bp|Canada.Ontario|BOLD:ABY9574
Hyppa xylinoides|[19031]|XAG993-05|2005-ONT-1577|629[0n]bp|Canada.Ontario|BOLD:ABY9574
Hyppa xylinoides|[19032]|XAH148-05|2005-ONT-1731|658[0n]bp|Canada.Ontario|BOLD:ABY9574
Hyppa xylinoides|[19033]|TTMNB367-06|MNBTT-367|658[1n]bp|Canada.New Brunswick|BOLD:ABY9574
Hyppa xylinoides|[19034]|TTMNB368-06|MNBTT-368|658[1n]bp|Canada.New Brunswick|BOLD:ABY9574
Hyppa xylinoides|[19035]|XAD367-04|04HBL007367|582[0n]bp|Canada.Ontario|BOLD:ABY9574
Hyppa xylinoides|[19036]|XAF741-05|2005-ONT-390|547[0n]bp|Canada.Ontario|BOLD:ABY9574
Hyppa xylinoides|[19037]|XAH005-05|2005-ONT-1588|637[0n]bp|Canada.Ontario|BOLD:ABY9574

[illegible]
Hyppa xylinoides|[19035]|XAD367-04|04HBL007367|582[0n]bp|Canada.Ontario|BOLD:ABY9574
Hyppa xylinoides|[19036]|XAF741-05|2005-ONT-390|547[0n]bp|Canada.Ontario|BOLD:ABY9574
Hyppa xylinoides|[19037]|XAH005-05|2005-ONT-1588|637[0n]bp|Canada.Ontario|BOLD:ABY9574
Hyppa xylinoides|[19038]|XAJ341-06|2006-ONT-0341|658[0n]bp|Canada.Ontario|BOLD:ABY9574
Hyppa xylinoides|[19039]|XAG347-05|2005-ONT-931|658[0n]bp|Canada.Ontario|BOLD:ABY9574
Hyppa xylinoides|[19040]|XAG876-05|2005-ONT-1460|658[0n]bp|Canada.Ontario|BOLD:ABY9574
Hyppa xylinoides|[19041]|LPSOB976-08|PPBP-1975|658[0n]bp|Canada.Ontario|BOLD:ABY9574
Hyppa xylinoides|[19042]|BLTIB223-08|BL404|658[0n]bp|Canada.Ontario|BOLD:ABY9574
Hyppa xylinoides|[19043]|XAJ405-06|2006-ONT-0405|658[0n]bp|Canada.Ontario|BOLD:ABY9574
Hyppa xylinoides|[19044]|BLTIB163-08|BL239|658[0n]bp|Canada.Ontario|BOLD:ABY9574
Hyppa xylinoides|[19045]|XAJ547-06|2006-ONT-0547|658[0n]bp|Canada.Ontario|BOLD:ABY9574
Hyppa xylinoides|[19046]|XAJ265-06|2006-ONT-0265|658[0n]bp|Canada.Ontario|BOLD:ABY9574
Hyppa xylinoides|[19047]|XAJ672-06|2006-ONT-0672|658[0n]bp|Canada.Ontario|BOLD:ABY9574
Hyppa xylinoides|[19048]|XAG805-05|2005-ONT-1389|658[0n]bp|Canada.Ontario|BOLD:ABY9574
Hyppa xylinoides|[19049]|XAB398-04|04HBL005398|658[0n]bp|Canada.Ontario|BOLD:ABY9574
Hyppa xylinoides|[19050]|JSMAY1452-11|BIOUG01497-G04|658[0n]bp|Canada.Ontario|BOLD:ABY9574
Hyppa xylinoides|[19051]|XAD355-04|04HBL007355|658[0n]bp|Canada.Ontario|BOLD:ABY9574
Hyppa xylinoides|[19052]|LPSOB326-08|PPBP-1325|658[0n]bp|Canada.Ontario|BOLD:ABY9574
Hyppa xylinoides|[19053]|XAJ318-06|2006-ONT-0318|658[0n]bp|Canada.Ontario|BOLD:ABY9574
Hyppa xylinoides|[19054]|XAJ681-06|2006-ONT-0681|656[0n]bp|Canada.Ontario|BOLD:ABY9574
Hyppa xylinoides|[19055]|TMG116-03|moth328.01|639[0n]bp|Canada.Ontario|BOLD:ABY9574
Hyppa xylinoides|[19056]|PMG120-03|moth329.01|617[0n]bp|Canada.Ontario|BOLD:ABY9574
Hyppa xylinoides|[19057]|XAD589-05|2005-ONT-4|642[0n]bp|Canada.Ontario|BOLD:ABY9574
Hyppa xylinoides|[19058]|RDLQ490-07|DH006301|612[0n]bp|Canada.Quebec|BOLD:ABY9574
Hyppa xylinoides|[19059]|XAF522-05|2005-ONT-171|658[0n]bp|Canada.Ontario|BOLD:ABY9574
Hyppa xylinoides|[19060]|XAB064-04|04HBL005064|658[0n]bp|Canada.Ontario|BOLD:ABY9574
Hyppa xylinoides|[19061]|XAJ548-06|2006-ONT-0548|658[0n]bp|Canada.Ontario|BOLD:ABY9574
Hyppa xylinoides|[19062]|LPSOC323-08|PPBP-2322|658[0n]bp|Canada.Ontario|BOLD:ABY9574
Hyppa xylinoides|[19063]|XAJ620-06|2006-ONT-0620|658[0n]bp|Canada.Ontario|BOLD:ABY9574
Hyppa xylinoides|[19064]|XAF547-05|2005-ONT-196|658[0n]bp|Canada.Ontario|BOLD:ABY9574
Hyppa xylinoides|[19065]|XAD235-04|04HBL007235|658[0n]bp|Canada.Ontario|BOLD:ABY9574
Hyppa xylinoides|[19066]|LPSOC086-08|PPBP-2085|658[0n]bp|Canada.Ontario|BOLD:ABY9574
Hyppa xylinoides|[19067]|XAJ546-06|2006-ONT-0546|658[0n]bp|Canada.Ontario|BOLD:ABY9574
Hyppa xylinoides|[19068]|LPSOB454-08|PPBP-1453|658[0n]bp|Canada.Ontario|BOLD:ABY9574
Hyppa xylinoides|[19069]|TTMNB375-06|MNBTT-375|658[0n]bp|Canada.New Brunswick|BOLD:ABY9574
Hyppa xylinoides|[19070]|XAJ583-06|2006-ONT-0583|658[0n]bp|Canada.Ontario|BOLD:ABY9574
Hyppa xylinoides|[19071]|MNBB360-05|05-NBSTA-276|658[0n]bp|Canada.New Brunswick|BOLD:ABY9574
Hyppa xylinoides|[19072]|PHMNB357-04|04HBL00583|658[0n]bp|Canada.New Brunswick|BOLD:ABY9574
Anathix aggressa|[19073]|RDNMB955-05|CNCNoctuoidea10730|658[0n]bp|Canada.Alberta|BOLD:ACF4918
Anathix aggressa|[19074]|RDNM730-05|CNCNoctuoidea7576|606[0n]bp|Canada.Alberta|BOLD:ACF4918
Anathix aggressa|[19075]|RDNMB954-05|CNCNoctuoidea10729|658[0n]bp|Canada.Alberta|BOLD:ABY4570
Anathix puta|[19076]|RDNMB957-05|CNCNoctuoidea10732|658[0n]bp|Canada.Alberta|BOLD:AAD2871
Anathix puta|[19077]|BBLPB432-10|10BBCLP-1431|658[0n]bp|Canada.Saskatchewan|BOLD:AAD2871
Anathix puta|[19078]|RDLQB514-05|DH010600|658[0n]bp|Canada.Quebec|BOLD:AAD2871
Anathix puta|[19079]|BBLPB365-10|10BBCLP-1364|658[0n]bp|Canada.Alberta|BOLD:AAD2871
Anathix puta|[19080]|RDLQ577-07|DH009071|586[0n]bp|Canada.Quebec|BOLD:AAD2871
Anathix puta|[19081]|RDLQB515-05|DH010601|658[0n]bp|Canada.Quebec|BOLD:AAD2871
Anathix puta|[19082]|BBLPB400-10|10BBCLP-1399|658[0n]bp|Canada.Alberta|BOLD:AAD2871
Anathix puta|[19083]|BBLPB436-10|10BBCLP-1435|658[0n]bp|Canada.Alberta|BOLD:AAD2871
Anathix puta|[19084]|BBLPB435-10|10BBCLP-1434|658[0n]bp|Canada.Alberta|BOLD:AAD2871
Anathix puta|[19085]|BBLPB433-10|10BBCLP-1432|658[0n]bp|Canada.Alberta|BOLD:AAD2871
Anathix puta|[19086]|BBLPB369-10|10BBCLP-1368|658[0n]bp|Canada.Saskatchewan|BOLD:AAD2871
Anathix puta|[19087]|BBLPB430-10|10BBCLP-1429|658[0n]bp|Canada.Saskatchewan|BOLD:AAD2871
Anathix puta|[19088]|BBLPB441-10|10BBCLP-1440|658[0n]bp|Canada.Saskatchewan|BOLD:AAD2871
Anathix puta|[19089]|BBLPB431-10|10BBCLP-1430|658[0n]bp|Canada.Saskatchewan|BOLD:AAD2871
Anathix puta|[19090]|XAH434-05|2005-ONT-2017|658[0n]bp|Canada.Ontario|BOLD:AAD2871
Anathix puta|[19091]|RDLQ578-07|DH009070|567[0n]bp|Canada.Quebec|BOLD:AAD2871
Anathix puta|[19092]|RDLQF174-06|DH011201|658[0n]bp|Canada.Quebec|BOLD:AAD2871
Anathix ralla|[19093]|RDLQB502-05|DH010588|658[0n]bp|Canada.Quebec|BOLD:AAC9569
Anathix ralla|[19094]|MNAC922-07|CNCLEP00027663|657[0n]bp|Canada.Quebec|BOLD:AAC9569
Anathix ralla|[19095]|RDNMB956-05|CNCNoctuoidea10731|658[0n]bp|Canada.Ontario|BOLD:AAC9569
Anathix ralla|[19096]|RDLQ575-07|DH011271|593[0n]bp|Canada.Quebec|BOLD:AAC9569
Anathix ralla|[19097]|RDLQ576-07|DH009067|594[0n]bp|Canada.Quebec|BOLD:AAC9569
Sunira bicolorago|[19098]|XAD497-04|04HBL007497|581[0n]bp|Canada.Ontario|BOLD:AAA4426
Sunira bicolorago|[19099]|XAH824-05|2005-ONT-2407|658[0n]bp|Canada.Ontario|BOLD:AAA4426
Sunira bicolorago|[19100]|XAB656-04|04HBL005656|658[0n]bp|Canada.Ontario|BOLD:AAA4426
Sunira bicolorago|[19101]|JSSEP1089-11|BIOUG01497-G02|658[0n]bp|Canada.Ontario|BOLD:AAA4426
Sunira bicolorago|[19102]|XAH633-05|2005-ONT-2216|658[0n]bp|Canada.Ontario|BOLD:AAA4426
Sunira bicolorago|[19103]|XAH785-05|2005-ONT-2368|658[0n]bp|Canada.Ontario|BOLD:AAA4426
Sunira bicolorago|[19104]|XAH636-05|2005-ONT-2219|658[0n]bp|Canada.Ontario|BOLD:AAA4426
Sunira bicolorago|[19105]|XAH764-05|2005-ONT-2347|658[0n]bp|Canada.Ontario|BOLD:AAA4426
Sunira bicolorago|[19106]|XAB658-04|04HBL005658|658[0n]bp|Canada.Ontario|BOLD:AAA4426
Sunira bicolorago|[19107]|XAB657-04|04HBL005657|658[0n]bp|Canada.Ontario|BOLD:AAA4426
Sunira bicolorago|[19108]|XAD495-04|04HBL007495|527[0n]bp|Canada.Ontario|BOLD:AAA4426
Sunira bicolorago|[19109]|PHSEP391-11|BIOUG01292-F02|658[0n]bp|Canada.Ontario|BOLD:AAA4426
Sunira bicolorago|[19110]|XAH708-05|2005-ONT-2291|650[0n]bp|Canada.Ontario|BOLD:AAA4426
Sunira bicolorago|[19111]|XAH603-05|2005-ONT-2186|658[0n]bp|Canada.Ontario|BOLD:AAA4426
Sunira bicolorago|[19112]|XAH627-05|2005-ONT-2210|658[0n]bp|Canada.Ontario|BOLD:AAA4426
Sunira bicolorago|[19113]|PHSEP397-11|BIOUG01292-F08|658[0n]bp|Canada.Ontario|BOLD:AAA4426
Sunira bicolorago|[19114]|XAH851-05|2005-ONT-2434|658[0n]bp|Canada.Ontario|BOLD:AAA4426
Sunira bicolorago|[19115]|XAH775-05|2005-ONT-2358|658[0n]bp|Canada.Ontario|BOLD:AAA4426
Sunira bicolorago|[19116]|XAH607-05|2005-ONT-2190|658[0n]bp|Canada.Ontario|BOLD:AAA4426
Sunira bicolorago|[19117]|XAB655-04|04HBL005655|658[0n]bp|Canada.Ontario|BOLD:AAA4426
Sunira bicolorago|[19118]|XAD494-04|04HBL007494|588[0n]bp|Canada.Ontario|BOLD:AAA4426
Sunira bicolorago|[19119]|XAB654-04|04HBL005654|658[0n]bp|Canada.Ontario|BOLD:AAA4426
Sunira bicolorago|[19120]|PHSEP400-11|BIOUG01292-F11|658[0n]bp|Canada.Ontario|BOLD:AAA4426
Sunira bicolorago|[19121]|RDNM740-05|CNCNoctuoidea7586|658[0n]bp|Canada.Ontario|BOLD:AAA4426
Sunira bicolorago|[19122]|XAH709-05|2005-ONT-2292|658[0n]bp|Canada.Ontario|BOLD:AAA4426
Sunira bicolorago|[19123]|PHNOV485-11|BIOUG01521-D07|658[0n]bp|Canada.Ontario|BOLD:AAA4426
Sunira bicolorago|[19124]|XAH790-05|2005-ONT-2373|658[0n]bp|Canada.Ontario|BOLD:AAA4426
Sunira bicolorago|[19125]|XAH789-05|2005-ONT-2372|658[0n]bp|Canada.Ontario|BOLD:AAA4426
Sunira bicolorago|[19126]|XAH710-05|2005-ONT-2293|645[0n]bp|Canada.Ontario|BOLD:AAA4426
Sunira bicolorago|[19127]|XAB700-04|04HBL005700|658[0n]bp|Canada.Ontario|BOLD:AAA4426
Sunira bicolorago|[19128]|XAH762-05|2005-ONT-2345|658[0n]bp|Canada.Ontario|BOLD:AAA4426
Sunira bicolorago|[19129]|XAB662-04|04HBL005662|658[0n]bp|Canada.Ontario|BOLD:AAA4426
Sunira bicolorago|[19130]|XAH612-05|2005-ONT-2195|633[0n]bp|Canada.Ontario|BOLD:AAA4426
Sunira bicolorago|[19131]|XAH748-05|2005-ONT-2331|658[0n]bp|Canada.Ontario|BOLD:AAA4426
Sunira bicolorago|[19132]|PHSEP407-11|BIOUG01292-G06|658[0n]bp|Canada.Ontario|BOLD:AAA4426
Sunira bicolorago|[19133]|XAB696-04|04HBL005696|658[0n]bp|Canada.Ontario|BOLD:AAA4426
Sunira bicolorago|[19134]|PHSEP393-11|BIOUG01292-F04|658[0n]bp|Canada.Ontario|BOLD:AAA4426
Sunira bicolorago|[19135]|XAH477-05|2005-ONT-2060|658[0n]bp|Canada.Ontario|BOLD:AAA4426
Sunira bicolorago|[19136]|XAH561-05|2005-ONT-2144|658[0n]bp|Canada.Ontario|BOLD:AAA4426
[illegible]

Sunira bicolorago|[19134]|PHSEP393-11|BIOUG01292-F04|658[0n]bp|Canada.Ontario|BOLD:AAA4426
Sunira bicolorago|[19135]|XAH477-05|2005-ONT-2060|658[0n]bp|Canada.Ontario|BOLD:AAA4426
Sunira bicolorago|[19136]|XAH561-05|2005-ONT-2144|658[0n]bp|Canada.Ontario|BOLD:AAA4426
Sunira bicolorago|[19137]|XAH836-05|2005-ONT-2419|658[0n]bp|Canada.Ontario|BOLD:AAA4426
Sunira bicolorago|[19138]|XAH535-05|2005-ONT-2118|658[0n]bp|Canada.Ontario|BOLD:AAA4426
Sunira bicolorago|[19139]|PHOCT920-11|BIOUG01497-E09|658[0n]bp|Canada.Ontario|BOLD:AAA4426
Sunira bicolorago|[19140]|PHSEP405-11|BIOUG01292-G04|658[0n]bp|Canada.Ontario|BOLD:AAA4426
Sunira bicolorago|[19141]|XAB698-04|04HBL005698|658[0n]bp|Canada.Ontario|BOLD:AAA4426
Sunira bicolorago|[19142]|XAH734-05|2005-ONT-2317|658[0n]bp|Canada.Ontario|BOLD:AAA4426
Sunira bicolorago|[19143]|RDLQB507-05|DH010593|658[0n]bp|Canada.Quebec|BOLD:AAA4426
Sunira bicolorago|[19144]|XAH788-05|2005-ONT-2371|658[0n]bp|Canada.Ontario|BOLD:AAA4426
Sunira bicolorago|[19145]|XAH802-05|2005-ONT-2385|658[0n]bp|Canada.Ontario|BOLD:AAA4426
Sunira bicolorago|[19146]|XAH662-05|2005-ONT-2245|658[0n]bp|Canada.Ontario|BOLD:AAA4426
Sunira bicolorago|[19147]|XAH747-05|2005-ONT-2330|658[0n]bp|Canada.Ontario|BOLD:AAA4426
Sunira bicolorago|[19148]|RDLQF246-06|DH011326|658[0n]bp|Canada.Quebec|BOLD:AAA4426
Sunira bicolorago|[19149]|XAH787-05|2005-ONT-2370|658[0n]bp|Canada.Ontario|BOLD:AAA4426
Sunira bicolorago|[19150]|XAH663-05|2005-ONT-2246|658[0n]bp|Canada.Ontario|BOLD:AAA4426
Sunira bicolorago|[19151]|XAH574-05|2005-ONT-2157|658[0n]bp|Canada.Ontario|BOLD:AAA4426
Sunira bicolorago|[19152]|XAB663-04|04HBL005663|658[0n]bp|Canada.Ontario|BOLD:AAA4426
Sunira bicolorago|[19153]|PHMNB287-04|04HBL007752|658[0n]bp|Canada.New Brunswick|BOLD:AAA4426
Sunira bicolorago|[19154]|XAB540-04|04HBL005540|658[0n]bp|Canada.Ontario|BOLD:AAA4426
Sunira bicolorago|[19155]|XAH637-05|2005-ONT-2220|658[0n]bp|Canada.Ontario|BOLD:AAA4426
Sunira bicolorago|[19156]|XAH698-05|2005-ONT-2281|658[0n]bp|Canada.Ontario|BOLD:AAA4426
Sunira bicolorago|[19157]|XAH635-05|2005-ONT-2218|658[0n]bp|Canada.Ontario|BOLD:AAA4426
Sunira bicolorago|[19158]|PHSEP403-11|BIOUG01292-G02|658[0n]bp|Canada.Ontario|BOLD:AAA4426
Sunira bicolorago|[19159]|XAH803-05|2005-ONT-2386|658[0n]bp|Canada.Ontario|BOLD:AAA4426
Sunira bicolorago|[19160]|XAH844-05|2005-ONT-2427|658[0n]bp|Canada.Ontario|BOLD:AAA4426
Sunira bicolorago|[19161]|XAH664-05|2005-ONT-2247|658[0n]bp|Canada.Ontario|BOLD:AAA4426
Sunira bicolorago|[19162]|XAB673-04|04HBL005673|658[0n]bp|Canada.Ontario|BOLD:AAA4426
Sunira bicolorago|[19163]|PHSEP399-11|BIOUG01292-F10|658[0n]bp|Canada.Ontario|BOLD:AAA4426
Sunira bicolorago|[19164]|XAB669-04|04HBL005669|658[0n]bp|Canada.Ontario|BOLD:AAA4426
Sunira bicolorago|[19165]|XAD492-04|04HBL007492|589[0n]bp|Canada.Ontario|BOLD:AAA4426
Sunira bicolorago|[19166]|XAD491-04|04HBL007491|583[0n]bp|Canada.Ontario|BOLD:AAA4426
Sunira bicolorago|[19167]|XAB672-04|04HBL005672|658[0n]bp|Canada.Ontario|BOLD:AAA4426
Sunira bicolorago|[19168]|RDLQ583-07|DH003125|592[1n]bp|Canada.Quebec|BOLD:AAA4426
Sunira bicolorago|[19169]|XAD493-04|04HBL007493|604[0n]bp|Canada.Ontario|BOLD:AAA4426
Sunira bicolorago|[19170]|RDLQ579-07|DH006168|599[0n]bp|Canada.Quebec|BOLD:AAA4426
Sunira bicolorago|[19171]|RDLQ580-07|DH007056|590[0n]bp|Canada.Quebec|BOLD:AAA4426
Sunira bicolorago|[19172]|PHMNB285-04|04HBL007750|616[0n]bp|Canada.New Brunswick|BOLD:AAA4426
Sunira bicolorago|[19173]|RDLQ581-07|DH003118|592[0n]bp|Canada.Quebec|BOLD:AAA4426
Sunira bicolorago|[19174]|XAH634-05|2005-ONT-2217|634[0n]bp|Canada.Ontario|BOLD:AAA4426
Sunira bicolorago|[19175]|RDLQ582-07|DH009884|592[0n]bp|Canada.Quebec|BOLD:AAA4426
Sunira bicolorago|[19176]|XAH632-05|2005-ONT-2215|658[0n]bp|Canada.Ontario|BOLD:AAA4426
Sunira bicolorago|[19177]|XAH582-05|2005-ONT-2165|658[0n]bp|Canada.Ontario|BOLD:AAA4426
Sunira bicolorago|[19178]|XAH786-05|2005-ONT-2369|658[0n]bp|Canada.Ontario|BOLD:AAA4426
Sunira bicolorago|[19179]|XAH689-05|2005-ONT-2272|658[0n]bp|Canada.Ontario|BOLD:AAA4426
Sunira bicolorago|[19180]|PHMNB303-04|04HBL007768|658[2n]bp|Canada.New Brunswick|BOLD:AAA4426
Sunira bicolorago|[19181]|XAH661-05|2005-ONT-2244|658[0n]bp|Canada.Ontario|BOLD:AAA4426
Sunira bicolorago|[19182]|RDNM741-05|CNCNoctuoidea7587|658[0n]bp|Canada.Ontario|BOLD:AAA4426
Sunira decipiens|[19183]|RDMAB944-09|UASM126463|620[0n]bp|Canada.British Columbia|BOLD:AAA4426
Sunira decipiens|[19184]|LALPA1321-12|AVBC 1323-11|601[0n]bp|Canada.British Columbia|BOLD:AAA4426
Sunira decipiens|[19185]|LALPA826-10|AVBC 828-10|658[0n]bp|Canada.British Columbia|BOLD:AAH9895
Sunira decipiens|[19186]|RDNM742-05|CNCNoctuoidea7588|565[3n]bp|Canada.British Columbia|BOLD:AAH9895
Sunira decipiens|[19187]|RDMAB943-09|UASM126464|634[0n]bp|Canada.British Columbia|BOLD:AAH9895
Sunira decipiens|[19188]|LALPA824-10|AVBC 826-10|658[0n]bp|Canada.British Columbia|BOLD:AAH9895
Sunira decipiens|[19189]|LALPA751-10|AVBC 753-10|658[0n]bp|Canada.British Columbia|BOLD:AAH9895
Sunira decipiens|[19190]|LALPA777-10|AVBC 779-10|658[0n]bp|Canada.British Columbia|BOLD:AAH9895
Sunira decipiens|[19191]|RDNM743-05|CNCNoctuoidea7589|529[0n]bp|Canada.British Columbia|BOLD:AAH9895
Sunira decipiens|[19192]|LALPA825-10|AVBC 827-10|658[0n]bp|Canada.British Columbia|BOLD:AAH9895
Sunira verberata|[19193]|LBCH2509-10|10-JDWBC-2509|658[0n]bp|Canada.British Columbia|BOLD:AAD2187
Sunira verberata|[19194]|RDNMC196-05|CNCNoctuoideaH008|658[0n]bp|Canada.New Brunswick|BOLD:AAD2187
Sunira verberata|[19195]|RDNMC195-05|CNCNoctuoideaH007|594[1n]bp|Canada.New Brunswick|BOLD:AAD2187
Sunira verberata|[19196]|LOWCE560-06|CGWC-4320|605[0n]bp|Canada.British Columbia|BOLD:AAD2187
Sunira verberata|[19197]|LBCH2510-10|10-JDWBC-2510|658[0n]bp|Canada.British Columbia|BOLD:AAD2187
Sunira verberata|[19198]|RDMAB580-06|UASM58546|658[0n]bp|Canada.Alberta|BOLD:AAD2187
Sunira verberata|[19199]|LBCH2638-10|10-JDWBC-2638|658[0n]bp|Canada.British Columbia|BOLD:AAD2187
Sunira verberata|[19200]|LBCH2508-10|10-JDWBC-2508|658[0n]bp|Canada.British Columbia|BOLD:AAD2187
Sunira verberata|[19201]|RDNMC199-05|CNCNoctuoideaH011|593[0n]bp|Canada.New Brunswick|BOLD:AAD2187
Sunira verberata|[19202]|LBCH2571-10|10-JDWBC-2571|632[0n]bp|Canada.British Columbia|BOLD:AAD2187
Sunira verberata|[19203]|RDNMC197-05|CNCNoctuoideaH009|658[0n]bp|Canada.New Brunswick|BOLD:AAD2187
Sunira verberata|[19204]|LBCH2536-10|10-JDWBC-2536|658[0n]bp|Canada.British Columbia|BOLD:AAD2187
Sunira verberata|[19205]|RDNMC198-05|CNCNoctuoideaH010|658[0n]bp|Canada.New Brunswick|BOLD:AAD2187
Agrochola pulchella|[19206]|LBCH2463-10|10-JDWBC-2463|658[0n]bp|Canada.British Columbia|BOLD:AAH6129
Agrochola pulchella|[19207]|LBCH2511-10|10-JDWBC-2511|658[0n]bp|Canada.British Columbia|BOLD:AAH6129
Agrochola pulchella|[19208]|RDNMF227-08|NOC14313|658[0n]bp|Canada.British Columbia|BOLD:AAF7887
Agrochola purpurea|[19209]|RDNMF492-08|NOC14578|658[0n]bp|Canada.British Columbia|BOLD:AAC1554
Agrochola purpurea|[19210]|RDNMF491-08|NOC14577|652[0n]bp|Canada.British Columbia|BOLD:AAC1554
Ipimorpha pleonectusa|[19211]|XAD089-04|04HBL007089|583[0n]bp|Canada.Ontario|BOLD:AAB4652
Ipimorpha pleonectusa|[19212]|XAG759-05|2005-ONT-1343|658[1n]bp|Canada.Ontario|BOLD:AAB4652
Ipimorpha pleonectusa|[19213]|BBLPA435-10|10BBCLP-0435|658[0n]bp|Canada.Alberta|BOLD:AAB4652
Ipimorpha pleonectusa|[19214]|RDMAB668-06|UASM7191|627[0n]bp|Canada.Alberta|BOLD:AAB4652
Ipimorpha pleonectusa|[19215]|RDMAB669-06|UASM58765|531[0n]bp|Canada.Alberta|BOLD:AAB4652
Ipimorpha pleonectusa|[19216]|LPABC990-09|08BBLEP-05401|658[0n]bp|Canada.Alberta|BOLD:AAB4652
Ipimorpha nanaimo|[19217]|RDNMF513-08|NOC14599|658[1n]bp|Canada.British Columbia|BOLD:AAB4652
Ipimorpha pleonectusa|[19218]|RDNMC021-05|CNCNoctuoidea10762|515[2n]bp|Canada.Alberta|BOLD:AAB4652
Ipimorpha pleonectusa|[19219]|XAG863-05|2005-ONT-1447|658[0n]bp|Canada.Ontario|BOLD:AAB4652
Ipimorpha pleonectusa|[19220]|XAG387-05|2005-ONT-971|656[0n]bp|Canada.Ontario|BOLD:AAB4652
Ipimorpha pleonectusa|[19221]|RDLQF253-06|DH011333|658[0n]bp|Canada.Quebec|BOLD:AAB4652
Ipimorpha pleonectusa|[19222]|LPMNB429-09|08BBLEP-05429|658[0n]bp|Canada.Manitoba|BOLD:AAB4652
Ipimorpha nanaimo|[19223]|LHLEP192-06|UBC-2006-1934|658[0n]bp|Canada.British Columbia|BOLD:AAB4652
Ipimorpha nanaimo|[19224]|RDNMF514-08|NOC14600|658[0n]bp|Canada.British Columbia|BOLD:AAB4652
Ipimorpha nanaimo|[19225]|LALPA1312-11|AVBC 1314-11|658[0n]bp|Canada.British Columbia|BOLD:AAB4652
Ipimorpha nanaimo|[19226]|RDNMF515-08|NOC14601|658[0n]bp|Canada.British Columbia|BOLD:AAB4652
Ipimorpha nanaimo|[19227]|LHLEP191-06|UBC-2006-1532|658[0n]bp|Canada.British Columbia|BOLD:AAB4652
Ipimorpha nanaimo|[19228]|RDNMF516-08|NOC14602|658[0n]bp|Canada.British Columbia|BOLD:AAB4652
Ipimorpha nanaimo|[19229]|LPVIB066-08|PFC-2006-1408|658[0n]bp|Canada.British Columbia|BOLD:AAB4652
Ipimorpha pleonectusa|[19230]|XAG974-05|2005-ONT-1558|510[1n]bp|Canada.Ontario|BOLD:AAB4652
Ipimorpha pleonectusa|[19231]|PHMO317-03|moth2385.02|639[0n]bp|Canada.Ontario|BOLD:AAB4652
Ipimorpha pleonectusa|[19232]|XAD301-04|04HBL007301|530[0n]bp|Canada.Ontario|BOLD:AAB4652
Ipimorpha pleonectusa|[19233]|RDMAB670-06|UASM2610|573[1n]bp|Canada.Alberta|BOLD:AAB4652
Ipimorpha pleonectusa|[19234]|XAG706-05|2005-ONT-1290|658[0n]bp|Canada.Ontario|BOLD:AAB4652
Ipimorpha pleonectusa|[19235]|LPMNB370-09|08BBLEP-05214|658[0n]bp|Canada.Manitoba|BOLD:AAB4652
Ipimorpha pleonectusa|[19236]|XAG551-05|2005-ONT-1135|586[0n]bp|Canada.Ontario|BOLD:AAB4652

Ipimorpha pleonectusa|[19234]|XAG706-05|2005-ONT-1290|658[0n]bp|Canada.Ontario|BOLD:AAB4652
Ipimorpha pleonectusa|[19235]|LPMNB370-09|08BBLEP-05214|658[0n]bp|Canada.Manitoba|BOLD:AAB4652
Ipimorpha pleonectusa|[19236]|XAG551-05|2005-ONT-1135|586[0n]bp|Canada.Ontario|BOLD:AAB4652
Ipimorpha pleonectusa|[19237]|LPMNB446-09|08BBLEP-05446|658[0n]bp|Canada.Manitoba|BOLD:AAB4652
Ipimorpha viridipallida|[19238]|RDNMF517-08|NOC14603|658[0n]bp|United States.Montana|BOLD:AAB4652
Ipimorpha nanaimo|[19239]|LPVIB063-08|PFC-2006-1405|658[0n]bp|Canada.British Columbia|BOLD:AAB4652
Ipimorpha nanaimo|[19240]|LPVIA305-08|PFC-2006-0471|658[0n]bp|Canada.British Columbia|BOLD:AAB4652
Ipimorpha nanaimo|[19241]|LPVIB276-08|PFC-2006-1666|658[0n]bp|Canada.British Columbia|BOLD:AAB4652
Ipimorpha pleonectusa|[19242]|LPABC152-09|08BBLEP-04371|614[1n]bp|Canada.Alberta|BOLD:AAB4652
Ipimorpha nanaimo|[19243]|LPVIB064-08|PFC-2006-1406|658[0n]bp|Canada.British Columbia|BOLD:AAB4652
Ipimorpha nanaimo|[19244]|LPVIB516-08|PFC-2006-1931|658[0n]bp|Canada.British Columbia|BOLD:AAB4652
Ipimorpha nanaimo|[19245]|BBLPA436-10|10BBCLP-0436|658[0n]bp|Canada.Saskatchewan|BOLD:AAB4652
Ipimorpha pleonectusa|[19246]|RDMAB667-06|UASM34687|555[0n]bp|Canada.Alberta|BOLD:AAB4652
Ipimorpha pleonectusa|[19247]|LBCH918-10|10-JDWBC-0918|658[0n]bp|Canada.British Columbia|BOLD:AAB4652
Ipimorpha pleonectusa|[19248]|BBLPA437-10|10BBCLP-0437|658[0n]bp|Canada.Saskatchewan|BOLD:AAB4652
Ipimorpha pleonectusa|[19249]|RDLQB630-05|DH010733|523[0n]bp|Canada.Quebec|BOLD:AAB4652
Ipimorpha pleonectusa|[19250]|XAG705-05|2005-ONT-1289|658[0n]bp|Canada.Ontario|BOLD:AAB4652
Ipimorpha pleonectusa|[19251]|BBLPA438-10|10BBCLP-0438|658[0n]bp|Canada.Saskatchewan|BOLD:AAB4652
Ipimorpha pleonectusa|[19252]|LPMNB431-09|08BBLEP-05431|658[0n]bp|Canada.Manitoba|BOLD:AAB4652
Ipimorpha pleonectusa|[19253]|BBLPC009-09|09BBELE-1009|658[0n]bp|Canada.New Brunswick|BOLD:AAB4652
Ipimorpha viridipallida|[19254]|RDNMG598-08|CNC LEP00052422|658[0n]bp|United States.Washington|BOLD:AA...
Ipimorpha nanaimo|[19255]|LPVIA840-08|PFC-2006-1140|658[0n]bp|Canada.British Columbia|BOLD:AAB4652
Ipimorpha nanaimo|[19256]|RDNMF512-08|NOC14598|658[0n]bp|Canada.British Columbia|BOLD:AAB4652
Ipimorpha nanaimo|[19257]|LPVIB517-08|PFC-2006-1932|658[0n]bp|Canada.British Columbia|BOLD:AAB4652
Ipimorpha nanaimo|[19258]|LPVIB518-08|PFC-2006-1933|658[0n]bp|Canada.British Columbia|BOLD:AAB4652
Ipimorpha nanaimo|[19259]|LPVIB519-08|PFC-2006-1934|658[0n]bp|Canada.British Columbia|BOLD:AAB4652
Ipimorpha nanaimo|[19260]|LPVIB661-08|PFC-2006-2110|576[0n]bp|Canada.British Columbia|BOLD:AAB4652
Ipimorpha pleonectusa|[19261]|LOWCD185-06|CGWC-3005|658[0n]bp|Canada.British Columbia|BOLD:AAB4652
Ipimorpha pleonectusa|[19262]|RDLQB631-05|DH010734|658[0n]bp|Canada.Quebec|BOLD:AAB4652
Ipimorpha pleonectusa|[19263]|LPVIB065-08|PFC-2006-1407|658[0n]bp|Canada.British Columbia|BOLD:AAB4652
Ipimorpha pleonectusa|[19264]|LBCH4670-10|10-JDWBC-4670|658[0n]bp|Canada.British Columbia|BOLD:AAB4652
Ipimorpha pleonectusa|[19265]|LBCH3789-10|10-JDWBC-3789|658[0n]bp|Canada.British Columbia|BOLD:AAB4652
Ipimorpha viridipallida|[19266]|RDNMG599-08|CNC LEP00052423|658[0n]bp|United States.Washington|BOLD:AA...
Dryotype opina|[19267]|LALPA772-10|AVBC 774-10|658[0n]bp|Canada.British Columbia|BOLD:AAF6858
Dryotype opina|[19268]|RDNMF796-08|UASM124970|658[0n]bp|Canada.British Columbia|BOLD:AAF6858
Dryotype opina|[19269]|RDNMF795-08|UASM124971|658[0n]bp|Canada.British Columbia|BOLD:AAF6858
Dryotype opina|[19270]|RDNMF797-08|UASM124972|658[0n]bp|Canada.British Columbia|BOLD:AAF6858
Mniotype ducta|[19271]|RDNMJ158-10|CNCLEP 70186|658[0n]bp|Canada.British Columbia|BOLD:ACE9804
Mniotype ducta|[19272]|RDLQ591-07|DH005611|594[0n]bp|Canada.Quebec|BOLD:ACE9804
Mniotype tenera|[19273]|BBLPB697-10|10BBCLP-1696|658[0n]bp|Canada.Ontario|BOLD:AAD0186
Mniotype tenera|[19274]|LALPA1215-11|AVBC 1217-11|658[0n]bp|Canada.British Columbia|BOLD:AAD0186
Mniotype tenera|[19275]|LALPA485-10|AVBC 487-10|658[0n]bp|Canada.British Columbia|BOLD:AAD0186
Mniotype tenera|[19276]|LALPA1241-11|AVBC 1243-11|658[0n]bp|Canada.British Columbia|BOLD:AAD0186
Mniotype tenera|[19277]|LCH257-04|04HBL003257|658[0n]bp|Canada.Manitoba|BOLD:AAD0186
Mniotype tenera|[19278]|BBLPC934-09|09BBELE-1934|658[0n]bp|Canada.Newfoundland and Labrador|BOLD:AAD0186
Mniotype tenera|[19279]|RDLQ590-07|DH008721|594[0n]bp|Canada.Quebec|BOLD:AAD0186
Mniotype tenera|[19280]|LBCC433-05|HLC-22313|658[0n]bp|Canada.British Columbia|BOLD:AAD0186
Mniotype tenera|[19281]|LALPA377-10|AVBC 379-10|658[0n]bp|Canada.British Columbia|BOLD:AAD0186
Mniotype tenera|[19282]|BBLPB677-10|10BBCLP-1676|658[0n]bp|Canada.British Columbia|BOLD:AAD0186
Mniotype tenera|[19283]|LPABC198-09|08BBLEP-04417|636[0n]bp|Canada.Alberta|BOLD:AAD0186
Mniotype tenera|[19284]|LBCB059-05|HLC-20999|658[0n]bp|Canada.British Columbia|BOLD:AAD0186
Mniotype tenera|[19285]|LPABC491-09|08BBLEP-04710|658[0n]bp|Canada.Alberta|BOLD:AAD0186
Mniotype tenera|[19286]|LBCG2471-09|08-JDWBC-2471|658[0n]bp|Canada.British Columbia|BOLD:AAD0186
Mniotype tenera|[19287]|RDNMG821-08|CNC LEP00052945|658[0n]bp|Canada.British Columbia|BOLD:AAD0186
Mniotype tenera|[19288]|LBCD046-05|HLC-22866|658[0n]bp|Canada.British Columbia|BOLD:AAD0186
Mniotype tenera|[19289]|LBCB404-05|HLC-21344|658[0n]bp|Canada.British Columbia|BOLD:AAD0186
Mniotype tenera|[19290]|LBCB939-05|HLC-21879|658[0n]bp|Canada.British Columbia|BOLD:AAD0186
Mniotype tenera|[19291]|RDMAB474-06|UASM77895|658[0n]bp|Canada.Alberta|BOLD:AAD0186
Mniotype tenera|[19292]|RDNMG822-08|CNC LEP00052946|658[0n]bp|Canada.British Columbia|BOLD:AAD0186
Fishia illocata|[19293]|LALPA779-10|AVBC 781-10|658[0n]bp|Canada.British Columbia|BOLD:AAA9136
Fishia illocata|[19294]|LALPA747-10|AVBC 749-10|644[0n]bp|Canada.British Columbia|BOLD:AAA9136
Fishia illocata|[19295]|LALPA757-10|AVBC 759-10|658[0n]bp|Canada.British Columbia|BOLD:AAA9136
Fishia illocata|[19296]|LALPA1300-11|AVBC 1302-11|658[0n]bp|Canada.British Columbia|BOLD:AAA9136
Fishia illocata|[19297]|LOWCD701-06|CGWC-3521|658[0n]bp|Canada.British Columbia|BOLD:AAA9136
Fishia illocata|[19298]|TMNBB184-06|MNBTT-1124|658[0n]bp|Canada.New Brunswick|BOLD:AAA9136
Fishia illocata|[19299]|TMNBB185-06|MNBTT-1125|658[0n]bp|Canada.New Brunswick|BOLD:AAA9136
Fishia illocata|[19300]|LOWCD700-06|CGWC-3520|589[0n]bp|Canada.British Columbia|BOLD:AAA9136
Fishia illocata|[19301]|LOWCD703-06|CGWC-3523|591[0n]bp|Canada.British Columbia|BOLD:AAA9136
Fishia illocata|[19302]|LOWCB454-05|CGWC-1394|658[0n]bp|Canada.British Columbia|BOLD:AAA9136
Fishia illocata|[19303]|LALPA771-10|AVBC 773-10|658[0n]bp|Canada.British Columbia|BOLD:AAA9136
Fishia illocata|[19304]|LALPA795-10|AVBC 797-10|658[0n]bp|Canada.British Columbia|BOLD:AAA9136
Fishia illocata|[19305]|LPVIB633-08|PFC-2006-2081|634[0n]bp|Canada.British Columbia|BOLD:AAA9136
Fishia illocata|[19306]|LALPA748-10|AVBC 750-10|658[0n]bp|Canada.British Columbia|BOLD:AAA9136
Fishia illocata|[19307]|LBCH2607-10|10-JDWBC-2607|658[0n]bp|Canada.British Columbia|BOLD:AAA9136
Fishia illocata|[19308]|RDLQB517-05|DH010603|658[0n]bp|Canada.Quebec|BOLD:AAA9136
Fishia illocata|[19309]|PHMO334-03|moth2598.02|637[5n]bp|Canada.Ontario|BOLD:AAA9136
Fishia illocata|[19310]|LOWCB459-05|CGWC-1399|658[0n]bp|Canada.British Columbia|BOLD:AAA9136
Fishia illocata|[19311]|LOWCD155-06|CGWC-2975|658[0n]bp|Canada.British Columbia|BOLD:AAA9136
Fishia illocata|[19312]|LOWCB455-05|CGWC-1395|658[0n]bp|Canada.British Columbia|BOLD:AAA9136
Fishia illocata|[19313]|LOWCB460-05|CGWC-1400|658[0n]bp|Canada.British Columbia|BOLD:AAA9136
Fishia illocata|[19314]|LOWCB452-05|CGWC-1392|658[0n]bp|Canada.British Columbia|BOLD:AAA9136
Fishia illocata|[19315]|RDLQF278-06|DH011370|658[0n]bp|Canada.Quebec|BOLD:AAA9136
Fishia illocata|[19316]|LBCH2789-10|10-JDWBC-2789|658[0n]bp|Canada.British Columbia|BOLD:AAA9136
Fishia illocata|[19317]|LOWCD704-06|CGWC-3524|658[0n]bp|Canada.British Columbia|BOLD:AAA9136
Fishia illocata|[19318]|LOWCD122-06|CGWC-2942|658[0n]bp|Canada.British Columbia|BOLD:AAA9136
Fishia illocata|[19319]|LBCH2704-10|10-JDWBC-2704|658[0n]bp|Canada.British Columbia|BOLD:AAA9136
Fishia illocata|[19320]|LBCH2861-10|10-JDWBC-2861|658[0n]bp|Canada.British Columbia|BOLD:AAA9136
Fishia illocata|[19321]|XAH565-05|2005-ONT-2148|658[0n]bp|Canada.Ontario|BOLD:AAA9136
Fishia illocata|[19322]|RDLQB518-05|DH010604|658[0n]bp|Canada.Quebec|BOLD:AAA9136
Fishia illocata|[19323]|RDNMC272-05|CNCNoctuoidea11906|617[0n]bp|Canada.New Brunswick|BOLD:AAA9136
Fishia illocata|[19324]|XAH312-05|2005-ONT-1895|639[0n]bp|Canada.Ontario|BOLD:AAA9136
Fishia illocata|[19325]|LOWCD699-06|CGWC-3519|588[0n]bp|Canada.British Columbia|BOLD:AAA9136
Fishia illocata|[19326]|LOWCD702-06|CGWC-3522|588[0n]bp|Canada.British Columbia|BOLD:AAA9136
Fishia illocata|[19327]|TTMNB353-06|MNBTT-353|613[0n]bp|Canada.New Brunswick|BOLD:AAA9136
Fishia illocata|[19328]|RDNMC271-05|CNCNoctuoidea11905|591[0n]bp|Canada.New Brunswick|BOLD:AAA9136
Fishia illocata|[19329]|RDLQF277-06|DH011369|658[0n]bp|Canada.Quebec|BOLD:AAA9136
Fishia illocata|[19330]|LOWCB461-05|CGWC-1401|658[0n]bp|Canada.British Columbia|BOLD:AAA9136
Fishia illocata|[19331]|LOWCB453-05|CGWC-1393|658[0n]bp|Canada.British Columbia|BOLD:AAA9136
Fishia discors|[19332]|LALPA810-10|AVBC 812-10|658[0n]bp|Canada.British Columbia|BOLD:AAB3715
Fishia discors|[19333]|RDMAB578-06|UASM58544|658[0n]bp|Canada.Alberta|BOLD:AAB3715
Fishia discors|[19334]|LALPA791-10|AVBC 793-10|658[0n]bp|Canada.British Columbia|BOLD:AAB3715
Fishia discors|[19335]|LOWCD933-06|CGWC-3753|584[0n]bp|Canada.British Columbia|BOLD:AAB3715
Fishia discors|[19336]|LOWCC026-05|CGWC-1906|658[0n]bp|Canada.British Columbia|BOLD:AAB3715

Fishia discors|[19334]|LALPA791-10|AVBC 793-10|658[0n]bp|Canada.British Columbia|BOLD:AAB3715
Fishia discors|[19335]|LOWCD933-06|CGWC-3753|584[0n]bp|Canada.British Columbia|BOLD:AAB3715
Fishia discors|[19336]|LOWCC026-05|CGWC-1906|658[0n]bp|Canada.British Columbia|BOLD:AAB3715
Fishia discors|[19337]|LOWCC037-05|CGWC-1917|658[0n]bp|Canada.British Columbia|BOLD:AAB3715
Fishia discors|[19338]|LOWCC025-05|CGWC-1905|658[0n]bp|Canada.British Columbia|BOLD:AAB3715
Fishia discors|[19339]|LOWCC031-05|CGWC-1911|658[0n]bp|Canada.British Columbia|BOLD:AAB3715
Fishia discors|[19340]|LOWCD930-06|CGWC-3750|658[0n]bp|Canada.British Columbia|BOLD:AAB3715
Fishia discors|[19341]|LOWCD936-06|CGWC-3756|658[0n]bp|Canada.British Columbia|BOLD:AAB3715
Fishia discors|[19342]|LOWCC030-05|CGWC-1910|658[0n]bp|Canada.British Columbia|BOLD:AAB3715
Fishia discors|[19343]|LOWCC034-05|CGWC-1914|658[0n]bp|Canada.British Columbia|BOLD:AAB3715
Fishia discors|[19344]|LOWCC036-05|CGWC-1916|658[0n]bp|Canada.British Columbia|BOLD:AAB3715
Fishia discors|[19345]|LOWCC029-05|CGWC-1909|658[0n]bp|Canada.British Columbia|BOLD:AAB3715
Fishia discors|[19346]|LOWCC038-05|CGWC-1918|658[0n]bp|Canada.British Columbia|BOLD:AAB3715
Fishia discors|[19347]|LOWCC028-05|CGWC-1908|658[0n]bp|Canada.British Columbia|BOLD:AAB3715
Fishia discors|[19348]|LOWCC039-05|CGWC-1919|658[0n]bp|Canada.British Columbia|BOLD:AAB3715
Fishia discors|[19349]|LOWCD932-06|CGWC-3752|658[0n]bp|Canada.British Columbia|BOLD:AAB3715
Fishia discors|[19350]|LOWCC027-05|CGWC-1907|658[0n]bp|Canada.British Columbia|BOLD:AAB3715
Fishia discors|[19351]|LOWCC032-05|CGWC-1912|658[0n]bp|Canada.British Columbia|BOLD:AAB3715
Fishia discors|[19352]|LOWCD929-06|CGWC-3749|658[0n]bp|Canada.British Columbia|BOLD:AAB3715
Fishia discors|[19353]|LOWCD931-06|CGWC-3751|658[0n]bp|Canada.British Columbia|BOLD:AAB3715
Fishia discors|[19354]|LOWCC033-05|CGWC-1913|658[0n]bp|Canada.British Columbia|BOLD:AAB3715
Fishia discors|[19355]|LOWCD934-06|CGWC-3754|658[0n]bp|Canada.British Columbia|BOLD:AAB3715
Fishia discors|[19356]|LOWCC035-05|CGWC-1915|658[0n]bp|Canada.British Columbia|BOLD:AAB3715
Fishia discors|[19357]|LOWCD935-06|CGWC-3755|596[0n]bp|Canada.British Columbia|BOLD:AAB3715
Fishia yosemitae|[19358]|RDMAB217-05|UASM41934|581[2n]bp|Canada.Alberta|BOLD:AAC0326
Fishia yosemitae|[19359]|RDMAB215-05|UASM41368|658[0n]bp|Canada.Alberta|BOLD:AAC0326
Fishia yosemitae|[19360]|RDMAB218-05|UASM58417|658[0n]bp|Canada.Alberta|BOLD:AAC0326
Fishia yosemitae|[19361]|LOWCB540-05|CGWC-1480|658[0n]bp|Canada.British Columbia|BOLD:AAC0326
Fishia yosemitae|[19362]|LOWCB539-05|CGWC-1479|658[0n]bp|Canada.British Columbia|BOLD:AAC0326
Fishia yosemitae|[19363]|LOWCB533-05|CGWC-1473|658[0n]bp|Canada.British Columbia|BOLD:AAC0326
Fishia yosemitae|[19364]|LOWCB532-05|CGWC-1472|658[0n]bp|Canada.British Columbia|BOLD:AAC0326
Fishia yosemitae|[19365]|RDNM548-05|CNCNoctuoidea6824|658[0n]bp|Canada.Ontario|BOLD:AAC0326
Fishia yosemitae|[19366]|LOWCB537-05|CGWC-1477|658[0n]bp|Canada.British Columbia|BOLD:AAC0326
Fishia yosemitae|[19367]|RDMAB216-05|UASM41367|658[0n]bp|Canada.Alberta|BOLD:AAC0326
Fishia yosemitae|[19368]|LOWCB535-05|CGWC-1475|658[0n]bp|Canada.British Columbia|BOLD:AAC0326
Fishia yosemitae|[19369]|RDNMF750-08|NOC14836|658[0n]bp|Canada.Ontario|BOLD:AAC0326
Fishia yosemitae|[19370]|RDNM547-05|CNCNoctuoidea6823|658[0n]bp|Canada.Ontario|BOLD:AAC0326
Fishia yosemitae|[19371]|LOWCB536-05|CGWC-1476|658[0n]bp|Canada.British Columbia|BOLD:AAC0326
Fishia yosemitae|[19372]|LOWCB531-05|CGWC-1471|611[0n]bp|Canada.British Columbia|BOLD:AAC0326
Fishia yosemitae|[19373]|LOWCB534-05|CGWC-1474|617[0n]bp|Canada.British Columbia|BOLD:AAC0326
Fishia yosemitae|[19374]|LOWCB538-05|CGWC-1478|629[0n]bp|Canada.British Columbia|BOLD:AAC0326
Fishia yosemitae|[19375]|RDNM546-05|CNCNoctuoidea6822|537[0n]bp|Canada.British Columbia|BOLD:AAC0326
Platypolia anceps|[19376]|LOWCB524-05|CGWC-1464|658[0n]bp|Canada.British Columbia|BOLD:AAD1074
Platypolia anceps|[19377]|LOWCB526-05|CGWC-1466|658[0n]bp|Canada.British Columbia|BOLD:AAD1074
Platypolia anceps|[19378]|LOWCB525-05|CGWC-1465|658[0n]bp|Canada.British Columbia|BOLD:AAD1074
Platypolia anceps|[19379]|LOWCB530-05|CGWC-1470|597[0n]bp|Canada.British Columbia|BOLD:AAD1074
Platypolia anceps|[19380]|LOWCC836-05|CGWC-2716|558[0n]bp|Canada.British Columbia|BOLD:AAD1074
Platypolia anceps|[19381]|LBCH2608-10|10-JDWBC-2608|658[0n]bp|Canada.British Columbia|BOLD:AAD1074
Platypolia anceps|[19382]|LBCH2591-10|10-JDWBC-2591|658[0n]bp|Canada.British Columbia|BOLD:AAD1074
Platypolia anceps|[19383]|LOWCB528-05|CGWC-1468|658[0n]bp|Canada.British Columbia|BOLD:AAD1074
Platypolia anceps|[19384]|LBCH2505-10|10-JDWBC-2505|658[0n]bp|Canada.British Columbia|BOLD:AAD1074
Platypolia anceps|[19385]|LOWCB529-05|CGWC-1469|658[0n]bp|Canada.British Columbia|BOLD:AAD1074
Platypolia anceps|[19386]|LOWCB527-05|CGWC-1467|658[0n]bp|Canada.British Columbia|BOLD:AAD1074
Platypolia contadina|[19387]|LOWCE596-06|CGWC-4356|656[0n]bp|Canada.British Columbia|BOLD:AAD4229
Platypolia contadina|[19388]|RDMAB611-06|UASM41716|658[0n]bp|Canada.Alberta|BOLD:AAD4229
Platypolia contadina|[19389]|RDMAB610-06|UASM56919|658[0n]bp|Canada.Alberta|BOLD:AAD4229
Platypolia contadina|[19390]|RDNM733-05|CNCNoctuoidea7579|658[0n]bp|Canada.British Columbia|BOLD:AAD4229
Platypolia contadina|[19391]|RDMAB609-06|UASM58667|658[0n]bp|Canada.Alberta|BOLD:AAD4229
Platypolia loda|[19392]|LOWCE585-06|CGWC-4345|658[0n]bp|Canada.British Columbia|BOLD:AAD1063
Platypolia loda|[19393]|LOWCE595-06|CGWC-4355|658[0n]bp|Canada.British Columbia|BOLD:AAD1063
Platypolia loda|[19394]|LOWCE587-06|CGWC-4347|658[0n]bp|Canada.British Columbia|BOLD:AAD1063
Platypolia loda|[19395]|LOWCE586-06|CGWC-4346|658[0n]bp|Canada.British Columbia|BOLD:AAD1063
Platypolia loda|[19396]|LOWCE582-06|CGWC-4342|658[0n]bp|Canada.British Columbia|BOLD:AAD1063
Platypolia loda|[19397]|LOWCE584-06|CGWC-4344|658[0n]bp|Canada.British Columbia|BOLD:AAD1063
Platypolia loda|[19398]|LOWCE583-06|CGWC-4343|658[0n]bp|Canada.British Columbia|BOLD:AAD1063
Platypolia loda|[19399]|LOWCE594-06|CGWC-4354|658[0n]bp|Canada.British Columbia|BOLD:AAD1063
Platypolia mactata|[19400]|LOWCE650-06|CGWC-4410|658[0n]bp|Canada.British Columbia|BOLD:AAC7837
Platypolia mactata|[19401]|LOWCE651-06|CGWC-4411|658[0n]bp|Canada.British Columbia|BOLD:AAC7837
Platypolia mactata|[19402]|LBCH2535-10|10-JDWBC-2535|658[0n]bp|Canada.British Columbia|BOLD:AAC7837
Platypolia mactata|[19403]|LOWCD161-06|CGWC-2981|658[0n]bp|Canada.British Columbia|BOLD:AAC7837
Platypolia mactata|[19404]|RDNMC273-05|CNCNoctuoidea11907|589[0n]bp|Canada.New Brunswick|BOLD:AAC7837
Platypolia mactata|[19405]|RDLQ463-07|DH002322|585[1n]bp|Canada.Quebec|BOLD:AAC7837
Platypolia mactata|[19406]|LOWCD191-06|CGWC-3011|616[0n]bp|Canada.British Columbia|BOLD:AAC7837
Platypolia mactata|[19407]|RDNMC274-05|CNCNoctuoidea11908|597[0n]bp|Canada.Alberta|BOLD:AAC7837
Platypolia mactata|[19408]|RDLQ464-07|DH002325|615[0n]bp|Canada.Quebec|BOLD:AAC7837
Sutyna privata|[19409]|TMNBB277-06|MNBTT-1217|656[0n]bp|Canada.New Brunswick|BOLD:AAB3593
Sutyna privata|[19410]|TTMNB377-06|MNBTT-377|658[0n]bp|Canada.New Brunswick|BOLD:AAB3593
Sutyna privata|[19411]|RDMAB953-09|UASM34582|620[0n]bp|Canada.Alberta|BOLD:AAB3593
Sutyna privata|[19412]|RDMAB952-09|UASM34724|602[0n]bp|Canada.Alberta|BOLD:AAB3593
Sutyna privata|[19413]|LOWCB175-05|CGWC-1115|658[0n]bp|Canada.British Columbia|BOLD:AAB3593
Sutyna privata|[19414]|RDLQ585-07|DH008791|588[1n]bp|Canada.Quebec|BOLD:AAB3593
Sutyna privata|[19415]|RDLQ586-07|DH003063|595[1n]bp|Canada.Quebec|BOLD:AAB3593
Sutyna privata|[19416]|RDLQ584-07|DH013449|584[0n]bp|Canada.Quebec|BOLD:AAB3593
Sutyna privata|[19417]|RDLQ587-07|DH003065|595[1n]bp|Canada.Quebec|BOLD:AAB3593
Sutyna privata|[19418]|RDLQ589-07|DH003064|590[0n]bp|Canada.Quebec|BOLD:AAB3593
Sutyna privata|[19419]|RDLQ588-07|DH013448|587[1n]bp|Canada.Quebec|BOLD:AAB3593
Sutyna privata|[19420]|TTMNB381-06|MNBTT-381|658[0n]bp|Canada.New Brunswick|BOLD:AAB3593
Sutyna privata|[19421]|RDMAB954-09|UASM2135|609[0n]bp|Canada.Alberta|BOLD:AAB3593
Sutyna privata|[19422]|TMNBB280-06|MNBTT-1220|658[0n]bp|Canada.New Brunswick|BOLD:AAB3593
Sutyna privata|[19423]|TTMNB379-06|MNBTT-379|658[0n]bp|Canada.New Brunswick|BOLD:AAB3593
Sutyna privata|[19424]|TMNBB275-06|MNBTT-1215|658[0n]bp|Canada.New Brunswick|BOLD:AAB3593
Sutyna privata|[19425]|TMNBB279-06|MNBTT-1219|658[0n]bp|Canada.New Brunswick|BOLD:AAB3593
Sutyna privata|[19426]|TMNBB281-06|MNBTT-1221|658[0n]bp|Canada.New Brunswick|BOLD:AAB3593
Sutyna privata|[19427]|TMNBB273-06|MNBTT-1213|658[0n]bp|Canada.New Brunswick|BOLD:AAB3593
Sutyna privata|[19428]|TTMNB383-06|MNBTT-383|658[0n]bp|Canada.New Brunswick|BOLD:AAB3593
Sutyna privata|[19429]|TMNBB272-06|MNBTT-1212|656[0n]bp|Canada.New Brunswick|BOLD:AAB3593
Sutyna privata|[19430]|TMNBB274-06|MNBTT-1214|656[0n]bp|Canada.New Brunswick|BOLD:AAB3593
Sutyna privata|[19431]|TTMNB380-06|MNBTT-380|658[0n]bp|Canada.New Brunswick|BOLD:AAB3593
Sutyna privata|[19432]|TTMNB384-06|MNBTT-384|658[0n]bp|Canada.New Brunswick|BOLD:AAB3593
Sutyna privata|[19433]|TTMNB382-06|MNBTT-382|658[0n]bp|Canada.New Brunswick|BOLD:AAB3593
Sutyna privata|[19434]|TMNBB278-06|MNBTT-1218|658[0n]bp|Canada.New Brunswick|BOLD:AAB3593
Sutyna privata|[19435]|TMNBB276-06|MNBTT-1216|658[0n]bp|Canada.New Brunswick|BOLD:AAB3593

Sutyna privata|[19433]|TTMNB382-06|MNBTT-382|658[0n]bp|Canada.New Brunswick|BOLD:AAB3593
Sutyna privata|[19434]|TMNBB278-06|MNBTT-1218|658[0n]bp|Canada.New Brunswick|BOLD:AAB3593
Sutyna privata|[19435]|TMNBB276-06|MNBTT-1216|658[0n]bp|Canada.New Brunswick|BOLD:AAB3593
Sutyna privata|[19436]|TTMNB378-06|MNBTT-378|658[1n]bp|Canada.New Brunswick|BOLD:AAB3593
Xylotype arcadia|[19437]|RDLQF275-06|DH011367|658[0n]bp|Canada.Quebec|BOLD:AAD4918
Xylotype arcadia|[19438]|RDLQF256-06|DH011336|658[0n]bp|Canada.Quebec|BOLD:AAD4918
Xylotype arcadia|[19439]|LOWCD120-06|CGWC-2940|610[0n]bp|Canada.British Columbia|BOLD:AAD4918
Xylotype arcadia|[19440]|RDLQF159-06|DH011186|658[0n]bp|Canada.Quebec|BOLD:AAD4918
Xylotype arcadia|[19441]|RDLQF274-06|DH011366|658[0n]bp|Canada.Quebec|BOLD:AAD4918
Xylotype arcadia|[19442]|LBCH2492-10|10-JDWBC-2492|658[0n]bp|Canada.British Columbia|BOLD:AAD4918
Xylotype arcadia|[19443]|TTMNB376-06|MNBTT-376|658[0n]bp|Canada.New Brunswick|BOLD:AAD4918
Xylotype arcadia|[19444]|DUNLP188-08|Dun-08-188|658[0n]bp|Canada.British Columbia|BOLD:AAD4918
Xylotype arcadia|[19445]|LOWCD119-06|CGWC-2939|565[0n]bp|Canada.British Columbia|BOLD:AAD4918
Zotheca tranquilla|[19446]|LBCH6014-10|10-JDWBC-6014|658[0n]bp|Canada.British Columbia|BOLD:AAD5204
Zotheca tranquilla|[19447]|LBCW047-08|08-JDWWI-0047|658[0n]bp|Canada.British Columbia|BOLD:AAD5204
Zotheca tranquilla|[19448]|LBCW048-08|08-JDWWI-0048|658[0n]bp|Canada.British Columbia|BOLD:AAD5204
Zotheca tranquilla|[19449]|LBCW049-08|08-JDWWI-0049|658[0n]bp|Canada.British Columbia|BOLD:AAD5204
Zotheca tranquilla|[19450]|RDNMF093-08|NOC14179|658[0n]bp|Canada.British Columbia|BOLD:AAD5204
Zotheca tranquilla|[19451]|LHLEP379-06|UBC-2006-1696|658[0n]bp|Canada.British Columbia|BOLD:AAD5204
Zotheca tranquilla|[19452]|RDNMF002-08|NOC14088|658[0n]bp|Canada.British Columbia|BOLD:AAD5204
Zotheca tranquilla|[19453]|LALPA419-10|AVBC 421-10|658[0n]bp|Canada.British Columbia|BOLD:AAD5204
Zotheca tranquilla|[19454]|LALPA439-10|AVBC 441-10|658[0n]bp|Canada.British Columbia|BOLD:AAD5204
Zotheca tranquilla|[19455]|LPVIA632-08|PFC-2006-0865|658[0n]bp|Canada.British Columbia|BOLD:AAD5204
Zotheca tranquilla|[19456]|LALPA568-10|AVBC 570-10|658[0n]bp|Canada.British Columbia|BOLD:AAD5204
Elaphria versicolor|[19457]|LPSO691-08|PPBP-0691|658[0n]bp|Canada.Ontario|BOLD:AAA4393
Elaphria versicolor|[19458]|RDLQG739-06|DH013032|658[0n]bp|Canada.Quebec|BOLD:AAA4393
Elaphria versicolor|[19459]|BBLPC171-09|09BBELE-1171|658[0n]bp|Canada.Nova Scotia|BOLD:AAA4393
Elaphria versicolor|[19460]|TMNBB218-06|MNBTT-1158|658[0n]bp|Canada.New Brunswick|BOLD:AAA4393
Elaphria versicolor|[19461]|MNBB142-05|05-NBSTA-058|658[0n]bp|Canada.New Brunswick|BOLD:AAA4393
Elaphria versicolor|[19462]|BBLPE273-09|09BBELE-2273|658[0n]bp|Canada.Nova Scotia|BOLD:AAA4393
Elaphria versicolor|[19463]|XAB309-04|04HBL005309|658[0n]bp|Canada.Ontario|BOLD:AAA4393
Elaphria versicolor|[19464]|LPSOC179-08|PPBP-2178|658[0n]bp|Canada.Ontario|BOLD:AAA4393
Elaphria versicolor|[19465]|LPSOD782-09|08BBLEP-00564|658[0n]bp|Canada.Ontario|BOLD:AAA4393
Elaphria versicolor|[19466]|BBLEC587-09|09BBELE-0587|658[0n]bp|Canada.Nova Scotia|BOLD:AAA4393
Elaphria versicolor|[19467]|BBLPC148-09|09BBELE-1148|658[0n]bp|Canada.Nova Scotia|BOLD:AAA4393
Elaphria versicolor|[19468]|RDLQG534-06|DH012827|621[4n]bp|Canada.Quebec|BOLD:AAA4393
Elaphria versicolor|[19469]|RDLQG699-06|DH012992|632[0n]bp|Canada.Quebec|BOLD:AAA4393
Elaphria versicolor|[19470]|BBLEC667-09|09BBELE-0667|643[0n]bp|Canada.Nova Scotia|BOLD:AAA4393
Elaphria versicolor|[19471]|RDLQG506-06|DH012799|634[0n]bp|Canada.Quebec|BOLD:AAA4393
Elaphria versicolor|[19472]|PHMO189-03|moth967.02|639[0n]bp|Canada.Ontario|BOLD:AAA4393
Elaphria versicolor|[19473]|RDLQG507-06|DH012800|620[0n]bp|Canada.Quebec|BOLD:AAA4393
Elaphria versicolor|[19474]|XAJ382-06|2006-ONT-0382|632[0n]bp|Canada.Ontario|BOLD:AAA4393
Elaphria versicolor|[19475]|RDLQG537-06|DH012830|658[0n]bp|Canada.Quebec|BOLD:AAA4393
Elaphria versicolor|[19476]|RDLQB925-05|AC000466|658[0n]bp|Canada.Quebec|BOLD:AAA4393
Elaphria versicolor|[19477]|XAI025-05|0102-ONT-0025|565[0n]bp|Canada.Ontario|BOLD:AAA4393
Elaphria versicolor|[19478]|MEC706-04|jflandry0706|658[0n]bp|Canada.Quebec|BOLD:AAA4393
Elaphria versicolor|[19479]|BBLPE278-09|09BBELE-2278|616[0n]bp|Canada.Nova Scotia|BOLD:AAA4393
Elaphria versicolor|[19480]|BBLPC566-09|09BBELE-1566|638[0n]bp|Canada.Nova Scotia|BOLD:AAA4393
Elaphria versicolor|[19481]|RDLQG510-06|DH012803|646[0n]bp|Canada.Quebec|BOLD:AAA4393
Elaphria versicolor|[19482]|XAB297-04|04HBL005297|614[0n]bp|Canada.Ontario|BOLD:AAA4393
Elaphria versicolor|[19483]|XAE499-04|Moth4499.03|574[1n]bp|Canada.Ontario|BOLD:AAA4393
Elaphria versicolor|[19484]|TTMNB370-06|MNBTT-370|519[0n]bp|Canada.New Brunswick|BOLD:AAA4393
Elaphria versicolor|[19485]|RDLQG509-06|DH012802|605[0n]bp|Canada.Quebec|BOLD:AAA4393
Elaphria versicolor|[19486]|RDLQG505-06|DH012798|605[0n]bp|Canada.Quebec|BOLD:AAA4393
Elaphria versicolor|[19487]|RDLQG508-06|DH012801|605[0n]bp|Canada.Quebec|BOLD:AAA4393
Elaphria versicolor|[19488]|XAB370-04|04HBL005370|658[0n]bp|Canada.Ontario|BOLD:AAA4393
Elaphria versicolor|[19489]|RDLQG533-06|DH012826|658[0n]bp|Canada.Quebec|BOLD:AAA4393
Elaphria versicolor|[19490]|PHMNB495-04|04HBL00721|658[0n]bp|Canada.New Brunswick|BOLD:AAA4393
Elaphria versicolor|[19491]|XAJ383-06|2006-ONT-0383|658[0n]bp|Canada.Ontario|BOLD:AAA4393
Elaphria versicolor|[19492]|PHMNB635-04|04HBL00861|658[0n]bp|Canada.New Brunswick|BOLD:AAA4393
Elaphria versicolor|[19493]|PHMNB386-04|04HBL00612|658[0n]bp|Canada.New Brunswick|BOLD:AAA4393
Elaphria versicolor|[19494]|RDLQG816-06|DH013109|655[0n]bp|Canada.Quebec|BOLD:AAA4393
Elaphria versicolor|[19495]|RDLQG839-06|DH013132|658[0n]bp|Canada.Quebec|BOLD:AAA4393
Elaphria versicolor|[19496]|PHMTV428-10|10PHMAL-2528|658[0n]bp|Canada.Ontario|BOLD:AAA4393
Elaphria versicolor|[19497]|RDLQB923-05|DH009159|658[0n]bp|Canada.Quebec|BOLD:AAA4393
Elaphria versicolor|[19498]|PHMNB643-04|04HBL00869|658[0n]bp|Canada.New Brunswick|BOLD:AAA4393
Elaphria versicolor|[19499]|RDLQG813-06|DH013106|658[0n]bp|Canada.Quebec|BOLD:AAA4393
Elaphria versicolor|[19500]|PHMNB497-04|04HBL00723|658[0n]bp|Canada.New Brunswick|BOLD:AAA4393
Elaphria versicolor|[19501]|PHMNB649-04|04HBL00875|658[0n]bp|Canada.New Brunswick|BOLD:AAA4393
Elaphria versicolor|[19502]|PHMNB546-04|04HBL00772|658[0n]bp|Canada.New Brunswick|BOLD:AAA4393
Elaphria versicolor|[19503]|PHMNB493-04|04HBL00719|658[0n]bp|Canada.New Brunswick|BOLD:AAA4393
Elaphria versicolor|[19504]|LPSOB865-08|PPBP-1864|658[0n]bp|Canada.Ontario|BOLD:AAA4393
Elaphria versicolor|[19505]|XAC325-04|04HBL006325|658[0n]bp|Canada.Ontario|BOLD:AAA4393
Elaphria versicolor|[19506]|RDLQG838-06|DH013131|658[0n]bp|Canada.Quebec|BOLD:AAA4393
Elaphria versicolor|[19507]|XAJ648-06|2006-ONT-0648|658[0n]bp|Canada.Ontario|BOLD:AAA4393
Chytonix palliatricula|[19508]|XAC596-04|04HBL006596|617[0n]bp|Canada.Ontario|BOLD:AAA6619
Chytonix palliatricula|[19509]|RDNMK219-11|CNCLEP 84129|658[0n]bp|Canada.Ontario|BOLD:AAA6619
Chytonix palliatricula|[19510]|XAI045-05|0102-ONT-0045|658[0n]bp|Canada.Ontario|BOLD:AAA6619
Chytonix palliatricula|[19511]|XAJ693-06|2006-ONT-0693|657[0n]bp|Canada.Ontario|BOLD:AAA6619
Chytonix palliatricula|[19512]|RDNMK220-11|CNCLEP 84130|658[0n]bp|Canada.Ontario|BOLD:AAA6619
Chytonix palliatricula|[19513]|PHMNB470-04|04HBL00696|658[0n]bp|Canada.New Brunswick|BOLD:AAA6619
Chytonix palliatricula|[19514]|LPMN010-08|08BBLEP-00808|658[0n]bp|Canada.Manitoba|BOLD:AAA6619
Chytonix palliatricula|[19515]|RDNMD300-06|CNCNoctuoidea12632|653[0n]bp|Canada.New Brunswick|BOLD:AAA6619
Chytonix palliatricula|[19516]|BBLEC579-09|09BBELE-0579|658[1n]bp|Canada.Nova Scotia|BOLD:AAA6619
Chytonix palliatricula|[19517]|LPSOB468-08|PPBP-1467|658[0n]bp|Canada.Ontario|BOLD:AAA6619
Chytonix palliatricula|[19518]|RDMAB077-05|UASM57604|571[0n]bp|Canada.Alberta|BOLD:AAA6619
Chytonix palliatricula|[19519]|PHMO231-03|moth1165.02|639[0n]bp|Canada.Ontario|BOLD:AAA6619
Chytonix palliatricula|[19520]|LPSOC138-08|PPBP-2137|658[0n]bp|Canada.Ontario|BOLD:AAA6619
Chytonix palliatricula|[19521]|TMNBB195-06|MNBTT-1135|657[0n]bp|Canada.New Brunswick|BOLD:AAA6619
Chytonix palliatricula|[19522]|XAK110-06|2006-ONT-1105|658[0n]bp|Canada.Ontario|BOLD:AAA6619
Chytonix palliatricula|[19523]|TMNBB194-06|MNBTT-1134|658[0n]bp|Canada.New Brunswick|BOLD:AAA6619
Chytonix palliatricula|[19524]|TMNBB193-06|MNBTT-1133|657[0n]bp|Canada.New Brunswick|BOLD:AAA6619
Chytonix palliatricula|[19525]|RDNMD301-06|CNCNoctuoidea12633|658[0n]bp|Canada.New Brunswick|BOLD:AAA6619
Chytonix palliatricula|[19526]|RDNMD298-06|CNCNoctuoidea12630|658[0n]bp|Canada.New Brunswick|BOLD:AAA6619
Chytonix palliatricula|[19527]|RDNMD299-06|CNCNoctuoidea12631|653[0n]bp|Canada.New Brunswick|BOLD:AAA6619
Chytonix palliatricula|[19528]|LPSOB599-08|PPBP-1598|658[0n]bp|Canada.Ontario|BOLD:AAA6619
Chytonix palliatricula|[19529]|LPSOD347-09|08BBLEP-00125|658[0n]bp|Canada.Ontario|BOLD:AAA6619
Chytonix palliatricula|[19530]|LPSOD321-09|08BBLEP-00099|658[0n]bp|Canada.Ontario|BOLD:AAA6619
Chytonix palliatricula|[19531]|LPSOD325-09|08BBLEP-00103|614[0n]bp|Canada.Ontario|BOLD:AAA6619
Chytonix palliatricula|[19532]|BBLPB409-10|10BBCLP-1408|658[0n]bp|Canada.Ontario|BOLD:AAA6619
Chytonix palliatricula|[19533]|RDLQB268-05|DH010354|658[0n]bp|Canada.Quebec|BOLD:AAA6619
Morrisonia latex|[19534]|RDLQG270-06|DH012478|658[0n]bp|Canada.Quebec|BOLD:AAB0781
Morrisonia latex|[19535]|XAE491-04|Moth4491.03|568[0n]bp|Canada.Ontario|BOLD:AAB0781

Chytonix palliatricula|[19533]|RDLQB268-05|DH010354|658[0n]bp|Canada.Quebec|BOLD:AAA6619
Morrisonia latex|[19534]|RDLQG270-06|DH012478|658[0n]bp|Canada.Quebec|BOLD:AAB0781
Morrisonia latex|[19535]|XAE491-04|Moth4491.03|568[0n]bp|Canada.Ontario|BOLD:AAB0781
Morrisonia latex|[19536]|LPSOC317-08|PPBP-2316|658[0n]bp|Canada.Ontario|BOLD:AAB0781
Morrisonia latex|[19537]|PHMNB453-04|04HBL00679|658[0n]bp|Canada.New Brunswick|BOLD:AAB0781
Morrisonia latex|[19538]|LPSOD504-09|08BBLEP-00283|658[0n]bp|Canada.Ontario|BOLD:AAB0781
Morrisonia latex|[19539]|PHJUL926-11|BIOUG01146-D07|658[0n]bp|Canada.Ontario|BOLD:AAB0781
Morrisonia latex|[19540]|XAI063-05|0102-ONT-0063|658[0n]bp|Canada.Ontario|BOLD:AAB0781
Morrisonia latex|[19541]|XAI066-05|0102-ONT-0066|658[0n]bp|Canada.Ontario|BOLD:AAB0781
Morrisonia latex|[19542]|XAK161-06|2006-ONT-1156|658[0n]bp|Canada.Ontario|BOLD:AAB0781
Morrisonia latex|[19543]|LPSOD322-09|08BBLEP-00100|658[0n]bp|Canada.Ontario|BOLD:AAB0781
Morrisonia latex|[19544]|LPSOD693-09|08BBLEP-00474|658[0n]bp|Canada.Ontario|BOLD:AAB0781
Morrisonia latex|[19545]|LPSOD255-09|08BBLEP-00033|658[0n]bp|Canada.Ontario|BOLD:AAB0781
Morrisonia latex|[19546]|XAE345-04|Moth4345.03|658[0n]bp|Canada.Ontario|BOLD:AAB0781
Morrisonia latex|[19547]|XAJ632-06|2006-ONT-0632|658[0n]bp|Canada.Ontario|BOLD:AAB0781
Morrisonia latex|[19548]|TTMNB410-06|MNBTT-410|658[0n]bp|Canada.New Brunswick|BOLD:AAB0781
Morrisonia latex|[19549]|LPSOB968-08|PPBP-1967|658[0n]bp|Canada.Ontario|BOLD:AAB0781
Morrisonia latex|[19550]|LPSOC306-08|PPBP-2305|654[0n]bp|Canada.Ontario|BOLD:AAB0781
Morrisonia latex|[19551]|XAB617-04|04HBL005617|619[0n]bp|Canada.Ontario|BOLD:AAB0781
Morrisonia latex|[19552]|XAC151-04|04HBL006151|619[0n]bp|Canada.Ontario|BOLD:AAB0781
Homorthodes carneola|[19553]|RDNMG467-08|CNC LEP00052291|658[0n]bp|United States.Colorado|BOLD:AAF1154
Homorthodes carneola|[19554]|RDNMG466-08|CNC LEP00052290|658[0n]bp|United States.Colorado|BOLD:AAF1154
Homorthodes carneola|[19555]|RDNMG017-08|NOC14958|658[0n]bp|United States.Arizona|BOLD:AAF1154
Orthosia pulchella|[19556]|LOWCD781-06|CGWC-3601|657[0n]bp|Canada.British Columbia|BOLD:AAF0758
Orthosia pulchella|[19557]|RDNMC463-05|CNCNoctuoidea12096|591[0n]bp|Canada.British Columbia|BOLD:AAF0758
Orthosia garmani|[19558]|RDNMF824-08|CNC LEP00053185|658[0n]bp|Canada.Ontario|BOLD:AAD0700
Egira dolosa|[19559]|XAF335-05|HLC-10376|658[0n]bp|Canada.Ontario|BOLD:AAA5658
Egira dolosa|[19560]|XAF278-05|HLC-10319|658[0n]bp|Canada.Ontario|BOLD:AAA5658
Egira dolosa|[19561]|LOWCE245-06|CGWC-4005|658[0n]bp|Canada.British Columbia|BOLD:AAA5658
Egira dolosa|[19562]|LOWCD631-06|CGWC-3451|658[0n]bp|Canada.British Columbia|BOLD:AAA5658
Egira dolosa|[19563]|PHMO031-03|moth117.02|639[0n]bp|Canada.Ontario|BOLD:AAA5658
Egira dolosa|[19564]|XAF429-05|HLC-10470|612[0n]bp|Canada.Ontario|BOLD:AAA5658
Egira dolosa|[19565]|XAJ235-06|2006-ONT-0235|658[0n]bp|Canada.Ontario|BOLD:AAA5658
Egira dolosa|[19566]|LOWCD630-06|CGWC-3450|658[0n]bp|Canada.British Columbia|BOLD:AAA5658
Egira dolosa|[19567]|LOWCD634-06|CGWC-3454|658[0n]bp|Canada.British Columbia|BOLD:AAA5658
Egira dolosa|[19568]|XAD644-05|2005-ONT-59|658[0n]bp|Canada.Ontario|BOLD:AAA5658
Egira dolosa|[19569]|XAF292-05|HLC-10333|658[0n]bp|Canada.Ontario|BOLD:AAA5658
Egira dolosa|[19570]|XAJ129-06|2006-ONT-0129|658[0n]bp|Canada.Ontario|BOLD:AAA5658
Egira dolosa|[19571]|XAF341-05|HLC-10382|658[0n]bp|Canada.Ontario|BOLD:AAA5658
Egira dolosa|[19572]|XAF413-05|HLC-10454|658[0n]bp|Canada.Ontario|BOLD:AAA5658
Egira dolosa|[19573]|XAJ138-06|2006-ONT-0138|658[0n]bp|Canada.Ontario|BOLD:AAA5658
Egira dolosa|[19574]|XAJ122-06|2006-ONT-0122|658[0n]bp|Canada.Ontario|BOLD:AAA5658
Egira dolosa|[19575]|XAJ140-06|2006-ONT-0140|658[0n]bp|Canada.Ontario|BOLD:AAA5658
Egira dolosa|[19576]|XAF347-05|HLC-10388|658[0n]bp|Canada.Ontario|BOLD:AAA5658
Egira dolosa|[19577]|XAF431-05|HLC-10472|658[0n]bp|Canada.Ontario|BOLD:AAA5658
Egira dolosa|[19578]|XAD648-05|2005-ONT-63|658[0n]bp|Canada.Ontario|BOLD:AAA5658
Egira dolosa|[19579]|LOWCD633-06|CGWC-3453|658[0n]bp|Canada.British Columbia|BOLD:AAA5658
Egira dolosa|[19580]|XAJ128-06|2006-ONT-0128|658[0n]bp|Canada.Ontario|BOLD:AAA5658
Egira dolosa|[19581]|XAJ185-06|2006-ONT-0185|658[0n]bp|Canada.Ontario|BOLD:AAA5658
Egira dolosa|[19582]|XAF418-05|HLC-10459|658[0n]bp|Canada.Ontario|BOLD:AAA5658
Egira dolosa|[19583]|XAJ234-06|2006-ONT-0234|658[0n]bp|Canada.Ontario|BOLD:AAA5658
Egira dolosa|[19584]|XAF382-05|HLC-10423|658[0n]bp|Canada.Ontario|BOLD:AAA5658
Egira dolosa|[19585]|LOWCD632-06|CGWC-3452|658[0n]bp|Canada.British Columbia|BOLD:AAA5658
Egira dolosa|[19586]|XAJ393-06|2006-ONT-0393|658[0n]bp|Canada.Ontario|BOLD:AAA5658
Egira dolosa|[19587]|XAJ225-06|2006-ONT-0225|658[0n]bp|Canada.Ontario|BOLD:AAA5658
Egira dolosa|[19588]|XAJ213-06|2006-ONT-0213|658[0n]bp|Canada.Ontario|BOLD:AAA5658
Egira dolosa|[19589]|XAF342-05|HLC-10383|658[0n]bp|Canada.Ontario|BOLD:AAA5658
Egira dolosa|[19590]|RDLQH104-06|DH013342|658[0n]bp|Canada.Quebec|BOLD:AAA5658
Egira dolosa|[19591]|XAJ132-06|2006-ONT-0132|658[0n]bp|Canada.Ontario|BOLD:AAA5658
Egira dolosa|[19592]|XAJ215-06|2006-ONT-0215|658[0n]bp|Canada.Ontario|BOLD:AAA5658
Egira dolosa|[19593]|XAF415-05|HLC-10456|658[0n]bp|Canada.Ontario|BOLD:AAA5658
Egira dolosa|[19594]|XAJ187-06|2006-ONT-0187|658[0n]bp|Canada.Ontario|BOLD:AAA5658
Egira dolosa|[19595]|XAD666-05|2005-ONT-81|658[0n]bp|Canada.Ontario|BOLD:AAA5658
Egira dolosa|[19596]|XAJ131-06|2006-ONT-0131|658[0n]bp|Canada.Ontario|BOLD:AAA5658
Egira dolosa|[19597]|XAB055-04|04HBL005055|658[0n]bp|Canada.Ontario|BOLD:AAA5658
Egira dolosa|[19598]|XAJ206-06|2006-ONT-0206|658[0n]bp|Canada.Ontario|BOLD:AAA5658
Egira dolosa|[19599]|XAJ443-06|2006-ONT-0443|658[0n]bp|Canada.Ontario|BOLD:AAA5658
Egira dolosa|[19600]|XAJ269-06|2006-ONT-0269|658[0n]bp|Canada.Ontario|BOLD:AAA5658
Egira dolosa|[19601]|XAJ134-06|2006-ONT-0134|658[0n]bp|Canada.Ontario|BOLD:AAA5658
Egira dolosa|[19602]|LOWCD629-06|CGWC-3449|658[0n]bp|Canada.British Columbia|BOLD:AAA5658
Egira dolosa|[19603]|XAF417-05|HLC-10458|647[0n]bp|Canada.Ontario|BOLD:AAA5658
Egira dolosa|[19604]|XAF430-05|HLC-10471|588[0n]bp|Canada.Ontario|BOLD:AAA5658
Egira dolosa|[19605]|XAJ186-06|2006-ONT-0186|607[0n]bp|Canada.Ontario|BOLD:AAA5658
Egira dolosa|[19606]|PMG109-03|moth135.01|617[0n]bp|Canada.Ontario|BOLD:AAA5658
Egira dolosa|[19607]|PHMO041-03|moth148.02|639[0n]bp|Canada.Ontario|BOLD:AAA5658
Egira dolosa|[19608]|TMG137-03|moth160.01|639[0n]bp|Canada.Ontario|BOLD:AAA5658
Egira dolosa|[19609]|PHMO042-03|moth149.02|639[0n]bp|Canada.Ontario|BOLD:AAA5658
Egira dolosa|[19610]|PHMO040-03|moth143.02|639[0n]bp|Canada.Ontario|BOLD:AAA5658
Egira dolosa|[19611]|TMG138-03|moth162.01|639[0n]bp|Canada.Ontario|BOLD:AAA5658
Egira dolosa|[19612]|TMG139-03|moth161.01|639[0n]bp|Canada.Ontario|BOLD:AAA5658
Egira dolosa|[19613]|PHMO043-03|moth150.02|639[0n]bp|Canada.Ontario|BOLD:AAA5658
Egira dolosa|[19614]|XAJ201-06|2006-ONT-0201|600[0n]bp|Canada.Ontario|BOLD:AAA5658
Egira dolosa|[19615]|XAJ090-06|2006-ONT-0090|618[0n]bp|Canada.Ontario|BOLD:AAA5658
Egira dolosa|[19616]|XAF416-05|HLC-10457|598[0n]bp|Canada.Ontario|BOLD:AAA5658
Egira dolosa|[19617]|XAC126-04|04HBL006126|525[0n]bp|Canada.Ontario|BOLD:AAA5658
Egira dolosa|[19618]|XAJ198-06|2006-ONT-0198|608[0n]bp|Canada.Ontario|BOLD:AAA5658
Egira dolosa|[19619]|XAJ199-06|2006-ONT-0199|607[0n]bp|Canada.Ontario|BOLD:AAA5658
Egira dolosa|[19620]|LOWCE368-06|CGWC-4128|577[0n]bp|Canada.British Columbia|BOLD:AAA5658
Egira dolosa|[19621]|XAF414-05|HLC-10455|650[0n]bp|Canada.Ontario|BOLD:AAA5658
Egira dolosa|[19622]|XAD614-05|2005-ONT-29|524[0n]bp|Canada.Ontario|
Egira dolosa|[19623]|LOWCE240-06|CGWC-4000|658[0n]bp|Canada.British Columbia|BOLD:AAA5658
Achatia distincta|[19624]|XAJ408-06|2006-ONT-0408|658[0n]bp|Canada.Ontario|BOLD:AAB7392
Achatia distincta|[19625]|KPOEC180-08|08OEC-223|658[0n]bp|Canada.Ontario|BOLD:AAB7392
Achatia distincta|[19626]|RDLQH107-06|DH013345|654[0n]bp|Canada.Quebec|BOLD:AAB7392
Achatia distincta|[19627]|XAJ130-06|2006-ONT-0130|658[0n]bp|Canada.Ontario|BOLD:AAB7392
Achatia distincta|[19628]|KPOEC043-08|08OEC-152|658[0n]bp|Canada.Ontario|BOLD:AAB7392
Achatia distincta|[19629]|XAJ230-06|2006-ONT-0230|658[0n]bp|Canada.Ontario|BOLD:AAB7392
Achatia distincta|[19630]|KPOEC177-08|08OEC-220|658[0n]bp|Canada.Ontario|BOLD:AAB7392
Achatia distincta|[19631]|RDNMH616-09|CNCLEP00062940|658[0n]bp|Canada.Quebec|BOLD:AAB7392
Achatia distincta|[19632]|XAJ337-06|2006-ONT-0337|658[0n]bp|Canada.Ontario|BOLD:AAB7392
Achatia distincta|[19633]|KPOEC123-08|08OEC-069|658[0n]bp|Canada.Ontario|BOLD:AAB7392
Achatia distincta|[19634]|RDNMH617-09|CNCLEP00062941|658[0n]bp|Canada.Ontario|BOLD:AAB7392
Achatia distincta|[19635]|KPOEC178-08|08OEC-221|658[0n]bp|Canada.Ontario|BOLD:AAB7392

[illegible]
Achatia distincta|[19633]|KPOEC123-08|08OEC-069|658[0n]bp|Canada.Ontario|BOLD:AAB7392
Achatia distincta|[19634]|RDNMH617-09|CNCLEP00062941|658[0n]bp|Canada.Ontario|BOLD:AAB7392
Achatia distincta|[19635]|KPOEC178-08|08OEC-221|658[0n]bp|Canada.Ontario|BOLD:AAB7392
Achatia distincta|[19636]|KPOEC073-08|08OEC-230|650[0n]bp|Canada.Ontario|BOLD:AAB7392
Achatia distincta|[19637]|KPOEC126-08|08OEC-072|658[0n]bp|Canada.Ontario|BOLD:AAB7392
Egira alternans|[19638]|TTMNB404-06|MNBTT-404|658[0n]bp|Canada.New Brunswick|BOLD:AAB6897
Egira alternans|[19639]|TTMNB405-06|MNBTT-405|658[0n]bp|Canada.New Brunswick|BOLD:AAB6897
Egira alternans|[19640]|TMNBB343-06|MNBTT-1283|652[0n]bp|Canada.New Brunswick|BOLD:AAB6897
Egira alternans|[19641]|TMNBB341-06|MNBTT-1281|658[0n]bp|Canada.New Brunswick|BOLD:AAB6897
Egira alternans|[19642]|TTMNB408-06|MNBTT-408|636[0n]bp|Canada.New Brunswick|BOLD:AAB6897
Egira alternans|[19643]|RDLQG319-06|DH012531|658[0n]bp|Canada.Quebec|BOLD:AAB6897
Egira alternans|[19644]|TMNBB340-06|MNBTT-1280|658[0n]bp|Canada.New Brunswick|BOLD:AAB6897
Egira alternans|[19645]|TMNBB344-06|MNBTT-1284|658[0n]bp|Canada.New Brunswick|BOLD:AAB6897
Egira alternans|[19646]|TTMNB406-06|MNBTT-406|658[0n]bp|Canada.New Brunswick|BOLD:AAB6897
Egira alternans|[19647]|TTMNB407-06|MNBTT-407|658[0n]bp|Canada.New Brunswick|BOLD:AAB6897
Egira alternans|[19648]|TTMNB409-06|MNBTT-409|658[0n]bp|Canada.New Brunswick|BOLD:AAB6897
Egira alternans|[19649]|TMNBB342-06|MNBTT-1282|658[0n]bp|Canada.New Brunswick|BOLD:AAB6897
Orthosia mys|[19650]|RDNMC470-05|CNCNoctuoidea12103|581[0n]bp|Canada.British Columbia|BOLD:AAD3143
Orthosia mys|[19651]|RDNMC469-05|CNCNoctuoidea12102|597[0n]bp|Canada.British Columbia|BOLD:AAD3143
Orthosia transparens|[19652]|LALPA023-10|AVBC 023-10|658[0n]bp|Canada.British Columbia|BOLD:AAB8764
Orthosia transparens|[19653]|LALPA048-10|AVBC 048-10|658[0n]bp|Canada.British Columbia|BOLD:AAB8764
Orthosia transparens|[19654]|LALPA1065-11|AVBC 875-11|658[0n]bp|Canada.British Columbia|BOLD:AAB8764
Orthosia transparens|[19655]|LALPA1103-11|AVBC 913-11|658[0n]bp|Canada.British Columbia|BOLD:AAB8764
Orthosia transparens|[19656]|LBCF006-07|07-JDWBC-0017|658[0n]bp|Canada.British Columbia|BOLD:AAB8764
Egira perlubens|[19657]|RDNMF251-08|NOC14337|658[0n]bp|Canada.British Columbia|BOLD:AAC9111
Egira perlubens|[19658]|LALPA070-10|AVBC 070-10|658[0n]bp|Canada.British Columbia|BOLD:AAC9111
Egira perlubens|[19659]|LALPA071-10|AVBC 071-10|658[0n]bp|Canada.British Columbia|BOLD:AAC9111
Egira perlubens|[19660]|LBCH5155-10|10-JDWBC-5155|658[0n]bp|Canada.British Columbia|BOLD:AAC9111
Egira perlubens|[19661]|LALPA072-10|AVBC 072-10|658[0n]bp|Canada.British Columbia|BOLD:AAC9111
Egira perlubens|[19662]|RDNMF247-08|NOC14333|658[0n]bp|Canada.British Columbia|BOLD:AAC9111
Egira perlubens|[19663]|LBCH5096-10|10-JDWBC-5096|658[0n]bp|Canada.British Columbia|BOLD:AAC9111
Egira perlubens|[19664]|LBCH4990-10|10-JDWBC-4990|658[0n]bp|Canada.British Columbia|BOLD:AAC9111
Egira perlubens|[19665]|DUNLP151-08|Dun-08-151|658[0n]bp|Canada.British Columbia|BOLD:AAC9111
Egira perlubens|[19666]|RDNMF249-08|NOC14335|658[0n]bp|Canada.British Columbia|BOLD:AAC9111
Egira perlubens|[19667]|LBCH4983-10|10-JDWBC-4983|658[0n]bp|Canada.British Columbia|BOLD:AAC9111
Egira perlubens|[19668]|LPVIC123-08|PFC-2006-2700|658[0n]bp|Canada.British Columbia|BOLD:AAC9111
Egira perlubens|[19669]|RDNMF248-08|NOC14334|658[0n]bp|Canada.British Columbia|BOLD:AAC9111
Egira perlubens|[19670]|DUNLP152-08|Dun-08-152|658[0n]bp|Canada.British Columbia|BOLD:AAC9111
Egira curialis|[19671]|LALPA1097-11|AVBC 907-11|658[0n]bp|Canada.British Columbia|BOLD:AAC7972
Egira curialis|[19672]|LBCH5013-10|10-JDWBC-5013|658[0n]bp|Canada.British Columbia|BOLD:AAC7972
Egira curialis|[19673]|LBCH5109-10|10-JDWBC-5109|658[0n]bp|Canada.British Columbia|BOLD:AAC7972
Egira curialis|[19674]|LBCH5158-10|10-JDWBC-5158|658[0n]bp|Canada.British Columbia|BOLD:AAC7972
Egira curialis|[19675]|LBCH4987-10|10-JDWBC-4987|658[0n]bp|Canada.British Columbia|BOLD:AAC7972
Egira curialis|[19676]|RDNMG479-08|CNC LEP00052303|658[0n]bp|Canada.British Columbia|BOLD:AAC7972
Egira curialis|[19677]|LBCH5161-10|10-JDWBC-5161|658[0n]bp|Canada.British Columbia|BOLD:AAC7972
Egira curialis|[19678]|LBCH4973-10|10-JDWBC-4973|658[0n]bp|Canada.British Columbia|BOLD:AAC7972
Egira curialis|[19679]|LBCH5002-10|10-JDWBC-5002|658[0n]bp|Canada.British Columbia|BOLD:AAC7972
Egira curialis|[19680]|LBCH5251-10|10-JDWBC-5251|658[0n]bp|Canada.British Columbia|BOLD:AAC7972
Egira curialis|[19681]|LBCG468-08|08-JDWBC-0468|658[0n]bp|Canada.British Columbia|BOLD:AAC7972
Egira curialis|[19682]|LBCG465-08|08-JDWBC-0465|658[0n]bp|Canada.British Columbia|BOLD:AAC7972
Egira curialis|[19683]|LBCH5014-10|10-JDWBC-5014|631[0n]bp|Canada.British Columbia|BOLD:AAC7972
Egira curialis|[19684]|LBCH5345-10|10-JDWBC-5345|658[0n]bp|Canada.British Columbia|BOLD:AAC7972
Egira curialis|[19685]|LBCG469-08|08-JDWBC-0469|658[0n]bp|Canada.British Columbia|BOLD:AAC7972
Egira curialis|[19686]|LBCH5287-10|10-JDWBC-5287|658[0n]bp|Canada.British Columbia|BOLD:AAC7972
Egira curialis|[19687]|LBCH4994-10|10-JDWBC-4994|658[0n]bp|Canada.British Columbia|BOLD:AAC7972
Egira rubrica|[19688]|LALPA069-10|AVBC 069-10|658[0n]bp|Canada.British Columbia|BOLD:ABY7939
Egira rubrica|[19689]|LALPA067-10|AVBC 067-10|658[0n]bp|Canada.British Columbia|BOLD:ABY7939
Egira rubrica|[19690]|LALPA068-10|AVBC 068-10|658[0n]bp|Canada.British Columbia|BOLD:ABY7939
Egira rubrica|[19691]|LBCH5000-10|10-JDWBC-5000|658[0n]bp|Canada.British Columbia|BOLD:ABY7939
Orthosia rubescens|[19692]|RDNMC465-05|CNCNoctuoidea12098|614[0n]bp|Canada.Ontario|BOLD:AAC0946
Orthosia rubescens|[19693]|KPOEC173-08|08OEC-214|655[0n]bp|Canada.Ontario|BOLD:AAC0946
Orthosia rubescens|[19694]|TMNBB332-06|MNBTT-1272|658[0n]bp|Canada.New Brunswick|BOLD:AAC0946
Orthosia rubescens|[19695]|RDLQ670-07|DH009523|655[0n]bp|Canada.Quebec|BOLD:AAC0946
Orthosia rubescens|[19696]|TMNBB330-06|MNBTT-1270|658[0n]bp|Canada.New Brunswick|BOLD:AAC0946
Orthosia rubescens|[19697]|PHMO032-03|moth123.02|639[0n]bp|Canada.Ontario|BOLD:AAC0946
Orthosia rubescens|[19698]|PMG145-03|moth299.01|617[0n]bp|Canada.Ontario|BOLD:AAC0946
Orthosia rubescens|[19699]|TMNBB331-06|MNBTT-1271|658[0n]bp|Canada.New Brunswick|BOLD:AAC0946
Orthosia rubescens|[19700]|XAE170-04|Moth4170.03|658[0n]bp|Canada.Ontario|BOLD:AAC0946
Orthosia ferrigera|[19701]|RDNMF259-08|NOC14345|658[0n]bp|United States.Oregon|BOLD:AAC8451
Orthosia ferrigera|[19702]|NAMUM248-08|RR-97-0748|658[0n]bp|United States.California|BOLD:AAC8451
Orthosia ferrigera|[19703]|RDNMF258-08|NOC14344|658[0n]bp|United States.Oregon|BOLD:AAC8451
Orthosia ferrigera|[19704]|RDNMF257-08|NOC14343|658[0n]bp|United States.Oregon|BOLD:AAC8451
Orthosia ferrigera|[19705]|LOCBB186-06|06-BLLOC-1126|658[0n]bp|United States.California|BOLD:AAC8451
Orthosia ferrigera|[19706]|LOCBB185-06|06-BLLOC-1125|658[0n]bp|United States.California|BOLD:AAC8451
Orthosia ferrigera|[19707]|LOCBB189-06|06-BLLOC-1129|626[0n]bp|United States.California|BOLD:AAC8451
Orthosia ferrigera|[19708]|LOCBB187-06|06-BLLOC-1127|658[0n]bp|United States.California|BOLD:AAC8451
Orthosia ferrigera|[19709]|LOCBB188-06|06-BLLOC-1128|658[0n]bp|United States.California|BOLD:AAC8451
Egira variabilis|[19710]|RDNMB361-05|CNCNoctuoidea10127|601[1n]bp|United States.California|BOLD:AAD0722
Egira variabilis|[19711]|RDNMD913-07|CNCNoctuoidea13244|655[0n]bp|United States.Wyoming|BOLD:AAD0722
Egira variabilis|[19712]|RDNMG993-08|CNC LEP00053117|658[0n]bp|United States.Wyoming|BOLD:AAD0722
Egira variabilis|[19713]|NAMUM394-09|RR-96-0076|658[0n]bp|United States.California|BOLD:AAD0722
Egira variabilis|[19714]|NAMUM385-09|RR-96-0066|658[0n]bp|United States.California|BOLD:AAD0722
Egira variabilis|[19715]|RDNMG992-08|CNC LEP00053116|658[0n]bp|United States.Wyoming|BOLD:AAD0722
Egira variabilis|[19716]|NAMUM386-09|RR-96-0067|658[0n]bp|United States.California|BOLD:AAD0722
Egira variabilis|[19717]|RDNMD912-07|CNCNoctuoidea13243|655[0n]bp|United States.Wyoming|BOLD:AAD0722
Orthosia praeses|[19718]|RDNMG519-08|CNC LEP00052343|658[0n]bp|Canada.British Columbia|BOLD:AAD6082
Orthosia praeses|[19719]|RDNMG520-08|CNC LEP00052344|658[0n]bp|Canada.British Columbia|BOLD:AAD6082
Orthosia praeses|[19720]|LALPA1042-11|AVBC 852-11|658[0n]bp|Canada.British Columbia|BOLD:AAD6082
Orthosia praeses|[19721]|LALPA011-10|AVBC 011-10|658[0n]bp|Canada.British Columbia|BOLD:AAD6082
Orthosia praeses|[19722]|LALPA1031-11|AVBC 841-11|658[0n]bp|Canada.British Columbia|BOLD:AAD6082
Orthosia praeses|[19723]|LALPA012-10|AVBC 012-10|658[0n]bp|Canada.British Columbia|BOLD:AAD6082
Orthosia praeses|[19724]|LALPA1032-11|AVBC 842-11|658[0n]bp|Canada.British Columbia|BOLD:AAD6082
Acerra normalis|[19725]|RDNMF242-08|NOC14328|609[1n]bp|Canada.British Columbia|BOLD:AAD6503
Acerra normalis|[19726]|RDNMF244-08|NOC14330|658[0n]bp|Canada.British Columbia|BOLD:AAD6503
Acerra normalis|[19727]|RDNMF243-08|NOC14329|640[0n]bp|Canada.British Columbia|BOLD:AAD6503
Acerra normalis|[19728]|RDNMF245-08|NOC14331|658[0n]bp|Canada.British Columbia|BOLD:AAD6503
Acerra normalis|[19729]|RDNMF246-08|NOC14332|609[0n]bp|Canada.British Columbia|BOLD:AAD6503
Acerra normalis|[19730]|LALPA005-10|AVBC 005-10|658[0n]bp|Canada.British Columbia|BOLD:AAD6503
Acerra normalis|[19731]|LALPA006-10|AVBC 006-10|658[0n]bp|Canada.British Columbia|BOLD:AAD6503
Orthosia segregata|[19732]|LOWC068-05|CGWC-0068|530[0n]bp|Canada.British Columbia|BOLD:AAB8772
Orthosia segregata|[19733]|LOWC067-05|CGWC-0067|525[0n]bp|Canada.British Columbia|BOLD:AAB8772
Orthosia segregata|[19734]|LOWC066-05|CGWC-0066|574[0n]bp|Canada.British Columbia|BOLD:AAB8772
[illegible]

Orthosia segregata|[19732]|LOWC068-05|CGWC-0068|530[0n]bp|Canada.British Columbia|BOLD:AAB8772
Orthosia segregata|[19733]|LOWC067-05|CGWC-0067|525[0n]bp|Canada.British Columbia|BOLD:AAB8772
Orthosia segregata|[19734]|LOWC066-05|CGWC-0066|574[0n]bp|Canada.British Columbia|BOLD:AAB8772
Orthosia segregata|[19735]|LOWCE239-06|CGWC-3999|658[0n]bp|Canada.British Columbia|BOLD:AAB8772
Orthosia segregata|[19736]|LOWCE407-06|CGWC-4167|599[0n]bp|Canada.British Columbia|BOLD:AAB8772
Orthosia segregata|[19737]|LOWCD626-06|CGWC-3446|657[0n]bp|Canada.British Columbia|BOLD:AAB8772
Orthosia segregata|[19738]|LBCH5255-10|10-JDWBC-5255|658[0n]bp|Canada.British Columbia|BOLD:AAB8772
Orthosia segregata|[19739]|LBCH5157-10|10-JDWBC-5157|658[0n]bp|Canada.British Columbia|BOLD:AAB8772
Orthosia segregata|[19740]|LOWCE024-06|CGWC-3784|658[0n]bp|Canada.British Columbia|BOLD:AAB8772
Orthosia segregata|[19741]|LBCH5209-10|10-JDWBC-5209|658[0n]bp|Canada.British Columbia|BOLD:AAB8772
Orthosia segregata|[19742]|LOWCD628-06|CGWC-3448|658[0n]bp|Canada.British Columbia|BOLD:AAB8772
Orthosia segregata|[19743]|LBCG470-08|08-JDWBC-0470|658[0n]bp|Canada.British Columbia|BOLD:AAB8772
Orthosia segregata|[19744]|RDLQ671-07|DH009711|658[0n]bp|Canada.Ontario|BOLD:AAB8772
Orthosia segregata|[19745]|LOWCD627-06|CGWC-3447|658[0n]bp|Canada.British Columbia|BOLD:AAB8772
Orthosia segregata|[19746]|LBCH5343-10|10-JDWBC-5343|658[0n]bp|Canada.British Columbia|BOLD:AAB8772
Orthosia segregata|[19747]|LOWCE233-06|CGWC-3993|658[0n]bp|Canada.British Columbia|BOLD:AAB8772
Orthosia segregata|[19748]|RDNMH391-09|JD2367|658[0n]bp|Canada.Alberta|BOLD:AAB8772
Orthosia segregata|[19749]|LOWC069-05|CGWC-0069|658[0n]bp|Canada.British Columbia|BOLD:AAB8772
Orthosia segregata|[19750]|LOWCD624-06|CGWC-3444|658[0n]bp|Canada.British Columbia|BOLD:AAB8772
Crocigrapha normani|[19751]|KPOEC125-08|08OEC-071|658[0n]bp|Canada.Ontario|BOLD:AAA6924
Crocigrapha normani|[19752]|XAF333-05|HLC-10374|658[0n]bp|Canada.Ontario|BOLD:AAA6924
Crocigrapha normani|[19753]|LMIS020-05|05-ONMIS-0020|658[0n]bp|Canada.Ontario|BOLD:AAA6924
Crocigrapha normani|[19754]|KPOEC124-08|08OEC-070|658[0n]bp|Canada.Ontario|BOLD:AAA6924
Crocigrapha normani|[19755]|TTMNB411-06|MNBTT-411|658[0n]bp|Canada.New Brunswick|BOLD:AAA6924
Crocigrapha normani|[19756]|TMNBB338-06|MNBTT-1278|656[0n]bp|Canada.New Brunswick|BOLD:AAA6924
Crocigrapha normani|[19757]|KPOEC045-08|08OEC-154|658[0n]bp|Canada.Ontario|BOLD:AAA6924
Crocigrapha normani|[19758]|LPSO550-08|PPBP-0550|658[0n]bp|Canada.Ontario|BOLD:AAA6924
Crocigrapha normani|[19759]|XAF434-05|HLC-10475|658[0n]bp|Canada.Ontario|BOLD:AAA6924
Crocigrapha normani|[19760]|KPOEC042-08|08OEC-151|658[0n]bp|Canada.Ontario|BOLD:AAA6924
Crocigrapha normani|[19761]|RDLQG463-06|DH012756|658[0n]bp|Canada.Quebec|BOLD:AAA6924
Crocigrapha normani|[19762]|KPOEC138-08|08OEC-142|658[0n]bp|Canada.Ontario|BOLD:AAA6924
Crocigrapha normani|[19763]|BLTIB091-08|BL0147|658[1n]bp|Canada.Ontario|BOLD:AAA6924
Crocigrapha normani|[19764]|PMG105-03|moth147.01|617[0n]bp|Canada.Ontario|BOLD:AAA6924
Crocigrapha normani|[19765]|KPOEC133-08|08OEC-134|658[0n]bp|Canada.Ontario|BOLD:AAA6924
Crocigrapha normani|[19766]|KPOEC175-08|08OEC-216|658[0n]bp|Canada.Ontario|BOLD:AAA6924
Crocigrapha normani|[19767]|RDLQH070-06|DH013307|658[0n]bp|Canada.Quebec|BOLD:AAA6924
Crocigrapha normani|[19768]|RDLQ665-07|DH009278|658[0n]bp|Canada.Quebec|BOLD:AAA6924
Crocigrapha normani|[19769]|XAJ410-06|2006-ONT-0410|658[0n]bp|Canada.Ontario|BOLD:AAA6924
Crocigrapha normani|[19770]|KPOEC041-08|08OEC-150|658[0n]bp|Canada.Ontario|BOLD:AAA6924
Crocigrapha normani|[19771]|KPOEC151-08|08OEC-192|658[0n]bp|Canada.Ontario|BOLD:AAA6924
Crocigrapha normani|[19772]|TMNBB339-06|MNBTT-1279|658[0n]bp|Canada.New Brunswick|BOLD:AAA6924
Crocigrapha normani|[19773]|LPSOD275-09|08BBLEP-00053|658[0n]bp|Canada.Ontario|BOLD:AAA6924
Crocigrapha normani|[19774]|XAJ508-06|2006-ONT-0508|658[0n]bp|Canada.Ontario|BOLD:AAA6924
Crocigrapha normani|[19775]|KPOEC154-08|08OEC-195|658[0n]bp|Canada.Ontario|BOLD:AAA6924
Crocigrapha normani|[19776]|XAF407-05|HLC-10448|658[0n]bp|Canada.Ontario|BOLD:AAA6924
Crocigrapha normani|[19777]|TMNBB335-06|MNBTT-1275|658[0n]bp|Canada.New Brunswick|BOLD:AAA6924
Crocigrapha normani|[19778]|XAJ216-06|2006-ONT-0216|658[0n]bp|Canada.Ontario|BOLD:AAA6924
Crocigrapha normani|[19779]|KPOEC141-08|08OEC-145|658[0n]bp|Canada.Ontario|BOLD:AAA6924
Crocigrapha normani|[19780]|TTMNB577-06|MNBTT-577|658[0n]bp|Canada.New Brunswick|BOLD:AAA6924
Crocigrapha normani|[19781]|LPSO258-08|PPBP-0258|658[0n]bp|Canada.Ontario|BOLD:AAA6924
Crocigrapha normani|[19782]|KPOEC114-08|08OEC-059|658[0n]bp|Canada.Ontario|BOLD:AAA6924
Crocigrapha normani|[19783]|BLTIB033-08|BL0060|658[0n]bp|Canada.Ontario|BOLD:AAA6924
Crocigrapha normani|[19784]|TMNBB336-06|MNBTT-1276|658[0n]bp|Canada.New Brunswick|BOLD:AAA6924
Crocigrapha normani|[19785]|TTMNB091-06|MNBTT-091|657[0n]bp|Canada.New Brunswick|BOLD:AAA6924
Crocigrapha normani|[19786]|TTMNB579-06|MNBTT-579|658[0n]bp|Canada.New Brunswick|BOLD:AAA6924
Crocigrapha normani|[19787]|XAJ456-06|2006-ONT-0456|658[0n]bp|Canada.Ontario|BOLD:AAA6924
Crocigrapha normani|[19788]|KPOEC070-08|08OEC-218|658[0n]bp|Canada.Ontario|BOLD:AAA6924
Crocigrapha normani|[19789]|TTMNB576-06|MNBTT-576|658[0n]bp|Canada.New Brunswick|BOLD:AAA6924
Crocigrapha normani|[19790]|TTMNB049-06|MNBTT-049|658[0n]bp|Canada.New Brunswick|BOLD:AAA6924
Crocigrapha normani|[19791]|KPOEC183-08|08OEC-226|655[0n]bp|Canada.Ontario|BOLD:AAA6924
Crocigrapha normani|[19792]|KPOEC082-08|08OEC-241|646[0n]bp|Canada.Ontario|BOLD:AAA6924
Crocigrapha normani|[19793]|TMG134-03|moth191.01|639[0n]bp|Canada.Ontario|BOLD:AAA6924
Crocigrapha normani|[19794]|TMG136-03|moth169.01|639[0n]bp|Canada.Ontario|BOLD:AAA6924
Crocigrapha normani|[19795]|TMG135-03|moth136.01|639[0n]bp|Canada.Ontario|BOLD:AAA6924
Crocigrapha normani|[19796]|TMNBB337-06|MNBTT-1277|658[0n]bp|Canada.New Brunswick|BOLD:AAA6924
Egira hiemalis|[19797]|DUNLP360-08|Dun-08-360|632[0n]bp|Canada.British Columbia|BOLD:ACE7810
Egira hiemalis|[19798]|LALPA1037-11|AVBC 847-11|658[0n]bp|Canada.British Columbia|BOLD:ACE7810
Egira hiemalis|[19799]|RDNMF253-08|NOC14339|658[0n]bp|Canada.British Columbia|BOLD:ACE7810
Egira hiemalis|[19800]|RDNMF254-08|NOC14340|658[0n]bp|Canada.British Columbia|BOLD:ACE7810
Egira hiemalis|[19801]|RDNMF255-08|NOC14341|658[0n]bp|Canada.British Columbia|BOLD:ACE7810
Egira cognata|[19802]|RDNMG475-08|CNC LEP00052299|658[1n]bp|United States.California|BOLD:AAD4439
Egira cognata|[19803]|RDNMG1014-08|CNC LEP00053138|658[0n]bp|United States.California|BOLD:AAD4439
Egira cognata|[19804]|RDNMG474-08|CNC LEP00052298|658[0n]bp|United States.California|BOLD:AAD4439
Egira cognata|[19805]|RDNMG476-08|CNC LEP00052300|658[0n]bp|United States.California|BOLD:AAD4439
Egira cognata|[19806]|RDNMD403-06|CNCNoctuoidea12735|658[0n]bp|United States.California|BOLD:AAD4439
Egira cognata|[19807]|RDNMD404-06|CNCNoctuoidea12736|658[0n]bp|United States.California|BOLD:AAD4439
Egira cognata|[19808]|GMLC153-09|2009GM-0022|658[0n]bp|United States.California|BOLD:ACF4391
Egira cognata|[19809]|RWWB504-10|RWWA-1503|633[0n]bp|United States.Washington|BOLD:ACF4391
Egira cognata|[19810]|RWWC898-12|RWWA-3387|658[0n]bp|United States.Washington|BOLD:ACF4391
Egira cognata|[19811]|RWWB522-10|RWWA-1521|658[0n]bp|United States.Washington|BOLD:ACF4391
Egira cognata|[19812]|RWWB594-10|RWWA-1593|658[0n]bp|United States.Washington|BOLD:ACF4391
Egira cognata|[19813]|RWWC172-11|RWWA-2149|658[0n]bp|United States.Washington|BOLD:ACF4391
Egira cognata|[19814]|RWWB496-10|RWWA-1495|658[0n]bp|United States.Washington|BOLD:ACF4391
Egira cognata|[19815]|RWWB526-10|RWWA-1525|658[0n]bp|United States.Washington|BOLD:ACF4391
Egira cognata|[19816]|RWWB527-10|RWWA-1526|658[0n]bp|United States.Washington|BOLD:ACF4391
Egira cognata|[19817]|RWWB514-10|RWWA-1513|658[0n]bp|United States.Washington|BOLD:ACF4391
Egira cognata|[19818]|RWWB550-10|RWWA-1549|658[0n]bp|United States.Washington|BOLD:ACF4391
Egira crucialis|[19819]|LALPA009-10|AVBC 009-10|658[0n]bp|Canada.British Columbia|BOLD:AAC1794
Egira crucialis|[19820]|RDNMF026-08|NOC14112|658[0n]bp|Canada.British Columbia|BOLD:AAC1794
Egira crucialis|[19821]|LALPA1049-11|AVBC 859-11|658[0n]bp|Canada.British Columbia|BOLD:AAC1794
Egira crucialis|[19822]|LBCH5284-10|10-JDWBC-5284|658[0n]bp|Canada.British Columbia|BOLD:AAC1794
Egira crucialis|[19823]|LBCF004-07|07-JDWBC-0015|658[0n]bp|Canada.British Columbia|BOLD:AAC1794
Egira crucialis|[19824]|LALPA007-10|AVBC 007-10|658[0n]bp|Canada.British Columbia|BOLD:AAC1794
Egira crucialis|[19825]|LBCF005-07|07-JDWBC-0016|658[0n]bp|Canada.British Columbia|BOLD:AAC1794
Egira crucialis|[19826]|LALPA008-10|AVBC 008-10|658[0n]bp|Canada.British Columbia|BOLD:AAC1794
Egira simplex|[19827]|RDNMF033-08|NOC14119|658[0n]bp|Canada.British Columbia|BOLD:AAC1794
Egira simplex|[19828]|RDNMG573-08|CNC LEP00052397|658[0n]bp|Canada.British Columbia|BOLD:ACE7118
Egira simplex|[19829]|LBCH4978-10|10-JDWBC-4978|658[0n]bp|Canada.British Columbia|BOLD:ACE7118
Egira simplex|[19830]|RDNMG472-08|CNC LEP00052296|658[0n]bp|Canada.British Columbia|BOLD:ACE7118
Egira simplex|[19831]|LBCH4974-10|10-JDWBC-4974|658[0n]bp|Canada.British Columbia|BOLD:ACE7118
Egira simplex|[19832]|DUNLP154-08|Dun-08-154|658[0n]bp|Canada.British Columbia|BOLD:ACE7118
Egira simplex|[19833]|LALPA064-10|AVBC 064-10|658[0n]bp|Canada.British Columbia|BOLD:ACE7118
Egira simplex|[19834]|LALPA063-10|AVBC 063-10|658[0n]bp|Canada.British Columbia|BOLD:ACE7118

Egira simplex|[19832]|DUNLP154-08|Dun-08-154|658[0n]bp|Canada.British Columbia|BOLD:ACE7118
Egira simplex|[19833]|LALPA064-10|AVBC 064-10|658[0n]bp|Canada.British Columbia|BOLD:ACE7118
Egira simplex|[19834]|LALPA063-10|AVBC 063-10|658[0n]bp|Canada.British Columbia|BOLD:ACE7118
Egira simplex|[19835]|DUNLP153-08|Dun-08-153|658[0n]bp|Canada.British Columbia|BOLD:ACE7118
Egira simplex|[19836]|LALPA066-10|AVBC 066-10|658[0n]bp|Canada.British Columbia|BOLD:ACE7118
Egira simplex|[19837]|LALPA065-10|AVBC 065-10|658[0n]bp|Canada.British Columbia|BOLD:ACE7118
Himella fidelis|[19838]|LPSO047-08|PPBP-0047|658[0n]bp|Canada.Ontario|BOLD:AAC5638
Himella fidelis|[19839]|LPSO677-08|PPBP-0677|658[0n]bp|Canada.Ontario|BOLD:AAC5638
Morrisonia confusa|[19840]|XAE281-04|Moth4281.03|658[0n]bp|Canada.Ontario|BOLD:AAA6652
Morrisonia confusa|[19841]|XAD669-05|2005-ONT-84|658[0n]bp|Canada.Ontario|BOLD:AAA6652
Morrisonia confusa|[19842]|RDLQ684-07|DH003997|592[0n]bp|Canada.Quebec|BOLD:AAA6652
Morrisonia confusa|[19843]|LMIS015-05|05-ONMIS-0015|658[0n]bp|Canada.Ontario|BOLD:AAA6652
Morrisonia confusa|[19844]|TMNBB353-06|MNBTT-1293|658[0n]bp|Canada.New Brunswick|BOLD:AAA6652
Morrisonia confusa|[19845]|TMNBB351-06|MNBTT-1291|658[0n]bp|Canada.New Brunswick|BOLD:AAA6652
Morrisonia confusa|[19846]|TMNBB352-06|MNBTT-1292|658[0n]bp|Canada.New Brunswick|BOLD:AAA6652
Morrisonia confusa|[19847]|BLTIB032-08|BL0059|658[0n]bp|Canada.Ontario|BOLD:AAA6652
Morrisonia confusa|[19848]|TMNBB357-06|MNBTT-1297|658[0n]bp|Canada.New Brunswick|BOLD:AAA6652
Morrisonia confusa|[19849]|TMNBB349-06|MNBTT-1289|658[0n]bp|Canada.New Brunswick|BOLD:AAA6652
Morrisonia confusa|[19850]|RDLQ683-07|DH009480|658[0n]bp|Canada.Quebec|BOLD:AAA6652
Morrisonia confusa|[19851]|TMNBB361-06|MNBTT-1301|658[0n]bp|Canada.New Brunswick|BOLD:AAA6652
Morrisonia confusa|[19852]|TMNBB356-06|MNBTT-1296|658[0n]bp|Canada.New Brunswick|BOLD:AAA6652
Morrisonia confusa|[19853]|TMNBB354-06|MNBTT-1294|658[0n]bp|Canada.New Brunswick|BOLD:AAA6652
Morrisonia confusa|[19854]|TMNBB348-06|MNBTT-1288|658[0n]bp|Canada.New Brunswick|BOLD:AAA6652
Morrisonia confusa|[19855]|MEC177-04|jflandry0177|658[0n]bp|Canada.Quebec|BOLD:AAA6652
Morrisonia confusa|[19856]|TMNBB359-06|MNBTT-1299|658[0n]bp|Canada.New Brunswick|BOLD:AAA6652
Morrisonia confusa|[19857]|TMNBB355-06|MNBTT-1295|658[0n]bp|Canada.New Brunswick|BOLD:AAA6652
Morrisonia confusa|[19858]|TMNBB350-06|MNBTT-1290|658[0n]bp|Canada.New Brunswick|BOLD:AAA6652
Morrisonia confusa|[19859]|XAJ411-06|2006-ONT-0411|656[0n]bp|Canada.Ontario|BOLD:AAA6652
Morrisonia confusa|[19860]|PMG135-03|moth148.01|617[0n]bp|Canada.Ontario|BOLD:AAA6652
Morrisonia confusa|[19861]|TMG141-03|moth314.01|639[0n]bp|Canada.Ontario|BOLD:AAA6652
Morrisonia confusa|[19862]|TMNBB360-06|MNBTT-1300|656[0n]bp|Canada.New Brunswick|BOLD:AAA6652
Morrisonia confusa|[19863]|TMNBB358-06|MNBTT-1298|656[0n]bp|Canada.New Brunswick|BOLD:AAA6652
Morrisonia confusa|[19864]|LPSO038-08|PPBP-0038|658[0n]bp|Canada.Ontario|BOLD:AAA6652
Morrisonia confusa|[19865]|XAJ355-06|2006-ONT-0355|658[0n]bp|Canada.Ontario|BOLD:AAA6652
Morrisonia confusa|[19866]|LPSO955-08|PPBP-0955|658[0n]bp|Canada.Ontario|BOLD:AAA6652
Morrisonia confusa|[19867]|XAC157-04|04HBL006157|658[0n]bp|Canada.Ontario|BOLD:AAA6652
Morrisonia confusa|[19868]|LPSO372-08|PPBP-0372|658[0n]bp|Canada.Ontario|BOLD:AAA6652
Morrisonia confusa|[19869]|XAJ404-06|2006-ONT-0404|658[0n]bp|Canada.Ontario|BOLD:AAA6652
Morrisonia confusa|[19870]|LPSO236-08|PPBP-0236|658[0n]bp|Canada.Ontario|BOLD:AAA6652
Morrisonia confusa|[19871]|XAB277-04|04HBL005277|658[0n]bp|Canada.Ontario|BOLD:AAA6652
Morrisonia confusa|[19872]|LPSO374-08|PPBP-0374|658[0n]bp|Canada.Ontario|BOLD:AAA6652
Morrisonia confusa|[19873]|LPSO049-08|PPBP-0049|658[0n]bp|Canada.Ontario|BOLD:AAA6652
Morrisonia evicta|[19874]|TMNBB347-06|MNBTT-1287|658[0n]bp|Canada.New Brunswick|BOLD:AAB1504
Morrisonia evicta|[19875]|TMNBB345-06|MNBTT-1285|658[0n]bp|Canada.New Brunswick|BOLD:AAB1504
Morrisonia evicta|[19876]|TTMNB587-06|MNBTT-587|658[0n]bp|Canada.New Brunswick|BOLD:AAB1504
Morrisonia evicta|[19877]|XAF323-05|HLC-10364|658[0n]bp|Canada.Ontario|BOLD:AAB1504
Morrisonia evicta|[19878]|RDLQG467-06|DH012760|658[0n]bp|Canada.Quebec|BOLD:AAB1504
Morrisonia evicta|[19879]|TMNBB346-06|MNBTT-1286|658[0n]bp|Canada.New Brunswick|BOLD:AAB1504
Morrisonia evicta|[19880]|XAF337-05|HLC-10378|658[0n]bp|Canada.Ontario|BOLD:AAB1504
Morrisonia evicta|[19881]|TTMNB056-06|MNBTT-056|627[0n]bp|Canada.New Brunswick|BOLD:AAB1504
Morrisonia evicta|[19882]|TMG140-03|moth116.01|639[0n]bp|Canada.Ontario|BOLD:AAB1504
Morrisonia evicta|[19883]|RDLQG464-06|DH012757|645[0n]bp|Canada.Quebec|BOLD:AAB1504
Morrisonia evicta|[19884]|XAJ229-06|2006-ONT-0229|658[0n]bp|Canada.Ontario|BOLD:AAB1504
Morrisonia evicta|[19885]|BLTIB054-08|BL0086|658[0n]bp|Canada.Ontario|BOLD:AAB1504
Morrisonia evicta|[19886]|LPSO251-08|PPBP-0251|658[0n]bp|Canada.Ontario|BOLD:AAB1504
Morrisonia evicta|[19887]|LPSO150-08|PPBP-0150|658[0n]bp|Canada.Ontario|BOLD:AAB1504
Morrisonia evicta|[19888]|LPSO253-08|PPBP-0253|657[0n]bp|Canada.Ontario|BOLD:AAB1504
Morrisonia evicta|[19889]|LPSO155-08|PPBP-0155|658[0n]bp|Canada.Ontario|BOLD:AAB1504
Morrisonia evicta|[19890]|XAJ226-06|2006-ONT-0226|658[0n]bp|Canada.Ontario|BOLD:AAB1504
Morrisonia evicta|[19891]|XAJ267-06|2006-ONT-0267|658[0n]bp|Canada.Ontario|BOLD:AAB1504
Morrisonia evicta|[19892]|XAJ111-06|2006-ONT-0111|658[0n]bp|Canada.Ontario|BOLD:AAB1504
Morrisonia evicta|[19893]|XAE154-04|Moth4154.03|658[0n]bp|Canada.Ontario|BOLD:AAB1504
Morrisonia evicta|[19894]|XAJ243-06|2006-ONT-0243|658[0n]bp|Canada.Ontario|BOLD:AAB1504
Morrisonia evicta|[19895]|PMG136-03|moth115.01|617[0n]bp|Canada.Ontario|BOLD:AAB1504
Orthosia alurina|[19896]|RDNMC466-05|CNCNoctuoidea12099|658[0n]bp|Canada.Ontario|BOLD:ACF2855
Orthosia alurina|[19897]|MEC135-04|jflandry0135|658[0n]bp|Canada.Quebec|BOLD:ACF2855
Orthosia alurina|[19898]|PMG142-03|moth114.01|617[0n]bp|Canada.Ontario|BOLD:ACF2855
Orthosia alurina|[19899]|MEC127-04|jflandry0127|593[2n]bp|Canada.Quebec|BOLD:ACF2855
Orthosia alurina|[19900]|RDLQ669-07|DH009427|646[0n]bp|Canada.Quebec|BOLD:ACF2855
Orthosia alurina|[19901]|MEC118-04|jflandry0118|658[0n]bp|Canada.Quebec|BOLD:ACF2855
Orthosia hibisci|[19902]|LALPA088-10|AVBC 088-10|658[0n]bp|Canada.British Columbia|BOLD:ACF3074
Orthosia hibisci|[19903]|LALPA050-10|AVBC 050-10|658[0n]bp|Canada.British Columbia|BOLD:ACF3074
Orthosia hibisci|[19904]|LALPA055-10|AVBC 055-10|658[0n]bp|Canada.British Columbia|BOLD:ACF3074
Orthosia hibisci|[19905]|LALPA051-10|AVBC 051-10|658[0n]bp|Canada.British Columbia|BOLD:ACF3074
Orthosia hibisci|[19906]|LALPA049-10|AVBC 049-10|658[0n]bp|Canada.British Columbia|BOLD:ACF3074
Orthosia hibisci|[19907]|LOWCD623-06|CGWC-3443|658[0n]bp|Canada.British Columbia|BOLD:AAA4128
Orthosia hibisci|[19908]|XAF308-05|HLC-10349|658[0n]bp|Canada.Ontario|BOLD:AAA4128
Orthosia hibisci|[19909]|LBCH2427-10|10-JDWBC-2427|658[0n]bp|Canada.British Columbia|BOLD:AAA4128
Orthosia hibisci|[19910]|XAE007-04|Moth4007.03|658[0n]bp|Canada.Ontario|BOLD:AAA4128
Orthosia hibisci|[19911]|LOWCE362-06|CGWC-4122|586[5n]bp|Canada.British Columbia|BOLD:AAA4128
Orthosia hibisci|[19912]|MEC129-04|jflandry0129|658[0n]bp|Canada.Quebec|BOLD:AAA4128
Orthosia hibisci|[19913]|RDLQ668-07|DH009587|583[0n]bp|Canada.Quebec|BOLD:AAA4128
Orthosia hibisci|[19914]|HEMAY1064-12|BIOUG02618-B06|583[0n]bp|Canada.Ontario|BOLD:AAA4128
Orthosia hibisci|[19915]|RDLQ667-07|DH004118|580[0n]bp|Canada.Quebec|BOLD:AAA4128
Orthosia hibisci|[19916]|RDLQ666-07|DH004096|647[0n]bp|Canada.Quebec|BOLD:AAA4128
Orthosia hibisci|[19917]|LBCH5001-10|10-JDWBC-5001|658[0n]bp|Canada.British Columbia|BOLD:AAA4128
Orthosia hibisci|[19918]|LOWCE018-06|CGWC-3778|658[0n]bp|Canada.British Columbia|BOLD:AAA4128
Orthosia hibisci|[19919]|XAE042-04|Moth4042.03|658[0n]bp|Canada.Ontario|BOLD:AAA4128
Orthosia hibisci|[19920]|XAC096-04|04HBL006096|658[0n]bp|Canada.Ontario|BOLD:AAA4128
Orthosia hibisci|[19921]|XAJ062-06|2006-ONT-0062|658[0n]bp|Canada.Ontario|BOLD:AAA4128
Orthosia hibisci|[19922]|LOWCE210-06|CGWC-3970|658[0n]bp|Canada.British Columbia|BOLD:AAA4128
Orthosia hibisci|[19923]|XAJ101-06|2006-ONT-0101|658[0n]bp|Canada.Ontario|BOLD:AAA4128
Orthosia hibisci|[19924]|LOWCD622-06|CGWC-3442|658[0n]bp|Canada.British Columbia|BOLD:AAA4128
Orthosia hibisci|[19925]|LOWCE019-06|CGWC-3779|658[0n]bp|Canada.British Columbia|BOLD:AAA4128
Orthosia hibisci|[19926]|LOWCE269-06|CGWC-4029|658[0n]bp|Canada.British Columbia|BOLD:AAA4128
Orthosia hibisci|[19927]|XAF261-05|HLC-10302|658[0n]bp|Canada.Ontario|BOLD:AAA4128
Orthosia hibisci|[19928]|LOWCE268-06|CGWC-4028|658[0n]bp|Canada.British Columbia|BOLD:AAA4128
Orthosia hibisci|[19929]|XAF291-05|HLC-10332|658[0n]bp|Canada.Ontario|BOLD:AAA4128
Orthosia hibisci|[19930]|XAJ189-06|2006-ONT-0189|658[0n]bp|Canada.Ontario|BOLD:AAA4128
Orthosia hibisci|[19931]|XAF433-05|HLC-10474|658[0n]bp|Canada.Ontario|BOLD:AAA4128
Orthosia hibisci|[19932]|MEC185-04|jflandry0185|658[0n]bp|Canada.Quebec|BOLD:AAA4128
Orthosia hibisci|[19933]|LOWCE010-06|CGWC-3770|658[0n]bp|Canada.British Columbia|BOLD:AAA4128
Orthosia hibisci|[19934]|LOWCE017-06|CGWC-3777|658[0n]bp|Canada.British Columbia|BOLD:AAA4128

[illegible]
Orthosia hibisci|[19932]|MEC185-04|jflandry0185|658[0n]bp|Canada.Quebec|BOLD:AAA4128
Orthosia hibisci|[19933]|LOWCE010-06|CGWC-3770|658[0n]bp|Canada.British Columbia|BOLD:AAA4128
Orthosia hibisci|[19934]|LOWCE017-06|CGWC-3777|658[0n]bp|Canada.British Columbia|BOLD:AAA4128
Orthosia hibisci|[19935]|LOWCE012-06|CGWC-3772|658[0n]bp|Canada.British Columbia|BOLD:AAA4128
Orthosia hibisci|[19936]|XAE150-04|Moth4150.03|658[0n]bp|Canada.Ontario|BOLD:AAA4128
Orthosia hibisci|[19937]|XAE132-04|Moth4132.03|658[0n]bp|Canada.Ontario|BOLD:AAA4128
Orthosia hibisci|[19938]|XAF194-05|HLC-10235|658[0n]bp|Canada.Ontario|BOLD:AAA4128
Orthosia hibisci|[19939]|LOWCE011-06|CGWC-3771|658[0n]bp|Canada.British Columbia|BOLD:AAA4128
Orthosia hibisci|[19940]|XAF196-05|HLC-10237|658[0n]bp|Canada.Ontario|BOLD:AAA4128
Orthosia hibisci|[19941]|XAF259-05|HLC-10300|658[0n]bp|Canada.Ontario|BOLD:AAA4128
Orthosia hibisci|[19942]|XAF297-05|HLC-10338|658[0n]bp|Canada.Ontario|BOLD:AAA4128
Orthosia hibisci|[19943]|LOWCE015-06|CGWC-3775|658[0n]bp|Canada.British Columbia|BOLD:AAA4128
Orthosia hibisci|[19944]|XAE041-04|Moth4041.03|658[0n]bp|Canada.Ontario|BOLD:AAA4128
Orthosia hibisci|[19945]|XAJ063-06|2006-ONT-0063|658[0n]bp|Canada.Ontario|BOLD:AAA4128
Orthosia hibisci|[19946]|XAJ061-06|2006-ONT-0061|658[0n]bp|Canada.Ontario|BOLD:AAA4128
Orthosia hibisci|[19947]|XAJ336-06|2006-ONT-0336|658[0n]bp|Canada.Ontario|BOLD:AAA4128
Orthosia hibisci|[19948]|MEC116-04|jflandry0116|658[0n]bp|Canada.Quebec|BOLD:AAA4128
Orthosia hibisci|[19949]|XAF195-05|HLC-10236|658[0n]bp|Canada.Ontario|BOLD:AAA4128
Orthosia hibisci|[19950]|XAF028-05|HBL008844|658[0n]bp|Canada.Ontario|BOLD:AAA4128
Orthosia hibisci|[19951]|LOWCE013-06|CGWC-3773|658[0n]bp|Canada.British Columbia|BOLD:AAA4128
Orthosia hibisci|[19952]|XAF257-05|HLC-10298|658[0n]bp|Canada.Ontario|BOLD:AAA4128
Orthosia hibisci|[19953]|MEC119-04|jflandry0119|658[0n]bp|Canada.Quebec|BOLD:AAA4128
Orthosia hibisci|[19954]|LMIS032-05|05-ONMIS-0032|658[0n]bp|Canada.Ontario|BOLD:AAA4128
Orthosia hibisci|[19955]|LOWCE014-06|CGWC-3774|658[0n]bp|Canada.British Columbia|BOLD:AAA4128
Orthosia hibisci|[19956]|LOWCD621-06|CGWC-3441|658[0n]bp|Canada.British Columbia|BOLD:AAA4128
Orthosia hibisci|[19957]|LOWCE220-06|CGWC-3980|658[0n]bp|Canada.British Columbia|BOLD:AAA4128
Orthosia hibisci|[19958]|LOWCE016-06|CGWC-3776|658[0n]bp|Canada.British Columbia|BOLD:AAA4128
Orthosia hibisci|[19959]|MEC117-04|jflandry0117|658[0n]bp|Canada.Quebec|BOLD:AAA4128
Orthosia hibisci|[19960]|XAJ087-06|2006-ONT-0087|658[0n]bp|Canada.Ontario|BOLD:AAA4128
Orthosia hibisci|[19961]|XAF285-05|HLC-10326|658[0n]bp|Canada.Ontario|BOLD:AAA4128
Orthosia hibisci|[19962]|PMG143-03|moth132.01|617[0n]bp|Canada.Ontario|BOLD:AAA4128
Orthosia hibisci|[19963]|LOWCE365-06|CGWC-4125|578[0n]bp|Canada.British Columbia|BOLD:AAA4128
Orthosia hibisci|[19964]|LOWCE455-06|CGWC-4215|617[0n]bp|Canada.British Columbia|BOLD:AAA4128
Orthosia hibisci|[19965]|RDLQH106-06|DH013344|621[0n]bp|Canada.Quebec|BOLD:AAA4128
Orthosia hibisci|[19966]|DUNLP178-08|Dun-08-178|604[0n]bp|Canada.British Columbia|BOLD:AAA4128
Orthosia hibisci|[19967]|PHMO034-03|moth132.02|639[0n]bp|Canada.Ontario|BOLD:AAA4128
Orthosia hibisci|[19968]|PHMO011-03|moth24.02|639[0n]bp|Canada.Ontario|BOLD:AAA4128
Orthosia hibisci|[19969]|PHMO035-03|moth133.02|639[0n]bp|Canada.Ontario|BOLD:AAA4128
Orthosia hibisci|[19970]|PHMO007-03|moth8.02|639[0n]bp|Canada.Ontario|BOLD:AAA4128
Orthosia hibisci|[19971]|PHMO039-03|moth142.02|639[0n]bp|Canada.Ontario|BOLD:AAA4128
Orthosia hibisci|[19972]|PHMO027-03|moth83.02|639[0n]bp|Canada.Ontario|BOLD:AAA4128
Orthosia hibisci|[19973]|PHMO026-03|moth81.02|639[0n]bp|Canada.Ontario|BOLD:AAA4128
Orthosia hibisci|[19974]|PHMO021-03|moth67.02|639[0n]bp|Canada.Ontario|BOLD:AAA4128
Orthosia hibisci|[19975]|TMG133-03|moth131.01|639[0n]bp|Canada.Ontario|BOLD:AAA4128
Orthosia hibisci|[19976]|LOWCE409-06|CGWC-4169|658[0n]bp|Canada.British Columbia|BOLD:AAA4128
Orthosia hibisci|[19977]|LOWCE457-06|CGWC-4217|658[0n]bp|Canada.British Columbia|BOLD:AAA4128
Orthosia hibisci|[19978]|XAF307-05|HLC-10348|658[0n]bp|Canada.Ontario|BOLD:AAA4128
Orthosia hibisci|[19979]|XAJ125-06|2006-ONT-0125|658[0n]bp|Canada.Ontario|BOLD:AAA4128
Orthosia pacifica|[19980]|RDNMG518-08|CNC LEP00052342|652[0n]bp|Canada.British Columbia|BOLD:AAE4149
Orthosia pacifica|[19981]|RDNMG517-08|CNC LEP00052341|658[0n]bp|Canada.British Columbia|BOLD:AAE4149
Orthosia pacifica|[19982]|RDMAB1042-09|UASM122734|658[0n]bp|Canada.British Columbia|BOLD:AAE4149
Orthosia pacifica|[19983]|RDNMC464-05|CNCNoctuoidea12097|584[1n]bp|Canada.British Columbia|BOLD:AAE4149
Orthosia pacifica|[19984]|RDMAB1043-09|UASM122735|635[0n]bp|Canada.British Columbia|BOLD:AAE4149
Orthosia revicta|[19985]|KPOEC160-08|08OEC-201|658[0n]bp|Canada.Ontario|BOLD:AAA9607
Orthosia revicta|[19986]|LBCH5346-10|10-JDWBC-5346|658[0n]bp|Canada.British Columbia|BOLD:AAA9607
Orthosia revicta|[19987]|LOWCE248-06|CGWC-4008|658[0n]bp|Canada.British Columbia|BOLD:AAA9607
Orthosia revicta|[19988]|LOWCE243-06|CGWC-4003|658[0n]bp|Canada.British Columbia|BOLD:AAA9607
Orthosia revicta|[19989]|LOWC070-05|CGWC-0070|600[0n]bp|Canada.British Columbia|BOLD:AAA9607
Orthosia revicta|[19990]|TTMNB050-06|MNBTT-050|658[0n]bp|Canada.New Brunswick|BOLD:AAA9607
Orthosia revicta|[19991]|TTMNB094-06|MNBTT-094|658[0n]bp|Canada.New Brunswick|BOLD:AAA9607
Orthosia revicta|[19992]|LALPA096-10|AVBC 096-10|658[0n]bp|Canada.British Columbia|BOLD:AAA9607
Orthosia revicta|[19993]|LOWCE251-06|CGWC-4011|658[0n]bp|Canada.British Columbia|BOLD:AAA9607
Orthosia revicta|[19994]|TMNBB334-06|MNBTT-1274|658[0n]bp|Canada.New Brunswick|BOLD:AAA9607
Orthosia revicta|[19995]|LOWCE232-06|CGWC-3992|658[0n]bp|Canada.British Columbia|BOLD:AAA9607
Orthosia revicta|[19996]|LOWCE249-06|CGWC-4009|658[0n]bp|Canada.British Columbia|BOLD:AAA9607
Orthosia revicta|[19997]|KPOEC076-08|08OEC-233|658[0n]bp|Canada.Ontario|BOLD:AAA9607
Orthosia revicta|[19998]|LOWCD620-06|CGWC-3440|658[0n]bp|Canada.British Columbia|BOLD:AAA9607
Orthosia revicta|[19999]|RDNMC467-05|CNCNoctuoidea12100|658[0n]bp|Canada.Quebec|BOLD:AAA9607
Orthosia revicta|[20000]|LOWCE252-06|CGWC-4012|658[0n]bp|Canada.British Columbia|BOLD:AAA9607
Orthosia revicta|[20001]|LALPA079-10|AVBC 079-10|658[0n]bp|Canada.British Columbia|BOLD:AAA9607
Orthosia revicta|[20002]|LBCH5038-10|10-JDWBC-5038|658[0n]bp|Canada.British Columbia|BOLD:AAA9607
Orthosia revicta|[20003]|RDNMC468-05|CNCNoctuoidea12101|658[0n]bp|Canada.British Columbia|BOLD:AAA9607
Orthosia revicta|[20004]|KPOEC127-08|08OEC-073|658[0n]bp|Canada.Ontario|BOLD:AAA9607
Orthosia revicta|[20005]|TTMNB582-06|MNBTT-582|658[0n]bp|Canada.New Brunswick|BOLD:AAA9607
Orthosia revicta|[20006]|LOWCE267-06|CGWC-4027|658[0n]bp|Canada.British Columbia|BOLD:AAA9607
Orthosia revicta|[20007]|LOWCE231-06|CGWC-3991|658[0n]bp|Canada.British Columbia|BOLD:AAA9607
Orthosia revicta|[20008]|TMNBB333-06|MNBTT-1273|658[0n]bp|Canada.New Brunswick|BOLD:AAA9607
Orthosia revicta|[20009]|LBCH5280-10|10-JDWBC-5280|658[0n]bp|Canada.British Columbia|BOLD:AAA9607
Orthosia revicta|[20010]|TTMNB093-06|MNBTT-093|656[0n]bp|Canada.New Brunswick|BOLD:AAA9607
Orthosia revicta|[20011]|RDLQG465-06|DH012758|656[0n]bp|Canada.Quebec|BOLD:AAA9607
Orthosia revicta|[20012]|TTMNB583-06|MNBTT-583|656[0n]bp|Canada.New Brunswick|BOLD:AAA9607
Orthosia revicta|[20013]|LOWCE456-06|CGWC-4216|658[0n]bp|Canada.British Columbia|BOLD:AAA9607
Orthosia revicta|[20014]|XAE068-04|Moth4068.03|575[0n]bp|Canada.Ontario|BOLD:AAA9607
Orthosia revicta|[20015]|LOWCD215-06|CGWC-3035|658[0n]bp|Canada.British Columbia|BOLD:AAA9607
Orthosia revicta|[20016]|PMG144-03|moth167.01|617[0n]bp|Canada.Ontario|BOLD:AAA9607
Orthosia revicta|[20017]|LOWCE371-06|CGWC-4131|622[0n]bp|Canada.British Columbia|BOLD:AAA9607
Orthosia revicta|[20018]|LOWCE462-06|CGWC-4222|616[0n]bp|Canada.British Columbia|BOLD:AAA9607
Orthosia revicta|[20019]|LOWCE266-06|CGWC-4026|526[0n]bp|Canada.British Columbia|BOLD:AAA9607
Orthosia revicta|[20020]|LOWCE370-06|CGWC-4130|586[0n]bp|Canada.British Columbia|BOLD:AAA9607
Stretchia muricina|[20021]|RDNMF268-08|NOC14354|658[0n]bp|Canada.British Columbia|BOLD:AAB2916
Stretchia muricina|[20022]|RDNMF267-08|NOC14353|658[0n]bp|Canada.British Columbia|BOLD:AAB2916
Stretchia muricina|[20023]|RDNMF266-08|NOC14352|658[0n]bp|Canada.British Columbia|BOLD:AAB2916
Stretchia muricina|[20024]|RDNMG346-08|NOC15193|543[0n]bp|Canada.Alberta|BOLD:AAB2916
Stretchia muricina|[20025]|LALPA1052-11|AVBC 862-11|658[0n]bp|Canada.British Columbia|BOLD:AAB2916
Stretchia muricina|[20026]|RDNMG345-08|NOC15192|599[1n]bp|Canada.Alberta|BOLD:AAB2916
Stretchia muricina|[20027]|RDNMG343-08|NOC15190|658[0n]bp|Canada.Alberta|BOLD:AAB2916
Stretchia muricina|[20028]|RDNMG347-08|NOC15194|658[0n]bp|Canada.Alberta|BOLD:AAB2916
Stretchia muricina|[20029]|RDNMG344-08|NOC15191|658[0n]bp|Canada.Alberta|BOLD:AAB2916
Stretchia plusiaeformis|[20030]|RDNMF261-08|NOC14347|658[0n]bp|Canada.British Columbia|BOLD:AAB6980
Stretchia plusiaeformis|[20031]|RDNMF264-08|NOC14350|658[0n]bp|Canada.British Columbia|BOLD:AAB6980
Stretchia plusiaeformis|[20032]|RDNMF260-08|NOC14346|658[0n]bp|Canada.British Columbia|BOLD:AAB6980
Stretchia plusiaeformis|[20033]|RDNMG351-08|NOC15198|658[0n]bp|Canada.Alberta|BOLD:ACE6587
[illegible]

Stretchia plusiaeformis|[20031]|RDNMF264-08|NOC14350|658[0n]bp|Canada.British Columbia|BOLD:AAB6980
Stretchia plusiaeformis|[20032]|RDNMF260-08|NOC14346|658[0n]bp|Canada.British Columbia|BOLD:AAB6980
Stretchia plusiaeformis|[20033]|RDNMG351-08|NOC15198|658[0n]bp|Canada.Alberta|BOLD:ACE6587
Stretchia plusiaeformis|[20034]|RDNMG349-08|NOC15196|658[0n]bp|Canada.Alberta|BOLD:ACE6587
Stretchia plusiaeformis|[20035]|RDNMG350-08|NOC15197|658[0n]bp|Canada.Alberta|BOLD:ACE6587
Stretchia plusiaeformis|[20036]|RDNMF293-08|NOC14379|640[0n]bp|Canada.Quebec|BOLD:ACE6587
Stretchia plusiaeformis|[20037]|RDNMG348-08|NOC15195|658[0n]bp|Canada.Alberta|BOLD:ACE6587
Cerapteryx graminis|[20038]|RDNMF760-08|UASM99470|658[0n]bp|Canada.Newfoundland and Labrador|BOLD:AAB4284
Cerapteryx graminis|[20039]|RDNMF762-08|UASM99472|658[0n]bp|Canada.Newfoundland and Labrador|BOLD:AAB4284
Cerapteryx graminis|[20040]|RDNMF759-08|UASM99469|658[0n]bp|Canada.Newfoundland and Labrador|BOLD:AAB4284
Cerapteryx graminis|[20041]|RDNMF761-08|UASM99471|658[0n]bp|Canada.Newfoundland and Labrador|BOLD:AAB4284
Nephelodes minians|[20042]|LOWC085-05|CGWC-0085|538[0n]bp|Canada.British Columbia|BOLD:AAA5081
Nephelodes minians|[20043]|BBLEC062-09|09BBELE-0062|658[0n]bp|Canada.New Brunswick|BOLD:AAA5081
Nephelodes minians|[20044]|BBLEC438-09|09BBELE-0438|658[0n]bp|Canada.New Brunswick|BOLD:AAA5081
Nephelodes minians|[20045]|BBLPC348-09|09BBELE-1348|658[0n]bp|Canada.New Brunswick|BOLD:AAA5081
Nephelodes minians|[20046]|BBLPC368-09|09BBELE-1368|658[0n]bp|Canada.New Brunswick|BOLD:AAA5081
Nephelodes minians|[20047]|BBLEC488-09|09BBELE-0488|658[0n]bp|Canada.New Brunswick|BOLD:AAA5081
Nephelodes minians|[20048]|RDLQB930-05|DH003930|530[0n]bp|Canada.Quebec|BOLD:AAA5081
Nephelodes minians|[20049]|BBLPC367-09|09BBELE-1367|658[0n]bp|Canada.New Brunswick|BOLD:AAA5081
Nephelodes minians|[20050]|BBLPC365-09|09BBELE-1365|658[0n]bp|Canada.New Brunswick|BOLD:AAA5081
Nephelodes minians|[20051]|RDLQB929-05|DH003937|658[0n]bp|Canada.Quebec|BOLD:AAA5081
Nephelodes minians|[20052]|BBLPC370-09|09BBELE-1370|658[0n]bp|Canada.New Brunswick|BOLD:AAA5081
Nephelodes minians|[20053]|XAB415-04|04HBL005415|658[0n]bp|Canada.Ontario|BOLD:AAA5081
Nephelodes minians|[20054]|BBLPB589-10|10BBCLP-1588|658[0n]bp|Canada.Saskatchewan|BOLD:AAA5081
Nephelodes minians|[20055]|NCCH062-11|BIOUG01573-F02|673[0n]bp|Canada.Ontario|BOLD:AAA5081
Nephelodes minians|[20056]|XAD358-04|04HBL007358|559[0n]bp|Canada.Ontario|BOLD:AAA5081
Nephelodes minians|[20057]|LOWC078-05|CGWC-0078|658[0n]bp|Canada.British Columbia|BOLD:AAA5081
Nephelodes minians|[20058]|BBLEC508-09|09BBELE-0508|658[0n]bp|Canada.New Brunswick|BOLD:AAA5081
Nephelodes minians|[20059]|XAH207-05|2005-ONT-1790|658[0n]bp|Canada.Ontario|BOLD:AAA5081
Nephelodes minians|[20060]|BBLPC355-09|09BBELE-1355|658[0n]bp|Canada.New Brunswick|BOLD:AAA5081
Nephelodes minians|[20061]|BBLEC443-09|09BBELE-0443|658[0n]bp|Canada.New Brunswick|BOLD:AAA5081
Nephelodes minians|[20062]|BBLPC027-09|09BBELE-1027|658[0n]bp|Canada.New Brunswick|BOLD:AAA5081
Nephelodes minians|[20063]|BBLPC371-09|09BBELE-1371|658[0n]bp|Canada.New Brunswick|BOLD:AAA5081
Nephelodes minians|[20064]|RDLQB884-05|DH010961|658[0n]bp|Canada.Quebec|BOLD:AAA5081
Nephelodes minians|[20065]|XAH241-05|2005-ONT-1824|658[0n]bp|Canada.Ontario|BOLD:AAA5081
Nephelodes minians|[20066]|BBLPC431-09|09BBELE-1431|658[0n]bp|Canada.New Brunswick|BOLD:AAA5081
Nephelodes minians|[20067]|XAB414-04|04HBL005414|611[0n]bp|Canada.Ontario|BOLD:AAA5081
Nephelodes minians|[20068]|XAB426-04|04HBL005426|658[0n]bp|Canada.Ontario|BOLD:AAA5081
Nephelodes minians|[20069]|RDLQB625-05|DH010728|515[1n]bp|Canada.Quebec|BOLD:AAA5081
Nephelodes minians|[20070]|RDLQB626-05|DH010729|607[0n]bp|Canada.Quebec|BOLD:AAA5081
Nephelodes minians|[20071]|XAD245-04|04HBL007245|658[0n]bp|Canada.Ontario|BOLD:AAA5081
Nephelodes minians|[20072]|BBLPB599-10|10BBCLP-1598|658[0n]bp|Canada.Saskatchewan|BOLD:AAA5081
Nephelodes minians|[20073]|BBLEC462-09|09BBELE-0462|658[0n]bp|Canada.New Brunswick|BOLD:AAA5081
Nephelodes minians|[20074]|LPSOD965-09|08BBLEP-05598|658[0n]bp|Canada.Ontario|BOLD:AAA5081
Nephelodes minians|[20075]|JSSEP1088-11|BIOUG01497-G01|658[0n]bp|Canada.Ontario|BOLD:AAA5081
Nephelodes minians|[20076]|TTMNB412-06|MNBTT-412|658[0n]bp|Canada.New Brunswick|BOLD:AAA5081
Nephelodes minians|[20077]|BBLEC038-09|09BBELE-0038|658[0n]bp|Canada.New Brunswick|BOLD:AAA5081
Nephelodes minians|[20078]|XAH392-05|2005-ONT-1975|658[0n]bp|Canada.Ontario|BOLD:AAA5081
Nephelodes minians|[20079]|TMNBB363-06|MNBTT-1303|658[0n]bp|Canada.New Brunswick|BOLD:AAA5081
Nephelodes minians|[20080]|XAB457-04|04HBL005457|658[0n]bp|Canada.Ontario|BOLD:AAA5081
Nephelodes minians|[20081]|XAD370-04|04HBL007370|658[0n]bp|Canada.Ontario|BOLD:AAA5081
Nephelodes minians|[20082]|BBLEC037-09|09BBELE-0037|658[0n]bp|Canada.New Brunswick|BOLD:AAA5081
Nephelodes minians|[20083]|BBLPC358-09|09BBELE-1358|658[0n]bp|Canada.New Brunswick|BOLD:AAA5081
Nephelodes minians|[20084]|TMNBB362-06|MNBTT-1302|636[0n]bp|Canada.New Brunswick|BOLD:AAA5081
Nephelodes minians|[20085]|XAH204-05|2005-ONT-1787|658[0n]bp|Canada.Ontario|BOLD:AAA5081
Nephelodes minians|[20086]|XAB436-04|04HBL005436|657[0n]bp|Canada.Ontario|BOLD:AAA5081
Nephelodes minians|[20087]|XAH294-05|2005-ONT-1877|658[0n]bp|Canada.Ontario|BOLD:AAA5081
Nephelodes minians|[20088]|XAD352-04|04HBL007352|658[0n]bp|Canada.Ontario|BOLD:AAA5081
Nephelodes minians|[20089]|XAH239-05|2005-ONT-1822|658[0n]bp|Canada.Ontario|BOLD:AAA5081
Nephelodes minians|[20090]|XAH266-05|2005-ONT-1849|658[0n]bp|Canada.Ontario|BOLD:AAA5081
Nephelodes minians|[20091]|XAD349-04|04HBL007349|592[0n]bp|Canada.Ontario|BOLD:AAA5081
Nephelodes minians|[20092]|BBLPC372-09|09BBELE-1372|658[0n]bp|Canada.New Brunswick|BOLD:AAA5081
Nephelodes minians|[20093]|BBLPB597-10|10BBCLP-1596|658[0n]bp|Canada.Saskatchewan|BOLD:AAA5081
Nephelodes minians|[20094]|BBLPB588-10|10BBCLP-1587|658[0n]bp|Canada.Alberta|BOLD:AAA5081
Nephelodes minians|[20095]|BBLPB603-10|10BBCLP-1602|658[0n]bp|Canada.Saskatchewan|BOLD:AAA5081
Nephelodes minians|[20096]|LPMNB229-09|08BBLEP-05073|658[0n]bp|Canada.Manitoba|BOLD:AAA5081
Nephelodes minians|[20097]|BBLPB498-10|10BBCLP-1497|658[0n]bp|Canada.Saskatchewan|BOLD:AAA5081
Nephelodes minians|[20098]|BBLPB600-10|10BBCLP-1599|658[0n]bp|Canada.Saskatchewan|BOLD:AAA5081
Nephelodes minians|[20099]|BBLPB591-10|10BBCLP-1590|658[1n]bp|Canada.Alberta|BOLD:AAA5081
Nephelodes minians|[20100]|LOWC080-05|CGWC-0080|658[0n]bp|Canada.British Columbia|BOLD:AAA5081
Nephelodes minians|[20101]|LOWC086-05|CGWC-0086|658[0n]bp|Canada.British Columbia|BOLD:AAA5081
Nephelodes minians|[20102]|LOWCD643-06|CGWC-3463|658[0n]bp|Canada.British Columbia|BOLD:AAA5081
Nephelodes minians|[20103]|LOWC083-05|CGWC-0083|658[0n]bp|Canada.British Columbia|BOLD:AAA5081
Nephelodes minians|[20104]|LOWCD182-06|CGWC-3002|658[0n]bp|Canada.British Columbia|BOLD:AAA5081
Nephelodes minians|[20105]|LOWCD647-06|CGWC-3467|658[0n]bp|Canada.British Columbia|BOLD:AAA5081
Nephelodes minians|[20106]|LOWCD648-06|CGWC-3468|658[0n]bp|Canada.British Columbia|BOLD:AAA5081
Nephelodes minians|[20107]|BBLPB605-10|10BBCLP-1604|658[0n]bp|Canada.Saskatchewan|BOLD:AAA5081
Nephelodes minians|[20108]|LOWC079-05|CGWC-0079|658[0n]bp|Canada.British Columbia|BOLD:AAA5081
Nephelodes minians|[20109]|BBLPB604-10|10BBCLP-1603|658[0n]bp|Canada.Saskatchewan|BOLD:AAA5081
Nephelodes minians|[20110]|LOWCD644-06|CGWC-3464|657[0n]bp|Canada.British Columbia|BOLD:AAA5081
Nephelodes minians|[20111]|LOWC081-05|CGWC-0081|658[0n]bp|Canada.British Columbia|BOLD:AAA5081
Nephelodes minians|[20112]|LOWC084-05|CGWC-0084|658[0n]bp|Canada.British Columbia|BOLD:AAA5081
Nephelodes minians|[20113]|BBLPB590-10|10BBCLP-1589|658[0n]bp|Canada.Alberta|BOLD:AAA5081
Nephelodes minians|[20114]|LOWCD645-06|CGWC-3465|658[0n]bp|Canada.British Columbia|BOLD:AAA5081
Nephelodes minians|[20115]|LOWC082-05|CGWC-0082|658[0n]bp|Canada.British Columbia|BOLD:AAA5081
Nephelodes minians|[20116]|XAH310-05|2005-ONT-1893|606[0n]bp|Canada.Ontario|BOLD:AAA5081
Nephelodes minians|[20117]|XAB434-04|04HBL005434|658[0n]bp|Canada.Ontario|BOLD:AAA5081
Nephelodes minians|[20118]|LPSOD984-09|08BBLEP-05617|658[0n]bp|Canada.Ontario|BOLD:AAA5081
Nephelodes minians|[20119]|LOWCD646-06|CGWC-3466|658[0n]bp|Canada.British Columbia|BOLD:AAA5081
Nephelodes minians|[20120]|BBLPB598-10|10BBCLP-1597|658[0n]bp|Canada.Saskatchewan|BOLD:AAA5081
Tholera americana|[20121]|LBCH7529-10|10-JDWBC-7529|636[0n]bp|Canada.British Columbia|BOLD:AAB9282
Tholera americana|[20122]|LOWCD636-06|CGWC-3456|658[0n]bp|Canada.British Columbia|BOLD:AAB9282
Tholera americana|[20123]|LOWC076-05|CGWC-0076|658[0n]bp|Canada.British Columbia|BOLD:AAB9282
Tholera americana|[20124]|LBCH6985-10|10-JDWBC-6985|658[0n]bp|Canada.British Columbia|BOLD:AAB9282
Tholera americana|[20125]|LBCH6930-10|10-JDWBC-6930|658[0n]bp|Canada.British Columbia|BOLD:AAB9282
Tholera americana|[20126]|LOWC077-05|CGWC-0077|658[0n]bp|Canada.British Columbia|BOLD:AAB9282
Tholera americana|[20127]|LBCH7072-10|10-JDWBC-7072|658[0n]bp|Canada.British Columbia|BOLD:AAB9282
Tholera americana|[20128]|LBCH7522-10|10-JDWBC-7522|658[0n]bp|Canada.British Columbia|BOLD:AAB9282
Tholera americana|[20129]|LBCH6872-10|10-JDWBC-6872|658[0n]bp|Canada.British Columbia|BOLD:AAB9282
Tholera americana|[20130]|LBCH7069-10|10-JDWBC-7069|658[0n]bp|Canada.British Columbia|BOLD:AAB9282
Tholera americana|[20131]|LOWC074-05|CGWC-0074|658[0n]bp|Canada.British Columbia|BOLD:AAB9282
Tholera americana|[20132]|LBCH7691-10|10-JDWBC-7691|658[0n]bp|Canada.British Columbia|BOLD:AAB9282
Tholera americana|[20133]|LOWC071-05|CGWC-0071|658[0n]bp|Canada.British Columbia|BOLD:AAB9282

Tholera americana|[20131]|LOWC074-05|CGWC-0074|658[0n]bp|Canada.British Columbia|BOLD:AAB9282
Tholera americana|[20132]|LBCH7691-10|10-JDWBC-7691|658[0n]bp|Canada.British Columbia|BOLD:AAB9282
Tholera americana|[20133]|LOWC071-05|CGWC-0071|658[0n]bp|Canada.British Columbia|BOLD:AAB9282
Tholera americana|[20134]|LOWCD640-06|CGWC-3460|657[0n]bp|Canada.British Columbia|BOLD:AAB9282
Tholera americana|[20135]|LBCH7523-10|10-JDWBC-7523|658[0n]bp|Canada.British Columbia|BOLD:AAB9282
Tholera americana|[20136]|LBCH7225-10|10-JDWBC-7225|658[0n]bp|Canada.British Columbia|BOLD:AAB9282
Tholera americana|[20137]|LOWC073-05|CGWC-0073|658[0n]bp|Canada.British Columbia|BOLD:AAB9282
Tholera americana|[20138]|LBCH7074-10|10-JDWBC-7074|658[0n]bp|Canada.British Columbia|BOLD:AAB9282
Tholera americana|[20139]|LOWC072-05|CGWC-0072|658[0n]bp|Canada.British Columbia|BOLD:AAB9282
Tholera americana|[20140]|LBCH7368-10|10-JDWBC-7368|658[0n]bp|Canada.British Columbia|BOLD:AAB9282
Tholera americana|[20141]|LBCH7916-10|10-JDWBC-7916|658[0n]bp|Canada.British Columbia|BOLD:AAB9282
Tholera americana|[20142]|LOWCD638-06|CGWC-3458|658[0n]bp|Canada.British Columbia|BOLD:AAB9282
Tholera americana|[20143]|LOWCD637-06|CGWC-3457|658[0n]bp|Canada.British Columbia|BOLD:AAB9282
Tholera americana|[20144]|LOWC075-05|CGWC-0075|658[0n]bp|Canada.British Columbia|BOLD:AAB9282
Tholera americana|[20145]|LBCH7071-10|10-JDWBC-7071|658[0n]bp|Canada.British Columbia|BOLD:AAB9282
Tholera americana|[20146]|LBCH6952-10|10-JDWBC-6952|658[0n]bp|Canada.British Columbia|BOLD:AAB9282
Tholera americana|[20147]|LOWCD635-06|CGWC-3455|658[0n]bp|Canada.British Columbia|BOLD:AAB9282
Tholera americana|[20148]|LBCH6874-10|10-JDWBC-6874|658[0n]bp|Canada.British Columbia|BOLD:AAB9282
Tholera americana|[20149]|LBCH7915-10|10-JDWBC-7915|658[0n]bp|Canada.British Columbia|BOLD:AAB9282
Tholera americana|[20150]|LOWCD639-06|CGWC-3459|658[0n]bp|Canada.British Columbia|BOLD:AAB9282
Tholera americana|[20151]|LBCH7073-10|10-JDWBC-7073|658[0n]bp|Canada.British Columbia|BOLD:AAB9282
Tholera americana|[20152]|LBCH7168-10|10-JDWBC-7168|658[0n]bp|Canada.British Columbia|BOLD:AAB9282
Tholera americana|[20153]|LOWCD642-06|CGWC-3462|600[0n]bp|Canada.British Columbia|BOLD:AAB9282
Tholera americana|[20154]|LBCH6917-10|10-JDWBC-6917|614[0n]bp|Canada.British Columbia|BOLD:AAB9282
Tholera americana|[20155]|RDMAB685-06|UASM58810|605[0n]bp|Canada.Alberta|BOLD:AAB9282
Tholera americana|[20156]|LOWCD641-06|CGWC-3461|601[0n]bp|Canada.British Columbia|BOLD:AAB9282
Tholera americana|[20157]|LBCH7526-10|10-JDWBC-7526|634[0n]bp|Canada.British Columbia|BOLD:AAB9282
Tholera americana|[20158]|LBCH7070-10|10-JDWBC-7070|658[0n]bp|Canada.British Columbia|BOLD:AAB9282
Tholera americana|[20159]|LBCH7068-10|10-JDWBC-7068|658[0n]bp|Canada.British Columbia|BOLD:AAB9282
Tholera americana|[20160]|LBCH7067-10|10-JDWBC-7067|658[0n]bp|Canada.British Columbia|BOLD:AAB9282
Tholera americana|[20161]|LBCH7524-10|10-JDWBC-7524|658[0n]bp|Canada.British Columbia|BOLD:AAB9282
Tholera americana|[20162]|LBCH6879-10|10-JDWBC-6879|658[0n]bp|Canada.British Columbia|BOLD:AAB9282
Tholera americana|[20163]|LBCH6948-10|10-JDWBC-6948|658[0n]bp|Canada.British Columbia|BOLD:AAB9282
Tholera americana|[20164]|LBCH6990-10|10-JDWBC-6990|658[0n]bp|Canada.British Columbia|BOLD:AAB9282
Tholera americana|[20165]|LBCH7527-10|10-JDWBC-7527|658[0n]bp|Canada.British Columbia|BOLD:AAB9282
Tholera americana|[20166]|LBCH7525-10|10-JDWBC-7525|658[0n]bp|Canada.British Columbia|BOLD:AAB9282
Tholera americana|[20167]|LBCH6794-10|10-JDWBC-6794|658[0n]bp|Canada.British Columbia|BOLD:AAB9282
Tholera americana|[20168]|LBCH6801-10|10-JDWBC-6801|658[0n]bp|Canada.British Columbia|BOLD:AAB9282
Tholera americana|[20169]|LBCH6798-10|10-JDWBC-6798|658[0n]bp|Canada.British Columbia|BOLD:AAB9282
Tholera americana|[20170]|LBCH6987-10|10-JDWBC-6987|658[0n]bp|Canada.British Columbia|BOLD:AAB9282
Tholera americana|[20171]|LBCH6945-10|10-JDWBC-6945|658[0n]bp|Canada.British Columbia|BOLD:AAB9282
Tholera americana|[20172]|LBCH7917-10|10-JDWBC-7917|658[0n]bp|Canada.British Columbia|BOLD:AAB9282
Tholera americana|[20173]|LBCH6986-10|10-JDWBC-6986|640[0n]bp|Canada.British Columbia|BOLD:AAB9282
Tholera americana|[20174]|LBCH6878-10|10-JDWBC-6878|635[0n]bp|Canada.British Columbia|BOLD:AAB9282
Tholera americana|[20175]|LBCH6944-10|10-JDWBC-6944|658[0n]bp|Canada.British Columbia|BOLD:AAB9282
Tholera americana|[20176]|LBCH6875-10|10-JDWBC-6875|636[0n]bp|Canada.British Columbia|BOLD:AAB9282
Tholera americana|[20177]|LBCH6797-10|10-JDWBC-6797|658[0n]bp|Canada.British Columbia|BOLD:AAB9282
Tholera americana|[20178]|LBCH7914-10|10-JDWBC-7914|658[0n]bp|Canada.British Columbia|BOLD:AAB9282
Tholera americana|[20179]|LBCH7745-10|10-JDWBC-7745|658[0n]bp|Canada.British Columbia|BOLD:AAB9282
Tholera americana|[20180]|LBCH7911-10|10-JDWBC-7911|658[0n]bp|Canada.British Columbia|BOLD:AAB9282
Tholera americana|[20181]|LBCH6877-10|10-JDWBC-6877|658[0n]bp|Canada.British Columbia|BOLD:AAB9282
Tholera americana|[20182]|LBCH6984-10|10-JDWBC-6984|658[0n]bp|Canada.British Columbia|BOLD:AAB9282
Tholera americana|[20183]|LBCH6991-10|10-JDWBC-6991|658[0n]bp|Canada.British Columbia|BOLD:AAB9282
Tholera americana|[20184]|LBCH7913-10|10-JDWBC-7913|658[0n]bp|Canada.British Columbia|BOLD:AAB9282
Tholera americana|[20185]|LBCH6762-10|10-JDWBC-6762|658[0n]bp|Canada.British Columbia|BOLD:AAB9282
Tholera americana|[20186]|LBCH6873-10|10-JDWBC-6873|658[0n]bp|Canada.British Columbia|BOLD:AAB9282
Tholera americana|[20187]|LBCH6800-10|10-JDWBC-6800|658[0n]bp|Canada.British Columbia|BOLD:AAB9282
Tholera americana|[20188]|LBCH6799-10|10-JDWBC-6799|658[0n]bp|Canada.British Columbia|BOLD:AAB9282
Tholera americana|[20189]|LBCH6989-10|10-JDWBC-6989|658[0n]bp|Canada.British Columbia|BOLD:AAB9282
Tholera americana|[20190]|LBCH7910-10|10-JDWBC-7910|658[0n]bp|Canada.British Columbia|BOLD:AAB9282
Tholera americana|[20191]|LBCH6950-10|10-JDWBC-6950|658[0n]bp|Canada.British Columbia|BOLD:AAB9282
Tholera americana|[20192]|LBCH6796-10|10-JDWBC-6796|658[0n]bp|Canada.British Columbia|BOLD:AAB9282
Tholera americana|[20193]|LBCH7912-10|10-JDWBC-7912|658[0n]bp|Canada.British Columbia|BOLD:AAB9282
Tholera americana|[20194]|LBCH6949-10|10-JDWBC-6949|658[0n]bp|Canada.British Columbia|BOLD:AAB9282
Tholera americana|[20195]|LBCH7528-10|10-JDWBC-7528|639[0n]bp|Canada.British Columbia|BOLD:AAB9282
Tholera americana|[20196]|LBCH6876-10|10-JDWBC-6876|634[0n]bp|Canada.British Columbia|BOLD:AAB9282
Tholera americana|[20197]|LBCH6795-10|10-JDWBC-6795|644[0n]bp|Canada.British Columbia|BOLD:AAB9282
Tholera americana|[20198]|LBCH6933-10|10-JDWBC-6933|658[0n]bp|Canada.British Columbia|BOLD:AAB9282
Tholera americana|[20199]|LBCH6988-10|10-JDWBC-6988|658[0n]bp|Canada.British Columbia|BOLD:AAB9282
Tholera americana|[20200]|LBCH6951-10|10-JDWBC-6951|658[0n]bp|Canada.British Columbia|BOLD:AAB9282
Scotogramma submarina|[20201]|RDNMC454-05|CNCNoctuoidea12087|657[0n]bp|Canada.Alberta|BOLD:AAE9851
Scotogramma submarina|[20202]|RDMAB106-05|UASM41990|658[0n]bp|Canada.Alberta|BOLD:AAE9851
Coranarta luteola|[20203]|RDNMG765-08|CNC LEP00052889|590[0n]bp|Canada.Yukon Territory|BOLD:ABY9453
Coranarta luteola|[20204]|RDMAB430-05|BCSC103|599[0n]bp|Canada.Alberta|BOLD:ABY9453
Coranarta luteola|[20205]|RDLQ634-07|DH003632|592[2n]bp|Canada.Quebec|BOLD:ABY9453
Coranarta luteola|[20206]|RDNMG766-08|CNC LEP00052890|658[0n]bp|Canada.Manitoba|BOLD:ABY9453
Coranarta luteola|[20207]|RDNMG767-08|CNC LEP00052891|658[0n]bp|Canada.Quebec|BOLD:ABY9453
Coranarta luteola|[20208]|BBLPB316-10|10BBCLP-1315|658[0n]bp|Canada.British Columbia|BOLD:ABY9453
Coranarta luteola|[20209]|RDNMG768-08|CNC LEP00052892|658[0n]bp|Canada.Yukon Territory|BOLD:ABY9453
Coranarta macrostigma|[20210]|RDMAB431-05|BCSC104|658[0n]bp|Canada.Alberta|BOLD:AAD9178
Coranarta macrostigma|[20211]|RDMAB090-05|UASM58036|652[0n]bp|Canada.Alberta|BOLD:AAD9178
Coranarta macrostigma|[20212]|RDMAB260-05|UASM58023|658[0n]bp|Canada.Alberta|BOLD:AAD9178
Coranarta macrostigma|[20213]|RDNMG824-08|CNC LEP00052948|658[0n]bp|Canada.Alberta|BOLD:AAD9178
Coranarta macrostigma|[20214]|RDMAB432-05|BCSC105|658[0n]bp|Canada.Alberta|BOLD:AAD9178
Coranarta macrostigma|[20215]|RDNMG825-08|CNC LEP00052949|658[0n]bp|Canada.Alberta|BOLD:AAD9178
Admetovis oxymorus|[20216]|LBCH6013-10|10-JDWBC-6013|658[0n]bp|Canada.British Columbia|BOLD:AAD7455
Admetovis oxymorus|[20217]|LBCH5440-10|10-JDWBC-5440|658[0n]bp|Canada.British Columbia|BOLD:AAD7455
Admetovis oxymorus|[20218]|LBCH5696-10|10-JDWBC-5696|658[0n]bp|Canada.British Columbia|BOLD:AAD7455
Admetovis oxymorus|[20219]|RDNMF540-08|NOC14626|658[0n]bp|Canada.British Columbia|BOLD:AAD7455
Admetovis similaris|[20220]|LBCH5083-10|10-JDWBC-5083|658[0n]bp|Canada.British Columbia|BOLD:AAB7673
Anarta edwardsii|[20221]|RDNMF343-08|NOC14429|658[0n]bp|Canada.British Columbia|BOLD:AAF3996
Anarta edwardsii|[20222]|RDNMF344-08|NOC14430|658[0n]bp|Canada.British Columbia|BOLD:AAF3996
Melanchra adjuncta|[20223]|XAF548-05|2005-ONT-197|658[0n]bp|Canada.Ontario|BOLD:ACF4823
Melanchra adjuncta|[20224]|PMG134-03|moth325.01|617[0n]bp|Canada.Ontario|BOLD:ACF4823
Melanchra adjuncta|[20225]|XAE562-04|Moth4562.03|530[0n]bp|Canada.Ontario|BOLD:ACF4823
Melanchra adjuncta|[20226]|PHMNB446-04|04HBL00672|658[0n]bp|Canada.New Brunswick|BOLD:ACF4823
Melanchra adjuncta|[20227]|RDLQ628-07|DH004656|602[1n]bp|Canada.Quebec|BOLD:AAA4742
Melanchra adjuncta|[20228]|LBCB940-05|HLC-21880|615[0n]bp|Canada.British Columbia|BOLD:AAA4742
Melanchra adjuncta|[20229]|PHMNB243-04|04HBL007708|609[0n]bp|Canada.New Brunswick|BOLD:AAA4742
Melanchra adjuncta|[20230]|PHMNB206-04|04HBL007671|609[0n]bp|Canada.New Brunswick|BOLD:AAA4742
Melanchra adjuncta|[20231]|XAI030-05|0102-ONT-0030|658[0n]bp|Canada.Ontario|BOLD:AAA4742
Melanchra adjuncta|[20232]|LBCA808-05|HLC-20808|658[0n]bp|Canada.British Columbia|BOLD:AAA4742
Melanchra adjuncta|[20233]|XAD343-04|04HBL007343|658[0n]bp|Canada.Ontario|BOLD:AAA4742

Melanchra adjuncta|[20231]|XAI030-05|0102-ONT-0030|658[0n]bp|Canada.Ontario|BOLD:AAA4742
Melanchra adjuncta|[20232]|LBCA808-05|HLC-20808|658[0n]bp|Canada.British Columbia|BOLD:AAA4742
Melanchra adjuncta|[20233]|XAD343-04|04HBL007343|658[0n]bp|Canada.Ontario|BOLD:AAA4742
Melanchra adjuncta|[20234]|BBLPC903-09|09BBELE-1903|658[0n]bp|Canada.Newfoundland and Labrador|BOLD:AA...
Melanchra adjuncta|[20235]|XAF469-05|2005-ONT-118|654[0n]bp|Canada.Ontario|BOLD:AAA4742
Melanchra adjuncta|[20236]|BLTIB001-08|BL0001|579[0n]bp|Canada.Ontario|BOLD:AAA4742
Melanchra adjuncta|[20237]|LPVIA228-08|PFC-2006-0310|636[0n]bp|Canada.British Columbia|BOLD:AAA4742
Melanchra adjuncta|[20238]|XAJ442-06|2006-ONT-0442|658[0n]bp|Canada.Ontario|BOLD:AAA4742
Melanchra adjuncta|[20239]|LOWCD275-06|CGWC-3095|593[0n]bp|Canada.British Columbia|BOLD:AAA4742
Melanchra adjuncta|[20240]|MEC586-04|jflandry0586|618[0n]bp|Canada.Quebec|BOLD:AAA4742
Melanchra adjuncta|[20241]|PHMNB538-04|04HBL00764|616[0n]bp|Canada.New Brunswick|BOLD:AAA4742
Melanchra adjuncta|[20242]|LBCB616-05|HLC-21556|658[0n]bp|Canada.British Columbia|BOLD:AAA4742
Melanchra adjuncta|[20243]|PMG133-03|moth309.01|617[0n]bp|Canada.Ontario|BOLD:AAA4742
Melanchra adjuncta|[20244]|TMG128-03|moth243.01|639[0n]bp|Canada.Ontario|BOLD:AAA4742
Melanchra adjuncta|[20245]|TMG130-03|moth251.01|639[0n]bp|Canada.Ontario|BOLD:AAA4742
Melanchra adjuncta|[20246]|TMG127-03|moth324.01|639[0n]bp|Canada.Ontario|BOLD:AAA4742
Melanchra adjuncta|[20247]|TTMNB085-06|MNBTT-085|658[0n]bp|Canada.New Brunswick|BOLD:AAA4742
Melanchra adjuncta|[20248]|XAI032-05|0102-ONT-0032|658[0n]bp|Canada.Ontario|BOLD:AAA4742
Melanchra adjuncta|[20249]|LBCB064-05|HLC-21004|658[0n]bp|Canada.British Columbia|BOLD:AAA4742
Melanchra adjuncta|[20250]|XAJ350-06|2006-ONT-0350|658[0n]bp|Canada.Ontario|BOLD:AAA4742
Melanchra adjuncta|[20251]|XAJ584-06|2006-ONT-0584|658[0n]bp|Canada.Ontario|BOLD:AAA4742
Melanchra adjuncta|[20252]|XAG970-05|2005-ONT-1554|658[0n]bp|Canada.Ontario|BOLD:AAA4742
Melanchra adjuncta|[20253]|BBLPE318-09|09BBELE-2318|658[0n]bp|Canada.Newfoundland and Labrador|BOLD:AA...
Melanchra adjuncta|[20254]|LHLEP383-06|UBC-2006-0751|658[0n]bp|Canada.British Columbia|BOLD:AAA4742
Melanchra adjuncta|[20255]|LBCB063-05|HLC-21003|658[0n]bp|Canada.British Columbia|BOLD:AAA4742
Melanchra adjuncta|[20256]|LBCC763-05|HLC-22643|658[0n]bp|Canada.British Columbia|BOLD:AAA4742
Melanchra adjuncta|[20257]|XAC160-04|04HBL006160|658[0n]bp|Canada.Ontario|BOLD:AAA4742
Melanchra adjuncta|[20258]|LBCA790-05|HLC-20790|658[0n]bp|Canada.British Columbia|BOLD:AAA4742
Melanchra adjuncta|[20259]|LPVIA227-08|PFC-2006-0309|658[0n]bp|Canada.British Columbia|BOLD:AAA4742
Melanchra adjuncta|[20260]|TTMNB394-06|MNBTT-394|658[0n]bp|Canada.New Brunswick|BOLD:AAA4742
Melanchra adjuncta|[20261]|LPVIA112-08|PFC-2006-0178|658[0n]bp|Canada.British Columbia|BOLD:AAA4742
Melanchra adjuncta|[20262]|XAE095-04|Moth4095.03|658[0n]bp|Canada.Ontario|BOLD:AAA4742
Melanchra adjuncta|[20263]|BBLPE119-09|09BBELE-2119|658[0n]bp|Canada.Nova Scotia|BOLD:AAA4742
Melanchra adjuncta|[20264]|XAC189-04|04HBL006189|658[0n]bp|Canada.Ontario|BOLD:AAA4742
Melanchra adjuncta|[20265]|RDLQ629-07|DH005381|644[0n]bp|Canada.Quebec|BOLD:AAA4742
Melanchra adjuncta|[20266]|LPABB073-08|08BBLEP-03338|658[0n]bp|Canada.Alberta|BOLD:AAA4742
Melanchra adjuncta|[20267]|PHMNB090-03|moth39.02|639[0n]bp|Canada.New Brunswick|BOLD:AAA4742
Melanchra adjuncta|[20268]|TMG129-03|moth344.01|639[0n]bp|Canada.Ontario|BOLD:AAA4742
Melanchra adjuncta|[20269]|KPOEC174-08|08OEC-215|647[0n]bp|Canada.Ontario|BOLD:AAA4742
Melanchra adjuncta|[20270]|PHMNB061-03|moth39.02SA|639[0n]bp|Canada.New Brunswick|BOLD:AAA4742
Melanchra adjuncta|[20271]|LBCB066-05|HLC-21006|658[0n]bp|Canada.British Columbia|BOLD:AAA4742
Melanchra adjuncta|[20272]|TMNBB312-06|MNBTT-1252|658[0n]bp|Canada.New Brunswick|BOLD:AAA4742
Melanchra adjuncta|[20273]|TTMNB395-06|MNBTT-395|658[0n]bp|Canada.New Brunswick|BOLD:AAA4742
Melanchra adjuncta|[20274]|JSAUG1677-11|BIOUG01497-F05|658[0n]bp|Canada.Ontario|BOLD:AAA4742
Melanchra adjuncta|[20275]|XAI031-05|0102-ONT-0031|658[0n]bp|Canada.Ontario|BOLD:AAA4742
Melanchra adjuncta|[20276]|PHMNB381-04|04HBL00607|658[0n]bp|Canada.New Brunswick|BOLD:AAA4742
Melanchra adjuncta|[20277]|XAJ676-06|2006-ONT-0676|658[0n]bp|Canada.Ontario|BOLD:AAA4742
Melanchra adjuncta|[20278]|XAF803-05|2005-ONT-452|658[0n]bp|Canada.Ontario|BOLD:AAA4742
Melanchra adjuncta|[20279]|LPSOC122-08|PPBP-2121|658[0n]bp|Canada.Ontario|BOLD:AAA4742
Melanchra adjuncta|[20280]|TMNBB311-06|MNBTT-1251|658[0n]bp|Canada.New Brunswick|BOLD:AAA4742
Melanchra adjuncta|[20281]|XAE094-04|Moth4094.03|658[0n]bp|Canada.Ontario|BOLD:AAA4742
Melanchra adjuncta|[20282]|RDLQ630-07|DH007531|658[0n]bp|Canada.Quebec|BOLD:AAA4742
Melanchra adjuncta|[20283]|PHMNB599-04|04HBL00825|658[0n]bp|Canada.New Brunswick|BOLD:AAA4742
Melanchra adjuncta|[20284]|LBCC434-05|HLC-22314|658[0n]bp|Canada.British Columbia|BOLD:AAA4742
Melanchra adjuncta|[20285]|BBLPB643-10|10BBCLP-1642|658[0n]bp|Canada.Alberta|BOLD:AAA4742
Melanchra adjuncta|[20286]|LBCC436-05|HLC-22316|658[0n]bp|Canada.British Columbia|BOLD:AAA4742
Melanchra adjuncta|[20287]|PHMNB600-04|04HBL00826|658[0n]bp|Canada.New Brunswick|BOLD:AAA4742
Melanchra adjuncta|[20288]|XAC161-04|04HBL006161|658[0n]bp|Canada.Ontario|BOLD:AAA4742
Melanchra adjuncta|[20289]|XAJ675-06|2006-ONT-0675|658[0n]bp|Canada.Ontario|BOLD:AAA4742
Melanchra adjuncta|[20290]|LOWCE838-06|CGWC-4598|658[0n]bp|Canada.British Columbia|BOLD:AAA4742
Melanchra adjuncta|[20291]|LPSOC101-08|PPBP-2100|658[0n]bp|Canada.Ontario|BOLD:AAA4742
Melanchra adjuncta|[20292]|XAF739-05|2005-ONT-388|658[0n]bp|Canada.Ontario|BOLD:AAA4742
Melanchra adjuncta|[20293]|LBCB065-05|HLC-21005|658[0n]bp|Canada.British Columbia|BOLD:AAA4742
Melanchra adjuncta|[20294]|KPOEC037-08|08OEC-140|658[0n]bp|Canada.Ontario|BOLD:AAA4742
Melanchra adjuncta|[20295]|TTMNB396-06|MNBTT-396|658[0n]bp|Canada.New Brunswick|BOLD:AAA4742
Melanchra adjuncta|[20296]|LBCC435-05|HLC-22315|658[0n]bp|Canada.British Columbia|BOLD:AAA4742
Melanchra adjuncta|[20297]|LPSOC313-08|PPBP-2312|658[0n]bp|Canada.Ontario|BOLD:AAA4742
Melanchra adjuncta|[20298]|LPSOC090-08|PPBP-2089|658[0n]bp|Canada.Ontario|BOLD:AAA4742
Melanchra adjuncta|[20299]|XAC179-04|04HBL006179|658[0n]bp|Canada.Ontario|BOLD:AAA4742
Melanchra adjuncta|[20300]|LBCC290-05|HLC-22170|658[0n]bp|Canada.British Columbia|BOLD:AAA4742
Melanchra adjuncta|[20301]|XAB604-04|04HBL005604|658[0n]bp|Canada.Ontario|BOLD:AAA4742
Melanchra adjuncta|[20302]|XAF779-05|2005-ONT-428|658[0n]bp|Canada.Ontario|BOLD:AAA4742
Melanchra adjuncta|[20303]|BBLPB642-10|10BBCLP-1641|658[0n]bp|Canada.Alberta|BOLD:AAA4742
Melanchra adjuncta|[20304]|PHMNB322-04|04HBL00548|658[0n]bp|Canada.New Brunswick|BOLD:AAA4742
Anarta crotchii ps1|[20305]|RDNM888-05|CNCNoctuoidea7728|577[3n]bp|Canada.British Columbia|BOLD:AAB9779
Anarta crotchii ps1|[20306]|RDNMC346-05|CNCNoctuoidea11980|559[0n]bp|Canada.British Columbia|BOLD:AAB9779
Anarta crotchii ps1|[20307]|RDNMC784-06|BOOG32|549[2n]bp|Canada.British Columbia|BOLD:AAB9779
Anarta crotchii ps1|[20308]|RDNMC777-06|BOOG25|574[0n]bp|Canada.British Columbia|BOLD:AAB9779
Anarta crotchii ps1|[20309]|RDNMC349-05|CNCNoctuoidea11983|579[0n]bp|Canada.British Columbia|BOLD:AAB9779
Anarta crotchii ps1|[20310]|RDNMC778-06|BOOG26|588[1n]bp|Canada.British Columbia|BOLD:AAB9779
Anarta crotchii|[20311]|LBCH4999-10|10-JDWBC-4999|658[0n]bp|Canada.British Columbia|BOLD:AAB9779
Anarta crotchii|[20312]|LBCH4993-10|10-JDWBC-4993|658[0n]bp|Canada.British Columbia|BOLD:AAB9779
Anarta crotchii|[20313]|LBCH4981-10|10-JDWBC-4981|658[0n]bp|Canada.British Columbia|BOLD:AAB9779
Anarta crotchii|[20314]|LBCH4977-10|10-JDWBC-4977|658[0n]bp|Canada.British Columbia|BOLD:AAB9779
Anarta crotchii|[20315]|LBCH5011-10|10-JDWBC-5011|658[0n]bp|Canada.British Columbia|BOLD:AAB9779
Anarta crotchii ps1|[20316]|RDNMC182-05|CNCNoctuoidea10923|658[0n]bp|Canada.British Columbia|BOLD:AAB9779
Anarta crotchii ps1|[20317]|RDNMC344-05|CNCNoctuoidea11978|582[3n]bp|Canada.British Columbia|BOLD:AAB9779
Anarta crotchii ps2|[20318]|RDNMC350-05|CNCNoctuoidea11984|585[0n]bp|Canada.British Columbia|BOLD:AAB8611
Anarta crotchii ps2|[20319]|RDNM889-05|CNCNoctuoidea7729|612[1n]bp|Canada.British Columbia|BOLD:AAB8611
Anarta crotchii ps2|[20320]|RDNMC780-06|BOOG28|604[0n]bp|Canada.British Columbia|BOLD:AAB8611
Anarta crotchii ps2|[20321]|RDNMC779-06|BOOG27|615[0n]bp|Canada.British Columbia|BOLD:AAB8611
Anarta crotchii ps2|[20322]|RDMAB471-06|UASM77913|658[0n]bp|Canada.Alberta|BOLD:AAB8611
Anarta crotchii ps2|[20323]|RDNMB965-05|CNCNoctuoidea10740|658[0n]bp|Canada.Alberta|BOLD:AAB8611
Anarta crotchii ps2|[20324]|RDNM890-05|CNCNoctuoidea7730|658[0n]bp|Canada.British Columbia|BOLD:AAB8611
Anarta crotchii ps2|[20325]|RDNMC345-05|CNCNoctuoidea11979|608[0n]bp|Canada.British Columbia|BOLD:AAB8611
Anarta crotchii ps2|[20326]|RDNMB116-05|CNCNoctuoidea10268|658[0n]bp|Canada.Alberta|BOLD:AAB8611
Anarta nigrolunata|[20327]|RDNMC352-05|CNCNoctuoidea11986|658[0n]bp|Canada.Yukon Territory|BOLD:AAD2342
Anarta nigrolunata|[20328]|RDNME419-08|LEP037843|658[0n]bp|Canada.Yukon Territory|BOLD:AAD2342
Anarta nigrolunata|[20329]|LPABB805-09|08BBLEP-04125|658[0n]bp|Canada.Alberta|BOLD:AAD2342
Anarta nigrolunata|[20330]|CHLEP123-09|09PROBE-09418|658[0n]bp|Canada.Manitoba|BOLD:AAD2342
Anarta nigrolunata|[20331]|LOWCD112-06|CGWC-2932|658[0n]bp|Canada.British Columbia|BOLD:AAD2342
Anarta nigrolunata|[20332]|LPABB806-09|08BBLEP-04126|658[0n]bp|Canada.Alberta|BOLD:AAD2342

Anarta nigrolunata|[20330]|CHLEP125-09|09PROBE-09418|658[0n]bp|Canada.Manitoba|BOLD:AAD2342
Anarta nigrolunata|[20331]|LOWCD112-06|CGWC-2932|658[0n]bp|Canada.British Columbia|BOLD:AAD2342
Anarta nigrolunata|[20332]|LPABB806-09|08BBLEP-04126|658[0n]bp|Canada.Alberta|BOLD:AAD2342
Anarta nigrolunata|[20333]|LCHQ867-08|07WNP-10759|658[0n]bp|Canada.Manitoba|BOLD:AAD2342
Anarta nigrolunata|[20334]|LPABB809-09|08BBLEP-04129|658[0n]bp|Canada.Alberta|BOLD:AAD2342
Anarta farnhami|[20335]|CHLEP095-09|09PROBE-09390|658[0n]bp|Canada.Manitoba|BOLD:AAA8739
Anarta farnhami|[20336]|LCHP391-07|07PROBE-10015|658[0n]bp|Canada.Manitoba|BOLD:AAA8739
Anarta farnhami|[20337]|LCH237-04|04HBL003237|658[0n]bp|Canada.Manitoba|BOLD:AAA8739
Anarta farnhami|[20338]|MHLEP086-07|CHU06-LEP-086|658[0n]bp|Canada.Manitoba|BOLD:AAA8739
Anarta farnhami|[20339]|CHLEP094-09|09PROBE-09389|658[0n]bp|Canada.Manitoba|BOLD:AAA8739
Anarta farnhami|[20340]|MHLEP055-07|CHU06-LEP-055|658[0n]bp|Canada.Manitoba|
Anarta farnhami|[20341]|CHLEP099-09|09PROBE-09394|658[0n]bp|Canada.Manitoba|BOLD:AAA8739
Anarta farnhami|[20342]|LCHP839-07|07PROBE-10596|658[0n]bp|Canada.Manitoba|BOLD:AAA8739
Anarta farnhami|[20343]|CHLEP092-09|09PROBE-09387|658[0n]bp|Canada.Manitoba|BOLD:AAA8739
Anarta farnhami|[20344]|CHLEP220-09|09PROBE-09515|658[0n]bp|Canada.Manitoba|BOLD:AAA8739
Anarta farnhami|[20345]|LCHP220-07|07PROBE-03790|658[0n]bp|Canada.Manitoba|BOLD:AAA8739
Anarta farnhami|[20346]|LCHP221-07|07PROBE-03791|658[0n]bp|Canada.Manitoba|BOLD:AAA8739
Anarta farnhami|[20347]|CHLEP100-09|09PROBE-09395|658[0n]bp|Canada.Manitoba|BOLD:AAA8739
Anarta farnhami|[20348]|LCHP787-07|07PROBE-10472|658[0n]bp|Canada.Manitoba|BOLD:AAA8739
Anarta farnhami|[20349]|CHLEP097-09|09PROBE-09392|658[0n]bp|Canada.Manitoba|BOLD:AAA8739
Anarta farnhami|[20350]|LCHQ123-07|07PROBE-10892|658[0n]bp|Canada.Manitoba|BOLD:AAA8739
Anarta farnhami|[20351]|CHLEP294-09|09PROBE-09589|658[0n]bp|Canada.Manitoba|BOLD:AAA8739
Anarta farnhami|[20352]|CHLEP096-09|09PROBE-09391|658[0n]bp|Canada.Manitoba|BOLD:AAA8739
Anarta farnhami|[20353]|HMCOL071-07|CHU05-COL-071|658[0n]bp|Canada.Manitoba|BOLD:AAA8739
Anarta farnhami|[20354]|CHLEP090-09|09PROBE-09385|658[0n]bp|Canada.Manitoba|BOLD:AAA8739
Anarta farnhami|[20355]|CHLEP098-09|09PROBE-09393|658[0n]bp|Canada.Manitoba|BOLD:AAA8739
Anarta farnhami|[20356]|CHLEP093-09|09PROBE-09388|658[0n]bp|Canada.Manitoba|BOLD:AAA8739
Anarta farnhami|[20357]|CHLEP102-09|09PROBE-09397|658[0n]bp|Canada.Manitoba|BOLD:AAA8739
Anarta farnhami|[20358]|LCHP829-07|07PROBE-10586|658[0n]bp|Canada.Manitoba|BOLD:AAA8739
Anarta farnhami|[20359]|CHLEP188-09|09PROBE-09483|658[0n]bp|Canada.Manitoba|BOLD:AAA8739
Anarta farnhami|[20360]|LCH238-04|04HBL003238|658[0n]bp|Canada.Manitoba|BOLD:AAA8739
Anarta farnhami|[20361]|CHLEP207-09|09PROBE-09502|658[0n]bp|Canada.Manitoba|BOLD:AAA8739
Anarta farnhami|[20362]|CHLEP103-09|09PROBE-09398|658[0n]bp|Canada.Manitoba|BOLD:AAA8739
Anarta farnhami|[20363]|CHLEP101-09|09PROBE-09396|658[0n]bp|Canada.Manitoba|BOLD:AAA8739
Anarta farnhami|[20364]|CHLEP212-09|09PROBE-09507|626[0n]bp|Canada.Manitoba|BOLD:AAA8739
Anarta farnhami|[20365]|CHLEP205-09|09PROBE-09500|634[0n]bp|Canada.Manitoba|BOLD:AAA8739
Anarta farnhami|[20366]|MHCOL362-07|CHU06-COL-362|645[0n]bp|Canada.Manitoba|BOLD:AAA8739
Anarta farnhami|[20367]|CHLEP156-09|09PROBE-09451|658[0n]bp|Canada.Manitoba|BOLD:AAA8739
Anarta farnhami|[20368]|CHLEP221-09|09PROBE-09516|658[0n]bp|Canada.Manitoba|BOLD:AAA8739
Anarta farnhami|[20369]|CHLEP291-09|09PROBE-09586|658[0n]bp|Canada.Manitoba|BOLD:AAA8739
Anarta farnhami|[20370]|MHLEP029-07|CHU06-LEP-029|658[0n]bp|Canada.Manitoba|BOLD:AAA8739
Anarta farnhami|[20371]|CHLEP091-09|09PROBE-09386|658[0n]bp|Canada.Manitoba|BOLD:AAA8739
Anarta farnhami|[20372]|CHLEP204-09|09PROBE-09499|658[0n]bp|Canada.Manitoba|BOLD:AAA8739
Anarta farnhami|[20373]|CHLEP206-09|09PROBE-09501|658[0n]bp|Canada.Manitoba|BOLD:AAA8739
Anarta farnhami|[20374]|CHLEP292-09|09PROBE-09587|658[0n]bp|Canada.Manitoba|BOLD:AAA8739
Anarta columbica|[20375]|LOWCD716-06|CGWC-3536|657[2n]bp|Canada.British Columbia|BOLD:AAA9985
Anarta columbica|[20376]|LOWCB724-05|CGWC-1664|658[0n]bp|Canada.British Columbia|BOLD:AAA9985
Anarta columbica|[20377]|LBCH5491-10|10-JDWBC-5491|658[0n]bp|Canada.British Columbia|BOLD:AAA9985
Anarta columbica|[20378]|LOWCD729-06|CGWC-3549|506[0n]bp|Canada.British Columbia|
Anarta columbica|[20379]|LOWCD727-06|CGWC-3547|587[0n]bp|Canada.British Columbia|BOLD:AAA9985
Anarta columbica|[20380]|LOWCD728-06|CGWC-3548|574[0n]bp|Canada.British Columbia|BOLD:AAA9985
Anarta columbica|[20381]|LBCH5094-10|10-JDWBC-5094|658[0n]bp|Canada.British Columbia|BOLD:AAA9985
Anarta columbica|[20382]|LOWCB316-05|CGWC-1256|581[0n]bp|Canada.British Columbia|BOLD:AAA9985
Anarta columbica|[20383]|LOWCD719-06|CGWC-3539|558[0n]bp|Canada.British Columbia|BOLD:AAA9985
Anarta columbica|[20384]|LBCG116-08|08-JDWBC-0116|658[0n]bp|Canada.British Columbia|BOLD:AAA9985
Anarta columbica|[20385]|LBCH5447-10|10-JDWBC-5447|658[0n]bp|Canada.British Columbia|BOLD:AAA9985
Anarta columbica|[20386]|LBCH5827-10|10-JDWBC-5827|658[0n]bp|Canada.British Columbia|BOLD:AAA9985
Anarta columbica|[20387]|LOWCD723-06|CGWC-3543|641[0n]bp|Canada.British Columbia|BOLD:AAA9985
Anarta columbica|[20388]|LBCH5444-10|10-JDWBC-5444|658[0n]bp|Canada.British Columbia|BOLD:AAA9985
Anarta columbica|[20389]|LBCH5160-10|10-JDWBC-5160|658[0n]bp|Canada.British Columbia|BOLD:AAA9985
Anarta columbica|[20390]|LBCH5482-10|10-JDWBC-5482|658[0n]bp|Canada.British Columbia|BOLD:AAA9985
Anarta columbica|[20391]|LOWCB719-05|CGWC-1659|658[0n]bp|Canada.British Columbia|BOLD:AAA9985
Anarta columbica|[20392]|RDNM871-05|CNCNoctuoidea7711|658[0n]bp|Canada.British Columbia|BOLD:AAA9985
Anarta columbica|[20393]|LBCH5423-10|10-JDWBC-5423|658[0n]bp|Canada.British Columbia|BOLD:AAA9985
Anarta columbica|[20394]|LBCH5486-10|10-JDWBC-5486|658[0n]bp|Canada.British Columbia|BOLD:AAA9985
Anarta columbica|[20395]|LBCH5212-10|10-JDWBC-5212|658[0n]bp|Canada.British Columbia|BOLD:AAA9985
Anarta columbica|[20396]|LBCG118-08|08-JDWBC-0118|658[0n]bp|Canada.British Columbia|BOLD:AAA9985
Anarta columbica|[20397]|RDNM872-05|CNCNoctuoidea7712|658[0n]bp|Canada.British Columbia|BOLD:AAA9985
Anarta columbica|[20398]|LBCH5830-10|10-JDWBC-5830|658[0n]bp|Canada.British Columbia|BOLD:AAA9985
Anarta columbica|[20399]|LBCH5286-10|10-JDWBC-5286|658[0n]bp|Canada.British Columbia|BOLD:AAA9985
Anarta columbica|[20400]|LBCG101-08|08-JDWBC-0101|658[0n]bp|Canada.British Columbia|BOLD:AAA9985
Anarta columbica|[20401]|LBCH5426-10|10-JDWBC-5426|658[0n]bp|Canada.British Columbia|BOLD:AAA9985
Anarta columbica|[20402]|LOWCD718-06|CGWC-3538|658[0n]bp|Canada.British Columbia|BOLD:AAA9985
Anarta columbica|[20403]|LBCG121-08|08-JDWBC-0121|658[0n]bp|Canada.British Columbia|BOLD:AAA9985
Anarta columbica|[20404]|LBCH5446-10|10-JDWBC-5446|658[0n]bp|Canada.British Columbia|BOLD:AAA9985
Anarta columbica|[20405]|LBCG335-08|08-JDWBC-0335|658[0n]bp|Canada.British Columbia|BOLD:AAA9985
Anarta columbica|[20406]|LBCH5809-10|10-JDWBC-5809|658[0n]bp|Canada.British Columbia|BOLD:AAA9985
Anarta columbica|[20407]|LBCG371-08|08-JDWBC-0371|658[0n]bp|Canada.British Columbia|BOLD:AAA9985
Anarta columbica|[20408]|LOWCB727-05|CGWC-1667|658[0n]bp|Canada.British Columbia|BOLD:AAA9985
Anarta columbica|[20409]|LBCH5213-10|10-JDWBC-5213|658[0n]bp|Canada.British Columbia|BOLD:AAA9985
Anarta columbica|[20410]|LBCH5252-10|10-JDWBC-5252|658[0n]bp|Canada.British Columbia|BOLD:AAA9985
Anarta columbica|[20411]|LBCH5443-10|10-JDWBC-5443|658[0n]bp|Canada.British Columbia|BOLD:AAA9985
Anarta columbica|[20412]|LBCH5494-10|10-JDWBC-5494|658[0n]bp|Canada.British Columbia|BOLD:AAA9985
Anarta columbica|[20413]|LBCH5424-10|10-JDWBC-5424|658[0n]bp|Canada.British Columbia|BOLD:AAA9985
Anarta columbica|[20414]|LOWCD714-06|CGWC-3534|658[0n]bp|Canada.British Columbia|BOLD:AAA9985
Anarta columbica|[20415]|LOWCB726-05|CGWC-1666|658[0n]bp|Canada.British Columbia|BOLD:AAA9985
Anarta columbica|[20416]|LBCH5427-10|10-JDWBC-5427|658[0n]bp|Canada.British Columbia|BOLD:AAA9985
Anarta columbica|[20417]|RDNM884-05|CNCNoctuoidea7724|658[0n]bp|Canada.British Columbia|BOLD:AAA9985
Anarta columbica|[20418]|LBCH5344-10|10-JDWBC-5344|658[0n]bp|Canada.British Columbia|BOLD:AAA9985
Anarta columbica|[20419]|LOWCD732-06|CGWC-3552|658[0n]bp|Canada.British Columbia|BOLD:AAA9985
Anarta columbica|[20420]|LBCH5825-10|10-JDWBC-5825|658[0n]bp|Canada.British Columbia|BOLD:AAA9985
Anarta columbica|[20421]|LOWCB725-05|CGWC-1665|658[0n]bp|Canada.British Columbia|BOLD:AAA9985
Anarta columbica|[20422]|LBCH5728-10|10-JDWBC-5728|658[0n]bp|Canada.British Columbia|BOLD:AAA9985
Anarta columbica|[20423]|LBCH5826-10|10-JDWBC-5826|658[0n]bp|Canada.British Columbia|BOLD:AAA9985
Anarta columbica|[20424]|LBCG343-08|08-JDWBC-0343|658[0n]bp|Canada.British Columbia|BOLD:AAA9985
Anarta columbica|[20425]|LBCH5601-10|10-JDWBC-5601|658[0n]bp|Canada.British Columbia|BOLD:AAA9985
Anarta columbica|[20426]|LBCH5445-10|10-JDWBC-5445|658[0n]bp|Canada.British Columbia|BOLD:AAA9985
Anarta columbica|[20427]|LOWCD726-06|CGWC-3546|658[0n]bp|Canada.British Columbia|BOLD:AAA9985
Anarta columbica|[20428]|LOWCB720-05|CGWC-1660|658[0n]bp|Canada.British Columbia|BOLD:AAA9985
Anarta columbica|[20429]|LBCH5449-10|10-JDWBC-5449|658[0n]bp|Canada.British Columbia|BOLD:AAA9985
Anarta columbica|[20430]|LBCH5448-10|10-JDWBC-5448|658[0n]bp|Canada.British Columbia|BOLD:AAA9985
Anarta columbica|[20431]|LOWCD725-06|CGWC-3545|658[0n]bp|Canada.British Columbia|BOLD:AAA9985
Anarta columbica|[20432]|LOWCD724-06|CGWC-3544|657[0n]bp|Canada.British Columbia|BOLD:AAA9985

Anarta columbica|[20430]|LBCH5448-10|10-JDWBC-5448|658[0n]bp|Canada.British Columbia|BOLD:AAA9985
Anarta columbica|[20431]|LOWCD725-06|CGWC-3545|658[0n]bp|Canada.British Columbia|BOLD:AAA9985
Anarta columbica|[20432]|LOWCD724-06|CGWC-3544|657[0n]bp|Canada.British Columbia|BOLD:AAA9985
Anarta columbica|[20433]|LBCG124-08|08-JDWBC-0124|640[0n]bp|Canada.British Columbia|BOLD:AAA9985
Anarta columbica|[20434]|LBCH5600-10|10-JDWBC-5600|644[0n]bp|Canada.British Columbia|BOLD:AAA9985
Anarta columbica|[20435]|LOWCD715-06|CGWC-3535|594[0n]bp|Canada.British Columbia|BOLD:AAA9985
Anarta columbica|[20436]|LBCH5159-10|10-JDWBC-5159|623[0n]bp|Canada.British Columbia|BOLD:AAA9985
Anarta columbica|[20437]|LOWCD721-06|CGWC-3541|657[0n]bp|Canada.British Columbia|BOLD:AAA9985
Anarta columbica|[20438]|LBCH5095-10|10-JDWBC-5095|658[0n]bp|Canada.British Columbia|BOLD:AAA9985
Anarta obesula|[20439]|RDNMF537-08|NOC14623|609[0n]bp|United States.Colorado|BOLD:AAA9985
Anarta obesula|[20440]|RDNMG830-08|CNC LEP00052954|609[0n]bp|United States.Wyoming|BOLD:AAA9985
Anarta obesula|[20441]|RDNMF536-08|NOC14622|609[0n]bp|United States.Wyoming|BOLD:AAA9985
Anarta obesula|[20442]|RDNMG829-08|CNC LEP00052953|592[0n]bp|United States.Wyoming|BOLD:AAA9985
Anarta hamata|[20443]|RDNM874-05|CNCNoctuoidea7714|575[1n]bp|United States.California|BOLD:ABZ1428
Anarta hamata|[20444]|RDNM877-05|CNCNoctuoidea7717|575[2n]bp|United States.Washington|BOLD:ABZ1428
Anarta hamata|[20445]|RDNM876-05|CNCNoctuoidea7716|658[0n]bp|United States.Washington|BOLD:ABZ1428
Anarta hamata|[20446]|RDNM875-05|CNCNoctuoidea7715|658[0n]bp|United States.Wyoming|BOLD:ABZ1428
Anarta hamata|[20447]|RDNM873-05|CNCNoctuoidea7713|658[0n]bp|United States.California|BOLD:ABZ1428
Anarta inconcinna|[20448]|LBCG3319-09|08-JDWBC-3319|658[0n]bp|Canada.British Columbia|BOLD:ABZ1962
Anarta inconcinna|[20449]|LBCH5492-10|10-JDWBC-5492|658[0n]bp|Canada.British Columbia|BOLD:ABZ1962
Anarta inconcinna|[20450]|LBCH5493-10|10-JDWBC-5493|658[0n]bp|Canada.British Columbia|BOLD:ABZ1962
Anarta inconcinna|[20451]|LOWCB721-05|CGWC-1661|658[0n]bp|Canada.British Columbia|BOLD:ABZ1962
Anarta inconcinna|[20452]|LOWCB723-05|CGWC-1663|658[0n]bp|Canada.British Columbia|BOLD:ABZ1962
Anarta inconcinna|[20453]|LOWCD733-06|CGWC-3553|658[0n]bp|Canada.British Columbia|BOLD:ABZ1962
Anarta inconcinna|[20454]|LOWCD717-06|CGWC-3537|658[0n]bp|Canada.British Columbia|BOLD:ABZ1962
Anarta inconcinna|[20455]|LBCH5051-10|10-JDWBC-5051|658[0n]bp|Canada.British Columbia|BOLD:ABZ1962
Anarta inconcinna|[20456]|LOWCD720-06|CGWC-3540|658[0n]bp|Canada.British Columbia|BOLD:ABZ1962
Anarta inconcinna|[20457]|LOWCC192-05|CGWC-2072|658[0n]bp|Canada.British Columbia|BOLD:ABZ1962
Anarta inconcinna|[20458]|LBCG2878-09|08-JDWBC-2878|658[0n]bp|Canada.British Columbia|BOLD:ABZ1962
Anarta inconcinna|[20459]|LOWCD731-06|CGWC-3551|568[0n]bp|Canada.British Columbia|BOLD:ABZ1962
Anarta inconcinna|[20460]|RDNM879-05|CNCNoctuoidea7719|658[0n]bp|Canada.Alberta|BOLD:ABZ1962
Anarta inconcinna|[20461]|RDNM878-05|CNCNoctuoidea7718|658[0n]bp|Canada.Alberta|BOLD:ABZ1962
Anarta inconcinna|[20462]|RDNM880-05|CNCNoctuoidea7720|658[0n]bp|Canada.British Columbia|BOLD:ABZ1962
Anarta alta|[20463]|RDNM891-05|CNCNoctuoidea7731|512[0n]bp|Canada.Alberta|BOLD:ACE5827
Anarta alta|[20464]|LPABB396-08|08BBLEP-03661|658[0n]bp|Canada.Alberta|BOLD:ACE5827
Anarta alta|[20465]|LPMN929-08|08BBLEP-02287|658[0n]bp|Canada.Alberta|BOLD:ACE5827
Anarta alta|[20466]|LPABB399-08|08BBLEP-03664|658[0n]bp|Canada.Alberta|BOLD:ACE5827
Anarta alta|[20467]|LPAB022-08|08BBLEP-02344|658[0n]bp|Canada.Alberta|BOLD:ACE5827
Anarta alta|[20468]|LPABB416-08|08BBLEP-03681|658[0n]bp|Canada.Alberta|BOLD:ACE5827
Anarta alta|[20469]|LPABB634-08|08BBLEP-03899|658[0n]bp|Canada.Alberta|BOLD:ACE5827
Anarta oregonica|[20470]|RDNM894-05|CNCNoctuoidea7734|505[0n]bp|Canada.British Columbia|BOLD:ACE5827
Anarta oregonica|[20471]|RDNM892-05|CNCNoctuoidea7732|658[0n]bp|Canada.British Columbia|BOLD:ACE5827
Anarta oregonica|[20472]|LBCD148-05|HLC-22968|658[0n]bp|Canada.British Columbia|BOLD:ACE5827
Anarta oregonica|[20473]|LBCG2367-09|08-JDWBC-2367|658[0n]bp|Canada.British Columbia|BOLD:ACE5827
Anarta oregonica|[20474]|LBCG2473-09|08-JDWBC-2473|658[0n]bp|Canada.British Columbia|BOLD:ACE5827
Anarta oregonica|[20475]|LBCC331-05|HLC-22211|658[0n]bp|Canada.British Columbia|BOLD:ACE5827
Anarta oregonica|[20476]|RDNM883-05|CNCNoctuoidea7723|658[0n]bp|Canada.British Columbia|BOLD:ACE5827
Anarta oregonica|[20477]|RDNM893-05|CNCNoctuoidea7733|570[1n]bp|Canada.British Columbia|BOLD:ACE5827
Anarta trifolii|[20478]|RDNMB963-05|CNCNoctuoidea10738|658[0n]bp|Canada.Alberta|BOLD:ACF4267
Anarta trifolii|[20479]|XAH268-05|2005-ONT-1851|658[0n]bp|Canada.Ontario|BOLD:ACF4267
Anarta trifolii|[20480]|RDLQG024-06|DH012155|658[0n]bp|Canada.Quebec|BOLD:ABZ7601
Anarta trifolii|[20481]|LCHP407-07|07PROBE-10031|658[0n]bp|Canada.Manitoba|BOLD:ABZ7601
Anarta trifolii|[20482]|RDLQG057-06|DH012188|658[0n]bp|Canada.Quebec|BOLD:ABZ7601
Anarta trifolii|[20483]|LCHQ118-07|07PROBE-10887|658[0n]bp|Canada.Manitoba|BOLD:ABZ7601
Anarta trifolii|[20484]|RDLQF229-06|DH011309|658[0n]bp|Canada.Quebec|BOLD:ABZ7601
Anarta trifolii|[20485]|RDNMC203-05|CNCNoctuoideaH015|658[0n]bp|Canada.New Brunswick|BOLD:ABZ7601
Anarta trifolii|[20486]|RDLQB765-05|DH010852|617[0n]bp|Canada.Quebec|BOLD:ABZ7601
Anarta trifolii|[20487]|RDLQB741-05|DH010656|658[0n]bp|Canada.Quebec|BOLD:ABZ7601
Anarta trifolii|[20488]|BLTIB673-08|BL956|658[1n]bp|Canada.Ontario|BOLD:ABZ7601
Anarta trifolii|[20489]|RDNMC204-05|CNCNoctuoideaH016|587[1n]bp|Canada.New Brunswick|BOLD:ABZ7601
Anarta trifolii|[20490]|RDNMB964-05|CNCNoctuoidea10739|658[0n]bp|Canada.British Columbia|BOLD:ABZ7601
Anarta antica|[20491]|RDMAB688-06|UASM58809|632[0n]bp|Canada.Alberta|BOLD:AAF3901
Anarta decepta|[20492]|LBCH5871-10|10-JDWBC-5871|658[0n]bp|Canada.British Columbia|BOLD:AAE1460
Anarta decepta|[20493]|LBCH7094-10|10-JDWBC-7094|658[0n]bp|Canada.British Columbia|BOLD:AAE1460
Anarta decepta|[20494]|LBCH5097-10|10-JDWBC-5097|658[0n]bp|Canada.British Columbia|BOLD:AAE1460
Anarta decepta|[20495]|RDNMF345-08|NOC14431|658[0n]bp|Canada.British Columbia|BOLD:AAE1460
Anarta decepta|[20496]|LBCH6154-10|10-JDWBC-6154|658[0n]bp|Canada.British Columbia|BOLD:AAE1460
Anarta decepta|[20497]|LBCH5170-10|10-JDWBC-5170|658[0n]bp|Canada.British Columbia|BOLD:AAE1460
Escaria homogena|[20498]|RDNMG1033-08|CNC LEP00053157|658[0n]bp|Canada.Alberta|BOLD:AAE2691
Escaria homogena|[20499]|RDMAB990-09|UASM57369|649[0n]bp|Canada.Alberta|BOLD:AAE2691
Escaria homogena|[20500]|RDMAB992-09|UASM24668|634[0n]bp|Canada.Alberta|BOLD:AAE2691
Escaria homogena|[20501]|RDMAB991-09|UASM57324|635[0n]bp|Canada.Alberta|BOLD:AAE2691
Escaria homogena|[20502]|RDMAB530-06|UASM58489|658[0n]bp|Canada.Alberta|BOLD:AAE2691
Anarta mutata|[20503]|LPSK499-08|08BBLEP-02067|658[0n]bp|Canada.Saskatchewan|BOLD:AAD7628
Anarta mutata|[20504]|LPSK511-08|08BBLEP-02079|658[0n]bp|Canada.Saskatchewan|BOLD:AAD7628
Anarta mutata|[20505]|LPSK562-08|08BBLEP-02130|658[0n]bp|Canada.Saskatchewan|BOLD:AAD7628
Anarta mutata|[20506]|LBCH6451-10|10-JDWBC-6451|642[0n]bp|Canada.British Columbia|BOLD:AAD7628
Afotella cylindrica|[20507]|RDMAB551-06|UASM58530|658[0n]bp|Canada.Alberta|BOLD:AAE6413
Afotella cylindrica|[20508]|RDNMG555-08|CNC LEP00052379|658[0n]bp|Canada.Alberta|BOLD:AAE6413
Afotella cylindrica|[20509]|RDNMG554-08|CNC LEP00052378|658[0n]bp|Canada.Alberta|BOLD:AAE6413
Hadenella pergentilis|[20510]|RDNMF532-08|NOC14618|658[0n]bp|United States.Colorado|BOLD:AAD7037
Hadenella pergentilis|[20511]|NAMUM146-08|RR-95-0165|657[0n]bp|United States.California|BOLD:AAD7036
Hadenella pergentilis|[20512]|RDNMF534-08|NOC14620|658[0n]bp|United States.California|BOLD:AAD7036
Hadenella pergentilis|[20513]|RDNMF531-08|NOC14617|658[0n]bp|United States.Oregon|BOLD:ACE6084
Hadenella pergentilis|[20514]|RDNMF535-08|NOC14621|658[1n]bp|United States.Oregon|BOLD:ACE6084
Hadenella pergentilis|[20515]|RDNMF533-08|NOC14619|658[0n]bp|United States.Oregon|BOLD:ACE6084
Scotogramma fervida|[20516]|RDNMB961-05|CNCNoctuoidea10736|658[0n]bp|Canada.Alberta|BOLD:AAC4952
Scotogramma fervida|[20517]|LPSK522-08|08BBLEP-02090|658[0n]bp|Canada.Saskatchewan|BOLD:AAC4952
Scotogramma fervida|[20518]|LPSK537-08|08BBLEP-02105|658[0n]bp|Canada.Saskatchewan|BOLD:AAC4952
Scotogramma fervida|[20519]|LPSK465-08|08BBLEP-02033|658[0n]bp|Canada.Saskatchewan|BOLD:AAC4952
Hada sutrina|[20520]|RDLQ636-07|DH008654|633[0n]bp|Canada.Quebec|BOLD:AAC2435
Hada sutrina|[20521]|RDLQ635-07|DH008652|633[0n]bp|Canada.Newfoundland and Labrador|BOLD:AAC2435
Hada sutrina|[20522]|RDMAB363-05|UASM77811|616[0n]bp|Canada.Alberta|BOLD:AAC2435
Hada sutrina|[20523]|RDNME521-08|LEP037945|658[0n]bp|Canada.Yukon Territory|BOLD:AAC2435
Hada sutrina|[20524]|BBLPB772-10|10BBCLP-1771|658[0n]bp|Canada.British Columbia|BOLD:AAC2435
Hada sutrina|[20525]|LBCA195-05|HLC-20195|658[0n]bp|Canada.British Columbia|BOLD:AAC2435
Hada sutrina|[20526]|BBLPB761-10|10BBCLP-1760|658[0n]bp|Canada.British Columbia|BOLD:AAC2435
Hada sutrina|[20527]|LBCH2196-10|10-JDWBC-2196|658[0n]bp|Canada.British Columbia|BOLD:AAC2435
Hada sutrina|[20528]|LBCH1921-10|10-JDWBC-1921|658[0n]bp|Canada.British Columbia|BOLD:AAC2435
Hada sutrina|[20529]|LBCG1345-09|08-JDWBC-1345|658[0n]bp|Canada.British Columbia|BOLD:AAC2435
Hada sutrina|[20530]|LBCG2395-09|08-JDWBC-2395|658[0n]bp|Canada.British Columbia|BOLD:AAC2435
Hada sutrina|[20531]|LBCH5490-10|10-JDWBC-5490|658[0n]bp|Canada.British Columbia|BOLD:AAC2435
Hada sutrina|[20532]|LBCH1717-10|10-JDWBC-1717|658[0n]bp|Canada.British Columbia|BOLD:AAC2435

[illegible]
Hada sutrina|[20530]|LBCG2395-09|08-JDWBC-2395|658[0n]bp|Canada.British Columbia|BOLD:AAC2435
Hada sutrina|[20531]|LBCH5490-10|10-JDWBC-5490|658[0n]bp|Canada.British Columbia|BOLD:AAC2435
Hada sutrina|[20532]|LBCH1717-10|10-JDWBC-1717|658[0n]bp|Canada.British Columbia|BOLD:AAC2435
Hada sutrina|[20533]|LALPA1088-11|AVBC 898-11|658[0n]bp|Canada.British Columbia|BOLD:AAC2435
Hada sutrina|[20534]|LALPA1139-11|AVBC 949-11|658[0n]bp|Canada.British Columbia|BOLD:AAC2435
Hada sutrina|[20535]|LBCG3043-09|08-JDWBC-3043|658[0n]bp|Canada.British Columbia|BOLD:AAC2435
Hada sutrina|[20536]|LALPA834-11|AVBC 1007-11|658[0n]bp|Canada.British Columbia|BOLD:AAC2435
Hada sutrina|[20537]|LBCC336-05|HLC-22216|658[0n]bp|Canada.British Columbia|BOLD:AAC2435
Hada sutrina|[20538]|LBCG2831-09|08-JDWBC-2831|658[0n]bp|Canada.British Columbia|BOLD:AAC2435
Hada sutrina|[20539]|LBCG2483-09|08-JDWBC-2483|658[0n]bp|Canada.British Columbia|BOLD:AAC2435
Hadena caelestis|[20540]|RDNMF240-08|NOC14326|658[0n]bp|Canada.British Columbia|BOLD:ACE5504
Hadena caelestis|[20541]|RDNMF241-08|NOC14327|658[0n]bp|Canada.British Columbia|BOLD:ACE5504
Hadena caelestis|[20542]|RDNMF239-08|NOC14325|658[0n]bp|Canada.British Columbia|BOLD:ACE5504
Hadena caelestis|[20543]|RDNMF238-08|NOC14324|658[0n]bp|Canada.British Columbia|BOLD:ACE5504
Hadena caelestis|[20544]|RDNMF237-08|NOC14323|658[0n]bp|Canada.British Columbia|BOLD:ACE5504
Hadena circumvadis|[20545]|LPMN946-08|08BBLEP-02304|658[0n]bp|Canada.Alberta|BOLD:ACE5504
Hadena circumvadis|[20546]|RDNMF235-08|NOC14321|658[0n]bp|Canada.Saskatchewan|BOLD:ACE5504
Hadena circumvadis|[20547]|RDNMF232-08|NOC14318|658[0n]bp|Canada.Alberta|BOLD:ACE5504
Hadena circumvadis|[20548]|RDNMF233-08|NOC14319|658[0n]bp|Canada.Saskatchewan|BOLD:ACE5504
Hadena ectrapela|[20549]|RDMAB574-06|UASM58564|658[0n]bp|Canada.Alberta|BOLD:AAB7648
Hadena ectrapela|[20550]|LBCH1578-10|10-JDWBC-1578|658[0n]bp|Canada.British Columbia|BOLD:AAB7648
Hadena capsularis|[20551]|RDNMB239-05|CNCNoctuoidea10005|603[0n]bp|Canada.Ontario|BOLD:AAD5556
Hadena capsularis|[20552]|RDLQ645-07|DH008651|657[0n]bp|Canada.Quebec|BOLD:AAD5556
Hadena capsularis|[20553]|RDNMF886-08|CNC LEP00053247|658[0n]bp|Canada.Ontario|BOLD:AAD5556
Hadena variolata|[20554]|LBCH5802-10|10-JDWBC-5802|658[0n]bp|Canada.British Columbia|BOLD:AAE6874
Hadena variolata|[20555]|RDMAB526-06|CBCC1067|658[0n]bp|Canada.Alberta|BOLD:AAE6874
Hadena variolata|[20556]|LBCH5611-10|10-JDWBC-5611|658[0n]bp|Canada.British Columbia|BOLD:AAE6874
Trachea delicata|[20557]|RDLQB695-05|DH010798|658[0n]bp|Canada.Quebec|BOLD:AAE8397
Trachea delicata|[20558]|RDMAB533-06|UASM58504|658[0n]bp|Canada.Alberta|BOLD:AAE8397
Trachea delicata|[20559]|RDMAB103-05|UASM41986|658[0n]bp|Canada.Alberta|BOLD:AAE8397
Sideridis rosea|[20560]|LPSK439-08|08BBLEP-02007|658[0n]bp|Canada.Saskatchewan|BOLD:AAC8783
Sideridis rosea|[20561]|LBCH5037-10|10-JDWBC-5037|658[0n]bp|Canada.British Columbia|BOLD:AAC8783
Sideridis rosea|[20562]|RDNMG835-08|CNC LEP00052959|658[0n]bp|Canada.New Brunswick|BOLD:AAC8783
Sideridis rosea|[20563]|RDNMG833-08|CNC LEP00052957|658[0n]bp|Canada.New Brunswick|BOLD:AAC8783
Sideridis rosea|[20564]|LBCH5416-10|10-JDWBC-5416|658[0n]bp|Canada.British Columbia|BOLD:AAC8783
Sideridis rosea|[20565]|LBCH5171-10|10-JDWBC-5171|658[0n]bp|Canada.British Columbia|BOLD:AAC8783
Sideridis rosea|[20566]|LBCH5340-10|10-JDWBC-5340|658[0n]bp|Canada.British Columbia|BOLD:AAC8783
Sideridis rosea|[20567]|LBCH5084-10|10-JDWBC-5084|658[0n]bp|Canada.British Columbia|BOLD:AAC8783
Sideridis rosea|[20568]|PHMO052-03|moth326.02|639[0n]bp|Canada.Ontario|BOLD:AAC8783
Sideridis rosea|[20569]|PHMO048-03|moth267.02|639[0n]bp|Canada.Ontario|BOLD:AAC8783
Sideridis rosea|[20570]|KPOEC140-08|08OEC-144|658[0n]bp|Canada.Ontario|BOLD:AAC8783
Sideridis rosea|[20571]|LPVIC125-08|PFC-2006-2702|658[0n]bp|Canada.British Columbia|BOLD:AAC8783
Sideridis rosea|[20572]|RDLQ619-07|DH008884|573[0n]bp|Canada.Quebec|BOLD:AAC8783
Sideridis congermana|[20573]|RDNMG846-08|CNC LEP00052970|658[0n]bp|Canada.Ontario|BOLD:AAF4037
Sideridis congermana|[20574]|RDLQ620-07|DH009921|658[0n]bp|Canada.Quebec|BOLD:AAF4037
Sideridis congermana|[20575]|LPSOB219-08|PPBP-1218|658[0n]bp|Canada.Ontario|BOLD:AAF4037
Sideridis maryx|[20576]|LPMN588-08|08BBLEP-01389|658[0n]bp|Canada.Manitoba|BOLD:AAB1581
Sideridis maryx|[20577]|LALPA471-10|AVBC 473-10|658[0n]bp|Canada.British Columbia|BOLD:AAB1581
Sideridis maryx|[20578]|LALPA841-11|AVBC 1014-11|658[0n]bp|Canada.British Columbia|BOLD:AAB1581
Sideridis maryx|[20579]|LOWCC204-05|CGWC-2084|658[0n]bp|Canada.British Columbia|BOLD:AAB1581
Sideridis maryx|[20580]|LOWCC203-05|CGWC-2083|585[0n]bp|Canada.British Columbia|BOLD:AAB1581
Sideridis maryx|[20581]|RDLQB225-05|DH010311|617[0n]bp|Canada.Quebec|BOLD:AAB1581
Sideridis maryx|[20582]|RDLQB229-05|DH010315|658[0n]bp|Canada.Quebec|BOLD:AAB1581
Sideridis maryx|[20583]|BBLPC816-09|09BBELE-1816|658[0n]bp|Canada.Newfoundland and Labrador|BOLD:AAB1581
Sideridis maryx|[20584]|TMNBB298-06|MNBTT-1238|658[0n]bp|Canada.New Brunswick|BOLD:AAB1581
Sideridis maryx|[20585]|TMNBB305-06|MNBTT-1245|658[0n]bp|Canada.New Brunswick|BOLD:AAB1581
Sideridis maryx|[20586]|RDLQB805-05|DH010892|658[0n]bp|Canada.Quebec|BOLD:AAB1581
Sideridis maryx|[20587]|TMNBB295-06|MNBTT-1235|658[0n]bp|Canada.New Brunswick|BOLD:AAB1581
Sideridis maryx|[20588]|TMNBB299-06|MNBTT-1239|658[0n]bp|Canada.New Brunswick|BOLD:AAB1581
Sideridis maryx|[20589]|TMNBB303-06|MNBTT-1243|658[0n]bp|Canada.New Brunswick|BOLD:AAB1581
Sideridis maryx|[20590]|RDLQB224-05|DH010310|658[0n]bp|Canada.Quebec|BOLD:AAB1581
Sideridis maryx|[20591]|TMNBB297-06|MNBTT-1237|658[0n]bp|Canada.New Brunswick|BOLD:AAB1581
Sideridis maryx|[20592]|TMNBB293-06|MNBTT-1233|658[0n]bp|Canada.New Brunswick|BOLD:AAB1581
Sideridis maryx|[20593]|BBLPB789-10|10BBCLP-1788|658[0n]bp|Canada.Ontario|BOLD:AAB1581
Sideridis maryx|[20594]|RDLQB227-05|DH010313|658[0n]bp|Canada.Quebec|BOLD:AAB1581
Sideridis maryx|[20595]|RDLQB228-05|DH010314|658[0n]bp|Canada.Quebec|BOLD:AAB1581
Sideridis maryx|[20596]|TMNBB304-06|MNBTT-1244|658[0n]bp|Canada.New Brunswick|BOLD:AAB1581
Sideridis maryx|[20597]|RDLQB222-05|DH010308|658[0n]bp|Canada.Quebec|BOLD:AAB1581
Sideridis maryx|[20598]|TMNBB294-06|MNBTT-1234|658[0n]bp|Canada.New Brunswick|BOLD:AAB1581
Sideridis maryx|[20599]|RDLQB226-05|DH010312|658[0n]bp|Canada.Quebec|BOLD:AAB1581
Sideridis maryx|[20600]|RDLQB223-05|DH010309|658[0n]bp|Canada.Quebec|BOLD:AAB1581
Sideridis maryx|[20601]|TMNBB296-06|MNBTT-1236|658[0n]bp|Canada.New Brunswick|BOLD:AAB1581
Sideridis maryx|[20602]|TTMNB549-06|MNBTT-549|658[0n]bp|Canada.New Brunswick|BOLD:AAB1581
Sideridis maryx|[20603]|TMNBB292-06|MNBTT-1232|658[0n]bp|Canada.New Brunswick|BOLD:AAB1581
Sideridis maryx|[20604]|RDLQB230-05|DH010316|658[1n]bp|Canada.Quebec|BOLD:AAB1581
Sideridis maryx|[20605]|TMNBB302-06|MNBTT-1242|656[0n]bp|Canada.New Brunswick|BOLD:AAB1581
Sideridis maryx|[20606]|TMNBB300-06|MNBTT-1240|656[0n]bp|Canada.New Brunswick|BOLD:AAB1581
Sideridis maryx|[20607]|TMNBB301-06|MNBTT-1241|656[0n]bp|Canada.New Brunswick|BOLD:AAB1581
Sideridis maryx|[20608]|RDLQB231-05|DH010317|658[0n]bp|Canada.Quebec|BOLD:AAB1581
Sideridis artesta|[20609]|RDNMG579-08|CNC LEP00052403|658[0n]bp|Canada.Alberta|BOLD:ABX6687
Sideridis artesta|[20610]|RDMAB538-06|UASM58521|658[0n]bp|Canada.Alberta|BOLD:ABX6687
Sideridis artesta|[20611]|RDNMG847-08|CNC LEP00052971|658[0n]bp|Canada.Alberta|BOLD:ABX6687
Sideridis uscripta|[20612]|RDNMD083-06|CNCNoctuoidea12469|658[0n]bp|United States|BOLD:ACE5150
Sideridis uscripta|[20613]|RDNMG447-08|CNC LEP00052271|658[0n]bp|United States.Wyoming|BOLD:ACE5146
Sideridis uscripta|[20614]|RDNMG448-08|CNC LEP00052272|658[0n]bp|United States.Wyoming|BOLD:ACE5146
Sideridis uscripta|[20615]|IAWLB125-10|IAWAZ-0915|658[0n]bp|United States.California|BOLD:ACE5146
Sideridis uscripta|[20616]|NAMUM259-08|RR-97-0782|658[0n]bp|United States.California|BOLD:ACE5146
Sideridis uscripta|[20617]|RDNMG446-08|CNC LEP00052270|658[0n]bp|United States.Wyoming|BOLD:ACE5146
Sideridis fuscolutea|[20618]|RDNMF287-08|NOC14373|658[0n]bp|United States.Oregon|BOLD:AAF4049
Sideridis fuscolutea|[20619]|RDNMF286-08|NOC14372|658[0n]bp|United States.Nevada|BOLD:AAF4049
Sideridis fuscolutea|[20620]|RDNMF288-08|NOC14374|658[0n]bp|United States.Oregon|BOLD:AAF4049
Melanchra picta|[20621]|XAC156-04|04HBL006156|639[0n]bp|Canada.Ontario|BOLD:AAC8574
Melanchra picta|[20622]|PHMO302-03|moth2210.02|639[0n]bp|Canada.Ontario|BOLD:AAC8574
Melanchra picta|[20623]|XAC159-04|04HBL006159|658[0n]bp|Canada.Ontario|BOLD:AAC8574
Melanchra picta|[20624]|LHLEP414-06|UBC-2006-1047|658[0n]bp|Canada.British Columbia|BOLD:AAC8574
Melanchra picta|[20625]|RDLQ631-07|DH009710|658[0n]bp|Canada.Ontario|BOLD:AAC8574
Melanchra assimilis|[20626]|RDNMB367-05|CNCNoctuoidea10133|658[0n]bp|Canada.Ontario|BOLD:ABZ8175
Melanchra assimilis|[20627]|RDLQG361-06|DH012595|658[0n]bp|Canada.Quebec|BOLD:ABZ8175
Melanchra assimilis|[20628]|BBLPE135-09|09BBELE-2135|658[0n]bp|Canada.Nova Scotia|BOLD:ABZ8175
Melanchra assimilis|[20629]|RDNMB366-05|CNCNoctuoidea10132|658[0n]bp|Canada.Ontario|BOLD:ABZ8175
Melanchra assimilis|[20630]|BBLPC196-09|09BBELE-1196|658[0n]bp|Canada.Nova Scotia|BOLD:ABZ8175
Melanchra assimilis|[20631]|RDLQB091-05|DH010177|658[0n]bp|Canada.Quebec|BOLD:ABZ8175
[illegible]

Melanchra assimilis|[20629]|RDNMB366-05|CNCNoctuoidea10132|658[0n]bp|Canada.Ontario|BOLD:ABZ8175
Melanchra assimilis|[20630]|BBLPC196-09|09BBELE-1196|658[0n]bp|Canada.Nova Scotia|BOLD:ABZ8175
Melanchra assimilis|[20631]|RDLQB091-05|DH010177|658[0n]bp|Canada.Quebec|BOLD:ABZ8175
Melanchra assimilis|[20632]|TMNBB315-06|MNBTT-1255|658[0n]bp|Canada.New Brunswick|BOLD:ABZ8175
Melanchra pulverulenta|[20633]|LCH248-04|04HBL003248|658[0n]bp|Canada.Manitoba|BOLD:AAB0758
Melanchra pulverulenta|[20634]|TMNBB313-06|MNBTT-1253|658[0n]bp|Canada.New Brunswick|BOLD:AAB0758
Melanchra pulverulenta|[20635]|LBCA447-05|HLC-20447|658[1n]bp|Canada.British Columbia|BOLD:AAB0758
Melanchra pulverulenta|[20636]|RDNMB362-05|CNCNoctuoidea10128|580[0n]bp|Canada.British Columbia|BOLD:A...
Melanchra pulverulenta|[20637]|LBCD096-05|HLC-22916|624[0n]bp|Canada.British Columbia|BOLD:AAB0758
Melanchra pulverulenta|[20638]|LOWCD159-06|CGWC-2979|616[0n]bp|Canada.British Columbia|BOLD:AAB0758
Melanchra pulverulenta|[20639]|BBLPE218-09|09BBELE-2218|658[0n]bp|Canada.Newfoundland and Labrador|BOLD ...
Melanchra pulverulenta|[20640]|LBCH2253-10|10-JDWBC-2253|658[0n]bp|Canada.British Columbia|BOLD:AAB0758
Melanchra pulverulenta|[20641]|LBCB186-05|HLC-21126|658[0n]bp|Canada.British Columbia|BOLD:AAB0758
Melanchra pulverulenta|[20642]|BBLPB338-10|10BBCLP-1337|658[0n]bp|Canada.British Columbia|BOLD:AAB0758
Melanchra pulverulenta|[20643]|RDLQB090-05|DH010176|658[0n]bp|Canada.Quebec|BOLD:AAB0758
Melanchra pulverulenta|[20644]|BBLPE242-09|09BBELE-2242|658[0n]bp|Canada.Newfoundland and Labrador|BOLD ...
Melanchra pulverulenta|[20645]|LBCD099-05|HLC-22919|658[0n]bp|Canada.British Columbia|BOLD:AAB0758
Melanchra pulverulenta|[20646]|LOWCC084-05|CGWC-1964|658[0n]bp|Canada.British Columbia|BOLD:AAB0758
Melanchra pulverulenta|[20647]|LBCB067-05|HLC-21007|658[0n]bp|Canada.British Columbia|BOLD:AAB0758
Melanchra pulverulenta|[20648]|LBCD097-05|HLC-22917|658[0n]bp|Canada.British Columbia|BOLD:AAB0758
Melanchra pulverulenta|[20649]|LBCC001-05|HLC-21881|658[0n]bp|Canada.British Columbia|BOLD:AAB0758
Melanchra pulverulenta|[20650]|BBLPC808-09|09BBELE-1808|658[0n]bp|Canada.Newfoundland and Labrador|BOLD ...
Melanchra pulverulenta|[20651]|RDLQB089-05|DH010175|658[0n]bp|Canada.Quebec|BOLD:AAB0758
Melanchra pulverulenta|[20652]|LBCH2197-10|10-JDWBC-2197|658[0n]bp|Canada.British Columbia|BOLD:AAB0758
Melanchra pulverulenta|[20653]|LBCD610-05|HLC-23430|658[0n]bp|Canada.British Columbia|BOLD:AAB0758
Melanchra pulverulenta|[20654]|LBCB185-05|HLC-21125|658[0n]bp|Canada.British Columbia|BOLD:AAB0758
Melanchra pulverulenta|[20655]|RDNMB363-05|CNCNoctuoidea10129|658[0n]bp|Canada.Quebec|BOLD:AAB0758
Melanchra pulverulenta|[20656]|LBCC295-05|HLC-22175|658[0n]bp|Canada.British Columbia|BOLD:AAB0758
Melanchra pulverulenta|[20657]|LBCB187-05|HLC-21127|658[0n]bp|Canada.British Columbia|BOLD:AAB0758
Melanchra pulverulenta|[20658]|RDNMB365-05|CNCNoctuoidea10131|658[0n]bp|Canada.British Columbia|BOLD:A...
Melanchra pulverulenta|[20659]|PHLCH306-10|10PROBE-25712|658[0n]bp|Canada.Manitoba|BOLD:AAB0758
Melanchra pulverulenta|[20660]|LBCH2254-10|10-JDWBC-2254|658[0n]bp|Canada.British Columbia|BOLD:AAB0758
Melanchra pulverulenta|[20661]|LBCC011-05|HLC-21891|658[0n]bp|Canada.British Columbia|BOLD:AAB0758
Melanchra pulverulenta|[20662]|LCH249-04|04HBL003249|658[0n]bp|Canada.Manitoba|BOLD:AAB0758
Melanchra pulverulenta|[20663]|BBLPC745-09|09BBELE-1745|658[0n]bp|Canada.Newfoundland and Labrador|BOLD ...
Melanchra pulverulenta|[20664]|BBLPE220-09|09BBELE-2220|658[0n]bp|Canada.Newfoundland and Labrador|BOLD ...
Melanchra pulverulenta|[20665]|LBCG2468-09|08-JDWBC-2468|658[0n]bp|Canada.British Columbia|BOLD:AAB0758
Melanchra pulverulenta|[20666]|LBCD098-05|HLC-22918|658[0n]bp|Canada.British Columbia|BOLD:AAB0758
Melanchra pulverulenta|[20667]|TMNBB314-06|MNBTT-1254|658[0n]bp|Canada.New Brunswick|BOLD:AAB0758
Melanchra pulverulenta|[20668]|LCHP822-07|07PROBE-10579|658[0n]bp|Canada.Manitoba|BOLD:AAB0758
Melanchra pulverulenta|[20669]|LBCA932-05|HLC-20932|658[0n]bp|Canada.British Columbia|BOLD:AAB0758
Melanchra pulverulenta|[20670]|LBCC557-05|HLC-22437|658[0n]bp|Canada.British Columbia|BOLD:AAB0758
Lacanobia grandis|[20671]|RDLQB278-05|DH010364|658[0n]bp|Canada.Quebec|BOLD:AAB6987
Lacanobia grandis|[20672]|RDLQB272-05|DH010358|658[0n]bp|Canada.Quebec|BOLD:AAB6987
Lacanobia grandis|[20673]|LPMN815-08|08BBLEP-01618|658[0n]bp|Canada.Manitoba|BOLD:AAB6987
Lacanobia grandis|[20674]|LPSOD430-09|08BBLEP-00209|658[0n]bp|Canada.Ontario|BOLD:AAB6987
Lacanobia grandis|[20675]|RDMAB537-06|UASM58522|658[0n]bp|Canada.Alberta|BOLD:AAB6987
Lacanobia grandis|[20676]|LPVIC118-08|PFC-2006-2695|617[0n]bp|Canada.British Columbia|BOLD:AAB6987
Lacanobia grandis|[20677]|RDMAB117-05|UASM41258|658[0n]bp|Canada.Alberta|BOLD:AAB6987
Lacanobia grandis|[20678]|TTMNB550-06|MNBTT-550|658[0n]bp|Canada.New Brunswick|BOLD:AAB6987
Lacanobia grandis|[20679]|XAC294-04|04HBL006294|658[0n]bp|Canada.Ontario|BOLD:AAB6987
Lacanobia grandis|[20680]|PHMNB537-04|04HBL00763|658[0n]bp|Canada.New Brunswick|BOLD:AAB6987
Lacanobia grandis|[20681]|LPSOB379-08|PPBP-1378|658[0n]bp|Canada.Ontario|BOLD:AAB6987
Lacanobia grandis|[20682]|PHMNB459-04|04HBL00685|658[0n]bp|Canada.New Brunswick|BOLD:AAB6987
Lacanobia grandis|[20683]|LPSOB316-08|PPBP-1315|658[0n]bp|Canada.Ontario|BOLD:AAB6987
Lacanobia grandis|[20684]|RDLQB275-05|DH010361|658[0n]bp|Canada.Quebec|BOLD:AAB6987
Lacanobia grandis|[20685]|RDLQB276-05|DH010362|658[0n]bp|Canada.Quebec|BOLD:AAB6987
Lacanobia grandis|[20686]|PHMNB445-04|04HBL00671|658[0n]bp|Canada.New Brunswick|BOLD:AAB6987
Lacanobia grandis|[20687]|RDLQB274-05|DH010360|658[0n]bp|Canada.Quebec|BOLD:AAB6987
Lacanobia grandis|[20688]|XAB242-04|04HBL005242|658[0n]bp|Canada.Ontario|BOLD:AAB6987
Lacanobia grandis|[20689]|RDLQB277-05|DH010363|658[0n]bp|Canada.Quebec|BOLD:AAB6987
Lacanobia grandis|[20690]|RDLQB273-05|DH010359|658[0n]bp|Canada.Quebec|BOLD:AAB6987
Papestra cristifera|[20691]|LPABC081-09|08BBLEP-04300|658[1n]bp|Canada.Alberta|BOLD:AAB4212
Papestra cristifera|[20692]|LALPA1098-11|AVBC 908-11|658[0n]bp|Canada.British Columbia|BOLD:AAB4212
Papestra cristifera|[20693]|LPABC475-09|08BBLEP-04694|658[0n]bp|Canada.Alberta|BOLD:AAB4212
Papestra cristifera|[20694]|LBCA603-05|HLC-20603|629[0n]bp|Canada.British Columbia|BOLD:AAB4212
Papestra cristifera|[20695]|BBLPB762-10|10BBCLP-1761|658[0n]bp|Canada.British Columbia|BOLD:AAB4212
Papestra cristifera|[20696]|LPABC323-09|08BBLEP-04542|658[0n]bp|Canada.Alberta|BOLD:AAB4212
Papestra cristifera|[20697]|LPSOD888-09|08BBLEP-00670|658[0n]bp|Canada.Ontario|BOLD:AAB4212
Papestra cristifera|[20698]|RDLQ642-07|DH008638|616[0n]bp|Canada.Newfoundland and Labrador|BOLD:AAB4212
Papestra cristifera|[20699]|LBCA738-05|HLC-20738|646[0n]bp|Canada.British Columbia|BOLD:AAB4212
Papestra cristifera|[20700]|LBCD292-05|HLC-23112|627[0n]bp|Canada.British Columbia|BOLD:AAB4212
Papestra cristifera|[20701]|LBCD047-05|HLC-22867|658[0n]bp|Canada.British Columbia|BOLD:AAB4212
Papestra brenda|[20702]|RDNMF560-08|NOC14646|609[0n]bp|Canada.British Columbia|BOLD:ABY8229
Papestra brenda|[20703]|LBCH5712-10|10-JDWBC-5712|658[0n]bp|Canada.British Columbia|BOLD:ABY8229
Papestra brenda|[20704]|RDMAB1058-09|UASM120801|600[0n]bp|Canada.British Columbia|BOLD:ABY8229
Papestra brenda|[20705]|RDMAB1059-09|UASM120804|658[0n]bp|Canada.British Columbia|BOLD:ABY8229
Papestra invalida|[20706]|LBCH5145-10|10-JDWBC-5145|658[0n]bp|Canada.British Columbia|BOLD:ACF1818
Papestra invalida|[20707]|RDNMF559-08|NOC14645|658[0n]bp|Canada.British Columbia|BOLD:ACF1818
Papestra biren|[20708]|RDLQ641-07|DH008636|658[0n]bp|Canada.Newfoundland and Labrador|BOLD:AAA9849
Papestra biren|[20709]|TMNBB318-06|MNBTT-1258|658[0n]bp|Canada.New Brunswick|BOLD:AAA9849
Papestra biren|[20710]|TMNBB320-06|MNBTT-1260|658[0n]bp|Canada.New Brunswick|BOLD:AAA9849
Papestra biren|[20711]|TMNBB316-06|MNBTT-1256|658[0n]bp|Canada.New Brunswick|BOLD:AAA9849
Papestra biren|[20712]|TMNBB319-06|MNBTT-1259|658[0n]bp|Canada.New Brunswick|BOLD:AAA9849
Papestra biren|[20713]|TMNBB321-06|MNBTT-1261|658[0n]bp|Canada.New Brunswick|BOLD:AAA9849
Papestra biren|[20714]|RDNMF561-08|NOC14647|658[0n]bp|Canada.British Columbia|BOLD:AAA9849
Papestra biren|[20715]|CHLEP310-09|09PROBE-09605|639[0n]bp|Canada.Manitoba|BOLD:AAA9849
Papestra biren|[20716]|TMNBB317-06|MNBTT-1257|658[0n]bp|Canada.New Brunswick|BOLD:AAA9849
Papestra quadrata|[20717]|LOWCC157-05|CGWC-2037|533[0n]bp|Canada.British Columbia|BOLD:AAA9849
Papestra quadrata|[20718]|LOWCD758-06|CGWC-3578|658[0n]bp|Canada.British Columbia|BOLD:AAA9849
Papestra quadrata|[20719]|LOWCD755-06|CGWC-3575|656[0n]bp|Canada.British Columbia|BOLD:AAA9849
Papestra quadrata|[20720]|LOWCD757-06|CGWC-3577|656[0n]bp|Canada.British Columbia|BOLD:AAA9849
Papestra quadrata|[20721]|LCHP465-07|07PROBE-10133|632[0n]bp|Canada.Manitoba|BOLD:AAA9849
Papestra quadrata|[20722]|RDLQ637-07|DH003635|618[0n]bp|Canada.Quebec|BOLD:AAA9849
Papestra quadrata|[20723]|CHLEP217-09|09PROBE-09512|658[0n]bp|Canada.Manitoba|BOLD:AAA9849
Papestra quadrata|[20724]|LCH259-04|04HBL003259|658[0n]bp|Canada.Manitoba|BOLD:AAA9849
Papestra quadrata|[20725]|LCH260-04|04HBL003260|658[0n]bp|Canada.Manitoba|BOLD:AAA9849
Papestra quadrata|[20726]|MHCOL149-07|CHU06-COL-149|658[0n]bp|Canada.Manitoba|BOLD:AAA9849
Papestra quadrata|[20727]|LCHP009-07|07PROBE-00067|658[0n]bp|Canada.Manitoba|BOLD:AAA9849
Papestra quadrata|[20728]|LCHP240-07|07PROBE-03808|658[0n]bp|Canada.Manitoba|BOLD:AAA9849
Papestra quadrata|[20729]|RDLQ638-07|DH008631|620[0n]bp|Canada.Newfoundland and Labrador|BOLD:AAA9849
Papestra quadrata|[20730]|CHLEP105-09|09PROBE-09400|658[0n]bp|Canada.Manitoba|BOLD:AAA9849
Papestra quadrata|[20731]|LCHP381-07|07PROBE-10005|658[0n]bp|Canada.Manitoba|BOLD:AAA9849

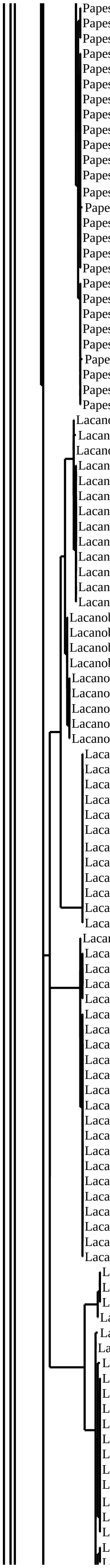

Papestra quadrata|[20729]|RDLQ638-07|DH008631|620[0n]bp|Canada.Newfoundland and Labrador|BOLD:AAA9849
Papestra quadrata|[20730]|CHLEP105-09|09PROBE-09400|658[0n]bp|Canada.Manitoba|BOLD:AAA9849
Papestra quadrata|[20731]|LCHP381-07|07PROBE-10005|658[0n]bp|Canada.Manitoba|BOLD:AAA9849
Papestra quadrata|[20732]|LBCG2474-09|08-JDWBC-2474|621[0n]bp|Canada.British Columbia|BOLD:AAA9849
Papestra quadrata|[20733]|LBCH1733-10|10-JDWBC-1733|658[0n]bp|Canada.British Columbia|BOLD:AAA9849
Papestra quadrata|[20734]|LBCH1853-10|10-JDWBC-1853|658[0n]bp|Canada.British Columbia|BOLD:AAA9849
Papestra quadrata|[20735]|LBCG2773-09|08-JDWBC-2773|658[0n]bp|Canada.British Columbia|BOLD:AAA9849
Papestra quadrata|[20736]|LBCG2774-09|08-JDWBC-2774|658[0n]bp|Canada.British Columbia|BOLD:AAA9849
Papestra quadrata|[20737]|LBCH1750-10|10-JDWBC-1750|658[0n]bp|Canada.British Columbia|BOLD:AAA9849
Papestra quadrata|[20738]|LBCH2255-10|10-JDWBC-2255|658[0n]bp|Canada.British Columbia|BOLD:AAA9849
Papestra quadrata|[20739]|LBCG3033-09|08-JDWBC-3033|658[0n]bp|Canada.British Columbia|BOLD:AAA9849
Papestra quadrata|[20740]|LBCG3032-09|08-JDWBC-3032|658[0n]bp|Canada.British Columbia|BOLD:AAA9849
Papestra quadrata|[20741]|LBCG2775-09|08-JDWBC-2775|658[0n]bp|Canada.British Columbia|BOLD:AAA9849
Papestra quadrata|[20742]|LBCH2265-10|10-JDWBC-2265|658[0n]bp|Canada.British Columbia|BOLD:AAA9849
Papestra quadrata|[20743]|LBCH2198-10|10-JDWBC-2198|658[0n]bp|Canada.British Columbia|BOLD:AAA9849
Papestra quadrata|[20744]|BBLPB873-10|10BBCLP-1872|658[0n]bp|Canada.British Columbia|BOLD:AAA9849
Papestra quadrata|[20745]|LBCG2487-09|08-JDWBC-2487|658[0n]bp|Canada.British Columbia|BOLD:AAA9849
Papestra quadrata|[20746]|LBCG2488-09|08-JDWBC-2488|658[0n]bp|Canada.British Columbia|BOLD:AAA9849
Papestra quadrata|[20747]|RDMAB608-06|UASM58664|658[0n]bp|Canada.Alberta|BOLD:AAA9849
Papestra quadrata|[20748]|CHLEP215-09|09PROBE-09510|658[0n]bp|Canada.Manitoba|BOLD:AAA9849
Papestra quadrata|[20749]|LCHP794-07|07PROBE-10480|658[0n]bp|Canada.Manitoba|BOLD:AAA9849
Papestra quadrata|[20750]|LCHP243-07|07PROBE-03811|658[0n]bp|Canada.Manitoba|BOLD:AAA9849
Papestra quadrata|[20751]|CHLEP157-09|09PROBE-09452|658[0n]bp|Canada.Manitoba|BOLD:AAA9849
Papestra quadrata|[20752]|LBCG2834-09|08-JDWBC-2834|658[0n]bp|Canada.British Columbia|BOLD:AAA9849
Papestra quadrata|[20753]|LCHP251-07|07PROBE-03819|656[0n]bp|Canada.Manitoba|BOLD:AAA9849
Papestra quadrata|[20754]|RDMAB607-06|UASM58663|624[0n]bp|Canada.Alberta|BOLD:AAA9849
Papestra quadrata|[20755]|CHLEP218-09|09PROBE-09513|643[0n]bp|Canada.Manitoba|BOLD:AAA9849
Lacanobia atlantica|[20756]|RDNM086-05|CNCNoctuoidea6355|500[0n]bp|Canada.British Columbia|
Lacanobia atlantica|[20757]|RDNM192-05|CNCNoctuoidea6374|658[0n]bp|Canada.British Columbia|BOLD:AAB7145
Lacanobia atlantica|[20758]|RDNM193-05|CNCNoctuoidea6375|658[0n]bp|Canada.British Columbia|BOLD:AAB7145
Lacanobia atlantica|[20759]|RDNM190-05|CNCNoctuoidea6372|658[0n]bp|Canada.Alberta|BOLD:AAB7145
Lacanobia atlantica|[20760]|BBLPB805-10|10BBCLP-1804|658[0n]bp|Canada.Saskatchewan|BOLD:AAB7145
Lacanobia atlantica|[20761]|RDLQ633-07|DH009404|658[0n]bp|Canada.Quebec|BOLD:AAB7145
Lacanobia atlantica|[20762]|BBLPB806-10|10BBCLP-1805|658[0n]bp|Canada.Ontario|BOLD:AAB7145
Lacanobia atlantica|[20763]|RDNM189-05|CNCNoctuoidea6371|658[0n]bp|Canada.Alberta|BOLD:AAB7145
Lacanobia atlantica|[20764]|LPMN338-08|08BBLEP-01137|658[0n]bp|Canada.Manitoba|BOLD:AAB7145
Lacanobia atlantica|[20765]|RDNM087-05|CNCNoctuoidea6356|658[0n]bp|Canada.Ontario|BOLD:AAB7145
Lacanobia atlantica|[20766]|RDLQ632-07|DH004922|658[0n]bp|Canada.Quebec|BOLD:AAB7145
Lacanobia atlantica|[20767]|RDNM191-05|CNCNoctuoidea6373|658[0n]bp|Canada.British Columbia|BOLD:AAB7145
Lacanobia atlantica|[20768]|BBLPB857-10|10BBCLP-1856|658[0n]bp|Canada.Ontario|BOLD:AAB7145
Lacanobia atlantica|[20769]|RDNM085-05|CNCNoctuoidea6354|566[0n]bp|Canada.British Columbia|BOLD:AAB7145
Lacanobia atlantica|[20770]|LPMN802-08|08BBLEP-01605|658[0n]bp|Canada.Manitoba|BOLD:AAB7145
Lacanobia atlantica|[20771]|RDNM084-05|CNCNoctuoidea6353|573[1n]bp|Canada.British Columbia|BOLD:AAB7145
Lacanobia atlantica|[20772]|BBLPB800-10|10BBCLP-1799|658[0n]bp|Canada.British Columbia|BOLD:AAB7145
Lacanobia atlantica|[20773]|TTMNB397-06|MNBTT-397|658[0n]bp|Canada.New Brunswick|BOLD:AAB7145
Lacanobia atlantica|[20774]|LPSOB230-08|PPBP-1229|646[0n]bp|Canada.Ontario|BOLD:AAB7145
Lacanobia atlantica|[20775]|LPSK124-08|08BBLEP-01692|658[0n]bp|Canada.Saskatchewan|BOLD:AAB7145
Lacanobia atlantica|[20776]|LPSOB243-08|PPBP-1242|658[0n]bp|Canada.Ontario|BOLD:AAB7145
Lacanobia atlantica|[20777]|RDNM088-05|CNCNoctuoidea6357|658[0n]bp|Canada.Ontario|BOLD:AAB7145
Lacanobia nevadae|[20778]|CHLEP280-09|09PROBE-09575|658[0n]bp|Canada.Manitoba|BOLD:ABY4611
Lacanobia nevadae|[20779]|LPABB078-08|08BBLEP-03343|658[0n]bp|Canada.Alberta|BOLD:ABY4611
Lacanobia nevadae|[20780]|LPABC479-09|08BBLEP-04698|623[0n]bp|Canada.Alberta|BOLD:ABY4611
Lacanobia nevadae|[20781]|BBLPC714-09|09BBELE-1714|547[0n]bp|Canada.Newfoundland and Labrador|BOLD:ABY...
Lacanobia nevadae|[20782]|LOWCD761-06|CGWC-3581|657[0n]bp|Canada.British Columbia|BOLD:ABY4611
Lacanobia nevadae|[20783]|LBCB825-05|HLC-21765|658[0n]bp|Canada.British Columbia|BOLD:ABY4611
Lacanobia nevadae|[20784]|RDLQF457-06|DH011564|658[0n]bp|Canada.Quebec|BOLD:ABY4611
Lacanobia nevadae|[20785]|LPABC010-09|08BBLEP-04229|658[0n]bp|Canada.Alberta|BOLD:ABY4611
Lacanobia nevadae|[20786]|LBCD454-05|HLC-23274|658[0n]bp|Canada.British Columbia|BOLD:ABY4611
Lacanobia nevadae|[20787]|BBLPE419-09|09BBELE-2419|658[0n]bp|Canada.Newfoundland and Labrador|BOLD:ABY...
Lacanobia nevadae|[20788]|LPAB235-08|08BBLEP-02557|658[0n]bp|Canada.Alberta|BOLD:ABY4611
Lacanobia nevadae|[20789]|LBCD105-05|HLC-22925|658[0n]bp|Canada.British Columbia|BOLD:ABY4611
Lacanobia radix|[20790]|LALPA412-10|AVBC 414-10|658[0n]bp|Canada.British Columbia|BOLD:AAB6755
Lacanobia radix|[20791]|LOWCC189-05|CGWC-2069|658[0n]bp|Canada.British Columbia|BOLD:AAB6755
Lacanobia radix|[20792]|LOWCC201-05|CGWC-2081|603[0n]bp|Canada.British Columbia|BOLD:AAB6755
Lacanobia radix|[20793]|LOWCC202-05|CGWC-2082|599[0n]bp|Canada.British Columbia|BOLD:AAB6755
Lacanobia radix|[20794]|LBCA600-05|HLC-20600|650[0n]bp|Canada.British Columbia|BOLD:AAB6755
Lacanobia radix|[20795]|LOWCE276-06|CGWC-4036|590[0n]bp|Canada.British Columbia|BOLD:AAB6755
Lacanobia radix|[20796]|LCHP835-07|07PROBE-10592|658[0n]bp|Canada.Manitoba|BOLD:AAB6755
Lacanobia radix|[20797]|LBCA547-05|HLC-20547|658[0n]bp|Canada.British Columbia|BOLD:AAB6755
Lacanobia radix|[20798]|LPABC396-09|08BBLEP-04615|658[0n]bp|Canada.Alberta|BOLD:AAB6755
Lacanobia radix|[20799]|RDLQB807-05|DH010894|658[0n]bp|Canada.Quebec|BOLD:AAB6755
Lacanobia radix|[20800]|LCHP965-07|07PROBE-10727|658[0n]bp|Canada.Manitoba|BOLD:AAB6755
Lacanobia radix|[20801]|LOWCC200-05|CGWC-2080|658[0n]bp|Canada.British Columbia|BOLD:AAB6755
Lacanobia radix|[20802]|LALPA956-11|AVBC 1129-11|658[0n]bp|Canada.British Columbia|BOLD:AAB6755
Lacanobia radix|[20803]|LPMN712-08|08BBLEP-01515|658[0n]bp|Canada.Manitoba|BOLD:AAB6755
Lacanobia radix|[20804]|BBLPB348-10|10BBCLP-1347|658[0n]bp|Canada.British Columbia|BOLD:AAB6755
Lacanobia radix|[20805]|LCH586-04|04HBL003586|658[0n]bp|Canada.Manitoba|BOLD:AAB6755
Lacanobia radix|[20806]|LPABB878-09|08BBLEP-04198|658[0n]bp|Canada.Alberta|BOLD:AAB6755
Lacanobia radix|[20807]|LCH247-04|04HBL003247|658[0n]bp|Canada.Manitoba|BOLD:AAB6755
Lacanobia radix|[20808]|LHLEP566-06|UBC-2006-1073|653[1n]bp|Canada.British Columbia|BOLD:AAB6755
Lacanobia radix|[20809]|LPABC251-09|08BBLEP-04470|625[1n]bp|Canada.Alberta|BOLD:AAB6755
Lacanobia radix|[20810]|RDLQG371-06|DH012605|658[0n]bp|Canada.Quebec|BOLD:AAB6755
Lacanobia radix|[20811]|LCHP543-07|07PROBE-10213|658[0n]bp|Canada.Manitoba|BOLD:AAB6755
Lacanobia sp.|[20812]|LBCH5673-10|10-JDWBC-5673|658[0n]bp|Canada.British Columbia|BOLD:ACF0859
Lacanobia sp.|[20813]|LALPA924-11|AVBC 1097-11|658[0n]bp|Canada.British Columbia|BOLD:ACF0859
Lacanobia sp.|[20814]|LBCH5912-10|10-JDWBC-5912|658[0n]bp|Canada.British Columbia|BOLD:ACF0859
Lacanobia sp.|[20815]|LBCH5697-10|10-JDWBC-5697|658[0n]bp|Canada.British Columbia|BOLD:ACF0859
Lacanobia subjuncta|[20816]|LPVIA231-08|PFC-2006-0313|658[0n]bp|Canada.British Columbia|BOLD:AAB0925
Lacanobia subjuncta|[20817]|LALPA1116-11|AVBC 926-11|658[0n]bp|Canada.British Columbia|BOLD:AAB0925
Lacanobia subjuncta|[20818]|LBCA583-05|HLC-20583|658[0n]bp|Canada.British Columbia|BOLD:AAB0925
Lacanobia subjuncta|[20819]|LALPA257-10|AVBC 258-10|658[0n]bp|Canada.British Columbia|BOLD:AAB0925
Lacanobia subjuncta|[20820]|PHMO342-03|moth2628.02|639[0n]bp|Canada.Ontario|BOLD:AAB0925
Lacanobia subjuncta|[20821]|XAH009-05|2005-ONT-1592|647[0n]bp|Canada.Ontario|BOLD:AAB0925
Lacanobia subjuncta|[20822]|LALPA338-10|AVBC 340-10|658[0n]bp|Canada.British Columbia|BOLD:AAB0925
Lacanobia subjuncta|[20823]|XAB103-04|04HBL005103|658[0n]bp|Canada.Ontario|BOLD:AAB0925
Lacanobia subjuncta|[20824]|LPSOC315-08|PPBP-2314|658[0n]bp|Canada.Ontario|BOLD:AAB0925
Lacanobia subjuncta|[20825]|XAB563-04|04HBL005563|658[0n]bp|Canada.Ontario|BOLD:AAB0925
Lacanobia subjuncta|[20826]|RDMAB124-05|UASM41270|658[0n]bp|Canada.Alberta|BOLD:AAB0925
Lacanobia subjuncta|[20827]|RDLQB508-05|DH010594|658[0n]bp|Canada.Quebec|BOLD:AAB0925
Lacanobia subjuncta|[20828]|LALPA838-11|AVBC 1011-11|658[0n]bp|Canada.British Columbia|BOLD:AAB0925
Lacanobia subjuncta|[20829]|RDLQF271-06|DH011363|658[0n]bp|Canada.Quebec|BOLD:AAB0925
Lacanobia subjuncta|[20830]|LOWCC823-05|CGWC-2703|588[0n]bp|Canada.British Columbia|BOLD:AAB0925
Lacanobia subjuncta|[20831]|LOWCC825-05|CGWC-2705|658[0n]bp|Canada.British Columbia|BOLD:AAB0925

[illegible]
Lacanobia subjuncta|[20829]|RDLQF271-06|DH011363|658[0n]bp|Canada.Quebec|BOLD:AAB0925
Lacanobia subjuncta|[20830]|LOWCC823-05|CGWC-2703|588[0n]bp|Canada.British Columbia|BOLD:AAB0925
Lacanobia subjuncta|[20831]|LOWCC825-05|CGWC-2705|658[0n]bp|Canada.British Columbia|BOLD:AAB0925
Lacanobia subjuncta|[20832]|LOWCC824-05|CGWC-2704|594[0n]bp|Canada.British Columbia|BOLD:AAB0925
Lacanobia subjuncta|[20833]|LBCB149-05|HLC-21089|658[0n]bp|Canada.British Columbia|BOLD:AAB0925
Lacanobia subjuncta|[20834]|LBCB184-05|HLC-21124|658[0n]bp|Canada.British Columbia|BOLD:AAB0925
Lacanobia subjuncta|[20835]|LOWCD750-06|CGWC-3570|658[0n]bp|Canada.British Columbia|BOLD:AAB0925
Lacanobia subjuncta|[20836]|XAH428-05|2005-ONT-2011|658[0n]bp|Canada.Ontario|BOLD:AAB0925
Lacanobia subjuncta|[20837]|LOWCD751-06|CGWC-3571|658[0n]bp|Canada.British Columbia|BOLD:AAB0925
Lacanobia subjuncta|[20838]|LOWCD752-06|CGWC-3572|658[0n]bp|Canada.British Columbia|BOLD:AAB0925
Lacanobia subjuncta|[20839]|LOWCC822-05|CGWC-2702|658[0n]bp|Canada.British Columbia|BOLD:AAB0925
Lacanobia subjuncta|[20840]|LOWCC828-05|CGWC-2708|658[0n]bp|Canada.British Columbia|BOLD:AAB0925
Lacanobia subjuncta|[20841]|LOWCD754-06|CGWC-3574|657[0n]bp|Canada.British Columbia|BOLD:AAB0925
Lacanobia subjuncta|[20842]|LOWCC827-05|CGWC-2707|658[0n]bp|Canada.British Columbia|BOLD:AAB0925
Lacanobia subjuncta|[20843]|LOWCD753-06|CGWC-3573|594[0n]bp|Canada.British Columbia|BOLD:AAB0925
Lacanobia subjuncta|[20844]|XAD287-04|04HBL007287|598[0n]bp|Canada.Ontario|BOLD:AAB0925
Lacanobia subjuncta|[20845]|PHMO257-03|moth1488.02|639[0n]bp|Canada.Ontario|BOLD:AAB0925
Lacanobia subjuncta|[20846]|LOWCC821-05|CGWC-2701|606[0n]bp|Canada.British Columbia|BOLD:AAB0925
Lacanobia subjuncta|[20847]|LBCA584-05|HLC-20584|652[0n]bp|Canada.British Columbia|BOLD:AAB0925
Lacanobia subjuncta|[20848]|LBCA581-05|HLC-20581|658[0n]bp|Canada.British Columbia|BOLD:AAB0925
Lacanobia subjuncta|[20849]|LOWCD749-06|CGWC-3569|658[0n]bp|Canada.British Columbia|BOLD:AAB0925
Lacanobia subjuncta|[20850]|LOWCC826-05|CGWC-2706|658[0n]bp|Canada.British Columbia|BOLD:AAB0925
Mamestra configurata|[20851]|LOWCD325-06|CGWC-3145|614[0n]bp|Canada.British Columbia|BOLD:ABY4746
Mamestra configurata|[20852]|LBCC755-05|HLC-22635|658[0n]bp|Canada.British Columbia|BOLD:ABY4746
Mamestra configurata|[20853]|LBCS263-07|UBC-2007-0771|658[0n]bp|Canada.British Columbia|BOLD:ABY4746
Mamestra configurata|[20854]|LPVIC124-08|PFC-2006-2701|658[0n]bp|Canada.British Columbia|BOLD:ABY4746
Mamestra configurata|[20855]|LALPA534-10|AVBC 536-10|658[0n]bp|Canada.British Columbia|BOLD:ABY4746
Mamestra configurata|[20856]|LHLEP066-06|UBC-2006-0272|658[0n]bp|Canada.British Columbia|BOLD:ABY4746
Mamestra curialis|[20857]|LOWCD274-06|CGWC-3094|598[0n]bp|Canada.British Columbia|BOLD:ABZ3096
Mamestra curialis|[20858]|RDMAB547-06|CBCC1033|606[0n]bp|Canada.Alberta|BOLD:ABZ3096
Mamestra curialis|[20859]|RDLQF453-06|DH011560|658[0n]bp|Canada.Quebec|BOLD:ABZ3096
Mamestra curialis|[20860]|LPMN816-08|08BBLEP-01619|658[0n]bp|Canada.Manitoba|BOLD:ABZ3096
Mamestra curialis|[20861]|LBCB361-05|HLC-21301|658[0n]bp|Canada.British Columbia|BOLD:ABZ3096
Spiramater lutra|[20862]|BBLPC963-09|09BBELE-1963|658[0n]bp|Canada.Newfoundland and Labrador|BOLD:AAA7346
Spiramater lutra|[20863]|LOWCD146-06|CGWC-2966|525[0n]bp|Canada.British Columbia|BOLD:AAA7346
Spiramater lutra|[20864]|LBCA215-05|HLC-20215|616[0n]bp|Canada.British Columbia|BOLD:AAA7346
Spiramater lutra|[20865]|LOWCC816-05|CGWC-2696|658[1n]bp|Canada.British Columbia|BOLD:AAA7346
Spiramater lutra|[20866]|LOWCD131-06|CGWC-2951|579[0n]bp|Canada.British Columbia|BOLD:AAA7346
Spiramater lutra|[20867]|LPVIA233-08|PFC-2006-0315|658[0n]bp|Canada.British Columbia|BOLD:AAA7346
Spiramater lutra|[20868]|LOWCC815-05|CGWC-2695|585[1n]bp|Canada.British Columbia|BOLD:AAA7346
Spiramater lutra|[20869]|BBLPE310-09|09BBELE-2310|658[0n]bp|Canada.Newfoundland and Labrador|BOLD:AAA7346
Spiramater lutra|[20870]|BBLPC644-09|09BBELE-1644|658[0n]bp|Canada.Newfoundland and Labrador|BOLD:AAA7346
Spiramater lutra|[20871]|BBLPE391-09|09BBELE-2391|658[0n]bp|Canada.Newfoundland and Labrador|BOLD:AAA7346
Spiramater lutra|[20872]|LOWCD273-06|CGWC-3093|658[0n]bp|Canada.British Columbia|BOLD:AAA7346
Spiramater lutra|[20873]|TTMNB554-06|MNBTT-554|658[0n]bp|Canada.New Brunswick|BOLD:AAA7346
Spiramater lutra|[20874]|LPSOD249-09|08BBLEP-00027|658[0n]bp|Canada.Ontario|BOLD:AAA7346
Spiramater lutra|[20875]|BBLPC855-09|09BBELE-1855|658[0n]bp|Canada.Newfoundland and Labrador|BOLD:AAA7346
Spiramater lutra|[20876]|RDLQB281-05|DH010367|658[0n]bp|Canada.Quebec|BOLD:AAA7346
Spiramater lutra|[20877]|BBLPC684-09|09BBELE-1684|658[0n]bp|Canada.Newfoundland and Labrador|BOLD:AAA7346
Spiramater lutra|[20878]|LOWCD271-06|CGWC-3091|658[0n]bp|Canada.British Columbia|BOLD:AAA7346
Spiramater lutra|[20879]|LALPA1184-11|AVBC 994-11|658[0n]bp|Canada.British Columbia|BOLD:AAA7346
Spiramater lutra|[20880]|LBCG034-08|08-JDWBC-0034|658[0n]bp|Canada.British Columbia|BOLD:AAA7346
Spiramater lutra|[20881]|TMNBB322-06|MNBTT-1262|658[0n]bp|Canada.New Brunswick|BOLD:AAA7346
Spiramater lutra|[20882]|LBCA800-05|HLC-20800|658[0n]bp|Canada.British Columbia|BOLD:AAA7346
Spiramater lutra|[20883]|LALPA892-11|AVBC 1065-11|658[0n]bp|Canada.British Columbia|BOLD:AAA7346
Spiramater lutra|[20884]|LBCB060-05|HLC-21000|658[0n]bp|Canada.British Columbia|BOLD:AAA7346
Spiramater lutra|[20885]|LOWCE816-06|CGWC-4576|658[0n]bp|Canada.British Columbia|BOLD:AAA7346
Spiramater lutra|[20886]|LALPA1167-11|AVBC 977-11|658[0n]bp|Canada.British Columbia|BOLD:AAA7346
Spiramater lutra|[20887]|LOWCC814-05|CGWC-2694|658[0n]bp|Canada.British Columbia|BOLD:AAA7346
Spiramater lutra|[20888]|LOWCD272-06|CGWC-3092|658[0n]bp|Canada.British Columbia|BOLD:AAA7346
Spiramater lutra|[20889]|TTMNB551-06|MNBTT-551|658[0n]bp|Canada.New Brunswick|BOLD:AAA7346
Spiramater lutra|[20890]|RDLQB279-05|DH010365|658[0n]bp|Canada.Quebec|BOLD:AAA7346
Spiramater lutra|[20891]|BBLPE349-09|09BBELE-2349|658[0n]bp|Canada.Newfoundland and Labrador|BOLD:AAA7346
Spiramater lutra|[20892]|TTMNB398-06|MNBTT-398|658[0n]bp|Canada.New Brunswick|BOLD:AAA7346
Spiramater lutra|[20893]|TTMNB553-06|MNBTT-553|658[0n]bp|Canada.New Brunswick|BOLD:AAA7346
Spiramater lutra|[20894]|BBLPC774-09|09BBELE-1774|658[0n]bp|Canada.Newfoundland and Labrador|BOLD:AAA7346
Spiramater lutra|[20895]|TMNBB323-06|MNBTT-1263|658[0n]bp|Canada.New Brunswick|BOLD:AAA7346
Spiramater lutra|[20896]|LALPA313-10|AVBC 315-10|658[0n]bp|Canada.British Columbia|BOLD:AAA7346
[truncated: 79,252 more chars]
